# Genomic and Metagenomic Insights into the Distribution of Nicotine-degrading Enzymes in Human Microbiota

**DOI:** 10.2174/0113892029302230240319042208

**Published:** 2024-03-20

**Authors:** Ying Guan, Zhouhai Zhu, Qiyuan Peng, Meng Li, Xuan Li, Jia-Wei Yang, Yan-Hong Lu, Meng Wang, Bin-Bin Xie

**Affiliations:** 1 Joint Institute of Tobacco and Health, Kunming, 650106, Yunnan, China;; 2 State Key Laboratory of Microbial Technology, Institute of Microbial Technology, Shandong University, Qingdao, 266237, China

**Keywords:** Nicotine-degrading enzymes, human microbiota, metagenomes, NicX, nicotine degradation, genome annotation

## Abstract

**Introduction:**

Nicotine degradation is a new strategy to block nicotine-induced pathology. The potential of human microbiota to degrade nicotine has not been explored.

**Aims:**

This study aimed to uncover the genomic potentials of human microbiota to degrade nicotine.

**Methods:**

To address this issue, we performed a systematic annotation of Nicotine-Degrading Enzymes (NDEs) from genomes and metagenomes of human microbiota. A total of 26,295 genomes and 1,596 metagenomes for human microbiota were downloaded from public databases and five types of NDEs were annotated with a custom pipeline. We found 959 NdhB, 785 NdhL, 987 NicX, three NicA1, and three NicA2 homologs.

**Results:**

Genomic classification revealed that six phylum-level taxa, including *Proteobacteria*, *Firmicutes*, *Firmicutes*_A, *Bacteroidota*, *Actinobacteriota*, and *Chloroflexota*, can produce NDEs, with *Proteobacteria* encoding all five types of NDEs studied. Analysis of NicX prevalence revealed differences among body sites. NicX homologs were found in gut and oral samples with a high prevalence but not found in lung samples. NicX was found in samples from both smokers and non-smokers, though the prevalence might be different.

**Conclusion:**

This study represents the first systematic investigation of NDEs from the human microbiota, providing new insights into the physiology and ecological functions of human microbiota and shedding new light on the development of nicotine-degrading probiotics for the treatment of smoking-related diseases.

## 1 INTRODUCTION

Tobacco smoke contains thousands of chemicals, including the well-known nicotine [1-4], and tobacco smoking is the leading cause of preventable death [5]. Smoking cessation is effective in improving the quality of life, but it is difficult to achieve for smokers. Nicotine degradation is considered a new strategy to block nicotine-induced pathology [6, 7]. Consistently, a recent study reported that smoking-related non-alcoholic steatohepatitis (NASH) can be alleviated by the degradation of gut nicotine with a gut bacterium *Bacteroides xylanisolvens* J1101, whose genome encodes a nicotine-degrading enzyme (NDE), NicX [8]. *B. xylanisolvens* is the first reported endogenous nicotine-degrading microbe from human microbiota, and its NicX is the first reported nicotine-degrading enzyme from human microbiota. Sequence and structural analyses have revealed that NicX from *B. xylanisolvens* is homologous to NicA1, an NDE from an environmental bacterium, *P. putida* S16 [8]. This observation implies that human microbiota may have NDEs that are also present in environmental microorganisms. It remains unclear whether other bacteria from human microbiota can also degrade nicotine. It is also unclear what kinds of NDEs human microbiota can produce.

Compared to the human microbiota, nicotine degradation by environmental bacteria has been studied more extensively. So far, three pathways for nicotine degradation have been identified from environmental bacteria: the pyridine pathway, the pyrrolidine pathway, and the variant of the pyridine and pyrrolidine pathway [9, 10]. The pyridine pathway has been systematically studied in *Paenarthrobacter nicotinovorans* pAO1, in which the first step is the hydroxylation of nicotine by nicotine dehydrogenase (Ndh), which contains three subunits, NdhM (medium subunit), NdhS (small subunit), and NdhL (large subunit), generating 6-hydroxynicotine [11, 12]. The pyrrolidine pathway has been systematically studied in *P. putida* S16, in which the nicotine is firstly converted to N-methylmyosmine by a nicotine oxidoreductase NicA1 or NicA2 [13, 14]. The variant of pyridine and pyrrolidine pathway was systematically studied in *Agrobacterium* sp. S33 [15]. Similar to the pyridine pathway, the first step of this pathway is also the hydroxylation of nicotine catalyzed by a nicotine dehydrogenase, which has a small subunit (NdhA) and a large subunit (NdhB) [15]. However, it is unclear whether the pathways existing in environmental bacteria also exist in human microbiota.

While nicotine degradation by bacteria seems a promising strategy to alleviate smoking-related diseases, only an endogenous nicotine-degrading bacterium has been identified from human microbiota, limiting the development of nicotine-degrading probiotics for alleviating smoking-induced diseases. To address this issue, we performed systematic annotations of nicotine-degrading enzymes in the human microbiota. By analyzing 26,295 genomes and 1,596 metagenomes from human microbiota, we found homologs of the current known NDEs, revealed bacterial groups producing these enzymes, and uncovered the distribution preference of NDE-producing microbiota in different body sites. This study provides insights into the nicotine-degrading potential of human microbiota, shedding new light on the development of nicotine-degrading probiotics for the treatment of smoking-related diseases.

## 2 MATERIALS AND METHODS

### 2.1 Genome and Metagenome Collection

Genome and metagenome sequences were downloaded from nine large projects, including (1) 2,820 assembled genomes from the Human Microbiome Project (HMP) [16] (NCBI BioProject accession PRJNA43021), (2) 8,622 genomes from the Expanded Human Oral Microbiome Database (eHOMD) [17] (https://www.homd.org/ftp//genomes/PROKKA/V10.1/), (3) 6,185 metagenome-assembled genomes from human reference gut microbiome catalog [18] (5,414 from https://www.mbiomenet.org/HRGM/listdir.php?directory=data, 280 from NCBI BioProject accession PRJNA678426, and 491 from NCBI BioProject accession PRJNA730993), (4) 3,324 genomes from the reference genomes of cultivated human gut bacteria project [19] (1,520 from NCBI BioProject PRJNA482748 and 1,804 from NCBI BioProject PRJNA903559), (5) 97 genomes from a cultured biobank of human gut microbiome [20] (NCBI BioProject PRJNA656402), (6) 216 genomes from a culture study by Browne *et al.* [21] (European Nucleotide Archive, ENA project accession PRJEB10915), (7) 452 metagenomes from Human Oral v1.0 [22] (https://www.ebi.ac.uk/metagenomics/genome-catalogues/human-oral-v1-0), (8) 4,579 metagenome-assembled genomes from the Unified Human Gastrointestinal Genome (UHGG) collection [23] (ENA project accession PRJEB33885), and (9) short reads for 558 metagenomes from Integrative Human Microbiome Project (iHMP) [24] (https://www.hmpdacc.org/).

In addition, the reads files for other eight metagenomic studies were downloaded, including 107 gut samples from Chen *et al.* [8], 162 gut samples from Wang *et al.* [25], 9 gut samples from Mas-Lloret *et al.* [26], 234 gut samples and 295 oral samples from Zhang *et al.* [27], 38 lung samples from Ren *et al.* [28], 47 lung samples from Zheng *et al.* [29], 18 lung samples from Feigelman *et al.* [30], and 128 gut samples from Ma *et al.* [31]. For these samples, the associated information (smoking status and the sampling body site) was obtained, if available.

### 2.2 Metagenome Assembly

The downloaded reads were firstly trimmed with trimmomatic v.0.39 [32] and then assembled using MEGAHIT v.1.2.9 [33]. Protein coding genes were predicted with MetaGeneMark v.3.38 [34].

### 2.3 Reference NDE Sequence Collection

Reference protein sequences for NDEs were downloaded from the NCBI nr database, including NdhL from strain pAO1 (CAD47952.1), NdhL from strain Y22 (AHK24899.1), NdhB from strain S33 (AMD56949.1), VppAL from strain SJY1 (AIH15806.1), NicA1 from strain S16 (AEJ10966.1), NicA2 from strain S16 (AEJ14620.1), and Nox from strain HZN6 (AGH68979.1). NicX sequence from *B. xylanisolvens* J1101 was obtained from the literature [8].

### 2.4 Outgroup Sequence Collection

To achieve high prediction accuracy, especially to reduce false positives, outgroup sequences of NDEs were obtained and used as negative controls. These outgroup sequences were not NDEs but showed detectable similarities to NDEs. The xanthine dehydrogenase large subunit sequences (XdhB, Uniprot accessions O54051, Q8RLC0, and Q9I3J0; the NCBI nr database accession ALT22391.1) were used as the outgroup for NdhB and NdhL. The group II intron reverse transcriptase sequences were used as the outgroup for NicA1 (PDB ID 5G2X) and NicX (PDB IDs 5HHJ and 6AR3). No outgroup sequences were found for NicA2.

### 2.5 NDE Annotation Pipeline

For each type of NDEs, the references were searched against the protein sequences of each downloaded (meta) genome with DIAMOND v.2.0.15.153 (parameters “--very-sensitive -e 0.001 -k 100000 --max-hsps 0 -f 6 qseqid sseqid pident qcovhsp qlen slen length mismatch gapopen qstart qend sstart send evalue bitscore”) [35]. Hits with the reference coverage < 40% or identity < 30% were excluded. Hits with sequence length < 50% of the query reference sequence length or > 150% of the query reference sequence length were also excluded. The remaining hits were mentioned as the candidate NDEs. The candidate NDEs were then filtered with the help of outgroup sequences based on the sequence search score and phylogenetic analyses as follows. Each candidate NDE sequence was searched against the NDE reference sequences and the corresponding outgroup sequence(s) with DIAMOND v.2.0.15.153 (parameters “--very-sensitive -e 0.001 -k 100000 --max-hsps 0 -f 6 qseqid sseqid pident qcovhsp qlen slen length mismatch gapopen qstart qend sstart send evalue bitscore”). The highest score with the reference sequences (ScoreRef) was compared with the highest score with the outgroup sequences (ScoreOut). The candidate sequence with ScoreRef < 1.2*ScoreOut was excluded. Next, the obtained sequences were aligned by using MAFFT v.7.520 (parameters “--maxiterate 1000 --globalpair --reorder”) [36] and a phylogenetic tree was reconstructed for each type of NDE with FastTree v.2.1.11 with default parameters [37]. A candidate sequence was excluded if its phylogenetic distance to the most closely related reference sequences was close to or bigger than that to the most closely related outgroup sequences. The resultant sequences were thought of as the annotated NDEs.

### 2.6 Taxonomic Classification

For each genome that contained genes for NDEs, the taxonomic classification was inferred using Genome Taxonomy Database Toolkit (GTDB-Tk) v.2.1.1 [38] with the workflow ‘classify_wf’ against the GTDB release R06-RS202 [39].

### 2.7 Phylogenetic Tree Reconstruction

The protein sequences for each type of NDEs were aligned using MAFFT v.7.520 (parameters “--maxiterate 1000 --globalpair --reorder”) [36]. The resultant alignment was inspected with the help of BioEdit v.7.0.9.0 [40] to remove sequences with too long insertions or deletions. The obtained alignment was used to reconstruct the phylogenetic tree with FastTree v.2.1.11 with default parameters [37]. The tree was visualized with the help of MEGA v.11.0.13 [41].

### 2.8 Three-dimensional Structure Prediction

The three-dimensional structures were predicted by using AlphaFold2 [42].

## 3 RESULTS

### 3.1 Collecting Genomes, Metagenomes, and Reference Sequences for NDEs

We collected a total of 26,295 assembled genomes for the human microbiome from public databases, including 3,637 genomes for pure cultures and 22,658 metagenome-assembled genomes. Moreover, 558 assembled metagenomes were downloaded. Short reads for 1,038 microbiota samples were also downloaded. For the genome and metagenome sequences without annotation, we predicted the protein-encoding genes. For the short reads from metagenome sequencing, we first assembled the metagenomes and then performed annotation. We also performed taxonomic classification for downloaded genomes (for both pure cultures and metagenome-assembled genomes) based on the genome sequence. Taken together, our final data sets contained protein sequences for a total of 26,295 genomes and 1,596 metagenomes.

To improve the annotation accuracy, we only included the protein sequences of the well-characterized NDEs as the reference. These sequences included (1) NicX from the gut bacterium *B. xylanisolvens* J1101, (2) Ndh large subunit L (NdhL) from *P. nicotinovorans* pAO1 and NdhL from *Rhodococcus* sp. Y22 (the pyridine pathway), (3) NicA1 and NicA2 from *P. putida* S16 (the pyrrolidine pathway), and (4) Ndh large subunit B (NdhB) from *Agrobacterium* sp. S33 and NdhB (designated as VppAL) from *Ochrobactrum* sp. SJY1 (the variant of pyridine and pyrrolidine pathway). In addition, we also collected the sequences for enzymes distantly related to NdhL, NdhB, NicX, or NicA1. These outgroup sequences were used to refine our prediction results, especially for excluding ambiguous predictions. No suitable outgroup sequence was found for NicA2.

### 3.2 Annotation of NDE Homologs

We developed an annotation pipeline to predict NDEs from genomes and metagenomes. To achieve high accuracy, ‘outgroup’ sequences were included in the pipeline as negative controls. These outgroup sequences were not from NDEs but showed detectable similarities to NDEs. In our pipeline, each obtained candidate NDE sequence was compared to the reference and outgroup, and those showing higher or close similarities to the outgroup than to the reference were excluded (Materials and Methods for details).

**Fig. (1):**
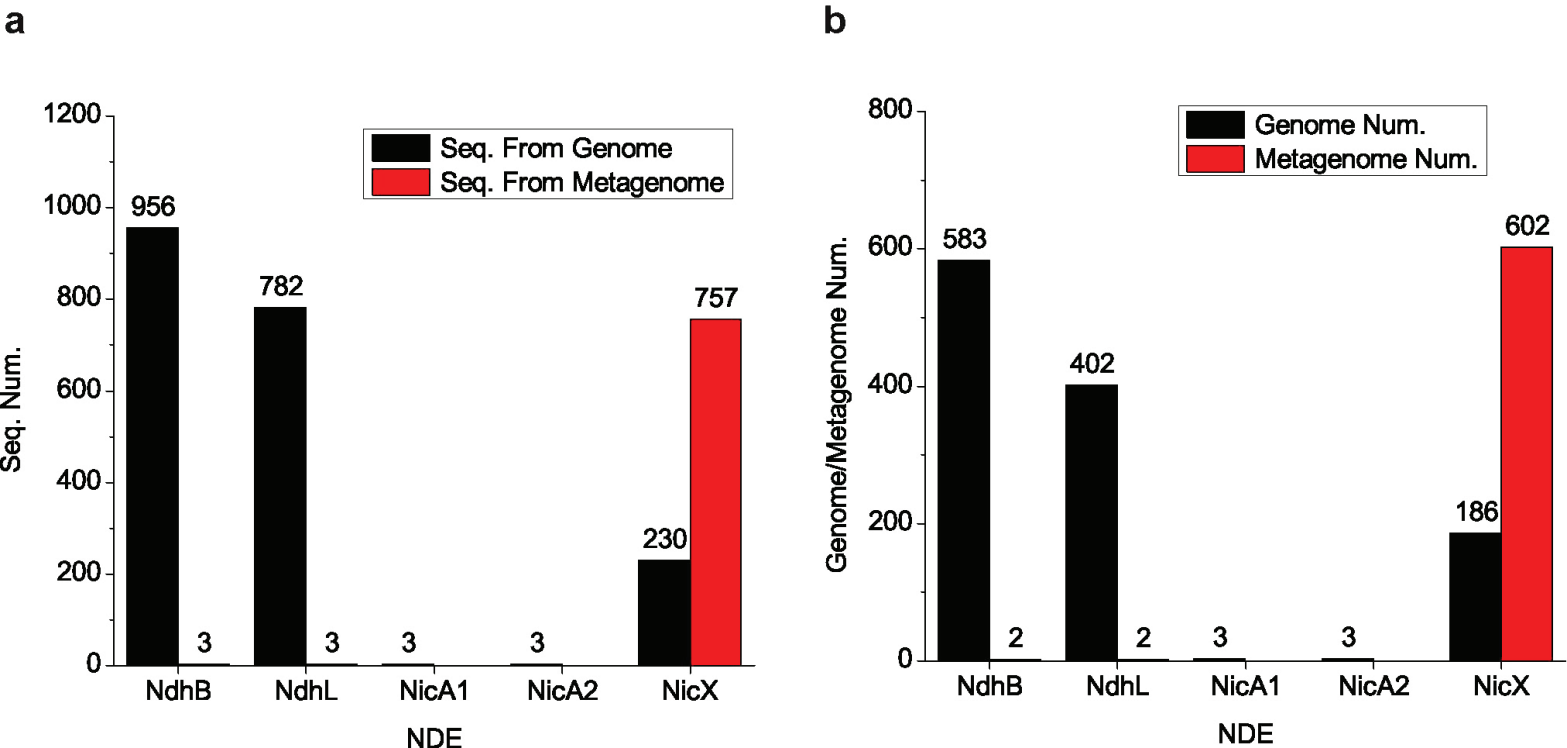
NDEs annotated from genomes and metagenomes. (**a**). Sequence number for each type of NDEs found from genomes (black) and metagenomes (red). (**b**). The number of genomes (black) and metagenomes (red) that contained NDEs.

With the above pipeline, we predicted 959 NdhB, 785 NdhL, 987 NicX, three NicA1, and three NicA2 homologs (Fig. **1a** and Supplementary materials **1-5**), clearly indicating that NDEs are widely present in human microbiota and that NicX is not the only type of NDEs present in human microbiota. The distribution of different types of NDEs was clearly different. NdhB, NdhL, and NicX were rich in human microbiota. A few NicA1 and NicA2 homologs were also found. Interestingly, it was noted that nearly all NdhB and NdhL homologs were found in genomes, and few were found in metagenomes (Fig. **1a**). In comparison, only a minor portion of NicX homologs were found in the genomes. Analyses of the (meta)genome number revealed the same distribution (Fig. **1b**). However, the reason for this observation is unclear. It might be related to the abundance of NDE-producing bacteria in the microbiota.

**Fig. (2):**
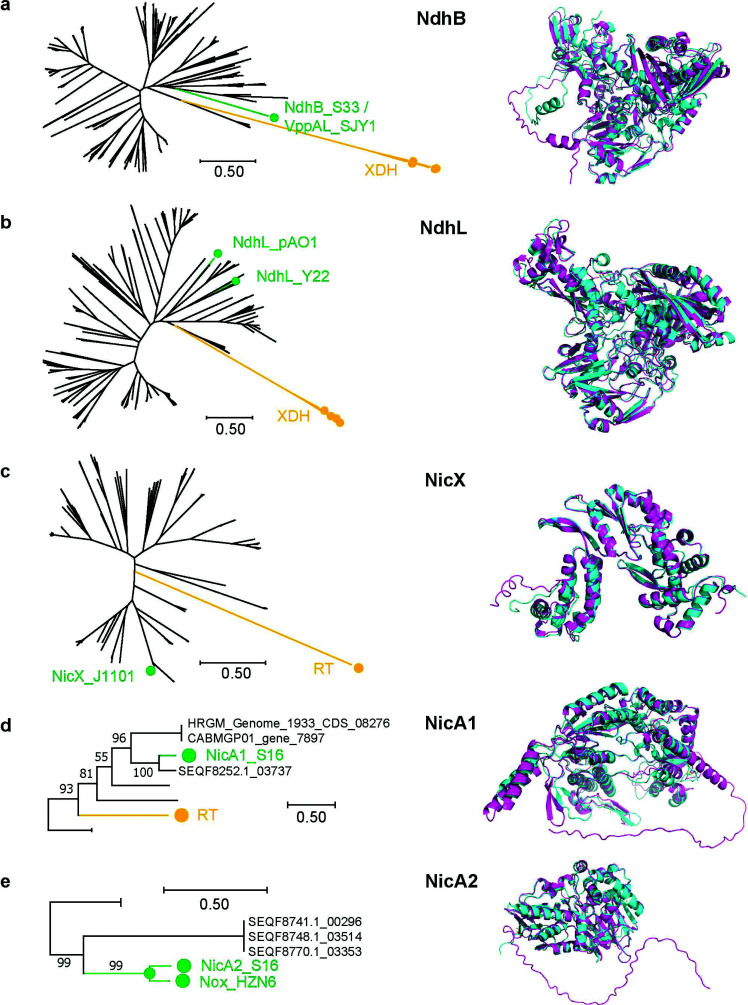
Phylogenetic trees for NDEs and structure superposition. (**a**). NdhB. Green, the reference sequences, including NdhB from strain S33 and VppAL from strain SJY1. Orange, the outgroup sequences. Right panel, structures of NdhB from S33 (magenta) and NdhB from genome HRGM_Genome_4330 (CDS_00127) (cyan). (**b**). NdhL. Green, references sequences, including NdhL from strain pAO1 and that from strain Y22. Orange, outgroup sequences. Right panel, structures of NdhL from strain pAO1 (magenta) and NdhB from genome SEQF5863.1 (gene_03686) (cyan). (**c**). NicX. Green, reference sequence, *i.e*., NicX from strain J1101. Orange, outgroup sequence. Right panel, structures of NicX from strain J1101 (magenta) and NicX from genome HRGM_Genome_1350 (CDS_00200) (cyan). (**d**). NicA1. Green, reference sequence, *i.e*., NicA1 from strain S16. Orange, outgroup sequence. Right panel, structures of NicA1 from S16 (magenta) and NicA1 from genome HRGM_Genome_1933 (CDS_08276) (cyan). (**e**). NicA2. Green, reference sequence, *i.e*., NicA2 from strain S16. Right panel, structures of NicA2 from S16 (magenta) and NicA2 from genome SEQF8741.1 (gene_00296) (cyan). The bar indicated 0.50 substitutions per site. RT, the group II intron reverse transcriptase. XDH, the xanthine dehydrogenase large subunit.

Since the annotation pipeline has excluded the candidate NDE homologs showing high similarities to the outgroup sequences, for all types of NDEs except for NicA2, the predicted NDEs had shorter distances to the reference NDEs than to the outgroup sequences on the phylogenetic trees (Fig. **2a**-**e**, green, reference, orange, outgroup). It was noted that while most NDE homologs were distantly related to the reference sequences, there were cases that predicted that homologs were highly similar to the references. For example, 67 NicX homologs were identical to the reference. Among them, four were from genomes, and taxonomic classification revealed that all four sequences (genome CABIXU02, gene_4266; genome HRGM_ Genome_1421, CDS_04332; genome AM23-12_scaffold, gene_567; genome GCA_001405055.1, gene_1552) were from the genus *Bacteroides*, including one (genome GCA_001405055.1, gene_1552) from *B. xylanisolvens*, the species in which the reference NicX was found. For NdhB, three homologs with identical sequences to the reference were found (genome SEQF8741.1, gene_00298; genome SEQF8748.1, gene_03516; and genome SEQF8770.1, gene_03351). Taxonomic classification revealed that these identical sequences were also from the genus *Agrobacterium*, to which the source organism of the reference NdhB also belongs. For the three homologs found for NicA1, one (genome SEQF8252.1, gene_03737) showed high identity (73.2%) to the reference sequence (Fig. **2d**). This NicA1 homolog was found in the genus *Pseudomonas*, to which the source organism of the reference NicA1 also belongs.

We predicted the three-dimensional structures of the NDE homologs distantly related to the reference. Structural superposition revealed that these distant NDE homologs had highly similar overall structures to the reference NDEs (Fig. **2**, right panels), except that the local structures of the short N-terminal stretches were not predicted for several sequences.

### 3.3 Taxonomic Classification of Bacteria Producing NDEs

**Fig. (3):**
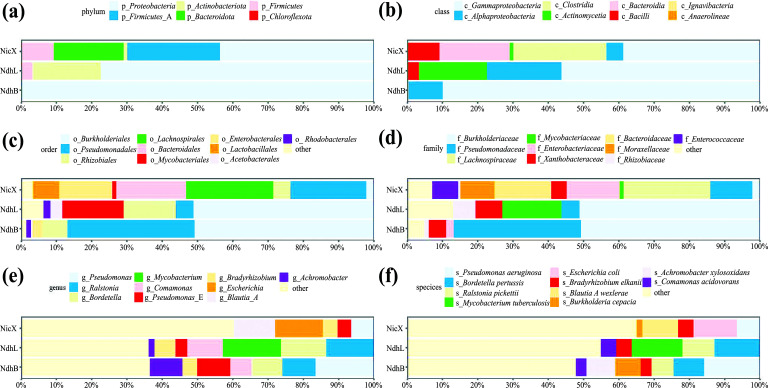
Taxonomic classification of genomes that encode NicX, NdhB, and NdhL. (**a**). The phylum level. (**b**). The class level. (**c**). The order level. (**d**). The family level. (**e**). The genus level. (**f**). The species level.

To reveal the potentials of different taxa to encode NDEs and the phylogenetic distribution of the NDEs, genomes harboring NDE genes were taxonomically classified based on the genome sequences (Figs. **3a**-**f**). It was clearly shown that different NDEs had different distributions. At the phylum level, NDEs were found in six taxa. NicX had the widest distribution, and it was found in five phyla, including *Proteobacteria* (43.5%), *Firmicutes*_A (26.3%), *Bacteroidota* (19.9%), *Firmicutes* (9.1%), and *Actinobacteriota* (1.1%). NdhL was found in four phyla, including *Proteobacteria* (77.4%), *Actinobacteriota* (19.4%), *Firmicutes* (3.0%), and *Chloroflexota* (0.2%). NdhB was found only in two phyla, including *Proteobacteria* (99.7%) and *Bacteroidota* (0.3%). For NicA1, only three homologs were found, and they were all from the same phylum, *Proteobacteria*. For NicA2, all three homologs were also from the same phylum, *Proteobacteria*. Generally, the phylum *Proteobacteria* showed the strongest potential to encode various types of NDEs, and it encoded all five types of NDEs studied here. In comparison, only two types of NDEs (NicX and NdhB) were found in the phylum *Bacteroidota*.

Comparison at different taxonomic levels revealed that NicX, NdhL, and NdhB shared a number of lower-rank taxa, though the ratios may vary. Generally, NdhL and NdhB had similar distributions. For example, at the genus level, all NDEs were found in a total of 77 genera. There were 26 genera containing both NdhL (found in 42 genera) and NdhB (found in 37 genera). In comparison, there were only six genera (*Bradyrhizobium*, *Burkholderia*, *Pseudomonas*, *Pseudomonas*_A, *Pseudomonas*_E, and *Variovorax*) containing both NicX (found in 31 genera) and NdhB, and only six genera (*Bradyrhizobium*, *Burkholderia*, *Pseudomonas*_A, *Pseudomonas*_E, *Rhizobium*, and *Variovorax*) containing both NicX and NdhL. Moreover, a remarkable difference in the distribution between NdhL and NdhB was also observed. The genus *Mycobacterium* was the most abundant genus possessing NdhL (16.4%). No NdhB was found in this genus.

### 3.4 Different Distributions Among Body Sites

**Fig. (4):**
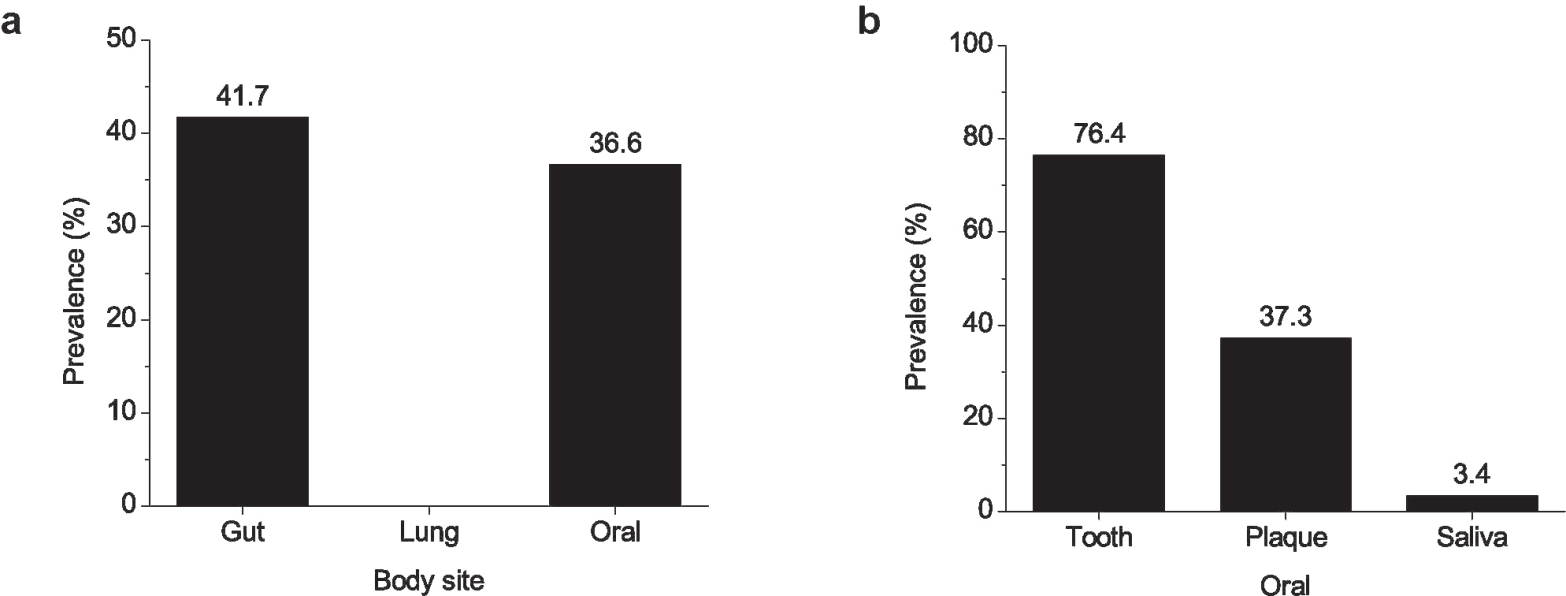
Prevalence of NicX in human microbiota samples from different body sites. A total of 640 gut samples, 103 lung samples, and 295 oral samples were analyzed (**a**). The oral samples included 72 tooth samples, 134 plaque samples, and 89 saliva samples (**b**).

The above analyses revealed that NicX homologs were found in a number of microbiota samples. To investigate whether NicX is associated with specific body sites, the proportions of microbiota samples encoding NicX (prevalence) were calculated. The collected samples could be classified into three categories, *i.e*., gut samples (n = 640), lung samples (n = 103), and oral samples (n = 295). It was clearly shown that NicX homologs were found in a high proportion of gut samples (41.7%) and oral samples (36.6%) (Fig. **4a**). Differently, no NicX homologs were found in lung samples (0%). The oral samples could be further classified into three sub- categories: tooth (n = 72), plaque (n = 134), and saliva (n = 89) [27]. Comparison of these sub-categories also revealed remarkable differences. NicX was found in up to 76.4% of tooth samples, which was 2.05 times the proportion of plaque samples (37.3%) (Fig. **4b**). In comparison, the proportion of saliva samples was very low (3.4%). These results demonstrated that NicX had different distributions among body sites.

### 3.5 Comparison of Distributions between Smokers and Non-smokers

**Fig. (5):**
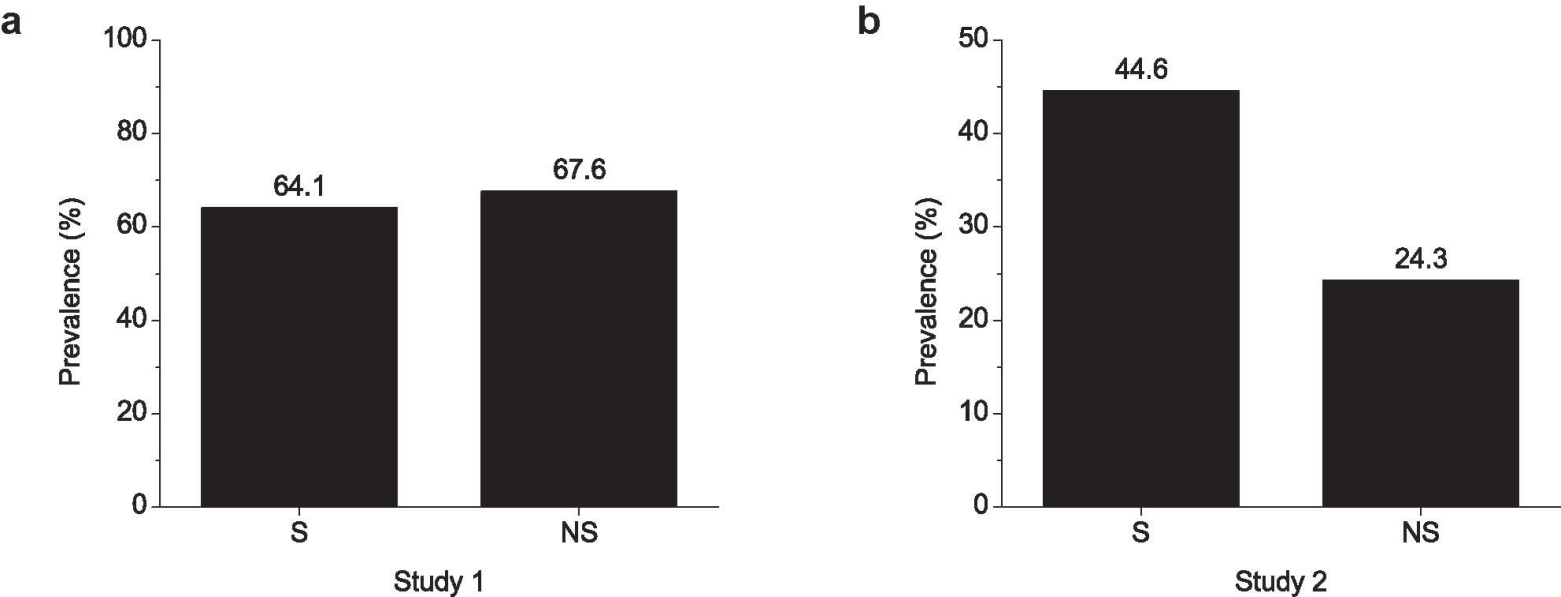
Prevalence of NicX in gut microbiota from smokers and non-smokers. (**a**). Prevalence based on samples from the study [8]. A total of 39 smoker samples and 68 non-smoker samples were analyzed. (**b**). Prevalence based on samples from the study [25]. A total of 92 smoker samples and 70 non-smoker samples were analyzed. S, smokers; NS, non-smokers.

To investigate whether the NDE distribution in microbiota is related to smoking status, the ratios of gut microbiota samples encoding NicX were compared between smokers and non-smokers. Datasets from two studies were included in our analyses [8, 25]. Results indicated that NDEs were present not only in the microbiota of smokers but also in that of non-smokers (Fig. **5**). Furthermore, it was noted that the NDE prevalence levels were different between the two studies (Fig. **5**). In the study of Chen *et al.* [8], smokers and non-smokers showed a high and similar NDE prevalence (64.1% *vs*. 67.6%). In the study of Wang *et al.* [25], both smokers and non-smokers showed NDE prevalence values lower than that in the study of Chen *et al.* [8]. Interestingly, the NDE prevalence in smokers was higher than that in non-smokers (44.6% *vs*. 24.3%).

## 4 DISCUSSION

Though NDEs from environmental bacteria have been extensively studied [9-15], NicX is the only NDE found in human microbiota so far [8]. The degradation of gut nicotine has been suggested as a strategy to reduce the harmful effects of smoking [6, 7]. A gut bacterium strain, *B. xylanisolvens* J1101, is able to alleviate smoking-related NASH by degrading gut nicotine microbiota with its NDE, NicX [8]. This study represented the first report on the systematic investigation of NDE-producing abilities of the human microbiota. The study uncovered a large number of bacterial taxa that could produce NDEs, providing new insights into the ecological functions of these taxa in human microbiota. This study also revealed thousands of NDE homologs from the human microbiota, representing a valuable resource of NDEs that merit further studies.

Most NDE homologs found in this study are distantly related to the reference NDE sequences from the environmental bacteria, suggesting that these NDEs encoded by human microbiota diverged from their possible common ancestors a long time ago. Similar to these observations, a great difference was also noted between the two NdhL reference sequences from the environmental bacteria (Fig. **2b**, green). The reference NicX from the gut bacterium *B. xylanisolvens* J1101 showed similarity to the NicA1 from the environmental bacterium *P. putida* S16 [8]. Furthermore, it was noted that several NdhB and NicA1 homologs showed high similarities to their respective references from environmental bacteria. Taxonomic annotation revealed that these NDE homologs were from the same genus or species as the reference sequences. These results indicated that, while maintaining a highly conserved three-dimensional structure, NDEs from distant taxa have less conserved sequences.

It was noted that the main types of NDE sequences annotated in genomes are different from those in metagenomes (NdhB + NdhL *vs*. NicX) (Fig. **1**). The reason for this observation remains unclear. One possible reason is that the number of genomes included in this dataset is small compared to the high diversity of the microorganisms in the human microbiota. As a result, those found in genomes may not be found in metagenomes due to the low abundances of the species harboring the NDE genes in microbiota samples. Similarly, those found in metagenomes may not be found in genomes since the microorganisms may not be included in the genome collection. It was also noted that NicX is the only NDE type that has a large number of sequences from both genomes and metagenomes. One possible reason is that the reference sequence of NicX is from a human gut bacterium [8] compared to other NDEs whose reference sequences are all from environmental bacteria. The relatively high similarities between the NicX reference sequences and homologs allow for the detection of more sequences from various microorganisms and microbiota.

The annotation pipeline used here was designed to achieve high prediction accuracy by including ‘outgroup’ sequences as negative controls. For each type of NDE, the reference sequence was searched against the NCBI nr database. The sequence names of the resultant top hits were checked. If the top hits have been consistently assigned a name different from the correct name of NDE, it means that the top hits have been assigned the wrong name in the database. For example, the top hits of NicX had the name ‘group II intron reverse transcriptase/group II intron reverse transcriptase/nicotine-degrading enzyme’ or ’group II intron reverse transcriptase/maturase’. These results suggested that the group II intron reverse transcriptase has a sequence similar to NicX, and NicX sequences were assigned the wrong name, ‘group II intron reverse transcriptase’, in the database. This readily happened when only ‘group II intron reverse transcriptase’ sequences were available in the reference database. Therefore, experimentally verified ‘group II intron reverse transcriptase’ sequences were used as outgroup sequences for NicX. By comparing the similarities to the reference and the outgroup, only sequences with remarkably higher similarities to NicX than to the outgroup ‘group II intron reverse transcriptase’ were kept in our results.

This study represents the first systematic investigation of NDEs from the human microbiota. Our results revealed that NDEs from the three major nicotine degradation pathways identified in environmental bacteria also exist in human microbiota. This study revealed a large number of taxa from human microbiota that have the ability to produce NDEs and uncovered different distributions of NDEs among body sites and between smokers and non-smokers, providing insights into the physiology and ecological functions of these taxa in human microbiota. The sequences of thousands of NDE homologs found in this study represent a valuable resource for the study of NDE enzymology.

Tobacco smoke contains various detrimental components, such as nicotine. Nicotine degradation is thought of as a new strategy to block nicotine-induced pathology [6, 7]. Chen *et al.* (2022) demonstrated the feasibility of this strategy [8]. In their study, a bacterium harboring an NDE gene *nicX* was used to degrade gut nicotine in mice to alleviate smoking-related NASH [8], suggesting that probiotics harboring NDEs may be used in the treatment of smoking-related diseases. So far, no other studies on screening nicotine-degrading gut bacteria have been reported and it remains unclear whether such bacteria are widely present in the human gut microbiota. This study showed that the NDE genes are widely present in human gut microbiota and thus demonstrated the feasibility of screening nicotine-degrading gut bacteria for the treatment of smoking-related diseases. There are two possible ways to screen the nicotine-degrading bacteria from the gut. One is the cultivation and screening of nicotine-degrading bacteria from gut samples. An increasing number of nicotine-degrading bacteria have been isolated from environmental samples [43]. Moreover, the media methods used in these studies may be helpful for the design of nicotine enrichment/selection media for gut bacteria. An alternative way is screening nicotine-degrading strains from species whose members have been found to harbor NDE genes. One such candidate species is *B. xylanisolvens,* which has been verified to be safe for *in vivo* use [44], and the strain *B. xylanisolvens* J1101 has the *nicX* gene [8]. Another candidate is *Blautia wexlerae*. It has been shown that *Blautia* is a new functional genus with potential probiotic properties [45], and our data showed that the *nicX* gene was widely present in *B. wexlerae* (Figs. **3e** and **f**).

## CONCLUSION

This study provides new insights into the association between human microbiota and health and sheds new light on the development of nicotine-degrading probiotics for the treatment of smoking-related diseases.

## Data Availability

The authors confirm that the data supporting the findings of this research are available within the article.

## LIST OF ABBREVIATIONS

eHOMD: Expanded Human Oral Microbiome Database
HMP: Human Microbiome Project
iHMP: Integrative Human Microbiome Project
NASH: Non-alcoholic Steatohepatitis
NDEs: Nicotine-degrading Enzymes
Ndh: Nicotine Dehydrogenase
UHGG: Unified Human Gastrointestinal Genome
