# Supplementary material for: Genomic and Metagenomic Insights into the Distribution of Nicotine-degrading Enzymes in Human Microbiota
Source: Curr Genomics. 2024 Mar 20;25(3):226–35. doi: 10.2174/0113892029302230240319042208 (PMC11288164; doi:10.2174/0113892029302230240319042208)
Supplement: Supplementary file 1 [file CG-25-226_SD1.zip › CG-25-226_SD1/Xie MS Suppl file 1.pdf]

>SEQF10162||SEQF10162.2_01767
MNAKTTKPRSGRRRFLLGALGIGGALVVGWGVMPPRSRVGDPGIFPEHNGEIALNGWIKITPEGDVVLAMPR
VEMGQGIHTALSMLAAEELDIPLSRVRIESAPVERIYGNVVAMGDSSLPLHPDSADKTWARALHWIMAKSARE
IGLIITGGSSSTADGWQPVREAAATARAALVQAAAREWNVPAADVSIREGQLIGPGGKQSTFGEMAKSARGISA
PSNVTLKPASQFRLIGKPAPRNDLAAKTDGSARFSIDTRLPGMLYAAVVMCPVFGGKLKTFQSKAALGMPGVR
YVVPFEGAGGGAPGVAVVADHYWQARQALATLEPVWDNGPHAKLDSAGIRQQLVSALDSDKGGFTYRSMG
DGLKAFDRADGATIVEAEYSAPYLAHATMEPINCTAQVTADGVHLWAPTQVATLAQLVAARAAGVSGDKVQID
IPLIGGGFGRRLESDFISQAVTIATKTEGKPVQVIWSREEDVRHDFYRPQAIARLKARVESGKVTAIASRSAGQSIL
AGELDRLFGAPSVGIDRYTAEGLFDLPYEIEHEHIAHLAVDLPVPVGFWRSVGHSYNGFFMEGFLNEVAAAAKL
DPLAMRRNLLKDHPRELKVLDTAAQAAGWGQPLAPAADGAPRARGIALHPSFGSVVAQVVEVSMKDGKPRV
HRVVCAVDCGTVVNPGIVAQQMESAVIFGLTAALYGRIDIKDGQVVQSNFPDYPALKMVETPVIETHIVPSTAEP
SGMGEVGVPPIAPAVAHAMAQLTGKPVRQLPMV
>SEQF10163||SEQF10163.1_01364
MNANATKPRRGRRRFLLGALGIGGALVVGWGVMPPRSRVGDPASFPEHHGEIALNGWIKITPDGSVVLAMP
RVEMGQGIHTALSMLAAEELDIPLARVSIESSPVERIYGNVVAMGIGSLPVHPDSEDKTWARALAWMMSKSA
REIGLIITGGSSSVADGWQPVREAAATARAALVEAAAREWNVPAAQVSIRDGQLIGPGGRQATFGSMAKTAQ
GISAPSSVRLKPASQYKWIGKPAPRNDLAAKTDGSARFSIDVRPPGMLYAAVVMCPVFGGKLKTFQSKAALSM
PGVRYVVPFEGSAGGAPGVAVVADHYWQARQALATVEPVWDNGPHAALDSAGIRQQLVNALNSDKGGFTY
RSMGDGLKAFDNTGGGTIVEAEYTAPYLAHATMEPMNCTAQVTKDSVQLWAPTQVATLAQMVAARAAGVD
RDKVRIDIPFIGGGFGRRLESDFVGQAVTIATRTEGRPVQVIWSREEDIRHDFYRPQAIARLKARVENGKVTAIAS
RSAGQSILAGELDRLFGAPSAGIDRYTVEGLFDLPYEIEHEHISHVSVDLPVPIGFWRSVGHSYAGFFLEGFLNEV
AAVARLDPLAMRRDLLRAHPRELKVLETAAQAAGWGQPLAPGADGAPRARGIALHPSFGSVVAQVVEVSLKD
GKPRVHRVVCAVDCGTVVNPRIVEQQMESAVIFGLTAALYGRIDIKDGQVVQSNFPDYPALKMAETPVIETHIV
PSAAAPTGVGEIGVPPIAPAVAHAVAQLTGKPVRQLPMA
>SEQF10164||SEQF10164.1_02963
MNAKTTKPRSGRRRFLLGALGIGGALVVGWGVMPPRSRVGDPGIFPEHNGEIALNGWIKITPEGDVVLAMPR
VEMGQGIHTALSMLAAEELDIPLSRVRIESAPVERIYGNVVAMGDSSLPLHPDSADKTWARALHWIMAKSARE
IGLIITGGSSSTADGWQPVREAAATARAALVQAAAREWNVPAADVSIREGQLIGPGGKQSTFGEMAKSARGISA
PSNVTLKPASQFRLIGKPAPRNDLAAKTDGSARFSIDTRLPGMLYAAVVMCPVFGGKLKTFQSKAALGMPGVR
YVVPFEGAGGGAPGVAVVADHYWQARQALATLEPVWDNGPHAKLDSAGIRQQLVSALDSDKGGFTYRSMG
DGLKAFDRADGATIVEAEYSAPYLAHATMEPINCTAQVTADGVHLWAPTQVATLAQLVAARAAGVSGDKVQID
IPLIGGGFGRRLESDFISQAVTIATKTEGKPVQVIWSREEDVRHDFYRPQAIARLKARVESGKVTAIASRSAGQSIL
AGELDRLFGAPSVGIDRYTAEGLFDLPYEIEHEHIAHLAVDLPVPVGFWRSVGHSYNGFFMEGFLNEVAAAAKL
DPLAMRRNLLKDHPRELKVLDTAAQAAGWGQPLAPAADGAPRARGIALHPSFGSVVAQVVEVSMKDGKPRV
HRVVCAVDCGTVVNPGIVAQQMESAVIFGLTAALYGRIDIKDGQVVQSNFPDYPALKMVETPVIETHIVPSTAEP
SGMGEVGVPPIAPAVAHAMAQLTGKPVRQLPMV
>SEQF10165||SEQF10165.1_05025
MNAKTTKPRSGRRRFLLGALGIGGALVVGWGVMPPRSRVGDPDIFPEHNGEIALNGWIKITPEGDVVLAMPR
VEMGQGIHTALSMLAAEELDIQLSRVRIESAPVERIYGNVVAMGDSSLPLHPDSADKTWARALHWIMAKSARE
IGLIITGGSSSTADGWQPVREAAATARAALVQAAAREWNVPAPDVSIREGQLIGPGGKQSTFGEMAKLARGISA
PSNVTLKPASQFRLIGKPAPRNDLAAKTDGSARFSIDTRLPGMLYAAVVMCPVFGGKLKTFQSKAALGMPGVR
YVVPFEGAGGGAPGVAVVADHYWQARQALATLEPVWDNGPHAKLDSAGIRQQLVSALDSDKGGFTYRSTGD
GLKAFDKTDGATIVEAEYTAPYLAHATMEPINCTAQVTSEGVHLWAPTQVATLAQLVAARAAGVSGDKVQIDIP
LIGGGFGRRLESDFISQAVTIATKTEGKPVQVIWSREEDVRHDFYRPQAIARLKARVESGKVTAIASRSAGQSILA

GELDRLFGAPSAGIDRYTAEGLFDLPYEIEHEHIAHLAVDLPVPVGFWRSVGHSYNGFFMEGFLNEVAAAAKLD
PLAMRRNLLKDHPRELKVLDTAAQAAGWGQPLAPAADGAPRARGIALHPSFGSVVAQVVEVSMKDGKPRVH
RVVCAVDCGTVVNPGIVAQQMESAVIFGLTAALYGRIDIKDGQVVQSNFTDYPALKMVETPVIETHIVPSTAEPS
GMGEVGVPPIAPAVAHAVAQLTGKPVRQLPMV
>SEQF10166||SEQF10166.1_05401
MNAKTTKPRSGRRRFLLGALGIGGALVVGWGVMPPRSRVGDPDIFPEHNGEIALNGWIKITPEGDVVLAMPR
VEMGQGIHTALSMLAAEELDIQLSRVRIESAPVERIYGNVVAMGDSSLPLHPDSADKTWARALHWIMAKSARE
IGLIITGGSSSTADGWQPVREAAATARAALVQAAAREWNVPAPDVSIREGQLIGPGGKQSTFGEMAKLARGISA
PSNVTLKPASQFRLIGKPAPRNDLAAKTDGSARFSIDTRLPGMLYAAVVMCPVFGGKLKTFQSKAALGMPGVR
YVVPFEGAGGGAPGVAVVADHYWQARQALATLEPVWDNGPHAKLDSAGIRQQLVSALDSDKGGFTYRSTGD
GLKAFDKTDGATIVEAEYTAPYLAHATMEPINCTAQVTSEGVHLWAPTQVATLAQLVAARAAGVSGDKVQIDIP
LIGGGFGRRLESDFISQAVTIATKTEGKPVQVIWSREEDVRHDFYRPQAIARLKARVESGKVTAIASRSAGQSILA
GELDRLFGAPSAGIDRYTAEGLFDLPYEIEHEHIAHLAVDLPVPVGFWRSVGHSYNGFFMEGFLNEVAAAAKLD
PLAMRRNLLKDHPRELKVLDTAAQAAGWGQPLAPAADGAPRARGIALHPSFGSVVAQVVEVSMKDGKPRVH
RVVCAVDCGTVVNPGIVAQQMESAVIFGLTAALYGRIDIKDGQVVQSNFTDYPALKMVETPVIETHIVPSTAEPS
GMGEVGVPPIAPAVAHAVAQLTGKPVRQLPMV
>SEQF10167||SEQF10167.1_01747
MNAKTTKPRSGRRRFLLGALGIGGALVVGWGVMPPRSRVGDPGIFPEHNGEIALNGWIKITPEGNVVLAMPR
VEMGQGIHTALSMLAAEELDIPLARVRIESAPVERIYGNVVAMGDSSLPLHPDSADKTWARALHWIMAKSARE
IGLIITGGSSSTADGWQPVREAAATARAALVEAAAREWNAPVAQVSIREGQLIGPGGKQSTFGEMAKSARGLS
APSNVTLKPASQFQLIGKPAPRNDLAAKTDGSARFSIDTRLPGMLYAAVVMCPAFGGKLKTFQSKAALGMPGV
RYVVPFEGTGGGAPGVAVVADHYWQARQALATLEPVWDNGPHAKLDSAGIRQQLVSALDSDKGGFTYRSM
GDGLKAFDKADGATLVEAEYSAPYLAHATMEPINCTAQVTPEGVHLWAPTQVATLAQLVAARAAGVSGDKVQI
DIPLIGGGFGRRLESDFIGQAVTIATKTDGKPVQVIWSREEDVRHDFYRPHAIARLKARVENGKVTAIASRSAGQ
SILAGELDRLFGAPSVGIDRYTAEGLFDLPYEIEHEHIAHLAVDLPVPVGFWRSVGHSYNGFFMEGFLNEVAAAA
KLDPLAMRRDLLKDHPRELKVLDTAAQAAGWGQPLAPAADGAPRARGIALHPSFGSVVAQVVEVSMKDGKP
RVHRVVCAVDCGTVVNPGIVAQQMESAVIFGLTAALYGRIDIKDGQVVQSNFPDYPALKMAETPVIETHIVPST
AEPSGMGEVGVPPIGPAVAHAVAQLTGKPVRQLPMA
>SEQF10168||SEQF10168.1_02221
MNAKTTKPRSGRRRFLLGALGIGGALVVGWGVMPPRSRVGDPDIFPEHNGEIALNGWIKITPEGDVVLAMPR
VEMGQGIHTALSMLAAEELDIQLSRVRIESAPVERIYGNVVAMGDSSLPLHPDSADKTWARALHWIMAKSARE
IGLIITGGSSSTADGWQPVREAAATARAALVQAAAREWNVPAPDVSIREGQLIGPGGKQSTFGEMAKLARGISA
PSNVTLKPASQFRLIGKPAPRNDLAAKTDGSARFSIDTRLPGMLYAAVVMCPVFGGKLKTFQSKAALGMPGVR
YVVPFEGAGGGAPGVAVVADHYWQARQALATLEPVWDNGPHAKLDSAGIRQQLVSALDSDKGGFTYRSTGD
GLKAFDKTDGATIVEAEYTAPYLAHATMEPINCTAQVTSEGVHLWAPTQVATLAQLVAARAAGVSGDKVQIDIP
LIGGGFGRRLESDFISQAVTIATKTEGKPVQVIWSREEDVRHDFYRPQAIARLKARVESGKVTAIASRSAGQSILA
GELDRLFGAPSAGIDRYTAEGLFDLPYEIEHEHIAHLAVDLPVPVGFWRSVGHSYNGFFMEGFLNEVAAAAKLD
PLAMRRNLLKDHPRELKVLDTAAQAAGWGQPLAPAADGAPRARGIALHPSFGSVVAQVVEVSMKDGKPRVH
RVVCAVDCGTVVNPGIVAQQMESAVIFGLTAALYGRIDIKDGQVVQSNFTDYPALKMVETPVIETHIVPSTAEPS
GMGEVGVPPIAPAVAHAVAQLTGKPVRQLPMV
>SEQF10169||SEQF10169.1_04059
MNAKTTKPRSGRRRFLLGALGIGGALVVGWGVMPPRSRVGDPGIFPEHNGEIALNGWIKITPEGNVVLAMPR
VEMGQGIHTALSMLAAEELDIPLARVRIESAPVERIYGNVVAMGDSSLPLHPDSADKTWARALHWIMAKSARE
IGLIITGGSSSTADGWQPVREAAATARAALVEAAAREWNAPVAQVSIREGQLIGPGGKQSTFGEMAKSARGLS

APSNVTLKPASQFQLIGKPAPRNDLAAKTDGSARFSIDTRLPGMLYAAVVMCPAFGGKLKTFQSKAALGMPGV
RYVVPFEGTGGGAPGVAVVADHYWQARQALATLEPVWDNGPHAKLDSAGIRQQLVSALDSDKGGFTYRSM
GDGLKAFDKADGATLVEAEYSAPYLAHATMEPINCTAQVTPEGVHLWAPTQVATLAQLVAARAAGVSGDKVQI
DIPLIGGGFGRRLESDFIGQAVTIATKTDGKPVQVIWSREEDVRHDFYRPHAIARLKARVENGKVTAIASRSAGQ
SILAGELDRLFGAPSVGIDRYTAEGLFDLPYEIEHEHIAHLAVDLPVPVGFWRSVGHSYNGFFMEGFLNEVAAAA
KLDPLAMRRDLLKDHPRELKVLDTAAQAAGWGQPLAPAADGAPRARGIALHPSFGSVVAQVVEVSMKDGKP
RVHRVVCAVDCGTVVNPGIVAQQMESAVIFGLTAALYGRIDIKDGQVVQSNFPDYPALKMAETPVIETHIVPST
AEPSGMGEVGVPPIGPAVAHAVAQLTGKPVRQLPMA
>SEQF10170||SEQF10170.1_00957
MNAKTTKPRSGRRRFLLGALGIGGALVVGWGVMPPRSRVGDPDIFPEHNGEIALNGWIKITPEGDVVLAMPR
VEMGQGIHTALSMLAAEELDIQLSRVRIESAPVERIYGNVVAMGDSSLPLHPDSADKTWARALHWIMAKSARE
IGLIITGGSSSTADGWQPVREAAATARAALVQAAAREWNVPAPDVSIREGQLIGPGGKQSTFGEMAKLARGISA
PSNVTLKPASQFRLIGKPAPRNDLAAKTDGSARFSIDTRLPGMLYAAVVMCPVFGGKLKTFQSKAALGMPGVR
YVVPFEGAGGGAPGVAVVADHYWQARQALATLEPVWDNGPHAKLDSAGIRQQLVSALDSDKGGFTYRSTGD
GLKAFDKTDGATIVEAEYTAPYLAHATMEPINCTAQVTSEGVHLWAPTQVATLAQLVAARAAGVSGDKVQIDIP
LIGGGFGRRLESDFISQAVTIATKTEGKPVQVIWSREEDVRHDFYRPQAIARLKARVESGKVTAIASRSAGQSILA
GELDRLFGAPSAGIDRYTAEGLFDLPYEIEHEHIAHLAVDLPVPVGFWRSVGHSYNGFFMEGFLNEVAAAAKLD
PLAMRRNLLKDHPRELKVLDTAAQAAGWGQPLAPAADGAPRARGIALHPSFGSVVAQVVEVSMKDGKPRVH
RVVCAVDCGTVVNPGIVAQQMESAVIFGLTAALYGRIDIKDGQVVQSNFTDYPALKMVETPVIETHIVPSTAEPS
GMGEVGVPPIAPAVAHAVAQLTGKPVRQLPMV
>SEQF10171||SEQF10171.1_02352
MNAKTTKPRSGRRRFLLGALGIGGALVVGWGVMPPRSRVGDPDIFPEHNGEIALNGWIKITPEGDVVLAMPR
VEMGQGIHTALSMLAAEELDIQLSRVRIESAPVERIYGNVVAMGDSSLPLHPDSADKTWARALHWIMAKSARE
IGLIITGGSSSTADGWQPVREAAATARAALVQAAAREWNVPAPDVSIREGQLIGPGGKQSTFGEMAKLARGISA
PSNVTLKPASQFRLIGKPAPRNDLAAKTDGSARFSIDTRLPGMLYAAVVMCPVFGGKLKTFQSKAALGMPGVR
YVVPFEGAGGGAPGVAVVADHYWQARQALATLEPVWDNGPHAKLDSAGIRQQLVSALDSDKGGFTYRSTGD
GLKAFDKTDGATIVEAEYTAPYLAHATMEPINCTAQVTSEGVHLWAPTQVATLAQLVAARAAGVSGDKVQIDIP
LIGGGFGRRLESDFISQAVTIATKTEGKPVQVIWSREEDVRHDFYRPQAIARLKARVESGKVTAIASRSAGQSILA
GELDRLFGAPSAGIDRYTAEGLFDLPYEIEHEHIAHLAVDLPVPVGFWRSVGHSYNGFFMEGFLNEVAAAAKLD
PLAMRRNLLKDHPRELKVLDTAAQAAGWGQPLAPAADGAPRARGIALHPSFGSVVAQVVEVSMKDGKPRVH
RVVCAVDCGTVVNPGIVAQQMESAVIFGLTAALYGRIDIKDGQVVQSNFTDYPALKMVETPVIETHIVPSTAEPS
GMGEVGVPPIAPAVAHAVAQLTGKPVRQLPMV
>SEQF10172||SEQF10172.1_02315
MNAKTTKPRSGRRRFLLGALGIGGALVVGWGVMPPRSRVGDPDIFPEHNGEIALNGWIKITPEGDVVLAMPR
VEMGQGIHTALSMLAAEELDIQLSRVRIESAPVERIYGNVVAMGDSSLPLHPDSADKTWARALHWIMAKSARE
IGLIITGGSSSTADGWQPVREAAATARAALVQAAAREWNVPAPDVSIREGQLIGPGGKQSTFGEMAKLARGISA
PSNVTLKPASQFRLIGKPAPRNDLAAKTDGSARFSIDTRLPGMLYAAVVMCPVFGGKLKTFQSKAALGMPGVR
YVVPFEGAGGGAPGVAVVADHYWQARQALATLEPVWDNGPHAKLDSAGIRQQLVSALDSDKGGFTYRSTGD
GLKAFDKTDGATIVEAEYTAPYLAHATMEPINCTAQVTSEGVHLWAPTQVATLAQLVAARAAGVSGDKVQIDIP
LIGGGFGRRLESDFISQAVTIATKTEGKPVQVIWSREEDVRHDFYRPQAIARLKARVESGKVTAIASRSAGQSILA
GELDRLFGAPSAGIDRYTAEGLFDLPYEIEHEHIAHLAVDLPVPVGFWRSVGHSYNGFFMEGFLNEVAAAAKLD
PLAMRRNLLKDHPRELKVLDTAAQAAGWGQPLAPAADGAPRARGIALHPSFGSVVAQVVEVSMKDGKPRVH
RVVCAVDCGTVVNPGIVAQQMESAVIFGLTAALYGRIDIKDGQVVQSNFTDYPALKMVETPVIETHIVPSTAEPS
GMGEVGVPPIAPAVAHAVAQLTGKPVRQLPMV

>SEQF10173||SEQF10173.1_04662
MNAKTTKPRSGRRRFLLGALGIGGALVVGWGVMPPRSRVGDPDIFPEHNGEIALNGWIKITPEGDVVLAMPR
VEMGQGIHTALSMLAAEELDIQLSRVRIESAPVERIYGNVVAMGDSSLPLHPDSADKTWARALHWIMAKSARE
IGLIITGGSSSTADGWQPVREAAATARAALVQAAAREWNVPAPDVSIREGQLIGPGGKQSTFGEMAKLARGISA
PSNVTLKPASQFRLIGKPAPRNDLAAKTDGSARFSIDTRLPGMLYAAVVMCPVFGGKLKTFQSKAALGMPGVR
YVVPFEGAGGGAPGVAVVADHYWQARQALATLEPVWDNGPHAKLDSAGIRQQLVSALDSDKGGFTYRSTGD
GLKAFDKTDGATIVEAEYTAPYLAHATMEPINCTAQVTSEGVHLWAPTQVATLAQLVAARAAGVSGDKVQIDIP
LIGGGFGRRLESDFISQAVTIATKTEGKPVQVIWSREEDVRHDFYRPQAIARLKARVESGKVTAIASRSAGQSILA
GELDRLFGAPSAGIDRYTAEGLFDLPYEIEHEHIAHLAVDLPVPVGFWRSVGHSYNGFFMEGFLNEVAAAAKLD
PLAMRRNLLKDHPRELKVLDTAAQAAGWGQPLAPAADGAPRARGIALHPSFGSVVAQVVEVSMKDGKPRVH
RVVCAVDCGTVVNPGIVAQQMESAVIFGLTAALYGRIDIKDGQVVQSNFTDYPALKMVETPVIETHIVPSTAEPS
GMGEVGVPPIAPAVAHAVAQLTGKPVRQLPMV
>SEQF10174||SEQF10174.1_01747
MNAKTTKPRSGRRRFLLGALGIGGALVVGWGVMPPRSRVGDPGIFPEHNGEIALNGWIKITPEGNVVLAMPR
VEMGQGIHTALSMLAAEELDIPLARVRIESAPVERIYGNVVAMGDSSLPLHPDSADKTWARALHWIMAKSARE
IGLIITGGSSSTADGWQPVREAAATARAALVEAAAREWNAPVAQVSIREGQLIGPGGKQSTFGEMAKSARGLS
APSNVTLKPASQFQLIGKPAPRNDLAAKTDGSARFSIDTRLPGMLYAAVVMCPAFGGKLKTFQSKAALGMPGV
RYVVPFEGTGGGAPGVAVVADHYWQARQALATLEPVWDNGPHAKLDSAGIRQQLVSALDSDKGGFTYRSM
GDGLKAFDKADGATLVEAEYSAPYLAHATMEPINCTAQVTPEGVHLWAPTQVATLAQLVAARAAGVSGDKVQI
DIPLIGGGFGRRLESDFIGQAVTIATKTDGKPVQVIWSREEDVRHDFYRPHAIARLKARVENGKVTAIASRSAGQ
SILAGELDRLFGAPSVGIDRYTAEGLFDLPYEIEHEHIAHLAVDLPVPVGFWRSVGHSYNGFFMEGFLNEVAAAA
KLDPLAMRRDLLKDHPRELKVLDTAAQAAGWGQPLAPAADGAPRARGIALHPSFGSVVAQVVEVSMKDGKP
RVHRVVCAVDCGTVVNPGIVAQQMESAVIFGLTAALYGRIDIKDGQVVQSNFPDYPALKMAETPVIETHIVPST
AEPSGMGEVGVPPIGPAVAHAVAQLTGKPVRQLPMA
>SEQF10175||SEQF10175.1_05479
MNAKTTKPRSGRRRFLLGALGIGGALVVGWGVMPPRSRVGDPDIFPEHNGEIALNGWIKITPEGDVVLAMPR
VEMGQGIHTALSMLAAEELDIQLSRVRIESAPVERIYGNVVAMGDSSLPLHPDSADKTWARALHWIMAKSARE
IGLIITGGSSSTADGWQPVREAAATARAALVQAAAREWNVPAPDVSIREGQLIGPGGKQSTFGEMAKLARGISA
PSNVTLKPASQFRLIGKPAPRNDLAAKTDGSARFSIDTRLPGMLYAAVVMCPVFGGKLKTFQSKAALGMPGVR
YVVPFEGAGGGAPGVAVVADHYWQARQALATLEPVWDNGPHAKLDSAGIRQQLVSALDSDKGGFTYRSTGD
GLKAFDKTDGATIVEAEYTAPYLAHATMEPINCTAQVTSEGVHLWAPTQVATLAQLVAARAAGVSGDKVQIDIP
LIGGGFGRRLESDFISQAVTIATKTEGKPVQVIWSREEDVRHDFYRPQAIARLKARVESGKVTAIASRSAGQSILA
GELDRLFGAPSAGIDRYTAEGLFDLPYEIEHEHIAHLAVDLPVPVGFWRSVGHSYNGFFMEGFLNEVAAAAKLD
PLAMRRNLLKDHPRELKVLDTAAQAAGWGQPLAPAADGAPRARGIALHPSFGSVVAQVVEVSMKDGKPRVH
RVVCAVDCGTVVNPGIVAQQMESAVIFGLTAALYGRIDIKDGQVVQSNFTDYPALKMVETPVIETHIVPSTAEPS
GMGEVGVPPIAPAVAHAVAQLTGKPVRQLPMV
>SEQF10176||SEQF10176.1_04354
MNAKTTKPRSGRRRFLLGALGIGGALVVGWGVMPPRSRVGDPGIFPEHNGEIALNGWIKITPEGNVVLAMPR
VEMGQGIHTALSMLAAEELDIPLARVRIESAPVERIYGNVVAMGDSSLPLHPDSADKTWARALHWIMAKSARE
IGLIITGGSSSTADGWQPVREAAATARAALVEAAAREWNAPVAQVSIREGQLIGPGGKQSTFGEMAKSARGLS
APSNVTLKPASQFQLIGKPAPRNDLAAKTDGSARFSIDTRLPGMLYAAVVMCPAFGGKLKTFQSKAALGMPGV
RYVVPFEGTGGGAPGVAVVADHYWQARQALATLEPVWDNGPHAKLDSAGIRQQLVSALDSDKGGFTYRSM
GDGLKAFDKADGATLVEAEYSAPYLAHATMEPINCTAQVTPEGVHLWAPTQVATLAQLVAARAAGVSGDKVQI
DIPLIGGGFGRRLESDFIGQAVTIATKTDGKPVQVIWSREEDVRHDFYRPHAIARLKARVENGKVTAIASRSAGQ

SILAGELDRLFGAPSVGIDRYTAEGLFDLPYEIEHEHIAHLAVDLPVPVGFWRSVGHSYNGFFMEGFLNEVAAAA
KLDPLAMRRDLLKDHPRELKVLDTAAQAAGWGQPLAPAADGAPRARGIALHPSFGSVVAQVVEVSMKDGKP
RVHRVVCAVDCGTVVNPGIVAQQMESAVIFGLTAALYGRIDIKDGQVVQSNFPDYPALKMAETPVIETHIVPST
AEPSGMGEVGVPPIGPAVAHAVAQLTGKPVRQLPMA
>SEQF10177||SEQF10177.1_02279
MNAKTTKPRSGRRRFLLGALGIGGALVVGWGVMPPRSRVGDPDIFPEHNGEIALNGWIKITPEGDVVLAMPR
VEMGQGIHTALSMLAAEELDIQLSRVRIESAPVERIYGNVVAMGDSSLPLHPDSADKTWARALHWIMAKSARE
IGLIITGGSSSTADGWQPVREAAATARAALVQAAAREWNVPAPDVSIREGQLIGPGGKQSTFGEMAKLARGISA
PSNVTLKPASQFRLIGKPAPRNDLAAKTDGSARFSIDTRLPGMLYAAVVMCPVFGGKLKTFQSKAALGMPGVR
YVVPFEGAGGGAPGVAVVADHYWQARQALATLEPVWDNGPHAKLDSAGIRQQLVSALDSDKGGFTYRSTGD
GLKAFDKTDGATIVEAEYTAPYLAHATMEPINCTAQVTSEGVHLWAPTQVATLAQLVAARAAGVSGDKVQIDIP
LIGGGFGRRLESDFISQAVTIATKTEGKPVQVIWSREEDVRHDFYRPQAIARLKARVESGKVTAIASRSAGQSILA
GELDRLFGAPSAGIDRYTAEGLFDLPYEIEHEHIAHLAVDLPVPVGFWRSVGHSYNGFFMEGFLNEVAAAAKLD
PLAMRRNLLKDHPRELKVLDTAAQAAGWGQPLAPAADGAPRARGIALHPSFGSVVAQVVEVSMKDGKPRVH
RVVCAVDCGTVVNPGIVAQQMESAVIFGLTAALYGRIDIKDGQVVQSNFTDYPALKMVETPVIETHIVPSTAEPS
GMGEVGVPPIAPAVAHAVAQLTGKPVRQLPMV
>SEQF10178||SEQF10178.1_05323
MNAKTTKPRSGRRRFLLGALGIGGALVVGWGVMPPRSRVGDPDIFPEHNGEIALNGWIKITPEGDVVLAMPR
VEMGQGIHTALSMLAAEELDIQLSRVRIESAPVERIYGNVVAMGDSSLPLHPDSADKTWARALHWIMAKSARE
IGLIITGGSSSTADGWQPVREAAATARAALVQAAAREWNVPAPDVSIREGQLIGPGGKQSTFGEMAKLARGISA
PSNVTLKPASQFRLIGKPAPRNDLAAKTDGSARFSIDTRLPGMLYAAVVMCPVFGGKLKTFQSKAALGMPGVR
YVVPFEGAGGGAPGVAVVADHYWQARQALATLEPVWDNGPHAKLDSAGIRQQLVSALDSDKGGFTYRSTGD
GLKAFDKTDGATIVEAEYTAPYLAHATMEPINCTAQVTSEGVHLWAPTQVATLAQLVAARAAGVSGDKVQIDIP
LIGGGFGRRLESDFISQAVTIATKTEGKPVQVIWSREEDVRHDFYRPQAIARLKARVESGKVTAIASRSAGQSILA
GELDRLFGAPSAGIDRYTAEGLFDLPYEIEHEHIAHLAVDLPVPVGFWRSVGHSYNGFFMEGFLNEVAAAAKLD
PLAMRRNLLKDHPRELKVLDTAAQAAGWGQPLAPAADGAPRARGIALHPSFGSVVAQVVEVSMKDGKPRVH
RVVCAVDCGTVVNPGIVAQQMESAVIFGLTAALYGRIDIKDGQVVQSNFTDYPALKMVETPVIETHIVPSTAEPS
GMGEVGVPPIAPAVAHAVAQLTGKPVRQLPMV
>SEQF10179||SEQF10179.1_05141
MNAKTTKPRSGRRRFLLGALGIGGALVVGWGVMPPRSRVGDPDIFPEHNGEIALNGWIKITPEGDVVLAMPR
VEMGQGIHTALSMLAAEELDIQLSRVRIESAPVERIYGNVVAMGDSSLPLHPDSADKTWARALHWIMAKSARE
IGLIITGGSSSTADGWQPVREAAATARAALVQAAAREWNVPAPDVSIREGQLIGPGGKQSTFGEMAKLARGISA
PSNVTLKPASQFRLIGKPAPRNDLAAKTDGSARFSIDTRLPGMLYAAVVMCPVFGGKLKTFQSKAALGMPGVR
YVVPFEGAGGGAPGVAVVADHYWQARQALATLEPVWDNGPHAKLDSAGIRQQLVSALDSDKGGFTYRSTGD
GLKAFDKTDGATIVEAEYTAPYLAHATMEPINCTAQVTSEGVHLWAPTQVATLAQLVAARAAGVSGDKVQIDIP
LIGGGFGRRLESDFISQAVTIATKTEGKPVQVIWSREEDVRHDFYRPQAIARLKARVESGKVTAIASRSAGQSILA
GELDRLFGAPSAGIDRYTAEGLFDLPYEIEHEHIAHLAVDLPVPVGFWRSVGHSYNGFFMEGFLNEVAAAAKLD
PLAMRRNLLKDHPRELKVLDTAAQAAGWGQPLAPAADGAPRARGIALHPSFGSVVAQVVEVSMKDGKPRVH
RVVCAVDCGTVVNPGIVAQQMESAVIFGLTAALYGRIDIKDGQVVQSNFTDYPALKMVETPVIETHIVPSTAEPS
GMGEVGVPPIAPAVAHAVAQLTGKPVRQLPMV
>SEQF10180||SEQF10180.1_04807
MNAKTTKPRSGRRRFLLGALGIGGALVVGWGVMPPRSRVGDPDIFPEHNGEIALNGWIKITPEGDVVLAMPR
VEMGQGIHTALSMLAAEELDIQLSRVRIESAPVERIYGNVVAMGDSSLPLHPDSADKTWARALHWIMAKSARE
IGLIITGGSSSTADGWQPVREAAATARAALVQAAAREWNVPAPDVSIREGQLIGPGGKQSTFGEMAKLARGISA

PSNVTLKPASQFRLIGKPAPRNDLAAKTDGSARFSIDTRLPGMLYAAVVMCPVFGGKLKTFQSKAALGMPGVR
YVVPFEGAGGGAPGVAVVADHYWQARQALATLEPVWDNGPHAKLDSAGIRQQLVSALDSDKGGFTYRSTGD
GLKAFDKTDGATIVEAEYTAPYLAHATMEPINCTAQVTSEGVHLWAPTQVATLAQLVAARAAGVSGDKVQIDIP
LIGGGFGRRLESDFISQAVTIATKTEGKPVQVIWSREEDVRHDFYRPQAIARLKARVESGKVTAIASRSAGQSILA
GELDRLFGAPSAGIDRYTAEGLFDLPYEIEHEHIAHLAVDLPVPVGFWRSVGHSYNGFFMEGFLNEVAAAAKLD
PLAMRRNLLKDHPRELKVLDTAAQAAGWGQPLAPAADGAPRARGIALHPSFGSVVAQVVEVSMKDGKPRVH
RVVCAVDCGTVVNPGIVAQQMESAVIFGLTAALYGRIDIKDGQVVQSNFTDYPALKMVETPVIETHIVPSTAEPS
GMGEVGVPPIAPAVAHAVAQLTGKPVRQLPMV
>SEQF10181||SEQF10181.1_05477
MNAKTTKPRSGRRRFLLGALGIGGALVVGWGVMPPRSRVGDPDIFPEHNGEIALNGWIKITPEGDVVLAMPR
VEMGQGIHTALSMLAAEELDIQLSRVRIESAPVERIYGNVVAMGDSSLPLHPDSADKTWARALHWIMAKSARE
IGLIITGGSSSTADGWQPVREAAATARAALVQAAAREWNVPAPDVSIREGQLIGPGGKQSTFGEMAKLARGISA
PSNVTLKPASQFRLIGKPAPRNDLAAKTDGSARFSIDTRLPGMLYAAVVMCPVFGGKLKTFQSKAALGMPGVR
YVVPFEGAGGGAPGVAVVADHYWQARQALATLEPVWDNGPHAKLDSAGIRQQLVSALDSDKGGFTYRSTGD
GLKAFDKTDGATIVEAEYTAPYLAHATMEPINCTAQVTSEGVHLWAPTQVATLAQLVAARAAGVSGDKVQIDIP
LIGGGFGRRLESDFISQAVTIATKTEGKPVQVIWSREEDVRHDFYRPQAIARLKARVESGKVTAIASRSAGQSILA
GELDRLFGAPSAGIDRYTAEGLFDLPYEIEHEHIAHLAVDLPVPVGFWRSVGHSYNGFFMEGFLNEVAAAAKLD
PLAMRRNLLKDHPRELKVLDTAAQAAGWGQPLAPAADGAPRARGIALHPSFGSVVAQVVEVSMKDGKPRVH
RVVCAVDCGTVVNPGIVAQQMESAVIFGLTAALYGRIDIKDGQVVQSNFTDYPALKMVETPVIETHIVPSTAEPS
GMGEVGVPPIAPAVAHAVAQLTGKPVRQLPMV
>SEQF10182||SEQF10182.1_02175
MNAKTTKPRSGRRRFLLGALGIGGALVVGWGVMPPRSRVGDPDIFPEHNGEIALNGWIKITPEGDVVLAMPR
VEMGQGIHTALSMLAAEELDIQLSRVRIESAPVERIYGNVVAMGDSSLPLHPDSADKTWARALHWIMAKSARE
IGLIITGGSSSTADGWQPVREAAATARAALVQAAAREWNVPAPDVSIREGQLIGPGGKQSTFGEMAKLARGISA
PSNVTLKPASQFRLIGKPAPRNDLAAKTDGSARFSIDTRLPGMLYAAVVMCPVFGGKLKTFQSKAALGMPGVR
YVVPFEGAGGGAPGVAVVADHYWQARQALATLEPVWDNGPHAKLDSAGIRQQLVSALDSDKGGFTYRSTGD
GLKAFDKTDGATIVEAEYTAPYLAHATMEPINCTAQVTSEGVHLWAPTQVATLAQLVAARAAGVSGDKVQIDIP
LIGGGFGRRLESDFISQAVTIATKTEGKPVQVIWSREEDVRHDFYRPQAIARLKARVESGKVTAIASRSAGQSILA
GELDRLFGAPSAGIDRYTAEGLFDLPYEIEHEHIAHLAVDLPVPVGFWRSVGHSYNGFFMEGFLNEVAAAAKLD
PLAMRRNLLKDHPRELKVLDTAAQAAGWGQPLAPAADGAPRARGIALHPSFGSVVAQVVEVSMKDGKPRVH
RVVCAVDCGTVVNPGIVAQQMESAVIFGLTAALYGRIDIKDGQVVQSNFTDYPALKMVETPVIETHIVPSTAEPS
GMGEVGVPPIAPAVAHAVAQLTGKPVRQLPMV
>SEQF10183||SEQF10183.1_02270
MNAKTTKPRSGRRRFLLGALGIGGALVVGWGVMPPRSRVGDPGIFPEHNGEIALNGWIKITPEGNVVLAMPR
VEMGQGIHTALSMLAAEELDIPLARVRIESAPVERIYGNVVAMGDSSLPLHPDSADKTWARALHWIMAKSARE
IGLIITGGSSSTADGWQPVREAAATARAALVEAAAREWNAPVAQVSIREGQLIGPGGKQSTFGEMAKSARGLS
APSNVTLKPASQFQLIGKPAPRNDLAAKTDGSARFSIDTRLPGMLYAAVVMCPAFGGKLKTFQSKAALGMPGV
RYVVPFEGTGGGAPGVAVVADHYWQARQALATLEPVWDNGPHAKLDSAGIRQQLVSALDSDKGGFTYRSM
GDGLKAFDKADGATLVEAEYSAPYLAHATMEPINCTAQVTPEGVHLWAPTQVATLAQLVAARAAGVSGDKVQI
DIPLIGGGFGRRLESDFIGQAVTIATKTDGKPVQVIWSREEDVRHDFYRPHAIARLKARVENGKVTAIASRSAGQ
SILAGELDRLFGAPSVGIDRYTAEGLFDLPYEIEHEHIAHLAVDLPVPVGFWRSVGHSYNGFFMEGFLNEVAAAA
KLDPLAMRRDLLKDHPRELKVLDTAAQAAGWGQPLAPAADGAPRARGIALHPSFGSVVAQVVEVSMKDGKP
RVHRVVCAVDCGTVVNPGIVAQQMESAVIFGLTAALYGRIDIKDGQVVQSNFPDYPALKMAETPVIETHIVPST
AEPSGMGEVGVPPIGPAVAHAVAQLTGKPVRQLPMA

>SEQF10184||SEQF10184.1_02593
MNAKTTKPRSGRRRFLLGALGIGGALVVGWGVMPPRSRVGDPDIFPEHNGEIALNGWIKITPEGDVVLAMPR
VEMGQGIHTALSMLAAEELDIQLSRVRIESAPVERIYGNVVAMGDSSLPLHPDSADKTWARALHWIMAKSARE
IGLIITGGSSSTADGWQPVREAAATARAALVQAAAREWNVPAPDVSIREGQLIGPGGKQSTFGEMAKLARGISA
PSNVTLKPASQFRLIGKPAPRNDLAAKTDGSARFSIDTRLPGMLYAAVVMCPVFGGKLKTFQSKAALGMPGVR
YVVPFEGAGGGAPGVAVVADHYWQARQALATLEPVWDNGPHAKLDSAGIRQQLVSALDSDKGGFTYRSTGD
GLKAFDKTDGATIVEAEYTAPYLAHATMEPINCTAQVTSEGVHLWAPTQVATLAQLVAARAAGVSGDKVQIDIP
LIGGGFGRRLESDFISQAVTIATKTEGKPVQVIWSREEDVRHDFYRPQAIARLKARVESGKVTAIASRSAGQSILA
GELDRLFGAPSAGIDRYTAEGLFDLPYEIEHEHIAHLAVDLPVPVGFWRSVGHSYNGFFMEGFLNEVAAAAKLD
PLAMRRNLLKDHPRELKVLDTAAQAAGWGQPLAPAADGAPRARGIALHPSFGSVVAQVVEVSMKDGKPRVH
RVVCAVDCGTVVNPGIVAQQMESAVIFGLTAALYGRIDIKDGQVVQSNFTDYPALKMVETPVIETHIVPSTAEPS
GMGEVGVPPIAPAVAHAVAQLTGKPVRQLPMV
>SEQF10185||SEQF10185.1_02504
MNAKTTKPRSGRRRFLLGALGIGGALVVGWGVMPPRSRVGDPDIFPEHNGEIALNGWIKITPEGDVVLAMPR
VEMGQGIHTALSMLAAEELDIQLSRVRIESAPVERIYGNVVAMGDSSLPLHPDSADKTWARALHWIMAKSARE
IGLIITGGSSSTADGWQPVREAAATARAALVQAAAREWNVPAPDVSIREGQLIGPGGKQSTFGEMAKLARGISA
PSNVTLKPASQFRLIGKPAPRNDLAAKTDGSARFSIDTRLPGMLYAAVVMCPVFGGKLKTFQSKAALGMPGVR
YVVPFEGAGGGAPGVAVVADHYWQARQALATLEPVWDNGPHAKLDSAGIRQQLVSALDSDKGGFTYRSTGD
GLKAFDKTDGATIVEAEYTAPYLAHATMEPINCTAQVTSEGVHLWAPTQVATLAQLVAARAAGVSGDKVQIDIP
LIGGGFGRRLESDFISQAVTIATKTEGKPVQVIWSREEDVRHDFYRPQAIARLKARVESGKVTAIASRSAGQSILA
GELDRLFGAPSAGIDRYTAEGLFDLPYEIEHEHIAHLAVDLPVPVGFWRSVGHSYNGFFMEGFLNEVAAAAKLD
PLAMRRNLLKDHPRELKVLDTAAQAAGWGQPLAPAADGAPRARGIALHPSFGSVVAQVVEVSMKDGKPRVH
RVVCAVDCGTVVNPGIVAQQMESAVIFGLTAALYGRIDIKDGQVVQSNFTDYPALKMVETPVIETHIVPSTAEPS
GMGEVGVPPIAPAVAHAVAQLTGKPVRQLPMV
>SEQF10186||SEQF10186.1_01646
MNAKTTKPRSGRRRFLLGALGIGGALVVGWGVMPPRSRVGDPGIFPEHNGEIALNGWIKITPEGNVVLAMPR
VEMGQGIHTALSMLAAEELDIPLARVRIESAPVERIYGNVVAMGDSSLPLHPDSADKTWARALHWIMAKSARE
IGLIITGGSSSTADGWQPVREAAATARAALVEAAAREWNAPVAQVSIREGQLIGPGGKQSTFGEMAKSARGLS
APSNVTLKPASQFQLIGKPAPRNDLAAKTDGSARFSIDTRLPGMLYAAVVMCPAFGGKLKTFQSKAALGMPGV
RYVVPFEGTGGGAPGVAVVADHYWQARQALATLEPVWDNGPHAKLDSAGIRQQLVSALDSDKGGFTYRSM
GDGLKAFDKADGATLVEAEYSAPYLAHATMEPINCTAQVTPEGVHLWAPTQVATLAQLVAARAAGVSGDKVQI
DIPLIGGGFGRRLESDFIGQAVTIATKTDGKPVQVIWSREEDVRHDFYRPHAIARLKARVENGKVTAIASRSAGQ
SILAGELDRLFGAPSVGIDRYTAEGLFDLPYEIEHEHIAHLAVDLPVPVGFWRSVGHSYNGFFMEGFLNEVAAAA
KLDPLAMRRDLLKDHPRELKVLDTAAQAAGWGQPLAPAADGAPRARGIALHPSFGSVVAQVVEVSMKDGKP
RVHRVVCAVDCGTVVNPGIVAQQMESAVIFGLTAALYGRIDIKDGQVVQSNFPDYPALKMAETPVIETHIVPST
AEPSGMGEVGVPPIGPAVAHAVAQLTGKPVRQLPMA
>SEQF10187||SEQF10187.1_04256
MNAKTTKPRSGRRRFLLGALGIGGALVVGWGVMPPRSRVGDPGIFPEHNGEIALNGWIKITPEGNVVLAMPR
VEMGQGIHTALSMLAAEELDIPLARVRIESAPVERIYGNVVAMGDSSLPLHPDSADKTWARALHWIMAKSARE
IGLIITGGSSSTADGWQPVREAAATARAALVEAAAREWNAPVAQVSIREGQLIGPGGKQSTFGEMAKSARGLS
APSNVTLKPASQFQLIGKPAPRNDLAAKTDGSARFSIDTRLPGMLYAAVVMCPAFGGKLKTFQSKAALGMPGV
RYVVPFEGTGGGAPGVAVVADHYWQARQALATLEPVWDNGPHAKLDSAGIRQQLVSALDSDKGGFTYRSM
GDGLKAFDKADGATLVEAEYSAPYLAHATMEPINCTAQVTPEGVHLWAPTQVATLAQLVAARAAGVSGDKVQI
DIPLIGGGFGRRLESDFIGQAVTIATKTDGKPVQVIWSREEDVRHDFYRPHAIARLKARVENGKVTAIASRSAGQ

SILAGELDRLFGAPSVGIDRYTAEGLFDLPYEIEHEHIAHLAVDLPVPVGFWRSVGHSYNGFFMEGFLNEVAAAA
KLDPLAMRRDLLKDHPRELKVLDTAAQAAGWGQPLAPAADGAPRARGIALHPSFGSVVAQVVEVSMKDGKP
RVHRVVCAVDCGTVVNPGIVAQQMESAVIFGLTAALYGRIDIKDGQVVQSNFPDYPALKMAETPVIETHIVPST
AEPSGMGEVGVPPIGPAVAHAVAQLTGKPVRQLPMA
>SEQF10188||SEQF10188.1_02183
MNAKTTKPRSGRRRFLLGALGIGGALVVGWGVMPPRSRVGDPDIFPEHKGEIALNGWIKITPEGDVVLAMPR
VEMGQGIHTALSMLAAEELDIPLSRVRIESAPVERIYGNVVAMGDSSLPLHPDSADKTWARALHWIMAKSARE
IGLIITGGSSSTADGWQPVREAAATARAALVQAAAREWNVPAADVSIREGQLIGPGGKQSTFGEMAKPPRGISA
PSNVTLKPASQFRLIGKPAPRNDLAAKTDGSARFSIDTRLPGMLYAAVVMCPVFGGKLKTFQSKAALGMPGVR
YVVPFEGAGGGAPGVAVVADHYWQARQALATLEPVWDNGPHAKLDSAGIRQQLVSALDSDKGGFTYRSMG
DGLKAFDKTDGTTIVEAEYTAPYLAHATMEPINCTAQVTSEGVHLWAPTQVATLAQLVAARAAGVSGDKVHIDI
PLIGGGFGRRLESDFIGQAVTIATKTEGKPVQVIWSREEDVRHDFYRPHAIARLKARVQNGKVTAIASRSAGQSIL
AGELDRLFGAPSAGIDRYTAEGLFDLPYEIEHEHIAHLAVDLPVPVGFWRSVGHSYNGFFMEGFLNEVATQAKL
DPLAIRRELLKDHPRELKVLDTAAQAAGWGQPLAPAADGAPRARGIALHPSFGSVVAQVVEVSMKDGKPRVH
RVVCAVDCGTVVNPGIVAQQMESAVIFGLSAALYGRIDIKDGQIVQSNFTDYPALKMAETPVIETHIVPSTAEPS
GMGEVGVPPIAPAVAHAVAQLTGKPVRQLPMV
>SEQF10189||SEQF10189.1_02311
MNAKTTKPRSGRRRFLLGALGIGGALVVGWGVMPPRSRVGDPDIFPEHNGEIALNGWIKITPEGDVVLAMPR
VEMGQGIHTALSMLAAEELDIQLSRVRIESAPVERIYGNVVAMGDSSLPLHPDSADKTWARALHWIMAKSARE
IGLIITGGSSSTADGWQPVREAAATARAALVQAAAREWNVPAPDVSIREGQLIGPGGKQSTFGEMAKLARGISA
PSNVTLKPASQFRLIGKPAPRNDLAAKTDGSARFSIDTRLPGMLYAAVVMCPVFGGKLKTFQSKAALGMPGVR
YVVPFEGAGGGAPGVAVVADHYWQARQALATLEPVWDNGPHAKLDSAGIRQQLVSALDSDKGGFTYRSTGD
GLKAFDKTDGATIVEAEYTAPYLAHATMEPINCTAQVTSEGVHLWAPTQVATLAQLVAARAAGVSGDKVQIDIP
LIGGGFGRRLESDFISQAVTIATKTEGKPVQVIWSREEDVRHDFYRPQAIARLKARVESGKVTAIASRSAGQSILA
GELDRLFGAPSAGIDRYTAEGLFDLPYEIEHEHIAHLAVDLPVPVGFWRSVGHSYNGFFMEGFLNEVAAAAKLD
PLAMRRNLLKDHPRELKVLDTAAQAAGWGQPLAPAADGAPRARGIALHPSFGSVVAQVVEVSMKDGKPRVH
RVVCAVDCGTVVNPGIVAQQMESAVIFGLTAALYGRIDIKDGQVVQSNFTDYPALKMVETPVIETHIVPSTAEPS
GMGEVGVPPIAPAVAHAVAQLTGKPVRQLPMV
>SEQF10190||SEQF10190.1_02596
MNAKTTKPRSGRRRFLLGALGIGGALVVGWGVMPPRSRVGDPDIFPEHNGEIALNGWIKITPEGDVVLAMPR
VEMGQGIHTALSMLAAEELDIQLSRVRIESAPVERIYGNVVAMGDSSLPLHPDSADKTWARALHWIMAKSARE
IGLIITGGSSSTADGWQPVREAAATARAALVQAAAREWNVPAPDVSIREGQLIGPGGKQSTFGEMAKLARGISA
PSNVTLKPASQFRLIGKPAPRNDLAAKTDGSARFSIDTRLPGMLYAAVVMCPVFGGKLKTFQSKAALGMPGVR
YVVPFEGAGGGAPGVAVVADHYWQARQALATLEPVWDNGPHAKLDSAGIRQQLVSALDSDKGGFTYRSTGD
GLKAFDKTDGATIVEAEYTAPYLAHATMEPINCTAQVTSEGVHLWAPTQVATLAQLVAARAAGVSGDKVQIDIP
LIGGGFGRRLESDFISQAVTIATKTEGKPVQVIWSREEDVRHDFYRPQAIARLKARVESGKVTAIASRSAGQSILA
GELDRLFGAPSAGIDRYTAEGLFDLPYEIEHEHIAHLAVDLPVPVGFWRSVGHSYNGFFMEGFLNEVAAAAKLD
PLAMRRNLLKDHPRELKVLDTAAQAAGWGQPLAPAADGAPRARGIALHPSFGSVVAQVVEVSMKDGKPRVH
RVVCAVDCGTVVNPGIVAQQMESAVIFGLTAALYGRIDIKDGQVVQSNFTDYPALKMVETPVIETHIVPSTAEPS
GMGEVGVPPIAPAVAHAVAQLTGKPVRQLPMV
>SEQF10191||SEQF10191.1_02107
MNAKTTKPRSGRRRFLLGALGIGGALVVGWGVMPPRSRVGDPDIFPEHNGEIALNGWIKITPEGDVVLAMPR
VEMGQGIHTALSMLAAEELDIQLSRVRIESAPVERIYGNVVAMGDSSLPLHPDSADKTWARALHWIMAKSARE
IGLIITGGSSSTADGWQPVREAAATARAALVQAAAREWNVPAPDVSIREGQLIGPGGKQSTFGEMAKLARGISA

PSNVTLKPASQFRLIGKPAPRNDLAAKTDGSARFSIDTRLPGMLYAAVVMCPVFGGKLKTFQSKAALGMPGVR
YVVPFEGAGGGAPGVAVVADHYWQARQALATLEPVWDNGPHAKLDSAGIRQQLVSALDSDKGGFTYRSTGD
GLKAFDKTDGATIVEAEYTAPYLAHATMEPINCTAQVTSEGVHLWAPTQVATLAQLVAARAAGVSGDKVQIDIP
LIGGGFGRRLESDFISQAVTIATKTEGKPVQVIWSREEDVRHDFYRPQAIARLKARVESGKVTAIASRSAGQSILA
GELDRLFGAPSAGIDRYTAEGLFDLPYEIEHEHIAHLAVDLPVPVGFWRSVGHSYNGFFMEGFLNEVAAAAKLD
PLAMRRNLLKDHPRELKVLDTAAQAAGWGQPLAPAADGAPRARGIALHPSFGSVVAQVVEVSMKDGKPRVH
RVVCAVDCGTVVNPGIVAQQMESAVIFGLTAALYGRIDIKDGQVVQSNFTDYPALKMVETPVIETHIVPSTAEPS
GMGEVGVPPIAPAVAHAVAQLTGKPVRQLPMV
>SEQF10192||SEQF10192.1_05401
MNAKTTKPRSGRRRFLLGALGIGGALVVGWGVMPPRSRVGDPDIFPEHNGEIALNGWIKITPEGDVVLAMPR
VEMGQGIHTALSMLAAEELDIQLSRVRIESAPVERIYGNVVAMGDSSLPLHPDSADKTWARALHWIMAKSARE
IGLIITGGSSSTADGWQPVREAAATARAALVQAAAREWNVPAPDVSIREGQLIGPGGKQSTFGEMAKLARGISA
PSNVTLKPASQFRLIGKPAPRNDLAAKTDGSARFSIDTRLPGMLYAAVVMCPVFGGKLKTFQSKAALGMPGVR
YVVPFEGAGGGAPGVAVVADHYWQARQALATLEPVWDNGPHAKLDSAGIRQQLVSALDSDKGGFTYRSTGD
GLKAFDKTDGATIVEAEYTAPYLAHATMEPINCTAQVTSEGVHLWAPTQVATLAQLVAARAAGVSGDKVQIDIP
LIGGGFGRRLESDFISQAVTIATKTEGKPVQVIWSREEDVRHDFYRPQAIARLKARVESGKVTAIASRSAGQSILA
GELDRLFGAPSAGIDRYTAEGLFDLPYEIEHEHIAHLAVDLPVPVGFWRSVGHSYNGFFMEGFLNEVAAAAKLD
PLAMRRNLLKDHPRELKVLDTAAQAAGWGQPLAPAADGAPRARGIALHPSFGSVVAQVVEVSMKDGKPRVH
RVVCAVDCGTVVNPGIVAQQMESAVIFGLTAALYGRIDIKDGQVVQSNFTDYPALKMVETPVIETHIVPSTAEPS
GMGEVGVPPIAPAVAHAVAQLTGKPVRQLPMV
>SEQF10193||SEQF10193.1_04966
MNAKATKPRSGRRRFLLGALGIGGALVVGWGVMPPRSRVGDPGVFPEHDGEIALNGWIKITPEGNVVLAMP
RVEMGQGIHTALSMLAAEELDIPLSRVSIESAPVERIYGNVVAMGDSSLPLHPDSADKTWARALHWIMAKSAR
EIGLIITGGSSSTADGWQPVREAAATARAALVEAAARAWNVSAADVSIREGQLIGPGGKQSTFGEMAKSARGIS
APSSVTLKPASQFQLIGKPAPRNDLAAKTDGSARFSIDTRLPGMLYAAVVMCPVFGGKLKTFQSKAALGMAGV
RYVVPFEGTDGGAPGVAVVADHYWQARQALATLEPVWDNGPHAKLDSAGIRQQLVSALDSDKGGFTYRSM
GDGLKAFDKADGATIVEAEYSAPYLAHATMEPINCTAQVTADGVHLWAPTQVATLAQLVAARAAGVSGDKVHI
DIPLIGGGFGRRLESDFVGQAVTIATKTEGKPVQVIWSREEDVRHDFYRPHAIARLKARVENGKVTAIASRSAGQ
SILAGELDRLFGAPSAGIDRYTAEGLFDLPYEIEHEHIAHLAVDLPVPVGFWRSVGHSYNGFFMEGFLNEVAAQA
KLDPLAMRRDLLKDHPRELKVLDTAAQAAGWGQPLAPAADGAPRARGIALHPSFGSVVAQVVEVSMKDGKP
RVHRVVCAVDCGTVVNPGIVAQQMESAVIFGLTAALYGRIDIKDGQVVQSNFPDYPALKMAETPVIETHIVPST
AEPSGMGEVGVPPIAPAVAHAVAQLTGKPVRQLPMV
>SEQF10193||SEQF10193.1_02630
MNAPRDLHAPSRRRFLQQSALLTAGLAVGFRLPDALAAGKDGAAGEFEPNAWVRVLADNTVKIVVHKHDSGT
GTQTALAACVAEELDVDPMRIAVVTPEDPFFQDYIHPIWKVYSTGGSTSVSLEYGRLRQAGATARAMLISAAAQ
RWGVPVAACSTADGTVVCNADGRKATYGELADAAGRLPAPADVALKKPADFKYIGKLRHKRDALAKATGRFQY
GIDVSLPGMLVAVVQRAPMPGARVRRIDSTEALKVRGVRQVMTIPMRSDVLGGNQEGVAVLADDYWAANK
GRAALKVDWDDSAFADFDSSDVAQHQAAWLASGKARVVHTVQTGNTDVAIAHGTKRIDAAYRMPYKQQNP
LEPVNITAWFKDGAMQYWGGIQVPSTAMEAAEVICGVRRDKVVLHEMVSGGSFGARESKYWLFEVTYLAKKT
GVPVKLMNSREDEMRALYGHPATYHRLEGALDAQGRLDALRIRAVSPASPEQWEPGYFDRPDRMDYSTTEAL
SKWDFPYRANHMDIGWVRHETGIPTGWYRAVSFIPNVFATESFIDELAVAARRDPVDFRVAHMADRPRHVDV
LRTAAQRAGWGRAAHGHALGVATHQAYDSYIAVVAQVARKDGRVVVEKLTCVADVGLAVSRTGVEEQLYGGL
MWGLGHALFDRIDIQGGAVQQSNFHDYPVMRMSDMPAIDIVIIDGNRDKPGGVGELANPPVAPAIANAIFRL
TGKRQRETPFNFDLKA

>SEQF10194||SEQF10194.1_02293
MNAKTTKPRSGRRRFLLGALGIGGALVVGWGVMPPRSRVGDPGIFPEHNGEIALNGWIKITPEGDVVLAMPR
VEMGQGIHTALSMLAAEELDIPLSRVRIESAPVERIYGNVVAMGDSSLPLHPDSADKTWARALHWIMAKSARE
IGLIITGGSSSTADGWQPVREAAATARAALVQAAAREWNVPAADVSIREGQLIGPGGKQSTFGEMAKSARGISA
PSNVTLKPASQFRLIGKPAPRNDLAAKTDGSARFSIDTRLPGMLYAAVVMCPVFGGKLKTFQSKAALGMPGVR
YVVPFEGAGGGAPGVAVVADHYWQARQALATLEPVWDNGPHAKLDSAGIRQQLVSALDSDKGGFTYRSMG
DGLKAFDRADGATIVEAEYSAPYLAHATMEPINCTAQVTADGVHLWAPTQVATLAQLVAARAAGVSGDKVQID
IPLIGGGFGRRLESDFISQAVTIATKTEGKPVQVIWSREEDVRHDFYRPQAIARLKARVESGKVTAIASRSAGQSIL
AGELDRLFGAPSVGIDRYTAEGLFDLPYEIEHEHIAHLAVDLPVPVGFWRSVGHSYNGFFMEGFLNEVAAAAKL
DPLAMRRNLLKDHPRELKVLDTAAQAAGWGQPLAPAADGAPRARGIALHPSFGSVVAQVVEVSMKDGKPRV
HRVVCAVDCGTVVNPGIVAQQMESAVIFGLTAALYGRIDIKDGQVVQSNFPDYPALKMVETPVIETHIVPSTAEP
SGMGEVGVPPIAPAVAHAMAQLTGKPVRQLPMV
>SEQF10195||SEQF10195.1_02310
MNAKTTKPRSGRRRFLLGALGIGGALVVGWGVMPPRSRVGDPDIFPEHNGEIALNGWIKITPEGDVVLAMPR
VEMGQGIHTALSMLAAEELDIQLSRVRIESAPVERIYGNVVAMGDSSLPLHPDSADKTWARALHWIMAKSARE
IGLIITGGSSSTADGWQPVREAAATARAALVQAAAREWNVPAPDVSIREGQLIGPGGKQSTFGEMAKLARGISA
PSNVTLKPASQFRLIGKPAPRNDLAAKTDGSARFSIDTRLPGMLYAAVVMCPVFGGKLKTFQSKAALGMPGVR
YVVPFEGAGGGAPGVAVVADHYWQARQALATLEPVWDNGPHAKLDSAGIRQQLVSALDSDKGGFTYRSTGD
GLKAFDKTDGATIVEAEYTAPYLAHATMEPINCTAQVTSEGVHLWAPTQVATLAQLVAARAAGVSGDKVQIDIP
LIGGGFGRRLESDFISQAVTIATKTEGKPVQVIWSREEDVRHDFYRPQAIARLKARVESGKVTAIASRSAGQSILA
GELDRLFGAPSAGIDRYTAEGLFDLPYEIEHEHIAHLAVDLPVPVGFWRSVGHSYNGFFMEGFLNEVAAAAKLD
PLAMRRNLLKDHPRELKVLDTAAQAAGWGQPLAPAADGAPRARGIALHPSFGSVVAQVVEVSMKDGKPRVH
RVVCAVDCGTVVNPGIVAQQMESAVIFGLTAALYGRIDIKDGQVVQSNFTDYPALKMVETPVIETHIVPSTAEPS
GMGEVGVPPIAPAVAHAVAQLTGKPVRQLPMV
>SEQF10196||SEQF10196.1_00200
MNAKTTKPRSGRRRFLLGALGIGGALVVGWGVMPPRSRVGDPDIFPEHNGEIALNGWIKITPEGDVVLAMPR
VEMGQGIHTALSMLAAEELDIQLSRVRIESAPVERIYGNVVAMGDSSLPLHPDSADKTWARALHWIMAKSARE
IGLIITGGSSSTADGWQPVREAAATARAALVQAAAREWNVPAPDVSIREGQLIGPGGKQSTFGEMAKLARGISA
PSNVTLKPASQFRLIGKPAPRNDLAAKTDGSARFSIDTRLPGMLYAAVVMCPVFGGKLKTFQSKAALGMPGVR
YVVPFEGAGGGAPGVAVVADHYWQARQALATLEPVWDNGPHAKLDSAGIRQQLVSALDSDKGGFTYRSTGD
GLKAFDKTDGATIVEAEYTAPYLAHATMEPINCTAQVTSEGVHLWAPTQVATLAQLVAARAAGVSGDKVQIDIP
LIGGGFGRRLESDFISQAVTIATKTEGKPVQVIWSREEDVRHDFYRPQAIARLKARVESGKVTAIASRSAGQSILA
GELDRLFGAPSAGIDRYTAEGLFDLPYEIEHEHIAHLAVDLPVPVGFWRSVGHSYNGFFMEGFLNEVAAAAKLD
PLAMRRNLLKDHPRELKVLDTAAQAAGWGQPLAPAADGAPRARGIALHPSFGSVVAQVVEVSMKDGKPRVH
RVVCAVDCGTVVNPGIVAQQMESAVIFGLTAALYGRIDIKDGQVVQSNFTDYPALKMVETPVIETHIVPSTAEPS
GMGEVGVPPIAPAVAHAVAQLTGKPVRQLPMV
>SEQF10197||SEQF10197.1_03413
MNAKTTKPRSGRRRFLLGALGIGGALVVGWGVMPPRSRVGDPGIFPEHNGEIALNGWIKITPEGDVVLAMPR
VEMGQGIHTALSMLAAEELDIPLSRVRIESAPVERIYGNVVAMGDSSLPLHPDSADKTWARALHWIMAKSARE
IGLIITGGSSSTADGWQPVREAAATARAALVQAAAREWNVPAADVSIREGQLIGPGGKQSTFGEMAKSARGISA
PSNVTLKPASQFRLIGKPAPRNDLAAKTDGSARFSIDTRLPGMLYAAVVMCPVFGGKLKTFQSKAALGMPGVR
YVVPFEGAGGGAPGVAVVADHYWQARQALATLEPVWDNGPHAKLDSAGIRQQLVSALDSDKGGFTYRSMG
DGLKAFDRADGATIVEAEYSAPYLAHATMEPINCTAQVTADGVHLWAPTQVATLAQLVAARAAGVSGDKVQID
IPLIGGGFGRRLESDFISQAVTIATKTEGKPVQVIWSREEDVRHDFYRPQAIARLKARVESGKVTAIASRSAGQSIL

AGELDRLFGAPSVGIDRYTAEGLFDLPYEIEHEHIAHLAVDLPVPVGFWRSVGHSYNGFFMEGFLNEVAAAAKL
DPLAMRRNLLKDHPRELKVLDTAAQAAGWGQPLAPAADGAPRARGIALHPSFGSVVAQVVEVSMKDGKPRV
HRVVCAVDCGTVVNPGIVAQQMESAVIFGLTAALYGRIDIKDGQVVQSNFPDYPALKMVETPVIETHIVPSTAEP
SGMGEVGVPPIAPAVAHAMAQLTGKPVRQLPMV
>SEQF10198||SEQF10198.1_04066
MNAKTTKPRSGRRRFLLGALGIGGALVVGWGVMPPRSRVGDPGIFPEHNGEIALNGWIKITPEGNVVLAMPR
VEMGQGIHTALSMLAAEELDIPLARVRIESAPVERIYGNVVAMGDSSLPLHPDSADKTWARALHWIMAKSARE
IGLIITGGSSSTADGWQPVREAAATARAALVEAAAREWNAPVAQVSIREGQLIGPGGKQSTFGEMAKSARGLS
APSNVTLKPASQFQLIGKPAPRNDLAAKTDGSARFSIDTRLPGMLYAAVVMCPAFGGKLKTFQSKAALGMPGV
RYVVPFEGTGGGAPGVAVVADHYWQARQALATLEPVWDNGPHAKLDSAGIRQQLVSALDSDKGGFTYRSM
GDGLKAFDKADGATLVEAEYSAPYLAHATMEPINCTAQVTPEGVHLWAPTQVATLAQLVAARAAGVSGDKVQI
DIPLIGGGFGRRLESDFIGQAVTIATKTDGKPVQVIWSREEDVRHDFYRPHAIARLKARVENGKVTAIASRSAGQ
SILAGELDRLFGAPSVGIDRYTAEGLFDLPYEIEHEHIAHLAVDLPVPVGFWRSVGHSYNGFFMEGFLNEVAAAA
KLDPLAMRRDLLKDHPRELKVLDTAAQAAGWGQPLAPAADGAPRARGIALHPSFGSVVAQVVEVSMKDGKP
RVHRVVCAVDCGTVVNPGIVAQQMESAVIFGLTAALYGRIDIKDGQVVQSNFPDYPALKMAETPVIETHIVPST
AEPSGMGEVGVPPIGPAVAHAVAQLTGKPVRQLPMA
>SEQF10199||SEQF10199.1_01066
MNAKTTKPRSGRRRFLLGALGIGGALVVGWGVMPPRSRVGDPDIFPEHKGEIALNGWIKITPESDVVLAMPRV
EMGQGIHTALSMLAAEELDIPLSRVRIESAPVERIYGNVVAMGDSSLPLHPDSADKTWARALHWIMAKSAREI
GLIITGGSSSTADGWQPVREAAATARAALVQAAAREWNVPAADVSIREGQLIGPGGKQSTFGEMAKSARGLSA
PSNVTLKPASQFQLIGKPAPRNDLAAKTDGSVRFSIDTRLPGMLYAAVVMCPVFGGKLKTFQSKAALGMPGVR
YVVPFEGTGGGAPGVAVVADHYWQARQALATLEPVWDNGPHAKLDSAGIRQQLVSALDSDKGGFTYRSMG
DGLKAFDKADGATLVEAEYSAPYLAHATMEPINCTAQVTKGGVHLWAPTQVATLAQLVAARAAGVSGDKVQID
IPLIGGGFGRRLESDFIGQAVTIATKTDGKPVQVIWSREEDVRHDFYRPHAIARLKARVENGKVTAIASRSAGQSI
LAGELDRLFGAPSVGIDRYTAEGLFDLPYEIEHEHIAHLAVDLPVPVGFWRSVGHSYNGFFMEGFLNEVAAAAK
LDPLAMRRDLLKDHPRELKVLDTAAQAAGWGQPLAPAADGAPRARGIALHPSFGSVVAQVVEVSMKDGKPR
VHRVVCAVDCGTVVNPGIVAQQMESAVIFGLTAALYGRIDIKDGQVVQSNFPDYPALKMAETPVIETHIVPSTAE
PSGMGEVGVPPIGPAVAHAVAQLTGKPVRQLPMA
>SEQF10200||SEQF10200.1_02521
MNAKTTKPRSGRRRFLLGALGIGGALVVGWGVMPPRSRVGDPDIFPEHNGEIALNGWIKITPEGDVVLAMPR
VEMGQGIHTALSMLAAEELDIQLSRVRIESAPVERIYGNVVAMGDSSLPLHPDSADKTWARALHWIMAKSARE
IGLIITGGSSSTADGWQPVREAAATARAALVQAAAREWNVPAPDVSIREGQLIGPGGKQSTFGEMAKLARGISA
PSNVTLKPASQFRLIGKPAPRNDLAAKTDGSARFSIDTRLPGMLYAAVVMCPVFGGKLKTFQSKAALGMPGVR
YVVPFEGAGGGAPGVAVVADHYWQARQALATLEPVWDNGPHAKLDSAGIRQQLVSALDSDKGGFTYRSTGD
GLKAFDKTDGATIVEAEYTAPYLAHATMEPINCTAQVTSEGVHLWAPTQVATLAQLVAARAAGVSGDKVQIDIP
LIGGGFGRRLESDFISQAVTIATKTEGKPVQVIWSREEDVRHDFYRPQAIARLKARVESGKVTAIASRSAGQSILA
GELDRLFGAPSAGIDRYTAEGLFDLPYEIEHEHIAHLAVDLPVPVGFWRSVGHSYNGFFMEGFLNEVAAAAKLD
PLAMRRNLLKDHPRELKVLDTAAQAAGWGQPLAPAADGAPRARGIALHPSFGSVVAQVVEVSMKDGKPRVH
RVVCAVDCGTVVNPGIVAQQMESAVIFGLTAALYGRIDIKDGQVVQSNFTDYPALKMVETPVIETHIVPSTAEPS
GMGEVGVPPIAPAVAHAVAQLTGKPVRQLPMV
>SEQF10201||SEQF10201.1_02691
MNAKTTKPRSGRRRFLLGALGIGGALVVGWGVMPPRSRVGDPDIFPEHNGEIALNGWIKITPEGDVVLAMPR
VEMGQGIHTALSMLAAEELDIQLSRVRIESAPVERIYGNVVAMGDSSLPLHPDSADKTWARALHWIMAKSARE
IGLIITGGSSSTADGWQPVREAAATARAALVQAAAREWNVPAPDVSIREGQLIGPGGKQSTFGEMAKLARGISA

PSNVTLKPASQFRLIGKPAPRNDLAAKTDGSARFSIDTRLPGMLYAAVVMCPVFGGKLKTFQSKAALGMPGVR
YVVPFEGAGGGAPGVAVVADHYWQARQALATLEPVWDNGPHAKLDSAGIRQQLVSALDSDKGGFTYRSTGD
GLKAFDKTDGATIVEAEYTAPYLAHATMEPINCTAQVTSEGVHLWAPTQVATLAQLVAARAAGVSGDKVQIDIP
LIGGGFGRRLESDFISQAVTIATKTEGKPVQVIWSREEDVRHDFYRPQAIARLKARVESGKVTAIASRSAGQSILA
GELDRLFGAPSAGIDRYTAEGLFDLPYEIEHEHIAHLAVDLPVPVGFWRSVGHSYNGFFMEGFLNEVAAAAKLD
PLAMRRNLLKDHPRELKVLDTAAQAAGWGQPLAPAADGAPRARGIALHPSFGSVVAQVVEVSMKDGKPRVH
RVVCAVDCGTVVNPGIVAQQMESAVIFGLTAALYGRIDIKDGQVVQSNFTDYPALKMVETPVIETHIVPSTAEPS
GMGEVGVPPIAPAVAHAVAQLTGKPVRQLPMV
>SEQF10202||SEQF10202.1_02593
MNAKTTKPRSGRRRFLLGALGIGGALVVGWGVMPPRSRVGDPDIFPEHNGEIALNGWIKITPEGDVVLAMPR
VEMGQGIHTALSMLAAEELDIQLSRVRIESAPVERIYGNVVAMGDSSLPLHPDSADKTWARALHWIMAKSARE
IGLIITGGSSSTADGWQPVREAAATARAALVQAAAREWNVPAPDVSIREGQLIGPGGKQSTFGEMAKLARGISA
PSNVTLKPASQFRLIGKPAPRNDLAAKTDGSARFSIDTRLPGMLYAAVVMCPVFGGKLKTFQSKAALGMPGVR
YVVPFEGAGGGAPGVAVVADHYWQARQALATLEPVWDNGPHAKLDSAGIRQQLVSALDSDKGGFTYRSTGD
GLKAFDKTDGATIVEAEYTAPYLAHATMEPINCTAQVTSEGVHLWAPTQVATLAQLVAARAAGVSGDKVQIDIP
LIGGGFGRRLESDFISQAVTIATKTEGKPVQVIWSREEDVRHDFYRPQAIARLKARVESGKVTAIASRSAGQSILA
GELDRLFGAPSAGIDRYTAEGLFDLPYEIEHEHIAHLAVDLPVPVGFWRSVGHSYNGFFMEGFLNEVAAAAKLD
PLAMRRNLLKDHPRELKVLDTAAQAAGWGQPLAPAADGAPRARGIALHPSFGSVVAQVVEVSMKDGKPRVH
RVVCAVDCGTVVNPGIVAQQMESAVIFGLTAALYGRIDIKDGQVVQSNFTDYPALKMVETPVIETHIVPSTAEPS
GMGEVGVPPIAPAVAHAVAQLTGKPVRQLPMV
>SEQF10203||SEQF10203.1_05099
MNAKTTKPRSGRRRFLLGALGIGGALVVGWGVMPPRSRVGDPDIFPEHNGEIALNGWIKITPEGDVVLAMPR
VEMGQGIHTALSMLAAEELDIQLSRVRIESAPVERIYGNVVAMGDSSLPLHPDSADKTWARALHWIMAKSARE
IGLIITGGSSSTADGWQPVREAAATARAALVQAAAREWNVPAPDVSIREGQLIGPGGKQSTFGEMAKLARGISA
PSNVTLKPASQFRLIGKPAPRNDLAAKTDGSARFSIDTRLPGMLYAAVVMCPVFGGKLKTFQSKAALGMPGVR
YVVPFEGAGGGAPGVAVVADHYWQARQALATLEPVWDNGPHAKLDSAGIRQQLVSALDSDKGGFTYRSTGD
GLKAFDKTDGATIVEAEYTAPYLAHATMEPINCTAQVTSEGVHLWAPTQVATLAQLVAARAAGVSGDKVQIDIP
LIGGGFGRRLESDFISQAVTIATKTEGKPVQVIWSREEDVRHDFYRPQAIARLKARVESGKVTAIASRSAGQSILA
GELDRLFGAPSAGIDRYTAEGLFDLPYEIEHEHIAHLAVDLPVPVGFWRSVGHSYNGFFMEGFLNEVAAAAKLD
PLAMRRNLLKDHPRELKVLDTAAQAAGWGQPLAPAADGAPRARGIALHPSFGSVVAQVVEVSMKDGKPRVH
RVVCAVDCGTVVNPGIVAQQMESAVIFGLTAALYGRIDIKDGQVVQSNFTDYPALKMVETPVIETHIVPSTAEPS
GMGEVGVPPIAPAVAHAVAQLTGKPVRQLPMV
>SEQF10204||SEQF10204.1_01948
MNAKTTKPRSGRRRFLLGALGIGGALVVGWGVMPPRSRVGDPGIFPEHNGEIALNGWIKITPEGNVVLAMPR
VEMGQGIHTALSMLAAEELDIPLARVRIESAPVERIYGNVVAMGDSSLPLHPDSADKTWARALHWIMAKSARE
IGLIITGGSSSTADGWQPVREAAATARAALVEAAAREWNAPVAQVSIREGQLIGPGGKQSTFGEMAKSARGLS
APSNVTLKPASQFQLIGKPAPRNDLAAKTDGSARFSIDTRLPGMLYAAVVMCPAFGGKLKTFQSKAALGMPGV
RYVVPFEGTGGGAPGVAVVADHYWQARQALATLEPVWDNGPHAKLDSAGIRQQLVSALDSDKGGFTYRSM
GDGLKAFDKADGATLVEAEYSAPYLAHATMEPINCTAQVTPEGVHLWAPTQVATLAQLVAARAAGVSGDKVQI
DIPLIGGGFGRRLESDFIGQAVTIATKTDGKPVQVIWSREEDVRHDFYRPHAIARLKARVENGKVTAIASRSAGQ
SILAGELDRLFGAPSVGIDRYTAEGLFDLPYEIEHEHIAHLAVDLPVPVGFWRSVGHSYNGFFMEGFLNEVAAAA
KLDPLAMRRDLLKDHPRELKVLDTAAQAAGWGQPLAPAADGAPRARGIALHPSFGSVVAQVVEVSMKDGKP
RVHRVVCAVDCGTVVNPGIVAQQMESAVIFGLTAALYGRIDIKDGQVVQSNFPDYPALKMAETPVIETHIVPST
AEPSGMGEVGVPPIGPAVAHAVAQLTGKPVRQLPMA

>SEQF10205||SEQF10205.1_04422
MNAKTTKPRSGRRRFLLGALGIGGALVVGWGVMPPRSRVGDPDIFPEHNGEIALNGWIKITPEGDVVLAMPR
VEMGQGIHTALSMLAAEELDIQLSRVRIESAPVERIYGNVVAMGDSSLPLHPDSADKTWARALHWIMAKSARE
IGLIITGGSSSTADGWQPVREAAATARAALVQAAAREWNVPAPDVSIREGQLIGPGGKQSTFGEMAKLARGISA
PSNVTLKPASQFRLIGKPAPRNDLAAKTDGSARFSIDTRLPGMLYAAVVMCPVFGGKLKTFQSKAALGMPGVR
YVVPFEGAGGGAPGVAVVADHYWQARQALATLEPVWDNGPHAKLDSAGIRQQLVSALDSDKGGFTYRSTGD
GLKAFDKTDGATIVEAEYTAPYLAHATMEPINCTAQVTSEGVHLWAPTQVATLAQLVAARAAGVSGDKVQIDIP
LIGGGFGRRLESDFISQAVTIATKTEGKPVQVIWSREEDVRHDFYRPQAIARLKARVESGKVTAIASRSAGQSILA
GELDRLFGAPSAGIDRYTAEGLFDLPYEIEHEHIAHLAVDLPVPVGFWRSVGHSYNGFFMEGFLNEVAAAAKLD
PLAMRRNLLKDHPRELKVLDTAAQAAGWGQPLAPAADGAPRARGIALHPSFGSVVAQVVEVSMKDGKPRVH
RVVCAVDCGTVVNPGIVAQQMESAVIFGLTAALYGRIDIKDGQVVQSNFTDYPALKMVETPVIETHIVPSTAEPS
GMGEVGVPPIAPAVAHAVAQLTGKPVRQLPMV
>SEQF10206||SEQF10206.1_00753
MNAKTTKPRSGRRRFLLGALGIGGALVVGWGVMPPRSRVGDPGIFPEHNGEIALNGWIKITPEGNVVLAMPR
VEMGQGIHTALSMLAAEELDIPLARVRIESAPVERIYGNVVAMGDSSLPLHPDSADKTWARALHWIMAKSARE
IGLIITGGSSSTADGWQPVREAAATARAALVEAAAREWNAPVAQVSIREGQLIGPGGKQSTFGEMAKSARGLS
APSNVTLKPASQFQLIGKPAPRNDLAAKTDGSARFSIDTRLPGMLYAAVVMCPAFGGKLKTFQSKAALGMPGV
RYVVPFEGTGGGAPGVAVVADHYWQARQALATLEPVWDNGPHAKLDSAGIRQQLVSALDSDKGGFTYRSM
GDGLKAFDKADGATLVEAEYSAPYLAHATMEPINCTAQVTPEGVHLWAPTQVATLAQLVAARAAGVSGDKVQI
DIPLIGGGFGRRLESDFIGQAVTIATKTDGKPVQVIWSREEDVRHDFYRPHAIARLKARVENGKVTAIASRSAGQ
SILAGELDRLFGAPSVGIDRYTAEGLFDLPYEIEHEHIAHLAVDLPVPVGFWRSVGHSYNGFFMEGFLNEVAAAA
KLDPLAMRRDLLKDHPRELKVLDTAAQAAGWGQPLAPAADGAPRARGIALHPSFGSVVAQVVEVSMKDGKP
RVHRVVCAVDCGTVVNPGIVAQQMESAVIFGLTAALYGRIDIKDGQVVQSNFPDYPALKMAETPVIETHIVPST
AEPSGMGEVGVPPIGPAVAHAVAQLTGKPVRQLPMA
>SEQF10207||SEQF10207.1_02523
MNAKTTKPRSGRRRFLLGALGIGGALVVGWGVMPPRSRVGDPDIFPEHNGEIALNGWIKITPEGDVVLAMPR
VEMGQGIHTALSMLAAEELDIQLSRVRIESAPVERIYGNVVAMGDSSLPLHPDSADKTWARALHWIMAKSARE
IGLIITGGSSSTADGWQPVREAAATARAALVQAAAREWNVPAPDVSIREGQLIGPGGKQSTFGEMAKLARGISA
PSNVTLKPASQFRLIGKPAPRNDLAAKTDGSARFSIDTRLPGMLYAAVVMCPVFGGKLKTFQSKAALGMPGVR
YVVPFEGAGGGAPGVAVVADHYWQARQALATLEPVWDNGPHAKLDSAGIRQQLVSALDSDKGGFTYRSTGD
GLKAFDKTDGATIVEAEYTAPYLAHATMEPINCTAQVTSEGVHLWAPTQVATLAQLVAARAAGVSGDKVQIDIP
LIGGGFGRRLESDFISQAVTIATKTEGKPVQVIWSREEDVRHDFYRPQAIARLKARVESGKVTAIASRSAGQSILA
GELDRLFGAPSAGIDRYTAEGLFDLPYEIEHEHIAHLAVDLPVPVGFWRSVGHSYNGFFMEGFLNEVAAAAKLD
PLAMRRNLLKDHPRELKVLDTAAQAAGWGQPLAPAADGAPRARGIALHPSFGSVVAQVVEVSMKDGKPRVH
RVVCAVDCGTVVNPGIVAQQMESAVIFGLTAALYGRIDIKDGQVVQSNFTDYPALKMVETPVIETHIVPSTAEPS
GMGEVGVPPIAPAVAHAVAQLTGKPVRQLPMV
>SEQF10208||SEQF10208.1_02107
MNAKTTKPRSGRRRFLLGALGIGGALVVGWGVMPPRSRVGDPGIFPEHNGEIALNGWIKITPEGNVVLAMPR
VEMGQGIHTALSMLAAEELDIPLARVRIESAPVERIYGNVVAMGDSSLPLHPDSADKTWARALHWIMAKSARE
IGLIITGGSSSTADGWQPVREAAATARAALVEAAAREWNAPVAQVSIREGQLIGPGGKQSTFGEMAKSARGLS
APSNVTLKPASQFQLIGKPAPRNDLAAKTDGSARFSIDTRLPGMLYAAVVMCPAFGGKLKTFQSKAALGMPGV
RYVVPFEGTGGGAPGVAVVADHYWQARQALATLEPVWDNGPHAKLDSAGIRQQLVSALDSDKGGFTYRSM
GDGLKAFDKADGATLVEAEYSAPYLAHATMEPINCTAQVTPEGVHLWAPTQVATLAQLVAARAAGVSGDKVQI
DIPLIGGGFGRRLESDFIGQAVTIATKTDGKPVQVIWSREEDVRHDFYRPHAIARLKARVENGKVTAIASRSAGQ

SILAGELDRLFGAPSVGIDRYTAEGLFDLPYEIEHEHIAHLAVDLPVPVGFWRSVGHSYNGFFMEGFLNEVAAAA
KLDPLAMRRDLLKDHPRELKVLDTAAQAAGWGQPLAPAADGAPRARGIALHPSFGSVVAQVVEVSMKDGKP
RVHRVVCAVDCGTVVNPGIVAQQMESAVIFGLTAALYGRIDIKDGQVVQSNFPDYPALKMAETPVIETHIVPST
AEPSGMGEVGVPPIGPAVAHAVAQLTGKPVRQLPMA
>SEQF10209||SEQF10209.1_03857
MNAKTTKPRSGRRRFLLGALGIGGALVVGWGVMPPRSRVGDPGIFPEHNGEIALNGWIKITPEGNVVLAMPR
VEMGQGIHTALSMLAAEELDIPLARVRIESAPVERIYGNVVAMGDSSLPLHPDSADKTWARALHWIMAKSARE
IGLIITGGSSSTADGWQPVREAAATARAALVEAAAREWNAPVAQVSIREGQLIGPGGKQSTFGEMAKSARGLS
APSNVTLKPASQFQLIGKPAPRNDLAAKTDGSARFSIDTRLPGMLYAAVVMCPAFGGKLKTFQSKAALGMPGV
RYVVPFEGTGGGAPGVAVVADHYWQARQALATLEPVWDNGPHAKLDSAGIRQQLVSALDSDKGGFTYRSM
GDGLKAFDKADGATLVEAEYSAPYLAHATMEPINCTAQVTPEGVHLWAPTQVATLAQLVAARAAGVSGDKVQI
DIPLIGGGFGRRLESDFIGQAVTIATKTDGKPVQVIWSREEDVRHDFYRPHAIARLKARVENGKVTAIASRSAGQ
SILAGELDRLFGAPSVGIDRYTAEGLFDLPYEIEHEHIAHLAVDLPVPVGFWRSVGHSYNGFFMEGFLNEVAAAA
KLDPLAMRRDLLKDHPRELKVLDTAAQAAGWGQPLAPAADGAPRARGIALHPSFGSVVAQVVEVSMKDGKP
RVHRVVCAVDCGTVVNPGIVAQQMESAVIFGLTAALYGRIDIKDGQVVQSNFPDYPALKMAETPVIETHIVPST
AEPSGMGEVGVPPIGPAVAHAVAQLTGKPVRQLPMA
>SEQF10210||SEQF10210.1_02415
MADNPNSDLDYPETSAPAAKPKRKGVKRRIFLAGSALVVGGGIFGVWWTDSNAKGRANTLIGGEGKHAFNSV
MTIAEDDTVTLFSPHIDFGQGSHTALGQMLADELDAAWEKVTIEQAPADMAFANAALAKGFLPTMVGDTVA
GLIPDAVIGLMARSMPLMITGGSSAIRFTGEVAMRRTGAAVRAALVAEAADRLGVPESELTTADSKVTHAKSGR
SLRYGELAAGAATRSLSSDPVLKTRDQWKLIGKPVPRRDIPSKVDGSAVYGIDFTLPDMRVATIAAAPVRGGKLE
SVDEAPALAISGVEKVVKLPDAVIVVAKGYWPATKGLAALSPKFTDGGHSAMSTPAIYAAQEKLRKASGEPDNV
GGEGDVDAAFAAAGVKLVEAEYRVPFLHHAMMEPFALTGHFKDGTLHLWGGLQDPLSTRAKAAKAAGLEVD
NVVFHPMIMGGGFGRRFPDLVEIIDQIAVLAKQVPYPVKLVWSREEEVRHGTYRPQSSAGLKASLKDGKITGW
RADYVQSGSAEGEVPFIYAIPATLRRHFAYQSNQIDGPWRSVNATQMGFYTESFMDELAAAAGEDPYQFRRKH
LATGSRHLAALDMVAKRSGWGTPLPKGVGRGIAIVESFDTIVAEVVEASVKEDGSPKVLKAWAVVDCGTTVNP
LNAEAQIAGGLIMGLSSAIGEQVTLDKGAVVESNFSDYPILKLADAPPAVDVHFIESGAKTGGIGEPGLPPASPAL
ANALSAATGKRIRNLPLLTQAKA
>SEQF10210||SEQF10210.1_01375
MLMRDLGIIPAPKVDGTGDGPSPYVNLSRRGFVGGAGLFVLGVTLAGCSSYVEPVIDADAFKLPDPGASPLTGV
KGGDATPSLWIAIDKDGAVKITCHRSEMGQQTWTAMAQIVADELEADWDKVAIVQAEGHERYGDQNTDGSR
SVRFNFHRLRVAGAAMRHMLVAAAALYWKLPQDQCSAKGGLVSNTKNDETLSYGNLAELAGRLAIPAEADIKL
KTPKEWRYIREEIPSLTVPRIVKGDSTFGIDVKRPGMVYAVVARPPQLFGRVGSVDDTKALAIAGVLSTMRLPDA
KPPALFQPLGGVAVVARDTWAAIEGRRALEIAWQDGPNAGYDSEAFAKQLQATARRSGKVRRSRGNVGAALA
AASKRVTAEYYAPHLNQSPMEPPSATAEWDGDRLECWACVQDPQNTRDTLAEALGIPKENIKVTPTWLGGAF
GRKSKPDFVIEAALIAREVGKPVKVTWTREDDIRHGYYHSVSAQYCEAGLDQDGKCTAWLHRTVFPPISSTFDN
TLAEPSDGEMSMGATDVPFAAPNLRVESGDAKGHMRIGWLRSVANIYHAFAVQSFAAELAHAAGRDQKDYLL
ELIGPPRMIDPESEGATYGNYGAELAEYPIDTARLRNVVEKASAMADWGRKLPAGRGLGIAVHRSFLSYIATVIEV
AVGKDGTLRIPGVWLAVDAGTVINPRHVRAQMEGGTIYGLSNALYGAITAKDGAVVQDNFPSWRLMRMGEA
PREFKVEIIASDAPPGGVGEPATPPAAPALANAIFAATGHRLRTLPLIGAEGDKLKLPATKTV
>SEQF1086||SEQF1086.1_02165
MRIAIQNLSRRRFVQGAGGLLLGLSLPPLARRAMAAGPQAGDGFAANAFVRIGADGRVTVLAKHLEMGQGA
YTGLATLLAEELDADWRQVRVEGAPADSARYGNQALGGLQGTGGSTAMFDSWEPMRRAGATARAMLVQAA
AQRWQVPADTIEVAEGVLSHPASGRRAGFGELAEAAARSPVPEDVPLKDPARFRLIGKHRLPHVDSAAKSDGS

ALYTQDMKLPGMLVAVVAHAPRLGAAVARVDDAAARAVPGVRAVVRFGGAALRHAGVAVLATNTWAARAG
RDALRIEWDEGPAYRQGSADILARYREAVGRPGSMAARKGDIDAAFAGAAKVIEAEYTYPYLAHAAMEPLNCL
VRLDDERCEIWNGEQFQTADQRAIAQYLGMPAERITLTQLYAGGSFGRRASSHADYLLEAVAIARTARAQGLNA
PVKLVWMREDDMRAGYYRPLNLHRARLALGADGALQAVHVRMAGQSILLGTPLADWVRDGVDPVSVEGLS
DLAYAVPNLQVELHTPTDVPVPVLWYRSVGHTHTAFSAETLIDEAAVAAGQDPVAYRLALLAAHPRHREVLQLA
AVRAGWREPLAAGAPGTRRGRGVAVHESFRSVMAQVVEVTIAADGALKVDRVVCAAVHCGLAVNPDVVRA
QMEGGIGFALSTALHGAITLKDGAVEQSNFHDYPVLRLAEMPAVEVHIAPSTQPPTGVGEPGVPPLAPALANAI
AQATGQRLRTLPLGTTVKA
>SEQF1295||SEQF1295.1_03304
MNSKIDLSNALPGSRRGFLKGAAVLGLSIGFQWSGARRALAAALPESGFAPNAFLRIAPDDSVTVIAKHVEMG
QGAYTGIATIVAEELDADWNKVRVESAPADAKRYANLAFGTMQGTGGSSAMANSWMQLREAGAKARAMLV
EAAARQWRVPAAELRTRDGFVEHPASQRKASYGSLAAAAAELPVPEKVQLKDSKDFRLIGHQAPRVDVPGKT
DGSAQFTLDVNLPGMLVALLQRPPLFGATVKAFDATAARAIPGVVEVVQVPHGVAVVAKGFWAAKQGRDALK
VEWDESKAEKRGSEALMAEYRKLAEQPGKPARRDGDAAGALAGAAKKVAASYEFPFLAHAPMEPLDAVVRLT
ADSCEIWAGDQFQTVDQGNAARTAGLKPEQVKINTLYAGGSFGRRANAWSDYIVEAVSIAKALGANGVPVKL
QWTREDDIHGGFYRPMYYHKLEAGLDADGKLVGWQHRIVGQSILEGTPFAAMMVKDGIDGTSVEGAANLPY
AVPNVSVELSTTQVGVPALWWRVVGSSHTVYAVEAFIDEAAQAAGKDPYLFRRDLLAEQPRLRGVLELAAEKA
GWDPAKPLPAGRGRGIAVTEAFKTFVAQVVEVSVDKDGKLKVERVVCAVDCGIPINPDVIAAQMEGGIGFGLG
AVLHSAITLKDGKVEQNNFDGYQVLRIAEMPRVEVHIVPSGEAPTGVGEPGVAPIGPALANAIFAATGQRLYHLP
FPTTFAKA
>SEQF1295||SEQF1295.1_02806
MKRSTLDDLVIGNLSRRGFLKGVGATGVLLVAANWGWRDALAAEQKAFGADAMPHGWVDNPKIYVSIDKDG
TVGIVCNRSEMGQGVRTSLAMVVADELEADWSRVKVIQAPGDEARYGNQDTDGSRSMRHWFEPMRRCGA
AARQMLEQAAANQWKVPLGECRAEQNRVLHAPSGRSLSFGELAEAAAGLDVPARDKLVLKKPEQFRYIGKDV
ARAIDGADIVNGRAGFGFDARFDDMLYAVVARPPIYGGKLKRYDAAAALKVPGVLKVIEIESRPIPSEFQPLGGV
AVVAKNTWAAIKGREALVLEWDAGVNGGYDSVAYRKQLEEAARKPGKVVRDSGDAAALFARGGDIVEAEYYL
PHLAQAPMEPPVSTAWYKDGACEVWAPTQAPQVTRERIAERLKLPFDKVTVNVTLLGGGFGRKSKPDYVLEA
AILAKAFPGRHLRVQWTREDDLHFSYFHTVSVERLQAVLGADGLPQAWLHRSVAPSITALFGPDSKHQGAFEL
GMGLTNLPFAIPNVRLENPEAPAHTRVGWFRSVSNIPHAFAIQSFVGELAAKAGQDPKDYLLKLLGPARRIDTAE
LGDSWNYGESPQRYPLDVGRLRGVIEEAARQSGWGGELPRGRARGIAAHYSFVTYVAVVIEVEVKDDGALLVH
KATIAADCGPQINPERIRSQLEGACVMGLGLAALGEISFKDGKVQQDNFHQYELARMPLAPKAVSVHLLKPDG
DLPLGGVGEPGVPPIAPALCNAIFAATGKRIRELPIRNQLQGWRKA
>SEQF1297||SEQF1297.1_01960
MNSKIDLSNALPGSRRGFLKGAAVVGLTIGFQWSGARRALAAALPDAGFAPNAFLRIAPDDSVTVIAKHVEMG
QGAYTGIATIVAEELDADWSKVRVESAPADAKRYANLAFGTMQGTGGSSAMANSWMQLREAGAKARAMLV
EAAARQWQVPAAELRTRDGFVEHPTSQRKASYGSLAAAAAELPVPEKVQLKDPKDFRLIGHQAPRVDVPGKT
DGSAQFTLDVSLPGMLVALLQRPPLFGATVKSFDATATRAIPGVVEVVQVPHGVAVVAKGFWAAKQGRDALKV
EWDESKAEKRGSEALMAEYRKLAEQPGKPARRDGDAAKAVAGATRRIAASYEFPFLAHAPMEPLDAVVRLTAD
SCEIWAGDQFQTVDQGNAARTAGLKPEQVKINTLYAGGSFGRRANAWSDYIVEAVSIAKALGANGVPVKLQW
TREDDIHGGFYRPMYYHRLEAGLDADGKLVGWQHRIVGQSILEGTPFAAVMVKDGIDATSVEGAANLPYAVP
NVSVELSTTQVGVPVLWWRVVGSSHTVYAVEAFIDEAAQAAGKDPYLFRRDLLAEQPRLRGVLELAAEKAGW
DPSRPLPAGRGRGIAVTEAFKTFVAQVVEVSVDKDGKLKVERVVCAVDCGIPINPDVIAAQMEGGIGFGLGAIL
HSAITLKDGKVEQNNFDGYQVLRIAEMPKVEVHIVPSGEAPTGVGEPGVAPIGPALANAIFAATGQRLYNLPFPT
SFAKA

>SEQF1297||SEQF1297.1_02473
MKRSYPDDLVIGNLSRRGFLKGVGATGVLLVAANWGWRDALAAEKKAFGADAMPHGWVDNPKIYVSIDRDG
TVGIVCNRSEMGQGVRTSLAMVVADELEADWSRVKVIQAPGDEARYGNQDTDGSRSMRHWFEPMRRCGA
AARQMLEQAAANQWKVPLGECRAEQNKVLHAPSGRSLSFGELAEAAAGLEVPARDKLLLKKPEQFRYIGKDV
ARAIDGADIVNGRAGFGFDARFDDMLYAVVARPPVYGGKLKRYDAAAALKVPGVVKVIEIEGRPIPSEFQPLGG
VAVVAQNTWAAIKGREALVVEWDAGVNGGYDSVAYRKQLEEAARKPGKVVRDSGDAAALFAKGGDIVEAEYY
LPHLAQAPMEPPVSTAWYKDGACEVWAPTQAPQVTRERIAERLKLPFDKVTVNVTLLGGGFGRKSKPDFVLEA
AILAKAFPGRHLRVQWTREDDLHFSYFHTVSVERLQAVLGADGLPQAWLHRSVAPSITALFGPDSKHQGAFEL
GMGLTNLPFAIPNVRLENPEAPAHTRVGWFRSVSNIPHAFAIQSFVGELAAKAGQDPKDYLLKLLGPARRIDTAE
LGDSWNYGESPERYPLDVGRLRGVIEEAARQSGWGGELPRGRARGIAAHYSFVTYVAVVIEVEVKDDGALLVH
KATIAADCGPQINPERIRSQLEGACVMGLGLAALGEISFKDGKVQQDNFHQYELARMPLAPKAVSVHLLKPDG
DLPLGGVGEPGVPPIAPALCNAIFAATGKRIRELPIRNQLQGWRKA
>SEQF1298||SEQF1298.1_03331
MNSKIDLSNALPGSRRGFLKGAAVVGLTIGFQWSGARRALAAALPDAGFAPNAFLRIAPDDSVTVIAKHVEMG
QGAYTGIATIVAEELDADWSKVRVESAPADAKRYANLAFGTMQGTGGSSAMANSWMQLREAGAKARAMLV
EAAARQWQVPAAELRTRDGFVEHPTSQRKASYGSLAAAAAELPVPENVQLKDPKDFRLIGHQAPRVDVPGKT
DGSAQFTLDVSLPGMLVALLQRPPLFGATVKSFDATATRAIPGVVEVVQVPHGVAVVAKGFWAAKQGRDALKV
EWDESKAEKRGSEALMAEYRKLAEQPGKPARRDGDAAKAVAGATRRIAASYEFPFLAHAPMEPLDAVVRLTAD
SCEIWAGDQFQTVDQGNAARTAGLKPEQVKINTLYAGGSFGRRANAWSDYIVEAVSIAKALGANGVPVKLQW
TREDDIHGGFYRPMYYHRLEAGLDADGKLVGWQHRIVGQSILEGTPFAAVMVKDGVDATSVEGAANLPYAVP
NVSVELSTTQVGVPVLWWRVVGSSHTVYAVEAFIDEAAQAAGKDPYLFRRDLLAEQPRLRGVLELAAEKAGW
DPSRPLPAGRGRGIAVTEAFKTFVAQVVEVSVDKDGKLKVERVVCAVDCGIPINPDVIAAQMEGGIGFGLGAIL
HSAITLKDGKVEQNNFDGYQVLRIAEMPKVEVHIVPSGEAPTGVGEPGVAPIGPALANAIFAATGQRLYNLPFPT
SFAKA
>SEQF1298||SEQF1298.1_02834
MKRSFPDDLVIGNLSRRGFLKGVGATGVLLVAANWGWRDALAAEKKAFGADAMPHGWVDNPKIYVSIDRDG
TVGIVCNRSEMGQGVRTSLAMVVADELEADWSRVKVIQAPGDEARYGNQDTDGSRSMRHWFEPMRRCGA
AARQMLEQAAANQWKVPLGECRAEQNKVLHAPSGRSLSFGELAEAAAGLEVPARDKLLLKKPEQFRYIGKDV
ARAIDGADIVNGRAGFGFDARFDDMLYAVVARPPVYGGKLKRYDAAAALKVPGVVKVIEIEGRPIPSEFQPLGG
VAVVAQNTWAAIKGREALVVEWDAGVNGGYDSVAYRKQLEEAARKPGKVVRDSGDAAALFAKGGDIVEAEYY
LPHLAQAPMEPPVSTAWYKDGACEVWVPTQAPQVTRERIAERLKLPFDKVTVNVTLLGGGFGRKSKPDFVLE
AAILAKAFPGRHLRVQWTREDDLHFSYFHTVSVERLQAVLGADGLPQAWLHRSVAPSITALFGPDSKHQGAFE
LGMGLTNLPFAIPNVRLENPEAPAHTRVGWFRSVSNIPHAFAIQSFVGELAAKAGQDPKDYLLKLLGPARRIDTA
ELGDSWNYGESPERYPLDVGRLRGVIEEAARQSGWGGELPRGRARGIAAHYSFVTYVAVVIEVEVKDDGALLV
HKATIAADCGPQINPERIRSQLEGACVMGLGLAALGEISFKDGKVQQDNFHQYELARMPLAPKAVSVHLLKPD
GDLPLGGVGEPGVPPIAPALCNAIFAATGKRIRELPIRNQLQGWRKA
>SEQF1300||SEQF1300.1_01826
MNRLPQDFALSNLSRRGFLKGVGATGALVLAASWGWQEAFAEDKKFGADGMPHGWVDDPKVYVSIASDGS
VTVICNRSEMGQGVRTSLSMVVADELDADWAQVKVRQAPGDEVRFGNQDTDGSRSMRHWYEPMRRCGA
AARTMLEQAAAAQWQVPLAQCRAQLHKVVHQPTGRELGYGALAAAAGALAVPARDSLRLKQPGEFRYIGKE
ATRAIDGQDIVNGRAVYGADVHFDGMLFAAVARPRVYGGKVKSVDDSAALKVPGVIKVMPIDSRPLPSEFQPL
GGVAVVASNTWAAIKGRDALRIEWEDGANAGYDSIQYRKQLEAAARQPGKVVRSTGNLDEALKGAESSLEASY
YLPHLAQAPMEPMVAVARFQDGRCEAWAPSQAPQVTRERIAERLGIGFEQVTVNVTLLGGGFGRKSKPDFILE
AAILAKAFPGKAVRVQWTREDDIHNSYFHTVSAEYLKAGLNKDGLPAAWLHRTVAPSITALFAPGMNHEGAFE

LGMGFTNMAYAIPNVRLENPEAAAHTRVGWYRSVSNIPHGFAIQSFVDELAHKAGQDPLQYQLKLLGPDRQID
PRTLSEEWNYGESPERYPIDTGRLRTVLETAAKAAGWGRSLPKGRGLGLAVHYSFVTYVAAVIEVEVKDDGTLIV
HQADIAVDCGPQINPERIRSQFEGACVMGLGNAVLGEISFKEGKVQQDNFHMYEVARMSLAPKQVQVHLVTP
PGDVPLGGVGEPGVPPIAPALCNAIFAATGKRIRNLPVRYQLQGWQQASA
>SEQF1301||SEQF1301.1_02251
MNSPVSRRGFLKGSAVLGGGLVVAFVVPGGNRFAMAAENEGKVFAPNAFLRIAADNSVTVLLGHSEMGQGI
WTGLTMLIAEELDADWSKIRVEHSPASAADYGMPAFGGMQITGGSTSTWMEFDRYRLAGATARQMLIEAAA
KRFNVAPSTIRTESGVVIAGDNRATYGELADAAGQLPVPDPKSITFKEAKDWKVIGKPTKRLDTPEKITGRAKFG
MDVQFEGLMTAMVARAPTFGATVKSFEGADALAIPGVHKVVQVPTGVAVVAEHYWAAKLGRDALKVDWDL
GPNADLSSEKLLASFRKLAATPGTSAAQAGDAKGNFGKAAKKIDVEYSVPYLAHAPMEPLNCTVKISADKCEIW
TGTQFQTLDQMVAGKITGLKPEQVEIHTEFLGGGFGRRANPTSDFVAEAVQVAKAAAMPVKTVWSREDDIRG
GYYRSMFLHQAKIGLGADGLPLAWQHVLVGQSIMAGTMLEKTMVKNGVDQTSVEGVSDSPYIKGLAHHQVD
LHSPSTGINVLWLRSVGHSHTAFVMESLIDEMATAAGQDPVEYRRTLLKEHARHLGVLNLAVEKANWKAPLPD
GHALGVAVHESFGSYVAQVAEVSQDNLAIRVHRVVCAVDCGVAVNPQSIAAQMESCITFGLGMALHSKLTLKD
GHVVQSNYHDYQVLRLNEMPLVEVHIVPSSEKPGGIGEAGVPPTAPAVANAVFALTGQRLRELPLQLSGV
>SEQF1301||SEQF1301.1_04295
MSRLPNDFALSNLSRRGFLKGVGATGALVLAASWGWQGALAEDKPKQFGADGMPNGWIDDPKVYVSIAAD
GTVTVVCNRSEMGQGVRTSLTMVIADELEADWAHVKVQQAPGDEVRFGNQDTDGSRSMRHWYEPMRRC
GAAARIMLEQAAAAQWKVPVGECRAQLHKVIHTPSGRELGYGELAAAASALTVPARDSLRLKQPSEFRYIGKQ
GTKAIDGADIVNGRAVYGADVHFDGMLFAVIARPAVYGGKVKSVDDSAALKVPGVLKVIQIEPRPLPSEFQPLG
GVAVVASNTWAALKGREALKIEWDDGPNATYDSVAYRKEIEAASLKPGKVVRNTGDIDKAIGSAASTLEASYYLP
HLAQAPMEPMVAIARYKDDTCEAWAPSQAPQVTRERIAERLGLPFDNVTFNVTLLGGGFGRKSKPDFVVEAA
VLAKEFPGKAVRVQWTREDDIHNSYFHTVSAEYLKAGVGKDGLPSAWLHRTVAPSITALFAPGMNHEAAFELG
MGFTNMAYAIPNVRLENPEATVHTRVGWYRSVSNIPHGFAIQSFVDELAHKAKEDPLKYQIKLLGPDRQIDPRT
LSEEWNYGESPERYPIDTGRMRTVLETAAKAAGWGRQLPKGRGLGLAVHYSFVTYVAAVIEVEVKDDGTLIVHK
ADIAVDCGPQINPERIRSQFEGACVMGLGNAVWGEISFKDGKVQQDNFHMYEVARMSLAPKEVAVHLVTPP
GEVPLGGVGEPGVPPIAPALCNAIFAATGQRIRNLPVRYQLQGWQKAQA
>SEQF1303||SEQF1303.1_00472
MSQPDTTLQSPSRRTLLKVGSLALGGLVIGFTLPFAGRSFAEQVLNEGPEDQPMSNATALDAFISIDRDGQVTFT
VPKIEMGQGAQSGLAVMVAEELEIGLEQITLKEAPPNEQIYNDKLLNFQATGGSTSIRSNWEPLRQAGAAARLL
LIQAAAQRWQLGADQLRAENGRVLGPDGQSLGYGELIEDAAKLPIPKDIPLKPADQFRLIGKPTRRLDTPAKVD
GTARFTIDLVVPGMKYASIRACPVLGGTLREVYERAARQIPGVIEVVRLDNAVAVIGEHTWAAFAGVRALEIDW
ALGDNAGIDSAQMEREIRAALDKPGAIANEQGDIDAALKDAARTFEAEYEMPFLAHAALEPMTCVAEVRADA
VELWVGTQVPVRAQTAAAEVAGRPAEQVIVNNQLIGGAFGRRLEVDFISQAVAIAAQVDYPIKLTWTREEDTT
HDMYRPHYIDRFAAALDAEGRLQGWRHTIAGASVLARFAPEAVPENGLDGDAVEVAMHPIYAMPNLRVNYV
PVPPRALHQSWWRGVGPLRSTYMLESFIDEVARSVEQDPVDYRMALLGSHPRAQGVLRLAAEKAGWGEPLE
AGHGRGVAVQEVFGSFLATVVELQVSEDKGIRLKRLVVAIDCGQVMNPVSVKSQIEGGTLFGLSAALFNEITVRE
GRVEQTNFHDYRQLRISDAPPVETYIVESREAPGGVGEAGTAMIAPALVNALAAANGTRIRRLPLARAGYYVI
>SEQF1395||SEQF1395.1_03866
MKPAAGISRRSALQAGGLALAFTWFGAGKAFAAISPRQQPGDAAAALADGNPAFAPNAFVRIDADGGVRLV
MPMAEMGQAIYTGSAMLLAEELGVELDQVRVEHSPPSEALYGMPLLGGQITGGSTSTRGTYGVLREAGAVAR
TLLVGAAAAQWKVDPAGCTVARGVVSHAASNRQLGFGALAGAAAKLPMPEKVTLKEPKDFKLIGQPLRRVDS
AGKVDGSTQFGIDVRLPGMKVATVRACPTLGGVLASVDDKAARAIRGVVDVLRIKDAVAVVGEHFWAAKRGL
DALKVQWTPGQNAALTTQQLRASLANALAKDKAIVGKETGKRPEGTLVQATYDLPMLAHATMEPLNTTVHVR

PDQCEIWVGTQVPTRCVSAAAKIAGVAEDKVVLHNQYLGGGFGRRLETDSVEQAVAFAKQVPYPLKVVWTRE
EDIRHDIVRPMYHDDISAVVDGDGQILWFGDRIAGGTVLGRWAPAFMGKDGMDSDLIECIAEPCYDLPNLKV
EWVRHDMPAGLNVGWWRGVGPTHNLFVMESFIDELAHRAKKDPVAYRRAMLKKNPRTLGVLDLAAGKIG
WGQGALAARVGRGVAVGDAFGSRVCAIVEAEVTPQGEVRMRRAVVAVDCGIAVNTGSIEAQIQGGLLFGLSA
ALFSEITLREGAIEQSNFHDYRMLRINEAPPVEVHTVKSGEAPGGLGEVGTAIAAPALANAIFAATGVRLRALPVN
RALLAQDKEALKKKIANAGLSGLGARSAA
>SEQF1395||SEQF1395.1_05910
MNASSHDVSRRVFLVAGGSLALGVAFNGAGARAATEKRAAFEPNAFIRIDADDTITLTMPRVEMGQGTYTALS
MLIAEELEIPLARVKLAHAPPDASRYGNPRAGGAQITGGSNSVQGAWEPLRTAGAMARVMLVEAAAQEWRV
AAAECSAHDGVVIHAPSGRQLAYGQLSQRAAGLPLPQQVTLKAPEAFKLIGRPVKRLDAAEKIDGRARYGIDAR
LPKMRYAAVAASPASGGKLVSVDDRAALALPGVRQVVRIDNAVAVIGDHSWAAKQGLAALKVVWDDGPAAS
MSTAGIRKKLEDGLARQDGAVARAQGDADAALRGAAKRLTAIYHSPFLAHAAMEPINCTVDLRADGCDVWVS
TQAPTRARDLAARASGLAADKVRIHNHLIGGGFGRRLEADYVEQSVALSRNVQGPVQFIWSREEDIQHDILRPA
YVDQLSAAIDAQGRPTAITHRVVGSSIMARVAPGLFRNGLDHDAVEAGLGPYEWPASRLDYVREEPGGGMVT
GWWRGVGPTHNCFVVESFVDELASMANKDPVAFRLALLPERSRARAVLELAVQKAGWDAPLRPAAAKGAKR
GRGVAVLAAFGSYLAQVAEVTVDASGDIVVDRVVCAVDCGMVVNPDTVEAQVEGGIHFGVSAALWGEITIKA
GRVEQSNFHDYRVLRLSEAPKVEVHIVPSREAPGGIGEPGTSVVLAALANAVSAATGQRLRSLPLRSNSA
>SEQF1395||SEQF1395.1_06333
MLPTHIDSTELPRILQRLMAASQSQPEDTTALPRRSFLKLAGAGGLALGAFPHMAMAQATGKQAAASTLKPTQ
QPSAFVQIAPNGEVTITHNRLEFGQGVQTGLPMILAEELDADWSLVRSKSGTNDAAYHDPVFGMHLTGGSNSI
KNSFTQYRELGARARAMLLSAAAARWNVDVSTLRTQAGTVLGPNGRKLGYGELAEAAMALPVPEKVSLKDPK
DFKLIGHPTTRLDARAKSSGRQDYGIDVKHAGQLTAVVAHPPVFGARLASVDDSAARAVKGVKAVLRVPLDRG
AEGVAVVADGYWPAKLGRDALKLQWDTAAVEKVDSEKQLVQYRELAKQPGARKFDADMAPLAKAPRQLEAE
FVFPYLAHAPMEPLNCTVKLSGDSAELWVGTQCAGLDGAAAARALGLKPEQVCVNVQMAGGGFGRRFVSTS
DVIVEACEIAKAARAAGLNAPVRLLWSREDDIKGGYYRPMHLHRARIGFDERGKVLAWDHVIVGQSITAGSVFE
PFQVKNGIDATATEGMRDPYPLPMRLTVHHPKVNVPVLWWRSVGSTHTAFVMETLLDEIARSTKQDPVAYRM
QLFGDKHPRHRAALQLAVDRSGYGKKQLPAGRAWGVAVHESFESVVAYVVEASVTDGQPVLHRATAGVHCNL
AVNPRSVEAQVQGAAVMGLSMCLPGGAITLKDGEVQQGNFGDFTVPRITDMPEFAVHIVPSAQPPKGIGEPG
LPPLAPAFANAIAQLTGKPLRQLPFDLADTKPA
>SEQF1413||SEQF1413.1_05107
MPQRPSNERGRTAGSAISLRRRHLLQSAAALLVAPAAGSLLIPLAQAAPAQAGAAAATASSIGDWVWIEPSGQV
VIGVSQCEVGQGIYTGLPQVLADELDADWASVTVRFVTGRDAYRNDAGEMPFQQFVGASMSMNYFYERMR
LAGAQARDVLLRAGAARLGVRASQCSTRAGRVLHSATGRSVGYGEIVADASRLSIAARPRMKSASEQGLIGRNL
RRVDTPAKVDGSAVFGIDVEVPGMLIGAVRMAPSVTGRIVRIRNEAEVRARTGVHAVVRTTQWPDPEPSTVV
VVADSYWIAKQAADALDIEFDAGAAASVDSERIHAQFVAGLASDKAVVARNLGKPREMLAAGKPITADYHSPYI
THATMEPLAATVHVRDGEVETWGPYQGQDFLRGELGKACGVPADKVIVHTTFLGGSFGRKYMPDFALHAAA
ASKAVGRPVKVIRSREDDIRHSYYRPGASGRLSAVLGADGLPAALHARISGQSLYGAINPKKMADAGGWDETM
VESIYDLIYGVPNLLVDAVDVQQPIPLSYLRSVGTTSSVFFLESFISELAHTAGVDDYQYRRRLLAGQPLALGVLDA
AARAARWEQPAPAGLHRAMTFNVYTGRGESFQTFVALVMELRVVEGRVRLERAICAIDAGRVVNPGLVKANV
EGGIGFALTNTFKSRLGFDKGVVQQSNFHDYPLLQLAEMPRVEVVLVESDRPPQGCGEVALGPTAPAVATALFH
ATGRRFRSMPLPQDIAST
>SEQF1619||SEQF1619.1_03624
MNSKIDLSNALPGSRRGFLKGAAVVGLTIGFQWSGARRALAAALPDAGFAPNAFLRIAPDDSVTVIAKHVEMG
QGAYTGIATIVAEELDADWSKVRVESAPADAKRYANLAFGTMQGTGGSSAMANSWMQLREAGAKARAMLV

EAAARQWRVPATELRTRDGFVEHPASQRKASYGSLAAAAAELPVPEKVQLKDPKDFRLIGHQAPRVDVPGKTD
GSAQFTLDVSLPGMLVALLQRPPLFGATVKSFDATATRAIPGVVEVVQVPHGVAVVAKGFWAAKQGRDALKVE
WDESKAEKRGSEALMAEYRKLAEQPGKPARRDGDAAKAVAGATRRIAASYEFPFLAHAPMEPLDAVVRLTADS
CEIWAGDQFQTVDQGNAARTAGLKPEQVKINTLYAGGSFGRRANAWSDYIVEAVSIAKALGANGVPVKLQWT
REDDIHGGFYRPMYYHRLEAGLDADGKLVGWQHRIVGQSILEGTPFAAVMVKDGIDATSVEGAANLPYAVPN
VSVELSTTQVGVPVLWWRVVGSSHTVYAVEAFIDEAAQAAGKDPYLFRRDLLAEQPRLRGVLELAAEKAGWD
PSRPLPAGRGRGIAVTEAFKTFVAQVVEVSVDKDGKLKVERVVCAVDCGIPINPDVIAAQMEGGIGFGLGAILHS
AITLKDGKVEQNNFDGYQVLRIAEMPKVEVHIVPSGEAPTGVGEPGVAPIGPALANAIFAATGQRLYNLPFPTSF
AKA
>SEQF1619||SEQF1619.1_03093
MKRSYPDDLVIGNLSRRGFLKGVGATGVLLVAANWGWRDALAAEKKAFGADAMPHGWVDNPKIYVSIDRDG
TVGIVCNRSEMGQGVRTSLAMVVADELEADWSRVKVIQAPGDEARYGNQDTDGSRSMRHWFEPMRRCGA
AARQMLEQAAANQWKVPLGECRAEQNKVLHAPSGRSLSFGELAEAAAGLEVPARDKLLLKKPEQFRYIGKDV
ARAIDGADIVNGRAGFGFDARFDDMLYAVVARPPVYGGKLKRYDAAAALKVPGVVKVIEIEGRPIPSEFQPLGG
VAVVAQNTWAAIKGREALVVEWDAGVNGGYDSVAYRKQLEEAARKPGKVVRDSGDAAALFARGGDIVEAEYY
LPHLAQAPMEPPVSTAWYKDGACEVWAPTQAPQVTRERIAERLKLPFDKVTVNVTLLGGGFGRKSKPDFVLEA
AILAKAFPGRHLRVQWTREDDLHFSYFHTVSVERLQAVLGADGLPQAWLHRSVAPSITALFGPDSKHQGAFEL
GMGLTNLPFAIPNVRLENPEAPAHTRVGWFRSVSNIPHAFAIQSFVGELAAKAGQDPKDYLLKLLGPARRIDTAE
LGDSWNYGESPERYPLDVGRLRGVIEEAARQSGWGGELPRGRARGIAAHYSFVTYVAVVIEVEVKDDGALLVH
KATIAADCGPQINPERIRSQLEGACVMGLGLAALGEISFKDGKVQQDNFHQYELARMPLAPKAVSVHLLKPDG
DLPLGGVGEPGVPPIAPALCNAIFAATGKRIRELPIRNQLQGWRKA
>SEQF1926||SEQF1926.1_04708
MTHTAQLSRRGFLLGALGAFTLTVTAKGLITTAWAAEPSAQKYGADSMPGGTVDDPLVFVSIAADGTVTIIAHR
AEMGTGVRTSLPMVVADEMEARWERVKVVQAQADEARYGNQNVDGSRSVRHFLMPMRRVGAAARQML
EAAAAARWSVPLAEVKAVQHEVLHQPSGRRLTYGELAADAAKQPVPTGDALKLKDRKEFRYIGKDQVRLVDLE
AIGKGQASYGMDMRLPGMVYAVVARPPVVGGKLRRLDSAKALAVPGVLKVVEIPPMQGAPAFQPLGGVAIV
ARNTWAARQGRDALEIEWDDGPNASYDSSAYRQTLESAARKPGKTMRSQGDAAQAWAKAPEAERVAAEYY
VPHLAHASMEPPAATVRIKDGRAEVWTSVQNPIAARDAVAIRLKLEPAKVTVNVLLLGGGFGRKSKPDFVDEA
AIVARAMPEGTPVKLVWTREDDIHHDYLHTVSVERLEAVLDQNGQVQSWLHRSAAPTIASLFAEGAKGQQLFE
SAMSAINMPYRIPNVQVETAEVEAHARIGWFRSVANIPHAYAAQCFIAELAHRAGKDPRDFALDLIGPARRIDP
GTMADTWNYSESPERYPYDTGRLRGVIEAACQGAEWGRTLPQGHGLGLAFCYSFMSYTATVVEVAVDAKGEV
RVVAVDMAMDCGPQINPERIRAQMEGGAIMGLGLALASEITFEQGRVKQSNFHDYEVLRHNASPRVIRTHLV
NDDHALPPGGVGEPPVPPVAPALCNAIFAATGKRIRSLPVRRVA
>SEQF1977||SEQF1977.1_01811
MNAKTTKPRSGRRRFLLGALGIGGALVVGWGVMPPRSRVGDPDIFPEHNGEIALNGWIKITPEGDVVLAMPR
VEMGQGIHTALSMLAAEELDIPLARVRIESAPVERIYGNVVAMGDSSLPLHPDSADKTWARALHWIMAKSARE
IGLIITGGSSSTADGWQPVREAAATARAALVQAAAREWNVPAADVSIREGQLIGPGGKQSTFGEMAKSARGISA
PSNVTLKPASQFRLIGKPAPRNDLAAKTDGSARFSIDTRLPGMLYAAVVMCPVFGGKLKTFQSKAALGMPGVR
YVVPFEGAGGGAPGVAVVADHYWQARQALATLEPVWDNGPHAKLDSAGIRQQLVSALDSDKGGFTYRSTGD
GLKAFDKTDGATIVEAEYTAPYLAHATMEPINCTAQVTSEGVHLWAPTQVATLAQLVAARAAGVSGDKVHIDIP
LIGGGFGRRLESDFIGQAVTIATKTEGKPVQVIWSREEDVRHDFYRPHAIARLKARVQNGKVTAIASRSAGQSIL
AGELDRLFGAPSAGIDRYTAEGLFDLPYEIEHEHIAHLAVDLPVPVGFWRSVGHSYNGFFMEGFLNEVAAQAKL
DPLAMRRELLKDHPRELKVLDTAAQAAGWGQPLAPAADGAPRARGIALHPSFGSVVAQVVEVSIKDGKPRVH
RVVCAVDCGTVVNPGIVAQQMESAVIFGLSAALYGRIDIKDGQIVQSNFTDYPALKMAETPVIETHIVPSTAEPS

GMGEVGVPPIAPAVAHAVAQLTGKPVRQLPMV
>SEQF1991||SEQF1991.5_00346
MKRSFPDDLVIGNLSRRGFLKGVGATGVLLVAANWGWRDALAAEKKAFGADAMPHGWVDNPKIYVSIDRDG
TVGIVCNRSEMGQGVRTSLAMVVADELEADWSRVKVIQAPGDEARYGNQDTDGSRSMRHWFDPMRRCGA
AARQMLEQAAANQWKVPLGECRAEQNKVLHAPSGRSLSFGELAEAAAGLEVPARDKLLLKKPEQFRYIGKDV
ARAIDGADIVNGRAGFGFDARFDDMLYAVVARPPVYGGKLKRYDAAAALKVPGVVKVIEIEGRPIPSEFQPLGG
VAVVAQNTWAAIKGREALVVEWDAGVNGGYDSVAYRKQLEEAARKPGKVVRDSGDAAALFAKGGDIVEAEYY
LPHLAQAPMEPPVSTAWYKDGACEVWAPTQAPQVTRERIAERLKLPFDKVTVNVTLLGGGFGRKSKPDFVLEA
AILAKAFPGRHLRVQWTREDDLHFSYFHTVSVERLQAVLGADGLPQAWLHRSVAPSITALFGPDSKHQGAFEL
GMGLTNLPFAIPNVRLENPEAPAHTRVGWFRSVSNIPHAFAIQSFVGELAAKAGQDPKDYLLKLLGPARRIDTAE
LGDSWNYGESPERYPLDVGRLRGVIEEAARQSGWGGELPRGRARGIAAHYSFVTYVAVVIEVEVKDDGALLVH
KATIAADCGPQINPERIRSQLEGACVMGLGLAALGEISFKDGKVQQDNFHQYELARMPLAPKAVSVHLLKPDG
DLPLGGVGEPGVPPIAPALCNAIFAATGKRIRELPIRNQLQGWRKA
>SEQF1991||SEQF1991.5_01338
MNSKIDLSNALPGSRRGFLKGAAVVGLTIGFQWSGARRALAAALPDAGFAPNAFLRIAPDDSVTVIAKHVEMG
QGAYTGIATIVAEELDADWSKVRVESAPADAKRYANLAFGTMQGTGGSSAMANSWMQLREAGAKARAMLV
EAAARQWRVPATELRTRDGFVEHPASQRKASYGSLAAAAAELPVPEKVQLKDPKDFRLIGHQAPRVDVPGKTD
GSAQFTLDVSLPGMLVALLQRPPLFGATVKSFDATATRAIPGVVEVVQVPHGVAVVAKGFWAAKQGRDALKVE
WDESKAEKRGSEALMAEYRKLAEQPGKPARRDGDAAKAVAGATRRIAASYEFPFLAHAPMEPLDAVVRLTADS
CEIWAGDQFQTVDQGNAARTAGLKPEQVKINTLYAGGSFGRRANAWSDYIVEAVSIAKALGANGVPVKLQWT
REDDIHGGFYRPMYYHRLEAGLDADGKMVGWQHRIVGQSILEGTPFAAVMVKDGVDATSVEGAANLPYAVH
NVSVELSTTQVGVPVLWWRVVGSSHTVYAVEAFIDEAAQAAGKDPYLFRRDLLAEQPRLRGVLELAAEKAGW
DPSRPLPAGRGRGIAVTEAFKTFVAQVVEVSVDKDGKLKVERVVCAVDCGIPINPDVIAAQMEGGIGFGLGAIL
HSAITLKDGKVEQNNFDGYQVLRIAEMPKVEVHIVPSGEAPTGVGEPGVAPIGPALANAIFAATGQRLYNLPFPT
SFAKA
>SEQF1992||SEQF1992.5_00481
MKRSYPDDLVIGNLSRRGFLKGVGATGVLLVAANWGWRDALAAEKKAFGADAMPHGWVDNPKIYVSIDRDG
TVGIVCNRSEMGQGVRTSLAMVVADELEADWSRVKVIQAPGDEARYGNQDTDGSRSMRHWFEPMRRCGA
AARQMLEQAAANQWKVPLGECRAEQNKVLHAPSGRSLSFGELAEAAAGLEVPARDNLLLKKPEQFRYIGKDV
ARAIDGADIVNGRAGFGFDARFDDMLYAVVARPPVYGGKLKRYDAAAALKVPGVVKVIEIEGRPIPSEFQPLGG
VAVVAQNTWAAIKGREALVVEWDAGVNGGYDSVAYRKQLEEAARKPGKVVRDSGDAAALFAKGGDIVEAEYY
LPHLAQAPMEPPVSTAWYKDGACEVWAPTQAPQVTRERIAERLKLPFDKVTVNVTLLGGGFGRKSKPDFVLEA
AILAKAFPGRHLRVQWTREDDLHFSYFHTVSVERLQAVLGADGLPQAWLHRSVAPSITALFGPDSKHQGAFEL
GMGLTNLPFAIPNVRLENPEAPAHTRVGWFRSVSNIPHAFAIQSFVGELTAKAGQDPKDYLLKLLGPARRIDTAE
LGDSWNYGESPERYPLDVGRLRGVIEEAARQSGWGGELPRGRARGIAAHYSFVTYVAVVIEVEVKDDGALLVH
KATIAADCGPQINPERIRSQLEGACVMGLGLAALGEISFKDGKVQQDNFHQYELARMPLAPKAVSVHLLKPDG
DLPLGGVGEPGVPPIAPALCNAIFAATGKRIRELPIRNQLQGWRKA
>SEQF1992||SEQF1992.5_01690
MNSKIDLSNALPGSRRGFLKGAAVVGLTIGFQWSGARRALAAALPDAGFAPNAFLRIAPDDSVTVIAKHVEMG
QGAYTGIATIVAEELDADWSKVRVESAPADAKRYANLAFGTMQGTGGSSAMANSWMQLREAGAKARAMLV
EAAARQWQVPAAELRTRDGFVEHPTSQRKASYGSLAAAAAELPVPEKVQLKDPKDFRLIGHQAPRVDVPGKT
DGSAQFTLDVSLPGMLVALLQRPPLFGATVKSFDATATRAIPGVVEVVQVPHGVAVVAKGFWAAKQGRDALKV
EWDESKAEKRGSEALMAEYRKLAEQPGKPARRDGDAAKAVAGATRRIAASYEFPFLAHAPMEPLDAVVRLTAD
SCEIWAGDQFQTVDQDNAARTAGLKPEQVKINTLYAGGSFGRRANAWSDYIVEAVSIAKALGANGVPVKLQW

TREDDIHGGFYRPMYYHRLEAGLDADGKLVGWQHRIVGQSILEGTPFAAVMVKDGVDATSVEGAANLPYAVP
NVSVELSTTQVGVPVLWWRVVGSSHTVYAVEAFIDEAAQAAGKDPYLFRRDLLAEQPRLRGVLELAAEKAGW
DPSRPLPAGRGRGIAVTEAFKTFVAQVVEVSVDKDGKLKVERVVCAVDCGIPINPDVIAAQMEGGIGFGLGAIL
HSAITLKDGKVEQNNFDGYQVLRIAEMPKVEVHIVPSGEAPTGVGEPGVAPISPALANAIFAATGQRLYNLPFPT
SFAKA
>SEQF2030||SEQF2030.1_01187
MPAGDAVKLKTRAEFRYIGKDEVRLVDLEAIGKGEAMYGIDMRLPGMVYAVVARPPVVGGKLRRVDSAKALAV
PGVLKVVEIPAMAGAPAFQPLGGVAVVASNTWAAMQGRAALAIEWDDGPNAAYDSVAYRETLTEASRKPGK
VVRDQGDAPQAWAKAGEAERFMAEYHVPHLAHASMETPVATVRIQDGAAEVWTSVQNPAAAQEAVAKRL
KLKPENVKVHVLLLGGGFGRKSKPDYVDEAAIVAQAMPAGTPVKLVWTREDDIHHDYLHTVSAEHLEAVVGKD
GKVQSWLHRSAAPTIASLFTEGAKGEQLFESAMSAINMPYVIPNVRVETAEVAAHARIGWFRSVANIPHAFAA
QCFIAELAHRAGRDHKQYALDLIGPARRIDPGTLADTWNYSESPERYPYDTGRLRGVIEAAASGAKWGRELPKG
HGLGLAFCYSFMSYTATVVEVAVDEKGEVRVVAVDMALDCGPQIKPERIRAQMEGGAIMGLSLALLGEITFEKG
RVKQNNFYDYEVLRHNASPRVIRTHLVNDDHALPPGGVGEPPVPPVAPALCNAIFAATGKRVRSLPVRSVA
>SEQF2047||SEQF2047.2_02125
MPIPNHLFADLPKGLRALAADSIDAGLKLERRDFLKLATASGFALGVFPAAVSAQAKGNEQVSTSALKPYQQPSA
FVKIDRDGAVTITINRLEFGQGVQTGLPMVLAEELDADWSKVQSAHGNADPAYLDPVMGMHLTGGSTAIKNS
YVQYRELGARTRSMLLATAAKRWSVDPQSLRTQAGQVIGPRGKKLGYGELADEAMKMPVPQQVKLKDAKDF
RIIGHATGRLDARAKSSGRQSYGIDMHLPGMLTAVVAHPPVYGSKIQSVDDAAAKAIKGVRAVLRVPSVWGGE
LVAVLADGYWPAKQGRDALKIQWDSAAVGKVDSARQLAQYRELAKKPGALKFNADVSALNGAVHKISAEYVF
PYLAHTPMEPLNCTVRVTGAGKDAKVELWLGTQAPGWEVATAARVLGVAPQNVRVNVQMAGGGFGRRAN
PRSDYVAEACEIAKAARTSGIKAPVRMIWSREDDVKGGYYRPMHVHRAEIGFDDKGKVIAWDHVIVGQSLAK
GTAFEGFMLKNGVDTTTVEGMKEPYDLPMRLSVHHPELNAPVLWWRSVGSTHTAYVMETLMDEIARAVKQ
DPVAYRLQQFGDRHPRHKAALQLAVEKSGYGKRQLAEGRAWGVAVHQSFDSVVAYVVEASMKDGAPKLHAV
TAGVHCNLAVNPRSVEAQVQGGALMGLGMCLPGAAITFKDGQVEQGNFNDYTVARLTDMPAISVHIVPSAD
APTGMGEPGVPPLAPAFANAIAKLSGHTPRELPFQKA
>SEQF2057||SEQF2057.2_01091
MPIPNHFLADMPKGLRALAADTIDAGTKLERRDFLKLATASGFALGVFPSAALAQAKGNEEVSTSALKPYQQPS
AFVKIDRDGTVTVTINRLEFGQGVQTGLPMVLAEELDADWSKLHSVHGNADPAYLDPVMGMHLTGGSTAIKN
SYVQYRELGARTRSMLLATAAKRWGVDPQSLRTQAGQVIGPRGKKLGYGELADEAMKMPVPQQVKLKGVKD
FRIIGRATGRLDARAKSSGSQSYGIDMHLPGMLTAVVAHPPVYGSKIQSVDDAATKAIKGVRAVLRVPSVWGGE
LVAVLADGYWPAKQGRDALKIQWDSAAVGKVDSARQLAQYRELAKKPGALKFDVDVSALNGAVHKISAEYVF
PYLAHTPMEPLNCTVRVTGAGKDAKVELWLGTQAPGWEVATAARVLGVAPQNVRVNVQMAGGGFGRRAN
PRSDYVAEACEIAKAARASGVDAPVRMIWSREDDVKGGYYRPMHVHRAEIGFDGKGKVIAWDHVIVGQSLAK
GTAFEGFMVKNGVDTTTVEGMKEPYDLPMRLSVHHPELNAPVLWWRSVGSTHTAYVMETLMDEIARAVGQ
DPVAYRLQQFGDRHPRHKAALQLAVEKSGYGKRRLAEGRAWGVAVHQSFDSVVAYVVEASMKDGTPKLHAVT
AGVHCNLAVNPRSVEAQVQGGALMGLGMCLPGAAITFKDGQVEQGNFNDYTVARLTDMPAITVHIVPSADA
PTGMGEPGVPPLAPAFANAIAKLSGHTPRELPFPKA
>SEQF2058||SEQF2058.1_00637
MPIPNHLFADLPKGLRALAADSIDAGLKLERRDFLKLATASGFALGVFPAAVSAQAKGNEQVSTSALKPYQQPSA
FVKIDRDGAVTITINRLEFGQGVQTGLPMVLAEELDADWSKVQSAHGNADPAYLDPVMGMHLTGGSTAIKNS
YVQYRELGARTRSMLLATAAKRWSVDPQSLRTQAGQVIGPRGKKLGYGELADEAMKMPVPQQVKLKDAKDF
RIIGHATGRLDARAKSSGRQSYGIDMHLPGMLTAVVAHPPVYGSKIQSVDDAAAKAIKGVRAVLRVPSVWGGE
LVAVLADGYWPAKQGRDALKIQWDSAAVGKVDSARQLAQYRELAKKPGALKFDADVSALNGAVHKISAEYVF

PYLAHTPMEPLNCTVRVTGAGKDAKVELWLGTQAPGWEVATAARVLGVAPQNVRVNVQMAGGGFGRRAN
PRSDYVAEACEIAKAARTSGIEAPVRMIWSREDDVKGGYYRPMHVHRAEIGFDGKGKVIAWDHVIVGQSLAK
GTAFEGFMVKNGVDTTTVEGMKEPYDLPMRLSVHHPELNAPVLWWRSVGSTHTAYVMETLMDEIARVVKQ
DPVAYRLQQFGDRHPRHKAALQLAVEKSGYGKRQLAEGRAWGVAVHQSFDSVVAYVVEASMKDGTPKLHAV
TAGVHCNLAVNPRSVEAQVQGGALMGLGMCLPGAAITFKDGQVEQGNFNDYTVARLTDMPAITVHIVPSAD
APTGMGEPGVPPLAPAFANAIAKLSGHTPRELPFPKA
>SEQF2167||SEQF2167.1_02831
MKRSYPDDLVIGNLSRRGFLKGVGATGVLLVAANWGWRDALAAEKKAFGADAMPHGWVDNPKIYVSIDRDG
TVGIVCNRSEMGQGVRTSLAMVVADELEADWSRVKVIQAPGDEARYGNQDTDGSRSMRHWFEPMRRCGA
AARQMLEQAAANQWKVPLGECRAEQNKVLHAPSGRSLSFGELAEAAAGLEVPARDKLLLKKPEQFRYIGKDV
ARAIDGADIVNGRAGFGFDARFDDMLYAVVARPPVYGGKLKRYDAAAALKVPGVVKVIEIEGRPIPSEFQPLGG
VAVVAQNTWAAIKGREALAVEWDAGVNGGYDSVAYRKQLEEAARKPGKVVRDSGDAAALFARGGDIVEAEYY
LPHLAQAPMEPPVSTAWYKDGACEVWAPTQAPQVTRERIAERLKLPFDKVTVNVTLLGGGFGRKSKPDFVLEA
AILAKAFPGRHLRVQWTREDDLHFSYFHTVSVERLQAVLGADGLPQAWLHRSVAPSITALFGPDSKHQGAFEL
GMGLTNLPFAIPNVRLENPEAPAHTRVGWFRSVSNIPHAFAIQSFVGELAAKAGQDPKDYLLKLLGPARRIDTAE
LGDSWNYGESPERYPLDVGRLRGVIEEAARQSGWGGELPRGRARGIAAHYSFVTYVAVVIEVEVKDDGALLVH
KATIAADCGPQINPERIRSQLEGACVMGLGLAALGEISFKDGKVQQDNFHQYELARMPLAPKAVSVHLLKPDG
DLPLGGVGEPGVPPIAPALCNAIFAATGKRIRELPIRNQLQGWRKA
>SEQF2167||SEQF2167.1_03333
MNSKIDLSNALPGSRRGFLKGAAVVGLTIGFQWSGARRALAAALPDAGFAPNAFLRIAPDDSVTVIAKHVEMG
QGAYTGIATIVAEELDADWSKVRVESAPADAKRYANLAFGTMQGTGGSSAMANSWMQLREAGAKARAMLV
EAAARQWRVPATELRTRDGFVEHPASQRKASYGSLAAAAAELPVPENVQLKDPKDFRLIGHQAPRVDVPGKT
DGSAQFTLDVSLPGMLVALLQRPPLFGATVKSFDATATRAIPGVVEVVQVPHGVAVVAKGFWAAKQGRDALKV
EWDESKAEKRGSEALMAEYRKLAEQPGKPARRDGDAAKAVAGATRRIAASYEFPFLAHAPMEPLDAVVRLTAD
SCEIWAGDQFQTVDQGNAARTAGLKPEQVKINTLYAGGSFGRRANAWSDYIVEAVSIAKALGANGVPVKLQW
TREDDIHGGFYRPMYYHRLEAGLDADGKLVGWQHRIVGQSILEGTPFAAVMVKDGIDATSVEGAANLPYAVP
NVSVELSTTQVGVPVLWWRVVGSSHTVYAVEAFIDEAAQAAGKDPYLFRRDLLAEQPRLRGVLELAAEKAGW
DPSRPLPAGRGRGIAVTEAFKTFVAQVVEVSVDKDGKLKVERVVCAVDCGIPINPDVIAAQMEGGIGFGLGAIL
HSAITLKDGKVEQNNFDGYQVLRIAEMPKVEVHIVPSGEAPTGVGEPGVAPIGPALANAIFAATGQRLYNLPFPT
SFAKA
>SEQF2168||SEQF2168.1_03407
MKRSYPDDLVIGNLSRRGFLKGVGATGVLLVAANWGWRDALAAEKKAFGADAMPHGWVDNPKIYVSIDRDG
TVGIVCNRSEMGQGVRTSLAMVVADELEADWSRVKVIQAPGDEARYGNQDTDGSRSMRHWFEPMRRCGA
AARQMLEHAAANQWKVPLGECRAEQNKVLHAPSGRSLSFGELAEAAAGLEVPARDNLLLKKPEQFRYIGKDV
ARAIDGADIVNGRAGFGFDARFDDMLYAVVARPPVYGGKLKRYDAAAALKVPGVVKVIEIEGRPIPSEFQPLGG
VAVVAQNTWAAIKGREALVVEWDAGVNGGYDSVAYRKQLEEAARKPGKVVRDSGDAAALFAKGGDIVEAEYY
LPHLAQAPMEPPVSTAWYKDGACEVWAPTQAPQVTRERIAERLKLPFDKVTVNVTLLGGGFGRKSKPDFVLEA
AILAKAFPGRHLRVQWTREDDLHFSYFHTVSVERLQAVLGADGLPQAWLHRSVAPSITALFGPDSKHQGAFEL
GMGLTNLPFAIPNVRLENPEAPAHTRVGWFRSVSNIPHAFAIQSFVGELAAKAGQDPKDYLLKLLGPARRIDTAE
LGDSWNYGESPERYPLDVGRLRGVIEEAARQSGWGGELPRGRARGIAAHYSFVTYVAVVIEVEVKDDGALLVH
KATIAADCGPQINPERIRSQLEGACVMGLGLAALGEISFKDGKVQQDNFHQYELARMPLAPKAVSVHLLKPDG
DLPLGGVGEPGVPPIAPALCNAIFAATGKRIRELPIRNQLQGWRKA
>SEQF2168||SEQF2168.1_02813
MNSKIDLSNALPGSRRGFLKGAAVVGLTIGFQWSGARRALAAALPDAGFAPNAFLRIAPDDSVTVIAKHVEMG

QGAYTGIATIVAEELDADWSKVRVESAPADAKRYANLAFGTMQGTGGSSAMANSWMQLREAGAKARAMLV
EAAARQWQIPAAELRTRDGFVEHPTSQRKASYGSLAAAAAELPVPEKVQLKDPKDFRLIGHQAPRVDVPGKTD
GSAQFTLDVSLPGMLVALLQRPPLFGATVKSFDATATRAIPGVVEVVQVPHGVAVVAKGFWAAKQGRDALKVE
WDESKAEKRGSEALMAEYRKLAEQPGKPARRDGDAAKAVAGATRRIAASYEFPFLAHAPMEPLDAVVRLTADS
CEFWAGDQFQTVDQGNAARTAGLKPEQVKINTLYAGGSFGRRANAWSDYIVEAVSIAKALGANGVPVKLQW
TREDDIHGGFYRPMYYHRLEAGLDADGKLVGWQHRIVGQSILEGTPFAAVMVKDGVDATSVEGAANLPYAVP
NVSVELSTTQVGVPVLWWRVVGSSHTVYAVEAFIDEAAQAAGKDPYLFRRDLLAEQPRLRGVLELAAEKAGW
DPSRPLPAGRGRGIAVTEAFKTFVAQVVEVSVDKDGKLKVERVVCAVDCGIPINPDVIAAQMEGGIGFGLGAIL
HSAITLKDGKVEQNNFDGYQVLRIAEMPKVEVHIVPSGEAPTGVGEPGVAPIGPALANAIFAATGQRLYNLPFPT
SFAKA
>SEQF2169||SEQF2169.1_03109
MLNEIFPNEQPRALQHMLERDEADGPAALPRRSFLKIVGIGGLALGAFPHLALAQEANGAAAAPLKPTQQPSA
FVQIAPNGEVTVTINRLEFGQGVQTGLPMILAEELDADWSQVRSRNGSNDAAYMDPAFGIHLTGGSNSIKNSY
TQYRELGARARAMLLSAAAARWNVDVASLSTQAGMVLGPAGRKASYGELAEAAMAMPVPEQITLKDPKDFR
IIGQATTRIDAKAKSSGQQNFGIDMHLPGQLTAVVARPPVFGARIASLDDGAARAAKGVKAVVRVPLDGGAEG
VAVVADSYWQAKLARDALKVEWDASAVEKVDSEKQLAQYRELAGKPGPLHFDADMTPLTSAPHQLDAEFVFP
YLAHAPMEPLNCTVQLAGDSAQLWVGTQFPGGDGAAAAKVLGLKPEQIQVNVQTAGGGFGRRGVPTNDFV
VLACEVAKAARTAGVNAPIRTLWSREDDIKGGYYRPMHLHRARIGFDDSGKVLAWDHALVGQSIITGTVFGGR
VKNGIDPTATEGLRNPYPLPMRLTVHHPKLNVPVLWWRSVGSTHTAFVMETLIDEIARTTKQDPVAYRMKLFG
EQSPRHREALQLAVDKSEYGKRQLPAGHAWGVAVHESFSSVVAYVVEASVQDGRPVLHNVTAGVHCNLAVNP
RSVEAQVQGAALMGLSMCLPGGAITLKDGVVQQSNFADFSVPRITDMPTFAVHIVPSAEPPTGMGEPGLPAL
APAFANAVASLTGKPLRELPFKLA
>SEQF2169||SEQF2169.1_04425
MSRLSDDFVLSNLSRRGFLKGASATGVLVLAATWGLPEAFAEEKKFGAEGMPHGAVDDPKVYVSIAADGSVTVI
CNRSEMGQGVRTSLSMVVADELEADWARVKVQQAPADEARFGNQDTDGSRSMRHWYEPMRRCGAAART
MLELAAAAQWKVPVSECHAQLHKVLHQPSGRELGYGELAAAASALPVPGRDSLRLKQPSEFRYIGKEASRAID
GADIVNGRAVFGADVHFDGMLYAVIARPPVYGGKVKSVDSSAALKVPGVVKVVQIEGRPLPSEFQPLGGVAVV
AKNTWAAIKGREALKIQWDDGPNAGYDSIVYRKELEAAALKPGKVVRSSGDLDDALAKADSTLEASYYLPHLSQ
SPMEPMVAVARFKDGQCEAWAPSQAPQVTRERVAERLGIPFEKVTVNITLLGGGFGRKSKPDFVVEAAVLAKE
FPGQAVRVQWTREDDIHHSYFHTVSAEYLKAGLNQDGMPSGWLHRTVAPSITALFAPGMTHEAPFEIGMGV
TNMAYAIPNLRLENPEAVAHARVGWYRSVSNIPHGFAIQSFIDELAHKAGQDPLKYQVRLLGPDRKIDPRTLSEE
WNYGESPERYPIDTARIRTVLETAAKAAGWGRELPKGRGLGLAVHYSFVTYVAAVIEVEVKDDGTVIVHKADIAV
DCGPQINPERIRSQFEGACVMGLGNAMVGEISFKDGKVQQDNFHMYEVARMSLAPKEVAVHLVTPPGEVPL
GGVGEPGVPPIAPALCNAIFAATGKRIRSLPVRYQLQGWQQAKA
>SEQF2170||SEQF2170.1_00496
MSQPDTTLQSPSRRTLLKVGSLALGGLVIGFTLPFAGRSFAEQVLNEGPEDQPMSNATALDAFISIDRDGQVTFT
VPKIEMGQGAQSGLAVMVAEELEIGLEQITLKEAPPNEQIYNDKLLNFQATGGSTSIRSNWEPLRQAGAAARLL
LIQAAAQHWQLGADQLRAENGRVLGPDGQSLGYGELVEDAAKLPVPEDIPLKPADQFRLIGKPTRRLDTPAKV
DGTARFTIDLVVPGMKYASIRACPVLGGTLREVDERAARQIPGVIEVVRLDNAVAVIGEHTWAAFAGVRALEID
WALGDNAGIDSAQMEREIREALDKPGAIANEQGDIDAALKDAARTFEAEYEMPFLAHAALEPMTCVAEVRAD
AVELWVGTQVPVRAQTAAAEAAGRPAEQVIVNNQLIGGAFGRRLEVDFISQAVAIAAQVDYPIKLTWTREEDT
THDMYRPHYIDRFAAALDAEGRLQGWRHTIAGASVLARFAPEAVPENGLDGDAVEVAMHPIYAMPNLRVNY
VPVPPRALHQSWWRGVGPLRSTYMLESFIDEVARSAEQDPVDYRMALLGSHPRAQGVLRLAAEKAGWGEPL
EAGHGRGVAVQEVFGSFLATVVELQVSEDKGIRLKRLVVAIDCGQVMNPVSVKSQIEGGTLFGLSAALFNEITVR

EGRVEQTNFHDYRQLRISDAPPVETYIVESREAPGGVGEAGTAMIAPALVNALAAANGTRIRRLPLARAGYYVI
>SEQF2171||SEQF2171.1_00520
MSQPDTTLQSPSRRTLLKVGSLALGGLVIGFTLPFAGRSFAEQILNEGPEDQPMSNATALDAFISIDRDGQVTFT
VPKIEMGQGAQSGLAVMVAEELEIGLEQITLKEAPPNEQIYNDKLLNFQATGGSTSIRSNWEPLRQAGAAARLL
LIQAAAQRWQLGADQLRAENGRVLGPDGQSLGYGELVEDAAKLPVPEDIPLKPADQFRLIGKPTRRLDTPAKV
DGTARFTIDLVVPGMKYASIRACPVLGGTLREVDERAARQIPGVIEVVRLDNAVAVIGEHTWAAFAGVRALEID
WALGDNAGIDSAQMEREIRGALDKPGAIANEQGDIDAALKDAANTFEAEYEMPFLAHAALEPMTCVAEVRA
DAVELWVGTQVPVRAQTAAAEAAGRLVEQVIVNNQLIGGAFGRRLEVDFISQAVAIAAQVDYPIKLTWTREED
TTHDMYRPHYIDRFAAALDAEGRLQGWRHTIAGASVLARFAPEAVPENGLDGDAVEVAMHPIYAMPNLRVN
YVPVPPRALHQSWWRGVGPLRSTYMLESFIDEVARSVEQDPVDYRMALLGSHPRAQGVLRLAAEKAGWGEP
LEAGHGRGVAVQEVFGSFLATVVELQVSEDKGIRLKRLVVAIDCGQVMNPVSVKSQIEGGTLFGLSAALFNEITV
REGRVEQTNFHDYRQLRISDAPPVETYIVESREAPGGVGEAGTAMIAPALVNALAAANGTRIRRLPLARAGYYVI
>SEQF2172||SEQF2172.1_02110
MNAKTTKPRSGRRRFLLGALGIGGALVVGWGVMPPRSRVGDPGIFPEHNGEIALNGWIKITPEGNVVLAMPR
VEMGQGIHTALSMLAAEELDIPLARVRIESAPVERIYGNVVAMGDSSLPLHPDSADKTWARALHWIMAKSARE
IGLIITGGSSSTADGWQPVREAAATARAALVEAAAREWNAPVAQVSIREGQLIGPGGKQSTFGEMAKSARGLS
APSNVTLKPASQFQLIGKPAPRNDLAAKTDGSARFSIDTRLPGMLYAAVVMCPAFGGKLKTFQSKAALGMPGV
RYVVPFEGTGGGAPGVAVVADHYWQARQALATLEPVWDNGPHAKLDSAGIRQQLVSALDSDKGGFTYRSM
GDGLKAFDKADGATLVEAEYSAPYLAHATMEPINCTAQVTPEGVHLWAPTQVATLAQLVAARAAGVSGDKVQI
DIPLIGGGFGRRLESDFIGQAVTIATKTDGKPVQVIWSREEDVRHDFYRPHAIARLKARVENGKVTAIASRSAGQ
SILAGELDRLFGAPSVGIDRYTAEGLFDLPYEIEHEHIAHLAVDLPVPVGFWRSVGHSYNGFFMEGFLNEVAAAA
KLDPLAMRRDLLKDHPRELKVLDTAAQAAGWGQPLAPAADGAPRARGIALHPSFGSVVAQVVEVSMKDGKP
RVHRVVCAVDCGTVVNPGIVAQQMESAVIFGLTAALYGRIDIKDGQVVQSNFPDYPALKMAETPVIETHIVPST
AEPSGMGEVGVPPIGPAVAHAVAQLTGKPVRQLPMA
>SEQF2183||SEQF2183.1_04025
MLPNIDYNDLPRALQRLMAQPSADEAATLPRRSFLKMAGAGGLVLGAFPHMAMAQADGAKPAAGGLKPTQ
QPSAFVQIAPNGEVMVTINRLEFGQGVQTGLPMILAEELDADWALVRSRSGTNDAAYADPLFGIHLTGGSNTIK
NSFTQYRELGARARAMLMSAAAARWKVDVATLRTQAGTVIGPGGRKLSYGELAEAAMALPVPEKVVLKDPKD
FRIIGRATTRLDARAKSSGRQDFGIDVKQPGQLTAVVAHPPVFGARLSSVDDSAARAVKGVKAVVRIPLDRGAE
GVAVVADGYWPAKLGRDALKLEWNTSAVEKVDSDKQLVQYRELAGRPGNRKFDADMAPLAKAPHKLEAEFV
FPYLAHAPMEPLNCTVKLSDGRAELWVGTQSADLDGQAAARTLKLKPEQVKVNVQMAGGGFGRRFVGSSDF
VVEACEIAKATRAAGLDAPVRLLWSREDDMKGGYYRPMHLHRASIGFDERGKILAWDHVIVGQSITAGTVFAG
MMVKDGIDATAVEGMRDPYPVPMRLTVHHPQVNVPVLWWRSVGSTHTAFVMETLIDEIARSTKQDPVAYR
MQLFGDKHPRHRAALQLAVDKSGYGKKKLADGRAWGVAVHESFESVVAYVVEASVKDGQPVLHRVTSGVHC
NLAVNPRSVEAQVQGAAVMGLSTCLQGSAITLKDGVVQQGNFGDFTVARITQVPEFDIHIVPSADAPKGMGE
PGLPPLAPAFANAIAQLTGKPLRQLPFNLA
>SEQF2183||SEQF2183.1_04195
MRPSIIGRPEIAPDDLDVIGHKNPTMPQLKRRHFVLGTFGAVGALVVGWSATPAASRLVGSEPLPAGPGQVAL
NGWVKVGGDNTVTLMMSQSEMGQGTHTGLAMLLAEEMDASLGQVRIESAGADAIYNNQAVILDALPFKPG
DEGRVKRATEHVVGKLLRSIPGLSGTGGSSSITDQWLPVREAGASARAMLIGAAAAAWSVPAGECRTEGGRVL
HPNGKSASFGELAQGASQQPLARNVALKKPADFKLIGQPSRRIENKAKLDGTAKFGMDTLPPGLLYASIAMCPTI
GGRVARFDAAAAQKLPGVRKVVALAPVGVTLIGTGSTSGGVAVIADTPYHAMRALKAVEIEWDHGPAASLSSR
EMIERLSQTLRTSEGNARLDEGDAAAALKSAAKTLDVEYRVPFLAHATMEPMNCTAQFKDGVATVWAPTQAP
GFARGAVASALGIDAKKVDLHVTFLGGGFGRRYSTDFVTQAAMLARETDGAPVQLIWSREEDMAHDYYRPAY

VARCKAGLDANGALVAWQTTTAGSSLGAPSFMDTSTDGAWNTAYDFPNARVAHEPVESAVTTGVWRSVAHS
QNGFFVESFIDECAAAAGKDPVAFRATLLAKDPRHLHVLQRAAELSGWGKPLADAPDGAKRARGVAIHRAFGS
IVAQVAEVSVSADKQIRVHRVVCVIDCGVPVNPNLIRQQMEGGIVYGLSAALHGEITIEKGQVQQSNFHDYMP
LRMNECPVIETEIVAGSDRPGGVGEPGTPPIAPAVANALFALTGQRLRSLPLKLA
>SEQF2193||SEQF2193.1_02636
MKRSYPDDLVIGNLSRRGFLKGVGATGVLLVAANWGWRDALAAEKKAFGADAMPHGWVDNPKIYVSIDRDG
TVGIVCNRSEMGQGVRTSLAMVVADELEADWSRVKVIQAPGDEARYGNQDTDGSRSMRHWFEPMRRCGA
AARQMLEHAAANQWKVPLGECRAEQNKVLHAPSGRSLSFGELAEAAAGLEVPARDNLLLKKPEQFRYIGKDV
ARAIDGADIVNGRAGFGFDARFDDMLYAVVARPPVYGGKLKRYDAAAALKVPGVVKVIEIEGRPIPSEFQPLGG
VAVVAQNTWAAIKGREALVVEWDAGVNGGYDSVAYRKQLEEAARKPGKVVRDSGDAAALFAKGGDIVEAEYY
LPHLAQAPMEPPVSTAWYKDGACEVWAPTQAPQVTRERIAERLKLPFDKVTVNVTLLGGGFGRKSKPDFVLEA
AILAKAFPGRHLRVQWTREDDLHFSYFHTVSVERLQAVLGADGLPQAWLHRSVAPSITALFGPDSKHQGAFEL
GMGLTNLPFAIPNVRLENPEAPAHTRVGWFRSVSNIPHAFAIQSFVGELAAKAGQDPKDYLLKLLGPARRIDTAE
LGDSWNYGESPERYPLDVGRLRGVIEEAARQSGWGGELPRGRARGIAAHYSFVTYVAVVIEVEVKDDGALLVH
KATIAADCGPQINPERIRSQLEGACVMGLGLAALGEISFKDGKVQQDNFHQYELARMPLAPKAVSVHLLKPDG
DLPLGGVGEPGVPPIAPALCNAIFAATGKRIRELPIRNQLQGWRKA
>SEQF2193||SEQF2193.1_02021
MNSKIDLSNALPGSRRGFLKGAAVVGLTIGFQWSGARRALAAALPDAGFAPNAFLRIAPDDSVTVIAKHVEMG
QGAYTGIATIVAEELDADWSKVRVESAPADAKRYANLAFGTMQGTGGSSAMANSWMQLREAGAKARAMLV
EAAARQWQIPAAELRTRDGFVEHPTSQRKASYGSLAAAAAELPVPEKVQLKDPKDFRLIGHQAPRVDVPGKTD
GSAQFTLDVSLPGMLVALLQRPPLFGATVKSFDATATRAIPGVVEVVQVPHGVAVVAKGFWAAKQGRDALKVE
WDESKAEKRGSEALMAEYRKLAEQPGKPARRDGDAAKAVAGATRRIAASYEFPFLAHAPMEPLDAVVRLTADS
CEFWAGDQFQTVDQGNAARTAGLKPEQVKINTLYAGGSFGRRANAWSDYIVEAVSIAKALGANGVPVKLQW
TREDDIHGGFYRPMYYHRLEAGLDADGKLVGWQHRIVGQSILEGTPFAAVMVKDGVDATSVEGAANLPYAVP
NVSVELSTTQVGVPVLWWRVVGSSHTVYAVEAFIDEAAQAAGKDPYLFRRDLLAEQPRLRGVLELAAEKAGW
DPSRPLPAGRGRGIAVTEAFKTFVAQVVEVSVDKDGKLKVERVVCAVDCGIPINPDVIAAQMEGGIGFGLGAIL
HSAITLKDGKVEQNNFDGYQVLRIAEMPKVEVHIVPSGEAPTGVGEPGVAPIGPALANAIFAATGQRLYNLPFPT
SFAKA
>SEQF2196||SEQF2196.1_02197
MNTPELIPGVPLGEPINLSRRRFLASTAVGALVIGFGLPLGSGRVQAATGVERGTQVPAFLEIRPDGTVRLLSPFM
EGGQGTHTAMAQIVGEELDADPATFIVEAAPPGDAYVVMDNGMRITGGSMSVRMSYPTMRRLGALARAML
LQAGAEQLRVPVSELTTQPGRVVHAASGRSVGYGELASRALDMPVPDPASITLRDPGQFRWIGKPIKRLDAYDK
STGKALYSIDLKVDGMLHAAVQHAPRLGMTVGSLRNQAQVETMKGVHSIHQLPGAVAVVAERWWHAKRAV
EAIQVDWLEPTADSKVRAMPADFSSDGYRDFLATQQGPARDDENEGDVAGALKNAKTQVEATYHNQYVNHA
QLEPPSALARFNADGSLDIWLPNQAPDMFRADIAKRTGLALEQINLHSPLLGGFFGRHFLYDSASPYPQAIALAK
AVGRPVKLIWSREEEFLRDVLRPVAVVKFRAALDADGLPIAIEAVSATEGPTEAIAGKQGDKIDPTALEGLSGKSY
AIPNKRIAQIYVKGPAMLGYWRSVGNSLNDFFYEAFLDELADTGKRDPYELRLHLLRDNPRLTTLLKAVAELSGG
WKRGPYTAEDGTRRARGVAMASPFGSHAAVIAEVSIEKGQVKVHHIWEAIDPGSVVNPAIVEAQVNGAVALG
LSQTLLEEAVYVDGKPRARNYDLYPILPPSRMAQVHVKIVESGEKMGGIGEPPLPAVAPAVANAVAQLTGQRIRS
LPLSRHTFS
>SEQF2266||SEQF2266.1_02188
MRIAIQNLSRRRFVQGAGGLLLGLSLPPLARRAMAAGPQAGDGFAANAFVRIGADGRVTVLAKHLEMGQGA
YTGLATLLAEELDADWRQVRVEGAPADSARYGNQALGGLQGTGGSTAMFDSWEPMRRAGATARAMLVQAA
AQRWQVPADTIEVAEGVLSHPASGRRAGFGELAEAAARSPVPEDVPLKDPARFRLIGKHRLPHVDSAAKSDGS

ALYTQDMKLPGMLVAVVAHAPRLGAAVARVDDAAARAVPGVRAVVRFGGAALRHAGVAVLATNTWAARAG
RDALRIEWDEGPAYRQGSADILARYREAVGRPGSMAARKGDIDAAFAGAAKVIEAEYTYPYLAHAAMEPLNCL
VRLDDERCEIWNGEQFQTADQRAIAQYLGMPAERITLTQLYAGGSFGRRASSHADYLLEAVAIARTARAQGLNA
PVKLVWMREDDMRAGYYRPLNLHRARLALGADGALQAVHVRMAGQSILLGTPLADWVRDGVDPVSVEGLS
DLAYAVPNLQVELHTPTDVPVPVLWYRSVGHTHTAFSAETLIDEAAVAAGQDPVAYRLALLAAHPRHREVLQLA
AVRAGWREPLAAGAPGTRRGRGVAVHESFRSVMAQVVEVTIAADGALKVDRVVCAAVHCGLAVNPDVVRA
QMEGGIGFALSTALHGAITLKDGAVEQSNFHDYPVLRLAEMPAVEVHIAPSTQPPTGVGEPGVPPLAPALANAI
AQATGQRLRTLPLGTTVKA
>SEQF2325||SEQF2325.2_02702
MNKHVKNLAPETTDLSRRSFLVGTAATGLVLGYAASGIDQALAAPAPASFEPSVWYSIAPDGLVTVTCGKADMG
QHVASTMAQIVAEELGANWKDMRVQLASNDPKFNDPVLGAQITGGSWSTMMNFDAMSRAGAAGRIALTE
AAAASMGVPAGELVVRDSRISHAKSKKSMSFAEVVKSGKATKTFTADDLKAIKLKTPDQYTMIGVSVPQLDIPSK
VNGTAKYGIDVMVPGMVYGALVTPPVRYGATVKSVDDSAAKKLPGFIKAVTLDDKTTTTTGWVVAVANTYAQ
AKKAAAALKISYDGGPNAKLSSESLFAEAKRLQGLSDSGEFFVKDGDPNAAYGSAAKVLEAEYTTNINIHAPMEP
MNATAEFKGDILHIYSGNQFATRSGAIAAGAAGIDPKFVVMHQMWLGGGFGRRLDADMMVPAVQAAKAVG
KPVKVIYTRENDMTMDFSRPLTYQKVKAGMDGDGKIVAMSHDVVSAWPTARWGIPDFLTPSVDKKGPLDSFT
VNGADFFYTVPNHYVRAIKNEMAHNATPSGQLRSVAPGWTFWAVESMIDEIAHATGKDPAQLRISLLDGKGK
NDGGAQRLRNTLLAAMGLAGYGTKQLPKGEGMGVACVSSQERATASWTACVAHVAVAPSGEVTVKKLTVAT
DVGTQVNPDGIRAQVEGAALWGMSLALFEKATLKDGGIEQTNFDSYTPLRMSQLPEVAVNVIANGEKATGVG
EPAVTVVAPAIGNAVFNAVGARVRGLPITAEAVKAAMKA
>SEQF2352||SEQF2352.3_01271
MGKFESRFRPEGGILNLSRRGFLKGTGGLALGVVFAPLLKGGEALAAAGDAFVPNAFVRLGTDGSVTVLAKHVE
MGQGTYTGLATLLAEELDADWNQVRVEGAPANAALYNNLAFGPIQGTGGSNAMANSWEQMRKAGASARA
MLVSAAAERWKVPASEIGVSQGVVSHAASGRKAGFGELAEAASRQAVPAEVKLKTPDQFVLIGRERLSRRDSQ
DKTDGTAVFTQDFKLPGMLVAVVAYPKRFGAVPAKVDSSKAKAVAGVVEVVEFTDTPHGRAGVAVLATNTWA
ARQGRDALVVEWDESRAFKLGSAEIFARYREAAGKAGAVAGKQGDISTAMAQPATLIEADFEFPYLAHAAMEP
MNCLVKISAGQCDIWNGEQFQTVDQLSIAKYLGIPVEKVSLTQLYAGGSFGRRANPRSDYLLEAVAITKAARAKG
HDVPVKLVWTREDDTHAGFFRPLFLHRAKLALDAEGNLQGWHQRLVGQSILKGSPFEGVMAKEGIDPTSVEG
VANLSYAVPNLLVELHSPDDIGVPVQWWRSVGHTHTAYSVETLIDQAATTAGKDPYEYRRALLGKHPRHLGVLQ
LAAEKAGWSQPLKAGAEGEKRGRGIAVHESFGSYVAQVAEVTVKADGSYRVDRVVCAVDCGVAINPDVIRAQ
MEGGIGFGLAAALHGAITLKDGVVEQSNFHDFQVLRINEMPVVEVHILPSAEAPTGVGEPGVPPLAPALGNAL
HAAVGQRFYSLPLPRQLKLA
>SEQF2408||SEQF2408.1_03680
MTIELDHPGSVRPSRRTFLKAAGATAVVSLTIGFEWAGLGRRALAATAPAADFAPNAFLRITPDGAVTVIAKHVEL
GQGAYTGIATIVAEELDADWSSVRVESAPADAKRYANRAFGTMQATGGSSAMSNSWQQLREAGGKARAMLV
SAAAARWKVPAGELATANGVVKHAKSGKTAAYGTLIADASKLPVPDKVTLKQPADFKLIGQRIPRVDAAAKSNG
TAHFTLDTTFPGMRVALLQRPPRFGATVKSFDASAAKAVPGVVSIVQVPRGIAVVATGFWAAKQGRDALKVEW
DETNAEKRSSDELMREYRELATKPGVSARKDGDADAAIAGAARKISATYAFPYLAHAPMEPLDAVVKLTADSCEI
WAGDQFQTVDQGNAAQVAGLKPEQVQIHTLYAGGSFGRRANAWSDYVVEAVSIAKALGADGKPLKLQWTR
EDDIQGGFYRPMYFHKLDAGLTADGRLVGWRHRIVGQSILAGTPFEPFMVKNGVDATSVEGATNLPYTVPNVS
VELTTTKVGVPVLWWRVVGSSHTAYAVEAFIDEAAHSAGKDPYLFRRDLLAKEPRMRAVLELAAQKAGWDPAK
PLPKGRGRGIAVAEAFGSYVAQVAEVSVDADGKVKVERVVCAVDCGIAINPDIVAAQMEGGIGFGLGAVLHSAI
TLKDGHVEQRNFDGYHVLRMAEMPKVEVHIVPSAEAPTGVGEPGVAPVGPAVANAIFAATGKRHYVLPFDSG
DTAKA

>SEQF2408||SEQF2408.1_02146
MNAPELSVHNESRRALLLGFASGGLLLAFGVPSLARAAAPVQPPVSPNPRYGGAGMPHGLRDDPHLFVAIAPD
GTVTVTCIRSEMGQGVRTSVALVVADELGADWTRVKVAQAVGDEPRYGNQNTDGSRSLRQSFAALRRAGAA
ARTMLEQAAAAAWGVDVRQVKATVHEVVDTKSGRKLGFGELAAKAAALPVPDTNSVALKAPAEFRYIGKGET
ALIDGRDIVGGRAHYGIDTRLDGMLYAVVARPPVYGDTVASFDGSAAEQLPGVVKVVQLVSTPLPSGFQPLGGV
AVIARDTWTAIQARAQLKIDWQHGPHAGYDSAAYRKTLEAAAEQPGDVIRNDGDAAAALAGAVKRVRATYYV
PHLAHATMEPPAAVARVADGRCEVWTCTQAPQTTRDEIAKALALPTERVTVNVTLLGGGFGRKSKPDYVVEA
ALLSKAVGAPVKLTFTREDDIAHDYFHAVSLESFDGGLDASGKVVAWQHRTVAPSIQSTFKAGVVHEQPGELA
QGIADLPFAVQNVRIENPAVEAHTRIGWFRSVYNIPHAFGIQSFVSELAHAAGRDPKDFLLDLIGPARRFEPHITV
KNVNYGEDPALYPVDTGRLRRVIETVAREAGWGRKLPKGHGLGIAAHRSFVSYTAAVCEVQIGADGAIAVPRVD
IAIDCGPQVNPERVRSQLEGAVVMGLGIALHGEITFKDGRPEQSNFNGFQVLRMSEAPREIRVHLVAPDDYATP
LGGVGEPGLPPVAPALTNAIFAATGTRIRSLPVADQLAKQKAG
>SEQF2408||SEQF2408.1_04282
MSRGLIEAGRAGAGVSRRSFLRFGVSLGAAAGGGLLLGFSMPAAGDDARRTVIGGDANESARAGVFAPNAFV
QIDRSGKVTLVMPKAEMGQGVYTALPMLIAEELEVPLSSVTLNHAPPNEKLFFDPLLGGQLTGGSTSVRYAWEP
MRRAGAIARTLLVSAAAKQWNVDPATCRAENGEVQHPPSGRRASYGQLADAAAKLPVPKDVALKKPADFKLV
GKPVKRLDSPEKVDGTAQFGLDVRVPGMLYAVIVNSPVFGGTVASVDDTAAKKIPGVRQVVRVDNAVAVVGD
HTWAAKRGASALVVKWNEGANAKVSTKDIVADLAHAAANGKGAVARKDGDVGKAFASAKTRVDAVYEQPFL
AHATMEPVNCTVHVRPDGCEVWLGTQVPTRVRDIVQRITAFPPERIVVHNHLLGGGFGRRLEADMAAQAVK
VGRQVNAPVKVMWTREEDIQHDMYRPYYYDKISAGLDANGKPVAWQHRIVGSSIMARFAPPAFQNGIDPDA
VEVSAELPYDLPNQLVDYVRQEPRHVPTAFWRGVGATRGTFVVESFIDELAAQARIDPVQYRRALLDKTPRARN
VLDVATKAAGWGSPLPKGQGRGVSVMHAFGSFFSIVIDVAVEDGEVQVKRAVCAVDCGMAVNPSTIDAQVQ
GGIIFGITGALYGEITIEDGRVVQSNFTDYRMMRINETPPIEVHLVKSGEAPGGIGEPGTAATAAALSNAIFAATGT
RLRKLPVGSQLKTA
>SEQF2411||SEQF2411.2_03198
MNLDHIASSIPDATLSRRSFLATAAAGGAFMLSLSLPLGKSEAASPEGFAPNAFIRIGRDGQVALTMPYVEMGQ
GTYTSVPMLIAEELDVSLKQVRLEHAPPNEKIYANPLLGVQATGNSNAMRGAWKPMREAGATARSMLVAAAA
KRWAVDPESCRTQDGEVVHPQTNRRLTYGELAAEAAKLPVPKAVALKPAADFKLIGTPAKRLDGPGKVNGTAV
YGIDVRPPGLKVATLVQSPVFGGRVKSVDDAEAKAVKGVRQIVRLDDAVAVVADHMGAAKKGLAALKIEWDD
GPNAGLATADIARELEQATLRSGPVAQNLGDADKAMAGAATKVEAIYQVPFLAHATMEPMNCTVHLRKDECEI
WIGNQAIARVQAMAAKAAGLPAEKVIVHNHLIGGGFGRRLDADGAVRAVEIAKHVDGPVKVVWTREEDIQQ
AMYRPYWFDRISAGLDDKGMPVAWKNRFAGSSVIARWLPPAFKDGLDPDTTEGAIDLVYNLPNFHVEYVRVE
PPGIPTAFWRSVGPSHNVFVTESFIDELAAAAKQDAVAYRRALLDKSPRAKAVLDLAAEKAGWGQALPKGSGR
GISLQFSFGSYMAHVAEVEVSKDGAVRVRRVICAVDCGTVVNPNTVQAQIQSGIVFGTTAALYGEITLKNGRVE
QGNFDTYQILRINEAPAIEVHVVKSTEPPGGMGETGTSAIVPAIANAIFAATGKRLRKMPIDTSVLKSA
>SEQF2423||SEQF2423.2_04526
MGKIETPDSATRGILNVSRRTLLKGAGGLALGIFFAPLMRGMDALAAGGPLEPNAFVRIDLDGTVTVLAKHLEM
GQGSYTGLATLLAEELDADWDKVRVEGAPADVKRYNNLAFGPMQGTGGSTAMANSWEQMRNAGATAKAM
LVAAAAQRWSVPVSEISVDKGVVSHAGSGRSAGFGDLVEAAASLPVPEQVQLKDPKDFKLIGKLELRRKDSTDK
TDGSAIFTQDFKLPGMLVAMVAYPPRFGGVPRSVDSSKAKAVRDVVEVVEFRDLPHGRSGVAVLAKNTWAAR
QGRDALVIEWDESQAFTLGSEEILAQYRDDAGKPGLPATSKGDTDAALAQAAKTVEADYEFPYLAHAAMEPM
NCLVKLSSDRCEIWNGEQFQTVDQTIISGYLGLTPEQVSLTQLYAGGSFGRRASSVSDYLLEAVAITKAARDKGVD
APVKMVWTREDDTRGGYFRPLYLHRVRIGLDQAGKLQAWHNRIVGQSIMAGTSMEPFMIKDGIDHTSVEGL
ANLSYAVPNLQVELSTPSNIKVPVLWWRSVGHTHTGYVAETMIDEAAVAAGQDPYAFRHALLESHPRHRGALE

LAAKQAGWDKPLAAGAEGEKRGRGIAVHESFGSFVAQVAEVTVKADGSYRLDRVVCAVDCGIAINPDVIKAQ
MEGGIGFALAAARHSAITLKEGRVEQSNFHDFQVLRMNEMPKVEVHIVPSAANPTGVGEPGVPPLAPALANA
LFAATGVRLRKLPFPAQIKA
>SEQF2487||SEQF2487.1_02899
MNIRTNIPTGSGKLRGFEKHVKVENVSRRSILKGLGIAGSFVLAAPVMTRQAFAYETGAGKMPHGVVVDPRVF
VAIAPDGIVTILAHRSEMGTGVRTSLPLIVAEEMEADWSRVHVQQAHGDEVKFGNQDTDGSRSTRHYLIPMR
QIGASARSMLEAAAAKKWGVPVTEVKAQNHEVVHSASGRKAGFGELAADAAKESVPSVEGLKLKDPKDFRYL
GKGQVSIVDLHDITTGKAHYGADVRLPGLKYAVIARPPVTGGKLKSFDDSAAKKVPGVEKVMEVKGWPWPSKF
QPLGGVAVIARNTGAAIKGRDALKIVWDDGANAKYDSVTYRASLEEAARKPGLVVRKEGDADAALKSADKVIT
GEYYLPHLAHVSMEPPVAVADVKGDKATIWAPVQSPGGTREDVAKTLGIPEDNVTVNVTLLGGGFGRKSKCDF
ALEAALLSKELGAPVKVQWTREDDVHHDFLHTVSVERIEAGLDKSGKVIAWRHRSVAPTIASTFAAGANHEAPF
ELGMGLIDNPFEIANLQCENPEAAAFTRIGWFRSVSNIPRAFAVQSMVGEIAHATGRDQKEMLLELIGSPRIVNL
SSVKDPWNYGEPYDSYPIDTARLRKVVELVAEKGEWGRKVPKGHGLGIAVHRSFVSYIATIVEVAVDDKGKFSVP
RVDTAIDCGTYVNPERIHSQIEGAAIMGLSLAKYGEISFKDGKVQQGNFDDFPVIRMDESPVITNVHIVPPGADT
PPSGVGEPGVPPFAPALINAIFAATGKRIRALPIGKQLET
>SEQF2487||SEQF2487.1_01290
MNKHVKNLAPETTDLSRRSFLVGTAATGLVLGYAASGIDQALAAPAPASFEPSVWYSIAPDGLVTVTCGKADMG
QHVASTMAQIVAEELGANWKDMRVQLASNDPKFNDPVLGAQITGGSWSTMMNFDAMSRAGAAGRIALTE
AAAASMGVPAGELVVRDSRISHAKSKKSMSFAEVVKSGKATKTFTADDLKAIKLKTPDQYTMIGVSVPQLDIPSK
VNGTAKYGIDVMVPGMVYGALVTPPVRYGATVKSVDDSAAKKLPGFIKAVTLDDKTTTTTGWVVAVANTYAQ
AKKAAAALKISYDGGPNAKLSSESLFAEAKRLQGLSDSGEFFVKDGDPNAAYGSAAKVLEAEYTTNINIHAPMEP
MNATAEFKGDILHIYSGNQFATRSGAIAAGAAGIDPKFVVMHQMWLGGGFGRRLDADMMVPAVQAAKAVG
KPVKVIYTRENDMTMDFSRPLTYQKVKAGMDGDGKIVAMSHDVVSAWPTARWGIPDFLTPSVDKKGPLDSFT
VNGADFFYTVPNHYVRAIKNEMAHNATPSGQLRSVAPGWTFWAVESMIDEIAHATGKDPAQLRISLLDGKGK
NDGGAQRLRNTLLAAMGLAGYGTKQLPKGEGMGVACVSSQERATASWTACVAHVAVAPSGEVTVKKLTVAT
DVGTQVNPDGIRAQVEGAALWGMSLALFEKATLKDGGIEQTNFDSYTPLRMSQLPEVAVNVIANGEKATGVG
EPAVTVVAPAIGNAVFNAVGARVRGLPITAEAVKAAMKA
>SEQF2487||SEQF2487.1_06318
MTLIDNLSERAADLSRRNFLRAGAIAGGGLLLSVSLPFAGRESEAAASDGFAPNAFVRIGGDGKVVLTMPYVEM
GQGTYTSIPMLIAEELEIGLTQVRLEHAPPSDKLYANPLLGVQATGNSNAMRGAWQPMRKAGATAKAMLVAA
AAKRWNVEPGTCRAENGEVHHAASGRKLGYGELATDAAQMPVPENVTLKSPSEFKLIGTPAKRLDTPSKINGT
AVYGIDARPPGVKIATLAQSPVFGGRVKRVDDAAAKAVNGVRQIVTLDDAVAVVADHMGAAKKGLAALTIEW
DEGAHAKLATSDIARELETATTKPGAVAQNIGDADKAMAGAATKVEATYQLPFLAHATMEPMNCTVHVRPDG
CEIWVGSQALSRAQAVAAKVLNMPPEKVVVHNHLLGGGFGRRLEVDGVIRAVQIAKQVDAPVKLVWTREEDI
QHDMYRPYWCDRIAVGLDASGKPVAWNNRFAGSSVLARWAPPAFRNGLDPDTTEGAIDLVYDIPNFHVEYV
RVEPPGIPTAFWRSVGPSHNVFVTESVIDELAAAAKQDPVDYRRALLGKSPRAKAALELAAAKAGWGGKLPAG
RGRGVSLQFVFGSYLAQVAEVEVARDGSVRVHRVVCAMDCGTVVNPDTVQAQLQSGINFGVTAALYGEITLK
DGRVEQSNFDSYQMLRIDQAPAIEVHIVPSTEPPGGMGETGTSGIVPAISNAIFAATGKRLRKMPVDPAVLKQT
>SEQF2487||SEQF2487.1_03804
MLPRTHKDAAMNQHVMPKLNRRAFVIGTATAGAGLALGLDLPFGGPAVVRAADGAPEVNAWVVIRPDDTV
VIRIARSEMGQGTLTGLAQLVAEELECDWSKVTTEYPTPGQSVARKRAWGDFSTGGSRGIRTSQDYVRRGGAT
ARVMLVQAAANEWKVPASECKAANSVITHTASGKTTTYGKVAEAAAKLEPPADVKLKDPKDWTIAGKGLKRLD
TSDKTTGKMVYGIDVKLPGMLNAAIKDCPVFGGKVKSFDEAKIAGMKGVKKVVQVGDSAVAVVADTWWHA
KTALDALPIVWDEGPNAKVSSETIATWLAEGLDNAQPAYIGNQNGDAKAAIAAAAKKVEAVYNYPYQNHATM

EPMNATVLYTPDKCEVWCGTQNGEAAFAAALEASGLPAEKVDVHKLMLGGGFGRRGMTDYVRQAVAIAKQ
MPGTPIKLLWSREEDMQHGKYHPITQCKLTGAFDADNNLVALHYRLSGQSILFSVRPEALQNGMDPAAFQGV
AQAGEAAIGYSVPNLLVEHSMRNPHVPPGFWRGVNVNHNAIYMECFMDELALSVGQDPLEFRRKLMGKHP
KHLAVLNAVAEKIGWGTPAPQGVYRGIAQVMGYGSYVAGAAEISVTDGSKIKVHRIVASTDPGYVVNPAQVER
QIAGSFVYGLSALFYGGCTVKDGRIEQTNFDTYNSMRINEMPKVEAVMVPSGGFWGGVGEPTIGVAAPAVLN
AYFAATGKRIRSFPLRNQNISFA
>SEQF2513||SEQF2513.1_03714
MSQHKDDFVVINLSRRGFLTGLAATGALVVAANWGWRDAFAEEKQYGAAGMPHGWVDNPKVFLSIGSDGL
VTFVCNRQEMGQGIRTSLALVVADELEADWEMMRVVQAQGDEERYGNQDTDGSRSMRHWFDPIRRCGAA
ARSMLEQAAARQWQVPVDECRAQGHKVVHQPSGRSLGYGQLAEAAAALEVPPRESLRLKPAGQFRYIGKDST
RAIDGADIVSGRAVYGADVRFDDMLYAVVARPPVYGGKLKNQDTAATLKVPGVVGVVEIASSPLPSGYQPLGGL
AVVAGNTWAAIKGCEALKLEWDDGPNAGYDSKAYRAELSRAARAPGKLVRDAGDVQAVFDKAERLLEAEYYIP
HLAQAPMEPMVATARFVDGACEVWAPVQAPQATRESVAERLGIPLDKVTVNVTLLGGGFGRKSKPDFVVEAA
LLAREFPGRALRVQWTREDDLRFSYFHTVSVEYLRGALTPSGKVDAWLHRSTAPSIAALFGPDPKHEMPLELGL
GFINLPYSLPNVRLENPEAAAHTRVGWYRSVSNIPHAFAVQSFIGELAAAAGRDHREFLFELIGPARKIDPRKLG
DEWNHGESPVLYPIDTGRLRSVVEVATQQAGWGGKLPKGRGLGLAVHYSFVTYVAVVLDVEVKDDGSLLLHKA
TVAVDCGPQINPDRIRSQMEGACIMGLGNAAVSEISFANGRVQQSNLHDYEVARMSLAPREIAVHAVRPEGE
VPLGGVGEPGVPPVAPALCNAIFAATGKRIRDLPIRDQLKGWNQA
>SEQF2514||SEQF2514.1_04227
MNAKTTKPRSGRRRFLLGALGIGGALVVGWGVMPPRSRVGDPGIFPEHNGEIALNGWIKITPEGNVVLAMPR
VEMGQGIHTALSMLAAEELDIPLARVRIESSPVERIYGNVVAMGDSSLPLHPDSADKTWARALHWIMAKSARE
IGLIITGGSSSTADGWQPVREAAATARAALVEAAAREWNAPVAQVSIREGQLIGPGGKQSTFGEMAKSARGLS
APSNVTLKPASQFQLIGKPAPRNDLAAKTDGSARFAIDTRLPGMLYAAVVMCPVFGGKLKTFQSKAALGMPGV
HYVVPFEGTGGGAPGVAVVADHYWQARQALATLEPVWDNGPHAKLDSAGIRQQLVSALDSDKGGFTYRSM
GDGLKAFDKADGATLVEAEYSAPYLAHATMEPINCTAQVTPEGVHLWAPTQVATLAQLVAARAAGVSGDKVQI
DIPFIGGGFGRRLESDFIGQAVTIATKTDGKPVQVIWSREEDVRHDFYRPHAIARLKARVENGKVTAIASRSAGQ
SILAGELDRLFGAPSVGIDRYTAEGLFDLPYEIEHEHIAHLAVDLPVPVGFWRSVGHSYNGFFMEGFLNEVAAAA
KLDPLAMRRDLLKDHPRELKVLDTAAQAAGWGQPLAPAADGAPRARGIALHPSFGSVVAQVVEVSMKDGKP
RVHRVVCAVDCGTVVNPGIVAQQMESAVIFGLTAALYGRIDIKDGQVVQSNFPDYPALKMAETPVIETHIVPST
AEPSGMGEVGVPPIGPAVAHAVAQLTGKPVRQLPMA
>SEQF2521||SEQF2521.1_02943
MTGTTELSRRRFLKGSVGVLLLAVTAKGLVTAAWAADPSPAFGADSMPGGTVDDPLVFVSIATDGTVTLVAHRA
EMGTGVRTSLPMVVADEMEARWDRVKVVQAEANETRYGNQNVDGSRSMRHFLMPMRRVGAAARQMLE
MAAAARWSVPLTEVKAVQHEVVHLPSGRRLGYGELAADAARQPVPNADALKLKARDEFRYIGKSQVRPVDLE
AIGKGQAIYGMDMRLPGMVYAVVARPPVVGGKLHRVDSSKALAIPGVLKVVEIPAFQGAPGFQPLGGVAVIAR
NTWAAMQGRAALQIEWDDGPNGRYDSAAYRQTLEAAARAPGKVMRNAGDAARAWAPETERLAAEYYVPH
LAHASMEPPVATVRIKGDGAEVWTSIQNPAAAQDAVAARLGLEPKNVTVNVLLLGGGFGRKSKPDFVDEAAIV
AMAMPEGTPVKLVWTREDDIHHDYLHTVSVERLEAVMGEDGQVRSWLHRSAAPTIASLFTEGAKGQQLFESA
MSAINMPYRIANVRVETAEVAAHTRIGWFRSVANIPHAFAAQCFIAELAHRAGKDHRQFALDLIGPARRIDPGT
LADTWNYSESPERYPYDTGRLRGVIEAACQGAGWGRELPEGHGLGLAFCYSFVSYTASVVEVAVNAEGEVRVV
AVDMALDCGPQINPERIRAQMEGGAIMGLSLALGSEITFENGRVKQSNFHDYEVLRHSASPRLIRTHLVNDDH
DLPPGGVGEPPVPPVAPALCNAIFAATGKRVRSLPVRRLA
>SEQF2536||SEQF2536.1_00918
MPQRPSNERGRVADSAISLRRRHLLQSAAALLVAPAAGSLLIPLAQAAPPAAGAAAAAAIGDWVWIEPSGQVV

IGVSQCEVGQGIYTGLPQVLADELDADWASVTVRFVTGRDAYRNDAGEMPFQQFVGASMSMNYFYERMRL
AGAQARDVLLRAGASRLGVRASQCMTRAGRVLHPATGRSVGYGEIVADASRLPLAARPRMKSASEQGLIGRNL
RRVDTPSKVDGSAVFGIDVEVPGMLIGAVRMVPSVTGRIVRIRNEAEVRARPGVHAVVRTTQWPDPEPSTVV
VVADSYWIAKQAADALDIEFDAGAAAGVDSERIHAQFVAGLSNDKAVVARSLGKPREVLAAGKTITADYHSPYI
THATMEPLAATVHVRDGEVETWGPYQGQDFLRGELGKACGVPADKVIVHTTFLGGSFGRKYMPDFALHAAA
ASKAVGRPVKVIRSREDDIRHSYYRPGASGRLSAVLGADGLPAALHARISGQSLYGAINPKKMADAGGWDETM
VESIYDLIYGVPNLLVDAVDVQQPIPLSYLRSVGTTSSVFFLESFISELAHTAGVDDYQYRRRLLAGQPLALGVLDA
AARAARWEQPAPAGLHRAMTFNVYTGRGESFQTFVALVMELRVVQGRVRLERAICAIDAGRVVNPGLVKANV
EGGIGFALTNTFKSRLDFDKGAVQQSNFHDYPLLQLSEMPRVEVVLVESDRPPQGCGEVALGPTAPAVATAMFH
ATGRRFRSMPLPQDIAST
>SEQF2537||SEQF2537.1_06242
MPQRPSNERGRVADSAISLRRRHLLQSAAALLVAPAAGSLLIPLAQAAPPAAGAAAAAAIGDWVWIEPSGQVV
IGVSQCEVGQGIYTGLPQVLADELDADWASVTVRFVTGRDAYRNDAGEMPFQQFVGASMSMNYFYERMRL
AGAQARDVLLRAGASRLGVRASQCMTRAGRVLHPATGRSVGYGEIVADASRLPLAARPRMKSASEQGLIGRNL
RRVDTPSKVDGSAVFGIDVEVPGMLIGAVRMAPSVTGRIVRIRNEAAVRARPGVHAVVRTTQWPDPEPSTVV
VVADSYWIAKQAADALDIEFDAGAAAGVDSERIHAQFVAGLSNDKAVVARSLGKPREVLAAGKTITADYHSPYI
THATMEPLAATVHVRDGEVETWGPYQGQDFLRGELGKACGVPADKVIVHTTFLGGSFGRKYMPDFALHAAA
ASKAVGRPVKVIRSREDDIRHSYYRPGASGRLSAVLGADGMPAALHARISGQSLYGAINPKKMADAGGWDET
MVESIYDLIYGVPNLLVDAVDVQQPIPLSYLRSVGTTSSVFFLESFISELAHTAGVDDYQYRRRLLAGQPLALGVLD
AAARAARWEQPAPAGLHRAMTFNVYTGRGESFQTFVALVMELRVVQGRVRLERAICAIDAGRVVNPGLVKAN
VEGGIGFALTNTFKSRLDFDKGAVQQSNFHDYPLLQLSEMPRVEVVLVESDRPPQGCGEVALGPTAPAVATAMF
HATGRRFRSMPLPQDIASI
>SEQF2546||SEQF2546.1_05959
MTTTMQVSRRGFLKGGLGALTLAVTGNGLVSAVWAADEPKKYGADSMPGGTVDDPLAFVSIAADGTVTIVAH
RAEMGTGVRTSLPMVVADEMEAAWDRVRVVQADADEARYGNQNVDGSRSVRHFLMPMRRVGAAARQM
LEAAAAARWSVPLAEVRATQHEVVHAPSGRRLGYGELAADAAKLPVPAGDAVKLKTRAEFRYIGKDEVRLVDLE
AIGKGEAMYGMDMRLPGMVYAVVARPPVVGGKLRRVDSAKALAVPGVLKVVEIPAMAGAPAFQPLGGVAVV
ASNTWAAMQGRAALAIEWDDGPNAAYDSVAYRETLTEASRKPGKVVRDQGDAPQAWAKAGETERFMAEY
HVPHLAHASMETPVATVRIQDGAAEVWTSVQNPAAAQEAVAKRLKLKPENVKVHVLLLGGGFGRKSKPDYVD
EAAIVAQAMPAGTPVKLVWTREDDIHHDYLHTVSAEHLEAVVGKDGKVQSWLHRSAAPTIASLFTEGAKGEQL
FESAMSAINMPYVIPNVRVETAEVAAHARIGWFRSVANIPHAFAAQCFIAELAHRAGQDHKQYALDLIGPARRI
DPGTLADTWNYSESPERYPYDTGRLRGVIEAAASGAKWGRELPKGHGLGLAFCYSFMSYTATVVEVAVDEKGE
VRVVAVDMALDCGPQIKPERIRAQMEGGAIMGLSLALLGEITFEKGRVKQNNFYDYEVLRHNASPRVIRTHLV
NDDHALPPGGVGEPPVPPVAPALCNAIFAATGKRVRSLPVRSVA
>SEQF2586||SEQF2586.1_03962
MKPAAGISRRSALQAGGLALAFTWFGAGKAFAAISPRQQPGDAAAALADGNPAFAPNAFVRIDADGGVRLV
MPMAEMGQAIYTGSAMLLAEELGVELDQVRVEHSPPNEALYGMPLLGGQITGGSTSTRGTYGVLREAGAVAR
TLLVSAAAEQWKVDPESCTVARGVVSHAASNRQLGFGALASAAAKLPMPEKVTLKEPKDFKLIGQPLRRVDSA
GKVNGSTQFGIDVRLPGMKVATVRACPTLGGVLASVDDKAARAIPGVIDVLRIKDAVAVVGEHFWAAKRGLDA
LTIQWTPGQNAALTTLQLRAALANALAKDKAILGKETGKRPEGTLVQATYDLPMLAHATMEPLNTTVHVRPDQ
CEIWVGTQVPTRCVSAAAKIAGVAEDKVVLHNQYLGGGFGRRLETDSVEQAVAFAKQVPYPLKVVWTREEDIR
HDIVRPMYHDDISAVVDSDGHILWFGDRIAGGTVLGRWAPAFMGKDGMDSDLIECVAEPCYDLPNLKVEWV
RHDMPSGLNVGWWRGVGPTHNLFVMESFIDELAHRAKKDPVAYRRAMLKKNPRTLAVLDLAAGKIGWGQG
ALAARVGRGVAVGDAFGSRVCAIVEAEVTPQGEVRMRRAVVAVDCGIAVNAGSIEAQIQGGLLFGLSAALFSEI

TLREGAIEQSNFHDYRMLRINEAPPVEVHTVKSGEAPGGLGEVGTAIAAPALANAIFAATGVRLRALPVNRALLA
QDKEALKKKIADSGFSGSNGLDARSAA
>SEQF2586||SEQF2586.1_06252
MLPTHIDPTELPRTLQRLMAAGQQQPEDTAALPRRSFLKLAGVGGLALGAFPHLAVAQATGKGPAASALKPAQ
QPSAFVQIAPNGEVTVTHNRLEFGQGVQTGLPMILAEELDADWSLVRSKSGTNDAAYHDPVFGMHITGGSNS
IKNSFTQYRELGARARAMLLSAAAARWNVDVATLRTQAGTVLGPNGRKLGYGELAEAAMALPVPEKVTLKDP
KDFRLIGRPTTRLDARAKCSGRQDFGIDVKHAGQLTAVVAHPPVFGARLASVDDSAARAVKGVKAVLRVPLDR
GAEGVAVVADGYWPAKLGRDALKLQWDTAAAEKVDSDKQLAQYRELARQPGARKFDADMAPLAKAPRQLE
AEFVFPYLAHAPMEPLNCTVKLSGDRAELWVGTQCAGLDGVAAARTLGLKPEQVRVNVQMAGGGFGRRFVS
TSDVIVEACEIAKAARAAGLNAPVRLLWSREDDVKGGYYRPMHLHHARIGFDERGKVLAWDHVIVGQSITTGT
VFGEFQVKNGIDATATEGMRDPYPLPMRLTVHHPKVNVPVLWWRSVGSTHTAFVMETLLDEIARSTRQDPVA
YRMQLFGDKHPRHRAALQLAVDQSGYGKKQLPAGRAWGVAVHESFESVVAYVVEASVKDGQPVLHRATAGV
HCNLAVNPRSVEAQVQGAAVMGLSMCLPGAAITLKDGEVQQSNFGDFTVARITDMPEFAVHIVPSAEPPKGI
GEPGLPPLAPAFANAIAQLTGKPLRQLPFNLA
>SEQF2603||SEQF2603.1_02566
MQALSNPSRRKFLAAGAVLGGGLVIGFTVPVARRQPGVEAAVSTTTFAPNAFLRIGADDSITVLLAHSEMGQG
VWTTLPLLIAEELDADWSRIHVEHAPAAPTYGNSAIGGMQATVGSSSIRTEFTRYRQAGAAARAMLLQAAAQR
LNVPAASLRTDTGAVVNGTQRIRYGELADDAGRLAVPDAASLALKAPGEWKWIGKGARRLDNLPKLTGRAQFG
IDVQFDGLLTAVVARAPTFGARVRSVDDGAARAVRGVRQVVQVPSGVAVVADHYWAARQGRDALKIEWEPA
AGAAPDSDRLLQEFARLAGAPGPVAAEAGDVAVAMRGAAQVVEREYRVPYLAHAAMEPLNCTVRLGAEGCD
VWVGTQMQTLAQRTAAQVSGLRPEQVRIYTTFLGGGFGRRAVQDFIGEAVHVAKAAGAPVKTVWSREDDM
RGGYYRSAFVHRVRVGLDRAGLPVAWRHGIAGQSIHPDTPGVHKTSVEGVADSPYVTRCPAHRVEAHSPRTSV
PVWYWRSVGHSHSGFVMEGMVDELARAARKDPLAYRRLLLKDEPRHLRVLELAADKFGWQRRPARGRGYGL
AVHASFGSFCAQAVEVSVSKGALRVHRVVCAIDCGIAVNPDNIRAQMEGAIVYGLSAALYGNLALKDGAALPSN
FHDYPVLRMPEMPRIDVHIVPGTDAPGGVGEPGTPPIAAAVANAVFALTGERLRTLPLQPRQA
>SEQF2603||SEQF2603.1_02446
MLPSFDHGEMPRNLQRLLARSQRDEPAMLPRRSFLKLAGSAGLALGAFPHLAITQAASAGGAASALKPTQQPL
AFVQIAPGGEVTVTVNRLEFGQGVQTALPMILAEELDADWSLVRSRHGSNDAAYVDPLFGIHLTGGSHSVKNS
FTQYRELGARARAMLLSAAATRWKVDVTTLRSRAGVVLGPGGSTLTYGELAEAAMALPVPEKVTLKDPKDFRII
GTPTTRIDARAKSSGRQDFGIDTRLPGQLTAVVAHPPVFGARLTTVDDSAARAIKGVKAVLRVPVDRGGEGVAV
VADGFWAARQGRDALKLQWNTAAVEKADSERLLAQYRELAARPGRRQFDADMAPLATAPRKLEAEFVFPYLA
HAPMEPLNCTVQLSDGRADLWVGSQCPGLDGAAAARALGLKPEQVKIHVQMAGGGFGRRFASTSDYVVEA
CAIARAARAAGMKAPIRTLWSREDDIKGGYYRPMHLHRARIGFDEQGKVLAWDHVIVGQSITSGTVFGEFQV
KNGIDATATEGMRDPYPLPMRLTVHHPTPNVPVLWWRSVGSTHTAYVMETLLDEIARVTRQDPVAYRMRLFG
DKHPRHRAALQLAVDKSGYGKKKLPAGRAWGVAVHESFDSVVAYVVEASVKEGRPQLHRVTAGVHCNLVVNP
RTVEAQVQGAAVMGLSMCLAGSAITLKDGVIEQSNFGDFTVARMTDTPAFDVHIVPSADPPTGMGEPGLPPL
APAFANAIARLTGKPLRQLPFKLT
>SEQF2603||SEQF2603.1_07037
MLTRRAFLLGGAGAAGVLVIGWAWLPPRQRLVGGEPLAVRDGQFALNGWVKVASDNTVTVVMNKAEMGQ
GVHTGAAMLLAEEMEADWSQIRVEPSPVDNLYNNIEGVVANLPFRPDDKGWPRHAAEWFTRKGVREVGM
MFTGGSTSIRDLWNPMREAGAAARMMLCAAAAKQWDVKAEECRAQGGKVVHPSGRSATFGELAAAAARE
GLPRKVELKDPASFRLIGTRTARLDSPAKLHGTASFGIDVVADAMVYASVQMCPVLGGKLQGFPPDDVKKLPGV
LDLVALPPFPGGSGGVAVIASDAWIAMQAVEKVQCKWDAGEAQNLSSAGIRDTLVKALDASNPRVWYEHGD
GRAMKQGKPAIQVLYSAPYLAHAALEPVNCTVLVQEDRAIVWAATQMPGLARRCVAKTLNLDAGKVELRQQSI

GGAFGRRLEVDFICQAAAIAAKRKGVPVQTIWSRPEDMRHDFYRPASVSRFEAWLDGNRKVIAVRNISASQSV
LESTAKRNFGLPDIFVSRLDKSTVEGAFDQAYDWPDMWVGHQTVDLAIPVGYWRSVGHSHHAFFMESLVDEL
AVTVGVDPLDYRMSLLKDDRQRAVLEQVRSMSAWGQPLRKKPGMVKVGRGVALHESFGSVVAQVAEVSVD
NAGQVRVDRVFCAIDCGMPVNPTLIEQQVEGGIVFGLSAALWQKITLERGKVQEDYYTAFPAIRLRDCPDIQVQ
VMPSMRPPQGVGESTTPPIAPAVANALFNATGERLRDLPLLKTLPPSGACDVKQSDQCQR
>SEQF2603||SEQF2603.1_00711
MRIRGIEALAGGQGGNDGEARQHGGIATLDRRSFLKLTGLAGGGLALGVAPLAQAQDAARPRTTAPPPQAFLII
APDNTVTVAVNRLEFGQGVHTALPMALAEELDADWRNVRATLAPAGDPYKDPAFGMQMTGGSTALNHSFE
QYRELGARARAMLVAAAAQQWKVDPASCKVEQGVITSGSHRATFGELAPAAMELPVPQRVTLKDPSQFRIVG
KPTPRLDSRGKMEATTPFGIDTRLDKMVVAVVARPPRFGGKVKSFNADKARAVQGVRGVMQVPVDRGGSGV
AVIASGYWPAKMGRDALDIQWEDAGSKVSSQALFDEYAKLAAQPGTVARPGEGDIGAAINGAARKIEADYRFP
YLAHAPMEPLNCTLQPEVAGGKVQAVKVWVGSQFQTVDQAAIARTLGLAPDKVVLNTMMAGGGFGRRAVP
TSDYIVEAANVLKAWVAAGHAEPLKVIWSREDDIRGGYYRPLHLHRARIGLDAQGKVVGWQHTIVGQSILKGT
PFEAFMVKNGVDATMTEGIVENDYDLPLQMSVHHPQVDVPVLWWRSVGNTHTAFVKETLADEMAAAAKQ
DPVAFRLARLDEKKHARHRAALQLAVDKSGYGKRKLPKGHAWGVAVHESFNTVVAYVVDVSVVKGEPRVHRV
TAGVHANRVVNPMSAEAQIQGACIFGLAMTKPGFAIEIEYGAVKNSNFPDYPPPRITDAPVVDVFFVPSQENPT
GLGEPGVPPLAPAVANALFTLTGKRQRQLPFVMA
>SEQF2610||SEQF2610.1_04218
MTTTMQVSRRGFLKGGLGALTLAVTGNGLVSAVWAADEPKKYGADSMPGGTVDDPLAFVSIAADGTVTIVAH
RAEMGTGVRTSLPMVVADEMEAAWDRVRVVQADADEARYGNQNVDGSRSVRHFLMPMRRVGAAARQM
LEAAAAARWSVPLAEVRATQHEVVHAPSGRRLGYGELAADAAKLPVPAGDAVKLKTRAEFRYIGKDEVRLVDLE
AIGKGEAMYGMDMRLPGMVYAVVARPPVVGGKLRRVDSAKALAVPGVLKVVEIPAMAGAPAFQPLGGVAVV
ASNTWAAMQGRAALAIEWDDGPNAAYDSVAYRETLTEASRKPGKVVRDQGDAPQAWAKAGETERFMAEY
HVPHLAHASMETPVATVRIQDGAAEVWTSVQNPAAAQEAVAKRLKLKPENVKVHVLLLGGGFGRKSKPDYVD
EAAIVAQAMPAGTPVKLVWTREDDIHHDYLHTVSAEHLEAVVGKDGKVQSWLHRSAAPTIASLFTEGAKGEQL
FESAMSAINMPYVIPNVRVETAEVAAHARIGWFRSVANIPHAFAAQCFIAELAHRAGRDHKQYALDLIGPARRI
DPGTLADTWNYSESPERYPYDTGRLRGVIEAAASGAKWGRELPKGHGLGLAFCYSFMSYTATVVEVAVDEKGE
VRVVAVDMALDCGPQIKPERIRAQMEGGAIMGLSLALLGEITFEKGRVKQNNFYDYEVLRHNASPRVIRTHLV
NDDHALPPGGVGEPPVPPVAPALCNAIFAATGKRVRSLPVRSVA
>SEQF2695||SEQF2695.1_01256
MKRSFPDDLVIGNLSRRGFLKGVGATGVLLVAANWGWRDALAAEKKAFGADAMPHGWVDNPKIYVSIDRDG
TVGIVCNRSEMGQGVRTSLAMVVADELEADWSRVKVIQAPGDEARYGNQDTDGSRSMRHWFEPMRRCGA
AARQMLEQAAANQWKVPLGECRAEQNKVLHAPSGRSLSFGELAEAAAGLEVPARDKLLLKKPEQFRYIGKDV
ARAIDGADIVNGRAGFGFDARFDDMLYAVVARPPVYGGKLKRYDAAAALKVPGVVKVIEIEGRPIPSEFQPLGG
VAVVAQNTWAAIKGREALVVEWDAGVNGGYDSVAYRKQLEEAARKPGKVVRDSGDAAALFAKGGDIVEAEYY
LPHLAQAPMEPPVSTAWYKDGACEVWAPTQAPQVTRERIAERLKLPFDKVTVNVTLLGGGFGRKSKPDFVLEA
AILAKAFPGRHLRVQWTREDDLHFSYFHTVSVERLQAVLGADELPQAWLHRSVAPSITALFGPDSKHQGAFELG
MGLTNLPFAIPNVRLENPEAPAHTRVGWFRSVSNIPHAFAIQSFVGELAAKAGQDPKDYLLKLLGPARRIDTAEL
GDSWNYGESPERYPLDVGRLRGVIEEAARQSGWGGELPRGRARGIAAHYSFVTYVAVVIEVEVKDDGALLVHK
ATIAADCGPQINPERIRSQLEGACVMGLGLAALGEISFKDGKVQQDNFHQYELARMPLAPKAVSVHLLKPDGD
LPLGGVGEPGVPPIAPALCNAIFAATGKRIRELPIRNQLQGWRKA
>SEQF2695||SEQF2695.1_00158
MNSKIDLSNALPGSRRGFLKGAAVVGLTIGFQWSGARRALAAALPDAGFAPNAFLRIAPDDSVTVIAKHVEMG
QGAYTGIATIVAEELDADWSKVRVESAPADAKRYANLAFGTMQGTGGSSAMANSWMQLREAGAKARAMLV

EAAARQWRVPATELRTRDGFVEHPASQRKASYGSLAAAAAELPVPENVQLKDPKDFRLIGHQAPRVDVPGKT
DGSAQFTLDVSLPGMLVALLQRPPLFGATVKSFDATATRAIPGVVEVVQVPHGVAVVAKGFWAAKQGRDALKV
EWDESKAEKRGSEALMAEYRKLAEQPGKPARRDGDAAKAVAGATRRIAASYEFPFLAHAPMEPLDAVVRLTAD
SCEIWAGDQFQTVDQGNAARTAGLKPEQVKINTLYAGGSFGRRANAWSDYIVEAVSIAKALGANGVPVKLQW
TREDDIHGGFYRPMYYHRLEAGLDADGKLVGWQHRIVGQSILEGTPFAAVMVKDGIDATSVEGAANLPYAVP
NVSVELSTTQVGVPVLWWRVVGSSHTVYAVEAFIDEAAQAAGKDPYLFRRDLLAEQPRLRGVLELAAEKAGW
DPSRPLPAGRGRGIAVTEAFKTFVAQVVEVSVDKDGKLKVERVVCAVDCGIPINPDVIAAQMEGGIGFGLGAIL
HSAITLKDGKVEQNNFDGYQVLRIAEMPKVEVHIVPSGEAPTGVGEPGVAPIGPALANAIFAATGQRLYNLPFPT
SFAKA
>SEQF2696||SEQF2696.1_03411
MNSKIDLSNALPGSRRGFLKGAAVVGLTIGFQWSGARRALAAALPDAGFAPNAFLRIAPDDSVTVIAKHVEMG
QGAYTGIATIVAEELDADWSKVRVESAPADAKRYANLAFGTMQGTGGSSAMANSWMQLREAGAKARAMLV
EAAARQWRVPATELRTRDGFVEHPASQRKASYGSLAAAAAELPVPEKVQLKDPKDFRLIGHQAPRVDVPGKTD
GSAQFTLDVSLPGMLVALLQRPPLFGATVKSFDATATRAIPGVVEVVQVPHGVAVVAKGFWAAKQGRDALKVE
WDESKAEKRGSEALMAEYRKLAEQPGKPARRDGDAAKAVAGATRRIAASYEFPFLAHAPMEPLDAVVRLTADS
CEIWAGDQFQTVDQGNAARTAGLKPEQVKINTLYAGGSFGRRANAWSDYIVEAVSIAKALGANGVPVKLQWT
REDDIHGGFYRPMYYHRLEAGLDADGKLVGWQHRIVGQSILEGTPFAAVMVKDGVDATSVEGAANLPYAVPN
VSVELSTTQVGVPVLWWRVVGSSHTVYAVEAFIDEAAQAAGKDPYLFRRDLLAEQPRLRGVLELAAEKAGWD
PSRPLPAGRGRGIAVTEAFKTFVAQVVEVSVDKDGKLKVERVVCAVDCGIPINPDVIAAQMEGGIGFGLGAILHS
AITLKDGKVEQNNFDGYQVLRIAEMPKVEVHIVPSGEAPTGVGEPGVAPIGPALANAIFAATGQRLYNLPFPTSF
AKA
>SEQF2696||SEQF2696.1_03178
MKRSYPDDLVIGNLSRRGFLKGVGATGVLLVAANWGWRDALAAEKKAFGADAMPHGWVDNPKIYVSIDRDG
TVGIVCNRSEMGQGVRTSLAMVVADELEADWSRVKVIQAPGDEARYGNQDTDGSRSMRHWFEPMRRCGA
AARQMLEQAAANQWKVPLGECRAEQNKVLHAPSGRSLSFGELAEAAAGLEVPARDKLLLKKPEQFRYIGKDV
ARAIDGADIVNGRAGFGFDARFDDMLYAVVARPPVYGGKLKRYDAAAALKVPGVVKVIEIEGRPIPSEFQPLGG
VAVVAQNTWAAIKGREALVVEWDAGVNGGYDSVAYRKQLEEAARKPGKVVRDSGDAAALFAKGGDIVEAEYY
LPHLAQAPMEPPVSTAWYKDGACEVWAPTQAPQVTRERIAERLKLPFDKVTVNVTLLGGGFGRKSKPDFVLEA
AILAKAFPGRHLRVQWTREDDLHFSYFHTVSVERLQAVLGADELPQAWLHRSVAPSITALFGPDSKHQGAFELG
MGLTNLPFAIPNVRLENPEAPAHTRVGWFRSVSNIPHAFAIQSFVGELAAKAGQDPKDYLLKLLGPARRIDTAEL
GDSWNYGESPERYPLDVGRLRGVIEEAARQSGWGGELPRGRARGIAAHYSFVTYVAVVIEVEVKDDGALLVHK
ATIAADCGPQINPERIRSQLEGACVMGLGLAALGEISFKDGKVQQDNFHQYELARMPLAPKAVSVHLLKPDGD
LPLGGVGEPGVPPIAPALCNAIFAATGKRIRELPIRNQLQGWRKA
>SEQF2697||SEQF2697.1_02707
MKRSYPDDLVIGNLSRRGFLKGVGATGVLLVAANWGWRDALAAEKKAFGADAMPHGWVDNPKIYVSIDRDG
TVGIVCNRSEMGQGVRTSLAMVVADELEADWSRVKVIQAPGDEARYGNQDTDGSRSMRHWFEPMRRCGA
AARQMLEQAAANQWKVPLGECRAEQNKVLHAPSGRSLSFGELAEAAAGLEVPARDKLLLKKPEQFRYIGKDV
ARAIDGADIVNGRAGFGFDARFDDMLYAVVARPPVYGGKLKRYDAAAALKVPGVVKVIEIEGRPIPSEFQPLGG
VAVVAQNTWAAIKGREALVVEWDAGVNGGYDSVAYRKQLEEAARKPGKVVRDSGDAAALFAKGGDVVEAEY
YLPHLAQAPMEPPVSTAWYKDGACEVWAPTQAPQVTRERIAERLKLPFDKVTVNVTLLGGGFGRKSKPDFVLE
AAILAKAFPGRHLRVQWTREDDLHFSYFHTVSVERLQAVLGADGLPQAWLHRSVAPSITALFGPDSKHQGAFE
LGMGLTNLPFAIPNVRLENPEAPAHTRVGWFRSVSNIPHAFAIQSFVGELAAKAGQDPKDYLLKLLGPARRIDTA
ELGDSWNYGESPERYPLDVGRLRGVIEEAARQSGWGGELPRGRARGIAAHYSFVTYVAVVIEVEVKDDGALLV
HKATIAADCGPQINPERIRSQLEGACVMGLGLAALGEISFKDGKVQQDNFHQYELARMPLAPKAVSVHLLKPD

GDLPLGGVGEPGVPPIAPALCNAIFAATGKRIRELPIRNQLQGWRKA
>SEQF2709||SEQF2709.1_04312
MPQRPSNERGRTAGSAISLRRRHLLQSAAALLVAPAAGSLLIPLAQAAPAEAGAAAATASSIGDWVWIEPSGQV
VIGVSQCEVGQGIYTGLPQVLADELDADWASVTVRFVTGRDAYRNDAGEMPFQQFVGASMSMNYFYERMR
LAGAQARDVLLRAGAARLGVRASQCSTRAGRVLHSATGRSVGYGEIVAEASRLPLAARPRMKSASEQGLIGRNL
RRVDTPAKVDGSAVFGIDVEVPGMLIGAVRMAPSVTGRIVRIRNEGEVSARPGVHAVVRTTQWPDPEPSTVV
VVADSYWIAKQAADALDIEFDAGAAAGVDSERIHAQFVAGLASDKAVVARSLGKPREMLAAGKPITADYHSPYI
THATMEPLAATVHVRDGEVETWGPYQGQDFLRGELGKACGVPADKVIVHTTFLGGSFGRKYMPDFALHAAA
ASKAVGRPVKVIRSREDDIRHSYYRPGASGRLSAVLGADGLPAALHARISGQSLYGAINPKKMADAGGWDETM
VESIYDLIYGVPNLLVDAVDVQQPIPLSYLRSVGTTSSVFFLESFISELAHTAGVDDYQYRRRLLAGQPLALGVLDA
AARAARWEQPVPAGLHRAMTFNVYTGRGESFQTFVALVMELRVVEGRVRLERAICAIDAGRVVNPGLVKANV
EGGIGFALTNTFKSRLGFDKGVVQQSNFHDYPLLQLSEMPRVEVVLVESDRPPQGCGEVALGPTAPAVATALFH
ATGRRFRSMPLPQDIAST
>SEQF2714||SEQF2714.1_02218
MGKIETPDSATRGILNVSRRTLLKGTGGLALGIFFAPLLRGLDALAAGGPLEPNAFVRIDLDGSVTVLAKHLEMG
QGSYTGLATLLAEELDADWDKVRVEGAPADVKRYNNLAFGPMQGTGGSTAMANSWEQMRNAGATAKAML
VAAAAQRWAVPASEISVDKGVVSHAGSGRSAGFGELVEAAASLPVPEQVQLKDPKDFKLIGKRELRRKDSLGKT
DGSAIFTQDFKLPGMLVAMVAYPPRFGGVPRSVDSSKAKAVRDVVEVVEFRDLPHGRSGVAVLAKNTWAARQ
GRDALVIEWDESQAFTLGSEEIFAQYRDSASKPGLPAASKGDTDAALAQAAKTVEADYEFPYLAHAAMEPMNC
LVKLSSDRCEIWNGEQFQTVDQAIISGYLGLTPEQVSLTQLYAGGSFGRRAGSVSDYLLEAVAITKAARDKGVDAP
VKMVWTREDDTRGGYFRPLYLHRVRIGLDEGGKLQAWHNRIVGQSIIAGTSMEPFLVKEGIDHTSVEGISNLSY
AVPNLQVELSTPSNIKVPVLWWRSVGHTHTGYVAETMIDEAAVAAGQDPYAFRHALLESHPRHRGALELAAKQ
AGWEKPLAAGAEGEKRGRGIAVHESFGSFVAQVAEVTVKADGSYRLDRVVCAVDCGIAINPDVIKAQMEGGIG
FALAAARHSAITLKEGRVEQSNFHDFQVLRLNEMPKVEVHIVPSAVNPTGVGEPGVPPLAPALANALFAATGVR
LRKLPFPSQIKA
>SEQF2716||SEQF2716.2_02602
MSAPELSAHNESRRALLLGFASGGLLLAFGVPSLARAAAPVQPPVSANPQYGGAGMPHGLRDDPNLFVAIAPD
GTVTITCIRSEMGQGVRTSVALVVADELGADWARVKVAQAVGDEPRYGNQNTDGSRSLRQSFAALRRAGAAA
RTMLEQAAATAWGVDVRQVKVTVHEVVDTKSGRKLGFGELASQAAALPAPDTKTLPLKAPAEFRYIGKGKTALI
DGRDLVGGSAHYGIDTRLDGMLYAVVARPPVYGDTVASFDASAAEKLPGVVKVVQLASTPLPSGFQPLGGVAVI
ARDTWTAIQARAQLKLDWKRGPHANYDSAEYRKTLEAAAAQPGDVIRNDGDATAALAGAAKRVRATYYIPHL
AHATMEPPAAVARVADGRCEVWTCTQAPQTTRDEIAKALGLPAERVTVNVTLLGGGFGRKSKPDYVVEAALLS
KAAGAPVKLTFTREDDIAHDYFHAVSLEAFDGGIDASGKVVAWQHRTVAPSIQSTFRAGVVHEQPGELAQGIV
DLPFAIPNVRIENPAAEAHTRIGWFRSVYNIPHAFGIQSFVSELAHAAGRDPKDFLLELIGPARRFEPHLAVKNVN
YGEDPALYPVDTGRLRRVVETVAREAGWGRTLPKGHGLGIAAHRSFVSYTAAVCEVQVGADGKITVPRVDIAID
CGPQVNPERVRSQLEGAVVMGLGIALHGEITFKDGHPEQRNFNGFQVLRMNEAPREIRVHLVAPDDFATPLG
GVGEPGLPPVAPALTNAIFAATGTRIRSLPVADQLAKPPAD
>SEQF2716||SEQF2716.2_05831
MTIELDNTDSVSPSRRTFLKAAGAAAAVSLTIGFEWAGLGRRALAANAPAAGFAPNAFLRITPDGTVTVIAKHV
ELGQGAYTGIATIVAEELDANWSNVRVESAPADAKRYANLAFGTMQGTGGSSAMANSWQQLREAGGKARA
MLVSAAAARWKVPAGELTTAKGVVTHAKSGKTAAYGTLVADASTLPVPDKVTLKQPAEFKLIGHRIPRVDASAKS
NGTAHFTLDTTFPGMRVALLQRPPRFGATVKSFDATAAKAVPGVVSVVQVPGGVAVVGTGFWAAKQGRDALK
VDWDETNAEKRGSDEIMREYRQLADKPGTSARKDGDADAAIAGSARKISATYEFPYLAHAPMEPLDAVVKLTP
NGCEIWAGDQFQTVDQANAAKTAGLKPEQVQIHTLYAGGSFGRRANAWSDYVVEAVSIAKALGADGKPVKL

QWTREDDIQGGFYRPMYFHKLDAGLTADGKLVGWRHRIVGQSILAGTPFEPFMVKNGVDATSVEGAANLPYA
VPNVSVELTTAKTGVPVLWWRVVGSSHTAYAVEAFIDEAAHTAGKDPYAFRHDLLEKEPRMRAVLDLAAQKAG
WDPAKPLPKGRGRGIAVAEAFKSYVAQVAEVSVDADGKVKVERVVCAVDCGIAINPDIVAAQMEGGIGFGLGA
VMHSAITLKDGQVEQRNFDGYHVLRMAEMPKVEVHIVPSAEAPTGVGEPGVAPVGPAVANAIFAATGKRHYV
LPFDSADSAKA
>SEQF2716||SEQF2716.2_06410
MSRGLIEAGRAGAGVSRRSFLKLGMSLGAAAGGGLLLGFSLPAAGDDARRSVIGGDAAEPAQPGVFAPNAFV
QIDRAGRVTLVMPKVEMGQGVYTSLPMLIAEELEVPLSSVTLDHAPPNEKLFLDPLLGGQLTGGSTSIRYAWEP
MRRAGATARTLLVAAAAKQWNVDPASCRAANGEVQHPPSGRRASYGQLADAAAKLPVPKDVALKKPEDFKLI
GTPAKRLDSPEKVDGTAQFGLDVRLPGMLYAVIVNSPVFGGTVASVDDTAAKKIPGVRQVVRVDNAVAVVGDH
TWAAKRGASALVVKWNEGAGAKVSTKDLFADLAQAAATGKGAVARKEGDVGKAFANAKTRVDAVYEQPLLA
HATMEPVNCTVHVRGDGCDIWVGTQVPTRVRDTAQQLTGLSPDKIVVHNHLLGGGFGRRLETDMVGQAVK
VGKQVSAPVKVIWTREEDVQHDMYRPCYYDKISAGLDANGKPVAWQHRIVGSSIMARFAPPAFQNGVDPDA
VEVAAELPYDLPNQLVDYVRQEPRHVPTAFWRGVGPTRGTFVVESFIDELATQTRTDPVQYRRALLDKTPRARN
VLDVATKAAGWGASLPKGQGRGVSVMHAFGSFFSIVIDVAVDGGEVQVKRVVCAVDCGMFVNPNTIEAQVQ
GGIIFGITGALYGEITIEDGRVVQGNFTDYRMMRINETPPIEVHLVKSGEAPGGIGEPGTAATAAALSNAIFAATG
TRLRKLPVGNQLKTA
>SEQF2719||SEQF2719.1_02571
MKKKQTRREFIKVVSVSGGGLILATYIPFNSLLAKAGDDPKIFSPSVYLKIDSNGIVTVIVHRSEMGQGVKTALPM
LIAEELEVDWEKIVIEQADADSKYGSQSTGGSTSIRRNWEPLRIAGATAREMLIVAAANKWSVDKSECYAENGF
VINKKSNQKISYGELVEDASKLPVPQNVKLKDPKDFKLIGKRIHRVDTPDKIYGKAKFGIDVVIPGMIYAALSRCPS
FGGRVKSFNPERAKKISGVIDVVQISNGVAVIADSTWNAFNGRDALEIEWDYGPNANVSTEDIRNEMMKHIKE
EGAEFESRGNIHQSISSEKFIEAIYEVPFMAHAPMEPMNCVAKYENGKIELWAPTQNPQNVKSEVAKALGLSEN
DVTVHVTLMGGGFGRRLVSDFAVEAAEISKTCGKAVKLTWTRKEDMKFGYYRPPSMHVLKGSVSSDGKPLKFY
HHVIAPSIRQMRFDKNLTAEKSEIKEGTVDLEYQIPNLKITGTLIPTHVPISWWRAVYNSQNPFAVESFIDELAYAA
GKDPYEFRKEMLPDDSRLKNVLIIAAEKSGWTNRMPKGKGRGLAISYGYESYCAQVAEVSVVNNKLKVEKFTAV
IDCGVVVNPDIVEAQMEGAIAFALSAAMKGEITIKNGGVEQNNFDDFEILTYDEMPIVDVHIVQNNFKVGGVG
EVGIAACAPALCNAIFNATGKRIRRLPVKI
>SEQF2720||SEQF2720.1_01101
MSVKQTRREFIKVFSMSGSALCLASFVPLNSLVASEGDKPKIFSPSVFIKIDTDGIVTIVVHRSEMGQGVRTALPM
IVAEELEVDWRKIKIEQAEGDPKYGDQITGGSTSVRKSWDPLRIAGATAREMLIAAAAARWNVKPSDCKAENG
YVVNKLNNKKVGYGDLVEEASKLPVPQNVKLKDPKDYKIIGKKLHRTDSPDKIYGKAKFGIDIVLPGMVYAAVGR
SPAFGATVKNYDDSVTKSLKGILDVKKVSTGVAVFAESTWQAFKGVENLSVEWNLGPSANVNSDTINNKLKEYL
KTDGDLVEIKGNPDDAEEKNYRTLEAIYEAPFQAHAPMEPMNCTAKFENNKVEVWAPTQSPQEAREAVIKTFG
LNEEDVVVYVTLMGGGFGRRTNPDFVVEAVELSKAVGKPVKVTWTREDDIKHDRYRPVSMHKLKGSVDKNN
KLYSFSHHVIAPSIAVQNWGAKLTPKQYDTLGGAIEKDYIIPNYKITASIVDIPVPIWYWRSVYHSQNPFAAECFID
ELAHAAGRDPYEFRMELLPDDSRMKAVLKTAAEKAGWGKKLPPGKGMGIAAFAGYDSFSAQVAEVTVTESKEI
IVDKITCAVDCGIVINPDIVEQQMDSGIAFALTAALKGEITIRDGSVVESNFDDFPMLQFSEMPVVETHIMQNTY
RVGGLGEVAVGPCAPAVGNAIFAATGKRLRKLPFRL
>SEQF2763||SEQF2763.1_03849
MADTQFAQSDAYLSALIREVRSVPPAGVSLTMGRRTFFKLAGASAAGLVLGFRLGGTAFAAETANVDEPDGKD
QAMNAFIRIAPDNTITIYSKCPEIGQGIKTSFGVIIADELDADWNHVVMEQADINPKVYGSQGAGGSTSIPHAW
DQLRQAGAGAKAMLVAVAAGQWAVDSSQITARDSVLTHAASGRSATYGSLAVAAAKMPVPDPKSLKLKSRAD
YRLIGKRYRGVDDPKVVSGQPLFGIDVQLPGMVYATYTKCPAAGGKVRSFNANEIKALHGVLDAFAVDGTGM

MVEVMPGVAIIARDTWSAFQAKDKLQVDWDLSEASRDSTSQFSAEARKQAANFPQKPDDNIGDVDKSFANA
AKTVEAYYEYPFAAHVPLEPMNTTAHWHDGVMEMWVPTQQPDRGLPIVAKVAGIAPEKVVMHQTRVGGG
FGRRLVNDYACEAAAIAKRVKDPVKLQWTREDDFAHDFYRPAGYHQFKGAIDKGGRLDAWQEHFITFTADGKK
EASGANLTDNLRYSIKAPNLRRAKTMFPLRIPTGAWRAPGDNAQVFAAQSFMHELSLASGRDHVEFLLDAVNR
DVPELVPKDRTVNFSPSRATGVIKMCADKAGWGKTLPKGSGLGLAWCYSHAGHVAQAVELSVDANKRIKIARIL
VVLDVGPIIDMAGSEAQAQGASTDALSTAMGLKINIENGLIQEQNYNAYPILRMPFAPMTIDPYFIQSDNPPTG
MGEPAFPALAPALGNAIFAATGERLRQLPLRDLGYSLAV
>SEQF2854||SEQF2854.1_02303
MAALSGAAASGSHDAPAAGIDRRTFLKLTGIAGGGLALGIVPPGGAHAEEAAAAVPAAKGPAAAPQAFIVIAPD
NTVTIAVNRLEFGQGVHTALPMMLAEDLDVDWRNVRAVLAPAGDPYKDPAMGIQMTGGSTAVKHSYQQYR
ELGARARAMLIAAAAQRWAVEPSACTTANGVVTAGNRRATYGELAQAAMEMPVPQQVTLKDPSRFALIGKP
TPRLDTRGKLDGSVGFGIDTQLDNLRVAVVARPPRFGGKVRRFNAEAARAIQGVVEVFEVPTDGGGTGVAVVA
TGYWPAKQGRDALQAEWDDSGSTVSSAALYEEYAKLARQPGRTPRADAFDLAGAVRTVEAEYRFPYLAHAPM
EPLNCTMQAEVAGGKPARVKVWAGTQFQTVDHGALAKAFGLPPEQVSVVTMMAGGGFGRRAVPTSDYLVE
AAQLMRAWVAKGHREPVKVIWSREDDIRGGYYRPLHLHHARIGIDAQGKVLGWEHAIVGQSLTMGSPFEAFL
VKDGVDHTMTEGIVDHDYGFPLRLSVHHPKVQVPVLWWRSVGHTHTAFVKETLIDELATVAKQDPVAYRLARL
DPVKGARQRAALQLAVEKSGYGKRRLPAGRAWGVAVHQSFDSAVAYVVEVSLRNGQPHVHRVTAGVHANRV
INPLSASAQIEGGCVFGLAMIRPGFAIEIDNGAVRNSNFGDFPPVRINDAPPVEVHFVPSNDPPTGLGEPGVPVI
APAVANAVFKLTGKRQRQLPFVMA
>SEQF2854||SEQF2854.1_04355
MRANDSTMTPAMTPDLSRRGFLQGTALGTLALAVGANGIIGLARADDDAPKKYGADSMPGGTVSDPLVFVSI
GADGTVTIVAHRAEMGTGVRTSLPMVVADEMEANWDRVKVVQAEANETRYGNQNVDGSRSVRHFLMPM
RRVGAAARQMLEAAAAARWSVPVGEVRAQRHEVVHLPTGRRLSYGELAADAAKQPVPAQGTLKLKTPDQFR
YIGKDRVRLVDLEAIGKGQATYGMDVRLPGMVYAVVARPPVVGGKVRAVRADKALAVPGVLKVVEIPPFSGAP
AFQPLGGVAVVARNTWAAMQGRAALEIEWDDGPNAAYDSVAYRRTLEAAARQPGKVVRNEGDAPAAWNK
SAESERYAAEYYLPHLAHASMEPPVATVRVSGKTAEVWTSVQNPVAAQTAVAKRLKLEPKDVRVNVVLLGGGF
GRKSKPDFVDEAAVVAQAMPGTPVKLVWTREDDIHHDYLHTVSVERLEAVVGKDGRPQTWLHRSAAPTIGSL
FTAGAKGQQPFELGMSAINMPYRIPNVRVETAEVEAHARIGWFRSVSNIPHAFAAQCFIAELAHRAGKDHKQF
ALDLIGPARKIDPRTMADSWNYTESPERYPYDTGRLRDVIEAATRGARWGRKLPRGHGLGLAFCYSFMSYTAAV
VEVAVNDKGEVQVVAVDMAMDCGPQINPERIRSQLEGGAIMGLSLALSSEITFEKGRVKQSNFHDYEVLRHHA
SPRTIRTHLVNGSHDVPPGGVGEPPVPPVAPALCNAIFAATGKRIRSLPVRTVA
>SEQF2867||SEQF2867.2_03702
MTVQTTRRGFLAGGTGLLLALALPVGRGRAQVTVAGPFAPNAFIRIGTDDLVTVMIKHLEMGQGPYTGLATLV
AEELDADWSQMRAEGAPADDTLYANLAFGAQGTGGSTAIANSFMQMRKAGAAARAMLVAAAAEEWGVPA
SEITVKAGVVAHEASGRSSGFGALSTAAARQPVPEDPPVKAPADFVLIGTDRPKLDSASKSTGTAQFTLDVYRDG
MLTVVVAHPPRFGATLARVDDSAALAVKGVEMVRTVPSGVAVYATNTYAALKGRDALILEWDDSKAETRSSIQ
MFAAFAEAAAQGGETVEEEGDLSVIDGAARVLTAEYRFPYLAHAPMEPLDAVIETKGGKAELWFGSQFPSFDKP
TIAGALGIAPEDVTINVLMAGGSFGRRAQGSAHLAAEVGEIAKAVGRDGAFKLVWTREDDVKGGYYRPMTVH
RLRAGLDADGTIIGWENVVANQSIMAGTPMEAMLKGGPDNSSFEGSTGLPYRLGARRIGWARMESPVSVLW
WRSVGHTHTAYAVETFLDEVLEAAGKDPLQGRLDLLPPEAARERAVIAEAARMADWQGPRRDGKGYGIAYAKS
FGTYVAEVVEVEDRGGTPRVTRVWCAVDCGIAVNPNVIRAQMEGGIGYALSAALHSRITLAPGGTVEQSNFHD
YPMLRIAEMPAVEVSIIRSDADPTGVGEPGVPPLAPAMANAWRALNGARQYQLPFGGAIS
>SEQF2889||SEQF2889.1_00689
MAITRRTLIKGSALAVAAMLLPISTRSPAAVVSQDVAPPDSQHELNDWIWIDRDGRIVIGVSQCEVGQGIYTGL

AEVVAAEMDADWAQVTVKFVTGRDAYRQVAGGEAFAQFVAASTSMTKFYQRARLAGAQARDFFLRAGAKH
FALSPSQCRTEKGWVLEKGGKRKVAYGDLVRYAAELPLDPQPALKSEAQERESVIGKPLLRVDTPEKVDGSAIYGI
DIDLPEMLIGVPWMVPDLSGKLVAVRNERQIRAMPGVVDLVLTRQWSMNNMVGLDHDMSLNTVIVVAASY
WQAKKAADLLEVDWLPGAGKALTDSAIAAENLAMLDGDTLVPAVDRGEASALIRGVEQGSRLHEARYSAPYV
AHATLEPCNATSHYGEGRIETWGPFQGQDMVRNVLAKMFGLKPTDVVVNNTTYLGGSFGRKYLPDAVMHATA
ASRAVGKPVKVIYPREIDMRHEYYRPACISHYRALLDENGYPQALWARYAGQSLFWQMRRETVNEAGGWDES
MVECVYNTPYRIPHLKVEAGIVEQPISLSYLRGVGSVASLFFLESFISELSHKANRDEYSFRRHLLQDSPEALRVLD
ATATAAGWQHEPPSGVSRGMACNIWVGRNNAFTTYVGLVVEIAIQEGRLRVLRAVCAIDCGKVINPNLVRANV
EGGIGFALTTCLHSELHFERGGVVEGNFDRYPLLAIAEMPKVEVVILDSARAPQGCGEVSTAVVAPAMASALHK
ATGKTYRTMPFPREFSSV
>SEQF2893||SEQF2893.1_02859
MHFDAQTARAHMPKGLVALMDQAQGAINTEANTAAEGVARRTFLKAAAASGFALGAYPLVATAQGAGTAPA
GLKPFEQPSAFVRIDTDGTVTVTINRLDFGQGVQTGLPMILAEELDADWAKVRSVHGDANPAYADPAFGMHLT
GGSNSLKNSYTQYRELGARTRAMLVSAAAAQWGVDASTLRTNAGFVVGPGGKKLAYGALAEAAMQQPVPEK
VTLKDPQQFRIIGKPTTRLDAQAKSSGQQDYGIDVRLPGMLTAVVARPPVFGAKLKSLDDSAAKAIKGVKAVLR
VPTDRGGEGVAVIAEGYWPAKQGRDALKVEWDTVSVEKPDTAQLLTQYRALAQKTGAIAIPADVAALANAPQK
ISAEFTFPYLAHAPMEPLNCTVKLDGDKAELWMGTQMPGLDAMAAAKVLGLQPQNMKVHTQMAGGGFGR
RAIPTSDYVVEACGVAKAARTAGINAPVRTLWSREDDIKGGYYRPMHVHRAEIGFDAQGNILAWDHVIVGQSI
LKGSPFEAFMVKNGVDTTAVEGMKEPYNIPMRLSVHHPQVNVPVLWWRSVGSTHTAYAMETLLDEVARATQ
QDPVAYRLRLMGDKHPRHKAALQLAVDQSGYGKKKLAAGRAWGVAVHESFSSVVAYVVEASVSKDGTPKLHS
VTAGVHCNLAVNPKSVEAQVQGGALMGLSMCLPGAAITLKDGVVEQSNFGDFAVPRITDMPQVAVHIVPSAE
PPTGMGEPGLPPLAPAFANAVARLTGKTPRELPFKLA
>SEQF3076||SEQF3076.1_01833
MTKNVSAQAISLSRRKLLQASGIAAGGLLLATALPFSRRSYAEQYVNKGPEDAPLDTPTALGAFLRIGHDGQITLIS
PKIEMGQGVQTGFAMMVAEELNVTLDQVRVQEAPPDEKLYGDKLLGFQATGGSSSTRSNWQPLREAAAAAR
VMLIQAAANQWKVSPDECRAENGKIIGPGNRELAYGALVDAAAKLPVPENVPLKKPEDYKVIGQPLRRLDTPG
KVDGSAKFTIDLHVPGMKIATVSACPVVGGTLASVDDRHARAVPGVRDVVKLENAVAVIGDHMWAALKGLKA
LEIQWDLGPNAGIDSAQIERALHAAFDREGAIAAEVGDINKAIAGASSKIEAEYEMPFLAHATLEPMTCVAQVR
EDACELWVGTQVPVMAQQAAAKATGLPPEKIIVNNQLIGGGFGRRLEADFIGQAAAIAKQVDYPIKLVWTREE
DTAHDLYRPHYIDRFSAGLDANGMPVGWSHTIVGASVMARFAPAAVPPNGLDADAVEVSNKPVYSLPNLRVR
YVPEAPKAILNSWWRGVGPLHGAYVMESFIDELAYAAKQDPVDYRMKLLGEHPRAQAVLKLAAEKANWSQKL
PAGHGRGIAVQEVFGSYLATVVEMQVDAQNGIHITRLICIADCGEVTNPTSVHSQLEGGTLFGLSAALYNEITVK
NGHVEQSNFHDYRQLRMSDAPPVETHIIPSHEIPGGIGEAGTAMIAPALVNALYAATGKRLRRLPVVRAGYHVA
>SEQF3080||SEQF3080.1_01508
MADNPNPNLEEIEAAPTDTTPVKKKGIKRRIFLAGAALLVGGGVFGLYWADNAAKSRARALTTGEGEHNFLTW
MKIAEDDTVTVFSPHIDFGQGTHTALGQMLADELDADWAKVKVEQAPSDVAFSNTALVEKFLGEMTGYPGLIA
ALPAAVMGALTRSQKIQITGGSSAVRFTGQGGMRVVGAAVRLALIEEAADRLGVPASELTTADSKVIHAASGRSL
RYGELAAGAAERSLRSDPVLKSRKDYRLIGKPVTRRDIPAKVDGSAVYGIDFTLPDMRVATIAAAPVRGGKLISVD
EAPALAVPGVEKVVKLDDAVIVVAKGYWQASKGLAALSPKFSDGGHGAMSTPAIYAAQDRLRETGKPDNTAGE
GDVAAAFKAAGGKVISADYRVPFLHHAMMEPFALTAHFKSGKLEMWGGLQDPIGARDRAAKAVGMDMEDV
IFHPMIMGGGFGRRFPGVAEIIDQAAQLARQVPYPVKLVWSREEDLRHGTYRPQSSARLAATLDDKGRIAAW
QSDYVQGDNAEGEVGFIYDVPATSRRHFAYTTNQIDGPWRSVNSTQHGFYNESFIDELAHAAGEDPYRFRRKH
LPDGSRHQKVLDEVARRSGWGTPLPAGVGRGIAIVESFGTIVAEVIEATVKDDGTPKVLKAFAVVDCGTTVNPL
NAEAQIAGGIIMGLSATIGEAITLDQGAVVESNFTDYPILRLADAPPLIDVHFIESDAPMGGIGEPGVPPASAALA

NALFMATGKRIRTLPLRQQAKA
>SEQF3148||SEQF3148.1_02416
MTVLSAPRSFRPSRRHILAGSAAVAGAFTFGFSIPAGAQQAGTPEINAWVVVHPDDKVVIRIARSEMGQGTLT
GLAQLVAEELNCDWSKVTTEYPTPGQNIARNRVWGNFSTGGSRGIRESHDYVRKGGAAARMMLIQAAANE
WKVPAAECSAEKSVITHKASGRSLRYGQVAAAAAKLEAPKEVPLKDPKDWTIAGKALPRLDTVDKTNGKKIYG
MDFKLPGMLNAAIKDCPVFGGKVKSFDAAKVRGMPGVKHVLPVGDSAVTVVAETWWQAKTALDALPIEWD
EGPNAKVTSESIAAWLKEGLDAPDAVVGNQNGDAKGALAGAARVVEATYSYPFQNHACMEVMNATALYTPE
KCEVWTPTQNGEAALAATAEASGLPLAKCEAYKIDLGGGFGRRGAVHDWVRQVVAIAKQIPGTPVKLIWSREE
DMLHGRFHPITQCKLRAGLDKDGNVTALHMRISGQSIVAGLFPQNIQNGRDPVVFQGLNAPGPEASIGYSFPN
LLIDHAMRNPHVPPGFWRGVNLNQNTIYLESFIDELAHETGKDPLEFRRSLMKSHPKHLAVLNAAAERAEWG
KPLPDGVFRGICQTMGFGSYVAAVAEVSVGADGKLTIHRIVAATDPGHAVNPAQIERQVEGSFVYGLSAALYGEI
TIKDGRVEQENFDTYEVMRLEAMPKVETVIVPSGGFWGGVGEPTIAVAAPAVLNAIFAATGKRVRSLPLKNADL
RKA
>SEQF3148||SEQF3148.1_02795
MTAHDPKLSRRSLLAGAGALVIGFHLPRAAKAQSGAGAAYRPGGNATFAPNAFIRVAPDSTVTVLIKHIEFGQG
PFTGLATLAAEEMDAAWSQMRAEHAPADVKLYANLAFGVQGTGGSTAIANSWEQMRKAGAAARMMLVQA
AADTWKVPAGEITVSDGVISHTSGRKGSFGEFAEAASKLPVPENPPLKPASAYKLIGKEGAVKRLDSADKSRGKA
QFTIDIHAPNMLTVVVARSPRFGGKVASFDAAEALKVKGVVDVKSIGYGVAVYANGMWPALKGREALKVTWD
DSAAEKRGSPELIAEYRKLVRQPGTVAGSHGDAEAALGKADKVIEAEFVFPYLAHAPMEPLDGYLEWDGEKAH
ARFGSQFQTTEHQTIAGILGLPPEKVTLETMLAGGSFGRRAQVSQHLAAELATVAKTIGPNRPVKVVWTREDDL
TGGYYRPLFVHRFRGAVKDGKIAAWSSTLVGQSFFLGTPFEAMVVKNGIDATSVEGANEIFYEVPDFRCEVHNP
KVGVPTLWWRSVGHTHTGYAVECFVDELLQAAGQDPVQGRLAMIGDRHPRAAGVLKAVADLAGWKGGAPV
DGRARGVAVVESFGSFVAQIAEVSMGPEGEPKVHKVWCAVDCGVAVNPDVIRAQVEGGIGFGLGHALYGQIT
LDGGRPVQTNFHEYRSLRINEMPEVEVRIIASTEKPSGIGEPGVPPIGPAVANALAALGHGRPRQLPMQGGNV
>SEQF3151||SEQF3151.1_01533
MADNPNSDLDYPETSAPAAKPKRKGVKRRIFLAGSALVVGGGIFGVWWTDSNAKGRANTLIGGEGKHAFNSV
MTIAEDDTVTLFSPHIDFGQGSHTALGQMLADELDAAWEKVTIEQAPADMAFANAALAKGFLPTMVGDTVA
GLIPDAVIGLMARSMPLMITGGSSAIRFTGEVAMRRTGAAVRAALVAEAADRLGVPESELTTADSKVTHAKSGR
SLRYGELAAGAATRSLSSDPVLKTRDQWKLIGKPVPRRDIPSKVDGSAVYGIDFTLPDMRVATIAAAPVRGGKLE
SVDEAPALAISGVEKVVKLPDAVIVVAKGYWPATKGLAALSPKFTDGGHSAMSTPAIYAAQEKLRKASGEPDNV
GGEGDVDAAFAAAGVKLVEAEYRVPFLHHAMMEPFALTGHFKDGTLHLWGGLQDPLSTRAKAAKAAGLEVD
NVVFHPMIMGGGFGRRFPDLVEIIDQIAVLAKQVPYPVKLVWSREEEVRHGTYRPQSSAGLKASLKDGKITGW
RADYVQSGSAEGEVPFIYAIPATLRRHFAYQSNQIDGPWRSVNATQMGFYTESFMDELAAAAGEDPYQFRRKH
LATGSRHLAALDMVAKRSGWGTPLPKGVGRGIAIVESFDTIVAEVVEASVKEDGSPKVLKAWAVVDCGTTVNP
LNAEAQIAGGLIMGLSSAIGEQVTLDKGAVVESNFSDYPILKLADAPPAVDVHFIESGAKTGGIGEPGLPPASPAL
ANALSAATGKRIRNLPLLTQAKA
>SEQF3151||SEQF3151.1_02575
MLMRDLGIIPAPKVDGTGDGPSPYVNLSRRGFVGGAGLFVLGVTLAGCSSYVEPVIDADAFKLPDPGASPLTGV
KGGDATPSLWIAIDKDGAVKITCHRSEMGQQTWTAMAQIVADELEADWDKVAIVQAEGHERYGDQNTDGSR
SVRFNFHRLRVAGAAMRHMLVAAAALYWKLPQDQCSAKGGLVSNTKNDETLSYGNLAELAGRLAIPAEADIKL
KTPKEWRYIREEIPSLTVPRIVKGDSTFGIDVKRPGMVYAVVARPPQLFGRVGSVDDTKALAIAGVLSTMRLPDA
KPPALFQPLGGVAVVARDTWAAIEGRRALEIAWQDGPNAGYDSEAFAKQLQATARRSGKVRRSRGNVGAALA
AASKRVTAEYYAPHLNQSPMEPPSATAEWDGDRLECWACVQDPQNTRDTLAEALGIPKENIKVTPTWLGGAF
GRKSKPDFVIEAALIAREVGKPVKVTWTREDDIRHGYYHSVSAQYCEAGLDQDGKCTAWLHRTVFPPISSTFDN

TLAEPSDGEMSMGATDVPFAAPNLRVESGDAKGHMRIGWLRSVANIYHAFAVQSFAAELAHAAGRDQKDYLL
ELIGPPRMIDPESEGATYGNYGAELAEYPIDTARLRNVVEKASAMADWGRKLPAGRGLGIAVHRSFLSYIATVIEV
AVGKDGTLRIPGVWLAVDAGTVINPRHVRAQMEGGTIYGLSNALYGAITAKDGAVVQDNFPSWRLMRMGEA
PREFKVEIIASDAPPGGVGEPATPPAAPALANAIFAATGHRLRTLPLIGAEGDKLKLPATKTV
>SEQF3155||SEQF3155.1_03374
MKRRTFLLSAAGTGGALVVGWGLLPPRSRLGSRNSLPVRDGQVGLNGWIKIDRRGRVILAMHRSEMGQGVH
TALPMLVAEELDVPLDQVLLEPAGPESIYGNVAMFVGSLPIHPAEREPDHGSAAVRLTEWMVTKLAREMGINVT
GGSSSVADAWDVLRTAAATARAQLLGAAALQWRLPVDELRIIDGVISHASGPSAHFGELARRAAALSVSEVRVK
PPQTWRLIGRSAPRTDVPAKVDGSARYGIDVRLPGMRYAAVRMCPMLGGSPGRIGVDEALRRPGVERVVRLG
PIAGSTAGVAVVARTYWHALQAVDAMPVEWRAPPHDPQRGLLDSSAIAAALEQAARQALAEDGGHAFHRR
GDVARAEQGAARVVEQVYHAPYLAHATMEPINCTARVQDGQVEVWAPTQVPTLARQVAARVAGVPPERVT
VHVTLVGGGFGRRLEVDHVGQAVRVAMETGGKPVQLIWPREEDLMHDFYRPAGAAVLRAALDAQGLPVALR
IASAGDAITPRWLERGLPAFAGPVDLPDKTASEGLFDLPYQVPHQRIAHAATHSGVPVGYWRSVGHSHNAFFSE
SFIDELAFEARQDPVAYRLALLRDKPRHQAVLRLAAERAGWDRPPPPGRARGVALHESFGSIVAEVVEVSARQG
RPQVHRVVCAIDCGAVVNPGIVAQQMESGVIFGLSAALHGRIDIRGGIVQQRNYPDHPVLTLADTPVIETHIVR
SQRAPGGVGEPGTPPVAPALANAWFALTGQRLRRLPLLGAASAPHPGN
>SEQF3155||SEQF3155.1_00724
MSTTTLENPARRRVLQAGAGLTLALYLPVHAARAAAGGQEAATPFVPNAFLRIGEDNRVTVIAKHLEMGQGSY
TGLATIVAEELDAAWSQVQVEGAPADARRYNNLGWGPVQGTGGSTAIANSWQQLREAGATARAMLVAAAA
AQWGVPAGEVTVSEGEVIHAASGRKASFGQLARAAAQQPVPQQVQLKDPKDFRLIGKRVPRVDGAGKTDGS
ARFTQDVQLPGMLVAVVAHPPRFGATLKSVDDRKARAVRGVVDVVRIPSGVAVLARDTWSAKKGRDALEIEW
DDSRAFRLGSAEILARYRELAATPGVVARREGDPDRALANAARTLEAAYDFPYLAHAAMEPMNCVIRLDADGC
EVWNGEQFQTVDQAAVAQVLGLRPEQVRLHMLYAGGSFGRRASKTSDYLVEAAHIVKAIGGRAPVKLVWLRE
DDMRAGYYRPAFHHRLWAGLDARGRLVGWRHRLVGQSILAGSPFEPMMVKDGIDPVSVEGAANLPYAIPNL
VVDLHSPQDIGVPVLWWRSVGSTHTAFSTECFIDELAQAAGQDPVAWRLALLAQHSRHAGVLRLAAERAGW
GRPLAPGAAGERRGRGVAVHESFHSYVAQVAEVTVREDGSYRVDRVVCAVDCGIAVNPDVIRAQVEGSIGFAL
STVLHGEITLRDGEVQQSNFHDYLVARITDMPQVEVHIVPSAANPTGIGEPAVPPLAPAVANALAAATGRRLRQ
LPIRPELLRV
>SEQF3156||SEQF3156.1_00054
MSTTTLENPARRRVLQAGAGLTLALYLPVHAARAAAGGQEAATPFVPNAFLRIGEDNRVTVIAKHLEMGQGSY
TGLATIVAEELDAAWSQVQVEGAPADARRYNNLGWGPVQGTGGSTAIANSWQQLREAGATARAMLVAAAA
AQWGVPAGEVTVSEGEVIHAASGRKASFGQLARAAAQQPVPQQVQLKDPKDFRLIGKRVPRVDGAGKTDGS
ARFTQDVQLPGMLVAVVAHPPRFGATLKSVDDRKARAVRGVVDVVRIPSGVAVLARDTWSAKKGRDALEIEW
DDSRAFRLGSAEILARYRELAATPGVVARREGDPDRALANAARTLEAAYDFPYLAHAAMEPMNCVIRLDADGC
EVWNGEQFQTVDQAAVAQVLGLRPEQVRLHMLYAGGSFGRRASKTSDYLVEAAHIVKAIGGRAPVKLVWLRE
DDMRAGYYRPAFHHRLWAGLDARGRLVGWRHRLVGQSILAGSPFEPMMVKDGIDPVSVEGAANLPYAIPNL
VVDLHSPQDIGVPVLWWRSVGSTHTAFSTECFIDELAQAAGQDPVAWRLALLAQHPRHAGVLRLAAERAGW
GRPLAPGAAGERRGRGVAVHESFHSYVAQVAEVTVREDGSYRVDRVVCAVDCGIAVNPDVIRAQVEGSIGFAL
STVLHGEITLRDGEVQQSNFHDYLVARITDMPQVEVHIVPSAANPTGIGEPAVPPLAPAVANALAAATGRRLRQ
LPIRPELLRA
>SEQF3156||SEQF3156.1_03041
MKRRTFLLSAAGTGGALVVGWGLLPPRSRLGSRNSLPVRDGQVGLNGWIKIDRRGRVILAMHRSEMGQGVH
TALPMLVAEELDVPLDQVLLEPAGPESIYGNVAMFVGSLPIHPAEREPDHGSAAVRLTEWMVTKLAREMGINVT
GGSSSVADAWDVLRTAAATARAQLLGAAALQWRLPVDELRIIDGVISHASGPSAHFGELARRAAALSVSEVRVK

PPQTWRLIGRSAPRTDVPAKVDGSARYGIDVRLPGMRYAAVRMCPMLGGSPGRIGVDEALRRPGVERVVRLG
PIAGSTAGVAVVARTYWHALQAVDAMPVEWRAPPHDPQRGLLDSSAIAAALEQAARQALAEDGGHAFHRR
GDVARAEQGAARVVEQVYHAPYLAHATMEPINCTARVQDGQVEVWAPTQVPTLARQVAARVAGVPPERVT
VHVTLVGGGFGRRLEVDHVGQAVRVAMETGGKPVQLIWPREEDLMHDFYRPAGAAVLRAALDAQGLPVTLR
IASAGDAITPRWLERGLPAFAGPVDLPDKTASEGLFDLPYQVPHQRIAHAATHSGVPVGYWRSVGHSHNAFFSE
SFIDELAFEARQDPVAYRLALLRDKPRHQAVLRLAAERAGWDRPPPPGRARGVALHESFGSIVAEVVEVSARQG
RPQVHRVVCAIDCGAVVNPGIVAQQMESGVIFGLSAALHGRIDIRGGIVQQRNYPDHPVLTLADTPVIETHIVR
SQRAPGGVGEPGTPPVAPALANAWFALTGQRLRRLPLLGAASAPHPGN
>SEQF3160||SEQF3160.1_00253
MSTTTLENPARRRVLQAGAGLTLALYLPVHAARAAAGGQEAATPFVPNAFLRIGEDNRVTVIAKHLEMGQGSY
TGLATIVAEELDAAWSQVQVEGAPADARRYNNLGWGPVQGTGGSTAIANSWQQLREAGATARAMLVAAAA
AQWGVPAGEVTVSEGEVIHAASGRKASFGQLARAAAQQPVPQQVQLKDPKDFRLIGKRVPRVDGAGKTDGS
ARFTQDVQLPGMLVAVVAHPPRFGATLKSVDDRKARAVRGVVDVVRIPSGVAVLARDTWSAKKGRDALEIEW
DDSRAFRLGSAEILARYRELAATPGVVARREGDPDRALANAARTLEAAYDFPYLAHAAMEPMNCVIRLDADGC
EVWNGEQFQTVDQAAVAQVLGLRPEQVRLHMLYAGGSFGRRASKTSDYLVEAAHIVKAIGGRAPVKLVWLRE
DDMRAGYYRPAFHHRLWAGLDARGRLVGWRHRLVGQSILAGSPFEPMMVKDGIDPVSVEGAANLPYAIPNL
VVDLHSPQDIGVPVLWWRSVGSTHTAFSTECFIDELAQAAGQDPVAWRLALLAQHSRHAGVLRLAAERAGW
GRPLAPGAAGERRGRGVAVHESFHSYVAQVAEVTVREDGSYRVDRVVCAVDCGIAVNPDVIRAQVEGSIGFAL
STVLHGEITLRDGEVQQSNFHDYLVARITDMPQVEVHIVPSAANPTGIGEPAVPPLAPAVANALAAATGRRLRQ
LPIRPELLRV
>SEQF3160||SEQF3160.1_01436
MKRRTFLLSAAGTGGALVVGWGLLPPRSRLGSRNSLPVRDGQVGLNGWIKIDRRGRVILAMHRSEMGQGVH
TALPMLVAEELDVPLDQVLLEPAGPESIYGNVAMFVGSLPIHPAEREPDHGSAAVRLTEWMVTKLAREMGINVT
GGSSSVADAWDVLRTAAATARAQLLGAAALQWRLPVDELRIIDGVISHASGPSAHFGELARRAAALSVSEVRVK
PPQTWRLIGRSAPRTDVPAKVDGSARYGIDVRLPGMRYAAVRMCPMLGGSPGRIGVDEALRRPGVERVVRLG
PIAGSTAGVAVVARTYWHALQAVDAMPVEWRAPPHDPQRGLLDSSAIAAALEQAARQALAEDGGHAFHRR
GDVARAEQGAARVVEQVYHAPYLAHATMEPINCTARVQDGQVEVWAPTQVPTLARQVAARVAGVPPERVT
VHVTLVGGGFGRRLEVDHVGQAVRVAMETGGKPVQLIWPREEDLMHDFYRPAGAAVLRAALDAQGLPVALR
IASAGDAITPRWLERGLPAFAGPVDLPDKTASEGLFDLPYQVPHQRIAHAATHSGVPVGYWRSVGHSHNAFFSE
SFIDELAFEARQDPVAYRLALLRDKPRHQAVLRLAAERAGWDRPPPPGRARGVALHESFGSIVAEVVEVSARQG
RPQVHRVVCAIDCGAVVNPGIVAQQMESGVIFGLSAALHGRIDIRGGIVQQRNYPDHPVLTLADTPVIETHIVR
SQRAPGGVGEPGTPPVAPALANAWFALTGQRLRRLPLLGAASAPHPGN
>SEQF3742||SEQF3742.1_02092
MLPTHIVPSELPRALQRLMAASQPDATAALPRRSFLKLAGVGGFAIGAFPHMAMAQADGAAKPAGGLKPSQQ
PLAFVQIAPDGEVTVTHNRLEFGQGVQTGLPMILAEELDADWSLVRSKSGTNDPAYHDPVFGMHLTGGSNSIK
NSFTQYRELGARARAMLLSAAAARWKVDVATLRTQAGTVLGPGGRKLGYGELAEAAMALPVPEKVTLKDSKD
FRIIGRPTTRLDARAKSSGRQDFGIDVKQPGQLTAVVAHPPVFGARLKSVDDAAARAVKGVKAVLRVPLDRGAE
GVAVVADGYWPAKMGRDALKLQWDTSAVEKVDSEKQLVQYRELATKPGPRKFDADMAPLARAPRQLEAEFV
FPYLAHAPMEPLNCTVKLSADRAELWVGTQSAGLDAAAAARTLGLKPEQVSVHVQMAGGGFGRRFVGSSDY
VVEACQIAKAARTAGLAGPVRLLWSREDDIKGGYYRPMHLHRARIGFDERGKILAWDHVIVGQSILTGTVFEPF
QVKDGIDASATEGMREPYPLPMRLTVHHPKVNVPVLWWRSVGSTHTAFVMETLIDEIAHSTKQDPVAYRMQL
FGDKHPRHRAALQLAVDKSGYGKKQLAQGRAWGVAVHESFESVVAYVVEASVKDGQPVLHRATAGVHCNLA
VNPRSVEAQVQGAAVMGLSMCLPGGAITLKDGEVQQSNFGDFAVARITSMPEFDVHVVPSADAPTGIGEPGL
PPLAPAFANALARLTGKPLRQMPFNLA

>SEQF3743||SEQF3743.1_03532
MLPNIDYNELPRALQRLMARPETDEADEVATLPRRSFLKMAGAGGFVLGAFPHMAMAQADGPAKAGGALKP
TQQPSAFVQIAPNGEVTVTINRLEFGQGVQTGLPMILAEELDADWALVRSRNGTNDAAYADPLFGMHLTGGS
NSIKNSYTQYRELGARARAMLLSAAAQRWKVDVATLRTQAGTVIGPGGRKATYGELAEAAMALPVPEKVTLKN
PKDFRIIGRPTTRLDARAKSSGRQDFGIDVKQPGQLTAVVAHPPVFGARLASVDDSAARAVKGVKAVLRIPLDR
GAEGVAVVADGYWPAKLGRDALKLQWDTAAVEKVDSEKQLAQYRELAGRPGNRKFDADMAPLANAPHKLD
AEFVFPYLAHAPMEPLNCTVKLTDGRAELWVGTQSADLDGQAAARTLKLKPEQVKVNVQMAGGGFGRRFVG
SSDFVVEACEIAKAARTAGLKDAPVRLLWSREDDIKGGYYRPMHLHRARIGFDARGKILAWDHVIVGQSITGGT
VFAGSMVKDGIDATAVEGMRDPYPVPMRLTVHHPQVNVPVLWWRSVGSTHTAFVMETLIDEIARTTKQDPV
AYRMNLFGDKQPRHRAALQLAVDKSGYGKKKLAEGRAWGVAVHESFESVVAYVVEASVKDGQPVLHRVTSGV
HCNLAVNPRSVEAQVQGAAVMGLSTCLQGSAITLKDGVVQQGNFGDFTVARITQMPEFDVHIVPSADAPKG
MGEPGLPPLAPAFANAVARLTGKPLRQLPFDLASA
>SEQF3743||SEQF3743.1_03343
MPQLKRRHFVLGTLGAAGALVVGWAATPAASRLVPGAPLPAGNGQVALNGWVKVGSDDTVTLVMTQSEMG
QGTHTGLAMLLAEEMDASMSQVRLEQAGFDAIYNNQAVILDALPFKPGDEGFGKRAAHQVVGKLLRTIPGLS
GTGGSSSVTDQWGPVREAGASARLMLVGAAAAQWQVPASECRTEAGRVLHAASNRSARFGELAAKAAQQP
LPTAVKLKAPADFRVIGQPQRRIDNAGKLNGTAIYGIDVLPPGLLYASIAMCPTLAGRVAHFDAKAAEALPGVRK
VVALEPVAASLVGTGSTAGGVAVIADTPYHAMRALKKVDITWDHGPAASLSSAEIIDRLARTLDADAGNARLDT
GDVAAALKSAARTIEVEYRVPFLAHATMEPMNCTVQFKDGAATVWAPTQAPGFARAAVAKTLGIDAEKVTLH
VTYLGGGFGRRYFIDFLVQAAQLAREAGGAPVQLIWSREEDMTHDFYRPAYVARCRAGFDAAGALVAWQTTS
AGSSLGAPSFMDNSTDGAWNTAYDFPQARVAHVPVESAVTTGIWRSVAHSQNGFFVESFIDECAVAAGKDPV
AFRAGLLAKDARHLAVLKRVAELSKWGEPLADGPDGAKRARGVAIHRAFGSIVAQVAEVSVTPERQIRVHRVV
CVIDCGLPVNPNLIRQQMEGGIVFGLSAALRGEITVARGQVQQSNFHDYTPLRIDECPVIEVDILAGTGAEVPPG
GVGEPGTPPIAPAVANAVFALTGQRLRSLPLRLA
>SEQF3744||SEQF3744.1_04234
MLPHIDYHDLPRSLQRLMARPAADEPAALPRRSFLKIVGASGFALGTFPHLALAQAGAAAPAAGGLKPTQQPLA
FVQIAPNGEVMVTINRLEFGQGVQTGLPMILAEELDADWALVRSRSGTNDAAYADPFFGMHLTGGSNTIKNSF
VQYRELGARARAMLLSAAAARWKVDASTLRTQAGTVLGPGGRKASYGELAEAAMALPVPEKVVLKDPKDFRII
GRPTQRLDAAAKSSGRQDFGIDVKHPGQLTAVVAHPPVFGARLASVDDSAARAVKGVKAVVRIPLDRGAEGVA
VVADGYWPAKLGRDALKLQWDTAAVEKVDSDKQLAQYRELADRPGNRKFDADMAPLASAPRKLEAEFVFPYL
AHAPMEPLNCTVKLSDGRAELWVGTQSADLDGFAAARALKLEPAQVKVNVQMAGGGFGRRFVSTSDFVVEA
CEIAKATRAAGLDVPVRLLWSREDDIKGGYYRPMHLHRARIGFDERGTILAWDHVIVGQSITAGTVFAGAMVK
DGIDATAVEGMRDPYPLPMRLTVHHPQLNVPVLWWRSVGSTHTAFVMETLIDEIARSTKQDPVAYRMQLFGD
KHPRHRAALQLAVDKSGYGKKKLADGRAWGVAVHESFESVVAYVVEASVKDGQPVLHRATSGVHCNLAVNPR
SVEAQVQGAAVMGLSTCLPGSAITLKDGVVQQGNFGDFTVARITQVPEFDIHIVPSADAPKGMGEPGLPPLAP
AFANAVAQLTGKPLRRLPFDLA
>SEQF3744||SEQF3744.1_03168
MLIDRLTEHAPADSPALSRRGFLSIGAALGGGLLIGVGLGAMPEAAEAAPAGTPPWTPNAFVKIAPDGRVTVT
MGYIEMGQGTYTSVPMLIAEELEVDLAAVRVEHAPPDDKLYGNPLLGFQVTGGSTTIRAAYEPLRRAGATARTV
LVQAAAQRWKVAPDSCKATRGEVVHAASGRRLKYGALVADAAKLPLPEKVALKAPADFRLIGTPAKRLDSPAKV
DGSALYGIDAKVPGMKVATLAQSPVFGGRLRSVDDSKAKAVRGVRQIVRLDDCVAVVADHMGAAKKGLAALA
IEWDGGANAGIDSAAIVESMMQASLQTGAVARNEGDDAAAMTGAAQRLEAVYEVPFLVHAAMEPLNCTVR
VRPDGCEVWVGTQVITRARAAAAAASGLPVEKVTVHNHLLGGGFGRRLEVDSITRAVQIAKQVDGPVKVVW
TREEDTQHDMYRPYFYDRVSAGLDAQGRPVAWRHRITGSSILKRWLPPAYNNGVDPETIDGAEKPPYALPNIR

VEYVNHEPPVPTAFWRGVGPTHNVFVVESFIDELAHAAKADPVDYRRALLGANPRAKAVLELAAQKAGWGTP
LPPQRGRGVSLQFAFGTFMALVAEVEVPKDGGEPVVRRVVCALDTGVVVNPDTVQAQVQSGVIFGISGALWG
EATLKDGRIEQSNFHDVRPLRINETPAIETHIVASGEAPGGMGEPGTSGIAPAITNAIFAVTGKRVRRLPVSRGLTS
A
>SEQF3745||SEQF3745.1_01823
MSAAAGISRRAALQAGGLALAFTWVGGGKAFAAISARQQPADAAAALADGNPAFAPNAFIRIDADGGVRLV
MPMVEMGQAIYTGSAMLLAEELGVELDQVRVEHSPPNEALYGMPLLGGQITGGSTSTRGTYAVLREAGAVAR
TLLVGAAAAQWSVDPTSCTVARGVVSHPASNRQLGFGALAAAAAKLPMPAKVALKEPKDFKLIGLPLRRVDSA
SKVDGSTQFGIDVRVPGMKVATVKASPTLGGVLASVDDKAARAIPGVLDVLRIKDAVAVVGEHFWAAKRGLDA
LKIQWTPGQNATLTTQQLRSALADALAKDKAIVGKETGKRPEGTLVQATYDLPMLAHATMEPLNTTVHVRPDG
CEIWVGTQVPARCVSVAAKITGLAADKVVLHNQYLGGGFGRRLETDSVEQAVAFARQVPYPLKVVWTREEDIR
HDIVRPMYHDDISAVVDGDGQILWFGDRISGGTVLGRWAPAFMGKDGMDGDLIECVAEPCYDLPNLKVEWV
RHDMPSGLNVGWWRGVGPTHNLFVLESFIDELAQRAKKDPVAYRRAMLKKNPRTLAVLDLAADKIGWGKGA
LPPRVGRGVAVGDPFGSRVCAMVEVEVTPQGEVRLRRAVVALDCGIAVNASSIEAQIQGGLLFGLSAALFSEITL
REGAIEQSNFHDYRMLRINESPPVEVHTVRSSEAPGGLGEVGTAIAAPALANAIFAATGVRLRALPVNRALLVQD
KEALKKKIADAGYAAPDERSAA
>SEQF3746||SEQF3746.1_02924
MNSLKHAGAGMDRRYFLTASAAIGGGLVVSLATGPLGHVAAATPAATAFQPDASVTIGSDGLVSFTLPLVEMGQ
GTYTSIPMLIAEELEVPLSQVRIVHAPSNPKLFTNPLLGVQATGGSTAVRAAWEPMRKAGASARTMLVAAAART
WNVPPASCRATRATVVHTPTGRVLGYGALAQRAAKEAVPEQVELKAPSQYRLIGTPAKRLDLSGKVNGSAVFGI
DVRLPGMKIAAVAACPVFGGKLASVDDTKARAVAGVRQIVKLDDAVAVIADHNGAARKGLAALQIQWDEGA
NAKFSTEVWAGQLKEASKGRGVSAAKIGNVDAALAAAAVKVQAEYWAPNLAHATLEPMNCTVHWRKTECE
VWLGTQALARAQEGVSRVTGLPVDKVIVHNHLIGGGFGRRLDVDYVEQAALIARQVEGPVKVIWSREEDMQ
HDVYRPFWYDQLSAALDASGKPTAFRHRVIGSSVVARWIPAWMKDGLDPDAVDAAQSPYEFENIDVEYVRHE
PPPPLTTGFWRGVGPTHNAFVVEGFIDELANAAKQDPMAFRRALLARQPRVMAVLDQVAQRSGWGTPLPAR
CGRGVALLAKESWESILAQVVEVQVGNDGLLTIKRITSALDCGQMINPDGVTAQVQGATVFGLTAALYGNITFK
DGRVVQSNFHDYQMVRMNEVSPMDISLITNHEKPGGMGEIGTTLVAPALVNAIHAATGVRLRKLPIDTDLLKLT
>SEQF3746||SEQF3746.1_05819
MLPNIDYDELPRALQRLMARPSTDEAATLPRRSFLKMAGAGGLVLGAFPHLAAAQADGAKPAAGGLKPTQQP
SAFVQIAPNGEVTVTINRLEFGQGVQTGLPMILAEELDADWALVRSRSGTNDAAYADPLFGMHLTGGSNSIKN
SFTQYRELGARARAMLMSAAAARWKVDVSTLRTQAGTVIGPGGRKLGYGELAEAAMALPVPEKVVLKDPKDF
RIIGRATTRLDARAKSSGRQDFGIDVRQPGQLTAVVAHPPVFGARLASVDDSAARAVKGVKAVVRIPLDRGAEG
VAVVADGYWPAKLGRDALKLQWDTAAVEKVDSDRQLVQYRELAGRPGNRKFDADMAPLANAPRKLEAEFVF
PYLAHAPMEPINCTVKLSDGRAELWVGTQSADLDGQAAARTLKLDAAQVKVNVQMAGGGFGRRFVGSSDF
VVEACEIAKATRAAGLDAPVRLLWSREDDIKGGYYRPMHLHRARIGFDERGKILAWDHVIVGQSITSGTVFGG
MMVKDGIDATAVEGMRDPYPVPMRLTVHHPEVNVPVLWWRSVGSTHTAFVMETLIDEIARSTKQDPVAYRM
QLFGDKHPRHRAALQLAVDKSGYGKKKLADGRAWGVAVHESFESVVAYVVEASVKDGQPVLHRVTSGVHCNL
VVNPRSVEAQVQGAAVMGLSTCLAGSAITLKDGVVQQGNFGDFTVARITQVPEFDIHVVPSADAPKGMGEP
GLPPLAPAFANAIAQLTGKPLRQLPFNLA
>SEQF3747||SEQF3747.1_04479
MLPTHIDPTELPRTLQRLMAAGQQQPEDTAALPRRSFLKLAGVGGLALGAFPHLAVAQATGKGPAASALKPAQ
QPSAFVQIAPNGEVTVTHNRLEFGQGVQTGLPMILAEELDADWSLVRSKSGTNDAAYHDPVFGMHITGGSNS
IKNSFTQYRELGARARAMLLSAAAARWNVDVATLRTQAGTVLGPNGRKLGYGELAEAAMALPVPEKVTLKDP
KDFRLIGRPTTRLDARAKCSGRQDFGIDVKHAGQLTAVVAHPPVFGARLASVDDSAARAVKGVKAVLRVPLDR

GAEGVAVVADGYWPAKLGRDALKLQWDTAAAEKVDSDKQLAQYRELARQPGARKFDADMAPLAKAPRQLE
AEFVFPYLAHAPMEPLNCTVKLSGDRAELWVGTQCAGLDGVAAARTLGLKPEQVRVNVQMAGGGFGRRFVS
TSDVIVEACEIAKAARAAGLNAPVRLLWSREDDVKGGYYRPMHLHHARIGFDERGKVLAWDHVIVGQSITTGT
VFGEFQVKNGIDATATEGMRDPYPLPMRLTVHHPKVNVPVLWWRSVGSTHTAFVMETLLDEIARSTRQDPVA
YRMQLFGDKHPRHRAALQLAVDQSGYGKKQLPAGRAWGVAVHESFESVVAYVVEASVKDGQPVLHRATAGV
HCNLAVNPRSVEAQVQGAAVMGLSMCLPGAAITLKDGEVQQSNFGDFTVARITDMPEFAVHIVPSAEPPKGI
GEPGLPPLAPAFANAIAQLTGKPLRQLPFNLA
>SEQF3747||SEQF3747.1_00374
MKPAAGISRRSALQAGGLALAFTWFGAGKAFAAISPRQQPGDAAAALADGNPAFAPNAFVRIDADGGVRLV
MPMAEMGQAIYTGSAMLLAEELGVELDQVRVEHSPPNEALYGMPLLGGQITGGSTSTRGTYGVLREAGAVAR
TLLVSAAAEQWKVDPESCTVARGVVSHAASNRRLGFGALASAAAKLPMPEKVTLKEPKDFKLIGQPLRRVDSA
GKVNGSTQFGIDVRLPGMKVATVRACPTLGGVLASVDDKAARAIPGVIDVLRIKDAVAVVGEHFWAAKRGLDA
LTIQWTPGQNAALTTLQLRAALANALAKDKAILGKETGKRPEGTLVQATYDLPMLAHATMEPLNTTVHVRPDQ
CEIWVGTQVPTRCVSAAAKIAGVAEDKVVLHNQYLGGGFGRRLETDSVEQAVAFAKQVPYPLKVVWTREEDIR
HDIVRPMYHDDISAVVDSDGHILWFGDRIAGGTVLGRWAPAFMGKDGMDSDLIECVAEPCYDLPNLKVEWV
RHDMPAGLNVGWWRGVGPTHNLFVMESFIDELAHRAKKDPVAYRRAMLKKNPRTLAVLDLAASKIGWGQG
ALAARVGRGVAVGDAFGSRVCAIVEAEVTPQGEVRMRRAVVAVDCGIAVNAGSIEAQIQGGLLFGLSAALFSEI
TLREGAIEQSNFHDYRMLRINEAPPVEVHTVKSGEAPGGLGEVGTAIAAPALANAIFAATGVRLRALPVNRALLA
QDKEALKKKIADSGFSGSNGLDARSAV
>SEQF3748||SEQF3748.1_04873
MKPAAGISRRSALQAGGLALAFTWFGAGKAFAAISPRQQPGDAAAALADGSPAFAPNAFVRIDADGGVRLVM
PMAEMGQAIYTGSAMLLAEELGVELDQVRVEHSPPSEALYGMPLLGGQITGGSTSTRGTYGVLREAGAVARTL
LVGAAAAQWKVDPAGCTVARGVVSHAPSGQQLGFGALAGAAAKLPMPEKVTLKEPKDFKLIGQPLRRVDSA
GKVDGSTQFGIDVRLPGMKVATVRACPTLGGVLASVDDKAARAIPGVVDVLRIKDAVAVVGEHFWAAKRGLD
ALKVQWTPGQNAALTTQQLRASLANALAKDKAIVGKETGKRPEGTLVQATYDLPMLAHATMEPLNTTVHVRP
DQCEIWVGTQVPTRCVSAAAKIAGVAEDKVVLHNQYLGGGFGRRLETDSVEQAVAFAKQVPYPLKVVWTREE
DIRHDIVRPMYHDDISAVVDGDGQILWFGDRIAGGTVLGRWAPAFMGKDGMDSDLIECVAEPCYDLPNLKVE
WVRHDMPAGLNVGWWRGVGPTHNLFVMESFIDELAHRAKKDPVAYRRAMLKKNPRTLAVLDLAAGKIGW
GQGALAARVGRGVAVGDAFGSRVCAIVEAEVTPQGEVRMRRAVVAVDCGIAVNTGSIEAQIQGGLLFGLSAAL
FSEITLREGAIEQSNFHDYRMLRINEAPTVEVHTVKSGEAPGGLGEVGTAIAAPALANAIFAATGVRLRALPVNR
ALLAQDKEALKKKIADAGPSGLGARSAA
>SEQF3748||SEQF3748.1_04064
MLPTHIDSTELPRILQRLMAASPQQPEDTAALPRRSFLKLAGAGGLALGAFPHMAMAQATGKQAAASTLKPTQ
QPSAFVQIAPNGEVTVTHNRLEFGQGVQTGLPMILAEELDADWNLVRSKSGTNDAAYHDPVFGMHLTGGSN
SIKNSFTQYRELGARARAMLLSAAAARWNVDVSTLRTQAGTVLGPNGRKLGYGELAEAAMALPVPEKVRLKD
PKDFKLIGHPTTRLDARAKSSGRQDYGIDVKHAGQLTAVVAHPPVFGARLASVDDSAARAVKGVKAVLRVPLDR
GAEGVAVVADGYWPAKLGRDALKLQWDTAAVEKVDSGRQLVQYRELAKQPGARKFDADMAPLAKAPRQLEA
EFVFPYLAHAPMEPLNCTVKLSGDSAELWVGTQCAGLDGAAAARALGLKPEQVRVNVQMAGGGFGRRFVST
SDVIVEACEIAKAARAAGLNAPVRLLWSREDDIKGGYYRPMHLHRARIGFDERGKVLAWDHVIVGQSITAGSVF
EPFQVKNGIDATATEGMRDPYPLPMRLTVHHPKVNVPVLWWRSVGSTHTAFVMETLLDEIARSTKQDPVAYR
MQLFGDKHPRHRAALQLAVDRSGYGKKQLPAGRAWGVAVHESFESVVAYVVEASVKDGQPVLHRATAGVHC
NLAVNPRSVEAQVQGAAVMGLSMCLPGGAITLKDGEVQQGNFGDFTVPRITDMPEFAVHIVPSAEPPKGIGE
PGLPPLAPAFANAIAQLTGKPLRQLPFDLADTKPA
>SEQF3749||SEQF3749.1_00820

MSAAAGISRRAALQAGGLALAFTWVGGSKAFAAISARQQPADAAAALADGNPAFAPNAFIRIDADGGVRLVM
PMVEMGQAIYTGSAMLLAEELGVELDQVRVEHSPPNEALYGMPLLGGQITGGSTSTRGTYAVLREAGAVARTL
LVGAAAAQWSVDPASCTVARGVVSHAASNRQLGFGALAAAAAKLPMPAKVALKEPKDFKLIGQPLRRVDSAG
KVNGSTQFGIDVRVPGMKVATVKASPTLGGVLASVDDKAARAIPGVIDVLRIKDAVAVVGEHFWAAKRGLDAL
KIRWTPGQNATLTTQQLRSALADALAKDKAIVGKETGKRPEGTLVQATYDLPMLAHATMEPLNTTVHVRPDGC
EIWVGTQVPARCVSVAAKITGLAEDKVVLHNQYLGGGFGRRLETDSVEQAVAFAKQVTYPLKVVWTREEDIRH
DIVRPMYHDDISAVVDGDGQILWFGDRISGGTVLGRWAPAFMGKDGMDGDLIECVAEPCYDLPNLKVEWVR
HDMPSGLNVGWWRGVGPTHNLFVLESFIDELAQRAKKDPVAYRRALLKKNPRTLAVLDLAAEKIGWGKGALP
ARVGRGVAVGDPFGSRVCAMVEVEVTPQGEVRLRRAVVALDCGIAVNASSIEAQIQGGLLFGLSAALFSEITLR
DGAIEQSNFHDYRMLRINESPPVEVHTIKSGEAPGGLGEVGTAIAAPALANAIFAATGVRLRALPVNRALLVQDK
EALKRKIADAPQAAPGERSTA
>SEQF3750||SEQF3750.1_03757
MKPAAGISRRSALQAGGLALAFTWFGAGKAFAAISPRQQPGDAAAALADGNPAFAPNAFVRIDADGGVRLV
MPMAEMGQAIYTGSAMLLAEELGVELDQVRVEHSPPSEALYGMPLLGGQITGGSTSTRGTYGVLREAGAVAR
TLLVGAAAAQWKVDPAGCTVARGVVSHAASNRQLGFGALASAAAKLPMPEKVTLKEPKDFKLIGQPLRRVDS
AGKVDGSTQFGIDVRLPGMKVATVRACPTLGGVLASVDDKAARAIPGVVDVLRIKDAVAVVGEHFWAAKRGL
DALKVQWTPGQNAALTTQQLRASLANALAKDKAIVGKETGKRPEGTLVQATYDLPMLAHATMEPLNTTVHVR
PDQCEIWVGTQVPTRCVSAAAKIAGVAEDKVVLHNQYLGGGFGRRLETDSVEQAVAFAKQVPYPLKVVWTRE
EDIRHDIVRPMYHDDISAVVDGDGQILWFGDRIAGGTVLGRWAPAFMGKDGMDSDLIECVAEPCYDLPNLKV
EWVRHDMPAGLNVGWWRGVGPTHNLFVMESFIDELAHRAKKDPVAYRRAMLKKNPRTLAVLDLAAGKIGW
GQGALAARVGRGVAVGDAFGSRVCAIVEAEVTPQGEVRMRRAVVAVDCGIAVNTGSIEAQIQGGLLFGLSAAL
FSEITLREGAIEQSNFHDYRMLRINEAPPVEVHTVKSGEAPGGLGEVGTAIAAPALANAIFAATGVRLRALPVNR
ALLAQDKEALKKKIANAGPSGLGARSAA
>SEQF3750||SEQF3750.1_05834
MLPTHIDSTELPRILQRLMAASQSQPEDTAALPRRSFLKLAGVGGLALGAFPHMAMAQATGRQAAASTLKPTQ
QPSAFVQIAPNGEVTVTHNRLEFGQGVQTGLPMILAEELDADWSLVRSKSGTNDPAYHDPVFGMHLTGGSNSI
KNSFTQYRELGARARAMLLSAAAARWKVDVATLRTQAGTVLGPNGRKLGYGELAEAAMALPVPEKVRLKDPK
DFKLIGHPTTRLDARAKSSGRQDFGIDVKHAGQLTAVVAHPPVFGARLASVDDSAARAVKGVKAVLRVPLDRG
AEGVAVVADGYWPAKLGRDALKLQWDTAAVEKVDSGRQLVQYRELAKQPGARKFDADMAPLAKAPRRLEAE
FVFPYLAHAPMEPLNCTVQLSGDSAELWVGTQCAGLDGAAAARALGLKPEQVRVNVQMAGGGFGRRFVSTS
DVIVEACEIAKAARAAGLNAPVRLLWSREDDIKGGYYRPMHLHHARIGFDERGKVLAWDHVIVGQSITAGSVF
EPFQVKNGIDATATEGMRDPYPLPMRLTVHHPKVNVPVLWWRSVGSTHTAFVMETLLDEIARSTRQDPVAYR
MQLFGDKHPRHRAALQLAVDRSGYGKKQLPAGRAWGVAVHESFESVVAYVVEASVKDGQPVLHRATAGVHC
NLAVNPRSVEAQVQGAAVMGLSMCLPGGAITLKDGEVQQGNFGDFTVPRITDMPEFAVHIVPSAEPPKGIGE
PGLPPLAPAFANAIAQLTGKPLRQLPFDLADTKPA
>SEQF3750||SEQF3750.1_04236
MSTSHESSGARQSVGLSRRSLLGLVATVGTAAGGGLLFGFSVPARSQGGARTSVIDGDGVEMPREGVFAPNAFI
QIDRRGKVTLVMPKVEMGQGVYTSIPMLLAEELEVPLASVTLAHAPPNEKLFADPMLAEQLTGASTSIRYAWEP
MRKAGASTRTLLVMAAAQRWKVDPSGCVAREGRVLHPQTSRSIGYGELVDVAARLQPPQNVRLKEPKDFKLI
GTRVKRLDSPEKVNGAAVFGLDVRVPGMVYAAIATCPVFGGRLAKFDDTHTRKIPDVQQVVSFDNGVAVIGA
HTWAARRGLQALEIQWDEGAGADVSTETIVTDLVKASQRSGAVARKEGDVEKAFANAKTRIDAVYHQPFLAH
ATMEPVNCTVHVRPDGCEVWVGTQVPGRVADAAAKVTGLPPEKIIVHNHLLGGGFGRRLEIDMVTQAVKVA
RQLNVPVKVVWTREEDIQHDMYRPYYYDRISAGLDANGMPVAWQHRIVGSSILARYAPAAMMKNGVDPDA
VAVAAELPYDLPNQLVDYVRQEPRGIPTAFWRGVGPTRSAFVVETFIDELATHAKVDPVKYRQALLGKSPRALN

VLNEAVRAARWAAGAPQVKGKGRGVSLMHAFGSFFAMVVDVTVDDGEVSVDHVTCAVDCGMAVNPDTIE
AQMQGGIVFGLTAALYGEITFKNGRVEQSNFNDYRVLRINETPDIDVHLVKSSEAPGGIGEPGTSALQPALANAI
FAATGKRLHALPIGDQLKSNASGGRR
>SEQF3751||SEQF3751.1_07537
MPALKRRHFLLGTAATLGALVVGWSASPTRQRLTTAEPLPATASGQVALNGWVKVSSDDTVTLMMAAAEMG
QGIHTGLAMLLAEEMDADLAQLRLEQAGYDPIYNNQAALLDNLPMFKPDDDGALKRGTRHVVSKLLREVPGL
WGSGGSSGIVDQWLPLREAGASARAMLVGAAAAAWGVPAAECEVATGRVSHPASQRSARFGELAARAAALP
LPAQPTLKDPSRYTLIGRPVHRLDSAAKLDGSARYGLDALPPDGLLHATLAMCPTLGGRVARFDDTAARAMPG
VRKVVALAPVAGGLGSGGMTSGAVAVIADTPWHAMRALDKVAIDWDHGAAASLSSRALIDQLAQSLDAPDQ
ADVHLETGDADAAMKSAERTIEAEYRVSFIAHATMEPMNCTVQFKDGAATVWVGVQGPGMLRGGIAKVLGI
EAAKVDIRLAYLGGGFGRRYLGDFVMQAAALARETDGMPVQLMWSREQDMAHDYYRPAYVARSKAGFDAQ
GRLVAWRVDTAGSSLGSPAFLSAAAEGTATTAYRFANARVAHRTMESAVTVGIWRSVNHSQNGFFTESFIDECA
HAAGQDPVAFRRALLAGSPAEARHLGVLQRVAAMSGWGTPLAPEADGTRRARGLALHRSFGSVVAQVAEVSL
TPERQIRVRRMYCAVDCGLAVNPNLVRQQMESAIVFGLSAALHGEITIERGQVQQSNFHDYAPLRMNECPQIE
IAIVPSSAPPTGVGEPGTPPVAPAVANAVFALTGQRLRSLPLRLA
>SEQF3751||SEQF3751.1_08286
MQIAPNGEVMVTINRLEFGQGVQTGLPMILAEELDADWALVRSRSGTNDAAYADPFFGMHLTGGSNTIKNSF
VQYRELGARARAMLLSAAAARWKVDASTLRTQAGTVLGPGGRQASYGELAEAAMALPVPEKVVLKDPKDFRI
IGRPTQRLDAAAKSSGRQDFGIDVKHPGQLTAVVAHPPVFGARLASVDDSAARAVKGVKAVVRIPLDRGAEGV
AVVADGYWPAKLGRDALKLQWDTAAVEKVDSDKQLAQYRELAGRPGNRKFDADMAPLANAPRKLEAEFVFP
YLAHAPMEPLNCTVKLSDGRAELWVGTQSADLDGIAAARALKLEPAQVKVNVQMAGGGFGRRFVSTSDFVV
EACEIAKATRAAGLDVPVRLLWSREDDIKGGYYRPMHLHRARIGFDERGRILAWDHVIVGQSITAGTVFAGAM
VKDGIDATAVEGMRDPYPLPMRLTVHHPQLNVPVLWWRSVGSTHTAFVMETLIDEIARSTKQDPVAYRMQLF
GDKHPRHRAALQLAVDKSGYGKKKLADGRAWGVAVHESFESVVAYVVEASVKDGQPVLHRVTSGVHCNLAV
NPRSVEAQVQGAAVMGLSTCLPGSAITLKDGVVQQGNFGDFTVARITQVPEFDIHIVPSADAPKGMGEPGLPP
LAPAFANAVAQLTGKPLRRLPFELA
>SEQF3752||SEQF3752.1_02467
MLPHIDYHDLPRSLQRLMARPAADEPAALPRRSFLKIVGASGFALGTFPHLALAQAGGAAPAAGGLKPTQQPL
AFVQIAPNGEVMVTINRLEFGQGVQTGLPMILAEELDADWALVRSRSGTNDAAYADPFFGMHLTGGSNTIKNS
FVQYRELGARARAMLLSAAAARWKVDASTLRTQAGTVLGPGGRQASYGELAEAAMALPVPEKVVLKDPKDFR
IIGRPTQRLDAAAKSSGRQDFGIDVKHPGQLTAVVAHPPVFGARLASVDDSAARAVKGVKAVVRIPLDRGAEGV
AVVADGYWPAKLGRDALKLQWDTAAVEKVDSDKQLAQYRELAGRPGNRKFDADMAPLANAPRKLEAEFVFP
YLAHAPMEPLNCTVKLSDGRAELWVGTQSADLDGIAAARALKLEPAQVKVNVQMAGGGFGRRFVSTSDFVV
EACEIAKATRAAGLDVPVRLLWSREDDIKGGYYRPMHLHRARIGFDERGRILAWDHVIVGQSITAGTVFAGAM
VKDGIDATAVEGMRDPYPLPMRLTVHHPQLNVPVLWWRSVGSTHTAFVMETLIDEIARSTKQDPVAYRMQLF
GDKHPRHRAALQLAVDKSGYGKKKLADGRAWGVAVHESFESVVAYVVEASVKDGQPVLHRVTSGVHCNLAV
NPRSVEAQVQGAAVMGLSTCLPGSAITLKDGVVQQGNFGDFTVARITQVPEFDIHIVPSADAPKGMGEPGLPP
LAPAFANAVAQLTGKPLRRLPFELA
>SEQF3752||SEQF3752.1_08649
MPALKRRHFLLGTAATLGALVVGWSASPTRQRLTTAEPLPATASGQVALNGWVKVSSDDTVTLMMAAAEMG
QGIHTGLAMLLAEEMDADLAQLRLEQAGYDPIYNNQAALLDNLPMFKPDDDGALKRGTRHVVSKLLREVPGL
WGSGGSSGIVDQWLPLREAGASARAMLVGAAAAAWGVPAAECEVATGRVSHPASQRSARFGELAARAAALP
LPAQPTLKDPSRYTLIGRPVHRLDSAAKLDGSARYGLDALPPDGLLHATLAMCPTLGGRVARFDDTAARAMPG
VRKVVALAPVAGGLGSGGMTSGAVAVIADTPWHAMRALDKVAIDWDHGAAASLSSRALIDQLAQSLDAPDQ

ADVHLETGDADAAMKSAERTIEAEYRVSFIAHATMEPMNCTVQFKDGAATVWVGVQGPGMLRGGIAKVLGI
EAAKVDIRLAYLGGGFGRRYLGDFVMQAAALARETDGMPVQLMWSREQDMAHDYYRPAYVARSKAGFDAQ
GRLVAWRVDTAGSSLGSPAFLSAAAEGTATTAYRFANARVAHRTMESAVTVGIWRSVNHSQNGFFTESFIDECA
HAAGQDPVAFRRALLAGSPAEARHLGVLQRVAAMSGWGTPLAPEADGTRRARGLALHRSFGSVVAQVAEVSL
TPERQIRVRRMYCAVDCGLAVNPNLVRQQMESAIVFGLSAALHGEITIERGQVQQSNFHDYAPLRMNECPQIE
IAIVPSSAPPTGVGEPGTPPVAPAVANAVFALTGQRLRSLPLRLA
>SEQF3753||SEQF3753.1_00609
MASTQASLKLSRRTVLKSTTGLVVAAYLAPFAGSALAATKARAAQAVFAPNAFIRVGTDNTVTVLVKHIEFGQGP
FTGLATIVAEEMDADWAQMRAEHAPADAKLYNNLAFGPVQGTGGSTAIANSYDQLRQAGATARALLVQAAAE
RWRVPAKDITVERGVLRHAASGKQGRFGEFALAASRLPAPAPTEVRLKDPSEFRLIGREGAVRKLDVPAKTNGT
AQFTIDIHEPNMLTVVVAHPPLFGAKVASFDAIEALAVKGVVEVKQVPTGVAVYAESTWPAIKGREKLRITWDES
AAEKRGSEQILAEYRAAARSPGAVADAHGNVDTTLASAERVIETEYAFPYLAHSPMEPLDGFLRWNDKGATARF
GSQIQTLDQAVIAGVLGLKPEQVAVETMLAGGSFGRRGEMGSDFANELAQVAKAIGPQRPVKLMWTRDDDV
QGGRYRPMFVHRMRGGLRGGKVVAWSDTIVGQSFIQGTMFESMLFKNGIDATMVEGANEIPYKIENFRCDL
HIQKVGVPTLSWRSVGSTHTGYAVECFVDQLLQTAGQDPVEGRMALFGDDPRFKTTLRAVADLAKWSGPAAG
SGRARGVSIVKAFGTYVAHIAEVSMGENGEPRVHKVWCAVDCGRVVNPDIVRAQMEGGIGFGLGHILYGEIRL
EGGRRVQRNFDTYRSLRIHEMPAIEVKIVDSTEKPTGVGEPGVPPIGPAVANALARLAGGERPRQLPMVREAA
>SEQF3753||SEQF3753.1_02520
MVTRRTVLLGSAGLVGALGALAVGWTALPPKPRLITTQPLPVGPGQSALNGWVKIGADNSVGIVMCKAEMG
QGIYTGLAMVLAEELDADWAQVRVERAPIDKIYQNIAATIDGLPFHPDDDGTLKAVASRVAGKAMREIGVMLT
GGSSSMRDLWLPMRQAGASARAMLVAAAAQQWKLPAAECRVEAGRVLHPSGKSVSFGELAAAAASQPLPD
HVLLKDPKTFKLIGQPRRRLEAASKMDGSARFGIDVLQPGMQYASVEMCPTLGGRVASFDAAEAQKLPGVKK
VLAVPGHNGGTGGVAVIADTPYHAMRAVKAVRVEWDHGPSASASTEEVYARLAQALDTDDGFNFYKHGDVD
AALKSAAKTVKAEYRAPYLAHATMEPMNCTAQFKDGRATVWAATQVPDLARSAAAKVLGLAPELVDVQVQLL
GGGFGRRCEVDVIAQAAAIARDAGGVPVQTIWSREQDMTHDFYRPACVSRFQAGLDASGNLLGWLNASAG
QAIVPQVMKRVFDLPGAGPDKTTSEGAFDQPYEWPAARIGHEAVELPVPVGFWRSVGHSHNAFFKESFLDEV
ATAAGRDPVALRAALLTKHPRHLKVLQRAAELSGWDRPLPASADGAKRARGVALHQSFGSIVAQVAEVSVSAD
KRIRVHRVVCVIDCGIAVNPNLIRQQMESAIVYGLSAALNDEVQIEKGQVQQTNFHNHPVLRLDECPVIETDIIA
SDESPEGVGEPGTPPIAPAVANALFALTGQRLRSLPLKLA
>SEQF3753||SEQF3753.1_00691
MNGHPLSRRRFLVSTSSAAGSLVIAFHLPGVAAAAQGKDFSPDAYLRVGADESVTIVVALVEMGQGTFTSIPMLI
AEELEVDLSRVRVEQAPADEKTYGHPLYGLQTTGGSASIQAAWAKLRQVGATARLMLVAAAAQTWSVPATECR
AENGTVRHLPSGRSASYGALAGTAATLPIPKDAPLKDPATFRLVGTPAHRLDTPSKVDGTAQFGIDVKVPGMKV
AAIAVCPFIGGRLISVDDARTRSVSGVRQVLTSSSAVAVVADHYGAARKGLAALRVRWDEGPSAAFSNKVVWGE
QLAQALSKQKGVVPVAEGSFEKANATAARRHRAVYESPPLAHSALEPLNCTIHVRKDGCDVWLGSQAPARVQS
LVAKAIGMAPEQVTVHNYLVGGGFGRKLDADYAETAAHLAKQVDFPLKVVFSREEDIQHDAYRPYFRDELAAVL
DRKGELVAFSHRSAGSAVIARYAPAWLSNGQDTDAVHTAETPYAVPNRYVEYVRHEPPAGLLTGNWRGVGPTH
HAFPNECFIDELAAMAKVDPLAFRERMLGKNPRALAMLRLAASKAGWGAAMPARRGRGIALVDAWGSYAAL
VTDITVGRDGSIKVDRMVCAVDCGLAVNPDGVEAQIESGIVYGLSAALYGTLTFEGGRVVQSNYHDYQPLRMH
EMPIVEVHILKSTLPPGGVGEMGTALVCPSLMNAVYSATGKRFRSYPVSADQLKTA
>SEQF3754||SEQF3754.1_01455
MVSRRTVLVGTAGVVGALMVGWTASPPRSRLVTADPLPVGPGQSALNGWVKIGADNSVGIVMCKAEMGQG
IYTGLAMVLAEELDADWAQVHVERAPVDKIYQNIVATIDGLPFHPDDDGTLKAVVERVAGKAMREIGLMLTGG
SSSMRDLWLPMRQAGASARAMLVAAAAQQWKVPAAECRVEAGRVAHPSGKSASFGELAAAAANQPLPEQV

VLKDPKTFKLIGQPRRRLDAASKMDGSARFGIDVLLPGMQYASVEMCPTLGGRVASFDAADAQKLPGVKKVLA
VPGYNGGTGGVAVIADTPYRAMRAVKAVRIEWDHGMTASVSTDEIYARLAQALDTDDSFSFYKHGDVDAALK
SAAKTVKAEYRAPYLAHATMEPMNCTVQFKDGRATVWAATQVPDVARSAAAKVLGIAPAEVDVQVTLLGGGF
GRRCEVDVIAQAAAIAREAGGVPVQTIWSREQDMTHDFYRPACVSRFQAGLDASGNLVGWLNTSAGQAIVP
QAMKRLFDLPGAGPDKTTSEGAFDQPYEWPAARIAHEVFELPVPVGFWRSVGHSHNAFFKESFLDEVAAAAG
RDPVAFRAALLARHPRHLKVLQRAAELSGWDRPLPAASDGTRRARGVALHQSFGSIVAQVAEVSVSADKRIRV
HRVTCVIDCGLAVNPNLVRQQMESGIVYGLSAALNDEVRIDKGQVQQTNFHNHPVLRLNECPVIETDIIASGES
PQGVGEPGTPPIAPAVANALFTLTGQRLRNLPLKLA
>SEQF3755||SEQF3755.1_08033
MPALKRRHFLLGTAATLGALVVGWSASPTRQRLTTAEPLPATASGQVALNGWVKVSSDDTVTLMMAAAEMG
QGIHTGLAMLLAEEMDADLAQLRLEQAGYDPIYNNQAALLDNLPMFKPDDDGALKRGTRHVVSKLLREVPGL
WGSGGSSGIVDQWLPLREAGASARAMLVGAAAAAWGVPAAECEVATGRVSHPASQRSARFGELAARAAALP
LPAQPTLKDPSRYTLIGRPVHRLDSAAKLDGSARYGLDALPPDGLLHATLAMCPTLGGRVARFDDTAARAMPG
VRKVVALAPVAGGLGSGGMTSGAVAVIADTPWHAMRALDKVAIDWDHGAAASLSSRALIDQLAQSLDAPDQ
ADVHLETGDADAAMKSAERTIEAEYRVSFIAHATMEPMNCTVQFKDGAATVWVGVQGPGMLRGGIAKVLGI
EAAKVDIRLAYLGGGFGRRYLGDFVMQAAALARETDGMPVQLMWSREQDMAHDYYRPAYVARSKAGFDAQ
GRLVAWRVDTAGSSLGSPAFLSAAAEGTATTAYRFANARVAHRTMESAVTVGIWRSVNHSQNGFFTESFIDECA
HAAGQDPVAFRRALLAGSPAEARHLGVLQRVAAMSGWGTPLAPEADGTRRARGLALHRSFGSVVAQVAEVSL
TPERQIRVRRMYCAVDCGLAVNPNLVRQQMESAIVFGLSAALHGEITIERGQVQQSNFHDYAPLRMNECPQIE
IAIVPSSAPPTGVGEPGTPPVAPAVANAVFALTGQRLRSLPLRLA
>SEQF3755||SEQF3755.1_06967
MLPHIDYHDLPRSLQRLMARPAADEPAALPRRSFLKIVGASGFALGTFPHLALAQAGGAAPAAGGLKPTQQPL
AFVQIAPNGEVMVTINRLEFGQGVQTGLPMILAEELDADWALVRSRSGTNDAAYADPFFGMHLTGGSNTIKNS
FVQYRELGARARAMLLSAAAARWKVDASTLRTQAGTVLGPGGRQASYGELAEAAMALPVPEKVVLKDPKDFR
IIGRPTQRLDAAAKSSGRQDFGIDVKHPGQLTAVVAHPPVFGARLASVDDSAARAVKGVKAVVRIPLDRGAEGV
AVVADGYWPAKLGRDALKLQWDTAAVEKVDSDKQLAQYRELAGRPGNRKFDADMAPLANAPRKLEAEFVFP
YLAHAPMEPLNCTVKLSDGRAELWVGTQSADLDGIAAARALKLEPAQVKVNVQMAGGGFGRRFVSTSDFVV
EACEIAKATRAAGLDVPVRLLWSREDDIKGGYYRPMHLHRARIGFDERGRILAWDHVIVGQSITAGTVFAGAM
VKDGIDATAVEGMRDPYPLPMRLTVHHPQLNVPVLWWRSVGSTHTAFVMETLIDEIARSTKQDPVAYRMQLF
GDKHPRHRAALQLAVDKSGYGKKKLADGRAWGVAVHESFESVVAYVVEASVKDGQPVLHRVTSGVHCNLAV
NPRSVEAQVQGAAVMGLSTCLPGSAITLKDGVVQQGNFGDFTVARITQVPEFDIHIVPSADAPKGMGEPGLPP
LAPAFANAVAQLTGKPLRRLPFELA
>SEQF3756||SEQF3756.1_08485
MLPHIDYHDLPRSLQRLMARPAADEPAALPRRSFLKIVGASGFALGTFPHLALAQAGGAAPAAGGLKPTQQPL
AFVQIAPNGEVMVTINRLEFGQGVQTGLPMILAEELDADWALVRSRSGTNDAAYADPFFGMHLTGGSNTIKNS
FVQYRELGARARAMLLSAAAARWKVDASTLRTQAGTVLGPGGRQASYGELAEAAMALPVPEKVVLKDPKDFR
IIGRPTQRLDAAAKSSGRQDFGIDVKHPGQLTAVVAHPPVFGARLASVDDSAARAVKGVKAVVRIPLDRGAEGV
AVVADGYWPAKLGRDALKLQWDTAAVEKVDSDKQLAQYRELAGRPGNRKFDADMAPLANAPRKLEAEFVFP
YLAHAPMEPLNCTVKLSDGRAELWVGTQSADLDGIAAARALKLEPAQVKVNVQMAGGGFGRRFVSTSDFVV
EACEIAKATRAAGLDVPVRLLWSREDDIKGGYYRPMHLHRARIGFDERGRILAWDHVIVGQSITAGTVFAGAM
VKDGIDATAVEGMRDPYPLPMRLTVHHPQLNVPVLWWRSVGSTHTAFVMETLIDEIARSTKQDPVAYRMQLF
GDKHPRHRAALQLAVDKSGYGKKKLADGRAWGVAVHESFESVVAYVVEASVKDGQPVLHRVTSGVHCNLAV
NPRSVEAQVQGAAVMGLSTCLPGSAITLKDGVVQQGNFGDFTVARITQVPEFDIHIVPSADAPKGMGEPGLPP
LAPAFANAVAQLTGKPLRRLPFELA

>SEQF3756||SEQF3756.1_06560
MPALKRRHFLLGTAATLGALVVGWSASPTRQRLTTAEPLPATASGQVALNGWVKVSSDDTVTLMMAAAEMG
QGIHTGLAMLLAEEMDADLAQLRLEQAGYDPIYNNQAALLDNLPMFKPDDDGALKRGTRHVVSKLLREVPGL
WGSGGSSGIVDQWLPLREAGASARAMLVGAAAAAWGVPAAECEVATGRVSHPASQRSARFGELAARAAALP
LPAQPTLKDPSRYTLIGRPVHRLDSAAKLDGSARYGLDALPPDGLLHATLAMCPTLGGRVARFDDTAARAMPG
VRKVVALAPVAGGLGSGGMTSGAVAVIADTPWHAMRALDKVAIDWDHGAAASLSSRALIDQLAQSLDAPDQ
ADVHLETGDADAAMKSAERTIEAEYRVSFIAHATMEPMNCTVQFKDGAATVWVGVQGPGMLRGGIAKVLGI
EAAKVDIRLAYLGGGFGRRYLGDFVMQAAALARETDGMPVQLMWSREQDMAHDYYRPAYVARSKAGFDAQ
GRLVAWRVDTAGSSLGSPAFLSAAAEGTATTAYRFANARVAHRTMESAVTVGIWRSVNHSQNGFFTESFIDECA
HAAGQDPVAFRRALLAGSPAEARHLGVLQRVAAMSGWGTPLAPEADGTRRARGLALHRSFGSVVAQVAEVSL
TPERQIRVRRMYCAVDCGLAVNPNLVRQQMESAIVFGLSAALHGEITIERGQVQQSNFHDYAPLRMNECPQIE
IAIVPSSAPPTGVGEPGTPPVAPAVANAVFALTGQRLRSLPLRLA
>SEQF3757||SEQF3757.1_06129
METTKPLQPSRRTVLKGAAGLVIGAYVAPFAGNALAATKAKAKAAQGVFAPNAFIRVAPDSTVTVLIKHIEFGQG
PFTGLATIVAEEMDADWSQMRAEHAPADAKLYNNLAFGPVQGTGGSTAVANSFDQLRQAGATARALLVQAA
AERWKVPAKEITVERGVLRHAASGKQGRFGDFAVAASRLPAPAEVKLKDPSEFRLIGREGAVRKLDVPAKTNGT
AQFTIDIHEPKMLTVVVAHAPLFGAKVASFDAIEALAVKGVVEVKQVPTGVAVYAESTWPAIKGREKLRITWDES
GAEKRGSEQILAEYRAAARSPGAVADAHGNLDTGFASADKVIEAEYAFPYLAHSPMEPLDGFLRFNDQGATARF
GSQIQTLDQGAIAGVLGLKPEQVALETMLAGGSFGRRGEMASDFATELAQVAKAIGPARPVKLMWTRDDDV
QGGRYRPLFVHRMRGGLRGGKVVAWSDTIVGQSFIQGTVFEPMLFKNGIDATMVEGANEIPYKIENFRCDLHI
QKVGVPTLSWRSVGSTHTGYAVECFVDELLQAAGQDPVEGRIALFGDDPRFKTTLRAVADIAKWSGPAAGSGR
SRGVSIVKSFGTYVAHIAEVSMGENGEPRVHKVWCAVDCGRVVNPDIVRAQMEGGIGFGLGHILYGEIRLEGG
RRVQRNFDTYRSLRIHEMPAIEVKIVESTEKPTGVGEPGVPPIGPAVANALARLTGGERPRQLPMVREAA
>SEQF3757||SEQF3757.1_02581
MVTRRTVLLGSAGVAGALGALMVGWTAMPPKPRLVTADPLPVGPGQSALNGWVKIGADNTVGIVMCKAE
MGQGIYTGLAMVLAEELDADWAQVRVERAPIDKIYQNIAATIDGLPFHPDDDGTLKAVASRVAGKAMREIGV
MLTGGSSSMRDLWLPMRQAGASARAMLVAAAAQQWKLPAAECRVEAGRVLHPSGKSVSFGELAAAAASQP
LPDHVLLKDPKTFKLIGQPRRRLEAASKMDGSARFGIDVLQPGMQYASVEMCPTLGGRVASFDAAEAQKLPGV
KKVLAVPGHNGGTGGVAVIADTPYHAMRAVKAVRVKWDHGPSASASTEEVYARLAQALDTDDGFNFYKHGD
VDAALKSAAKTVRAEYRAPYLAHATMEPMNCTAQFKDGRATVWAATQVPDLARSAAAKVLGLAPELVDVQV
QLLGGGFGRRCEVDVIAQAAAIARDAGGVPVQTIWSREQDMTHDFYRPACVSRFQAGLDASGNLLGWLNAS
AGQAIVPQVMKRVFDLPGAGPDKTTSEGAFDQPYEWPAARIGHEAVELPVPVGFWRSVGHSHNAFFKESFLD
EVATAAGRDPVALRAALLTKHPRHLKVLQRAAELSGWDRPLPASADGVKRARGVALHQSFGSIVAQVAEVSVS
ADKRIRVHRVVCVIDCGIAVNPNLIRQQMESAIVYGLSAALNDEVQIEKGQVQQTNFHNHPVLRLDECPVIETD
IIASDESPEGVGEPGTPPIAPAVANALFALTGQRLRSLPLKLA
>SEQF3758||SEQF3758.1_07719
MPALKRRHFLLGTAATLGALVVGWSASPTRQRLTTAEPLPATASGQVALNGWVKVSSDDTVTLMMAAAEMG
QGIHTGLAMLLAEEMDADLAQLRLEQAGYDPIYNNQAALLDNLPMFKPDDDGALKRGTRHVVSKLLREVPGL
WGSGGSSGIVDQWLPLREAGASARAMLVGAAAAAWGVPAAECEVATGRVSHPASQRSARFGELAARAAALP
LPAQPTLKDPSRYTLIGRPVHRLDSAAKLDGSARYGLDALPPDGLLHATLAMCPTLGGRVARFDDTAARAMPG
VRKVVALAPVAGGLGSGGMTSGAVAVIADTPWHAMRALDKVAIDWDHGAAASLSSRALIDQLAQSLDAPDQ
ADVHLETGDADAAMKSAERTIEAEYRVSFIAHATMEPMNCTVQFKDGAATVWVGVQGPGMLRGGIAKVLGI
EAAKVDIRLAYLGGGFGRRYLGDFVMQAAALARETDGMPVQLMWSREQDMAHDYYRPAYVARSKAGFDAQ
GRLVAWRVDTAGSSLGSPAFLSAAAEGTATTAYRFANARVAHRTMESAVTVGIWRSVNHSQNGFFTESFIDECA

HAAGQDPVAFRRALLAGSPAEARHLGVLQRVAAMSGWGTPLAPEADGTRRARGLALHRSFGSVVAQVAEVSL
TPERQIRVRRMYCAVDCGLAVNPNLVRQQMESAIVFGLSAALHGEITIERGQVQQSNFHDYAPLRMNECPQIE
IAIVPSSAPPTGVGEPGTPPVAPAVANAVFALTGQRLRSLPLRLA
>SEQF3758||SEQF3758.1_07656
MLPHIDYHDLPRSLQRLMARPAADEPAALPRRSFLKIVGASGFALGTFPHLALAQAGGAAPAAGGLKPTQQPL
AFVQIAPNGEVMVTINRLEFGQGVQTGLPMILAEELDADWALVRSRSGTNDAAYADPFFGMHLTGGSNTIKNS
FVQYRELGARARAMLLSAAAARWKVDASTLRTQAGTVLGPGGRQASYGELAEAAMALPVPEKVVLKDPKDFR
IIGRPTQRLDAAAKSSGRQDFGIDVKHPGQLTAVVAHPPVFGARLASVDDSAARAVKGVKAVVRIPLDRGAEGV
AVVADGYWPAKLGRDALKLQWDTAAVEKVDSDKQLAQYRELAGRPGNRKFDADMAPLANAPRKLEAEFVFP
YLAHAPMEPLNCTVKLSDGRAELWVGTQSADLDGIAAARALKLEPAQVKVNVQMAGGGFGRRFVSTSDFVV
EACEIAKATRAAGLDVPVRLLWSREDDIKGGYYRPMHLHRARIGFDERGRILAWDHVIVGQSITAGTVFAGAM
VKDGIDATAVEGMRDPYPLPMRLTVHHPQLNVPVLWWRSVGSTHTAFVMETLIDEIARSTKQDPVAYRMQLF
GDKHPRHRAALQLAVDKSGYGKKKLADGRAWGVAVHESFESVVAYVVEASVKDGQPVLHRVTSGVHCNLAV
NPRSVEAQVQGAAVMGLSTCLPGSAITLKDGVVQQGNFGDFTVARITQVPEFDIHIVPSADAPKGMGEPGLPP
LAPAFANAVAQLTGKPLRRLPFELA
>SEQF3759||SEQF3759.1_05618
MLIDRLTEHAPVDSPALSRRGFLSIGAALGGGLLIGVGLGAVPDAAEAAGASAGTPPWTPNAFVKIAPDGRVTV
TMGYIEMGQGTYTSVPMLIAEELEVDMAAVRVEHAPPDDKLYGNPLLGFQVTGGSTTIRAAYEPLRRAGATAR
TVLVQAAAQRWKVAPDSCKAARGEVVHAASGRRLKYGALVADAAKLPLPEKVALKAPADFRLIGTPAKRLDSPA
KVNGSALYGIDAKVPGMKVATLAQSPVFGGRLRSVDDSKAKAVRGVRQIVRLDDCVAVVADHMGAAKKGLAA
LAIEWDGGANAGVDSAAIVESMKQASLQTGAVARNEGDNAAAMTGAAQRLEAVYEVPFLVHAAMEPLNCT
VHVRPDGCEVWVGTQVITRARAAAAAASGLPVEKVTVHNHLLGGGFGRRLEVDSITRAVQIAKQVDGPVKVV
WTREEDTQHDMYRPYFYDRVSAGLDAQGRPVAWRHRITGSSILKRWLPPAYNNGFDPETIDGAEKPPYALPNI
RIEYVNHEPPVPTAFWRGVGPTHNVFVVESFIDELAHAAKADPVDYRRALLGANPRAKAVLELAAQKAGWGT
PLPPQRGRGVSLQFAFGTFMALVAEVEVPKDGGEPVVRRVVCALDTGVVVNPDTMQAQVQSGVIFGISGAL
WGEATVKDGRIEQSNFHDVRPLRINETPAIETHIVASGEAPGGMGEPGTSGIAPAITNAIFAVTGKRVRKLPVSR
GLTSA
>SEQF3759||SEQF3759.1_08283
MLPHIDYHDLPRSLQRLMARPAADEAAALPRRSFLKIVGASGFALGTFPHLALAQTGGAAPAAGGLKPTQQPL
AFVQIAPNGEVMVTINRLEFGQGVQTGLPMILAEELDADWALVRSRSGTNDAAYADPFFGMHLTGGSNTIKNS
FVQYRELGARARAMLLSAAAARWKVDPSTLRTQAGTVLGPGGRKASYGELAEAAMALPVPEKVVLKDPKDFR
IIGRPTQRLDAAAKSSGRQDFGIDVKHPGQLTAVVAHPPVFGARLASVDDSAARAVKGVKAVVRIPLDRGAEGV
AVVADGYWPAKLGRDALKLQWDTAAVEKVDSDKQLAQYRELAGRPGNRKFDADMAPLASAPRKLEAEFVFPY
LAHAPMEPLNCTVKLSDGRAELWVGTQSADLDGLAAARALKLEPAQVKVNVQMAGGGFGRRFVSTSDFVVE
ACEIAKATRAAGLDVPVRLLWSREDDIKGGYYRPMHLHRARIGFDERGRILAWDHVIVGQSITAGTVFAGAMV
KDGIDATAVEGMRDPYPLPMRLTVHHPQLNVPVLWWRSVGSTHTAFVMETLIDEIARSTKQDPVAYRMQLFG
DKHPRHRAALQLAVDKSGYGKKKLADGRAWGVAVHESFESVVAYVVEASVKDGQPVLHRVTSGVHCNLAVN
PRSVEAQVQGAAVMGLSTCLPGSAITLKDGVVQQGNFGDFTVARITQVPEFDIHIVPSGDAPKGMGEPGLPPL
APAFANAVAQLTGKPLRQLPFELA
>SEQF3759||SEQF3759.1_08861
MKHDRMPMADTASTHGLTRRAFVATALAAGGGLALTVSLPPLEAKAAGAQPEAFAPNAVVRIGTDGLVTLVM
PRVEMGQGIYTAVALLIAEELEIDPRRVRLEHAPADETRYANPLTGGQITGGSTSVRSTWEPMRRAGATARLLLV
EAAAHQWKVEASTCRASDGAVEHVPTRRKLAYGQLARAAAGLPVPAEVPLKDLATLKRIGQPLLRLDGPDKVN
GRARYGLDASVAGMRIAAIAHCPVIGGRLASVDDARAMAVKGVRQVVRIDNAVAVVADHMGAARKGLAALE

IRWDEGANADYSTEKLIAALADASSRSGAVARKEGDTVAAARSAARTVDAVYQQPLLAHAPMEPVNCTVHVR
PDACELWVGTQVPARAQAAAAELTGLPIERVEVHNHLLGGGFGRRLDIDFVLQAVKIAKQVNGPVKVVWTREE
DTRHSTFRPYHYNRLSAALDARGRPVAWHHRVTASSILARWAPARFQNELDGDAIRDAAGPYGFANVLVEYVR
EEPPAGITTAFWRGVGHMQNAFPVECFVDELAHLAGSDAIAYRHDLLEKHPRARHVLELVARKSGWGGALPA
GKGRGVALTFCFGSYAAQVTEVSVDADGAVRVDRVVIAVDCGRLISPDTVVAQMQGGTAFGLSAVLYGNISIKD
GRVEQGNFDTYRVLRMDEMPVIETHLVPSTETPGGVGEVGTVLTAPSVLNAVFAATGKRIRRLPFAAEELKRA
>SEQF3760||SEQF3760.1_00074
MLPTHIDSTELPRILQRLMAASPQQPEDTAALPRRSFLKLAGAGGLALGAFPHMAMAQATGKQAAASTLKPTQ
QPSAFVQIAPNGEVTVTHNRLEFGQGVQTGLPMILAEELDADWNLVRSKSGTNDAAYHDPVFGMHLTGGSN
SIKNSFTQYRELGARARAMLLSAAAARWNVDVSTLRTQAGTVLGPNGRKLGYGELAEAAMALPVPEKVRLKD
PKDFKLIGHPTTRLDARAKSSGRQDYGIDVKHAGQLTAVVAHPPVFGARLASVDDSAARAVKGVKAVLRVPLDR
GAEGVAVVADGYWPAKLGRDALKLQWDTAAVEKVDSGRQLVQYRELAKQPGARKFDADMAPLAKAPRQLEA
EFVFPYLAHAPMEPLNCTVKLSGDSAELWVGTQCAGLDGAAAARALGLKPEQVRVNVQMAGGGFGRRFVST
SDVIVEACEIAKAARAAGLNAPVRLLWSREDDIKGGYYRPMHLHRARIGFDERGKVLAWDHVIVGQSITAGSVF
EPFQVKNGIDATATEGMRDPYPLPMRLTVHHPKVNVPVLWWRSVGSTHTAFVMETLLDEIARSTKQDPVAYR
MQLFGDKHPRHRAALQLAVDRSGYGKKQLPAGRAWGVAVHESFESVVAYVVEASVKDGQPVLHRATAGVHC
NLAVNPRSVEAQVQGAAVMGLSMCLPGGAITLKDGEVQQGNFGDFTVPRITDMPEFAVHIVPSAEPPKGIGE
PGLPPLAPAFANAIAQLTGKPLRQLPFDLADTKPA
>SEQF3760||SEQF3760.1_06135
MKPAAGISRRSALQAGGLALAFTWFGAGKAFAAISPRQQPGDAAAALADGSPAFAPNAFVRIDADGGVRLVM
PMAEMGQAIYTGSAMLLAEELGVELDQVRVEHSPPSEALYGMPLLGGQITGGSTSTRGTYGVLREAGAVARTL
LVGAAAAQWKVDPAGCTVARGVVSHAPSGQQLGFGALAGAAAKLPMPEKVTLKEPKDFKLIGQPLRRVDSA
GKVDGSTQFGIDVRLPGMKVATVRACPTLGGVLASVDDKAARAIPGVVDVLRIKDAVAVVGEHFWAAKRGLD
ALKVQWTPGQNAALTTQQLRASLANALAKDKAIVGKETGKRPEGTLVQATYDLPMLAHATMEPLNTTVHVRP
DQCEIWVGTQVPTRCVSAAAKIAGVAEDKVVLHNQYLGGGFGRRLETDSVEQAVAFAKQVPYPLKVVWTREE
DIRHDIVRPMYHDDISAVVDGDGQILWFGDRIAGGTVLGRWAPAFMGKDGMDSDLIECVAEPCYDLPNLKVE
WVRHDMPAGLNVGWWRGVGPTHNLFVMESFIDELAHRAKKDPVAYRRAMLKKNPRTLAVLDLAAGKIGW
GQGALAARVGRGVAVGDAFGSRVCAIVEAEVTPQGEVRMRRAVVAVDCGIAVNTGSIEAQIQGGLLFGLSAAL
FSEITLREGAIEQSNFHDYRMLRINEAPTVEVHTVKSGEAPGGLGEVGTAIAAPALANAIFAATGVRLRALPVNR
ALLAQDKEALKKKIADAGPSGLGARSAA
>SEQF3761||SEQF3761.1_05268
MLPHIDYHDLPRSLQRLMARPAADEPAALPRRSFLKIVGASGFALGTFPHLALAQAGGAAPAAGGLKPTQQPL
AFVQIAPNGEVMVTINRLEFGQGVQTGLPMILAEELDADWALVRSRSGTNDAAYADPFFGMHLTGGSNTIKNS
FVQYRELGARARAMLLSAAAARWKVDASTLRTQAGTVLGPGGRQASYGELAEAAMALPVPEKVVLKDPKDFR
IIGRPTQRLDAAAKSSGRQDFGIDVKHPGQLTAVVAHPPVFGARLASVDDSAARAVKGVKAVVRIPLDRGAEGV
AVVADGYWPAKLGRDALKLQWDTAAVEKVDSDKQLAQYRELAGRPGNRKFDADMAPLANAPRKLEAEFVFP
YLAHAPMEPLNCTVKLSDGRAELWVGTQSADLDGIAAARALKLEPAQVKVNVQMAGGGFGRRFVSTSDFVV
EACEIAKATRAAGLDVPVRLLWSREDDIKGGYYRPMHLHRARIGFDERGRILAWDHVIVGQSITAGTVFAGAM
VKDGIDATAVEGMRDPYPLPMRLTVHHPQLNVPVLWWRSVGSTHTAFVMETLIDEIARSTKQDPVAYRMQLF
GDKHPRHRAALQLAVDKSGYGKKKLADGRAWGVAVHESFESVVAYVVEASVKDGQPVLHRVTSGVHCNLAV
NPRSVEAQVQGAAVMGLSTCLPGSAITLKDGVVQQGNFGDFTVARITQVPEFDIHIVPSADAPKGMGEPGLPP
LAPAFANAVAQLTGKPLRRLPFELA
>SEQF3761||SEQF3761.1_03030
MPALKRRHFLLGTAATLGALVVGWSASPTRQRLTTAEPLPATASGQVALNGWVKVSSDDTVTLMMAAAEMG

QGIHTGLAMLLAEEMDADLAQLRLEQAGYDPIYNNQAALLDNLPMFKPDDDGALKRGTRHVVSKLLREVPGL
WGSGGSSGIVDQWLPLREAGASARAMLVGAAAAAWGVPAAECEVATGRVSHPASQRSARFGELAARAAALP
LPAQPTLKDPSRYTLIGRPVHRLDSAAKLDGSARYGLDALPPDGLLHATLAMCPTLGGRVARFDDTAARAMPG
VRKVVALAPVAGGLGSGGMTSGAVAVIADTPWHAMRALDKVAIDWDHGAAASLSSRALIDQLAQSLDAPDQ
ADVHLETGDADAAMKSAERTIEAEYRVSFIAHATMEPMNCTVQFKDGAATVWVGVQGPGMLRGGIAKVLGI
EAAKVDIRLAYLGGGFGRRYLGDFVMQAAALARETDGMPVQLMWSREQDMAHDYYRPAYVARSKAGFDAQ
GRLVAWRVDTAGSSLGSPAFLSAAAEGTATTAYRFANARVAHRTMESAVTVGIWRSVNHSQNGFFTESFIDECA
HAAGQDPVAFRRALLAGSPAEARHLGVLQRVAAMSGWGTPLAPEADGTRRARGLALHRSFGSVVAQVAEVSL
TPERQIRVRRMYCAVDCGLAVNPNLVRQQMESAIVFGLSAALHGEITIERGQVQQSNFHDYAPLRMNECPQIE
IAIVPSSAPPTGVGEPGTPPVAPAVANAVFALTGQRLRSLPLRLA
>SEQF3762||SEQF3762.1_03171
MPKLKRRHFVLGTAGAVGALVVGWAATPVASRLTGSQPLPAAPGQVALNGWVKVGSDNTVTLMMSQSEMG
QGTHTGLSMLLAEEMGASLEQIRLEMAGSDAIYNNQAAILDALPFRPGDEGAMKRSAEHVMGKLLRAIPGLS
GTGGSSSITDQWVPLREAGASARTMLLGAAAVLWQVPVAECRAEAGRVLHQASGRSATFGELAPKAAQQPLP
TQVALKKPADFKLIGQPVRRMDGAAKLDGSATFGLDVLPPGLLYASIAMCPTTGGRVASFDATAAQKLPGVRKV
MALEPVGATLIGTGATPGGVAVIADTPYHAMRAVKALAIEWDHGPAASLSSSEMIERLSQTLRTRPGNARLDD
GDVAGAFRSAAKTIEAEYRVPFLAHATMEPMNCTVQFKDGKATVWAPTQAPGFTRGAAAKALGIDADKVELH
VTYLGGGFGRRYSTDFVTQAATLARETGGAPVQLFWSREEDMAHDFYRPAYVARCRAGFDAAGALVAWQTVT
AGSSMGAPSLMDTATDGAWNTAYAFPNARVAHVPVESAMPTGVWRSVAHSQNGFFVESFIDECAAAAGKD
PVAFRAALLAKDERHLRVLRRVAELSNWSQPPAPGPDGAKRARGLAIHRSFGSIVAQVAEVSVSPDRQVRVHR
VTCVVDCGVAVNPNLIRQQMEGAIVYGLSAALHGEITVEKGRVQQSNFHDYMPLRMNECPAIEVEIAASGEAP
GGVGEPGTPPIAPAVANAVFALTGQRLRSLPLRLA
>SEQF3762||SEQF3762.1_05261
MKTLDRPASGIGIGGISRRAALQAGGLALAFTWLGTGKAFAAINPRQQPGDAAAALADGNPAFAPNAFIRIDA
DGGVRLVMPMAEMGQAIYTGSAMLLAEELGVDLDQVRVEHSPANEALYGMPLLGGQITGGSTSTRGTFGVLR
EAGAVARTLLVSAAAAQWKVDPASCTVERGVVSHAASGRQLGFGALAGAAAKLPMPAKVALKEPKDFRLIGQ
PLRRVDSAGKVNGTAQFGIDVRVPGMKVATVKASPTLGGVLASVDDKAARAIPGVIDVLRIKDAVAVVGEHFW
AAKCGLEALKLKWTPGRNATLTTQQLRAALADALAKDKAIVGKETGKRPEGTLVQATYDLPMLAHATMEPLNT
TVHVRPDGCDMWVGTQVPARCVGVAAKITGLAEDKVVLHNQYLGGGFGRRLETDSVEQAVAFAKQVPYPLK
VVWTREEDIRHDIVRPMYHDEISAVVDGEGRIQWFGDRISGGTVLGRWAPAFMGKDGMDGDLIECVAEPCY
DLPNLKVEWVRHDMPAGLNVGWWRGVGPTHNLFVMESFIDELAQRAKKDPVAYRRAMLQKNPRTLAVLDL
AASRIGWGQGSLPARVGRGVAVGDAFGSRVCAIIEAEVSPQGEVRMRRAVVAVDCGIAVNASSIEAQIQGGLL
FGLSAALYNEITLRDGAIEQSNFHDYRTLRINEAPAIEVHLVKSAEAPGGLGEVGTAIAAPALANAIFAATGVRLRA
LPVNRALLAQDKEALKKKVADAGPGKGFDEGRSA
>SEQF3763||SEQF3763.1_02377
MTTRNPPLDAPLGEPINLSRRRFLASTAVGALVIGFGLPLGASRAYAATGGPAERGTQVPAFLEIRPDGSVRLLSPF
MEGGQGTHTAMAQIVGEELDADPATFVVEAAPPGEAYVVMENGMRITGGSMSIRMSYPTMRRLGALARAM
LLQAGAEQLGVPLAQLTTQPGRVVHAASGRSLGYGELAGRALDMPVPDPATITLRDPSQFRWIGKPVKRLDAY
DKSTGKAQYSIDLKVDGMLHAAVQHAPRLGMTVGSLRNQSQVEAMKGVHSVHQLPGAVAVVAERWWHAK
RAVEAIQVEWLEAAAGSSVRAMPADFSSDKYRDFLAAQQGPARDDENEGDVAGALTNAKTKIEATYHNQYLN
HAQLEPPSALARFNPDGSLEVWLPNQAPDMFRADMAKRTGLSIEQITLHSPLLGGFFGRHFLYDSANPYPQAI
ALAKAVGRPIKLIWSREEEFLRDVLRPVAVVKFRAALDDKGLPVAIEAVSATEGPSEAIAGKQGEKLDPTALEGLS
GKSYAIPNKRIAQIYVKGPAMLGYWRSVGNSLNDFFYEAFLDELADKGGHDPYELRLHLLRDNKRLTTLLQAAGE
LSGGWKRGPYTAEDGTRRARGVAMASPFGSHAAVIAEVSIESGQVKVHHIWEAIDPGSIVNPAIVEAQVNGAV

ALGLSQTLLEEAVYVDGKPRARNYDLYPILPPSRMAQVHVRIVESGEKMGGIGEPPLPAVAPAVANAVAHLTGQ
RIRSLPLSRYTFS
>SEQF3764||SEQF3764.1_02272
MTTRNPPLDAPLGEPINLSRRRFLASTAVGALVIGFGLPLGASRAYAATGGPAERGTQVPAFLEIRPDGSVRLLSPF
MEGGQGTHTAMAQIVGEELDADPATFVVEAAPPGEAYVVMENGMRITGGSMSIRMSYPTMRRLGALARAM
LLQAGAEQLGVPLAQLTTQPGRVVHAASGRSLGYGELAGRALDMPVPDPATITLRDPSQFRWIGKPVKRLDAY
DKSTGKAQYSIDLKVDGMLHAAVQHAPRLGMTVGSLRNQSQVEAMKGVHSVHQLPGAVAVVAERWWHAK
RAVEAIQVEWLEAAAGSSVRAMPADFSSDKYRDFLAAQQGPARDDENEGDVAGALTNAKTKIEATYHNQYLN
HAQLEPPSALARFNPDGSLEVWLPNQAPDMFRADMAKRTGLSIEQITLHSPLLGGFFGRHFLYDSANPYPQAI
ALAKAVGRPIKLIWSREEEFLRDVLRPVAVVKFRAALDDKGLPVAIEAVSATEGPSEAIAGKQGEKLDPTALEGLS
GKSYAIPNKRIAQIYVKGPAMLGYWRSVGNSLNDFFYEAFLDELADKGGHDPYELRLHLLRDNKRLTTLLQAAGE
LSGGWKRGPYTAEDGTRRARGVAMASPFGSHAAVIAEVSIESGQVKVHHIWEAIDPGSIVNPAIVEAQVNGAV
ALGLSQTLLEEAVYVDGKPRARNYDLYPILPPSRMAQVHVRIVESGEKMGGIGEPPLPAVAPAVANAVAHLTGQ
RIRSLPLSRYTFS
>SEQF3765||SEQF3765.1_04506
MSRLPNDFALSNLSRRGFLKGVGATGALVLAASWGWQDALAEDKPKQFGADGMPNGWIDDPKVYVSIAAD
GTVTVVCNRSEMGQGVRTSLTMVVADELEADWAHVKVQQAPGDEVRFGNQDTDGSRSMRHWYEPMRRC
GAAARTMLEQAAAAQWKVPVGECRAQLHKVIHTPSGRELGYGELAAAASALTVPARDSLRLKQPSEFRYIGKE
GTKAIDGADIVNGRAVYGADVHFDGMLFAVIARPAVYGGKVKSVDDSAALKVPGVLKVIQIEPRPLPSEFQPLG
GVAVVASNTWAALKGREALKIEWDDGPNATYDSVAYRKEIEGASLKPGKVVRNTGDIDKAIGSAASTLEASYYLP
HLAQAPMEPMVAIARYKDGTCEAWAPSQAPQVTRERIAERLGLPFDNVTFNVTLLGGGFGRKSKPDFVVEAA
VLAKEFPGKAVRVQWTREDDIHNSYFHTVSAEYLKAGVGKDGLPSAWLHRTVAPSITALFAPGMNHEAAFELG
MGFTNMAYAIPNVRLENPEATVHTRVGWYRSVSNIPHGFAIQSFVDELAHKAKEDPLKYQIKLLGPDRQIDPRT
LSEEWNYGESPERYPIDTGRMRTVLETAAKAAGWGRQLPKGRGLGLAVHYSFVTYVAAVIEVEVKDDGTLIVHK
ADIAVDCGPQINPERIRSQFEGACVMGLGNAVWGEISFKDGKVQQDNFHMYEVARMSLAPKEVAVHLVTPP
GEVPLGGVGEPGVPPIAPALCNAIFAATGQRIRNLPVRYQLQGWQKAQA
>SEQF3765||SEQF3765.1_02539
MNSPVSRRGFLKGSAVLGGGLVVAFVVPGGNRFAMAAENEGKVFAPNAFLRIAADNSVTVLLGHSEMGQGI
WTGLTMLIAEELDADWSKIRVEHSPASAADYGMPGFGGMQITGGSTSTWMEFDRYRLAGATARQMLIEAAA
KRFNVAPSTIRTEAGVVIAGDNRATYGELADAAGQLPVPDPKSITFKEAKDWKVIGKPTKRLDTPEKITGRAKFG
MDVQFDGLMTAMVARAPAFGATVKSFEGAEALAIPGVHKVVQVPTGVAVVAEHYWAAKLGRDALKVDWDL
GPNADLSSEKLLASFRKLAATPGTSAAQAGDAKGNFGKAAKKIDVEYSVPYLAHAPMEPLNCTVKISADKCEIW
TGTQFQTLDQMVAGKITGLKPEQVEIHTEFLGGGFGRRANPTSDFVAEAVQVAKAAAMPVKTVWSREDDIRG
GYYRSMFLHQAKIGLGADGLPLAWQHVLVGQSIMAGTMLEKTMVKNGVDQTSVEGVSDSPYIKGLAHHQVD
LHSPTTGINVLWLRSVGHSHTAFVMESLIDEMATAAGKDPVEYRRTLLKDHARHLGVLNLAVEKANWKAPLPD
GHALGVAVHESFGSYVAQVAEVSQDNLAIRVHRVVCAVDCGIAVNPQSIAAQMESCITFGLGMALHSKLTLKD
GHVVQSNYHDYQVLRLNEMPLVEVHIVPSSEKPGGIGEAGVPPTAPAVANAVFALTGQRLRELPLQMSGV
>SEQF3766||SEQF3766.1_04566
MSALPADFVLNNLSRRGFLKGVGATGALVVAASWGLPDAFAADAVKKYGGDAMPHGLIDDPKVYVSIATDGT
VTVICNRSEMGQGVRTSLSLVVADELDADWAHVKVQQAPGDEVRYGNQDTDGSRSMRHWYEPMRRCGAS
VRAMLEQAAAEQWNVPVGECRAQLHKVIHQPSARELGYGELATAAGALAVPARDSLRLKQPSEFRYIGKEGVK
AIDGADIVNGRAVYGADVHFDGMLYATVARPKVYGGKVKSFDASAALKVPGVVKVLEIESRPLPSEFQPLGGIA
VIASNTWAAIKGREALTIVWDDGVNAGYNSVDYRKTLEAAAQKPGKVVRNTGNLDQALGESDSTLEAAYYLPH
LAQSPMEPMVAIARFDNGQCEAWAPSQAPQVTRERIAERLGLPFDNVTVNVTLLGGGFGRKSKPDFVVEAAI

LAKAFPGKAVRVQWTREDDIHNAYFHTVSVEYLKAGLNKDGLPSAWLHRTVAPSITALFAPGMNHEAAFELG
MGFTNMAYAIPNVRLENPEAAAHTRVGWYRSVSNIPHGFAIQCFVDELAHKAGQDPLQYQLKLLGPDRQIDP
KTLSDEWNYGESPERYPIDTARLRGVLETAAKAAGWGRTLPKGRGLGLAVHYSFVTYVAAVIEVEVQGDGTLIV
HKADIAVDCGPQINPERIRSQFEGACVMGLGNAVLGEISFKDGKVQQDNFHMYEVARMSLAPRQVAVHLVTP
KGEVPLGGVGEPGVPPIAPALCNAIFAATGKRIRELPVRYQLQGWQEVKA
>SEQF3767||SEQF3767.1_05980
MLNEIFPNELPRALQHSLERDQADGPATLPRRSFLKIVGIGGLALGAFPHLALAQEANGSAATPLKPTQQPSAFV
QIAPDGVVTVTLNRLEFGQGVQTSLPMILAEELDADWSLVRSRNGNSDAAYLDPNLGIHLTGGSSSIKNSYTQY
RELGARARAMLLAAAAARWNVDVASLSTQAGTVFGPGGRKASYGELAEAAMAMPVPEKVTLKDPKDFRIIG
QATTRIDAKAKSSGQQDFGIDIHLPGQLTAVVARPPVFGARIASLDDSAARATKGVKAVFRVPLDRGAEGVAVV
ADGYWQAKLARDALKLEWDLTNVEKVDSEKQLTQYRELATQPGPRQFDADMTPLASAPHQLQAEFVFPYLAH
APMEPLNCTVQLAQGRAQLWVGTQFPGGDGAAAARVLNLQPEQVQVNVQTAGGGFGRRGVPTNDFVVLA
CEVAKAAHAAGLDVPIRTLWSREDDIKGGYYRPMHLHRAHIGFDDSGKVLAWDHALVGQSIVSGTPFEGMIK
NGIDATATEGMRNPYPLPMRLTVHHPKLNVPVLWWRSVGSTHTAFVMETLIDEIARTTKQDPVAYRMKLFGD
QNPRHRAALQLAVDKSEYGKRQLAAGRAWGVAVHESFSSVVAYVVEASVKDGRPVLHNVTAGVHCNLVVNP
RSIEAQVQGAALMGLSMCLPGGAVTLKDGVVQQSNFADFSVPRITDMPEFAVHIVPSAEPPTGMGEPGLPAL
APAFANAVASLTGKPLRELPFKLA
>SEQF3767||SEQF3767.1_04715
MSRLPNDFVLSNLSRRGFLKGASATGVLVLAASWGMPDAFAEEKKFGAEGMPHGAVDDPKVYVSIAADGSVT
VICNRSEMGQGVRTSLSMVVADELEADWARVKVQQAPADEARFGNQDTDGSRSMRHWYEPMRRCGAAA
RTMLELAAAAQWKVPVGECHAQLHKVLHQPSGRELAYGELAAAASALPVPSRDSLRLKQPSEFRYIGKEASRAI
DGADIVNGRAVFGADVHLDGMLYAVIARPPVYGGKVKSVDSSAALKVAGVVKVVQIEGRPLPSEFQPLGGVAV
VAKNTWAAIKGREALKIQWDDGPNAAYDSIAYRKELEAAALKPGKVVRSSGDLDDALAKADSTLEAAYYLPHLS
QSPMEPMVAVARFKDGQCEAWAPSQAPQVTRERVAERLGIPFEKVTVNITLLGGGFGRKSKPDFVVEAAVLA
KEFPGQPIRVQWTREDDIHHSYFHTVSAEYLKAGLNQDGMPSGWLHRTVAPSITALFAPGMTHEAPFEIGMG
VTNMAYAIPNLRLENPEAVAHARVGWYRSVSNIPHGFAIQSFIDELAHKAGQDPLKYQVKLLGPDRKIDPRTLSE
EWNYGESPERYPIDTARIRTVLETAAKAAGWGRELPKGRGLGLAVHYSFVTYVAAVIEVEVKDDGTVIVHKADIA
VDCGPQINPERIRSQFEGACVMGLGNAMVGEISFKDGKVQQDNFHMYEVARMSLAPKEVAVHLVTPPGEVP
LGGVGEPGVPPIAPALCNAIFAATGKRIRNLPVRYQLQGWQQAKA
>SEQF3767||SEQF3767.1_05930
MNPVLVPSRRGFLKSGAVLGSGLIVGFSVPGAKRLMAQTTDSEFAPNAFLRIAPDDSVTILLAHSEMGQGVWT
TLTMLIAEELDADWSRLKVEHAPAAAAYASPQRGLQATVGSSSIREEFERYRRAGAMARTLLIQAAAIRLGQAPS
ALRAENGVVIIGNTRLRYGELAEQAALLPAPDPATLTLKEPGAWTLIGKGALRLDSPEKISGRAQYGIDIQLDGLLC
AVVARAPMLGGTVRAFDATQARALPGVRNVVQIPSGIAVIADHYWAAKRGCDVLQVEWEAASGAVIFDSDR
QREDLVRLTHTPGLVATQNGDVEEAQKAAHKVVEMQYCVPYLAHAATEPLNCTVRLSADGCDIWVGSQMQT
FNQRTAAKITGLAPEQIRIHTTFLGGGFGRRAVQDFVAEAVQVAKAAGVPVKTLWSRENDMQGGYYRSAFAQ
RLKVGLGRGGQPLSWSQTSAGQSIFPDAEGIHPTSIEGMSDSPYIKHTPAYRVEAHSPRSNVPVWYWRSVGHS
HNAFVMESAVDELAHAAGQDPLAYRRLLLRTEPRHLGVLNLAAERFGWGRKAAAGRGQGIAVHQSFGSFCA
QAVEISIERDEIRVHRVVCAIDCGIPVNPDNIKAQMEGAIVFGLSAALFGQISIKEGRVQQSNFHDYRVLRMNE
MPQIEVHVLPSTEGPGGVGEPATPPVAPAVANALFALTGQRLRELPLRLSSHA
>SEQF3768||SEQF3768.1_05530
MLNEIFPNELPRALQHMLERDEADGPAALPRRSFLKIVGIGGLALGAFPHLALAQEANGAVAAPLKPTQQPSAF
VQIAPNGEVTVTINRLEFGQGVQTGLPMILAEELDADWSLVRSRNGSNDAAYMDPAFGIHLTGGSNTIKNSYT
QYRELGARARAMLLSAAAARWNVDVASLSTQAGMVLGPAGRKASYGELAQAAMAMPVPEQITLKDPKDFRII

GQATTRIDAKAKSSGQQDFGIDMHLPGQLTAVVARPPVFGARIAALDDSAARATKGVKAVLRVPLDGGAEGVA
VVADSYWQAKLARDALKVEWNASAVEKLDSEKQLAQYRELASQPGPLHFDADMTPLATAPHRLEAEFLFPYLA
HAPMEPLNCTVQLAGKNGAQLWVGTQFPGGDAAAAAKVLDLKPEQIQVNVQTAGGGFGRRGVPTNDFVVL
ACEVAKAARTAGVDAPIRTLWSREDDIKGGYYRPMHLHRARIGFDDSGKVLAWDHALVGQSIITGTVFGGRVK
NGIDPTATEGLRDPYPLPMRLTVHHPKLNVPVLWWRSVGSTHTAFVMETLIDEIARTTKQDPVAYRMKLFGDQ
SPRHRAALQLAVDKSEYGKRQLPAGRAWGVAVHESFSSVVAYVVEASVQDGRPVLHNVTAGVHCNLAVNPRS
VEAQVQGAALMGLSMCLPGGAITLKDGVVQQSNFADFSVPRITDMPEFAVHIVPSAEPPTGMGEPGLPALAP
AFANAVASLTGKPMRELPFKLA
>SEQF3768||SEQF3768.1_04147
MSRLPDDFMLSNLSRRGFLKGASATGVLVLAATWGLPDAFAEEKKFGAEGMPHGAVDDPKVYVSIASDGSVT
VICNRSEMGQGVRTSLSMVVADELDADWALVKVKQAPADEARFGNQDTDGSRSMRHWYEPMRRCGAAAR
TMLELAAAAQWKVPVGECHAQLHKVLHQPSGRELGYGELAAAASALAVPARDSLRLKQPSEFRYIGKEASRAI
DGADIVNGRAVFGADVHFDGMLYAVIARPPVYGGKVKSVDSSAALKVPGVVKVVQIEGRPLPSEFQPLGGVAV
VAKNTWAAIKGREALKIQWDDGPNAGYDSIAYRKELEAAALKPGKVVRSSGDLDDALAKADSTLEASYYLPHLS
QSPMEPMVAVARFKDGQCEAWAPSQAPQVTRERVAERLGIPFEKVTVNITLLGGGFGRKSKPDFVVEAAVLA
KEFPGQAVRVQWTREDDIHHSYFHTVSAEYLKAGLNQDGMPSGWLHRTVAPSITALFAPGMTHEAPFEIGM
GVTNMAYAIPNLRLENPEAVAHARVGWYRSVSNIPHGFAIQSFIDELAHKAGQDPLKYQVKLLGPDRKIDPRTL
SEEWNYGESPERYPIDTARIRTVLETAAKAAGWGRELPKGRGLGLAVHYSFVTYVAAVLEVEVKDDGTVIVHKA
DIAVDCGPQINPERIRSQFEGACVMGLGNAMVGEISFKDGKVQQDNFHMYEVARMSLAPKEVAVHLVTPPG
EVPLGGVGEPGVPPIAPALCNAIFAATGQRIRNLPVRYQLQGWQQAKA
>SEQF3769||SEQF3769.1_01953
MNMPGLIPGLSLDEPVNLSRRRFLASTAVGALVIGFGLPLGSGRVQAATGATTERGTQVPAFLEIRPDGTVRLLS
PFMEGGQGTHTAMAQIVGEELDADPATFIVEAAPPGEAYVVMDNGMRITGGSMSVRMSYPTMRRLGALAR
AMLLQAGAEQLGVPVAQLTTQPGRVVHAASGRSLGYGELASRALDMPVPDAAGITLRDPSQFRWIGKPVKRV
DAYDKSTGKALYSIDLKVDDMLHAAVQHAPRLGMTVGSLRNQAQVEAMPGVHSVHQLPGAVAVVAERWW
HAKRAVEAIQVDWLEAAADAKVRAMPADFSSDGFRDFLATQQGPARDDENEGDVAGALKNAKTQIEATYHN
QYVNHAQLEPPSALARFNPDGSLDIWLPNQAPDMFRADIAKRTGLAPAQINLHSPLLGGFFGRHFLYDSASPYP
QAIALAKAVGRPVKLIWSREEEFLRDVLRPVAVVKFRAALDADGLPVAIEAVSATEGPSEAIAGKQGEKLDPTALE
GLTGKSYAIPNKRIAQIYVKGPAMLGYWRSVGNSLNDFFYEAFLDELADQGKRDPYELRLHLLRDNPRLTTLLKA
VAELSGGWKRGPYTAEDGTRRARGVAMASPFGSHAAVIAEVSIEHGQVKVHHIWEAIDPGSIVNPAIVEAQVN
GAVALGLSQTLLEEAVYVDGKPRARNYDLYPILPPSRMAQVHVKIIESGEKMGGIGEPPLPAVAPAVVNAVAQLT
GQRVRSLPLSRHTFS
>SEQF3771||SEQF3771.1_02296
MNSPVSRRGFLKGSAVLGGGLVVAFVVPGGNRFAMAAENEGKVFAPNAFLRIAADNSVTVLLGHSEMGQGI
WTGLTMLIAEELDADWSKIRVEHSPASAADYGMPAFGGMQITGGSTSTWMEFDRYRLAGATARQMLIEAAA
KRFNVAPSTIRTESGVVIAGDNRATYGELADAAGQLPVPDPKSITFKEAKDWKVIGKPTKRLDTPEKITGRAKFG
MDVQFDGLMTAMVARAPAFGATVKSFEGAEALAIPGVHKVVQVPTGMAVVAEHYWAAKLGRDALKVDWD
LGPNADLSSEKLLASFRKLAATPGTSAAQAGDAKGNFGKAAKKIDVEYSVPYLAHAPMEPLNCTVKISADKCEI
WTGTQFQTLDQMVAGKITGLKPEQVEIHTEFLGGGFGRRANPTSDFVAEAVQVAKAAAMPVKTVWSREDDI
RGGYYRSMFLHQAKIGLGPDGLPLAWQHVLVGQSIMAGTMLEKTMVKNSVDQTSVEGVSDSPYIKGLAHHQ
VDLHSPTTGINVLWLRSVGHSHTAFVMESLIDEMATAAGKDPVEYRRTLLKDHARHLGVLNLAVEKANWKAPL
PDGHALGVAVHESFGSYVAQVAEVSQDNLAIRVHRVVCAVDCGIAVNPQSIAAQMESCITFGLGMALHSKLTL
KDGHVVQSNYHDYQVLRLNEMPLVEVHIVPSSEKPGGIGEAGVPPTAPAVANAVFALTGQRLRELPLQLSGV
>SEQF3771||SEQF3771.1_04068

MSRLPNDFALSNLSRRGFLKCVGATGALVLAASWGWQDALAEDKPKKFGADGMPNGWIDDPKVYVSIAADG
TVTVVCNRSEMGQGVRTSLTMVVADELEADWTHVKVQQAPGDEVRFGNQDTDGSRSMRHWYEPMRRCG
AAARTMLEQAAAAQWKVPVGECRAQLHKVIHTPSGRELGYGELAAAASALTVPPRDSLRLKQSSEFRYIGKEG
TKAIDGADIVNGRAVYGADVHFDGMLFAVIARPAVYGGKVKSVDDSVALKVPGVLKVIQIEPRPLPSEFQPLGG
VAVVASNTWAALKGREALKIEWDDGPNATYDSVAYRKEIEAASLKPGKVVRNTGDIDKAIGSAASTLEASYYLPH
LAQAPMEPMVAIARYKDGTCEAWAPSQAPQVTRERIAERLGLPFDNVTFNVTLLGGGFGRKSKPDFVVEAAVL
AKEFPGKAVRVQWTREDDIHNSYFHTVSAEYLKAGVGKDGLPSAWLHRTVAPSITALFAPGMNHEAAFELGM
GFTNMAYAIPNVRLENPEATVHTRVGWYRSVSNIPHGFAIQSFVDELAHKAKEDPLKYQIKLLGPDRQIDPRTLS
EEWNYGESPERYPIDTGRMRTVLETAAKAAGWGRQLPKGRGLGLAVHYSFVTYVAAVIEVEVKDDGTLIVHKA
DIAVDCGPQINPERIRSQFEGACVMGLGNAVWGEISFKDGKVQQDNFHMYEVARMSLAPKEVAVHLVTPPG
EVPLGGVGEPGVPPIAPALCNAIFAATGQRIRNLPVRYQLQGWQKAQA
>SEQF3772||SEQF3772.1_00295
MSQLPNDFALSNLSRRGFLKGVSATGALVLAASWGWQDAFAEEKKFGAAGMPNGWIDDPKVYVSIAADGSV
TVVCNRSEMGQGVRTSLSMVVADELDADWAMVKVRQAPGDEVRFGNQDTDGSRSMRHWYEPMRRCGA
AARTMLEQAAADQWKVPVGECRAQLHKVIHQPTGRELGYGALAVAAGALAVPARESLRLKQSSEFRYIGKEGT
KAIDGADIVNGRAVYGADVHFDGMLFATVARPAVYGGKVKSFDASAALKVPGVVKVIQIESRPLPSEFQPLGGV
AVIASNTWAAIKGREALKIEWDDGANAGYDSIVYRKELEAAALKPGKVVRSTGNIEEAMNSADSSLEAAYYLPH
LAQSPMEPMVAVARFKDGHCEAWAPSQAPQVTRERIAERLGIPFDKVTVNVTLLGGGFGRKSKPDFILEAAILA
KEFPGKAVRVQWTREDDIHCSYFHTVSAEYLKASLNKDGMPSGWLHRTVAPSITALFAPGMNHEAAFELGMG
FTNMAYAIPNVRLENPDAAAHTRVGWYRSVSNIPHGFAIQSFVDELAHKAGQDPLKYQIKLLGPDRQIDPRTLS
EEWNYGESPERYPIDTARLRGVLETAAKGAGWGRKLPKGRGLGLAVHYSFVTYVAAVIEVEVKDDGTLIVHKADI
AVDCGPQINPERIRSQFEGACVMGLGNAVLGEISFKDGKVQQDNFHMYEVARMSLAPKEVAVHLVTPAGNVP
LGGVGEPGVPPIAPALCNAIFAATGKRIRSLPVRYQLQGWQQSKA
>SEQF3772||SEQF3772.1_04840
MNAKIETSRRDFLKSTAVLGAGLVVAFVIPGANRFAQAASTPDAVFAPNAFLRIAPDGSVTILLGHSEMGQGIW
TGLTMLIAEELDADWTKIRVEHAPASAADYGLPAFGGMQITGGSTSTWMEFDRYRQAGAAARLMLIEAAAKR
FNVAPSKIQTEPGVVIAGDQRATYGELANDAGQLPVPDPASIKLKDAKDWKIIGKPTKRLDTPEKITGQAKFGM
DVQFDGLLTAVVARPPVFGGSVKSFDGAAALALPGVHKVVQVPTGIAVVADHYWAAKLGRDALKIDWDLGPN
AGLDSDALLQNFRKLAAIPGTPASQAGDISAALSKAVKTIDVEYSVPYLAHAPMEPLNCTVKITPDKCEIWTGTQ
FQTLDQMIAAKITGLKPEQVEIHTEFLGGGFGRRANPTSDFVSEAVQVAKAAVAPVKTVWSREDDIRGGYYRSA
FLHHARIGLDAKGMPQAWQHVLVGQSIMDGTPFEATMVKNGVDATSVEGVADSPYIKGLANHLVDLHSPKT
GISVLWMRSVGHTHTAFVVESLIDELATAAGKDPVEYRRTLLKEHPRHLGVLNLAVEKANWKAPLPAGHALGVA
VHESFGSYVAQVAEVSQDNLKIRVHRVVCAVDCGVVVNPASIAAQMESGITFGLGFTLHSKLTFKDGKVVQSNY
HDFQVLRLNEMPVVEVHIVPSTEKPGGIGETGVPPTAPAVANAVFALTGQRLRELPLQLAGV
>SEQF3773||SEQF3773.1_02477
MNSPVSRRGFLKGSAVLGGGLVVAFVVPGGNRFAMAAENEGKVFAPNAFLRIAADNSVTVLLGHSEMGQGI
WTGLTMLIAEELDADWSKIRVEHSPASAADYGMPGFGGMQITGGSTSTWMEFDRYRLAGATARQMLIEAAA
KRFNVAPSTIRTESGVVIAGDNRATYGELADAAGQLPVPDPKSITFKEAKDWKVIGKPTKRLDTPEKITGRAKFG
MDVQFDGLMTAMVARAPAFGATVKSFEGAEALAIPGVHKVVQVPTGVAVVAEHYWAAKLGRDALKVDWDL
GPNADLSSEKLLASFRKLAATPGTSAAQAGDAKGNFGKAAKKIDVEYSVPYLAHAPMEPLNCTVKISADKCEIW
TGTQFQTLDQMVAGKITGLKPEQVEIHTEFLGGGFGRRANPTSDFVAEAVQVAKAAAMPVKTVWSREDDIRG
GYYRSMFLHQAKIGLGADGMPLAWQHMLVGQSIMAGTMLEQTMVKNGVDQTSVEGVSDSPYIRGLAHHQ
VDLHSPTTGINVLWLRSVGHSHTAFVMESLIDEMATAAGKDPVEYRRTLLKDHARHLGVLNLAVEKANWKAPL
PDGHALGVAVHESFGSYVAQVAEVSQDNLAIRVHRVVCAVDCGIAVNPQSIAAQMESCITFGLGMALHSKLTL

KDGHVVQSNYHDYQVLRLNEMPVVEVHILPSTDKPGGIGEAGVPPTAPAVANAVFALTGQRLRELPLLLSGV
>SEQF3773||SEQF3773.1_04498
MSRLPNDFALSNLSRRGFLKGVGATGALVLAASWGWQDALAEDKPKQFGADGMPNGWIDDPKVYVSIAAD
GTVTVICNRSEMGQGVRTSLTMVVADELEADWAHVKVQQAPGDEVRFGNQDTDGSRSMRHWYEPMRRC
GAAARTMLEQAAAAQWKAPVGECRAQLHKVIHTPSGRELGYGELAAAASALTVPARDSLRLKQPSEFRYIGKE
GTKAIDGADIVNGRAVYGADVHFDGMLFAVIARPAVYGGKVKSVGDSAALKVPGVLKVIQIEPRPLPSEFQPLG
GVAVVASNTWAALKGREALKIEWDDGPNATYDSVAYRKEIEAASLKPGKVVRNTGDIDKAIGSAASTLEASYYLP
HLAQAPMEPMVAIARYKDGTCEAWAPSQAPQVTRERIAERLGLPFDNVTFNVTLLGGGFGRKSKPDFVVEAA
VLAKEFPGKAVRVQWTREDDIHNSYFHTVSAEYLKAGVGKDGLPSAWLHRTVAPSITALFAPGMNHEAAFELG
MGFTNMAYAIPNVRLENPEATVHTRVGWYRSVSNIPHGFAIQSFVDELAHKAKEDPLQYQIKLLGPDRQIDPRT
LSEEWNYGESPERYPIDTGRMRTVLETAAKAAGWGRQLPKGRGLGLAVHYSFVTYVAAVIEVEVKDDGTLIVHK
ADIAVDCGPQINPERIRSQFEGACVMGLGNAVWGEISFKDGKVQQDNFHMYEVARMSLAPKEVAVHLVTPP
GEVPLGGVGEPGVPPIAPALCNAIFAATGQRIRNLPVRYQLQGWQKAQA
>SEQF3775||SEQF3775.1_05065
MSALPADFVLNNLSRRGFLKGVGATGALVVAASWGLPDAFAADAVKKYGGDAMPHGLIDDPKVYVSIATDGT
VTVICNRSEMGQGVRTSLSLVVADELDADWAHVKVQQAPGDEVRYGNQDTDGSRSMRHWYEPMRRCGAS
VRAMLEQAAAEQWNVPVGECRAQLHKVIHQPSARELGYGELATAAGALAVPARDSLRLKQPSEFRYIGKEGVK
AIDGADIVNGRAVYGADVHFDGMLYATVARPKVYGGKVKSFDASAALKVPGVVKVLEIESRPLPSEFQPLGGIA
VIASNTWAAIKGREALTIVWDDGVNAGYNSVDYRKTLEAAAQKPGKVVRNTGNLDQALGESDSTLEAAYYLPH
LVQSPMEPMVAIARFDNGQCEAWAPSQAPQVTRERIAERLGLPFDNVTVNVTLLGGGFGRKSKPDFVVEAAIL
AKAFPGKAVRVQWTREDDIHNAYFHTVSVEYLKAGLNKDGLPSAWLHRTVAPSITALFAPGMNHEAAFELGM
GFTNMAYAIPNVRLENPEAAAHTRVGWYRSVSNIPHGFAIQCFVDELAHKAGQDPLQYQLKLLGPDRQIDPKT
LSDEWNYGEPPERYPIDTARLRGVLETAAKAAGWGRTLPKGRGLGLAVHYSFVTYVAAVIEVEVQGDGTLIVHK
ADIAVDCGPQINPERIRSQFEGACVMGLGNAVLGEISFKDGKVQQDNFHMYEVARMSLAPRQVAVHLVTPKG
EVPLGGVGEPGVPPIAPALCNAIFAATGKRIRELPVRYQLQGWQEVKA
>SEQF3777||SEQF3777.1_01826
MSRLPSNFVLNNLSRRGFLKGIGATAALVVAASWGVPGAFAEEVKKYGAEAMPNGVIDDPKVYVSIAADGSVT
VICNRSEMGQGVRTSLAMVVADELEADWAQVKVEQAPGDEVRFGNQDTDGSRSMRHWYEPMRRCGAAV
RSMLEQAAAEQWKVPLGECRAQLHKVIHQPSGRELGYGALAAAAGALAVPARDSLRLKQPSEFRYIGKEATKAI
DGADIVNGRALYGADVHFDGMLFATIARPKVYGGKVKSFDASAALKVPGAIKVLSIESRPLPSEFQPLGGVAVVA
SNTWAAIKGREALQIVWEDGANAGYNSIDYRKTLEAAALQPGKVVRNTGSIDQALSDADSTFDAAYYLPHLAQ
SPMEPMVAVARYQDGQCEAWAPSQAPQVTRERVAERLGLPFDNVTIHVTLLGGGFGRKSKPDFIIEAAVLAKE
FPGKAVRVQWTREDDIHNSYFHTVSAEYLKAGLNKDGMPSGWLHRTVAPSITALFAPGMNHEAAFELGMGF
TNMAYAIPNVRLENPEAAAHTRVGWYRSVSNIPHGFAIQSFVDELAHKAGQDPLKYQLKLLGPDRQIDPRTLSE
EWNYGESPERYPIDTARMRGVLETAAKAAGWGRALPKGRGLGLAVHYSFVTYVAAVIEVEVKGDGTLIVHKADI
AVDCGPQINPERIRSQFEGACVMGLGNAVLGEISFKDGKVQQDNFHMYEVARMSLAPREIVIHLVTPPGEVPL
GGVGEPGVPPIAPALCNAIFAATGKRIRSLPVRYQLQGWQQEAKA
>SEQF3777||SEQF3777.1_02765
MKKTIEMNVDMSRRRLLQGSGIAVGGLVLSTWLPPLVSKSAASEAAALGRLGDRSAEGFGAFVRVGPDGVVT
VISPKIEMGQGAQTGIAMMVAEELEVDLDKVVIQEAPPNSALYTDTLLQFQATGGSTSTRYTWEPLRRAGATAR
ILLIQAAALQWRVAPSLCHAQNGQVFGPKGLQADYGDLVEAAATLPLPDAVPLKTPEQFKLLGTPAQRLDTPAK
VNGKARFTIDLQIPGMLVASSITCPVYGGRLRSVDETEARRVLGVRDIVRLDNAVAVTASNFWACQQAIKALKIE
WELGSNATIGSKQLDQELLAASSRDGVVAKRTGDIEQAKQGSSSQFEAVYEQALLSHSPLEPMSCVAHVRKDA
CELWVGTQVPVFAQQTAAQVTGLPLEKIQVHNQLIGGAFGRRLEFDFITQAVAIARQVDYPIKLVWSREEDMT

HDLYRPLYADRMQAALDKQGRPLGWEHRIAGASILARYAGSLPPSGVDADAVEVAVEPIYRLPHLQVRYIRQEP
SVVPVSWWRGVGPLRGTYALECFIDELAHNAKADPVVYRLELLADQPRAQAVLRLLAEKSDWYQSLPAGQGR
GVAVSSVFGSYVATLVELEMQGEFGLRIKRLISVVDCGFATNPTSVLAQVEGGTLFGLSASLFNEILIENGQVQQT
NFHNYRQLRISEAPAVEVHLLPSLEAPGGVGEAGTALIGPALVNALYAASGTRIRRLPLSRAGYYPV
>SEQF3778||SEQF3778.1_03060
MNPVLVPSRRGFLKSGAVLGSGLIVGFSVPGAKRLMAQTTDSEFAPNAFLRIAPDDSVTILLAHSEMGQGVWT
TLTMLIAEELDADWSRLKVEHAPAAAAYASPQRGLQATVGSSSIREEFERYRRAGAMARTLLIQAAAIRLGQAPS
GLRTENGVVITGNTRLRYGELAEQAALLPAPDPATLTLKEPGAWTLIGKGALRLDSPEKISGRAQYGIDIQLDGLLC
AVVARAPMLGGTVRAFDATQARALPGVRNVVQIPSGIAVIADHYWAAKRGCDVLQVEWEAASGAVILDSDR
QREDLVRLTHTPGLVATQNGDVEKAQKAAHKVVEMQYCVPYLAHAAMEPLNCTVRLSADGCDIWVGSQMQ
TFNQRTAAKITGLAPEQIRIHTTFLGGGFGRRAVQDFVAEAVQVAKAAGVPVKTLWSRENDMQGGYYRSAFA
QRLKVGLGRDGRPLSWSQTSAGQSIFPDAEGIHPTSIEGMSDSPYIKHTPAYRVEAHSPRSNVPVWYWRSVGH
SHNAFVMESAVDELAHAAGQDPLAYRRLLLRTEPRHLGVLNLAAERFGWGRKAAAGRGQGIAVHQSFGSFCA
QAVEISIERDEIRVHRVVCAIDCGIPVNPDNIKAQMEGAIVFGLSAALFGQISIKEGRVQQSNFHDYRVLRINEM
PQIEVHVLPSTEGPGGVGEPATPPVAPAVANALFALTGQRLRELPLRLSSHA
>SEQF3778||SEQF3778.1_04274
MSRLPDDFVLSNLSRRGFLKGASATGVLVLAATWGLPDAFAEEKKFGAEGMPHGAVDDPKVYVSIAADGSVTV
ICNRSEMGQGVRTSLSMVVADEMEADWARVKVQQAPADEARFGNQDTDGSRSMRHWYEPMRRCGAAAR
TMLEQAAAAQWKVPVGECHAQLHKVLHQPSGRELSYGELAAAASALPVPSRDSLRLKQPSEFRYIGKEASRAI
DGADIVNGRAVFGADVHLDGMLYAVIARPPVYGGKVKSVDSSAALKVPGVVKVVQIEGRPLPSEFQPLGGVAV
VAKNTWAAIKGREALKIQWDDGPNAAYDSIAYRKELEAAALKPGKVVRSSGDLDDALAKADSTLEASYYLPHLS
QSPMEPMVAVARFKDGQCEAWAPSQAPQVTRERIAERLGIPFDKVTVNITLLGGGFGRKSKPDFVVEAAVLAK
EFPGQPIRVQWTREDDIHHSYFHTVSAEYLKAGLNQDGMPSGWLHRTVAPSITALFAPGMTHEAPFEIGMGV
TNMAYAIPNLRLENPEAVAHARVGWYRSVSNIPHGFAIQSFIDELAHKAGQDPLKYQVKLLGPDRKIDPRTLSEE
WNYGESPERYPIDTARIRTVLETAAKAAGWGRELPKGRGLGLAVHYSFVTYVAAVIEVEVKDDGTVIVHKADIAV
DCGPQINPERIRSQFEGACVMGLGNAMVGEISFKDGKVQQDNFHMYEVARMSLAPKEVAVHLVTPPGEVPL
GGVGEPGVPPIAPALCNAIFAATGKRIRNLPVRYQLQGWQQAKA
>SEQF3779||SEQF3779.1_04185
MSRLPNDFALSNLSRRGFLKGVGATGALVLAASWGWQDALAEDKPKKFGADGMPNGWIDDPKVYVSIAADG
TVTVVCNRSEMGQGVRTSLTMVVADELEADWAHVKVQQAPGDEVRFGNQDTDGSRSMRHWYEPMRRCG
AAARTMLEQAAAAQWKVPVSECHAQLHKVIHTPSGRELGYGELAAAASALPVPGRDSLRLKQPSEFRYIGKEG
TKAIDGADIVNGRAVYGADVHFDDMLFAVIARPAVYGGKVKSVDDSAALKVPGVLKVIQIEPRPLPSEFQPLGG
VAVVASNTWAALKGREALKIEWDDGPNASYDSVAYRKEIEAASLKPGKVVRNTGDIDKAIGSAASTLEASYYLPH
LAQAPMEPMVAIARYKEGVCEAWAPSQAPQVTRERIAERLGLPFDNVTFNVTLLGGGFGRKSKPDFVVEAAIL
AKEFPGKAVRVQWTREDDIHNSYFHTVSAEYLKAGVGKDGLPSAWLHRTVAPSITALFAPGMNHEAAFELGM
GFTNMAYAIPNVRLENPEATVHTRVGWYRSVSNIPHGFAIQSFVDELAHKAKEDPLKYQIKLLGPDRQIDPRTLS
EEWNYGESPERYPIDTGRMRTVLETAAKAAGWGRKLPKGRGLGLAVHYSFVTYVAAVIEVEVKDDGTLIVHKA
DIAVDCGPQINPERIRSQFEGACVMGLGNAVWGEISFKDGKVQQDNFHMYEVARMSLAPKEVAVHLVSPPG
EVPLGGVGEPGVPPIAPALCNAIFAATGQRIRNLPVRYQLQGWQKAQA
>SEQF3779||SEQF3779.1_02248
MNSPVSRRGFLKGSAVLGGGLVVAFVVPGGNRFATAAENEGKVFAPNAFLRIAPDNSVTVLLGHSEMGQGIW
TGLTMLIAEELDADWSKIRVEHSPASAADYGMPAFGGMQITGGSTSTWMEFDRYRLAGAMARQMLIEAAAK
RFNVAPSTIRTESGVVIAGDNRATYGELADAAGQLPVPDPKSITFKEAKDWKVIGKPTKRLDTPEKITGRAKFGM
DVQFDGLMTAMVARAPVFGASVKSFEGAQALAVPGVHKVVQVPTGVAVIADHYWAAKLGRDALKVDWDLG

PHADLSSEKLLESFRKLAATPGISASKAGDAKGSFGKAAKKIDVEYSVPYLAHAPMEPLNCTVKISAEKCEIWTGT
QFQTLDQMIAGKITGLKPEQVEIHTEFLGGGFGRRANPTSDFVAEAVQVAKAAAMPVKTVWSREDDIRGGYY
RSMFLHQARIGLGADGLPLAWQHALVGQSIMAGTMLEKTMVKNGVDQTSVEGVADSPYIQGLADHQVDLH
SPATGINVLWLRSVGHSHTAFVMESLIDEMATAAGKDPVEYRRTLLKEHARHLGVLNLAVEKANWKAPLPDGH
ALGVAVHESFGSYVAQVAEVSQDNLKIRVHRVVCAVDCGIAVNPQSIAAQMESCITFGLSMALHSKLTLKDGQV
VQSNYHDYQVLRLNEMPVVEVHIVPSTDKPGGIGEAGVPPTAPAVANALFALTGQRLRELPLQLSGV
>SEQF3779||SEQF3779.1_03059
MNVRIDPSQHASVLDLHEPINVSRRRFLTGTAVGALVLGFGLPLGVTRVQAAAATATAERGTQVPAFLEIRPDNR
VRLLCPFMEGGQGTFTAMAQIVGEELDADPATFLVEAAPPGEAYVVMENGMRITGGSMSVRMSYPVMRRLG
ALARAMLLQAGAQQLGVPVSELSTEPGKVVHAKSGRSLAYGELAERAMDLPVPDPASVKLRDPSQFRWIGKP
VKRLDAYDKSTGKALYSIDLKVDDMLHAAVQHAPRLGMTVSNLRNEDQVKAMKGVHSVHRLPGAVAVVAER
WWHAKRAVEAIQVDWQEPTADSQVRPMPADFSSDAWFKRLAEDKSPAKDDEHEGDVASILKETKTRIDATYH
NQYLNHGQLEPPSALARFNPDGSLEVWLPNQAPDMFRADIAKRTGLDPSRITLHSPLLGGFFGRHFLYDSASPY
PQAITLAKAVGRPVKLIWSREEEFLRDVLRPVAAVNFRAALDSDGWPLAIEAISATEGPTEALAGKQGEKLDPTA
LEGLSGKSYAIPNKRIAQIYVKGPAMLGYWRSVGNSLNDFFYESFLDELADKGGKDPFDLRLHLLRDNKRLTTLLQ
AVGELSGGWKRGPFTAEDGSRRARGVAMASPFGTETAVIAEVSIENGQVKVHDIWQAIDPGSIVNPAIVEAQV
NGAVALGLSQTLVEEAVWIDGKPRARNYDLYPILPPARMARVHVRVVESGEKMGGIGEPPLPAVAPAVANAVA
TLTGQRVRSLPLSRHTFT
>SEQF3780||SEQF3780.1_02323
MNSPVSRRGFLKGSAVLGGGLVVAFVVPGAHRFALAAENEGKVFAPNAFLRIAADNSVTVLLGHSEMGQGIW
TGLTMLIAEELDADWSTIRVEHSPASAANYGMPAFGGMQITGGSTSTWMEFDRYRLAGATARQMLVEAAAKR
FNVAASTIRTESGVVIAGDKRATYGELADAAGQLPVPDPKSITFKEAKDWKVIGKPTKRLDTPEKITGRAKFGMD
VQFEGLMTAMVARAPVFGATVKSFEGAEALAVPGVHKVVQVPTGVAVIADHYWAAKLGRDALKVDWDLGP
HADLSSQGLLDSFRKLAATPGTSASQAGDPKGNFDKAAKKIDVEYSVPYLAHAPMEPLNCTVKISAEKCEIWTG
TQFQTLDQMVAGKITGLKPEQVEIHTEFLGGGFGRRANPTSDFVAEAVQVAKAAGLPVKTVWSREDDIRGGYY
RSMFLHQARIGLDGQGMPLSWQHVLVGQSIMAGTMLEQTMVKNGIDATSVEGVADSPYIKGLAHQQVELHS
PQTGINVLWLRSVGHSHTAFVMESLIDEMATAAGKDPIEYRRTLLKDHARHLGVLNLAVEKANWTAPLPDGHA
LGVAVHESFGSYVAQVAEVSQDNLAIRVHRVVCAVDCGIAVNPQSIAAQMESCITFGLGMALHSKLTLKDGAV
VQSNYHDYQVLRLNEMPLVEVHIVPSSDKPGGIGEAGVPPTAPAVANAVYALTGQRLRELPLQLSGG
>SEQF3780||SEQF3780.1_03972
MSRLPNDFALSNLSRRGFLKGVGATGALVLAASWGWQDALAEDAPKKFGADGMPNGWIDDPKVYVSIAAD
GTVTVVCNRSEMGQGVRTSLTMVVADELDADWAKVKVRQAPGDEVRFGNQDTDGSRSMRHWYEPMRRC
GAAARTMLEQAAAAQWQVPVGECHAQLHKVIHKPSGRELGYGELAAAASALAVPARDSLRLKQPSEFRYIGKE
GTKAIDGADIVNGRAVYGADVHFDGMLYATIARPAVYGGKVKSLDDSAALKVPGVLKIMQIESRPLPSEFQPLG
GVAVIASNTWAAIKGREALKIEWDDGPNASYDSIAYRKELEAASRKPGKVVRNTGDIDKALSGAASSLEASYYLP
HLAQAPMEPMVAIARYDKGVCEAWAPSQAPQVTRERIAERLGLPFDNVTFNVTLLGGGFGRKSKPDFVVEAAI
LAKEFPGKAVRVQWTREDDIHNSYFHTVSAEYLKAAVGKDGMPSGWLHRTVAPSITALFAPGMNHEAAFELG
MGFTNMAYAIPSVRLENPEATVHTRVGWYRSVSNIPHGFAIQSFVDELAHKAGVDPLKYQIKLLGPDRQIDPRT
LSEEWNYGESLERYPIDTGRMRTVLETAAKAAGWGRQLPKGRGLGLAVHYSFVTYVAAVIEVEVKDDGTLIVHK
ADIAVDCGPQINPERIRSQFEGACVMGLGNAVLGEISFKDGKVQQDNFHMYEVARMSLAPKEVAVHLVTPPG
DVPLGGVGEPGVPPIAPALCNAIFAATGQRIRNLPVRYQLQGWQKAQA
>SEQF3781||SEQF3781.3_05934
MSQLPNDFALSNLSRRGFLKGVGATSALVIAASWGWQDAFAAEKEKKFGADGMPNGWIDDPKVYVSIATDG
TVTVVCNRSEMGQGVRTSLTMVVADELDADWALVKVQQAPGDEVRFGNQDTDGSRSMRHWYEPMRRCG

AAARTMLEQAAAEQWKVPVGECHAQLHKVIHKPTGRELGYGALAAAAGALAVPARDSLRLKEPSQFRYIGKE
GTKAIDGADIVNGRAIYGADVHFDGMLYATIARPSVYGGKVKSVDDSAALKVPGVIKVIQIEGRPLPSEFQPLGG
VAVVASNTWAAIKGRDALKIEWDDGPNASYDSIAYRKEIEAASLKPGKVVRNTGNIDEAMGSANSTLEASYYLP
HLTQAPMEPMVAIARFKDGVCEAWAPSQAPQVTRERIGERLGIPFDNVTFNVTLLGGGFGRKSKPDFIIEAAIL
AKEFPGKAVRVQWTREDDIHCSYFHTVSAEYLKASLNKDGLPSGWLHRTVAPSITALFAPGMNHEAAFELGM
GFTNMAYAIPNVRLENPEAAIHTRVGWYRSVSNIPHGFAIQSFVDELAHKAGEDPLKYQISLLGPDRQIDPRTLS
EEWNYGESPERYPIDTGRMRTVLETAAKAAGWGRQLPKGRGLGLAVHYSFVTYVAAVIEVEVKDDGTLIVHKA
DIAVDCGPQINPERIRSQFEGACVMGLGNAVLGEISFKDGKVQQDNFHMYEVARMSLAPKEVAVHLVTPPGN
VPLGGVGEPGVPPIAPALCNAIFAATGKRIRNLPVRYQLQGWQKAEA
>SEQF3781||SEQF3781.3_04747
MNSINPLSRRGFLKGSALLGGGLVVAFVIPGAHRFAMGAENQGNVFAPNAFLRIGNDNSVTVLLGHSEMGQG
IWTGLTMLIAEELDADWSKIRVEHSPASAADYGLAGFGGMQITGGSTSTWMEFDRYRQAGAAARLMLVEAA
AKRFNVAPSSIRTESGVVIAGDQRATYGELADDAGQLPVPAPASIAFKEAKDWKVIGKPTKRLDTPEKITGRTKF
GMDVQFDGLMTAMVARPPVLGGHVKSFEGAEALAIPGVHKVVQVPSGVAVIADHYWAAKLGRDALKVDWD
LGPNAGLDSQNLLESFRKLAATPGAPASKAGDAEGALSKAAKAIDVEYSVPYLAHAPMEPLNCTVKITQDKCEI
WTGTQFQTLDQMIAGKITGLKPEQVEIHTEFLGGGFGRRANPTSDFVAEAVEVAKAAGGPVKTVWSREDDIR
GGYYRSAFLHQARIGLDAGGMPMAWKHVMVGQSIMAGTMLEATMVKDGIDKTSVEGVADSPYLAGMAN
HQVDLHSPKTGITVLWLRSVGHTHTGFVMESLIDELAEAAGKDPVEYRRTLLKAHPRHLGVLNLAVEKANWTA
PLPDGHALGVAVHESFGSYVAQVAEVSQDNLAIRVHRVVCAVDCGIAVNPLSIAAQMESAITFGLGFTLHSKLTF
KNGEVQQSNYHDYQVLRLNEMPVVEVHIVPSTDKPGGIGEAGVPPTAPAVANAVFALTGQRLRELPLQLSGV
>SEQF3783||SEQF3783.1_02740
MIRSIENESRRRFLKGTAGLTLAVYMPWSLADSTARVGKKEGEFEPNAFLRIGEDNVVTVISKHLEMGQGAYTG
LATLVAEELDADWAQVRVESAPAESRYKNSLLGIQGTGGSSSIANSWEQLRMTGATARAMLVSAAAWLWKVP
AQEIEVVDGVLRHAASGKQATFGQLAKLAADQPVPAQVKLKDPSAFKLIGRQQLARKDSADKVNGKAIYTQDI
HLPGMLTAVVAHPPRIGAKVKSFDAKAALAIPGVVHVVQIPSGVAVVAKDTWSAKKGRDAVAIQWDESGAFK
QSSEQLFKRYRELAKTEGTVVRNDGDVNTGLAGATRTLEAEYDFPFLAHSAMEPMNCVVALTKYGCETWHGA
QMQSHVQTVLADLFGLEPEQVRVHTLYAGGSFGRRASKDADYVLENAHIVKAIGGKAPVKLVWLREDDMQA
GHYRPMYHHRLTAGLDEQGKLVAWQHRLVGQSVFAGSPFAAFIKDGIDSTSVEGGSTLPYRIPNLRVDLHTPSD
IPVPIQWWRVVGHSHNAFSTECFFDELAQMAGQDPVAWRLAMLEGHSRHIETLKLVAQKAAWDTPLAAGK
QGERRGRGVALHEAFGSVVAQVVEVTVQSDGKFKVDRVVCSVECGIAVNPDVIRAQVEGSVGFALSAAMHEA
ITLTDGKIDQSNFHNNEPLRIYDMPKVEVHIVPSASPPSGIGEPPVAPLAPALVNALAAATGKRIRRLPISDQLSA
>SEQF3783||SEQF3783.1_04058
MSQLPSNFALSNLSRRGFLKGVGATGALVVAASWGLPQAFADEVKQYGGAAMPNGLVDDPKVYVSIASDGTV
TVICNRSEMGQGVRTSLTMVVADELEADWALVKVAQAPGDEARFGNQDTDGSRSMRHWFEPMRRCGAAV
RTMLEQAAAEQWKVPLGECRAQLHKVIHQPSGRELGYGALAAAASALAVPARDSLRLKQPSEFRYIGKEATKAI
DGADIVNGRAVYGADVHFDGMLFATIARPKVYGGKVKSFDASAALKVPGVIKVLQIESRPIPSEFQPLGGVAVV
ASNTWAAIKGREALQIVWDDGANAGYNSTDYRKTLEAAALQPGKVVRNTGSIEQALNDADSTLEAAYYLPHLA
QSPMEPMVAVARYQDGQCEAWAPSQAPQVTRERVAERLGLPFDNVTIHVTLLGGGFGRKSKPDFIIEAALLAK
EFPGKAVRVQWTREDDIHNSYFHTVSAEYLKAGLNKDGLPSGWLHRTVAPSITALFAPGMNHEAAFELGMGF
TNMAYAIPNVRLENPEAAAHTRVGWYRSVSNIPHGFAIQSFVDELAHKAGQDPLKYQLKLLGPDRQIDPRTLSE
EWNYGESPERYPIDTARMRGVLETAAKAAGWGRTLPKGRGLGLAVHYSFVTYVAAVIEVEVKDDGTLIVHKADI
AVDCGPQINPERIRSQFEGACVMGLGNAVLGEISFKDGKVQQDNFHMYEVARMSLAPREIAIHLVTPEAEVPL
GGVGEPGVPPIAPALCNAIFAATGKRIRSLPVRYQLQGWQQAAKA
>SEQF3784||SEQF3784.1_03284

MSRLPNDFVLSNLSRRGFLKGASATGVLVLAASWGMPDAFAEEKKFGAEGMPHGAVDDPKVYVSIAADGSVT
VICNRSEMGQGVRTSLSMVVADELEADWARVKVQQAPADEARFGNQDTDGSRSMRHWYEPMRRCGAAA
RTMLELAAAAQWKVPVGECHAQLHKVLHRPSGRELAYGELAAAASALPVPSRDSLRLKQPSEFRYIGKEASRAI
DGADIVNGRAVFGADVHLDGMLYAVIARPPVYGGKVKSVDSSAALKVAGVVKVVQIEGRPLPSEFQPLGGVAV
VAKNTWAAIKGREALKIQWDDGPNAAYDSIAYRKELEAAALKPGKVVRSSGDLDDALAKADSTLEAAYYLPHLS
QSPMEPMVAVARFKDGQCEAWAPSQAPQVTRERVAERLGIPFEKVTVNITLLGGGFGRKSKPDFVVEAAVLA
KEFPGQPIRVQWTREDDIHHSYFHTVSAEYLKAGLNQDGMPSGWLHRTVAPSITALFAPGMTHEAPFEIGMG
VTNMAYAIPNLRLENPEAVAHARVGWYRSVSNIPHGFAIQSFIDELAHKAGQDPLKYQVKLLGPDRKIDPRTLSE
EWNYGESPERYPIDTARIRTVLETAAKAAGWGRELPKGRGLGLAVHYSFVTYVAAVIEVEVKDDGTVIVHKADIA
VDCGPQINPERIRSQFEGACVMGLGNAMVGEISFKDGKVQQDNFHMYEVARMSLAPKEVAVHLVTPPGEVP
LGGVGEPGVPPIAPALCNAIFAATGKRIRNLPVRYQLQGWQQAKA
>SEQF3784||SEQF3784.1_02004
MLNEIFPNELPRALQHSLERDQADGPATLPRRSFLKIVGIGGLALGAFPHLALAQEANGSAATPLKPTQQPSAFV
QIAPDGVVTVTLNRLEFGQGVQTSLPMILAEELDADWSLVRSRNGNSDAAYLDPNLGIHLTGGSSSIKNSYTQY
RELGARARAMLLAAAAARWNVDVASLSTQAGTVFGPGGRKASYGELAEAAMAMPVPEKVTLKDPKDFRIIG
QATTRIDAKAKSSGQQDFGIDMHLPGQLTAVVARPPVFGARIASLDDSAARATKGVKAVFRVPLDRGAEGVAV
VADGYWQAKLARDALKLEWDLTNVEKVDSEKQLIQYRELATQPGPRQFDADMTPLASAPHQLQAEFVFPYLA
HAPMEPLNCTVQLAQGRAQLWVGTQFPGGDGAAAARVLNLQPEQVQVNVQTAGGGFGRRGVPTNDFVVL
ACEVAKAAHAAGLDVPIRTLWSREDDIKGGYYRPMHLHRAHIGFDDSGKVLAWDHALVGQSIVSGTPFEGMI
KNGIDATATEGMRNPYPLPMRLTVHHPKLNVPVLWWRSVGSTHTAFVMETLIDEIARTTKQDPVAYRMKLFG
DENPRHRAALQLAVDKSEYGKRQLAAGRAWGVAVHESFSSVVAYVVEASVKDGRPVLHNVTAGVHCNLVVNP
RSIEAQVQGAALMGLSMCLPGGAVTLKDGVVQQSNFADFSVPRITDMPEFAVHIVPSAEPPTGMGEPGLPAL
APAFANAVASLTGKPLRELPFKLA
>SEQF3785||SEQF3785.1_04528
MSALPADFVLNNLSRRGFLKGVGATGALVVAASWGLPDAFAADAVKKYGGDAMPHGLIDDPKVYVSIATDGT
VTVICNRSEMGQGVRTSLSLVVADELDADWAHVKVQQAPGDEVRYGNQDTDGSRSMRHWYEPMRRCGAS
VRAMLEQAAAEQWNVPVGECRAQLHKVIHQPSARELGYGELATAAGALAVPARDSLRLKQPSEFRYIGKEGVK
AIDGADIVNGRAVYGADVHFDGMLYATVARPKVYGGKVKSFDASAALKVPGVVKVLEIESRPLPSEFQPLGGIA
VIASNTWAAIKGREALTIVWDDGVNAGYNSVDYRKTLEAAAQKPGKVVRNTGNLDQALGESDSTLEAAYYLPH
LAQSPMEPMVAIARFDNGQCEAWAPSQAPQVTRERIAERLGLPFDNVTVNVTLLGGGFGRKSKPDFVVEAAI
LAKAFPGKAVRVQWTREDDIHNAYFHTVSVEYLKAGLNKDGLPSAWLHRTVAPSITALFAPGMNHEAAFELG
MGFTNMAYAIPNVRLENPEAAAHTRVGWYRSVSNIPHGFAIQCFVDELAHKAGQDPLQYQLKLLGPDRQIDP
KTLSDEWNYGESPERYPIDTARLRGVLETAAKAAGWGRTLPKGRGLGLAVHYSFVTYVAAVIEVEVQGDGTLIV
HKADIAVDCGPQINPERIRSQFEGACVMGLGNAVLGEISFKDGKVQQDNFHMYEVARMSLAPRQVAVHLVTP
KGEVPLGGVGEPGVPPIAPALCNAIFAATGKRIRELPVRYQLQGWQEVKA
>SEQF3786||SEQF3786.1_03937
MSRVPNDFALSNLSRRGFLKGVGATSALVLAASWGWHDALAEDAPKKFGADGMPNGWIDDPKVYVSIAADG
SVTVVCNRSEMGQGVRTSLSMVVADELEADWAQVKVQQAPGDEVRFGNQDTDGSRSMRHWYEPMRRCG
AAARTMLEQAAAAQWKVPVGECHAQLHKVIHKPSGRELGYGELAAAASALAVPARDSLRLKQPSEFRYIGKEG
SKAIDGVDIVNGRAIYGADVHFDGMLYATIARPAVYGGKVKSLDDSAALKVPGVLKVLQIEGRPLPSEFQPLGGV
AVVASNTWAAIKGREALKIEWDDGPNASYDSIAYRKELEAASLTPGKVVRNTGDIDKALSGAASTLEASYYLPHL
AQAPMEPMVAIARFKDGVCEAWAPSQAPQVTRERIAERLGVAFEKVTFNVTLLGGGFGRKSKPDFVVEAAILA
KEFPGKAVRVQWTREDDIHNSYFHTVSAEYLKASVDNNGMPSGWLHRTVAPSITALFAPNMNHEAAFELGM
GFTNMAYAIPNVRLENPEAKVHTRVGWYRSVSNIPHGFAIQSFVDELAHNAGEDPLKYQIKLLGPDRQIDPRTL

SEEWNYGESPERYPIDTGRMRTVLETAAKAAGWGRKLPKGRGLGLAVHYSFVTYVAAVIEVEVKDDGSLIVHKA
DIAVDCGPQINPERIRSQFEGACVMGLGNAVLGEISFKDGKVQQDNFHMYEVARMSLAPKEVAVHLVTPPGD
VPLGGVGEPGVPPIAPALCNAIFAATGKRIRDLPVRYQLQGWQKTEA
>SEQF3788||SEQF3788.1_04410
MTTRNPPLDAPLGEPINLSRRRFLASTAVGALVIGFGLPLGASRAYAATGGPAERGTQVPAFLEIRPDGSVRLLSPF
MEGGQGTHTAMAQIVGEELDADPATFVVEAAPPGEAYVVMENGMRITGGSMSIRMSYPTMRRLGALARAM
LLQAGAEQLGVPLAQLTTQPGRVVHAASGRSLGYGELAGRALDMPVPDPATITLRDPSQFRWIGKPVKRLDAY
DKSTGKAQYSIDLKVDGMLHAAVQHAPRLGMTVGSLRNQSQVEAMKGVHSVHQLPGAVAVVAERWWHAK
RAVEAIQVEWLEAAAGSSVRAMPADFSSDKYRDFLAAQQGPARDDENEGDVAGALTNAKTKIEATYHNQYLN
HAQLEPPSALARFNPDGSLEVWLPNQAPDMFRADMAKRTGLSIEQITLHSPLLGGFFGRHFLYDSANPYPQAI
ALAKAVGRPIKLIWSREEEFLRDVLRPVAVVKFRAALDDKGLPVAIEAVSATEGPSEAIAGKQGEKLDPTALEGLS
GKSYAIPNKRIAQIYVKGPAMLGYWRSVGNSLNDFFYEAFLDELADKGGHDPYELRLHLLRDNKRLTTLLQAAGE
LSGGWKRGPYTAEDGTRRARGVAMASPFGSHAAVIAEVSIESGQVKVHHIWEAIDPGSIVNPAIVEAQVNGAV
ALGLSQTLLEEAVYVDGKPRARNYDLYPILPPSRMAQVHVRIVESGEKMGGIGEPPLPAVAPAVANAVAHLTGQ
RIRSLPLSRYTFS
>SEQF3789||SEQF3789.1_04058
MSRLPNDFALSNLSRRGFLKGVGATGALVLAASWGWQDALAEDKPKQFGADGMPNGWIDDPKVYVSIAAD
GTVTVVCNRSEMGQGVRTSLTMVVADELEADWAHVKVQQAPGDEVRFGNQDTDGSRSMRHWYEPMRRC
GAAARTMLEQAAAAQWKVPVGECRAQLHKVIHTPSGRELGYGELAAAASALTVPARDSLRLKQPSEFRYIGKE
GTKAIDGADIVNGRAVYGADVHFDGMLFAVIARPAVYGGKVKSVDDSAALKVPGVLKVIQIEPRPLPSEFQPLG
GVAVVAANTWAALKGREALKIEWDDGPNATYDSVAYRKEIEAASLKPGKVVRNNGDIDKAIGSAASTLEASYYL
PHLAQAPMEPMVAIARYKDGTCEAWAPSQAPQVTRERIAERLGLPFDNVTFNVTLLGGGFGRKSKPDFVVEA
AVLAKEFPGKAVRVQWTREDDIHNSYFHTVSAEYLKAGVGKDGLPSAWLHRTVAPSITALFAPGMNHEAAFEL
GMGFTNMAYAIPNVRLENPEATVHTRVGWYRSVSNIPHGFAIQSFVDELAHKAKEDPLKYQIRLLGPDRQIDPR
TLSEEWNYGESPERYPIDTGRMRTVLETAAKAAGWGRQLPKGRGLGLAVHYSFVTYVAAVIEVEVKDDGTLIVH
KADIAVDCGPQINPERIRSQFEGACVMGLGNAVWGEISFKDGKVQQDNFHMYEVARMSLAPKEVAVHLVTP
PGEVPLGGVGEPGVPPIAPALCNAIFAATGQRIRNLPVRYQLQGWQKAQA
>SEQF3789||SEQF3789.1_02120
MNSPVSRRGFLKGSAVLGGGLVVAFVVPGGNRFAMAAENEGKVFAPNAFLRIAADNSVTVLLGHSEMGQGI
WTGLTMLIAEELDADWSKIRVEHSPASAADYGMPGFGGMQITGGSTSTWMEFDRYRLAGATARQMLIEAAA
KRFNVAPSTIRTESGVVIAGDNRATYGELADAAGQLPVPDPKSITFKEAKDWKVIGKPTKRLDTPEKITGRAKFG
MDVQFEGLMTAMVARAPAFGATVKSFEGAEALAIPGVHKVVQVPTGVAVVAEHYWAAKLGRDALKVDWDL
GPNADLSSEKLLASFRKLAATPGTSAAQAGDAKGNFGKAAKKVDVEYSVPYLAHAPMEPLNCTVKISADKCEI
WTGTQFQTLDQMVAGKITGLKPEQVEIHTEFLGGGFGRRANPTSDFVAEAVQVAKAAAMPVKTVWSREDDI
RGGYYRSMFLHQAKIGLGADGMPLAWQHVLVGQSIMAGTMLEKTMVKNGVDQTSVEGVSDSPYIKGLAHH
QVDLHSPTTGINVLWLRSVGHSHTAFVMESLIDEMATAAGKDPVEYRRTLLKDHARHLGVLNLAVEKANWKA
PLPDGHALGVAVHESFGSYVAQVAEVSQDNLAIRVHRVVCAVDCGIAVNPQSIAAQMESCITFGLGMALHSKL
TLKDGHVVQSNYHDYQVLRLNEMPVVEVHIVPSTDKPGGIGEAGVPPTAPAVANAVFALTGQRLRELPLLLSGV
>SEQF3790||SEQF3790.2_02964
MLNEIFPNEHPRALQHLLERDEADGPATLPRRSFLKIVGVGGLALGAFPHLALAQEANGAAAPLKPTQQPSAFV
QIAPSGEVTVTINRLEFGQGVQTGLPMILAEELDADWSLVRSRNGNSDAAYMDPAFGIHLTGGSNSIKNSYTQY
RELGARARAMLLSAAAARWNVDVASLSTQAGMVLGPAGRKASYGELAEAAMAMPVPEQITLKDPKDFRIIGQ
ATTRIDAKAKSSGQQDFGIDMHLPGQLTAVVARPPVFGARIASLDDSAARATKGVKAVLRIPLDGGAEGVAVVA
DSYWQAKLARDALKVEWDASAVEKVDSEKQLAQYRELAKQPGPLHFDADMTPLASAPHQLDAEFVFPYLAH

APMEPLNCTVQLAGDGAQLWVGTQFPGGDGAAAAKVLNLKPEQIQVNVQTAGGGFGRRGVPTNDFVVLAC
EVAKAARTAGVNAPIRTLWSREDDIKGGYYRPMHLHRARIGFDDSGKVLAWDHALVGQSIITGTVFGGRVKNG
IDPTATEGLRNPYPLPMRLTVHHPKLNVPVLWWRSVGSTHTAFVMETLIDEIARTTRQDPVAYRMKLFGEQSPR
HREALQLAVDKSEYGKRQLPAGHAWGVAVHESFSSVVAYVVEASVQDGRPVLHNVTAGVHCNLAVNPRSVEA
QVQGAALMGLSMCLPGGAITLKDGVVQQSNFADFSVPRITDMPAFAVHIVPSAEPPTGMGEPGLPALAPAFA
NAVASLTGKPLRELPFKLT
>SEQF3790||SEQF3790.2_04232
MSRLPDDFVLSNLSRRGFLKGASATGVLVLAATWGLPEAFAEEKKFGAEGMPHGAVDDPKVYVSIAADGSVTV
ICNRSEMGQGVRTSLSMVVADELEADWARVKVQQAPADEARFGNQDTDGSRSMRHWYEPMRRCGAAART
MLEQAAAAQWKVPVGECHAQLHKVLHQPSGRELGYGELAAAASALPVPGRDSLRLKQPSEFRYIGKEASRAID
GADIVNGRAVFGADVHFDGMLYAVIARPPVYGGKVKSVDSSAALKVPGVIKVVQIEGRPLPSEFQPLGGVAVVA
KNTWAAIKGREALKIQWDDGPNAGYDSIAYRKELEAAALKPGKVVRSSGDLDDALAKADSTLEAAYYLPHLSQS
PMEPMVAVARFKDGQCEAWAPSQAPQVTRERVAERLGIPFDKVTVNITLLGGGFGRKSKPDFVVEAAVLAKEF
PGQPIRVQWTREDDIHHSYFHTVSAEYLKAGLNQDGMPSGWLHRTVAPSITALFAPGMTHEAPFEIGMGVTN
MAYAIPNLRLENPEATAHTRVGWYRSVSNIPHGFAIQSFIDELAHKAGQDPLKYQVKLLGPDRKIDPRTLSEEWN
YGESPERYPIDTARIRTVLETAAKAAGWGRELPKGRGLGLAVHYSFVTYVAAVIEVEVKDDGTVIVHKADIAVDC
GPQINPERIRSQFEGACVMGLGNAMVGEISFKDGKVQQDNFHMYEVARMSLAPKEVAVHLVTPPGEVPLGG
VGEPGVPPIAPALCNAIFAATGKRIRSLPVRYQLQGWQQAKA
>SEQF3791||SEQF3791.1_06094
MSRLPDDFVLSNLSRRGFLKGASATGVLVLAATWGLPDAFAEEKKFGAEGMPHGAVDDPKVYVSIATDGSVTVI
CNRSEMGQGVRTSLSMVVADELEADWARVKVQQAPADEARFGNQDTDGSRSMRHWYEPMRRCGAAART
MLELAAAAQWKVPVGECHAQLHKVLHQPSGRELGYGELAAAASALAVPSRGSLRLKQPSEFRYIGKEASRAID
GADIVNGRAVFGADVHFDGMLYAVIARPPVYGGKVKSVDSSAALKVPGVVKVVQIEGRPLPSEFQPLGGVAVV
AKNTWAAIKGREALKIQWDDGPNAGYDSIAYRKELEAAALKPGKVVRSSGDLDDALAKADSTLEASYYLPHLSQ
SPMEPMVAVARFKDGQCEAWAPSQAPQVTRERVAERLGIPFEKVTVNITLLGGGFGRKSKPDFVVEAAVLAKE
FPGQAIRVQWTREDDIHHSYFHTVSAEYLKAGLNQDGMPSGWLHRTVAPSITALFAPGMTHEAPFEIGMGVT
NMAYAIPNLRLENPEAVAHARVGWYRSVSNIPHGFAIQSFIDELAHKAGQDPLKYQVKLLGPDRKIDPRSLSEE
WNYGESPERYPIDTARIRTVLETAAKAAGWGRELPKGRGLGLAVHYSFVTYVAAVIEVEVKDDGTVIVHKADIAV
DCGPQINPERIRSQFEGACVMGLGNAMVGEISFKDGKVQQDNFHMYEVARMSLAPKEVAVHLVTPPGEVPL
GGVGEPGVPPIAPALCNAIFAATGKRIRSLPVRYQLQGWQQAKA
>SEQF3791||SEQF3791.1_01234
MLNEIFPNERPRALQLLLERDEADSPATLPRRSFLKIVGIGGLALGAFPHLALAQETNGAAVPLKPSQQPSAFVQI
APSGEVTVTINRLEFGQGVQTGLPMILAEELDADWSLVRSRNGNSDAAYMDPNFGIHLTGGSNSIKNSYTQYR
ELGARARAMLLSAAAARWNVDVASLSTQAGMVLGPAGRKASYGELAEAAMAMPVPEQITLKDPKDFRIIGQA
TTRIDAKAKSSGQQDFGIDMHLPGQLTAVVARPPVFGARIASLDDSAARATKGVKAVLRVPLDGGAEGVAVVA
DSYWQAKLARDALKVEWDASAVEKVDSEKQLAQYRELANQPGPLHFDADMTPLASAPHQLDAEFVFPYLAH
APMEPLNCTVQLAGDGAQLWVGTQFPGGDGAAAAKVLGLKPEQIQVNVQTAGGGFGRRGVPTNDFVVLAC
EVAKAARTAGVNAPIRTLWSREDDIKGGYYRPMHLHRARIGFDDSGKVLAWDHALVGQSIITGTVFGGRVKNG
IDPTATEGLRNPYPLPMRLTVHHPKLNVPVLWWRSVGSTHTAFVMETLIDEIARTTKQDPVAYRMKLFGDQSP
RHREALQLAVDKSEYGKRQLPAGHAWGVAVHESFSSVVAYVVEASVQDGRPVLHNVTAGVHCNLAVNPRSVE
AQVQGAALMGLSMCLPGGAITLKDGVVQQSNFADFSVPRITDMPTFAVHIVPSAEPPTGMGEPGLPALAPAF
ANAVASLTGKPLRELPFKLA
>SEQF3792||SEQF3792.1_02989
MKDPIDSSRRAFVKGGAVLGAGLVVAFVIPGGNRFARAASTADGIFAPNAFLRIAPDSSVTILLGHSEMGQGIW

TGLSMLVAEELDADWASIRVEHGPASAADYGLPGFGGMQITGGSTSTWMEFDRYRLAGAAARLMLIEAAAKR
FNVAPSQIRTEPGVVIAGEQRATYGELANDAGQLPMPDAATITLKQPKDWTLIGKPTPRLDTPEKITGQARFGID
VQFDGLLTAMVARPPMFGGSVASFDAQPALAIPGVRKVLQVPTGVAVVADHYWAAKLGRDALKIDWNPGPN
AGLDSVSLLQRFRQLATTPGTPASQAGDVDAGLAKASKTIDVEYSVPYLAHAPMEPLNCTVKISAEQCEIWTGT
QFQTLDQMVAAKITGLKPEQVVIHTEFLGGGFGRRANPTSDFVSEAVQVAHAAGAAVKTMWAREDDIRGGY
YRSAFLHHARIGLGDDGMPVAWKQVMVGQSIMAGTSLAATMVKDGIDKTSVEGVVDSPYLTGLADHQVQLH
SPQTGISVLWLRSVGHSHTAFVMESLVDELAAAAGKDPVEYRRTLLKDQPRHLGVLNLAVEKANWKAPLPAGH
ALGVAVHESFGSYVAQVSEVSQDNLKIRVHRVVCAVDCGIAVNPLSIVAQMESCITFGLGFTLHSKLTIKEGQVE
QSNYHDYQVLRLNEMPRVEVHILPSSEKPGGIGEAGVPPTAPAVANAVYALTGQRLRELPLQLEGV
>SEQF3792||SEQF3792.1_02596
MSVRIDPALADVALSLTDPVNLSRRRFLTGTAIGALVLGFGLPLGSVRVQAAVAASVERGTQVPAFLEIRPDSSVR
LLCPFMEGGQGIFTAMAQIVGEELDVDPASFLVEAAPPGEAYVVMANGMRITGGSMSVRMSYPVMRRLGAL
ARAMLLQAGAQQLQVPLSELTTEPGKVLHAASGRSLAYGELAERAMDLPVPDVDSVQLRDPSQFRWIGKPVK
RIDAYDKSTGKALYSIDQKVDGMLHAAVQHAPRLGMTVGQLRNEEQVKAMKGVHSVHRLPGAVAVVAERW
WHARRAVEAMQVDWQEPTADSKVRPMPADFSSDAYLTQLAAAKGSARDDENEGDVAASLASAKARVEATY
HNQYLNHAQLEPPSALARFNPDGSLEVWLPNQAPDMFRADIAKRTGLDPAQITVHSPLLGGFFGRHFLYDSAS
PYPQAIALAKAVGRPVKLIWSREEEFLRDVLRPVAAVHFRGALGNDGWPVAIEAISTTEGPTEAIAGKQGEKLDP
SALEGLSGKSYAIANKRIAQLYVKGPAMLGYWRSVGNSLNDFFYESFLDELADKGGKDPFELRLHLLRDNPRLTTL
LQAVGELSGGWKRGPFTAEDGSKRARGVAMASPFGSQAAVIAEVSIENGQVKVHDIWQAIDPGSIVNPAIVEA
QVNGAVALGLSQTLVEEAVWVDGQPRARNFDRYPILPPARMARVHVRVVESGEKMGGIGEPPLPAVAPAVAN
AVAALTGQRIRSLPMSRHTFT
>SEQF3792||SEQF3792.1_01876
MNQLPSDFALSNLSRRGFLKGLGATGALVLAASWGWQDALAEGKQFGADGMPNGWVDDPKVYVSIASDGR
VTVVCNRSEMGQGVRTSLSMVVADELEADWAQVSVRQAPGDEVRFGNQDTDGSRSMRHWYEPMRRCGA
AARSMLEQAAADQWQVPVGECRAQLHKVVHQPSGRELGYGALAEAAAALPVPARDSLRLKQPGEFRYIGKES
VRAIDGEDIVNGRAVYGADVHFEGMLFATVARPAVYGGTVKSFDASAAIKVPGVIKVMQIESRPIPSEFQPLGGI
AVVASNTWAAIKGREALKIVWNDGPNAGYDSIAYRKELESASLKPGKVVRSTGNIDEALNAADSVLEASYYLPHL
AQSPMEPMVAVARFKDGHCEAWAPSQAPQVTRERIAERLGIAFDKVTVNVTLLGGGFGRKSKPDFVLEAAILA
KAFPGKALRVQWTREDDIHNSYFHTVSVEYLKASLNKDGMPSGWLHRTVAPSITALFAPGMNHEAAFELGMG
FTNMAYAIPNVRLENPEAAAHTRVGWYRSVSNIPHGFAIQSFVDELAHKAGQDPLKYQIKLLGPDRQIDPRTLS
EEWNYGESPERYPIDTARLRGVLETAAKAAGWGRDLPKGRGLGLAVHYSFVTYVAVVIEVEVKDDGTVLVHKA
DIAVDCGPQINPERIRSQFEGACVMGLGNAVLGEISFKDGKVQQDNFHMYEVARMSLAPKEVAVHLVTPPGT
VPLGGVGEPGVPPIAPALCNAIFAATGKRIRSLPVRWQLQGWQQAKA
>SEQF3793||SEQF3793.1_02375
MNSPVSRRGFLKGSAIVAGGLVVAFVVPGGHRFARAAENAGKVFAPNAFLRIAADNSVTVLLGHSEMGQGIW
TGLTMLIAEELDADWSKIRVEHSPASAADYGMPAFGGMQITGGSTTTWMEFDRYRLAGATARQMLVQAAAK
RFDVAPSAIRTESGVVIAGDQRATYGELADAAGQLPVPDPQTITLKQARDWKIIGKPTRRLDTPEKITGRAKFGM
DVQFEGLMTAMVARAPAFGATVKSFEGAAALAIPGVHKVLQVPSGVAVIADHYWAAKLGRDALKVNWDLGP
HADMSSESLLDSFRKLAATPGTSAGKAGDAQGSFGKAAKKIEVEYSVPYLAHAPMEPLNCTVKISAGKCEIWTG
TQFQTLDQMVAGKITGLKPEQVEIHTEFLGGGFGRRANPTSDFVAEAVQVAKAAGMPVKTVWAREDDIRGGY
YRSMFLHQARIGLGADGLPSSWEHVLVGQSIMAGTLLEKTMVKDGIDATSVEGVADSPYIKDLAHHQVDLHSP
QTGINVLWLRSVGHTHSGFVMESLVDELATAAGQDPVEYRRTLLKNHPRHLGVLNLAVEKANWKAPLPDGHA
LGVAVHESFGSYVAQVAEVSQDNLAIRVHRVVCAVDCGIAVNPQSIVAQMESCITFGLGMALHSKLTLKNGGV
VQSNYHDYQVLRLNEMPVVEVHIVPSSEKPGGIGEPGVPPTAPAVANAVFALTGQRLRELPLQLSGV

>SEQF3793||SEQF3793.1_01618
MSRLPNDFALSNLSRRGFLKGVGATGALVLAASWGWQDALAEDKPKQFGADGMPNGWIDDPKVYVSIAAD
GTVTVVCNRSEMGQGVRTSLTMVVADELEADWARVKVQQAPGDEVRFGNQDTDGSRSMRHWYEPMRRC
GAAARTMLEQAAAAQWQVPVGECHAQLHKVIHKPSGRELGYGELAAAASALAVPARDSLRLKQPAEFRYIGKE
GNKAIDGDDIVNGRAVYGADVHFDGMLYATIARPAVYGGKVKSLDDSAALKVPGVLKVIQIEPRPLPSEFQPLG
GVAVVASNTWAAIKGREALKIEWDDGPNASYDSVAYRKELEAASLKPGKVVRNTGDIDKALSGAASTLEASYYL
PHLAQAPMEPMVAIARFDKGLCEAWAPSQAPQVTRERIAERLGVPFDNVTFNVTLLGGGFGRKSKPDFVVEA
AILAKEFPGKAVRVQWTREDDIHNSYFHTVSAEYLKAGVGKDGMPSGWLHRTVAPSITALFAPDMNHEAAFEL
GMGFTNMAYAIPNVRLENPEAKVHTRVGWYRSVSNIPHGFAIQSFVDELAHKAGEDPLKYQIKLLGADRQIDP
RTLSEEWNYGESPERYPIDTGRMRTVLETAAKAAGWGRKLPKGRGLGLAVHYSFVTYVAAVIEVEVKDDGTLIV
HKADIAVDCGPQINPERIRSQFEGACVMGLGNAVLGEISFKDGKVQQDNFHMYEVARMSLAPKEVAVHLVTP
PGNVPLGGVGEPGVPPIAPALCNAIFAATGKRIRNLPVRYQLQDWQKAQA
>SEQF3795||SEQF3795.1_01547
MSRLPNDFVLSNLSRRGFLKGASATGVLVLAASWGLPDAFAEEKKYGAEGMPHGAVDDPKVYVSIATDGSVTV
ICNRSEMGQGVRTSLSMVVADELEADWALVKVQQAPADEARFGNQDTDGSRSMRHWYEPMRRCGAAARS
MLEQAAAAQWNVPVGECRAQLHKVVHQPSGRELAYGALATAASALPVPARDSLRLKQPADFRYIGKEATRAV
DGADIVNGRAVFGADVHFEGMLYAVIARPPVYGGKVKTVDSSAALKVPGVVKVVQIEGRPLPSEFQPLGGVAV
LAKNTWAAIKGREALKIEWDDGPNAGYDSIAYRKELEAAALKPGKVLRNTGDLDDALAKADSSLEATYYLPHLS
QSPMEPMVAVARFKDGQCEAWAPSQAPQVTRERIAERLGIAFDKVTVNITLLGGGFGRKSKPDFVVEAAVLAK
EFPGQAIRVQWTREDDIHHSYFHTVSAEYLKAGLNQDGMPSGWLHRTVAPSITALFAPGMTHEAPFEVGMGL
TNMAYAIPNLRLENPEAVAHTRVGWYRSVSNIPHGFAIQSFIDELAHKAGQDPLKYHVKLLGPDRKIDPRTLSEE
WNYGESPERYPIDTARIRVVLETAAKAAGWGRQLPKGRGLGLAVHYSFVTYVAAAIEVEVKDDGTVIVHKADIA
VDCGPQINPERIRSQFEGACVMGLGNAMVGEISFKDGKVQQDNFHMYEVARMSLAPKEVAVHLVTPPGEVP
LGGVGEPGVPPIAPALCNAIFAATGKRIRNLPVRYQLQGWQQAKA
>SEQF3795||SEQF3795.1_02827
MLNEIFPQETPRALQQMLERDAGDGPATLARRSFLKIVGIGGLALGAFPHLALAQQAGDGQAVLKPTQQPAAF
VQITPSGEVTVTINRLEFGQGVQTALPMILAEELDADWSLVRTRNGSNDAAYMDPSFGIHLTGGSNSIKNSYTQ
YRELGARARAMLLAAAAARWNVDVASLTTEAGKVLGPGGRQASYGELAEAAMAMPVPEQVTLKDPKDFRIIG
QATTRIDAKAKSSGRQDFGIDMHLPGQLTAVVARPPVFGARIASLDDSAARAAKGVKAVFRVPLDRGAEGVAV
VAESYWQAKTARDALKVEWDVSRVEKVDSDKQLLQYRELADQPGPLHFDADMAPLATAPHRLDAEFLFPYLA
HAPMEPLNCTVQLAEDGAQLWVGTQFPGGDGAAAAKVLGLKPEQVQVNVQTAGGGFGRRGVPTNDFAVL
ACEVAKGARAAGFNAPVRTLWSREDDIKGGYYRPMHLHRARIGFDDSGKVLAWDHALVGQSIITGTVFGGRV
KNGIDPTATEGLRDPYPLPMRLTVHHPELNVPVLWWRSVGSTHTAFVMETLIDEIARTTQQDPVAYRMKLFGD
QSPRHREALQLAVDKSEYGKRQLAVGHAWGVAVHESFSSVVAYVVEASVQDGRPVLHQVTAGVHCNLVVNPR
SVEAQVQGAALMGLSMCLPGGAITLKDGVVQQSNFADFSVPRITDMPAFAVHIVPSAEPPTGMGEPGLPALA
PAFANAIASLTGKPIRQLPFKLA
>SEQF3796||SEQF3796.1_02324
MNSPVSRRGFLKGSAVLGGGLVVAFVVPGAHRFAMAAENEGKVFAPNAFLRIAADNSVTVLLGHSEMGQGI
WTGLTMLIAEELDADWSTIRVEHSPASAADYGMPAFGGMQITGGSTSTWMEFDRYRLAGATARQMLVEAAA
KRFDVAPSAIRTESGVVIAGDKRATYGELADAAGQLPVPDPKSITFKEAKDWKVIGKPTKRLDTPEKITGRAKFG
MDVQFDGLMTAMVARAPVFGATVKSFEGAEALAVPGVHKVVQVPTGVAVIADHYWAAKLGRDALKVDWDL
GPHTDLSSQGLLDSFRKLAATPGTSASQAGDAKGNFDKAAKKIDVEYSVPYLAHAPMEPLNCTVKISTEKCEIW
TGTQFQTLDQMVAGKITGLKPEHVEIHTEFLGGGFGRRANPTSDFVAEAVQVAKAAGMPVKTVWSREDDIRG
GYYRSMFLHQARIGLDGQGLPLSWQHVLVGQSIMTGTLLEATMVKNGIDPTSVEGVADSPYIKGLAHQQVEL

HSPQTGINVLWLRSVGHSHTAFVMESLIDEMAAAANKDPVEYRRTLLKDHARHLGVLNLAVEKANWQAPLPD
GHALGVAVHESFGSYVAQVAEVSQDNLAIRVHRVVCAVDCGIAVNPQSIAAQMESCITFGLGMALHSKLTVKD
GAVVQSNYHDYQVLRLNEMPLVEVHIVPSSDKPGGIGEAGVPPTAPAVANAVYALTGQRLRELPLQLAGV
>SEQF3796||SEQF3796.1_04066
MSRLPNDFALSNLSRRGFLKGVGATGALVLAASWGWQDALAADAPKKFGADGMPNGWIDDPKVYVSIAAD
GTVTVVCNRSEMGQGVRTSLSMVVADELDADWAKVKVQQAPGDEVRFGNQDTDGSRSMRHWYEPMRRC
GAAARTMLEQAAAAHWQVPVGECQAQLHKVLHKPSGRELGYGELAAAASALAVPARDSLRLKQPSEFRYIGK
EGTKAIDGADIVNGRAVYGADVHFDGMLYATIARPAVYGGKVKSLDDSAALKVPGVLKVMQIESRPLPSEFQPL
GGVAVIASNTWAAIKGREALKIEWDDGPNASYDSIAYRKELEAASRKPGKVVRNTGDIDKALSGAASSLEASYYL
PHLAQAPMEPMVAIARYDKGVCEAWAPSQAPQVTRERIAERLGLPFDNVTFNVTLLGGGFGRKSKPDFVVEA
AILAKEFPGKAVRVQWTREDDIHNSYFHTVSAEYLKAAIGKDGMPSGWLHRTVAPSITALFAPGMNHEAAFEL
GMGFTNMAYAIPNVRLENPEATVHTRVGWYRSVSNIPHGFAIQSFVDELAHKAGVDPLKYQIKLLGPDRQIDP
RTLSEEWNYGESPERYPIDTGRMRTVLETAAKAAGWGRQLPKGRGLGLAVHYSFVTYVAAVIEVEVKDDGTLIV
HKADIAVDCGPQINPERIRSQFEGACVMGLGNAVLGEISFKDGKVQQDNFHMYEVARMSLAPKEIAVHLVTPP
GDVPLGGVGEPGVPPIAPALCNAIFAATGQRIRNLPVRYQLQGWHKAHA
>SEQF3797||SEQF3797.1_04566
MNSPVSRRGFLKGSALLGGGLVVAFAVPGGHRFAMAAEGEGKVFAPNAFLRIGADNSVTVLLGHSEMGQGI
WTGLTMLIAEELDADWSTIRVEHSPASAADYGMPAFGGMQITGGSTSTWMEFDRYRLAGATARQMLVEAAA
KRFDVAPSTIRTESGVVIAGDKRATYGELADAAGQLPVPDPKSITFKEAKDWKVIGKPTKRLDTPEKITGRAKFG
MDVQFEGLMTAMVARAPAFGATVKSFEGAQALAIPGVHKVLQVPSGVAVIAEHYWAAKLGRDALKVDWDLG
PHADLSSEKLLESFRKLATTPGTSASQAGDAKGNFSKAAKKIDVEYSVPYLAHAPMEPLNCTVKISEQKCEIWTG
TQFQTLDQMVAGKITGLKPEQVEIHTEFLGGGFGRRANPTSDFVAEAVQVAKAAGLPVKTVWSREDDIRGGYY
RSMFLHQARIGLGADGLPLSWQHVLVGQSIMTGTLLEATMVKNGIDPTSVEGVSDSPYVKGLAHHQVDLHSP
QTGINVLWLRSVGHSHTAFVMESLIDEMATAAGKDPVEYRRTLLKEHPRHLGVLNLAVEKANWTAPLPEGHAL
GVAVHESFGSYVAQVAQVSQDNLAIRVHRVVCAVDCGIAVNPQSIAAQMESCITFGLGMALHSKLTLKDGAVV
QSNYHDYQVLRLNEMPVVEVHIVPSSDKPGGIGEAGVPPTAPAVANAVYALTGQRLRELPLQLSGV
>SEQF3797||SEQF3797.1_00705
MKKTIEMNVDMSRRRLLQGSGIAVGGLVLSTWLPPLVSKSAASEAAALGRLGDRSAEGFGAFVRVGPDGVVT
VISPKIEMGQGAQTGIAMMVAEELEVDLDKVVIQEAPPNSALYTDTLLQFQATGGSTSTRYTWEPLRRAGATAR
ILLIQAASLQWRVAPSLCHAQNGQVFGPKGLQADYGDLVEAAATLPLPDAVPLKTPEQFKLLGTPAQRLDTPAK
VNGKARFTIDLQIPGMLVASSITCPVYGGRLRSVDETEARRVLGVRDIVRLDNAVAVTASNFWACQQAIKALKIE
WELGSNATIGSKQLDQELLAASSRDGVVAKRTGDIEQAKQGSSSQFEAVYEQALLSHSPLEPMSCVAHVRKDA
CELWVGTQVPVFAQQTAAQVTGLPLEKIQVHNQLIGGAFGRRLEFDFITQAVAIARQVDYPIKLVWSREEDMT
HDLYRPLYADRMQAALDKQGRPLGWEHRIAGASILARYAGSLPPSGVDADAVEVAVEPIYRLPHLQVRYIRQEP
SVVPVSWWRGVGPLRGTYALECFIDELAHNAKADPVVYRLELLADQPRAQAVLRLLAEKSDWYQSLPAGQGR
GVAVSSVFGSYVATLVELEMQGEFGLRIKRLISVVDCGFATNPTSVLAQVEGGTLFGLSASLFNEILIENGQVQQT
NYHNYRQLRISEAPAVEVHLLSSLEAPGGVGEAGTALIGPALVNALYAASGTRIRRLPLSRAGYYPV
>SEQF3797||SEQF3797.1_00357
MSRLPNDFALSNLSRRGFLKGATATGALVLAASWGWQDALAEDKPKQFGADGMPNGWIDDPKVYVSIATDG
TVTVVCNRSEMGQGVRTSLTMVVADELEADWAHVKVQQAPGDEVRFGNQDTDGSRSMRHWYEPMRRCG
AAARTMLEQAAAAQWKVPVGECHAQLHKVIHKPSGRELGYGELAAAASALAVPARDSLRLKQPSEFRYIGKEG
TKAIDGDDIVKGRAVYGADVHFDGMLYATIARPAVYGGKVKSLDDSAALKVPGVIKVMQIEGRPLPSEFQPLGG
VAVVASNTWAAIKGREALKIEWDDGPNASYDSIAYRKELEAASLKPGKVVRNTGDIGKALSSAASSLEASYYLPH
LAQAPMEPMVAIARFNNGVCEAWAPSQAPQVTRERIAERLGLPFDNVTVNVTLLGGGFGRKSKPDFVIEAAIL

AKEFPGKAVRVQWTREDDVHNSYFHTVSAEYLKAGVGKDGMPSGWLHRTVAPSITALFAPGMNHEAVFELG
MGFTNMAYAIPNVRLENPEARVHTRVGWYRSVSNIPHGFAIQSFVDELAHKAGVDPLKYQIKLLGPDRQIDPR
TLSEEWNYGESPERYPIDTGRMRAVLETAAKAAGWGRQLPKGRGLGLAVHYSFVTYVAAVIEVEVKDDGTLIVH
KADIAVDCGPQINPERIRSQFEGACVMGLGNAVLGEISFKDGKVQQDNFHMYEVARMSLAPKEVAVHLVTPP
GDVPLGGVGEPGVPPIAPALCNAIFAATGRRIRNLPVRYQLQGWQKAEA
>SEQF3799||SEQF3799.1_04968
MNILNELLDAPVNLSRRRFLASTAVGALVIGFGLPLGSGRVQAATAAERGTQVPAFLEIRPDGTVRLLSPFMEGG
QGTHTAMAQIVGEELDADPATFIVEAAPPGEAYVVMENGMRITGGSMSVRMSYPTMRRLGALARAMLLQA
GAKQLGVPVGELTTQPGQVVHAASGRSLGYGELASSALDMPVPDPASITLRDPSQFRWIGKPVKRLDAYDKST
GKALYSIDLKVDNMLHAAVQHAPRLGMTVGSLRNQAQVEGMKGVHSVHVLPGAVAVVAERWWHAKRAVE
AVQVDWQEAAADSTLRVMPADFSSDKHFEFLAAQQGPARDDENEGDVAAALKGAKTQVEATYHNQYLNHG
QLEPPSALARFNTDGSLDIWLPNQAPDMFRADIAKRTGLDPAQINLHSPLLGGFFGRHFLYESANPYPQAIALA
KAVGRPIKLIWSREEEFLRDVLRPVAVVKFRAALDDKGLPVAIEAVSATEGPTEALAGKQGDKIDPTAVEGLSGKS
YAIPNKRIAQIYVKGAPMLGYWRSVGNSLNDFFYESFLDELADKGKQDPYELRLHLLRDNPRLTTLLKAAVELAG
GWKRGPFTAEDGSRRARGVAMASPFGSHTAAIAEVSIEKGKVRVHDIWQAIDPGSIVNPAIVEAQVNGAVALG
LSQTLLEEAVYVDGKPRARNYDLYPILAPAQMARVHVRIVESGEKMGGIGEPPLPAVAPAVANAVAQLTGQRVR
SLPLSRHTFS
>SEQF3802||SEQF3802.1_00643
MTTRNPPLDAPLGEPINLSRRRFLASTAVGALVIGFGLPLGASRAYAATGGPAERGTQVPAFLEIRPDGSVRLLSPF
MEGGQGTHTAMAQIVGEELDADPATFVVEAAPPGEAYVVMENGMRITGGSMSIRMSYPTMRRLGALARAM
LLQAGAEQLGVPLAQLTTQPGRVVHAASGRSLGYGELAGRALDMPVPDPATITLRDPSQFRWIGKPVKRLDAY
DKSTGKAQYSIDLKVDGMLHAAVQHAPRLGMTVGSLRNQAQVEAMKGVHSVHQLPGAVAVVAERWWHAK
RAVEAIQVEWLEAAAGSSVRAMPADFSSDKYRDFLAAQQGPARDDENEGDVAGALTNAKTKIEATYHNQYLN
HAQLEPPSALARFNPDGSLEVWLPNQAPDMFRADMAKRTGLSIEQITLHSPLLGGFFGRHFLYDSANPYPQAI
ALAKAVGRPIKLIWSREEEFLRDVLRPVAVVKFRAALDDKGLPVAIEAVSATEGPSEAIAGKQGEKLDPTALEGLS
GKSYAIPNKRIAQIYVKGPAMLGYWRSVGNSLNDFFYEAFLDELADKGGHDPYELRLHLLRDNKRLTTLLQAAGE
LSGGWKRGPYTAEDGTRRARGVAMASPFGSHAAVIAEVSIESGQVKVHHIWEAIDPGSIVNPAIVEAQVNGAV
ALGLSQTLLEEAVYVDGKPRARNYDLYPILPPSRMAQVHVRIVESGEKMGGIGEPPLPAVAPAVANAVAHLTGQ
RIRSLPLSRYTFS
>SEQF3803||SEQF3803.1_01636
MNSPVSRRGFLKGSALLSGGLMVAFVVPGANRFARAAENQNKTFAPNAFLRIAADNSVTVLLGHSEMGQGI
WTGLTMLIAEELDADWSKIRVEHSPASAADYGLPAFGGMQITGGSTSTWMEFDRYRLAGATARQMLVEAAAK
RFDVAPSTIRTESGVVIAGDKRATYGELADAAGQLPVPDPKTITFKEAKDWKVIGKPTKRLDTPEKITGRAKFGM
DVQFEGLMTAMVARAPVFGATVKSFEGAAALAIPGVHKVVQVPSGVAVVAEHFWAAKLGRDALKVDWDLGP
LAGMSSEKLLESFRKLAATPGTSATQAGDAKANFGKAAKKIEVEYSVPYLAHAPMEPLNCTVKISAGKCEIWTGT
QFQTLDQMVASKITGLKPEQVEIHTEFLGGGFGRRANPTSDFVAEAVQVAKAADLPVKTVWAREDDIRGGYYR
SMYLHKAQVGLGADGLPMSWQHVLVGQSIMAGTMLEAAMVKNGIDATSVEGVADSPYIKDLPHHQVELHS
PQTGINVLWLRSVGHTHTGFVMESLIDELATAAGKDPVEYRRTLLKAHPRHLGVLNLAVEKANWGAPLPDGHA
LGVAVHESFGSYVAQVAEVSQDNLAIRVHRVVCAVDCGIAVNPQSIAAQMESCITFGLGMALHSKLTVKDGGV
VQSNYHDYQVLRLNEMPVVEVHIVPSSEKPGGIGEPGVPPTAPAVANAVFALTGQRLRELPLQLSGV
>SEQF3803||SEQF3803.1_04017
MSRVPNDFALSNLSRRGFLKGVGATSALVLAASWGWQDALADDAAKKFGADGMPNGWIDDPKVYVSIAAD
GSVTVVCNRSEMGQGVRTSLSMVVADELEADWAQVKVQQAPGDEVRFGNQDTDGSRSMRHWYEPMRRC
GAAARTMLEQAAAAQWKVPVGECHAQLHKVIHKPSGRELGYGELAAAASALAVPARDSLRLKQPSEFRYIGKE

GTKAIDGADIVNGRAVYGADVHFDGMLYATIARPAVYGGKVKSLDDSAALKVPGVLKVMQIEGRPLPSEFQPL
GGVAVVASNTWAAIKGREALKIEWDDGPNASYDSIAYRKELEAASLKAGKVVRNTGDIDKALSGAASTLEASYY
LPHLAQAPMEPMVAIARYKDGVCEAWAPSQAPQVTRERIAERLGVPFEKVTVNVTLLGGGFGRKSKPDFVVE
AAILAKEFPGKAVRVQWTREDDIHNSYFHTVSAEYLKAGIDKNGMPSGWLHRTVAPSITALFAPDMNHEAAFE
LGMGFTNMAYAIPNVRLENPEAKVHTRVGWYRSVSNIPHGFAIQSFVDELAHKAGEDPLKYQIKLLGPDRQIDP
RTLSEEWNYGESPERYPIDTGRMRTVLETAAKAAGWGRKLPKGRGLGLAVHYSFVTYVAAVIEVEVKDDGSLIV
HKADIAVDCGPQINPERIRSQFEGACVMGLSNAVLGEISFKDGKVQQDNFHMYEVARMSLAPKEVAVHLVTP
PGDVPLGGVGEPGVPPIAPALCNAIFAATGKRIRNLPVRYQLQGWQKAEA
>SEQF3804||SEQF3804.1_02029
MSRLPNDFALSNLSRRGFLKGVGATGALVLAASWGWQDALAEDKPKQFGADGMPNGWIDDPKVYVSIAAD
GTVTVVCNRSEMGQGVRTSLTMVVADELEADWAHVKVQQAPGDEVRFGNQDTDGSRSMRHWYEPMRRC
GAAARTMLEQAAAAQWKVPVGECRAQLHKIIHKPSGRELGYGELAAAASALPVPARDSLRLKQPSEFRYIGKE
GSKAIDGDDIVNGRAVYGADVHFDGMLYATIARPAVYGGKVKSVDDSAALKVPGVLKVIQIETRPLPSEFQPLG
GVAVVASNTWAAIKGREALKIEWDDGPNASYDSIAYRKELEAASLKPGKVVRNTGDIDKALSDAVSTLEASYYLP
HLAQAPMEPMVAIARYQDGVCEAWAPSQAPQVTRERIGERLGLPFDNVTFNVTLLGGGFGRKSKPDFVVEAA
ILAKEFPGKAVRVQWTREDDIHNSYFHTVSAEYVKAGVGKDGMPSGWLHRTVAPSITALFAPGMNHEAAFEL
GMGFTNMAYAIPNVRLENPEATVHTRVGWYRSVSNIPHGFAIQSFVDELAHKAGEDPLKYQIKLLGPDRQIDP
RTLSEEWNYGESPERYPIDTGRMRTVLETAAKAAGWGRKLPKGRGLGLAVHYSFVTYVAAVIEVEVKDDGTLIV
HKADIAVDCGPQINPERIRSQFEGACVMGLGNAVLGEISFKDGKVQQDNFHMYEVARMSLAPKEIAVHLVTPP
GDVPLGGVGEPGVPPIAPALCNAIFAATGKRIRNLPVRYQLQGWQKAEA
>SEQF3804||SEQF3804.1_03680
MNSPVSRRGFLKGSAVLGGGLVVAFVVPGGHKFAYAAENEGKVFAPNAFLRIAADNSVTVLLGHSEMGQGIW
TGLTMLIAEELDADWSKIRVEHSPASAADYGMPGFGGMQITGGSTSTWMEFDRYRLAGATARQMLVEAAAK
RFEVAPSAIRTESGVVIAGDKRATYGELADAAGQLPVPDPKSITFKEAKDWKVIGKPTKRLDTPEKITGRAKFGM
DVQFEGLMTAMVARPPVFGASVKSFEGAEALAVPGVHKVLQVPTGVAVIADHYWAAKLGRDALKIDWDLGP
NADLSSEKLLASFRKLATTQGTSASQAGDAKGSFGKAAKKIDVEYSVPYLAHAPMEPLNCTVKISAEKCEIWTGT
QFQTLDQMVAGKITGLKPEQVEIHTEFLGGGFGRRANPTSDFVAEAVQVAKAAAMPVKTVWSREDDIRGGYY
RSMFLHQAKIGLGADGLPSSWQHVLVGQSIMTGTLMEATMVKNGIDPTSVEGVSDSPYVKGLANHQVDLHS
PQTGINVLWLRSVGHSHTGFVMESLIDEMATAAGKDPVEYRRTLLKDHPRHLGVLNLAVEKANWKAPLPDGH
ALGVAVHESFGSYVAQVAEVSQDNLKIRVHRVVCAVDCGIAVNPQSIAAQMESCITFGLGMALHSKLTLKDGAV
LQSNYHDYQVLRLNEMPVVEVHIVPSSDKPGGIGEAGVPPTAPAVANAVFALTGQRLRELPLQLSGV
>SEQF4072||SEQF4072.1_05959
MTIELDNTGSVRPSRRTFLKAAGAAAAVSLTIGFEWAGLGRRALAATAPAADFAPNAFLRITPDGAVTVIAKHVE
MGQGAYTGIATIVAEELDADWSTVRVESAPADAKRYANLAFGTMQGTGGSSAMANSWQQLREAGGKARA
MLVSAAAARWKVPAGELTTANGIVSHAKSGKKAAYGTLVADASKLPVPDKVALKQPADFKLIGQRIPRVDASPK
SNGTAHFTLDTTFPGMRVALLQRPPRFGATVKSFDATAAKAVPGVVSVVQVPGGVAVVGTGFWAAKQGRDAL
KVEWDEAHAEKRSSDEIMREYRQLADKPGTSARKDGDADAAIAGAARKIGATYEFPYLAHAPMEPLDAVVKLT
ADSCEIWAGDQFQTVDQANAARTAGLKPEQVQIHTLYAGGSFGRRANAWSDYVVEAVSIAKALGADGKPVKL
QWTREDDIQGGFYRPMYFHKLDAGLTADGKLVGWRHRIVGQSILAGTPFEAFMVKNGIDATSVEGAANLPYA
VPNVSVELTTTKVGLPVLWWRVVGSSHTAYAVEAFIDEAAHTAGKDPYAFRRDLLAKEPRMRAVLDLAAQKAG
WDPAKPLPKGRGRGIAVAEAFKSYVAQVAEVSVDADGKVKVERVVCAVDCGIAINPDIVAAQMEGGIGFGLGA
VMHSAITLKDGQVEQRNFDGYHVLRIAEMPKVEVHIVPSAEAPTGVGEPGVAPVGPAVANAIFAATGKRHYVL
PFDSADSAKA
>SEQF4072||SEQF4072.1_01361

MSAPELSVHNESRRALLLGFASGGLLLAFGVPSLVRAAVPSQPPVSADPRYGGAGMPHGLRDDPHLFVAIAPD
GTVTVTCIRSEMGQGVRTSVALVVADELGADWARVKVEQAVGDEPRYGNQNTDGSRSLRQSFAALRRAGAA
ARTMLEQAAAAAWGVDARQVKATVHEVVDTKSGRKLGFGELAAKAAALPAPDPATVPLKAPAEFRYIGKGQT
ALIDGRDIVAGRAHYGIDTRLDGMLYAVVARPPTYGDTVASFDASAAEKLPGVVKVVPLAPTPLPSGFQPLGGV
AVVARDTWTAIQARAQLKIDWKHGPNANYDSAAYRKTLEAAAAQPGDVIRNDGDAAAALAGAAKRVRATYYI
PHLAHATMEPPAAVARVADGRCEVWTCTQAPQTTRDEVAKALGLPGERVTVNVTLLGGGFGRKSKPDYVVEA
ALLSKAVGAPVKLTFTREDDLAHDYFHAVSLEAFDGGIDASGKVVAWQHRTVAPSIQSTFRAGIVHEQPGELAQ
GIADLPFAIPNVRIENPAAQAHTRIGWFRSVYNIPHAFGIQSFVSELAHAAGRDPKDFLLELIGPARRFEPHITVK
NVNYGEDPALYPVDTGRLRRVVETVAREAGWGRKLPKGHGLGIAAHRSFVSYTAAVCEVQVDADGKITVPRVD
IAIDCGPQVNPERVRSQLEGAVVMGLGIALHGEITFKDGHPEQSNFNGFQVLRMNEAPREIRVHLVAPDDFAT
PLGGVGEPGLPPVAPALTNAIFAATGTRIRSLPVADQLAKPRAG
>SEQF4072||SEQF4072.1_05327
MSRGLIEAGKVAGQAAGAGVSRRSFLKLGMSLGAAAGGGLLLGFSLPAAGDDARRSVIGGDGDETARAGVFA
PNAFVQIDRAGKVTLVMPKVEMGQGVYTALPMLIAEELEVPLSSVTLDHAPPNEKLFLDPLLGGQLTGGSTSVR
YAWEPLRRAGATARTLLVAAAAKQWNVDPASCRAVNGEVQHPPSGRRVSYGQLADAAAKLPVPKDVALKKPA
DFKLIGTPAKRLDSPEKVDGTAQFGLDVRLPGMLYAVIVNSPVFGGTVASIDDTAAKKIPGVRQVVRADNAVAV
VGDHTWAAKRGASALVVKWNEGAGAKVSTKDIVADLAQAAANGKGAVARKDGDVGKGFADAKTRIDAVYE
QPLLAHATMEPVNCTVHVRADGCEIWVGTQVPTRAVDTVQKLTSFPPERIVVHNHLLGGGFGRRLETDMIGQ
AVKIAKQVNAPVKVIWTREEDIQHDMYRPYYYDRISAGLDANGKPIAWQHRIVGSSILARFAPPAFQHGVDPD
AVEVATDLPYDLPNQLIDYVRQEPRHVPTAFWRGVGPTRSTFVVESFIDELAAQTKTDPVQYRRALLGKTPRAL
NVLDVATKAAGWGPSLPTGQGRGVSVMHAFGSFFSIVIDVAVDNGEVQVKRVVCAVDCGMSVNPNTIEAQV
QGGIIFGITGALYGEITIEDGRVVQSNFTDYRMLRINETPPIEVHLVKSGEAPGGIGEPGTAATAAALSNAIFAATG
KRLRKLPVGDQLKTA
>SEQF4073||SEQF4073.1_03640
MTIELDNTGSVRPSRRTFLKAAGAAAAVSLTIGFEWAGLGRRALAATAPAADFAPNAFLRITPDGAVTVIAKHVE
MGQGAYTGIATIVAEELDADWSTVRVESAPADAKRYANLAFGTMQGTGGSSAMANSWQQLREAGGKARA
MLVSAAAARWKVPAGELTTANGIVSHAKSGKKAAYGTLVADASKLPVPDKVALKQPADFKLIGQRIPRVDASPK
SNGTAHFTLDTTFPGMRVALLQRPPRFGATVKSFDATAAKAVPGVVSVVQVPGGVAVVGTGFWAAKQGRDAL
KVEWDEAHAEKRSSDEIMREYRQLADKPGTSARKDGDADAAIAGAARKIGATYEFPYLAHAPMEPLDAVVKLT
ADSCEIWAGDQFQTVDQANAARTAGLKPEQVQIHTLYAGGSFGRRANAWSDYVVEAVSIAKALGADGKPVKL
QWTREDDIQGGFYRPMYFHKLDAGLTADGKLVGWRHRIVGQSILAGTPFEAFMVKNGIDATSVEGAANLPYA
VPNVSVELTTTKVGLPVLWWRVVGSSHTAYAVEAFIDEAAHTAGKDPYAFRRDLLAKEPRMRAVLDLAAQKAG
WDPAKPLPKGRGRGIAVAEAFKSYVAQVAEVSVDADGKVKVERVVCAVDCGIAINPDIVAAQMEGGIGFGLGA
VMHSAITLKDGQVEQRNFDGYHVLRIAEMPKVEVHIVPSAEAPTGVGEPGVAPVGPAVANAIFAATGKRHYVL
PFDSADSAKA
>SEQF4073||SEQF4073.1_06059
MSRGLIEAGKVAGQAAGAGVSRRSFLKLGMSLGAAAGGGLLLGFSLPAAGDDARRSVIGGDGDETARAGVFA
PNAFVQIDRAGKVTLVMPKVEMGQGVYTALPMLIAEELEVPLSSVTLDHAPPNEKLFLDPLLGGQLTGGSTSVR
YAWEPLRRAGATARTLLVAAAAKQWNVDPASCRAVNGEVQHPPSGRRVSYGQLADAAAKLPVPKDVALKKPA
DFKLIGTPAKRLDSPEKVDGTAQFGLDVRLPGMLYAVIVNSPVFGGTVASIDDTAAKKIPGVRQVVRADNAVAV
VGDHTWAAKRGASALVVKWNEGAGAKVSTKDIVADLAQAAANGKGAVARKDGDVGKGFADAKTRIDAVYE
QPLLAHATMEPVNCTVHVRADGCEIWVGTQVPTRAVDTVQKLTSFPPERIVVHNHLLGGGFGRRLETDMIGQ
AVKIAKQVNAPVKVIWTREEDIQHDMYRPYYYDRISAGLDANGKPIAWQHRIVGSSILARFAPPAFQHGVDPD
AVEVATDLPYDLPNQLIDYVRQEPRHVPTAFWRGVGPTRSTFVVESFIDELAAQTKTDPVQYRRALLGKTPRAL

NVLDVATKAAGWGPSLPTGQGRGVSVMHAFGSFFSIVIDVAVDNGEVQVKRVVCAVDCGMSVNPNTIEAQV
QGGIIFGITGALYGEITIEDGRVVQSNFTDYRMLRINETPPIEVHLVKSGEAPGGIGEPGTAATAAALSNAIFAATG
KRLRKLPVGDQLKTA
>SEQF4073||SEQF4073.1_02633
MSAPELSVHNESRRALLLGFASGGLLLAFGVPSLVRAAVPSQPPVSADPRYGGAGMPHGLRDDPHLFVAIAPD
GTVTVTCIRSEMGQGVRTSVALVVADELGADWARVKVEQAVGDEPRYGNQNTDGSRSLRQSFAALRRAGAA
ARTMLEQAAAAAWGVDARQVKATVHEVVDTKSGRKLGFGELAAKAAALPAPDPATVPLKAPAEFRYIGKGQT
ALIDGRDIVAGRAHYGIDTRLDGMLYAVVARPPTYGDTVASFDASAAEKLPGVVKVVPLAPTPLPSGFQPLGGV
AVVARDTWTAIQARAQLKIDWKHGPNANYDSAAYRKTLEAAAAQPGDVIRNDGDAAAALAGAAKRVRATYYI
PHLAHATMEPPAAVARVADGRCEVWTCTQAPQTTRDEVAKALGLPGERVTVNVTLLGGGFGRKSKPDYVVEA
ALLSKAVGAPVKLTFTREDDLAHDYFHAVSLEAFDGGIDASGKVVAWQHRTVAPSIQSTFRAGIVHEQPGELAQ
GIADLPFAIPNVRIENPAAQAHTRIGWFRSVYNIPHAFGIQSFVSELAHAAGRDPKDFLLELIGPARRFEPHITVK
NVNYGEDPALYPVDTGRLRRVVETVAREAGWGRKLPKGHGLGIAAHRSFVSYTAAVCEVQVDADGKITVPRVD
IAIDCGPQVNPERVRSQLEGAVVMGLGIALHGEITFKDGHPEQSNFNGFQVLRMNEAPREIRVHLVAPDDFAT
PLGGVGEPGLPPVAPALTNAIFAATGTRIRSLPVADQLAKPRAG
>SEQF4074||SEQF4074.1_05686
MTIELDNTGSVRPSRRTFLKAAGAAAVSLTIGFDFTGFGRRALAATAPAAGFAPNAFLRITPDGAVTVIAKHVEM
GQGAYTGIATIVAEELDADWSSVRVESAPADAKRYANLAFGTMQGTGGSSAMANSWQQLREAGGKARAML
VSAAAARWKVPAGELTTKDGFVIHAKTGRQAGYGTLVAQAAKLPVPDKVVLKQPAEFRLIGHKIPRVDAPAKSD
GTAHFTLDTTFPGMRVALLQRPPRFGATVKSFDATAAKAVPGVVSVVQVPGGVAVVGTGFWAAKQGRDALKV
EWDEAHAEKRGSDEIMREYRRLANQPGAPARTDGDAGAAIAGAARKIGATYEFPYLAHAPMEPLDAVVKLTA
DSCEIWAGDQFQTVDQANAARTAGLKPEQVQIHTLYAGGSFGRRANAWSDYVVEAVSIAKALGADGKPVKLQ
WTREDDIQGGFYRPMYFHKLDAGLTADGKLVGWRHRIVGQSILAGTPFEAFMVKNGVDATSVEGAANLPYAV
PNVSVELTTTKVGLPVLWWRVVGSSHTAYAVEAFIDEAAHTAGKDPYAFRRDLLAKEPRMRAVLDLAAQKAG
WDPAKPLPKGRGRGIAVAEAFKSYVAQVAEVSVDADGKVKVERVVCAVDCGIAINPDIVAAQMEGGIGFGLGA
VMHSAITLKDGQVEQRNFDGYHVLRIAEMPKVEVHIVPSAEAPTGVGEPGVAPVGPAVANAIFAATGKRHYVL
PFDSADSAKA
>SEQF4074||SEQF4074.1_04945
MSRGLIEAGQAGAGVSRRSFLKLGMSLGAAAGGGLLLGFSLPAAGDDARRSVIGGDGDETARAGVFAPNAFV
QIDRAGKVTLVMPKVEMGQGVYTALPMLIAEELEVPLSNVTLDHAPPNEKLFLDPLLGGQLTGGSTSVRYAWE
PLRRAGATARTLLVAAAAKQWNVDPASCRASNGEVQHPPSGRRASYGQLADAAAKLPVPKDVALKKPADFKLI
GTPAKRLDSPEKVDGTAQFGLDVRLPGMLYAVIVNSPVFGGTVASVDDTAAKKIPGVRQVVRADNAVAVVGD
HTWAAKRGASALVVKWNEGADAKVSTKDIVADLAQAAANGKGAVARKDGDVGKGFADAKTRIDAVYEQPFL
AHATMEPVNCTVHMRADGCEIWVGTQVPTRARDTVQQLTSFPPEKIVVHNHLLGGGFGRRLETDMIGQAVK
IAKQVNAPVKVIWTREEDIQHDMYRPYYYDRISAGLDANGKPIAWQHRIVGSSILARFAPPAFQHGVDPDAVE
VATDLPYDLPNQLIDYVRQEPRHVPTAFWRGVGPTRSTFVVESFIDELAAQTKTDPVQYRRALLGKTPRALNVL
DVATKAAGWGPSLPKGQGRGVSVMHAFGSFFSIVIDVAVDDGEVQVKRVVCAVDCGMFVNPNTIEAQVQG
GIIFGITGALYGEITIEDGRVVQSNFTDYRMLRINETPPIEVHLVKSAEAPGGIGEPGTAALAAALANAIFAATGKRL
RKLPVGDQLKTA
>SEQF4075||SEQF4075.1_06208
MSRGLIEAGKVAGQAAGAGVSRRSFLKLGMSLGAAAGGGLLLGFSLPAAGDDARRSVIGGDGDETARAGVFA
PNAFVQIDRAGKVTLVMPKVEMGQGVYTALPMLIAEELEVPLSSVTLDHAPPNEKLFLDPLLGGQLTGGSTSIRY
AWEPLRRAGATARTLLVAAAAKQWNVDPASCRAVNGEVQHPPSGRRVSYGQLADAAAKLPVPKDVALKKPAD
FKLIGTPAKRLDSPEKVDGTAQFGLDVRLPGMLYAVIVNSPVFGGTVASIDDTAAKKIPGVRQVVRADNAVAVV

GDHTWAAKRGASALVVKWNEGAGAKVSTKDIVADLAQAAANGKGAVARKDGDVGKGFADAKTRVDAVYEQ
PLLAHATMEPVNCTVHVRADGCEIWVGTQVPTRAVDTVQQLTSFPPEKIVVHNHLLGGGFGRRLETDMIGQA
VKIAKQVNAPVKVIWTREEDIQHDMYRPYYYDRISAGLDANGKPIAWQHRIVGSSILARFAPPAFQHGVDPDA
VEVATDLPYDLPNQLIDYVRQEPRHVPTAFWRGVGPTRSTFVVESFIDELAAQTKTDPVQYRRALLGKTPRALN
VLDVATKAAGWGPSLPTGQGRGVSVMHAFGSFFSIVIDVAVDNGEVQVKRVVCAVDCGMSVNPNTIEAQVQ
GGIIFGITGALYGEITIEDGRVVQSNFTDYRMLRINETPPIEVHLVKSGEAPGGIGEPGTAATAAALSNAIFAATGK
RLRKLPVGDQLKTA
>SEQF4075||SEQF4075.1_00865
MSAPELSVHNESRRALLLGFASGGLLLAFGVPSLVRAAVPNQPPVSADPRYGGAGMPHGLRDDPHLFVAIAPD
GTVTVTCIRSEMGQGVRTSVALVVADELGADWARVKVAQAVGDEPRYGNQNTDGSRSLRQSFAALRRAGAA
ARTMLEQAAAAAWGVDARQVKATVHEVVDTKSGRKLGFGELAAKAAALPAPDPATVPLKAPAEFRYIGKGETA
LIDGRDIVAGRAHYGIDTRLDGMLYAVVARPPAYGDTMTSFDASAAEKLPGVVKVVPLASTPLPSGFQPLGGVA
VVARDTWTAIQARAQLKIDWKHGPNANYDSAAYRKTLEAAAAQPGDVIRNDGDAAAALAGAAKRVRATYYIP
HLAHATMEPPAAVARVADGRCEVWTCTQAPQTTRDEVAKALGLPGERVTVNVTLLGGGFGRKSKPDYVVEAA
LLSKAVGAPVKLTFTREDDLAHDYFHAVSLEAFDGGIDASGKVVAWQHRTVAPSIQSTFRAGVVHEQPGELAQ
GIADLPFAIPNVRIENPAAQAHTRIGWFRSVYNIPHAFGIQSFVSELAHAAGRDPKDFLLELIGPARRFEPHITVK
NVNYGEDPALYPVDTGRLRRVVETVAREAGWGRRLPKGHGLGIAAHRSFVSYTAAVCEVQVDADGKITVPRVD
IAIDCGPQVNPERVRSQLEGAVVMGLGIALHGEITFKDGYPEQSNFNGFQVLRMNEAPREIRVHLVAPDDFATP
LGGVGEPGLPPVAPALTNAIFAATGTRIRSLPVADQLAKPRAG
>SEQF4075||SEQF4075.1_03777
MTIELDNTGSVRPSRRTFLKAAGAAAAVSLTIGFEWAGLGRRALAATAPAADFAPNAFLRITPDGAVTVIAKHVE
MGQGAYTGIATIVAEELDADWSTVRVESAPADAKRYANLAFGTMQGTGGSSAMANSWQQLREAGGKARA
MLVSAAAARWKVPAGELTTANGVVTHAKSGKKAAYGTLVADASKLPVPDKVVLKQPADFKLIGQRIPRVDASPK
SNGTAHFTLDTTLPGMRVALLQRPPRFGATVKSFDATAARAVPGVVSVVQVPGGIAVVGTGFWAAKQGRDAL
KVEWDEAHAEKRGSDEIMREYRQLADKPGTSARKDGDADAAIAGAARKIGATYEFPYLAHAPMEPLDAVVKLT
ADSCEIWAGDQFQTVDQANAARTAGLKPEQVQIHTLYAGGSFGRRANAWSDYVVEAVSIAKALGADGKPVKL
QWTREDDIQGGFYRPMYFHKLDAGLTEDGKLVGWRHRIVGQSILAGTPFEAFMVKNGIDATSVEGAANLPYA
VPNVSVELTTTKVGLPVLWWRVVGSSHTAYAVEAFIDEAAHTAGKDPYAFRRDLLAKEPRMRAVLDLAAQKAG
WDPAKPLPKGRGRGIAVAEAFKSYVAQVAEVSVDADGKVKVERVVCAVDCGIAINPDIVAAQMEGGIGFGLGA
VMHSAITLKDGQVEQRNFDGYHVLRIAEMPKVEVHIVPSAEAPTGVGEPGVAPVGPAVANAIFAATGKRHYVL
PFDSADSAKA
>SEQF4076||SEQF4076.2_04689
MSRGLIEAGKVAGQAAGAGVSRRSFLKLGMSLGAAAGGGLLLGFSLPAAGDDARRSVIGGDGDETARAGVFA
PNAFVQIDRAGKVTLVMPKVEMGQGVYTALPMLIAEELEVPLSSVTLDHAPPNEKLFLDPLLGGQLTGGSTSVR
YAWEPLRRAGATARTLLVAAAAKQWNVDPASCRAVNGEVQHPPSGRRVSYGQLADAAAKLPVPKDVALKKPA
DFKLIGTPAKRLDSPEKVDGTAQFGLDVRLPGMLYAVIVNSPVFGGTVASIDDTAAKKIPGVRQVVRADNAVAV
VGDHTWAAKRGASALVVKWNEGAGAKVSTKDIVADLAQAAANGKGAVARKDGDVGKGFADAKTRIDAVYE
QPLLAHATMEPVNCTVHVRADGCEIWVGTQVPTRAVDTVQKLTSFPPERIVVHNHLLGGGFGRRLETDMIGQ
AVKIAKQVNAPVKVIWTREEDIQHDMYRPYYYDRISAGLDANGKPIAWQHRIVGSSILARFAPPAFQHGVDPD
AVEVATDLPYDLPNQLIDYVRQEPRHVPTAFWRGVGPTRSTFVVESFIDELAAQTKTDPVQYRRALLGKTPRAL
NVLDVATKAAGWGPSLPTGQGRGVSVMHAFGSFFSIVIDVAVDNGEVQVKRVVCAVDCGMSVNPNTIEAQV
QGGIIFGITGALYGEITIEDGRVVQSNFTDYRMLRINETPPIEVHLVKSGEAPGGIGEPGTAATAAALSNAIFAATG
KRLRKLPVGDQLKTA
>SEQF4076||SEQF4076.2_03498

MSAPELSVHNESRRALLLGFASGGLLLAFGVPSLVRAAVPSQPPVSADPRYGGAGMPHGLRDDPHLFVAIAPD
GTVTVTCIRSEMGQGVRTSVALVVADELGADWARVKVEQAVGDEPRYGNQNTDGSRSLRQSFAALRRAGAA
ARTMLEQAAAAAWGVDARQVKATVHEVVDTKSGRKLGFGELAAKAAALPAPDPATVPLKAPAEFRYIGKGQT
ALIDGRDIVAGRAHYGIDTRLDGMLYAVVARPPTYGDTVASFDASAAEKLPGVVKVVPLAPTPLPSGFQPLGGV
AVVARDTWTAIQARAQLKIDWKHGPNANYDSAAYRKTLEAAAAQPGDVIRNDGDAAAALAGAAKRVRATYYI
PHLAHATMEPPAAVARVADGRCEVWTCTQAPQTTRDEVAKALGLPGERVTVNVTLLGGGFGRKSKPDYVVEA
ALLSKAVGAPVKLTFTREDDLAHDYFHAVSLEAFDGGIDASGKVVAWQHRTVAPSIQSTFRAGIVHEQPGELAQ
GIADLPFAIPNVRIENPAAQAHTRIGWFRSVYNIPHAFGIQSFVSELAHAAGRDPKDFLLELIGPARRFEPHITVK
NVNYGEDPALYPVDTGRLRRVVETVAREAGWGRKLPKGHGLGIAAHRSFVSYTAAVCEVQVDADGKITVPRVD
IAIDCGPQVNPERVRSQLEGAVVMGLGIALHGEITFKDGHPEQSNFNGFQVLRMNEAPREIRVHLVAPDDFAT
PLGGVGEPGLPPVAPALTNAIFAATGTRIRSLPVADQLAKPRAG
>SEQF4076||SEQF4076.2_05321
MTIELDNTGSVRPSRRTFLKAAGAAAAVSLTIGFEWAGLGRRALAATAPAADFAPNAFLRITPDGAVTVIAKHVE
MGQGAYTGIATIVAEELDADWSTVRVESAPADAKRYANLAFGTMQGTGGSSAMANSWQQLREAGGKARA
MLVSAAAARWKVPAGELTTANGIVSHAKSGKKAAYGTLVADASKLPVPDKVALKQPADFKLIGQRIPRVDASPK
SNGTAHFTLDTTFPGMRVALLQRPPRFGATVKSFDATAAKAVPGVVSVVQVPGGVAVVGTGFWAAKQGRDAL
KVEWDEAHAEKRSSDEIMREYRQLADKPGTSARKDGDADAAIAGAARKIGATYEFPYLAHAPMEPLDAVVKLT
ADSCEIWAGDQFQTVDQANAARTAGLKPEQVQIHTLYAGGSFGRRANAWSDYVVEAVSIAKALGADGKPVKL
QWTREDDIQGGFYRPMYFHKLDAGLTADGKLVGWRHRIVGQSILAGTPFEAFMVKNGIDATSVEGAANLPYA
VPNVSVELTTTKVGLPVLWWRVVGSSHTAYAVEAFIDEAAHTAGKDPYAFRRDLLAKEPRMRAVLDLAAQKAG
WDPAKPLPKGRGRGIAVAEAFKSYVAQVAEVSVDADGKVKVERVVCAVDCGIAINPDIVAAQMEGGIGFGLGA
VMHSAITLKDGQVEQRNFDGYHVLRIAEMPKVEVHIVPSAEAPTGVGEPGVAPVGPAVANAIFAATGKRHYVL
PFDSADSAKA
>SEQF4077||SEQF4077.1_01602
MSRGLIEAGKVAGQAAGAGVSRRSFLKLGMSLGAAAGGGLLLGFSLPAAGDDARRSVIGGDGDETARAGVFA
PNAFVQIDRAGKVTLVMPKVEMGQGVYTALPMLIAEELEVPLSSVTLDHAPPNEKLFLDPLLGGQLTGGSTSIRY
AWEPLRRAGATARTLLVAAAAKQWNVDPASCRAVNGEVQHPPSGRRTSYGQLADAAAKLPVPKDVALKKPAD
FKLIGTPAKRLDSPEKVDGTAQFGLDVRLPGMLYAVIVNSPVFGGTVASIDDTAAKKIPGVRQVVRADNAVAVV
GDHTWAAKRGASALVVKWNEGAGAKVSTKDIVADLAQAAANGKGAVARKDGDVGKGFADAKTRIDAVYEQ
PLLAHATMEPVNCTVHVRADGCEIWVGTQVPTRAVDTVQQLTSFPPEKIVVHNHLLGGGFGRRLETDMIGQA
VKIAKQVNAPVKVIWTREEDIQHDMYRPYYYDRISAGLDANGKPIAWQHRIVGSSILARFAPPAFQHGVDPDA
VEVATDLPYDLPNQLIDYVRQEPRHVPTAFWRGVGPTRSTFVVESFIDELAAQTKTDPVQYRRALLGKTPRALN
VLDVATKAAGWGPSLPTGQGRGVSVMHAFGSFFSIVIDVAVDNGEVQVKRVVCAVDCGMSVNPNTIEAQVQ
GGIIFGITGALYGEITIEDGRVVQSNFTDYRMLRINETPPIEVHLVKSGEAPGGIGEPGTAATAAALSNAIFAATGK
RLRKLPVGDQLKTA
>SEQF4077||SEQF4077.1_05242
MSAPDLSVHNESRRALLLGFASGGLLLAFGVPSLVRAAVPNQPPVSADPRYGGAGMPHGLRDDPHLFVAIAPD
GTVTVTCIRSEMGQGVRTSVALVVADELGADWARVKVAQAVGDEPRYGNQNTDGSRSLRQSFAALRRAGAA
ARTMLEQAAAAAWGVDARQVKATVHEVVDTKSGRKLGFGELAAKAAALPAPDPATVPLKAPAEFRYIGKGETA
LIDGRDIVAGRAHYGIDTRLDGMLYAVVARPPAYGDTMASFDASAAEKLPGVVKVMPLAPTPLPSGFQPLGGV
AVVARDTWTAIQARAQLKIDWKHGPNANYDSAAYRKTLEAAAAQPGDVIRNDGDAAAALAGAAKRVRATYYI
PHLAHATMEPPAAVARVADGRCEVWTCTQAPQTTRDEVAKALGLPGERVTVNVTLLGGGFGRKSKPDYVVEA
ALLSKAVGAPVKLTFTREDDLAHDYFHAVSLEAFDGGIDASGKVVAWQHRTVAPSIQSTFRAGVVHEQPGELA
QGIADLPFAIPNVRIENPAAQAHTRIGWFRSVYNIPHAFGIQSFVSELAHAAGRDPKDFLLELIGPARRFEPHITV

KNVNYGEDPALYPVDTGRLRRVVETVAREAGWGRRLPKGHGLGIAAHRSFVSYTAAVCEVQVDADGKITVPRV
DIAIDCGPQVNPERVRSQLEGAVVMGLGIALHGEITFKDGHPEQSNFNGFQVLRMNEAPREIRVHLVAPDDFA
TPLGGVGEPGLPPVAPALTNAIFAATGTRIRSLPVADQLAKPRAG
>SEQF4077||SEQF4077.1_00857
MTIELDNTGSVRPSRRTFLKAAGAAAAVSLTIGFEWAGLGRRALAATAPAADFAPNAFLRITPDGAVTVIAKHVE
MGQGAYTGIATIVAEELDADWSTVRVESAPADAKRYANLAFGTMQGTGGSSAMANSWQQLREAGGKARA
MLVSAAAARWKVPAGELTTANGVVTHAKSGKKAAYGTLVADASKLPVPDKVVLKQPADFKLIGHRIPRVDASPK
SNGTAHFTLDTTFPGMRVALLQRPPRFGATVKSFDATAARAVPGVVSVVQVPGGIAVVGTGFWAAKQGRDAL
KVEWDEAHAEKRGSDEIMREYRQLADKPGTSARKDGDADAAIAGAARKIGATYEFPYLAHAPMEPLDAVVKLT
ADSCEIWAGDQFQTVDQANAARTAGLKPEQVQIHTLYAGGSFGRRANAWSDYVVEAVSIAKALGADGKPVKL
QWTREDDIQGGFYRPMYFHKLDAGLTEDGKLVGWRHRIVGQSILAGTPFEAFMVKNGIDATSVEGAANLPYA
VPNVSVELTTTKVGLPVLWWRVVGSSHTAYAVEAFIDEAAHTAGKDPYAFRRDLLAKEPRMRAVLDLAAQKAG
WDPAKPLPKGRGRGIAVAEAFKSYVAQVAEVSVDADGKVKVERVVCAVDCGIAINPDIVAAQMEGGIGFGLGA
VMHSAITLKDGQVEQRNFDGYHVLRIAEMPKVEVHIVPSAEAPTGVGEPGVAPVGPAVANAIFAATGKRHYVL
PFDSADSAKA
>SEQF4078||SEQF4078.1_04278
MTIELDNTGSVRPSRRTFLKAAGAAAAVSLTIGFEWAGLGRRALAATAPAADFAPNAFLRITPDGAVTVIAKHVE
MGQGAYTGIATIVAEELDADWSTVRVESAPADAKRYANLAFGTMQGTGGSSAMANSWQQLREAGGKARA
MLVSAAAARWKVPAGELTTANGVVTHAKSGKKAAYGTLVADASKLPVPDKVVLKQPADFKLIGHRIPRVDASPK
SNGTAHFTLDTTFPGMRVALLQRPPRFGATVKSFDATAARAVPGVVSVVQVPGGIAVVGTGFWAAKQGRDAL
KVEWDEAHAEKRGSDEIMREYRQLADKPGTSARKDGDADAAIAGAARKIGATYEFPYLAHAPMEPLDAVVKLT
ADSCEIWAGDQFQTVDQANAARTAGLKPEQVQIHTLYAGGSFGRRANAWSDYVVEAVSIAKALGADGKPVKL
QWTREDDIQGGFYRPMYFHKLDAGLTEDGKLVGWRHRIVGQSILAGTPFEAFMVKNGIDATSVEGAANLPYA
VPNVSVELTTTKVGLPVLWWRVVGSSHTAYAVEAFIDEAAHTAGKDPYAFRRDLLAKEPRMRAVLDLAAQKAG
WDPAKPLPKGRGRGIAVAEAFKSYVAQVAEVSVDADGKVKVERVVCAVDCGIAINPDIVAAQMEGGIGFGLGA
VMHSAITLKDGQVEQRNFDGYHVLRIAEMPKVEVHIVPSAEAPTGVGEPGVAPVGPAVANAIFAATGKRHYVL
PFDSADSAKA
>SEQF4078||SEQF4078.1_05023
MSRGLIEAGKVAGQAAGAGVSRRSFLKLGMSLGAAAGGGLLLGFSLPAAGDDARRSVIGGDGDETARAGVFA
PNAFVQIDRAGKVTLVMPKVEMGQGVYTALPMLIAEELEVPLSSVTLDHAPPNEKLFLDPLLGGQLTGGSTSIRY
AWEPLRRAGATARTLLVAAAAKQWNVDPASCRAVNGEVQHPPSGRRTSYGQLADAAAKLPVPKDVALKKPAD
FKLIGTPAKRLDSPEKVDGTAQFGLDVRLPGMLYAVIVNSPVFGGTVASIDDTAAKKIPGVRQVVRADNAVAVV
GDHTWAAKRGASALVVKWNEGAGAKVSTKDIVADLAQAAANGKGAVARKDGDVGKGFADAKTRIDAVYEQ
PLLAHATMEPVNCTVHVRADGCEIWVGTQVPTRAVDTVQQLTSFPPEKIVVHNHLLGGGFGRRLETDMIGQA
VKIAKQVNAPVKVIWTREEDIQHDMYRPYYYDRISAGLDANGKPIAWQHRIVGSSILARFAPPAFQHGVDPDA
VEVATDLPYDLPNQLIDYVRQEPRHVPTAFWRGVGPTRSTFVVESFIDELAAQTKTDPVQYRRALLGKTPRALN
VLDVATKAAGWGPSLPTGQGRGVSVMHAFGSFFSIVIDVAVDNGEVQVKRVVCAVDCGMSVNPNTIEAQVQ
GGIIFGITGALYGEITIEDGRVVQSNFTDYRMLRINETPPIEVHLVKSGEAPGGIGEPGTAATAAALSNAIFAATGK
RLRKLPVGDQLKTA
>SEQF4078||SEQF4078.1_02143
MSAPDLSVHNESRRALLLGFASGGLLLAFGVPSLVRAAVPNQPPVSADPRYGGAGMPHGLRDDPHLFVAIAPD
GTVTVTCIRSEMGQGVRTSVALVVADELGADWARVKVAQAVGDEPRYGNQNTDGSRSLRQSFAALRRAGAA
ARTMLEQAAAAAWGVDARQVKATVHEVVDTKSGRKLGFGELAAKAAALPAPDPATVPLKAPAEFRYIGKGETA
LIDGRDIVAGRAHYGIDTRLDGMLYAVVARPPAYGDTMASFDASAAEKLPGVVKVMPLAPTPLPSGFQPLGGV

AVVARDTWTAIQARAQLKIDWKHGPNANYDSAAYRKTLEAAAAQPGDVIRNDGDAAAALAGAAKRVRATYYI
PHLAHATMEPPAAVARVADGRCEVWTCTQAPQTTRDEVAKALGLPGERVTVNVTLLGGGFGRKSKPDYVVEA
ALLSKAVGAPVKLTFTREDDLAHDYFHAVSLEAFDGGIDASGKVVAWQHRTVAPSIQSTFRAGVVHEQPGELA
QGIADLPFAIPNVRIENPAAQAHTRIGWFRSVYNIPHAFGIQSFVSELAHAAGRDPKDFLLELIGPARRFEPHITV
KNVNYGEDPALYPVDTGRLRRVVETVAREAGWGRRLPKGHGLGIAAHRSFVSYTAAVCEVQVDADGKITVPRV
DIAIDCGPQVNPERVRSQLEGAVVMGLGIALHGEITFKDGHPEQSNFNGFQVLRMNEAPREIRVHLVAPDDFA
TPLGGVGEPGLPPVAPALTNAIFAATGTRIRSLPVADQLAKPRAG
>SEQF4079||SEQF4079.1_03713
MTIELDNTGSVRPSRRTFLKAAGAAAAVSLTIGFEWAGLGRRALAATAPAADFAPNAFLRITPDGAVTVIAKHVE
MGQGAYTGIATIVAEELDADWSTVRVESAPADAKRYANLAFGTMQGTGGSSAMANSWQQLREAGGKARA
MLVSAAAARWKVPAGELTTANGVVTHAQSGKKAAYGTLVADASKLPVPDKVVLKQPADFKLIGQRIPRVDASP
KSNGTAHFTLDTTFPGMRVALLQRPPRFGATVKSFDATAARAVPGVVSVVQVPGGVAVVGTGFWAAKQGRD
ALKVEWDEAHAEKRGSDEIMREYRQLADKPGTSARKDGDADAAIAGAARKIGATYEFPYLAHAPMEPLDAVV
KLTADSCEIWAGDQFQTVDQANAARTAGLKPEQVQIHTLYAGGSFGRRANAWSDYVVEAVSIAKALGADGKP
VKLQWTREDDIQGGFYRPMYFHKLDAGLTADGKLVGWRHRIVGQSILAGTPFEAFMVKNGIDATSVEGAANL
PYAVPNVSVELTTTKVGLPVLWWRVVGSSHTAYAVEAFIDEAAHTAGKDPYAFRRDLLAKEPRMRAVLDLAAQK
AGWDPAKPLPKGRGRGIAVAEAFKSYVAQVAEVSVDADGKVKVERVVCAVDCGIAINPDIVAAQMEGGIGFGL
GAVMHSAITLKDGQVEQRNFDGYHVLRIAEMPKVEVHIVPSAEAPTGVGEPGVAPVGPAVANAIFAATGKRHY
VLPFDSADSAKA
>SEQF4079||SEQF4079.1_04312
MSRGLIEAGKVAGQAAGAGVSRRSFLKLGMSLGAAAGGGLLLGFSLPAAGDDARRSVIGGDGDETARAGVFA
PNAFVQIDRVGKVTLVMPKVEMGQGVYTALPMLIAEELEVPLSSVTLDHAPPNEKLFLDPLLGGQLTGGSTSIRY
AWEPLRRAGATARTLLVAAAAKQWNVDPASCRAVNGEVQHPPSGRRVSYGQLADAAAKLPVPKDVALKKPAD
FKLIGTPAKRLDSPEKVDGTAQFGLDVRLPGMLYAVIVNSPVFGGTVASIDDTAAKKIPGVRQVVRADNAVAVV
GDHTWAAKRGASALVVKWNEGAGAKVSTKDIVADLAQAAANGKGAVARKDGDVGKGFADAKTRIDAVYEQ
PLLAHATMEPVNCTVHVRADGCEIWVGTQVPTRAVDTVQQLTSFPPEKIVVHNHLLGGGFGRRLETDMIGQA
VKIAKQVNAPVKVIWTREEDIQHDMYRPYYYDRISAGLDANGKPIAWQHRIVGSSILARFAPPAFQHGVDPDA
VEVATDLPYDLPNQLIDYVRQEPRHVPTAFWRGVGPTRSTFVVESFIDELAAQTKTDPVQYRRALLGKTPRALN
VLDVATKAAGWGPSLPTGQGRGVSVMHAFGSFFSIVIDVAVDNGEVQVKRVVCAVDCGMSVNPNTIEAQVQ
GGIIFGITGALYGEITIEDGRVVQSNFTDYRMLRINETPPIEVHLVKSGEAPGGIGEPGTAATAAALSNAIFAATGK
RLRKLPVGDQLKTA
>SEQF4079||SEQF4079.1_01319
MSAPELSVHNESRRALLLGFASGGLLLAFGVPSLVRAAVPNQPPVSADPRYGGAGMPHGLRDDPHLFVAIAPD
GTVTVTCIRSEMGQGVRTSVALVVADELGADWARVKVAQAVGDEPRYGNQNTDGSRSLRQSFAALRRAGAA
ARTMLEQAAAAAWGVDARQVRATVHEVVDTKSGRKLGFGELAAKAAALPAPDPATVPLKAPAEFRYIGKGETA
LIDGRDIVAGRAHYGIDTRLDGMLYAVVARPPAYGDTMASFDASAAEKLPGVVKVVPLASTPLPSGFQPLGGVA
VVARDTWTAIQARAQLKIDWKHGPNANYDSAAYRKTLEAAAAQPGDVIRNDGDAAAALAGAAKRVRATYYIP
HLAHATMEPPAAVARVADGQCEVWTCTQAPQTTRDEVAKALGLPGERVTVNVTLLGGGFGRKSKPDYVVEA
ALLSKAVGAPVKLTFTREDDLAHDYFHAVSLEAFDGGIDASGKVVAWQHRTVAPSIQSTFRAGVVHEQPGELA
QGIADLPFAIPNVRIENPAAQAHTRIGWFRSVYNIPHAFGIQSFVSELAHAAGRDPKDFLLELIGPARRFEPHITV
KNVNYGEDPALYPVDTGRLRRVVETVAREAGWGRRLPKGHGLGIAAHRSFVSYTAAVCEVQVDADGKITVPRV
DIAIDCGPQVNPERVRSQLEGAVVMGLGIALHGEITFKDGHPEQSNFNGFQVLRMNEAPREIRVHLVAPDDFA
TPLGGVGEPGLPPVAPALTNAIFAATGTRIRSLPVADQLAKPRAG
>SEQF4080||SEQF4080.1_05732

MTIELDNTGSVRPSRRTFLKAAGAAAAVSLTIGFEWAGLGRRALAATAPAADFAPNAFLRITPDGAVTVIAKHVE
MGQGAYTGIATIVAEELDADWSTVRVESAPADAKRYANLAFGTMQGTGGSSAMANSWQQLREAGGKARA
MLVSAAAARWKVPAGELTTANGVVTHAKSGKKAAYGTLVADASKLPVPDKVVLKQPADFKLIGQRIPRVDASPK
SNGTAHFTLDTTLPGMRVALLQRPPRFGATVKSFDATAAKAVPGVVSVVQVPGGIAVVGTGFWAAKQGRDAL
KVEWDEAHAEKRGSDEIMREYRQLADKPGTSARKDGDADAAIAGAARKIGATYEFPYLAHAPMEPLDAVVKLT
ADSCEIWAGDQFQTVDQANAARTAGLKPEQVRIHTLYAGGSFGRRANAWSDYVVEAVSIAKALGADGKPVKL
QWTREDDIQGGFYRPMYFHKLDAGLTEDGKLVGWRHRIVGQSILAGTPFEAFMVKNGIDATSVEGAANLPYA
VPNVSVELTTTKVGLPVLWWRVVGSSHTAYAVEAFIDEAAHTAGKDPYAFRRDLLAKEPRMRAVLDLAAQKAG
WDPAKPLPKGRGRGIAVAEAFKSYVAQVAEVSVDADGKVKVERVVCAVDCGIAINPDIVAAQMEGGIGFGLGA
VMHSAITLKDGQVEQRNFDGYHVLRIAEMPKVEVHIVPSAEAPTGVGEPGVAPVGPAVANAIFAATGKRHYVL
PFDSADSAKA
>SEQF4080||SEQF4080.1_01343
MSAPELSVHNESRRALLLGFASGGLLLAFGVPSLVRAAVPNQPPVSADPRYGGAGMPHGLRDDPHLFVTIAPD
GTVTVTCIRSEMGQGVRTSVALVVADELGADWARVKVAQAVGDEPRYGNQNTDGSRSLRQSFAALRRAGAA
ARTMLEQAAAAAWGVDARQVRATVHEVVDTKSGRKLGFGELAAKAAALPAPDPATVPLKAPAEFRYIGKGETA
LIDGRDIVAGRAHYGIDTRLDGMLYAVVARPPAYGDTMASFDASAAEKLPGVVKVVPLASTPLPSGFQPLGGVA
VVARDTWTAIQARAQLKIDWKHGPNANYDSAAYRKTLEAAAAQPGDVIRNDGDAAAALAGAAKRVRATYYIP
HLAHATMEPPAAVARVADGRCEVWTCTQAPQTTRDEVAKALGLPGERVTVNVTLLGGGFGRKSKPDYVVEAA
LLSKAVGAPVKLTFTREDDLAHDYFHAVSLEAFDGGIDASGKVVAWQHRTVAPSIQSTFRAGVVHEQPGELAQ
GIADLPFAIPNVRIENPAAQAHTRIGWFRSVYNIPHAFGIQSFVSELAHAAGRDPKDFLLELIGPARRFEPHITVK
NVNYGEDPALYPVDTGRLRRVVETVAREAGWGRRLPKGHGLGIAAHRSFVSYTAAVCEVQVDADGKITVPRVD
IAIDCGPQVNPERVRSQLEGAVVMGLGIALHGEITFKDGHPEQSNFNGFQVLRMNEAPREIRVHLVAPDDFAT
PLGGVGEPGLPPVAPALTNAIFAATGTRIRSLPVADQLAKPRAG
>SEQF4080||SEQF4080.1_04991
MSRGLIEAGKVAGQAAGAGVSRRSFLKLGMSLGAAAGGGLLLGFSLPAAGDDARRSVIGGDGDEIARAGVFAP
NAFVQIDRAGKVTLVMPKVEMGQGVYTALPMLIAEELEVPLSSVTLDHAPPNEKLFLDPLLGGQLTGGSTSIRYA
WEPLRRAGATARTLLVAAAAKQWSVDPASCRAVNGEVQHPPSGRRTSYGQLADAAAKLPVPKDVALKKPADF
KLIGTPAKRLDSPEKVDGTAQFGLDVRLPGMLYAVIVNSPVFGGTVASIDDTAAKKIPGVRQVVRADNAVAVVG
DHTWAAKRGASALVVKWNEGAGAKVSTKDIVADLAQAAANGKGAVARKDGDVGKGFADAKTRVDAVYEQP
LLAHATMEPVNCTVHVRADGCEIWVGTQVPTRAVDTVQQLTSFPPEKIVVHNHLLGGGFGRRLETDMIGQAV
KIAKQVNAPVKVIWTREEDIQHDMYRPYYYDRISAGLDANGKPIAWQHRIVGSSILARFAPPAFQHGVDPDAV
EVATDLPYDLPNQLIDYVRQEPRHVPTAFWRGVGPTRSTFVVESFIDELAAQTKTDPVQYRRALLGKTPRALNVL
DVATKAAGWGPSLPTGQGRGVSVMHAFGSFFSIVIDVAVDNGEVQVKRVVCAVDCGMSVNPNTIEAQVQG
GIIFGITGALYGEITIEDGRVVQSNFTDYRMLRINETPPIEVHLVKSGEAPGGIGEPGTAATAAALSNAIFAATGKRL
RKLPVGDQLKTA
>SEQF4081||SEQF4081.1_01208
MSRPADAVRAQPVPNAARRVFLKGGVALAGSLLLPLAFGDVVQAAGDGARFREINDWVRVDADGRTIIGLSQ
AEVGQGVHTGLPQVLADEMDADWRSVTVEFVTGRDAYRIDAANEAPQQFVGASMSATMFYTRLRIAGAQA
RSAFLRAGAARLGVRDTQCVTRDGRVIHPPSGRSLSYGALVDDAARLPHDPQPRLKPASAHTLIGRPLHKLDVP
AKVDGSAIFGIDVQVPDMLVGALTMAPTLNGKPSAVKNRDALRAMPGVADVVVAKDAVIVVAQTYWQAKKA
CDAADIVWDAGPTPAFDSTTILAQRKGALQAEHAVVATQVGEPGRHLAESGNVVEADYHTPYIAHATMEPVN
ATVHVRAGEIEVWGPIQGQDKVRWTLSALFGVPAERVIVNTTFLGGSFGRKYVPDFVVHAAVASKAVGRPVKV
IRSREDDIRHGFYRPCASARFRAALGRDGLPVALHARVAGHSLYAAIKRDRYDKAGGWDETMLDGLYDLCYDV
PNLLVDSVTVMQPIPVSFMRSVGSTSTVFFLESFVNELAHTVRADPVQYRRALLKHDALALRVLDATAARANWF

GRAPAGLSRGVAYSLYTGRGGAFSTYVAAVAEVRVTRGNVKLERIVCGIDCGRAINPLLIREMVEGGVGFALTNT
FRSEITFEHGAVVQRNFADYPLLGLAAMPKIEVVIVDSDRDPQGCGEVALPPVAPAVADAIWRATGERPRSMPF
DTPLAT
>SEQF4081||SEQF4081.1_03933
MTIELDNPGSVRPSRRTFLKAAGAAAAVSLTIGFDWAGLGRRALAATAPAGDFAPNAFLRITPDGALTVIAKHVE
MGQGAYTGIATIVAEELDADWSSVRVESAPADAKRYANLAFGTMQGTGGSSAMANSWQQLREAGGKARAM
LVSAAAARWKVPAGELTTANGVVTHTKSGKTAAYGTLVADASKLPVPDKVTLKQPADFKLIGHRIPRVDGSSKSN
GTAHFTLDTTFPGMRVALLQRPPRFGATVKSFDATAAKAVPGVVSVVQVPGGVAVVATGFWAAKQGRDALKV
EWDETKAEKRGSDELMREYRQLAEKPGASARKDGDADAAIAGAARKISATYEFPYLAHAPMEPLDAVVKLTPT
SCEIWAGDQFQTVDQGNAAKTAGLKPEQVKIHTLYAGGSFGRRANAWSDYVVEAVSIAKALGADGKPVKLQW
TREDDIQGGFYRPMYFHKLDAGLTADGKLVGWRHRIVGQSILAGTPFEAFMVKNGIDATSVEGAANLPYAVPN
VSVELTTTQVGLPVLWWRVVGSSHTAYAVEAFIDEAAHAAGKDPYAFRRDLLAKEPRMRAVLELAAQKAGWD
PAKPLPKGRGRGIAVAEAFKSYVAQVAEVSVDADGKVKVERVVCAVDCGIAINPDIVAAQMEGGIGFGLGAAM
HSAITLKDGQVEQRNFDGYHVLRMAEMPKVEVHIVPSAEAPTGVGEPGVAPVGPAVANAIFAATGKRHYVLPF
DSADSAKA
>SEQF4081||SEQF4081.1_01360
MSAPELSVHNESRRALLLGFASGGLLLAFGVPSLARAAAPVQPPVSANPQYGGAGMPHGLRDDPNLFVAIAPD
GTVTVTCIRSEMGQGVRTSVALVVADELGADWARVKVAQAVGDEPRYGNQNTDGSRSLRQSFAALRRAGAA
ARTMLAQAAAAEWGVDVRQVKVTVHEVVDSKSGRKLGFGELAAKAAALPVPDTKSVPLKAPAEFRYIGKGKT
ALIDGRDIVGGRAQYGIDTRLDGMLYAVVARPPAYGDTVASFDASAAGKLPGVVKVAQLAATPLPSGFQPLGGV
AVIARDTWTAIQARAQLKIDWKHGPNAGYDSTEYRKTLEAAAAQPGDVIRNDGDVAAALAGAAKRVRATYYV
PHLAHATMEPPAAVARVADGRCEVWTCTQAPQTTRDELAKALGLPAERVTVNVTLLGGGFGRKSKPDYVVEA
ALLSKAVGAPVKLTFTREDDIAHDYFHAVSLEAFDGGIDASGKVVAWQHRTVAPSIQSTFRAGVVHEQPGELAQ
GIADLPFAIPNVRLENPAAEAHTRIGWFRSVYNIPHAFGIQSFVSELAHAAGRDPKDFLLELIGPARRFEPHITVK
NVNYGEDPALYPVDTGRLRRVVETVAREAGWGRTLPNGHGLGIAAHRSFVSYTAAVCEVQVDADGKISVPRVD
IAIDCGPQVNPERVRSQLEGAVVMGLGIALHGEITFKDGHPEQSNFNGFQVLRMNEAPREIRVHLVAPDDFAT
PLGGVGEPGLPPVAPALTNAIFAATGTRIRSLPVADQLAKPQAG
>SEQF4081||SEQF4081.1_03873
MSADTRIARPGRRRFLLGAVGLGGALVVGWGVMPPRSRLGDPAVFPETSGQIALNGWIKITSEGNAILAMPRV
EMGQGIHTGLSMLAAEELDIPLTRVSIESSPIERIYGNVVAMGDSSLPLHPDDADKLWARALHWIMAKSAREIG
LIITGGSSSMADGWQPVREAAATARATLVEAAAREWGVQSTLVTVREGQLIGPGGKQMPFSAVAQKAREIAPP
SNVTLKPASQYQLVGKPAPRNDIAAKTDGSARFAIDVRPSGMLYAAVVMCPIFGGKLKTFNAKAALVMPGVRY
VVPFDGSTGGAPGVAVVADHYWQARQALGALEPEWDNGPHATLDSADIHQQLVGALNSDKGSFTYRSTGDG
LKAFENAQGATIVEAEYSAPYLAHATMEPINCTAQVTKNRVQLWAPTQVATLAQLVAARAAGVSRDQVHIEIPLI
GGGFGRRLESDFVGQAVTIAARTAGRPVQVIWSREDDIKHDFYRPQVIARLKARVENGRVTAIASRSAGQSILA
GELERLFGAPSLDIDRYTAEGLFDQPYEIEHEHIAHLVVDLPVPIGFWRSVGHSYTGFFLESFLNDVAAAAKLDPL
AMRRDLLKAHPRERKVLDIAAQTAGWGQPLAPAADGAPRARGLALHNAFGSVVAQVVEVSLKDGKPRVHRV
VCVADCGTVVNPGIVAQQMEGGIIFGLSAALYGQIQIKDGRVIQSNFPDYPVLKMAEAPAIEIHLVPSTAEPTGV
GEIAVPPVAPAVAHAVAQLTGKSVRQLPMV
>SEQF4081||SEQF4081.1_04400
MSRGLLEAGRAGAGVSRRGFLKLGMSLGAAAGGGLLLGFSLPAAGDDARRSVIGGDAAEPAAPGVFAPNAFV
QIDRAGKVTLVMPKVEMGQGVYTALPMLIAEELEVPLSSVTLDHAPPNEKLFLDPLLGGQLTGGSTSVRYAWEP
MRRAGATARTLLVTAAAKQWNVDPATCRAANGEVQHPPSGRRASYGQLADAAAKLPVPKDVALKQPADFKLI
GTPAKRLDSPEKVDGTAQFGLDVRLPGMLYAVIVNSPVFGGTVASVDDTAARKIPGVRQVVRVDNAVAVVGD

HTWAAKRGASALVVKWNEGAGAKVSTKDLFADLAQAAANGKGAVARKEGDVGKAFANAKTRVDAVYEQPLL
AHATMEPVNCTVHVRGDGCEIWVGTQVPTRARDTAQQLTGLPPDKIVVHNHLLGGGFGRRLETDMVGQAV
KVGKQVNAPVKVIWTREEDVQHDMYRPCYYDKISAGLDANGKPVAWQHRIVGSSIMARFAPPAFKDGVDPD
AVEVAAELPYDLPNQLVDYVRQEPRHVPTAFWRGVGPTRGTFVVESFIDELAAQTKTDPVQYRRALLDKTPRA
RNVLDVATKAAGWGAPLPKGQGRGVSVMHAFGSFFSIVIDVAVDGGEVQVKRAVCAVDCGMFVNPNTIEAQ
VQGGIIFGITGALYGEITIEDGRVVQTNFTDYRMMRINEVPPIEVHLVKSGEAPGGIGEPGTAATAAALSNAIFAA
TGTRLRKLPVGNQLKTA
>SEQF4082||SEQF4082.3_04435
MSRGLIEAGKVAGQAAGAGVSRRSFLKLGMSLGAAAGGGLLLGFSLPAAGDDARRSVIGGDGDETARAGVFA
PNAFVQIDRAGKVTLVMPKVEMGQGVYTALPMLIAEELEVPLSSVTLDHAPPNEKLFLDPLLGGQLTGGSTSVR
YAWEPLRRAGATARTLLVAAAAKQWNVDPASCRAVNGEVQHPPSGRRVSYGQLADAAAKLPVPKDVALKKPA
DFKLIGTPAKRLDSPEKVDGTAQFGLDVRLPGMLYAVIVNSPVFGGTVASIDDTAAKKIPGVRQVVRADNAVAV
VGDHTWAAKRGASALVVKWNEGAGAKVSTKDIVADLAQAAANGKGAVARKDGDVGKGFADAKTRIDAVYE
QPLLAHATMEPVNCTVHVRADGCEIWVGTQVPTRAVDTVQKLTSFPPERIVVHNHLLGGGFGRRLETDMIGQ
AVKIAKQVNAPVKVIWTREEDIQHDMYRPYYYDRISAGLDANGKPIAWQHRIVGSSILARFAPPAFQHGVDPD
AVEVATDLPYDLPNQLIDYVRQEPRHVPTAFWRGVGPTRSTFVVESFIDELAAQTKTDPVQYRRALLGKTPRAL
NVLDVATKAAGWGPSLPTGQGRGVSVMHAFGSFFSIVIDVAVDNGEVQVKRVVCAVDCGMSVNPNTIEAQV
QGGIIFGITGALYGEITIEDGRVVQSNFTDYRMLRINETPPIEVHLVKSGEAPGGIGEPGTAATAAALSNAIFAATG
KRLRKLPVGDQLKTA
>SEQF4082||SEQF4082.3_03803
MTIELDNTGSVRPSRRTFLKAAGAAAAVSLTIGFEWAGLGRRALAATAPAADFAPNAFLRITPDGAVTVIAKHVE
MGQGAYTGIATIVAEELDADWSTVRVESAPADAKRYANLAFGTMQGTGGSSAMANSWQQLREAGGKARA
MLVSAAAARWKVPAGELTTANGIVSHAKSGKKAAYGTLVADASKLPVPDKVALKQPADFKLIGQRIPRVDASPK
SNGTAHFTLDTTFPGMRVALLQRPPRFGATVKSFDATAAKAVPGVVSVVQVPGGVAVVGTGFWAAKQGRDAL
KVEWDEAHAEKRSSDEIMREYRQLADKPGTSARKDGDADAAIAGAARKIGATYEFPYLAHAPMEPLDAVVKLT
ADSCEIWAGDQFQTVDQANAARTAGLKPEQVQIHTLYAGGSFGRRANAWSDYVVEAVSIAKALGADGKPVKL
QWTREDDIQGGFYRPMYFHKLDAGLTADGKLVGWRHRIVGQSILAGTPFEAFMVKNGIDATSVEGAANLPYA
VPNVSVELTTTKVGLPVLWWRVVGSSHTAYAVEAFIDEAAHTAGKDPYAFRRDLLAKEPRMRAVLDLAAQKAG
WDPAKPLPKGRGRGIAVAEAFKSYVAQVAEVSVDADGKVKVERVVCAVDCGIAINPDIVAAQMEGGIGFGLGA
VMHSAITLKDGQVEQRNFDGYHVLRIAEMPKVEVHIVPSAEAPTGVGEPGVAPVGPAVANAIFAATGKRHYVL
PFDSADSAKA
>SEQF4082||SEQF4082.3_02970
MSAPELSVHNESRRALLLGFASGGLLLAFGVPSLVRAAVPSQPPVSADPRYGGAGMPHGLRDDPHLFVAIAPD
GTVTVTCIRSEMGQGVRTSVALVVADELGADWARVKVEQAVGDEPRYGNQNTDGSRSLRQSFAALRRAGAA
ARTMLEQAAAAAWGVDARQVKATVHEVVDTKSGRKLGFGELAAKAAALPAPDPATVPLKAPAEFRYIGKGQT
ALIDGRDIVAGRAHYGIDTRLDGMLYAVVARPPTYGDTVASFDASAAEKLPGVVKVVPLAPTPLPSGFQPLGGV
AVVARDTWTAIQARAQLKIDWKHGPNANYDSAAYRKTLEAAAAQPGDVIRNDGDAAAALAGAAKRVRATYYI
PHLAHATMEPPAAVARVADGRCEVWTCTQAPQTTRDEVAKALGLPGERVTVNVTLLGGGFGRKSKPDYVVEA
ALLSKAVGAPVKLTFTREDDLAHDYFHAVSLEAFDGGIDASGKVVAWQHRTVAPSIQSTFRAGIVHEQPGELAQ
GIADLPFAIPNVRIENPAAQAHTRIGWFRSVYNIPHAFGIQSFVSELAHAAGRDPKDFLLELIGPARRFEPHITVK
NVNYGEDPALYPVDTGRLRRVVETVAREAGWGRKLPKGHGLGIAAHRSFVSYTAAVCEVQVDADGKITVPRVD
IAIDCGPQVNPERVRSQLEGAVVMGLGIALHGEITFKDGHPEQSNFNGFQVLRMNEAPREIRVHLVAPDDFAT
PLGGVGEPGLPPVAPALTNAIFAATGTRIRSLPVADQLAKPRAG
>SEQF4083||SEQF4083.1_06037

MTIELDNPGSVRPSRRTFLKAAGAAAAVSLTIGFDWAGLGRRALAATAPAGDFAPNAFLRITPDGAVTVIAKHV
EMGQGAYTGIATIVAEELDADWSSVRVESAPADAKRYANLAFGTMQGTGGSSAMANSWQQLREAGGKARA
MLVSAAAARWKVPAGELTTANGVVSHAKSGKTAAYGTLVADASKLPVPDKVTLKQPADFKLIGHRIPRVDGSSK
SNGTAHFTLDTTFPGMRVALLQRPPRFGATVKSFDATAAKAVPGVVSVVQVPGGVAVVATGFWAAKQGRDAL
KVEWDETKAEKRGSDELMREYRQLAEKPGASARKDGDADAAIAGAARKISATYEFPYLAHAPMEPLDAVVKLT
PTSCEIWAGDQFQTVDQGNAAKTAGLKPEQVKIHTLYAGGSFGRRANAWSDYVVEAVSIAKALGADGKPVKL
QWTREDDIQGGFYRPMYFHKLDAGLTADGRLVGWRHRIVGQSILAGTPFEAFMVKNGIDATSVEGAANLPYA
VPNVSVELTTTRVGLPVLWWRVVGSSHTAYAVEAFIDEAAHAAGKDPYAFRRDLLAKEPRMRAVLELAAQKAG
WDPAKPLPKGRGRGIAVAEAFKSYVAQVAEVSVDADGKVKVERVVCAVDCGIAINPDIVAAQMEGGIGFGLGA
AMHSAITLKDGQVEQRNFDGYHVLRMAEMPKVEVHIVPSAEAPTGVGEPGVAPVGPAVANAIFAATGKRHYV
LPFDSADSAKA
>SEQF4083||SEQF4083.1_00386
MSAPELSVHNESRRALLLGFASGGLLLAFGVPSLARAAAPVQPPVSANPQYGGAGMPHGLRDDPNLFVAIAPD
GTVTVTCIRSEMGQGVRTSVALVVADELGADWARVKVAQAVGDEPRYGNQNTDGSRSLRQSFAALRRAGAA
ARTMLAQAAAGEWGVDVRQVKVTVHEVVDSKSGRKLGFGELAAKAAALPVPDTKSVPLKAPAEFRYIGKGKT
ALIDGRDIVGGRAQYGIDTRLDGMLYAVVARPPAYGDTVASFDASAAGKLPGVVKVVQLAATPLPSGFQPLGGV
AVIARDTWTAIQARAQLKIDWKHGPNAGYDSTEYRKTLEAAAAQPGDVIRNDGDVAAALAGAAKRVRATYYV
PHLAHATMEPPAAVARVADGRCEVWTCTQAPQTTRDELAKALGLPAERVTVNVTLLGGGFGRKSKPDYVVEA
ALLSKAVGAPVKLTFTREDDIAHDYFHAVSLEAFDGGIDASGKVVAWQHRTVAPSIQSTFRAGVVHEQPGELAQ
GIADLPFAIPNVRLENPAAEAHTRIGWFRSVYNIPHAFGIQSFVSELAHAAGRDPKDFLLELIGPARRFEPHITVK
NVNYGEDPALYPVDTGRLRRVVETVAREAGWGRKLPNGHGLGIAAHRSFVSYTAAVCEVQVDADGKISVPRVD
IAIDCGPQVNPERVRSQLEGAVVMGLGIALHGEITFKDGHPEQSNFNGFQVLRMNEAPREIRVHLVAPDDFAT
PLGGVGEPGLPPVAPALTNAIFAATGTRIRSLPVADQLAKPQAG
>SEQF4083||SEQF4083.1_03607
MSRGLLEAGRAGAGVSRRGFLKLGMSLGAAAGGGLLLGFSLPAAGDDARRSVIGGDAAEPAAPGVFAPNAFV
QIDRAGKVTLVMPKVEMGQGVYTALPMLIAEELEVPLSSVTLDHAPPNEKLFLDPLLGGQLTGGSTSVRYAWEP
MRRAGATARTLLVTAAAKQWNVDPATCRAANGEVQHPPSGRRASYGQLADAAAKLPVPKDVALKQPADFKLI
GTPAKRLDSPEKVDGTAQFGLDVRLPGMLYAVIVNSPVFGGTVASVDDTAARKIPGVRQVVRVDNAVAVVGD
HTWAAKRGASALVVKWNEGAGAKVSTKDLFADLAQAAANGKGAVARKEGDVGKAFANAKTRVDAVYEQPLL
AHATMEPVNCTVHVRGDGCEIWVGTQVPTRARDTAQQLTGLPPDKIVVHNHLLGGGFGRRLETDMVGQAV
KVGKQVNAPVKVIWTREEDVQHDMYRPCYYDKISAGLDANGKPVAWQHRIVGSSIMARFAPPAFKDGVDPD
AVEVAAELPYDLPNQLVDYVRQEPRHVPTAFWRGVGPTRGTFVVESFIDELAAQTKTDPVQYRRALLDKTPRA
RNVLDVATKAAGWGAPLPKGQGRGVSVMHAFGSFFSIVIDVAVDGGEVQVKRAVCAVDCGMFVNPNTIEAQ
VQGGIIFGITGALYGEITIEDGRVVQTNFTDYRMMRINEVPPIEVHLVKSGEAPGGIGEPGTAATAAALSNAIFAA
TGTRLRKLPVGNQLKTA
>SEQF4083||SEQF4083.1_00541
MSRPADAVRAQPVPNAARRVFLKGGVALAGSLLLPLAFGDVVQAAGDGARFREINDWVRVDADGRTIIGLSQ
AEVGQGVHTGLPQVLADEMDADWRSVTVEFVTGRDAYRIDAANEAPQQFVGASMSATMFYMRLRIAGAQA
RSAFLRAGAARLGVRDTQCVTRDGRVIHPPSGRSLSYGALVDDAARLPHDPQPRLKPASAHTLIGRPLHKLDVP
AKVDGSAIFGIDVQVPDMLVGALTMAPTLNGKPSAVKNRDALRAMPGVADVVVAKDAVIVVAQTYWQAKKA
CDAADIVWDAGPTPAFDSTTILAQRKGALQAEHAVVATQVGEPGRHLAESGNVVEADYHTPYIAHATMEPVN
ATVHVRAGEIEVWGPIQGQDKVRWTLSALFGVPAERVIVNTTFLGGSFGRKYVPDFVVHAAVASKAVGRPVKV
IRSREDDIRHGFYRPCASARFRAALGRDGLPVALHARVAGHSLYAAIKRDRYDKAGGWDETMLDGLYDLCYDV
PNLLVDSVTVMQPIPVSFMRSVGSTSTVFFLESFVNELAHTVRADPVQYRRALLKHDALALRVLDATAARANWF

GRAPAGLSRGVAYSLYTGRGGAFSTYVAAVAEVRVTRGNVKLERIVCGIDCGRAINPLLIREMVEGGVGFALTNT
FRSEITFEHGAVVQRNFADYPLLGLAAMPKIEVVIVDSDRDPQGCGEVALPPVAPAVADAIWRATGERPRSMPF
DTPLAT
>SEQF4084||SEQF4084.1_01317
MSAPELSVHNESRRALLLGFASGGLLLAFGVPSLVRAAAPVQPPVSADPRYGGAGMPHGLRDDPHLFVAIAPD
GTVTVTCIRSEMGQGVRTSVALVVADELGADWARVKVAQAVGDEPRYGNQNTDGSRSLRQSFAALRRAGAA
ARTMLEQAAAAAWGVDARQVKATVHEVVDTKSGRKLGFGELAAKAAALPAPDPATVPLKAPAEFRYIGKGQT
ALIDGCDIVAGRAHYGIDTRLDGMLYAVVARPPAYGDTVASFDASAAEKLPGVVKVVPLTPTPLPSGFQPLGGVA
VVARDTWTAIQARAQLKIDWKHGPNANYDSAAYRKTLEAAAAQPGDVIRNDGDAAAALAGAAKRVRATYYIP
HLAHATMEPPAAVARVADGRCEVWTCTQAPQTTRDEVAKALGLPGERVTVNVTLLGGGFGRKSKPDYVVEAA
LLSKAVGAPVKLTFTREDDLAHDYFHAVSLEAFDGGIDASGKVVAWQHRTVAPSIQSTFRAGIVHEQPGELAQG
IADLPFAIPNVRIENPAAQAHTRIGWFRSVYNIPHAFGIQSFVSELAHAAGRDPKDFLLELIGPARRFEPHITVKN
VNYGEDPALYPVDTGRLRRVVETVAREAGWGRKLPKGRGLGIAAHRSFVSYTAAVCEVQVDADGKITVPRVDIA
IDCGPQVNPERVRSQLEGAVVMGLGIALHGEITFKDGHPEQSNFNGFQVLRMNEAPREIRVHLVAPDDFATPL
GGVGEPGLPPVAPALTNAIFAATGTRIRSLPVADQLAKPRAG
>SEQF4084||SEQF4084.1_06342
MTIELDNTGSVRPSRRTFLKAAGAAAAVSLTIGFEWAGLGRRALAATAPAADFAPNAFLRITPDGAVTVIAKHVE
MGQGAYTGIATIVAEELDADWSTVRVESAPADAKRYANLAFGTMQGTGGSSAMANSWQQLREAGGKARA
MLVSAAAARWKVPAGELTTANGVVTHAKSGKKAAYGTLVADASKLPVPDKVVLKQPADFKLIGQRIPRVDASPK
SNGTAHFTLDTTFPGMRVALLQRPPRFGATVKSFDATAAKAVPGVVSVVQVPGGVAVVGTGFWAAKQGRDAL
KVEWDEAHAEKRSSDEIMREYRQLADKPGTSARKDGDADAAIAGAARKIGATYEFPYLAHAPMEPLDAVVKLT
ADSCEIWAGDQFQTVDQANAARTAGLKPEQVQIHTLYAGGSFGRRANAWSDYVVEAVSIAKALGADGKPVKL
QWTREDDIQGGFYRPMYFHKLDAGLTADGKLVGWRHRIVGQSILAGTPFEAFMVKNGIDATSVEGAANLPYA
VPNVSVELTTTKVGLPVLWWRVVGSSHTAYAVEAFIDEAAHTAGKDPYAFRRDLLAKEPRMRAVLDLAAQKAG
WDPAKPLPKGRGRGIAVAEAFKSYVAQVAEVSVDADGKVKVERVVCAVDCGIAINPDIVAAQMEGGIGFGLGA
VMHSAITLKDGQVEQRNFDGYHVLRIAEMPKVEVHIVPSAEAPTGVGEPGVAPVGPAVANAIFAATGKRHYVL
PFDSADSAKA
>SEQF4084||SEQF4084.1_03961
MSRGLIEAGKVAGQAAGAGVSRRSFLKLGMSLGAAAGGGLLLGFSLPAAGDDARRSVIGGDGDETARAGVFA
PNAFVQIDRAGKVTLVMPKVEMGQGVYTALPMLIAEELEVPLSSVTLDHAPPNEKLFLDPLLGGQLTGGSTSVR
YAWEPLRRAGATARTLLVAAAAKQWNVDPASCRAVNGEVQHPPSGRRVSYGQLADAAAKLPVPKDVALKKPA
DFKLIGTPAKRLDSPEKVDGTAQFGLDVRLPGMLYAVIVNSPVFGGTVASIDDTAAKKIPGVRQVVRADNAVAV
VGDHTWAAKRGASALVVKWNEGAGAKVSTKDIVADLAQAAANGKGAVARKDGDVGKGFADAKTRIDAVYE
QPLLAHATMEPVNCTVHVRADGCEIWVGTQVPTRAVDTVQKLTSFPPEKIVVHNHLLGGGFGRRLETDMIGQ
AVKIAKQVNAPVKVIWTREEDIQHDMYRPYYYDRISAGLDANGKPIAWQHRIVGSSILARFAPPAFQHGVDPD
AVEVATDLPYDLPNQLIDYVRQEPRHVPTAFWRGVGPTRSTFVVESFIDELAAQTKTDPVQYRRALLGKTPRAL
NVLDVATKAAGWGPSLPTGQGRGVSVMHAFGSFFSIVIDVAVDNGEVQVKRVVCAVDCGMSVNPNTIEAQV
QGGIIFGITGALYGEITIEDGRVVQSNFTDYRMLRINETPPIEVHLVKSGEAPGGIGEPGTAATAAALSNAIFAATG
KRLRKLPVGDQLKTA
>SEQF4085||SEQF4085.1_03621
MTIELDNTGSVRPSRRTFLKAAGAAAAVSLTIGFEWAGLGRRALAATAPAADFAPNAFLRITPDGAVTVIAKHVE
MGQGAYTGIATIVAEELDADWSTVRVESAPADAKRYANLAFGTMQGTGGSSAMANSWQQLREAGGKARA
MLVSAAAARWKVPAGELTTANGVVTHAKSGKKAAYGTLVADASKLPVPDKVALKQPADFKLIGKRIPRVDASPK
SNGTAHFTLDTTFPGMRVALLQRPPRFGATVKSFDATAAKAVPGVVSVVQVPGGVAVVGTGFWAAKQGRDAL

KVEWDEAHAEKRSSDEIMREYRQLADKPGTSARKDGDADAAIAGAARKIGATYEFPYLAHAPMEPLDAVVKLT
ADSCEIWAGDQFQTVDQANAARTAGLKPEQVQIHTLYAGGSFGRRANAWSDYVVEAVSIAKALGADGKPVKL
QWTREDDIQGGFYRPMYFHKLDAGLTADGKLVGWRHRIVGQSILAGTPFEAFMVKNGIDATSVEGAANLPYA
VPNVSVELTTTKVGLPVLWWRVVGSSHTAYAVEAFIDEAAHTAGKDPYAFRRDLLAKEPRMRAVLDLAAQKAG
WDPAKPLPKGRGRGIAVAEAFKSYVAQVAEVSVDADGKVKVERVVCAVDCGIAINPDIVAAQMEGGIGFGLGA
VMHSAITLKDGQVEQRNFDGYHVLRIAEMPKVEVHIVPSAEAPTGVGEPGVAPVGPAVANAIFAATGKRHYVL
PFDSADSAKA
>SEQF4085||SEQF4085.1_02979
MSRGLIEAGKAAGQAAGAGVSRRSFLKLGMSLGAAAGGGLLLGFSLPAAGDDARRSVIGGDGDETARAGVFA
PNAFVQIDRAGKVTLVMPKVEMGQGVYTALPMLIAEELEVPLSSVTLDHAPPNEKLFLDPLLGGQLTGGSTSVR
YAWEPLRRAGATARTLLVAAAAKQWNVDPASCRAVNGEVQHPPSGRRVSYGQLADAAAKLPVPKDVVLKKPA
DFKLIGTPAKRLDSPEKVDGTAQFGLDVRLPGMLYAVIVNSPVFGGTVASIDDTAAKKIPGVRQVVRADNAVAV
VGDHTWAAKRGASALVVKWNEGAGAKVSTKDIVADLAQAAANGKGAVARKDGDVGKGFADAKTRVDAVYE
QPLLAHATMEPVNCTVHVRADGCEIWVGTQVPTRAVDTVQKLTSFPPEKIVVHNHLLGGGFGRRLETDMIGQ
AVKIAKQVNAPVKVIWTREEDIQHDMYRPYYYDRISAGLDANGKPIAWQHRIVGSSILARFAPPAFQHGVDPD
AVEVAIDLPYDLPNQLIDYVRQEPRHVPTAFWRGVGPTRSTFVVESFIDELAAQTKTDPVQYRRALLGKTPRALN
VLDVATKAAGWGPSLPTGQGRGVSVMHAFGSFFSIVIDVAVDNGEVQVKRVVCAVDCGMSVNPNTIEAQVQ
GGIIFGITGALYGEITIEDGRVVQSNFTDYRMLRINETPPIEVHLVKSGEAPGGIGEPGTAATAAALSNAIFAATGK
RLRKLPVGDQLKTA
>SEQF4085||SEQF4085.1_05893
MSAPELSVHNESRRALLLGFASGGLLLAFGVPSLVRAAVPSQPPVSADPRYGGAGMPHGLRDDPHLFVAIAPD
GTVTVTCIRSEMGQGVRTSVALVVADELGADWARVKVAQAVGDEPRYGNQNTDGSRSLRQSFAALRRAGAA
ARTMLEQAAAAAWGVDARQVKATVHEVVDTKSGRKLGFGELAAKAAALPAPDPATVPLKAPAEFRYIGKGQT
ALIDGRDIVAGRAHYGIDTRLDGMLYAVVARPPAYGDTVASFDASAAEKLPGVVKAVPLTPTPLPSGFQPLGGVA
VVARDTWTAIQARAQLKIDWKHGPNANYDSAAYRKTLEAAAAQPGDVIRNDGDAAAALAGAAKRVRATYYIP
HLAHATMEPPAAVARVADGRCEVWTCTQAPQTTRDEVAKALGLPGERVTVNVTLLGGGFGRKSKPDYVVEAA
LLSKAVGAPVKLTFTREDDLAHDYFHAVSLEAFDGGIDASGKVVAWRHRTVAPSIQSTFRAGIVHEQPGELAQG
IADLPFAIPNVRIENPAAQAHTRIGWFRSVYNIPHAFGIQSFVSELAHAAGRDPKDFLLELIGPARRFEPHITVKN
VNYGEDPALYPVDTGRLRRVVETVAREAGWGRKLPKGHGLGIAAHRSFVSYTAAVCEVQVDADGKITVPRVDI
AIDCGPQVNPERVRSQLEGAVVMGLGIALHGEITFKDGHPEQSNFNGFQVLRMNEAPREIRVHLVAPDDFATP
LGGVGEPGLPPVAPALTNAIFAATGTRIRSLPVADQLAKPRAG
>SEQF4086||SEQF4086.1_02508
MSAPDLSVHNESRRALLLGFASGGLLLAFGVPSLVRAAVPNQPPVSADPRYGGAGMPHGLRDDPHLFVAIAPD
GTVTVTCIRSEMGQGVRTSVALVVADELGADWARVKVAQAVGDEPRYGNQNTDGSRSLRQSFAALRRAGAA
ARTMLEQAAAAAWGVDARQVKATVHEVVDTKSGRKLGFGELAAKAAALPAPDPATVPLKAPAEFRYIGKGETA
LIDGRDIVAGRAHYGIDTRLDGMLYAVVARPPAYGDTMASFDASAAEKLPGVVKVMPLAPTPLPSGFQPLGGV
AVVARDTWTAIQARAQLKIDWKHGPNANYDSAAYRKTLEAAAAQPGDVIRNDGDAAAALAGAAKRVRATYYI
PHLAHATMEPPAAVARVADGRCEVWTCTQAPQTTRDEVAKALGLPGERVTVNVTLLGGGFGRKSKPDYVVEA
ALLSKAVGAPVKLTFTREDDLAHDYFHAVSLEAFDGGIDASGKVVAWQHRTVAPSIQSTFRAGVVHEQPGELA
QGIADLPFAIPNVRIENPAAQAHTRIGWFRSVYNIPHAFGIQSFVSELAHAAGRDPKDFLLELIGPARRFEPHITV
KNVNYGEDPALYPVDTGRLRRVVETVAREAGWGRRLPKGHGLGIAAHRSFVSYTAAVCEVQVDADGKITVPRV
DIAIDCGPQVNPERVRSQLEGAVVMGLGIALHGEITFKDGHPEQSNFNGFQVLRMNEAPREIRVHLVAPDDFA
TPLGGVGEPGLPPVAPALTNAIFAATGTRIRSLPVADQLAKPRAG
>SEQF4086||SEQF4086.1_06219

MSRGLIEAGKVAGQAAGAGVSRRSFLKLGMSLGAAAGGGLLLGFSLPAAGDDARRSVIGGDGDETARAGVFA
PNAFVQIDRAGKVTLVMPKVEMGQGVYTALPMLIAEELEVPLSSVTLDHAPPNEKLFLDPLLGGQLTGGSTSIRY
AWEPLRRAGATARTLLVAAAAKQWNVDPASCRAVNGEVQHPPSGRRTSYGQLADAAAKLPVPKDVALKKPAD
FKLIGTPAKRLDSPEKVDGTAQFGLDVRLPGMLYAVIVNSPVFGGTVASIDDTAAKKIPGVRQVVRADNAVAVV
GDHTWAAKRGASALVVKWNEGAGAKVSTKDIVADLAQAAANGKGAVARKDGDVGKGFADAKTRIDAVYEQ
PLLAHATMEPVNCTVHVRADGCEIWVGTQVPTRAVDTVQQLTSFPPEKIVVHNHLLGGGFGRRLETDMIGQA
VKIAKQVNAPVKVIWTREEDIQHDMYRPYYYDRISAGLDANGKPIAWQHRIVGSSILARFAPPAFQHGVDPDA
VEVATDLPYDLPNQLIDYVRQEPRHVPTAFWRGVGPTRSTFVVESFIDELAAQTKTDPVQYRRALLGKTPRALN
VLDVATKAAGWGPSLPTGQGRGVSVMHAFGSFFSIVIDVAVDNGEVQVKRVVCAVDCGMSVNPNTIEAQVQ
GGIIFGITGALYGEITIEDGRVVQSNFTDYRMLRINETPPIEVHLVKSGEAPGGIGEPGTAATAAALSNAIFAATGK
RLRKLPVGDQLKTA
>SEQF4086||SEQF4086.1_05475
MTIELDNTGSVRPSRRTFLKAAGAAAAVSLTIGFEWAGLGRRALAATAPAADFAPNAFLRITPDGAVTVIAKHVE
MGQGAYTGIATIVAEELDADWSTVRVESAPADAKRYANLAFGTMQGTGGSSAMANSWQQLREAGGKARA
MLVSAAAARWKVPAGELTTANGVVTHAKSGKKAAYGTLVADASKLPVPDKVVLKQPADFKLIGHRIPRVDASPK
SNGTAHFTLDTTFPGMRVALLQRPPRFGATVKSFDATAARAVPGVVSVVQVPGGIAVVGTGFWAAKQGRDAL
KVEWDEAHAEKRGSDEIMREYRQLADKPGTSARKDGDADAAIAGAARKIGATYEFPYLAHAPMEPLDAVVKLT
ADSCEIWAGDQFQTVDQANAARTAGLKPEQVQIHTLYAGGSFGRRANAWSDYVVEAVSIAKALGADGKPVKL
QWTREDDIQGGFYRPMYFHKLDAGLTEDGKLVGWRHRIVGQSILAGTPFEAFMVKNGIDATSVEGAANLPYA
VPNVSVELTTTKVGLPVLWWRVVGSSHTAYAVEAFIDEAAHTAGKDPYAFRRDLLAKEPRMRAVLDLAAQKAG
WDPAKPLPKGRGRGIAVAEAFKSYVAQVAEVSVDADGKVKVERVVCAVDCGIAINPDIVAAQMEGGIGFGLGA
VMHSAITLKDGQVEQRNFDGYHVLRIAEMPKVEVHIVPSAEAPTGVGEPGVAPVGPAVANAIFAATGKRHYVL
PFDSADSAKA
>SEQF4087||SEQF4087.1_05232
MSRGLIEAGKVAGQAAGAGVSRRSFLKLGMSLGAAAGGGLLLGFSLPAAGDDARRSVIGGDGDETARAGVFA
PNAFVQIDRAGKVTLVMPKVEMGQGVYTALPMLIAEELEVPLSSVTLDHAPPNEKLFLDPLLGGQLTGGSTSVR
YAWEPLRRAGATARTLLVAAAAKQWNVDPASCRAVNGEVQHPPSGRRVSYGQLADAAAKLPVPKDVALKKPA
DFKLIGTPAKRLDSPEKVDGTAQFGLDVRLPGMLYAVIVNSPVFGGTVASIDDTAAKKIPGVRQVVRADNAVAV
VGDHTWAAKRGASALVVKWNEGAGAKVSTKDIVADLAQAAANGKGAVARKDGDVGKGFADAKTRIDAVYE
QPLLAHATMEPVNCTVHVRADGCEIWVGTQVPTRAVDTVQKLTSFPPEKIVVHNHLLGGGFGRRLETDMIGQ
AVKIAKQVNAPVKVIWTREEDIQHDMYRPYYYDRISAGLDANGKPIAWQHRIVGSSILARFAPPAFQHGVDPD
AVEVATDLPYDLPNQLIDYVRQEPRHVPTAFWRGVGPTRSTFVVESFIDELAAQTKTDPVQYRRALLGKTPRAL
NVLDVATKAAGWGPSLPTGQGRGVSVMHAFGSFFSIVIDVAVDNGEVQVKRVVCAVDCGMSVNPNTIEAQV
QGGIIFGITGALYGEITIEDGRVVQSNFTDYRMLRINETPPIEVHLVKSGEAPGGIGEPGTAATAAALSNAIFAATG
KRLRKLPVGDQLKTA
>SEQF4087||SEQF4087.1_08835
MSAPELSVHNESRRALLLGFASGGLLLAFGVPSLVRAAAPVQPPVSADPRYGGAGMPHGLRDDPHLFVAIAPD
GTVTVTCIRSEMGQGVRTSVALVVADELGADWARVKVAQAVGDEPRYGNQNTDGSRSLRQSFAALRRAGAA
ARTMLEQAAAAAWGVDARQVKATVHEVVDTKSGRKLGFGELAAKAAALPAPDPATVPLKAPAEFRYIGKGQT
ALIDGRDIVAGRAHYGIDTRLDGMLYAVVARPPAYGDTVASFDASAAEKLPGVVKVVPLAPTPLPSGFQPLGGV
AVVARDTWTAIQARAQLKIDWKHGPNANYDSAAYRKTLEAAAAQPGDVIRNDGDAAAALAGAAKRVRATYYI
PHLAHATMEPPAAVARVADGRCEVWTCTQAPQTTRDEVAKALGLPGERVTVNVTLLGGGFGRKSKPDYVVEA
ALLSKAVGAPVKLTFTREDDLAHDYFHAVSLEAFDGGIDASGKVVAWQHRTVAPSIQSTFRAGIVHEQPGELAQ
GIADLPFAIPNVRIENPAAQAHTRIGWFRSVYNIPHAFGIQSFVSELAHAAGRDPKDFLLELIGPARRFEPHITVK

NVNYGEDPALYPVDTGRLRRVVETVAREAGWGRKLPKGHGLGIAAHRSFVSYTAAVCEVQVDADGKITVPRVD
IAIDCGPQVNPERVRSQLEGAVVMGLGIALHGEITFKDGHPEQSNFNGFQVLRMNEAPREIRVHLVAPDDFAT
PLGGVGEPGLPPVAPALTNAIFAATGTRIRSLPVADQLAKPRAG
>SEQF4087||SEQF4087.1_02013
MTIELDNTGSVRPSRRTFLKAAGAAAAVSLTIGFEWAGLGRRALAATAPAADFAPNAFLRITPDGAVTVIAKHVE
MGQGAYTGIATIVAEELDADWSTVRVESAPADAKRYANLAFGTMQGTGGSSAMANSWQQLREAGGKARA
MLVSAAAARWKVPAGELTTANGVVTHAKSGKKAAYGTLVADASKLPVPDKVVLKQPADFKLIGQRIPRVDASPK
SNGTAHFTLDTTFPGMRVALLQRPPRFGATVKSFDATAAKAVPGVVSVVQVPGGVAVVGTGFWAAKQGRDAL
KVEWDEAHAEKRSSDEIMREYRQLADKPGTSARKDGDADAAIAGAARKIGATYEFPYLAHAPMEPLDAVVKLT
ADSCEIWAGDQFQTVDQANAARTAGLKPEQVQIHTLYAGGSFGRRANAWSDYVVEAVSIAKALGADGKPVKL
QWTREDDIQGGFYRPMYFHKLDAGLTADGKLVGWRHRIVGQSILAGTPFEAFMVKNGIDATSVEGAANLPYA
VPNVSVELTTTKVGLPVLWWRVVGSSHTAYAVEAFIDEAAHTAGKDPYAFRRDLLAKEPRMRAVLDLAAQKAG
WDPAKPLPKGRGRGIAVAEAFKSYVAQVAEVSVDADGKVKVERVVCAVDCGIAINPDIVAAQMEGGIGFGLGA
VLHSAITLKDGQVEQRNFDGYHVLRIAEMPKVEVHIVPSAEAPTGVGEPGVAPVGPAVANAIFAATGRRHYVLP
FDSADSAKA
>SEQF4088||SEQF4088.1_00536
MSAPDLSVHNESRRALLLGFASGGLLLAFGVPSLVRAAVPNQPPVSADPRYGGAGMPHGLRDDPHLFVAIAPD
GTVTVTCIRSEMGQGVRTSVALVVADELGADWARVKVAQAVGDEPRYGNQNTDGSRSLRQSFAALRRAGAA
ARTMLEQAAAAAWGVDARQVKATVHEVVDTKSGRKLGFGELAAKAAALRAPDPATVPLKAPAEFRYIGKGET
ALIDGRDIVAGRAHYGIDTRLDGMLYAVVARPPAYGDTMASFDASAAEKLPGVVKVVPLASTPLPSGFQPLGGV
AVVARDTWTAIQARAQLKIDWKHGPNANYDSAAYRKTLEAAAAQPGDVIRNDGDAAAALAGAAKRVRATYYI
PHLAHATMEPPAAVARVADGRCEVWTCTQAPQTTRDEVAKALGLPGERVTVNVTLLGGGFGRKSKPDYVVEA
ALLSKAVGAPVKLTFTREDDLAHDYFHAVSLEAFDGGIDASGKVVAWQHRTVAPSIQSTFRAGVVHEQPGELA
QGIADLPFAIPNVRIENPAAQAHTRIGWFRSVYNIPHAFGIQSFVSELAHAAGRDPKDFLLELIGPARRFEPHITV
KNVNYGEDPALYPVDTGRLRRVVETVAREAGWGRRLPKGHGLGIAAHRSFVSYTAAVCEVQVDADGKITVPRV
DIAIDCGPQVNPERVRSQLEGAVVMGLGIALHGEITFKDGHPEQSNFNGFQVLRMNEAPREIRVHLVAPDDFA
TPLGGVGEPGLPPVAPALTNAIFAATGTRIRSLPVADQLAKPRAG
>SEQF4088||SEQF4088.1_03072
MTIELDNTGSVRPSRRTFLKAAGAAAAVSLTIGFEWAGLGRRALAATAPAADFAPNAFLRITPDGAVTVIAKHVE
MGQGAYTGIATIVAEELDADWSTVRVESAPADAKRYANLAFGTMQGTGGSSAMANSWQQLREAGGKARA
MLVSAAAARWKVPAGELTTANGVVTHAKSGKKAAYGTLVADASKLPVPDKVVLKQPADFKLIGHRIPRVDASPK
SNGTAHFTLDTTFPGMRVALLQRPPRFGATVKSFDATAARAVPGVVSVVQVPGGIAVVGTGFWAAKQGRDAL
KVEWDEAHAEKRGSDEIMREYRQLADKPGTSARKDGDADAAIAGAARKIGATYEFPYLAHAPMEPLDAVVKLT
ADSCEIWAGDQFQTVDQANAARTAGVKPEQVRIHTLYAGGSFGRRANAWSDYVVEAVSIAKALGADGKPVKL
QWTREDDIQGGFYRPMYFHKLDAGLTEDGKLVGWRHRIVGQSILAGTPFEAFMVKNGIDATSVEGAANLPYA
VPNVSVELTTTKVGLPVLWWRVVGSSHTAYAVEAFIDEAAHTAGKDPYAFRRDLLAKEPRMRAVLDLAAQKAG
WDPAKPLPKGRGRGIAVAEAFKSYVAQVAEVSVDADGKVKVERVVCAVDCGIAINPDIVAAQMEGGIGFGLGA
VMHSAITLKDGQVEQRNFDGYHVLRIAEMPKVEVHIVPSAEAPTGVGEPGVAPVGPAVANAIFAATGKRHYVL
PFDSADSAKA
>SEQF4088||SEQF4088.1_05065
MSRGLIEAGKVAGQAAGAGVSRRSFLKLGMSLGAAAGGGLLLGFSLPAAGDDARRSVIGGDGDETARAGVFA
PNAFVQIDRAGKVTLVMPKVEMGQGVYTALPMLIAEELEVPLSSVTLDHAPPNEKLFLDPLLGGQLTGGSTSIRY
AWEPLRRAGATARTLLVAAAAKQWNVDPASCRAVNGEVQHPPSGRRTSYGQLADAAAKLPVPKDVALKKPAD
FKLIGTPAKRLDSPEKVDGTAQFGLDVRLPGMLYAVIVNSPVFGGTVASIDDTATKKIPGVRQVVRADNAVAVVG

DHTWAAKRGASALVVKWNEGAGAKVSTKDIVADLAQAAANGKGAVARKDGDVGKGFADAKTRIDAVYEQPL
LAHATMEPVNCTVHVRADGCEIWVGTQVPTRAVDTVQQLTSFPPEKIVVHNHLLGGGFGRRLETDMIGQAVK
IAKQVNAPVKVIWTREEDIQHDMYRPYYYDRISAGLDANGKPIAWQHRIVGSSILARFAPPAFQHGVDPDAVE
VATDLPYDLPNQLIDYVRQEPRHVPTAFWRGVGPTRSTFVVESFIDELAAQTKTDPVQYRRALLGKTPRALNVL
DVATKAAGWGPSLPTGQGRGVSVMHAFGSFFSIVIDVAVDNGEVQVKRVVCAVDCGMSVNPNTIEAQVQG
GIIFGITGALYGEITIEDGRVVQSNFTDYRMLRINETPPIEVHLVKSGEAPGGIGEPGTAATAAALSNAIFAATGKRL
RKLPVGDQLKTA
>SEQF4088||SEQF4088.1_02164
MSADVTTPRRGRRRFLLGALGLGGALVVGWGVMPPRSRLGDPAAFPETSGQIALNGWIKITPEGNVILAMPR
VEMGQGIHTALSMLAAEELDIPLARVSIESSPIERIYGNVVAMGDSSLPLHQDDADKTWARALHWIMAKSAREI
GLIITGGSSSVADGWQPVREAAATARATLVEAAAREWGVQPTRVMVREGQLIGPDGQQMPFSAVAQKARNI
APPSSVTLKPASQYQLVGKPAPRNDIAGKTDGSARFAIDARPPGMLYAAVVMCPVLGGKLKAFQSKAALVMPG
VRHVVPFDGSTGGAPGVAVVADHYWQARQALATLEPEWDNGPHATLDSAGIHQQLVGALDSDKGGFTYRST
GDGLKAFENAQGATIVEAEYGAPYLAHAAMEPINCTAQVTKDRVQLWAPTQVATLAQLVAARAAGVSRDQVH
IDIPLIGGGFGRRLESDFVGQAVTIATKTEGHPVQVIWAREDDIRHDFYRPQAIARLKARVENGKVTAIASRSAG
QSILAGELERLFGAPSLGIDRYTAEGLFDLPYEIEHEHIAHLVVDLPVPIGFWRSVGHSYTGFFLEGFLNDVAAAAK
LDPLAMRRDLLKAHPRELKVLDIAAQAAGWGQPLAPAADGAPRARGLALHNSFGSVVAQVVEVSLQDGKPR
VHRVVCAADCGTVVNPGIVAQQLESGIIFGLSAALYSQIQIKDGRVVQSNFPDYPVLKMAEAPVIETHVVPSTAE
PTGVGEIAVPPIAPAVAHAVAQLTGKPVRQLPMV
>SEQF4089||SEQF4089.1_07876
MTIELDNTGSVRPSRRTFLKAAGAAAAVSLTIGFEWAGLGRRALAATAPAADFAPNAFLRITPDGAVTVIAKHVE
MGQGAYTGIATIVAEELDADWSTVRVESAPADAKRYANLAFGTMQGTGGSSAMANSWQQLREAGGKARA
MLVSAAAARWKVPAGELTTANGVVTHAKSGKKAAYGTLVADASKLPVPDKVVLKQPADFKLIGHRIPRVDAAP
KSNGTAHFTLDTTFPGMRVALLQRPPRFGATVKSFDATAARAVPGVVSVVQVPGGVAVVGTGFWAAKQGRD
ALKVEWDEAHAEKRGSDEIMREYRQLADKPGTSARKDGDADAAIAGAARKIGATYEFPYLAHAPMEPLDAVV
KLTADSCEIWAGDQFQTVDQANAARTAGLKPEQVQIHTLYAGGSFGRRANAWSDYVVEAVSIAKALGADGKP
VKLQWTREDDIQGGFYRPMYFHKLDAGLTEDGKLVGWRHRIVGQSILAGTPFEAFMVKNGIDATSVEGAANL
PYAVPNVSVELTTTKVGLPVLWWRVVGSSHTAYAVEAFIDEAAHTAGKDPYAFRRDLLAKEPRMRAVLDLAAQK
AGWDPAKPLPKGRGRGIAVAEAFKSYVAQVAEVSVDADGKVKVERVVCAVDCGIAINPDIVAAQMEGGIGFGL
GAVMHSAITLKDGQVEQRNFDGYHVLRIAEMPKVEVHIVPSAEAPTGVGEPGVAPVGPAVANAIFAATGKRHY
VLPFDSADSAKA
>SEQF4089||SEQF4089.1_02963
MSRGLIEAGKVAGQAAGAGVSRRSFLKLGMSLGAAAGGGLLLGFSLPAAGDDARRSVIGGDGDETARAGVFA
PNAFVQIDRAGKVTLVMPKVEMGQGVYTALPMLIAEELEVPLSSVTLDHAPPNEKLFLDPLLGGQLTGGSTSIRY
AWEPLRRAGATARTLLVAAAAKQWSVDPASCRAVNGEVQHPPSGRRVSYGQLADAAAKLPVPKDVALKKPAD
FKLIGTPAKRLDSPEKVDGTAQFGLDVRLPGMLYAVIVNSPVFGGTVASIDDTAAKKIPGVRQIVRADNAVAVVG
DHTWAAKRGASALVVKWNEGAGAKVSTKDIVADLAQAAANGKGAVARKDGDVGKGFADAKTRVDAVYEQP
LLAHATMEPVNCTVHVRADGCEIWVGTQVPTRAVDTVQQLTSFPPEKIVVHNHLLGGGFGRRLETDMIGQAV
KIAKQVNAPVKVIWTREEDIQHDMYRPYYYDRISAGLDANGKPIAWQHRIVGSSILARFAPPAFQHGVDPDAV
EVATDLPYDLPNQLIDYVRQEPRHVPTAFWRGVGPTRSTFVVESFIDELAAQTKTDPVQYRRALLGKTPRALNVL
DVATKAAGWGPSLPTGQGRGVSVMHAFGSFFSIVIDVAVDNGEVQVKRVVCAVDCGMSVNPNTIEAQVQG
GIIFGITGALYGEITIEDGRVVQSNFTDYRMLRINETPPIEVHLVKSGEAPGGIGEPGTAATAAALSNAIFAATGKRL
RKLPVGDQLKTA
>SEQF4089||SEQF4089.1_06591

MSAPELSVHNESRRALLLGFASGGLLLAFGVPSLVRAAVPNQPPVSVDPRYGGAGMPHGLRDDPHLFVAIAPD
GTVTVTCIRSEMGQGVRTSVALVVADELGADWARVKVAQAVGDEPRYGNQNTDGSRSLRQSFAALRRAGAA
ARTMLEQAAAAAWGVDARQVRAIVHEVVDTKSGRKLGFGELAAKAAALPAPDPATVPLKAPAEFRYIGKGETA
LIDGRDIVAGRAHYGIDTRLDGMLYAVVARPPAYGDTMASFDASAAEKLPGVVKVVPLASTPLPSGFQPLGGVA
VVARDTWTAIQARAQLKIDWKHGPNANYDSAAYRKTLEAAAAQPGDVIRNDGDAAAALAGAAKRVRATYYIP
HLAHATMEPPAAVARVADGRCEVWTCTQAPQTTRDEVAKALGLPGERVTVNVTLLGGGFGRKSKPDYVVEAA
LLSKAVGAPVKLTFTREDDLAHDYFHAVSLEAFDGGIDASGKVVAWQHRTVAPSIQSTFRAGVVHEQPGELAQ
GIADLPFAIPNVRIENPAAQAHTRIGWFRSVYNIPHAFGIQSFVSELAHAAGRDPKDFLLELIGPARRFEPHITVK
NVNYGEDPALYPVDTGRLRRVVETVAREAGWGRRLPKGHGLGIAAHRSFVSYTAAVCEVQVDADGKITVPRVD
IAIDCGPQVNPERVRSQLEGAVVMGLGIALHGEITFKDGHPEQSNFNGFQVLRMNEAPREIRVHLVAPDDFAT
PLGGVGEPGLPPAAPALTNAIFAATGTRIRSLPVADQLAKPRAG
>SEQF4090||SEQF4090.1_02043
MSAPELSVHNESRRALLLGFASGGLLLAFGVPSLVRAAVPNQPPVSADPRYGGAGMPHGLRDDPHLFVAIAPD
GTVTVTCIRSEMGQGVRTSVALVVADELGADWARVKVAQAVGDEPRYGNQNTDGSRSLRQSFAALRRAGAA
ARTMLEQAAAAAWGVDARQVKATVHEVVDTKSGRKLGFGELAAKAAALPAPDPATVPLKAPAEFRYIGKGETA
LIDGRDIVAGRAHYGIDTRLDGMLYAVVARPPAYGDTMASFDASAAEKLPGVVKVVPLASTPLPSGFQPLGGVA
VVARDTWTAIQARAQLKIDWKHGPNANYDSAAYRKTLEAAAAQPGDVIRNDGDAAAALAGAAKRVRATYYIP
HLAHATMEPPAAVARVADGRCEVWTCTQAPQTTRDEVAKALGLPSERVMVNVTLLGGGFGRKSKPDYVVEA
ALLSKAVGAPVKLTFTREDDLAHDYFHAVSLEAFDGGIDASGKVVAWQHRTVAPSIQSTFRAGVVHEQPGELA
QGIADLPFAIPNVRIENPAAQAHTRIGWFRSVYNIPHAFGIQSFVSELAHAAGRDPKDFLLELIGPARRFEPHITV
KNVNYGEDPALYPVDTGRLRRVVETVAREAGWGRRLPKGHGLGIAAHRSFVSYTAAVCEVQVDADGKITVPRV
DIAIDCGPQVNPERVRSQLEGAVVMGLGIALHGEITFKDGHPEQRNFNGFQVLRMNEAPREIRVHLVAPDDFA
TPLGGVGEPGLPPVAPALTNAIFAATGTRIRSLPVADQLAKPRAG
>SEQF4090||SEQF4090.1_05333
MTIELDNTGSVRPSRRTFLKAAGAAAAVSLTIGFEWAGLGRRALAATAPAADFAPNAFLRITPDGAVTVIAKHVE
MGQGAYTGIATIVAEELDADWSTVRVESAPADAKRYANLAFGTMQGTGGSSAMANSWQQLREAGGKARA
MLVSAAAARWKVPAGELTTANGVVTHAKSGKKAAYGTLVADASKLPVPDKVVLKQPADFKLIGHRIPRVDASPK
SNGTAHFTLDTTFPGMRVALLQRPPRFGATVKSFDATAAKAVPGVVSVVQVPGGIAVVGTGFWAAKQGRDAL
KVEWDEAHAEKRGSDEIMREYRQLADKPGTSARKDGDADAAIAGAARKIGATYEFPYLAHAPMEPLDAVVKLT
ADSCEIWAGDQFQTVDQANAARTAGLKPEQVQIHTLYAGGSFGRRANAWSDYVVEAVSIAKALGADGKPVKL
QWTREDDIQGGFYRPMYFHKLDAGLTEDGKLVGWRHRIVGQSILAGTPFEAFMVKNGIDATSVEGAANLPYA
VPNVSVELTTTKVGLPVLWWRVVGSSHTAYAVEAFIDEAAHTAGKDPYAFRRDLLAKEPRMRAVLDLAAQKAG
WDPAKPLPKGRGRGIAVAEAFKSYVAQVAEVSVDADGKVKVERVVCAVDCGIAINPDIVAAQMEGGIGFGLGA
VMHSAITLKDGQVEQRNFDGYHVLRIAEMPKVEVHIVPSAEAPTGVGEPGVAPVGPAVANAIFAATGKRHYVL
PFDSADSAKA
>SEQF4090||SEQF4090.1_06234
MSRGLIEAGKVAGQAADAGVSRRSFLKLGMSLGAAVGGGLLLGFSLPAAGDDARRSVIGGDGDETARAGVFAP
NAFVQIDRAGKVTLVMPKVEMGQGVYTALPMLIAEELEVPLSSVTLDHAPPNEKLFLDPLLGGQLTGGSTSIRYA
WEPLRRAGATARTLLVAAAAKQWNVDPASCRAVNGEVQHPPSGRRTSYGQLADAAAKLPVPKDVALKKPADF
KLIGTPAKRLDSPEKVDGTAQFGLDVRLPGMLYAVIVNSPVFGGTVASIDDTAAKKIPGVRQVVRADNAVAVVG
DHTWAAKRGASALVVKWNEGAGAKVSTKDIVADLAQAAANGKGAVARKDGDVGKGFADAKTRVDAVYEQP
LLAHATMEPVNCTVHVRADGCEIWVGTQVPTRAVDTVQQLTSFPPEKIVVHNHLLGGGFGRRLETDMIGQAV
KIAKQVNAPVKVIWTREEDIQHDMYRPYYYDRISAGLDANGKPIAWQHRIVGSSILARFAPPAFQHGVDPDAV
EVATDLPYDLPNQLIDYVRQEPRHVPTAFWRGVGPTRSTFVVESFIDELAAQTKTDPVQYRRALLGKTPRALNVL

DVATKAAGWGPSLPTGQGRGVSVMHAFGSFFSIVIDVAVDNGEVQVKRVVCAVDCGMSVNPNTIEAQVQG
GIIFGITGALYGEITIEDGRVVQSNFTDYRMLRINETPPIEVHLVKSGEAPGGIGEPGTAATAAALSNAIFAATGKRL
RKLPVGDQLKTA
>SEQF4091||SEQF4091.1_04994
MTIELDNTGSVRPSRRTFLKAAGAAAAVSLTIGFEWAGLGRRALAATAPAADFAPNAFLRITPDGAVTVIAKHVE
MGQGAYTGIATIVAEELDADWSTVRVESAPADAKRYANLAFGTMQGTGGSSAMANSWQQLREAGGKARA
MLVSAAAARWKVPAGELTTANGIVSHAKSGKKAAYGTLVADASKLPVPDKVALKQPADFKLIGQRIPRVDASPK
SNGTAHFTLDTTFPGMRVALLQRPPRFGATVKSFDATAAKAVPGVVSVVQVPGGVAVVGTGFWAAKQGRDAL
KVEWDEAHAEKRSSDEIMREYRQLADKPGTSARKDGDADAAIAGAARKIGATYEFPYLAHAPMEPLDAVVKLT
ADSCEIWAGDQFQTVDQANAARTAGLKPEQVQIHTLYAGGSFGRRANAWSDYVVEAVSIAKALGADGKPVKL
QWTREDDIQGGFYRPMYFHKLDAGLTADGKLVGWRHRIVGQSILAGTPFEAFMVKNGIDATSVEGAANLPYA
VPNVSVELTTTKVGLPVLWWRVVGSSHTAYAVEAFIDEAAHTAGKDPYAFRRDLLAKEPRMRAVLDLAAQKAG
WDPAKPLPKGRGRGIAVAEAFKSYVAQVAEVSVDADGKVKVERVVCAVDCGIAINPDIVAAQMEGGIGFGLGA
VMHSAITLKDGQVEQRNFDGYHVLRIAEMPKVEVHIVPSAEAPTGVGEPGVAPVGPAVANAIFAATGKRHYVL
PFDSADSAKA
>SEQF4091||SEQF4091.1_06287
MSAPELSVHNESRRALLLGFASGGLLLAFGVPSLVRAAVPSQPPVSADPRYGGAGMPHGLRDDPHLFVAIAPD
GTVTVTCIRSEMGQGVRTSVALVVADELGADWARVKVEQAVGDEPRYGNQNTDGSRSLRQSFAALRRAGAA
ARTMLEQAAAAAWGVDARQVKATVHEVVDTKSGRKLGFGELAAKAAALPAPDPATVPLKAPAEFRYIGKGQT
ALIDGRDIVAGRAHYGIDTRLDGMLYAVVARPPTYGDTVASFDASAAEKLPGVVKVVPLAPTPLPSGFQPLGGV
AVVARDTWTAIQARAQLKIDWKHGPNANYDSAAYRKTLEAAAAQPGDVIRNDGDAAAALAGAAKRVRATYYI
PHLAHATMEPPAAVARVADGRCEVWTCTQAPQTTRDEVAKALGLPGERVTVNVTLLGGGFGRKSKPDYVVEA
ALLSKAVGAPVKLTFTREDDLAHDYFHAVSLEAFDGGIDASGKVVAWQHRTVAPSIQSTFRAGIVHEQPGELAQ
GIADLPFAIPNVRIENPAAQAHTRIGWFRSVYNIPHAFGIQSFVSELAHAAGRDPKDFLLELIGPARRFEPHITVK
NVNYGEDPALYPVDTGRLRRVVETVAREAGWGRKLPKGHGLGIAAHRSFVSYTAAVCEVQVDADGKITVPRVD
IAIDCGPQVNPERVRSQLEGAVVMGLGIALHGEITFKDGHPEQSNFNGFQVLRMNEAPREIRVHLVAPDDFAT
PLGGVGEPGLPPVAPALTNAIFAATGTRIRSLPVADQLAKPRAG
>SEQF4091||SEQF4091.1_01922
MSRGLIEAGKVAGQAAGAGVSRRSFLKLGMSLGAAAGGGLLLGFSLPAAGDDARRSVIGGDGDETARAGVFA
PNAFVQIDRAGKVTLVMPKVEMGQGVYTALPMLIAEELEVPLSSVTLDHAPPNEKLFLDPLLGGQLTGGSTSVR
YAWEPLRRAGATARTLLVAAAAKQWNVDPASCRAVNGEVQHPPSGRRVSYGQLADAAAKLPVPKDVALKKPA
DFKLIGTPAKRLDSPEKVDGTAQFGLDVRLPGMLYAVIVNSPVFGGTVASIDDTAAKKIPGVRQVVRADNAVAV
VGDHTWAAKRGASALVVKWNEGAGAKVSTKDIVADLAQAAANGKGAVARKDGDVGKGFADAKTRIDAVYE
QPLLAHATMEPVNCTVHVRADGCEIWVGTQVPTRAVDTVQKLTSFPPERIVVHNHLLGGGFGRRLETDMIGQ
AVKIAKQVNAPVKVIWTREEDIQHDMYRPYYYDRISAGLDANGKPIAWQHRIVGSSILARFAPPAFQHGVDPD
AVEVATDLPYDLPNQLIDYVRQEPRHVPTAFWRGVGPTRSTFVVESFIDELAAQTKTDPVQYRRALLGKTPRAL
NVLDVATKAAGWGPSLPTGQGRGVSVMHAFGSFFSIVIDVAVDNGEVQVKRVVCAVDCGMSVNPNTIEAQV
QGGIIFGITGALYGEITIEDGRVVQSNFTDYRMLRINETPPIEVHLVKSGEAPGGIGEPGTAATAAALSNAIFAATG
KRLRKLPVGDQLKTA
>SEQF4092||SEQF4092.1_02013
MSRGLIEAGKVAGQAAGAGVSRRSFLKLGMSLGAAAGGGLLLGFSLPAAGDDARRSVIGGDGDETARAGVFA
PNAFVQIDRAGKVTLVMPKVEMGQGVYTALPMLIAEELEVPLSSVTLDHAPPNEKLFLDPLLGGQLTGGSTSIRY
AWEPLRRAGATARTLLVAAAAKQWSVDPASCRAVNGEVQHPPSGRRTSYGQLADAAAKLPVPKDVALKKPAD
FKLIGTPAKRLDSPEKVDGTAQFGLDVRLPGMLYAVIVNSPVFGGTVASIDDTAAKKIPGVRQVVRADNAVAVV

GDHTWAAKRGASALVVKWNEGAGAKVSTKDIVADLAQAAANGKGAVARKDGDVGKGFADAKTRVDAVYEQ
PLLAHATMEPVNCTVHVRADGCEIWVGTQVPTRAVDTVQQLTSFPPEKIVVHNHLLGGGFGRRLETDMIGQA
VKIAKQVNAPVKVIWTREEDIQHDMYRPYYYDRISAGLDANGKPIAWQHRIVGSSILARFAPPAFQHGVDPDA
VEVATDLPYDLPNQLIDYVRQEPRHVPTAFWRGVGPTRSTFVVESFIDELAAQTKTDPVQYRRALLGKTPRALN
VLDVATKAAGWGPSLPTGQGRGVSVMHAFGSFFSIVIDVAVDNGEVQVKRVVCAVDCGMSVNPNTIEAQVQ
GGIIFGITGALYGEITIEDGRVVQSNFTDYRMLRINETPPIEVHLVKSGEAPGGIGEPGTAATAAALSNAIFAATGK
RLRKLPVGDQLKTA
>SEQF4092||SEQF4092.1_06449
MSAPELSVHNESRRALLLGFASGGLLLAFGVPSLVRAAVPNQPPVSADPRYGGAGMPHGLRDDPHLFVAIAPD
GTVTVTCIRSEMGQGVRTSVALVVADELGADWARVKVAQAVGDEPRYGNQNTDGSRSLRQSFAALRRAGAA
ARTMLEQAAAAAWGVDARQVKATVHEVVDTKSGRKLGFGELAAKAAALPAPDPATVPLKAPAEFRYIGKGETA
LIDGRDIVAGRAHYGIDTRLDGMLYAVVARPPAYGDTMASFDASAAEKLPGVVKVVPLASTPLPSGFQPLGGVA
VVARDTWTAIQARAQLKIDWKHGPNANYDSAAYRKTLEAAAAQPGDVIRNDGDAAAALAGAAKRVRATYYIP
HLAHATMEPPAAVARVADGRCEVWTCTQAPQTTRDEVAKALGLPSERVTVNVTLLGGGFGRKSKPDYVVEAA
LLSKAVGAPVKLTFTREDDLAHDYFHAVSLEAFDGGIDASGKVVAWQHRTVAPSIQSTFRAGVVHEQPGELAQ
GIADLPFAIPNVRIENPAAQAHTRIGWFRSVYNIPHAFGIQSFVSELAHAAGRDPKDFLLELIGPARRFEPHITVK
NVNYGEDPALYPVDTGRLRRVVETVAREAGWGRRLPKGHGLGIAAHRSFVSYTAAVCEVQVDADGKITVPRVD
IAIDCGPQVNPERVRSQLEGAVVMGLGIALHGEITFKDGHPEQRNFNGFQVLRMNEAPREIRVHLVAPDDFAT
PLGGVGEPGLPPVAPALTNAIFAATGTRIRSLPVADQLAKPRAG
>SEQF4092||SEQF4092.1_07330
MTIELDNTGSVRPSRRTFLKAAGAAAAVSLTIGFEWAGLGRRALAATAPAADFAPNAFLRITPDGAVTVIAKHVE
MGQGAYTGIATIVAEELDADWSTVRVESAPADAKRYANLAFGTMQGTGGSSAMANSWQQLREAGGKARA
MLVSAAAARWKVPAGELTTANGVVTHAKSGKKAAYGTLVADASKLPVPDKVVLKQPADFKLIGQRIPRVDASPK
SNGTAHFTLDTTFPGMRVALLQRPPRFGATVKSFDATAARAVPGVVSVVQVPGGVAVVGTGFWAAKQGRDAL
KVEWDEAHAEKRGSDEIMREYRQLADKPGTSARKDGDADAAIAGAARKIGATYEFPYLAHAPMEPLDAVVKLT
ADSCEIWAGDQFQTVDQANAARTAGLKPEQVRIHTLYAGGSFGRRANAWSDYVVEAVSIAKALGADGKPVKL
QWTREDDIQGGFYRPMYFHKLDAGLTEDGKLVGWRHRIVGQSILAGTPFEAFMVKNGIDATSVEGAANLPYA
VPNVSVELTTTKVGLPVLWWRVVGSSHTAYAVEAFIDEAAHTAGKDPYAFRRDLLAKEPRMRAVLDLAAQKAG
WDPAKPLPKGRGRGIAVAEAFKSYVAQVAEVSVDADGKVKVERVVCAVDCGIAINPDIVAAQMEGGIGFGLGA
VMHSAITLKDGQVEQRNFDGYHVLRIAEMPKVEVHIVPSAEAPTGVGEPGVAPVGPAVANAIFAATGKRHYVL
PFDSADSAKA
>SEQF4093||SEQF4093.1_02319
MSRGLIEAGKVAGQAAGAGVSRRSFLKLGMSLGAAAGGGLLLGFSLPAAGDDARRSVIGGDGDETARAGVFA
PNAFVQIDRAGKVTLVMPKVEMGQGVYTALPMLIAEELEVPLSSVTLDHAPPNEKLFLDPLLGGQLTGGSTSVR
YAWEPLRRAGATARTLLVAAAAKQWNVDPASCRAVNGEVQHPPSGRRVSYGQLADAAAKLPVPKDVALKKPA
DFKLIGTPAKRLDSPEKVDGTAQFGLDVRLPGMLYAVIVNSPVFGGTVASIDDTAAKKIPGVRQVVRADNAVAV
VGDHTWAAKRGASALVVKWNEGAGAKVSTKDIVADLAQAAANGKGAVARKDGDVGKGFADAKTRIDAVYE
QPLLAHATMEPVNCTVHVRADGCEIWVGTQVPTRAVDTVQKLTSFPPEKIVVHNHLLGGGFGRRLETDMIGQ
AVKIAKQVNAPVKVIWTREEDIQHDMYRPYYYDRISAGLDANGKPIAWQHRIVGSSILARFAPPAFQHGVDPD
AVEVATDLPYDLPNQLIDYVRQEPRHVPTAFWRGVGPTRSTFVVESFIDELAAQTKTDPVQYRRALLGKTPRAL
NVLDVATKAAGWGPSLPTGQGRGVSVMHAFGSFFSIVIDVAVDNGEVQVKRVVCAVDCGMSVNPNTIEAQV
QGGIIFGITGALYGEITIEDGRVVQSNFTDYRMLRINETPPIEVHLVKSGEAPGGIGEPGTAATAAALSNAIFAATG
KRLRKLPVGDQLKTA
>SEQF4093||SEQF4093.1_06021

MTIELDNTGSVRPSRRTFLKAAGAAAAVSLTIGFEWAGLGRRALAATAPAADFAPNAFLRITPDGAVTVIAKHVE
MGQGAYTGIATIVAEELDADWSTVRVESAPADAKRYANLAFGTMQGTGGSSAMANSWQQLREAGGKARA
MLVSAAAARWKVPAGELTTANGVVTHAKSGKKAAYGTLVADASKLPVPDKVVLKQPADFKLIGQRIPRVDASPK
SNGTAHFTLDTTFPGMRVALLQRPPRFGATVKSFDATAAKAVPGVVSVVQVPGGVAVVGTGFWAAKQGRDAL
KVEWDEAHAEKRSSDEIMREYRQLADKPGTSARKDGDADAAIAGAARKIGATYEFPYLAHAPMEPLDAVVKLT
ADSCEIWAGDQFQTVDQANAARTAGLKPEQVQIHTLYAGGSFGRRANAWSDYVVEAVSIAKALGADGKPVKL
QWTREDDIQGGFYRPMYFHKLDAGLTADGKLVGWRHRIVGQSILAGTPFEAFMVKNGIDATSVEGAANLPYA
VPNVSVELTTTKVGLPVLWWRVVGSSHTAYAVEAFIDEAAHTAGKDPYAFRRDLLAKEPRMRAVLDLAAQKAG
WDPAKPLPKGRGRGIAVAEAFKSYVAQVAEVSVDADGKVKVERVVCAVDCGIAINPDIVAAQMEGGIGFGLGA
VMHSAITLKDGQVEQRNFDGYHVLRIAEMPKVEVHIVPSAEAPTGVGEPGVAPVGPAVANAIFAATGKRHYVL
PFDSADSAKA
>SEQF4093||SEQF4093.1_03130
MSAPELSVHNESRRALLLGFASGGLLLAFGVPSLVRAAAPVQPPVSADPRYGGAGMPHGLRDDPHLFVAIAPD
GTVTVTCIRSEMGQGVRTSVALVVADELGADWARVKVAQAVGDEPRYGNQNTDGSRSLRQSFAALRRAGAA
ARTMLEQAAAAAWGVDARQVKATVHEVVDTKSGRKLGFGELAAKAAALPAPDPATVPLKAPAEFRYIGKGQT
ALIDGCDIVAGRAHYGIDTRLDGMLYAVVARPPAYGDTVASFDASAAEKLPGVVKVVPLTPTPLPSGFQPLGGVA
VVARDTWTAIQARAQLKIDWKHGPNANYDSAAYRKTLEAAAAQPGDVIRNDGDAAAALAGAAKRVRATYYIP
HLAHATMEPPAAVARVADGRCEVWTCTQAPQTTRDEVAKALGLPGERVTVNVTLLGGGFGRKSKPDYVVEAA
LLSKAVGAPVKLTFTREDDLAHDYFHAVSLEAFDGGIDASGKVVAWQHRTVAPSIQSTFRAGIVHEQPGELAQG
IADLPFAIPNVRIENPAAQAHTRIGWFRSVYNIPHAFGIQSFVSELAHAAGRDPKDFLLELIGPARRFEPHITVKN
VNYGEDPALYPVDTGRLRRVVETVAREAGWGRKLPKGRGLGIAAHRSFVSYTAAVCEVQVDADGKITVPRVDIA
IDCGPQVNPERVRSQLEGAVVMGLGIALHGEITFKDGHPEQSNFNGFQVLRMNEAPREIRVHLVAPDDFATPL
GGVGEPGLPPVAPALTNAIFAATGTRIRSLPVADQLAKPRAG
>SEQF4094||SEQF4094.1_02298
MSAPELSVHNESRRALLLGFASGGLLLAFGVPSLVRAAVPNQPPVSADPRYGGAGMPHGLRDDPHLFVAIAPD
GTVTVTCIRSEMGQGVRTSVALVVADELGADWARVKVAQAVGDEPRYGNQNTDGSRSLRQSFAALRRAGAA
ARTMLEQAAAAAWGVDARQVKATVHEVVDTKSGRKLGFGELAAKAAALPAPDPATVPLKAPAEFRYIGKGETA
LIDGRDIVAGRAHYGIDTRLDGMLYAVVARPPAYGDTMASFDASAAEKLPGVVKVVPLASTPLPSGFQPLGGVA
VVARDTWTAIQARAQLKIDWKHGPNANYDSAAYRKTLEAAAAQPGDVIRNDGDAAAALAGAAKRVRATYYIP
HLAHATMEPPAAVARVADGRCEVWTCTQAPQTTRDEVAKALGLPGERVTVNVTLLGGGFGRKSKPDYVVEAA
LLSKAVGAPVKLTFTREDDLAHDYFHAVSLEAFDGGIDASGKVVAWQHRTVAPSIQSTFRAGIVHEQPGELAQG
IADLPFAIPNVRIENPAAQAHTRIGWFRSVYNIPHAFGIQSFVSELAHAAGRDPKDFLLELIGPARRFEPHITVKN
VNYGEDPALYPVDTGRLRRVVETVAREAGWGRRLPKGHGLGIAAHRSFVSYTAAVCEVQVDADGKIAVPRVDI
AIDCGPQVNPERVRSQLEGAVVMGLGIALHGEITFKDGHPEQSNFNGFQVLRMNEAPREIRVHLVAPDDFATP
LGGVGEPGLPPVAPALTNAIFAATGTRIRSLPVADQLAKPRAG
>SEQF4094||SEQF4094.1_02036
MTIELDNTGSVRPSRRTFLKAAGAAAAVSLTIGFEWAGLGRRALAATAPAADFAPNAFLRITPDGAVTVIAKHVE
MGQGAYTGIATIVAEELDADWSTVRVESAPADAKRYANLAFGTMQGTGGSSAMANSWQQLREAGGKARA
MLVSAAAASWKVPAGELTTANGVVTHAKSGKKAAYGTLVADASKLPVPDKVVLKQPADFKLIGHRIPRVDASPK
SNGTAHFTLDTTFPGMRVALLQRPPRFGATVKSFDATAAKAVPGVVSVVQVPGGVAVVGTGFWAAKQGRDAL
KVEWDEAHAEKRGSDEIMREYRQLADKPGTSARKDGDADAAIAGAARKIGATYEFPYLAHAPMEPLDAVVKLT
ADSCEIWAGDQFQTVDQANAARTAGLKPEQVRIHTLYAGGSFGRRANAWSDYVVEAVSIAKALGADGKPVKL
QWTREDDIQGGFYRPMYFHKLDAGLTEDGKLVGWRHRIVGQSILAGTPFEAFMVKNGIDATSVEGAANLPYA
VPNVSVELTTTKVGLPVLWWRVVGSSHTAYAVEAFIDEAAHTAGKDPYAFRRDLLAKEPRMRAVLDLAAQKAG

WDPAKPLPKGRGRGIAVAEAFKSYVAQVAEVSVDADGKVKVERVVCAVDCGIAINPDIVAAQMEGGIGFGLGA
VMHSAITLKDGQVEQRNFDGYHVLRIAEMPKVEVHIVPSAEAPTGVGEPGVAPVGPAVANAIFAATGKRHYVL
PFDSADSAKA
>SEQF4094||SEQF4094.1_00993
MSRGLIEAGKVAGQAAGAGVSRRSFLKLGMSLGAAAGGGLLLGFSLPAAGDDARRSVIGGDGDETARAGVFA
PNAFVQIDRAGKVTLVMPKVEMGQGVYTALPMLIAEELEVPLSSVTLDHAPPNEKLFLDPLLGGQLTGGSTSIRY
AWEPLRRAGATARTLLVAAAAKQWSVDPAFCRAVNGEVQHPPSGRRTSYGQLADAAAKLPVPKDVALKKPAD
FKLIGTPAKRLDSPEKVDGTAQFGLDVRLPGMLYAVIVNSPVFGGTVASIDDTAAKKIPGVRQVVRADNAVAVV
GDHTWAAKRGASALVVKWNEGAGAKVSTKDIVADLAQAAANGKGAVARKDGDVGKGFADAKTRVDAVYEQ
PLLAHATMEPVNCTVHVRADGCEIWVGTQVPTRAVDTVQQLTSFPPEKIVVHNHLLGGGFGRRLETDMIGQA
VKIAKQVNAPVKVIWTREEDIQHDMYRPYYYDRISAGLDANGKPIAWQHRIVGSSILARFAPPAFQHGVDPDA
VEVATDLPYDLPNQLIDYVRQEPRHVPTAFWRGVGPTRSTFVVESFIDELAAQTKTDPVQYRRALLGKTPRALN
VLDVATKAAGWGPSLPTGQGRGVSVMHAFGSFFSIVIDVAVDNGEVQVKRVVCAVDCGMSVNPNTIEAQVQ
GGIIFGITGALYGEITIEDGRVVQSNFTDYRMLRINETPPIEVHLVKSGEAPGGIGEPGTAATAAALSNAIFAATGK
RLRKLPVGDQLKTA
>SEQF4095||SEQF4095.1_00483
MSRGLIEAGKAAGQAAGAGVSRRSFLKLGMSLGAAAGGGLLLGFSLPAAGDDARRSVIGGDGDETARAGVFA
PNAFVQIDRAGKVTLVMPKVEMGQGVYTALPMLIAEELEVPLSSVTLDHAPPNEKLFLDPLLGGQLTGGSTSIRY
AWEPLRRAGATARTLLVAAAAKQWSVDPASCRAVNGEVQHPPSGRRTSYGQLADAAAKLPVPKDVALKKPAD
FKLIGTPAKRLDSPEKVDGTAQFGLDVRLPGMLYAVIVNSPVFGGTVASIDDTAAKKIPGVRQVVRADNAVAVV
GDHTWAAKRGASALVVKWNEGAGAKVSTKDIVADLAQAAANGKGAVARKDGDVGKGFADAKTRVDAVYEQ
PLLAHATMEPVNCTVHVRADGCEIWVGTQVPTRAVDTVQKLTSFPPEKIVVHNHLLGGGFGRRLETDMIGQA
VKIAKQVNAPVKVIWTREEDIQHDMYRPYYYDRISAGLDANGKPIAWQHRIVGSSILARFAPPAFQHGVDPDA
VEVATDLPYDLPNQLIDYVRQEPRHVPTAFWRGVGPTRSTFVVESFIDELAAQTKTDPVQYRRALLGKTPRALN
VLDVATKAAGWGPSLPTGQGRGVSVMHAFGSFFSIVIDVAVDNGEVQVKRVVCAVDCGMSVNPNTIEAQVQ
GGIIFGITGALYGEITIEDGRVVQSNFTDYRMLRINETPPIEVHLVKSGEAPGGIGEPGTAATAAALSNAIFAATGK
RLRKLPVGDQLKTA
>SEQF4095||SEQF4095.1_03791
MTIELDNTGSVRPSRRTFLKAAGAAAAVSLTIGFEWAGLGRRALAATAPAADFAPNAFLRITPDGAVTVIAKHVE
MGQGAYTGIATIVAEELDADWSTVRVESAPADAKRYANLAFGTMQGTGGSSAMANSWQQLREAGGKARA
MLVSAAAARWKVPAGELTTANGVVTHAKSGKKAAYGTLVADASKLPVPDKVALKQPADFKLIGQRIPRVDASPK
SNGTAHFTLDTTFPGMRVALLQRPPRFGATVKSFDATAAKAVPGVVSVVQVPGGVAVVGTGFWAAKQGRDAL
KVEWDEAHTEKRSSDEIMREYRQLADKPGTSARKDGDADAAIAGAARKIGATYEFPYLAHAPMEPLDAVVKLT
ADSCEIWAGDQFQTVDQANAARTAGLKPEQVQIHTLYAGGSFGRRANAWSDYVVEAVSIAKALGADGKPVKL
QWTREDDIQGGFYRPMYFHKLDAGLTADGKLVGWRHRIVGQSILAGTPFEAFMVKNGIDATSVEGAANLPYA
VPNVSVELTTTKVGLPVLWWRVVGSSHTAYAVEAFIDEAAHTAGKDPYAFRRDLLAKEPRMRAVLDLAAQKAG
WDPAKPLPKGRGRGIAVAEAFKSYVAQVAEVSVDADGKVKVERVVCAVDCGIAINPDIVAAQMEGGIGFGLGA
VMHSAITLKDGQVEQRNFDGYHVLRIAEMPKVEVHIVPSAEAPTGVGEPGVAPVGPAVANAIFAATGKRHYVL
PFDSADSAKA
>SEQF4095||SEQF4095.1_04401
MSAPELSVHNESRRALLLGFASGGLLLAFGVPSLVRAAVPSQPPVSADPRYGGAGMPHGLRDDPHLFVAIAPD
GTVTVTCIRSEMGQGVRTSVALVVADELGADWARVKVAQAVGDEPRYGNQNTDGSRSLRQSFAALRRAGAA
ARTMLEQAAAAAWGVDARQVKATVHEVVDTKSGRKLGFGELAAKAAALPAPDPATVPLKAPAEFRYIGKGQT
ALIDGRDIVAGRAHYGIDTRLDGMLYAVVARPPAYGDTVASFDASAAEKLPGVVKVVPLTPTPLPSGFQPLGGVA

VVARDTWTAIQARAQLKIDWKHGPNANYDSAAYRKTLEAAAAQPGDVIRNDGDAAAALAGAAKRVRATYYIP
HLAHATMEPPAAVARVADGRCEVWTCTQAPQTTRDEVAKALGLPGERVTVNVTLLGGGFGRKSKPDYVVEAA
LLSKAVGAPVKLTFTREDDLAHDYFHAVSLEAFDGGIDASGKVVAWQHRTVAPSIQSTFRAGIVHEQPGELAQG
IADLPFAIPNVRIENPAAQAHTRIGWFRSVYNIPHAFGIQSFVSELAHAAGRDPKDFLLELIGPARRFEPHITVKN
VNYGEDPALYPVDTGRLRRVVETVAREAGWGRKLPKGRGLGIAAHRSFVSYTAAVCEVQVDADGKITVPRVDIA
IDCGPQVNPERVRSQLEGAVVMGLGIALHGEITFKDGHPEQSNFNGFQVLRMNEAPREIRVHLVAPDDFATPL
GGVGEPGLPPVAPALTNAIFAATGTRIRSLPVADQLAKPRAG
>SEQF4096||SEQF4096.1_02684
MTIELDNTGSVRPSRRTFLKAAGAAAAVSLTIGFEWAGLGRRALAATAPAADFAPNAFLRITPDGAVTVIAKHVE
MGQGAYTGIATIVAEELDADWSTVRVESAPADAKRYANLAFGTMQGTGGSSAMANSWQQLREAGGKARA
MLVSAAAARWKVPAGELTTANGVVTHAKSGKKAAYGTLVADASKLPVPDKVVLKQPADFKLIGQRIPRVDASPK
SNGTAHFTLDTTFPGMRVALLQRPPRFGATVKSFDATAARAVPGVVSVVQVPGGIAVVGTGFWAAKQGRDAL
KVEWDEAHAEKRSSDEIMREYRQLADKPGTSARKDGDADAAIAGAARKIGATYEFPYLAHAPMEPLDAVVKLT
ADSCEIWAGDQFQTVDQANAARTAGLKPEQVQIHTLYAGGSFGRRANAWSDYVVEAVSIAKALGADGKPVKL
QWTREDDIQGGFYRPMYFHKLDAGLTEDGKLVGWRHRIVGQSILAGTPFEAFMVKNGIDATSVEGAANLPYA
VPNVSVELTTTKVGLPVLWWRVVGSSHTAYAVEAFIDEAAHTAGKDPYAFRRDLLAKEPRMRAVLDLAAQKAG
WDPAKPLPKGRGRGIAVAEAFKSYVAQVAEVSVDADGKVKVERVVCAVDCGIAINPDIVAAQMEGGIGFGLGA
VMHSAITLKDGQVEQRNFDGYHVLRIAEMPKVEVHIVPSAEAPTGVGEPGVAPVGPAVANAIFAATGKRHYVL
PFDSADSAKA
>SEQF4096||SEQF4096.1_07192
MSAPELSVHNESRRALLLGFASGGLLLAFGVPSLVRAAVPNQPPVSADPRYGGAGMPHGLRDDPHLFVAIAPD
GTVTVTCIRSEMGQGVRTSVALVVADELGADWARVKVAQAVGDEPRYGNQNTDGSRSLRQSFAALRRAGAA
ARTMLEQAAAAAWGVDARQVKATVHEVVDTKSGRKLGFGELAAKAAALPAPDPATVPLKASAEFRYIGKGETA
LIDGRDIVAGRAHYGIDTRLDGMLYAVVARPPAYGDTMASFDASAAEKLPGVVKVVPLASTPLPSGFQPLGGVA
VVARDTWTAIQARAQLKIDWKHGPNANYDSAAYRKTLEAAAAQPGDVIRNDGDAAAALAGAAKRVRATYYIP
HLAHATMEPPAAVARVADGRCEVWTCTQAPQTTRDEVAKALGLPGERVTVNVTLLGGGFGRKSKPDYVVEAA
LLSKAVGAPVKLTFTREDDLAHDYFHAVSLEAFDGGIDASGKVVAWQHRTVAPSIQSTFRAGVVHEQPGELAQ
GIADLPFAIPNVRIENPAAQAHTRIGWFRSVYNIPHAFGIQSFVSELAHAAGRDPKDFLLELIGPARRFEPHITVK
NMNYGEDPALYPVDTGRLRRVVETVAREASWGRRLPKGHGLGIAAHRSFVSYTAAVCEVQVDAGGKITVPRVD
IAIDCGPQVNPERVRSQLEGAVVMGLGIALHGEITFKDGHPEQSNFNGFQVLRMNEAPREIRVHLVAPDDFAT
PLGGVGEPGLPPVAPALTNAIFAATGTRIRSLPVADQLAKPRAG
>SEQF4096||SEQF4096.1_00993
MSRGLIEAGKVAGQAAGAGVSRRSFLKLGMSLGAAAGGGLLLGFSLPAAGDDARRSVIGGDGDETARAGVFA
PNAFVQIDRAGKVTLVMPKVEMGQGVYTALPMLIAEELEVPLSSVTLDHAPPNEKLFLDPLLGGQLTGGSTSIRY
AWEPLRRAGATARTLLVAAAAKQWSVDPASCRAVNGEVQHPPSGQRVSYGQLADAAAKLPVPKDVALKKPAD
FKLIGTPAKRLDSPEKVDGAAQFGLDVRLPGMLYAVIVNSPVFGGTVASIDDTAAKKIPGVRQVVRADNAVAVV
GDHTWAAKRGASALVVKWNEGAGAKVSTKDIVADLAQAAANGKGAVARKDGDVGKGFADAKTRIDAVYEQ
PLLAHATMEPVNCTVHVRADGCEIWVGTQVPTRAVDTVQQLTSFPPEKIVVHNHLLGGGFGRRLETDMIGQA
VKIAKQVNAPVKVIWTREEDIQHDMYRPYYYDRISAGLDANGKPIAWQHRIVGSSILARFAPPAFQHGVDPDA
VEVATDLPYDLPNQLIDYVRQEPRHVPTAFWRGVGPTRSTFVVESFIDELAAQTKTDPVQYRRALLGKTPRALN
VLDVATKAAGWGPSLPTGQGRGVSVMHAFGSFFSIVIDVAVDNGEVQVKRVVCAVDCGMSVNPNTIEAQVQ
GGIIFGITGALYGEITIEDGRVVQSNFTDYRMLRINETPPIEVHLVKSGEAPGGIGEPGTAATAAALSNAIFAATGK
RLRKLPVGDQLKTA
>SEQF4097||SEQF4097.1_02950

MSAPELSVHNESRRALLLGFVSGGLLLAFGVPSLVRAAVPNQPPVSADPRYGGAGMPHGLRDDPHLFVAIAPD
GTVTVTCIRSEMGQGVRTSVALVVADELGADWARVKVAQAVGDEPRYGNQNTDGSRSLRQSFAALRRAGAA
ARTMLEQAAAAAWGVDARQVRATVHEVVDTKSGRKLGFGELAAKAAALPAPDPATVPLKAPAEFRYIGKGETA
LIDGRDIVAGRAHYGIDTRLDGMLYAVVARPPAYGDTIASFDASAAEKLPGVVKVVPLAPTPLPSGFQPLGGVAV
VARDTWTAIQARAQLKIDWKHGPNANYDSAAYRKTLEAAAAQPGDVIRNDGDAAAALAGAAKRVRATYYIPH
LAHATMEPPAAVARVADGQCEVWTCTQAPQTTRDEVAKALGLPGERVTVNVTLLGGGFGRKSKPDYVVEAAL
LSKAVAAPVKLTFTREDDLAHDYFHAVSLEAFDGGIDASGKVVAWQHRTVAPSIQSTFRAGIVHEQPGELAQGI
ADLPFAIPNVRIENPAAQAHTRIGWFRSVFNIPHAFGIQSFVSELAHAAGRDPKDFLLELIGPARRFEPHITVKNV
NYGEDPALYPVDTGRLRRVVETVAREAGWGRRLPKGRGLGIAAHRSFVSYTAAVCEVQVDADGKITVPRVDIAI
DCGPQVNPERVRSQLEGAVVMGLGIALHGEITFKNGHPEQSNFNGFQVLRMNEAPREIRVHLVAPDDFATPL
GGVGEPGLPPVAPALTNAIFAATGTRIRSLPVADQLAKPRAG
>SEQF4097||SEQF4097.1_04608
MTIELDNTGSVRPSRRTFLKAAGAAAAVSLTIGFEWAGLGRRALAATAPAADFAPNAFLRITPDGAVTVIAKHVE
MGQGAYTGIATIVAEELDADWSTVRVESAPADAKRYANLAFGTMQGTGGSSAMANSWQQLREAGGKARA
MLVSAAAARWKVPAGELTTANGVVTHAKSGKKASYGTLVADASKLPVPDKVVLKQPADFKLIGQRIPRVDASPK
SNGTAHFTLDTTFPGMRVALLQRPPRFGATVKSFDATAARAVPGVVSVVQVPGGVAVVGTGFWAAKQGRDAL
KVEWDEAHAEKRGSDEIMREYRQLADKPGTSARKDGDADAAIAGAARKIGATYEFPYLAHAPMEPLDAVVKLT
ADSCEIWAGDQFQTVDQANAARTAGLKPEQVQIHTLYAGGSFGRRANAWSDYVVEAVSIAKALGADGKPVKL
QWTREDDIQGGFYRPMYFHKLDAGLTADGKLVGWRHRIVGQSILAGTPFEAFMVKNGIDATSVEGAANLPYA
VPNVSVELTTTKVGLPVLWWRVVGSSHTAYAVEAFIDEAAHTAGKDPYAFRRDLLAKEPRMRAVLDLAAQKAG
WDPAKPLPKGRGRGIAVAEAFKSYVAQVAEVSVDADGKVKVERVVCAVDCGIAINPDIVAAQMEGGIGFGLGA
VMHSAITLKDGQVEQRNFDGYHVLRIAEMPKVEVHIVPSAEAPTGVGEPGVAPVGPAVANAIFAATGKRHYVL
PFDSADSAKA
>SEQF4097||SEQF4097.1_01393
MSRGLIEAGKVAGQAAGAGVSRRSFLKLGMSLGAAAGGGLLLGFSLPAAGDDARRSVIGGDGDETARAGVFA
PNAFVQIDRAGKVTLVMPKVEMGQGVYTALPMLIAEELEVPLSSVTLDHAPPNEKLFLDPLLGGQLTGGSTSIRY
AWEPLRRAGATARTLLVAAAAKQWNVDPASCRAVNGEVQHPPSGRRVSYGQLADAAAKLPVPKDVALKKPAD
FKLIGTPAKRLDSPEKVDGTAQFGLDVRLPGMLYAVIVNSPVFGGTVASIDDTAAKKIPGVRQVVRADNAVAVV
GDHTWAAKRGASALVVKWNEGAGAKVSTKDIVADLAQAAANGKGAVARKDGDVGKGFADAKTRIDAVYEQ
PLLAHATMEPVNCTVHVRADGCEIWVGTQVPTRAVDTVQQLTSFPPEKIVVHNHLLGGGFGRRLETDMIGQA
VKIAKQVNAPVKVIWTREEDIQHDMYRPYYYDRISAGLDANGKPIAWQHRIVGSSILARFAPPAFQHGVDPDA
VEVATDLPYDLPNQLIDYVRQEPRHVPTAFWRGVGPTRSTFVVESFIDELAAQTKTDPVQYRRALLGKTPRALN
VLDVATKAAGWGPSLPTGQGRGVSVMHAFGSFFSIVIDVAVDNGEVQVKRVVCAVDCGMSVNPNTIEAQVQ
GGIIFGITGALYGEITIEDGRVVQSNFTDYRMLRINETPPIEVHLVKSGEAPGGIGEPGTAATAAALSNAIFAATGK
RLRKLPVGDQLKTA
>SEQF4098||SEQF4098.1_03398
MTIELDNTGSVRPSRRTFLKAAGAAAAVSLTIGFEWAGLGRRALAATAPAADFAPNAFLRITPDGAVTVIAKHVE
MGQGAYTGIATIVAEELDADWSTVRVESAPADAKRYANLAFGTMQGTGGSSAMANSWQQLREAGGKARA
MLVSAAAARWKVPAGELTTANGVVTHAKSGKKAAYGTLVADASKLPVPDKVVLKQPADFKLIGQRIPRVDASPK
SNGTAHFTLDTTFPGMRVALLQRPPRFGATVKSFDATAARAVPGVVSVVQVPGGIAVVGTGFWAAKQGRDAL
KVEWDEAHAEKRSSDEIMREYRQLADKPGTSARKDGDADAAIAGAARKIGATYEFPYLAHAPMEPLDAVVKLT
ADSCEIWAGDQFQTVDQANAARTAGLKPEQVQIHTLYAGGSFGRRANAWSDYVVEAVSIAKALGADGKPVKL
QWTREDDIQGGFYRPMYFHKLDAGLTEDGKLVGWRHRIVGQSILAGTPFEAFMVKNGIDATSVEGAANLPYA
VPNVSVELTTTKVGLPVLWWRVVGSSHTAYAVEAFIDEAAHTAGKDPYAFRRDLLAKEPRMRAVLDLAAQKAG

WDPAKPLPKGRGRGIAVAEAFKSYVAQVAEVSVDADGKVKVERVVCAVDCGIAINPDIVAAQMEGGIGFGLGA
VMHSAITLKDGQVEQRNFDGYHVLRIAEMPKVEVHIVPSAEAPTGVGEPGVAPVGPAVANAIFAATGKRHYVL
PFDSADSAKA
>SEQF4098||SEQF4098.1_00448
MSAPELSVHNESRRALLLGFASGGLLLAFGVPSLVRAAVPNQPPVSADPRYGGAGMPHGLRDDPHLFVAIAPD
GTVTVTCIRSEMGQGVRTSVALVVADELGADWARVKVAQAVGDEPRYGNQNTDGSRSLRQSFAALRRAGAA
ARTMLEQAAAAAWGVDARQVKATVHEVVDTKSGRKLGFGELAAKAAALPAPDPATVPLKASAEFRYIGKGETA
LIDGRDIVAGRAHYGIDTRLDGMLYAVVARPPAYGDTMASFDASAAEKLPGVVKVVPLASTPLPSGFQPLGGVA
VVARDTWTAIQARAQLKIDWKHGPNANYDSAAYRKTLEAAAAQPGDVIRNDGDAAAALAGAAKRVRATYYIP
HLAHATMEPPAAVARVADGRCEVWTCTQAPQTTRDEVAKALGLPGERVTVNVTLLGGGFGRKSKPDYVVEAA
LLSKAVGAPVKLTFTREDDLAHDYFHAVSLEAFDGGIDASGKVVAWQHRTVAPSIQSTFRAGVVHEQPGELAQ
GIADLPFAIPNVRIENPAAQAHTRIGWFRSVYNIPHAFGIQSFVSELAHAAGRDPKDFLLELIGPARRFEPHITVK
NMNYGEDPALYPVDTGRLRRVVETVAREASWGRRLPKGHGLGIAAHRSFVSYTAAVCEVQVDAGGKITVPRVD
IAIDCGPQVNPERVRSQLEGAVVMGLGIALHGEITFKDGHPEQSNFNGFQVLRMNEAPREIRVHLVAPDDFAT
PLGGVGEPGLPPVAPALTNAIFAATGTRIRSLPVADQLAKPRAG
>SEQF4098||SEQF4098.1_01168
MSRGLIEAGKVAGQAAGAGVSRRSFLKLGMSLGAAAGGGLLLGFSLPAAGDDARRSVIGGDGDETARAGVFA
PNAFVQIDRAGKVTLVMPKVEMGQGVYTALPMLIAEELEVPLSSVTLDHAPPNEKLFLDPLLGGQLTGGSTSIRY
AWEPLRRAGATARTLLVAAAAKQWSVDPASCRAVNGEVQHPPSGQRVSYGQLADAAAKLPVPKDVALKKPAD
FKLIGTPAKRLDSPEKVDGAAQFGLDVRLPGMLYAVIVNSPVFGGTVASIDDTAAKKIPGVRQVVRADNAVAVV
GDHTWAAKRGASALVVKWNEGAGAKVSTKDIVADLAQAAANGKGAVARKDGDVGKGFADAKTRIDAVYEQ
PLLAHATMEPVNCTVHVRADGCEIWVGTQVPTRAVDTVQQLTSFPPEKIVVHNHLLGGGFGRRLETDMIGQA
VKIAKQVNAPVKVIWTREEDIQHDMYRPYYYDRISAGLDANGKPIAWQHRIVGSSILARFAPPAFQHGVDPDA
VEVATDLPYDLPNQLIDYVRQEPRHVPTAFWRGVGPTRSTFVVESFIDELAAQTKTDPVQYRRALLGKTPRALN
VLDVATKAAGWGPSLPTGQGRGVSVMHAFGSFFSIVIDVAVDNGEVQVKRVVCAVDCGMSVNPNTIEAQVQ
GGIIFGITGALYGEITIEDGRVVQSNFTDYRMLRINETPPIEVHLVKSGEAPGGIGEPGTAATAAALSNAIFAATGK
RLRKLPVGDQLKTA
>SEQF4099||SEQF4099.1_04108
MSRGLIEAGKVAGQAAGAGVSRRSFLKLGMSLGAAAGGGLLLGFSLPAAGDDARRSVIGGDGDETARAGVFA
PNAFVQIDRAGKVTLVMPKVEMGQGVYTALPMLIAEELEVPLSSVTLDHAPPNEKLFLDPLLGGQLTGGSTSIRY
AWEPLRRAGATARTLLVAAAAKQWSVDPASCRAVNGEVQHPPSGRRTSYGQLADAAAKLPVPKDVALKKPAD
FKLIGTPAKRLDSPEKVDGTAQFGLDVRLPGMLYAVIVNSPVFGGTVASIDDTAAKKIPGVRQVVRADNAVAVV
GDHTWAAKRGASALVVKWNEGAGAKVSTKDIVADLAQAAANGKGAVARKDGDVGKGFADAKTRVDAVYEQ
PLLAHATMEPVNCTVHVRADGCEIWVGTQVPTRAVDTVQQLTSFPPEKIVVHNHLLGGGFGRRLETDMIGQA
VKIAKQVNAPVKVIWTREEDIQHDMYRPYYYDRISAGLDANGKPIAWQHRIVGSSILARFAPPAFQHGVDPDA
VEVATDLPYDLPNQLIDYVRQEPRHVPTAFWRGVGPTRSTFVVESFIDELAAQTKTDPVQYRRALLGKTPRALN
VLDVATKAAGWGPSLPTGQGRGVSVMHAFGSFFSIVIDVAVDNGEVQVKRVVCAVDCGMSVNPNTIEAQVQ
GGIIFGITGALYGEITIEDGRVVQSNFTDYRMLRINETPPIEVHLVKSGEAPGGIGEPGTAATAAALSNAIFAATGK
RLRKLPVGDQLKTA
>SEQF4099||SEQF4099.1_01238
MSAPELSVHNESRRALLLGFASGGLLLAFGVPSLVRAAVPNQPPVSADPRYGGAGMPHGLRDDPHLFVAIAPD
GTVTVTCIRSEMGQGVRTSVALVVADELGADWARVKVAQAVGDEPRYGNQNTDGSRSLRQSFAALRRAGAA
ARTMLEQAAAAAWGVDARQVKATVHEVVDTKSGRKLGFGELAAKAAALPAPDPATVPLKAPAEFRYIGKGETA
LIDGRDIVAGRAHYGIDTRLDGMLYAVVARPPAYGDTMASFDASAAEKLPGVVKVVPLASTPLPSGFQPLGGVA

VVARDTWTAIQARAQLKIDWKHGPNANYDSAAYRKTLEAAAAQPGDVIRNDGDAAAALAGAAKRVRATYYIP
HLAHATMEPPAAVARVADGRCEVWTCTQAPQTTRDEVAKALGLPSERVTVNVTLLGGGFGRKSKPDYVVEAA
LLSKAVGAPVKLTFTREDDLAHDYFHAVSLEAFDGGIDASGKVVAWQHRTVAPSIQSTFRAGVVHEQPGELAQ
GIADLPFAIPNVRIENPAAQAHTRIGWFRSVYNIPHAFGIQSFVSELAHAAGRDPKDFLLELIGPARRFEPHITVK
NVNYGEDPALYPVDTGRLRRVVETVAREAGWGRRLPKGHGLGIAAHRSFVSYTAAVCEVQVDADGKITVPRVD
IAIDCGPQVNPERVRSQLEGAVVMGLGIALHGEITFKDGHPEQRNFNGFQVLRMNEAPREIRVHLVAPDDFAT
PLGGVGEPGLPPVAPALTNAIFAATGTRIRSLPVADQLAKPRAG
>SEQF4099||SEQF4099.1_04602
MTIELDNTGSVRPSRRTFLKAAGAAAAVSLTIGFEWAGLGRRALAATAPAADFAPNAFLRITPDGAVTVIAKHVE
MGQGAYTGIATIVAEELDADWSTVRVESAPADAKRYANLAFGTMQGTGGSSAMANSWQQLREAGGKARA
MLVSAAAARWKVPAGELTTANGVVTHAKSGKKAAYGTLVADASKLPVPDKVVLKQPADFKLIGQRIPRVDASPK
SNGTAHFTLDTTFPGMRVALLQRPPRFGATVKSFDATAARAVPGVVSVVQVPGGVAVVGTGFWAAKQGRDAL
KVEWDEAHAEKRGSDEIMREYRQLADKPGTSARKDGDADAAIAGAARKIGATYEFPYLAHAPMEPLDAVVKLT
ADSCEIWAGDQFQTVDQANAARTAGLKPEQVRIHTLYAGGSFGRRANAWSDYVVEAVSIAKALGADGKPVKL
QWTREDDIQGGFYRPMYFHKLDAGLTEDGKLVGWRHRIVGQSILAGTPFEAFMVKNGIDATSVEGAANLPYA
VPNVSVELTTTKVGLPVLWWRVVGSSHTAYAVEAFIDEAAHTAGKDPYAFRRDLLAKEPRMRAVLDLAAQKAG
WDPAKPLPKGRGRGIAVAEAFKSYVAQVAEVSVDADGKVKVERVVCAVDCGIAINPDIVAAQMEGGIGFGLGA
VMHSAITLKDGQVEQRNFDGYHVLRIAEMPKVEVHIVPSAEAPTGVGEPGVAPVGPAVANAIFAATGKRHYVL
PFDSADSAKA
>SEQF4100||SEQF4100.1_05773
MSRGLIEAGRAGTGVSRRSFLKLGMSLGAAAGGGLLLGFSLPAAGDDARRSVIGGDAAEPAAPGVFAPNAFVQ
IDRAGKITLVMPKVEMGQGVYTALPMLIAEELEVPLSSVTLDHAPPNEKLFLDPLLGGQLTGGSTSVRYAWEPLR
RAGATARTLLVAAAAKQWNVDPASCRAANGEVQHPPSGRRASYGQLADAAAKLPVPKDVVLKKPADFKLIGTP
AKRLDSPEKVDGTAQFGLDVRVPGMLYAVIVNSPVFGGTVASVDDTAARKIPGVRQVVRVDNAVAVVGDHTW
AAKRGASALVVKWNEGADAKVSTKDLFADLAHAAANGKGAVARKEGDVGKAFANAKTRVDAVYEQPLLAHA
TMEPVNCTVHVRGDGCEIWVGTQVPTRVRDTAQQLTGLSPDKIVVHNHLLGGGFGRRLETDMVGQAVKVAK
QVDAPVKVIWTREEDVQHDMYRPCYYDAISAGLDANGKPVAWQHRIVGSSILARFAPPAVKDGVDPDAVEVA
AELPYDLPNQLVDYVRQEPRHVPTAFWRGVGPTRSTFVVESFIDELAAQTKTDPVQYRRALLDKTPRARNVLDV
ATKAAGWGASLPKGQGRGVSVMHAFGSFFSIVIDVAVDGGEVQVKRVVCAVDCGMYVNPNTIEAQVQGGIIF
GITGALYGEITIEDGRVVQTNFTDYRMMRINEVPPIEVHLVKSGEAPGGIGEPGTAATAAALSNAIFAATGTRLRK
LPVGNQLKTA
>SEQF4100||SEQF4100.1_03655
MTIELDNTDSVRPSRRTFLKAAGAAAAVSLTIGFDWAGLGRRALAATAPASDFAPNAFLRITPDGAVTVIAKHVE
MGQGAYTGIATIVAEELDADWSSVRVESAPADAKRYANLAFGTMQGTGGSSAMSNSWQQLREAGGKARAM
LVQAAAARWKVPAAELTTANGVVTHTKSGKTAAYGTLVADASKLPVPDKVTLKQPADFKLIGHRIPRVDASSKS
NGTAHFTLDTTFPGMRVALLQRPPRFGATVKSFDATAAKAVPGVVSVVQVPGGVAVVANGFWAAKQGRDAL
KVEWDETKAEKRGSDELMREYRQLAEKPGTSARKDGDADAAIAGAARKISATYEFPYLAHAPMEPLDAVVKLT
ANSCEIWAGDQFQTVDQGNAARTAGLKPEQVQIHTLYAGGSFGRRANAWSDYVVEAVSIAKALGADGKPVKL
QWTREDDIQGGFYRPMYFHKLDAGLTADGRLVGWRHRIVGQSILAGTPFEAFMVKNGVDATSVEGAANLPYA
VPNVSVELTTTKVGLPVLWWRVVGSSHTAYAVEAFIDEAAHAAGKDPYAFRRDLLAKEPRMRAVLELAAQKAG
WDPAKPLPKGRGRGIAVAEAFKSYVAQVAEVSVDADGKVKVERVVCAVDCGIAINPDIVAAQMEGGIGFGLGA
AMHSAITLKDGQVEQRNFDGYHVLRMAEMPKVEVHIVPSAEAPTGVGEPGVAPVGPAVANAIFAATGKRHYV
LPFDSAESAKA
>SEQF4101||SEQF4101.1_01144

MSRGLIEAGKVAGQAAGAGVSRRSFLKLGMSLGAAAGGGLLLGFSLPAAGDDARRSVIGGDGDETARAGVFA
PNAFVQIDRAGKVTLVMPKVEMGQGVYTALPMLIAEELEVPLSSVTLDHAPPNEKLFLDPLLGGQLTGGSTSIRY
AWEPLRRAGATARTLLVAAAAKQWSVDPASCRAVNGEVQHPPSGQRVSYGQLADAAAKLPVPKDVALKKPAD
FKLIGTPAKRLDSPEKVDGAAQFGLDVRLPGMLYAVIVNSPVFGGTVASIDDTAAKKIPGVRQVVRADNAVAVV
GDHTWAAKRGASALVVKWNEGAGAKVSTKDIVADLAQAAANGKGAVARKDGDVGKGFADAKTRIDAVYEQ
PLLAHATMEPVNCTVHVRADGCEIWVGTQVPTRAVDTVQQLTSFPPEKIVVHNHLLGGGFGRRLETDMIGQA
VKIAKQVNAPVKVIWTREEDIQHDMYRPYYYDRISAGLDANGKPIAWQHRIVGSSILARFAPPAFQHGVDPDA
VEVATDLPYDLPNQLIDYVRQEPRHVPTAFWRGVGPTRSTFVVESFIDELAAQTKTDPVQYRRALLGKTPRALN
VLDVATKAAGWGPSLPTGQGRGVSVMHAFGSFFSIVIDVAVDNGEVQVKRVVCAVDCGMSVNPNTIEAQVQ
GGIIFGITGALYGEITIEDGRVVQSNFTDYRMLRINETPPIEVHLVKSGEAPGGIGEPGTAATAAALSNAIFAATGK
RLRKLPVGDQLKTA
>SEQF4101||SEQF4101.1_03973
MSAPELSVHNESRRALLLGFASGGLLLAFGVPSLVRAAVPNQPPVSADPRYGGAGMPHGLRDDPHLFVAIAPD
GTVTVTCIRSEMGQGVRTSVALVVADELGADWARVKVAQAVGDEPRYGNQNTDGSRSLRQSFAALRRAGAA
ARTMLEQAAAAAWGVDARQVKATVHEVVDTKSGRKLGFGELAAKAAALPAPDPATVPLKASAEFRYIGKGETA
LIDGRDIVAGRAHYGIDTRLDGMLYAVVARPPAYGDTMASFDASAAEKLPGVVKVVPLASTPLPSGFQPLGGVA
VVARDTWTAIQARAQLKIDWKHGPNANYDSAAYRKTLEAAAAQPGDVIRNDGDAAAALAGAAKRVRATYYIP
HLAHATMEPPAAVARVADGRCEVWTCTQAPQTTRDEVAKALGLPGERVTVNVTLLGGGFGRKSKPDYVVEAA
LLSKAVGAPVKLTFTREDDLAHDYFHAVSLEAFDGGIDASGKVVAWQHRTVAPSIQSTFRAGVVHEQPGELAQ
GIADLPFAIPNVRIENPAAQAHTRIGWFRSVYNIPHAFGIQSFVSELAHAAGRDPKDFLLELIGPARRFEPHITVK
NMNYGEDPALYPVDTGRLRRVVETVAREASWGRRLPKGHGLGIAAHRSFVSYTAAVCEVQVDAGGKITVPRVD
IAIDCGPQVNPERVRSQLEGAVVMGLGIALHGEITFKDGHPEQSNFNGFQVLRMNEAPREIRVHLVAPDDFAT
PLGGVGEPGLPPVAPALTNAIFAATGTRIRSLPVADQLAKPRAG
>SEQF4101||SEQF4101.1_07384
MTIELDNTGSVRPSRRTFLKAAGAAAAVSLTIGFEWAGLGRRALAATAPAADFAPNAFLRITPDGAVTVIAKHVE
MGQGAYTGIATIVAEELDADWSTVRVESAPADAKRYANLAFGTMQGTGGSSAMANSWQQLREAGGKARA
MLVSAAAARWKVPAGELTTANGVVTHAKSGKKAAYGTLVADASKLPVPDKVVLKQPADFKLIGQRIPRVDASPK
SNGTAHFTLDTTFPGMRVALLQRPPRFGATVKSFDATAARAVPGVVSVVQVPGGIAVVGTGFWAAKQGRDAL
KVEWDEAHAEKRSSDEIMREYRQLADKPGTSARKDGDADAAIAGAARKIGATYEFPYLAHAPMEPLDAVVKLT
ADSCEIWAGDQFQTVDQANAARTAGLKPEQVQIHTLYAGGSFGRRANAWSDYVVEAVSIAKALGADGKPVKL
QWTREDDIQGGFYRPMYFHKLDAGLTEDGKLVGWRHRIVGQSILAGTPFEAFMVKNGIDATSVEGAANLPYA
VPNVSVELTTTKVGLPVLWWRVVGSSHTAYAVEAFIDEAAHTAGKDPYAFRRDLLAKEPRMRAVLDLAAQKAG
WDPAKPLPKGRGRGIAVAEAFKSYVAQVAEVSVDADGKVKVERVVCAVDCGIAINPDIVAAQMEGGIGFGLGA
VMHSAITLKDGQVEQRNFDGYHVLRIAEMPKVEVHIVPSAEAPTGVGEPGVAPVGPAVANAIFAATGKRHYVL
PFDSADSAKA
>SEQF4102||SEQF4102.1_04173
MTIELDNTGSVRPSRRTFLKAAGAAAAVSLTIGFEWAGLGRRALAATAPAADFAPNAFLRITPDGAVTVIAKHVE
MGQGAYTGIATIVAEELDADWSTVRVESAPADAKRYANLAFGTMQGTGGSSAMANSWQQLREAGGKARA
MLVSAAAARWKVPAGELTTANGVVTHAKSGKKAAYGTLVADASKLPVPDKVVLKQPADFKLIGQRIPRVDASPK
SNGTAHFTLDTTFPGMRVALLQRPPRFGATVKSFDATAAKAVPGVVSVVQVPGGIAVVGTGFWAAKQGRDAL
KVEWDEAHAEKRGSDEIMREYRQLADKPGTSARKDGDADAAIAGAARKIGATYEFPYLAHAPMEPLDAVVKLT
ADSCEIWAGDQFQTVDQANAARTAGLKPEQVQIHTLYAGGSFGRRANAWSDYVVEAVSIAKALGADGKPVKL
QWTREDDIQGGFYRPMYFHKLDAGLTEDGKLVGWRHRIVGQSILAGTPFEAFMVKNGIDATSVEGAANLPYA
VPNVSVELTTTKVGLPVLWWRVVGSSHTAYAVEAFIDEAAHTAGKDPYAFRRDLLAKEPRMRAVLDLAAQKAG

WDPAKPLPKGRGRGIAVAEAFKSYVAQVAEVSVDADGKVKVERVVCAVDCGIAINPDIVAAQMEGGIGFGLGA
VMHSAITLKDGQVEQRNFDGYHVLRIAEMPKVEVHIVPSAEAPTGVGEPGVAPVGPAVANAIFAATGKRHYVL
PFDSADSAKA
>SEQF4102||SEQF4102.1_03383
MSAPELSVHNESRRALLLGFASGGLLLAFGVPSLVRAAVPNQPPVSADPRYGGAGMPHGLRDDPHLFVAIAPD
GTVTVTCIRSEMGQGVRTSVALVVADELGADWARVKVAQAVGDEPRYGNQNTDGSRSLRQSFAALRRAGAA
ARTMLEQAAAAAWGVDARQVKATVHEVVDTKSGRKLGFGELAAKAAALPAPDPATVPLKAPAEFRYIGKGETA
LIDGRDIVAGRAHYGIDTRLDGMLYAVVARPPAYGDTMTSFDASAAEKLPGVVKVVPLASTPLPSGFQPLGGVA
VVARDTWTAIQARAQLKIDWKHGPNANYDSAAYRKTLEAAAAQPGDVIRNDGDAAAALAGAAKRVRATYYIP
HLAHATMEPPAAVARVADGRCEVWTCTQAPQTTRDEVAKALGLPGERVTANVTLLGGGFGRKSKPDYVVEAA
LLSKAVGAPVKLTFTREDDLAYDYFHAVSLEAFDGGIDASGKVVAWQHRTVAPSIQSTFRAGVVHEQPGELAQG
IADLPFAIPNVRIENPAAQAHTRIGWFRSVYNIPHAFGIQSFVSELAHAAGRDPKDFLLELIGPARRFEPHITVKN
VNYGEDPALYPVDTGRLRRVVETVAREAGWGRRLPKGHGLGIAAHRSFVSYTAAVCEVQVDADGKITVPRVDI
AIDCGPQVNPERVRSQLEGAVVMGLGIALHGEITFKDGHPEQSNFNGFQVLRMNEAPREIRVHLVAPDDFATP
LGGVGEPGLPPVAPALTNAIFAATGTRIRSLPVADQLAKPRAG
>SEQF4102||SEQF4102.1_05519
MSRGLIEAGKVAGQAAGAGVSRRSFLKLGMSLGAAAGGGLLLGFSLPAAGDDARRSVIGGDGDETARAGVFA
PNAFVQIDRAGKVTLVMPKVEMGQGVYTALPMLIAEELEVPLSSVTLDHAPPNEKLFLDPLLGGQLTGGSTSIRY
AWEPLRRAGATARTLLVAAAAKQWNVDPASCRAVNGEVQHPPSGRRTSYGQLADAAAKLPVPKDVALKKPAD
FKLIGTPARRLDSPEKVDGTAQFGLDVRLPGMLYAVIVNSPVFGGTVASIDDTAAKKIPGVRQVVRADNAVAVV
GDHTWAAKRGASALVVKWNEGAGAKVSTKDIVADLAQAAANGKGAVARKDGDVGKGFADAKTRVDAVYEQ
PLLAHATMEPVNCTVHVRADGCEIWVGTQVPTRAVDTVQQLTSFPPEKIVVHNHLLGGGFGRRLETDMIGQA
VKIAKQVNAPVKVIWTREEDIQHDMYRPYYYDRISAGLDANGKPIAWQHRIVGSSILARFAPPAFQHGVDPDA
VEVATDLPYDLPNQLIDYVRQEPRHVPTAFWRGVGPTRSTFVVESFIDELAAQTKTDPVQYRRALLGKTPRALN
VLDVATKAAGWGPSLPTGQGRGVSVMHAFGSFFSIVIDVAVDNGEVQVKRVVCAVDCGMSVNPNTIEAQVQ
GGIIFGITGALYGEITIEDGRVVQSNFTDYRMLRINETPPIEVHLVKSGEAPGGIGEPGTAATAAALSNAIFAATGK
RLRKLPVGDQLKTA
>SEQF4103||SEQF4103.1_02051
MSAPELSVHNESRRALLLGFASGGLLLAFGVPSLVRAAVPNQPPVSADPRYGGAGMPHGLRDDPHLFVAIAPD
GTVTVTCIRSEMGQGVRTSVAIVVADELGADWARVKVAQAVGDEPRYGNQNTDGSRSLRQSFAALRRAGAA
ARTMLEQAAAAAWGVDARQVKATVHEVVDTKSGRKLGFGELAAKAAALPAPDPATVPLKAPAEFRYIGKGETA
LIDGRDIVAGRAHYGIDTRLDGMLYAVVARPPAYGDTMASFDASAAEKLPGVVKVVPLAPTPLPSGFQPLGGVA
VVARDTWTAIQARAQLKIDWKHGPNANYDSAAYRKTLEAAAAQPGDVIRNDGDAAAALAGAAKRVRATYYIP
HLAHATMEPPAAVARVADGRCEVWTCTQAPQTTRDEVAKALGLPGERVTVNVTLLGGGFGRKSKPDYVVEAA
LLSKAVGAPVKLTFTREDDLAHDYFHAVSLEAFDGGIDASGKVVAWQHRTVAPSIQSTFRAGIVHEQPGELAQG
IADLPFAIPNVRIENPAAQAHTRIGWFRSVYNIPHAFGIQSFVSELAHAAGRDPKDFLLELIGPARRFEPHITVKN
VNYGEDPALYPVDTGRLRRVVETVAREAGWGRTLPKGHGLGIAAHRSFVSYTAAVCEVQVDADGKITVPRVDIA
IDCGPQVNPERVRSQLEGAVVMGLGIALHGEITFKDGHPEQSNFNGFQVLRMNEAPREIRVHLVAPDDFATPL
GGVGEPGLPPVAPALTNAIFAATGTRIRSLPVADQLAKPRAG
>SEQF4103||SEQF4103.1_07642
MTIELDNTGSVRPSRRTFLKAAGAAAAVSLTIGFEWAGLGRRALAATAPAADFAPNAFLRITPDGAVTVIAKHVE
MGQGAYTGIATIVAEELDADWSTVRVESAPADAKRYANLAFGTMQGTGGSSAMANSWQQLREAGGKARA
MLVSAAAARWKVPAGELTTANGVVTHAKSGKKAAYGTLVADASKLPVPDKVVLKQPADFKLIGHRIPRVDASPK
SNGTAHFTLDTTFPGMRVALLQRPPRFGATVKSFDATAARAVPGVVSVVQVPGGVAVVGTGFWAAKQGRDAL

KVEWDEAHAEKRGSDEIMREYRQLADKPGTSARKDGDADAAIAGAARKIGATYEFPYLAHAPMEPLDAVVKLT
ADSCEIWAGDQFQTVDQANAARTAGLKPEQVRIHTLYAGGSFGRRANAWSDYVVEAVSIAKALGADGKPVKL
QWTREDDIQGGFYRPMYFHKLDAGLTEDGKLVGWRHRIVGQSILAGTPFEAFMVKNGIDATSVEGAANLPYA
VPNVSVELTTTKVGLPVLWWRVVGSSHTAYAVEAFIDEAAHTAGKDPYAFRRDLLAKEPRMRAVLDLAAQKAG
WDPAKPLPKGRGRGIAVAEAFKSYVAQVAEVSVDADGKVKVERVVCAVDCGIAINPDIVAAQMEGGIGFGLGA
VMHSAITLKDGQVEQRNFDGYHVLRIAEMPKVEVHIVPSAEAPTGVGEPGVAPVGPAVANAIFAATGKRHYVL
PFDSADSAKA
>SEQF4103||SEQF4103.1_05075
MSRGLIEAGKVAGQAAGAGVSRRSFLKLGMSLGAAAGGGLLLGFSLPAAGDDARRSVIGGDGDETARAGVFA
PNAFVQIDRAGKVTLVMPKVEMGQGVYTALPMLIAEELEVPLSSVTLDHAPPNEKLFLDPLLGGQLTGGSTSIRY
AWEPLRRAGATARTLLVVAAAKQWNVDPASCRAVNGEVQHPSSGRRVSYGQLADAAAKLPVPKDVALKKPAD
FKLIGTPAKRLDSPEKVDGTAQFGLDVRLPGMLYAVIVNSPVFGGTVASIDDTAAKKIPGVRQVVRADNAVAVV
GDHTWAAKRGASALVVKWNEGAGAKVSTKDIVADLAQAAANGKGAVARKDGDVGKGFADAKTRVDAVYEQ
PLLAHATMEPVNCTVHVRADGCEIWVGTQVPTRAVDTVQQLTSFPPEKIVVHNHLLGGGFGRRLETDMIGQA
VKIAKQVNAPVKVIWTREEDIQHDMYRPYYYDRISAGLDANGKPIAWQHRIVGSSILARFAPPAFQHGVDPDA
VEVATDLPYDLPNQLIDYVRQEPRHVPTAFWRGVGPTRSTFVVESFIDELAAQTKTDPVQYRRALLGKTPRALN
VLDVATKAAGWGPSLPTGQGRGVSVMHAFGSFFSIVIDVAVDNGEVQVKRVVCAVDCGMSVNPNTIEAQVQ
GGIIFGITGALYGEITIEDGRVVQSNFTDYRMLRINETPPIEVHLVKSGEAPGGIGEPGTAATAAALSNAIFAATGK
RLRKLPVGDQLKTA
>SEQF4104||SEQF4104.1_00960
MSRGLIEAGKVAGQAAGAGVSRRSFLKLGMSLGAAAGGGLLLGFSLPAAGDDARRSVIGGDGDETARAGVFA
PNAFVQIDRAGKVTLVMPKVEMGQGVYTALPMLIAEELEVPLSSVTLDHAPPNEKLFLDPLLGGQLTGGSTSIRY
AWEPLRRAGATARTLLVAAAAKQWNVDPASCRAVNGEVQHPPSGRRVSYGQLADAAAKLPVPKDVALKKPAD
FKLIGTPAKRLDSPEKVDGTAQFGLDVRLPGMLYAVIVNSPVFGGTVASIDDTAAKKIPGVRQVVRADNAVAVV
GDHTWAAKRGASALVVKWNEGAGAKVSTKDIVADLAQAAANGKGAVARKDGDVGKGFADAKTRIDAVYEQ
PLLAHATMEPVNCTVHVRADGCEIWVGTQVPTRAVDTVQKLTSFPPEKIVVHNHLLGGGFGRRLETDMIGQA
VKIAKQVNAPVKVIWTREEDIQHDMYRPYYYDRISAGLDANGKPIAWQHRIVGSSILARFAPPAFQHGVDPDA
VEVAIDLPYDLPNQLIDYVRQEPRHVPTAFWRGVGPTRSTFVVESFIDELAAQTKTDPVQYRRALLGKTPRALNV
LDVATKAAGWGPSLPTGQGRGVSVMHAFGSFFSIVIDVAVDNGEVQVKRVVCAVDCGMSVNPNTIEAQVQG
GIIFGITGALYGEITIEDGRVVQSNFTDYRMLRINETPPIEVHLVKSGEAPGGIGEPGTAATAAALSNAIFAATGKRL
RKLPVGDQLKTA
>SEQF4104||SEQF4104.1_06822
MSAPELSVHNESRRALLLGFASGGLLLAFGVPSLVRAAAPVQPPVSADPRYGGAGMPHGLRDDPHLFVAIAPD
GTVTVTCIRSEMGQGVRTSVALVVADELGADWARVKVAQAVGDEPRYGNQNTDGSRSLRQSFAALRRAGAA
ARTMLEQAAAAAWGVDARQVKATVHEVVDTKSGRKLGFGELAAKAAALPAPDPATVPLKAPAEFRYIGKGQT
ALIDGRDIVAGRAHYGIDTRLDGMLYAVVARPPAYGDTVASFDASAAEKLPGVVKVVPLTPTPLPSGFQPLGGVA
VVARDTWTAIQARAQLKIDWKHGPNANYDSAAYRKTLEAAAAQPGDVIRNDGDAAAALAGATKRVRATYYIP
HLAHATMEPPAAVARVADGRCEVWTCTQAPQTTRDEVAKALGLPGERVTVNVTLLGGGFGRKSKPDYVVEAA
LLSKAVGAPVKLTFTREDDLAHDYFHAVSLEAFDGGIDASGKVVGWQHRTVAPSIQSTFRAGIVHEQPGELAQ
GIADLPFAIPNVRIENPAAQAHTRIGWFRSVYNIPHAFGIQSFVSELAHAAGRDPKDFLLELIGPARRFEPHITVK
NVNYGEDPALYPVDTGRLRRVVETVAREAGWGRKLPKGHGLGIAAHRSFVSYTAAVCEVQVDADGKITVPRVD
IAIDCGPQVNPERVRSQLEGAVVMGLGIALHGEITFKDGHPEQSNFNGFQVLRMNEAPREIRVHLVAPDDFAT
PLGGVGEPGLPPVAPALTNAIFAATGTRIRSLPVADQLAKPRAG
>SEQF4104||SEQF4104.1_01683

MTIELDNTGSVRPSRRTFLKAAGAAAAVSLTIGFEWAGLGRRALAATAPAADFAPNAFLRITPDGAVTVIAKHVE
MGQGAYTGIATIVAEELDADWSTVRVESAPADAKRYANLAFGTMQGTGGSSAMANSWQQLREAGGKARA
MLVSAAAARWKVPAGELTTANGVVTHAKSGKKAAYGTLVADASKLPVPDKVVLKQPADFKLIGQRIPRVDASPK
SNGTAHFTLDTTFPGMRVALLQRPPRFGATVKSFDATAAKAVPGVVSVVQVPGGVAVVGTGFWAAKQGRDAL
KVEWDEAHAEKRSSDEIMREYRQLADKPGTSARKDGDADAAISGAARKIGATYEFPYLAHAPMEPLDAVVKLT
ADSCEIWAGDQFQTVDQANAARTAGLKPEQVQIHTLYAGGSFGRRANAWSDYVVEAVSIAKALGADGKPVKL
QWTREDDIQGGFYRPMYFHKLDAGLTADGKLVGWRHRIVGQSILAGTPFEAFMVKNGIDATSVEGAANLPYA
VPNVSVELTTTKVGLPVLWWRVVGSSHTAYAVEAFIDEAAHTAGKDPYAFRRDLLAKEPRMRAVLDLAAQKAG
WDPAKPLPKGRGRGIAVAEAFKSYVAQVAEVSVDADGKVKVERVVCAVDCGIAINPDIVAAQMEGGIGFGLGA
VMHSAITLKDGQVEQRNFDGYHVLRIAEMPKVEVHIVPSAEAPTGVGEPGVAPVGPAVANAIFAATGKRHYVL
PFDSADSAKA
>SEQF4105||SEQF4105.1_01042
MSAPELSVHNESRRALLLGFASGGLLLAFGVPSLVRAAAPVQPPVSADPRYGGAGMPHGLRDDPHLFVAIAPD
GTVTVTCIRSEMGQGVRTSVALVVADELGADWARVKVAQAVGDEPRYGNQNTDGSRSLRQSFAALRRAGAA
ARTMLEQAAAAAWGVDARQVKATVHEVVDTKSGRKLGFGELAAKAAALPAPDPATVPLKAPAEFRYIGKGQT
ALIDGRDIVAGRAHYGIDTRLDGMLYAVVARPPAYGDTVASFDASAAEKLPGVVKVVPLTPTPLPSGFQPLGGVA
VVARDTWTAIQARAQLKIDWKHGPNANYDSAAYRKTLEAAAAQPGDVIRNDGDAAAALAGATKRVRATYYIP
HLAHATMEPPAAVARVADGRCEVWTCTQAPQTTRDEVAKALGLPGERVTVNVTLLGGGFGRKSKPDYVVEAA
LLSKAVGAPVKLTFTREDDLAHDYFHAVSLEAFDGGIDASGKVVAWQHRTVAPSIQSTFRAGIVHEQPGELAQG
IADLPFAIPNVRIENPAAQAHTRIGWFRSVYNIPHAFGIQSFVSELAHAAGRDPKDFLLELIGPARRFEPHITVKN
VNYGEDPALYPVDTGRLRRVVETVAREAGWGRKLPKGRGLGIAAHRSFVSYTAAVCEVQVDADGKITVPRVDIA
IDCGPQVNPERVRSQLEGAVVMGLGIALHGEITFKDGHPEQSNFNGFQVLRMNEAPREIRVHLVAPDDFATPL
GGVGEPGLPPVAPALTNAIFAATGTRIRSLPVADQLAKPRAG
>SEQF4105||SEQF4105.1_07021
MSRGLIEAGKVAGQAAGAGVSRRSFLKLGMSLGAAAGGGLLLGFSLPAAGDDARRSVIGGDGDETARAGVFA
PNAFVQIDRAGKVTLVMPKVEMGQGVYTALPMLIAEELEVPLSSVTLDHAPPNEKLFLDPLLGGQLTGGSTSIRY
AWEPLRRAGATARTLLVAAAAKQWNVDPASCRAVNGEVQHPPSGRRTSYGQLADAAAKLPVPKDVALKKPAD
FKLIGTPAKRLDSPEKVDGTAQFGLDVRLPGMLYAVIVNSPVFGGTVASIDDTAAKKIPGVRQVVRADNAVAVV
GDHTWAAKRGASALVVKWNEGAGAKVSTKDIVADLAQAAVSGKGAVARKEGDVGHAFANAKTRVDAVYEQ
PFLAHATMEPVNCTVHVRGDGCEIWVGTQVPTRAVDTVQQLTGLSPDKIVVHNHLLGGGFGRRLETDMIDQ
AVKVGKQVNAPVKVIWTREEDIQHDMYRPYYYDKISAGLDANGKPIAWQHRIVGSSLMARFAPPAFRNGIDP
DAVEVSADLPYDLPNQLVDYVRQEPRHVPTAFWRGVGPTRGTFVVESFIDELAAQTKTDPVQYRRALLGKAPR
ALNVLDVATKAAGWGPSLPKGQGRGVSVMYAFGTFFSMVVDVAVDNGEVQVKRVVCAVDCGMSVNPNTIE
AQVQGGIIFGITGALYGEITIEDGRVVQSNFTDYRMLRINETPPIEVHLVKSGEAPGGIGEPGTAATAAALSNAIFA
ATGKRLRKLPVGDQLKTA
>SEQF4105||SEQF4105.1_01985
MSRGLIEAGKVAGQAAGAGVSRRSFLKLGMSLGAAAGGGLLLGFSLPAAGDDARRSVIGGDGDETARAGVFA
PNAFVQIDRAGKVTLVMPKVEMGQGVYTALPMLIAEELEVPLSSVTLDHAPPNEKLFLDPLLGGQLTGGSTSVR
YAWEPLRRAGATARTLLVTAAAKQWNVDPASCRAVNGEVQHPPSGRRVSYGQLADAAAKLPVPKDVALKKPA
DFKLIGTPAKRLDSPEKVDGTAQFGLDVRLPGMLYAVIVNSPVFGGTVASIDDTAAKKIPGVRQVVRADNAVAV
VGDHTWAAKRGASALVVKWNEGAGAKVSTKDIVADLAQAAANGKGAVARKDGDVGKGFADAKTRIDAVYE
QPLLAHATMEPVNCTVHVRADGCEIWVGTQVPTRAVDTVQKLTSFPPEKIVVHNHLLGGGFGRRLETDMIGQ
AVKIAKQVNAPVKVIWTREEDIQHDMYRPYYYDRISAGLDANGKPIAWQHRIVGSSILARFAPPAFQHGVDPD
AVEVATDLPYDLPNQLIDYVRQEPRHVPTAFWRGVGPTRSTFVVESFIDELAAQTKTDPVQYRRALLGKTPRAL

NVLDVATKAAGWGPSLPTGQGRGVSVMHAFGSFFSIVIDVAVDNGEVQVKRVVCAVDCGMSVNPNTIEAQV
QGGIIFGITGALYGEITIEDGRVVQSNFTDYRMLRINETPPIEVHLVKSGEAPGGIGEPGTAATAAALSNAIFAATG
KRLRKLPVGDQLKTA
>SEQF4105||SEQF4105.1_00663
MTIELDNTGSVRPSRRTFLKAAGAAAAVSLTIGFEWTGLGRRALAATAPAADFAPNAFLRITPDGAVTVIAKHVE
MGQGAYTGIATIVAEELDADWSTVRVESAPADAKRYANLAFGTMQGTGGSSAMANSWQQLREAGGKARA
MLVSAAAARWKVPAGELTTANGVVTHAKSGKKAAYGTLVADASKLPVPDKVVLKQPADFKLIGQRIPRVDASPK
SNGTAHFTLDTTFPGMRVALLQRPPRFGATVKSFDATAAKAVPGVVSVVQVPGGVAVVGTGFWAAKQGRDAL
KVEWDEAHAEKRSSDEIMREYRQLADKPGTSARKDGDADAAISGAARKIGATYEFPYLAHAPMEPLDAVVKLT
ADSCEIWAGDQFQTVDQANAARTAGLKPEQVQIHTLYAGGSFGRRANAWSDYVVEAVSIAKALGADGKPVKL
QWTREDDIQGGFYRPMYFHKLDAGLTADGKLVGWRHRIVGQSILAGTPFEAFMVKNGIDATSVEGAANLPYA
VPNVSVELTTTKVGLPVLWWRVVGSSHTAYAVEAFIDEAAHTAGKDPYAFRRDLLAKEPRMRAVLDLAAQKAG
WDPAKPLPKGRGRGIAVAEAFKSYVAQVAEVSVDADGKVKVERVVCAVDCGIAINPDIVAAQMEGGIGFGLGA
VMHSAITLKDGQVEQRNFDGYHVLRIAEMPKVEVHIVPSAEAPTGVGEPGVAPVGPAVANAIFAATGKRHYVL
PFDSADSAKA
>SEQF4106||SEQF4106.1_05346
MTIELDNTGSVRPSRRTFLKAAGAAAAVSLTIGFEWAGLGRRALAATAPAADFAPNAFLRITPDGAVTVIAKHVE
MGQGAYTGIATIVAEELDADWSTVRVESAPADAKRYANLAFGTMQGTGGSSAMANSWQQLREAGGKARA
MLVSAAAARWKVPAGELTTANGVVTHAKSGKKAAYGTLVADASKLPVPDKVVLKQPADFKLIGQRIPRVDASPK
SNGTAHFTLDTTFPGMRVALLQRPPRFGATVKSFDATAARAVPGVVSVVQVPGGVAVVGTGFWAAKQGRDAL
KVEWDEAHAEKRSSDEIMREYRQLADKPGTSARKDGDADAAIAGAARKIGATYEFPYLAHAPMEPLDAVVKLT
ADSCEIWAGDQFQTVDQANAARTAGLKPEQVRIHTLYAGGSFGRRANAWSDYVVEAVSIAKALGADGKPVKL
QWTREDDIQGGFYRPMYFHKLDAGLTADGKLVGWRHRIVGQSILAGTPFEAFMVKNGIDATSVEGAANLPYA
VPNVSVELTTTKVGLPVLWWRVVGSSHTAYAVEAFIDEAAHTAGKDPYAFRRDLLAKEPRMRAVLDLAAQKAG
WDPAKPLPKGRGRGIAVAEAFKSYVAQVAEVSVDADGKVKVERVVCAVDCGIAINPDIVAAQMEGGIGFGLGA
VMHSAITLKDGQVEQRNFDGYHVLRIAEMPKVEVHIVPSAEAPTGVGEPGVAPVGPAVANAIFAATGKRHYVL
PFDSADSAKA
>SEQF4106||SEQF4106.1_00276
MSRGLIEAGKVAGQAAGAGVSRRSFLKLGMSLGAAAGGGLLLGFSLPAAGDDARRSVIGGDGDETARAGVFA
PNAFVQIDRAGKVTLVMPKVEMGQGVYTALPMLIAEELEVPLSSVTLDHAPPNEKLFLDPLLGGQLTGGSTSIRY
AWEPLRRAGATARTLLVAAAAKQWNVDPASCRAVNGEVQHPPSGRRVSYGQLADAAAKLPVPKDVALKKPAD
FKLIGTPAKRLDSPEKVDGTAQFGLDVRLPGMLYAVIVNSPVFGGTVASIDDTAAKKIPGVRQVVRADNAVAVV
GDHTWAAKRGASALVVKWNEGAGAKVSTKDIVADLAQAAANGKGAVARKDGDVGKGFADAKTRIDAVYEQ
PLLAHATMEPVNCTVHVRADGCEIWVGTQVPTRAVDTVQQLTSFPPEKIVVHNHLLGGGFGRRLETDMIGQA
VKIAKQVNAPVKVIWTREEDIQHDMYRPYYYDRISAGLDANGKPIAWQHRIVGSSILARFAPPAFQHGVDPDA
VEVATDLPYDLPNQLIDYVRQEPRHVPTAFWRGVGPTRSTFVVESFIDELAAQTKTDPVQYRRALLGKTPRALN
VLDVATKAAGWGPSLPTGQGRGVSVMHAFGSFFSIVIDVAVDNGEVQVKRVVCAVDCGMSVNPNTIEAQVQ
GGIIFGITGALYGEITIEDGRVVQSNFTDYRMLRINETPPIEVHLVKSGEAPGGIGEPGTAATAAALSNAIFAATGK
RLRKLPVGDQLKTA
>SEQF4106||SEQF4106.1_03609
MNAPELSVHNESRRALLLGFASGGLLLAFGVPSLVRAAVPNQPPVSADPRYGGAGMPHGLRDDPHLFVAIAPD
GTVTVTCIRSEMGQGVRTSVALVVADELGADWARVKVAQAVGDEPRYGNQNTDGSRSLRQSFAALRRAGAA
ARTMLEQAAAAAWGVDARQVRATVHEVVDTKSGRKLGFGELAAKAAALPAPDPATVPLKAPAEFRYIGKGETA
LIDGRDIVAGRAHYGIDTRLDGMLYAVIARPPAYGDTMASFDASAAEKLPGVVKVVPLASTPLPSGFQPLGGVA

VVARDTWTAIQARAQLKIDWKHGPNANYDSAAYRKTLEAAAAQPGDVIRNDGDAAAALAGAAKRVRATYYIP
HLAHATMEPPAAVARVADGQCEVWTCTQAPQTTRDEVAKALGLPGERVTVNVTLLGGGFGRKSKPDYVVEA
ALLSKAVGAPVKLTFTREDDLAHDYFHAVSLEAFDGGIDASGKVVAWQHRTVAPSIQSTFRAGIVHEQPGELAQ
GIADLPFAIPNVRIENPAAQAHTRIGWFRSVYNIPHAFGIQSFVSELAHAAGRDPKDFLLELIGPARRFEPHITVK
NVNYGEDPALYPVDTGRLRRVVETVAREAGWGRRLPKGRGLGIAAHRSFVSYTAAVCEVQVDADGKITVPRVD
IAIDCGPQVNPERVRSQLEGAVVMGLGIALHGEITFKDGHPEQSNFNGFQVLRMNEAPREIRVHLVAPDDFAT
PLGGVGEPGLPPVAPALTNAIFAATGTRIRSLPVADQLAKPRAG
>SEQF4107||SEQF4107.1_02806
MSRGLIEAGKVAGQTAGAGVSRRSFLKLGMSLGAAAGGGLLLGFSLPAAGDDARRSVIGGDGDETARAGVFAP
NAFVQIDRAGKVTLVMPKVEMGQGVYTALPMLIAEELEVPLSSVTLDHAPPNEKLFLDPLLGGQLTGGSTSVRY
AWEPLRRAGATARTLLVAAAAKQWNVDPASCRAVNGEVQHPPSGRRVSYGQLADAAAKLPVPKDVVLKKPA
DFKLIGTPAKRLDSPEKVDGTAQFGLDVRLPGMLYAVIVNSPVFGGTVASIDDTAAKKIPGVRQVVRADNAVAV
VGDHTWAAKRGASALVVKWNEGAGAKVSTKDIVADLAQAAANGKGAVARKDGDVGKGFADAKTRIDAVYE
QPLLAHATMEPVNCTVHVRADGCEIWVGTQVPTRAVDTVQKLTSFPPEKIVVHNHLLGGGFGRRLETDMIGQ
AVKIAKQVNAPVKVIWTREEDIQHDMYRPYYYDRISAGLDANGKPIAWQHRIVGSSILARFAPPAFQHGVDPD
AVEVATDLPYDLPNQLIDYVRQEPRHVPTAFWRGVGPTRSTFVVESFIDELAAQTKTDPVQYRRALLGKTPRAL
NVLDVATKAAGWGPSLPTGQGRGVSVMHAFGSFFSIVIDVAVDNGEVQVKRVVCAVDCGMSVNPNTIEAQV
QGGIIFGITGALYGEITIEDGRVVQSNFTDYRMLRINETPPIEVHLVKSGEAPGGIGEPGTAATAAALSNAIFAATG
KRLRKLPVGDQLKTA
>SEQF4107||SEQF4107.1_06918
MSAPELSVHNESRRALLLGFASGGLLLAFGVPSLVRAAVPSQPPVSADPRYGGAGMPHGLRDDPHLFVAIAPD
GTVTVTCIRSEMGQGVRTSVALVVADELGADWARVKVAQAVGDEPRYGNQNTDGSRSLRQSFAALRRAGAA
ARTMLEQAAAAAWGVDARQVKATVHEVVDTKSGRKLGFGELAAKAAALPAPDPATVPLKAPAEFRYIGKGQT
ALIDGRDIVAGRAHYGIDTRLDGMLYAVVARPPAYGDTVASFDASAAEKLPGVVKVVPLTPTPLPSGFQPLGGVA
VVARDTWTAIQARAQLKIDWKHGPNANYDSAAYRKTLEAAAAQPGDVIRNDGDAAAALAGAAKRVRATYYIP
HLAHATMEPPAAVARVADGRCEVWTCTQAPQTTRDEVAKALGLPGERVTVNVTLLGGGFGRKSKPDYVVEAA
LLSKAVGAPVKLTFTREDDLAHDYFHAVSLEAFDGGIDASGKVVAWQHRTVAPSIQSTFRAGIVHEQPGELAQG
IADLPFAIPNVRIENPAAQAHTRIGWFRSVYNIPHAFGIQSFVSELAHAAGRDPKDFLLELIGPARRFEPHITVKN
VNYGEDPALYPVDTGRLRRVVETVAREAGWGRKRPKGHGLGIAAHRSFVSYTAAVCEVQVDADGKITVPRVDI
AIDCGPQVNPERVRSQLEGAVVMGLGIALHGEITFKDGHPEQSNFNGFQVLRMNEAPREIRVHLVAPDDFATP
LGGVGEPGLPPVAPALTNAIFAATGTRIRSLPVADQLAKPRAG
>SEQF4107||SEQF4107.1_04399
MTIELDNTGSVRPSRRTFLKAAGAAAAVSLTIGFEWAGLGRRALAATAPAADFAPNAFLRITPDGAVTVIAKHVE
MGQGAYTGIATIVAEELDADWSTVRVESAPADAKRYANLAFGTMQGTGGSSAMANSWQQLREAGGKARA
MLVSAAAARWKVPAGELTTANGVVTHAKSGKKAAYGTLVADASKLPVPDKVALKQPADFKLIGKRIPRVDASPK
SNGTAHFTLDTTFPGMRVALLQRPPRFGATVKSFDATAAKAVPGVVSVVQVPGGVAVVGTGFWAAKQGRDAL
KVEWDEAHAEKRSSDEIMREYRQLADKPGTSARKDGDADAAIAGAARKIGATYEFPYLAHAPMEPLDAVVKLT
ADSCEIWAGDQFQTVDQANAARTAGLKPEQVQIHTLYAGGSFGRRANAWSDYVVEAVSIAKALGADGKPVKL
QWTREDDIQGGFYRPMYFHKLDAGLTADGKLVGWRHRIVGQSILAGTPFEAFMVKNGIDATSVEGAANLPYA
VPNVSVELTTTKVGLPVLWWRVVGSSHTAYAVEAFIDEAAHTAGKDPYAFRRDLLAKEPRMRAVLDLAAQKAG
WDPAKPLPKGRGRGIAVAEAFKSYVAQVAEVSVDADGKVKVERVVCAVDCGIAINPDIVAAQMEGGIGFGLGA
VMHSAITLKDGQVEQRNFDGYHVLRIAEMPKVEVHIVPSAEAPTGVGEPGVAPVGPAVANAIFAATGKRHYVL
PFDSADSAKA
>SEQF4108||SEQF4108.1_07718

MSAPELSVHNESRRALLLGFASGGLLLAFGVPSLVRAAVPNQPPVSADPRYGGAGMPHGLRDDPHLFVAIAPD
GTVTVTCIRSEMGQGVRTSVALVVADELGADWARVKVAQAVGDEPRYGNQNTDGSRSLRQSFAALRRAGAA
ARTMLEQAAAAAWGVDARQVKATVHEVVDTKSGRKLGFGELAAKAAALPAPDPATVPLKASAEFRYIGKGETA
LIDGRDIVAGRAHYGIDTRLDGMLYAVVARPPAYGDTMASFDASAAEKLPGVVKVVPLASTPLPSGFQPLGGVA
VVARDTWTAIQARAQLKIDWKHGPNANYDSAAYRKTLEAAAAQPGDVIRNDGDAAAALAGAAKRVRATYYIP
HLAHATMEPPAAVARVADGRCEVWTCTQAPQTTRDEVAKALGLPGERVTVNVTLLGGGFGRKSKPDYVVEAA
LLSKAVGAPVKLTFTREDDLAHDYFHAVSLEAFDGGIDASGKVVAWQHRTVAPSIQSTFRAGVVHEQPGELAQ
GIADLPFAIPNVRIENPAAQAHTRIGWFRSVYNIPHAFGIQSFVSELAHAAGRDPKDFLLELIGPARRFEPHITVK
NMNYGEDPALYPVDTGRLRRVVETVAREASWGRRLPKGHGLGIAAHRSFVSYTAAVCEVQVDAGGKITVPRVD
IAIDCGPQVNPERVRSQLEGAVVMGLGIALHGEITFKDGHPEQSNFNGFQVLRMNEAPREIRVHLVAPDDFAT
PLGGVGEPGLPPVAPALTNAIFAATGTRIRSLPVADQLAKPRAG
>SEQF4108||SEQF4108.1_01313
MSRGLIEAGKVAGQAAGAGVSRRSFLKLGMSLGAAAGGGLLLGFSLPAAGDDARRSVIGGDGDETARAGVFA
PNAFVQIDRAGKVTLVMPKVEMGQGVYTALPMLIAEELEVPLSSVTLDHAPPNEKLFLDPLLGGQLTGGSTSIRY
AWEPLRRAGATARTLLVAAAAKQWSVDPASCRAVNGEVQHPPSGQRVSYGQLADAAAKLPVPKDVALKKPAD
FKLIGTPAKRLDSPEKVDGAAQFGLDVRLPGMLYAVIVNSPVFGGTVASIDDTAAKKIPGVRQVVRADNAVAVV
GDHTWAAKRGASALVVKWNEGAGAKVSTKDIVADLAQAAANGKGAVARKDGDVGKGFADAKTRIDAVYEQ
PLLAHATMEPVNCTVHVRADGCEIWVGTQVPTRAVDTVQQLTSFPPEKIVVHNHLLGGGFGRRLETDMIGQA
VKIAKQVNAPVKVIWTREEDIQHDMYRPYYYDRISAGLDANGKPIAWQHRIVGSSILARFAPPAFQHGVDPDA
VEVATDLPYDLPNQLIDYVRQEPRHVPTAFWRGVGPTRSTFVVESFIDELAAQTKTDPVQYRRALLGKTPRALN
VLDVATKAAGWGPSLPTGQGRGVSVMHAFGSFFSIVIDVAVDNGEVQVKRVVCAVDCGMSVNPNTIEAQVQ
GGIIFGITGALYGEITIEDGRVVQSNFTDYRMLRINETPPIEVHLVKSGEAPGGIGEPGTAATAAALSNAIFAATGK
RLRKLPVGDQLKTA
>SEQF4108||SEQF4108.1_07035
MTIELDNTGSVRPSRRTFLKAAGAAAAVSLTIGFEWAGLGRRALAATAPAADFAPNAFLRITPDGAVTVIAKHVE
MGQGAYTGIATIVAEELDADWSTVRVESAPADAKRYANLAFGTMQGTGGSSAMANSWQQLREAGGKARA
MLVSAAAARWKVPAGELTTANGVVTHAKSGKKAAYGTLVADASKLPVPDKVVLKQPADFKLIGQRIPRVDASPK
SNGTAHFTLDTTFPGMRVALLQRPPRFGATVKSFDATAARAVPGVVSVVQVPGGIAVVGTGFWAAKQGRDAL
KVEWDEAHAEKRSSDEIMREYRQLADKPGTSARKDGDADAAIAGAARKIGATYEFPYLAHAPMEPLDAVVKLT
ADSCEIWAGDQFQTVDQANAARTAGLKPEQVQIHTLYAGGSFGRRANAWSDYVVEAVSIAKALGADGKPVKL
QWTREDDIQGGFYRPMYFHKLDAGLTEDGKLVGWRHRIVGQSILAGTPFEAFMVKNGIDATSVEGAANLPYA
VPNVSVELTTTKVGLPVLWWRVVGSSHTAYAVEAFIDEAAHTAGKDPYAFRRDLLAKEPRMRAVLDLAAQKAG
WDPAKPLPKGRGRGIAVAEAFKSYVAQVAEVSVDADGKVKVERVVCAVDCGIAINPDIVAAQMEGGIGFGLGA
VMHSAITLKDGQVEQRNFDGYHVLRIAEMPKVEVHIVPSAEAPTGVGEPGVAPVGPAVANAIFAATGKRHYVL
PFDSADSAKA
>SEQF4109||SEQF4109.1_05908
MSRGLIEAGKVAGQAAGAGVSRRSFLKLGMSLGAAAGGGLLLGFSLPAAGDDARRSVIGGDGDETARAGVFA
PNAFVQIDRAGKVTLVMPKVEMGQGVYTALPMLIAEELEVPLSSVTLDHAPPNEKLFLDPLLGGQLTGGSTSIRY
AWEPLRRAGATARTLLVAAAAKQWNVDPASCRAVNGEVQHPPSGRRVSYGQLADAAAKLPVPKDVALKKPAD
FKLIGTPAKRLDSPEKVDGTAQFGLDVRLPGMLYAVIVNSPVFGGTVASIDDTAAKKIPGVRQVVRADNAVAVV
GDHTWAAKRGASALVVKWNEGAGAKVSTKDIVADLAQAAANGKGAVARKDGDVGKGFADAKTRIDAVYEQ
PLLAHATMEPVNCTVHVRADGCEIWVGTQVPTRAVDTVQKLTSFPPEKIVVHNHLLGGGFGRRLETDMIGQA
VKIAKQVNAPVKVIWTREEDIQHDMYRPYYYDRISAGLDANGKPIAWQHRIVGSSILARFAPPAFQHGVDPDA
VEVATDLPYDLPNQLIDYVRQEPRHVPTAFWRGVGPTRSTFVVESFIDELAAQTKTDPVQYRRALLGKTPRALN

VLDVATKAAGWGPSLPTGQGRGVSVMHAFGSFFSIVIDVAVDNGEVQVKRVVCAVDCGMSVNPNTIEAQVQ
GGIIFGITGALYGEITIEDGRVVQSNFTDYRMLRINETPPIEVHLVKSGEAPGGIGEPGTAATAAALSNAIFAATGK
RLRKLPVGDQLKTA
>SEQF4109||SEQF4109.1_07787
MTIELDNTGSVRPSRRTFLKAAGAAAAVSLTIGFEWAGLGRRALAATAPAADFAPNAFLRITPDGAVTVIAKHVE
MGQGAYTGIATIVAEELDADWSTVRVESAPADAKRYANLAFGTMQGTGGSSAMANSWQQLREAGGKARA
MLVSAAAARWKVPAGELTTANGVVTHAKSGKKAAYGTLVADASKLPVPDKVVLKQPADFKLIGKRIPRVDASPK
SNGTAHFTLDTTFPGMRVALLQRPPRFGATVKSFDATAAKAVPGVVSVVQVPGGVAVVGTGFWAAKQGRDAL
KVEWDEAHAEKRGSDEIMREYRQLADKPGTSARKDGDADAAIAGAARKIGATYEFPYLAHAPMEPLDAVVKLT
ADSCEIWAGDQFQTVDQANAARTAGLKPEQVQIHTLYAGGSFGRRANAWSDYVVEAVSIAKALGADGKPVKL
QWTREDDIQGGFYRPMYFHKLDAGLTADGKLVGWRHRIVGQSILAGTPFEAFMVKNGIDATSVEGAANLPYA
VPNVSVELTTTKVGLPVLWWRVVGSSHTAYAVEAFIDEAAHTAGKDPYAFRRDLLAKEPRMRAVLDLAAQKAG
WDPAKPLPKGRGRGIAVAEAFKSYVAQVAEVSVDADGKVKVERVVCAVDCGIAINPDIVAAQMEGGIGFGLGA
VMHSAITLKDGQVEQRNFDGYHVLRIAEMPKVEVHIVPSAEAPTGVGEPGVAPVGPAVANAIFAATGKRHYVL
PFDSADSAKA
>SEQF4109||SEQF4109.1_06425
MSAPELSVHNESRRALLLGFASGGLLLAFGVPSLVRAAVPSQPPVSADPRYGGAGMPHGLRDDPHLFVAIAPD
GTVTVTCIRSEMGQGVRTSVALVVADELGADWARVKVAQAVGDEPRYGNQNTDGSRSLRQSFAALRRAGAA
ARTMLEQAAAAAWGVDARQVKTTVHEVVDTKSGRKLGFGELAAKAAALPAPDPATVPLKAPAEFRYIGKGQT
ALIDGRDIVAGRAHYGIDTRLDGMLYAVVARPPAYGDTVASFDASAAEKLPGVVKVVPLTPTPLPSGFQPLGGVA
VVARDTWTAIQARAQLKIDWKHGPNANYDSAAYRKTLEAAAAQPGDVIRNDGDAAAALAGAAKRVRATYYIP
HLAHATMEPPAAVARVADGRCEVWTCTQAPQTTRDEVAKALGLPGERVTVNVTLLGGGFGRKSKPDYVVEAA
LLSKAVGAPVKLTFTREDDLAHDYFHAVSLEAFDGGIDASGKVVAWQHRTVAPSIQSTFRAGIVHEQPGELAQG
IADLPFAIPNVRIENPAAQAHTRIGWFRSVYNIPHAFGIQSFVSELAHAAGRDPKDFLLELIGPARRFEPHITVKN
VNYGEDPALYPVDTGRLRRVVETVAREAGWGRKLSKGHGLGIAAHRSFVSYTAAVCEVQVDADGKITVPRVDIA
IDCGPQVNPERVRSQLEGAVVMGLGIALHGEITFKDGHPEQSNFNGFQVLRMNEAPREIRVHLVAPDDFATPL
GGVGEPGLPPVAPALTNAIFAATGTRIRSLPVADQLAKPRAG
>SEQF4110||SEQF4110.1_04031
MTIELDNTGSVRPSRRTFLKAAGAAAVSLTIGFDFTGFGRRALAATAPAAGFAPNAFLRITPDGAVTVIAKHVEM
GQGAYTGIATIVAEELDADWSSVRVESAPADAKRYANLAFGTMQGTGGSSAMANSWQQLREAGGKARAML
VSAAAARWKVPAGELTTANGVVTHAKSGKTAAYGTLVADASKLPVPDKVVLKQPADFKLIGQRIPRVDASAKSD
GTAHFTLDTTFPGMRVALLQRPPRFGATVKSFDATAAKAVPGVVSVVQVPGGVAVVGTGFWAAKQGRDALKV
EWDEAHAEKRGSDEIMREYRRLADQPGTSARKDGDADAAIAGAARKIGATYEFPYLAHAPMEPLDAVVKLTA
DSCEIWAGDQFQTVDQANAARTAGLKPEQVQIHTLYAGGSFGRRANAWSDYVVEAVSIAKALGADGKPVKLQ
WTREDDIQGGFYRPMYFHKLDAGLTADGKLVGWRHRIVGQSILAGTPFEAFMVKNGVDATSVEGAANLPYAV
PNVSVELTTTKVGLPVLWWRVVGSSHTAYAVEAFIDEAAHTAGKDPYAFRRDLLAKEPRMRAVLDLAAQKAG
WDPAKPLPKGRGRGIAVAEAFKSYVAQVAEVSVDADGKVKVERVVCAVDCGIAINPDIVAAQMEGGIGFGLGA
VMHSAITLKDGQVEQRNFDGYHVLRIAEMPKVEVHIVPSAEAPTGVGEPGVAPVGPAVANAIFAATGKRHYVL
PFDSADSAKA
>SEQF4110||SEQF4110.1_04714
MSAPELCVQNESRRALLLGFASGGLLLAFGVPSLVRAAVPVQPPVSANPQYGGAGMPHGLRDDPHLFVAIAPD
GTVTVTCIRSEMGQGVRTSVALVVADELGADWARVKVAQAVGDEPRYGNQNTDGSRSLRQSFAALRRAGAA
ARTMLEQAAAAAWGVDARQVKAAVHEVVDTKSGRKLGFGELATKAAALPAPDPATVVLKAPAEFRYIGKGETA
LIDGRDIVAGRAHYGIDTRLDGMLYAVVARPPAYGDTVTSFDASAAEKLPGVVKVVPLAPTPLPSGFQPLGGVAV

VARDTWTAIQARAQLKIDWKRGPNADYDSAAYRKTLEAAAAQPGDVIRNDGDAAAALAGAAKRVRATYYVP
HLAHATMEPPAAVARVADGRCEVWTCTQAPQTTRDEVAKALGLPGERVTVNVTLLGGGFGRKSKPDYVVEAA
LLSKAVGAPVKLTFTREDDLAHDYFHAVSLEAFDGGIDASGKVVAWQHRTVAPSIQSTFRAGIVHEQPGELAQG
IADLPFAIPNVRIENPAAQAHTRIGWFRSVYNIPHAFGIQSFVSELAHAAGRDPKDFLLELIGPARRFEPHITVKN
VNYGEDPALYPVDTGRLRRVVETVAREAGWGRKLPKGHGLGIAAHRSFVSYTAAVCEVQVDADGKITVPRVDI
AIDCGPQVNPERVRSQLEGAVVMGLGIALHGEITFKDGHPEQSNFNGFQVLRMNEAPREIRVHLVAPDDFATP
LGGVGEPGLPPVAPALTNAIFAATGTRIRSLPVADQLAKPRAG
>SEQF4110||SEQF4110.1_01348
MSRGLIEAGKVVGQAAGAGVSRRSFLKLGMSLGAAAGGGLLLGFSLPAAGDDARRSVIGGDGDETARAGVFA
PNAFVQIDRAGKVTLVMPKVEMGQGVYTALPMLIAEELEVPLSNVTLDHAPPNEKLFLDPLLGGQLTGGSTSV
RYAWEPLRRAGATARTLLVAAAAKQWNVDPASCRASNGEVQHPPSGRRASYGQLADAAAKLPVPKDVALKKP
ADFKLIGTPAKRLDSPEKVDGTAQFGLDVRLPGMLYAVIVNSPVFGGTVASVDDTAAKKIPGVRQVVRADNAVA
VVGDHTWAAKRGASALVVKWNEGADAKVSTKDIVADLAQAAANGKGAVARKDGDVGKGFADAKTRIDAVYE
QPFLAHATMEPVNCTVHVRADGCEIWVGTQVPTRARDTVQQLTSFPPEKIVVHNHLLGGGFGRRLETDMIGQ
AVKIAKQVNAPVKVIWTREEDIQHDMYRPYYYDRISAGLDANGKLIAWQHRIVGSSILARFAPPAFQHGVDPD
AVEVATDLPYDLPNQLIDYVRQEPRHVPTAFWRGVGPTRSTFVVESFIDELAAQTKTDPVQYRRALLGKTPRAL
NVLDVATKAAGWGPSLPKGQGRGVSVMHAFGSFFSIVIDVAVDDGEVQVKRVVCAVDCGMFVNPNTIEAQV
QGGIIFGITGALYGEITIEDGRVVQSNFTDYRMLRINETPPIEVHLVKSAEAPGGIGEPGTAATAAALSNAIFAATG
KRLRKLPVGDQLKTA
>SEQF4111||SEQF4111.1_01660
MSRGLIEAGKVAGQAAGAGVSRRSFLKLGMSLGAAAGGGLLLGFSLPAAGDDARRSVIGGDGDETARAGVFA
PNAFVQIDRAGKVTLVMPKVEMGQGVYTALPMLIAEELEVPLSSVTLDHAPPNEKLFLDPLLGGQLTGGSTSIRY
AWEPLRRAGATARTLLVAAAAKQWNVDPASCRAVNGEVQHPPSGRRTSYGQLADAAAKLPVPKDVALKKPAD
FKLIGTPARRLDSPEKVDGTAQFGLDVRLPGMLYAVIVNSPVFGGTVASIDDTAAKKIPGVRQVVRADNAVAVV
GDHTWAAKRGASALVVKWNEGAGAKVSTKDIVADLAQAAANGKGAVARKDGDVGKGFADAKTRVDAVYEQ
PLLAHATMEPVNCTVHVRADGCEIWVGTQVPTRAVDTVQQLTSFPPEKIVVHNHLLGGGFGRRLETDMIGQA
VKIAKQVNAPVKVIWTREEDIQHDMYRPYYYDRISAGLDANGKPIAWQHRIVGSSILARFAPPAFQHGVDPDA
VEVATDLPYDLPNQLIDYVRQEPRHVPTAFWRGVGPTRSTFVVESFIDELAAQTKTDPVQYRRALLGKTPRALN
VLDVATKAAGWGPSLPTGQGRGVSVMHAFGSFFSIVIDVAVDNGEVQVKRVVCAVDCGMSVNPNTIEAQVQ
GGIIFGITGALYGEITIEDGRVVQSNFTDYRMLRINETPPIEVHLVKSGEAPGGIGEPGTAATAAALSNAIFAATGK
RLRKLPVGDQLKTA
>SEQF4111||SEQF4111.1_02357
MSAPELSVHNESRRALLLGFASGGLLLAFGVPSLVRAAVPNQPPVSADPRYGGAGMPHGLRDDPHLFVAIAPD
GTVTVTCIRSEMGQGVRTSVALVVADELGADWARVKVAQAVGDEPRYGNQNTDGSRSLRQSFAALRRAGAA
ARTMLEQAAAAAWGVDARQVKATVHEVVDTKSGRKLGFGELAAKAAALPAPDPATVPLKAPAEFRYIGKGETA
LIDGRDIVAGRAHYGIDTRLDGMLYAVVARPPAYGDTMTSFDASAAEKLPGVVKVVPLASTPLPSGFQPLGGVA
VVARDTWTAIQARAQLKIDWKHGPNANYDSAAYRKTLEAAAAQPGDVIRNDGDAAAALAGAAKRVRATYYIP
HLAHATMEPPAAVARVADGRCEVWTCTQAPQTTRDEVAKALGLPGERVTANVTLLGGGFGRKSKPDYVVEAA
LLSKAVGAPVKLTFTREDDLAYDYFHAVSLEAFDGGIDASGKVVAWQHRTVAPSIQSTFRAGVVHEQPGELAQG
IADLPFAIPNVRIENPAAQAHTRIGWFRSVYNIPHAFGIQSFVSELAHAAGRDPKDFLLELIGPARRFEPHITVKN
VNYGEDPALYPVDTGRLRRVVETVAREAGWGRRLPKGHGLGIAAHRSFVSYTAAVCEVQVDADGKITVPRVDI
AIDCGPQVNPERVRSQLEGAVVMGLGIALHGEITFKDGHPEQSNFNGFQVLRMNEAPREIRVHLVAPDDFATP
LGGVGEPGLPPVAPALTNAIFAATGTRIRSLPVADQLAKPRAG
>SEQF4111||SEQF4111.1_05416

MTIELDNTGSVRPSRRTFLKAAGAAAAVSLTIGFEWAGLGRRALAATAPAADFAPNAFLRITPDGAVTVIAKHVE
MGQGAYTGIATIVAEELDADWSTVRVESAPADAKRYANLAFGTMQGTGGSSAMANSWQQLREAGGKARA
MLVSAAAARWKVPAGELTTANGVVTHAKSGKKAAYGTLVADASKLPVPDKVVLKQPADFKLIGQRIPRVDASPK
SNGTAHFTLDTTFPGMRVALLQRPPRFGATVKSFDATAAKAVPGVVSVVQVPGGIAVVGTGFWAAKQGRDAL
KVEWDEAHAEKRGSDEIMREYRQLADKPGTSARKDGDADAAIAGAARKIGATYEFPYLAHAPMEPLDAVVKLT
ADSCEIWAGDQFQTVDQANAARTAGLKPEQVQIHTLYAGGSFGRRANAWSDYVVEAVSIAKALGADGKPVKL
QWTREDDIQGGFYRPMYFHKLDAGLTEDGKLVGWRHRIVGQSILAGTPFEAFMVKNGIDATSVEGAANLPYA
VPNVSVELTTTKVGLPVLWWRVVGSSHTAYAVEAFIDEAAHTAGKDPYAFRRDLLAKEPRMRAVLDLAAQKAG
WDPAKPLPKGRGRGIAVAEAFKSYVAQVAEVSVDADGKVKVERVVCAVDCGIAINPDIVAAQMEGGIGFGLGA
VMHSAITLKDGQVEQRNFDGYHVLRIAEMPKVEVHIVPSAEAPTGVGEPGVAPVGPAVANAIFAATGKRHYVL
PFDSADSAKA
>SEQF4112||SEQF4112.1_00153
MSAPELSVHNESRRALLLGFASGGLLLAFGVPSLVRAAVPNQPPVSADPRYGGAGMPHGLRDDPHLFVAIAPD
GTVTVTCIRSEMGQGVRTSVALVVADELGADWARVKVAQAVGDEPRYGNQNTDGSRSLRQSFAALRRAGAA
ARTMLEQAAAAAWGVDARQVKATVHEVVDTKSGRKLGFGELAAKAAALPAPDPATVPLKAPAEFRYIGKGETA
LIDGRDIVAGRAHYGIDTRLDGMLYAVVARPPAYGDTMTSFDASAAEKLPGVVKVVPLASTPLPSGFQPLGGVA
VVARDTWTAIQARAQLKIDWKHGPNANYDSAAYRKTLEAAAAQPGDVIRNDGDAAAALAGAAKRVRATYYIP
HLAHATMEPPAAVARVADGRCEVWTCTQAPQTTRDEVAKALGLPGERVTANVTLLGGGFGRKSKPDYVVEAA
LLSKAVGAPVKLTFTREDDLAYDYFHAVSLEAFDGGIDASGKVVAWQHRTVAPSIQSTFRAGVVHEQPGELAQG
IADLPFAIPNVRIENPAAQAHTRIGWFRSVYNIPHAFGIQSFVSELAHAAGRDPKDFLLELIGPARRFEPHITVKN
VNYGEDPALYPVDTGRLRRVVETVAREAGWGRRLPKGHGLGIAAHRSFVSYTAAVCEVQVDADGKITVPRVDI
AIDCGPQVNPERVRSQLEGAVVMGLGIALHGEITFKDGHPEQSNFNGFQVLRMNEAPREIRVHLVAPDDFATP
LGGVGEPGLPPVAPALTNAIFAATGTRIRSLPVADQLAKPRAG
>SEQF4112||SEQF4112.1_02441
MTIELDNTGSVRPSRRTFLKAAGAAAAVSLTIGFEWAGLGRRALAATAPAADFAPNAFLRITPDGAVTVIAKHVE
MGQGAYTGIATIVAEELDADWSTVRVESAPADAKRYANLAFGTMQGTGGSSAMANSWQQLREAGGKARA
MLVSAAAARWKVPAGELTTANGVVTHAKSGKKAAYGTLVADASKLPVPDKVVLKQPADFKLIGQRIPRVDASPK
SNGTAHFTLDTTFPGMRVALLQRPPRFGATVKSFDATAAKAVPGVVSVVQVPGGIAVVGTGFWAAKQGRDAL
KVEWDEAHAEKRGSDEIMREYRQLADKPGTSARKDGDADAAIAGAARKIGATYEFPYLAHAPMEPLDAVVKLT
ADSCEIWAGDQFQTVDQANAARTAGLKPEQVQIHTLYAGGSFGRRANAWSDYVVEAVSIAKALGADGKPVKL
QWTREDDIQGGFYRPMYFHKLDAGLTEDGKLVGWRHRIVGQSILAGTPFEAFMVKNGIDATSVEGAANLPYA
VPNVSVELTTTKVGLPVLWWRVVGSSHTAYAVEAFIDEAAHTAGKDPYAFRRDLLAKEPRMRAVLDLAAQKAG
WDPAKPLPKGRGRGIAVAEAFKSYVAQVAEVSVDADGKVKVERVVCAVDCGIAINPDIVAAQMEGGIGFGLGA
VMHSAITLKDGQVEQRNFDGYHVLRIAEMPKVEVHIVPSAEAPTGVGEPGVAPVGPAVANAIFAATGKRHYVL
PFDSADSAKA
>SEQF4112||SEQF4112.1_04200
MSRGLIEAGKVAGQAAGAGVSRRSFLKLGMSLGAAAGGGLLLGFSLPAAGDDARRSVIGGDGDETARAGVFA
PNAFVQIDRAGKVTLVMPKVEMGQGVYTALPMLIAEELEVPLSSVTLDHAPPNEKLFLDPLLGGQLTGGSTSIRY
AWEPLRRAGATARTLLVAAAAKQWNVDPASCRAVNGEVQHPPSGRRTSYGQLADAAAKLPVPKDVALKKPAD
FKLIGTPARRLDSPEKVDGTAQFGLDVRLPGMLYAVIVNSPVFGGTVASIDDTAAKKIPGVRQVVRADNAVAVV
GDHTWAAKRGASALVVKWNEGAGAKVSTKDIVADLAQAAANGKGAVARKDGDVGKGFADAKTRVDAVYEQ
PLLAHATMEPVNCTVHVRADGCEIWVGTQVPTRAVDTVQQLTSFPPEKIVVHNHLLGGGFGRRLETDMIGQA
VKIAKQVNAPVKVIWTREEDIQHDMYRPYYYDRISAGLDANGKPIAWQHRIVGSSILARFAPPAFQHGVDPDA
VEVATDLPYDLPNQLIDYVRQEPRHVPTAFWRGVGPTRSTFVVESFIDELAAQTKTDPVQYRRALLGKTPRALN

VLDVATKAAGWGPSLPTGQGRGVSVMHAFGSFFSIVIDVAVDNGEVQVKRVVCAVDCGMSVNPNTIEAQVQ
GGIIFGITGALYGEITIEDGRVVQSNFTDYRMLRINETPPIEVHLVKSGEAPGGIGEPGTAATAAALSNAIFAATGK
RLRKLPVGDQLKTA
>SEQF4113||SEQF4113.1_05753
MSAPELSVHNESRRALLLGFASGGLLLAFGVPSLVRAAVPNQPPVSVDPRYGGAGMPHGLRDDPHLFVAIAPD
GTVTVTCIRSEMGQGVRTSVALVVADELGADWARVKVAQAVGDEPRYGNQNTDGSRSLRQSFAALRRAGAA
ARTMLEQAAAAAWGVDARQVKATVHEVVDTKSGRKLGFGELAAKAAALPAPDPATVPLKAPAEFRYIGKGETA
LIDGRDIVAGRAHYGIDTRLDGMLYAVVARPPAYGDTMASFDASAAEKLPGVVKVVPLASTPLPSGFQPLGGVA
VVARDTWTAIQARAQLKIDWKHGPNANYDSAAYRKTLEAAAAQPGDVIRNDGDAAAALAGAAKRVRATYYIP
HLAHATMEPPAAVARVADGRCEVWTCTQAPQTTRDEVAKALGLPGERVTVNVTLLGGGFGRKSKPDYVVEAA
LLSKAVGAPVKLTFTREDDLAHDYFHAVSLEAFDGGIDASGKVVAWQHRTVAPSIQSTFRAGVVHEQPGELAQ
GIADLPFAIPNVRIENPAAQAHTRIGWFRSVYNIPHAFGIQSFVSELAHAAGRDPKDFLLELIGPARRFEPHITVK
NVNYGEDPALYPVDTGRLRRVVETVAREAGWGRRLPKGHGLGIAAHRSFVSYTAAVCEVQVDADGKITVPRVD
IAIDCGPQVNPERVRSQLEGAVVMGLGIALHGEITFKDGHPEQSNFNGFQVLRMNEAPREIRVHLVAPDDFAT
PLGGVGEPGLPPVAPALTNAIFAATGTRIRSLPVADQLAKPRAG
>SEQF4113||SEQF4113.1_01855
MTIELDNTGSVRPSRRTFLKAAGAAAAVSLTIGFEWAGLGRRALAATAPAADFAPNAFLRITPDGAVTVIAKHVE
MGQGAYTGIATIVAEELDADWSTVRVESAPADAKRYANLAFGTMQGTGGSSAMANSWQQLREAGGKARA
MLVSAAAARWKVPAGELTTANGVVTHAKSGKKAAYGTLVADASKLPVPDKVVLKQPADFKLIGHRIPRVDASPK
SNGTAHFTLDTTFPGMRVALLQRPPRFGATVKSFDATAARAVPGVVSVVQVPGGIAVVGTGFWAAKQGRDAL
KVEWDEAHAEKRGSDEIMREYRQLADKPGTSARKDGDADAAIAGAARKIGATYEFPYLAHAPMEPLDAVVKLT
ADSCEIWAGDQFQTVDQANAARTAGLKPEQVRIHTLYAGGSFGRRANAWSDYVVEAVSIAKALGADGKPVKL
QWTREDDIQGGFYRPMYFHKLDAGLTEDGKLVGWRHRIVGQSILAGTPFEAFMVKNGIDATSVEGAANLPYA
VPNVSVELTTTKVGLPVLWWRVVGSSHTAYAVEAFIDEAAHTAGKDPYAFRRDLLAKEPRMRAVLDLAAQKAG
WDPAKPLPKGRGRGIAVAEAFKSYVAQVAEVSVDADGKVKVERVVCAVDCGIAINPDIVAAQMEGGIGFGLGA
VMHSAITLKDGQVEQRNFDGYHVLRIAEMPKVEVHIVPSAEAPTGVGEPGVAPVGPAVANAIFAATGKRHYVL
PFDSADSAKA
>SEQF4113||SEQF4113.1_03096
MSRGLIEAGKVAGQAAGAGVSRRSFLKLGMSLGAAAGGGLLLGFSLPAAGDDARRSVIGGDGDETARAGVFA
PNAFVQIDRAGKVTLVMPKVEMGQGVYTALPMLIAEELEVPLSSVTLDHAPPNEKLFLDPLLGGQLTGGSTSIRY
AWEPLRRAGATARTLLVAAAAKQWSVDPASCRAVNGEVQHPPSGRRVSYGQLADAAAKLPVPKDVALKKPAD
FKLIGTPAKRLDSPEKVDGTAQFGLDVRLPGMLYAVIVNSPVFGGTVASIDDTAAKKIPGVRQVVRADNAVAVV
GDHTWAAKRGASALVVKWNEGAGAKVSTKDIVADLAQAAANGKGAVARKDGDVGKGFADAKTRVDAVYEQ
PLLAHATMEPVNCTVHVRADGCEIWVGTQVPTRAVDTVQQLTSFPPEKIVVHNHLLGGGFGRRLETDMIGQA
VKIAKQVNAPVKVIWTREEDIQHDMYRPYYYDRISAGLDANGKPIAWQHRIVGSSILARFAPPAFQHGVDPDA
VEVATDLPYDLPNQLIDYVRQEPRHVPTAFWRGVGPTRSTFVVESFIDELAAQTKTDPVQYRRALLGKTPRALN
VLDVATKAAGWGPSLPTGQGRGVSVMHAFGSFFSIVIDVAVDNGEVQVKRVVCAVDCGMSVNPNTIEAQVQ
GGIIFGITGALYGEITIEDGRVVQSNFTDYRMLRINETPPIEVHLVKSGEAPGGIGEPGTAATAAALSNAIFAATGK
RLRKLPVGDQLKTA
>SEQF4114||SEQF4114.1_07112
MSRGLIEAGKVAGQAAGAGVSRRSFLKLGMSLGAAAGGGLLLGFSLPAAGDDARRSVIGGDGDETARAGVFA
PNAFVQIDRAGKVTLVMPKVEMGQGVYTALPMLIAEELEVPLSSVTLDHAPPNEKLFLDPLLGGQLTGGSTSIRY
AWEPLRRAGATARTLLVAAAAKQWSVDPASCRAVNGEVQHPPSGRRTSYGQLADAAAKLPVPKDVALKKPAD
FKLIGTPAKRLDSPEKVDGTAQFGLDVRLPGMLYAVIVNSPVFGGTVASIDDTAAKKIPGVRQVVRADNAVAVV

GDHTWAAKRGASALVVKWNEGAGAKVSTKDIVADLAQAAANGKGAVARKDGDVGKGFADAKTRVDAVYEQ
PLLAHATMEPVNCTVHVRADGCEIWVGTQVPTRAVDTVQQLTSFPPEKIVVHNHLLGGGFGRRLETDMIGQA
VKIAKQVNAPVKVIWTREEDIQHDMYRPYYYDRISAGLDANGKPIAWQHRIVGSSILARFAPPAFQHGVDPDA
VEVATDLPYDLPNQLIDYVRQEPRHVPTAFWRGVGPTRSTFVVESFIDELAAQTKTDPVQYRRALLGKTPRALN
VLDVATKAAGWGPSLPTGQGRGVSVMHAFGSFFSIVIDVAVDNGEVQVKRVVCAVDCGMSVNPNTIEAQVQ
GGIIFGITGALYGEITIEDGRVVQSNFTDYRMLRINETPPIEVHLVKSGEAPGGIGEPGTAATAAALSNAIFAATGK
RLRKLPVGDQLKTA
>SEQF4114||SEQF4114.1_03049
MSAPELSVHNESRRALLLGFASGGLLLAFGVPSLVRAAVPNQPPVSADPRYGGAGMPHGLRDDPHLFVAIAPD
GTVTVTCIRSEMGQGVRTSVALVVADELGADWARVKVAQAVGDEPRYGNQNTDGSRSLRQSFAALRRAGAA
ARTMLEQAAAAAWGVDARQVKATVHEVVDTKSGRKLGFGELAAKAAALPAPDPATVPLKAPAEFRYIGKGETA
LIDGRDIVAGRAHYGIDTRLDGMLYAVVARPPAYGDTMASFDASAAEKLPGVVKVVPLASTPLPSGFQPLGGVA
VVARDTWTAIQARAQLKIDWKHGPNANYDSAAYRKTLEAAAAQPGDVIRNDGDAAAALAGAAKRVRATYYIP
HLAHATMEPPAAVARVADGRCEVWTCTQAPQTTRDEVAKALGLPSERVTVNVTLLGGGFGRKSKPDYVVEAA
LLSKAVGAPVKLTFTREDDLAHDYFHAVSLEAFDGGIDASGKVVAWQHRTVAPSIQSTFRAGVVHEQPGELAQ
GIADLPFAIPNVRIENPAAQAHTRIGWFRSVYNIPHAFGIQSFVSELAHAAGRDPKDFLLELIGPARRFEPHITVK
NVNYGEDPALYPVDTGRLRRVVETVAREAGWGRRLPKGHGLGIAAHRSFVSYTAAVCEVQVDADGKITVPRVD
IAIDCGPQVNPERVRSQLEGAVVMGLGIALHGEITFKDGHPEQRNFNGFQVLRMNEAPREIRVHLVAPDDFAT
PLGGVGEPGLPPVAPALTNAIFAATGTRIRSLPVADQLAKPRAG
>SEQF4114||SEQF4114.1_05549
MTIELDNTGSVRPSRRTFLKAAGAAAAVSLTIGFEWAGLGRRALAATAPAADFAPNAFLRITPDGAVTVIAKHVE
MGQGAYTGIATIVAEELDADWSTVRVESAPADAKRYANLAFGTMQGTGGSSAMANSWQQLREAGGKARA
MLVSAAAARWKVPAGELTTANGVVTHAKSGKKAAYGTLVADASKLPVPDKVVLKQPADFKLIGQRIPRVDASPK
SNGTAHFTLDTTFPGMRVALLQRPPRFGATVKSFDATAARAVPGVVSVVQVPGGVAVVGTGFWAAKQGRDAL
KVEWDEAHAEKRGSDEIMREYRQLADKPGTSARKDGDADAAIAGAARKIGATYEFPYLAHAPMEPLDAVVKLT
ADSCEIWAGDQFQTVDQANAARTAGLKPEQVRIHTLYAGGSFGRRANAWSDYVVEAVSIAKALGADGKPVKL
QWTREDDIQGGFYRPMYFHKLDAGLTEDGKLVGWRHRIVGQSILAGTPFEAFMVKNGIDATSVEGAANLPYA
VPNVSVELTTTKVGLPVLWWRVVGSSHTAYAVEAFIDEAAHTAGKDPYAFRRDLLAKEPRMRAVLDLAAQKAG
WDPAKPLPKGRGRGIAVAEAFKSYVAQVAEVSVDADGKVKVERVVCAVDCGIAINPDIVAAQMEGGIGFGLGA
VMHSAITLKDGQVEQRNFDGYHVLRIAEMPKVEVHIVPSAEAPTGVGEPGVAPVGPAVANAIFAATGKRHYVL
PFDSADSAKA
>SEQF4115||SEQF4115.1_00266
MSAPELSVHNESRRALLLGFASGGLLLAFGVPSLVRAAAPVQPPVSADPRYGGAGMPHGLRDDPHLFVAIAPD
GTVTVTCIRSEMGQGVRTSVALVVADELGADWARVKVAQAVGDEPRYGNQNTDGSRSLRQSFAALRRAGAA
ARTMLEQAAAAAWGVDARQVKATVHEVVDTKSGRKLGFGELAAKAAALPAPDPATVPLKAPAEFRYIGKGQT
ALIDGRDIVAGRAHYGIDTRLDGMLYAVVARPPAYGDTVASFDASAAEKLPGVVKVVPLAPTPLPSGFQPLGGV
AVVARDTWTAIQARAQLKIDWKHGPNANYDSAAYRKTLEAAAAQPGDVIRNDGDAAAALAGAAKRVRATYYI
PHLAHATMEPPAAVARVADGRCEVWTCTQAPQTTRDEVAKALGLPGERVTVNVTLLGGGFGRKSKPDYVVEA
ALLSKAVGAPVKLTFTREDDLAHDYFHAVSLEAFDGGIDASGKVVAWQHRTVAPSIQSTFRAGIVHEQPGELAQ
GIADLPFAIPNVRIENPAAQAHTRIGWFRSVYNIPHAFGIQSFVSELAHAAGRDPKDFLLELIGPARRFEPHITVK
NVNYGEDPALYPVDTGRLRRVVETVAREAGWGRKLPKGHGLGIAAHRSFVSYTAAVCEVQVDADGKITVPRVD
IAIDCGPQVNPERVRSQLEGAVVMGLGIALHGEITFKDGHPEQSNFNGFQVLRMNEAPREIRVHLVAPDDFAT
PLGGVGEPGLPPVAPALTNAIFAATGTRIRSLPVADQLAKPRAG
>SEQF4115||SEQF4115.1_03292

MTIELDNTGSVRPSRRTFLKAAGAAAAVSLTIGFEWAGLGRRALAATAPAADFAPNAFLRITPDGAVTVIAKHVE
MGQGAYTGIATIVAEELDADWSTVRVESAPADAKRYANLAFGTMQGTGGSSAMANSWQQLREAGGKARA
MLVSAAAARWKVPAGELTTANGVVTHAKSGKKAAYGTLVADASKLPVPDKVVLKQPADFKLIGQRIPRVDASPK
SNGTAHFTLDTTFPGMRVALLQRPPRFGATVKSFDATAAKAVPGVVSVVQVPGGVAVVGTGFWAAKQGRDAL
KVEWDEAHAEKRSSDEIMREYRQLADKPGTSARKDGDADAAIAGAARKIGATYEFPYLAHAPMEPLDAVVKLT
ADSCEIWAGDQFQTVDQANAARTAGLKPEQVQIHTLYAGGSFGRRANAWSDYVVEAVSIAKALGADGKPVKL
QWTREDDIQGGFYRPMYFHKLDAGLTADGKLVGWRHRIVGQSILAGTPFEAFMVKNGIDATSVEGAANLPYA
VPNVSVELTTTKVGLPVLWWRVVGSSHTAYAVEAFIDEAAHTAGKDPYAFRRDLLAKEPRMRAVLDLAAQKAG
WDPAKPLPKGRGRGIAVAEAFKSYVAQVAEVSVDADGKVKVERVVCAVDCGIAINPDIVAAQMEGGIGFGLGA
VLHSAITLKDGQVEQRNFDGYHVLRIAEMPKVEVHIVPSAEAPTGVGEPGVAPVGPAVANAIFAATGRRHYVLP
FDSADSAKA
>SEQF4115||SEQF4115.1_08037
MSRGLIEAGKVAGQAAGAGVSRRSFLKLGMSLGAAAGGGLLLGFSLPAAGDDARRSVIGGDGDETARAGVFA
PNAFVQIDRAGKVTLVMPKVEMGQGVYTALPMLIAEELEVPLSSVTLDHAPPNEKLFLDPLLGGQLTGGSTSVR
YAWEPLRRAGATARTLLVAAAAKQWNVDPASCRAVNGEVQHPPSGRRVSYGQLADAAAKLPVPKDVALKKPA
DFKLIGTPAKRLDSPEKVDGTAQFGLDVRLPGMLYAVIVNSPVFGGTVASIDDTAAKKIPGVRQVVRADNAVAV
VGDHTWAAKRGASALVVKWNEGAGAKVSTKDIVADLAQAAANGKGAVARKDGDVGKGFADAKTRIDAVYE
QPLLAHATMEPVNCTVHVRADGCEIWVGTQVPTRAVDTVQKLTSFPPEKIVVHNHLLGGGFGRRLETDMIGQ
AVKIAKQVNAPVKVIWTREEDIQHDMYRPYYYDRISAGLDANGKPIAWQHRIVGSSILARFAPPAFQHGVDPD
AVEVATDLPYDLPNQLIDYVRQEPRHVPTAFWRGVGPTRSTFVVESFIDELAAQTKTDPVQYRRALLGKTPRAL
NVLDVATKAAGWGPSLPTGQGRGVSVMHAFGSFFSIVIDVAVDNGEVQVKRVVCAVDCGMSVNPNTIEAQV
QGGIIFGITGALYGEITIEDGRVVQSNFTDYRMLRINETPPIEVHLVKSGEAPGGIGEPGTAATAAALSNAIFAATG
KRLRKLPVGDQLKTA
>SEQF4116||SEQF4116.1_01263
MSRGLIEAGKVAGQAAGAGVSRRSFLKLGMSLGAAAGGGLLLGFSLPAAGDDARRSVIGGDGDETARAGVFA
PNAFVQIDRAGKVTLVMPKVEMGQGVYTALPMLIAEELEVPLSSVTLDHAPPNEELFLDPLLGGQLTGGSTSIRY
AWEPLRRAGATARTLLVAAAAKQWNVDPASCRAVNGEVQHPPSGRRTSYGQLADAAAKLPVPKDVALKKPAD
FKLIGTPAKRLDSPEKVDGTAQFGLDVRLPGMLYAVIVNSPVFGGTVASIDDTATKKIPGVRQVVRADNAVAVVG
DHTWAAKRGASALVVKWNEGAGAKVSTKDIVADLAQAAANGKGAVARKDGDVGKGFADAKTRVDAVYEQP
LLAHATMEPVNCTVHVRADGCEIWVGTQVPTRAVDTVQQLTSFPPEKIVVHNHLLGGGFGRRLETDMIGQAV
KIAKQVNAPVKVIWTREEDIQHDMYRPYYYDRISAGLDANGKPIAWQHRIVGSSILARFAPPAFQHGVDPDAV
EVATDLPYDLPNQLIDYVRQEPRHVPTAFWRGVGPTRSTFVVESFIDELAAQTKTDPVQYRRALLGKTPRALNVL
DVATKAAGWGPSLPTGQGRGVSVMHAFGSFFSIVIDVAVDNGEVQVKRVVCAVDCGMSVNPNTIEAQVQG
GIIFGITGALYGEITIEDGRVVQSNFTDYRMLRINETPPIEVHLVKSGEAPGGIGEPGTAATAAALSNAIFAATGKRL
RKLPVGDQLKTA
>SEQF4116||SEQF4116.1_04057
MTIELDNTGSVRPSRRTFLKAAGAAAAVSLTIGFEWAGLGRRALAATAPAADFAPNAFLRITPDGAVTVIAKHVE
MGQGAYTGIATIVAEELDADWSTVRVESAPADAKRYANLAFGTMQGTGGSSAMANSWQQLREAGGKARA
MLVSAAAARWKVPAGELTTANGVVTHAKSGKKAAYGTLVADASKLPVPDKVVLKQPADFKLIGHRIPRVDASPK
SNGTAHFTLDTTFPGMRVALLQRPPRFGATVKSFDATAARAVPGVVSVVQVPGGIAVVGTGFWAAKQGRDAL
KVEWDEAHAEKRGSDEIMREYRQLADKPGTSARKDGDADAAIAGAARKIGATYEFPYLAHAPMEPLDAVVKLT
ADSCEIWAGDQFQTVDQANAARTAGLKPEQVQIHTLYAGGSFGRRANAWSDYVVEAVSIAKALGADGKPVKL
QWTREDDIQGGFYRPMYFHKLDAGLTEDGKLVGWRHRIVGQSILAGTPFEAFMVKNGIDATSVEGAANLPYA
VPNVSVELTTTKVGLPVLWWRVVGSSHTAYAVEAFIDEAAHTAGKDPYAFRRDLLEKEPRMRAVLDLAAQKAG

WDPAKPLPKGRGRGIAVAEAFKSYVAQVAEVSVDADGKVKVERVVCAVDCGIAINPDIVAAQMEGGIGFGLGA
VMHSAITLKDGQVEQRNFDGYHVLRIAEMPKVEVHIVPSAEAPTGVGEPGVAPVGPAVANAIFAATGKRHYVL
PFDSADSAKA
>SEQF4116||SEQF4116.1_04242
MSAPNLSVHNESRRALLLGFASGGLLLAFGVPSLVRAAVPNQPPVSADPRYGGAGMPHGLRDDPHLFVAIAPD
GTVTVTCIRSEMGQGVRTSVALVVADELGADWARVKVAQAFGDEPRYGNQNTDGSRSLRQSFAALRRAGAA
ARTMLEQAAAAAWGVDARQVKATVHEVVDTKSGRKLGFGELAAKAAALPAPDPATVPLKAPAEFRYIGKGETT
LIDGRDIVAGRAHYGIDTRLDGMLYAVVARPPAYGDTMASFDASAAEKLPGVVKVVPLAPTPLPSGFQPLGGVA
VVARDTWTAIQARAQLKIDWKHGPNANYDSAAYRKTLEAAAAQPGDVIRNDGDAAAALAGAAKRVRATYYIP
HLAHATMEPPAAVARVADGRCEVWTCTQAPQTTRDEVAKALGLPGERVTVNVTLLGGGFGRKSKPDYVVEAA
LLSKAVGAPVKLTFTREDDLAHDYFHAVSLEAFDGGIDASGKVVAWQHRTVAPSIQSTFRAGVVHEQPGELAQ
GIADLPFAIPNVRIENPAAQAHTRIGWFRSVYNIPHAFGIQSFVSELAHAAGRDPKDFLLELIGPARRFEPHITVK
NVNYGEDPALYPVDTGRLRRVVETVAREAGWGRRLPKGHGLGIAAHRSFVSYTAAVCEVQVDADGKITVPRVD
IAIDCGPQVNPERVRSQLEGAVVMGLGIALHGEITFKDGHPEQSNFNGFQVLRMNEAPREIRVHLVAPDDFAT
PLGGVGEPGLPPVAPALTNAIFAATGTRIRSLPVADQLAKPRAG
>SEQF4117||SEQF4117.1_06858
MSRGLIEAGKVAGQAAGAGVSRRSFLKLGMSLGAAAGGGLLLGFSLPAAGDDARRSVIGGDGDETARAGVFA
PNAFVQIDRAGKVTLVMPKVEMGQGVYTALPMLIAEELEVPLSSVTLDHAPPNEKLFLDPLLGGQLTGGSTSIRY
AWEPLRRAGATARTLLVAAAAKQWSVDPASCRAVNGEVQHPPSGRRTSYGQLADAAAKLPVPKDVALKKPAD
FKLIGTPAKRLDSPEKVDGTAQFGLDVRLPGMLYAVIVNSPVFGGTVASIDDTAAKKIPGVRQVVRADNAVAVV
GDHTWAAKRGASALVVKWNEGAGAKVSTKDIVADLAQAAANGKGAVARKDGDVGKGFADAKTRVDAVYEQ
PLLAHATMEPVNCTVHVRADGCEIWVGTQVPTRAVDTVQQLTSFPPEKIVVHNHLLGGGFGRRLETDMIGQA
VKIAKQVNAPVKVIWTREEDIQHDMYRPYYYDRISAGLDANGKPIAWQHRIVGSSILARFAPPAFQHGVDPDA
VEVATDLPYDLPNQLIDYVRQEPRHVPTAFWRGVGPTRSTFVVESFIDELAAQTKTDPVQYRRALLGKTPRALN
VLDVATKAAGWGPSLPTGQGRGVSVMHAFGSFFSIVIDVAVDNGEVQVKRVVCAVDCGMSVNPNTIEAQVQ
GGIIFGITGALYGEITIEDGRVVQSNFTDYRMLRINETPPIEVHLVKSGEAPGGIGEPGTAATAAALSNAIFAATGK
RLRKLPVGDQLKTA
>SEQF4117||SEQF4117.1_05754
MTIELDNTGSVRPSRRTFLKAAGAAAAVSLTIGFEWAGLGRRALAATAPAADFAPNAFLRITPDGAVTVIAKHVE
MGQGAYTGIATIVAEELDADWSTVRVESAPADAKRYANLAFGTMQGTGGSSAMANSWQQLREAGGKARA
MLVSAAAARWKVPAGELTTANGVVTHAKSGKKAAYGTLVADASKLPVPDKVVLKQPADFKLIGQRIPRVDASPK
SNGTAHFTLDTTFPGMRVALLQRPPRFGATVKSFDATAARAVPGVVSVVQVPGGVAVVGTGFWAAKQGRDAL
KVEWDEAHAEKRGSDEIMREYRQLADKPGTSARKDGDADAAIAGAARKIGATYEFPYLAHAPMEPLDAVVKLT
ADSCEIWAGDQFQTVDQANAARTAGLKPEQVRIHTLYAGGSFGRRANAWSDYVVEAVSIAKALGADGKPVKL
QWTREDDIQGGFYRPMYFHKLDAGLTEDGKLVGWRHRIVGQSILAGTPFEAFMVKNGIDATSVEGAANLPYA
VPNVSVELTTTKVGLPVLWWRVVGSSHTAYAVEAFIDEAAHTAGKDPYAFRRDLLAKEPRMRAVLDLAAQKAG
WDPAKPLPKGRGRGIAVAEAFKSYVAQVAEVSVDADGKVKVERVVCAVDCGIAINPDIVAAQMEGGIGFGLGA
VMHSAITLKDGQVEQRNFDGYHVLRIAEMPKVEVHIVPSAEAPTGVGEPGVAPVGPAVANAIFAATGKRHYVL
PFDSADSAKA
>SEQF4117||SEQF4117.1_01573
MSAPELSVHNESRRALLLGFASGGLLLAFGVPSLVRAAVPNQPPVSADPRYGGAGMPHGLRDDPHLFVAIAPD
GTVTVTCIRSEMGQGVRTSVALVVADELGADWARVKVAQAVGDEPRYGNQNTDGSRSLRQSFAALRRAGAA
ARTMLEQAAAAAWGVDARQVKATVHEVVDTKSGRKLGFGELAAKAAALPAPDPATVPLKAPAEFRYIGKGETA
LIDGRDIVAGRAHYGIDTRLDGMLYAVVARPPAYGDTMASFDASAAEKLPGVVKVVPLASTPLPSGFQPLGGVA

VVARDTWTAIQARAQLKIDWKHGPNANYDSAAYRKTLEAAAAQPGDVIRNDGDAAAALAGAAKRVRATYYIP
HLAHATMEPPAAVARVADGRCEVWTCTQAPQTTRDEVAKALGLPSERVTVNVTLLGGGFGRKSKPDYVVEAA
LLSKAVGAPVKLTFTREDDLAHDYFHAVSLEAFDGGIDASGKVVAWQHRTVAPSIQSTFRAGVVHEQPGELAQ
GIADLPFAIPNVRIENPAAQAHTRIGWFRSVYNIPHAFGIQSFVSELAHAAGRDPKDFLLELIGPARRFEPHITVK
NVNYGEDPALYPVDTGRLRRVVETVAREAGWGRRLPKGHGLGIAAHRSFVSYTAAVCEVQVDADGKITVPRVD
IAIDCGPQVNPERVRSQLEGAVVMGLGIALHGEITFKDGHPEQRNFNGFQVLRMNEAPREIRVHLVAPDDFAT
PLGGVGEPGLPPVAPALTNAIFAATGTRIRSLPVADQLAKPRAG
>SEQF4982||SEQF4982.1_00124
MKRRTFLLSAAGTGGALVVGWGLLPPRSRLGSRNSLPVRDGQVGLNGWIKIDRRGRVILAMHRSEMGQGVH
TALPMLVAEELDVPLDQVLLEPAGPESIYGNVAMFVGSLPIHPAEREPDHGSAAVRLTEWMVTKLAREMGINVT
GGSSSVADAWDVLRTAAATARAQLLGAAALQWRLPVDELRIIDGVISHASGPSAHFGELARRAAALSVSEVRVK
PPQTWRLIGRSAPRTDVPAKVDGSARYGIDVRLPGMRYAAVRMCPMLGGSPGRIGVDEALRRPGVERVVRLG
PIAGSTAGVAVVARTYWHALQAVDAMPVEWRAPPHDPQRGLLDSSAIAAALEQAARQALAEDGGHAFHRR
GDVARAEQGAARVVEQVYHAPYLAHATMEPINCTARVQDGQVEVWAPTQVPTLARQVAARVAGVPPERVT
VHVTLVGGGFGRRLEVDHVGQAVRVAMETGGKPVQLIWPREEDLMHDFYRPAGAAVLRAALDAQGLPVALR
IASAGDAITPRWLERGLPAFAGPVDLPDKTASEGLFDLPYQVPHQRIAHAATHSGVPVGYWRSVGHSHNAFFSE
SFIDELAFEARQDPVAYRLALLRDKPRHQAVLRLAAERAGWDRPPPPGRARGVALHESFGSIVAEVVEVSARQG
RPQVHRVVCAIDCGAVVNPGIVAQQMESGVIFGLSAALHGRIDIRGGIVQQRNYPDHPVLTLADTPVIETHIVR
SQRAPGGVGEPGTPPVAPALANAWFALTGQRLRRLPLLGAASAPHPGN
>SEQF4982||SEQF4982.1_01314
MSTTTLENPARRRVLQAGAGLTLALYLPVHAARAAAGGQEAATPFVPNAFLRIGEDNRVTVIAKHLEMGQGSY
TGLATIVAEELDAAWSQVQVEGAPADARRYNNLGWGPVQGTGGSTAIANSWQQLREAGATARAMLVAAAA
AQWGVPAGEVTVSEGEVIHAASGRKASFGQLARAAAQQPVPQQVQLKDPKDFRLIGKRVPRVDGAGKTDGS
ARFTQDVQLPGMLVAVVAHPPRFGATLKSVDDRKARAVRGVVDVVRIPSGVAVLARDTWSAKKGRDALEIEW
DDSRAFRLGSAEILARYRELAATPGVVARREGDPDRALANAARTLEAAYDFPYLAHAAMEPMNCVIRLDADGC
EVWNGEQFQTVDQAAVAQVLGLRPEQVRLHMLYAGGSFGRRASKTSDYLVEAAHIVKAIGGRAPVKLVWLRE
DDMRAGYYRPAFHHRLWAGLDARGRLVGWRHRLVGQSILAGSPFEPMMVKDGIDPVSVEGAANLPYAIPNL
VVDLHSPQDIGVPVLWWRSVGSTHTAFSTECFIDELAQAAGQDPVAWRLALLAQHSRHAGVLRLAAERAGW
GRPLAPGAAGERRGRGVAVHESFHSYVAQVAEVTVREDGSYRVDRVVCAVDCGIAVNPDVIRAQVEGSIGFAL
STVLHGEITLRDGEVQQSNFHDYLVARITDMPQVEVHIVPSAANPTGIGEPAVPPLAPAVANALAAATGRRLRQ
LPIRPELLRV
>SEQF5378||SEQF5378.1_03910
MTTTMQVSRRGFLKGGLGALTLAVTGNGLVSAVWAADEPKKYGADSMPGGTVDDPLAFVSIAADGTVTIVAH
RAEMGTGVRTSLPMVVADEMEAAWDRVRVVQADADEARYGNQNVDGSRSVRHFLMPMRRVGAAARQM
LEAAAAARWSVPLAEVRATQHEVVHAPTGRRLGYGELAADAAKLPVPAGDAVKLKTRAEFRYIGKDEVRLVDLE
AIGKGEAMYGIDMRLPGMVYAVVARPPVVGGKLRRVDSAKALAVPGVLKVVEIPAMAGAPAFQPLGGVAVVA
SNTWAAMQGRAALAIEWDDGPNAAYDSVAYRETLTEASRKPGKVVRDQGDAPQAWAKAGEAERFMAEYH
VPHLAHASMETPVATVRIQDGAAEVWTSVQNPAAAQEAVAKRLKLKPENVEVHVLLLGGGFGRKSKPDYVDE
AAIVAQAMPAGTPVKLVWTREDDIHHDYLHTVSAEHLEAVVGKDGKVQSWLHRSAAPTIASLFTEGAKGEQLF
ESAMSAINMPYVIPNVRVETAEVAAHARIGWFRSVANIPHAFAAQCFIAELAHRAGQDHKQYALDLIGPARRID
PGTLADTWNYSESPERYPYDTGRLRGVIEAAASGAKWGRELPKGHGLGLAFCYSFMSYTATVVEVAVDEKGEV
RVVAVDMALDCGPQIKPERIRAQMEGGAIMGLSLALLGEITFEKGRVKQNNFYDYEVLRHNASPRVIRTHLVN
DDHALPPGGVGEPPVPPVAPALCNAIFAATGKRVRSLPVRSVA
>SEQF5379||SEQF5379.1_04209

MTTTMQVSRRGFLKGGLGALTLAVTGNGLVSAVWAADEPKKYGADSMPGGTVDDPLAFVSIAADGTVTIVAH
RAEMGTGVRTSLPMVVADEMEAAWDRVRVVQADADEARYGNQNVDGSRSVRHFLMPMRRVGAAARQM
LEAAAAARWSVPLAEVRATQHEVVHAPSGRRLGYGELAADAAKLPVPAGDAVKLKTRAEFRYIGKDEVRLVDLE
AIGKGEAMYGMDMRLPGMVYAVVARPPVVGGKLRRVDSAKALAVPGVLKVVEIPAMAGAPAFQPLGGVAVV
ASNTWAAMQGRAALAIEWDDGPNAAYDSVAYRETLTEASRKPGKVVRDQGDAPQAWAKAGETERFMAEY
HVPHLAHASMETPVATVRIQDGAAEVWTSVQNPAAAQEAVAKRLKLKPENVKVHVLLLGGGFGRKSKPDYVD
EAAIVAQAMPAGTPVKLVWTREDDIHHDYLHTVSAEHLEAVVGKDGKVQSWLHRSAAPTIASLFTEGAKGEQL
FESAMSAINMPYVIPNVRVETAEVAAHARIGWFRSVANIPHAFAAQCFIAELAHRAGRDHKQYALDLIGPARRI
DPGTLADTWNYSESPERYPYDTGRLRGVIEAAASGAKWGRELPKGHGLGLAFCYSFMSYTATVVEVAVDEKGE
VRVVAVDMALDCGPQIKPERIRAQMEGGAIMGLSLALLGEITFEKGRVKQNNFYDYEVLRHNASPRVIRTHLV
NDDHALPPGGVGEPPVPPVAPALCNAIFAATGKRVRSLPVRSVA
>SEQF5380||SEQF5380.1_01369
MTTTMQVSRRGFLKGGLGALTLAVTGNGLVSAVWAADEPKKYGADSMPGGTVDDPLAFVSIAADGTVTIVAH
RAEMGTGVRTSLPMVVADEMEAAWDRVRVVQADADEARYGNQNVDGSRSVRHFLMPMRRVGAAARQM
LEAAAAARWSVPLAEVRATQHEVVHAPSGRRLGYGELAADAAKLPVPAGDAVKLKTRAEFRYIGKDEVRLVDLE
AIGKGEAMYGMDMRLPGMVYAVVARPPVVGGKLRRVDSAKALAVPGVLKVVEIPAMAGAPAFQPLGGVAVV
ASNTWAAMQGRAALAIEWDDGPNAAYDSVAYRETLTEASRKPGKVVRDQGDAPQAWAKAGETERFMAEY
HVPHLAHASMETPVATVRIQDGAAEVWTSVQNPAAAQEAVAKRLKLKPENVKVHVLLLGGGFGRKSKPDYVD
EAAIVAQAMPAGTPVKLVWTREDDIHHDYLHTVSAEHLEAVVGKDGKVQSWLHRSAAPTIASLFTEGAKGEQL
FESAMSAINMPYVIPNVRVETAEVAAHARIGWFRSVANIPHAFAAQCFIAELAHRAGRDHKQYALDLIGPARRI
DPGTLADTWNYSESPERYPYDTGRLRGVIEAAASGAKWGRELPKGHGLGLAFCYSFMSYTATVVEVAVDEKGE
VRVVAVDMALDCGPQIKPERIRAQMEGGAIMGLSLALLGEITFEKGRVKQNNFYDYEVLRHNASPRVIRTHLV
NDDHALPPGGVGEPPVPPVAPALCNAIFAATGKRVRSLPVRSVA
>SEQF5381||SEQF5381.1_01647
MTTTMQVSRRGFLKGGLGALTLAVTGNGLVSAVWAADEPKKYGADSMPGGTVDDPLAFVSIAADGTVTIVAH
RAEMGTGVRTSLPMVVADEMEAAWDRVRVVQADADEARYGNQNVDGSRSVRHFLMPMRRVGAAARQM
LEAAAAARWSVPLAEVRATQHEVVHAPSGRRLGYGELAADAAKLPVPAGDAVKLKTRAEFRYIGKDEVRLVDLE
AIGKGEAMYGMDMRLPGMVYAVVARPPVVGGKLRRVDSAKALAVPGVLKVVEIPAMAGAPAFQPLGGVAVV
ASNTWAAMQGRAALAIEWDDGPNAAYDSVAYRETLTEASRKPGKVVRDQGDAPQAWAKAGETERFMAEY
HVPHLAHASMETPVATVRIQDGAAEVWTSVQNPAAAQEAVAKRLKLKPENVKVHVLLLGGGFGRKSKPDYVD
EAAIVAQAMPAGTPVKLVWTREDDIHHDYLHTVSAEHLEAVVGKDGKVQSWLHRSAAPTIASLFTEGAKGEQL
FESAMSAINMPYVIPNVRVETAEVAAHARIGWFRSVANIPHAFAAQCFIAELAHRAGRDHKQYALDLIGPARRI
DPGTLADTWNYSESPERYPYDTGRLRGVIEAAASGAKWGRELPKGHGLGLAFCYSFMSYTATVVEVAVDEKGE
VRVVAVDMALDCGPQIKPERIRAQMEGGAIMGLSLALLGEITFEKGRVKQNNFYDYEVLRHNASPRVIRTHLV
NDDHALPPGGVGEPPVPPVAPALCNAIFAATGKRVRSLPVRSVA
>SEQF5382||SEQF5382.1_05163
MTAAAQLSRRAFLQGSLGVLTLAVTARGWVTTAVAAEPAAKAYGADSMPGGTVDDPLVFVSIAADGAVTIVAH
RAEMGTGVRTSLPMVVADEMEARWERVKVVQAPGDEARYGNQNVDGSRSMRHFLMPMRRVGAAARQM
LEAAAAARWAVPVAEVKAEQHEVLHPPTGRRLSYGDLAADAAKQPVPAGDALKLKDRSAFRYIGKDQVRLVDL
EAIGKGQATYGMDMRLPGMVYAVVARPPVVGGRLRRFDSAKALAVPGVLKVVEIPPMQGAPAFQPLGGVAV
VARNTWAARQGRDALEIEWDDGPNGSYDSAAYRQTLQAAARAPGKTMRNQGDAANAWAKAPEAERVAAE
YYVPHLAHASMEPPVATVQIKGNSAEVWTSVQNPAAAKSAVAARLKLEPANVKVNVLLLGGGFGRKSKPDFVD
EAAIVARAMPDGTPVKLVWTREDDIHHDYLHTVSVERLEAVMDAQGQVQSWLHRSAAPTIASLFAQGAKGQ
QMFESAMSAINMPYRIPNVRVETAEVDAHARIGWFRSVANIPHAFAAQCFIDELAHRAGKDPQAFALDLIGPA

RQIDPGTMADTWNYTESPERYPYDTGRLRGVIEAACKGAGWGRTLPKGHGLGLAFCYSFMSYTATVVEVAVD
DKGEVRVVAVDMAMDCGPQINPERIRAQMEGGAIMGLGLALTSEITFEKGRVKQSNFHDYEVLRHNASPRMI
RTHLVNDDHALPPGGVGEPPVPPVAPALCNAIFAATGKRIRSLPVRKVA
>SEQF5384||SEQF5384.1_00805
MTTTMQVSRRGFLKGGLGALTLAVTGNGLVSAVWAADEPKKYGADSMPGGTVDDPLAFVSIAADGTVTIVAH
RAEMGTGVRTSLPIVVADEMEAAWDRVRVVQADADEARYGNQNVDGSRSVRHFLMPMRRVGAAARQML
EAAAAARWSVPLAEVRATQHEVVHAPSGRRLGYGELAADAAKLPVPAGDAVKLKTRAEFRYIGKDEVRLVDLE
AIGKGEAMYGIDMRLPGMVYAVVARPPVVGGKLRRVDSAKALAVPGVLKVVEIPAMAGAPAFQPLGGVAVVA
SNTWAAMQGRAALAIEWDDGPNAAYDSVAYRETLTEASRKPGKVVRDQGDAPQAWAKAGEAERFMAEYH
VPHLAHASMETPVATVRIQDGAAEVWTSVQNPAAAQEAVAKRLKLKPENVKVHVLLLGGGFGRKSKPDYVDE
AAIVAQAMPAGTPVKLVWTREDDIHHDYLHTVSAEHLEAVVGKDGKVQSWLHRSAAPTIASLFTEGAKGEQLF
ESAMSAINMPYVIPNVRVETAEVAAHARIGWFRSVANIPHAFAAQCFIAELAHRAGQDHKQYALDLIGPARRID
PGTLADTWNYSESPERYPYDTGRLRGVIEAAASGAKWGRELPKGHGLGLAFCYSFMSYTATVVEVAVDEKGEV
RVVAVDMALDCGPQIKPERIRAQMEGGAIMGLSLALLGEITFEKGRVKQNNFYDYEVLRHNASPRVIRTHLVN
DDHALPPGGVGEPPVPPVAPALCNAIFAATGKRVRSLPVRSVA
>SEQF5385||SEQF5385.1_03119
MTTTMQVSRRGFLKGGLGALTLAVTGNGLVSAVWAADEPKKYGADSMPGGTVDDPLAFVSIAADGTVTIVAH
RAEMGTGVRTSLPMVVADEMEAAWDRVRVVQADADEARYGNQNVDGSRSVRHFLMPMRRVGAAARQM
LEAAAAARWSVPLAEVRATQHEVVHAPSGRRLGYGELAADAAKLPVPAGDAVKLKTRAEFRYIGKDEVRLVDLE
AIGKGEAMYGMDMRLPGMVYAVVARPPVVGGKLRRVDSAKALAVPGVLKVVEIPAMAGAPAFQPLGGVAVV
ASNTWAAMQGRAALAIEWDDGPNAAYDSVAYRETLTEASRKPGKVVRDQGDAPQAWAKAGETERFMAEY
HVPHLAHASMETPVATVRIQDGAAEVWTSVQNPAAAQEAVAKRLKLKPENVKVHVLLLGGGFGRKSKPDYVD
EAAIVAQAMPAGTPVKLVWTREDDIHHDYLHTVSAEHLEAVVGKDGKVQSWLHRSAAPTIASLFTEGAKGEQL
FESAMSAINMPYVIPNVRVETAEVAAHARIGWFRSVANIPHAFAAQCFIAELAHRAGRDHKQYALDLIGPARRI
DPGTLADTWNYSESPERYPYDTGRLRGVIEAAASGAKWGRELPKGHGLGLAFCYSFMSYTATVVEVAVDEKGE
VRVVAVDMALDCGPQIKPERIRAQMEGGAIMGLSLALLGEITFEKGRVKQNNFYDYEVLRHNASPRVIRTHLV
NDDHALPPGGVGEPPVPPVAPALCNAIFAATGKRVRSLPVRSVA
>SEQF5386||SEQF5386.1_00078
MTTTMQVSRRGFLKGGLGALTLAVTGNGLVSAVWAADEPKKYGADSMPGGTVDDPLAFVSIAADGTVTIVAH
RAEMGTGVRTSLPMVVADEMEAAWDRVRVVQADADEARYGNQNVDGSRSVRHFLMPMRRVGAAARQM
LEAAAAARWSVPLAEVRATQHAVVHAPSGRRLGYGELAADAAKLPVPAGDAVKLKTRAEFRYIGKDEVRLVDLE
AIGKGEAMYGIDMRLPGMVYAVVARPPVVGGKLRRVDSAKALAVPGVLKVVEIPAMAGAPAFQPLGGVAVVA
SNTWAAMQGRAALAIEWDDGPNAAYDSVAYRETLTEASRKPGKVVRDQGDAPQAWAKAGEAERFMAEYH
VPHLAHASMETPVATVRIQDGAAEVWTSVQNPAAAQEAVAKRLKLKPENVEVHVLLLGGGFGRKSKPDYVDE
AAIVAQAMPAGTPVKLVWTREDDIHHDYLHTVSAEHLEAVVGKDGKVQSWLHRSAAPTIASLFTEGAKGEQLF
ESAMSAINMPYVIPNVRVETAEVAAHARIGWFRSVANIPHAFAAQCFIAELAHRAGQDHKQYALDLIGPARRID
PGTLADTWNYSESPERYPYDTGRLRGVIEAAASGAKWGRELPKGHGLGLAFCYSFMSYTATVVEVAVDEKGEV
RVVAVDMALDCGPQIKPERIRAQMEGGAIMGLSLALLGEITFEKGRVKQNNFYDYEVLRHNASPRVIRTHLVN
DDHALPPGGVGEPPVPPVAPALCNAIFAATGKRVRSLPVRSVA
>SEQF5387||SEQF5387.1_05389
MTTTMQVSRRGFLKGGLGALTLAVSGNGLVSAVWAADEPKKYGADSMPGGTVDDPLAFVSIAADGTVTIVAH
RAEMGTGVRTSLPMVVADEMEAAWDRVRVVQADADEARYGNQNVDGSRSVRHFLMPMRRVGAAARQM
LEAAAAARWSVPLAEVRATQHEVVHAPSGRRLGYGELAADAAKLPVPAGDAVKLKTRAEFRYIGKDEVRLVDLE
AIGKGEAMYGMDMRLPGMVYAVVARPPVVGGKLRRVDSAKALAVPGVLKVVEIPAMAGAPAFQPLGGVAVV

ASNTWAAMQGRAALAIEWDDGPNAAYDSVAYRETLTEASRKPGKVVRDQGDAPQAWAKAGETERFMAEY
HVPHLAHASMETPVATVRIQDGAAEVWTSVQNPAAAQEAVAKRLKLKPENVKVHVLLLGGGFGRKSKPDYVD
EAAIVAQAMPAGTPVKLVWTREDDIHHDYLHTVSAEHLEAVVGKDGKVQSWLHRSAAPTIASLFTEGAKGEQL
FESAMSAINMPYVIPNVRVETAEVAAHARIGWFRSVANIPHAFAAQCFIAELAHRAGQDHKQYALDLIGPARRI
DPGTLADTWNYSESPERYPYDTGRLRGVIEAAASGAKWGRELPKGHGLGLAFCYSFMSYTATVVEVAVDEKGE
VRVVAVDMALDCGPQIKPERIRAQMEGGAIMGLSLALLGEITFEKGRVKQNNFYDYEVLRHNASPRVIRTHLV
NDDHALPPGGVGEPPVPPVAPALCNAIFAATGKRVRSLPVRSVA
>SEQF5388||SEQF5388.2_00721
MTTTMQVSRRGFLKGGLGALTLAVTGNGLVSAVWAADEPKKYGADSMPGGTVDDPLAFVSIAADGTVTIVAH
RAEMGTGVRTSLPMVVADEMEAAWDRVRVVQADADEARYGNQNVDGSRSVRHFLMPMRRVGAAARQM
LEAAAAARWSVPLAEVRATQHEVVHAPSGRRLGYGELAADAAKLPVPAGDAVKLKTRAEFRYIGKDEVRLVDLE
AIGKGEAMYGIDMRLPGMVYAVVARPPVVGGKLRRVDSAKALAVPGVLKVVEIPAMAGAPAFQPLGGVAVVA
SNTWAAMQGRAALAIEWDDGPNAAYDSVAYRETLTEASRKPGKVVRDQGDAPQAWAKAGEAERFMAEYH
VPHLAHASMETPVATVRIQDGAAEVWTSVQNPAAAQEAVAKRLKLKPENVKVHVLLLGGGFGRKSKPDYVDE
AAIVAQAMPAGTPVKLVWTREDDIHHDYLHTVSAEHLEAVVGKDGKVQSWLHRSAAPTIASLFTEGAKGEQLF
ESAMSAINMPYVIPNVRVETAEVAAHARIGWFRSVANIPHAFAAQCFIAELAHRAGQDHKQYALDLIGPARRID
PGTLADTWNYSESPERYPYDTGRLRGVIEAAASGAKWGRELPKGHGLGLAFCYSFMSYTATVVEVAVDEKGEV
RVVAVDMALDCGPQIKPERIRAQMEGGAIMGLSLALLGEITFEKGRVKQNNFYDYEVLRHNASPRVIRTHLVN
DDHALPPGGVGEPPVPPVAPALCNAIFAATGKRVRSLPVRSVA
>SEQF5389||SEQF5389.1_05592
MTTTMQVSRRGFLKGGLGALTLAVTGNGLVSAVWAADEPKKYGADSMPGGTVDDPLAFVSIAADGTVTIVAH
RAEMGTGVRTSLPMVVADEMEAAWDRVRVVQADADEARYGNQNVDGSRSVRHFLMPMRRVGAAARQM
LEAAAAARWSVPLAEVRATQHEVVHAPSGRRLGYGELAADAAKLPVPAGDAVKLKTRAEFRYIGKDEVRLVDLE
AIGKGEAMYGMDMRLPGMVYAVVARPPVVGGKLRRVDSAKALAVPGVLKVVEIPAMAGAPAFQPLGGVAVV
ASNTWAAMQGRAALAIEWDDGPNAAYDSVAYRETLTEASRKPGKVVRDQGDAPQAWAKAGEAERFMAEY
HVPHLAHASMETPVATVRIQDGAAEVWTSVQNPAAAQEAVAKRLKLKPENVKVHVLLLGGGFGRKSKPDYVD
EAAIVAQAMPAGTPVKLVWTREDDIHHDYLHTVSAEHLEAVVGKDGKVQSWLHRSAAPTIASLFTEGAKGEQL
FESAMSAINMPYVIPNVRVETAEVAAHARIGWFRSVANIPHAFAAQCFIAELAHRAGQDHKQYALDLIGPARRI
DPGTLADTWNYSESPERYPYDTGRLRGVIEAAASGAKWGRELPKGHGLGLAFCYSFMSYTATVVEVAVDEKGE
VRVVAVDMALDCGPQIKPERIRAQMEGGAIMGLSLALLGEITFEKGRVKQNNFYDYEVLRHNASPRVIRTHLV
NDDHALPPGGVGEPPVPPVAPALCNAIFAATGKRVRSLPVRSVA
>SEQF5390||SEQF5390.2_03557
MTTTMQASRRGFLKGGLGALTLAVTGNGLVSAVWAADAPRKYGADSMPGGTVDDPLAFVSIAADGAVTIIAH
RAEMGTGVRTSLPMVVADEMEAAWDRVRVVQADADEARYGNQNVDGSRSMRHFLMPMRRVGAAARQ
MLEAAAAARWAVPVAEVRATQHQVVHVPTDRKLGYGELAADAAKLPVPKGDTLKLKTRAEFRYIGKEQVRLVD
LEAIGKGQAIYGMDMRLPGMVYAVVARPPVVGGKPRRVDSAKALAVPGVLKVVDIPAMAGAPAFQPLGGVA
VVASNTWAAMQGRAALQIEWDDGPNADYDSAAYRETLTAASRKPGKVVRDQGDAPQAWAKAGEAGRFSA
EYHVPHLAHASMETPVATVRIQDGGAEVWTSVQNPAAAQEAVANRLKLKPENVKVHVLLLGGGFGRKSKPDY
VDEAAIVAQAMPAGTPVKLVWTREDDIHHDYLHTVSAEHLEAVVGQDGKVQSWLHRSAAPTIASLFTEGAKG
EQLFESAMSAINMPYVIPNVRVETAEVAAHARIGWFRSVANIPHAFAAQCFIAELAHRAGQDHKQYALDLIGPA
RRIDPGTLADTWNYSESPERYPYDTGRLRGVIEAAASGARWGREMPKGHGLGLAFCYSFMSYTATVVEVAVDE
KGEVRVLAVDMAMDCGPQINPERIRAQMEGGAIMGLSLALLGEITFEKGRVRQNNFYDYEVLRHNASPRVIRT
HLVNDDHALPPGGVGEPPVPPVAPALCNAIFAATGKRVRSLPVRSVA
>SEQF5391||SEQF5391.1_01854

MTHIAQLSRRGFLQGALGALTLTVTARGLITAAWAAESPAQKYGADSMPGGTVDDPLVFVSIAADGTVTIVAHR
AEMGTGVRTSLPMVVADEMEARWDRVKVIQAQADEARYGNQNVDGSRSVRHFLMPMRRVGAAARQMLE
AAAAARWSVPVAEVKAVQHEVLHQPSGRRLTYGELAADAAKQPVPAGDALKLKDRAEFRYIGKEHVRLVDLEA
IGKGQASYGMDMRLPGMVYAVVARPPVVGGKLRRLDSAKALAVPGVLKVVEIPPMQGAPAFQPLGGVAIVAR
NTWAARQGRDALQIEWDDGPNGSYDSSAYRQTLESAARKPGKTMRSQGDAAQAWAKAPEAERVAAEYYVP
HLAHASMEPPTATVRIKDGRAEVWTSIQNPIAARDAVAARLKLEPADVAVNVLLLGGGFGRKSKPDFVDEAAIV
ARAMPEGTPVKLVWTREDDIHHDYLHTVSVERLEAVLDKNGQVQSWLHRSAAPTIASLFSEGAKGQQLFESA
MSAINMPYRIPNVRVETAEVEAHARIGWFRSVANIPHAYAAQCFIAELAHRAGKDPKDFALDLIGPARRIDPST
MADTWNYSESPELYPYDTGRLRGVIEAACKGAEWGRTLPQGHGLGLAFCYSFMSYTATVVEVAVDAKGEVRV
VAVDMAMDCGPQINPERIRAQMEGGAIMGLGLALASEISFERGRVKQSNFHDYEVLRHNASPRVIRTHLVND
DHTLPPGGVGEPPVPPVAPALCNAIFAATGKRIRSLPVRRVA
>SEQF5392||SEQF5392.1_05255
MTTTMQVSRRGFLKGGLGALTLAVTGNGLVSAVWAADEPKKYGADSMPGGTVDDPLAFVSIAADGTVTIVAH
RAEMGTGVRTSLPMVVADEMEAAWDRVRVVQADADEARYGNQNVDGSRSVRHFLMPMRRVGAAARQM
LEAAAAARWSVPLAEVRATQHEVVHAPSGRRLGYGELAADAAKLPVPAGDAVKLKTRAEFRYIGKDEVRLVDLE
AIGKGEAMYGMDMRLPGMVYAVVARPPVVGGKLRRVDSAKALAVPGVLKVVEIPAMAGAPAFQPLGGVAVV
ASNTWAAMQGRAALAIEWDDGPNAAYDSVAYRETLTEASRKPGKVVRDQGDAPQAWAKAGEAERFMAEY
HVPHLAHASMETPVATVRIQDGAAEVWTSVQNPAAAQEAVAKRLKLKPENVKVHVLLLGGGFGRKSKPDYVD
EAAIVAQAMPAGTPVKLVWTREDDIHHDYLHTVSAEHLEAVVGKDGKVQSWLHRSAAPTIASLFTEGAKGEQL
FESAMSAINMPYVIPNVRVETAEVAAHARIGWFRSVANIPHAFAAQCFIAELAHRAGRDHKQYALDLIGPARRI
DPGTLADTWNYSESPERYPYDTGRLRGVIEAAASGAKWGRELPKGHGLGLAFCYSFMSYTATVVEVAVDEKGE
VRVVAVDMALDCGPQIKPERIRAQMEGGAIMGLSLALLGEITFEKGRVKQNNFYDYEVLRHNASPRVIRTHLV
NDDHALPPGGVGEPPVPPVAPALCNAIFAATGKRVRSLPVRSVA
>SEQF5393||SEQF5393.1_05675
MTTTMQVSRRGFLKGGLGALTLAVTGNGLVSAVWAADEPKKYGADSMPGGTVDDPLAFVSIAADGTVTIVAH
RAEMGTGVRTSLPMVVADEMEAAWDRVRVVQADADEARYGNQNVDGSRSVRHFLMPMRRVGAAARQM
LEAAAAARWSVPLAEVRATQHEVVHAPSGRRLGYGELAADAAKLPVPAGDAVKLKTRAEFRYIGKDEVRLVDLE
AIGKGEAMYGMDMRLPGMVYAVVARPPVVGGKLRRVDSAKALAVPGVLKVVEIPAMAGAPAFQPLGGVAVV
ASNTWAAMQGRAALAIEWDDGPNAAYDSVAYRETLTEASRKPGKVVRDQGDAPQAWAKAGEAERFMAEY
HVPHLAHASMETPVATVRIQDGAAEVWTSVQNPAAAQEAVAKRLKLKPENVKVHVLLLGGGFGRKSKPDYVD
EAAIVAQAMPAGTPVKLVWTREDDIHHDYLHTVSAEHLEAVVGKDGKVQSWLHRSAAPTIASLFTEGAKGEQL
FESAMSAINMPYVIPNVRVETAEVAAHARIGWFRSVANIPHAFAAQCFIAELAHRAGQDHKQYALDLIGPARRI
DPGTLADTWNYSESPERYPYDTGRLRGVIEAAASGAKWGRELPKGHGLGLAFCYSFMSYTATVVEVAVDEKGE
VRVVAVDMALDCGPQIKPERIRAQMEGGAIMGLSLALLGEITFEKGRVKQNNFYDYEVLRHNASPRVIRTHLV
NDDHALPPGGVGEPPVPPVAPALCNAIFAATGKRVRSLPVRSVA
>SEQF5394||SEQF5394.1_04143
MTTTMQVSRRGFLKGGLGALTLAVTGNGLVSAVWAADEPKKYGADSMPGGTVDDPLAFVSIAADGTVTIVAH
RAEMGTGVRTSLPMVVADEMEAAWDRVRVVQADADEARYGNQNVDGSRSVRHFLMPMRRVGAAARQM
LEAAAAARWSVPLAEVRATQHEVVHAPSGRRLGYGELAADAAKLPVPAGDAVKLKTRAEFRYIGKDEVRLVDLE
AIGKGEAMYGMDMRLPGMVYAVVARPPVVGGKLRRVDSAKALAVPGVLKVVEIPAMAGAPAFQPLGGVAVV
ASNTWAAMQGRAALAIEWDDGPNAAYDSVAYRETLTEASRKPGKVVRDQGDAPQAWAKAGETERFMAEY
HVPHLAHASMETPVATVRIQDGAAEVWTSVQNPAAAQEAVAKRLKLKPENVKVHVLLLGGGFGRKSKPDYVD
EAAIVAQAMPAGTPVKLVWTREDDIHHDYLHTVSAEHLEAVVGKDGKVQSWLHRSAAPTIASLFTEGAKGEQL
FESAMSAINMPYVIPNVRVETAEVAAHARIGWFRSVANIPHAFAAQCFIAELAHRAGRDHKQYALDLIGPARRI

DPGTLADTWNYSESPERYPYDTGRLRGVIEAAASGAKWGRELPKGHGLGLAFCYSFMSYTATVVEVAVDEKGE
VRVVAVDMALDCGPQIKPERIRAQMEGGAIMGLSLALLGEITFEKGRVKQNNFYDYEVLRHNASPRVIRTHLV
NDDHALPPGGVGEPPVPPVAPALCNAIFAATGKRVRSLPVRSVA
>SEQF5396||SEQF5396.1_04290
MTTTMQVSRRGFLKGGLGALTLAVTGNGLVSAVWAADEPKKYGADSMPGGTVDDPLAFVSIAADGTVTIVAH
RAEMGTGVRTSLPMVVADEMEAAWDRVRVVQADADEARYGNQNVDGSRSVRHFLMPMRRVGAAARQM
LEAAAAARWSVPLAEVRATQHEVVHAPSGRRLGYGELAPDAAKLPVPAGDAVKLKTRAEFRYIGKDEVRLVDLE
AIGKGEAMYGMDMRLPGMVYAVVARPPVVGGKLRRVDSAKALAVPGVLKVVEIPAMAGAPAFQPLGGVAVV
ASNTWAAMQGRAALAIEWDDGPNAAYDSVAYRETLTEASRKPGKVVRDQGDAPQAWAKAGEAERFMAEY
HVPHLAHASMETPVATVRIQDGAAEVWTSVQNPAAAQEAVAKRLKLKPENVKVHVLLLGGGFGRKSKPDYVD
EAAIVAQAMPAGTPVKLVWTREDDIHHDYLHTVSAEHLEAVVGKDGKVQSWLHRSAAPTIASLFTEGAKGEQL
FESAMSAINMPYVIPNVRVETAEVAAHARIGWFRSVANIPHAFAAQCFIAELAHRAGQDHKQYALDLIGPARRI
DPGTLADTWNYSESPERYPYDTGRLRGVIEAAASGAKWGRELPKGHGLGLAFCYSFMSYTATVVEVAVDEKGE
VRVVAVDMALDCGPQIKPERIRAQMEGGAIMGLSLALLGEISFEKGRVKQNNFYDYEVLRHNASPRVIRTHLVN
DDHALPPGGVGEPPVPPVAPALCNAIFAATGKRVRSLPVRSVA
>SEQF5397||SEQF5397.1_02247
MTTTMQVSRRGFLKGGLGALTLAVTGNGLVSAVWAADEPKKYGADSMPGGTVDDPLAFVSIAADGTVTIVAH
RAEMGTGVRTSLPMVVADEMEAAWDRVRVVQADADEARYGNQNVDGSRSVRHFLMPMRRVGAAARQM
LEAAAAARWSVPLAEVRATQHEVVHAPSGRRLGYGELAADAAKLPVPAGDAVKLKTRAEFRYIGKDEVRLVDLE
AIGKGEAMYGMDMRLPGMVYAVVARPPVVGGKLRRVDSAKALAVPGVLKVVEIPAMAGAPAFQPLGGVAVV
ASNTWAAMQGRAALAIEWDDGPNAAYDSVAYRETLTEASRKPGKVVRDQGDAPQAWAKAGEAERFMAEY
HVPHLAHASMETPVATVRIQDGAAEVWTSVQNPAAAQEAVAKRLKLKPENVKVHVLLLGGGFGRKSKPDYVD
EAAIVAQAMPAGTPVKLVWTREDDIHHDYLHTVSAEHLEAVVGKDGKVQSWLHRSAAPTIASLFTEGAKGEQL
FESAMSAINMPYVIPNVRVETAEVAAHARIGWFRSVANIPHAFAAQCFIAELAHRAGRDHKQYALDLIGPARRI
DPGTLADTWNYSESPERYPYDTGRLRGVIEAAASGAKWGRELPKGHGLGLAFCYSFMSYTATVVEVAVDEKGE
VRVVAVDMALDCGPQIKPERIRAQMEGGAIMGLSLALLGEITFEKGRVKQNNFYDYEVLRHNASPRVIRTHLV
NDDHALPPGGVGEPPVPPVAPALCNAIFAATGKRVRSLPVRSVA
>SEQF5398||SEQF5398.1_03389
MTTTMQVSRRGFLKGGLGALTLAVTGNGLVSAVWAADEPKKYGADSMPGGTVDDPLAFVSIAADGTVTIVAH
RAEMGTGVRTSLPMVVADEMEAAWDRVRVVQADADEARYGNQNVDGSRSVRHFLMPMRRVGAAARQM
LEAAAAARWSVPLAEVRATQHEVVHAPTGRRLGYGELAADAAKLPVPAGDAVKLKTRAEFRYIGKDEVRLVDLE
AIGKGEAMYGMDMRLPGMVYAVVARPPVVGGKLRRVDSAKALAVPGVLKVVEIPAMAGAPAFQPLGGVAVV
ASNTWAAMQGRAALAIEWDDGPNAAYDSVAYRETLTEASRKPGKVVRDQGDAPQAWAKAGETERFMAEY
HVPHLAHASMETPVATVRIQDGAAEVWTSVQNPAAAQEAVAKRLKLKPENVKVHVLLLGGGFGRKSKPDYVD
EAAIVAQAMPAGTPVKLVWTREDDIHHDYLHTVSAEHLEAVVGKDGKVQSWLHRSAAPTIASLFTEGAKGEQL
FESAMSAINMPYVIPNVRVETAEVAAHARIGWFRSVANIPHAFAAQCFIAELAHRAGQDHKQYALDLIGPARRI
DPGTLADTWNYSESPERYPYDTGRLRGVIEAAASGAKWGRELPKGHGLGLAFCYSFMSYTATVVEVAVDEKGE
VRVVAVDMALDCGPQIKPERIRAQMEGGAIMGLSLALLGEITFEKGRVKQNNFYDYEVLRHNASPRVIRTHLV
NDDHALPPGGVGEPPVPPVAPALCNAIFAATGKRVRSLPVRSVA
>SEQF5399||SEQF5399.1_01228
MTTTMQVSRRGFLKGGLGALTLAVTGNGLVSAVWAADEPKKYGADSMPGGTVDDPLAFVSIAADGTVTIVAH
RAEMGTGVRTSLPMVVADEMEAAWDRVRVVQADADEARYGNQNVDGSRSVRHFLMPMRRVGAAARQM
LEAAAAARWSVPLAEVRATQHEVVHAPSGRRLGYGELAADAAKLPVPAGDAVKLKTRAEFRYIGKDEVRLVDLE
AIGKGEAMYGMDMRLPGMVYAVVARPPVVGGKLRRVDSAKALAVPGVLKVVEIPAMAGAPAFQPLGGVAVV

ASNTWAAMQGRAALAIEWDDGPNAAYDSVAYRETLTEASRKPGKVVRDQGDAPQAWAKAGETERFMAEY
HVPHLAHASMETPVATVRIQDGAAEVWTSVQNPAAAQEAVAKRLKLKPENVKVHVLLLGGGFGRKSKPDYVD
EAAIVAQAMPAGTPVKLVWTREDDIHHDYLHTVSAEHLEAVVGKDGKVQSWLHRSAAPTIASLFTEGAKGEQL
FESAMSAINMPYVIPNVRVETAEVAAHARIGWFRSVANIPHAFAAQCFIAELAHRAGQDHKQYALDLIGPARRI
DPGTLADTWNYSESPERYPYDTGRLRGVIEAAASGAKWGRELPKGHGLGLAFCYSFMSYTATVVEVAVDEKGE
VRVVAVDMALDCGPQIKPERIRAQMEGGAIMGLSLALLGEITFEKGRVKQNNFYDYEVLRHNASPRVIRTHLV
NDDHALPPGGVGEPPVPPVAPALCNAIFAATGKRVRSLPVRSVA
>SEQF5400||SEQF5400.1_05615
MTHIAQLSRRGFLQGALGALTLTVTARGLITAAWAAESPAQKYGADSMPGGTVDDPLVFVSIAADGTVTIVAHR
AEMGTGVRTSLPMVVADEMEARWDRVKVIQAQADEARYGNQNVDGSRSVRHFLMPMRRVGAAARQMLE
AAAAARWSVPVAEVKAVQHEVLHQPSGRRLTYGELAADAAKQPVPAGDALKLKDRAEFRYIGKEHVRLVDLEA
IGKGQASYGMDMRLPGMVYAVVARPPVVGGKLRRLDSAKALAVPGVLKVVEIPPMQGAPAFQPLGGVAIVAR
NTWAARQGRDALQIEWDDGPNGSYDSSAYRQTLESAARKPGKTMRSQGDAAQAWAKAPEAERVAAEYYVP
HLAHASMEPPTATVRIKDGRAEVWTSIQNPIAARDAVAARLKLEPADVAVNVLLLGGGFGRKSKPDFVDEAAIV
ARAMPEGTPVKLVWTREDDIHHDYLHTVSVERLEAVLDKNGQVQSWLHRSAAPTIASLFSEGAKGQQLFESA
MSAINMPYRIPNVRVETAEVEAHARIGWFRSVANIPHAYAAQCFIAELAHRAGKDPKDFALDLIGPARRIDPST
MADTWNYSESPELYPYDTGRLRGVIEAACKGAEWGRTLPQGHGLGLAFCYSFMSYTATVVEVAVDAKGEVRV
VAVDMAMDCGPQINPERIRAQMEGGAIMGLGLALASEISFERGRVKQSNFHDYEVLRHNASPRVIRTHLVND
DHTLPPGGVGEPPVPPVAPALCNAIFAATGKRIRSLPVRRVA
>SEQF5402||SEQF5402.1_06013
MTTTMQVSRRGFLKGGLGALTLAVTGNGLVSAVWAADEPKKYGADSMPGGTVDDPLAFVSIAADGTVTIVAH
RAEMGTGVRTSLPMVVADEMEAAWDRVRVVQADADEARYGNQNVDGSRSVRHFLMPMRRVGAAARQM
LEAAAAARWSVPLAEVRATQHEVVHAPSGRRLGYGELAPDAAKLPVPAGDAVKLKTRAEFRYIGKDEVRLVDLE
AIGKGEAMYGMDMRLPGMVYAVVARPPVVGGKLRRVDSAKALAVPGVLKVVEIPAMAGAPAFQPLGGVAVV
ASNTWAAMQGRAALAIEWDDGPNAAYDSVAYRETLTEASRKPGKVVRDQGDAPQAWAKAGEAERFMAEY
HVPHLAHASMETPVATVRIQDGAAEVWTSVQNPAAAQEAVAKRLKLKPENVKVHVLLLGGGFGRKSKPDYVD
EAAIVAQAMPAGTPVKLVWTREDDIHHDYLHTVSAEHLEAVVGKDGKVQSWLHRSAAPTIASLFTEGAKGEQL
FESAMSAINMPYVIPNVRVETAEVAAHARIGWFRSVANIPHAFAAQCFIAELAHRAGQDHKQYALDLIGPARRI
DPGTLADTWNYSESPERYPYDTGRLRGVIEAAASGAKWGRELPKGHGLGLAFCYSFMSYTATVVEVAVDEKGE
VRVVAVDMALDCGPQIKPERIRAQMEGGAIMGLSLALLGEISFEKGRVKQNNFYDYEVLRHNASPRVIRTHLVN
DDHALPPGGVGEPPVPPVAPALCNAIFAATGKRVRSLPVRSVA
>SEQF5403||SEQF5403.1_01984
MTTTMQVSRRGFLKGGLGALTLAVTGNGLVSAVWAADEPKKYGADSMPGGTVDDPLAFVSIAADGTVTIVAH
RAEMGTGVRTSLPMVVADEMEAAWDRVRVVQADADEARYGNQNVDGSRSVRHFLMPMRRVGAAARQM
LEAAAAARWSVPLAEVRATQHEVVHAPSGRRLGYGELAADAAKLPVPAGDAVKLKTRAEFRYIGKDEVRLVDLE
AIGKGEAMYGMDMRLPGMVYAVVARPPVVGGKLRRVDSAKALAVPGVLKVVEIPAMAGAPAFQPLGGVAVV
ASNTWAAMQGRAALAIEWDDGPNAAYDSVAYRETLTEASRKPGKVVRDQGDAPQAWAKAGETERFMAEY
HVPHLAHASMETPVATVRIQDGAAEVWTSVQNPAAAQEAVAKRLKLKPENVKVHVLLLGGGFGRKSKPDYVD
EAAIVAQAMPAGTPVKLVWTREDDIHHDYLHTVSAEHLEAVVGKDGKVQSWLHRSAAPTIASLFTEGAKGEQL
FESAMSAINMPYVIPNVRVETAEVAAHARIGWFRSVANIPHAFAAQCFIAELAHRAGQDHKQYALDLIGPARRI
DPGTLADTWNYSESPERYPYDTGRLRGVIEAAASGAKWGRELPKGHGLGLAFCYSFMSYTATVVEVAVDEKGE
VRVVAVDMALDCGPQIKPERIRAQMEGGAIMGLSLALLGEISFEKGRVKQNNFYDYEVLRHNASPRVIRTHLVN
DDHALPPGGVGEPPVPPVAPALCNAIFAATGKRVRSLPVRSVA
>SEQF5404||SEQF5404.1_02383

MTTTMQVSRRGFLKGGLGALTLAVSGNGLVSAVWAADEPKKYGADSMPGGTVDDPLAFVSIAADGTVTIVAH
RAEMGTGVRTSLPMVVADEMEAAWDRVRVVQADADEARYGNQNVDGSRSVRHFLMPMRRVGAAARQM
LEAAAAARWSVPLAEVRATQHEVVHAPSGRRLGYGELAADAAKLPVPAGDAVKLKTRAEFRYIGKDEVRLVDLE
AIGKGEAMYGMDMRLPGMVYAVVARPPVVGGKLRRVDSAKALAVPGVLKVVEIPAMAGAPAFQPLGGVAVV
ASNTWAAMQGRAALAIEWDDGPNAAYDSVAYRETLTEASRKPGKVVRDQGDAPQAWAKAGETERFMAEY
HVPHLAHASMETPVATVRIQDGAAEVWTSVQNPAAAQEAVAKRLKLKPENVKVHVLLLGGGFGRKSKPDYVD
EAAIVAQAMPAGTPVKLVWTREDDIHHDYLHTVSAEHLEAVVGKDGKVQSWLHRSAAPTIASLFTEGAKGEQL
FESAMSAINMPYVIPNVRVETAEVAAHARIGWFRSVANIPHAFAAQCFIAELAHRAGQDHKQYALDLIGPARRI
DPGTLADTWNYSESPERYPYDTGRLRGVIEAAASGAKWGRELPKGHGLGLAFCYSFMSYTATVVEVAVDEKGE
VRVVAVDMALDCGPQIKPERIRAQMEGGAIMGLSLALLGEISFEKGRVKQNNFYDYEVLRHNASPRVIRTHLVN
DDHALPPGGVGEPPVPPVAPALCNAIFAATGKRVRSLPVRSVA
>SEQF5405||SEQF5405.1_06282
MTHTAQLSRRGFLQGALGALTLTVTARGLVSTAWAAEPPAQKYGADSMPGGTVDDPLVFVSIAADGTVTIVAH
RAEMGTGVRTSLPMVVADEMEARWERVKVIQAQADEVRYGNQNVDGSRSVRHFLMPMRRVGAAARQML
EAAAAARWSVPVAEVKAVQHEVLHQPSGRRLTYGELAADAAKQPVPAGDALKLKDRAEFRYIGKDQVRLVDLE
AIGKGQASYGMDMRLPGMVYAVVARPPVVGGKLRRLDSAKALAVPGVLKVVEIPPMQGAPAFQPLGGVAIV
ARNTWAARQGRDALEIEWDDGPNGSYDSSAYRQTLELAARKPGKTMRSQGDAAQAWAKAPEAERVAAEYY
VPHLAHASMEPPAATVRIRDGRAEVWTSIQNPIAARDAVAARLKLEPAKVTVNVLLLGGGFGRKSKPDFVDEA
AIVARAMPEGTPVKLVWTREDDIHHDYLHTVSVERLEAVLDQSGQVQSWLHRSAAPTIASLFSEGAKGQQLFE
SAMSAINMPYRIPNVRVETAEVEAHARIGWFRSVANIPHAYAAQCFIAELAHRAGKDPKDFALDLIGPARRIDP
GTMADTWNYSESPERYPYDTGRLRGVIEAACKGAEWGRTLPQGHGLGLAFCYSFMSYTATVVEVAVDAKGEV
RVVAVDMAMDCGPQINPERIRAQMEGGAIMGLGLALASEITFERGRVKQSNFHDYEVLRHNASPRVIRTHLV
NDDHTLPPGGVGEPPVPPVAPALCNAIFAATGKRIRSLPVRRVA
>SEQF5406||SEQF5406.1_03988
MTTTMQVSRRGFLKGGLGALTLAVTGNGLVSAVWAADEPKKYGADSMPGGTVDDPLAFVSIAADGTVTIVAH
RAEMGTGVRTSLPMVVADEMEAAWDRVRVVQADADEARYGNQNVDGSRSVRHFLMPMRRVGAAARQM
LEAAAAARWSVPLAEVRATQHEVVHAPSGRRLGYGELAADAAKLPVPAGDAVKLKTRAEFRYIGKDEVRLVDLE
AIGKGEAMYGMDMRLPGMVYAVVARPPVVGGKLRRVDSAKALAVPGVLKVVEIPAMAGAPAFQPLGGVAVV
ASNTWAAMQGRAALAIEWDDGPNAAYDSVAYRETLTEASRKPGKVVRDQGDAPQAWAKAGETERFMAEY
HVPHLAHASMETPVATVRIQDGAAEVWTSVQNPAAAQEAVAKRLKLKPENVKVHVLLLGGGFGRKSKPDYVD
EAAIVAQAMPAGTPVKLVWTREDDIHHDYLHTVSAEHLEAVVGKDGKVQSWLHRSAAPTIASLFTEGAKGEQL
FESAMSAINMPYVIPNVRVETAEVAAHARIGWFRSVANIPHAFAAQCFIAELAHRAGQDHKQYALDLIGPARRI
DPGTLADTWNYSESPERYPYDTGRLRGVIEAAASGAKWGRELPKGHGLGLAFCYSFMSYTATVVEVAVDEKGE
VRVVAVDMALDCGPQIKPERIRAQMEGGAIMGLSLALLGEITFEKGRVKQNNFYDYEVLRHNASPRVIRTHLV
NDDHALPPGGVGEPPVPPVAPALCNAIFAATGKRVRSLPVRSVA
>SEQF5407||SEQF5407.1_03107
MTTTMQVSRRGFLKGGLGALTLAVTGNGLVSAVWAADEPKKYGADSMPGGTVDDPLAFVSIAADGTVTIVAH
RAEMGTGVRTSLPMVVADEMEAAWDRVRVVQADADEARYGNQNVDGSRSVRHFLMPMRRVGAAARQM
LEAAAAARWSVPFAEVRATQHEVVHAPTGRRLGYGELAADAAKLPVPAGDAVKLKTRAEFRYIGKDEVRLVDLE
AIGKGEAMYGMDMRLPGMVYAVVARPPVVGGKLRRVDSAKALAVPGVLKVVEIPAMAGAPAFQPLGGVAVV
ASNTWAAMQGRAALAIEWDDGPNAAYDSVAYRETLTEASRKPGKVVRDQGDAPQAWAKAGETERFMAEY
HVPHLAHASMETPVATVRIQDGAAEVWTSVQNPAAAQEAVAKRLKLKPENVKVHVLLLGGGFGRKSKPDYVD
EAAIVAQAMPAGTPVKLVWTREDDIHHDYLHTVSAEHLEAVVGKDGKVQAWLHRSAAPTIASLFTEGAKGEQL
FESAMSAINMPYVIPNVRVETAEVAAHARIGWFRSVANIPHAFAAQCFIAELAHRAGQDHKQYALDLIGPARRI

DPGTLADTWNYSESPERYPYDTGRLRGVIEAAASGAKWGRELPKGHGLGLAFCYSFMSYTATVVEVAVDEKGE
VRVVAVDMALDCGPQIKPERIRAQMEGGAIMGLSLALLGEITFEKGRVKQNNFYDYEVLRHNASPRVIRTHLV
NDDHALPPGGVGEPPVPPVAPALCNAIFAATGKRVRSLPVRSVA
>SEQF5408||SEQF5408.1_05077
MTTTMQVSRRGFLKGGLGALTLAVTGNGLVSAVWAADEPKKYGADSMPGGTVDDPLAFVSIAADGTVTIVAH
RAEMGTGVRTSLPMVVADEMEAAWDRVRVVQADADEARYGNQNVDGSRSVRHFLMPMRRVGAAARQM
LEAAAAARWSVPLAEVRATQHEVVHAPSGRRLGYGELAADAAKLPVPAGDAVKLKTRAEFRYIGKDEVRLVDLE
AIGKGEAMYGMDMRLPGMVYAVVARPPVVGGKLRRVDSAKALAVPGVLKVVEIPAMAGAPAFQPLGGVAVV
ASNTWAAMQGRAALAIEWDDGPNAAYDSVAYRETLTEASRKPGKVVRDQGDAPQAWAKAGETERFMAEY
HVPHLAHASMETPVATVRIQDGAAEVWTSVQNPAAAQEAVAKRLKLKPENVKVHVLLLGGGFGRKSKPDYVD
EAAIVAQAMPAGTPVKLVWTREDDIHHDYLHTVSAEHLEAVVGKDGKVQSWLHRSAAPTIASLFTEGAKGEQL
FESAMSAINMPYVIPNVRVETAEVAAHARIGWFRSVANIPHAFAAQCFIAELAHRAGQDHKQYALDLIGPARRI
DPGTLADTWNYSESPERYPYDTGRLRGVIEAAASGAKWGRELPKGHGLGLAFCYSFMSYTATVVEVAVDEKGE
VRVVAVDMALDCGPQIKPERIRAQMEGGAIMGLSLALLGEITFEKGRVKQNNFYDYEVLRHNASPRVIRTHLV
NDDHALPPGGVGEPPVPPVAPALCNAIFAATGKRVRSLPVRSVA
>SEQF5409||SEQF5409.1_01266
MTTTMQVSRRGFLKGGLGALTLAVTGNGLVSAVWAADEPKKYGADSMPGGTVDDPLAFVSIAADGTVTIVAH
RAEMGTGVRTSLPMVVADEMEAAWDRVRVVQADADEARYGNQNVDGSRSVRHFLMPMRRVGAAARQM
LEAAAAARWSVPLAEVRATQHEVVHAPSGRRLGYGELAADAAKLPVPAGDAVKLKTRAEFRYIGKDEVRLVDLE
AIGKGEAMYGMDMRLPGMVYAVVARPPVVGGKLRRVDSAKALAVPGVLKVVEIPAMAGAPAFQPLGGVAVV
ASNTWAAMQGRAALAIEWDDGPNAAYDSVAYRETLTEASRKPGKVVRDQGDAPQAWAKAGEAERFMAEY
HVPHLAHASMETPVATVRIQDGAAEVWTSVQNPAAAQEAVAKRLKLKPENVKVHVLLLGGGFGRKSKPDYVD
EAAIVAQAMPAGTPVKLVWTREDDIHHDYLHTVSAEHLEAVVGKDGKVQSWLHRSAAPTIASLFTEGAKGEQL
FESAMSAINMPYVIPNVRVETAEVAAHARIGWFRSVANIPHAFAAQCFIAELAHRAGRDHKQYALDLIGPARRI
DPGTLADTWNYSESPERYPYDTGRLRGVIEAAASGAKWGRELPKGHGLGLAFCYSFMSYTATVVEVAVDEKGE
VRVVAVDMALDCGPQIKPERIRAQMEGGAIMGLSLALLGEITFEKGRVKQNNFYDYEVLRHNASPRVIRTHLV
NDDHALPPGGVGEPPVPPVAPALCNAIFAATGKRVRSLPVRSVA
>SEQF5410||SEQF5410.1_01914
MTTTMHVSRRGFLKGGLGALTLAVTGNGLVSAVWAADEPKKYGADSMPGGTVDDPLAFVSIAADGTVTIVAH
RAEMGTGVRTSLPMVVADEMEAAWDRVRVVQADADEARYGNQNVDGSRSVRHFLMPMRRVGAAARQM
LEAAAAARWSVPLAEVRATQHEVVHAPSGRRLGYGELAADAAKLPVPAGDTVKLKTRAEFRYIGKDEVRLVDLE
AIGKGEAMYGMDMRLPGMVYAVVARPPVVGGKLRRVDSAKALAVPGVLKVVEIPAMAGAPAFQPLGGVAVV
ASNTWAAMQGRAALAIEWDDGPNAAYDSVAYRETLTEASRKPGKVVRDQGDAPQAWAKAGETERFMAEY
HVPHLAHASMETPVATVRIQDGAAEVWTSVQNPAAAQEAVAKRLKLKPENVKVHVLLLGGGFGRKSKPDYVD
EAAIVAQAMPAGTPVKLVWTREDDIHHDYLHTVSAEHLEAVVGKDGKVQSWLHRSAAPTIASLFTEGAKGEQL
FESAMSAINMPYVIPNVRVETAEVAAHARIGWFRSVANIPHAFAAQCFIAELAHRAGQDHKQYALDLIGPARRI
DPGTLADTWNYSESPERYPYDTGRLRGVIEAAASGAKWGRELPKGHGLGLAFCYSFMSYTATVVEVAVDEKGE
VRVVAVDMALDCGPQIKPERIRAQMEGGAIMGLSLALLGEISFEKGRVKQNNFYDYEVLRHNASPRVIRTHLVN
DDHALPPGGVGEPPVPPVAPALCNAIFAATGKRVRSLPVRSVA
>SEQF5411||SEQF5411.1_01812
MTTTMQVSRRGFLKGGLGALTLAVSGNGLVSAVWAADEPKKYGADSMPGGTVDDPLAFVSIAADGTVTIVAH
RAEMGTGVRTSLPMVVADEMEAAWDRVRVVQADADEARYGNQNVDGSRSVRHFLMPMRRVGAAARQM
LEAAAAARWSVPLAEVRATQHEVVHAPSGRRLGYGELAADAAKLPVPAGDAVKLKTRAEFRYIGKDEVRLVDLE
AIGKGEAMYGMDMRLPGMVYAVVARPPVVGGKLRRVDSAKALAVPGVLKVVEIPAMAGAPAFQPLGGVAVV

ASNTWAAMQGRAALAIEWDDGPNAAYDSVAYRETLTEASRKPGKVVRDQGDAPQAWAKAGETERFMAEY
HVPHLAHASMETPVATVRIQDGAAEVWTSVQNPAAAQEAVAKRLKLKPENVKVHVLLLGGGFGRKSKPDYVD
EAAIVAQAMPAGTPVKLVWTREDDIHHDYLHTVSAEHLEAVVGKDGKVQSWLHRSAAPTIASLFTEGAKGEQL
FESAMSAINMPYVIPNVRVETAEVAAHARIGWFRSVANIPHAFAAQCFIAELAHRAGQDHKQYALDLIGPARRI
DPGTLADTWNYSESPERYPYDTGRLRGVIEAAASGAKWGRELPKGHGLGLAFCYSFMSYTATVVEVAVDEKGE
VRVVAVDMALDCGPQIKPERIRAQMEGGAIMGLSLALLGEISFEKGRVKQNNFYDYEVLRHNASPRVIRTHLVN
DDHALPPGGVGEPPVPPVAPALCNAIFAATGKRVRSLPVRSVA
>SEQF5412||SEQF5412.1_01384
MTTTMQVSRRGFLKGGLGALTLAVTGNGLVSAVWAADEPRKYGADSMPGGTVDDPLAFVSIAADGTVTIVAH
RAEMGTGVRTSLPMVVADEMEAAWDRVRVVQADADEARYGNQNVDGSRSVRHFLMPMRRVGAAARQM
LEAAAAARWSVPLAEVRATQHEVVHASTGRRLGYGELAADAAKLPVPAGDALKLKTRAEFRYIGKDEVRLVDLE
AIGKGEAAYGMDMRLPGMVYAVVARPPVVGGKLRRVDSAKALAVPGVLKVVEIPAMAGAPAFQPLGGVAVV
ASNTWAAMQGRAALAIEWDDGPNAAYDSVAYRETLTEASRKPGKVVRDQGDAPQAWAKAGEAERFMAEY
HVPHLAHASMETPVATVRIQDGAAEVWTSVQNPAAAQEAVAKRLKLKPENVKVHVLLLGGGFGRKSKPDYVD
EAAIVAQAMPAGTPVKLVWTREDDIHHDYLHTVSAEHLEAVVGKDGKVRSWLHRSAAPTIASLFTEGAKGEQL
FESAMSAINMPYVIPNVRVETAEVAAHARIGWFRSVANIPHAFAAQCFIAELAHRAGQDHKQYALDLIGPARRI
DPGTLADTWNYSESPERYPYDTGRLRGVIEAAASGAKWGRALPKGHGLGLAFCYSFMSYTATVVEVAVDEKGE
VRVVAVDMALDCGPQIKPERIRAQMEGGAIMGLSLALLGEITFEKGRVKQNNFYDYEVLRHNASPRVIRTHLV
NDDHALPPGGVGEPPVPPVAPALCNAIFAATGKRVRSLPVRSVA
>SEQF5413||SEQF5413.1_01684
MTTTMQVSRRGFLKGGLGALTLAVSGNGLVSAVWAADEPKKYGADSMPGGTVDDPLAFVSIAADGTVTIVAH
RAEMGTGVRTSLPMVVADEMEAAWDRVRVVQADADEARYGNQNVDGSRSVRHFLMPMRRVGAAARQM
LEAAAAARWSVPLAEVRATQHEVVHAPSGRRLGYGELAADAAKLPVPAGDAVKLKTRAEFRYIGKDEVRLVDLE
AIGKGEAMYGMDMRLPGMVYAVVARPPVVGGKLRRVDSAKALAVPGVLKVVEIPAMAGAPAFQPLGGVAVV
ASNTWAAMQGRAALAIEWDDGPNAAYDSVAYRETLTEASRKPGKVVRDQGDAPQAWAKAGETERFMAEY
HVPHLAHASMETPVATVRIQDGAAEVWTSVQNPAAAQEAVAKRLKLKPENVKVHVLLLGGGFGRKSKPDYVD
EAAIVAQAMPAGTPVKLVWTREDDIHHDYLHTVSAEHLEAVVGKDGKVQSWLHRSAAPTIASLFTEGAKGEQL
FESAMSAINMPYVIPNVRVETAEVAAHARIGWFRSVANIPHAFAAQCFIAELAHRAGQDHKQYALDLIGPARRI
DPGTLADTWNYSESPERYPYDTGRLRGVIEAAASGAKWGRELPKGHGLGLAFCYSFMSYTATVVEVAVDEKGE
VRVVAVDMALDCGPQIKPERIRAQMEGGAIMGLSLALLGEITFEKGRVKQNNFYDYEVLRHNASPRVIRTHLV
NDDHALPPGGVGEPPVPPVAPALCNAIFAATGKRVRSLPVRSVA
>SEQF5414||SEQF5414.1_04510
MTTTMQVSRRGFLKGGLGALTLAVTGNGLVSAVWAADEPKKYGADSMPGGTVDDPLAFVSIAADGTVTIVAH
RAEMGTGVRTSLPMVVADEMEAAWDRVRVVQADADEARYGNQNVDGSRSVRHFLMPMRRVGAAARQM
LEAAAAARWSVPLAEVRATQHEVVHAPSGRRLGYGELAADAAKLPVPAGDAVKLKTRAEFRYIGKDEVRLVDLE
AIGKGEAMYGMDMRLPGMVYAVVARPPVVGGKLRRVDSAKALAVPGVLKVVEIPAMAGAPAFQPLGGVAVV
ASNTWAAMQGRAALAIEWDDGPNAAYDSVAYRETLTEASRKPGKVVRDQGDAPQAWAKAGETERFMAEY
HVPHLAHASMETPVATVRIQDGAAEVWTSVQNPAAAQEAVAKRLKLKPENVKVHVLLLGGGFGRKSKPDYVD
EAAIVAQAMPAGTPVKLVWTREDDIHHDYLHTVSAEHLEAVVGKDGKVQSWLHRSAAPTIASLFTEGAKGEQL
FESAMSAINMPYVIPNVRVETAEVAAHARIGWFRSVANIPHAFAAQCFIAELAHRAGQDHKQYALDLIGPARRI
DPGTLADTWNYSESPERYPYDTGRLRGVIEAAASGAKWGRELPKGHGLGLAFCYSFMSYTATVVEVAVDEKGE
VRVVAVDMALDCGPQIKPERIRAQMEGGAIMGLSLALLGEITFEKGRVKQNNFYDYEVLRHNASPRVIRTHLV
NDDHALPPGGVGEPPVPPVAPALCNAIFAATGKRVRSLPVRSVA
>SEQF5415||SEQF5415.1_04705

MTTTMQVSRRGFLKGGLGALTLAVTGNGLVSAVWAADEPKKYGADSMPGGTVDDPLAFVSIAADGTVTIVAH
RAEMGTGVRTSLPMVVADEMEAAWDRVRVVQADADEARYGNQNVDGSRSVRHFLMPMRRVGAAARQM
LEAAAAARWSVPLAEVRATQHEVVHAPSGRRLGYGELAADAAKLPVPAGDAVKLKTRAEFRYIGKDEVRLVDLE
AIGKGEAMYGMDMRLPGMVYAVVARPPVVGGKLRRVDSAKALAVPGVLKVVEIPAMAGAPAFQPLGGVAVV
ASNTWAAMQGRAALAIEWDDGPNAAYDSVAYRETLTEASRKPGKVVRDQGDAPQAWAKAGETERFMAEY
HVPHLAHASMETPVATVRIQDGAAEVWTSVQNPAAAQEAVAKRLKLKPENVKVHVLLLGGGFGRKSKPDYVD
EAAIVAQAMPAGTPVKLVWTREDDIHHDYLHTVSAEHLEAVVGKDGKVQSWLHRSAAPTIASLFTEGAKGEQL
FESAMSAINMPYVIPNVRVETAEVAAHARIGWFRSVANIPHAFAAQCFIAELAHRAGQDHKQYALDLIGPARRI
DPGTLADTWNYSESPERYPYDTGRLRGVIEAAASGAKWGRELPKGHGLGLAFCYSFMSYTATVVEVAVDEKGE
VRVVAVDMALDCGPQIKPERIRAQMEGGAIMGLSLALLGEISFEKGRVKQNNFYDYEVLRHNASPRVIRTHLVN
DDHALPPGGVGEPPVPPVAPALCNAIFAATGKRVRSLPVRSVA
>SEQF5416||SEQF5416.1_05536
MTHIAQLSRRGFLQGALGALTLTVTARGLITAAWAAESPAQKYGADSMPGGTVDDPLVFVSIAADGTVTIVAHR
AEMGTGVRTSLPMVVADEMEARWDRVKVIQAQADEARYGNQNVDGSRSVRHFLMPMRRVGAAARQMLE
AAAAARWSVPVAEVKAVQHEVLHQPSGRRLTYGELAADAAKQPVPAGDALKLKDRAEFRYIGKEHVRLVDLEA
IGKGQASYGMDMRLPGMVYAVVARPPVVGGKLRRLDSAKALAVPGVLKVVEIPPMQGAPAFQPLGGVAIVAR
NTWAARQGRDALQIEWDDGPNGSYDSSAYRQTLESAARKPGKTMRSQGDAAQAWAKAPEAERVAAEYYVP
HLAHASMEPPTATVRIKDGRAEVWTSIQNPIAARDAVAARLKLEPADVAVNVLLLGGGFGRKSKPDFVDEAAIV
ARAMPEGTPVKLVWTREDDIHHDYLHTVSVERLEAVLDKNGQVQSWLHRSAAPTIASLFSEGAKGQQLFESA
MSAINMPYRIPNVRVETAEVEAHARIGWFRSVANIPHAYAAQCFIAELAHRAGKDPKDFALDLIGPARRIDPST
MADTWNYSESPELYPYDTGRLRGVIEAACKGAEWGRTLPQGHGLGLAFCYSFMSYTATVVEVAVDAKGEVRV
VAVDMAMDCGPQINPERIRAQMEGGAIMGLGLALASEISFERGRVKQSNFHDYEVLRHNASPRVIRTHLVND
DHTLPPGGVGEPPVPPVAPALCNAIFAATGKRIRSLPVRRVA
>SEQF5417||SEQF5417.1_02825
MTTTMQVSRRGFLKGGLGALTLAVSGNGLVSAVWAADEPKKYGADSMPGGTVDDPLAFVSIAADGTVTIVAH
RAEMGTGVRTSLPMVVADEMEAAWDRVRVVQADADEARYGNQNVDGSRSVRHFLMPMRRVGAAARQM
LEAAAAARWSVPLAEVRATQHEVVHAPSGRRLGYGELAADAAKLPVPAGDAVKLKTRAEFRYIGKDEVRLVDLE
AIGKGEAMYGMDMRLPGMVYAVVARPPVVGGKLRRVDSAKALAVPGVLKVVEIPAMAGAPAFQPLGGVAVV
ASNTWAAMQGRAALAIEWDDGPNAAYDSVAYRETLTEASRKPGKVVRDQGDAPQAWAKAGETERFMAEY
HVPHLAHASMETPVATVRIQDGAAEVWTSVQNPAAAQEAVAKRLKLKPENVKVHVLLLGGGFGRKSKPDYVD
EAAIVAQAMPAGTPVKLVWTREDDIHHDYLHTVSAEHLEAVVGKDGKVQSWLHRSAAPTIASLFTEGAKGEQL
FESAMSAINMPYVIPNVRVETAEVAAHARIGWFRSVANIPHAFAAQCFIAELAHRAGQDHKQYALDLIGPARRI
DPGTLADTWNYSESPERYPYDTGRLRGVIEAAASGAKWGRELPKGHGLGLAFCYSFMSYTATVVEVAVDEKGE
VRVVAVDMALDCGPQIKPERIRAQMEGGAIMGLSLALLGEISFEKGRVKQNNFYDYEVLRHNASPRVIRTHLVN
DDHALPPGGVGEPPVPPVAPALCNAIFAATGKRVRSLPVRSVA
>SEQF5418||SEQF5418.1_00653
MTTTMQVSRRGFLKGGLGALTLAVTGNGLVSAVWAADEPKKYGADSMPGGTVDDPLAFVSIAADGTVTIVAH
RAEMGTGVRTSLPMVVADEMEAAWDRVRVVQADADEARYGNQNVDGSRSVRHFLMPMRRVGAAARQM
LEAAAAARWSVPLAEVRATQHEVVHAPTGRRLGYGELAADAAKLPVPAGDAVKLKTRAEFRYIGKDEVRLVDLE
AIGKGEAMYGIDMRLPGMVYAVVARPPVVGGKLRRVDSAKALAVPGVLKVVEIPAMAGAPAFQPLGGVAVVA
SNTWAAMQGRAALAIEWDDGPNAAYDSVAYRETLTEASRKPGKVVRDQGDAPQAWAKAGEAERFMAEYH
VPHLAHASMETPVATVRIQDGAAEVWTSVQNPAAAQEAVAKRLKLKPENVKVHVLLLGGGFGRKSKPDYVDE
AAIVAQAMPAGTPVKLVWTREDDIHHDYLHTVSAEHLEAVVGKDGKVQSWLHRSAAPTIASLFTEGAKGEQLF
ESAMSAINMPYVIPNVRVETAEVAAHARIGWFRSVANIPHAFAAQCFIAELAHRAGRDHKQYALDLIGPARRID

PGTLADTWNYSESPERYPYDTGRLRGVIEAAASGAKWGRELPKGHGLGLAFCYSFMSYTATVVEVAVDEKGEV
RVVAVDMALDCGPQIKPERIRAQMEGGAIMGLSLALLGEITFEKGRVKQNNFYDYEVLRHNASPRVIRTHLVN
DDHALPPGGVGEPPVPPVAPALCNAIFAATGKRVRSLPVRSVA
>SEQF5419||SEQF5419.1_00055
MTTTMQVSRRGFLKGGLGALTLAVTGNGLVSAVWAADEPKKYGADSMPGGTVDDPLAFVSIAADGTVTIVAH
RAEMGTGVRTSLPMVVADEMEAAWDRVRVVQADADEARYGNQNVDGSRSVRHFLMPMRRVGAAARQM
LEAAAAARWSVPLAEVRATQHEVVHAPSGRRLGYGELAADAAKLPVPAGDAVKLKTRAEFRYIGKDEVRLVDLE
AIGKGEAMYGMDMRLPGMVYAVVARPPVVGGKLRRVDSAKALAVPGVLKVVEIPAMAGAPAFQPLGGVAVV
ASNTWAAMQGRAALAIEWDDGPNAAYDSVAYRETLTEASRKPGKVVRDQGDAPQAWAKAGETERFMAEY
HVPHLAHASMETPVATVRIQDGAAEVWTSVQNPAAAQEAVAKRLKLKPENVKVHVLLLGGGFGRKSKPDYVD
EAAIVAQAMPAGTPVKLVWTREDDIHHDYLHTVSAEHLEAVVGKDGKVQSWLHRSAAPTIASLFTEGAKGEQL
FESAMSAINMPYVIPNVRVETAEVAAHARIGWFRSVANIPHAFAAQCFIAELAHRAGQDHKQYALDLIGPARRI
DPGTLADTWNYSESPERYPYDTGRLRGVIEAAASGAKWGRELPKGHGLGLAFCYSFMSYTATVVEVAVDEKGE
VRVVAVDMALDCGPQIKPERIRAQMEGGAIMGLSLALLGEITFEKGRVKQNNFYDYEVLRHNASPRVIRTHLV
NDDHALPPGGVGEPPVPPVAPALCNAIFAATGKRVRSLPVRSVA
>SEQF5420||SEQF5420.1_04377
MTTTMQVSRRGFLKGGLGALTLAVTGNGLVSAVWAADEPKKYGADSMPGGTVDDPLAFVSIAADGTVTIVAH
RAEMGTGVRTSLPMVVADEMEAAWDRVRVVQADADEARYGNQNVDGSRSVRHFLMPMRRVGAAARQM
LEAAAAARWSVPLAEVRATQHEVVHAPSGRRLGYGELAADAAKLPVPAGDAVKLKTRAEFRYIGKDEVRLVDLE
AIGKGEAMYGMDMRLPGMVYAVVARPPVVGGKLRRVDSAKALAVPGVLKVVEIPAMAGAPAFQPLGGVAVV
ASNTWAAMQGRAALAIEWDDGPNAAYDSVAYRETLTEASRKPGKVVRDQGDAPQAWAKAGEAERFMAEY
HVPHLAHASMETPVATVRIQDGAAEVWTSVQNPAAAQEAVAKRLKLKPENVKVHVLLLGGGFGRKSKPDYVD
EAAIVAQAMPAGTPVKLVWTREDDIHHDYLHTVSAEHLEAVVGKDGKVQSWLHRSAAPTIASLFTEGAKGEQL
FESAMSAINMPYVIPNVRVETAEVAAHARIGWFRSVANIPHAFAAQCFIAELAHRAGQDHKQYALDLIGPARRI
DPGTLADTWNYSESPERYPYDTGRLRGVIEAAASGAKWGRELPKGHGLGLAFCYSFMSYTATVVEVAVDEKGE
VRVVAVDMALDCGPQIKPERIRAQMEGGAIMGLSLALLGEITFEKGRVKQNNFYDYEVLRHNASPRVIRTYLVN
DDHALPPGGVGEPPVPPVAPALCNAIFAATGKRVRSLPVRSVA
>SEQF5421||SEQF5421.1_00529
MTTTMQVSRRGFLKGGLGALTLAVTGNGLVSAVWAADEPKKYGADSMPGGTVDDPLAFVSIAADGTVTIVAH
RAEMGTGVRTSLPMVVADEMEAAWDRVRVVQADADEARYGNQNVDGSRSVRHFLMPMRRVGAAARQM
LEAAAAARWSVPLAEVRATQHEVVHAPSGRRLGYGELAADAAKLPVPAGDAVKLKTRAEFRYIGKDEVRLVDLE
AIGKGEAMYGMDMRLPGMVYAVVARPPVVGGKLRRVDSAKALAVPGVLKVVEIPAMAGAPAFQPLGGVAVV
ASNTWAAMQGRAALAIEWDDGPNAAYDSVAYRETLTEASRKPGKVVRDQGDAPQAWAKAGEAERFMAEY
HVPHLAHASMETPVATVRIQDGAAEVWTSVQNPAAAQEAVAKRLKLKPENVKVHVLLLGGGFGRKSKPDYVD
EAAIVAQAMPAGTPVKLVWTREDDIHHDYLHTVSAEHLEAVVGKDGKVQSWLHRSAAPTIASLFTEGAKGEQL
FESAMSAINMPYVIPNVRVETAEVAAHARIGWFRSVANIPHAFAAQCFIAELAHRAGQDHKQYALDLIGPARRI
DPGTLADTWNYSESPERYPYDTGRLRGVIEAAASGAKWGRELPKGHGLGLAFCYSFMSYTATVVEVAVDEKGE
VRVVAVDMALDCGPQIKPERIRAQMEGGAIMGLSLALLGEITFEKGRVKQNNFYDYEVLRHNASPRVIRTHLV
NDDHALPPGGVGEPPVPPVAPALCNAIFAATGKRVRSLPVRSVA
>SEQF5422||SEQF5422.1_00805
MTTTMQVSRRGFLKGGLGALTLAVTGNGLVSAVWAADEPKKYGADSMPGGTVDDPLAFVSIAADGTVTIVAH
RAEMGTGVRTSLPMVVADEMEAAWDRVRVVQADADEARYGNQNVDGSRSVRHFLMPMRRVGAAARQM
LEAAAAARWSVPLAEVRATQHEVVHAPSGRRLGYGELAADAAKLPVPAGDAVKLKTRAEFRYIGKDEVRLVDLE
AIGKGEAMYGMDMRLPGMVYAVVARPPVVGGKLRRVDSAKALAVPGVLKVVEIPAMAGAPAFQPLGGVAVV

ASNTWAAMQGRAALAIEWDDGPNAAYDSVAYRETLTEASRKPGKVVRDQGDAPQAWAKAGEAERFMAEY
HVPHLAHASMETPVATVRIQDGAAEVWTSVQNPAAAQEAVAKRLKLKPENVKVHVLLLGGGFGRKSKPDYVD
EAAIVAQAMPAGTPVKLVWTREDDIHHDYLHTVSAEHLEAVVGKDGKVQSWLHRSAAPTIASLFTEGAKGEQL
FESAMSAINMPYVIPNVRVETAEVAAHARIGWFRSVANIPHAFAAQCFIAELAHRAGQDHKQYALDLIGPARRI
DPGTLADTWNYSESPERYPYDTGRLRGVIEAAASGAKWGRELPKGHGLGLAFCYSFMSYTATVVEVAVDEKGE
VRVVAVDMALDCGPQIKPERIRAQMEGGAIMGLSLALLGEITFEKGRVKQNNFYDYEVLRHNASPRVIRTHLV
NDDHALPPGGVGEPPVPPVAPALCNAIFAATGKRVRSLPVRSVA
>SEQF5423||SEQF5423.1_03417
MTTTMQVSRRGFLKGGLGALTLTVTGNGLVSAVWAADEPKKYGADSMPGGTVDDPLAFVSIAADGTVTIVAH
RAEMGTGVRTSLPMVVADEMEAAWDRVRVVQADADEARYGNQNVDGSRSVRHFLMPMRRVGAAARQM
LEAAAAARWSVPLAEVRATQHEVVHAPSGRRLGYGELAADAAKLPVPAGDAVKLKTRAEFRYIGKDEVRLVDLE
AIGKGEAMYGMDMRLPGMVYAVVARPPVVGGKLRRVDSAKALAVPGVLKVVEIPAMAGAPAFQPLGGVAVV
ASNTWAAMQGRAALAIEWDDGPNAAYDSVAYRETLTEASRKPGKVVRDQGDAPQAWAKAGEAERFMAEY
HVPHLAHASMETPVATVRIQDGAAEVWTSVQNPAAAQEAVAKRLKLKPENVKVHVLLLGGGFGRKSKPDYVD
EAAIVAQAMPAGTPVKLVWTREDDIHHDYLHTVSAEHLEAVVGKDGKVQSWLHRSAAPTIASLFTEGAKGEQL
FESAMSAINMPYVIPNVRVETAEVAAHARIGWFRSVANIPHAFAAQCFIAELAHRAGRDHKQYALDLIGPARRI
DPGTLADTWNYSESPERYPYDTGRLRGVIEAAASGAKWGRELPKGHGLGLAFCYSFMSYTATVVEVAVDEKGE
VRVVAVDMALDCGPQIKPERIRAQMEGGAIMGLSLALLGEITFEKGRVKQNNFYDYEVLRHNASPRVIRTHLV
NDDHALPPGGVGEPPVPPVAPALCNAIFAATGKRVRSLPVRSVA
>SEQF5475||SEQF5475.1_02290
MSTFEQDAAPETGIFNVSRRTFLRGSGGLALGVLFSPLLDAAELLAGNGSFEPNAFVRIDRSGQVTVIAKHVEM
GQGAYTGLATLVAEELDADWSRVVVEGAPADAKRYNNLAFGPFQGTGGSSSIANAYEQMRKAGATARAMLVA
AAARQWQVPADSLRVEQGVISHAASGRKAGFGELAEAAARESVPASVPLKEPKDFRLIGQAVPRKDSPDKTDG
RAVFTQDFKLPGMLVAVVAYPPRFGAVPSKVDAARAKAVPGVVEVVEFRDLPHGRAGVAVLAHNTWAARSG
RDALAVEWDEQRAFTLGSEEIFARYRDAAAQPGTVATRQGEVEPVLAKAHQRIEAEFEFPYLAHSAMEPMNCL
VRLSEGACELWNGEQWQTGDQASVARLLGIAPEKVTITQLYAGGSFGRRANPVSDYPLEAVAIAKAAWDKGVK
APVKLVWTREDDTRGGYYRPAYLHRARLALDAEGRLTAWHHRIVGQSIIKGTGFEAVMIKDGVDQTSVEGLSNL
SYAVPNLQVELSTPSDIGVPVQWWRSVGHTHTGFAAEVLVDEAATTAGKDPYAFRHGLLEKHPRHRGVLELVA
DKAGWSQPLKPGAEGEKRGRGIAVHESFGSYVAQVAEVTVKADGSFRVDRVVCAVDCGLAINPDVIKAQMEG
GIGFGLAAALHGAITLREGRVEQSNFHDYQVLRMNEMPVVEVHILPSAEKPTGVGEPGVPPLAPAVANALYAAT
GVRLRKLPFPSQVKA
>SEQF5476||SEQF5476.1_02289
MSTFEQDAAPETGIFNVSRRTFLRGSGGLALGVLFSPLLDAAELLAGNGSFEPNAFVRIDRSGQVTVIAKHVEM
GQGAYTGLATLVAEELDADWSRVVVEGAPADAKRYNNLAFGPFQGTGGSSSIANAYEQMRKAGATARAMLVA
AAARQWQVPADSLRVEQGVISHAASGRKAGFGELAEAAARESVPASVPLKEPKDFRLIGQAVPRKDSPDKTDG
RAVFTQDFKLPGMLVAVVAYPPRFGAVPAKVDATRAKAVPGVVEVVEFRDLPHGRAGVAVLAHNTWAARSGR
DALAVEWDEQRAFTLGSEEIFARYRDAAAQPGTVATRQGEVEPVLAKAHQRIEAEFEFPYLAHSAMEPMNCLV
RLSEGACELWNGEQWQTGDQASVARLLGIAPEKVTITQLYAGGSFGRRANPVSDYPLEAVAIAKAAWDKGVKA
PVKLVWTREDDTRGGYYRPAYLHRARLALDAEGRLTAWHHRIVGQSIIKGTGFEAVMIKDGVDQTSVEGLSNLS
YAVPNLQVELSTPSDIGVPVQWWRSVGHTHTGFAAEVLVDEAATTAGKDPYAFRHGLLEKHPRHRGVLELVAD
KAGWSQPLKPGAEGEKRGRGIAVHESFGSYVAQVAEVTVKADGSFRVDRVVCAVDCGLAINPDVIKAQMEGG
IGFGLAAALHGAITLREGRVEQSNFHDYQVLRMNEMPVVEVHILPSAEKPTGVGEPGVPPLAPAVANALYAATG
VRLRKLPFPTQVKA
>SEQF5477||SEQF5477.1_02304

MSTFEQDAAPETGIFNVSRRTFLRGSGGLALGVLFSPLLDAAELLAGNGSFEPNAFVRIDRSGQVTVIAKHVEM
GQGAYTGLATLVAEELDADWSRVVVEGAPADAKRYNNLAFGPFQGTGGSSSIANAYEQMRKAGATARAMLVA
AAARQWQVPADSLRVEQGVISHAASNRKAGFGELAEAAARESVPASVPLKEPKDFRLIGQAVPRKDSPDKTDG
RAVFTQDFKLPGMLVAVVAYPPRFGAVPAKVDATRAKAVPGVVEVVEFRDLPHGRAGVAVLAHNTWAARSGR
DALAVEWDEQRAFTLGSEEIFARYRDAAAQPGTVATRQGDVEPVLAKAHQRIEAEFEFPYLAHSAMEPMNCLV
RLSEGACELWNGEQWQTGDQASVARLLGIAPEKVTITQLYAGGSFGRRANPVSDYPLEAVAIAKAAWDKGVKA
PVKLVWTREDDTRGGYYRPAYLHRARLALDAEGRLTAWHHRIVGQSIIKGTGFEAVMIKDGVDQTSVEGLSNLS
YAVPNLQVELSTPSDIGVPVQWWRSVGHTHTGFAAEVLADEAATTAGKDPYAFRHGLLEKHPRHRGVLELVAD
KAGWSQPLKPGAEGEKRGRGIAVHESFGSYVAQVAEVTVKADGSFRVDRVVCAVDCGLAINPDVIKAQMEGG
IGFGLAAALHGAITLREGRVEQSNFHDYQVLRMNEMPVVEVHILPSAEKPTGVGEPGVPPLAPAVANALYAATG
VRLRKLPFPSQVKA
>SEQF5478||SEQF5478.1_03645
MSTFEQDAAPETGIFNVSRRTFLRGSGGLALGVLFSPLLDAAELLAGNGSFEPNAFVRIDSSGQVTVIAKHVEM
GQGAYTGLATLVAEELDADWSRVVVEGAPADAKRYNNLAFGPFQGTGGSSSIANAYEQMRKAGATARAMLVA
AAARQWQVPADSLRVEQGVISHAASGRKAGFGELAEAAARESVPASVTLKAPKDFRLIGQAVPRKDSPDKTDG
RAVFTQDFKLPGMLVAVVAYPPRFGAVPAKVDATRAKAVPGVVEVVEFRDLPHGRAGVAVLAHNTWAARSGR
DALTVEWDEQRAFTLGSEEIFARYRDAAAQPGTVATRQGDVEPVLAKAHQRIEAEFEFPYLAHSAMEPMNCLV
RLSEGACELWNGEQWQTGDQASVARLLGIVPEKVTITQLYAGGSFGRRANPVSDYPLEAVAIAKAAWDKGVKA
PVKLVWTREDDTRGGYYRPAYLHRARLALDAEGRLTAWHHRIVGQSIIKGTGFEAVMIKDGVDQTSVEGLANLS
YAVPNLQVELSTPSDIGVPVQWWRSVGHTHTGFAAEVLVDEAATTAGKDPYAFRHGLLEKHPRHRGVLELVAD
KAGWSKPLKPGAEGEKRGRGIAVHESFGSYVAQVAEVTVKADGSFRVDRVVCAVDCGLAINPDVIKAQMEGG
IGFGLAAALHGAITLKEGRVEQSNFHDYQVLRMNEMPVVEVHILPSAEKPTGVGEPGVPPLAPAVANALYAATG
VRLRKLPFPTQVKA
>SEQF5479||SEQF5479.1_02696
MSTFEQDAAPETGIFNVSRRTFLRGSGGLALGVLFSPLLDAAELLAGNGSFEPNAFVRIDSSGQVTVIAKHVEM
GQGAYTGLATLVAEELDADWSRVVVEGAPADAKRYNNLAFGPFQGTGGSSSIANAYEQMRKAGATARAMLVA
AAARQWQVPADSLRVEQGVISHAASGRKAGFGELAEAAARESVPASVTLKEPKDFRLIGQAVPRKDSPDKTDG
RAVFTQDFKLPGMLVAVVAYPPRFGAVPAKVDATRAKAVPGVVEVVEFRDLPHGRAGVAVLAHNTWAARSGR
DALTVEWDEQRAFTLGSEEIFARYRDAAAQPGTVATRQGDVEPVLAKAHQRIEAEFEFPYLAHSAMEPMNCLV
RLSEGACELWNGEQWQTGDQASVARLLGIVPEKVTITQLYAGGSFGRRANPVSDYPLEAVAIAKAAWDKGVKA
PVKLVWTREDDTRGGYYRPAYLHRARLALDAEGRLTAWHHRIVGQSIIKGTGFEAVMIKDGVDQTSVEGLANLS
YAVPNLQVELSTPSDIGVPVQWWRSVGHTHTGFAAEVLVDEAATTAGKDPYAFRHGLLEKHPRHRGVLELVAD
KAGWSRPLKPGAEGEKRGRGIAVHESFGSYVAQVAEVTVKADGSFRVDRVVCAVDCGLAINPDVIKAQMEGG
IGFGLAAALHGAITLKEGRVEQSNFHDYQVLRMNEMPVVEVHILPSAEKPTGVGEPGVPPLAPAVANALYAATG
VRLRKLPFPTQVKA
>SEQF5480||SEQF5480.1_04208
MSTFEQDAAPETGIFNVSRRTFLRGSGGLALGVLFSPLLDAAELLAGNGSFEPNAFVRIDRSGQVTVIAKHVEM
GQGAYTGLATLVAEELDADWSRVVVEGAPADAKRYNNLAFGPFQGTGGSSSIANAYEQMRKAGATARAMLVA
AAARQWQVPADSLRVEQGVISHAASGRKAGFGELAEAAARESVPASVPLKEPKDFRLIGQAVPRKDSPDKTDG
RAVFTQDFKLPGMLVAVVAYPPRFGAVPAKVDATRAKAVPGVVEVVEFRDLPHGRAGVAVLAHNTWAARSGR
DALAVEWDEQRAFTLGSEEIFARYRDVAAQPGTVATRQGEVEPVLAKAHQRIEAEFEFPYLAHSAMEPMNCLV
RLSEGACELWNGEQWQTGDQASVARLLGIAPEKVTITQLYAGGSFGRRANPVSDYPLEAVAIAKAAWDKGVKA
PVKLVWTREDDTRGGYYRPAYLHRARLALDAEGRLTAWHHRIVGQSIIKGTGFEAVMIKDGVDQTSVEGLSNLS
YAVPNLQVELSTPSDIGVPVQWWRSVGHTHTGFAAEVLVDEAATTAGKDPYAFRHGLLEKHPRHRGVLELVAD

KAGWSRPLKPGAEGEKRGRGIAVHESFGSYVAQVAEVTVKADGSFRVDRVVCAVDCGLAINPDVIKAQMEGG
IGFGLAAALHGAITLREGRVEQSNFHDYQVLRMNEMPVVEVHILPSAEKPTGVGEPGVPPLAPAVANALYAATG
VRLRKLPFPSQVKA
>SEQF5481||SEQF5481.1_00210
MSTFEQDAAPETGIFNVSRRTFLRGSGGLALGVLFSPLLDAAELLAGNGSFEPNAFVRIDSSGQVTVIAKHVEM
GQGAYTGLATLVAEELDADWSRVVVEGAPADAKRYNNLAFGPFQGTGGSSSIANAYEQMRKAGATARAMLVA
AAARQWQVPADSLRVEQGVISHAASGRKAGFGELAEAAARESVPASVPLKAPKDFRLIGQAVPRKDSPDKTDG
RAVFTQDFKLPGMLVAVVAYPPRFGAVPAKVDATRAKAVPGVVEVVEFRDLPHGRAGVAVLAHNTWAARSGR
DALTVEWDEQRAFTLGSEEIFARYRDAAAQPGTVATRQGDVEPVLAKAHQRIEAEFEFPYLAHSAMEPMNCLV
RLSEGACELWNGEQWQTGDQASVARLLGIAPEKVTITQLYAGGSFGRRANPVSDYPLEAVAIAKAAWDKGVKA
PVKLVWTREDDTRGGYYRPAYLHRARLALDAEGRLTAWHHRIVGQSIIKGTGFEAVMIKDGVDQTSVEGLANLS
YAVPNLQVELSTPSDIGVPVQWWRSVGHTHTGFAAEVLVDEAATTAGKDPYAFRHGLLEKHPRHRGVLELVAD
KAGWSKPLKPGAEGEKRGRGIAVHESFGSYVAQVAEVTVKADGSFRVDRVVCAVDCGLAINPDVIKAQMEGG
IGFGLAAALHGAITLKEGRVEQSNFHDYQVLRMNEMPVVEVHILPSAEKPTGVGEPGVPPLAPAVANALYAATG
VRLRKLPFPTQVKA
>SEQF5482||SEQF5482.1_00025
MSTFEQDAAPETGIFNVSRRTFLRGSGGLALGVLFSPLLDAAELLAGNGSFEPNAFVRIDRSGQVTVIAKHVEM
GQGAYTGLATLVAEELDADWSRVVVEGAPADAKRYNNLAFGPFQGTGGSSSIANAYEQMRKAGATARAMLVA
AAARQWQVPADSLRVEQGVISHAASGRKAGFGELAEAAARESVPASVPLKEPKDFRLIGQAVPRKDSPDKTDG
RAVFTQDFKLPGMLVAVVAYPPRFGAVPAKVDATRAKAVPGVVEVVEFRDLPHGRAGVAVLAHNTWAARSGR
DALAVEWDEQRAFTLGSEEIFARYRDAAAQPGTVATRQGEVEPVLAKAHQRIEAEFEFPYLAHSAMEPMNCLV
RLSEGACELWNGEQWQTGDQASVARLLGIAPEKVTITQLYAGGSFGRRANPVSDYPLEAVAIAKAAWDKGVKA
PVKLVWTREDDTRGGYYRPAYLHRARLALDAEGRLTAWHHRIVGQSIIKGTGFEAVMIKDGVDQTSVEGLSNLS
YAVPNLQVELSTPSDIGVPVQWWRSVGHTHTGFAAEVLVDEAATTAGKDPYAFRHGLLEKHPRHRGVLELVAD
KAGWSRPLKPGGEGEKRGRGIAVHESFGSYVAQVAEVTVKADGSFRVDRVVCAVDCGLAINPDVIKAQMEGG
IGFGLAAALHGAITLREGRVEQSNFHDYQVLRMNEMPVVEVHILPSAEKPTGVGEPGVPPLAPAVANALYAATG
VRLRKLPFPSQVKA
>SEQF5484||SEQF5484.1_00619
MSTFEQDAAPETGIFNVSRRTFLRGSGGLALGVLFSPLLDAAELLAGNGSFEPNAFVRIDSSGQVTVIAKHVEM
GQGAYTGLATLVAEELDADWSRVVVEGAPADAKRYNNLAFGPFQGTGGSSSIANAYEQMRKAGATARAMLVA
AAARQWQVPADSLRVEQGVISHAASGRKAGFGELAEAAARESVPASVTLKAPKDFRLIGQAVPRKDSPDKTDG
RAVFTQDFKLPGMLVAVVAYPPRFGAVPAKVDATRAKAVPGVVEVVEFRDLPHGRAGVAVLAHNTWAARSGR
DALTVEWDEQRAFTLGSEEIFARYRDAAAQPGTVATRQGDVEPVLAKAHQRIEAEFEFPYLAHSAMEPMNCLV
RLSEGACELWNGEQWQTGDQASVARLLGIAPEKVTITQLYAGGSFGRRANPVSDYPLEAVAIAKAAWDKGVKA
PVKLVWTREDDTRGGYYRPAYLHRARLALDAEGRLTAWHHRIVGQSIIKGTGFEAVMIKDGVDQTSVEGLANLS
YAVPNLQVELSTPSDVGVPVQWWRSVGHTHTGFAAEVLVDEAATTAGKDPYAFRHGLLEKHPRHRGVLELVAD
KAGWSKPLKPGAEGEKRGRGIAVHESFGSYVAQVAEVTVMADGSFRVDRVVCAVDCGLAINPDVIKAQMEG
GIGFGLAAALHGAITLKEGRVEQSNFHDYQVLRMNEMPVVEVHILPSAEKPTGVGEPGVPPLAPAVANALYAAT
GVRLRKLPFPTQVKA
>SEQF5485||SEQF5485.1_01758
MSTFEQDAAPETGIFNVSRRTFLRGSGGLALGVLFSPLLDAAELLAGNGSFEPNAFVRIDSSGQVTVIAKHVEM
GQGAYTGLATLVAEELDADWSRVVVEGAPADAKRYNNLAFGPFQGTGGSSSIANAYEQMRKAGATARAMLVA
AAARQWQVPADSLRVEQGVISHAASGRKAGFGELAEAAARESVPASVTLKAPKDFRLIGQAVPRKDSPDKTDG
RAVFTQDFKLPGMLVAVVAYPPRFGAVPAKVDATRAKAVPGVVEVVEFRDLPHGRAGVAVLAHNTWAARSGR

DALTVEWDEQRAFTLGSEEIFARYRDAAAQPGTVATRQGDVEPVLAKAHQRIEAEFEFPYLAHSAMEPMNCLV
RLSEGACELWNGEQWQTGDQASVARLLGIVPEKVTITQLYAGGSFGRRANPVSDYPLEAVAIAKAAWDKGVKA
PVKLVWTREDDTRGGYYRPAYLHRARLALDAEGRLTAWHHRIVGQSIIKGTGFEAVMIKDGVDQTSVEGLANLS
YAVPNLQVELSTPSDIGVPVQWWRSVGHTHTGFAAEVLVDEAATTAGKDPYAFRHGLLEKHPRHRGVLELVAD
KAGWSKPLKPGAEGEKRGRGIAVHESFGSYVAQVAEVTVKADGSFRVDRVVCAVDCGLAINPDVIKAQMEGG
IGFGLAAALHGAITLKEGRVEQSNFHDYQVLRMNEMPVVEVHILPSAEKPTGVGEPGVPPLAPAVANALYAATG
VRLRKLPFPTQVKA
>SEQF5486||SEQF5486.1_01345
MSTFEQDAAPETGIFNVSRRTFLRGSGGLALGVLFSPLLDAAELLAGNGSFEPNAFVRIDSSGQVTVIAKHVEM
GQGAYTGLATLVAEELDADWSRVVVEGAPADAKRYNNLAFGPFQGTGGSSSIANAYEQMRKAGATARAMLVA
AAARQWQVPADSLRVEQGVISHAASGRKAGFGELAEAAARESVPASVTLKAPKDFRLIGQAVPRKDSPDKTDG
RAVFTQDFKLPGMLVAVVAYPPRFGAVPAKVDATRAKAVPGVVEVVEFRDLPHGRAGVAVLAHNTWAARSGR
DALTVEWDEQRAFTLGSEEIFARYRDAAAQPGTVATRQGDVEPVLAKAHQRIEAEFEFPYLAHSAMEPMNCLV
RLSEGACELWNGEQWQTGDQASVARLLGIVPEKVTITQLYAGGSFGRRANPVSDYPLEAVAIAKAAWDKGVKA
PVKLVWTREDDTRGGYYRPAYLHRARLALDAEGRLTAWHHRIVGQSIIKGTGFEAVMIKDGVDQTSVEGLANLS
YAVPNLQVELSTPSDIGVPVQWWRSVGHTHTGFAAEVLVDEAATTAGKDPYAFRHGLLEKHPRHRGVLELVAD
KAGWSKPLKPGAEGEKRGRGIAVHESFGSYVAQVAEVTVKADGSFRVDRVVCAVDCGLAINPDVIKAQMEGG
IGFGLAAALHGAITLKEGRVEQSNFHDYQVLRMNEMPVVEVHILPSAEKPTGVGEPGVPPLAPAVANALYAATG
VRLRKLPFPTQVKA
>SEQF5487||SEQF5487.1_01120
MSTFEQDAAPETGIFNVSRRTFLRGSGGLALGVLFSPLLDAAELLAGNGSFEPNAFVRIDSSGQVTVIAKHVEM
GQGAYTGLATLVAEELDADWSRVVVEGAPADAKRYNNLAFGPFQGTGGSSSIANAYEQMRKAGATARAMLVA
AAARQWQVPADSLRVEQGVISHAASGRKAGFGELAEAAARESVPASVTLKAPKDFRLIGQAVPRKDSPDKTDG
RAVFTQDFKLPGMLVAVVAYPPRFGAVPAKVDATRAKAVPGVVEVVEFRDLPHGRAGVAVLAHNTWAARSGR
DALTVEWDEQRAFTLGSEEIFARYRDAAAQPGTVATRQGDVEPVLAKAHQRIEAEFEFPYLAHSAMEPMNCLV
RLSEGACELWNGEQWQTGDQASVARLLGIVPEKVTITQLYAGGSFGRRANPVSDYPLEAVAIAKAAWDKGVKA
PVKLVWTREDDTRGGYYRPAYLHRARLALDAEGRLTAWHHRIVGQSIIKGTGFEAVMIKDGVDQTSVEGLANLS
YAVPNLQVELSTPSDIGVPVQWWRSVGHTHTGFAAEVLVDEAATTAGKDPYAFRHGLLEKHPRHRGVLELVAD
KAGWSKPLKPGAEGEKRGRGIAVHESFGSYVAQVAEVTVKADGSFRVDRVVCAVDCGLAINPDVIKAQMEGG
IGFGLAAALHGAITLKEGRVEQSNFHDYQVLRMNEMPVVEVHILPSAEKPTGVGEPGVPPLAPAVANALYAATG
VRLRKLPFPTQVKA
>SEQF5488||SEQF5488.1_01544
MSTFEQDAAPETGIFNVSRRTFLRGSGGLALGVLFSPLLDAAELLAGNGSFEPNAFVRIDRSGQVTVIAKHVEM
GQGAYTGLATLVAEELDADWSRVVVEGAPADAKRYNNLAFGPFQGTGGSSSIANAYEQMRKAGATARAMLVA
AAARQWQVPADSLRVEQGVISHAASGRKAGFGELAEAAARESVPASVPLKEPKDFRLIGQAVPRKDSPDKTDG
RAVFTQDFKLPGMLVAVVAYPPRFGAVPAKVDATRAKAVPGVVEVVEFRDLPHGRAGVAVLAHNTWAARSGR
DALAVEWDEQRAFTLGSEEIFARYRDAAAQPGTVATRQGEVEPVLAKAHQRIEAEFEFPYLAHSAMEPMNCLV
RLSEGACELWNGEQWQTGDQASVARLLGIAPEKVTITQLYAGGSFGRRANPVSDYPLEAVAIAKAAWDKGVKA
PVKLVWTREDDTRGGYYRPAYLHRARLALDAEGRLTAWHHRIVGQSIIKGTGFEAVMIKDGVDQTSVEGLSNLS
YAVPNLQVELSTPSDIGVPVQWWRSVGHTHTGFAAEVLVDEAATIAGKDPYAFRHGLLEKHPRHRGVLELVAD
KAGWSQPLKPGAEGEKRGRGIAVHESFGSYVAQVAEVTVKADGSFRVDRVVCAVDCGLAINPDVIKAQMEGG
IGFGLAAALHGAITLREGRVEQSNFHDYQVLRMNEMPVVEVHILPSAEKPTGVGEPGVPPLAPAVANALYAATG
VRLRKLPFPTQVKA
>SEQF5489||SEQF5489.1_05250

MSTFEQDAAPETGIFNVSRRTFLRGSGGLALGVLFSPLLDAAELLAGSGSFEPNAFVRIDSSGQVTVIAKHVEMG
QGAYTGLATLVAEELDADWSRVVVEGAPADAKRYNNLAFGPFQGTGGSSSIANAYEQMRKAGATARAMLVAA
AARQWQVPADSLRVEQGVISHAASGRKAGFGELAEAAARESVPASVTLKAPKDFRLIGQAVPRKDSPDKTDGR
AVFTQDFKLPGMLVAVVAYPPRFGAVPAKVDATRAKAVPGVVEVVEFRDLPHGRAGVAVLAHNTWAARSGRD
ALTVEWDEQRAFTLGSEEIFARYRDAAAQPGTVATRQGDVEPVLAKAHQRIEAEFEFPYLAHSAMEPMNCLVR
LSEGACELWNGEQWQTGDQASVARLLGIAPEKVTITQLYAGGSFGRRANPVSDYPLEAVAIAKAAWDKGVKAP
VKLVWTREDDTRGGYYRPAYLHRARLALDAEGRLTAWHHRIVGQSIIKGTGFEAVMIKDGVDQTSVEGLSNLSY
AIPNLQVELSTPSDIGVPVQWWRSVGHTHTGFAAEVLVDEAATTAGKDPYAFRHGLLEKHPRHRGVLELVADK
AGWSKPLKPGAEGEKRGRGIAVHESFGSYVAQVAEVTVKADGSFRVDRVVCAVDCGLAINPDVIKAQMEGGI
GFGLAAALHGAITLKEGRVEQSNFHDYQVLRMNEMPVVEVHILPSAEKPTGVGEPGVPPLAPAVANALYAATG
VRLRKLPFPTQVKA
>SEQF5490||SEQF5490.1_00634
MSTFEQDAAPETGIFNVSRRTFLRGSGGLALGVLFSPLLDAAELLAGNGSFEPNAFVRIDSSGQVTVIAKHVEM
GQGAYTGLATLVAEELDADWSRVVVEGAPADAKRYNNLAFGPFQGTGGSSSIANAYEQMRKAGATARAMLVA
AAARQWQVPADSLRVEQGVISHAASGRKAGFGELAESAARESVPASVTLKEPKDFRLIGQAVPRKDSPDKTDG
RAVFTQDFKLPGMLVAVVAYPPRFGAVPAKVDATRAKAVPGVVEVVEFRDLPHGRAGVAVLAHNTWAARSGR
DALTVEWDEQRAFTLGSEEIFARYRDAAAQPGTVATRQGDVEPVLAKAHQRIEAEFEFPYLAHSAMEPMNCLV
RLSEGACELWNGEQWQTGDQASVARLLGIAPEKVTITQLYAGGSFGRRANPVSDYPLEAVAIAKAAWDKGVKA
PVKLVWTREDDTRGGYYRPAYLHRARLALDAEGRLTAWHHRIVGQSIIKGTGFEAVMIKDGVDQTSVEGLANLS
YAVPNLQVELSTPSDIGVPVQWWRSVGHTHTGFAAEVLVDEAATTAGKDPYAFRHGLLEKHPRHRGVLELVAD
KAGWSKPLKPGAEGEKRGRGIAVHESFGSYVAQVAEVTVKADGSFRVDRVVCAVDCGLAINPDVIKAQMEGG
IGFGLAAALHGAITLKEGRVEQSNFHDYQVLRMNEMPVVEVHILPSAEKPTGVGEPGVPPLAPAVANALYAATG
VRLRKLPFPTQVKA
>SEQF5491||SEQF5491.1_00413
MSTFEQDAAPETGIFNISRRTFLRGSGGLALGVLFSPLLDAAELLAGNGSFEPNAFVRIDRSGQVTVIAKHVEMG
QGAYTGLATLVAEELDADWSRVVVEGAPADAKRYNNLAFGPFQGTGGSSSIANAYEQMRKAGATARAMLVAA
AARQWQVPADSLRVEQGVISHAASGRKAGFGELAEAAARESVPASVSLKVPKDFRLIGQAVPRKDSPDKTDGR
AVFTQDFKLPGMLVAVVAYPPRFGAVPAKVDATRAKAVPGVVEVVEFRDLPHGRAGVAVLAHNTWAARSGRD
ALAVEWDEQRAFTLGSEEIFARYRDAAAQPGTVATRQGDVEPVLAKAHQRIEAEFEFPYLAHSAMEPMNCLVR
LSEGACELWNGEQWQTGDQASVARLLGIAPEKVTITQLYAGGSFGRRANPVSDYPLEAVAIAKAAWDKGVKAP
VKLVWTREDDTRGGYYRPAYLHRARLALDAEGRLTAWHHRIVGQSIIKGTGFEAVMIKDGVDQTSVEGLANLSY
AVPNLQVELSTPSDIGVPVQWWRSVGHTHTGFAAEVLVDEAATTAGKDPYAFRHGLLEKHPRHRGVLELVADK
AGWSQPLKPGAEGEKRGRGIAVHESFGSYVAQVAEVTVKADGSFRVDRVVCAVDCGLAINPDVIKAQMEGGI
GFGLAAALHGAITLREGRVEQSNFHDYQVLRMNEMPVVEVHILPSAEKPTGVGEPGVPPLAPAVANALYAATG
VRLRKLPFPSQVKA
>SEQF5492||SEQF5492.1_01068
MSTFEQDAAPETGIFNVSRRTFLRGSGGLALGVLFSPLLDAAELLAGNGSFEPNAFVRIDRSGQVTVIAKHVEM
GQGAYTGLATLVAEELDADWSRVVVEGAPADAKRYNNLAFGPFQGTGGSSSIANAYEQMRKAGATARAMLVA
AAARQWQVPADSLRVEQGVISHAASGRKAGFGELAEAAARESVPASVPLKEPKDFRLIGQAVPRKDSPDKTDG
RAVFTQDFKLPGMLVAVVAYPPRFGAVPAKVDATRAKAVPGVVEVVEFRDLPHGRAGVAVLAHNTWAARSGR
DALAVEWDEQRAFTLGSEEIFARYRDAAAQPGTVATRQGEVEPVLAKAHQRIEAEFEFPYLAHSAMEPMNCLV
RLSEGACELWNGEQWQTGDQASVARLLGIAPEKVTITQLYAGGSFGRRANPVSDYPLEAVAIAKAAWDKGVKA
PVKLVWTREDDTRGGYYRPAYLHRARLALDAEGRLTAWHHRIVGQSIIKGTGFEAVMIKDGVDQTSVEGLSNLS
YAVPNLQVELSTPSDIGVPVQWWRSVGHTHTGFAAEVLVDEAATTAGKDPYAFRHGLLEKHPRHRGVLELVAD

KAGWSQPLKPGAEGEKRGRGIAVHESFGSYVAQVAEVTVKADGGFRVDRVVCAVDCGLAINPDVIKAQMEG
GIGFGLAAALHGAITLREGRVEQSNFHDYQVLRMNEMPVVEVHILPSAEKPTGVGEPGVPPLAPAVANALYAAT
GVRLRKLPFPTQVKA
>SEQF5493||SEQF5493.1_05342
MSTFEQDAAPETGIFNVSRRTFLRGSGGLALGVLFSPLLDAAELLAGNGSFEPNAFVRIDSSGQVTVIAKHVEM
GQGAYTGLATLVAEELDADWSRVVVEGAPADAKRYNNLAFGPFQGTGGSSSIANAYEQMRKAGATARAMLVA
AAARQWQVPADSLRVEQGVISHAASGRKAGFGELAEAAARESVPASVPLKAPKDFRLIGQAVPRKDSPDKTDG
RAVFTQDFKLPGMLVAVVAYPPRFGAVPAKVDATRAKAVPGVVEVVEFRDLPHGRAGVAVLAHNTWAARSGR
DALTVEWDEQRAFTLGSEEIFARYRDAAAQPGTVATRQGDVEPVLAKAHQRIEAEFEFPYLAHSAMEPMNCLV
RLSEGACELWNGEQWQTGDQASVARLLGIAPEKVTITQLYAGGSFGRRANPVSDYPLEAVAIAKAAWDKGVKA
PVKLVWTREDDTRGGYYRPAYLHRARLALDAEGRLTAWHHRIVGQSIIKGTGFEAVMIKDGVDQTSVEGLANLS
YAVPNLQVELSTPSDIGVPVQWWRSVGHTHTGFAAEVLVDEAATTAGKDPYAFRHGLLEKHPRHRGVLELVAD
KAGWSKPLKPGAEGEKRGRGIAVHESFGSYVAQVAEVTVKADGSFRVDRVVCAVDCGLAINPDVIKAQMEGG
IGFGLAAALHGAITLKEGRVEQSNFHDYQVLRMNEMPVVEVHILPSAEKPTGVGEPGVPPLAPAVANALYAATG
VRLRKLPFPTQVKA
>SEQF5494||SEQF5494.1_01336
MSTFEQDAAPETGIFNVSRRTFLRGSGGLALGVLFSPLLDAAELLAGNGSFEPNAFVRIDSSGQVTVIAKHVEM
GQGAYTGLATLVAEELDADWSRVVVEGAPADAKRYNNLAFGPFQGTGGSSSIANAYEQMRKAGATARAMLVA
AAARQWQVPADSLRVEQGVISHAASGRKAGFGELAEAAARESVPASVTLKAPKDFRLIGQAVPRKDSPDKTDG
RAVFTQDFKLPGMLVAVVAYPPRFGAVPAKVDATRAKAVPGVVEVVEFRDLPHGRAGVAVLAHNTWAARSGR
DALTVEWDEQRAFTLGSEEIFARYRDAAAQPGTVATRQGDVEPVLAKAHQRIEAEFEFPYLAHSAMEPMNCLV
RLSEGACELWNGEQWQTGDQASVARLLGIVPEKVTITQLYAGGSFGRRANPVSDYPLEAVAIAKAAWDKGVKA
PVKLVWTREDDTRGGYYRPAYLHRARLALDAEGRLTAWHHRIVGQSIIKGTGFEAVMIKDGVDQTSVEGLANLS
YAVPNLQVELSTPSDIGVPVQWWRSVGHTHTGFAAEVLVDEAATTAGKDPYAFRHGLLEKHPRHRGVLELVAD
KAGWSKPLKPGAEGEKRGRGIAVHESFGSYVAQVAEVTVKADGSFRVDRVVCAVDCGLAINPDVIKAQMEGG
IGFGLAAALHGAITLKEGRVEQSNFHDYQVLRMNEMPVVEVHILPSAEKPTGVGEPGVPPLAPAVANALYAATG
VRLRKLPFPTQVKA
>SEQF5495||SEQF5495.1_03963
MSTFEQDAAPETGIFNVSRRTFLRGSGGLALGVLFSPLLDAAELLAGNGSFEPNAFVRIDRSGQVTVIAKHVEM
GQGAYTGLATLVAEELDADWSRVVVEGAPADAKRYNNLAFGPFQGTGGSSSIANAYEQMRKAGATARAMLVA
AAARQWQVPADSLRVEQGVISHAASGRKAGFGELAEAAARESVPASVPLKAPKDFRLIGQAVPRKDSPDKTDG
RAVFTQDFKLPGMLVAVVAYPPRFGAVPAKVDATRAKAVPGVVEVVEFRDLPHGRAGVAVLAHNTWAARSGR
DALAVEWDEQRAFTLGSEEIFARYRDAAAQPGTVATRQGDVEPVLAKAHQRIEAEFEFPYLAHSAMEPMNCLV
RLSEGACELWNGEQWQTGDQASVARLLGIAPEKVTITQLYAGGSFGRRANPVSDYPLEAVAIAKAAWDKGVKA
PVKLVWTREDDTRGGYYRPAYLHRARLALDAEGRLTAWHHRIVGQSIIKGTGFEAVMIKDGVDQTSVEGLSNLS
YAVPNLQVELSTPSDIGVPVQWWRSVGHTHTGFAAEVLVDEAATTAGKDPYAFRHGLLEKHPRHRGVLELVAD
KAGWSQPLKPGAEGEKRGRGIAVHESFGSYVAQVAEVTVKADGSFRVDRVVCAVDCGLAINPDVIKAQMEGG
IGFGLAAALHGAITLREGRVEQSNFHDYQVLRMNEMPVVEVHILPSAEKPTGVGEPGVPPLAPAVANALYAATG
VRLRKLPFPTQVKA
>SEQF5855||SEQF5855.1_03927
MPQRPSNERGRTAGSAISLRRRHLLQSAAALLVAPAAGSLLIPLAQAAPAEAGAAAATASSIGDWVWIEPSGQV
VIGVSQCEVGQGIYTGLPQVLADELDADWASVTVRFVTGRDAYRNDAGEMPFQQFVGASMSMNYFYERMR
LAGAQARDVLLRAGAARLGVRASQCSTRAGRVLHSATGRSVGYGEIVAEASRLPLAARPRMKSASEQGLIGRNL
RRVDTPAKVDGSAVFGIDVEVPGMLIGAVRMAPSVTGRIVRIRNEGEVSARPGVHAVVRTTQWPDPEPSTVV

VVADSYWIAKQAADALDIEFDAGAAAGVDSERIHAQFVAGLASDKAVVARSLGKPREMLAAGKPITADYHSPYI
THATMEPLAATVHVRDGEVETWGPYQGQDFLRGELGKACGVPADKVIVHTTFLGGSFGRKYMPDFALHAAA
ASKAVGRPVKVIRSREDDIRHSYYRPGASGRLSAVLGADGLPAALHARISGQSLYGAINPKKMADAGGWDETM
VESIYDLIYGVPNLLVDAVDVQQPIPLSYLRSVGTTSSVFFLESFISELAHTAGVDDYQYRRRLLAGQPLALGVLDA
AARAARWEQPVPAGLHRAMTFNVYTGRGESFQTFVALVMELRVVEGRVRLERAICAIDAGRVVNPGLVKANV
EGGIGFALTNTFKSRLGFDKGVVQQSNFHDYPLLQLSEMPRVEVVLVESDRPPQGCGEVALGPTAPAVATALFH
ATGRRFRSMPLPQDIAST
>SEQF5856||SEQF5856.1_01958
MPQRPSNERGRTAGSAISLRRRHLLQSAAALLVAPAAGSLLIPLAQAAPAEAGAAAATASSIGDWVWIEPSGQV
VIGVSQCEVGQGIYTGLPQVLADELDADWASVTVRFVTGRDAYRNDAGEMPFQQFVGASMSMNYFYERMR
LAGAQARDVLLRAGAARLGVRASQCSTRAGRVLHSATGRSVGYGEIVADASRLSIAARPRMKSASEQGLIGRNL
RRVDTPAKVDGSAVFGIDVEVPGMLIGAVRMAPSVTGRIVRIRNEGEVRTRPGVHAIVRTTQWPDPEPSTVVV
VADSYWIAKQAADALDIEFDAGAAAGVDSERIHAQFVAGLASDKAVVARSLGKPREMLAAGKPITADYHSPYIT
HATMEPLAATVHVRDGEVETWGPYQGQDFLRGELGKACGVPADKVIVHTTFLGGSFGRKYMPDFALHAAAA
SKAVGRPVKVIRSREDDIRHSYYRPGASGRLSAVLGADGLPAALHARISGQSLYGAINPKKMADAGGWDETMV
ESIYDLIYGVPNLLVDAVDVQQPIPLSYLRSVGTTSSVFFLESFISELAHTAGVDDYQYRRRLLAGQPLALGVLDAA
ARAARWEQPAPAGLHRAMTFNVYTGRGESFQTFVALVMELRVVEGRVRLERAICAIDAGRVVNPGLVKANVE
GGIGFALTNTFKSRLGFDKGVVQQSNFHDYPLLQLSEMPRVEVVLVESDRPPQGCGEVALGPTAPAVATALFHA
TGRRFRSMPLPQDIAST
>SEQF5857||SEQF5857.1_05568
MPQRPSNERGRTAGSAISLRRRHLLQSAAALLVAPAAGSLLIPLAQAAPAEAGAAAATASSIGDWVWIEPSGQV
VIGVSQCEVGQGIYTGLPQVLADELDADWASVTVRFVTGRDAYRNDAGEMPFQQFVGASMSMNYFYERMR
LAGAQARDVLLRAGAARLGVRASQCSTGAGRVLHSATGRSVGYGEIVAEASRLSIAARPRMKSASEQGLIGRNL
RRVDTPAKVDGSAVFGIDVEVPGMLIGAVRMAPSVTGRIVRIRNEAEVRARPGVHAVVRTTQWPDPEPSTVV
VVADSYWIAKQAADALDIEFDAGAAAGVDSERIHAQFVAGLSNDKAVVARSLGKPREMLAAGKPITADYHSPYI
THATMEPLAATVHVRDGEVETWGPYQGQDFLRGELGKACGVPADKVIVHTTFLGGSFGRKYMPDFALHAAA
ASKAVGRPVKVIRSREDDIRHSYYRPGASGRLSAVLGADGLPAALHARISGQSLYGAINPKKMADAGGWDETM
VESIYDLIYGVPNLLVDAVDVQQPIPLSYLRSVGSTSSVFFLESFISELAHTAGVDDYQYRRRLLAGQPLALGVLDA
AAKAARWEQPAPAGLHRAMTFNVYTGRGESFQTFVALVMELRVVEGRVRLERAICAIDAGRVVNPGLVKANV
EGGIGFALTNTFKSRLGFDKGVVQQSNFHDYPLLQLSEMPRVEVVLVESDRPPQGCGEVALGPTAPAVATALFH
ATGRRFRSMPLPQDIAST
>SEQF5858||SEQF5858.1_05719
MPQRPSNERGRTAGSAISLRRRHLLQGAAALLVAPAAGSLLVPLAQAAPAGAGAAAATASSIGDWLWIEPSGQ
VVIGVSQCEVGQGIYTGLPQVLADELDADWASVTVRFVTGRDAYRNDAGEMPFQQFVGASMSMNYFYERM
RLAGAQARDVLLRAGAARLGVRPSQCSTRAGRVLHSATGRSVGYGEIVAEASRLSIAARPRMKSASEQGLIGRN
LRRVDTPAKVDGSAVFGIDVEVPGMLIGAVRMAPSVTGRIVRIRNEAEVRARPGVHAIVRTTQWPDPEPSTVV
VVADSYWIAKQAADALDIEFDAGAAAGVHSERIHAQFVAGLASDKAVVARSLGKPREMLAAGKPITADYHSPYI
THATMEPLAATVHVRDGEVETWGPYQGQDFLRGELGKACGVPADKVIVHTTFLGGSFGRKYMPDFALHAAA
ASKAVGRPVKVIRSREDDIRHSYYRPGASGRLSAVLGADGMPAALHARISGQSLYGAINPKKMADAGGWDET
MVESIYDLIYGVPNLLVDAVDVQQPIPLSYLRSVGTTSSVFFLESFISELAHTAGVDDYQYRRRLLAGQPLALGVLD
AAAQAARWEQPAPAGLHRAMSFNVYTGRGESFQTFVALVMELRVVEGRVRLERAICAIDAGRVVNPGLVKAN
VEGGIGFALTNTFKSRLGFDKGVVQQSNFHDYPLLQLAEMPRVEVVLVESDRPPQGCGEVALGPTAPAVATALF
HATGRRFRSMPLPQDIASA
>SEQF5859||SEQF5859.1_04376

MPQRPSNERGRTAGSAISLRRRHLLQSAAALLVAPAAGSLLIPLAQAAPAQAGAAAATASSIGDWVWIEPSGQV
VIGVSQCEVGQGIYTGLPQVLADELDADWASVTVRFVTGRDAYRNDAGEMPFQQFVGASMSMNYFYERMR
LAGAQARDVLLRAGAARLGVRASQCSTRAGRVLHSATGRSVGYGEIVADASRLSIAARPRMKSASEQGLIGRNL
RRVDTPAKVDGSAVFGIDVEVPGMLIGAVRMAPSVTGRIVRIRNEAEVRARTGVHAVVRTTQWPDPEPSTVV
VVADSYWIAKQAADALDIEFDAGAAASVDSERIHAQFVAGLASDKAVVARNLGKPREMLAAGKPITADYHSPYI
THATMEPLAATVHVRDGEVETWGPYQGQDFLRGELGKACGVPADKVIVHTTFLGGSFGRKYMPDFALHAAA
ASKAVGRPVKVIRSREDDIRHSYYRPGASGRLSAVLGADGLPAALHARISGQSLYGAINPKKMADAGGWDETM
VESIYDLIYGVPNLLVDAVDVQQPIPLSYLRSVGTTSSVFFLESFISELAHTAGVDDYQYRRRLLAGQPLALGVLDA
AARAARWEQPAPAGLHRAMTFNVYTGRGESFQTFVALVMELRVVEGRVRLERAICAIDAGRVVNPGLVKANV
EGGIGFALTNTFKSRLGFDKGVVQQSNFHDYPLLQLAEMPRVEVVLVESDRPPQGCGEVALGPTAPAVATALFH
ATGRRFRSMPLPQDIAST
>SEQF5860||SEQF5860.1_00375
MPQRPSNERGRTAGSAISLRRRHLLQSAAALLVAPAAGSLLIPLAQAAPAEAGAAAATASSIGDWVWIEPSGQV
VIGVSQCEVGQGIYTGLPQVLADELDADWASVTVRFVTGRDAYRNDAGEMPFQQFVGASMSMNYFYERMR
LAGAQARDVLLRAGAARLGVRASQCSTRAGRVLHSATGRSVGYGEIVADASRLSIAARPRMKSASEQGLIGRNL
RRVDTPAKVDGSAVFGIDVEVPGMLIGAVRMAPSVTGRIVRIRNEGEVRTRPGVHAIVRTTQWPDPEPSTVVV
VADSYWIAKQAADALDIEFDAGAAAGVDSERIHAQFVAGLASDKAVVARSLGKPREMLAAGKPITADYHSPYIT
HATMEPLAATVHVRDGEVETWGPYQGQDFLRGELGKACGVPADKVIVHTTFLGGSFGRKYMPDFALHAAAA
SKAVGRPVKVIRSREDDIRHSYYRPGASGRLSAVLGADGLPAALHARISGQSLYGAINPKKMADAGGWDETMV
ESIYDLIYGVPNLLVDAVDVQQPIPLSYLRSVGTTSSVFFLESFISELAHTAGVDDYQYRRRLLAGQPLALGVLDAA
AKAARWEQPAPAGLHRAMTFNVYTGRGESFQTFVALVMELRVVEGRVRLERAICAIDAGRVVNPGLVKANVE
GGIGFALTNTFKSRLGFDKGVVQQSNFHDYPLLQLSEMPRVEVVLVESDRPPQGCGEVALGPTAPAVATALFHA
TGRRFRSMPLPQDITST
>SEQF5861||SEQF5861.2_02730
MPQRPSNERGRTAGSAISLRRRHLLQSAAALLVAPAAGSLLIPLAQAAPAGAGAAAATASSIGDWVWIEPSGQV
VIGVSQCEVGQGIYTGLPQVLADELDADWASVTVRFVTGRDAYRNDAGEMPFQQFVGASMSMNYFYERMR
LAGAQARDVLLRAGAARLGVRASQCSTRAGRVLHSATGRSVGYGEIVADASRLSIAARPRMKSASEQGLIGRNL
RRVDTPAKVDGSAVFGIDVEVPGMLIGAVRMAPSVTGRIVRIRNEAEVRARPGVHAIVRTTQWPDPEPSTVVV
VADSYWIAKQAADALDIEFDAGAAAGVDSERIHAQFVAGLSNDKAVVARSLGKPREMLAAGKPITADYHSPYIT
HATMEPLAATVHVRDGEVETWGPYQGQDFLRGELGKACGVPADKVIVHTTFLGGSFGRKYMPDFALHAAAA
SKAVGRPVKVIRSREDDIRHSYYRPGASGRLSAVLGADGLPAALHARISGQSLYGAINPKKMADAGGWDETMV
ESIYDLIYGVPNLLVDAVDVQQPIPLSYLRSVGTTSSVFFLESFISELAHTAGVDDYQYRRRLLAGQPLALGVLDAA
AKAARWEQPAPAGLHRAMTFNVYTGRGESFQTFVALVMELRVVEGRVRLERAICAIDAGRVVNPGLVKANVE
GGIGFALTNTFKSRLGFDKGVVQQSNFHDYPLLQLSEMPRVEVVLVESDRPPQGCGEVALGPTAPAVATALFHA
TGRRFRSMPLPQDIAST
>SEQF5862||SEQF5862.1_01897
MPQRPSNERGRTAGSAISLRRRHLLQSAAALLVAPAAGSLLIPLAQAAPAEAGAAAATASSIGDWVWIEPSGQV
VIGVSQCEVGQGIYTGLPQVLADELDADWASVTVRFVTGRDAYRNDAGEMPFQQFVGASMSMNYFYERMR
LAGAQARDVLLRAGAARLGVRASQCSTRAGRVLHSATGRSVGYGEIVADASRLPLAARPRMKSASEQGLIGRN
LRRVDTPAKVDGSAVFGIDVEVPGMLIGAVRMAPSVTGRIVRIRNEAEVRARPGVHAIVRTTQWPDPEPSTVV
VVADSYWIAKQAADALDIEFDAGAAAGVDSERIHAQFVAGLSNDKAVVARSLGKPREMLAAGKPITADYHSPYI
THATMEPLAATVHVRDGEVETWGPYQGQDFLRGELGKACGVPADKVIVHTTFLGGSFGRKYMPDFALHAAA
ASKAVGRPVKVIRSREDDIRHSYYRPGASGRMSAVLGADGLPAALHARISGQSLYGAINPKKMADAGGWDET
MVESIYDLIYGVPNLLVDAVDVQQPIPLSYLRSVGSTSSVFFLESFISELAHTAGVDDYQYRRRLLAGQPLALGVLD

AAAKAARWEQPAPAGLHRAMTFNVYTGRGESFQTFVALVMELRVVEGRVRLERAICAIDAGRVVNPGLVKAN
VEGGIGFALTNTFKSRLGFDKGVVQQSNFHDYPLLQLSEMPRVEVVLVESDRPPQGCGEVALGPTAPAVATALF
HATGRRFRSMPLPQDIAPT
>SEQF5863||SEQF5863.1_01173
MPQRPSNERGRTAGSAISLRRRHLLQSAAALLVAPAAGSLLIPLAQAAPAGGGAAAATASSIGDWVWIEPSGQV
VIGVSQCEVGQGIYTGLPQVLADELDADWASVTVRFVTGRDAYRNDAGEMPFQQFVGASMSMNYFYERMR
LAGAQARDVLLRAGAARLGVRASQCSTRAGRVLHSATGRSVGYGEIVADASRLSIAARPRMKSASEQGLIGRNL
RRVDTPAKVDGSAVFGIDVEVPGMLIGAVRMAPSVTGRIVRIRNEAEVRARPGVHAVVRTTQWPDPEPSTVV
VVADSYWIAKQAADALDIEFDAGAAAGVDSERIHAQFVAGLASDKAVVARSLGKPREMLAAGKPITADYHSPYI
THATMEPLAATVHVRDGEVETWGPYQGQDFLRGELGKACGVPADKVIVHTTFLGGSFGRKYMPDFALHAAA
ASKVVGRPVKVIRSREDDIRHSYYRPGASGRLSAVLGADGMPAALHARISGQSLYGAINPKKMADAGGWDET
MVESIYDLIYGVPNLLVDAVDVQQPIPLSYLRSVGTTSSVFFLESFISELAHTAGVDDYQYRRRLLAGQPLALGVLD
AAAKAARWEQPAPAGLHRAMTFNVYTGRGESFQTFVALVMELRVVEGRVRLERAICAIDAGRVVNPGLVKAN
VEGGIGFALTNTFKSRLGFDKGVVQQSNFHDYPLLQLSEMPRVEVVLVESDRPPQGCGEVALGPTAPAVATALF
HATGRRFRSMPLPQDIAST
>SEQF5864||SEQF5864.2_02013
MPQRPSNERGRVADSAISLRRRHLLQSAAALLVAPAAGSLLIPLAQAAPPAAGAAAAAAIGDWVWIEPSGQVV
IGVSQCEVGQGIYTGLPQVLADELDADWASVTVRFVTGRDAYRNDAGEMPFQQFVGASMSMNYFYERMRL
AGAQARDVLLRAGASRLGVRASQCMTRAGRVLHPATGRSVGYGEIVADASRLPLAARPRMKSASEQGLIGRNL
RRVDTPSKVDGSAVFGIDVEVPGMLIGAVRMAPSVTGRIVRIHNEAEVRARPGVHAVVRTTQWPDPEPSTVV
VVADSYWIAKQAADALDIEFDAGAAAGVDSERIHAQFVAGLSNDKAVVARSLGKPREVLAAGKTITADYHSPYI
THATMEPLAATVHVRDGEVETWGPYQGQDFLRGELGKACGVPADKVIVHTTFLGGSFGRKYMPDFALHAAA
ASKAVGRPVKVIRSREDDIRHSYYRPGASGRLSAVLGADGLPAALHARISGQSLYGAINPKKMADAGGWDETM
VESIYDLIYGVPNLLVDAVDVQQPIPLSYLRSVGTTSSVFFLESFISELAHTAGVDDYQYRRRLLAGQPLALGVLDA
AARAARWEQPAPAGLHRAMTFNVYTGRGESFQTFVALVMELRVVQGRVRLERAICAIDAGRVVNPGLVKANV
EGGIGFALTNTFKSRLDFDKGAVQQSNFHDYPLLQLSEMPRVEVVLVESDRPPQGCGEVALGPTAPAVATAMFH
ATGRRFRSMPLPQDIAST
>SEQF5865||SEQF5865.1_00919
MPQRPSNERGRTAGSAISLRRRHLLQSAAALLLAPAAGSLLIPLAQAAPAGAGAAAATASSIGDWVWIEPSGQV
VIGVSQCEVGQGIYTGLPQVLADELDADWASVTVRFVTGRDAYRNDAGEMPFQQFVGASMSMNYFYERMR
LAGAQARDVLLRAGAARLGVRASQCSTRAGRVLHSATGRSVGYGEIVADASRLSIAARPRMKSASEQGLIGRNL
RRVDTPAKVDGSAVFGIDVEVPGMLIGAVRMAPSVTGRIVRIRNEAEVRARRGVHAVVRTTQWPDPEPSTVV
VVADSYWIAKQAADALDIEFDAGAAAGVDSERIHAQFVAGLASDKAVVARSLGKPREMLAAGKPITADYHSPYI
THATMEPLAATVHVRDGEVETWGPYQGQDFLRGELGKACGVPADKVIVHTTFLGGSFGRKYMPDFALHAAA
ASKAVGRPVKVIRSREDDIRHSYYRPGASGRLSAVLGADGLPAALHARISGQSLYGAINPKKMADAGGWDETM
VESIYDLIYGVPNLLVDAVDVQQPIPLSYLRSVGTTSSVFFLESFISELAHTAGVDDYQYRRRLLAGQPLALGVLDA
AARAARWEQPAPAGLHRAMTFNVYTGRGESFQTFVALVMELRVVEGRVRLERAICAIDAGRVVNPGLVKANV
EGGIGFALTNTFKSRLGFDKGVVQQSNFHDYPLLQLSEMPRVEVVLVESDRPPQGCGEVALGPTAPAVATALFH
ATGRRFRSMPLPQDIAST
>SEQF5866||SEQF5866.1_04991
MPQRPSNERGRTAGSAISLRRRHLLQSAAALLVAPAAGSLLIPLAQAAPAEAGAAAATASSIGDWVWIEPSGQV
VIGVSQCEVGQGIYTGLPQVLADELDADWASVTVRFVTGRDAYRNDAGEMPFQQFVGASMSMNYFYERMR
LAGAQARDVLLRAGAARLGVRASQCSTRAGRVLHSATGRSVGYGEIVAEASRLSIAARPRMKSASEQGLIGRNL
RRVDTPAKVDGSAVFGIDVEVPGMLIGAVRMAPSVTGRIVRIRNEAEVRARPGVHAVVRTTQWPDPEPSTVV

VVADSYWIAKQAADALDIEFDAGAAAGVDSERIHAQFVAGLSNDKAVVARSLGKPREMLAAGKPITADYHSPYI
THATMEPLAATVHVRDGEVETWGPYQGQDFLRGELGKACGVPADKVIVHTTFLGGSFGRKYMPDFALHAAA
ASKAVGRPVKVIRSREDDIRHSYYRPGASGRLSAVLGADGLPAALHARISGQSLYGAINPKKMADAGGWDETM
VESIYDLIYGVPNLLVDAVDVQQPIPLSYLRSVGTTSSVFFLESFISELAHTAGVDDYQYRRRLLAGQPLALGVLDA
AAKAARWEQPAPAGLHRAMTFNVYTGRGESFQTFVALVMELRVVEGRVRLERAICAIDAGRVVNPGLVKANV
EGGIGFALTNTFKSRLGFDKGVVQQSNFHDYPLLQLSEMPRVEVVLVESDRPPQGCGEVALGPTAPAVATALFH
ATGRRFRSMPLPQDIAST
>SEQF5867||SEQF5867.1_02079
MPQRPSNERGRTAGSAISLRRRHLLQSAAALLVAPAAGSLLIPLAQAAPAEAGAAAATASSIGDWVWIEPSGQV
VIGVSQCEVGQGIYTGLPQVLADELDADWASVTVRFVTGRDAYRNDAGEMPFQQFVGASMSMNYFYERMR
LAGAQARDVLLRAGAARLGVRASQCSTRAGRVLHSATGRSVGYGEIVADASRLSIAARPRMKSASEQGLIGRNL
RRVDTPAKVDGSAVFGIDVEVPGMLIGAVRMAPSVTGRIVRIRNEGEVRARPGVHAVVRTTQWPDPEPSTVV
VVADSYWIAKQAADALDIEFDAGAAAGVDSERIHAQFVAGLSNDKAVVARSLGKPREMLAAGKPITADYHSPYI
THATMEPLAATVHVRDGEVETWGPYQGQDFLRGELGKACGVPADKVIVHTTFLGGSFGRKYMPDFALHAAA
ASKAVGRPVKVIRSREDDIRHSYYRPGASGRLSAVLGADGLPAALHARISGQSLYGAINPKKMADAGGWDETM
VESIYDLIYGVPNLLVDAVDVQQPIPLSYLRSVGTTSSVFFLESFISELAHTAGVDDYQYRRRLLAGQPLALGVLDA
AAKAARWEQPAPAGLHRAMTFNVYTGRGESFQTFVALVMELRVVEGRVRLERAICAIDAGRVVNPGLVKANV
EGGIGFALTNTFKSRLGFDKGVVQQSNFHDYPLLQLSEMPRVEVVLVESDRPPQGCGEVALGPTAPAVATALFH
ATGRRFRSMPLPQDIAST
>SEQF5868||SEQF5868.1_05114
MPQRPSNERGRTAGSAISLRRRHLLQSAAALLVAPAAGSLLIPLAQAAPAEAGAAAATASSIGDWVWIEPSGQV
VIGVSQCEVGQGIYTGLPQVLADELDADWASVTVRFVTGRDAYRNDAGEMPFQQFVGASMSMNYFYERMR
LAGAQARDVLLRAGAARLGVRASQCSTRAGRVLHSATGRSVGYGEIVAEASRLPLAARPRMKSASEQGLIGRNL
RRVDTPAKVDGSAVFGIDVEVPGMLIGAVRMAPSVTGRIVRIRNEGEVSARPGVHAVVRTTQWPDPEPSTVV
VVADSYWIAKQAADALDIEFDAGAAAGVDSERIHAQFVAGLASDKAVVARSLGKPREMLAAGKPITADYHSPYI
THATMEPLAATVHVRDGEVETWGPYQGQDFLRGELGKACGVPADKVIVHTTFLGGSFGRKYMPDFALHAAA
ASKAVGRPVKVIRSREDDIRHSYYRPGASGRLSAVLGADGLPAALHARISGQSLYGAINPKKMADAGGWDETM
VESIYDLIYGVPNLLVDAVDVQQPIPLSYLRSVGTTSSVFFLESFISELAHTAGVDDYQYRRRLLAGQPLALGVLDA
AARAARWEQPVPAGLHRAMTFNVYTGRGESFQTFVALVMELRVVEGRVRLERAICAIDAGRVVNPGLVKANV
EGGIGFALTNTFKSRLGFDKGVVQQSNFHDYPLLQLSEMPRVEVVLVESDRPPQGCGEVALGPTAPAVATALFH
ATGRRFRSMPLPQDIAST
>SEQF5869||SEQF5869.1_04525
MPQRPSNERGRTAGSAISLRRRHLLQSAAALLVAPAAGSLLIPLAQAAPAEAGAAAATASSIGDWVWIEPSGQV
VIGVSQCEVGQGIYTGLPQVLADELDADWASVTVRFVTGRDAYRNDAGEMPFQQFVGASMSMNYFYERMR
LAGAQARDVLLRAGAARLGVRASQCSTRAGRVLHSATGRSVGYGEIVAEASRLSIAARPRMKSASEQGLIGRNL
RRVDTPAKVDGSAVFGIDVEVPGMLIGAVRMAPSVTGRIVRIRNEGEVRARPGVHAVVRTTQWPDPEPSTVV
VVADSYWIAKQAADALDIEFDAGAAAGVDSERIHAQFVAGLASDKAVVARSLGKPREMLAAGKPITADYHSPYI
THATMEPLAATVHVRDGEVETWGPYQGQDFLRGELGKACGVPADKVIVHTTFLGGSFGRKYMPDFALHAAA
ASKAVGRPVKVIRSREDDIRHSYYRPGASGRLSAVLGADGMPAALHARISGQSLYGAINPKKMADAGGWDET
MVESIYDLIYGVPNLLVDAVDVQQPIPLSYLRSVGTTSSVFFLESFISELAHTAGVDDYQYRRRLLAGQPLALGVLD
AAARAARWEQPAPAGLHRAMTFNVYTGRGESFQTFVALVMELRVVEGRVRLERAICAIDAGRVVNPGLVKAN
VEGGIGFALTNTFKSRLGFDKGVVQQSNFHDYPLLQLSEMPRVEVVLVESDRPPQGCGEVALGPTAPAVATALF
HATGRRFRSMPLPQDIAST
>SEQF5870||SEQF5870.2_02233

MPQRPSNERGRTAGSAISLRRRHLLQSAAALLVAPAAGSLLIPLAQAAPAEAGAAAATASSIGDWVWIEPSGQV
VIGVSQCEVGQGIYTGLPQVLADELDADWASVTVRFVTGRDAYRNDAGEMPFQQFVGASMSMNYFYERMR
LAGAQARDVLLRAGAARLGVRASQCSTRAGRVLHSATGRSVGYGEIVAEASRLSIAARPRMKSASEQGLIGRNL
RRVDTPAKVDGSAVFGIDVEVPGMLIGAVRMAPSVTGRIVRIRNEAEVRARPGVHAIVRTTQWPDPEPSTVVV
VADSYWIAKQAADALDIEFDAGAAAGVDSERIHAQFVAGLASDKAVVARSLGKPREMLAAGKPITADYHSPYIT
HATMEPLAATVHVRDGEVETWGPYQGQDFLRGELGKACGVPADKVIVHTTFLGGSFGRKYMPDFALHAAAA
SKAVGRPVKVIRSREDDIRHSYYRPGASGRLSAVLGADGLPAALHARISGQSLYGAINPKKMADAGGWDETMV
ESIYDLIYGVPNLLVDAVDVQQPIPLSYLRSVGTTSSVFFLESFISELAHTAGVDDYQYRRRLLAGQPLALGVLDAA
AKAAHWEQPAPAGLHRAMTFNVYTGRGESFQTFVALVMELRVVEGRVRLERAICAIDAGRVVNPGLVKANVE
GGIGFALTNTFKSRLRFDKGVVQQSNFHDYPLLQLSEMPRVEVVLVESDRPPQGCGEVALGPTAPAVATALFHAT
GRRFRSMPLPQDIAST
>SEQF5945||SEQF5945.1_00341
MAITRRTLLKGSALAVAAMLLPISTRSLAAVVSQDAAPPDGQYELNDWIWIDSGGRIVIGVSQCEVGQGIYTGL
AEVVAAEMDADWAQVTVKFVTGRDAYRQVAGGEAFAQFVAASTSMTKFYQRTRLAGAQARDFFLRAGARH
LALSPRQCRTEKGWVIEEGGQRKVAYGDLVCYAAELPLDPQPALKSEAQEQESFIGKPLLRVDTPEKVDGSAIYGI
DIDLPEMLIGVPWMVPDLSGKLVAVRNERQIRAMPGVVDLVLTRQWSMNNMVGLDHDMSLNTVIVVAASY
WQAKKAADLLEVDWLPGAGQALSGSAIAAENLAMLDGDTLVPAVDRGEAPALIRGAEQDSRLHEARYGAPYV
AHATLEPCNATSHYEEGRIETWGPFQGQDMVRNVLAKMFGLKPTDVVVNTTYLGGSFGRKYLPDAVMHATA
ASRAVGKPVKVIYPREIDIRHEYYRPGCISHYRALLDENGYPQALWARYAGQSLFWQMRRETVHEAGGWDES
MVECVYSTPYRIPHLKVEAGIVEQPISLSYLRGVGSVASLFFLESFISELSHKAKRDEYQYRRYLLQGNPEALRVLEA
TAAAAGWQREPPPGVYRGMSCNIWLGRNNAFTTYVGLVVEIAIQEGRLRVLRAVCAIDCGKVINPNLVRANVE
GGIGFALTTCLHSELHFERGGVVEGNFDRYPLLAIAEMPKVEVVVLDSARAPQGCGEVSTAVVAPAIASALHKAT
GKTYRTMPFPREFSSV
>SEQF5947||SEQF5947.1_00824
MAITRRTLLKGSALAVAAMLLPISTRSLAAVVSQDAAPPDGQYELNDWIWIDSDGRIVIGVSQCEVGQGIYTGL
AEVVAAEMDADWAQVTVKFVTGRDAYRQVAGGEAFAQFVAASTSMTKFYQRTRLAGAQARDFFLRAGARH
LALSPRQCRTEKGWVIEEGGQRKVAYGDLVRYAAELPLDPQPALKSETQEQESFIGKPLLRVDTPEKVDGSAIYGI
DIDLPEMLIGVPWMVPDLSGKLVAVRNERQIRAMPGVVDLVLTRQWSMNNMVGLDHDMSLNTVIVVATSY
WQAKKAADLLEVDWLPGAGQALTDSAIAAENLAMLDGDTLVSAVDRGEAPALICGAEQDSRLHEARYGAPYV
AHATLEPCNATSHYEEGRIETWGPFQGQDMVRNVLAKMFGLKPTDVVVNTTYLGGSFGRKYLPDAVMHATA
ASRAVGKPVKVIYPREIDIRHEYYRPGCISHYRALLDENGYPQALWARYAGQSLFWQMRRETVHEAGGWDES
MVECVYSTPYRIPHLKVEAGIVEQPISLSYLRGVGSVASLFFLESFISELSHKAKRDEYQYRRYLLQGNPEALRVLEA
TAAAAGWQREPSPGVYRGMSCNIWLGRNNAFTTYVGLVVEIAIQEGRLRVLRAVCAIDCGKVINPNLVRANVE
GGIGFALTTCLHSELHFERGGVVEGNFDRYPLLAIAEMPKVEVVVLDSARAPQGCGEVSTAVVAPAMASALHKA
TGKTYRTMPFPREFSSV
>SEQF5948||SEQF5948.1_01433
MAITRRTLLKGSALAVAAMLLPISTRSLAAVVSQDTAPPDNQYELNDWIWIDREGRIVIGVSQCEVGQGIYTGL
AEVVAAEMDADWAQVTVKFVTGRDAYRQVAGGEAFAQFVAASTSMTKFYQRTRLAGAQARDFFLRAGAKHF
ALSPQQCRTEKGWVLEEGGQRKIAYGDLVRYAAELPLDPQPALKSEAQEQESLIGKPLLRVDTPEKVDGSAIYGI
DIDLPEMLIGVPWMVPDLSGKLVAVRNERQIRAMPGVVDLVLTRQWSMNNMVGLDHDMSLNTVIVVAASY
WQAKKAADLLEVDWLPGAGQALTGSAIAAENLAMLDGDTLVPAVDRGEALALIHNAEQDSRLHEARYSAPYV
AHATLEPCNATSHYEEGRIETWGPFQGQDMVRNVLAKMFGLQPTDVVVNTTYLGGSFGRKYLPDAVMHATA
ASRAVGKPVKVIYPREIDMRHEYYRPACISHYRALLDENGYPQALWARYAGQSLFWQMRRETVHEAGGWDES
MVECVYSTPYCIPHLKVEAGIVEQPISLSYLRGVGSVASLFFLESFISELSHKANLDEYQYRRHLLQDTPEALRVLDA

TAAASGWQHEPPPGVYRGMSCNIWVGRNNAFTTYVGLVVEIAIQEGRLRVLRAICAIDCGKVINPNLVRANVE
GGIGFALTTCLHSELHFERGGVVEGNFDRYPLLAIAEMPKVEVVVLDSARAPQGCGEVSTAVVAPAVASALHKA
TGKTYRTMPFPRQFSSV
>SEQF5949||SEQF5949.1_01441
MAITRRTLIKGSALAVAAMLLPISTRSLAAVVSQDVAPPDSQHELNDWIWIDRDGRIVIGVSQCEVGQGIYTGL
AEVVAAEMDADWAQVTVKFVTGRDAYRQVAGGEAFAQFVAASTSMTKFYQRARLAGAQARDFFLRAGAKH
FALSPSQCRTEKGWVLEKGGKRKVAYGDLVRYAAELPLDPQPALKSEAQEQESFIGKPLLRVDTPEKVDGSAIYGI
DIDLPEMLIGVPWMVPDLSGKLVAVRNERQIRAMPGVVDLVLTRQWSMNNMVGLDHDMSLNTVIVVAASY
WQAKKAADLLEVDWLPGAGKALTGSVIAAENLTMLDGDTLVPAVDRGEASALIRGAESGSRLHEARYSAPYVA
HATLEPCNATSHYGEGRIETWGPFQGQDMVRNVLAKMFGLKPTDVVVNTTYLGGSFGRKYLPDAVMHATAA
SRAVGKPVKVVYPREIDMRHEYYRPACISHYRALLDENGYPQALWARYAGQSLFWQMRRETVNEAGGWDES
MVECVYSTPYRIPHLKVEAGIVEQPISLSYLRGVGSVASLFFLESFISELSHKSNRDEYSYRRHLLQDSPEALRVLDA
TATAAGWQHEPPSGVSRGMACNIWVGRNNAFTTYVGLVVEIAIQEGRLRVLRAVCAIDCGKVINPNLVRANVE
GGIGFALTTCLHSELHFERGGVVEGNFDRYPLLAIAEMPKVEVVILDSARAPQGCGEVSTAVVAPAMASALHKA
TGKTYRTMPFPREFSSV
>SEQF5950||SEQF5950.1_03561
MAITRRTLLKGSALAVAAMLLPISTRSLAAVVSQDTVPPDNQYELNDWIWIDREGRIVIGVSQCEVGQGIYTGL
AEVVAAEMDADWAQVTVKFVTGRDAYRQVAGGEAFAQFVAASTSMTKFYQRTRLAGAQARDFFLRAGAKHF
ALSPQQCRTEKGWVLEEGGQRKIAYGDLVRYAAELPLDPQPALKSEAQEQESLIGKPLLRVDTPEKVDGSAIYGI
DIDLPEMLIGVPWMVPDLSGKLVAVRNERQIRAMPGVVDLVLTRQWSMNNMVGLDHDMSLNTVIVVAASY
WQAKKAADLLEVDWLPGAGQALTGSAIAAENLAMLDGDTLVPAVDRGEASALIHNAEQDSRLHEARYSAPYV
AHATLEPCNATSHYEEGRIETWGPFQGQDMVRNVLAKMFGLKPTDVVVNTTYLGGSFGRKYLPDAVMHATA
ASRAVGKPVKVIYPREIDMRHEYYRPACISHYRALLDENGYPQALWARYAGQSLFWQMRRETVHEAGGWDES
MVECVYSTPYCIPHLKVEAGIVEQPISLSYLRGVGSVASLFFLESFISELSHKANLDEYQYRRHLLQDTPEALRVLDA
TAAASGWQHEPPPGVYRGMSCNIWVGRNNAFTTYVGLVVEIAIQEGRLRVLRAICAIDCGKVINPNLVRANVE
GGIGFALTTCLHSELHFERGGVVEGNFDRYPLLAIAEMPKVEVVVLDSARAPQGCGEVSTAVVAPAVASALHKA
TGKTYRTMPFPRQFSSV
>SEQF5952||SEQF5952.1_04545
MAITRRTLLKGSALAVAAMLLPISTRSLAAVVSQDAAPPDGQYELNDWIWIDSGGRIVIGVSQCEVGQGIYTGL
AEVVAAEMDADWAQVTVKFVTGRDAYRQVAGGEAFAQFVAASTSMTKFYQRTRLAGAQARDFFLRAGARH
LALSPRQCRTEKGWVIEEGGQRKVAYGDLVRYAAELPLDPQPALKSEAQEQESFIGKPLLRVDTPEKVDGSAIYGI
DIDLPEMLIGVPWMVPDLSGKLVAVRNERQIRAMPGVVDLVLTRQWSMNNMVGLDHDMSLNTVIVVAASY
WQAKKAADLLEVDWLPGAGQALSGSAIAAENLAMLDGDTLVPAVDRGEAPALIRGAEQDSRLHEARYGAPYV
AHATLEPCNATSHYEEGRIETWGPFQGQDMVRNVLAKMFGLKPTDVVVNTTYLGGSFGRKYLPDAVMHATA
ASRAVGKPVKVIYPREIDIRHEYYRPGCISHYRALLDENGYPQALWARYAGQSLFWQMRRETVHEAGGWDES
MVECVYSTPYRIPHLKVEAGIVEQPISLSYLRGVGSVASLFFLESFISELSHKAKRDEYQYRRYLLQGNPEALRVLEA
TAAAAGWQREPPPGVYRGMSCNIWLGRNNAFTTYVGLVVEIAIQEGRLRVLRAVCAIDCGKVINPNLVRANVE
GGIGFALTTCLHSELHFERGGVVEGNFDRYPLLAIAEMPKVEVVVLDSARAPQGCGEVSTAVVAPAIASALHKAT
GKTYRTMPFPREFSSV
>SEQF5953||SEQF5953.1_02831
MAITRRTLLKGSALAVAAMLLPISTRSLAAVVSQDAAPPDGQYELNDWIWIDSGGRIVIGVSQCEVGQGIYTGL
AEVVAAEMDADWAQVTVKFVTGRDAYRQVAGGEAFAQFVAASTSMTKFYQRTRLAGAQARDFFLRAGARH
LALSPRQCRTEKGWVIEEGGQRKVAYGDLVRYAAELPLDPQPALKSEAQEQESFIGKPLLRVDTPEKVDGSAIYGI
DIDLPEMLIGVPWMVPDLSGKLVAVRNERQIRAMPGVVDLVLTRQWSMNNMVGLDHDMSLNTVIVVAASY

WQAKKAADLLEVDWLPGAGQALSGSAIAAENLAMLDGDTLVPAVDRGEAPALIRGAEQDSRLHEARYGAPYV
AHATLEPCNATSHYEEGRIETWGPFQGQDMVRNVLAKMFGLKPTDVVVNTTYLGGSFGRKYLPDAVMHATA
ASRAVGKPVKVIYPREIDIRHEYYRPGCISHYRALLDENGYPQALWARYAGQSLFWQMRRETVHEAGGWDES
MVECVYSTPYRIPHLKVEAGIVEQPISLSYLRGVGSVASLFFLESFISELSHKAKRDEYQYRRYLLQGNPEALRVLEA
TAAAAGWQREPPPGVYRGMSCNIWLGRNNAFTTYVGLVVEIAIQEGRLRVLRAVCAIDCGKVINPNLVRANVE
GGIGFALTTCLHSELHFERGGVVEGNFDRYPLLAIAEMPKVEVVVLDSARAPQGCGEVSTAVVAPAIASALHKAT
GKTYRTMPFPREFSSV
>SEQF5959||SEQF5959.1_04399
MAITRRALIKGSALAVAAMLLPISTRSLAAVVSQDVAPPDGQHELNDWIWIDRDGRIVIGVSQCEVGQGIYTGL
AEVVAAEMDADWAQVTVKFVTGRDAYRQVAGGEAFAQFVAASTSMTKFYQRARLAGAQARDFFLRAGAKH
FALSPAQCRTEKGWVLEKGGQRKVAYGDLVRYAAELPLDPQPGLKSEAQERESVIGKPLLRVDTPEKVDGSAIYG
IDIDLPEMLIGVPWMVPDLSGKLVAVRNERQIRAMPGVVDLVLTRQWSMNNMVGLDHDMSLNTVIVVAASY
WQAKKAADLLEVDWLPGVGKALTDSAIAAENLAMLDGDTLVPAVDRGEASALIRGAEQGSRLHEARYSAPYV
AHATLEPCNATSHYGEGRIETWGPFQGQDMVRNVLAKMFGLKPTDVVVNTTYLGGSFGRKYLPDAVMHATA
ASRAVGKPVKVIYPREIDMRHEYYRPACISHYRALLDENDYPQALWARYAGQSLFWQMRRETVNEAGGWDES
MVECVYSTPYRIPHLKVEAGIVDQPISLSYLRGVGSVASLFFLESFISELSHKANRDEYSYRRHLLQDSPEALRVLD
ATAAAAGWQHEPPLGVYRGMACNIWVGRNNAFTTYVGLVVEIAIQGGRLRVLRAVCAIDCGKVINPNLVRAN
VEGGIGFALTTCLHSELHFERGGVVEGNFDRYPLLAIAEMPKVEVVVLDSARAPQGCGEVSTAVVAPAIASALHK
ATGKTYRTMPFPREFSSV
>SEQF6035||SEQF6035.1_07221
MLPRTHKDAAMNQHIMPKLNRRAFVIGTATAGAGLALGLDLPFGGPAVVRAADGAPEVNAWVVIRPDDTVV
IRIARSEMGQGTLTGLAQLVAEELECDWSKVTTEYPTPGQSVARKRAWGDFSTGGSRGIRTSQDYVRKGGATA
RVMLIQAAANEWKVPASECKAANGVITHTPSGKTTTYGKVAEAAAKLEPPADVKLKDPKDWTIAGKGLKRLDT
VDKTTGKMVYGIDVKLPGMLNAAIKDCPVFGGKVKSFDEAKIAGMKGVRKVVQVGDSAVAVVADTWWHAK
TALDALPIVWDEGPNAKVSSETIATWLAEGLDNAQPAYIGNQNGDAKAAIAAAAKKVEAVYNYPYQNHATME
PMNATVLYTPDKCEVWCGTQNGEAAFAAALEASGLPAEKVDVHKLMLGGGFGRRGMTDYVRQAVAIAKQM
PGTPIKLLWSREEDMQHGKYHPITQCKLTGAFDADNNLVALHYRLSGQSILFSVRPEALQNGMDPAAFQGVAQ
AGEAAIGYSVPNLLVEHSMRNPHVPPGFWRGVNVNHNAIYMECFMDELALSVGQDPLEFRRKLMGKHPKHL
AVLNAVAEKIGWGTPAPQGVYRGIAQVMGYGSYVAGAAEISVTDGSKIKVHRIVASTDPGYVVNPAQVERQIA
GSFVYGLSALFYGGCTVKDGRIEQTNFDTYNSMRINEMPKVEAVMVPSGGFWGGVGEPTIGVAAPAVLNAYF
AATGKRIRSFPLRNQNISFA
>SEQF6035||SEQF6035.1_04466
MTLIDNLSERAADLSRRNFLRAGAIAGGGLLLSVSLPFAGRESEAAASDGFAPNAFVRIGGDGKVVLTMPYVEM
GQGTYTSIPMLIAEELEIGLTQVRLEHAPPSDKLYANPLLGVQATGNSNAMRGAWQPMRKAGATAKAMLVAA
AAKRWNVEPGTCRAENGEVHHAASGRKLGYGELATDAAQMPVPENVTLKSPSEFKLIGTPAKRLDTPSKINGT
AVYGIDARPPGVKIATLAQSPVFGGRVKRVDDAAAKAVNGVRQIVTLDDAVAVVADHMGAAKKGLAALTIEW
DEGAHAKLATSDIARELETATTKPGAVAQNIGDADKAMAGAATKVEATYQLPFLAHATMEPMNCTVHVRPDG
CEIWVGSQALSRAQAVAAKVLNMPPEKVVVHNHLLGGGFGRRLEVDGVIRAVQIAKQVDAPVKLVWTREEDI
QHDMYRPYWCDRIAVGLDASGKPVAWNNRFAGSSVLARWAPPAFRNGLDPDTTEGAIDLVYDIPNFHVEYV
RVEPPGIPTAFWRSVGPSHNVFVTESVIDELAAAAKQDPVDYRRALLGKSPRAKAALELAAAKAGWGGKLPAG
RGRGVSLQFVFGSYLAQVAEVEVARDGSVRVHRVVCAMDCGTVVNPDTVQAQLQSGINFGVTAALYGEITLK
DGRVEQSNFDSYQMLRIDQAPAIEVHIVPSTEPPGGMGETGTSGIVPAISNAIFAATGKRLRKMPVDPAVLKQT
>SEQF6035||SEQF6035.1_00975
MNKHVKNLAPETTDLSRRSFLVGTAATGLVLGYAASGIDQALAAPATANFEPSVWYSIAPDGLVTVTCGKADMG

QHVASTMAQIVAEELGANWKDMRVQLASNDPKFNDPVLGAQITGGSWSTMMNFDAMSRAGAAGRIALTE
AAAASMGVPAAELVVRDSRISHPKSKKSMSFAEVVKSGKATKTFTADDLKAIKLKTPDQYTMIGVSVPQLDIPSK
VNGTAKYGIDVMVPGMVYGALVTPPVRYGATVKSVDDSAAKKLPGFIKAVTLDDKTTTTTGWVVAVANTYAQ
AKKAAAALKISYDGGPNAKLSSESLFAEAKRLQGLSDSGEFFVKDGDPNAAYGSAAKVLEAEYTTNINIHAPMEP
MNATAEFKGDILHIYSGNQFATRSGAIAAGAAGIDPKFVVMHQMWLGGGFGRRLDADMMVPAVQAAKAVG
KPVKVIYSRENDMTMDFSRPLTCQKIKAGMDGDGKIVALSHDVVSAWPTQRWGIPDFLTPSVDKKGPLDSFTV
NGADFFYTVPNHYVRAIKNEMAHNATPSGQLRSVAPGWTFWAVESMIDEIAHATGKDPAQLRISLLDGKGKN
DGGAQRLRNTLLAAMGLAGYGTKQLPKGEGMGVACVSSQERATASWTACVAHVAVAPSGEVTVKKLTVATDV
GTQVNPDGIRAQVEGAALWGMSLALFEKATLKDGGIEQTNFDSYTPLRMSQLPEVAVNVIANGEKATGVGEPA
VTVVAPAIGNAVFNAVGARVRGLPITAEAVKAAMKA
>SEQF6035||SEQF6035.1_08098
MNIRTNIPTDSGKLRGFEKHVKVENVSRRSILKGLGIAGSFVLAAPVMTRQAFAYETGAGKMPHGVVVDPRVF
VAIAPDGIVTILAHRSEMGTGVRTSLPLIVAEEMEADWSRVHVQQAHGDEVKFGNQDTDGSRSTRHYLIPMR
QIGASARSMLEAAAAKKWGVPVTEVKAQNHEVVHSASGRKAGFGELAADAAKESVPSVEGLKLKDPKDFRYL
GKGQVSIVDLHDITTGKAPYGADVRLPGLKYAVIARPPVTGGKLKSFDDSAAKKVPGVEKVMEVKGWPWPSKF
QPLGGVAVIARNTGAAIKGRDALKIVWDDGANAKYDSVTYRASLEEAARKPGLVVRKEGDADAALKSADKVIT
GEYYLPHLAHVSMEPPVAVADVKGDKATIWAPVQSPGGTREDVAKTLGIPEDNVTVNVTLLGGGFGRKSKCDF
ALEAALLSKELGAPVKVQWTREDDVHHDFLHTVSVERIEAGLDKSGKVIAWRHRSVAPTIASTFAAGANHEAPF
ELGMGLIDNPFEIANLQCENPEAAAFTRIGWFRSVSNIPRAFAVQSMVGEIAHATGRDQKEMLLELIGSPRIVNL
SSVKDPWNYGEPYDSYPIDTARLRKVVELVADKGEWGRKVPKGHGLGIAVHRSFVSYIATIVEVAVDDKGKFSVP
RVDTAIDCGTYVNPERIHSQIEGAAIMGLSLAKYGEISFKDGKVQQGNFDDFPVIRMDESPVITNVHIVPPGADT
PPSGVGEPGVPPFAPALINAIFAATGKRIRALPIGKQLET
>SEQF6036||SEQF6036.1_07279
MNIRTNIPTGSGKLRGFEKHVKVENVSRRSILKGLGIAGSFVLAAPVMTRQAFAYETGAGKMPHGVVVDPRVF
VAIAPDGIVTILAHRSEMGTGVRTSLPLIVAEEMEADWSRVHVQQAHGDEVKFGNQDTDGSRSTRHYLIPMR
QIGASARSMLEAAAAKKWGVPVTEVKAQNHEVVHSASGRKAGFGELAADAAKESVPSVEGLKLKDPKDFRYL
GKGQVSIVDLHDITTGKAHYGADVRLPGLKYAVIARPPVTGGKLKSFDDSAAKKVPGVEKVMEVKGWPWPSKF
QPLGGVAVIARNTGAAIKGRDALKIVWDDGANAKYDSVTYRASLEEAARKPGLVVRKEGDADAALKSADKVIT
GEYYLPHLAHVSMEPPVAVADVKGDKATIWAPVQSPGGTREDVAKTLGIPEDNVTVNVTLLGGGFGRKSKCDF
ALEAALLSKELGAPVKVQWTREDDVHHDFLHTVSVERIEAGLDKSGKVIAWRHRSVAPTIASTFAAGANHEAPF
ELGMGLIDNPFEIANLQCENPEAAAFTRIGWFRSVSNIPRAFAVQSMVGEIAHATGRDQKEMLLELIGSPRIVNL
SSVKDPWNYGEPYDSYPIDTARLRKVVELVAEKGEWGRKVPKGHGLGIAVHRSFVSYIATIVEVAVDDKGKFSVP
RVDTAIDCGTYVNPERIHSQIEGAAIMGLSLAKYGEISFKDGKVQQGNFDDFPVIRMDESPVITNVHIVPPGADT
PPSGVGEPGVPPFAPALINAIFAATGKRIRALPIGKQLET
>SEQF6036||SEQF6036.1_03868
MTLIDNLSERAADLSRRNFLRAGAIAGGGLLLSVSLPFAGRESEAAASDGFAPNAFVRIGGDGKVVLTMPYVEM
GQGTYTSIPMLIAEELEIGLTQVRLEHAPPSDKLYANPLLGVQATGNSNAMRGAWQPMRKAGATAKAMLVAA
AAKRWNVEPGTCRAENGEVHHAASGRKLGYGELATDAAQMPVPENVTLKSPSEFKLIGTPAKRLDTPSKINGT
AVYGIDARPPGVKIATLAQSPVFGGRVKRVDDAAAKAVNGVRQIVTLDDAVAVVADHMGAAKKGLAALTIEW
DEGAHAKLATSDIARELETATTKPGAVAQNIGDADKAMAGAATKVEATYQLPFLAHATMEPMNCTVHVRPDG
CEIWVGSQALSRAQAVAAKVLNMPPEKVVVHNHLLGGGFGRRLEVDGVIRAVQIAKQVDAPVKLVWTREEDI
QHDMYRPYWCDRIAVGLDASGKPVAWNNRFAGSSVLARWAPPAFRNGLDPDTTEGAIDLVYDIPNFHVEYV
RVEPPGIPTAFWRSVGPSHNVFVTESVIDELAAAAKQDPVDYRRALLGKSPRAKAALELAAAKAGWGGKLPAG
RGRGVSLQFVFGSYLAQVAEVEVARDGSVRVHRVVCAMDCGTVVNPDTVQAQLQSGINFGVTAALYGEITLK

DGRVEQSNFDSYQMLRIDQAPAIEVHIVPSTEPPGGMGETGTSGIVPAISNAIFAATGKRLRKMPVDPAVLKQT
>SEQF6036||SEQF6036.1_06382
MLPRTHKDAAMNQHVMPKLNRRAFVIGTATAGAGLALGLDLPFGGPAVVRAADGAPEVNAWVVIRPDDTV
VIRIARSEMGQGTLTGLAQLVAEELECDWSKVTTEYPTPGQSVARKRAWGDFSTGGSRGIRTSQDYVRRGGAT
ARVMLVQAAANEWKVPASECKAANSVITHTASGKTTTYGKVAEAAAKLEPPADVKLKDPKDWTIAGKGLKRLD
TSDKTTGKMVYGIDVKLPGMLNAAIKDCPVFGGKVKSFDEAKIAGMKGVKKVVQVGDSAVAVVADTWWHA
KTALDALPIVWDEGPNAKVSSETIATWLAEGLDNAQPAYIGNQNGDAKAAIAAAAKKVEAVYNYPYQNHATM
EPMNATVLYTPDKCEVWCGTQNGEAAFAAALEASGLPAEKVDVHKLMLGGGFGRRGMTDYVRQAVAIAKQ
MPGTPIKLLWSREEDMQHGKYHPITQCKLTGAFDADNNLVALHYRLSGQSILFSVRPEALQNGMDPAAFQGV
AQAGEAAIGYSVPNLLVEHSMRNPHVPPGFWRGVNVNHNAIYMECFMDELALSVGQDPLEFRRKLMGKHP
KHLAVLNAVAEKIGWGTPAPQGVYRGIAQVMGYGSYVAGAAEISVTDGSKIKVHRIVASTDPGYVVNPAQVER
QIAGSFVYGLSALFYGGCTVKDGRIEQTNFDTYNSMRINEMPKVEAVMVPSGGFWGGVGEPTIGVAAPAVLN
AYFAATGKRIRSFPLRNQNISFA
>SEQF6036||SEQF6036.1_00229
MNKHVKNLAPETTDLSRRSFLVGTAATGLVLGYAASGIDQALAAPAPASFEPSVWYSIAPDGLVTVTCGKADMG
QHVASTMAQIVAEELGANWKDMRVQLASNDPKFNDPVLGAQITGGSWSTMMNFDAMSRAGAAGRIALTE
AAAASMGVPAGELVVRDSRISHAKSKKSMSFAEVVKSGKATKTFTADDLKAIKLKTPDQYTMIGVSVPQLDIPSK
VNGTAKYGIDVMVPGMVYGALVTPPVRYGATVKSVDDSAAKKLPGFIKAVTLDDKTTTTTGWVVAVANTYAQ
AKKAAAALKISYDGGPNAKLSSESLFAEAKRLQGLSDSGEFFVKDGDPNAAYGSAAKVLEAEYTTNINIHAPMEP
MNATAEFKGDILHIYSGNQFATRSGAIAAGAAGIDPKFVVMHQMWLGGGFGRRLDADMMVPAVQAAKAVG
KPVKVIYTRENDMTMDFSRPLTYQKVKAGMDGDGKIVAMSHDVVSAWPTARWGIPDFLTPSVDKKGPLDSFT
VNGADFFYTVPNHYVRAIKNEMAHNATPSGQLRSVAPGWTFWAVESMIDEIAHATGKDPAQLRISLLDGKGK
NDGGAQRLRNTLLAAMGLAGYGTKQLPKGEGMGVACVSSQERATASWTACVAHVAVAPSGEVTVKKLTVAT
DVGTQVNPDGIRAQVEGAALWGMSLALFEKATLKDGGIEQTNFDSYTPLRMSQLPEVAVNVIANGEKATGVG
EPAVTVVAPAIGNAVFNAVGARVRGLPITAEAVKAAMKA
>SEQF6037||SEQF6037.1_04639
MNIRTNIPTDSGKLRGFEKHVKVENVSRRSILKGLGIAGSFVLAAPVMTRQAFAYETGAGKMPHGVVVDPRVF
VAIAPDGIVTILAHRSEMGTGVRTSLPLIVAEEMEADWSRVHVQQAHGDEVKFGNQDTDGSRSTRHYLIPMR
QIGASARSMLEAAAAKKWGVPVTEVKAQNHEVVHSASGRKIGFGELAADAAKEPVPSVAGLKLKDPKDFRYL
GKGQVSIVDLHDITTGKAHYGADVRLPGLKYAVIARPPVTGGKLKSFDDSAAKKVPGVEKVMEVKGWPWPSKF
QPLGGVAVIARNTGAAIKGRDALKIVWDDGANAKYDSVTYRASLEEAARKPGLVVRKEGDVDAALKSADKVIT
GEYYLPHLAHVSMEPPVAVADVKGDKATIWAPVQSPGGTREDVAKTLGIPEDNVTVNVTLLGGGFGRKSKCDF
ALEAALLSKELGAPVKVQWTREDDVHHDFLHTVSVERIEAGLDKSGKVIAWRHRSVAPTIASTFAAGANHEAPF
ELGMGLIDNPFEIANLQCENPEAAAFTRIGWFRSVSNIPRAFAVQSMVGEIAHATGRDQKEMLLELIGSPRIVNL
SSVKDPWNYGEPYDSYPIDTARLRKVVELVAEKGEWGRKVPKGHGLGIAVHRSFVSYIATIVEVAVDDKGKFSVP
RVDTAIDCGTYVNPERIHSQIEGAAIMGLSLAKYGEISFKDGKVQQGNFDDFPVIRMDESPVITNVHIVPPGADT
PPSGVGEPGVPPFAPALINAIFAATGKRIRALPIGKQLET
>SEQF6037||SEQF6037.1_03358
MTLIDNLSERAADLSRRNFLRAGAIAGGGLLLSVSLPFASRESEAAASDGFAPNAFVRIGGDGKVVLTMPYVEM
GQGTYTSIPMLIAEELEIGLAQVRLEHAPPSDKLYANPLLGVQATGNSNAMRGAWQPMRKAGATAKAMLVAA
AAKRWNVEPGTCRAENGEVHHAASGRKLGYGELATDAAQMPVPENVTLKSPSEFKLIGTPAKRLDTPSKINGT
AVYGIDARPPGVKVATLAQSPVFGGRVKRVDDAAAKAVKGVRQIVTLDDAVAVVADHMGAAKKGLAALTIEW
DEGAHAKLATSDIARELEAATTKPGTVAQNIGDADKAMAGAATKVEATYQLPFLAHATMEPMNCTVHVRPDG
CEIWVGSQALSRAQAVAAKVLNMPPEKVVVHNHLLGGGFGRRLEVDGVIRAVQIAKQVDAPVKVVWTREEDI

QHDMYRPYWCDRIAVGLDASGKPVAWNNRFAGSSVLARWAPPAFRNGLDPDTTEGAIDLVYDIPNFHVEYV
RVEPPGIPTAFWRSVGPSHNVFVTESVIDELAAAAKQDPLDYRRALLSKSPRAKAALELAAAKAGWGGKLPAGR
GRGVSLQFVFGSYLAQVAEVEVARDGSVRVHRVVCAMDCGTVVNPDTVQAQLQSGINFGITAALYGEITLKDG
RVEQSNFDSYQMLRIDQAPAIEVHIVPSTEPPGGMGETGTSGIVPAISNAIFAATGKRLRKMPVDPAVLKQT
>SEQF6037||SEQF6037.1_04728
MLPRTHKDAAMNQHVMPKLNRRAFVIGTATAGAGLALGLDLPFGGPAVVRAADGAPEVNAWVVIRPDDTV
VIRIARSEMGQGTLTGLAQLVAEELECDWSKVTTEYPTPGQSVARKRAWGDFSTGGSRGIRTSQDYVRKGGAT
ARVMLIQAAANEWKVPASECKVSNGVITHTASGKTTTYGKVAEAAAKLEPPADVKLKDPKDWTIAGKGLKRLD
TSDKTTGKMVYGIDVKLPGMLNAAIKDCPVFGGKVKSFDEAKISGMKGVKKVVQVGDSAVAVVADTWWHAK
TALDALPIVWDEGPNAKVSSETIATWLAEGLDNAQPAYIGNQNGDAKAAIAAAAKKVEAVYSYPYQNHATMEP
MNATVLYTPDKCEVWCGTQNGEAAFAAALEASGLPAEKVDVHKLMLGGGFGRRGMTDYVRQAVAIAKQMP
GTPIKLLWSREEDMQHGKYHPITQCKLTGAFDADNNLVALHYRLSGQSILFSVRPEALQNGMDPAAFQGVAQA
GEAAIGYSVPNLLVEHSMRNPHVPPGFWRGVNVNHNAIYMECFMDELALSVGQDPLEFRRKLMGKHPKHLA
VLNAVAEKIGWGTPAPQGVYRGIAQVMGYGSYVAGAAEISVTDGSKIKVHRIVASTDPGYVVNPAQVERQIAG
SFVYGLSALFYGGCTVKDGRIEQTNFDTYNSMRINEMPKVEAVMVPSGGFWGGVGEPTIGVAAPAVLNAYFA
ATGKRIRSFPLRNQNISFA
>SEQF6037||SEQF6037.1_07416
MDRQILKGRSDETAMSRRSLLQGTGLLLGFALTGTSTKSVFAASASQVVENEVMGTFAPNGFIRINPTGAVTLIM
PMVEMGQGVYTSLSMLLAEELEVKLDQIQVQHAPPNHALYVNSIIGLQNTGGSASVRAFWTPLRQAGAVGR
NLMIAAAAKRWNIDPATCRAASGVVFDRSGSKHLNYGELVEAAAKLPVPAAANIKLKDPKDFVLIGTRAKRVDS
SIKVDGRALYGIDVRLPGMKVAAVGISPVLGGKAKTVNEKAALTVMGVRQVVNIDEAVAVVADHMGAAKKGL
EAAAITWDDGPNSKVNNADIVKQLEAESKKPGVVARNHGDAGKALAAAAQRLDAIYQVPFLAHAAMEPMN
CTVHLQKDRCDIWVGTQAPTITQALVVELTGLPKDSIKIHNHLIGGGFGRRLEADGTVLAVKIAKHVDSPVKVIW
SREEDIQHDMYRPYYLDRLSAGLDAAGKPVAWTHRIAGSSIFARYYPPYIKDGLDPDAVEAAAEPPYALPNIHVD
YVQVEPRGVRTSWWRGVGPTHNVFVVESFMDELAYAAKQDPVAYRKGLLGHNPRALAVLSLAAEKAGWGSP
LPARHGRGISVQFAYGSYVSQVAEVEVAADGSVKVKRIVCALDCGMYVNPDTIEAQVQGGTLFGLTAALRGSITF
KDGRVEQSNFDSYLPMRIDEAPVVETHLIKNAEAPGGVGEAPTAIVNAAVTNAVFAATGKRVRSLPIDADSLKSS
S
>SEQF6037||SEQF6037.1_02793
MNKHVKNLAPETTDLSRRSFLVGTAATGLVLGYAASGIDHALAAPATANFEPSVWYSIAPDGLVTVTCGKADMG
QHVASTMAQIVAEELGANWKDMRVQLASNDPKFNDPVLGAQITGGSWSTMMNFDAMSRAGAAGRIALTE
AAAASMGVPAGELVVRDSRISHPKSKKSMSFAEVVKSGKATKTFTADDLKAIKLKTPDQYTMIGVSVPQLDIPSK
VNGTAKYGIDVMVPGMVYGALVTPPVRYGATVKSVDDSAAKKLPGFIKAVTLDDKTTTTTGWVVAVANTYAQ
AKKAAAALKISYDGGPNAKLSSESLFAEAKRLQGLSDSGEFFVKDGDPNAAYGSAAKVLEAEYTTNINIHAPMEP
MNATAEFKGDILHIYSGNQFATRSGAIAAGAAGIDPKFVVMHQMWLGGGFGRRLDADMMVPAVQAAKAVG
KPVKVIYTRENDMTMDFSRPLTYQKVKAGMDGDGKIVAMSHDVVSAWPTARWGIPDFLTPSVDKKGPLDSFT
VNGADFFYTVPNHYVRAIKNEMAHNATPSGQLRSVAPGWTFWAVESMIDEIAHATGKDPAQLRISLLDGKGK
NDGGAQRLRNTLLAAMGLAGYGTKQLPKGEGMGVACVSSQERATASWTACVAHVAVAPSGEVTVKKLTVAT
DVGTQVNPDGIRAQVEGAALWGMSLALFEKATLKDGGIEQTNFDSYTPLRMSQLPEVAVNVIANGEKATGVG
EPAVTVVAPAIGNAVFNAVGARVRGLPITAEAVKAAMKA
>SEQF6038||SEQF6038.1_02566
MLPRTHKDAAMNQHVMPKLNRRAFVIGTATAGLALGLDLPFGGPAVVRAADGAPEVNAPEVNAWVVIRPD
DTVVIRIARSEMGQGTLTGLAQLVAEELECDWSKVTTEYPTPGQSVARKRAWGDFSTGGSRGIRTSQDYVRKG
GATARVMLIQAAANEWKVPASECKAANSVITHTPSGKTTTYGKVAEAAAKLEPPADVKLKDPKDWTIAGKGLK

RLDTVDKTTGKMVYGIDVKLPGMLNAAIKDCPVFGGKVKSFDEAKIAGMKGVKKVVQVGDSAVAVVADTW
WHAKTALDALPVVWDEGPNAKVSSETIATWLAEGLDNAQPAYVGNQNGDAKAAIAAAAKKVEAVYNYPYQN
HATMEPMNATVLYTPDKCEVWCGTQNGEAAFAAALEASGLPAEKVDVHKLMLGGGFGRRGMTDYVRQAVA
IAKQMPGTPIKLLWSREEDMQHGKYHPITQCKLTGAFDADNNLVALHYRLSGQSILFSVRPEALQNGMDPAAF
QGVAQAGEAAIGYSVPNLLVEHSMRNPHVPPGFWRGVNVNHNAIYMECFMDELALSVGQDPLEFRRKLMG
KHPKHLAVLNAVAEKIGWGTPAPQGVYRGIAQVMGYGSYVAGAAEISVTDGSKIKVHRIVASTDPGYVVNPAQ
VERQIAGSFVYGLSALFYGGCTVKDGRIEQTNFDTYNSMRINEMPKVEAVMVPSGGFWGGVGEPTIGVAAPA
VLNAYFAATGKRIRSFPLRNQNISFA
>SEQF6038||SEQF6038.1_00582
MTLIDNLSERAADLSRRNFLRAGAIAGGGLLLSVSLPFAGRESEAAASDGFAPNAFVRIGGDGKVVLTMPYVEM
GQGTYTSIPMLIAEELEIGLTQVRLEHAPPSDKLYANPLLGVQATGNSNAMRGAWQPMRKAGATAKAMLVAA
AAKRWNVEPGTCRAENGEVHHAASGRKLGYGELATDAAQMPVPENVTLKSPSEFKLIGTPAKRLDTPSKINGT
AVYGIDARPPGVKIATLAQSPVFGGRVKRVDDAAAKAVKGVRQIVTLDDAVAVVADHMGAAKKGLAALTIEW
DEGTHAKLATSDIARELEAATTKPGAVAQNIGDADKAMAGAATKVEATYQLPFLAHATMEPMNCTVHVRPDG
CEIWVGSQALSRAQAVAAKVLNMPPEKVVVHNHLLGGGFGRRLEVDGVIRAVQIAKQVDAPVKLVWTREEDI
QHDMYRPYWCDRIAVGLDASGKPVAWNNRFAGSSVLARWAPPAFRNGLDPDTTEGAIDLVYDIPNFHVEYV
RVEPPGIPTAFWRSVGPSHNVFVTESMIDELAAAAKQDPVDYRRALLGKSPRAKAALELAAAKAGWGGKLPA
GRGRGVSLQFVFGSYLAQVAEVEVAKDGSVRVHRVVCAMDCGTVVNPDTVQAQLQSGINFGVTAALYGEITL
KDGRVEQTNFDSYQMLRIDQAPAIEVHIVPSTEPPGGMGETGTSGIVPAISNAIFAATGKRLRKMPVDPAVLKQ
T
>SEQF6038||SEQF6038.1_07335
MNIRTNIPTDSGKLRGFEKHVKVENVSRRSILKGLGIAGSFVLAAPVMTRRAFAYETGAGKMPHGVVVDPRVF
VAIAPDGIVTILAHRSEMGTGVRTSLPLIVAEEMEADWSRVHVQQAHGDEVKFGNQDTDGSRSTRHYLIPMR
QIGASARSMLEAAAAKKWGVPVTEVKAQNHEVVHSASGRKAGFGELAADAAKESVPSVEGLKLKDPKDFRYL
GKGQVSIVDLHDITTGKAPYGADVRLPGLKYAVIARPPVTGGKLKSFDDSAAKKVPGVEKVMEVKGWPWPSKF
QPLGGVAVIARNTGAAIKGRDALKIVWDDGANAKYDSVTYRASLEEAARKPGLVVRKEGDADAALKSADKVIT
GEYYLPHLAHVSMEPPVAVADVKGDKATIWAPVQSPGGTREDVAKTLGIPEDNVTVNVTLLGGGFGRKSKCDF
ALEAALLSKELGAPVKVQWTREDDVHHDFLHTVSVERIEAGLDKSGKVIAWRHRSVAPTIASTFAAGANHEAPF
ELGMGLIDNPFEIANLQCENPEAAAFTRIGWFRSVSNIPRAFAVQSMVGEIAHATGRDQKEMLLELIGSPRIVNL
SSVKDPWNYGEPYDSYPIDTARLRKVVELVAEKGEWGRKVPKGHGLGIAVHRSFVSYIATIVEVAVDDKGKFSVP
RVDTAIDCGTYVNPERIHSQIEGAAIMGLSLAKYGEISFKDGKVQQGNFDDFPVIRMDESPVITNVYIVPPGADT
PPSGVGEPGVPPFAPALINAIFAATGKRIRALPIGKQLET
>SEQF6038||SEQF6038.1_05264
MMDLETERLPGPVASRYLAQPSRRSLLKVGASALGGLALTVALPSLSKAASEESAPDLNAFVRIEADGRVHLTIPS
VEMGQGIYTAMSMLLAEELEVGLDAVTVEHAPPNDALYANPIPILRQQTTGASASIRGFWRPLRLAGAAARLM
LVAAAAKQWHVAALACSVRNGVVFDPSGTKSLPYRDLLRSAAAERPPAPDQIKLKSPEQFKLIGTSPKRIEAADK
VSGRTQFGIDVMLPGLRVAALAISPVMGGRPKDVNRAAALATKGVRQVVTIDRAVAIVADHMGAAKKGLVAA
AVAWDDGPNGSVDSKMLVEQLERASENPGAVARSEGHFEAAFAGAARRIESTYELPFLAHAAMEPMNCTVH
VRKESCEIWVGTQIPTVTQAAVAALLGMPQSAVVIHNQYIGGGFGRRLEPDGTLLAVEIGKQVDGPVKVIWSR
EEDIQHDIYRPYYYDRISAGLDAAGLPVAWHHRVCGSSIIARAVPALFKDGLDFDAVEGAAEPPYALPNILVDYVR
AEPPGVTTGFWRGVGPAHNVFVVESFVDELAAAAGKDPVAYRRRTLLGHNPRALAVLNLAAEKADWGKPMSA
GKGRGVAVQFAFGSYLALVADVAVDDRGAVKVERVVCAVDCGLPVNPHMIDAQVQSGTIFGLTAALRGAITFK
NGRVEQSNFDSYLPMRIEETPRIETYIVSSTAEPGGLGEAATAIVAPAVTNAIFAATGRRVRRLPIEAT
>SEQF6038||SEQF6038.1_04227

MDELVLKEQIVDEATMSRRAFLQGTGLLLGFSLTGARAERVFAAPASQVVEHEVAGTFAPNGFIRINPTGAVTLV
MPMIEMGQGVYTSLSMLLAEELEVTLDQIQVQHAPPNHALYVNSIIGIQNTGGSASVRAFWTPLRQAGAVGR
NLLIAAAAKRWNVDPATCRAKDGFVFDAPGLKHLSYGELAAAAAKLPVPLAADVKLKDPKDFTLIGTRAKRVDS
SIKVDGRALYGIDTRLPGMTVAAVAISPVLGGKAKTVDENAALMVKGVRQVVNIGEAVAVVANHMGAARKGL
EAAAITWDDGPNGKVSSSDIVKQLEEESRKPGAVARNDGDVGKALAGAAQRVDAIYQVPFLAHAAMEPMNC
TVHLQKDRCDIWVGTQAPTITQSLVTELTGLPKEAITIHNHLIGGGFGRRLEADGTILAVKIAKHVDGPVKVIWSR
EEDIQHDMYRPYYLDRLSAGLDAAGKPVAWTHRIAGSSVMARYYPPYVKNGLDPDAVEAAAEPPYALPNIHV
DFVRVEPPGVRTSWWRGVGPTHNVFVVESFIDELAHAAKQDPVTYRKGLLGHNPRALAVLSLAAEKAGWGSP
LPARHGRGISVQFAYGSFTSQVAEVEVAADGSVKVWRIVCAIDCGMYINPDTIEAQVQGGTLFGLTAALHGSITF
KDGRVEQSNFDTYLPMRIDEVPLVETHLIKNAEAPGGVGEAPTAIVSAAVTNAIFAATGKRVRSLPIDRNSLKSSS
>SEQF6038||SEQF6038.1_06067
MNKHVKNLAPETTDLSRRSFLVGTAATGLVLGYAASGIDQALAASATANFEPSVWYSIAPDGLVTVTCGKADMG
QHVASTMAQIVAEELGASWKDMRVQLASNDPKFNDPVLGAQITGGSWSTMMNFDAMSRAGAAGRIALTE
AAAASMGVPAGELVVRDSRISHAKSKKSMSFAEVVKSGKATKTFTADDLKAIKLKTPDQYTMIGVSVPQLDIPSK
VNGTAKYGIDVMVPGMVYGALVTPPVRYGATVKSVDDSAAKKLPGFIKAVTLDDKTTTTTGWVVAVANTYAQ
AKKAAAALKISYDGGPNAKLSSESLFAEAKRLQGLSDSGEFFVKDGDPNAAYGSAAKVLEAEYTTNINIHAPMEP
MNATAEFKGDILHIYSGNQFATRSGAIAAGAAGIDPKFVVMHQMWLGGGFGRRLDADMMVPAVQAAKAVG
KPVKVIYTRENDMTMDFSRPLTYQKVKAGMDGDGKIVAMSHDVVSAWPTARWGIPDFLTPSVDKKGPLDSFT
VNGADFFYTVPNHYVRAIKNEMAHNATPSGQLRSVAPGWTFWAVESMIDEIAHATGKDPAQLRISLLDGKGK
NDGGAQRLRNTLLAAMGLAGYGTKQLPKGEGMGVACVSSQERATASWTACVAHVAVAPSGEVTVKKLTVAT
DVGTQVNPDGIRAQVEGAALWGMSLALFEKATLKDGGIEQTNFDSYTPLRMSQLPEVAVNVIANGEKATGVG
EPAVTVVAPAIGNAVFNAVGARVRGLPITAEAVKAAMKA
>SEQF6039||SEQF6039.1_03949
MNKHVKNLAPETTDLSRRSFLVGTAATGLVLGYAASGIDQALAAPAPASFEPSVWYSIAPDGLVTVTCGKADMG
QHVASTMAQIVAEELGANWKDMRVQLASNDPKFNDPVLGAQITGGSWSTMMNFDAMSRAGAAGRIALTE
AAAASMGVPAGELVVRDSRISHAKSKKSMSFAEVVKSGKATKTFTADDLKAIKLKTPDQYTMIGVSVPQLDIPSK
VNGTAKYGIDVMVPGMVYGALVTPPVRYGATVKSVDDSAAKKLPGFIKAVTLDDKTTTTTGWVVAVANTYAQ
AKKAAAALKISYDGGPNAKLSSESLFAEAKRLQGLSDSGEFFVKDGDPNAAYGSAAKVLEAEYTTNINIHAPMEP
MNATAEFKGDILHIYSGNQFATRSGAIAAGAAGIDPKFVVMHQMWLGGGFGRRLDADMMVPAVQAAKAVG
KPVKVIYTRENDMTMDFSRPLTYQKVKAGMDGDGKIVAMSHDVVSAWPTARWGIPDFLTPSVDKKGPLDSFT
VNGADFFYTVPNHYVRAIKNEMAHNATPSGQLRSVAPGWTFWAVESMIDEIAHATGKDPAQLRISLLDGKGK
NDGGAQRLRNTLLAAMGLAGYGTKQLPKGEGMGVACVSSQERATASWTACVAHVAVAPSGEVTVKKLTVAT
DVGTQVNPDGIRAQVEGAALWGMSLALFEKATLKDGGIEQTNFDSYTPLRMSQLPEVAVNVIANGEKATGVG
EPAVTVVAPAIGNAVFNAVGARVRGLPITAEAVKAAMKA
>SEQF6039||SEQF6039.1_06865
MNIRTNIPTGSGKLRGFEKHVKVENVSRRSILKGLGIAGSFVLAAPVMTRQAFAYETGAGKMPHGVVVDPRVF
VAIAPDGIVTILAHRSEMGTGVRTSLPLIVAEEMEADWSRVHVQQAHGDEVKFGNQDTDGSRSTRHYLIPMR
QIGASARSMLEAAAAKKWGVPVTEVKAQNHEVVHSASGRKAGFGELAADAAKESVPSVEGLKLKDPKDFRYL
GKGQVSIVDLHDITTGKAHYGADVRLPGLKYAVIARPPVTGGKLKSFDDSAAKKVPGVEKVMEVKGWPWPSKF
QPLGGVAVIARNTGAAIKGRDALKIVWDDGANAKYDSVTYRASLEEAARKPGLVVRKEGDADAALKSADKVIT
GEYYLPHLAHVSMEPPVAVADVKGDKATIWAPVQSPGGTREDVAKTLGIPEDNVTVNVTLLGGGFGRKSKCDF
ALEAALLSKELGAPVKVQWTREDDVHHDFLHTVSVERIEAGLDKSGKVIAWRHRSVAPTIASTFAAGANHEAPF
ELGMGLIDNPFEIANLQCENPEAAAFTRIGWFRSVSNIPRAFAVQSMVGEIAHATGRDQKEMLLELIGSPRIVNL
SSVKDPWNYGEPYDSYPIDTARLRKVVELVAEKGEWGRKVPKGHGLGIAVHRSFVSYIATIVEVAVDDKGKFSVP

RVDTAIDCGTYVNPERIHSQIEGAAIMGLSLAKYGEISFKDGKVQQGNFDDFPVIRMDESPVITNVHIVPPGADT
PPSGVGEPGVPPFAPALINAIFAATGKRIRALPIGKQLET
>SEQF6039||SEQF6039.1_04082
MTLIDNLSERAADLSRRNFLRAGAIAGGGLLLSVSLPFAGRESEAAASDGFAPNAFVRIGGDGKVVLTMPYVEM
GQGTYTSIPMLIAEELEIGLTQVRLEHAPPSDKLYANPLLGVQATGNSNAMRGAWQPMRKAGATAKAMLVAA
AAKRWNVEPGTCRAENGEVHHAASGRKLGYGELATDAAQMPVPENVTLKSPSEFKLIGTPAKRLDTPSKINGT
AVYGIDARPPGVKIATLAQSPVFGGRVKRVDDAAAKAVNGVRQIVTLDDAVAVVADHMGAAKKGLAALTIEW
DEGAHAKLATSDIARELETATTKPGAVAQNIGDADKAMAGAATKVEATYQLPFLAHATMEPMNCTVHVRPDG
CEIWVGSQALSRAQAVAAKVLNMPPEKVVVHNHLLGGGFGRRLEVDGVIRAVQIAKQVDAPVKLVWTREEDI
QHDMYRPYWCDRIAVGLDASGKPVAWNNRFAGSSVLARWAPPAFRNGLDPDTTEGAIDLVYDIPNFHVEYV
RVEPPGIPTAFWRSVGPSHNVFVTESVIDELAAAAKQDPVDYRRALLGKSPRAKAALELAAAKAGWGGKLPAG
RGRGVSLQFVFGSYLAQVAEVEVARDGSVRVHRVVCAMDCGTVVNPDTVQAQLQSGINFGVTAALYGEITLK
DGRVEQSNFDSYQMLRIDQAPAIEVHIVPSTEPPGGMGETGTSGIVPAISNAIFAATGKRLRKMPVDPAVLKQT
>SEQF6039||SEQF6039.1_08383
MLPRTHKDAAMNQHVMPKLNRRAFVIGTATAGAGLALGLDLPFGGPAVVRAADGAPEVNAWVVIRPDDTV
VIRIARSEMGQGTLTGLAQLVAEELECDWSKVTTEYPTPGQSVARKRAWGDFSTGGSRGIRTSQDYVRRGGAT
ARVMLVQAAANEWKVPASECKAANSVITHTASGKTTTYGKVAEAAAKLEPPADVKLKDPKDWTIAGKGLKRLD
TSDKTTGKMVYGIDVKLPGMLNAAIKDCPVFGGKVKSFDEAKIAGMKGVKKVVQVGDSAVAVVADTWWHA
KTALDALPIVWDEGPNAKVSSETIATWLAEGLDNAQPAYIGNQNGDAKAAIAAAAKKVEAVYNYPYQNHATM
EPMNATVLYTPDKCEVWCGTQNGEAAFAAALEASGLPAEKVDVHKLMLGGGFGRRGMTDYVRQAVAIAKQ
MPGTPIKLLWSREEDMQHGKYHPITQCKLTGAFDADNNLVALHYRLSGQSILFSVRPEALQNGMDPAAFQGV
AQAGEAAIGYSVPNLLVEHSMRNPHVPPGFWRGVNVNHNAIYMECFMDELALSVGQDPLEFRRKLMGKHP
KHLAVLNAVAEKIGWGTPAPQGVYRGIAQVMGYGSYVAGAAEISVTDGSKIKVHRIVASTDPGYVVNPAQVER
QIAGSFVYGLSALFYGGCTVKDGRIEQTNFDTYNSMRINEMPKVEAVMVPSGGFWGGVGEPTIGVAAPAVLN
AYFAATGKRIRSFPLRNQNISFA
>SEQF6040||SEQF6040.1_02932
MNKHVKNLAPETTDLSRRSFLVGTAATGLVLGYAGSGIDQALAAPAPANFEPSVWYSIAPDGLVTVTCGKADM
GQHVASTMAQIVAEELGANWKDMRVQLASNDPKFNDPVLGAQITGGSWSTMMNFDAMSRAGAAGRIALT
EAAAASMGVPAGELVVRDSKISHAKSKKSMSFAEVVKSGKATKTFTADDLKAIKLKTPDQYTMIGVSVPQLDIPS
KTNGTAKYGIDVMVPGMVHGALVTPPVRYGATVKSVDDSAARKLPGFIKAVTLDDKTNTTTGWVVAVANTYA
QAKKAAAALKISYDGGPNAKLSSESLLTEAKRLQGLDDSGQFFVKDGDPKAAFGSAAKVLEAEYTTNINIHAPM
EPMNATAEFKGDILHIYSGNQFATRSGAIAAGAAGIDPKFVVMHQMWLGGGFGRRLDADMMVPAVQAAKA
VGKPVKVIYTRENDMTMDFSRPLTYQKVKAGMDGGGKIVAMSHDVVSAWPTARWGIPDFLTPSVDKKGPLD
SFTVNGADFFYTVPNHYVRAIKNEMAHNATPSGQLRSVAPGWTFWAVESMIDEIAHATGKDPAQLRISLLDGK
GKNDGGAQRLRNTLLAAMGLAGYGTKQLPKGEGMGVACVSSQERATASWTACVAHVAVAPSGEVTVKKLTV
ATDVGTQVNPDGIRAQVEGAALWGMSLALFEKATLKDGGIEQTNFDSYTPLRMSQLPEVAVNVIANGEKATG
VGEPAVTVVAPAIGNAVFNAVGARVRGLPITAEAVKAAMKA
>SEQF6040||SEQF6040.1_01457
MLPRTHKDAAMNQHVMPKLNRRAFVIGTATAGAGLALGLDLPFGGPAVVRAADGAPEVNAWVVIRPDDTV
VIRIARSEMGQGTLTGLAQLVAEELECDWSKVTTEYPTPGQSVARKRAWGDFSTGGSRGIRTSQDYVRKGGAT
ARVMLIQAAANEWKVPASECKAANSIITHTPSGKTTTYGKVAEAAAKLEPPADVKLKDPKDWTIAGKGLKRLDT
VDKTTGKMVYGIDVKLPGMLNAAIKDCPVFGGKVKSFDEAKIAGMKGVKKVVQVGDTAVAVVADTWWHAK
TALDALPIVWDEGPNAKVSSETIATWLAEGLDNAQPAYVGNQNGDAKAAIASAAKKVEAVYSYPYQNHATME
PMNATVLYTPDKCEVWCGTQNGEAAFAAALEASGLPAEKVDVHKLMLGGGFGRRGMTDYVRQAVAIAKQM

PGTPIKLLWSREEDMQHGKYHPITQCKLTGAFDADNNLVALHYRLSGQSILFSVRPEALQNGMDPAAFQGVAQ
AGEAAIGYSVPNLLVEHSMRNPHVPPGFWRGVNVNHNAIYMECFMDELALAVGQDPLEFRRKLMGKHPKHL
AVLNAVAEKIGWGTPAPQGVYRGIAQVMGYGSYVAGAAEISVTDGSKIKVHRIVASTDPGYVVNPAQVERQIA
GSFVYGLSALFYGGCTVKDGRIEQTNFDTYNSMRINEMPKVESVMVPSGGFWGGVGEPTIGVAAPAVLNAYF
AATGKRIRSFPLRNQNISFA
>SEQF6040||SEQF6040.1_05839
MSILINPSKLRGFEKHVKIENVSRRSVLKGLGLAGGFVLAAPVMTRRAFAYETGAGQMPHGTVVDPRVFVSIAS
DGVVTIIAHRAEMGTGVRTSLPMIVAEEMEADWKRVRVQQAHGDEVKYGNQDTDGSRSTRHYLIPMRQIGA
SARTMLEQAAAKRWGVPATEVKAVDHEVVHTASGRKLGFGELAADAAKQSVPSVEGLKLKDPKDFRYLGKGEI
SMVDLHDITTGAARYGADVRLPSMKYAVIARPPVTGGKVVSFDGSEAMKVSGVEKVLEVKGWPWPSKFQPL
GGVAVIARNTGAAIKGRDVLKITWDDGPNAKYDSIAYRGQLEEAARKPGLVVRKEGDVDAALKGADKVIVGEY
YLPHLAHVAMEPPVAVADVKGDKAEIWAPVQSAGGTREDVAKTLDIPQENVTVNVTLLGGGFGRKSKCDFALE
AALLSKELGAPVKVQWTREDDIRNGFLHTVSVERVEAGLDKGGKVIAWRHRSVAPSIASTFAAGTVHQAPFEL
GMGLVDMPFEIANVQCENPEAAAHTRVGWFRSVSNIPRAFAVQSMVAEIAQATNRDPKDMLLELVGAPRILN
LTSVKDLWNYGEPYDSYPIDTGRLRNVVELVADKGGWGRAVPKGHGLGIAAHRSFVSYIATIVEVAVDEKGKLTV
PRVDTAIDCGTYVNPERIQSQIEGTAIMGLSLAKHGAITFKDGKVQQGNFDDFPVIRIDESPSVTNVYIVPPGRD
TPPSGVGEPGVPPFAPALINAIFAATGKRIRALPIGKQLEA
>SEQF6040||SEQF6040.1_04688
MNQHIRSAPVVEPQLDLSRRAFLIGAGTVGLAFGYAAIPGAEAIAAPAVPSGFEPSVWYSIGSDGLITVTSGKAD
MGQHIASAMAQMIAEELGAAWKDVRLQLASNDPKFNDPVLGAQITGGSWSTLMNFDAMSRAGAAGRIALT
EAAAAIMGVRVDELIVQESTIKHAKSGRQISFADVVKSGKPLRSFTPEQLKAIALKTSDQYQLIGVSVPQLDIPSKT
NGTAKYGIDTFLPGMVYGKLVLPPVRYGAKVKSVDDTAARKIPGFVRAVVLDDKTETTSGWVVVVAATYAAAR
KAAELLKVEWDKGPYANVSSETLLAEAKRLHAREDTGLLFVKKGDAAAALGSAAKVIEAEYSTNINIHAPLEPMN
ATAHLQGDIWHVYTGNQFVTRTGAIAAAAAGVDPKYVVMHQAWLGGGFGRRLDSDMVIPAILAAKAVGKPV
KVIYSREDDMAMDFSRPLTYQPIKGGLDKDGNLIAISHDVVSAWPTKRWGIPGFLTPSVDKKGALDGFAISGAD
HFYTVPNHTVRTILNELAQGATPSGQLRSVAPGWTFWAVESMIDELAFAAGKDPAQFRIAMLDGQGENSGGA
QRLRNALLAAMGIAGYGSLSLPKGEGMGVACVSSQERATASWTACVAHVAVAPSGDVKVKKLTVATDVSTAVH
PDNIKAQVEGAALWGLSLALYEKATLKDGGFEQTNFDTYTPLRMSQLPEVAVNIIANGGKPTGVGEPATTVVAP
AVGNAIFSAVGARVRSLPITPEAVKAAMKS
>SEQF6040||SEQF6040.1_05044
MMNEPFLRERVVDETAISRRGFLQGTGLLLGFVLSGASTGSVFAAATSQVVENEITGTFAPNGFIRINPTGAVTLV
IPMIEMGQGVYTSLSMLLAEELEVTLDQIQVQHAPPNHALYVNSIIGIQNTGGSASVRAFWTPLRQAGAVGRN
LLIAAAAKRWNVDPATCRAKNGVVFDATGSMHLSYGELATAAAKFPVPPAANVKLKDPKDFSLIGTSAKRVDSP
IKVDGRALYGIDTRLPGMKVAAVAISPVLGGKAKKVDEKAALAVKGVRQLINIDEAVAVVADHMGAAKKGLEAA
AIAWDDGPNGKVNSADIVRQLEEESRKPGAVARNVGDVAKALAKAAQRVDAVYQVPFLAHAAMEPMNCTV
HLQKDRCDIWVGTQAPTITQSQVAELTGLPKDAIKIHNHLIGGGFGRRLEADGTILAVKIAKHVDGPVKVIWSRE
EDIQHDMYRPYYLDRLSAGLDTAGQPVAWMHRIAGSSVMARYYPPYFKDGLDPDAVEAAAEPPYALPSIHVD
FVRVEPPGVRTSWWRGVGPTHNVFVVESFIDELAHAAKQDPVAYRKGLLGHNPRALTVLSLAAEKAGWGSPL
PARHGRGISVQFAYGSYTSQVAEVEVAADGSVKVKRIVCVIDCGVYVNPDTIEAQIQGGTLFGLTAALHGSITFKD
GRVEQSNFDSYLPMRIDEVPVVETHLIKNAEAPGGVGEAPTAIVSAAVTNAIFAATGKRVRSLPIETDVLKSSS
>SEQF6040||SEQF6040.1_02559
MTLIDNLSERAADLSRRNFLRAGAIAGGGLLLSVSLPFASRESEAAASDGFAPNAFVRIGGDGKVVLTMPYVEM
GQGTYTSIPMLIAEELEIGLTQVRLEHAPPSDKLYANPLLGVQATGNSNAMRGAWQPMRKAGATAKAMLVAA
AAKRWNVEPGTCRAENGEVHHAASGRKLGYGELATDAAQMPVPENVTLKSPSEFKLIGTPAKRLDTPSKINGT

AVYGIDARPPGVKVATLAQSPVFGGRVKRVDDAAAKAVKGVRQIVTLDDAVAVVADHMGAAKKGLAALTIEW
DEGPHAKLATSDIARELEAVTTKPGAVAQNIGDADKAMAGAGTKVEATYQLPFLAHATMEPMNCTVHVRSDG
CEIWVGSQALSRAQAVAAKVLNMAPEKVVVHNHLLGGGFGRRLEVDGVIRAVQIAKQVDAPVKVVWTREED
IQHDMYRPYWCDRIAVGLDASGKPVAWNNRFAGSSVLARWAPPAFRNGLDPDTTEGAIDLVYDIPNFHVEYV
RVEPPGIPTAFWRSVGPSHNVFVTESVIDELAAAAKQDPVDYRRALLGKSPRAKAALELAAAKAGWGGKLPAG
RGRGVSLQFVFGSYLAQVAEVEVAKDGSVRVHRVVCAMDCGTVVNPDTVQAQLQSGINFGVTAALYGEITLK
DGRVEQSNFDSYQMLRIDQAPAIEVHIVPSTEPPGGMGETGTSGIVPAISNAIFAATGKRLRKMPVDPALLKQT
>SEQF6040||SEQF6040.1_03158
MNIRTNPSKLRGFEKHVKVENVSRRSILKGLGIAGSFVLAAPVMTRQAFAYETGAGKMPHGVVVDPRVFVAIA
PDGIVTILAHRSEMGTGVRTSLPLIVAEEMEADWSRVHVQQAHGDEVKFGNQDTDGSRSTRHYLIPMRQIGA
SARSMLEAAAAKRWGVPVTEVKAQNHEVVHSASGRKAGFGELAADAAKESVPSVEGLKLKDPKDFRYLGKGQ
VSIVDLHDITTGKAHYGADVRLPGLKYAVIARPPVTGGKLKSFDDSAAKKVPGVEKVMEVKGWPWPSKFQPLG
GVAVIARNTGAAIKGRDALKIVWDDGANAKYDSVTYRASLEEAARKPGLVVRKEGDVDAALKSADKVITGEYYL
PHLAHVSMEPPVAVADVKGDKATIWAPVQSPGGTREDVAKTLGIPEDNVTVNVTLLGGGFGRKSKCDFALEAA
LLSKELGAPVKVQWTREDDVHHDFLHTVSVERIEAGLDKSGKVIAWRHRSVAPTIASTFAAGANHEAPFELGM
GLIDNPFEIANLQCENPEAAAFTRIGWFRSVSNIPRAFAVQSMVGEIAHATGRDQKEMLLELIGSPRIVNLSSVK
DPWNYGEPYDSYPIDTARLRKVVELVAEKGEWGRKVPKGHGLGIAVHRSFVSYIATIVEVAVDDKGKFSVPRVDT
AIDCGTYVNPERIHSQIEGAAIMGLSLAKYGEISFKDGKVQQGNFDDFPVIRMDESPAITNVHIVPPGADTPPSG
VGEPGVPPFAPALINAIFAATGKRIRALPIGKQLET
>SEQF6041||SEQF6041.1_05382
MNIRTNIPTGSGKLRGFEKHVKVENVSRRSILKGLGIAGSFVLAAPVMTRQAFAYETGAGKMPHGVVVDPRVF
VAIAPDGIVTILAHRSEMGTGVRTSLPLIVAEEMEADWSRVHVQQAHGDEVKFGNQDTDGSRSTRHYLIPMR
QIGASARSMLEAAAAKKWGVPVTEVKAQNHEVVHSASGRKAGFGELAADAAKESVPSVEGLKLKDPKDFRYL
GKGQVSIVDLHDITTGKAHYGADVRLPGLKYAVIARPPVTGGKLKSFDDSAAKKVPGVEKVMEVKGWPWPSKF
QPLGGVAVIARNTGAAIKGRDALKIVWDDGANAKYDSVTYRASLEEAARKPGLVVRKEGDADAALKSADKVIT
GEYYLPHLAHVSMEPPVAVADVKGDKATIWAPVQSPGGTREDVAKTLGIPEDNVTVNVTLLGGGFGRKSKCDF
ALEAALLSKELGAPVKVQWTREDDVHHDFLHTVSVERIEAGLDKSGKVIAWRHRSVAPTIASTFAAGANHEAPF
ELGMGLIDNPFEIANLQCENPEAAAFTRIGWFRSVSNIPRAFAVQSMVGEIAHATGRDQKEMLLELIGSPRIVNL
SSVKDPWNYGEPYDSYPIDTARLRKVVELVAEKGEWGRKVPKGHGLGIAVHRSFVSYIATIVEVAVDDKGKFSVP
RVDTAIDCGTYVNPERIHSQIEGAAIMGLSLAKYGEISFKDGKVQQGNFDDFPVIRMDESPVITNVHIVPPGADT
PPSGVGEPGVPPFAPALINAIFAATGKRIRALPIGKQLET
>SEQF6041||SEQF6041.1_06683
MNKHVKNLAPETTDLSRRSFLVGTAATGLVLGYAASGIDQALAAPAPASFEPSVWYSIAPDGLVTVTCGKADMG
QHVASTMAQIVAEELGANWKDMRVQLASNDPKFNDPVLGAQITGGSWSTMMNFDAMSRAGAAGRIALTE
AAAASMGVPAGELVVRDSRISHAKSKKSMSFAEVVKSGKATKTFTADDLKAIKLKTPDQYTMIGVSVPQLDIPSK
VNGTAKYGIDVMVPGMVYGALVTPPVRYGATVKSVDDSAAKKLPGFIKAVTLDDKTTTTTGWVVAVANTYAQ
AKKAAAALKISYDGGPNAKLSSESLFAEAKRLQGLSDSGEFFVKDGDPNAAYGSAAKVLEAEYTTNINIHAPMEP
MNATAEFKGDILHIYSGNQFATRSGAIAAGAAGIDPKFVVMHQMWLGGGFGRRLDADMMVPAVQAAKAVG
KPVKVIYTRENDMTMDFSRPLTYQKVKAGMDGDGKIVAMSHDVVSAWPTARWGIPDFLTPSVDKKGPLDSFT
VNGADFFYTVPNHYVRAIKNEMAHNATPSGQLRSVAPGWTFWAVESMIDEIAHATGKDPAQLRISLLDGKGK
NDGGAQRLRNTLLAAMGLAGYGTKQLPKGEGMGVACVSSQERATASWTACVAHVAVAPSGEVTVKKLTVAT
DVGTQVNPDGIRAQVEGAALWGMSLALFEKATLKDGGIEQTNFDSYTPLRMSQLPEVAVNVIANGEKATGVG
EPAVTVVAPAIGNAVFNAVGARVRGLPITAEAVKAAMKA
>SEQF6041||SEQF6041.1_01085

MLPRTHKDAAMNQHVMPKLNRRAFVIGTATAGAGLALGLDLPFGGPAVVRAADGAPEVNAWVVIRPDDTV
VIRIARSEMGQGTLTGLAQLVAEELECDWSKVTTEYPTPGQSVARKRAWGDFSTGGSRGIRTSQDYVRRGGAT
ARVMLVQAAANEWKVPASECKAANSVITHTASGKTTTYGKVAEAAAKLEPPADVKLKDPKDWTIAGKGLKRLD
TSDKTTGKMVYGIDVKLPGMLNAAIKDCPVFGGKVKSFDEAKIAGMKGVKKVVQVGDSAVAVVADTWWHA
KTALDALPIVWDEGPNAKVSSETIATWLAEGLDNAQPAYIGNQNGDAKAAIAAAAKKVEAVYNYPYQNHATM
EPMNATVLYTPDKCEVWCGTQNGEAAFAAALEASGLPAEKVDVHKLMLGGGFGRRGMTDYVRQAVAIAKQ
MPGTPIKLLWSREEDMQHGKYHPITQCKLTGAFDADNNLVALHYRLSGQSILFSVRPEALQNGMDPAAFQGV
AQAGEAAIGYSVPNLLVEHSMRNPHVPPGFWRGVNVNHNAIYMECFMDELALSVGQDPLEFRRKLMGKHP
KHLAVLNAVAEKIGWGTPAPQGVYRGIAQVMGYGSYVAGAAEISVTDGSKIKVHRIVASTDPGYVVNPAQVER
QIAGSFVYGLSALFYGGCTVKDGRIEQTNFDTYNSMRINEMPKVEAVMVPSGGFWGGVGEPTIGVAAPAVLN
AYFAATGKRIRSFPLRNQNISFA
>SEQF6041||SEQF6041.1_01776
MTLIDNLSERAADLSRRNFLRAGAIAGGGLLLSVSLPFAGRESEAAASDGFAPNAFVRIGGDGKVVLTMPYVEM
GQGTYTSIPMLIAEELEIGLTQVRLEHAPPSDKLYANPLLGVQATGNSNAMRGAWQPMRKAGATAKAMLVAA
AAKRWNVEPGTCRAENGEVHHAASGRKLGYGELATDAAQMPVPENVTLKSPSEFKLIGTPAKRLDTPSKINGT
AVYGIDARPPGVKIATLAQSPVFGGRVKRVDDAAAKAVNGVRQIVTLDDAVAVVADHMGAAKKGLAALTIEW
DEGAHAKLATSDIARELETATTKPGAVAQNIGDADKAMAGAATKVEATYQLPFLAHATMEPMNCTVHVRPDG
CEIWVGSQALSRAQAVAAKVLNMPPEKVVVHNHLLGGGFGRRLEVDGVIRAVQIAKQVDAPVKLVWTREEDI
QHDMYRPYWCDRIAVGLDASGKPVAWNNRFAGSSVLARWAPPAFRNGLDPDTTEGAIDLVYDIPNFHVEYV
RVEPPGIPTAFWRSVGPSHNVFVTESVIDELAAAAKQDPVDYRRALLGKSPRAKAALELAAAKAGWGGKLPAG
RGRGVSLQFVFGSYLAQVAEVEVARDGSVRVHRVVCAMDCGTVVNPDTVQAQLQSGINFGVTAALYGEITLK
DGRVEQSNFDSYQMLRIDQAPAIEVHIVPSTEPPGGMGETGTSGIVPAISNAIFAATGKRLRKMPVDPAVLKQT
>SEQF6042||SEQF6042.1_02842
MNIRTNIPTGSGKLRGFEKHVKVENVSRRSILKGLGIAGSFVLAAPVMTRQAFAYETGAGKMPHGVVVDPRVF
VAIAPDGIVTILAHRSEMGTGVRTSLPLIVAEEMEADWSRVHVQQAHGDEVKFGNQDTDGSRSTRHYLIPMR
QIGASARSMLEAAAAKKWGVPVTEVKAQNHEVVHSASGRKAGFGELAADAAKESVPSVEGLKLKDPKDFRYL
GKGQVSIVDLHDITTGKAHYGADVRLPGLKYAVIARPPVTGGKLKSFDDSAAKKVPGVEKVMEVKGWPWPSKF
QPLGGVAVIARNTGAAIKGRDALKIVWDDGANAKYDSVTYRASLEEAARKPGLVVRKEGDADAALKSADKVIT
GEYYLPHLAHVSMEPPVAVADVKGDKATIWAPVQSPGGTREDVAKTLGIPEDNVTVNVTLLGGGFGRKSKCDF
ALEAALLSKELGAPVKVQWTREDDVHHDFLHTVSVERIEAGLDKSGKVIAWRHRSVAPTIASTFAAGANHEAPF
ELGMGLIDNPFEIANLQCENPEAAAFTRIGWFRSVSNIPRAFAVQSMVGEIAHATGRDQKEMLLELIGSPRIVNL
SSVKDPWNYGEPYDSYPIDTARLRKVVELVAEKGEWGRKVPKGHGLGIAVHRSFVSYIATIVEVAVDDKGKFSVP
RVDTAIDCGTYVNPERIHSQIEGAAIMGLSLAKYGEISFKDGKVQQGNFDDFPVIRMDESPVITNVHIVPPGADT
PPSGVGEPGVPPFAPALINAIFAATGKRIRALPIGKQLET
>SEQF6042||SEQF6042.1_03675
MLPRTHKDAAMNQHVMPKLNRRAFVIGTATAGAGLALGLDLPFGGPAVVRAADGAPEVNAWVVIRPDDTV
VIRIARSEMGQGTLTGLAQLVAEELECDWSKVTTEYPTPGQSVARKRAWGDFSTGGSRGIRTSQDYVRRGGAT
ARVMLVQAAANEWKVPASECKAANSVITHTASGKTTTYGKVAEAAAKLEPPADVKLKDPKDWTIAGKGLKRLD
TSDKTTGKMVYGIDVKLPGMLNAAIKDCPVFGGKVKSFDEAKIAGMKGVKKVVQVGDSAVAVVADTWWHA
KTALDALPIVWDEGPNAKVSSETIATWLAEGLDNAQPAYIGNQNGDAKAAIAAAAKKVEAVYNYPYQNHATM
EPMNATVLYTPDKCEVWCGTQNGEAAFAAALEASGLPAEKVDVHKLMLGGGFGRRGMTDYVRQAVAIAKQ
MPGTPIKLLWSREEDMQHGKYHPITQCKLTGAFDADNNLVALHYRLSGQSILFSVRPEALQNGMDPAAFQGV
AQAGEAAIGYSVPNLLVEHSMRNPHVPPGFWRGVNVNHNAIYMECFMDELALSVGQDPLEFRRKLMGKHP
KHLAVLNAVAEKIGWGTPAPQGVYRGIAQVMGYGSYVAGAAEISVTDGSKIKVHRIVASTDPGYVVNPAQVER

QIAGSFVYGLSALFYGGCTVKDGRIEQTNFDTYNSMRINEMPKVEAVMVPSGGFWGGVGEPTIGVAAPAVLN
AYFAATGKRIRSFPLRNQNISFA
>SEQF6042||SEQF6042.1_01151
MNKHVKNLAPETTDLSRRSFLVGTAATGLVLGYAASGIDQALAAPAPASFEPSVWYSIAPDGLVTVTCGKADMG
QHVASTMAQIVAEELGANWKDMRVQLASNDPKFNDPVLGAQITGGSWSTMMNFDAMSRAGAAGRIALTE
AAAASMGVPAGELVVRDSRISHAKSKKSMSFAEVVKSGKATKTFTADDLKAIKLKTPDQYTMIGVSVPQLDIPSK
VNGTAKYGIDVMVPGMVYGALVTPPVRYGATVKSVDDSAAKKLPGFIKAVTLDDKTTTTTGWVVAVANTYAQ
AKKAAAALKISYDGGPNAKLSSESLFAEAKRLQGLSDSGEFFVKDGDPNAAYGSAAKVLEAEYTTNINIHAPMEP
MNATAEFKGDILHIYSGNQFATRSGAIAAGAAGIDPKFVVMHQMWLGGGFGRRLDADMMVPAVQAAKAVG
KPVKVIYTRENDMTMDFSRPLTYQKVKAGMDGDGKIVAMSHDVVSAWPTARWGIPDFLTPSVDKKGPLDSFT
VNGADFFYTVPNHYVRAIKNEMAHNATPSGQLRSVAPGWTFWAVESMIDEIAHATGKDPAQLRISLLDGKGK
NDGGAQRLRNTLLAAMGLAGYGTKQLPKGEGMGVACVSSQERATASWTACVAHVAVAPSGEVTVKKLTVAT
DVGTQVNPDGIRAQVEGAALWGMSLALFEKATLKDGGIEQTNFDSYTPLRMSQLPEVAVNVIANGEKATGVG
EPAVTVVAPAIGNAVFNAVGARVRGLPITAEAVKAAMKA
>SEQF6042||SEQF6042.1_06265
MTLIDNLSERAADLSRRNFLRAGAIAGGGLLLSVSLPFAGRESEAAASDGFAPNAFVRIGGDGKVVLTMPYVEM
GQGTYTSIPMLIAEELEIGLTQVRLEHAPPSDKLYANPLLGVQATGNSNAMRGAWQPMRKAGATAKAMLVAA
AAKRWNVEPGTCRAENGEVHHAASGRKLGYGELATDAAQMPVPENVTLKSPSEFKLIGTPAKRLDTPSKINGT
AVYGIDARPPGVKIATLAQSPVFGGRVKRVDDAAAKAVNGVRQIVTLDDAVAVVADHMGAAKKGLAALTIEW
DEGAHAKLATSDIARELETATTKPGAVAQNIGDADKAMAGAATKVEATYQLPFLAHATMEPMNCTVHVRPDG
CEIWVGSQALSRAQAVAAKVLNMPPEKVVVHNHLLGGGFGRRLEVDGVIRAVQIAKQVDAPVKLVWTREEDI
QHDMYRPYWCDRIAVGLDASGKPVAWNNRFAGSSVLARWAPPAFRNGLDPDTTEGAIDLVYDIPNFHVEYV
RVEPPGIPTAFWRSVGPSHNVFVTESVIDELAAAAKQDPVDYRRALLGKSPRAKAALELAAAKAGWGGKLPAG
RGRGVSLQFVFGSYLAQVAEVEVARDGSVRVHRVVCAMDCGTVVNPDTVQAQLQSGINFGVTAALYGEITLK
DGRVEQSNFDSYQMLRIDQAPAIEVHIVPSTEPPGGMGETGTSGIVPAISNAIFAATGKRLRKMPVDPAVLKQT
>SEQF6043||SEQF6043.1_05097
MTLIDNLSERAADLSRRNFLRAGAVAGGGLLLSVGLPFASRESEAAASDGFAPNAFVRIGSDGKVVLTMPYVEM
GQGTYTSIPMLIAEELEIGLTQVRLEHAPPSDKLYANPLLGVQATGNSNAMRGAWQPMRKAGATAKAMLVAA
AAKRWNVEPGTCRAENGEVHHAASGRKLGYGELATDAAQMPVPENVTLKSPSEFKLIGTPAKRLDTPSKINGT
AVYGIDARPPGVKIATLAQSPVFGGRVKRVDDAAAKAVKGVRQIVTLDDAVAVVADHMGAAKKGLAALTIEW
DEGPHAKLATADIARELEAATTKPGTIAQNIGDADKAMAGAATKVEATYQLPFLAHATMEPMNCTVHVRPDG
CEIWVGSQALSRAQAVAAKVLNMPPEKVVVHNHLLGGGFGRRLEVDGVIRAVQIAKQVDAPVKLVWTREEDI
QHDMYRPYWCDRISVGLDASGKPVAWNNRFAGSSVLARWAPPAFRNGLDPDTTEGAIDLVYDIPNFHVEYVR
VEPPGIPTAFWRSVGPSHNVFVTESVIDELAAAAKQDPVDYRRALLGKSPRAKAALELAAAKAGWGGKLPAGR
GRGVSLQFVFGSYLAQIAEVEVARDGSVRVHRVVCAMDCGTVVNPDTVQAQLQSGINFGVTAALYGEITLKDG
RVEQSNFDSYQMLRIDQAPAIEVHIVPSTEPPGGMGETGTSGIVPAISNAIFAATGKRLRKMPVDPAALKQT
>SEQF6043||SEQF6043.1_04606
MLPRTHKDAAMNQHVMPKLNRRAFVIGTATAGAGLALGLDLPFGGPAVVRAADGAPEVNAWVVIRPDDTV
VIRIARSEMGQGTLTGLAQLVAEELECDWSKVTTEYPTPGQSVARKRAWGDFSTGGSRGIRTSQDYVRKGGAT
ARVMLIQAAANEWKVPASECKVSNGVVTHTPSGKTTTYGKVAEAAAKLEPPADVKLKDPKDWTIAGKGLKRL
DTSDKTTGKMVYGIDVKLPGMLNAAIKDCPVFGGKVKSFDEAKIAGMKGVKKVVQVGDSAVAVVADTWWH
AKTALDALPIVWDEGPNAKVSSETIATWLAEGLDNAQPAYIGNQNGDAKAAIASAAKKVEAVYSYPYQNHATM
EPMNATVLYTPDKCEVWCGTQNGEAAFAAALEASGLPAEKVDVHKLMLGGGFGRRGMTDYVRQAVAIAKQ
MPGTPIKLLWSREEDMQHGKYHPITQCKLTGAFDTDNNLVALHYRLSGQSILFSVRPEALQNGMDPAAFQGVA

QAGEAAIGYSVPNLLVEHSMRNPHVPPGFWRGVNVNHNAIYMECFMDELALAVGQDPLEFRRKLMRKHPK
HLAVLNAVAEKIGWGTPAPQGVYRGIAQVMGYGSYVAGAAEISVTDGSKIKVHRIVASTDPGYVVNPAQVERQ
IAGSFVYGLSALFYGGCTVKDGRIEQTNFDTYNSMRINEMPKVESVMVPSGGFWGGVGEPTIGVAAPAVLNAY
FAATGKRIRSFPLRNQNISFA
>SEQF6043||SEQF6043.1_03868
MDRQILKGRGNETAMSRRSFLQGTGLLLGFAFTGTSPESVFAASASQVVENEVVGTFAPNGFIRINPTGAVRLI
MPMVEMGQGVYTSLSMLLAEELEVRLDQIQVQHAPPNHALYVNSIIGIQNTGGSASVRAFWTPLRQAGAVG
RNLLIAAAAKRWNVDPATCRAKGGIVFDKSGSKHLSYGELADDAAKLPVPAAASVKLKDPKDFALIGTRAKRVD
SSIKVDGRALYGIDVRLPGMTVAAVAISPVLGGKAKTVNEKAALAVKGVRQVVNIDEAVAVVADHMGAAKKGL
EAASITWDDGPNGKVNNADIVRQLEEESSKPGVVARSDGDAGKALAAAAQRLDAIYQVPFLAHAAMEPMNC
TVHLQKDRCDIWVGTQAPTITQALVVELTGLPKDAIKIHNHLIGGGFGRRLEADGTVLAVKIAKHVDGPVKVIW
SREEDIQHDMYRPYYLDRLSAGLDVAGRPVAWTHRIAGSSIFARYYPPYIKDGLDPDAVEAAAEPPYALPNIHVD
YVQVEPRGVKTSWWRGVGPTHNVFVVESFMDELAHAAKRDPVAYRKELLSHNPRALAVLSLAAEKAGWGSP
LPPRHGRGISVQFAYGSYVSQVAEVEVAADGSVKVKRIICALDCGMHVNPDTIEAQIQGGTLFGLTAALHGSITF
KDGRVEQSNFDSYLPMRIDEVPEVETYLIKNAEAPGGVGEAPTAIVSAAVTNAIFAATGKRVRSLPIDTDSLKSSS
>SEQF6043||SEQF6043.1_06795
MNIRTNPSKLRGFEKHVKVENVSRRSILKGLGIAGSFVLAAPVMTRQAFAYETGAGKMPHGVVVDPRVFVAIA
PDGIVTILAHRSEMGTGVRTSLPLIVAEEMEADWSRVHVQQAHGDEVKFGNQDTDGSRSTRHYLIPMRQIGA
SARSMLEAAAAKKWGVPVTEVKAQNHEVVHSASGRKAGFGELAADAAKESVPSVEGLKLKDPKDFRYLGKGQ
VSIVDLHDITTGKAHYGADVRLPGLKYAVIARPPVTGGKLKSFDDSAAKKVPGVEKVMEVKGWPWPSKFQPLG
GVAVIARNTGAAIKGRDALKIVWDDGANAKYDSVTYRASLEEAARKPGLVVRKEGDADAALKSADKVITGEYYL
PHLAHVSMEPPVAVADVKGDKATIWAPVQSPGGTREDVAKTLGIPEDNVTVNVTLLGGGFGRKSKCDFALEAA
LLSKELGAPVKVQWTREDDVHHDFLHTVSVERIEAGLDKSGKVIAWRHRSVAPTIASTFAAGANHEAPFELGM
GLIDNPFEIANLQCENPEAAAFTRIGWFRSVSNIPRAFAVQSMVGEIAHATGRDQKEMLLELIGSPRIVNLSSVK
DPWNYGEPYDSYPIDTARLRKVVELVAEKGEWGRKVPKGHGLGIAVHRSFVSYIATIVEVAVDDKGKFSVPRVDT
AIDCGTYVNPERIHSQIEGAAIMGLSLAKYGEISFKDGKVQQGNFDDFPVIRMDESPIITNVYIVPPGADTPPSG
VGEPGVPPFAPALINAIFAATGKRIRALPIGKQLET
>SEQF6043||SEQF6043.1_03219
MNKHVKNLAPETTDLSRRSFLVGTAATGLVLGYSASGIDQALAAPAPANFEPSVWYSIAPDGLVTVTCGKADM
GQHVASTMAQIVAEELGANWKDMRVQLASNDPKFNDPVLGAQITGGSWSTMMNFDAMSRAGAAGRIALT
EAAAASMGVPAGELVVRDSKISHAKSKKSMSFAEVVKSGKATKTFTADDLKAIKLKTPDQYTMIGVSVPQLDIPS
KVNGTAKYGIDVMVPGMVYGALVTPPVRYGATVKSVDDSAAKKLPGFIKAVTLDDKTTTTTGWVVAVANTYA
QAKKAAAALKISYDGGPNAKLSSESLFAEAKRLQGLSDSGEFFVKDGDPNAAFGSAAKLLEAEYTTNINIHAPM
EPMNATAEFKGDILHIYSGNQFATRSGAIAAGAAGIDPKFVVMHQMWLGGGFGRRLDADMMVPAVQAAKA
VGKPVKVIYTRENDMTMDFSRPLTYQKVKAGMDGDGKIVAMSHDVVSAWPTARWGIPDFLTPSVDKKGPLD
SFTVNGADFFYTVPNHYVRAIKNEMAHNATPSGQLRSVAPGWTFWAVESMIDEIAHATGKDPAQLRISLLDGK
GKNDGGAQRLRNTLLAAMGLAGYGTKQLPKGEGMGVACVSSQERATASWTACVAHVAVAPSGEVTVKKLTV
ATDVGTQVNPDGIRAQVEGAALWGMSLALFEKATLKDGGIEQTNFDSYTPLRMSQLPEVAVNVIANGEKATG
VGEPAVTVVAPAIGNAVFNAVGARVRGLPITAEAVKAAMKA
>SEQF6044||SEQF6044.1_03060
MNIRTNIPTGSGKLRGFEKHVKVENVSRRSILKGLGIAGSFVLAAPVMTRQAFAYETGAGKMPHGVVVDPRVF
VAIAPDGIVTILAHRSEMGTGVRTSLPLIVAEEMEADWSRVHVQQAHGDEVKFGNQDTDGSRSTRHYLIPMR
QIGASARSMLEAAAAKKWGVPVTEVKAQNHEVVHSASGRKAGFGELAADAAKESVPSVEGLKLKDPKDFRYL
GKGQVSIVDLHDITTGKAHYGADVRLPGLKYAVIARPPVTGGKLKSFDDSAAKKVPGVEKVMEVKGWPWPSKF

QPLGGVAVIARNTGAAIKGRDALKIVWDDGANAKYDSVTYRASLEEAARKPGLVVRKEGDADAALKSADKVIT
GEYYLPHLAHVSMEPPVAVADVKGDKATIWAPVQSPGGTREDVAKTLGIPEDNVTVNVTLLGGGFGRKSKCDF
ALEAALLSKELGAPVKVQWTREDDVHHDFLHTVSVERIEAGLDKSGKVIAWRHRSVAPTIASTFAAGANHEAPF
ELGMGLIDNPFEIANLQCENPEAAAFTRIGWFRSVSNIPRAFAVQSMVGEIAHATGRDQKEMLLELIGSPRIVNL
SSVKDPWNYGEPYDSYPIDTARLRKVVELVAEKGEWGRKVPKGHGLGIAVHRSFVSYIATIVEVAVDDKGKFSVP
RVDTAIDCGTYVNPERIHSQIEGAAIMGLSLAKYGEISFKDGKVQQGNFDDFPVIRMDESPVITNVHIVPPGADT
PPSGVGEPGVPPFAPALINAIFAATGKRIRALPIGKQLET
>SEQF6044||SEQF6044.1_04775
MNKHVKNLAPETTDLSRRSFLVGTAATGLVLGYAASGIDQALAAPAPASFEPSVWYSIAPDGLVTVTCGKADMG
QHVASTMAQIVAEELGANWKDMRVQLASNDPKFNDPVLGAQITGGSWSTMMNFDAMSRAGAAGRIALTE
AAAASMGVPAGELVVRDSRISHAKSKKSMSFAEVVKSGKATKTFTADDLKAIKLKTPDQYTMIGVSVPQLDIPSK
VNGTAKYGIDVMVPGMVYGALVTPPVRYGATVKSVDDSAAKKLPGFIKAVTLDDKTTTTTGWVVAVANTYAQ
AKKAAAALKISYDGGPNAKLSSESLFAEAKRLQGLSDSGEFFVKDGDPNAAYGSAAKVLEAEYTTNINIHAPMEP
MNATAEFKGDILHIYSGNQFATRSGAIAAGAAGIDPKFVVMHQMWLGGGFGRRLDADMMVPAVQAAKAVG
KPVKVIYTRENDMTMDFSRPLTYQKVKAGMDGDGKIVAMSHDVVSAWPTARWGIPDFLTPSVDKKGPLDSFT
VNGADFFYTVPNHYVRAIKNEMAHNATPSGQLRSVAPGWTFWAVESMIDEIAHATGKDPAQLRISLLDGKGK
NDGGAQRLRNTLLAAMGLAGYGTKQLPKGEGMGVACVSSQERATASWTACVAHVAVAPSGEVTVKKLTVAT
DVGTQVNPDGIRAQVEGAALWGMSLALFEKATLKDGGIEQTNFDSYTPLRMSQLPEVAVNVIANGEKATGVG
EPAVTVVAPAIGNAVFNAVGARVRGLPITAEAVKAAMKA
>SEQF6044||SEQF6044.1_02186
MLPRTHKDAAMNQHVMPKLNRRAFVIGTATAGAGLALGLDLPFGGPAVVRAADGAPEVNAPEVNAWVVIR
PDDTVVIRIARSEMGQGTLTGLAQLVAEELECDWSKVTTEYPTPGQSVARKRAWGDFSTGGSRGIRTSQDYVR
KGGATARVMLIQAAANEWKVPASECKAANSVITHTPSGKTTTYGKVAEAAAKLEPPADVKLKDPKDWTIAGKG
LKRLDTVDKTTGKMVYGIDVKLPGMLNAAIKDCPVFGGKVKSFDEAKIAGMKGVKKVVQVGDSAVAVVADT
WWHAKTALDALPVVWDEGPNAKVSSETIATWLAEGLDNAQPAYVGNQNGDAKAAIAAAAKKVEAVYNYPY
QNHATMEPMNATVLYTPDKCEVWCGTQNGEAAFAAALEASGLPAEKVDVHKLMLGGGFGRRGMTDYVRQ
AVAIAKQMPGTPIKLLWSREEDMQHGKYHPITQCKLTGAFDADNNLVALHYRLSGQSILFSVRPEALQNGMDP
AAFQGVAQAGEAAIGYSVPNLLVEHSMRNPHVPPGFWRGVNVNHNAIYMECFMDELALSVGQDPLEFRRKL
MGKHPKHLAVLNAVAEKIGWGTPAPQGVYRGIAQVMGYGSYVAGAAEISVTDGSKIKVHRIVASTDPGYVVN
PAQVERQIAGSFVYGLSALFYGGCTVKDGRIEQTNFDTYNSMRINEMPKVEAVMVPSGGFWGGVGEPTIGVA
APAVLNAYFAATGKRIRSFPLRNQNISFA
>SEQF6044||SEQF6044.1_08492
MTLIDNLSERAADLSRRNFLRAGAIAGGGLLLSVSLPFAGRESEAAASDGFAPNAFVRIGGDGKVVLTMPYVEM
GQGTYTSIPMLIAEELEIGLTQVRLEHAPPSDKLYANPLLGVQATGNSNAMRGAWQPMRKAGATAKAMLVAA
AAKRWNVEPGTCRAENGEVHHAASGRKLGYGELATDAAQMPVPENVTLKSPSEFKLIGTPAKRLDTPSKINGT
AVYGIDARPPGVKIATLAQSPVFGGRVKRVDDAAAKAVNGVRQIVTLDDAVAVVADHMGAAKKGLAALTIEW
DEGAHAKLATSDIARELETATTKPGAVAQNIGDADKAMAGAATKVEATYQLPFLAHATMEPMNCTVHVRPDG
CEIWVGSQALSRAQAVAAKVLNMPPEKVVVHNHLLGGGFGRRLEVDGVIRAVQIAKQVDAPVKLVWTREEDI
QHDMYRPYWCDRIAVGLDASGKPVAWNNRFAGSSVLARWAPPAFRNGLDPDTTEGAIDLVYDIPNFHVEYV
RVEPPGIPTAFWRSVGPSHNVFVTESVIDELAAAAKQDPVDYRRALLGKSPRAKAALELAAAKAGWGGKLPAG
RGRGVSLQFVFGSYLAQVAEVEVARDGSVRVHRVVCAMDCGTVVNPDTVQAQLQSGINFGVTAALYGEITLK
DGRVEQSNFDSYQMLRIDQAPAIEVHIVPSTEPPGGMGETGTSGIVPAISNAIFAATGKRLRKMPVDPAVLKQT
>SEQF6045||SEQF6045.1_00940
MNKHVKNLAPETTDLSRRSFLVGTAATGLVLGYAASGIDQALAAPAPASFEPSVWYSIAPDGLVTVTCGKADMG

QHVASTMAQIVAEELGANWKDMRVQLASNDPKFNDPVLGAQITGGSWSTMMNFDAMSRAGAAGRIALTE
AAAASMGVPAGELVVRDSRISHAKSKKSMSFAEVVKSGKATKTFTADDLKAIKLKTPDQYTMIGVSVPQLDIPSK
VNGTAKYGIDVMVPGMVYGALVTPPVRYGATVKSVDDSAAKKLPGFIKAVTLDDKTTTTTGWVVAVANTYAQ
AKKAAAALKISYDGGPNAKLSSESLFAEAKRLQGLSDSGEFFVKDGDPNAAYGSAAKVLEAEYTTNINIHAPMEP
MNATAEFKGDILHIYSGNQFATRSGAIAAGAAGIDPKFVVMHQMWLGGGFGRRLDADMMVPAVQAAKAVG
KPVKVIYTRENDMTMDFSRPLTYQKVKAGMDGDGKIVAMSHDVVSAWPTARWGIPDFLTPSVDKKGPLDSFT
VNGADFFYTVPNHYVRAIKNEMAHNATPSGQLRSVAPGWTFWAVESMIDEIAHATGKDPAQLRISLLDGKGK
NDGGAQRLRNTLLAAMGLAGYGTKQLPKGEGMGVACVSSQERATASWTACVAHVAVAPSGEVTVKKLTVAT
DVGTQVNPDGIRAQVEGAALWGMSLALFEKATLKDGGIEQTNFDSYTPLRMSQLPEVAVNVIANGEKATGVG
EPAVTVVAPAIGNAVFNAVGARVRGLPITAEAVKAAMKA
>SEQF6045||SEQF6045.1_04275
MLPRTHKDAAMNQHVMPKLNRRAFVIGTATAGAGLALGLDLPFGGPAVVRAADGAPEVNAWVVIRPDDTV
VIRIARSEMGQGTLTGLAQLVAEELECDWSKVTTEYPTPGQSVARKRAWGDFSTGGSRGIRTSQDYVRRGGAT
ARVMLVQAAANEWKVPASECKAANSVITHTASGKTTTYGKVAEAAAKLEPPADVKLKDPKDWTIAGKGLKRLD
TSDKTTGKMVYGIDVKLPGMLNAAIKDCPVFGGKVKSFDEAKIAGMKGVKKVVQVGDSAVAVVADTWWHA
KTALDALPIVWDEGPNAKVSSETIATWLAEGLDNAQPAYIGNQNGDAKAAIAAAAKKVEAVYNYPYQNHATM
EPMNATVLYTPDKCEVWCGTQNGEAAFAAALEASGLPAEKVDVHKLMLGGGFGRRGMTDYVRQAVAIAKQ
MPGTPIKLLWSREEDMQHGKYHPITQCKLTGAFDADNNLVALHYRLSGQSILFSVRPEALQNGMDPAAFQGV
AQAGEAAIGYSVPNLLVEHSMRNPHVPPGFWRGVNVNHNAIYMECFMDELALSVGQDPLEFRRKLMGKHP
KHLAVLNAVAEKIGWGTPAPQGVYRGIAQVMGYGSYVAGAAEISVTDGSKIKVHRIVASTDPGYVVNPAQVER
QIAGSFVYGLSALFYGGCTVKDGRIEQTNFDTYNSMRINEMPKVEAVMVPSGGFWGGVGEPTIGVAAPAVLN
AYFAATGKRIRSFPLRNQNISFA
>SEQF6045||SEQF6045.1_07517
MTLIDNLSERAADLSRRNFLRAGAIAGGGLLLSVSLPFAGRESEAAASDGFAPNAFVRIGGDGKVVLTMPYVEM
GQGTYTSIPMLIAEELEIGLTQVRLEHAPPSDKLYANPLLGVQATGNSNAMRGAWQPMRKAGATAKAMLVAA
AAKRWNVEPGTCRAENGEVHHAASGRKLGYGELATDAAQMPVPENVTLKSPSEFKLIGTPAKRLDTPSKINGT
AVYGIDARPPGVKIATLAQSPVFGGRVKRVDDAAAKAVNGVRQIVTLDDAVAVVADHMGAAKKGLAALTIEW
DEGAHAKLATSDIARELETATTKPGAVAQNIGDADKAMAGAATKVEATYQLPFLAHATMEPMNCTVHVRPDG
CEIWVGSQALSRAQAVAAKVLNMPPEKVVVHNHLLGGGFGRRLEVDGVIRAVQIAKQVDAPVKLVWTREEDI
QHDMYRPYWCDRIAVGLDASGKPVAWNNRFAGSSVLARWAPPAFRNGLDPDTTEGAIDLVYDIPNFHVEYV
RVEPPGIPTAFWRSVGPSHNVFVTESVIDELAAAAKQDPVDYRRALLGKSPRAKAALELAAAKAGWGGKLPAG
RGRGVSLQFVFGSYLAQVAEVEVARDGSVRVHRVVCAMDCGTVVNPDTVQAQLQSGINFGVTAALYGEITLK
DGRVEQSNFDSYQMLRIDQAPAIEVHIVPSTEPPGGMGETGTSGIVPAISNAIFAATGKRLRKMPVDPAVLKQT
>SEQF6045||SEQF6045.1_05972
MNIRTNIPTGSGKLRGFEKHVKVENVSRRSILKGLGIAGSFVLAAPVMTRQAFAYETGAGKMPHGVVVDPRVF
VAIAPDGIVTILAHRSEMGTGVRTSLPLIVAEEMEADWSRVHVQQAHGDEVKFGNQDTDGSRSTRHYLIPMR
QIGASARSMLEAAAAKKWGVPVTEVKAQNHEVVHSASGRKAGFGELAADAAKESVPSVEGLKLKDPKDFRYL
GKGQVSIVDLHDITTGKAHYGADVRLPGLKYAVIARPPVTGGKLKSFDDSAAKKVPGVEKVMEVKGWPWPSKF
QPLGGVAVIARNTGAAIKGRDALKIVWDDGANAKYDSVTYRASLEEAARKPGLVVRKEGDADAALKSADKVIT
GEYYLPHLAHVSMEPPVAVADVKGDKATIWAPVQSPGGTREDVAKTLGIPEDNVTVNVTLLGGGFGRKSKCDF
ALEAALLSKELGAPVKVQWTREDDVHHDFLHTVSVERIEAGLDKSGKVIAWRHRSVAPTIASTFAAGANHEAPF
ELGMGLIDNPFEIANLQCENPEAAAFTRIGWFRSVSNIPRAFAVQSMVGEIAHATGRDQKEMLLELIGSPRIVNL
SSVKDPWNYGEPYDSYPIDTARLRKVVELVAEKGEWGRKVPKGHGLGIAVHRSFVSYIATIVEVAVDDKGKFSVP
RVDTAIDCGTYVNPERIHSQIEGAAIMGLSLAKYGEISFKDGKVQQGNFDDFPVIRMDESPVITNVHIVPPGADT

PPSGVGEPGVPPFAPALINAIFAATGKRIRALPIGKQLET
>SEQF6046||SEQF6046.1_04844
MMNEPFLRERVVDETAISRRGFLQGTGLLLGFVLSGASTGSVFAAATSQVVENEITGTFAPNGFIRINPTGAVTLV
IPMIEMGQGVYTSLSMLLAEELEVTLDQIQVQHAPPNHALYVNSIIGIQNTGGSASVRAFWTPLRQAGAVGRN
LLIAAAAKRWNVDPATCRAKNGVVFDATGSMHLSYGELATAAAKFPVPPAANVKLKDPKDFSLIGTSAKRVDSP
IKVDGRALYGIDTRLPGMKVAAVAISPVLGGKAKKVDEKAALAVKGVRQLINIDEAVAVVADHMGAAKKGLEAA
AIAWDDGPNGKVNSADIVRQLEEESRKPGAVARNVGDVAKALAKAAQRVDAVYQVPFLAHAAMEPMNCTV
HLQKDRCDIWVGTQAPTITQSQVAELTGLPKDAIKIHNHLIGGGFGRRLEADGTILAVKIAKHVDGPVKVIWSRE
EDIQHDMYRPYYLDRLSAGLDTAGQPVAWMHRIAGSSVMARYYPPYFKDGLDPDAVEAAAEPPYALPSIHVD
FVRVEPPGVRTSWWRGVGPTHNVFVVESFIDELAHAAKQDPVAYRKGLLGHNPRALTVLSLAAEKAGWGSPL
PARHGRGISVQFAYGSYTSQVAEVEVAADGSVKVKRIVCVIDCGVYVNPDTIEAQIQGGTLFGLTAALHGSITFKD
GRVEQSNFDSYLPMRIDEVPVVETHLIKNAEAPGGVGEAPTAIVSAAVTNAIFAATGKRVRSLPIETDVLKSSS
>SEQF6046||SEQF6046.1_02040
MNKHVKNLAPETTDLSRRSFLVGTAATGLVLGYAGSGIDQALAAPAPANFEPSVWYSIAPDGLVTVTCGKADM
GQHVASTMAQIVAEELGANWKDMRVQLASNDPKFNDPVLGAQITGGSWSTMMNFDAMSRAGAAGRIALT
EAAAASMGVPAGELVVRDSKISHAKSKKSMSFAEVVKSGKATKTFTADDLKAIKLKTPDQYTMIGVSVPQLDIPS
KTNGTAKYGIDVMVPGMVHGALVTPPVRYGATVKSVDDSAARKLPGFIKAVTLDDKTNTTTGWVVAVANTYA
QAKKAAAALKISYDGGPNAKLSSESLLTEAKRLQGLDDSGQFFVKDGDPKAAFGSAAKVLEAEYTTNINIHAPM
EPMNATAEFKGDILHIYSGNQFATRSGAIAAGAAGIDPKFVVMHQMWLGGGFGRRLDADMMVPAVQAAKA
VGKPVKVIYTRENDMTMDFSRPLTYQKVKAGMDGGGKIVAMSHDVVSAWPTARWGIPDFLTPSVDKKGPLD
SFTVNGADFFYTVPNHYVRAIKNEMAHNATPSGQLRSVAPGWTFWAVESMIDEIAHATGKDPAQLRISLLDGK
GKNDGGAQRLRNTLLAAMGLAGYGTKQLPKGEGMGVACVSSQERATASWTACVAHVAVAPSGEVTVKKLTV
ATDVGTQVNPDGIRAQVEGAALWGMSLALFEKATLKDGGIEQTNFDSYTPLRMSQLPEVAVNVIANGEKATG
VGEPAVTVVAPAIGNAVFNAVGARVRGLPITAEAVKAAMKA
>SEQF6046||SEQF6046.1_04665
MNIRTNPSKLRGFEKHVKVENVSRRSILKGLGIAGSFVLAAPVMTRQAFAYETGAGKMPHGVVVDPRVFVAIA
PDGIVTILAHRSEMGTGVRTSLPLIVAEEMEADWSRVHVQQAHGDEVKFGNQDTDGSRSTRHYLIPMRQIGA
SARSMLEAAAAKRWGVPVTEVKAQNHEVVHSASGRKAGFGELAADAAKESVPSVEGLKLKDPKDFRYLGKGQ
VSIVDLHDITTGKAHYGADVRLPGLKYAVIARPPVTGGKLKSFDDSAAKKVPGVEKVMEVKGWPWPSKFQPLG
GVAVIARNTGAAIKGRDALKIVWDDGANAKYDSVTYRASLEEAARKPGLVVRKEGDVDAALKSADKVITGEYYL
PHLAHVSMEPPVAVADVKGDKATIWAPVQSPGGTREDVAKTLGIPEDNVTVNVTLLGGGFGRKSKCDFALEAA
LLSKELGAPVKVQWTREDDVHHDFLHTVSVERIEAGLDKSGKVIAWRHRSVAPTIASTFAAGANHEAPFELGM
GLIDNPFEIANLQCENPEAAAFTRIGWFRSVSNIPRAFAVQSMVGEIAHATGRDQKEMLLELIGSPRIVNLSSVK
DPWNYGEPYDSYPIDTARLRKVVELVAEKGEWGRKVPKGHGLGIAVHRSFVSYIATIVEVAVDDKGKFSVPRVDT
AIDCGTYVNPERIHSQIEGAAIMGLSLAKYGEISFKDGKVQQGNFDDFPVIRMDESPAITNVHIVPPGADTPPSG
VGEPGVPPFAPALINAIFAATGKRIRALPIGKQLET
>SEQF6046||SEQF6046.1_07705
MLPRTHKDAAMNQHVMPKLNRRAFVIGTATAGAGLALGLDLPFGGPAVVRAADGAPEVNAWVVIRPDDTV
VIRIARSEMGQGTLTGLAQLVAEELECDWSKVTTEYPTPGQSVARKRAWGDFSTGGSRGIRTSQDYVRKGGAT
ARVMLIQAAANEWKVPASECKAANSIITHTPSGKTTTYGKVAEAAAKLEPPADVKLKDPKDWTIAGKGLKRLDT
VDKTTGKMVYGIDVKLPGMLNAAIKDCPVFGGKVKSFDEAKIAGMKGVKKVVQVGDTAVAVVADTWWHAK
TALDALPIVWDEGPNAKVSSETIATWLAEGLDNAQPAYVGNQNGDAKAAIASAAKKVEAVYSYPYQNHATME
PMNATVLYTPDKCEVWCGTQNGEAAFAAALEASGLPAEKVDVHKLMLGGGFGRRGMTDYVRQAVAIAKQM
PGTPIKLLWSREEDMQHGKYHPITQCKLTGAFDADNNLVALHYRLSGQSILFSVRPEALQNGMDPAAFQGVAQ

AGEAAIGYSVPNLLVEHSMRNPHVPPGFWRGVNVNHNAIYMECFMDELALAVGQDPLEFRRKLMGKHPKHL
AVLNAVAEKIGWGTPAPQGVYRGIAQVMGYGSYVAGAAEISVTDGSKIKVHRIVASTDPGYVVNPAQVERQIA
GSFVYGLSALFYGGCTVKDGRIEQTNFDTYNSMRINEMPKVESVMVPSGGFWGGVGEPTIGVAAPAVLNAYF
AATGKRIRSFPLRNQNISFA
>SEQF6046||SEQF6046.1_01784
MTLIDNLSERAADLSRRNFLRAGAIAGGGLLLSVSLPFASRESEAAASDGFAPNAFVRIGGDGKVVLTMPYVEM
GQGTYTSIPMLIAEELEIGLTQVRLEHAPPSDKLYANPLLGVQATGNSNAMRGAWQPMRKAGATAKAMLVAA
AAKRWNVEPGTCRAENGEVHHAASGRKLGYGELATDAAQMPVPENVTLKSPSEFKLIGTPAKRLDTPSKINGT
AVYGIDARPPGVKVATLAQSPVFGGRVKRVDDAAAKAVKGVRQIVTLDDAVAVVADHMGAAKKGLAALTIEW
DEGPHAKLATSDIARELEAVTTKPGAVAQNIGDADKAMAGAGTKVEATYQLPFLAHATMEPMNCTVHVRSDG
CEIWVGSQALSRAQAVAAKVLNMAPEKVVVHNHLLGGGFGRRLEVDGVIRAVQIAKQVDAPVKVVWTREED
IQHDMYRPYWCDRIAVGLDASGKPVAWNNRFAGSSVLARWAPPAFRNGLDPDTTEGAIDLVYDIPNFHVEYV
RVEPPGIPTAFWRSVGPSHNVFVTESVIDELAAAAKQDPVDYRRALLGKSPRAKAALELAAAKAGWGGKLPAG
RGRGVSLQFVFGSYLAQVAEVEVAKDGSVRVHRVVCAMDCGTVVNPDTVQAQLQSGINFGVTAALYGEITLK
DGRVEQSNFDSYQMLRIDQAPAIEVHIVPSTEPPGGMGETGTSGIVPAISNAIFAATGKRLRKMPVDPALLKQT
>SEQF6047||SEQF6047.1_00358
MNIRTNIPTDSGKLRGFEKHVKVENVSRRSILKGLGIAGSFVLAAPVMTRQAFAYETGAGKMPHGVVVDPRVF
VAIAPDGIVTILAHRSEMGTGVRTSLPLIVAEEMEADWSRVHVQQAHGDEVKFGNQDTDGSRSTRHYLIPMR
QIGASARSMLEAAAAKKWGVPVTEVKAQNHEVVHSASGRKAGFGELAADAAKESVPSVEGLKLKDPKDFRYL
GKGQVSIVDLHDITTGKAPYGADVRLPGLKYAVIARPPVTGGKLKSFDDSAAKKVPGVEKVMEVKGWPWPSKF
QPLGGVAVIARNTGAAIKGRDALKIVWDDGANAKYDSVTYRASLEEAARKPGLVVRKEGDADAALKSADKVIT
GEYYLPHLAHVSMEPPVAVADVKGDKATIWAPVQSPGGTREDVAKTLGIPEDNVTVNVTLLGGGFGRKSKCDF
ALEAALLSKELGAPVKVQWTREDDVHHDFLHTVSVERIEAGLDKSGKVIAWRHRSVAPTIASTFAAGANHEAPF
ELGMGLIDNPFEIANLQCENPEAAAFTRIGWFRSVSNIPRAFAVQSMVGEIAHATGRDQKEMLLELIGSPRIVNL
SSVKDPWNYGEPYDSYPIDTARLRKVVELVADKGEWGRKVPKGHGLGIAVHRSFVSYIATIVEVAVDDKGKFSVP
RVDTAIDCGTYVNPERIHSQIEGAAIMGLSLAKYGEISFKDGKVQQGNFDDFPVIRMDESPVITNVHIVPPGADT
PPSGVGEPGVPPFAPALINAIFAATGKRIRALPIGKQLET
>SEQF6047||SEQF6047.1_05895
MTLIDNLSERAADLSRRNFLRAGAIAGGGLLLSVSLPFAGRESEAAASDGFAPNAFVRIGGDGKVVLTMPYVEM
GQGTYTSIPMLIAEELEIGLTQVRLEHAPPSDKLYANPLLGVQATGNSNAMRGAWQPMRKAGATAKAMLVAA
AAKRWNVEPGTCRAENGEVHHAASGRKLGYGELATDAAQMPVPENVTLKSPSEFKLIGTPAKRLDTPSKINGT
AVYGIDARPPGVKIATLAQSPVFGGRVKRVDDAAAKAVNGVRQIVTLDDAVAVVADHMGAAKKGLAALTIEW
DEGAHAKLATSDIARELETATTKPGAVAQNIGDADKAMAGAATKVEATYQLPFLAHATMEPMNCTVHVRPDG
CEIWVGSQALSRAQAVAAKVLNMPPEKVVVHNHLLGGGFGRRLEVDGVIRAVQIAKQVDAPVKLVWTREEDI
QHDMYRPYWCDRIAVGLDASGKPVAWNNRFAGSSVLARWAPPAFRNGLDPDTTEGAIDLVYDIPNFHVEYV
RVEPPGIPTAFWRSVGPSHNVFVTESVIDELAAAAKQDPVDYRRALLGKSPRAKAALELAAAKAGWGGKLPAG
RGRGVSLQFVFGSYLAQVAEVEVARDGSVRVHRVVCAMDCGTVVNPDTVQAQLQSGINFGVTAALYGEITLK
DGRVEQSNFDSYQMLRIDQAPAIEVHIVPSTEPPGGMGETGTSGIVPAISNAIFAATGKRLRKMPVDPAVLKQT
>SEQF6047||SEQF6047.1_08765
MLPRTHKDAAMNQHIMPKLNRRAFVIGTATAGAGLALGLDLPFGGPAVVRAADGAPEVNAWVVIRPDDTVV
IRIARSEMGQGTLTGLAQLVAEELECDWSKVTTEYPTPGQSVARKRAWGDFSTGGSRGIRTSQDYVRKGGATA
RVMLIQAAANEWKVPASECKAANGVITHTPSGKTTTYGKVAEAAAKLEPPADVKLKDPKDWTIAGKGLKRLDT
VDKTTGKMVYGIDVKLPGMLNAAIKDCPVFGGKVKSFDEAKIAGMKGVRKVVQVGDSAVAVVADTWWHAK
TALDALPIVWDEGPNAKVSSETIATWLAEGLDNAQPAYIGNQNGDAKAAIAAAAKKVEAVYNYPYQNHATME

PMNATVLYTPDKCEVWCGTQNGEAAFAAALEASGLPAEKVDVHKLMLGGGFGRRGMTDYVRQAVAIAKQM
PGTPIKLLWSREEDMQHGKYHPITQCKLTGAFDADNNLVALHYRLSGQSILFSVRPEALQNGMDPAAFQGVAQ
AGEAAIGYSVPNLLVEHSMRNPHVPPGFWRGVNVNHNAIYMECFMDELALSVGQDPLEFRRKLMGKHPKHL
AVLNAVAEKIGWGTPAPQGVYRGIAQVMGYGSYVAGAAEISVTDGSKIKVHRIVASTDPGYVVNPAQVERQIA
GSFVYGLSALFYGGCTVKDGRIEQTNFDTYNSMRINEMPKVEAVMVPSGGFWGGVGEPTIGVAAPAVLNAYF
AATGKRIRSFPLRNQNISFA
>SEQF6047||SEQF6047.1_02287
MNKHVKNLAPETTDLSRRSFLVGTAATGLVLGYAASGIDQALAAPATANFEPSVWYSIAPDGLVTVTCGKADMG
QHVASTMAQIVAEELGANWKDMRVQLASNDPKFNDPVLGAQITGGSWSTMMNFDAMSRAGAAGRIALTE
AAAASMGVPAAELVVRDSRISHPKSKKSMSFAEVVKSGKATKTFTADDLKAIKLKTPDQYTMIGVSVPQLDIPSK
VNGTAKYGIDVMVPGMVYGALVTPPVRYGATVKSVDDSAAKKLPGFIKAVTLDDKTTTTTGWVVAVANTYAQ
AKKAAAALKISYDGGPNAKLSSESLFAEAKRLQGLSDSGEFFVKDGDPNAAYGSAAKVLEAEYTTNINIHAPMEP
MNATAEFKGDILHIYSGNQFATRSGAIAAGAAGIDPKFVVMHQMWLGGGFGRRLDADMMVPAVQAAKAVG
KPVKVIYSRENDMTMDFSRPLTCQKIKAGMDGDGKIVALSHDVVSAWPTQRWGIPDFLTPSVDKKGPLDSFTV
NGADFFYTVPNHYVRAIKNEMAHNATPSGQLRSVAPGWTFWAVESMIDEIAHATGKDPAQLRISLLDGKGKN
DGGAQRLRNTLLAAMGLAGYGTKQLPKGEGMGVACVSSQERATASWTACVAHVAVAPSGEVTVKKLTVATDV
GTQVNPDGIRAQVEGAALWGMSLALFEKATLKDGGIEQTNFDSYTPLRMSQLPEVAVNVIANGEKATGVGEPA
VTVVAPAIGNAVFNAVGARVRGLPITAEAVKAAMKA
>SEQF6048||SEQF6048.1_07537
MNIRTNPSKLRGFEKHVKVENVSRRSILKGLGIAGSFVLAAPVMTRQAFAYETGAGKMPHGVVVDPRVFVAIA
PDGIVTILAHRSEMGTGVRTSLPLIVAEEMEADWSRVHVQQAHGDEVKFGNQDTDGSRSTRHYLIPMRQIGA
SARSMLEAAAAKKWGVPATEVKAQNHEVVHSASGRKIGFGELAADAAKGSVPSVEGLKLKDPKDFRYLGKGQ
VSIVDLHDITTGKAHYGADVRLPGLKYAVIARPPVTGGKLKSFDDSAAKKVPGVEKVMEVKGWPWPSKFQPLG
GVAVIARNTGAAIKGRDALKIVWDDGANAKYDSVTYRASLEEAARKPGLVVRKEGDADAALKSADKVITGEYYL
PHLAHVSMEPPVAVADVKGDKATIWAPVQSPGGTREDVAKTLGIPEDNVTVNVTLLGGGFGRKSKCDFALEAA
LLSKELGAPVKVQWTREDDVHHDFLHTVSVERIEAGLDKSGKVIAWRHRSVAPTIASTFAAGANHEAPFELGM
GLIDNPFEIANLQCENPEAAAFTRIGWFRSVSNIPRAFAVQSMAAEIAHATGRDQKDMLLELIGSPRIVNLSSVK
DPWNYGEPYDSYPIDTARLRKVVELVADKGEWGRKVPKGHGLGIAVHRSFVSYIATVVEVAVDDKGKFSVPRVD
TAIDCGTYVNPERIHSQIEGAAIMGLSLAKYGEISFKDGKVQQGNFDDFPVIRMDESPVITNVYIVPPGADTPPS
GVGEPGVPPFAPALINAIFAATGKRIRALPIGKQLET
>SEQF6048||SEQF6048.1_07048
MDGSIATKSPRADLSRRRFIVTGLTAAGGFAIGFGARPARSASVRNTPWTDHETLSTDIDAWISIEPDDAVLIRYA
RSEMGQGSMTALPMMINEELAADWGRVKVEYASANRSFRENNVYGDMSSVGSHSVRDSHEKMQQVGASA
RVRLIQAAATRWGVSPAECTADKSLVTHGPTKRSFRYGELAADAAKTALDKEPAIKSPAEYTFIGSEVRRLDVPYK
VDGSAQFAMDTRLPGMVFAAIQACPVHGGKLKSVDESPLSGDPNILKVVKLPNAVAVVAKGTFWRAKQGLAK
LHPEWETGEAGATDTPQFNKEYRAELDRVGKIARNDGDIDKVIPAAYRQMSAVYEVPYLAHATMEPLNATIWI
QGDRIDAWVGSQAAHRTLALAAKVSGLKPENIYIHNTYVGGGFGRKSTSDEVGQAIQIAMAMGSTPVKMIW
TREEDMRQGHYRPQGACSFTAGLDANGMPQGWRIHFAVGSIMRSLGLSKVENGIEPFATEGLANNPYLVPAT
RVECSLKNTHIEVGFWRSVGASQNAFWIESFMDEMAAAGGHDPYQFRRTLLKGNQEWLAVLDKAAEMGD
WGKPLPPGKARGIAIHMCYNTIAAEVVEITMDNVRGNFSVDRVTVAVDPGHVVNALGVQEQMEGGVIFALS
AALYGKITVKNGVVEQGNFDSYRMVRYAQAPRIDVHMVPSRGNRWGGCGEPGAAPLTPALCNAIFSATGKRI
RSLPIMDHDLSSSA
>SEQF6048||SEQF6048.1_01673
MTLIDNLSERAADLSRRNFLRAGAIAGGGLLLSVSLPFASRESEAAASDGFVPNAFIRIGSDGKVVLTMPYVEMG

QGTYTSIPMLIAEELEIGLTQVRLEHAPPSDKLYANPLLGVQATGNSNAMRGAWQPMRKAGATAKAMLVAAA
AKRWNVEPGTCRADNGEVHHAASGRKLGYGELATDAAQMPVPENVTLKSPSEFKLIGTPAKRLDTPSKINGTA
VYGIDARPPGVKVATLAQSPVFGGRVKRVDDAAAKAVKGVRQIVTLDDAVAVVADHMGAAKKGLAALAIEWD
EGPHAKLATSDIARELEAATTKPGTVAQNIGDADKAMAGAATKVEATYQLPFLAHATMEPMNCTVHVRPDGC
EIWVGSQALSRAQAVAAKVLNMPPEKVVVHNHLLGGGFGRRLEVDGVIRAVQIAKQVDAPVKLVWTREEDIQ
HDMYRPYWCDRIAVGLDASGKPVAWNNRFAGSSVLARWAPPAFRNGLDPDTTEGAIDLVYDIPNFHVEYVRV
EPPGIPTAFWRSVGPSHNVFVTESVIDELAAAVKQDPVDYRRALLGKSPRAKAALELAAAKAGWGGKLPAGHG
RGVSLQFVFGSYLAQVAEVEVARDGSVRVHRVVCAMDCGTVVNPDTVQAQLQSGINFGVTAALYGEITLKDG
RVEQSNFDSYQMLRIDQAPAIEVHIVPSTEPPGGMGETGTSGIVPAISNAIFAATGKRLRKMPVDPAVLKQT
>SEQF6048||SEQF6048.1_00843
MNKHVKNLASETTDLSRRSFLVGTAATGLVLGYSASGIDQALAAPAPANFEPSVWYSIAPDGLVTVTCGKADMG
QHVASTMAQIVAEELGANWKDMRVQLASNDPKFNDPVLGAQITGGSWSTMMNFDAMSRAGAAGRIALTE
AAAASMGVPAGELVVRDSKISHAKSKKSMSFAEVVKSGKATKTFTADDLKAIKLKTPDQYTMIGVSVPQLDIPSK
VNGTAKYGIDVMVPGMVHGALVTPPVRYGATVKSVDDSAAKKLPGFIKAVTLDDKTTTTTGWVVAVANTYAQ
AKKAAAALKISYDGGPNAKLSSESLFTEAKRLQGLSDSGEFFVKDGDPNAAFGSAAKLLEAEYTTNINIHAPMEP
MNATAEFKGDILHIYSGNQFATRSGAIAAGAAGIDPKFVVMHQMWLGGGFGRRLDADMMVPAVQAAKAVG
KPVKVIYTRENDMTMDFSRPLTYQKVKAGMDGDGKIVAMSHDVVSAWPTARWGIPDFLTPSVDKKGPLDSFT
VNGADFFYTVPNHYVRAIKNEMAHNATPSGQLRSVAPGWTFWAVESMIDEIAHATGKDPAQLRISLLDGKGK
NDGGAQRLRNTLLAAMGLAGYGTKQLPKGEGMGVACVSSQERATASWTACVAHVAVAPSGEVTVKKLTVAT
DVGTQVNPDGIRAQVEGAALWGMSLALFEKATLKDGGIEQTNFDSYTPLRMSQLPEVAVNVIANGEKATGVG
EPAVTVVAPAIGNAVFNAVGARVRGLPITAEAVKAAMKA
>SEQF6048||SEQF6048.1_06871
MNILTNPKKLRGFERYVKVDNVSRRSILKGLGLAGGFVLAAPVMSRPGFAAYQTGADKMPHGTVVDPRVFVAI
APDGIVTIVAHRAEMGTGVRTSLPLIVAEELEADWKRVRVQQAHGDEVKFGNQDTDGSRSTRHYLIPMRQIG
ASARTMLEQAAAKRWGVPASEVKAVNHEVVHSASGRKFGFGQLAADAAEQSVPSVEGLKLKDPKDFRYLGKG
EIGIVDLHDITTGAARYGADVRLPGMKYAVIARPPVTGGKVVSFDGSEAMKVSGVEKVLEAKAWPWPSKFQPL
GGVAVIARNTGAAIKGRDALKIVWDDGANGKYESVAYRAELEAAARKPGLVVRKEGDVEAALKGADKVIVGEY
YLPHLAHVAMEPPAAVADVKGDKVEVWAPVQSPGGTREDVAKTLDIPQEKVTVNVTLLGGGFGRKSKCDFAL
EAALLSKELGAPVKVQWTREDDIRNGFLHTVSVERIEAGLDKSGKVTAWRHRSVAPSIASTFAANTVHQAPFEL
GMGLVDMPFEIANVQCENPEAAAHTRIGWFRSVSNIPRAFAVQSMVAEIAHATKRDQKDMLLELIGSPRIVKL
DSVKDLWNYGEPYDSYPIDTARLRKVVELVADKGGWGRPVPKGRGLGIAAHRSFVSYVATIVEVAVDDKGKFTV
PRVDTAIDCGTYVNPERIQSQIEGTAIMGMSLAKHGAITFKDGKVQQGNFDDFPVIRIDESPAITNVYIVPPGPD
MPPSGVGEPGVPPFAPALTNAIFAATGKRIRTLPIGKQLEA
>SEQF6048||SEQF6048.1_02545
MLPRTHKDAAMNQYVMPKLNRRAFVIGTATAGLALGLDLPFGGPAVVRAADGAPEVNAPEVNAWVVIRPDD
TVVIRIARSEMGQGTLTGLAQLVAEELECDWSKVTTEYPTPGQSVARKRAWGDFSTGGSRGIRTSQDYVRKGG
ATARVMLIQAAANEWKVPASECKVSSGVITHTASGKTTTYGKVAEAAAKLEPPADVKLKDPKDWTIAGKGLKRL
DTSDKTTGKMVYGIDVKLPGMLNAAIKDCPVFGGKVKSFDEAKIAGMKGVKKVVQVGDSAVAVVADTWWH
AKTALDALPIVWDEGPNAKVSSETIATWLAEGLDNAQPAYVGNQNGDAKAAIAAAAKKVEAVYSYPYQNHAT
MEPMNATVLYTPDKCEVWCGTQNGEAAFAAALEASGLPAEKVDVHKLMLGGGFGRRGMTDYVRQAVAIAK
QMPGTPIKLLWSREEDMQHGKYHPITQCKLTGAFDADNNLVALHYRLSGQSILFSVRPEALQNGMDPAAFQG
VAQAGEAAIGYSVPNLLVEHSMRNPHVPPGFWRGVNVNHNAIYMECFMDELALAVGQDPLEFRRKLMGKH
PKHLAVLNAVAEKIGWGTPAPQGVYRGIAQVMGYGSYVAGAAEISVTDGSKIKVHRIVASTDPGYVVNPAQVE
RQIAGSFVYGLSALFYGGCTVKDGRIEQTNFDTYNSMRINEMPKVEAVMVPSGGFWGGVGEPTIGVAAPAVL

NAYFAATGKRIRSFPLRNQNISFA
>SEQF6049||SEQF6049.1_07333
MNIRTNIPTDSGKLRGFEKHVKVENVSRRSILKGLGIAGSFVLAAPVMTRRAFAYETGAGKMPHGVVVDPRVF
VAIAPDGIVTILAHRSEMGTGVRTSLPLIVAEEMEADWSRVHVQQAHGDEVKFGNQDTDGSRSTRHYLIPMR
QIGASARSMLEAAAAKKWGVPVTEVKAQNHEVVHSASGRKAGFGELAADAAKESVPSVEGLKLKDPKDFRYL
GKGQVSIVDLHDITTGKAPYGADVRLPGLKYAVIARPPVTGGKLKSFDDSAAKKVPGVEKVMEVKGWPWPSKF
QPLGGVAVIARNTGAAIKGRDALKIVWDDGANAKYDSVTYRASLEEAARKPGLVVRKEGDADAALKSADKVIT
GEYYLPHLAHVSMEPPVAVADVKGDKATIWAPVQSPGGTREDVAKTLGIPEDNVTVNVTLLGGGFGRKSKCDF
ALEAALLSKELGAPVKVQWTREDDVHHDFLHTVSVERIEAGLDKSGKVIAWRHRSVAPTIASTFAAGANHEAPF
ELGMGLIDNPFEIANLQCENPEAAAFTRIGWFRSVSNIPRAFAVQSMVGEIAHATGRDQKEMLLELIGSPRIVNL
SSVKDPWNYGEPYDSYPIDTARLRKVVELVAEKGEWGRKVPKGHGLGIAVHRSFVSYIATIVEVAVDDKGKFSVP
RVDTAIDCGTYVNPERIHSQIEGAAIMGLSLAKYGEISFKDGKVQQGNFDDFPVIRMDESPVITNVYIVPPGADT
PPSGVGEPGVPPFAPALINAIFAATGKRIRALPIGKQLET
>SEQF6049||SEQF6049.1_02193
MNKHVKNLAPETTDLSRRSFLVGTAATGLVLGYAASGIDQALAASATANFEPSVWYSIAPDGLVTVTCGKADMG
QHVASTMAQIVAEELGASWKDMRVQLASNDPKFNDPVLGAQITGGSWSTMMNFDAMSRAGAAGRIALTE
AAAASMGVPAGELVVRDSRISHAKSKKSMSFAEVVKSGKATKTFTADDLKAIKLKTPDQYTMIGVSVPQLDIPSK
VNGTAKYGIDVMVPGMVYGALVTPPVRYGATVKSVDDSAAKKLPGFIKAVTLDDKTTTTTGWVVAVANTYAQ
AKKAAAALKISYDGGPNAKLSSESLFAEAKRLQGLSDSGEFFVKDGDPNAAYGSAAKVLEAEYTTNINIHAPMEP
MNATAEFKGDILHIYSGNQFATRSGAIAAGAAGIDPKFVVMHQMWLGGGFGRRLDADMMVPAVQAAKAVG
KPVKVIYTRENDMTMDFSRPLTYQKVKAGMDGDGKIVAMSHDVVSAWPTARWGIPDFLTPSVDKKGPLDSFT
VNGADFFYTVPNHYVRAIKNEMAHNATPSGQLRSVAPGWTFWAVESMIDEIAHATGKDPAQLRISLLDGKGK
NDGGAQRLRNTLLAAMGLAGYGTKQLPKGEGMGVACVSSQERATASWTACVAHVAVAPSGEVTVKKLTVAT
DVGTQVNPDGIRAQVEGAALWGMSLALFEKATLKDGGIEQTNFDSYTPLRMSQLPEVAVNVIANGEKATGVG
EPAVTVVAPAIGNAVFNAVGARVRGLPITAEAVKAAMKA
>SEQF6049||SEQF6049.1_01349
MDELVLKEQIVDEATMSRRAFLQGTGLLLGFSLTGARAERVFAAPASQVVEHEVAGTFAPNGFIRINPTGAVTLV
MPMIEMGQGVYTSLSMLLAEELEVTLDQIQVQHAPPNHALYVNSIIGIQNTGGSASVRAFWTPLRQAGAVGR
NLLIAAAAKRWNVDPATCRAKDGFVFDAPGLKHLSYGELAAAAAKLPVPLAADVKLKDPKDFTLIGTRAKRVDS
SIKVDGRALYGIDTRLPGMTVAAVAISPVLGGKAKTVDENAALMVKGVRQVVNIGEAVAVVANHMGAARKGL
EAAAITWDDGPNGKVSSSDIVKQLEEESRKPGAVARNDGDVGKALAGAAQRVDAIYQVPFLAHAAMEPMNC
TVHLQKDRCDIWVGTQAPTITQSLVTELTGLPKEAITIHNHLIGGGFGRRLEADGTILAVKIAKHVDGPVKVIWSR
EEDIQHDMYRPYYLDRLSAGLDAAGKPVAWTHRIAGSSVMARYYPPYVKNGLDPDAVEAAAEPPYALPNIHV
DFVRVEPPGVRTSWWRGVGPTHNVFVVESFIDELAHAAKQDPVTYRKGLLGHNPRALAVLSLAAEKAGWGSP
LPARHGRGISVQFAYGSFTSQVAEVEVAADGSVKVWRIVCAIDCGMYINPDTIEAQVQGGTLFGLTAALHGSITF
KDGRVEQSNFDTYLPMRIDEVPLVETHLIKNAEAPGGVGEAPTAIVSAAVTNAIFAATGKRVRSLPIDRNSLKSSS
>SEQF6049||SEQF6049.1_00054
MMDLETERLPGPVASRYLAQPSRRSLLKVGASALGGLALTVALPSLSKAASEESAPDLNAFVRIEADGRVHLTIPS
VEMGQGIYTAMSMLLAEELEVGLDAVTVEHAPPNDALYANPIPILRQQTTGASASIRGFWRPLRLAGAAARLM
LVAAAAKQWHVAALACSVRNGVVFDPSGTKSLPYRDLLRSAAAERPPAPDQIKLKSPEQFKLIGTSPKRIEAADK
VSGRTQFGIDVMLPGLRVAALAISPVMGGRPKDVNRAAALATKGVRQVVTIDRAVAIVADHMGAAKKGLVAA
AVAWDDGPNGSVDSKMLVEQLERASENPGAVARSEGHFEAAFAGAARRIESTYELPFLAHAAMEPMNCTVH
VRKESCEIWVGTQIPTVTQAAVAALLGMPQSAVVIHNQYIGGGFGRRLEPDGTLLAVEIGKQVDGPVKVIWSR
EEDIQHDIYRPYYYDRISAGLDAAGLPVAWHHRVCGSSIIARAVPALFKDGLDFDAVEGAAEPPYALPNILVDYVR

AEPPGVTTGFWRGVGPAHNVFVVESFVDELAAAAGKDPVAYRRTLLGHNPRALAVLNLAAEKADWGKPMSA
GKGRGVAVQFAFGSYLALVADVAVDDRGAVKVERVVCAVDCGLPVNPHMIDAQVQSGTIFGLTAALRGAITFK
NGRVEQSNFDSYLPMRIEETPRIETYIVSSTAEPGGLGEAATAIVAPAVTNAIFAATGRRVRRLPIEAT
>SEQF6049||SEQF6049.1_00231
MLPRTHKDAAMNQHVMPKLNRRAFVIGTATAGLALGLDLPFGGPAVVRAADGAPEVNAPEVNAWVVIRPD
DTVVIRIARSEMGQGTLTGLAQLVAEELECDWSKVTTEYPTPGQSVARKRAWGDFSTGGSRGIRTSQDYVRKG
GATARVMLIQAAANEWKVPASECKAANSVITHTPSGKTTTYGKVAEAAAKLEPPADVKLKDPKDWTIAGKGLK
RLDTVDKTTGKMVYGIDVKLPGMLNAAIKDCPVFGGKVKSFDEAKIAGMKGVKKVVQVGDSAVAVVADTW
WHAKTALDALPVVWDEGPNAKVSSETIATWLAEGLDNAQPAYVGNQNGDAKAAIAAAAKKVEAVYNYPYQN
HATMEPMNATVLYTPDKCEVWCGTQNGEAAFAAALEASGLPAEKVDVHKLMLGGGFGRRGMTDYVRQAVA
IAKQMPGTPIKLLWSREEDMQHGKYHPITQCKLTGAFDADNNLVALHYRLSGQSILFSVRPEALQNGMDPAAF
QGVAQAGEAAIGYSVPNLLVEHSMRNPHVPPGFWRGVNVNHNAIYMECFMDELALSVGQDPLEFRRKLMG
KHPKHLAVLNAVAEKIGWGTPAPQGVYRGIAQVMGYGSYVAGAAEISVTDGSKIKVHRIVASTDPGYVVNPAQ
VERQIAGSFVYGLSALFYGGCTVKDGRIEQTNFDTYNSMRINEMPKVEAVMVPSGGFWGGVGEPTIGVAAPA
VLNAYFAATGKRIRSFPLRNQNISFA
>SEQF6049||SEQF6049.1_06272
MTLIDNLSERAADLSRRNFLRAGAIAGGGLLLSVSLPFAGRESEAAASDGFAPNAFVRIGGDGKVVLTMPYVEM
GQGTYTSIPMLIAEELEIGLTQVRLEHAPPSDKLYANPLLGVQATGNSNAMRGAWQPMRKAGATAKAMLVAA
AAKRWNVEPGTCRAENGEVHHAASGRKLGYGELATDAAQMPVPENVTLKSPSEFKLIGTPAKRLDTPSKINGT
AVYGIDARPPGVKIATLAQSPVFGGRVKRVDDAAAKAVKGVRQIVTLDDAVAVVADHMGAAKKGLAALTIEW
DEGTHAKLATSDIARELEAATTKPGAVAQNIGDADKAMAGAATKVEATYQLPFLAHATMEPMNCTVHVRPDG
CEIWVGSQALSRAQAVAAKVLNMPPEKVVVHNHLLGGGFGRRLEVDGVIRAVQIAKQVDAPVKLVWTREEDI
QHDMYRPYWCDRIAVGLDASGKPVAWNNRFAGSSVLARWAPPAFRNGLDPDTTEGAIDLVYDIPNFHVEYV
RVEPPGIPTAFWRSVGPSHNVFVTESMIDELAAAAKQDPVDYRRALLGKSPRAKAALELAAAKAGWGGKLPA
GRGRGVSLQFVFGSYLAQVAEVEVAKDGSVRVHRVVCAMDCGTVVNPDTVQAQLQSGINFGVTAALYGEITL
KDGRVEQTNFDSYQMLRIDQAPAIEVHIVPSTEPPGGMGETGTSGIVPAISNAIFAATGKRLRKMPVDPAVLKQ
T
>SEQF6050||SEQF6050.1_00690
MLPRTHKDAAMNQHVMPKLNRRAFVIGTATAGAGLALGLDLPFGGPAVVRAADGAPEVNAWVVIRPDDTV
VIRIARSEMGQGTLTGLAQLVAEELECDWSKVTTEYPTPGQSVARKRAWGDFSTGGSRGIRTSQDYVRRGGAT
ARVMLVQAAANEWKVPASECKAANSVITHTASGKTTTYGKVAEAAAKLEPPADVKLKDPKDWTIAGKGLKRLD
TSDKTTGKMVYGIDVKLPGMLNAAIKDCPVFGGKVKSFDEAKIAGMKGVKKVVQVGDSAVAVVADTWWHA
KTALDALPIVWDEGPNAKVSSETIATWLAEGLDNAQPAYIGNQNGDAKAAIAAAAKKVEAVYNYPYQNHATM
EPMNATVLYTPDKCEVWCGTQNGEAAFAAALEASGLPAEKVDVHKLMLGGGFGRRGMTDYVRQAVAIAKQ
MPGTPIKLLWSREEDMQHGKYHPITQCKLTGAFDADNNLVALHYRLSGQSILFSVRPEALQNGMDPAAFQGV
AQAGEAAIGYSVPNLLVEHSMRNPHVPPGFWRGVNVNHNAIYMECFMDELALSVGQDPLEFRRKLMGKHP
KHLAVLNAVAEKIGWGTPAPQGVYRGIAQVMGYGSYVAGAAEISVTDGSKIKVHRIVASTDPGYVVNPAQVER
QIAGSFVYGLSALFYGGCTVKDGRIEQTNFDTYNSMRINEMPKVEAVMVPSGGFWGGVGEPTIGVAAPAVLN
AYFAATGKRIRSFPLRNQNISFA
>SEQF6050||SEQF6050.1_03031
MNIRTNIPTGSGKLRGFEKHVKVENVSRRSILKGLGIAGSFVLAAPVMTRQAFAYETGAGKMPHGVVVDPRVF
VAIAPDGIVTILAHRSEMGTGVRTSLPLIVAEEMEADWSRVHVQQAHGDEVKFGNQDTDGSRSTRHYLIPMR
QIGASARSMLEAAAAKKWGVPVTEVKAQNHEVVHSASGRKAGFGELAADAAKESVPSVEGLKLKDPKDFRYL
GKGQVSIVDLHDITTGKAHYGADVRLPGLKYAVIARPPVTGGKLKSFDDSAAKKVPGVEKVMEVKGWPWPSKF

QPLGGVAVIARNTGAAIKGRDALKIVWDDGANAKYDSVTYRASLEEAARKPGLVVRKEGDADAALKSADKVIT
GEYYLPHLAHVSMEPPVAVADVKGDKATIWAPVQSPGGTREDVAKTLGIPEDNVTVNVTLLGGGFGRKSKCDF
ALEAALLSKELGAPVKVQWTREDDVHHDFLHTVSVERIEAGLDKSGKVIAWRHRSVAPTIASTFAAGANHEAPF
ELGMGLIDNPFEIANLQCENPEAAAFTRIGWFRSVSNIPRAFAVQSMVGEIAHATGRDQKEMLLELIGSPRIVNL
SSVKDPWNYGEPYDSYPIDTARLRKVVELVAEKGEWGRKVPKGHGLGIAVHRSFVSYIATIVEVAVDDKGKFSVP
RVDTAIDCGTYVNPERIHSQIEGAAIMGLSLAKYGEISFKDGKVQQGNFDDFPVIRMDESPVITNVHIVPPGADT
PPSGVGEPGVPPFAPALINAIFAATGKRIRALPIGKQLET
>SEQF6050||SEQF6050.1_01189
MNKHVKNLAPETTDLSRRSFLVGTAATGLVLGYAASGIDQALAAPAPASFEPSVWYSIAPDGLVTVTCGKADMG
QHVASTMAQIVAEELGANWKDMRVQLASNDPKFNDPVLGAQITGGSWSTMMNFDAMSRAGAAGRIALTE
AAAASMGVPAGELVVRDSRISHAKSKKSMSFAEVVKSGKATKTFTADDLKAIKLKTPDQYTMIGVSVPQLDIPSK
VNGTAKYGIDVMVPGMVYGALVTPPVRYGATVKSVDDSAAKKLPGFIKAVTLDDKTTTTTGWVVAVANTYAQ
AKKAAAALKISYDGGPNAKLSSESLFAEAKRLQGLSDSGEFFVKDGDPNAAYGSAAKVLEAEYTTNINIHAPMEP
MNATAEFKGDILHIYSGNQFATRSGAIAAGAAGIDPKFVVMHQMWLGGGFGRRLDADMMVPAVQAAKAVG
KPVKVIYTRENDMTMDFSRPLTYQKVKAGMDGDGKIVAMSHDVVSAWPTARWGIPDFLTPSVDKKGPLDSFT
VNGADFFYTVPNHYVRAIKNEMAHNATPSGQLRSVAPGWTFWAVESMIDEIAHATGKDPAQLRISLLDGKGK
NDGGAQRLRNTLLAAMGLAGYGTKQLPKGEGMGVACVSSQERATASWTACVAHVAVAPSGEVTVKKLTVAT
DVGTQVNPDGIRAQVEGAALWGMSLALFEKATLKDGGIEQTNFDSYTPLRMSQLPEVAVNVIANGEKATGVG
EPAVTVVAPAIGNAVFNAVGARVRGLPITAEAVKAAMKA
>SEQF6051||SEQF6051.1_01133
MTLIDNLSERAADLSRRNFLRAGAIAGGGLLLSVSLPFASRESEAATADGFAPNAFVRIGGDGKVVLTMPYVEM
GQGTYTSIPMLIAEELEIGLTQVRLEHAPPSDKLYANPLLGVQATGNSNAMRGAWQPMRKAGATAKAMLVAA
AAKRWNVEPGTCRAENGEVHHAASGRKLGYGELATDAAQMPVPENVTLKSPSEFKLIGTPAKRLDTPSKINGT
TVYGIDARPPGVKVATLAQSPVFGGRVKRVDDAAAKAVKGVRQIVTLDDAVAVVADHMGAAKKGLAALTIEW
DEGVHAKLATSDIARELEAATTKPGAVAQNIGDADKAMTGAATKVEATYQLPFLAHATMEPMNCTVHVRADG
CEIWVGNQALSRVQAVAAKMLDLPPEKVVVHNHLLGGGFGRRLEVDGVIRAVQIAKQVDTPVKVVWTREEDI
QHDMYRPYWCDRIAVGLDASGKPVAWNNRFAGSSVLARWAPPAFRNGLDPDTTEGAIDLVYDIPNFHVEYV
RVEPPGIPTAFWRSVGPSHNVFVTESMIDELAAAAKQDPLDYRRALLGKSPRAKAALELAAAKAGWGGKLPAG
RGRGVSLQFVFGSYLAQVAEVEVARDGSVRVHRVVCAMDCGTVVNPDTVQAQLQSGINFGVTAALYGEITLK
DGRVEQGNFDSYQMLRIDQAPAIEVHIVPSTEPPGGMGETGTSGIVPAISNAIFAATGKRLRKMPVDPAVLKQT
>SEQF6051||SEQF6051.1_03262
MNILTNPSKLRGFEKHVKVENVSRRSILKGLGIAGSFVLAAPVMTRQAFAYETGAGKMPHGVVVDPRVFVAIAP
DGIVTILAHRSEMGTGVRTSLPLIVAEEMEADWSRVHVQQAHGDEVKFGNQDTDGSRSTRHYLIPMRQIGAS
ARSMLEAAAAKKWGVPVAEVKAQNHEVVHSASGRKAGFGELAADAAKEPVPSIEGLKLKDPKDFRYLGKGQV
SIVDLHDITTGKAQYGADVRLPGLKYAVIARPPVTGGKLKSFDDSAAKKVPGVEKVMEVKGWPWPSKFQPLGG
VAVIARNTGAAIKGRDALKIVWDDGANAKYDSVTYRASLEEAARKPGLVVRKEGDVDAALKSADKVITGEYYLP
HLAHVSMEPPVAVADVKGDKATIWAPVQSPGGTREDVAKTLGIPEDNVTVNVTLLGGGFGRKSKCDFALEAAL
LSKELGAPVKVQWTREDDVHHDFLHTVSVERIEAGLDKSGKVIAWRHRSVAPTIASTFAAGANHEAPFELGMG
LIDNPFEIANLQCENPEAAAFTRIGWFRSVSNIPRAFAVQSMVGEIAHATGRDQKEMLLELIGSPRIVNLSSVKD
PWNYGEPYDSYPIDTARLRKVVELVADKGEWGRKVPKGHGLGIAVHRSFVSYIATIVEVAVDDKGKFSVPRVDTA
IDCGTFVNPERIHSQIEGAAIMGLSLAKYGEISFKDGKVQQGNFDDFPVIRMDESPVITNVYIVPPGADTPPSGV
GEPGVPPFAPALINAIFAATGKRIRALPIGKQLET
>SEQF6051||SEQF6051.1_07105
MNKHVKNLAPETTDLSRRSFLVGTAATGLVLGYAASGIDQALAAPATTNFEPSVWYSIAPDGLVTVTCGKADMG

QHVASTMAQIVAEELGASWKDMRVQLASNDPKFNDPVLGAQITGGSWSTMMNFDAMSRAGAAGRIALTE
AAAASMGVPAGELVVRDSRISHAKSKKSMSFAEVVKSGKATKTFTADDLKAIKLKTPDQYTMIGVSVPQLDIPSK
VNGTAKYGIDVMVPGMVYGALVTPPVRYGATVKSVDDSAAKKLPGFIKAVTLDDKTTTTTGWVVAVANTYAQ
AKKAAAALKISYDGGPNAKLSSESLFAEAKRLQGLSDSGEFFVKDGDPNAAYGSAAKVLEAEYTTNINIHAPMEP
MNATAEFKGDILHIYSGNQFATRSGAIAAGAAGIDPKFVVMHQMWLGGGFGRRLDADMMVPAVQAAKAVG
KPVKVIYTRENDMTMDFSRPLTYQKVKAGMDGDGKIVAMSHDVVSAWPTARWGIPDFLTPSVDKKGPLDSFT
VNGADFFYTVPNHYVRAIKNEMAHNATPSGQLRSVAPGWTFWAVESMIDEIAHATGKDPAQLRISLLDGKGK
NDGGAQRLRNTLLAAMGLAGYGTKQLPKGEGMGVACVSSQERATASWTACVAHVAVAPSGEVTVKKLTVAT
DVGTQVNPDGIRAQVEGAALWGMSLALFEKATLKDGGIEQTNFDSYTPLRMSQLPEVAVNVIANGEKATGVG
EPAVTVVAPAIGNAVFNAVGARVRGLPITAEAVKAAMKA
>SEQF6051||SEQF6051.1_04528
MLPRTHKDAAMNQHVMPKLNRRAFVIGTATAGAGLALGLDLPFGGPAVVRAADGAPEVNAWVVIRPDDTV
VIRIARSEMGQGTLTGLAQLVAEELECDWSKVTTEYPTPGQSVARKRAWGDFSTGGSRGIRTSQDYVRKGGAT
ARVMLIQAAANEWKVPASECKVSNGVITHTALGKTTTYGKVAEAAAKLEPPADVKLKDPKDWTIAGKGLKRLD
TVDKTTGKMIYGIDVKLPGMLNAAIKDCPVFGGKVKSFDEAKIAGMKGVKQVMQVGDSAVAVVADTWWHA
KTALDALPIVWDEGPNAKVSSETIATWLAEGLDNAQPAYIGNQNGDAKAAIAAAAKKVEAVYSYPYQNHATME
PMNATVLYTPDKCEVWCGTQNGEAAFAAALEASGLPAEKVDVHKLMLGGGFGRRGMTDYVRQAVAIAKQM
PGTPIKLLWSREEDMQHGKYHPITQCKLTGAFDADNNLVALHYRLSGQSILFSVRPEALQNGMDPAAFQGVAQ
AGEAAIGYSVPNLLVEHSMRNPHVPPGFWRGVNVNHNAIYMECFMDELALSVGQDPLEFRRKLMGKHPKHL
AVLNAVAEKIGWGTPAPQGVYRGIAQVMGYGSYVAGAAEISVTDGSKIKVHRIVASTDPGYVVNPAQVERQIA
GSFVYGLSALFYGGCTVKDGRIEQTNFDTYNSMRINEMPKVEAVMVPSGGFWGGVGEPTIGVAAPAVLNAYF
AATGKRIRSFPLRNQNISFA
>SEQF6052||SEQF6052.1_06199
MNIRTNIPTGSGKLRGFEKHVKVENVSRRSILKGLGIAGSFVLAAPVMTRQAFAYETGAGKMPHGVVVDPRVF
VAIAPDGIVTILAHRSEMGTGVRTSLPLIVAEEMEADWSRVHVQQAHGDEVKFGNQDTDGSRSTRHYLIPMR
QIGASARSMLEAAAAKKWGVPVTEVKAQNHEVVHSASGRKAGFGELAADAAKESVPSVEGLKLKDPKDFRYL
GKGQVSIVDLHDITTGKAHYGADVRLPGLKYAVIARPPVTGGKLKSFDDSAAKKVPGVEKVMEVKGWPWPSKF
QPLGGVAVIARNTGAAIKGRDALKIVWDDGANAKYDSVTYRASLEEAARKPGLVVRKEGDADAALKSADKVIT
GEYYLPHLAHVSMEPPVAVADVKGDKATIWAPVQSPGGTREDVAKTLGIPEDNVTVNVTLLGGGFGRKSKCDF
ALEAALLSKELGAPVKVQWTREDDVHHDFLHTVSVERIEAGLDKSGKVIAWRHRSVAPTIASTFAAGANHEAPF
ELGMGLIDNPFEIANLQCENPEAAAFTRIGWFRSVSNIPRAFAVQSMVGEIAHATGRDQKEMLLELIGSPRIVNL
SSVKDPWNYGEPYDSYPIDTARLRKVVELVAEKGEWGRKVPKGHGLGIAVHRSFVSYIATIVEVAVDDKGKFSVP
RVDTAIDCGTYVNPERIHSQIEGAAIMGLSLAKYGEISFKDGKVQQGNFDDFPVIRMDESPVITNVHIVPPGADT
PPSGVGEPGVPPFAPALINAIFAATGKRIRALPIGKQLET
>SEQF6052||SEQF6052.1_02937
MTLIDNLSERAADLSRRNFLRAGAIAGGGLLLSVSLPFAGRESEAAASDGFAPNAFVRIGGDGKVVLTMPYVEM
GQGTYTSIPMLIAEELEIGLTQVRLEHAPPSDKLYANPLLGVQATGNSNAMRGAWQPMRKAGATAKAMLVAA
AAKRWNVEPGTCRAENGEVHHAASGRKLGYGELATDAAQMPVPENVTLKSPSEFKLIGTPAKRLDTPSKINGT
AVYGIDARPPGVKIATLAQSPVFGGRVKRVDDAAAKAVNGVRQIVTLDDAVAVVADHMGAAKKGLAALTIEW
DEGAHAKLATSDIARELETATTKPGAVAQNIGDADKAMAGAATKVEATYQLPFLAHATMEPMNCTVHVRPDG
CEIWVGSQALSRAQAVAAKVLNMPPEKVVVHNHLLGGGFGRRLEVDGVIRAVQIAKQVDAPVKLVWTREEDI
QHDMYRPYWCDRIAVGLDASGKPVAWNNRFAGSSVLARWAPPAFRNGLDPDTTEGAIDLVYDIPNFHVEYV
RVEPPGIPTAFWRSVGPSHNVFVTESVIDELAAAAKQDPVDYRRALLGKSPRAKAALELAAAKAGWGGKLPAG
RGRGVSLQFVFGSYLAQVAEVEVARDGSVRVHRVVCAMDCGTVVNPDTVQAQLQSGINFGVTAALYGEITLK

DGRVEQSNFDSYQMLRIDQAPAIEVHIVPSTEPPGGMGETGTSGIVPAISNAIFAATGKRLRKMPVDPAVLKQT
>SEQF6052||SEQF6052.1_05700
MLPRTHKDAAMNQHVMPKLNRRAFVIGTATAGAGLALGLDLPFGGPAVVRAADGAPEVNAWVVIRPDDTV
VIRIARSEMGQGTLTGLAQLVAEELECDWSKVTTEYPTPGQSVARKRAWGDFSTGGSRGIRTSQDYVRRGGAT
ARVMLVQAAANEWKVPASECKAANSVITHTASGKTTTYGKVAEAAAKLEPPADVKLKDPKDWTIAGKGLKRLD
TSDKTTGKMVYGIDVKLPGMLNAAIKDCPVFGGKVKSFDEAKIAGMKGVKKVVQVGDSAVAVVADTWWHA
KTALDALPIVWDEGPNAKVSSETIATWLAEGLDNAQPAYIGNQNGDAKAAIAAAAKKVEAVYNYPYQNHATM
EPMNATVLYTPDKCEVWCGTQNGEAAFAAALEASGLPAEKVDVHKLMLGGGFGRRGMTDYVRQAVAIAKQ
MPGTPIKLLWSREEDMQHGKYHPITQCKLTGAFDADNNLVALHYRLSGQSILFSVRPEALQNGMDPAAFQGV
AQAGEAAIGYSVPNLLVEHSMRNPHVPPGFWRGVNVNHNAIYMECFMDELALSVGQDPLEFRRKLMGKHP
KHLAVLNAVAEKIGWGTPAPQGVYRGIAQVMGYGSYVAGAAEISVTDGSKIKVHRIVASTDPGYVVNPAQVER
QIAGSFVYGLSALFYGGCTVKDGRIEQTNFDTYNSMRINEMPKVEAVMVPSGGFWGGVGEPTIGVAAPAVLN
AYFAATGKRIRSFPLRNQNISFA
>SEQF6052||SEQF6052.1_07757
MNKHVKNLAPETTDLSRRSFLVGTAATGLVLGYAASGIDQALAAPAPASFEPSVWYSIAPDGLVTVTCGKADMG
QHVASTMAQIVAEELGANWKDMRVQLASNDPKFNDPVLGAQITGGSWSTMMNFDAMSRAGAAGRIALTE
AAAASMGVPAGELVVRDSRISHAKSKKSMSFAEVVKSGKATKTFTADDLKAIKLKTPDQYTMIGVSVPQLDIPSK
VNGTAKYGIDVMVPGMVYGALVTPPVRYGATVKSVDDSAAKKLPGFIKAVTLDDKTTTTTGWVVAVANTYAQ
AKKAAAALKISYDGGPNAKLSSESLFAEAKRLQGLSDSGEFFVKDGDPNAAYGSAAKVLEAEYTTNINIHAPMEP
MNATAEFKGDILHIYSGNQFATRSGAIAAGAAGIDPKFVVMHQMWLGGGFGRRLDADMMVPAVQAAKAVG
KPVKVIYTRENDMTMDFSRPLTYQKVKAGMDGDGKIVAMSHDVVSAWPTARWGIPDFLTPSVDKKGPLDSFT
VNGADFFYTVPNHYVRAIKNEMAHNATPSGQLRSVAPGWTFWAVESMIDEIAHATGKDPAQLRISLLDGKGK
NDGGAQRLRNTLLAAMGLAGYGTKQLPKGEGMGVACVSSQERATASWTACVAHVAVAPSGEVTVKKLTVAT
DVGTQVNPDGIRAQVEGAALWGMSLALFEKATLKDGGIEQTNFDSYTPLRMSQLPEVAVNVIANGEKATGVG
EPAVTVVAPAIGNAVFNAVGARVRGLPITAEAVKAAMKA
>SEQF7361||SEQF7361.1_02954
MTKNVSAQAVILSRRKLLQSSGIAAGGLLLATALPFSRRSYAEQYVNKGPEDAPLDTPTALGAFLRIGHDGQITLIS
PKIEMGQGVQTGFAMMVAEELNVTLDQVRVQEAPPDEKLYGDKLLGFQATGGSSSTRSNWQPLREAAAAAR
VMLIQAAANQWKVSPDECRAENGKIIGPGNRELAYGALVDAAAKLPVPENVPLKKPEDYKVIGQPLRRLDTPG
KVDGSAKFTIDLHVPGMKIATVSACPVVGGTLASVDDRHARAVPGVRDVVKLENAVAVIGDHMWAALKGLKA
LEIQWDLGPNAGIDSAQIERALHAAFDREGAIAAEVGDINKAIAGASSKIEAEYEMPFLAHATLEPMTCVAQVR
EDACELWVGTQVPVMAQQAAAKATGLPPEKIIVNNQLIGGGFGRRLEADFIGQAAAIAKQVDYPIKLVWTREE
DTAHDLYRPHYIDRFSAGLDANGMPVGWSHTIVGASVMARFAPAAVPPNGLDADAVEVSNKPVYSLPNLRVR
YVPEAPKAILNSWWRGVGPLHGAYVMESFIDELAYAAKQDPVDYRMKLLGEHPRAQAVLKLAAEKANWSQKL
PAGHGRGIAVQEVFGSYLATVVEMQVDAQNGIHITRLICIADCGEVTNPTSVHSQLEGGTLFGLSAALYNEITVK
DGHVEQSNFHDYRQLRMSDAPPVETHIIPSHEIPGGIGEAGTAMIAPALVNALYAATGKRIRRLPVVRAGYHVA
>SEQF7362||SEQF7362.1_02698
MTKNVSAQAISLSRRKLLQASGIAAGGLLLATALPFSRRSYAEQYVNKGPEDAPLDTPTALGAFLRIGHDGQITLIS
PKIEMGQGVQTGFAMMVAEELNVTLDQVRVQEAPPDEKLYGDKLLGFQATGGSSSTRSNWQPLREAAAAAR
VMLIQAAANQWKVSPDECRAENGKIIGPGNRELAYGALVDAAAKLPVPENVPLKKPEDYKVIGQPLRRLDTPG
KVDGSAKFTIDLHVPGMKIATVSACPVVGGTLASVDDRHARAVPGVRDVVKLENAVAVIGDHMWAALKGLKA
LEIQWDLGPNAGIDSAQIERALHAAFDREGAIAAEVGDINKAIAGASSKIEAEYEMPFLAHATLEPMTCVAQVR
EDACELWVGTQVPVMAQQAAAKATGLPPEKIIVNNQLIGGGFGRRLEADFIGQAAAIAKQVDYPIKLVWTREE
DTAHDLYRPHYIDRFSAGLDANGMPVGWSHTIVGASVMARFAPAAVPPNGLDADAVEVSNKPVYSLPNLRVR

YVPEAPKAILNSWWRGVGPLHGAYVMESFIDELAYAAKQDPVDYRMKLLGEHPRAQAVLKLAAEKANWSQKL
PAGHGRGIAVQEVFGSYLATVVEMQVDAQNGIHITRLICIADCGEVTNPTSVHSQLEGGTLFGLSAALYNEITVK
DGHVEQSNFHDYRQLRMSDAPPVETHIIPSHEIPGGIGEAGTAMIAPALVNALYAATGKRLRRLPVVRAGYHVA
>SEQF7364||SEQF7364.1_01406
MTKNVSAQAISLSRRKLLQASGIAAGGLLLATALPFSRRSYAEQYVNKGPEDAPLDTPTALGAFLRIGHDGQITLIS
PKIEMGQGVQTGFAMMVAEELNVTLDQVRVQEAPPDEKLYGDKLLGFQATGGSSSTRSNWQPLREAAAAAR
VMLIQAAANQWKVSPDECRAENGKIIGPGNRELAYGALVDAAAKLPVPENVPLKKPEDYKVIGQPLRRLDTPG
KVDGSAKFTIDLHVPGMKIATVSACPVVGGTLASVDDRHARAVPGVRDVVKLDNAVAVIGDHMWAALKGLK
ALEIQWDLGPNAGIDSAQIERALHAAFDREGAIAAEVGDINKAIAGASSKIEAEYEMPFLAHATLEPMTCVAQV
REDACELWVGTQVPVMAQQAAAKATGLPPEKIIVNNQLIGGGFGRRLEADFIGQAAAIAKQVDYPIKLVWTRE
EDTAHDLYRPHYIDRFSAGLDANGMPVGWSHTIVGASVMARFAPAAVPPNGLDADAVEVSNKPVYSLPNLRV
RYVPEAPKAILNSWWRGVGPLHGAYVMESFIDELAYAAKQDPVDYRMKLLGEHPRAQAVLKLAAEKANWSQ
KLPAGHGRGIAVQEVFGSYLATVVEMQVDAQNGIHITRLICIADCGEVTNPTSVHSQLEGGTLFGLSAALYNEIT
VKDGHVEQSNFHDYRQLRMSDAPPVETHIIPSHEIPGGIGEAGTAMIAPALVNALYAATGKRIRRLPVVRAGYH
VA
>SEQF7365||SEQF7365.1_04985
MTKNVSAQAISLSRRKLLQASGIAAGGLLLATALPFSRRSYAEQYVNKGPEDAPLDTPTALGAFLRIGHDGQITLIS
PKIEMGQGVQTGFAMMVAEELNVTLDQVRVQEAPPDEKLYGDKLLGFQATGGSSSTRSNWQPLREAAAAAR
VMLIQAAANQWKVSPDECRAENGKIIGPGNRELAYGALVDAAAKLPVPENVPLKKPEDYKVIGQPLRRLDTPG
KVDGSAKFTIDLHVPGMKIATVSACPVVGGTLASVDDRHARAVPGVRDVVKLENAVAVIGDHMWAALKGLKA
LEIQWDLGPNAGIDSAQIERALHAAFDREGAIAAEVGDINKAIAGASSKIEAEYEMPFLAHATLEPMTCVAQVR
EDACELWVGTQVPVMAQQAAAKATGLPPEKIIVNNQLIGGGFGRRLEADFIGQAAAIAKQVDYPIKLVWTREE
DTAHDLYRPHYIDRFSAGLDANGMPVGWSHTIVGASVMARFAPAAVPPNGLDADAVEVSNKPVYSLPNLRVR
YVPEAPKAILNSWWRGVGPLHGAYVMESFIDELAYAAKQDPVDYRMKLLGEHPRAQAVLKLAAEKANWSQKL
PAGHGRGIAVQEVFGSYLATVVEMQVDAQNGIHITRLICIADCGEVTNPTSVHSQLEGGTLFGLSAALYNEITVK
NGHVEQSNFHDYRQLRMSDAPPVETHIIPSHEIPGGIGEAGTAMIAPALVNALYAATGKRLRRLPVVRAGYHVA
>SEQF7366||SEQF7366.1_01230
MTKNVSAQAISLSRRKLLQASGIAAGGLLLATALPFSRRSYAEQYVNKGPEDAPLDTPTALGAFLRIGHDGQITLIS
PKIEMGQGVQTGFAMMVAEELNVTLDQVRVQEAPPDEKLYGDKLLGFQATGGSSSTRSNWQPLREAAAAAR
VMLIQAAANQWKVSPDECRAENGKIIGPGNRELAYGALVDAAAKLPVPENVPLKKPEDYKVIGQPLRRLDTPG
KVDGSAKFTIDLHVPGMKIATVSACPVVGGTLASVDDRHARAVPGVRDVVKLDNAVAVIGDHMWAALKGLK
ALEIQWDLGPNAGIDSAQIERALHAAFDREGAIAAEVGDINKAIAGASSKIEAEYEMPFLAHATLEPMTCVAQV
REDACELWVGTQVPVMAQQAAAKATGLPPEKIIVNNQLIGGGFGRRLEADFIGQAAAIAKQVDYPIKLVWTRE
EDTAHDLYRPHYIDRFSAGLDANGMPVGWSHTIVGASVMARFAPAAVPPNGLDADAVEVSNKPVYSLPNLRV
RYVPEAPKAILNSWWRGVGPLHGAYVMESFIDELAYAAKQDPVDYRMKLLGEHPRAQAVLKLAAEKANWSQ
KLPAGHGRGIAVQEVFGSYLATVVEMQVDAQNGIHITRLICIADCGEVTNPTSVHSQLEGGTLFGLSAALYNEIT
VKDGHVEQSNFHDYRQLRMSDAPPVETHIIPSHEIPGGIGEAGTAMIAPALVNALYAATGKRIRRLPVVRAGYH
VA
>SEQF7737||SEQF7737.1_03262
MNSKIDLSNALPGSRRGFLKGAAVVGLTIGFQWSGARRALAAALPDAGFAPNAFLRIAPDDSVTVIAKHVEMG
QGAYTGIATIVAEELDADWSKVRVESAPADAKRYANLAFGTMQGTGGSSAMANSWMQLREAGAKARAMLV
EAAARQWRVPATELRTRDGFVEHPASQRKASYGSLAAAAAELPVPEKVQLKDPKDFRLIGHQAPRVDVPGKTD
GSAQFTLDVSLPGMLVALLQRPPLFGATVKSFDATATRAIPGVVEVVQVPHGVAVVAKGFWAAKQGRDALKVE
WDESKAEKRGSEALMAEYRKLAEQPGKPARRDGDAAKAVAGATRRIAASYEFPFLAHAPMEPLDAVVRLTADS

CEIWAGDQFQTVDQGNAARTAGLKPEQVKINTLYAGGSFGRRANAWSDYIVEAVSIAKALGANGVPVKLQWT
REDDIHGGFYRPMYYHRLEAGLDADGKLVGWQHRIVGQSILEGTPFAAVMVKDGIDATSVEGAANLPYAVPN
VSVELSTTQVGVPVLWWRVVGSSHTVYAVEAFIDEAAQAAGKDPYLFRRDLLAEQPRLRGVLELAAEKAGWD
PSRPLPAGRGRGIAVTEAFKTFVAQVVEVSVDKDGKLKVERVVCAVDCGIPINPDVIAAQMEGGIGFGLGAILHS
AITLKDGKVEQNNFDGYQVLRIAEMPKVEVHIVPSGEAPTGVGEPGVAPIGPALANAIFAATGQRLYNLPFPTSF
AKA
>SEQF7737||SEQF7737.1_02733
MKRSYPDDLVIGNLSRRGFLKGVGATGVLLVAANWGWRDALAAEKKAFGADAMPHGWVDNPKIYVSIDRDG
TVGIVCNRSEMGQGVRTSLAMVVADELEADWSRVKVIQAPGDEARYGNQDTDGSRSMRHWFEPMRRCGA
AARQMLEQAAANQWKVPLGECRAEQNKVLHAPSGRSLSFGELAEAAAGLEVPARDKLLLKKPEQFRYIGKDV
ARAIDGADIVNGRAGFGFDARFDDMLYAVVARPPVYGGKLKRYDAAAALKVPGVVKVIEIEGRPIPSEFQPLGG
VAVVAQNTWAAIKGREALVVEWDAGVNGGYDSVAYRKQLEEAARKPGKVVRDSGDAAALFAKGGDIVEAEYY
LPHLAQAPMEPPVSTAWYKDGACEVWAPTQAPQVTRERIAERLKLPFDKVTVNVTLLGGGFGRKSKPDFVLEA
AILAKAFPGRHLRVQWTREDDLHFSYFHTVSVERLQAVLGADGLPQAWLHRSVAPSITALFGPDSKHQGAFEL
GMGLTNLPFAIPNVRLENPEAPAHTRVGWFRSVSNIPHAFAIQSFVGELAAKAGQDPKDYLLKLLGPARRIDTAE
LGDSWNYGESPERYPLDVGRLRGVIEEAARQSGWGGELPRGRARGIAAHYSFVTYVAVVIEVEVKDDGALLVH
KATIAADCGPQINPERIRSQLEGACVMGLGLAALGEISFKDGKVQQDNFHQYELARMPLAPKAVSVHLLKPDG
DLPLGGVGEPGVPPIAPALCNAIFAATGKRIRELPIRNQLQGWRKA
>SEQF7738||SEQF7738.1_01178
MKRSYPDDLVIGNLSRRGFLKGVGATGVLLVAANWGWRDALAAEKKAFGADAMPHGWVDNPKIYVSIDRDG
TVGIVCNRSEMGQGVRTSLAMVVADELEADWSRVKVIQAPGDEARYGNQDTDGSRSMRHWFEPMRRCGA
AARQMLEQAAANQWKVPLGECRAEQNKVLHAPSGRSLSFGELAEAAAGLEVPARDKLLLKKPEQFRYIGKDV
ARAIDGADIVNGRAGFGFDARFDDMLYAVVARPPVYGGKLKRYDAAAALKVPGVVKVIEIEGRPIPSEFQPLGG
VAVVAQNTWAAIKGREALVVEWDAGVNGGYDSVAYRKQLEEAARKPGKVVRDSGDAAALFAKGGDIVEAEYY
LPHLAQAPMEPPVSTAWYKDGACEVWAPTQAPQVTRERIAERLKLPFDKVTVNVTLLGGGFGRKSKPDFVLEA
AILAKAFPGRHLRVQWTREDDLHFSYFHTVSVERLQAVLGADGLPQAWLHRSVAPSITALFGPDSKHQGAFEL
GMGLTNLPFAIPNVRLENPEAPAHTRVGWFRSVSNIPHAFAIQSFVGELAAKAGQDPKDYLLKLLGPARRIDTAE
LGDSWNYGESPERYPLDVGRLRGVIEEAARQSGWGGELPRGRARGIAAHYSFVTYVAVVIEVEVKDDGALLVH
KATIAADCGPQINPERIRSQLEGACVMGLGLAALGEISFKDGKVQQDNFHQYELARMPLAPKAVSVHLLKPDG
DLPLGGVGEPGVPPIAPALCNAIFAATGKRIRELPIRNQLQGWRKA
>SEQF7738||SEQF7738.1_01706
MNSKIDLSNALPGSRRGFLKGAAVVGLTIGFQWSGARRALAAALPDAGFAPNAFLRIAPDDSVTVIAKHVEMG
QGAYTGIATIVAEELDADWSKVRVESAPADAKRYANLAFGTMQGTGGSSAMANSWMQLREAGAKARAMLV
EAAARQWRVPATELRTRDGFVEHPASQRKASYGSLAAAAAELPVPEKVQLKDPKDFRLIGHQAPRVDVPGKTD
GSAQFTLDVSLPGMLVALLQRPPLFGATVKSFDATATRAIPGVVEVVQVPHGVAVVAKGFWTAKQGRDALKVE
WDESKAEKRGSEALMAEYRKLAEQPGKPARRDGDAAKAVAGATRRIAASYEFPFLAHAPMEPLDAVVRLTADS
CEIWAGDQFQTVDQGNAARTAGLKPEQVKINTLYAGGSFGRRANAWSDYIVEAVSIAKALGANGVPVKLQWT
REDDIHGGFYRPMYYHRLEAGLDADGKLVGWQHRIVGQSILEGTPFAAVMVKDGIDATSVEGAANLPYAVPN
VSVELSTTQVGVPVLWWRVVGSSHTVYAVEAFIDEAAQAAGKDPYLFRRDLLAEQPRLRGVLELAAEKAGWD
PSRPLPAGRGRGIAVTEAFKTFVAQVVEVSVDKDGKLKVERVVCAVDCGIPINPDVIAAQMEGGIGFGLGAILHS
AITLKDGKVEQNNFDGYQVLRIAEMPKVEVHIVPSGEAPTGVGEPGVAPIGPALANAIFAATGQRLYNLPFPTSF
AKA
>SEQF7739||SEQF7739.1_03213
MKRSYPDDLVIGNLSRRGFLKGVGATGVLLVAANWGWRDALAAEKKAFGADAMPHGWVDNPKIYVSIDRDG

TVGVVCNRSEMGQGVRTSLAMVVADELEADWSRVKVIQAPGDEARYGNQDTDGSRSMRHWFEPMRRCG
AAARQMLEQAAANQWKVPLGECRAEQNKVLHAPSGRSLSFGELAEAAAGLEVPARDKLLLKKPEQFRYIGKD
VARAIDGADIVNGRAGFGFDARFDDMLYAVVARPPVYGGKLKRYDAAAALKVPGVVKVIEIEGRPIPSEFQPLG
GVAVVAQNTWAAIKGREALVVEWDAGVNGGYDSVAYRKQLEEAARKPGKVVRDSGDAAALFAKGGDIVEAE
YYLPHLAQAPMEPPVSTAWYKDGACEVWAPTQAPQVTRERIAERLKLPFDKVTVNVTLLGGGFGRKSKPDFVL
EAAILAKAFPGRHLRVQWTREDDLHFSYFHTVSVERLQAVLGADGLPQAWLHRSVAPSITALFGPDSKHQGAF
ELGMGLTNLPFAIPNVRLENPEAPAHTRVGWFRSVSNIPHAFAIQSFVGELAAKAGQDPKDYLLKLLGPARRIDT
AELGDSWNYGESPERYPLDVGRLRGVIEEAARQSGWGGELPRGRARGIAAHYSFVTYVAVVIEVEVKDDGALL
VHKATIAADCGPQINPERIRSQLEGACVMGLGLAALGEISFKDGKVQQDNFHQYELARMPLAPKAVSVHLLKP
DGDLPLGGVGEPGVPPIAPALCNAIFAATGKRIRELPIRNQLQGWRKA
>SEQF7739||SEQF7739.1_03801
MNSKIDLSNALPGSRRGFLKGAAVVGLTIGFQWSGARRALAAALPDAGFAPNAFLRIAPDDSVTVIAKHVEMG
QGAYTGIATIVAEELDADWSKVRVESAPADAKRYANLAFGTMQGTGGSSAMANSWMQLREAGAKARAMLV
EAAARQWRVPATELRTRDGFVEHPASQRKASYGSLAAAAAELPVPEKVQLKDPKDFRLIGHQAPRVDVPGKTD
GSAQFTLDVSLPGMLVALLQRPPLFGATVKSFDATATRAIPGVVEVVQVPHGVAVVAKGFWAAKQGRDALKVE
WDESKAEKRGSEALMAEYRKLAEQPGKPARRDGDAAKAVAGATRRIAASYEFPFLAHAPMEPLDAVVRLTADS
CEIWAGDQFQTVDQGNAARTAGLKPEQVKINTLYAGGSFGRRANAWSDYIVEAVSIAKALGANGVPVKLQWT
REDDIHGGFYRPMYYHRLEAGLDADGKLVGWQHRIVGQSILEGTPFAAVMVKDGVDATSVEGAANLPYAVPN
VSVELSTTQVGVPVLWWRVVGSSHTVYAVEAFIDEAAQAAGKDPYLFRRDLLAEQPRLRGVLELAAEKAGWD
PSRPLPAGRGRGIAVTEAFKTFVAQVVEVSVDKDGKLKVERVVCAVDCGIPINPDVIAAQMEGGIGFGLGAILHS
AITLKDGKVEQNNFDGYQVLRIAEMPKVEVHIVPSGEAPTGVGEPGVAPIGPALANAIFAATGQRLYNLPFPTSF
AKA
>SEQF7740||SEQF7740.1_03321
MNSKIDLSNALPGSRRGFLKGAAVVGLTIGFQWSGARRALAAALPDAGFAPNAFLRIAPDDSVTVIAKHVEMG
QGAYTGIATIVAEELDADWSKVRVESAPADAKRYANLAFGTMQGTGGSSAMANSWMQLREAGAKARAMLV
EAAARQWRVPATELRTRDGFVEHPASQRKASYGSLAAAAAELPVPEKVQLKDPKDFRLIGHQAPRVDVPGKTD
GSAQFTLDVSLPGMLVALLQRPPLFGATVKSFDATATRAIPGVVEVVQVPHGVAVVAKGFWAAKQGRDALKVE
WDESKAEKRGSEALMAEYRKLAEQPGKPARRDGDAAKAVAGATRRIAASYEFPFLAHAPMEPLDAVVRLTADS
CEIWAGDQFQTVDQGNAARTAGLKPEQVKINTLYAGGSFGRRANAWSDYIVEAVSIAKALGANGVPVKLQWT
REDDIHGGFYRPMYYHRLEAGLDADGKLVGWQHRIVGQSILEGTPFAAVMVKNGIDATSVEGAANLPYAVPN
VSVELSTTQVGVPVLWWRVVGSSHTVYAVEAFIDEAAQAAGKDPYLFRHDLLAEQPRLRGVLELAAEKAGWD
PSRPLPAGRGRGIAVTEAFKTFVAQVVEVSVDKDGKLKVERVVCAVDCGIPINPDVIAAQMEGGIGFGLGAILHS
AITLKDGKVEQNNFDGYQVLRIAEMPKVEVHIVPSGEAPTGVGEPGVAPIGPALANAIFAATGQRLYNLPFPTSF
AKA
>SEQF7740||SEQF7740.1_02815
MKRSYPDDLVIGNLSRRGFLKGVGATGVLLVAANWGWRDALAAEKKAFGADAMPHGWVDNPKIYVSIDRDG
TVGIVCNRSEMGQGVRTSLAMVVADELEADWSRVKVIQAPGDEARYGNQDTDGSRSMRHWFEPMRRCGA
AARQMLEQAAANQWKVPLGECRAEQNKVLHAPSGRSLSFGELAEAAAGLEVPARDKLLLKKPEQFRYIGKDV
ARAIDGADIVNGRAGFGFDARFDDMLYAVVARPPVYGGKLKRYDAAAALKVPGVVKVIEIEGRPIPSEFQPLGG
VAVVAQNTWAAIKGREALVVEWDAGVNGGYDSVAYRKQLEEAARKPGKVVRDSGDAAALFAKGGDIVEAEYY
LPHLAQAPMEPPVSTAWYKDGACEVWAPTQAPQVTRERIAERLKLPFDKVTVNVTLLGGGFGRKSKPDFVLEA
AILAKAFPGRHLRVQWTREDDLHFSYFHTVSVERLQAVLGADGLPQAWLHRSVAPSITALFGPDSKHQGAFEL
GMGLTNLPFAIPNVRLENPEAPAHTRVGWFRSVSNIPHAFAIQSFVGELAAKAGQDPKDYLLKLLGPARRIDTAE
LGDSWNYGESPERYPLDVGRLRGVIEEAARQSGWGGELPRGRARGIAAHYSFVTYVAVVIEVEVKDDGALLVH

KATIAADCGPQINPERIRSQLEGACVMGLGLAALGEISFKDGKVQQDNFHQYELARMPLAPKAVSVHLLKPDG
DLPLGGVGEPGVPPIAPALCNAIFAATGKRIRELPIRNQLQGWRKA
>SEQF7741||SEQF7741.1_03491
MKRSYPDDLVIGNLSRRGFLKGVGATGVLLVAANWGWRDALAAEKKAFGADAMPHGWVDNPKIYVSIDRDG
TVGIVCNRSEMGQGVRTSLAMVVADELEADWSRVKVIQAPGDEARYGNQDTDGSRSMRHWFEPMRRCGA
AARQMLEQAAANQWKVPLGECRAEQNKVLHAPSGRSLSFGELAEAAAGLEVPARDKLLLKKPEQFRYIGKDV
ARAIDGADIVNGRAGFGFDARFDDMLYAVVARPPVYGGKLKRYDAAAALKVPGVVKVIEIEGRPIPSEFQPLGG
VAVVAQNTWAAIKGREALVVEWDAGVNGGYDSVAYRKQLEEAARKPGKVVRDSGDAAALFAKGGDIVEAEYY
LPHLAQAPMEPPVSTAWYKDGACEVWAPTQAPQVTRERIAERLKLPFDKVTVNVTLLGGGFGRKSKPDFVLEA
AILAKAFPGRHLRVQWTREDDLHFSYFHTVSVERLQAVLGADGLPQAWLHRSVAPSITALFGPDSKHQGAFEL
GMGLTNLPFAIPNVRLENPEAPAHTRVGWFRSVSNIPHAFAIQSFVGELAAKAGQDPKDYLLKLLGPARRIDTAE
LGDSWNYGESPERYPLDVGRLRGVIEEAARQSGWGGELPRGRARGIAAHYSFVTYVAVVIEVEVKDDGALLVH
KATIAADCGPQINPERIRSQLEGACVMGLGLAALGEISFKDGKVQQDNFHQYELARMPLAPKAVSVHLLKPDG
DLPLGGVGEPGVPPIAPALCNAIFAATGKRIRELPIRNQLQGWRKA
>SEQF7741||SEQF7741.1_02825
MNSKIDLSNALPGSRRGFLKGAAVVGLTIGFQWSGARRALAAALPDAGFAPNAFLRIAPDDSVTVIAKHVEMG
QGAYTGIATIVAEELDADWSKVRVESAPADAKRYANLAFGTMQGTGGSSAMANSWMQLREAGAKARAMLV
EAAARQWRVPATELRTRDGFVEHPASQRKASYGSLAAAAAELPVPEKVQLKDPKDFRLIGHQAPRVDVPGKTD
GSAQFTLDVSLPGMLVALLQRPPLFGATVKSFDATATRAIPGVVEVVQVPHGVAVVAKGFWAAKQGRDALKVE
WDESKAEKRGSEALMAEYRKLAEQPGKPARRDGDAAKAVAGATRRIAASYEFPFLAHAPMEPLDAVVRLTADS
CEIWAGDQFQTVDQGNAARTAGLKPEQVKINTLYAGGSFGRRANAWSDYIVEAVSIAKALGANGVPVKLQWT
REDDIHGGFYRPMYYHRLEAGLDADGKLVGWQHRIVGQSILEGTPFAAVMVKDGIDATSVEGAANLPYAVPN
VSVELSTTQVGVPVLWWRVVGSSHTVYAVEAFIDEAAQAAGKDPYLFRRDLLAEQPRLRGVLELAAEKAGWD
PSRPLPAGRGRGIAVTEAFKTFVAQVVEVSVDKDGKLKVERVVCAVDCGIPINPDVIAAQMEGGIGFGLGAILHS
AITLKDGKVEQNNFDGYQVLRIAEMPKVEVHIVPSGEAPTGVGEPGVAPIGPALANAIFAATGQRLYNLPFPTSF
AKA
>SEQF7742||SEQF7742.1_01685
MKRSYPDDLVIGNLSRRGFLKGVGATGVLLVAANWGWRDALAAEKKAFGADAMPHGWVDNPKIYVSIDRDG
TVGIVCNRSEMGQGVRTSLAMVVADELEADWSRVKVIQAPGDEARYGNQDTDGSRSMRHWFEPMRRCGA
AARQMLEQAAANQWKVPLGECRAEQNKVLHAPSGRSLSFGELAEAAAGLEVPARDKLLLKKPEQFRYIGKDV
ARAIDGADIVNGRAGFGFDARFDDMLYAVVARPPVYGGKLKRYDAAAALKVPGVVKVIEIEGRPIPSEFQPLGG
VAVVAQNTWAAIKGREALVVEWDAGVNGGYDSVAYRKQLEEAARKPGKVVRDSGDAAALFAKGGDIVEAEYY
LPHLAQAPMEPPVSTAWYKDGACEVWAPTQAPQVTRERIAERLKLPFDKVTVNVTLLGGGFGRKSKPDFVLEA
AILAKAFPGRHLRVQWTREDDLHFSYFHTVSVERLQAVLGADGLPQAWLHRSVAPSITALFGPDSKHQGAFEL
GMGLTNLPFAIPNVRLENPEAPAHTRVGWFRSVSNIPHAFAIQSFVGELAAKAGQDPKDYLLKLLGPARRIDTAE
LGDSWNYGESPERYPLDVGRLRGVIEEAARQSGWGGELPRGRARGIAAHYSFVTYVAVVIEVEVKDDGALLVH
KATIAADCGPQINPERIRSQLEGACVMGLGLAALGEISFKDGKVQQDNFHQYELARMPLAPKAVSVHLLKPDG
DLPLGGVGEPGVPPIAPALCNAIFAATGKRIRELPIRNQLQGWRKA
>SEQF7742||SEQF7742.1_02198
MNSKIDLSNALPGSRRGFLKGAAVVGLTIGFQWSGARRALAAALPDAGFAPNAFLRIAPDDSVTVIAKHVEMG
QGAYTGIATIVAEELDADWSKVRVESAPADAKRYANLAFGTMQGTGGSSAMANSWMQLREAGAKARAMLV
EAAARQWQVPAAELRTRDGFVEHPTSQRKASYGSLAAAAAELPVPEKVQLKDPKDFRLIGHQAPRVDVPGKT
DGSAQFTLDVSLPGMLVALLQRPPLFGATVKSFDATATRAIPGVVEVVQVPHGVAVVAKGFWAAKQGRDALKV
EWDESKAEKRGSEALMAEYRKLAEQPGKPARRDGDAAKAVAGATRRIAASYEFPFLAHAPMEPLDAVVRLTAD

SCEIWAGDQFQTVDQGNAARTAGLKPEQVKINTLYAGGSFGRRANAWSDYIVEAVSIAKALGANGVPVKLQW
TREDDIHGGFYRPMYYHRLEAGLDADGKLVGWQHRIVGQSILEGTPFAAVMVKDGIDATSVEGAANLPYAVP
NVSVELSTTQVGVPVLWWRVVGSSHTVYAVEAFIDEAAQAAGKDPYLFRRDLLAEQPRLRGVLELAAEKAGW
DPSRPLPAGRGRGIAVTEAFKTFVAQVVEVSVDKDGKLKVERVVCAVDCGIPINPDVIAAQMEGGIGFGLGAIL
HSAITLKDGKVEQNNFDGYQVLRIAEMPKVEVHIVPSGEAPTGVGEPGVAPIGPALANAIFAATGQRLYNLPFPT
SFAKA
>SEQF7743||SEQF7743.1_03240
MNSKIDLSNALPGSRRGFLKGAAVVGLTIGFQWSGARRALAAALPDAGFAPNAFLRIAPDDSVTVIAKHVEMG
QGAYTGIATIVAEELDADWSKVRVESAPADAKRYANLAFGTMQGTGGSSAMANSWMQLREAGAKARAMLV
EAAARQWRVPATELRTRDGFVEHPASQRKASYGSLAAAAAELPVPEKVQLKDPKDFRLIGHQAPRVDVPGKTD
GSAQFTLDVSLPGMLVALLQRPPLFGATVKSFDATATRAIPGVVEVVQVPHGVAVVAKGFWAAKQGRDALKVE
WDESKAEKRGSEALMAEYRKLAEQPGKPARRDGDAAKAVAGATRRIAASYEFPFLAHAPMEPLDAVVRLTADS
CEIWAGDQFQTVDQGNAARTAGLKPEQVKINTLYAGGSFGRRANAWSDYIVEAVSIAKALGANGVPVKLQWT
REDDIHGGFYRPMYYHRLEAGLDADGKLVGWQHRIVGQSILEGTPFAAVMVKDGIDATSVEGAANLPYAVPN
VSVELSTTQVGVPVLWWRVVGSSHTVYAVEAFIDEAAQAAGKDPYLFRRDLLAEQPRLRGVLELAAEKAGWD
PSRPLPAGRGRGIAVTEAFKTFVAQVVEVSVDKDGKLKVERVVCAVDCGIPINPDVIAAQMEGGIGFGLGAILHS
AITLKDGKVEQNNFDGYQVLRIAEMPKVEVHIVPSGEAPTGVGEPGVAPIGPALANAIFAATGQRLYNLPFPTSF
AKA
>SEQF7743||SEQF7743.1_02735
MKRSYPDDLVIGNLSRRGFLKGVGATGVLLVAANWGWRDALAAEKKAFGADAMPHGWVDNPKIYVSIDRDG
TVGIVCNRSEMGQGVRTSLAMVVADELEADWSRVKVIQAPGDEARYGNQDTDGSRSMRHWFEPMRRCGA
AARQMLEQAAANQWKVPLGECRAEQNKVLHAPSGRSLSFGELAEAAAGLEVPARDKLLLKKPEQFRYIGKDV
ARAIDGADIVNGRAGFGFDARFDDMLYAVVARPPVYGGKLKRYDAAAALKVPGVVKVIEIEGRPIPSEFQPLGG
VAVVAQNTWAAIKGREALVVEWDAGVNGGYDSVAYRKQLEEAARKPGKVVRDSGDAAALFARGGDIVEAEYY
LPHLAQAPMEPPVSTAWYKDGACEVWAPTQAPQVTRERIAERLKLPFDKVTVNVTLLGGGFGRKSKPDFVLEA
AILAKAFPGRHLRVQWTREDDLHFSYFHTVSVERLQAVLGADGLPQAWLHRSVAPSITALFGPDSKHQGAFEL
GMGLTNLPFAIPNVRLENPEAPAHTRVGWFRSVSNIPHAFAIQSFVGELAAKAGQDPKDYLLKLLGPARRIDTAE
LGDSWNYGESPERYPLDVGRLRGVIEEAARQSGWGGELPRGRARGIAAHYSFVTYVAVVIEVEVKDDGALLVH
KATIAADCGPQINPERIRSQLEGACVMGLGLAALGEISFKDGKVQQDNFHQYELARMPLAPKAVSVHLLKPDG
DLPLGGVGEPGVPPIAPALCNAIFAATGKRIRELPIRNQLQGWRKA
>SEQF7744||SEQF7744.1_02779
MKRSYPDDLVIGNLSRRGFLKGVGATGVLLVAANWGWRDALAAEKKAFGADAMPHGWVDNPKIYVSIDRDG
TVGIVCNRSEMGQGVRTSLAMVVADELEADWSRVKVIQAPGDEARYGNQDTDGSRSMRHWFEPMRRCGA
AARQMLEQAAANQWKVPLGECRAEQNKVLHAPSGRSLSFGELAEAAAGLEVPARDKLLLKKPEQFRYIGKDV
ARAIDGADIVNGRAGFGFDARFDDMLYAVVARPPVYGGKLKRYDAAAALKVPGVVKVIEIEGRPIPSEFQPLGG
VAVVAQNTWAAIKGREALVVEWDAGVNGGYDSVAYRKQLEEAARKPGKVVRDSGDAAALFARGGDIVEAEYY
LPHLAQAPMEPPVSTAWYKDGACEVWAPTQAPQVTRERIAERLKLPFDKVTVNVTLLGGGFGRKSKPDFVLEA
AILAKAFPGRHLRVQWTREDDLHFSYFHTVSVERMQAVLGADELPQAWLHRSVAPSITALFGPDSKHQGAFEL
GMGLTNLPFAIPNVRLENPEAPAHTRVGWFRSVSNIPHAFAIQSFVGELAAKAGQDPKDYLLKLLGPARRIDTAE
LGDSWNYGESPERYPLDVGRLRGVIEEAARQSGWGGELPRGRARGIAAHYSFVTYVAVVIEVEVKDDGALLVH
KATIAADCGPQINPERIRSQLEGACVMGLGLAALGEISFKDGKVQQDNFHQYELARMPLAPKAVSVHLLKPDG
DLPLGGVGEPGVPPIAPALCNAIFAATGKRIRELPIRNQLQGWRKA
>SEQF7744||SEQF7744.1_02244
MNSKIDLSNALPGSRRGFLKGAAVVGLTIGFQWSGARRALAAALPDAGFAPNAFLRIAPDDSVTVIAKHVEMG

QGAYTGIATIVAEELDADWSKVRVESAPADAKRYANLAFGTMQGTGGSSAMANSWMQLREAGAKARAMLV
EAAARQWRVPATELRTRDGFVEHPASQRKASYGSLAAAAAELPVPEKVQLKDPKDFRLIGHQAPRVDVPGKTD
GSAQFTLDVSLPGMLVALLQRPPLFGATVKSFDATATRAIPGVVEVVQVPHGVAVVAKGFWAAKQGRDALKVE
WDESKAEKRGSEALMAEYRKLAEQPGKPARRDGNAAKAVAGATRRIAASYEFPFLAHAPMEPLDAVVRLTADS
CEIWAGDQFQTVDQGNAARTAGLKPEQVKINTLYAGGSFGRRANAWSDYIVEAVSIAKALGANGVPVKLQWT
REDDIHGGFYRPMYYHRLEAGLDADGKLVGWQHRIVGQSILEGTPFAAVMVKDGIDATSVEGAANLPYAVPN
VSVELSTTQVGVPVLWWRVVGSSHTVYAVEAFIDEAAQAAGKDPYLFRRDLLAEQPRLRGVLELAAEKAGWD
PSRPLPAGRGRGIAVTEAFKTFVAQVVEVSVDKDGKLKVERVVCAVDCGIPINPDVIAAQMEGGIGFGLGAILHS
AITLKDGKVEQNNFDGYQVLRIAEMPKVEVHIVPSGEAPTGVGEPGVAPIGPALANAIFAATGQRLYNLPFPTSF
AKA
>SEQF7745||SEQF7745.1_02742
MKRSYPDDLVIGNLSRRGFLKGVGATGVLLVAANWGWRDALAAEKKAFGADAMPHGWVDNPKIYVSIDRDG
TVGIVCNRSEMGQGVRTSLAMVVADELEADWSRVKVIQAPGDEARYGNQDTDGSRSMRHWFEPMRRCGA
AARQMLEQAAANQWKVPLGECRAEQNKVLHAPSGRSLSFGELAEAAAGLEVPARDKLLLKKPEQFRYIGKDV
ARAIDGADIVNGRAGFGFDARFDDMLYAVVARPPVYGGKLKRYDAAAALKVPGVVKVIEIEGRPIPSEFQPLGG
VAVVAQNTWAAIKGREALAVEWDAGVNGGYDSVAYRKQLEEAARKPGKVVRDSGDAAALFARGGDIVEAEYY
LPHLAQAPMEPPVSTAWYKDGACEVWAPTQAPQVTRERIAERLKLPFDKVTVNVTLLGGGFGRKSKPDFVLEA
AILAKAFPGRHLRVQWTREDDLHFSYFHTVSVERLQAVLGADGLPQAWLHRSVAPSITALFGPDSKHQGAFEL
GMGLTNLPFAIPNVRLENPEAPAHTRVGWFRSVSNIPHAFAIQSFVGELAAKAGQDPKDYLLKLLGPARRIDTAE
LGDSWNYGESPERYPLDVGRLRGVIEEAARQSGWGGELPRGRARGIAAHYSFVTYVAVVIEVEVKDDGALLVH
KATIAADCGPQINPERIRSQLEGACVMGLGLAALGEISFKDGKVQQDNFHQYELARMPLAPKAVSVHLLKPDG
DLPLGGVGEPGVPPIAPALCNAIFAATGKRIRELPIRNQLQGWRKA
>SEQF7745||SEQF7745.1_03249
MNSKIDLSNALPGSRRGFLKGAAVVGLTIGFQWSGARRALAAALPDAGFAPNAFLRIAPDDSVTVIAKHVEMG
QGAYTGIATIVAEELDADWSKVRVESAPADAKRYANLAFGTMQGTGGSSAMANSWMQLREAGAKARAMLV
EAAARQWRVPATELRTRDGFVEHPASQRKASYGSLAAAAAELPVPENVQLKDPKDFRLIGHQAPRVDVPGKT
DGSAQFTLDVSLPGMLVALLQRPPLFGATVKSFDATATRAIPGVVEVVQVPHGVAVVAKGFWAAKQGRDALKV
EWDESKAEKRGSEALMAEYRKLAEQPGKPARRDGDAAKAVAGATRRIAASYEFPFLAHAPMEPLDAVVRLTAD
SCEIWAGDQFQTVDQGNAARTAGLKPEQVKINTLYAGGSFGRRANAWSDYIVEAVSIAKALGANGVPVKLQW
TREDDIHGGFYRPMYYHRLEAGLDADGKLVGWQHRIVGQSILEGTPFAAVMVKDGIDATSVEGAANLPYAVP
NVSVELSTTQVGVPVLWWRVVGSSHTVYAVEAFIDEAAQAAGKDPYLFRRDLLAEQPRLRGVLELAAEKAGW
DPSRPLPAGRGRGIAVTEAFKTFVAQVVEVSVDKDGKLKVERVVCAVDCGIPINPDVIAAQMEGGIGFGLGAIL
HSAITLKDGKVEQNNFDGYQVLRIAEMPKVEVHIVPSGEAPTGVGEPGVAPIGPALANAIFAATGQRLYNLPFPT
SFAKA
>SEQF7746||SEQF7746.1_03240
MNSKIDLSNALPGSRRGFLKGAAVVGLSIGFQWSGARRALAAALPESGFAPNAFLRIAPDDSVTVIAKHVEMG
QGAYTGIATIVAEELDADWNKVRVESAPADAKRYANLAFGTMQGTGGSSAMANSWMQLREAGAKARAMLV
EAAARQWRVPAAELRTRDGFVEHPASQRKASYGSLAAAAAELPVPEKVQLKDPKDFRLIGHQAPRVDVPGKT
DGSAQFTLDVSLPGMLVALLQRPPLFGATVKAFDATAARAIPGVVEVVQVPHGVAVVAKGFWAAKQGRDALK
VEWDESKAEKRGSEALMAEYRKLAEQPGKPVRRDGDAAGALAGAAKKVAASYEFPFLAHAPMEPLDAVVRLT
ADSCEIWAGDQFQTVDQGNAARTAGLKPEQVKINTLYAGGSFGRRANAWSDYIVEAVSIAKALGANGVPVKL
QWTREDDIHGGFYRPMYYHKLEAGLDADGKLVGWQHRIVGQSILEGTPFAAMMVKDGIDGTSVEGAANLPY
AVPNVSVELSTTQVGVPVLWWRVVGSSHTVYAVEAFIDEAAQAAGKDPYLFRRDLLAEQPRLRGVLELAAEKA
GWDPAKPLPAGRGRGIAVTEAFKTFVAQVVEVSVDKDGKLKVERVVCAVDCGIPINPDVIAAQMEGGIGFGLG

AVLHSAITLKDGKVEQNNFDGYQVLRIAEMPRVEVHIVPSGEAPTGVGEPGVAPIGPALANAIFAATGQRLYHLP
FPTTFAKA
>SEQF7746||SEQF7746.1_02812
MKRSTLDDLVIGNLSRRGFLKGVGATGVLLVAANWGWRDALAAEQKAFGADAMPHGWVDNPKIYVSIDKDG
TVGIVCNRSEMGQGVRTSLAMVVADELEADWSRVRVIQAPGDEARYGNQDTDGSRSMRHWFEPMRRCGA
AARQMLEQAAANQWKVPLDECRAEQNRVLHAPSGRSLSFGELAEAAAGLDVPARDKLVLKKPEQFRYIGKDV
ARAIDGADIVNGRAGFGFDARFDDMLYAVVARPPVYGGKLKRYDAAAALKVPGVLKVIEIESRPIPSEFQPLGGV
AVVARNTWAAIKGREALVLEWDAGVNGGYDSAAYRKQLEEAARKPGKVVRDSGDAAALFARGGDIVEAEYYL
PHLAQAPMEPPVSTAWYKDGACEVWAPTQAPQVTRERIAERLELPFDKVTVNVTLLGGGFGRKSKPDYVLEA
AILAKEFPGRHLRVQWTREDDLHFSYFHTVSVERLQAVLGADGLPQAWLHRSVAPSITALFGPDSKHQGAFELG
MGLTNLPFAIPNVRLENPEAPAHTRVGWFRSVSNIPHAFAIQSFVGELAAKAGQDPKDYLLKLLGPARRIDTAEL
GDSWNYGESPQRYPLDVGRLRGVIEEAARQSGWGGELPRGRARGIAAHYSFVTYVAVVIEVEVKDDGALLVHK
ATIAADCGPQINPERIRSQLEGACVMGLGLAALGEISFKDGKVQQDNFHQYELARMPLAPKAVSVHLLKPDGD
LPLGGVGEPGVPPIAPALCNAIFAATGKRIRELPIRNQLQGWRKA
>SEQF7747||SEQF7747.1_02730
MKRSYPDDLVIGNLSRRGFLKGVGATGVLLVAANWGWRDALAAEKKAFGADAMPHGWVDNPKIYVSIDRDG
TVGIVCNRSEMGQGVRTSLAMVVADELEADWSRVKVIQAPGDEARYGNQDTDGSRSMRHWFEPMRRCGA
AARQMLEQAAANQWKVPLGECRAEQNKVLHAPSGRSLSFGELAEAAAGLEVPARDKLLLKKPEQFRYIGKDV
ARAIDGADIVNGRAGFGFDARFDDMLYAVVARPPLYGGKLKRYDAAAALKVPGVVKVIEIEGRPIPSEFQPLGG
VAVVAQNTWAAIKGREALVVEWDAGVNGGYDSVAYRKQLEEAARKPGKVVRDSGDAAALFAKGGDIVEAEYY
LPHLAQAPMEPPVSTAWYKDGACEVWAPTQAPQVTRERIAERLKLPFDKVTVNVTLLGGGFGRKSKPDFVLEA
AILAKAFPGRHLRVQWTREDDLHFSYFHTVSVERLQAVLGADGLPQAWLHRSVAPSITALFGPDSKHQGAFEL
GMGLTNLPFAIPNVRLENPEAPAHTRVGWFRSVSNIPHAFAIQSFVGELAAKAGQDPKDYLLKLLGPARRIDTAE
LGDSWNYGESPERYPLDVGRLRGVIEEAARQSGWGGELPRGRARGIAAHYSFVTYVAVVIEVEVKDDGALLVH
KATIAADCGPQINPERIRSQLEGACVMGLGLAALGEISFKDGKVQQDNFHQYELARMPLAPKAVSVHLLKPDG
DLPLGGVGEPGVPPIAPALCNAIFAATGKRIRELPIRNQLQGWRKA
>SEQF7747||SEQF7747.1_03261
MNSKIDLSNALPGSRRGFLKGAAVVGLTIGFQWSGARRALAAALPDAGFAPNAFLRIAPDDSVTVIAKHVEMG
QGAYTGIATIVAEELDADWSKVRVESAPADAKRYANLAFGTMQGTGGSSAMANSWMQLREAGAKARAMLV
EAAARQWRVPATELRTRDGFVEHPASQRKASYGSLAAAAAELPVPENVQLKDPKDFRLIGHQAPRVDVPGKT
DGSAQFTLDVSLPGMLVALLQRPPLFGATVKSFDATATRAIPGVVEVVQVPHGVAVVAKGFWAAKQGRDALKV
EWDESKAEKRGSEALMAEYRKLAEQPGKPARRDGDAAKAVAGATRRIAASYEFPFLAHAPMEPLDAVVRLTAD
SCEIWAGDQFQTVDQGNAARTAGLKPEQVKINTLYAGGSFGRRANAWSDYIVEAVSIAKALGANGVPVKLQW
TREDDIHGGFYRPMYYHRLEAGLDADGKLVGWQHRIVGQSILEGTPFAAVMVKDGIDATSVEGAANLPYAVP
NVSVELSTTQVGVPVLWWRVVGSSHTVYAVEAFIDEAAQAAGKDPYLFRRDLLAEQPRLRGVLELAAEKAGW
DPSRPLPAGRGRGIAVTEAFKTFVAQVVEVSVDKDGKLKVERVVCAVDCGIPINPDVIAAQMEGGIGFGLGAIL
HSAITLKDGKVEQNNFDGYQVLRIAEMPKVEVHIVPSGEAPTGVGEPGVAPIGPALANAIFAATGQRLYNLPFPT
SFAKA
>SEQF7748||SEQF7748.1_03977
MKRSYPDDLVIGNLSRRGFLKGVGATGVLLVAANWGWRDALAAEKKAFGADAMPHGWVDNPKIYVSIDRDG
TVGIVCNRSEMGQGVRTSLAMVVADELEADWSRVKVIQAPGDEARYGNQDTDGSRSMRHWFEPMRRCGA
AARQMLEQAAANQWKVPLGECRAEQNKVLHAPSGRSLSFGELAEAAAGLEVPARDKLLLKKPEQFRYIGKDV
ARAIDGADIVNGRAGFGFDARFDDMLYAVVARPPVYGGKLKRYDAAAALKVPGVVKVIEIEGRPIPSEFQPLGG
VAVVAQNTWAAIKGREALVVEWDAGVNGGYDSVAYRKQLEEAARKPGKVVRDSGDAAALFARGGDIVEAEYY

LPHLAQAPMEPPVSTAWYKDGACEVWAPTQAPQVTRERIAERLKLPFDKVTVNVTLLGGGFGRKSKPDFVLEA
AILAKAFPGRHLRVQWTREDDLHFSYFHTVSVERLQAVLGADGLPQAWLHRSVAPSITALFGPDSKHQGAFEL
GMGLTNLPFAIPNVRLENPEAPAHTRVGWFRSVSNIPHAFAIQSFVGELAAKAGQDPKDYLLKLLGPARRIDTAE
LGDSWNYGESPERYPLDVGRLRGVIEEAARQSGWGGELPRGRARGIAAHYSFVTYVAVVIEVEVKDDGALLVH
KATIAADCGPQINPERIRSQLEGACVMGLGLAALGEISFKDGKVQQDNFHQYELARMPLAPKAVSVHLLKPDG
DLPLGGVGEPGVPPIAPALCNAIFAATGKRIRELPIRNQLQGWRKA
>SEQF7748||SEQF7748.1_03415
MNSKIDLSNALPGSRRGFLKGAAVVGLTIGFQWSGARRALAAALPDAGFAPNAFLRIAPDDSVTVIAKHVEMG
QGAYTGIATIVAEELDADWSKVRVESAPADAKRYANLAFGTMQGTGGSSAMANSWMQLREAGAKARAMLV
EAAARQWRVPATELRTRDGFVEHPASQRKASYGSLAAAAAELPVPEKVQLKDPKDFRLIGHQAPRVDVPGKTD
GSAQFTLDVSLPGMLVALLQRPPLFGATVKSFDATATRAIPGVLEVVQVPHGVAVVAKGFWAAKQGRDALKVE
WDESKAEKRGSEALMAEYRKLAEQPGKPARRDGDAAKAVAGATRRIAASYEFPFLAHAPMEPLDAVVRLTADS
CEIWAGDQFQTVDQGNAARTAGLKPEQVKINTLYAGGSFGRRANAWSDYIVEAVSIAKALGANGVPVKLQWT
REDDIHGGFYRPMYYHRLEAGLDADGKLVGWQHRIVGQSILEGTPFAAVMVKDGVDATSVEGAANLPYAVPN
VSVELSTTQVGVPVLWWRVVGSSHTVYAVEAFIDEAAQAAGKDPYLFRRDLLAEQPRLRGVLELAAEKAGWD
PSRPLPAGRGRGIAVTEAFKTFVAQVVEVSVDKDGKLKVERVVCAVDCGIPINPDVIAAQMEGGIGFGLGAILHS
AITLKDGKVEQNNFDGYQVLRIAEMPKVEVHIVPSGEAPTGVGEPGVAPIGPALANAIFAATGQRLYNLPFPTSF
AKA
>SEQF7749||SEQF7749.1_05928
MNSKIDLSNALPGSRRGFLKGAAVVGLTIGFQWSGARRALAAALPDAGFAPNAFLRIAPDDSVTVIAKHVEMG
QGAYTGIATIVAEELDADWSKVRVESAPADAKRYANLAFGTMQGTGGSSAMANSWMQLREAGAKARAMLV
EAAARQWQVPAAELRTRDGFVEHPASQRKASYGSLAAAAAELPVPENVQLKDPKDFRLIGHQAPRVDVPGKT
DGSAQFTLDVSLPGMLVALLQRPPLFGATVKSFDATATRAIPGVVEVVQVPHGVAVVAKGFWAAKQGRDALKV
EWDESKAEKRGSEALMAEYRKLAEQPGKPARRDGDAAKAVAGATRRIAASYEFPFLAHAPMEPLDAVVRLTAD
SCEIWAGDQFQTVDQGNAARTAGLKPEQVKINTLYAGGSFGRRANAWSDYIVEAVSIAKALGANGVPVKLQW
TREDDIHGGFYRPMYYHRLEAGLDADGKLVGWQHRIVGQSILEGTPFAAVMVKDGVDATSVEGAANLPYAVP
NVSVELSTTQVGVPVLWWRVVGSSHTVYAVEAFIDEAAQAAGKDPYLFRRDLLAEQPRLRGVLELAAEKAGW
DPSRPLPAGRGRGIAVTEAFKTFVAQVVEVSVDKDGKLKVERVVCAVDCGIPINPDVIAAQMEGGIGFGLGAIL
HSAITLKDGKVEQNNFDGYQVLRIAEMPKVEVHIVPSGEAPTGVGEPGVAPIGPALANAIFAATGQRLYNLPFPT
SFAKA
>SEQF7749||SEQF7749.1_05375
MKRSFPDDLIIGNLSRRGFLKGVGATGVLLVAANWGWRDALAAEKKAFGADAMPHGWVDNPKIYVSIDRDG
TVGIVCNRSEMGQGVRTSLAMVVADELEADWSRVKVIQAPGDEARYGNQDTDGSRSMRHWFEPMRRCGA
AARQMLEQAAANQWKVPLGECRAEQNKVLHAPSGRSLSFGELAEAAAGLEVPARDKLLLKKPEQFRYIGKDV
ARAIDGADIVNGRAGFGFDARFDDMLYAVVARPPVYGGKLKRYDAAAALKVPGVVKVIEIEGRPIPSEFQPLGG
VAVVAQNTWAAIKGREALVVEWDAGVNGGYDSVAYRKQLEEAARKPGKVVRDSGDAAALFAKGGDVVEAEY
YLPHLAQAPMEPPVSTAWYKDGACEVWAPTQAPQVTRERIAERLKLPFDKVTVNVTLLGGGFGRKSKPDFVLE
AAILAKAFPGRYLRVQWTREDDLHFSYFHTVSVERLQAVLGADGLPQAWLHRSVAPSITALFGPDSKHQGAFEL
GMGLTNLPFAIPNVRLENPEAPAHTRVGWFRSVSNIPHAFAIQSFVGELAAKAGQDPKDYLLKLLGPARRIDTAE
LGDSWNYGESPERYPLDVGRLRGVIEEAARQSGWGGELPRGRARGIAAHYSFVTYVAVVIEVEVKDDGALLVH
KATIAADCGPQINPERIRSQLEGACVMGLGLAALGEISFKDGKVQQDNFHQYELARMPLAPKAVSVHLLKPDG
DLPLGGVGEPGVPPIAPALCNAIFAATGKRIRELPIRNQLQGWRKA
>SEQF7750||SEQF7750.1_03493
MNSKIDLSNALPGSRRGFLKGAAVVGLTIGFQWSGARRALAAALPDAGFAPNAFLRIAPDDSVTVIAKHVEMG

QGAYTGIATIVAEELDADWSKVRVESAPADAKRYANLAFGTMQGTGGSSAMANSWMQLREAGAKARAMLV
EAAARQWRVPATELRTRDGFVEHPASQRKASYGSLAAAAAELPVPEKVQLKDPKDFRLIGHQAPRVDVPGKTD
GSAQFTLDVSLPGMLVALLQRPPLFGATVKSFDATATRAIPGVVEVVQVPHGVAVVAKGFWAAKQGRDALKVE
WDESKAEKRGSEALMAEYRKLAEQPGKPARRDGDAAKAVAGATRRIAASYEFPFLAHAPMEPLDAVVRLTADS
CEIWAGDQFQTVDQGNAARTAGLKPEQVKINTLYAGGSFGRRANAWSDYIVEAVSIAKALGANGVPVKLQWT
REDDIHGGFYRPMYYHRLEAGLDADGKLVGWQHRIVGQSILEGTPFAAVMVKDGVDATSVEGAANLPYAVPN
VSVELSTTQVGVPVLWWRVVGSSHTVYAVEAFIDEAAQAAGKDPYLFRRDLLAEQPRLRGVLELAAEKAGWD
PSRPLPAGRGRGIAVTEAFKTFVAQVVEVSVDKDGKLKVERVVCAVDCGIPINPDVIAAQMEGGIGFGLGAILHS
AITLKDGKVEQNNFDGYQVLRIAEMPKVEVHIVPSGEAPTGVGEPGVAPIGPALANAIFAATGQRLYNLPFPTSF
AKA
>SEQF7750||SEQF7750.1_02903
MKRSYPDDLVIGNLSRRGFLKGVGATGVLLVAANWGWRDALAAEKKAFGADAMPHGWVDNPKIYVSIDRDG
TVGIVCNRSEMGQGVRTSLAMVVADELEADWSRVKVIQAPGDEARYGNQDTDGSRSMRHWFEPMRRCGA
AARQMLEQAAANQWKVPLGECRAEQNKVLHAPSGRSLSFGELAEAAAGLEVPARDKLLLKKPEQFRYIGKDV
ARAIDGADIVNGRAGFGFDARFDDMLYAVVARPPLYGGKLKRYDAAAALKVPGVVKVIEIEGRPIPSEFQPLGG
VAVVAQNTWAAIKGREALVVEWDAGVNGGYDSVAYRKQLEEAARKPGKVVRDSGDAAALFAKGGDIVEAEYY
LPHLAQAPMEPPVSTAWYKDGACEVWAPTQAPQVTRERIAERLKLPFDKVTVNVTLLGGGFGRKSKPDFVLEA
AILAKAFPGRHLRVQWTREDDLHFSYFHTVSVERLQAVLGADGLPQAWLHRSVAPSITALFGPDSKHQGAFEL
GMGLTNLPFAIPNVRLENPEAPAHTRVGWFRSVSNIPHAFAIQSFVGELAAKAGQDPKDYLLKLLGPARRIDTAE
LGDSWNYGESPERYPLDVGRLRGVIEEAARQSGWGGELPRGRARGIAAHYSFVTYVAVVIEVEVKDDGALLVH
KATIAADCGPQINPERIRSQLEGACVMGLGLAALGEISFKDGKVQQDNFHQYELARMPLAPKAVSVHLLKPDG
DLPLGGVGEPGVPPIAPALCNAIFAATGKRIRELPIRNQLQGWRKA
>SEQF7751||SEQF7751.1_03214
MNSKIDLSNALPGSRRGFLKGAAVVGLTIGFQWSGARRALAAALPDAGFAPNAFLRIAPDDSVTVIAKHVEMG
QGAYTGIATIVAEELDADWSKVRVESAPADAKRYANLAFGTMQGTGGSSAMANSWMQLREAGAKARAMLV
EAAARQWRVPATELRTRDGFVEHPASQRKASYGSLAAAAAELPVPEKVQLKDPKDFRLIGHQAPRVDVPGKTD
GSAQFTLDVSLPGMLVALLQRPPLFGATVKSFDATATRAIPGVVEVVQVPHGVAVVAKGFWAAKQGRDALKVE
WDESKAEKRGSEALMAEYRKLAEQPGKPARRDGDAAKAVAGATRRIAASYEFPFLAHAPMEPLDAVVRLTADS
CEIWAGDQFQTVDQGNAARTAGLKPEQVKINTLYAGGSFGRRANAWSDYIVEAVSIAKALGANGVPVKLQWT
REDDIHGGFYRPMYYHRLEAGLDADGKLVGWQHRIVGQSILEGTPFAAVMVKNGIDATSVEGAANLPYAVPN
VSVELSTTQVGVPVLWWRVVGSSHTVYAVEAFIDEAAQAAGKDPYLFRHDLLAEQPRLRGVLELAAEKAGWD
PSRPLPAGRGRGIAVTEAFKTFVAQVVEVSVDKDGKLKVERVVCAVDCGIPINPDVIAAQMEGGIGFGLGAILHS
AITLKDGKVEQNNFDGYQVLRIAEMPKVEVHIVPSGEAPTGVGEPGVAPIGPALANAIFAATGQRLYNLPFPTSF
AKA
>SEQF7751||SEQF7751.1_02817
MKRSYPDDLVIGNLSRRGFLKGVGATGVLLVAANWGWRDALAAEKKAFGADAMPHGWVDNPKIYVSIDRDG
TVGIVCNRSEMGQGVRTSLAMVVADELEADWSRVKVIQAPGDEARYGNQDTDGSRSMRHWFEPMRRCGA
AARQMLEQAAANQWKVPLGECRAEQNKVLHAPSGRSLSFGELAEAAAGLEVPARDKLLLKKPEQFRYIGKDV
ARAIDGADIVNGRAGFGFDARFDDMLYAVVARPPVYGGKLKRYDAAAALKVPGVVKVIEIEGRPIPSEFQPLGG
VAVVAQNTWAAIKGREALVVEWDAGVNGGYDSVAYRKQLEEAARKPGKVVRDSGDAAALFAKGGDIVEAEYY
LPHLAQAPMEPPVSTAWYKDGACEVWAPTQAPQVTRERIAERLKLPFDKVTVNVTLLGGGFGRKSKPDFVLEA
AILAKAFPGRHLRVQWTREDDLHFSYFHTVSVERLQAVLGADGLPQAWLHRSVAPSITALFGPDSKHQGAFEL
GMGLTNLPFAIPNVRLENPEAPAHTRVGWFRSVSNIPHAFAIQSFVGELAAKAGQDPKDYLLKLLGPARRIDTAE
LGDSWNYGESPERYPLDVGRLRGVIEEAARQSGWGGELPRGRARGIAAHYSFVTYVAVVIEVEVKDDGALLVH

KATIAADCGPQINPERIRSQLEGACVMGLGLAALGEISFKDGKVQQDNFHQYELARMPLAPKAVSVHLLKPDG
DLPLGGVGEPGVPPIAPALCNAIFAATGKRIRELPIRNQLQGWRKA
>SEQF7752||SEQF7752.1_03111
MKRSYPDDLVIGNLSRRGFLKGVGATGVLLVAANWGWRDALAAEKKAFGADAMPHGWVDNPKIYVSIDRDG
TVGIVCNRSEMGQGVRTSLAMVVADELEADWSRVKVIQAPGDEARYGNQDTDGSRSMRHWFEPMRRCGA
AARQMLEQAAANQWKVPLGECRAEQNKVLHAPSGRSLSFGELAEAAAGLEVPARDKLLLKKPEQFRYIGKDV
ARAIDGADIVNGRAGFGFDARFDDMLYAVVARPPVYGGKLKRYDAAAALKVPGVVKVIEIEGRPIPSEFQPLGG
VAVVAQNTWAAIKGREALVVEWDAGVNGGYDSVAYRKQLEEAARKPGKVVRDSGDAAALFAKGGDIVEAEYY
LPHLAQAPMEPPVSTAWYKDGACEVWAPTQAPQVTRERIAERLKLPFDKVTVNVTLLGGGFGRKSKPDFVLEA
AILAKAFPGRHLRVQWTREDDLHFSYFHTVSVERLQAVLGADGLPQAWLHRSVAPSITALFGPDSKHQGAFEL
GMGLTNLPFAIPNVRLENPEAPAHTRVGWFRSVSNIPHAFAIQSFVGELAAKAGQDPKDYLLKLLGPARRIDTAE
LGDSWNYGESPERYPLDVGRLRGVIEEAARQSGWGGELPRGRARGIAAHYSFVTYVAVVIEVEVKDDGALLVH
KATIAADCGPQINPERIRSQLEGACVMGLGLAALGEISFKDGKVQQDNFHQYELARMPLAPKAVSVHLLKPDG
DLPLGGVGEPGVPPIAPALCNAIFAATGKRIRELPIRNQLQGWRKA
>SEQF7752||SEQF7752.1_03719
MNSKIDLSNALPGSRRGFLKGAAVVGLTIGFQWSGARRALAAALPDAGFAPNAFLRIAPDDSVTVIAKHVEMG
QGAYTGIATIVAEELDADWSKVRVESAPADAKRYANLAFGTMQGTGGSSAMANSWMQLREAGAKARAMLV
EAAARQWRVPATELRTRDGFVEHPASQRKASYGSLAAAAAELPVPEKVQLKDPKDFRLIGHQAPRVDVPGKTD
GSAQFTLDVSLPGMLVALLQRPPLFGATVKSFDATATRAIPGVVEVVQVPHGVAVVAKGFWAAKQGRDALKVE
WDESKAEKRGSEALMAEYRKLAEQPGKPARRDGDAAKAVAGATRRIAASYEFPFLAHAPMEPLDAVVRLTADS
CEIWAGDQFQTVDQGNAARTAGLKPEQVKINTLYAGGSFGRRANAWSDYIVEAVSIAKALGANGVPVKLQWT
REDDIHGGFYRPMYYHRLEAGLDADGKLVGWQHRIVGQSILEGTPFAAVMVKNGIDATSVEGAANLPYAVPN
VSVELSTTQVGVPVLWWRVVGSSHTVYAVEAFIDEAAQAAGKDPYLFRHDLLAEQPRLRGVLELAAEKAGWD
PSRPLPAGRGRGIAVTEAFKTFVAQVVEVSVDKDGKLKVERVVCAVDCGIPINPDVIAAQMEGGIGFGLGAILHS
AITLKDGKVEQNNFDGYQVLRIAEMPKVEVHIVPSGEAPTGVGEPGVAPIGPALANAIFAATGQRLYNLPFPTSF
AKA
>SEQF7753||SEQF7753.1_01696
MKRSYPDDLVIGNLSRRGFLKGVGATGVLLVAANWGWRDALAAEKKAFGADAMPHGWVDNPKIYVSIDRDG
TVGIVCNRSEMGQGVRTSLAMVVADELEADWSRVKVIQAPGDEARYGNQDTDGSRSMRHWFEPMRRCGA
AARQMLEQAAANQWKVPLGECRAEQNKVLHAPSGRSLSFGELAEAAAGLEVPARDKLLLKKPEQFRYIGKDV
ARAIDGADIVNGRAGFGFDARFDDMLYAVVARPPVYGGKLKRYDAAAALKVPGVVKVIEIEGRPIPSEFQPLGG
VAVVAQNTWAAIKGREALVVEWDAGVNGGYDSVAYRKQLEEAARKPGKVVRDSGDAAALFARGGDIVEAEYY
LPHLAQAPMEPPVSTAWYKDGACEVWAPTQAPQVTRERIAERLKLPFDKVTVNVTLLGGGFGRKSKPDFVLEA
AILAKAFPGRHLRVQWTREDDLHFSYFHTVSVERLQAVLGADGLPQAWLHRSVAPSITALFGPDSKHQGAFEL
GMGLTNLPFAIPNVRLENPEAPAHTRVGWFRSVSNIPHAFAIQSFVGELAAKAGQDPKDYLLKLLGPARRIDTAE
LGDSWNYGESPERYPLDVGRLRGVIEEAARQSGWGGELPRGRARGIAAHYSFVTYVAVVIEVEVKDDGALLVH
KATIAADCGPQINPERIRSQLEGACVMGLGLAALGEISFKDGKVQQDNFHQYELARMPLAPKAVSVHLLKPDG
DLPLGGVGEPGVPPIAPALCNAIFAATGKRIRELPIRNQLQGWRKA
>SEQF7753||SEQF7753.1_02368
MNSKIDLSNALPGSRRGFLKGAAVVGLTIGFQWSGARRALAAALPDAGFAPNAFLRIAPDDSVTVIAKHVEMG
QGAYTGIATIVAEELDADWSKVRVESAPADAKRYANLAFGTMQGTGGSSAMANSWMQLREAGAKARAMLV
EAAARQWRVPATELRTRDGFVEHPASQRKASYGSLAAAAAELPVPEKVQLKDPKDFRLIGHQAPRVDVPGKTD
GSAQFTLDVSLPGMLVALLQRPPLFGATVKSFDATATRAIPGVVEVVQVPHGVAVVAKGFWAAKQGRDALKVE
WDESKAEKRGSEALMAEYRKLAEQPGKPARRDGDAAKAVAGATRRIAASYEFPFLAHAPMEPLDAVVRLTADS

CEIWAGDQFQTVDQGNAARTAGLKPEQVKINTLYAGGSFGRRANAWSDYIVEAVSIAKALGANGVPVKLQWT
REDDIHGGFYRPMYYHRLEAGLDADGKLVGWQHRIVGQSILEGTPFAAVMVKDGVDATSVEGAANLPYAVPN
VSVELSTTQVGVPVLWWRVVGSSHTVYAVEAFIDEAAQAAGKDPYLFRRDLLAEQPRLRGVLELAAEKAGWD
PSRPLPAGRGRGIAVTEAFKTFVAQVVEVSVDKDGKLKVERVVCAVDCGIPINPDVIAAQMEGGIGFGLGAILHS
AITLKDGKVEQNNFDGYQVLRIAEMPKVEVHIVPSGEAPTGVGEPGVAPIGPALANAIFAATGQRLYNLPFPTSF
AKA
>SEQF7754||SEQF7754.1_04195
MKRSYPDDLVIGNLSRRGFLKGVGATGVLLVAANWGWRDALAAEKKAFGADAMPHGWVDNPKIYVSIDRDG
TVGIVCNRSEMGQGVRTSLAMVVADELEADWSRVKVIQAPGDEARYGNQDTDGSRSMRHWFEPMRRCGA
AARQMLEQAAANQWKVPLGECRAEQNKVLHAPSGRSLSFGELAEAAAGLEVPARDKLLLKKPEQFRYIGKDV
ARAIDGADIVNGRAGFGFDARFDDMLYAVVARPPLYGGKLKRYDAAAALKVPGVVKVIEIEGRPIPSEFQPLGG
VAVVAQNTWAAIKGREALVVEWDAGVNGGYDSVAYRKQLEEAARKPGKVVRDSGDAAALFAKGGDIVEAEYY
LPHLAQAPMEPPVSTAWYKDGACEVWAPTQAPQVTRERIAERLKLPFDKVTVNVTLLGGGFGRKSKPDFVLEA
AILAKAFPGRHLRVQWTREDDLHFSYFHTVSVERLQAVLGADGLPQAWLHRSVAPSITALFGPDSKHQGAFEL
GMGLTNLPFAIPNVRLENPEAPAHTRVGWFRSVSNIPHAFAIQSFVGELAAKAGQDPKDYLLKLLGPARRIDTAE
LGDSWNYGESPERYPLDVGRLRGVIEEAARQSGWGGELPRGRARGIAAHYSFVTYVAVVIEVEVKDDGALLVH
KATIAADCGPQINPERIRSQLEGACVMGLGLAALGEISFKDGKVQQDNFHQYELARMPLAPKAVSVHLLKPDG
DLPLGGVGEPGVPPIAPALCNAIFAATGKRIRELPIRNQLQGWRKA
>SEQF7754||SEQF7754.1_04742
MNSKIDLSNALPGSRRGFLKGAAVVGLTIGFQWSGARRALAAALPDAGFAPNAFLRIAPDDSVTVIAKHVEMG
QGAYTGIATIVAEELDADWSKVRVESAPADAKRYANLAFGTMQGTGGSSAMANSWMQLREAGAKARAMLV
EAAARQWRVPATELRTRDGFVEHPASQRKASYGSLAAAAAELPVPEKVQLKDPKDFRLIGHQAPRVDVPGKTD
GSAQFTLDVSLPGMLVALLQRPPLFGATVKSFDATATRAIPGVVEVVQVPHGVAVVAKGFWAAKQGRDALKVE
WDESKAEKRGSEALMAEYRKLAEQPGKPARRDGDAAKAVAGATRRIAASYEFPFLAHAPMEPLDAVVRLTADS
CEIWAGDQFQTVDQGNAARTAGLKPEQVKINTLYAGGSFGRRANAWSDYIVEAVSIAKALGANGVPVKLQWT
REDDIHGGFYRPMYYHRLEAGLDADGKLVGWQHRIVGQSILEGTPFAAVMVKDGIDATSVEGAANLPYAVPN
VSVELSTTQVGVPVLWWRVVGSSHTVYAVEAFIDEAAQAAGKDPYLFRRDLLAEQPRLRGVLELAAEKAGWD
PSRPLPAGRGRGIAVTEAFKTFVAQVVEVSVDKDGKLKVERVVCAVDCGIPINPDVIAAQMEGGIGFGLGAILHS
AITLKDGKVEQNNFDGYQVLRIAEMPKVEVHIVPSGEAPTGVGEPGVAPIGPALANAIFAATGQRLYNLPFPTSF
AKA
>SEQF7755||SEQF7755.1_02893
MKRSYPDDLVIGNLSRRGFLKGVGATGVLLVAANWGWRDALAAEKKAFGADAMPHGWVDNPKIYVSIDRDG
TVGIVCNRSEMGQGVRTSLAMVVADELEADWSRVKVIQAPGDEARYGNQDTDGSRSMRHWFEPMRRCGA
AARQMLEQAAANQWKVPLGECRAEQNKVLHAPSGRSLSFGELAEAAAGLEVPARDKLLLKKPEQFRYIGKDV
ARAIDGADIVNGRAGFGFDARFDDMLYAVVARPPVYGGKLKRYDAAAALKVPGVVKVIEIEGRPIPSEFQPLGG
VAVVAQNTWAAIKGREALVVEWDAGVNGGYDSVAYRKQLEEAARKPGKVVRDGGDAAALFAKGGDIVEAEY
YLPHLAQAPMEPPVSTAWYKDGACEVWAPTQAPQVTRERIAERLKLPFDKVTVNVTLLGGGFGRKSKPDFVLE
AAILAKAFPGRHLRVQWTREDDLHFSYFHTVSVERLQAVLGADGLPQAWLHRSVAPSITALFGPNSKHQGAFE
LGMGLTNLPFAIPNVRLENPEAPAHTRVGWFRSVSNIPHAFAIQSFVGELAAKAGQDPKDYLLKLLGPARRIDTA
ELGDSWNYGESPERYPLDVGRLRGVIEEAARQSGWGGELPRGRARGIAAHYSFVTYVAVVIEVEVKDDGALLV
HKATIAADCGPQINPERIRSQLEGACVMGLGLAALGEISFKDGKVQQDNFHQYELARMPLAPKAVSVHLLKPD
GDLPLGGVGEPGVPPIAPALCNAIFAATGKRIRELPIRNQLQGWRKA
>SEQF7755||SEQF7755.1_03429
MNSKIDLSNALPGSRRGFLKGAAVVGLTIGFQWSGARRALAAALPDAGFAPNAFLRIAPDDSVTVIAKHVEMG

QGAYTGIATIVAEELDADWSKVRVESAPADAKRYANLAFGTMQGTGGSSAMANSWMQLREAGAKARAMLV
EAAARQWRVPATELRTRDGFVEHPASQRKASYGSLAAAAAELPVPEKVQLKDPKDFRLIGHQAPRVDVPGKTD
GSAQFTLDVSLPGMLVALLQRPPLFGATVKSFDATATRAIPGVVEVVQVPHGVAVVAKGFWAAKQGRDALKVE
WDESKAEKRGSEALMAEYRKLAEQPGKPARRDGNAAKAVAGATRRIAASYEFPFLAHAPMEPLDAVVRLTADS
CEIWAGDQFQTVDQGNAARTAGLKPEQVKINTLYAGGSFGRRANAWSDYIVEAVSIAKALGANGVPVKLQWT
REDDIHGGFYRPMYYHRLEAGLDADGKLVGWQHRIVGQSILEGTPFAAVMVKDGIDATSVEGAANLPYAVPN
VSVELSTTQVGVPVLWWRVVGSSHTVYAVEAFIDEAAQAAGKDPYLFRRDLLAEQPRLRGVLELAAEKAGWD
PSRPLPAGRGRGIAVTEAFKTFVAQVVEVSVDKDGKLKVERVVCAVDCGIPINPDVIAAQMEGGIGFGLGAILHS
AITLKDGKVEQNNFDGYQVLRIAEMPKVEVHIVPSGEAPTGVGEPGVAPIGPALANAIFAATGQRLYNLPFPTSF
AKA
>SEQF7756||SEQF7756.1_00533
MNSKIDLSNALPGSRRGFLKGAAVVGLTIGFQWSGARRALAAALPDAGFAPNAFLRIAPDDSVTVIAKHVEMG
QGAYTGIATIVAEELDADWSKVRVESAPADAKRYANLAFGTMQGTGGSSAMANSWMQLREAGAKARAMLV
EAAARQWRVPATELRTRDGFVEHPASQRKASYGSLAAAAAELPVPENVQLKDPKDFRLIGHQAPRVDVPGKT
DGSAQFTLDVSLPGMLVALLQRPPLFGATVKSFDATATRAIPGVVEVVQVPHGVAVVAKGFWAAKQGRDALKV
EWDESKAEKRGSEALMAEYRKLAEQPGKPARRDGDAAKAVAGATRRIAASYEFPFLAHAPMEPLDAVVRLTAD
SCEIWAGDQFQTVDQGNAARTAGLKPEQVKINTLYAGGSFGRRANAWSDYIVEAVSIAKALGANGVPVKLQW
TREDDIHGGFYRPMYYHRLEAGLDADGKLVGWQHRIVGQSILEGTPFAAVMVKDGIDATSVEGAANLPYAVP
NVSVELSTTQVGVPVLWWRVVGSSHTVYAVEAFIDEAAQAAGKDPYLFRRDLLAEQPRLRGVLELAAEKAGW
DPSRPLPAGRGRGIAVTEAFKTFVAQVVEVSVDKDGKLKVERVVCAVDCGIPINPDVIAAQMEGGIGFGLGAIL
HSAITLKDGKVEQNNFDGYQVLRIAEMPKVEVHIVPSGEAPTGVGEPGVAPIGPALANAIFAATGQRLYNLPFPT
SFAKA
>SEQF7756||SEQF7756.1_01079
MKRSYPDDLVIGNLSRRGFLKGVGATGVLLVAANWGWRDALAAEKKAFGADAMPHGWVDNPKIYVSIDRDG
TVGIVCNRSEMGQGVRTSLAMVVADELEADWSRVKVIQAPGDEARYGNQDTDGSRSMRHWFEPMRRCGA
AARQMLEQAAANQWKVPLGECRAEQNKVLHAPSGRSLSFGELAEAAAGLEVPARDKLLLKKPEQFRYIGKDV
ARAIDGADIVNGRAGFGFDARFDDMLYAVVARPPVYGGKLKRYDAAAALKVPGVVKVIEIEGRPIPSEFQPLGG
VAVVAQNTWAAIKGREALVVEWDAGVNGGYDSVAYRKQLEEAARKPGKVVRDSGDAAALFAKGGDIVEAEYY
LPHLAQAPMEPPVSTAWYKDGACEVWAPTQAPQVTRERIAERLKLPFDKVTVNVTLLGGGFGRKSKPDFVLEA
AILAKAFPGRHLRVQWTREDDLHFSYFHTVSVERLQAVLGADGLPQAWLHRSVAPSITALFGPDSKHQGAFEL
GMGLTNLPFAIPNVRLENPEAPAHTRVGWFRSVSNIPHAFAIQSFVGELAAKAGQDPKDYLLKLLGPARRIDTAE
LGDSWNYGESPERYPLDVGRLRGVIEEAARQSGWGGELPRGRARGIAAHYSFVTYVAVVIEVEVKDDGALLVH
KATIAADCGPQINPERIRSQLEGACVMGLGLAALGEISFKDGKVQQDNFHQYELARMPLAPKAVSVHLLKPDG
DLPLGGVGEPGVPPIAPALCNAIFAATGKRIRELPIRNQLQGWRKA
>SEQF7757||SEQF7757.1_03350
MNSKIDLSNALPGSRRGFLKGAAVVGLTIGFQWSGARRALAAALPDAGFAPNAFLRIAPDDSVTVIAKHVEMG
QGAYTGIATIVAEELDADWSKVRVESAPADAKRYANLAFGTMQGTGGSSAMANSWMQLREAGAKARAMLV
EAAARQWQIPAAELRTRDGFVEHPTSQRKASYGSLAAAAAELPVPEKVQLKDPKDFRLIGHQAPRVDVPGKTD
GSAQFTLDVSLPGMLVALLQRPPLFGATVKSFDATATRAIPGVVEVVQVPHGVAVVAKGFWAAKQGRDALKVE
WDESKAEKRGSEALMAEYRKLAEQPGKPARRDGDAAKAVAGATRRIAASYEFPFLAHAPMEPLDAVVRLTADS
CEFWAGDQFQTVDQGNAARTAGLKPEQVKINTLYAGGSFGRRANAWSDYIVEAVSIAKALGANGVPVKLQW
TREDDIHGGFYRPMYYHRLEAGLDADGKLVGWQHRIVGQSILEGTPFAAVMVKDGVDATSVEGAANLPYAVP
NVSVELSTTQVGVPVLWWRVVGSSHTVYAVEAFIDEAAQAAGKDPYLFRRDLLAEQPRLRGVLELAAEKAGW
DPSRPLPAGRGRGIAVTEAFKTFVAQVVEVSVDKDGKLKVERVVCAVDCGIPINPDVIAAQMEGGIGFGLGAIL

HSAITLKDGKVEQNNFDGYQVLRIAEMPKVEVHIVPSGEAPTGVGEPGVAPIGPALANAIFAATGQRLYNLPFPT
SFAKA
>SEQF7757||SEQF7757.1_02905
MKRSYPDDLVIGNLSRRGFLKGVGATGVLLVAANWGWRDALAAEKKAFGADAMPHGWVDNPKIYVSIDRDG
TVGIVCNRSEMGQGVRTSLAMVVADELEADWSRVKVIQAPGDEARYGNQDTDGSRSMRHWFEPMRRCGA
AARQMLEHAAANQWKVPLGECRAEQNKVLHAPSGRSLSFGELAEAAAGLEVPARDNLLLKKPEQFRYIGKDV
ARAIDGADIVNGRAGFGFDARFDDMLYAVVARPPVYGGKLKRYDAAAALKVPGVVKVIEIEGRPIPSEFQPLGG
VAVVAQNTWAAIKGREALVVEWDAGVNGGYDSVAYRKQLEEAARKPGKVVRDSGDAAALFAKGGDIVEAEYY
LPHLAQAPMEPPVSTAWYKDGACEVWAPTQAPQVTRERIAERLKLPFDKVTVNVTLLGGGFGRKSKPDFVLEA
AILAKAFPGRHLRVQWTREDDLHFSYFHTVSVERLQAVLGADGLPQAWLHRSVAPSITALFGPDSKHQGAFEL
GMGLTNLPFAIPNVRLENPEAPAHTRVGWFRSVSNIPHAFAIQSFVGELAAKAGQDPKDYLLKLLGPARRIDTAE
LGDSWNYGESPERYPLDVGRLRGVIEEAARQSGWGGELPRGRARGIAAHYSFVTYVAVVIEVEVKDDGALLVH
KATIAADCGPQINPERIRSQLEGACVMGLGLAALGEISFKDGKVQQDNFHQYELARMPLAPKAVSVHLLKPDG
DLPLGGVGEPGVPPIAPALCNAIFAATGKRIRELPIRNQLQGWRKA
>SEQF7758||SEQF7758.1_03111
MKRSYPDDLVIGNLSRRGFLKGVGATGVLLVAANWGWRDALAAEKKAFGADAMPHGWVDNPKIYVSIDRDG
TVGIVCNRSEMGQGVRTSLAMVVADELEADWSRVKVIQAPGDEARYGNQDTDGSRSMRHWFEPMRRCGA
AARQMLEQAAANQWKVPLGECRAEQNKVLHAPSGRSLSFGELAEAAAGLEVPARDKLLLKKPEQFRYIGKDV
ARAIDGADIVNGRAGFGFDARFDDMLYAVVARPPVYGGKLKRYDAAAALKVPGVVKVIEIEGRPIPSEFQPLGG
VAVVAQNTWAAIKGREALVVEWDAGVNGGYDSVAYRKQLEEAARKPGKVVRDSGDAAALFAKGGDIVEAEYY
LPHLAQAPMEPPVSTAWYKDGACEVWAPTQAPQVTRERIAERLKLPFDKVTVNVTLLGGGFGRKSKPDFVLEA
AILAKAFPGRHLRVQWTREDDLHFSYFHTVSVERLQAVLGADGLPQAWLHRSVAPSITALFGPDSKHQGAFEL
GMGLTNLPFAIPNVRLENPEAPAHTRVGWFRSVSNIPHAFAIQSFVGELAAKAGQDPKDYLLKLLGPARRIDTAE
LGDSWNYGESPERYPLDVGRLRGVIEEAARQSGWGGELPRGRARGIAAHYSFVTYVAVVIEVEVKDDGALLVH
KATIAADCGPQINPERIRSQLEGACVMGLGLAALGEISFKDGKVQQDNFHQYELARMPLAPKAVSVHLLKPDG
DLPLGGVGEPGVPPIAPALCNAIFAATGKRIRELPIRNQLQGWRKA
>SEQF7758||SEQF7758.1_02516
MIAKHVEMGQGAYTGIATIVAEELDADWSKVRVESAPADAKRYANLAFGTMQGTGGSSAMANSWMQLREA
GAKARAMLVEAAARQWQVPAAELRTRDGFVEHPTSQRKASYGSLAAAAAELPVPENVQLKDPKDFRLIGHQA
PRVDVPGKTDGSAQFTLDVSLPGMLVALLQRPPLFGATVKSFDATATRAIPGVVEVVQVPHGVAVVAKGFWAA
KQGRDALKVEWDESKAEKRGSEALMAEYRKLAEQPGKPARRDGDAAKAVAGATRRIAASYEFPFLAHAPMEP
LDAVVRLTADSCEIWAGDQFQTVDQGNAARTAGLKPEQVKINTLYAGGSFGRRANAWSDYIVEAVSIAKALGA
NGVPVKLQWTREDDIHGGFYRPMYYHRLEAGLDADGKLVGWQHRIVGQSILEGTPFAAVMVKDGVDATSVE
GAANLPYAVPNVSVELSTTQVGVPVLWWRVVGSSHTVYAVEAFIDEAAQAAGKDPYLFRRDLLAEQPRLRGVL
ELAAEKAGWDPSRPLPAGRGRGIAVTEAFKTFVAQVVEVSVDKDGKLKVERVVCAVDCGIPINPDVIAAQMEG
GIGFGLGAILHSAITLKDGKVEQNNFDGYQVLRIAEMPKVEVHIVPSGEAPTGVGEPGVAPIGPALANAIFAATG
QRLYNLPFPTSFAKA
>SEQF7759||SEQF7759.1_03407
MNSKIDLSNALPGSRRGFLKGAAVVGLTIGFQWSGARRALAAALPDAGFAPNAFLRIAPDDSVTVIAKHVEMG
QGAYTGIATIVAEELDADWSKVRVESAPADAKRYANLAFGTMQGTGGSSAMANSWMQLREAGAKARAMLV
EAAARQWRVPATELRTRDGFVEHPASQRKASYGSLAAAAAELPVPEKVQLKDPKDFRLIGHQAPRVDVPGKTD
GSAQFTLDVSLPGMLVALLQRPPLFGATVKSFDATATRAIPGVVEVVQVPHGVAVVAKGFWAAKQGRDALKVE
WDESKAEKRGSEALMAEYRKLAEQPGKPARRDGDAAKAVAGATRRIAASYEFPFLAHAPMEPLDAVVRLTADS
CEIWAGDQFQTVDQGNAARTAGLKPEQVKINTLYAGGSFGRRANAWSDYIVEAVSIAKALGANGVPVKLQWT

REDDIHGGFYRPMYYHRLEAGLDADGKMVGWQHRIVGQSILEGTPFAAVMVKDGVDATSVEGAANLPYAVH
NVSVELSTTQVGVPVLWWRVVGSSHTVYAVEAFIDEAAQAAGKDPYLFRRDLLAEQPRLRGVLELAAEKAGW
DPSRPLPAGRGRGIAVTEAFKTFVAQVVEVSVDKDGKLKVERVVCAVDCGIPINPDVIAAQMEGGIGFGLGAIL
HSAITLKDGKVEQNNFDGYQVLRIAEMPKVEVHIVPSGEAPTGVGEPGVAPIGPALANAIFAATGQRLYNLPFPT
SFAKA
>SEQF7759||SEQF7759.1_02811
MKRSFPDDLVIGNLSRRGFLKGVGATGVLLVAANWGWRDALAAEKKAFGADAMPHGWVDNPKIYVSIDRDG
TVGIVCNRSEMGQGVRTSLAMVVADELEADWSRVKVIQAPGDEARYGNQDTDGSRSMRHWFDPMRRCGA
AARQMLEQAAANQWKVPLGECRAEQNKVLHAPSGRSLSFGELAEAAAGLEVPARDKLLLKKPEQFRYIGKDV
ARAIDGADIVNGRAGFGFDARFDDMLYAVVARPPVYGGKLKRYDAAAALKVPGVVKVIEIEGRPIPSEFQPLGG
VAVVAQNTWAAIKGREALVVEWDAGVNGGYDSVAYRKQLEEAARKPGKVVRDSGDAAALFAKGGDIVEAEYY
LPHLAQAPMEPPVSTAWYKDGACEVWAPTQAPQVTRERIAERLKLPFDKVTVNVTLLGGGFGRKSKPDFVLEA
AILAKAFPGRHLRVQWTREDDLHFSYFHTVSVERLQAVLGADGLPQAWLHRSVAPSITALFGPDSKHQGAFEL
GMGLTNLPFAIPNVRLENPEAPAHTRVGWFRSVSNIPHAFAIQSFVGELAAKAGQDPKDYLLKLLGPARRIDTAE
LGDSWNYGESPERYPLDVGRLRGVIEEAARQSGWGGELPRGRARGIAAHYSFVTYVAVVIEVEVKDDGALLVH
KATIAADCGPQINPERIRSQLEGACVMGLGLAALGEISFKDGKVQQDNFHQYELARMPLAPKAVSVHLLKPDG
DLPLGGVGEPGVPPIAPALCNAIFAATGKRIRELPIRNQLQGWRKA
>SEQF7760||SEQF7760.1_02902
MKRSFPDDLVIGNLSRRGFLKGVGATGVLLVAANWGWRDALAAEKKAFGADAMPHGWVDNPKIYVSIDRDG
TVGIVCNRSEMGQGVRTSLAMVVADELEADWSRVKVIQAPGDEARYGNQDTDGSRSMRHWFEPMRRCGA
AARQMLEQAAANQWKVPLGECRAEQNKVLHAPSGRSLSFGELAEAAAGLEVPARDKLLLKKPEQFRYIGKDV
ARAIDGADIVNGRAGFGFDARFDDMLYAVVARPPVYGGKLKRYDAAAALKVPGVVKVIEIEGRPIPSEFQPLGG
VAVVAQNTWAAIKGREALVVEWDAGVNGGYDSVAYRKQLEEAARKPGKVVRDSGDAAALFAKGGDIVEAEYY
LPHLAQAPMEPPVSTAWYKDGACEVWAPTQAPQVTRERIAERLKLPFDKVTVNVTLLGGGFGRKSKPDFVLEA
AILAKAFPGRHLRVQWTREDDLHFSYFHTVSVERLQAVLGADGLPQAWLHRSVAPSITALFGPDSKHQGAFEL
GMGLTNLPFAIPNVRLENPEAPAHTRVGWFRSVSNIPHAFAIQSFVGELAAKAGQDPKDYLLKLLGPARRIDTAE
LGDSWNYGESPERYPLDVGRLRGVIEEAARQSGWGGELPRGRARGIAAHYSFVTYVAVVIEVEVKDDGALLVH
KATIAADCGPQINPERIRSQLEGACVMGLGLAALGEISFKDGKVQQDNFHQYELARMPLAPKAVSVHLLKPDG
DLPLGGVGEPGVPPIAPALCNAIFAATGKRIRELPIRNQLQGWRKA
>SEQF7760||SEQF7760.1_03682
MNSKIDLSNALPGSRRGFLKGAAVVGLTIGFQWSGARRALAAALPDAGFAPNAFLRIAPDDSVTVIAKHVEMG
QGAYTGIATIVAEELDADWSKVRVESAPADAKRYANLAFGTMQGTGGSSAMANSWMQLREAGAKARAMLV
EAAARQWQVPAAELRTRDGFVEHPTSQRKASYGSLAAAAAELPVPENVQLKDPKDFRLIGHQAPRVDVPGKT
DGSAQFTLDISLPGMLVALLQRPPLFGATVKSFDATATRAIPGVVEVVQVPHGVAVVAKGFWAAKQGRDALKV
EWDESKAEKRGSEALMAEYRKLAEQPGKPARRDGDAAKAVAGATRRIAASYEFPFLAHAPMEPLDAVVRLTAD
SCEIWAGDQFQTVDQGNAARTAGLKPEQVKINTLYAGGSFGRRANAWSDYIVEAVSIAKALGANGVPVKLQW
TREDDIHGGFYRPMYYHRLEAGLDADGKLVGWQHRIVGQSILEGTPFAAVMVKDGVDATSVEGAANLPYAVP
NVSVELSTTQVGVPVLWWRVVGSSHTVYAVEAFIDEAAQAADKDPYLFRRDLLAEQPRLRGVLELAAEKAGW
DPSRPLPAGRGRGIAVTEAFKTFVAQVVEVSVDKDGKLKVERVVCAVDCGIPINPDVIAAQMEGGIGFGLGAIL
HSAITLKDGKVEQNNFDGYQVLRIAEMPKVEVHIVPSGEAPTGVGEPGVAPIGPALANAIFAATGQRLYNLPFPT
SFAKA
>SEQF7761||SEQF7761.1_02852
MKRSYPDDLVIGNLSRRGFLKGVGATGVLLVAANWGWRDALAAEKKAFGADAMPHGWVDNPKIYVSIDRDG
TVGIVCNRSEMGQGVRTSLAMVVADELEADWSRVKVIQAPGDEARYGNQDTDGSRSMRHWFEPMRRCGA

AARQMLEQAAANQWKVPLGECRAEQNKVLHAPSGRSLSFGELAEAAAGLEVPARDKLLLKKPEQFRYIGKDV
ARAIDGADIVNGRAGFGFDARFDDMLYAVVARPPVYGGKLKRYDAAAALKVPGVVKVIEIEGRPIPSEFQPLGG
VAVVAQNTWAAIKGREALVVEWDAGVNGGYDSVAYRKQLEEAARKPGKVVRDSGDAAALFAKGGDIVEAEYY
LPHLAQAPMEPPVSTAWYKDGACEVWAPTQAPQVTRERIAERLKLPFDKVTVNVTLLGGGFGRKSKPDFVLEA
AILAKAFPGRHLRVQWTREDDLHFSYFHTVSVERLQAVLGADELPQAWLHRSVAPSITALFGPDSKHQGAFELG
MGLTNLPFAIPNVRLENPEAPAHTRVGWFRSVSNIPHAFAIQSFVGELAAKAGQDPKDYLLKLLGPARRIDTAEL
GDSWNYGESPERYPLDVGRLRGVIEEAARQSGWGGELPRGRARGIAAHYSFVTYVAVVIEVEVKDDGALLVHK
ATIAADCGPQINPERIRSQLEGACVMGLGLAALGEISFKDGKVQQDNFHQYELARMPLAPKAVSVHLLKPDGD
LPLGGVGEPGVPPIAPALCNAIFAATGKRIRELPIRNQLQGWRKA
>SEQF7761||SEQF7761.1_03375
MNSKIDLSNALPGSRRGFLKGAAVVGLTIGFQWSGARRALAAALPDAGFAPNAFLRIAPDDSVTVIAKHVEMG
QGAYTGIATIVAEELDADWSKVRVESAPADAKRYANLAFGTMQGTGGSSAMANSWMQLREAGAKARAMLV
EAAARQWRVPATELRTRDGFVEHPASQRKASYGSLAAAAAELPVPEKVQLKDPKDFRLIGHQAPRVDVPGKTD
GSAQFTLDVSLPGMLVALLQRPPLFGATVKSFDATATRAIPGVVEVVQVPHGVAVVAKGFWAAKQGRDALKVE
WDESKAEKRGSEALMAEYRKLAEQPGKPARRDGDAAKAVAGATRRIAASYEFPFLAHAPMEPLDAVVRLTADS
CEIWAGDQFQTVDQGNAARTAGLKPEQVKINTLYAGGSFGRRANAWSDYIVEAVSIAKALGANGVPVKLQWT
REDDIHGGFYRPMYYHRLEAGLDADGKLVGWQHRIVGQSILEGTPFAAVMVKDGVDATSVEGAANLPYAVPN
VSVELSTTQVGVPVLWWRVVGSSHTVYAVEAFIDEAAQAAGKDPYLFRRDLLAEQPRLRGVLELAAEKAGWD
PSRPLPAGRGRGIAVTEAFKTFVAQVVEVSVDKDGKLKVERVVCAVDCGIPINPDVIAAQMEGGIGFGLGAILHS
AITLKDGKVEQNNFDGYQVLRIAEMPKVEVHIVPSGEAPTGVGEPGVAPIGPALANAIFAATGQRLYNLPFPTSF
AKA
>SEQF7762||SEQF7762.1_03578
MNSKIDLSNALPGSRRGFLKGAAVVGLTIGFQWSGARRALAAALPDAGFAPNAFLRIAPDDSVTVIAKHVEMG
QGAYTGIATIVAEELDADWSKVRVESAPADAKRYANLAFGTMQGTGGSSAMANSWMQLREAGAKARAMLV
EAAARQWQIPAAELRTRDGFVEHPTSQRKASYGSLAAAAAELPVPEKVQLKDPKDFRLIGHQAPRVDVPGKTD
GSAQFTLDVSLPGMLVALLQRPPLFGATVKSFDATATRAIPGVVEVVQVPHGVAVVAKGFWAAKQGRDALKVE
WDESKAEKRGSEALMAEYRKLAEQPGKPARRDGDAAKAVAGATRRIAASYEFPFLAHAPMEPLDAVVRLTADS
CEFWAGDQFQTVDQGNAARTAGLKPEQVKINTLYAGGSFGRRANAWSDYIVEAVSIAKALGANGVPVKLQW
TREDDIHGGFYRPMYYHRLEAGLDADGKLVGWQHRIVGQSILEGTPFAAVMVKDGVDATSVEGAANLPYAVP
NVSVELSTTQVGVPVLWWRVVGSSHTVYAVEAFIDEAAQAAGKDPYLFRRDLLAEQPRLRGVLELAAEKAGW
DPSRPLPAGRGRGIAVTEAFKTFVAQVVEVSVDKDGKLKVERVVCAVDCGIPINPDVIAAQMEGGIGFGLGAIL
HSAITLKDGKVEQNNFDGYQVLRIAEMPKVEVHIVPSGEAPTGVGEPGVAPIGPALANAIFAATGQRLYNLPFPT
SFAKA
>SEQF7762||SEQF7762.1_02998
MKRSYPDDLVIGNLSRRGFLKGVGATGVLLVAANWGWRDALAAEKKAFGADAMPHGWVDNPKIYVSIDRDG
TVGIVCNRSEMGQGVRTSLAMVVADELEADWSRVKVIQAPGDEARYGNQDTDGSRSMRHWFEPMRRCGA
AARQMLEHAAANQWKVPLGECRAEQNKVLHAPSGRSLSFGELAEAAAGLEVPARDNLLLKKPEQFRYIGKDV
ARAIDGADIVNGRAGFGFDARFDDMLYAVVARPPVYGGKLKRYDAAAALKVPGVVKVIEIEGRPIPSEFQPLGG
VAVVAQNTWAAIKGREALVVEWDAGVNGGYDSVAYRKQLEEAARKPGKVVRDSGDAAALFAKGGDIVEAEYY
LPHLAQAPMEPPVSTAWYKDGACEVWAPTQAPQVTRERIAERLKLPFDKVTVNVTLLGGGFGRKSKPDFVLEA
AILAKAFPGRHLRVQWTREDDLHFSYFHTVSVERLQAVLGADGLPQAWLHRSVAPSITALFGPDSKHQGAFEL
GMGLTNLPFAIPNVRLENPEAPAHTRVGWFRSVSNIPHAFAIQSFVGELAAKAGQDPKDYLLKLLGPARRIDTAE
LGDSWNYGESPERYPLDVGRLRGVIEEAARQSGWGGELPRGRARGIAAHYSFVTYVAVVIEVEVKDDGALLVH
KATIAADCGPQINPERIRSQLEGACVMGLGLAALGEISFKDGKVQQDNFHQYELARMPLAPKAVSVHLLKPDG

DLPLGGVGEPGVPPIAPALCNAIFAATGKRIRELPIRNQLQGWRKA
>SEQF7763||SEQF7763.1_02348
MNSKIDLSNALPGSRRGFLKGAAVVGLTIGFQWSGARRALAAALPDAGFAPNAFLRIAPDDSVTVIAKHVEMG
QGAYTGIATIVAEELDADWSKVRVESAPADAKRYANLAFGTMQGTGGSSAMANSWMQLREAGAKARAMLV
EAAARQWRVPATELRTRDGFVEHPASQRKASYGSLAAAAAELPVPEKVQLKDPKDFRLIGHQAPRVDVPGKTD
GSAQFTLDVSLPGMLVALLQRPPLFGATVKSFDATATRAIPGVVEVVQVPHGVAVVAKGFWAAKQGRDALKVE
WDESKAEKRGSEALMAEYRKLAEQPGKPARRDGDAAKAVAGATRRIAASYEFPFLAHAPMEPLDAVVRLTADS
CEIWAGDQFQTVDQGNAARTAGLKPEQVKINTLYAGGSFGRRANAWSDYIVEAVSIAKALGANGVPVKLQWT
REDDIHGGFYRPMYYHRLEAGLDADGKLVGWQHRIVGQSILEGTPFAAVMVKDGVDATSVEGAANLPYAVPN
VSVELSTTQVGVPVLWWRVVGSSHTVYAVEAFIDEAAQAAGKDPYLFRRDLLAEQPRLRGVLELAAEKAGWD
PSRPLPAGRGRGIAVTEAFKTFVAQVVEVSVDKDGKLKVERVVCAVDCGIPINPDVIAAQMEGGIGFGLGAILHS
AITLKDGKVEQNNFDGYQVLRIAEMPKVEVHIVPSGEAPTGVGEPGVAPIGPALANAIFAATGQRLYNLPFPTSF
AKA
>SEQF7763||SEQF7763.1_01742
MKRSYPDDLVIGNLSRRGFLKGVGATGVLLVAANWGWRDALAAEKKAFGADAMPHGWVDNPKIYVSIDRDG
TVGIVCNRSEMGQGVRTSLAMVVADELEADWSRVKVIQAPGDEARYGNQDTDGSRSMRHWFEPMRRCGA
AARQMLEQAAANQWKVPLGECRAEQNKVLHAPSGRSLSFGELAEAAAGLEVPARDKLLLKKPEQFRYIGKDV
ARAIDGADIVNGRAGFGFDARFDDMLYAVVARPPVYGGKLKRYDAAAALKVPGVVKVIEIEGRPIPSEFQPLGG
VAVVAQNTWAAIKGREALVVEWDAGVNGGYDSVAYRKQLEEAARKPGKVVRDSGDAAALFARGGDIVEAEYY
LPHLAQAPMEPPVSTAWYKDGACEVWAPTQAPQVTRERIAERLKLPFDKVTVNVTLLGGGFGRKSKPDFVLEA
AILAKAFPGRHLRVQWTREDDLHFSYFHTVSVERLQAVLGADGLPQAWLHRSVAPSITALFGPDSKHQGAFEL
GMGLTNLPFAIPNVRLENPEAPAHTRVGWFRSVSNIPHAFAIQSFVGELAAKAGQDPKDYLLKLLGPARRIDTAE
LGDSWNYGESPERYPLDVGRLRGVIEEAARQSGWGGELPRGRARGIAAHYSFVTYVAVVIEVEVKDDGALLVH
KATIAADCGPQINPERIRSQLEGACVMGLGLAALGEISFKDGKVQQDNFHQYELARMPLAPKAVSVHLLKPDG
DLPLGGVGEPGVPPIAPALCNAIFAATGKRIRELPIRNQLQGWRKA
>SEQF7764||SEQF7764.3_03525
MKRSFPDDLVIGNLSRRGFLKGVGATGVLLVAANWGWRDALAAEKKAFGADAMPHGWVDNPKIYVSIDRDG
TVGIVCNRSEMGQGVRTSLAMVVADELEADWSRVKVIQAPGDEARYGNQDTDGSRSMRHWFEPMRRCGA
AARQMLEQAAANQWKVPLGECRAEQNKVLHAPSGRSLSFGELAEAAAGLEVPARDKLLLKKPEQFRYIGKDV
ARAIDGADIVNGRAGFGFDARFDDMLYAVVARPPVYGGKLKRYDAAAALKVPGVVKVIEIEGRPIPSEFQPLGG
VAVVAQNTWAAIKGREALVVEWDAGVNGGYDSVAYRKQLEEAARKPGKVVRDSGDAAALFAKGGDVVEAEY
YLPHLAQAPMEPPVSTAWYKDGACEVWAPTQAPQVTRERIAERLKLPFDKVTVNVTLLGGGFGRKSKPDFVLE
AAILAKALPGRHLRVQWTREDDLHFSYFHTVSVERLQAVLGADGLPQAWLHRSVAPSITALFGPDSKHQGAFEL
GMGLTNLPFAIPNVRLENPEAPAHTRVGWFRSVSNIPHAFAIQSFVGELAAKAGQDPKDYLLKLLGPARRIDTAE
LGDSWNYGESPERYPLDVGRLRGVIEEAARQSGWGGELPRGRARGIAAHYSFVTYVAVVIEVEVKDDGALLVH
KATIAADCGPQINPERIRSQLEGACVMGLGLAALGEISFKDGKVQQDNFHQYELARMPLAPKAVSVHLLKPDG
DLPLGGVGEPGVPPIAPALCNAIFAATGKRIRELPIRNQLQGWRKA
>SEQF7764||SEQF7764.3_03007
MNSKIDLSNALPGSRRGFLKGAAVVGLTIGFQWSGARRALAAALPDAGFAPNAFLRIAPDDSVTVIAKHVEMG
QGAYTGIATIVAEELDADWSKVRVESAPADAKRYANLAFGTMQGTGGSSAMANSWMQLREAGAKARAMLV
EAAARQWQVPAAELRTRDGFVEHPTSQRKASYGSLAAAAAELPVPENVQLKDPKDFRLIGHQAPRVDVPGKT
DGSAQFTLDVSLPGMLVALLQRPPLFGATVKSFDATATRAIPGVVEVVQVPHGVAVVAKGFWAAKQGRDALKV
EWDESKAEKRGSEALMAEYRKLAEQPGKPARRDGDAAKAVAGATRRIAASYEFPFLAHAPMEPLDAVVRLTAD
SCEIWAGDQFQTVDQGNAARTAGLKPEQVKINTLYAGGSFGRRANAWSDYIVEAVSIAKALGANGVPVKLQW

TREDDIHGGFYRPMYYHRLEAGLDADGKLVGWQHRIVGQSILEGTPFAAVMVKDGVDATSVEGAANLPYAVP
NVSVELSTTQVGVPVLWWRVVGSSHTVYAVEAFIDEAAQAAGKDPYLFRRDLLAEQPRLRGVLELAAEKAGW
DPSRPLPAGHGRGIAVTEAFKTFVAQVVEVSVDKDGKLKVERVVCAVDCGIPINPDVIAAQMEGGIGFGLGAIL
HSAITLKDGKVEQNNFDGYQVLRIAEMPKVEVHIVPSGEAPTGVGEPGVAPIGPALANAIFAATGQRLYNLPFPT
SFAKA
>SEQF7765||SEQF7765.1_02855
MKRSYPDDLVIGNLSRRGFLKGVGATGVLLVAANWGWRDALAAEKKAFGADAMPHGWVDNPKIYVSIDRDG
TVGIVCNRSEMGQGVRTSLAMVVADELEADWSRVKVIQAPGDEARYGNQDTDGSRSMRHWFEPMRRCGA
AARQMLEQAAANQWKVPLGECRAEQNKVLHAPSGRSLSFGELAEAAAGLEVPARDKLLLKKPEQFRYIGKDV
ARAIDGADIVNGRAGFGFDARFDDMLYAVVARPPVYGGKLKRYDAAAALKVPGVVKVIEIEGRPIPSEFQPLGG
VAVVAQNTWAAIKGREALVVEWDAGVNGGYDSVAYRKQLEEAARKPGKVVRDSGDAAALFAKGGDIVEAEYY
LPHLAQAPMEPPVSTAWYKDGACEVWAPTQAPQVTRERIAERLKLPFDKVTVNVTLLGGGFGRKSKPDFVLEA
AILAKAFPGRHLRVQWTREDDLHFSYFHTVSVERLQAVLGADGLPQAWLHRSVAPSITALFGPDSKHQGAFEL
GMGLTNLPFAIPNVRLENPEAPAHTRVGWFRSVSNIPHAFAIQSFVGELAAKAGQDPKDYLLKLLGPARRIDTAE
LGDSWNYGESPERYPLDVGRLRGVIEEAARQSGWGGELPRGRARGIAAHYSFVTYVAVVIEVEVKDDGALLVH
KATIAADCGPQINPERIRSQLEGACVMGLGLAALGEISFKDGKVQQDNFHQYELARMPLAPKAVSVHLLEPDG
DLPLGGVGEPGVPPIAPALCNAIFAATGKRIRELPIRNQLQGWRKA
>SEQF7765||SEQF7765.1_03375
MNSKIDLSNALPGSRRGFLKGAAVVGLTIGFQWSGARRALAAALPDAGFAPNAFLRIAPDDSVTVIAKHVEMG
QGAYTGIATIVAEELDADWSKVRVESAPADAKRYANLAFGTMQGTGGSSAMANSWMQLREAGAKARAMLV
EAAARQWQVPAAELRTRDGFVEHPASQRKASYGSLAAAAAELPVPENVQLKDPKDFRLIGHQAPRVDVPGKT
DGSAQFTLDVSLPGMLVALLQRPPLFGATVKSFDATATRAIPGVVEVVQVPHGVAVVAKGFWAAKQGRDALKV
EWDESKAEKRGSEALMAEYRKLAEQPGKPARRDGDAAKAVAGATRRIAASYEFPFLAHAPMEPLDAVVRLTAD
SCEIWAGDQFQTVDQGNAARTAGLKPEQVKINTLYAGGSFGRRANAWSDYIVEAVSIAKALGANGVPVKLQW
TREDDIHGGFYRPMYYHRLEAGLDADGKLVGWQHRIVGQSILEGTPFAAVMVKDGVDATSVEGAANLPYAVP
NVSVELSTTQVGVPVLWWRVVGSSHTVYAVEAFIDEAAQAAGKDPYLFRRDLLAEQPRLRGVLELAAEKAGW
DPSRPLPAGRGRGIAVTEAFKTFVAQVVEVSVDKDGKLKVERVVCAVDCGIPINPDVIAAQMEGGIGFGIGAILH
SAITLKDGKVEQNNFDGYQVLRIAEMPKVEVHIVPSGEAPTGVGEPGVAPIGPALANAIFAATGQRLYNLPFPTS
FAKA
>SEQF7766||SEQF7766.2_02479
MKKPNEVTVDMSRRRLLQGSGIALSGLVLSTWLPPLVAKSAAAEAAAAGRLGDHSAEGYGAFVRIGPDGVVTV
ISPKIEMGQGAQTGIAMMVAEELEVPLDQVVIQEAPPNSALYTDSLMQFQATGGSTSTRVTWEPLRRAGATAR
ILLIQAAALQWRVAPSLCHAQNGQVFGPNGLQAAYGDLVEAAATLPLPDAVPLKTPEQFKLLGTPAQRLDTPAK
VNGKAHFTIDLQIPGMLVASSITCPVYGGRLLNVDDSQARRVPGVRDIVRLDNAVAVTASNFWTCQQAIRALKI
EWDLGPHAAIGSAQLDQELLVASSRDGVVAKRSGDIEQAKQQSASQFEAVYEQALLSHSPFEPMSCVAHVRKD
ACELWVGTQVPVFAQQTAAQVTGLPVEKIQVHNQLIGGAFGRRLEFDFITQAVAIARQVDYPIKLIWSREEDMT
HDLYRPLYADRMQAALDEQGRPLGWEHRIAGASILARYVGSLPPNGVDSDAVEVAVDPIYSLVHLQVRYIRQEP
SVVPVSWWRGVGPLRGTYALECFIDELAHNAKADPVAYRLELMAEQPRAQAVLRLLAEKTDWNKPLPAGQGR
GVAVSAVFGSYVATLVELEMHGDLGIRIKRLVSVVDCGFATNPTSVKAQIEGGTLFGLSASLFNEILIENGQVQQT
NFHNYRQLRISEAPAVEVHLLPSLEAPGGVGEAGTALIGPALVNALYAASGQRIRRLPLSRFGYYPV
>SEQF7766||SEQF7766.2_03596
MNSKIDLSNALPGSRRGFLKGAAVVGLTIGFQWSGARRALAAALPDAGFAPNAFLRIAPDDSVTVIAKHVEMG
QGAYTGIATIVAEELDADWSKVRVESAPADAKRYANLAFGTMQGTGGSSAMANSWMQLREAGAKARAMLV
EAAARQWQVPAAELRTRDGFVEHPASQRKASYGSLAAAAAELPVPENVQLKDPKDFRLIGHQAPRVDVPGKT

DGSAQFTLDVSLPGMLVALLQRPPLFGATVKSFDATATRAIPGVVEVVQVPHGVAVVAKGFWAAKQGRDALKV
EWDESKAEKRGSEALMAEYRKLAEQPGKPARRDGDAAKAVAGATRRIAASYEFPFLAHAPMEPLDAVVRLTAD
SCEIWAGDQFQTVDQGNAARTAGLKPEQVKINTLYAGGSFGRRANAWSDYIVEAVSIAKALGANGVPVKLQW
TREDDIHGGFYRPMYYHRLEAGLDADGKLVGWQHRIVGQSILEGTPFAAVMVKDGVDATSVEGAANLPYAVP
NVSVELSTTQVGVPVLWWRVVGSSHTVYAVEAFIDEAAQAAGKDPYLFRRDLLAEQPRLRGVLELAAEKAGW
DPSRPLPAGRGRGIAVTEAFKTFVAQVVEVSVDKDGKLKVERVVCAVDCGIPINPDVIAAQMEGGIGFGLGAIL
HSAITLKDGKVEQNNFDGYQVLRIAEMPKVEVHIVPSGEAPTGVGEPGVAPIGPALANAIFAATGQRLYNLPFPT
SFAKA
>SEQF7766||SEQF7766.2_03054
MKRSFPDDLIIGNLSRRGFLKGVGATGVLLVAANWGWRDALAAEKKAFGADAMPHGWVDNPKIYVSIDRDG
TVGIVCNRSEMGQGVRTSLAMVVADELEADWSRVKVIQAPGDEARYGNQDTDGSRSMRHWFEPMRRCGA
AARQMLEQAAANQWKVPLGECRAEQNKVLHAPSGRSLSFGELAEAAAGLEVPARDKLLLKKPEQFRYIGKDV
ARAIDGADIVNGRAGFGFDARFDDMLYAVVARPPVYGGKLKRYDAAAALKVPGVVKVIEIEGRPIPSEFQPLGG
VAVVAQNTWAAIKGREALVVEWDAGVNGGYDSVAYRKQLEEAARKPGKVVRDSGDAAALFAKGGDVVEAEY
YLPHLAQAPMEPPVSTAWYKDGACEVWAPTQAPQVTRERIAERLKLPFDKVTVNVTLLGGGFGRKSKPDFVLE
AAILAKAFPGRYLRVQWTREDDLHFSYFHTVSVERLQAVLGADGLPQAWLHRSVAPSITALFGPDSKHQGAFEL
GMGLTNLPFAIPNVRLENPEAPAHTRVGWFRSVSNIPHAFAIQSFVGELAAKAGQDPKDYLLKLLGPARRIDTAE
LGDSWNYGESPERYPLDVGRLRGVIEEAARQSGWGGELPRGRARGIAAHYSFVTYVAVVIEVEVKDDGALLVH
KATIAADCGPQINPERIRSQLEGACVMGLGLAALGEISFKDGKVQQDNFHQYELARMPLAPKAVSVHLLKPDG
DLPLGGVGEPGVPPIAPALCNAIFAATGKRIRELPIRNQLQGWRKA
>SEQF7767||SEQF7767.1_05327
MKRSYPDDLVIGNLSRRGFLKGVGATGVLLVAANWGWRDALAAEKKAFGADAMPHGWVDNPKIYVSIDRDG
TVGIVCNRSEMGQGVRTSLAMVVADELEADWSRVKVIQAPGDEARYGNQDTDGSRSMRHWFEPMRRCGA
AARQMLEQAAANQWKVPLGECRAEQNKVLHAPSGRSLSFGELAEAAAGLEVPARDKLLLKKPEQFRYIGKDV
ARAIDGADIVNGRAGFGFDARFDDMLYAVVARPPVYGGKLKRYDAAAALKVPGVVKVIEIEGRPIPSEFQPLGG
VAVVAQNTWAAIKGREALVVEWDAGVNGGYDSVAYRKQLEEAARKPGKVVRDSGDAAALFARGGDIVEAEYY
LPHLAQAPMEPPVSTAWYKDGACEVWAPTQAPQVTRERIAERLKLPFDKVTVNVTLLGGGFGRKSKPDFVLEA
AILAKAFPGRHLRVQWTREDDLHFSYFHTVSVERLQAVLGADGLPQAWLHRSVAPSITALFGPDSKHQGAFEL
GMGLTNLPFAIPNVRLENPEAPAHTRVGWFRSVSNIPHAFAIQSFVGELAAKAGQDPKDYLLKLLGPARRIDTAE
LGDSWNYGESPERYPLDVGRLRGVIEEAARQSGWGGELPRGRARGVAAHYSFVTYVAVVIEVEVKDDGALLVH
KATIAADCGPQINPERIRSQLEGACVMGLGLAALGEISFKDGKVQQDNFHQYELARMPLAPKAVSVHLLKPDG
DLPLGGVGEPGVPPIAPALCNAIFAATGKRIRELPIRNQLQGWRKA
>SEQF7767||SEQF7767.1_05872
MNSKIDLSNALPGSRRGFLKGAAVVGLTIGFQWSGARRALAAALPDAGFAPNAFLRIAPDDSVTVIAKHVEMG
QGAYTGIATIVAEELDADWSKVRVESAPADAKRYANLAFGTMQGTGGSSAMANSWMQLREAGAKARAMLV
EAAARQWRVPATELRTRDGFVEHPASQRKASYGSLAAAAAELPVPENVQLKDPKDFRLIGHQAPRVDVPGKT
DGSAQFTLDVSLPGMLVALLQRPPLFGATVKSFDATATRAIPGVVEVVQVPHGVAVVAKGFWAAKQGRDALKV
EWDESKAEKRGSEALMAEYRKLAEQPGKPARRDGDAAKAVAGATRRIAASYEFPFLAHAPMEPLDAVVRLTAD
SCEIWAGDQFQTVDQGNAARTAGLKPEQVKINTLYAGGSFGRRANAWSDYIVEAVSIAKALGANGVPVKLQW
TREDDIHGGFYRPMYYHRLEAGLDADGKLVGWQHRIVGQSILEGTPFAAVMVKDGIDATSVEGAANLPYAVP
NVSVELSTTQVGVPVLWWRVVGSSHTVYAVEAFIDEAAQAAGKDPYLFRRDLLAEQPRLRGVLELAAEKAGW
DPSRPLPAGRGRGIAVTEAFKTFVAQVVEVSVDKDGKLKVERVVCAVDCGIPINPDVIAAQMEGGIGFGLGAIL
HSAITLKDGKVEQNNFDGYQVLRIAEMPKVEVHIVPSGEAPTGVGEPGVAPIGPALANAIFAATGQRLYNLPFPT
SFAKA

>SEQF7768||SEQF7768.2_01316
MKRSFPDDLIIGNLSRRGFLKGVGATGVLLVAANWGWRDALAAEKKAFGADAMPHGWVDNPKIYVSIDRDG
TVGIVCNRSEMGQGVRTSLAMVVADELEADWSRVKVIQAPGDEARYGNQDTDGSRSMRHWFEPMRRCGA
AARQMLEQAAANQWKVPLGECRAEQNKVLHAPSGRSLSFGELAEAAAGLEVPARDKLLLKKPEQFRYIGKDV
ARAIDGADIVNGRAGFGFDARFDDMLYAVVARPPVYGGKLKRYDAAAALKVPGVVKVIEIEGRPIPSEFQPLGG
VAVVAQNTWAAIKGREALVVEWDAGVNGGYDSVAYRKQLEEAARKPGKVVRDSGDAAALFAKGGDVVEAEY
YLPHLAQAPMEPPVSTAWYKDGACEVWAPTQAPQVTRERIAERLKLPFDKVTVNVTLLGGGFGRKSKPDFVLE
AAILAKAFPGRYLRVQWTREDDLHFSYFHTVSVERLQAVLGADGLPQAWLHRSVAPSITALFGPDSKHQGAFEL
GMGLTNLPFAIPNVRLENPEAPAHTRVGWFRSVSNIPHAFAIQSFVGELAAKAGQDPKDYLLKLLGPARRIDTAE
LGDSWNYGESPERYPLDVGRLRGVIEEAARQSGWGGELPRGRARGIAAHYSFVTYVAVVIEVEVKDDGALLVH
KATIAADCGPQINPERIRSQLEGACVMGLGLAALGEISFKDGKVQQDNFHQYELARMPLAPKAVSVHLLKPDG
DLPLGGVGEPGVPPIAPALCNAIFAATGKRIRELPIRNQLQGWRKA
>SEQF7768||SEQF7768.2_03470
MNSKIDLSNALPGSRRGFLKGAAVVGLTIGFQWSGARRALAAALPDAGFAPNAFLRIAPDDSVTVIAKHVEMG
QGAYTGIATIVAEELDADWSKVRVESAPADAKRYANLAFGTMQGTGGSSAMANSWMQLREAGAKARAMLV
EAAARQWQVPAAELRTRDGFVEHPASQRKASYGSLAAAAAELPVPENVQLKDPKDFRLIGHQAPRVDVPGKT
DGSAQFTLDVSLPGMLVALLQRPPLFGATVKSFDATATRAIPGVVEVVQVPHGVAVVAKGFWAAKQGRDALKV
EWDESKAEKRGSEALMAEYRKLAEQPGKPARRDGDAAKAVAGATRRIAASYEFPFLAHAPMEPLDAVVRLTAD
SCEIWAGDQFQTVDQGNAARTAGLKPEQVKINTLYAGGSFGRRANAWSDYIVEAVSIAKALGANGVPVKLQW
TREDDIHGGFYRPMYYHRLEAGLDADGKLVGWQHRIVGQSILEGTPFAAVMVKDGVDATSVEGAANLPYAVP
NVSVELSTTQVGVPVLWWRVVGSSHTVYAVEAFIDEAAQAAGKDPYLFRRDLLAEQPRLRGVLELAAEKAGW
DPSRPLPAGRGRGIAVTEAFKTFVAQVVEVSVDKDGKLKVERVVCAVDCGIPINPDVIAAQMEGGIGFGLGAIL
HSAITLKDGKVEQNNFDGYQVLRIAEMPKVEVHIVPSGEAPTGVGEPGVAPIGPALANAIFAATGQRLYNLPFPT
SFAKA
>SEQF7769||SEQF7769.1_02740
MKRSYPDDLVIGNLSRRGFLKGVGATGVLLVAANWGWRDALAAEKKAFGADAMPHGWVDNPKIYVSIDRDG
TVGIVCNRSEMGQGVRTSLAMVVADELEADWSRVKVIQAPGDEARYGNQDTDGSRSMRHWFEPMRRCGA
AARQMLEQAAANQWKVPLGECRAEQNKVLHAPSGRSLSFGELAEAAAGLEVPARDKLLLKKPEQFRYIGKDV
ARAIDGADIVNGRAGFGFDARFDDMLYAVVARPPVYGGKLKRYDAAAALKVPGVVKVIEIEGRPIPSEFQPLGG
VAVVAQNTWAAIKGREALMVEWDAGVNGGYDSVAYRKQLEEAARKPGKVVRDSGDAAALFAKGGDVVEAE
YYLPHLAQAPMEPPVSTAWYKDGACEVWAPTQAPQVTRERIAERLKLPFDKVTVNVTLLGGGFGRKSKPDFVL
EAAILAKAFPGRHLRVQWTREDDLHFSYFHTVSVERLQAVLGADGLPQAWLHRSVAPSITALFGPDSKHQGAF
ELGMGLTNLPFAIPNVRLENPEAPAHTRVGWFRSVSNIPHAFAIQSFVGELAAKADQDPKDYLLKLLGPARRIDT
AELGDSWNYGESPERYPLDVGRLRGVIEEAARQSGWGGELPRGRARGIAAHYSFVTYVAVVIEVEVKDDGALL
VHKATIAADCGPQINPERIRSQLEGACVMGLGLAALGEISFKDGKVQQDNFHQYELARMPLAPKAVSVHLLEP
DGDLPLGGVGEPGVPPIAPALCNAIFAATGKRIRELPIRNQLQGWRKA
>SEQF7769||SEQF7769.1_03333
MNSKIDLSNALPGSRRGFLKGAAVVGLTIGFQWSGARRALAAALPDAGFAPNAFLRIAPDDSVTVIAKHVEMG
QGAYTGIATIVAEELDADWSKVRVESAPADAKRYANLAFGTMQGTGGSSAMANSWMQLREAGAKARAMLV
EAAARQWRVPATELRTRDGFVEHPASQRKASYGSLAAAAAELPVPEKVQLKDPKDFRLIGHQAPRVDVPGKTD
GSAQFTLDVSLPGMLVALLQRPPLFGATVKSFDATATRAIPGVVEVVQVPHGVAVVAKGFWAAKQGRDALKVE
WDESKAEKRGSEALMAEYRKLAEQPGKPARRDGDAAKAVAGATRRIAASYEFPFLAHAPMEPLDAVVRLTADS
CEIWAGDQFQTVDQGNAARTAGLKPEQVKINTLYAGGSFGRRANAWSDYIVEAVSIAKALGANGVPVKLQWT
REDDIHGGFYRPMYYHRLEAGLDADGKLVGWQHRIVGQSILEGTPFAAVMVKDGVDATSVEGAANLPYAVPN

VSVELSTTQVGVPVLWWRVVGSSHTVYAVEAFIDEAAQAAGKDPYLFRRDLLAEQPRLRGVLELAAEKAGWD
PSRPLPAGRGRGIAVTEAFKTFVAQVVEVSVDKDGKLKVERVVCAVDCGIPINPDVIAAQMEGGIGFGLGAILHS
AITLKDGKVEQNNFDGYQVLRIAEMPKVEVHIVPSGEAPTGVGEPGVAPIGPALANAIFAATGQRLYNLPFPTSF
AKA
>SEQF7770||SEQF7770.1_03133
MNSKIDLSNALPGSRRGFLKGAAVVGLTIGFQWSGARRALAAALPDAGFAPNAFLRIAPDDSVTVIAKHVEMG
QGAYTGIATIVAEELDADWSKVRVESAPADAKRYANLAFGTMQGTGGSSAMANSWMQLREAGAKARAMLV
EAAARQWQIPAAELRTRDGFVEHPTSQRKASYGSLAAAAAELPVPEKVQLKDPKDFRLIGHQAPRVDVPGKTD
GSAQFTLDVSLPGMLVALLQRPPLFGATVKSFDATATRAIPGVVEVVQVPHGVAVVAKGFWAAKQGRDALKVE
WDESKAEKRGSEALMAEYRKLAEQPGKPARRDGDAAKAVAGATRRIAASYEFPFLAHAPMEPLDAVVRLTADS
CEFWAGDQFQTVDQGNAARTAGLKPEQVKINTLYAGGSFGRRANAWSDYIVEAVSIAKALGANGVPVKLQW
TREDDIHGGFYRPMYYHRLEAGLDADGKLVGWQHRIVGQSILEGTPFAAVMVKDGVDATSVEGAANLPYAVP
NVSVELSTTQVGVPVLWWRVVGSSHTVYAVEAFIDEAAQAAGKDPYLFRRDLLAEQPRLRGVLELAAEKAGW
DPSRPLPAGRGRGIAVTEAFKTFVAQVVEVSVDKDGKLKVERVVCAVDCGIPINPDVIAAQMEGGIGFGLGAIL
HSAITLKDGKVEQNNFDGYQVLRIAEMPKVEVHIVPSGEAPTGVGEPGVAPIGPALANAIFAATGQRLYNLPFPT
SFAKA
>SEQF7770||SEQF7770.1_03658
MKRSYPDDLVIGNLSRRGFLKGVGATGVLLVAANWGWRDALAAEKKAFGADAMPHGWVDNPKIYVSIDRDG
TVGIVCNRSEMGQGVRTSLAMVVADELEADWSRVKVIQAPGDEARYGNQDTDGSRSMRHWFEPMRRCGA
AARQMLEHAAANQWKVPLGECRAEQNKVLHAPSGRSLSFGELAEAAAGLEVPARDNLLLKKPEQFRYIGKDV
ARAIDGADIVNGRAGFGFDARFDDMLYAVVARPPVYGGKLKRYDAAAALKVPGVVKVIEIEGRPIPSEFQPLGG
VAVVAQNTWAAIKGREALVVEWDAGVNGGYDSVAYRKQLEEAARKPGKVVRDSGDAAALFAKGGDIVEAEYY
LPHLAQAPMEPPVSTAWYKDGACEVWAPTQAPQVTRERIAERLKLPFDKVTVNVTLLGGGFGRKSKPDFVLEA
AILAKAFPGRHLRVQWTREDDLHFSYFHTVSVERLQAVLGADGLPQAWLHRSVAPSITALFGPDSKHQGAFEL
GMGLTNLPFAIPNVRLENPEAPAHTRVGWFRSVSNIPHAFAIQSFVGELAAKAGQDPKDYLLKLLGPARRIDTAE
LGDSWNYGESPERYPLDVGRLRGVIEEAARQSGWGGELPRGRARGIAAHYSFVTYVAVVIEVEVKDDGALLVH
KATIAADCGPQINPERIRSQLEGACVMGLGLAALGEISFKDGKVQQDNFHQYELARMPLAPKAVSVHLLKPDG
DLPLGGVGEPGVPPIAPALCNAIFAATGKRIRELPIRNQLQGWRKA
>SEQF7771||SEQF7771.1_03461
MNSKIDLSNALPGSRRGFLKGAAVVGLTIGFQWSGARRALAAALPDAGFAPNAFLRIAPDDSVTVIAKHVEMG
QGAYTGIATIVAEELDADWSKVRVESAPADAKRYANLAFGTMQGTGGSSAMANSWMQLREAGAKARAMLV
EAAARQWQVPAAELRTRDGFVEHPTSQRKASYGSLAAAAAELPVPEKVQLKDPKDFRLIGHQAPRVDVPGKT
DGSAQFTLDVSLPGMLVALLQRPPLFGATVKSFDATATRAIPGVVEVVQVPHGVAVVAKGFWAAKQGRDALKV
EWDESKAEKRGSEALMAEYRKLAEQPGKPARRDGDAAKAVAGATRRIAASYEFPFLAHAPMEPLDAVVRLTAD
SCEIWAGDQFQTVDQGNAARTAGLKPEQVKINTLYAGGSFGRRANAWSDYIVEAVSIAKALGANGVPVKLQW
TREDDIHGGFYRPMYYHRLEAGLDADGKLVGWQHRIVGQSILEGTPFAAVMVKDGVDATSVEGAANLPYAVP
NVSVELSTTQVGVPVLWWRVVGSSHTVYAVEAFIDEAAQAAGKDPYLFRRDLLAEQPRLRGVLELAAEKAGW
DPSRPLPAGRGRGIAVTEAFKTFVAQVVEVSVDKDGKLKVERVVCAVDCGIPINPDVIAAQMEGGIGFGLGAIL
HSAITLKDGKVEQNNFDGYQVLRIAEMPKVEVHIVPSGEAPTGVGEPGVAPIGPALANAIFAATGQRLYNLPFPT
SFAKA
>SEQF7771||SEQF7771.1_02942
MKRSYPDDLVIGNLSRRGFLKGVGATGVLLVAANWGWRDALAAEKKAFGADAMPHGWVDNPKIYVSIDRDG
TVGIVCNRSEMGQGVRTSLAMVVADELEADWSRVKVIQAPGDEARYGNQDTDGSRSMRHWFEPMRRCGA
AARQMLEQAAANQWKVPLGECRAEQNKVLHAPSGRSLSFGELAEAAAGLEVPARDKLLLKKPEQFRYIGKDV

ARAIDGADIVNGRAGFGFDARFDDMLYAVVARPPVYGGKLKRYDAAAALKVPGVVKVIEIEGRPIPSEFQPLGG
VAVVAQNTWAAIKGREALVVEWDAGVNGGYDSVAYRKQLEEAARKPGKVVRDSGDAAALFARGGDIVEAEYY
LPHLAQAPMEPPVSTAWYKDGACEVWAPTQAPQVTRERIAERLKLPFDKVTVNVTLLGGGFGRKSKPDFVLEA
AILAKAFPGRHLRVQWTREDDLHFSYFHTVSVERLQAVLGADGLPQAWLHRSVAPSITALFGPDSKHQGAFEL
GMGLTNLPFAIPNVRLENPEAPAHTRVGWFRSVSNIPHAFAIQSFVGELAAKAGQDPKDYLLKLLGPARRIDTAE
LGDSWNYGESPERYPLDVGRLRGVIEEAARQSGWGGELPMGRARGIAAHYSFVTYVAVVIEVEVKDDGALLVH
KATIAADCGPQINPERIRSQLEGACVMGLGLAALGEISFKDGKVQQDNFHQYELARMPLAPKAVSVHLLKPDG
DLPLGGVGEPGVPPIAPALCNAIFAATGKRIRELPIRNQLQGWRKA
>SEQF7772||SEQF7772.1_02781
MKRSYPDDLVIGNLSRRGFLKGVGATGVLLVAANWGWRDALAAEKKAFGADAMPHGWVDNPKIYVSIDRDG
TVGIVCNRSEMGQGVRTSLAMVVADELEADWSRVKVIQAPGDEARYGNQDTDGSRSMRHWFEPMRRCGA
AARQMLEQAAANQWKVPLGECRAEQNKVLHAPSGRSLSFGELAEAAAGLEVPARDKLLLKKPEQFRYIGKDV
ARAIDGADIVNGRAGFGFDARFDDMLYAVVARPPVYGGKLKRYDAAAALKVPGVVKVIEIEGRPIPSEFQPLGG
VAVVAQNTWAAIKGREALVVEWDAGVNGGYDSVAYRKQLEEAARKPGKVVRDSGDAAALFAKGGDIVEAEYY
LPHLAQAPMEPPVSTAWYKDGACEVWAPTQAPQVTRERIAERLKLPFDKVTVNVTLLGGGFGRKSKPDFVLEA
AILAKAFPGRHLRVQWTREDDLHFSYFHTVSVERLQAVLGADGLPQAWLHRSVAPSITALFGPDSKHQGAFEL
GMGLTNLPFAIPNVRLENPEAPAHTRVGWFRSVSNIPHAFAIQSFVGELAAKAGQDPKDYLLKLLGPARRIDTAE
LGDSWNYGESPERYPLDVGRLRGVIEEAARQSGWGGELPRGRARGIAAHYSFVTYVAVVIEVEVKDDGALLVH
KATIAADCGPQINPERIRSQLEGACVMGLGLAALGEISFKDGKVQQDNFHQYELARMPLAPKAVSVHLLKPDG
DLPLGGVGEPGVPPIAPALCNAIFAATGKRIRELPIRNQLQGWRKA
>SEQF7772||SEQF7772.1_03283
MNSKIDLSNALPGSRRGFLKGAAVVGLTIGFQWSGARRALAAALPDAGFAPNAFLRIAPDDSVTVIAKHVEMG
QGAYTGIATIVAEELDADWSKVRVESAPADAKRYANLAFGTMQGTGGSSAMANSWMQLREAGAKARAMLV
EAAARQWQVPAAELRTRDGFVEHPTSQRKASYGSLAAAAAELPVPEKVQLKDPKDFRLIGHQAPRVDVPGKT
DGSAQFTLDVSLPGMLVALLQRPPLFGATVKSFDATATRAIPGVVEVVQVPHGVAVVAKGFWAAKQGRDALKV
EWDESKAEKRGSEALMAEYRKLAEQPGKPARRDGDAAKAVAGATRRIAASYEFPFLAHAPMEPLDAVVRLTAD
SCEIWAGDQFQTVDQGNAARTAGLKPEQVKINTLYAGGSFGRRANAWSDYIVEAVSIAKALGANGVPVKLQW
TREDDIHGGFYRPMYYHRLEAGLDADGKLVGWQHRIVGQSILEGTPFAAVMVKDGIDATSVEGAANLPYAVP
NVSVELSTTQVGVPVLWWRVVGSSHTVYAVEAFIDEAAQAAGKDPYLFRRDLLAEQPRLRGVLELAAEKAGW
DPSRPLPAGRGRGIAVTEAFKTFVAQVVEVSVDKDGKLKVERVVCAVDCGIPINPDVIAAQMEGGIGFGLGAIL
HSAITLKDGKVEQNNFDGYQVLRIAEMPKVEVHIVPSGEAPTGVGEPGVAPIGPALANAIFAATGQRLYNLPFPT
SFAKA
>SEQF7773||SEQF7773.1_02811
MKRSYPDDLVIGNLSRRGFLKGVGATGVLLVAANWGWRDALAAEKKAFGADAMPHGWVDNPKIYVSIDRDG
TVGIVCNRSEMGQGVRTSLAMVVADELEADWSRVKVIQAPGDEARYGNQDTDGSRSMRHWFEPMRRCGA
AARQMLEQAAANQWKVPLGECRAEQNKVLHAPSGRSLSFGELAEAAAGLEVPARDKLLLKKPEQFRYIGKDV
ARAIDGADIVNGRAGFGFDARFDDMLYAVVARPPVYGGKLKRYDAAAALKVPGVVKVIEIEGRPIPSEFQPLGG
VAVVAQNTWAAIKGREALVVEWDAGVNGGYDSVAYRKQLEEAARKPGKVVRDSGDAAALFAKGGDIVEAEYY
LPHLAQAPMEPPVSTAWYKDGACEVWAPTQAPQVTRERIAERLKLPFDKVTVNVTLLGGGFGRKSKPDFVLEA
AILAKAFPGRHLRVQWTREDDLHFSYFHTVSVERLQAVLGADGLPQAWLHRSVAPSITALFGPDSKHQGAFEL
GMGLTNLPFAIPNVRLENPEAPAHTRVGWFRSVSNIPHAFAIQSFVGELAAKAGQDPKDYLLKLLGPARRIDTAE
LGDSWNYGESPERYPLDVGRLRGVIEEAARQSGWGGELPRGRARGIAAHYSFVTYVAVVIEVEVKDDGALLVH
KATIAADCGPQINPERIRSQLEGACVMGLGLAALGEISFKDGKVQQDNFHQYELARMPLAPKAVSVHLLKPDG
DLPLGGVGEPGVPPIAPALCNAIFAATGKRIRELPIRNQLQGWRKA

>SEQF7773||SEQF7773.1_03208
MNSKIDLSNALPGSRRGFLKGAAVVGLTIGFQWSGARRALAAALPDAGFAPNAFLRIAPDDSVTVIAKHVEMG
QGAYTGIATIVAEELDADWSKVRVESAPADAKRYANLAFGTMQGTGGSSAMANSWMQLREAGAKARAMLV
EAAARQWRVPATELRTRDGFVEHPASQRKASYGSLAAAAAELPVPEKVQLKDPKDFRLIGHQAPRVDVPGKTD
GSAQFTLDVSLPGMLVALLQRPPLFGATVKSFDATATRAIPGVVEVVQVPHGVAVVAKGFWAAKQGRDALKVE
WDESKAEKRGSEALMAEYRKLAEQPGKPARRDGDAAKAVAGATRRIAASYEFPFLAHAPMEPLDAVVRLTADS
CEIWAGDQFQTVDQGNAARTAGLKPEQVKINTLYAGGSFGRRANAWSDYIVEAVSIAKALGANGVPVKLQWT
REDDIHGGFYRPMYYHRLEAGLDADGKLVGWQHRIVGQSILEGTPFAAVMVKNGIDATSVEGAANLPYAVPN
VSVELSTTQVGVPVLWWRVVGSSHTVYAVEAFIDEAAQAAGKDPYLFRHDLLAEQPRLRGVLELAAEKAGWD
PSRPLPAGRGRGIAVTEAFKTFVAQVVEVSVDKDGKLKVERVVCAVDCGIPINPDVIAAQMEGGIGFGLGAILHS
AITLKDGKVEQNNFDGYQVLRIAEMPKVEVHIVPSGEAPTGVGEPGVAPIGPALANAIFAATGQRLYNLPFPTSF
AKA
>SEQF7774||SEQF7774.1_02935
MKRSYPDDLVIGNLSRRGFLKGVGATGVLLVAANWGWRDALAAEKKAFGADAMPHGWVDNPKIYVSIDRDG
TVGIVCNRSEMGQGVRTSLAMVVADELEADWSRVKVIQAPGDEARYGNQDTDGSRSMRHWFEPMRRCGA
AARQMLEQAAANQWKVPLGECRAEQNKVLHAPSGRSLSFGELAEAAAGLEVPARDKLLLKKPEQFRYIGKDV
ARAIDGADIVNGRAGFGFDARFDDMLYAVVARPPVYGGKLKRYDAAAALKVPGVVKVIEIEGRPIPSEFQPLGG
VAVVAQNTWAAIKGREALVVEWDAGVNGGYDSVAYRKQLEEAARKPGKVVRDSGDAAALFAKGGDIVEAEYY
LPHLAQAPMEPPVSTAWYKDGACEVWAPTQAPQVTRERIAERLKLPFDKVTVNVTLLGGGFGRKSKPDFVLEA
AILAKAFPGRHLRVQWTREDDLHFSYFHTVSVERLQAVLGADGLPQAWLHRSVAPSITALFGPDSKHQGAFEL
GMGLTNLPFAIPNVRLENPEAPAHTRVGWFRSVSNIPHAFAIQSFVGELAAKAGQDPKDYLLKLLGPARRIDTAE
LGDSWNYGESPERYPLDVGRLRGVIEEAARQSGWGGELPRGRARGIAAHYSFVTYVAVVIEVEVKDDGALLVH
KATIAADCGPQINPERIRSQLEGACVMGLGLAALGEISFKDGKVQQDNFHQYELARMPLAPKAVSVHLLKPDG
DLPLGGVGEPGVPPIAPALCNAIFAATGKRIRELPIRNQLQGWRKA
>SEQF7774||SEQF7774.1_03484
MNSKIDLSNALPGSRRGFLKGAAVVGLTIGFQWSGARRALAAALPDAGFAPNAFLRIAPDDSVTVIAKHVEMG
QGAYTGIATIVAEELDADWSKVRVESAPADAKRYANLAFGTMQGTGGSSAMANSWMQLREAGAKARAMLV
EAAARQWRVPATELRTRDGFVEHPASQRKASYGSLAAAAAELPVPEKVQLKDPKDFRLIGHQAPRVDVPGKTD
GSAQFTLDVSLPGMLVALLQRPPLFGATVKSFDATATRAIPGVVEVVQVPHGVAVVAKGFWAAKQGRDALKVE
WDESKAEKRGSEALMAEYRKLAEQPGKPARRDGDAAKAVAGATRRIAASYEFPFLAHAPMEPLDAVVRLTADS
CEIWAGDQFQTVDQGNAARTAGLKPEQVKINTLYAGGSFGRRANAWSDYIVEAVSIAKALGANGVPVKLQWT
REDDIHGGFYRPMYYHRLEAGLDADGKLVGWQHRIVGQSILEGTPFAAVMVKDGIDATSVEGAANLPYAVPN
VSVELSTTQVGVPVLWWRVVGSSHTVYAVEAFIDEAAQAAGKDPYLFRRDLLAEQPRLRGVLELAAEKAGWD
PSRPLPAGRGRGIAVTEAFKTFVAQVVEVSVDKDGKLKVERVVCAVDCGIPINPDVIAAQMEGGIGFGLGAILHS
AITLKDGKVEQNNFDGYQVLRIAEMPKVEVHIVPSGEAPTGVGEPGVAPIGPALANAIFAATGQRLYNLPFPTSF
AKA
>SEQF7775||SEQF7775.1_00219
MNSKIDLSNALPGSRRGFLKGAAVVGLTIGFQWSGARRALAAALPDAGFAPNAFLRIAPDDSVTVIAKHVEMG
QGAYTGIATIVAEELDADWSKVRVESAPADAKRYANLAFGTMQGTGGSSAMANSWMQLREAGAKARAMLV
EAAARQWRVPATELRTRDGFVEHPASQRKASYGSLAAAAAELPVPEKVQLKDPKDFRLIGHQAPRVDVPGKTD
GSAQFTLDVSLPGMLVALLQRPPLFGATVKSFDATATRAIPGVVEVVQVPHGVAVVAKGFWAAKQGRDALKVE
WDESKAEKRGSEALMAEYRKLAEQPGKPARRDGDAAKAVAGATRRIAASYEFPFLAHAPMEPLDAVVRLTADS
CEIWAGDQFQTVDQGNAARTAGLKPEQVKINTLYAGGSFGRRANAWSDYIVEAVSIAKALGANGVPVKLQWT
REDDIHGGFYRPMYYHRLEAGLDADGKLVGWQHRIVGQSILEGTPFAAVMVKDGVDATSVEGAANLPYAVPN

VSVELSTTQVGVPVLWWRVVGSSHTVYAVEAFIDEAAQAAGKDPYLFRRDLLAEQPRLRGVLELAAEKAGWD
PSRPLPAGRGRGIAVTEAFKTFVAQVVEVSVDKDGKLKVERVVCAVDCGIPINPDVIAAQMEGGIGFGLGAILHS
AITLKDGKVEQNNFDGYQVLRIAEMPKVEVHIVPSGEAPTGVGEPGVAPIGPALANAIFAATGQRLYNLPFPTSF
AKA
>SEQF7775||SEQF7775.1_06385
MKRSYPDDLVIGNLSRRGFLKGVGATGVLLVAANWGWRDALAAEKKAFGADAMPHGWVDNPKIYVSIDRDG
TVGIVCNRSEMGQGVRTSLAMVVADELEADWSRVKVIQAPGDEARYGNQDTDGSRSMRHWFEPMRRCGA
AARQMLEQAAANQWKVPLGECRAEQNKVLHAPSGRSLSFGELAEAAAGLEVPARDKLLLKKPEQFRYIGKDV
ARAIDGADIVNGRAGFGFDARFDDMLYAVVARPPVYGGKLKRYDAAAALKVPGVVKVIEIEGRPIPSEFQPLGG
VAVVAQNTWAAIKGREALVVEWDAGVNGGYDSVAYRKQLEEAARKPGKVVRDSGDAAALFARGGDIVEAEYY
LPHLAQAPMEPPVSTAWYKDGACEVWAPTQAPQVTRERIAERLKLPFDKVTVNVTLLGGGFGRKSKPDFVLEA
AILAKAFPGRHLRVQWTREDDLHFSYFHTVSVERLQAVLGADGLPQAWLHRSVAPSITALFGPDSKHQGAFEL
GMGLTNLPFAIPNVRLENPEAPAHTRVGWFRSVSNIPHAFAIQSFVGELAAKAGQDPKDYLLKLLGPARRIDTAE
LGDSWNYGESPERYPLDVGRLRGVIEEAARQSGWGGELPRGRARGIAAHYSFVTYVAVVIEVEVKDDGALLVH
KATIAADCGPQINPERIRSQLEGACVMGLGLAALGEISFKDGKVQQDNFHQYELARMPLAPKAVSVHLLKPDG
DLPLGGVGEPGVPPIAPALCNAIFAATGKRIRELPIRNQLQGWRKA
>SEQF7776||SEQF7776.2_03647
MNSKIDLSNALPGSRRGFLKGAAVVGLTIGFQWSGARRALAAALPDAGFAPNAFLRIAPDDSVTVIAKHVEMG
QGAYTGIATIVAEELDADWSKVRVESAPADAKRYANLAFGTMQGTGGSSAMANSWMQLREAGAKARAMLV
EAAARQWRVPATELRTRDGFVEHPASQRKASYGSLAAAAAELPVPEKVQLKDPKDFRLIGHQAPRVDVPGKTD
GSAQFTLDVSLPGMLVALLQRPPLFGATVKSFDATATRAIPGVVEVVQVPHGVAVVAKGFWAAKQGRDALKVE
WDESKAEKRGSEALMAEYRKLAEQPGKPARRDGDAAKAVAGATRRIAASYEFPFLAHAPMEPLDAVVRLTADS
CEIWAGDQFQTVDQGNAARTAGLKPEQVKINTLYAGGSFGRRANAWSDYIVEAVSIAKALGANGVPVKLQWT
REDDIHGGFYRPMYYHRLEAGLDADGKLVGWQHRIVGQSILEGTPFAAVMVKDGIDATSVEGAANLPYAVPN
VSVELSTTQVGVPVLWWRVVGSSHTVYAVEAFIDEAAQAAGKDPYLFRRDLLAEQPRLRGVLELAAEKAGWD
PSRPLPAGRGRGIAVTEAFKTFVAQVVEVSVDKDGKLKVERVVCAVDCGIPINPDVIAAQMEGGIGFGLGAILHS
AITLKDGKVEQNNFDGYQVLRIAEMPKVEVHIVPSGEAPTGVGEPGVAPIGPALANAIFAATGQRLYNLPFPTSF
AKA
>SEQF7777||SEQF7777.1_04885
MKRSYPDDLVIGNLSRRGFLKGVGATGVLLVAANWGWRDALAAEKKAFGADAMPHGWVDNPKIYVSIDRDG
TVGIVCNRSEMGQGVRTSLAMVVADELEADWSRVKVIQAPGDEARYGNQDTDGSRSMRHWFEPMRRCGA
AARQMLEQAAANQWKVPLGECRAEQNKVLHAPSGRSLSFGELAEAAAGLEVPARDKLLLKKPEQFRYIGKDV
ARAIDGADIVNGRAGFGFDARFDDMLYAVVARPPVYGGKLKRYDAAAALKVPGVVKVIEIEGRPIPSEFQPLGG
VAVVAQNTWAAIKGREALVVEWDAGVNGGYDSVAYRKQLEEAARKPGKVVRDSGDAAALFAKGGDIVEAEYY
LPHLAQAPMEPPVSTAWYKDGACEVWAPTQAPQVTRERIAERLKLPFDKVTVNVTLLGGGFGRKSKPDFVLEA
AILAKAFPGRHLRVQWTREDDLHFSYFHTVSVERLQAVLGADELPQAWLHRSVAPSITALFGPDSKHQGAFELG
MGLTNLPFAIPNVRLENPEAPAHTRVGWFRSVSNIPHAFAIQSFVGELAAKAGQDPKDYLLKLLGPARRIDTAEL
GDSWNYGESPERYPLDVGRLRGVIEEAARQSGWGGELPRGRARGIAAHYSFVTYVAVVIEVEVKDDGALLVHK
ATIAADCGPQINPERIRSQLEGACVMGLGLAALGEISFKDGKVQQDNFHQYELARMPLAPKAVSVHLLKPDGD
LPLGGVGEPGVPPIAPALCNAIFAATGKRIRELPIRNQLQGWRKA
>SEQF7777||SEQF7777.1_04360
MNSKIDLSNALPGSRRGFLKGAAVVGLTIGFQWSGARRALAAALPDAGFAPNAFLRIAPDDSVTVIAKHVEMG
QGAYTGIATIVAEELDADWSKVRVESAPADAKRYANLAFGTMQGTGGSSAMANSWMQLREAGAKARAMLV
EAAARQWRVPATELRTRDGFVEHPASQRKASYGSLAAAAAELPVPEKVQLKDPKDFRLIGHQAPRVDVPGKTD

GSAQFTLDVSLPGMLVALLQRPPLFGATVKSFDATATRAIPGVVEVVQVPHGVAVVAKGFWAAKQGRDALKVE
WDESKAEKRGSEALMAEYRKLAEQPGKPARRDGDAAKAVAGATRRIAASYEFPFLAHAPMEPLDAVVRLTADS
CEIWAGDQFQTVDQGNAARTAGLKPEQVKINTLYAGGSFGRRANAWSDYIVEAVSIAKALGANGVPVKLQWT
REDDIHGGFYRPMYYHRLEAGLDADGKLVGWQHRIVGQSILEGTPFAAVMVKDGVDATSVEGAANLPYAVPN
VSVELSTTQVGVPVLWWRVVGSSHTVYAVEAFIDEAAQAAGKDPYLFRRDLLAEQPRLRGVLELAAEKAGWD
PSRPLPAGRGRGIAVTEAFKTFVAQVVEVSVDKDGKLKVERVVCAVDCGIPINPDVIAAQMEGGIGFGLGAILHS
AITLKDGKVEQNNFDGYQVLRIAEMPKVEVHIVPSGEAPTGVGEPGVAPIGPALANAIFAATGQRLYNLPFPTSF
AKA
>SEQF7778||SEQF7778.1_02490
MNSKIDLSNALPGSRRGFLKGAAVVGLTIGFQWSGARRALAAALPDAGFAPNAFLRIAPDDSVTVIAKHVEMG
QGAYTGIATIVAEELDADWSKVRVESAPADAKRYANLAFGTMQGTGGSSAMANSWMQLREAGAKARAMLV
EAAARQWRVPATELRTRDGFVEHPASQRKASYGSLAAAAAELPVPENVQLKDPKDFRLIGHQAPRVDVPGKT
DGSAQFTLDVSLPGMLVALLQRPPLFGATVKSFDATATRAIPGVVEVVQVPHGVAVVAKGFWAAKQGRDALKV
EWDESKAEKRGSEALMAEYRKLAEQPGKPARRDGDAAKAVAGATRRIAASYEFPFLAHAPMEPLDAVVRLTAD
SCEIWAGDQFQTVDQGNAARTAGLKPEQVKINTLYAGGSFGRRANAWSDYIVEAVSIAKALGANGVPVKLQW
TREDDIHGGFYRPMYYHRLEAGLDADGKLVGWQHRIVGQSILEGTPFAAVMVKDDIDATSVEGAANLPYAVP
NVSVELSTTQVGVPVLWWRVVGSSHTVYAVEAFIDEAAQAAGKDPYLFRRDLLAEQPRLRGVLELAAEKAGW
DPSRPLPAGRGRGIAVTEAFKTFVAQVVEVSVDKDGKLKVERVVCAVDCGIPINPDVIAAQMEGGIGFGLGAIL
HSAITLKDGKVEQNNFDGYQVLRIAEMPKVEVHIVPSGEAPTGVGEPGVAPIGPALANAIFAATGQRLYNLPFPT
SFAKA
>SEQF7778||SEQF7778.1_03054
MKRSFPDDLVIGNLSRRGFLKGVGATGVLLVAANWGWRDALAAEKKAFGADAMPHGWVDNPKIYVSIDRDG
TVGIVCNRSEMGQGVRTSLAMVVADELEADWSRVKVIQAPGDEARYGNQDTDGSRSMRHWFEPMRRCGA
AARQMLEQAAANQWKVPLGECRAEQNKVLHAPSGRSLSFGELAEAAAGLEVPARDKLLLKKPEQFRYIGKDV
ARAIDGADIVNGRAGFGFDARFDDMLYAVVARPPVYGGKLKRYDAAAALKVPGVVKVIEIEGRPIPSEFQPLGG
VAVVAQNTWAAIKGREALVVEWDAGVNGGYDSVAYRKQLEEAARKPGKVVRDSGDAAALFAKGGDVVEAEY
YLPHLAQAPMEPPVSTAWYKDGACEVWAPTQAPQVTRERIAERLKLPFDKVTVNVTLLGGGFGRKSKPDFVLE
AAILAKAFPGRHLRVQWTREDDLHFSYFHTVSVERLQAVLGADGLPQAWLHRSVAPSITALFGPDSKHQGAFE
LGMGLTNLPFAIPNVRLENPEAPAHTRVGWFRSVSNIPHAFAIQSFVGELAAKAGQDPKDYLLKLLGPARRIDTA
ELGDSWNYGESPERYPLDVGRLRGVIEEAARQSGWGGELPRGRARGIAAHYSFVTYVAVVIEVEVKDDGALLV
HKATIAADCGPQINPERIRSQLEGACVMGLGLAALGEISFKDGKVQQDNFHQYELARMPLAPKAVSVHLLKPD
GDLPLGGVGEPGVPPIAPALCNAIFAATGKRIRELPIRNQLQGWRKA
>SEQF7779||SEQF7779.1_02896
MKRSYPDDLVIGNLSRRGFLKGVGATGVLLVAANWGWRDALAAEKKAFGADAMPHGWVDNPKIYVSIDRDG
TVGIVCNRSEMGQGVRTSLAMVVADELEADWSRVKVIQAPGDEARYGNQDTDGSRSMRHWFEPMRRCGA
AARQMLEQAAANQWKVPLGECRAEQNKVLHAPSGRSLSFGELAEPAAGLEVPARDKLLLKKPEQFRYIGKDVA
RAIDGADIVNGRAGFGFDARFDDMLYAVVARPPVYGGKLKRYDAAAALKVPGVVKVIEIEGRPIPSEFQPLGGV
AVVAQNTWAAIKGREALVVEWDAGVNGGYDSVAYRKQLEEAARKPGKVVRDSGDAAALFAKGGDVVEAEYY
LPHLAQAPMEPPVSTAWYKDGACEVWAPTQAPQVTRERIAERLKLPFDKVTVNVTLLGGGFGRKSKPDFVLEA
AILAKAFPGRHLRVQWTREDDLHFSYFHTVSVERLQAVLGADGLPQAWLHRSVAPSITALFGPDSKHQGAFEL
GMGLTNLPFAIPNVRLENPEAPAHTRVGWFRSVSNIPHAFAIQSFVGELAAKAGQDPKDYLLKLLGPARRIDTAE
LGDSWNYGESPERYPLDVGRLRGVIEEAARQSGWGGELPRGRARGIAAHYSFVTYVAVVIEVEVKDDGALLVH
KATIAADCGPQINPERIRSQLEGACVMGLGLAALGEISFKDGKVQQDNFHQYELARMPLAPKAVSVHLLKPDG
DLPLGGVGEPGVPPIAPALCNAIFAATGKRIRELPIRNQLQGWRKA

>SEQF7779||SEQF7779.1_03420
MNSKIDLSNALPGSRRGFLKGAAVVGLTIGFQWSGARRALAAALPDAGFAPNAFLRIAPDDSVTVIAKHVEMG
QGAYTGIATIVAEELDADWSKVRVESAPADAKRYANLAFGTMQGTGGSSAMANSWMQLREAGAKARAMLV
EAAARQWQVPAAELRTRDGFVEHPASQRKASYGSLAAAAAELPVPENVQLKDPKDFRLIGHQAPRVDVPGKT
DGSAQFTLDVSLPGMLVALLQRPPLFGATVKSFDATATRAIPGVVEVVQVPHGVAVVAKGFWAAKQGRDALKV
EWDESKAEKRGSEALMAEYRKLAEQPGKPARRDGDAAKAVAGATRRIAASYEFPFLAHAPMEPLDAVVRLTAD
SCEIWAGDQFQTVDQGNAARTAGLKPEQVKINTLYAGGSFGRRANAWSDYIVEAVSIAKALGANGVPVKLQW
TREDDIHGGFYRPMYYHRLEAGLDADGKLVGWQHRIVGQSILEGTPFAAVMVKDGVDATSVEGAANLPYAVP
NVSVELSTTQVGVPVLWWRVVGSSHTVYAVEAFIDEAAQAAGKDPYLFRRDLLAEQPRLRGVLELAAEKAGW
DPSRPLPAGRGRGIAVTEAFKTFVAQVVEVSVDKDGKLKVERVVCAVDCGIPINPDVIAAQMEGGIGFGIGAILH
SAITLKDGKVEQNNFDGYQVLRIAEMPKVEVHIVPSGEAPTGVGEPGVAPIGPALANAIFAATGQRLYNLPFPTS
FAKA
>SEQF7780||SEQF7780.1_01989
MNSKIDLSNALPGSRRGFLKGAAVVGLTIGFQWSGARRALAAALPDAGFAPNAFLRIAPDDSVTVIAKHVEMG
QGAYTGIATIVAEELDADWSKVRVESAPADAKRYANLAFGTMQGTGGSSAMANSWMQLREAGAKARAMLV
EAAARQWRVPATELRTRDGFVEHPASQRKASYGSLAAAAAELPVPEKVQLKDPKDFRLIGHQAPRVDVPGKTD
GSAQFTLDVSLPGMLVALLQRPPLFGATVKSFDATATRAIPGVLEVVQVPHGVAVVAKGFWAAKQGRDALKVE
WDESKAEKRGSEALMAEYRKLAEQPGKPARRDGDAAKAVAGATRRIAASYEFPFLAHAPMEPLDAVVRLTADS
CEIWAGDQFQTVDQGNAARTAGLKPEQVKINTLYAGGSFGRRANAWSDYIVEAVSIAKALGANGVPVKLQWT
REDDIHGGFYRPMYYHRLEAGLDADGKLVGWQHRIVGQSILEGTPFAAVMVKDGIDATSVEGAANLPYAVPN
VSVELSTTQVGVPVLWWRVVGSSHTVYAVEAFIDEAAQAAGKDPYLFRRDLLAEQPRLRGVLELAAEKAGWD
PSRPLPAGRGRGIAVTEAFKTFVAQVVEVSVDKDGKLKVERVVCAVDCGIPINPDVIAAQMEGGIGFGLGAILHS
AITLKDGKVEQNNFDGYQVLRIAEMPKVEVHIVPSGEAPTGVGEPGVAPIGPALANAIFAATGQRLYNLPFPTSF
AKA
>SEQF7780||SEQF7780.1_02513
MKRSYPDDLVIGNLSRRGFLKGVGATGVLLVAANWGWRDALAAEKKAFGADAMPHGWVDNPKIYVSIDRDG
TVGIVCNRSEMGQGVRTSLAMVVADELEADWSRVKVIQAPGDEARYGNQDTDGSRSMRHWFEPMRRCGA
AARQMLEQAAANQWKVPLGECRAEQNKVLHAPSGRSLSFGELAEAAAGLEVPARDKLLLKKPEQFRYIGKDV
ARAIDGADIVNGRAGFGFDARFDDMLYAVVARPPVYGGKLKRYDAAAALKVPGVVKVIEIEGRPIPSEFQPLGG
VAVVAQNTWAAIKGREALVVEWDAGVNGGYDSVAYRKQLEEAARKPGKVVRDSGDAAALFAKGGDIVEAEYY
LPHLAQAPMEPPVSTAWYKDGACEVWAPTQAPQVTRERIAERLKLPFDKVTVNVTLLGGGFGRKSKPDFVLEA
AILAKAFPGRHLRVQWTREDDLHFSYFHTVSVERLQAVLGADGLPQAWLHRSVAPSITALFGPDSKHQGAFEL
GMGLTNLPFAIPNVRLENPEAPAHTRVGWFRSVSNIPHAFAIQSFVGELAAKAGQDPKDYLLKLLGPARRIDTAE
LGDSWNYGESPERYPLDVGRLRGVIEEAARQSGWGGELPRGRARGIAAHYSFVTYVAVVIEVEVKDDGALLVH
KATIAADCGPQINPERIRSQLEGACVMGLGLAALGEISFKDGKVQQDNFHQYELARMPLAPKAVSVHLLKPDG
DLPLGGVGEPGVPPIAPALCNAIFAATGKRIRELPIRNQLQGWRKA
>SEQF7781||SEQF7781.1_03886
MKRSYPDDLVIGNLSRRGFLKGVGATGVLLVAANWGWRDALAAEKKAFGADAMPHGWVDNPKIYVSIDRDG
TVGIVCNRSEMGQGVRTSLAMVVADELEADWSRVKVIQAPGDEARYGNQDTDGSRSMRHWFEPMRRCGA
AARQMLEQAAANQWKVPLGECRAEQNKVLHAPSGRSLSFGELAEAAAGLEVPARDKLLLKKPEQFRYIGKDV
ARAIDGADIVNGRAGFGFDARFDDMLYAVVARPPVYGGKLKRYDAAAALKVPGVVKVIEIEGRPIPSEFQPLGG
VAVVAQNTWAAIKGREALVVEWDAGVNGGYDSVAYRKQLEEAARKPGKVVRDSGDAAALFAKGGDIVEAEYY
LPHLAQAPMEPPVSTAWYKDGACEVWAPTQAPQVTRERIAERLKLPFDKVTVNVTLLGGGFGRKSKPDFVLEA
AILAKAFPGRHLRVQWTREDDLHFSYFHTVSVERLQAVLGADGLPQAWLHRSVAPSITALFGPDSKHQGAFEL

GMGLTNLPFAIPNVRLENPEAPAHTRVGWFRSVSSIPHAFAIQSFVGELAAKAGQDPKDYLLKLLGPARRIDTAE
LGDSWNYGESPERYPLDVGRLRGVIEEAARQSGWGGELPRGRARGIAAHYSFVTYVAVVIEVEVKDDGALLVH
KATIAADCGPQINPERIRSQLEGACVMGLGLAALGEISFKDGKVQQDNFHQYELARMPLAPKAVSVHLLKPDG
DLPLGGVGEPGVPPIAPALCNAIFAATGKRIRELPIRNQLQGWRKA
>SEQF7781||SEQF7781.1_03318
MNSKIDLSNALPGSRRGFLKGAAVVGLTIGFQWSGARRALAAALPDAGFAPNAFLRIAPDDSVTVIAKHVEMG
QGAYTGIATIVAEELDADWSKVRVESAPADAKRYANLAFGTMQGTGGSSAMANSWMQLREAGAKARAMLV
EAAARQWRVPATELRTRDGFVEHPASQRKASYGSLAAAAAELPVPEKVQLKDPKDFRLIGHQAPRVDVPGKTD
GSAQFTLDVSLPGMLVALLQRPPRFGATVKSFDATATRAIPGVLEVVQVPHGVAVVAKGFWAAKQGRDALKVE
WDESKAEKRGSEALMAEYRKLAEQPGKPARRDGDAAKAVAGATRRIAASYEFPFLAHAPMEPLDAVVRLTADS
CEIWAGDQFQTVDQGNAARTAGLKPEQVKINTLYAGGSFGRRANAWSDYIVEAVSIAKALGANGVPVKLQWT
REDDIHGGFYRPMYYHRLEAGLDADGKLVGWQHRIVGQSILEGTPFAAVMVKDGVDATSVEGAANLPYAVPN
VSVELSTTQVGVPVLWWRVVGSSHTVYAVEAFIDEAAQAAGKDPYLFRRDLLAEQPRLRGVLELAAEKAGWD
PSRPLPAGRGRGIAVTEAFKTFVAQVVEVSVDKDGKLKVERVVCAVDCGIPINPDVIAAQMEGGIGFGLGAILHS
AITLKDGKVEQNNFDGYQVLRIAEMPKVEVHIVPSGEAPTGVGEPGVAPIGPALANAIFAATGQRLYNLPFPTSF
AKA
>SEQF7782||SEQF7782.1_03199
MNSKIDLSNALPGSRRGFLKGAAVVGLTIGFQWSGARRALAAALPDAGFAPNAFLRIAPDDSVTVIAKHVEMG
QGAYTGIATIVAEELDADWSKVRVESAPADAKRYANLAFGTMQGTGGSSAMANSWMQLREAGAKARAMLV
EAAARQWQVPAAELRTRDGFVEHPTSQRKASYGSLAAAAAELPVPEKVQLKDPKDFRLIGHQAPRVDVPGKT
DGSAQFTLDVSLPGMLVALLQRPPLFGATVKSFDATATRAIPGVVEVVQVPHGVAVVAKGFWAAKQGRDALKV
EWDESKAEKRGSEALMAEYRKLAEQPGKPARRDGDAAKAVAGATRRIAASYEFPFLAHAPMEPLDAVVRLTAD
SCEIWAGDQFQTVDQGNAARTAGLKPEQVKINTLYAGGSFGRRANAWSDYIVEAVSIAKALGANGVPVKLQW
TREDDIHGGFYRPMYYHRLEAGLDADGKLVGWQHRIVGQSILEGTPFAAVMVKDGIDATSVEGAANLPYAVP
NVSVELSTTQVGVPVLWWRVVGSSHTVYAVEAFIDEAAQAAGKDPYLFRRDLLAEQPRLRGVLELAAEKAGW
DPSRPLPAGRGRGIAVTEAFKTFVAQVVEVSVDKDGKLKVERVVCAVDCGIPINPDVIAAQMEGGIGFGLGAIL
HSAITLKDGKVEQNNFDGYQVLRIAEMPKVEVHIVPSGEAPTGVGEPGVAPIGPALANAIFAATGQRLYNLPFPT
SFAKA
>SEQF7782||SEQF7782.1_02686
MKRSYPDDLVIGNLSRRGFLKGVGATGVLLVAANWGWRDALAAEKKAFGADAMPHGWVDNPKIYVSIDRDG
TVGIVCNRSEMGQGVRTSLAMVVADELEADWSRVKVIQAPGDEARYGNQDTDGSRSMRHWFEPMRRCGA
AARQMLEQAAANQWKVPLGECRAEQNKVLHAPSGRSLSFGELAEAAAGLEVPARDKLLLKKPEQFRYIGKDV
ARAIDGADIVNGRAGFGFDARFDDMLYAVVARPPVYGGKLKRYDAAAALKVPGVVKVIEIEGRPIPSEFQPLGG
VAVVAQNTWAAIKGREALVVEWDAGVNGGYDSVAYRKQLEEAARKPGKVVRDSGDAAALFAKGGDIVEAEYY
LPHLAQAPMEPPVSTAWYKDGACEVWAPTQAPQVTRERIAERLKLPFDKVTVNVTLLGGGFGRKSKPDFVLEA
AILAKAFPGRHLRVQWTREDDLHFSYFHTVSVERLQAVLGADGLPQAWLHRSVAPSITALFGPDSKHQGAFEL
GMGLTNLPFAIPNVRLENPEAPAHTRVGWFRSVSNIPHAFAIQSFVGELAAKAGQDPKDYLLKLLGPARRIDTAE
LGDSWNYGESPERYPLDVGRLRGVIEEAARQSGWGGELPRGRARGIAAHYSFVTYVAVVIEVEVKDDGALLVH
KATIAADCGPQINPERIRSQLEGACVMGLGLAALGEISFKDGKVQQDNFHQYELARMPLAPKAVSVHLLKPDG
DLPLGGVGEPGVPPIAPALCNAIFAATGKRIRELPIRNQLQGWRKA
>SEQF7783||SEQF7783.1_03226
MNSKIDLSNALPGSRRGFLKGAAVVGLTIGFQWSGARRALAAALPDAGFAPNAFLRIAPDDSVTVIAKHVEMG
QGAYTGIATIVAEELDADWSKVRVESAPADAKRYANLAFGTMQGTGGSSAMANSWMQLREAGAKARAMLV
EAAARQWQIPAAELRTRDGFVEHPASQRKASYGSLAAAAAELPVPEKVQLKDPKDFRLIGHQAPRVDVPGKTD

GSAQFTLDVSLPGMLVALLQRPPLFGATVKSFDATATRAIPGVVEVVQVPHGVAVVAKGFWAAKQGRDALKVE
WDESKAEKRGSEALMAEYRKLAEQPGKPARRDGDAAKAVAGATRRIAASYEFPFLAHAPMEPLDAVVRLTADS
CEIWAGDQFQTVDQGNAARTAGLKPEQVKINTLYAGGSFGRRANAWSDYIVEAVSIAKALGANGVPVKLQWT
REDDIHGGFYRPMYYHRLEAGLDADGKLVGWQHRIVGQSILEGTPFAAVMVKDGVDATSVEGAANLPYAVPN
VSVELSTTQVGVPVLWWRVVGSSHTVYAVEAFIDEAAQAAGKDPYLFRRDLLAEQPRLRGVLELAAEKAGWD
PSRPLPAGRGRGIAVTEAFKTFVAQVVEVSVDKDGKLKVERVVCAVDCGIPINPDVIAAQMEGGIGFGLGAILHS
AITLKDGKVEQNNFDGYQVLRIAEMPKVEVHIVPSGEAPTGVGEPGVAPIGPALANAIFAATGQRLYNLPFPTSF
AKA
>SEQF7783||SEQF7783.1_02718
MKRSFPDDLVIGNLSRRGFLKGVGATGVLLVAANWGWRDALAAEKKAFGADAMPHGWVDNPKIYVSIDRDG
TVGIVCNRSEMGQGVRTSLAMVVADELEADWSRVKVIQAPGDEARYGNQDTDGSRSMRHWFEPMRRCGA
AARQMLEQAAANQWKVPLGECRAEQNKVLHAPSGRSLSFGELAEAAAGLEVPARDKLLLKKPEQFRYIGKDV
ARAIDGADIVNGRAGFGFDARFDDMLYAVVARPPVYGGKLKRYDAAAALKVPGVVKVIEIEGRPIPSEFQPLGG
VAVVAQNTWAAIKGREALVVEWDAGVNGGYDSVAYRKQLEEAARKPGKVVRDSGDAAALFAKGGDIVEAEYY
LPHLAQAPMEPPVSTAWYKDGACEVWAPTQAPQVTRERIAERLKLPFDKVTVNVTLLGGGFGRKSKPDFVLEA
AILAKAFPGRHLRVQWTREDDLHFSYFHTVSVERLQAVLGADGLPQAWLHRSVAPSITALFGPDSKHQGAFEL
GMGLTNLPFAIPNVRLENPEAPAHTRVGWFRSVSNIPHAFAIQSFVGELAAKAGQDPKDYLLKLLGPARRIDTAE
LGDSWNYGESPERYPLDVGRLRGVIEEAARQSGWGGELPRGRARGIAAHYSFVTYVAVVIEVEVKDDGALLVH
KATIAADCGPQINPERIRSQLEGACVMGLGLAALGEISFKDGKVQQDNFHQYELARMPLAPKAVSVHLLKPDG
DLPLGGVGEPGVPPIAPALCNAIFAATGKRIRELPIRNQLQGWRKA
>SEQF7784||SEQF7784.1_03389
MNSKIDLSNALPGSRRGFLKGAAVVGLTIGFQWSGARRALAAALPDAGFAPNAFLRIAPDDSVTVIAKHVEMG
QGAYTGIATIVAEELDADWSKVRVESAPADAKRYANLAFGTMQGTGGSSAMANSWMQLREAGAKARAMLV
EAAARQWRVPATELRTRDGFVEHPASQRKASYGSLAAAAAELPVPEKVQLKDPKDFRLIGHQAPRVDVPGKTD
GSAQFTLDVSLPGMLVALLQRPPLFGATVKSFDATATRAIPGVVEVVQVPHGVAVVAKGFWAAKQGRDALKVE
WDESKAEKRGSEALMAEYRKLAEQPGKPARRDGDAAKAVAGATRRIAASYEFPFLAHAPMEPLDAVVRLTADS
CEIWAGDQFQTVDQGNAARTAGLKPEQVKINTLYAGGSFGRRANAWSDYIVEAVSIAKALGANGVPVKLQWT
REDDIHGGFYRPMYYHRLEAGLDADGKLVGWQHRIVGQSILEGTPFAAVMVKDGVDATSVEGAANLPYAVPN
VSVELSTTQVGVPVLWWRVVGSSHTVYAVEAFIDEAAQAAGKDPYLFRRDLLAEQPRLRGVLELAAEKAGWD
PSRPLPAGRGRGIAVTEAFKTFVAQVVEVSVDKDGKLKVERVVCAVDCGIPINPDVIAAQMEGGIGFGLGAILHS
AITLKDGKVEQNNFDGYQVLRIAEMPKVEVHIVPSGEAPTGVGEPGVAPIGPALANAIFAATGQRLYNLPFPTSF
AKA
>SEQF7784||SEQF7784.1_02851
MKRSYPDDLVIGNLSRRGFLKGVGATGVLLVAANWGWRDALAAEKKAFGADAMPHGWVDNPKIYVSIDRDG
TVGVVCNRSEMGQGVRTSLAMVVADELEADWSRVKVIQAPGDEARYGNQDTDGSRSMRHWFEPMRRCG
AAARQMLEQAAANQWKVPLGECRAEQNKVLHAPSGRSLSFGELAEAAAGLEVPARDKLLLKKPEQFRYIGKD
VARAIDGADIVNGRAGFGFDARFDDMLYAVVARPPVYGGKLKRYDAAAALKVPGVVKVIEIEGRPIPSEFQPLG
GVAVVAQNTWAAIKGREALVVEWDAGVNGGYDSVAYRKQLEEAARKPGKVVRDSGDAAALFAKGGDIVEAE
YYLPHLAQAPMEPPVSTAWYKDGACEVWAPTQAPQVTRERIAERLKLPFDKVTVNVTLLGGGFGRKSKPDFVL
EAAILAKAFPGRHLRVQWTREDDLHFSYFHTVSVERLQAVLGADGLPQAWLHRSVAPSITALFGPDSKHQGAF
ELGMGLTNLPFAIPNVRLENPEAPAHTRVGWFRSVSNIPHAFAIQSFVGELAAKAGQDPKDYLLKLLGPARRIDT
AELGDSWNYGESPERYPLDVGRLRGVIEEAARQSGWGGELPRGRARGIAAHYSFVTYVAVVIEVEVKDDGALL
VHKATIAADCGPQINPERIRSQLEGACVMGLGLAALGEISFKDGKVQQDNFHQYELARMPLAPKAVSVHLLKP
DGDLPLGGVGEPGVPPIAPALCNAIFAATGKRIRELPIRNQLQGWRKA

>SEQF7923||SEQF7923.1_02054
MRIAIQNLSRRRFVQGAGGLLLGLSLPPLARRAMAAGPQAGDGFAANAFVRIGADGRVTVLAKHLEMGQGA
YTGLATLLAEELDADWRQVRVEGAPADSARYGNQALGGLQGTGGSTAMFDSWEPMRRAGATARAMLVQAA
AQRWQVPADTIEVAEGVLSHPASGRRAGFGELAEAAARSPVPEDVPLKDPARFRLIGKHRLPHVDSAAKSDGS
ALYTQDMKLPGMLVAVVAHAPRLGAAVARVDDAAARAVPGVRAVVRFGGAALRHAGVAVLATNTWAARAG
RDALRIEWDEGPAYRQGSADILARYREAVGRPGSMAARKGDIDAAFAGAAKVIEAEYTYPYLAHAAMEPLNCL
VRLDDERCEIWNGEQFQTADQRAIAQYLGMPAERITLTQLYAGGSFGRRASSHADYLLEAVAIARTARAQGLNA
PVKLVWMREDDMRAGYYRPLNLHRARLALGADGALQAVHVRMAGQSILLGTPLADWVRDGVDPVSVEGLS
DLAYAVPNLQVELHTPTDVPVPVLWYRSVGHTHTAFSAETLIDEAAVAAGQDPVAYRLALLAAHPRHREVLQLA
AVRAGWREPLAAGAPGTRRGRGVAVHESFRSVMAQVVEVTIAADGALKVDRVVCAAVHCGLAVNPDVVRA
QMEGGIGFALSTALHGAITLKDGAVEQSNFHDYPVLRLAEMPAVEVHIAPSTQPPTGVGEPGVPPLAPALANAI
AQATGQRLRTLPLGTTVKA
>SEQF7924||SEQF7924.1_01046
MRIAIQNLSRRRFVQGAGGLLLGLSLPPLARRAMAAGPQAGDGFAANAFVRIGADGRVTVLAKHLEMGQGA
YTGLATLLAEELDADWRQVRVEGAPADSARYGNQALGGLQGTGGSTAMFDSWEPMRRAGATARAMLVQAA
AQRWQVPADTIEVAEGVLSHPASGRRAGFGELAEAAARSPVPEDVPLKDPARFRLIGKHRLPRVDSAAKSDGS
ALYTQDMKLPGMLVAVVAHAPRLGAAVARVDDAAARAVPGVRAVVRFGGAALRHAGVAVLATNTWAARAG
RDALRIEWDEGPAYRQGSADILARYREAVGRPGSMAARKGDIDAAFAGAAKVIEAEYTYPYLAHAAMEPLNCL
VRLDDERCEIWNGEQFQTADQRAIAQYLGMPAERITLTQLYAGGSFGRRASSHADYLLEAVAIARTARAQGLNA
PVKLVWMREDDMRAGYYRPLNLHRARLALGADGALQAVHVRMAGQSILLGTPLADWVRDGVDPVSVEGLS
DLAYAVPNLQVELHTPTDVPVPVLWYRSVGHTHTAFSAETLIDEAAVAAGQDPVAYRLALLAAHPRHREVLQLA
AARAGWREPLAAGAPGTRRGRGVAVHESFRSVMAQVVEVTIAADGALKVDRVVCAAVHCGLAVNPDVVRA
QMEGGIGFALSTALHGAITLKDGAVEQSNFHDYPVLRLAEMPAVEVHIAPSTQPPTGVGEPGVPPLAPALANAI
AQATGQRLRTLPLGTTVKA
>SEQF7925||SEQF7925.1_01465
MRIAIQNLSRRRFVQGAGGLLLGLSLPPLARRAMAAGPQAGDGFAANAFVRIGADGRVTVLAKHLEMGQGA
YTGLATLLAEELDADWRQVRVEGAPADSARYGNQALGGLQGTGGSTAMFDSWEPMRRAGATARAMLVQAA
AQRWQVPADTIEVAEGVLSHPASGRRAGFGELAEAAARSPVPEDVPLKDPARFRLIGKHRLPRVDSAAKSDGS
ALYTQDMKLPGMLVAVVAHAPRLGAAVARVDDAAARAVPGVRAVVRFGGAALRHAGVAVLATNTWAARAG
RDALRIEWDEGPAYRQGSADILARYREAVGRPGSMAARKGDIDAAFAGAAKVIEAEYTYPYLAHAAMEPLNCL
VRLDDERCEIWNGEQFQTADQRAIAQYLGMPAERITLTQLYAGGSFGRRASSHADYLLEAVAIARTARAQGLNA
PVKLVWMREDDMRAGYYRPLNLHRARLALGADGALQAVHVRMAGQSILLGTPLADWVRDGVDPVSVEGLS
DLAYAVPNLQVELHTPTDVPVPVLWYRSVGHTHTAFSAETLIDEAAVAAGQDPVAYRLALLAAHPRHREVLQLA
AARAGWREPLAAGAPGTRRGRGVAVHESFRSVMAQVVEVTIAADGALKVDRVVCAAVHCGLAVNPDVVRA
QMEGGIGFALSTALHGAITLKDGAVEQSNFHDYPVLRLAEMPAVEVHIAPSTQPPTGVGEPGVPPLAPALANAI
AQATGQRLRTLPLGTTVKA
>SEQF7926||SEQF7926.1_01978
MRIAIQNLSRRRFVQGAGGLLLGLSLPPLARRAMAAGPQAGDGFAANAFVRIGADGRVTVLAKHLEMGQGA
YTGLATLLAEELDADWRQVRVEGAPADSARYGNQALGGLQGTGGSTAMFDSWEPMRRAGATARAMLVQAA
AQRWQVPADTIEVAEGVLSHPASGRRAGFGELAEAAARSPVPEDVPLKDPARFRLIGKHRLPHVDSAAKSDGS
ALYTQDMKLPGMLVAVVAHAPRLGAAVARVDDAAARAVPGVRAVVRFGGAALRHAGVAVLATNTWAARAG
RDALRIEWDEGPAYRQGSADILARYREAVGRPGSMAARKGDIDAAFAGAAKVIEAEYTYPYLAHAAMEPLNCL
VRLDDERCEIWNGEQFQTADQRAIAQYLGMPAERITLTQLYAGGSFGRRASSHADYLLEAVAIARTARAQGLNA
PVKLVWMREDDMRAGYYRPLNLHRARLALGADGALQAVHVRMAGQSILLGTPLADWVRDGVDPVSVEGLS

DLAYAVPNLQVELHTPTDVPVPVLWYRSVGHTHTAFSAETLIDEAAVAAGQDPVAYRLALLAAHPRHREVLQLA
AVRAGWREPLAAGAPGTRRGRGVAVHESFRSVMAQVVEVTIAADGALKVDRVVCAAVHCGLAVNPDVVRA
QMEGGIGFALSTALHGAITLKDGAVEQSNFHDYPVLRLAEMPAVEVHIAPSTQPPTGVGEPGVPPLAPALANAI
AQATGQRLRTLPLGTTVKA
>SEQF7927||SEQF7927.1_01973
MRIAIQNLSRRRFVQGAGGLLLGLSLPPLARRAMAAGPQAGDGFAANAFVRIGADGRVTVLAKHLEMGQGA
YTGLATLLAEELDADWRQVRVEGAPADSARYGNQALGGLQGTGGSTAMFDSWEPMRRAGATARAMLVQAA
AQRWQVPADTIEVAEGVLSHPASGRRAGFGELAEAAARSPVPEDVPLKDPARFRLIGKHRLPHVDSAAKSDGS
ALYTQDMKLPGMLVAVVAHAPRLGAAVARVDDAAARAVPGVRAVVRFGGAALRHAGVAVLATNTWAARAG
RDALRIEWDEGPAYRQGSADILARYREAVGRPGSMAARKGDIDAAFAGAAKVIEAEYTYPYLAHAAMEPLNCL
VRLDDERCEIWNGEQFQTADQRAIAQYLGMPAERITLTQLYAGGSFGRRASSHADYLLEAVAIARTARAQGLNA
PVKLVWMREDDMRAGYYRPLNLHRARLALGADGALQAVHVRMAGQSILLGTPLADWVRDGVDPVSVEGLS
DLAYAVPNLQVELHTPTDVPVPVLWYRSVGHTHTAFSAETLIDEAAVAAGQDPVAYRLALLAAHPRHREVLQLA
AVRAGWREPLAAGAPGTRRGRGVAVHESFRSVMAQVVEVTIAADGALKVDRVVCAAVHCGLAVNPDVVRA
QMEGGIGFALSTALHGAITLKDGAVEQSNFHDYPVLRLAEMPAVEVHIAPSTQPPTGVGEPGVPPLAPALANAI
AQATGQRLRTLPLGTTVKA
>SEQF7928||SEQF7928.1_02132
MRIAIQNLSRRRFVQGAGGLLLGLSLPPLARRAMAAGPQAGDGFAANAFVRIGADGRVTVLAKHLEMGQGA
YTGLATLLAEELDADWRQVRVEGAPADSARYGNQALGGLQGTGGSTAMFDSWEPMRRAGATARAMLVQAA
AQRWQVPADTIEVAEGVLSHPASGRRAGFGELAEAAARSPVPEDVPLKDPARFRLIGKHRLPHVDSAAKSDGS
ALYTQDMKLPGMLVAVVAHAPRLGAAVARVDDAAARAVPGVRAVVRFGGAALRHAGVAVLATNTWAARAG
RDALRIEWDEGPAYRQGSADILARYREAVGRPGSMAARKGDIDAAFAGAAKVIEAEYTYPYLAHAAMEPLNCL
VRLDDERCEIWNGEQFQTADQRAIAQYLGMPAERITLTQLYAGGSFGRRASSHADYLLEAVAIARTARAQGLNA
PVKLVWMREDDMRAGYYRPLNLHRARLALGADGALQAVHVRMAGQSILLGTPLADWVRDGVDPVSVEGLS
DLAYAVPNLQVELHTPTDVPVPVLWYRSVGHTHTAFSAETLIDEAAVAAGQDPVAYRLALLAAHPRHREVLQLA
AVRAGWREPLAAGAPGTRRGRGVAVHESFRSVMAQVVEVTIAADGALKVDRVVCAAVHCGLAVNPDVVRA
QMEGGIGFALSTALHGAITLKDGAVEQSNFHDYPVLRLAEMPAVEVHIAPSTQPPTGVGEPGVPPLAPALANAI
AQATGQRLRTLPLGTTVKA
>SEQF7929||SEQF7929.1_01991
MRIAIQNLSRRRFVQGAGGLLLGLSLPPLARRAMAAGPQAGDGFAANAFVRIGADGRVTVLAKHLEMGQGA
YTGLATLLAEELDADWRQVRVEGAPADSARYGNQALGGLQGTGGSTAMFDSWEPMRRAGATARAMLVQAA
AQRWQVPADTIEVAEGVLSHPASGRRAGFGELAEAAARSPVPEDVPLKDPARFRLIGKHRLPHVDSAAKSDGS
ALYTQDMKLPGMLVAVVAHAPRLGAAVARVDDAAARAVPGVRAVVRFGGAALRHAGVAVLATNTWAARAG
RDALRIEWDEGPAYRQGSADILARYREAVGRPGSMAARKGDIDAAFAGAAKVIEAEYTYPYLAHAAMEPLNCL
VRLDDERCEIWNGEQFQTADQRAIAQYLGMPAERITLTQLYAGGSFGRRASSHADYLLEAVAIARTARAQGLNA
PVKLVWMREDDMRAGYYRPLNLHRARLALGADGALQAVHVRMAGQSILLGTPLADWVRDGVDPVSVEGLS
DLAYAVPNLQVELHTPTDVPVPVLWYRSVGHTHTAFSAETLIDEAAVAAGQDPVAYRLALLAAHPRHREVLQLA
AVRAGWREPLAAGAPGTRRGRGVAVHESFRSVMAQVVEVTIAADGALKVDRVVCAAVHCGLAVNPDVVRA
QMEGGIGFALSTALHGAITLKDGAVEQSNFHDYPVLRLAEMPAVEVHIAPSTQPPTGVGEPGVPPLAPALANAI
AQATGQRLRTLPLGTTVKA
>SEQF7930||SEQF7930.1_01968
MRIAIQNLSRRRFVQGAGGLLLGLSLPPLARRAMAAGPQAGDGFAANAFVRIGADGRVTVLAKHLEMGQGA
YTGLATLLAEELDADWRQVRVEGAPADSARYGNQALGGLQGTGGSTAMFDSWEPMRRAGATARAMLVQAA
AQRWQVPADTIEVAEGVLSHPASGRRAGFGELAEAAARSPVPEDVPLKDPARFRLIGKHRLPHVDSAAKSDGS

ALYTQDMKLPGMLVAVVAHAPRLGAAVARVDDAAARAVPGVRAVVRFGGAALRHAGVAVLATNTWAARAG
RDALRIEWDEGPAYRQGSADILARYREAVGRPGSMAARKGDIDAAFAGAAKVIEAEYTYPYLAHAAMEPLNCL
VRLDDERCEIWNGEQFQTADQRAIAQYLGMPAERITLTQLYAGGSFGRRASSHADYLLEAVAIARTARAQGLNA
PVKLVWMREDDMRAGYYRPLNLHRARLALGADGALQAVHVRMAGQSILLGTPLADWVRDGVDPVSVEGLS
DLAYAVPNLQVELHTPTDVPVPVLWYRSVGHTHTAFSAETLIDEAAVAAGQDPVAYRLALLAAHPRHREVLQLA
AVRAGWREPLAAGAPGTRRGRGVAVHESFRSVMAQVVEVTIAADGALKVDRVVCAAVHCGLAVNPDVVRA
QMEGGIGFALSTALHGAITLKDGAVEQSNFHDYPVLRLAEMPAVEVHIAPSTQPPTGVGEPGVPPLAPALANAI
AQATGQRLRTLPLGTTVKA
>SEQF7931||SEQF7931.1_02017
MRIAIQNLSRRRFVQGAGGLLLGLSLPPLARRAMAAGPQAGDGFAANAFVRIGADGRVTVLAKHLEMGQGA
YTGLATLLAEELDADWRQVRVEGAPADSARYGNQALGGLQGTGGSTAMFDSWEPMRRAGATARAMLVQAA
AQRWQVPADTIEVAEGVLSHPASGRRAGFGELAEAAARSPVPEDVPLKDPARFRLIGKHRLPHVDSAAKSDGS
ALYTQDMKLPGMLVAVVAHAPRLGAAVARVDDAAARAVPGVRAVVRFGGAALRHAGVAVLATNTWAARAG
RDALRIEWDEGPAYRQGSADILARYREAVGRPGSMAARKGDIDAAFAGAAKVIEAEYTYPYLAHAAMEPLNCL
VRLDDERCEIWNGEQFQTADQRAIAQYLGMPAERITLTQLYAGGSFGRRASSHADYLLEAVAIARTARAQGLNA
PVKLVWMREDDMRAGYYRPLNLHRARLALGADGALQAVHVRMAGQSILLGTPLADWVRDGVDPVSVEGLS
DLAYAVPNLQVELHTPTDVPVPVLWYRSVGHTHTAFSAETLIDEAAVAAGQDPVAYRLALLAAHPRHREVLQLA
AVRAGWREPLAAGAPGTRRGRGVAVHESFRSVMAQVVEVTIAADGALKVDRVVCAAVHCGLAVNPDVVRA
QMEGGIGFALSTALHGAITLKDGAVEQSNFHDYPVLRLAEMPAVEVHIAPSTQPPTGVGEPGVPPLAPALANAI
AQATGQRLRTLPLGTTVKA
>SEQF7932||SEQF7932.1_01893
MRIAIQNLSRRRFVQGAGGLLLGLSLPPLARRAMAAGPQAGDGFAANAFVRIGADGRVTVLAKHLEMGQGA
YTGLATLLAEELDADWRQVRVEGAPADSARYGNQALGGLQGTGGSTAMFDSWEPMRRAGATARAMLVQAA
AQRWQVPADTIEVAEGVLSHPASGRRAGFGELAEAAARSPVPEDVPLKDPARFRLIGKHRLPHVDSAAKSDGS
ALYTQDMKLPGMLVAVVAHAPRLGAAVARVDDAAARAVPGVRAVVRFGGAALRHAGVAVLATNTWAARAG
RDALRIEWDEGPAYRQGSADILARYREAVGRPGSMAARKGDIDAAFAGAAKVIEAEYTYPYLAHAAMEPLNCL
VRLDDERCEIWNGEQFQTADQRAIAQYLGMPAERITLTQLYAGGSFGRRASSHADYLLEAVAIARTARAQGLNA
PVKLVWMREDDMRAGYYRPLNLHRARLALGADGALQAVHVRMAGQSILLGTPLADWVRDGVDPVSVEGLS
DLAYAVPNLQVELHTPTDVPVPVLWYRSVGHTHTAFSAETLIDEAAVAAGQDPVAYRLALLAAHPRHREVLQLA
AVRAGWREPLAAGAPGTRRGRGVAVHESFRSVMAQVVEVTIAADGALKVDRVVCAAVHCGLAVNPDVVRA
QMEGGIGFALSTALHGAITLKDGAVEQSNFHDYPVLRLAEMPAVEVHIAPSTQPPTGVGEPGVPPLAPALANAI
AQATGQRLRTLPLGTTVKA
>SEQF7933||SEQF7933.1_01972
MRIAIQNLSRRRFVQGAGGLLLGLSLPPLARRAMAAGPQAGDGFAANAFVRIGADGRVTVLAKHLEMGQGA
YTGLATLLAEELDADWRQVRVEGAPADSARYGNQALGGLQGTGGSTAMFDSWEPMRRAGATARAMLVQAA
AQRWQVPADTIEVAEGVLSHPASGRRAGFGELAEAAARSPVPEDVPLKDPARFRLIGKHRLPHVDSAAKSDGS
ALYTQDMKLPGMLVAVVAHAPRLGAAVARVDDAAARAVPGVRAVVRFGGAALRHAGVAVLATNTWAARAG
RDALRIEWDEGPAYRQGSADILARYREAVGRPGSMAARKGDIDAAFAGAAKVIEAEYTYPYLAHAAMEPLNCL
VRLDDERCEIWNGEQFQTADQRAIAQYLGMPAERITLTQLYAGGSFGRRASSHADYLLEAVAIARTARAQGLNA
PVKLVWMREDDMRAGYYRPLNLHRARLALGADGALQAVHVRMAGQSILLGTPLADWVRDGVDPVSVEGLS
DLAYAVPNLQVELHTPTDVPVPVLWYRSVGHTHTAFSAETLIDEAAVAAGQDPVAYRLALLAAHPRHREVLQLA
AVRAGWREPLAAGAPGTRRGRGVAVHESFRSVMAQVVEVTIAADGALKVDRVVCAAVHCGLAVNPDVVRA
QMEGGIGFALSTALHGAITLKDGAVEQSNFHDYPVLRLAEMPAVEVHIAPSTQPPTGVGEPGVPPLAPALANAI
AQATGQRLRTLPLGTTVKA

>SEQF7934||SEQF7934.1_01926
MRIAIQNLSRRRFVQGAGGLLLGLSLPPLARRAMAAGPQAGDGFAANAFVRIGADGRVTVLAKHLEMGQGA
YTGLATLLAEELDADWRQVRVEGAPADSARYGNQALGGLQGTGGSTAMFDSWEPMRRAGATARAMLVQAA
AQRWQVPADTIEVAEGVLSHPASGRRAGFGELAEAAARSPVPEDVPLKDPARFRLIGKHRLPHVDSAAKSDGS
ALYTQDMKLPGMLVAVVAHAPRLGAAVARVDDAAARAVPGVRAVVRFGGAALRHAGVAVLATNTWAARAG
RDALRIEWDEGPAYRQGSADILARYREAVGRPGSMAARKGDIDAAFAGAAKVIEAEYTYPYLAHAAMEPLNCL
VRLDDERCEIWNGEQFQTADQRAIAQYLGMPAERITLTQLYAGGSFGRRASSHADYLLEAVAIARTARAQGLNA
PVKLVWMREDDMRAGYYRPLNLHRARLALGADGALQAVHVRMAGQSILLGTPLADWVRDGVDPVSVEGLS
DLAYAVPNLQVELHTPTDVPVPVLWYRSVGHTHTAFSAETLIDEAAVAAGQDPVAYRLALLAAHPRHREVLQLA
AVRAGWREPLAAGAPGTRRGRGVAVHESFRSVMAQVVEVTIAADGALKVDRVVCAAVHCGLAVNPDVVRA
QMEGGIGFALSTALHGAITLKDGAVEQSNFHDYPVLRLAEMPAVEVHIAPSTQPPTGVGEPGVPPLAPALANAI
AQATGQRLRTLPLGTTVKA
>SEQF7935||SEQF7935.1_01975
MRIAIQNLSRRRFVQGAGGLLLGLSLPPLARRAMAAGPQAGDGFAANAFVRIGADGRVTVLAKHLEMGQGA
YTGLATLLAEELDADWRQVRVEGAPADSARYGNQALGGLQGTGGSTAMFDSWEPMRRAGATARAMLVQAA
AQRWQVPADTIEVAEGVLSHPASGRRAGFGELAEAAARSPVPEDVPLKDPARFRLIGKHRLPHVDSAAKSDGS
ALYTQDMKLPGMLVAVVAHAPRLGAAVARVDDAAARAVPGVRAVVRFGGAALRHAGVAVLATNTWAARAG
RDALRIEWDEGPAYRQGSADILARYREAVGRPGSMAARKGDIDAAFAGAAKVIEAEYTYPYLAHAAMEPLNCL
VRLDDERCEIWNGEQFQTADQRAIAQYLGMPAERITLTQLYAGGSFGRRASSHADYLLEAVAIARTARAQGLNA
PVKLVWMREDDMRAGYYRPLNLHRARLALGADGALQAVHVRMAGQSILLGTPLADWVRDGVDPVSVEGLS
DLAYAVPNLQVELHTPTDVPVPVLWYRSVGHTHTAFSAETLIDEAAVAAGQDPVAYRLALLAAHPRHREVLQLA
AVRAGWREPLAAGAPGTRRGRGVAVHESFRSVMAQVVEVTIAADGALKVDRVVCAAVHCGLAVNPDVVRA
QMEGGIGFALSTALHGAITLKDGAVEQSNFHDYPVLRLAEMPAVEVHIAPSTQPPTGVGEPGVPPLAPALANAI
AQATGQRLRTLPLGTTVKA
>SEQF7936||SEQF7936.1_01970
MRIAIQNLSRRRFVQGAGGLLLGLSLPPLARRAMAAGPQAGDGFAANAFVRIGADGRVTVLAKHLEMGQGA
YTGLATLLAEELDADWRQVRVEGAPADSARYGNQALGGLQGTGGSTAMFDSWEPMRRAGATARAMLVQAA
AQRWQVPADTIEVAEGVLSHPASGRRAGFGELAEAAARSPVPEDVPLKDPARFRLIGKHRLPHVDSAAKSDGS
ALYTQDMKLPGMLVAVVAHAPRLGAAVARVDDAAARAVPGVRAVVRFGGAALRHAGVAVLATNTWAARAG
RDALRIEWDEGPAYRQGSADILARYREAVGRPGSMAARKGDIDAAFAGAAKVIEAEYTYPYLAHAAMEPLNCL
VRLDDERCEIWNGEQFQTADQRAIAQYLGMPAERITLTQLYAGGSFGRRASSHADYLLEAVAIARTARAQGLNA
PVKLVWMREDDMRAGYYRPLNLHRARLALGADGALQAVHVRMAGQSILLGTPLADWVRDGVDPVSVEGLS
DLAYAVPNLQVELHTPTDVPVPVLWYRSVGHTHTAFSAETLIDEAAVAAGQDPVAYRLALLAAHPRHREVLQLA
AVRAGWREPLAAGAPGTRRGRGVAVHESFRSVMAQVVEVTIAADGALKVDRVVCAAVHCGLAVNPDVVRA
QMEGGIGFALSTALHGAITLKDGAVEQSNFHDYPVLRLAEMPAVEVHIAPSTQPPTGVGEPGVPPLAPALANAI
AQATGQRLRTLPLGTTVKA
>SEQF7937||SEQF7937.1_02020
MRIAIQNLSRRRFVQGAGGLLLGLSLPPLARRAMAAGPQAGDGFAANAFVRIGADGRVTVLAKHLEMGQGA
YTGLATLLAEELDADWRQVRVEGAPADSARYGNQALGGLQGTGGSTAMFDSWEPMRRAGATARAMLVQAA
AQRWQVPADTIEVAEGVLSHPASGRRAGFGELAEAAARSPVPEDVPLKDPARFRLIGKHRLPHVDSAAKSDGS
ALYTQDMKLPGMLVAVVAHAPRLGAAVARVDDAAARAVPGVRAVVRFGGAALRHAGVAVLATNTWAARAG
RDALRIEWDEGPAYRQGSADILARYREAVGRPGSMAARKGDIDAAFAGAAKVIEAEYTYPYLAHAAMEPLNCL
VRLDDERCEIWNGEQFQTADQRAIAQYLGMPAERITLTQLYAGGSFGRRASSHADYLLEAVAIARTARAQGLNA
PVKLVWMREDDMRAGYYRPLNLHRARLALGADGALQAVHVRMAGQSILLGTPLADWVRDGVDPVSVEGLS

DLAYAVPNLQVELHTPTDVPVPVLWYRSVGHTHTAFSAETLIDEAAVAAGQDPVAYRLALLAAHPRHREVLQLA
AVRAGWREPLAAGAPGTRRGRGVAVHESFRSVMAQVVEVTIAADGALKVDRVVCAAVHCGLAVNPDVVRA
QMEGGIGFALSTALHGAITLKDGAVEQSNFHDYPVLRLAEMPAVEVHIAPSTQPPTGVGEPGVPPLAPALANAI
AQATGQRLRTLPLGTTVKA
>SEQF7938||SEQF7938.1_01968
MRIAIQNLSRRRFVQGAGGLLLGLSLPPLARRAMAAGPQAGDGFAANAFVRIGADGRVTVLAKHLEMGQGA
YTGLATLLAEELDADWRQVRVEGAPADSARYGNQALGGLQGTGGSTAMFDSWEPMRRAGATARAMLVQAA
AQRWQVPADTIEVAEGVLSHPASGRRAGFGELAEAAARSPVPEDVPLKDPARFRLIGKHRLPHVDSAAKSDGS
ALYTQDMKLPGMLVAVVAHAPRLGAAVARVDDAAARAVPGVRAVVRFGGAALRHAGVAVLATNTWAARAG
RDALRIEWDEGPAYRQGSADILARYREAVGRPGSMAARKGDIDAAFAGAAKVIEAEYTYPYLAHAAMEPLNCL
VRLDDERCEIWNGEQFQTADQRAIAQYLGMPAERITLTQLYAGGSFGRRASSHADYLLEAVAIARTARAQGLNA
PVKLVWMREDDMRAGYYRPLNLHRARLALGADGALQAVHVRMAGQSILLGTPLADWVRDGVDPVSVEGLS
DLAYAVPNLQVELHTPTDVPVPVLWYRSVGHTHTAFSAETLIDEAAVAAGQDPVAYRLALLAAHPRHREVLQLA
AVRAGWREPLAAGAPGTRRGRGVAVHESFRSVMAQVVEVTIAADGALKVDRVVCAAVHCGLAVNPDVVRA
QMEGGIGFALSTALHGAITLKDGAVEQSNFHDYPVLRLAEMPAVEVHIAPSTQPPTGVGEPGVPPLAPALANAI
AQATGQRLRTLPLGTTVKA
>SEQF7940||SEQF7940.1_02286
MRIAIQNLSRRRFVQGAGGLLLGLSLPPLARRAMAAGPQAGDGFAANAFVRIGADGRVTVLAKHLEMGQGA
YTGLATLLAEELDADWRQVRVEGAPADSARYGNQALGGLQGTGGSTAMFDSWEPMRRAGATARAMLVQAA
AQRWQVPADTIEVAEGVLSHPASGRRAGFGELAEAAARSPVPEDVPLKDPARFRLIGKHRLPHVDSAAKSDGS
ALYTQDMKLPGMLVAVVAHAPRLGAAVARVDDAAARAVPGVRAVVRFGGAALRHAGVAVLATNTWAARAG
RDALRIEWDEGPAYRQGSADILARYREAVGRPGSMAARKGDIDAAFAGAAKVIEAEYTYPYLAHAAMEPLNCL
VRLDDERCEIWNGEQFQTADQRAIAQYLGMPAERITLTQLYAGGSFGRRASSHADYLLEAVAIARTARAQGLNA
PVKLVWMREDDMRAGYYRPLNLHRARLALGADGALQAVHVRMAGQSILLGTPLADWVRDGVDPVSVEGLS
DLAYAVPNLQVELHTPTDVPVPVLWYRSVGHTHTAFSAETLIDEAAVAAGQDPVAYRLALLAAHPRHREVLQLA
AVRAGWREPLAAGAPGTRRGRGVAVHESFRSVMAQVVEVTIAADGALKVDRVVCAAVHCGLAVNPDVVRA
QMEGGIGFALSTALHGAITLKDGAVEQSNFHDYPVLRLAEMPAVEVHIAPSTQPPTGVGEPGVPPLAPALANAI
AQATGQRLRTLPLGTTVKA
>SEQF7941||SEQF7941.1_01968
MRIAIQNLSRRRFVQGAGGLLLGLSLPPLARRAMAAGPQAGDGFAANAFVRIGADGRVTVLAKHLEMGQGA
YTGLATLLAEELDADWRQVRVEGAPADSARYGNQALGGLQGTGGSTAMFDSWEPMRRAGATARAMLVQAA
AQRWQVPADTIEVAEGVLSHPASGRRAGFGELAEAAARSPVPEDVPLKDPARFRLIGKHRLPHVDSAAKSDGS
ALYTQDMKLPGMLVAVVAHAPRLGAAVARVDDAAARAVPGVRAVVRFGGAALRHAGVAVLATNTWAARAG
RDALRIEWDEGPAYRQGSADILARYREAVGRPGSMAARKGDIDAAFAGAAKVIEAEYTYPYLAHAAMEPLNCL
VRLDDERCEIWNGEQFQTADQRAIAQYLGMPAERITLTQLYAGGSFGRRASSHADYLLEAVAIARTARAQGLNA
PVKLVWMREDDMRAGYYRPLNLHRARLALGADGALQAVHVRMAGQSILLGTPLADWVRDGVDPVSVEGLS
DLAYAVPNLQVELHTPTDVPVPVLWYRSVGHTHTAFSAETLIDEAAVAAGQDPVAYRLALLAAHPRHREVLQLA
AVRAGWREPLAAGAPGTRRGRGVAVHESFRSVMAQVVEVTIAADGALKVDRVVCAAVHCGLAVNPDVVRA
QMEGGIGFALSTALHGAITLKDGAVEQSNFHDYPVLRLAEMPAVEVHIAPSTQPPTGVGEPGVPPLAPALANAI
AQATGQRLRTLPLGTTVKA
>SEQF7942||SEQF7942.1_01303
MRIAIQNLSRRRFVQGAGGLLLGLSLPPLARRAMAAGPQAGDGFAANAFVRIGADGRVTVLAKHLEMGQGA
YTGLATLLAEELDADWRQVRVEGAPADSARYGNQALGGLQGTGGSTAMFDSWEPMRRAGATARAMLVQAA
AQRWQVPADTIEVAEGVLSHPASGRRAGFGELAEAAARSPVPEDVPLKDPARFRLIGKHRLPHVDSAAKSDGS

ALYTQDMKLPGMLVAVVAHAPRLGAAVARVDDAAARAVPGVRAVVRFGGAALRHAGVAVLATNTWAARAG
RDALRIEWDEGPAYRQGSADILARYREAVGRPGSMAARKGDIDAAFAGAAKVIEAEYTYPYLAHAAMEPLNCL
VRLDDERCEIWNGEQFQTADQRAIAQYLGMPAERITLTQLYAGGSFGRRASSHADYLLEAVAIARTARAQGLNA
PVKLVWMREDDMRAGYYRPLNLHRARLALGADGALQAVHVRMAGQSILLGTPLADWVRDGVDPVSVEGLS
DLAYAVPNLQVELHTPTDVPVPVLWYRSVGHTHTAFSAETLIDEAAVAAGQDPVAYRLALLAAHPRHREVLQLA
AVRAGWREPLAAGAPGTRRGRGVAVHESFRSVMAQVVEVTIAADGALKVDRVVCAAVHCGLAVNPDVVRA
QMEGGIGFALSTALHGAITLKDGAVEQSNFHDYPVLRLAEMPAVEVHIAPSTQPPTGVGEPGVPPLAPALANAI
AQATGQRLRTLPLGTTVKA
>SEQF7943||SEQF7943.1_01983
MRIAIQNLSRRRFVQGAGGLLLGLSLPPLARRAMAAGPQAGDGFAANAFVRIGADGRVTVLAKHLEMGQGA
YTGLATLLAEELDADWRQVRVEGAPADSARYGNQALGGLQGTGGSTAMFDSWEPMRRAGATARAMLVQAA
AQRWQVPADTIEVAEGVLSHPASGRRAGFGELAEAAARSPVPEDVPLKDPARFRLIGKHRLPHVDSAAKSDGS
ALYTQDMKLPGMLVAVVAHAPRLGAAVARVDDAAARAVPGVRAVVRFGGAALRHAGVAVLATNTWAARAG
RDALRIEWDEGPAYRQGSADILARYREAVGRPGSMAARKGDIDAAFAGAAKVIEAEYTYPYLAHAAMEPLNCL
VRLDDERCEIWNGEQFQTADQRAIAQYLGMPAERITLTQLYAGGSFGRRASSHADYLLEAVAIARTARAQGLNA
PVKLVWMREDDMRAGYYRPLNLHRARLALGADGALQAVHVRMAGQSILLGTPLADWVRDGVDPVSVEGLS
DLAYAVPNLQVELHTPTDVPVPVLWYRSVGHTHTAFSAETLIDEAAVAAGQDPVAYRLALLAAHPRHREVLQLA
AVRAGWREPLAAGAPGTRRGRGVAVHESFRSVMAQVVEVTIAADGALKVDRVVCAAVHCGLAVNPDVVRA
QMEGGIGFALSTALHGAITLKDGAVEQSNFHDYPVLRLAEMPAVEVHIAPSTQPPTGVGEPGVPPLAPALANAI
AQATGQRLRTLPLGTTVKA
>SEQF7944||SEQF7944.1_02012
MRIAIQNLSRRRFVQGAGGLLLGLSLPPLARRAMAAGPQAGDGFAANAFVRIGADGRVTVLAKHLEMGQGA
YTGLATLLAEELDADWRQVRVEGAPADSARYGNQALGGLQGTGGSTAMFDSWEPMRRAGATARAMLVQAA
AQRWQVPADTIEVAEGVLSHPASGRRAGFGELAEAAARSPVPEDVPLKDPARFRLIGKHRLPHVDSAAKSDGS
ALYTQDMKLPGMLVAVVAHAPRLGAAVARVDDAAARAVPGVRAVVRFGGAALRHAGVAVLATNTWAARAG
RDALRIEWDEGPAYRQGSADILARYREAVGRPGSMAARKGDIDAAFAGAAKVIEAEYTYPYLAHAAMEPLNCL
VRLDDERCEIWNGEQFQTADQRAIAQYLGMPAERITLTQLYAGGSFGRRASSHADYLLEAVAIARTARAQGLNA
PVKLVWMREDDMRAGYYRPLNLHRARLALGADGALQAVHVRMAGQSILLGTPLADWVRDGVDPVSVEGLS
DLAYAVPNLQVELHTPTDVPVPVLWYRSVGHTHTAFSAETLIDEAAVAAGQDPVAYRLALLAAHPRHREVLQLA
AVRAGWREPLAAGAPGTRRGRGVAVHESFRSVMAQVVEVTIAADGALKVDRVVCAAVHCGLAVNPDVVRA
QMEGGIGFALSTALHGAITLKDGAVEQSNFHDYPVLRLAEMPAVEVHIAPSTQPPTGVGEPGVPPLAPALANAI
AQATGQRLRTLPLGTTVKA
>SEQF7945||SEQF7945.1_01893
MRIAIQNLSRRRFVQGAGGLLLGLSLPPLARRAMAAGPQAGDGFAANAFVRIGADGRVTVLAKHLEMGQGA
YTGLATLLAEELDADWRQVRVEGAPADSARYGNQALGGLQGTGGSTAMFDSWEPMRRAGATARAMLVQAA
AQRWQVPADTIEVAEGVLSHPASGRRAGFGELAEAAARSPVPEDVPLKDPARFRLIGKHRLPHVDSAAKSDGS
ALYTQDMKLPGMLVAVVAHAPRLGAAVARVDDAAARAVPGVRAVVRFGGAALRHAGVAVLATNTWAARAG
RDALRIEWDEGPAYRQGSADILARYREAVGRPGSMAARKGDIDAAFAGAAKVIEAEYTYPYLAHAAMEPLNCL
VRLDDERCEIWNGEQFQTADQRAIAQYLGMPAERITLTQLYAGGSFGRRASSHADYLLEAVAIARTARAQGLNA
PVKLVWMREDDMRAGYYRPLNLHRARLALGADGALQAVHVRMAGQSILLGTPLADWVRDGVDPVSVEGLS
DLAYAVPNLQVELHTPTDVPVPVLWYRSVGHTHTAFSAETLIDEAAVAAGQDPVAYRLALLAAHPRHREVLQLA
AVRAGWREPLAAGAPGTRRGRGVAVHESFRSVMAQVVEVTIAADGALKVDRVVCAAVHCGLAVNPDVVRA
QMEGGIGFALSTALHGAITLKDGAVEQSNFHDYPVLRLAEMPAVEVHIAPSTQPPTGVGEPGVPPLAPALANAI
AQATGQRLRTLPLGTTVKA

>SEQF7946||SEQF7946.1_01975
MRIAIQNLSRRRFVQGAGGLLLGLSLPPLARRAMAAGPQAGDGFAANAFVRIGADGRVTVLAKHLEMGQGA
YTGLATLLAEELDADWRQVRVEGAPADSARYGNQALGGLQGTGGSTAMFDSWEPMRRAGATARAMLVQAA
AQRWQVPADTIEVAEGVLSHPASGRRAGFGELAEAAARSPVPEDVPLKDPARFRLIGKHRLPHVDSAAKSDGS
ALYTQDMKLPGMLVAVVAHAPRLGAAVARVDDAAARAVPGVRAVVRFGGAALRHAGVAVLATNTWAARAG
RDALRIEWDEGPAYRQGSADILARYREAVGRPGSMAARKGDIDAAFAGAAKVIEAEYTYPYLAHAAMEPLNCL
VRLDDERCEIWNGEQFQTADQRAIAQYLGMPAERITLTQLYAGGSFGRRASSHADYLLEAVAIARTARAQGLNA
PVKLVWMREDDMRAGYYRPLNLHRARLALGADGALQAVHVRMAGQSILLGTPLADWVRDGVDPVSVEGLS
DLAYAVPNLQVELHTPTDVPVPVLWYRSVGHTHTAFSAETLIDEAAVAAGQDPVAYRLALLAAHPRHREVLQLA
AVRAGWREPLAAGAPGTRRGRGVAVHESFRSVMAQVVEVTIAADGALKVDRVVCAAVHCGLAVNPDVVRA
QMEGGIGFALSTALHGAITLKDGAVEQSNFHDYPVLRLAEMPAVEVHIAPSTQPPTGVGEPGVPPLAPALANAI
AQATGQRLRTLPLGTTVKA
>SEQF7947||SEQF7947.1_02344
MRIAIQNLSRRRFVQGAGGLLLGLSLPPLARRAMAAGPQAGDGFAANAFVRIGADGRVTVLAKHLEMGQGA
YTGLATLLAEELDADWRQVRVEGAPADSARYGNQALGGLQGTGGSTAMFDSWEPMRRAGATARAMLVQAA
AQRWQVPADTIEVAEGVLSHPASGRRAGFGELAEAAARSPVPEDVPLKDPARFRLIGKHRLPHVDSAAKSDGS
ALYTQDMKLPGMLVAVVAHAPRLGAAVARVDDAAARAVPGVRAVVRFGGAALRHAGVAVLATNTWAARAG
RDALRIEWDEGPAYRQGSADILARYREAVGRPGSMAARKGDIDAAFAGAAKVIEAEYTYPYLAHAAMEPLNCL
VRLDDERCEIWNGEQFQTADQRAIAQYLGMPAERITLTQLYAGGSFGRRASSHADYLLEAVAIARTARAQGLNA
PVKLVWMREDDMRAGYYRPLNLHRARLALGADGALQAVHVRMAGQSILLGTPLADWVRDGVDPVSVEGLS
DLAYAVPNLQVELHTPTDVPVPVLWYRSVGHTHTAFSAETLIDEAAVAAGQDPVAYRLALLAAHPRHREVLQLA
AVRAGWREPLAAGAPGTRRGRGVAVHESFRSVMAQVVEVTIAADGALKVDRVVCAAVHCGLAVNPDVVRA
QMEGGIGFALSTALHGAITLKDGAVEQSNFHDYPVLRLAEMPAVEVHIAPSTQPPTGVGEPGVPPLAPALANAI
AQATGQRLRTLPLGTTVKA
>SEQF7948||SEQF7948.1_02026
MRIAIQNLSRRRFVQGAGGLLLGLSLPPLARRAMAAGPQAGDGFAANAFVRIGADGRVTVLAKHLEMGQGA
YTGLATLLAEELDADWRQVRVEGAPADSARYGNQALGGLQGTGGSTAMFDSWEPMRRAGATARAMLVQAA
AQRWQVPADTIEVAEGVLSHPASGRRAGFGELAEAAARSPVPEDVPLKDPARFRLIGKHRLPHVDSAAKSDGS
ALYTQDMKLPGMLVAVVAHAPRLGAAVARVDDAAARAVPGVRAVVRFGGAALRHAGVAVLATNTWAARAG
RDALRIEWDEGPAYRQGSADILARYREAVGRPGSMAARKGDIDAAFAGAAKVIEAEYTYPYLAHAAMEPLNCL
VRLDDERCEIWNGEQFQTADQRAIAQYLGMPAERITLTQLYAGGSFGRRASSHADYLLEAVAIARTARAQGLNA
PVKLVWMREDDMRAGYYRPLNLHRARLALGADGALQAVHVRMAGQSILLGTPLADWVRDGVDPVSVEGLS
DLAYAVPNLQVELHTPTDVPVPVLWYRSVGHTHTAFSAETLIDEAAVAAGQDPVAYRLALLAAHPRHREVLQLA
AVRAGWREPLAAGAPGTRRGRGVAVHESFRSVMAQVVEVTIAADGALKVDRVVCAAVHCGLAVNPDVVRA
QMEGGIGFALSTALHGAITLKDGAVEQSNFHDYPVLRLAEMPAVEVHIAPSTQPPTGVGEPGVPPLAPALANAI
AQATGQRLRTLPLGTTVKA
>SEQF7949||SEQF7949.1_01673
MRIAIQNLSRRRFVQGAGGLLLGLSLPPLARRAMAAGPQAGDGFAANAFVRIGADGRVTVLAKHLEMGQGA
YTGLATLLAEELDADWRQVRVEGAPADSARYGNQALGGLQGTGGSTAMFDSWEPMRRAGATARAMLVQAA
AQRWQVPADTIEVAEGVLSHPASGRRAGFGELAEAAARSPVPEDVPLKDPARFRLIGKHRLPHVDSAAKSDGS
ALYTQDMKLPGMLVAVVAHAPRLGAAVARVDDAAARAVPGVRAVVRFGGAALRHAGVAVLATNTWAARAG
RDALRIEWDEGPAYRQGSADILARYREAVGRPGSMAARKGDIDAAFAGAAKVIEAEYTYPYLAHAAMEPLNCL
VRLDDERCEIWNGEQFQTADQRAIAQYLGMPAERITLTQLYAGGSFGRRASSHADYLLEAVAIARTARAQGLNA
PVKLVWMREDDMRAGYYRPLNLHRARLALGADGALQAVHVRMAGQSILLGTPLADWVRDGVDPVSVEGLS

DLAYAVPNLQVELHTPTDVPVPVLWYRSVGHTHTAFSAETLIDEAAVAAGQDPVAYRLALLAAHPRHREVLQLA
AVRAGWREPLAAGAPGTRRGRGVAVHESFRSVMAQVVEVTIAADGALKVDRVVCAAVHCGLAVNPDVVRA
QMEGGIGFALSTALHGAITLKDGAVEQSNFHDYPVLRLAEMPAVEVHIAPSTQPPTGVGEPGVPPLAPALANAI
AQATGQRLRTLPLGTTVKA
>SEQF7950||SEQF7950.1_02015
MRIAIQNLSRRRFVQGAGGLLLGLSLPPLARRAMAAGPQAGDGFAANAFVRIGADGRVTVLAKHLEMGQGA
YTGLATLLAEELDADWRQVRVEGAPADSARYGNQALGGLQGTGGSTAMFDSWEPMRRAGATARAMLVQAA
AQRWQVPADTIEVAEGVLSHPASGRRAGFGELAEAAARSPVPEDVPLKDPARFRLIGKHRLPHVDSAAKSDGS
ALYTQDMKLPGMLVAVVAHAPRLGAAVARVDDAAARAVPGVRAVVRFGGAALRHAGVAVLATNTWAARAG
RDALRIEWDEGPAYRQGSADILARYREAVGRPGSMAARKGDIDAAFAGAAKVIEAEYTYPYLAHAAMEPLNCL
VRLDDERCEIWNGEQFQTADQRAIAQYLGMPAERITLTQLYAGGSFGRRASSHADYLLEAVAIARTARAQGLNA
PVKLVWMREDDMRAGYYRPLNLHRARLALGADGALQAVHVRMAGQSILLGTPLADWVRDGVDPVSVEGLS
DLAYAVPNLQVELHTPTDVPVPVLWYRSVGHTHTAFSAETLIDEAAVAAGQDPVAYRLALLAAHPRHREVLQLA
AVRAGWREPLAAGAPGTRRGRGVAVHESFRSVMAQVVEVTIAADGALKVDRVVCAAVHCGLAVNPDVVRA
QMEGGIGFALSTALHGAITLKDGAVEQSNFHDYPVLRLAEMPAVEVHIAPSTQPPTGVGEPGVPPLAPALANAI
AQATGQRLRTLPLGTTVKA
>SEQF7951||SEQF7951.1_01933
MRIAIQNLSRRRFVQGAGGLLLGLSLPPLARRAMAAGPQAGDGFAANAFVRIGADGRVTVLAKHLEMGQGA
YTGLATLLAEELDADWRQVRVEGAPADSARYGNQALGGLQGTGGSTAMFDSWEPMRRAGATARAMLVQAA
AQRWQVPADTIEVAEGVLSHPASGRRAGFGELAEAAARSPVPEDVPLKDPARFRLIGKHRLPHVDSAAKSDGS
ALYTQDMKLPGMLVAVVAHAPRLGAAVARVDDAAARAVPGVRAVVRFGGAALRHAGVAVLATNTWAARAG
RDALRIEWDEGPAYRQGSADILARYREAVGRPGSMAARKGDIDAAFAGAAKVIEAEYTYPYLAHAAMEPLNCL
VRLDDERCEIWNGEQFQTADQRAIAQYLGMPAERITLTQLYAGGSFGRRASSHADYLLEAVAIARTARAQGLNA
PVKLVWMREDDMRAGYYRPLNLHRARLALGADGALQAVHVRMAGQSILLGTPLADWVRDGVDPVSVEGLS
DLAYAVPNLQVELHTPTDVPVPVLWYRSVGHTHTAFSAETLIDEAAVAAGQDPVAYRLALLAAHPRHREVLQLA
AVRAGWREPLAAGAPGTRRGRGVAVHESFRSVMAQVVEVTIAADGALKVDRVVCAAVHCGLAVNPDVVRA
QMEGGIGFALSTALHGAITLKDGAVEQSNFHDYPVLRLAEMPAVEVHIAPSTQPPTGVGEPGVPPLAPALANAI
AQATGQRLRTLPLGTTVKA
>SEQF7952||SEQF7952.1_02017
MRIAIQNLSRRRFVQGAGGLLLGLSLPPLARRAMAAGPQAGDGFAANAFVRIGADGRVTVLAKHLEMGQGA
YTGLATLLAEELDADWRQVRVEGAPADSARYGNQALGGLQGTGGSTAMFDSWEPMRRAGATARAMLVQAA
AQRWQVPADTIEVAEGVLSHPASGRRAGFGELAEAAARSPVPEDVPLKDPARFRLIGKHRLPHVDSAAKSDGS
ALYTQDMKLPGMLVAVVAHAPRLGAAVARVDDAAARAVPGVRAVVRFGGAALRHAGVAVLATNTWAARAG
RDALRIEWDEGPAYRQGSADILARYREAVGRPGSMAARKGDIDAAFAGAAKVIEAEYTYPYLAHAAMEPLNCL
VRLDDERCEIWNGEQFQTADQRAIAQYLGMPAERITLTQLYAGGSFGRRASSHADYLLEAVAIARTARAQGLNA
PVKLVWMREDDMRAGYYRPLNLHRARLALGADGALQAVHVRMAGQSILLGTPLADWVRDGVDPVSVEGLS
DLAYAVPNLQVELHTPTDVPVPVLWYRSVGHTHTAFSAETLIDEAAVAAGQDPVAYRLALLAAHPRHREVLQLA
AVRAGWREPLAAGAPGTRRGRGVAVHESFRSVMAQVVEVTIAADGALKVDRVVCAAVHCGLAVNPDVVRA
QMEGGIGFALSTALHGAITLKDGAVEQSNFHDYPVLRLAEMPAVEVHIAPSTQPPTGVGEPGVPPLAPALANAI
AQATGQRLRTLPLGTTVKA
>SEQF7953||SEQF7953.1_01984
MRIAIQNLSRRRFVQGAGGLLLGLSLPPLARRAMAAGPQAGDGFAANAFVRIGADGRVTVLAKHLEMGQGA
YTGLATLLAEELDADWRQVRVEGAPADSARYGNQALGGLQGTGGSTAMFDSWEPMRRAGATARAMLVQAA
AQRWQVPADTIEVAEGVLSHPASGRRAGFGELAEAAARSPVPEDVPLKDPARFRLIGKHRLPHVDSAAKSDGS

ALYTQDMKLPGMLVAVVAHAPRLGAAVARVDDAAARAVPGVRAVVRFGGAALRHAGVAVLATNTWAARAG
RDALRIEWDEGPAYRQGSADILARYREAVGRPGSMAARKGDIDAAFAGAAKVIEAEYTYPYLAHAAMEPLNCL
VRLDDERCEIWNGEQFQTADQRAIAQYLGMPAERITLTQLYAGGSFGRRASSHADYLLEAVAIARTARAQGLNA
PVKLVWMREDDMRAGYYRPLNLHRARLALGADGALQAVHVRMAGQSILLGTPLADWVRDGVDPVSVEGLS
DLAYAVPNLQVELHTPTDVPVPVLWYRSVGHTHTAFSAETLIDEAAVAAGQDPVAYRLALLAAHPRHREVLQLA
AVRAGWREPLAAGAPGTRRGRGVAVHESFRSVMAQVVEVTIAADGALKVDRVVCAAVHCGLAVNPDVVRA
QMEGGIGFALSTALHGAITLKDGAVEQSNFHDYPVLRLAEMPAVEVHIAPSTQPPTGVGEPGVPPLAPALANAI
AQATGQRLRTLPLGTTVKA
>SEQF7954||SEQF7954.1_01895
MRIAIQNLSRRRFVQGAGGLLLGLSLPPLARRAMAAGPQAGDGFAANAFVRIGADGRVTVLAKHLEMGQGA
YTGLATLLAEELDADWRQVRVEGAPADSARYGNQALGGLQGTGGSTAMFDSWEPMRRAGATARAMLVQAA
AQRWQVPADTIEVAEGVLSHPASGRRAGFGELAEAAARSPVPEDVPLKDPARFRLIGKHRLPHVDSAAKSDGS
ALYTQDMKLPGMLVAVVAHAPRLGAAVARVDDAAARAVPGVRAVVRFGGAALRHAGVAVLATNTWAARAG
RDALRIEWDEGPAYRQGSADILARYREAVGRPGSMAARKGDIDAAFAGAAKVIEAEYTYPYLAHAAMEPLNCL
VRLDDERCEIWNGEQFQTADQRAIAQYLGMPAERITLTQLYAGGSFGRRASSHADYLLEAVAIARTARAQGLNA
PVKLVWMREDDMRAGYYRPLNLHRARLALGADGALQAVHVRMAGQSILLGTPLADWVRDGVDPVSVEGLS
DLAYAVPNLQVELHTPTDVPVPVLWYRSVGHTHTAFSAETLIDEAAVAAGQDPVAYRLALLAAHPRHREVLQLA
AVRAGWREPLAAGAPGTRRGRGVAVHESFRSVMAQVVEVTIAADGALKVDRVVCAAVHCGLAVNPDVVRA
QMEGGIGFALSTALHGAITLKDGAVEQSNFHDYPVLRLAEMPAVEVHIAPSTQPPTGVGEPGVPPLAPALANAI
AQATGQRLRTLPLGTTVKA
>SEQF7955||SEQF7955.1_02233
MRIAIQNLSRRRFVQGAGGLLLGLSLPPLARRAMAAGPQAGDGFAANAFVRIGADGRVTVLAKHLEMGQGA
YTGLATLLAEELDADWRQVRVEGAPADSARYGNQALGGLQGTGGSTAMFDSWEPMRRAGATARAMLVQAA
AQRWQVPADTIEVAEGVLSHPASGRRAGFGELAEAAARSPVPEDVPLKDPARFRLIGKHRLPHVDSAAKSDGS
ALYTQDMKLPGMLVAVVAHAPRLGAAVARVDDAAARAVPGVRAVVRFGGAALRHAGVAVLATNTWAARAG
RDALRIEWDEGPAYRQGSADILARYREAVGRPGSMAARKGDIDAAFAGAAKVIEAEYTYPYLAHAAMEPLNCL
VRLDDERCEIWNGEQFQTADQRAIAQYLGMPAERITLTQLYAGGSFGRRASSHADYLLEAVAIARTARAQGLNA
PVKLVWMREDDMRAGYYRPLNLHRARLALGADGALQAVHVRMAGQSILLGTPLADWVRDGVDPVSVEGLS
DLAYAVPNLQVELHTPTDVPVPVLWYRSVGHTHTAFSAETLIDEAAVAAGQDPVAYRLALLAAHPRHREVLQLA
AVRAGWREPLAAGAPGTRRGRGVAVHESFRSVMAQVVEVTIAADGALKVDRVVCAAVHCGLAVNPDVVRA
QMEGGIGFALSTALHGAITLKDGAVEQSNFHDYPVLRLAEMPAVEVHIAPSTQPPTGVGEPGVPPLAPALANAI
AQATGQRLRTLPLGTTVKA
>SEQF7956||SEQF7956.1_01895
MRIAIQNLSRRRFVQGAGGLLLGLSLPPLARRAMAAGPQAGDGFAANAFVRIGADGRVTVLAKHLEMGQGA
YTGLATLLAEELDADWRQVRVEGAPADSARYGNQALGGLQGTGGSTAMFDSWEPMRRAGATARAMLVQAA
AQRWQVPADTIEVAEGVLSHPASGRRAGFGELAEAAARSPVPEDVPLKDPARFRLIGKHRLPHVDSAAKSDGS
ALYTQDMKLPGMLVAVVAHAPRLGAAVARVDDAAARAVPGVRAVVRFGGAALRHAGVAVLATNTWAARAG
RDALRIEWDEGPAYRQGSADILARYREAVGRPGSMAARKGDIDAAFAGAAKVIEAEYTYPYLAHAAMEPLNCL
VRLDDERCEIWNGEQFQTADQRAIAQYLGMPAERITLTQLYAGGSFGRRASSHADYLLEAVAIARTARAQGLNA
PVKLVWMREDDMRAGYYRPLNLHRARLALGADGALQAVHVRMAGQSILLGTPLADWVRDGVDPVSVEGLS
DLAYAVPNLQVELHTPTDVPVPVLWYRSVGHTHTAFSAETLIDEAAVAAGQDPVAYRLALLAAHPRHREVLQLA
AVRAGWREPLAAGAPGTRRGRGVAVHESFRSVMAQVVEVTIAADGALKVDRVVCAAVHCGLAVNPDVVRA
QMEGGIGFALSTALHGAITLKDGAVEQSNFHDYPVLRLAEMPAVEVHIAPSTQPPTGVGEPGVPPLAPALANAI
AQATGQRLRTLPLGTTVKA

>SEQF7957||SEQF7957.1_01972
MRIAIQNLSRRRFVQGAGGLLLGLSLPPLARRAMAAGPQAGDGFAANAFVRIGADGRVTVLAKHLEMGQGA
YTGLATLLAEELDADWRQVRVEGAPADSARYGNQALGGLQGTGGSTAMFDSWEPMRRAGATARAMLVQAA
AQRWQVPADTIEVAEGVLSHPASGRRAGFGELAEAAARSPVPEDVPLKDPARFRLIGKHRLPHVDSAAKSDGS
ALYTQDMKLPGMLVAVVAHAPRLGAAVARVDDAAARAVPGVRAVVRFGGAALRHAGVAVLATNTWAARAG
RDALRIEWDEGPAYRQGSADILARYREAVGRPGSMAARKGDIDAAFAGAAKVIEAEYTYPYLAHAAMEPLNCL
VRLDDERCEIWNGEQFQTADQRAIAQYLGMPAERITLTQLYAGGSFGRRASSHADYLLEAVAIARTARAQGLNA
PVKLVWMREDDMRAGYYRPLNLHRARLALGADGALQAVHVRMAGQSILLGTPLADWVRDGVDPVSVEGLS
DLAYAVPNLQVELHTPTDVPVPVLWYRSVGHTHTAFSAETLIDEAAVAAGQDPVAYRLALLAAHPRHREVLQLA
AVRAGWREPLAAGAPGTRRGRGVAVHESFRSVMAQVVEVTIAADGALKVDRVVCAAVHCGLAVNPDVVRA
QMEGGIGFALSTALHGAITLKDGAVEQSNFHDYPVLRLAEMPAVEVHIAPSTQPPTGVGEPGVPPLAPALANAI
AQATGQRLRTLPLGTTVKA
>SEQF7958||SEQF7958.1_01999
MRIAIQNLSRRRFVQGAGGLLLGLSLPPLARRAMAAGPQAGDGFAANAFVRIGADGRVTVLAKHLEMGQGA
YTGLATLLAEELDADWRQVRVEGAPADSARYGNQALGGLQGTGGSTAMFDSWEPMRRAGATARAMLVQAA
AQRWQVPADTIEVAEGVLSHPASGRRAGFGELAEAAARSPVPEDVPLKDPARFRLIGKHRLPHVDSAAKSDGS
ALYTQDMKLPGMLVAVVAHAPRLGAAVARVDDAAARAVPGVRAVVRFGGAALRHAGVAVLATNTWAARAG
RDALRIEWDEGPAYRQGSADILARYREAVGRPGSMAARKGDIDAAFAGAAKVIEAEYTYPYLAHAAMEPLNCL
VRLDDERCEIWNGEQFQTADQRAIAQYLGMPAERITLTQLYAGGSFGRRASSHADYLLEAVAIARTARAQGLNA
PVKLVWMREDDMRAGYYRPLNLHRARLALGADGALQAVHVRMAGQSILLGTPLADWVRDGVDPVSVEGLS
DLAYAVPNLQVELHTPTDVPVPVLWYRSVGHTHTAFSAETLIDEAAVAAGQDPVAYRLALLAAHPRHREVLQLA
AVRAGWREPLAAGAPGTRRGRGVAVHESFRSVMAQVVEVTIAADGALKVDRVVCAAVHCGLAVNPDVVRA
QMEGGIGFALSTALHGAITLKDGAVEQSNFHDYPVLRLAEMPAVEVHIAPSTQPPTGVGEPGVPPLAPALANAI
AQATGQRLRTLPLGTTVKA
>SEQF7959||SEQF7959.1_01970
MRIAIQNLSRRRFVQGAGGLLLGLSLPPLARRAMAAGPQAGDGFAANAFVRIGADGRVTVLAKHLEMGQGA
YTGLATLLAEELDADWRQVRVEGAPADSARYGNQALGGLQGTGGSTAMFDSWEPMRRAGATARAMLVQAA
AQRWQVPADTIEVAEGVLSHPASGRRAGFGELAEAAARSPVPEDVPLKDPARFRLIGKHRLPHVDSAAKSDGS
ALYTQDMKLPGMLVAVVAHAPRLGAAVARVDDAAARAVPGVRAVVRFGGAALRHAGVAVLATNTWAARAG
RDALRIEWDEGPAYRQGSADILARYREAVGRPGSMAARKGDIDAAFAGAAKVIEAEYTYPYLAHAAMEPLNCL
VRLDDERCEIWNGEQFQTADQRAIAQYLGMPAERITLTQLYAGGSFGRRASSHADYLLEAVAIARTARAQGLNA
PVKLVWMREDDMRAGYYRPLNLHRARLALGADGALQAVHVRMAGQSILLGTPLADWVRDGVDPVSVEGLS
DLAYAVPNLQVELHTPTDVPVPVLWYRSVGHTHTAFSAETLIDEAAVAAGQDPVAYRLALLAAHPRHREVLQLA
AVRAGWREPLAAGAPGTRRGRGVAVHESFRSVMAQVVEVTIAADGALKVDRVVCAAVHCGLAVNPDVVRA
QMEGGIGFALSTALHGAITLKDGAVEQSNFHDYPVLRLAEMPAVEVHIAPSTQPPTGVGEPGVPPLAPALANAI
AQATGQRLRTLPLGTTVKA
>SEQF7960||SEQF7960.1_01970
MRIAIQNLSRRRFVQGAGGLLLGLSLPPLARRAMAAGPQAGDGFAANAFVRIGADGRVTVLAKHLEMGQGA
YTGLATLLAEELDADWRQVRVEGAPADSARYGNQALGGLQGTGGSTAMFDSWEPMRRAGATARAMLVQAA
AQRWQVPADTIEVAEGVLSHPASGRRAGFGELAEAAARSPVPEDVPLKDPARFRLIGKHRLPHVDSAAKSDGS
ALYTQDMKLPGMLVAVVAHAPRLGAAVARVDDAAARAVPGVRAVVRFGGAALRHAGVAVLATNTWAARAG
RDALRIEWDEGPAYRQGSADILARYREAVGRPGSMAARKGDIDAAFAGAAKVIEAEYTYPYLAHAAMEPLNCL
VRLDDERCEIWNGEQFQTADQRAIAQYLGMPAERITLTQLYAGGSFGRRASSHADYLLEAVAIARTARAQGLNA
PVKLVWMREDDMRAGYYRPLNLHRARLALGADGALQAVHVRMAGQSILLGTPLADWVRDGVDPVSVEGLS

DLAYAVPNLQVELHTPTDVPVPVLWYRSVGHTHTAFSAETLIDEAAVAAGQDPVAYRLALLAAHPRHREVLQLA
AVRAGWREPLAAGAPGTRRGRGVAVHESFRSVMAQVVEVTIAADGALKVDRVVCAAVHCGLAVNPDVVRA
QMEGGIGFALSTALHGAITLKDGAVEQSNFHDYPVLRLAEMPAVEVHIAPSTQPPTGVGEPGVPPLAPALANAI
AQATGQRLRTLPLGTTVKA
>SEQF7961||SEQF7961.1_01984
MRIAIQNLSRRRFVQGAGGLLLGLSLPPLARRAMAAGPQAGDGFAANAFVRIGADGRVTVLAKHLEMGQGA
YTGLATLLAEELDADWRQVRVEGAPADSARYGNQALGGLQGTGGSTAMFDSWEPMRRAGATARAMLVQAA
AQRWQVPADTIEVAEGVLSHPASGRRAGFGELAEAAARSPVPEDVPLKDPARFRLIGKHRLPHVDSAAKSDGS
ALYTQDMKLPGMLVAVVAHAPRLGAAVARVDDAAARAVPGVRAVVRFGGAALRHAGVAVLATNTWAARAG
RDALRIEWDEGPAYRQGSADILARYREAVGRPGSMAARKGDIDAAFAGAAKVIEAEYTYPYLAHAAMEPLNCL
VRLDDERCEIWNGEQFQTADQRAIAQYLGMPAERITLTQLYAGGSFGRRASSHADYLLEAVAIARTARAQGLNA
PVKLVWMREDDMRAGYYRPLNLHRARLALGADGALQAVHVRMAGQSILLGTPLADWVRDGVDPVSVEGLS
DLAYAVPNLQVELHTPTDVPVPVLWYRSVGHTHTAFSAETLIDEAAVAAGQDPVAYRLALLAAHPRHREVLQLA
AVRAGWREPLAAGAPGTRRGRGVAVHESFRSVMAQVVEVTIAADGALKVDRVVCAAVHCGLAVNPDVVRA
QMEGGIGFALSTALHGAITLKDGAVEQSNFHDYPVLRLAEMPAVEVHIAPSTQPPTGVGEPGVPPLAPALANAI
AQATGQRLRTLPLGTTVKA
>SEQF7962||SEQF7962.1_02024
MRIAIQNLSRRRFVQGAGGLLLGLSLPPLARRAMAAGPQAGDGFAANAFVRIGADGRVTVLAKHLEMGQGA
YTGLATLLAEELDADWRQVRVEGAPADSARYGNQALGGLQGTGGSTAMFDSWEPMRRAGATARAMLVQAA
AQRWQVPADTIEVAEGVLSHPASGRRAGFGELAEAAARSPVPEDVPLKDPARFRLIGKHRLPHVDSAAKSDGS
ALYTQDMKLPGMLVAVVAHAPRLGAAVARVDDAAARAVPGVRAVVRFGGAALRHAGVAVLATNTWAARAG
RDALRIEWDEGPAYRQGSADILARYREAVGRPGSMAARKGDIDAAFAGAAKVIEAEYTYPYLAHAAMEPLNCL
VRLDDERCEIWNGEQFQTADQRAIAQYLGMPAERITLTQLYAGGSFGRRASSHADYLLEAVAIARTARAQGLNA
PVKLVWMREDDMRAGYYRPLNLHRARLALGADGALQAVHVRMAGQSILLGTPLADWVRDGVDPVSVEGLS
DLAYAVPNLQVELHTPTDVPVPVLWYRSVGHTHTAFSAETLIDEAAVAAGQDPVAYRLALLAAHPRHREVLQLA
AVRAGWREPLAAGAPGTRRGRGVAVHESFRSVMAQVVEVTIAADGALKVDRVVCAAVHCGLAVNPDVVRA
QMEGGIGFALSTALHGAITLKDGAVEQSNFHDYPVLRLAEMPAVEVHIAPSTQPPTGVGEPGVPPLAPALANAI
AQATGQRLRTLPLGTTVKA
>SEQF7963||SEQF7963.1_01993
MRIAIQNLSRRRFVQGAGGLLLGLSLPPLARRAMAAGPQAGDGFAANAFVRIGADGRVTVLAKHLEMGQGA
YTGLATLLAEELDADWRQVRVEGAPADSARYGNQALGGLQGTGGSTAMFDSWEPMRRAGATARAMLVQAA
AQRWQVPADTIEVAEGVLSHPASGRRAGFGELAEAAARSPVPEDVPLKDPARFRLIGKHRLPHVDSAAKSDGS
ALYTQDMKLPGMLVAVVAHAPRLGAAVARVDDAAARAVPGVRAVVRFGGAALRHAGVAVLATNTWAARAG
RDALRIEWDEGPAYRQGSADILARYREAVGRPGSMAARKGDIDAAFAGAAKVIEAEYTYPYLAHAAMEPLNCL
VRLDDERCEIWNGEQFQTADQRAIAQYLGMPAERITLTQLYAGGSFGRRASSHADYLLEAVAIARTARAQGLNA
PVKLVWMREDDMRAGYYRPLNLHRARLALGADGALQAVHVRMAGQSILLGTPLADWVRDGVDPVSVEGLS
DLAYAVPNLQVELHTPTDVPVPVLWYRSVGHTHTAFSAETLIDEAAVAAGQDPVAYRLALLAAHPRHREVLQLA
AVRAGWREPLAAGAPGTRRGRGVAVHESFRSVMAQVVEVTIAADGALKVDRVVCAAVHCGLAVNPDVVRA
QMEGGIGFALSTALHGAITLKDGAVEQSNFHDYPVLRLAEMPAVEVHIAPSTQPPTGVGEPGVPPLAPALANAI
AQATGQRLRTLPLGTTVKA
>SEQF7964||SEQF7964.3_01966
MRIAIQNLSRRRFVQGAGGLLLGLSLPPLARRAMAAGPQAGDGFAANAFVRIGADGRVTVLAKHLEMGQGA
YTGLATLLAEELDADWRQVRVEGAPADSARYGNQALGGLQGTGGSTAMFDSWEPMRRAGATARAMLVQAA
AQRWQVPADTIEVAEGVLSHPASGRRAGFGELAEAAARSPVPEDVPLKDPARFRLIGKHRLPHVDSAAKSDGS

ALYTQDMKLPGMLVAVVAHAPRLGAAVARVDDAAARAVPGVRAVVRFGGAALRHAGVAVLATNTWAARAG
RDALRIEWDEGPAYRQGSADILARYREAVGRPGSMAARKGDIDAAFAGAAKVIEAEYTYPYLAHAAMEPLNCL
VRLDDERCEIWNGEQFQTADQRAIAQYLGMPAERITLTQLYAGGSFGRRASSHADYLLEAVAIARTARAQGLNA
PVKLVWMREDDMRAGYYRPLNLHRARLALGADGALQAVHVRMAGQSILLGTPLADWVRDGVDPVSVEGLS
DLAYAVPNLQVELHTPTDVPVPVLWYRSVGHTHTAFSAETLIDEAAVAAGQDPVAYRLALLAAHPRHREVLQLA
AVRAGWREPLAAGAPGTRRGRGVAVHESFRSVMAQVVEVTIAADGALKVDRVVCAAVHCGLAVNPDVVRA
QMEGGIGFALSTALHGAITLKDGAVEQSNFHDYPVLRLAEMPAVEVHIAPSTQPPTGVGEPGVPPLAPALANAI
AQATGQRLRTLPLGTTVKA
>SEQF7965||SEQF7965.1_02020
MRIAIQNLSRRRFVQGAGGLLLGLSLPPLARRAMAAGPQAGDGFAANAFVRIGADGRVTVLAKHLEMGQGA
YTGLATLLAEELDADWRQVRVEGAPADSARYGNQALGGLQGTGGSTAMFDSWEPMRRAGATARAMLVQAA
AQRWQVPADTIEVAEGVLSHPASGRRAGFGELAEAAARSPVPEDVPLKDPARFRLIGKHRLPHVDSAAKSDGS
ALYTQDMKLPGMLVAVVAHAPRLGAAVARVDDAAARAVPGVRAVVRFGGAALRHAGVAVLATNTWAARAG
RDALRIEWDEGPAYRQGSADILARYREAVGRPGSMAARKGDIDAAFAGAAKVIEAEYTYPYLAHAAMEPLNCL
VRLDDERCEIWNGEQFQTADQRAIAQYLGMPAERITLTQLYAGGSFGRRASSHADYLLEAVAIARTARAQGLNA
PVKLVWMREDDMRAGYYRPLNLHRARLALGADGALQAVHVRMAGQSILLGTPLADWVRDGVDPVSVEGLS
DLAYAVPNLQVELHTPTDVPVPVLWYRSVGHTHTAFSAETLIDEAAVAAGQDPVAYRLALLAAHPRHREVLQLA
AVRAGWREPLAAGAPGTRRGRGVAVHESFRSVMAQVVEVTIAADGALKVDRVVCAAVHCGLAVNPDVVRA
QMEGGIGFALSTALHGAITLKDGAVEQSNFHDYPVLRLAEMPAVEVHIAPSTQPPTGVGEPGVPPLAPALANAI
AQATGQRLRTLPLGTTVKA
>SEQF7966||SEQF7966.1_02020
MRIAIQNLSRRRFVQGAGGLLLGLSLPPLARRAMAAGPQAGDGFAANAFVRIGADGRVTVLAKHLEMGQGA
YTGLATLLAEELDADWRQVRVEGAPADSARYGNQALGGLQGTGGSTAMFDSWEPMRRAGATARAMLVQAA
AQRWQVPADTIEVAEGVLSHPASGRRAGFGELAEAAARSPVPEDVPLKDPARFRLIGKHRLPHVDSAAKSDGS
ALYTQDMKLPGMLVAVVAHAPRLGAAVARVDDAAARAVPGVRAVVRFGGAALRHAGVAVLATNTWAARAG
RDALRIEWDEGPAYRQGSADILARYREAVGRPGSMAARKGDIDAAFAGAAKVIEAEYTYPYLAHAAMEPLNCL
VRLDDERCEIWNGEQFQTADQRAIAQYLGMPAERITLTQLYAGGSFGRRASSHADYLLEAVAIARTARAQGLNA
PVKLVWMREDDMRAGYYRPLNLHRARLALGADGALQAVHVRMAGQSILLGTPLADWVRDGVDPVSVEGLS
DLAYAVPNLQVELHTPTDVPVPVLWYRSVGHTHTAFSAETLIDEAAVAAGQDPVAYRLALLAAHPRHREVLQLA
AVRAGWREPLAAGAPGTRRGRGVAVHESFRSVMAQVVEVTIAADGALKVDRVVCAAVHCGLAVNPDVVRA
QMEGGIGFALSTALHGAITLKDGAVEQSNFHDYPVLRLAEMPAVEVHIAPSTQPPTGVGEPGVPPLAPALANAI
AQATGQRLRTLPLGTTVKA
>SEQF7967||SEQF7967.1_01940
MRIAIQNLSRRRFVQGAGGLLLGLSLPPLARRAMAAGPQAGDGFAANAFVRIGADGRVTVLAKHLEMGQGA
YTGLATLLAEELDADWRQVRVEGAPADSARYGNQALGGLQGTGGSTAMFDSWEPMRRAGATARAMLVQAA
AQRWQVPADTIEVAEGVLSHPASGRRAGFGELAEAAARSPVPEDVPLKDPARFRLIGKHRLPHVDSAAKSDGS
ALYTQDMKLPGMLVAVVAHAPRLGAAVARVDDAAARAVPGVRAVVRFGGAALRHAGVAVLATNTWAARAG
RDALRIEWDEGPAYRQGSADILARYREAVGRPGSMAARKGDIDAAFAGAAKVIEAEYTYPYLAHAAMEPLNCL
VRLDDERCEIWNGEQFQTADQRAIAQYLGMPAERITLTQLYAGGSFGRRASSHADYLLEAVAIARTARAQGLNA
PVKLVWMREDDMRAGYYRPLNLHRARLALGADGALQAVHVRMAGQSILLGTPLADWVRDGVDPVSVEGLS
DLAYAVPNLQVELHTPTDVPVPVLWYRSVGHTHTAFSAETLIDEAAVAAGQDPVAYRLALLAAHPRHREVLQLA
AVRAGWREPLAAGAPGTRRGRGVAVHESFRSVMAQVVEVTIAADGALKVDRVVCAAVHCGLAVNPDVVRA
QMEGGIGFALSTALHGAITLKDGAVEQSNFHDYPVLRLAEMPAVEVHIAPSTQPPTGVGEPGVPPLAPALANAI
AQATGQRLRTLPLGTTVKA

>SEQF7968||SEQF7968.1_01927
MRIAIQNLSRRRFVQGAGGLLLGLSLPPLARRAMAAGPQAGDGFAANAFVRIGADGRVTVLAKHLEMGQGA
YTGLATLLAEELDADWRQVRVEGAPADSARYGNQALGGLQGTGGSTAMFDSWEPMRRAGATARAMLVQAA
AQRWQVPADTIEVAEGVLSHPASGRRAGFGELAEAAARSPVPEDVPLKDPARFRLIGKHRLPHVDSAAKSDGS
ALYTQDMKLPGMLVAVVAHAPRLGAAVARVDDAAARAVPGVRAVVRFGGAALRHAGVAVLATNTWAARAG
RDALRIEWDEGPAYRQGSADILARYREAVGRPGSMAARKGDIDAAFAGAAKVIEAEYTYPYLAHAAMEPLNCL
VRLDDERCEIWNGEQFQTADQRAIAQYLGMPAERITLTQLYAGGSFGRRASSHADYLLEAVAIARTARAQGLNA
PVKLVWMREDDMRAGYYRPLNLHRARLALGADGALQAVHVRMAGQSILLGTPLADWVRDGVDPVSVEGLS
DLAYAVPNLQVELHTPTDVPVPVLWYRSVGHTHTAFSAETLIDEAAVAAGQDPVAYRLALLAAHPRHREVLQLA
AVRAGWREPLAAGAPGTRRGRGVAVHESFRSVMAQVVEVTIAADGALKVDRVVCAAVHCGLAVNPDVVRA
QMEGGIGFALSTALHGAITLKDGAVEQSNFHDYPVLRLAEMPAVEVHIAPSTQPPTGVGEPGVPPLAPALANAI
AQATGQRLRTLPLGTTVKA
>SEQF7969||SEQF7969.1_01900
MRIAIQNLSRRRFVQGAGGLLLGLSLPPLARRAMAAGPQAGDGFAANAFVRIGADGRVTVLAKHLEMGQGA
YTGLATLLAEELDADWRQVRVEGAPADSARYGNQALGGLQGTGGSTAMFDSWEPMRRAGATARAMLVQAA
AQRWQVPADTIEVAEGVLSHPASGRRAGFGELAEAAARSPVPEDVPLKDPARFRLIGKHRLPHVDSAAKSDGS
ALYTQDMKLPGMLVAVVAHAPRLGAAVARVDDAAARAVPGVRAVVRFGGAALRHAGVAVLATNTWAARAG
RDALRIEWDEGPAYRQGSADILARYREAVGRPGSMAARKGDIDAAFAGAAKVIEAEYTYPYLAHAAMEPLNCL
VRLDDERCEIWNGEQFQTADQRAIAQYLGMPAERITLTQLYAGGSFGRRASSHADYLLEAVAIARTARAQGLNA
PVKLVWMREDDMRAGYYRPLNLHRARLALGADGALQAVHVRMAGQSILLGTPLADWVRDGVDPVSVEGLS
DLAYAVPNLQVELHTPTDVPVPVLWYRSVGHTHTAFSAETLIDEAAVAAGQDPVAYRLALLAAHPRHREVLQLA
AVRAGWREPLAAGAPGTRRGRGVAVHESFRSVMAQVVEVTIAADGALKVDRVVCAAVHCGLAVNPDVVRA
QMEGGIGFALSTALHGAITLKDGAVEQSNFHDYPVLRLAEMPAVEVHIAPSTQPPTGVGEPGVPPLAPALANAI
AQATGQRLRTLPLGTTVKA
>SEQF7970||SEQF7970.1_02017
MRIAIQNLSRRRFVQGAGGLLLGLSLPPLARRAMAAGPQAGDGFAANAFVRIGADGRVTVLAKHLEMGQGA
YTGLATLLAEELDADWRQVRVEGAPADSARYGNQALGGLQGTGGSTAMFDSWEPMRRAGATARAMLVQAA
AQRWQVPADTIEVAEGVLSHPASGRRAGFGELAEAAARSPVPEDVPLKDPARFRLIGKHRLPHVDSAAKSDGS
ALYTQDMKLPGMLVAVVAHAPRLGAAVARVDDAAARAVPGVRAVVRFGGAALRHAGVAVLATNTWAARAG
RDALRIEWDEGPAYRQGSADILARYREAVGRPGSMAARKGDIDAAFAGAAKVIEAEYTYPYLAHAAMEPLNCL
VRLDDERCEIWNGEQFQTADQRAIAQYLGMPAERITLTQLYAGGSFGRRASSHADYLLEAVAIARTARAQGLNA
PVKLVWMREDDMRAGYYRPLNLHRARLALGADGALQAVHVRMAGQSILLGTPLADWVRDGVDPVSVEGLS
DLAYAVPNLQVELHTPTDVPVPVLWYRSVGHTHTAFSAETLIDEAAVAAGQDPVAYRLALLAAHPRHREVLQLA
AVRAGWREPLAAGAPGTRRGRGVAVHESFRSVMAQVVEVTIAADGALKVDRVVCAAVHCGLAVNPDVVRA
QMEGGIGFALSTALHGAITLKDGAVEQSNFHDYPVLRLAEMPAVEVHIAPSTQPPTGVGEPGVPPLAPALANAI
AQATGQRLRTLPLGTTVKA
>SEQF7971||SEQF7971.1_01978
MRIAIQNLSRRRFVQGAGGLLLGLSLPPLARRAMAAGPQAGDGFAANAFVRIGADGRVTVLAKHLEMGQGA
YTGLATLLAEELDADWRQVRVEGAPADSARYGNQALGGLQGTGGSTAMFDSWEPMRRAGATARAMLVQAA
AQRWQVPADTIEVAEGVLSHPASGRRAGFGELAEAAARSPVPEDVPLKDPARFRLIGKHRLPHVDSAAKSDGS
ALYTQDMKLPGMLVAVVAHAPRLGAAVARVDDAAARAVPGVRAVVRFGGAALRHAGVAVLATNTWAARAG
RDALRIEWDEGPAYRQGSADILARYREAVGRPGSMAARKGDIDAAFAGAAKVIEAEYTYPYLAHAAMEPLNCL
VRLDDERCEIWNGEQFQTADQRAIAQYLGMPAERITLTQLYAGGSFGRRASSHADYLLEAVAIARTARAQGLNA
PVKLVWMREDDMRAGYYRPLNLHRARLALGADGALQAVHVRMAGQSILLGTPLADWVRDGVDPVSVEGLS

DLAYAVPNLQVELHTPTDVPVPVLWYRSVGHTHTAFSAETLIDEAAVAAGQDPVAYRLALLAAHPRHREVLQLA
AVRAGWREPLAAGAPGTRRGRGVAVHESFRSVMAQVVEVTIAADGALKVDRVVCAAVHCGLAVNPDVVRA
QMEGGIGFALSTALHGAITLKDGAVEQSNFHDYPVLRLAEMPAVEVHIAPSTQPPTGVGEPGVPPLAPALANAI
AQATGQRLRTLPLGTTVKA
>SEQF7972||SEQF7972.1_01926
MRIAIQNLSRRRFVQGAGGLLLGLSLPPLARRAMAAGPQAGDGFAANAFVRIGADGRVTVLAKHLEMGQGA
YTGLATLLAEELDADWRQVRVEGAPADSARYGNQALGGLQGTGGSTAMFDSWEPMRRAGATARAMLVQAA
AQRWQVPADTIEVAEGVLSHPASGRRAGFGELAEAAARSPVPEDVPLKDPARFRLIGKHRLPHVDSAAKSDGS
ALYTQDMKLPGMLVAVVAHAPRLGAAVARVDDAAARAVPGVRAVVRFGGAALRHAGVAVLATNTWAARAG
RDALRIEWDEGPAYRQGSADILARYREAVGRPGSMAARKGDIDAAFAGAAKVIEAEYTYPYLAHAAMEPLNCL
VRLDDERCEIWNGEQFQTADQRAIAQYLGMPAERITLTQLYAGGSFGRRASSHADYLLEAVAIARTARAQGLNA
PVKLVWMREDDMRAGYYRPLNLHRARLALGADGALQAVHVRMAGQSILLGTPLADWVRDGVDPVSVEGLS
DLAYAVPNLQVELHTPTDVPVPVLWYRSVGHTHTAFSAETLIDEAAVAAGQDPVAYRLALLAAHPRHREVLQLA
AVRAGWREPLAAGAPGTRRGRGVAVHESFRSVMAQVVEVTIAADGALKVDRVVCAAVHCGLAVNPDVVRA
QMEGGIGFALSTALHGAITLKDGAVEQSNFHDYPVLRLAEMPAVEVHIAPSTQPPTGVGEPGVPPLAPALANAI
AQATGQRLRTLPLGTTVKA
>SEQF8249||SEQF8249.1_00489
MSQPDTTLQSPSRRTLLKVGSLALGGLVIGFALPFAGRSFAEQVLNEGPEDQPMSNATALDAFISIDRDGQVTFT
VPKIEMGQGAQSGLAVMVAEELEIGLEQITLKEAPPNEQIYNDKLLNFQATGGSTSIRSNWEPLRQAGAAARLL
LIQAAAQRWQLGAGQLRAENGRVLGPDGQSLGYGELVEDAAKLPVPEDIPLKPADQFRLIGKPTRRLDTPAKV
DGTARFTIDLVVPGMKYASIRACPVLGGTLREVDERAARQIPGVIEVVRLDNAVAVIGEHTWAAFAGVRALEID
WALGDNAGIDSAQMEREIREALDKPGAIANEQGDIDAALKDAARTFEAEYEMPFLAHAALEPMTCVAEVRAD
AVELWVGTQVPVRAQTAAAEVAGRPAEQVIVNNQLIGGAFGRRLEVDFISQAVAIAAQVDYPIKLTWTREEDTT
HDMYRPHYIDRFAAALDAEGRLQGWRHTIAGASVLARFAPEAVPENGLDGDAVEVAMHPIYAMPNLRVNYV
PVPPRALHQSWWRGVGPLRSTYMLESFIDEVARSVEQDPVDYRMALLGSHPRAQGVLRLAAEKAGWGEPLE
AGHGRGVAVQEVFGSFLATVVELQVSEDKGIRLKRLVVAIDCGQVMNPVSVKSQIEGGTLFGLSAALFNEITVRE
GRVEQTNFHDYRQLRISDAPPVETYIVESREAPGGVGEAGTAMIAPALVNALAAANGTRIRRLPLARAGYYVI
>SEQF8250||SEQF8250.1_02796
MSQPDTTLQSPSRRTLLKVGSLALGGLVIGFTLPFAGRSFAEQVLNEGPEDQPMSNATALDAFISIDRDGQVTFT
VPKIEMGQGAQSGLAVMVAEELEIGLEQITLKEAPPNEQIYNDKLLNFQATGGSTSIRSNWEPLRQAGAAARLL
LIQAAAQHWQLGADQLRAENGRVLGPDGQSLGYGELVEDAAKLPVPEDIPLKPADQFRLIGKPTRRLDTPAKV
DGTARFTIDLVVPGMKYASIRACPVLGGTLREVDERAARQIPGVIEVVRLDNAVAVIGEHTWAAFAGVRALEID
WALGDNAGIDSAQMEREIREALDKPGAIANEQGDIDAALKDAARTFEAEYEMPFLAHAALEPMTCVAEVRAD
AVELWVGTQVPVRAQTAAAEAAGRPAEQVIVNNQLIGGAFGRRLEVDFISQAVAIAAQVDYPIKLTWTREEDT
THDMYRPHYIDRFAAALDAEGRLQGWRHTIAGASVLARFAPEAVPENGLDGDAVEVAMHPIYAMPNLRVNY
VPVPPRALHQSWWRGVGPLRSTYMLESFIDEVARSAEQDPVDYRMALLGSHPRAQGVLRLAAEKAGWGEPL
EAGHGRGVAVQEVFGSFLATVVELQVSEDKGIRLKRLVVAIDCGQVMNPVSVKSQIEGGTLFGLSAALFNEITVR
EGRVEQTNFHDYRQLRISDAPPVETYIVESREAPGGVGEAGTAMIAPALVNALAAANGTRIRRLPLARAGYYVI
>SEQF8256||SEQF8256.1_00515
MSQPDTTLQSPSRRTLLKVGSLALGGLVIGFTLPFAGRSFAEQVLNEGPEDQPMSNATALDAFISIDRDGQVTFT
VPKIEMGQGAQSGLAVMVAEELEIGLEQITLKEAPPNEQIYNDKLLNFQATGGSTSIRSNWEPLRQAGAAARLL
LIQAAAQRWQLGADQLRAENGRVLGPDGQSLGYGELVEDAAKLPVPEDIPLKPADQYRLIGKPTRRLDTPAKV
DGTARFTIDLVVPGMKYASIRACPVLGGTLREVDERAARQIPGVIEVVRLDNAVAVIGEHTWAAFAGVRALEID
WALGDNAGIDSAQMEREIRAALDKPGAIANEQGDIDAALKDAARTFEAEYEMPFLAHAALEPMTCVAEVRAD

AVELWVGTQVPVRAQTAAAEAAGRPAEQVIVNNQLIGGAFGRRLEVDFISQAVAIAAQVDYPIKLTWTREEDT
THDMYRPHYIDRFAAALDAEGRLQGWRHTIAGASVLARFAPEAVPENGLDGDAVEVAMHPSYAMPNLRVNY
VPVPPRALHQSWWRGVGPLRSTYMLESFIDEVARSVEQDPVDYRMALLGSHPRAQGVLRLAAEKAGWGEPL
EAGHGRGVAVQEVFGSFLATVVELQVSEDKGIRLKRLVVAIDCGQVMNPVSVKSQIEGGTLFGLSAALFNEITVR
EGRVEQTNFHDYRQLRISDAPPVETYIVESREAPGGVGEAGTAMIAPALVNALAAANGTRIRRLPLARAGYYVI
>SEQF8257||SEQF8257.1_00482
MSQPDTTLQSPSRRTLLKVGSLALGGLVIGFTLPFAGRSFAEQILNEGPEDQPMSNATALDAFISIDRDGQVTFT
VPKIEMGQGAQSGLAVMVAEELEIGLEQITLKEAPPNEQIYNDKLLNFQATGGSTSIRSNWEPLRQAGAAARLL
LIQAAAQRWQLGADQLRAENGRVLGPDGQSLGYGELVEDAAKLPVPEDIPLKPADQFRLIGKPTRRLDTPAKV
DGTARFTIDLVVPGMKYASIRACPVLGGTLREVDERAARQIPGVIEVVRLDNAVAVIGEHTWAAFAGVRALEID
WALGDNAGIDSAQMEREIRAALDKPGAIANEQGDIDAALKDAARTFEAEYEMPFLAHAALEPMTCVAEVRAD
AVELWVGTQVPVRAQTAAAEAAGRPAEQVIVNNQLIGGAFGRRLEVDFISQAVAIAAQVDYPIKLTWTREEDT
THDMYRPHYIDRFAAALDAEGRLQGWRHTIAGASVLARFAPEAVPENGLDGDAVEVAMHPSYAMPNLRVNY
VPVPPRALHQSWWRGVGPLRSTYMLESFIDEVARSVEQDPVDYRMALLGSHPRAQGVLRLAAEKAGWGEPL
EAGHGRGVAVQEVFGSFLATVVELQVSEDKGIRLKRLVVAIDCGQVMNPVSVKSQIEGGTLFGLSAALFNEITVR
EGRVEQTNFHDYRQLRISDAPPVETYIVESREAPGGVGEAGTAMIAPALVNALAAANGTRIRRLPLARAGYYVI
>SEQF8259||SEQF8259.1_01082
MANPDNTLQSPSRRHLLKVGSLALGGLVIGFSLPFSGRSFAEQVLNEGPEDQPMSNATALDAFISIDRDGQVTF
TVPKIEMGQGAQSGLAVMVAEELEIGLDQIRLKEAPPNEAIYNDKLLNFQATGGSTSIRSNWEPLRQAGAAARL
LLIQAAAQRWQVGADQLRAENGRVLGPGGQSLGYGELAEDASKLPVPENIPLKPAEQFKLIGKPTRRLDTPAKV
DGTARFTIDLVLPGMKYASARACPVLGGTVRHLDDRAARQVPGVIDVIRLDDAVAVIGEHTWASFAGLRALEIE
WDYGEHASIDSSQMERDIQDALDKPGAVANEQGDIDQALKDAAKTFEAEYEMPFLAHAALEPMSCVAQVRP
DAVELWVGTQVPVRAQTAAAEASGRPAEQVIVNNQLIGGAFGRRLEVDFISQAVTIAAKVDYPIKLTWTREEDT
THDLYRPHYIDRLAAALDDQGRLLGWRHTIAGASVLARFAPEAVPENGLDGDAVEVAQHPIYALANLRVNYVP
VPPRALRQSWWRGVGPLRSTYMLESFIDEVARSVDRDPVQYRMALLGSQPRAQQVLRLAAEKAGWGDPLEA
GHGRGVAVQEVFGSFLATVVELQVTEDKGIKLKRLVVAIDCGQVMNPVSVKSQIEGGTLFGLSAALFNEITVREG
RVEQSNFHDYRQLRISDAPPVETHIVDSREAPGGVGESGTAMIAPALVNALAAANGTRIRRLPLARAGYYVI
>SEQF8260||SEQF8260.1_00516
MSQPDTTLQSPSRRTLLKVGSLALGGLVIGFTLPFAGRSFAEQILNEGPEDQPMSNATALDAFISIDRDGQVTFT
VPKIEMGQGAQSGLAVMVAEELEIGLEQISLKEAPPNEQIYNDKLLNFQATGGSTSIRSNWEPLRQAGAAARLL
LIQAAAQSWQLGADQLRAENGRVLGPDGQSLGYGELVEDAAKLPVPEDIPLKPADQFRLIGKPTRRLDTPAKVD
GTARFTIDLVVQGMKYASIRACPVLGGTLREVDERAARQIPGVIEVVRLDNAVAVIGEHTWAAFAGVRALEIDW
ALGDNAGIDSAQMEREIRAALDKPGAIANEQGDIDAALKDAARTFEAGYEMPFLAHAALEPMTCVAEVRADA
VELWVGTQVPVRAQTAAAEAAGRPVEQVIVNNQLIGGAFGRRLEVDFISQAVAIAAQVDYPIKLTWTREEDTT
HDMYRPHYIDRFAAALDAEGRLQGWRHTIAGASVLARFAPEAVPENGLDGDAVEVAMHPIYAMPNLRVNYV
PVPPRALHQSWWRGVGPLRSTYMLESFIDEVARSVEQDPVDYRMALLGNHPRAQGVLRLAAEKAGWGEPLE
AGHGRGVAVQEVFGSFLATVVELQVSEDKGIRLKRLVVAIDCGQVMNPVSVKSQIEGGTLFGLSAALFNEITVRE
GRVEQTNFHDYRQLRISDAPPVETYIVESREAPGGVGEAGTAMIAPALVNALAAANGTRIRRLPLARAGYYVI
>SEQF8261||SEQF8261.2_00499
MSQPDTTLQSPSRRTLLKVGSLALGGLVIGFTLPFAGRSFAEQVLNEGPEDQPMSNATALDAFISIDRDGQVTFT
VPKIEMGQGAQSGLAVMVAEELEIGLEQITLKEAPPNEQIYNDKLLNFQATGGSTSIRSNWEPLRQAGAAARLL
LIQAATQRWQLGADQLRAENGRVLGPDGQSLGYGELVEDAAKLPVPEDIPLKPADQFRLIGKPTRRLDTPAKVD
GTARFTIDLVVPGMKYASIRACPVLGGTLREVDERAARQIPGVIEVVRLDNAVAVIGEHTWAAFAGVRALEIDW
ALGDNAGIDSAQMEREIRAALDKPGAIANEQGDIDAALKDAARTFEAEYEMPFLAHAALEPMTCVAEVRADA

VELWVGTQVPVRAQTAAAEAAGRPVEQVIVNNQLIGGAFGRRLEVDFISQAVAIAAQVDYPIKLTWTREEDTT
HDMYRPHYIDRFAAALDAEGRLQGWRHTIAGASVLARFAPEAVPENGLDGDAVEVAMHPIYAMPNLRVHYV
PVPPRALHQSWWRGVGPLRSTYMLESFIDEVARSVEQDPVDYRMALLGSHPRAQGVLRLAAEKAGWGEPLE
AGHGRGVAVQEVFGSFLATVVELQVSEDKGIRLKRLVVAIDCGQVMNPVSVKSQIEGGTLFGLSAALFNEITVRE
GRVEQTNFHDYRQLRISDAPPVETYIVESREAPGGVGEAGTAMIAPALVNALAAANGTRIRRLPLARAGYYVI
>SEQF8262||SEQF8262.1_00527
MSQPDTTLQSPSRRTLLKVGSLALGGLVIGFTLPFAGRSFAEQVLNEGPEDQPMSNATALDAFISIDRDGQATFT
VPKIEMGQGAQSGLAVMVAEELEIGLEQITLKEAPPNEQIYNDKLLNFQATGGSTSIRSNWEPLRQAGAAARLL
LIQAAAQRWQLGADQLRAENGRVLGPDGQSLSYGELVEDAAKLPVPEDIPLKPADQFRLIGKPTRRLDTPAKVD
GTARFTIDLVVPGMKYASIRACPVLGGTLREVDERAARQIPGVIEVVRLDNAVAVIGEHTWAAFAGVRALEIDW
ALGDNAGIDSAQMEREIRAALDKPGAIANEQGDIDAALKDAANTFEAEYEMPFLAHAALEPMTCVAEVRADA
VELWVGTQVPVRAQTAAAEAAGRPAEQVIVNNQLIGGAFGRRLEVDFISQAVAIAAQVDYPIKLTWTREEDTT
HDMYRPHYIDRFAAALDAEGRLQGWRHTIAGASVLARFAPEAVPENGLDGDAVEVAMHPIYAMPNLRVNYV
PVPPRALHQSWWRGVGPLRSTYMLESFIDEVARSVEQDPVDYRMALLGSHPRAQGVLRLAAEKAGWGEPLE
AGHGRGVAVQEVFGSFLATVVELQVSEDKGIRLKRLVVAIDCGQVMNPVSVKSQIEGGTLFGLSAALFNEITVRE
GRVEQTNFHDYRQLRISDAPPVETYIVESREAPGGVGEAGTAMIAPALVNALAAANGTRIRRLPLARAGYYVI
>SEQF8263||SEQF8263.1_01979
MAHPDTTLESRSRRNLLKVGSLALGGLVIGFSLPFPGRSFAEQVLSEGPEDQPMSNATALDAFIGIDRDGKVIFTV
PKIEMGQGAQSGLAVMIAEELEIGLDQITLKEAPPNEAIYNDKLLNFQATGGSTSIRSNWEPLRQAGAAARLLLI
QAAAQRWQVGADQLRAENGRVLGPDGQSFGYGELVDDASKLPVPENIPLKPAEQFKLIGKPTRRLDTPAKVN
GTARFTIDLVVPGMKYASARACPVIGGTVRSVDDRAARKVPGVIDVIRLGDAVAVIGEHTWATFAGVRALEIEW
DYGDNADIDSAQMEREIKEALDKPGALANEQGDIDKALKDAAKTFEAEYQMPFLAHAALEPMTCVAQVRPDA
VELWVGTQVPVRAQTAAAEASGRPAEQIIVNNQLIGGAFGRRLEVDFIHQAAAIAAKVDYPIKLTWLREEDTTH
DMYRPHYIDRFAAAMDAEGRLLGWRHTIAGASVLARFAPAAVPDSGLDGDAVEVAIHPIYAMQNLRVNYVPV
PPKALRQSWWRGVGPLRSTYMLESFIDEVARSVDRDPVEYRMELLGSHPRAQAVLRLAAEQAGWGEPLEAG
HGRGVAVQEVFGSFLATVVELQVTEDKGIKLKRLVVAVDCGQVMNPVSVKSQIEGGTLFGLSAALFNEITVSKG
RVEQSNFHDYRQLRISEAPPVETFIVESREAPGGVGEAGTAMIAPALVNALASANGTRIRRLPLARAGYYVI
>SEQF8266||SEQF8266.1_01813
MSQPDTTLQSPSRRTLLKVGSLALGGLVIGFTLPFAGRSFAEQVLNEGPEDQPMSNATALDAFISIDRDGQVTFT
VPKIEMGQGAQSGLAVMVAEELEIGLEQITLKEAPPNEQIYNDKLLNFQATGGSTSIRSNWEPLRQAGAAARLL
LIQAAAQRWQLGADQLRAENGRVLGPDGQSLGYGELVEDAAKLPVPEDIPLKPADQYRLIGKPTRRLDTPAKV
DGTARFTIDLAVPGMKYASIRACPVLGGTLREVDERAARQIPGVIEVVRLDNAVAVIGEHTWAAFAGVRALEID
WALGDNAGIDSAQMEREIRAALDKPGAIANEQGDIDAALKDAARTFEAEYEMPFLAHAALEPMTCVAEVRAD
AVELWVGTQVPVRAQTAAAEAAGRPVEQVIVNNQLIGGAFGRRLEVDFISQAVAIAAQVDYPIKLTWTREEDT
THDMYRPHYIDRFAAALDAEGRLQGWRHTIAGASVLARFAPEAVPENGLDGDAVEVAMHPIYAMPNLRVNY
VPVPPRALHQSWWRGVGPLRSTYMLESFIDEVARSVEQDPVDYRMALLGNHPRAQGVLRLAAEKAGWGEPL
EAGHGRGVAVQEVFGSFLATVVELQVSEDKGIRLKRLVVAIDCGQVMNPVSVKSQIEGGTLFGLSAALFNEITVR
EGRVEQTNFHDYRQLRISDAPPVETYIVESREAPGGVGEAGTAMIAPALVNALAAANGTRIRRLPLARAGYYVI
>SEQF8267||SEQF8267.1_03735
MSQPDTTLQSPSRRTLLKVGSLALGGLVIGFTLPFAGRSFAEQVLNEGPEDQPMSNATALDAFISIDRDGQVTFT
VPKIEMGQGAQSGLAVMVAEELEIGLEQITLKEAPPNEQIYNDKLLNFQATGGSTSIRSNWEPLRQAGAAARLL
LIQAAAQRWQLGADQLRAENGRVLGPDGQSLGYGELVEDAAKLPVPEDIPLKPADQFRLIGKPTRRLDTPAKV
DGTARFTIDLVVPGMKYASIRACPVLGGTLREVDERAARQIPGVIEVVRLDNAVAVIGEHTWAAFAGVRALEID
WALGDNAGIDSAQMEREIRAALDKPGAIANEQGDIDAALKDAARTFEAEYEMPFLAHAALEPMTCVAEVRAD

AVELWVGTQVPVRAQTAAAEAAGRPVEQVIVNNQLIGGAFGRRLEVDFISQAVAIAAQVDYPIKLTWTREEDT
THDMYRPHYIDRFAAALDAEGRLQGWRHTIAGASVLARFAPEAVPENGLDGDAVEVAMHPIYAMPNLRVHY
VPVPPRALHQSWWRGVGPLRSTYMLESFIDEVARSVEQDPVDYRMALLGSHPRAQRVLRLAAEKAGWGEPL
EAGHGRGVAVQEVFGSFLATVVELQVSEDKGIRLKRLVVAIDCGQVMNPVSVKSQIEGGTLFGLSAALFNEITVR
EGRVEQTNFHDYRQLRISDAPPVETYIVESREAPGGVGEAGTAMIAPALVNALAAANGTRIRRLPLARAGYYVI
>SEQF8271||SEQF8271.1_04044
MSQSDTTLQSPSRRTLLKVGSLALGGLVIGFTLPFAGRSFAEQVLNEGPEDQPMSNATALDAFISIDRDGQVTFT
VPKIEMGQGAQSGLAVMVAEELEIGLEQITLKEAPPNEQIYNDKLLNFQATGGSTSIRSNWEPLRQAGAAARLL
LIQAATQRWQLGADQLRAENGRVLGPDGQSLGYGELVEDAAKLPVPEDIPLKPADQFRLIGKPTRRLDTPAKVD
GTARFTIDLVVPGMKYASIRACPVLGGTLREVNERAARQIPGVIEVVRLDNAVAVIGEHTWAAFAGVRALEIDW
ALGDNAGIDSAQMEREIRAALDKPGAIANEQGDIDAALKDAARTFEAEYEMPFLAHAALEPMTCVAEVRADA
VELWVGTQVPVRAQTAAAEAAGRPAEQVIVNNQLIGGAFGRRLEVDFISQAVAIAAQVDYPIKLTWTREEDTT
HDMYRPHYIDRFAAALDAEGRLQGWRHTIAGASVLARFAPEAVPENGLDGDAVEVAMHPIYAMPNLRVNYV
PVPPRALHQSWWRGVGPLRSTYMLESFIDEVARSVEQDPVDYRMALLGSHPRAQGVLRLAAEKAGWGEPLE
AGHGRGVAVQEVFGSFLATVVELQVSEDKGIRLKRLVVAIDCGQVMNPVSVKSQIEGGTLFGLSAALFNEITVRE
GRVEQTNFHDYRQLRISDAPPVETYIVESREAPGGVGEAGTAMIAPALVNALAAANGTRIRRLPLARAGYYVI
>SEQF8273||SEQF8273.1_02917
MSQPDTTLQSPSRRTLLKVGSLALGGLVIGFTLPFAGRSFAEQILNEGPEDQPMSNATALDAFISIDRDGQVTFT
VPKIEMGQGAQSGLAVMVAEELEIGLEQITLKEAPPNEQIYNDKLLNFQATGGSTSIRSNWEPLRQAGAAARLL
LIQAAAQRWQLGADQLRAENGRVLGPDGQSLGYGELVEDAAKLPVPEDIPLKPADQFRLIGKPTRRLDTPAKV
DGTARFTIDLVVPGMKYASIRACPVLGGTLREVDERAARQIPGVIEVVRLDNAVAVIGEHTWAAFAGVRALEID
WALGDNAGIDSAQMEREIRGALDKPGAIANEQGDIDAALKDAANTFEAEYEMPFLAHAALEPMTCVAEVRA
DAVELWVGTQVPVRAQTAAAEAAGRLVEQVIVNNQLIGGAFGRRLEVDFISQAVAIAAQVDYPIKLTWTREED
TTHDMYRPHYIDRFAAALDAEGRLQGWRHTIAGASVLARFAPEAVPENGLDGDAVEVAMHPIYAMPNLRVN
YVPVPPRALHQSWWRGVGPLRSTYMLESFIDEVARSVEQDPVDYRMALLGSHPRAQGVLRLAAEKAGWGEP
LEAGHGRGVAVQEVFGSFLATVVELQVSEDKGIRLKRLVVAIDCGQVMNPVSVKSQIEGGTLFGLSAALFNEITV
REGRVEQTNFHDYRQLRISDAPPVETYIVESREAPGGVGEAGTAMIAPALVNALAAANGTRIRRLPLARAGYYVI
>SEQF8276||SEQF8276.1_01585
MSQPDTTLQSPSRRNLLKVGSLALGGLVIGFTLPFAGRSFAEQVLNEGPEDQPMSNATALDAFISIDRDGQVTFT
VPKIEMGQGAQSGLAVMVAEELEIGLEQITLKEAPPNEQIYNDKLLNFQATGGSTSIRSNWEPLRQAGAAARLL
LIQAAAQRWQLGADQLRAENGRVLGPDGQSLGYGELVEDAAKLPVPEDIPLKPADQFRLIGKPTRRLDTPAKV
DGTARFTIDLVVPGMKYASIRACPVLGGTLREADERAARQIPGVIEVVRLDNAVAVIGEHTWAAFAGVRALEID
WALGDNAGIDSAQMEREIRGALDKPGAIANEQGDIDAALKDAANTFEAEYEMPFLAHAALEPMTCVAEVRA
DAVELWVGTQVPVRAQTAAAEAAGRPVEQVIVNNQLIGGAFGRRLEVDFISQAVAIAAQVDYPIKLTWTREED
TTHDMYRPHYIDRFAAALDAEGRLQGWRHTIAGASVLARFAPEAVPENGLDGDAVEVAMHPIYAMPNLRVN
YVPVPPRALHQSWWRGVGPLRSTYMLESFIDEVARSVEQDPVDYRMALLGSHPRAQGVLRLAAEKAGWGEP
LEAGHGRGVAVQEVFGSFLATVVELQVSEDKGIRLKRLVVAIDCGQVMNPVSVKSQIEGGTLFGLSAALFNEITV
REGRVEQTNFHDYRQLRISDAPPVETYIVESREAPGGVGEAGTAMIAPALVNALAAANGTRIRRLPLARAGYYVI
>SEQF8278||SEQF8278.1_04175
MAHPDTTLESRSRRNLLKVGSLALGGLVIGFSLPFPGRSFAEQVLSEGPEDQPMSNAKALDAFIGIDRDGKVIFT
VPKIEMGQGAQSGLAVMIAEELEIGLDQITLKEAPPNEAIYNDKLLNFQATGGSTSIRSNWEPLRQAGAAARLLL
IQAAAQRWQVGADQLRAENGRVLGPDGQSFGYGELVDDASKLPVPENIPLKPAEQFKLIGKPTRRLDTPAKVN
GTARFTIDLVVPGMKYASARACPVIGGTVRSVDDRAARKVPGVIDVIRLGDAVAVIGEHTWATFAGVRALEIEW
DYGDNADIDSAQMEREIKEALDKPGALANEQGDIDKALKDAAKTFEAEYQMPFLAHAALEPMTCVAQVRPDA

VELWVGTQVPVRAQTAAAEASGRPAEQIIVNNQLIGGAFGRRLEVDFIHQAAAIAAKVDYPIKLTWLREEDTTH
DMYRPHYIDRFAAAMDAEGRLLGWRHTIAGASVLARFAPAAVPDSGLDGDAVEVAIHPIYAMQNLRVNYVPV
PPKALRQSWWRGVGPLRSTYMLESFIDEVARSVDRDPVEYRMELLGSHPRAQAVLRLAAEQAGWGEPLEAG
HGRGVAVQEVFGSFLATVVELQVTEDKGIKLKRLVVAVDCGQVINPVSVKSQIEGGTLFGLSAALFNEITVSKGRV
EQSNFHDYRQLRISEAPPVETFIVESREAPGGVGEAGTAMIAPALVNALASANGTRIRRLPLARAGYYVI
>SEQF8280||SEQF8280.1_01281
MSQPDTTLQSPSRRTLLKVGSLALGGLVIGFTLPFAGRSFAEQVLNEGPEDQPMSNATALDAFISIDRDGQVTFT
VPKIEMGQGAQSGLAVMVAEELEIGLEQITLKEAPPNEQIYNDKLLNFQATGGSTSIRSNWEPLRQAGAAARLL
LIQAAAQRWQLGADQLRAENGRVLGPDGQSLGYGELVEDAAKLPVPEDIPLKPADQYRLIGKPTRRLDTPAKV
DGTARFTIDLAVPGMKYASIRACPVLGGTLREVDERAARQIPGVIEVVRLDNAVAVIGEHTWAAFAGVRALEID
WALGDNAGIDSAQMEREIRAALDKPGAIANEQGDIDAALKDAARTFEAEYEMPFLAHAALEPMTCVAEVRAD
AVELWVGTQVPVRAQTAAAEAAGRPVEQVIVNNQLIGGAFGRRLEVDFISQAVAIAAQVDYPIKLTWTREEDT
THDMYRPHYIDRFAAALDAEGRLQGWRHTIAGASVLARFAPEAVPENGLDGDAVEVAMHPIYAMPNLRVNY
VPVPPRALHQSWWRGVGPLRSTYMLESFIDEVARSVEQDPVDYRMALLGNHPRAQGVLRLAAEKAGWGEPL
EAGHGRGVAVQEVFGSFLATVVELQVSEDKGIRLKRLVVAIDCGQVMNPVSVKSQIEGGTLFGLSAALFNEITVR
EGRVEQTNFHDYRQLRISDAPPVETYIVESREAPGGVGEAGTAMIAPALVNALAAANGTRIRRLPLARAGYYVI
>SEQF8281||SEQF8281.1_00567
MTGSALSRRRFLQGSLGLLTLAVTARGLVSVAWADDAPRYGADGMPGGTVDDPLVFVSIATDGTVTIVAHRAE
MGTGVRTSLPMVVADEMEARWDRVKVVQAEADEARYGNQNVDGSRSVRHFLMPMRRVGAAARQMLEA
AAAARWAVPVAEVRATQHEVLHQPSGRRLGYGELAADAARQPVPTGDALRLKTRAEFRYIGKDRVRLVDLEDI
GTGQAIYGMDMRLPGMVYAVVARPPVVGGTLRRLDSRKALALPGVLQVIEIPAFQGAPAFQPLGGVAVVARN
TWAAMQGRAALELEWDDGANAGYDSAAYRQALEEASRAPGKVMRDQGDAPGSWAQAAERERFAAEYYVP
HLAHASMEPPVATVRIQDGRADVWTSVQNPVAARDAVAARLGLQPEKVKVNVLLLGGGFGRKSKPDFVDEA
AIVARAMPEGTPVKLVWTREDDIRHDYLHTVSCERLEAVVDRQGQVRSWLHRSAAPTIASLFAEGAKGQQLFE
SAMSAINMPYRIPNVRVETAEVPAHARIGWFRSVANIPHAFAAQCFIAELAHRAGKDHRQFALDLIGPARRLDP
GTLADTWNYTESPERYPYDTGRLRGVIEAACEGAGWGRELPEGHGLGLAFCYSFMSYTASVVEVAVDDEGEVR
VLAVDMALDCGPQINPERIRAQMEGGAIMGLGLALASEITFESGRVQQSNFHDYEVLRHSASPRLIRTHLVND
DHDLPPGGVGEPPVPPVAPALCNAIFAATGKRVRSLPVRRVA
>SEQF8282||SEQF8282.1_01779
MTRSIENHSRRRFLKGAAGLTLAIYFPWSFAQDAQKQAVSATFEPNAFLRIGEDDSVTVIAKHLEMGQGTYTGL
ATIVAEELDADWSQVRVQSAPADASRYNNLQWGPMQGTGGSSAIANSWEQLRQAGATARAMLVAAAAQR
WQVPAESITMAEGVLRHPPSGRQARFGELAGAAAEQAVPEQVRLKEPSEFKLIGQRIARKDSPEKINGSAIYTQ
DIHLPDMLTAVVAHPPRFGALVASFDPAPALAIDGVVEVVEIPNGVAVLARDTWTAKRGRDALSVEWDERAAF
RLSSAQAFERFRTLAETPGTVAREEGDSEAALDGAAHTLEAEYDFPYLAHAAMEPMNCVIRLDREGCEAWFGA
QIQTYDQAMLAGLFGLEPEQVRIHTLYAGGSFGRRASKGADYILETAHIVKAIEGRAPVKLVWLREDDMQAGN
YRPMFHHRLRAGLDAQGRLVAWQHRLVGQSVAAGSPFEGAIQNGIDATSVEGAANLPYAIANMRVDLHTPED
IAVPVQWWRAVGSTHTAFSTECFIDELAQTAGQDPMTWRLAMLEAHPRHAGVLKLAAEKAGWDRPLEPGK
DGERRGRGVAVHEAFGSFVAQVAEVTVQSDGRYRVDRVVCAVDCGIVVNPDVVRAQTEGGVGFALSAAMSE
AITFTDGKVDQSNFHDYSPLRIADMPEVEVHLVPSTAAPTGIGEPPVAPLAPALVNALAAATGKRVRRLPIGQQL
AS
>SEQF8282||SEQF8282.1_00122
MNAITPQTTGLSRRGFLKASAAVGGGLLISFTLPDLMRGAQAAEAGSFAPNAYVRIDHEGRIFLTIQPVEMGQG
TYTSMPALIAEELEVGLDQVTIEHAPADDRRYANPMLGFQVTGGSTSVPANWTPLREAGAAARMLLVAAAAQ
RWGVPADACQAQRGEVLHPASGRRLGYGELVDAAAKLPLPETLPLKEPKDFKLIGTPAKRTDSPDKVNGKALFG

IDVRPEGMKVAAIMLCPVVGGSLDSVDPAPAMAVRGVHQVLRTDNSVAVVAEHMGAARKGLAALQIEWNG
GANAQVSSAQMLEEIKAASRQPGALARSEGDVEAALAKATRKIEATYQVPFLAHACLEPVNCTVHVRKDACDL
WLGTQVPARAKAVAAQLTGLPEEAVQVHNHLIGGGFGRRLDVDFVADAVKIAKQVDYPLKVIWSREEDTRHST
LRPFHYNHLSVGLDEQNRPIAWTHRVTGGSILARWLPARFTNNVDGDAVRDACGPYAFPNLAVHYVRHEPPS
GILPAFWRGVGHTQNAFMVEGLLDEVIQLVEADPFEYRYPLLQQHPRALRVLELLREKSAWDTPLEPGRGRGL
ALTHCFGTFAAQVAEVTVSPSGEVKVDKVTSVVDCGIAINPDSVVAQMQGGTIFGLTAALFGDITFKDGRVEQG
NFDSYRMLRINEAPLLETHVVVSEESPGGLGEVPTVLIAPAVVNAIFAATGKRLRRLPIDPQELRTV
>SEQF8283||SEQF8283.1_03468
MTGSALSRRRFLQGSLGLLTLAVTARGLVSVAWADDAPRYGADSMPGGTVDDPLVFVSIATDGTVTIVAHRAE
MGTGVRTSLPMVVADEMEARWDRVEVVQAEADEARYGNQNVDGSRSVRHFLMPMRRVGAAARQMLEA
AAAARWAVPVAEVRATQHEVLHQPSGRRLGYGELAADAARQPVPTGDALRLKTRAEFRYIGKDQVRLVDLEDI
GTGQAIYGMDMRLPGMVYAVVARPPVVGGTLRRLDSRKALALPGVLQVIEIPAFQGAPAFQPLGGVAVVARN
TWAAMQGRAALELEWDDGANAGYDSAAYRQALEEASRAPGKVMRDQGDAPGSWAQAAERERFAAEYYVP
HLAHASMEPPVATVRIQDGRADVWTSVQNPVAARDAVAARLELQPEKVKVNVLLLGGGFGRKSKPDFVDEAA
IVARAMPEGTPVKLVWTREDDIRHDYLHTVSCERLEAVVDRQGQVRSWLHRSAAPTIASLFAEGAKGQQLFES
AMSAINMPYRIPNVRVETAEVPAHARIGWFRSVANIPHAFAAQCFIAELAHRTGQDHRQFALDLIGPARRLDP
GTLADTWNYTESPERYPYDTGRLRGVIEAACEGAGWGRELPEGHGLGLAFCYSFMSYTASVVEVAVDDEGEVR
VLAVDMALDCGPQINPERIRAQMEGGAIMGLGLALASEITFESGRVQQSNFHDYEVLRHSASPRLIRTHLVND
DHDLPPGGVGEPPVPPVAPALCNAIFAATGKRVRSLPVRRVV
>SEQF8284||SEQF8284.1_00189
MSQPDTTLQSPSRRTLLKVGSLALGGLVIGFTLPFAGRSFAEQVLNEGPEDQPMSNATALDAFISIDRDGQVTFT
VPKIEMGQGAQSGLAVMVAEELEIGLEQITLKEAPPNEQIYNDKLLNFQATGGSTSIRSNWEPLRQAGAAARLL
LIQAAAQRWQLGADQLRAENGRVLGPDGQSLGYGELVEDAAKLPVPEDIPLKPADQFRLIGKPTRRLDTPAKV
DGTARFTIDLVVPGMKYASIRACPVLGGTLREADERAARQIPGVIEVVRLDTAVAVIGEHTWAAFAGVRALEID
WALGDNAGIDSAQMEREIRGALDKPGAIANEQGDIDAALKDAANTFEAEYEMPFLAHAALEPMTCVAEVRA
DAVELWVGTQVPVRAQTAAAEAAGRPVEQVIVNNQLIGGAFGRRLEVDFISQAVAIAAQVDYPIKLTWTREED
TTHDMYRPHYIDRFAAALDAEGRLQGWRHTIAGASVLARFAPEAVPENGLDGDAVEVAMHPIYAMPNLRVN
YVPVPPRALHQSWWRGVGPLRSTYMLESFIDEVARSVEQDPVDYRMALLGSHPRAQGVLRLAAEKAGWGEP
LEAGHGRGVAVQEVFGSFLATVVELQVSEDKGIRLKRLVVAIDCGQVMNPVSVKSQIEGGTLFGLSAALFNEITV
REGRVEQTNFHDYRQLRISDAPPVETYIVESREAPGGVGEAGTAMIAPALVNALAAANGTRIRRLPLARAGYYVI
>SEQF8285||SEQF8285.1_03483
MSQPDTTLQSPSRRTLLKVGSLALGGLVIGFTLPFAGRSFAEQVLNEGPEDQPMSNATALDAFISIDRDGQVTFT
VPKIEMGQGAQSGLAVMVAEELEIGLEQITLKEAPPNEQIYNDKLLNFQATGGSTSIRSNWEPLRQAGAAARLL
LIQAAAQRWQLGADQLRAENGRVLGPDGQSLGYGELVEDAAKLPVPEDIPLKPADQYRLIGKPTRRLDTPAKV
DGTARFTIDLAVPGMKYASIRACPVLGGTLREVDERAARQIPGVIEVVRLDNAVAVIGEHTWAAFAGVRALEID
WALGDNAGIDSAQMEREIRAALDKPGAIANEQGDIDAALKDAARTFEAEYEMPFLAHAALEPMTCVAEVRAD
AVELWVGTQVPVRAQTAAAEAAGRPVEQVIVNNQLIGGAFGRRLEVDFISQAVAIAAQVDYPIKLTWTREEDT
THDMYRPHYIDRFAAALDAEGRLQGWRHTIAGASVLARFAPEAVPENGLDGDAVEVAMHPIYAMPNLRVNY
VPVPPRALHQSWWRGVGPLRSTYMLESFIDEVARSVEQDPVDYRMALLGNHPRAQGVLRLAAEKAGWGEPL
EAGHGRGVAVQEVFGSFLATVVELQVSEDKGIRLKRLVVAIDCGQVMNPVSVKSQIEGGTLFGLSAALFNEITVR
EGRVEQTNFHDYRQLRISDAPPVETYIVESREAPGGVGEAGTAMIAPALVNALAAANGTRIRRLPLARAGYYVI
>SEQF8286||SEQF8286.1_02607
MSQPDTTLQSPSRRTLLKVGSLALGGLVIGFTLPFAGRSFAEQILNEGPEDQPMSNATALDAFISIDRDGQVTFT
VPKIEMGQGAQSGLAVMVAEELEIGLEQITLKEAPPNEQIYNDKLLNFQATGGSTSIRSNWEPLRQAGAAARLL

LIQAAAQRWQLGADQLRAENGRVLGPDGQSLGYGELVEDAAKLPVPEDIPLKPADQFRLIGKPTRRLDTPAKV
DGTARFTIDLVVPGMKYASIRACPVLGGTLREVNERAARQIPGVIEVVRLDNAVAVIGEHTWAAFAGVRALEID
WALGDNAGIDSAQMEREIRAALDKPGAIANEQGDIDAALKDAARTFEAEYEMPFLAHAALEPMTCVAEVRAD
AVELWVGTQVPVRAQTAAAEAAGRPVEQVIVNNQLIGGAFGRRLEVDFISQAVAIAAQVDYPIKLTWTREEDT
THDMYRPHYIDRFAAALDAEGRLQGWRHTIAGASVLARFAPEAVPENGLDGDAVEVAMHPIYAMPNLRVNY
VPVPPRALHQSWWRGVGPLRSTYMLESFIDEVARSVEQDPVDYRMALLGNHPRAQGVLRLAAEKAGWGEPL
EAGHGRGVAVQEVFGSFLATVVELQVSEDKGIRLKRLVVAIDCGQVMNPVSVKTQIEGGTLFGLSAALFNEITVR
EGRVEQTNFHDYRQLRISDAPPVETYIVESREAPGGVGEAGTAMIAPALVNALAAANGTRIRRLPLARAGYYVI
>SEQF8604||SEQF8604.1_00086
MHFDAHTARAHMPKGLVALMDQAQGAINTEANAAATEGVARRTFLKAAAASGFALGAYPLVATAQGAGTAP
AGLKPFEQPSAFVRIDTDGTVTVTINRLDFGQGVQTGLPMILAEELDADWAKVRSVHGDANPAYADPAFGMH
LTGGSNSLKNSYTQYRELGARTRAMLVSAAAAQWGVDASTLRTNAGFVVGPGGKKLAYGALAEAAMQQPVP
EKVTLKDPKQFRIIGKPTTRLDAQAKSSGQQDYGIDVRLPGILTAVVARPPVFGAKLKSLDDSTAKAIKGVKAVLR
VPTDRGGEGVAIIAEGYWPAKQGRDALKVEWDTASVEKPDTAQLLTQYRALAQKTGSIAIPADVAALANAPQKI
SAEFTFPYLAHAPMEPLNCTVKLDGDKAELWMGTQMPGLDAMAAAKVLGLQPQNVKVHTQMAGGGFGRR
AIPTSDYVVEACGVAKAARTAGINAPVRTLWSREDDIKGGYYRPMHVHRAEIGFDAQGNILAWDHVIVGQSIV
KGTPFEGFMVKNGVDSTAVEGMKEPYNIPMRLSVHHPQVNVPVLWWRSVGSTHTAYAMETLLDEVARATKQ
DPVAYRLRLMGDKHPRHKAALQLAVDQSGYGKKKLAAGRAWGVAVHESFSSVVAYVVEASVSKDGTPKLHSV
TAGVHCNLAVNPKSVEAQVQGGALMGLSMCLPGAAITLKDGVVEQSNFGDFAVPRITDMPQVAVHIVPSAEP
PTGMGEPGLPPLAPAFANAVARLTGKTPRELPFKLA
>SEQF8605||SEQF8605.1_03694
MHFDAHTARAHMPKGLVALMDQAQGAINIEANAAATEGVARRTFLKAAAASGFALGAYPLVATAKGAGTAPA
GLKPFEQPSAFVRIDTDGTVTVTINRLDFGQGVQTGLPMILAEELDADWAKVRSVHGDANPAYADPAFGMHLT
GGSNSLKNSYTQYRELGARTRAMLVSAAAAQWGVDASTLRTNAGFVVGPGGKKLAYGALAEAAMQQPVPEK
VTLKDPKQFRIIGKPTTRLDAQAKSSGQQDYGIDVRLPGILTAVVARPPVFGAKLKSLDDSTAKAIKGVKAVLRVP
TDRGGEGVAIIAEGYWPAKQGRDALKVEWDTASVEKPDTAQLLTQYRALAQKTGSIAIPADVAALANAPQKISA
EFTFPYLAHAPMEPLNCTVKLDGDKAELWMGTQMPGLDAMAAAKVLGLQPQNVKVHTQMAGGGFGRRAI
PTSDYVVEACGVAKAARTAGINAPVRTLWSREDDIKGGYYRPMHVHRAEIGFDAQGNILAWDHVIVGQSIVKG
TPFEGFMVKNGVDSTAVEGMKEPYNIPMRLSVHHPQVNVPVLWWRSVGSTHTAYAMETLLDEVARATQQDP
VAYRLRLMGDKHPRHKAALQLAVDQSGYGKKKLAAGRAWGVAVHESFSSVVAYVVEASVSKDGTPKLHSVTA
GVHCNLAVNPKSVEAQVQGGALMGLSMCLPGAAITLKDGVVEQSNFGDFAVPRITDMPQVAVHIVPSAEPPT
GMGEPGLPPLAPAFANAVARLTGKTPRELPFKLG
>SEQF8609||SEQF8609.1_02238
MPIPNHFLADMPKGLRALAADTIDAGTKLERRDFLTLATASGFALGVFPSAALAQAKGNEQVSANVLKPTQQPS
AFVKIDRDGTVTVTINRLEFGQGVQTGLPMVLAEELDADWSKMNSVHGNADPAYMDPVMGMHLTGGSTAI
KNSYVQYRELGARTRSMLLATAAKRWGVDPQSLRTQTGQVIGPRGKRLGYGELADEAMKMPVPQQVKLKDV
KDFRIIGHATGRLDARTKSSGKQSYGIDMHLPGMLTALVAHPPVYGSKIQSVDDSATKAIKGVRAVLRVPSVWG
GELVAVLADGYWPAKQGRDALKIQWDSAAVGKVDSARQLAQYRDLAKKPGALKFDADVSAMNGAVHKISAE
YVFPYLAHTPMEPLNCTVRVTGAGKDAKAELWLGTQAPGWEAATAARVLGVAPQNVRVNVQMAGGGFGRR
ANPRSDYVAEACEIAKAARASGIDAPVRMIWSREDDVKGGYYRPMHVHRAEIGFDGKGKVIAWDHVIVGQSL
AKGTAFEGFMVKNGVDTTTVEGMKEPYDLPMRLSVHHPELNAPVLWWRSVGSTHTAYVMETLMDEIARAVK
QDPVAYRLQQFGDRHPRHKAALQLAVEKSGYGKRQLAEGRAWGVAVHQSFDSVVAYVVEASMKDGTPKLHA
VTAGVHCNLAVNPRSVEAQVQGGALMGLGMCLPGAAITFKDGQVEQGNFNDYTVARLTDMPAITVHIVPSA
DAPTGMGEPGVPPLAPAFANAIAKLSGHTPRELPFPKA

>SEQF8609||SEQF8609.1_00153
MQALQVDRRDFLKAGGAAASLVLGFKLPIAAAKGQPARDPDDINAWLRIAPDGTVTIMVPSAEIGQGVYTSAP
MLIAEELECDWRQVRAQIAPTDPVYANRMFKVQATASSTSSRWSFEPLRRIGAAARLMLVEAAAREWRVDAA
GCTAVRGRVLHAASGRSLGYGELAPKAAQLPRPDVARIVLKARPEWRLIGQPVQRLDIPLKTNGSAVFGVDVKV
GGMLIGTVAACPVRGGRLKPFDDKPALAVKGVHKVVPLAGSAVAVLGESYWPARQGLARLQLDWQLPEGPLV
DSKAMLAQLRGAAAQTDTVAKRTGDPEAALREAAQRVEAEYEVPYLAHATLEPINATADVRADRAEIWGPTQ
VCGEIAERLAPHLGLPAERIAVHSTFVGGGFGRREEFDVFIQAALASQAARRPVKLIWSREEDIQQDFYRPAAAA
RFAAVLGEGGSVQALEARLACSSIYIRNFPDRVKNGVDPKSVEGVVDVPYALPHFGVRYAMVNSAIPAGFWRG
VGYTQNCFFFESFIDELAHQARRNPLDFRLALLKDQPRHAGLLRRLASRAGWGQAPSPSGGDRHLGLALSEAW
GSICGTAVELSVKDKRITIHRVVCVVDCGTVINPATVQRQLEGATIWGLAAAAFGEISIEQGKVLQSNFHDYPML
HLAQTPPIETELIESGAKIGGVGESGVPPLAPALANALFAATGERLRSLPLSRHGYTLA
>SEQF8611||SEQF8611.1_04226
MPIPNHFLADMPKGLRALAADTIDAGTKLERRDFLKLATASGFALGVFPSAALAQAKGNEEVSTSALKPYQQPS
AFVKIDRDGTVTVTINRLEFGQGVQTGLPMVLAEELDADWSKLHSVHGNADPAYLDPVMGMHLTGGSTAIKN
SYVQYRELGARTRSMLLATAAKRWGVDPQSLRTQAGQVIGPRGKKLGYGELADEAMKMPVPQQVKLKGVKD
FRIIGRATGRLDARAKSSGSQSYGIDMHLPGMLTAVVAHPPVYGSKIQSVDDAATKAIKGVRAVLRVPSVWGGE
LVAVLADGYWPAKQGRDALKIQWDSAAVGKVDSARQLAQYRELAKKPGALKFDVDVSALNGAVHKISAEYVF
PYLAHTPMEPLNCTVRVTGAGKDAKVELWLGTQAPGWEVATAARVLGVAPQNVRVNVQMAGGGFGRRAN
PRSDYVAEACEIAKAARASGVDAPVRMIWSREDDVKGGYYRPMHVHRAEIGFDGKGKVIAWDHVIVGQSLAK
GTAFEGFMVKNGVDTTTVEGMKEPYDLPMRLSVHHPELNAPVLWWRSVGSTHTAYVMETLMDEIARAVGQ
DPVAYRLQQFGDRHPRHKAALQLAVEKSGYGKRRLAEGRAWGVAVHQSFDSVVAYVVEASMKDGTPKLHAVT
AGVHCNLAVNPRSVEAQVQGGALMGLGMCLPGAAITFKDGQVEQGNFNDYTVARLTDMPAITVHIVPSADA
PTGMGEPGVPPLAPAFANAIAKLSGHTPRELPFPKA
>SEQF8612||SEQF8612.1_02558
MPIPNHFLADMPKGLRALAADTIDAGAKLERRDFLKLATASGFALGVFPSAALAQAKGNEEVSTSALKPYQQPS
AFVKIDRDGAVTITINRLEFGQGVQTGLPMVLAEELDADWSKVHSVHGNADPAYLDPVMGMHLTGGSTAIKN
SYVQYRELGARARSMLLATAAKRWGVDPQSLRTQAGQVIGPRGKKLGYGELADEAMKMPVPQQVKLKDVKD
FRIIGHATGRLDARAKSSGRQSYGIDMHLPGMLTAVVAHPPVYGSKIQSVDDAAAKAIKGVRAVLRVPSVWGG
ELVAVLADGYWAAKQGRDALKIQWDSSAVGKVDSARQLAQYRELVKKPGALKFDADVSALNGAAHKISAEYVF
PYLAHTPMEPLNCTVRVTGAGKDAKVELWLGTQAPGWEVATAARVLGVAPQNVRVNVQMAGGGFGRRAN
PRSDYVAEACEIAKAARASGIEAPVRMIWSREDDVKGGYYRPMHVHRAEIGFDGKGKVIAWDHVIVGQSLAK
GTAFEGFMVKNGVDTTTVEGMKEPYDLPMRLSVHHPELNAPVLWWRSVGSTHTAYVMETLMDEIARAVKQ
DPVAYRLQQFGDRHPRHKAALQLAVEKSGYGKRQLAEGRAWGVAVHQSFDSVVAYVVEASMKDGAPKLHAV
TAGVHCNLAVNPRSVEAQVQGGALMGLGMCLPGAAITFKDGQVEQGNFNDYTVARLTDMPAISVHIVPSAD
APTGMGEPGVPPLAPAFANAIAKLSGHTPRELPFLKA
>SEQF8613||SEQF8613.1_02825
MPIPNHFLADMPKGLRALAADTIDAGTKLERRDFLKLATASGFALGVFPSAALAQAKGNEEVSTSALKPYQQPS
AFVKIDRDGTVTVTINRLEFGQGVQTGLPMVLAEELDADWSKLHSVHGNADPAYLDPVMGMHLTGGSTAIKN
SYVQYRELGARTRSMLLATAAKRWGVDPQSLRTQAGQVIGPRGKKLGYGELADEAMKMPVPQQVKLKGVKD
FRIIGRATGRLDARAKSSGSQSYGIDMHLPGMLTAVVAHPPVYGSKIQSVDDAATKAIKGVRAVLRVPSVWGGE
LVAVLADGYWPAKQGRDALKIQWDSAAVGKVDSARQLAQYRELAKKPGALKFDVDVSALNGAVHKISAEYVF
PYLAHTPMEPLNCTVRVTGAGKDAKVELWLGTQAPGWEVATAARVLGVAPQNVRVNVQMAGGGFGRRAN
PRSDYVAEACEIAKAARASGVDAPVRMIWSREDDVKGGYYRPMHVHRAEIGFDGKGKVIAWDHVIVGQSLAK
GTAFEGFMVKNGVDTTTVEGMKEPYDLPMRLSVHHPELNAPVLWWRSVGSTHTAYVMETLMDEIARAVGQ

DPVAYRLQQFGDRHPRHKAALQLAVEKSGYGKRRLAEGRAWGVAVHQSFDSVVAYVVEASMKDGTPKLHAVT
AGVHCNLAVNPRSVEAQVQGGALMGLGMCLPGAAITFKDGQVEQGNFNDYTVARLTDMPAITVHIVPSADA
PTGMGEPGVPPLAPAFANAIAKLSGHTPRELPFPKA
>SEQF8614||SEQF8614.1_05022
MPIPQHFFADLPKGLRALAADTLDTGSKLERREFLKLATASGFALGIFPAAATAQTRGDEQVAASALKPTQQPSAF
VKIGRDGVVTITINRLEFGQGVQTGLPMVLAEELDADWSKVQGVHGNADPAYVDPVMGMHLTGGSTAIKNS
YVQYRELGARTRAMLLATAARRWGVDPQSLRTQAGQVIGPKGRKLGYGELADEAMKMPVPQQVVLKDAKDF
RIIGRATGRLDARAKSSGRQSYGIDMHLPGMLTAVVAHPPVYGSKIQSVDDAAAKAIKGVRAVLRVPSVWGGEL
VAVLADGYWPAKQGRDALKVQWDSSAVGKVDSVQQLAQYRELSKRPGAPKFDADVSALGGAAHKISAEYVF
PYLAHTPMEPLNCTVRVTGAGKDARVELWLGTQAPGWEVATAARVLGVTPQNVRVNVQMAGGGFGRRAN
PRSDYVAEACEIAKAARASGIDAPVRMIWSREDDVKGGYYRPMHVHRAEIGFDEKGRVLAWDHVIVGQSLAK
GTAFEGFMLKNGVDTTTVEGMKEPYDLPMRLSVHHPELNAPVLWWRSVGSTHTAYVMETLIDEIARAVKKDP
VAYRLEQFDDRHPRHKAALQLAVEKSGYGKRQLAEGRAWGVAVHQSFDSVVAYVVEASVTEGVPKLHAVTAG
VHCNLAVNPRSVEAQVQGGALMGLGMCLPGAAITFKDGQVEQGNFNDYTVARLTDMPAITVHIVPSADAPT
GMGEPGVPPLAPAFANAIAKLSGQTPRELPFPRA
>SEQF8615||SEQF8615.1_00961
MPIPNHFLADMPKGLRALAADTIDAGAKLERRDFLKLATASGFALGVFPSAALAQAKGNEQVSTNALKPYQQP
SAFVKIDRDGAVTITINRLEFGQGVQTGLPMVLAEELDADWSKVHSVHGNADPAYLDPVMGMHLTGGSTAIK
NSYVQYRELGARTRSMLLATAAKRWGVDLQSLRTQAGQVIGPRGKKLGYGELADEAMKMPVPQQVKLKDVK
EFRIIGHATGRLDARAKSSGRQYYGIDMHLPGMLTAVVAHPPVYGSKIQSVDDAAAKAIKGVRAVLRVPSVWG
GELVAVLADGYWPAKQGRDALKIQWDSSAVGKVDSARQLAQYRELAKKPGALKFDADVSALNGAAHKISAEY
VFPYLAHTPMEPLNCTVRVTGAGKDAKVELWLGTQAPGWEVATAARVLGVAPQNVRVNVQMAGGGFGRR
ANPRSDYVAEACEIAKAARASGIEAPVRMIWSREDDVKGGYYRPMHVHRAEIGFDGKGKVVAWDHVIVGQSL
AKGTAFEGFMVKNGVDTTTVEGMKEPYDLPMRLSVHHPELNAPVLWWRSVGSTHTAYVMETLMDEIARAVK
QDPVAYRLQQFGDRHPRHKAALQLAVEKSGYGKRQLAEGRAWGVAVHQSFDSVVAYVVEASMKDGAPKLHA
VTAGVHCNLAVNPRSVEAQVQGGALMGLGMCLPGAAITFKDGQVEQGNFNDYTVARLTDMPAISVHIVPSA
DAPTGMGEPGVPPLAPAFANAIAKLSGHTPRELPFQKA
>SEQF8616||SEQF8616.1_02284
MPIPNHFLADMPKGLRALAADTIDAGAKLERRDFLKLATASGFALGVFPSAALAQAKGNEQVSTNALKPYQQP
SAFVKIDRDGAVTITINRLEFGQGVQTGLPMVLAEELDADWSKVHSVHGNADPAYLDPVMGMHLTGGSTAIK
NSYVQYRELGARTRSMLLATAAKRWGVDLQSLRTQAGQVIGPRGKKLGYGELADEAMKMPVPQQVKLKDVK
EFRIIGHATGRLDARAKSSGRQYYGIDMHLPGMLTAVVAHPPVYGSKIQSVDDAAAKAIKGVRAVLRVPSVWG
GELVAVLADGYWPAKQGRDALKIQWDSSAVGKVDSARQLAQYRELAKKPGALKFDADVSALNGAAHKISAEY
VFPYLAHTPMEPLNCTVRVTGAGKDAKVELWLGTQAPGWEVATAARVLGVAPQNVRVNVQMAGGGFGRR
ANPRSDYVAEACEIAKAARASGIEAPVRMIWSREDDVKGGYYRPMHVHRAEIGFDGKGKVVAWDHVIVGQSL
AKGTAFEGFMVKNGVDTTTVEGMKEPYDLPMRLSVHHPELNAPVLWWRSVGSTHTAYVMETLMDEIARAVK
QDPVAYRLQQFGDRHPRHKAALQLAVEKSGYGKRQLAEGRAWGVAVHQSFDSVVAYVVEASMKDGAPKLHA
VTAGVHCNLAVNPRSVEAQVQGGALMGLGMCLPGAAITFKDGQVEQGNFNDYTVARLTDMPAISVHIVPSA
DAPTGMGEPGVPPLAPAFANAIAKLSGHTPRELPFQKA
>SEQF8618||SEQF8618.2_03193
MPIPQHFFADLPKGLRALAADTLDTGSKLERREFLKLATASGFALGIFPAAATAQTRGDEQVAASALKPTQQPSAF
VKIGRDGVVTITINRLEFGQGVQTGLPMVLAEELDADWSKVQGVHGNADPAYVDPVMGMHLTGGSTAIKNS
YVQYRELGARTRAMLLATAARRWGVDPQSLRTQAGQVIGPKGRKLGYGELADEAMKMPVPQQVVLKDAKDF
RIIGRATGRLDARAKSSGRQSYGIDMHLPGMLTAVVAHPPVYGSKIQSVDDAAAKAIKGVRAVLRVPSVWGGEL

VAVLADGYWPAKQGRDALKVQWDSSAVGKVDSVQQLAQYRELAKRPGAPKFDADVSALGGAAHKISAEYVF
PYLAHAPMEPLNCTVRVTGAGKDARVELWLGTQAPGWEVATAARVLGVTPQNVRVNVQMAGGGFGRRAN
PRSDYVAEACEIVKAARASGIDAPVRMIWSREDDVKGGYYRPMHVHRAEIGFDEKGRVLAWDHVIVGQSLAK
GTAFEGFMLKNGVDTTTVEGMKEPYDLPMRLSVHHPELNAPVLWWRSVGSTHTAYVMETLIDEIARAVKKDP
VAYRLEQFGDSHPRHKAALQLAVEKSGYGKRQLAEGRAWGVAVHQSFDSVVAYVVEASVTEGVPKLHAVTAG
VHCNLAVNPRSVEAQVQGGALMGLGMCLPGAAITFKDGQVEQGNFNDYTVARLTDMPAITVHIVPSADAPT
GMGEPGVPPLAPAFANAIAKLSGQTPRELPFPRA
>SEQF8620||SEQF8620.1_03074
MPIPNHLFADLPKGLRALAADSIDADLKLERRDFLKLATASGFALGVFPAAVPAQAKGNEQVSTSSLKPYQQPSA
FVKIDRDGAVTITINRLEFGQGVQTGLPMVLAEELDADWSKVQSVHGNADPAYLDPVMGMHLTGGSTAIKNS
YVQYRELGARTRSMLLATAAKRWGVDPQSLRTQAGQVIGPRGKKLSYGELADEAMKMPVPQQVKLKDVKDF
RIIGHATGRLDARAKSSGRQSYGIDMHLPGMLTAVVAHPPVYGSKIQAVDDAAAKAIKGVRAVLRVPSVWGGE
LVAVLADGYWPAKQGRDALKIQWDSAAVGKVDSAQQLAQYRELAKKPGALKFDADVSALNGAPHKISAEYVF
PYLAHTPMEPLNCTVRVTGAGKDAKVELWLGTQAPGWEVATAARVLGVAPQNVRVNVQMAGGGFGRRAN
PRSDYVAEACEIAKAARTSGIGAPVRMIWSREDDVKGGYYRPMHVHRAEIGFDGKGKVIAWDHVIVGQSLAK
GTAFEGFMLKNGVDTTTVEGMKEPYELPMRLSVHHPELNAPVLWWRSVGSTHTAYVMETLMDEIARAVKQD
PVAYRLQQFGDRHPRHKAALQLAVEKSGYGKRQLAEGRAWGVAVHQSFDSVVAYVVEASMKDGAPKLHAVT
AGVHCNLAVNPRSVEAQVQGGALMGLGMCLPGAAITFKDGQVEQGNFNDYTVARLTDMPAISVHIVPSADA
PTGMGEPGVPPLAPAFANAIAKLSGHTPRELPFQKA
>SEQF8621||SEQF8621.1_02463
MPIPNHFLADMPKGLRALAADTIDAGTKLERRDFLKLATASGFALGVFPSAALAQAKGNEEVSTSALKPYQQPS
AFVKIDRDGTVTVTINRLEFGQGVQTGLPMVLAEELDADWSKLHSVHGNADPAYLDPVMGMHLTGGSTAIKN
SYVQYRELGARTRSMLLATAAKRWGVDPQSLRTQAGQVIGPRGKKLGYGELADEAMKMPVPQQVKLKGVKD
FRIIGRATGRLDARAKSSGSQSYGIDMHLPGMLTAVVAHPPVYGSKIQSVDDAATKAIKGVRAVLRVPSVWGGE
LVAVLADGYWPAKQGRDALKIQWDSAAVGKVDSARQLAQYRELAKKPGALKFDVDVSALNGAVHKISAEYVF
PYLAHTPMEPLNCTVRVTGAGKDAKVELWLGTQAPGWEVATAARVLGVAPQNVRVNVQMAGGGFGRRAN
PRSDYVAEACEIAKAARASGVDAPVRMIWSREDDVKGGYYRPMHVHRAEIGFDGKGKVIAWDHVIVGQSLAK
GTAFEGFMVKNGVDTTTVEGMKEPYDLPMRLSVHHPELNAPVLWWRSVGSTHTAYVMETLMDEIARAVGQ
DPVAYRLQQFGDRHPRHKAALQLAVEKSGYGKRRLAEGRAWGVAVHQSFDSVVAYVVEASMKDGTPKLHAVT
AGVHCNLAVNPRSVEAQVQGGALMGLGMCLPGAAITFKDGQVEQGNFNDYTVARLTDMPAITVHIVPSADA
PTGMGEPGVPPLAPAFANAIAKLSGHTPRELPFPKA
>SEQF8622||SEQF8622.1_03923
MPIPNHLFADLPKGLRALAADSIDAGLKLERRDFLKLATASGFALGVFPAAVSAQAKGNEEVSTSALKPYQQPSA
FVKIDRDGAVTITINRLEFGQGVQTGLPMVLAEELDADWSKVQSAHGNADPAYLDPVMGMHLTGGSTAIKNS
YVQYRELGARTRSMLLATAAKRWGVDPQSLRTQAGQVIGPRGKKLGYGELADEAMKMPVPQQVKLKDAKDF
RIIGHATGRLDARAKSSGRQSYGIDMHLPGMLTAVVAHPPVYGSKIQSVDDAAAKAIKGVRAVLRVPSVWGGE
LVAVLADGYWPAKQGRDALKIQWDSAAVGKVDSARQLAQYRELAKKPGALKFDADVSALSGAVHKISAEYVFP
YLAHTPMEPLNCTVRVTGAGKDAKVELWLGTQAPGWEVATAARVLGVAPQNVRVNVQMAGGGFGRRANP
RSDYVAEACEIAKAARTSGIEAPVRMIWSREDDVKGGYYRPMHVHRAEIGFDDKGKVIAWDHVIVGQSLAKGT
AFEGFMVKNGVDTTTVEGMKEPYDLPMRLSVHHPELNAPVLWWRSVGSTHTAYVMETLMDEIARAVKQDP
VAYRLQQFGDRHPRHKAALQLAVEKSGYGKRQLAEGRAWGVAVHQSFDSVVAYVVEASMKDGAPKLHAVTA
GVHCNLAVNPRSVEAQVQGGALMGLGMCLPGAAITFKDGQVEQGNFNDYTVARLTDMPAISVHIVPSADAP
TGMGEPGVPPLAPAFANAIAKLSGHTPRELPFPKA
>SEQF8717||SEQF8717.1_04136

MQMQRLGARIAALSGAAASGSHDAPAAGIDRRTFLKLTGIAGGGLALGIVPPGAAHAEEAAAAVPAAKGPAAA
PQAFIVIAPDNTVTIAVNRLEFGQGVHTALPMMLAEDLDVDWRNVRAVLAPAGDPYKDPAMGIQMTGGSTA
VKHSYQQYRELGARARAMLIAAAAQRWAVEPSACTTANGVVTAGNRRATYGELAQAAMEMPVPQQVTLKD
PSRFALIGKPTPRLDTRGKLDGSVGFGIDTQLDNLRVAVVARPPRFGGKVRRFNAEAARAIQGVVEVFEVPTDG
GGTGVAVVATGYWPAKQGRDALQAEWDDSGSTVSSAALYEEYAKLARQPGRTPRADAFDLAGAVRTVEAEYR
FPYLAHAPMEPLNCTMQAEVAGGKPARVKVWAGTQFQTVDHGALAKAFGLPPEQVSVVTMMAGGGFGRR
AVPTSDYLVEAAQLMRAWVAKGHREPVKVIWSREDDIRGGYYRPLHLHHARIGIDAQGKVLGWEHAIVGQSL
TMGSPFEAFLVKDGVDHTMTEGIVDHDYGFPLRLSVHHPKVQVPVLWWRSVGHTHTAFVKETLIDELATVAK
QDPVAYRLARLDPVKGARQRAALQLAVEKSGYGKRRLPAGRAWGVAVHQSFDSAVAYVVEVSLRNGQPHVHR
VTAGVHANRVINPLSASAQIEGGCVFGLAMIRPGFAIEIDNGAVRNSNFGDFPPVRINDAPPVEVHFVPSDDPP
TGLGEPGVPVIAPAVANAVFKLTGKRQRQLPFVMA
>SEQF8717||SEQF8717.1_01087
MRANDSTMTPAMTPDLSRRGFLQGTALGTLALAVGANGIIGLARADDDAPKKYGADSMPGGTVSDPLVFVSI
GADGTVTIVAHRAEMGTGVRTSLPMVVADEMEANWDRVKVVQAEANETRYGNQNVDGSRSVRHFLMPM
RRVGAAARQMLEAAAAARWSVPVGEVRAQRHEVVHLPTGRRLSYGELAADAAKQPVPAQGTLKLKTPDQFR
YIGKDRVRLVDLEAIGKGQATYGMDVRLPGMVYAVVARPPVVGGKVRAVRADKALAVPGVLKVVEIPPFAGAP
AFQPLGGVAVVARNTWAAMQGRAALEIEWDDGPNAAYDSVAYRRTLEAAARQPGKVVRNEGDAPAAWNK
SAESERYAAEYYLPHLAHASMEPPVATVRVSGKTAEVWTSVQNPVAAQTAVAKRLKLEPKDVRVNVVLLGGGF
GRKSKPDFVDEAAVVAQAMPGTPVKLVWTREDDIHHDYLHTVSVERLEAVVGKDGRPQTWLHRSAAPTIGSL
FTAGAKGQQPFELGMSAINMPYRIPNVRVETAEVEAHARIGWFRSVSNIPHAFAAQCFIAELAHRAGKDHKQF
ALDLIGPARKIDPRTMADSWNYTESPERYPYDTGRLRDVIEAATRGARWGRKLPRGHGLGLAFCYSFMSYTAAV
VEVAVNDKGEVQVVAVDMAMDCGPQINPERIRSQLEGGAIMGLSLALSSEITFEKGRVKQSNFHDYEVLRHHA
SPRTIRTHLVNGSHDVPPGGVGEPPVPPVAPALCNAIFAATGKRIRSLPVRTVA
>SEQF8717||SEQF8717.1_01316
MKAPLDVSRRRFLAAGAVLGGGLVIGFTVPSVRRLADGSVEGAVAAEPVPWAPNAFLRIGADDSVTVLLAHSE
MGQGVWTSLALLIADELDADWMRIRVEHAPAAPAYGIAMLGGMQGTVGSRSVRSEFLRYRQAGAAARAML
LQAAAARWQVPVDALRTDSGAVLHDGRRLRYGELVHEAAALPVPAAEALRLKDARDWKWIGKGARRLDAAD
KITGRARFGIDVKLDGLLTAVVARAPVFGAKLASFDDSAARAVPGVRQVLAVPSGVAVLADHYWAAKRGRDAL
KVEWQGGDKALGSAALIADFARLAREPGPIALEAGDVDAGMAQAHRTVEAEYRLPYLAHTPMEPLNCTVRIA
PDGCDVWVGTQMQTLAQRTVAQVAGLEPAQVRIHTTFLGGGFGRRAVQDFVAEATHVARAAGLPVKTIWSR
EDDVRGGYYRSGFVHRIRVGLDRDGRPLAWRHGIAGQSNHPEREGVHPTSVEGVVDSPYVLGTSAHRVVAHS
PRTAVPVWYWRSVGHSHAAFAMESMVDELAHASGQDPVAYRLRLLHDAPRHRRVLELAAQRFGWGRRPQS
GRGHGIAVHACFGSVTAQAVEVSVRDGAIRVHRVVCAIDCGVAVNPDNVRAQMESAIVYGLSAALHGRVTIV
DGAVRESNFHDYPALRIAEMPVIEVHVVDSGESPGGAGEPGTPPIAPAVANAVFQLTGARLRELPLTLPARARTR
PTA
>SEQF8718||SEQF8718.1_02875
MQMQRLGARIAALSGAAASGSHDAPAAGIDRRTFLKLTGIAGGGLALGIVPPGAAHAEEAAAAVPAAKGPAAA
PQAFIVIAPDNTVTIAVNRLEFGQGVHTALPMMLAEDLDVDWRNVRAVLAPAGDPYKDPAMGIQMTGGSTA
VKHSYQQYRELGARARAMLIAAAAQRWAVEPSACTTANGVVTAGNRRATYGELAQAAMEMPVPQQVTLKD
PSRFALIGKPTPRLDARGKLDGSVGFGIDTQLDNLRVAVVARPPRFGGKVRRFNAEAARAIQGVVEVFEVPTDG
GGTGVAVVATGYWPAKQGRDALQAEWDDSGSTVSSAALYEEYAKLARQPGRTPRADAFDLAGAARTVEAEYR
FPYLAHAPMEPLNCTMQAEVAGGKPARVKVWAGTQFQTVDHGALAKAFGLPPEQVSVVTMMAGGGFGRR
AVPTSDYLVEAAQLMRAWVAKGHREPVKVIWSREDDIRGGYYRPLHLHHARIGIDAQGKVLGWEHAIVGQSL
TMGSPFEAFLVKDGVDHTMTEGIVDHDYGFPLRLSVHHPKVQVPVLWWRSVGHTHTAFVKETLIDELATVAK

QDPVAYRLARLDPVKGARQRAALQLAVEKSGYGKRRLPAGRAWGVAVHQSFDSAVAYVVEVSLRNGQPHVHR
VTAGVHANRVINPLSASAQIEGGCVFGLAMIRPGFAIEIDNGAVRNSNFGDFPPVRISDAPPVEVHFVPSDDPP
TGLGEPGVPVIAPAVANAVFKLTGKRQRQLPFVMA
>SEQF8718||SEQF8718.1_00037
MKAPLDLSRRRFLAAGAVLGGGLVIGFTVPSVRRLADGSVEGAVAAEPVPWVPNAFLRIGADDSVTVLLAHSE
MGQGVWTSLALLIADELDADWMRIRVEHAPAAPAYGIAMLGGMQGTVGSRSVRSEFLRYRQAGAAARAML
VQAAAARWQVPVDVLRTDSGAVLHDGRRLRYGELVHEAAALPVPAAEALRLKDARDWKWIGKGARRLDAAD
KITGRARFGIDVKLDGLLTAVVARAPMFGAKLASFDDSAARAVPGVRQVLAVPSGVAVQADHYWAAKRGRDA
LKVEWQGGDKALGSAALIADFARLAREPGPIALEAGDVDAGMAQAHRTVEAEYRLPYLAHTPMEPLNCTVRIA
PDGCDVWVGTQMQTLAQRTVAQVAGLEPAQVRIHTTFLGGGFGRRAVQDFVAEATHVARAAGLPVKTIWSR
EDDVRGGYYRSGFVHRIRVGLDRDGRPLAWRHGIAGQSNHPEREGVHPTSVEGVVDSPYVLGTSAHRVVAHS
PRTAVPVWYWRSVGHSHAAFAMESMVDELAHASGQDPVAYRLRLLHDAPRHRRVLELAAQRFGWGRRPQS
GRGHGIAVHACFGSVTAQAVEVSVRDGAIRVHRVVCAIDCGVAVNPDNVRAQMESAIVYGLSAALHGRVTIV
DGAVRESNFHDYPALRIAEMPVIEVHVVDSGESPGGAGEPGTPPIAPAVANAVFQLTGARLRELPLTLPARARTR
PTA
>SEQF8718||SEQF8718.1_00324
MRANDSTMTPAMTPDLSRRGFLQGTALGTLALAVGANGIIRLARADDDAPKKYGADSMPGGTVSDPLVFVSI
GADGTVTIVAHRAEMGTGVRTSLPMVVADEMEANWDRVKVVQAEANETRYGNQNVDGSRSVRHFLMPM
RRVGAAARQMLEAAAAARWSVPVGEVRAQRHEVVHLPTGRRLSYGELAADAAKQPVPAQGTLKLKTPDQFR
YIGKDRVRLVDLEAIGKGQATYGMDVRLPGMVYAVVARPPVVGGKVRAVRADKALAVPGVLKVVEIPPFSGAP
AFQPLGGVAVVARNTWAAMQGRAALEIEWDDGPNAAYDSVAYRRTLEAAARQPGKVVRNEGDAPAAWNK
SAESERYAAEYYLPHLAHASMEPPVATVRVSGKTAEVWTSVQNPVAAQTAVAKRLKLEPKDVRVNVVLLGGGF
GRKSKPDFVDEAAVVAQAMPGTPVKLVWTREDDIHHDYLHTVSVERLEAVVGKDGRPQTWLHRSAAPTIGSL
FTAGAKGQQPFELGMSAINMPYRIPNVRVETAEVEAHARIGWFRSVSNIPHAFAAQCFIAELAHRAGKDHKQF
ALDLIGPARKIDPRTMADSWNYTESPERYPYDTGRLRDVIEAATRGARWGRKLPRGHGLGLAFCYSFMSYTAAV
VEVAVNDKGEVQVVAVDMAMDCGPQINPERIRSQLEGGAIMGLSLALSSEITFEKGRVKQSNFHDYEVLRHHA
SPRTIRTHLVNGSHDVPPGGVGEPPVPPVAPALCNAIFAATGKRIRSLPVRTVA
>SEQF8719||SEQF8719.1_01129
MQMQRLGARIAALSGGAGDGPHDGHGAHGAHDGHDAPAAGIDRRTFLKLTGLAGGGLALGIAPLGEVRADE
AAAPTAKGPAAAPQAFIVIAPDNTVTVAVNRLEFGQGVHTALPMALAEELDVDWRNVRAVLAPAGDPYKDPA
MGIQMTGGSTAVKHSYQQYRELGARARAMLVAAAAQQWQVEPSACTAAQGVVTAGNRRATYGELAQAAM
ALPVPQQVTLKDPSRFTLIGKPTPRLDTRGKLDGSVGFGIDTQLDNLMVAVVARPPRFGGKVRRFNADAARAIK
GVAEVLEVPTDGGGTGVAVIANGYWPAKQGRDALQAEWDDSGSTVSTAALYEEYAKLARQPGRTPRADAFDL
AGAARTIEAEYRFPYLAHAPMEPLNCTMQAEVAGGKPSRVKVWAGTQFQTVDQGALAKAFGLPPEQVSVVT
MMAGGGFGRRAVPTSDYLVEAAQVMRAWVAKGHREPVKVIWSREDDIRGGYYRPLHLHRARIGVDAQGRV
LGWEHAIVGQSLTMGSPFEAFLVKDGVDHTMTEGIVDHDYGFPLRLSVHHPKVQVPVLWWRSVGHTHTAFV
KETLIDELATVAKQDPVAYRLAQLDPVKGARQRAALQLAVEKSGYGKRRLPTGRAWGVAVHQSFDSAVAYVVE
VSLQNGQPHVHRVTAGVHANRVVNPLSARAQIEGGCVFGLAMIRPGFAIEIDNGAVRNSNFGDFPPVRINDA
PPVEVHFVPSDDPPTGLGEPGVPVIAPAVANAVFKLTGKRQRQLPFVMA
>SEQF8719||SEQF8719.1_00073
MRNDDSIQAPDLSRRGFLQGTLLGTLSLAVGANGVIGLAYADDDAPKKYGADSMPGGTVSDPLVFVSIGADGT
VTIVAHRAEMGTGVRTSLPMVVADEMEANWDRVKIVQAEANETRYGNQNVDGSRSVRHFLMPMRRVGAA
ARQMLEAAAAARWAVPVGEVRAQRHEVVHLPTGRRLPYGELAADAAKQPVPAEGALKLKTPDQFRYIGKDRV
RMVDLEAIGKGQATYGMDVRLPDMVYAVVARPPVVGGKVRAVRAEKALAVPGVLKVVEIPPFSGAPAFQPLG

GVAVVARNTWAAMQGRAALEIDWDDGPNAGYDSAAYRRTLEAAARQPGKVVRNEGDAPAAWNNSAEAER
YAAEYYVPHLAHASMEPPVATVRVSGKSAEVWTSIQNPVAAQTAVAKRLKLEPKDVRVNVLLLGGGFGRKSKP
DFVDEAAIVAQAMPGTPVKLVWTREDDIHHDYLHTVSVERIEAVVGKDGRPQTWLHRSAAPTIGSLFTQGAK
GQQLFESGMSAINMPYRIPNVRVETAEVEAHARIGWFRSVANIPHAFAAQCFIAELAHRAGKDHKQFALDLIGP
ARKIDPRTMADTWNYTESPERYPYDTGRLRDVIEAAARGARWGRKLPRGHGLGLAFCYSFMSYTATVVEVAV
NDKGEVQVVAVDMAMDCGPQINPERIRAQMEGGAIMGLSLALTSEISFEKGRVKQSNFHDYEVLRHHASPRV
IRTHLVNGSHDTPPGGVGEPPVPPVAPALCNAIFAATGKRIRSLPVRTVA
>SEQF8720||SEQF8720.1_02425
MQMQRLGARLAALSGGAADRGHDGHDGHDDHDGHDAPRAGIDRRTFLKLTGMAGGGLALGIAPLSAAAA
EEAAAPVAKGPAAAPQAFIVIAPDNTVTIAVNRLEFGQGVHTALPMALAEELDVDWRNVRAVLAPAGDPYKDP
AMGIQMTGGSTAVKHSYQQYRELGARARAMLVAAAAQRWQVDPSACTTAQGVVTAGNRRATYGELAPAA
MEMPVPQQVTLKDPSRFTLIGKPTPRLDTRGKLDGSVGFGIDTQLDNLMVAVVARPPRFGGKVRRFNADAAR
AVKGVVEVLEVPTDGGGTGVAVVANGYWPARQGRDALQVEWDDSGSTVSSAALYEEYAKLARQPGKTPRAD
AFDLGGAARTVEAEYRFPYLAHAPMEPLNCTMQAEVADGKPARVKVWAGTQFQTVDHGALARTFGLPPEQI
SIVTMMAGGGFGRRAVPSADYLVEAAQVMRAWVAKGHREPVKVIWSREDDIRGGYYRPLHLHHARIGVDAR
GKVLGWEHAIVGQSLVMGSPFEAFLVKDGVDHTMTEGIVDHDYGFPLRLSVHHPKVQVPVLWWRSVGHTHT
AFVKETLIDELATVARQDPVAYRLAQLDPVKGARQRAALQLAVEKSGYGKRKLPAGRAWGVAVHQSFDSAVAY
VVEVSLQNGQPHVHRVTAGVHANRVVNPLSARAQIEGGCVFGLAMIRPGFAIEIENGAVKNSNFGDFPPVRIN
DAPPVEVHFVPSEDPPTGLGEPGVPVIAPAVANAVFKLTGKRQRQLPFVMA
>SEQF8720||SEQF8720.1_03650
MRAKDSSMTPNLSRRGFLQGTALGTLSLAVGANGVIGLAYADDDAQKKYGADSMPGGTVSDPLVFVSIASDGT
VTIVAHRAEMGTGVRTSLPMVVADEMEANWDRVKVVQAEANETRYGNQNVDGSRSVRHFLMPMRRVGA
AARQMLEATAAARWSVPVGEVRAQRHEVVHLPTGRRLAYGELAADAAKQPVPAEGTLKLKTPDQFRYIGKDR
VRLVDLEAIGKGQATYGMDVRLPGMVYAVVARPPVVGGKVRVFRAEKALAVPGVLKVVEIPAFSGAPAFQPLG
GVAVVARNTWAAMQGRAALEIDWDDGPNAGYDSVAYRRTLEEAARRPGKVVRNEGDAAAAWSKSAQAER
YAAEYYVPHLAHASMEPPVATVRVSGKTAEVWTSVQNPVAAQAAVAKRLKLEPKDVRVNVLLLGGGFGRKSKP
DFVDEAAIVAQAMPGTPVKLVWTREDDIHHDYLHTVSVERLEAVVGKDGRPQTWLHRSAAPTIGSLFTAGAK
GQQPFELGMSAINMPYRIPNVRVETAEVEAHARIGWFRSVSNIPHAFAAQCFIAELAHRAGKDHKQFALDLIGP
ARKIDPRTMADTWNYTESPERYPYDTGRLRDVIEAATRGARWGRKLPRGHGLGLAFCYSFMSYTAAVVEVAVN
DKGEVQVVAVDMAIDCGPQINPERIRSQLEGGAIMGLSLALTSEISFEKGRVKQSNFHDYEVLRHHASPRVIRTH
LVNGNHDVPPGGVGEPPVPPVAPALCNAIFAATGKRIRALPVRTVA
>SEQF8720||SEQF8720.1_03454
MKAPLDLSRRRFLAAGAVLGGGLVIGFSVPSVRRLLDGDPVEVAMAAEPAPFIPNAFLRIGADDSVTVLLAHSE
MGQGVWTSLALLIAEELDADWTRIRVEHAPAAPAYGIAMLGGMQGTVGSRSVRSEFLRYRQAGAAARAMLL
QAAAARWQVPVDVLRTDSGAVVHQEQRLRYGELVHEAATLPVPAADALRLKDAHDWKWIGKGARRLDAAD
KITGRARFGIDVKLDGLLTAVVARAPAFGARMVSFDDSAARAVPGVRQVVAVPSGVAVLADHYWAAKRGRDA
LKIEWQGADTALESAGLLRDFARLAREPGPIALEAGDVDAAIAQAQRVVEAEYRLPYLAHTPMEPLNCTVRIGP
DGCDVWVGTQMQTLAQRTVAQITGLEPAQVRIHTTFLGGGFGRRAVQDFVAEATHVARAAGAPVKTMWSR
EEDVRGGYYRSGFVHRIRVGLDRAGRPLAWRHGMAGQSNHPEREGLHPTSVEGVVDSPYVLGAAAHRVVAH
SPRTAVPVWYWRSVGHSHSAFAMESMVDELAHASRQDPLAYRMLLLKDAPRHRRVLEVAAQRFGWGRRAQ
AGRGHGIAVHACFGSVAAQAVEVSLQDGAIRVHRVVCAIDCGVAVNPDNVRAQMESAIVYGLSAALHGRVTIE
RGAVRESNFHDYPALRMAEMPVIEVHVIDSGESPGGAGEPGTPPIAPAVANALFQLTGTRLRELPLQLAQRART
RSSA
>SEQF8721||SEQF8721.1_04125

MRPPLDVSRRRFLAAGAVLGGGLVIGFSVPSVRRLLDGQPVEVATAAEPAPFIPNAFLRIGADDSVTVLLAHSEM
GQGVWTSLALLIAEELDADWTRIRVEHAPAAPAYGIAMLGGMQGTVGSRSVRAEFLRYRQAGAAARAMLVQ
AAAARWQVPVDALRTDSGAVLHDGRRLRYGELVHEAAALAVPAADALRLKDAHDWKWIGKGARRLDAADKI
TGRARFGIDVKLDGLLTAVVARAPAFGAKMVSFDDSAARAVPGVRQVVAVPSGVAVLADHYWAAKRGRDALK
VEWQGADTALESAGLLRDFARLAREPGPIALEAGDVDTAIAQAHRVVEAEYRLPYLAHTPMEPLNCTVRIGPD
GCDVWVGTQMQTLAQRTVAQITGQEAAQVRIHTTFLGGGFGRRAVQDFVAEATHVARAAGAPVKTIWSRED
DVRGGYYRSGFVHRIRVGLDRAGRPLVWRHGMAGQSNHPEREGLHPSSVEGVVDSPYVLGAAAHRVVAHSP
RTAVPVWYWRSVGNSHSAFAMESMVDELAHASRQDPVAYRMLLLKDAPRHRRVLEVAAQRFGWDRRPQA
GRGRGIAVHACFGSVAAQAVEVSLQDGAIRVHRVVCAIDCGVAVNPDNVRAQMESAIVYGLSAALHGRVTIER
GAVRESNFHDYPALRMAQMPVIEAHVVDSGESPGGAGEPGTPPIAPAVANAVFQLTGTRLRELPLQLPQPRRT
RSST
>SEQF8721||SEQF8721.1_04068
MGKHVMANDEMSQRGAVAAPNLSRRGFLQGTALGTLSLAVGANGVIGLAYADDDAPKKYGADSMPGGTVS
DPLVFVSIAADGTVTIVAHRAEMGTGVRTSLPMVVADEMEANWDRVKVVQAEANETRYGNQNVDGSRSVR
HFLMPMRRVGAAARQMLEATAAARWSVPVGEVRAQRHEVIHLPTGRRLPYGELAGDAAKQPVPAEGTLKLK
TPDQFRYIGKDRVRLVDLEAIGKGQATYGMDVRLPNMVYAVVARPPVVGGKVRAVRAEKALAVPGVLKVVEIP
AFSGAPAFQPLGGVAVVARNTWAAMQGRAALEIDWDDGPNAGYDSVAYRRTLEEAARRPGKVVRNEGDAA
AAWSKSAEAERYAAEYYVPHLAHASMEPPVATVRVSGKTAEVWTSVQNPVAAQTAVAKRLKLEPKDVRVNVLL
LGGGFGRKSKPDFVDEAAIVAQAMPGTPVKLVWTREDDIHHDYLHTVSVERLEAVVGKDGRPQTWLHRSAAP
TIGSLFTAGAKGQQPFELGMSAINMPYRIPNVRVETAEVEAHARIGWFRSVSNIPHAFAAQCFIAELAHRAGKD
HKQFALELIGPARKIDPRSMADSWNYTESPERYPYDTGRLRDVIEAATRGARWGRKLPRGHGLGLAFCYSFMSY
TAAVVEVAVNAKGEVQVVAVDMAMDCGPQINPERIRSQLEGGAIMGLSLALSSEISFEKGRVKQSNFHDYEVL
RHHASPRVIRTHLVNGSHDVPPGGVGEPPVPPVAPALCNAIFAATGKRIRSLPVRTVA
>SEQF8721||SEQF8721.1_04646
MQMQRLGARLAALSGGAADDAHDVPRAGIDRRTFLKLTGMAGGGLALGIAPLTAAAAEPAAAPVAKGPAAA
PQAFIVIAPDNTVTIAVNRLEFGQGVHTALPMALAEELDVDWRNVRAVLAPAGDPYKDPAMGIQMTGGSTAV
KHSYQQYRELGARARAMLVAAAAQRWQVDPSACTTAQGVVIAGNRRATYGELAPAAMEMPVPQQVTLKDP
SRFTLIGKPTPRLDTRGKLDGSVGFGIDTQLDNLMVAVVARPPRFGGKVRRFNADAARAVKGVAEVLEVPTDG
GGTGVAVVANGYWPAKQGRDALQVEWDDSGSTASSAALYEEYAKLARQPGKTPRADAFDLSGAARTIEAEYR
FPYLAHAPMEPLNCTMQAEVAGDKPARVKVWAGTQFQTVDHGALAKTFGLPPEQISIVTMMAGGGFGRRA
VPSADYLVEAAQVMRAWVARGHREPVKVIWSREDDIRGGYYRPLHLHRARIGVDAQGKVLGWEHAIVGQSL
VMGSPFEAFLVKDGVDHTMTEGIVDHDYGFPLRLSVHHPKVQVPVLWWRSVGHTHTAFVKETLIDELATVAR
QDPAAYRLAQLDPVKGARQRAALQLAVEKSGYGKRKLPAGRAWGVAVHQSFDSAVAYVVEVSLQNGQPHVH
RVTAGVHANRVINPLSARAQIEGGCVFGLAMIRPGFAIEIENGAVKNSNFGDFPPVRINDAPPVEVHFVPSDDP
PTGLGEPGVPVIAPAVANAVFKLTGKRQRQLPFVMA
>SEQF8722||SEQF8722.1_03228
MRANEPTMTSTMTSAVTPNLSRRGFLQGTALGTLSLVVGANGVIGLARADDDAPKKYGADSMPGGTVSDPLV
FVSIAADGTVTIVAHRAEMGTGVRTSLPMVVADEMEANWDRVKIVQAEANETRYGNQNVDGSRSVRHFLM
PMRRVGAAARQMLEAAAAARWSVPVGEVRAQRHEVVHLPTGRRLPYGELAADAAKQPVPAQGTLKLKTPD
QFRYIGKDRVRMVDLEAIGKGQATYGMDVRLPGMVYAVVARPPVVGGKARAVRADKALAVPGVLKVVEIPPF
AGAPAFQPLGGVAVVARNTWAAMQGRAALEIEWDDGPNAGYDSVAYRRTLEAAARQPGKVVRNEGDAPAA
WNKSAETERYAAEYYLPHLAHASMEPPVATVRVSGKTAEVWTSVQNPVAAQTAVAKRLKLEPKDVRVNVVLLG
GGFGRKSKPDFVDEAAVVAQAMPGTPVKLVWTREDDIHHDYLHTVSVERLEAVVGKDGRPQTWLHRSAAPTI
GSLFTAGAKGQQPFELGMSAINMPYRIPNVRVETAEVEAHARIGWFRSVSNIPHAFAAQCFIAELAHRAGKDH

KQFALDLIGPARKIDPRTMADSWNYTESPERYPYDTGRLRDVIEAATRGARWGRKLPRGHGLGLAFCYSFMSYT
AAVIEVAVNDKGEVQVVAVDMAMDCGPQINPERIRSQLEGGAIMGLSLALSSEISFEKGRVKQSNFHDYEVLR
HHASPRVIRTHLVNGSHDVPPGGVGEPPVPPVAPALCNAIFAATGKRIRSLPVRTVA
>SEQF8722||SEQF8722.1_03633
MQMQRLGARIAALSGAAAAGSHDAPAAGIDRRTFLKLSGIAGGGLALGIAPLAAVHADEAAVPAAKGPAAAP
QAFIVIAPDNTVTIAVNRLEFGQGVHTALPMMLAEELDADWRNVRAVLAPAGDPYKDPAMGIQMTGGSTAV
KHSYQQYRELGARARAMLIAAAAQRWAVEPSACTAANGVVTAGNRRATYGELAQAAMEMPVPQQVTLKDP
SRFTLIGKPTPRLDTRGKLDGAVGFGIDTQLDDLRVAVVARPPRFGGKVRRFNAEAARAIQGVIEVLEVPTDGG
GTGVAVVANGYWPAKQGRDALQAEWDDSGSTVSSAALFEEYAKLARQPGRTPRADAFDLAGAARTVEAEYRF
PYLAHAPMEPLNCTMQAEVAGGKPTRVKVWAGTQFQTVDHGALAKAFGLPPEQVSIVTMMAGGGFGRRA
VPTADYLVEAAQLMRAWVAKGHREPVKVIWSREDDIRGGYYRPLHLHHARIGIDAQGRVLGWEHAIVGQSLT
MGSPFEAFLVKDGVDHTMTEGIVDHDYGFPLRLSVHHPKVQVPVLWWRSVGHTHTAFVKETLIDELATVAKQ
DPVAYRLARLDPVKGARQRAALQLAVDKSGYGKRRLPAGRAWGVAVHQSFDSAVAYVVEVSLQNGQPHVHR
VTAGVHANRVINPLSARAQIEGGCVFGLAMIRPGFAIEIDNGAVRNSNFGDFPPVRINDAPPVEVHFVPSDDPP
TGLGEPGVPVIAPAVANAVFKLTGKRQRQLPFVMA
>SEQF8722||SEQF8722.1_03438
MKAPLDVSRRRFLAAGAVLGGGLVIGFSVPSMRRLADGAVDDAMAAEPAPWTPNAFLRIGADDSVTVLLAHS
EMGQGVWTSLALLIAEELDADWTRIRVEHAPAATAYGIAMLGGMQGTVGSRSVRSEFLRYRQAGAAARAML
VQAAAARWQVPVDVLRTDSGAVVHDGRRLRYGELVHEAAALPVPAAEALRLKNAGDWKWIGKGARRLDAA
DKITGRARFGIDVKLDGLLTAVVARAPMFGAKLVSFDDSAARAVPGVRQVLAVPSGVAVLADHYWAAKRGRD
ALKVEWQGGDKALGSAALMADFARLAREPGPIALEAGDVDAVMGQARRTVEAEYRLPYLAHTPMEPLNCTV
RFGPDGCDVWVGTQMQTLAQRTVAQIAGVEPAQVRIHTTFLGGGFGRRAVQDFIAEATHVARAAGAPVKTV
WSREDDVRGGYYRSGFVHRIRVGLDRDGRPLAWRHGIAGQSNHPEREGVHPTSVEGVVDSPYVLGATAHRV
VAHSPRTAVPVWYWRSVGHSHAAFAMESMVDELAHASGQDPVAYRLRLLKDAPRHRRVLELAAQRFGWGR
RPQPGRGHGIAVHACFGSVAAQAVEVSVRDGAIRVHRVVCAIDCGVAVNPDNVRAQMESAIVYGLSAALHGR
VTIVDGAVRESNFHDYPALRMAEMPVIEVHIVDSAESPGGAGEPGTPPIAPAVANAVFQLTGARLRELPLTLPPR
ARTRPNA
>SEQF8723||SEQF8723.1_03895
MKAPLDLSRRRFLAAGAVLGGGLVIGFSVPSVRRLLDGDPVEVAMAAEPAPFIPNAFLRIGADDSVTVLLAHSE
MGQGVWTSLALLIAEELDADWTRIRVEHAPAAPAYGIAMLGGMQGTVGSRSVRSEFLRYRQAGAAARAMLL
QAAAARWQVPVDVLRTDSGAVVHQEQRLRYGELVHEAATLPVPAADALRLKDAHDWKWIGKGARRLDAAD
KITGRARFGIDVKLDGLLTAVVARAPAFGARMVSFDDSAARAVPGVRQVVAVPSGVAVLADHYWAAKRGRDA
LKIEWQGADTALESAGLLRDFARLAREPGPIALEAGDVDAAIAQAQRVVEAEYRLPYLAHTPMEPLNCTVRIGA
DGCDVWVGTQMQTLAQRTVAQITGLEPAQVRIHTTFLGGGFGRRAVQDFVAEATHVARAAGAPVKTMWSR
EDDVRGGYYRSGFVHRIRVGLDHAGRPLAWRHGMAGQSNHPEREGLHPTSVEGVVDSPYVLGAAAHRVVA
HSPRTAVPVWYWRSVGHSHSAFAMESMVDELAHASRQDPLAYRMLLLKDAPRHRRVLEVAAQRFGWGRRA
QAGRGHGIAVHACFGSVAAQAVEVSLQDGAIRVHRVVCAIDCGVAVNPDNVRAQMESAIVYGLSAALHGRVT
IERGAVRESNFHDYPALRMAEMPVIEVHVIDSGESPGGAGEPGTPPIAPAVANALFQLTGTRLRELPLQLPQPRR
ARSSA
>SEQF8723||SEQF8723.1_01272
MRAKDSSMTPNLSRRGFLQGTALGTLSLAVCANGVIGLAYADDDAQKKYGADSMPGGTVSDPLVFVSIASDGT
VTIVAHRAEMGTGVRTSLPMVVADEMEANWDRVKVVQAEANETRYGNQNVDGSRSVRHFLMPMRRVGA
AARQMLEATAAARWSVPVGEVRAQRHEVVHLPTGRRLPYGELAADAAKQPVPAEGTLRLKTPDQFRYIGKDR
VRLVDLEAIGKGQATYGMDVRLPGMVYAVVARPPVVGGKVRAVRAEKALAVPGVLKVVEIPAFSGAPAFQPLG

GVAVVARNTWAAMQGRAALEIDWDDGPNAGYDSVAYRRTLEEAARRPGKVVRNEGDAAAAWSKSAQAER
YAAEYYVPHLAHASMEPPVATVRVSGKTAEVWTSVQNPVAAQAAVAKRLKLEPKDVRVNVLLLGGGFGRKSKP
DFVDEAAVVAQAMPGTPVKLVWTREDDIHHDYLHTVSVERLEAVVGKDGRPQTWLHRSAAPTIGSLFTAGAK
GQQPFELGMSAINMPYRIPNVRVETAEVEAHARIGWFRSVSNIPHAFAAQCFIAELAHRAGKDHKQFALDLIGP
ARKIDPRTMADTWNYTESPERYPYDTGRLRDVIEAATRGARWGRKLPRGHGLGLAFCYSFMSYTAAVVEVAVN
DKGEVQVVAVDMAIDCGPQINPERIRSQLEGGAIMGLSLALTSEISFEKGRVKQSNFHDYEVLRHHASPRVIRTH
LVNGSHDVPPGGVGEPPVPPVAPALCNAIFAATGKRIRALPVRTVA
>SEQF8723||SEQF8723.1_01905
MQMQRLGARLAALSGGAADRGHDGHDAHDGHDAPRAGIDRRTFLKLTGMAGGGLALGIAPLSAAAAEEAA
APVAKGPAAAPQAFIVIAPDNTVTIAVNRLEFGQGVHTALPMALAEELDVDWRNVRAVLAPAGDPYKDPAMG
IQMTGGSTAVKHSYQQYRELGARARAMLVAAAAQRWQVDPSACTTAQGVVTAGNRRATYGELAPAAMEMP
VLQQVTLKDPSRFTLIGKPTPRLDTRGKLDGSVGFGIDTQLDNLMVAVVARPPRFGGKVRRFNADAARAVKGV
VEVLEVPTDGGGTGVAVVANGYWPARQGRDALQVEWDDSGSTVSSAALYEEYAKLARQPGKMPRADAFDLG
GAARTVEAEYRFPYLAHAPMEPLNCTMQAEVADGKPARVKVWAGTQFQTVDHGALARTFGLPPEQISIVTM
MAGGGFGRRAVPSADYLVEAAQVMRAWVAKGHREPVKVIWSREDDIRGGYYRPLHLHRARIGVDARGKVLG
WEHAIVGQSLVMGSPFEAFLVKDGVDHTMTEGIVDHDYGFPLRLSVHHPKVQVPVLWWRSVGHTHTAFVKE
TLIDELATVARQDPVAYRLSQLDPVKGARQRAALQLAVEKSGYGKRKLPAGRAWGVAVHQSFDSAVAYVVEVSL
QNGQPHVHRVTAGVHANRVVNPLSARAQIEGGCVFGLAMIRPGFAIEIENGAVKNSNFGDFPPVRINDAPPV
EVHFVPSEDPPTGLGEPGVPVIAPAVANAVFKLTGKRQRQLPFVMA
>SEQF8724||SEQF8724.1_04821
MAALSGAAASGSHDAPAAGIDRRTFLKLTGIAGGGLALGIVPPGGAHAEEAAAAVPAAKGPAAAPQAFIVIAPD
NTVTIAVNRLEFGQGVHTALPMMLAEDLDVDWRNVRAVLAPAGDPYKDPAMGIQMTGGSTAVKHSYQQYR
ELGARARAMLIAAAAQRWAVEPSACTTANGVVTAGNRRATYGELAQAAMEMPVPQQVTLKDPSRFALIGKP
TPRLDTRGKLDGSVGFGIDTQLDNLRVAVVARPPRFGGKVRRFNAEAARAIQGVVEVFEVPTDGGGTGVAVVA
TGYWPAKQGRDALQAEWDDSGSTVSSAALYEEYAKLARQPGRTPRADAFDLAGAVRTVEAEYRFPYLAHAPM
EPLNCTMQAEVAGGKPARVKVWAGTQFQTVDHGALAKAFGLPPEQVSVVTMMAGGGFGRRAVPTSDYLVE
AAQLMRAWVAKGHREPVKVIWSREDDIRGGYYRPLHLHHARIGIDAQGKVLGWEHAIVGQSLTMGSPFEAFL
VKDGVDHTMTEGIVDHDYGFPLRLSVHHPKVQVPVLWWRSVGHTHTAFVKETLIDELATVAKQDPVAYRLARL
DPVKGARQRAALQLAVEKSGYGKRRLPAGRAWGVAVHQSFDSAVAYVVEVSLRNGQPHVHRVTAGVHANRV
INPLSASAQIEGGCVFGLAMIRPGFAIEIDNGAVRNSNFGDFPPVRINDAPPVEVHFVPSNDPPTGLGEPGVPVI
APAVANAVFKLTGKRQRQLPFVMA
>SEQF8724||SEQF8724.1_00502
MRANDSTMTPAMTPDLSRRGFLQGTALGTLALAVGANGIIGLARADDDAPKKYGADSMPGGTVSDPLVFVSI
GADGTVTIVAHRAEMGTGVRTSLPMVVADEMEANWDRVKVVQAEANETRYGNQNVDGSRSVRHFLMPM
RRVGAAARQMLEAAAAARWSVPVGEVRAQRHEVVHLPTGRRLSYGELAADAAKQPVPAQGTLKLKTPDQFR
YIGKDRVRLVDLEAIGKGQATYGMDVRLPGMVYAVVARPPVVGGKVRAVRADKALAVPGVLKVVEIPPFSGAP
AFQPLGGVAVVARNTWAAMQGRAALEIEWDDGPNAAYDSVAYRRTLEAAARQPGKVVRNEGDAPAAWNK
SAESERYAAEYYLPHLAHASMEPPVATVRVSGKTAEVWTSVQNPVAAQTAVAKRLKLEPKDVRVNVVLLGGGF
GRKSKPDFVDEAAVVAQAMPGTPVKLVWTREDDIHHDYLHTVSVERLEAVVGKDGRPQTWLHRSAAPTIGSL
FTAGAKGQQPFELGMSAINMPYRIPNVRVETAEVEAHARIGWFRSVSNIPHAFAAQCFIAELAHRAGKDHKQF
ALDLIGPARKIDPRTMADSWNYTESPERYPYDTGRLRDVIEAATRGARWGRKLPRGHGLGLAFCYSFMSYTAAV
VEVAVNDKGEVQVVAVDMAMDCGPQINPERIRSQLEGGAIMGLSLALSSEITFEKGRVKQSNFHDYEVLRHHA
SPRTIRTHLVNGSHDVPPGGVGEPPVPPVAPALCNAIFAATGKRIRSLPVRTVA
>SEQF8724||SEQF8724.1_01811

MKAPLDVSRRRFLAAGAVLGGGLVIGFTVPSVRRLADGSVEGAVAAEPVPWAPNAFLRIGADDSVTVLLAHSE
MGQGVWTSLALLIADELDADWMRIRVEHAPAAPAYGIAMLGGMQGTVGSRSVRSEFLRYRQAGAAARAML
LQAAAARWQVPVDALRTDSGAVLHDGRRLRYGELVHEAAALPVPAAEALRLKDARDWKWIGKGARRLDAAD
KITGRARFGIDVKLDGLLTAVVARAPVFGAKLASFDDSAARAVPGVRQVLAVPSGVAVLADHYWAAKRGRDAL
KVEWQGGDKALGSAALIADFARLAREPGPIALEAGDVDAGMAQAHRTVEAEYRLPYLAHTPMEPLNCTVRIA
PDGCDVWVGTQMQTLAQRTVAQVAGLEPAQVRIHTTFLGGGFGRRAVQDFVAEATHVARAAGLPVKTIWSR
EDDVRGGYYRSGFVHRIRVGLDRDGRPLAWRHGIAGQSNHPEREGVHPTSVEGVVDSPYVLGTSAHRVVAHS
PRTAVPVWYWRSVGHSHAAFAMESMVDELAHASGQDPVAYRLRLLHDAPRHRRVLELAAQRFGWGRRPQS
GRGHGIAVHACFGSVTAQAVEVSVRDGAIRVHRVVCAIDCGVAVNPDNVRAQMESAIVYGLSAALHGRVTIV
DGAVRESNFHDYPALRIAEMPVIEVHVVDSGESPGGAGEPGTPPIAPAVANAVFQLTGARLRELPLTLPARARTR
PTA
>SEQF8725||SEQF8725.1_03478
MAALSGAAASGSHDAPAAGIDRRTFLKLTGIAGGGLALGIVPPGGAHAEEAAAAVPAAKGPAAAPQAFIVIAPD
NTVTIAVNRLEFGQGVHTALPMMLAEDLDVDWRNVRAVLAPAGDPYKDPAMGIQMTGGSTAVKHSYQQYR
ELGARARAMLIAAAAQRWAVEPSACTTANGVVTAGNRRATYGELAQAAMEMPVPQQVTLKDPSRFALIGKP
TPRLDTRGKLDGSVGFGIDTQLDNLRVAVVARPPRFGGKVRRFNAEAARAIQGVVEVFEVPTDGGGTGVAVVA
TGYWPAKQGRDALQAEWDDSGSTVSSAALYEEYAKLARQPGRTPRADAFDLAGAVRTVEAEYRFPYLAHAPM
EPLNCTMQAEVAGGKPARVKVWAGTQFQTVDHGALAKAFGLPPEQVSVVTMMAGGGFGRRAVPTSDYLVE
AAQLMRAWVAKGHREPVKVIWSREDDIRGGYYRPLHLHHARIGIDAQGKVLGWEHAIVGQSLTMGSPFEAFL
VKDGVDHTMTEGIVDHDYGFPLRLSVHHPKVQVPVLWWRSVGHTHTAFVKETLIDELATVAKQDPVAYRLARL
DPVKGARQRAALQLAVEKSGYGKRRLPAGRAWGVAVHQSFDSAVAYVVEVSLRNGQPHVHRVTAGVHANRV
INPLSASAQIEGGCVFGLAMIRPGFAIEIDNGAVRNSNFGDFPPVRINDAPPVEVHFVPSNDPPTGLGEPGVPVI
APAVANAVFKLTGKRQRQLPFVMA
>SEQF8725||SEQF8725.1_00397
MRANDSTMTPAMTPDLSRRGFLQGTALGTLALAVGANGIIGLARADDDAPKKYGADSMPGGTVSDPLVFVSI
GADGTVTIVAHRAEMGTGVRTSLPMVVADEMEANWDRVKVVQAEANETRYGNQNVDGSRSVRHFLMPM
RRVGAAARQMLEAAAAARWSVPVGEVRAQRHEVVHLPTGRRLSYGELAADAAKQPVPAQGTLKLKTPDQFR
YIGKDRVRLVDLEAIGKGQATYGMDVRLPGMVYAVVARPPVVGGKVRAVRADKALAVPGVLKVVEIPPFSGAP
AFQPLGGVAVVARNTWAAMQGRAALEIEWDDGPNAAYDSVAYRRTLEAAARQPGKVVRNEGDAPAAWNK
SAESERYAAEYYLPHLAHASMEPPVATVRVSGKTAEVWTSVQNPVAAQTAVAKRLKLEPKDVRVNVVLLGGGF
GRKSKPDFVDEAAVVAQAMPGTPVKLVWTREDDIHHDYLHTVSVERLEAVVGKDGRPQTWLHRSAAPTIGSL
FTAGAKGQQPFELGMSAINMPYRIPNVRVETAEVEAHARIGWFRSVSNIPHAFAAQCFIAELAHRAGKDHKQF
ALDLIGPARKIDPRTMADSWNYTESPERYPYDTGRLRDVIEAATRGARWGRKLPRGHGLGLAFCYSFMSYTAAV
VEVAVNDKGEVQVVAVDMAMDCGPQINPERIRSQLEGGAIMGLSLALSSEITFEKGRVKQSNFHDYEVLRHHA
SPRTIRTHLVNGSHDVPPGGVGEPPVPPVAPALCNAIFAATGKRIRSLPVRTVA
>SEQF8725||SEQF8725.1_03737
MKAPLDVSRRRFLAAGAVLGGGLVIGFTVPSVRRLADGSVEGAVAAEPVPWAPNAFLRIGADDSVTVLLAHSE
MGQGVWTSLALLIADELDADWMRIRVEHAPAAPAYGIAMLGGMQGTVGSRSVRSEFLRYRQAGAAARAML
LQAAAARWQVPVDALRTDSGAVLHDGRRLRYGELVHEAAALPVPAAEALRLKDARDWKWIGKGARRLDAAD
KITGRARFGIDVKLDGLLTAVVARAPVFGAKLASFDDSAARAVPGVRQVLAVPSGVAVLADHYWAAKRGRDAL
KVEWQGGDKALGSAALIADFARLAREPGPIALEAGDVDAGMAQAHRTVEAEYRLPYLAHTPMEPLNCTVRIA
PDGCDVWVGTQMQTLAQRTVAQVAGLEPAQVRIHTTFLGGGFGRRAVQDFVAEATHVARAAGLPVKTIWSR
EDDVRGGYYRSGFVHRIRVGLDRDGRPLAWRHGIAGQSNHPEREGVHPTSVEGVVDSPYVLGTSAHRVVAHS
PRTAVPVWYWRSVGHSHAAFAMESMVDELAHASGQDPVAYRLRLLHDAPRHRRVLELAAQRFGWGRRPQS

GRGHGIAVHACFGSVTAQAVEVSVRDGAIRVHRVVCAIDCGVAVNPDNVRAQMESAIVYGLSAALHGRVTIV
DGAVRESNFHDYPALRIAEMPVIEVHVVDSGESPGGAGEPGTPPIAPAVANAVFQLTGARLRELPLTLPARARTR
PTA
>SEQF8741||SEQF8741.1_00298
MSVSVNRRAFLLGTGALAGAVLFPITRSGAQSSTAPATEGVEILSWIVINPDNTVTYTVPQTEVGQGVTTTIPQ
MLAEELDADWEKMSVAFYDPAISKARGTPYVWTTTLGSLSAHYLFAPSRLAAAQVRAMLLEAAAERLSVPTEEL
TTGGNQVHHGKSGRSLNYADVAAAAVKLTPPLAEKVVYKSVADRRFIGKPISPLSTVPATRGEIVYGIDIDLPGM
RYAAVQQSPVFGGKLVRVDDEALKELPGNPQIVQIKGGFVGYNSPVPDGEDPDLWAAAVNIDDTVAVVADSW
WEAKSALESLEIEWDGGSHAGLTSDKLKTALSTQASGELPVLVETGDVGKRIKEAKLSLKAEYVFPFMDPAPLEPL
NCTVLIEKSKATVWAGSQYADDAHRIATELTGLAPENVKFNLMQCGGGFGRRVQNDFVYQAVQIGKAMSGTP
VKVLWSREECIKHSTYTPLTVVKFAGALDEDENIDTWSCRIASAQAALQSYGGTHFPFFFPNMHIAYQRDKTTPI
PFGWMRGVGLTQHLWMNFGFLNELAVLAHKDSVDLYRALLDPSLIPKDLEQYDIAVARSKTLRRVLDAAAEKA
GWGGRAKNGSGRGIAVSDTAYYLGYESSSKAAVVDVTIKDASVRVDKVAITIDAGTIINPDIVRQQLEGCISYALT
NAFYSEITLENGQVQQNNFYDYPILRIAQVPEIEINLLPSEGSPQSVGEDAVPITIAALVNAIADAGGPRIRSLPIKE
LSLV
>SEQF8748||SEQF8748.1_03516
MSVSVNRRAFLLGTGALAGAVLFPITRSGAQSSTAPATEGVEILSWIVINPDNTVTYTVPQTEVGQGVTTTIPQ
MLAEELDADWEKMSVAFYDPAISKARGTPYVWTTTLGSLSAHYLFAPSRLAAAQVRAMLLEAAAERLSVPTEEL
TTGGNQVHHGKSGRSLNYADVAAAAVKLTPPLAEKVVYKSVADRRFIGKPISPLSTVPATRGEIVYGIDIDLPGM
RYAAVQQSPVFGGKLVRVDDEALKELPGNPQIVQIKGGFVGYNSPVPDGEDPDLWAAAVNIDDTVAVVADSW
WEAKSALESLEIEWDGGSHAGLTSDKLKTALSTQASGELPVLVETGDVGKRIKEAKLSLKAEYVFPFMDPAPLEPL
NCTVLIEKSKATVWAGSQYADDAHRIATELTGLAPENVKFNLMQCGGGFGRRVQNDFVYQAVQIGKAMSGTP
VKVLWSREECIKHSTYTPLTVVKFAGALDEDENIDTWSCRIASAQAALQSYGGTHFPFFFPNMHIAYQRDKTTPI
PFGWMRGVGLTQHLWMNFGFLNELAVLAHKDSVDLYRALLDPSLIPKDLEQYDIAVARSKTLRRVLDAAAEKA
GWGGRAKNGSGRGIAVSDTAYYLGYESSSKAAVVDVTIKDASVRVDKVAITIDAGTIINPDIVRQQLEGCISYALT
NAFYSEITLENGQVQQNNFYDYPILRIAQVPEIEINLLPSEGSPQSVGEDAVPITIAALVNAIADAGGPRIRSLPIKE
LSLV
>SEQF8753||SEQF8753.1_05191
MINNLHSRRNFLKGTVLATGGLVLSVQLPRLGRAAVAAGQENQKSFFPNAFIKIDSDGITLIMAHHEVGQGIFTS
SAMLMAEELVVGLDQVKLQPAPPDLSKYLDPLLFDQSTGGSTSTRADWMRLRQAGAAARMMLVQAAASEW
SVPALECRVELGVVHHDATGRSMTYLEVAPKAALLSVPENVVLKEPPEFRVIGTRAKRLDTPHKVNGSAIYGIDIII
PNMKFATLAISPVVGGLISKMNEDAARAVKGVHDIVKLPDAVAVIGDHMWAAKAGVDALEIEWEAGPNGSM
NLNSIIKKLEQASQQQGVIIRNDKDAASVISNAAVKLSAVYESPFLSHSPLEPLNCTLHIQDNKAEMWVGTQVP
VRAQKAVAEATGLPQESVVVNNQLIGGAFGRRLDVDSIELAARLLKDIRYPVKLVWTREQDMTHDYYRPYYYD
RVAAGLDKDGRLIGRTHRVTGSSIFARYAPAAFIKGVDPDAIECASETPYDEDAVFVDYVRNEPDGVNTSWWR
GVGPSHNLFVVESFVDEMAAAIEQDPVEFRRRMLTKNPRALAVLNEAAEKAGWGEKLPAGRGRGVSLQVAFG
SYLAHVLDIEITKEEVKLLRSVIAVDCGITINPDTVEAQMQGGVIFGLSSAMYNQVTFTDGAVDQTNFDMYRAL
RINEIPKIEIYHIKSQENPGGVGEAATAAAAGALANAIFAASGKRIRSLPLAEAALS
>SEQF8757||SEQF8757.1_05005
MRREFLKTGAFCVAFSLASKNRQAFAKSATPVDAGNAAFSPNAFIQVQRDGRVRLTIPNVEMGQGIYTSEAAIL
AEEMDLGLDQIIVDHAPASPDRYTMPLQATQLTGGSTSIRGSWDVLRSAGATARLMLINAAASRWNVRPDSCE
AKRGVVTHIQSGRSLDYAMLIDDAQRQTVPEVVPLKDRSKFTIIGKPWRRIDTQDKVLGATRFGIDAQPTGHKC
AAVIMDPVWHARPRVENVPLPAGIHEIVALDDCLAVIADDFWTASEGARALPVEWDYGSCIPFDTAQLRKAM
DHAASTAEALTARHDASWSGRPVTQAAEYEFPVLAHAALEPMNATVAYRKDELEIWIGTQAPGRVLDAAHRLT

GFPRERIQVHNHYFGGGFGRRVETDQVEQAIRIAMKVDYPVKVIWSRATDIQRDLPRPPFLDRISAALADDGFP
IEWTDHITGCSIFSRFAPQFMRENGVDPDTVDGADNLPYSIPSVHVQWTPFDMPDQRPIGWWRGVGPTHNL
FRVECFLDELANHAGIDPYLYRTRLLQNNPRLLKVLHLAADKADWHRPLKQGVGRGIAVGKAYGSYVCAVVEVD
ATSTSEIRLTRCVCAVDCGIVINPNTIEAQIEGGLIFGWTAALWGELTYASGAVEQSNFHDYRLMRMNEVPSIAV
HLVESDEHPSGVGELGTAIASPALANAVFHATGFRCRRLPLMPSLAEHLQSSSGGVLRRMNHLLSSFIRQGGKP
>SEQF8766||SEQF8766.1_04362
MINNLHSRRNFLKGTVLATAGLVLAVQLPRLGRAAVAAGQESQKSFFPNAFIKIDTDGITLIMAHHEVGQGIFTS
STMMIAEELEVGLDQVKLQPAPPDLSKYLDPLLFDQSTGGSTSTRADWMRLRQAGAAARMMLVQAAASEW
SVPAFECRVELGVVHHDAAGRSMTYLEAAPKAALLPVPENVVLKVPSEFRVIGTRAKRLDTPQKVNGSAIYGIDII
IPDMKFATLAISPVVGGLISKMNEDAARAVKGVHDIVKLPDAVAVIGDHMWAAKAGVDALEIEWEAGPNGSM
NLNSIVEKLEQASQQQGVIIRNDKDAASVISNAAVKLSAVYESPFLSHSPLEPLNCTLHIQDHKAEMWVGTQVP
VRAQKAVAEATGLPQESVVVNNQLIGGAFGRRLDVDSIELAARLLKDIRYPVKLVWTREQDMTHDYYRPYYYD
RVAAGLDKDGRLIGRTHRVTGSSIFARYAPAAFINGVDPDAIDCASETPYDEDAVFVDYVRSEPDGVNTSWWR
GVGPSHNLFVVESFVDEMAAATKQDPVEFRRRMLTKNPRALAVLNEAAKKAGWGEKLPAGRGRGVSLQVAF
GSYLAHVLDIEITKEEVKLLRSVIAVDCGITINPDTVDAQMQGGVIFGLSSAMYNQVTFTDGAVDQTNFDMYR
ALRINEIPKIEIYQVKSQENPGGVGEAATAAAAAALANAIFAASGKRIRSLPLAEAALS
>SEQF8770||SEQF8770.1_03351
MSVSVNRRAFLLGTGALAGAVLFPITRSGAQSSTAPATEGVEILSWIVINPDNTVTYTVPQTEVGQGVTTTIPQ
MLAEELDADWEKMSVAFYDPAISKARGTPYVWTTTLGSLSAHYLFAPSRLAAAQVRAMLLEAAAERLSVPTEEL
TTGGNQVHHGKSGRSLNYADVAAAAVKLTPPLAEKVVYKSVADRRFIGKPISPLSTVPATRGEIVYGIDIDLPGM
RYAAVQQSPVFGGKLVRVDDEALKELPGNPQIVQIKGGFVGYNSPVPDGEDPDLWAAAVNIDDTVAVVADSW
WEAKSALESLEIEWDGGSHAGLTSDKLKTALSTQASGELPVLVETGDVGKRIKEAKLSLKAEYVFPFMDPAPLEPL
NCTVLIEKSKATVWAGSQYADDAHRIATELTGLAPENVKFNLMQCGGGFGRRVQNDFVYQAVQIGKAMSGTP
VKVLWSREECIKHSTYTPLTVVKFAGALDEDENIDTWSCRIASAQAALQSYGGTHFPFFFPNMHIAYQRDKTTPI
PFGWMRGVGLTQHLWMNFGFLNELAVLAHKDSVDLYRALLDPSLIPKDLEQYDIAVARSKTLRRVLDAAAEKA
GWGGRAKNGSGRGIAVSDTAYYLGYESSSKAAVVDVTIKDASVRVDKVAITIDAGTIINPDIVRQQLEGCISYALT
NAFYSEITLENGQVQQNNFYDYPILRIAQVPEIEINLLPSEGSPQSVGEDAVPITIAALVNAIADAGGPRIRSLPIKE
LSLV
>SEQF8771||SEQF8771.1_00485
MSNKLAGPQLNRRNFLKAGAALGGGLVVSLGPIATTALAATDFVPNAFIRIDRRGLVTLVMPQVEMGQGIYTA
QAMLIAEELDVSLEDVKIEHAPVDEALYGHPMLGRQMTGGSTSIRAFWTPLRTAGATARTLLTQAAAREWNVD
VAACRNEQGFVVNPDGRTRRAYGELVDMAASLPIPPADTIILKQPSEFRLIGTSAKRLDTRGKTNGALKYGIDAS
PTGTKIAAIAISPALGGKPTSVNEAAALAIKGVRQVVITEDSVAVVADHTGAAKKGLEAASIEWEDGPNGNVSQ
ADILRQLEEQSQQAGAVARSEGDVNGALSSAAVRIDAVYQLPFLAHTAMEPMNCTVHVREDACELWVGTQN
MSSAKQAAAAVTGLPPEKIIINNHIVGGGFGRRLEVDGVAHAVKIAKQVTSPVKVIWSREEDIQHGYYRPYYYD
RISGGIDSDGNPVAWSHRVSGSSIFARIAPGAMQNGVDPDGVEGAAHPPYKLPAIHVEFKQVEPQGVLTSWW
RGVGPSHNIFVVESFIDELAAAAKADPVDYRRRLLSENPRALAVLELAAEKAGWGAGLPERHGRGVAVQFAFG
SYLAMIADVSVATDGTVRVTRIVTAVDCGLTVNPDTIAAQMEGGALYGLTGALYGAITFAQGRVEQSNFDTYLPL
RIDEAPHVETHIVRSAEAPGGIGEAATSAAFPAVTNAIFAATGKRVRILPINPEELKA
>SEQF8797||SEQF8797.1_03694
MTVQTTRRGFLAGGTGLLLALALPVGRGRAQMTVAGPFAPNAFIRIGTDDLVTVMIKHLEMGQGPYTGLATLV
AEELDADWSQMRAEGAPADDTLYANLAFGAQGTGGSTAIANSFMQMRKAGAAARAMLVAAAAEEWGVPA
SEITVKAGVVAHEASGRSSGFGALSTAAARQPVPEDPPVKAPADFVLIGTDRPKLDSASKSTGTAQFTMDVYRD
GMLTVVVAHPPRFGATLARVDDSAALAVKGVEMVRTVPSGVAVYATNTYAALKGRDALILEWDDSKAETRSST

QMFAAFAEAAAQGGETVEEEGDLSAIDGAARVLTAEYRFPYLAHAPMEPLDAVIETKGGKAELWFGSQFPSFD
KPTIAGALGIAPEDVRINVLMAGGSFGRRAQGSAHLAAEVGAIAKAAGRDGAFKLVWTREDDVKGGYYRPMT
VHRLRAGLDADGAIVAWENVVANQSIMAGTPMEAMLKGGPDNSSFEGSTGLPYRLGAKRIGWARMESPVSV
LWWRSVGHTHTAYAVETFLDEVLEAAGKDPVQGRLDLLPPEAARERAVIAEVARMSGWQGARRDGKGYGIAY
AKSFGTYVAEVVEVENRGGTPRVTRVWCAVDCGIAVNPNVIRAQMEGGIGYALSAALHSRITLAPGGTVEQSN
FHDYPMLRIAEMPAVEVSIIRSAADPTGVGEPGVPPLAPAMANAWRALNGARQYQLPFGGAIS
>SEQF8798||SEQF8798.1_03496
MTVQTTRRGFLAGGTGLLLALALPVGRGRAQVTVAGPFAPNAFVRIGTDDLVTVMIKHLEMGQGPYTGLATLV
AEELDADWSQMRAEGAPADDRLYANLAFGAQGTGGSTAIANSFMQMRKAGAAARAMLVAAAAEEWGVPA
SEITVKAGVVAHEASGRSSGFGALSTAAARQPVPEDPPVKAPADFVLIGTDRPKLDSASKSTGTAQFTLDVYRDG
MLTVVVAHPPRFGATLARVDDSAALAVKGVEMVRTVPSGVAVYATNTYAALKGRDALILEWDDSKAETRSSTQ
MFAAFAEAAAQGGETVEEEGDLSAIDGAARVLTAEYRFPYLAHAPMEPLDAVIETKGGKAELWFGSQFPSFDK
PTIAGALGIAPEDVTINVLMAGGSFGRRAQGSAHLAAEVGEIARAAGRDGAFKLVWTREDDVKGGYYRPMTV
HRLRAGLDADGAIVGWENVIANQSIMAGTPMEAMLKGGPDNSSFEGSTGLPYRLGARRIGWARMESPVSVL
WWRSVGHTHTAYAVETFLDEVLEAAGKDPVQGRLDLLPPDAARERAVIAEAARMSGWQGPRRDGKGYGIAY
AKSFGTYVAEVVEVEDRAGTPRVTRVWCAVDCGIAVNPNVIRAQMEGGIGYALSAALHSRITLAPGGTVEQSN
FHDYPMLRIAEMPAVEVSIIRSDADPTGVGEPGVPPLAPAMANAWRALNGARQYQLPFGGAIS
>SEQF8799||SEQF8799.1_04206
MTVQTTRRGFLAGGTGLLLALALPVGRGRAQVTVAGPFAPNAFIRIGTDDLVTVMIKHLEMGQGPYTGLATLV
AEELDADWSQMRAEGAPADDTLYANLAFGAQGTGGSTAIANSFMQMRKAGAAARAMLVAAAAEEWGVPA
SEITVKAGVVAHEASGRSSGFGALSTAAARQPVPEDPPVKAPADFVLIGTNRPKLDSASKSTGTAQFTLDVYRDG
MLTVVVAHPPRFGATLARVDDSAALAVKGVEMVRTVPSGVAVYATNTYAALKGRDALILEWDDSKAETRSSTQ
MFAAFAEAAAQGGETVEEEGDLSAIDGAARVLTAEYRFPYLAHAPMEPLDAVIETKGGKAELWFGSQFPSFDK
PTIAGALGIAPEDVTINVLMAGGSFGRRAQGSAHLAAEVGEIAKAAGRDGAFKLVWTREDDVKGGYYRPMTV
HRLRAGLDADGTIIGWENVVANQSIMAGTPMEAMLKGGPDNSSFEGSTGLPYRLGARRIGWARMESPVSVL
WWRSVGHTHTTYAVETFLDEVLEAAGKDPVQGRLDLLPPEAARERAVIAEAARMAGWQGPRRDGKGYGIAY
AKSFGTYVAEVVEVEDRGGTPRVTRVWCAIDCGIAVNPNVIRAQMEGGIGYALSAALHSRITLAPGGTVEQSNF
HDYPMLRIAEMPAVEVSIIRSAADPTGVGEPGVPPLAPAMANAWRALNGARQYQLPFGGAIS
>SEQF8800||SEQF8800.1_03369
MTVQTTRRGFLAGGTGLLLALALPVGRGRAQMTVAGPFAPNAFIRIGTDDLVTVMIKHLEMGQGPYTGLATLV
AEELDADWSQMRAEGAPADDTLYANLAFGAQGTGGSTAIANSFMQMRKAGAAARAMLVAAAAEEWGVPA
SEITVKAGVVAHEASGRSSGFGALSTAAARQPVPEDPPVKAPADFVLIGTDRPKLDSASKSTGTAQFTMDVYRD
GMLTVVVAHPPRFGATLARVDDSAALAVKGVEMVRTVPSGVAVYATNTYAALKGRDALILEWDDSKAETRSST
QMFAAFAEAAAQGGETVEEEGDLSAIDGAARVLTAEYRFPYLAHAPMEPLDAVIETKGGKAELWFGSQFPSFD
KPTIAGALGIAPEDVRINVLMAGGSFGRRAQGSAHLAAEVGAIAKAAGRDGAFKLVWTREDDVKGGYYRPMT
VHRLRAGLDADGAIVAWENVVANQSIMAGTPMEAMLKGGPDNSSFEGSTGLPYRLGAKRIGWARMESPVSV
LWWRSVGHTHTAYAVETFLDEVLEAAGKDPVQGRLDLLPPEAARERAVIAEVARMSGWQGARRDGKGYGIAY
AKSFGTYVAEVVEVENRGGTPRVTRVWCAVDCGIAVNPNVIRAQMEGGIGYALSAALHSRITLAPGGTVEQSN
FHDYPMLRIAEMPAVEVSIIRSAADPTGVGEPGVPPLAPAMANAWRALNGARQYQLPFGGAIS
>SEQF8801||SEQF8801.1_00247
MTVQTTRRGFLAGGTGLLLALALPVGRGRAQVTVAGPFAPNAFIRIGTDDLVTVMIKHLEMGQGPYTGLATLV
AEELDADWSQMRAEGAPADDRLYANLAFGAQGTGGSTAIANSFMQMRKAGAAARAMLVAAAAEEWGVPA
SEITVKAGVVAHEASGRSSGFGALSTAAARQPVPEDPPVKAPADFVLIGTDRPKLDSASKSTGTAQFTLDVYRDG
MLTVVVAHPPRFGATLARVDDSAALAVKGVEMVRTVPSGVAVYATNTYAAMKGRDALILEWDDSKAETRSST

QMFAAFAEAAAQGGETVEEEGDLSAIDGAARVLTAEYRFPYLAHAPMEPLDAVIETKGGKAELWFGSQFPFFD
KPTIAGALGIAPEDVTINVLMAGGSFGRRAQGSAHLAAEVGAIAKAAGRDGAFKLVWTREDDVKGGYYRPMT
VHRLRAGLDTDGAIIGWENVVANQSIMAGTPMEAMLKGGPDNSSFEGSTGLPYRLGAKRIGWARMESPVSV
LWWRSVGHTHTAYAVETFLDEVLEAAGKDPVQGRLDLLPPEAARERAVIAEAARMAGWQGPRRDGKGYGIAY
AKSFGTYVAEVVEVEDRGGTPRVTRVWCAVDCGIAVNPNLIRAQMEGGIGYALSAALHSRITLAPGGTVEQSN
FHDYPMLRIAEMPAVEVSIIRSAADPTGVGEPGVPPLAPAMANAWRALNGARQYQLPFGGAIS
>SEQF8802||SEQF8802.1_03007
MTVQTTRRGFLAGGTGLLLALALPVGRGRAQVTVAGPFAPNAFIRIGTDNLVTVMIKHLEMGQGPYTGLATLV
AEELDADWSQMRAEGAPADDRLYANLAFGAQGTGGSTAIANSFMQMRKAGAAARAMLVAAAAEEWGVPA
SEITVKAGVVAHEASGRSSGFGALSTAAARQPVPEDPPVKAPADFVLIGTDRPKLDSASKSTGTAQFTLDVYRDG
MLTVVVAHPPRFGATLARVDDSAALAVKGVEMVRTVPSGVAVYATNTYAALKGRDALILEWDDSKAETRSSTQ
MFAAFAEAAAQGGETVEEEGDLSAIDGAARVLTAEYRFPYLAHAPMEPLDAVIETKGGKAELWFGSQFPSFDK
PTIADALGIAPEDVTINVLMAGGSFGRRAQGSAHLAAEVGEIAKAAGRDGAFKLVWTREDDVKGGYYRPMTV
HRLRAGLDADGTIIAWENVVANQSIMAGTPMEAMLKGGPDNSSFEGSTGLPYRLGAKRIGWARMESPVSVL
WWRSVGHTHTAYAVETFLDEVLEAAGKDPVQGRLDLLPPDAARERAVIAEAARMADWQGPRRDGKGYGIAY
AKSFGTYVAEVVEVEDRGGTPRITRVWCAVDCGIAVNPNVIRAQMEGGIGYALSAALHSRITLAPGGTVEQSNF
HDYPMLRIAEMPAVEVSIIRSDADPTGVGEPGVPPLAPAMANAWRALNGARQYQLPFGGAIS
>SEQF8803||SEQF8803.1_03147
MTVQTTRRGFLAGGTGLLLALALPVGRGRAQVTVAGPFAPNAFIRIGTDDLVTVMIKHLEMGQGPYTGLATLV
AEELDADWSQMRAEGAPADDRLYANLAFGAQGTGGSTAIANSFMQMRKAGAAARAMLVAAAAEEWGVPA
SEITVKAGVVAHEASGRSSGFGALSTAAARQPVPEDPPVKAPADFVLIGTDRPKLDSASKSTGTAQFTLDVYRDG
MLTVVVAHPPRFGATLARVDDSAALAVKGVEMVRTVPSGVAVYATNTYAAMKGRDALILEWDDSKAETRSST
QMFAAFAEAAAQGGETVEEEGDLSAIDGAARVLTAEYRFPYLAHAPMEPLDAVIETKGGKAELWFGSQFPFFD
KPTIAGALGIAPEDVTINVLMAGGSFGRRAQGSAHLAAEVGAIAKAAGRDGAFKLVWTREDDVKGGYYRPMT
VHRLRAGLDTDGAIIGWENVVANQSIMAGTPMEAMLKGGPDNSSFEGSTGLPYRLGAKRIGWARMESPVSV
LWWRSVGHTHTAYAVETFLDEVLEAAGKDPVQGRLDLLPPEAARERAVIAEAARMAGWQGPRRDGKGYGIAY
AKSFGTYVAEVVEVEDRGGTPRVTRVWCAVDCGIAVNPNLIRAQMEGGIGYALSAALHSRITLAPGGTVEQSN
FHDYPMLRIAEMPAVEVSIIRSAADPTGVGEPGVPPLAPAMANAWRALNGARQYQLPFGGAIS
>SEQF9565||SEQF9565.1_01540
MDAKITLAVDDASPIPAADDANAPASAGRRTFIKAGGGLALAVWMGLPPALAADKKTAAGATLTPNAFVRIAP
DNTVTVVVKHLEMGQGTFTGLPTLVAEELDAAWSQIKVEGAPADAKRYNNLFWGPTQGTGGSTAMANSFEQ
MRKAGATARAMLVSAAAKRWNVPAGEITVKDGVLRHAASGRQASFGELADAAAKEAVPETVVLKDPKDFVLI
GKRVGRTDSAAKVNGSAIYTQDLKLPGMLTAVVAHPPRFGARMARYDAAPALAVKGVVAVLPIPSGVAVVAKD
FWSARQGREKLQVQWDETGAFAGGSDSIIADYRQLAQKPGLVARNDGNSAQALGNADKVVEATYTFPYLAH
AAMEPMNAVAHFTGDALEIWNGEQFQTVDQITLGKAVGLPPEKVTLHMLFAGGSFGRRANPHSDYLLEATHI
AKGLADQGKRLPVKLVWTREDDTSAGWYRPIYVHKLVAGLDKAGQIVAWQHRIVGQSIIAGTAFEGALVKNGI
DGTSVEGAANLPYRIPNLTVDLHSPTQPVPVQWWRSVGATHTAYSTETFLDRLFKEAGTDPVAGRRALLTGHPR
HLAALELAAKQAGWGTPLAPGKAGEQRARGVAVHESFNTMVAQVAEVTRRGDGSFTVDKVTCAVECGLAIN
PDVVRAQMEGGIGYGLSGALFGAIHVDKGAVRERNFDAYRVLRIDEMPHIDVHIVPSAGAPTGVGEPGTPVIA
PAVANALASLTGEWTTEQPFIKAKA
>SEQF9896||SEQF9896.1_04530
MGKIETPDSATRGILNVSRRTLLKGAGGLALGIFFAPLMRGMDALAAGGPLEPNAFVRIDLDGTVTVLAKHLEM
GQGSYTGLATLLAEELDADWDKVRVEGAPADVKRYNNLAFGPMQGTGGSTAMANSWEQMRNAGATAKAM
LVAAAAQRWSVPVSEISVDKGVVSHAGSGRSAGFGDLVEAAASLPVPEQVQLKDPKDFKLIGKLELRRKDSTDK

TDGSAIFTQDFKLPGMLVAMVAYPPRFGGVPRSVDSSKAKAVRDVVEVVEFRDLPHGRSGVAVLAKNTWAAR
QGRDALVIEWDESQAFTLGSEEILAQYRDDAGKPGLPATSKGDTDAALAQAAKTVEADYEFPYLAHAAMEPM
NCLVKLSSDRCEIWNGEQFQTVDQTIISGYLGLTPEQVSLTQLYAGGSFGRRASSVSDYLLEAVAITKAARDKGVD
APVKMVWTREDDTRGGYFRPLYLHRVRIGLDQAGKLQAWHNRIVGQSIMAGTSMEPFMIKDGIDHTSVEGL
ANLSYAVPNLQVELSTPSNIKVPVLWWRSVGHTHTGYVAETMIDEAAVAAGQDPYAFRHALLESHPRHRGALE
LAAKQAGWDKPLAAGAEGEKRGRGIAVHESFGSFVAQVAEVTVKADGSYRLDRVVCAVDCGIAINPDVIKAQ
MEGGIGFALAAARHSAITLKEGRVEQSNFHDFQVLRMNEMPKVEVHIVPSAANPTGVGEPGVPPLAPALANA
LFAATGVRLRKLPFPAQIKA
>SEQF9897||SEQF9897.1_04641
MGKIETPDSATRGILNVSRRTLLKGAGGLALGIFFAPLMRGMDALAAGGPLEPNAFVRIDLDGTVTVLAKHLEM
GQGSYTGLATLLAEELDADWDKVRVEGAPADVKRYNNLAFGPMQGTGGSTAMANSWEQMRNAGATAKAM
LVAAAAQRWSVPVSEISVDKGVVSHAGSGRSAGFGDLVEAAASLPVPEQVQLKDPKDFKLIGKLELRRKDSTDK
TDGSAIFTQDFKLPGMLVAMVAYPPRFGGVPRSVDSSKAKAVRDVVEVVEFRDLPHGRSGVAVLAKNTWAAR
QGRDALVIEWDESQAFTLGSEEILAQYRDDAGKPGLPATSKGDTDAALAQAAKTVEADYEFPYLAHAAMEPM
NCLVKLSSDRCEIWNGEQFQTVDQTIISGYLGLTPEQVSLTQLYAGGSFGRRASSVSDYLLEAVAITKAARDKGVD
APVKMVWTREDDTRGGYFRPLYLHRVRIGLDEAGKLQAWHNRIVGQSIMTGTSMEPFMIKEGIDHTSVEGLS
NLSYAVPNLQVELSTPSNIKVPVLWWRSVGHTHTGYVAETMIDEAAVAAGQDPYSFRHALLDSHPRHRGALEL
AAKQAGWDKPLAAGAEGEKRGRGIAVHESFGSFVAQVAEVTVKADGSYRLDRVVCAVDCGIAINPDVIKAQM
EGGIGFALAAAQHSAITLKEGRVEQSNFHDFQVLRMNEMPKVEVHIVPSAANPTGVGEPGVPPLAPALANALF
AATGVRLRKLPFPAQIKA
>SEQF9898||SEQF9898.1_00996
MGKIETPDSATRGILNVSRRTLLKGAGGLALGIFFAPLMRGMDALAAGGPLEPNAFVRIDLDGTVTVLAKHLEM
GQGSYTGLATLLAEELDADWDKVRVEGAPADVKRYNNLAFGPMQGTGGSTAMANSWEQMRNAGATAKAM
LVAAAAQRWSVPASEISVSQGVVSHAGSGRSAGFGELVEAAASLPVPEQVQLKDPKDFKLIGKLELRRKDSTDK
TDGSAIFTQDFKLPGMLVAMVAYPPRFGGVPRSVDSSKAKAVRDVVEVVEFRDLPHGRSGVAVLAKNTWAAR
QGRDALVIEWDESQAFTLGSEEILAQYRDDAGKPGLPATSKGDTDAALAQAAKTVEAEYEFPYLAHAAMEPM
NCLVKLSSDRCEIWNGEQFQTVDQAIISGYLGMTPEQVSLTQLYAGGSFGRRAGSVSDYLLEAVAITKAARDKGV
DVPVKMVWTREDDTRGGYFRPLYLHRVRIGLDQAGKLQAWHNRIVGQSIMAGTSMEPFMIKDGIDHTSVEG
LSNLSYAVPNLQVELSTPSNIKVPVLWWRSVGHTHTGYVAETMIDEAAVAAGQDPYAFRHALLDSHPRHRGAL
ELAAKEAGWDKPLAAGGEGEKRGRGIAVHESFGSFVAQVAEVTVKADGSYRLDRVVCAVDCGIAINPDVIKAQ
MEGGIGFALAAARHSAITLKEGRVEQSNFHDFQVLRLNEMPKVEVHIVPSAANPTGVGEPGVPPLAPALANAL
FAATGVRLRKLPFPAQIKA
>SEQF9899||SEQF9899.1_02681
MGKIETPDSATRGILNVSRRTLLKGAGGLALGIFFAPLMRGMDALAAGGPLEPNAFVRIDLDGTVTVLAKHLEM
GQGSYTGLATLLAEELDADWDKVRVEGAPADVKRYNNLAFGPMQGTGGSTAMANSWEQMRNAGATAKAM
LVAAAAQRWSVPVSEISVDKGVVSHAGSGRSAGFGDLVEAAASLPVPEQVQLKDPKDFKLIGKLELRRKDSTDK
TDGSAIFTQDFKLPGMLVAMVAYPPRFGGVPRSVDSSKAKAVRDVVEVVEFRDLPHGRSGVAVLAKNTWAAR
QGRDALVIEWDESQAFTLGSEEILAQYRDDAGKPGLPATSKGDADAALAQAAKTVEADYEFPYLAHAAMEPM
NCLVKLSSDRCEIWNGEQFQTVDQTIISGYLGLTPEQVSLTQLYAGGSFGRRASSVSDYLLEAVAITKAARDKGVD
APVKMVWTREDDTRGGYFRPLYLHRVRIGLDQAGKLQAWHNRIVGQSIMAGTSMEPFMIKNGIDHTSVEGL
ANLSYAVRNLQVELSTPSNIKVPVLWWRSVGHTHTGYVVETMIDEAAVAAGQDPYAFRHALLESHPRHRGALE
LAAKQAGWDKPLAAGAEGEKRGRGIAVHESFGSFVAQVAEVTVKADGSYRLDRVVCAVDCGIAINPDVIKAQ
MEGGIGFALAAARHSAITLKEGRVEQSNFHDFQVLRMNEMPKVEVHIVPSAANPTGVGEPGVPPLAPALANA
LFAATGVRLRKLPFPAQIKA

>SEQF9900||SEQF9900.1_00574
MGKIETPDSATRGILNVSRRTLLKGAGGLALGIFFAPLMRGMDALAAGGPLEPNAFVRIDLDGTVTVLAKHLEM
GQGSYTGLATLLAEELDADWDKVRVEGAPADVKRYNNLAFGPMQGTGGSTAMANSWEQMRNAGATAKAM
LVAAAAQRWSVPVSEISVDKGVVSHAGSGRSAGFGDLVEAAASLPVPEQVQLKDPKDFKLIGKLELRRKDSTDK
TDGSAIFTQDFKLPGMLVAMVAYPPRFGGVPRSVDSSKAKAVRDVVEVVEFRDLPHGRSGVAVLAKNTWAAR
QGRDALVIEWDESQAFTLGSEEILAQYRDDAGKPGLPATSKGDTDAALAQAAKTVEADYEFPYLAHAAMEPM
NCLVKLSSDRCEIWNGEQFQTVDQTIISGYLGLTPEQVSLTQLYAGGSFGRRASSVSDYLLEAVAITKAARDKGVD
APVKMVWTREDDTRGGYFRPLYLHRVRIGLDQAGKLQAWHNRIVGQSIMAGTSMEPFMIKDGIDHTSVEGL
ANLSYAVPNLQVELSTPSNIKVPVLWWRSVGHTHTGYVAETMIDEAAVAAGQDPYAFRHALLESHPRHRGALE
LAAKQAGWDKPLAAGAEGEKRGRGIAVHESFGSFVAQVAEVTVKADGSYRLDRVVCAVDCGIAINPDVIKAQ
MEGGIGFALAAARHSAITLKEGRVEQSNFHDFQVLRMNEMPKVEVHIVPSAANPTGVGEPGVPPLAPALANA
LFAATGVRLRKLPFPAQIKA
>SEQF9901||SEQF9901.1_03622
MGKIETPDSATRGILNVSRRTLLKGAGGLALGIFFAPLMRGMDALAAGGPLEPNAFVRIDLDGTVTVLAKHLEM
GQGSYTGLATLLAEELDADWDKVRVEGAPADVKRYNNLAFGPMQGTGGSTAMANSWEQMRNAGATAKAM
LVAAAAQRWSVPVSEISVDKGVVSHAGSGRSAGFGDLVEAAASLPVPEQVQLKDPKDFKLIGKLELRRKDSTDK
TDGSAIFTQDFKLPGMLVAMVAYPPRFGGVPRSVDSSKAKAVRDVVEVVEFRDLPHGRSGVAVLAKNTWAAR
QGRDALVIEWDESQAFTLGSEEILAQYRDDAGKPGLPATSKGDTDAALAQAAKTVEADYEFPYLAHAAMEPM
NCLVKLSSDRCEIWNGEQFQTVDQTIISGYLGLTPEQVSLTQLYAGGSFGRRASSVSDYLLEAVAITKAARDKGVD
APVKMVWTREDDTRGGYFRPLYLHRVRIGLDQAGKLQAWHNRIVGQSIMAGTSMEPFMIKDGIDHTSVEGL
ANLSYAVPNLQVELSTPSNIKVPVLWWRSVGHTHTGYVAETMIDEAAVAAGQDPYAFRHALLESHPRHRGALE
LAAKQAGWDKPLAAGAEGEKRGRGIAVHESFGSFVAQVAEVTVKADGSYRLDRVVCAVDCGIAINPDVIKAQ
MEGGIGFALAAARHSAITLKEGRVEQSNFHDFQVLRMNEMPKVEVHIVPSAANPTGVGEPGVPPLAPALANA
LFAATGVRLRKLPFPAQIKA
>SEQF9902||SEQF9902.1_02826
MGKIETPDSATRGILNVSRRTLLKGAGGLALGIFFAPLMRGMDALAAGGPLEPNAFVRIDLDGTVTVLAKHLEM
GQGSYTGLATLLAEELDADWDKVRVEGAPADVKRYNNLAFGPMQGTGGSTAMANSWEQMRNAGATAKAM
LVAAAAQRWSVPVSEISVDKGVVSHAGSGRSAGFGDLVEAAASLPVPEQVQLKDPKDFKLIGKLELRRKDSTDK
TDGSAIFTQDFKLPGMLVAMVAYPPRFGGVPRSVDSSKAKAVRDVVEVVEFRDLPHGRSGVAVLAKNTWAAR
QGRDALVIEWDESQAFTLGSEEILAQYRDDAGKPGLPATSKGDTDAALAQAAKTVEADYEFPYLAHAAMEPM
NCLVKLSSDRCEIWNGEQFQTVDQTIISGYLGLTPEQVSLTQLYAGGSFGRRASSVSDYLLEAVAITKAARDKGVD
APVKMVWTREDDTRGGYFRPLYLHRVRIGLDQAGKLQAWHNRIVGQSIMAGTSMEPFMIKDGIDHTSVEGL
ANLSYAVPNLQVELSTPSNIKVPVLWWRSVGHTHTGYVAETMIDEAAVAAGQDPYAFRHALLESHPRHRGALE
LAAKQAGWDKPLAAGAEGEKRGRGIAVHESFGSFVAQVAEVTVKADGSYRLDRVVCAVDCGIAINPDVIKAQ
MEGGIGFALAAARHSAITLKEGRVEQSNFHDFQVLRMNEMPKVEVHIVPSAANPTGVGEPGVPPLAPALANA
LFAATGVRLRKLPFPAQIKA
>SEQF9903||SEQF9903.1_03089
MGKIETPDSATRGILNVSRRTLLKGAGGLALGIFFAPLMRGMDALAAGGPLEPNAFVRIDLDGTVTVLAKHLEM
GQGSYTGLATLLAEELDADWDKVRVEGAPADVKRYNNLAFGPMQGTGGSTAMANSWEQMRNAGATAKAM
LVAAAAQRWSVPVSEISVDKGVVSHAGSGRSAGFGDLVEAAASLPVPEQVQLKDPKDFKLIGKLELRRKDSTDK
TDGSAIFTQDFKLPGMLVAMVAYPPRFGGVPRSVDSSKAKAVRDVVEVVEFRDLPHGRSGVAVLAKNTWAAR
QGRDALVIEWDESQAFTLGSEEILAQYRDDAGKPGLPATSKGDTDAALAQAAKTVEADYEFPYLAHAAMEPM
NCLVKLSSDRCEIWNGEQFQTVDQTIISGYLGLTPEQVSLTQLYAGGSFGRRASSVSDYLLEAVAITKAARDKGVD
APVKMVWTREDDTRGGYFRPLYLHRVRIGLDQAGKLQAWHNRIVGQSIMAGTSMEPFMIKDGIDHTSVEGL

ANLSYAVPNLQVELSTPSNIKVPVLWWRSVGHTHTGYVAETMIDEAAVAAGQDPYAFRHALLESHPRHRGALE
LAAKQAGWDKPLAAGAEGEKRGRGIAVHESFGSFVAQVAEVTVKADGSYRLDRVVCAVDCGIAINPDVIKAQ
MEGGIGFALAAARHSAITLKEGRVEQSNFHDFQVLRMNEMPKVEVHIVPSAANPTGVGEPGVPPLAPALANA
LFAATGVRLRKLPFPAQIKA
>SEQF9904||SEQF9904.1_04627
MGKIETPDSAARGILNVSRRSLLKGTGGLALGFFLAPLMRGGEALAATASQAFGPNAFVRIDSDGMVTVLAKHL
EMGQGSYTGLATLLAEELDADWSKVRVEGAPADAKLYNNTAFGPMQGTGGSTAMANSWEQMRNAGAAAK
AMLVAAAAQRWGVPASEIRVSQGVVSHAGSGRQAGFGELVEAAAQLPVPQQVQLKDPQDFKLIGKLDLRRK
DSHGKTDGSAVFTQDFKLPGMLVAMVAYPPRFGGVPRSVDSSKTKAVRDVVDVVELRDLPHGRTGVAVLAKN
TWAARQGRDALVIDWDESQAFTLGSEEILAQYRESAGKPGLPATRNGDAEAALAKAAKVVEADYEFPYLAHAA
MEPMNCLVKLSDDRCEIWNGEQFQTVDQNLIGAYLGLPPEKITLNQLYAGGSFGRRASSVSDYLLEAVAITKAA
RGKGVDAPVKLVWTREDDTRAGSYRPLYLHRVRIGLDEAGNLQAWHNRLVGQSIIAGTSLEAALVHDGIDHTS
VEGVSNLSYAVPNLQVELSTPTNIKVPVLWWRSVGHTHTGYVAETMIDEAAVAAGKDPYAFRDALLEKHPRHR
GALKLAAEQAGWDKPLAAGADGEKRGRGIAVHESFGSFVAQVAEVTVKADGSYRLDRVVCAVDCGIAINPDVI
KAQMEGGIGFALAMARHSAITLKEGRVEQSNFHDFQVLRMNEMPKVEVHIVPSAANPSGVGEPGVPPLAPAL
ANALFAATGVRLRKLPFPAQIKA
>SEQF9905||SEQF9905.1_02193
MGKIETPDSATRGILNVSRRTLLKGAGGLALGIFFAPLMRGMDALAAGGPLEPNAFVRIDLDGTVTVLAKHLEM
GQGSYTGLATLLAEELDADWDKVRVEGAPADVKRYNNLAFGPMQGTGGSTAMANSWEQMRNAGATAKAM
LVAAAAQRWSVPVSEISVDKGVVSHAGSGRSAGFGDLVEAAASLPVPEQVQLKDPKDFKLIGKLELRRKDSTDK
TDGSAIFTQDFKLPGMLVAMVAYPPRFGGVPRSVDSSKAKAVRDVVEVVEFRDLPHGRSGVAVLAKNTWAAR
QGRDALVIEWDESQAFTLGSEEILAQYRDDAGKPGLPATSKGDTDAALAQAAKTVEADYEFPYLAHAAMEPM
NCLVKLSSDRCEIWNGEQFQTVDQTIISGYLGLTPEQVSLTQLYAGGSFGRRASSVSDYLLEAVAITKAARDKGVD
APVKMVWTREDDTRGGYFRPLYLHRVRIGLDQAGKLQAWHNRIVGQSIMAGTSMEPFMIKDGIDHTSVEGL
ANLSYAVPNLQVELSTPSNIKVPVLWWRSVGHTHTGYVAETMIDEAAVAAGQDPYAFRHALLESHPRHRGALE
LAAKQAGWDKPLAAGAEGEKRGRGIAVHESFGSFVAQVAEVTVKADGSYRLDRVVCAVDCGIAINPDVIKAQ
MEGGIGFALAAARHSAITLKEGRVEQSNFHDFQVLRMNEMPKVEVHIVPSAANPTGVGEPGVPPLAPALANA
LFAATGVRLRKLPFPAQIKA
>SEQF9906||SEQF9906.1_03498
MGKIETPDSAARGILNVSRRSLLKGTGGLALGIFFAPLIRGMDAVAAGGPLEPNAFVRIDLDGSVTVLAKHLEM
GQGSYTGLATLLAEELDADWETVRVEGAPADVKRYNNLAFGPMQGTGGSTAMANSWEQMRNAGATAKAM
LVAAAAQRWAVPASEISVSKGVVSHPGSGRSAGFGELVEAAAALPVPEQVQLKQPQDFKLIGKVELRRKDSPGK
TDGSAVFTQDFKLPGMLVAMVAYPPRFGGVPRSVDSSKAKAARDVVDVVEFRDLPHGRAGVAVLAKNTWAA
RQGRDALVIDWDESQAFTLGSEEILAQYRESAGKPGLPAAARGDADAALAKAAKTIEADYEFPYLAHAAMEPM
NCLVKLSADRCEIWNGEQFQTVDQAIISGYLGFKPEQVSLTQLYAGGSFGRRASSRSDYLLEAVAITKAARDKGV
DAPVKMVWAREDDTRGGFFRPMYLHRARIGLDEAGKLQAWHNRIVGQSIIAGTSMEPFLVKEGVDHTSVEG
VSNLSYAVPNLKVELSTPSNIKVPVLWWRSVGHTHTGYVAETMIDEAAVAAGQDPYAFRHALLENHPRHRGAL
ELAARQAGWDKPLAAGAAGEKRGRGIAVHESFGSFVAQVAEVTVKADGSYRLDRVVCAVDCGIAINPDVIKA
QMEGGIGFALAAARHSAITLKDGRVEQSNFHDFQVLRMNEMPRVDVHIVPSAQNPTGVGEPGVPPLAPALA
NALFAATGVRLRKLPFPAQIKA
>SEQF9907||SEQF9907.1_00527
MGKIETPDSATRGILNVSRRTLLKGAGGLALGIFFAPLMRGMDALAAGGPLEPNAFVRIDLDGTVTVLAKHLEM
GQGSYTGLATLLAEELDADWDKVRVEGAPADVKRYNNLAFGPMQGTGGSTAMANSWEQMRNAGATAKAM
LVAAAAQRWSVPVSEISVDKGVVSHAGSGRSAGFGDLVEAAASLPVPEQVQLKDPKDFKLIGKLELRRKDSTDK

TDGSAIFTQDFKLPGMLVAMVAYPPRFGGVPRSVDSSKAKAVRDVVEVVEFRDLPHGRSGVAVLAKNTWAAR
QGRDALVIEWDESQAFTLGSEEILAQYRDDAGKPGLPATSKGDTDAALAQAAKTVEADYEFPYLAHAAMEPM
NCLVKLSSDRCEIWNGEQFQTVDQTIISGYLGLTPEQVSLTQLYAGGSFGRRASSVSDYLLEAVAITKAARDKGVD
APVKMVWTREDDTRGGYFRPLYLHRVRIGLDQAGKLQAWHNRIVGQSIMAGTSMEPFMIKDGIDHTSVEGL
ANLSYAVPNLQVELSTPSNIKVPVLWWRSVGHTHTGYVAETMIDEAAVAAGQDPYAFRHALLESHPRHRGALE
LAAKQAGWDKPLAAGAEGEKRGRGIAVHESFGSFVAQVAEVTVKADGSYRLDRVVCAVDCGIAINPDVIKAQ
MEGGIGFALAAARHSAITLKEGRVEQSNFHDFQVLRMNEMPKVEVHIVPSAANPTGVGEPGVPPLAPALANA
LFAATGVRLRKLPFPAQIKA
>SEQF9908||SEQF9908.1_02754
MGKIETPDSATRGILNVSRRTLLKGAGGLALGIFFAPLMRGMDALAAGGPLEPNAFVRIDLDGTVTVLAKHLEM
GQGSYTGLATLLAEELDADWDKVRVEGAPADVKRYNNLAFGPMQGTGGSTAMANSWEQMRNAGATAKAM
LVAAAAQRWSVPVSEISVDKGVVSHAGSGRSAGFGDLVEAAASLPVPEQVQLKDPKDFKLIGKLELRRKDSTDK
TDGSAIFTQDFKLPGMLVAMVAYPPRFGGVPRSVDSSKAKAVRDVVEVVEFRDLPHGRSGVAVLAKNTWAAR
QGRDALVIEWDESQAFTLGSEEILAQYRDDAGKPGLPATSKGDTDAALAQAAKTVEADYEFPYLAHAAMEPM
NCLVKLSSDRCEIWNGEQFQTVDQTIISGYLGLTPEQVSLTQLYAGGSFGRRASSVSDYLLEAVAITKAARDKGVD
APVKMVWTREDDTRGGYFRPLYLHRVRIGLDQAGKLQAWHNRIVGQSIMAGTSMEPFMIKDGIDHTSVEGL
ANLSYAVPNLQVELSTPSNIKVPVLWWRSVGHTHTGYVAETMIDEAAVAAGQDPYAFRHALLESHPRHRGALE
LAAKQAGWDKPLAAGAEGEKRGRGIAVHESFGSFVAQVAEVTVKADGSYRLDRVVCAVDCGIAINPDVIKAQ
MEGGIGFALAAARHSAITLKEGRVEQSNFHDFQVLRMNEMPKVEVHIVPSAANPTGVGEPGVPPLAPALANA
LFAATGVRLRKLPFPAQIKA
>SEQF9909||SEQF9909.1_02392
MGKIETPDSATRGILNVSRRTLLKGAGGLALGIFFAPLMRGMDALAAGGPLEPNAFVRIDLDGTVTVLAKHLEM
GQGSYTGLATLLAEELDADWDKVRVEGAPADVKRYNNLAFGPMQGTGGSTAMANSWEQMRNAGATAKAM
LVAAAAQRWNVPVSEISVDKGVVSHAGSGRSAGFGDLVEAAASLPVPEQVQLKDPKDFKLIGKLELRRKDSTDK
TDGSAIFTQDFKLPGMLVAMVAYPPRFGGVPRSVDSSKAKAVRDVVEVVEFRDLPHGRSGVAVLAKNTWAAR
QGRDALVIEWDESQAFTLGSEEILAQYRDDAGKPGLPATSKGDTDAALAQAAKTVEADYEFPYLAHAAMEPM
NCLVKLSSDRCEIWNGEQFQTVDQTIISGYLGLTPEQVSLTQLYAGGSFGRRASSVSDYLLEAVAITKAARDKGVD
APVKMVWTREDDTRGGYFRPLYLHRVRIGLDQAGKLQAWHNRIVGQSIMAGTSMEPFMIKNGIDHTSVEGL
ANLSYAVPNLQVELSTPSNIKVPVLWWRSVGHTHTGYVVETMIDEAAVAAGQDPYAFRHALLESHPRHRGALE
LAAKQAGWDKPLAAGAEGEKRGRGIAVHESFGSFVAQVAEVTVKADGSYRLDRVVCAVDCGIAINPDVIKAQ
MEGGIGFALAAARHSAITLKEGRVEQSNFHDFQVLRMNEMPKVEVHIVPSAANPTGVGEPGVPPLAPALANA
LFAATGVRLRKLPFPAQIKA
>HRGM_Genome_0200||HRGM_Genome_0200_CDS_03839
MSAHIIDMESTGLPTNPSRRSLLKAAGATGLMIAVTPAGHVFAQTAAAADKFGGDRMPGGVVDNPLIFVSIAP
NGIVTVTCHRSEMGQGVRTSVPLIVAEELEADLNRVRVRQADGDEARYGNQNTDGSRSVRHWLEPGRRVGA
AARAMLEAAAAAQWGVPAAEVQAQNHVLVHKPTGRKLPFGQVAEAAAKLPVPARDSLKLKNPAQFRYIGKD
GRKLIDGNDIVTGRAEFGIDVRLDGMVYAVIARPPVVGGKVKSYDATDTLKVPGVLKVAEIAPAPAPVVFNPLG
GIAVVAKNTWAAIKGREALKVEWDDGPHASYDSEAYKTELEAAARAPAKPARDDGKTMEVLASAKRKVVADY
YLPHIAHATMEPPVAVARIVDGRCEAWAPIQAPENARKTVADKLGLKADQVTVHVTLLGGGFGRKSKPDFVAE
AALVSKAMDGRPVKLQWTRDDDLHHDYFHAVSVQHLEAALDDKGMPVAWLHRSAAPTIRSTFVAGAKGLGV
NELGHTALNVPFQIPNVRVEAPEVEAHTRIGWFRSVYNIPHAFGVQCFVAELAHAAGRDPKDYLLELIGPARRIN
PTALGDTSNYGENPALYPIDTGRMRRVVELAAKGANWGRKLPKGHGLGIATAYSFMTYTAVAIEVAVDDKGEFQ
VVSADIAIDCGPQVNPERIRSQVEGAVVMGIGLAKHGEISFKEGRVVQTNFHDHVLLRHAERPQALRVHLAPS
DHSVPPGGVGEPGLPPVAPALANALFAATGKRIRRLPIRDQLSSAA

>HRGM_Genome_0384||HRGM_Genome_0384_CDS_02676
MTKNVSAQAISLSRRKLLQASGIAAGGLLLATALPFSRRSYAEQYVNKGPEDAPLDTPTALGAFLRIGHDGQITLIS
PKIEMGQGVQTGFAMMVAEELNVTLDQVRVQEAPPDEKLYGDKLLGFQATGGSSSTRSNWQPLREAAAAAR
VMLIQAAANQWKVSPDECRAENGKIIGPGNRELAYGALVDAAAKLPVPENVPLKKPEDYKVIGQPLRRLDTPG
KVDGSAKFTIDLHVPGMKIATVSACPVVGGTLASVDDRHARAVPGVRDVVKLENAVAVIGDHMWAALKGLKA
LEIQWGLGPNAGIDSAQIERALHAAFDREGAIAAEVGDINKAIAGASSKIEAEYEMPFLAHATLEPMTCVAQVR
EDACELWVGTQVPVMAQQAAAKATGLPPEKIIVNNQLIGGGFGRRLEADFIGQAAAIAKQVDYPIKLVWTREE
DTAHDLYRPHYIDRFSAGLDANGMPVGWSHTIVGASVMARFAPAAVPPNGLDADAVEVSNKPVYSLPNLRVR
YVPEAPKAILNSWWRGVGPLHGAYVMESFIDELAYAAKQDPVDYRMKLLGEHPRAQAVLKLAAEKANWSQKL
PAGHGRGIAVQEVFGSYLATVVEMQVDAQNGIHITRLICIADCGEVTNPTSVHSQLEGGTLFGLSAALYNEITVK
NGHVEQSNFHDYRQLRMSDAPPVETHIIPSHEIPGGIGEAGTAMIAPALVNALYAATGKRLRRLPVVRAGYHVA
>HRGM_Genome_1533||HRGM_Genome_1533_CDS_03386
MNAKTTKPRSGRRRFLLGALGIGGALVVGWGVMPPRSRVGDPGIFPEHNGEIALNGWIKITPEGDVVLAMPR
VEMGQGIHTALSMLAAEELDIPLSRVRIESAPVERIYGNVVAMGDSSLPLHPDSADKTWARALHWIMAKSARE
IGLIITGGSSSTADGWQPVREAAATARAALVQAAAREWNVPAADVSIREGQLIGPGGKQSTFGEMAKSARGISA
PSNVTLKPASQFRLIGKPAPRNDLAAKTDGSARFSIDTRLPGMLYAAVVMCPVFGGKLKTFQSKAALGMPGVR
YVVPFEGAGGGAPGVAVVADHYWQARQALATLEPVWDNGPHAKLDSAGIRQQLVSALDSDKGGFTYRSMG
DGLKAFDRADGATIVEAEYSAPYLAHATMEPINCTAQVTADGVHLWAPTQVATLAQLVAARAAGVSGDKVQID
IPLIGGGFGRRLESDFISQAVTIATKTEGKPVQVIWSREEDVRHDFYRPQAIARLKARVESGKVTAIASRSAGQSIL
AGELDRLFGAPSVGIDRYTAEGLFDLPYEIEHEHIAHLAVDLPVPVGFWRSVGHSYNGFFMEGFLNEVAAAAKL
DPLAMRRNLLKDHPRELKVLDTAAQAAGWGQPLAPAADGAPRARGIALHPSFGSVVAQVVEVSMKDGKPRV
HRVVCAVDCGTVVNPGIVAQQMESAVIFGLTAALYGRIDIKDGQVVQSNFPDYPALKMVETPVIETHIVPSTAEP
SGMGEVGVPPIAPAVAHAMAQLTGKPVRQLPMV
>HRGM_Genome_1580||HRGM_Genome_1580_CDS_03395
MKRSFPDDLIIGNLSRRGFLKGVGATGVLLVAANWGWRDALAAEKKAFGADAMPHGWVDNPKIYVSIDRDG
TVGIVCNRSEMGQGVRTSLAMVVADELEADWSRVKVIQAPGDEARYGNQDTDGSRSMRHWFEPMRRCGA
AARQMLEQAAANQWKVPLGECRAEQNKVLHAPSGRSLSFGELAEAAAGLEVPARDKLLLKKPEQFRYIGKDV
ARAIDGADIVNGRAGFGFDARFDDMLYAVVARPPVYGGKLKRYDAAAALKVPGVVKVIEIEGRPIPSEFQPLGG
VAVVAQNTWAAIKGREALVVEWDAGVNGGYDSVAYRKQLEEAARKPGKVVRDSGDAAALFAKGGDVVEAEY
YLPHLAQAPMEPPVSTAWYKDGACEVWAPTQAPQVTRERIAERLKLPFDKVTVNVTLLGGGFGRKSKPDFVLE
AAILAKAFPGRYLRVQWTREDDLHFSYFHTVSVERLQAVLGADGLPQAWLHRSVAPSITALFGPDSKHQGAFEL
GMGLTNLPFAIPNVRLENPEAPAHTRVGWFRSVSNIPHAFAIQSFVGELAAKAGQDPKDYLLKLLGPARRIDTAE
LGDSWNYGESPERYPLDVGRLRGVIEEAARQSGWGGELPRGRARGIAAHYSFVTYVAVVIEVEVKDDGALLVH
KATIAADCGPQINPERIRSQLEGACVMGLGLAALGEISFKDGKVQQDNFHQYELARMPLAPKAVSVHLLEPDG
DLPLGGVGEPGVPPIAPALCNAIFAATGKRIRELPIRNQLQGWRKA
>HRGM_Genome_1580||HRGM_Genome_1580_CDS_04978
MNSKIDLSNALPGSRRGFLKGAAVVGLTIGFQWSGARRALAAALPDAGFAPNAFLRIAPDDSVTVIAKHVEMG
QGAYTGIATIVAEELDADWSKVRVESAPADAKRYANLAFGTMQGTGGSSAMANSWMQLREAGAKARAMLV
EAAARQWQVPAAELRTRDGFVEHPTSQRKASYGSLAAAAAELPVPEKVQLKDPKDFRLIGHQAPRVDVPGKT
DGSAQFTLDVSLPGMLVALLQRPPLFGATVKSFDATATRAIPGVVEVVQVPHGVAVVAKGFWAAKQGRDALKV
EWDESKAEKRGSEALMAEYRKLAEQPGKPARRDGDAAKAVAGATRRIAASYEFPFLAHAPMEPLDAVVRLTAD
SCEIWAGDQFQTVDQGNAARTAGLKPEQVKINTLYAGGSFGRRANAWSDYIVEAVSIAKALGANGVPVKLQW
TREDDIHGGFYRPMYYHRLEAGLDADGKLVGWQHRIVGQSILEDTPFAAVMVKDGVDATSVEGAANLPYAVP
NVSVELSTTQVGVPVLWWRVVGSSHTVYAVEAFIDEAAQAAGKDPYLFRRDLLAEQPRLRGVLELAAEKAGW

DPSRPLPAGRGRGIAVTEAFKTFVAQVVEVSVDKDGKLKVERVVCAVDCGIPINPDVIAAQMEGGIGFGLGAIL
HSAITLKDGKVEQNNFDGYQVLRIAEMPKVEVHIVPSGEAPTGVGEPGVAPIGPALANAIFAATGQRLYNLPFPT
SFAKA
>HRGM_Genome_1581||HRGM_Genome_1581_CDS_00719
MADLQQRASREPEDAGDGILNVSRRHFLRGAGGLALGIYFAPLLGRFGDPQAAAAKAFEPNAFVSIAPDGTVT
VIAKHVEMGQGSYTGLATLLAEELDADWSKVRVEGAPADAKRYANLAFGTLQGTGGSSAMANSFEQMRKAG
ATARAMLVAAAAQQWKVPAEQIEVHDGVVEHKASGHKAGFGQLAEAAAQQAVPTDVKLKAPEDFKLIGQVK
LPRKDSQDKTDGQARFTQDVHLPDMLVAVVAHPPRFGGVPAKVDDSKARAVPGVVAVVQFPGSDSRFAGVA
VLAKNTWAARQGRDALQVAWDESNAFRMGSAEIFARYQELAAKPGVVARNEGDIAKALDKPAKLIEAQYQFP
FLAHASMEPLNCVVRLSDGACEIWNGEQWQTGDQMAVGQLLGIAPEKVSITQLYAGGSFGRRANPHSDYVLE
AVSIAKAAREHGHKGPVKMVWTREDDTRGGYYRPAFLHSARLALDGQGNLVGWEQHLVGQSFIVGTPFEKA
MVKDGIDQVAVEGAADLPYAVPNLRVEQTLVPEVKVPTQWWRSVGHTHTAYSTETLIDEAAVAAGKDPYEFRR
ALLEKHPRHLGVLDLVADKAGWKQPLKAGGEGEKRGRGIAVHESFGSFVAQVVEVTVKADKSFRIDRVVCAVD
CGLAINPDVIRAQMEGGIGYGLAMALHSAITLKEGVVEQSNFHDFQVLRINEMPAVEVHIVPSSEAPTGVGEPG
VPPAAPALANALAAATGKRIRNLPIGNQLQA
>HRGM_Genome_1582||HRGM_Genome_1582_CDS_03427
MNEQPFSPSRRQLLKVAGLGFGALVIGFSLPFAGRSFAEQVLPEGPEDGTLPTATALDAFISIDRDGKVTFTVPKIE
MGQGAQSGLAMMIAEELEVPLEGITLKEAPPNEAIYNDSLLNFQATGGSTSIRANWEPLRRAGAAARLMLIQA
AAQRWQVPADGLRAENGRVHGPDGQSLGYGELVEAASQLPIPEDIPLKPASEFKVIGKPLRRLDTPSKVDGRAR
FTIDLAVPGMKYASIRACPVVGGSVANVDDSAARRIPGVVEVVRLGNAVAVIGEHTWAAFSGVRALQIEWDFG
EHAGLDSEQMERAIRDALDQPGALANEVGDIQNALDNAARTVEAEYEMPFLAHAALEPMGCVAQVRPDAVE
LWVGTQVPVRAQTAAAEEAGRPPEQVIVNNQLIGGAFGRRLEVDFIAQAVAIAKQVDYPVKLTWTREEDTAHD
MYRPHYIDRFAAALDGDGRLLGWRHSIAGASVLARFAPEAVPENGLDGDAVEVAQHPIYALEHLRVNYIPVPPK
AIHQSWWRGVGPLRSTYMLESFIDEVARSVERDPVDYRLQLLGQQPRAQAVLRLAAEKSGWGEALPTGHGRG
VAVQEVFGSYLATVVELEVTEGKGIRIKRLVVAIDCGLVMNPVSVKSQIEGGTLFGLSAALFNEITVRDGRVEQSN
FHDYRQLRISDAPPVETYIIDSTEAPGGVGEAGTAMIAPALVNALHSASGERIRRLPLARAGYHVLGGRPA
>HRGM_Genome_1705||HRGM_Genome_1705_CDS_02351
MRKQPISSKFALGEEPTNLSRRRFLLASGSVAGALVLGIGLPSGQARAQAAQQPLSPGTRVPAFLEIRPDSSVRFL
SPFIEGGQGIFTAMAQIVGEELDADPATFVVENAPAGDEYKVMDSGMRITGGSQSVRTSYTTMRRLGALARH
MLLQAAASALAVPLASLRTEPGKVVHDASGQTLTYGELASRARDLPVPPADAVSLKDPAQFRWIGKPVKRLDA
WEKSTGQAVYTIDCRVDDMLHAAVQHAPRFGMTPGAWRNEAQVRAMKGVHSVHRLPDAVAVVAERWW
QAKRAVEALQVEWLAPADAELMPPDFSSDDFARTLANEPGDGQEAEVKGDLAAGMSAAKTTLSATYHSQYLN
HAQLEPPSALARFNDDGTLELWIPNQAPEMFQGEVAKRTGLDASKILIHSPLLGGFFGRHFVYDGAMAWPQA
VELAKAVGRPVKLIWSREEEFLCDTHRPMAAVRFRAGLDDEGYPLALEAVSICEGPTEGISGKQGDKLDPTATEG
LTGKSYAIPHLRIAQIYKKGPVKLGYWRSVGNSMNDFLYESFLDEIADHGRLDPFELRLRLLKGNARLTTLLHAVA
DLSGGWKPGPYTAEDGSRRARGVAMASPFGTEAAAIAEVSIENGQVRVHRIWEAIDPGSIVNPAIVEAQVNGA
VALGLSQVLLEEALFEKGQPVARNFDLYPILPPSRMAQVEVRIIESGAKMGGIGEPPLPAVPPAVTNAVSRLTGQR
IRSMPLSRYNFT
>HRGM_Genome_1715||HRGM_Genome_1715_CDS_05038
MAFTLDNHADRHQASLVNVSRRRFVQGAGVLVLGWQLPGVLAAQPADKADPFVPNAFLSIDTQGRVTVYAK
HVEMGQGAYTGLATLVADELDADWAQVRVEGAPADTARYKNLAMGIQGTGGSTAMANSFKQMREAGAAA
RAMLVGAAAQAWKVDPATITVREGVVRHAASGRQAGFGELAAAAAAQPVPARPRLKSPKDFRLIGKASLRRT
DSADKVHGRARFTQDVKLPGMLVAIAAHAPRFGATVAGYDASAALAVPGVVAAIPFEGSAHRSAGVAVLARNT
WAARTGRDALKVRWDESRAYTEGSDELLARYRQAASRPGLPAATRGNVETAFAKAAKVIEADYEFPFLAHASM

EPLNCVVQLGDGRCEIWNGEQFQTMDQKAVADYLGIAPGHVTINQLYAGGSFGRRASPHADYVVEAVAIAQG
ARKAGFDVPVKMVWTREDDMRGGYYRPAYLHRARLALDERGRLQGWQVRVVGQSLIKGSVFEEFMVKNGI
DATSVEGLSDLPYGIPNLSVELHTPDDVRVPVQWYRSVGHTHTAFTAETLVDEAAHAARRDPVALRRELLKGHP
RHLAALETAVRAAQWDRPLAAGKSGVRRGRGVAVHESFNSVVAQVVEVSVDAGGELKVDRVVCAVACGLAIN
PDVVKAQMEGGIGFALSTALHGAITLDKGAVVQSNFHDYPVLRINEMPRVEVHIVPSQDDPTGVGEPGVPPLA
PALANAIFEATGERVRTLPIRTPLRKAV
>HRGM_Genome_1736||HRGM_Genome_1736_CDS_03191
MRRMSFSTVADEVVPGRRKFLKLMGGVGAGLTLGFSLPVSNQAQAADMTSSAVPFEPNAFVRIGSDNKVTV
MIKHIEMGQGTYTGLTTLVAEELDADWAQMVAEGAPADASRYNNTFWGPMQGTGGSTAIANSYIQMRTAG
AAAKAMLVAAAAQRWNVDASQIQVRAGIVSHGNKKATFGELALDAAKQPVPDEASVKLKTPEQFVFIGKRKV
SRKDTGKNNGTAIFTQDIKLPGMLTAMVLHAPKFGAKVKTVDATQAKASPGVVDVVTIPTGVAVLAKDYWSAK
KGRDLLSVTWDESQAFTKSSAQIMTEYKTLSQKEGLSARNEGDAVSALKQADSVIDNTYEFPFLSHSPMEPMN
CVAQVSDQGCEIWNGEQFQTVDQMNIAQLLGLKPEQVTLHMLLAGGSFGRRANPHSDYLIETVEIARQKKGT
PIKMVWSREDDTQVGYYRPAYVHHIQAGLDANGNISSWKQHIVGQSILTGTAFEAFAVKEGIDGTSVEGASNIP
YAIPNLSIQLTTVKDGPTVQWWRSVGSTHTAFAVETMIDELAANAGKDPVEMRMQLLAKHPRWQGVLKLAA
EKAGWGKTLPAGSGMGVAVHESFNTFVAQVAQVSVKNGQISVDKVVCAVDCGVAVNPDVIAAQMEGGIGF
GLSPTLMSAITLGEGGMVEQSNFHNYQVLRMNQMPDVEVHIVPSAEAPSGVGEPGVPPIAPAVANAVAAAT
GQRLHTLPLKLATS
>HRGM_Genome_1871||HRGM_Genome_1871_CDS_00541
MAITRRTLIKGSALAVAAMLLPISTRSLAAVVSQDVAPPDSQHELNDWIWIDRDGRIVIGVSQCEVGQGIYTGL
AEVVAAEMDADWAQVTVKFVTGRDAYRQVAGGEAFAQFVAASTSMTKFYQRARLAGAQARDFFLRAGAKH
FALSPSQCRTEKGWVLEKGGKRKVAYGDLVRYAAELPLDPQPALKSEAQERESVIGKPLLRVDTPEKVDGSAIYGI
DIDLPEMLIGVPWMVPDLSGKLVAVRNERQIRAMPGVVDLVLTRQWSMNNMVGLDHDMSLNTVIVVAASY
WQAKKAADLLEVDWLPGAGKALTDSAIAAENLAMLDGDTLVPAVDRGEASALIRGVEQGSRLHEARYSAPYV
AHATLEPCNATSHYGEGRIETWGPFQGQDMVRNVLAKMFGLKPTDVVVNTTYLGGSFGRKYLPDAVMHATA
ASRAVGKPVKVVYPREIDMRHEYYRPACISHYRALLDENGYPQALWARYAGQSLFWQMRRETVNEAGGWDE
SMVECVYSTPYRIPHLKVEAGIVEQPISLSYLRGVGSVASLFFLESFISELSHKANRDEYSFRRHLLQDSPEALRVLD
ATATAAGWQHEPPSGVSRGMACNIWVGRNNAFTTYVGLVVEIAIQEGRLRVLRAVCAIDCGKVINPNLVRANV
EGGIGFALTTCLHSELHFERGGVVEGNFDRYPLLAIAEMPKVEVVILDSARAPQGCGEVSTAVVAPAMASALHK
ATGKTYRTMPFPREFSSV
>HRGM_Genome_1904||HRGM_Genome_1904_CDS_01701
MRDFLNGYVPEEALLKNTTQSGTSRRNFIKLMSTAGAGLTLGVHLPSVAAGDKETAGTSFVPNAFVRVGSDDT
VTILIKHLDMGQGITTGLATIAADELDADWSKVTTEAAPANANQYNNLLFGPVQGTGGSTAIANSFMQLRMA
GAKAKAMLINAAAKVWEVSPSEIMTSSNTLSHNASGKSASYGEMAELAALQTVPSDEQVTLKTPEQFTIIGKTG
TLRKDQGKSNGTATFTQDVQLEGMLTAVVAHPPAFGAQVVSFNATAAKQSPGVVDVVQIPNGVAVIAKDFWS
AKTGRDKLTIEWARSAFNKSTEQLKKEYTALAQRPGQPARNEGDANKVLGASDDVIEATYYFPYLAHAPMEPM
NCVVLVGDGKAELWYGAQLQTIDQYAVAKVLGIKPENVTINTLFAGGSFGRRGNPHSDYVVEATMIAKTKPGV
PVKLVWTREDDMQAGYYRPMYVHKIRGAVDADGNLSAWEQRIVGQSIAAGTAFEQFMVKDGVDSSSVEGA
STLPYAIPNLAVELHTVQQPVPVVWWRSVGHTHNAFSTETFFDELAHKAGKDPVELRRTLLKNHPRHLGVLNL
AVEKSDWGKPLAKGKGRGVAVHESFRTFVAQVVEVTVENGQITVDRVVCAVDCGVAINPDIIKTQMQGGIGF
GLSPALVSEITLQNGATVQSNFHDYQVLREMQMPEIEVHIVPSAEPPTGVGEPGTPPIAPAVANAVFAATGKRLY
DLPMKMS
>HRGM_Genome_1933||HRGM_Genome_1933_CDS_00864
MTHHSDSATRIVNVSRRGFLAGMAAGAFILAVTGAQRVRAQEKKFGADAMPHGWRDDPKIFIAIDPDGTVTV

TCHRQEMGQGVRTSIAMVIADELEADWSKVRVVQAYADEAHFGNQDTDGSRSLRHQFMPMRRAGAAART
MLEQAAAKQWGVPIAQVKAGHHAVVHEATQRTLGYGELAAAAADLPVPSRESLKLKDPSAFRYIGRDTISLVD
NRDITTGKAIYGIDARAEGMLHAVIARPPVYGGAVASYDATETMKVPGVVKVVKIDRPAVPSEFQPIGGVAVIAR
NTWAAIRGREKLKITWDDGPNAGYSSDSYKAELEAAARKPGKVIRNEGDVDEAMAKAAKRVTAEYYIPHLAQA
PMEPPAALVQIKDGRCEAWACVQAPQVTRTRLAEKLGLPEDKVKVNVTLLGGGFGRKSKPDFVLEAGLLSQA
MDGAPVQVTWTREDDLHHSFYHTVSIEHLEAGLDERGLPAAWLHRTVAPTIGSIFAPDPKHELPMELGMGVT
NMPFAIPNIRSENPEAQAHVRIGWYRSVSNIPHAFAIQCFVSELAHAAGRDPKDYLLDVIGPARLIDPTEQGDV
WNYGEDPRLYPIDTGRLRRVVETAAKGIGWGRQLPKGRGLGIAGHYSFVTYAAVAAEVDVGGDGKLAIPRVDIA
VDCGPQVNPDRIRAQMQGAVVMGVGMANASEISFRNGRAEQDNFDSYMVPRIFDAPKEINVHLIPGESYAQ
PLGGVGEPGLPPVMPAILNAVFAATGKRIRQVPIGDQLRA
>HRGM_Genome_1933||HRGM_Genome_1933_CDS_07409
MIRESLSSPVTGASRSVLSRRQFLQAGAAAGGGLMLSLSLPFGYGDAVASGADAFVPNAFIRIDSDGRIVLTMPS
VEMGQGTYTAIPMLIAEELEADLSQVRLEHAPPNERLYGNPLLGGIQATGNSNAIRAAWQPLRHAGATARTML
VAAAAQRWNIDPASCRAQSGEVLHPATGRRFTYGELAPDAARLPVPESVVLKRSEEFRLIGTPAKRLDTPAKVN
GTAVYGIDVRPPGVKIATLAQSPVFGGRVRGVDDTAAMAVKGVRQIVRLDDAVAVVADHMGAAKKGLAALVIE
WDDGPHAGLTTREIVAELETATLNPGAVAQKIGNVDQAMASAVTRIEAIYQVPFLAHATMEPVNCTAHVRPDG
CEVWVGTQAIARVQAAAAKAAGLPLDKVVVHNYLIGGGFGRRLEADYVVRAIQIAQHVDGPVKVVWTREEDI
QHDMYRPYWLDRISAGLDEKGMPVAWSNRFAGSSVLARWAPPTFNNGLDPDTIEGAIDLVYDLPNLHVEYVR
VEPPGIPTAFWRSVGPSHNVFVTESFMDELAAAAKQDPVAYRLALLGKTPRARAVLELAAQKADWEQPLPERV
GRGVSLQHVFATYMAHVAEVEVAKDGVVRVRRVVCAVDCGTVVNPDTVRAQIQSAVMFGITAALHGEITVKD
GRVEQSNFDTYPILRMNEAPAVEVHIVPSSEPPGGMGEAGTSAIVPAVANAVFAATGKRLRKMPIDVAAQEGT
K
>HRGM_Genome_1933||HRGM_Genome_1933_CDS_05588
MTIEHSLSRRQFLVSSAAVGGLALGFTLPLGAQPAAQTESEVNAWVVIKPDETVVVRIGRVEMGQGTLTGLAQL
VAEELDCDWDKVTTEYPTPGQNLARSRVWGNFQTAGSRGIRDSHDYMRKGGATARHMLIQAAANGWQVPA
TECTVSKGVITHAPSGRTTTYGKVADAAAKLEAPKDVQLKDTKDWMIAGKPVKRLDTAPKINGSQIYSIDFTRP
GMLNAAIRACPVFGGKLKGFQATAVERMPGVKKVVQVGDNAVAVVADTWWRAKTALDALPVEWDPGPNA
ALSSETITAMLNEGLDAQQTFIGNQAGDVKAAIAGAAKKVEAVYGYPYQHHATMEPMNATALWTSDRCEVW
TATQNGEAALATAASAADLPVGKCDVYRLHLGGGFGRRATSHDFVRQAVLIAKQMPGTPVKLIWSREEDMLH
GWYHPVTQCKLTGALDDQGNLTGLHMRISGQSILSTVNPQGLQNGRDPATFQGLNPSGTEGVFGYSIPNLLID
HAMRNPPIPPGFWRGVNNNQNALYLECFMDELAHAAGQDPLAFRRKLLVQHPKHLAVLNAVAERAGWGSP
APQGVYRGLAQHMGYGSYVAACAEVSVANDGKLKIHRIVAATDCGHAVNPQQIAAQVEGSFVYGLTAMLYGEI
TIKDGKVEQENFDTYPMMRMDEMPEVETIVMPSGDFWGGVGEPTIFVAAPAVLNAIFAATGKRIRSVPLSKA
DLRKA
>HRGM_Genome_1933||HRGM_Genome_1933_CDS_04204
MTTALRVSRRAFLAGSAAAVGGLSFGFHIPFSDAVAQEAAPEINAWVVVRPDDTVLIRIARSEMGQGTLTGLAQ
LVAEELDCDWSKVTTEYPTPGQNLARSRVWGNFSTGGSRGIRDSHDYVRKGGATARQMLIQAAADSWGVPV
SECSAANSVIKHGPSGRTARYGEVAEKAAKITPPSDVTLKDPKDWKIAGKPLKRLDTAEKLTGKQVYGIDLKLPG
MLNAAIKDCPVFGGRVKSFDAAAIQGRPGIKKVVPVGESAVAVVADTWWRAKSALDELPIVWDEGPNATVSS
ATIAEMLKEGLDAEHAVDGNRVGDARAAIASAAKKVEAVYSYPFQNHATMEPMNATALWTPDRCEVWTPTQ
NGEAALAATAEAAGLHPSKCDVHKIHLGGGFGRRGAVHDWVTQAVTIARQLPGTPVKLIWSREEDMLHGRYH
PITQCKLTGALDDQGNLTGLHMRISGQSIIASIFPQNIQNGKDPAVFQGLNPGGAEAAIGYTIPNLLIDHAMRNT
HVPPGFWRGVNLNQNAIYLECFIDELAHTTGQDPLEFRRKLMTQHPRHLAVLNAVAERVGWGTPPPAGIYRG
LAQTMGYGSYVAACAEISVSDDGKLKIHRIVAATDPGHVVNPQQVAAQVEGSFVYGLSAALYGECTVASGRIEQ

ENFDTYPVMRIDEMPAVEALIVPSGGFWGGVGEPTIAVAAPAVLNAIFAATGKRVRSIPIKHADLRKA
>HRGM_Genome_4330||HRGM_Genome_4330_CDS_05893
MNLQVNQHVKLNRRAFVIGTATAGAGLALGLDLPFGGPAVVRAADGAPEVNAWVVIRPDDTVVIRIARSEMG
QGTLTGLAQLVAEELECDWSKVTTEYPTPGQSVARKRAWGDFSTGGSRGIRTSQDYVRKGGATARVMLIQAAA
NEWKVPAAECKVSNGVITHTASGKTTTYGKVAEAAARLEPPADVKLKDPKDWTIAGKGLKRLDTVDKTTGKMI
YGIDVKLPGMLNAAIKDCPVFGGKVKSFDEAKIANMKGVKKVVPVGDSAVAVVADTWWRAKTALDALPIVW
DEGDNAKVSSETIAKWLAEGLDNAQPAYVGNQNGDAKAAIASAAKKVEAVYSYPYQNHATMEPMNATVLYTP
DKCEVWCGTQNGEAAFAAALEASGLPAEKVDVHKLMLGGGFGRRGMTDYVRQAVAIAKQMPGTPIKLLWSR
EEDMQHGKYHPVTQCKLTGAFDADDNLVALHYRLSGQSILFSVRPEALQNGMDPAAFQGVAQSGEAAIGYSV
PNLLVEHSMRNPHVPPGFWRGVNVNHNAIYMECFMDELAQAVGQDPLEFRRKLMGKNPKHLAVLNAVAEK
IGWGTPAPQGVYRGIAQVMGYGSYVAGAAEISVTDGSKIKVHRIVASTDPGYVVNPAQVERQIAGSFVYGLSAL
FYGGCTVKDGRIEQTNFDTYNSMRINEMPKVEAVMVPSGGVWGGVGEPTIRDAAAGGVNA
>HRGM_Genome_4330||HRGM_Genome_4330_CDS_00127
MNKHVKNLAPETTDLSRRSFLVGTAATGLVLGYAGSGIDQALAAPAPANFEPSVWYSIAPDGLVTVTCGKADM
GQHVASTMAQIVAEELGANWKDMRVQLASNDPKFNDPVLGAQITGGSWSTMMNFDAMSRAGAAGRIALT
EAAAASMGVPASELVVRASTISHPKSKKSMSFADVVKSGKATKTFTADDLKAIKLKTPDQYTMIGVSVPQLDIPL
KVNGTAKYGIDTMLPGMVYGALVTPPVRYGAAVKSVDDSAAKKLPGFIKAVTLDDKTMTTTGWVVAVANTYA
QAKKAAEALKISYDGGPNAKASSEALLAEAKRLQGLSDSGQFFVKDGDPDAAFGTAAKVLEAEYTTNINIHAPM
EPMNATAEFKGDILHIYSGNQFATRSGAIAAGAAGIDPKFVVMHQTWLGGGFGRRLDADMMVPAVQAAKAV
GKPVKVIYSRENDMTMDFSRPLTFQKVKAGVDGDGKLVALSHDVVSAWPTQRWGIPDFLSPSVDKKGPLDAF
TVNGADFFYTVPNHYVRAIKNEMAHNATPSGQLRSVAPGWTFWAVESMVDEIAHATGKDPALLRVELLDGKG
KNDGGAQRLRNTLLAAMGLAGYGTKKLPKGEGMGVACVSSQERATASWTACVAHVAVAPSGEVTVKKLTVAT
DVGTQVNPDGIRAQVEGAALWGMSLALFEKATLKDGGIEQTNFDSYTPLRMSQLPEVAVSVIANGEKATGVGE
PAVTVVAPAIGNAVFNAVGARVRGLPITAEAVKAAMKA
>HRGM_Genome_4330||HRGM_Genome_4330_CDS_01871
MTLMDNLSERAADLSRRNFLRASAIAGGGLLLSVNLPFAGRESEAAAAAGDFAPNAFVRIGGDGKVVLTMPYV
EMGQGTYTSIPMLIAEELEIGLNQVQLEHAPPSDKLYANPLLGVQATGNSNAMRGAWQPMRKAGATAKAML
VAAAAKRWNVEPATCRAENGEVHHAASGRKLGYGELAADAAQMPVPENVTLKSPAEFKLIGTAAKRLDTPSKV
NGTAVYGIDARPPGVKVATLAQSPVFGGRVKRVDDAAAKAVNGVRQIVTLDDAVAVVADHMGAAKKGLAALT
IEWDEGPHAKLATADIARELEAATTKPGAVAQNIGDADNALAGAATKVEATYQLPFLAHATMEPMNCTVHVRP
DGCEIWVGNQALSRVQAVTAKLLNLPPEKVVVHNHLLGGGFGRRLEVDGVIRAVQIARQVDAPLKLVWTREE
DIQHDMYRPYWCDRISVGLDASGKPIAWNNRFAGSSVIARWAPPAFRNGLDPDTTEGAIDLVYDIPNFHVEYV
RVEPPGIPTAFWRSVGPSHNVFVTESMIDEMAAAAKQDPVEYRKALLGKSPRAKAALELAAAKAGWGGKLPA
GRGRGVSLQFVFGSYLALIAEVEVAKDGSVRVHRVVCAMDCGTVVNPDTVQAQLQSGINFGVTAALYGEITLK
DGRVEQTNFDSYQMLRIDQAPAIEVHIVPSTEPPGGMGETGTSGIVPAISNAIFAATGKRLRKMPVDPAVLKQA
>HRGM_Genome_4330||HRGM_Genome_4330_CDS_01596
MISEISRRSVLRAGVAIGGGLLLEMRLPEFAYGSERLVDGGEFSPNAFIRIDGRGAISFIMRSVEMGQGIYTAAA
MLIAEELEVRLDQIEALAAPANEALYTDPILGQQETGGSASTRSSWIPLRQAGATARVMLIMAAAKRWGVVFE
DCVARQGMVTHSASGRSASYGELAEDAARQPVPDNVPLKPASEFRLIGSSAHRLDSAAKANGTATFGIDIKVPG
MKIGTVAACPVRGGRLVSIDEAAAKRVSGVRDVVRLDDLVSVIGDNMWAATQGLAAANPHWDEGPNKSVST
EDLIKDLDTASRQPGVVAKQEGDPVSAIAGSVTRIDAVYQLPFLAHAPMEPINTTIHIRADGADVWVGTQVPVR
AQLAVALVTGLPQESINIHNQYLGGGFGRRLDVDSIHQAARIAKQLPYPVKLIWTREEDIQHDLYRPYYYDRVSA
GLDAQGNISGWTHRVTGSSVAARWRPSRMQKDGHLDPDAVLGATETPYELPATLVDYVRCEPRVFDTLWWR
GVGQTHNVFVVESLMDELAAAASQDPVDFRRKFLKKNSRGRSVLDLAVEKSGWGKPLPKGWGRGVALQFSF

NTHVASVLEAEISGGEIRLRRVHIAVDCGPVVNPNIIEAQMEGGMIFGLTMALYGEITVTNGRVDQSNFNDYR
MLRMNQAPEISVHLVNNPDAPIGGIGETGTVAAAPALANAIYSATGRRLRRIPFAQQVAQTQ
>HRGM_Genome_4331||HRGM_Genome_4331_CDS_05548
MNALLKIDLSAVMPLIPDQGVTNLSRRRLLGLGAGAFVLGTLLPAFGARAQAAAAVKPGTRVPAFLVIGQDNTV
KLLSPFVEGGQGINTGLAQIIGEELDVHPSRFEVECAPPGPDYAILNGLRLTGGSFSTRSSYEVMRRLGASARDM
LIRAAAAKLNVTADTLNTDDGYVVHAGSNRRVTYGELAEQALTLTPAENVALRDPATFRYIRQPMARLDVRAKS
TGKAVYAIDQKLDGMLYAAIQHAPVLGTEPERVSNESAVAAMPGVHAVHRLPGAVAVTADSWYRARKAVETLE
VTWSKAPATGFDAVAADYSSTGILAALKASDASGLSAEKDGDVAAAFANAAKIVEAEYDAPYLAHGQLEPPSSM
ARFNADGTLELFVPNQMPELFQSIAAKVGDVAPDRVILHSPMLGGFFGRHFAYGSSNPFQQAILLAKATKRPVK
VLWSREEEFKMDALRPLSFSRFKAALDKDGIPIAIKVRTVGEGPIGRWFGVTVGGKVDSSAVEGLVEKPYAIANR
SMEYVKFAHPVTIAFWRSVGHSMNDYFYEGFLDEIADAGGKDPYQLRLALLENKPRHLKLLETVARMSGGWK
RGPYEAEGGKRARGVALASPFGSETATIAEVSLQKGEVRVHNLWIAFDPGSIVNPAIITSQVESAAALGLSAALFE
ELVYKDGRRQQHNFDDYPILSRSAMPSVHVEIVESGAPMGGVGEPGLPGIAPAVVNAVAALTGRHVRSLPLAK
AKLGV
>HRGM_Genome_4331||HRGM_Genome_4331_CDS_07067
MRFNRTTSLGSAATLSRRNFLVNTAVAGGGLLLSFSLPSGQSMGSVAGAFEPNAFIRIGRDGLVVLTMPYVEMG
QGTYTSIPMLVAEELEVGLAQVRLEHAPPDEKVYANPLLGVQATGNSNAIRGAWKPLRQAGATARVMLVEAAA
RRFGDDARHCRAEAGEVIHMPSGRRLKYGDLVADAAMVPPPGKVVLKNADEFKLIGTPASRLDVSGKVNGSAI
YGIDVRPPGLKIATLAQSPVFGGRLKNVDDTAARAVKGVSQIVRLNDAVAVVADHMGAAKKGLEALEIEWEEG
THAGLSTQDIARGLEDATLKAGPVAQSVGDAARAMATAATRVQANYHLPFLAHAAMEPMNCTVHFRGSECEI
WVGTQAIARVQAAAAKAAGLAVDKVIVHNHLIGGGFGRRLEADGAVRAVEIARHVDGPVKVVWTREEDIQH
DMYRPYWVDRIEAGLDKSGRPIAWINRFAGSSVIARWLPPAFSNGLDPDTTEGAIDLVYSLPNFHVEYVRVEPP
GIPTAFWRSVGPSHNVFVTESFIDELAAAARQDAVAYRRALLDHNTRARAVLDLAAEKAGWGTALKKGHGRGV
ALQNVFGSYLAQVAEVEVANDGTVRVHRVVCAMDCGIVINPDTVQAQVQGGVMFGITAALYGEITLQSGRVK
QANFDTYQMLRIDQAPAIEVYVVNSKESPGGMGEAGTSGIVPAVANAVFAATGKRLRKMPITPDALKA
>HRGM_Genome_4331||HRGM_Genome_4331_CDS_07593
MNQHVKAAPSMAPDLSRRSFLVGTAATGLVLGYAGLADGALAATTPASFEPSVWYSIAPDGLVTVTCGKADM
GQHIASTMAQIICEELGAAWKDMRVQLASNDPKFNDPVLGAQITGGSWSTMMNFDAMSRAGAAGRIALTE
AAASVMGVTAKELVVRDGVVMHPKSKKQMSYAEIVKSGKITKSFTADELKALTLKTPDQYTMIGVSVPQLDIPA
KTNGTAKYGIDTMLPGMVYGKVVTPPVRFGATVKSVDDSEAKKVPGFIKAVVLDDKTGSTSGWVVAVASTFAN
AKKAADALKISYDKGPYANVSTDSIITEAMRLQAQDDAGQFFVKDGDANAALAGAAKVLEAEYTTSINIHAPM
EPMNATAEFKGDILHIYSGNQFATRSGAIAAGAAGIDPKYVVMHQAWLGGGFGRRLDADMMVPAVQAAKAV
GKPVKVIYSRENDMTMDYSRPLTYQKVKAGLDSNGKLIALSHDVVSAWPTARWGIPDFLTPSVDKKGPLDSFT
VNGADFFYTVPNHHVRAIKNELAHNATPSGQLRSVAPGWTFWAVESMIDEIAAASGQDPAQFRIALLDGKGK
NDGGAQRLRNTLLAAMGLSGYGAKKLPKGEGMGVACVSSQERATASWTACVAHVAVADNGAVTVKKLTVAT
DVGTQVHPDNIRAQVEGAALWGLSLAMYEKATLKDGGIEQTNFDSYTPLRMSQVPEVAIAVIANGEKATGVGE
PAVTVVAPALGNAIYNACGARLRSLPITAEAVKANMKA
>HRGM_Genome_4331||HRGM_Genome_4331_CDS_02394
MNKHVSPRLNRRSFIIGTAALGGGLALGLDLPFGGPQVVRAADGSPEVNAWVVIRPDDTVVIRIARSEMGQGS
LTGLAQLVAEELACDWSKVTTEYPTPGQNVARKRVWGDFSTGGSRGIRSSQDYVRKGGAAARMMLIQAAAD
QWKVPVAECTAANSVITHKASGRTTTYGKVAEAAAKLTPPADVKLKDPKDWTLIGKGVKRLDTADKVTGAMIY
GADIKLPGMLNAAIKDCPVTGGKLKSYDEAKIAGMKGVKKVVAVDGTAVAVVADTWWHAKTALDALPIVWD
EGENAKVSSASIAKWLAEGLESGPAFVGNENGDAKAALASAAKKVEATYNYPYQNHATMEPMNATALYTPERC
EVWCGTQNGEAAFAAVLEASGLPADKCEVHKLILGGGFGRRGQTDYVRQAVQIAKAMPGTPVKLLWSREED

MTHGRYHPITQCKLTGGFDADNNLTALHMRISGQSILFSLRPDALVNGKDPATFQGLAPSGEATIGYSVPNLLIE
HSMRNPHINPGFWRGVNVNQNAIYLECFMDELANAVGQDPLEFRRRLMSKNPKHLAVLDAVAEKIGWGSPA
PEGVYRGLAQLHGYGSYVAGAAEISVIDGTKIKIHRIVASTDPGYVVNPAQVERQIAGSFVYGLSALFYGGCTVK
DGRIEQTNFDTYNSMRIAEMPKVEAIMIPSGGFWGGVGEPTIGVAAPAVLNAYFAATGKRIRSVPLRDQNITFA
>HRGM_Genome_4331||HRGM_Genome_4331_CDS_04615
MNRIIRDHSSTTSAGRPVNLSRRGFLGAAAGALVLGFDLPGTKAVAQGAAPVAKAPNVAAFLEIRPDSTILLRSP
FIEGGQGISTALAQIVGEELDADPATFVVECAPPGADYLVVGGARFTGGSFSVRASYATMRRVGASARQMLLQA
AAARWRVPADSLSTEPGRVRHAASNRTLAYGELAADAAKLPVPETVALRPEKDFRWIGKPFARLDARDKSTGK
VSYGIDLKVDGMLQAAVQHAPRRGQEPGAFANEAEVKAMPGVHSIHRLAGAVAVVAERWWYARRAVETLKV
TWTEPAAGAQGVVPANFSSEGRKAQLAAMPGPGFDAEKVGDVTAGLAGAGRVITASYDAPYLVHGQLEPPSA
IARWNADGTLDLWLPNQAPEMFQAAAAKVAGITPDKVKLHSPPLGGFFGRHFVYPPANPFPQVIQLAKAVGR
PVKVIWSREEEFSRDAMRPLGFARFKGGLDKDGIPVAIEAEAVGDGPLARWFGRKPDTADSSAVEGIAEKPYAIP
HRRVAHVLVEDPNVLGFWRSVGHSMNDFFYETFFDELADAGRQDPYQLRLRLLADKPRHKTLLEAVGELSGG
WKRGPFTAADGSKRARGVAMASPFGSEVATIAEVSLKDGAVRVHDVWVAIDPGRIVNPAIIESQVNSAVALGLS
SALLEEIVFVDGAPQARNFDAYPILPPDRMPRVHVRIVESGAPMGGIGEPGLPGVPPAVANAVAALTGQRVRSL
PLSKVKFEEAAGRT
>HRGM_Genome_4331||HRGM_Genome_4331_CDS_00990
MTIIANPDSATSASRRGFERHLKVENVSRRAILQTLGLAGGFVLAAPLLSRPAFAAYETGAGKMPHGTVVDPKIF
VSIAPDGIVSILAHRSEMGTGVRTSLPLIVAEEMEADWTKVRVVQAPGDEVKFGNQDTDGSRSTRHYLLPMRQ
IGAMARAMLEAAAAKRLGVPASEVKAVNHEVVHSASGKRLGFGELAADAANQPVPAVDSVQLKSPKDFRYLG
KGQVSIVDLHDITVGKARYGADVRLPGMKYAVIARPPVTGGKVKSFDSAEALKVPGVEQVLEVKGWPWPSKF
QPLGGVAVVARNTGAAIKGRDALKVEWDDGPNAAYDSVAYRAELEAAARQPGLVVRQEGDVEAALKSADKIV
TGEYYLPHFAHASMEPPVAVADVKGDKAEIWAPVQSPGGTREDVAKTLQLPPENVTVNVTLLGGGFGRKSKC
DFALEAALLSKTLGAPVKVQWTRDDDIQHDFLHTVSVERIEAGLDKSGKVVAWRHRSVAPTILSTFAAGADHAA
PFELGMGLVDMPFEIANIQCENPAAKAMTRIGWFRSVSNIPRAFAVQSMVGELAHATGRDQKDMLLELIGTP
RVVKLASVKDLWNYGEPYESYPIDTGRLRRVVEFVAEKGNWGRSLPKGHGLGIAAHRSFVSYIATIVEVSVDDKG
KLLVHQVDSAIDCGVFVNPERIQSQLEGAAIMGLSLAKYGEVSFKNGRVQQRNFDDHPVVRIDEAPLVTNVHIV
PADADTPPSGVGEPGVPPFAPALANAIFAATGKRLRALPIGNQLAT
>HRGM_Genome_4332||HRGM_Genome_4332_CDS_01586
MGFLLERRQFLAGISATAALAYLDLPVLAAQSETSLGETGLGAWIRIGKNGAVTILQPQAEMGQGVNTSISMLIA
EELEVDVRRVTVEFPPAAAAYANKVYGFQTTAESTSIRSFFDKCRTVGAQAREMLVSAAAKKWRLDPATLRAEA
GYVVDPQSGNRLGYGELAEAASKLPAPEKPRLKSKSEWKIIGKPVKRLDTAAKTNGKAVYGIDVKVPGMLTAAV
MQCPVPGGKLKSVDEKPALAVTGVKQVVKLDNLVAVLADGYWQAKAGLDALSIEWDRGVGDGFTTEMAFAQ
FRQALDKKPGSKAEETGNVDAAFTGAAKVIAAEYTAPYLAHATMEPQNATAHYTPDKLTIWAPTQAQGLVGIVI
GPLVGLKADQVECHTAFLGGGFGRRFELDVPIQAALISKAAKVPVKVIWSREEDMSHDFYRPGAVVRLEAAIDG
DRKITGLRTKIASSSILSRAVPDLVKDGIDITAVDGVKGTDYEFGSRTLHYNLENTPIPVGFWRSVAHSINGWVME
GFVNELAIELNEDPVALRRELLAGKARNISVLDALAERSDWTKKTAGRYKGIAIHHSFDAIIGHVVEISVPKLGQIR
IEKITTVADVGTAINPDTIRAQIQSAIVYGLSAAMTGETVFAGGEAVQKNFDSFEVLRLASMPQIDVHILELGGAI
AGVGEPGLPPLAPALVAAVNVAFSRRIRSLPLAGHGVALA
>HRGM_Genome_4332||HRGM_Genome_4332_CDS_00898
MNLDHIASSIPDATLSRRSFLATAAAGGAFMLSLSLPLGKGEAASPEGFAPNAFIRIGSDGQVALTMPYVEMGQ
GTYTSIPMLIAEELDVSLKQVRLEHAPPNEKIYANPLLGVQATGNSNAMRGAWKPMREAGATARSMLVAAAAK
RWAVDPESCRTQDGEVVHPQTNRRLTYGELAAEAAKLPVPKAVALKPAADFKLIGTPAKRLDGPGKVNGTAVY
GIDVRPPGLKVATLVQSPVFGGRVKSVDDAEAKAVKGVRQIVRLDDAVAVVADHMGAAKKGLAALKIEWDDG

PNAGLATADIARELEQATLRSGPVAQNLGDADKAMAGAATKVEAIYQVPFLAHATMEPMNCTVHLRKDECEI
WIGNQAIARVQAMAAKAAGLPAEKVIVHNHLIGGGFGRRLDADGAVRAVEIAKHVDGPVKVVWTREEDIQH
AMYRPYWFDRISAGLDDKGMPVAWKNRFAGSSVIARWLPPAFKDGLDPDTTEGAIDLVYNLPNFHVEYVRVE
PQGIPTAFWRSVGPSHNVFVTESFIDELAAEAKRDAVAYRRALLDKSPRAKAVLDLAAEKAGWGQALPKGSGR
GISLQFSFGSYMAHVAEVEVSKDGAVRVRRVICAVDCGTVVNPNTVQAQIQSGIVFGTTAALYGEITLKNGRVE
QGNFDTYQILRINEAPAIEVHVVKSTEPPGGMGETGTSAIVPAIANAIFAATGKRLRKMPIDTSVLKSA
>HRGM_Genome_4334||HRGM_Genome_4334_CDS_01405
MRIRGIEALAGGSGEAVKTGDAVAPLTRRGFLKLTGMAGGGLALGIGSVVESAHAQGAAKPASSPQAFLQIAPD
NTVTVAVNRLEFGQGVHTALPMALAEELDVDWKNVRAMLAPAGDPYKDPMFGIQMTGGSTAVNHSFEQYR
ELGARARAMLIAAAAQQWKVDPASCATSLGVVTSGANRATYGELAQAAMAQPVPAQVKLKDPAQFRIVGKP
TPRLDAASKLHGDGVFGLDVKLKDMKVAVVAHPPRFGGKVKSFNADKARKIKGVADVFLVPVDRGGTGVAVV
ADGYWPAKTAREALEIVWEDAGSKVSSAALFDEYSKLAAQPGTVARALEGGNIDTALSGAAKVIEAEYRVPYLA
HAPMEPLNCTMQAEIAANKATAVKVWVGSQFQTIDQAAIARTLGLTPDKVTLNTMMAGGGFGRRAVPTSDY
LVESANVMRSWIAAGHTEPLKVMWSREDDIKGGYYRPLHVHRARVGVDAQGKVVGWQHTIVGQSIITGTPF
EPMMVKNGVDATMVEGIIENDYGLPLQLNVHHPKTDVPVLWWRSVGNTHTAFVKETLADEMATAAKQDPV
AWRMARLDEKAHARHRAALQLAVDKSGYGKKKLPKGHAWGVAVHESFGSVVAYVVDVSIEKGQPKVHRVTA
GVHANRVVNPLTAEAQVQGGCVFGISMTKPGFAIEIENGVVKNSNFPDYPPPRITDAPVVDVFFVPSNDNPTG
LGEPGVPAISPAIANALFRLTGKRQRQMPFVLT
>HRGM_Genome_4807||HRGM_Genome_4807_CDS_02421
MVKRRRVLLGGAATAGALVIGWGVVPPRQRLHTAQPLPQGAGQVALNGWLKVAADNTVTVMMAKSEMG
QGAHTGLAAILAEELDADWAQVRLEMAPIDDIYNNLATVVDGLPFHPDNDGSIKAVAGWLTAKTMREVGVM
MTGGSSSIKDLWRPMREAGAHARAMLINAAAVQWKVQAAECKASAGVVEHAAGQRATFGELAALAAQQP
PPGKVTLKEPAEFKLIGKPLTRIEAPSKLDGTAVFGIDARPPGLLHASVVMCPTRGGKVAQFDGAAAQKMPGVK
QVLAVAPYHGGTGGVAVIADTPWHAKKAAAAVTVQWDHGPAAMLSSAAVMTQLTQALDQAQGFGYHSVG
DVDQALQGAATTLTADYRAPYLAHATMEPINCTVQFKDGAARVWTSTQVPGLARMAAAKALGIDEDKVTVH
QLLLGGGFGRRLEVDYVAQAAAIAKAAGGAPVQTLWSREQDMQHDFYRPACVARFKAGLDAQGQLVAWHN
VSAGQAIVPQVLSRTFGLPGAGPDKTASEGAFDQPYEWPHARIAHEIIDLPLPVGFWRSVGHSHQAFFKESFV
DEAAHAAKKDPMAFRAALLQKHPRHRKVLQKAAELAGWGQPLPPDASGVQRARGVALHQSFGSIVAQVAEV
SVGADKAIRVHRVVCVIDCGAAINPNLIAQQMESAVVYGLTAALWGEVTIKDGQVQQSNLHDYPLLRMADCP
RVETHIMASAEPPEGVGEPGVPPIAPAVANALFALTGQRLRALPLKLS
>HRGM_Genome_4807||HRGM_Genome_4807_CDS_02103
MLHNIAPHELPRALQHLIERDRPTDLAALPRRSFLKLAGVSGFALGAFPLLADAQANPAAPVGLKPTQQPSNFV
RIAPNGEVTVTVNRLEFGQGVQTGLAMILAEELDADWKLVRSTHGSGDAAFVDPLFGIHLTGGSHSIKNSYVQY
RELGARARAMLLSAAAARWKVDVSTLRTQAGTVLGPSGRKASYGELAQAAMALPVPEQVTLKDAKDFRLIGR
ATGRLDARAKSSGQQDFGIDVRRPGQLTAVVARPPVFGARLASVDDSAARAVPGVKAVLRIPGDQGGEGVAV
VADGYWPAKQGRDALKLQWDTSKVEKVDSVQLLAQYRTLAGQPGPRAFDADMAPLATAPKQLEAEFVFPYLA
HAPMEPLNCTVQLTDGSAELWLGSQCPGLDAGAVATALSLKPEQVKVNVLMAGGGFGRRFSSTSDYVVEAAQ
VAKAARTAGLNAPVRTLWSREDDIKGGYYRPMHLHRARIGFDERGNVLAWDHVIVGQSITTGTVFGGFQVKN
GIDATATEGMRDPYDLPMRLTVHHPKVNVPVLWWRSVGSTHTAYVMETLLDNIARATKQDPVAYRMRLFGDK
HPRHRAALQLAVDKSGYGKKQLPAGRAWGVAVHESFSSVVAYVVEASVKDGNPVLHRVTSGVHCNLAVNPRSI
EAQVQGAAMMGLSMCLPGAAITFKDGVVEQSNFGDYPVARMTDAPAFDVHIVPSADAPTGMGEPGLPPLA
PAFANAIAQLTGQPLRELPFKLA
>HRGM_Genome_4807||HRGM_Genome_4807_CDS_00954
MHFEPHALQGQAQHFMPKHLQALVDKAQGAITSGAIDVSDGVARRTFLKVSAASGFALGAFPLVSAAQGAAA

AAPTGLKPHEQPSAFVRIDADGTVTVTINRLDFGQGVQTGLPMILAEELDADWSKVRSVHGDANPAYADPAFG
MHLTGGSNSIKNSYTQYRELGARTRAMLVGAAAAQWGVDASALRTSNGFVVGPGGKKLGYGQLAEAAMKQ
PVPEKVTLKDPKQFRLIGKPTGRLDAKAKSSGQQDYGIDVRLPGMLTAVVARPPVFGAKVKSLDDSAAKAIKGV
KAVLRVPTDRGGEGIAIVADGYWPAKQGRDALKVEWDTSAVTKPDTTALLAQYRELAAKPGNVAMQADMAP
LAGAPHKISAEFVFPYLAHAPMEPLNCTVKLDGDKAELWMGTQMPGLDAMAAAKTLGLQPQNVKVNTQM
AGGGFGRRAIPTSDYVVEACGVAKAARTAGITAPVRTLWSREDDIKGGYYRPMHVHRAEIGFDAKGKILAWDH
TIVGQSITKGSPFEAFMIKNGIDATAIEGMKEPYDVPMKLSVHHPDVNVPVLWWRSVGSTHTAYAMETLIDEVA
RATKQDPVAYRLALMGDKHPRHKAALQLAVAQSGYGKKKLAAGRAWGVAVHESFESVVAYVVEASVKDGTPK
LHSITAGVHCNLVVNPKSVEAQVQGAALMGLGTCLPGAAITLKDGVVEQSNFGDYAVPRITDMPQVTVHIVPS
ADPPKGMGEPGLPPLAPAFANAIAQITGKTPRELPFKLA
>HRGM_Genome_4809||HRGM_Genome_4809_CDS_03197
MIHEAKLMAELASAPEASESGAPILSNLSRRGILGGMGALVLALSVREGRAEEKSEPKKFGADGMPHGWQDD
PTIFVAIAADGTVTVTNHRSEMGQGVRTSIALTVADELDADWSKVKVVQAWGDETRFGNQDTDGSRSLRHFF
RHFRHTGAAARAMLIQAAAAKWNVPASEITTDKHVLVHAKSGRRIGYGEVAAAAAELPVPARESVRLKDPKAF
RYIGKGEIGIIDNRDITTGRAVYGLDVKRDGMLYALVARPPVYGGKVASYDDAETLKVPGVVKVFKIDGGAIPSEF
MPLGGVAVVAKNTWAAMKGREALKITWDDGPNANYDTDVFRGELEKAARAPGKVVRTAGDVDGAMAKAK
RKVEAEYFVPHLVQAPMEPPAAVAQVKDGACEAWACTQGPQAAHDRLMKALGLPGDKVRVNVTLLGGGFG
RKSKPDYVVEAALCSREVGGAPVKLVWTREDDLHHGYYHTISVERLEAGLDEKGMPVAWLHRSVAPTIGSIFAP
DPKHELPFELGMGLVNTPFALPNIRLENPEAAAHVRIGWFRSVSNIPHAFAIQSFVAELAHAAGRDPKDYLLDLI
GPARRIDPTTIGDVWNHGEDPALYPIDTGRLRRVIETAAKGAGWGRKLAKGRGLGLAGHYSFVTYTAVAAEVEV
GPKGEVKVNAVDIAVDCGPQVNPERIRSQMEGAVVMGMGLALTSQMTFKNGRAEQGNFDGFEVVRLDAAP
KEVRVHLVPSGTWDGPLGGVGEPGVPPVAPAIANAVFQATGRRVRQLPIRDQLSA
>HRGM_Genome_5019||HRGM_Genome_5019_CDS_04777
MSKPSSFPKTATSRRAFLQGGGLLLGFALFGAGIKPVLAASTAQPVDGDVTAAFAPDAFIRIGTDGLITLILPNIEM
GQGTHTGEATLIAEELEVSLDQVKAVDAPPNDKLYATAALGGQATGGSTSMRATWEPLRKAGATARMMLVAA
AAAEWSVPVSECVARLGVVTHQPTGRQLAYGALADAAARQPVPAEVKLKDQKDFQLIGKSSRRLDTPGKVNG
AVKYGIDIRVPDMKVATVAACPVLGGKLGDVDDKAARAIPGVRDVVRLDNAVAVIGDHFWAAKKGLEALDIT
WVEGANANLASANIMAALKAASERKKPIMARQEGDVDGRMKAAVKKVEATYELPFLAHAPMEPINCVVHVR
PDECEIWVGTQVPAIAQGLAAKVTGFPLEKVILHNQLIGGGFGRRLVAESVAQAVAIAKQVSYPVKVIWTREEDI
QHDLYRPAYYDRIAAGLGADGLPTVWVDHVAGGSVLGNYIPGGWPEDKLDDDAVEGAAKPPYDLPVIQVDW
VREDPPVPITWWRGVGPTHNVFVVESFMDELAHAAGKDPVEYRRALTRNQPRAAGVLELVAEKSGWGTPLA
AGMGRGISLHDAFGSYMAAVLEISVSPAGEITLHRAVVAVDCGITINPNTVEAQIEGGLIFGLSAALYSGITFTDG
RVDQSNFHDYRILRNNEAPKIEIHHVKSSESPGGIGETATVSAAPALANAIFAATGKRLRTLPFNRDALKTDGTDK
KSVSMIPPLAAPLAAALASAKPADTELEKL
>HRGM_Genome_5144||HRGM_Genome_5144_CDS_04186
MTTTMQVSRRGFLKGGLGALTLAVTGNGLVSAVWAADEPKKYGADSMPGGTVDDPLAFVSIAADGTVTIVAH
RAEMGTGVRTSLPMVVADEMEAAWDRVRVVQADADEARYGNQNVDGSRSVRHFLMPMRRVGAAARQM
LEAAAAARWSVPLAEVRATQHEVVHAPSGRRLGYGELAADAAKLPVPAGDAVKLKTRAEFRYIGKDEVRLVDLE
AIGKGEAMYGMDMRLPGMVYAVVARPPVVGGKLRRVDSAKALAVPGVLKVVEIPAMAGAPAFQPLGGVAVV
ASNTWAAMQGRAALAIEWDDGPNAAYDSVAYRETLTEASRKPGKVVRDQGDAPQAWAKAGETERFMAEY
HVPHLAHASMETPVATVRIQDGAAEVWTSVQNPAAAQEAVAKRLKLKPENVKVHVLLLGGGFGRKSKPDYVD
EAAIVAQAMPAGTPVKLVWTREDDIHHDYLHTVSAEHLEAVVGKDGKVQSWLHRSAAPTIASLFTEGAKGEQL
FESAMSAINMPYVIPNVRVETAEVAAHARIGWFRSVANIPHAFAAQCFIAELAHRAGQDHKQYALDLIGPARRI
DPGTLADTWNYSESPERYPYDTGRLRGVIEAAASGAKWGRELPKGHGLGLAFCYSFMSYTATVVEVAVDEKGE

VRVVAVDMALDCGPQIKPERIRAQMEGGAIMGLSLALLGEITFEKGRVKQNNFYDYEVLRHNASPRVIRTHLV
NDDHALPPGGVGEPPVPPVAPALCNAIFAATGKRVRSLPVRSVA
>CABIYZ02||gene_4204|GeneMark.hmm|763_aa|+|542925|545216
MMTTTMQVSRRGFLKGGLGALTLAVTGNGLVSAVWAADEPKKYGADSMPGGTVDDPLAFVSIAADGTVTIV
AHRAEMGTGVRTSLPMVVADEMEAAWDRVRVVQADADEARYGNQNVDGSRSVRHFLMPMRRVGAAAR
QMLEAAAAARWSVPLAEVRATQHEVVHAPSGRRLGYGELAADAAKLPVPAGDAVKLKTRAEFRYIGKDEVRLV
DLEAIGKGEAMYGMDMRLPGMVYAVVARPPVVGGKLRRVDSAKALAVPGVLKVVEIPAMAGAPAFQPLGGV
AVVASNTWAAMQGRAALAIEWDDGPNAAYDSVAYRETLTEASRKPGKVVRDQGDAPQAWAKAGETERFM
AEYHVPHLAHASMETPVATVRIQDGAAEVWTSVQNPAAAQEAVAKRLKLKPENVKVHVLLLGGGFGRKSKPD
YVDEAAIVAQAMPAGTPVKLVWTREDDIHHDYLHTVSAEHLEAVVGKDGKVQSWLHRSAAPTIASLFTEGAKG
EQLFESAMSAINMPYVIPNVRVETAEVAAHARIGWFRSVANIPHAFAAQCFIAELAHRAGQDHKQYALDLIGPA
RRIDPGTLADTWNYSESPERYPYDTGRLRGVIEAAASGAKWGRELPKGHGLGLAFCYSFMSYTATVVEVAVDEK
GEVRVVAVDMALDCGPQIKPERIRAQMEGGAIMGLSLALLGEITFEKGRVKQNNFYDYEVLRHNASPRVIRTHL
VNDDHALPPGGVGEPPVPPVAPALCNAIFAATGKRVRSLPVRSVA
>CABKLU02||gene_2470|GeneMark.hmm|756_aa|-|7163|9433
MVKRRRVLLGGAATAGALVIGWGVVPPRQRLHTAQPLPQGAGQVALNGWLKVAADNTVTVMMAKSEMG
QGAHTGLAAILAEELDADWAQVRLEMAPIDDIYNNLATVVDGLPFHPDNDGSIKAVAGWLTAKTMREVGVM
MTGGSSSIKDLWRPMREAGAHARAMLINAAAVQWKVQAAECKASAGVVEHAAGQRATFGELAALAAQQP
PPGKVTLKEPAEFKLIGKPLTRIEAPSKLDGTAVFGIDARPPGLLHASVVMCPTRGGKVAQFDGAAAQKMPGVK
QVLAVAPYHGGTGGVAVIADTPWHAKKAAAAVTVQWDHGPAAMLSSAAVMTQLTQALDQAQGFGYHSVG
DVDQALQGAATTLTADYRAPYLAHATMEPINCTVQFKDGAARVWTSTQVPGLARMAAAKALGIDEDKVTVH
QLLLGGGFGRRLEVDYVAQAAAIAKAAGGAPVQTLWSREQDMQHDFYRPACVARFKAGLDAQGQLVAWHN
VSAGQAIVPQVLSRTFGLPGAGPDKTASEGAFDQPYEWPHARIAHEIIDLPLPVGFWRSVGHSHQAFFKESFV
DEAAHAAKKDPMAFRAALLQKHPRHRKVLQKAAELAGWGQPLPPDASGVQRARGVALHQSFGSIVAQVAEV
SVGADKAIRVHRVVCVIDCGAAINPNLIAQQMESAVVYGLTAALWGEVTIKDGQVQQSNLHDYPLLRMADCP
RVETHIMASAEPPEGVGEPGVPPIAPAVANALFALTGQRLRALPLKLS
>CABKLU02||gene_2149|GeneMark.hmm|746_aa|-|784|3024
MLHNIAPHELPRALQHLIERDRPTDLAALPRRSFLKLAGVSGFALGAFPLLADAQANPAAPVGLKPTQQPSNFV
RIAPNGEVTVTVNRLEFGQGVQTGLAMILAEELDADWKLVRSTHGSGDAAFVDPLFGIHLTGGSHSIKNSYVQY
RELGARARAMLLSAAAARWKVDVSTLRTQAGTVLGPSGRKASYGELAQAAMALPVPEQVTLKDAKDFRLIGR
ATGRLDARAKSSGQQDFGIDVRRPGQLTAVVARPPVFGARLASVDDSAARAVPGVKAVLRIPGDQGGEGVAV
VADGYWPAKQGRDALKLQWDTSKVEKVDSVQLLAQYRTLAGQPGPRAFDADMAPLATAPKQLEAEFVFPYLA
HAPMEPLNCTVQLTDGSAELWLGSQCPGLDAGAVATALSLKPEQVKVNVLMAGGGFGRRFSSTSDYVVEAAQ
VAKAARTAGLNAPVRTLWSREDDIKGGYYRPMHLHRARIGFDERGNVLAWDHVIVGQSITTGTVFGGFQVKN
GIDATATEGMRDPYDLPMRLTVHHPKVNVPVLWWRSVGSTHTAYVMETLLDNIARATKQDPVAYRMRLFGDK
HPRHRAALQLAVDKSGYGKKQLPAGRAWGVAVHESFSSVVAYVVEASVKDGNPVLHRVTSGVHCNLAVNPRSI
EAQVQGAAMMGLSMCLPGAAITFKDGVVEQSNFGDYPVARMTDAPAFDVHIVPSADAPTGMGEPGLPPLA
PAFANAIAQLTGQPLRELPFKLA
>CABKLU02||gene_961|GeneMark.hmm|765_aa|-|9759|12056
LKETTMHFEPHALQGQAQHFMPKHLQALVDKAQGAITSGAIDVSDGVARRTFLKVSAASGFALGAFPLVSAAQ
GAAAAAPTGLKPHEQPSAFVRIDADGTVTVTINRLDFGQGVQTGLPMILAEELDADWSKVRSVHGDANPAYA
DPAFGMHLTGGSNSIKNSYTQYRELGARTRAMLVGAAAAQWGVDASALRTSNGFVVGPGGKKLGYGQLAEA
AMKQPVPEKVTLKDPKQFRLIGKPTGRLDAKAKSSGQQDYGIDVRLPGMLTAVVARPPVFGAKVKSLDDSAAK
AIKGVKAVLRVPTDRGGEGIAIVADGYWPAKQGRDALKVEWDTSAVTKPDTTALLAQYRELAAKPGNVAMQA

DMAPLAGAPHKISAEFVFPYLAHAPMEPLNCTVKLDGDKAELWMGTQMPGLDAMAAAKTLGLQPQNVKV
NTQMAGGGFGRRAIPTSDYVVEACGVAKAARTAGITAPVRTLWSREDDIKGGYYRPMHVHRAEIGFDAKGKIL
AWDHTIVGQSITKGSPFEAFMIKNGIDATAIEGMKEPYDVPMKLSVHHPDVNVPVLWWRSVGSTHTAYAMET
LIDEVARATKQDPVAYRLALMGDKHPRHKAALQLAVAQSGYGKKKLAAGRAWGVAVHESFESVVAYVVEASVK
DGTPKLHSITAGVHCNLVVNPKSVEAQVQGAALMGLGTCLPGAAITLKDGVVEQSNFGDYAVPRITDMPQVT
VHIVPSADPPKGMGEPGLPPLAPAFANAIAQITGKTPRELPFKLA
>CABKMH02||gene_3467|GeneMark.hmm|780_aa|-|27|2369
VIHEAKLMAELASAPEASESGAPILSNLSRRGILGGMGALVLALSVREGRAEEKSEPKKFGADGMPHGWQDDP
TIFVAIAADGTVTVTNHRSEMGQGVRTSIALTVADELDADWSKVKVVQAWGDETRFGNQDTDGSRSLRHFFR
HFRHTGAAARAMLIQAAAAKWNVPASEITTDKHVLVHAKSGRRIGYGEVAAAAAELPVPARESVRLKDPKAFR
YIGKGEIGIIDNRDITTGRAVYGLDVKRDGMLYALVARPPVYGGKVASYDDAETLKVPGVVKVFKIDGGAIPSEF
MPLGGVAVVAKNTWAAMKGREALKITWDDGPNANYDTDVFRGELEKAARAPGKVVRTAGDVDGAMAKAK
RKVEAEYFVPHLVQAPMEPPAAVAQVKDGACEAWACTQGPQAAHDRLMKALGLPGDKVRVNVTLLGGGFG
RKSKPDYVVEAALCSREVGGAPVKLVWTREDDLHHGYYHTISVERLEAGLDEKGMPVAWLHRSVAPTIGSIFAP
DPKHELPFELGMGLVNTPFALPNIRLENPEAAAHVRIGWFRSVSNIPHAFAIQSFVAELAHAAGRDPKDYLLDLI
GPARRIDPTTIGDVWNHGEDPALYPIDTGRLRRVIETAAKGAGWGRKLAKGRGLGLAGHYSFVTYTAVAAEVEV
GPKGEVKVNAVDIAVDCGPQVNPERIRSQMEGAVVMGMGLALTSQMTFKNGRAEQGNFDGFEVVRLDAAP
KEVRVHLVPSGTWDGPLGGVGEPGVPPVAPAIANAVFQATGRRVRQLPIRDQLSA
>CABKQE02||gene_3372|GeneMark.hmm|765_aa|-|272210|274507
MNAKTTKPRSGRRRFLLGALGIGGALVVGWGVMPPRSRVGDPGIFPEHNGEIALNGWIKITPEGDVVLAMPR
VEMGQGIHTALSMLAAEELDIPLSRVRIESAPVERIYGNVVAMGDSSLPLHPDSADKTWARALHWIMAKSARE
IGLIITGGSSSTADGWQPVREAAATARAALVQAAAREWNVPAADVSIREGQLIGPGGKQSTFGEMAKSARGISA
PSNVTLKPASQFRLIGKPAPRNDLAAKTDGSARFSIDTRLPGMLYAAVVMCPVFGGKLKTFQSKAALGMPGVR
YVVPFEGAGGGAPGVAVVADHYWQARQALATLEPVWDNGPHAKLDSAGIRQQLVSALDSDKGGFTYRSMG
DGLKAFDRADGATIVEAEYSAPYLAHATMEPINCTAQVTADGVHLWAPTQVATLAQLVAARAAGVSGDKVQID
IPLIGGGFGRRLESDFISQAVTIATKTEGKPVQVIWSREEDVRHDFYRPQAIARLKARVESGKVTAIASRSAGQSIL
AGELDRLFGAPSVGIDRYTAEGLFDLPYEIEHEHIAHLAVDLPVPVGFWRSVGHSYNGFFMEGFLNEVAAAAKL
DPLAMRRNLLKDHPRELKVLDTAAQAAGWGQPLAPAADGAPRARGIALHPSFGSVVAQVVEVSMKDGKPRV
HRVVCAVDCGTVVNPGIVAQQMESAVIFGLTAALYGRIDIKDGQVVQSNFPDYPALKMVETPVIETHIVPSTAEP
SGMGEVGVPPIAPAVAHAMAQLTGKPVRQLPMV
>CABKRR02||gene_3842|GeneMark.hmm|773_aa|+|949069|951390
MSAHIIDMESTGLPTNPSRRSLLKAAGATGLMIAVTPAGHVFAQTAAAADKFGGDRMPGGVVDNPLIFVSIAP
NGIVTVTCHRSEMGQGVRTSVPLIVAEELEADLNRVRVRQADGDEARYGNQNTDGSRSVRHWLEPGRRVGA
AARAMLEAAAAAQWGVPAAEVQAQNHVLVHKPTGRKLPFGQVAEAAAKLPVPARDSLKLKNPAQFRYIGKD
GRKLIDGNDIVTGRAEFGIDVRLDGMVYAVIARPPVVGGKVKSYDATDTLKVPGVLKVAEIAPAPAPVVFNPLG
GIAVVAKNTWAAIKGREALKVEWDDGPHASYDSEAYKTELEAAARAPAKPARDDGKTMEVLASAKRKVVADY
YLPHIAHATMEPPVAVARIVDGRCEAWAPIQAPENARKTVADKLGLKADQVTVHVTLLGGGFGRKSKPDFVAE
AALVSKAMDGRPVKLQWTRDDDLHHDYFHAVSVQHLEAALDDKGMPVAWLHRSAAPTIRSTFVAGAKGLGV
NELGHTALNVPFQIPNVRVEAPEVEAHTRIGWFRSVYNIPHAFGVQCFVAELAHAAGRDPKDYLLELIGPARRIN
PTALGDTSNYGENPALYPIDTGRMRRVVELAAKGANWGRKLPKGHGLGIATAYSFMTYTAVAIEVAVDDKGEFQ
VVSADIAIDCGPQVNPERIRSQVEGAVVMGIGLAKHGEISFKEGRVVQTNFHDHVLLRHAERPQALRVHLAPS
DHSVPPGGVGEPGLPPVAPALANALFAATGKRIRRLPIRDQLSSAA
>CABKSW02||gene_2542|GeneMark.hmm|733_aa|-|2788410|2790611
MTKNVSAQAISLSRRKLLQASGIAAGGLLLATALPFSRRSYAEQYVNKGPEDAPLDTPTALGAFLRIGHDGQITLIS

PKIEMGQGVQTGFAMMVAEELNVTLDQVRVQEAPPDEKLYGDKLLGFQATGGSSSTRSNWQPLREAAAAAR
VMLIQAAANQWKVSPDECRAENGKIIGPGNRELAYGALVDAAAKLPVPENVPLKKPEDYKVIGQPLRRLDTPG
KVDGSAKFTIDLHVPGMKIATVSACPVVGGTLASVDDRHARAVPGVRDVVKLENAVAVIGDHMWAALKGLKA
LEIQWGLGPNAGIDSAQIERALHAAFDREGAIAAEVGDINKAIAGASSKIEAEYEMPFLAHATLEPMTCVAQVR
EDACELWVGTQVPVMAQQAAAKATGLPPEKIIVNNQLIGGGFGRRLEADFIGQAAAIAKQVDYPIKLVWTREE
DTAHDLYRPHYIDRFSAGLDANGMPVGWSHTIVGASVMARFAPAAVPPNGLDADAVEVSNKPVYSLPNLRVR
YVPEAPKAILNSWWRGVGPLHGAYVMESFIDELAYAAKQDPVDYRMKLLGEHPRAQAVLKLAAEKANWSQKL
PAGHGRGIAVQEVFGSYLATVVEMQVDAQNGIHITRLICIADCGEVTNPTSVHSQLEGGTLFGLSAALYNEITVK
NGHVEQSNFHDYRQLRMSDAPPVETHIIPSHEIPGGIGEAGTAMIAPALVNALYAATGKRLRRLPVVRAGYHVA
>CABLBX02||gene_3186|GeneMark.hmm|728_aa|-|121893|124079
MSFSTVADEVVPGRRKFLKLMGGVGAGLTLGFSLPVSNQAQAADMTSSAVPFEPNAFVRIGSDNKVTVMIKHI
EMGQGTYTGLTTLVAEELDADWAQMVAEGAPADASRYNNTFWGPMQGTGGSTAIANSYIQMRTAGAAAKA
MLVAAAAQRWNVDASQIQVRAGIVSHGNKKATFGELALDAAKQPVPDEASVKLKTPEQFVFIGKRKVSRKDT
GKNNGTAIFTQDIKLPGMLTAMVLHAPKFGAKVKTVDATQAKASPGVVDVVTIPTGVAVLAKDYWSAKKGRDL
LSVTWDESQAFTKSSAQIMTEYKTLSQKEGLSARNEGDAVSALKQADSVIDNTYEFPFLSHSPMEPMNCVAQV
SDQGCEIWNGEQFQTVDQMNIAQLLGLKPEQVTLHMLLAGGSFGRRANPHSDYLIETVEIARQKKGTPIKMV
WSREDDTQVGYYRPAYVHHIQAGLDANGNISSWKQHIVGQSILTGTAFEAFAVKEGIDGTSVEGASNIPYAIPNL
SIQLTTVKDGPTVQWWRSVGSTHTAFAVETMIDELAANAGKDPVEMRMQLLAKHPRWQGVLKLAAEKAGW
GKTLPAGSGMGVAVHESFNTFVAQVAQVSVKNGQISVDKVVCAVDCGVAVNPDVIAAQMEGGIGFGLSPTL
MSAITLGEGGMVEQSNFHNYQVLRMNQMPDVEVHIVPSAEAPSGVGEPGVPPIAPAVANAVAAATGQRLHT
LPLKLATS
>CABMGP01||gene_5325|GeneMark.hmm|723_aa|+|69750|71921
MTIEHSLSRRQFLVSSAAVGGLALGFTLPLGAQPAAQTESEVNAWVVIKPDETVVVRIGRVEMGQGTLTGLAQL
VAEELDCDWDKVTTEYPTPGQNLARSRVWGNFQTAGSRGIRDSHDYMRKGGATARHMLIQAAANGWQVPA
TECTVSKGVITHAPSGRTTTYGKVADAAAKLEAPKDVQLKDTKDWMIAGKPVKRLDTAPKINGSQIYSIDFTRP
GMLNAAIRACPVFGGKLKGFQATAVERMPGVKKVVQVGDNAVAVVADTWWRAKTALDALPVEWDPGPNA
ALSSETITAMLNEGLDAQQTFIGNQAGDVKAAIAGAAKKVEAVYGYPYQHHATMEPMNATALWTSDRCEVW
TATQNGEAALATAASAADLPVGKCDVYRLHLGGGFGRRATSHDFVRQAVLIAKQMPGTPVKLIWSREEDMLH
GWYHPVTQCKLTGALDDQGNLTGLHMRISGQSILSTVNPQGLQNGRDPATFQGLNPSGTEGVFGYSIPNLLID
HAMRNPPIPPGFWRGVNNNQNALYLECFMDELAHAAGQDPLAFRRKLLVQHPKHLAVLNAVAERAGWGSP
APQGVYRGLAQHMGYGSYVAACAEVSVANDGKLKIHRIVAATDCGHAVNPQQIAAQVEGSFVYGLTAMLYGEI
TIKDGKVEQENFDTYPMMRMDEMPEVETIVMPSGDFWGGVGEPTIFVAAPAVLNAIFAATGKRIRSVPLSKA
DLRKA
>CABMGP01||gene_4003|GeneMark.hmm|733_aa|-|320213|322414
LRQEAQEDIMTTALRVSRRAFLAGSAAAVGGLSFGFHIPFSDAVAQEAAPEINAWVVVRPDDTVLIRIARSEMG
QGTLTGLAQLVAEELDCDWSKVTTEYPTPGQNLARSRVWGNFSTGGSRGIRDSHDYVRKGGATARQMLIQAA
ADSWGVPVSECSAANSVIKHGPSGRTARYGEVAEKAAKITPPSDVTLKDPKDWKIAGKPLKRLDTAEKLTGKQV
YGIDLKLPGMLNAAIKDCPVFGGRVKSFDAAAIQGRPGIKKVVPVGESAVAVVADTWWRAKSALDELPIVWDE
GPNATVSSATIAEMLKEGLDAEHAVDGNRVGDARAAIASAAKKVEAVYSYPFQNHATMEPMNATALWTPDRC
EVWTPTQNGEAALAATAEAAGLHPSKCDVHKIHLGGGFGRRGAVHDWVTQAVTIARQLPGTPVKLIWSREED
MLHGRYHPITQCKLTGALDDQGNLTGLHMRISGQSIIASIFPQNIQNGKDPAVFQGLNPGGAEAAIGYTIPNLLI
DHAMRNTHVPPGFWRGVNLNQNAIYLECFIDELAHTTGQDPLEFRRKLMTQHPRHLAVLNAVAERVGWGTP
PPAGIYRGLAQTMGYGSYVAACAEISVSDDGKLKIHRIVAATDPGHVVNPQQVAAQVEGSFVYGLSAALYGECT
VASGRIEQENFDTYPVMRIDEMPAVEALIVPSGGFWGGVGEPTIAVAAPAVLNAIFAATGKRVRSIPIKHADLRK

A
>CABMGP01||gene_7069|GeneMark.hmm|731_aa|+|236649|238844
MIRESLSSPVTGASRSVLSRRQFLQAGAAAGGGLMLSLSLPFGYGDAVASGADAFVPNAFIRIDSDGRIVLTMPS
VEMGQGTYTAIPMLIAEELEADLSQVRLEHAPPNERLYGNPLLGGIQATGNSNAIRAAWQPLRHAGATARTML
VAAAAQRWNIDPASCRAQSGEVLHPATGRRFTYGELAPDAARLPVPESVVLKRSEEFRLIGTPAKRLDTPAKVN
GTAVYGIDVRPPGVKIATLAQSPVFGGRVRGVDDTAAMAVKGVRQIVRLDDAVAVVADHMGAAKKGLAALVIE
WDDGPHAGLTTREIVAELETATLNPGAVAQKIGNVDQAMASAVTRIEAIYQVPFLAHATMEPVNCTAHVRPDG
CEVWVGTQAIARVQAAAAKAAGLPLDKVVVHNYLIGGGFGRRLEADYVVRAIQIAQHVDGPVKVVWTREEDI
QHDMYRPYWLDRISAGLDEKGMPVAWSNRFAGSSVLARWAPPTFNNGLDPDTIEGAIDLVYDLPNLHVEYVR
VEPPGIPTAFWRSVGPSHNVFVTESFMDELAAAAKQDPVAYRLALLGKTPRARAVLELAAQKADWEQPLPERV
GRGVSLQHVFATYMAHVAEVEVAKDGVVRVRRVVCAVDCGTVVNPDTVRAQIQSAVMFGITAALHGEITVKD
GRVEQSNFDTYPILRMNEAPAVEVHIVPSSEPPGGMGEAGTSAIVPAVANAVFAATGKRLRKMPIDVAAQEGT
K
>CABMGP01||gene_814|GeneMark.hmm|765_aa|+|885701|887998
MTHHSDSATRIVNVSRRGFLAGMAAGAFILAVTGAQRVRAQEKKFGADAMPHGWRDDPKIFIAIDPDGTVTV
TCHRQEMGQGVRTSIAMVIADELEADWSKVRVVQAYADEAHFGNQDTDGSRSLRHQFMPMRRAGAAART
MLEQAAAKQWGVPIAQVKAGHHAVVHEATQRTLGYGELAAAAADLPVPSRESLKLKDPSAFRYIGRDTISLVD
NRDITTGKAIYGIDARAEGMLHAVIARPPVYGGAVASYDATETMKVPGVVKVVKIDRPAVPSEFQPIGGVAVIAR
NTWAAIRGREKLKITWDDGPNAGYSSDSYKAELEAAARKPGKVIRNEGDVDEAMAKAAKRVTAEYYIPHLAQA
PMEPPAALVQIKDGRCEAWACVQAPQVTRTRLAEKLGLPEDKVKVNVTLLGGGFGRKSKPDFVLEAGLLSQA
MDGAPVQVTWTREDDLHHSFYHTVSIEHLEAGLDERGLPAAWLHRTVAPTIGSIFAPDPKHELPMELGMGVT
NMPFAIPNIRSENPEAQAHVRIGWYRSVSNIPHAFAIQCFVSELAHAAGRDPKDYLLDVIGPARLIDPTEQGDV
WNYGEDPRLYPIDTGRLRRVVETAAKGIGWGRQLPKGRGLGIAGHYSFVTYAAVAAEVDVGGDGKLAIPRVDIA
VDCGPQVNPDRIRAQMQGAVVMGVGMANASEISFRNGRAEQDNFDSYMVPRIFDAPKEINVHLIPGESYAQ
PLGGVGEPGLPPVMPAILNAVFAATGKRIRQVPIGDQLRA
>CABMKO01||gene_2324|GeneMark.hmm|744_aa|-|2481888|2484122
VRKQPISSKFALGEEPTNLSRRRFLLASGSVAGALVLGIGLPSGQARAQAAQQPLSPGTRVPAFLEIRPDSSVRFLS
PFIEGGQGIFTAMAQIVGEELDADPATFVVENAPAGDEYKVMDSGMRITGGSQSVRTSYTTMRRLGALARHM
LLQAAASALAVPLASLRTEPGKVVHDASGQTLTYGELASRARDLPVPPADAVSLKDPAQFRWIGKPVKRLDAWE
KSTGQAVYTIDCRVDDMLHAAVQHAPRFGMTPGAWRNEAQVRAMKGVHSVHRLPDAVAVVAERWWQAK
RAVEALQVEWLAPADAELMPPDFSSDDFARTLANEPGDGQEAEVKGDLAAGMSAAKTTLSATYHSQYLNHA
QLEPPSALARFNDDGTLELWIPNQAPEMFQGEVAKRTGLDASKILIHSPLLGGFFGRHFVYDGAMAWPQAVEL
AKAVGRPVKLIWSREEEFLCDTHRPMAAVRFRAGLDDEGYPLALEAVSICEGPTEGISGKQGDKLDPTATEGLTG
KSYAIPHLRIAQIYKKGPVKLGYWRSVGNSMNDFLYESFLDEIADHGRLDPFELRLRLLKGNARLTTLLHAVADLS
GGWKPGPYTAEDGSRRARGVAMASPFGTEAAAIAEVSIENGQVRVHRIWEAIDPGSIVNPAIVEAQVNGAVAL
GLSQVLLEEALFEKGQPVARNFDLYPILPPSRMAQVEVRIIESGAKMGGIGEPPLPAVPPAVTNAVSRLTGQRIRS
MPLSRYNFT
>CABMKW01||gene_5072|GeneMark.hmm|768_aa|-|35314|37620
MSVSGPQSIAPPRRWLEGEGIMAFTLDNHADRHQASLVNVSRRRFVQGAGVLVLGWQLPGVLAAQPADKA
DPFVPNAFLSIDTQGRVTVYAKHVEMGQGAYTGLATLVADELDADWAQVRVEGAPADTARYKNLAMGIQGT
GGSTAMANSFKQMREAGAAARAMLVGAAAQAWKVDPATITVREGVVRHAASGRQAGFGELAAAAAAQPV
PARPRLKSPKDFRLIGKASLRRTDSADKVHGRARFTQDVKLPGMLVAIAAHAPRFGATVAGYDASAALAVPGVV
AAIPFEGSAHRSAGVAVLARNTWAARTGRDALKVRWDESRAYTEGSDELLARYRQAASRPGLPAATRGNVETA
FAKAAKVIEADYEFPFLAHASMEPLNCVVQLGDGRCEIWNGEQFQTMDQKAVADYLGIAPGHVTINQLYAGG

SFGRRASPHADYVVEAVAIAQGARKAGFDVPVKMVWTREDDMRGGYYRPAYLHRARLALDERGRLQGWQV
RVVGQSLIKGSVFEEFMVKNGIDATSVEGLSDLPYGIPNLSVELHTPDDVRVPVQWYRSVGHTHTAFTAETLVDE
AAHAARRDPVALRRELLKGHPRHLAALETAVRAAQWDRPLAAGKSGVRRGRGVAVHESFNSVVAQVVEVSVD
AGGELKVDRVVCAVACGLAINPDVVKAQMEGGIGFALSTALHGAITLDKGAVVQSNFHDYPVLRINEMPRVEV
HIVPSQDDPTGVGEPGVPPLAPALANAIFEATGERVRTLPIRTPLRKAV
>CABMLY01||gene_5044|GeneMark.hmm|731_aa|+|20294|22489
MNSKIDLSNALPGSRRGFLKGAAVVGLTIGFQWSGARRALAAALPDAGFAPNAFLRIAPDDSVTVIAKHVEMG
QGAYTGIATIVAEELDADWSKVRVESAPADAKRYANLAFGTMQGTGGSSAMANSWMQLREAGAKARAMLV
EAAARQWQVPAAELRTRDGFVEHPTSQRKASYGSLAAAAAELPVPEKVQLKDPKDFRLIGHQAPRVDVPGKT
DGSAQFTLDVSLPGMLVALLQRPPLFGATVKSFDATATRAIPGVVEVVQVPHGVAVVAKGFWAAKQGRDALKV
EWDESKAEKRGSEALMAEYRKLAEQPGKPARRDGDAAKAVAGATRRIAASYEFPFLAHAPMEPLDAVVRLTAD
SCEIWAGDQFQTVDQGNAARTAGLKPEQVKINTLYAGGSFGRRANAWSDYIVEAVSIAKALGANGVPVKLQW
TREDDIHGGFYRPMYYHRLEAGLDADGKLVGWQHRIVGQSILEDTPFAAVMVKDGVDATSVEGAANLPYAVP
NVSVSVELSTTQVGVPVLWWRVVGSSHTVYAVEAFIDEAAQAAGKDPYLFRRDLLAEQPRLRGVLELAAEKAGW
DPSRPLPAGRGRGIAVTEAFKTFVAQVVEVSVDKDGKLKVERVVCAVDCGIPINPDVIAAQMEGGIGFGLGAIL
HSAITLKDGKVEQNNFDGYQVLRIAEMPKVEVHIVPSGEAPTGVGEPGVAPIGPALANAIFAATGQRLYNLPFPT
SFAKA
>CABMLY01||gene_3404|GeneMark.hmm|771_aa|-|47160|49475
MKRSFPDDLIIGNLSRRGFLKGVGATGVLLVAANWGWRDALAAEKKAFGADAMPHGWVDNPKIYVSIDRDG
TVGIVCNRSEMGQGVRTSLAMVVADELEADWSRVKVIQAPGDEARYGNQDTDGSRSMRHWFEPMRRCGA
AARQMLEQAAANQWKVPLGECRAEQNKVLHAPSGRSLSFGELAEAAAGLEVPARDKLLLKKPEQFRYIGKDV
ARAIDGADIVNGRAGFGFDARFDDMLYAVVARPPVYGGKLKRYDAAAALKVPGVVKVIEIEGRPIPSEFQPLGG
VAVVAQNTWAAIKGREALVVEWDAGVNGGYDSVAYRKQLEEAARKPGKVVRDSGDAAALFAKGGDVVEAEY
YLPHLAQAPMEPPVSTAWYKDGACEVWAPTQAPQVTRERIAERLKLPFDKVTVNVTLLGGGFGRKSKPDFVLE
AAILAKAFPGRYLRVQWTREDDLHFSYFHTVSVERLQAVLGADGLPQAWLHRSVAPSITALFGPDSKHQGAFEL
GMGLTNLPFAIPNVRLENPEAPAHTRVGWFRSVSNIPHAFAIQSFVGELAAKAGQDPKDYLLKLLGPARRIDTAE
LGDSWNYGESPERYPLDVGRLRGVIEEAARQSGWGGELPRGRARGIAAHYSFVTYVAVVIEVEVKDDGALLVH
KATIAADCGPQINPERIRSQLEGACVMGLGLAALGEISFKDGKVQQDNFHQYELARMPLAPKAVSVHLLEPDG
DLPLGGVGEPGVPPIAPALCNAIFAATGKRIRELPIRNQLQGWRKA
>CABMOB01||gene_1683|GeneMark.hmm|734_aa|-|1882653|1884857
MRDFLNGYVPEEALLKNTTQSGTSRRNFIKLMSTAGAGLTLGVHLPSVAAGDKETAGTSFVPNAFVRVGSDDT
VTILIKHLDMGQGITTGLATIAADELDADWSKVTTEAAPANANQYNNLLFGPVQGTGGSTAIANSFMQLRMA
GAKAKAMLINAAAKVWEVSPSEIMTSSNTLSHNASGKSASYGEMAELAALQTVPSDEQVTLKTPEQFTIIGKTG
TLRKDQGKSNGTATFTQDVQLEGMLTAVVAHPPAFGAQVVSFNATAAKQSPGVVDVVQIPNGVAVIAKDFWS
AKTGRDKLTIEWARSAFNKSTEQLKKEYTALAQRPGQPARNEGDANKVLGASDDVIEATYYFPYLAHAPMEPM
NCVVLVGDGKAELWYGAQLQTIDQYAVAKVLGIKPENVTINTLFAGGSFGRRGNPHSDYVVEATMIAKTKPGV
PVKLVWTREDDMQAGYYRPMYVHKIRGAVDADGNLSAWEQRIVGQSIAAGTAFEQFMVKDGVDSSSVEGA
STLPYAIPNLAVELHTVQQPVPVVWWRSVGHTHNAFSTETFFDELAHKAGKDPVELRRTLLKNHPRHLGVLNL
AVEKSDWGKPLAKGKGRGVAVHESFRTFVAQVVEVTVENGQITVDRVVCAVDCGVAINPDIIKTQMQGGIGF
GLSPALVSEITLQNGATVQSNFHDYQVLREMQMPEIEVHIVPSAEPPTGVGEPGTPPIAPAVANAVFAATGKRLY
DLPMKMS
>CABSQF01||gene_4821|GeneMark.hmm|768_aa|+|8830|11136
MEIASMSKPSSFPKTATSRRAFLQGGGLLLGFALFGAGIKPVLAASTAQPVDGDVTAAFAPDAFIRIGTDGLITLIL
PNIEMGQGTHTGEATLIAEELEVSLDQVKAVDAPPNDKLYATAALGGQATGGSTSMRATWEPLRKAGATARM

MLVAAAAAEWSVPVSECVARLGVVTHQPTGRQLAYGALADAAARQPVPAEVKLKDQKDFQLIGKSSRRLDTP
GKVNGAVKYGIDIRVPDMKVATVAACPVLGGKLGDVDDKAARAIPGVRDVVRLDNAVAVIGDHFWAAKKGLE
ALDITWVEGANANLASANIMAALKAASERKKPIMARQEGDVDGRMKAAVKKVEATYELPFLAHAPMEPINCV
VHVRPDECEIWVGTQVPAIAQGLAAKVTGFPLEKVILHNQLIGGGFGRRLVAESVAQAVAIAKQVSYPVKVIWT
REEDIQHDLYRPAYYDRIAAGLGADGLPTVWVDHVAGGSVLGNYIPGGWPEDKLDDDAVEGAAKPPYDLPVI
QVDWVREDPPVPITWWRGVGPTHNVFVVESFMDELAHAAGKDPVEYRRALTRNQPRAAGVLELVAEKSGW
GTPLAAGMGRGISLHDAFGSYMAAVLEISVSPAGEITLHRAVVAVDCGITINPNTVEAQIEGGLIFGLSAALYSGI
TFTDGRVDQSNFHDYRILRNNEAPKIEIHHVKSSESPGGIGETATVSAAPALANAIFAATGKRLRTLPFNRDALKT
DGTDKKSVSMIPPLAAPLAAALASAKPADTELEKL
>CABTAY01||gene_4700|GeneMark.hmm|746_aa|+|110719|112959
MNRIIRDHSSTTSAGRPVNLSRRGFLGAAAGALVLGFDLPGTKAVAQGAAPVAKAPNVAAFLEIRPDSTILLRSP
FIEGGQGISTALAQIVGEELDADPATFVVECAPPGADYLVVGGARFTGGSFSVRASYATMRRVGASARQMLLQA
AAARWRVPADSLSTEPGRVRHAASNRTLAYGELAADAAKLPVPETVALRPEKDFRWIGKPFARLDARDKSTGK
VSYGIDLKVDGMLQAAVQHAPRRGQEPGAFANEAEVKAMPGVHSIHRLAGAVAVVAERWWYARRAVETLKV
TWTEPAAGAQGVVPANFSSEGRKAQLAAMPGPGFDAEKVGDVTAGLAGAGRVITASYDAPYLVHGQLEPPSA
IARWNADGTLDLWLPNQAPEMFQAAAAKVAGITPDKVKLHSPPLGGFFGRHFVYPPANPFPQVIQLAKAVGR
PVKVIWSREEEFSRDAMRPLGFARFKGGLDKDGIPVAIEAEAVGDGPLARWFGRKPDTADSSAVEGIAEKPYAIP
HRRVAHVLVEDPNVLGFWRSVGHSMNDFFYETFFDELADAGRQDPYQLRLRLLADKPRHKTLLEAVGELSGG
WKRGPFTAADGSKRARGVAMASPFGSEVATIAEVSLKDGAVRVHDVWVAIDPGRIVNPAIIESQVNSAVALGLS
SALLEEIVFVDGAPQARNFDAYPILPPDRMPRVHVRIVESGAPMGGIGEPGLPGVPPAVANAVAALTGQRVRSL
PLSKVKFEEAAGRT
>CABTAY01||gene_5639|GeneMark.hmm|745_aa|+|49887|52124
MNALLKIDLSAVMPLIPDQGVTNLSRRRLLGLGAGAFVLGTLLPAFGARAQAAAAVKPGTRVPAFLVIGQDNTV
KLLSPFVEGGQGINTGLAQIIGEELDVHPSRFEVECAPPGPDYAILNGLRLTGGSFSTRSSYEVMRRLGASARDM
LIRAAAAKLNVTADTLNTDDGYVVHAGSNRRVTYGELAEQALTLTPAENVALRDPATFRYIRQPMARLDVRAKS
TGKAVYAIDQKLDGMLYAAIQHAPVLGTEPERVSNESAVAAMPGVHAVHRLPGAVAVTADSWYRARKAVETLE
VTWSKAPATGFDAVAADYSSTGILAALKASDASGLSAEKDGDVAAAFANAAKIVEAEYDAPYLAHGQLEPPSSM
ARFNADGTLELFVPNQMPELFQSIAAKVGDVAPDRVILHSPMLGGFFGRHFAYGSSNPFQQAILLAKATKRPVK
VLWSREEEFKMDALRPLSFSRFKAALDKDGIPIAIKVRTVGEGPIGRWFGVTVGGKVDSSAVEGLVEKPYAIANR
SMEYVKFAHPVTIAFWRSVGHSMNDYFYEGFLDEIADAGGKDPYQLRLALLENKPRHLKLLETVARMSGGWK
RGPYEAEGGKRARGVALASPFGSETATIAEVSLQKGEVRVHNLWIAFDPGSIVNPAIITSQVESAAALGLSAALFE
ELVYKDGRRQQHNFDDYPILSRSAMPSVHVEIVESGAPMGGVGEPGLPGIAPAVVNAVAALTGRHVRSLPLAK
AKLGV
>CABTAY01||gene_7758|GeneMark.hmm|760_aa|+|450|2732
MNQHVKAAPSMAPDLSRRSFLVGTAATGLVLGYAGLADGALAATTPASFEPSVWYSIAPDGLVTVTCGKADM
GQHIASTMAQIICEELGAAWKDMRVQLASNDPKFNDPVLGAQITGGSWSTMMNFDAMSRAGAAGRIALTE
AAASVMGVTAKELVVRDGVVMHPKSKKQMSYAEIVKSGKITKSFTADELKALTLKTPDQYTMIGVSVPQLDIPA
KTNGTAKYGIDTMLPGMVYGKVVTPPVRFGATVKSVDDSEAKKVPGFIKAVVLDDKTGSTSGWVVAVASTFAN
AKKAADALKISYDKGPYANVSTDSIITEAMRLQAQDDAGQFFVKDGDANAALAGAAKVLEAEYTTSINIHAPM
EPMNATAEFKGDILHIYSGNQFATRSGAIAAGAAGIDPKYVVMHQAWLGGGFGRRLDADMMVPAVQAAKAV
GKPVKVIYSRENDMTMDYSRPLTYQKVKAGLDSNGKLIALSHDVVSAWPTARWGIPDFLTPSVDKKGPLDSFT
VNGADFFYTVPNHHVRAIKNELAHNATPSGQLRSVAPGWTFWAVESMIDEIAAASGQDPAQFRIALLDGKGK
NDGGAQRLRNTLLAAMGLSGYGAKKLPKGEGMGVACVSSQERATASWTACVAHVAVADNGAVTVKKLTVAT
DVGTQVHPDNIRAQVEGAALWGLSLAMYEKATLKDGGIEQTNFDSYTPLRMSQVPEVAIAVIANGEKATGVGE

PAVTVVAPALGNAIYNACGARLRSLPITAEAVKANMKA
>CABTAY01||gene_2433|GeneMark.hmm|737_aa|-|176543|178756
MPPRTPEEVGMNKHVSPRLNRRSFIIGTAALGGGLALGLDLPFGGPQVVRAADGSPEVNAWVVIRPDDTVVIR
IARSEMGQGSLTGLAQLVAEELACDWSKVTTEYPTPGQNVARKRVWGDFSTGGSRGIRSSQDYVRKGGAAAR
MMLIQAAADQWKVPVAECTAANSVITHKASGRTTTYGKVAEAAAKLTPPADVKLKDPKDWTLIGKGVKRLDT
ADKVTGAMIYGADIKLPGMLNAAIKDCPVTGGKLKSYDEAKIAGMKGVKKVVAVDGTAVAVVADTWWHAKT
ALDALPIVWDEGENAKVSSASIAKWLAEGLESGPAFVGNENGDAKAALASAAKKVEATYNYPYQNHATMEPM
NATALYTPERCEVWCGTQNGEAAFAAVLEASGLPADKCEVHKLILGGGFGRRGQTDYVRQAVQIAKAMPGTP
VKLLWSREEDMTHGRYHPITQCKLTGGFDADNNLTALHMRISGQSILFSLRPDALVNGKDPATFQGLAPSGEATI
GYSVPNLLIEHSMRNPHINPGFWRGVNVNQNAIYLECFMDELANAVGQDPLEFRRRLMSKNPKHLAVLDAVA
EKIGWGSPAPEGVYRGLAQLHGYGSYVAGAAEISVIDGTKIKIHRIVASTDPGYVVNPAQVERQIAGSFVYGLSAL
FYGGCTVKDGRIEQTNFDTYNSMRIAEMPKVEAIMIPSGGFWGGVGEPTIGVAAPAVLNAYFAATGKRIRSVPL
RDQNITFA
>CABTAY01||gene_1008|GeneMark.hmm|777_aa|-|251405|253738
MTIIANPDSATSASRRGFERHLKVENVSRRAILQTLGLAGGFVLAAPLLSRPAFAAYETGAGKMPHGTVVDPKIF
VSIAPDGIVSILAHRSEMGTGVRTSLPLIVAEEMEADWTKVRVVQAPGDEVKFGNQDTDGSRSTRHYLLPMRQ
IGAMARAMLEAAAAKRLGVPASEVKAVNHEVVHSASGKRLGFGELAADAANQPVPAVDSVQLKSPKDFRYLG
KGQVSIVDLHDITVGKARYGADVRLPGMKYAVIARPPVTGGKVKSFDSAEALKVPGVEQVLEVKGWPWPSKF
QPLGGVAVVARNTGAAIKGRDALKVEWDDGPNAAYDSVAYRAELEAAARQPGLVVRQEGDVEAALKSADKIV
TGEYYLPHFAHASMEPPVAVADVKGDKAEIWAPVQSPGGTREDVAKTLQLPPENVTVNVTLLGGGFGRKSKC
DFALEAALLSKTLGAPVKVQWTRDDDIQHDFLHTVSVERIEAGLDKSGKVVAWRHRSVAPTILSTFAAGADHAA
PFELGMGLVDMPFEIANIQCENPAAKAMTRIGWFRSVSNIPRAFAVQSMVGELAHATGRDQKDMLLELIGTP
RVVKLASVKDLWNYGEPYESYPIDTGRLRRVVEFVAEKGNWGRSLPKGHGLGIAAHRSFVSYIATIVEVSVDDKG
KLLVHQVDSAIDCGVFVNPERIQSQLEGAAIMGLSLAKYGEVSFKNGRVQQRNFDDHPVVRIDEAPLVTNVHIV
PADADTPPSGVGEPGVPPFAPALANAIFAATGKRLRALPIGNQLAT
>CABTAY01||gene_7206|GeneMark.hmm|723_aa|-|7866|10037
MRFNRTTSLGSAATLSRRNFLVNTAVAGGGLLLSFSLPSGQSMGSVAGAFEPNAFIRIGRDGLVVLTMPYVEMG
QGTYTSIPMLVAEELEVGLAQVRLEHAPPDEKVYANPLLGVQATGNSNAIRGAWKPLRQAGATARVMLVEAAA
RRFGDDARHCRAEAGEVIHMPSGRRLKYGDLVADAAMVPPPGKVVLKNADEFKLIGTPASRLDVSGKVNGSAI
YGIDVRPPGLKIATLAQSPVFGGRLKNVDDTAARAVKGVSQIVRLNDAVAVVADHMGAAKKGLEALEIEWEEG
THAGLSTQDIARGLEDATLKAGPVAQSVGDAARAMATAATRVQANYHLPFLAHAAMEPMNCTVHFRGSECEI
WVGTQAIARVQAAAAKAAGLAVDKVIVHNHLIGGGFGRRLEADGAVRAVEIARHVDGPVKVVWTREEDIQH
DMYRPYWVDRIEAGLDKSGRPIAWINRFAGSSVIARWLPPAFSNGLDPDTTEGAIDLVYSLPNFHVEYVRVEPP
GIPTAFWRSVGPSHNVFVTESFIDELAAAARQDAVAYRRALLDHNTRARAVLDLAAEKAGWGTALKKGHGRGV
ALQNVFGSYLAQVAEVEVANDGTVRVHRVVCAMDCGIVINPDTVQAQVQGGVMFGITAALYGEITLQSGRVK
QANFDTYQMLRIDQAPAIEVYVVNSKESPGGMGEAGTSGIVPAVANAVFAATGKRLRKMPITPDALKA
>CABTKA01||gene_3415|GeneMark.hmm|734_aa|-|179932|182136
MNEQPFSPSRRQLLKVAGLGFGALVIGFSLPFAGRSFAEQVLPEGPEDGTLPTATALDAFISIDRDGKVTFTVPKIE
MGQGAQSGLAMMIAEELEVPLEGITLKEAPPNEAIYNDSLLNFQATGGSTSIRANWEPLRRAGAAARLMLIQA
AAQRWQVPADGLRAENGRVHGPDGQSLGYGELVEAASQLPIPEDIPLKPASEFKVIGKPLRRLDTPSKVDGRAR
FTIDLAVPGMKYASIRACPVVGGSVANVDDSAARRIPGVVEVVRLGNAVAVIGEHTWAAFSGVRALQIEWDFG
EHAGLDSEQMERAIRDALDQPGALANEVGDIQNALDNAARTVEAEYEMPFLAHAALEPMGCVAQVRPDAVE
LWVGTQVPVRAQTAAAEEAGRPPEQVIVNNQLIGGAFGRRLEVDFIAQAVAIAKQVDYPVKLTWTREEDTAHD
MYRPHYIDRFAAALDGDGRLLGWRHSIAGASVLARFAPEAVPENGLDGDAVEVAQHPIYALEHLRVNYIPVPPK

AIHQSWWRGVGPLRSTYMLESFIDEVARSVERDPVDYRLQLLGQQPRAQAVLRLAAEKSGWGEALPTGHGRG
VAVQEVFGSYLATVVELEVTEGKGIRIKRLVVAIDCGLVMNPVSVKSQIEGGTLFGLSAALFNEITVRDGRVEQSN
FHDYRQLRISDAPPVETYIIDSTEAPGGVGEAGTAMIAPALVNALHSASGERIRRLPLARAGYHVLGGRPA
>CABTKS01||gene_537|GeneMark.hmm|747_aa|+|515864|518107
MAITRRTLIKGSALAVAAMLLPISTRSLAAVVSQDVAPPDSQHELNDWIWIDRDGRIVIGVSQCEVGQGIYTGL
AEVVAAEMDADWAQVTVKFVTGRDAYRQVAGGEAFAQFVAASTSMTKFYQRARLAGAQARDFFLRAGAKH
FALSPSQCRTEKGWVLEKGGKRKVAYGDLVRYAAELPLDPQPALKSEAQERESVIGKPLLRVDTPEKVDGSAIYGI
DIDLPEMLIGVPWMVPDLSGKLVAVRNERQIRAMPGVVDLVLTRQWSMNNMVGLDHDMSLNTVIVVAASY
WQAKKAADLLEVDWLPGAGKALTDSAIAAENLAMLDGDTLVPAVDRGEASALIRGVEQGSRLHEARYSAPYV
AHATLEPCNATSHYGEGRIETWGPFQGQDMVRNVLAKMFGLKPTDVVVNTTYLGGSFGRKYLPDAVMHATA
ASRAVGKPVKVVYPREIDMRHEYYRPACISHYRALLDENGYPQALWARYAGQSLFWQMRRETVNEAGGWDE
SMVECVYSTPYRIPHLKVEAGIVEQPISLSYLRGVGSVASLFFLESFISELSHKANRDEYSFRRHLLQDSPEALRVLD
ATATAAGWQHEPPSGVSRGMACNIWVGRNNAFTTYVGLVVEIAIQEGRLRVLRAVCAIDCGKVINPNLVRANV
EGGIGFALTTCLHSELHFERGGVVEGNFDRYPLLAIAEMPKVEVVILDSARAPQGCGEVSTAVVAPAMASALHK
ATGKTYRTMPFPREFSSV
>CABTWY01||gene_128|GeneMark.hmm|761_aa|-|129132|131417
MNKHVKNLAPETTDLSRRSFLVGTAATGLVLGYAGSGIDQALAAPAPANFEPSVWYSIAPDGLVTVTCGKADM
GQHVASTMAQIVAEELGANWKDMRVQLASNDPKFNDPVLGAQITGGSWSTMMNFDAMSRAGAAGRIALT
EAAAASMGVPASELVVRASTISHPKSKKSMSFADVVKSGKATKTFTADDLKAIKLKTPDQYTMIGVSVPQLDIPL
KVNGTAKYGIDTMLPGMVYGALVTPPVRYGAAVKSVDDSAAKKLPGFIKAVTLDDKTMTTTGWVVAVANTYA
QAKKAAEALKISYDGGPNAKASSEALLAEAKRLQGLSDSGQFFVKDGDPDAAFGTAAKVLEAEYTTNINIHAPM
EPMNATAEFKGDILHIYSGNQFATRSGAIAAGAAGIDPKFVVMHQTWLGGGFGRRLDADMMVPAVQAAKAV
GKPVKVIYSRENDMTMDFSRPLTFQKVKAGVDGDGKLVALSHDVVSAWPTQRWGIPDFLSPSVDKKGPLDAF
TVNGADFFYTVPNHYVRAIKNEMAHNATPSGQLRSVAPGWTFWAVESMVDEIAHATGKDPALLRVELLDGKG
KNDGGAQRLRNTLLAAMGLAGYGTKKLPKGEGMGVACVSSQERATASWTACVAHVAVAPSGEVTVKKLTVAT
DVGTQVNPDGIRAQVEGAALWGMSLALFEKATLKDGGIEQTNFDSYTPLRMSQLPEVAVSVIANGEKATGVGE
PAVTVVAPAIGNAVFNAVGARVRGLPITAEAVKAAMKA
>CABTWY01||gene_6113|GeneMark.hmm|718_aa|+|7277|9433
MLPRTREEAAMNLQVNQHVKLNRRAFVIGTATAGAGLALGLDLPFGGPAVVRAADGAPEVNAWVVIRPDDT
VVIRIARSEMGQGTLTGLAQLVAEELECDWSKVTTEYPTPGQSVARKRAWGDFSTGGSRGIRTSQDYVRKGGA
TARVMLIQAAANEWKVPAAECKVSNGVITHTASGKTTTYGKVAEAAARLEPPADVKLKDPKDWTIAGKGLKRL
DTVDKTTGKMIYGIDVKLPGMLNAAIKDCPVFGGKVKSFDEAKIANMKGVKKVVPVGDSAVAVVADTWWRA
KTALDALPIVWDEGDNAKVSSETIAKWLAEGLDNAQPAYVGNQNGDAKAAIASAAKKVEAVYSYPYQNHATM
EPMNATVLYTPDKCEVWCGTQNGEAAFAAALEASGLPAEKVDVHKLMLGGGFGRRGMTDYVRQAVAIAKQ
MPGTPIKLLWSREEDMQHGKYHPVTQCKLTGAFDADDNLVALHYRLSGQSILFSVRPEALQNGMDPAAFQGV
AQSGEAAIGYSVPNLLVEHSMRNPHVPPGFWRGVNVNHNAIYMECFMDELAQAVGQDPLEFRRKLMGKNP
KHLAVLNAVAEKIGWGTPAPQGVYRGIAQVMGYGSYVAGAAEISVTDGSKIKVHRIVASTDPGYVVNPAQVER
QIAGSFVYGLSALFYGGCTVKDGRIEQTNFDTYNSMRINEMPKVEAVMVPSGGVWGGVGEPTIRDAAAGGV
NA
>CABTWY01||gene_1947|GeneMark.hmm|726_aa|+|3898|6078
MTLMDNLSERAADLSRRNFLRASAIAGGGLLLSVNLPFAGRESEAAAAAGDFAPNAFVRIGGDGKVVLTMPYV
EMGQGTYTSIPMLIAEELEIGLNQVQLEHAPPSDKLYANPLLGVQATGNSNAMRGAWQPMRKAGATAKAML
VAAAAKRWNVEPATCRAENGEVHHAASGRKLGYGELAADAAQMPVPENVTLKSPAEFKLIGTAAKRLDTPSKV
NGTAVYGIDARPPGVKVATLAQSPVFGGRVKRVDDAAAKAVNGVRQIVTLDDAVAVVADHMGAAKKGLAALT

IEWDEGPHAKLATADIARELEAATTKPGAVAQNIGDADNALAGAATKVEATYQLPFLAHATMEPMNCTVHVRP
DGCEIWVGNQALSRVQAVTAKLLNLPPEKVVVHNHLLGGGFGRRLEVDGVIRAVQIARQVDAPLKLVWTREE
DIQHDMYRPYWCDRISVGLDASGKPIAWNNRFAGSSVIARWAPPAFRNGLDPDTTEGAIDLVYDIPNFHVEYV
RVEPPGIPTAFWRSVGPSHNVFVTESMIDEMAAAAKQDPVEYRKALLGKSPRAKAALELAAAKAGWGGKLPA
GRGRGVSLQFVFGSYLALIAEVEVAKDGSVRVHRVVCAMDCGTVVNPDTVQAQLQSGINFGVTAALYGEITLK
DGRVEQTNFDSYQMLRIDQAPAIEVHIVPSTEPPGGMGETGTSGIVPAISNAIFAATGKRLRKMPVDPAVLKQA
>CABTWY01||gene_1664|GeneMark.hmm|695_aa|+|9512|11599
MRLPEFAYGSERLVDGGEFSPNAFIRIDGRGAISFIMRSVEMGQGIYTAAAMLIAEELEVRLDQIEALAAPANEAL
YTDPILGQQETGGSASTRSSWIPLRQAGATARVMLIMAAAKRWGVVFEDCVARQGMVTHSASGRSASYGELA
EDAARQPVPDNVPLKPASEFRLIGSSAHRLDSAAKANGTATFGIDIKVPGMKIGTVAACPVRGGRLVSIDEAAAK
RVSGVRDVVRLDDLVSVIGDNMWAATQGLAAANPHWDEGPNKSVSTEDLIKDLDTASRQPGVVAKQEGDPV
SAIAGSVTRIDAVYQLPFLAHAPMEPINTTIHIRADGADVWVGTQVPVRAQLAVALVTGLPQESINIHNQYLGG
GFGRRLDVDSIHQAARIAKQLPYPVKLIWTREEDIQHDLYRPYYYDRVSAGLDAQGNISGWTHRVTGSSVAAR
WRPSRMQKDGHLDPDAVLGATETPYELPATLVDYVRCEPRVFDTLWWRGVGQTHNVFVVESLMDELAAAAS
QDPVDFRRKFLKKNSRGRSVLDLAVEKSGWGKPLPKGWGRGVALQFSFNTHVASVLEAEISGGEIRLRRVHIAV
DCGPVVNPNIIEAQMEGGMIFGLTMALYGEITVTNGRVDQSNFNDYRMLRMNQAPEISVHLVNNPDAPIGGI
GETGTVAAAPALANAIYSATGRRLRRIPFAQQVAQTQ
>CABTWZ01||gene_1395|GeneMark.hmm|750_aa|-|16089|18341
MRIRGIEALAGGSGEAVKTGDAVAPLTRRGFLKLTGMAGGGLALGIGSVVESAHAQGAAKPASSPQAFLQIAPD
NTVTVAVNRLEFGQGVHTALPMALAEELDVDWKNVRAMLAPAGDPYKDPMFGIQMTGGSTAVNHSFEQYR
ELGARARAMLIAAAAQQWKVDPASCATSLGVVTSGANRATYGELAQAAMAQPVPAQVKLKDPAQFRIVGKP
TPRLDAASKLHGDGVFGLDVKLKDMKVAVVAHPPRFGGKVKSFNADKARKIKGVADVFLVPVDRGGTGVAVV
ADGYWPAKTAREALEIVWEDAGSKVSSAALFDEYSKLAAQPGTVARALEGGNIDTALSGAAKVIEAEYRVPYLA
HAPMEPLNCTMQAEIAANKATAVKVWVGSQFQTIDQAAIARTLGLTPDKVTLNTMMAGGGFGRRAVPTSDY
LVESANVMRSWIAAGHTEPLKVMWSREDDIKGGYYRPLHVHRARVGVDAQGKVVGWQHTIVGQSIITGTPF
EPMMVKNGVDATMVEGIIENDYGLPLQLNVHHPKTDVPVLWWRSVGNTHTAFVKETLADEMATAAKQDPV
AWRMARLDEKAHARHRAALQLAVDKSGYGKKKLPKGHAWGVAVHESFGSVVAYVVDVSIEKGQPKVHRVTA
GVHANRVVNPLTAEAQVQGGCVFGISMTKPGFAIEIENGVVKNSNFPDYPPPRITDAPVVDVFFVPSNDNPTG
LGEPGVPAISPAIANALFRLTGKRQRQMPFVLT
>CABTXE01||gene_1609|GeneMark.hmm|711_aa|-|39840|41975
MGFLLERRQFLAGISATAALAYLDLPVLAAQSETSLGETGLGAWIRIGKNGAVTILQPQAEMGQGVNTSISMLIA
EELEVDVRRVTVEFPPAAAAYANKVYGFQTTAESTSIRSFFDKCRTVGAQAREMLVSAAAKKWRLDPATLRAEA
GYVVDPQSGNRLGYGELAEAASKLPAPEKPRLKSKSEWKIIGKPVKRLDTAAKTNGKAVYGIDVKVPGMLTAAV
MQCPVPGGKLKSVDEKPALAVTGVKQVVKLDNLVAVLADGYWQAKAGLDALSIEWDRGVGDGFTTEMAFAQ
FRQALDKKPGSKAEETGNVDAAFTGAAKVIAAEYTAPYLAHATMEPQNATAHYTPDKLTIWAPTQAQGLVGIVI
GPLVGLKADQVECHTAFLGGGFGRRFELDVPIQAALISKAAKVPVKVIWSREEDMSHDFYRPGAVVRLEAAIDG
DRKITGLRTKIASSSILSRAVPDLVKDGIDITAVDGVKGTDYEFGSRTLHYNLENTPIPVGFWRSVAHSINGWVME
GFVNELAIELNEDPVALRRELLAGKARNISVLDALAERSDWTKKTAGRYKGIAIHHSFDAIIGHVVEISVPKLGQIR
IEKITTVADVGTAINPDTIRAQIQSAIVYGLSAAMTGETVFAGGEAVQKNFDSFEVLRLASMPQIDVHILELGGAI
AGVGEPGLPPLAPALVAAVNVAFSRRIRSLPLAGHGVALA
>CABTXE01||gene_918|GeneMark.hmm|723_aa|-|43687|45858
MNLDHIASSIPDATLSRRSFLATAAAGGAFMLSLSLPLGKGEAASPEGFAPNAFIRIGSDGQVALTMPYVEMGQ
GTYTSIPMLIAEELDVSLKQVRLEHAPPNEKIYANPLLGVQATGNSNAMRGAWKPMREAGATARSMLVAAAAK
RWAVDPESCRTQDGEVVHPQTNRRLTYGELAAEAAKLPVPKAVALKPAADFKLIGTPAKRLDGPGKVNGTAVY

GIDVRPPGLKVATLVQSPVFGGRVKSVDDAEAKAVKGVRQIVRLDDAVAVVADHMGAAKKGLAALKIEWDDG
PNAGLATADIARELEQATLRSGPVAQNLGDADKAMAGAATKVEAIYQVPFLAHATMEPMNCTVHLRKDECEI
WIGNQAIARVQAMAAKAAGLPAEKVIVHNHLIGGGFGRRLDADGAVRAVEIAKHVDGPVKVVWTREEDIQH
AMYRPYWFDRISAGLDDKGMPVAWKNRFAGSSVIARWLPPAFKDGLDPDTTEGAIDLVYNLPNFHVEYVRVE
PQGIPTAFWRSVGPSHNVFVTESFIDELAAEAKRDAVAYRRALLDKSPRAKAVLDLAAEKAGWGQALPKGSGR
GISLQFSFGSYMAHVAEVEVSKDGAVRVRRVICAVDCGTVVNPNTVQAQIQSGIVFGTTAALYGEITLKNGRVE
QGNFDTYQILRINEAPAIEVHVVKSTEPPGGMGETGTSAIVPAIANAIFAATGKRLRKMPIDTSVLKSA
>CABUNC01||gene_742|GeneMark.hmm|752_aa|-|106070|108328
MADLQQRASREPEDAGDGILNVSRRHFLRGAGGLALGIYFAPLLGRFGDPQAAAAKAFEPNAFVSIAPDGTVT
VIAKHVEMGQGSYTGLATLLAEELDADWSKVRVEGAPADAKRYANLAFGTLQGTGGSSAMANSFEQMRKAG
ATARAMLVAAAAQQWKVPAEQIEVHDGVVEHKASGHKAGFGQLAEAAAQQAVPTDVKLKAPEDFKLIGQVK
LPRKDSQDKTDGQARFTQDVHLPDMLVAVVAHPPRFGGVPAKVDDSKARAVPGVVAVVQFPGSDSRFAGVA
VLAKNTWAARQGRDALQVAWDESNAFRMGSAEIFARYQELAAKPGVVARNEGDIAKALDKPAKLIEAQYQFP
FLAHASMEPLNCVVRLSDGACEIWNGEQWQTGDQMAVGQLLGIAPEKVSITQLYAGGSFGRRANPHSDYVLE
AVSIAKAAREHGHKGPVKMVWTREDDTRGGYYRPAFLHSARLALDGQGNLVGWEQHLVGQSFIVGTPFEKA
MVKDGIDQVAVEGAADLPYAVPNLRVEQTLVPEVKVPTQWWRSVGHTHTAYSTETLIDEAAVAAGKDPYEFRR
ALLEKHPRHLGVLDLVADKAGWKQPLKAGGEGEKRGRGIAVHESFGSFVAQVVEVTVKADKSFRIDRVVCAVD
CGLAINPDVIRAQMEGGIGYGLAMALHSAITLKEGVVEQSNFHDFQVLRINEMPAVEVHIVPSSEAPTGVGEPG
VPPAAPALANALAAATGKRIRNLPIGNQLQA
>ACRC01P||EFV86562.1
TGRRLGYGELAADAAKLPVPAGDAVKLKTRAEFRYIGKDEVRLVDLEAIGKGEAMYGIDMRLPGMVYAVVARP
PVVGGKLRRVDSAKALAVPGVLKVVEIPAMAGAPAFQPLGGVAVVASNTWAAMQGRAALAIEWDDGPNAA
YDSVAYRETLTEASRKPGKVVRDQGDAPQAWAKAGEAERFMAEYHVPHLAHASMETPVATVRIQDGAAEVW
TSVQNPAAAQEAVAKRLKLKPENVKVHVLLLGGGFGRKSKPDYVDEAAIVAQAMPAGTPVKLVWTREDDIHH
DYLHTVSAEHLEAVVGKDGKVQSWLHRSAAPTIASLFTEGAKGEQLFESAMSAINMPYVIPNVRVETAEVAAH
ARIGWFRSVANIPHAFAAQCFIAELAHRAGRDHKQYALDLIGPARRIDPGTLADTWNYSESPERYPYDTGRLRG
VIEAAASGAKWGRELPKGHGLGLAFCYSFMSYTATVVEVAVDEKGEVRVVAVDMALDCGPQIKPERIRAQME
GGAIMGLSLALLGEITFEKGRVKQNNFYDYEVLRHNASPRVIRTHLVNDDHALPPGGVGEPPVPPVAPALCNAIF
AATGKRVRSLPVRSVA
>ACUF01P||EFP64414.1
MNAKTTKPRSGRRRFLLGALGIGGALVVGWGVMPPRSRVGDPGIFPEHNGEIALNGWIKITPEGDVVLAMPR
VEMGQGIHTALSMLAAEELDIPLSRVRIESAPVERIYGNVVAMGDSSLPLHPDSADKTWARALHWIMAKSARE
IGLIITGGSSSTADGWQPVREAAATARAALVQAAAREWNVPAADVSIREGQLIGPGGKQSTFGEMAKSARGISA
PSNVTLKPASQFRLIGKPAPRNDLAAKTDGSARFSIDTRLPGMLYAAVVMCPVFGGKLKTFQSKAALGMPGVR
YVVPFEGAGGGAPGVAVVADHYWQARQALATLEPVWDNGPHAKLDSAGIRQQLVSALDSDKGGFTYRSMG
DGLKAFDRADGATIVEAEYSAPYLAHATMEPINCTAQVTADGVHLWAPTQVATLAQLVAARAAGVSGDKVQID
IPLIGGGFGRRLESDFISQAVTIATKTEGKPVQVIWSREEDVRHDFYRPQAIARLKARVESGKVTAIASRSAGQSIL
AGELDRLFGAPSVGIDRYTAEGLFDLPYEIEHEHIAHLAVDLPVPVGFWRSVGHSYNGFFMEGFLNEVAAAAKL
DPLAMRRNLLKDHPRELKVLDTAAQAAGWGQPLAPAADGAPRARGIALHPSFGSVVAQVVEVSMKDGKPRV
HRVVCAVDCGTVVNPGIVAQQMESAVIFGLTAALYGRIDIKDGQVVQSNFPDYPALKMVETPVIETHIVPSTAEP
SGMGEVGVPPIAPAVAHAMAQLTGKPVRQLPMV
>ACWU01P||EHF09422.1
MKRSYPDDLVIGNLSRRGFLKGVGATGVLLVAANWGWRDALAAEKKAFGADAMPHGWVDNPKIYVSIDRDG
TVGIVCNRSEMGQGVRTSLAMVVADELEADWSRVKVIQAPGDEARYGNQDTDGSRSMRHWFEPMRRCGA

AARQMLEQAAANQWKVPLGECRAEQNKVLHAPSGRSLSFGELAEAAAGLEVPARDKLLLKKPEQFRYIGKDV
ARAIDGADIVNGRAGFGFDARFDDMLYAVVARPPVYGGKLKRYDAAAALKVPGVVKVIEIEGRPIPSEFQPLGG
VAVVAQNTWAAIKGREALVVEWDAGVNGGYDSVAYRKQLEEAARKPGKVVRDSGDAAALFAKGGDVVEAEY
YLPHLAQAPMEPPVSTAWYKDGACEVWAPTQAPQVTRERIAERLKLPFDKVTVNVTLLGGGFGRKSKPDFVLE
AAILAKAFPGRHLRVQWTREDDLHFSYFHTVSVERLQAVLGADGLPQAWLHRSVAPSITALFGPDSKHQGAFE
LGMGLTNLPFAIPNVRLENPEAPAHTRVGWFRSVSNIPHAFAIQSFVGELAAKAGQDPKDYLLKLLGPARRIDTA
ELGDSWNYGESPERYPLDVGRLRGVIEEAARQSGWGGELPRGRARGIAAHYSFVTYVAVVIEVEVKDDGALLV
HKATIAADCGPQINPERIRSQLEGACVMGLGLAALGEISFKDGKVQQDNFHQYELARMPLAPKAVSVHLLKPD
GDLPLGGVGEPGVPPIAPALCNAIFAATGKRIRELPIRNQLQGWRKA
>ACWU01P||EHF13557.1
MNSKIDLSNALPGSRRGFLKGAAVVGLTIGFQWSGARRALAAALPDAGFAPNAFLRIAPDDSVTVIAKHVEMG
QGAYTGIATIVAEELDADWSKVRVESAPADAKRYANLAFGTMQGTGGSSAMANSWMQLREAGAKARAMLV
EAAARQWQIPAAELRTRDGFVEHPASQRKASYGSLAAAAAELPVPEKVQLKDPKDFRLIGHQAPRVDVPGKTD
GSAQFTLDVSLPGMLVALLQRPPLFGATVKSFDATATRAIPGVVEVVQVPHGVAVVAKGFWAAKQGRDALKVE
WDESKAEKRGSEALMAEYRKLAEQPGKPARRDGDAAKAVAGATRRIAASYEFPFLAHAPMEPLDAVVRLTADS
CEIWAGDQFQTVDQGNAARTAGLKPEQVKINTLYAGGSFGRRANAWSDYIVEAVSIAKALGANGVPVKLQWT
REDDIHGGFYRPMYYHRLEAGLDADGKLVGWQHRIVGQSILEGTPFAAVMVKDGVDATSVEGAANLPYAVPN
VSVELSTTQVGVPVLWWRVVGSSHTVYAVEAFIDEAAQAAGKDPYLFRRDLLAEQPRLRGVLELAAEKAGWD
PSRPLPAGRGRGIAVTEAFKTFVAQVVEVSVDKDGKLKVERVVCAVDCGIPINPDVIAAQMEGGIGFGLGAILHS
AITLKDGKVEQNNFDGYQVLRIAEMPKVEVHIVPSGEAPTGVGEPGVAPIGPALANAIFAATGQRLYNLPFPTSF
AKA
>ADVL01||EFH12486.1
AAPRPGSRVAAFLEIRPDNTVLLKSPFIEGGQGVDTAMAQILGEELDVEPARFTVECAPPGADYALVNGMRITG
GSYSVRSAYPVLRQLGARARQMLLQAAAARLGVAADSLATEPGRVIHAASGRSLAYGALAAEAASLPVPESAPL
RAERDFRWIGKPVARLDVRAKSTGQAVYAIDLTVEGMLQAAVQHAPRLGQEPRALANEAEVRAMPGVHSIHR
LPGAVAVLADRWWRARRAVEALQVDWAEPAAGARRAMPADFSSNAMREALAKARGNALPAESHGDAEAAL
KTATRTLEAQYDAPYLAHGQLEPPSAIAHWKGDGTLELWLPNQAPEMFQRAAARVAGIAPEKVVIHSPLLGGF
FGRHFLYDNANPFPQAILLSKAVGRPVKLIWSREEEFLRDALRPMGLARFRAGLGPDGLPVAFAAEAVGEGPVG
RYFGGQPGRADPSAVEGISEKPYAIPNRLVVQVPHAHPAIIGFWRSVGHSMNDFFYEAFLDEIAEAGGQDPYAL
RLRLLADKPRHKALLEEAVGELSGGWRRGPYDAPDGTRRARGVAMASPFGSEVATIAEVSLREGEVVVHEVWV
AVDPGRMVNPAIIEAQVNSAVALGLSSALLEEVVYENGIPQARNFDGYPILPPGRMPRVQVRIIESGAPMGGIG
EPGLPGVPPAVVNAVAALTGQRIRSLPLSKARLGAA
>AGEZ01||EHM01677.1
MAAPKPAEEADMTVQSNLSRRRFVIGSAAAAGGLSLGFGLPDAPSALAQASSSLPEVNAWVVIRPDETVVIRIA
RSEMGQGTLTGLAQLVAEELECDWARVTTEFPGPSESLRRNRVWGNFSTGGSRGIRESERLVREGGAAARHML
VAAAAADWGVPAEQCRVAKGVITHPASNRSVTYGAVASAASRMTPPSSITLKDPKDWTIAGQPLPRLDTAEKLT
GKLVYGADLQFPGMLNAAVKACPYSGGKIASYDEAAVANMPGVRKVIRIGDATVAVVADTWWRAKTALDALP
ITWDEGPYRDLTSASIAETLNEGLDADEAFIGNRAGDAKAVLARSQRKVTATYAFPFQNHATMEPMNATALFTG
DRCEVWVPTQNGEASLAAAAEAAGLPARQCEVHKLMLGGGFGRRGFQDYVTQAVTIARHFQGTHVKLLWSR
EEDMLQGYYHPTTQCRMSGALDDQGNLIALHMRISGQSILASVRPEGMQGGMDPVVFQGLTPNSPEGMLG
YSIPDLLIDHAMRNPPIRPGFWRGVNLNQNAVYVECFMDELAHEAGIDPLAFRRKLMANHPKHLATLNAVAE
RIGWDTPPAAGVGRGLAQIMGFGAYVAAAAEVSVENGELKVHRIIAATDPGHVVNPAQVERQVEGSFAYGLSA
GLLGECTVTGGRIDQENFDTYDVVRMVQMPKVETIMLPSGGFWGGVGEPTIAVATPAVLNALFAATGKRIRM
LPLKNHSLA

>AGWX01||EKS36728.1
MNLDHIASSIPDATLSRRSFLATAAAGGAFMLSLSLPLGKSEAASPEGFAPNAFIRIGRDGQVALTMPYVEMGQ
GTYTSVPMLIAEELDVSLKQVRLEHAPPNEKIYANPLLGVQATGNSNAMRGAWKPMREAGATARSMLVAAAA
KRWAVDPESCRTQDGEVVHPQTNRRLTYGELAAEAAKLPVPKAVALKPAADFKLIGTPAKRLDGPGKVNGTAV
YGIDVRPPGLKVATLVQSPVFGGRVKSVDDAEAKAVKGVRQIVRLDDAVAVVADHMGAAKKGLAALKIEWDD
GPNAGLATADIARELEQATLRSGPVAQNLGDADKAMAGAATKVEAIYQVPFLAHATMEPMNCTVHLRKDECEI
WIGNQAIARVQAMAAKAAGLPAEKVIVHNHLIGGGFGRRLDADGAVRAVEIAKHVDGPVKVVWTREEDIQQ
AMYRPYWFDRISAGLDDKGMPVAWKNRFAGSSVIARWLPPAFKDGLDPDTTEGAIDLVYNLPNFHVEYVRVE
PPGIPTAFWRSVGPSHNVFVTESFIDELAAAAKQDAVAYRRALLDKSPRAKAVLDLAAEKAGWGQALPKGSGR
GISLQFSFGSYMAHVAEVEVSKDGAVRVRRVICAVDCGTVVNPNTVQAQIQSGIVFGTTAALYGEITLKNGRVE
QGNFDTYQILRINEAPAIEVHVVKSTEPPGGMGETGTSAIVPAIANAIFAATGKRLRKMPIDTSVLKSA
>AGYX01||EPD39766.1
MPQRPSNERGRVADSAISLRRRHLLQSAAALLVAPAAGSLLIPLAQAAPPAAGAAAAAAIGDWVWIEPSGQVV
IGVSQCEVGQGIYTGLPQVLADELDADWASVTVRFVTGRDAYRNDAGEMPFQQFVGASMSMNYFYERMRL
AGAQARDVLLRAGASRLGVRASQCMTRAGRVLHPATGRSVGYGEIVADASRLPLAARPRMKSASEQGLIGRNL
RRVDTPSKVDGSAVFGIDVEVPGMLIGAVRMAPSVTGRIVRIRNEAAVRARPGVHAVVRTTQWPDPEPSTVV
VVADSYWIAKQAADALDIEFDAGAAAGVDSERIHAQFVAGLSNDKAVVARSLGKPREVLAAGKTITADYHSPYI
THATMEPLAATVHVRDGEVETWGPYQGQDFLRGELGKACGVPADKVIVHTTFLGGSFGRKYMPDFALHAAA
ASKAVGRPVKVIRSREDDIRHSYYRPGASGRLSAVLGADGMPAALHARISGQSLYGAINPKKMADAGGWDET
MVESIYDLIYGVPNLLVDAVDVQQPIPLSYLRSVGTTSSVFFLESFISELAHTAGVDDYQYRRRLLAGQPLALGVLD
AAARAARWEQPAPAGLHRAMTFNVYTGRGESFQTFVALVMELRVVQGRVRLERAICAIDAGRVVNPGLVKAN
VEGGIGFALTNTFKSRLDFDKGAVQQSNFHDYPLLQLSEMPRVEVVLVESDRPPQGCGEVALGPTAPAVATAMF
HATGRRFRSMPLPQDIASI
>AGYY01||EPD46882.1
MPQRPSNERGRVADSAISLRRRHLLQSAAALLVAPAAGSLLIPLAQAAPPAAGAAAAAAIGDWVWIEPSGQVV
IGVSQCEVGQGIYTGLPQVLADELDADWASVTVRFVTGRDAYRNDAGEMPFQQFVGASMSMNYFYERMRL
AGAQARDVLLRAGASRLGVRASQCMTRAGRVLHPATGRSVGYGEIVADASRLPLAARPRMKSASEQGLIGRNL
RRVDTPSKVDGSAVFGIDVEVPGMLIGAVRMVPSVTGRIVRIRNEAEVRARPGVHAVVRTTQWPDPEPSTVV
VVADSYWIAKQAADALDIEFDAGAAAGVDSERIHAQFVAGLSNDKAVVARSLGKPREVLAAGKTITADYHSPYI
THATMEPLAATVHVRDGEVETWGPYQGQDFLRGELGKACGVPADKVIVHTTFLGGSFGRKYMPDFALHAAA
ASKAVGRPVKVIRSREDDIRHSYYRPGASGRLSAVLGADGLPAALHARISGQSLYGAINPKKMADAGGWDETM
VESIYDLIYGVPNLLVDAVDVQQPIPLSYLRSVGTTSSVFFLESFISELAHTAGVDDYQYRRRLLAGQPLALGVLDA
AARAARWEQPAPAGLHRAMTFNVYTGRGESFQTFVALVMELRVVQGRVRLERAICAIDAGRVVNPGLVKANV
EGGIGFALTNTFKSRLDFDKGAVQQSNFHDYPLLQLSEMPRVEVVLVESDRPPQGCGEVALGPTAPAVATAMFH
ATGRRFRSMPLPQDIAST
>AGZI01||EKU81967.1
MTLASTSRRGFLRAGAAGGAFLIGMSAQGVLAAVSDTKAGAASPDFVPNAFIRIARDGSVILISKQPEIGQGIKT
SLPMVIAEELDVAWDSVRVIQGDLNLEAYGSQGAGGSNSTPNNYENFRRLGATARAVLVQAAAQAWKAPARE
CTTDGGHVLHAASKRRLAYGDLVEAAARLPLPDAASVAVKDPATFKLLGKRIGGVDNPAIVSGKPLFGIDVQLPG
MLHAQYVKCPVFGGKPLRANLDAVRALPGVKDAFIIEGTSNLNGLRPGVAIVATSTWAAIRARRALEVTWDEG
GAAKHSWADFTAQAKAAASQPGATVLRKDGDVAAALKGAARTVEAAYSYPFISHASMEPQNCTAWIKPDGAL
ELWAPTQNPNAGQALVSGTCGIPKEKITMHIIRSGGGFGRRLSSDFIVEATAIAQRMKDKAPVKLTWTREDDLQ
HDHFRPGGFHFLRGGVDEAGKVVGWHNHFVTFANRVEQDGKSTLRNGSGGSLSPDEFPGRWLANCQLEQT
ALECSVPMGPWRAPGSCVFAWAFHSFIDELAHAGGRDPLAFRLELLGDKDIMAPSVERGQPYNVARMRAVL

QTAADKAGWGKKKFARGQGQGIAFHFSHRGYVAQVAEVTVSQDGKVKVDRVVVALDVGPTIINLSGAENQV
EGSVIDGLSTLMFPELNLENGRIVQSNFHDYQLLRIGDAPAKIEMHFVKSDQPVTGLGEPPLPPLAPAVCNAIFA
ATGKRVRELPLSKADLSWS
>AGZI01||EKU80642.1
MHERMRKPTRRSFLLAGLAAGGALLIGWGAQPPRQRLHPARPLALAGDEVALNGWVALAPDGTVSVVVPRS
EMGQGVHTALPMLVAEELDVGLDAVRVIQPPIDKIYANVTVLRENLPFHPDDAGRTAQGAQWLMAKVGREL
GIMFTGGSSSVRDAWLPMREAGALARAMLVKAAAQEWGARVEDCRTEDGFVIHLDGRSAGYGALAALAAQ
AGAGLTARDVRLKPPSAFRLIGRPQPRLDSRAKADGSARFGIDARPARMVYAALAMAPTIGATVAAFDADAVR
RMPGVLAVVDVSSALAGRTGAGAGVAVVASTWWQARQAAQALPVTWQPGEGAALSSAAILAGLARALDEE
RGYVYHETGAQEVEGAVRTLRAEYRAPFLAHAALEPVNCTAQVARGKVRLWASTQVPSIAVDCAARVAGVARE
DVAIEVMLLGGGFGRRLEVDMVAQAVAIAKALDGQPVQLIWSREQDTMHDVYRPAALARFTAQLDEGGNILA
WDNKSASGAIGHQYFERSLGLPGVGPDKTTAEGEYDHQYAIPNQRVAHVTVDSAVPIGYWRSVGHSHNAFFK
ESFLDEVAHAGGQDPVALRRKLLARHPRALAVLDAAVAAAGQPPPRRAHGVALHRSFGSMVAQVAEVSVDGR
EIRVHRVVCAIDCGLVVNPTIVAQQVESSVVFGLSAALAGEITIEQGKVRQSNFGDYPLLRIGQAPQVEIIVMASR
AHPEGVGEPAVPPVAPAVAAAVFKLTGQRLRSLPLTLN
>AGZU01||EKU73731.1
MNAPILSRRRFLSASLVAGGGLLFDLNIPLAGAAEGAPQILTAFVRILPDNRVVIGAKNAEIGQGAKTMLPMLIA
EELDVDWAQVTIEQTHADQKIFGGQTAGGSRTTPREWLPTRKAGAAARAMLVAAAAQLWGVAPATLKTGSG
KVSDPASGKSISYAALAAAAAQQPAPDPATLKLKDPKDFRIIGQSIGGVDTPAIVAGKPLFGIDFKLPGMLYAVLET
CPAFGGTFQSANLDAVKALPGVAHVLTIKGDGTPESLFDGVAILSSSWWSANQAREALKVEWDMSAVSGFSTE
GYAAQAAERLKGKADGDIVRAGDVDAAFATAAKTVSAEYDYPFLAHGTLEPQNCTALFKDGAIEIWAPTQNPES
GRGLVAKALNLPPDKIRINFTRIGGGFGRRLMNDYMVQAAAIAAQLPGVPVKLLYNRQQDMQRDFYRPAGW
HGFRAALDKTGKLTAFHDHFVTFGKDSKPVRSAEMPATEIPAGLIDTVLLEQSFLSSNMPTGWLRAPGSNALAF
VTQAFLDEVAQAAGKDLPTLMLELLGEPRELPRGPNAQPFVTGRARGVIEKVVAMSGWADRGKLPKGRGKGF
AFYYSHMGYFAEVLEVALVDGMPKVATVWVAGDVGSQIINPMNALHQAQGSVIEGLGQALAGQKITQVAGA
VEQANFDTHPLQRIPDTPQIIVEFVKTDYPPTGMGEPALPPVIPALVNALHAATGKRIRTLPIVPEMFA
>AQFN01||EMZ59144.1
MKRSYPDDLVIGNLSRRGFLKGVGATGVLLVAANWGWRDALAAEKKAFGADAMPHGWVDNPKIYVSIDRDG
TVGIVCNRSEMGQGVRTSLAMVVADELEADWSRVKVIQAPGDEARYGNQDTDGSRSMRHWFEPMRRCGA
AARQMLEQAAANQWKVPLGECRAEQNKVLHAPSGRSLSFGELAEAAAGLEVPARDKLLLKKPEQFRYIGKDV
ARAIDGADIVNGRAGFGFDARFDDMLYAVVARPPVYGGKLKRYDAAAALKVPGVVKVIEIEGRPIPSEFQPLGG
VAVVAQNTWAAIKGREALAVEWDAGVNGGYDSVAYRKQLEEAARKPGKVVRDSGDAAALFAKGGDIVEAEYY
LPHLAQAPMEPPVSTAWYKDGACEVWAPTQAPQVTRERIAERLKLPFDKVTVNVTLLGGGFGRKSKPDFVLEA
AILAKAFPGRHLRVQWTREDDLHFSYFHTVSVERLQAVLGADGLPQAWLHRSVAPSITALFGPDSKHQGAFEL
GMGLTNLPFAIPNVRLENPEAPAHTRVGWFRSVSNIPHAFAIQSFVGELAAKAGQDPKDYLLKLLGPARRIDTAE
LGDSWNYGESPERYPLDVGRLRGVIEEAARQSGWGGELPRGRARGIAAHYSFVTYVAVVIEVEVKDDGALLVH
KATIAADCGPQINPERIRSQLEGACVMGLGLAALGEISFKDGKVQQDNFHQYELARMPLAPKAVSVHLLKPDG
DLPLGGVGEPGVPPIAPALCNAIFAATGKRIRELPIRNQLQGWRKA
>AQFN01||EMZ49708.1
MNSKIDLSNALPGSRRGFLKGAAVVGLTIGFQWSGARRALAAALPDAGFAPNAFLRIAPDDSVTVIAKHVEMG
QGAYTGIATIVAEELDADWSKVRVESAPADAKRYANLAFGTMQGTGGSSAMANSWMQLREAGAKARAMLV
EAAARQWRVPATELRTRDGFVEHPASQRKASYGSLAAAAAELPVPEKVQLKDPKDFRLIGHQAPRVDVPGKTD
GSAQFTLDVSLPGMLVALLQRPPLFGATVKSFDATATRAIPGVVEVVQVPHGVAVVAKGFWAAKQGRDALKVE
WDESKAEKRGSEALMAEYRKLAEQPGKPARRDGDAAKAVAGATRRIAASYEFPFLAHAPMEPLDAVVRLTADS

CEIWAGDQFQTVDQGNAARTAGLKPEQVKINTLYAGGSFGRRANAWSDYIVEAVSIAKALGANGVPVKLQWT
REDDIHGGFYRPMYYHRLEAGLDADGKLVGWQHRIVGQSILEGTPFAAVMVKDGIDATSVEGAANLPYAVPN
VSVELSTTQVGVPVLWWRVVGSSHTVYAVEAFIDEAAQAAGKDPYLFRHDLLAEQPRLRGVLELAAEKAGWD
PSRPLPAGRGRGIAVTEAFKTFVAQVVEVSVDKDGKLKVERVVCAVDCGIPINPDVIAAQMEGGIGFGLGAILHS
AITLKDGKVEQNNFDGYQVLRIAEMPKVEVHIVPSGEAPTGVGEPGVAPIGPALANAIFAATGQRLYNLPFPTSF
AKA
>AQFO01||EMZ49109.1
MNSKIDLSNALPGSRRGFLKGAAVVGLTIGFQWSGARRALAAALPDAGFAPNAFLRIAPDDSVTVIAKHVEMG
QGAYTGIATIVAEELDADWSKVRVESAPADAKRYANLAFGTMQGTGGSSAMANSWMQLREAGAKARAMLV
EAAARQWRVPATELRTRDGFVEHPASQRKASYGSLAAAAAELPVPEKVQLKDPKDFRLIGHQAPRVDVPGKTD
GSAQFTLDVSLPGMLVALLQRPPLFGATVKSFDATATRAIPGVVEVVQVPHGVAVVAKGFWAAKQGRDALKVE
WDESKAEKRGSEALMAEYRKLAEQPGKPARRDGDAAKAVAGATRRIAASYEFPFLAHAPMEPLDAVVRLTADS
CEIWAGDQFQTVDQGNAARTAGLKPEQVKINTLYAGGSFGRRANAWSDYIVEAVSIAKALGANGVPVKLQWT
REDDIHGGFYRPMYYHRLEAGLDADGKLVGWQHRIVGQSILEGTPFAAVMVKNGIDATSVEGAANLPYAVPN
VSVELSTTQVGVPVLWWRVVGSSHTVYAVEAFIDEAAQAAGKDPYLFRHDLLAEQPRLRGVLELAAEKAGWD
PSRPLPAGRGRGIAVTEAFKTFVAQVVEVSVDKDGKLKVERVVCAVDCGIPINPDVIAAQMEGGIGFGLGAILHS
AITLKDGKVEQNNFDGYQVLRIAEMPKVEVHIVPSGEAPTGVGEPGVAPIGPALANAIFAATGQRLYNLPFPTSF
AKA
>AQFO01||EMZ59427.1
MKRSYPDDLVIGNLSRRGFLKGVGATGVLLVAANWGWRDALAAEKKAFGADAMPHGWVDNPKIYVSIDRDG
TVGIVCNRSEMGQGVRTSLAMVVADELEADWSRVKVIQAPGDEARYGNQDTDGSRSMRHWFEPMRRCGA
AARQMLEQAAANQWKVPLGECRAEQNKVLHAPSGRSLSFGELAEAAAGLEVPARDKLLLKKPEQFRYIGKDV
ARAIDGADIVNGRAGFGFDARFDDMLYAVVARPPVYGGKLKRYDAAAALKVPGVVKVIEIEGRPIPSEFQPLGG
VAVVAQNTWAAIKGREALVVEWDAGVNGGYDSVAYRKQLEEAARKPGKVVRDSGDAAALFAKGGDIVEAEYY
LPHLAQAPMEPPVSTAWYKDGACEVWAPTQAPQVTRERIAERLKLPFDKVTVNVTLLGGGFGRKSKPDFVLEA
AILAKAFPGRHLRVQWTREDDLHFSYFHTVSVERLQAVLGADGLPQAWLHRSVAPSITALFGPDSKHQGAFEL
GMGLTNLPFAIPNVRLENPEAPAHTRVGWFRSVSNIPHAFAIQSFVGELAAKAGQDPKDYLLKLLGPARRIDTAE
LGDSWNYGESPERYPLDVGRLRGVIEEAARQSGWGGELPRGRARGIAAHYSFVTYVAVVIEVEVKDDGALLVH
KATIAADCGPQINPERIRSQLEGACVMGLGLAALGEISFKDGKVQQDNFHQYELARMPLAPKAVSVHLLKPDG
DLPLGGVGEPGVPPIAPALCNAIFAATGKRIRELPIRNQLQGWRKA
>AQFP01||ENA33221.1
MTKNVSAQAISLSRRKLLQASGIAAGGLLLATALPFSRRSYAEQYVNKGPEDAPLDTPTALGAFLRIGHDGQITLIS
PKIEMGQGVQTGFAMMVAEELNVTLDQVRVQEAPPDEKLYGDKLLGFQATGGSSSTRSNWQPLREAAAAAR
VMLIQAAANQWKVSPDECRAENGKIIGPGNRELAYGALVDAAAKLPVPENVPLKKPEDYKVIGQPLRRLDTPG
KVDGSAKFTIDLHVPGMKIATVSACPVVGGTLASVDDRHARAVPGVRDVVKLENAVAVIGDHMWAALKGLKA
LEIQWGLGPNAGIDSAQIERALHAAFDREGAIAAEVGDINKAIAGASSKIEAEYEMPFLAHATLEPMTCVAQVR
EDACELWVGTQVPVMAQQAAAKATGLPPEKIIVNNQLIGGGFGRRLEADFIGQAAAIAKQVDYPIKLVWTREE
DTAHDLYRPHYIDRFSAGLDANGMPVGWSHTIVGASVMARFAPAAVPPNGLDADAVEVSNKPVYSLPNLRVR
YVPEAPKAILNSWWRGVGPLHGAYVMESFIDELAYAAKQDPVDYRMKLLGEHPRAQAVLKLAAEKANWSQKL
PAGHGRGIAVQEVFGSYLATVVEMQVDAQNGIHITRLICIADCGEVTNPTSVHSQLEGGTLFGLSAALYNEITVK
NGHVEQSNFHDYRQLRMSDAPPVETHIIPSHEIPGGIGEAGTAMIAPALVNALYAATGKRLRRLPVVRAGYHVA
>JZWM01||KJJ13135.1
MNSKIDLSNALPGSRRGFLKGAAVVGLTIGFQWSGARRALAAALPDAGFAPNAFLRIAPDDSVTVIAKHVEMG
QGAYTGIATIVAEELDADWSKVRVESAPADAKRYANLAFGTMQGTGGSSAMANSWMQLREAGAKARAMLV

EAAARQWRVPATELRTRDGFVEHPASQRKASYGSLAAAAAELPVPEKVQLKDPKDFRLIGHQAPRVDVPGKTD
GSAQFTLDVSLPGMLVALLQRPPLFGATVKSFDATATRAIPGVVEVVQVPHGVAVVAKGFWAAKQGRDALKVE
WDESKAEKRGSEALMAEYRKLAEQPGKPARRDGDAAKAVAGATRRIAASYEFPFLAHAPMEPLDAVVRLTADS
CEIWAGDQFQTVDQGNAARTAGLKPEQVKINTLYAGGSFGRRANAWSDYIVEAVSIAKALGANGVPVKLQWT
REDDIHGGFYRPMYYHRLEAGLDADGKLVGWQHRIVGQSILEGTPFAAVMVKDGIDATSVEGAANLPYAVPN
VSVELSTTQVGVPVLWWRVVGSSHTVYAVEAFIDEAAQAAGKDPYLFRRDLLAEQPRLRGVLELAAEKAGWD
PSRPLPAGRGRGIAVTEAFKTFVAQVVEVSVDKDGKLKVERVVCAVDCGIPINPDVIAAQMEGGIGFGLGAILHS
AITLKDGKVEQNNFDGYQVLRIAEMPKVEVHIVPSGEAPTGVGEPGVAPIGPALANAIFAATGQRLYNLPFPTSF
AKA
>JZWM01||KJJ18253.1
MKRSYPDDLVIGNLSRRGFLKGVGATGVLLVAANWGWRDALAAEKKAFGADAMPHGWVDNPKIYVSIDRDG
TVGIVCNRSEMGQGVRTSLAMVVADELEADWSRVKVIQAPGDEARYGNQDTDGSRSMRHWFEPMRRCGA
AARQMLEQAAANQWKVPLGECRAEQNKVLHAPSGRSLSFGELAEAAAGLEVPARDKLLLKKPEQFRYIGKDV
ARAIDGADIVNGRAGFGFDARFDDMLYAVVARPPVYGGKLKRYDAAAALKVPGVVKVIEIEGRPIPSEFQPLGG
VAVVAQNTWAAIKGREALVVEWDAGVNGGYDSVAYRKQLEEAARKPGKVVRDSGDAAALFARGGDIVEAEYY
LPHLAQAPMEPPVSTAWYKDGACEVWAPTQAPQVTRERIAERLKLPFDKVTVNVTLLGGGFGRKSKPDFVLEA
AILAKAFPGRHLRVQWTREDDLHFSYFHTVSVERLQAVLGADGLPQAWLHRSVAPSITALFGPDSKHQGAFEL
GMGLTNLPFAIPNVRLENPEAPAHTRVGWFRSVSNIPHAFAIQSFVGELAAKAGQDPKDYLLKLLGPARRIDTAE
LGDSWNYGESPERYPLDVGRLRGVIEEAARQSGWGGELPRGRARGIAAHYSFVTYVAVVIEVEVKDDGALLVH
KATIAADCGPQINPERIRSQLEGACVMGLGLAALGEISFKDGKVQQDNFHQYELARMPLAPKAVSVHLLKPDG
DLPLGGVGEPGVPPIAPALCNAIFAATGKRIRELPIRNQLQGWRKA
>LTER01||OFJ83419.1
MNSKIDLSNALPGSRRGFLKGAAVVGLTIGFQWSGARRALAAALPDAGFAPNAFLRIAPDDSVTVIAKHVEMG
QGAYTGIATIVAEELDADWSKVRVESAPADAKRYANLAFGTMQGTGGSSAMANSWMQLREAGAKARAMLV
EAAARQWRVPATELRTRDGFVEHPASQRKASYGSLAAAAAELPVPEKVQLKDPKDFRLIGHQAPRVDVPGKTD
GSAQFTLDVSLPGMLVALLQRPPLFGATVKSFDATATRAIPGVVEVVQVPHGVAVVAKGFWAAKQGRDALKVE
WDESKAEKRGSEALMAEYRKLAEQPGKPARRDGDAAKAVAGATRRIAASYEFPFLAHAPMEPLDAVVRLTADS
CEIWAGDQFQTVDQGNAARTAGLKPEQVKINTLYAGGSFGRRANAWSDYIVEAVSIAKALGANGVPVKLQWT
REDDIHGGFYRPMYYHRLEAGLDADGKLVGWQHRIVGQSILEGTPFAAVMVKDGIDATSVEGAANLPYAVPN
VSVELSTTQVGVPVLWWRVVGSSHTVYAVEAFIDEAAQAAGKDPYLFRRDLLAEQPRLRGVLELATEKAGWDP
SRPLPAGRGRGIAVTEAFKTFVAQVVEVSVDKDGKLKVERVVCAVDCGIPINPDVIAAQMEGGIGFGLGAILHS
AITLKDGKVEQNNFDGYQVLRIAEMPKVEVHIVPSGEAPTGVGEPGVAPIGPALANAIFAATGQRLYNLPFPTSF
AKA
>LTES01||OFJ87703.1
MNSKIDLSNALPGSRRGFLKGAAVVGLTIGFQWSGARRALAAALPDAGFAPNAFLRIAPDDSVTVIAKHVEMG
QGAYTGIATIVAEELDADWSKVRVESAPADAKRYANLAFGTMQGTGGSSAMANSWMQLREAGAKARAMLV
EAAARQWRVPATELRTRDGFVEHPASQRKASYGSLAAAAAELPVPENVQLKDPKDFRLIGHQAPRVDVPGKT
DGSAQFTLDVSLPGMLVALLQRPPLFGATVKSFDATATRAIPGVVEVVQVPHGVAVVAKGFWAAKQGRDALKV
EWDESKAEKRGSEALMAEYRKLAEQPGKPARRDGDAAKAVAGATRRIAASYEFPFLAHAPMEPLDAVVRLTAD
SCEIWAGDQFQTVDQGNAARTAGLKPEQVKINTLYAGGSFGRRANAWSDYIVEAVSIAKALGANGVPVKLQW
TREDDIHGGFYRPMYYHRLEAGLDADGKLVGWQHRIVGQSILEGTPFAAVMVKDGIDATSVEGAANLPYAVP
NVSVELSTTQVGVPVLWWRVVGSSHTVYAVEAFIDEAAQAAGKDPYLFRRDLLAEQPRLRGVLELAAEKAGW
DPSRPLPAGRGRGIAVTEAFKTFVAQVVEVSVDKDGKLKVERVVCAVDCGIPINPDVIAAQMEGGIGFGLGAIL
HSAITLKDGKVEQNNFDGYQVLRIAEMPKVEVHIVPSGEAPTGVGEPGVAPIGPALANAIFAATGQRLYNLPFPT

SFAKA
>LTES01||OFJ90593.1
MKRSYPDDLVIGNLSRRGFLKGVGATGVLLVAANWGWRDALAAEKKAFGADAMPHGWVDNPKIYVSIDRDG
TVGIVCNRSEMGQGVRTSLAMVVADELEADWSRVKVIQAPGDEARYGNQDTDGSRSMRHWFEPMRRCGA
AARQMLEQAAANQWKVPLGECRAEQNKVLHAPSGRSLSFGELAEAAAGLEVPARDKLLLKKPEQFRYIGKDV
ARAIDGADIVNGRAGFGFDARFDDMLYAVVARPPLYGGKLKRYDAAAALKVPGVVKVIEIEGRPIPSEFQPLGG
VAVVAQNTWAAIKGREALVVEWDAGVNGGYDSVAYRKQLEEAARKPGKVVRDSGDAAALFAKGGDIVEAEYY
LPHLAQAPMEPPVSTAWYKDGACEVWAPTQAPQVTRERIAERLKLPFDKVTVNVTLLGGGFGRKSKPDFVLEA
AILAKAFPGRHLRVQWTREDDLHFSYFHTVSVERLQAVLGADGLPQAWLHRSVAPSITALFGPDSKHQGAFEL
GMGLTNLPFAIPNVRLENPEAPAHTRVGWFRSVSNIPHAFAIQSFVGELAAKAGQDPKDYLLKLLGPARRIDTAE
LGDSWNYGESPERYPLDVGRLRGVIEEAARQSGWGGELPRGRARGIAAHYSFVTYVAVVIEVEVKDDGALLVH
KATIAADCGPQINPERIRSQLEGACVMGLGLAALGEISFKDGKVQQDNFHQYELARMPLAPKAVSVHLLKPDG
DLPLGGVGEPGVPPIAPALCNAIFAATGKRIRELPIRNQLQGWRKA
>LTFB01||OFK13753.1
MKRSYPDDLVIGNLSRRGFLKGVGATGVLLVAANWGWRDALAAEKKAFGADAMPHGWVDNPKIYVSIDRDG
TVGIVCNRSEMGQGVRTSLAMVVADELEADWSRVKVIQAPGDEARYGNQDTDGSRSMRHWFEPMRRCGA
AARQMLEQAAANQWKVPLGECRAEQNKVLHAPSGRSLSFGELAEAAAGLEVPARDKLLLKKPEQFRYIGKDV
ARAIDGADIVNGRAGFGFDARFDDMLYAVVARPPVYGGKLKRYDAAAALKVPGVVKVIEIEGRPIPSEFQPLGG
VAVVAQNTWAAIKGREALVVEWDAGVNGGYDSVAYRKQLEEAARKPGKVVRDSGDAAALFAKGGDIVEAEYY
LPHLAQAPMEPPVSTAWYKDGACEVWAPTQAPQVTRERIAERLKLPFDKVTVNVTLLGGGFGRKSKPDFVLEA
AILAKAFPGRHLRVQWTREDDLHFSYFHTVSVERLQAVLGADELPQAWLHRSVAPSITALFGPDSKHQGAFELG
MGLTNLPFAIPNVRLENPEAPAHTRVGWFRSVSNIPHAFAIQSFVGELAAKAGQDPKDYLLKLLGPARRIDTAEL
GDSWNYGESPERYPLDVGRLRGVIEEAARQSGWGGELPRGRARGIAAHYSFVTYVAVVIEVEVKDDGALLVHK
ATIAADCGPQINPERIRSQLEGACVMGLGLAALGEISFKDGKVQQDNFHQYELARMPLAPKAVSVHLLKPDGD
LPLGGVGEPGVPPIAPALCNAIFAATGKRIRELPIRNQLQGWRKA
>LTFB01||OFK12839.1
MNSKIDLSNALPGSRRGFLKGAAVVGLTIGFQWSGARRALAAALPDAGFAPNAFLRIAPDDSVTVIAKHVEMG
QGAYTGIATIVAEELDADWSKVRVESAPADAKRYANLAFGTMQGTGGSSAMANSWMQLREAGAKARAMLV
EAAARQWRVPATELRTRDGFVEHPASQRKASYGSLAAAAAELPVPEKVQLKDPKDFRLIGHQAPRVDVPGKTD
GSAQFTLDVSLPGMLVALLQRPPLFGATVKSFDATATRAIPGVVEVVQVPHGVAVVAKGFWAAKQGRDALKVE
WDESKAEKRGSEALMAEYRKLAEQPGKPARRDGDAAKAVAGATRRIAASYEFPFLAHAPMEPLDAVVRLTADS
CEIWAGDQFQTVDQGNAARTAGLKPEQVKINTLYAGGSFGRRANAWSDYIVEAVSIAKALGANGVPVKLQWT
REDDIHGGFYRPMYYHRLEAGLDADGKMVGWQHRIVGQSILEGTPFAAVMVKDGVDATSVEGAANLPYAVP
NVSVELSTTQVGVPVLWWRVVGSSHTVYAVEAFIDEAAQAAGKDPYLFRRDLLAEQPRLRGVLELAAEKAGW
DPSRPLPAGRGRGIAVTEAFKTFVAQVVEVSVDKDGKLKVERVVCAVDCGIPINPDVIAAQMEGGIGFGLGAIL
HSAITLKDGKVEQNNFDGYQVLRIAEMPKVEVHIVPSGEAPTGVGEPGVAPIGPALANAIFAATGQRLYNLPFPT
SFAKA
>LTGL01||OFL03435.1
MNSKIDLSNALPGSRRGFLKGAAVVGLTIGFQWSGARRALAAALPDAGFAPNAFLRIAPDDSVTVIAKHVEMG
QGAYTGIATIVAEELDADWSKVRVESAPADAKRYANLAFGTMQGTGGSSAMANSWMQLREAGAKARAMLV
EAAARQWRVPATELRTRDGFVEHPASQRKASYGSLAAAAAELPVPEKVQLKDPKDFRLIGHQAPRVDVPGKTD
GSAQFTLDVSLPGMLVALLQRPPLFGATVKSFDATATRAIPGVVEVVQVPHGVAVVAKGFWAAKQGRDALKVE
WDESKAEKRGSEALMAEYRKLAEQPGKPARRDGDAAKAVAGATRRIAASYEFPFLAHAPMEPLDAVVRLTADS
CEIWAGDQFQTVDQGNAARTAGLKPEQVKINTLYAGGSFGRRANAWSDYIVEAVSIAKALGANGVPVKLQWT

REDDIHGGFYRPMYYHRLEAGLDADGKLVGWQHRIVGQSILEGTPFAAVMVKDGIDATSVEGAANLPYAVPN
VSVELSTTQVGVPVLWWRVVGSSHTVYAVEAFIDEAAQAAGKDPYLFRRDLLAEQPRLRGVLELAAEKAGWD
PSRPLPAGRGRGIAVTEAFKTFVAQVVEVSVDKDGKLKVERVVCAVDCGIPINPDVIAAQMEGGIGFGLGAILHS
AITLKDGKVEQNNFDGYQVLRIAEMPKAEVHIVPSGEAPTGVGEPGVAPIGPALANAIFAATGQRLYNLPFPTSF
AKA
>LTGL01||OFL07387.1
MKRSFPDDLVIGNLSRRGFLKGVGATGVLLVAANWGWRDALAAEKKAFGADAMPHGWVDNPKIYVSIDRDG
TVGIVCNRSEMGQGVRTSLAMVVADELEADWSRVKVIQAPGDEARYGNQDTDGSRSMRHWFEPMRRCGA
AARQMLEQAAANQWKVPLGECRAEQNKVLHAPSGRSLSFGELAEAAAGLEVPARDKLLLKKPEQFRYIGKDV
ARAIDGADIVNGRAGFGFDARFDDMLYAVVARPPVYGGKLKRYDAAAALKVPGVVKVIEIEGRPIPSEFQPLGG
VAVVAQNTWAAIKGREALVVEWDAGVNGGYDSVAYRKQLEEAARKPGKVVRDSGDAAALFAKGGDIVEAEYY
LPHLAQAPMEPPVSTAWYKDGACEVWAPTQAPQVTRERIAERLKLPFDKVTVNVTLLGGGFGRKSKPDFVLEA
AILAKAFPGRHLRVQWTREDDLHFSYFHTVSVERLQAVLGADGLPQAWLHRSVAPSITALFGPDSKHQGAFEL
GMGLTNLPFAIPNVRLENPEAPAHTRVGWFRSVSNIPHAFAIQSFVGELAAKAGQDPKDYLLKLLGPARRIDTAE
LGDSWNYGESPERYPLDVGRLRGVIEEAARQSGWGGELPRGRARGIAAHYSFVTYVAVVIEVEVKDDGALLVH
KATIAADCGPQINPERIRSQLEGACVMGLGLAALGEISFKDGKVQQDNFHQYELARMPLAPKAVSVHLLKPDG
DLPLGGVGEPGVPPIAPALCNAIFAATGKRIRELPIRNQLQGWRKA
>LTGM01||OFL06078.1
MNSKIDLSNALPGSRRGFLKGAAVVGLTIGFQWSGARRALAAALPDAGFAPNAFLRIAPDDSVTVIAKHVEMG
QGAYTGIATIVAEELDADWSKVRVESAPADAKRYANLAFGTMQGTGGSSAMANSWMQLREAGAKARAMLV
EAAARQWRVPATELRTRDGFVEHPASQRKASYGSLAAAAAELPVPEKVQLKDPKDFRLIGHQAPRVDVPGKTD
GSAQFTLDVSLPGMLVALLQRPPLFGATVKSFDATATRAIPGVVEVVQVPHGVAVVAKGFWAAKQGRDALKVE
WDESKAEKRGSEALMAEYRKLAEQPGKPARRDGDAAKAVAGATRRIAASYEFPFLAHAPMEPLDAVVRLTADS
CEIWAGDQFQTVDQGNAARTAGLKPEQVKINTLYAGGSFGRRANAWSDYIVEAVSIAKALGANGVPVKLQWT
REDDIHGGFYRPMYYHRLEAGLDADGKLVGWQHRIVGQSILEGTPFAAVMVKDGIDATSVEGAANLPYAVPN
VSVELSTTQVGVPVLWWRVVGSSHTVYAVEAFIDEAAQAAGKDPYLFRRDLLAEQPRLRGVLELAAEKAGWD
PSRPLPAGRGRGIAVTEAFKTFVAQVVEVSVDKDGKLKVERVVCAVDCGIPINPDVIAAQMEGGIGFGLGAILHS
AITLKDGKVEQNNFDGYQVLRIAEMPKVEVHIVPSGEAPTGVGEPGVAPIGPALANAIFAATGQRLYNLPFPTSF
AKA
>LTGM01||OFL00967.1
MKRSYPDDLVIGNLSRRGFLKGVGATGVLLVAANWGWRDALAAEKKAFGADAMPHGWVDNPKIYVSIDRDG
TVGIVCNRSEMGQGVRTSLAMVVADELEADWSRVKVIQAPGDEARYGNQDTDGSRSMRHWFEPMRRCGA
AARQMLEQAAANQWKVPLGECRAEQNKVLHAPSGRSLSFGELAEAAAGLEVPARDKLLLKKPEQFRYIGKDV
ARAIDGADIVNGRAGFGFDARFDDMLYAVVARPPVYGGKLKRYDAAAALKVPGVVKVIEIEGRPIPSEFQPLGG
VAVVAQNTWAAIKGREALVVEWDAGVNGGYDSVAYRKQLEEAARKPGKVVRDSGDAAALFAKGGDIVEAEYY
LPHLAQAPMEPPVSTAWYKDGACEVWAPTQAPQVTRERIAERLKLPFDKVTVNVTLLGGGFGRKSKPDFVLEA
AILAKAFPGRHLRVQWTREDDLHFSYFHTVSVERLQAVLGADGLPQAWLHRSVAPSITALFGPDSKHQGAFEL
GMGLTNLPFAIPNVRLENPEAPAHTRVGWFRSVSNIPHAFAIQSFVGELAAKAGQDPKDYLLKLLGPARRIDTAE
LGDSWNYGESPERYPLDVGRLRGVIEEAARQSGWGGELPRGRARGIAAHYSFVTYVAVVIEVEVKDDGALLVH
KATIAADCGPQINPERIRSQLEGACVMGLGLAALGEISFKDGKVQQDNFHQYELARMPLAPKAVSVHLLKPDG
DLPLGGVGEPGVPPIAPALCNAIFAATGKRIRELPIRNQLQGWRKA
>LTGY01P||OFL33910.1
MTTTMQVSRRGFLKGGLGALTLAVTGNGLVSAVWAADEPKKYGADSMPGGTVDDPLAFVSIAADGTVTIVAH
RAEMGTGVRTSLPMVVADEMEAAWDRVRVVQADADEARYGNQNVDGSRSVRHFLMPMRRVGAAARQM

LEAAAAARWSVPLAEVRATQHEVVHAPSGRRLGYGELAADAAKLPVPAGDAVKLKTRAEFRYIGKDEVRLVDLE
AIGKGEAMYGMDMRLPGMVYAVVARPPVVGGKLRRVDSAKALAVPGVLKVVEIPAMAGAPAFQPLGGVAVV
ASNTWAAMQGRAALAIEWDDGPNAAYDSVAYRETLTEASRKPGKVVRDQGDAPQAWAKAGEAERFMAEY
HVPHLAHASMETPVATVRIQDGAAEVWTSVQNPAAAQEAVAKRLKLKPENVKVHVLLLGGGFGRKSKPDYVD
EAAIVAQAMPAGTPVKLVWTREDDIHHDYLHTVSAEHLEAVVGKDGKVQSWLHRSAAPTIASLFTEGAKGEQL
FESAMSAINMPYVIPNVRVETAEVAAHARIGWFRSVANIPHAFAAQCFIAELAHRAGQDHKQYALDLIGPARRI
DPGTLADTWNYSESPERYPYDTGRLRGVIEAAASGAKWGRELPKGHGLGLAFCYSFMSYTATVVEVAVDEKGE
VRVVAVDMALDCGPQIKPERIRAQMEGGAIMGLSLALLGEITFEKGRVKQNNFYDYEVLRHNASPRVIRTHLV
NDDHALPPGGVGEPPVPPVAPALCNAIFAATGKRVRSLPVRSVA
>LTIA01||OFL96130.1
MNSKIDLSNALPGSRRGFLKGAAVVGLTIGFQWSGARRALAAALPDAGFAPNAFLRIAPDDSVTVIAKHVEMG
QGAYTGIATIVAEELDADWSKVRVESAPADAKRYANLAFGTMQGTGGSSAMANSWMQLREAGAKARAMLV
EAAARQWRVPATELRTRDGFVEHPASQRKASYGSLAAAAAELPVPEKVQLKDPKDFRLIGHQAPRVDVPGKTD
GSAQFTLDVSLPGMLVALLQRPPLFGATVKSFDATATRAIPGVVEVVQVPHGVAVVAKGFWAAKQGRDALKVE
WDESKAEKRGSEALMAEYRKLAEQPGKPARRDGDAAKAVAGATRRIAASYEFPFLAHAPMEPLDAVVRLTADS
CEIWAGDQFQTVDQGNAARTAGLKPEQVKINTLYAGGSFGRRANAWSDYIVEAVSIAKALGANGVPVKLQWT
REDDIHGGFYRPMYYHRLEAGLDADGKLVGWQHRIVGQSILEGTPFAAVMVKDGIDATSVEGAANLPYAVPN
VSVELSTTQVGVPVLWWRVVGSSHTVYAVEAFIDEAAQAAGKDPYLFRRDLLAEQPRLRGVLELAAEKAGWD
PSRPLPAGRGRGIAVTEAFKTFVAQVVEVSVDKDGKLKVERVVCAVDCGIPINPDVIAAQMEGGIGFGLGAILHS
AITLKDGKVEQNNFDGYQVLRIAEMPKVEVHIVPSGEAPTGVGEPGVAPIGPALANAIFAATGQRLYNLPFPTSF
AKA
>LTIA01||OFM04390.1
MKRSFPDDLVIGNLSRRGFLKGVGATGVLLVAANWGWRDALAAEKKAFGADAMPHGWVDNPKIYVSIDRDG
TVGIVCNRSEMGQGVRTSLAMVVADELEADWSRVKVIQAPGDEARYGNQDTDGSRSMRHWFEPMRRCGA
AARQMLEQAAANQWKVPLGECRAEQNKVLHAPSGRSLSFGELAEAAAGLEVPARDKLLLKKPEQFRYIGKDV
ARAIDGADIVNGRAGFGFDARFDDMLYAVVARPPVYGGKLKRYDAAAALKVPGVVKVIEIEGRPIPSEFQPLGG
VAVVAQNTWAAIKGREALVVEWDAGVNGGYDSVAYRKQLEEAARKPGKVVRDSGDAAALFAKGGDIVEAEYY
LPHLAQAPMEPPVSTAWYKDGACEVWAPTQAPQVTRERIAERLKLPFDKVTVNVTLLGGGFGRKSKPDFVLEA
AILAKAFPGRHLRVQWTREDDLHFSYFHTVSVERLQAVLGADGLPQAWLHRSVAPSITALFGPDSKHQGAFEL
GMGLTNLPFAIPNVRLENPEAPAHTRVGWFRSVSNIPHAFAIQSFVGELAAKAGQDPKDYLLKLLGPARRIDTAE
LGDSWNYGESPERYPLDVGRLRGVIEEAARQSGWGGELPRGRARGIAAHYSFVTYVAVVIEVEVKDDGALLVH
KATIAADCGPQINPERIRSQLEGACVMGLGLAALGEISFKDGKVQQDNFHQYELARMPLAPKAVSVHLLKPDG
DLPLGGVGEPGVPPIAPALCNAIFAATGKRIRELPIRNQLQGWRKA
>LTIF01||OFM27240.1
MNSKIDLSNALPGSRRGFLKGAAVVGLTIGFQWSGARRALAAALPDAGFAPNAFLRIAPDDSVTVIAKHVEMG
QGAYTGIATIVAEELDADWSKVRVESAPADAKRYANLAFGTMQGTGGSSAMANSWMQLREAGAKARAMLV
EAAARQWRVPATELRTRDGFVEHPASQRKASYGSLAAAAAELPVPEKVQLKDPKDFRLIGHQAPRVDVPGKTD
GSAQFTLDVSLPGMLVALLQRPPLFGATVKSFDATATRAIPGVVEVVQVPHGVAVVAKGFWAAKQGRDALKVE
WDESKAEKRGSEALMAEYRKLAEQPGKPARRDGDAAKAVAGATRRIAASYEFPFLAHAPMEPLDAVVRLTADS
CEIWAGDQFQTVDQGNAARTAGLKPEQVKINTLYAGGSFGRRANAWSDYIVEAVSIAKALGANGVPVKLQWT
REDDIHGGFYRPMYYHRLEAGLDADGKLVGWQHRIVGQSILEGTPFAAVMVKDGIDATSVEGAANLPYAVPN
VSVELSTTQVGVPVLWWRVVGSSHTVYAVEAFIDEAAQAAGKDPYLFRRDLLAEQPRLRGVLELATEKAGWDP
SRPLPAGRGRGIAVTEAFKTFVAQVVEVSVDKDGKLKVERVVCAVDCGIPINPDVIAAQMEGGIGFGLGAILHS
AITLKDGKVEQNNFDGYQVLRIAEMPKVEVHIVPSGEAPTGVGEPGVAPIGPALANAIFAATGQRLYNLPFPTSF

AKA
>LTIT01||OFM48717.1
MNSKIDLSNALPGSRRGFLKGAAVVGLTIGFQWSGARRALAAALPDAGFAPNAFLRIAPDDSVTVIAKHVEMG
QGAYTGIATIVAEELDADWSKVRVESAPADAKRYANLAFGTMQGTGGSSAMANSWMQLREAGAKARAMLV
EAAARQWRVPATELRTRDGFVEHPASQRKASYGSLAAAAAELPVPEKVQLKDPKDFRLIGHQAPRVDVPGKTD
GSAQFTLDVSLPGMLVALLQRPPLFGATVKSFDATATRAIPGVLEVVQVPHGVAVVAKGFWAAKQGRDALKVE
WDESKAEKRGSEALMAEYRKLAEQPGKPARRDGDAAKAVAGATRRIAASYEFPFLAHAPMEPLDAVVRLTADS
CEIWAGDQFQTVDQGNAARTAGLKPEQVKINTLYAGGSFGRRANAWSDYIVEAVSIAKALGANGVPVKLQWT
REDDIHGGFYRPMYYHRLEAGLDADGKLVGWQHRIVGQSILEGTPFAAVMVKDGIDATSVEGAANLPYAVPN
VSVELSTTQVGVPVLWWRVVGSSHTVYAVEAFIDEAAQAAGKDPYLFRRDLLAEQPRLRGVLELAAEKAGWD
PSRPLPAGRGRGIAVTEAFKTFVAQVVEVSVDKDGKLKVERVVCAVDCGIPINPDVIAAQMEGGIGFGLGAILHS
AITLKDGKVEQNNFDGYQVLRIAEMPKVEVHIVPSGEAPTGVGEPGVAPIGPALANAIFAATGQRLYNLPFPTSF
AKA
>LTIT01||OFM48866.1
MKRSFPDDLVIGNLSRRGFLKGVGATGVLLVAANWGWRDALAAEKKAFGADAMPHGWVDNPKIYVSIDRDG
TVGIVCNRSEMGQGVRTSLAMVVADELEADWSRVKVIQAPGDEARYGNQDTDGSRSMRHWFEPMRRCGA
AARQMLEQAAANQWKVPLGECRAEQNKVLHAPSGRSLSFGELAEAAAGLEVPARDKLLLKKPEQFRYIGKDV
ARAIDGADIVNGRAGFGFDARFDDMLYAVVARPPVYGGKLKRYDAAAALKVPGVVKVIEIEGRPIPSEFQPLGG
VAVVAQNTWAAIKGREALVVEWDAGVNGGYDSVAYRKQLEEAARKPGKVVRDSGDAAALFAKDGDIVEAEYY
LPHLAQAPMEPPVSTAWYKDGACEVWAPTQAPQVTRERIAERLKLPFDKVTVNVTLLGGGFGRKSKPDFVLEA
AILAKAFPGRHLRVQWTREDDLHFSYFHTVSVERLQAVLGADGLPQAWLHRSVAPSITALFGPDSKHQGAFEL
GMGLTNLPFAIPNVRLENPEAPAHTRVGWFRSVSNIPHAFAIQSFVGELAAKAGQDPKDYLLKLLGPARRIDTAE
LGDSWNYGESPERYPLDVGRLRGVIEEAARQSGWGGELPRGRARGIAAHYSFVTYVAVVIEVEVKDDGALLVH
KATIAADCGPQINPERIRSQLEGACVMGLGLAALGEISFKDGKVQQDNFHQYELARMPLAPKAVSVHLLKPDG
DLPLGGVGEPGVPPIAPALCNAIFAATGKRIRELPIRNQLQGWRKA
>LTJA01||OFM63570.1
MNSKIDLSNALPGSRRGFLKGAAVVGLTIGFQWSGARRALAAALPDAGFAPNAFLRIAPDDSVTVIAKHVEMG
QGAYTGIATIVAEELDADWSKVRVESAPADAKRYANLAFGTMQGTGGSSAMANSWMQLREAGAKARAMLV
EAAARQWQVPAAELRTRDGFVEHPTSQRKASYGSLAAAAAELPVPENVQLKDPKDFRLIGHQAPRVDVPGKT
DGSAQFTLDVSLPGMLVALLQRPPLFGATVKSFDATATRAIPGVVEVVQVPHGVAVVAKGFWAAKQGRDALKV
EWDESKAEKRGSEALMAEYRKLAEQPGKPARRDGDAAKAVAGATRRIAASYEFPFLAHAPMEPLDAVVRLTAD
SCEIWAGDQFQTVDQGNAARTAGLKPEQVKINTLYAGGSFGRRANAWSDYIVEAVSIAKALGANGVPVKLQW
TREDDIHGGFYRPMYYHRLEAGLDADGKLVGWQHRIVGQSILEGTPFAAVMVKDGVDATSVEGAANLPYAVP
NVSVELSTTQVGVPVLWWRVVGSSHTVYAVEAFIDEAAQAAGKDPYLFRRDLLAEQPRLRGVLELAAEKAGW
DPSRPLPAGRGRGIAVTEAFKTFVAQVVEVSVDKDGKLKVERVVCAVDCGIPINPDVIAAQMEGGIGFGLGAIL
HSAITLKDGKVEQNNFDGYQVLRIAEMPKVEVHIVPSGEAPTGVGEPGVAPIGPALANAIFAATGQRLYNLPFPT
SFAKA
>LTJA01||OFM68936.1
MKRSFPDDLVIGNLSRRGFLKGVGATGVLLVAANWGWRDALAAEKKAFGADAMPHGWVDNPKIYVSIDRDG
TVGIVCNRSEMGQGVRTSLAMVVADELEADWSRVKVIQAPGDEARYGNQDTDGSRSMRHWFEPMRRCGA
AARQMLEQAAANQWKVPLGECRAEQNKVLHAPSGRSLSFGELAEAAAGLEVPARDKLLLKKPEQFRYIGKDV
ARAIDGADIVNGRAGFGFDARFDDMLYAVVARPPVYGGKLKRYDAAAALKVPGVVKVIEIEGRPIPSEFQPLGG
VAVVAQNTWAAIKGREALVVEWDAGVNGGYDSVAYRKQLEEAARKPGKVVRDSGDAAALFAKGGDIVEAEYY
LPHLAQAPMEPPVSTAWYKDGACEVWVPTQAPQVTRERIAERLKLPFDKVTVNVTLLGGGFGRKSKPDFVLE

AAILAKAFPGRHLRVQWTREDDLHFSYFHTVSVERLQAVLGADGLPQAWLHRSVAPSITALFGPDSKHQGAFE
LGMGLTNLPFAIPNVRLENPEAPAHTRVGWFRSVSNIPHAFAIQSFVGELAAKAGQDPKDYLLKLLGPARRIDTA
ELGDSWNYGESPERYPLDVGRLRGVIEEAARQSGWGGELPRGRARGIAAHYSFVTYVAVVIEVEVKDDGALLV
HKATIAADCGPQINPERIRSQLEGACVMGLGLAALGEISFKDGKVQQDNFHQYELARMPLAPKAVSVHLLKPD
GDLPLGGVGEPGVPPIAPALCNAIFAATGKRIRELPIRNQLQGWRKA
>LTJC01||OFM72123.1
MKRSYPDDLVIGNLSRRGFLKGVGATGVLLVAANWGWRDALAAEKKAFGADAMPHGWVDNPKIYVSIDRDG
TVGIVCNRSEMGQGVRTSLAMVVADELEADWSRVKVIQAPGDEARYGNQDTDGSRSMRHWFEPMRRCGA
AARQMLEQAAANQWKVPLGECRAEQNKVLHAPSGRSLSFGELAEAAAGLEVPARDKLLLKKPEQFRYIGKDV
ARAIDGADIVNGRAGFGFDARFDDMLYAVVARPPVYGGKLKRYDVAAALKVPGVVKVIEIEGRPIPSEFQPLGG
VAVVAQNTWAAIKGREALVVEWDAGVNGGYDSVAYRKQLEEAARKPGKVVRDSGDAAALFAKGGDIVEAEYY
LPHLAQAPMEPPVSTAWYKDGACEVWAPTQAPQVTRERIAERLKLPFDKVTVNVTLLGGGFGRKSKPDFVLEA
AILAKAFPGRHLRVQWTREDDLHFSYFHTVSVERLQAVLGADGLPQAWLHRSVAPSITALFGPDSKHQGAFEL
GMGLTNLPFAIPNVRLENPEAPAHTRVGWFRSVSNIPHAFAIQSFVGELAAKAGQDPKDYLLKLLGPARRIDTAE
LGDSWNYGESPERYPLDVGRLRGVIEEAARQSGWGGELPRGRARGIAAHYSFVTYVAVVIEVEVKDDGALLVH
KATIAADCGPQINPERIRSQLEGACVMGLGLAALGEISFKDGKVQQDNFHQYELARMPLAPKAVSVHLLKPDG
DLPLGGVGEPGVPPIAPALCNAIFAATGKRIRELPIRNQLQGWRKA
>LTJC01||OFM68056.1
MNSKIDLSNALPGSRRGFLKGAAVVGLTIGFQWSGARRALAAALPDAGFAPNAFLRIAPDDSVTVIAKHVEMG
QGAYTGIATIVAEELDADWSKVRVESAPADAKRYANLAFGTMQGTGGSSAMANSWMQLREAGAKARAMLV
EAAARQWRVPATELRTRDGFVEHPASQRKASYGSLAAAAAELPVPEKVQLKDPKDFRLIGHQAPRVDVPGKTD
GSAQFTLDVSLPGMLVALLQRPPRFGATVKSFDATATRAIPGVLEVVQVPHGVAVVAKGFWAAKQGRDALKVE
WDESKAEKRGSEALMAEYRKLAEQPGKPARRDGDAAKAVAGATRRIAASYEFPFLAHAPMEPLDAVVRLTADS
CEIWAGDQFQTVDQGNAARTAGLKPEQVKINTLYAGGSFGRRANAWSDYIVEAVSIAKALGANGVPVKLQWT
REDDIHGGFYRPMYYHRLEAGLDADGKLVGWQHRIVGQSILEGTPFAAVMVKDGVDATSVEGAANLPYAVPN
VSVELSTTQVGVPVLWWRVVGSSHTVYAVEAFIDEAAQAAGKDPYLFRRDLLAEQPRLRGVLELAAEKAGWD
PSRPLPAGRGRGIAVTEAFKTFVAQVVEVSVDKDGKLKVERVVCAVDCGIPINPDVIAAQMEGGIGFGLGAILHS
AITLKDGKVEQNNFDGYQVLRIAEMPKVEVHIVPSGEAPTGVGEPGVAPIGPALANAIFAATGQRLYNLPFPTSF
AKA
>LTNB01||OHR95860.1
MADLQQRASREPEDAGDGILNVSRRHFLRGAGGLALGIYFAPLLGRFGDPQAAAAKAFEPNAFVRIAPDGTVT
VIAKHVEMGQGSYTGLATLLAEELDADWSKVRVEGAPADAKRYANLAFGTLQGTGGSSAMANSFEQMRKAG
ATARAMLVAAAAQQWKVPAEQIEVHDGVVEHKASGHKAGFGQLAEAAAKQPVPAEVKLKAPEDFKLIGQVKL
PRKDSQDKTDGQARFTQDVHLPDMLVAVVAHPPRFGGVPKKVDDSKAKAVPGVVAVVQFPGSDSRFAGVAV
LAKNTWAARQGRDALQVEWDESNAFRMGSAEIFARYQELAAKPGVVARNEGDIAKALDKPAKLIEAQYQFPF
LAHASMEPLNCVVKLSDGACEIWNGEQWQTGDQMAVGQLLGIAPEKVSITQLYAGGSFGRRANPHSDYVLEA
VSIAKAAREHGHKGPVKMVWTREDDTRGGYYRPAFLHSARLALDGQGNLVGWEQHLVGQSFIVGTPFEKVM
VKDGIDKVAVEGAADLPYAVPNLRVEQTLVPEIKVPTQWWRSVGHTHTAYSTETLIDEAAVAAGKDPYEFRRAL
LEKHPRHLGVLDLVADKAGWKQPLKAGGEGEKRGRGIAVHESFGSFVAQVVEVTVKADKSFRIDRVVCAVDCG
LAINPDVIRAQMEGGIGYGLAMALHSAITLKEGVVEQSNFHDFQVLRINEMPAVEVHIVPSSEAPTGVGEPGVP
PAAPALANALAAATGKRIRNLPIGNQLQA
>LTOT01||OHQ63127.1
MKRSYPDDLVIGNLSRRGFLKGVGATGVLLVAANWGWRDALAAEKKAFGADAMPHGWVDNPKIYVSIDRDG
TVGIVCNRSEMGQGVRTSLAMVVADELEADWSRVKVIQAPGDEARYGNQDTDGSRSMRHWFEPMRRCGA

AARQMLEQAAANQWKVPLGECRAEQNKVLHAPSGRSLSFGELAEAAAGLEVPARDKLLLKKPEQFRYIGKDV
ARAIDGADIVNGRAGFGFDARFDDMLYAVVARPPVYGGKLKRYDAAAALKVPGVVKVIEIEGRPIPSEFQPLGG
VAVVAQNTWAAIKGREALVVEWDAGVNGGYDSVAYRKQLEEAARKPGKVVRDSGDAAALFAKGGDIVEAEYY
LPHLAQAPMEPPVSTAWYKDGACEVWAPTQAPQVTRERIAERLKLPFDKVTVNVTLLGGGFGRKSKPDFVLEA
AILAKAFPGRHLRVQWTREDDLHFSYFHTVSVERLQAVLGADGLPQAWLHRSVAPSITALFGPDSKHQGAFEL
GMGLTNLPFAIPNVRLENPEAPAHTRVGWFRSVSNIPHAFAIQSFVGELAAKAGQDPKDYLLKLLGPARRIDTAE
LGDSWNYGESPERYPLDVGRLRGVIEEAARQSGWGGELPRGRARGIAAHYSFVTYVAVVIEVEVKDDGALLVH
KATIAADCGPQINPERIRSQLEGACVMGLGLAALGEISFKDGKVQQDNFHQYELARMPLAPKAVSVHLLKPDG
DLPLGGVGEPGVPPIAPALCNAIFAATGKRIRELPIRNQLQGWRKA
>LTOT01||OHQ67461.1
MNSKIDLSNALPGSRRGFLKGAAVVGLTIGFQWSGARRALAAALPDAGFAPNAFLRIAPDDSVTVIAKHVEMG
QGAYTGIATIVAEELDADWSKVRVESAPADAKRYANLAFGTMQGTGGSSAMANSWMQLREAGAKARAMLV
EAAARQWRVPATELRTRDGFVEHPASQRKASYGSLAAAAAELPVPEKVQLKDPKDFRLIGHQAPRVDVPGKTD
GSAQFTLDVSLPGMLVALLQRPPLFGATVKSFDATATRAIPGVVEVVQVPHGVAVVAKGFWAAKQGRDALKVE
WDESKAEKRGSEALMAEYRKLAEQPGKPARRDGDAAKAVAGATRRIAASYEFPFLAHAPMEPLDAVVRLTADS
CEIWAGDQFQTVDQGNAARTAGLKPEQVKINTLYAGGSFGRRANAWSDYIVEAVSIAKALGANGVPVKLQWT
REDDIHGGFYRPMYYHRLEAGLDADGKLVGWQHRIVGQSILEGTPFAAVMVKDGIDATSVEGAANLPYAVPN
VSVELSTTQVGVPVLWWRVVGSSHTVYAVEAFIDEAAQAAGKDPYLFRRDLLAEQPRLRGVLELAAEKAGWD
PSRPLPAGRGRGIAVTEAFKTFVAQVVEVSVDKDGKLKVERVVCAVDCGIPINPDVIAAQMEGGIGFGLGAILHS
AITLKDGKVEQNNFDGYQVLRIAEMPKVEVHIVPSGEAPTGVGEPGVAPIGPALANAIFAATGQRLYNLPFPTSF
AKA
>LTOY01||OHQ43994.1
MNSKIDLSNALPGSRRGFLKGAAVVGLTIGFQWSGARRALAAALPDAGFAPNAFLRIAPDDSVTVIAKHVEMG
QGAYTGIATIVAEELDADWSKVRVESAPADAKRYANLAFGTMQGTGGSSAMANSWMQLREAGAKARAMLV
EAAARQWRVPATELRTRDGFVEHPASQRKASYGSLAAAAAELPVPENVQLKDPKDFRLIGHQAPRVDVPGKT
DGSAQFTLDVSLPGMLVALLQRPPLFGATVKSFDATATRAIPGVVEVVQVPHGVAVVAKGFWAAKQGRDALKV
EWDESKAEKRGSEALMAEYRKLAEQPGKPARRDGDAAKAVAGATRRIAASYEFPFLAHAPMEPLDAVVRLTAD
SCEIWAGDQFQTVDQGNAARTAGLKPEQVKINTLYAGGSFGRRANAWSDYIVEAVSIAKALGANGVPVKLQW
TREDDIHGGFYRPMYYHRLEAGLDADGKMVGWQHRIVGQSILEGTPFAAVMVKDGIDATSVEGAANLPYAVP
NVSVELSTTQVGVPVLWWRVVGSSHTVYAVEAFIDEAAQAAGKDPYLFRRDLLAEQPRLRGVLELAAEKAGW
DPSRPLPAGRGRGIAVTEAFKTFVAQVVEVSVDKDGKLKVERVVCAVDCGIPINPDVIAAQMEGGIGFGLGAIL
HSAITLKDGKVEQNNFDGYQVLRIAEMPKVEVHIVPSGEAPTGVGEPGVAPIGPALANAIFAATGQRLYNLPFPT
SFAKA
>LTOY01||OHQ51985.1
MKRSYPDDLVIGNLSRRGFLKGVGATGVLLVAANWGWRDALAAEKKAFGADAMPHGWVDNPKIYVSIDRDG
TVGIVCNRSEMGQGVRTSLAMVVADELEADWSRVKVIQAPGDEARYGNQDTDGSRSMRHWFEPMRRCGA
AARQMLEQAAANQWKVPLGECRAEQNKVLHAPSGRSLSFGELAEAAAGLEVPARDKLLLKKPEQFRYIGKDV
ARAIDGADIVNGRAGFGFDARFDDMLYAVVARPPVYGGKLKRYDAAAALKVPGVVKVIEIEGRPIPSEFQPLGG
VAVVAQNTWAAIKGREALVVEWDAGVNGGYDSVAYRKQLEEAARKPGKVVRDSGDAAALFAKGGDIVEAEYY
LPHLAQAPMEPPVSTAWYKDGACEVWAPTQAPQVTRERIAERLKLPFDKVTVNVTLLGGGFGRKSKPDFVLEA
AILAKAFPGRHLRVQWTREDDLHFSYFHTVSVERLQAVLGADGLPQAWLHRSVAPSITALFGPDSKHQGAFEL
GMGLTNLPFAIPNVRLENPEAPAHTRVGWFRSVSNIPHAFAIQSFVGELAAKAGQDPKDYLLKLLGPARRIDTAE
LGDSWNYGESPERYPLDVGRLRGVIEEAARQSGWGGELPRGRARGIAAHYSFVTYVAVVIEVEVKDDGALLVH
KATIAADCGPQINPERIRSQLEGACVMGLGLAALGEISFKDGKVQQDNFHQYELARMPLAPKAVSVHLLKPDG

DLPLGGVGEPGVPPIAPALCNAIFAATGKRIRELPIRNQLQGWRKA
>LTQM01||OHP44384.1
MNSKIDLSNALPGSRRGFLKGAAVVGLTIGFQWSGARRALAAALPDAGFAPNAFLRIAPDDSVTVIAKHVEMG
QGAYTGIATIVAEELDADWSKVRVESAPADAKRYANLAFGTMQGTGGSSAMANSWMQLREAGAKARAMLV
EAAARQWRVPATELRTRDGFVEHPASQRKASYGSLAAAAAELPVPEKVQLKDPKDFRLIGHQAPRVDVPGKTD
GSAQFTLDVSLPGMLVALLQRPPLFGATVKSFDATATRAIPGVVEVVQVPHGVAVVAKGFWAAKQGRDALKVE
WDESKAEKRGSEALMAEYRKLAEQPGKPARRDGDAAKAVAGATRRIAASYEFPFLAHAPMEPLDAVVRLTADS
CEIWAGDQFQTVDQGNAARTAGLKPEQVKINTLYAGGSFGRRANAWSDYIVEAVSIAKALGANGVPVKLQWT
REDDIHGGFYRPMYYHRLEAGLDADGKLVGWQHRIVGQSILEGTPFAAVMVKDGIDATSVEGAANLPYAVPN
VSVELSTTQVGVPVLWWRVVGSSHTVYAVEAFIDEAAQAAGKDPYLFRRDLLAEQPRLRGVLELAAEKAGWD
PSRPLPAGRGRGIAVTEAFKTFVAQVVEVSVDKDGKLKVERVVCAVDCGIPINPDVIAAQMEGGIGFGLGAILHS
AITLKDGKVEQNNFDGYQVLRIAEMPKVEVHIVPSGEAPTGVGEPGVAPIGPALANAIFAATGQRLYNLPFPTSF
AKA
>LTQM01||OHP42709.1
MKRSYPDDLVIGNLSRRGFLKGVGATGVLLVAANWGWRDALAAEKKAFGADAMPHGWVDNPKIYVSIDRDG
TVGIVCNRSEMGQGVRTSLAMVVADELEADWSRVKVIQAPGDEARYGNQDTDGSRSMRHWFEPMRRCGA
AARQMLEQAAANQWKVPLGECRAEQNKVLHAPSGRSLSFGELAEAAAGLEVPARDKLLLKKPEQFRYIGKDV
ARAIDGADIVNGRAGFGFDARFDDMLYAVVARPPVYGGKLKRYDAAAALKVPGVVKVIEIEGRPIPSEFQPLGG
VAVVAQNTWAAIKGREALAVEWDAGVNGGYDSVAYRKQLEEAARKPGKVVRDSGDAAALFARGGDIVEAEYY
LPHLAQAPMEPPVSTAWYKDGACEVWAPTQAPQVTRERIAERLKLPFDKVTVNVTLLGGGFGRKSKPDFVLEA
AILAKAFPGRHLRVQWTREDDLHFSYFHTVSVERLQAVLGADGLPQAWLHRSVAPSITALFGPDSKHQGAFEL
GMGLTNLPFAIPNVRLENPEAPAHTRVGWFRSVSNIPHAFAIQSFVGELAAKAGQDPKDYLLKLLGPARRIDTAE
LGDSWNYGESPERYPLDVGRLRGVIEEAARQSGWGGELPRGRARGIAAHYSFVTYVAVVIEVEVKDDGALLVH
KATIAADCGPQINPERIRSQLEGACVMGLGLAALGEISFKDGKVQQDNFHQYELARMPLAPKAVSVHLLKPDG
DLPLGGVGEPGVPPIAPALCNAIFAATGKRIRELPIRNQLQGWRKA
>LTQN01||OHP51523.1
MNSKIDLSNALPGSRRGFLKGAAVVGLTIGFQWSGARRALAAALPDAGFAPNAFLRIAPDDSVTVIAKHVEMG
QGAYTGIATIVAEELDADWSKVRVESAPADAKRYANLAFGTMQGTGGSSAMANSWMQLREAGAKARAMLV
EAAARQWRVPATELRTRDGFVEHPASQRKASYGSLAAAAAELPVPEKVQLKDPKDFRLIGHQAPRVDVPGKTD
GSAQFTLDVSLPGMLVALLQRPPLFGATVKSFDATATRAIPGVVEVVQVPHGVAVVAKGFWAAKQGRDALKVE
WDESKAEKRGSEALMAEYRKLAEQPGKPARRDGDAAKAVAGATRRIAASYEFPFLAHAPMEPLDAVVRLTADS
CEIWAGDQFQTVDQGNAARTAGLKPEQVKINTLYAGGSFGRRANAWSDYIVEAVSIAKALGANGVPVKLQWT
REDDIHGGFYRPMYYHRLEAGLDADGKLVGWQHRIVGQSILEGTPFAAVMVKDGIDATSVEGAANLPYAVPN
VSVELSTTQVGVPVLWWRVVGSSHTVYAVEAFIDEAAQAAGKDPYLFRRDLLAEQPRLRGVLELAAEKAGWD
PSRPLPAGRGRGIAVTEAFKTFVAQVVEVSVDKDGKLKVERVVCAVDCGIPINPDVIAAQMEGGIGFGLGAILHS
AITLKDGKVEQNNFDGYQVLRIAEMPKVEVHIVPSGEAPTGVGEPGVAPIGPALANAIFAATGQRLYNLPFPTSF
AKA
>LTQN01||OHP42628.1
MKRSFPDDLVIGNLSRRGFLKGVGATGVLLVAANWGWRDALAAEKKAFGADAMPHGWVDNPKIYVSIDRDG
TVGIVCNRSEMGQGVRTSLAMVVADELEADWSRVKVIQAPGDEARYGNQDTDGSRSMRHWFEPMRRCGA
AARQMLEQAAANQWKVPLGECRAEQNKVLHAPSGRSLSFGELAEAAAGLEVPARDKLLLKKPEQFRYIGKDV
ARAIDGADIVNGRAGFGFDARFDDMLYAVVARPPVYGGKLKRYDAAAALKVPGVVKVIEIEGRPIPSEFQPLGG
VAVVAQNTWAAIKGREALVVEWDAGVNGGYDSVAYRKQLEEAARKPGKVVRDSGDAAALFAKGGDIVEAEYY
LPHLAQAPMEPPVSTAWYKDGACEVWAPTQAPQVTRERIAERLKLPFDKVTVNVTLLGGGFGRKSKPDFVLEA

AILAKAFPGRHLRVQWTREDDLHFSYFHTVSVERLQAVLGADGLPQAWLHRSVAPSITALFGPDSKHQGAFEL
GMGLTNLPFAIPNVRLENPEAPAHTRVGWFRSVSNIPHAFAIQSFVGELAAKAGQDPKDYLLKLLGPARRIDTAE
LGDSWNYGESPERYPLDVGRLRGVIEEAARQSGWGGELPRGRARGIAAHYSFVTYVAVVIEVEVKDDGALLVH
KATIAADCGPQINPERIRSQLEGACVMGLGLAALGEISFKNGKVQQDNFHQYELARMPLAPKAVSVHLLKPDG
DLPLGGVGEPGVPPIAPALCNAIFAATGKRIRELPIRNQLQGWRKA
>LTQO01||OHP33394.1
MNSKIDLSNALPGSRRGFLKGAAVVGLTIGFQWSGARRALAAALPDAGFAPNAFLRIAPDDSVTVIAKHVEMG
QGAYTGIATIVAEELDADWSKVRVESAPADAKRYANLAFGTMQGTGGSSAMANSWMQLREAGAKARAMLV
EAAARQWRVPATELRTRDGFVEHPASQRKASYGSLAAAAAELPVPEKVQLKDPKDFRLIGHQAPRVDVPGKTD
GSAQFTLDVSLPGMLVALLQRPPLFGATVKSFDATATRAIPGVVEVVQVPHGVAVVAKGFWAAKQGRDALKVE
WDESKAEKRGSEALMAEYRKLAEQPGKPARRDGDAAKAVAGATRRIAASYEFPFLAHAPMEPLDAVVRLTADS
CEIWAGDQFQTVDQGNAARTAGLKPEQVKINTLYAGGSFGRRANAWSDYIVEAVSIAKALGANGVPVKLQWT
REDDIHGGFYRPMYYHRLEAGLDADGKLVGWQHRIVGQSILEGTPFAAVMVKDGIDATSVEGAANLPYAVPN
VSVELSTTQVGVPVLWWRVVGSSHTVYAVEAFIDEAAQAAGKDPYLFRRDLLAEQPRLRGVLELAAKKAGWD
PSRPLPAGRGRGIAVTEAFKTFVAQVVEVSVDKDGKLKVERVVCAVDCGIPINPDVIAAQMEGGIGFGLGAILHS
AITLKDGKVEQNNFDGYQVLRIAEMPKVEVHIVPSGEAPTGVGEPGVAPIGPALANAIFAATGQRLYNLPFPTSF
AKA
>LTQO01||OHP34390.1
MKRSYPDDLVIGNLSRRGFLKGVGATGVLLVAANWGWRDALAAEKKAFGADAMPHGWVDNPKIYVSIDRDG
TVGIVCNRSEMGQGVRTSLAMVVADELEADWSRVKVIQAPGDEARYGNQDTDGSRSMRHWFEPMRRCGA
AARQMLEQAAANQWKVPLGECRAEQNKVLHAPSGRSLSFGELAEAAAGLEVPARDKLLLKKPEQFRYIGKDV
ARAIDGADIVNGRAGFGFDARFDDMLYAVVARPPVYGGKLKRYDAAAALKVPGVVKVIEIEGRPIPSEFQPLGG
VAVVAQNTWAAIKGREALVVEWDAGVNGGYDSVAYRKQLEEAARKPGKVVRDSGDAAALFARGGDIVEAEYY
LPHLAQAPMEPPVSTAWYKDGACEVWAPTQAPQVTRERIAERLKLPFDKVTVNVTLLGGGFGRKSKPDFVLEA
AILAKAFPGRHLRVQWTREDDLHFSYFHTVSVERLQAVLGADGLPQAWLHRSVAPSITALFGPDSKHQGAFEL
GMGLTNLPFAIPNVRLENPEAPAHTRVGWFRSVSNIPHAFAIQSFVGELAAKAGQDPKDYLLKLLGPARRIDTAE
LGDSWNYGESPERYPLDVGRLRGVIEEAARQSGWGGELPRGRARGIAAHYSFVTYVAVVIEVEVKDDGALLVH
KATIAADCGPQINPERIRSQLEGACVMGLGLAALGEISFKDGKVQQDNFHQYELARMPLAPKAVSVHLLKPDG
DLPLGGVGEPGVPPIAPALCNAIFAATGKRIRELPIRNQLQGWRKA
>LTQP01||OHP30733.1
MKRSYPDDLVIGNLSRRGFLKGVGATGVLLVAANWGWRDALAAEKKAFGADAMPHGWVDNPKIYVSIDRDG
TVGIVCNRSEMGQGVRTSLAMVVADELEADWSRVKVIQAPGDEARYGNQDTDGSRSMRHWFEPMRRCGA
AARQMLEQAAANQWKVPLGECRAEQNKVLHAPSGRSLSFGELAEAAAGLEVPARDKLLLKKPEQFRYIGKDV
ARAIDGADIVNGRAGFGFDARFDDMLYAVVARPPVYGGKLKRYDAAAALKVPGVVKVIEIEGRPIPSEFQPLGG
VAVVAQNTWAAIKGREALVVEWDAGVNGGYDSVAYRKQLEEAARKPGKVVRDSGDAAALFARGGDIVEAEYY
LPHLAQAPMEPPVSTAWYKDGACEVWAPTQAPQVTRERIAERLKLPFDKVTVNVTLLGGGFGRKSKPDFVLEA
AILAKAFPGRHLRVQWTREDDLHFSYFHTVSVERLQAVLGADGLPQAWLHRSVAPSITALFGPDSKHQGAFEL
GMGLTNLPFAIPNVRLENPEAPAHTRVGWFRSVSNIPHAFAIQSFVGELAAKAGQDPKDYLLKLLGPARRIDTAE
LGDSWNYGESPERYPLDVGRLRGVIEEAARQSGWGGELPRGRARGIAAHYSFVTYVAVVIEVEVKDDGALLVH
KATIAADCGPQINPERIRSQLEGACVMGLGLAALGEISFKDGKVQQDNFHQYELARMPLAPKAVSVHLLKPDG
DLPLGGVGEPGVPPIAPALCNAIFAATGKRIRELPIRNQLQGWRKA
>LTQP01||OHP27829.1
MNSKIDLSNALPGSRRGFLKGAAVVGLTIGFQWSGARRALAAALPDAGFAPNAFLRIAPDDSVTVIAKHVEMG
QGAYTGIATIVAEELDADWSKVRVESAPADAKRYANLAFGTMQGTGGSSAMANSWMQLREAGAKARAMLV

EAAARQWRVPATELRTRDGFVEHPASQRKASYGSLAAAAAELPVPEKVQLKDPKDFRLIGHQAPRVDVPGKTD
GSAQFTLDVSLPGMLVALLQRPPLFGATVKSFDATATRAIPGVVEVVQVPHGVAVVAKGFWAAKQGRDALKVE
WDESKAEKRGSEALMAEYRKLAEQPGKPARRDGDAAKAVAGATRRIAASYEFPFLAHAPMEPLDAVVRLTADS
CEIWAGDQFQTVDQGNAARTAGLKPEQVKINTLYAGGSFGRRANAWSDYIVEAVSIAKALGANGVPVKLQWT
REDDIHGGFYRPMYYHRLEAGLDADGKLVGWQHRIVGQSILEGTPFAAVMVKDGIDATSVEGAANLPYAVPN
VSVELSTTQVGVPVLWWRVVGSSHTVYAVEAFIDEAAQAAGKDPYLFRRDLLAEQPRLRGVLELAAKKAGWD
PSRPLPAGRGRGIAVTEAFKTFVAQVVEVSVDKDGKLKVERVVCAVDCGIPINPDVIAAQMEGGIGFGLGAILHS
AITLKDGKVEQNNFDGYQVLRIAEMPKVEVHIVPSGEAPTGVGEPGVAPIGPALANAIFAATGQRLYNLPFPTSF
AKA
>LTQU01||OHP14529.1
MNSKIDLSNALPGSRRGFLKGAAVVGLTIGFQWSGARRALAAALPDAGFAPNAFLRIAPDDSVTVIAKHVEMG
QGAYTGIATIVAEELDADWSKVRVESAPADAKRYANLAFGTMQGTGGSSAMANSWMQLREAGAKARAMLV
EAAARQWRVPATELRTRDGFVEHPASQRKASYGSLAAAAAELPVPEKVQLKDPKDFRLIGHQAPRVDVPGKTD
GSAQFTLDVSLPGMLVALLQRPPLFGATVKSFDATATRAIPGVVEVVQVPHGVAVVAKGFWAAKQGRDALKVE
WDESKAEKRGSEALMAEYRKLAEQPGKPARRDGDAAKAVAGATRRIAASYEFPFLAHAPMEPLDAVVRLTADS
CEIWAGDQFQTVDQGNAARTAGLKPEQVKINTLYAGGSFGRRANAWSDYIVEAVSIAKALGANGVPVKLQWT
REDDIHGGFYRPMYYHRLEAGLDADGKLVGWQHRIVGQSILEGTPFAAVMVKDGIDATSVEGAANLPYAVPN
VSVELSTTQVGVPVLWWRVVGSSHTVYAVEAFIDEAAQAAGKDPYLFRRDLLAEQPRLRGVLELAAEKAGWD
PSRPLPAGRGRGIAVTEAFKTFVAQVVEVSVDKDGKLKVERVVCAVDCGIPINPDVIAAQMEGGIGFGLGAILHS
AITLKDGKVEQNNFDGYQVLRIAEMPKVEVHIVPSGEAPTGVGEPGVAPIGPALANAIFAATGQRLYNLPFPTSF
AKA
>LTQU01||OHP12554.1
MKRSYPDDLVIGNLSRRGFLKGVGATGVLLVAANWGWRDALAAEKKAFGADAMPHGWVDNPKIYVSIDRDG
TVGIVCNRSEMGQGVRTSLAMVVADELEADWSRVKVIQAPGDEARYGNQDTDGSRSMRHWFEPMRRCGA
AARQMLEQAAANQWKVPLGECRAEQNKVLHAPSGRSLSFGELAEAAAGLEVPARDKLLLKKPEQFRYIGKDV
ARAIDGADIVNGRAGFGFDARFDDMLYAVVARPPVYGGKLKRYDAAAALKVPGVVKVIEIEGRPIPSEFQPLGG
VAVVAQNTWAAIKGREALAVEWDAGVNGGYDSVAYRKQLEEAARKPGKVVRDSGDAAALFARGGDIVEAEYY
LPHLAQAPMEPPVSTAWYKDGACEVWAPTQAPQVTRERIAERLKLPFDKVTVNVTLLGGGFGRKSKPDFVLEA
AILAKAFPGRHLRVQWTREDDLHFSYFHTVSVERLQAVLGADGLPQAWLHRSVAPSITALFGPDSKHQGAFEL
GMGLTNLPFAIPNVRLENPEAPAHTRVGWFRSVSNIPHAFAIQSFVGELAAKAGQDPKDYLLKLLGPARRIDTAE
LGDSWNYGESPERYPLDVGRLRGVIEEAARQSGWGGELPRGRARGIAAHYSFVTYVAVVIEVEVKDDGALLVH
KATIAADCGPQINPERIRSQLEGACVMGLGLAALGEISFKDGKVQQDNFHQYELARMPLAPKAVSVHLLKPDG
DLPLGGVGEPGVPPIAPALCNAIFAATGKRIRELPIRNQLQGWRKA
>LTQY01||OHO95877.1
MNSKIDLSNALPGSRRGFLKGAAVVGLTIGFQWSGARRALAAALPDAGFAPNAFLRIAPDDSVTVIAKHVEMG
QGAYTGIATIVAEELDADWSKVRVESAPADAKRYANLAFGTMQGTGGSSAMANSWMQLREAGAKARAMLV
EAAARQWQIPAAELRTRDGFVEHPASQRKASYGSLAAAAAELPVPEKVQLKDPKDFRLIGHQAPRVDVPGKTD
GSAQFTLDVSLPGMLVALLQRPPLFGATVKSFDATATRAIPGVVEVVQVPHGVAVVAKGFWAAKQGRDALKVE
WDESKAEKRGSEALMAEYRKLAEQPGKPARRDGDAAKAVAGATRRIAASYEFPFLAHAPMEPLDAVVRLTADS
CEIWAGDQFQTVDQGNAARTAGLKPEQVKINTLYAGGSFGRRANAWSDYIVEAVSIAKALGANGVPVKLQWT
REDDIHGGFYRPMYYHRLEAGLDADGKLVGWQHRIVGQSILEGTPFAAVMVKDGVDATSVEGAANLPYAVPN
VSVELSTTQVGVPVLWWRVVGSSHTVYAVEAFIDEAAQAAGKDPYLFRRDLLAEQPRLRGVLELAAEKAGWD
PSRPLPAGRGRGIAVTEAFKTFVAQVVEVSVDKDGKLKVERVVCAVDCGIPINPDVIAAQMEGGIGFGLGAILHS
AITLKDGKVEQNNFDGYQVLRIAEMPKVEVHIVPSGEAPTGVGEPGVAPIGPALANAIFAATGQRLYNLPFPTSF

AKA
>LTQY01||OHO99530.1
MKRSFPDDLVIGNLSRRGFLKGVGATGVLLVAANWGWRDALAAEKKAFGADAMPHGWVDNPKIYVSIDRDG
TVGIVCNRSEMGQGVRTSLAMVVADELEADWSRVKVIQAPGDEARYGNQDTDGSRSMRHWFEPMRRCGA
AARQMLEQAAANQWKVPLGECRAEQNKVLHAPSGRSLSFGELAEAAAGLEVPARDKLLLKKPEQFRYIGKDV
ARAIDGADIVNGRAGFGFDARFDDMLYAVVARPPVYGGKLKRYDAAAALKVPGVVKVIEIEGRPIPSEFQPLGG
VAVVAQNTWAAIKGREALVVEWDAGVNGGYDSVAYRKQLEEAARKPGKVVRDSGDAAALFAKGGDIVEAEYY
LPHLAQAPMEPPVSTAWYKDGACEVWAPTQAPQVTRERIAERLKLPFDKVTVNVTLLGGGFGRKSKPDFVLEA
AILAKAFPGRHLRVQWTREDDLHFSYFHTVSVERLQAVLGADGLPQAWLHRSVAPSITALFGPDSKHQGAFEL
GMGLTNLPFAIPNVRLENPEAPAHTRVGWFRSVSNIPHAFAIQSFVGELAAKAGQDPKDYLLKLLGPARRIDTAE
LGDSWNYGESPERYPLDVGRLRGVIEEAARQSGWGGELPRGRARGIAAHYSFVTYVAVVIEVEVKDDGALLVH
KATIAADCGPQINPERIRSQLEGACVMGLGLAALGEISFKDGKVQQDNFHQYELARMPLAPKAVSVHLLKPDG
DLPLGGVGEPGVPPIAPALCNAIFAATGKRIRELPIRNQLQGWRKA
>LTSZ01P||OFO62184.1
MTTTMQVSRRGFLKGGLGALTLAVTGNGLVSAVWAADEPKKYGADSMPGGTVDDPLAFVSIAADGTVTIVAH
RAEMGTGVRTSLPMVVADEMEAAWDRVRVVQADADEARYGNQNVDGSRSVRHFLMPMRRVGAAARQM
LEAAAAARWSVPLAEVRATQHEVVHAPSGRRLGYGELAADAAKLPVPAGDAVKLKTRAEFRYIGKDEVRLVDLE
AIGKGEAMYGMDMRLPGMVYAVVARPPVVGGKLRRVDSAKALAVPGVLKVVEIPAMAGAPAFQPLGGVAVV
ASNTWAAMQGRAALAIEWDDGPNAAYDSVAYRETLTEASRKPGKVVRDQGDAPQAWAKAGEAERFMAEY
HVPHLAHASMETPVATVRIQDGAAEVWTSVQNPAAAQEAVAKRLKLKPENVKVHVLLLGGGFGRKSKPDYVD
EAAIVAQAMPAGTPVKLVWTREDDIHHDYLHTVSAEHLEAVVGKDGKVQSWLHRSAAPTIASLFTEGAKGEQL
FESAMSAINMPYVIPNVRVETAEVAAHARIGWFRSVANIPHAFAAQCFIAELAHRAGQDHKQYALDLIGPARRI
DPGTLADTWNYSESPERYPYDTGRLRGVIEAAASGAKWGRELPKGHGLGLAFCYSFMSYTATVVEVAVDEKGE
VRVVAVDMALDCGPQIKPERIRAQMEGGAIMGLSLALLGEITFEKGRVKQNNFYDYEVLRHNASPRVIRTYLVN
DDHALPPGGVGEPPVPPVAPALCNAIFAATGKRVRSLPVRSVA
>LTTI01||OFO88005.1
MNSKIDLSNALPGSRRGFLKGAAVVGLTIGFQWSGARRALAAALPDAGFAPNAFLRIAPDDSVTVIAKHVEMG
QGAYTGIATIVAEELDADWSKVRVESAPADAKRYANLAFGTMQGTGGSSAMANSWMQLREAGAKARAMLV
EAAARRWRVPATELRTRDGFVEHPASQRKASYGSLAAAAAELPVPEKVQLKDPKDFRLIGHQAPRVDVPGKTD
GSAQFTLDVSLPGMLVALLQRPPLFGATVKSFDATATRAIPGVVEVVQVPHGVAVVAKGFWAAKQGRDALKVE
WDESKAEKRGSEALMAEYRKLAEQPGKPARRDGDAAKAVAGATRRIAASYEFPFLAHAPMEPLDAVVRLTADS
CEIWAGDQFQTVDQGNAARTAGLKPEQVKINTLYAGGSFGRRANAWSDYIVEAVSIAKALGANGVPVKLQWT
REDDIHGGFYRPMYYHRLEAGLDADGKLVGWQHRIVGQSILEGTPFAAVMVKDGIDATSVEGAANLPYAVPN
VSVELSTTQVGVPVLWWRVVGSSHTVYAVEAFIDEAAQAAGKDPYLFRRDLLAEQPRLRGVLELAAEKAGWD
PSRPLPAGRGRGIAVTEAFKTFVAQVVEVSVDKDGKLKVERVVCAVDCGIPINPDVIAAQMEGGIGFGLGAILHS
AITLKDGKVEQNNFDGYQVLRIAEMPKVEVHIVPSGEAPTGVGEPGVAPIGPALANAIFAATGQRLYNLPFPTSF
AKA
>LTTI01||OFO80642.1
MKRSYPDDLVIGNLSRRGFLKGVGATGVLLVAANWGWRDALAAEKKAFGADAMPHGWVDNPKIYVSIDRDG
TVGIVCNRSEMGQGVRTSLAMVVADELEADWSRVKVIQAPGDEARYGNQDTDGSRSMRHWFEPMRRCGA
AARQMLEQAAANQWKVPLGECRAEQNKVLHAPSGRSLSFGELAEAAAGLEVPARDKLLLKKPEQFRYIGKDV
ARAIDGADIVNGRAGFGFDARFDDMLYAVVARPPVYGGKLKRYDAAAALKVPGVVKVIEIEGRPIPSEFQPLGG
VAVVAQNTWAAIKGREALVVEWDAGVNGGYDSVAYRKQLEEAARKPGKVVRDSGDAAALFARGGDIVEAEYY
LPHLAQAPMEPPVSTAWYKDGACEVWAPTQAPQVTRERIAERLKLPFDKVTVNVTLLGGGFGRKSKPDFVLEA

AILAKAFPGRHLRVQWTREDDLHFSYFHTVSVERLQAVLGADGLPQAWLHRSVAPSITALFGPDSKHQGAFEL
GMGLTNLPFAIPNVRLENPEAPAHTRVGWFRSVSNIPHAFAIQSFVGELAAKAGQDPKDYLLKLLGPARRIDTAE
LGDSWNYGESPERYPLDVGRLRGVIEEAARQSGWGGELPRGRARGIAAHYSFVTYVAVVIEVEVKDDGALLVH
KATIAADCGPQINPERIRSQLEGACVMGLGLAALGEISFKDGKVQQDNFHQYELARMPLAPKAVSVHLLKPDG
DLPLGGVGEPGVPPIAPALCNAIFAATGKRIRELPIRNQLQGWRKA
>LTUG01||OFP48667.1
MKRSFPDDLVIGNLSRRGFLKGVGATGVLLVAANWGWRDALAAEKKAFGADAMPHGWVDNPKIYVSIDRDG
TVGIVCNRSEMGQGVRTSLAMVVADELEADWSRVKVIQAPGDEARYGNQDTDGSRSMRHWFEPMRRCGA
AARQMLEQAAANQWKVPLGECRAEQNKVLHAPSGRSLSFGELAEAAAGLEVPARDKLLLKKPEQFRYIGKDV
ARAIDGADIVNGRAGFGFDARFDDMLYAVVARPPVYGGKLKRYDAAAALKVPGVVKVIEIEGRPIPSEFQPLGG
VAVVAQNTWAAIKGREALVVEWDAGVNGGYDSVAYRKQLEEAARKPGKVVRDSGDAAALFAKGGDIVEAEYY
LPHLAQAPMEPPVSTAWYKDGACEVWAPTQAPQVTRERIAERLKLPFDKVTVNVTLLGGGFGRKSKPDFVLEA
AILAKAFPGRHLRVQWTREDDLHFSYFHTVSVERLQAVLGADGLPQAWLHRSVAPSITALFGPDSKHQGAFEL
GMGLTNLPFAIPNVRLENPEAPAHTRVGWFRSVSNIPHAFAIQSFVGELAAKAGQDPKDYLLKLLGPARRIDTAE
LGDSWNYGESPERYPLDVGRLRGVIEEAARQSGWGGELPRGRARGIAAHYSFVTYVAVVIEVEVKDDGALLVH
KATIAADCGPQINPERIRSQLEGACVMGLGLAALGEISFKDGKVQQDNFHQYELARMPLAPKAVSVHLLKPDG
DLPLGGVGEPGVPPIAPALCNAIFAATGKRIRELPIRNQLQGWRKA
>LTUG01||OFP44280.1
MNSKIDLSNALPGSRRGFLKGAAVVGLTIGFQWSGARRALAAALPDAGFAPNAFLRIAPDDSVTVIAKHVEMG
QGAYTGIATIVAEELDADWSKVRVESAPADAKRYANLAFGTMQGTGGSSAMANSWMQLREAGAKARAMLV
EAAARQWRVPATELRTRDGFVEHPASQRKASYGSLAAAAAELPVPEKVQLKDPKDFRLIGHQAPRVDVPGKTD
GSAQFTLDVSLPGMLVALLQRPPLFGATVKSFDATATRAIPGVVEVVQVPHGVAVVAKGFWAAKQGRDALKVE
WDESKAEKRGSEALMAEYRKLAEQPGKPARRDGDAAKAVAGATRRIAASYEFPFLAHAPMEPLDAVVRLTADS
CEIWAGDQFQTVDQGNAARTAGLKPEQVKINTLYAGGSFGRRANAWSDYIVEAVSIAKALGANGVPVKLQWT
REDDIHGGFYRPMYYHRLEAGLDADGKLVGWQHRIVGQSILEGTPFAAVMVKDGIDATSVEGAANLPYAVPN
VSVELSTTQVGVPVLWWRVVGSSHTVYAVEAFIDEAAQAAGKDPYLFRRDLLAEQPRLRGVLELAAEKAGWD
PSRPLPAGRGRGIAVTEAFKTFVAQVVEVSVDKDGKLKVERVVCAVDCGIPINPDVIAAQMEGGIGFGLGAILHS
AITLKDGKVEQNNFDGYQVLRIAEMPKVEVHIVPSGEAPTGVGEPGVAPIGPALANAIFAATGQRLYNLPFPTSF
AKA
>LTVO01||OFQ22251.1
MNSKIDLSNALPGSRRGFLKGAAVVGLTIGFQWSGARRALAAALPDAGFAPNAFLRIAPDDSVTVIAKHVEMG
QGAYTGIATIVAEELDADWSKVRVESAPADAKRYANLAFGTMQGTGGSSAMANSWMQLREAGAKARAMLV
EAAARQWRVPATELRTRDGFVEHPASQRKASYGSLAAAAAELPVPEKVQLKDPKDFRLIGHQAPRVDVPGKTD
GSAQFTLDVSLPGMLVALLQRPPLFGATVKSFDATATRAIPGVVEVVQVPHGVAVVAKGFWAAKQGRDALKVE
WDESKAEKRGSEALMAEYRKLAEQPGKPARRDGDAAKAVAGATRRIAASYEFPFLAHAPMEPLDAVVRLTADS
CEIWAGDQFQTVDQGNAARTAGLKPEQVKINTLYAGGSFGRRANAWSDYIVEAVSIAKALGANGVPVKLQWT
REDDIHGGFYRPMYYHRLEAGLDADGKLVGWQHRIVGQSILEGTPFAAVMVKDGVDATSVEGAANLPYAVPN
VSVELSTTQVGVPVLWWRVVGSSHTVYAVEAFIDEAAQAAGKDPYLFRRDLLAEQPRLRGVLELAAEKAGWD
PSRPLPAGRGRGIAVTEAFKTFVAQVVEVSVDKDGKLKVERVVCAVDCGIPINPDVIAAQMEGGIGFGLGAILHS
AITLKDGKVEQNNFDGYQVLRIAEMPKVEVHIVPSGEAPTGVGEPGVAPIGPALANAIFAATGQRLYNLPFPTSF
AKA
>LTVO01||OFQ23220.1
MKRSYPDDLVIGNLSRRGFLKGVGATGVLLVAANWGWRDALAAEKKAFGADAMPHGWVDNPKIYVSIDRDG
TVGIVCNRSEMGQGVRTSLAMVVADELEADWSRVKVIQAPGDEARYGNQDTDGSRSMRHWFEPMRRCGA

AARQMLEQAAANQWKVPLGECRAEQNKVLHAPSGRSLSFGELAEAAAGLEVPARDKLLLKKPEQFRYIGKDV
ARAIDGADIVNGRAGFGFDARFDDMLYAVVARPPVYGGKLKRYDAAAALKVPGVVKVIEIEGRPIPSEFQPLGG
VAVVAQNTWAAIKGREALAVEWDAGVNGGYDSVAYRKQLEEAARKPGKVVRDSGDAAALFARGGDIVEAEYY
LPHLAQAPMEPPVSTAWYKDGACEVWAPTQAPQVTRERIAERLKLPFDKVTVNVTLLGGGFGRKSKPDFVLEA
AILAKAFPGRHLRVQWTREDDLHFSYFHTVSVERLQAVLGADGLPQAWLHRSVAPSITALFGPDSKHQGAFEL
GMGLTNLPFAIPNVRLENPEAPAHTRVGWFRSVSNIPHAFAIQSFVGELAAKAGQDPKDYLLKLLGPARRIDTAE
LGDSWNYGESPERYPLDVGRLRGVIEEAARQSGWGGELPRGRARGIAAHYSFVTYVAVVIEVEVKDDGALLVH
KATIAADCGPQINPERIRSQLEGACVMGLGLAALGEISFKDGKVQQDNFHQYELARMPLAPKAVSVHLLKPDG
DLPLGGVGEPGVPPIAPALCNAIFAATGKRIRELPIRNQLQGWRKA
>LTVV01P||OFQ49801.1
MTTTMHVSRRGFLKGGLGALTLAVTGNGLVSAVWAADEPKKYGADSMPGGTVDDPLAFVSIAADGTVTIVAH
RAEMGTGVRTSLPMVVADEMEAAWDRVRVVQADADEARYGNQNVDGSRSVRHFLMPMRRVGAAARQM
LEAAAAARWSVPLAEVRATQHEVVHAPSGRRLGYGELAADAAKLPVPAGDTVKLKTRAEFRYIGKDEVRLVDLE
AIGKGEAMYGMDMRLPGMVYAVVARPPVVGGKLRRVDSAKALAVPGVLKVVEIPAMAGAPAFQPLGGVAVV
ASNTWAAMQGRAALAIEWDDGPNAAYDSVAYRETLTEASRKPGKVVRDQGDAPQAWAKAGETERFMAEY
HVPHLAHASMETPVATVRIQDGAAEVWTSVQNPAAAQEAVAKRLKLKPENVKVHVLLLGGGFGRKSKPDYVD
EAAIVAQAMPAGTPVKLVWTREDDIHHDYLHTVSAEHLEAVVGKDGKVQSWLHRSAAPTIASLFTEGAKGEQL
FESAMSAINMPYVIPNVRVETAEVAAHARIGWFRSVANIPHAFAAQCFIAELAHRAGQDHKQYALDLIGPARRI
DPGTLADTWNYSESPERYPYDTGRLRGVIEAAASGAKWGRELPKGHGLGLAFCYSFMSYTATVVEVAVDEKGE
VRVVAVDMALDCGPQIKPERIRAQMEGGAIMGLSLALLGEISFEKGRVKQNNFYDYEVLRHNASPRVIRTHLVN
DDHALPPGGVGEPPVPPVAPALCNAIFAATGKRVRSLPVRSVA
>LTWH01||OFQ77461.1
MNSKIDLSNALPGSRRGFLKGAAVVGLTIGFQWSGARRALAAALPDAGFAPNAFLRIAPDDSVTVIAKHVEMG
QGAYTGIATIVAEELDADWSKVRVESAPADAKRYANLAFGTMQGTGGSSAMANSWMQLREAGAKARAMLV
EAAARQWQVPAAELRTRDGFVEHPTSQRKASYGSLAAAAAELPVPEKVQLKDPKDFRLIGHQAPRVDVPGKT
DGSAQFTLDVSLPGMLVALLQRPPLFGATVKSFDATATRAIPGVVEVVQVPHGVAVVAKGFWAAKQGRDALKV
EWDESKAEKRGSEALMAEYRKLAEQPGKPARRDGDAAKAVAGATRRIAASYEFPFLAHAPMEPLDAVVRLTAD
SCEIWAGDQFQTVDQGNAARTAGLKPEQVKINTLYAGGSFGRRANAWSDYIVEAVSIAKALGANGVPVKLQW
TREDDIHGGFYRPMYYHRLEAGLDADGKLVGWQHRIVGQSILEGTPFAAVMVKDGVDATSVEGAANLPYAVP
NVSVELSTTQVGVPVLWWRVVGSSHTVYAVEAFIDEAAQAAGKDPYLFRRDLLAEQPRLRGVLELAAEKAGW
DPSRPLPAGRGRGIAVTEAFKTFVAQVVEVSVDKDGKLKVERVVCAVDCGIPINPDVIAAQMEGGIGFGLGAIL
HSAITLKDGKVEQNNFDGYQVLRIAEMPKVEVHIVPSGEAPTGVGEPGVAPIGPALANAIFAATGQRLYNLPFPT
TFAKA
>LTWH01||OFQ79036.1
MKRSYPDDLVIGNLSRRGFLKGVGATGVLLVAANWGWRDALAAEKKAFGADAMPHGWVDNPKIYVSIDRDG
TVGIVCNRSEMGQGVRTSLAMVVADELEADWSRVKVIQAPGDEARYGNQDTDGSRSMRHWFEPMRRCGA
AARQMLEQAAANQWKVPLGECRAEQNKVLHAPSGRSLSFGELAEAAAGLEVPARDKLLLKKPEQFRYIGKDV
ARAIDGADIVNGRAGFGFDARFDDMLYAVVARPPVYGGKLKRYDAAAALKVPGVVKVIEIEGRPIPSEFQPLGG
VAVVAQNTWAAIKGREALVVEWDAGVNGGYDSVAYRKQLEEAARKPGKVVRDSGDAAALFAKGGDVVEAEY
YLPHLAQAPMEPPVSTAWYKDGACEVWAPTQAPQVTRERIAERLKLPFDKVTVNVTLLGGGFGRKSKPDFVLE
AAILAKAFPGRHLRVQWTREDDLHFSYFHTVSVERLQAVLGADGLPQAWLHRSVAPSITALFGPDSKHQGAFE
LGMGLTNLPFAIPNVRLENPEAPAHTRVGWFRSVSNIPHAFAIQSFVGELAAKAGQDPKDYLLKLLGPARRIDTA
ELGDSWNYGESPERYPLDVGRLRGVIEEAARQSGWGGELPRGRARGIAAHYSFVTYVAVVIEVEVKDDGALLV
HKATIAADCGPQINPERIRSQLEGACVMGLGLAALGEISFKDGKVQQDNFHQYELARMPLAPKAVSVHLLKPD

GDLPLGGVGEPGVPPIAPALCNAIFAATGKRIRELPIRNQLQGWRKA
>LTWO01||OFQ92323.1
MNSKIDLSNALPGSRRGFLKGAAVVGLTIGFQWSGARRALAAALPDAGFAPNAFLRIAPDDSVTVIAKHVEMG
QGAYTGIATIVAEELDADWSKVRVESAPADAKRYANLAFGTMQGTGGSSAMANSWMQLREAGAKARAMLV
EAAARQWQVPAAELRTRDGFVEHPASQRKASYGSLAAAAAELPVPENVQLKDPKDFRLIGHQAPRVDVPGKT
DGSAQFTLDVSLPGMLVALLQRPPLFGATVKSFDATATRAIPGVVEVVQVPHGVAVVAKGFWAAKQGRDALKV
EWDESKAEKRGSEALMAEYRKLAEQPGKPARRDGDAAKAVAGATRRIAASYEFPFLAHAPMEPLDAVVRLTAD
SCEIWAGDQFQTVDQGNAARTAGLKPEQVKINTLYAGGSFGRRANAWSDYIVEAVSIAKALGANGVPVKLQW
TREDDIHGGFYRPMYYHRLEAGLDADGKLVGWQHRIVGQSILEGTPFAAVMVKDGVDATSVEGAANLPYAVP
NVSVELSTTQVGVPVLWWRVVGSSHTVYAVEAFIDEAAQAAGKDPYLFRRDLLAEQPRLRGVLELAAEKAGW
DPSRPLPAGRGRGIAVTEAFKTFVAQVVEVSVDKDGKLKVERVVCAVDCGIPINPDVIAAQMEGGIGFGLGAIL
HSAITLKDGKVEQNNFDGYQVLRIAEMPKVEVHIVPSGEAPTGVGEPGVAPIGPALANAIFAATGQRLYNLPFPT
SFAKA
>LTWO01||OFR00156.1
MKRSYPDDLVIGNLSRRGFLKGVGATGVLLVAANWGWRDALAAEKKAFGADAMPHGWVDNPKIYVSIDRDG
TVGIVCNRSEMGQGVRTSLAMVVADELEADWSRVKVIQAPGDEARYGNQDTDGSRSMRHWFEPMRRCGA
AARQMLEQAAANQWKVPLGECRAEQNKVLHAPSGRSLSFGELAEAAAGLEVPARDKLLLKKPEQFRYIGKDV
ARTIDGADIVNGRAGFGFDARFDDMLYAVVARPPVYGGKLKRYDAAAALKVPGVVKVIEIEGRPIPSEFQPLGG
VAVVAQNTWAAIKGREALVVEWDAGVNGGYDSVAYRKQLEEAARKPGKVVRDSGDAAALFAKGGDIVEAEYY
LPHLAQAPMEPPVSTAWYKDGACEVWAPTQAPQVTRERIAERLKLPFDKVTVNVTLLGGGFGRKSKPDFVLEA
AILAKAFPGRHLRVQWTREDDLHFSYFHTVSVERLQAVLGADGLPQAWLHRSVAPSITALFGPDSKHQGAFEL
GMGLTNLPFAIPNVRLENPEAPAHTRVGWFRSVSNIPHAFAIQSFVGELAAKAGQDPKDYLLKLLGPARRIDTAE
LGDSWNYGESPERYPLDVGRLRGVIEEAARQSGWGGELPRGRARGIAAHYSFVTYVAVVIEVEVKDDGALLVH
KATIAADCGPQINPERIRSQLEGACVMGLGLAALGEISFKDGKVQQDNFHQYELARMPLAPKAVSVHLLKPDG
DLPLGGVGEPGVPPIAPALCNAIFAATGKRIRELPIRNQLQGWRKA
>LTWV01||OFR08407.1
MKRSYPDDLVIGNLSRRGFLKGVGATGVLLVAANWGWRDALAAEKKAFGADAMPHGWVDNPKIYVSIDRDG
TVGIVCNRSEMGQGVRTSLAMVVADELEADWSRVKVIQAPGDEARYGNQDTDGSRSMRHWFEPMRRCGA
AARQMLEQAAANQWKVPLGECRAEQNKVLHAPSGRSLSFGELAEAAAGLEVPARDKLLLKKPEQFRYIGKDV
ARAIDGADIVNGRAGFGFDARFDDMLYAVVARPPVYGGKLKRYDAAAALKVPGVVKVIEIEGRPIPSEFQPLGG
VAVVAQNTWAAIKGREALVVEWDAGVNGGYDSVAYRKQLEEAARKPGKVVRDSGDAAALFARGGDIVEAEYY
LPHLAQAPMEPPVSTAWYKDGACEVWAPTQAPQVTRERIAERLKLPFDKVTVNVTLLGGGFGRKSKPDFVLEA
AILAKAFPGRHLRVQWTREDDLHFSYFHTVSVERLQAVLGADGLPQAWLHRSVAPSITALFGPDSKHQGAFEL
GMGLTNLPFAIPNVRLENPEAPAHTRVGWFRSVSNIPHAFAIQSFVGELAAKAGQDPKDYLLKLLGPARRIDTAE
LGDSWNYGESPERYPLDVGRLRGVIEEAARQSGWGGELPRGRARGIAAHYSFVTYVAVVIEVEVKDDGALLVH
KATIAADCGPQINPERIRSQLEGACVMGLGLAALGEISFKDGKVQQDNFHQYELARMPLAPKAVSVHLLKPDG
DLPLGGVGEPGVPPIAPALCNAIFAATGKRIRELPIRNQLQGWRKA
>LTWV01||OFR17085.1
MNSKIDLSNALPGSRRGFLKGAAVVGLTIGFQWSGARRALAAALPDAGFAPNAFLRIAPDDSVTVIAKHVEMG
QGAYTGIATIVAEELDADWSKVRVESAPADAKRYANLAFGTMQGTGGSSAMANSWMQLREAGAKARAMLV
EAAARRWRVPATELRTRDGFVEHPASQRKASYGSLAAAAAELPVPEKVQLKDPKDFRLIGHQAPRVDVPGKTD
GSAQFTLDVSLPGMLVALLQRPPLFGATVKSFDATATRAIPGVVEVVQVPHGVAVVAKGFWAAKQGRDALKVE
WDESKAEKRGSEALMAEYRKLAEQPGKPARRDGDAAKAVAGATRRIAASYEFPFLAHAPMEPLDAVVRLTADS
CEIWAGDQFQTVDQGNAARTAGLKPEQVKINTLYAGGSFGRRANAWSDYIVEAVSIAKALGANGVPVKLQWT

REDDIHGGFYRPMYYHRLEAGLDADGKLVGWQHRIVGQSILEGTPFAAVMVKDGIDATSVEGAANLPYAVPN
VSVELSTTQVGVPVLWWRVVGSSHTVYAVEAFIDEAAQAAGKDPYLFRRDLLAEQPRLRGVLELAAEKAGWD
PSRPLPAGRGRGIAVTEAFKTFVAQVVEVSVDKDGKLKVERVVCAVDCGIPINPDVIAAQMEGGIGFGLGAILHS
AITLKDGKVEQNNFDGYQVLRIAEMPKVEVHIVPSGEAPTGVGEPGVAPIGPALANAIFAATGQRLYNLPFPTSF
AKA
>LTXL01||OFR58412.1
PRQWRVPAAELRTRDGFVEHPASQRKASYGSLAAAAAELPVPEKVQLKDPKDFRLIGHQAPRVDVPGKTDGS
AQFTLDVSLPGMLVALLQRPPLFGATLKSFDATATRAIPGVVEVVQVPHGVAVVAKGFWAAKQGRDALKVEW
DESKAEKRGSEALMAEYRKLAEQPGKPARRDGDAAKALAGATRRIAASYEFPFLAHAPMEPLDAVVRLTADSCE
IWAGDQFQTVDQGNAARTAGLQPEQVKINTLYAGGSFGRRANAWSDYIVEAVSIAKALGANGVPVKLQWTRE
DDIHGGFYRPMYYHRLEAGLDADGKLVGWQHRIVGQSILEGTPFAAVMVKDGVDATSVEGAANLPYAVPNVS
VELSTTQVGVPVLWWRVVGSSHTVYAVEAFIDEAAQAAGKDPYLFRRDLLAEQPRLRGVLELAAEKAGWDPS
RPLPAGRGRGIAVTEAFKTFVAQVVEVSVDKDGKLKVERVVCAVDCGIPINPDVIAAQMEGGIGFGLGAILHSAI
TLKDGKVEQNNFDGYQVLRIAEMPKVEVHIVPSGEAPTGVGEPGVAPIGPALANAIFAATGQRLYNLPFPTSFAK
A
>LTXL01||OFR58469.1
MKRSFPDDLVIGNLSRRGFLKGVGATGVLLVAANWGWRDALAAEKKAFGADAMPHGWVDNPKIYVSIDRDG
TVGIVCNRSEMGQGVRTSLAMVVADELEADWSRVKVIQAPGDEARYGNQDTDGSRSMRHWFEPMRRCGA
AARQMLEQAAANQWKVPLGECRAEQNKVLHAPSGRSLSFGELAEAAAGLEVPARDKLLLKKPEQFRYIGKDV
ARAIDGADIVNGRAGFGFDARFDDMLYAVVARPPVYGGKLKRYDAAAALKVPGVVKVIEIEGRPIPSEFQPLGG
VAVVAQNTWAAIKGREALVVEWDAGVNGGYDSAAYRKQLEEAARKPGKVVRDSGDAAALFARGGDIVEAEY
YLPHLAQAPMEPPVSTAWYKDGACEVWAPTQAPQVTRERIAERLELPFDKVTVNVTLLGGGFGRKSKPDYVLE
AAILAKAFPGRHLRVQWTREDDLHFSYFHTVSVERLQAVLGADGLPQAWLHRSVAPSITALFGPDSKHQGAFE
LGMGLTNLPFAIPNVRLENPEAAAHTRVGWFRSVSNIPHAFAIQSFVGELAAKAGQDPKDYLLKLLGPARRIDTA
ELGDGWNYGESPERYPLDVGRLRGVIEEAARQSGWGGELPRGRARGIAAHYSFVTYVAVVIEVEVKDDGALLV
HKATIAADCGPQINPERIRSQLEGACVMGLGLAALGEISFKEGKVQQDNFHQYELARMPLAPKAVSVHLLKPD
GDLPLGGVGEPGVPPIAPALCNAIFAATGKRIRELPIRNQLQGWRKA
>LTZT01P||OFU79905.1
MTTTMQVSRRGFLKGGLGALTLAVTGNGLVSAVWAADEPKKYGADSMPGGTVDDPLAFVSIAADGTVTIVAH
RAEMGTGVRTSLPMVVADEMEAAWDRVRVVQADADEARYGNQNVDGSRSVRHFLMPMRRVGAAARQM
LEAAAAARWSVPLAEVRATQHEVVHAPSGRRLGYGELAADAAKLPVPAGDAVKLKTRAEFRYIGKDEVRLVDLE
AIGKGEAMYGMDMRLPGMVYAVVARPPVVGGKLRRVDSAKALAVPGVLKVVEIPAMAGAPAFQPLGGVAVV
ASNTWAAMQGRAALAIEWDDGPNAAYDSVAYRETLTEASRKPGKVVRDQGDAPQAWAKAGEAERFMAEY
HVPHLAHASMETPVATVRIQDGAAEVWTSVQNPAAAQEAVAKRLKLKPENVKVHVLLLGGGFGRKSKPDYVD
EAAIVAQAMPAGTPVKLVWTREDDIHHDYLHTVSAEHLEAVVGKDGKVQSWLHRSAAPTIASLFTEGAKGEQL
FESAMSAINMPYVIPNVRVETAEVAAHARIGWFRSVANIPHAFAAQCFIAELAHRAGRDHKQYALDLIGPARRI
DPGTLADTWNYSESPERYPYDTGRLRGVIEAAASGAKWGRELPKGHGLGLAFCYSFMSYTATVVEVAVDEKGE
VRVVAVDMALDCGPQIKPERIRAQMEGGAIMGLSLALLGEITFEKGRVKQNNFYDYEVLRHNASPRVIRTHLV
NDDHALPPGGVGEPPVVPPVAPALCNAIFAATGKRVRSLPVRSVA
>LUAB01||OFV04264.1
MNSKIDLSNALPGSRRGFLKGAAVVGLTIGFQWSGARRALAAALPDAGFAPNAFLRIAPDDSVTVIAKHVEMG
QGAYTGIATIVAEELDADWSKVRVESAPADAKRYANLAFGTMQGTGGSSAMANSWMQLREAGAKARAMLV
EAAARQWRVPATELRTRDGFVEHPASQRKASYGSLAAAAAELPVPEKVQLKDPKDFRLIGHQAPRVDVPGKTD
GSAQFTLDVSLPGMLVALLQRPPLFGATVKSFDATATRAIPGVVEVVQVPHGVAVVAKGFWAAKQGRDALKVE

WDESKAEKRGSEALMAEYRKLAEQPGKPARRDGDAAKAVAGATRRIAASYEFPFLAHAPMEPLDAVVRLTADS
CEIWAGDQFQTVDQGNAARTAGLKPEQVKINTLYAGGSFGRRANAWSDYIVEAVSIAKALGANGVPVKLQWT
REDDIHGGFYRPMYYHRLEAGLDADGKMVGWQHRIVGQSILEGTPFAAVMVKDGIDATSVEGAANLPYAVP
NVSVELSTTQVGVPVLWWRVVGSSHTVYAVEAFIDEAAQAAGKDPYLFRRDLLAEQPRLRGVLELAAEKAGW
DPSRPLPAGRGRGIAVTEAFKTFVAQVVEVSVDKDGKLKVERVVCAVDCGIPINPDVIAAQMEGGIGFGLGAIL
HSAITLKDGKVEQNNFDGYQVLRIAEMPKVEVHIVPSGEAPTGVGEPGVAPIGPALANAIFAATGQRLYNLPFPT
SFAKA
>LUAB01||OFU89337.1
MKRSYPDDLVIGNLSRRGFLKGVGATGVLLVAANWGWRDALAAEKKAFGADAMPHGWVDNPKIYVSIDRDG
TVGIVCNRSEMGQGVRTSLAMVVADELEADWSRVKVIQAPGDEARYGNQDTDGSRSMRHWFEPMRRCGA
AARQMLEQAAANQWKVPLGECRAEQNKVLHAPSGRSLSFGELAEAAAGLEVPARDKLLLKTPEQFRYIGKDV
ARAIDGADIVNGRAGFGFDARFDDMLYAVVARPPVYGGKLKRYDAAAALKVPGVVKVIEIEGRPIPSEFQPLGG
VAVVAQNTWAAIKGREALVVEWDAGVNGGYDSVAYRKQLEEAARKPGKVVRDSGDAAALFAKGGDIVEAEYY
LPHLAQAPMEPPVSTAWYKDGACEVWAPTQAPQVTRERIAERLKLPFDKVTVNVTLLGGGFGRKSKPDFVLEA
AILAKAFPGRHLRVQWTREDDLHFSYFHTVSVERLQAVLGADGLPQAWLHRSVAPSITALFGPDSKHQGAFEL
GMGLTNLPFAIPNVRLENPEAPAHTRVGWFRSVSNIPHAFAIQSFVGELAAKAGQDPKDYLLKLLGPARRIDTAE
LGDSWNYGESPERYPLDVGRLRGVIEEAARQSGWGGELPRGRARGIAAHYSFVTYVAVVIEVEVKDDGALLVH
KATIAADCGPQINPERIRSQLEGACVMGLGLAALGEISFKDGKVQQDNFHQYELARMPLAPKAVSVHLLKPDG
DLPLGGVGEPGVPPIAPALCNAIFAATGKRIRELPIRNQLQGWRKA
>LWMZ01||OFS77261.1
MKTPLELPEDLLSLTPGETVNLSRRRFLAGTAVGALVLGFGLPLGSPRVQAATATGTERGTQVPAFLEIRPDNTVR
LLSPFMEGGQGTYTAMAQIVGEELDADPATFVVDSAPPGEAYVVMENGMRITGGSMSVRMSYPTMRRLGA
MARAMLLQAGAEHFDVPVGELSTEPGKVVHGASGRSITYGELAGRAMDLPVPDPARVTLRDPSQFRWIGKPV
KRVDAYDKSTGKAQYSIDIHVDGMLQAAVQHAPRLGMTPGGIRNEAQVKAMKGVHSVHLLPGAVAVVAER
WWYAKRAAEALQVDWQEPGPDATVRPMPKDFSSDAWREHLAAQPGPGRDDEKLGDVAAALASAKTTVEAT
YHNQYLNHAQLEPPSATARFNPDGSLEVWLPNQAPDMFRDDIAKRTGLDPARITLHSPLLGGFFGRHFLYDSA
NPYPQAIALAKAVGRPVKVIWSREEEFLRDVLRPVAVVKFRAGLDDKGLPVALEAVSATEGPTEAIAGKQGEQL
DPTALEGLSGKAYAIANTRVAQIYVKGPAMLGYWRSVGNSLNDFFYEAFLDELADKGGQDPYALRLHLLQGNPR
LTTLLQAVGELSGGWKRGPFTAEDGSTRARGVAMASPFGSEAAVIAEVSIENGQVKVHDIWQAIDPGSIVNPAI
IEAQVNGAVALGLSQVLLEEAVWQNGMPRARNYDLYPVLPPSRMARVHVRIIESGAKMGGIGEPPLPAVAPAV
ANAVARLTGQRIRSMPLSRYTFS
>LWNF01||OFS84617.1
MNSKIDLSNALPGSRRGFLKGAAVVGLTIGFQWSGARRALAAALPDAGFAPNAFLRIAPDDSVTVIAKHVEMG
QGAYTGIATIVAEELDADWSKVRVESAPADAKRYANLAFGTMQGTGGSSAMANSWMQLREAGAKARAMLV
EAAARQWRVPATELRTRDGFVEHPASQRKASYGSLAAAAAELPVPEKVQLKDPKDFRLIGHQAPRVDVPGKTD
GSAQFTLDVSLPGMLVALLQRPPRFGATVKSFDATATRAIPGVLEVVQVPHGVAVVAKGFWAAKQGRDALKVE
WDESKAEKRGSEALMAEYRKLAEQPGKPARRDGDAAKAVAGATRRIAASYEFPFLAHAPMEPLDAVVRLTADS
CEIWAGDQFQTVDQGNAARTAGLKPEQVKINTLYAGGSFGRRANAWSDYIVEAVSIAKALGANGVPVKLQWT
REDDIHGGFYRPMYYHRLEAGLDADGKLVGWQHRIVGQSILEGTPFAAVMVKDGVDATSVEGAANLPYAVPN
VSVELSTTQVGVPVLWWRVVGSSHTVYAVEAFIDEAAQAAGKDPYLFRRDLLAEQPRLRGVLELAAEKAGWD
PSRPLPAGRGRGIAVTEAFKTFVAQVVEVSVDKDGKLKVERVVCAVDCGIPINPDVIAAQMEGGIGFGLGAILHS
AITLKDGKVEQNNFDGYQVLRIAEMPKVEVHIVPSGEAPTGVGEPGVAPIGPALANAIFAATGQRLYNLPFPTSF
AKA
>LWNF01||OFS91956.1

MKRSYPDDLVIGNLSRRGFLKGVGATGVLLVAANWGWRDALAAEKKAFGADAMPHGWVDNPKIYVSIDRDG
TVGIVCNRSEMGQGVRTSLAMVVADELEADWSRVKVIQAPGDEARYGNQDTDGSRSMRHWFEPMRRCGA
AARQMLEQAAANQWKVPLGECRAEQNKVLHAPSGRSLSFGELAEAAAGLEVPARDKLLLKKPEQFRYIGKDV
ARAIDGADIVNGRAGFGFDARFDDMLYAVVARPPVYGGKLKRYDAAAALKVPGVVKVIEIEGRPIPSEFQPLGG
VAVVAQNTWAAIKGREALVVEWDAGVNGGYDSVAYRKQLEEAARKPGKVVRDSGDAAALFAKGGDIVEAEYY
LPHLAQAPMEPPVSTAWYKDGACEVWAPTQAPQVTRERIAERLKLPFDKVTVNVTLLGGGFGRKSKPDFVLEA
AILAKAFPGRHLRVQWTREDDLHFSYFHTVSVERLQAVLGADGLPQAWLHRSVAPSITALFGPDSKHQGAFEL
GMGLTNLPFAIPNVRLENPEAPAHTRVGWFRSVSNIPHAFAIQSFVGELAAKAGQDPKDYLLKLLGPARRIDTAE
LGDSWNYGESPERYPLDVGRLRGVIEEAARQSGWGGELPRGRARGIAAHYSFVTYVAVVIEVEVKDDGALLVH
KATIAADCGPQINPERIRSQLEGACVMGLGLAALGEISFKDGKVQQDNFHQYELARMPLAPKAVSVHLLKPDG
DLPLGGVGEPGVPPIAPALCNAIFAATGKRIRELPIRNQLQGWRKA
>LWOO01||OFT75522.1
MSRGLIEAGHAGAAMSRRSFLRFGMSLGAAAGGGLLLGFSLPAAGDHTRRSVIGGDADEPARAGVFAPNAFV
QIDRSGRVTLVMPKVEMGQGVYTALPMLIAEELEVPLSSVTLDHAPPNEKLFLDPLLGGQLTGGSTSVRYAWEP
LRRAGATARVLLVSAAAKQWNVEPTACHAENGEVRHPPSGRRASYGELADAAAKLPVPADVALKKPEQFKLIGT
PAKRLDSPEKVDGVAQFGLDVRLPGMLYAVIVNSPVFGGTVASVDDTAAKKIPGVRQIVRVDDAVAVVGDHTW
AAKRGASALVVKWNEGANANVSTKDLFADLAQAAANGKGAVARKDGDVDHAFSNAKTRVDAVYEQPLLAHA
TMEPVNCTVHVRSDACEVWVGTQVPTRARDTAQRITGLPAERIVVHNHLLGGGFGRRLETDMIGQAVKIGKQ
VGAPVKVVWTREEDIQHDMYRPCYHDRISAALDANGKPIAWRHRIVGSSILARFAPPAFKDGVDPDAVEVAID
LPYDVPNQLIDYVRQEPRHVPTAFWRGVGPTRSTFVVESFIDELAAQAKTDPVQYRRALLDKTPRARNVLDIAT
KAAGWGAALPQGQGRGVSVMHAFGSFFAIVADVAVDDGEVRVTRVVCAVDCGMTVNPNTIEAQVQGGIIF
GITGALYGEVTIENGRVMQRNFNDYRVLRINETPPIDVHIVKSGEAPGGIGEPGTAATAAAVANAIFAATGKRLR
KLPIGNQLKTA
>LWOO01||OFT83163.1
MNAQPLSVCNESRRALLLGFASGGLLLAFGVPSLARAAAGQPPVSANPQYGGAGMPHGLRDDPNLFVAIAPD
GTVTVTCIRSEMGQGVRTSVALVVADELGADWARVKVAQAVGDEARYGNQNTDGSRSLRQSFAALRRAGAA
ARTMLEQAAAATWGIDVRQVKATVHQVVDTKSGRRLGYGELAAKAAALPVPDSAALVLKAPTEFRYIGKGETA
LIDGRDIVAGRAQYGIDTRLDGMLYAVVARPPAYGDTVASFDAGAAEKLPGVVKIVPLASTPLPAGFQPLGGVAV
VARDTWTAIQARKQLKIDWKRGPNAGYDSAAYRKTLEAAAAQPGDVIRNDGDTAAALAGAAKRVRATYYVPH
LAHATMEPPAALARVADGRCEVWTCTQAPQTTRDEVAKALGLPAERVTVNVTLLGGGFGRKSKPDYVVEAALL
SKAVGAPVKLTFTREDDIAHDYFHAVSLEAFDGALDAAGKVVAWQHRTVAPSIQSTFKAGVVHEQPGELAQGI
ADLPFAIPNVRIENPAAPAHTRIGWFRSVYNIPHAFGIQSFVAELAHAAGRDPKDFLLDLIGPARRFEPHIAVKNV
NYGEDPALYPVDTGRLRRVVETVAREAGWGRTLPKGHGLGIAAHRSFVSYTAVACEVQVGDDGAIAVPRVDIAI
DCGPQVNPERVRSQLEGAVVMGLGLALHGEITFKDGQPEQSNFNGFQVLRMNEAPREIRVHLVAPDDYATPL
GGVGEPGLPPVAPALTNAIFAATGKRIRSLPIADQLRDSKVA
>LWOO01||OFT77929.1
MTIELDTRDSVRPSRRTFLKAAGAAAAVSLTIGFEWAGLGRRAAAATAAPEAGAFAPNAFLRVTPDGAVTVIAK
HVELGQGAYTGIATIVAEELDADWSSVRVESAPADAKRYANLAFGTMQGTGGSSAMSNSWQQLREAGGKAR
AMLVSAAAARWNVPASELTTANGVVTHAKSGKTAAYGTLVADASKLPVPDKVVLKQPADFKLIGHRIPRVDASA
KSNGTAHFTLDTTFPGMRVALLQRPPRFGATVKSFDATAAKAVPGVVSVVQVPGGIAVVGTGFWAAKQGRDA
LKVEWDETNAEKRGSDEIMREYRLLAAKPGASARKDGDADAAIAGAARRISASYEFPYLAHAPMEPLDAVVKL
TKDSCEIWAGDQFQTVDQGNAAKVAGLKPEQVQIHTLYAGGSFGRRANAWSDYVVEAVSIAKALGADGKPVK
LQWTREDDIQGGFYRPMYFHKLDAGLTADGRLVGWRHRIVGQSILAGTPFEAFMVKNGIDATSVEGAANLPY
AVPNVSVELTTTKVGLPVLWWRVVGSSHTAYAVEAFIDEAAYAAGKDPYLFRRDLLAKEPRMRAVLELAAQKAG

WDPAKPLPKGRGRGIAVAEAFKSYVAQVAEVSVDADGKVKVERVVCAVDCGIAINPDIVAAQMEGGIGFGLGA
AMHSAITLKDGRVEQRNFDGYQVLRMAEMPKVEVHIVPSAEAPTGVGEPGVAPVGPAVANAIFAATGKRHYV
LPFDSGRNASA
>LWPU01P||OFS67913.1
MTTTMQVSRRGFLKGGLGALTLAVTGNGLVSAVWAADEPKKYGADSMPGGTVDDPLAFVSIAADGTVTIVAH
RAEMGTGVRTSLPMVVADEMEAAWDRVRVVQADADEARYGNQNVDGSRSVRHFLMPMRRVGAAARQM
LEAAAAARWSVPLAEVRATQHEVVHAPSGRRLGYGELAADAAKLPVPAGDAVKLKTRAEFRYIGKDEVRLVDLE
AIGKGEAMYGMDMRLPGMVYAVVARPPVVGGKLRRVDSAKALAVPGVLKVVEIPAMAGAPAFQPLGGVAVV
ASNTWAAMQGRAALAIEWDDGPNAAYDSVAYRETLTEASRKPGKVVRDQGDAPQAWAKAGEAERFMAEY
HVPHLAHASMETPVATVRIQDGAAEVWTSVQNPAAAQEAVAKRLKLKPENVKVHVLLLGGGFGRKSKPDYVD
EAAIVAQAMPAGTPVKLVWTREDDIHHDYLHTVSAEHLEAVVGKDGKVQSWLHRSAAPTIASLFTEGAKGEQL
FESAMSAINMPYVIPNVRVETAEVAAHARIGWFRSVANIPHAFAAQCFIAELAHRAGQDHKQYALDLIGPARRI
DPGTLADTWNYSESPERYPYDTGRLRGVIEAAASGAKWGRELPKGHGLGLAFCYSFMSYTATVVEVAVDEKGE
VRVVAVDMALDCGPQIKPERIRAQMEGGAIMGLSLALLGEITFEKGRVKQNNFYDYEVLRHNASPRVIRTHLV
NDDHALPPGGVGEPPVPPVAPALCNAIFAATGKRVRSLPVRSVA
>LWQQ01||OFS62398.1
MNSKIDLSNALPGSRRGFLKGAAVVGLTIGFQWSGARRALAAALPDAGFAPNAFLRIAPDDSVTVIAKHVEMG
QGAYTGIATIVAEELDADWSKVRVESAPADAKRYANLAFGTMQGTGGSSAMANSWMQLREAGAKARAMLV
EAAARQWRVPATELRTRDGFVEHPASQRKASYGSLAAAAAELPVPEKVQLKDPKDFRLIGHQAPRVDVPGKTD
GSAQFTLDVSLPGMLVALLQRPPLFGATVKSFDATATRAIPGVVEVVQVPHGVAVVAKGFWAAKQGRDALKVE
WDESKAEKRGSEALMAEYRKLAEQPGKPARRDGDAAKAVAGATRRIAASYEFPFLAHAPMEPLDAVVRLTADS
CEIWAGDQFQTVDQGNAARTAGLKPEQVKINTLYAGGSFGRRANAWSDYIVEAVSIAKALGANGVPVKLQWT
REDDIHGGFYRPMYYHRLEAGLDADGKLVGWQHRIVGQSILEGTPFAAVMVKDGIDATSVEGAANLPYAVPN
VSVELSTTQVGVPVLWWRVVGSSHTVYAVEAFIDEAAQAAGKDPYLFRRDLLAEQPRLRGVLELAAEKAGWD
PSRPLPAGRGRGIAVTEAFKTFVAQVVEVSVDKDGKLKVERVVCAVDCGIPINPDVIAAQMEGGIGFGLGAILHS
AITLKDGKVEQNNFDGYQVLRIAEMPKVEVHIVPSGEAPTGVGEPGVAPIGPALANAIFAATGQRLYNLPFPTSF
AKA
>LWQQ01||OFS62948.1
MKRSYPDDLVIGNLSRRGFLKGVGATGVLLVAANWGWRDALAAEKKAFGADAMPHGWVDNPKIYVSIDRDG
TVGIVCNRSEMGQGVRTSLAMVVADELEADWSRVKVIQAPGDEARYGNQDTDGSRSMRHWFEPMRRCGA
AARQMLEQAAANQWKVPLGECRAEQNKVLHAPSGRSLSFGELAEAAAGLEVPARDKLLLKKPEQFRYIGKDV
ARAIDGADIVNGRAGFGFDARFDDMLYAVVARPPVYGGKLKRYDAAAALKVPGVVKVIEIEGRPIPSEFQPLGG
VAVVAQNTWAAIKGREALVVEWDAGVNGGYDSVAYRKQLEEAARKPGKVVRDSGDAAALFARGGDIVEAEYY
LPHLAQAPMEPPVSTAWYKDGACEVWAPTQAPQVTRERIAERLKLPFDKVTVNVTLLGGGFGRKSKPDFVLEA
AILAKAFPGRHLRVQWTREDDLHFSYFHTVSVERLQAVLGADGLPQAWLHRSVAPSITALFGPDSKHQGAFEL
GMGLTNLPFAIPNVRLENPEAPAHTRVGWFRSVSNIPHAFAIQSFVGELAAKAGQDPKDYLLKLLGPARRIDTAE
LGDSWNYGESPERYPLDVGRLRGVIEEAARQSGWGGELPRGRARGIAAHYSFVTYVAVVIEVEVKDDGALLVH
KATIAADCGPQINPERIRSQLEGACVMGLGLAALGEISFKDGKVQQDNFHQYELARMPLAPKAVSVHLLKPDG
DLPLGGVGEPGVPPIAPALCNAIFAATGKRIRELPIRNQLQGWRKA
>N221356||gene_213670|GeneMark.hmm|715_aa|-|92|2239
MNVSTTRRGFLAGGAGLMIALSLPMGRARAQGAAATAPFAPNAFIRIGTDDLVTVMIKHIEMGQGPYTGLSTL
VAEELDADWSQMRAEGAPADAKLYANLAFGAQGTGGSTAMANSYMQMRKAGAAARAMLVAAAAAEWGV
PAEEITVKAGVVAHEASGKTSGFGALTEAAAKQAVPEDPPLKTAKDFVLIGKDLPRLDTKSKTNGTAIFTMDVYR
DGMLTVLVAHPPKFGAKVKSVDDKAALAVKGVEMVRPISSGVAVYATNTYAAMKGREALVVEWDDTGAETRG

TDEIYASLAQMVAEGGKTVEEQGDIAAVTDPSKVLEAEFRFPYLAHAPLEPLDAVIETKGGKAEMWYGCQFPTF
DHMAVAATLGIPIEDVSINVLMAGGSFGRRAQGSSHLAIEAAEIAKAAGRDGAFKLVWTREDDLKGGYYRPITV
HKLRAGISDDGKILSWENVVANQSILTGTPMAAMLKGAPDSTSFEGSSGLPYQFGARRIGWGQMESPVSVLW
WRSVGHTHTAYAVEAFLDEVLEEMGKDSIAGRLELLPPEATRERGVIEKVAEISGWTGRTRDGKGYGMAYTKSF
NTYVAEVVEVEDRGGKPHVTKVWCAVDCGVAVNPDVIRGQMEGGIGYALSAVLHSKITMAPGGEVEQSNFH
DYPMLRISEMPEVEVAIIQSDADPTGVGEPGVPPLGPALANAWRALSGQKQHQLPFLAGVIS
>R8H2||gene_95683|GeneMark.hmm|771_aa|-|10421|12736
MKRSYPDDLVIGNLSRRGFLKGVGATGVLLVAANWGWRDALAAEKKAFGADAMPHGWVDNPKIYVSIDRDG
TVGIVCNRSEMGQGVRTSLAMVVADELEADWSRVKVIQAPGDEARYGNQDTDGSRSMRHWFEPMRRCGA
AARQMLEQAAANQWKVPLGECRAEQNKVLHAPSGRSLSFGELAEAAAGLEVPARDKLLLKKPEQFRYIGKDV
ARAIDGADIVNGRAGFGFDARFDDMLYAVVARPPVYGGKLKRYDAAAALKVPGVVKVIEIEGRPIPSEFQPLGG
VAVVAQNTWAAIKGREALMVEWDAGVNGGYDSVAYRKQLEEAARKPGKVVRDSGDAAALFAKGGDVVEAE
YYLPHLAQAPMEPPVSTAWYKDGACEVWAPTQAPQVTRERIAERLKLPFDKVTVNVTLLGGGFGRKSKPDFVL
EAAILAKAFPGRHLRVQWTREDDLHFSYFHTVSVERLQAVLGADGLPQAWLHRSVAPSITALFGPDSKHQGAF
ELGMGLTNLPFAIPNVRLENPEAPAHTRVGWFRSVSNIPHAFAIQSFVGELAAKAGQDPKDYLLKLLGPARRIDT
AELGDSWNYGESPERYPLDVGRLRGVIEEAARQSGWGGELPRGRARGIAAHYSFVTYVAVVIEVEVKDDGALL
VHKATIAADCGPQINPERIRSQLEGACVMGLGLAALGEISFKDGKVQQDNFHQYELARMPLAPKAVSVHLLEP
DGDLPLGGVGEPGVPPIAPALCNAIFAATGKRIRELPIRNQLQGWRKA
>R8H2||gene_443333|GeneMark.hmm|589_aa|-|3276|5045
VEAAARQWRVPATELRTRDGFVEHPASQRKASYGSLAAAAAELPVPEKVQLKDPKDFRLIGHQAPRVDVPGKT
DGSAQFTLDVSLPGMLVALLQRPPLFGATVKSFDATATRAIPGVVEVVQVPHGVAVVAKGFWAAKQGRDALKV
EWDESKAEKRGSEALMAEYRKLAEQPGKPARRDGDAAKAVAGATRRIAASYEFPFLAHAPMEPLDAVVRLTAD
SCEIWAGDQFQTVDQGNAARTAGLKPEQVKINTLYAGGSFGRRANAWSDYIVEAVSIAKALGANGVPVKLQW
TREDDIHGGFYRPMYYHRLEAGLDADGKLVGWQHRIVGQSILEGTPFAAVMVKDGIDATSVEGAANLPYAVP
NVSVELSTTQVGVPVLWWRVVGSSHTVYAVEAFIDEAAQAAGKDPYLFRRDLLAEQPRLRGVLELAAEKAGW
DPSRPLPAGRGRGIAVTEAFKTFVAQVVEVSVDKDGKLKVERVVCAVDCGIPINPDVIAAQMEGGIGFGLGAIL
HSAITLKDGKVEQNNFDGYQVLRIAEMPKVEVHIVPSGEAPTGVGEPGVAPIGPALANAIFAATGQRLYNLPFPT
SFAKA
