# Supplementary material for: Genomic and Metagenomic Insights into the Distribution of Nicotine-degrading Enzymes in Human Microbiota
Source: Curr Genomics. 2024 Mar 20;25(3):226–35. doi: 10.2174/0113892029302230240319042208 (PMC11288164; doi:10.2174/0113892029302230240319042208)
Supplement: Supplementary file 1 [file CG-25-226_SD1.zip › CG-25-226_SD1/Xie MS Suppl file 2.pdf]

>scaffold_AM104-69||gene_1106|GeneMark.hmm|783_aa|-|99829|102180
MGIEGIGARVARKEDKRFLTGKGRYTDDMVVPGMKYAFFIRSPHAYAKLISIDANAALEMPGVIAVLDGKQLQA
DGIGNLICGWMIHSKDGSPMNMGPWRPLAVDTVRYVGDAVAVVIADTLGEARDAAEAVAVEYETLDAVTEA
VDALKPGAPQVHDNAPGNLIFDWALGNPEAEVDAAISASAHVTEIEIHNNRLSPNPMEPRAALGIYDSAEDHY
TCYTTSQNPHVARLVMSAFYNVAPENKLRVIAPDVGGGFGSKIYIYPEEIVCLWASKKTGVPVKWTADRTEAFM
TDAHGRDHISKVKMAFDADHRITALKVDTIANLGAYMSLFSSAVPTYLYATLLSGQYAIPAINANVRTVYTNTVPV
DAYRGAGRPEATYLLERTMETAARELGLSPAELRRINFIRSFPYQTPVIMNYDAGDYEASLNAAMDAADWAGFA
ARRTEAEGRGKKRGIGMSCYIEACGIAPSAAVGSLGAGVGLWESAEVRVNAVGTIEVMTGSHSHGQGHETTFA
QLVADRLGVSIDSVNIVHGDTDKVQMGMGTYGSRSGAVGMSAIVKALDKVEAKAKKIAAHLMEADESDIVIE
GGELKVAGTDKSLPWFQVALAAYTAHNLPAGMEPGLKEGAFYDPANFTFPAGCYICEVEIDPETGTTKIVQFVA
ADDFGNIINPMIVEGQVHGGIAQGVGQALLEGVHYDKDTGQLLTASFMDYAMPRADDLPSFKLSHQNTPCPS
NPLGIKGCGEAGAIGSPPALMNAITDAIGHNDLTMPATPQKVWKALNALH
>scaffold_TM114-134||gene_2605|GeneMark.hmm|780_aa|-|63949|66291
MNWKTPVPLRKEDTRFLTGKGRFISDITMDGMHHVYIVRSEKAHAEITSVECEDAWLTGVIGIYSCEDLSHDLG
EVPMVWPVEGLKNPGHPLLADKIVRYIGEPILIIIADHLYTAKQAAKRIRVDYKELPSITSLNMAIKNEDRTRIHPDI
KDNIAYEWKRLYGDEGSKNKGSSFIEQRLVIPRVAPLPLETRGIIAYYDKSNDLLSIWSSTQMPHVLRMCLANCLQ
HKEQRIEVTAPDIGGAFGSKMNVYREEILVAYFAKTLKLPLKYIETRSENFTASTHGRDQIQDIKLYHTDGLIQGME
ITIHANMGAYLQAATTGIPIYTTQMLSGCYDIPYINVKIKAYYTNQTPTDAYRGAGRPEATYLVESMMDRLAKEC
RLDPADIRRKNFIKAGAFPKKVVTGLTYDSGNYEKTLNKLIEVSELEKWRRLQKERRERNSRKQIGIGLSTYVEFCG
TGPSKINKKIGLQTGGFESAVVRMLPTGMVEVISGVCSSGQGHQTSLSLIVERKLGCLADEIEVTTSRASLSPWG
GGTYGSRSIAVGGSAIDLACDKLIQKGKKFLAVHWKSSLENICFYKGIFLEKGGRKMSLKELTGILYLAHDLPEGFE
PGLEATVFFEPSNYTFPFGAHLCVIEIDSETGDRSIVAYTAVDDFGNVIHEKIAEGQILGGIMQGIGESLYEEIKHDK
DTGSMCTHNLRTYEISKTVDAPYIHLFRTKTPTSINPLGAKGVGEAGAIAAPPAVMNAVKDALKIYRVKDINMPA
TKRKIWEAIHLSQEKDDE
>SEQF10162||SEQF10162.2_01718
MKRFDAGETTGTAQETQQTTGQPYVGRPMQRVEDAAILTGRGRYADDLGVKPGTLHAAILRSPHAHAELGLID
FAAALKAPGVRAVLTGADLPAWSKPFVVGVKAPMEQWALAMDRVRYVGEPVAVVVAESRALAEDALDLVRV
DYRVLPPVVSIEAAIADAAPQLHSGLGSNVASDRHFRYGDPEAAFATAPHRVSLTVHYPRNTCTPIECGVVIAEHL
PGNEGYQVTSNFMGPFSLHAVMAMALQVSANHLRHIAPRDSGGSFGVKQAVFPYVVLMCLASRKAGAPVK
WVEDRLEHLSAATSATARLSTIEAAVEADGRIVALDYDQLEDCGGYLRAPEPATFYRMHGVLTGAYAIPNLRVRN
RVVLTNKTPTGLVRGFGGPQVYFALERLVQRISIELNLDPLDVYRRNFVPSNAFPYRAAAGALLDSGNYQLAMS
RALETGAYDELKRRRDIARAEGRLYGIGFAAIVEPSVSNMGYITTATPAEARRKAGPKNGAIASATVSVDLLGGVV
VTIASTPAGQGHMTVCAQVVADVLGIDPAEVIVNVEFDTHKDAWSVAAGNYSSRFAGAVAGTVHLAATRVRD
KLARIVASQLDCDPAELIFAEGRITRRDAPETAVVFARAASNAPHWSPQLLPAGEEPGLRETVFWSPPNLDAPDE
QDRINTSACYGFAFDLCGLEIDRATGRVRIDRYVTAHDAGKLLNPALADGQIRGAFAQGLGAALMEEFRYGPDG
SFQSGTLADYLLPTTCEVPDPMIVHLETPSPFTPLGAKGLGEGNNMSTPPCIANAVADALGVRDIRLPLTPAKVM
AMIGLEDPPPSRLELAETATAAATGGKERSKGAKALSARGTVDLDAPPEAVFAVLLDPQALAQVVPGCHVLEPIG
DNRYRADVTVGVGMIKARYEAEIALSDLEPPHRLRLSGAGLSSLGSARGSGMVELAPHEGGTRLTYDYEAEVSG
KVAAVGGRMLEGAAKVVLRQLFESLGRQAGGKPVRKPGWIARLLALFGARR
>SEQF10162||SEQF10162.2_01332
MNAPAEPNNHLIGASVKRKEDFRFLTGAGQYTDDVVQAHQSYAVFLRSPYAHARIKHINTDAARNHPGVLAVL
TGDDLAADKVNGLPCGWLIHSIDGTPMKEPPHPVLAQGKVRHVGDQVALVVAESVKIAKDAVEMIDVEYDEL
PAVVDTATADTAGTAVHDDVPNNTCYTWGHGDKAATDAAFARAAHVTRLDIVNNRLIPNAIEPRAVNASYSRQ
DDSYTLYVANQNPHVERLLMSAFVLGLTEAKVRVIAPDVGGGFGSKIFLYPEDVALTWASKKVGRPIKWTAERSE

SFLTDAHGRDHVTHAELALDAQGNFLAMRVHTTANMGAYLSTFASSVPTILYATLLAGQYKTPAIYAEVKAVFTN
TAPVDAYRGAGRPEATYVVERLVETAAHELQIDPAELRRRNFIRTFPYATPVGLTYDTGDYEPCLDRAIELADVKGF
AARRDASRAKGRLRGMGYSCYIEACGLAPSNIAGALGARAGLFEAGEIRVHPTGSVTVFTGSHSHGQGHETTFA
QVVADRLGVPIDNIEIVHGDTGRIPFGMGTYGSRSIAVGGSAIMKALDKIEAKAKKIAAHLLEASAEDIEFKDGVF
RVAGTDRTKTFGEVALTAYVPHNYPLDKLEPGLDENAFYDPTNFTYPAGAYICEVEVDPDTGEVHIDRFVAVDDF
GNIINPMIVEGQVHGGLGQGIGQALLEACVYDENGQLLTGSYMDYAMPRANDLPSFTVETAKGTPCTHNPLG
VKGCGEAGAIGSPPALINAIVDALAPLGVKDIQMPATPHRVWQTIQAAKA
>SEQF10163||SEQF10163.1_01671
MNAPAEPNHHLIGAAVKRKEDFRFLTGAGQYTDDVVQAHQSYAAFLRSPYAHARIKHINADAARRHPGVLAVL
TGDDLAADKVNGLPCGWLIHSIDGTPMKEPPHPVLAQGKVRHVGDQVALVVAESVKIAKDAVEMIDVEYDEL
PAVVDTATADTAGTAVHDDVPNNTCYIWGHGDKAATDAAFAKAAHVTQLDIVNNRLIPNAIEPRAVNASYSRQ
DDSYTLYVANQNPHVERLLMSAFVLGLSEAKVRVIAPDVGGGFGSKIFLYPEDVALTWASKKVGRPIKWTAERSE
SFLTDAHGRDHVTHAELALDAQGNFLAMRVHTTANMGAYLSTFASSVPTILYATLLAGQYKTPAIYAEVKAVFTN
TAPVDAYRGAGRPEATYVVERLVETAARELQIDPAELRRRNFIRTFPYATPVGLTYDTGDYEPCLDRAIELADVKGF
AARRDASRAKGRLRGMGYSCYIEACGLAPSNIAGALGARAGLFEAGEIRVHPTGSVTVFTGSHSHGQGHETTFA
QVVADRLGVPIDNIEIVHGDTGRIPFGMGTYGSRSIAVGGSAIMKALDKIEAKAKKIAAHLLEASAEDIEFKDGVF
RVAGTDRTKTFGEVALTAYVPHNYPLDKLEPGLDENAFYDPTNFTYPAGAYICEVEVDPDTGEVHIDRFVAVDDF
GNIINPMIVEGQVHGGLGQGIGQALLEACVYDENGQLLTGSYMDYAMPRANDLPSFTVETAKGTPCTHNPLG
VKGCGEAGAIGSPPALINAIVDALAPLGVKDIQMPATPHRVWQAIQAAKA
>SEQF10164||SEQF10164.1_03012
MKRFDAGETTGTAQETQQTTGQPYVGRPMQRVEDAAILTGRGRYADDLGVKPGTLHAAILRSPHAHAELGLID
FAAALKAPGVRAVLTGADLPAWSKPFVVGVKAPMEQWALAMDRVRYVGEPVAVVVAESRALAEDALDLVRV
DYRVLPPVVSIEAAIADAAPQLHSGLGSNVASDRHFRYGDPEAAFATAPHRVSLTVHYPRNTCTPIECGVVIAEHL
PGNEGYQVTSNFMGPFSLHAVMAMALQVSANHLRHIAPRDSGGSFGVKQAVFPYVVLMCLASRKAGAPVK
WVEDRLEHLSAATSATARLSTIEAAVEADGRIVALDYDQLEDCGGYLRAPEPATFYRMHGVLTGAYAIPNLRVRN
RVVLTNKTPTGLVRGFGGPQVYFALERLVQRISIELNLDPLDVYRRNFVPSNAFPYRAAAGALLDSGNYQLAMS
RALETGAYDELKRRRDIARAEGRLYGIGFAAIVEPSVSNMGYITTATPAEARRKAGPKNGAIASATVSVDLLGGVV
VTIASTPAGQGHMTVCAQVVADVLGIDPAEVIVNVEFDTHKDAWSVAAGNYSSRFAGAVAGTVHLAATRVRD
KLARIVASQLDCDPAELIFAEGRITRRDAPETAVVFARAASNAPHWSPQLLPAGEEPGLRETVFWSPPNLDAPDE
QDRINTSACYGFAFDLCGLEIDRATGRVRIDRYVTAHDAGKLLNPALADGQIRGAFAQGLGAALMEEFRYGPDG
SFQSGTLADYLLPTTCEVPDPMIVHLETPSPFTPLGAKGLGEGNNMSTPPCIANAVADALGVRDIRLPLTPAKVM
AMIGLEDPPPSRLELAETATAAATGGKERSKGAKALSARGTVDLDAPPEAVFAVLLDPQALAQVVPGCHVLEPIG
DNRYRADVTVGVGMIKARYEAEIALSDLEPPHRLRLSGAGLSSLGSARGSGMVELAPHEGGTRLTYDYEAEVSG
KVAAVGGRMLEGAAKVVLRQLFESLGRQAGGKPVRKPGWIARLLALFGARR
>SEQF10164||SEQF10164.1_00148
MNAPAEPNNHLIGASVKRKEDFRFLTGAGQYTDDVVQAHQSYAVFLRSPYAHARIKHINTDAARNHPGVLAVL
TGDDLAADKVNGLPCGWLIHSIDGTPMKEPPHPVLAQGKVRHVGDQVALVVAESVKIAKDAVEMIDVEYDEL
PAVVDTATADTAGTAVHDDVPNNTCYTWGHGDKAATDAAFARAAHVTRLDIVNNRLIPNAIEPRAVNASYSRQ
DDSYTLYVANQNPHVERLLMSAFVLGLTEAKVRVIAPDVGGGFGSKIFLYPEDVALTWASKKVGRPIKWTAERSE
SFLTDAHGRDHVTHAELALDAQGNFLAMRVHTTANMGAYLSTFASSVPTILYATLLAGQYKTPAIYAEVKAVFTN
TAPVDAYRGAGRPEATYVVERLVETAAHELQIDPAELRRRNFIRTFPYATPVGLTYDTGDYEPCLDRAIELADVKGF
AARRDASRAKGRLRGMGYSCYIEACGLAPSNIAGALGARAGLFEAGEIRVHPTGSVTVFTGSHSHGQGHETTFA
QVVADRLGVPIDNIEIVHGDTGRIPFGMGTYGSRSIAVGGSAIMKALDKIEAKAKKIAAHLLEASAEDIEFKDGVF
RVAGTDRTKTFGEVALTAYVPHNYPLDKLEPGLDENAFYDPTNFTYPAGAYICEVEVDPDTGEVHIDRFVAVDDF

GNIINPMIVEGQVHGGLGQGIGQALLEACVYDENGQLLTGSYMDYAMPRANDLPSFTVETAKGTPCTHNPLG
VKGCGEAGAIGSPPALINAIVDALAPLGVKDIQMPATPHRVWQTIQAAKA
>SEQF10165||SEQF10165.1_05048
MKRFDAGETTGTAQETQQTTGQPYVGRPMQRVEDAAILTGRGRYADDLGVKPGTLHAAILRSPHAHAELGLID
FAAALKAPGVRAVLTGADLPAWSKPFVVGVKAPMEQWALAMDRVRYVGEPVAVVVAESRALAEDALDLVRV
DYRVLPPVVSIEAAIADAAPQLHSGLGSNVASDRHFRYGDPEAAFATAPHRVSLTVHYPRNTCTPIECGVVIAEHL
PGNEGYQVTSNFMGPFSLHAVMAMALQVSANHLRHIAPRDSGGSFGVKQAVFPYVVLMCLASRKAGAPVK
WVEDRLEHLSAATSATARLSTIEAAVEADGRIVALDYDQLEDCGGYLRAPEPATFYRMHGVLTGAYAIPNLRVRN
RVVLTNKTPTGLVRGFGGPQVYFALERLVQRISIELNLDPLDVYRRNFVPSNAFPYRAAAGALLDSGNYQLAMS
RALETGAYDELKRRRDIARAEGRLYGIGFAAIVEPSVSNMGYITTATPAEARRKAGPKNGAIASATVSVDLLGGVV
VTIASTPAGQGHMTVCAQVVADVLGIDPAEVIVNVEFDTHKDAWSVAAGNYSSRFAGAVAGTVHLAATRVRD
KLARIVASQLDCDPAELIFAEGRITRRDAPETAVVFARAASNAPHWSPQLLPAGEEPGLRETVFWSPPNLDAPDE
QDRINTSACYGFAFDLCGLEIDRATGRVRIDRYVTAHDAGKLLNPALADGQIRGAFAQGLGAALMEEFRYGPDG
SFQSGTLADYLLPTTCEVPDPMIVHLETPSPFTPLGAKGLGEGNNMTTPPCIANAVADALGVRDIRLPLTPAKV
MAMIGLEDPPPSRLELAETATAAATGGKERSKGAKALSARGTVDLDAPPEAVFAVLLDPQALAQVVPGCHVLEP
IGDNRYRADVTVGVGMIKARYEAEIALSDLEPPHRLRLSGAGLSSLGSARGSGMVELAPHEGGTRLTYDYEAEV
SGKVAAVGGRMLEGAAKVVLRQLFESLGRQAGGKPVRKPGWIARLLALFGARR
>SEQF10165||SEQF10165.1_04175
MNAPAEPNNHLIGASVKRKEDFRFLTGAGQYTDDVVQAHQSYAVFLRSPYAHARIKHINTDAARNHPGVLAVL
TGDDLAADKVNGLPCGWLIHSIDGTPMKEPPHPVLAQGKVRHVGDQVALVVAESVKIAKDAVEMIDVEYDEL
AAVVDTATADTAGTAVHDDVPNNTCYTWGHGDKAATDAAFAKAAHVTHLDIVNNRLIPNAIEPRAVNASYSR
QDDSYTLYVANQNPHVERLLMSAFVLGLTEAKVRVIAPDVGGGFGSKIFLYPEDVALTWASKKVGRPIKWTAER
SESFLTDAHGRDHVTHAELALDAQGNFLAMRVHTTANMGAYLSTFASSVPTILYATLLAGQYKTPAIYAEVKAVF
TNTAPVDAYRGAGRPEATYVVERLVETAARELQIDPAELRRRNFIRTFPYATPVGLTYDTGDYEPCLDRAIELADVK
GFAARRDASRAKGRLRGLGYSCYIEACGLAPSNIAGALGARAGLFEAGEIRVHPTGSVTVFTGSHSHGQGHETT
FAQVLADRLGVPIDNIEIVHGDTGRIPFGMGTYGSRSIAVGGSAIMKALDKIEAKAKKIAAHLLEASAEDIEFKDG
VFRVAGTDRTKTFGEVALTAYVPHNYPLDKLEPGLDENAFYDPTNFTYPAGAYICEVEVDPDTGEVHIDRFVAVD
DFGNIINPMIVEGQVHGGLGQGIGQALLEACVYDENGQLLTGSYMDYAMPRANDLPSFTVETAKGTPCTHNP
LGVKGCGEAGAIGSPPALINAIVDALAPLGVKDIQMPATPHRVWQTIQAAKA
>SEQF10166||SEQF10166.1_04582
MNAPAEPNNHLIGASVKRKEDFRFLTGAGQYTDDVVQAHQSYAVFLRSPYAHARIKHINTDAARNHPGVLAVL
TGDDLAADKVNGLPCGWLIHSIDGTPMKEPPHPVLAQGKVRHVGDQVALVVAESVKIAKDAVEMIDVEYDEL
AAVVDTATADTAGTAVHDDVPNNTCYTWGHGDKAATDAAFAKAAHVTHLDIVNNRLIPNAIEPRAVNASYSR
QDDSYTLYVANQNPHVERLLMSAFVLGLTEAKVRVIAPDVGGGFGSKIFLYPEDVALTWASKKVGRPIKWTAER
SESFLTDAHGRDHVTHAELALDAQGNFLAMRVHTTANMGAYLSTFASSVPTILYATLLAGQYKTPAIYAEVKAVF
TNTAPVDAYRGAGRPEATYVVERLVETAARELQIDPAELRRRNFIRTFPYATPVGLTYDTGDYEPCLDRAIELADVK
GFAARRDASRAKGRLRGLGYSCYIEACGLAPSNIAGALGARAGLFEAGEIRVHPTGSVTVFTGSHSHGQGHETT
FAQVLADRLGVPIDNIEIVHGDTGRIPFGMGTYGSRSIAVGGSAIMKALDKIEAKAKKIAAHLLEASAEDIEFKDG
VFRVAGTDRTKTFGEVALTAYVPHNYPLDKLEPGLDENAFYDPTNFTYPAGAYICEVEVDPDTGEVHIDRFVAVD
DFGNIINPMIVEGQVHGGLGQGIGQALLEACVYDENGQLLTGSYMDYAMPRANDLPSFTVETAKGTPCTHNP
LGVKGCGEAGAIGSPPALINAIVDALAPLGVKDIQMPATPHRVWQTIQAAKA
>SEQF10166||SEQF10166.1_05378
MKRFDAGETTGTAQETQQTTGQPYVGRPMQRVEDAAILTGRGRYADDLGVKPGTLHAAILRSPHAHAELGLID
FAAALKAPGVRAVLTGADLPAWSKPFVVGVKAPMEQWALAMDRVRYVGEPVAVVVAESRALAEDALDLVRV

DYRVLPPVVSIEAAIADAAPQLHSGLGSNVASDRHFRYGDPEAAFATAPHRVSLTVHYPRNTCTPIECGVVIAEHL
PGNEGYQVTSNFMGPFSLHAVMAMALQVSANHLRHIAPRDSGGSFGVKQAVFPYVVLMCLASRKAGAPVK
WVEDRLEHLSAATSATARLSTIEAAVEADGRIVALDYDQLEDCGGYLRAPEPATFYRMHGVLTGAYAIPNLRVRN
RVVLTNKTPTGLVRGFGGPQVYFALERLVQRISIELNLDPLDVYRRNFVPSNAFPYRAAAGALLDSGNYQLAMS
RALETGAYDELKRRRDIARAEGRLYGIGFAAIVEPSVSNMGYITTATPAEARRKAGPKNGAIASATVSVDLLGGVV
VTIASTPAGQGHMTVCAQVVADVLGIDPAEVIVNVEFDTHKDAWSVAAGNYSSRFAGAVAGTVHLAATRVRD
KLARIVASQLDCDPAELIFAEGRITRRDAPETAVVFARAASNAPHWSPQLLPAGEEPGLRETVFWSPPNLDAPDE
QDRINTSACYGFAFDLCGLEIDRATGRVRIDRYVTAHDAGKLLNPALADGQIRGAFAQGLGAALMEEFRYGPDG
SFQSGTLADYLLPTTCEVPDPMIVHLETPSPFTPLGAKGLGEGNNMTTPPCIANAVADALGVRDIRLPLTPAKV
MAMIGLEDPPPSRLELAETATAAATGGKERSKGAKALSARGTVDLDAPPEAVFAVLLDPQALAQVVPGCHVLEP
IGDNRYRADVTVGVGMIKARYEAEIALSDLEPPHRLRLSGAGLSSLGSARGSGMVELAPHEGGTRLTYDYEAEV
SGKVAAVGGRMLEGAAKVVLRQLFESLGRQAGGKPVRKPGWIARLLALFGARR
>SEQF10167||SEQF10167.1_01771
MKRFDAGETTGTAQETQQTTGQPYVGRPMQRVEDAAILTGRGRYADDLGVKPGTLHAAILRSPHAHAELGVI
DFAAALKAPGVRAVLTGADLPAWSKPFVVGVKAPMEQWALAMDRVRYVGEPVAVVVAESRALAEDALDLVR
VDYRVLPPVVSIEAAIADAAPQLHSGLGSNVASDRHFRYGDPEAAFATAPHRVSLTAHYPRNTCTPIECGVVIAE
HLPGNEGYQVTSNFMGPFSLHAVMAMALQVSANHLRHIAPRDSGGSFGVKQAVFPYVVLMCLASRKAGAPV
KWVEDRLEHLSASTSATARLSTIEAAVEADGRIVALDYDQLEDCGGYLRAPEPATFYRMHGVLTGAYAIPNLRVR
NRVVLTNKTPTGLVRGFGGPQVYFALERLVQRISIELNLDPLDVYRRNFVPTNAFPYRAAAGALLDSGNYQLAM
SRALETGAYDELKRRRDIARAEGRLYGIGFAAIVEPSVSNMGYITTATPAEARRKAGPKNGAIASATVSVDLLGGV
VVTIASTPAGQGHMTVCAQVVADVLGIDPAEVIVNVEFDTHKDAWSVAAGNYSSRFAGAVAGTVHLAATRVR
DKLARIVASHLDCDPAELIFAEGRITRRDAPETAMVFARAASNAPHWSPQLLPAGEEPGLRETVFWSPPNLDAP
DEQDRINTSACYGFAFDLCGLEIDRATGRVRIDRYVTAHDAGKLLNPALADGQIRGAFAQGLGAALMEEFRYGP
DGSFQSGTLADYLLPTTCEVPDPIIVHLETPSPFTPLGAKGLGEGNNMSTPPCIANAVADALGVRDIRLPLTPAKV
MAMIGLEDPPPSRLELAETATAAATGGKERSKGAKALSARGTVDLDAPPEAVFAVLLDPQALAQVVPGCHVLEP
IGDNRYRADVTVGVGMIKARYEAEIALSDLEPPHRLRLSGAGLSSLGSARGSGMVELAPHEGGTRLTYDYEAEV
SGKVAAVGGRMLEGAAKVVLRQLFESLGRQAGGKPVRKPGWIARLLALFGARR
>SEQF10167||SEQF10167.1_02370
MNAPAEPNNHLIGASVKRKEDFRFLTGAGQYTDDVVQAHQSYAVFLRSPYAHARIKHINTDAARNHPGVLAVL
TGDDLAADKVNGLPCGWLIHSIDGTPMKEPPHPVLAQGKVRHVGDQVALVVAESVKIAKDAVEMIDVEYDEL
PAVVDTATADTAGTAVHDDVPNNTCYTWGHGDKAATDAAFAKAAHVTHLDIVNNRLIPNAIEPRAVNASYSRQ
DDSYTLYVANQNPHVERLLMSAFVLGLTEAKVRVIAPDVGGGFGSKIFLYPEDVALTWASKKVGRPIKWTAERSE
SFLTDAHGRDHVTHAELALDAQGNFLAMRVHTTANMGAYLSTFASSVPTILYATLLAGQYKTPAIYAEVKAVFTN
TAPVDAYRGAGRPEATYVVERLVETAARELQVDPAELRRRNFIRTFPYATPVGLTYDTGDYEPCLDRAIELADVKG
FAARRDASRAKGRLRGLGYSCYIEACGLAPSNIAGALGARAGLFEAGEIRVHPTGSVTVFTGSHSHGQGHETTFA
QVVADRLGVPIDNIEIVHGDTGRIPFGMGTYGSRSIAVGGSAIMKALDKIEAKAKKIAAHLLEASAEDIEFKDGVF
RVAGTDRTKTFGEVALTAYVPHNYPLDKLEPGLDENAFYDPTNFTYPAGAYICEVEVDPDTGEVHIDRFVAVDDF
GNIINPMIVEGQVHGGLGQGIGQALLEACVYDENGQLLTGSYMDYAMPRANDLPSFTVETAKGTPCTHNPLG
VKGCGEAGAIGSPPALINAIVDALAPLGVKDIQMPATPHRVWQTIQAAKA
>SEQF10168||SEQF10168.1_02244
MKRFDAGETTGTAQETQQTTGQPYVGRPMQRVEDAAILTGRGRYADDLGVKPGTLHAAILRSPHAHAELGLID
FAAALKAPGVRAVLTGADLPAWSKPFVVGVKAPMEQWALAMDRVRYVGEPVAVVVAESRALAEDALDLVRV
DYRVLPPVVSIEAAIADAAPQLHSGLGSNVASDRHFRYGDPEAAFATAPHRVSLTVHYPRNTCTPIECGVVIAEHL
PGNEGYQVTSNFMGPFSLHAVMAMALQVSANHLRHIAPRDSGGSFGVKQAVFPYVVLMCLASRKAGAPVK

WVEDRLEHLSAATSATARLSTIEAAVEADGRIVALDYDQLEDCGGYLRAPEPATFYRMHGVLTGAYAIPNLRVRN
RVVLTNKTPTGLVRGFGGPQVYFALERLVQRISIELNLDPLDVYRRNFVPSNAFPYRAAAGALLDSGNYQLAMS
RALETGAYDELKRRRDIARAEGRLYGIGFAAIVEPSVSNMGYITTATPAEARRKAGPKNGAIASATVSVDLLGGVV
VTIASTPAGQGHMTVCAQVVADVLGIDPAEVIVNVEFDTHKDAWSVAAGNYSSRFAGAVAGTVHLAATRVRD
KLARIVASQLDCDPAELIFAEGRITRRDAPETAVVFARAASNAPHWSPQLLPAGEEPGLRETVFWSPPNLDAPDE
QDRINTSACYGFAFDLCGLEIDRATGRVRIDRYVTAHDAGKLLNPALADGQIRGAFAQGLGAALMEEFRYGPDG
SFQSGTLADYLLPTTCEVPDPMIVHLETPSPFTPLGAKGLGEGNNMTTPPCIANAVADALGVRDIRLPLTPAKV
MAMIGLEDPPPSRLELAETATAAATGGKERSKGAKALSARGTVDLDAPPEAVFAVLLDPQALAQVVPGCHVLEP
IGDNRYRADVTVGVGMIKARYEAEIALSDLEPPHRLRLSGAGLSSLGSARGSGMVELAPHEGGTRLTYDYEAEV
SGKVAAVGGRMLEGAAKVVLRQLFESLGRQAGGKPVRKPGWIARLLALFGARR
>SEQF10168||SEQF10168.1_01680
MNAPAEPNNHLIGASVKRKEDFRFLTGAGQYTDDVVQAHQSYAVFLRSPYAHARIKHINTDAARNHPGVLAVL
TGDDLAADKVNGLPCGWLIHSIDGTPMKEPPHPVLAQGKVRHVGDQVALVVAESVKIAKDAVEMIDVEYDEL
AAVVDTATADTAGTAVHDDVPNNTCYTWGHGDKAATDAAFAKAAHVTHLDIVNNRLIPNAIEPRAVNASYSR
QDDSYTLYVANQNPHVERLLMSAFVLGLTEAKVRVIAPDVGGGFGSKIFLYPEDVALTWASKKVGRPIKWTAER
SESFLTDAHGRDHVTHAELALDAQGNFLAMRVHTTANMGAYLSTFASSVPTILYATLLAGQYKTPAIYAEVKAVF
TNTAPVDAYRGAGRPEATYVVERLVETAARELQIDPAELRRRNFIRTFPYATPVGLTYDTGDYEPCLDRAIELADVK
GFAARRDASRAKGRLRGLGYSCYIEACGLAPSNIAGALGARAGLFEAGEIRVHPTGSVTVFTGSHSHGQGHETT
FAQVLADRLGVPIDNIEIVHGDTGRIPFGMGTYGSRSIAVGGSAIMKALDKIEAKAKKIAAHLLEASAEDIEFKDG
VFRVAGTDRTKTFGEVALTAYVPHNYPLDKLEPGLDENAFYDPTNFTYPAGAYICEVEVDPDTGEVHIDRFVAVD
DFGNIINPMIVEGQVHGGLGQGIGQALLEACVYDENGQLLTGSYMDYAMPRANDLPSFTVETAKGTPCTHNP
LGVKGCGEAGAIGSPPALINAIVDALAPLGVKDIQMPATPHRVWQTIQAAKA
>SEQF10169||SEQF10169.1_04682
MNAPAEPNNHLIGASVKRKEDFRFLTGAGQYTDDVVQAHQSYAVFLRSPYAHARIKHINTDAARNHPGVLAVL
TGDDLAADKVNGLPCGWLIHSIDGTPMKEPPHPVLAQGKVRHVGDQVALVVAESVKIAKDAVEMIDVEYDEL
PAVVDTATADTAGTAVHDDVPNNTCYTWGHGDKAATDAAFAKAAHVTHLDIVNNRLIPNAIEPRAVNASYSRQ
DDSYTLYVANQNPHVERLLMSAFVLGLTEAKVRVIAPDVGGGFGSKIFLYPEDVALTWASKKVGRPIKWTAERSE
SFLTDAHGRDHVTHAELALDAQGNFLAMRVHTTANMGAYLSTFASSVPTILYATLLAGQYKTPAIYAEVKAVFTN
TAPVDAYRGAGRPEATYVVERLVETAARELQVDPAELRRRNFIRTFPYATPVGLTYDTGDYEPCLDRAIELADVKG
FAARRDASRAKGRLRGLGYSCYIEACGLAPSNIAGALGARAGLFEAGEIRVHPTGSVTVFTGSHSHGQGHETTFA
QVVADRLGVPIDNIEIVHGDTGRIPFGMGTYGSRSIAVGGSAIMKALDKIEAKAKKIAAHLLEASAEDIEFKDGVF
RVAGTDRTKTFGEVALTAYVPHNYPLDKLEPGLDENAFYDPTNFTYPAGAYICEVEVDPDTGEVHIDRFVAVDDF
GNIINPMIVEGQVHGGLGQGIGQALLEACVYDENGQLLTGSYMDYAMPRANDLPSFTVETAKGTPCTHNPLG
VKGCGEAGAIGSPPALINAIVDALAPLGVKDIQMPATPHRVWQTIQAAKA
>SEQF10169||SEQF10169.1_04083
MKRFDAGETTGTAQETQQTTGQPYVGRPMQRVEDAAILTGRGRYADDLGVKPGTLHAAILRSPHAHAELGVI
DFAAALKAPGVRAVLTGADLPAWSKPFVVGVKAPMEQWALAMDRVRYVGEPVAVVVAESRALAEDALDLVR
VDYRVLPPVVSIEAAIADAAPQLHSGLGSNVASDRHFRYGDPEAAFATAPHRVSLTAHYPRNTCTPIECGVVIAE
HLPGNEGYQVTSNFMGPFSLHAVMAMALQVSANHLRHIAPRDSGGSFGVKQAVFPYVVLMCLASRKAGAPV
KWVEDRLEHLSASTSATARLSTIEAAVEADGRIVALDYDQLEDCGGYLRAPEPATFYRMHGVLTGAYAIPNLRVR
NRVVLTNKTPTGLVRGFGGPQVYFALERLVQRISIELNLDPLDVYRRNFVPTNAFPYRAAAGALLDSGNYQLAM
SRALETGAYDELKRRRDIARAEGRLYGIGFAAIVEPSVSNMGYITTATPAEARRKAGPKNGAIASATVSVDLLGGV
VVTIASTPAGQGHMTVCAQVVADVLGIDPAEVIVNVEFDTHKDAWSVAAGNYSSRFAGAVAGTVHLAATRVR
DKLARIVASHLDCDPAELIFAEGRITRRDAPETAMVFARAASNAPHWSPQLLPAGEEPGLRETVFWSPPNLDAP

DEQDRINTSACYGFAFDLCGLEIDRATGRVRIDRYVTAHDAGKLLNPALADGQIRGAFAQGLGAALMEEFRYGP
DGSFQSGTLADYLLPTTCEVPDPIIVHLETPSPFTPLGAKGLGEGNNMSTPPCIANAVADALGVRDIRLPLTPAKV
MAMIGLEDPPPSRLELAETATAAATGGKERSKGAKALSARGTVDLDAPPEAVFAVLLDPQALAQVVPGCHVLEP
IGDNRYRADVTVGVGMIKARYEAEIALSDLEPPHRLRLSGAGLSSLGSARGSGMVELAPHEGGTRLTYDYEAEV
SGKVAAVGGRMLEGAAKVVLRQLFESLGRQAGGKPVRKPGWIARLLALFGARR
>SEQF10170||SEQF10170.1_00980
MKRFDAGETTGTAQETQQTTGQPYVGRPMQRVEDAAILTGRGRYADDLGVKPGTLHAAILRSPHAHAELGLID
FAAALKAPGVRAVLTGADLPAWSKPFVVGVKAPMEQWALAMDRVRYVGEPVAVVVAESRALAEDALDLVRV
DYRVLPPVVSIEAAIADAAPQLHSGLGSNVASDRHFRYGDPEAAFATAPHRVSLTVHYPRNTCTPIECGVVIAEHL
PGNEGYQVTSNFMGPFSLHAVMAMALQVSANHLRHIAPRDSGGSFGVKQAVFPYVVLMCLASRKAGAPVK
WVEDRLEHLSAATSATARLSTIEAAVEADGRIVALDYDQLEDCGGYLRAPEPATFYRMHGVLTGAYAIPNLRVRN
RVVLTNKTPTGLVRGFGGPQVYFALERLVQRISIELNLDPLDVYRRNFVPSNAFPYRAAAGALLDSGNYQLAMS
RALETGAYDELKRRRDIARAEGRLYGIGFAAIVEPSVSNMGYITTATPAEARRKAGPKNGAIASATVSVDLLGGVV
VTIASTPAGQGHMTVCAQVVADVLGIDPAEVIVNVEFDTHKDAWSVAAGNYSSRFAGAVAGTVHLAATRVRD
KLARIVASQLDCDPAELIFAEGRITRRDAPETAVVFARAASNAPHWSPQLLPAGEEPGLRETVFWSPPNLDAPDE
QDRINTSACYGFAFDLCGLEIDRATGRVRIDRYVTAHDAGKLLNPALADGQIRGAFAQGLGAALMEEFRYGPDG
SFQSGTLADYLLPTTCEVPDPMIVHLETPSPFTPLGAKGLGEGNNMTTPPCIANAVADALGVRDIRLPLTPAKV
MAMIGLEDPPPSRLELAETATAAATGGKERSKGAKALSARGTVDLDAPPEAVFAVLLDPQALAQVVPGCHVLEP
IGDNRYRADVTVGVGMIKARYEAEIALSDLEPPHRLRLSGAGLSSLGSARGSGMVELAPHEGGTRLTYDYEAEV
SGKVAAVGGRMLEGAAKVVLRQLFESLGRQAGGKPVRKPGWIARLLALFGARR
>SEQF10170||SEQF10170.1_04199
MNAPAEPNNHLIGASVKRKEDFRFLTGAGQYTDDVVQAHQSYAVFLRSPYAHARIKHINTDAARNHPGVLAVL
TGDDLAADKVNGLPCGWLIHSIDGTPMKEPPHPVLAQGKVRHVGDQVALVVAESVKIAKDAVEMIDVEYDEL
AAVVDTATADTAGTAVHDDVPNNTCYTWGHGDKAATDAAFAKAAHVTHLDIVNNRLIPNAIEPRAVNASYSR
QDDSYTLYVANQNPHVERLLMSAFVLGLTEAKVRVIAPDVGGGFGSKIFLYPEDVALTWASKKVGRPIKWTAER
SESFLTDAHGRDHVTHAELALDAQGNFLAMRVHTTANMGAYLSTFASSVPTILYATLLAGQYKTPAIYAEVKAVF
TNTAPVDAYRGAGRPEATYVVERLVETAARELQIDPAELRRRNFIRTFPYATPVGLTYDTGDYEPCLDRAIELADVK
GFAARRDASRAKGRLRGLGYSCYIEACGLAPSNIAGALGARAGLFEAGEIRVHPTGSVTVFTGSHSHGQGHETT
FAQVLADRLGVPIDNIEIVHGDTGRIPFGMGTYGSRSIAVGGSAIMKALDKIEAKAKKIAAHLLEASAEDIEFKDG
VFRVAGTDRTKTFGEVALTAYVPHNYPLDKLEPGLDENAFYDPTNFTYPAGAYICEVEVDPDTGEVHIDRFVAVD
DFGNIINPMIVEGQVHGGLGQGIGQALLEACVYDENGQLLTGSYMDYAMPRANDLPSFTVETAKGTPCTHNP
LGVKGCGEAGAIGSPPALINAIVDALAPLGVKDIQMPATPHRVWQTIQAAKA
>SEQF10171||SEQF10171.1_01622
MNAPAEPNNHLIGASVKRKEDFRFLTGAGQYTDDVVQAHQSYAVFLRSPYAHARIKHINTDAARNHPGVLAVL
TGDDLAADKVNGLPCGWLIHSIDGTPMKEPPHPVLAQGKVRHVGDQVALVVAESVKIAKDAVEMIDVEYDEL
AAVVDTATADTAGTAVHDDVPNNTCYTWGHGDKAATDAAFAKAAHVTHLDIVNNRLIPNAIEPRAVNASYSR
QDDSYTLYVANQNPHVERLLMSAFVLGLTEAKVRVIAPDVGGGFGSKIFLYPEDVALTWASKKVGRPIKWTAER
SESFLTDAHGRDHVTHAELALDAQGNFLAMRVHTTANMGAYLSTFASSVPTILYATLLAGQYKTPAIYAEVKAVF
TNTAPVDAYRGAGRPEATYVVERLVETAARELQIDPAELRRRNFIRTFPYATPVGLTYDTGDYEPCLDRAIELADVK
GFAARRDASRAKGRLRGLGYSCYIEACGLAPSNIAGALGARAGLFEAGEIRVHPTGSVTVFTGSHSHGQGHETT
FAQVLADRLGVPIDNIEIVHGDTGRIPFGMGTYGSRSIAVGGSAIMKALDKIEAKAKKIAAHLLEASAEDIEFKDG
VFRVAGTDRTKTFGEVALTAYVPHNYPLDKLEPGLDENAFYDPTNFTYPAGAYICEVEVDPDTGEVHIDRFVAVD
DFGNIINPMIVEGQVHGGLGQGIGQALLEACVYDENGQLLTGSYMDYAMPRANDLPSFTVETAKGTPCTHNP
LGVKGCGEAGAIGSPPALINAIVDALAPLGVKDIQMPATPHRVWQTIQAAKA

>SEQF10171||SEQF10171.1_02329
MKRFDAGETTGTAQETQQTTGQPYVGRPMQRVEDAAILTGRGRYADDLGVKPGTLHAAILRSPHAHAELGLID
FAAALKAPGVRAVLTGADLPAWSKPFVVGVKAPMEQWALAMDRVRYVGEPVAVVVAESRALAEDALDLVRV
DYRVLPPVVSIEAAIADAAPQLHSGLGSNVASDRHFRYGDPEAAFATAPHRVSLTVHYPRNTCTPIECGVVIAEHL
PGNEGYQVTSNFMGPFSLHAVMAMALQVSANHLRHIAPRDSGGSFGVKQAVFPYVVLMCLASRKAGAPVK
WVEDRLEHLSAATSATARLSTIEAAVEADGRIVALDYDQLEDCGGYLRAPEPATFYRMHGVLTGAYAIPNLRVRN
RVVLTNKTPTGLVRGFGGPQVYFALERLVQRISIELNLDPLDVYRRNFVPSNAFPYRAAAGALLDSGNYQLAMS
RALETGAYDELKRRRDIARAEGRLYGIGFAAIVEPSVSNMGYITTATPAEARRKAGPKNGAIASATVSVDLLGGVV
VTIASTPAGQGHMTVCAQVVADVLGIDPAEVIVNVEFDTHKDAWSVAAGNYSSRFAGAVAGTVHLAATRVRD
KLARIVASQLDCDPAELIFAEGRITRRDAPETAVVFARAASNAPHWSPQLLPAGEEPGLRETVFWSPPNLDAPDE
QDRINTSACYGFAFDLCGLEIDRATGRVRIDRYVTAHDAGKLLNPALADGQIRGAFAQGLGAALMEEFRYGPDG
SFQSGTLADYLLPTTCEVPDPMIVHLETPSPFTPLGAKGLGEGNNMTTPPCIANAVADALGVRDIRLPLTPAKV
MAMIGLEDPPPSRLELAETATAAATGGKERSKGAKALSARGTVDLDAPPEAVFAVLLDPQALAQVVPGCHVLEP
IGDNRYRADVTVGVGMIKARYEAEIALSDLEPPHRLRLSGAGLSSLGSARGSGMVELAPHEGGTRLTYDYEAEV
SGKVAAVGGRMLEGAAKVVLRQLFESLGRQAGGKPVRKPGWIARLLALFGARR
>SEQF10172||SEQF10172.1_02338
MKRFDAGETTGTAQETQQTTGQPYVGRPMQRVEDAAILTGRGRYADDLGVKPGTLHAAILRSPHAHAELGLID
FAAALKAPGVRAVLTGADLPAWSKPFVVGVKAPMEQWALAMDRVRYVGEPVAVVVAESRALAEDALDLVRV
DYRVLPPVVSIEAAIADAAPQLHSGLGSNVASDRHFRYGDPEAAFATAPHRVSLTVHYPRNTCTPIECGVVIAEHL
PGNEGYQVTSNFMGPFSLHAVMAMALQVSANHLRHIAPRDSGGSFGVKQAVFPYVVLMCLASRKAGAPVK
WVEDRLEHLSAATSATARLSTIEAAVEADGRIVALDYDQLEDCGGYLRAPEPATFYRMHGVLTGAYAIPNLRVRN
RVVLTNKTPTGLVRGFGGPQVYFALERLVQRISIELNLDPLDVYRRNFVPSNAFPYRAAAGALLDSGNYQLAMS
RALETGAYDELKRRRDIARAEGRLYGIGFAAIVEPSVSNMGYITTATPAEARRKAGPKNGAIASATVSVDLLGGVV
VTIASTPAGQGHMTVCAQVVADVLGIDPAEVIVNVEFDTHKDAWSVAAGNYSSRFAGAVAGTVHLAATRVRD
KLARIVASQLDCDPAELIFAEGRITRRDAPETAVVFARAASNAPHWSPQLLPAGEEPGLRETVFWSPPNLDAPDE
QDRINTSACYGFAFDLCGLEIDRATGRVRIDRYVTAHDAGKLLNPALADGQIRGAFAQGLGAALMEEFRYGPDG
SFQSGTLADYLLPTTCEVPDPMIVHLETPSPFTPLGAKGLGEGNNMTTPPCIANAVADALGVRDIRLPLTPAKV
MAMIGLEDPPPSRLELAETATAAATGGKERSKGAKALSARGTVDLDAPPEAVFAVLLDPQALAQVVPGCHVLEP
IGDNRYRADVTVGVGMIKARYEAEIALSDLEPPHRLRLSGAGLSSLGSARGSGMVELAPHEGGTRLTYDYEAEV
SGKVAAVGGRMLEGAAKVVLRQLFESLGRQAGGKPVRKPGWIARLLALFGARR
>SEQF10172||SEQF10172.1_01655
MNAPAEPNNHLIGASVKRKEDFRFLTGAGQYTDDVVQAHQSYAVFLRSPYAHARIKHINTDAARNHPGVLAVL
TGDDLAADKVNGLPCGWLIHSIDGTPMKEPPHPVLAQGKVRHVGDQVALVVAESVKIAKDAVEMIDVEYDEL
AAVVDTATADTAGTAVHDDVPNNTCYTWGHGDKAATDAAFAKAAHVTHLDIVNNRLIPNAIEPRAVNASYSR
QDDSYTLYVANQNPHVERLLMSAFVLGLTEAKVRVIAPDVGGGFGSKIFLYPEDVALTWASKKVGRPIKWTAER
SESFLTDAHGRDHVTHAELALDAQGNFLAMRVHTTANMGAYLSTFASSVPTILYATLLAGQYKTPAIYAEVKAVF
TNTAPVDAYRGAGRPEATYVVERLVETAARELQIDPAELRRRNFIRTFPYATPVGLTYDTGDYEPCLDRAIELADVK
GFAARRDASRAKGRLRGLGYSCYIEACGLAPSNIAGALGARAGLFEAGEIRVHPTGSVTVFTGSHSHGQGHETT
FAQVLADRLGVPIDNIEIVHGDTGRIPFGMGTYGSRSIAVGGSAIMKALDKIEAKAKKIAAHLLEASAEDIEFKDG
VFRVAGTDRTKTFGEVALTAYVPHNYPLDKLEPGLDENAFYDPTNFTYPAGAYICEVEVDPDTGEVHIDRFVAVD
DFGNIINPMIVEGQVHGGLGQGIGQALLEACVYDENGQLLTGSYMDYAMPRANDLPSFTVETAKGTPCTHNP
LGVKGCGEAGAIGSPPALINAIVDALAPLGVKDIQMPATPHRVWQTIQAAKA
>SEQF10173||SEQF10173.1_04685
MKRFDAGETTGTAQETQQTTGQPYVGRPMQRVEDAAILTGRGRYADDLGVKPGTLHAAILRSPHAHAELGLID

FAAALKAPGVRAVLTGADLPAWSKPFVVGVKAPMEQWALAMDRVRYVGEPVAVVVAESRALAEDALDLVRV
DYRVLPPVVSIEAAIADAAPQLHSGLGSNVASDRHFRYGDPEAAFATAPHRVSLTVHYPRNTCTPIECGVVIAEHL
PGNEGYQVTSNFMGPFSLHAVMAMALQVSANHLRHIAPRDSGGSFGVKQAVFPYVVLMCLASRKAGAPVK
WVEDRLEHLSAATSATARLSTIEAAVEADGRIVALDYDQLEDCGGYLRAPEPATFYRMHGVLTGAYAIPNLRVRN
RVVLTNKTPTGLVRGFGGPQVYFALERLVQRISIELNLDPLDVYRRNFVPSNAFPYRAAAGALLDSGNYQLAMS
RALETGAYDELKRRRDIARAEGRLYGIGFAAIVEPSVSNMGYITTATPAEARRKAGPKNGAIASATVSVDLLGGVV
VTIASTPAGQGHMTVCAQVVADVLGIDPAEVIVNVEFDTHKDAWSVAAGNYSSRFAGAVAGTVHLAATRVRD
KLARIVASQLDCDPAELIFAEGRITRRDAPETAVVFARAASNAPHWSPQLLPAGEEPGLRETVFWSPPNLDAPDE
QDRINTSACYGFAFDLCGLEIDRATGRVRIDRYVTAHDAGKLLNPALADGQIRGAFAQGLGAALMEEFRYGPDG
SFQSGTLADYLLPTTCEVPDPMIVHLETPSPFTPLGAKGLGEGNNMTTPPCIANAVADALGVRDIRLPLTPAKV
MAMIGLEDPPPSRLELAETATAAATGGKERSKGAKALSARGTVDLDAPPEAVFAVLLDPQALAQVVPGCHVLEP
IGDNRYRADVTVGVGMIKARYEAEIALSDLEPPHRLRLSGAGLSSLGSARGSGMVELAPHEGGTRLTYDYEAEV
SGKVAAVGGRMLEGAAKVVLRQLFESLGRQAGGKPVRKPGWIARLLALFGARR
>SEQF10173||SEQF10173.1_04238
MNAPAEPNNHLIGASVKRKEDFRFLTGAGQYTDDVVQAHQSYAVFLRSPYAHARIKHINTDAARNHPGVLAVL
TGDDLAADKVNGLPCGWLIHSIDGTPMKEPPHPVLAQGKVRHVGDQVALVVAESVKIAKDAVEMIDVEYDEL
AAVVDTATADTAGTAVHDDVPNNTCYTWGHGDKAATDAAFAKAAHVTHLDIVNNRLIPNAIEPRAVNASYSR
QDDSYTLYVANQNPHVERLLMSAFVLGLTEAKVRVIAPDVGGGFGSKIFLYPEDVALTWASKKVGRPIKWTAER
SESFLTDAHGRDHVTHAELALDAQGNFLAMRVHTTANMGAYLSTFASSVPTILYATLLAGQYKTPAIYAEVKAVF
TNTAPVDAYRGAGRPEATYVVERLVETAARELQIDPAELRRRNFIRTFPYATPVGLTYDTGDYEPCLDRAIELADVK
GFAARRDASRAKGRLRGLGYSCYIEACGLAPSNIAGALGARAGLFEAGEIRVHPTGSVTVFTGSHSHGQGHETT
FAQVLADRLGVPIDNIEIVHGDTGRIPFGMGTYGSRSIAVGGSAIMKALDKIEAKAKKIAAHLLEASAEDIEFKDG
VFRVAGTDRTKTFGEVALTAYVPHNYPLDKLEPGLDENAFYDPTNFTYPAGAYICEVEVDPDTGEVHIDRFVAVD
DFGNIINPMIVEGQVHGGLGQGIGQALLEACVYDENGQLLTGSYMDYAMPRANDLPSFTVETAKGTPCTHNP
LGVKGCGEAGAIGSPPALINAIVDALAPLGVKDIQMPATPHRVWQTIQAAKA
>SEQF10174||SEQF10174.1_01771
MKRFDAGETTGTAQETQQTTGQPYVGRPMQRVEDAAILTGRGRYADDLGVKPGTLHAAILRSPHAHAELGVI
DFAAALKAPGVRAVLTGADLPAWSKPFVVGVKAPMEQWALAMDRVRYVGEPVAVVVAESRALAEDALDLVR
VDYRVLPPVVSIEAAIADAAPQLHSGLGSNVASDRHFRYGDPEAAFATAPHRVSLTAHYPRNTCTPIECGVVIAE
HLPGNEGYQVTSNFMGPFSLHAVMAMALQVSANHLRHIAPRDSGGSFGVKQAVFPYVVLMCLASRKAGAPV
KWVEDRLEHLSASTSATARLSTIEAAVEADGRIVALDYDQLEDCGGYLRAPEPATFYRMHGVLTGAYAIPNLRVR
NRVVLTNKTPTGLVRGFGGPQVYFALERLVQRISIELNLDPLDVYRRNFVPTNAFPYRAAAGALLDSGNYQLAM
SRALETGAYDELKRRRDIARAEGRLYGIGFAAIVEPSVSNMGYITTATPAEARRKAGPKNGAIASATVSVDLLGGV
VVTIASTPAGQGHMTVCAQVVADVLGIDPAEVIVNVEFDTHKDAWSVAAGNYSSRFAGAVAGTVHLAATRVR
DKLARIVASHLDCDPAELIFAEGRITRRDAPETAMVFARAASNAPHWSPQLLPAGEEPGLRETVFWSPPNLDAP
DEQDRINTSACYGFAFDLCGLEIDRATGRVRIDRYVTAHDAGKLLNPALADGQIRGAFAQGLGAALMEEFRYGP
DGSFQSGTLADYLLPTTCEVPDPIIVHLETPSPFTPLGAKGLGEGNNMSTPPCIANAVADALGVRDIRLPLTPAKV
MAMIGLEDPPPSRLELAETATAAATGGKERSKGAKALSARGTVDLDAPPEAVFAVLLDPQALAQVVPGCHVLEP
IGDNRYRADVTVGVGMIKARYEAEIALSDLEPPHRLRLSGAGLSSLGSARGSGMVELAPHEGGTRLTYDYEAEV
SGKVAAVGGRMLEGAAKVVLRQLFESLGRQAGGKPVRKPGWIARLLALFGARR
>SEQF10174||SEQF10174.1_02487
MNAPAEPNNHLIGASVKRKEDFRFLTGAGQYTDDVVQAHQSYAVFLRSPYAHARIKHINTDAARNHPGVLAVL
TGDDLAADKVNGLPCGWLIHSIDGTPMKEPPHPVLAQGKVRHVGDQVALVVAESVKIAKDAVEMIDVEYDEL
PAVVDTATADTAGTAVHDDVPNNTCYTWGHGDKAATDAAFAKAAHVTHLDIVNNRLIPNAIEPRAVNASYSRQ

DDSYTLYVANQNPHVERLLMSAFVLGLTEAKVRVIAPDVGGGFGSKIFLYPEDVALTWASKKVGRPIKWTAERSE
SFLTDAHGRDHVTHAELALDAQGNFLAMRVHTTANMGAYLSTFASSVPTILYATLLAGQYKTPAIYAEVKAVFTN
TAPVDAYRGAGRPEATYVVERLVETAARELQVDPAELRRRNFIRTFPYATPVGLTYDTGDYEPCLDRAIELADVKG
FAARRDASRAKGRLRGLGYSCYIEACGLAPSNIAGALGARAGLFEAGEIRVHPTGSVTVFTGSHSHGQGHETTFA
QVVADRLGVPIDNIEIVHGDTGRIPFGMGTYGSRSIAVGGSAIMKALDKIEAKAKKIAAHLLEASAEDIEFKDGVF
RVAGTDRTKTFGEVALTAYVPHNYPLDKLEPGLDENAFYDPTNFTYPAGAYICEVEVDPDTGEVHIDRFVAVDDF
GNIINPMIVEGQVHGGLGQGIGQALLEACVYDENGQLLTGSYMDYAMPRANDLPSFTVETAKGTPCTHNPLG
VKGCGEAGAIGSPPALINAIVDALAPLGVKDIQMPATPHRVWQTIQAAKA
>SEQF10175||SEQF10175.1_04529
MNAPAEPNNHLIGASVKRKEDFRFLTGAGQYTDDVVQAHQSYAVFLRSPYAHARIKHINTDAARNHPGVLAVL
TGDDLAADKVNGLPCGWLIHSIDGTPMKEPPHPVLAQGKVRHVGDQVALVVAESVKIAKDAVEMIDVEYDEL
AAVVDTATADTAGTAVHDDVPNNTCYTWGHGDKAATDAAFAKAAHVTHLDIVNNRLIPNAIEPRAVNASYSR
QDDSYTLYVANQNPHVERLLMSAFVLGLTEAKVRVIAPDVGGGFGSKIFLYPEDVALTWASKKVGRPIKWTAER
SESFLTDAHGRDHVTHAELALDAQGNFLAMRVHTTANMGAYLSTFASSVPTILYATLLAGQYKTPAIYAEVKAVF
TNTAPVDAYRGAGRPEATYVVERLVETAARELQIDPAELRRRNFIRTFPYATPVGLTYDTGDYEPCLDRAIELADVK
GFAARRDASRAKGRLRGLGYSCYIEACGLAPSNIAGALGARAGLFEAGEIRVHPTGSVTVFTGSHSHGQGHETT
FAQVLADRLGVPIDNIEIVHGDTGRIPFGMGTYGSRSIAVGGSAIMKALDKIEAKAKKIAAHLLEASAEDIEFKDG
VFRVAGTDRTKTFGEVALTAYVPHNYPLDKLEPGLDENAFYDPTNFTYPAGAYICEVEVDPDTGEVHIDRFVAVD
DFGNIINPMIVEGQVHGGLGQGIGQALLEACVYDENGQLLTGSYMDYAMPRANDLPSFTVETAKGTPCTHNP
LGVKGCGEAGAIGSPPALINAIVDALAPLGVKDIQMPATPHRVWQTIQAAKA
>SEQF10175||SEQF10175.1_05456
MKRFDAGETTGTAQETQQTTGQPYVGRPMQRVEDAAILTGRGRYADDLGVKPGTLHAAILRSPHAHAELGLID
FAAALKAPGVRAVLTGADLPAWSKPFVVGVKAPMEQWALAMDRVRYVGEPVAVVVAESRALAEDALDLVRV
DYRVLPPVVSIEAAIADAAPQLHSGLGSNVASDRHFRYGDPEAAFATAPHRVSLTVHYPRNTCTPIECGVVIAEHL
PGNEGYQVTSNFMGPFSLHAVMAMALQVSANHLRHIAPRDSGGSFGVKQAVFPYVVLMCLASRKAGAPVK
WVEDRLEHLSAATSATARLSTIEAAVEADGRIVALDYDQLEDCGGYLRAPEPATFYRMHGVLTGAYAIPNLRVRN
RVVLTNKTPTGLVRGFGGPQVYFALERLVQRISIELNLDPLDVYRRNFVPSNAFPYRAAAGALLDSGNYQLAMS
RALETGAYDELKRRRDIARAEGRLYGIGFAAIVEPSVSNMGYITTATPAEARRKAGPKNGAIASATVSVDLLGGVV
VTIASTPAGQGHMTVCAQVVADVLGIDPAEVIVNVEFDTHKDAWSVAAGNYSSRFAGAVAGTVHLAATRVRD
KLARIVASQLDCDPAELIFAEGRITRRDAPETAVVFARAASNAPHWSPQLLPAGEEPGLRETVFWSPPNLDAPDE
QDRINTSACYGFAFDLCGLEIDRATGRVRIDRYVTAHDAGKLLNPALADGQIRGAFAQGLGAALMEEFRYGPDG
SFQSGTLADYLLPTTCEVPDPMIVHLETPSPFTPLGAKGLGEGNNMTTPPCIANAVADALGVRDIRLPLTPAKV
MAMIGLEDPPPSRLELAETATAAATGGKERSKGAKALSARGTVDLDAPPEAVFAVLLDPQALAQVVPGCHVLEP
IGDNRYRADVTVGVGMIKARYEAEIALSDLEPPHRLRLSGAGLSSLGSARGSGMVELAPHEGGTRLTYDYEAEV
SGKVAAVGGRMLEGAAKVVLRQLFESLGRQAGGKPVRKPGWIARLLALFGARR
>SEQF10176||SEQF10176.1_05098
MNAPAEPNNHLIGASVKRKEDFRFLTGAGQYTDDVVQAHQSYAVFLRSPYAHARIKHINTDAARNHPGVLAVL
TGDDLAADKVNGLPCGWLIHSIDGTPMKEPPHPVLAQGKVRHVGDQVALVVAESVKIAKDAVEMIDVEYDEL
PAVVDTATADTAGTAVHDDVPNNTCYTWGHGDKAATDAAFAKAAHVTHLDIVNNRLIPNAIEPRAVNASYSRQ
DDSYTLYVANQNPHVERLLMSAFVLGLTEAKVRVIAPDVGGGFGSKIFLYPEDVALTWASKKVGRPIKWTAERSE
SFLTDAHGRDHVTHAELALDAQGNFLAMRVHTTANMGAYLSTFASSVPTILYATLLAGQYKTPAIYAEVKAVFTN
TAPVDAYRGAGRPEATYVVERLVETAARELQVDPAELRRRNFIRTFPYATPVGLTYDTGDYEPCLDRAIELADVKG
FAARRDASRAKGRLRGLGYSCYIEACGLAPSNIAGALGARAGLFEAGEIRVHPTGSVTVFTGSHSHGQGHETTFA
QVVADRLGVPIDNIEIVHGDTGRIPFGMGTYGSRSIAVGGSAIMKALDKIEAKAKKIAAHLLEASAEDIEFKDGVF

RVAGTDRTKTFGEVALTAYVPHNYPLDKLEPGLDENAFYDPTNFTYPAGAYICEVEVDPDTGEVHIDRFVAVDDF
GNIINPMIVEGQVHGGLGQGIGQALLEACVYDENGQLLTGSYMDYAMPRANDLPSFTVETAKGTPCTHNPLG
VKGCGEAGAIGSPPALINAIVDALAPLGVKDIQMPATPHRVWQTIQAAKA
>SEQF10176||SEQF10176.1_04378
MKRFDAGETTGTAQETQQTTGQPYVGRPMQRVEDAAILTGRGRYADDLGVKPGTLHAAILRSPHAHAELGVI
DFAAALKAPGVRAVLTGADLPAWSKPFVVGVKAPMEQWALAMDRVRYVGEPVAVVVAESRALAEDALDLVR
VDYRVLPPVVSIEAAIADAAPQLHSGLGSNVASDRHFRYGDPEAAFATAPHRVSLTAHYPRNTCTPIECGVVIAE
HLPGNEGYQVTSNFMGPFSLHAVMAMALQVSANHLRHIAPRDSGGSFGVKQAVFPYVVLMCLASRKAGAPV
KWVEDRLEHLSASTSATARLSTIEAAVEADGRIVALDYDQLEDCGGYLRAPEPATFYRMHGVLTGAYAIPNLRVR
NRVVLTNKTPTGLVRGFGGPQVYFALERLVQRISIELNLDPLDVYRRNFVPTNAFPYRAAAGALLDSGNYQLAM
SRALETGAYDELKRRRDIARAEGRLYGIGFAAIVEPSVSNMGYITTATPAEARRKAGPKNGAIASATVSVDLLGGV
VVTIASTPAGQGHMTVCAQVVADVLGIDPAEVIVNVEFDTHKDAWSVAAGNYSSRFAGAVAGTVHLAATRVR
DKLARIVASHLDCDPAELIFAEGRITRRDAPETAMVFARAASNAPHWSPQLLPAGEEPGLRETVFWSPPNLDAP
DEQDRINTSACYGFAFDLCGLEIDRATGRVRIDRYVTAHDAGKLLNPALADGQIRGAFAQGLGAALMEEFRYGP
DGSFQSGTLADYLLPTTCEVPDPIIVHLETPSPFTPLGAKGLGEGNNMSTPPCIANAVADALGVRDIRLPLTPAKV
MAMIGLEDPPPSRLELAETATAAATGGKERSKGAKALSARGTVDLDAPPEAVFAVLLDPQALAQVVPGCHVLEP
IGDNRYRADVTVGVGMIKARYEAEIALSDLEPPHRLRLSGAGLSSLGSARGSGMVELAPHEGGTRLTYDYEAEV
SGKVAAVGGRMLEGAAKVVLRQLFESLGRQAGGKPVRKPGWIARLLALFGARR
>SEQF10177||SEQF10177.1_02302
MKRFDAGETTGTAQETQQTTGQPYVGRPMQRVEDAAILTGRGRYADDLGVKPGTLHAAILRSPHAHAELGLID
FAAALKAPGVRAVLTGADLPAWSKPFVVGVKAPMEQWALAMDRVRYVGEPVAVVVAESRALAEDALDLVRV
DYRVLPPVVSIEAAIADAAPQLHSGLGSNVASDRHFRYGDPEAAFATAPHRVSLTVHYPRNTCTPIECGVVIAEHL
PGNEGYQVTSNFMGPFSLHAVMAMALQVSANHLRHIAPRDSGGSFGVKQAVFPYVVLMCLASRKAGAPVK
WVEDRLEHLSAATSATARLSTIEAAVEADGRIVALDYDQLEDCGGYLRAPEPATFYRMHGVLTGAYAIPNLRVRN
RVVLTNKTPTGLVRGFGGPQVYFALERLVQRISIELNLDPLDVYRRNFVPSNAFPYRAAAGALLDSGNYQLAMS
RALETGAYDELKRRRDIARAEGRLYGIGFAAIVEPSVSNMGYITTATPAEARRKAGPKNGAIASATVSVDLLGGVV
VTIASTPAGQGHMTVCAQVVADVLGIDPAEVIVNVEFDTHKDAWSVAAGNYSSRFAGAVAGTVHLAATRVRD
KLARIVASQLDCDPAELIFAEGRITRRDAPETAVVFARAASNAPHWSPQLLPAGEEPGLRETVFWSPPNLDAPDE
QDRINTSACYGFAFDLCGLEIDRATGRVRIDRYVTAHDAGKLLNPALADGQIRGAFAQGLGAALMEEFRYGPDG
SFQSGTLADYLLPTTCEVPDPMIVHLETPSPFTPLGAKGLGEGNNMTTPPCIANAVADALGVRDIRLPLTPAKV
MAMIGLEDPPPSRLELAETATAAATGGKERSKGAKALSARGTVDLDAPPEAVFAVLLDPQALAQVVPGCHVLEP
IGDNRYRADVTVGVGMIKARYEAEIALSDLEPPHRLRLSGAGLSSLGSARGSGMVELAPHEGGTRLTYDYEAEV
SGKVAAVGGRMLEGAAKVVLRQLFESLGRQAGGKPVRKPGWIARLLALFGARR
>SEQF10177||SEQF10177.1_01739
MNAPAEPNNHLIGASVKRKEDFRFLTGAGQYTDDVVQAHQSYAVFLRSPYAHARIKHINTDAARNHPGVLAVL
TGDDLAADKVNGLPCGWLIHSIDGTPMKEPPHPVLAQGKVRHVGDQVALVVAESVKIAKDAVEMIDVEYDEL
AAVVDTATADTAGTAVHDDVPNNTCYTWGHGDKAATDAAFAKAAHVTHLDIVNNRLIPNAIEPRAVNASYSR
QDDSYTLYVANQNPHVERLLMSAFVLGLTEAKVRVIAPDVGGGFGSKIFLYPEDVALTWASKKVGRPIKWTAER
SESFLTDAHGRDHVTHAELALDAQGNFLAMRVHTTANMGAYLSTFASSVPTILYATLLAGQYKTPAIYAEVKAVF
TNTAPVDAYRGAGRPEATYVVERLVETAARELQIDPAELRRRNFIRTFPYATPVGLTYDTGDYEPCLDRAIELADVK
GFAARRDASRAKGRLRGLGYSCYIEACGLAPSNIAGALGARAGLFEAGEIRVHPTGSVTVFTGSHSHGQGHETT
FAQVLADRLGVPIDNIEIVHGDTGRIPFGMGTYGSRSIAVGGSAIMKALDKIEAKAKKIAAHLLEASAEDIEFKDG
VFRVAGTDRTKTFGEVALTAYVPHNYPLDKLEPGLDENAFYDPTNFTYPAGAYICEVEVDPDTGEVHIDRFVAVD
DFGNIINPMIVEGQVHGGLGQGIGQALLEACVYDENGQLLTGSYMDYAMPRANDLPSFTVETAKGTPCTHNP

LGVKGCGEAGAIGSPPALINAIVDALAPLGVKDIQMPATPHRVWQTIQAAKA
>SEQF10178||SEQF10178.1_04726
MNAPAEPNNHLIGASVKRKEDFRFLTGAGQYTDDVVQAHQSYAVFLRSPYAHARIKHINTDAARNHPGVLAVL
TGDDLAADKVNGLPCGWLIHSIDGTPMKEPPHPVLAQGKVRHVGDQVALVVAESVKIAKDAVEMIDVEYDEL
AAVVDTATADTAGTAVHDDVPNNTCYTWGHGDKAATDAAFAKAAHVTHLDIVNNRLIPNAIEPRAVNASYSR
QDDSYTLYVANQNPHVERLLMSAFVLGLTEAKVRVIAPDVGGGFGSKIFLYPEDVALTWASKKVGRPIKWTAER
SESFLTDAHGRDHVTHAELALDAQGNFLAMRVHTTANMGAYLSTFASSVPTILYATLLAGQYKTPAIYAEVKAVF
TNTAPVDAYRGAGRPEATYVVERLVETAARELQIDPAELRRRNFIRTFPYATPVGLTYDTGDYEPCLDRAIELADVK
GFAARRDASRAKGRLRGLGYSCYIEACGLAPSNIAGALGARAGLFEAGEIRVHPTGSVTVFTGSHSHGQGHETT
FAQVLADRLGVPIDNIEIVHGDTGRIPFGMGTYGSRSIAVGGSAIMKALDKIEAKAKKIAAHLLEASAEDIEFKDG
VFRVAGTDRTKTFGEVALTAYVPHNYPLDKLEPGLDENAFYDPTNFTYPAGAYICEVEVDPDTGEVHIDRFVAVD
DFGNIINPMIVEGQVHGGLGQGIGQALLEACVYDENGQLLTGSYMDYAMPRANDLPSFTVETAKGTPCTHNP
LGVKGCGEAGAIGSPPALINAIVDALAPLGVKDIQMPATPHRVWQTIQAAKA
>SEQF10178||SEQF10178.1_05346
MKRFDAGETTGTAQETQQTTGQPYVGRPMQRVEDAAILTGRGRYADDLGVKPGTLHAAILRSPHAHAELGLID
FAAALKAPGVRAVLTGADLPAWSKPFVVGVKAPMEQWALAMDRVRYVGEPVAVVVAESRALAEDALDLVRV
DYRVLPPVVSIEAAIADAAPQLHSGLGSNVASDRHFRYGDPEAAFATAPHRVSLTVHYPRNTCTPIECGVVIAEHL
PGNEGYQVTSNFMGPFSLHAVMAMALQVSANHLRHIAPRDSGGSFGVKQAVFPYVVLMCLASRKAGAPVK
WVEDRLEHLSAATSATARLSTIEAAVEADGRIVALDYDQLEDCGGYLRAPEPATFYRMHGVLTGAYAIPNLRVRN
RVVLTNKTPTGLVRGFGGPQVYFALERLVQRISIELNLDPLDVYRRNFVPSNAFPYRAAAGALLDSGNYQLAMS
RALETGAYDELKRRRDIARAEGRLYGIGFAAIVEPSVSNMGYITTATPAEARRKAGPKNGAIASATVSVDLLGGVV
VTIASTPAGQGHMTVCAQVVADVLGIDPAEVIVNVEFDTHKDAWSVAAGNYSSRFAGAVAGTVHLAATRVRD
KLARIVASQLDCDPAELIFAEGRITRRDAPETAVVFARAASNAPHWSPQLLPAGEEPGLRETVFWSPPNLDAPDE
QDRINTSACYGFAFDLCGLEIDRATGRVRIDRYVTAHDAGKLLNPALADGQIRGAFAQGLGAALMEEFRYGPDG
SFQSGTLADYLLPTTCEVPDPMIVHLETPSPFTPLGAKGLGEGNNMTTPPCIANAVADALGVRDIRLPLTPAKV
MAMIGLEDPPPSRLELAETATAAATGGKERSKGAKALSARGTVDLDAPPEAVFAVLLDPQALAQVVPGCHVLEP
IGDNRYRADVTVGVGMIKARYEAEIALSDLEPPHRLRLSGAGLSSLGSARGSGMVELAPHEGGTRLTYDYEAEV
SGKVAAVGGRMLEGAAKVVLRQLFESLGRQAGGKPVRKPGWIARLLALFGARR
>SEQF10179||SEQF10179.1_04766
MNAPAEPNNHLIGASVKRKEDFRFLTGAGQYTDDVVQAHQSYAVFLRSPYAHARIKHINTDAARNHPGVLAVL
TGDDLAADKVNGLPCGWLIHSIDGTPMKEPPHPVLAQGKVRHVGDQVALVVAESVKIAKDAVEMIDVEYDEL
AAVVDTATADTAGTAVHDDVPNNTCYTWGHGDKAATDAAFAKAAHVTHLDIVNNRLIPNAIEPRAVNASYSR
QDDSYTLYVANQNPHVERLLMSAFVLGLTEAKVRVIAPDVGGGFGSKIFLYPEDVALTWASKKVGRPIKWTAER
SESFLTDAHGRDHVTHAELALDAQGNFLAMRVHTTANMGAYLSTFASSVPTILYATLLAGQYKTPAIYAEVKAVF
TNTAPVDAYRGAGRPEATYVVERLVETAARELQIDPAELRRRNFIRTFPYATPVGLTYDTGDYEPCLDRAIELADVK
GFAARRDASRAKGRLRGLGYSCYIEACGLAPSNIAGALGARAGLFEAGEIRVHPTGSVTVFTGSHSHGQGHETT
FAQVLADRLGVPIDNIEIVHGDTGRIPFGMGTYGSRSIAVGGSAIMKALDKIEAKAKKIAAHLLEASAEDIEFKDG
VFRVAGTDRTKTFGEVALTAYVPHNYPLDKLEPGLDENAFYDPTNFTYPAGAYICEVEVDPDTGEVHIDRFVAVD
DFGNIINPMIVEGQVHGGLGQGIGQALLEACVYDENGQLLTGSYMDYAMPRANDLPSFTVETAKGTPCTHNP
LGVKGCGEAGAIGSPPALINAIVDALAPLGVKDIQMPATPHRVWQTIQAAKA
>SEQF10179||SEQF10179.1_05164
MKRFDAGETTGTAQETQQTTGQPYVGRPMQRVEDAAILTGRGRYADDLGVKPGTLHAAILRSPHAHAELGLID
FAAALKAPGVRAVLTGADLPAWSKPFVVGVKAPMEQWALAMDRVRYVGEPVAVVVAESRALAEDALDLVRV
DYRVLPPVVSIEAAIADAAPQLHSGLGSNVASDRHFRYGDPEAAFATAPHRVSLTVHYPRNTCTPIECGVVIAEHL

PGNEGYQVTSNFMGPFSLHAVMAMALQVSANHLRHIAPRDSGGSFGVKQAVFPYVVLMCLASRKAGAPVK
WVEDRLEHLSAATSATARLSTIEAAVEADGRIVALDYDQLEDCGGYLRAPEPATFYRMHGVLTGAYAIPNLRVRN
RVVLTNKTPTGLVRGFGGPQVYFALERLVQRISIELNLDPLDVYRRNFVPSNAFPYRAAAGALLDSGNYQLAMS
RALETGAYDELKRRRDIARAEGRLYGIGFAAIVEPSVSNMGYITTATPAEARRKAGPKNGAIASATVSVDLLGGVV
VTIASTPAGQGHMTVCAQVVADVLGIDPAEVIVNVEFDTHKDAWSVAAGNYSSRFAGAVAGTVHLAATRVRD
KLARIVASQLDCDPAELIFAEGRITRRDAPETAVVFARAASNAPHWSPQLLPAGEEPGLRETVFWSPPNLDAPDE
QDRINTSACYGFAFDLCGLEIDRATGRVRIDRYVTAHDAGKLLNPALADGQIRGAFAQGLGAALMEEFRYGPDG
SFQSGTLADYLLPTTCEVPDPMIVHLETPSPFTPLGAKGLGEGNNMTTPPCIANAVADALGVRDIRLPLTPAKV
MAMIGLEDPPPSRLELAETATAAATGGKERSKGAKALSARGTVDLDAPPEAVFAVLLDPQALAQVVPGCHVLEP
IGDNRYRADVTVGVGMIKARYEAEIALSDLEPPHRLRLSGAGLSSLGSARGSGMVELAPHEGGTRLTYDYEAEV
SGKVAAVGGRMLEGAAKVVLRQLFESLGRQAGGKPVRKPGWIARLLALFGARR
>SEQF10180||SEQF10180.1_04784
MKRFDAGETTGTAQETQQTTGQPYVGRPMQRVEDAAILTGRGRYADDLGVKPGTLHAAILRSPHAHAELGLID
FAAALKAPGVRAVLTGADLPAWSKPFVVGVKAPMEQWALAMDRVRYVGEPVAVVVAESRALAEDALDLVRV
DYRVLPPVVSIEAAIADAAPQLHSGLGSNVASDRHFRYGDPEAAFATAPHRVSLTVHYPRNTCTPIECGVVIAEHL
PGNEGYQVTSNFMGPFSLHAVMAMALQVSANHLRHIAPRDSGGSFGVKQAVFPYVVLMCLASRKAGAPVK
WVEDRLEHLSAATSATARLSTIEAAVEADGRIVALDYDQLEDCGGYLRAPEPATFYRMHGVLTGAYAIPNLRVRN
RVVLTNKTPTGLVRGFGGPQVYFALERLVQRISIELNLDPLDVYRRNFVPSNAFPYRAAAGALLDSGNYQLAMS
RALETGAYDELKRRRDIARAEGRLYGIGFAAIVEPSVSNMGYITTATPAEARRKAGPKNGAIASATVSVDLLGGVV
VTIASTPAGQGHMTVCAQVVADVLGIDPAEVIVNVEFDTHKDAWSVAAGNYSSRFAGAVAGTVHLAATRVRD
KLARIVASQLDCDPAELIFAEGRITRRDAPETAVVFARAASNAPHWSPQLLPAGEEPGLRETVFWSPPNLDAPDE
QDRINTSACYGFAFDLCGLEIDRATGRVRIDRYVTAHDAGKLLNPALADGQIRGAFAQGLGAALMEEFRYGPDG
SFQSGTLADYLLPTTCEVPDPMIVHLETPSPFTPLGAKGLGEGNNMTTPPCIANAVADALGVRDIRLPLTPAKV
MAMIGLEDPPPSRLELAETATAAATGGKERSKGAKALSARGTVDLDAPPEAVFAVLLDPQALAQVVPGCHVLEP
IGDNRYRADVTVGVGMIKARYEAEIALSDLEPPHRLRLSGAGLSSLGSARGSGMVELAPHEGGTRLTYDYEAEV
SGKVAAVGGRMLEGAAKVVLRQLFESLGRQAGGKPVRKPGWIARLLALFGARR
>SEQF10180||SEQF10180.1_03709
MNAPAEPNNHLIGASVKRKEDFRFLTGAGQYTDDVVQAHQSYAVFLRSPYAHARIKHINTDAARNHPGVLAVL
TGDDLAADKVNGLPCGWLIHSIDGTPMKEPPHPVLAQGKVRHVGDQVALVVAESVKIAKDAVEMIDVEYDEL
AAVVDTATADTAGTAVHDDVPNNTCYTWGHGDKAATDAAFAKAAHVTHLDIVNNRLIPNAIEPRAVNASYSR
QDDSYTLYVANQNPHVERLLMSAFVLGLTEAKVRVIAPDVGGGFGSKIFLYPEDVALTWASKKVGRPIKWTAER
SESFLTDAHGRDHVTHAELALDAQGNFLAMRVHTTANMGAYLSTFASSVPTILYATLLAGQYKTPAIYAEVKAVF
TNTAPVDAYRGAGRPEATYVVERLVETAARELQIDPAELRRRNFIRTFPYATPVGLTYDTGDYEPCLDRAIELADVK
GFAARRDASRAKGRLRGLGYSCYIEACGLAPSNIAGALGARAGLFEAGEIRVHPTGSVTVFTGSHSHGQGHETT
FAQVLADRLGVPIDNIEIVHGDTGRIPFGMGTYGSRSIAVGGSAIMKALDKIEAKAKKIAAHLLEASAEDIEFKDG
VFRVAGTDRTKTFGEVALTAYVPHNYPLDKLEPGLDENAFYDPTNFTYPAGAYICEVEVDPDTGEVHIDRFVAVD
DFGNIINPMIVEGQVHGGLGQGIGQALLEACVYDENGQLLTGSYMDYAMPRANDLPSFTVETAKGTPCTHNP
LGVKGCGEAGAIGSPPALINAIVDALAPLGVKDIQMPATPHRVWQTIQAAKA
>SEQF10181||SEQF10181.1_04524
MNAPAEPNNHLIGASVKRKEDFRFLTGAGQYTDDVVQAHQSYAVFLRSPYAHARIKHINTDAARNHPGVLAVL
TGDDLAADKVNGLPCGWLIHSIDGTPMKEPPHPVLAQGKVRHVGDQVALVVAESVKIAKDAVEMIDVEYDEL
AAVVDTATADTAGTAVHDDVPNNTCYTWGHGDKAATDAAFAKAAHVTHLDIVNNRLIPNAIEPRAVNASYSR
QDDSYTLYVANQNPHVERLLMSAFVLGLTEAKVRVIAPDVGGGFGSKIFLYPEDVALTWASKKVGRPIKWTAER
SESFLTDAHGRDHVTHAELALDAQGNFLAMRVHTTANMGAYLSTFASSVPTILYATLLAGQYKTPAIYAEVKAVF

TNTAPVDAYRGAGRPEATYVVERLVETAARELQIDPAELRRRNFIRTFPYATPVGLTYDTGDYEPCLDRAIELADVK
GFAARRDASRAKGRLRGLGYSCYIEACGLAPSNIAGALGARAGLFEAGEIRVHPTGSVTVFTGSHSHGQGHETT
FAQVLADRLGVPIDNIEIVHGDTGRIPFGMGTYGSRSIAVGGSAIMKALDKIEAKAKKIAAHLLEASAEDIEFKDG
VFRVAGTDRTKTFGEVALTAYVPHNYPLDKLEPGLDENAFYDPTNFTYPAGAYICEVEVDPDTGEVHIDRFVAVD
DFGNIINPMIVEGQVHGGLGQGIGQALLEACVYDENGQLLTGSYMDYAMPRANDLPSFTVETAKGTPCTHNP
LGVKGCGEAGAIGSPPALINAIVDALAPLGVKDIQMPATPHRVWQTIQAAKA
>SEQF10181||SEQF10181.1_05454
MKRFDAGETTGTAQETQQTTGQPYVGRPMQRVEDAAILTGRGRYADDLGVKPGTLHAAILRSPHAHAELGLID
FAAALKAPGVRAVLTGADLPAWSKPFVVGVKAPMEQWALAMDRVRYVGEPVAVVVAESRALAEDALDLVRV
DYRVLPPVVSIEAAIADAAPQLHSGLGSNVASDRHFRYGDPEAAFATAPHRVSLTVHYPRNTCTPIECGVVIAEHL
PGNEGYQVTSNFMGPFSLHAVMAMALQVSANHLRHIAPRDSGGSFGVKQAVFPYVVLMCLASRKAGAPVK
WVEDRLEHLSAATSATARLSTIEAAVEADGRIVALDYDQLEDCGGYLRAPEPATFYRMHGVLTGAYAIPNLRVRN
RVVLTNKTPTGLVRGFGGPQVYFALERLVQRISIELNLDPLDVYRRNFVPSNAFPYRAAAGALLDSGNYQLAMS
RALETGAYDELKRRRDIARAEGRLYGIGFAAIVEPSVSNMGYITTATPAEARRKAGPKNGAIASATVSVDLLGGVV
VTIASTPAGQGHMTVCAQVVADVLGIDPAEVIVNVEFDTHKDAWSVAAGNYSSRFAGAVAGTVHLAATRVRD
KLARIVASQLDCDPAELIFAEGRITRRDAPETAVVFARAASNAPHWSPQLLPAGEEPGLRETVFWSPPNLDAPDE
QDRINTSACYGFAFDLCGLEIDRATGRVRIDRYVTAHDAGKLLNPALADGQIRGAFAQGLGAALMEEFRYGPDG
SFQSGTLADYLLPTTCEVPDPMIVHLETPSPFTPLGAKGLGEGNNMTTPPCIANAVADALGVRDIRLPLTPAKV
MAMIGLEDPPPSRLELAETATAAATGGKERSKGAKALSARGTVDLDAPPEAVFAVLLDPQALAQVVPGCHVLEP
IGDNRYRADVTVGVGMIKARYEAEIALSDLEPPHRLRLSGAGLSSLGSARGSGMVELAPHEGGTRLTYDYEAEV
SGKVAAVGGRMLEGAAKVVLRQLFESLGRQAGGKPVRKPGWIARLLALFGARR
>SEQF10182||SEQF10182.1_02152
MKRFDAGETTGTAQETQQTTGQPYVGRPMQRVEDAAILTGRGRYADDLGVKPGTLHAAILRSPHAHAELGLID
FAAALKAPGVRAVLTGADLPAWSKPFVVGVKAPMEQWALAMDRVRYVGEPVAVVVAESRALAEDALDLVRV
DYRVLPPVVSIEAAIADAAPQLHSGLGSNVASDRHFRYGDPEAAFATAPHRVSLTVHYPRNTCTPIECGVVIAEHL
PGNEGYQVTSNFMGPFSLHAVMAMALQVSANHLRHIAPRDSGGSFGVKQAVFPYVVLMCLASRKAGAPVK
WVEDRLEHLSAATSATARLSTIEAAVEADGRIVALDYDQLEDCGGYLRAPEPATFYRMHGVLTGAYAIPNLRVRN
RVVLTNKTPTGLVRGFGGPQVYFALERLVQRISIELNLDPLDVYRRNFVPSNAFPYRAAAGALLDSGNYQLAMS
RALETGAYDELKRRRDIARAEGRLYGIGFAAIVEPSVSNMGYITTATPAEARRKAGPKNGAIASATVSVDLLGGVV
VTIASTPAGQGHMTVCAQVVADVLGIDPAEVIVNVEFDTHKDAWSVAAGNYSSRFAGAVAGTVHLAATRVRD
KLARIVASQLDCDPAELIFAEGRITRRDAPETAVVFARAASNAPHWSPQLLPAGEEPGLRETVFWSPPNLDAPDE
QDRINTSACYGFAFDLCGLEIDRATGRVRIDRYVTAHDAGKLLNPALADGQIRGAFAQGLGAALMEEFRYGPDG
SFQSGTLADYLLPTTCEVPDPMIVHLETPSPFTPLGAKGLGEGNNMTTPPCIANAVADALGVRDIRLPLTPAKV
MAMIGLEDPPPSRLELAETATAAATGGKERSKGAKALSARGTVDLDAPPEAVFAVLLDPQALAQVVPGCHVLEP
IGDNRYRADVTVGVGMIKARYEAEIALSDLEPPHRLRLSGAGLSSLGSARGSGMVELAPHEGGTRLTYDYEAEV
SGKVAAVGGRMLEGAAKVVLRQLFESLGRQAGGKPVRKPGWIARLLALFGARR
>SEQF10182||SEQF10182.1_01308
MNAPAEPNNHLIGASVKRKEDFRFLTGAGQYTDDVVQAHQSYAVFLRSPYAHARIKHINTDAARNHPGVLAVL
TGDDLAADKVNGLPCGWLIHSIDGTPMKEPPHPVLAQGKVRHVGDQVALVVAESVKIAKDAVEMIDVEYDEL
AAVVDTATADTAGTAVHDDVPNNTCYTWGHGDKAATDAAFAKAAHVTHLDIVNNRLIPNAIEPRAVNASYSR
QDDSYTLYVANQNPHVERLLMSAFVLGLTEAKVRVIAPDVGGGFGSKIFLYPEDVALTWASKKVGRPIKWTAER
SESFLTDAHGRDHVTHAELALDAQGNFLAMRVHTTANMGAYLSTFASSVPTILYATLLAGQYKTPAIYAEVKAVF
TNTAPVDAYRGAGRPEATYVVERLVETAARELQIDPAELRRRNFIRTFPYATPVGLTYDTGDYEPCLDRAIELADVK
GFAARRDASRAKGRLRGLGYSCYIEACGLAPSNIAGALGARAGLFEAGEIRVHPTGSVTVFTGSHSHGQGHETT

FAQVLADRLGVPIDNIEIVHGDTGRIPFGMGTYGSRSIAVGGSAIMKALDKIEAKAKKIAAHLLEASAEDIEFKDG
VFRVAGTDRTKTFGEVALTAYVPHNYPLDKLEPGLDENAFYDPTNFTYPAGAYICEVEVDPDTGEVHIDRFVAVD
DFGNIINPMIVEGQVHGGLGQGIGQALLEACVYDENGQLLTGSYMDYAMPRANDLPSFTVETAKGTPCTHNP
LGVKGCGEAGAIGSPPALINAIVDALAPLGVKDIQMPATPHRVWQTIQAAKA
>SEQF10183||SEQF10183.1_02246
MKRFDAGETTGTAQETQQTTGQPYVGRPMQRVEDAAILTGRGRYADDLGVKPGTLHAAILRSPHAHAELGVI
DFAAALKAPGVRAVLTGADLPAWSKPFVVGVKAPMEQWALAMDRVRYVGEPVAVVVAESRALAEDALDLVR
VDYRVLPPVVSIEAAIADAAPQLHSGLGSNVASDRHFRYGDPEAAFATAPHRVSLTAHYPRNTCTPIECGVVIAE
HLPGNEGYQVTSNFMGPFSLHAVMAMALQVSANHLRHIAPRDSGGSFGVKQAVFPYVVLMCLASRKAGAPV
KWVEDRLEHLSASTSATARLSTIEAAVEADGRIVALDYDQLEDCGGYLRAPEPATFYRMHGVLTGAYAIPNLRVR
NRVVLTNKTPTGLVRGFGGPQVYFALERLVQRISIELNLDPLDVYRRNFVPTNAFPYRAAAGALLDSGNYQLAM
SRALETGAYDELKRRRDIARAEGRLYGIGFAAIVEPSVSNMGYITTATPAEARRKAGPKNGAIASATVSVDLLGGV
VVTIASTPAGQGHMTVCAQVVADVLGIDPAEVIVNVEFDTHKDAWSVAAGNYSSRFAGAVAGTVHLAATRVR
DKLARIVASHLDCDPAELIFAEGRITRRDAPETAMVFARAASNAPHWSPQLLPAGEEPGLRETVFWSPPNLDAP
DEQDRINTSACYGFAFDLCGLEIDRATGRVRIDRYVTAHDAGKLLNPALADGQIRGAFAQGLGAALMEEFRYGP
DGSFQSGTLADYLLPTTCEVPDPIIVHLETPSPFTPLGAKGLGEGNNMSTPPCIANAVADALGVRDIRLPLTPAKV
MAMIGLEDPPPSRLELAETATAAATGGKERSKGAKALSARGTVDLDAPPEAVFAVLLDPQALAQVVPGCHVLEP
IGDNRYRADVTVGVGMIKARYEAEIALSDLEPPHRLRLSGAGLSSLGSARGSGMVELAPHEGGTRLTYDYEAEV
SGKVAAVGGRMLEGAAKVVLRQLFESLGRQAGGKPVRKPGWIARLLALFGARR
>SEQF10183||SEQF10183.1_04198
MNAPAEPNNHLIGASVKRKEDFRFLTGAGQYTDDVVQAHQSYAVFLRSPYAHARIKHINTDAARNHPGVLAVL
TGDDLAADKVNGLPCGWLIHSIDGTPMKEPPHPVLAQGKVRHVGDQVALVVAESVKIAKDAVEMIDVEYDEL
PAVVDTATADTAGTAVHDDVPNNTCYTWGHGDKAATDAAFAKAAHVTHLDIVNNRLIPNAIEPRAVNASYSRQ
DDSYTLYVANQNPHVERLLMSAFVLGLTEAKVRVIAPDVGGGFGSKIFLYPEDVALTWASKKVGRPIKWTAERSE
SFLTDAHGRDHVTHAELALDAQGNFLAMRVHTTANMGAYLSTFASSVPTILYATLLAGQYKTPAIYAEVKAVFTN
TAPVDAYRGAGRPEATYVVERLVETAARELQVDPAELRRRNFIRTFPYATPVGLTYDTGDYEPCLDRAIELADVKG
FAARRDASRAKGRLRGLGYSCYIEACGLAPSNIAGALGARAGLFEAGEIRVHPTGSVTVFTGSHSHGQGHETTFA
QVVADRLGVPIDNIEIVHGDTGRIPFGMGTYGSRSIAVGGSAIMKALDKIEAKAKKIAAHLLEASAEDIEFKDGVF
RVAGTDRTKTFGEVALTAYVPHNYPLDKLEPGLDENAFYDPTNFTYPAGAYICEVEVDPDTGEVHIDRFVAVDDF
GNIINPMIVEGQVHGGLGQGIGQALLEACVYDENGQLLTGSYMDYAMPRANDLPSFTVETAKGTPCTHNPLG
VKGCGEAGAIGSPPALINAIVDALAPLGVKDIQMPATPHRVWQTIQAAKA
>SEQF10184||SEQF10184.1_02570
MKRFDAGETTGTAQETQQTTGQPYVGRPMQRVEDAAILTGRGRYADDLGVKPGTLHAAILRSPHAHAELGLID
FAAALKAPGVRAVLTGADLPAWSKPFVVGVKAPMEQWALAMDRVRYVGEPVAVVVAESRALAEDALDLVRV
DYRVLPPVVSIEAAIADAAPQLHSGLGSNVASDRHFRYGDPEAAFATAPHRVSLTVHYPRNTCTPIECGVVIAEHL
PGNEGYQVTSNFMGPFSLHAVMAMALQVSANHLRHIAPRDSGGSFGVKQAVFPYVVLMCLASRKAGAPVK
WVEDRLEHLSAATSATARLSTIEAAVEADGRIVALDYDQLEDCGGYLRAPEPATFYRMHGVLTGAYAIPNLRVRN
RVVLTNKTPTGLVRGFGGPQVYFALERLVQRISIELNLDPLDVYRRNFVPSNAFPYRAAAGALLDSGNYQLAMS
RALETGAYDELKRRRDIARAEGRLYGIGFAAIVEPSVSNMGYITTATPAEARRKAGPKNGAIASATVSVDLLGGVV
VTIASTPAGQGHMTVCAQVVADVLGIDPAEVIVNVEFDTHKDAWSVAAGNYSSRFAGAVAGTVHLAATRVRD
KLARIVASQLDCDPAELIFAEGRITRRDAPETAVVFARAASNAPHWSPQLLPAGEEPGLRETVFWSPPNLDAPDE
QDRINTSACYGFAFDLCGLEIDRATGRVRIDRYVTAHDAGKLLNPALADGQIRGAFAQGLGAALMEEFRYGPDG
SFQSGTLADYLLPTTCEVPDPMIVHLETPSPFTPLGAKGLGEGNNMTTPPCIANAVADALGVRDIRLPLTPAKV
MAMIGLEDPPPSRLELAETATAAATGGKERSKGAKALSARGTVDLDAPPEAVFAVLLDPQALAQVVPGCHVLEP

IGDNRYRADVTVGVGMIKARYEAEIALSDLEPPHRLRLSGAGLSSLGSARGSGMVELAPHEGGTRLTYDYEAEV
SGKVAAVGGRMLEGAAKVVLRQLFESLGRQAGGKPVRKPGWIARLLALFGARR
>SEQF10184||SEQF10184.1_01675
MNAPAEPNNHLIGASVKRKEDFRFLTGAGQYTDDVVQAHQSYAVFLRSPYAHARIKHINTDAARNHPGVLAVL
TGDDLAADKVNGLPCGWLIHSIDGTPMKEPPHPVLAQGKVRHVGDQVALVVAESVKIAKDAVEMIDVEYDEL
AAVVDTATADTAGTAVHDDVPNNTCYTWGHGDKAATDAAFAKAAHVTHLDIVNNRLIPNAIEPRAVNASYSR
QDDSYTLYVANQNPHVERLLMSAFVLGLTEAKVRVIAPDVGGGFGSKIFLYPEDVALTWASKKVGRPIKWTAER
SESFLTDAHGRDHVTHAELALDAQGNFLAMRVHTTANMGAYLSTFASSVPTILYATLLAGQYKTPAIYAEVKAVF
TNTAPVDAYRGAGRPEATYVVERLVETAARELQIDPAELRRRNFIRTFPYATPVGLTYDTGDYEPCLDRAIELADVK
GFAARRDASRAKGRLRGLGYSCYIEACGLAPSNIAGALGARAGLFEAGEIRVHPTGSVTVFTGSHSHGQGHETT
FAQVLADRLGVPIDNIEIVHGDTGRIPFGMGTYGSRSIAVGGSAIMKALDKIEAKAKKIAAHLLEASAEDIEFKDG
VFRVAGTDRTKTFGEVALTAYVPHNYPLDKLEPGLDENAFYDPTNFTYPAGAYICEVEVDPDTGEVHIDRFVAVD
DFGNIINPMIVEGQVHGGLGQGIGQALLEACVYDENGQLLTGSYMDYAMPRANDLPSFTVETAKGTPCTHNP
LGVKGCGEAGAIGSPPALINAIVDALAPLGVKDIQMPATPHRVWQTIQAAKA
>SEQF10185||SEQF10185.1_02481
MKRFDAGETTGTAQETQQTTGQPYVGRPMQRVEDAAILTGRGRYADDLGVKPGTLHAAILRSPHAHAELGLID
FAAALKAPGVRAVLTGADLPAWSKPFVVGVKAPMEQWALAMDRVRYVGEPVAVVVAESRALAEDALDLVRV
DYRVLPPVVSIEAAIADAAPQLHSGLGSNVASDRHFRYGDPEAAFATAPHRVSLTVHYPRNTCTPIECGVVIAEHL
PGNEGYQVTSNFMGPFSLHAVMAMALQVSANHLRHIAPRDSGGSFGVKQAVFPYVVLMCLASRKAGAPVK
WVEDRLEHLSAATSATARLSTIEAAVEADGRIVALDYDQLEDCGGYLRAPEPATFYRMHGVLTGAYAIPNLRVRN
RVVLTNKTPTGLVRGFGGPQVYFALERLVQRISIELNLDPLDVYRRNFVPSNAFPYRAAAGALLDSGNYQLAMS
RALETGAYDELKRRRDIARAEGRLYGIGFAAIVEPSVSNMGYITTATPAEARRKAGPKNGAIASATVSVDLLGGVV
VTIASTPAGQGHMTVCAQVVADVLGIDPAEVIVNVEFDTHKDAWSVAAGNYSSRFAGAVAGTVHLAATRVRD
KLARIVASQLDCDPAELIFAEGRITRRDAPETAVVFARAASNAPHWSPQLLPAGEEPGLRETVFWSPPNLDAPDE
QDRINTSACYGFAFDLCGLEIDRATGRVRIDRYVTAHDAGKLLNPALADGQIRGAFAQGLGAALMEEFRYGPDG
SFQSGTLADYLLPTTCEVPDPMIVHLETPSPFTPLGAKGLGEGNNMTTPPCIANAVADALGVRDIRLPLTPAKV
MAMIGLEDPPPSRLELAETATAAATGGKERSKGAKALSARGTVDLDAPPEAVFAVLLDPQALAQVVPGCHVLEP
IGDNRYRADVTVGVGMIKARYEAEIALSDLEPPHRLRLSGAGLSSLGSARGSGMVELAPHEGGTRLTYDYEAEV
SGKVAAVGGRMLEGAAKVVLRQLFESLGRQAGGKPVRKPGWIARLLALFGARR
>SEQF10185||SEQF10185.1_01677
MNAPAEPNNHLIGASVKRKEDFRFLTGAGQYTDDVVQAHQSYAVFLRSPYAHARIKHINTDAARNHPGVLAVL
TGDDLAADKVNGLPCGWLIHSIDGTPMKEPPHPVLAQGKVRHVGDQVALVVAESVKIAKDAVEMIDVEYDEL
AAVVDTATADTAGTAVHDDVPNNTCYTWGHGDKAATDAAFAKAAHVTHLDIVNNRLIPNAIEPRAVNASYSR
QDDSYTLYVANQNPHVERLLMSAFVLGLTEAKVRVIAPDVGGGFGSKIFLYPEDVALTWASKKVGRPIKWTAER
SESFLTDAHGRDHVTHAELALDAQGNFLAMRVHTTANMGAYLSTFASSVPTILYATLLAGQYKTPAIYAEVKAVF
TNTAPVDAYRGAGRPEATYVVERLVETAARELQIDPAELRRRNFIRTFPYATPVGLTYDTGDYEPCLDRAIELADVK
GFAARRDASRAKGRLRGLGYSCYIEACGLAPSNIAGALGARAGLFEAGEIRVHPTGSVTVFTGSHSHGQGHETT
FAQVLADRLGVPIDNIEIVHGDTGRIPFGMGTYGSRSIAVGGSAIMKALDKIEAKAKKIAAHLLEASAEDIEFKDG
VFRVAGTDRTKTFGEVALTAYVPHNYPLDKLEPGLDENAFYDPTNFTYPAGAYICEVEVDPDTGEVHIDRFVAVD
DFGNIINPMIVEGQVHGGLGQGIGQALLEACVYDENGQLLTGSYMDYAMPRANDLPSFTVETAKGTPCTHNP
LGVKGCGEAGAIGSPPALINAIVDALAPLGVKDIQMPATPHRVWQTIQAAKA
>SEQF10186||SEQF10186.1_02387
MNAPAEPNNHLIGASVKRKEDFRFLTGAGQYTDDVVQAHQSYAVFLRSPYAHARIKHINTDAARNHPGVLAVL
TGDDLAADKVNGLPCGWLIHSIDGTPMKEPPHPVLAQGKVRHVGDQVALVVAESVKIAKDAVEMIDVEYDEL

PAVVDTATADTAGTAVHDDVPNNTCYTWGHGDKAATDAAFAKAAHVTHLDIVNNRLIPNAIEPRAVNASYSRQ
DDSYTLYVANQNPHVERLLMSAFVLGLTEAKVRVIAPDVGGGFGSKIFLYPEDVALTWASKKVGRPIKWTAERSE
SFLTDAHGRDHVTHAELALDAQGNFLAMRVHTTANMGAYLSTFASSVPTILYATLLAGQYKTPAIYAEVKAVFTN
TAPVDAYRGAGRPEATYVVERLVETAARELQVDPAELRRRNFIRTFPYATPVGLTYDTGDYEPCLDRAIELADVKG
FAARRDASRAKGRLRGLGYSCYIEACGLAPSNIAGALGARAGLFEAGEIRVHPTGSVTVFTGSHSHGQGHETTFA
QVVADRLGVPIDNIEIVHGDTGRIPFGMGTYGSRSIAVGGSAIMKALDKIEAKAKKIAAHLLEASAEDIEFKDGVF
RVAGTDRTKTFGEVALTAYVPHNYPLDKLEPGLDENAFYDPTNFTYPAGAYICEVEVDPDTGEVHIDRFVAVDDF
GNIINPMIVEGQVHGGLGQGIGQALLEACVYDENGQLLTGSYMDYAMPRANDLPSFTVETAKGTPCTHNPLG
VKGCGEAGAIGSPPALINAIVDALAPLGVKDIQMPATPHRVWQTIQAAKA
>SEQF10186||SEQF10186.1_01670
MKRFDAGETTGTAQETQQTTGQPYVGRPMQRVEDAAILTGRGRYADDLGVKPGTLHAAILRSPHAHAELGVI
DFAAALKAPGVRAVLTGADLPAWSKPFVVGVKAPMEQWALAMDRVRYVGEPVAVVVAESRALAEDALDLVR
VDYRVLPPVVSIEAAIADAAPQLHSGLGSNVASDRHFRYGDPEAAFATAPHRVSLTAHYPRNTCTPIECGVVIAE
HLPGNEGYQVTSNFMGPFSLHAVMAMALQVSANHLRHIAPRDSGGSFGVKQAVFPYVVLMCLASRKAGAPV
KWVEDRLEHLSASTSATARLSTIEAAVEADGRIVALDYDQLEDCGGYLRAPEPATFYRMHGVLTGAYAIPNLRVR
NRVVLTNKTPTGLVRGFGGPQVYFALERLVQRISIELNLDPLDVYRRNFVPTNAFPYRAAAGALLDSGNYQLAM
SRALETGAYDELKRRRDIARAEGRLYGIGFAAIVEPSVSNMGYITTATPAEARRKAGPKNGAIASATVSVDLLGGV
VVTIASTPAGQGHMTVCAQVVADVLGIDPAEVIVNVEFDTHKDAWSVAAGNYSSRFAGAVAGTVHLAATRVR
DKLARIVASHLDCDPAELIFAEGRITRRDAPETAMVFARAASNAPHWSPQLLPAGEEPGLRETVFWSPPNLDAP
DEQDRINTSACYGFAFDLCGLEIDRATGRVRIDRYVTAHDAGKLLNPALADGQIRGAFAQGLGAALMEEFRYGP
DGSFQSGTLADYLLPTTCEVPDPIIVHLETPSPFTPLGAKGLGEGNNMSTPPCIANAVADALGVRDIRLPLTPAKV
MAMIGLEDPPPSRLELAETATAAATGGKERSKGAKALSARGTVDLDAPPEAVFAVLLDPQALAQVVPGCHVLEP
IGDNRYRADVTVGVGMIKARYEAEIALSDLEPPHRLRLSGAGLSSLGSARGSGMVELAPHEGGTRLTYDYEAEV
SGKVAAVGGRMLEGAAKVVLRQLFESLGRQAGGKPVRKPGWIARLLALFGARR
>SEQF10187||SEQF10187.1_04679
MNAPAEPNNHLIGASVKRKEDFRFLTGAGQYTDDVVQAHQSYAVFLRSPYAHARIKHINTDAARNHPGVLAVL
TGDDLAADKVNGLPCGWLIHSIDGTPMKEPPHPVLAQGKVRHVGDQVALVVAESVKIAKDAVEMIDVEYDEL
PAVVDTATADTAGTAVHDDVPNNTCYTWGHGDKAATDAAFAKAAHVTHLDIVNNRLIPNAIEPRAVNASYSRQ
DDSYTLYVANQNPHVERLLMSAFVLGLTEAKVRVIAPDVGGGFGSKIFLYPEDVALTWASKKVGRPIKWTAERSE
SFLTDAHGRDHVTHAELALDAQGNFLAMRVHTTANMGAYLSTFASSVPTILYATLLAGQYKTPAIYAEVKAVFTN
TAPVDAYRGAGRPEATYVVERLVETAARELQVDPAELRRRNFIRTFPYATPVGLTYDTGDYEPCLDRAIELADVKG
FAARRDASRAKGRLRGLGYSCYIEACGLAPSNIAGALGARAGLFEAGEIRVHPTGSVTVFTGSHSHGQGHETTFA
QVVADRLGVPIDNIEIVHGDTGRIPFGMGTYGSRSIAVGGSAIMKALDKIEAKAKKIAAHLLEASAEDIEFKDGVF
RVAGTDRTKTFGEVALTAYVPHNYPLDKLEPGLDENAFYDPTNFTYPAGAYICEVEVDPDTGEVHIDRFVAVDDF
GNIINPMIVEGQVHGGLGQGIGQALLEACVYDENGQLLTGSYMDYAMPRANDLPSFTVETAKGTPCTHNPLG
VKGCGEAGAIGSPPALINAIVDALAPLGVKDIQMPATPHRVWQTIQAAKA
>SEQF10187||SEQF10187.1_04232
MKRFDAGETTGTAQETQQTTGQPYVGRPMQRVEDAAILTGRGRYADDLGVKPGTLHAAILRSPHAHAELGVI
DFAAALKAPGVRAVLTGADLPAWSKPFVVGVKAPMEQWALAMDRVRYVGEPVAVVVAESRALAEDALDLVR
VDYRVLPPVVSIEAAIADAAPQLHSGLGSNVASDRHFRYGDPEAAFATAPHRVSLTAHYPRNTCTPIECGVVIAE
HLPGNEGYQVTSNFMGPFSLHAVMAMALQVSANHLRHIAPRDSGGSFGVKQAVFPYVVLMCLASRKAGAPV
KWVEDRLEHLSASTSATARLSTIEAAVEADGRIVALDYDQLEDCGGYLRAPEPATFYRMHGVLTGAYAIPNLRVR
NRVVLTNKTPTGLVRGFGGPQVYFALERLVQRISIELNLDPLDVYRRNFVPTNAFPYRAAAGALLDSGNYQLAM
SRALETGAYDELKRRRDIARAEGRLYGIGFAAIVEPSVSNMGYITTATPAEARRKAGPKNGAIASATVSVDLLGGV

VVTIASTPAGQGHMTVCAQVVADVLGIDPAEVIVNVEFDTHKDAWSVAAGNYSSRFAGAVAGTVHLAATRVR
DKLARIVASHLDCDPAELIFAEGRITRRDAPETAMVFARAASNAPHWSPQLLPAGEEPGLRETVFWSPPNLDAP
DEQDRINTSACYGFAFDLCGLEIDRATGRVRIDRYVTAHDAGKLLNPALADGQIRGAFAQGLGAALMEEFRYGP
DGSFQSGTLADYLLPTTCEVPDPIIVHLETPSPFTPLGAKGLGEGNNMSTPPCIANAVADALGVRDIRLPLTPAKV
MAMIGLEDPPPSRLELAETATAAATGGKERSKGAKALSARGTVDLDAPPEAVFAVLLDPQALAQVVPGCHVLEP
IGDNRYRADVTVGVGMIKARYEAEIALSDLEPPHRLRLSGAGLSSLGSARGSGMVELAPHEGGTRLTYDYEAEV
SGKVAAVGGRMLEGAAKVVLRQLFESLGRQAGGKPVRKPGWIARLLALFGARR
>SEQF10188||SEQF10188.1_03537
MNAPAEPNNHLIGASVKRKEDFRFLTGAGQYTDDVVQAHQSYAVFLRSPYAHARIKHINADAARNHPGVLAVL
TGDDLAADKVNGLPCGWLIHSIDGTPMKEPPHPVLAQGKVRHVGDQVALVVAESVKIAKDAVEMIDVEYDEL
PAVVDTATADTAGTAVHDDVPNNTCYTWGHGDKAATDAAFAKAAHVTHLDIVNNRLIPNAIEPRAVNASYSRQ
DDSYTLYVANQNPHVERLLMSAFVLGLTEAKVRVIAPDVGGGFGSKIFLYPEDVALTWASKKVGRPIKWTAERSE
SFLTDAHGRDHVTHAELALDAQGNFLAMRVHTTANMGAYLSTFASSVPTILYATLLAGQYKTPAIYAEVKAVFTN
TAPVDAYRGAGRPEATYVVERLVETAARELQVDPAELRRRNFIRTFPYATPVGLTYDTGDYEPCLDRAIELADVKG
FAARRDASRAKGRLRGLGYSCYIEACGLAPSNIAGALGARAGLFEAGEIRVHPTGSVTVFTGSHSHGQGHETTFA
QVVADRLGVPIDNIEIVHGDTGRIPFGMGTYGSRSIAVGGSAIMKALDKIEAKAKKIAAHLLEASAEDIEFKDGVF
RVAGTDRTKTFGEVALTAYVPHNYPLDKLEPGLDENAFYDPTNFTYPAGAYICEVEVDPDTGEVHIDRFVAVDDF
GNIINPMIVEGQVHGGLGQGIGQALLEACVYDENGQLLTGSYMDYAMPRANDLPSFTVETAKGTPCTHNPLG
VKGCGEAGAIGSPPALINAIVDALAPLGVKDIQMPATPHRVWQTIQAAKA
>SEQF10189||SEQF10189.1_01771
MNAPAEPNNHLIGASVKRKEDFRFLTGAGQYTDDVVQAHQSYAVFLRSPYAHARIKHINTDAARNHPGVLAVL
TGDDLAADKVNGLPCGWLIHSIDGTPMKEPPHPVLAQGKVRHVGDQVALVVAESVKIAKDAVEMIDVEYDEL
AAVVDTATADTAGTAVHDDVPNNTCYTWGHGDKAATDAAFAKAAHVTHLDIVNNRLIPNAIEPRAVNASYSR
QDDSYTLYVANQNPHVERLLMSAFVLGLTEAKVRVIAPDVGGGFGSKIFLYPEDVALTWASKKVGRPIKWTAER
SESFLTDAHGRDHVTHAELALDAQGNFLAMRVHTTANMGAYLSTFASSVPTILYATLLAGQYKTPAIYAEVKAVF
TNTAPVDAYRGAGRPEATYVVERLVETAARELQIDPAELRRRNFIRTFPYATPVGLTYDTGDYEPCLDRAIELADVK
GFAARRDASRAKGRLRGLGYSCYIEACGLAPSNIAGALGARAGLFEAGEIRVHPTGSVTVFTGSHSHGQGHETT
FAQVLADRLGVPIDNIEIVHGDTGRIPFGMGTYGSRSIAVGGSAIMKALDKIEAKAKKIAAHLLEASAEDIEFKDG
VFRVAGTDRTKTFGEVALTAYVPHNYPLDKLEPGLDENAFYDPTNFTYPAGAYICEVEVDPDTGEVHIDRFVAVD
DFGNIINPMIVEGQVHGGLGQGIGQALLEACVYDENGQLLTGSYMDYAMPRANDLPSFTVETAKGTPCTHNP
LGVKGCGEAGAIGSPPALINAIVDALAPLGVKDIQMPATPHRVWQTIQAAKA
>SEQF10189||SEQF10189.1_02334
MKRFDAGETTGTAQETQQTTGQPYVGRPMQRVEDAAILTGRGRYADDLGVKPGTLHAAILRSPHAHAELGLID
FAAALKAPGVRAVLTGADLPAWSKPFVVGVKAPMEQWALAMDRVRYVGEPVAVVVAESRALAEDALDLVRV
DYRVLPPVVSIEAAIADAAPQLHSGLGSNVASDRHFRYGDPEAAFATAPHRVSLTVHYPRNTCTPIECGVVIAEHL
PGNEGYQVTSNFMGPFSLHAVMAMALQVSANHLRHIAPRDSGGSFGVKQAVFPYVVLMCLASRKAGAPVK
WVEDRLEHLSAATSATARLSTIEAAVEADGRIVALDYDQLEDCGGYLRAPEPATFYRMHGVLTGAYAIPNLRVRN
RVVLTNKTPTGLVRGFGGPQVYFALERLVQRISIELNLDPLDVYRRNFVPSNAFPYRAAAGALLDSGNYQLAMS
RALETGAYDELKRRRDIARAEGRLYGIGFAAIVEPSVSNMGYITTATPAEARRKAGPKNGAIASATVSVDLLGGVV
VTIASTPAGQGHMTVCAQVVADVLGIDPAEVIVNVEFDTHKDAWSVAAGNYSSRFAGAVAGTVHLAATRVRD
KLARIVASQLDCDPAELIFAEGRITRRDAPETAVVFARAASNAPHWSPQLLPAGEEPGLRETVFWSPPNLDAPDE
QDRINTSACYGFAFDLCGLEIDRATGRVRIDRYVTAHDAGKLLNPALADGQIRGAFAQGLGAALMEEFRYGPDG
SFQSGTLADYLLPTTCEVPDPMIVHLETPSPFTPLGAKGLGEGNNMTTPPCIANAVADALGVRDIRLPLTPAKV
MAMIGLEDPPPSRLELAETATAAATGGKERSKGAKALSARGTVDLDAPPEAVFAVLLDPQALAQVVPGCHVLEP

IGDNRYRADVTVGVGMIKARYEAEIALSDLEPPHRLRLSGAGLSSLGSARGSGMVELAPHEGGTRLTYDYEAEV
SGKVAAVGGRMLEGAAKVVLRQLFESLGRQAGGKPVRKPGWIARLLALFGARR
>SEQF10190||SEQF10190.1_01769
MNAPAEPNNHLIGASVKRKEDFRFLTGAGQYTDDVVQAHQSYAVFLRSPYAHARIKHINTDAARNHPGVLAVL
TGDDLAADKVNGLPCGWLIHSIDGTPMKEPPHPVLAQGKVRHVGDQVALVVAESVKIAKDAVEMIDVEYDEL
AAVVDTATADTAGTAVHDDVPNNTCYTWGHGDKAATDAAFAKAAHVTHLDIVNNRLIPNAIEPRAVNASYSR
QDDSYTLYVANQNPHVERLLMSAFVLGLTEAKVRVIAPDVGGGFGSKIFLYPEDVALTWASKKVGRPIKWTAER
SESFLTDAHGRDHVTHAELALDAQGNFLAMRVHTTANMGAYLSTFASSVPTILYATLLAGQYKTPAIYAEVKAVF
TNTAPVDAYRGAGRPEATYVVERLVETAARELQIDPAELRRRNFIRTFPYATPVGLTYDTGDYEPCLDRAIELADVK
GFAARRDASRAKGRLRGLGYSCYIEACGLAPSNIAGALGARAGLFEAGEIRVHPTGSVTVFTGSHSHGQGHETT
FAQVLADRLGVPIDNIEIVHGDTGRIPFGMGTYGSRSIAVGGSAIMKALDKIEAKAKKIAAHLLEASAEDIEFKDG
VFRVAGTDRTKTFGEVALTAYVPHNYPLDKLEPGLDENAFYDPTNFTYPAGAYICEVEVDPDTGEVHIDRFVAVD
DFGNIINPMIVEGQVHGGLGQGIGQALLEACVYDENGQLLTGSYMDYAMPRANDLPSFTVETAKGTPCTHNP
LGVKGCGEAGAIGSPPALINAIVDALAPLGVKDIQMPATPHRVWQTIQAAKA
>SEQF10190||SEQF10190.1_02573
MKRFDAGETTGTAQETQQTTGQPYVGRPMQRVEDAAILTGRGRYADDLGVKPGTLHAAILRSPHAHAELGLID
FAAALKAPGVRAVLTGADLPAWSKPFVVGVKAPMEQWALAMDRVRYVGEPVAVVVAESRALAEDALDLVRV
DYRVLPPVVSIEAAIADAAPQLHSGLGSNVASDRHFRYGDPEAAFATAPHRVSLTVHYPRNTCTPIECGVVIAEHL
PGNEGYQVTSNFMGPFSLHAVMAMALQVSANHLRHIAPRDSGGSFGVKQAVFPYVVLMCLASRKAGAPVK
WVEDRLEHLSAATSATARLSTIEAAVEADGRIVALDYDQLEDCGGYLRAPEPATFYRMHGVLTGAYAIPNLRVRN
RVVLTNKTPTGLVRGFGGPQVYFALERLVQRISIELNLDPLDVYRRNFVPSNAFPYRAAAGALLDSGNYQLAMS
RALETGAYDELKRRRDIARAEGRLYGIGFAAIVEPSVSNMGYITTATPAEARRKAGPKNGAIASATVSVDLLGGVV
VTIASTPAGQGHMTVCAQVVADVLGIDPAEVIVNVEFDTHKDAWSVAAGNYSSRFAGAVAGTVHLAATRVRD
KLARIVASQLDCDPAELIFAEGRITRRDAPETAVVFARAASNAPHWSPQLLPAGEEPGLRETVFWSPPNLDAPDE
QDRINTSACYGFAFDLCGLEIDRATGRVRIDRYVTAHDAGKLLNPALADGQIRGAFAQGLGAALMEEFRYGPDG
SFQSGTLADYLLPTTCEVPDPMIVHLETPSPFTPLGAKGLGEGNNMTTPPCIANAVADALGVRDIRLPLTPAKV
MAMIGLEDPPPSRLELAETATAAATGGKERSKGAKALSARGTVDLDAPPEAVFAVLLDPQALAQVVPGCHVLEP
IGDNRYRADVTVGVGMIKARYEAEIALSDLEPPHRLRLSGAGLSSLGSARGSGMVELAPHEGGTRLTYDYEAEV
SGKVAAVGGRMLEGAAKVVLRQLFESLGRQAGGKPVRKPGWIARLLALFGARR
>SEQF10191||SEQF10191.1_02130
MKRFDAGETTGTAQETQQTTGQPYVGRPMQRVEDAAILTGRGRYADDLGVKPGTLHAAILRSPHAHAELGLID
FAAALKAPGVRAVLTGADLPAWSKPFVVGVKAPMEQWALAMDRVRYVGEPVAVVVAESRALAEDALDLVRV
DYRVLPPVVSIEAAIADAAPQLHSGLGSNVASDRHFRYGDPEAAFATAPHRVSLTVHYPRNTCTPIECGVVIAEHL
PGNEGYQVTSNFMGPFSLHAVMAMALQVSANHLRHIAPRDSGGSFGVKQAVFPYVVLMCLASRKAGAPVK
WVEDRLEHLSAATSATARLSTIEAAVEADGRIVALDYDQLEDCGGYLRAPEPATFYRMHGVLTGAYAIPNLRVRN
RVVLTNKTPTGLVRGFGGPQVYFALERLVQRISIELNLDPLDVYRRNFVPSNAFPYRAAAGALLDSGNYQLAMS
RALETGAYDELKRRRDIARAEGRLYGIGFAAIVEPSVSNMGYITTATPAEARRKAGPKNGAIASATVSVDLLGGVV
VTIASTPAGQGHMTVCAQVVADVLGIDPAEVIVNVEFDTHKDAWSVAAGNYSSRFAGAVAGTVHLAATRVRD
KLARIVASQLDCDPAELIFAEGRITRRDAPETAVVFARAASNAPHWSPQLLPAGEEPGLRETVFWSPPNLDAPDE
QDRINTSACYGFAFDLCGLEIDRATGRVRIDRYVTAHDAGKLLNPALADGQIRGAFAQGLGAALMEEFRYGPDG
SFQSGTLADYLLPTTCEVPDPMIVHLETPSPFTPLGAKGLGEGNNMTTPPCIANAVADALGVRDIRLPLTPAKV
MAMIGLEDPPPSRLELAETATAAATGGKERSKGAKALSARGTVDLDAPPEAVFAVLLDPQALAQVVPGCHVLEP
IGDNRYRADVTVGVGMIKARYEAEIALSDLEPPHRLRLSGAGLSSLGSARGSGMVELAPHEGGTRLTYDYEAEV
SGKVAAVGGRMLEGAAKVVLRQLFESLGRQAGGKPVRKPGWIARLLALFGARR

>SEQF10191||SEQF10191.1_01216
MNAPAEPNNHLIGASVKRKEDFRFLTGAGQYTDDVVQAHQSYAVFLRSPYAHARIKHINTDAARNHPGVLAVL
TGDDLAADKVNGLPCGWLIHSIDGTPMKEPPHPVLAQGKVRHVGDQVALVVAESVKIAKDAVEMIDVEYDEL
AAVVDTATADTAGTAVHDDVPNNTCYTWGHGDKAATDAAFAKAAHVTHLDIVNNRLIPNAIEPRAVNASYSR
QDDSYTLYVANQNPHVERLLMSAFVLGLTEAKVRVIAPDVGGGFGSKIFLYPEDVALTWASKKVGRPIKWTAER
SESFLTDAHGRDHVTHAELALDAQGNFLAMRVHTTANMGAYLSTFASSVPTILYATLLAGQYKTPAIYAEVKAVF
TNTAPVDAYRGAGRPEATYVVERLVETAARELQIDPAELRRRNFIRTFPYATPVGLTYDTGDYEPCLDRAIELADVK
GFAARRDASRAKGRLRGLGYSCYIEACGLAPSNIAGALGARAGLFEAGEIRVHPTGSVTVFTGSHSHGQGHETT
FAQVLADRLGVPIDNIEIVHGDTGRIPFGMGTYGSRSIAVGGSAIMKALDKIEAKAKKIAAHLLEASAEDIEFKDG
VFRVAGTDRTKTFGEVALTAYVPHNYPLDKLEPGLDENAFYDPTNFTYPAGAYICEVEVDPDTGEVHIDRFVAVD
DFGNIINPMIVEGQVHGGLGQGIGQALLEACVYDENGQLLTGSYMDYAMPRANDLPSFTVETAKGTPCTHNP
LGVKGCGEAGAIGSPPALINAIVDALAPLGVKDIQMPATPHRVWQTIQAAKA
>SEQF10192||SEQF10192.1_05378
MKRFDAGETTGTAQETQQTTGQPYVGRPMQRVEDAAILTGRGRYADDLGVKPGTLHAAILRSPHAHAELGLID
FAAALKAPGVRAVLTGADLPAWSKPFVVGVKAPMEQWALAMDRVRYVGEPVAVVVAESRALAEDALDLVRV
DYRVLPPVVSIEAAIADAAPQLHSGLGSNVASDRHFRYGDPEAAFATAPHRVSLTVHYPRNTCTPIECGVVIAEHL
PGNEGYQVTSNFMGPFSLHAVMAMALQVSANHLRHIAPRDSGGSFGVKQAVFPYVVLMCLASRKAGAPVK
WVEDRLEHLSAATSATARLSTIEAAVEADGRIVALDYDQLEDCGGYLRAPEPATFYRMHGVLTGAYAIPNLRVRN
RVVLTNKTPTGLVRGFGGPQVYFALERLVQRISIELNLDPLDVYRRNFVPSNAFPYRAAAGALLDSGNYQLAMS
RALETGAYDELKRRRDIARAEGRLYGIGFAAIVEPSVSNMGYITTATPAEARRKAGPKNGAIASATVSVDLLGGVV
VTIASTPAGQGHMTVCAQVVADVLGIDPAEVIVNVEFDTHKDAWSVAAGNYSSRFAGAVAGTVHLAATRVRD
KLARIVASQLDCDPAELIFAEGRITRRDAPETAVVFARAASNAPHWSPQLLPAGEEPGLRETVFWSPPNLDAPDE
QDRINTSACYGFAFDLCGLEIDRATGRVRIDRYVTAHDAGKLLNPALADGQIRGAFAQGLGAALMEEFRYGPDG
SFQSGTLADYLLPTTCEVPDPMIVHLETPSPFTPLGAKGLGEGNNMTTPPCIANAVADALGVRDIRLPLTPAKV
MAMIGLEDPPPSRLELAETATAAATGGKERSKGAKALSARGTVDLDAPPEAVFAVLLDPQALAQVVPGCHVLEP
IGDNRYRADVTVGVGMIKARYEAEIALSDLEPPHRLRLSGAGLSSLGSARGSGMVELAPHEGGTRLTYDYEAEV
SGKVAAVGGRMLEGAAKVVLRQLFESLGRQAGGKPVRKPGWIARLLALFGARR
>SEQF10192||SEQF10192.1_04732
MNAPAEPNNHLIGASVKRKEDFRFLTGAGQYTDDVVQAHQSYAVFLRSPYAHARIKHINTDAARNHPGVLAVL
TGDDLAADKVNGLPCGWLIHSIDGTPMKEPPHPVLAQGKVRHVGDQVALVVAESVKIAKDAVEMIDVEYDEL
AAVVDTATADTAGTAVHDDVPNNTCYTWGHGDKAATDAAFAKAAHVTHLDIVNNRLIPNAIEPRAVNASYSR
QDDSYTLYVANQNPHVERLLMSAFVLGLTEAKVRVIAPDVGGGFGSKIFLYPEDVALTWASKKVGRPIKWTAER
SESFLTDAHGRDHVTHAELALDAQGNFLAMRVHTTANMGAYLSTFASSVPTILYATLLAGQYKTPAIYAEVKAVF
TNTAPVDAYRGAGRPEATYVVERLVETAARELQIDPAELRRRNFIRTFPYATPVGLTYDTGDYEPCLDRAIELADVK
GFAARRDASRAKGRLRGLGYSCYIEACGLAPSNIAGALGARAGLFEAGEIRVHPTGSVTVFTGSHSHGQGHETT
FAQVLADRLGVPIDNIEIVHGDTGRIPFGMGTYGSRSIAVGGSAIMKALDKIEAKAKKIAAHLLEASAEDIEFKDG
VFRVAGTDRTKTFGEVALTAYVPHNYPLDKLEPGLDENAFYDPTNFTYPAGAYICEVEVDPDTGEVHIDRFVAVD
DFGNIINPMIVEGQVHGGLGQGIGQALLEACVYDENGQLLTGSYMDYAMPRANDLPSFTVETAKGTPCTHNP
LGVKGCGEAGAIGSPPALINAIVDALAPLGVKDIQMPATPHRVWQTIQAAKA
>SEQF10193||SEQF10193.1_04269
MNAPAEPNNHLIGAAVKRKEDFRFLTGAGQYTDDVVQAHQSYAVFLRSPYAHARIKHINAEAARNHPGVLAVL
TGDDLAADKVNGLPCGWLIHSIDGTPMKEPPHPVLAQGKVRHVGDQVALVVAESVKIAKDAVEMIDVEYDEL
PAVVDTATADTAGTAVHDDVPNNTCYTWGHGDKAATDAAFAKAAHVTQLDIVNNRLIPNAIEPRAVNASYSR
QDDSYTLYVANQNPHVERLLMSAFVLGLTEAKVRVIAPDVGGGFGSKIFLYPEDVALTWASKKVGRPIKWTAER

SESFLTDAHGRDHVTHAELALDAQGNFLAMRVHTTANMGAYLSTFASSVPTILYATLLAGQYKTPAIYAEVKAVF
TNTAPVDAYRGAGRPEATYVVERLVETAARELQIDPAELRRRNFIRTFPYATPVGLTYDTGDYEPCLDRAIELADVK
GFAARRDASRAKGRLRGMGYSCYIEACGLAPSNIAGALGARAGLFEAGEIRVHPTGSVTVFTGSHSHGQGHET
TFAQVVADRLGVPIDNIEIVHGDTGRIPFGMGTYGSRSIAVGGSAIMKALDKIEAKAKKIAAHLLEASAEDIEFKD
GVFRVAGTDRTKTFGEVALTAYVPHNYPLDKLEPGLDENAFYDPTNFTYPAGAYICEVEVDPDTGEVHIDRFVAV
DDFGNIINPMIVEGQVHGGLGQGIGQALLEACVYDENGQLLTGSYMDYAMPRANDLPSFTVETAKGTPCTHN
PLGVKGCGEAGAIGSPPALINAIVDALAPLGVKDIQMPATPHRVWQTIQAAKA
>SEQF10194||SEQF10194.1_02730
MNAPAEPNNHLIGASVKRKEDFRFLTGAGQYTDDVVQAHQSYAVFLRSPYAHARIKHINTDAARNHPGVLAVL
TGDDLAADKVNGLPCGWLIHSIDGTPMKEPPHPVLAQGKVRHVGDQVALVVAESVKIAKDAVEMIDVEYDEL
PAVVDTATADTAGTAVHDDVPNNTCYTWGHGDKAATDAAFARAAHVTRLDIVNNRLIPNAIEPRAVNASYSRQ
DDSYTLYVANQNPHVERLLMSAFVLGLTEAKVRVIAPDVGGGFGSKIFLYPEDVALTWASKKVGRPIKWTAERSE
SFLTDAHGRDHVTHAELALDAQGNFLAMRVHTTANMGAYLSTFASSVPTILYATLLAGQYKTPAIYAEVKAVFTN
TAPVDAYRGAGRPEATYVVERLVETAAHELQIDPAELRRRNFIRTFPYATPVGLTYDTGDYEPCLDRAIELADVKGF
AARRDASRAKGRLRGMGYSCYIEACGLAPSNIAGALGARAGLFEAGEIRVHPTGSVTVFTGSHSHGQGHETTFA
QVVADRLGVPIDNIEIVHGDTGRIPFGMGTYGSRSIAVGGSAIMKALDKIEAKAKKIAAHLLEASAEDIEFKDGVF
RVAGTDRTKTFGEVALTAYVPHNYPLDKLEPGLDENAFYDPTNFTYPAGAYICEVEVDPDTGEVHIDRFVAVDDF
GNIINPMIVEGQVHGGLGQGIGQALLEACVYDENGQLLTGSYMDYAMPRANDLPSFTVETAKGTPCTHNPLG
VKGCGEAGAIGSPPALINAIVDALAPLGVKDIQMPATPHRVWQTIQAAKA
>SEQF10194||SEQF10194.1_02342
MKRFDAGETTGTAQETQQTTGQPYVGRPMQRVEDAAILTGRGRYADDLGVKPGTLHAAILRSPHAHAELGLID
FAAALKAPGVRAVLTGADLPAWSKPFVVGVKAPMEQWALAMDRVRYVGEPVAVVVAESRALAEDALDLVRV
DYRVLPPVVSIEAAIADAAPQLHSGLGSNVASDRHFRYGDPEAAFATAPHRVSLTVHYPRNTCTPIECGVVIAEHL
PGNEGYQVTSNFMGPFSLHAVMAMALQVSANHLRHIAPRDSGGSFGVKQAVFPYVVLMCLASRKAGAPVK
WVEDRLEHLSAATSATARLSTIEAAVEADGRIVALDYDQLEDCGGYLRAPEPATFYRMHGVLTGAYAIPNLRVRN
RVVLTNKTPTGLVRGFGGPQVYFALERLVQRISIELNLDPLDVYRRNFVPSNAFPYRAAAGALLDSGNYQLAMS
RALETGAYDELKRRRDIARAEGRLYGIGFAAIVEPSVSNMGYITTATPAEARRKAGPKNGAIASATVSVDLLGGVV
VTIASTPAGQGHMTVCAQVVADVLGIDPAEVIVNVEFDTHKDAWSVAAGNYSSRFAGAVAGTVHLAATRVRD
KLARIVASQLDCDPAELIFAEGRITRRDAPETAVVFARAASNAPHWSPQLLPAGEEPGLRETVFWSPPNLDAPDE
QDRINTSACYGFAFDLCGLEIDRATGRVRIDRYVTAHDAGKLLNPALADGQIRGAFAQGLGAALMEEFRYGPDG
SFQSGTLADYLLPTTCEVPDPMIVHLETPSPFTPLGAKGLGEGNNMSTPPCIANAVADALGVRDIRLPLTPAKVM
AMIGLEDPPPSRLELAETATAAATGGKERSKGAKALSARGTVDLDAPPEAVFAVLLDPQALAQVVPGCHVLEPIG
DNRYRADVTVGVGMIKARYEAEIALSDLEPPHRLRLSGAGLSSLGSARGSGMVELAPHEGGTRLTYDYEAEVSG
KVAAVGGRMLEGAAKVVLRQLFESLGRQAGGKPVRKPGWIARLLALFGARR
>SEQF10195||SEQF10195.1_01769
MNAPAEPNNHLIGASVKRKEDFRFLTGAGQYTDDVVQAHQSYAVFLRSPYAHARIKHINTDAARNHPGVLAVL
TGDDLAADKVNGLPCGWLIHSIDGTPMKEPPHPVLAQGKVRHVGDQVALVVAESVKIAKDAVEMIDVEYDEL
AAVVDTATADTAGTAVHDDVPNNTCYTWGHGDKAATDAAFAKAAHVTHLDIVNNRLIPNAIEPRAVNASYSR
QDDSYTLYVANQNPHVERLLMSAFVLGLTEAKVRVIAPDVGGGFGSKIFLYPEDVALTWASKKVGRPIKWTAER
SESFLTDAHGRDHVTHAELALDAQGNFLAMRVHTTANMGAYLSTFASSVPTILYATLLAGQYKTPAIYAEVKAVF
TNTAPVDAYRGAGRPEATYVVERLVETAARELQIDPAELRRRNFIRTFPYATPVGLTYDTGDYEPCLDRAIELADVK
GFAARRDASRAKGRLRGLGYSCYIEACGLAPSNIAGALGARAGLFEAGEIRVHPTGSVTVFTGSHSHGQGHETT
FAQVLADRLGVPIDNIEIVHGDTGRIPFGMGTYGSRSIAVGGSAIMKALDKIEAKAKKIAAHLLEASAEDIEFKDG
VFRVAGTDRTKTFGEVALTAYVPHNYPLDKLEPGLDENAFYDPTNFTYPAGAYICEVEVDPDTGEVHIDRFVAVD

DFGNIINPMIVEGQVHGGLGQGIGQALLEACVYDENGQLLTGSYMDYAMPRANDLPSFTVETAKGTPCTHNP
LGVKGCGEAGAIGSPPALINAIVDALAPLGVKDIQMPATPHRVWQTIQAAKA
>SEQF10195||SEQF10195.1_02333
MKRFDAGETTGTAQETQQTTGQPYVGRPMQRVEDAAILTGRGRYADDLGVKPGTLHAAILRSPHAHAELGLID
FAAALKAPGVRAVLTGADLPAWSKPFVVGVKAPMEQWALAMDRVRYVGEPVAVVVAESRALAEDALDLVRV
DYRVLPPVVSIEAAIADAAPQLHSGLGSNVASDRHFRYGDPEAAFATAPHRVSLTVHYPRNTCTPIECGVVIAEHL
PGNEGYQVTSNFMGPFSLHAVMAMALQVSANHLRHIAPRDSGGSFGVKQAVFPYVVLMCLASRKAGAPVK
WVEDRLEHLSAATSATARLSTIEAAVEADGRIVALDYDQLEDCGGYLRAPEPATFYRMHGVLTGAYAIPNLRVRN
RVVLTNKTPTGLVRGFGGPQVYFALERLVQRISIELNLDPLDVYRRNFVPSNAFPYRAAAGALLDSGNYQLAMS
RALETGAYDELKRRRDIARAEGRLYGIGFAAIVEPSVSNMGYITTATPAEARRKAGPKNGAIASATVSVDLLGGVV
VTIASTPAGQGHMTVCAQVVADVLGIDPAEVIVNVEFDTHKDAWSVAAGNYSSRFAGAVAGTVHLAATRVRD
KLARIVASQLDCDPAELIFAEGRITRRDAPETAVVFARAASNAPHWSPQLLPAGEEPGLRETVFWSPPNLDAPDE
QDRINTSACYGFAFDLCGLEIDRATGRVRIDRYVTAHDAGKLLNPALADGQIRGAFAQGLGAALMEEFRYGPDG
SFQSGTLADYLLPTTCEVPDPMIVHLETPSPFTPLGAKGLGEGNNMTTPPCIANAVADALGVRDIRLPLTPAKV
MAMIGLEDPPPSRLELAETATAAATGGKERSKGAKALSARGTVDLDAPPEAVFAVLLDPQALAQVVPGCHVLEP
IGDNRYRADVTVGVGMIKARYEAEIALSDLEPPHRLRLSGAGLSSLGSARGSGMVELAPHEGGTRLTYDYEAEV
SGKVAAVGGRMLEGAAKVVLRQLFESLGRQAGGKPVRKPGWIARLLALFGARR
>SEQF10196||SEQF10196.1_00223
MKRFDAGETTGTAQETQQTTGQPYVGRPMQRVEDAAILTGRGRYADDLGVKPGTLHAAILRSPHAHAELGLID
FAAALKAPGVRAVLTGADLPAWSKPFVVGVKAPMEQWALAMDRVRYVGEPVAVVVAESRALAEDALDLVRV
DYRVLPPVVSIEAAIADAAPQLHSGLGSNVASDRHFRYGDPEAAFATAPHRVSLTVHYPRNTCTPIECGVVIAEHL
PGNEGYQVTSNFMGPFSLHAVMAMALQVSANHLRHIAPRDSGGSFGVKQAVFPYVVLMCLASRKAGAPVK
WVEDRLEHLSAATSATARLSTIEAAVEADGRIVALDYDQLEDCGGYLRAPEPATFYRMHGVLTGAYAIPNLRVRN
RVVLTNKTPTGLVRGFGGPQVYFALERLVQRISIELNLDPLDVYRRNFVPSNAFPYRAAAGALLDSGNYQLAMS
RALETGAYDELKRRRDIARAEGRLYGIGFAAIVEPSVSNMGYITTATPAEARRKAGPKNGAIASATVSVDLLGGVV
VTIASTPAGQGHMTVCAQVVADVLGIDPAEVIVNVEFDTHKDAWSVAAGNYSSRFAGAVAGTVHLAATRVRD
KLARIVASQLDCDPAELIFAEGRITRRDAPETAVVFARAASNAPHWSPQLLPAGEEPGLRETVFWSPPNLDAPDE
QDRINTSACYGFAFDLCGLEIDRATGRVRIDRYVTAHDAGKLLNPALADGQIRGAFAQGLGAALMEEFRYGPDG
SFQSGTLADYLLPTTCEVPDPMIVHLETPSPFTPLGAKGLGEGNNMTTPPCIANAVADALGVRDIRLPLTPAKV
MAMIGLEDPPPSRLELAETATAAATGGKERSKGAKALSARGTVDLDAPPEAVFAVLLDPQALAQVVPGCHVLEP
IGDNRYRADVTVGVGMIKARYEAEIALSDLEPPHRLRLSGAGLSSLGSARGSGMVELAPHEGGTRLTYDYEAEV
SGKVAAVGGRMLEGAAKVVLRQLFESLGRQAGGKPVRKPGWIARLLALFGARR
>SEQF10196||SEQF10196.1_04193
MNAPAEPNNHLIGASVKRKEDFRFLTGAGQYTDDVVQAHQSYAVFLRSPYAHARIKHINTDAARNHPGVLAVL
TGDDLAADKVNGLPCGWLIHSIDGTPMKEPPHPVLAQGKVRHVGDQVALVVAESVKIAKDAVEMIDVEYDEL
AAVVDTATADTAGTAVHDDVPNNTCYTWGHGDKAATDAAFAKAAHVTHLDIVNNRLIPNAIEPRAVNASYSR
QDDSYTLYVANQNPHVERLLMSAFVLGLTEAKVRVIAPDVGGGFGSKIFLYPEDVALTWASKKVGRPIKWTAER
SESFLTDAHGRDHVTHAELALDAQGNFLAMRVHTTANMGAYLSTFASSVPTILYATLLAGQYKTPAIYAEVKAVF
TNTAPVDAYRGAGRPEATYVVERLVETAARELQIDPAELRRRNFIRTFPYATPVGLTYDTGDYEPCLDRAIELADVK
GFAARRDASRAKGRLRGLGYSCYIEACGLAPSNIAGALGARAGLFEAGEIRVHPTGSVTVFTGSHSHGQGHETT
FAQVLADRLGVPIDNIEIVHGDTGRIPFGMGTYGSRSIAVGGSAIMKALDKIEAKAKKIAAHLLEASAEDIEFKDG
VFRVAGTDRTKTFGEVALTAYVPHNYPLDKLEPGLDENAFYDPTNFTYPAGAYICEVEVDPDTGEVHIDRFVAVD
DFGNIINPMIVEGQVHGGLGQGIGQALLEACVYDENGQLLTGSYMDYAMPRANDLPSFTVETAKGTPCTHNP
LGVKGCGEAGAIGSPPALINAIVDALAPLGVKDIQMPATPHRVWQTIQAAKA

>SEQF10197||SEQF10197.1_03366
MKRFDAGETTGTAQETQQTTGQPYVGRPMQRVEDAAILTGRGRYADDLGVKPGTLHAAILRSPHAHAELGLID
FAAALKAPGVRAVLTGADLPAWSKPFVVGVKAPMEQWALAMDRVRYVGEPVAVVVAESRALAEDALDLVRV
DYRVLPPVVSIEAAIADAAPQLHSGLGSNVASDRHFRYGDPEAAFATAPHRVSLTVHYPRNTCTPIECGVVIAEHL
PGNEGYQVTSNFMGPFSLHAVMAMALQVSANHLRHIAPRDSGGSFGVKQAVFPYVVLMCLASRKAGAPVK
WVEDRLEHLSAATSATARLSTIEAAVEADGRIVALDYDQLEDCGGYLRAPEPATFYRMHGVLTGAYAIPNLRVRN
RVVLTNKTPTGLVRGFGGPQVYFALERLVQRISIELNLDPLDVYRRNFVPSNAFPYRAAAGALLDSGNYQLAMS
RALETGAYDELKRRRDIARAEGRLYGIGFAAIVEPSVSNMGYITTATPAEARRKAGPKNGAIASATVSVDLLGGVV
VTIASTPAGQGHMTVCAQVVADVLGIDPAEVIVNVEFDTHKDAWSVAAGNYSSRFAGAVAGTVHLAATRVRD
KLARIVASQLDCDPAELIFAEGRITRRDAPETAVVFARAASNAPHWSPQLLPAGEEPGLRETVFWSPPNLDAPDE
QDRINTSACYGFAFDLCGLEIDRATGRVRIDRYVTAHDAGKLLNPALADGQIRGAFAQGLGAALMEEFRYGPDG
SFQSGTLADYLLPTTCEVPDPMIVHLETPSPFTPLGAKGLGEGNNMSTPPCIANAVADALGVRDIRLPLTPAKVM
AMIGLEDPPPSRLELAETATAAATGGKERSKGAKALSARGTVDLDAPPEAVFAVLLDPQALAQVVPGCHVLEPIG
DNRYRADVTVGVGMIKARYEAEIALSDLEPPHRLRLSGAGLSSLGSARGSGMVELAPHEGGTRLTYDYEAEVSG
KVAAVGGRMLEGAAKVVLRQLFESLGRQAGGKPVRKPGWIARLLALFGARR
>SEQF10197||SEQF10197.1_00177
MNAPAEPNNHLIGASVKRKEDFRFLTGAGQYTDDVVQAHQSYAVFLRSPYAHARIKHINTDAARNHPGVLAVL
TGDDLAADKVNGLPCGWLIHSIDGTPMKEPPHPVLAQGKVRHVGDQVALVVAESVKIAKDAVEMIDVEYDEL
PAVVDTATADTAGTAVHDDVPNNTCYTWGHGDKAATDAAFARAAHVTRLDIVNNRLIPNAIEPRAVNASYSRQ
DDSYTLYVANQNPHVERLLMSAFVLGLTEAKVRVIAPDVGGGFGSKIFLYPEDVALTWASKKVGRPIKWTAERSE
SFLTDAHGRDHVTHAELALDAQGNFLAMRVHTTANMGAYLSTFASSVPTILYATLLAGQYKTPAIYAEVKAVFTN
TAPVDAYRGAGRPEATYVVERLVETAAHELQIDPAELRRRNFIRTFPYATPVGLTYDTGDYEPCLDRAIELADVKGF
AARRDASRAKGRLRGMGYSCYIEACGLAPSNIAGALGARAGLFEAGEIRVHPTGSVTVFTGSHSHGQGHETTFA
QVVADRLGVPIDNIEIVHGDTGRIPFGMGTYGSRSIAVGGSAIMKALDKIEAKAKKIAAHLLEASAEDIEFKDGVF
RVAGTDRTKTFGEVALTAYVPHNYPLDKLEPGLDENAFYDPTNFTYPAGAYICEVEVDPDTGEVHIDRFVAVDDF
GNIINPMIVEGQVHGGLGQGIGQALLEACVYDENGQLLTGSYMDYAMPRANDLPSFTVETAKGTPCTHNPLG
VKGCGEAGAIGSPPALINAIVDALAPLGVKDIQMPATPHRVWQTIQAAKA
>SEQF10198||SEQF10198.1_04828
MNAPAEPNNHLIGASVKRKEDFRFLTGAGQYTDDVVQAHQSYAVFLRSPYAHARIKHINTDAARNHPGVLAVL
TGDDLAADKVNGLPCGWLIHSIDGTPMKEPPHPVLAQGKVRHVGDQVALVVAESVKIAKDAVEMIDVEYDEL
PAVVDTATADTAGTAVHDDVPNNTCYTWGHGDKAATDAAFAKAAHVTHLDIVNNRLIPNAIEPRAVNASYSRQ
DDSYTLYVANQNPHVERLLMSAFVLGLTEAKVRVIAPDVGGGFGSKIFLYPEDVALTWASKKVGRPIKWTAERSE
SFLTDAHGRDHVTHAELALDAQGNFLAMRVHTTANMGAYLSTFASSVPTILYATLLAGQYKTPAIYAEVKAVFTN
TAPVDAYRGAGRPEATYVVERLVETAARELQVDPAELRRRNFIRTFPYATPVGLTYDTGDYEPCLDRAIELADVKG
FAARRDASRAKGRLRGLGYSCYIEACGLAPSNIAGALGARAGLFEAGEIRVHPTGSVTVFTGSHSHGQGHETTFA
QVVADRLGVPIDNIEIVHGDTGRIPFGMGTYGSRSIAVGGSAIMKALDKIEAKAKKIAAHLLEASAEDIEFKDGVF
RVAGTDRTKTFGEVALTAYVPHNYPLDKLEPGLDENAFYDPTNFTYPAGAYICEVEVDPDTGEVHIDRFVAVDDF
GNIINPMIVEGQVHGGLGQGIGQALLEACVYDENGQLLTGSYMDYAMPRANDLPSFTVETAKGTPCTHNPLG
VKGCGEAGAIGSPPALINAIVDALAPLGVKDIQMPATPHRVWQTIQAAKA
>SEQF10198||SEQF10198.1_04042
MKRFDAGETTGTAQETQQTTGQPYVGRPMQRVEDAAILTGRGRYADDLGVKPGTLHAAILRSPHAHAELGVI
DFAAALKAPGVRAVLTGADLPAWSKPFVVGVKAPMEQWALAMDRVRYVGEPVAVVVAESRALAEDALDLVR
VDYRVLPPVVSIEAAIADAAPQLHSGLGSNVASDRHFRYGDPEAAFATAPHRVSLTAHYPRNTCTPIECGVVIAE
HLPGNEGYQVTSNFMGPFSLHAVMAMALQVSANHLRHIAPRDSGGSFGVKQAVFPYVVLMCLASRKAGAPV

KWVEDRLEHLSASTSATARLSTIEAAVEADGRIVALDYDQLEDCGGYLRAPEPATFYRMHGVLTGAYAIPNLRVR
NRVVLTNKTPTGLVRGFGGPQVYFALERLVQRISIELNLDPLDVYRRNFVPTNAFPYRAAAGALLDSGNYQLAM
SRALETGAYDELKRRRDIARAEGRLYGIGFAAIVEPSVSNMGYITTATPAEARRKAGPKNGAIASATVSVDLLGGV
VVTIASTPAGQGHMTVCAQVVADVLGIDPAEVIVNVEFDTHKDAWSVAAGNYSSRFAGAVAGTVHLAATRVR
DKLARIVASHLDCDPAELIFAEGRITRRDAPETAMVFARAASNAPHWSPQLLPAGEEPGLRETVFWSPPNLDAP
DEQDRINTSACYGFAFDLCGLEIDRATGRVRIDRYVTAHDAGKLLNPALADGQIRGAFAQGLGAALMEEFRYGP
DGSFQSGTLADYLLPTTCEVPDPIIVHLETPSPFTPLGAKGLGEGNNMSTPPCIANAVADALGVRDIRLPLTPAKV
MAMIGLEDPPPSRLELAETATAAATGGKERSKGAKALSARGTVDLDAPPEAVFAVLLDPQALAQVVPGCHVLEP
IGDNRYRADVTVGVGMIKARYEAEIALSDLEPPHRLRLSGAGLSSLGSARGSGMVELAPHEGGTRLTYDYEAEV
SGKVAAVGGRMLEGAAKVVLRQLFESLGRQAGGKPVRKPGWIARLLALFGARR
>SEQF10199||SEQF10199.1_01090
MKRFDAGETTGTAQETQQTTGQPYVGRPMQRVEDAAILTGRGRYADDLGVKPGTLHAAILRSPHAHAELGLID
FAAALKAPGVRAVLTGADLPAWSKPFVVGVKAPMEQWALAMDRVRYVGEPVAVVVAESRALAEDALDFVRV
DYRVLPPVVSIEAAIADAAPQLHSGLGSNVASDRHFRYGDPEAAFATAPHRVSLTAHYPRNTCTPIECGVVIAEHL
PGNEGYQVTSNFMGPFSLHAVMAMALQVSANHLRHIAPRDSGGSFGVKQAVFPYVVLMCLASRKAGAPVK
WVEDRLEHLSASTSATARLSTIEAAVEADGRIVALDYDQLEDCGGYLRAPEPATFYRMHGVLTGAYAIPNLRVRN
RVVLTNKTPTGLVRGFGGPQVYFALERLVQRISIELNLDPLDVYRRNFVPSNAFPYRAAAGALLDSGNYQLAMS
RALETGAYDELKRRRDIARAEGRLYGIGFAAIVEPSVSNMGYITTATPAEARRKAGPKNGAIASATVSVDLLGGVV
VTIASTPAGQGHMTVCAQVVADVLGIDPAEVIVNVEFDTHKDAWSVAAGNYSSRFAGAVAGTVHLAATRVRD
KLARIVASHLDCDPAELIFAEGRITRRDAPETAVVFARAASNAPHWSPQLLPAGEEPGLRETVFWSPPNLDAPDE
QDRINTSACYGFAFDLCGLEIDRATGRVRIDRYVTAHDAGKLLNPALADGQIRGAFAQGLGAALMEEFRYGPDG
SFQSGTLADYLLPTTCEVPDPIIVHLETPSPFTPLGAKGLGEGNNMSTPPCIANAVADALGVRDIRLPLTPAKVMA
MIGLEDPPPSRLELAETATAAATGGKERSKGAKALSARGTVDLDAPPEAVFAVLLDPQALAQVVPGCHVLEPIGD
NRYRADVTVGVGMIKARYEAEIALSDLEPPHRLRLSGAGLSSLGSARGSGMVELAPHEGGTRLTYDYEAEVSGK
VAAVGGRMLEGAAKVVLRQLFESLGRQAGGKPVRKPGWIARLLALFGARR
>SEQF10199||SEQF10199.1_01779
MNAPAEPNNHLIGASVKRKEDFRFLTGAGQYTDDVVQAHQSYAVFLRSPYAHARIKHINTDAARNHPGVLAVL
TGDDLAADKVNGLPCGWLIHSIDGTPMKEPPHPVLAQGKVRHVGDQVALVVAESVKIAKDAVEMIDVEYDEL
PAVVDTATADTAGTAVHDDVPNNTCYTWGHGDKAATDAAFAKAAHVTHLDIVNNRLIPNAIEPRAVNASYSRQ
DDSYTLYVANQNPHVERLLMSAFVLGLTEAKVRVIAPDVGGGFGSKIFLYPEDVALTWASKKVGRPIKWTAERSE
SFLTDAHGRDHVTHAELALDAQGNFLAMRVHTTANMGAYLSTFASSVPTILYATLLAGQYKTPAIYAEVKAVFTN
TAPVDAYRGAGRPEATYVVERLVETAARELQVDPAELRRRNFIRTFPYATPVGLTYDTGDYEPCLDRAIELADVKG
FAARRDASRAKGRLRGLGYSCYIEACGLAPSNIAGALGARAGLFEAGEIRVHPTGSVTVFTGSHSHGQGHETTFA
QVVADRLGVPIDNIEIVHGDTGRIPFGMGTYGSRSIAVGGSAIMKALDKIEAKAKKIAAHLLEASAEDIEFKDGVF
RVAGTDRTKTFGEVALTAYVPHNYPLDKLEPGLDENAFYDPTNFTYPAGAYICEVEVDPDTGEVHIDRFVAVDDF
GNIINPMIVEGQVHGGLGQGIGQALLEACVYDENGQLLTGSYMDYAMPRANDLPSFTVETAKGTPCTHNPLG
VKGCGEAGAIGSPPALINAIVDALAPLGVKDIQMPATPHRVWQTIQAAKA
>SEQF10200||SEQF10200.1_01676
MNAPAEPNNHLIGASVKRKEDFRFLTGAGQYTDDVVQAHQSYAVFLRSPYAHARIKHINTDAARNHPGVLAVL
TGDDLAADKVNGLPCGWLIHSIDGTPMKEPPHPVLAQGKVRHVGDQVALVVAESVKIAKDAVEMIDVEYDEL
AAVVDTATADTAGTAVHDDVPNNTCYTWGHGDKAATDAAFAKAAHVTHLDIVNNRLIPNAIEPRAVNASYSR
QDDSYTLYVANQNPHVERLLMSAFVLGLTEAKVRVIAPDVGGGFGSKIFLYPEDVALTWASKKVGRPIKWTAER
SESFLTDAHGRDHVTHAELALDAQGNFLAMRVHTTANMGAYLSTFASSVPTILYATLLAGQYKTPAIYAEVKAVF
TNTAPVDAYRGAGRPEATYVVERLVETAARELQIDPAELRRRNFIRTFPYATPVGLTYDTGDYEPCLDRAIELADVK

GFAARRDASRAKGRLRGLGYSCYIEACGLAPSNIAGALGARAGLFEAGEIRVHPTGSVTVFTGSHSHGQGHETT
FAQVLADRLGVPIDNIEIVHGDTGRIPFGMGTYGSRSIAVGGSAIMKALDKIEAKAKKIAAHLLEASAEDIEFKDG
VFRVAGTDRTKTFGEVALTAYVPHNYPLDKLEPGLDENAFYDPTNFTYPAGAYICEVEVDPDTGEVHIDRFVAVD
DFGNIINPMIVEGQVHGGLGQGIGQALLEACVYDENGQLLTGSYMDYAMPRANDLPSFTVETAKGTPCTHNP
LGVKGCGEAGAIGSPPALINAIVDALAPLGVKDIQMPATPHRVWQTIQAAKA
>SEQF10200||SEQF10200.1_02544
MKRFDAGETTGTAQETQQTTGQPYVGRPMQRVEDAAILTGRGRYADDLGVKPGTLHAAILRSPHAHAELGLID
FAAALKAPGVRAVLTGADLPAWSKPFVVGVKAPMEQWALAMDRVRYVGEPVAVVVAESRALAEDALDLVRV
DYRVLPPVVSIEAAIADAAPQLHSGLGSNVASDRHFRYGDPEAAFATAPHRVSLTVHYPRNTCTPIECGVVIAEHL
PGNEGYQVTSNFMGPFSLHAVMAMALQVSANHLRHIAPRDSGGSFGVKQAVFPYVVLMCLASRKAGAPVK
WVEDRLEHLSAATSATARLSTIEAAVEADGRIVALDYDQLEDCGGYLRAPEPATFYRMHGVLTGAYAIPNLRVRN
RVVLTNKTPTGLVRGFGGPQVYFALERLVQRISIELNLDPLDVYRRNFVPSNAFPYRAAAGALLDSGNYQLAMS
RALETGAYDELKRRRDIARAEGRLYGIGFAAIVEPSVSNMGYITTATPAEARRKAGPKNGAIASATVSVDLLGGVV
VTIASTPAGQGHMTVCAQVVADVLGIDPAEVIVNVEFDTHKDAWSVAAGNYSSRFAGAVAGTVHLAATRVRD
KLARIVASQLDCDPAELIFAEGRITRRDAPETAVVFARAASNAPHWSPQLLPAGEEPGLRETVFWSPPNLDAPDE
QDRINTSACYGFAFDLCGLEIDRATGRVRIDRYVTAHDAGKLLNPALADGQIRGAFAQGLGAALMEEFRYGPDG
SFQSGTLADYLLPTTCEVPDPMIVHLETPSPFTPLGAKGLGEGNNMTTPPCIANAVADALGVRDIRLPLTPAKV
MAMIGLEDPPPSRLELAETATAAATGGKERSKGAKALSARGTVDLDAPPEAVFAVLLDPQALAQVVPGCHVLEP
IGDNRYRADVTVGVGMIKARYEAEIALSDLEPPHRLRLSGAGLSSLGSARGSGMVELAPHEGGTRLTYDYEAEV
SGKVAAVGGRMLEGAAKVVLRQLFESLGRQAGGKPVRKPGWIARLLALFGARR
>SEQF10201||SEQF10201.1_03266
MNAPAEPNNHLIGASVKRKEDFRFLTGAGQYTDDVVQAHQSYAVFLRSPYAHARIKHINTDAARNHPGVLAVL
TGDDLAADKVNGLPCGWLIHSIDGTPMKEPPHPVLAQGKVRHVGDQVALVVAESVKIAKDAVEMIDVEYDEL
AAVVDTATADTAGTAVHDDVPNNTCYTWGHGDKAATDAAFAKAAHVTHLDIVNNRLIPNAIEPRAVNASYSR
QDDSYTLYVANQNPHVERLLMSAFVLGLTEAKVRVIAPDVGGGFGSKIFLYPEDVALTWASKKVGRPIKWTAER
SESFLTDAHGRDHVTHAELALDAQGNFLAMRVHTTANMGAYLSTFASSVPTILYATLLAGQYKTPAIYAEVKAVF
TNTAPVDAYRGAGRPEATYVVERLVETAARELQIDPAELRRRNFIRTFPYATPVGLTYDTGDYEPCLDRAIELADVK
GFAARRDASRAKGRLRGLGYSCYIEACGLAPSNIAGALGARAGLFEAGEIRVHPTGSVTVFTGSHSHGQGHETT
FAQVLADRLGVPIDNIEIVHGDTGRIPFGMGTYGSRSIAVGGSAIMKALDKIEAKAKKIAAHLLEASAEDIEFKDG
VFRVAGTDRTKTFGEVALTAYVPHNYPLDKLEPGLDENAFYDPTNFTYPAGAYICEVEVDPDTGEVHIDRFVAVD
DFGNIINPMIVEGQVHGGLGQGIGQALLEACVYDENGQLLTGSYMDYAMPRANDLPSFTVETAKGTPCTHNP
LGVKGCGEAGAIGSPPALINAIVDALAPLGVKDIQMPATPHRVWQTIQAAKA
>SEQF10201||SEQF10201.1_02714
MKRFDAGETTGTAQETQQTTGQPYVGRPMQRVEDAAILTGRGRYADDLGVKPGTLHAAILRSPHAHAELGLID
FAAALKAPGVRAVLTGADLPAWSKPFVVGVKAPMEQWALAMDRVRYVGEPVAVVVAESRALAEDALDLVRV
DYRVLPPVVSIEAAIADAAPQLHSGLGSNVASDRHFRYGDPEAAFATAPHRVSLTVHYPRNTCTPIECGVVIAEHL
PGNEGYQVTSNFMGPFSLHAVMAMALQVSANHLRHIAPRDSGGSFGVKQAVFPYVVLMCLASRKAGAPVK
WVEDRLEHLSAATSATARLSTIEAAVEADGRIVALDYDQLEDCGGYLRAPEPATFYRMHGVLTGAYAIPNLRVRN
RVVLTNKTPTGLVRGFGGPQVYFALERLVQRISIELNLDPLDVYRRNFVPSNAFPYRAAAGALLDSGNYQLAMS
RALETGAYDELKRRRDIARAEGRLYGIGFAAIVEPSVSNMGYITTATPAEARRKAGPKNGAIASATVSVDLLGGVV
VTIASTPAGQGHMTVCAQVVADVLGIDPAEVIVNVEFDTHKDAWSVAAGNYSSRFAGAVAGTVHLAATRVRD
KLARIVASQLDCDPAELIFAEGRITRRDAPETAVVFARAASNAPHWSPQLLPAGEEPGLRETVFWSPPNLDAPDE
QDRINTSACYGFAFDLCGLEIDRATGRVRIDRYVTAHDAGKLLNPALADGQIRGAFAQGLGAALMEEFRYGPDG
SFQSGTLADYLLPTTCEVPDPMIVHLETPSPFTPLGAKGLGEGNNMTTPPCIANAVADALGVRDIRLPLTPAKV

MAMIGLEDPPPSRLELAETATAAATGGKERSKGAKALSARGTVDLDAPPEAVFAVLLDPQALAQVVPGCHVLEP
IGDNRYRADVTVGVGMIKARYEAEIALSDLEPPHRLRLSGAGLSSLGSARGSGMVELAPHEGGTRLTYDYEAEV
SGKVAAVGGRMLEGAAKVVLRQLFESLGRQAGGKPVRKPGWIARLLALFGARR
>SEQF10202||SEQF10202.1_02570
MKRFDAGETTGTAQETQQTTGQPYVGRPMQRVEDAAILTGRGRYADDLGVKPGTLHAAILRSPHAHAELGLID
FAAALKAPGVRAVLTGADLPAWSKPFVVGVKAPMEQWALAMDRVRYVGEPVAVVVAESRALAEDALDLVRV
DYRVLPPVVSIEAAIADAAPQLHSGLGSNVASDRHFRYGDPEAAFATAPHRVSLTVHYPRNTCTPIECGVVIAEHL
PGNEGYQVTSNFMGPFSLHAVMAMALQVSANHLRHIAPRDSGGSFGVKQAVFPYVVLMCLASRKAGAPVK
WVEDRLEHLSAATSATARLSTIEAAVEADGRIVALDYDQLEDCGGYLRAPEPATFYRMHGVLTGAYAIPNLRVRN
RVVLTNKTPTGLVRGFGGPQVYFALERLVQRISIELNLDPLDVYRRNFVPSNAFPYRAAAGALLDSGNYQLAMS
RALETGAYDELKRRRDIARAEGRLYGIGFAAIVEPSVSNMGYITTATPAEARRKAGPKNGAIASATVSVDLLGGVV
VTIASTPAGQGHMTVCAQVVADVLGIDPAEVIVNVEFDTHKDAWSVAAGNYSSRFAGAVAGTVHLAATRVRD
KLARIVASQLDCDPAELIFAEGRITRRDAPETAVVFARAASNAPHWSPQLLPAGEEPGLRETVFWSPPNLDAPDE
QDRINTSACYGFAFDLCGLEIDRATGRVRIDRYVTAHDAGKLLNPALADGQIRGAFAQGLGAALMEEFRYGPDG
SFQSGTLADYLLPTTCEVPDPMIVHLETPSPFTPLGAKGLGEGNNMTTPPCIANAVADALGVRDIRLPLTPAKV
MAMIGLEDPPPSRLELAETATAAATGGKERSKGAKALSARGTVDLDAPPEAVFAVLLDPQALAQVVPGCHVLEP
IGDNRYRADVTVGVGMIKARYEAEIALSDLEPPHRLRLSGAGLSSLGSARGSGMVELAPHEGGTRLTYDYEAEV
SGKVAAVGGRMLEGAAKVVLRQLFESLGRQAGGKPVRKPGWIARLLALFGARR
>SEQF10202||SEQF10202.1_01767
MNAPAEPNNHLIGASVKRKEDFRFLTGAGQYTDDVVQAHQSYAVFLRSPYAHARIKHINTDAARNHPGVLAVL
TGDDLAADKVNGLPCGWLIHSIDGTPMKEPPHPVLAQGKVRHVGDQVALVVAESVKIAKDAVEMIDVEYDEL
AAVVDTATADTAGTAVHDDVPNNTCYTWGHGDKAATDAAFAKAAHVTHLDIVNNRLIPNAIEPRAVNASYSR
QDDSYTLYVANQNPHVERLLMSAFVLGLTEAKVRVIAPDVGGGFGSKIFLYPEDVALTWASKKVGRPIKWTAER
SESFLTDAHGRDHVTHAELALDAQGNFLAMRVHTTANMGAYLSTFASSVPTILYATLLAGQYKTPAIYAEVKAVF
TNTAPVDAYRGAGRPEATYVVERLVETAARELQIDPAELRRRNFIRTFPYATPVGLTYDTGDYEPCLDRAIELADVK
GFAARRDASRAKGRLRGLGYSCYIEACGLAPSNIAGALGARAGLFEAGEIRVHPTGSVTVFTGSHSHGQGHETT
FAQVLADRLGVPIDNIEIVHGDTGRIPFGMGTYGSRSIAVGGSAIMKALDKIEAKAKKIAAHLLEASAEDIEFKDG
VFRVAGTDRTKTFGEVALTAYVPHNYPLDKLEPGLDENAFYDPTNFTYPAGAYICEVEVDPDTGEVHIDRFVAVD
DFGNIINPMIVEGQVHGGLGQGIGQALLEACVYDENGQLLTGSYMDYAMPRANDLPSFTVETAKGTPCTHNP
LGVKGCGEAGAIGSPPALINAIVDALAPLGVKDIQMPATPHRVWQTIQAAKA
>SEQF10203||SEQF10203.1_05076
MKRFDAGETTGTAQETQQTTGQPYVGRPMQRVEDAAILTGRGRYADDLGVKPGTLHAAILRSPHAHAELGLID
FAAALKAPGVRAVLTGADLPAWSKPFVVGVKAPMEQWALAMDRVRYVGEPVAVVVAESRALAEDALDLVRV
DYRVLPPVVSIEAAIADAAPQLHSGLGSNVASDRHFRYGDPEAAFATAPHRVSLTVHYPRNTCTPIECGVVIAEHL
PGNEGYQVTSNFMGPFSLHAVMAMALQVSANHLRHIAPRDSGGSFGVKQAVFPYVVLMCLASRKAGAPVK
WVEDRLEHLSAATSATARLSTIEAAVEADGRIVALDYDQLEDCGGYLRAPEPATFYRMHGVLTGAYAIPNLRVRN
RVVLTNKTPTGLVRGFGGPQVYFALERLVQRISIELNLDPLDVYRRNFVPSNAFPYRAAAGALLDSGNYQLAMS
RALETGAYDELKRRRDIARAEGRLYGIGFAAIVEPSVSNMGYITTATPAEARRKAGPKNGAIASATVSVDLLGGVV
VTIASTPAGQGHMTVCAQVVADVLGIDPAEVIVNVEFDTHKDAWSVAAGNYSSRFAGAVAGTVHLAATRVRD
KLARIVASQLDCDPAELIFAEGRITRRDAPETAVVFARAASNAPHWSPQLLPAGEEPGLRETVFWSPPNLDAPDE
QDRINTSACYGFAFDLCGLEIDRATGRVRIDRYVTAHDAGKLLNPALADGQIRGAFAQGLGAALMEEFRYGPDG
SFQSGTLADYLLPTTCEVPDPMIVHLETPSPFTPLGAKGLGEGNNMTTPPCIANAVADALGVRDIRLPLTPAKV
MAMIGLEDPPPSRLELAETATAAATGGKERSKGAKALSARGTVDLDAPPEAVFAVLLDPQALAQVVPGCHVLEP
IGDNRYRADVTVGVGMIKARYEAEIALSDLEPPHRLRLSGAGLSSLGSARGSGMVELAPHEGGTRLTYDYEAEV

SGKVAAVGGRMLEGAAKVVLRQLFESLGRQAGGKPVRKPGWIARLLALFGARR
>SEQF10203||SEQF10203.1_04263
MNAPAEPNNHLIGASVKRKEDFRFLTGAGQYTDDVVQAHQSYAVFLRSPYAHARIKHINTDAARNHPGVLAVL
TGDDLAADKVNGLPCGWLIHSIDGTPMKEPPHPVLAQGKVRHVGDQVALVVAESVKIAKDAVEMIDVEYDEL
AAVVDTATADTAGTAVHDDVPNNTCYTWGHGDKAATDAAFAKAAHVTHLDIVNNRLIPNAIEPRAVNASYSR
QDDSYTLYVANQNPHVERLLMSAFVLGLTEAKVRVIAPDVGGGFGSKIFLYPEDVALTWASKKVGRPIKWTAER
SESFLTDAHGRDHVTHAELALDAQGNFLAMRVHTTANMGAYLSTFASSVPTILYATLLAGQYKTPAIYAEVKAVF
TNTAPVDAYRGAGRPEATYVVERLVETAARELQIDPAELRRRNFIRTFPYATPVGLTYDTGDYEPCLDRAIELADVK
GFAARRDASRAKGRLRGLGYSCYIEACGLAPSNIAGALGARAGLFEAGEIRVHPTGSVTVFTGSHSHGQGHETT
FAQVLADRLGVPIDNIEIVHGDTGRIPFGMGTYGSRSIAVGGSAIMKALDKIEAKAKKIAAHLLEASAEDIEFKDG
VFRVAGTDRTKTFGEVALTAYVPHNYPLDKLEPGLDENAFYDPTNFTYPAGAYICEVEVDPDTGEVHIDRFVAVD
DFGNIINPMIVEGQVHGGLGQGIGQALLEACVYDENGQLLTGSYMDYAMPRANDLPSFTVETAKGTPCTHNP
LGVKGCGEAGAIGSPPALINAIVDALAPLGVKDIQMPATPHRVWQTIQAAKA
>SEQF10204||SEQF10204.1_01924
MKRFDAGETTGTAQETQQTTGQPYVGRPMQRVEDAAILTGRGRYADDLGVKPGTLHAAILRSPHAHAELGVI
DFAAALKAPGVRAVLTGADLPAWSKPFVVGVKAPMEQWALAMDRVRYVGEPVAVVVAESRALAEDALDLVR
VDYRVLPPVVSIEAAIADAAPQLHSGLGSNVASDRHFRYGDPEAAFATAPHRVSLTAHYPRNTCTPIECGVVIAE
HLPGNEGYQVTSNFMGPFSLHAVMAMALQVSANHLRHIAPRDSGGSFGVKQAVFPYVVLMCLASRKAGAPV
KWVEDRLEHLSASTSATARLSTIEAAVEADGRIVALDYDQLEDCGGYLRAPEPATFYRMHGVLTGAYAIPNLRVR
NRVVLTNKTPTGLVRGFGGPQVYFALERLVQRISIELNLDPLDVYRRNFVPTNAFPYRAAAGALLDSGNYQLAM
SRALETGAYDELKRRRDIARAEGRLYGIGFAAIVEPSVSNMGYITTATPAEARRKAGPKNGAIASATVSVDLLGGV
VVTIASTPAGQGHMTVCAQVVADVLGIDPAEVIVNVEFDTHKDAWSVAAGNYSSRFAGAVAGTVHLAATRVR
DKLARIVASHLDCDPAELIFAEGRITRRDAPETAMVFARAASNAPHWSPQLLPAGEEPGLRETVFWSPPNLDAP
DEQDRINTSACYGFAFDLCGLEIDRATGRVRIDRYVTAHDAGKLLNPALADGQIRGAFAQGLGAALMEEFRYGP
DGSFQSGTLADYLLPTTCEVPDPIIVHLETPSPFTPLGAKGLGEGNNMSTPPCIANAVADALGVRDIRLPLTPAKV
MAMIGLEDPPPSRLELAETATAAATGGKERSKGAKALSARGTVDLDAPPEAVFAVLLDPQALAQVVPGCHVLEP
IGDNRYRADVTVGVGMIKARYEAEIALSDLEPPHRLRLSGAGLSSLGSARGSGMVELAPHEGGTRLTYDYEAEV
SGKVAAVGGRMLEGAAKVVLRQLFESLGRQAGGKPVRKPGWIARLLALFGARR
>SEQF10204||SEQF10204.1_02371
MNAPAEPNNHLIGASVKRKEDFRFLTGAGQYTDDVVQAHQSYAVFLRSPYAHARIKHINTDAARNHPGVLAVL
TGDDLAADKVNGLPCGWLIHSIDGTPMKEPPHPVLAQGKVRHVGDQVALVVAESVKIAKDAVEMIDVEYDEL
PAVVDTATADTAGTAVHDDVPNNTCYTWGHGDKAATDAAFAKAAHVTHLDIVNNRLIPNAIEPRAVNASYSRQ
DDSYTLYVANQNPHVERLLMSAFVLGLTEAKVRVIAPDVGGGFGSKIFLYPEDVALTWASKKVGRPIKWTAERSE
SFLTDAHGRDHVTHAELALDAQGNFLAMRVHTTANMGAYLSTFASSVPTILYATLLAGQYKTPAIYAEVKAVFTN
TAPVDAYRGAGRPEATYVVERLVETAARELQVDPAELRRRNFIRTFPYATPVGLTYDTGDYEPCLDRAIELADVKG
FAARRDASRAKGRLRGLGYSCYIEACGLAPSNIAGALGARAGLFEAGEIRVHPTGSVTVFTGSHSHGQGHETTFA
QVVADRLGVPIDNIEIVHGDTGRIPFGMGTYGSRSIAVGGSAIMKALDKIEAKAKKIAAHLLEASAEDIEFKDGVF
RVAGTDRTKTFGEVALTAYVPHNYPLDKLEPGLDENAFYDPTNFTYPAGAYICEVEVDPDTGEVHIDRFVAVDDF
GNIINPMIVEGQVHGGLGQGIGQALLEACVYDENGQLLTGSYMDYAMPRANDLPSFTVETAKGTPCTHNPLG
VKGCGEAGAIGSPPALINAIVDALAPLGVKDIQMPATPHRVWQTIQAAKA
>SEQF10205||SEQF10205.1_04445
MKRFDAGETTGTAQETQQTTGQPYVGRPMQRVEDAAILTGRGRYADDLGVKPGTLHAAILRSPHAHAELGLID
FAAALKAPGVRAVLTGADLPAWSKPFVVGVKAPMEQWALAMDRVRYVGEPVAVVVAESRALAEDALDLVRV
DYRVLPPVVSIEAAIADAAPQLHSGLGSNVASDRHFRYGDPEAAFATAPHRVSLTVHYPRNTCTPIECGVVIAEHL

PGNEGYQVTSNFMGPFSLHAVMAMALQVSANHLRHIAPRDSGGSFGVKQAVFPYVVLMCLASRKAGAPVK
WVEDRLEHLSAATSATARLSTIEAAVEADGRIVALDYDQLEDCGGYLRAPEPATFYRMHGVLTGAYAIPNLRVRN
RVVLTNKTPTGLVRGFGGPQVYFALERLVQRISIELNLDPLDVYRRNFVPSNAFPYRAAAGALLDSGNYQLAMS
RALETGAYDELKRRRDIARAEGRLYGIGFAAIVEPSVSNMGYITTATPAEARRKAGPKNGAIASATVSVDLLGGVV
VTIASTPAGQGHMTVCAQVVADVLGIDPAEVIVNVEFDTHKDAWSVAAGNYSSRFAGAVAGTVHLAATRVRD
KLARIVASQLDCDPAELIFAEGRITRRDAPETAVVFARAASNAPHWSPQLLPAGEEPGLRETVFWSPPNLDAPDE
QDRINTSACYGFAFDLCGLEIDRATGRVRIDRYVTAHDAGKLLNPALADGQIRGAFAQGLGAALMEEFRYGPDG
SFQSGTLADYLLPTTCEVPDPMIVHLETPSPFTPLGAKGLGEGNNMTTPPCIANAVADALGVRDIRLPLTPAKV
MAMIGLEDPPPSRLELAETATAAATGGKERSKGAKALSARGTVDLDAPPEAVFAVLLDPQALAQVVPGCHVLEP
IGDNRYRADVTVGVGMIKARYEAEIALSDLEPPHRLRLSGAGLSSLGSARGSGMVELAPHEGGTRLTYDYEAEV
SGKVAAVGGRMLEGAAKVVLRQLFESLGRQAGGKPVRKPGWIARLLALFGARR
>SEQF10205||SEQF10205.1_04090
MNAPAEPNNHLIGASVKRKEDFRFLTGAGQYTDDVVQAHQSYAVFLRSPYAHARIKHINTDAARNHPGVLAVL
TGDDLAADKVNGLPCGWLIHSIDGTPMKEPPHPVLAQGKVRHVGDQVALVVAESVKIAKDAVEMIDVEYDEL
AAVVDTATADTAGTAVHDDVPNNTCYTWGHGDKAATDAAFAKAAHVTHLDIVNNRLIPNAIEPRAVNASYSR
QDDSYTLYVANQNPHVERLLMSAFVLGLTEAKVRVIAPDVGGGFGSKIFLYPEDVALTWASKKVGRPIKWTAER
SESFLTDAHGRDHVTHAELALDAQGNFLAMRVHTTANMGAYLSTFASSVPTILYATLLAGQYKTPAIYAEVKAVF
TNTAPVDAYRGAGRPEATYVVERLVETAARELQIDPAELRRRNFIRTFPYATPVGLTYDTGDYEPCLDRAIELADVK
GFAARRDASRAKGRLRGLGYSCYIEACGLAPSNIAGALGARAGLFEAGEIRVHPTGSVTVFTGSHSHGQGHETT
FAQVLADRLGVPIDNIEIVHGDTGRIPFGMGTYGSRSIAVGGSAIMKALDKIEAKAKKIAAHLLEASAEDIEFKDG
VFRVAGTDRTKTFGEVALTAYVPHNYPLDKLEPGLDENAFYDPTNFTYPAGAYICEVEVDPDTGEVHIDRFVAVD
DFGNIINPMIVEGQVHGGLGQGIGQALLEACVYDENGQLLTGSYMDYAMPRANDLPSFTVETAKGTPCTHNP
LGVKGCGEAGAIGSPPALINAIVDALAPLGVKDIQMPATPHRVWQTIQAAKA
>SEQF10206||SEQF10206.1_02233
MNAPAEPNNHLIGASVKRKEDFRFLTGAGQYTDDVVQAHQSYAVFLRSPYAHARIKHINTDAARNHPGVLAVL
TGDDLAADKVNGLPCGWLIHSIDGTPMKEPPHPVLAQGKVRHVGDQVALVVAESVKIAKDAVEMIDVEYDEL
PAVVDTATADTAGTAVHDDVPNNTCYTWGHGDKAATDAAFAKAAHVTHLDIVNNRLIPNAIEPRAVNASYSRQ
DDSYTLYVANQNPHVERLLMSAFVLGLTEAKVRVIAPDVGGGFGSKIFLYPEDVALTWASKKVGRPIKWTAERSE
SFLTDAHGRDHVTHAELALDAQGNFLAMRVHTTANMGAYLSTFASSVPTILYATLLAGQYKTPAIYAEVKAVFTN
TAPVDAYRGAGRPEATYVVERLVETAARELQVDPAELRRRNFIRTFPYATPVGLTYDTGDYEPCLDRAIELADVKG
FAARRDASRAKGRLRGLGYSCYIEACGLAPSNIAGALGARAGLFEAGEIRVHPTGSVTVFTGSHSHGQGHETTFA
QVVADRLGVPIDNIEIVHGDTGRIPFGMGTYGSRSIAVGGSAIMKALDKIEAKAKKIAAHLLEASAEDIEFKDGVF
RVAGTDRTKTFGEVALTAYVPHNYPLDKLEPGLDENAFYDPTNFTYPAGAYICEVEVDPDTGEVHIDRFVAVDDF
GNIINPMIVEGQVHGGLGQGIGQALLEACVYDENGQLLTGSYMDYAMPRANDLPSFTVETAKGTPCTHNPLG
VKGCGEAGAIGSPPALINAIVDALAPLGVKDIQMPATPHRVWQTIQAAKA
>SEQF10206||SEQF10206.1_00729
MKRFDAGETTGTAQETQQTTGQPYVGRPMQRVEDAAILTGRGRYADDLGVKPGTLHAAILRSPHAHAELGVI
DFAAALKAPGVRAVLTGADLPAWSKPFVVGVKAPMEQWALAMDRVRYVGEPVAVVVAESRALAEDALDLVR
VDYRVLPPVVSIEAAIADAAPQLHSGLGSNVASDRHFRYGDPEAAFATAPHRVSLTAHYPRNTCTPIECGVVIAE
HLPGNEGYQVTSNFMGPFSLHAVMAMALQVSANHLRHIAPRDSGGSFGVKQAVFPYVVLMCLASRKAGAPV
KWVEDRLEHLSASTSATARLSTIEAAVEADGRIVALDYDQLEDCGGYLRAPEPATFYRMHGVLTGAYAIPNLRVR
NRVVLTNKTPTGLVRGFGGPQVYFALERLVQRISIELNLDPLDVYRRNFVPTNAFPYRAAAGALLDSGNYQLAM
SRALETGAYDELKRRRDIARAEGRLYGIGFAAIVEPSVSNMGYITTATPAEARRKAGPKNGAIASATVSVDLLGGV
VVTIASTPAGQGHMTVCAQVVADVLGIDPAEVIVNVEFDTHKDAWSVAAGNYSSRFAGAVAGTVHLAATRVR

DKLARIVASHLDCDPAELIFAEGRITRRDAPETAMVFARAASNAPHWSPQLLPAGEEPGLRETVFWSPPNLDAP
DEQDRINTSACYGFAFDLCGLEIDRATGRVRIDRYVTAHDAGKLLNPALADGQIRGAFAQGLGAALMEEFRYGP
DGSFQSGTLADYLLPTTCEVPDPIIVHLETPSPFTPLGAKGLGEGNNMSTPPCIANAVADALGVRDIRLPLTPAKV
MAMIGLEDPPPSRLELAETATAAATGGKERSKGAKALSARGTVDLDAPPEAVFAVLLDPQALAQVVPGCHVLEP
IGDNRYRADVTVGVGMIKARYEAEIALSDLEPPHRLRLSGAGLSSLGSARGSGMVELAPHEGGTRLTYDYEAEV
SGKVAAVGGRMLEGAAKVVLRQLFESLGRQAGGKPVRKPGWIARLLALFGARR
>SEQF10207||SEQF10207.1_01768
MNAPAEPNNHLIGASVKRKEDFRFLTGAGQYTDDVVQAHQSYAVFLRSPYAHARIKHINTDAARNHPGVLAVL
TGDDLAADKVNGLPCGWLIHSIDGTPMKEPPHPVLAQGKVRHVGDQVALVVAESVKIAKDAVEMIDVEYDEL
AAVVDTATADTAGTAVHDDVPNNTCYTWGHGDKAATDAAFAKAAHVTHLDIVNNRLIPNAIEPRAVNASYSR
QDDSYTLYVANQNPHVERLLMSAFVLGLTEAKVRVIAPDVGGGFGSKIFLYPEDVALTWASKKVGRPIKWTAER
SESFLTDAHGRDHVTHAELALDAQGNFLAMRVHTTANMGAYLSTFASSVPTILYATLLAGQYKTPAIYAEVKAVF
TNTAPVDAYRGAGRPEATYVVERLVETAARELQIDPAELRRRNFIRTFPYATPVGLTYDTGDYEPCLDRAIELADVK
GFAARRDASRAKGRLRGLGYSCYIEACGLAPSNIAGALGARAGLFEAGEIRVHPTGSVTVFTGSHSHGQGHETT
FAQVLADRLGVPIDNIEIVHGDTGRIPFGMGTYGSRSIAVGGSAIMKALDKIEAKAKKIAAHLLEASAEDIEFKDG
VFRVAGTDRTKTFGEVALTAYVPHNYPLDKLEPGLDENAFYDPTNFTYPAGAYICEVEVDPDTGEVHIDRFVAVD
DFGNIINPMIVEGQVHGGLGQGIGQALLEACVYDENGQLLTGSYMDYAMPRANDLPSFTVETAKGTPCTHNP
LGVKGCGEAGAIGSPPALINAIVDALAPLGVKDIQMPATPHRVWQTIQAAKA
>SEQF10207||SEQF10207.1_02546
MKRFDAGETTGTAQETQQTTGQPYVGRPMQRVEDAAILTGRGRYADDLGVKPGTLHAAILRSPHAHAELGLID
FAAALKAPGVRAVLTGADLPAWSKPFVVGVKAPMEQWALAMDRVRYVGEPVAVVVAESRALAEDALDLVRV
DYRVLPPVVSIEAAIADAAPQLHSGLGSNVASDRHFRYGDPEAAFATAPHRVSLTVHYPRNTCTPIECGVVIAEHL
PGNEGYQVTSNFMGPFSLHAVMAMALQVSANHLRHIAPRDSGGSFGVKQAVFPYVVLMCLASRKAGAPVK
WVEDRLEHLSAATSATARLSTIEAAVEADGRIVALDYDQLEDCGGYLRAPEPATFYRMHGVLTGAYAIPNLRVRN
RVVLTNKTPTGLVRGFGGPQVYFALERLVQRISIELNLDPLDVYRRNFVPSNAFPYRAAAGALLDSGNYQLAMS
RALETGAYDELKRRRDIARAEGRLYGIGFAAIVEPSVSNMGYITTATPAEARRKAGPKNGAIASATVSVDLLGGVV
VTIASTPAGQGHMTVCAQVVADVLGIDPAEVIVNVEFDTHKDAWSVAAGNYSSRFAGAVAGTVHLAATRVRD
KLARIVASQLDCDPAELIFAEGRITRRDAPETAVVFARAASNAPHWSPQLLPAGEEPGLRETVFWSPPNLDAPDE
QDRINTSACYGFAFDLCGLEIDRATGRVRIDRYVTAHDAGKLLNPALADGQIRGAFAQGLGAALMEEFRYGPDG
SFQSGTLADYLLPTTCEVPDPMIVHLETPSPFTPLGAKGLGEGNNMTTPPCIANAVADALGVRDIRLPLTPAKV
MAMIGLEDPPPSRLELAETATAAATGGKERSKGAKALSARGTVDLDAPPEAVFAVLLDPQALAQVVPGCHVLEP
IGDNRYRADVTVGVGMIKARYEAEIALSDLEPPHRLRLSGAGLSSLGSARGSGMVELAPHEGGTRLTYDYEAEV
SGKVAAVGGRMLEGAAKVVLRQLFESLGRQAGGKPVRKPGWIARLLALFGARR
>SEQF10208||SEQF10208.1_02068
MKRFDAGETTGTAQETQQTTGQPYVGRPMQRVEDAAILTGRGRYADDLGVKPGTLHAAILRSPHAHAELGVI
DFAAALKAPGVRAVLTGADLPAWSKPFVVGVKAPMEQWALAMDRVRYVGEPVAVVVAESRALAEDALDLVR
VDYRVLPPVVSIEAAIADAAPQLHSGLGSNVASDRHFRYGDPEAAFATAPHRVSLTAHYPRNTCTPIECGVVIAE
HLPGNEGYQVTSNFMGPFSLHAVMAMALQVSANHLRHIAPRDSGGSFGVKQAVFPYVVLMCLASRKAGAPV
KWVEDRLEHLSASTSATARLSTIEAAVEADGRIVALDYDQLEDCGGYLRAPEPATFYRMHGVLTGAYAIPNLRVR
NRVVLTNKTPTGLVRGFGGPQVYFALERLVQRISIELNLDPLDVYRRNFVPTNAFPYRAAAGALLDSGNYQLAM
SRALETGAYDELKRRRDIARAEGRLYGIGFAAIVEPSVSNMGYITTATPAEARRKAGPKNGAIASATVSVDLLGGV
VVTIASTPAGQGHMTVCAQVVADVLGIDPAEVIVNVEFDTHKDAWSVAAGNYSSRFAGAVAGTVHLAATRVR
DKLARIVASHLDCDSAELIFAEGRITRRDAPETAVVFARAASNAPHWSPQLLPAGEEPGLRETVFWSPPNLDAP
DEQDRINTSACYGFAFDLCGLEIDRATGRVRIDRYVTAHDAGKLLNPALADGQIRGAFAQGLGAALMEEFRYGP

DGSFQSGTLADYLLPTTCEVPDPIIVHLETPSPFTPLGAKGLGEGNNMSTPPCIANAVADALGVRDIRLPLTPAKV
MAMIGLEDPPPSRLELAETATAAATGGKERSKGAKALSARGTVDLDAPPEAVFAVLLDPQALAQVVPGCHVLEP
IGDNRYRADVTVGVGMIKARYEAEIALSDLEPPHRLRLSGAGLSSLGSARGSGMVELAPHEGGTRLTYDYEAEV
SGKVAAVGGRMLEGAAKVVLRQLFESLGRQAGGKPVRKPGWIARLLALFGARR
>SEQF10208||SEQF10208.1_02923
MNAPAEPNNHLIGASVKRKEDFRFLTGAGQYTDDVVQAHQSYAVFLRSPYAHARIKHINTDAACNHPGVLAVL
TGDDLAADKVNGLPCGWLIHSIDGTPMKEPPHPVLAQGKVRHVGDQVALVVAESVKIAKDAVEMIDVEYDEL
PAVVDTATADTAGTAVHDDVPNNTCYTWGHGDKAATDAAFAKAAHVTHLDIVNNRLIPNAIEPRAVNASYSRQ
DDSYTLYVANQNPHVERLLMSAFVLGLTEAKVRVIAPDVGGGFGSKIFLYPEDVALTWASKKVGRPIKWTAERSE
SFLTDAHGRDHVTHAELALDAQGNFLAMRVHTTANMGAYLSTFASSVPTILYATLLAGQYKTPAIYAEVKAVFTN
TAPVDAYRGAGRPEATYVVERLVETAARELQVDPAELRRRNFIRTFPYATPVGLTYDTGDYEPCLDRAIELADVKG
FAARRDASRAKGRLRGLGYSCYIEACGLAPSNIAGALGARAGLFEAGEIRVHPTGSVTVFTGSHSHGQGHETTFA
QVVADRLGVPIDNIEIVHGDTGRIPFGMGTYGSRSIAVGGSAIMKALDKIEAKAKKIAAHLLEASAEDIEFKDGVF
RVAGTDRTKTFGEVALTAYVPHNYPLDKLEPGLDENAFYDPTNFTYPAGAYICEVEVDPDTGEVHIDRFVAVDDF
GNIINPMIVEGQVHGGLGQGIGQALLEACVYDENGQLLTGSYMDYAMPRANDLPSFTVETAKGTPCTHNPLG
VKGCGEAGAIGSPPALINAIVDALAPLGVKDIQMPATPHRVWQTIQAAKA
>SEQF10209||SEQF10209.1_04599
MNAPAEPNNHLIGASVKRKEDFRFLTGAGQYTDDVVQAHQSYAVFLRSPYAHARIKHINTDAARNHPGVLAVL
TGDDLAADKVNGLPCGWLIHSIDGTPMKEPPHPVLAQGKVRHVGDQVALVVAESVKIAKDAVEMIDVEYDEL
PAVVDTATADTAGTAVHDDVPNNTCYTWGHGDKAATDAAFAKAAHVTHLDIVNNRLIPNAIEPRAVNASYSRQ
DDSYTLYVANQNPHVERLLMSAFVLGLTEAKVRVIAPDVGGGFGSKIFLYPEDVALTWASKKVGRPIKWTAERSE
SFLTDAHGRDHVTHAELALDAQGNFLAMRVHTTANMGAYLSTFASSVPTILYATLLAGQYKTPAIYAEVKAVFTN
TAPVDAYRGAGRPEATYVVERLVETAARELQVDPAELRRRNFIRTFPYATPVGLTYDTGDYEPCLDRAIELADVKG
FAARRDASRAKGRLRGLGYSCYIEACGLAPSNIAGALGARAGLFEAGEIRVHPTGSVTVFTGSHSHGQGHETTFA
QVVADRLGVPIDNIEIVHGDTGRIPFGMGTYGSRSIAVGGSAIMKALDKIEAKAKKIAAHLLEASAEDIEFKDGVF
RVAGTDRTKTFGEVALTAYVPHNYPLDKLEPGLDENAFYDPTNFTYPAGAYICEVEVDPDTGEVHIDRFVAVDDF
GNIINPMIVEGQVHGGLGQGIGQALLEACVYDENGQLLTGSYMDYAMPRANDLPSFTVETAKGTPCTHNPLG
VKGCGEAGAIGSPPALINAIVDALAPLGVKDIQMPATPHRVWQTIQAAKA
>SEQF10209||SEQF10209.1_03881
MKRFDAGETTGTAQETQQTTGQPYVGRPMQRVEDAAILTGRGRYADDLGVKPGTLHAAILRSPHAHAELGVI
DFAAALKAPGVRAVLTGADLPAWSKPFVVGVKAPMEQWALAMDRVRYVGEPVAVVVAESRALAEDALDLVR
VDYRVLPPVVSIEAAIADAAPQLHSGLGSNVASDRHFRYGDPEAAFATAPHRVSLTAHYPRNTCTPIECGVVIAE
HLPGNEGYQVTSNFMGPFSLHAVMAMALQVSANHLRHIAPRDSGGSFGVKQAVFPYVVLMCLASRKAGAPV
KWVEDRLEHLSASTSATARLSTIEAAVEADGRIVALDYDQLEDCGGYLRAPEPATFYRMHGVLTGAYAIPNLRVR
NRVVLTNKTPTGLVRGFGGPQVYFALERLVQRISIELNLDPLDVYRRNFVPTNAFPYRAAAGALLDSGNYQLAM
SRALETGAYDELKRRRDIARAEGRLYGIGFAAIVEPSVSNMGYITTATPAEARRKAGPKNGAIASATVSVDLLGGV
VVTIASTPAGQGHMTVCAQVVADVLGIDPAEVIVNVEFDTHKDAWSVAAGNYSSRFAGAVAGTVHLAATRVR
DKLARIVASHLDCDPAELIFAEGRITRRDAPETAMVFARAASNAPHWSPQLLPAGEEPGLRETVFWSPPNLDAP
DEQDRINTSACYGFAFDLCGLEIDRATGRVRIDRYVTAHDAGKLLNPALADGQIRGAFAQGLGAALMEEFRYGP
DGSFQSGTLADYLLPTTCEVPDPIIVHLETPSPFTPLGAKGLGEGNNMSTPPCIANAVADALGVRDIRLPLTPAKV
MAMIGLEDPPPSRLELAETATAAATGGKERSKGAKALSARGTVDLDAPPEAVFAVLLDPQALAQVVPGCHVLEP
IGDNRYRADVTVGVGMIKARYEAEIALSDLEPPHRLRLSGAGLSSLGSARGSGMVELAPHEGGTRLTYDYEAEV
SGKVAAVGGRMLEGAAKVVLRQLFESLGRQAGGKPVRKPGWIARLLALFGARR
>SEQF1025||SEQF1025.1_00939

MGIEGVGARVARKEDKRFITGAGRYVDDMVVPGMKHAAFVRSPHAHAQIKKIDVRRAQAMPGVIGVLTGKE
LKADGIGNLICGWMIHSKDGSPMKMGAWSPLAVDKVRYVGDAVVVVVAETKGQARDAAEAVEITYKELKAV
VEATKALEKGAPQIHAEAENNLIFDWEIGDAKATDAAIKAAAHVTRMKIVNNRLVPNAMEPRAALGHYDKAE
DHYTCWTTSQNPHVARLVMSAFYNVAPENKLRVIAPDVGGGFGSKIYIYPEEIVCLWASKKTGVPVKWVADRT
ESFLSDAHGRDHVSTVEMAFDKNNRITGLKVDTIANLGAYMSLFSSCVPTYLYATLLSGQYDIPAIHANVRTVYT
NTAPVDAYRGAGRPEATYLLERTMEAAARELGVSPAELRRKNFITSFPHQTPVIMNYDAGDYGASLDAAMKAS
DYAGFAKRKAAAAKKGLLRGIGMSCYIEACGIAPSAAVGSLGAGVGLWESAEVRVNAVGTIEVLTGSHSHGQG
HETTFAQLVNQRFGVPIDSVSIVHGDTDKVQMGMGTYGSRSGAVGMSAISKALDKVEAKAKKIAAHLLEADEG
DIVIENGALKVAGTDKNVPWFQVALAAYTAHNLPAGMEPGLKETAFYDPSNFTFPAGCYICEVEIEPETGTTEIV
QFVAADDFGNIINPMIVEGQVHGGIAQGIGQALLEGAHYDASGQLLTASYMDYTMPRAGDLPSFKVSTSNTPC
PGNPLGIKGCGEAGAIGSPPAVINAITDAIGIADIAMPASPPTVWAAIRAAKH
>SEQF1025||SEQF1025.1_06216
MTVVTPKFGMGASVLRREDAAFIQGQGRYTDDIQPAGVLHGYVLRSPIAKASFTIGSIEAAKAAPGVHLVLTGG
DLTHLRDLKSGVMQPQPDGTKAPTRDIPILCRDRVNYVGDAVAFVVADSRALAQDAAELIEVDYDGEDAASGT
ATALAEGTPLVWPELGSNRAFTYHMGDKKKTDAAFAGAAHVTRIEFVNNRLVCNYMEPRSAIGEWNVGENRF
VLTTGSQGVHSMQYILASVFKIKKDQLRVITPDVGGGFGPKSFVYREYPLVLEAAKRLGRPVKWAGDRTEHFLTD
AQGRDNAVTAEMALDKDGRFLGMRVDLLANIGAYISQYGPFIPYIGVTMSTGVYDIRALDVSVTGLYTNTCPVD
AYRGAGRPEAAFLLEKLVDACAHDLGLPVEEIRRRNFIRPEQFPYRTQTGRLYDNGEFEGHMDRAIERSQWKAF
PQRLEQSKADGKIRGIGMATYIEACAFPGSEPAFVELNGDGTVTLKIGTQTNGQGHATAYAQFLSEKLNLDIDKI
HVRQGDTDELKAGGGTGGSRSIPLGGVSASRAGEDLANKIKRIAADELEASAGDIELSDGVARIVGTDRSIDFSSI
AKAAKTPDDLKGFGEFVQDECTYPNGTHICEVEIDPDTGATEIVRYTIVDDFGVTVNPILLAGQVHGGVVQGIG
QALTENTIHGEDGQLLTASFMDYAMPRADNFPFFHFETRNVPSTTNALGIKGAGEAGTIGSTPAALNAVTDAL
WRAYGIRHIEMPATPARIWAAIRGASPT
>SEQF1032||SEQF1032.1_00400
MTTIESRPPSPEDLADNAQQPCGHGRMMRKEDPRFIRGRGTYVDDVALPGMLHLAILRSPYAHARIVRIDVTA
AQAHPKVKAVVTGADLAAKGLAWMPTLANDVQAVLATDKTRFQGQEVAFVVAEDRYSARDACELVDVDYEP
RDPVVDARTALDPSAPVIRTDLEGKSDNHIFDWETGDAAATEAVFAKADVVVQQEIVYPRVHPAPMETCGAVA
DLDPVTGKLTLWTTSQAPHAHRTLYALVAGLPEHKIRVISPDIGGGFGNKVPIYPGYVCAIVASLLLDKPVKWME
DRSENLTSTGFARDYIMVGEIAANRDGKILAIRSNVLADHGAFNAQAAPAKYPAGFFGVFTGSYDIEAAYCHMT
AVYTNKAPGGVAYACSFRITEAVYFVERLVDCLAFELKMDPAELRLRNLLRPNQFPYQSKTGWVYDSGDYETTM
RKAMNMIGYEALRAEQKQRRARGELMGIGMSFFTEAVGAGPRKDMDILGLGMADGCELRVHPTGKAVLRLS
VQTQGQGHETTFAQIVAEELGIAPDDIEVVHGDTDQTPFGLGTYGSRSTPVSGGAAALVARKVRDKAKIIASGM
LEVSVADLQWEKGKFHVKGDPSAAVTIADIAMRAHGAGDLPEGIEGGLDAEVCYNPSNLTYPYGAYFCVVDIDP
GTAVVKVRRFLAVDDCGTRINPMIIEGQVHGGIVDGIGMALMEMIAFDEDGNCLGGSLMDYLIPTALEVPHLE
TGHTVTPSPHHPIGAKGIGESATVGSPPAVVNAVVDALAPFGVRHADMPLTPSRVWEAMQGRATPPI
>SEQF1034||SEQF1034.2_00399
MTTIESRPPSPEDLADNAQQPCGHGRMMRKEDPRFIRGRGTYVDDVALPGMLHLAILRSPYAHARIVRIDVTA
AQAHPKVKAVVTGADLAAKGLAWMPTLANDVQAVLATDKTRFQGQEVAFVVAEDRYSARDACELVDVDYEP
RDPVVDARTALDPSAPVIRTDLEGKSDNHIFDWETGDAAATEAVFAKADVVVQQEIVYPRVHPAPMETCGAVA
DLDPVTGKLTLWTTSQAPHAHRTLYALVAGLPEHKIRVISPDIGGGFGNKVPIYPGYVCAIVASLLLDKPVKWME
DRSENLTSTGFARDYIMVGEIAANRDGKILAIRSNVLADHGAFNAQAAPAKYPAGFFGVFTGSYDIEAAYCHMT
AVYTNKAPGGVAYACSFRITEAVYFVERLVDCLAFELKMDPAELRLRNLLRPNQFPYQSKTGWVYDSGDYETTM
RKAMNMIGYEALRAEQKQRRARGELMGIGMSFFTEAVGAGPRKDMDILGLGMADGCELRVHPTGKAVLRLS
VQTQGQGHETTFAQIVAEELGIAPDDIEVVHGDTDQTPFGLGTYGSRSTPVSGGAAALVARKVRDKAKIIASGM

LEVSVADLQWEKGKFHVKGDPSAAVTIADIAMRAHGAGDLPEGIEGGLDAEVCYNPSNLTYPYGAYFCVVDIDP
GTAVVKVRRFLAVDDCGTRINPMIIEGQVHGGIVDGIGMALMEMIAFDEDGNCLGGSLMDYLIPTALEVPHLE
TGHTVTPSPHHPIGAKGIGESATVGSPPAVVNAVVDALAPFGVRHADMPLTPSRVWEAMQGRATPPI
>SEQF1086||SEQF1086.1_00597
MTTEHTGAEVGAGSRQGVGARVPRKEDARHLHGKGNFVADMAMPGLCEVAFLRSPLAHARITDVRVPESVR
DKVVLRSMMGDARDIAADSTLPTYQPSVQPPLASGKVRFVGEPVAMTFAPTRAEAEDHAELVEVDYDDLPVY
ADVAGAQQATSDLVHEHWRDNVFVTLNADRDFDEHAARAEVVVRRKIDLARQCMVPMEGKAVLAYWDHQ
ADQLVVISATQVPHMIRSVLAQCLDLEQGRVRVVSPDVGGAFGYKCVLQQEELCVAWLAKTYKRPFRFIEDRRE
HLTAGANSREHHYEMTAYADKRGKLLALDARITIDGGAYSVWPFTIGLEPDQAVGNLPGPYGFRGYRCETRCVA
TNKPGFVPYRGRTGVCFAIELTMDAVAREVGREPWEVRLENLVQPEQMPYVNVTNKHFDSGDYPASLRKALE
MIDIDGVRTRQARGEADGRRIGVGVATYTEQAAHGTSVFAAWGTPVIPGFDQATVRVTPDGGLEVRVGVHSH
GQGMETTFAQIAHEILGIDVARIKVLHGDTGQTPFSTGTYASRSLVMSGGAVSQACKRLLPRMRHIAAHMLGV
ADDAVTLQDGIYRAGEKSVATGDVADAWYLRPQLLPPDVDPAGLEVNVGYKPKVDTGCFTYASHAAVVAVDP
DTGAVEILDYVVVEDCGTMINPMVVEGQTIGGIAQGIGTAFYEETPYDANGQPLASTLADYMLPGPTEVPNM
RLHHFETPSPHTEFGAKGMGEGGAIAPPAVLFNAVNDALRGLGAAELSRTPLTPIRVLQAIAQGAGEAA
>SEQF1086||SEQF1086.1_00693
MTDATSPGLGRSAPRIEDDALLRGQARFLDDIEVEGVLHACFVRSPHAHARLVSIDLSAARAVPGVAAVYGARD
LFGQLTSWRMPLGFPLAALPDDTTPFVLAEREVAFVGEAIAVVVADSRHIAEDAAARVAIEYEVLGAVVDCRDA
LRPDAPLVRGELASNILQQYTLAYGDCETAFAQAHRVLEDDFWVHRGCAHPMEGRGVLARMDRATDTLTVWS
STQMAHELHYTLALMLGQPEDRLRVVTPDVGGGFGAKFMIYPEEMAIPAAARKLGRPVKWVEDRREHFTTSI
QERDQYWKVAMAIDDQGHVLGIRGNFVHDNGAYTPQGTNVPYNAASSMTGPYVVPAFSLDVSVAYTNKVPV
ATVRGAGYPQAAFVMERMMDRVAAELGIDPGECRRRNLIGPAKIPYTKPLKSRAGMPLTIDSGDFPALQACAL
QASDYDGFAVRRDAALARGRWRGIAVANSVEPTGRGPFEVARVRVQPSGQVSIYTGALAMGQGIKTTLAQIC
AGHLGVPVAAVEVQAGDTAYVGYGMGGFASRQAIMAGSAVDQAAAQVRRQALETAAAVLKAEAETLELADG
EVRAPDGQSVSLARLAMLRKGVPGYALVSPGDPGLDATAYFHCDAQTYAGASHVCEVEVDPATGAIEIVRYVAA
QDSGRIINPQLAEGQVHGGVVHGIGNALFEWMGYDAAGQPLSTTFAEYLLPTAPEVPPIEVVFQPSPTPLNPL
GVKGVGECATIPVAVAVVGAVEHAVAHCGVRVTEFPLTPVRLLELLTQAEARAAAPTDSGEFVDGVH
>SEQF1087||SEQF1087.1_03400
MGVEGIGARVTRKEDKRFLTGKGRYTDDMVTPGMKYAYFVRSPHAHAKITGLDTSAALAMPGVIGVLDGKQL
LADGIGNLICGWMIHSKDGSPMKMGAWRPLAVDTVRYVGDAVAIVVADSLGEARDAAEAVVVDYDELPAVV
EAVEALKPDAPQIHPEAPSNLIFDWEIGDGAATDSAIAAAAHVTEIEIHNNRLSPNAMEPRATLGIYDSAEDHYT
CYTTSQNPHVARLVMSAFYNVAPENKLRVIAPDVGGGFGSKIYIYPEEIVCLWASKKTGVPVKWTADRTEAFLTD
AHGRDHVSKVKMAFDAEHRIIGLKVDTIANLGAYMSLFSSAVPTYLYATLLSGQYAIPAIHANVRTVYTNTVPVD
AYRGAGRPEATYLLERTMETAARELGISPAELRRANFIRSFPYQTQVIMNYDAGDYEASLNAAMQQADWSNFP
ARKAEAAARGMKRGIGMSCYIEACGIAPSQAVGSLGAGVGLWESAEVRVNAVGTIEVLTGSHSHGQGHETTF
AQLVADRLGVPIDSISIVHGDTDKVQMGMGTYGSRSGAVGMSAVVKALDKVEAKAKKIAAHLMEADESDIVIE
NGEVKVAGTDKSLPWFQVALSAYTAHNLPAGMEPGLKETAFYDPANFTFPAGCYICEVEVDPETGKTEIIQFVAA
DDFGNIINPMIVEGQVHGGIAQGIGQALLEGVHYDENGQLLTASFMDYTMPRADDLPSFKLSHQNTPCPSNPL
GIKGCGEAGAIGSPPALINAITDAIGNNTLTMPATPLKVWEATRAVA
>SEQF1096||SEQF1096.1_00397
MTTIESRPPSPEDLADNAQQPCGHGRMMRKEDPRFIRGRGTYVDDVALPGMLHLAILRSPYAHARIVRIDVTA
AQAHPKVKAVVTGADLAAKGLAWMPTLANDVQAVLATDKTRFQGQEVAFVVAEDRYSARDACELVDVDYEP
RDPVVDARTALDPSAPVIRTDLEGKSDNHIFDWETGDAAATEAVFAKADVVVQQEIVYPRVHPAPMETCGAVA
DLDPVTGKLTLWTTSQAPHAHRTLYALVAGLPEHKIRVISPDIGGGFGNKVPIYPGYVCAIVASLLLDKPVKWME

DRSENLTSTGFARDYIMVGEIAANRDGKILAIRSNVLADHGAFNAQAAPAKYPAGFFGVFTGSYDIEAAYCHMT
AVYTNKAPGGVAYACSFRITEAVYFVERLVDCLAFELKMDPAELRLRNLLRPNQFPYQSKTGWVYDSGDYETTM
RKAMNMIGYEALRAEQKQRRARGELMGIGMSFFTEAVGAGPRKDMDILGLGMADGCELRVHPTGKAVLRLS
VQTQGQGHETTFAQIVAEELGIAPDDIEVVHGDTDQTPFGLGTYGSRSTPVSGGAAALVARKVRDKAKIIASGM
LEVSVADLQWEKGKFHVKGDPSAAVTIADIAMRAHGAGDLPEGIEGGLDAEVCYNPSNLTYPYGAYFCVVDIDP
GTAVVKVRRFLAVDDCGTRINPMIIEGQVHGGIVDGIGMALMEMIAFDEDGNCLGGSLMDYLIPTALEVPHLE
TGHTVTPSPHHPIGAKGIGESATVGSPPAVVNAVVDALAPFGVRHADMPLTPSRVWEAMQGRATPPI
>SEQF1129||SEQF1129.1_00401
MTTIESRPPSPEDLADNAQQPCGHGRMMRKEDPRFIRGRGTYVDDVALPGMLHLAILRSPYAHARIVRIDVTA
AQAHPKVKAVVTGADLAAKGLAWMPTLANDVQAVLATDKTRFQGQEVAFVVAEDRYSARDACELVDVDYEP
RDPVVDARTALDPSAPVIRTDLEGKSDNHIFDWETGDAAATEAVFAKADVVVQQEIVYPRVHPAPMETCGAVA
DLDPVTGKLTLWTTSQAPHAHRTLYALVAGLPEHKIRVISPDIGGGFGNKVPIYPGYVCAIVASLLLDKPVKWME
DRSENLTSTGFARDYIMVGEIAANRDGKILAIRSNVLADHGAFNAQAAPAKYPAGFFGVFTGSYDIEAAYCHMT
AVYTNKAPGGVAYACSFRITEAVYFVERLVDCLAFELKMDPAELRLRNLLRPNQFPYQSKTGWVYDSGDYETTM
RKAMNMIGYEALRAEQKQRRARGELMGIGMSFFTEAVGAGPRKDMDILGLGMADGCELRVHPTGKAVLRLS
VQTQGQGHETTFAQIVAEELGIAPDDIEVVHGDTDQTPFGLGTYGSRSTPVSGGAAALVARKVRDKAKIIASGM
LEVSVADLQWEKGKFHVKGDPSAAVTIADIAMRAHGAGDLPEGIEGGLDAEVCYNPSNLTYPYGAYFCVVDIDP
GTAVVKVRRFLAVDDCGTRINPMIIEGQVHGGIVDGIGMALMEMIAFDEDGNCLGGSLMDYLIPTALEVPHLE
TGHTVTPSPHHPIGAKGIGESATVGSPPAVVNAVVDALAPFGVRHADMPLTPSRVWEAMQGRATPPI
>SEQF1130||SEQF1130.1_00399
MTTIESRPPSPEDLADNAQQPCGHGRMMRKEDPRFIRGRGTYVDDVALPGMLHLAILRSPYAHARIVRIDVTA
AQAHPKVKAVVTGADLAAKGLAWMPTLANDVQAVLATDKTRFQGQEVAFVVAEDRYSARDACELVDVDYEP
RDPVVDARTALDPSAPVIRTDLEGKSDNHIFDWETGDAAATEAVFAKADVVVQQEIVYPRVHPAPMETCGAVA
DLDPVTGKLTLWTTSQAPHAHRTLYALVAGLPEHKIRVISPDIGGGFGNKVPIYPGYVCAIVASLLLDKPVKWME
DRSENLTSTGFARDYIMVGEIAANRDGKILAIRSNVLADHGAFNAQAAPAKYPAGFFGVFTGSYDIEAAYCHMT
AVYTNKAPGGVAYACSFRITEAVYFVERLVDCLAFELKMDPAELRLRNLLRPNQFPYQSKTGWVYDSGDYETTM
RKAMNMIGYEALRAEQKQRRARGELMGIGMSFFTEAVGAGPRKDMDILGLGMADGCELRVHPTGKAVLRLS
VQTQGQGHETTFAQIVAEELGIAPDDIEVVHGDTDQTPFGLGTYGSRSTPVSGGAAALVARKVRDKAKIIASGM
LEVSVADLQWEKGKFHVKGDPSAAVTIADIAMRAHGAGDLPEGIEGGLDAEVCYNPSNLTYPYGAYFCVVDIDP
GTAVVKVRRFLAVDDCGTRINPMIIEGQVHGGIVDGIGMALMEMIAFDEDGNCLGGSLMDYLIPTALEVPHLE
TGHTVTPSPHHPIGAKGIGESATVGSPPAVVNAVVDALAPFGVRHADMPLTPSRVWEAMQGRATPPI
>SEQF1132||SEQF1132.2_00400
MTTIESRPPSPEDLADNAQQPCGHGRMMRKEDPRFIRGRGTYVDDVALPGMLHLAILRSPYAHARIVRIDVTA
AQAHPKVKAVVTGADLAAKGLAWMPTLANDVQAVLATDKTRFQGQEVAFVVAEDRYSARDACELVDVDYEP
RDPVVDARTALDPSAPVIRTDLEGKSDNHIFDWETGDAAATEAVFAKADVVVQQEIVYPRVHPAPMETCGAVA
DLDPVTGKLTLWTTSQAPHAHRTLYALVAGLPEHKIRVISPDIGGGFGNKVPIYPGYVCAIVASLLLDKPVKWME
DRSENLTSTGFARDYIMVGEIAANRDGKILAIRSNVLADHGAFNAQAAPAKYPAGFFGVFTGSYDIEAAYCHMT
AVYTNKAPGGVAYACSFRITEAVYFVERLVDCLAFELKMDPAELRLRNLLRPNQFPYQSKTGWVYDSGDYETTM
RKAMNMIGYEALRAEQKQRRARGELMGIGMSFFTEAVGAGPRKDMDILGLGMADGCELRVHPTGKAVLRLS
VQTQGQGHETTFAQIVAEELGIAPDDIEVVHGDTDQTPFGLGTYGSRSTPVSGGAAALVARKVRDKAKIIASGM
LEVSVADLQWEKGKFHVKGDPSAAVTIADIAMRAHGAGDLPEGIEGGLDAEVCYNPSNLTYPYGAYFCVVDIDP
GTAVVKVRRFLAVDDCGTRINPMIIEGQVHGGIVDGIGMALMEMIAFDEDGNCLGGSLMDYLIPTALEVPHLE
TGHTVTPSPHHPIGAKGIGESATVGSPPAVVNAVVDALAPFGVRHADMPLTPSRVWEAMQGRATPPI
>SEQF1395||SEQF1395.1_02390

MTTDSIPTRFGSGQAVRRLEDESLLSGAGRYTDDVALPGQTHLVFLRSPYPHARIVSIDTAAAAAMPGVLRVITG
AELAEAGVKPMPGAVGFKRADGSDCASPPRLALAHGTARFVGEAVAAVVAETVQQARDAAEAVVVDYEALP
MVVDLASATADGAPLLCEEATGNVAAEMRHGSSDAATAAFAKAKHVVALDVVNQRVVALTIEPRSVLAAPDAE
SGRLTIRMSTQMPSGVRDSVCAAIGLAKEKVRVVVGDVGGGFGMKTGAYPEDIAVAFAALQVGRPVKWVAD
RSEEFLSSAHGRDIEARAELALDADGKILALRIRTLANVGAYATGTGVAIQLLIGPWVQTSVYDIQTIDFHFKAVLT
NTAPTGAYRGAGRPEAIFTIERLMDEAARQIGIDRIALRRRNFIRPEQMPYKNPMAQTYDTGKFESVMDQALAL
ADWQGFDARAAESANNGKHRGLGIATFLEWTGGNVFEERVTVSVQADGVIEVFSAVNAMGQGIATSLAQLA
VDAFGVPIEKVRVVLGDTDRGDGFGSAGSRSLFTGGSAVRIGAERTIDKARALAAQEFEAAIDDITYTRGVFTVA
GTDLELDLFALAGKQPEREIFVDSTSTVAGPTWPNGCHVCEIEIDPPTGEISVVAYSSVNDVGRVINPMIVRGQL
EGGAVQGIGQALYEQVVYDNETGQPLTGSLMDYAAPRADIVQAMFHMEMDESTPCANNPLGVKGVGELGTI
GATPAIVNAVADAFARNGLAASAPRLHMPLSPARVWQAMHTVD
>SEQF1395||SEQF1395.1_01609
MGASDFSNLPHIGEALRRKEDYRFLTGAGNYTDDITLANQSHAVFVRSPHAHAVIKSVDTAEALKMPGVVGIFS
GKDIEGKMGGLPCGWLINNPDGTPMKEPMHPILAIHKVRYVGDHVAMVVAETVEQAKNAAEAVVVDYDVLP
AVVSVADAAKKASGVTLHDEAPDNQCYKWALGDKAAVDAAFANAAHVTKLDLVNNRLIPNPIEPRVAIGSYSR
GTDDYTLYVSNQNPHVERLLMTAFVLGLPEHKVRVIAPDVGGGFGSKIFLYAEDVCLTWAAKQLNRNIKWTAER
SECFLSDAHGRDHVSHAEMAMDKDGKFLAMRVHTDANLGAYLSTFSTAVPTILYATLLAGQYTTPQIYVEVDA
WFTNTAPVDAYRGAGRPEATYLLERLVSRCAWEMNLGQDEIRKRNFITTFPYQTPVALQYDTGDFHACMDKA
RVLADVDGYAQRKSASEAAGKLRGIGYSSYIEACGIAPSNIAGALGARAGLFECGEIRVHPTGSVTVFTGSHSHG
QGHETTFAQVVAARLGIPVDNVDVVHGDTGRVPFGMGTYGSRSISVGGAAIMKALDKIETKAKKIAAHLMEAS
DADIEFANGEFTVKGTDKKIPFGQVALTAYVPHNYPLDKLEPGLNETAFYDPTNFTFPGGTYICEVEVDKQTGEV
RVDRFTAVDDFGTIINPMIVEGQVHGGLVQGIGQALLENCVYDNETGQLLTGSFMDYAMPRAGDFPQFKLDT
VCTPCTHNPLGTKGCGEAGAIGSPPAVINAVLDALAPLGVKDFDMPASASRVWEAMQAAASH
>SEQF1395||SEQF1395.1_04316
MNQADIPARVLKREGIGARVPRKEDARHMVGKGNFVGDFVLPGLQEVAFLRSPLAHATITGVEIPEALAGKVFL
REMMLDAADIGSPSSLPTYQYSALPPLASGKVRHVGEAVAMAVAPTRAVAEDLLEEVQVSYDELPVVYHGAESSL
AATGDFLHPGWKDNVFLTLRANRDFDELAAKAEVKVSRTVELSRQCIVPLEGKSVLAYWDFQADQLVVVSATQ
VPHLLRVGIAQHLSMNEEAVRVVSPDVGGAFGYKGQLYPEDLCIAWLAKTYRTPFRYLEDRREHLIVGANTRQH
HYQLTAYADRTGKLLALDAVITIDGGAYSYYPFFVGLEPGQAIGNLPGPYTFRGYRCETLCVATNKPGFMAYRGV
ARTGVCFAIELLMDAVAREVGREPWEVRMDNLVPAAAMPYVNVANKHFDSGDFPASLRRAVEMIGLATVRER
QARGEPDGRRIGFGTATYTEQSAHGTTVFANWGLPVVPGFDQAVVRMTADGGLEVRVGVHSHGQGMETSF
AQIANDVLGIPVARIRVVHGDTALTPFSSGTYASRATVMSGGAISTACKELLPRIQSIAGYLMGVDPQTVALVEGF
AVAGEKSIPLSQVGGAWYLTPQDLPENVHLGGLEVSRAYKPRVDTGTFTYATHAVVVAVDTQTGEVEILDYVVV
EDCGTMVNPMIVEGQTIGGIAQGIGTAMYEESPYDDQGQPLASTLADYILPGATEVPRIRIEHFETPSPHTEFGA
KGVGEGGAIAPPAVIFNAVNDALRGTGAAEVLMTPLTPRRLLKALESAKPEQGLVK
>SEQF1413||SEQF1413.1_02357
MGASDFSKLPHIGEPVKRKEDYRFLTGAGQYTDDIALAAQAHAVFVRSPHAHARVRSVSTDAAKAAPGVIGVLT
GADVAADKINGLPCGWLITSTNGEPMKEPPHPILALDTVRYVGDQVAMVVAETLEQARDAAELVEVDYDPLPA
VVLVADAAAGSVPGAVVHDIAPDNHCYKWAIGDKAAVDAVFAGAAHIAQLDLVNNRLIPNAMEPRAAIGSYS
RANDEYTLYVANQNPHVERLLMTAFVMGLPEHKVRVIAPDVGGGFGSKIFLYAEDVCLTWGARKLNRNIKWTA
DRSESFLTDAHGRDHVSHAEMAMDASGKFLAMRVHTDANLGAYLSTFASAVPTILYATLLAGQYATPQVYVEV
DAWFTHTAPVDAYRGAGRPEATYLLERLVSRCAWQLNLSQAEIRRRNFVTSFPYQTPVALQYDVGDYGACMDK
AEALADVAGFAARRADSEARGLRRGLGYSSYIEACGLAPSNIAGALGARAGLFECGEVRVHPTGSVTVFTGSHS
HGQGHETTFAQVVAARLGIPVENVDIVHGDTGRVPFGMGTYGSRSISVGGAAIMKALDKIEAKAKKIAAHLME

ASDADIDFADGEFTVRGTDKKIPFAQIALTAYVPHNYPLDKLEPGLNETAFYDPTNFTFPAGTYICEVEVDPATGV
VRVDRFSAVDDFGTIINPMIVEGQVHGGVAQGIGQALLENCVYDRETGQLLTGSFMDYAMPRADDFPEFKLG
TVCTPCTHNPLGTKGCGEAGAIGSPPAVINAVLDALHPLGVRDLDMPASPHRVWSAIDAAATS
>SEQF1926||SEQF1926.1_02475
MTTANADTHGRPGKPQGVGARVPRKEDARHLHGKGNFVADMAMPGLSEVAFLRSPLAHARIASVRVAEQIA
DKAFVRQAMADARDIVADSTLPSYQVSAQPPLASGKVRFVGEPIAMVFAPTRAEAEDYAELIEVDYDDLPVYAD
VVSAQAAQGDFVHEQWRDNVFVTLNVDKQFDELAAQADVVVRRKIDLSRQCMVPMEGKAVLAYWDHQA
DQLVVVSATQVPHMIRSVLAQCLDLEQGRVRVVSPDVGGAFGYKCVLQQEELCVAWLAKTFKKPFRFIEDRRE
HLTAGANSREHHYEMVAYADRRGKLLALDAKITIDGGAYSVWPFTIGLEPGQAIGNLPGPYAFKGYRCVTRAVAT
NKPGFVPYRGVARTGVCFAIELTMDAIAREVGREPWEVRQENLVQGEQMPYVNVTGKHLDSGDYPASLQQA
MDMIGVDAIRERQRQGEADGRLIGVGLATYTEQAAHGTSVFAAWGTPVIPGFDQATARVTPDGGLELRVGVH
SHGQGMETTFAQIANEILGVDVASIKLLHGDTGQTPFSTGTYASRSLVMSGGAVSQACKRLLPRLTHIGAHLLQA
DPANVAWNGDRLEAGGKSVSVKDVADAWYLRPQLLPSDVDPSGLEVTVGYKPKVDTGCFTYATHAAVVAVDP
GTGGVEILDYVVVEDCGTMINPMVVEGQTIGGVAQGIGTAFYEETPYDDNGQPLASTLADYMLPGATEVPNM
RLHHFETPSPHTEFGAKGMGEGGAIAPPAVLFNAVNDALRPLGAAELLRTPLSPTRVLAAIAEGSRKTATVAAEV
TA
>SEQF1977||SEQF1977.1_01786
MKRFDAGETTGTAQETQQTTGQPYVGRPMQRVEDAAILTGRGRYADDIGVKPGTLHAAILRSPHAHAELGLID
FAAALKAPGVRAVLTGADLPAWSKPFVVGVKAPMEQWALAMDRVRYVGEPVAVVVAESRALAEDALDLVRV
DYRVLPPVVSIEAAIADAAPQLHSGLGSNVASDRHFRYGDPEAAFATAPHRVSLTVHYPRNTCTPIECGVVIAEHL
PGNEGYQVTSNFMGPFSLHAVMAMALQVSANHLRHIAPRDSGGSFGVKQAVFPYVVLMCLASRKAGAPVK
WVEDRLEHLSAATSATARLSTIEAAVEADGRIVALDYDQLEDCGGYLRAPEPATFYRMHGVLTGAYAIPNLRVRN
RVVLTNKTPTGLVRGFGGPQVYFALERLVQRIAIELNLDPLDVYRRNFVPTNAFPYRAAAGALLDSGDYQLAMS
RALETGAYDELKRRRDVARAEGRLYGIGFAAIVEPSVSNMGYITTATPAEARRKAGPKNGAIASATVSVDLLGGV
VVTIASTPAGQGHMTVCAQVVADVLGIDPAEVIVNVEFDTHKDAWSVAAGNYSSRFAGAVAGTVHLAATRVR
DKLARIVASQLDCDPAELIFAEGRITRRDAPETAVVFARAASNAPHWSPQLLPAGEEPGLRETVFWSPPNLDAP
DEQDRINTSACYGFAFDLCGLEIDRATGRVRIDRYVTAHDAGKLLNPALADGQIRGAFAQGLGAALMEEFRYGP
DGSFQSGTLADYLLPTTCEVPDPIIVHLETPSPFTPLGAKGLGEGNNMSTPPCIANAVADALGVRDIRLPLTPAKV
MAMIGFEDPPPSRLELAETATAAATGGKERTKGAKALSARGTVDLDAPPEAVFAVLLDPQALAQVVPGCHVLEP
IGDNRYRADVTVGVGMIKARYEAEIALSDLEPPHRLRLSGAGLSSLGSARGSGMVELAPHEGGTRLTYDYEAEV
SGKVAAVGGRMLEGAAKVVLRQLFESLGRQAGGKPVRKPGWIARLLALFGARR
>SEQF1977||SEQF1977.1_01446
MNAPAEPNNHLIGASVKRKEDFRFLTGAGQYTDDVVQAHQSYAVFLRSPYAHARIKHINTDAARNHPGVLAVL
TGDDLAADKVNGLPCGWLIHSIDGTPMKEPPHPVLAQGKVRHVGDQVALVVAESVKIAKDAVEMIDVEYDEL
PAVVDTATADTAGTAVHDDVPNNTCYTWGHGDKAATDAAFARAAHVTHLDIVNNRLIPNAIEPRAVNASYSR
QDDSYTLYVANQNPHVERLLMSAFVLGLTEAKVRVIAPDVGGGFGSKIFLYPEDVALTWASKKVGRPIKWTAER
SESFLTDAHGRDHVTHAELALDAQGNFLAMRVHTTANMGAYLSTFASSVPTILYATLLAGQYKTPAIYAEVKAVF
TNTAPVDAYRGAGRPEATYVVERLVETAARELQIDPAELRRRNFIRTFPYATPVGLTYDTGDYEPCLDRAIELADVK
GFAARRDASRAKGRLRGLGYSCYIEACGLAPSNIAGALGARAGLFEAGEIRVHPTGSVTVFTGSHSHGQGHETT
FAQVVADRLGVPIDNIEIVHGDTGRIPFGMGTYGSRSIAVGGSAIMKALDKIEAKAKKIAAHLLEASAEDIEFKDG
VFRVAGTDRTKTFGEVALTAYVPHNYPLDKLEPGLDENAFYDPTNFTYPAGAYICEVEVDPDTGEVHIDRFVAVD
DFGNIINPMIVEGQVHGGLGQGIGQALLEACVYDENGQLLTGSYMDYAMPRANDLPSFTVETAKGTPCTHNP
LGVKGCGEAGAIGSPPALINAIVDALAPLGVKDIQMPATPHRVWQTIQAAKA
>SEQF1981||SEQF1981.1_01121

MGASDFSKLPHIGEAVLRREDDRFLTGAGQYTDDITLGAQAYAVFVRSPHAHARLRSVNVDAAKAAPGVVEVL
TGADVAAAGINGLPCGWLITSTNGEPMKEPPHPILALDTVRYVGDQVAMVVAETLQQARDAAELVEVDYAVLP
AVVNVADAAGGKKPGAVVHDIAPDNRCYQWAIGDKAAVDAVFAGAAHVTRLDLVNNRLIPNAMEPRVAIGS
YNRAMDEYTLYVANQNPHVERLLMTAFVMGLPESKVRVIAPDVGGGFGSKIFLYAEDVCLTWAAKKLNRNIKW
TADRSESFLTDAHGRDHVSHAEMAMDADGKFLAMRVHTDANLGAYLSTFASAVPTILYATLLAGQYTTPQIHIE
VDAWFTNTSPVDAYRGAGRPEATYLLERLVSRCAWEMNLSQAEIRRRNFITSFPYQTPVALQYDIGDYQACMD
QAEQLADVAGFAARRAASEARGLKRGLGYSSYIEACGLAPSNIAGALGARAGLFECGEVRVHPTGSVTVFTGSH
SHGQGHETTFAQVVAARLGIPVEHVEIVHGDTGRVPFGMGTYGSRSISVGGAAIMKALDKIEAKAKKIAAHLM
EASDADIDFAGGEFTVRGTDRKLPFAQIALTAYVPHNYPLDKLEPGLDETAFYDPTNFTFPAGTYICEVEVDPATG
VVRVDRFSAVDDFGTIINPMIVEGQVHGGLAQGIGQALLENCVYDRDSGQLLTGSFMDYAMPRADDLPDFKL
GTVCTPCTHNPLGTKGCGEAGAIGSPPAVINAVLDALHSLGVKDLDMPASPHRVWEAIDAARP
>SEQF2047||SEQF2047.2_03662
MGASDFSKLPHIGEAVLRREDDRFLTGSGQYTDDITLGAQAYAVFVRSPHAHARLRSVNVDAAKAAPGVVGVL
TGADVAAAGINGLPCGWLITSTNGEPMKEPPHPILALDTVRYVGDQVAMVVAETLQQARDAAELVEVDYAVLP
AVVNVADAAGGKKPGAVVHDIAPDNRCYQWAIGDKAAVDAVFAGAAHVTRLDLVNNRLIPNAMEPRVAIGS
YNRAMDEYTLYVANQNPHVERLLMTAFVMGLPESKVRVIAPDVGGGFGSKIFLYAEDVCLTWAAKKLNRNIKW
TADRSESFLTDAHGRDHVSHAEMAMDADGKFLAMRVHTDANLGAYLSTFASAVPTILYATLLAGQYTTPQIHIE
VDAWFTNTSPVDAYRGAGRPEATYLLERLVSRCAWEMNLSQAEIRRRNFITSFPYQTPVALQYDTGDYKACMD
QAEQLADVAGFATRRAASEARGLKRGLGYSSYIEACGLAPSNIAGALGARAGLFECGEVRVHPTGSVTVFTGSHS
HGQGHETTFAQVVAARLGIPVEHVEIVHGDTGRVPFGMGTYGSRSISVGGAAIMKALDKIEAKAKKIAAHLME
ASDADIDFAGGEFTVRGTDRKLPFAQIALTAYVPHNYPLDKLEPGLDETAFYDPTNFTFPAGTYICEVEVDPATGV
VRVDRFSAVDDFGTIINPMIVEGQVHGGLAQGIGQALLENCVYDRDSGQLLTGSFMDYAMPRADDLPDFKLG
TVCTPCTHNPLGTKGCGEAGAIGSPPAVINAVLDALHSLGVKDLDMPASPHRVWEAIDAARP
>SEQF2057||SEQF2057.2_01322
MGASDFSKLPHIGEPLLRREDERFLTGAGQYTDDITLGAQTYAVFVRSPHAHARLRSVNIDAAKAAEGVIGVLTG
ADVAAAGINGLPCGWLITSTNGEPMKEPPHPILALDTVRYVGDQVAMVVAETLQQARDAAELVEVDYEVLPA
VVNVADAAGGKKPGAVVHDIAPDNHCYKWAIGDKAAVDAVFAGAAHVTRLDLVNNRLIPNAMEPRVAIGSY
NRAMDEYTLYVANQNPHVERLLMTAFVMGLPESKVRVIAPDVGGGFGSKIFLYAEDVCLTWAAKKLNRNIKWT
ADRSESFLTDAHGRDHVSHAEMAMDADGKFLAMRVHTDANLGAYLSTFASAVPTILYATLLAGQYTTPQIHIEV
DAWFTNTSPVDAYRGAGRPEATYLLERLVSRCAWEMNLSQAEIRRRNFITSFPYQTPVALQYDIGDYKACMDQ
AEQLADVAGFAARRAASEAKGLRRGLGYSSYIEACGLAPSNIAGALGARAGLFECGEVRVHPTGSVTVFTGSHS
HGQGHETTFAQVVAARLGIPVEHVEIVHGDTGRVPFGMGTYGSRSISVGGAAIMKALDKIEAKAKKIAAHLME
ASDADIDFAGGEFTVRGTDRKLPFAQIALTAYVPHNYPLDKLEPGLDETAFYDPTNFTFPAGTYICEVEVDLATGV
VRVDRFSAVDDFGTIINPMIVEGQVHGGLAQGIGQALLENCVYDRDSGQLLTGSFMDYAMPRADDLPDFKLG
TVCTPCTHNPLGTKGCGEAGAIGSPPAVINAVLDALHSLGVKDLDMPASPHRVWEAIDAARP
>SEQF2058||SEQF2058.1_02294
MNARMHPELRDELGAQGQNHGSPAARAFGKSMPRKEDERLLRGDGKFVDDVQYAHQFEMAVLRCPFPHA
RIRSVDTEAALALPGVRHILTAKSVRDLSDPLTVLRPVPNAPSLPYYALAQDVALYEGQPVVSVVATSRAVAEDAL
ELIDVDYEPLPHVSDALASLEEGAPVLHPGSMKSNLMARQTDAAGDATARMQEAVHIIEDTMRVNRVTPLPM
ETRGIVASWRPGAHELLVYLSTQTPHLVRKQLAESLRLSESCIQVVARDVGGGFGQKLGAFPEDVLACLHAMILR
RPVKWIEDRMEHFRASTHGRESVHRFRLAADEEGNFTGIINDYVTDIGGWNSPFGSAQLASVVFTGPYKVPDA
SVTREVVLTNKTPVGAYRGYGQPEVNFALEVLVDRMARKMGRDPFDLRMQNLLQPQDLPWTTPSGAVYDSG
DYPRSLRMAAEAVSYEAHRMTSRLPRADGRIVGIGFSSFVERTGYASARFLAKRGSQFGAHESVTLRANRSGGV
DVYTGVSSIGQSGETAIAQLCSEALGIDYEHICVHSGDTATSPLNTGAFASRTLIAVAGAVREAAQAFQAKILRLAA

WTLETSPDKLSVDGDVVRFKSGPTQSVPLSQVFTRAIVGQGIPADESPGLEATSHYEPPDAAFAFGAAAAVVAV
DPITGEFQIERFLIVHDCGVVVNPKVVDGQVRGALVQGLGAALGEELRYDEETGQLVSGSMLDYFVPIAADVPP
IDMLHTEVPSPVTPYGVRGVGEVGTIPPGAAIVNAICDALSAQGVEISSLPITPEAVWRAMERAKAQQSGVNA
>SEQF2058||SEQF2058.1_01019
MGASDFSKLPHIGEAVLRREDDRFLTGSGQYTDDITLGAQAYAVFVRSPHAHARLRSVNVDAAKAAPGVVGVL
TGADVAAAGINGLPCGWLITSTNGEPMKEPPHPILALDTVRYVGDQVAMVVAETLQQARDAAELVEVDYAVLP
AVVNVADAAGGKKPGAVVHDIAPDNRCYQWAIGDKAAVDAVFAGAAHVTRLDLVNNRLIPNAMEPRVAIGS
YNRAMDEYTLYVANQNPHVERLLMTAFVMGLPESKVRVIAPDVGGGFGSKIFLYAEDVCLTWAAKKLNRNIKW
TADRSESFLTDAHGRDHVSHAEMAMDADGKFLAMRVHTDANLGAYLSTFASAVPTILYATLLAGQYTTPQIHIE
VDAWFTNTSPVDAYRGAGRPEATYLLERLVSRCAWEMNLSQAEIRRRNFITSFPYQTPVALQYDTGDYKACMD
QAEQLADVAGFATRRAASEARGLKRGLGYSSYIEACGLAPSNIAGALGARAGLFECGEVRVHPTGSVTVFTGSHS
HGQGHETTFAQVVAARLGIPVEHVEIVHGDTGRVPFGMGTYGSRSISVGGAAIMKALDKIEAKAKKIAAHLME
ASDADIDFAGGEFTVRGTDRKLPFAQIALTAYVPHNYPLDKLEPGLDETAFYDPTNFTFPAGTYICEVEVDPATGV
VRVDRFSAVDDFGTIINPMIVEGQVHGGLAQGIGQALLENCVYDRDSGQLLTGSFMDYAMPRADDLPDFKLG
TVCTPCTHNPLGTKGCGEAGAIGSPPAVINAVLDALHSLGVKDLDMPASPHRVWEAIDAARP
>SEQF2172||SEQF2172.1_01371
MNAPAEPNNHLIGASVKRKEDFRFLTGAGQYTDDVVQAHQSYAVFLRSPYAHARIKHINTDAACNHPGVLAVL
TGDDLAADKVNGLPCGWLIHSIDGTPMKEPPHPVLAQGKVRHVGDQVALVVAESVKIAKDAVEMIDVEYDEL
PAVVDTATADTAGTAVHDDVPNNTCYTWGHGDKAATDAAFAKAAHVTHLDIVNNRLIPNAIEPRAVNASYSRQ
DDSYTLYVANQNPHVERLLMSAFVLGLTEAKVRVIAPDVGGGFGSKIFLYPEDVALTWASKKVGRPIKWTAERSE
SFLTDAHGRDHVTHAELALDAQGNFLAMRVHTTANMGAYLSTFASSVPTILYATLLAGQYKTPAIYAEVKAVFTN
TAPVDAYRGAGRPEATYVVERLVETAARELQVDPAELRRRNFIRTFPYATPVGLTYDTGDYEPCLDRAIELADVKG
FAARRDASRAKGRLRGLGYSCYIEACGLAPSNIAGALGARAGLFEAGEIRVHPTGSVTVFTGSHSHGQGHETTFA
QVVADRLGVPIDNIEIVHGDTGRIPFGMGTYGSRSIAVGGSAIMKALDKIEAKAKKIAAHLLEASAEDIEFKDGVF
RVAGTDRTKTFGEVALTAYVPHNYPLDKLEPGLDENAFYDPTNFTYPAGAYICEVEVDPDTGEVHIDRFVAVDDF
GNIINPMIVEGQVHGGLGQGIGQALLEACVYDENGQLLTGSYMDYAMPRANDLPSFTVETAKGTPCTHNPLG
VKGCGEAGAIGSPPALINAIVDALAPLGVKDIQMPATPHRVWQTIQAAKA
>SEQF2172||SEQF2172.1_02070
MKRFDAGETTGTAQETQQTTGQPYVGRPMQRVEDAAILTGRGRYADDLGVKPGTLHAAILRSPHAHAELGVI
DFAAALKAPGVRAVLTGADLPAWSKPFVVGVKAPMEQWALAMDRVRYVGEPVAVVVAESRALAEDALDLVR
VDYRVLPPVVSIEAAIADAAPQLHSGLGSNVASDRHFRYGDPEAAFATAPHRVSLTAHYPRNTCTPIECGVVIAE
HLPGNEGYQVTSNFMGPFSLHAVMAMALQVSANHLRHIAPRDSGGSFGVKQAVFPYVVLMCLASRKAGAPV
KWVEDRLEHLSASTSATARLSTIEAAVEADGRIVALDYDQLEDCGGYLRAPEPATFYRMHGVLTGAYAIPNLRVR
NRVVLTNKTPTGLVRGFGGPQVYFALERLVQRISIELNLDPLDVYRRNFVPTNAFPYRAAAGALLDSGNYQLAM
SRALETGAYDELKRRRDIARAEGRLYGIGFAAIVEPSVSNMGYITTATPAEARRKAGPKNGAIASATVSVDLLGGV
VVTIASTPAGQGHMTVCAQVVADVLGIDPAEVIVNVEFDTHKDAWSVAAGNYSSRFAGAVAGTVHLAATRVR
DKLARIVASHLDCDSAELIFAEGRITRRDAPETAVVFARAASNAPHWSPQLLPAGEEPGLRETVFWSPPNLDAP
DEQDRINTSACYGFAFDLCGLEIDRATGRVRIDRYVTAHDAGKLLNPALADGQIRGAFAQGLGAALMEEFRYGP
DGSFQSGTLADYLLPTTCEVPDPIIVHLETPSPFTPLGAKGLGEGNNMSTPPCIANAVADALGVRDIRLPLTPAKV
MAMIGLEDPPPSRLELAETATAAATGGKERSKGAKALSARGTVDLDAPPEAVFAVLLDPQALAQVVPGCHVLEP
IGDNRYRADVTVGVGMIKARYEAEIALSDLEPPHRLRLSGAGLSSLGSARGSGMVELAPHEGGTRLTYDYEAEV
SGKVAAVGGRMLEGAAKVVLRQLFESLGRQAGGKPVRKPGWIARLLALFGARR
>SEQF2183||SEQF2183.1_02594
MTTELNPTRFGSGQAVRRLEDESLLAGAGRYTDDVTLPDQAHLVFLRSPYPHARIVSIDTSTAAAMPGVLRVIT

GAELAAAGVKPMPGAAGFKRADGSDSASPPRYAMANERTRFVGEAVAAVIAETVQQARDAAEAVMVDYEDL
PMVVDLASATADGAPQLCEEATGNIAAEMRHGNSEAATAAFAKASHVVALDVINQRVVALTIEPRSVLAVHDA
KTDRLTIRMSTQMPSGVRDSVCAAIGLAKEKVRVVVGDVGGGFGMKTGAYPEDIAVAFAALQVKRPVKWVAD
RSEEFLSSAHGRDIEARAELALDAEGKILALRIKTLANVGAYATGTGVAIQLLIGPWVQTSVYDIQTIDFHFKAVLT
NTAPTGAYRGAGRPEAIFTIERLMDEAARQTGIDRIILRRMNFIQPDQMPYKNPMAQVYDTGNFESVMDQAL
TLADWQGFEARAAESAKNGKHRGLGIATFLEWTGGNVFEERVTVSVQADGVIEVFSAVNAMGQGIATSLAQL
AVDAFGVPIEKVRVVLGDTDRGDGFGSAGSRSLFTGGSAVRIGAERTIDKARELAAQEFEAAIDDIIYSRGSFSVA
GTDLELDLFTLAGKQPEREIFVDSTSTVAGPTWPNGCHICEIELDPPTGEISVVAYSSVNDVGRVINPMIVRGQLE
GGAVQGIGQALYEQVVYDQETGQPVTGSLMDYVAPRADIVDTMFHMEMDESTPCTNNPLGVKGVGELGTIG
ATPAIVNAVADAFARNGLAATAPRLHMPLSPSRVWAAMHTVD
>SEQF2183||SEQF2183.1_01778
MGASDFSNLPHIGEALRRKEDYRFLTGAGNYTDDITLANQSHAVFVRSPHAHAVIKSIDIAEASKMPGVVGIFSG
KDIEGKMGGLPCGWLINNPDGTPMKEPMHPILAIKKVRYVGDHVAMVVAETVEQAKNAAEAVVVDYEVLPA
LVSVADAAKKTGSTTLHDEAPDNQCYKWVLGDKAAVDNVFTTAAHITKLDLVNNRLIPNPIEPRVAIGSYSRGT
DDYTLYVSNQNPHVERLLMTAFVLGLPEHKVRVIAPDVGGGFGSKIFLYAEDVCLTWAAKQLNRNIKWTAERSE
CFLSDAHGRDHVSHAEMAMDKDGKFLAMRVHTDANLGAYLSTFSTAVPTILYATLLAGQYTTPQIYVEVDAWF
TNTAPVDAYRGAGRPEATYLLERLVSRCAWEMNLGQDEIRKRNFITTFPYQTPVALQYDTGDFHACMDKARVL
ADVEGYAQRKSATEAKGKLRGIGYSSYIEACGIAPSNIAGALGARAGLFECGEIRVHPTGSVTVFTGSHSHGQGH
ETTFAQVVAARLGIPVENVDVVHGDTGRVPFGMGTYGSRSISVGGAAIMKALDKIETKAKKIAAHLMEASDADI
EFANGEFTVKGTDKKIPFGQVALTAYVPHNYPLDKLEPGLNETAFYDPTNFTFPGGTYICEVEIDKQTGEVKVDRF
TAVDDFGTIINPMIVEGQVHGGLVQGIGQALLENCVYDNETGQLLTGSFMDYAMPRAGDFPQFKLDTVCTPCT
HNPLGTKGCGEAGAIGSPPAVINAVLDALAPLGVKDFDMPASSSRVWEAMQKGGTPQDAQAPSLAASTQGR
PAP
>SEQF2266||SEQF2266.1_00621
MTTEHTGAEVGAGSRQGVGARVPRKEDARHLHGKGNFVADMAMPGLCEVAFLRSPLAHARITDVRVPESVR
DKVVLRSMMGDARDIAADSTLPTYQPSVQPPLASGKVRFVGEPVAMTFAPTRAEAEDHAELVEVDYDDLPVY
ADVAGAQQATSDLVHEHWRDNVFVTLNADRDFDEHAARAEVVVRRKIDLARQCMVPMEGKAVLAYWDHQ
ADQLVVISATQVPHMIRSVLAQCLDLEQGRVRVVSPDVGGAFGYKCVLQQEELCVAWLAKTYKRPFRFIEDRRE
HLTAGANSREHHYEMTAYADKRGKLLALDARITIDGGAYSVWPFTIGLEPDQAVGNLPGPYGFRGYRCETRCVA
TNKPGFVPYRGRTGVCFAIELTMDAVAREVGREPWEVRLENLVQPEQMPYVNVTNKHFDSGDYPASLRKALE
MIDIDGVRTRQARGEADGRRIGVGVATYTEQAAHGTSVFAAWGTPVIPGFDQATVRVTPDGGLEVRVGVHSH
GQGMETTFAQIAHEILGIDVARIKVLHGDTGQTPFSTGTYASRSLVMSGGAVSQACKRLLPRMRHIAAHMLGV
ADDAVTLQDGIYRAGEKSVATGDVADAWYLRPQLLPPDVDPAGLEVNVGYKPKVDTGCFTYASHAAVVAVDP
DTGAVEILDYVVVEDCGTMINPMVVEGQTIGGIAQGIGTAFYEETPYDANGQPLASTLADYMLPGPTEVPNM
RLHHFETPSPHTEFGAKGMGEGGAIAPPAVLFNAVNDALRGLGAAELSRTPLTPIRVLQAIAQGAGEAA
>SEQF2266||SEQF2266.1_00717
MTDATSPGLGRSAPRIEDDALLRGQARFLDDIEVEGVLHACFVRSPHAHARLVSIDLSAARAVPGVAAVYGARD
LFGQLTSWRMPLGFPLAALPDDTTPFVLAEREVAFVGEAIAVVVADSRHIAEDAAARVAIEYEVLGAVVDCRDA
LRPDAPLVRGELASNILQQYTLAYGDCETAFAQAHRVLEDDFWVHRGCAHPMEGRGVLARMDRATDTLTVWS
STQMAHELHYTLALMLGQPEDRLRVVTPDVGGGFGAKFMIYPEEMAIPAAARKLGRPVKWVEDRREHFTTSI
QERDQYWKVAMAIDDQGHVLGIRGNFVHDNGAYTPQGTNVPYNAASSMTGPYVVPAFSLDVSVAYTNKVPV
ATVRGAGYPQAAFVMERMMDRVAAELGIDPGECRRRNLIGPAKIPYTKPLKSRAGMPLTIDSGDFPALQACAL
QASDYDGFAVRRDAALARGRWRGIAVANSVEPTGRGPFEVARVRVQPSGQVSIYTGALAMGQGIKTTLAQIC
AGHLGVPVAAVEVQAGDTAYVGYGMGGFASRQAIMAGSAVDQAAAQVRRQALETAAAVLKAEAETLELADG

EVRAPDGQSVSLARLAMLRKGVPGYALVSPGDPGLDATAYFHCDAQTYAGASHVCEVEVDPATGAIEIVRYVAA
QDSGRIINPQLAEGQVHGGVVHGIGNALFEWMGYDAAGQPLSTTFAEYLLPTAPEVPPIEVVFQPSPTPLNPL
GVKGVGECATIPVAVAVVGAVEHAVAHCGVRVTEFPLTPVRLLELLTQAEARAAAPTDSGEFVDGVH
>SEQF2283||SEQF2283.1_00400
MTTIESRPPSPEDLADNAQQPCGHGRMMRKEDPRFIRGRGTYVDDVALPGMLHLAILRSPYAHARIVRIDVTA
AQAHPKVKAVVTGADLAAKGLAWMPTLANDVQAVLATDKTRFQGQEVAFVVAEDRYSARDACELVDVDYEP
RDPVVDARTALDPSAPVIRTDLEGKSDNHIFDWETGDAAATEAVFAKADVVVQQEIVYPRVHPAPMETCGAVA
DLDPVTGKLTLWTTSQAPHAHRTLYALVAGLPEHKIRVISPDIGGGFGNKVPIYPGYVCAIVASLLLDKPVKWME
DRSENLTSTGFARDYIMVGEIAANRDGKILAIRSNVLADHGAFNAQAAPAKYPAGFFGVFTGSYDIEAAYCHMT
AVYTNKAPGGVAYACSFRITEAVYFVERLVDCLAFELKMDPAELRLRNLLRPNQFPYQSKTGWVYDSGDYETTM
RKAMNMIGYEALRAEQKQRRARGELMGIGMSFFTEAVGAGPRKDMDILGLGMADGCELRVHPTGKAVLRLS
VQTQGQGHETTFAQIVAEELGIAPDDIEVVHGDTDQTPFGLGTYGSRSTPVSGGAAALVARKVRDKAKIIASGM
LEVSVADLQWEKGKFHVKGDPSAAVTIADIAMRAHGAGDLPEGIEGGLDAEVCYNPSNLTYPYGAYFCVVDIDP
GTAVVKVRRFLAVDDCGTRINPMIIEGQVHGGIVDGIGMALMEMIAFDEDGNCLGGSLMDYLIPTALEVPHLE
TGHTVTPSPHHPIGAKGIGESATVGSPPAVVNAVVDALAPFGVRHADMPLTPSRVWEAMQGRATPPI
>SEQF2299||SEQF2299.1_00405
MTTIESRPPSPEDLADNAQQPCGHGRMMRKEDPRFIRGRGTYVDDVALPGMLHLAILRSPYAHARIVRIDVTA
AQAHPKVKAVVTGADLAAKGLAWMPTLANDVQAVLATDKTRFQGQEVAFVVAEDRYSARDACELVDVDYEP
RDPVVDARTALDPSAPVIRTDLEGKSDNHIFDWETGDAAATEAVFAKADVVVQQEIVYPRVHPAPMETCGAVA
DLDPVTGKLTLWTTSQAPHAHRTLYALVAGLPEHKIRVISPDIGGGFGNKVPIYPGYVCAIVASLLLDKPVKWME
DRSENLTSTGFARDYIMVGEIAANRDGKILAIRSNVLADHGAFNAQAAPAKYPAGFFGVFTGSYDIEAAYCHMT
AVYTNKAPGGVAYACSFRITEAVYFVERLVDCLAFELKMDPAELRLRNLLRPNQFPYQSKTGWVYDSGDYETTM
RKAMNMIGYEALRAEQKQRRARGELMGIGMSFFTEAVGAGPRKDMDILGLGMADGCELRVHPTGKAVLRLS
VQTQGQGHETTFAQIVAEELGIAPDDIEVVHGDTDQTPFGLGTYGSRSTPVSGGAAALVARKVRDKAKIIASGM
LEVSVADLQWEKGKFHVKGDPSAAVTIADIAMRAHGAGDLPEGIEGGLDAEVCYNPSNLTYPYGAYFCVVDIDP
GTAVVKVRRFLAVDDCGTRINPMIIEGQVHGGIVDGIGMALMEMIAFDEDGNCLGGSLMDYLIPTALEVPHLE
TGHTVTPSPHHPIGAKGIGESATVGSPPAVVNAVVDALAPFGVRHADMPLTPSRVWEAMQGRATPPI
>SEQF2325||SEQF2325.2_06089
MAAPIKFGVGQSVRRKEDDALIRGKGRYTDDVAPSPALHALMLRSPHAHATYTIDAGKARGMPGVALILTAAD
VAELGGLPCLFNLETDPFTAPPYPILAKDEVRHVGDAVAFVVADTVDHARDAIEAIDVKWTPLPAAVGLVNAVK
KDAPQVWPDKPGNVLFDVSIGDKKAAEDAFAKAHAVAEITIVNPRVITNFMETRAAVAEYDAKKDHLTLTIGSQ
GSHRLREILCDMILKMPKENMRVICPDVGGGFGTKLFPYREYALISVAARKLKKSIKWTAERSDHFMGDAQGR
DNLTTAKMALAEDGKFLGMDVDLMGDMGAYLSTFAPYIPHGGAGMLPGLYDIQAFHCRVRTVFTNTVPVDA
YRGAGRPEAAYVIERLVDAAARKLGMTPDSIRRKNFIPPKSLPYTTATGKVYDSGDFVAHMKRAMEIANWKEFP
KRAKAARKDGLVRGIGMASYVEVCGTMGEETANVALDANGDISVLIGTQSSGQGHQTAYAQIVAEQFGVPPER
VHVLQGDTDKIATGLGTGGSASIPSGGVSVQRATHDLGNKLKELAAQALEAGAGDLEIADGRIRIAGTDRSISFA
DLAKRPGGDTSKMNASATFASADGTYPNGTHLAEVEIDPATGIIKIVSYVIVDDFGVTLNPLMLAGQVHGGAM
QGIGQALMEQAVYSPTDGQLVTGTFMDYALPRASDGPSFVFETHNVPCTTNPMGVKGAGEAGAIGSCPAVV
NAIVEGLHREYKIDHIDMPATPERVWIAIREAQRRHNL
>SEQF2325||SEQF2325.2_03923
MTVTRAPADTLSVLDRPNSYIGKTVPRPNLDRLLQGRGQYVSDLELPRMAHVVFLRSPHAHAKIAAIDADAATR
MPGVISIVTGRELEAVITPWVGVLSHLKGLKSAPQHAIAVDRVCWQGEAVAAIVATSRAAAEDAMEHISVDYEE
LEAVTDMRAALDPAASVIHAALGDNLAFERTLDAGDVDLALSGSELVEADFVFGRHTGVTLEPRAVVADWNPA
EARLTIYQGTQAPHMVQNIAALHLGLREAQVRVVCKDVGGSFGIKVHIYADEMATYALSKLLRRPVKFVADRVE

SFSTDIHARDHRCRGRIGVRPDGTITAFEIDDLTGIGPYSMYPRTSAIEANQVVNLVGGPYATANYRARARVVFQ
NKNVMCQYRAVGHPIACSVTEGLVDLAAAKIGMDPVEIRRRNLIADDAYPCASPSGMKFERLSHHASLTKLLQ
MMDYDALRAEQAALRARNVLRGIGIASFIEVTNPSAAFYGVGGAKISSQDGVAVRLDAQGSVICQTSITEQGQ
GSESLTAQIVGSVLGVSMERVRVTLGDTDNTPYGGGTWASRGAGIGGEAALQAAKALRENILNVAAAILQSTPA
ELDIVDNGIVNAADGAPRIELHELARIVYFRPDTLPPGIQPELMATRHFVPRQYPFAFTNGVQASWLEVDTETG
FVQLLKHWVVEDCGTIINPQLVDEQIRGGVVQGLGAALFEKCIYDERGQLTNANMADYLVPMSGEMPDIEIGH
VVSPTQESELGAKGAGEAGTAGAAAAVANAVNDALRPFGATITEIPLTPQVILTALGRI
>SEQF2325||SEQF2325.2_03307
MGVEGIGARVVRKEDRRFITGQGRYVDDVKMVGMSYAHFIRSPHAHAKVKGIDSAEAMKMPGVIAVLTGQQ
IVDDKVGNLICGWAITSKDGSPMKMGAWPAMAPETVRFVGQAVAVVIAETKNQARDAAEAVVVDYEELPAA
ADIKAAIKPGAPQLHPEAPGNIVYDWHLGDEAAVKDAFSKAANVVTLDLTNNRLVPNAMEPRAAVAHYDQAE
EHYTLYTTSQNPHVARLVLSAFYNIAPEHKLRVIAPDVGGGFGSKIYIYPEEMVALWASKKVGRPVKWTGDRSEA
FLTDAHGRDHVSKAEMAFDKDNKILGLRVKTHANFGAYMSLFSSAVPTYLYATLLSGQYVIPAIYAEVIGVYTNTT
PVDAYRGAGRPEASYLIERMMETAARQLKVDPTELRKKNFITQFPYQTPVIMAYDIGDFHASIDAAMKAIDYAG
FPARKAKAKADGKLRGIGISCYIEACGIAPSKAVGSLGAGVGLWESAEIRVNPVGTIEVLTGSHSHGQGHETTFC
QIIAERLGVPISQVSIVHGDTDKVQFGMGTYGSRSLAVGGTAIVKAMEKVEAKAKKIAAHALEASESDIVIENGE
FKVTGTDKSIALPMVALAAYTAHNLPDGMEPGLKETAFYDPTNFTFPAGAYICELEVDPGTGKTSFVNFVAADD
FGRLINPMIVEGQVHGGLAQGIGQALLEGAIYDDTGQLVTASFMDYTMPRADDLPSFQLSHTTTLCPGNPLGV
KGCGEAGAIGASAAVINAITDAISNNKLEMPATPDRVWHAIHG
>SEQF2325||SEQF2325.2_04889
MKASNSYIGRPMERVEDLRLLRGRGTYVADVNRPNQLYAVILRSSTAHGLIKSIDSSAALALPGVHRVLTGKDLG
DNVPRIPLRLQPLPQLETFHQPVIADGKVRYVGEPVAVVIADSAAMAEDALEHIILDIEPLPAVANREHAEAKRS
VLFEDHGSNVAITWKAFRGDADDAFKNADYIRRETFKVQRHAAMFMEPRGFVAEWDAVAGKLTVWGAAKT
AWHNRRTLAAQLDLPIEAVDLIEVDVGGGFGSRGEFYPEDYLIPAAARIAGRPVKWTEDRREHLMSANHARD
MECDVEFATTRDGRFIGLRGQIWSDIGAYVRTNGSVGPRNIAQYMCGPYCMEHVDLKSSMLTTNKTPCGTYR
GPGRYETDFVRERMIDLAAQDLKIDRIELRRRNLVADAQMPYPLPSITPYDSSTELDSGDYHTVFDRCLKEFDWE
EKKKLSGKLIDGYYHGIAIGSFIEGGAAGPKEEVRLVLETDGALSVYMGSSSVGQGLETIMAQIAADAMEMPYH
KITILHGSTAYVKDGYGAYHSRSTVMGGSAILLGAEKLKELIRKTAAERLGCKPEEVIVDSERASFDGNQLSFADLS
EAPLEVEAEFFNKKYTWAYGTQAAHVAVDPGTGHVKVIEVMSVEDVGRMINPLTLHGQAIGSMVQGLGGAFL
EHLVYDDEGQLLTGSFADYLLPTASDFPKLDSVTLELRPCPNNPLGAKGAGEGGLIPVGGLMANAVADALSHLN
VQPMQLPLSPPRIWQLVEEAEASHAKT
>SEQF2411||SEQF2411.2_01509
MLDHPSRVSSDNDIALQKFGVGQPVRRKEDDTLVRGKGTYTDDISLPGQAYAWIVRSTHAHGIIKGIDVEAARA
MPGVLGVWTGKDLAAAGYNPFTVGLPMKNRDGSPLLQTNRLPLMTDKVRFVGDPVAFVVAETLAQARDAG
EAVVVDIEPLPSVTSAEDAAKPGAPLLYDHIPSNVVLDYHAGDDAALDAAFARAAHVTRLDIVNTRLAVVAMEP
RAALASYDKKSQRFTIEIPTQGVAGNRTSLAKTLNVPNDKVHLRTHHVGGSFGMKNANYPEYICILHAARELGR
AVKWTDERSTSFLSDSHGRAQDVHCELALAADGAFLGVRVRGFGNLGAYITGVAPLPLSLNIAKNINSVYRMPL
IGVDIKCVLTNTTLMGAYRGAGRPEANYFMERLIDRAAEEMGIDRLAMRKRNFIKPAQIPYAAANGLTYDSGDF
PGVFSKAVEQADVAGFAKRRKESRKRGKLRGIAVGSYLEITAPPSAELGKIVFEADGTVRLITGTLDYGQGHATPF
AQVLAAQLGVPFDAIKLTQGDSDIVHTGNGTGGSRSITASGAAIVASSRLVIEKGKRAAAHVLEASEGDMEFAN
GRFTIAGTDRGIGIMELAAKLRNSKMPEGVPSSLDVDHTVQGVPSTFPNGCHVAEVEVDPDTGNVQIVRYSGV
NDFGTVVNPMIVAGQIHGGVAQGIGQALMECVTYDDNGQPVTGSFMDYALPRAGDIPSMTLGSHPSPATSN
PLGTKGCGEAGCAGSLATIVNAVLDALSDEGVTSIDMPLTSEKVWRAIQDGRKAKKSA
>SEQF2411||SEQF2411.2_00841

MTEAAAIRYMGQPLRRREDFKFITGKGRYTDDMKSPGMLHMAILRSPHAHAVIKHVDLSTAQSAPGVHLVLS
GADLVGKMGSIEPNWVIPGTKVPDRPVVAVDRVRFVGECVAVVVAETQALAHDAVGLIEVDYETLPAVIDEEA
AIREGAPQLHDNVPKNITTLYKIGGGDYKKAASEADHVIKLRVINNRLIPTCMETRSILAEPNVDGTLTVNIQSQV
PHMHRRWIADTVRIPEHQLRIVAPDIGGGFGAKMHLYPEELLCPYLARQLGVPVKWWESRSESHQSTNHGRA
HTETIEVAFRNDGKILGLRVETLGNVGAYLSNMASGGPTVNTVNFGTGTYKIDNYEAFSRVVVTNTVPVDAYRG
YGRPEGGYIAERAIDAVARHLKLDQVEVRKRNFIQRADFPHRPYNGPAVIYDSGNYQGLLAKALEVFKYDERIAE
RDQLRSQGRYRGIGVAAYTHMCGMAPSRRLSLMGFNRGGWESARVSIDSSGRATIFSGSMSQGHGHNTSLA
QIAADVLQIPIESIDIVQGDTRQVQAGHGTFNSRSMAVGGSSVHVTSQRIVAKARKIAASMLEVDEKDVSYRAG
EFSVPGTDIAPLSFGKIARMAYVGHKLPDGMEPGLDETTFYDPAGMGSPSGIHMAYIEVDPETGMVDILDYVA
VDDVGTIINPLLAAGQIHGGVVQGIAQALYEEVSYDPDTGQLMTGSLLDYAVPRAEHVPNIRSSFQETPSPTNPI
GVKGVGESGSIAAPPCMVHAVLDALSPFEILHLDMPMTPPRIWSAVQQARAGVTQ
>SEQF2411||SEQF2411.2_03184
MNDSSMKQRGLSVLDRPNSYIGKTVPRPNLGRLMQGRGQYVSDLTLPRMAHVVFLRSPYAHARILKIDATEAK
AMPGVIAVVTGVELAKVISPWVGVLSHLKGLKSAPQHAIAVDRACWQGEAVAAIIATSRALAEDAAELVSVDYE
ELVPVTDMRTALDPATPVIHPDLGDNLAFERNLDAGAVDQAFRESDEVVEAEFVFGRHTGVTLEPRAVLADWN
SGDERLTVYLGTQAPHMVQNIAAKHLGLDEPQVRVVCKDVGGSFGIKVHIYADEMATLALSKLLRRPIKFVADR
VESFNTDIHARDHVCKARIGVSKNGTINAFEIDDVTGIGPYSMYPRTSAIEANQVVNLVGGPYTTQNYRARTRV
VFQNKNVTCQYRGVGHPIACSITEGLVDLAAQRIGMDPFEIRRRNLIRDDAYPCGSPSGLKFEALSHHASLDKLY
AMMNYGALRAEQAALRKQGIYRGIGIASFIEVTNPSAAFYGVGGARISSQDGVAVRLDATGRVICQTSITEQGQ
GSESLTAQIVGSVLGVSMERVRVILGDTDNTPYGGGTWASRGAGIGGEAALQAAKVLRNNILDVAAAILQAKPA
DLDIVDNAVVDVHGGQQRIELHELARIVYFRPDTLPPGFQPELMATRHFVPREYPFAFTNGIQASWLEVDTRTG
FIKLLKHWVVEDCGTIINPQLVDEQIRGGVVQGLGAALYEHCIYDERGQLTNANMADYLVPMSGEMPDIDVGH
IVSPTLDGELGAKGAGEAGTAAAAAVVANAVNDALSPFNVTVTEIPLTPRVILTALGRI
>SEQF2411||SEQF2411.2_00830
MHNVGDDLATRPKMVGARVRRTEDPRLLTGNGNYVDDRQSAGMLHVAFRRSDHSHARIVSINCEAARRSPG
VIGIFTGADLEGRIKPLIATSRMANYHATPLVALAQGKVRYVGEPVVAVVAESRYLAEDAIELIEIDFEALPVVIDPE
AAVQPDSPLLHKEIGTNVLLSREFKRGDVDAEMENAVVRVSGRFRMRRKTPLAMENRSYVAEYSAGQDALTLY
SATQIPGIIRDALSEALDMPGNRLRVIAPDVGGGFGGKASLYPEELFVTFAARHLGRAVKWTSDRLEDLASTSQG
FDEIIDAELGFDKAGFAVALRADVIGDVGAYSIYPWTAVLEPVQVVSFLPGPYKIQHYRGRVRGVATSKPPMGPY
RGVGRPASTFVTERLMDMAARKFNVDPKLLRLKNMIQPDEFPHKIGSGIVWDQCSFTECLEAACERIGYATLRE
RQAQARAEGRWFGIGMACYAELTGIGSRISVAPGMPINTGTETAKILIDSTGAVTVAVGTAAHGQGHETTFAQV
VAEHLGARLQDIRVIHGDSAAVPNATGSYASRSAVLAGGAATLAAQDVKEKVLRAASHLLEASVADLTIEDGRIA
VAGTDRAMTFRELARAVYSEMGRIPSDQREELSSTKTYDPVFGTAATAAHVAVLEIDPETYQVKLERFLVAEDCG
KLINPMIVDGQVHGGVAQGIGAALYEEVIYNDEGQNVTASLVDYVVPSASEVPSIDVVHIEAASKTTLGGFRGM
GEGGTIGAPAAVANAISDALASLNIDVFELPVTPERLFRLIENAKAHS
>SEQF2411||SEQF2411.2_00703
MDFQGVGASLLRKEDDRFLRGRGQYVGDLKLPGLKDVAFVRSPLAHAKIRGVRIPPEFQDRVFTASDLTDVKPI
VAASGLPGFKHSEQPLLAHEKVRQVGELVAMCIGGTRAEAEDIAGAVELDLDELPVVSEMLTARRPGAPLVHD
HWGDNVFLETYIDVNMEAAYDAPIKVTREIRTARQCMAPLEGRATVAYWDKRLDQLVLYTGTQQPHIIRTGISE
CLGLDQSKVRVISPDVGGGFGYKALMPPEDVCLAWLAMHCGHPVRWIEDRREHLTASANCREHHYVITAYAD
RDGTLRGIECEATVDSGAYSSYPFSACLEAAQIASILPGPYDFPSYRCRTWSVATNKCPILPYRGVARTGVCYAME
LMVDAVAREAGLDPLEVRLKNLVKPEQMPFDNITKKHFDSGDYPEALKRAMARIDLDAIRKQQENTHRLIGIGL
SIYCEQGAHGTSVYSGWGIPMIPGHEQATARVTPDGGLELRIGVHSHGQSMETTLPQIAHEILGIETPKIKLVHG
DTEYTPYSTGTWGSRSAVMAGGAVSRASQEIAGLIKGIGAHLLQTDVANVRLEDGCVIGPSGSVTVKEVAHTW

YRRPQDLPASVDPRGLETTIGYKPVRDSGTFSYAAHIAVVAVDPEMGDIEILDYVIVEDGGKLINPMVVDGQIYG
GLAQGIGTALYEEMPFDPSAQPLASTFADYLLPGPTEVPAPQVEHMETLSPYTEFGVKGLGEGGAIAPPAAIGNA
VNDALKSLGVELLCCPMTPRRVLEAIHRAKGTRSVRCT
>SEQF2411||SEQF2411.2_00232
MTQPAAAEVELNRPWVGRSIQRVEDFALLMGRGRFIDDLGTRPGTLHAAILRSPHAHADIVAIDTSAALKSRGV
AAVLVGEDVKKLTSSLVVGVKAPVECWPIAVGRVRYVGEPVAVVVASDRYLAEDAIDLVEVQYSVRPAVIDPLVA
VRADAPVLHDGFSGNVASDRRFRYGDPEKAFAEAAHRISIDIKYPRNSCTPIETYGLIADYDPAENAFDVLANFQ
GPFSIHAVISRALKVPGNRLRLRTPPDSGGSFGVKQGIFPYIVLIAAASRVVGRPVKWIEDRLEHLTASVSATNRAT
TLAAAVTAEGKILALDWDQVEDCGAHLRAPEPATLYRMHGNLTGAYDIRHVAVRNRVVVTNKMPTGLNRGFG
GPQVYFALERLLHRIAIELGLDPLEVIRLNLVPADAFPYRTATGALLDSGNYQEALIRGARDGQLAELKARRDQAR
AEGRLYGIGYTAVVEPSVSNMGYITTVLTAEERRKAGPKNGAQATATIGLDPVGGVTVHVASVPQGQGHRTVLS
QVVADVFGLLPQDIRVNTEIDTAKDAWSIASGNYASRFAPAVAGTAKLAAQRLAAKLARIAASQLNVDASDIVFA
GGSVGSKHNPENKISFSRLAALSHWSPGSLPDDVGDTIRETVFWTPPELTPPDDHDLINSSLCHGFIFDFCGVEI
DRTTLQTRIDRYVTMHDCGTILHPGMVDGQIRGGFVQALGAALYEEYAYGPDGSYLTGTLADYLLPTTTEVPEP
VILHMETPSPFTPLGSKGVGEGNCMSTPVCIANAVADALDVKDLTLPLVPARLAEIVRGAEPSAPAGRAVSGPKA
GGNDRRLRGEGNASVNVPPERVWDMLLDPETLRAVVPGCQSVEKVSDTHFRADVTLGIGPVTGRYRANVML
SDLDPPHAVTLSGSAEGVLGFGGGEGRITLTPDGNGGTTMHYVYHAAIGGKVASIGGRLLDGATKVIIGQFFSA
LARQAEGGGSGRGGLSLVVRLKRLIGIGS
>SEQF2411||SEQF2411.2_02829
MAPVKFGVGQSVLRKEDDALIRGKGRYTDDYTPAAAMHALVLRSPHAHAKFKIDVSRARGLPGVGAILTADDI
QELGSLPCLFNLPDEPFTGPPYPILARDVVRHVGDAIAFVVADTVEQARDAIEAIEVEWTALPAAIGLVNAVKKD
APQVWPDHAGNILFDTSIGDKKAAEAAFAKAHAVAEVTIVNPRIVTNYMETRAVVCEYDAKRDHLTLTIGSQGS
HRLRDILCQNVLNIPVEKMRVICPDVGGGFGTKLFPYREYALAAVAARKLKKTVKWTAERADHFVGDAQGRDN
VTTARMALAEDGKFIGMDVDLMGDMGAYLSTFGPYIPHGGAGMLPGLYDLQAFHCRVRTVFTNTVPVDAYR
GAGRPEAAYVVERLVDAAARKLGMTPDAIRRKNFISPRAMPYTTATGKIYDSGDFAAHMKRAMDIAEWKEFP
RRAKAAKKQGLVRGIGMGTYVEVCGTMGEETAQVRLDPDGDVTILIGTQSSGQGHQTAYAQLVAEQFGIAPER
VHIHQGDTDEIPTGLGTGGSSSIPSGGVSVERATRKLGENLKEIAADALETGVGDLEFRDGAIRIVGTDRTISFADL
AKRADPSKLNASATFSSADGTYPNGTHIAEVEIDPATGVIHVVNYVIVDDFGVTLNPLLLAGQVHGGTMQGIG
QALMEAAVSGAADGQLVTGTFMDYALPRAADGPSIKFETHNVPCKTNPLGVKGAGEAGAIGSCPAVVNAIIDG
LYREYGIDHIDMPATAERVWMAIAKSRREHRL
>SEQF2411||SEQF2411.2_04438
MSVEGIGARVTRKEDKRFITGKGKYTDDVRLHGMTYASFVRSPHAHAKIKSINVDAAKAMPGVVDVLTGQQLV
DDKIGNLICGWMIHSKDGSPMKMGAWPAMAPETVRFVGNAVAVVIAETRNQARDAAEAVEVTYQELPAVA
DIRSAIAPGAPQLHPEAPGNVIYDWSIGDEAATGEAFKKAANVVAMDITNNRLVPNAMEPRAAVAEYDSAEEH
FTLYTTSQNPHVARLVLSAFYNVAAENKLRVIAPDVGGGFGSKIFIYPEEMVALWASKRTGRPVKWTSDRTEAFL
TDAHGRDHITRAEMAFDKDNKIIGLRVKTHANLGAYMSLFSSSVPTYLYATLLSGQYNIPNIYAEVVSVYTNTTPV
DAYRGAGRPEASFVMERMMETAARQLKVDPAELRRKNFITSFPHQTPVIMAYDAGDFNASLDAALKAIDYAGF
PARKAKAKAEGKLRGIGFSCYIEACGIAPSKAVGSLGAGVGLWESCEVRVNPVGTIEILTGSHSHGQGHETTFAQ
VVADRLGIPIGQVSIVHGDTDKVQFGMGTYGSRSGAVGMSAIVKAMEKVEAKAKKIVAHQLEASENDIVIENG
EFKVTGTDKAIALPMVALAAYTAHNLPDGMEPGLKETAFYDPTNFTFPAGAYVCEVDVDPGTGKTDIVNFVAA
DDFGRLINPMIVEGQVHGGLAQGIGQAMLEGAVYDKSGQLVTASFMDYAMPRADDLPSFKVSHTMTPCPSN
PLGIKGCGEAGAIGSTPAVINAITDAIGNNKLEMPASPDRVWHAIHQQQAAE
>SEQF2411||SEQF2411.2_04413
MNILPSNLRFGAGQSVKRLEDQRLLTGQGHFIDDKAQDGALWLYVLRSPHAHARITAIDTSAALAMPGVAAVY

TGADLVADDIGTIPTLPIFKRPDGSPMAAPPRRLLAHDVVRFAGEPVAAVLAPSRADAQTAAEAIVVDYEILPAV
VSPADALAPGAPVVWPDAPDNIAAAMSYGDAAATEAAFAKAKHVVSLDITSQRLVPSALEPRSTMAEVDKKT
GRLTLHVQSQTPTATRDILSDVVLKRPKDSVHVMVGDIGGGFGHKVNLYPEDGIVAYAATKLKRTVRWRGDRID
DFIGGSHGRDLTSTGEFALDEKGRVLAFRVRSLGGTGAYLTGAGVIIPLVLGPFVATGVYDLPLVHFDIKAVLTHTA
PTGPYRGAGRPEAVFIVERLMDAAARQIGMDPRQIRKVNYIKPSQLPYKNAVGEVYDSGAFAHMMERASKLA
DWDGFNARKKAAKKKGLLYGRGLTSYIEWTGGRVHTEKVTLSATAEGRIVLQSGTQAMGQGLQTSYSQMVAG
ALGIPLDRIDVIQGDTDKATGGGSVGSRSLFVGGTATVVSAGDLIQKAREKASHVLEASVGDIEYGDGVLTVVGT
DRRVSLFDLAKDEKDSRLSVESKGEVDGPTWPNGTHICEVEIDPETGVTRVVRYTTVDDVGVAINPMLVTGQV
HGGVAQGIGQALYEGVVYDSSGQLLTASYQDYCIPRASDMPHIDVTLDDSAPCKTNPLGAKGCGESGAIGGPP
CIVNGVMDALSDLGITSLQTPLTPVKIWNAIQQAKATKAA
>SEQF2411||SEQF2411.2_00778
MVEQGVGARLLRKEDDRLMRGRGEFVGDIRLPGMRDVAFVRSPLAHARIKGIRIPPEYRDTVFIAADLEGVKPI
RAVSGLPGFKVSEQPILAFEKVRQVGELIAMCVADTRAEAEDIAAAVEVDFEELPAVHDMLLAKRPDSALLHEH
WGDNIFLETFVDVNMEAAYDAPIKITREISTARQSMAPMEGRGTVAMWHKRMDQLVLYTGNQQPHIVRNGL
SECLGLEQIQVRIVSPDVGGGFGYKGIVLTEDVCLGWLAKRCGYPVRWIEDRREHLTAGANCREHHYNITVYAD
RDGKLRGVECEATVDSGAYSSYPFSACLEAAQVASILPGPYDFPSYRCRTWSVATNKCPILPYRGVARTGVCFAIE
LMMDAIARETGLEPYEVRLKNLVQPEQMPFDNITKKHFDSGDYPESLRQALAKIDLPALRERQKKPEADGRLIG
VGLSVYCEQGAHGTSVYAGWGIPMVPGHEQATARMTPDGGLELRVGVHSHGQSMETTLPQVAHEILGIDTA
KIKLVHGDTEYTPYSTGSWGSRCAVMAGGAVATASRELGKLVLGIGAHLLQTDISNVKLSDGVVVGPSGSVTLQ
EVAYTWYRRPQDLPPSVDPRGLEVTIGYKPVRDSGTFSYASHIAVVAVDPEMGDIELLDYVIVEDGGKLINPMV
VDGQIYGGLAQGIGTALYEEMNFDTSGQPLASTFADYLLPGPTEVPEPKLGHMETLAPYTEFGVKGLGEGGAIA
PPGAIGNAVNDALKSLGAEIRHSPITPRRVLEAIEEAKSTARAKAGEGVLA
>SEQF2423||SEQF2423.2_02375
MNLYQPDPVTQHSGHIGARQTRVEDAALLRGLGCYADDAATPPGTLHAAIVRSPHPHARITAVDFSKALLMKG
VHGVLIGEDIKRWALPFPVGVRQPMEHWCIAVDKVRYVGEPVAVVIAESRYLAEDALEGVRVDYEPLPPIIDPE
AATTEQAPVLHEAVGSNVVNERHFRYGEPEQAFAQAPHRVALKVSFPRSSCTPIECYVVLAQYERATGIYDVLA
NFQGPYALHTVMARALNVPSNRLRLRTPKDSGGSFGIKQGVFPYVVMMGLAARKVGAPVKWVEDRLEHLQG
ASSATNRVTEIEAAVQADGRITALRYDQIDDCGAYLRAPEPATFYRMHGNLTGAYAIRNLLVRNRVVLTNKTPTGL
NRGFGGPQVYFALERLLQHIAVQLDLDPLEVIRRNLVPADAFPYRAAAGALLDSGNYQAGIDLAVKDGGLDELL
RRRDQARGEGRLYGIGYAAVIEPSISNMGYITTAMMPEERRKAGPKNGAVSTATINVGPLGDVSVHVSSTPQG
QGHQTAVAQIVADVLGVALESIVVNVELDTQKDAWSIASGNYSSRFAGAVAGVVYQAALKVRDRLAAIAAAQL
QAKPEDIRFGGGKIFVANGGPSAAFHRIAGATHWTPGLLPQGEPGGLRETAFWSPPQLMAPDDDDLINSSLCY
GFVFDICGLEIDRVTGAIHLDRYVTCHDAGRLLNPALVDGQIRGGFTQGLGAALMEEFAYGEDGSFLSGTFADYL
VPTAPEVLEPVILHLETPSPFTPLGAKGVGEGNNMSTPVCIANAVADALGRADIRLPLTPSKVRSMIGIDEPPRPA
GMQQDDAFAAEPAGGSALRANDAVVIPAPPQQVFDTLLDPATLAAIIPGCHALELQGENRYRADVTVGVGMI
RARFEAKVALSELDPPHSLRLSGSGTSSMGSAEGHARVRFVALENGHTRLEYQYQVAVSGKVAAVGSRMMQG
ASKVIIGQIFTRLSQRVSGQAITAGWWSRLRALFAGLLGKGGAQ
>SEQF2487||SEQF2487.1_07023
MTVTRAPADTLSVLDRPNSYIGKTVPRPNLDRLLQGRGQYVSDLELPRMAHVVFLRSPHAHAKIAAIDADAATR
MPGVISIVTGRELEAVITPWVGVLSHLKGLKSAPQHAIAVDRVCWQGEAVAAIVATSRAAAEDAMEHISVDYEE
LEAVTDMRAALDPAASVIHAALGDNLAFERTLDAGDVDLALSGSELVEADFVFGRHTGVTLEPRAVVADWNPA
EARLTIYQGTQAPHMVQNIAALHLGLREAQVRVVCKDVGGSFGIKVHIYADEMATYALSKLLRRPVKFVADRVE
SFNTDIHARDHRCRGRIGVRPDGTITAFEIDDLTGIGPYSMYPRTSAIEANQVVNLVGGPYATANYRARARVVFQ
NKNVMCQYRAVGHPIACSVTEGLVDLAAAKIGMDPVEIRRRNLIADDAYPCASPSGMKFERLSHHASLTKLLQ

MMDYDALRAEQAALRARNVHRGIGIASFIEVTNPSAAFYGVGGAKISSQDGVAVRLDAQGSVICQTSITEQGQ
GSESLTAQIVGSVLGVSMERVRVTLGDTDNTPYGGGTWASRGAGIGGEAALQAAKALRENILNVAAAILQSTPA
ELDIVDNGVVNAADGAPRIDLNELARIVYFRPDTLPPGIQPELMATRHFVPRQYPFAFTNGVQASWLEVDTET
GFVQLLKHWVVEDCGTIINPQLVDEQIRGGVVQGLGAALFEKCIYDERGQLTNANMADYLVPMSGEMPDIEIG
HVVSPTQESELGAKGAGEAGTAGAAAAVANAVNDALRPFGATITEIPLTPQVILTALGRI
>SEQF2487||SEQF2487.1_06807
MAAPIKFGVGQSVRRKEDDALIRGRGRYTDDVAPSPALHALMLRSPHAHATYTIDAGKARGMPGVALILTAAD
VADLGGLPCLFNLETDPFTAPPYPILAKDEVRHVGDAVAFVVADTVDHARDAIEAIDVKWTPLPAAVGLVNAVK
KDAPQVWPDKPGNVLFDVSIGDKKAAEDAFAKAHAVAEITIVNPRVITNFMETRAAVAEYDAKKDHLTLTIGSQ
GSHRLREILCDMILKMPKENMRVICPDVGGGFGTKLFPYREYALISVAARKLKKSIKWTAERSDHFMGDAQGR
DNLTTAKMALAEDGKFLGMDVDLMGDMGAYLSTFAPYIPHGGAGMLPGLYDIQAFHCRVRTVFTNTVPVDA
YRGAGRPEAAYVIERLVDAAARKLGKTPDAIRRKNFIPPKSLPYTTATGKVYDSGDFVAHMKRAMEIANWKEFP
KRAKAARKDGLVRGIGMASYVEVCGTMGEETANVALDPNGDISVLIGTQSSGQGHQTAYAQIVAEQFGVPPER
VHVLQGDTDKIATGLGTGGSASIPSGGVSVQRATHDLGNKLKELAAQALEAGAGDLEIADGRIRIAGTDRSISFA
DLAKRPGGDTSKMNASATFASADGTYPNGTHLAEVEIDPATGIIKIVSYVIVDDFGVTLNPLMLAGQVHGGAM
QGIGQALMEQAVYSPADGQLVTGTFMDYALPRASDGPSFVFETHNVPCTTNPMGVKGAGEAGAIGSCPAVV
NAIVEGLHREYKIDHIDMPATPERIWIAIREAQRRHNL
>SEQF2487||SEQF2487.1_01848
MGVEGIGARVVRKEDRRFITGQGRYVDDVKMVGMSYAHFIRSPHAHAKVKGIDSAEAMKMPGVIAVLTGQQ
IVDDKVGNLICGWAITSKDGSPMKMGAWPAMAPETVRFVGQAVAVVIAETKNQARDAAEAVVVDYEELPAA
ADIKAAIKPGAPQLHPEAPGNIVYDWHLGDEAAVKDAFSKAANVVTLDLTNNRLVPNAMEPRAAVAHYDQAE
EHYTLYTTSQNPHVARLVLSAFYNIAPEHKLRVIAPDVGGGFGSKIYIYPEEMVALWASKKVGRPVKWTGDRSEA
FLTDAHGRDHVSKAEMAFDKDNKILGLRVKTHANFGAYMSLFSSAVPTYLYATLLSGQYVIPAIYAEVIGVYTNTT
PVDAYRGAGRPEASYLIERMMETAARQLKVDPTELRKKNFITQFPYQTPVIMAYDIGDFHASIDAAMKAIDYAG
FPARKAKAKADGKLRGIGISCYIEACGIAPSKAVGSLGAGVGLWESAEIRVNPVGTIEVLTGSHSHGQGHETTFC
QIIAERLGVPISQVSIVHGDTDKVQFGMGTYGSRSLAVGGTAIVKAMEKVEAKAKKIAAHALEASESDIVIENGE
FKVTGTDKSIALPMVALAAYTAHNLPDGMEPGLKETAFYDPTNFTFPAGAYICELEVDPGTGKTSFVNFVAADD
FGRLINPMIVEGQVHGGLAQGIGQALLEGAIYDDTGQLVTASFMDYTMPRADDLPSFQLSHTTTLCPGNPLGV
KGCGEAGAIGASAAVINAITDAISNNKLEMPATPDRVWHAIHG
>SEQF2487||SEQF2487.1_07832
MNILPGNMRFGAGQPVKRLEDQRLVTGKGHFIDDKPQDGALWLHVLRSPHAHANIKSIDAKTALEMPGVKAV
YTGADLVKDDIGTLPTLAIFKRPDGSPMTVPPRRLLAHEVVRYAGEGVAAVVATSRVLAQTAAEAIEIDYEVLPSV
VDPVEAIKPGAPAVWPEAPDNIVAAMSYGDAAKVEEAFANAAHKVSLDLVSQRLVPSAMEPRSTIAEIEKKTGR
LILHVQSQTPGSTRDLLAESILKRPKDSVRVLVGDIGGGFGQKTSLYPEDGIVAYAATKLNEKIRWRGDRTDEFVG
GTHGRDLTSTGEFALDAKGRVLAYRVRSIGGTGAYSSGTANIIPLVLGPFVQTGVYDLPLVHFEVKSVMTHTAPVG
AYRGAGRPEAVFIVERLFDAAARQIGMDPRTIRKVNYIKPAQLPYTNAVGQVYDSGAFAHMLERASDLADWN
GFAARKKAAKKKGLLYGRGLTSYIEWTGGRAHTEKVSLHATAEGRVILWSGTMAMGQGLATTYTQMVADTLGI
SMDKIDVVQGDTDLATGFGSVGSRSLFVGGTAVAVSTNDMINKARDKASNLLEASVEDIEYRDGFLTVVGTDR
RISLFEIAAKENGAKLSVESEGNVDGPSWPNGTHICEVEIDPETGVTRVVRYTTVDDVGVAVNPMLVTGQVHG
GVVQGIGQALYEGVAYSEEGQLLTASYQDYCIPRADDIPPITVTLDGSAPCKTNPLGAKGCGESGAIGGPPCITNG
VMDALSEVGITQLNTPLTPAKIWQAIRDAKVGAA
>SEQF2487||SEQF2487.1_04790
MQDHTPPASLENAIALQKYGVGQPVRRKEDDTLVRGKGKYTDDFSLPGQAYCWMVRSSHAHGLIKGIDTAAA
KAMPGVLGVWTGADLAAAGYNPFTCGLPLKNRDGSPLKQTNRPALVTDKVRFVGDPVAFVVAETAAQARDA

AEAVEVDIEPLPAVTDAAEAAKPGAPQLYDDIPDNVALDYHYGDTAKIEAAFAGAAHVTKLDIVNTRVAVVSME
PRVALAHYDKKTERFTLQVPTQGVSGNKAILARLLNVPADKVRILTGNVGGSFGMKNLNYPEYTCIAHAARELG
RPVKWLDERSTSFLSDSQGRAQLIHAELALDADGKFLAVRLSGYGNLGAYITGVAPGPLSLNTGKNLASVYRTPL
LGVDIKTVLTNTTLMGAYRGAGRPEANYYMERLIDAAADEMGINRFTLRKRNFIKPSQLPFPAASGVTYDSGDF
AAVFQKALEISDYDNFAKRKKESKKSGKLRGIAVGSYLEVTAPPSGELGKISFEPDGSVKLTTGTLDYGQGHATPFA
QVLSDQLGVPFEKITLEQGDSDLVRFGNGTGGSRSITATGQAIVEASALVVEKGKKAAAHMLEASEADIEFGAGR
FTIAGTDRSIGIMELAERMRAGKMPEGTPETLDVDHATKETASTFPNGCHVAEVEIDPDTGVTRIVRYSAVNDF
GVVVNPMIVAGQLHGGVAQGIGQALMEEVSYDASGQPITGSFMDYALPRAGDVPSMLVGDHPSPAKSNPLG
TKGCGEAGCAGSLVCIVNAVVDALSDYGIKHINMPLTPERVWRAIQDAKAKAA
>SEQF2514||SEQF2514.1_00441
MNAPAEPNNHLIGASVKRKEDFRFLTGAGQYTDDVVQAHQSYAVFLRSPYAHARIKHINTDAARNHPGVLAVL
TGDDLAADKVNGLPCGWLIHSIDGTPMKEPPHPVLAQGKVRHVGDQVALVVAESVKIAKDAVEMIDVEYDEL
PAVVDTATADTAGTAVHDDVPNNTCYTWGHGDKAATDAAFAKAAHVTHLDIVNNRLIPNAIEPRAVNASYSRQ
DDSYTLYVANQNPHVERLLMSAFVLGLTEAKVRVIAPDVGGGFGSKIFLYPEDVALTWASKKVGRPIKWTAERSE
SFLTDAHGRDHVTHAELALDAQGNFLAMRVHTTANMGAYLSTFASSVPTILYATLLAGQYKTPAIYAEVKAVFTN
TAPVDAYRGAGRPEATYVVERLVETAARELQVDPAELRRRNFIRTFPYATPVGLTYDTGDYEPCLDRAIELADVKG
FAARRDASRAKGRLRGLGYSCYIEACGLAPSNIAGALGARAGLFEAGEIRVHPTGSVTVFTGSHSHGQGHETTFA
QVVADRLGVPIDNIEIVHGDTGRIPFGMGTYGSRSIAVGGSAIMKALDKIEAKAKKIAAHLLEASAEDIEFKDGVF
RVAGTDRTKTFGEVALTAYVPHNYPLDKLEPGLDENAFYDPTNFTYPAGAYICEVEVDPDTGEVHIDRFVAVDDF
GNIINPMIVEGQVHGGLGQGIGQALLEACVYDENGQLLTGSYMDYAMPRANDLPSFTVETAKGTPCTHNPLG
VKGCGEAGAIGSPPALINAIVDALAPLGVKDIQMPATPHRVWQTIQAAKA
>SEQF2536||SEQF2536.1_05415
MGASDFSKLPHIGEPVKRKEDYRFLTGAGQYTDDIALAAQAHAVFVRSPHAHARVRSVSTDAAKAAPGVIGVLT
GADVAADKINGLPCGWLITSTNGEPMKEPPHPILALDTVRYVGDQVAMVVAETLEQARDAAELVEVDYDPLPA
VVLVADAAAGSVPGAVVHDIAPDNHCYKWAIGDKAAVDAVFAGAAHIAQLDLVNNRLVPNAMEPRAAIGSYS
RANDEYTLYVANQNPHVERLLMTAFVMGLPEHKVRVIAPDVGGGFGSKIFLYAEDVCLTWGARKLNRSIKWTA
DRSESFLTDAHGRDHVSHAEMAMDASGKFLAMRVHTDANLGAYLSTFASAVPTILYATLLAGQYATPQVYVEV
DAWFTHTAPVDAYRGAGRPEATYLLERLVSRCAWQLNLSQAEIRRRNFVTSFPYQTPVALQYDVGDYGACMDK
AEALADVAGFAARRADSEARGLRRGLGYSSYIEACGLAPSNIAGALGARAGLFECGEVRVHPTGSVTVFTGSHS
HGQGHETTFAQVVAARLGIPVENVDIVHGDTGRVPFGMGTYGSRSISVGGAAIMKALDKIEAKAKKIAAHLME
ASDADIDFAGGEFTVRGTDKKIPFAQIALTAYVPHNYPLDKLEPGLNETAFYDPTNFTFPAGTYICEVEVDPATGV
VRVDRFSAVDDFGTIINPMIVEGQVHGGVAQGIGQALLENCVYDRETGQLLTGSFMDYAMPRADDFPEFKLG
TVCTPCTHNPLGTKGCGEAGAIGSPPAVINAVLDALHPLGVRDLDMPASPHRVWSAIDAAATS
>SEQF2537||SEQF2537.1_01966
MGASDFSKLPHIGEPVKRKEDYRFLTGAGQYTDDIALAAQAHAVFVRSPHAHARVRSVSTDAAKAAPGVIGVLT
GADVAADKINGLPCGWLITSTNGEPMKEPPHPILALDTVRYVGDQVAMVVAETLEQARDAAELVEVDYDPLPA
VVLVADAAAGSVPGAVVHDIAPDNHCYKWAIGDKAAVDAVFAGAAHIAQLDLVNNRLVPNAMEPRAAIGSYS
RANDEYTLYVANQNPHVERLLMTAFVMGLPEHKVRVIAPDVGGGFGSKIFLYAEDVCLTWGARKLNRSIKWTA
DRSESFLTDAHGRDHVSHAEMAMDASGKFLAMRVHTDANLGAYLSTFASAVPTILYATLLAGQYATPQVYVEV
DAWFTHTAPVDAYRGAGRPEATYLLERLVSRCAWQLNLSQAEIRRRNFVTSFPYQTPVALQYDVGDYGACMDK
AEALADVAGFAARRADSEARGLRRGLGYSSYIEACGLAPSNIAGALGARAGLFECGEVRVHPTGSVTVFTGSHS
HGQGHETTFAQVVAARLGIPVENVDIVHGDTGRVPFGMGTYGSRSISVGGAAIMKALDKIEAKAKKIAAHLME
ASDADIDFAGGEFTVRGTDKKIPFAQIALTAYVPHNYPLDKLEPGLNETAFYDPTNFTFPAGTYICEVEVDPATGV
VRVDRFSAVDDFGTIINPMIVEGQVHGGVAQGIGQALLENCVYDRETGQLLTGSFMDYAMPRADDFPEFKLG

TVCTPCTHNPLGTKGCGEAGAIGSPPAVINAVLDALHPLGVRDLDMPASPHRVWSAIDAAATS
>SEQF2586||SEQF2586.1_01695
MGASDFSNLPHIGEALRRKEDYRFLTGAGNYTDDITLANQSHAVFVRSPHAHAVIKSVDTAEALKMPGVVGIFS
GKDIEGKMGGLPCGWLINNPDGTPMKEPMHPILAINKVRYVGDHVAMVVAETVEQAKNAAEAVVVDYDVL
PALVSVADAAKKASGVTLHDEAPDNQCYKWTLGDKAAVDAVFTTAAHVTKLDLVNNRLIPNPIEPRVAIGSYSR
GTDDYTLYVSNQNPHVERLLMTAFVLGLPEHKVRVIAPDVGGGFGSKIFLYAEDVCLTWAAKQLNRNIKWTAER
SECFLSDAHGRDHVSHAEMALDKDGKFLAMRVHTDANLGAYLSTFSTAVPTILYATLLAGQYTTPQIYVEVDAW
FTNTAPVDAYRGAGRPEATYLLERLVSRCAWEMNLGQDEIRKRNFITTFPYQTPVALQYDTGDFHACMDKARV
LADVGGYAKRKSASEAAGKLRGIGYSSYIEACGIAPSNIAGALGARAGLFECGEIRVHPTGSVTVFTGSHSHGQG
HETTFAQVVAARLGIPVDNVDVVHGDTGRVPFGMGTYGSRSISVGGAAIMKALDKIETKAKKIAAHLMEASDA
DIEFANGEFTVKGTDKKIPFGQVALTAYVPHNYPLDKLEPGLNETAFYDPTNFTFPGGTYICEVEVDKQTGEVRV
DRFTAVDDFGTIINPMIVEGQVHGGLVQGIGQALLENCVYDNETGQLLTGSFMDYAMPRAGDFPQFNLDTVC
TPCTHNPLGTKGCGEAGAIGSPPAVINAVLDALAPLGVKDFDMPASASRVWEAMQAAASH
>SEQF2586||SEQF2586.1_02521
MTTDSTPTRFGSGQAVRRLEDESLLSGAGRYTDDVTLPGQTYLVFLRSPYPHARIVSIDTAAAAAMPGVLRVITG
AELAEAGVKPMPGAVGFKRADGSDCASPPRLAMAYDRARFVGEAVAAVVAETVQQARDAAEAVVVDYEALP
MVVDLASATADDAPLLCEEASGNVAAEMRHGSSDAATAAFAKAKHVVALDVVNQRVVALTIEPRSVLAAPDAE
SGRLTIRMSTQMPSGVRDSICAAIGLAKEKVRVVVGDVGGGFGMKTGAYPEDIAVAFAALQVKRPVKWVADR
SEEFLSSAHGRDIEAKAELALDADGKILALRIRTLANVGAYATGTGVAIQLLIGPWVQTSVYDIQTIDFHFKAVLTN
TAPTGAYRGAGRPEAIFTIERLMDEAARQTGIDRIALRRRNFIRPEQMPYKNPMAQTYDTGKFESVMDQALAL
ADWQGFEARAAESAKNGKHRGLGIATFLEWTGGNVFEERVTVSVQADGVIEVFSAVNAMGQGIATSLAQLAV
DAFGVPIEKVRVVLGDTDRGDGFGSAGSRSLFTGGSAVRIGAERTIDKARALAAQEFEAAIDDITYTRGIFSVTGT
DLELDLFALAGKQPEREIFVDSTSTVAGPTWPNGCHVCEIEIDPPTGEISVVAYSSVNDVGRVINPMIVRGQLEG
GAVQGIGQALYEQVVYDNETGQPLTGSLMDYAAPRADIVETMFHMEMDESTPCANNPLGVKGVGELGTIGAT
PTIVNAVADAFARNGLAASAPRLHMPLSPARVWQAMHTVD
>SEQF2603||SEQF2603.1_04682
MSEAQNGSATKYIGKSVRRREDKRLMSGRGKFTDDHQFPGMVFAAFVRSPFAHAKVRGIDTADARALPGVIG
VLTYADIEGKVGDIRPNWVVGNSKVPPHPPLAADRVRYAGEAVAMVVAQTREIAADAAELVTVDYEELPTVVD
AEAALAESAPQLHDNVPGNRIGVFRLKGGNYEDAVKQADRVVSIRLVNQRLIPSPLEPRALCATYDPTDERLTFI
VPTQVPHMSRRWLAETLRWPEHRIRLVAPDIGGGFGAKMHFYAEEVLVAFASRHFGLPVSWTETRSENHVATT
HGRAHTEYVDAPVTKDGKVLGIRLRSFANLGAYLSNMATGIPTINTANYITGNYQISSVDAEVNLVTTNTTPVDA
YRGAGRPEAAYIIERLMDAVAGELDIDPVELRRRNLVRADQFPYQPHDGARGKWDSGDYEACLAKATELIGYQR
WREEQATLRQQGKYIGIGVICYLELVGMGISPMLEKVGFDRGGWESAHVRVHSDGKVTLFSGSMPQGHGHA
TSFAQIVAEALQLPMDDIDVVQGDTDRVLAGHGTFNSRSMPVGGSAAYLAASKILAKARRIAGIMLEVPEAEV
HYADGQFTSAANPTVIPFAKVARMAYLAHKLPRDVEAGLDERVFYEPVALGAPNGCHAVVVEVDAETGTVAIL
DYVAVDDVGVMINPMLCHGQMHGGIAQGIGQALFEEAKYDEAGQLLSGSLLDYGFPRIEQIPRMRTGFHVSP
APSNPLGVKGIGEAGCVGAPPAIVAAVCDALKPFGISHLDMPLTPPKVWRAIHETSRSAT
>SEQF2603||SEQF2603.1_04960
MTSSTEPDHPDAVRGQGVGARVARKEDARHLHGKGRFVADIAMPDLQEVAFLRSPVAHARLLSVTKPARHAA
AVIVREDMAAASDIVADSAFPSYQPSAQPPLASGKVRFVGEPVAMAFAPTRAEAEDIAEQVAVEFDELTALTDV
TQARQLSEEVRVHDHWRDNTFLTLNADKNFEARRHEASVVVRRSIDLARQCMVPMEGKAVLAYWDHQFDQ
LVVYSATQVPHMIRTILGHCLGLEQERVRVISPDVGGAFGYKCVLQQEELCIAWLAMTYKRPFRFIEDRREHLTA
GANSREHHYDLTAYADARGRLLALDAKIVIDGGAYSVWPFTIGLEPGQAIGNLPGPYAFDGYRCETTCVATNKPG
FVPYRGVARTGVCFAMELMIDAIAREVQREPWEVRCENLVPPEAMPYVNVSNKHFDGGDYPASVRKAMEMI

GLPEIRARQRAGPQGSRYVGVGFGTYTEQSAHGTSVFAAWGTPVIPGYDSATVRITPDGGLEVRVGVHSHGQ
GMETTFAQIAHEILGIDIGRIKLVHGDTGLTPFSTGTYASRSLVMSGGAVSRACKALLPRLQKIGAHMLGQPVEA
VAFAAGTVRAGDAGGVDIVRIANAWYINPHLLPPDVDAQGLEVTMGFKPAVDTGSFTYATHAVAVEVDVDSG
HVEILDYVVVEDCGTMINPMVVEGQTYGGVAQGIGTAMFEEMRYDGNGQPLASTLADYMLPGPTEIPAIRIH
HFETPSPHTEFGAKGMGEGGAIAPPAAIFNAVNDALRGFGVELKETPLTPRKILEALDAAEAGAQNMKAA
>SEQF2603||SEQF2603.1_00426
MNAPENQRLVGAPVKRKEDFRYLTGSGQYTDDIVLPQQSYAFFLRSPHAHARIRSIDKSEALAAPGVIAVLTGDE
LAADKVGGLPCGWLIHSMDGSPMKEPPHPVIAHDKVRHVGDQVALVIAETLQQARDAAEKIDVDYEELPAVV
NAADAASASTLVHDDVPANTCYTWGHGDKAATDAAFAKAAHVTTLDIVNNRLIPNAIEPRAVNASYTRQDDS
YTVYVANQNPHVERLLMGAFVLGLPESRLRIIAPDVGGGFGSKIFLYPEDVALTWASKKVGRPVKWTADRSESFL
TDAHGRDHVTKAELALDADGKFLALRVHTVANMGAYLSTFASSVPTILYATLLAGQYATPAIYAEVQAVFTNTAP
VDAYRGAGRPEATYVVERLVETAAHELNTDPAELRRKNFIRQFPYATPVGLTYDTGDYEPCLARAQELADVKGFP
ARRDEAKQRGKLRGLGYSCYIEACGLAPSNIAGALGARAGLFEVGEIRVHPTGTVTVFTGSHSHGQGHETTFAQI
VADRLGLQLDQVEIVHGDTGRVPFGMGTYGSRSLAVGGSAIMKALDKIEAKAKKIAAHLLEASDTDIEFSNGVF
RVAGTDRTKTFGEVALTAYVPHNYPLDKLEPGLNENAFYDPTNFTYPSGAYVCEVEVDPDTGESKVVKFTAVDD
FGNIINPMIVEGQVHGGIGQGLGQAMLEQCVYDPDSGQLLTGSYMDYAMPRAADLPDFTVETASGTPCTHN
PLGVKGCGEAGAIGSPPAFINALVDALSPLGVKDVQMPATPHRVWQAIHAARP
>SEQF2603||SEQF2603.1_07337
MTKMMEPTPDLAALAGQPWIGRRLERREDARHLEARAQFVADVELPGCKEVAFVRSPVAHGHLRGIRLPQEY
PAGTFWTASDLAGDVLPISCALLRDEFNGAPWPLLAVDRVRYVGEAVVMVVAPTRAQAETLAEEVELDIEVLPP
VVDAVAELATPGPVLHAGLSSNVVMRVARTLGDFDAVLAQADAGGLRRVTRQFAMDRVLASPLETRGCVASA
DRRSGHLTLYLSTQRPHLVRSFVAEQLTRLSEAELRVIVPDVGGGFGAKSNLYPEEVLLAALALRVGHPVRWIEDR
YEHFVASNHSRQHAQEVSAWFDASGRIHGIDARIVVDSGAYSAKTSTGAIEANMAANVLLGPYDVRNYRFEAV
SVYTNKSPLGPYRGVGRPGGCFAMERIVDEVAHALRLDPVAVRRRNLIPPNAFPYTTATGLVYDSGNYAEALDA
AVERVAAKWGTPEPGSGRWREGIGYAMLVEQAAHGAEEWRRRGSPAVYGHESARATLHSDGSLEIEVGTLSH
GQGHQTSLAQIAAQVTTLDPAGIHIRQGDTAAAPFGMGTVASRSIVMAGGAVRQACAALVAKARSIGAVLVG
CPAEALRLQDGNLMAPDGRTVTLAEVARVAYIGIHQLPREIAPGMSVEAAYRPEVETGTFSYAVHAARVRVDID
TGLVRVTGYVVAEDCGTVVNPLIVDGQIIGGVAQGIGQALYEAMRYNADGQPETVTFGDYHVPSAVEIPPVDII
HLCTPSPFSAFGVKGMGEGGSVPPPAAIANAVRAALKEFDVQVCATPITPDGLLEQLLAHPDYAEVMA
>SEQF2603||SEQF2603.1_07345
MKRFDAGETTGTVQETQEIRRAPHVGRPMQRVEDAAILTGRGRYADDLGVRPGTLHAAILRSPHAHAELGAID
YAAALKAPGVHAVLTGADLPGWSRPFVVGVKAPMEQWALAMERVRYVGEPVAVLVAESRALAEDALDLVRVE
YRVLPPVVSIEAAVADDASVLHSGLGSNVASDRHFRYGDPEAAFAAAPHRVALTAHYPRNTCTPIECGVVIAEHL
PANEGYQVTSNFMGPFSLHAVMAMALQVPANRLRHVAPRDSGGSFGVKQAVFPYVVLMCLASRKAGAPVK
WVEDRLEHLSAATSATARLSTLEAAVEADGRIVALSYDQLEDCGGYLRAPEPATFYRMHGLLTGAYAIPNLLVRNR
VVLTNKTPTGLVRGFGGPQVYFALERLVQRIAVELGLDPLDVYRRNFVPADAFPYRAAAGALLDSGNYQLAMSR
ALDAGGYGELKRRRDAARAEGRLYGIGFAAIVEPSVSNMGYITTATPAEARRRAGPKNGAIASATVSVDLLGGVV
VTIASTPAGQGHMTVSAQVVADVLGIDPGEVVVNVEFDTHKDAWSVAAGNYSSRFAGAVAGTVHLAATRVR
DKLARIVASHLGCDPAELAFADGRIQRRGAPDTALPFARAASNAPHWSPQLLPAGEEPGLRETVFWSPPNLDA
PDEQDRVNTSACYGFAFDLCGVEIDRATGRVRIDRYVTAHDAGKLLNPALADGQIRGAFAQGLGAALMEEFRY
GPDGSFQSGTLADYLMPTTCEVPDPLIVHLETPSPFTPLGAKGLGEGNNMSTPPCVANAVADALGVRDIRLPLT
PARVMAMIGFDDPPPSRPELAEAASAASASGKGGGKSGKALSARGTVDLDATPAAVFAVLLDPQALANVVPG
CHALVPIGDNRYRADVTVGVGMIKARYEAEIALSDLEPPHRLRLAGAGLSSLGSARGAGLVELAPHGAGTRLTYD
YEAEVSGKVAAVGGRMLEGAAKVVLRQLFESLGRQAGGKPVRKPGWIARLLALLGVRH

>SEQF2709||SEQF2709.1_01480
MGASDFSKLPHIGEPVKRKEDYRFLTGAGQYTDDIALAAQAHAVFVRSPHAHARVRSVSTDAARAAPGVIGVLT
GADVAADKINGLPCGWLITSTNGEPMKEPPHPILALDTVRYVGDQVAMVVAETLEQARDAAELVEVDYDPLPA
VVLVADAAAGSVPGAVVHDIAPDNHCYKWAIGDKAAVDAVFAGAAHVAQLDLVNNRLIPNAMEPRAAIGSYS
RANDEYTLYVANQNPHVERLLMTAFVMGLPEHKVRVIAPDVGGGFGSKIFLYAEDVCLTWGARKLNRSIKWTA
DRSESFLTDAHGRDHVSHAEMAMDASGKFLAMRVHTDANLGAYLSTFASAVPTILYATLLAGQYATPQVYVEV
DAWFTHTAPVDAYRGAGRPEATYLLERLVSRCAWQLNLTQAEIRRRNFVTSFPYQTPVALQYDVGDYGACMDK
AEALADVAGFAARRADSEARGLRRGLGYSSYIEACGLAPSNIAGALGARAGLFECGEVRVHPTGSVTVFTGSHS
HGQGHETTFAQVVAARLGIPVENVDIVHGDTGRVPFGMGTYGSRSISVGGAAIMKALDKIEAKAKKIAAHLME
ASDADIDFAGGEFTVRGTDKKIPFAQIALTAYVPHNYPLDKLEPGLNETAFYDPTNFTFPAGTYICEVEVDPATGV
VRVDRFSAVDDFGTIINPMIVEGQVHGGVAQGIGQALMENCVYDRETGQLLTGSFMDYAMPRADDFPEFKLG
TVCTPCTHNPLGTKGCGEAGAIGSPPAVINAVLDALHPLGVRDLDMPASPHRVWSAIDAAATS
>SEQF2715||SEQF2715.1_00578
MAGRVFGQSIPRNEDPYLLTGQAKFIDDIELPGMLHAAFYRSDYAHARIKKIDVSEARKLPGVVAVYTAEDFGEF
VRPGPLQVPPPTAIKGSVFHARPLMPIAKDKVRYSGEPLAVIIAESRYIAEDAMDYIVADLEPLPAVTDLEKALEPG
APLVHEDLESNLAALVKQERGNYEEAAAKADVVIKKRIVVDRGAGAAMENRGLVVNWDDRAREMTIWATTQ
APIPLRNSIAGRLGLFEEQVRVITPFIGGGFGPKIMTSQPDDVLLPIIAMWLKRPIKWIEDRRENFLGTTSERDQI
HYAEIALTKDGIILGVKDVFYHNTGAYDPYGMTVPLNTQTHTVSCYEVPNFYTEIRMVFTNEYVVTPVRGAGRP
EGVFVMERLMDYAAQELGMDPAEIRRRNLLRKERFPYPTGIIGQDFVEGVLDSGDYPETLRRVMEVIEYEKFRK
EIQPQLRAQGKHKGIGIVCFTEGTAVGPYEGARVTVGANGKVTVATGISTQGQGHFTVFAQIAAEQLGVDVKD
VKVVTGDTGHFHWGAGTFASRGMGVAGTAVYNAAVKVRKKALEFGARFLGVSEDEVELCDGKVCVKGDPSR
SMPLGEIALKANPTRGTIPPGVEPGLEATAYFGPPYGATGAGAVAMIVDVDPETMAVNIEKFAIVHDCGTVVNP
LLLEGQVMGGVHMGIGNAFYEKIKYDENGQLLTASLMDYLIPQATDMPKEMVVEHLETPSPLNPLGMKGVGE
AGAIPTPACFMQALEDALYEYRLQIDETPLNPSLLWEMVQKAPKK
>SEQF2716||SEQF2716.2_07571
MNEAPNTEAPVDTGGVGARVRRREDERHLNGKGRFVADYTFPDLQEVAFLRSPLAHAIVRRIGKPERYAAQVI
VRDDMAGATDIVADSSLPSYRSSAHPPLASGKVRFVGEPVAMCFAATRAEAEDIAEEIELDLDELPAFANAFSAR
ERTDTRVHDHWPDNLFLDLKADVDFDAHAQRATVVVKQKVDLARQCMVPMEGKAALAYWDHTQAQLVVIT
STQVPHMIRTILAQCLGLDQAQVRVISPDVGGAFGYKCVLQQEELCVAWLALTYKRPFRFIEDRREHLIAGANTR
QHHYELTAYADDRGRLLALDAQLLIDGGAYSAWPFTIGLEAGQALGNLPGPYDFRGYRCRTQCAATNKPGFLPY
RGVARTGVCFAIELTMDAIARAVGREAWEVRHDNLVRGADMPYTNVVKKHYDSGDFQASLKQAVEQIDVSR
WRQRQALGEPDGRLIGVGFATFTEQSAHGTSVFASWGLPVVPGYDLATARITADGGIELRAGIHSHGQGMETT
LAQIASEVLGVPVQKIRLVHGDTALTPYSTGTYASRSIVMAGGAVAAACRELVPRLIRIGAHLLNEAESAVRFDAG
RVVGASGSVTLADVAAAWYLRPDTLPKGVDLAGLETTQGFKPAVDTGAFSYATHAAVVAVDTQTGQVEILDYVI
VEDCGTMINPMVVEGQTIGGTAQGIGTAFYEETLYDDNAQPLTSTLADYMLPGPTELPSLRIHHMETPSPYTAF
GAKGVGEGGAIAPPAALFNAVNDALRPLGARVSETPLTPRRLLQAIEAAKGGHAAGTHSAVAAGEAIR
>SEQF2763||SEQF2763.1_07144
MSNDKPIVRREDGRLIHGRGRFVDDEGSRALHCKLVRSPYAHARILGMDTRRAESLPGVVCVLTGAEVRELTNR
FPQMAPEPTSRISDYCMATERVRFQGEPVAAVVATSAAIASDGVELVRVDYDPLPVVVDAVASLGVDAPILHDE
SGTNQTWGSLFDWGDVDGAFDSADVVVEIDRLKFHRFSSTPLEGFAGLVTWEPTGRVDMLCNLIQPGVAMK
FIAPALRISPEQLRIRTQDIGGGFGIKQNLYPYLMIAALCSRKVGYRPVKWIEERREHLQGSAHGNERTFLDIRVAL
TADGEITAVDARHVDDCGAYPRYEPLGAVIWSQVAPASYRLQNLRIDFRQAMTNKCPVGPNRGFSRMQNVW
FLERVIDICAHEMGIAPDVMRKRNYVPEMPWTTPNGCVYDSGDYCAMLDMAKDLIGWDDWIAKRDAWRA
EGRIVGIGIGTALDSGTNNFGQSRIVNPNSPLSGNSEVANVRLGVDGTVTVSVGSVPQGQSHETIAAQVVGQE

LGISSDLVLVAIGFDSERMSHNNHSGTYASQFAVTSLGAIHGAVEKLRQELLRAGSYFLGQPAETLRVGTFDGVAS
VAAPDGQSVSLARISNLVNAQSAELPPELMDVSLNCRHVYRAPFQVPDIEKKFGNLTLTYAAQVHVAVIELDPLT
YNIHVHDYSIVDDCGKVINHQIVKGQVIGAAAHGFGAALMECLKYDEAGNLLTATFSDYCPLTIMNMPNLHYG
NRESPSPFTYNGAKGMGEGGGGPIFAISSALQDAVATTGMRLDQSHYSPSDIHALLSGIAERRGAKVSMTVLTP
SREPLQREGGRLRWPMCGRANGT
>SEQF2763||SEQF2763.1_02159
MGIEGVGARVARKEDKRFITGAGRYVDDMVVPGMKHAAFVRSPHAHAQIKKIDVKRAQAMPGVIGVLTGKE
LKADGIGNLICGWMIHSKDGSPMKMGAWSPLAVDKVRYVGDAVVVVVAETKGQARDAAEAVEITYKELKAV
VEATKALEKGAPQIHAEAENNLIFDWEIGDAKATDAAIKAAAHVTRMKIVNNRLVPNAMEPRAALGHYDKAE
DHYTCWTTSQNPHVARLVMSAFYNVAPENKLRVIAPDVGGGFGSKIYIYPEEIVCLWASKKTGVPVKWVADRT
ESFLSDAHGRDHVSTVEMAFDKNNRITGLKVDTIANLGAYMSLFSSCVPTYLYATLLSGQYDIPAIHANVRTVYT
NTAPVDAYRGAGRPEATYLLERTMEAAARELGVSPAELRRKNFITSFPHQTPVIMNYDAGDYGASLDAAMKAS
DYAGFAKRKAAAAKKGLLRGIGMSCYIEACGIAPSAAVGSLGAGVGLWESAEVRVNAVGTIEVLTGSHSHGQG
HETTFAQLVNQRFGVPIDSVSIVHGDTDKVQMGMGTYGSRSGAVGMSAISKALDKVEAKAKKIAAHLLEADEG
DIVIENGALKVAGTDKNVPWFQVALAAYTAHNLPAGMEPGLKETAFYDPSNFTFPAGCYICEVEIEPETGTTEIV
QFVAADDFGNIINPMIVEGQVHGGIAQGIGQALLEGAHYDASGQLLTASYMDYTMPRAGDLPSFKVSTSNTPC
PGNPLGIKGCGEAGAIGSPPAVINAITDAIGIADIAMPASPPTVWAAIRAAKH
>SEQF2763||SEQF2763.1_00736
MTVVTPKFGMGASVLRREDTAFIQGQGRYTDDIKPDGVLHGYVLRSPIAKASFTIGSIDAAKAAPGVHLVLTGG
DVTHLRDLKSGVMQAQPDGTKAPTRDIPILCRDRVNYVGDAVAFVVADSRALAQDAAELIEVDYDGEDAASGT
ATALAEGTPLVWPELGSNRAFTYHIGDKKKTDAAFAGAAHVTRIEFVNNRLVCNYMEPRSAIGEWNIGENRFVL
TTGSQGVHSMQYILASVFKIKKDQLRVITPDVGGGFGPKSFVYREYPLVLEAAKRLGRPVKWAGDRTEHFLTDA
QGRDNAVTAEMALDKDGRFLGMRVELLANIGAYISQYGPFIPYIGVTMSTGVYDIRALDVSVTGLYTNTCPVDA
YRGAGRPEAAFLLEKLVDACAHDLGLPVEEIRRRNFIRPKQFPYRTQTGRLYDNGEFEGHMDRAIERSQWKAFP
QRLEQSKADGKIRGIGMATYIEACAFPGSEPAFVELNGDGTVTLKIGTQTNGQGHATAYAQFLSEKLNLDIDKIH
VRQGDTDELKAGGGTGGSRSIPLGGVSASRAGEDLANKIKRIAADELEASAGDIELSDGIARIVGTDRSIDFSSIA
KAAKTTDDLKGFGEFVQDECTYPNGTHICEVEIDPDTGATEIVRYTIVDDFGVTVNPILLAGQVHGGVVQGIGQ
ALTENTIHGEDGQLLTASFMDYAMPRADNFPFFHFETRNVPSTTNALGIKGAGEAGTIGSTPAALNAVTDALWR
AYGIRHIEMPATPARIWAAIRGASPT
>SEQF2763||SEQF2763.1_07139
MNSFERREDARLVRGTGRFVDDDHSVSHLHLVFVRSPYAHANIISIDLSAALAVEGVVTAFTGEDVKALTVPYQE
FSQGPLGPIIDYGIAVEKVRFQGEPVVCIVARSPEIGVDAGELVHIEYEPLPVVIDVEDAIADNVVIHKESGTNLVW
GGKFDWGDVDGAFASAAHVVEIDKLYFHRFSSTPLECCGVIARWDRDDGVDFLTGLMQPGITQQYIAPVLGLP
PEKIRIRTRDIGGAFGVRQGIFTYMTLAAIASRKVGGRSVKWAETRSEHLVSSSHGSDRTFLNTRVALDENGVILA
IDSTHIDDGGAYPRYEPLGGVIWSQVAPGNYRLQNLRIDFSQVSTNKCPTGSNRGYSRMQHIWFLERVVDICG
HQLGIPADEIRRRNYVPEMPWTTPNGCVYDSGDYGKMLESAKALVGWDEWRAKQKTMRAEGKLIGIGIGTTL
DSGTNNFGQSKIITPYSPFSGNSEGAGVRIDGGGMVNVAMGSVPQGQSHETVAAQVVAAELGIPLEFVHVES
GFDTQRDVHTPFSGTYASQFVVSSLSAVHGAVDKLRAELVELASIVTGNDAAGLKVGTIDGQPVVMTEDGSTAL
TFGQLWARLYLEGGTLPKEAGHLTLACKHVYRPPFEIPDLDRKYGNLTLTYAAQLHIVVVEIDELTSAVRILDYAAM
DDCGRVINHTIVRGQVNGAIAHGIGAALMESLRYDENGNLLTATFSDYTPITIMNMPRVKLGNMESPSPFTYN
GAKGMGEGGGCALHALSSALQDALASNGIVVQCSHYSPSELYDLISSHVGSAVVRKRTAAVGGSGSAVCAIEQH
ESPQQ
>SEQF2764||SEQF2764.1_05630
MGIEGVGARVARKEDKRFITGAGRYVDDMVVPGMKHAAFVRSPHAHAQIKKIDVKKAQAMPGVIGVLTGKE

LKADGIGNLICGWMIHSKDGSPMKMGAWSPLAFDKVRYVGDAVVIVVAETKGQARDAAEAVEITYKELKAVV
EATKALEKGAPQIHPEADNNLIFDWEIGDAKATDAAIKAAAHVTRMKIVNNRLVPNAMEPRAALGHYDKADD
HYTCWTTSQNPHVARLVMSAFYNVAPENKLRVIAPDVGGGFGSKIYIYPEEIVCLWASKKTGVPVKWVADRTE
SFLTDAHGRDHVSTVEMAFDTNNRITGFKVDTIANLGAYMSLFSSCVPTYLYATLLSGQYDIPTIHANVRTVYTNT
APVDAYRGAGRPEATYLLERTMEAAARELGVSPAELRRKNFITTFPHQTPVIMNYDAGDYGASLDAAMNVSDY
AGFAKRKAAAAKQGKLRGIGMSCYIEACGIAPSAAVGSLGAGVGLWESAEVRVNAVGTIEVLTGSHSHGQGHE
TTFAQLVNQRFGVPIDSVSIVHGDTDKVQMGMGTYGSRSGAVGMSAISKALDKVEAKAKKIAAHLLEADEGDI
VIENGALKVAGTDKNVPWFQVALAAYTAHNLPAGMEPGLKETAFYDPSNFTFPAGCYICEVEIDPETGTTEIVQF
VAADDFGNIINPMIVEGQVHGGIAQGIGQALLEGAHYDASGQLLTASYMDYTMPRAGDLPSFKVSTSNTPCPG
NPLGIKGCGEAGAIGSPPAVINAITDAIGIADIAMPASPSTVWAAIRAAKH
>SEQF2853||SEQF2853.1_01998
MQGIAQTAHRIEDRRFLRGEGRYLADIRPEGCTAMVVVRSPHGNARIGGIDTAVALAMPGVLAVLTAADVATA
GAKALPCLAEIGKWDWARPVLPSRPLLAADQVRHVGEPVAVVIAETPDMARDAADMVVVDYEPLETCATVE
QALAGETSIWPEAPDNIGFDWHSGDAERVADAFARAANVVSVDLHNNRVAGMSLETRGAIGEWDTREERFT
LYVSSQGGHAIRRILCQNVFGIPEGRMRVITPDVGGGFGPKIFTYQEYALVLLAAARLNRPVKWVSDRGESLISD
TQGRAQTCRAELALDDTGRFLAMRFDCLSDMGAYPGQHGPNIATVAGDGLHPATYDLEAVHVRVRGVFTNTL
PTDSYRGAGRPEVIYAVERLVDAAARELGFDPVDLRLRNFVPRGKMPFTTVTGQVYDDGDFAQLTARAVALSDF
TGRESLREAARECGKLSGMGLAYFIDRCGRGLDEFAELRFDPSGSAVLLVGSQNNGQGHETAYANIVAQGLGLD
RNLIRVVQGDTDQVAFGRGTGGSRALAVGGNAVHLATGRIRAKLEAIAAHLLEGDISALRQEEGRFHLTGTNHS
VTLADCVRAAFAPSLLPPGMEPGLSIAAHYRPERPTFPNGCHIARVEIDIETAEIRLTRYVAVNDVGTILNPRLVEG
QFHGGLAQGISQALLEEVVYDPETAQPLTGSLMDYCYPKADDFVAFETDFVEIPCRTNPLGVKGCGEAGTIAAP
PAIANAVMDALRDYDTEGLQMPFTAPKLFAVLARGPRAAGK
>SEQF2854||SEQF2854.1_00414
MNAPETPRLVGASVKRKEDYRYLTGIGQYTDDIVQPQQSYGYFVRSPYAHARIRSIDTREAMASPGVIGVFTGD
DMAADKVGGLPCGWLIHSIDGSPMKEPPHPVLAQGKVRHVGDQVALVVAETLQQARDAAEKIEVDYEELPAV
VRTADATSAATLVHDDVPANTCYVWGHGDRAATDAAFAKAAHVTTLEIVNNRLVPNAIEPRAVNASYNRQDD
SYTLYVASQNPHVERLLMGAFVLGLPESRLRVIAPDVGGGFGSKIFLYPEDVALTWASKKVKRPIKWTAERSESFL
TDAHGRDHVTRAELALDADGKFLAMRVHTTANMGAYLSTFASSVPTILYATLLAGQYATPAIYAEVTAVFTNTAP
VDAYRGAGRPEATYVVERLVETAARELGIDPAELRRRNFIRSFPYATPVGLTYDTGDYEPCLQRAQELADVAGFPA
RRDEAQRRGKLRGLGYSCYIEACGLAPSNIAGALGARAGLFEVGEIRVHPTGTVTVFTGSHSHGQGHETTFAQV
VADRLGIPLDAVEVVHGDTGRVPFGMGTYGSRSLAVGGSAIVKALDKIEAKARQIAAHLLEASADDIEFSNGVFR
VAGTDRSKTFGEVALSAYVPHNYPLDRLEPGLNENAFYDPTNFTYPSGAYVCEVEVDPDTGETRVVKFTAVDDF
GNIINPMIVEGQVHGGIGQGLGQAMLEQCVYDGDSGQLLTGSYMDYAMPRAADLPDFTVETAQGTPCTHNP
LGVKGCGEAGAIGSPPAFINALIDALSPLGVSDLQMPATPHRVWQAIHAARQGDGALGASPAAGQTADQTATA
ARTI
>SEQF2854||SEQF2854.1_03370
MTTPAAHDDRIGNGGNNGGNNGSKTSGIGARLRRKEDDRFLRGRGDYVANLRMVGMRDVAFVRSPLAHAR
IRGIEKPEGLADAVFTLADLDGVRPIVADSGLAGFKSSAQPVLADAKVRQVGEMIAMCVAPTRAQAEDIAAQV
FVDFDELPAVVDMLAAREPGAPLVHEHWGDNVFLSTAVGTDRDNEAALAAVRASAPIHVVRTLRTARQSMAP
MEGRGVVAWWDRRLAQLVVVTAAQMPHINRTGLAGCLGLDEGQVRVISPDVGGGFGYKGILLPEEVCVAWL
AMRLGRPVRWIEDRREQLTANANCREHVYRIEAWADRDGRLLAVDCEATVDSGCYSSYPFSACLEAAQVGSIL
PGPYMMERYRCRTWSVATNKPPILPYRGVARTGVCFAIESVMDAIAVEAGLEPHQVRLRNLVPPERMPYDNIT
GKHFDSGDYPECVRRAVAAIDLPGLRARQQRGEPDGRRIGVGFAVFCEQGAHGTSVYHGWGIPMVPGREPA
VVRLTPDGVLEIRAGVHSHGQGMETTLAQIAHEVLGVDTGQVRVLLGDTAITPYSTGTWGSRSIVMAGGAVG

QACKQLRSRLLRIGAHLLGMPEPAASWQDGAVVAGGSRATLAELAHVWYRQPQKLPADVDPAGLEVMTTYQ
ATRDTGTFSYACHAVALAVDTELGKVELLDYAIVEDGGVLINPMVVDGQVYGGTAQGIGTALYEEMRYSEDGQP
LASTLADYLLPGATEVPAIRIDHMETPAPYTEFGQKGIGESGAIGSPAAIVNAINDALRPLGAELRELPASPRAILAA
LANARAGDAGHSGHARPMPSSPAEVVA
>SEQF2885||SEQF2885.1_00384
MTAEVTDRPATTEIGRDRRRKEDQRLITGRTRWTDNIVLPGMLHMAMVRSPFAHARIASVDTSEAASATNVVA
VYSGADLDESVGVLINAWPVSPDQVAPVHPPMPADRVTFAGEIVAVVVARSAAEARDAAELIDVDYEELPAAL
DLKEAAEDTVLAHPDLGTNKSALWTFDSAEAGTGGDVEKAIATARQDGIVIEREYRQQRLIPAFMEPRSVVVDP
TGEQLTMWTATQIPHIVRFALAATTGVPESKIRVIAPDVGGGFGGKLQVTPEEWLAWWVARQLGRPVKYTETR
SESLMAAHHGRDQWQRLTLAADKQGKVTGLKVDLLADLGSYVALIGGGVPVLGAFMFNAIYKFPAYHFSMQS
VLTNKAWTDAYRGAGRPEATFGIERIMDELAHELGEDPLVVRERNWITHEEFPFTSVAGLEYDSGNYEAATDLA
KQMFGYDELREEQRRRRESKDPVQLGIGISTFTEMCGLAPSRVLGSLDYGAGGWEHASVRMLATGKVEVVVG
VSPHGQGHETAFSQIVADRLGVPFEDVEILHGDTQISHKGLDTYGSRSLVVGGEAVVRAADKVIDKARRYAAHV
LEANPDDLEFTAGRFGVAGTDQSVGITDIAAAAFASHDYPDDIEPGIDAEATFDPVNFNYPHGTHLSAIEIDTET
GEVRLRKYTCVDDVGQVINPLIVEGQIHGGLVQGISQALWEEAVYDDSGTLVSGSFVDYLVPTAADTISFDTATTS
TAATGNTLGTKGVGEAGTIASTPCIVNGVVDALRIFGVDDVQMPCTPERVWQAIRSGSGGEDTTGAAAPHFDE
ATGDQGQTDRMAGADGPTGPAMSTEGEVR
>SEQF2893||SEQF2893.1_01735
MGASDFSKLPYIGEALKRKEDYRFLTGAGQYTDDVVLAAQCHAVFVRSPHAHAKINSINIDAAKAAPGVLGVFT
GADVAADNINGLPCGWLITSTNGEPMKEPPHPILAQGKVRYVGDHVAMVVAHTQQQARDAAELVEVDYDVL
PAVVNVADAASGAGAGAVVHDIAQDNHCFKWAIGDKGGVDAAFANAAHVTKLDLVNNRLIPNAMEPRAAIG
SYNRASDEYTLYVSNQNPHVERLLMTAFVMGLPEHKVRVIAPDVGGGFGSKIYLYAEDVCLTWASKKLNRNIK
WVADRSEAFLSDAHGRDHVSHAEMAMDKDGKFLALRVHTHANLGAYLSTFASAVPTILYATLLAGQYSTPQVY
VEVDSWFTNTAPVDAYRGAGRPEATYLLERLVTRCAWEMGLSQDEIRRRNFIQTFPYQTPVALQYDTGDFHAC
MDGANKLADVAGFEQRKAASAAKGLLRGIGYSSYIEACGIAPSNIAGALGARAGLFECGEVRVHPTGSVTVFTG
SHSHGQGHETTFAQVVAARLGIPVENVDIVHGDTGRVPFGMGTYGSRSISVGGAAIMKALDKIEAKAKKIAAH
LMEASDADIDFANGEFTVRGTDKKIPFGQVALTAYVPHNYPLDKLEPGLNETAFYDPTNFTFPAGTYICEVEIDPQ
TGVTRVDKFTAVDDFGTIINPMIVEGQVHGGLVQGIGQALLENCVYDRETGQLLTGSFMDYAMPRADDFPEFK
LGHVCTPCTHNPLGTKGCGEAGAIGSPPAVINAVLDALRPLGVKDFDMPASPHRVWEAIQSAKA
>SEQF2894||SEQF2894.2_00718
MPDTNMPDPSRMRFAVGQPVTRREDPMLLRGEGHYTDDLQAEGQAHAWMVRSPYAHGVINGIDTAEAKS
MLGVLAVYTGADLAEYGPIRCAMPLKNRDGSPLASPVRPALATDRVRYVGDPVAIVIAETPAEARDAAEAVML
DIDPLDAVTEASAAAAPGAPQLYDDVPGNVVLDWHSGDAEKVAAAFATAAHRVKLPLRNNRIVVAAMEPRAA
IAEYDAESGRYTLRACSQGVFGLRRQIAEDILRVPVEKVHVLTTQVGGSFGMKGTVYPEYPGMFHAARLLGRPV
KWTDERSGSFVSDQHGRDHETVAELALDAEGRFLAVRLTSFANMGAYLTTVGPLMGTMGFVKNVQSNYATPL
IEVDTKSVFTNTSPVGAYRGAGRPEGNYYMERLVEAAAAQIGMDPLELRRRNHIRPEQMPYDAASGSRYDGG
EFTALMEEALRQADWDGFAARREESASRGLLRGRGLGNYLECTAPAGKEHGGVRFEEDGGVTIITGTLDYGQG
HWTPFAQLLGSRLGVPFERIRLIQGDSDLLKHGGGTGGSRSMMSSGTAIIEASEKVIEKGREAASHLLEAAVADIE
FDPENGRFAIAGTDRGIGILELAARMRAAPELPEDLRSLDVTHVTEDKPMAYPNGCHVAEVEVDPETGHVHLV
RYLAVSDFGVEVNPLMVQGQVHGGVAQGIGQALMERVSYDEGGQILSASFMDYALPRAEDLPPILTASRPVPA
KTNPVGAKGCGEAGCAGSLPAVMNALADALRAAGAEPVDMPATPEKVWMALQRASRG
>SEQF2894||SEQF2894.2_00438
MAKFGLSQSLRRVEDPRLLKGEGRYTDDLAVAGTATGYLLRSPHAHATILSIDTADARAMPGVLAIYTAEDLAAD
GLGPLPHAGSLKNRDGSRSANAPRFLLASGKVRHVGDPVAFVVAESLDAAKDAAEAILVDYDPLPACTDLATAN

DPGQPLVFDEVPENRVFDWAIGDKAKTDALFDAAAHVTRLTVVNNRVVVASMEGRAALAEYDSATERFTLQA
TSQGAWHLKDMLAEHVFKLPKEKFRVVTHDVGGGFGMKLFCYVEYGLACYAARKLGRPVKWTAERSEAFVSD
THGRDNITLGELALDRDGKFLALRTRNLANMGAYLSQFAGFIPTAAGTKVLASVYGFQAIYANVIGALTHTTPVD
AYRGAGRPESNYLVERLIDAAARELGIDRAELRRRNMVPPSAMPHTTPVGQKYDSGDFAQVLDEALRRADWA
GFPARQAEARARGKRRGIGLAYYLEATGGDPSERAEVRFTEDGHVEVLVGTQSTGQGHETAYTMIIAERLGVPV
DRIRVRQGDSDEIPTGGGTGGARSLYSEGTALLATASTVLERGRQAAGEALEASPADIEFTTTGALDGGGRFSIAG
TDRGIGILELAARQRAAAARGEQATLLDAAEVAEVPFGTFPNGCHIAEVEVDPETGLIGIARYLVVDDVGHAINP
LIVRGQVHGGVAQGVGQALHEHTVYDPESGQLLSASFMDYALPRAEDLPAIEVDLVEIPCETNPLGVKGAGEA
GAVGSPPAVMNALVDALAPDGVTHLDMPATPERVWRALGQALSRAA
>SEQF2908||SEQF2908.1_02446
MPDTTMPDPTRMRFAVGQPVTRREDPMLLRGEGRYTDDLQAEGQAYAWMVRSPYAHGVINGIDTAEAKSM
PGVLAIYTGADLAEYGPIKCAMPLKNRDGSPLSSPVRPALAMDRVRYVGDPVAIVIAETPAEARDAAEAVMLDI
DPLDAVTEASAAAAPGAPQLYDDVPGNVVLDWHSGDAEKVAAAFAGAAHRVKLSLRNNRIVVAAMEPRAAI
AEYDAESGRYTLRACSQGVFGLRRQIAEDILRVPVEKVHVLTTQVGGSFGMKGTVYPEYPGMFHAARLLGRPV
KWTDERSGSFVSDQHGRDHETVAELALDAEGRFLAVRLTSFANMGAYLTTVGPLMGTMGFVKNIQSNYATPLI
EVDTKSVFTNTSPVGAYRGAGRPEGNYYMERLVEAAAAQIGMDPLELRRRNHIQPGQMPYSTAAGTQYDGG
EFTALMEEALRQADWDSFAARREDSASRGLLRGRGLGNYLECTAPAGKEHGGVRFEEDGGVTIITGTLDYGQG
HWTPFAQLLGSRLGVPFDRIRLIQGDSDLLKHGGGTGGSRSMMSSGTAIIEASEKVIEKGREAASHLLEAAVADI
EFDPENGRFAIAGTDRGIGILELAARMRTAPELPEELRSLDVTHVTEDKPMAYPNGCHVAEVEVDPETGHVHLV
RYLAVSDFGVEVNPMMVQGQVHGGVAQGIGQALMERVSYDEGGQILSASFMDYALPRAEDLPPILTASRPVP
AKTNPVGAKGCGEAGCAGSLPAVMNALADALRAAGAEPVDMPATPEKVWMALQRASRG
>SEQF2908||SEQF2908.1_02241
MAKFGLSQSLRRVEDPRLLKGEGRYTDDLAVAGTATGYLLRSPHAHATILSIDTADARAMPGVLAIYTAEDLAAD
GLGPLPHAGSLKNRDGTRSANAPRFLLASGKVRHVGDPVAFVVAESLDAAKDAAEAILVDYDPLPACTDLATAN
EPGQPLVFDDVPENRVFDWAIGDKAKTDALFDTAAHVTRLTVVNNRVVVASMEGRAALAEYDAATERFTLQA
TSQGAWHLKDMLAEHVFKLPKEKFRVVTHDVGGGFGMKLFCYVEYGLACYATRRLGRPVKWTAERSEAFVSD
THGRDNITLGELALDRDGKFLALRTRNLANMGAYLSQFAGFIPTAAGTKVLASVYGFQAIYANVIGALTHTTPVD
AYRGAGRPESNYLVERLIDAAARELGIDRAELRRRNMVPPSAMPHTTPVGQKYDSGDFARVLDEALERAGWA
GFPARQAEARARGKRRGIGLAYYLEATGGDPSERAEVRFTEDGNVEVLVGTQSTGQGHETAYTMIIAERLGVPV
DRIRVRQGDSDEIPTGGGTGGARSLYSEGTALLATASTVLERGRQAAGEALEAAPADIEFTTTGALDGGGRFTIA
GTDRGIGILELAARQRAAVAKGESATLLDAAEVAEVPFGTFPNGCHVAEVEVDPETGLIEIARYLVVDDVGHAIN
PLIVRGQVHGGVAQGVGQALHERTVYDPESGQLLSASFMDYALPRAEDLPAIEVDLVEIPCETNPLGVKGAGEA
GAVGSPPAVMNALVDALAADGVTHLDMPATPERVWQALGQSMARAA
>SEQF3086||SEQF3086.1_05024
MNAEISGHGLGASRWIGKRVPRKEDPRLLTGRGQFVDDVVLPGMLHVAFARSPVACGRIISIDREAAQQMPG
VHAVLTAADIAARPIRMLSFFFTEAEIAIAPLADGHVSYLGEPVALVVAESRAMAEDAAALVEIACEEGQPIVSIAQ
ARTGAPIHPGMDSNIAAEIGDEEIDEELEQRLAASALRIRQKVVHQRISQAPMETRGVIASRDGPEELTLWITCQ
SPHLVARWVSLALDLPDSAIRVIAKDVGGAFGLKNHPWKEECAVIVAALLLGKPLKWIEDRYEALTASNQAREAE
MTLEAGFDAEGRLTGSHGIYDCNNGAFPQGADSNIAVHMFVWAAYRQPAYAFVSRGWYTNTNGLAAYRGP
WAMESLVRETLLDKAGRRLGIDPVEIRRRNLVYLADQPATSSMGIPLEDITPGECLEKLLQHFDMAQFRREQAK
ARSEGRYLGVGIAAYVEPTGSAGSMAPMTGELAQVRIEPTGKVTATMSTHSQGHGTATTMAQCIADRLGVRYE
DVTVFEGDSSRGGFGPGAAGSRQGVIAGGAAIRCSELLADKVRTVAAHLLNASPDSVVLSDGMVHVEGAPEM
TRPLSEIAAIAYGEPDRLPPDLETGLEMQFRYQPPPMTFTSAAHLAIVEVDAETGFVTILRWISSEDCGTVINPAV
VEGQISGGLAQAIGMVLLEDMPYDAHGNPLAATFKDYLLPGIGDVPVFEFVHANTPSRTIGGMRGVGEGGAII

GPPTLVNAIADALSPFGEIEELVLPLTPARILDVIEQRDVSGTSKANSPVAETPVTLSPEPVPAEDVVQGVSAAGPA
GDWDMVLKTPMGPQAMQAHFDVEGSALRGYLAAPEGRQEFRGGIVEGNRLRFDLKVEKPMKITLKYDLEVS
GDAIAGKCKMGMFGSAKVTGKRAP
>SEQF3148||SEQF3148.1_04776
MNMRVSQAAHSATPVGIGQSLPRNEDAVLVRGEGRYTDDLSLEGQLHAAFVRSPHAHGIIREIDVSAARAMK
GVVAVYTADDLEGQGYGTLKCQLDMTSRDGTPLRKPVRKALAIDKVRYVGDPVAMVVARTPAQARDAAETVA
LDIDILPAVTSAEEALEPGAPQLFDDVPGNLVLDYHFGDAAKVAAAFAEAAHVTKLRIVNNRIVVNPIEPRAAIGA
YDRKTGRYTLHAPSQGAFGMRNNLAAAMGVPAGRMRLVTGHVGGSFGMKASVFPEYVVLLHAARLLKKPVK
WTDQRAESFVSDHHGRDMAFEAELALDARGRFLATRFTGFGNMGGYLSSVGPMMATLNIGKNSVGMYRTP
LVEVATRCAVTNTVPIAAYRGAGRPEGNYFMERLIDAAAREMKIDKAVLRQRNLIPTDKLPWTTPIGTQYDSGD
FPALFARALEAADWKGYRARERASRKAGKLRGRGIGCYLEVTAPPTNEMGGIHFEPDGTVTIVTGTLDYGQGH
WTPFAQVLTSQLGVPFDKIRLVQGDSDRLIAGGGTGGSKSIMASGSAIIEASEKVVEKGKVLAAHFLEAGLPDIEF
AEGRFGIAGTDRSIGIMELAARVRTSASLPDDLPATLDVDHVFKAAPSAYPNGCHIAEVEIDPDTGQVEIAGYVM
VNDFGTLVNPMIVEGQLHGGVVQGIGQAIYEMTSYDEAGQLMTGSFMDYAMPRAADVPFFTFISQGVPTRT
NRVGAKGCGEAGCAGSLPSVMNAIVDALSPYGVDHVDMPATPLAIWRIIHERGGHA
>SEQF3148||SEQF3148.1_02918
MRPMKFGFGQPVRRVEDQRLTTGTGRYTDDIAVEGALHAYVLRSQYAHARFTIRDKETARRMKGVKLVLTGED
VAEYGDLPCKGHIKTVSGEMSQSLPVPVLPRDTVRHVGEAVAFIVAETLAQARDAAEAIEIDWQPLPAVTGIEEA
LAKGAPQVWADRPGNVAFEAEQGDREKTEKAFAKAARTVSLTIVNNRLASNYMETRACVAEYDKAEKRWTLT
LGSQGSHGMRDLIATYVLKVDPKRIRVVTPDVGGGFGTKIFLYREYPLAMIAAEKLKRPVRWVADRTEHFLADT
HGRANLATATMALDAKGKFIGLKVDLSAEMGAWLSQYGPFIPWVGTTMTPGCYNIPAVHVLFRGVLTHTTPID
AYRGAGRPEAAYLIERLVDAIASETGKTPDAVRAQNFVKPSEMPHKTQTGPVYDSGEFEGHMRHAMQVADW
KGFKARHKAAAKAGKIRGIGMASYIEACGGGGPESSTVILEKDGMVTVLIGTQSNGQGHETAYSQLVSQHLDIP
MDRIRVVQGDTDRVETGSGTGGSRSIPVGGAALNKATEILTDNLKQLASETLEAAVGDLEIANGAVRIVGTDKQ
LDLAAIAALPGATPTMLKVHQSWTPPEATYPNGTHVCELEIDPGTGQTEILNYVVVDDFGVTLNPLMLQGQVH
GGAVQGIGQALMEEIRFDPDGQMLTATFMDYALPRAIDVPNFHFETRNVRCLTNAIGVKGAGEAGAIGACPAV
MNAMVDALDRAAGIKAIDMPATPVKVFNALKEAGYRL
>SEQF3148||SEQF3148.1_01393
MHARIPGHRAETLGPALTPTDPRSLRRFEDERFLTGQGFYLDDEAVPDALHAMVLRSPHAHAAILSIDAEAARG
LPGVHAVYTAAEIADLAPLPCSLAVAGEAPLIVPPRSPLAGERVRHVGDPVAFIVAETAELAREASELIAVDYDILPS
VTELAAANAPDAPEIWPQAPGNLALRYRLGDGAAVATAFAQAAHVVTCRVVNNRIAAAALEPRAALGRFDKA
SGRYRLSLSGASVHDIRRELAAVLRIAPERIDVACPDVGGGFGMKNVTYPEYALVLVAAERLGRPVRWLAERIED
FSGGVHARDNLTTGKLALDAKGRFLALQVETTANLGAYVSSLGPGSATTAPTPAMGGLYDIPAMTMDVRCVFT
NTVPIDAYRGAGKPEANYLIERLVEAAANRLRIDSATLRKRNFIRRFPYRKPFGAVLDCGSFSSNLDQVLAAADRA
GFRRRRAESRRRGKLRGFGIGCFLETSRGAPNEDAWLSFRADGGIDMTVGTQSNGQGHETSFAQLLASRFGLP
VERFRLIQGDTARVPSGGGHGGARSLHMGGGALALAADALLEKARPAAAELLQAAAEAVSFHNGRFLAPGAG
EVDLGSVARHVAETTGKPLASHGARRNAPLTFPNGAQAAEVEVDPQTGEVTLLRYVAVDDYGTLINPLLTEAQV
HGGLAQGIGQALMEEARYDPDGGQLLSATFMDYAMPRAMDLPAFELSMVELPTEANPLGAKGAGQAGCIG
APQTVIHAILDALRPLGVTHLDMPAIPARIWRAIRDAQEAQTKD
>SEQF3148||SEQF3148.1_04043
MSATGIGAAVRRKEDHRFITGQGRYTDDINRPGQAHAYFLRSPHAHAKIKSIDTKAAAGMPGVIGIFTGADLAN
DKIGGLICGWMIHNKDGSAMRAGPHPALAQGKVRYVGDHVAVVVAETLAQAKDAAEAIAVDYQELPAVVDT
AKAVGAKTVVHDEAPDNSVFNWHLGDKDATEKAFKKAAKHVTKIDLVNNRLVPNAMEPRAAVGDYDDGEGIF
TLYTTSQNPHVARLVLSAFIGIAPENKLRVIAPDVGGGFGSKIFIYAEETVCVWAAKKVGRPVKWTADRTEAFLSD

AHGRDHVTHAELATDAEGKILGMKVHTTANLGAYLSTFSSSVPTYLYAPLLSGQYDIPAIYCEVDAVYTNTAPVDA
YRGAGRPEATFVVERLIEITARQLGKDPAKFRMQNYIKKFPHQTPVIMMYDAGNYGASLKKALEMIDYKGLGKR
KRDSARNGKLRGIGFSSYIEACGIAPSAAVGSLGAGVGLWESAEVRVNPVGTVEVLTGSHSHGQGHETTFAQLV
SDRLGIPIDNVSIIHGDTDKVQMGMGTYGSRSGAVGMSAIFKAIDKVIAKGKKVAAHVLEADEADIDFADGHFA
VKGTDRKLDFGSCALQAYVAHKFNGQELEPGLKEGAFYDPTNFTFPAGVHICEVEIDPQTGVTTIERWAAVDDF
GNIINPMIVEGQVHGGIAQGVGQALLEGARYNADGQLVTASFMDYTMPRADDLPSFDVGMTVTPCPSNPLG
IKGCGEAGAIASPPAVINAITDALGHEDIAMPATPQAVWRAAQKSISRMAAE
>SEQF3155||SEQF3155.1_01821
MGASDFAKLPYIGEALRRKEDARFLTGAGQYTDDIVLANQRYAVFVRSPHAHARINSIDTSQAKSMPGVRAVFT
GADLAGKVNGLPCGWLITSTDGTPMKEPPHPVLAIGKVRYVGDPVAMVVADSVEQAKNAAEAVMVDYEVLG
ACVDVRDARNAPALHDEAPDNHCYKWAIGDKAQVDAAFAKAAHVTRIDLVNNRLVPNAIEPRSAIGVYNRGS
DEYTLYVANQNPHVERLLMTAFVLGLPEHKVRVIAPDVGGGFGSKIFLYAEDVAVTWAARQLNCAVKWTAERS
ESFLTDAHGRDHVTHAEMAMDKDGRFLAMRVHTDANLGAYLSTFSTAVPTILYATLLAGQYTTPQIYVEVDAW
FTNTAPVDAYRGAGRPEATYLLERLVTRCGWEMGLSQDEIRRRNLITQFPYQTPVALQYDTGDYVACLDKAMQ
LADVAGFEARRQASEAKGLKRGIGFSSYIEACGLAPSNIAGALGARAGLFECGEVRVHPTGSVTVFTGAHSHGQ
GHETTFAQVVAARLGIPVENVDVVHGDTGRIPFGMGTYGSRSISVGGAAIMKALDKIEAKARKIAAHLMEASE
ADIEFTNGEFRVKGTDKKVPFAQVALTAYVPHNYPLDQLEPGLNETAFYDPTNFTFPAGTYICEVEVDPATGQVR
VDRFTAVDDFGVIINPMIVEGQVHGGIAQGIGQALLENCVYDKETGQLLTGSFMDYAMPRADDLPDFQLGTV
CTPCTHNALGTKGCGEAGAIGAPPAVINAVLDALRDLGVKDFDMPATPARVWEAIQQARAQ
>SEQF3155||SEQF3155.1_03607
MKRDVVNLEPGVESTPPIAPVTEDPRYIGARLPRHGIERLTQGQGQYVDDIELPRLAHVVYWRSPVAHMRIKGI
DDRIARSMPGVLMVATGQDLAKVCKPWVAVLGHLTGMKSAPQYPLAVERACWQGEPVVAVVAETRAQAED
ALQHLVVDWEELPLTVEMERALAPDEPVIHPELGDNVCFSRRLDVGEVDRVFAEADVVAETTFEFGRHTGVTLE
PRSQIAHWQPAEQRLTVYHSCQAPHMMQDLYARQFDLPAHAVRVICKDVGGSFGVKVHAYPDDFATVALSM
LLGRPVKFVADRLESFTSDIHARHHRVKGRIAVNRDGEILGFEIDDLTGIGPYSMFPRTSAIEGNQVVNLVGGPYR
HQHYRAQLHVVFQNKTPTCQYRGVGHPIACAVTEGLVDLAAQRIGMDPLEIRARNVIPDDAYPATGASGIKLEV
LSHEACLRKLREMMNYDALRAEQAALRKQGIWRGIGLATVIELTNPSAAFYGIGGARIASQDGATVRLNPEGHV
TVLVGVGEQGQGTEAIYRQIAADAVGVDIDQVRVITGDTDVTPYGGGTWASRGAGIGGEAVLLAGQALRGNIL
KTAAVILNREAGQLGLHRGQVVDAVTGEALLPLSEVGRIAYFRTDTLPQGFTPELMVTRHYAQRDYPFIFTNGV
QASYVEVDPDTGFVTLLKHWAVEDCGRVLNPMLVDEQMRGAIVQGIGGALYEECLYDEQGQMLNANMADY
LVPMAAEMPDIEVAHVMTPTRSSRLGAKGAGEAGTAGAPAAVMNAINDALAPFGAHVHSQPITPEKILRALG
KVK
>SEQF3156||SEQF3156.1_02550
MKRDVVNLEPGVESTPPIAPVTEDPRYIGARLPRHGIERLTQGQGQYVDDIELPRLAHVVYWRSPVAHMRIKGI
DDRIARSMPGVLMVATGQDLAKVCKPWVAVLGHLTGMKSAPQYPLAVERACWQGEPVVAVVAETRAQAED
ALQHLVVDWEELPLTVEMERALAPDEPVIHPELGDNVCFSRRLDVGEVDRVFAEADVVAETTFEFGRHTGVTLE
PRSQIAHWQPAEQRLTVYHSCQAPHMMQDLYARQFDLPAHAVRVICKDVGGSFGVKVHAYPDDFATVALSM
LLGRPVKFVADRLESFTSDIHARHHRVKGRIAVNRDGEILGFEIDDLTGIGPYSMFPRTSAIEGNQVVNLVGGPYR
HQHYRAQLHVVFQNKTPTCQYRGVGHPIACAVTEGLVDLAAQRIGMDPLEIRARNVIPDDAYPATGASGIKLEV
LSHEACLRKLREMMNYDALRAEQAALRKQGIWRGIGLATVIELTNPSAAFYGIGGARIASQDGATVRLNPEGHV
TVLVGVGEQGQGTEAIYRQIAADAVGVDIDQVRVITGDTDVTPYGGGTWASRGAGIGGEAVLLAGQALRGNIL
KTAAVILNREAGQLGLHRGQVVDAVTGEALLPLSEVGRIAYFRTDTLPQGFTPELMVTRHYAQRDYPFIFTNGV
QASYVEVDPDTGFVTLLKHWAVEDCGRVLNPMLVDEQMRGAIVQGIGGALYEECLYDEQGQMLNANMADY
LVPMAAEMPDIEVAHVMTPTRSSRLGAKGAGEAGTAGAPAAVMNAINDALAPFGAHVHSQPITPEKILRALG

KVK
>SEQF3156||SEQF3156.1_01705
MGASDFAKLPYIGEALRRKEDARFLTGAGQYTDDIVLANQRYAVFVRSPHAHARINSIDTSQAKSMPGVRAVFT
GADLAGKVNGLPCGWLITSTDGTPMKEPPHPVLAIGKVRYVGDPVAMVVADSVEQAKNAAEAVMVDYEVLG
ACVDVRDARNAPALHDEAPDNHCYKWAIGDKAQVDAAFAKAAHVTRIDLVNNRLVPNAIEPRSAIGVYNRGS
DEYTLYVANQNPHVERLLMTAFVLGLPEHKVRVIAPDVGGGFGSKIFLYAEDVAVTWAARQLNCAVKWTAERS
ESFLTDAHGRDHVTHAEMAMDKDGRFLAMRVHTDANLGAYLSTFSTAVPTILYATLLAGQYTTPQIYVEVDAW
FTNTAPVDAYRGAGRPEATYLLERLVTRCGWEMGLSQDEIRRRNLITQFPYQTPVALQYDTGDYVACLDKAMQ
LADVAGFEARRQASEAKGLKRGIGFSSYIEACGLAPSNIAGALGARAGLFECGEVRVHPTGSVTVFTGAHSHGQ
GHETTFAQVVAARLGIPVENVDVVHGDTGRIPFGMGTYGSRSISVGGAAIMKALDKIEAKARKIAAHLMEASE
ADIEFTNGEFRVKGTDKKVPFAQVALTAYVPHNYPLDQLEPGLNETAFYDPTNFTFPAGTYICEVEVDPATGQVR
VDRFTAVDDFGVIINPMIVEGQVHGGIAQGIGQALLENCVYDKETGQLLTGSFMDYAMPRADDLPDFQLGTV
CTPCTHNALGTKGCGEAGAIGAPPAVINAVLDALRDLGVKDFDMPATPARVWEAIQQARAQ
>SEQF3160||SEQF3160.1_01145
MGASDFAKLPYIGEALRRKEDARFLTGAGQYTDDIVLANQRYAVFVRSPHAHARINSIDTSQAKSMPGVRAVFT
GADLAGKVNGLPCGWLITSTDGTPMKEPPHPVLAIGKVRYVGDPVAMVVADSVEQAKNAAEAVMVDYEVLG
ACVDVRDARNAPALHDEAPDNHCYKWAIGDKAQVDAAFAKAAHVTRIDLVNNRLVPNAIEPRSAIGVYNRGS
DEYTLYVANQNPHVERLLMTAFVLGLPEHKVRVIAPDVGGGFGSKIFLYAEDVAVTWAARQLNCAVKWTAERS
ESFLTDAHGRDHVTHAEMAMDKDGRFLAMRVHTDANLGAYLSTFSTAVPTILYATLLAGQYTTPQIYVEVDAW
FTNTAPVDAYRGAGRPEATYLLERLVTRCGWEMGLSQDEIRRRNLITQFPYQTPVALQYDTGDYVACLDKAMQ
LADVAGFEARRQASEAKGLKRGIGFSSYIEACGLAPSNIAGALGARAGLFECGEVRVHPTGSVTVFTGAHSHGQ
GHETTFAQVVAARLGIPVENVDVVHGDTGRIPFGMGTYGSRSISVGGAAIMKALDKIEAKARKIAAHLMEASE
ADIEFTNGEFRVKGTDKKVPFAQVALTAYVPHNYPLDQLEPGLNETAFYDPTNFTFPAGTYICEVEVDPATGQVR
VDRFTAVDDFGVIINPMIVEGQVHGGIAQGIGQALLENCVYDKETGQLLTGSFMDYAMPRADDLPDFQLGTV
CTPCTHNALGTKGCGEAGAIGAPPAVINAVLDALRDLGVKDFDMPATPARVWEAIQQARAQ
>SEQF3160||SEQF3160.1_00103
MKRDVVNLEPGVESTPPIAPVTEDPRYIGARLPRHGIERLTQGQGQYVDDIELPRLAHVVYWRSPVAHMRIKGI
DDRIARSMPGVLMVATGQDLAKVCKPWVAVLGHLTGMKSAPQYPLAVERACWQGEPVVAVVAETRAQAED
ALQHLVVDWEELPLTVEMERALAPDEPVIHPELGDNVCFSRRLDVGEVDRVFAEADVVAETTFEFGRHTGVTLE
PRSQIAHWQPAEQRLTVYHSCQAPHMMQDLYARQFDLPAHAVRVICKDVGGSFGVKVHAYPDDFATVALSM
LLGRPVKFVADRLESFTSDIHARHHRVKGRIAVNRDGEILGFEIDDLTGIGPYSMFPRTSAIEGNQVVNLVGGPYR
HQHYRAQLHVVFQNKTPTCQYRGVGHPIACAVTEGLVDLAAQRIGMDPLEIRARNVIPDDAYPATGASGIKLEV
LSHEACLRKLREMMNYDALRAEQAALRKQGIWRGIGLATVIELTNPSAAFYGIGGARIASQDGATVRLNPEGHV
TVLVGVGEQGQGTEAIYRQIAADAVGVDIDQVRVITGDTDVTPYGGGTWASRGAGIGGEAVLLAGQALRGNIL
KTAAVILNREAGQLGLHRGQVVDAVTGEALLPLSEVGRIAYFRTDTLPQGFTPELMVTRHYAQRDYPFIFTNGV
QASYVEVDPDTGFVTLLKHWAVEDCGRVLNPMLVDEQMRGAIVQGIGGALYEECLYDEQGQMLNANMADY
LVPMAAEMPDIEVAHVMTPTRSSRLGAKGAGEAGTAGAPAAVMNAINDALAPFGAHVHSQPITPEKILRALG
KVK
>SEQF3742||SEQF3742.1_02498
MTTESNPTRFGSGQAVRRLEDESLLAGAGRYTDDVTLPGQAHLVFLRSPYPHARIVSIDTVAAAAMPGVLRVIT
GTEMAEAGVKPMPGAAGFKRADGGDCASPPRLAMAHERVRFVGEAVAAVVAETVQQARDAAEVVMVDYD
ELPMVVDLASATADGAPQLCAEATGNIAAEMKHGSSEAATAAFAKASHVVALDVVNQRVVALTIEPRSVLAAY
DGESDRLTIRMSTQMPSGVRDSVCGAIGLAKEKVRVVVGDVGGGFGMKTGAYPEDIAVAFAALQVKRPLKWV
AERSEEFLSSAHGRDIEARAELALDADGKILALRIKTLANVGAYATGTGVAIQLLIGPWVQTSVYDIQTIDFHFKAV

LTNTAPTGAYRGAGRPEAIFTIERLMDEAARQTGIDRVELRRRNFIRPEQMPYKNPMAQTYDTGKFESVMDQA
LALADWQGFETRAAESARQGKHRGLGIATFLEWTGGNVFEERVTVSVQADGVIEVFSAVNAMGQGIATSLAQ
LAVDAFGVPIEKVRVVLGDTDRGDGFGSAGSRSLFTGGSAVRIGAERTIDKARELAAQEFEASVDDIVYSRGVFN
VAGTDLELDLFALAGKQPEGEIFVDSTSTVAGPTWPNGCHICEIEIDPPTGEISVVAYSSVNDVGRVINPMIVRG
QLEGGAVQGIGQALYEQVVYDHDTGQPVTGSLMDYAAPRADIVQTMFHMEMDESTPCINNPLGVKGVGELG
TIGATPAIVNAVADAFARNGFAASAPRLHMPLSPARVWQAMHTVD
>SEQF3742||SEQF3742.1_01636
MGASDFSNLPHIGEALRRKEDYRFLTGAGNYTDDITLANQSHAVFVRSPHAHAVVKSVDTAEALKMPGVVGIF
TGKDIEGKMGGLPCGWLINNPDGTPMKEPMHPILAINKVRYVGDHVAMVVAETVEQAKNAAEAVVVDYDV
LPALVSVADAAKTASGVTLHDEAPDNRCYKWALGDKAAVDAVFANAAHVTKLDLVNNRLVPNPIEPRVAIGSYS
RGTDDYTLYVSNQNPHVERLLMTAFVLGLPEHKVRVIAPDVGGGFGSKIFLYAEDVCLTWAAKQLNRNIKWTAE
RSECFLSDAHGRDHVSHAEMAMDKDGKFLAMRVHTDANLGAYLSTFSTAVPTILYATLLAGQYTTPQIYVEVDA
WFTNTAPVDAYRGAGRPEATYLLERLVSRCAWEMNLGQDEIRKRNFITTFPYQTPVALQYDTGDFHACMDKAK
ALAEVEGYAQRKSASEAQGKLRGIGYSSYIEACGIAPSNIAGALGARAGLFECGEIRVHPTGSVTVFTGSHSHGQ
GHETTFAQVVAARLGIPVDNVDVVHGDTGRVPFGMGTYGSRSISVGGAAIMKALDKIETKAKKIAAHLMEASD
ADIEFANGEFTVKGTDKKIPFGQVALTAYVPHNYPLDKLEPGLNETAFYDPTNFTFPGGTYICEVEVDKQTGEVRV
DRFTAVDDFGTIINPMIVEGQVHGGLVQGIGQALLENCVYDNETGQLLTGSFMDYAMPRAGDFPQFKLDTVC
TPCTHNPLGTKGCGEAGAIGSPPAVINAVLDALAPLGVKDFDMPASASRVWEAMQKGSGSAAADAQPQEPAL
SASTQGRPAP
>SEQF3743||SEQF3743.1_04581
MTTEPNPTRFGSGQAVRRLEDESLLAGAGRYTDDVTLSEQAHLVFLRSPYPHARIASIDTSAAVGMPGVLRVIT
GADLAEAGVKPMPGAAGFKRADGSDSASPPRRAMAHERARFVGEPVACVIADTVQQARDAAEAVMVEYEE
LPMVVDLPGATADGAPLLCDEATGNIAAEMRHGSSEAATAAFARAAHVIALDVNNQRVVALTIEPRAVLATHDS
KTDRLTIRMSTQMPSGVRDSVCAAIGLAKEKVRVVVGDVGGGFGMKTGAYPEDIAVAYAALQVGRPVKWVAD
RSEEFLSSAHGRDIEARAEMALDADGKILALRIKTLANVGAYATGTGVAIQLLIGPWVQTSVYDIQTIDFHFKAVL
TNTAPTGAYRGAGRPEAIFTIERLMDEAARQTGIDRIALRRRNFIRPEQMPYKNPMAQTYDTGQFESVMDQAL
QLADWQGFEARAAESAARGKQRGLGIATFLEWTGGNVFEERVTVSVQADGVIEVFSAVNAMGQGIATSLAQL
AVDAFGVPIEKVRVVLGDTDRGDGFGSAGSRSLFTGGSAVRIGAERTIDKARELAAQEFEASIDDIVYSRGTFIVA
GTDLELDLFTLAAKQPGREIFVDSTSTVAGPSWPNGCHICEIELDPPTGEISVVAYSSVNDVGRVVNPMIVRGQL
EGGAVQGIGQALYEQVVYDTETGQPVTGSLMDYAAPRADIVDTMFQMEMDESTPCKNNPLGVKGVGELGTI
GATPAIVNAVADAFARNGLGARAPRLHMPLSPSRVWQAMQTID
>SEQF3743||SEQF3743.1_06213
MGASDFSNLPHIGEALRRKEDYRFLTGAGNYTDDITLANQSHAVFVRSPHAHATIKSIDITEASKMPGVVGIFSG
KDIEGKMGGLPCGWLINNPDGTPMKEPMHPILAIKKVRYVGDHVAMVVAETVEQAKNAAEAVVVDYDVLPA
LVSVADAAKKTGSTTLHDEAPDNQCYKWVLGDKAAVDNVFTTAAHVTKLDLVNNRLIPNPIEPRVAIGSYSRGT
DDYTLYVSNQNPHVERLLMTAFVLGLPEHKVRVIAPDVGGGFGSKIFLYAEDVCLTWAAKQLNRNIKWTAERSE
CFLSDAHGRDHVSHAEMAMDSQGKFLAMRVHTDANLGAYLSTFSTAVPTILYATLLAGQYTTPQIYVEVDAWF
TNTAPVDAYRGAGRPEATYLLERLVSRCAWEMNLGQDEIRKRNFITSFPYQTPVALQYDTGDFHACMDKARVL
ADVEGYAQRKSATEAKGKLRGMGYSSYIEACGIAPSNIAGALGARAGLFECGEIRVHPTGSVTVFTGSHSHGQG
HETTFAQVVAARLGIPVDNVDVVHGDTGRVPFGMGTYGSRSISVGGAAIMKALDKIETKAKKIAAHLMEASDA
DIEFANGEFTVKGTDKKIPFGQVALTAYVPHNYPLDKLEPGLNETAFYDPTNFTFPGGTYICEVEIDKETGEVKVD
RFTAVDDFGTIINPMIVEGQVHGGLVQGIGQALLENCVYDNETGQLLTGSFMDYAMPRAADFPQFKLDTVCTP
CTHNPLGTKGCGEAGAIGSPPAVINAVLDALAPLGVKDFDMPASSSRVWEAMQKGSTPQDAQAPSLAASTQG
RPAP

>SEQF3744||SEQF3744.1_08624
MSFIGKRVERLEDERLLRGNGCYVDDIRLPDMWHVGFVRSSHAHARIMNVDVSRALDTPGVREVLLAADLGD
AGERRMPQAFPSPAITLSKTQYLLARGEVCYVGEAIAVVIADSRYAAEDGVNAVDIDYEPLPVVTDCQSALGADS
ARAHDGADSNLAGRLQAGYGDIDGAFSKADVKLTRHIVMHRGGCHSMEGRGVVTALQPGHAGLTLWTSTQS
PHAVRRQVAAFLGWEEPRVRVMTPDVGGGFGPKAAVYPEELLLAVLALRLGVPLKWTEDRREHFLSTTQQRD
QVWDIEIAATAEGRLLALRGKGAHDNGAYLPYGLVLPQSSLWPFPGPYAMEALDLTLDVAYTNLVPTSPVRGAG
RPNAAFVMERCIDAVARHLQMDPAAVREVNFVRPDQFPYATGMRYRDGSMASYDSGDFGACLRKALEAAKY
DEFARRREASAEAGLLRGIGVASYVDDTGSGPFEGATVQVLSTGSALVVTGAAGQGQGHATILAQICAERLGIEL
GRVEVVSGDTGRMPFGLGTFGSRIAVAAGSSVNQAAGEVAAKAIRFAAQIWKTDAGALGLAGGHVVEKAGEC
RSIGLGDIARALAGVPGMPLPGGLSPGLEATAYMPISGPTTASGTQVAEVEIDPQTGHVDIVDYVVAHDCGRML
NPMLVEGQIIGGVVHGIGNAFYERMVYDAEGQPLSMNYGEYLLPTAPEIPAIRIVHLETPSPLNPLEAKGAGEG
GTIPAAAALIAAVEHALSEHGAHVDHHPVSPQDILRWVEAER
>SEQF3744||SEQF3744.1_07271
MDITDAKQIGTGHDYVGKRVQRTEDRKLLQGQGSFVDDLKIPGTLHAAVLRSPFAHARVLRIDVSAARTMAGV
VAVYTFAELQESARVGIPILVPNPAIRQMHAQRLLAHEEVFTVGDPVAFVVADNRYLAEDACALIEVEYEELPVSA
NAIDALKPGAALAHAGAPDNIAAHVTMAYGDAAAAFENAPVVVTDTFWQNRGSAHPMEARGYMAESHAST
GQLTVWSSGQAPHLEKRVLVDLLEWDPEKLRVIMNDVGGGFGPKGMFYPEEALVCIAARDLRRPVKWIEDRR
EHFLTAMQERDQWWTISLALDLDGGIRGAKLEMVHDNGAYLPWGIIMPWIGVTTTPGPYVIPSMSVDLKVVL
TNKGGTSPVRGAARPQAVFAMERILDRAAARLGMDRAQIRERNFIQPEQMPYDVGFIYRDGKPMRYDSGDY
PATQAAALARANYAGFPERQRQARAEGRYIGIGVANYVEGTGLGPFEGATVRVQQNGRVSVLTGAAPQGQGH
HTTFMQICAQELNLPMDRVDVITADTGVISMGVGTFASRITVTAGNSVYLAARKVRERLLSLAAFIMQCDVSSL
ALGEARVHLVADPARSMGFGELARIAQGMPGFSFPEGLKGGLEETQYFSPAQSTFCNGTAVVELEVDKHTGQT
RIINYVMAHDCGNLINPLIVDGQVQGAVAHGIGNATLEWMQYDENAQPVTTNFGEYLMPMATDVPRVDTV
HLETPSPLNPLGVKGAGEGGTIPAAAAIISAIENALGEFRLELKNSPTVPQDLFEQLLAAGAYSEGSAA
>SEQF3744||SEQF3744.1_02718
MRFGSGQAVRRLEDQSLLSGAGRYTDDVSLPSQSHLVFLRSPYPHARIAAIDTSAALALPGVLAVITGADLAAAG
VKPMPGAAGFKRADGSDSASPARRAIAHERVRFVGEAVAVVVADTVQQARDAAEAVMVEYEELPMVVDLAA
ATAEGAPQLCDEAGGNIAAEMRHGSSEAATAAFAKAAHVVALDVVNQRVVALTIEPRSVIAAPDADTGRLTIR
MSTQMPSGVRDSICAAIGLAKDKVRVVVGDVGGGFGMKTGAYPEDIAVAFAALQVKRPVKWVADRSEEFLSS
AHGRDIEAKAELALDADGRILALRIKTLANVGAYATGTGVAIQLLIGPWVQTSVYDIQTIDFHFKAVLTNTAPTGA
YRGAGRPEAIFTIERLMDEAARQTGIDRIQLRRRNFIRPEQMPYKNPMAQTYDTGKFESVMDQALGLADWNG
FEGRAAESARNGRHRGLGIATFLEWTGGNVFEERVTVSVQADGVIEVFSAVNAMGQGIATSLAQLAVDAFGV
PIEKVRVVLGDTDRGDGFGSAGSRSLFTGGSAVRIGAERTIDKARELAAQEFEVAIDDVTYSRGAFAVIGTDLELD
LFTLAGKQPEREIFVDSTSTVAGPTWPNGCHICEIEIDPPTGEISVVAYSSVNDVGRVVNPMIVRGQLEGGAVQ
GIGQALYEQVVYDHETGQPVTGSLMDYAAPRADIVSTMFHMEMDESTPCKNNPLGVKGVGELGTIGATPAIV
NAVADAFARNGHAARAPRLHMPLSPSRVWQAMQTID
>SEQF3744||SEQF3744.1_01940
MGASDFANLPHIGDALRRKEDYRFLTGAGNYTDDITLANQSHAVFVRSPHAHARVVSVDIADALTMPGVVGIF
SGKDIEGKMGGLPCGWLINNPDGTPMKEPMHPILAINKVRYVGDHVAMVVAETVEQAKNAAEAVVVEYEVL
PALVGVADAAKKSGGVTIHDEAPDNQCYKWALGDKAAVDAAFAGAAHVTKLDLVNNRLIPNPIEPRVAIGSYSR
GTDDYTLYVSNQNPHVERLLMTAFVLGLPEHKVRVIAPDVGGGFGSKIFLYAEDVCLTWAAKQLNRNIKWTAER
SECFLSDAHGRDHVSHAEMAMDKDGKFLAMRVHTDANLGAYLSTFSTAVPTILYATLLAGQYTTPQIYVEVDA
WFTNTAPVDAYRGAGRPEATYLLERLVSRCAWEMNLGQDEIRKRNFITAFPYQTPVALQYDTGDFHACMDKA
RKLAEVEGYAQRKSASEAAGKLRGIGYSSYIEACGIAPSNIAGALGARAGLFECGEIRVHPTGSVTVFTGSHSHGQ

GHETTFAQVVAARLGIAVDAVDVVHGDTGRVPFGMGTYGSRSISVGGAAIMKALDKIEAKAKKIAAHLMEASD
ADIEFANGEFTVKGTDKKIPFGQVALTAYVPHNYPLDKLEPGLNETAFYDPTNFTFPGGTYICEVEVDRQTGEVR
VDRFTAVDDFGTIINPMIVEGQVHGGLVQGIGQALLENCVYDNETGQLLTGSFMDYAMPRAAEFPQFKLDTVC
TPCTHNPLGTKGCGEAGAIGSPPAVINAVLDALAPLGVKDFDMPASSSRVWEAMQKGSGSAASDPVAPQPAL
AASTQGRPAA
>SEQF3744||SEQF3744.1_06973
MDHADIPARLLKREGIGARVPRKEDARHLVGKGNFVGDFVLPGLQEVAFLRSSLAHAVVTGVEVPETLAGKVFL
REMMSDAADIGSPSSLPTYQYSALPPLASGKVRHVGEAIAMAVAPTRAMAEDLLEEVEVSYDELPVYHSAESAL
AATKEFMHPEWKDNVFLTLRANRDFEELAAKAEVKVSRTVELSRQCIVPLEGKSVLAYWDFQTDQLVVVSATQ
VPHLLRVGIAQHLSMNEEAVRVVSPDVGGAFGYKGQLYPEDLCVAWLAKTYRTPFRYLEDRREHLIVGANTRQ
HHYQLTAYADRTGKLLALDAVITIDGGAYSYYPFFVGLEPGQAIGNLPGPYTFRGYRCETLCVATNKPGFMAYRG
VARTGVCFAIELLMDAVAREVGREPWEVRMENLVPAAAMPYVNVANKHFDSGDFPASLRRAVEMIGLDAVRE
RQARGEPDGRLIGFGTATYTEQSAHGTTVFANWGLPVVPGFDQAVVRMTADGGLEVRVGVHSHGQGMETS
FAQIANDVLGIPVARIRVVHGDTALTPFSSGTYASRATVMSGGAISVACKELLPRLQSIAGYLMGVDAPTVALIEG
RAVAGDKSIPLSDVGGAWYLTPQDLPANVYLGGLEVSRAYKPNVDTGTFTYATHAVVVAVDTETGEVEILDYVV
VEDCGTMVNPMIVEGQTIGGIAQGIGTAMYEESPYDDQGQPLASTLADYILPGATEVPRIRIEHFETPSPHTEFG
AKGVGEGGAIAPPAVIFNAVNDALRGTGAAEVLMTPLTPRRLLTALEGAKREQEHAR
>SEQF3745||SEQF3745.1_02336
MGASDFSNLPHIGEALRRKEDYRFLTGAGNYTDDITLANQSHAVFVRSPHAHATVKSVDIAEAMKMPGVVGIF
SGKDIEGKMGGLPCGWLINNPDGTPMKEPMHPILAINKVRYVGDHVAMVVAETVEQARNAAEAVVVDYDV
LPALVSVADAAKKAGGVTLHDEAPDNQCYKWALGDKAAVDAAFANAAHVTKLDLVNNRLVPNPIEPRVAIGSY
SRGTDDYTLYVSNQNPHVERLLMTAFVLGLPEHKVRVIAPDVGGGFGSKIFLYAEDVCLTWAAKQLNRNIKWTA
ERSECFLSDAHGRDHVSHAEMAMDKDGKFLAMRVHTDANLGAYLSTFATAVPTILYATLLAGQYTTPQIYVEVD
AWFTNTAPVDAYRGAGRPEATYLLERLVSRCAWEMNLGQDEIRKRNFISTFPYQTPVALQYDTGDFHACMDKA
RVLAEVDGYAARKAASEAGGKLRGMGYSSYIEACGIAPSNIAGALGARAGLFECGEIRVHPTGSVTVFTGSHSH
GQGHETTFAQVVAARLGIPVENVDVVHGDTGRVPFGMGTYGSRSISVGGAAIMKALDKIETKAKKIAAHLME
ASDADIEFANGEFTVKGTDKKIPFGQVALTAYVPHNYPLDKLEPGLNETAFYDPTNFTFPGGTYICEVEIDRQTGE
VRVDRFTAVDDFGTIINPMIVEGQVHGGLVQGIGQALLENCVYDRETGQLLTGSFMDYAMPRAAEFPQFKLDT
VCTPCTHNPLGTKGCGEAGAIGSPPAVINAVLDALAPLGVKDFDMPASSSRVWEAMQAASGANH
>SEQF3745||SEQF3745.1_03990
MTTEPNPTRFGSGQAVRRLEDESLLAGAGRYTDDVTLPDQAHLVFLRSSYPHARIRAMDTSAAAAMPGVLCVI
TGADMAQAGVKPMPGAAGFKRADGSDSASPPRHAMAHERTRFVGEAVALVVADTVQQARDACEAIMVDY
EDLPMVVDLASATAEGAPQLCEEASGNVAAEMRHGSSEAATAAFAKASHVVALDVNNQRVVALTIEPRSVLAA
QDNETGRLTIRMSTQMPSGVRDSLCAAIGLPKEKVRVVVGDVGGGFGMKTGAYPEDIAVAFAALQVHRPVK
WVADRSEEFLSSAHGRDIEARAEMALDANGKILALRIKTLANVGAYATGTGVAIQLLIGPWVQTSVYDIQTIDFH
FKAVLTNTAPTGAYRGAGRPEAIFTIERLMDEAARQTGIDRIALRRRNFIRPEQMPYKNPMAQTYDTGNFESVM
DQALMLADWQGFEGRAAASASQGKHRGLGIATFLEWTGGNVFEERVTVSVQAEGVIEVFSAVNAMGQGIAT
SLAQLAVDAFGVPIDKVRVVLGDTDRGDGFGSAGSRSLFTGGSAVRIGAERTIDKARELAAQEFEAAVDDITYTR
GVFMVAGTDLELDLFTLAGKQPDREIFVDSTSTVAGPTWPNGCHICEIEIDPPTGEISVVAYSSVNDVGRVINPM
IVRGQLEGGAVQGIGQALYEQVVYDHETGQPVTGSLMDYAAPRADIVDTMFNMEMDESTPCKNNPLGVKGV
GELGTIGATPAIVNAVADAFARNGLATRAPRLHMPLSPARVWQAMQTVD
>SEQF3746||SEQF3746.1_03274
MTTELNPTRFGSGQAVRRLEDESLLAGAGRYTDDVTLPDQAHLVFLRSPYPHARIVSIDTSTAAGMPGVLRVIT
GADLVAAEVKPMPGAAGFKRADGGDSASPPRHALASERTRFVGEAVAAVIADTVQQARDAAEAVMVDYEDL

PMVVDLASATAEGAPQLCEEATGNIAAEMRHGSSEAATAAFAEAAHVVALDVINQRVVALTIEPRSVLAAHDAK
TDRLTIRMSTQMPSGVRDSVCAAIGLAKEKVRVVVGDVGGGFGMKTGAYPEDIAVAFAALQVQRPVKWVAER
SEEFLSSAHGRDIEARAELALDAEGKILALRIKTLANVGAYATGTGVAIQLLIGPWVQTSVYDIQTIDFHFKAVLTNT
APTGAYRGAGRPEAIFTIERLMDEAARQTGIDRIILRRMNFIQPDQMPYKNPMAQVYDTGNFESVMDQALTL
ADWQGFEARAADSAKNGKHRGLGIATFLEWTGGNVFEERVTVSVQADGVIEVFSAVNAMGQGIATSLAQLA
VDAFGVPIEKVRVVLGDTDRGDGFGSAGSRSLFTGGSAVRIGAERTIDKARELAAQEFEAAIDDIIYSRGSFSVAG
TDLELDLFTLAGKQPDREIFVDSTSTVAGPTWPNGCHICEIELDPPTGEISVVAYSSVNDVGRVINPMIVRGQLE
GGAVQGIGQALYEQVVYDQETGQPVTGSLMDYVAPRADIVDTMFNMEMDESTPCTNNPLGVKGVGELGTIG
ATPAIVNAVADAFARNGLAATAPRLHMPLSPSRVWAAMHTVD
>SEQF3746||SEQF3746.1_00547
MGASDFSNLPHIGEALRRKEDYRFLTGAGNYTDDITLANQSHAVFVRSPHAHAAIKSIDIAEALKMPGVVGIFSG
KDIEGKMGGLPCGWLINNPDGTPMKEPMHPILAIKKVRYVGDHVAMVVAETVEQAKNAAEAVVVDYDVLPA
LVSVADAAKKTGSTTLHDEAPDNQCYKWVLGDKAAVDNVFTTAAHITRLDLVNNRLIPNPIEPRVAIGSYSRGT
DDYTLYVSNQNPHVERLLMTAFVLGLPEHKVRVIAPDVGGGFGSKIFLYAEDVCLTWAAKQLNRNIKWTAERSE
CFLSDAHGRDHVSHAEMAMDKDGKFLAMRVHTDANLGAYLSTFSTAVPTILYATLLAGQYTTPQIYVEVDAWF
TNTAPVDAYRGAGRPEATYLLERLVSRCAWEMNLGQDEIRKRNFITTFPYQTPVALQYDTGDFHACMDKARVL
ADVEGYAQRKSATEAKGKLRGIGYSSYIEACGIAPSNIAGALGARAGLFECGEIRVHPTGSVTVFTGSHSHGQGH
ETTFAQVVAARLGIPVDNVDVVHGDTGRVPFGMGTYGSRSISVGGAAIMKALDKIETKAKKIAAHLMEASDAD
IEFANGEFTVKGTDKKIPFGQVALTAYVPHNYPLDKLEPGLNETAFYDPTNFTFPGGTYICEVEIDKQTGEVRVDR
FTAVDDFGTIINPMIVEGQVHGGLVQGIGQALLENCVYDNETGQLLTGSFMDYAMPRAGDFPQFKLDTVCTPC
THNPLGTKGCGEAGAIGSPPAVINAVLDALAPLGVKDFDMPASPSRVWEAMQKGGTPEDVQAPSLAASTQGR
PAP
>SEQF3747||SEQF3747.1_03629
MNARTPSVALERPADTDQGIGGCPRRKEDRRLLLGRGQYVGDIRMPDMLDVAFVRSPVAHAKLGRIDKPEGL
ASMVYTMDDLSGVHPILAASALPGFKRSVQHPLAKDKVRFVGELVAACVAPTRARAEDIAAQVSVDYADLPVL
ADMEAAIDSATRVHDEWSDNVFAETNTDRDVSAPGATATRTVRRRLRTSRQCMMPLEGRAVLCYWDHRLDQ
LVMYSAAQVPHINRTGLAECLGLDQGQIRVVSPDVGGGFGYKGLLLPEEICCAWLARHLGRPVRWLEDRREQL
GANANCREHAYDIAVEVEPDGRLVGIECDAMVDSGAYSSYPFSACLEAAQIGSILPGPYVMDRFVCRTRSVATN
KPPILPYRGVARTGVCFALELMLDAAAREIGIEPYELRARSLVPASAMPYTNITNKYFDSGDYLACMTKAVEALD
WQVWRERQPKAPAGKRIGLGLAVFCEQGAHGTSVYHGWGIPMVPGYEQCAARFTPDGILEIRLGVHSHGQG
METSMAQVAHTVLGIDIDRVRVQHGDTANSPYSTGTWGSRCAVMAGGAVGTACQALATRMTALAAALLGVP
AATLRLEGGRIGVPGSPGLALDEIAHTWYRAPQKIPLGIDPSGLEVVVGYKTAPDTGTFSYACHACAVEVDIATG
RVILLDYAICEDGGVLLNPQIVEGQLIGGLAQGIGTALYEEMPFDGEGQPLASTLADYLMPGAGEMPPLKIIHME
TPSPLTLFGQKGIGEGGAIAPPAAIVNAVNDALHALGAELTECPASPERVLRALAAARTGALA
>SEQF3747||SEQF3747.1_06438
MNQADIPARVLKREGIGARVPRKEDARHMVGKGNFVGDFVLPGLQEVAFLRSPLAHATITGVEIPEELAGKVFL
REMMMDAADIGSPSSLPTYQYSALPPLASGKVRHVGEAVAMAVAPTRAVAEDLLEEVQVSYDELPVYHGAESS
LAAKGDFLHPEWKDNVFLTLRANRDFDELAAKAEVKVSRTVELSRQCIVPLEGKSVLAYWDFQADQLVVVSAT
QVPHLLRVGIAQHLSMNEEAVRVVSPDVGGAFGYKGQLYPEDLCIAWLAKTYRTPFRYLEDRREHLIVGANTRQ
HHYQLTAYADRTGKLLALDAVITIDGGAYSYYPFFVGLEPGQAIGNLPGPYTFRGYRCETLCVATNKPGFMAYRG
VARTGVCFAIELLMDAVAREVGREPWEVRMDNLVPAAAMPYVNVANKHFDSGDFPASLRRAVEMIGLAAVR
ERQARGEPDGRRIGFGTATYTEQSAHGTTVFANWGLPVVPGFDQAVVRMTADGGLEVRVGVHSHGQGMET
SFAQIANDVLGIPVARIRVVHGDTALTPFSSGTYASRATVMSGGAISTACKELLPRIQSIAGYLMGVEPQTVALVE
GFAVAGEKSIPLSEVGGAWYLTPQDLPENVHLGGLEVSRAYKPRVDTGTFTYATHAVVVAVDTQTGEVEILDYVV

VEDCGTMVNPMIVEGQTIGGIAQGIGTAMYEESPYDDQGQPLASTLADYILPGATEVPRIRIEHFETPSPHTEFG
AKGVGEGGAIAPPAVIFNAVNDALRGTGASEVLMTPLTPRRLLKALESAKPQQERVK
>SEQF3747||SEQF3747.1_00999
MTTDSTPTRFGSGQAVRRLEDESLLSGAGRYTDDVTLPGQTYLVFLRSPYPHARIVSIDTAAAAAMPGVLRVITG
AELAEAGVKPMPGAVGFKRADGSDCASPPRLAMAHDRARFVGEAVAAVVAETVQQARDAAEAVVVDYEALP
MVVDLASATADDAPLLCEEASGNVAAEMRHGSSDAATAAFAKAKHVVALDVVNQRVVALTIEPRSVLAAPDAE
SGRLTIRMSTQMPSGVRDSICAAIGLAKEKVRVVVGDVGGGFGMKTGAYPEDIAVAFAALQVKRPVKWVADR
SEEFLSSAHGRDIEAKAELALDADGKILALRIRTLANVGAYATGTGVAIQLLIGPWVQTSVYDIQTIDFHFKAVLTN
TAPTGAYRGAGRPEAIFTIERLMDEAARQTGIDRIALRRRNFIRPEQMPYKNPMAQTYDTGKFESVMDQALAL
ADWQGFEARAAESAKNGKHRGLGIATFLEWTGGNVFEERVTVSVQADGVIEVFSAVNAMGQGIATSLAQLAV
DAFGVPIEKVRVVLGDTDRGDGFGSAGSRSLFTGGSAVRIGAERTIDKARALAAQEFEAAIDDITYTRGVFSVAG
TDLELDLFALAGKQPEREIFVDSTSTVAGPTWPNGCHVCEIEIDPPTGEISVVAYSSVNDVGRVINPMIVRGQLE
GGAVQGIGQALYEQVVYDNETGQPLTGSLMDYAAPRADIVETMFHMEMDESTPCANNPLGVKGVGELGTIG
ATPTIVNAVADAFARNGLAASAPRLHMPLSPARVWQAMHTVD
>SEQF3747||SEQF3747.1_03795
MGASDFSNLPHIGEALRRKEDYRFLTGAGNYTDDITLANQSHAVFVRSPHAHAVIKSVDTTEALKMPGVVGIFS
GKDIEGKMGGLPCGWLINNPDGTPMKEPMHPILAINKVRYVGDHVAMVVAETVEQAKNAAEAVVVDYDVL
PALVSVADAAKKASGVTLHDEAPDNQCYKWTLGDKAAVDAVFTTAAHVTKLDLVNNRLIPNPIEPRVAIGSYSR
GTDDYTLYVSNQNPHVERLLMTAFVLGLPEHKVRVIAPDVGGGFGSKIFLYAEDVCLTWAAKQLNRNIKWTAER
SECFLSDAHGRDHVSHAEMALDKDGKFLAMRVHTDANLGAYLSTFSTAVPTILYATLLAGQYTTPQIYVEVDAW
FTNTAPVDAYRGAGRPEATYLLERLVSRCAWEMNLGQDEIRKRNFITTFPYQTPVALQYDTGDFHACMDKARV
LADVGGYAKRKSASEAAGKLRGIGYSSYIEACGIAPSNIAGALGARAGLFECGEIRVHPTGSVTVFTGSHSHGQG
HETTFAQVVAARLGIPVDNVDVVHGDTGRVPFGMGTYGSRSISVGGAAIMKALDKIETKAKKIAAHLMEASDA
DIEFANGEFTVKGTDKKIPFGQVALTAYVPHNYPLDKLEPGLNETAFYDPTNFTFPGGTYICEVEVDKQTGEVRV
DRFTAVDDFGTIINPMIVEGQVHGGLVQGIGQALLENCVYDNETGQLLTGSFMDYAMPRAGDFPQFNLDTVC
TPCTHNPLGTKGCGEAGAIGSPPAVINAVLDALAPLGVKDFDMPASASRVWEAMQAAASH
>SEQF3748||SEQF3748.1_00126
MTTDSIPTRFGSGQAVRRLEDESLLSGAGRYTDDVALPGQTHLVFLRSPYPHARIVSIDTAAAAAMPGVLRVITG
AELAEAGVKPMPGAVGFKRADGSDCASPPRLALAHGTARFVGEAVAAVVAETVQQARDAAEAVVVDYEALP
MVVDLASATADGAPLLCEEATGNVAAEMRHGSSDAATAAFAKAKHVVALDVVNQRVVALTIEPRSVLAAPDAE
SGRLTIRMSTQMPSGVRDSVCAAIGLAKEKVRVVVGDVGGGFGMKTGAYPEDIAVAFAALQVGRPVKWVAD
RSEEFLSSAHGRDIEARAELALDADGKILALRIRTLANVGAYATGTGVAIQLLIGPWVQTSVYDIQTIDFHFKAVLT
NTAPTGAYRGAGRPEAIFTIERLMDEAARQIGIDRIALRRRNFIRPEQMPYKNPMAQTYDTGKFESVMDQALAL
ADWQGFDARAAESANNGKHRGLGIATFLEWTGGNVFEERVTVSVQADGVIEVFSAVNAMGQGIATSLAQLA
VDAFGVPIEKVRVVLGDTDRGDGFGSAGSRSLFTGGSAVRIGAERTIDKARALAAQEFEAAIDDITYTRGVFTVA
GTDLELDLFALAGKQPEREIFVDSTSTVAGPTWPNGCHVCEIEIDPPTGEISVVAYSSVNDVGRVINPMIVRGQL
EGGAVQGIGQALYEQVVYDNETGQPLTGSLMDYAAPRADIVQAMFHMEMDESTPCANNPLGVKGVGELGTI
GATPAIVNAVADAFARNGLAASAPRLHMPLSPARVWQAMHTVD
>SEQF3748||SEQF3748.1_04417
MNQADIPARVLKREGIGARVPRKEDARHMVGKGNFVGDFVLPGLQEVAFLRSPLAHATITGVEIPEALAGKVFL
REMMLDAADIGSPSSLPTYQYSALPPLASGKVRHVGEAVAMAVAPTRAVAEDLLEEVQVSYDELPVVYHGAESSL
AATGDFLHPGWKDNVFLTLRANRDFDELAAKAEVKVSRTVELSRQCIVPLEGKSVLAYWDFQADQLVVVSATQ
VPHLLRVGIAQHLSMNEEAVRVVSPDVGGAFGYKGQLYPEDLCIAWLAKTYRTPFRYLEDRREHLIVGANTRQH
HYQLTAYADRTGKLLALDAVITIDGGAYSYYPFFVGLEPGQAIGNLPGPYTFRGYRCETLCVATNKPGFMAYRGV

ARTGVCFAIELLMDAVAREVGREPWEVRMDNLVPAAAMPYVNVANKHFDSGDFPASLRRAVEMIGLATVRER
QARGEPDGRRIGFGTATYTEQSAHGTTVFANWGLPVVPGFDQAVVRMTADGGLEVRVGVHSHGQGMETSF
AQIANDVLGIPVARIRVVHGDTALTPFSSGTYASRATVMSGGAISTACKELLPRIQSIAGYLMGVDPQAVALVEGF
AVAGEKSIPLSQVGGAWYLTPQDLPENVHLGGLEVSRAYKPRVDTGTFTYATHAVVVAVDTQTGEVEILDYVVV
EDCGTMVNPMIVEGQTIGGIAQGIGTAMYEESPYDDQGQPLASTLADYILPGATEVPRIRIEHFETPSPHTEFGA
KGVGEGGAIAPPAVIFNAVNDALRGTGAAEVLMTPLTPRRLLKALESAKPEQGLVK
>SEQF3748||SEQF3748.1_00371
MLMSAPPFVDAPARPHVGRPAQRVEDRALLTGRGRYGDDAPVRSDTLHAAVLRSPHAHARVLAVRTARAEA
MSGVRAVLTGADVQRWSKPFIVGVKQPMELWALAVDKVRYAGEPIAVVVAESRYLAEDALELLEADYEQLPAV
VDIESAAAEGAPVLHERVGSNVVSDRSFCYGDPDAAFAAAPHRVALSVHYPRNSCTPIETAVVIAEFLSEQDGYD
IQSNFMGPFSLHTVMAMALKVPGNRLRHRTFADSGGSFGVKQAVMAPAVLMCLAARKAGSPVKWVEDRLE
HLTAATSATGRLCHAEAAVEADGRITALSLDQFDDCGGYLRAPEPATFYRMHGCLTGAYAIEHLRVRNRVVLTNK
TPSGLVRGFGGPQVYFALERLVQRIAVELSLDPLDVYRRNFIASDAFPYRAAAGALIDSGDYQAALAMAVRDGG
LAELRQRQETARAEGRLYGIGFAAIVEPSVSNMGYITTVMPREQRTKAGPKNGAIAAATVGIDPLGGLSVVIASS
PAGQGHRTVCAQIVADVFGVEPSEVSVNVEFDTQKDAWSVAAGNYSSRFAGAVAGTLHLACTRLRDKLAGIAA
ASLDCAAADVVFAYGKVYARHAPERSLPFARFAASPHWAPGLLPAGTDPGLRETAFWTPPQLTAPDDQDRVNT
SAAYGFAFDICAVEVDRDTGRVRIDRYVTTHDAGTLLNPALADGQIRGAFAQGLGAALMEEFRYGADGSFQSG
TFADYLVPTTCEVPDPVILHLETPSPFTPLGAKGLGEGNNMSTPPCIANAVADALGRADIVLPLTPSRVMHLLGM
DDPPPSAGSAAAAMPASEPPANPKGGKGKALSARGEILLPATPEAVFAVLLDPVALARVIPGCNALESVGPNRH
RADVTVGVGMIKARYAAEIALSEIEAPRRLRLAGSGLSSVGAAKGSGLVTLEPKDGGTLLRYDYEAEVSGKVAAV
GGRMLEGAAKIVLKQLFEQLGRQAGGQGAAPVADPWWRRLLRMLGGAK
>SEQF3748||SEQF3748.1_00990
MGASDFSNLPHIGEALRRKEDYRFLTGAGNYTDDITLANQSHAVFVRSPHAHAVIKSVDTAEALKMPGVVGIFS
GKDIEGKMGGLPCGWLINNPDGTPMKEPMHPILAIHKVRYVGDHVAMVVAETVEQAKNAAEAVVVDYDVLP
AVVSVADAAKKASGVTLHDEAPDNQCYKWALGDKAAVDAAFANAAHVTKLDLVNNRLIPNPIEPRVAIGSYSR
GTDDYTLYVSNQNPHVERLLMTAFVLGLPEHKVRVIAPDVGGGFGSKIFLYAEDVCLTWAAKQLNRNIKWTAER
SECFLSDAHGRDHVSHAEMAMDKDGKFLAMRVHTDANLGAYLSTFSTAVPTILYATLLAGQYTTPQIYVEVDA
WFTNTAPVDAYRGAGRPEATYLLERLVSRCAWEMNLGQDEIRKRNFITTFPYQTPVALQYDTGDFHACMDKA
RVLADVDGYAQRKSASEAAGKLRGIGYSSYIEACGIAPSNIAGALGARAGLFECGEIRVHPTGSVTVFTGSHSHG
QGHETTFAQVVAARLGIPVDNVDVVHGDTGRVPFGMGTYGSRSISVGGAAIMKALDKIETKAKKIAAHLMEAS
DADIEFANGEFTVKGTDKKIPFGQVALTAYVPHNYPLDKLEPGLNETAFYDPTNFTFPGGTYICEVEVDKQTGEV
RVDRFTAVDDFGTIINPMIVEGQVHGGLVQGIGQALLENCVYDNETGQLLTGSFMDYAMPRAGDFPQFKLDT
VCTPCTHNPLGTKGCGEAGAIGSPPAVINAVLDALAPLGVKDFDMPASASRVWEAMQAAASH
>SEQF3749||SEQF3749.1_00737
MTTESNPTRFGSGQAVRRLEDESLLAGAGRYTDDVTLPGQAHLVFLRSSYPHARILSVDTSTAAAMPGVLSVIT
GADMARAGVKPMPGAAGFKRADGSDSASPPRYAMAHERTRFVGEAVALVVADTVQQARDACEAIMVDYED
LPMVVDLASATAEGAPQLCEAASGNIAAEMRHGSSEAATAAFAKASHVVALDVNNQRVVALTIEPRSVLAAQD
ADTGRLTIRMSTQMPSGVRDSLCAAIGLPKEKVRVVVGDVGGGFGMKTGAYPEDIAVAFAALQVRRPVKWVA
DRSEEFLSSAHGRDIEARAEMALDADGKILALRIKTLANVGAYATGTGVAIQLLIGPWVQTSVYDIQTIDFHFKAV
LTNTAPTGAYRGAGRPEAIFTIERLMDEAARQTGIDRIALRRRNFIRPEQMPYKNPMAQTYDTGNFESVMDQA
LALADWQGFEGRAAASASQGKHRGLGIATFLEWTGGNVFEERVTVSVQADGVIEVFSAVNAMGQGIATSLAQ
LAVDAFGVPIEKVRVVLGDTDRGDGFGSAGSRSLFTGGSAVRIGAERTIDKARELAAQEFEAAVDDITYTRGVF
MVAGTDLELDLFTLAGKQPGREIFVDSTSTVAGPTWPNGCHICEIEIDPPTGEISVVAYSSVNDVGRVINPMIVR
GQLEGGAVQGIGQALYEQVVYDHETGQPVTGSLMDYAAPRADIVDTMFNMEMDESTPCKNNPLGVKGVGE

LGTIGATPAIVNAVADAFARNGLATRAPRLHMPLSPARVWQAMQTVD
>SEQF3749||SEQF3749.1_01274
MGASDFSNLPHIGEALRRKEDYRFLTGAGNYTDDITLANQSHAVFVRSPHAHATVKSVDISEAMKMPGVIGIFS
GKDIEGKMGGLPCGWLINNPDGTPMKEPMHPILAINKVRYVGDHVAMVVAETVEQAKNAAEAVVVDYDVL
PALVSVADAARKAGGVTLHDEAPDNQCYKWALGDKAAVDAAFANAAHVTKLDLVNNRLIPNPIEPRVAIGSYS
RGTDDYTLYVSNQNPHVERLLMTAFVLGLPEHKVRVIAPDVGGGFGSKIFLYAEDVCLTWAAKQLNRNIKWTAE
RSECFLSDAHGRDHVSHAEMAMDKDGKFLAMRVHTDANLGAYLSTFSTAVPTILYATLLAGQYTTPQIYVEVDA
WFTNTAPVDAYRGAGRPEATYLLERLVSRCAWEMNLGQDEIRKRNFITAFPYQTPVALQYDTGDFHACMDKA
RVLAEVDGYAARKAASEAGGKLRGIGYSSYIEACGIAPSNIAGALGARAGLFECGEIRVHPTGSVTVFTGSHSHG
QGHETTFAQVVAARLGIPVENVDVVHGDTGRVPFGMGTYGSRSISVGGAAIMKALDKIETKAKKIAAHLMEAS
DADIEFANGEFTVKGTDKKIPFGQVALTAYVPHNYPLDKLEPGLNETAFYDPTNFTFPGGTYICEVEIDRQTAEVR
VDRFTAVDDFGTIINPMIVEGQVHGGLVQGIGQALLENCVYDKETGQLLTGSFMDYAMPRAAEFPQFKLDTVC
TPCTHNPLGTKGCGEAGAIGSPPAVINAVLDALAPLGVKDFDMPASSSRVWEAMQKGSGSAAADTLPQEPAL
AASTQGRPAP
>SEQF3749||SEQF3749.1_04871
MNQADIPARVLKPEGIGARVPRKEDARHMVGKGNFVGDFVLPGLQEVAFLRSPLAHATITGVEIPQEHAGKIFV
REMMTDAADIGSPSSLPTYQYSALPPLASGKVRHVGEAIAMAVAPTRAVAEDLLEEVQVSYDELPVYHGAESSL
AATGDFLHPGWKDNVFLTLRANRDFDELAAKAEVKVSRTVELSRQCIVPLEGKSVLAYWDFQADQLVVVSATQ
VPHLLRVGIAQHLSMNEEAVRVVSPDVGGAFGYKGQLYPEDLCIAWLAKTYRTPFRYLEDRREHLIVGANTRQH
HYKLTAYADRTGKLLALDAVITIDGGAYSYYPFFVGLEPGQAIGNLPGPYTFRGYRCETLCVATNKPGFMAYRGVA
RTGVCFAIELLMDAVAREVGREPWEVRMDNLVPADAMPYVNVANKHFDSGDFPASLRRAVEMVGVAAVRER
QTRGEPDGRRIGFGTATYTEQSAHGTTVFANWGLPVVPGFDQAVVRMTADGGLEVRVGVHSHGQGMETSF
AQIANDVLGIPVARIRVVHGDTALTPFSSGTYASRATVMSGGAIATACKELLPRIQSIAAYLMGVDPQTVALVEGF
AVAGEKSIPLSQVGGAWYLTPQDLPENVHLGGLEVSRAYKPKVDTGTFTYATHAVVVAVDTQTGEVEILDYVVV
EDCGTMVNPMIVEGQTIGGIAQGIGTAMYEESPYDDQGQPLASTLADYILPGATEVPRIRIEHFETPSPHTEFGA
KGVGEGGAIAPPAVIFNAVNDALRGTGAAEVLMTPLTPRRLLKALESAKPQQELVK
>SEQF3750||SEQF3750.1_06154
MNQADIPARVLKREGIGARVPRKEDARHMVGKGNFVGDFVLPGLQEVAFLRSPLAHATITGVEIPEALAGKVFL
REMMLDAADIGSPSSLPTYQYSALPPLASGKVRHVGEAVAMAVAPTRAVAEDLLEEVQVSYDELPVYHGAESSL
AATGDFLHPGWKDNVFLRLRANRDFDELAAKAEVKVSRTVELSRQCIVPLEGKSVLAYWDFQADQLVVVSATQ
VPHLLRVGIAQHLSMNEEAVRVVSPDVGGAFGYKGQLYPEDLCIAWLAKTYRTPFRYLEDRREHLIVGANTRQH
HYQLTAYADRTGKLLALDAVITIDGGAYSYYPFFVGLEPGQAIGNLPGPYTFRGYRCETLCVATNKPGFMAYRGV
ARTGVCFAIELLMDAVAREVGREPWEVRMDNLVPAAAMPYVNVANKHFDSGDFPASLRRAVEMIGLAAVRE
RQARGEPDGRRIGFGTATYTEQSAHGTTVFANWGLPVVPGFDQAVVRMTADGGLEVRVGVHSHGQGMETS
FAQIANDVLGIPVARIRVVHGDTALTPFSSGTYASRATVMSGGAISTACKELLPRIQSIAGYLMGVDPQTVALVEG
FAVAGEKSIPLSQVGGAWYLTPQDLPENVHLGGLEVSRAYKPKVDTGTFTYATHAVVVAVDTQTGEVEILDYVV
VEDCGTMVNPMIVEGQTIGGIAQGIGTAMYEESPYDDQGQPLASTLADYILPGATEVPRIRIEHFETPSPHTEFG
AKGVGEGGAIAPPAVIFNAVNDALRGTGAAEVLMTPLTPRRLLKALESAKPEQGLVK
>SEQF3750||SEQF3750.1_04595
MTTDSIPTRFGSGQAVRRLEDESLLSGAGRYTDDVALPGQTHLVFLRSPYPHARIVSIDTAAAAAMPGVLRVITG
AELAEAGVKPMPGAVGFKRADGSDCASPPRLALAHGTARFVGEAVAAVVAETVQQARDAAEAVVVDYEALP
MVVDLASATADGAPLLCEEATGNVAAEMRHGSSDAATAAFAKAKHVVALDVVNQRVVALTIEPRSVLAAPDAE
SGRLTIRMSTQMPSGVRDSVCAAIGLAKEKVRVVVGDVGGGFGMKTGAYPEDIAVAFAALQVGRPVKWVAD
RSEEFLSSAHGRDIEARAELALDADGKILALRIRTLANVGAYATGTGVAIQLLIGPWVQTSVYDIQTIDFHFKAVLT

NTAPTGAYRGAGRPEAIFTIERLMDEAARQIGIDRIALRRRNFIRPEQMPYKNPMAQTYDTGKFESVMDQALAL
ADWQGFDARAAESANNGKHRGLGIATFLEWTGGNVFEERVTVSVQADGVIEVFSAVNAMGQGIATSLAQLA
VDAFGVPIEKVRVVLGDTDRGDGFGSAGSRSLFTGGSAVRIGAERTIDKARALAAQEFEAAIDDITYTRGVFTVA
GTDLELDLFALAGKQPEREIFVDSTSTVAGPTWPNGCHVCEIEIDPPTGEISVVAYSSVNDVGRVINPMIVRGQL
EGGAVQGIGQALYEQVVYDNETGQPLTGSLMDYAAPRADIVQAMFHMEMDESTPCANNPLGVKGVGELGTI
GATPAIVNAVADAFARNGLAASAPRLHMPLSPARVWQAMHTVD
>SEQF3750||SEQF3750.1_00602
MGASDFSNLPHIGEALRRKEDYRFLTGAGNYTDDITLANQSHAVFVRSPHAHAVIKSVDTAEALKMPGVVGIFS
GKDIEGKMGGLPCGWLINNPDGTPMKEPMHPILAINKVRYVGDHVAMVVAETVEQAKNAAEAVVVDYDVL
PAVVSVADAAKKASGVTLHDEAPDNQCYKWALGDKAAVDAVFAGAAHVTQLDLVNNRLVPNPIEPRVAIGSYS
RGTDDYTLYVSNQNPHVERLLMTAFVLGLPEHKVRVIAPDVGGGFGSKIFLYAEDVCLTWAAKQLNRNIKWTAE
RSECFLSDAHGRDHVSHAEMAMDKDGKFLAMRVHTDANLGAYLSTFSTAVPTILYATLLAGQYTTPQIYVEVDA
WFTNTAPVDAYRGAGRPEATYLLERLVSRCAWEMNLGQDEIRKRNFITTFPYQTPVALQYDTGDFHACMDKA
RVLADVDGYAQRKSASEAAGKLRGIGYSSYIEACGIAPSNIAGALGARAGLFECGEIRVHPTGSVTVFTGSHSHG
QGHETTFAQVVAARLGIPVDNVDVVHGDTGRVPFGMGTYGSRSISVGGAAIMKALDKIETKAKKIAAHLMEAS
DADIEFANGEFTVKGTDKKIPFGQVALTAYVPHNYPLDKLEPGLNETAFYDPTNFTFPGGTYICEVEVDKQTGEV
RVDRFTAVDDFGTIINPMIVEGQVHGGLVQGIGQALLENCVYDNETGQLLTGSFMDYAMPRAGDFPQFKLDT
VCTPCTHNPLGTKGCGEAGAIGSPPAVINAVLDALAPLGVKDFDMPASASRVWEAMQAAASH
>SEQF3751||SEQF3751.1_01896
MRFGSGQAVRRLEDQSLLSGAGRYTDDVSLPNQSHLVFLRSPYPHARLVSVDTSAALALPGVLAVITGADLAAA
GVKPMPGAAGFKRADGGDSASPARHAIAHERVRFVGEAVAVVVADTVQQARDAAEAVMVEYEELPMVVDL
AAATAEGAPQLCEAASGNVAAEMRHGSSEAATAAFAKAAHVVALDVVNQRVVALTIEPRSVLAAPDADTGRLT
IRMSTQMPSGVRDSICAAIGLAKDKVRVVVGDVGGGFGMKTGAYPEDIAVAFAALQVKRPVKWVADRSEEFL
SSAHGRDIEAKAELALDAKGRILALRIKTLANVGAYATGTGVAIQLLIGPWVQTSVYDIQTIDFHFKAVLTNTAPTG
AYRGAGRPEAIFTIERLMDEAARQTGIDRIELRRRNFIRPEQMPYKNPMAQTYDTGKFESVMDQALGLADWN
GFEARAAESARNGRHRGLGIATFLEWTGGNVFEERVTVSVQADGVIEVFSAVNAMGQGIATSLAQLAVDAFG
VPIEKVRVVLGDTDRGDGFGSAGSRSLFTGGSAVRIGAERTIDKARELAAQEFEVAIDDVTYSRGAFAVIGTDLEL
DLFTLAGKQPEREIFVDSTSTVAGPTWPNGCHICEIEIDPPTGEISVVAYSSVNDVGRVVNPMIVRGQLEGGAV
QGIGQALYEQVVYDHETGQPVTGSLMDYAAPRADIVSTMFHMEMDESTPCKNNPLGVKGVGELGTIGATPAI
VNAVADAFARNGHAARAPRLHMPLSPSRVWQAMQTVD
>SEQF3751||SEQF3751.1_00513
MGASDFANLPHIGNALRRKEDYRFLTGAGNYTDDITLANQSHAVFVRSPHAHARIVSVDIADALTMPGVVGIFS
GKDIEGKMGGLPCGWLINNPDGTPMKEPMHPILAINKVRYVGDHVAMVVAETVEQAKNAAEAVVVEYEVLP
ALVGVADAAKKAGGVTIHDEAPDNQCYKWALGDKAAVDAAFAGAAHVTKLDLVNNRLIPNPIEPRVAIGSYSR
GTDDYTLYVSNQNPHVERLLMTAFVLGLPEHKVRVIAPDVGGGFGSKIFLYAEDVCLTWAAKQLNRNIKWTAER
SECFLSDAHGRDHVSHAEMAMDKDGKFLAMRVHTDANLGAYLSTFSTAVPTILYATLLAGQYTTPQIYVEVDA
WFTNTAPVDAYRGAGRPEATYLLERLVSRCAWEMNLGQDEIRQRNFITAFPYQTPVALQYDTGDFHACMDKA
RKLAEVEGYAQRKSASEAAGKLRGIGYSSYIEACGIAPSNIAGALGARAGLFECGEIRVHPTGSVTVFTGSHSHGQ
GHETTFAQVVAARLGIAVEAVDVVHGDTGRVPFGMGTYGSRSISVGGAAIMKALDKIEAKAKKIAAHLMEASD
ADIEFANGEFTVKGTDKKIPFGQVALTAYVPHNYPLDKLEPGLNETAFYDPTNFTFPGGTYICEVEIDKQTGEVRV
DRFTAVDDFGTIINPMIVEGQVHGGLVQGIGQALLENCVYDNETGQLLTGSFMDYAMPRAAEFPQFKLDTVCT
PCTHNPLGTKGCGEAGAIGSPPAVINAVLDALAPLGVKDFDMPASSSRVWEAMQKAGAAA
>SEQF3751||SEQF3751.1_00589
MTANPATRTADLSQRPHLGRPARRVEDRALLTGQGRFGDDAPVRAGTLHAAIVRSPHAHAKLLALRTARAEAS

PGVRAVLTGADVQRWSKPFIVGVKQPMELWALAIDRVRYVGEPVAVVIAENRYLAEDASDLVEADYETLPAVID
IESAAADDAPVLHERVGSNVVSDRSFRYGDPEAAFALAPHRVSITVHYPRNSCTPIETGVAIAEFISEQEGYELQS
NFMGPFSLHTVMAMALKVPGNRLRHRTFPDSGGSFGVKQAVMAPAVLMCLAARKAGAPVKWVEDRLEHLS
AATSATGRLCHAEAAVDPDGRITALSLDQFDDCGGYLRAPEPATFYRMHGCLTGAYAIANLRVRNRVVLTNKTPT
GLVRGFGGPQVYFALERLVQRIAVELCLDPLDVYRRNFIPRDAFPYHAAAGALIDSGDYHRALEMVVAEGELDM
LRRRQQEARAQGRLYGIGFAAIVEPSVSNMGYITTVLPAEQRAKAGPKNGATAAATVSIDPLGGINAVIASAPAG
QGHRTVCAQVIADAFGVNPAEVTVNVEFDTQKDAWSVAAGNYSSRFAGAVAGTLHLACLKLRDKLARIAAPTL
GCAPEEVVFAGGKIHVRQAPDKGLPFARFAASPHWAPALLPPGEDPGLRETAFWTAPQLAAPDEDDRVNTSA
AYGFAFDLCAVEVDRDTGRIRIDRYVTAHDAGTLLNPALADGQIRGAFAQGLGAALMEEFRYGTDGSFQSGTFA
DYLVPTTCEVPDPLILHLETPSPFTPLGAKGLGEGNNMSTPPCIANAVADALGVSDIRLPLKPSRVLELLGIDDPFP
SSGSRTATDAARTGVEHGDEHSLPDASPRKSGKALSARGEITLPATPEAVFAVLLDPKALARVIPGCHALEETAPH
RYRADVTVGVGMIKARYAAEVSLSDLDPPRRLRLAGSGISSMGSARGEGLVTLSATDGGTLLKYDYEAEVSGKV
AAVGARMLEGAAKIVLRQLFEQLGRQAGGQEGIDAAAASTSLWQRLLRRLGVGGHR
>SEQF3752||SEQF3752.1_05179
MNDLTLPAPVREIEAQRYIGQSVERPNMQRLTQGRANYVDDLELPRMLHVVYLRSPHAHARIVRIDAEAARR
MPGIAAIVDGRQLAAVCAPWVGTLKHLAGMRSPPQHALAIERATWQGEAVLAILGRTRALAEDALDAVQVE
WEPLPAAVHMDTALDAATPVIHPELGSNLCFHREIDTGGVEAVFEGADVVVEDEFRFGRHTGVTLEPRAVLADF
TPTGRRLVVHHSHQAPHMMADLYARQLGLQEADVRVVCRDVGGSFGIKVHAYADDFATVALSVLLGRPVKFV
ADRLESFVTDVHAREHRVKARLALARDGRMLAFQIDDLTGIGPFSMFPRTSAIEGNQVINITGGPYKHAHYKAT
LDVAFLNKVQTSQYRGVGHPIACTVTEGLVDLAAAKLNIDPLELRRRNVMEDGSYPRQGASGILLEGLSHQACL
DRIETLMDYGALRREQATLRERGVHRGIGLAALIELTNPGPAFYGVGGARIAAQDGATVRLDPTGVVHCATGVG
EQGQGTETIFAQIVADTLGLPMASVRVLTGDTAATPYGGGTWASRGAGIGGEAVLLASRDLRRHILALAAAHLK
RPAEGLTIAHGEVRCLATGDTLSDLAELGRLAHFRPDLLPAGFQPELVVTRHYAQRDYPFVFTNGVQASHVEVD
VDTGFVRLLRHWVVEDCGRVLNPMLVDEQIRGAVVQGIGGVLYEECLYDDHGLMRNGSMADYLVPMAFEM
PDIEVAHIETPTRTSQLGAKGAGEAGTAGAPAAVLNAINDALRPFGAHVSEQPVTPEKVLRALGRI
>SEQF3752||SEQF3752.1_03151
MGASDFANLPHIGNALRRKEDYRFLTGAGNYTDDITLANQSHAVFVRSPHAHARIVSVDIADALTMPGVVGIFS
GKDIEGKMGGLPCGWLINNPDGTPMKEPMHPILAINKVRYVGDHVAMVVAETVEQAKNAAEAVVVEYEVLP
ALVGVADAAKKAGGVTIHDEAPDNQCYKWALGDKAAVDAAFAGAAHVTKLDLVNNRLIPNPIEPRVAIGSYSR
GTDDYTLYVSNQNPHVERLLMTAFVLGLPEHKVRVIAPDVGGGFGSKIFLYAEDVCLTWAAKQLNRNIKWTAER
SECFLSDAHGRDHVSHAEMAMDKDGKFLAMRVHTDANLGAYLSTFSTAVPTILYATLLAGQYTTPQIYVEVDA
WFTNTAPVDAYRGAGRPEATYLLERLVSRCAWEMNLGQDEIRQRNFITAFPYQTPVALQYDTGDFHACMDKA
RKLAEVEGYAQRKSASEAAGKLRGIGYSSYIEACGIAPSNIAGALGARAGLFECGEIRVHPTGSVTVFTGSHSHGQ
GHETTFAQVVAARLGIAVEAVDVVHGDTGRVPFGMGTYGSRSISVGGAAIMKALDKIEAKAKKIAAHLMEASD
ADIEFANGEFTVKGTDKKIPFGQVALTAYVPHNYPLDKLEPGLNETAFYDPTNFTFPGGTYICEVEIDKQTGEVRV
DRFTAVDDFGTIINPMIVEGQVHGGLVQGIGQALLENCVYDNETGQLLTGSFMDYAMPRAAEFPQFKLDTVCT
PCTHNPLGTKGCGEAGAIGSPPAVINAVLDALAPLGVKDFDMPASSSRVWEAMQKAGAAA
>SEQF3752||SEQF3752.1_01717
MTANPATRTADLSQRPHLGRPARRVEDRALLTGQGRFGDDAPVRAGTLHAAIVRSPHAHAKLLALRTARAEAS
PGVRAVLTGADVQRWSKPFIVGVKQPMELWALAIDRVRYVGEPVAVVIAENRYLAEDASDLVEADYETLPAVID
IESAAADDAPVLHERVGSNVVSDRSFRYGDPEAAFALAPHRVSITVHYPRNSCTPIETGVAIAEFISEQEGYELQS
NFMGPFSLHTVMAMALKVPGNRLRHRTFPDSGGSFGVKQAVMAPAVLMCLAARKAGAPVKWVEDRLEHLS
AATSATGRLCHAEAAVDPDGRITALSLDQFDDCGGYLRAPEPATFYRMHGCLTGAYAIANLRVRNRVVLTNKTPT
GLVRGFGGPQVYFALERLVQRIAVELCLDPLDVYRRNFIPRDAFPYHAAAGALIDSGDYHRALEMVVAEGELDM

LRRRQQEARAQGRLYGIGFAAIVEPSVSNMGYITTVLPAEQRAKAGPKNGATAAATVSIDPLGGINAVIASAPAG
QGHRTVCAQVIADAFGVNPAEVTVNVEFDTQKDAWSVAAGNYSSRFAGAVAGTLHLACLKLRDKLARIAAPTL
GCAPEEVVFAGGKIHVRQAPDKGLPFARFAASPHWAPALLPPGEDPGLRETAFWTAPQLAAPDEDDRVNTSA
AYGFAFDLCAVEVDRDTGRIRIDRYVTAHDAGTLLNPALADGQIRGAFAQGLGAALMEEFRYGTDGSFQSGTFA
DYLVPTTCEVPDPLILHLETPSPFTPLGAKGLGEGNNMSTPPCIANAVADALGVSDIRLPLKPSRVLELLGIDDPFP
SSGSRTATDAARTGVEHGDEHSLPDASPRKSGKALSARGEITLPATPEAVFAVLLDPKALARVIPGCHALEETAPH
RYRADVTVGVGMIKARYAAEVSLSDLDPPRRLRLAGSGISSMGSARGEGLVTLSATDGGTLLKYDYEAEVSGKV
AAVGARMLEGAAKIVLRQLFEQLGRQAGGQEGIDAAAASTSLWQRLLRRLGVGGHR
>SEQF3752||SEQF3752.1_02941
MRFGSGQAVRRLEDQSLLSGAGRYTDDVSLPNQSHLVFLRSPYPHARLVSVDTSAALALPGVLAVITGADLAAA
GVKPMPGAAGFKRADGGDSASPARHAIAHERVRFVGEAVAVVVADTVQQARDAAEAVMVEYEELPMVVDL
AAATAEGAPQLCEAASGNVAAEMRHGSSEAATAAFAKAAHVVALDVVNQRVVALTIEPRSVLAAPDADTGRLT
IRMSTQMPSGVRDSICAAIGLAKDKVRVVVGDVGGGFGMKTGAYPEDIAVAFAALQVKRPVKWVADRSEEFL
SSAHGRDIEAKAELALDAKGRILALRIKTLANVGAYATGTGVAIQLLIGPWVQTSVYDIQTIDFHFKAVLTNTAPTG
AYRGAGRPEAIFTIERLMDEAARQTGIDRIELRRRNFIRPEQMPYKNPMAQTYDTGKFESVMDQALGLADWN
GFEARAAESARNGRHRGLGIATFLEWTGGNVFEERVTVSVQADGVIEVFSAVNAMGQGIATSLAQLAVDAFG
VPIEKVRVVLGDTDRGDGFGSAGSRSLFTGGSAVRIGAERTIDKARELAAQEFEVAIDDVTYSRGAFAVIGTDLEL
DLFTLAGKQPEREIFVDSTSTVAGPTWPNGCHICEIEIDPPTGEISVVAYSSVNDVGRVVNPMIVRGQLEGGAV
QGIGQALYEQVVYDHETGQPVTGSLMDYAAPRADIVSTMFHMEMDESTPCKNNPLGVKGVGELGTIGATPAI
VNAVADAFARNGHAARAPRLHMPLSPSRVWQAMQTVD
>SEQF3753||SEQF3753.1_02768
MGAPDFASLPHIGEALKRKEDYRFLTGAGQYTDDVTMANQSHAIFLRSPHAHADIRSIDTAAAEKMPGVVGIFS
GKDIDGKMGGLPCGWLINNPDGTPMKEPPHPILAIGKVRYVGDHVAMVVAETVEQAKNAAEAIVVDYEVLPA
LVSVADAAKKASGVTIHEAAPDNQCYKWALGDKAAVDAAFAKAAHVTKLDLVNNRLVPNPMEPRVAIGSYSRA
TEEYTLYVANQNPHVERLLMTAFVLGLPEHKVRVIAPDVGGGFGSKIFLYAEDVALTWAAKQLNRSIKWTGDRS
ECFLSDAHGRDHVSHAEMALDKDGKFLALRVHTDANLGAYLSTFSTAVPTILYATLLAGQYTTPQIYVEVDAWFT
NTAPVDAYRGAGRPEATYLLERLVSRCAWEMNLGQDEIRKRNFITTFPYQTPVALQYDTGDFHASMDKAKDLA
EVASFAERKAATEAEGKLRGIGYSSYIEACGIAPSNIAGALGARAGLFECGEIRVHPTGSVTVFTGSHSHGQGHET
TFAQVVAARLGIAVEAVDVVHGDTGRVPFGMGTYGSRSISVGGAAIMKALDKIETKAKKIAAHLMEASDADVE
FANGEFTVKGTDKKIPFGQVALTAYVPHNYPLDKLEPGLNETAFYDPTNFTFPGGTYICEVEIDKGTGEVKIDRFTA
VDDFGTIINPMIVEGQVHGGLVQGLGQALMENCVYDKESGQLLTGSFMDYAMPRADDFPIFKVATTCTPCTH
NPLGTKGCGEAGAIGSPPALINAVLDALAPLGVKDFDMPASPHRVWEAMQKGSTSPTQEPQQPPLTASTQGRP
AA
>SEQF3753||SEQF3753.1_06565
MSTETNPMRFGSGQAVRRLEDESLLAGAGRFTDDVTLPNQTFLAFVRSPYPHARLASVDTAPAAAMPGVLRVL
TGAELDGAGVKPLPGAAGFKRADGTDCASPPRHVLALDRVRFVGEPVAAVIAESAREARDAAEAVMVDYEAL
PMVVDLAGATAEGAPLVWDEATGNIAAEMRHGSRDATSAAFAKASHVVALDVVNQRVVALTIEPRSVLADFD
TVSDRITLRMSTQMPSGVRTSVCDAIGLPQEQVRVVVGDVGGGFGMKTSAYPEDIVVAFAARAVRRPVKWVA
DRSEEFLSSWHGRDIEARAELALDASGKILGLRIRTLANVGAYAGGTGVAIQLLIGPWVQTSVYDIQTIDFHFHAV
LTHTAPTGAYRGAGRPEAIYTIERLMDEAARQTGIDRIELRRRNFIQPAQMPYTNPMQQTYDTGKFESVMDQA
QQLADWRGFDARAAESKRQGKLRGLGIATFLEWTGGNVFEERVTVSVQVDGIIEVFSAVNAMGQGIATTLAQ
LAVDAFGVPIEKVRIVLGDTDRGDGFGSAGSRSLFTGGSAVRVGAERTIDKARELAAQELEVAPADVEYAAGVFK
VAGTDLQLDLFTLAGRQPDRQIFVDSTSAVAGPTWPNGCHICEVEIDPPTGDVNVLAYASVNDVGRVVNPMIV
RGQLEGGAVQGIGQALCERVVYDVETGQPLTGSLMDYAAPRADIIASMFKTEMDQSTPCLNNPLGVKGVGEL

GTIGATPAVVNAVADALARAGMAGLTPRLDMPLTPARMWALMQPGP
>SEQF3753||SEQF3753.1_05400
MSNDHEARTGQGVGARVERKEDARHLHGRGRFVADMSMPELSEVAFLRSPLAHARIVSARVPQEGAERVVL
RESMRDALDIVADSTLPTYQSSAQPPLASGKVRFVGEPVGMVFAPTRAEAEDLAEQVEVEFEDLPVYADAASSR
AATGELIHPGWRDNTFVTLNADTDFELHAKQASLVVRRSIDLARHCMVPMEGKAVLAYWDHQADQLVVVSAT
QVPHMIRTVIAQCLGLEQGRVRVISPDVGGAFGYKCVLQQEELCVAWLAKTHRKPFRYIEDRREHLIAGANTRE
HHYEMTAYADARGRLLALDARITIDGGAYSVWPFTIGLEPGQAVGNLPGPYAFRGYRCVTECIATNKPGFVPYR
GVARTGVCFAMELTMDAIARAVGREPWEVRLENLVPATAMPHVNVANKHFDSGDYPASLQKARDMIGLDAV
RERQRKAEADGRLIGVGFATYTEQSAHGTSVFATWGTPVIPGFDSAMVRITPDGGLEIRVGVHSHGQGMETTF
AQIAHEVLGIDIASIKLVHGDTGLTPFSTGTYASRSLVMSGGAVSKACKALAPRVRHIGAHLLGAKPEEVAIETGRV
VGSAGSVSIAEVAQAWYLRPDRLPADVDPQGLEVTTGFKPKVDTGAFSYASHAVTVAVDPDTGHVEILDYVIVE
DCGTMINPMVVEGQTYGGVAQGIGTALYEETPYDRNGQPLASTLADYMLPGATEVPNLRIHHFETPSPHTEFG
AKGMGEGGAIAPPAAIFNAVNDALRPLGAEVAHTPLTPRRLLEAIAAAQGAEKQKAA
>SEQF3754||SEQF3754.1_01072
MTTDSTPTRFGSGQAVRRLEDESLLSGAGRYTDDVTLAGQTHLVFLRSPYPHARIVSIDTAAAAAMPGVLRVIT
GAELAEAGVKPLPGAVGFKRADGGDCASPARLALAHDRTRFVGEAVAAVVAETVQQARDAAEAVVVDYEALP
MVVDLASAAADGAPLLCEEASGNVAAEMRHGSSDAATAAFAKAKHVVALDVVNQRVVALTIEPRSVLAAPDA
ESGRLTIRMSTQMPSGVRDSICAAIGLAKEKVRVVVGDVGGGFGMKTGAYPEDIAVAFAALQVKRPVKWVAD
RSEEFLSSAHGRDIEARAELALDADGKILALRIRTLANVGAYATGTGVAIQLLIGPWVQTSVYDIQTIDFHFKAVLT
NTAPTGAYRGAGRPEAIFTIERLMDEAARQTGIDRIALRRRNFIRPEQMPYKNPMAQTYDTGKFESVMDQALV
LADWQGFDARAADSAKNGKHRGLGIATFLEWTGGNVFEERVTVSVQADGVIEVFSAVNAMGQGIATSLAQL
AVDAFGVPIEKVRVVLGDTDRGDGFGSAGSRSLFTGGSAVRIGAERTIDKARELAAQEFEASIDDITYTRGVFTV
AGTDLELDLFALAGKQPEREIFVDSTSTVAGPTWPNGCHVCEIEIDPPTGEISVVAYSSVNDVGRVINPMIVRGQ
LEGGAVQGIGQALYEQVVYDNETGQPLTGSLMDYAAPRADIVEAMFHMEMDESTPCANNPLGVKGVGELGTI
GATPAIVNAVADAFARNGFASTTPRLHMPLSPARVWQAMHTVD
>SEQF3754||SEQF3754.1_00406
MGASDFSNLPHIGEALRRKEDYRFLTGAGNYTDDITLANQSHAVFVRSPHAHAVIKSVDTAQALKMPGVVGIFS
GKDIEGKMGGLPCGWLINNPDGTPMKEPMHPILAINKVRYVGDHVAMVVAETVEQAKNAAEAVVVDYDVL
PALVSVADAAKKASGVTLHDEAPDNQCYKWTLGDKAAVDTVFATAAHVTRLDLVNNRLIPNPIEPRVAIGSYSR
GTDDYTLYVSNQNPHVERLLMTAFVLGLPEHKVRVIAPDVGGGFGSKIFLYAEDVCLTWAAKQLNRNIKWTAER
SECFLSDAHGRDHVSHAEMAMDKDGKFLAMRVHTDANLGAYLSTFSTAVPTILYATLLAGQYTTPQIYVEVDA
WFTNTAPVDAYRGAGRPEATYLLERLVSRCAWEMNLGQDEIRKRNFITTFPYQTPVALQYDTGDFHACMDKA
RVLADVEGYAQRKAATEAAGKLRGMGYSSYIEACGIAPSNIAGALGARAGLFECGEIRVHPTGSVTVFTGSHSH
GQGHETTFAQVVAARLGIPVDNVDVVHGDTGRVPFGMGTYGSRSISVGGAAIMKALDKIETKAKKIAAHLME
ASDADIEFANGEFTVKGTDKKIPFGQVALTAYVPHNYPLDKLEPGLNETAFYDPTNFTFPGGTYICEVEVDRQTG
EVTIDRFTAVDDFGTIINPMIVEGQVHGGLVQGIGQALLENCVYDNETGQLLTGSFMDYAMPRAGDFPQFNLD
TVCTPCTHNPLGTKGCGEAGAIGSPPAVINAVLDALAPLGVKDFDMPASASRVWEAMQKGSVSATQDAQTQK
PSLAASTQGRPAP
>SEQF3755||SEQF3755.1_04563
MNDLTLPAPVREIEAQRYIGQSVERPNMQRLTQGRANYVDDLELPRMLHVVYLRSPHAHARIVRIDAEAARR
MPGIAAIVDGRQLAAVCAPWVGTLKHLAGMRSPPQHALAIERATWQGEAVLAILGRTRALAEDALDAVQVE
WEPLPAAVHMDTALDAATPVIHPELGSNLCFHREIDTGGVEAVFEGADVVVEDEFRFGRHTGVTLEPRAVLADF
TPTGRRLVVHHSHQAPHMMADLYARQLGLQEADVRVVCRDVGGSFGIKVHAYADDFATVALSVLLGRPVKFV
ADRLESFVTDVHAREHRVKARLALARDGRMLAFQIDDLTGIGPFSMFPRTSAIEGNQVINITGGPYKHAHYKAT

LDVAFLNKVQTSQYRGVGHPIACTVTEGLVDLAAAKLNIDPLELRRRNVMEDGSYPRQGASGILLEGLSHQACL
DRIETLMDYGALRREQATLRERGVHRGIGLAALIELTNPGPAFYGVGGARIAAQDGATVRLDPTGVVHCATGVG
EQGQGTETIFAQIVADTLGLPMASVRVLTGDTAATPYGGGTWASRGAGIGGEAVLLASRDLRRHILALAAAHLK
RPAEGLTIAHGEVRCLATGDTLSDLAELGRLAHFRPDLLPAGFQPELVVTRHYAQRDYPFVFTNGVQASHVEVD
VDTGFVRLLRHWVVEDCGRVLNPMLVDEQIRGAVVQGIGGVLYEECLYDDHGLMRNGSMADYLVPMAFEM
PDIEVAHIETPTRTSQLGAKGAGEAGTAGAPAAVLNAINDALRPFGAHVSEQPVTPEKVLRALGRI
>SEQF3755||SEQF3755.1_01740
MRFGSGQAVRRLEDQSLLSGAGRYTDDVSLPNQSHLVFLRSPYPHARLVSVDTSAALALPGVLAVITGADLAAA
GVKPMPGAAGFKRADGGDSASPARHAIAHERVRFVGEAVAVVVADTVQQARDAAEAVMVEYEELPMVVDL
AAATAEGAPQLCEAASGNVAAEMRHGSSEAATAAFAKAAHVVALDVVNQRVVALTIEPRSVLAAPDADTGRLT
IRMSTQMPSGVRDSICAAIGLAKDKVRVVVGDVGGGFGMKTGAYPEDIAVAFAALQVKRPVKWVADRSEEFL
SSAHGRDIEAKAELALDAKGRILALRIKTLANVGAYATGTGVAIQLLIGPWVQTSVYDIQTIDFHFKAVLTNTAPTG
AYRGAGRPEAIFTIERLMDEAARQTGIDRIELRRRNFIRPEQMPYKNPMAQTYDTGKFESVMDQALGLADWN
GFEARAAESARNGRHRGLGIATFLEWTGGNVFEERVTVSVQADGVIEVFSAVNAMGQGIATSLAQLAVDAFG
VPIEKVRVVLGDTDRGDGFGSAGSRSLFTGGSAVRIGAERTIDKARELAAQEFEVAIDDVTYSRGAFAVIGTDLEL
DLFTLAGKQPEREIFVDSTSTVAGPTWPNGCHICEIEIDPPTGEISVVAYSSVNDVGRVVNPMIVRGQLEGGAV
QGIGQALYEQVVYDHETGQPVTGSLMDYAAPRADIVSTMFHMEMDESTPCKNNPLGVKGVGELGTIGATPAI
VNAVADAFARNGHAARAPRLHMPLSPSRVWQAMQTVD
>SEQF3755||SEQF3755.1_02281
MGASDFANLPHIGNALRRKEDYRFLTGAGNYTDDITLANQSHAVFVRSPHAHARIVSVDIADALTMPGVVGIFS
GKDIEGKMGGLPCGWLINNPDGTPMKEPMHPILAINKVRYVGDHVAMVVAETVEQAKNAAEAVVVEYEVLP
ALVGVADAAKKAGGVTIHDEAPDNQCYKWALGDKAAVDAAFAGAAHVTKLDLVNNRLIPNPIEPRVAIGSYSR
GTDDYTLYVSNQNPHVERLLMTAFVLGLPEHKVRVIAPDVGGGFGSKIFLYAEDVCLTWAAKQLNRNIKWTAER
SECFLSDAHGRDHVSHAEMAMDKDGKFLAMRVHTDANLGAYLSTFSTAVPTILYATLLAGQYTTPQIYVEVDA
WFTNTAPVDAYRGAGRPEATYLLERLVSRCAWEMNLGQDEIRQRNFITAFPYQTPVALQYDTGDFHACMDKA
RKLAEVEGYAQRKSASEAAGKLRGIGYSSYIEACGIAPSNIAGALGARAGLFECGEIRVHPTGSVTVFTGSHSHGQ
GHETTFAQVVAARLGIAVEAVDVVHGDTGRVPFGMGTYGSRSISVGGAAIMKALDKIEAKAKKIAAHLMEASD
ADIEFANGEFTVKGTDKKIPFGQVALTAYVPHNYPLDKLEPGLNETAFYDPTNFTFPGGTYICEVEIDKQTGEVRV
DRFTAVDDFGTIINPMIVEGQVHGGLVQGIGQALLENCVYDNETGQLLTGSFMDYAMPRAAEFPQFKLDTVCT
PCTHNPLGTKGCGEAGAIGSPPAVINAVLDALAPLGVKDFDMPASSSRVWEAMQKAGAAA
>SEQF3755||SEQF3755.1_01182
MTANPATRTADLSQRPHLGRPARRVEDRALLTGQGRFGDDAPVRAGTLHAAIVRSPHAHAKLLALRTARAEAS
PGVRAVLTGADVQRWSKPFIVGVKQPMELWALAIDRVRYVGEPVAVVIAENRYLAEDASDLVEADYETLPAVID
IESAAADDAPVLHERVGSNVVSDRSFRYGDPEAAFALAPHRVSITVHYPRNSCTPIETGVAIAEFISEQEGYELQS
NFMGPFSLHTVMAMALKVPGNRLRHRTFPDSGGSFGVKQAVMAPAVLMCLAARKAGAPVKWVEDRLEHLS
AATSATGRLCHAEAAVDPDGRITALSLDQFDDCGGYLRAPEPATFYRMHGCLTGAYAIANLRVRNRVVLTNKTPT
GLVRGFGGPQVYFALERLVQRIAVELCLDPLDVYRRNFIPRDAFPYHAAAGALIDSGDYHRALEMVVAEGELDM
LRRRQQEARAQGRLYGIGFAAIVEPSVSNMGYITTVLPAEQRAKAGPKNGATAAATVSIDPLGGINAVIASAPAG
QGHRTVCAQVIADAFGVNPAEVTVNVEFDTQKDAWSVAAGNYSSRFAGAVAGTLHLACLKLRDKLARIAAPTL
GCAPEEVVFAGGKIHVRQAPDKGLPFARFAASPHWAPALLPPGEDPGLRETAFWTAPQLAAPDEDDRVNTSA
AYGFAFDLCAVEVDRDTGRIRIDRYVTAHDAGTLLNPALADGQIRGAFAQGLGAALMEEFRYGTDGSFQSGTFA
DYLVPTTCEVPDPLILHLETPSPFTPLGAKGLGEGNNMSTPPCIANAVADALGVSDIRLPLKPSRVLELLGIDDPFP
SSGSRTATDAARTGVEHGDEHSLPDASPRKSGKALSARGEITLPATPEAVFAVLLDPKALARVIPGCHALEETAPH
RYRADVTVGVGMIKARYAAEVSLSDLDPPRRLRLAGSGISSMGSARGEGLVTLSATDGGTLLKYDYEAEVSGKV

AAVGARMLEGAAKIVLRQLFEQLGRQAGGQEGIDAAAASTSLWQRLLRRLGVGGHR
>SEQF3756||SEQF3756.1_01055
MTANPATRTADLSQRPHLGRPARRVEDRALLTGQGRFGDDAPVRAGTLHAAIVRSPHAHAKLLALRTARAEAS
PGVRAVLTGADVQRWSKPFIVGVKQPMELWALAIDRVRYVGEPVAVVIAENRYLAEDASDLVEADYETLPAVID
IESAAADDAPVLHERVGSNVVSDRSFRYGDPEAAFALAPHRVSITVHYPRNSCTPIETGVAIAEFISEQEGYELQS
NFMGPFSLHTVMAMALKVPGNRLRHRTFPDSGGSFGVKQAVMAPAVLMCLAARKAGAPVKWVEDRLEHLS
AATSATGRLCHAEAAVDPDGRITALSLDQFDDCGGYLRAPEPATFYRMHGCLTGAYAIANLRVRNRVVLTNKTPT
GLVRGFGGPQVYFALERLVQRIAVELCLDPLDVYRRNFIPRDAFPYHAAAGALIDSGDYHRALEMVVAEGELDM
LRRRQQEARAQGRLYGIGFAAIVEPSVSNMGYITTVLPAEQRAKAGPKNGATAAATVSIDPLGGINAVIASAPAG
QGHRTVCAQVIADAFGVNPAEVTVNVEFDTQKDAWSVAAGNYSSRFAGAVAGTLHLACLKLRDKLARIAAPTL
GCAPEEVVFAGGKIHVRQAPDKGLPFARFAASPHWAPALLPPGEDPGLRETAFWTAPQLAAPDEDDRVNTSA
AYGFAFDLCAVEVDRDTGRIRIDRYVTAHDAGTLLNPALADGQIRGAFAQGLGAALMEEFRYGTDGSFQSGTFA
DYLVPTTCEVPDPLILHLETPSPFTPLGAKGLGEGNNMSTPPCIANAVADALGVSDIRLPLKPSRVLELLGIDDPFP
SSGSRTATDAARTGVEHGDEHSLPDASPRKSGKALSARGEITLPATPEAVFAVLLDPKALARVIPGCHALEETAPH
RYRADVTVGVGMIKARYAAEVSLSDLDPPRRLRLAGSGISSMGSARGEGLVTLSATDGGTLLKYDYEAEVSGKV
AAVGARMLEGAAKIVLRQLFEQLGRQAGGQEGIDAAAASTSLWQRLLRRLGVGGHR
>SEQF3756||SEQF3756.1_01483
MRFGSGQAVRRLEDQSLLSGAGRYTDDVSLPNQSHLVFLRSPYPHARLVSVDTSAALALPGVLAVITGADLAAA
GVKPMPGAAGFKRADGGDSASPARHAIAHERVRFVGEAVAVVVADTVQQARDAAEAVMVEYEELPMVVDL
AAATAEGAPQLCEAASGNVAAEMRHGSSEAATAAFAKAAHVVALDVVNQRVVALTIEPRSVLAAPDADTGRLT
IRMSTQMPSGVRDSICAAIGLAKDKVRVVVGDVGGGFGMKTGAYPEDIAVAFAALQVKRPVKWVADRSEEFL
SSAHGRDIEAKAELALDAKGRILALRIKTLANVGAYATGTGVAIQLLIGPWVQTSVYDIQTIDFHFKAVLTNTAPTG
AYRGAGRPEAIFTIERLMDEAARQTGIDRIELRRRNFIRPEQMPYKNPMAQTYDTGKFESVMDQALGLADWN
GFEARAAESARNGRHRGLGIATFLEWTGGNVFEERVTVSVQADGVIEVFSAVNAMGQGIATSLAQLAVDAFG
VPIEKVRVVLGDTDRGDGFGSAGSRSLFTGGSAVRIGAERTIDKARELAAQEFEVAIDDVTYSRGAFAVIGTDLEL
DLFTLAGKQPEREIFVDSTSTVAGPTWPNGCHICEIEIDPPTGEISVVAYSSVNDVGRVVNPMIVRGQLEGGAV
QGIGQALYEQVVYDHETGQPVTGSLMDYAAPRADIVSTMFHMEMDESTPCKNNPLGVKGVGELGTIGATPAI
VNAVADAFARNGHAARAPRLHMPLSPSRVWQAMQTVD
>SEQF3756||SEQF3756.1_02423
MNDLTLPAPVREIEAQRYIGQSVERPNMQRLTQGRANYVDDLELPRMLHVVYLRSPHAHARIVRIDAEAARR
MPGIAAIVDGRQLAAVCAPWVGTLKHLAGMRSPPQHALAIERATWQGEAVLAILGRTRALAEDALDAVQVE
WEPLPAAVHMDTALDAATPVIHPELGSNLCFHREIDTGGVEAVFEGADVVVEDEFRFGRHTGVTLEPRAVLADF
TPTGRRLVVHHSHQAPHMMADLYARQLGLQEADVRVVCRDVGGSFGIKVHAYADDFATVALSVLLGRPVKFV
ADRLESFVTDVHAREHRVKARLALARDGRMLAFQIDDLTGIGPFSMFPRTSAIEGNQVINITGGPYKHAHYKAT
LDVAFLNKVQTSQYRGVGHPIACTVTEGLVDLAAAKLNIDPLELRRRNVMEDGSYPRQGASGILLEGLSHQACL
DRIETLMDYGALRREQATLRERGVHRGIGLAALIELTNPGPAFYGVGGARIAAQDGATVRLDPTGVVHCATGVG
EQGQGTETIFAQIVADTLGLPMASVRVLTGDTAATPYGGGTWASRGAGIGGEAVLLASRDLRRHILALAAAHLK
RPAEGLTIAHGEVRCLATGDTLSDLAELGRLAHFRPDLLPAGFQPELVVTRHYAQRDYPFVFTNGVQASHVEVD
VDTGFVRLLRHWVVEDCGRVLNPMLVDEQIRGAVVQGIGGVLYEECLYDDHGLMRNGSMADYLVPMAFEM
PDIEVAHIETPTRTSQLGAKGAGEAGTAGAPAAVLNAINDALRPFGAHVSEQPVTPEKVLRALGRI
>SEQF3756||SEQF3756.1_00519
MGASDFANLPHIGNALRRKEDYRFLTGAGNYTDDITLANQSHAVFVRSPHAHARIVSVDIADALTMPGVVGIFS
GKDIEGKMGGLPCGWLINNPDGTPMKEPMHPILAINKVRYVGDHVAMVVAETVEQAKNAAEAVVVEYEVLP
ALVGVADAAKKAGGVTIHDEAPDNQCYKWALGDKAAVDAAFAGAAHVTKLDLVNNRLIPNPIEPRVAIGSYSR

GTDDYTLYVSNQNPHVERLLMTAFVLGLPEHKVRVIAPDVGGGFGSKIFLYAEDVCLTWAAKQLNRNIKWTAER
SECFLSDAHGRDHVSHAEMAMDKDGKFLAMRVHTDANLGAYLSTFSTAVPTILYATLLAGQYTTPQIYVEVDA
WFTNTAPVDAYRGAGRPEATYLLERLVSRCAWEMNLGQDEIRQRNFITAFPYQTPVALQYDTGDFHACMDKA
RKLAEVEGYAQRKSASEAAGKLRGIGYSSYIEACGIAPSNIAGALGARAGLFECGEIRVHPTGSVTVFTGSHSHGQ
GHETTFAQVVAARLGIAVEAVDVVHGDTGRVPFGMGTYGSRSISVGGAAIMKALDKIEAKAKKIAAHLMEASD
ADIEFANGEFTVKGTDKKIPFGQVALTAYVPHNYPLDKLEPGLNETAFYDPTNFTFPGGTYICEVEIDKQTGEVRV
DRFTAVDDFGTIINPMIVEGQVHGGLVQGIGQALLENCVYDNETGQLLTGSFMDYAMPRAAEFPQFKLDTVCT
PCTHNPLGTKGCGEAGAIGSPPAVINAVLDALAPLGVKDFDMPASSSRVWEAMQKAGAAA
>SEQF3757||SEQF3757.1_04125
MTTETQPMRFGSGQAVRRLEDESLLAGAGRFTDDVTLPNQTFLAFVRSPYPHARLASIDTASAAAMPGVLRILT
GAELEGAGVQPLPGAAGFRRADGDYAAPPRHVLALDRVRFVGEPVAAVIAETAREARDAAEAVMVDYEELPM
VVDLAGATSEGAPRVWDEASGNIAAEMRHGSTEAASAAFAKASHVVALDVVNQRVVALTIEPRSVLADFDAA
SERITLRMSTQMPSGVRTSVCDAIGLPQEQVRVVVGDVGGGFGMKTSIYPEDIVVAFAARAVRRPVKWVAERS
EEFLSSWHGRDIEAHAELALDADGKILALRIKTLANVGAYAGGTGVAIQLLIGPWVQTSVYDIQTIDFHFLAVLTH
TAPTGAYRGAGRPEAIYTIERLMDEAARQIGIDRIELRRRNFIQPAQMPYTNPMQQTYDTGKFESVMEQALALA
DWQGFEARAAESKRQGRLRGLGIATFLEWTGGNVFEERVTVSVQADGIIEVFSAVNAMGQGIATTLAQLAVD
AFGVPIEKVRIVLGDTDRGDGFGSAGSRSLFTGGSAVRVGAERTIDRARELAARELEVAPADVEYAAGVFKVAGT
DLQLDLFALAGRQPDRQIFVDSTSAVAGPTWPNGCHICELEIDPPTGEVSILAYASVNDVGRVVNPMIVRGQLE
GGAVQGIGQALCERVVYDIETGQPLTGSLMDYAAPRADIVASMFKTEMDQSTPCLNNPLGVKGVGELGTIGAT
PAVVNAVADALARNGLAGRTPHLDMPLTPARLWQWMQPAG
>SEQF3757||SEQF3757.1_02265
MGAPDFAKLPHIGEALKRKEDYRFLTGAGQYTDDVTLANQSHAVFLRSPHAHANIKSIDTAAAEKMPGVVGIFS
GKDIDGKMGGLPCGWLINNPDGTPMKEPPHPILAIGKVRYVGDHVAMVVAETVEQAKNAAEAIVVDYDVLP
ALVSVADAAKKAGGVTLHDAAPDNQCYKWALGDKAAVDAAFAKAAHVTKLDLVNNRLVPNPMEPRVAIGSYS
RATEEYTLYVANQNPHVERLLMTAFVLGLPEHKVRVIAPDVGGGFGSKIFLYAEDVALTWAAKQLNRSIKWTGD
RSECFLSDAHGRDHVSHAEMAMDKDGKFLALRVHTDANLGAYLSTFSTAVPTILYATLLAGQYTTPQIYVEVDA
WFTNTAPVDAYRGAGRPEATYLLERLVSRCAWEMNLGQDEIRKRNFITAFPYQTPVALQYDTGDFHASMNRA
QELAEVAGFAQRKAASEAQGKLRGIGYSSYIEACGIAPSNIAGALGARAGLFECGEIRVHPTGSVTVFTGSHSHG
QGHETTFAQVVAARLGIPVENIDVVHGDTGRVPFGMGTYGSRSISVGGAAIMKALDKIEIKAKKIAAHLMEASD
ADVEFANGEFTVKGTDKKIPFGQVALTAYVPHNYPLDKLEPGLNETAFYDPTNFTFPGGTYICEVEIDKGTGEVKI
DRFTAVDDFGTIINPMIVEGQVHGGLVQGIGQALMENCVYDNESGQLLTGSFMDYAMPRADDFPNFKLDTTC
TPCTHNPLGTKGCGEAGAIGSPPAVINAVLDALAPLGVKDFDMPASPHRVWEAMQKGSTSPTQEPQQPSLTAS
TQGRPAA
>SEQF3758||SEQF3758.1_00260
MTANPATRTADLSQRPHLGRPARRVEDRALLTGQGRFGDDAPVRAGTLHAAIVRSPHAHAKLLALRTARAEAS
PGVRAVLTGADVQRWSKPFIVGVKQPMELWALAIDRVRYVGEPVAVVIAENRYLAEDASDLVEADYETLPAVID
IESAAADDAPVLHERVGSNVVSDRSFRYGDPEAAFALAPHRVSITVHYPRNSCTPIETGVAIAEFISEQEGYELQS
NFMGPFSLHTVMAMALKVPGNRLRHRTFPDSGGSFGVKQAVMAPAVLMCLAARKAGAPVKWVEDRLEHLS
AATSATGRLCHAEAAVDPDGRITALSLDQFDDCGGYLRAPEPATFYRMHGCLTGAYAIANLRVRNRVVLTNKTPT
GLVRGFGGPQVYFALERLVQRIAVELCLDPLDVYRRNFIPRDAFPYHAAAGALIDSGDYHRALEMVVAEGELDM
LRRRQQEARAQGRLYGIGFAAIVEPSVSNMGYITTVLPAEQRAKAGPKNGATAAATVSIDPLGGINAVIASAPAG
QGHRTVCAQVIADAFGVNPAEVTVNVEFDTQKDAWSVAAGNYSSRFAGAVAGTLHLACLKLRDKLARIAAPTL
GCAPEEVVFAGGKIHVRQAPDKGLPFARFAASPHWAPALLPPGEDPGLRETAFWTAPQLAAPDEDDRVNTSA
AYGFAFDLCAVEVDRDTGRIRIDRYVTAHDAGTLLNPALADGQIRGAFAQGLGAALMEEFRYGTDGSFQSGTFA

DYLVPTTCEVPDPLILHLETPSPFTPLGAKGLGEGNNMSTPPCIANAVADALGVSDIRLPLKPSRVLELLGIDDPFP
SSGSRTATDAARTGVEHGDEHSLPDASPRKSGKALSARGEITLPATPEAVFAVLLDPKALARVIPGCHALEETAPH
RYRADVTVGVGMIKARYAAEVSLSDLDPPRRLRLAGSGISSMGSARGEGLVTLSATDGGTLLKYDYEAEVSGKV
AAVGARMLEGAAKIVLRQLFEQLGRQAGGQEGIDAAAASTSLWQRLLRRLGVGGHR
>SEQF3758||SEQF3758.1_04983
MNDLTLPAPVREIEAQRYIGQSVERPNMQRLTQGRANYVDDLELPRMLHVVYLRSPHAHARIVRIDAEAARR
MPGIAAIVDGRQLAAVCAPWVGTLKHLAGMRSPPQHALAIERATWQGEAVLAILGRTRALAEDALDAVQVE
WEPLPAAVHMDTALDAATPVIHPELGSNLCFHREIDTGGVEAVFEGADVVVEDEFRFGRHTGVTLEPRAVLADF
TPTGRRLVVHHSHQAPHMMADLYARQLGLQEADVRVVCRDVGGSFGIKVHAYADDFATVALSVLLGRPVKFV
ADRLESFVTDVHAREHRVKARLALARDGRMLAFQIDDLTGIGPFSMFPRTSAIEGNQVINITGGPYKHAHYKAT
LDVAFLNKVQTSQYRGVGHPIACTVTEGLVDLAAAKLNIDPLELRRRNVMEDGSYPRQGASGILLEGLSHQACL
DRIETLMDYGALRREQATLRERGVHRGIGLAALIELTNPGPAFYGVGGARIAAQDGATVRLDPTGVVHCATGVG
EQGQGTETIFAQIVADTLGLPMASVRVLTGDTAATPYGGGTWASRGAGIGGEAVLLASRDLRRHILALAAAHLK
RPAEGLTIAHGEVRCLATGDTLSDLAELGRLAHFRPDLLPAGFQPELVVTRHYAQRDYPFVFTNGVQASHVEVD
VDTGFVRLLRHWVVEDCGRVLNPMLVDEQIRGAVVQGIGGVLYEECLYDDHGLMRNGSMADYLVPMAFEM
PDIEVAHIETPTRTSQLGAKGAGEAGTAGAPAAVLNAINDALRPFGAHVSEQPVTPEKVLRALGRI
>SEQF3758||SEQF3758.1_00089
MGASDFANLPHIGNALRRKEDYRFLTGAGNYTDDITLANQSHAVFVRSPHAHARIVSVDIADALTMPGVVGIFS
GKDIEGKMGGLPCGWLINNPDGTPMKEPMHPILAINKVRYVGDHVAMVVAETVEQAKNAAEAVVVEYEVLP
ALVGVADAAKKAGGVTIHDEAPDNQCYKWALGDKAAVDAAFAGAAHVTKLDLVNNRLIPNPIEPRVAIGSYSR
GTDDYTLYVSNQNPHVERLLMTAFVLGLPEHKVRVIAPDVGGGFGSKIFLYAEDVCLTWAAKQLNRNIKWTAER
SECFLSDAHGRDHVSHAEMAMDKDGKFLAMRVHTDANLGAYLSTFSTAVPTILYATLLAGQYTTPQIYVEVDA
WFTNTAPVDAYRGAGRPEATYLLERLVSRCAWEMNLGQDEIRQRNFITAFPYQTPVALQYDTGDFHACMDKA
RKLAEVEGYAQRKSASEAAGKLRGIGYSSYIEACGIAPSNIAGALGARAGLFECGEIRVHPTGSVTVFTGSHSHGQ
GHETTFAQVVAARLGIAVEAVDVVHGDTGRVPFGMGTYGSRSISVGGAAIMKALDKIEAKAKKIAAHLMEASD
ADIEFANGEFTVKGTDKKIPFGQVALTAYVPHNYPLDKLEPGLNETAFYDPTNFTFPGGTYICEVEIDKQTGEVRV
DRFTAVDDFGTIINPMIVEGQVHGGLVQGIGQALLENCVYDNETGQLLTGSFMDYAMPRAAEFPQFKLDTVCT
PCTHNPLGTKGCGEAGAIGSPPAVINAVLDALAPLGVKDFDMPASSSRVWEAMQKAGAAA
>SEQF3758||SEQF3758.1_03122
MRFGSGQAVRRLEDQSLLSGAGRYTDDVSLPNQSHLVFLRSPYPHARLVSVDTSAALALPGVLAVITGADLAAA
GVKPMPGAAGFKRADGGDSASPARHAIAHERVRFVGEAVAVVVADTVQQARDAAEAVMVEYEELPMVVDL
AAATAEGAPQLCEAASGNVAAEMRHGSSEAATAAFAKAAHVVALDVVNQRVVALTIEPRSVLAAPDADTGRLT
IRMSTQMPSGVRDSICAAIGLAKDKVRVVVGDVGGGFGMKTGAYPEDIAVAFAALQVKRPVKWVADRSEEFL
SSAHGRDIEAKAELALDAKGRILALRIKTLANVGAYATGTGVAIQLLIGPWVQTSVYDIQTIDFHFKAVLTNTAPTG
AYRGAGRPEAIFTIERLMDEAARQTGIDRIELRRRNFIRPEQMPYKNPMAQTYDTGKFESVMDQALGLADWN
GFEARAAESARNGRHRGLGIATFLEWTGGNVFEERVTVSVQADGVIEVFSAVNAMGQGIATSLAQLAVDAFG
VPIEKVRVVLGDTDRGDGFGSAGSRSLFTGGSAVRIGAERTIDKARELAAQEFEVAIDDVTYSRGAFAVIGTDLEL
DLFTLAGKQPEREIFVDSTSTVAGPTWPNGCHICEIEIDPPTGEISVVAYSSVNDVGRVVNPMIVRGQLEGGAV
QGIGQALYEQVVYDHETGQPVTGSLMDYAAPRADIVSTMFHMEMDESTPCKNNPLGVKGVGELGTIGATPAI
VNAVADAFARNGHAARAPRLHMPLSPSRVWQAMQTVD
>SEQF3759||SEQF3759.1_02922
MDHADIPARLLKREGIGARVPRKEDARHLVGKGNFVGDFVLPGLQEVAFLRSSLAHAVVTGVEIPETLAGKVFLR
EMMSDAADIGSPSSLPTYQYSALPPLASGKVRHVGEAIAMAVAPTRAMAEDLLEEVEVSYDELPVYHSAESALA
ATKEFMHPEWKDNVFLTLRANRDFEELAAKAEVKVSRTVELSRQCIVPLEGKSVLAYWDFQTDQLVVVSATQV

PHLLRVGIAQHLSMNEEAVRVVSPDVGGAFGYKGQLYPEDLCVAWLAKTYRTPFRYLEDRREHLIVGANTRQH
HYQLTAYADRTGKLLALDAVITIDGGAYSYYPFFVGLEPGQAIGNLPGPYTFRGYRCETLCVATNKPGFMAYRGV
ARTGVCFAIELLMDAVAREVGREPWEVRMENLVPAAAMPYVNVANKHFDSGDFPASLRRAVEMIRLDAVRER
QARGEPDGRLIGFGTATYTEQSAHGTTVFANWGLPVVPGFDQAVVRMTADGGLEVRVGVHSHGQGMETSF
AQIANDVLGIPVARIRVMHGDTALTPFSSGTYASRATVMSGGAISVACKELLPRIQSIAGYLMGVDAPTVALIEGR
AVAGDKSIPLSDVGGAWYLTPQDLPANVHLGGLEVSRAYKPNVDTGTFTYATHAVVVAVDTETGEVEILDYVVV
EDCGTMVNPMIVEGQTIGGIAQGIGTAMYEESPYDDQGQPLASTLADYILPGATEVPRIRIEHFETPSPHTEFGA
KGVGEGGAIAPPAVIFNAVNDALRGTGAAEVLMTPLTPRRLLTALEGAKREQEHAR
>SEQF3759||SEQF3759.1_06044
MGASDFANLPHIGDALRRKEDYRFLTGAGNYTDDITLANQSHAVFVRSPHAHARVVSVDIADALTMPGVVGIF
SGKDIEGKMGGLPCGWLINNPDGTPMKEPMHPILAINKVRYVGDHVAMVVAETVEQAKNAAEAVVVEYEVL
PALVGVADAAKKAGGVTIHDEAPDNQCYKWALGDKAAVDAAFAGAAHVTKLDLVNNRLIPNPIEPRVAIGSYS
RGTDDYTLYVSNQNPHVERLLMTAFVLGLPEHKVRVIAPDVGGGFGSKIFLYAEDVCLTWAAKQLNRNIKWTAE
RSECFLSDAHGRDHVSHAEMAMDKDGKFLAMRVHTDANLGAYLSTFSTAVPTILYATLLAGQYTTPQIYVEVDA
WFTNTAPVDAYRGAGRPEATYLLERLVSRCAWEMNLGQDEIRQRNFITAFPYQTPVALQYDTGDFHACMDKA
RKLAEVEGYGQRKSASEAAGKLRGIGYSSYIEACGIAPSNIAGALGARAGLFECGEIRVHPTGSVTVFTGSHSHG
QGHETTFAQVVAARLGIAVEAVDVVHGDTGRVPFGMGTYGSRSISVGGAAIMKALDKIEAKAKKIAAHLMEAS
DADIEFANGEFTVKGTDKKIPFGQVALTAYVPHNYPLDKLEPGLNETAFYDPTNFTFPGGTYICEVEIDRQTGEVR
VDRFTAVDDFGTIINPMIVEGQVHGGLVQGIGQALLENCVYDNETGQLLTGSFMDYAMPRAAEFPQFKLDTVC
TPCTHNPLGTKGCGEAGAIGSPPAVINAVLDALAPLGVKDFDMPASSSRVWEAMQKGSGSAASDPVAQQPAL
AASTQGRPAA
>SEQF3759||SEQF3759.1_05874
MTVSARQRCARARYKRTTLTTDPNPMRFGSGQAVRRLEDQSLLSGAGRYTDDVSLPSQSHLVFLRSPYPHARI
ASVDASAALALPGVLAVITGADLAAAGVKPMPGAAGFKRADGGDSASPARHAIAHERVRFVGEAVAVVVAET
VQQARDAAEAVMVEYEELPMVVDLAAATADGAPQLCDEAGGNIAAEMRHGSSEAATAAFAKAAHVVALDV
VNQRVVALTIEPRSVLAAPDADTGRLTIRMSTQMPSGVRDSICAAIGLAKDKVRVVVGDVGGGFGMKTGAYPE
DIAVAFAALQVKRPVKWVADRSEEFLSSAHGRDIEAKAELALDAKGRILALRIKTLANVGAYATGTGVAIQLLIGP
WVQTSVYDIQTIDFHFKAVLTNTAPTGAYRGAGRPEAIFTIERLMDEAARQTGIDRIELRRRNFIRPEQMPYKNP
MAQTYDTGKFESVMDQALGLADWNGFEGRAAESARNGRHRGLGIATFLEWTGGNVFEERVTVSVQADGVIE
VFSAVNAMGQGIATSLAQLAVDAFGVPIEKVRVVLGDTDRGDGFGSAGSRSLFTGGSAVRIGAERTIDKARELA
AQEFEVAIDDVTYSRGAFAVIGTDLELDLFTLAGKQPEREIFVDSTSTVAGPTWPNGCHICEIEIDPPTGEISVVAY
SSVNDVGRVVNPMIVRGQLEGGAVQGIGQALYEQVVYDHETGQPVTGSLMDYAAPRADIVSTMFHMEMDE
STPCKNNPLGVKGVGELGTIGATPAIVNAVADAFARNGLAARAPRLHMPLSPSRVWQAMQTVD
>SEQF3760||SEQF3760.1_01831
MGASDFSNLPHIGEALRRKEDYRFLTGAGNYTDDITLANQSHAVFVRSPHAHAVIKSVDTAEALKMPGVVGIFS
GKDIEGKMGGLPCGWLINNPDGTPMKEPMHPILAIHKVRYVGDHVAMVVAETVEQAKNAAEAVVVDYDVLP
AVVSVADAAKKASGVTLHDEAPDNQCYKWALGDKAAVDAAFANAAHVTKLDLVNNRLIPNPIEPRVAIGSYSR
GTDDYTLYVSNQNPHVERLLMTAFVLGLPEHKVRVIAPDVGGGFGSKIFLYAEDVCLTWAAKQLNRNIKWTAER
SECFLSDAHGRDHVSHAEMAMDKDGKFLAMRVHTDANLGAYLSTFSTAVPTILYATLLAGQYTTPQIYVEVDA
WFTNTAPVDAYRGAGRPEATYLLERLVSRCAWEMNLGQDEIRKRNFITTFPYQTPVALQYDTGDFHACMDKA
RVLADVDGYAQRKSASEAAGKLRGIGYSSYIEACGIAPSNIAGALGARAGLFECGEIRVHPTGSVTVFTGSHSHG
QGHETTFAQVVAARLGIPVDNVDVVHGDTGRVPFGMGTYGSRSISVGGAAIMKALDKIETKAKKIAAHLMEAS
DADIEFANGEFTVKGTDKKIPFGQVALTAYVPHNYPLDKLEPGLNETAFYDPTNFTFPGGTYICEVEVDKQTGEV
RVDRFTAVDDFGTIINPMIVEGQVHGGLVQGIGQALLENCVYDNETGQLLTGSFMDYAMPRAGDFPQFKLDT

VCTPCTHNPLGTKGCGEAGAIGSPPAVINAVLDALAPLGVKDFDMPASASRVWEAMQAAASH
>SEQF3760||SEQF3760.1_05003
MTTDSIPTRFGSGQAVRRLEDESLLSGAGRYTDDVALPGQTHLVFLRSPYPHARIVSIDTAAAAAMPGVLRVITG
AELAEAGVKPMPGAVGFKRADGSDCASPPRLALAHGTARFVGEAVAAVVAETVQQARDAAEAVVVDYEALP
MVVDLASATADGAPLLCEEATGNVAAEMRHGSSDAATAAFAKAKHVVALDVVNQRVVALTIEPRSVLAAPDAE
SGRLTIRMSTQMPSGVRDSVCAAIGLAKEKVRVVVGDVGGGFGMKTGAYPEDIAVAFAALQVGRPVKWVAD
RSEEFLSSAHGRDIEARAELALDADGKILALRIRTLANVGAYATGTGVAIQLLIGPWVQTSVYDIQTIDFHFKAVLT
NTAPTGAYRGAGRPEAIFTIERLMDEAARQIGIDRIALRRRNFIRPEQMPYKNPMAQTYDTGKFESVMDQALAL
ADWQGFDARAAESANNGKHRGLGIATFLEWTGGNVFEERVTVSVQADGVIEVFSAVNAMGQGIATSLAQLA
VDAFGVPIEKVRVVLGDTDRGDGFGSAGSRSLFTGGSAVRIGAERTIDKARALAAQEFEAAIDDITYTRGVFTVA
GTDLELDLFALAGKQPEREIFVDSTSTVAGPTWPNGCHVCEIEIDPPTGEISVVAYSSVNDVGRVINPMIVRGQL
EGGAVQGIGQALYEQVVYDNETGQPLTGSLMDYAAPRADIVQAMFHMEMDESTPCANNPLGVKGVGELGTI
GATPAIVNAVADAFARNGLAASAPRLHMPLSPARVWQAMHTVD
>SEQF3760||SEQF3760.1_03410
MLMSAPPFVDAPARPHVGRPAQRVEDRALLTGRGRYGDDAPVRSDTLHAAVLRSPHAHARVLAVRTARAEA
MSGVRAVLTGADVQRWSKPFIVGVKQPMELWALAVDKVRYAGEPIAVVVAESRYLAEDALELLEADYEQLPAV
VDIESAAAEGAPVLHERVGSNVVSDRSFCYGDPDAAFAAAPHRVALSVHYPRNSCTPIETAVVIAEFLSEQDGYD
IQSNFMGPFSLHTVMAMALKVPGNRLRHRTFADSGGSFGVKQAVMAPAVLMCLAARKAGSPVKWVEDRLE
HLTAATSATGRLCHAEAAVEADGRITALSLDQFDDCGGYLRAPEPATFYRMHGCLTGAYAIEHLRVRNRVVLTNK
TPSGLVRGFGGPQVYFALERLVQRIAVELSLDPLDVYRRNFIASDAFPYRAAAGALIDSGDYQAALAMAVRDGG
LAELRQRQETARAEGRLYGIGFAAIVEPSVSNMGYITTVMPREQRTKAGPKNGAIAAATVGIDPLGGLSVVIASS
PAGQGHRTVCAQIVADVFGVEPSEVSVNVEFDTQKDAWSVAAGNYSSRFAGAVAGTLHLACTRLRDKLAGIAA
ASLDCAAADVVFAYGKVYARHAPERSLPFARFAASPHWAPGLLPAGTDPGLRETAFWTPPQLTAPDDQDRVNT
SAAYGFAFDICAVEVDRDTGRVRIDRYVTTHDAGTLLNPALADGQIRGAFAQGLGAALMEEFRYGADGSFQSG
TFADYLVPTTCEVPDPVILHLETPSPFTPLGAKGLGEGNNMSTPPCIANAVADALGRADIVLPLTPSRVMHLLGM
DDPPPSAGSAAAAMPASEPPANPKGGKGKALSARGEILLPATPEAVFAVLLDPVALARVIPGCNALESVGPNRH
RADVTVGVGMIKARYAAEIALSEIEAPRRLRLAGSGLSSVGAAKGSGLVTLEPKDGGTLLRYDYEAEVSGKVAAV
GGRMLEGAAKIVLKQLFEQLGRQAGGQGAAPVADPWWRRLLRMLGGAK
>SEQF3760||SEQF3760.1_05375
MNQADIPARVLKREGIGARVPRKEDARHMVGKGNFVGDFVLPGLQEVAFLRSPLAHATITGVEIPEALAGKVFL
REMMLDAADIGSPSSLPTYQYSALPPLASGKVRHVGEAVAMAVAPTRAVAEDLLEEVQVSYDELPVYHGAESSL
AATGDFLHPGWKDNVFLTLRANRDFDELAAKAEVKVSRTVELSRQCIVPLEGKSVLAYWDFQADQLVVVSATQ
VPHLLRVGIAQHLSMNEEAVRVVSPDVGGAFGYKGQLYPEDLCIAWLAKTYRTPFRYLEDRREHLIVGANTRQH
HYQLTAYADRTGKLLALDAVITIDGGAYSYYPFFVGLEPGQAIGNLPGPYTFRGYRCETLCVATNKPGFMAYRGV
ARTGVCFAIELLMDAVAREVGREPWEVRMDNLVPAAAMPYVNVANKHFDSGDFPASLRRAVEMIGLATVRER
QARGEPDGRRIGFGTATYTEQSAHGTTVFANWGLPVVPGFDQAVVRMTADGGLEVRVGVHSHGQGMETSF
AQIANDVLGIPVARIRVVHGDTALTPFSSGTYASRATVMSGGAISTACKELLPRIQSIAGYLMGVDPQAVALVEGF
AVAGEKSIPLSQVGGAWYLTPQDLPENVHLGGLEVSRAYKPRVDTGTFTYATHAVVVAVDTQTGEVEILDYVVV
EDCGTMVNPMIVEGQTIGGIAQGIGTAMYEESPYDDQGQPLASTLADYILPGATEVPRIRIEHFETPSPHTEFGA
KGVGEGGAIAPPAVIFNAVNDALRGTGAAEVLMTPLTPRRLLKALESAKPEQGLVK
>SEQF3761||SEQF3761.1_08246
MNDLTLPAPVREIEAQRYIGQSVERPNMQRLTQGRANYVDDLELPRMLHVVYLRSPHAHARIVRIDAEAARR
MPGIAAIVDGRQLAAVCAPWVGTLKHLAGMRSPPQHALAIERATWQGEAVLAILGRTRALAEDALDAVQVE
WEPLPAAVHMDTALDAATPVIHPELGSNLCFHREIDTGGVEAVFEGADVVVEDEFRFGRHTGVTLEPRAVLADF

TPTGRRLVVHHSHQAPHMMADLYARQLGLQEADVRVVCRDVGGSFGIKVHAYADDFATVALSVLLGRPVKFV
ADRLESFVTDVHAREHRVKARLALARDGRMLAFQIDDLTGIGPFSMFPRTSAIEGNQVINITGGPYKHAHYKAT
LDVAFLNKVQTSQYRGVGHPIACTVTEGLVDLAAAKLNIDPLELRRRNVMEDGSYPRQGASGILLEGLSHQACL
DRIETLMDYGALRREQATLRERGVHRGIGLAALIELTNPGPAFYGVGGARIAAQDGATVRLDPTGVVHCATGVG
EQGQGTETIFAQIVADTLGLPMASVRVLTGDTAATPYGGGTWASRGAGIGGEAVLLASRDLRRHILALAAAHLK
RPAEGLTIAHGEVRCLATGDTLSDLAELGRLAHFRPDLLPAGFQPELVVTRHYAQRDYPFVFTNGVQASHVEVD
VDTGFVRLLRHWVVEDCGRVLNPMLVDEQIRGAVVQGIGGVLYEECLYDDHGLMRNGSMADYLVPMAFEM
PDIEVAHIETPTRTSQLGAKGAGEAGTAGAPAAVLNAINDALRPFGAHVSEQPVTPEKVLRALGRI
>SEQF3761||SEQF3761.1_01276
MRFGSGQAVRRLEDQSLLSGAGRYTDDVSLPNQSHLVFLRSPYPHARLVSVDTSAALALPGVLAVITGADLAAA
GVKPMPGAAGFKRADGGDSASPARHAIAHERVRFVGEAVAVVVADTVQQARDAAEAVMVEYEELPMVVDL
AAATAEGAPQLCEAASGNVAAEMRHGSSEAATAAFAKAAHVVALDVVNQRVVALTIEPRSVLAAPDADTGRLT
IRMSTQMPSGVRDSICAAIGLAKDKVRVVVGDVGGGFGMKTGAYPEDIAVAFAALQVKRPVKWVADRSEEFL
SSAHGRDIEAKAELALDAKGRILALRIKTLANVGAYATGTGVAIQLLIGPWVQTSVYDIQTIDFHFKAVLTNTAPTG
AYRGAGRPEAIFTIERLMDEAARQTGIDRIELRRRNFIRPEQMPYKNPMAQTYDTGKFESVMDQALGLADWN
GFEARAAESARNGRHRGLGIATFLEWTGGNVFEERVTVSVQADGVIEVFSAVNAMGQGIATSLAQLAVDAFG
VPIEKVRVVLGDTDRGDGFGSAGSRSLFTGGSAVRIGAERTIDKARELAAQEFEVAIDDVTYSRGAFAVIGTDLEL
DLFTLAGKQPEREIFVDSTSTVAGPTWPNGCHICEIEIDPPTGEISVVAYSSVNDVGRVVNPMIVRGQLEGGAV
QGIGQALYEQVVYDHETGQPVTGSLMDYAAPRADIVSTMFHMEMDESTPCKNNPLGVKGVGELGTIGATPAI
VNAVADAFARNGHAARAPRLHMPLSPSRVWQAMQTVD
>SEQF3761||SEQF3761.1_01436
MGASDFANLPHIGNALRRKEDYRFLTGAGNYTDDITLANQSHAVFVRSPHAHARIVSVDIADALTMPGVVGIFS
GKDIEGKMGGLPCGWLINNPDGTPMKEPMHPILAINKVRYVGDHVAMVVAETVEQAKNAAEAVVVEYEVLP
ALVGVADAAKKAGGVTIHDEAPDNQCYKWALGDKAAVDAAFAGAAHVTKLDLVNNRLIPNPIEPRVAIGSYSR
GTDDYTLYVSNQNPHVERLLMTAFVLGLPEHKVRVIAPDVGGGFGSKIFLYAEDVCLTWAAKQLNRNIKWTAER
SECFLSDAHGRDHVSHAEMAMDKDGKFLAMRVHTDANLGAYLSTFSTAVPTILYATLLAGQYTTPQIYVEVDA
WFTNTAPVDAYRGAGRPEATYLLERLVSRCAWEMNLGQDEIRQRNFITAFPYQTPVALQYDTGDFHACMDKA
RKLAEVEGYAQRKSASEAAGKLRGIGYSSYIEACGIAPSNIAGALGARAGLFECGEIRVHPTGSVTVFTGSHSHGQ
GHETTFAQVVAARLGIAVEAVDVVHGDTGRVPFGMGTYGSRSISVGGAAIMKALDKIEAKAKKIAAHLMEASD
ADIEFANGEFTVKGTDKKIPFGQVALTAYVPHNYPLDKLEPGLNETAFYDPTNFTFPGGTYICEVEIDKQTGEVRV
DRFTAVDDFGTIINPMIVEGQVHGGLVQGIGQALLENCVYDNETGQLLTGSFMDYAMPRAAEFPQFKLDTVCT
PCTHNPLGTKGCGEAGAIGSPPAVINAVLDALAPLGVKDFDMPASSSRVWEAMQKAGAAA
>SEQF3761||SEQF3761.1_00414
MTANPATRTADLSQRPHLGRPARRVEDRALLTGQGRFGDDAPVRAGTLHAAIVRSPHAHAKLLALRTARAEAS
PGVRAVLTGADVQRWSKPFIVGVKQPMELWALAIDRVRYVGEPVAVVIAENRYLAEDASDLVEADYETLPAVID
IESAAADDAPVLHERVGSNVVSDRSFRYGDPEAAFALAPHRVSITVHYPRNSCTPIETGVAIAEFISEQEGYELQS
NFMGPFSLHTVMAMALKVPGNRLRHRTFPDSGGSFGVKQAVMAPAVLMCLAARKAGAPVKWVEDRLEHLS
AATSATGRLCHAEAAVDPDGRITALSLDQFDDCGGYLRAPEPATFYRMHGCLTGAYAIANLRVRNRVVLTNKTPT
GLVRGFGGPQVYFALERLVQRIAVELCLDPLDVYRRNFIPRDAFPYHAAAGALIDSGDYHRALEMVVAEGELDM
LRRRQQEARAQGRLYGIGFAAIVEPSVSNMGYITTVLPAEQRAKAGPKNGATAAATVSIDPLGGINAVIASAPAG
QGHRTVCAQVIADAFGVNPAEVTVNVEFDTQKDAWSVAAGNYSSRFAGAVAGTLHLACLKLRDKLARIAAPTL
GCAPEEVVFAGGKIHVRQAPDKGLPFARFAASPHWAPALLPPGEDPGLRETAFWTAPQLAAPDEDDRVNTSA
AYGFAFDLCAVEVDRDTGRIRIDRYVTAHDAGTLLNPALADGQIRGAFAQGLGAALMEEFRYGTDGSFQSGTFA
DYLVPTTCEVPDPLILHLETPSPFTPLGAKGLGEGNNMSTPPCIANAVADALGVSDIRLPLKPSRVLELLGIDDPFP

SSGSRTATDAARTGVEHGDEHSLPDASPRKSGKALSARGEITLPATPEAVFAVLLDPKALARVIPGCHALEETAPH
RYRADVTVGVGMIKARYAAEVSLSDLDPPRRLRLAGSGISSMGSARGEGLVTLSATDGGTLLKYDYEAEVSGKV
AAVGARMLEGAAKIVLRQLFEQLGRQAGGQEGIDAAAASTSLWQRLLRRLGVGGHR
>SEQF3762||SEQF3762.1_02910
MGASDFSNLPHIGEAVRRKEDYRFLTGSGNYTDDITLANQSHAVFVRSPHAHAVVKSVDTAEALKMPGVIGIFS
GKDIEGKMGGLPCGWLINNPDGTPMKEPMHPILAIKKVRYVGDHVAMVVAETVEQARNAAEAVVVDYEVLP
ALVSVADAAKKAGGVTLHDEAPDNQCYKWALGDKAAVDAVFAGAAHVTKLDLVNNRLVPNPIEPRVAIGSYN
RAGDDYTLYVSNQNPHVERLLMTAFVLGLPEHKVRVIAPDVGGGFGSKIFLYAEDVCLTWAARQLNRNIKWTA
DRSECFLSDAHGRDHVSHAEMAMDKDGKFLAMRVHTDANLGAYLSTFSTAVPTILYATLLAGQYTTPQIYVEVD
AWFTNTAPVDAYRGAGRPEATYLLERLVSRCAWEMNLGQDEIRKRNFITAFPYQTPVALQYDTGDFHACMDK
ARVLAEVDGYAARKAASEANGKLRGIGYSSYIEACGIAPSNIAGALGARAGLFECGEIRVHPTGSVTVFTGSHSH
GQGHETTFAQVVAARLGIPVENVDVVHGDTGRVPFGMGTYGSRSISVGGAAIMKALDKIETKAKKIAAHLME
ASDADVEFANGEFTVKGTDKKIPFGQVALTAYVPHNYPLDKLEPGLNETAFYDPTNFTFPGGTYICEVEVDKQTG
EVRVDRFTAVDDFGTIINPMIVEGQVHGGLVQGIGQALLENCVYDKETGQLLTGSFMDYAMPRAGDFPQFKLD
TVCTPCTHNPLGTKGCGEAGAIGSPPAVINAVLDALAPLGVKDFDMPASPARVWEAMQAAAANP
>SEQF3762||SEQF3762.1_01094
MTTEPNPTRFGSGQAVRRLEDESLLSGAGRYTDDVTLPEQAHLVFLRSSYPHARIVSIDTSAAAAMPGVLRVITG
AEMADAGVKPLPGAAGFKRADGSDSASPPRRAIAHERTRFVGEVVAAVVAETVQQARDAAEAIMVEYEELP
MVVDLASATAEGAPLLCEEATGNISAEMRHGSSEAATAAFARASHVIALDVINQRVVALTIEPRSVLAARDPKTG
RLTIRMSTQMPSGVRDAICAAIGLPKEKVRVVVGDVGGGFGMKTGAYPEDIAVAYAALQVDRPVKWVADRSE
EFLSSAHGRDIEARAEMALDADGRILALRIKTLANVGAYATGTGVAIQLLIGPWVQTSVYDIQTIDFHFKAVLTNT
APTGAYRGAGRPEAIFTIERLMDEAARQTGIDRVALRRRNFIRPEQMPYKNPMAQTYDTGNFESVMDQALTLA
DWQGFEARAAESAGRGKHRGLGIATFLEWTGGNVFEERVTVSVQADGVIEVFSAVNAMGQGIATSLAQLAVD
AFGVPIEKVRVVLGDTDRGDGFGSAGSRSLFTGGSAVRIGAERTIDKARELAAQEFEASLDDIVYSRGVFNVAGT
DLELDLFTLAAKQPGREIFVDSTSTVAGPTWPNGCHICEIEIDPPTGEISVVAYSSVNDVGRVINPMIVRGQLEGG
AVQGIGQALYEQVVYDHETGQPVTGSLMDYAAPRADIVDTMFNMEMDESTPCKNNPLGVKGVGELGTIGAT
PAIVNAVADAFARNGLATRAPRLHMPLSPARVWQAMQTVD
>SEQF3762||SEQF3762.1_05300
MDRADIPARLLKREGIGARVPRKEDARHLVGKGNFVGDFVLPGLQEVAFLRSPLAHAVVTGVEIPEALAGKVFL
RDMMSDAADIGSPSSVPTYQYSALPPLASGKVRHVGEAIAMAVAPTRAMAEDLLEEVEVSYDELPVYHSADSA
LAATGEFMHPEWKDNVFLTLRADRDFEELAAKAEVKVSRTVELSRQCIVPMEGKSVLAYWDFQTDQLVVVSAT
QVPHLLRVGIAQHLAMNEEAVRVVSPDVGGAFGYKGQLYPEDLCVAWLAKTYRTPFRYLEDRREHLIVGANTR
QHHYRLTAYADRTGKLLALDAVITIDGGAYSYYPFFVGLEPGQAIGNLPGPYSFRGYRCETLCVATNKPGFMAYR
GVARTGVCFAIELLMDAVAREVGREPWEVRLENLVPASAMPYVNVANKHFDSGDFPASLRRAVEMIGLKAVRE
RQARGEPDGRLIGFGTATYTEQSAHGTTVFANWGLPVVPGFDQAVVRMTADGGLEVRVGVHSHGQGMETS
FAQIANDVLGIPVSRIRVVHGDTALTPFSSGTYASRATVMSGGAISVACKELLPRIQSIAAYLMGVDAQTVALIEG
RAVSGDKSISLSEVGGAWYLTPQDLPADVHLGGLEVSRAYKPKVDTGTFTYATHAVVVAVDTETGEVEILDYVVV
EDCGTMVNPMIVEGQTIGGIAQGIGTAMYEESPYDEQGQPLASTLADYILPGATEVPRIRIDHFETPSPHTEFGA
KGVGEGGAIAPPAVIFNAVNDALRGTGAAEVLMTPLTPRRLLAALESAKREEEHAR
>SEQF3768||SEQF3768.1_05481
MTSFQPATESQTGHIGARQTRVEDAALLRGLGCYADDAAIPPGTLHAAIIRSPHAHARITSVDFSSALLMKGVHG
VLVGEDVKRWALPFPVGVRQPMEHWCVAVDKVRYVGEPVAVVIAESRYLAEDAIEGVRVEYEPLPPIIDPERAT
AEQAPILHEAVGSNVVNERRFRYGEPEQAFEQAPHKVSLKVKFPRSSCTPIECYVVLAQYERATGIYDVLANFQG
PYALHTVMARALNVPGNRLRLRTPKDSGGSFGIKQGVFPYVVMMGLASRKVGAPVKWVEDRLEHLQGASSA

TNRVTEIEAAVEADGRITALRYDQIDDCGAYLRAPEPATFYRMHGNLTGAYAIRNLQVRNRVVLTNKTPSGLNRG
FGGPQVYFALERLMQHIAVQLKLDPLDVIRRNLVPADAFPYRAAAGALLDSGNYQAGIALAAADGGLDELLKRR
DQARAEGRIYGIGYAAVIEPSISNMGYITTAMTPEERRKAGPKNGAVATATINVGPLGDVSVHVSSAPQGQGHQ
TTVAQVVAEVLGVALESIVVNVELDTQKDAWSIASGNYSSRFAGAVAGAVYKAALKIRDRLAAIAAEQLQASPE
DIRFAGGKIFVVNGGAVAPFHRIAGATHWSPGLLPEGESGGLRETAFWSPPQLVAPDDQDQVNSSLCYGFVFDI
CGLEIDRMTGEIHIDRYVTCHDAGRLLNPALVDGQIRGGFTQGLGAALMEEFAYGEDGSFLSGTFADYLVPTAPE
AIEPVILHMETPSPFTPLGAKGVGEGNNMSTPVCIANAVADALGRSDIRLPLTPSKVRTLIGIDEPPRPAGMEPD
EDLDAAPAGGPALRANDSVVIPASPQQVFDTLLDPQTLAAIIPGCHDLVLEGENRYRADVTVGVGMIRARFEAK
VALSDLDPPHSLRLSGSGSSSMGSAQGQAKVRFVELENGHTRLEYQYQVAVSGKVAAVGGRMLQGASKVIIG
QIFTRLSQRVSGQAISTSWWARLRASLGAMFGKGGAQ
>SEQF3781||SEQF3781.3_04067
MTSFQPATKSQTGHIGARQTRVEDAALLRGLGCYADDAAIPPGTLHAAIIRSPHAHARITSVDFSKALLMKGVH
GVLVGEDVKRWALPFPVGVRQPMEHWCVAVDKVRYVGEPVAVVIAESRYLAEDAIEGVRVEYEPLPPIIDPELA
TAEQAPILHEAVGSNVVNERHFRYGEPEQAFEQAPHKVSLKVKFPRSSCTPIECYVVLAQYERATGIYDVLANFQ
GPYALHTVMARALNVPGNRLRLRTPKDSGGSFGIKQGVFPYVVMMGLASRKVGAPVKWVEDRLEHLQGASS
ATNRVTEIEAAVEADGRITALRYDQIDDCGAYLRAPEPATFYRMHGNLTGAYAIRHLQVRNRVVLTNKTPSGLNR
GFGGPQVYFALERLLQHIAVQLKLDPLDVIRRNLVPADAFPYRAAAGALLDSGNYQAGIALAAADGGLDELLER
RDQARAEGRIYGIGYAAVIEPSISNMGYITTAMTPEERRKAGPKNGAVATATINVGPLGDVSVHVSSAPQGQGH
QTTVAQVVAEVLGVALESIVVNVELDTQKDAWSIASGNYSSRFAGAVAGAVYKAALKIRDRLAAIAAEQLQASP
EDIRFAGGKIFVVNGGAVAPFHRIAGATHWSPGLLPEGESGGLRETAFWSPPQLEAPDDQDQVNSSLCYGFVF
DICGLEIDRMTGEIHIDRYVTCHDAGRLLNPALVDGQIRGGFTQGLGAALMEEFAYGEDGSFLSGTFADYLVPTA
PEVIEPMILHMETPSPFTPLGAKGVGEGNNMSTPVCIANAVADALGRSDIRLPLTPSKVRTLIGIDEPPRPAGME
ADDNLDAAPAGGPALRANDSVVIPASPQQVFDTLLDPQTLAAIIPGCHDLVLEGENRYRADVTVGVGMIRARF
EAKVALSDLDPPHSLRLSGSGSSSMGSAQGQAKVRFVELENGHTRLEYQYQVAVSGKVAAVGGRMLQGASKV
IIGQIFTRLSQRVSGQAISTGWWARLRASLGALFGKGGAQ
>SEQF3991||SEQF3991.1_01844
MQGIAQTAHRIEDRRFLRGEGRYLADIRPEGCTAMVVVRSPHGNARIGGIDTAVALAMPGVLAVLTAADVATA
GAKALPCLAEIGKWDWARPVLPSRPLLAADQVRHVGEPVAVVIAETPDMARDAADMVVVDYEPLETCATVE
QALAGETSIWPEAPDNIGFDWHSGDAERVADAFARAANVVSVDLHNNRVAGMSLETRGAIGEWDTREERFT
LYVSSQGGHAIRRILCQNVFGIPEGRMRVITPDVGGGFGPKIFTYQEYALVLLAAARLNRPVKWVSDRGESLISD
TQGRAQTCRAELALDDTGRFLAMRFDCLSDMGAYPGQHGPNIATVAGDGLHPATYDLEAVHVRVRGVFTNTL
PTDSYRGAGRPEVIYAVERLVDAAARELGFDPVDLRLRNFVPRGKMPFTTVTGQVYDDGDFAQLTARAVALSDF
TGRESLREAARECGKLSGMGLAYFIDRCGRGLDEFAELRFDPSGSAVLLVGSQNNGQGHETAYANIVAQGLGLD
RNLIRVVQGDTDQVAFGRGTGGSRALAVGGNAVHLATGRIRAKLEAIAAHLLEGDISALRQEEGRFHLTGTNHS
VTLADCVRAAFAPSLLPPGMEPGLSIAAHYRPERPTFPNGCHIARVEIDIETAEIRLTRYVAVNDVGTILNPRLVEG
QFHGGLAQGISQALLEEVVYDPETAQPLTGSLMDYCYPKADDFVAFETDFVEIPCRTNPLGVKGCGEAGTIAAP
PAIANAVMDALRDYDTEGLQMPFTAPKLFAVLARGPRAAGK
>SEQF3992||SEQF3992.1_01981
MQGIAQTAHRIEDRRFLRGEGRYLADIRPEGCTAMVVVRSPHGNARIGGIDTAVALAMPGVLAVLTAADVATA
GAKALPCLAEIGKWDWARPVLPSRPLLAADQVRHVGEPVAVVIAETPDMARDAADMVVVDYEPLETCATVE
QALAGETSIWPEAPDNIGFDWHSGDAERVADAFARAANVVSVDLHNNRVAGMSLETRGAIGEWDTREERFT
LYVSSQGGHAIRRILCQNVFGIPEGRMRVITPDVGGGFGPKIFTYQEYALVLLAAARLNRPVKWVSDRGESLISD
TQGRAQTCRAELALDDTGRFLAMRFDCLSDMGAYPGQHGPNIATVAGDGLHPATYDLEAVHVRVRGVFTNTL
PTDSYRGAGRPEVIYAVERLVDAAARELGFDPVDLRLRNFVPRGKMPFTTVTGQVYDDGDFAQLTARAVALSDF

TGRESLREAARECGKLSGMGLAYFIDRCGRGLDEFAELRFDPSGSAVLLVGSQNNGQGHETAYANIVAQGLGLD
RNLIRVVQGDTDQVAFGRGTGGSRALAVGGNAVHLATGRIRAKLEAIAAHLLEGDISALRQEEGRFHLTGTNHS
VTLADCVRAAFAPSLLPPGMEPGLSIAAHYRPERPTFPNGCHIARVEIDIETAEIRLTRYVAVNDVGTILNPRLVEG
QFHGGLAQGISQALLEEVVYDPETAQPLTGSLMDYCYPKADDFVAFETDFVEIPCRTNPLGVKGCGEAGTIAAP
PAIANAVMDALRDYDTEGLQMPFTAPKLFAVLARGPRAAGK
>SEQF4982||SEQF4982.1_00416
MGASDFAKLPYIGEALRRKEDARFLTGAGQYTDDIVLANQRYAVFVRSPHAHARINSIDTSQAKSMPGVRAVFT
GADLAGKVNGLPCGWLITSTDGTPMKEPPHPVLAIGKVRYVGDPVAMVVADSVEQAKNAAEAVMVDYEVLG
ACVDVRDARNAPALHDEAPDNHCYKWAIGDKAQVDAAFAKAAHVTRIDLVNNRLVPNAIEPRSAIGVYNRGS
DEYTLYVANQNPHVERLLMTAFVLGLPEHKVRVIAPDVGGGFGSKIFLYAEDVAVTWAARQLNCAVKWTAERS
ESFLTDAHGRDHVTHAEMAMDKDGRFLAMRVHTDANLGAYLSTFSTAVPTILYATLLAGQYTTPQIYVEVDAW
FTNTAPVDAYRGAGRPEATYLLERLVTRCGWEMGLSQDEIRRRNLITQFPYQTPVALQYDTGDYVACLDKAMQ
LADVAGFEARRQASEAKGLKRGIGFSSYIEACGLAPSNIAGALGARAGLFECGEVRVHPTGSVTVFTGAHSHGQ
GHETTFAQVVAARLGIPVENVDVVHGDTGRIPFGMGTYGSRSISVGGAAIMKALDKIEAKARKIAAHLMEASE
ADIEFTNGEFRVKGTDKKVPFAQVALTAYVPHNYPLDQLEPGLNETAFYDPTNFTFPAGTYICEVEVDPATGQVR
VDRFTAVDDFGVIINPMIVEGQVHGGIAQGIGQALLENCVYDKETGQLLTGSFMDYAMPRADDLPDFQLGTV
CTPCTHNALGTKGCGEAGAIGAPPAVINAVLDALRDLGVKDFDMPATPARVWEAIQQARAQ
>SEQF4982||SEQF4982.1_01164
MKRDVVNLEPGVESTPPIAPVTEDPRYIGARLPRHGIERLTQGQGQYVDDIELPRLAHVVYWRSPVAHMRIKGI
DDRIARSMPGVLMVATGQDLAKVCKPWVAVLGHLTGMKSAPQYPLAVERACWQGEPVVAVVAETRAQAED
ALQHLVVDWEELPLTVEMERALAPDEPVIHPELGDNVCFSRRLDVGEVDRVFAEADVVAETTFEFGRHTGVTLE
PRSQIAHWQPAEQRLTVYHSCQAPHMMQDLYARQFDLPAHAVRVICKDVGGSFGVKVHAYPDDFATVALSM
LLGRPVKFVADRLESFTSDIHARHHRVKGRIAVNRDGEILGFEIDDLTGIGPYSMFPRTSAIEGNQVVNLVGGPYR
HQHYRAQLHVVFQNKTPTCQYRGVGHPIACAVTEGLVDLAAQRIGMDPLEIRARNVIPDDAYPATGASGIKLEV
LSHEACLRKLREMMNYDALRAEQAALRKQGIWRGIGLATVIELTNPSAAFYGIGGARIASQDGATVRLNPEGHV
TVLVGVGEQGQGTEAIYRQIAADAVGVDIDQVRVITGDTDVTPYGGGTWASRGAGIGGEAVLLAGQALRGNIL
KTAAVILNREAGQLGLHRGQVVDAVTGEALLPLSEVGRIAYFRTDTLPQGFTPELMVTRHYAQRDYPFIFTNGV
QASYVEVDPDTGFVTLLKHWAVEDCGRVLNPMLVDEQMRGAIVQGIGGALYEECLYDEQGQMLNANMADY
LVPMAAEMPDIEVAHVMTPTRSSRLGAKGAGEAGTAGAPAAVMNAINDALAPFGAHVHSQPITPEKILRALG
KVK
>SEQF5083||SEQF5083.2_00398
MTTIESRPPSPEDLADNAQQPCGHGRMMRKEDPRFIRGRGTYVDDVALPGMLHLAILRSPYAHARIVRIDVTA
AQAHPKVKAVVTGADLAAKGLAWMPTLANDVQAVLATDKTRFQGQEVAFVVAEDRYSARDACELVDVDYEP
RDPVVDARTALDPSAPVIRTDLEGKSDNHIFDWETGDAAATEAVFAKADVVVQQEIVYPRVHPAPMETCGAVA
DLDPVTGKLTLWTTSQAPHAHRTLYALVAGLPEHKIRVISPDIGGGFGNKVPIYPGYVCAIVASLLLDKPVKWME
DRSENLTSTGFARDYIMVGEIAANRDGKILAIRSNVLADHGAFNAQAAPAKYPAGFFGVFTGSYDIEAAYCHMT
AVYTNKAPGGVAYACSFRITEAVYFVERLVDCLAFELKMDPAELRLRNLLRPNQFPYQSKTGWVYDSGDYETTM
RKAMNMIGYEALRAEQKQRRARGELMGIGMSFFTEAVGAGPRKDMDILGLGMADGCELRVHPTGKAVLRLS
VQTQGQGHETTFAQIVAEELGIAPDDIEVVHGDTDQTPFGLGTYGSRSTPVSGGAAALVARKVRDKAKIIASGM
LEVSVADLQWEKGKFHVKGDPSAAVTIADIAMRAHGAGDLPEGIEGGLDAEVCYNPSNLTYPYGAYFCVVDIDP
GTAVVKVRRFLAVDDCGTRINPMIIEGQVHGGIVDGIGMALMEMIAFDEDGNCLGGSLMDYLIPTALEVPHLE
TGHTVTPSPHHPIGAKGIGESATVGSPPAVVNAVVDALAPFGVRHADMPLTPSRVWEAMQGRATPPI
>SEQF5084||SEQF5084.1_00397
MTTIESRPPSPEDLADNAQQPCGHGRMMRKEDPRFIRGRGTYVDDVALPGMLHLAILRSPYAHARIVRIDVTA

AQAHPKVKAVVTGADLAAKGLAWMPTLANDVQAVLATDKTRFQGQEVAFVVAEDRYSARDACELVDVDYEP
RDPVVDARTALDPSAPVIRTDLEGKSDNHIFDWETGDAAATEAVFAKADVVVQQEIVYPRVHPAPMETCGAVA
DLDPVTGKLTLWTTSQAPHAHRTLYALVAGLPEHKIRVISPDIGGGFGNKVPIYPGYVCAIVASLLLDKPVKWME
DRSENLTSTGFARDYIMVGEIAANRDGKILAIRSNVLADHGAFNAQAAPAKYPAGFFGVFTGSYDIEAAYCHMT
AVYTNKAPGGVAYACSFRITEAVYFVERLVDCLAFELKMDPAELRLRNLLRPNQFPYQSKTGWVYDSGDYETTM
RKAMNMIGYEALRAEQKQRRARGELMGIGMSFFTEAVGAGPRKDMDILGLGMADGCELRVHPTGKAVLRLS
VQTQGQGHETTFAQIVAEELGIAPDDIEVVHGDTDQTPFGLGTYGSRSTPVSGGAAALVARKVRDKAKIIASGM
LEVSVADLQWEKGKFHVKGDPSAAVTIADIAMRAHGAGDLPEGIEGGLDAEVCYNPSNLTYPYGAYFCVVDIDP
GTAVVKVRRFLAVDDCGTRINPMIIEGQVHGGIVDGIGMALMEMIAFDEDGNCLGGSLMDYLIPTALEVPHLE
TGHTVTPSPHHPIGAKGIGESATVGSPPAVVNAVVDALAPFGVRHADMPLTPSRVWEAMQGRATPPI
>SEQF5085||SEQF5085.1_00402
MTTIESRPPSPEDLADNAQQPCGHGRMMRKEDPRFIRGRGTYVDDVALPGMLHLAILRSPYAHARIVRIDVTA
AQAHPKVKAVVTGADLAAKGLAWMPTLANDVQAMLATDKTRFQGQEVAFVVAEDRYSARDACELVDVDYE
PRDPVVDARTALDPSAPVIRTDLEGKSDNHIFDWETGDAAATEAVFAKADVVVQQEIVYPRVHPAPMETCGAV
ADLDPVTGKLTLWTTSQAPHAHRTLYALVAGLPEHKIRVISPDIGGGFGNKVPIYPGYVCAIVASLLLDKPVKWM
EDRSENLTSTGFARDYIMVGEIAANRDGKILAIRSNVLADHGAFNAQAAPAKYPAGFFGVFTGSYDIEAAYCHM
TAVYTNKAPGGVAYACSFRITEAVYFVERLVDCLAFELKMDPAELRLRNLLRPNQFPYQSKTGWVYDSGDYETT
MRKAMNMIGYEALRAEQKQRRARGELMGIGMSFFTEAVGAGPRKDMDILGLGMADGCELRVHPTGKAVLR
LSVQTQGQGHETTFAQIVAEELGIAPDDIEVVHGDTDQTPFGLGTYGSRSTPVSGGAAALVARKVRDKAKIIASG
MLEVSVADLQWEKGKFHVKGDPSAAVTIADIAMRAHGAGDLPEGIEGGLDAEVCYNPSNLTYPYGAYFCVVDI
DPGTAVVKVRRFLAVDDCGTRINPMIIEGQVHGGIVDGIGMALMEMIAFDEDGNCLGGSLMDYLIPTALEVPH
LETGHTVTPSPHHPIGAKGIGESATVGSPPAVVNAVVDALAPFGVRHADMPLTPSRVWEAMQGRATPPI
>SEQF5086||SEQF5086.1_00401
MTTIESRPPSPEDLADNAQQPCGHGRMMRKEDPRFIRGRGTYVDDVALPGMLHLAILRSPYAHARIVRIDVTA
AQAHPKVKAVVTGADLAAKGLAWMPTLANDVQAVLATDKTRFQGQEVAFVVAEDRYSARDACELVDVDYEP
RDPVVDARTALDPSAPVIRTDLEGKSDNHIFDWETGDAAATEAVFAKADVVVQQEIVYPRVHPAPMETCGAVA
DLDPVTGKLTLWTTSQAPHAHRTLYALVAGLPEHKIRVISPDIGGGFGNKVPIYPGYVCAIVASLLLDKPVKWME
DRSENLTSTGFARDYIMVGEIAANRDGKILAIRSNVLADHGAFNAQAAPAKYPAGFFGVFTGSYDIEAAYCHMT
AVYTNKAPGGVAYACSFRITEAVYFVERLVDCLAFELKMDPAELRLRNLLRPNQFPYQSKTGWVYDSGDYETTM
RKAMNMIGYEALRAEQKQRRARGELMGIGMSFFTEAVGAGPRKDMDILGLGMADGCELRVHPTGKAVLRLS
VQTQGQGHETTFAQIVAEELGIAPDDIEVVHGDTDQTPFGLGTYGSRSTPVSGGAAALVARKVRDKAKIIASGM
LEVSVADLQWEKGKFHVKGDPSAAVTIADIAMRAHGAGDLPEGIEGGLDAEVCYNPSNLTYPYGAYFCVVDIDP
GTAVVKVRRFLAVDDCGTRINPMIIEGQVHGGIVDGIGMALMEMIAFDEDGNCLGGSLMDYLIPTALEVPHLE
TGHTVTPSPHHPIGAKGIGESATVGSPPAVVNAVVDALAPFGVRHADMPLTPSRVWEAMQGRATPPI
>SEQF5087||SEQF5087.1_00400
MTTIESRPPSPEDLADNAQQPCGHGRMMRKEDPRFIRGRGTYVDDVALPGMLHLAILRSPYAHARIVRIDVTA
AQAHPKVKAVVTGADLAAKGLAWMPTLANDVQAVLATDKTRFQGQEVAFVVAEDRYSARDACELVDVDYEP
RDPVVDARTALDPSAPVIRTDLEGKSDNHIFDWETGDAAATEAVFAKADVVVQQEIVYPRVHPAPMETCGAVA
DLDPVTGKLTLWTTSQAPHAHRTLYALVAGLPEHKIRVISPDIGGGFGNKVPIYPGYVCAIVASLLLDKPVKWME
DRSENLTSTGFARDYIMVGEIAANRDGKILAIRSNVLADHGAFNAQAAPAKYPAGFFGVFTGSYDIEAAYCHMT
AVYTNKAPGGVAYACSFRITEAVYFVERLVDCLAFELKMDPAELRLRNLLRPNQFPYQSKTGWVYDSGDYETTM
RKAMNMIGYEALRAEQKQRRARGELMGIGMSFFTEAVGAGPRKDMDILGLGMADGCELRVHPTGKAVLRLS
VQTQGQGHETTFAQIVAEELGIAPDDIEVVHGDTDQTPFGLGTYGSRSTPVSGGAAALVARKVRDKAKIIASGM
LEVSVADLQWEKGKFHVKGDPSAAVTIADIAMRAHGAGDLPEGIEGGLDAEVCYNPSNLTYPYGAYFCVVDIDP

GTAVVKVRRFLAVDDCGTRINPMIIEGQVHGGIVDGIGMALMEMIAFDEDGNCLGGSLMDYLIPTALEVPHLE
TGHTVTPSPHHPIGAKGIGESATVGSPPAVVNAVVDALAPFGVRHADMPLTPSRVWEAMQGRATPPI
>SEQF5088||SEQF5088.1_00400
MTTIESRPPSPEDLADNAQQPCGHGRMMRKEDPRFIRGRGTYVDDVALPGMLHLAILRSPYAHARIVRIDVTA
AQAHPKVKAVVTGADLAAKGLAWMPTLANDVQAVLATDKTRFQGQEVAFVVAEDRYSARDACELVDVDYEP
RDPVVDARTALDPSAPVIRTDLEGKSDNHIFDWETGDAAATEAVFAKADVVVQQEIVYPRVHPAPMETCGAVA
DLDPVTGKLTLWTTSQAPHAHRTLYALVAGLPEHKIRVISPDIGGGFGNKVPIYPGYVCAIVASLLLDKPVKWME
DRSENLTSTGFARDYIMVGEIAANRDGKILAIRSNVLADHGAFNAQAAPAKYPAGFFGVFTGSYDIEAAYCHMT
AVYTNKAPGGVAYACSFRITEAVYFVERLVDCLAFELKMDPAELRLRNLLRPNQFPYQSKTGWVYDSGDYETTM
RKAMNMIGYEALRAEQKQRRARGELMGIGMSFFTEAVGAGPRKDMDILGLGMADGCELRVHPTGKAVLRLS
VQTQGQGHETTFAQIVAEELGIAPDDIEVVHGDTDQTPFGLGTYGSRSTPVSGGAAALVARKVRDKAKIIASGM
LEVSVADLQWEKGKFHVKGDPSAAVTIADIAMRAHGAGDLPEGIEGGLDAEVCYNPSNLTYPYGAYFCVVDIDP
GTAVVKVRRFLAVDDCGTRINPMIIEGQVHGGIVDGIGMALMEMIAFDEDGNCLGGSLMDYLIPTALEVPHLE
TGHTVTPSPHHPIGAKGIGESATVGSPPAVVNAVVDALAPFGVRHADMPLTPSRVWEAMQGRATPPI
>SEQF5089||SEQF5089.1_00397
MTTIESRPPSPEDLADNAQQPCGHGRMMRKEDPRFIRGRGTYVDDVALPGMLHLAILRSPYAHARIVRIDVTA
AQAHPKVKAVVTGADLAAKGLAWMPTLANDVQAVLATDKTRFQGQEVAFVVAEDRYSARDACELVDVDYEP
RDPVVDARTALDPSAPVIRTDLEGKSDNHIFDWETGDAAATEAVFAKADVVVQQEIVYPRVHPAPMETCGAVA
DLDPVTGKLTLWTTSQAPHAHRTLYALVAGLPEHKIRVISPDIGGGFGNKVPIYPGYVCAIVASLLLDKPVKWME
DRSENLTSTGFARDYIMVGEIAANRDGKILAIRSNVLADHGAFNAQAAPAKYPAGFFGVFTGSYDIEAAYCHMT
AVYTNKAPGGVAYACSFRITEAVYFVERLVDCLAFELKMDPAELRLRNLLRPNQFPYQSKTGWVYDSGDYETTM
RKAMNMIGYEALRAEQKQRRARGELMGIGMSFFTEAVGAGPRKDMDILGLGMADGCELRVHPTGKAVLRLS
VQTQGQGHETTFAQIVAEELGIAPDDIEVVHGDTDQTPFGLGTYGSRSTPVSGGAAALVARKVRDKAKIIASGM
LEVSVADLQWEKGKFHVKGDPSAAVTIADIAMRAHGAGDLPEGIEGGLDAEVCYNPSNLTYPYGAYFCVVDIDP
GTAVVKVRRFLAVDDCGTRINPMIIEGQVHGGIVDGIGMALMEMIAFDEDGNCLGGSLMDYLIPTALEVPHLE
TGHTVTPSPHHPIGAKGIGESATVGSPPAVVNAVVDALAPFGVRHADMPLTPSRVWEAMQGRATPPI
>SEQF5090||SEQF5090.1_00396
MTTIESRPPSPEDLADNAQQPCGHGRMMRKEDPRFIRGRGTYVDDVALPGMLHLAILRSPYAHARIVRIDVTA
AQAHPKVKAVVTGADLAAKGLAWMPTLANDVQAVLATDKTRFQGQEVAFVVAEDRYSARDACELVDVDYEP
RDPVVDARTALDPSAPVIRTDLEGKSDNHIFDWETGDAAATEAVFAKADVVVQQEIVYPRVHPAPMETCGAVA
DLDPVTGKLTLWTTSQAPHAHRTLYALVAGLPEHKIRVISPDIGGGFGNKVPIYPGYVCAIVASLLLDKPVKWME
DRSENLTSTGFARDYIMVGEIAANRDGKILAIRSNVLADHGAFNAQAAPAKYPAGFFGVFTGSYDIEAAYCHMT
AVYTNKAPGGVAYACSFRITEAVYFVERLVDCLAFELKMDPAELRLRNLLRPNQFPYQSKTGWVYDSGDYETTM
RKAMNMIGYEALRAEQKQRRARGELMGIGMSFFTEAVGAGPRKDMDILGLGMADGCELRVHPTGKAVLRLS
VQTQGQGHETTFAQIVAEELGIAPDDIEVVHGDTDQTPFGLGTYGSRSTPVSGGAAALVARKVRDKAKIIASGM
LEVSVADLQWEKGKFHVKGDPSAAVTIADIAMRAHGAGDLPEGIEGGLDAEVCYNPSNLTYPYGAYFCVVDIDP
GTAVVKVRRFLAVDDCGTRINPMIIEGQVHGGIVDGIGMALMEMIAFDEDGNCLGGSLMDYLIPTALEVPHLE
TGHTVTPSPHHPIGAKGIGESATVGSPPAVVNAVVDALAPFGVRHADMPLTPSRVWEAMQGRATPPI
>SEQF5091||SEQF5091.1_00397
MTTIESRPPSPEDLADNAQQPCGHGRMMRKEDPRFIRGRGTYVDDVALPGMLHLAILRSPYAHARIVRIDVTA
AQAHPKVKAVVTGADLAAKGLAWMPTLANDVQAVLATDKTRFQGQEVAFVVAEDRYSARDACELVDVDYEP
RDPVVDARTALDPSAPVIRTDLEGKSDNHIFDWETGDAAATEAVFAKADVVVQQEIVYPRVHPAPMETCGAVA
DLDPVTGKLTLWTTSQAPHAHRTLYALVAGLPEHKIRVISPDIGGGFGNKVPIYPGYVCAIVASLLLDKPVKWME
DRSENLTSTGFARDYIMVGEIAANRDGKILAIRSNVLADHGAFNAQAAPAKYPAGFFGVFTGSYDIEAAYCHMT

AVYTNKAPGGVAYACSFRITEAVYFVERLVDCLAFELKMDPAELRLRNLLRPNQFPYQSKTGWVYDSGDYETTM
RKAMNMIGYEALRAEQKQRRARGELMGIGMSFFTEAVGAGPRKDMDILGLGMADGCELRVHPTGKAVLRLS
VQTQGQGHETTFAQIVAEELGIAPDDIEVVHGDTDQTPFGLGTYGSRSTPVSGGAAALVARKVRDKAKIIASGM
LEVSVADLQWEKGKFHVKGDPSAAVTIADIAMRAHGAGDLPEGIEGGLDAEVCYNPSNLTYPYGAYFCVVDIDP
GTAVVKVRRFLAVDDCGTRINPMIIEGQVHGGIVDGIGMALMEMIAFDEDGNCLGGSLMDYLIPTALEVPHLE
TGHTVTPSPHHPIGAKGIGESATVGSPPAVVNAVVDALAPFGVRHADMPLTPSRVWEAMQGRATPPI
>SEQF5092||SEQF5092.1_00401
MTTIESRPPSPEDLADNAQQPCGHGRMMRKEDPRFIRGRGTYVDDVALPGMLHLAILRSPYAHARIVRIDVTA
AQAHPKVKAVVTGADLAAKGLAWMPTLANDVQAVLATDKTRFQGQEVAFVVAEDRYSARDACELVDVDYEP
RDPVVDARTALDPSAPVIRTDLEGKSDNHIFDWETGDAAATEAVFAKADVVVQQEIVYPRVHPAPMETCGAVA
DLDPVTGKLTLWTTSQAPHAHRTLYALVAGLPEHKIRVISPDIGGGFGNKVPIYPGYVCAIVASLLLDKPVKWME
DRSENLTSTGFARDYIMVGEIAANRDGKILAIRSNVLADHGAFNAQAAPAKYPAGFFGVFTGSYDIEAAYCHMT
AVYTNKAPGGVAYACSFRITEAVYFVERLVDCLAFELKMDPAELRLRNLLRPNQFPYQSKTGWVYDSGDYETTM
RKAMNMIGYEALRAEQKQRRARGELMGIGMSFFTEAVGAGPRKDMDILGLGMADGCELRVHPTGKAVLRLS
VQTQGQGHETTFAQIVAEELGIAPDDIEVVHGDTDQTPFGLGTYGSRSTPVSGGAAALVARKVRDKAKIIASGM
LEVSVADLQWEKGKFHVKGDPSAAVTIADIAMRAHGAGDLPEGIEGGLDAEVCYNPSNLTYPYGAYFCVVDIDP
GTAVVKVRRFLAVDDCGTRINPMIIEGQVHGGIVDGIGMALMEMIAFDEDGNCLGGSLMDYLIPTALEVPHLE
TGHTVTPSPHHPIGAKGIGESATVGSPPAVVNAVVDALAPFGVRHADMPLTPSRVWEAMQGRATPPI
>SEQF5093||SEQF5093.1_00400
MTTIESRPPSPEDLADNAQQPCGHGRMMRKEDPRFIRGRGTYVDDVALPGMLHLAILRSPYAHARIVRIDVTA
AQAHPKVKAVVTGADLAAKGLAWMPTLANDVQAVLATDKTRFQGQEVAFVVAEDRYSARDACELVDVDYEP
RDPVVDARTALDPSAPVIRTDLEGKSDNHIFDWETGDAAATEAVFAKADVVVQQEIVYPRVHPAPMETCGAVA
DLDPVTGKLTLWTTSQAPHAHRTLYALVAGLPEHKIRVISPDIGGGFGNKVPIYPGYVCAIVASLLLDKPVKWME
DRSENLTSTGFARDYIMVGEIAANRDGKILAIRSNVLADHGAFNAQAAPAKYPAGFFGVFTGSYDIEAAYCHMT
AVYTNKAPGGVAYACSFRITEAVYFVERLVDCLAFELKMDPAELRLRNLLRPNQFPYQSKTGWVYDSGDYETTM
RKAMNMIGYEALRAEQKQRRARGELMGIGMSFFTEAVGAGPRKDMDILGLGMADGCELRVHPTGKAVLRLS
VQTQGQGHETTFAQIVAEELGIAPDDIEVVHGDTDQTPFGLGTYGSRSTPVSGGAAALVARKVRDKAKIIASGM
LEVSVADLQWEKGKFHVKGDPSAAVTIADIAMRAHGAGDLPEGIEGGLDAEVCYNPSNLTYPYGAYFCVVDIDP
GTAVVKVRRFLAVDDCGTRINPMIIEGQVHGGIVDGIGMALMEMIAFDEDGNCLGGSLMDYLIPTALEVPHLE
TGHTVTPSPHHPIGAKGIGESATVGSPPAVVNAVVDALAPFGVRHADMPLTPSRVWEAMQGRATPPI
>SEQF5094||SEQF5094.1_00399
MTTIESRPPSPEDLADNAQQPCGHGRMMRKEDPRFIRGRGTYVDDVALPGMLHLAILRSPYAHARIVRIDVTA
AQAHPKVKAVVTGADLAAKGLAWMPTLANDVQAVLATDKTRFQGQEVAFVVAEDRYSARDACELVDVDYEP
RDPVVDARTALDPSAPVIRTDLEGKSDNHIFDWETGDAAATEAVFAKADVVVQQEIVYPRVHPAPMETCGAVA
DLDPVTGKLTLWTTSQAPHAHRTLYALVAGLPEHKIRVISPDIGGGFGNKVPIYPGYVCAIVASLLLDKPVKWME
DRSENLTSTGFARDYIMVGEIAANRDGKILAIRSNVLADHGAFNAQAAPAKYPAGFFGVFTGSYDIEAAYCHMT
AVYTNKAPGGVAYACSFRITEAVYFVERLVDCLAFELKMDPAELRLRNLLRPNQFPYQSKTGWVYDSGDYETTM
RKAMNMIGYEALRAEQKQRRARGELMGIGMSFFTEAVGAGPRKDMDILGLGMADGCELRVHPTGKAVLRLS
VQTQGQGHETTFAQIVAEELGIAPDDIEVVHGDTDQTPFGLGTYGSRSTPVSGGAAALVARKVRDKAKIIASGM
LEVSVADLQWEKGKFHVKGDPSAAVTIADIAMRAHGAGDLPEGIEGGLDAEVCYNPSNLTYPYGAYFCVVDIDP
GTAVVKVRRFLAVDDCGTRINPMIIEGQVHGGIVDGIGMALMEMIAFDEDGNCLGGSLMDYLIPTALEVPHLE
TGHTVTPSPHHPIGAKGIGESATVGSPPAVVNAVVDALAPFGVRHADMPLTPSRVWEAMQGRATPPI
>SEQF5095||SEQF5095.1_00394
MTTIESRPPSPEDLADNAQQPCGHGRMMRKEDPRFIRGRGTYVDDVALPGMLHLAILRSPYAHARIVRIDVTA

AQAHPKVKAVVTGADLAAKGLAWMPTLANDVQAVLATDKTRFQGQEVAFVVAEDRYSARDACELVDVDYEP
RDPVVDARTALDPSAPVIRTDLEGKSDNHIFDWETGDAAATEAVFAKADVVVQQEIVYPRVHPAPMETCGAVA
DLDPVTGKLTLWTTSQAPHAHRTLYALVAGLPEHKIRVISPDIGGGFGNKVPIYPGYVCAIVASLLLDKPVKWME
DRSENLTSTGFARDYIMVGEIAANRDGKILAIRSNVLADHGAFNAQAAPAKYPAGFFGVFTGSYDIEAAYCHMT
AVYTNKAPGGVAYACSFRITEAVYFVERLVDCLAFELKMDPAELRLRNLLRPNQFPYQSKTGWVYDSGDYETTM
RKAMNMIGYEALRAEQKQRRARGELMGIGMSFFTEAVGAGPRKDMDILGLGMADGCELRVHPTGKAVLRLS
VQTQGQGHETTFAQIVAEELGIAPDDIEVVHGDTDQTPFGLGTYGSRSTPVSGGAAALVARKVRDKAKIIASGM
LEVSVADLQWEKGKFHVKGDPSAAVTIADIAMRAHGAGDLPEGIEGGLDAEVCYNPSNLTYPYGAYFCVVDIDP
GTAVVKVRRFLAVDDCGTRINPMIIEGQVHGGIVDGIGMALMEMIAFDEDGNCLGGSLMDYLIPTALEVPHLE
TGHTVTPSPHHPIGAKGIGESATVGSPPAVVNAVVDALAPFGVRHADMPLTPSRVWEAMQGRATPPI
>SEQF5096||SEQF5096.1_00398
MTTIESRPPSPEDLADNAQQPCGHGRMMRKEDPRFIRGRGTYVDDVALPGMLHLAILRSPYAHARIVRIDVTA
AQAHPKVKAVVTGADLAAKGLAWMPTLANDVQAVLATDKTRFQGQEVAFVVAEDRYSARDACELVDVDYEP
RDPVVDARTALDPSAPVIRTDLEGKSDNHIFDWETGDAAATEAVFAKADVVVQQEIVYPRVHPAPMETCGAVA
DLDPVTGKLTLWTTSQAPHAHRTLYALVAGLPEHKIRVISPDIGGGFGNKVPIYPGYVCAIVASLLLDKPVKWME
DRSENLTSTGFARDYIMVGEIAANRDGKILAIRSNVLADHGAFNAQAAPAKYPAGFFGVFTGSYDIEAAYCHMT
AVYTNKAPGGVAYACSFRITEAVYFVERLVDCLAFELKMDPAELRLRNLLRPNQFPYQSKTGWVYDSGDYETTM
RKAMNMIGYEALRAEQKQRRARGELMGIGMSFFTEAVGAGPRKDMDILGLGMADGCELRVHPTGKAVLRLS
VQTQGQGHETTFAQIVAEELGIAPDDIEVVHGDTDQTPFGLGTYGSRSTPVSGGAAALVARKVRDKAKIIASGM
LEVSVADLQWEKGKFHVKGDPSAAVTIADIAMRAHGAGDLPEGIEGGLDAEVCYNPSNLTYPYGAYFCVVDIDP
GTAVVKVRRFLAVDDCGTRINPMIIEGQVHGGIVDGIGMALMEMIAFDEDGNCLGGSLMDYLIPTALEVPHLE
TGHTVTPSPHHPIGAKGIGESATVGSPPAVVNAVVDALAPFGVRHADMPLTPSRVWEAMQGRATPPI
>SEQF5097||SEQF5097.1_00400
MTTIESRPPSPEDLADNAQQPCGHGRMMRKEDPRFIRGRGTYVDDVALPGMLHLAILRSPYAHARIVRIDVTA
AQAHPKVKAVVTGADLAAKGLAWMPTLANDVQAVLATDKTRFQGQEVAFVVAEDRYSARDACELVDVDYEP
RDPVVDARTALDPSAPVIRTDLEGKSDNHIFDWETGDAAATEAVFAKADVVVQQEIVYPRVHPAPMETCGAVA
DLDPVTGKLTLWTTSQAPHAHRTLYALVAGLPEHKIRVISPDIGGGFGNKVPIYPGYVCAIVASLLLDKPVKWME
DRSENLTSTGFARDYIMVGEIAANRDGKILAIRSNVLADHGAFNAQAAPAKYPAGFFGVFTGSYDIEAAYCHMT
AVYTNKAPGGVAYACSFRITEAVYFVERLVDCLAFELKMDPAELRLRNLLRPNQFPYQSKTGWVYDSGDYETTM
RKAMNMIGYEALRAEQKQRRARGELMGIGMSFFTEAVGAGPRKDMDILGLGMADGCELRVHPTGKAVLRLS
VQTQGQGHETTFAQIVAEELGIAPDDIEVVHGDTDQTPFGLGTYGSRSTPVSGGAAALVARKVRDKAKIIASGM
LEVSVADLQWEKGKFHVKGDPSAAVTIADIAMRAHGAGDLPEGIEGGLDAEVCYNPSNLTYPYGAYFCVVDIDP
GTAVVKVRRFLAVDDCGTRINPMIIEGQVHGGIVDGIGMALMEMIAFDEDGNCLGGSLMDYLIPTALEVPHLE
TGHTVTPSPHHPIGAKGIGESATVGSPPAVVNAVVDALAPFGVRHADMPLTPSRVWEAMQGRATPPI
>SEQF5098||SEQF5098.1_00402
MTTIESRPPSPEDLADNAQQPCGHGRMMRKEDPRFIRGRGTYVDDVALPGMLHLAILRSPYAHARIVRIDVTA
AQAHPKVKAVVTGADLAAKGLAWMPTLANDVQAVLATDKTRFQGQEVAFVVAEDRYSARDACELVDVDYEP
RDPVVDARTALDPSAPVIRTDLEGKSDNHIFDWETGDAAATEAVFAKADVVVQQEIVYPRVHPAPMETCGAVA
DLDPVTGKLTLWTTSQAPHAHRTLYALVAGLPEHKIRVISPDIGGGFGNKVPIYPGYVCAIVASLLLDKPVKWME
DRSENLTSTGFARDYIMVGEIAANRDGKILAIRSNVLADHGAFNAQAAPAKYPAGFFGVFTGSYDIEAAYCHMT
AVYTNKAPGGVAYACSFRITEAVYFVERLVDCLAFELKMDPAELRLRNLLRPNQFPYQSKTGWVYDSGDYETTM
RKAMNMIGYEALRAEQKQRRARGELMGIGMSFFTEAVGAGPRKDMDILGLGMADGCELRVHPTGKAVLRLS
VQTQGQGHETTFAQIVAEELGIAPDDIEVVHGDTDQTPFGLGTYGSRSTPVSGGAAALVARKVRDKAKIIASGM
LEVSVADLQWEKGKFHVKGDPSAAVTIADIAMRAHGAGDLPEGIEGGLDAEVCYNPSNLTYPYGAYFCVVDIDP

GTAVVKVRRFLAVDDCGTRINPMIIEGQVHGGIVDGIGMALMEMIAFDEDGNCLGGSLMDYLIPTALEVPHLE
TGHTVTPSPHHPIGAKGIGESATVGSPPAVVNAVVDALAPFGVRHADMPLTPSRVWEAMQGRATPPI
>SEQF5099||SEQF5099.1_00398
MTTIESRPPSPEDLADNAQQPCGHGRMMRKEDPRFIRGRGTYVDDVALPGMLHLAILRSPYAHARIVRIDVTA
AQAHPKVKAVVTGADLAAKGLAWMPTLANDVQAVLATDKTRFQGQEVAFVVAEDRYSARDACELVDVDYEP
RDPVVDARTALDPSAPVIRTDLEGKSDNHIFDWETGDAAATEAVFAKADVVVQQEIVYPRVHPAPMETCGAVA
DLDPVTGKLTLWTTSQAPHAHRTLYALVAGLPEHKIRVISPDIGGGFGNKVPIYPGYVCAIVASLLLDKPVKWME
DRSENLTSTGFARDYIMVGEIAANRDGKILAIRSNVLADHGAFNAQAAPAKYPAGFFGVFTGSYDIEAAYCHMT
AVYTNKAPGGVAYACSFRITEAVYFVERLVDCLAFELKMDPAELRLRNLLRPNQFPYQSKTGWVYDSGDYETTM
RKAMNMIGYEALRAEQKQRRARGELMGIGMSFFTEAVGAGPRKDMDILGLGMADGCELRVHPTGKAVLRLS
VQTQGQGHETTFAQIVAEELGIAPDDIEVVHGDTDQTPFGLGTYGSRSTPVSGGAAALVARKVRDKAKIIASGM
LEVSVADLQWEKGKFHVKGDPSAAVTIADIAMRAHGAGDLPEGIEGGLDAEVCYNPSNLTYPYGAYFCVVDIDP
GTAVVKVRRFLAVDDCGTRINPMIIEGQVHGGIVDGIGMALMEMIAFDEDGNCLGGSLMDYLIPTALEVPHLE
TGHTVTPSPHHPIGAKGIGESATVGSPPAVVNAVVDALAPFGVRHADMPLTPSRVWEAMQGRATPPI
>SEQF5100||SEQF5100.1_00402
MTTIESRPPSPEDLADNAQQPCGHGRMMRKEDPRFIRGRGTYVDDVALPGMLHLAILRSPYAHARIVRIDVTA
AQAHPKVKAVVTGADLAAKGLAWMPTLANDVQAVLATDKTRFQGQEVAFVVAEDRYSARDACELVDVDYEP
RDPVVDARTALDPSAPVIRTDLEGKSDNHIFDWETGDAAATEAVFAKADVVVQQEIVYPRVHPAPMETCGAVA
DLDPVTGKLTLWTTSQAPHAHRTLYALVAGLPEHKIRVISPDIGGGFGNKVPIYPGYVCAIVASLLLDKPVKWME
DRSENLTSTGFARDYIMVGEIAANRDGKILAIRSNVLADHGAFNAQAAPAKYPAGFFGVFTGSYDIEAAYCHMT
AVYTNKAPGGVAYACSFRITEAVYFVERLVDCLAFELKMDPAELRLRNLLRPNQFPYQSKTGWVYDSGDYETTM
RKAMNMIGYEALRAEQKQRRARGELMGIGMSFFTEAVGAGPRKDMDILGLGMADGCELRVHPTGKAVLRLS
VQTQGQGHETTFAQIVAEELGIAPDDIEVVHGDTDQTPFGLGTYGSRSTPVSGGAAALVARKVRDKAKIIASGM
LEVSVADLQWEKGKFHVKGDPSAAVTIADIAMRAHGAGDLPEGIEGGLDAEVCYNPSNLTYPYGAYFCVVDIDP
GTAVVKVRRFLAVDDCGTRINPMIIEGQVHGGIVDGIGMALMEMIAFDEDGNCLGGSLMDYLIPTALEVPHLE
TGHTVTPSPHHPIGAKGIGESATVGSPPAVVNAVVDALAPFGVRHADMPLTPSRVWEAMQGRATPPI
>SEQF5101||SEQF5101.1_00398
MTTIESRPPSPEDLADNAQQPCGHGRMMRKEDPRFIRGRGTYVDDVALPGMLHLAILRSPYAHARIVRIDVTA
AQAHPKVKAVVTGADLAAKGLAWMPTLANDVQAVLATDKTRFQGQEVAFVVAEDRYSARDACELVDVDYEP
RDPVVDARTALDPSAPVIRTDLEGKSDNHIFDWETGDAAATEAVFAKADVVVQQEIVYPRVHPAPMETCGAVA
DLDPVTGKLTLWTTSQAPHAHRTLYALVAGLPEHKIRVISPDIGGGFGNKVPIYPGYVCAIVASLLLDKPVKWME
DRSENLTSTGFARDYIMVGEIAANRDGKILAIRSNVLADHGAFNAQAAPAKYPAGFFGVFTGSYDIEAAYCHMT
AVYTNKAPGGVAYACSFRITEAVYFVERLVDCLAFELKMDPAELRLRNLLRPNQFPYQSKTGWVYDSGDYETTM
RKAMNMIGYEALRAEQKQRRARGELMGIGMSFFTEAVGAGPRKDMDILGLGMADGCELRVHPTGKAVLRLS
VQTQGQGHETTFAQIVAEELGIAPDDIEVVHGDTDQTPFGLGTYGSRSTPVSGGAAALVARKVRDKAKIIASGM
LEVSVADLQWEKGKFHVKGDPSAAVTIADIAMRAHSAGDLPEGIEGGLDAEVCYNPSNLTYPYGAYFCVVDIDP
GTAVVKVRRFLAVDDCGTRINPMIIEGQVHGGIVDGIGMALMEMIAFDEDGNCLGGSLMDYLIPTALEVPHLE
TGHTVTPSPHHPIGAKGIGESATVGSPPAVVNAVVDALAPFGVRHADMPLTPSRVWEAMQGRATPPI
>SEQF5102||SEQF5102.1_00398
MTTIESRPPSPEDLADNAQQPCGHGRMMRKEDPRFIRGRGTYVDDVALPGMLHLAILRSPYAHARIVRIDVTA
AQAHPKVKAVVTGADLAAKGLAWMPTLANDVQAVLATDKTRFQGQEVAFVVAEDRYSARDACELVDVDYEP
RDPVVDARTALDPSAPVIRTDLEGKSDNHIFDWETGDAAATEAVFAKADVVVQQEIVYPRVHPAPMETCGAVA
DLDPVTGKLTLWTTSQAPHAHRTLYALVAGLPEHKIRVISPDIGGGFGNKVPIYPGYVCAIVASLLLDKPVKWME
DRSENLTSTGFARDYIMVGEIAANRDGKILAIRSNVLADHGAFNAQAAPAKYPAGFFGVFTGSYDIEAAYCHMT

AVYTNKAPGGVAYACSFRITEAVYFVERLVDCLAFELKMDPAELRLRNLLRPNQFPYQSKTGWVYDSGDYETTM
RKAMNMIGYEALRAEQKQRRARGELMGIGMSFFTEAVGAGPRKDMDILGLGMADGCELRVHPTGKAVLRLS
VQTQGQGHETTFAQIVAEELGIAPDDIEVVHGDTDQTPFGLGTYGSRSTPVSGGAAALVARKVRDKAKIIASGM
LEVSVADLQWEKGKFHVKGDPSAAVTIADIAMRAHGAGDLPEGIEGGLDAEVCYNPSNLTYPYGAYFCVVDIDP
GTAVVKVRRFLAVDDCGTRINPMIIEGQVHGGIVDGIGMALMEMIAFDEDGNCLGGSLMDYLIPTALEVPHLE
TGHTVTPSPHHPIGAKGIGESATVGSPPAVVNAVVDALAPFGVRHADMPLTPSRVWEAMQGRATPPI
>SEQF5103||SEQF5103.1_00399
MTTIESRPPSPEDLADNAQQPCGHGRMMRKEDPRFIRGRGTYVDDVALPGMLHLAILRSPYAHARIVRIDVTA
AQAHPKVKAVVTGADLAAKGLAWMPTLANDVQAVLATDKTRFQGQEVAFVVAEDRYSARDACELVDVDYEP
RDPVVDARTALDPSAPVIRTDLEGKSDNHIFDWETGDAAATEAVFAKADVVVQQEIVYPRVHPAPMETCGAVA
DLDPVTGKLTLWTTSQAPHAHRTLYALVAGLPEHKIRVISPDIGGGFGNKVPIYPGYVCAIVASLLLDKPVKWME
DRSENLTSTGFARDYIMVGEIAANRDGKILAIRSNVLADHGAFNAQAAPAKYPAGFFGVFTGSYDIEAAYCHMT
AVYTNKAPGGVAYACSFRITEAVYFVERLVDCLAFELKMDPAELRLRNLLRPNQFPYQSKTGWVYDSGDYETTM
RKAMNMIGYEALRAEQKQRRARGELMGIGMSFFTEAVGAGPRKDMDILGLGMADGCELRVHPTGKAVLRLS
VQTQGQGHETTFAQIVAEELGIAPDDIEVVHGDTDQTPFGLGTYGSRSTPVSGGAAALVARKVRDKAKIIASGM
LEVSVADLQWEKGKFHVKGDPSAAVTIADIAMRAHGAGDLPEGIEGGLDAEVCYNPSNLTYPYGAYFCVVDIDP
GTAVVKVRRFLAVDDCGTRINPMIIEGQVHGGIVDGIGMALMEMIAFDEDGNCLGGSLMDYLIPTALEVPHLE
TGHTVTPSPHHPIGAKGIGESATVGSPPAVVNAVVDALAPFGVRHADMPLTPSRVWEAMQGRATPPI
>SEQF5104||SEQF5104.1_00395
MTTIESRPPSPEDLADNAQQPCGHGRMMRKEDPRFIRGRGTYVDDVALPGMLHLAILRSPYAHARIVRIDVTA
AQAHPKVKAVVTGADLAAKGLAWMPTLANDVQAVLATDKTRFQGQEVAFVVAEDRYSARDACELVDVDYEP
RDPVVDARTALDPSAPVIRTDLEGKSDNHIFDWETGDAAATEAVFAKADVVVQQEIVYPRVHPAPMETCGAVA
DLDPVTGKLTLWTTSQAPHAHRTLYALVAGLPEHKIRVISPDIGGGFGNKVPIYPGYVCAIVASLLLDKPVKWME
DRSENLTSTGFARDYIMVGEIAANRDGKILAIRSNVLADHGAFNAQAAPAKYPAGFFGVFTGSYDIEAAYCHMT
AVYTNKAPGGVAYACSFRITEAVYFVERLVDCLAFELKMDPAELRLRNLLRPNQFPYQSKTGWVYDSGDYETTM
RKAMNMIGYEALRAEQKQRRARGELMGIGMSFFTEAVGAGPRKDMDILGLGMADGCELRVHPTGKAVLRLS
VQTQGQGHETTFAQIVAEELGIAPDDIEVVHGDTDQTPFGLGTYGSRSTPVSGGAAALVARKVRDKAKIIASGM
LEVSVADLQWEKGKFHVKGDPSAAVTIADIAMRAHGAGDLPEGIEGGLDAEVCYNPSNLTYPYGAYFCVVDIDP
GTAVVKVRRFLAVDDCGTRINPMIIEGQVHGGIVDGIGMALMEMIAFDEDGNCLGGSLMDYLIPTALEVPHLE
TGHTVTPSPHHPIGAKGIGESATVGSPPAVVNAVVDALAPFGVRHADMPLTPSRVWEAMQGRATPPI
>SEQF5105||SEQF5105.1_00401
MTTIESRPPSPEDLADNAQQPCGHGRMMRKEDPRFIRGRGTYVDDVALPGMLHLAILRSPYAHARIVRIDVTA
AQAHPKVKAVVTGADLAAKGLAWMPTLANDVQAVLATDKTRFQGQEVAFVVAEDRYSARDACELVDVDYEP
RDPVVDARTALDPSAPVIRTDLEGKSDNHIFDWETGDAAATEAVFAKADVVVQQEIVYPRVHPAPMETCGAVA
DLDPVNGKLTLWTTSQAPHAHRTLYALVAGLPEHKIRVISPYIGGGFGNKVPIYPGYVCAIVASLLLDKPVKWME
DRSENLTSTGFARDYIMVGEIAANRDGKILAIRSNVLADHGAFNAQAAPAKYPAGFFGVFTGSYDIEAAYCHMT
AVYTNKAPGGVAYACSFRITEAVYFVERLVDCLAFELKMDPAELRLRNLLRPNQFPYQSKTGWVYDSGDYETTM
RKAMNMIGYEALRAEQKQRRARGELMGIGMSFFTEAVGAGPRKDMDILGLGMADGCELRVHPTGKAVLRLS
VQTQGQGHETTFAQIVAEELGIAPDDIEVVHGDTDQTPFGLGTYGSRSTPVSGGAAALVARKVRDKAKIIASGM
LEVSVADLQWEKGKFHVKGDPSAAVTIADIAMRAHGAGDLPEGIEGGLDAEVCYNPSNLTYPYGAYFCVVDIDP
GTAVVKVRRFLAVDDCGTRINPMIIEGQVHGGIVDGIGMALMEMIAFDEDGNCLGGSLMDYLIPTALEVPHLE
TGHTVTPSPHHPIGAKGIGESATVGSPPAVVNAVVDALAPFGVRHADMPLTPSRVWEAMQGRATPPI
>SEQF5106||SEQF5106.1_00393
MTTIESRPPSPEDLADNAQQPCGHGRMMRKEDPRFIRGRGTYVDDVALPGMLHLAILRSPYAHARIVRIDVTA

AQAHPKVKAVVTGADLAAKGLAWMPTLANDVQAVLATDKTRFQGQEVAFVVAEDRYSARDACELVDVDYEP
RDPVVDARTALDPSAPVIRTDLEGKSDNHIFDWETGDAAATEAVFAKADVVVQQEIVYPRVHPAPMETCGAVA
DLDPVTGKLTLWTTSQAPHAHRTLYALVAGLPEHKIRVISPDIGGGFGNKVPIYPGYVCAIVASLLLDKPVKWME
DRSENLTSTGFARDYIMVGEIAANRDGKILAIRSNVLADHGAFNAQAAPAKYPAGFFGVFTGSYDIEAAYCHMT
AVYTNKAPGGVAYACSFRITEAVYFVERLVDCLAFELKMDPAELRLRNLLRPNQFPYQSKTGWVYDSGDYETTM
RKAMNMIGYEALRAEQKQRRARGELMGIGMSFFTEAVGAGPRKDMDILGLGMADGCELRVHPTGKAVLRLS
VQTQGQGHETTFAQIVAEELGIAPDDIEVVHGDTDQTPFGLGTYGSRSTPVSGGAAALVARKVRDKAKIIASGM
LEVSVADLQWEKGKFHVKGDPSAAVTIADIAMRAHGAGDLPEGIEGGLDAEVCYNPSNLTYPYGAYFCVVDIDP
GTAVVKVRRFLAVDDCGTRINPMIIEGQVHGGIVDGIGMALMEMIAFDEDGNCLGGSLMDYLIPTALEVPHLE
TGHTVTPSPHHPIGAKGIGESATVGSPPAVVNAVVDALAPFGVRHADMPLTPSRVWEAMQGRATPPI
>SEQF5107||SEQF5107.1_00397
MTTIESRPPSPEDLADNAQQPCGHGRMMRKEDPRFIRGRGTYVDDVALPGMLHLAILRSPYAHARIVRIDVTA
AQAHPKVKAVVTGADLAAKGLAWMPTLANDVQAVLATDKTRFQGQEVAFVVAEDRYSARDACELVDVDYEP
RDPVVDARTALDPSAPVIRTDLEGKSDNHIFDWETGDAAATEAVFAKADVVVQQEIVYPRVHPAPMETCGAVA
DLDPVTGKLTLWTTSQAPHAHRTLYALVAGLPEHKIRVISPDIGGGFGNKVPIYPGYVCAIVASLLLDKPVKWME
DRSENLTSTGFARDYIMVGEIAANRDGKILAIRSNVLADHGAFNAQAAPAKYPAGFFGVFTGSYDIEAAYCHMT
AVYTNKAPGGVAYACSFRITEAVYFVERLVDCLAFELKMDPAELRLRNLLRPNQFPYQSKTGWVYDSGDYETTM
RKAMNMIGYEALRAEQKQRRARGELMGIGMSFFTEAVGAGPRKDMDILGLGMADGCELRVHPTGKAVLRLS
VQTQGQGHETTFAQIVAEELGIAPDDIEVVHGDTDQTPFGLGTYGSRSTPVSGGAAALVARKVRDKAKIIASGM
LEVSVADLQWEKGKFHVKGDPSAAVTIADIAMRAHGAGDLPEGIEGGLDAEVCYNPSNLTYPYGAYFCVVDIDP
GTAVVKVRRFLAVDDCGTRINPMIIEGQVHGGIVDGIGMALMEMIAFDEDGNCLGGSLMDYLIPTALEVPHLE
TGHTVTPSPHHPIGAKGIGESATVGSPPAVVNAVVDALAPFGVRHADMPLTPSRVWEAMQGRATPPI
>SEQF5108||SEQF5108.1_00397
MTTIESRPPSPEDLADNAQQPCGHGRMMRKEDPRFIRGRGTYVDDVALPGMLHLAILRSPYAHARIVRIDVTA
AQAHPKVKAVVTGADLAAKGLAWMPTLANDVQAVLATDKTRFQGQEVAFVVAEDRYSARDACELVDVDYEP
RDPVVDARTALDPSAPVIRTDLEGKSDNHIFDWETGDAAATEAVFAKADVVVQQEIVYPRVHPAPMETCGAVA
DLDPVTGKLTLWTTSQAPHAHRTLYALVAGLPEHKIRVISPDIGGGFGNKVPIYPGYVCAIVASLLLDKPVKWME
DRSENLTSTGFARDYIMVGEIAANRDGKILAIRSNVLADHGAFNAQAAPAKYPAGFFGVFTGSYDIEAAYCHMT
AVYTNKAPGGVAYACSFRITEAVYFVERLVDCLAFELKMDPAELRLRNLLRPNQFPYQSKTGWVYDSGDYETTM
RKAMNMIGYEALRAEQKQRRARGELMGIGMSFFTEAVGAGPRKDMDILGLGMADGCELRVHPTGKAVLRLS
VQTQGQGHETTFAQIVAEELGIAPDDIEVVHGDTDQTPFGLGTYGSRSTPVSGGAAALVARKVRDKAKIIASGM
LEVSVADLQWEKGKFHVKGDPSAAVTIADIAMRAHGAGDLPEGIEGGLDAEVCYNPSNLTYPYGAYFCVVDIDP
GTAVVKVRRFLAVDDCGTRINPMIIEGQVHGGIVDGIGMALMEMIAFDEDGNCLGGSLMDYLIPTALEVPHLE
TGHTVTPSPHHPIGAKGIGESATVGSPPAVVNAVVDALAPFGVRHADMPLTPSRVWEAMQGRATPPI
>SEQF5109||SEQF5109.1_00404
MTTIESRPPSPEDLADNAQQPCGHGRMMRKEDPRFIRGRGTYVDDVALPGMLHLAILRSPYAHARIVRIDVTA
AQAHPKVKAVVTGADLAAKGLAWMPTLANDVQAVLATDKTRFQGQEVAFVVAEDRYSARDACELVDVDYEP
RDPVVDARTALDPSAPVIRTDLEGKSDNHIFDWETGDAAATEAVFAKADVVVQQEIVYPRVHPAPMETCGAVA
DLDPVTGKLTLWTTSQAPHAHRTLYALVAGLPEHKIRVISPDIGGGFGNKVPIYPGYVCAIVASLLLDKPVKWME
DRSENLTSTGFARDYIMVGEIAANRDGKILAIRSNVLADHGAFNAQAAPAKYPAGFFGVFTGSYDIEAAYCHMT
AVYTNKAPGGVAYACSFRITEAVYFVERLVDCLAFELKMDPAELRLRNLLRPNQFPYQSKTGWVYDSGDYETTM
RKAMNMIGYEALRAEQKQRRARGELMGIGMSFFTEAVGAGPRKDMDILGLGMADGCELRVHPTGKAVLRLS
VQTQGQGHETTFAQIVAEELGIAPDDIEVVHGDTDQTPFGLGTYGSRSTPVSGGAAALVARKVRDKAKIIASGM
LEVSVADLQWEKGKFHVKGDPSAAVTIADIAMRAHGAGDLPEGIEGGLDAEVCYNPSNLTYPYGAYFCVVDIDP

GTAVVKVRRFLAVDDCGTRINPMIIEGQVHGGIVDGIGMALMEMIAFDEDGNCLGGSLMDYLIPTALEVPHLE
TGHTVTPSPHHPIGAKGIGESATVGSPPAVVNAVVDALAPFGVRHADMPLTPSRVWEAMQGRATPPI
>SEQF5110||SEQF5110.1_00410
MTTIESRPPSPEDLADNAQQPCGHGRMMRKEDPRFIRGRGTYVDDVALPGMLHLAILRSPYAHARIVRIDVTA
AQAHPKVKAVVTGADLAAKGLAWMPTLANDVQAVLATDKTRFQGQEVAFVVAEDRYSARDACELVDVDYEP
RDPVVDARTALDPSAPVIRTDLEGKSDNHIFDWETGDAAATEAVFAKADVVVQQEIVYPRVHPAPMETCGAVA
DLDPVTGKLTLWTTSQAPHAHRTLYALVAGLPEHKIRVISPDIGGGFGNKVPIYPGYVCAIVASLLLDKPVKWME
DRSENLTSTGFARDYIMVGEIAANRDGKILAIRSNVLADHGAFNAQAAPAKYPAGFFGVFTGSYDIEAAYCHMT
AVYTNKAPGGVAYACSFRITEAVYFVERLVDCLAFELKMDPAELRLRNLLRPNQFPYQSKTGWVYDSGDYETTM
RKAMNMIGYEALRAEQKQRRARGELMGIGMSFFTEAVGAGPRKDMDILGLGMADGCELRVHPTGKAVLRLS
VQTQGQGHETTFAQIVAEELGIAPDDIEVVHGDTDQTPFGLGTYGSRSTPVSGGAAALVARKVRDKAKIIASGM
LEVSVADLQWEKGKFHVKGDPSAAVTIADIAMRAHGAGDLPEGIEGGLDAEVCYNPSNLTYPYGAYFCVVDIDP
GTAVVKVRRFLAVDDCGTRINPMIIEGQVHGGIVDGIGMALMEMIAFDEDGNCLGGSLMDYLIPTALEVPHLE
TGHTVTPSPHHPIGAKGIGESATVGSPPAVVNAVVDALAPFGVRHADMPLTPSRVWEAMQGRATPPI
>SEQF5111||SEQF5111.1_00400
MTTIESRPPSPEDLADNAQQPCGHGRMMRKEDPRFIRGRGTYVDDVALPGMLHLAILRSPYAHARIVRIDVTA
AQVHPKVKAVVTGADLAAKGLAWMPTLANDVQAVLATDKTRFQGQEVAFVVAEDRYSARDACELVDVDYEP
RDPVVDARTALDPSAPVIRTDLEGKSDNHIFDWETGDAAATEAVFAKADVVVQQEIVYPRVHPAPMETCGAVA
DLDPVTGKLTLWTTSQAPHAHRTLYALVAGLPEHKIRVISPDIGGGFGNKVPIYPGYVCAIVASLLLDKPVKWME
DRSENLTSTGFARDYIMVGEIAANRDGKILAIRSNVLADHGAFNAQAAPAKYPAGFFGVFTGSYDIEAAYCHMT
AVYTNKAPGGVAYACSFRITEAVYFVERLVDCLAFELKMDPAELRLRNLLRPNQFPYQSKTGWVYDSGDYETTM
RKAMNMIGYEALRAEQKQRRARGELMGIGMSFFTEAVGAGPRKDMDILGLGMADGCELRVHPTGKAVLRLS
VQTQGQGHETTFAQIVAEELGIAPDDIEVVHGDTDQTPFGLGTYGSRSTPVSGGAAALVARKVRDKAKIIASGM
LEVSVADLQWEKGKFHVKGDPSAAVTIADIAMRAHGAGDLPEGIEGGLDAEVCYNPSNLTYPYGAYFCVVDIDP
GTAVVKVRRFLAVDDCGTRINPMIIEGQVHGGIVDGIGMALMEMIAFDEDGNCLGGSLMDYLIPTALEVPHLE
TGHTVTPSPHHPIGAKGIGESATVGSPPAVVNAVVDALAPFGVRHADMPLTPSRVWEAMQGRATPPI
>SEQF5112||SEQF5112.1_00399
MTTIESRPPSPEDLADNAQQPCGHGRMMRKEDPRFIRGRGTYVDDVALPGMLHLAILRSPYAHARIVRIDVTA
AQAHPKVKAVVTGADLAAKGLAWMPTLANDVQAVLATDKTRFQGQEVAFVVAEDRYSARDACELVDVDYEP
RDPVVDARTALDPSAPVIRTDLEGKSDNHIFDWETGDAAATEAVFAKADVVVQQEIVYPRVHPAPMETCGAVA
DLDPVTGKLTLWTTSQAPHAHRTLYALVAGLPEHKIRVISPDIGGGFGNKVPIYPGYVCAIVASLLLDKPVKWME
DRSENLTSTGFARDYIMVGEIAANRDGKILAIRSNVLADHGAFNAQAAPAKYPAGFFGVFTGSYDIEAAYCHMT
AVYTNKAPGGVAYACSFRITEAVYFVERLVDCLAFELKMDPAELRLRNLLRPNQFPYQSKTGWVYDSGDYETTM
RKAMNMIGYEALRAEQKQRRARGELMGIEMSFFTEAVGAGPRKDMDILGLGMADGCELRVHPTGKAVLRLS
VQTQGQGHETTFAQIVAEELGIAPDDIEVVHGDTDQTPFGLGTYGSRSTPVSGGAAALVARKVRDKAKIIASGM
LEVSVADLQWEKGKFHVKGDPSAAVTIADIAMRAHGAGDLPEGIEGGLDAEVCYNPSNLTYPYGAYFCVVDIDP
GTAVVKVRRFLAVDDCGTRINPMIIEGQVHGGIVDGIGMALMEMIAFDEDGNCLGGSLMDYLIPTALEVPHLE
TGHTVTPSPHHPIGAKGIGESATVGSPPAVVNAVVDALAPFGVRHADMPLTPSRVWEAMQGRATPPI
>SEQF5113||SEQF5113.1_00404
MTTIESRPPSPEDLADNAQQPCGHGRMMRKEDPRFIRGRGTYVDDVALPGMLHLAILRSPYAHARIVRIDVTA
AQAHPKVKAVVTGADLAAKGLAWMPTLANDVQAVLATDKTRFQGQEVAFVVAEDRYSARDACELVDVDYEP
RDPVVDARTALDPSAPVIRTDLEGKSDNHIFDWETGDAAATEAVFAKADVVVQQEIVYPRVHPAPMETCGAVA
DLDPVTGKLTLWTTSQAPHAHRTLYALVAGLPEHKIRVISPDIGGGFGNKVPIYPGYVCAIVASLLLDKPVKWME
DRSENLTSTGFARDYIMVGEIAANRDGKILAIRSNVLADHGAFNAQAAPAKYPAGFFGVFTGSYDIEAAYCHMT

AVYTNKAPGGVAYACSFRITEAVYFVERLVDCLAFELKMDPAELRLRNLLRPNQFPYQSKTGWVYDSGDYETTM
RKAMNMIGYEALRAEQKQRRARGELMGIGMSFFTEAVGAGPRKDMDILGLGMADGCELRVHPTGKAVLRLS
VQTQGQGHETTFAQIVAEELGIAPDDIEVVHGDTDQTPFGLGTYGSRSTPVSGGAAALVARKVRDKAKIIASGM
LEVSVADLQWEKGKFHVKGDPSAAVTIADIAMRAHGAGDLPEGIEGGLDAEVCYNPSNLTYPYGAYFCVVDIDP
GTAVVKVRRFLAVDDCGTRINPMIIEGQVHGGIVDGIGMALMEMIAFDEDGNCLGGSLMDYLIPTALEVPHLE
TGHTVTPSPHHPIGAKGIGESATVGSPPAVVNAVVDALAPFGVRHADMPLTPSRVWEAMQGRATPPI
>SEQF5114||SEQF5114.1_00398
MTTIESRPPSPEDLADNAQQPCGHGRMMRKEDPRFIRGRGTYVDDVALPGMLHLAILRSPYAHARIVRIDVTA
AQAHPKVKAVVTGADLAAKGLAWMPTLANDVQAVLATDKTRFQGQEVAFVVAEDRYSARDACELVDVDYEP
RDPVVDARTALDPSAPVIRTDLEGKSDNHIFDWETGDAAATEAVFAKADVVVQQEIVYPRVHPAPMETCGAVA
DLDPVTGKLTLWTTSQAPHAHRTLYALVAGLPEHKIRVISPDIGGGFGNKVPIYPGYVCAIVASLLLDKPVKWME
DRSENLTSTGFARDYIMVGEIAANRDGKILAIRSNVLADHGAFNAQAAPAKYPAGFFGVFTGSYDIEAAYCHMT
AVYTNKAPGGVAYACSFRITEAVYFVERLVDCLAFELKMDPAELRLRNLLRPNQFPYQSKTGWVYDSGDYETTM
RKAMNMIGYEALRAEQKQRRARGELMGIEMSFFTEAVGAGPRKDMDILGLGMADGCELRVHPTGKAVLRLS
VQTQGQGHETTFAQIVAEELGIAPDDIEVVHGDTDQTPFGLGTYGSRSTPVSGGAAALVARKVRDKAKIIASGM
LEVSVADLQWEKGKFHVKGDPSAAVTIADIAMRAHGAGDLPEGIEGGLDAEVCYNPSNLTYPYGAYFCVVDIDP
GTAVVKVRRFLAVDDCGTRINPMIIEGQVHGGIVDGIGMALMEMIAFDEDGNCLGGSLMDYLIPTALEVPHLE
TGHTVTPSPHHPIGAKGIGESATVGSPPAVVNAVVDALAPFGVRHADMPLTPSRVWEAMQGRATPPI
>SEQF5115||SEQF5115.1_00402
MTTIESRPPSPEDLADNAQQPCGHGRMMRKEDPRFIRGRGTYVDDVALPGMLHLAILRSPYAHARIVRIDVTA
AQAHPKVKAVVTGADLAAKGLAWMPTLANDVQAVLATDKTRFQGQEVAFVVAEDRYSARDACELVDVDYEP
RDPVVDARTALDPSAPVIRTDLEGKSDNHIFDWETGDAAATEAVFAKADVVVQQEIVYPRVHPAPMETCGAVA
DLDPVTGKLTLWTTSQAPHAHRTLYALVAGLPEHKIRVISPDIGGGFGNKVPIYPGYVCAIVASLLLDKPVKWME
DRSENLTSTGFARDYIMVGEIAANRDGKILAIRSNVLADHGAFNAQAAPAKYPAGFFGVFTGSYDIEAAYCHMT
AVYTNKAPGGVAYACSFRITEAVYFVERLVDCLAFELKMDPAELRLRNLLRPNQFPYQSKTGWVYDSGDYETTM
RKAMNMIGYEALRAEQKQRRARGELMGIGMSFFTEAVGAGPRKDMDILGLGMADGCELRVHPTGKAVLRLS
VQTQGQGHETTFAQIVAEELGIAPDDIEVVHGDTDQTPFGLGTYGSRSTPVSGGAAALVARKVRDKAKIIASGM
LEVSVADLQWEKGKFHVKGDPSAAVTIADIAMRAHGAGDLPEGIEGGLDAEVCYNPSNLTYPYGAYFCVVDIDP
GTAVVKVRRFLAVDDCGTRINPMIIEGQVHGGIVDGIGMALMEMIAFDEDGNCLGGSLMDYLIPTALEVPHLE
TGHTVTPSPHHPIGAKGIGESATVGSPPAVVNAVVDALAPFGVRHADMPLTPSRVWEAMQGRATPPI
>SEQF5116||SEQF5116.1_00405
MTTIESRPPSPEDLADNAQQPCGHGRMMRKEDPRFIRGRGTYVDDVALPGMLHLAILRSPYAHARIVRIDVTA
AQAHPKVKAVVTGADLAAKGLAWMPTLANDVQAVLATDKTRFQGQEVAFVVAEDRYSARDACELVDVDYEP
RDPVVDARTALDPSAPVIRTDLEGKSDNHIFDWETGDAAATEAVFAKADVVVQQEIVYPRVHPAPMETCGAVA
DLDPVTGKLTLWTTSQAPHAHRTLYALVAGLPEHKIRVISPDIGGGFGNKVPIYPGYVCAIVASLLLDKPVKWME
DRSENLTSTGFARDYIMVGEIAANRDGKILAIRSNVLADHGAFNAQAAPAKYPAGFFGVFTGSYDIEAAYCHMT
AVYTNKAPGGVAYACSFRITEAVYFVERLVDCLAFELKMDPAELRLRNLLRPNQFPYQSKTGWVYDSGDYETTM
RKAMNMIGYEALRAEQKQRRARGELMGIGMSFFTEAVGAGPRKDMDILGLGMADGCELRVHPTGKAVLRLS
VQTQGQGHETTFAQIVAEELGIAPDDIEVVHGDTDQTPFGLGTYGSRSTPVSGGAAALVARKVRDKAKIIASGM
LEVSVADLQWEKGKFHVKGDPSAAVTIADIAMRAHGAGDLPEGIEGGLDAEVCYNPSNLTYPYGAYFCVVDIDP
GTAVVKVRRFLAVDDCGTRINPMIIEGQVHGGIVDGIGMALMEMIAFDEDGNCLGGSLMDYLIPTALEVPHLE
TGHTVTPSPHHPIGAKGIGESATVGSPPAVVNAVVDALAPFGVRHADMPLTPSRVWEAMQGRATPPI
>SEQF5117||SEQF5117.1_00398
MTTIESRPPSPEDLADNAQQPCGHGRMMRKEDPRFIRGRGTYVDDVALPGMLHLAILRSPYAHARIVRIDVTA

AQAHPKVKAVVTGADLAAKGLAWMPTLANDVQAVLATDKTRFQGQEVAFVVAEDRYSARDACELVDVDYEP
RDPVVDARTALDPSAPVIRTDLEGKSDNHIFDWETGDAAATEAVFAKADVVVQQEIVYPRVHPAPMETCGAVA
DLDPVTGKLTLWTTSQAPHAHRTLYALVAGLPEHKIRVISPDIGGGFGNKVPIYPGYVCAIVASLLLDKPVKWME
DRSENLTSTGFARDYIMVGEIAANRDGKILAIRSNVLADHGAFNAQAAPAKYPAGFFGVFTGSYDIEAAYCHMT
AVYTNKAPGGVAYACSFRITEAVYFVERLVDCLAFELKMDPAELRLRNLLRPNQFPYQSKTGWVYDSGDYETTM
RKAMNMIGYEALRAEQKQRRARGELMGIGMSFFTEAVGAGPRKDMDILGLGMADGCELRVHPTGKAVLRLS
VQTQGQGHETTFAQIVAEELGIAPDDIEVVHGDTDQTPFGLGTYGSRSTPVSGGAAALVARKVRDKAKIIASGM
LEVSVADLQWEKGKFHVKGDPSAAVTIADIAMRAHGAGDLPEGIEGGLDAEVCYNPSNLTYPYGAYFCVVDIDP
GTAVVKVRRFLAVDDCGTRINPMIIEGQVHGGIVDGIGMALMEMIAFDEDGNCLGGSLMDYLIPTALEVPHLE
TGHTVTPSPHHPIGAKGIGESATVGSPPAVVNAVVDALAPFGVRHADMPLTPSRVWEAMQGRATPPI
>SEQF5118||SEQF5118.1_00400
MTTIESRPPSPEDLADNAQQPCGHGRMMRKEDPRFIRGRGTYVDDVALPGMLHLAILRSPYAHARIVRIDVTA
AQAHPKVKAVVTGADLAAKGLAWMPTLANDVQAVLATDKTRFQGQEVAFVVAEDRYSARDACELVDVDYEP
RDPVVDARTALDPSAPVIRTDLEGKSDNHIFDWETGDAAATEAVFAKADVVVQQEIVYPRVHPAPMETCGAVA
DLDPVTGKLTLWTTSQAPHAHRTLYALVAGLPEHKIRVISPDIGGGFGNKVPIYPGYVCAIVASLLLDKPVKWME
DRSENLTSTGFARDYIMVGEIAANRDGKILAIRSNVLADHGAFNAQAAPAKYPAGFFGVFTGSYDIEAAYCHMT
AVYTNKAPGGVAYACSFRITEAVYFVERLVDCLAFELKMDPAELRLRNLLRPNQFPYQSKTGWVYDSGDYETTM
RKAMNMIGYEALRAEQKQRRARGELMGIGMSFFTEAVGAGPRKDMDILGLGMADGCELRVHPTGKAVLRLS
VQTQGQGHETTFAQIVAEELGIAPDDIEVVHGDTDQTPFGLGTYGSRSTPVSGGAAALVARKVRDKAKIIASGM
LEVSVADLQWEKGKFHVKGDPSAAVTIADIAMRAHGAGDLPEGIEGGLDAEVCYNPSNLTYPYGAYFCVVDIDP
GTAVVKVRRFLAVDDCGTRINPMIIEGQVHGGIVDGIGMALMEMIAFDEDGNCLGGSLMDYLIPTALEVPHLE
TGHTVTPSPHHPIGAKGIGESATVGSPPAVVNAVVDALAPFGVRHADMPLTPSRVWEAMQGRATPPI
>SEQF5119||SEQF5119.2_00397
MTTIESRPPSPEDLADNAQQPCGHGRMMRKEDPRFIRGRGTYVDDVALPGMLHLAILRSPYAHARIVRIDVTA
AQAHPKVKAVVTGADLAAKGLAWMPTLANDVQAVLATDKTRFQGQEVAFVVAEDRYSARDACELVDVDYEP
RDPVVDARTALDPSAPVIRTDLEGKSDNHIFDWETGDAAATEAVFAKADVVVQQEIVYPRVHPAPMETCGAVA
DLDPVTGKLTLWTTSQAPHAHRTLYALVAGLPEHKIRVISPDIGGGFGNKVPIYPGYVCAIVASLLLDKPVKWME
DRSENLTSTGFARDYIMVGEIAANRDGKILAIRSNVLADHGAFNAQAAPAKYPAGFFGVFTGSYDIEAAYCHMT
AVYTNKAPGGVAYACSFRITEAVYFVERLVDCLAFELKMDPAELRLRNLLRPNQFPYQSKTGWVYDSGDYETTM
RKAMNMIGYEALRAEQKQRRARGELMGIGMSFFTEAVGAGPRKDMDILGLGMADGCELRVHPTGKAVLRLS
VQTQGQGHETTFAQIVAEELGIAPDDIEVVHGDTDQTPFGLGTYGSRSTPVSGGAAALVARKVRDKAKIIASGM
LEVSVADLQWEKGKFHVKGDPSAAVTIADIAMRAHGAGDLPEGIEGGLDAEVCYNPSNLTYPYGAYFCVVDIDP
GTAVVKVRRFLAVDDCGTRINPMIIEGQVHGGIVDGIGMALMEMIAFDEDGNCLGGSLMDYLIPTALEVPHLE
TGHTVTPSPHHPIGAKGIGESATVGSPPAVVNAVVDALAPFGVRHADMPLTPSRVWEAMQGRATPPI
>SEQF5120||SEQF5120.1_00399
MTTIESRPPSPEDLADNAQQPCGHGRMMRKEDPRFIRGRGTYVDDVALPGMLHLAILRSPYAHARIVRIDVTA
AQAHPKVKAVVTGADLAAKGLAWMPTLANDVQAVLATDKTRFQGQEVAFVVAEDRYSARDACELVDVDYEP
RDPVVDARTALDPSAPVIRTDLEGKSDNHIFDWETGDAAATEAVFAKADVVVQQEIVYPRVHPAPMETCGAVA
DLDPVTGKLTLWTTSQAPHAHRTLYALVAGLPEHKIRVISPDIGGGFGNKVPIYPGYVCAIVASLLLDKPVKWME
DRSENLTSTGFARDYIMVGEIAANRDGKILAIRSNVLADHGAFNAQAAPAKYPAGFFGVFTGSYDIEAAYCHMT
AVYTNKAPGGVAYACSFRITEAVYFVERLVDCLAFELKMDPAELRLRNLLRPNQFPYQSKTGWVYDSGDYETTM
RKAMNMIGYEALRAEQKQRRARGELMGIGMSFFTEAVGAGPRKDMDILGLGMADGCELRVHPTGKAVLRLS
VQTQGQGHETTFAQIVAEELGIAPDDIEVVHGDTDQTPFGLGTYGSRSTPVSGGAAALVARKVRDKAKIIASGM
LEVSVADLQWEKGKFHVKGDPSAAVTIADIAMRAHGAGDLPEGIEGGLDAEVCYNPSNLTYPYGAYFCVVDIDP

GTAVVKVRRFLAVDDCGTRINPMIIEGQVHGGIVDGIGMALMEMIAFDEDGNCLGGSLMDYLIPTALEVPHLE
TGHTVTPSPHHPIGAKGIGESATVGSPPAVVNAVVDALAPFGVRHADMPLTPSRVWEAMQGRATPPI
>SEQF5121||SEQF5121.1_00396
MTTIESRPPSPEDLADNAQQPCGHGRMMRKEDPRFIRGRGTYVDDVALPGMLHLAILRSPYAHARIVRIDVTA
AQAHPKVKAVVTGADLAAKGLAWMPTLANDVQAVLATDKTRFQGQEVAFVVAEDRYSARDACELVDVDYEP
RDPVVDARTALDPSAPVIRTDLEGKSDNHIFDWETGDAAATEAVFAKADVVVQQEIVYPRVHPAPMETCGAVA
DLDPVTGKLTLWTTSQAPHAHRTLYALVAGLPEHKIRVISPDIGGGFGNKVPIYPGYVCAIVASLLLDKPVKWME
DRSENLTSTGFARDYIMVGEIAANRDGKILAIRSNVLADHGAFNAQAAPAKYPAGFFGVFTGSYDIEAAYCHMT
AVYTNKAPGGVAYACSFRITEAVYFVERLVDCLAFELKMDPAELRLRNLLRPNQFPYQSKTGWVYDSGDYETTM
RKAMNMIGYEALRAEQKQRRARGELMGIGMSFFTEAVGAGPRKDMDILGLGMADGCELRVHPTGKAVLRLS
VQTQGQGHETTFAQIVAEELGIAPDDIEVVHGDTDQTPFGLGTYGSRSTPVSGGAAALVARKVRDKAKIIASGM
LEVSVADLQWEKGKFHVKGDPSAAVTIADIAMRAHGAGDLPEGIEGGLDAEVCYNPSNLTYPYGAYFCVVDIDP
GTAVVKVRRFLAVDDCGTRINPMIIEGQVHGGIVDGIGMALMEMIAFDEDGNCLGGSLMDYLIPTALEVPHLE
TGHTVTPSPHHPIGAKGIGESATVGSPPAVVNAVVDALAPFGVRHADMPLTPSRVWEAMQGRATPPI
>SEQF5122||SEQF5122.1_00401
MTTIESRPPSPEDLADNAQQPCGHGRMMRKEDPRFIRGRGTYVDDVALPGMLHLAILRSPYAHARIVRIDVTA
AQAHPKVKAVVTGADLAAKGLAWMPTLANDVQAVLATDKTRFQGQEVAFVVAEDRYSARDACELVDVDYEP
RDPVVDARTALDPSAPVIRTDLEGKSDNHIFDWETGDAAATEAVFAKADVVVQQEIVYPRVHPAPMETCGAVA
DLDPVTGKLTLWTTSQAPHAHRTLYALVAGLPEHKIRVISPDIGGGFGNKVPIYPGYVCAIVASLLLDKPVKWME
DRSENLTSTGFARDYIMVGEIAANRDGKILAIRSNVLADHGAFNAQAAPAKYPAGFFGVFTGSYDIEAAYCHMT
AVYTNKAPGGVAYACSFRITEAVYFVERLVDCLAFELKMDPAELRLRNLLRPNQFPYQSKTGWVYDSGDYETTM
RKAMNMIGYEALRAEQKQRRARGELMGIGMSFFTEAVGAGPRKDMDILGLGMADGCELRVHPTGKAVLRLS
VQTQGQGHETTFAQIVAEELGIAPDDIEVVHGDTDQTPFGLGTYGSRSTPVSGGAAALVARKVRDKAKIIASGM
LEVSVADLQWEKGKFHVKGDPSAAVTIADIAMRAHGAGDLPEGIEGGLDAEVCYNPSNLTYPYGAYFCVVDIDP
GTAVVKVRRFLAVDDCGTRINPMIIEGQVHGGIVDGIGMALMEMIAFDEDGNCLGGSLMDYLIPTALEVPHLE
TGHTVTPSPHHPIGAKGIGESATVGSPPAVVNAVVDALAPFGVRHADMPLTPSRVWEAMQGRATPPI
>SEQF5123||SEQF5123.1_00398
MTTIESRPPSPEDLADNAQQPCGHGRMMRKEDPRFIRGRGTYVDDVALPGMLHLAILRSPYAHARIVRIDVTA
AQAHPKVKAVVTGADLAAKGLAWMPTLANDVQAVLATDKTRFQGQEVAFVVAEDRYSARDACELVDVDYEP
RDPVVDARTALDPSAPVIRTDLEGKSDNHIFDWETGDAAATEAVFAKADVVVQQEIVYPRVHPAPMETCGAVA
DLDPVTGKLTLWTTSQAPHAHRTLYALVAGLPEHKIRVISPDIGGGFGNKVPIYPGYVCAIVASLLLDKPVKWME
DRSENLTSTGFARDYIMVGEIAANRDGKILAIRSNVLADHGAFNAQAAPAKYPAGFFGVFTGSYDIEAAYCHMT
AVYTNKAPGGVAYACSFRITEAVYFVERLVDCLAFELKMDPAELRLRNLLRPNQFPYQSKTGWVYDSGDYETTM
RKAMNMIGYEALRAEQKQRRARGELMGIGMSFFTEAVGAGPRKDMDILGLGMADGCELRVHPTGKAVLRLS
VQTQGQGHETTFAQIVAEELGIAPDDIEVVHGDTDQTPFGLGTYGSRSTPVSGGAAALVARKVRDKAKIIASGM
LEVSVADLQWEKGKFHVKGDPSAAVTIADIAMRAHGAGDLPEGIEGGLDAEVCYNPSNLTYPYGAYFCVVDIDP
GTAVVKVRRFLAVDDCGTRINPMIIEGQVHGGIVDGIGMALMEMIAFDEDGNCLGGSLMDYLIPTALEVPHLE
TGHTVTPSPHHPIGAKGIGESATVGSPPAVVNAVVDALAPFGVRHADMPLTPSRVWEAMQGRATPPI
>SEQF5124||SEQF5124.1_00399
MTTIESRPPSPEDLADNAQQPCGHGRMMRKEDPRFIRGRGTYVDDVALPGMLHLAILRSPYAHARIVRIDVTA
AQAHPKVKAVVTGADLAAKGLAWMPTLANDVQAVLATDKTRFQGQEVAFVVAEDRYSARDACELVDVDYEP
RDPVVDARTALDPSAPVIRTDLEGKSDNHIFDWETGDAAATEAVFAKADVVVQQEIVYPRVHPAPMETCGAVA
DLDPVTGKLTLWTTSQAPHAHRTLYALVAGLPEHKIRVISPDIGGGFGNKVPIYPGYVCAIVASLLLDKPVKWME
DRSENLTSTGFARDYIMVGEIAANRDGKILAIRSNVLADHGAFNAQAAPAKYPAGFFGVFTGSYDIEAAYCHMT

AVYTNKAPGGVAYACSFRITEAVYFVERLVDCLAFELKMDPAELRLRNLLRPNQFPYQSKTGWVYDSGDYETTM
RKAMNMIGYEALRAEQKQRRARGELMGIGMSFFTEAVGAGPRKDMDILGLGMADGCELRVHPTGKAVLRLS
VQTQGQGHETTFAQIVAEELGIAPDDIEVVHGDTDQTPFGLGTYGSRSTPVSGGAAALVARKVRDKAKIIASGM
LEVSVADLQWEKGKFHVKGDPSAAVTIADIAMRAHGAGDLPEGIEGGLDAEVCYNPSNLTYPYGAYFCVVDIDP
GTAVVKVRRFLAVDDCGTRINPMIIEGQVHGGIVDGIGMALMEMIAFDEDGNCLGGSLMDYLIPTALEVPHLE
TGHTVTPSPHHPIGAKGIGESATVGSPPAVVNAVVDALAPFGVRHADMPLTPSRVWEAMQGRATPPI
>SEQF5125||SEQF5125.1_00400
MTTIESRPPSPEDLADNAQQPCGHGRMMRKEDPRFIRGRGTYVDDVALPGMLHLAILRSPYAHARIVRIDVTA
AQAHPKVKAVVTGADLAAKGLAWMPTLANDVQAVLATDKTRFQGQEVAFVVAEDRYSARDACELVDVDYEP
RDPVVDARTALDPSAPVIRTDLEGKSDNHIFDWETGDAAATEAVFAKADVVVQQEIVYPRVHPAPMETCGAVA
DLDPVTGKLTLWTTSQAPHAHRTLYALVAGLPEHKIRVISPDIGGGFGNKVPIYPGYVCAIVASLLLDKPVKWME
DRSENLTSTGFARDYIMVGEIAANRDGKILAIRSNVLADHGAFNAQAAPAKYPAGFFGVFTGSYDIEAAYCHMT
AVYTNKAPGGVAYACSFRITEAVYFVERLVDCLAFELKMDPAELRLRNLLRPNQFPYQSKTGWVYDSGDYETTM
RKAMNMIGYEALRAEQKQRRARGELMGIGMSFFTEAVGAGPRKDMDILGLGMADGCELRVHPTGKAVLRLS
VQTQGQGHETTFAQIVAEELGIAPDDIEVVHGDTDQTPFGLGTYGSRSTPVSGGAAALVARKVRDKAKIIASGM
LEVSVADLQWEKGKFHVKGDPSAAVTIADIAMRAHGAGDLPEGIEGGLDAEVCYNPSNLTYPYGAYFCVVDIDP
GTAVVKVRRFLAVDDCGTRINPMIIEGQVHGGIVDGIGMALMEMIAFDEDGNCLGGSLMDYLIPTALEVPHLE
TGHTVTPSPHHPIGAKGIGESATVGSPPAVVNAVVDALAPFGVRHADMPLTPSRVWEAMQGRATPPI
>SEQF5126||SEQF5126.1_00404
MTTIESRPPSPEDLADNAQQPCGHGRMMRKEDPRFIRGRGTYVDDVALPGMLHLAILRSPYAHARIVRIDVTA
AQAHPKVKAVVTGADLAAKGLAWMPTLANDVQAVLATDKTRFQGQEVAFVVAEDRYSARDACELVDVDYEP
RDPVVDARTALDPSAPVIRTDLEGKSDNHIFDWETGDAAATEAVFAKADVVVQQEIVYPRVHPAPMETCGAVA
DLDPVTGKLTLWTTSQAPHAHRTLYALVAGLPEHKIRVISPDIGGGFGNKVPIYPGYVCAIVASLLLDKPVKWME
DRSENLTSTGFARDYIMVGEIAANRDGKILAIRSNVLADHGAFNAQAAPAKYPAGFFGVFTGSYDIEAAYCHMT
AVYTNKAPGGVAYACSFRITEAVYFVERLVDCLAFELKMDPAELRLRNLLRPNQFPYQSKTGWVYDSGDYETTM
RKAMNMIGYEALRAEQKQRRARGELMGIGMSFFTEAVGAGPRKDMDILGLGMADGCELRVHPTGKAVLRLS
VQTQGQGHETTFAQIVAEELGIAPDDIEVVHGDADQTPFGLGTYGSRSTPVSGGAAALVARKVRDKAKIIASGM
LEVSVADLQWEKGKFHVKGDPSAAVTIADIAMRAHGAGDLPEGIEGGLDAEVCYNPSNLTYPYGAYFCVVDIDP
GTAVVKVRRFLAVDDCGTRINPMIIEGQVHGGIVDGIGMALMEMIAFDEDGNCLGGSLMDYLIPTALEVPHLE
TGHTVTPSPHHPIGAKGIGESATVGSPPAVVNAVVDALAPFGVRHADMPLTPSRVWEAMQGRATPPI
>SEQF5127||SEQF5127.1_00398
MTTIESRPPSPEDLADNAQQPCGHGRMMRKEDPRFIRGRGTYVDDVALPGMLHLAILRSPYAHARIVRIDVTA
AQAHPKVKAVVTGADLAAKGLAWMPTLANDVQAVLATDKTRFQGQEVAFVVAEDRYSARDACELVDVDYEP
RDPVVDARTALDPSAPVIRTDLEGKSDNHIFDWETGDAAATEAVFAKADVVVQQEIVYPRVHPAPMETCGAVA
DLDPVTGKLTLWTTSQAPHAHRTLYALVAGLPEHKIRVISPDIGGGFGNKVPIYPGYVCAIVASLLLDKPVKWME
DRSENLTSTGFARDYIMVGEIAANRDGKILAIRSNVLADHGAFNAQAAPAKYPAGFFGVFTGSYDIEAAYCHMT
AVYTNKAPGGVAYACSFRITEAVYFVERLVDCLAFELKMDPAELRLRNLLRPNQFPYQSKTGWVYDSGDYETTM
RKAMNMIGYEALRAEQKQRRARGELMGIGMSFFTEAVGAGPRKDMDILGLGMADGCELRVHPTGKAVLRLS
VQTQGQGHETTFAQIVAEELGIAPDDIEVVHGDTDQTPFGLGTYGSRSTPVSGGAAALVARKVRDKAKIIASGM
LEVSVADLQWEKGKFHVKGDPSAAVTIADIAMRAHGAGDLPEGIEGGLDAEVCYNPSNLTYPYGAYFCVVDIDP
GTAVVKVRRFLAVDDCGTRINPMIIEGQVHGGIVDGIGMALMEMIAFDEDGNCLGGSLMDYLIPTALEVPHLE
TGHTVTPSPHHPIGAKGIGESATVGSPPAVVNAVVDALAPFGVRHADMPLTPSRVWEAMQGRATPPI
>SEQF5128||SEQF5128.1_00398
MTTIESRPPSPEDLADNAQQPCGHGRMMRKEDPRFIRGRGTYVDDVALPGMLHLAILRSPYAHARIVRIDVTA

AQAHPKVKAVVTGADLAAKGLAWMPTLANDVQAVLATDKTRFQGQEVAFVVAEDRYSARDACELVDVDYEP
RDPVVDARTALDPSAPVIRTDLEGKSDNHIFDWETGDAAATEAVFAKADVVVQQEIVYPRVHPAPMETCGAVA
DLDPVTGKLTLWTTSQAPHAHRTLYALVAGLPEHKIRVISPDIGGGFGNKVPIYPGYVCAIVASLLLDKPVKWME
DRSENLTSTGFARDYIMVGEIAANRDGKILAIRSNVLADHGAFNAQAAPAKYPAGFFGVFTGSYDIEAAYCHMT
AVYTNKAPGGVAYACSFRITEAVYFVERLVDCLAFELKMDPAELRLRNLLRPNQFPYQSKTGWVYDSGDYETTM
RKAMNMIGYEALRAEQKQRRARGELMGIGMSFFTEAVGAGPRKDMDILGLGMADGCELRVHPTGKAVLRLS
VQTQGQGHETTFAQIVAEELGIAPDDIEVVHGDTDQTPFGLGTYGSRSTPVSGGAAALVARKVRDKAKIIASGM
LEVSVADLQWEKGKFHVKGDPSAAVTIADIAMRAHGAGDLPEGIEGGLDAEVCYNPSNLTYPYGAYFCVVDIDP
GTAVVKVRRFLAVDDCGTRINPMIIEGQVHGGIVDGIGMALMEMIAFDEDGNCLGGSLMDYLIPTALEVPHLE
TGHTVTPSPHHPIGAKGIGESATVGSPPAVVNAVVDALAPFGVRHADMPLTPSRVWEAMQGRATPPI
>SEQF5129||SEQF5129.1_00395
MTTIESRPPSPEDLADNAQQPCGHGRMMRKEDPRFIRGRGTYVDDVALPGMLHLAILRSPYAHARIVRIDVTA
AQAHPKVKAVVTGADLAAKGLAWMPTLANDVQAVLATDKTRFQGQEVAFVVAEDRYSARDACELVDVDYEP
RDPVVDARTALDPSAPVIRTDLEGKSDNHIFDWETGDAAATEAVFAKADVVVQQEIVYPRVHPAPMETCGAVA
DLDPVTGKLTLWTTSQAPHAHRTLYALVAGLPEHKIRVISPDIGGGFGNKVPIYPGYVCAIVASLLLDKPVKWME
DRSENLTSTGFARDYIMVGEIAANRDGKILAIRSNVLADHGAFNAQAAPAKYPAGFFGVFTGSYDIEAAYCHMT
AVYTNKAPGGVAYACSFRITEAVYFVERLVDCLAFELKMDPAELRLRNLLRPNQFPYQSKTGWVYDSGDYETTM
RKAMNMIGYEALRAEQKQRRARGELMGIGMSFFTEAVGAGPRKDMDILGLGMADGCELRVHPTGKAVLRLS
VQTQGQGHETTFAQIVAEELGIAPDDIEVVHGDTDQTPFGLGTYGSRSTPVSGGAAALVARKVRDKAKIIASGM
LEVSVADLQWEKGKFHVKGDPSAAVTIADIAMRAHGAGDLPEGIEGGLDAEVCYNPSNLTYPYGAYFCVVDIDP
GTAVVKVRRFLAVDDCGTRINPMIIEGQVHGGIVDGIGMALMEMIAFDEDGNCLGGSLMDYLIPTALEVPHLE
TGHTVTPSPHHPIGAKGIGESATVGSPPAVVNAVVDALAPFGVRHADMPLTPSRVWEAMQGRATPPI
>SEQF5130||SEQF5130.1_00397
MTTIESRPPSPEDLADNAQQPCGHGRMMRKEDPRFIRGRGTYVDDVALPGMLHLAILRSPYAHARIVRIDVTA
AQAHPKVKAVVTGADLAAKGLAWMPTLANDVQAVLATDKTRFQGQEVAFVVAEDRYSARDACELVDVDYEP
RDPVVDARTALDPSAPVIRTDLEGKSDNHIFDWETGDAAATEAVFAKADVVVQQEIVYPRVHPAPMETCGAVA
DLDPVTGKLTLWTTSQAPHAHRTLYALVAGLPEHKIRVISPDIGGGFGNKVPIYPGYVCAIVASLLLDKPVKWME
DRSENLTSTGFARDYIMVGEIAANRDGKILAIRSNVLADHGAFNAQAAPAKYPAGFFGVFTGSYDIEAAYCHMT
AVYTNKAPGGVAYACSFRITEAVYFVERLVDCLAFELKMDPAELRLRNLLRPNQFPYQSKTGWVYDSGDYETTM
RKAMNMIGYEALRAEQKQRRARGELMGIGMSFFTEAVGAGPRKDMDILGLGMADGCELRVHPTGKAVLRLS
VQTQGQGHETTFAQIVAEELGIAPDDIEVVHGDTDQTPFGLGTYGSRSTPVSGGAAALVARKVRDKAKIIASGM
LEVSVADLQWEKGKFHVKGDPSAAVTIADIAMRAHGAGDLPEGIEGGLDAEVCYNPSNLTYPYGAYFCVVDIDP
GTAVVKVRRFLAVDDCGTRINPMIIEGQVHGGIVDGIGMALMEMIAFDEDGNCLGGSLMDYLIPTALEVPHLE
TGHTVTPSPHHPIGAKGIGESATVGSPPAVVNAVVDALAPFGVRHADMPLTPSRVWEAMQGRATPPI
>SEQF5225||SEQF5225.1_04814
MTVVTPKFGMGASVLRREDAAFIQGQGRYTDDIQPAGVLHGYVLRSPIAKASFTIGSIEAAKAAPGVHLVLTGG
DLTHLRDLKSGVMQPQPDGTKAPTRDIPILCRDRVNYVGDAVAFVVADSRALAQDAAELIEVDYDGEDAASGT
ATALAEGTPLVWPELGSNRAFTYHMGDKKKTDAAFAGAAHVTRIEFVNNRLVCNYMEPRSAIGEWNVGENRF
VLTTGSQGVHSMQYILASVFKIKKDQLRVITPDVGGGFGPKSFVYREYPLVLEAAKRLGRPVKWAGDRTEHFLTD
AQGRDNAVTAEMALDKDGRFLGMRVDLLANIGAYISQYGPFIPYIGVTMSTGVYDIRALDVSVTGLYTNTCPVD
AYRGAGRPEAAFLLEKLVDACAHDLGLPVEEIRRRNFIRPEQFPYRTQTGRLYDNGEFEGHMDRAIERSQWKAF
PQRLEQSKADGKIRGIGMATYIEACAFPGSEPAFVELNGDGTVTLKIGTQTNGQGHATAYAQFLSEKLNLDIDKI
HVRQGDTDELKAGGGTGGSRSIPLGGVSASRAGEDLANKIKRIAADELEASAGDIELSDGVARIVGTDRSIDFSSI
AKAAKTPDDLKGFGEFVQDECTYPNGTHICEVEIDPDTGATEIVRYTIVDDFGVTVNPILLAGQVHGGVVQGIG

QALTENTIHGEDGQLLTASFMDYAMPRADNFPFFHFETRNVPSTTNALGIKGAGEAGTIGSTPAALNAVTDAL
WRAYGIRHIEMPATPARIWAAIRGASPT
>SEQF5225||SEQF5225.1_03386
MGIEGVGVRVARKEDKRFITGAGRYVDDMVVPGMKHAAFVRSPHAHAQIKKIDVRRAQAMPGVIGVLTGKE
LKADGIGNLICGWMIHSKDGSPMKMGAWSPLAVDKVRYVGDAVVVVVAETKGQARDAAEAVEITYKELKAV
VEATKALEKGAPQIHAEAENNLIFDWEIGDAKATDAAIKAAAHVTRMKIVNNRLVPNAMEPRAALGHYDKAE
DHYTCWTTSQNPHVARLVMSAFYNVAPENKLRVIAPDVGGGFGSKIYIYPEEIVCLWASKKTGVPVKWVADRT
ESFLSDAHGRDHVSTVEMAFDKNNRITGLKVDTIANLGAYMSLFSSCVPTYLYATLLSGQYDIPAIHANVRTVYT
NTAPVDAYRGAGRPEATYLLERTMEAAARELGVSPAELRRKNFITSFPHQTPVIMNYDAGDYGASLDAAMKAS
DYAGFAKRKAAAAKKGLLRGIGMSCYIEACGIAPSAAVGSLGAGVGLWESAEVRVNAVGTIEVLTGSHSHGQG
HETTFAQLVNQRFGVPIDSVSIVHGDTDKVQMGMGTYGSRSGAVGMSAISKALDKVEAKAKKIAAHLLEADEG
DIVIENGALKVAGTDKTVPWFQVALAAYTAHNLPAGMEPGLKETAFYDPSNFTFPAGCYICEVEIEPETGTTEIV
QFVAADDFGNIINPMIVEGQVHGGIAQGIGQALLEGAHYDASGQLLTASYMDYTMPRAGDLPSFKVSTSNTPC
PGNPLGIKGCGEAGAIGSPPAVINAITDAIGIADIAMPASPPTVWAAIRAAKH
>SEQF5226||SEQF5226.1_04814
MTVVTPKFGMGASVLRREDAAFIQGQGRYTDDIQPAGVLHGYVLRSPIAKASFTIGSIEAAKAAPGVHLVLTGG
DLTHLRDLKSGVMQPQPDGTKAPTRDIPILCRDRVNYVGDAVAFVVADSRALAQDAAELIEVDYDGEDAASGT
ATALAEGTPLVWPELGSNRAFTYHMGDKKKTDAAFAGAAHVTRIEFVNNRLVCNYMEPRSAIGEWNVGENRF
VLTTGSQGVHSMQYILASVFKIKKDQLRVITPDVGGGFGPKSFVYREYPLVLEAAKRLGRPVKWAGDRTEHFLTD
AQGRDNAVTAEMALDKDGRFLGMRVDLLANIGAYISQYGPFIPYIGVTMSTGVYDIRALDVSVTGLYTNTCPVD
AYRGAGRPEAAFLLEKLVDACAHDLGLPVEEIRRRNFIRPEQFPYRTQTGRLYDNGEFEGHMDRAIERSQWKAF
PQRLEQSKADGKIRGIGMATYIEACAFPGSEPAFVELNGDGTVTLKIGTQTNGQGHATAYAQFLSEKLNLDIDKI
HVRQGDTDELKAGGGTGGSRSIPLGGVSASRAGEDLANKIKRIAADELEASAGDIELSDGVARIVGTDRSIDFSSI
AKAAKTPDDLKGFGEFVQDECTYPNGTHICEVEIDPDTGATEIVRYTIVDDFGVTVNPILLAGQVHGGVVQGIG
QALTENTIHGEDGQLLTASFMDYAMPRADNFPFFHFETRNVPSTTNALGIKGAGEAGTIGSTPAALNAVTDAL
WRAYGIRHIEMPATPARIWAAIRGASPT
>SEQF5226||SEQF5226.1_03386
MGIEGVGVRVARKEDKRFITGAGRYVDDMVVPGMKHAAFVRSPHAHAQIKKIDVRRAQAMPGVIGVLTGKE
LKADGIGNLICGWMIHSKDGSPMKMGAWSPLAVDKVRYVGDAVVVVVAETKGQARDAAEAVEITYKELKAV
VEATKALEKGAPQIHAEAENNLIFDWEIGDAKATDAAIKAAAHVTRMKIVNNRLVPNAMEPRAALGHYDKAE
DHYTCWTTSQNPHVARLVMSAFYNVAPENKLRVIAPDVGGGFGSKIYIYPEEIVCLWASKKTGVPVKWVADRT
ESFLSDAHGRDHVSTVEMAFDKNNRITGLKVDTIANLGAYMSLFSSCVPTYLYATLLSGQYDIPAIHANVRTVYT
NTAPVDAYRGAGRPEATYLLERTMEAAARELGVSPAELRRKNFITSFPHQTPVIMNYDAGDYGASLDAAMKAS
DYAGFAKRKAAAAKKGLLRGIGMSCYIEACGIAPSAAVGSLGAGVGLWESAEVRVNAVGTIEVLTGSHSHGQG
HETTFAQLVNQRFGVPIDSVSIVHGDTDKVQMGMGTYGSRSGAVGMSAISKALDKVEAKAKKIAAHLLEADEG
DIVIENGALKVAGTDKTVPWFQVALAAYTAHNLPAGMEPGLKETAFYDPSNFTFPAGCYICEVEIEPETGTTEIV
QFVAADDFGNIINPMIVEGQVHGGIAQGIGQALLEGAHYDASGQLLTASYMDYTMPRAGDLPSFKVSTSNTPC
PGNPLGIKGCGEAGAIGSPPAVINAITDAIGIADIAMPASPPTVWAAIRAAKH
>SEQF5227||SEQF5227.1_04801
MTVVTPKFGMGASVLRREDAAFIQGQGRYTDDIQPAGVLHGYVLRSPIAKASFTIGSIEAAKAAPGVHLVLTGG
DLTHLRDLKSGVMQPQPDGTKAPTRDIPILCRDRVNYVGDAVAFVVADSRALAQDAAELIEVDYDGEDAASGT
ATALAEGTPLVWPELGSNRAFTYHMGDKKKTDAAFAGAAHVTRIEFVNNRLVCNYMEPRSAIGEWNVGENRF
VLTTGSQGVHSMQYILASVFKIKKDQLRVITPDVGGGFGPKSFVYREYPLVLEAAKRLGRPVKWAGDRTEHFLTD
AQGRDNAVTAEMALDKDGRFLGMRVDLLANIGAYISQYGPFIPYIGVTMSTGVYDIRALDVSVTGLYTNTCPVD

AYRGAGRPEAAFLLEKLVDACAHDLGLPVEEIRRRNFIRPEQFPYRTQTGRLYDNGEFEGHMDRAIERSQWKAF
PQRLEQSKADGKIRGIGMATYIEACAFPGSEPAFVELNGDGTVTLKIGTQTNGQGHATAYAQFLSEKLNLDIDKI
HVRQGDTDELKAGGGTGGSRSIPLGGVSASRAGEDLANKIKRIAADELEASAGDIELSDGVARIVGTDRSIDFSSI
AKAAKTPDDLKGFGEFVQDECTYPNGTHICEVEIDPDTGATEIVRYTIVDDFGVTVNPILLAGQVHGGVVQGIG
QALTENTIHGEDGQLLTASFMDYAMPRADNFPFFHFETRNVPSTTNALGIKGAGEAGTIGSTPAALNAVTDAL
WRAYGIRHIEMPATPARIWAAIRGASPT
>SEQF5227||SEQF5227.1_03380
MGIEGVGVRVARKEDKRFITGAGRYVDDMVVPGMKHAAFVRSPHAHAQIKKIDVRRAQAMPGVIGVLTGKE
LKADGIGNLICGWMIHSKDGSPMKMGAWSPLAVDKVRYVGDAVVVVVAETKGQARDAAEAVEITYKELKAV
VEATKALEKGAPQIHAEAENNLIFDWEIGDAKATDAAIKAAAHVTRMKIVNNRLVPNAMEPRAALGHYDKAE
DHYTCWTTSQNPHVARLVMSAFYNVAPENKLRVIAPDVGGGFGSKIYIYPEEIVCLWASKKTGVPVKWVADRT
ESFLSDAHGRDHVSTVEMAFDKNNRITGLKVDTIANLGAYMSLFSSCVPTYLYATLLSGQYDIPAIHANVRTVYT
NTAPVDAYRGAGRPEATYLLERTMEAAARELGVSPAELRRKNFITSFPHQTPVIMNYDAGDYGASLDAAMKAS
DYAGFAKRKAAAAKKGLLRGIGMSCYIEACGIAPSAAVGSLGAGVGLWESAEVRVNAVGTIEVLTGSHSHGQG
HETTFAQLVNQRFGVPIDSVSIVHGDTDKVQMGMGTYGSRSGAVGMSAISKALDKVEAKAKKIAAHLLEADEG
DIVIENGALKVAGTDKTVPWFQVALAAYTAHNLPAGMEPGLKETAFYDPSNFTFPAGCYICEVEIEPETGTTEIV
QFVAADDFGNIINPMIVEGQVHGGIAQGIGQALLEGAHYDASGQLLTASYMDYTMPRAGDLPSFKVSTSNTPC
PGNPLGIKGCGEAGAIGSPPAVINAITDAIGIADIAMPASPPTVWAAIRAAKH
>SEQF5228||SEQF5228.1_04814
MTVVTPKFGMGASVLRREDAAFIQGQGRYTDDIQPAGVLHGYVLRSPIAKASFTIGSIEAAKAAPGVHLVLTGG
DLTHLRDLKSGVMQPQPDGTKAPTRDIPILCRDRVNYVGDAVAFVVADSRALAQDAAELIEVDYDGEDAASGT
ATALAEGTPLVWPELGSNRAFTYHMGDKKKTDAAFAGAAHVTRIEFVNNRLVCNYMEPRSAIGEWNVGENRF
VLTTGSQGVHSMQYILASVFKIKKDQLRVITPDVGGGFGPKSFVYREYPLVLEAAKRLGRPVKWAGDRTEHFLTD
AQGRDNAVTAEMALDKDGRFLGMRVDLLANIGAYISQYGPFIPYIGVTMSTGVYDIRALDVSVTGLYTNTCPVD
AYRGAGRPEAAFLLEKLVDACAHDLGLPVEEIRRRNFIRPEQFPYRTQTGRLYDNGEFEGHMDRAIERSQWKAF
PQRLEQSKADGKIRGIGMATYIEACAFPGSEPAFVELNGDGTVTLKIGTQTNGQGHATAYAQFLSEKLNLDIDKI
HVRQGDTDELKAGGGTGGSRSIPLGGVSASRAGEDLANKIKRIAADELEASAGDIELSDGVARIVGTDRSIDFSSI
AKAAKTPDDLKGFGEFVQDECTYPNGTHICEVEIDPDTGATEIVRYTIVDDFGVTVNPILLAGQVHGGVVQGIG
QALTENTIHGEDGQLLTASFMDYAMPRADNFPFFHFETRNVPSTTNALGIKGAGEAGTIGSTPAALNAVTDAL
WRAYGIRHIEMPATPARIWAAIRGASPT
>SEQF5228||SEQF5228.1_03386
MGIEGVGVRVARKEDKRFITGAGRYVDDMVVPGMKHAAFVRSPHAHAQIKKIDVRRAQAMPGVIGVLTGKE
LKADGIGNLICGWMIHSKDGSPMKMGAWSPLAVDKVRYVGDAVVVVVAETKGQARDAAEAVEITYKELKAV
VEATKALEKGAPQIHAEAENNLIFDWEIGDAKATDAAIKAAAHVTRMKIVNNRLVPNAMEPRAALGHYDKAE
DHYTCWTTSQNPHVARLVMSAFYNVAPENKLRVIAPDVGGGFGSKIYIYPEEIVCLWASKKTGVPVKWVADRT
ESFLSDAHGRDHVSTVEMAFDKNNRITGLKVDTIANLGAYMSLFSSCVPTYLYATLLSGQYDIPAIHANVRTVYT
NTAPVDAYRGAGRPEATYLLERTMEAAARELGVSPAELRRKNFITSFPHQTPVIMNYDAGDYGASLDAAMKAS
DYAGFAKRKAAAAKKGLLRGIGMSCYIEACGIAPSAAVGSLGAGVGLWESAEVRVNAVGTIEVLTGSHSHGQG
HETTFAQLVNQRFGVPIDSVSIVHGDTDKVQMGMGTYGSRSGAVGMSAISKALDKVEAKAKKIAAHLLEADEG
DIVIENGALKVAGTDKTVPWFQVALAAYTAHNLPAGMEPGLKETAFYDPSNFTFPAGCYICEVEIEPETGTTEIV
QFVAADDFGNIINPMIVEGQVHGGIAQGIGQALLEGAHYDASGQLLTASYMDYTMPRAGDLPSFKVSTSNTPC
PGNPLGIKGCGEAGAIGSPPAVINAITDAIGIADIAMPASPPTVWAAIRAAKH
>SEQF5229||SEQF5229.1_04814
MTVVTPKFGMGASVLRREDAAFIQGQGRYTDDIQPAGVLHGYVLRSPIAKASFTIGSIEAAKAAPGVHLVLTGG

DLTHLRDLKSGVMQPQPDGTKAPTRDIPILCRDRVNYVGDAVAFVVADSRALAQDAAELIEVDYDGEDAASGT
ATALAEGTPLVWPELGSNRAFTYHMGDKKKTDAAFAGAAHVTRIEFVNNRLVCNYMEPRSAIGEWNVGENRF
VLTTGSQGVHSMQYILASVFKIKKDQLRVITPDVGGGFGPKSFVYREYPLVLEAAKRLGRPVKWAGDRTEHFLTD
AQGRDNAVTAEMALDKDGRFLGMRVDLLANIGAYISQYGPFIPYIGVTMSTGVYDIRALDVSVTGLYTNTCPVD
AYRGAGRPEAAFLLEKLVDACAHDLGLPVEEIRRRNFIRPEQFPYRTQTGRLYDNGEFEGHMDRAIERSQWKAF
PQRLEQSKADGKIRGIGMATYIEACAFPGSEPAFVELNGDGTVTLKIGTQTNGQGHATAYAQFLSEKLNLDIDKI
HVRQGDTDELKAGGGTGGSRSIPLGGVSASRAGEDLANKIKRIAADELEASAGDIELSDGVARIVGTDRSIDFSSI
AKAAKTPDDLKGFGEFVQDECTYPNGTHICEVEIDPDTGATEIVRYTIVDDFGVTVNPILLAGQVHGGVVQGIG
QALTENTIHGEDGQLLTASFMDYAMPRADNFPFFHFETRNVPSTTNALGIKGAGEAGTIGSTPAALNAVTDAL
WRAYGIRHIEMPATPARIWAAIRGASPT
>SEQF5229||SEQF5229.1_03387
MGIEGVGVRVARKEDKRFITGAGRYVDDMVVPGMKHAAFVRSPHAHAQIKKIDVRRAQAMPGVIGVLTGKE
LKADGIGNLICGWMIHSKDGSPMKMGAWSPLAVDKVRYVGDAVVVVVAETKGQARDAAEAVEITYKELKAV
VEATKALEKGAPQIHAEAENNLIFDWEIGDAKATDAAIKAAAHVTRMKIVNNRLVPNAMEPRAALGHYDKAE
DHYTCWTTSQNPHVARLVMSAFYNVAPENKLRVIAPDVGGGFGSKIYIYPEEIVCLWASKKTGVPVKWVADRT
ESFLSDAHGRDHVSTVEMAFDKNNRITGLKVDTIANLGAYMSLFSSCVPTYLYATLLSGQYDIPAIHANVRTVYT
NTAPVDAYRGAGRPEATYLLERTMEAAARELGVSPAELRRKNFITSFPHQTPVIMNYDAGDYGASLDAAMKAS
DYAGFAKRKAAAAKKGLLRGIGMSCYIEACGIAPSAAVGSLGAGVGLWESAEVRVNAVGTIEVLTGSHSHGQG
HETTFAQLVNQRFGVPIDSVSIVHGDTDKVQMGMGTYGSRSGAVGMSAISKALDKVEAKAKKIAAHLLEADEG
DIVIENGALKVAGTDKTVPWFQVALAAYTAHNLPAGMEPGLKETAFYDPSNFTFPAGCYICEVEIEPETGTTEIV
QFVAADDFGNIINPMIVEGQVHGGIAQGIGQALLEGAHYDASGQLLTASYMDYTMPRAGDLPSFKVSTSNTPC
PGNPLGIKGCGEAGAIGSPPAVINAITDAIGIADIAMPASPPTVWAAIRAAKH
>SEQF5230||SEQF5230.1_06630
MGIEGVGARVARKEDKRFITGAGRYVDDMVVPGMKHAAFVRSPHAHAQIKKIDVRRAQAMPGVIGVLTGKE
LKADGIGNLICGWMIHSKDGSPMKMGAWSPLAVDKVRYVGDAVVVVVAETKGQARDAAEAVEITYKELKAV
VEATKALEKGAPQIHAEAENNLIFDWEIGDAKATDAAIKAAAHVTRMKIVNNRLVPNAMEPRAALGHYDKAE
DHYTCWTTSQNPHVARLVMSAFYNVAPENKLRVIAPDVGGGFGSKIYIYPEEIVCLWASKKTGVPVKWVADRT
ESFLSDAHGRDHVSTVEMAFDKNNRITGLKVDTIANLGAYMSLFSSCVPTYLYATLLSGQYDIPAIHANVRTVYT
NTAPVDAYRGAGRPEATYLLERTMEAAARELRVSPAELRRENFITSFPHQTPVIMNYDAGDYGASLDAAMKAS
DYAGFAKRKAAAAKKGLLRGIGMSCYIEACGIAPSAAVGSLGAGVGLWESAEVRVNAVGTIEVLTGSHSHGQG
HETTFAQLVNQRFGVPIDSVSIVHGDTDKVQMGMGTYGSRSGAVGMSAISKALDKVEAKAKKIAAHLLEADEG
DIVIENGALKVAGTDKNVPWFQVALAAYTAHNLPAGMEPGLKETAFYDPSNFTFPAGCYICEVEIEPETGTTEIV
QFVAADDFGNIINPMIVEGQVHGGIAQGIGQALLEGAHYDASGQLLTASYMDYTMPRAGDLPSFKVSTSNTPC
PGNPLGIKGCGEAGAIGSPPAVINAITDAIGIADIAMPASPPTVWAAIRAAKH
>SEQF5230||SEQF5230.1_04173
MTVVTPKFGMGASVLRREDAAFIQGQGRYTDDIQPAGVLHGYVLRSPIAKASFTIGSIEAAKAAPGVHLVLTGG
DLTHLHDLKSGVMQPQPDGTKAPTRDIPILCRDRVNYVGDAVAFVVADSRALAQDAAELIEVDYDGEDAASGT
ATALAEGTPLVWPELGSNRAFTYHMGDKKKTDAAFAGAAHVTRIEFVNNRLVCNYMEPRSAIGEWNVGENRF
VLTTGSQGVHSMQYILASVFKIKKDQLRVITPDVGGGFGPKSFVYREYPLVLEAAKRLGRPVKWAGDRTEHFLTD
AQGRDNAVTAEMALDKDGRFLGMRVDLLANIGAYISQYGPFIPYIGVTMSTGVYDIRALDVSVTGLYTNTCPVD
AYRGAGRPEAAFLLEKLVDACAHDLGLPVEEIRRRNFIRPEQFPYRTQTGRLYDNGEFEGHMDRAIERSQWKAF
PQRLEQSKADGKIRGIGMATYIEACAFPGSEPAFVELNGDGTVTLKIGTQTNGQGHATAYAQFLSEKLNLDIDKI
HVRQGDTDELKAGGGTGGSRSIPLGGVSASRAGEDLANKIKRIAADELEASAGDIELSDGVARIVGTDRSIDFSSI
AKAAKTPDDLKGFGEFVQDECTYPNGTHICEVEIDPDTGATEIVRYTIVDDFGVTVNPILLAGQVHGGVVQGIG

QALTENTIHGEDGQLLTASFMDYAMPRADNFPFFHFETRNVPSTTNALGIKGAGEAGTIGSTPAALNAVTDAL
WRAYGIRHIEMPATPARIWAAIRGASPT
>SEQF5231||SEQF5231.1_00672
MTVVTPKFGMGASVLRREDAAFIQGQGRYTDDIQPAGVLHGYVLRSPIAKASFTIGSIEAAKAAPGVHLVLTGG
DLTHLRDLKSGVMQPQPDGTKAPTRDIPILCRDRVNYVGDAVAFVVADSRALAQDAAELIEVDYDGEDAASGT
ATALAEGTPLVWPELGSNRAFTYHMGDKKKTDAAFAGAAHVTRIEFVNNRLVCNYMEPRSAIGEWNVGENRF
VLTTGSQGVHSMQYILASVFKIKKDQLRVITPDVGGGFGPKSFVYREYPLVLEAAKRLGRPVKWAGDRTEHFLTD
AQGRDNAVTAEMALDKDGRFLGMRVDLLANIGAYISQYGPFIPYIGVTMSTGVYDIRALDVSVTGLYTNTCPVD
AYRGAGRPEAAFLLEKLVDACAHDLGLPVEEIRRRNFIRPEQFPYRTQTGRLYDNGEFEGHMDRAIERSQWKAF
PQRLEQSKADGKIRGIGMATYIEACAFPGSEPAFVELNGDGTVTLKIGTQTNGQGHATAYAQFLSEKLNLDIDKI
HVRQGDTDELKAGGGTGGSRSIPLGGVSASRAGEDLANKIKRIAADELEASAGDIELSDGVARIVGTDRSIDFSSI
AKAAKTPDDLKGFGEFVQDECTYPNGTHICEVEIDPDTGATEIVRYTIVDDFGVTVNPILLAGQVHGGVVQGIG
QALTENTIHGEDGQLLTASFMDYAMPRADNFPFFHFETRNVPSTTNALGIKGAGEAGTIGSTPAALNAVTDAL
WRAYGIRHIEMPATPARIWAAIRGASPT
>SEQF5231||SEQF5231.1_01247
MGIEGVGVRVARKEDKRFITGAGRYVDDMVVPGMKHAAFVRSPHAHAQIKKIDVRRAQAMPGVIGVLTGKE
LKADGIGNLICGWMIHSKDGSPMKMGAWSPLAVDKVRYVGDAVVVVVAETKGQARDAAEAVEITYKELKAV
VEATKALEKGAPQIHAEAENNLIFDWEIGDAKATDAAIKAAAHVTRMKIVNNRLVPNAMEPRAALGHYDKAE
DHYTCWTTSQNPHVARLVMSAFYNVAPENKLRVIAPDVGGGFGSKIYIYPEEIVCLWASKKTGVPVKWVADRT
ESFLSDAHGRDHVSTVEMAFDKNNRITGLKVDTIANLGAYMSLFSSCVPTYLYATLLSGQYDIPAIHANVRTVYT
NTAPVDAYRGAGRPEATYLLERTMEAAARELGVSPAELRRKNFITSFPHQTPVIMNYDAGDYGASLDAAMKAS
DYAGFAKRKAAAAKKGLLRGIGMSCYIEACGIAPSAAVGSLGAGVGLWESAEVRVNAVGTIEVLTGSHSHGQG
HETTFAQLVNQRFGVPIDSVSIVHGDTDKVQMGMGTYGSRSGAVGMSAISKALDKVEAKAKKIAAHLLEADEG
DIVIENGALKVAGTDKTVPWFQVALAAYTAHNLPAGMEPGLKETAFYDPSNFTFPAGCYICEVEIEPETGTTEIV
QFVAADDFGNIINPMIVEGQVHGGIAQGIGQALLEGAHYDASGQLLTASYMDYTMPRAGDLPSFKVSTSNTPC
PGNPLGIKGCGEAGAIGSPPAVINAITDAIGIADIAMPASPPTVWAAIRAAKH
>SEQF5232||SEQF5232.1_01031
MGIEGVGVRVARKEDKRFITGAGRYVDDMVVPGMKHAAFVRSPHAHAQIKKIDVRRAQAMPGVIGVLTGKE
LKADGIGNLICGWMIHSKDGSPMKMGAWSPLAVDKVRYVGDAVVVVVAETKGQARDAAEAVEITYKELKAV
VEATKALEKGAPQIHAEAENNLIFDWEIGDAKATDAAIKAAAHVTRMKIVNNRLVPNAMEPRAALGHYDKAE
DHYTCWTTSQNPHVARLVMSAFYNVAPENKLRVIAPDVGGGFGSKIYIYPEEIVCLWASKKTGVPVKWVADRT
ESFLSDAHGRDHVSTVEMAFDKNNRITGLKVDTIANLGAYMSLFSSCVPTYLYATLLSGQYDIPAIHANVRTVYT
NTAPVDAYRGAGRPEATYLLERTMEAAARELGVSPAELRRKNFITSFPHQTPVIMNYDAGDYGASLDAAMKAS
DYAGFAKRKAAAAKKGLLRGIGMSCYIEACGIAPSAAVGSLGAGVGLWESAEVRVNAVGTIEVLTGSHSHGQG
HETTFAQLVNQRFGVPIDSVSIVHGDTDKVQMGMGTYGSRSGAVGMSAISKALDKVEAKAKKIAAHLLEADEG
DIVIENGALKVAGTDKTVPWFQVALAAYTAHNLPAGMEPGLKETAFYDPSNFTFPAGCYICEVEIEPETGTTEIV
QFVAADDFGNIINPMIVEGQVHGGIAQGIGQALLEGAHYDASGQLLTASYMDYTMPRAGDLPSFKVSTSNTPC
PGNPLGIKGCGEAGAIGSPPAVINAITDAIGIADIAMPASPPTVWAAIRAAKH
>SEQF5232||SEQF5232.1_04613
MTVVTPKFGMGASVLRREDAAFIQGQGRYTDDIQPAGVLHGYVLRSPIAKASFTIGSIEAAKAAPGVHLVLTGG
DLTHLRDLKSGVMQPQPDGTKAPTRDIPILCRDRVNYVGDAVAFVVADSRALAQDAAELIEVDYDGEDAASGT
ATALAEGTPLVWPELGSNRAFTYHMGDKKKTDAAFAGAAHVTRIEFVNNRLVCNYMEPRSAIGEWNVGENRF
VLTTGSQGVHSMQYILASVFKIKKDQLRVITPDVGGGFGPKSFVYREYPLVLEAAKRLGRPVKWAGDRTEHFLTD
AQGRDNAVTAEMALDKDGRFLGMRVDLLANIGAYISQYGPFIPYIGVTMSTGVYDIRALDVSVTGLYTNTCPVD

AYRGAGRPEAAFLLEKLVDACAHDLGLPVEEIRRRNFIRPEQFPYRTQTGRLYDNGEFEGHMDRAIERSQWKAF
PQRLEQSKADGKIRGIGMATYIEACAFPGSEPAFVELNGDGTVTLKIGTQTNGQGHATAYAQFLSEKLNLDIDKI
HVRQGDTDELKAGGGTGGSRSIPLGGVSASRAGEDLANKIKRIAADELEASAGDIELSDGVARIVGTDRSIDFSSI
AKAAKTPDDLKGFGEFVQDECTYPNGTHICEVEIDPDTGATEIVRYTIVDDFGVTVNPILLAGQVHGGVVQGIG
QALTENTIHGEDGQLLTASFMDYAMPRADNFPFFHFETRNVPSTTNALGIKGAGEAGTIGSTPAALNAVTDAL
WRAYGIRHIEMPATPARIWAAIRGASPT
>SEQF5233||SEQF5233.1_00672
MTVVTPKFGMGASVLRREDAAFIQGQGRYTDDIQPAGVLHGYVLRSPIAKASFTIGSIEAAKAAPGVHLVLTGG
DLTHLRDLKSGVMQPQPDGTKAPTRDIPILCRDRVNYVGDAVAFVVADSRALAQDAAELIEVDYDGEDAASGT
ATALAEGTPLVWPELGSNRAFTYHMGDKKKTDAAFAGAAHVTRIEFVNNRLVCNYMEPRSAIGEWNVGENRF
VLTTGSQGVHSMQYILASVFKIKKDQLRVITPDVGGGFGPKSFVYREYPLVLEAAKRLGRPVKWAGDRTEHFLTD
AQGRDNAVTAEMALDKDGRFLGMRVDLLANIGAYISQYGPFIPYIGVTMSTGVYDIRALDVSVTGLYTNTCPVD
AYRGAGRPEAAFLLEKLVDACAHDLGLPVEEIRRRNFIRPEQFPYRTQTGRLYDNGEFEGHMDRAIERSQWKAF
PQRLEQSKADGKIRGIGMATYIEACAFPGSEPAFVELNGDGTVTLKIGTQTNGQGHATAYAQFLSEKLNLDIDKI
HVRQGDTDELKAGGGTGGSRSIPLGGVSASRAGEDLANKIKRIAADELEASAGDIELSDGVARIVGTDRSIDFSSI
AKAAKTPDDLKGFGEFVQDECTYPNGTHICEVEIDPDTGATEIVRYTIVDDFGVTVNPILLAGQVHGGVVQGIG
QALTENTIHGEDGQLLTASFMDYAMPRADNFPFFHFETRNVPSTTNALGIKGAGEAGTIGSTPAALNAVTDAL
WRAYGIRHIEMPATPARIWAAIRGASPT
>SEQF5233||SEQF5233.1_00951
MGIEGVGVRVARKEDKRFITGAGRYVDDMVVPGMKHAAFVRSPHAHAQIKKIDVRRAQAMPGVIGVLTGKE
LKADGIGNLICGWMIHSKDGSPMKMGAWSPLAVDKVRYVGDAVVVVVAETKGQARDAAEAVEITYKELKAV
VEATKALEKGAPQIHAEAENNLIFDWEIGDAKATDAAIKAAAHVTRMKIVNNRLVPNAMEPRAALGHYDKAE
DHYTCWTTSQNPHVARLVMSAFYNVAPENKLRVIAPDVGGGFGSKIYIYPEEIVCLWASKKTGVPVKWVADRT
ESFLSDAHGRDHVSTVEMAFDKNNRITGLKVDTIANLGAYMSLFSSCVPTYLYATLLSGQYDIPAIHANVRTVYT
NTAPVDAYRGAGRPEATYLLERTMEAAARELGVSPAELRRKNFITSFPHQTPVIMNYDAGDYGASLDAAMKAS
DYAGFAKRKAAAAKKGLLRGIGMSCYIEACGIAPSAAVGSLGAGVGLWESAEVRVNAVGTIEVLTGSHSHGQG
HETTFAQLVNQRFGVPIDSVSIVHGDTDKVQMGMGTYGSRSGAVGMSAISKALDKVEAKAKKIAAHLLEADEG
DIVIENGALKVAGTDKTVPWFQVALAAYTAHNLPAGMEPGLKETAFYDPSNFTFPAGCYICEVEIEPETGTTEIV
QFVAADDFGNIINPMIVEGQVHGGIAQGIGQALLEGAHYDASGQLLTASYMDYTMPRAGDLPSFKVSTSNTPC
PGNPLGIKGCGEAGAIGSPPAVINAITDAIGIADIAMPASPPTVWAAIRAAKH
>SEQF5234||SEQF5234.1_05921
MGIEGVGVRVARKEDKRFITGAGRYVDDMVVPGMKHAAFVRSPHAHAQIKKIDVRRAQAMPGVIGVLTGKE
LKADGIGNLICGWMIHSKDGSPMKMGAWSPLAVDKVRYVGDAVVVVVAETKGQARDAAEAVEITYKELKAV
VEATKALEKGAPQIHAEAENNLIFDWEIGDAKATDAAIKAAAHVTRMKIVNNRLVPNAMEPRAALGHYDKAE
DHYTCWTTSQNPHVARLVMSAFYNVAPENKLRVIAPDVGGGFGSKIYIYPEEIVCLWASKKTGVPVKWVADRT
ESFLSDAHGRDHVSTVEMAFDKNNRITGLKVDTIANLGAYMSLFSSCVPTYLYATLLSGQYDIPAIHANVRTVYT
NTAPVDAYRGAGRPEATYLLERTMEAAARELGVSPAELRRKNFITSFPHQTPVIMNYDAGDYGASLDAAMKAS
DYAGFAKRKAAAAKKGLLRGIGMSCYIEACGIAPSAAVGSLGAGVGLWESAEVRVNAVGTIEVLTGSHSHGQG
HETTFAQLVNQRFGVPIDSVSIVHGDTDKVQMGMGTYGSRSGAVGMSAISKALDKVEAKAKKIAAHLLEADEG
DIVIENGALKVAGTDKTVPWFQVALAAYTAHNLPAGMEPGLKETAFYDPSNFTFPAGCYICEVEIEPETGTTEIV
QFVAADDFGNIINPMIVEGQVHGGIAQGIGQALLEGAHYDASGQLLTASYMDYTMPRAGDLPSFKVSTSNTPC
PGNPLGIKGCGEAGAIGSPPAVINAITDAIGIADIAMPASPPTVWAAIRAAKH
>SEQF5234||SEQF5234.1_02268
MTVVTPKFGMGASVLRREDAAFIQGQGRYTDDIQPAGVLHGYVLRSPIAKASFTIGSIEAAKAAPGVHLVLTGG

DLTHLRDLKSGVMQPQPDGTKAPTRDIPILCRDRVNYVGDAVAFVVADSRALAQDAAELIEVDYDGEDAASGT
ATALAEGTPLVWPELGSNRAFTYHMGDKKKTDAAFAGAAHVTRIEFVNNRLVCNYMEPRSAIGEWNVGENRF
VLTTGSQGVHSMQYILASVFKIKKDQLRVITPDVGGGFGPKSFVYREYPLVLEAAKRLGRPVKWAGDRTEHFLTD
AQGRDNAVTAEMALDKDGRFLGMRVDLLANIGAYISQYGPFIPYIGVTMSTGVYDIRALDVSVTGLYTNTCPVD
AYRGAGRPEAAFLLEKLVDACAHDLGLPVEEIRRRNFIRPEQFPYRTQTGRLYDNGEFEGHMDRAIERSQWKAF
PQRLEQSKADGKIRGIGMATYIEACAFPGSEPAFVELNGDGTVTLKIGTQTNGQGHATAYAQFLSEKLNLDIDKI
HVRQGDTDELKAGGGTGGSRSIPLGGVSASRAGEDLANKIKRIAADELEASAGDIELSDGVARIVGTDRSIDFSSI
AKAAKTPDDLKGFGEFVQDECTYPNGTHICEVEIDPDTGATEIVRYTIVDDFGVTVNPILLAGQVHGGVVQGIG
QALTENTIHGEDGQLLTASFMDYAMPRADNFPFFHFETRNVPSTTNALGIKGAGEAGTIGSTPAALNAVTDAL
WRAYGIRHIEMPATPARIWAAIRGASPT
>SEQF5235||SEQF5235.1_00955
MTVVTPKFGMGASVLRREDAAFIQGQGRYTDDIQPAGVLHGYVLRSPIAKASFTIGSIEAAKAAPGVHLVLTGG
DLTHLRDLKSGVMQPQPDGTKAPTRDIPILCRDRVNYVGDAVAFVVADSRALAQDAAELIEVDYDGEDAASGT
ATALAEGTPLVWPELGSNRAFTYHMGDKKKTDAAFAGAAHVTRIEFVNNRLVCNYMEPRSAIGEWNVGENRF
VLTTGSQGVHSMQYILASVFKIKKDQLRVITPDVGGGFGPKSFVYREYPLVLEAAKRLGRPVKWAGDRTEHFLTD
AQGRDNAVTAEMALDKDGRFLGMRVDLLANIGAYISQYGPFIPYIGVTMSTGVYDIRALDVSVTGLYTNTCPVD
AYRGAGRPEAAFLLEKLVDACAHDLGLPVEEIRRRNFIRPEQFPYRTQTGRLYDNGEFEGHMDRAIERSQWKAF
PQRLEQSKADGKIRGIGMATYIEACAFPGSEPAFVELNGDGTVTLKIGTQTNGQGHATAYAQFLSEKLNLDIDKI
HVRQGDTDELKAGGGTGGSRSIPLGGVSASRAGEDLANKIKRIAADELEASAGDIELSDGVARIVGTDRSIDFSSI
AKAAKTPDDLKGFGEFVQDECTYPNGTHICEVEIDPDTGATEIVRYTIVDDFGVTVNPILLAGQVHGGVVQGIG
QALTENTIHGEDGQLLTASFMDYAMPRADNFPFFHFETRNVPSTTNALGIKGAGEAGTIGSTPAALNAVTDAL
WRAYGIRHIEMPATPARIWAAIRGASPT
>SEQF5235||SEQF5235.1_02385
MGIEGVGVRVARKEDKRFITGAGRYVDDMVVPGMKHAAFVRSPHAHAQIKKIDVRRAQAMPGVIGVLTGKE
LKADGIGNLICGWMIHSKDGSPMKMGAWSPLAVDKVRYVGDAVVVVVAETKGQARDAAEAVEITYKELKAV
VEATKALEKGAPQIHAEAENNLIFDWEIGDAKATDAAIKAAAHVTRMKIVNNRLVPNAMEPRAALGHYDKAE
DHYTCWTTSQNPHVARLVMSAFYNVAPENKLRVIAPDVGGGFGSKIYIYPEEIVCLWASKKTGVPVKWVADRT
ESFLSDAHGRDHVSTVEMAFDKNNRITGLKVDTIANLGAYMSLFSSCVPTYLYATLLSGQYDIPAIHANVRTVYT
NTAPVDAYRGAGRPEATYLLERTMEAAARELGVSPAELRRKNFITSFPHQTPVIMNYDAGDYGASLDAAMKAS
DYAGFAKRKAAAAKKGLLRGIGMSCYIEACGIAPSAAVGSLGAGVGLWESAEVRVNAVGTIEVLTGSHSHGQG
HETTFAQLVNQRFGVPIDSVSIVHGDTDKVQMGMGTYGSRSGAVGMSAISKALDKVEAKAKKIAAHLLEADEG
DIVIENGALKVAGTDKTVPWFQVALAAYTAHNLPAGMEPGLKETAFYDPSNFTFPAGCYICEVEIEPETGTTEIV
QFVAADDFGNIINPMIVEGQVHGGIAQGIGQALLEGAHYDASGQLLTASYMDYTMPRAGDLPSFKVSTSNTPC
PGNPLGIKGCGEAGAIGSPPAVINAITDAIGIADIAMPASPPTVWAAIRAAKH
>SEQF5391||SEQF5391.1_03889
MTTANADTHGRPGKPQGVGARVPRKEDARHLHGKGNFVADMAMPGLCEVAFLRSPLAHARIASVRVAEQIA
DKAFVRQAMADARDIVADSTLPSYQVSAQPPLASGKVRFVGEPVAMVFAPTRAEAEDYAEMIEVDYDDLPVY
ADVVSAQAAQGDFVHEQWRDNVFVTLNVDKQFDELAAQADVVVRRKIDLSRQCMVPMEGKAVLAYWDH
QADQLVVVSATQVPHMIRSVLAQCLDLEQGRVRVVSPDVGGAFGYKCVLQQEELCVAWLAKTFKKPFRFIEDR
REHLTAGANSREHHYEMVAYADRRGKLLALDAKITIDGGAYSVWPFTIGLEPGQAIGNLPGPYAFKGYRCVTRA
VATNKPGFVPYRGVARTGVCFAMELTMDAIAREVGREPWEIRQENLVQGEQMPYVNVTGKHLDSGDYPASL
QQAMDMIGVDAVRERQRQGEADGRLIGVGLATYTEQAAHGTSVFAAWGTPVIPGFDQATARVTPDGGLELR
VGVHSHGQGMETTFAQIANEILGVDVASIKLLHGDTGQTPFSTGTYASRSLVMSGGAVSQACKRLLPRLTHIGA
HLLQADPASVAWNGDRLEAGGKSVSVKDVADAWYLRPQLLPPDVDPAGLEVTVGYKPKVDTGCFTYATHAAV

VAVDPGTGGVEILDYVVVEDCGTMINPMVVEGQTIGGVAQGIGTAFYEETPYDDNGQPLASTLADYMLPGAT
EVPNMRLHHFETPSPHTEFGAKGMGEGGAIAPPAVLFNAVNDALRPLGAAELLRTPLSPTRVLAAIAQGSRKTA
TVAAEVTA
>SEQF5400||SEQF5400.1_03036
MTTANADTHGRPGKPQGVGARVPRKEDARHLHGKGNFVADMAMPGLCEVAFLRSPLAHARIASVRVAEQIA
DKAFVRQAMADARDIVADSTLPSYQVSAQPPLASGKVRFVGEPVAMVFAPTRAEAEDYAEMIEVDYDDLPVY
ADVVSAQAAQGDFVHEQWRDNVFVTLNVDKQFDELAAQADVVVRRKIDLSRQCMVPMEGKAVLAYWDH
QADQLVVVSATQVPHMIRSVLAQCLDLEQGRVRVVSPDVGGAFGYKCVLQQEELCVAWLAKTFKKPFRFIEDR
REHLTAGANSREHHYEMVAYADRRGKLLALDAKITIDGGAYSVWPFTIGLEPGQAIGNLPGPYAFKGYRCVTRA
VATNKPGFVPYRGVARTGVCFAMELTMDAIAREVGREPWEIRQENLVQGEQMPYVNVTGKHLDSGDYPASL
QQAMDMIGVDAVRERQRQGEADGRLIGVGLATYTEQAAHGTSVFAAWGTPVIPGFDQATARVTPDGGLELR
VGVHSHGQGMETTFAQIANEILGVDVASIKLLHGDTGQTPFSTGTYASRSLVMSGGAVSQACKRLLPRLTHIGA
HLLQADPASVAWNGDRLEAGGKSVSVKDVADAWYLRPQLLPPDVDPAGLEVTVGYKPKVDTGCFTYATHAAV
VAVDPGTGGVEILDYVVVEDCGTMINPMVVEGQTIGGVAQGIGTAFYEETPYDDNGQPLASTLADYMLPGAT
EVPNMRLHHFETPSPHTEFGAKGMGEGGAIAPPAVLFNAVNDALRPLGAAELLRTPLSPTRVLAAIAQGSRKTA
TVAAEVTA
>SEQF5401||SEQF5401.1_01589
MSNKEETEGPVEDGGVGARVRRREDERHLHGKGRFVADYKFPDLQEVAFLRSPVAHARISRVGKPQAWADRV
FVRADMPEAADIVADSSLPSYKSSAHPPLASGKVRFVGEPIAMCVAATRAEAEDIAEEVELDLDELPAFANAYAA
RDRQDVRVHDHWDDNLFLDLKADVDFDTHAGRAAVVVKQKVDLARQCMAPMEGKAVLAYWDHPQSQLV
VITSTQVPHMIRTVLAQCLGIEQAQVRVISPDVGGAFGYKCVLQQEELCIAWLALRFKRPFRFIEDRREHLIAGA
NTRQHHYELTAYADETGRLLALDANLLIDGGAYSAWPFTIGLETGQALGNLPGPYDFHGYRCRTQCAATNKPGF
LPYRGVARTGVCFAIELTMDAIARAVGREAWEVRHANLVKGADMPYTNVVKKHYDSGDFQESLRRAVEQIDVE
KWRRRQQAGEDDGRLVGVGFATFTEQSAHGTAVFASWGLPVVPGYDLATVRVTADGGLELRAGIHSHGQGM
ETTLPQIAHEILGVPIAKIKLIHGDTGLTPYSTGTYASRSIVMAGGAVAATCRELVPRIVAIGAHLLGEPEQDVKFER
GQVVGRTGSVALADVAAAWYLRPEKLPAGVNLAGLEATQGFKPKVDTGAFSYATHAAAVAVDTQTGHVEILDY
VIVEDCGRMINPMVVEGQTIGGTAQGIGTAFYEESLYDDNAQPLTSTLADYMLPGPTELPALKIIHMETPSPYTE
FGAKGVGEGGAIAPPAALFNAVNDALRPLKAQVSETPLTPRRLLAAIEAARGGAESAGRAARIAEAA
>SEQF5405||SEQF5405.1_03959
MTTANADTHGRPGKPQGVGARVPRKEDARHLHGKGNFVADMAMPGLCEVAFLRSPLAHARIASVRVAEQIA
DKAFVRQAMADARDIVADSTLPSYQVSAQPPLASGKVRFVGEPVAMVFAPTRAEAEDYAEMIEVDYDDLPVY
ADVVSAQAAQGDFVHEQWRDNVFVTLNVDKQFDELAAQADVVVRRKIDLSRQCMVPMEGKAVLAYWDH
QADQLVVVSATQVPHMIRSVLAQCLDLEQGRVRVVSPDVGGAFGYKCVLQQEELCVAWLAKTFKKPFRFIEDR
REHLTAGANSREHHYEMVAYADRRGKLLALDAKITIDGGAYSVWPFTIGLEPGQAIGNLPGPYAFKGYRCVTRA
VATNKPGFVPYRGVARTGVCFAMELTMDAIAREVGREPWEIRQENLVQGEQMPYVNVTGKHLDSGDYPASL
QQAMDMIGVDAIRERQRQGEADDRLIGVGLATYTEQAAHGTSVFAAWGTPVIPGFDQATARVTPDGGLELR
VGVHSHGQGMETTFAQIANEILGVDVASIKLLHGDTGQTPFSTGTYASRSLVMSGGAVSQACKRLLPRLTHIGA
HLLQADPASVAWNGDRLEAGGKSVSVKDVADAWYLRPQLLPPDVDPAGLEVTVGYKPKVDTGCFTYATHAAV
VAVDPGTGGVEILDYVVVEDCGTMINPMVVEGQTIGGVAQGIGTAFYEETPYDDNGQPLASTLADYMLPGAT
EVPNMRLHHFETPSPHTEFGAKGMGEGGAIAPPAVLFNAVNDALRPLGAAELLRTPLSPTRVLAAIAQGSRRTA
PVAAEVTA
>SEQF5416||SEQF5416.1_04069
MTTANADTHGRPGKPQGVGARVPRKEDARHLHGKGNFVADMAMPGLCEVAFLRSPLAHARIASVRVAEQIA
DKAFVRQAMADARDIVADSTLPSYQVSAQPPLASGKVRFVGEPVAMVFAPTRAEAEDYAEMIEVDYDDLPVY

ADVVSAQAAQGDFVHEQWRDNVFVTLNVDKQFDELAAQADVVVRRKIDLSRQCMVPMEGKAVLAYWDH
QADQLVVVSATQVPHMIRSVLAQCLDLEQGRVRVVSPDVGGAFGYKCVLQQEELCVAWLAKTFKKPFRFIEDR
REHLTAGANSREHHYEMVAYADRRGKLLALDAKITIDGGAYSVWPFTIGLEPGQAIGNLPGPYAFKGYRCVTRA
VATNKPGFVPYRGVARTGVCFAMELTMDAIAREVGREPWEIRQENLVQGEQMPYVNVTGKHLDSGDYPASL
QQAMDMIGVDAVRERQRQGEADGRLIGVGLATYTEQAAHGTSVFAAWGTPVIPGFDQATARVTPDGGLELR
VGVHSHGQGMETTFAQIANEILGVDVASIKLLHGDTGQTPFSTGTYASRSLVMSGGAVSQACKRLLPRLTHIGA
HLLQADPASVAWNGDRLEAGGKSVSVKDVADAWYLRPQLLPPDVDPAGLEVTVGYKPKVDTGCFTYATHAAV
VAVDPGTGGVEILDYVVVEDCGTMINPMVVEGQTIGGVAQGIGTAFYEETPYDDNGQPLASTLADYMLPGAT
EVPNMRLHHFETPSPHTEFGAKGMGEGGAIAPPAVLFNAVNDALRPLGAAELLRTPLSPTRVLAAIAQGSRKTA
TVAAEVTA
>SEQF5855||SEQF5855.1_01105
MGASDFSKLPHIGEPVKRKEDYRFLTGAGQYTDDIALAAQAHAVFVRSPHAHARVRSVSTDAARAAPGVIGVLT
GADVAADKINGLPCGWLITSTNGEPMKEPPHPILALDTVRYVGDQVAMVVAETLEQARDAAELVEVDYDPLPA
VVLVADAAAGSVPGAVVHDIAPDNHCYKWAIGDKAAVDAVFAGAAHVAQLDLVNNRLIPNAMEPRAAIGSYS
RANDEYTLYVANQNPHVERLLMTAFVMGLPEHKVRVIAPDVGGGFGSKIFLYAEDVCLTWGARKLNRSIKWTA
DRSESFLTDAHGRDHVSHAEMAMDASGKFLAMRVHTDANLGAYLSTFASAVPTILYATLLAGQYATPQVYVEV
DAWFTHTAPVDAYRGAGRPEATYLLERLVSRCAWQLNLTQAEIRRRNFVTSFPYQTPVALQYDVGDYGACMDK
AEALADVAGFAARRADSEARGLRRGLGYSSYIEACGLAPSNIAGALGARAGLFECGEVRVHPTGSVTVFTGSHS
HGQGHETTFAQVVAARLGIPVENVDIVHGDTGRVPFGMGTYGSRSISVGGAAIMKALDKIEAKAKKIAAHLME
ASDADIDFAGGEFTVRGTDKKIPFAQIALTAYVPHNYPLDKLEPGLNETAFYDPTNFTFPAGTYICEVEVDPATGV
VRVDRFSAVDDFGTIINPMIVEGQVHGGVAQGIGQALMENCVYDRETGQLLTGSFMDYAMPRADDFPEFKLG
TVCTPCTHNPLGTKGCGEAGAIGSPPAVINAVLDALHPLGVRDLDMPASPHRVWSAIDAAATS
>SEQF5856||SEQF5856.1_04526
MGASDFSKLPHIGEPVKRKEDYRFLTGAGQYTDDIALAAQAHAVFVRSPHAHARVRSVSTDAARAAPGVIGVLT
GADVAADKINGLPCGWLITSTNGEPMKEPPHPILALDTVRYVGDQVAMVVAETLEQARDAAELVEVDYDPLPA
VVLVADAAAGSVSGAVVHDIAPDNHCYKWAIGDKAAVDAVFAGAAHVAQLDLVNNRLIPNAMEPRAAIGSYS
RANDEYTLYVANQNPHVERLLMTAFVMGLPEHKVRVIAPDVGGGFGSKIFLYAEDVCLTWGARKLNRNIKWTA
DRSESFLTDAHGRDHVSHAEMAMDASGKFLAMRVHTDANLGAYLSTFASAVPTILYATLLAGQYATPQVYVEV
DAWFTHTAPVDAYRGAGRPEATYLLERLVSRCAWQLNLSQAEIRRRNFVTSFPYQTPVALQYDVGDYGACMDK
AEALADVAGFAARRADSEARGLRRGLGYSSYIEACGLAPSNIAGALGARAGLFECGEVRVHPTGSVTVFTGSHS
HGQGHETTFAQVVAARLGIPVENVDIVHGDTGRVPFGMGTYGSRSISVGGAAIMKALDKIEAKAKKIAAHLME
ASDADIDFAGGEFTVRGTDKKIPFAQIALTAYVPHNYPLDKLEPGLNETAFYDPTNFTFPAGTYICEVEVDPATGV
VRVDRFSAVDDFGTIINPMIVEGQVHGGVAQGIGQALLENCVYDRETGQLLTGSFMDYAMPRADDFPEFKLG
TVCTPCTHNPLGTKGCGEAGAIGSPPAVINAVLDALHPLGVRDLDMPASPHRVWSAIDAAATS
>SEQF5857||SEQF5857.1_03172
MGASDFSKLPHIGEPVKRKEDYRFLTGAGQYTDDIALAAQAHAVFVRSPHAHARVRSVSTDAARAAPGVIGVLT
GADVAADKINGLPCGWLITSTNGEPMKEPPHPILALDTVRYVGDQVAMVVAETLEQARDAAELVEVDYDPLPA
VVLVADAAAGSVPGAVVHDIAPDNHCYKWAIGDKAAVDAVFAGAAHVAQLDLVNNRLIPNAMEPRAAIGSYS
RANDEYTLYVANQNPHVERLLMTAFVMGLPEHKVRVIAPDVGGGFGSKIFLYAEDVCLTWGARKLNRSIKWTA
DRSESFLTDAHGRDHVSHAEMAMDASGKFLAMRVHTDANLGAYLSTFASAVPTILYATLLAGQYATPQVYVEV
DAWFTNTAPVDAYRGAGRPEATYLLERLVSRCAWQLNLSQAEIRRRNFVTSFPYQTPVALQYDVGDYGACMDK
AEALADVAGFAARRADSEARGLRRGLGYSSYIEACGLAPSNIAGALGARAGLFECGEVRVHPTGSVTVFTGSHS
HGQGHETTFAQVVAARLGIPVENVDIVHGDTGRVPFGMGTYGSRSISVGGAAIMKALDKIEAKAKKIAAHLME
ASDADIDFAGGEFTVRGTDKKIPFAQIALTAYVPHNYPLDKLEPGLNETAFYDPTNFTFPAGTYICEVEVDPATGV

VRVDRFSAVDDFGTIINPMIVEGQVHGGVAQGIGQALLENCVYDRETGQLLTGSFMDYAMPRADDFPEFKLG
TVCTPCTHNPLGTKGCGEAGAIGSPPAVINAVLDALHPLGVRDLDMPASPHRVWSAIDAAATS
>SEQF5858||SEQF5858.1_02946
MSDQGVGASPLRKEDDRLMRGRGQFVADIRLAGLQDVVFVRSPLAHARIAGVQVPPGHSDRVYCAQHLDGV
KPVRAVSGLPGFKVSEQSVLAVGKVRHVGENVAICVAPTRAEAEDIAALVRLDLEELPVVHDMLAARESASGLL
HEHWDDNVFLETQVHVNMEAVSDAAFQVTREIRTSRQCMAPMEGRGAVAHWDSRLEQLVLYTSTQIPHIVR
SGLAECLGLEEGQVRVITPDVGGGFGYKGILLTEEVALGWLAMRRGHPVRWIEDRREHLTASANCREHHYHITG
YADAQGRLLGLQCEATVDSGAYSSYPFSACLEAAQVASILPGPYDFAAYSCKTWSVATNKCPILPYRGVARTGVCF
ALELLLDDLAREAGVEPYELRLRSLVRPEQMPFDNITNKHFDSGDYPEALSQAVAAIDVTAIRARQRRGEPDGRL
IGVGLAVYCEQAAHGTSVYAGWGIPMVPGHEQATARLTPDGGLELRIGAHSHGQSLETTLPQVAHEILGIDIARI
RLVHGDTAYTPYSTGTWGSRCMVMSGGAVARACRTLARRLAHIGASMLQVAPEQVCVRDGLVAGPQGSVTIA
DVARLWYRRPQDVPPGADAGGLEVTEGYKPQRDSGTFGYGVHAVLLALDPETGDVEILDYVIVEDGGKLVNP
MVVDGQIYGGTAQGIGTALYEEMAFDASGQPQASTFADYLLPGPTEVPEPRLIHMETLSPYTEFGVKGIGESGA
IPPPAAIGNAINDALHPLGAQLHVSPATPRRILEAIARAQGASGIAAARAEAAARMDKEFA
>SEQF5858||SEQF5858.1_02233
MGASDFSKLPHIGEPVKRKEDYRFLTGAGQYTDDIALAAQAHAVFVRSPHAHARVRSVSTDAAKAAPGVIGVLT
GADVAADKINGLPCGWLITSTNGEPMKEPPHPILALDTVRYVGDQVAMVVAETLEQARDAAELVEVDYDPLPA
VVLVADAAAGRVPGAVVHDIAPDNHCYKWAIGDKAAVDAVFAGAAHIAQLDLVNNRLIPNAMEPRAAIGSYS
RANDEYTLYVANQNPHVERLLMTAFVMGLPEHKVRVIAPDVGGGFGSKIFLYAEDVCLTWGARKLNRSIKWTA
DRSESFLTDAHGRDHVSHAEMAMDASGKFLAMRVHTDANLGAYLSTFASAVPTILYATLLAGQYATPQVYVEV
DAWFTNTAPVDAYRGAGRPEATYLLERLVSRCAWQLNLSQAEIRRRNFVTSFPYQTPVALQYDVGDYGACMDK
AEALADVAGFAARRADSEARGLRRGLGYSSYIEACGLAPSNIAGALGARAGLFECGEVRVHPTGSVTVFTGSHS
HGQGHETTFAQVVAARLGIPVENVDIVHGDTGRVPFGMGTYGSRSISVGGAAIMKALDKIEAKAKKIAAHLME
ASDADIDFAGGEFTVRGTDKKIPFAQIALTAYVPHNYPLDKLEPGLNETAFYDPTNFTFPAGTYICEVEVDPATGV
VRVDRFSAVDDFGTIINPMIVEGQVHGGVAQGIGQALLENCVYDRETGQLLTGSFMDYAMPRADDFPEFRLG
TVCTPCTHNPLGTKGCGEAGAIGSPPAVINAVLDALHPLGVRDLDMPASPHRVWSAIDAAATS
>SEQF5859||SEQF5859.1_00813
MGASDFSKLPHIGEPVKRKEDYRFLTGAGQYTDDIALAAQAHAVFVRSPHAHARVRSVSTDAAKAAPGVIGVLT
GADVAADKINGLPCGWLITSTNGEPMKEPPHPILALDTVRYVGDQVAMVVAETLEQARDAAELVEVDYDPLPA
VVLVADAAAGSVPGAVVHDIAPDNHCYKWAIGDKAAVDAVFAGAAHIAQLDLVNNRLIPNAMEPRAAIGSYS
RANDEYTLYVANQNPHVERLLMTAFVMGLPEHKVRVIAPDVGGGFGSKIFLYAEDVCLTWGARKLNRNIKWTA
DRSESFLTDAHGRDHVSHAEMAMDASGKFLAMRVHTDANLGAYLSTFASAVPTILYATLLAGQYATPQVYVEV
DAWFTHTAPVDAYRGAGRPEATYLLERLVSRCAWQLNLSQAEIRRRNFVTSFPYQTPVALQYDVGDYGACMDK
AEALADVAGFAARRADSEARGLRRGLGYSSYIEACGLAPSNIAGALGARAGLFECGEVRVHPTGSVTVFTGSHS
HGQGHETTFAQVVAARLGIPVENVDIVHGDTGRVPFGMGTYGSRSISVGGAAIMKALDKIEAKAKKIAAHLME
ASDADIDFADGEFTVRGTDKKIPFAQIALTAYVPHNYPLDKLEPGLNETAFYDPTNFTFPAGTYICEVEVDPATGV
VRVDRFSAVDDFGTIINPMIVEGQVHGGVAQGIGQALLENCVYDRETGQLLTGSFMDYAMPRADDFPEFKLG
TVCTPCTHNPLGTKGCGEAGAIGSPPAVINAVLDALHPLGVRDLDMPASPHRVWSAIDAAATS
>SEQF5860||SEQF5860.1_03816
MGASDFSKLPHIGEPVKRKEDYRFLTGAGQYTDDIALAAQAHAVFVRSPHAHARVRSVSTDAAKAAPGVIGVLT
GADVAADKINGLPCGWLITSTNGEPMKEPPHPILALDTVRYVGDQVAMVVAETLEQARDAAELVEVDYDPLPA
VVLVADAAAGSVPGAVVHDIAPDNHCYKWAIGDKAAVDAVFAGAAHIAQLDLVNNRLIPNAMEPRAAIGSYS
RANDEYTLYVANQNPHVERLLMTAFVMGLPEHKVRVIAPDVGGGFGSKIFLYAEDVCLTWGARKLNRSIKWTA
DRSESFLTDAHGRDHVSHAEMAMDASGKFLAMRVHTDANLGAYLSTFASAVPTILYATLLAGQYATPQVYVEV

DAWFTHTAPVDAYRGAGRPEATYLLERLVSRCAWQLNLSQAEIRRRNFVTSFPYQTPVALQYDVGDYGACMDK
AEALADVAGFAARRADSEARGLRRGLGYSSYIEACGLAPSNIAGALGARAGLFECGEVRVHPTGSVTVFTGSHS
HGQGHETTFAQVVAARLGIPVENVDIVHGDTGRVPFGMGTYGSRSISVGGAAIMKALDKIEAKAKKIAAHLME
ASDADIDFAGGEFTVRGTDKKIPFAQIALTAYVPHNYPLDKLEPGLNETAFYDPTNFTFPAGTYICEVEVDPATGV
VRVDRFSAVDDFGTIINPMIVEGQVHGGVAQGIGQALLENCVYDRETGQLLTGSFMDYAMPRADDFPEFKLG
TVCTPCTHNPLGTKGCGEAGAIGSPPAVINAVLDALHPLGVRDLDMPASPHRVWSAIDAAATS
>SEQF5861||SEQF5861.2_04409
MGASDFSKLPHIGEPVKRKEDYRFLTGAGQYTDDIALAAQAHAVFVRSPHAHARVRSVSTDAAKAAPGVIGVLT
GADVAADKINGLPCGWLITSTNGEPMKEPPHPILALDTVRYVGDQVAMVVAETLEQARDAAELVEVDYDPLPA
VVLVADAAAGSVPGAVVHDIAPDNHCYKWAIGDKAAVDAVFAGAAHIVQLDLVNNRLIPNAMEPRAAIGSYS
RANDEYTLYVANQNPHVERLLMTAFVMGLPEHKVRVIAPDVGGGFGSKIFLYAEDVCLTWGARKLNRSIKWTA
DRSESFLTDAHGRDHVSHAEMAMDASGKFLAMRVHTDANLGAYLSTFASAVPTILYATLLAGQYATPQVYVEV
DAWFTNTAPVDAYRGAGRPEATYLLERLVSRCAWQLNLTQAEIRRRNFVTSFPYQTPVALQYDVGDYGACMDK
AEALADVAGFAARRADSEARGLRRGLGYSSYIEACGLAPSNIAGALGARAGLFECGEVRVHPTGSVTVFTGSHS
HGQGHETTFAQVVAARLGIPVENVDIVHGDTGRVPFGMGTYGSRSISVGGAAIMKALDKIEAKAKKIAAHLME
ASDADIDFAGGEFTVRGTDKKIPFAQIALTAYVPHNYPLDKLEPGLNETAFYDPTNFTFPAGTYICEVEVDPATGV
VRVDRFSAVDDFGTIINPMIVEGQVHGGVAQGIGQALLENCVYDRETGQLLTGSFMDYAMPRADDFPEFKLG
TVCTPCTHNPLGTKGCGEAGAIGSPPAVINAVLDALHPLGVRDLDMPASPHRVWSAIDAAATS
>SEQF5862||SEQF5862.1_05178
MGASDFSKLPHIGEPVKRKEDYRFLTGAGQYTDDIALAAQAHAVFVRSPHAHARLRSVSTDAAKAAPGVIGVLT
GADVAADKINGLPCGWLITSTNGEPMKEPPHPILALDTVRYVGDQVAMVVAETLEQARDAAELVEVDYDPLPA
VVLVADAAAGSVPGAVVHDIAPDNHCYKWAIGDKAAVDAVFAGAAHIAQLDLVNNRLIPNAMEPRAAIGSYS
RANDEYTLYVANQNPHVERLLMTAFVMGLPEHKVRVIAPDVGGGFGSKIFLYAEDVCLTWGARKLNRSIKWTA
DRSESFLTDAHGRDHVSHAEMAMDASGKFLAMRVHTDANLGAYLSTFASAVPTILYATLLAGQYATPQVYVEV
DAWFTHTAPVDAYRGAGRPEATYLLERLVSRCAWQLNLSQAEIRRRNFVTSFPYQTPVALQYDVGDYGACMDK
AEALADVAGFAARRADSEARGLRRGLGYSSYIEACGLAPSNIAGALGARAGLFECGEVRVHPTGSVTVFTGSHS
HGQGHETTFAQVVAARLGIPVENVDIVHGDTGRVPFGMGTYGSRSISVGGAAIMKALDKIEAKAKKIAAHLME
ASDADIDFAGGEFTVRGTDKKIPFAQIALTAYVPHNYPLDKLEPGLNETAFYDPTNFTFPAGTYICEVEVDPATGV
VRVDRFSAVDDFGTIINPMIVEGQVHGGVAQGIGQALLENCVYDRETGQLLTGSFMDYAMPRADDFPEFKLG
TVCTPCTHNPLGTKGCGEAGAIGSPPAVINAVLDALHPLGVRDLDMPASPHRVWSAIDAAATS
>SEQF5863||SEQF5863.1_03686
MGASDFSKLPHIGEPVKRKEDYRFLTGAGQYTDDIALAAQAHAVFVRSPHAHARVRSVSTDVAKAAPGVIGVLT
GADVAADKINGLPCGWLITSTNGEPMKEPPHPILALDTVRYVGDQVAMVVAETLEQARDAAELVEVDYDPLPA
VVLVADAAAGSVPGAVVHDIAPDNHCYKWAIGDKAAVDAVFAGAAHVAQLDLVNNRLIPNAMEPRAAIGSYS
RANDEYTLYVANQNPHVERLLMTAFVMGLPEHKVRVIAPDVGGGFGSKIFLYAEDVCLTWGARKLNRNIKWTA
DRSESFLTDAHGRDHVSHAEMAMDASGKFLAMRVHTDANLGAYLSTFASAVPTILYATLLAGQYATPQVYVEV
DAWFTHTAPVDAYRGAGRPEATYLLERLVSRCAWQLNLTQAEIRRRNFVTSFPYQTPVALQYDVGDYGACMDK
AEALADVAGFAARRAYSEARGLRRGLGYSSYIEACGLAPSNIAGALGARAGLFECGEVRVHPTGSVTVFTGSHSH
GQGHETTFAQVVAARLGIPVENVDIVHGDTGRVPFGMGTYGSRSISVGGAAIMKALDKIEAKAKKIAAHLMEA
SDADIDFAGGEFTVRGTDKKIPFAQIALTAYVPHNYPLDKLEPGLNETAFYDPTNFTFPAGTYICEVEVDPATGVV
RVDRFSAVDDFGTIINPMIVEGQVHGGVAQGIGQALLENCVYDRETGQLLTGSFMDYAMPRADDFPEFKLGT
VCTPCTHNPLGTKGCGEAGAIGSPPAVINAVLDALHPLGVRDLDMPASPHRVWSAIDAAATS
>SEQF5864||SEQF5864.2_02639
MGASDFSKLPHIGEPVKRKEDYRFLTGAGQYTDDIALAAQAHAVFVRSPHAHARVRSVSTDAARAAPGVIGVLT

GADVAADKINGLPCGWLITSTNGEPMKEPPHPILALDTVRYVGDQVAMVVAETLEQARDAAELVEVDYDPLPA
VVLVADAAAGSVPGAVVHDIAPDNHCYKWAIGDKAAVDAVFAGAAHIAQLDLVNNRLVPNAMEPRAAIGSYS
RANDEYTLYVANQNPHVERLLMTAFVMGLPEHKVRVIAPDVGGGFGSKIFLYAEDVCLTWGARKLNRSIKWTA
DRSESFLTDAHGRDHVSHAEMAMDASGKFLAMRVHTDANLGAYLSTFASAVPTILYATLLAGQYATPQVYVEV
DAWFTHTAPVDAYRGAGRPEATYLLERLVSRCAWQLNLSQAEIRRRNFVTSFPYQTPVALQYDVGDYGACMDK
AEALADVAGFAARRADSEARGLRRGLGYSSYIEACGLAPSNIAGALGARAGLFECGEVRVHPTGSVTVFTGSHS
HGQGHETTFAQVVAARLGIPVENVDIVHGDTGRVPFGMGTYGSRSISVGGAAIMKALDKIEAKAKKIAAHLME
ASDADIDFAGGEFTVRGTDKKIPFAQIALTAYVPHNYPLDKLEPGLNETAFYDPTNFTFPAGTYICEVEVDPATGV
VRVDRFSAVDDFGTIINPMIVEGQVHGGVAQGIGQALLENCVYDRETGQLLTGSFMDYAMPRADDFPEFKLG
TVCTPCTHNPLGTKGCGEAGAIGSPPAVINAVLDALHPLGVRDLDMPASPHRVWSAIDAAATS
>SEQF5865||SEQF5865.1_02283
MGASDFSKLPHIGEPVKRKEDYRFLTGAGQYTDDIALAAQAHAVFVRSPHAHARVRSVSTDAAKAAPGVIGVLT
GADVAADKINGLPCGWLITSTNGEPMKEPPHPILALDTVRYVGDQVAMVVAETLEQARDAAELVEVDYDPLPA
VVLVADAAAGSVSGAVVHDIAPDNHCYKWAIGDKAAVDAVFAGAAHIAQLDLVNNRLIPNAMEPRAAIGSYS
RANDEYTLYVANQNPHVERLLMTAFVMGLPEHKVRVIAPDVGGGFGSKIFLYAEDVCLTWGARKLNRNIKWTA
DRSESFLTDAHGRDHVSHAEMAMDASGKFLAMRVHTDANLGAYLSTFASAVPTILYATLLAGQYATPQVYVEV
DAWFTHTAPVDAYRGAGRPEATYLLERLVSRCAWQLNLTQAEIRRRNFVTSFPYQTPVALQYDVGDYGACMDK
AEALADVAGFAARRADSEARGLRRGLGYSSYIEACGLAPSNIAGALGARAGLFECGEVRVHPTGSVTVFTGSHS
HGQGHETTFAQVVAARLGIPVENVDIVHGDTGRVPFGMGTYGSRSISVGGAAIMKALDKIEAKAKKIAAHLME
ASDADIDFAGGEFTVRGTDKKIPFAQIALTAYVPHNYPLDKLEPGLNETAFYDPTNFTFPAGTYICEVEVDPATGV
VRVDRFSAVDDFGTIINPMIVEGQVHGGVAQGIGQALLENCVYDRETGQLLTGSFMDYAMPRADDFPEFKLG
TVCTPCTHNPLGTKGCGEAGAIGSPPAVINAVLDALHPLGVRDLDMPASPHRVWSAIDAAATS
>SEQF5866||SEQF5866.1_01506
MGASDFSKLPHIGEPVKRKEDYRFLTGAGQYTDDIALAAQAHAVFVRSPHAHARVRSVSTDAARAAPGVIGVLT
GADVAADKINGLPCGWLITSTNGEPMKEPPHPILALDTVRYVGDQVAMVVAETLEQARDAAELVEVDYDPLPA
VVLVADAAAGSVPGAVVHDIAPDNHCYKWAIGDKAAVDAVFAGAAHVAQLDLVNNRLIPNAMEPRAAIGSYS
RANDEYTLYVANQNPHVERLLMTAFVMGLPEHKVRVIAPDVGGGFGSKIFLYAEDVCLTWGARKLNRSIKWTA
DRSESFLTDAHGRDHVSHAEMAMDASGKFLAMRVHTDANLGAYLSTFASAVPTILYATLLAGQYATPQVYVEV
DAWFTHTAPVDAYRGAGRPEATYLLERLVSRCAWQLNLTQAEIRRRNFVTSFPYQTPVALQYDVGDYGACMDK
AEALADVAGFAARRADSEARGLRRGLGYSSYIEACGLAPSNIAGALGARAGLFECGEVRVHPTGSVTVFTGSHS
HGQGHETTFAQVVAARLGIPVENVDIVHGDTGRVPFGMGTYGSRSISVGGAAIMKALDKIEAKAKKIAAHLME
ASDADIDFAGGEFTVRGTDKKIPFAQIALTAYVPHNYPLDKLEPGLNETAFYDPTNFTFPAGTYICEVEVDPATGV
VRVDRFSAVDDFGTIINPMIVEGQVHGGVAQGIGQALLENCVYDRETGQLLTGSFMDYAMPRADDFPEFKLG
TVCTPCTHNPLGTKGCGEAGAIGSPPAVINAVLDALHPLGVRDLDMPASPHRVWSAIDAAATS
>SEQF5867||SEQF5867.1_03163
MGASDFSKLPHIGEPVKRKEDYRFLTGAGQYTDDIALAAQAHAVFVRSPHAHARVRSVSTDAAKAAPGVIGVLT
GADVAADKINGLPCGWLITSTNGEPMKEPPHPILALDTVRYVGDQVAMVVAETLEQARDAAELVEVDYDPLPA
VVLVADAAAGSVSGAVVHDIAPDNHCYKWAIGDKAAVDAVFAGAAHIAQLDLVNNRLIPNAMEPRAAIGSYS
RANDEYTLYVANQNPHVERLLMTAFVMGLPEHKVRVIAPDVGGGFGSKIFLYAEDVCLTWGARKLNRNIKWTA
DRSESFLTDAHGRDHVSHAEMAMDASGKFLAMRVHTDANLGAYLSTFASAVPTILYATLLAGQYATPQVYVEV
DAWFTHTAPVDAYRGAGRPEATYLLERLVSRCAWQLNLSQAEIRRRNFVTSFPYQTPVALQYDVGDYGACMDK
AEALADVAGFAARRADSEARGLRRGLGYSSYIEACGLAPSNIAGALGARAGLFECGEVRVHPTGSVTVFTGSHS
HGQGHETTFAQVVAARLGIPVENVDIVHGDTGRVPFGMGTYGSRSISVGGAAIMKALDKIEAKAKKIAAHLME
ASDADIDFAGGEFTVRGTDKKIPFAQIALTAYVPHNYPLDKLEPGLNETAFYDPTNFTFPAGTYICEVEVDPATGV

VRVDRFSAVDDFGTIINPMIVEGQVHGGVAQGIGQALLENCVYDRETGQLLTGSFMDYAMPRADDFPEFKLG
TVCTPCTHNPLGTKGCGEAGAIGSPPAVINAVLDALHPLGVRDLDMPASPHRVWSAIDAAATS
>SEQF5868||SEQF5868.1_01086
MGASDFSKLPHIGEPVKRKEDYRFLTGAGQYTDDIALAAQAHAVFVRSPHAHARVRSVSTDAARAAPGVIGVLT
GADVAADKINGLPCGWLITSTNGEPMKEPPHPILALDTVRYVGDQVAMVVAETLEQARDAAELVEVDYDPLPA
VVLVADAAAGSVPGAVVHDIAPDNHCYKWAIGDKAAVDAVFAGAAHVAQLDLVNNRLIPNAMEPRAAIGSYS
RANDEYTLYVANQNPHVERLLMTAFVMGLPEHKVRVIAPDVGGGFGSKIFLYAEDVCLTWGARKLNRSIKWTA
DRSESFLTDAHGRDHVSHAEMAMDASGKFLAMRVHTDANLGAYLSTFASAVPTILYATLLAGQYATPQVYVEV
DAWFTHTAPVDAYRGAGRPEATYLLERLVSRCAWQLNLTQAEIRRRNFVTSFPYQTPVALQYDVGDYGACMDK
AEALADVAGFAARRADSEARGLRRGLGYSSYIEACGLAPSNIAGALGARAGLFECGEVRVHPTGSVTVFTGSHS
HGQGHETTFAQVVAARLGIPVENVDIVHGDTGRVPFGMGTYGSRSISVGGAAIMKALDKIEAKAKKIAAHLME
ASDADIDFAGGEFTVRGTDKKIPFAQIALTAYVPHNYPLDKLEPGLNETAFYDPTNFTFPAGTYICEVEVDPATGV
VRVDRFSAVDDFGTIINPMIVEGQVHGGVAQGIGQALMENCVYDRETGQLLTGSFMDYAMPRADDFPEFKLG
TVCTPCTHNPLGTKGCGEAGAIGSPPAVINAVLDALHPLGVRDLDMPASPHRVWSAIDAAATS
>SEQF5869||SEQF5869.1_01006
MGASDFSKLPHIGEPVKRKEDYRFLTGAGQYTDDIALAAQAHAVFVRSPHAHARVRSVSTDAARAAPGVIGVLT
GADVAADKINGLPCGWLITSTNGEPMKEPPHPILALDTVRYVGDQVAMVVAETLEQARDAAELVEVDYDPLPA
VVLVADAAAGSVSGAVVHDIAPDNHCYKWAIGDKAAVDAVFAGAAHIAQLDLVNNRLIPNAMEPRAAIGSYS
RANDEYTLYVANQNPHVERLLMTAFVMGLPEHKVRVIAPDVGGGFGSKIFLYAEDVCLTWGARKLNRSIKWTA
DRSESFLTDAHGRDHVSHAEMAMDASGKFLAMRVHTDANLGAYLSTFASAVPTILYATLLAGQYATPQVYVEV
DAWFTHTAPVDAYRGAGRPEATYLLERLVSRCAWQLNLTQAEIRRRNFVTSFPYQTPVALQYDVGDYGACMDK
AEALADVAGFAARRADSEARGLRRGLGYSSYIEACGLAPSNIAGALGARAGLFECGEVRVHPTGSVTVFTGSHS
HGQGHETTFAQVVAARLGIPVENVDIVHGDTGRVPFGMGTYGSRSISVGGAAIMKALDKIEAKAKKIAAHLME
ASDADIDFAGGEFTVRGTDKKIPFAQIALTAYVPHNYPLDKLEPGLNETAFYDPTNFTFPAGTYICEVEVDPATGV
VRVDRFSAVDDFGTIINPMIVEGQVHGGVAQGIGQALLENCVYDRETGQLLTGSFMDYAMPRADDFPEFKLG
TVCTPCTHNPLGTKGCGEAGAIGSPPAVINAVLDALHPLGVRDLDMPASPHRVWSAIDAAATS
>SEQF5870||SEQF5870.2_03378
MGASDFSKLPHIGEPVKRKEDYRFLTGAGQYTDDIALAAQAHAVFVRSPHAHARVRSVSTDAAKAAPGVIGVLT
GADVAADKINGLPCGWLITSTNGEPMKEPPHPILALDTVRYVGDQVAMVVAETLEQARDAAELVEVDYDPLPA
VVLVADAAAGSVPGAVVHDIAPDNHCYKWAIGDKAAVDAVFAGAAHIAQLDLVNNRLIPNAMEPRAAIGSYS
RANDEYTLYVANQNPHVERLLMTAFVMGLPEHKVRVIAPDVGGGFGSKIFLYAEDVCLTWGARKLNRSIKWTA
DRSESFLTDAHGRDHVSHAEMAMDASGKFLAMRVHTDANLGAYLSTFASAVPTILYATLLAGQYATPQVYVEV
DAWFTHTAPVDAYRGAGRPEATYLLERLVSRCAWQLNLSQAEIRRRNFVTSFPYQTPVALQYDVGDYGACMDK
AEALADVAGFAARRADSEARGLRRGLGYSSYIEACGLAPSNIAGALGARAGLFECGEVRVHPTGSVTVFTGSHS
HGQGHETTFAQVVAARLGIPVENVDIVHGDTGRVPFGMGTYGSRSISVGGAAIMKALDKIEAKAKKIAAHLME
ASDADIDFAGGEFTVRGTDKKIPFAQIALTAYVPHNYPLDKLEPGLNETAFYDPTNFTFPAGTYICEVEVDPATGV
VRVDRFSAVDDFGTIINPMIVEGQVHGGVAQGIGQALLENCVYDRETGQLLTGSFMDYAMPRADDFPEFKLG
TVCTPCTHNPLGTKGCGEAGAIGSPPAVINAVLDALHPLGVRDLDMPASPHRVWSAIDAAATS
>SEQF6035||SEQF6035.1_03056
MTGDEATNPLRLEDRALLTGRASFVDDIHLDRMAHAVFVRSPVAHARIASINIQPALAAGACAVLVGSDLPFIEK
RLAARYWHPSARKVLPTFLAVDVVRFVGEAVAMVLAESRYLAEDIAELVEIDYEPLEVSADAKQSLLAQPPSLHA
EWPNNIAAEFRHIIGDAEAALRQSPNRIRRRFCFNRQGGMPLETRGCVADYDTANERLNIWTSTQTHYAVRAN
VADVLELPEQSVRVLTGDVGGGFGAKSRPYNEEVLVSYASRLVGRPVKWIEDRLEHMQATTQSRGIQTELELGY
DSEGQISALSGRLVVDIGAYIFTSGIITAEVAAAHCCGPYKIPHVALDVFCIGTNKTPLATCRGAGQPEATFPLECLL

DLIATERGLTAFELRRRNIVAPQDMPYDPSISYGGAKGIFESGDFPQLVRRAVQASGYHESVENGPGRERIAWGL
ACGIEGTGLINYETARIQIDASGVIHIHCGLSSQGQGQATALREVCVQAIGADPRYVKVTLGDTGLLGFGRGTFAS
RGAVMGGNATLGAAEKIRQTLLAGAGQLLQCAPESLRISKGQVLRSDGTATTLSLRDLAQAYQPNGPLFTGNVT
ALDETFVFDNKNTVTMAVSVHAAKVAVDERTGACKVLDYLVVHDAGRMLVPKIVEGQIVGGAAEGIGCALFSE
FVHDDQGQLLTGSLSDYLLISAPETPRIRVDHLETHASTNPLGVRGVGEGGTIAAPPAIVNAVRRAINPESVELEE
QLFRLPLRPDSVLRAMGQL
>SEQF6035||SEQF6035.1_00420
MGVEGIGARVVRKEDRRFITGQGRYVDDVKMVGMSYAHFIRSPHAHAKVKGIDSAEAMKMPGVIAVLTGQQ
IVDDKVGNLICGWAITSKDGSPMKMGAWPAMAPETVRFVGQAVAVVIAETKNQARDAAEAVVVDYEELPAA
ADIKAAIKPGAPQLHPEAPGNIVYDWHLGDEAAVKDAFSKAANVVTLDLTNNRLVPNAMEPRAAVAHYDQAE
EHYTLYTTSQNPHVARLVLSAFYNIAPEHKLRVIAPDVGGGFGSKIYIYPEEMVALWASKKVGRPVKWTGDRSEA
FLTDAHGRDHVSKAEMAFDKDNKILGLRVKTHANFGAYMSLFSSAVPTYLYATLLSGQYVIPAIYAEVIGVYTNTT
PVDAYRGAGRPEASYLIERMMETAARQLKVDPTELRKKNFITQFPYQTPVIMAYDIGDFHASIDAAMKAIDYAG
FPARKAKAKADGKLRGIGISCYIEACGIAPSKAVGSLGAGVGLWESAEIRVNPVGTIEVLTGSHSHGQGHETTFC
QIIAERLGVPISQVSIVHGDTDKVQFGMGTYGSRSLAVGGTAIVKAMEKVEAKAKKIAAHALEASESDIVIENGE
FKVTGTDKSIALPMVALAAYTAHNLPDGMEPGLKETAFYDPTNFTFPAGAYICELEVDPGTGKTSFVNFVAADD
FGRLINPMIVEGQVHGGLAQGIGQALLEGAVYDDTGQLVTASFMDYTMPRADDLPSFQLSHTTTLCPGNPLG
VKGCGEAGAIGASAAVINAITDAIGNNKLEMPATPDRVWHAIHG
>SEQF6035||SEQF6035.1_06228
MQDHTPPASLENAIALQKYGVGQPVRRKEDDTLVRGKGKYTDDFSLPGQAYCWMVRSSHAHGLIKGIDTAAA
KAMPGVLGVWTGADLAAAGYNPFTCGLPLKNRDGSPLKQTNRPALVTDKVRFVGDPVAFVVAETAAQARDA
AEAVEVDIEPLPAVTDAAEAAKPGAPQLYDDIPDNVALDYHYGDTAKIEAAFAGAAHVTKLDIVNTRVAVVSME
PRVALAHYDKKTERFTLQVPTQGVSGNKAILARLLNVPADKVRILTGNVGGSFGMKNLNYPEYTCIAHAARELG
RPVKWLDERSTSFLSDSQGRAQLIHAELALDADGKFLAVRLSGYGNLGAYITGVAPGPLSLNTGKNLASVYRTPL
LGVDIKTVLTNTTLMGAYRGAGRPEANYYMERLIDAAADEMGINRFTLRKRNFIKPSQLPFPAASGVTYDSGDF
AAVFQKALEISDYDNFAKRKKESKKSGKLRGIAVGSYLEVTAPPSGELGKISFEPDGSVKLTTGTLDYGQGHATPFA
QVLSDQLGVPFEKITLEQGDSDLVRFGNGTGGSRSITATGQAIVEASALVVEKGKKAAAHMLEASEADIEFGAGR
FTIAGTDRSIGIMELAERMRAGKMPEGTPETLDVDHATKETASTFPNGCHVAEVEIDPDTGVTRIVRYSAVNDF
GVVVNPMIVAGQLHGGVAQGIGQALMEEVSYDASGQPITGSFMDYALPRAGDVPSMLVGDHPSPAKSNPLG
TKGCGEAGCAGSLVCIVNAVVDALSDYGIKHINMPLTPERVWRAIQDAKAKAA
>SEQF6035||SEQF6035.1_03162
MNILPGNMRFGAGQPVKRLEDQRLVTGKGHFIDDKPQDGALWLHVLRSPHAHANIKSIDAKAALEMPGVKA
VVYTGADLVKDDIGTLPTLAIFKRPDGSPMTVPPRRLLAHEVVRYAGEGVAAVVATSRVLAQTAAEAIEIDYEVLPS
VVDPVEAIKPGAPAVWPEAPDNIVAAMSYGDAAKVEEAFANAAHKVSLDLVSQRLVPSAMEPRSTIAEIEKKTG
RLILHVQSQTPGSTRDLLAESILKRPKDSVRVLVGDIGGGFGQKTSLYPEDGIVAYAATKLNEKIRWRGDRTDEFV
GGTHGRDLTSTGEFALDAKGRVLAYRVRSIGGTGAYSSGTANIIPLVLGPFVQTGVYDLPLVHFEVKSVMTHTAPV
GAYRGAGRPEAVFIVERLFDAAARQIGMDPRTIRKVNYIKPAQLPYTNAVGQVYDSGAFAHMLERASDLADW
NGFAARKKAAKKKGLLYGRGLTSYIEWTGGRAHTEKVSLHATAEGRVILWSGTMAMGQGLATTYTQMVADTL
GISMDKIDVVQGDTDLATGFGSVGSRSLFVGGTAVAVSTNDMINKARDKASNLLEASVEDIEYRDGFLTVVGTD
RRISLFEIAAKENGAKLSVESEGNVDGPSWPNGTHICEVEINPETGVTRVVRYTTVDDVGVAVNPMLVTGQVH
GGVVQGIGQALYEGVAYSEEGQLLTASYQDYCIPRADDIPPITVTLDGSAPCKTNPLGAKGCGESGAIGGPPCITN
GVMDALSEVGITQLNTPLTPAKIWQAIRDAKVGAA
>SEQF6035||SEQF6035.1_03834
MTVTRAPADTLSVLDRPNSYIGKTVPRPNLDRLLQGRGQYVSDLELPRMAHVVFLRSPHAHAKIAAIDADAATR

MPRVISIVTGRELEAVITPWVGVLSHLKGLKSAPQHAIAVDRVCWQGEAVAAIVATSRAAAEDAMEHISVDYEE
LEAVTDMRAALDPAAPVIHAALGDNLAFERTLDAGDVDHALSGSELVEADFVFGRHTGVTLEPRAVVADWNA
AEARLTIYQGTQAPHMVQNIAALHLGLREAQVRVVCKDVGGSFGIKVHIYADEMATYALSKLLRRPVKFVADRV
ESFNTDIHARDHRCRGRIGVRPDGTITAFEIDDLTGIGPYSMYPRTSAIEANQVVNLVGGPYVTANYRARARVVF
QNKNMMCQYRAVGHPIACSVTEGLVDLAAAKIGMDPVEIRRRNLIADDAYPCASPSGMKFEQLSHHASLTKLL
QMMDYDALRAEQAAWRARNVHRGIGIASFIEVTNPSAAFYGVGGAKISSQDGVAVRLDAQGSVICQTSITEQ
GQGSESLTAQIVGSVLGVSMERVRVILGDTDNTPYGGGTWASRGAGIGGEAALQAAKALRENILNVAAAILQST
PAELDIVDNGIVNAADGAPRIELHELARIVYFRPDTLPPGIQPELMATRHFVPRQYPFAFTNGVQASWLEVDTET
GFVKLLKHWVVEDCGTIINPQLVDEQIRGGVVQGLGAALFEKCIYDERGQLTNANMADYLVPMSGEMPDIEIG
HVVSPTQESELGAKGAGEAGTAGAAAAVANAVNDALRPFGATITEIPLTPQVILTALGRI
>SEQF6035||SEQF6035.1_04041
MAAPIKFGVGQSVRRKEDDALIRGKGRYTDDVAPSPALHALMLRSPHAHATYTIDAGKARGMPGVALILTAAD
VAELGGLPCLFNLETDPFTAPPYPILAKDEVRHVGDAVAFVVADTVDHARDAIEAIDVKWTPLPAAVGLVNAVK
KDAPQVWPDKPGNVLFDVSIGDKKAAEDAFAKAHAVAEITIVNPRVITNFMETRAAVAEYDAKKDHLTLTIGSQ
GSHRLREILCDMILKMPKENMRVICPDVGGGFGTKLFPYREYALISVAARKLKKSIKWTAERSDHFMGDAQGR
DNLTTAKMALAEDGKFLGMDVDLMGDMGAYLSTFAPYIPHGGAGMLPGLYDIQAFHCRVRTVFTNTVPVDA
YRGAGRPEAAYVIERLVDAAARKLGMTPDSIRRKNFIPPKSLPYTTATGKVYDSGDFVAHMKRAMEIANWKEFP
KRAKAARKDGLVRGIGMASYVEVCGTMGEETANVALDANGDISVLIGTQSSGQGHQTAYAQIVAEQFGVPPER
VHVLQGDTDKIATGLGTGGSASIPSGGVSVQRATHDLGNKLKELAAQALEAGAGDLEIADGRIRIAGTDRSISFA
DLAKRPGGDTSKMNASATFASADGTYPNGTHLAEVEIDPATGIIKIVSYVIVDDFGVTLNPLMLAGQVHGGAM
QGIGQALMEQAVYSPTDGQLVTGTFMDYALPRASDGPSFVFETHNVPCTTNPMGVKGAGEAGAIGSCPAVV
NAIVEGLHREYKIDHIDMPATPERVWIAIREAQRRHNL
>SEQF6036||SEQF6036.1_02354
MNILPGNMRFGAGQPVKRLEDQRLVTGKGHFIDDKPQDGALWLHVLRSPHAHANIKSIDAKTALEMPGVKAV
YTGADLVKDDIGTLPTLAIFKRPDGSPMTVPPRRLLAHEVVRYAGEGVAAVVATSRVLAQTAAEAIEIDYEVLPSV
VDPVEAIKPGAPAVWPEAPDNIVAAMSYGDAAKVEEAFANAAHKVSLDLVSQRLVPSAMEPRSTIAEIEKKTGR
LILHVQSQTPGSTRDLLAESILKRPKDSVRVLVGDIGGGFGQKTSLYPEDGIVAYAATKLNEKIRWRGDRTDEFVG
GTHGRDLTSTGEFALDAKGRVLAYRVRSIGGTGAYSSGTANIIPLVLGPFVQTGVYDLPLVHFEVKSVMTHTAPVG
AYRGAGRPEAVFIVERLFDAAARQIGMDPRTIRKVNYIKPAQLPYTNAVGQVYDSGAFAHMLERASDLADWN
GFAARKKAAKKKGLLYGRGLTSYIEWTGGRAHTEKVSLHATAEGRVILWSGTMAMGQGLATTYTQMVADTLGI
SMDKIDVVQGDTDLATGFGSVGSRSLFVGGTAVAVSTNDMINKARDKASNLLEASVEDIEYRDGFLTVVGTDR
RISLFEIAAKENGAKLSVESEGNVDGPSWPNGTHICEVEIDPETGVTRVVRYTTVDDVGVAVNPMLVTGQVHG
GVVQGIGQALYEGVAYSEEGQLLTASYQDYCIPRADDIPPITVTLDGSAPCKTNPLGAKGCGESGAIGGPPCITNG
VMDALSEVGITQLNTPLTPAKIWQAIRDAKVGAA
>SEQF6036||SEQF6036.1_03160
MTVTRAPADTLSVLDRPNSYIGKTVPRPNLDRLLQGRGQYVSDLELPRMAHVVFLRSPHAHAKIAAIDADAATR
MPGVISIVTGRELEAVITPWVGVLSHLKGLKSAPQHAIAVDRVCWQGEAVAAIVATSRAAAEDAMEHISVDYEE
LEAVTDMRAALDPAASVIHAALGDNLAFERTLDAGDVDLALSGSELVEADFVFGRHTGVTLEPRAVVADWNPA
EARLTIYQGTQAPHMVQNIAALHLGLREAQVRVVCKDVGGSFGIKVHIYADEMATYALSKLLRRPVKFVADRVE
SFNTDIHARDHRCRGRIGVRPDGTITAFEIDDLTGIGPYSMYPRTSAIEANQVVNLVGGPYATANYRARARVVFQ
NKNVMCQYRAVGHPIACSVTEGLVDLAAAKIGMDPVEIRRRNLIADDAYPCASPSGMKFERLSHHASLTKLLQ
MMDYDALRAEQAALRARNVHRGIGIASFIEVTNPSAAFYGVGGAKISSQDGVAVRLDAQGSVICQTSITEQGQ
GSESLTAQIVGSVLGVSMERVRVTLGDTDNTPYGGGTWASRGAGIGGEAALQAAKALRENILNVAAAILQSTPA
ELDIVDNGVVNAADGAPRIDLNELARIVYFRPDTLPPGIQPELMATRHFVPRQYPFAFTNGVQASWLEVDTET

GFVQLLKHWVVEDCGTIINPQLVDEQIRGGVVQGLGAALFEKCIYDERGQLTNANMADYLVPMSGEMPDIEIG
HVVSPTQESELGAKGAGEAGTAGAAAAVANAVNDALRPFGATITEIPLTPQVILTALGRI
>SEQF6036||SEQF6036.1_05395
MQDHTPPASLENAIALQKYGVGQPVRRKEDDTLVRGKGKYTDDFSLPGQAYCWMVRSSHAHGLIKGIDTAAA
KAMPGVLGVWTGADLAAAGYNPFTCGLPLKNRDGSPLKQTNRPALVTDKVRFVGDPVAFVVAETAAQARDA
AEAVEVDIEPLPAVTDAAEAAKPGAPQLYDDIPDNVALDYHYGDTAKIEAAFAGAAHVTKLDIVNTRVAVVSME
PRVALAHYDKKTERFTLQVPTQGVSGNKAILARLLNVPADKVRILTGNVGGSFGMKNLNYPEYTCIAHAARELG
RPVKWLDERSTSFLSDSQGRAQLIHAELALDADGKFLAVRLSGYGNLGAYITGVAPGPLSLNTGKNLASVYRTPL
LGVDIKTVLTNTTLMGAYRGAGRPEANYYMERLIDAAADEMGINRFTLRKRNFIKPSQLPFPAASGVTYDSGDF
AAVFQKALEISDYDNFAKRKKESKKSGKLRGIAVGSYLEVTAPPSGELGKISFEPDGSVKLTTGTLDYGQGHATPFA
QVLSDQLGVPFEKITLEQGDSDLVRFGNGTGGSRSITATGQAIVEASALVVEKGKKAAAHMLEASEADIEFGAGR
FTIAGTDRSIGIMELAERMRAGKMPEGTPETLDVDHATKETASTFPNGCHVAEVEIDPDTGVTRIVRYSAVNDF
GVVVNPMIVAGQLHGGVAQGIGQALMEEVSYDASGQPITGSFMDYALPRAGDVPSMLVGDHPSPAKSNPLG
TKGCGEAGCAGSLVCIVNAVVDALSDYGIKHINMPLTPERVWRAIQDAKAKAA
>SEQF6036||SEQF6036.1_08330
MGVEGIGARVVRKEDRRFITGQGRYVDDVKMVGMSYAHFIRSPHAHAKVKGIDSAEAMKMPGVIAVLTGQQ
IVDDKVGNLICGWAITSKDGSPMKMGAWPAMAPETVRFVGQAVAVVIAETKNQARDAAEAVVVDYEELPAA
ADIKAAIKPGAPQLHPEAPGNIVYDWHLGDEAAVKDAFSKAANVVTLDLTNNRLVPNAMEPRAAVAHYDQAE
EHYTLYTTSQNPHVARLVLSAFYNIAPEHKLRVIAPDVGGGFGSKIYIYPEEMVALWASKKVGRPVKWTGDRSEA
FLTDAHGRDHVSKAEMAFDKDNKILGLRVKTHANFGAYMSLFSSAVPTYLYATLLSGQYVIPAIYAEVIGVYTNTT
PVDAYRGAGRPEASYLIERMMETAARQLKVDPTELRKKNFITQFPYQTPVIMAYDIGDFHASIDAAMKAIDYAG
FPARKAKAKADGKLRGIGISCYIEACGIAPSKAVGSLGAGVGLWESAEIRVNPVGTIEVLTGSHSHGQGHETTFC
QIIAERLGVPISQVSIVHGDTDKVQFGMGTYGSRSLAVGGTAIVKAMEKVEAKAKKIAAHALEASESDIVIENGE
FKVTGTDKSIALPMVALAAYTAHNLPDGMEPGLKETAFYDPTNFTFPAGAYICELEVDPGTGKTSFVNFVAADD
FGRLINPMIVEGQVHGGLAQGIGQALLEGAIYDDTGQLVTASFMDYTMPRADDLPSFQLSHTTTLCPGNPLGV
KGCGEAGAIGASAAVINAITDAISNNKLEMPATPDRVWHAIHG
>SEQF6036||SEQF6036.1_03379
MAAPIKFGVGQSVRRKEDDALIRGRGRYTDDVAPSPALHALMLRSPHAHATYTIDAGKARGMPGVALILTAAD
VADLGGLPCLFNLETDPFTAPPYPILAKDEVRHVGDAVAFVVADTVDHARDAIEAIDVKWTPLPAAVGLVNAVK
KDAPQVWPDKPGNVLFDVSIGDKKAAEDAFAKAHAVAEITIVNPRVITNFMETRAAVAEYDAKKDHLTLTIGSQ
GSHRLREILCDMILKMPKENMRVICPDVGGGFGTKLFPYREYALISVAARKLKKSIKWTAERSDHFMGDAQGR
DNLTTAKMALAEDGKFLGMDVDLMGDMGAYLSTFAPYIPHGGAGMLPGLYDIQAFHCRVRTVFTNTVPVDA
YRGAGRPEAAYVIERLVDAAARKLGKTPDAIRRKNFIPPKSLPYTTATGKVYDSGDFVAHMKRAMEIANWKEFP
KRAKAARKDGLVRGIGMASYVEVCGTMGEETANVALDPNGDISVLIGTQSSGQGHQTAYAQIVAEQFGVPPER
VHVLQGDTDKIATGLGTGGSASIPSGGVSVQRATHDLGNKLKELAAQALEAGAGDLEIADGRIRIAGTDRSISFA
DLAKRPGGDTSKMNASATFASADGTYPNGTHLAEVEIDPATGIIKIVSYVIVDDFGVTLNPLMLAGQVHGGAM
QGIGQALMEQAVYSPADGQLVTGTFMDYALPRASDGPSFVFETHNVPCTTNPMGVKGAGEAGAIGSCPAVV
NAIVEGLHREYKIDHIDMPATPERIWIAIREAQRRHNL
>SEQF6037||SEQF6037.1_00643
MNILPGNMRFGAGQPVKRLEDQRLVTGKGHFIDDKPQDGALWLHVLRSPHAHANIKSIDAKAALEMPGVKA
VYTGADLVKDGIGTLPTLAIFKRPDGSPMTVPPRRLLAHEVVRYAGEGVAAVVATSRVLAQTAAEAIEIDYEVLPS
VVDPVEAIKPGAPAVWPEAPDNIVAAMSYGDAAKVEEAFANAAHKVSLDLVSQRLVPSAMEPRSTIAEIEKKTG
RLILHVQSQTPGSTRDLLAESILKRPKDSVRVLVGDIGGGFGQKTSLYPEDGIVAYAATKLNEKIRWRGDRTDEFV
GGTHGRDLTSTGEFALDAKGRVLAYRVRSIGGTGAYSSGTANIIPLVLGPFVQTGVYDLPLVHFEVKSVMTHTAPV

GAYRGAGRPEAVFIVERLFDAAARQIGMDPRTIRKVNYIKPAQLPYTNAVGQVYDSGAFAHMLERASDLADW
NGFAARKKAAKKKGLLYGRGLTSYIEWTGGRAHTENVSLHATAEGRVILHSGTMAMGQGLATTYTQMIADTLG
IAMDKIDVIQGDTDLATGFGSVGSRSLFVGGTAVAVSSNDMINKARDKASNLLEASVEDIEYRDGFLTVVGTDR
RISLFEIAAKESGAKLSVESEGNVDGPSWPNGTHICEVEIDPETGVTRVVRYTTVDDVGVAVNPMLVTGQVHG
GVVQGIGQALYEGVAYSEEGQLLTASYQDYCIPRADDIPPITVTLDGSAPCKTNPLGAKGCGESGAIGGPPCITNG
VMDALSEVGIKQLNTPLTPSKIWQAIRDAKVGAA
>SEQF6037||SEQF6037.1_01340
MGVEGIGARVVRKEDRRFITGQGRYVDDVKMVGMSYAHFIRSPHAHAKVKGIDSAEAMKMPGVIAVLTGQQ
IVGDKVGNLICGWAITSKDGSPMKMGAWPAMAPETVRFVGQAVAVVIAETKNQARDAAEAVVVDYEELPAA
ADIKAAIKPGAPQLHPEAPGNIVYDWHLGDEAAVKDAFSKAANVVTLDLTNNRLVPNAMEPRAAVAHYDQAE
EHYTLYTTSQNPHVARLVLSAFYNIAPEHKLRVIAPDVGGGFGSKIYIYPEEMVALWASKKVGRPVKWTGDRSEA
FLTDAHGRDHVSKAELAFDKDNKILGLRVKTHANFGAYMSLFSSAVPTYLYATLLSGQYVIPAIYAEVIGVYTNTTP
VDAYRGAGRPEASYLIERMMETAARQLKVDPTELRKKNFITQFPYQTPVIMAYDIGDFHASIDAAMNAIDYAGF
PARKAKAKADGKLRGIGISCYIEACGIAPSKAVGSLGAGVGLWESAEIRVNPVGTIEVLTGSHSHGQGHETTFCQI
IAERLGVPISQVSVVHGDTDKVQFGMGTYGSRSLAVGGTAIVKAMEKVEAKAKKIAAHALEASESDIVIENGEFK
VTGTDKSIALPMVALAAYTAHNLPDGMEPGLKETAFYDPTNFTFPAGAYICELEVDPGTGKTSFVNFVAADDFG
RLINPMIVEGQVHGGLAQGIGQALLEGAIYDDTGQLVTASFMDYTMPRADDLPSFQLSHTTTLCPGNPLGVKG
CGEAGAIGASAAVINAITDAIGNNKLEMPATPDRVWHAIHG
>SEQF6037||SEQF6037.1_03016
MQDHTPPASLENAIALQKYGVGQPVRRKEDDTLVRGKGKYTDDFSLPGQAYCWMVRSSHAHGLIKGIDTAAA
KAMPGVLGVWTGADLAAAGYNPFTCGLPLKNRDGSPLKQTNRPALVTDKVRFVGDPVAFVVAETAAQARDA
AEAVEVDIEPLPAVTDAAEAAKPGAPQLYDDIPDNVALDYHYGDTAKIEAAFAGAAHVTKLDIVNTRVAVVSME
PRVALAHYDKKTERFTLQVPTQGVSGNKAILARLLNVPADKVRILTGNVGGSFGMKNLNYPEYTCIAHAARELG
RPVKWLDERSTSFLSDSQGRAQLIHAELALDADGKFLAVRLSGYGNLGAYITGVAPGPLSLNTGKNLASVYRTPL
LGVDIKTVLTNTTLMGAYRGAGRPEANYYMERLIDAAADEMGINRFTLRKRNFIKPSQLPFPAASGVIYDSGDF
AGVFQKALEISDYDNFAKRKKESKKSGKLRGIAVGSYLEVTAPPSGELGKISFEPDGSVKLTTGTLDYGQGHATPF
AQVLSDQLGVPFEKITLEQGDSDLVRFGNGTGGSRSITATGQAIVEASALVVEKGKKAAAHMLEASEADIEFGAG
RFTIAGTDRSIGIMELAERMRAGKMPEGTPETLDVDHATKETASTFPNGCHVAEVEIDPDTGVTRIVRYSAVND
FGVVVNPMIVAGQLHGGVAQGIGQALMEEVSYDASGQPITGSFMDYALPRAGDVPSMLVGDHPSPAKSNPL
GTKGCGEAGCAGSLVCIVNAVVDALSEYGIKHINMPLTPERVWRAIQDAKAKAA
>SEQF6037||SEQF6037.1_05555
MAAPIKFGVGQSVRRKEDDALIRGKGRYTDDVAPSPALHALMLRSPHAHATYTIDAGKARGMPGVALILTAAD
VAELGGLPCLFNLETDPFTAPPYPILAKDEVRHVGDAVAFVVADTVDHARDAIEAIDVKWTPLPAAVGLVNAVK
KDAPQVWPDKPGNVLFDVSIGDKKAAEDAFAKAHAVAEITIVNPRVITNFMETRAAVAEYDAKKDHLTLTIGSQ
GSHRLREILCDMILKMPKENMRVICPDVGGGFGTKLFPYREYALISVAARKLKKSIKWTAERSDHFMGDAQGR
DNLTTAKMALAEDGKFLGMDVDLMGDMGAYLSTFAPYIPHGGAGMLPGLYDIQAFHCRVRTVFTNTVPVDA
YRGAGRPEAAYVIERLVDAAARKLGMTPDAIRRKNFIPPKSLPYTTATGKVYDSGDFVAHMKRAMEIANWKEF
PKRAKAARKDGLVRGIGMASYVEVCGTMGEETANVALDANGDISVLIGTQSSGQGHQTAYAQIVAEQFGVPPE
RVHVLQGDTDKIATGLGTGGSASIPSGGVSVQRATHDLGNKLKELAAQALEAGAGDLEIADGRIRIAGTDRSISF
ADLAKRPGSDTSKMNASATFASADGTYPNGTHLAEVEIDPATGIIKIVSYVIVDDFGVTLNPLMLAGQVHGGA
MQGIGQALMEQAVYSPTDGQLVTGTFMDYALPRASDGPSFVFETHNVPCTTNPMGVKGAGEAGAIGSCPAV
VNAIVEGLHREYKIDHIDMPATPERVWIAIREAQRRHNL
>SEQF6037||SEQF6037.1_07640
MTVTRAPADTLSVLDRPNSYIGKTVPRPNLDRLLQGRGQYVSDLELPRMAHLVFLRSPHAHAKIAAIDADAARR

MPGVISIVTGRELEAVITPWVGVLSHLKGLKSAPQHAIAVDRVCWQGEAVAAIVATSRAAAEDAMEHISVDYEE
LEAVTDMRAALDPAAPVIHAALGDNLAFERTLDAGDVDHALSGSELVEADFVFGRHTGVTLEPRAVVADWNA
AEARLTIYQGTQAPHMVQNIAALHLGLREAQVRVVCKDVGGSFGIKVHIYADEMATYALSKLLRRPVKFVADRV
ESFNTDIHARDHRCRGRIGVRPDGTITAFQIDDLTGIGPYSMYPRTSAIEANQVVNLVGGPYATANYRARARVVF
QNKNVMCQYRAVGHPIACSVTEGLVDLAAARIGMDPVEIRRRNLIADDAYPCASPSGMKFEQLSHHASLTKLL
QMMDYDALRAEQAALRARNVHRGIGIASFIEVTNPSAAFYGVGGAKISSQDGVAVRLDAQGSVICQTSITEQG
QGSESLTAQIVGSVLGVSMERVRVILGDTDNTPYGGGTWASRGAGIGGEAALRAAKALRENILNVAAAILQSTP
AELDIVDNGIVNAADGAPRIELHELARIVYFRPDTLPPGIQPELMATRHFVPRQYPFAFTNGVQASWLEVDTET
GFVTLLKHWVVEDCGTIINPQLVDEQIRGGVVQGLGAALFEKCIYDERGQLTNANMADYLVPMSGEMPDIEV
GHVVSPTQESELGAKGAGEAGTAGAAAAVANAVNDALRPFGATITEIPLTPQVILTALGRI
>SEQF6038||SEQF6038.1_04035
MQDHTPPASLENAIALQKYGVGQPVRRKEDDTLVRGKGKYTDDFSLPGQAYCWMVRSSHAHGLIKGIDTAAA
KAMPGVLGVWTGADLAAAGYNPFTCGLPLKNRDGSPLKQTNRPALVTDKVRFVGDPVAFVVAETAAQARDA
AEAVEVDIEPLPAVTDAAEAAKPGAPQLYDDIPDNVALDYHYGDTAKIEAAFAGAAHVTKLDIVNTRVAVVSME
PRVALAHYDKKTERFTLQVPTQGVSGNKAILARLLNVPADKVRILTGNVGGSFGMKNLNYPEYTCIAHAARELG
RPVKWLDERSTSFLSDSQGRAQLIHAELALDADGKFLAVRLSGYGNLGAYITGVAPGPLSLNTGKNLASVYRTPL
LGVDIKTVLTNTTLMGAYRGAGRPEANYYMERLIDAAADEMGINRFTLRKRNFIKPSQLPFPAASGVTYDSGDF
AAVFQKALEISDYDNFAKRKKESKKSGKLRGIAVGSYLEVTAPPSGELGKISFEPDGSVKLTTGTLDYGQGHATPFA
QVLSDQLGVPFEKITLEQGDSDLVRFGNGTGGSRSITATGQAIVEASALVVEKGKKAAAHMLEASEADIEFGAGR
FTIAGTDRSIGIMELAERMRAGKMPEGTPETLDVDHATKETASTFPNGCHVAEVEIDPDTGVTRIVRYSAVNDF
GVVVNPMIVAGQLHGGVAQGIGQALMEEVSYDASGQPITGSFMDYALPRAGDVPSMLVGDHPSPAKSNPLG
TKGCGEAGCAGSLVCIVNAVVDALSDYGIKHINMPLTPERVWRAIQDAKAKAA
>SEQF6038||SEQF6038.1_00211
MTVTRAPADTLSVLDRPNSYIGKTVPRPNLDRLLQGRGQYVSDLELPRMAHVVFLRSPHAHAKIAAIDADAATR
MPGVISIVTGRELEAVITPWVGVLSHLKGLKSAPQHAIAVDRVCWQGEAVAAIVATSRAAAEDAMEHISVDYEE
LEAVTDMRAALDPAASVIHAALGDNLAFERTLDAGDVDLALSGSELVEADFVFGRHTGVTLEPRAVVADWNPA
EARLTIYQGTQAPHMVQNIAALHLGLREAQVRVVCKDVGGSFGIKVHIYADEMATYALSKLLRRPVKFMADRV
ESFNTDIHARDHRCRGRIGVRPDGTITAFEIDDLTGIGPYSMYPRTSAIEANQVVNLVGGPYATANYRARARVVF
QNKNVMCQYRAVGHPIACSVTEGLVDLAAAKIGMDPVEIRRRNLIADDAYPCASPSGMKFERLSHHASLTKLL
QMMDYDALRAEQAALRARNVHRGIGIASFIEVTNPSAAFYGVGGAKISSQDGVAVRLDGQGSVICQTSITEQG
QGSESLTAQIVGSVLGVSMERVRVTLGDTDNTPYGGGTWASRGAGIGGEAALQAAKGLRENILNVAAAILQST
PDGLDIVNNTIVNADDGAPRIELNELARIVYFRPDTLPPGIQPELMATRHFVPRQYPFAFTNGVQASWLEVDTE
TGFVQLLKHWVVEDCGTIINPQLVDEQIRGGVVQGLGAALFEKCIYDERGQLTNANMADYLVPMSGEMPDIEI
GHVVSPTQESELGAKGAGEAGTAGAAAAVANAVNDALRPFGATITEIPLTPQVILTALGRI
>SEQF6038||SEQF6038.1_05520
MGVEGIGARVVRKEDRRFITGQGRYVDDVKMVGMSYAHFIRSPHAHAKVKGIDSAEAMKMPGVIAVLTGQQ
IVDDKVGNLICGWAITSKDGSPMKMGAWPAMAPETVRFVGQAVAVVIAETKNQARDAAEAVVVDYEELPAA
ADIKAAIKPGAPQLHPEAPGNIVYDWHLGDEAAVKDALSKAANVVTLDLTNNRLVPNAMEPRAAVAHYDQAE
EHYTLYTTSQNPHVARLVLSAFYNIAPEHKLRVIAPDVGGGFGSKIYIYPEEMVALWASKKVGRPVKWTGDRSEA
FLTDAHGRDHVSKAEMAFDKDNKILGLRVKTHANFGAYMSLFSSAVPTYLYATLLSGQYVIPAIYAEVIGVYTNTT
PVDAYRGAGRPEASYLIERMMETAARQLKVDPTELRKKNFITQFPYQTPVIMAYDIGDFHASIDAAMKAIDYAG
FPARKAKAKADGKLRGIGISCYIEACGIAPSKAVGSLGAGVGLWESAEIRVNPVGTIEVLTGSHSHGQGHETTFC
QIIAERLGVPISQVSIVHGDTDKVQFGMGTYGSRSLAVGGTAIVKAMEKVEAKAKKIAAHALEASESDIVIENGE
FKVTGTDKSIALPMVALAAYTAHNLPDGMEPGLKETAFYDPTNFTFPAGAYICELEVDPGTGKTSFVNFVAADD

FGRLINPMIVEGQVHGGLAQGIGQALLEGAVYDDTGQLVTASFMDYTMPRADDLPSFQLSHTTTLCPGNPLG
VKGCGEAGAIGASAAVINAITDAIGNNKLEMPATPDRVWHAIHG
>SEQF6038||SEQF6038.1_06942
MNILPGNMRFGAGQPVKRLEDQRLVTGKGHFIDDKPQDGALWLHVLRSPHAHANIKSIDAKTALEMPGVKAV
YTGADLVKDDIGTLPTLAIFKRPDGSPMTVPPRRLLAHEVVRYAGEGVAAVVATSRVLAQTAAEAIEIDYEVLPSV
VDPVEAIKPGAPAVWPEAPDNIVAAMSYGDAAKVEEAFANAAHKVSLDLVSQRLVPSAMEPRSTIAEIEKKTGR
LILHVQSQTPGSTRDLLAESILKRPKDSVRVLVGDIGGGFGQKTSLYPEDGIVAYAATKLNEKIRWRGDRTDEFVG
GTHGRDLTSTGEFALDAKGRVLAYRVRSIGGTGAYSSGTANIIPLVLGPFVQTGVYDLPLVHFEVKSVMTHTAPVG
AYRGAGRPEAVFIVERLFDAAARQIGMDPRTIRKVNYIKPAQLPYTNAVGQVYDSGAFAHMLERASDLADWN
GFAARKKAAKKKGLLYGRGLTSYIEWTGGRAHTEKVSLHATAEGRVILWSGTMAMGQGLATTYTQMVADTLGI
SMDKIDVVQGDTDLATGFGSVGSRSLFVGGTAVAVSTNDMINKARDKASNLLEASVEDIEYRDGFLTVVGTDR
RISLFEIAAKENGAKLSVESEGNVDGPSWPNGTHICEVEIDPETGVTRVVRYTTVDDVGVAVNPMLVTGQVHG
GVVQGIGQALYEGVAYSEEGQLLTASYQDYCIPRADDIPPITVTLDGSAPCKTNPLGAKGCGESGAIGGPPCITNG
VMDALSEVGITQLNTPLTPAKIWQAIRDAKVGAA
>SEQF6038||SEQF6038.1_04185
MAAPIKFGVGQSVRRKEDDALIRGKGRYTDDVAPSPALHALMLRSPHAHATYTIDAGKARGMPGVALILTAAD
VAELGGLPCLFNLETDPFTAPPYPILAKDEVRHVGDAVAFVVADTVDHARDAIEAIDVKWTPLPAAVGLVNAVK
KDAPQVWPDKPGNVLFDVSIGDKKAAEDAFAKAHAVAEITIVNPRVITNFMETRAAVAEYDAKKDHLTLTIGSQ
GSHRLREILCDMILKMPKENMRVICPDVGGGFGTKLFPYREYALISVAARKLKKSIKWTAERSDHFMGDAQGR
DNLTTAKMALAEDGKFLGMDVDLMGDMGAYLSTFAPYIPHGGAGMLPGLYDIQAFHCRVRTVFTNTVPVDA
YRGAGRPEAAYVIERLVDAAARKLGMTPDAIRRKNFIPPKSLPYTTATGKVYDSGDFVAHMKRAMEIANWKEF
PKRAKAARKDGLVRGIGMASYVEVCGTMGEETANVALDANGDISVLIGTQSSGQGHQTAYAQIVAEQFGVPPE
RVHVLQGDTDKIATGLGTGGSASIPSGGVSVQRATHDLGNKLKELAAQALEAGAGDLEIADGRIRIAGTDRSISF
ADLAKRPGGDTSKMNASATFASADGTYPNGTHLAEVEIDPATGIIKIVSYVIVDDFGVTLNPLMLAGQVHGGA
MQGIGQALMEQAVYSPTDGQLVTGTFMDYALPRASDGPSFVFETHNVPCTTNPMGVKGAGEAGAIGSCPAV
VNAIVEGLHREYKIDHIDMPATPERVWIAIREAQRRHNL
>SEQF6038||SEQF6038.1_03297
MEDTAISNEGIGARVLRKEDARHLRGRGQFAGDIKIAGMQEIAFVRSPVAHALITSRRKPAGRETDVLFAEDLAS
VLPITTRSSIPGYKVSDYPALATDRVRFVGEIVAMCVAATRAQAEDLSELAEIEYAELEPIVSCEAGRRPDAPLIHER
WGDNLFLATSFDQGMEEVVASAPVRVDLELSTARQVMHPMEGKGLVAHWDHRAGQLVVQTSTQVPHMIRI
GLAECLGIPQAMIRVIAPDVGGGFGYKCMLTPEEVAVSWLALTRKGAFRWLEDRREHLTAGANARQHEYKITAY
ADRTGRLLGLDAEVAIDCGAYSVWPFSACLEAAQAGGNLPGPYDLKAYRCRTYSVATNKPPFAPYRGVARPGVC
FAIEATIDAIARAIGREPWEVRLENLVAGAAMPYTNITGKHYDSGDYPASLKAVKEMIGLDRLRARGGRDERGRY
LGVGFATYTEQSAHGTKVFATWGLPLVPGFDQAQVKLTPDGALEVRAGIHTIGQGLETTLAQIAHERTGVPLQH
IRVTLGDTALTPFSTGAYASRGIVMSGGAVSVAAGIVADRIRAIAAHLLQARSEDVIFQAGRIVAGEASVGYEDVG
RAWYMRPDQLPDNVDRGGLEAVGAYKPAVDGGVFSYASHAASIAVDPEAGDVEILDYAIVEDCGRMVNPMIV
DGQTFGGAAQGIGTALFEESPYDDNGQPLASTLIDYLLPGPTELPRFRIQHMETPSPYSAHGLKGVGEGGAIAPS
AAIVNAINDALKPLGAVIGQIPASPERILAAIAAAKPGAAA
>SEQF6039||SEQF6039.1_09097
MKASNSYIGRPMERVEDLRLLRGRGTYVADVNRPNQLYAVILRSSTAHGLIKSIDSSAALALPGVHRVLTGKDLG
DNVPRIPLRLQPLPQLETFHQPVIADGKVRYVGEPVAVVIADSAAMAEDALEHIILDIEPLPAVANREHAEAKRS
VLFEDHGSNVAITWKAFRGDADDAFKNADYIRRETFKVQRHAAMFMEPRGFVAEWDAVAGKLTVWGAAKT
AWHNRRTLAAQLDLPIEAVDLIEVDVGGGFGSRGEFYPEDYLIPAAARIAGRPVKWTEDRREHLMSANHARD
MECDVEFATTRDGRFIGLRGQIWSDIGAYVRTNGSVGPRNIAQYMCGPYCMEHVDLKSSMLTTNKTPCGTYR

GPGRYETDFVRERMIDLAAQDLKIDRIELRRRNLVADAQMPYPLPSITPYDSSTELDSGDYHTVFDRCLKEFDWE
EKKKLSGKLIDGYYHGIAIGSFIEGGAAGPKEEVRLVLETDGALSVYMGSSSVGQGLETIMAQIAADAMEMPYH
KITILHGSTAYVKDGYGAYHSRSTVMGGSAILLGAEKLKELIRKTAAERLGCKPEEVIVDSERASFDGNQLSFADLS
EAPLEVEAEFFNKKYTWAYGTQAAHVAVDPGTGHVKVIEVMSVEDVGRMINPLTLHGQAIGSMVQGLGGAFL
EHLVYDDEGQLLTGSFADYLLPTASDFPKLDSVTLELRPCPNNPLGAKGAGEGGLIPVGGLMANAVADALSHLN
VQPMQLPLSPPRIWQLVEEAEASHAKT
>SEQF6039||SEQF6039.1_02269
MNNETRPHSSDVLRPRNVGAAIKRTEDPRLLTGRGEYSADRKPDRLLHVAFLRSGQPHALITRIDTVAAQEAPG
VIAVLTAEGIAGDFKPVIPFSRMPNYYATPIVPLAIGKVRYVGEAVAAVIATSRYLAEDALELIEVDYEPLGAIARAEL
AVVDDAVLLHEEAGTNVLISREFKKGDVVADLKAAAVRVSGRFEMTRKAPLAMEPRSYTAEYDKRRDAVTLYTS
SNIPGIVRDAICESLDLPGHRLRVVAPDVGGSFGSKGSLYPEELLICIAARKLGRSLKWTADRLEEVSSSSQAFAEIV
DADMGFDANGIATSLQADVIGDVGAYSIYPWTCGLEPVQVVSFLPGPYKIASYRGSVRGVATCKPPTGPYRGVG
RPISTFVAERLMDLGAKALGLDPLEIRRRNLIRAEEFPYRIASGIIWDKTGFVECLEAAAEVVGYEQLRAQQADAR
KHGRLFGIGIASYAELTGIGSRIAVAPGMPINTGSETAKITIDSTGAITAAFGVASHGQGLETTLAQIVADDLGARF
EDVRVIQGDSDEVPMSTGTYASRSAVLGGGAAKQASRIVQHKIKRVASHLLEANQDDIEVLDGKAVVIGTDRAV
TFKQVAKAVYSDMKTLPVEAREELTATYTYDPINGTTAAATHIAVVEVDPATCFVKILKYVVAEDCGRIINPMIVV
GQVHGGVAQGIGAALFEELVYDDDGQLLSASLVDYVIPSAPEVPAMDIVHIESESAVAGGFRGMGEGGTIGAP
AAIANAIADALSPLGIDVSILPMTPERIFKLMEQTRLNARGKADE
>SEQF6039||SEQF6039.1_03159
MAAPIKFGVGQSVRRKEDDALIRGKGRYTDDVAPSPALHALMLRSPHAHATYTIDAGKARGMPGVALILTAAD
VAELGGLPCLFNLETDPFTAPPYPILAKDEVRHVGDAVAFVVADTVDHARDAIEAIDVKWTPLPAAVGLVNAVK
KDAPQVWPDKPGNVLFDVSIGDKKAAEDAFAKAHAVAEITIVNPRVITNFMETRAAVAEYDAKKDHLTLTIGSQ
GSHRLREILCDMILKMPKENMRVICPDVGGGFGTKLFPYREYALISVAARKLKKSIKWTAERSDHFMGDAQGR
DNLTTAKMALAEDGKFLGMDVDLMGDMGAYLSTFAPYIPHGGAGMLPGLYDIQAFHCRVRTVFTNTVPVDA
YRGAGRPEAAYVIERLVDAAARKLGMTPDSIRRKNFIPPKSLPYTTATGKVYDSGDFVAHMKRAMEIANWKEFP
KRAKAARKDGLVRGIGMASYVEVCGTMGEETANVALDANGDISVLIGTQSSGQGHQTAYAQIVAEQFGVPPER
VHVLQGDTDKIATGLGTGGSASIPSGGVSVQRATHDLGNKLKELAAQALEAGAGDLEIADGRIRIAGTDRSISFA
DLAKRPGGDTSKMNASATFASADGTYPNGTHLAEVEIDPATGIIKIVSYVIVDDFGVTLNPLMLAGQVHGGAM
QGIGQALMEQAVYSPTDGQLVTGTFMDYALPRASDGPSFVFETHNVPCTTNPMGVKGAGEAGAIGSCPAVV
NAIVEGLHREYKIDHIDMPATPERVWIAIREAQRRHNL
>SEQF6039||SEQF6039.1_05804
MGVEGIGARVVRKEDRRFITGQGRYVDDVKMVGMSYAHFIRSPHAHAKVKGIDSAEAMKMPGVIAVLTGQQ
IVDDKVGNLICGWAITSKDGSPMKMGAWPAMAPETVRFVGQAVAVVIAETKNQARDAAEAVVVDYEELPAA
ADIKAAIKPGAPQLHPEAPGNIVYDWHLGDEAAVKDAFSKAANVVTLDLTNNRLVPNAMEPRAAVAHYDQAE
EHYTLYTTSQNPHVARLVLSAFYNIAPEHKLRVIAPDVGGGFGSKIYIYPEEMVALWASKKVGRPVKWTGDRSEA
FLTDAHGRDHVSKAEMAFDKDNKILGLRVKTHANFGAYMSLFSSAVPTYLYATLLSGQYVIPAIYAEVIGVYTNTT
PVDAYRGAGRPEASYLIERMMETAARQLKVDPTELRKKNFITQFPYQTPVIMAYDIGDFHASIDAAMKAIDYAG
FPARKAKAKADGKLRGIGISCYIEACGIAPSKAVGSLGAGVGLWESAEIRVNPVGTIEVLTGSHSHGQGHETTFC
QIIAERLGVPISQVSIVHGDTDKVQFGMGTYGSRSLAVGGTAIVKAMEKVEAKAKKIAAHALEASESDIVIENGE
FKVTGTDKSIALPMVALAAYTAHNLPDGMEPGLKETAFYDPTNFTFPAGAYICELEVDPGTGKTSFVNFVAADD
FGRLINPMIVEGQVHGGLAQGIGQALLEGAIYDDTGQLVTASFMDYTMPRADDLPSFQLSHTTTLCPGNPLGV
KGCGEAGAIGASAAVINAITDAISNNKLEMPATPDRVWHAIHG
>SEQF6039||SEQF6039.1_00206
MTVTRAPADTLSVLDRPNSYIGKTVPRPNLDRLLQGRGQYVSDLELPRMAHVVFLRSPHAHAKIAAIDADAATR

MPGVISIVTGRELEAVITPWVGVLSHLKGLKSAPQHAIAVDRVCWQGEAVAAIVATSRAAAEDAMEHISVDYEE
LEAVTDMRAALDPAASVIHAALGDNLAFERTLDAGDVDLALSGSELVEADFVFGRHTGVTLEPRAVVADWNPA
EARLTIYQGTQAPHMVQNIAALHLGLREAQVRVVCKDVGGSFGIKVHIYADEMATYALSKLLRRPVKFVADRVE
SFSTDIHARDHRCRGRIGVRPDGTITAFEIDDLTGIGPYSMYPRTSAIEANQVVNLVGGPYATANYRARARVVFQ
NKNVMCQYRAVGHPIACSVTEGLVDLAAAKIGMDPVEIRRRNLIADDAYPCASPSGMKFERLSHHASLTKLLQ
MMDYDALRAEQAALRARNVLRGIGIASFIEVTNPSAAFYGVGGAKISSQDGVAVRLDAQGSVICQTSITEQGQ
GSESLTAQIVGSVLGVSMERVRVTLGDTDNTPYGGGTWASRGAGIGGEAALQAAKALRENILNVAAAILQSTPA
ELDIVDNGIVNAADGAPRIELHELARIVYFRPDTLPPGIQPELMATRHFVPRQYPFAFTNGVQASWLEVDTETG
FVQLLKHWVVEDCGTIINPQLVDEQIRGGVVQGLGAALFEKCIYDERGQLTNANMADYLVPMSGEMPDIEIGH
VVSPTQESELGAKGAGEAGTAGAAAAVANAVNDALRPFGATITEIPLTPQVILTALGRI
>SEQF6039||SEQF6039.1_08700
MQDHTPPASLENAIALQKYGVGQPVRRKEDDTLVRGKGKYTDDFSLPGQAYCWMVRSSHAHGLIKGIDTAAA
KAMPGVLGVWTGADLAAAGYNPFTCGLPLKNRDGSPLKQTNRPALVTDKVRFVGDPVAFVVAETAAQARDA
AEAVEVDIEPLPAVTDAAEAAKPGAPQLYDDIPDNVALDYHYGDTAKIEAAFAGAAHVTKLDIVNTRVAVVSME
PRVALAHYDKKTERFTLQVPTQGVSGNKAILARLLNVPADKVRILTGNVGGSFGMKNLNYPEYTCIAHAARELG
RPVKWLDERSTSFLSDSQGRAQLIHAELALDADGKFLAVRLSGYGNLGAYITGVAPGPLSLNTGKNLASVYRTPL
LGVDIKTVLTNTTLMGAYRGAGRPEANYYMERLIDAAADEMGINRFTLRKRNFIKPSQLPFPAASGVTYDSGDF
AAVFQKALEISDYDNFAKRKKESKKSGKLRGIAVGSYLEVTAPPSGELGKISFEPDGSVKLTTGTLDYGQGHATPFA
QVLSDQLGVPFEKITLEQGDSDLVRFGNGTGGSRSITATGQAIVEASALVVEKGKKAAAHMLEASEADIEFGAGR
FTIAGTDRSIGIMELAERMRAGKMPEGTPETLDVDHATKETSSTFPNGCHVAEVEIDPDTGVTRIVRYSAVNDF
GVVVNPMIVAGQLHGGVAQGIGQALMEEVSYDASGQPITGSFMDYALPRAGDVPSMLVGDHPSPAKSNPLG
TKGCGEAGCAGSLVCIVNAVVDALSDYGIKHINMPLTPERVWRAIQDAKAKAA
>SEQF6040||SEQF6040.1_02092
MNILPGNMRFGAGQPVKRLEDQRLVTGKGHFIDDKPQDGALWLHVLRSPHAHANIKSINAKAALEMPGVKA
VVYTGADLVKDDIGTLPTLAIFKRPDGSPMTVPPRRLLAHEVVRYAGEGVAAVVATSRVLAQTAAEAIEIDYEVLPS
VVDPVEAIKPGAPAVWPEAPDNVVAAMSYGDAAKVEEAFANAAHKVSLDLVSQRLVPSAMEPRSTIAEIEKKT
GRLILHVQSQTPGSTRDLLAESILKRPKDSVRVLVGDIGGGFGQKTSLYPEDGIVAYAATKLNEKIRWRGDRTDEF
VGGTHGRDLTSTGEFALDAKGRVLAYRVRSIGGTGAYSSGTANIIPLVLGPFVQTGVYDLPLVHFEVKSVMTHTAP
VGAYRGAGRPEAVFIVERLFDAAARQIGMDPRTIRKVNYIKPAQLPYTNAVGQVYDSGAFAHMLERASDLAD
WNGFTARKKAAKKKGLLYGRGLTSYIEWTGGRAHTENVSLHATAEGRVILHSGTMAMGQGLATTYTQMIADTL
GIAMDKIDVIQGDTDLATGFGSVGSRSLFVGGTAVAVSSNDMINKARDKASNLLEASVEDIEYRDGFLTVVGTD
RRISLFEIAAKENGAKLSVESEGNVDGPSWPNGTHICEVEIDPETGVTRVARYTTVDDVGVAVNPMLVTGQVH
GGVVQGIGQALYEGVAYSEEGQLLTASYQDYCIPRADDIPPITVTLDGSAPCKTNPLGAKGCGESGAIGGPPCITN
GVMDALSEVGIKQLNTPLTPSKIWQAIQNAKAGA
>SEQF6040||SEQF6040.1_04707
MGVEGIGARVVRKEDRRFITGQGRYVDDVKMVGMSYAHFIRSPHAHAKVKGIDSAEAMKMPGVIAVLTGQQ
IVDDKVGNLICGWAITSKDGSPMKMGAWPAMAPETVRFVGQAVAVVIAETKNLAKDAAEAVVVDYEELPAA
ADIKAAIKPGAPQLHPEAPGNIVYDWHIGDEAAVGDAFSKAANVVTLDLTNNRLVPNAMEPRAAVAHYDQAE
EHYTLYTTSQNPHVARLVLSAFYNIAPEHKLRVIAPDVGGGFGSKIYIYPEEMVALWASKKVNRPVKWTGDRTE
AFLTDAHGRDHVSKAEMAFDKDNKILGLRVKTHANFGAYMSLFSSAVPTYLYATLLSGQYVIPAIYAEVIGVYTNT
TPVDAYRGAGRPEASYLVERMMETAARQLKVDPAELRKKNFITQFPYQTPVIMAYDIGDFNASIDAAMKAIDYA
GFPARKAKAKADGKLRGIGVSCYIEACGIAPSKAVGSLGAGVGLWESAEIRVNPVGTIEVLTGSHSHGQGHETTF
AQLISERLGVPISQVQIVHGDTDKVQFGMGTYGSRSLAVGGTAIVKAMEKVEAKAKKIAAHALEASESDIVIENG
EFKVTGIDKSIALPMVALAAYTAHNLPDGMEPGLKETAFYDPTNFTFPAGAYICELEVDPGTGKTSFVNFVAADD

FGRLINPMIVEGQVHGGLAQGIGQALLEGAVYDGTGQLVTASFMDYSMPRADDLPSFQLSHTTTLCPGNPLG
VKGCGEAGAIGASAAVINAITDAIGNNKLEMPATPDRVWHAIHG
>SEQF6040||SEQF6040.1_04015
MAAPIKFGVGQSVRRKEDDALIRGKGRYTDDVAPSPALHALMLRSPHAHATYTIDAGKARGMPGVALILTAAD
VAELGGLPCLFNLETDPFTAPPYPILAKDEVRHVGDAVAFVVADTVDHARDAIEAIDVKWTPLPAAVGLVNAVK
KDAPQVWPDKPGNVLFDVSIGDKGAAEAAFAKAHAVAEITIVNPRVITNFMETRAAVAEYDAKKDHLTLTIGSQ
GSHRLREILCDMILKMPKENMRVICPDVGGGFGTKLFPYREYALISVAARKLKKSIKWTAERSDHFMGDAQGR
DNLTTAKMALAEDGKFLGMDVDLMGDMGAYLSTFAPYIPHGGAGMLPGLYDIQAFHCRVRTVFTNTVPVDA
YRGAGRPEAAYVIERLVDAAARKLGMTPDAIRRKNFIPPKSLPYTTATGKVYDSGDFVAHMKRAMEIANWKEF
PKRAKAARKEGLVRGIGMASYVEVCGTMGEETANVALDPNGDIAVLIGTQSSGQGHQTAYAQIVAEQFGVPPE
RVHVLQGDTDKIATGLGTGGSASIPSGGVSVQRATHDLGNKLKELAAQALEAGAGDLEIADGRIRIAGTDRSISF
ADLAKRPGGDTSKMNASATFASADGTYPNGTHLAEVEIDPATGIIKIINYVIVDDFGVTLNPLMLAGQVHGGA
MQGIGQALMEQAVYSPTDGQLVTGTFMDYALPRASDGPSFVFETHNVPCTTNPMGVKGAGEAGAIGSCPAV
VNAIVEGLHREYKIDHIDMPATPERVWIAIREAQRRHNL
>SEQF6040||SEQF6040.1_01715
MMVTRAPADALSVLDRPNSYIGKTVPRPNLDRLLQGRGQYVSDLELPRMAHVVFLRSPHAHAKIAAIDADAA
RRMPGVVSIVTGRELEAVITPWVGVLSHLKGLKSAPQHAIAVDRVCWQGEAVAAIVATSRAAAEDAMERISVD
YEELEAVTDMRAALDPAAPVIHAALGDNLAFERTLDAGDVDHALSGSELVEADFVFGRHTGVTLESRAVVADW
NAAETRLTIYQGTQAPHMVQNIAALHLGLREAQVRVVCKDVGGSFGIKVHIYADEMATYALSKLLRRPVKFVAD
RVESFNTDIHARDHRCRGRIGVRPDGTITAFEIDDLTGIGPYSMYPRTSAIEANQVVNLVGGPYATANYRARARV
VFQNKNVMCQYRAVGHPIACSVTEGLVDLAAAKIGMDPVEIRRRNLIADDAYPCASPSGMKFEQLSHHASLTK
LLQMMDYDALRAEQAALRARNVHRGIGIASFIEVTNPSAAFYGVGGAKISSQDGVAVRLDAQGSVICQTSITEQ
GQGSESLTAQIVGSVLGVSMERVRVILGDTEHTPYGGGTWASRGAGIGGEAALRAAKALRQNILNVAAAILQST
PAELDIVDNGIVNAADGAPRIELNELARIVYFRPDTLPPGIQPELMATRHFVPRQYPFAFTNGVQASWLEVDTET
GFVKLLKHWVVEDCGTIINPQLVDEQIRGGVVQGLGAALFEKCIYDERGQLTNANMADYLVPMSGEMPDIEV
GHVVSPTQESELGAKGAGEAGTAGAAAAVANAVNDALRPFGAIITEIPLTPQVILTALGRI
>SEQF6040||SEQF6040.1_02311
MQDHTPPASLENAIALQKYGVGQPVRRKEDDTLVRGKGKYTDDFSLPGQATCWMVRSSHAHGIIKGIDTAAA
RAMPGVLGVWTGADLAAAGYNPFTCGLPLKNRDGSPLKQTNRPALVTDKVRFVGDPVAFVVAETAAQARDA
AEAVGVDIEPLPAVTDAAEAAKPGAPQLYDDIPDNVALDYHYGDTAKIEAAFAGAAHVTKLDIVNTRVAVVSME
PRVALAHYDKKTERFTLQVPTQGVSGNKAILARLLNVPADKVRILTGNVGGSFGMKNLNYPEYTCIAHAARELG
RPVKWLDERSTSFLSDSQGRAQLIHAELALDADGKFLAVRLSGYGNLGAYITGVAPGPLSLNTGKNLASVYRTPL
LGVDIKTVLTNTTLMGAYRGAGRPEANYYMERLIDAAADEMGINRFTLRKRNFIKPSQLPFPAASGVTYDSGDF
AGVFHKALEISDYDNFAKRKKESKKSGKLRGIAVGSYLEVTAPPSGELGKITFEPDGSVKLTTGTLDYGQGHATPF
AQVLSDQLGVPFEKITLEQGDSDLVRFGNGTGGSRSITATGQAIVEASALVVEKGKKAAAHMLEASEADIEFGAG
RFTIAGTDRSIGIMELAERMRAGKMPEGTPETLDVDHSTKETASTFPNGCHVAEVEIDPDTGVTRIVRYSAVND
FGVVVNPMIVAGQLHGGVAQGIGQALMEEISYDASGQPITGSFMDYALPRAGDVPAMLVGDHPSPAKSNPLG
TKGCGEAGCAGSLVCIVNAVVDALSEYGIKHINMPLTPERVWRAIQDAKASAA
>SEQF6040||SEQF6040.1_06377
MGVEGIGARVVRKEDRRFITGQGRYVDDVKMVGMSYAHFIRSPHAHAKVKGIDSAEAMKMPGVIAVLTGQQ
IVDDKVGNLICGWAITSKDGSPMKMGAWPAMAPETVRFVGQAVAVVIAETKNQARDAAEAVVVNYEELPAV
PDIKAAIKPGAAQLHPEAPGNIVYDWHLGDEAAVKDAFSKAANVVTLDLTNNRLVPNAMEPRAAIAHYDQAE
EHYTLYTTSQNPHVARLVLSAFYNIAPEHKLRVIAPDVGGGFGSKIYIYPEEMVALWASKKVGRPVKWTGDRSEA
FLTDAHGRDHVSKAELAFDKDNKILGLRVKTHANFGAYMSLFSSAVPTYLYATLLSGQYVIPAIYAEVIGVYTNTTP

VDAYRGAGRPEASYLIERMMETAARQLKVDPTELRKKNFITQFPYQTPVIMAYDIGDFHASIDAAMKAIDYAGF
PARKAKAKADGKLRGIGISCYIEACGIAPSKAVGSLGAGVGLWESAEIRVNPVGTIEVLTGSHSHGQGHETTFCQI
IAERLGVPISQVSIVHGDTDKVQFGMGTYGSRSLAVGGTAIVKAMEKVEAKAKKIAAHALEASESDIVIENGEFK
VTGTDKSIALPMVALAAYTAHNLPEGMEPGLKETAFYDPTNFTFPAGAYICELEVDPGTGKTSFVNFVAADDFG
RLINPMIVEGQVHGGLAQGIGQALLEGVVYDGTGQLMTASFMDYTMPRADDLPSFQLSHTTTLCPGNPLGVK
GCGEAGAIGASAAVINAITDAIGNNKLEMPATPDRVWHAIHG
>SEQF6041||SEQF6041.1_07088
MAAPIKFGVGQSVRRKEDDALIRGRGRYTDDVAPSPALHALMLRSPHAHATYTIDAGKARGMPGVALILTAAD
VADLGGLPCLFNLETDPFTAPPYPILAKDEVRHVGDAVAFVVADTVDHARDAIEAIDVKWTPLPAAVGLVNAVK
KDAPQVWPDKPGNVLFDVSIGDKKAAEDAFAKAHAVAEITIVNPRVITNFMETRAAVAEYDAKKDHLTLTIGSQ
GSHRLREILCDMILKMPKENMRVICPDVGGGFGTKLFPYREYALISVAARKLKKSIKWTAERSDHFMGDAQGR
DNLTTAKMALAEDGKFLGMDVDLMGDMGAYLSTFAPYIPHGGAGMLPGLYDIQAFHCRVRTVFTNTVPVDA
YRGAGRPEAAYVIERLVDAAARKLGKTPDAIRRKNFIPPKSLPYTTATGKVYDSGDFVAHMKRAMEIANWKEFP
KRAKAARKDGLVRGIGMASYVEVCGTMGEETANVALDPNGDISVLIGTQSSGQGHQTAYAQIVAEQFGVPPER
VHVLQGDTDKIATGLGTGGSASIPSGGVSVQRATHDLGNKLKELAAQALEAGAGDLEIADGRIRIAGTDRSISFA
DLAKRPGGDTSKMNASATFASADGTYPNGTHLAEVEIDPATGIIKIVSYVIVDDFGVTLNPLMLAGQVHGGAM
QGIGQALMEQAVYSPADGQLVTGTFMDYALPRASDGPSFVFETHNVPCTTNPMGVKGAGEAGAIGSCPAVV
NAIVEGLHREYKIDHIDMPATPERIWIAIREAQRRHNL
>SEQF6041||SEQF6041.1_03648
MNILPGNMRFGAGQPVKRLEDQRLVTGKGHFIDDKPQDGALWLHVLRSPHAHANIKSIDAKTALEMPGVKAV
YTGADLVKDDIGTLPTLAIFKRPDGSPMTVPPRRLLAHEVVRYAGEGVAAVVATSRVLAQTAAEAIEIDYEVLPSV
VDPVEAIKPGAPAVWPEAPDNIVAAMSYGDAAKVEEAFANAAHKVSLDLVSQRLVPSAMEPRSTIAEIEKKTGR
LILHVQSQTPGSTRDLLAESILKRPKDSVRVLVGDIGGGFGQKTSLYPEDGIVAYAATKLNEKIRWRGDRTDEFVG
GTHGRDLTSTGEFALDAKGRVLAYRVRSIGGTGAYSSGTANIIPLVLGPFVQTGVYDLPLVHFEVKSVMTHTAPVG
AYRGAGRPEAVFIVERLFDAAARQIGMDPRTIRKVNYIKPAQLPYTNAVGQVYDSGAFAHMLERASDLADWN
GFAARKKAAKKKGLLYGRGLTSYIEWTGGRAHTEKVSLHATAEGRVILWSGTMAMGQGLATTYTQMVADTLGI
SMDKIDVVQGDTDLATGFGSVGSRSLFVGGTAVAVSTNDMINKARDKASNLLEASVEDIEYRDGFLTVVGTDR
RISLFEIAAKENGAKLSVESEGNVDGPSWPNGTHICEVEIDPETGVTRVVRYTTVDDVGVAVNPMLVTGQVHG
GVVQGIGQALYEGVAYSEEGQLLTASYQDYCIPRADDIPPITVTLDGSAPCKTNPLGAKGCGESGAIGGPPCITNG
VMDALSEVGITQLNTPLTPAKIWQAIRDAKVGAA
>SEQF6041||SEQF6041.1_07671
MGVEGIGARVVRKEDRRFITGQGRYVDDVKMVGMSYAHFIRSPHAHAKVKGIDSAEAMKMPGVIAVLTGQQ
IVDDKVGNLICGWAITSKDGSPMKMGAWPAMAPETVRFVGQAVAVVIAETKNQARDAAEAVVVDYEELPAA
ADIKAAIKPGAPQLHPEAPGNIVYDWHLGDEAAVKDAFSKAANVVTLDLTNNRLVPNAMEPRAAVAHYDQAE
EHYTLYTTSQNPHVARLVLSAFYNIAPEHKLRVIAPDVGGGFGSKIYIYPEEMVALWASKKVGRPVKWTGDRSEA
FLTDAHGRDHVSKAEMAFDKDNKILGLRVKTHANFGAYMSLFSSAVPTYLYATLLSGQYVIPAIYAEVIGVYTNTT
PVDAYRGAGRPEASYLIERMMETAARQLKVDPTELRKKNFITQFPYQTPVIMAYDIGDFHASIDAAMKAIDYAG
FPARKAKAKADGKLRGIGISCYIEACGIAPSKAVGSLGAGVGLWESAEIRVNPVGTIEVLTGSHSHGQGHETTFC
QIIAERLGVPISQVSIVHGDTDKVQFGMGTYGSRSLAVGGTAIVKAMEKVEAKAKKIAAHALEASESDIVIENGE
FKVTGTDKSIALPMVALAAYTAHNLPDGMEPGLKETAFYDPTNFTFPAGAYICELEVDPGTGKTSFVNFVAADD
FGRLINPMIVEGQVHGGLAQGIGQALLEGAIYDDTGQLVTASFMDYTMPRADDLPSFQLSHTTTLCPGNPLGV
KGCGEAGAIGASAAVINAITDAISNNKLEMPATPDRVWHAIHG
>SEQF6041||SEQF6041.1_02055
MTVTRAPADTLSVLDRPNSYIGKTVPRPNLDRLLQGRGQYVSDLELPRMAHVVFLRSPHAHAKIAAIDADAATR

MPGVISIVTGRELEAVITPWVGVLSHLKGLKSAPQHAIAVDRVCWQGEAVAAIVATSRAAAEDAMEHISVDYEE
LEAVTDMRAALDPAASVIHAALGDNLAFERTLDAGDVDLALSGSELVEADFVFGRHTGVTLEPRAVVADWNPA
EARLTIYQGTQAPHMVQNIAALHLGLREAQVRVVCKDVGGSFGIKVHIYADEMATYALSKLLRRPVKFVADRVE
SFNTDIHARDHRCRGRIGVRPDGTITAFEIDDLTGIGPYSMYPRTSAIEANQVVNLVGGPYATANYRARARVVFQ
NKNVMCQYRAVGHPIACSVTEGLVDLAAAKIGMDPVEIRRRNLIADDAYPCASPSGMKFERLSHHASLTKLLQ
MMDYDALRAEQAALRARNVHRGIGIASFIEVTNPSAAFYGVGGAKISSQDGVAVRLDAQGSVICQTSITEQGQ
GSESLTAQIVGSVLGVSMERVRVTLGDTDNTPYGGGTWASRGAGIGGEAALQAAKALRENILNVAAAILQSTPA
ELDIVDNGVVNAADGAPRIDLNELARIVYFRPDTLPPGIQPELMATRHFVPRQYPFAFTNGVQASWLEVDTET
GFVQLLKHWVVEDCGTIINPQLVDEQIRGGVVQGLGAALFEKCIYDERGQLTNANMADYLVPMSGEMPDIEIG
HVVSPTQESELGAKGAGEAGTAGAAAAVANAVNDALRPFGATITEIPLTPQVILTALGRI
>SEQF6041||SEQF6041.1_00228
MQDHTPPASLENAIALQKYGVGQPVRRKEDDTLVRGKGKYTDDFSLPGQAYCWMVRSSHAHGLIKGIDTAAA
KAMPGVLGVWTGADLAAAGYNPFTCGLPLKNRDGSPLKQTNRPALVTDKVRFVGDPVAFVVAETAAQARDA
AEAVEVDIEPLPAVTDAAEAAKPGAPQLYDDIPDNVALDYHYGDTAKIEAAFAGAAHVTKLDIVNTRVAVVSME
PRVALAHYDKKTERFTLQVPTQGVSGNKAILARLLNVPADKVRILTGNVGGSFGMKNLNYPEYTCIAHAARELG
RPVKWLDERSTSFLSDSQGRAQLIHAELALDADGKFLAVRLSGYGNLGAYITGVAPGPLSLNTGKNLASVYRTPL
LGVDIKTVLTNTTLMGAYRGAGRPEANYYMERLIDAAADEMGINRFTLRKRNFIKPSQLPFPAASGVTYDSGDF
AAVFQKALEISDYDNFAKRKKESKKSGKLRGIAVGSYLEVTAPPSGELGKISFEPDGSVKLTTGTLDYGQGHATPFA
QVLSDQLGVPFEKITLEQGDSDLVRFGNGTGGSRSITATGQAIVEASALVVEKGKKAAAHMLEASEADIEFGAGR
FTIAGTDRSIGIMELAERMRAGKMPEGTPETLDVDHATKETASTFPNGCHVAEVEIDPDTGVTRIVRYSAVNDF
GVVVNPMIVAGQLHGGVAQGIGQALMEEVSYDASGQPITGSFMDYALPRAGDVPSMLVGDHPSPAKSNPLG
TKGCGEAGCAGSLVCIVNAVVDALSDYGIKHINMPLTPERVWRAIQDAKAKAA
>SEQF6042||SEQF6042.1_06879
MTVTRAPADTLSVLDRPNSYIGKTVPRPNLDRLLQGRGQYVSDLELPRMAHVVFLRSPHAHAKIAAIDADAATR
MPGVISIVTGRELEAVITPWVGVLSHLKGLKSAPQHAIAVDRVCWQGEAVAAIVATSRAAAEDAMEHISVDYEE
LEAVTDMRAALDPAASVIHAALGDNLAFERTLDAGDVDLALSGSELVEADFVFGRHTGVTLEPRAVVADWNPA
EARLTIYQGTQAPHMVQNIAALHLGLREAQVRVVCKDVGGSFGIKVHIYADEMATYALSKLLRRPVKFVADRVE
SFNTDIHARDHRCRGRIGVRPDGTITAFEIDDLTGIGPYSMYPRTSAIEANQVVNLVGGPYATANYRARARVVFQ
NKNVMCQYRAVGHPIACSVTEGLVDLAAAKIGMDPVEIRRRNLIADDAYPCASPSGMKFERLSHHASLTKLLQ
MMDYDALRAEQAALRARNVHRGIGIASFIEVTNPSAAFYGVGGAKISSQDGVAVRLDAQGSVICQTSITEQGQ
GSESLTAQIVGSVLGVSMERVRVTLGDTDNTPYGGGTWASRGAGIGGEAALQAAKALRENILNVAAAILQSTPA
ELDIVDNGVVNAADGAPRIDLNELARIVYFRPDTLPPGIQPELMATRHFVPRQYPFAFTNGVQASWLEVDTET
GFVQLLKHWVVEDCGTIINPQLVDEQIRGGVVQGLGAALFEKCIYDERGQLTNANMADYLVPMSGEMPDIEIG
HVVSPTQESELGAKGAGEAGTAGAAAAVANAVNDALRPFGATITEIPLTPQVILTALGRI
>SEQF6042||SEQF6042.1_06672
MAAPIKFGVGQSVRRKEDDALIRGRGRYTDDVAPSPALHALMLRSPHAHATYTIDAGKARGMPGVALILTAAD
VADLGGLPCLFNLETDPFTAPPYPILAKDEVRHVGDAVAFVVADTVDHARDAIEAIDVKWTPLPAAVGLVNAVK
KDAPQVWPDKPGNVLFDVSIGDKKAAEDAFAKAHAVAEITIVNPRVITNFMETRAAVAEYDAKKDHLTLTIGSQ
GSHRLREILCDMILKMPKENMRVICPDVGGGFGTKLFPYREYALISVAARKLKKSIKWTAERSDHFMGDAQGR
DNLTTAKMALAEDGKFLGMDVDLMGDMGAYLSTFAPYIPHGGAGMLPGLYDIQAFHCRVRTVFTNTVPVDA
YRGAGRPEAAYVIERLVDAAARKLGTTPDAIRRKNFIPPKSLPYTTATGKVYDSGDFVAHMKRAMEIANWKEFP
KRAKAARKDGLVRGIGMASYVEVCGTMGEETANVALDPNGDISVLIGTQSSGQGHQTAYAQIVAEQFGVPPER
VHVLQGDTDKIATGLGTGGSASIPSGGVSVQRATHDLGNKLKELAAQALEAGAGDLEIADGRIRIAGTDRSISFA
DLAKRPGGDTSKMNASATFASADGTYPNGTHLAEVEIDPATGIIKIVSYVIVDDFGVTLNPLMLAGQVHGGAM

QGIGQALMEQAVYSPADGQLVTGTFMDYALPRASDGPSFVFETHNVPCTTNPMGVKGAGEAGAIGSCPAVV
NAIVEGLHREYKIDHIDMPATPERIWIAIREAQRRHNL
>SEQF6042||SEQF6042.1_01722
MGVEGIGARVVRKEDRRFITGQGRYVDDVKMVGMSYAHFIRSPHAHAKVKGIDSAEAMKMPGVIAVLTGQQ
IVDDKVGNLICGWAITSKDGSPMKMGAWPAMAPETVRFVGQAVAVVIAETKNQARDAAEAVVVDYEELPAA
ADIKAAIKPGAPQLHPEAPGNIVYDWHLGDEAAVKDAFSKAANVVTLDLTNNRLVPNAMEPRAAVAHYDQAE
EHYTLYTTSQNPHVARLVLSAFYNIAPEHKLRVIAPDVGGGFGSKIYIYPEEMVALWASKKVGRPVKWTGDRSEA
FLTDAHGRDHVSKAEMAFDKDNKILGLRVKTHANFGAYMSLFSSAVPTYLYATLLSGQYVIPAIYAEVIGVYTNTT
PVDAYRGAGRPEASYLIERMMETAARQLKVDPTELRKKNFITQFPYQTPVIMAYDIGDFHASIDAAMKAIDYAG
FPARKAKAKADGKLRGIGISCYIEACGIAPSKAVGSLGAGVGLWESAEIRVNPVGTIEVLTGSHSHGQGHETTFC
QIIAERLGVPISQVSIVHGDTDKVQFGMGTYGSRSLAVGGTAIVKAMEKVEAKAKKIAAHALEASESDIVIENGE
FKVTGTDKSIALPMVALAAYTAHNLPDGMEPGLKETAFYDPTNFTFPAGAYICELEVDPGTGKTSFVNFVAADD
FGRLINPMIVEGQVHGGLAQGIGQALLEGAIYDDTGQLVTASFMDYTMPRADDLPSFQLSHTTTLCPGNPLGV
KGCGEAGAIGASAAVINAITDAISNNKLEMPATPDRVWHAIHG
>SEQF6042||SEQF6042.1_07801
MNILPGNMRFGAGQPVKRLEDQRLVTGKGHFIDDKPQDGALWLHVLRSPHAHANIKSIDAKTALEMPGVKAV
YTGADLVKDDIGTLPTLAIFKRPDGSPMTVPPRRLLAHEVVRYAGEGVAAVVATSRVLAQTAAEAIEIDYEVLPSV
VDPVEAIKPGAPAVWPEAPDNIVAAMSYGDAAKVEEAFANAAHKVSLDLVSQRLVPSAMEPRSTIAEIEKKTGR
LILHVQSQTPGSTRDLLAESILKRPKDSVRVLVGDIGGGFGQKTSLYPEDGIVAYAATKLNEKIRWRGDRTDEFVG
GTHGRDLTSTGEFALDAKGRVLAYRVRSIGGTGAYSSGTANIIPLVLGPFVQTGVYDLPLVHFEVKSVMTHTAPVG
AYRGAGRPEAVFIVERLFDAAARQIGMDPRTIRKVNYIKPAQLPYTNAVGQVYDSGAFAHMLERASDLADWN
GFAARKKAAKKKGLLYGRGLTSYIEWTGGRAHTEKVSLHATAEGRVILWSGTMAMGQGLATTYTQMVADTLGI
SMDKIDVVQGDTDLATGFGSVGSRSLFVGGTAVAVSTNDMINKARDKASNLLEASVEDIEYRDGFLTVVGTDR
RISLFEIAAKENGAKLSVESEGNVDGPSWPNGTHICEVEIDPETGVTRVVRYTTVDDVGVAVNPMLVTGQVHG
GVVQGIGQALYEGVAYSEEGQLLTASYQDYCIPRADDIPPITVTLDGSAPCKTNPLGAKGCGESGAIGGPPCITNG
VMDALSEVGITQLNTPLTPAKIWQAIRDAKVGAA
>SEQF6042||SEQF6042.1_04690
MQDHTPPASLENAIALQKYGVGQPVRRKEDDTLVRGKGKYTDDFSLPGQAYCWMVRSSHAHGLIKGIDTAAA
KAMPGVLGVWTGADLAAAGYNPFTCGLPLKNRDGSPLKQTNRPALVTDKVRFVGDPVAFVVAETAAQARDA
AEAVEVDIEPLPAVTDAAEAAKPGAPQLYDDIPDNVALDYHYGDTAKIEAAFAGAAHVTKLDIVNTRVAVVSME
PRVALAHYDKKTERFTLQVPTQGVSGNKAILARLLNVPADKVRILTGNVGGSFGMKNLNYPEYTCIAHAARELG
RPVKWLDERSTSFLSDSQGRAQLIHAELALDADGKFLAVRLSGYGNLGAYITGVAPGPLSLNTGKNLASVYRTPL
LGVDIKTVLTNTTLMGAYRGAGRPEANYYMERLIDAAADEMGINRFTLRKRNFIKPSQLPFPAASGVTYDSGDF
AAVFQKALEISDYDNFAKRKKESKKSGKLRGIAVGSYLEVTAPPSGELGKISFEPDGSVKLTTGTLDYGQGHATPFA
QVLSDQLGVPFEKITLEQGDSDLVRFGNGTGGSRSITATGQAIVEASALVVEKGKKAAAHMLEASEADIEFGAGR
FTIAGTDRSIGIMELAERMRAGKMPEGTPETLDVDHATKETASTFPNGCHVAEVEIDPDTGVTRIVRYSAVNDF
GVVVNPMIVAGQLHGGVAQGIGQALMEEVSYDASGQPITGSFMDYALPRAGDVPSMLVGDHPSPAKSNPLG
TKGCGEAGCAGSLVCIVNAVVDALSDYGIKHINMPLTPERVWRAIQDAKAKAA
>SEQF6043||SEQF6043.1_06085
MTVTRAPVDTLSVLDRPNSYIGKTVPRPNLDRLLQGRGQYVSDLELPRMAHVVFLRSPHAHAKIAAIDVDTARR
MPGVISIVTGRELEAVITPWVGVLSHLKGLKSAPQHAIAVDRVCWQGEAVAAIVATSRAAAEDAMEHISVDYEE
LEAVTDMRAALDPAAPVIHAALGDNLAFERTLDAGDVDHALAGSELVEADFVFGRHTGVTLEPRAVVADWNA
AEARLTIYQGTQAPHMVQNIAALHLGLREAQVRVVCKDVGGSFGIKVHIYADEMATYALSKLLRRPVKFVADRV
ESFNTDIHARDHRCRGRIGVRPDGTITAFEIDDLTGIGPYSMYPRTSAIEANQVVNLVGGPYATANYRARARVVF

QNKNVMCQYRAVGHPIACSVTEGLVDLAAAKIGMDPVEIRRRNLIADDAYPCASPSGMKFEQLSHHASLTKLL
QMMDYDVLRAEQAALRARNVHRGIGIASFIEVTNPSAAFYGVGGAKISSQDGVAVRLDAQGSVICQTSITEQG
QGSESLTAQIVGSVLGVSMERVRVILGDTDNTPYGGGTWASRGAGIGGEAALQAAKALRENILNVAAAILQSTP
AELDIVDNGIVNTADGAPRIELHELARIVYFRPDTLPPGIQPELMATRHFVPRQYPFAFTNGVQASWLEVDTETG
FVKLLKHWVVEDCGTIINPQLVDEQIRGGVVQGLGAALFEKCIYDERGQLTNANMADYLVPMSGEMPDIEIGH
VVSPTRESELGAKGAGEAGTAGAAAAVANAVNDALRPFGVTITEIPLTPQVILTALGRI
>SEQF6043||SEQF6043.1_00181
MQDHTPPASLENAIALQKYGVGQPVRRKEDDTLVRGKGKYTDDFSLPGQAYCWMVRSSHAHGVIKGIDTAAA
KAMPGVLGVWTGADLTAAGYNPFTCGLPLKNRDGSPLKQTNRPALVTDKVRFVGDPVAFVVAETAAQARDAA
EAVEVDIEPLPAVTDAAEAARPGAPQLYDDIPDNVALDYHYGDTAKIEAAFAGAAHVTKLDIVNTRVAVVSMEP
RVALAHYDKTTERFTLQVPTQGVSGNKAILARLLNVPADKVRILTGNVGGSFGMKNLNYPEYTCIAHAARELGR
PVKWLDERSTSFLSDSQGRAQLIHAELALDADGKFLAVRLSGHGNLGAYITGVAPGPLSLNTGKNLASVYRTPLL
GVDIKTVLTNTTLMGAYRGAGRPEANYYMERLIDAAADEMGINRFTLRKRNFIKPSQLPFPAASGVTYDSGDFA
GVFQKALEISDYENFTKRKKESKKSGKLRGIAVGSYLEVTAPPSGELGKISFEPDGSVKLTTGTLDYGQGHATPFAQ
VLSDQLGVPFEKITLEQGDSDLVRFGNGTGGSRSITATGQAIVEASALVVEKGKRAAAHMLEASEADIEFGAGRF
TIAGTDRSIGIMELAERMRAGKMPEGTPETLDVDHATKETASTFPNGCHVAEVEIDPDTGVTRIVRYSAVNDFG
VVVNPMIVAGQLHGGVAQGIGQALMEEVSYDASGQPITGSFMDYALPRAGDVPSMLVGDHPSPAKSNPLGT
KGCGEAGCAGSLVCIVNAVVDALSEYGIKHINMPLTPERVWRAIQDAKAKAA
>SEQF6043||SEQF6043.1_05170
MKASNSYIGRPMERVEDLRLLRGRGTYVADVNRPNQLYAVILRSSTAHGLIKSIDSSAALALPGVHRVLTGKDLG
DNVPRIPLRLQPLPQLETFHQPVIADGKVRYVGEPVAVVIADSAAMAEDALEHIILDIEPLPAVANREHAEAKRS
VLFEDHGSNVAITWKAFRGDADDAFKNADYIRRETFKVQRHAAMFMEPRGFVAEWDAVAGKLTVWGAAKT
AWHNRRTLAAQLDLPIEAVDLIEVDVGGGFGSRGEFYPEDYLIPAAARIVGRPVKWTEDRREHLMSANHARD
MECDVEFATTRDGRFIGLRGQIWSDIGAYVRTNGSVGPRNIAQYMCGPYCMEHVDLKSSMLTTNKTPCGTYR
GPGRYETDFVRERMIDLAAQDLKIDRIELRRRNLVADAQMPYPLPSITPYDSSTELDSGDYHTVFDRCLKEFDWE
EKKKLSGKLIDGYYHGIAIGSFIEGGAAGPKEEVRLVLETDGALSVYMGSSSVGQGLETIMAQIAADAMEMPYH
KITILHGSTAYVKDGYGAYHSRSTVMGGSAILLGAEKLKELIRKTAAERLGCKPEEVIVDSERASFDGNQLSFADLS
EAPLEVEAEFFNKKYTWAYGTQAAHVAVDPGTGHVKVIEVMSVEDVGRMINPLTLHGQAIGSMVQGLGGAFL
EHLVYDDEGQLLTGSFADYLLPTASDFPKLDSVTLELRPCPNNPLGAKGAGEGGLIPVGGLMANAVADALSHLN
VQPMQLPLSPPRIWQLVEEAEASHAKT
>SEQF6043||SEQF6043.1_08242
MGVEGIGARVVRKEDRRFITGQGRYVDDVKMVGMSYAHFIRSPHAHAKVKGIDSAEAMKMPGVIAVLTGQQ
IVDDKVGNLICGWAITSKDGSPMKMGAWPAMAPETVRFVGQAVAVVIAETKNQARDAAEAVVVDYEELPAA
ADIKAAIKPGAPQLHPEAPGNIVYDWHLGDEAAVKDAFSKAANVVTLDLTNNRLVPNAMEPRAAIAHYDQAE
EHYTLYTTSQNPHVARLVLSAFYNIAPEHKLRVIAPDVGGGFGSKIYIYPEEMVALWASKKVGRPVKWTGDRSEA
FLTDAHGRDHVSKAELAFDKDNKILGLRVKTHANFGAYMSLFSSAVPTYLYATLLSGQYVIPAIYAEVIGVYTNTTP
VDAYRGAGRPEASYLIERMMETAARQLKVDPTELRKKNFITQFPYQTPVIMAYDIGDFHASIDAAMKAIDYAGF
PARKAKAKADGKLRGIGISCYIEACGIAPSKAVGSLGAGVGLWESAEIRVNPVGTIEVLTGSHSHGQGHETTFCQI
IAERLGVPISQVSIVHGDTDKVQFGMGTYGSRSLAVGGTAIVKAMEKVEAKAKKIAAHALEASESDIVIENGEFK
VTGTDKSIALPMVALAAYTAHNLPDGMEPGLKETAFYDPTNFTFPAGAYICELEVDPGTGKTSFVNFVAADDFG
RLINPMIVEGQVHGGLAQGIGQALLEGAVYDDTGQLVTASFMDYTMPRADDLPSFQLSHTTTLCPGNPLGVK
GCGEAGAIGASAAVINAITDAIGNNKLEMPATPDRVWHAIHG
>SEQF6043||SEQF6043.1_03481
MAAPIKFGVGQSVRRKEDDALIRGRGRYTDDVAPSPALHALMLRSPHAHATYTIDAGKARGMPGVALILTAAD

VAELGGLPCLFNLETDPFTAPPYPILAKDEVRHVGDAVAFVVADTVDHARDAIEAIDVKWTPLPAAVGLVNAVK
KDAPQVWPDKPGNVLFDVSIGDKKAAEDAFAKAHAVAEITIVNPRVITNFMETRAAVAEYDAKKDHLTLTIGSQ
GSHRLREILCDMILKMPKENMRVICPDVGGGFGTKLFPYREYALISVAARKLKKSIKWTAERSDHFMGDAQGR
DNLTTAKMALAEDGKFLGMDVDLMGDMGAYLSTFAPYIPHGGAGMLPGLYDIQAFHCRVRTVFTNTVPVDA
YRGAGRPEAAYVIERLVDAAARKLGMTPDAIRRKNFIPPKSLPYTTATGKVYDSGDFVAHMKRAMEIANWKEF
PKRAKAARKDGLVRGIGMASYVEVCGTMGEETANVALDPNGDISVLIGTQSSGQGHQTAYAQIVAEQFGVPPE
RVHVLQGDTDKIATGLGTGGSASIPSGGVSVQRATHDLGNKLKELAAQALEAGAGDLEIADGRIRIAGTDRSISF
ADLAKRPGGDTSKMNANATFASADGTYPNGTHLAEVEIDPATGIIKIVSYVIVDDFGVTLNPLMLAGQVHGGA
MQGIGQALMEQAVYSPTDGQLVTGTFMDYALPRASDGPSFVFETHNVPCTTNPMGVKGAGEAGAIGSCPAV
VNAIVEGLHREYKIDHIDMPATPERVWIAIREAQRRHNL
>SEQF6043||SEQF6043.1_04930
MNILPGNMRFGAGQPVKRLEDQRLVTGKGHFIDDKPQDGALWLHVLRSPHAHAYIKSIDAKAALEMPGVKAV
YTGADLVKDDIGTLPTLAIFKRPDGSPMTVPPRRLLAHEVVRYAGEGVAAVVATSRVLAQTATEAIEIDYEVLPSV
VDPVEAIKPGAPAVWPEAPDNIVAAMSYGDAAKVEEAFANAAHTVSLDLVSQRLVPSAMEPRSTIAEIEKKTGR
LILHVQSQTPGSTRDLLAESILKRPKDSVRVLVGDIGGGFGQKTSLYPEDGIVAYAATKLNEKIRWRGDRTDEFVG
GTHGRDLTSTGEFALDAKGRVLAYRVRSIGGTGAYSSGTANIIPLVLGPFVQTGVYDLPLVHFEVKSVMTHTAPVG
AYRGAGRPEAVFIVERLFDAAARQIGMDPRTIRKVNYIKPAQLPYTNAVGQVYDSGAFAHMLERASDLADWN
GFAARKKAAKKKGLLYGRGLTSYIEWTGGRAHTENVSLHATAEGRIILHSGTMAMGQGLATTYTQMIADTLGIA
MDKIDVIQGDTDLATGFGSVGSRSLFVGGTAVAVSSNDMINKARDKASNLLEASVEDIEYRDGFLTVVGTDRRIS
LFEIAAKESGAKLSVESEGNVDGPSWPNGTHICEVEIDPETGVTRVVRYTTVDDVGVAVNPMLVTGQVHGGVV
QGIGQALYEGVAYNEEGQLLTASYQDYCIPRADDIPPITVTLDGSAPCKTNPLGAKGCGESGAIGGPPCITNGVM
DALSEVGIIQLNTPLTPSKIWQAIRDAKVGAA
>SEQF6044||SEQF6044.1_08088
MAAPIKFGVGQSVRRKEDDALIRGRGRYTDDVAPSPALHALMLRSPHAHATYTIDAGKARGMPGVALVLTAAD
VAELGGLPCLFNLETDPFTAPPYPILAKDEVRHVGDAVAFVVADTVDHARDAIEAIDVKWTPLPAAVGLVNAVK
KDAPQVWPDKPGNVLFDVSIGDKKAAEDALAKAHAVAEITIVNPRVITNFMETRAAVAEYDAKKDHLTLTIGSQ
GSHRLREILCDMILKMPKENMRVICPDVGGGFGTKLFPYREYALISVAARKLKKSIKWTAERSDHFMGDAQGR
DNLTTAKMALAEDGKFLGMDVDLMGDMGAYLSTFAPYIPHGGAGMLPGLYDIQAFHCRVRTVFTNTVPVDA
YRGAGRPEAAYVIERLVDAAARKLGMTPDAIRRKNFIPPKSLPYTTATGKVYDSGDFVAHMKRAMEIANWKEF
PKRAKAARKDGLVRGIGMASYVEVCGTMGEETANVALDPNGDISVLIGTQSSGQGHQTAYAQIVAEQFGVPPE
RVHVLQGDTDKIATGLGTGGSASIPSGGVSVQRATHDLGNKLKELAAQALEAGAGDLEIADGRIRIAGTDRSISF
ADLAKRPGGDTSKMNASATFASADGTYPNGTHLAEVEIDPATGIIKIVGYVIVDDFGVTLNPLMLAGQVHGGA
MQGIGQALMEQAVYSPADGQLVTGTFMDYALPRASDGPSFVFETHNVPCTTNPMGVKGAGEAGAIGSCPAV
VNAIVEGLHREYKIDHIDMPATPERVWIAIREAQRRHNL
>SEQF6044||SEQF6044.1_07657
MTVTGAPADTLSVLDRPNSYIGKTVPRPNLDRLLQGRGQYVSDLELPRMAHVVFLRSPHAHAKITAIDADAARR
MPGVISIVTGRELEAVITPWVGVLSHLKGLKSAPQHAIAVDRVCWQGEAVAAIVATSRAAAEDAVEHVSVDYEE
LEAVTDMRAALDPAAPVIHAALGDNLAFERTLDAGDVDHALSGSELVEADFVFGRHTGVTLEPRAVVADWNA
AEARLTIYQGTQAPHMVQNIAALHLGLREAQVRVVCKDVGGSFGIKVHIYADEMATYALSKLLRRPVKFVADRV
ESFNTDIHARDHRCRGRIGVRSDGTITAFEIDDLTGIGPYSMYPRTSAIEANQVVNLVGGPYATANYRARARVVF
QNKNVMCQYRAVGHPIACSVTEGLVDLAAAKIGMDPVEIRWRNLIADDAYPCASPSGMKFEQLSHHASLTKLL
QMMDYDALRAEQAALRARNVHRGIGIASFIEVTNPSAAFYGVGGAKISSQDGVALRLDAQGSVICQTSITEQG
QGSESLTAQIVGSVLGVSMERVRVILGDTDNTPYGGGTWASRGAGIGGEAALQAAKALRENILNVAAAILQSTP
AELDIVDNGIVNTADGAPRIELHELARIVYFRPDTLPPGIQPELMATRHFVPRQYPFAFTNGVQASWLEVDTETG

FIKLLKHWVVEDCGTIINPQLVDEQIRGGVVQGLGAALFEKCTYDERGQLTNANMADYLVPMSGEMPDIEIGH
VVSPTQESELGAKGAGEAGTAGAAAAVANAVNDALRPFGATITEIPLTPQVILTALGRI
>SEQF6044||SEQF6044.1_04225
MGVEGIGARVVRKEDRRFITGQGRYVDDVKMVGMSYAHFIRSPHAHAKVKGIDSAEAMKMPGVIAVLTGQQ
IVDDKVGNLICGWAITSKDGSPMKMGAWPAMAPETVRFVGQAVAVVIAETKNQARDAAEAVVVDYEELPAA
ADIKAAIKPGAPQLHPEAPGNIVYDWHLGDEAAVKDAFSKAANVVTLDLTNNRLVPNAMEPRAAVAHYDQAE
EHYTLYTTSQNPHVARLVLSAFYNIAPEHKLRVIAPDVGGGFGSKIYIYPEEMVALWASKKVGRPVKWTGDRSEA
FLTDAHGRDHVSKAEMAFDKDNKILGLRVKTHANFGAYMSLFSSAVPTYLYATLLSGQYVIPAIYAEVIGVYTNTT
PVDAYRGAGRPEASYLIERMMETAARQLKVDPTELRKKNFITQFPYQTPVIMAYDIGDFHASIDAAMKAIDYAG
FPARKAKAKADGKLRGIGISCYIEACGIAPSKAVGSLGAGVGLWESAEIRVNPVGTIEVLTGSHSHGQGHETTFC
QIIAERLGVPISQVSIVHGDTDKVQFGMGTYGSRSLAVGGTAIVKAMEKVEAKAKKIAAHALEASESDIVIENGE
FKVTGTDKSIALPMVALAAYTAHNLPDGMEPGLKETAFYDPTNFTFPAGAYICELEVDPGTGKTSFVNFVAADD
FGRLINPMIVEGQVHGGLAQGIGQALLEGAIYDDTGQLVTASFMDYTMPRADDLPSFQLSHTTTLCPGNPLGV
KGCGEAGAIGASAAVINAITDAISNNKLEMPATPDRVWHAIHG
>SEQF6044||SEQF6044.1_01176
MQDHTPPASLENAIALQKYGVGQPVRRKEDDTLVRGKGKYTDDFSLPGQAYCWMVRSSHAHGLIKGIDTAAA
KAMPGVLGVWTGADLAAAGYNPFTCGLPLKNRDGSPLKQTNRPALVTDKVRFVGDPVAFVVAETAAQARDA
AEAVEVDIEPLPAVTDAAEAAKPGAPQLYDDIPDNVALDYHYGDTAKIEAAFAGAAHVTKLDIVNTRVAVVSME
PRVALAHYDKKTERFTLQVPTQGVSGNKAILARLLNVPADKVRILTGNVGGSFGMKNLNYPEYTCIAHAARELG
RPVKWLDERSTSFLSDSQGRAQLIHAELALDADGKFLAVRLSGYGNLGAYITGVAPGPLSLNTGKNLASVYRTPL
LGVDIKTVLTNTTLMGAYRGAGRPEANYYMERLIDAAADEMGINRFTLRKRNFIKPSQLPFPAASGVTYDSGDF
AAVFQKALEISDYDNFAKRKKESKKSGKLRGIAVGSYLEVTAPPSGELGKISFEPDGSVKLTTGTLDYGQGHATPFA
QVLSDQLGVPFEKITLEQGDSDLVRFGNGTGGSRSITATGQAIVEASALVVEKGKKAAAHMLEASEADIEFGAGR
FTIAGTDRSIGIMELAERMRAGKMPEGTPETLDVDHATKETASTFPNGCHVAEVEIDPDTGVTRIVRYSAVNDF
GVVVNPMIVAGQLHGGVAQGIGQALMEEVSYDASGQPITGSFMDYALPRAGDVPSMLVGDHPSPAKSNPLG
TKGCGEAGCAGSLVCIVNAVVDALSDYGIKHINMPLTPERVWRAIQDAKAKAA
>SEQF6044||SEQF6044.1_06918
MNILPGNMRFGAGQPVKRLEDQRLVTGKGHFIDDKPQDGALWLHVLRSPHAHANIKSIDAKTALEMPGVKAV
YTGADLVKDDIGTLPTLAIFKRPDGSPMTVPPRRLLAHEVVRYAGEGVAAVVATSRVLAQTAAEAIEIDYEVLPSV
VDPVEAIKPGAPAVWPEAPDNIVAAMSYGDAAKVEEAFANAAHKVSLDLVSQRLVPSAMEPRSTIAEIEKKTGR
LILHVQSQTPGSTRDLLAESILKRPKDSVRVLVGDIGGGFGQKTSLYPEDGIVAYAATKLNEKIRWRGDRTDEFVG
GTHGRDLTSTGEFALDAKGRVLAYRVRSIGGTGAYSSGTANIIPLVLGPFVQTGVYDLPLVHFEVKSVMTHTAPVG
AYRGAGRPEAVFIVERLFDAAARQIGMDPRTIRKVNYIKPAQLPYTNAVGQVYDSGAFAHMLERASDLADWN
GFAARKKAAKKKGLLYGRGLTSYIEWTGGRAHTEKVSLHATAEGRVILWSGTMAMGQGLATTYTQMVADTLGI
SMDKIDVVQGDTDLATGFGSVGSRSLFVGGTAVAVSTNDMINKARDKASNLLEASVEDIEYRDGFLTVVGTDR
RISLFEIAAKENGAKLSVESEGNVDGPSWPNGTHICEVEIDPETGVTRVVRYTTVDDVGVAVNPMLVTGQVHG
GVVQGIGQALYEGVAYSEEGQLLTASYQDYCIPRADDIPPITVTLDGSAPCKTNPLGAKGCGESGAIGGPPCITNG
VMDALSEVGITQLNTPLTPAKIWQAIRDAKVGAA
>SEQF6045||SEQF6045.1_07950
MAAPIKFGVGQSVRRKEDDALIRGKGRYTDDVAPSPALHALMLRSPHAHATYMIDAGKARGMPGVALILTAAD
VAELGGLPCLFNLETDPFTAPPYPILAKDEVRHVGDAVAFVVADTVDHARDAIEAIDVKWTPLPAAVGLVNAVK
KDAPQVWPDKPGNVLFDVSIGDKKAAEDAFAKAHAVAEITIVNPRVITNFMETRAAVAEYDAKKDHLTLTIGSQ
GSHRLREILCDMILKMPKENMRVICPDVGGGFGTKLFPYREYALISVAARKLKKSIKWTAERSDHFMGDAQGR
DNLTTAKMALAEDGKFLGMDVDLMGDMGAYLSTFAPYIPHGGAGMLPGLYDIQAFHCRVRTVFTNTVPVDA

YRGAGRPEAAYVIERLVDAAARKLGMTPDAIRRKNFIPPKSLPYTTATGKVYDSGDFVAHMKRAMEIANWKEF
PKRAKAARKDGLVRGIGMASYVEVCGTMGEETANVALDANGDISVLIGTQSSGQGHQTAYAQIVAEQFGVPPE
RVHVLQGDTDKIATGLGTGGSASIPSGGVSVQRATHDLGNKLKELAAQALEAGAGDLEIADGRIRIAGTDRSISF
ADLAKRPGGDTSKMNASATFASADGTYPNGTHLAEVEIDPATGIIKIVNYVIVDDFGVTLNPLMLAGQVHGGA
MQGIGQALMEQAVYSPTDGQLVTGTFMDYALPRASDGPSFVFETHNVPCTTNPMGVKGAGEAGAIGSCPAV
VNAIVEGLHREYKIDHIDMPATPERVWIAIREAQRRHNL
>SEQF6045||SEQF6045.1_02833
MNILPGNMRFGAGQPVKRLEDQRLVTGKGHFIDDKPQDGALWLHVLRSPHAHANIKSIDAKTALEMPGVKAV
YTGADLVKDDIGTLPTLAIFKRPDGSPMTVPPRRLLAHEVVRYAGEGVAAVVATSRVLAQTAAEAIEIDYEVLPSV
VDPVEAIKPGAPAVWPEAPDNIVAAMSYGDAAKVEEAFANAAHKVSLDLVSQRLVPSAMEPRSTIAEIEKKTGR
LILHVQSQTPGSTRDLLAESILKRPKDSVRVLVGDIGGGFGQKTSLYPEDGIVAYAATKLNEKIRWRGDRTDEFVG
GTHGRDLTSTGEFALDAKGRVLAYRVRSIGGTGAYSSGTANIIPLVLGPFVQTGVYDLPLVHFEVKSVMTHTAPVG
AYRGAGRPEAVFIVERLFDAAARQIGMDPRTIRKVNYIKPAQLPYTNAVGQVYDSGAFAHMLERASDLADWN
GFAARKKAAKKKGLLYGRGLTSYIEWTGGRAHTEKVSLHATAEGRVILWSGTMAMGQGLATTYTQMVADTLGI
SMDKIDVVQGDTDLATGFGSVGSRSLFVGGTAVAVSTNDMINKARDKASNLLEASVEDIEYRDGFLTVVGTDR
RISLFEIAAKENGAKLSVESEGNVDGPSWPNGTHICEVEIDPETGVTRVVRYTTVDDVGVAVNPMLVTGQVHG
GVVQGIGQALYEGVAYSEEGQLLTASYQDYCIPRADDIPPITVTLDGSAPCKTNPLGAKGCGESGAIGGPPCITNG
VMDALSEVGITQLNTPLTPAKIWQAIRDAKVGAA
>SEQF6045||SEQF6045.1_00319
MGVEGIGARVVRKEDRRFITGQGRYVDDVKMVGMSYAHFIRSPHAHAKVKGIDSAEAMKMPGVIAVLTGQQ
IVDDKVGNLICGWAITSKDGSPMKMGAWPAMAPETVRFVGQAVAVVIAETKNQARDAAEAVVVDYEELPAA
ADIKAAIKPGAPQLHPEAPGNIVYDWHLGDEAAVKDAFSKAANVVTLDLTNNRLVPNAMEPRAAVAHYDQAE
EHYTLYTTSQNPHVARLVLSAFYNIAPEHKLRVIAPDVGGGFGSKIYIYPEEMVALWASKKVGRPVKWTGDRSEA
FLTDAHGRDHVSKAEMAFDKDNKILGLRVKTHANFGAYMSLFSSAVPTYLYATLLSGQYVIPAIYAEVIGVYTNTT
PVDAYRGAGRPEASYLIERMMETAARQLKVDPTELRKKNFITQFPYQTPVIMAYDIGDFHASIDAAMKAIDYAG
FPARKAKAKADGKLRGIGISCYIEACGIAPSKAVGSLGAGVGLWESAEIRVNPVGTIEVLTGSHSHGQGHETTFC
QIIAERLGVPISQVSIVHGDTDKVQFGMGTYGSRSLAVGGTAIVKAMEKVEAKAKKIAAHALEASESDIVIENGE
FKVTGTDKSIALPMVALAAYTAHNLPDGMEPGLKETAFYDPTNFTFPAGAYICELEVDPGTGKTSFVNFVAADD
FGRLINPMIVEGQVHGGLAQGIGQALLEGAIYDDTGQLVTASFMDYTMPRADDLPSFQLSHTTTLCPGNPLGV
KGCGEAGAIGASAAVINAITDAISNNKLEMPATPDRVWHAIHG
>SEQF6045||SEQF6045.1_03590
MTVTRAPADTLSVLDRPNSYIGKTVPRPNLDRLLQGRGQYVSNLELPRMAHVVFLRSPHAHAKIAAIDADAATR
MPGVISIVTGRELEAVITPWVGVLSHLKGLKSAPQHAIAVDRVCWQGEAVAAIVATSRAAAEDAMEHISVDYEE
LEAVTDMRAALDPAASVIHAALGDNLAFERTLDAGDVDLALSGSELVEADFVFGRHTGVTLEPRAVVADWNPA
EARLTIYQGTQAPHMVQNIAALHLGLREAQVRVVCKDVGGSFGIKVHIYADEMATYALSKLLRRPVKFVADRVE
SFNTDIHARDHRCRGRIGVRPDGTITAFEIDDLTGIGPYSMYPRTSAIEANQVVNLVGGPYATANYRARARVVFQ
NKNVMCQYRAVGHPIACSVTEGLVDLAAAKIGMDPVEIRRRNLIADDAYPCASPSGMKFERLSHHASLTKLLQ
MMDYDALRAEQAALRARNVLRGIGIASFIEVTNPSAAFYGVGGAKISSQDGVAVRLDAQGSVICQTSITEQGQ
GSESLTAQIVGSVLGVSMERVRVTLGDTDNTPYGGGTWASRGAGIGGEAALQAAKALRENILNVAAAILQSTPA
ELDIVDNGVVNAADGAPRIDLNELARIVYFRPDTLPPGIQPELMATRHFVPRQYPFAFTNGVQASWLEVDTET
GFVQLLKHWVVEDCGTIINPQLVDEQIRGGVVQGLGAALFEKCIYDERGQLTNANMADYLVPMSGEMPDIEIG
HVVSPTQESELGAKGAGEAGTAGAAAAVANAVNDALRPFGATITEIPLTPQVILTALGRI
>SEQF6046||SEQF6046.1_04190
MMVTRAPADALSVLDRPNSYIGKTVPRPNLDRLLQGRGQYVSDLELPRMAHVVFLRSPHAHAKIAAIDADAA

RRMPGVVSIVTGRELEAVITPWVGVLSHLKGLKSAPQHAIAVDRVCWQGEAVAAIVATSRAAAEDAMERISVD
YEELEAVTDMRAALDPAAPVIHAALGDNLAFERTLDAGDVDHALSGSELVEADFVFGRHTGVTLESRAVVADW
NAAETRLTIYQGTQAPHMVQNIAALHLGLREAQVRVVCKDVGGSFGIKVHIYADEMATYALSKLLRRPVKFVAD
RVESFNTDIHARDHRCRGRIGVRPDGTITAFEIDDLTGIGPYSMYPRTSAIEANQVVNLVGGPYATANYRARARV
VFQNKNVMCQYRAVGHPIACSVTEGLVDLAAAKIGMDPVEIRRRNLIADDAYPCASPSGMKFEQLSHHASLTK
LLQMMDYDALRAEQAALRARNVHRGIGIASFIEVTNPSAAFYGVGGAKISSQDGVAVRLDAQGSVICQTSITEQ
GQGSESLTAQIVGSVLGVSMERVRVILGDTEHTPYGGGTWASRGAGIGGEAALRAAKALRQNILNVAAAILQST
PAELDIVDNGIVNAADGAPRIELNELARIVYFRPDTLPPGIQPELMATRHFVPRQYPFAFTNGVQASWLEVDTET
GFVKLLKHWVVEDCGTIINPQLVDEQIRGGVVQGLGAALFEKCIYDERGQLTNANMADYLVPMSGEMPDIEV
GHVVSPTQESELGAKGAGEAGTAGAAAAVANAVNDALRPFGAIITEIPLTPQVILTALGRI
>SEQF6046||SEQF6046.1_01625
MGVEGIGARVVRKEDRRFITGQGRYVDDVKMVGMSYAHFIRSPHAHAKVKGIDSAEAMKMPGVIAVLTGQQ
IVDDKVGNLICGWAITSKDGSPMKMGAWPAMAPETVRFVGQAVAVVIAETKNQARDAAEAVVVNYEELPAV
PDIKAAIKPGAAQLHPEAPGNIVYDWHLGDEAAVKDAFSKAANVVTLDLTNNRLVPNAMEPRAAIAHYDQAE
EHYTLYTTSQNPHVARLVLSAFYNIAPEHKLRVIAPDVGGGFGSKIYIYPEEMVALWASKKVGRPVKWTGDRSEA
FLTDAHGRDHVSKAELAFDKDNKILGLRVKTHANFGAYMSLFSSAVPTYLYATLLSGQYVIPAIYAEVIGVYTNTTP
VDAYRGAGRPEASYLIERMMETAARQLKVDPTELRKKNFITQFPYQTPVIMAYDIGDFHASIDAAMKAIDYAGF
PARKAKAKADGKLRGIGISCYIEACGIAPSKAVGSLGAGVGLWESAEIRVNPVGTIEVLTGSHSHGQGHETTFCQI
IAERLGVPISQVSIVHGDTDKVQFGMGTYGSRSLAVGGTAIVKAMEKVEAKAKKIAAHALEASESDIVIENGEFK
VTGTDKSIALPMVALAAYTAHNLPEGMEPGLKETAFYDPTNFTFPAGAYICELEVDPGTGKTSFVNFVAADDFG
RLINPMIVEGQVHGGLAQGIGQALLEGVVYDGTGQLMTASFMDYTMPRADDLPSFQLSHTTTLCPGNPLGVK
GCGEAGAIGASAAVINAITDAIGNNKLEMPATPDRVWHAIHG
>SEQF6046||SEQF6046.1_01436
MQDHTPPASLENAIALQKYGVGQPVRRKEDDTLVRGKGKYTDDFSLPGQATCWMVRSSHAHGIIKGIDTAAA
RAMPGVLGVWTGADLAAAGYNPFTCGLPLKNRDGSPLKQTNRPALVTDKVRFVGDPVAFVVAETAAQARDA
AEAVGVDIEPLPAVTDAAEAAKPGAPQLYDDIPDNVALDYHYGDTAKIEAAFAGAAHVTKLDIVNTRVAVVSME
PRVALAHYDKKTERFTLQVPTQGVSGNKAILARLLNVPADKVRILTGNVGGSFGMKNLNYPEYTCIAHAARELG
RPVKWLDERSTSFLSDSQGRAQLIHAELALDADGKFLAVRLSGYGNLGAYITGVAPGPLSLNTGKNLASVYRTPL
LGVDIKTVLTNTTLMGAYRGAGRPEANYYMERLIDAAADEMGINRFTLRKRNFIKPSQLPFPAASGVTYDSGDF
AGVFHKALEISDYDNFAKRKKESKKSGKLRGIAVGSYLEVTAPPSGELGKITFEPDGSVKLTTGTLDYGQGHATPF
AQVLSDQLGVPFEKITLEQGDSDLVRFGNGTGGSRSITATGQAIVEASALVVEKGKKAAAHMLEASEADIEFGAG
RFTIAGTDRSIGIMELAERMRAGKMPEGTPETLDVDHSTKETASTFPNGCHVAEVEIDPDTGVTRIVRYSAVND
FGVVVNPMIVAGQLHGGVAQGIGQALMEEISYDASGQPITGSFMDYALPRAGDVPAMLVGDHPSPAKSNPLG
TKGCGEAGCAGSLVCIVNAVVDALSEYGIKHINMPLTPERVWRAIQDAKASAA
>SEQF6046||SEQF6046.1_02548
MAAPIKFGVGQSVRRKEDDALIRGKGRYTDDVAPSPALHALMLRSPHAHATYTIDAGKARGMPGVALILTAAD
VAELGGLPCLFNLETDPFTAPPYPILAKDEVRHVGDAVAFVVADTVDHARDAIEAIDVKWTPLPAAVGLVNAVK
KDAPQVWPDKPGNVLFDVSIGDKGAAEAAFAKAHAVAEITIVNPRVITNFMETRAAVAEYDAKKDHLTLTIGSQ
GSHRLREILCDMILKMPKENMRVICPDVGGGFGTKLFPYREYALISVAARKLKKSIKWTAERSDHFMGDAQGR
DNLTTAKMALAEDGKFLGMDVDLMGDMGAYLSTFAPYIPHGGAGMLPGLYDIQAFHCRVRTVFTNTVPVDA
YRGAGRPEAAYVIERLVDAAARKLGMTPDAIRRKNFIPPKSLPYTTATGKVYDSGDFVAHMKRAMEIANWKEF
PKRAKAARKEGLVRGIGMASYVEVCGTMGEETANVALDPNGDIAVLIGTQSSGQGHQTAYAQIVAEQFGVPPE
RVHVLQGDTDKIATGLGTGGSASIPSGGVSVQRATHDLGNKLKELAAQALEAGAGDLEIADGRIRIAGTDRSISF
ADLAKRPGGDTSKMNASATFASADGTYPNGTHLAEVEIDPATGIIKIINYVIVDDFGVTLNPLMLAGQVHGGA

MQGIGQALMEQAVYSPTDGQLVTGTFMDYALPRASDGPSFVFETHNVPCTTNPMGVKGAGEAGAIGSCPAV
VNAIVEGLHREYKIDHIDMPATPERVWIAIREAQRRHNL
>SEQF6046||SEQF6046.1_01315
MNILPGNMRFGAGQPVKRLEDQRLVTGKGHFIDDKPQDGALWLHVLRSPHAHANIKSINAKAALEMPGVKA
VYTGADLVKDDIGTLPTLAIFKRPDGSPMTVPPRRLLAHEVVRYAGEGVAAVVATSRVLAQTAAEAIEIDYEVLPS
VVDPVEAIKPGAPAVWPEAPDNVVAAMSYGDAAKVEEAFANAAHKVSLDLVSQRLVPSAMEPRSTIAEIEKKT
GRLILHVQSQTPGSTRDLLAESILKRPKDSVRVLVGDIGGGFGQKTSLYPEDGIVAYAATKLNEKIRWRGDRTDEF
VGGTHGRDLTSTGEFALDAKGRVLAYRVRSIGGTGAYSSGTANIIPLVLGPFVQTGVYDLPLVHFEVKSVMTHTAP
VGAYRGAGRPEAVFIVERLFDAAARQIGMDPRTIRKVNYIKPAQLPYTNAVGQVYDSGAFAHMLERASDLAD
WNGFTARKKAAKKKGLLYGRGLTSYIEWTGGRAHTENVSLHATAEGRVILHSGTMAMGQGLATTYTQMIADTL
GIAMDKIDVIQGDTDLATGFGSVGSRSLFVGGTAVAVSSNDMINKARDKASNLLEASVEDIEYRDGFLTVVGTD
RRISLFEIAAKENGAKLSVESEGNVDGPSWPNGTHICEVEIDPETGVTRVARYTTVDDVGVAVNPMLVTGQVH
GGVVQGIGQALYEGVAYSEEGQLLTASYQDYCIPRADDIPPITVTLDGSAPCKTNPLGAKGCGESGAIGGPPCITN
GVMDALSEVGIKQLNTPLTPSKIWQAIQNAKAGA
>SEQF6047||SEQF6047.1_07818
MQDHTPPASLENAIALQKYGVGQPVRRKEDDTLVRGKGKYTDDFSLPGQAYCWMVRSSHAHGLIKGIDTAAA
KAMPGVLGVWTGADLAAAGYNPFTCGLPLKNRDGSPLKQTNRPALVTDKVRFVGDPVAFVVAETAAQARDA
AEAVEVDIEPLPAVTDAAEAAKPGAPQLYDDIPDNVALDYHYGDTAKIEAAFAGAAHVTKLDIVNTRVAVVSME
PRVALAHYDKKTERFTLQVPTQGVSGNKAILARLLNVPADKVRILTGNVGGSFGMKNLNYPEYTCIAHAARELG
RPVKWLDERSTSFLSDSQGRAQLIHAELALDADGKFLAVRLSGYGNLGAYITGVAPGPLSLNTGKNLASVYRTPL
LGVDIKTVLTNTTLMGAYRGAGRPEANYYMERLIDAAADEMGINRFTLRKRNFIKPSQLPFPAASGVTYDSGDF
AAVFQKALEISDYDNFTKRKKESKKSGKLRGIAVGSYLEVTAPPSGELGKISFEPDGSVKLTTGTLDYGQGHATPFA
QVLSDQLGVPFEKITLEQGDSDLVRFGNGTGGSRSITATGQAIVEASALVVEKGKKAAAHMLEASEADIEFGAGR
FTIAGTDRSIGIMELAERMRAGKMPEGTPETLDVDHATKETASTFPNGCHVAEVEIDPDTGVTRIVRYSAVNDF
GVVVNPMIVAGQLHGGVAQGIGQALMEEVSYDASGQPITGSFMDYALPRAGDVPSMLVGDHPSPAKSNPLG
TKGCGEAGCAGSLVCIVNAVVDALSEYGIKHINMPLTPERVWRAIQDAKAKAA
>SEQF6047||SEQF6047.1_01735
MGVEGIGARVVRKEDRRFITGQGRYVDDVKMVGMSYAHFIRSPHAHAKVKGIDSAEAMKMPGVIAVLTGQQ
IVDDKVGNLICGWAITSKDGSPMKMGAWPAMAPETVRFVGQAVAVVIAETKNQARDAAEAVVVDYEELPAA
ADIKAAIKPGAPQLHPEAPGNIVYDWHLGDEAAVKDAFSKAANVVTLDLTNNRLVPNAMEPRAAVAHYDQAE
EHYTLYTTSQNPHVARLVLSAFYNIAPEHKLRVIAPDVGGGFGSKIYIYPEEMVALWASKKVGRPVKWTGDRSEA
FLTDAHGRDHVSKAEMAFDKDNKILGLRVKTHANFGAYMSLFSSAVPTYLYATLLSGQYVIPAIYAEVIGVYTNTT
PVDAYRGAGRPEASYLIERMMETAARQLKVDPTELRKKNFITQFPYQTPVIMAYDIGDFHASIDAAMKAIDYAG
FPARKAKAKADGKLRGIGISCYIEACGIAPSKAVGSLGAGVGLWESAEIRVNPVGTIEVLTGSHSHGQGHETTFC
QIIAERLGVPISQVSIVHGDTDKVQFGMGTYGSRSLAVGGTAIVKAMEKVEAKAKKIAAHALEASESDIVIENGE
FKVTGTDKSIALPMVALAAYTAHNLPDGMEPGLKETAFYDPTNFTFPAGAYICELEVDPGTGKTSFVNFVAADD
FGRLINPMIVEGQVHGGLAQGIGQALLEGAVYDDTGQLVTASFMDYTMPRADDLPSFQLSHTTTLCPGNPLG
VKGCGEAGAIGASAAVINAITDAIGNNKLEMPATPDRVWHAIHG
>SEQF6047||SEQF6047.1_04605
MNILPGNMRFGAGQPVKRLEDQRLVTGKGHFIDDKPQDGALWLHVLRSPHAHANIKSIDAKAALEMPGVKA
VYTGADLVKDDIGTLPTLAIFKRPDGSPMTVPPRRLLAHEVVRYAGEGVAAVVATSRVLAQTAAEAIEIDYEVLPS
VVDPVEAIKPGAPAVWPEAPDNIVAAMSYGDAAKVEEAFANAAHKVSLDLVSQRLVPSAMEPRSTIAEIEKKTG
RLILHVQSQTPGSTRDLLAESILKRPKDSVRVLVGDIGGGFGQKTSLYPEDGIVAYAATKLNEKIRWRGDRTDEFV
GGTHGRDLTSTGEFALDAKGRVLAYRVRSIGGTGAYSSGTANIIPLVLGPFVQTGVYDLPLVHFEVKSVMTHTAPV

GAYRGAGRPEAVFIVERLFDAAARQIGMDPRTIRKVNYIKPAQLPYTNAVGQVYDSGAFAHMLERASDLADW
NGFAARKKAAKKKGLLYGRGLTSYIEWTGGRAHTEKVSLHATAEGRVILWSGTMAMGQGLATTYTQMVADTL
GISMDKIDVVQGDTDLATGFGSVGSRSLFVGGTAVAVSTNDMINKARDKASNLLEASVEDIEYRDGFLTVVGTD
RRISLFEIAAKENGAKLSVESEGNVDGPSWPNGTHICEVEIDPETGVTRVVRYTTVDDVGVAVNPMLVTGQVH
GGVVQGIGQALYEGVAYSEEGQLLTASYQDYCIPRADDIPPITVTLDGSAPCKTNPLGAKGCGESGAIGGPPCITN
GVMDALSEVGITQLNTPLTPAKIWQAIRDAKVGAA
>SEQF6047||SEQF6047.1_05280
MTVTRAPADTLSVLDRPNSYIGKTVPRPNLDRLLQGRGQYVSDLELPRMAHVVFLRSPHAHAKIAAIDADAATR
MPRVISIVTGRELEAVITPWVGVLSHLKGLKSAPQHAIAVDRVCWQGEAVAAIVATSRAAAEDAMEHISVDYEE
LEAVTDMRAALDPAAPVIHAALGDNLAFERTLDAGDVDHALSGSELVEADFVFGRHTGVTLEPRAVVADWNA
AEARLTIYQGTQAPHMVQNIAALHLGLREAQVRVVCKDVGGSFGIKVHIYADEMATYALSKLLRRPVKFVADRV
ESFNTDIHARDHRCRGRIGVRPDGTITAFEIDDLTGIGPYSMYPRTSAIEANQVVNLVGGPYVTANYRARARVVF
QNKNMMCQYRAVGHPIACSVTEGLVDLAAAKIGMDPVEIRRRNLIADDAYPCASPSGMKFEQLSHHASLTKLL
QMMDYDALRAEQAAWRARNVHRGIGIASFIEVTNPSAAFYGVGGAKISSQDGVAVRLDAQGSVICQTSITEQ
GQGSESLTAQIVGSVLGVSMERVRVILGDTDNTPYGGGTWASRGAGIGGEAALQAAKALRENILNVAAAILQST
PAELDIVDNGIVNAADGAPRIELHELARIVYFRPDTLPPGIQPELMATRHFVPRQYPFAFTNGVQASWLEVDTET
GFVKLLKHWVVEDCGTIINPQLVDEQIRGGVVQGLGAALFEKCIYDERGQLTNANMADYLVPMSGEMPDIEIG
HVVSPTQESELGAKGAGEAGTAGAAAAVANAVNDALRPFGATITEIPLTPQVILTALGRI
>SEQF6047||SEQF6047.1_05486
MAAPIKFGVGQSVRRKEDDALIRGKGRYTDDVAPSPALHALMLRSPHAHATYTIDAGKARGMPGVALILTAAD
VAELGGLPCLFNLETDPFTAPPYPILAKDEVRHVGDAVAFVVADTVDHARDAIEAIDVKWTPLPAAVGLVNAVK
KDAPQVWPDKPGNVLFDVSIGDKKAAEDAFAKAHAVAEITIVNPRVITNFMETRAAVAEYDAKKDHLTLTIGSQ
GSHRLREILCDMILKMPKENMRVICPDVGGGFGTKLFPYREYALISVAARKLKKSIKWTAERSDHFMGDAQGR
DNLTTAKMALAEDGKFLGMDVDLMGDMGAYLSTFAPYIPHGGAGMLPGLYDIQAFHCRVRTVFTNTVPVDA
YRGAGRPEAAYVIERLVDAAARKLGMTPDSIRRKNFIPPKSLPYTTATGKVYDSGDFVAHMKRAMEIANWKEFP
KRAKAARKDGLVRGIGMASYVEVCGTMGEETANVALDANGDISVLIGTQSSGQGHQTAYAQIVAEQFGVPPER
VHVLQGDTDKIATGLGTGGSASIPSGGVSVQRATHDLGNKLKELAAQALEAGAGDLEIADGRIRIAGTDRSISFA
DLAKRPGGDTSKMNASATFASADGTYPNGTHLAEVEIDPATGIIKIVSYVIVDDFGVTLNPLMLAGQVHGGAM
QGIGQALMEQAVYSPTDGQLVTGTFMDYALPRASDGPSFVFETHNVPCTTNPMGVKGAGEAGAIGSCPAVV
NAIVEGLHREYKIDHIDMPATPERVWIAIREAQRRHNL
>SEQF6048||SEQF6048.1_04767
MGVEGIGARVVRKEDRRFITGQGRYVDDVKMVGMSYAHFIRSPHAHAKVKGIDSAEAMKMPGVIAVLTGQQ
IVDDKVGNLICGWAITSKDGSPMKMGAWPAMAPETVRFVGQAVAVVIAETKNQARDAAEAVVVDYEELPAA
ADIKAAIKPGAPQLHPEAPGNIVYDWHLGDEAAVKDAFSKAANVVTLDLTNNRLVPNAMEPRAAIAHYDQAE
EHYTLYTTSQNPHVARLVLSAFYNIAPEHKLRVIAPDVGGGFGSKIYIYPEEMVALWASKKVGRPVKWTGDRSEA
FLTDAHGRDHVSKAELAFDKDNKILGLRVKTHANFGAYMSLFSSAVPTYLYATLLSGQYVIPAIYAEVIGVYTNTTP
VDAYRGAGRPEASYLIERMMETAARQLKVDPTELRKKNFITQFPYQTPVIMAYDIGDFHASIDAAMKAIDYAGF
PARKAKAKADGKLRGIGISCYIEACGIAPSKAVGSLGAGVGLWESAEIRVNPVGTIEVLTGSHSHGQGHETTFCQI
IAERLGVPISQVSIVHGDTDKVQFGMGTYGSRSLAVGGTAIVKAMEKVEAKAKKIAAHALEASESDIVIENGEFK
VTGTDKSIALPMVALAAYTAHNLPDGMEPGLKETAFYDPTNFTFPAGAYICELEVDPGTGKTSFVDFVAADDFG
RLINPMIVEGQVHGGLAQGIGQALLEGVVYDGTGQLMTASFMDYTMPRADDLPSFQLSHTTTLCPGNPLGVK
GCGEAGAIGASAAVINAITDAIGNNKLEMPATPDRVWHAIHG
>SEQF6048||SEQF6048.1_02797
MQDHTPPASLENAIALQKYGVGQPVRRKEDDTLVRGKGKYTDDFSLPGQATCWMVRSSHAHGLIKGIDTAAA

KAMPGVLGVWTGADLAAAGYNPFTCGLPLKNRDGSPLKQTNRPALVTDKVRFVGDPVAFVVAETAAQARDA
AEAVEVDIEPLPAVTDAAEAARPGAPQLYDDIPDNVALDYHYGDTAKIEAAFAGAAHVTKLDIVNTRVAVVSME
PRVALAHYDKKTERFTLQVPTQGVSGNKAILARLLNVPADKVRILTGNVGGSFGMKNLNYPEYTCIAHAARELG
RPVKWLDERSTSFLSDSQGRAQLIHAELALDADGKFLAVRLSGYGNLGAYITGVAPGPLSLNTGKNLASVYRTPL
LGVDIKTVLTNTTLMGAYRGAGRPEANYYMERLIDAAADEMGINRFTLRKRNFIKPSQLPFPAASGVTYDSGDF
TGVFQKALEISDYENFAKRKKESKKSGKLRGIAVGSYLEVTAPPSGELGKISFGPDGSVKLTTGTLDYGQGHATPFA
QVLSDQLGVPFEKITLEQGDSDLVRFGNGTGGSRSITATGQAIVEASALVVEKGKKAAAHMLEASEADIEFGAGR
FTIAGTDRSIGIMELAERMRAGKMPEGTPETLDVDHATKETASTFPNGCHVAEVEIDPDTGVTRIVRYSAVNDF
GVVVNPMIVAGQLHGGVAQGIGQALMEEVSYDASGQPITGSFMDYALPRAGDVPSMLVGDHPSPAKSNPLG
TKGCGEAGCAGSLVCIVNAVVDALSEYGIKHINMPLTPERVWRAIQDAKAKAA
>SEQF6048||SEQF6048.1_08451
MNNETRPHSSDVLRPRNVGAAIKRTEDPRLLTGRGEYSADRKPDRLLHVAFLRSGQPHALITRIDTVAAQEAPG
VIAVLTAEGIAGDFKPVIPFSRMPNYYATPIVPLAIGKVRYVGEAVAAVIATSRYLAEDALELIEVDYEPLGAIARAEL
AVVDDAVLLHEEAGTNVLISREFKKGDVVADLKAAAVRVSGRFEMTRKAPLAMEPRSYTAEYDKRRDAVTLYTS
SNIPGIVRDAICESLDLPGHRLRVVAPDVGGSFGSKGSLYPEELLICIAARKLGRSLKWTADRLEEVSSSSQAFAEIV
DADMGFDANGIATSLQADVIGDVGAYSIYPWTCGLEPVQVVSFLPGPYKIASYRGSVRGVATCKPPTGPYRGVG
RPISTFVAERLMDLGAKALGLDPLEIRRRNLIRAEEFPYRIASGIIWDKTGFVECLEAAAEVVGYEQLRAQQADAR
KHGRLFGIGIASYAELTGIGSRIAVAPGMPINTGSETAKITIDSTGAITAAFGVASHGQGLETTLAQIVADDLGARF
EDVRVIQGDSDEVPMSTGTYASRSAVLGGGAAKQASRIVQHKIKRVASHLLEANQDDIEVLDGKAVVIGTDRAV
TFKQVAKAVYSDMKTLPVEAREELTATYTYDPINGTTAAATHIAVVEVDPATCFVKILKYVVAEDCGRIINPMIVV
GQVHGGVAQGIGAALFEELVYDDDGQLLSASLVDYVIPSAPEVPAMDIVHIESESAVAGGFRGMGEGGTIGAP
AAIANAIADALSPLGIDVSILPMTPERIFKLMEQTRLNARGKADE
>SEQF6048||SEQF6048.1_08022
MKASNSYIGRPMERVEDLRLLRGRGTYVADVNRPNQLYAVILRSSTAHGLIKSIDSSAALALPGVHRVLTGKDLG
DNVPRIPLRLQPLPQLETFHQPVIADGKVRYVGEPVAVVIADSAAMAEDALEHIVLDIEPLPAVANREHAEAKRS
VLFEDHGSNVAITWKAFRGDADDAFKNADYIRRETFKVQRHAAMFMEPRGFVAEWDAVAGKLTVWGAAKT
AWHNRRTLAAQLDLPIEAVDLIEVDVGGGFGSRGEFYPEDYLIPAAARIVGRPVKWTEDRREHLMSANHARD
MECDVEFATTRDGRFIGLRGQIWSDIGAYVRTNGSVGPRNIAQYMCGPYCMEHVDLKSSMLTTNKTPCGTYR
GPGRYETDFVRERMIDLAAQDLKIDRIELRRRNLVADAQMPYPLPSITPYDSSTELDSGDYHTVFDRCLKEFDWE
EKKKLSGKLIDGYYHGIAIGSFIEGGAAGPKEEVRLVLETDGALSVYMGSSSVGQGLETIMAQIAADAMEMPYH
KITILHGSTAYVKDGYGAYHSRSTVMGGSAILLGAEKLKELIRKTAAERLGCKPEEVIVDSERASFDGNQLSFADLS
EAPLEVEAEFFNKKYTWAYGTQAAHVAVDPGTGHVKVMEVMSVEDVGRMINPLTLHGQAIGSMVQGLGGA
FLEHLVYDDEGQLLTGSFADYLLPTASDFPKLDSVTLELRPCPNNPLGAKGAGEGGLIPVGGLMANAVADALSHL
NVQPMQLPLSPPRIWQLVEEAEASHAKT
>SEQF6048||SEQF6048.1_02047
MTVTRAPADTLSVLDRPNSYIGKTVPRPNLDRLLQGRGQYVSDLELPRMAHVVFLRSPHAHAKIAAIDADAATR
MPGVISIVTGRELEAVITPWVGVLSHLKGLKSAPQHAIAVDRVCWQGEAVAAIVATSRAAAEDAMEHISVDYEE
LEAVTDMRAALDPAAPVIHAALGDNLAFERTLDAGDVDHALSGSELVEADFVFGRHTGVTLEPRAVVADWNA
AEARLTIYQGTQAPHMVQNIAALHLGLREAQVRVVCKDVGGSFGIKVHIYADEMATYALSKLLRRPVKFVADRIE
SFNTDIHARDHRCRGRIGVRPDGMITAFEIDDLTGIGPYSMYPRTSAIEANQVVNLVGGPYATANYRARARVVF
QNKNVMCQYRAVGHPIACSVTEGLVDLAAAKIGMDPVEIRRRNLIADDAYPCASPSGMKFEQLSHHASLTKLL
QMMDYDALRAEQAALRARNVHRGIGIASFIEVTNPSAAFYGVGGAKISSQDGVAVRLDAQGSVICQTSITEQG
QGSESLTAQIVGSVLGVSMERVRVILGDTDNTPYGGGTWASRGAGIGGEAALQAAKALRENILNVAAAILQSTP
TELDIVDNGIVNAADGAPRIELHELARIVYFRPDTLPPGIQPELMATRHFVPRQYPFAFTNGVQASWLEVDTET

GFVKLLKHWVVEDCGTIINPQLVDEQIRGGVVQGLGAALFEKCIYDERGQLTNANMADYLVPMSGEMPDIEIG
HVVSPTQESELGAKGAGEAGTAGAAAAVANAVNDALRPFGATITEIPLTPQVILTALGRI
>SEQF6048||SEQF6048.1_05384
MNILPGNMRFGAGQPVKRLEDQRLVTGKGHFLDDKPQDGALWLHVLRSPHAHANIKSIDAKAALEMPGVKA
VVYTGADLVKDDIGTLPTLAIFKRPDGSPMTVPPRRLLAHEVVRYAGEGVAAVVATSRVLAQTATEAIEIDYEVLPS
VVDPVEAIKPGAPAVWPEAPDNIVAAMSYGDAAKVEEAFANAAHKVSLDLVSQRLVPSAMEPRSTIAEIEKKTG
RLILHVQSQTPGSTRDLLAESILKRPKDSVRVLVGDIGGGFGQKTSLYPEDGIVAYAATKLNEKIRWRGDRTDEFV
GGTHGRDLTSTGEFALDAKGRVLAYRVRSIGGTGAYSSGTANIIPLVLGPFVQTGVYDLPLVHFEVKSVMTHTAPV
GAYRGAGRPEAVFIVERLFDAAARQIGMDPRTIRKVNYIKPAQLPYTNAVGQVYDSGAFAHMLERASDLADW
NGFAARKKAAKKKGLLYGRGLTSYIEWTGGRAHTENVSLHATAEGRVILHSGTMAMGQGLATTYTQMISDTLGI
AMDRIDVIQGDTDLATGFGSVGSRSLFVGGTAVAVSSNDMINKARDKASNLLEASVEDIEYRDGFLAVVGTDRR
ISLFEIAAKENGAKLSVESEGNVDGPSWPNGTHICEVEIDPETGVTRVVRYTTVDDVGVAVNPMLVTGQVHGG
VVQGIGQALYEGVAYSEEGQLLTASYQDYCIPRADDIPPITVTLDGSAPCKTNPLGAKGCGESGAIGGPPCITNGV
MDALSEVGITQLNTPLTPSKIWQAIRDAKVTAA
>SEQF6048||SEQF6048.1_04486
MAAPIKFGVGQSVRRKEDDALIRGRGRYTDDVAPSPALHALMLRSPHAHATYTIDAGKARGMPGVALILTAAD
VAELGGLPCLFNLETDPFTAPPYPILAKDEVRHVGDAVAFVVADTVDHARDAIEAIDVKWTPLPAAVGLVNAVK
KDAPQVWPDKPGNVLFDVSIGDKNAAEDAFAKAHAVAEITIVNPRVITNFMETRAAVAEYDAKKDHLTLTIGSQ
GSHRLREILCDMILKMPKENMRVICPDVGGGFGTKLFPYREYALISVAARKLKKSIKWTAERSDHFMGDAQGR
DNLTTAKMALAEDGKFLGMDVDLMGDMGAYLSTFAPYIPHGGAGMLPGLYDIQAFHCRVRTVFTNTVPVDA
YRGAGRPEAAYVIERLVDAAARKLGMTPDAIRRKNFIPPKSLPYTTATGKVYDSGDFVAHMKRAMEIANWKEF
PKRAKAARKDGLVRGIGMASYVEVCGTMGEETANVALDPNGDISVLIGTQSSGQGHQTAYAQIVAEQFGVPPE
RVHVLQGDTDKIATGLGTGGSASIPSGGVSVQRATHDLGNKLKELAAQALEAGAGDLEIADGRIRIAGTDRSISF
ADLAKRPGGDTSKMNASATFASADGTYPNGTHLAEVEIDPATGIIKIVSYVIVDDFGVTLNPLMLAGQVHGGA
MQGIGQALMEQAVYSPADGQLVTGTFMDYALPRASDGPSFVFETHNVPCTTNPMGVKGAGEAGAIGSCPAV
VNAIVEGLHREYKIDHIDMPATPERVWIAIREAQRRHNL
>SEQF6049||SEQF6049.1_05605
MNILPGNMRFGAGQPVKRLEDQRLVTGKGHFIDDKPQDGALWLHVLRSPHAHANIKSIDAKTALEMPGVKAV
YTGADLVKDDIGTLPTLAIFKRPDGSPMTVPPRRLLAHEVVRYAGEGVAAVVATSRVLAQTAAEAIEIDYEVLPSV
VDPVEAIKPGAPAVWPEAPDNIVAAMSYGDAAKVEEAFANAAHKVSLDLVSQRLVPSAMEPRSTIAEIEKKTGR
LILHVQSQTPGSTRDLLAESILKRPKDSVRVLVGDIGGGFGQKTSLYPEDGIVAYAATKLNEKIRWRGDRTDEFVG
GTHGRDLTSTGEFALDAKGRVLAYRVRSIGGTGAYSSGTANIIPLVLGPFVQTGVYDLPLVHFEVKSVMTHTAPVG
AYRGAGRPEAVFIVERLFDAAARQIGMDPRTIRKVNYIKPAQLPYTNAVGQVYDSGAFAHMLERASDLADWN
GFAARKKAAKKKGLLYGRGLTSYIEWTGGRAHTEKVSLHATAEGRVILWSGTMAMGQGLATTYTQMVADTLGI
SMDKIDVVQGDTDLATGFGSVGSRSLFVGGTAVAVSTNDMINKARDKASNLLEASVEDIEYRDGFLTVVGTDR
RISLFEIAAKENGAKLSVESEGNVDGPSWPNGTHICEVEIDPETGVTRVVRYTTVDDVGVAVNPMLVTGQVHG
GVVQGIGQALYEGVAYSEEGQLLTASYQDYCIPRADDIPPITVTLDGSAPCKTNPLGAKGCGESGAIGGPPCITNG
VMDALSEVGITQLNTPLTPAKIWQAIRDAKVGAA
>SEQF6049||SEQF6049.1_01565
MTVTRAPADTLSVLDRPNSYIGKTVPRPNLDRLLQGRGQYVSDLELPRMAHVVFLRSPHAHAKIAAIDADAATR
MPGVISIVTGRELEAVITPWVGVLSHLKGLKSAPQHAIAVDRVCWQGEAVAAIVATSRAAAEDAMEHISVDYEE
LEAVTDMRAALDPAASVIHAALGDNLAFERTLDAGDVDLALSGSELVEADFVFGRHTGVTLEPRAVVADWNPA
EARLTIYQGTQAPHMVQNIAALHLGLREAQVRVVCKDVGGSFGIKVHIYADEMATYALSKLLRRPVKFMADRV
ESFNTDIHARDHRCRGRIGVRPDGTITAFEIDDLTGIGPYSMYPRTSAIEANQVVNLVGGPYATANYRARARVVF

QNKNVMCQYRAVGHPIACSVTEGLVDLAAAKIGMDPVEIRRRNLIADDAYPCASPSGMKFERLSHHASLTKLL
QMMDYDALRAEQAALRARNVHRGIGIASFIEVTNPSAAFYGVGGAKISSQDGVAVRLDGQGSVICQTSITEQG
QGSESLTAQIVGSVLGVSMERVRVTLGDTDNTPYGGGTWASRGAGIGGEAALQAAKGLRENILNVAAAILQST
PDGLDIVNNTIVNADDGAPRIELNELARIVYFRPDTLPPGIQPELMATRHFVPRQYPFAFTNGVQASWLEVDTE
TGFVQLLKHWVVEDCGTIINPQLVDEQIRGGVVQGLGAALFEKCIYDERGQLTNANMADYLVPMSGEMPDIEI
GHVVSPTQESELGAKGAGEAGTAGAAAAVANAVNDALRPFGATITEIPLTPQVILTALGRI
>SEQF6049||SEQF6049.1_08250
MGVEGIGARVVRKEDRRFITGQGRYVDDVKMVGMSYAHFIRSPHAHAKVKGIDSAEAMKMPGVIAVLTGQQ
IVDDKVGNLICGWAITSKDGSPMKMGAWPAMAPETVRFVGQAVAVVIAETKNQARDAAEAVVVDYEELPAA
ADIKAAIKPGAPQLHPEAPGNIVYDWHLGDEAAVKDALSKAANVVTLDLTNNRLVPNAMEPRAAVAHYDQAE
EHYTLYTTSQNPHVARLVLSAFYNIAPEHKLRVIAPDVGGGFGSKIYIYPEEMVALWASKKVGRPVKWTGDRSEA
FLTDAHGRDHVSKAEMAFDKDNKILGLRVKTHANFGAYMSLFSSAVPTYLYATLLSGQYVIPAIYAEVIGVYTNTT
PVDAYRGAGRPEASYLIERMMETAARQLKVDPTELRKKNFITQFPYQTPVIMAYDIGDFHASIDAAMKAIDYAG
FPARKAKAKADGKLRGIGISCYIEACGIAPSKAVGSLGAGVGLWESAEIRVNPVGTIEVLTGSHSHGQGHETTFC
QIIAERLGVPISQVSIVHGDTDKVQFGMGTYGSRSLAVGGTAIVKAMEKVEAKAKKIAAHALEASESDIVIENGE
FKVTGTDKSIALPMVALAAYTAHNLPDGMEPGLKETAFYDPTNFTFPAGAYICELEVDPGTGKTSFVNFVAADD
FGRLINPMIVEGQVHGGLAQGIGQALLEGAVYDDTGQLVTASFMDYTMPRADDLPSFQLSHTTTLCPGNPLG
VKGCGEAGAIGASAAVINAITDAIGNNKLEMPATPDRVWHAIHG
>SEQF6049||SEQF6049.1_02742
MEDTAISNEGIGARVLRKEDARHLRGRGQFAGDIKIAGMQEIAFVRSPVAHALITSRRKPAGRETDVLFAEDLAS
VLPITTRSSIPGYKVSDYPALATDRVRFVGEIVAMCVAATRAQAEDLSELAEIEYAELEPIVSCEAGRRPDAPLIHER
WGDNLFLATSFDQGMEEVVASAPVRVDLELSTARQVMHPMEGKGLVAHWDHRAGQLVVQTSTQVPHMIRI
GLAECLGIPQAMIRVIAPDVGGGFGYKCMLTPEEVAVSWLALTRKGAFRWLEDRREHLTAGANARQHEYKITAY
ADRTGRLLGLDAEVAIDCGAYSVWPFSACLEAAQAGGNLPGPYDLKAYRCRTYSVATNKPPFAPYRGVARPGVC
FAIEATIDAIARAIGREPWEVRLENLVAGAAMPYTNITGKHYDSGDYPASLKAVKEMIGLDRLRARGGRDERGRY
LGVGFATYTEQSAHGTKVFATWGLPLVPGFDQAQVKLTPDGALEVRAGIHTIGQGLETTLAQIAHERTGVPLQH
IRVTLGDTALTPFSTGAYASRGIVMSGGAVSVAAGIVADRIRAIAAHLLQARSEDVIFQAGRIVAGEASVGYEDVG
RAWYMRPDQLPDNVDRGGLEAVGAYKPAVDGGVFSYASHAASIAVDPEAGDVEILDYAIVEDCGRMVNPMIV
DGQTFGGAAQGIGTALFEESPYDDNGQPLASTLIDYLLPGPTELPRFRIQHMETPSPYSAHGLKGVGEGGAIAPS
AAIVNAINDALKPLGAVIGQIPASPERILAAIAAAKPGAAA
>SEQF6049||SEQF6049.1_04643
MQDHTPPASLENAIALQKYGVGQPVRRKEDDTLVRGKGKYTDDFSLPGQAYCWMVRSSHAHGLIKGIDTAAA
KAMPGVLGVWTGADLAAAGYNPFTCGLPLKNRDGSPLKQTNRPALVTDKVRFVGDPVAFVVAETAAQARDA
AEAVEVDIEPLPAVTDAAEAAKPGAPQLYDDIPDNVALDYHYGDTAKIEAAFAGAAHVTKLDIVNTRVAVVSME
PRVALAHYDKKTERFTLQVPTQGVSGNKAILARLLNVPADKVRILTGNVGGSFGMKNLNYPEYTCIAHAARELG
RPVKWLDERSTSFLSDSQGRAQLIHAELALDADGKFLAVRLSGYGNLGAYITGVAPGPLSLNTGKNLASVYRTPL
LGVDIKTVLTNTTLMGAYRGAGRPEANYYMERLIDAAADEMGINRFTLRKRNFIKPSQLPFPAASGVTYDSGDF
AAVFQKALEISDYDNFAKRKKESKKSGKLRGIAVGSYLEVTAPPSGELGKISFEPDGSVKLTTGTLDYGQGHATPFA
QVLSDQLGVPFEKITLEQGDSDLVRFGNGTGGSRSITATGQAIVEASALVVEKGKKAAAHMLEASEADIEFGAGR
FTIAGTDRSIGIMELAERMRAGKMPEGTPETLDVDHATKETASTFPNGCHVAEVEIDPDTGVTRIVRYSAVNDF
GVVVNPMIVAGQLHGGVAQGIGQALMEEVSYDASGQPITGSFMDYALPRAGDVPSMLVGDHPSPAKSNPLG
TKGCGEAGCAGSLVCIVNAVVDALSDYGIKHINMPLTPERVWRAIQDAKAKAA
>SEQF6049||SEQF6049.1_01848
MAAPIKFGVGQSVRRKEDDALIRGKGRYTDDVAPSPALHALMLRSPHAHATYTIDAGKARGMPGVALILTAAD

VAELGGLPCLFNLETDPFTAPPYPILAKDEVRHVGDAVAFVVADTVDHARDAIEAIDVKWTPLPAAVGLVNAVK
KDAPQVWPDKPGNVLFDVSIGDKKAAEDAFAKAHAVAEITIVNPRVITNFMETRAAVAEYDAKKDHLTLTIGSQ
GSHRLREILCDMILKMPKENMRVICPDVGGGFGTKLFPYREYALISVAARKLKKSIKWTAERSDHFMGDAQGR
DNLTTAKMALAEDGKFLGMDVDLMGDMGAYLSTFAPYIPHGGAGMLPGLYDIQAFHCRVRTVFTNTVPVDA
YRGAGRPEAAYVIERLVDAAARKLGMTPDAIRRKNFIPPKSLPYTTATGKVYDSGDFVAHMKRAMEIANWKEF
PKRAKAARKDGLVRGIGMASYVEVCGTMGEETANVALDANGDISVLIGTQSSGQGHQTAYAQIVAEQFGVPPE
RVHVLQGDTDKIATGLGTGGSASIPSGGVSVQRATHDLGNKLKELAAQALEAGAGDLEIADGRIRIAGTDRSISF
ADLAKRPGGDTSKMNASATFASADGTYPNGTHLAEVEIDPATGIIKIVSYVIVDDFGVTLNPLMLAGQVHGGA
MQGIGQALMEQAVYSPTDGQLVTGTFMDYALPRASDGPSFVFETHNVPCTTNPMGVKGAGEAGAIGSCPAV
VNAIVEGLHREYKIDHIDMPATPERVWIAIREAQRRHNL
>SEQF6050||SEQF6050.1_05669
MNNETRPHSSDVLRPRNVGAAIKRTEDPRLLTGRGEYSADRKPDRLLHVAFLRSGQPHALITRIDTVAAQEAPG
VIAVLTAEGIAGDFKPVIPFSRMPNYYATPIVPLAIGKVRYVGEAVAAVIATSRYLAEDALELIEVDYEPLGAIARAEL
AVVDDAVLLHEEAGTNVLISREFKKGDVVADLKAAAVRVSGRFEMTRKAPLAMEPRSYTAEYDKRRDAVTLYTS
SNIPGIVRDAICESLDLPGHRLRVVAPDVGGSFGSKGSLYPEELLICIAARKLGRSLKWTADRLEEVSSSSQAFAEIV
DADMGFDANGIATSLQADVIGDVGAYSIYPWTCGLEPVQVVSFLPGPYKIASYRGSVRGVATCKPPTGPYRGVG
RPISTFVAERLMDLGAKALGLDPLEIRRRNLIRAEEFPYRIASGIIWDKTGFVECLEAAAEVVGYEQLRAQQADAR
KHGRLFGIGIASYAELTGIGSRIAVAPGMPINTGSETAKITIDSTGAITAAFGVASHGQGLETTLAQIVADDLGARF
EDVRVIQGDSDEVPMSTGTYASRSAVLGGGAAKQASRIVQHKIKRVASHLLEANQDDIEVLDGKAVVIGTDRAV
TFKQVAKAVYSDMKTLPVEAREELTATYTYDPINGTTAAATHIAVVEVDPATCFVKILKYVVAEDCGRIINPMIVV
GQVHGGVAQGIGAALFEELVYDDDGQLLSASLVDYVIPSAPEVPAMDIVHIESESAVAGGFRGMGEGGTIGAP
AAIANAIADALSPLGIDVSILPMTPERIFKLMEQTRLNARGKADE
>SEQF6050||SEQF6050.1_02975
MAAPIKFGVGQSVRRKEDDALIRGKGRYTDDVAPSPALHALMLRSPHAHATYTIDAGKARGMPGVALILTAAD
VAELGGLPCLFNLETDPFTAPPYPILAKDEVRHVGDAVAFVVADTVDHARDAIEAIDVKWTPLPAAVGLVNAVK
KDAPQVWPDKPGNVLFDVSIGDKKAAEDAFAKAHAVAEITIVNPRVITNFMETRAAVAEYDAKKDHLTLTIGSQ
GSHRLREILCDMILKMPKENMRVICPDVGGGFGTKLFPYREYALISVAARKLKKSIKWTAERSDHFMGDAQGR
DNLTTAKMALAEDGKFLGMDVDLMGDMGAYLSTFAPYIPHGGAGMLPGLYDIQAFHCRVRTVFTNTVPVDA
YRGAGRPEAAYVIERLVDAAARKLGMTPDSIRRKNFIPPKSLPYTTATGKVYDSGDFVAHMKRAMEIANWKEFP
KRAKAARKDGLVRGIGMASYVEVCGTMGEETANVALDANGDISVLIGTQSSGQGHQTAYAQIVAEQFGVPPER
VHVLQGDTDKIATGLGTGGSASIPSGGVSVQRATHDLGNKLKELAAQALEAGAGDLEIADGRIRIAGTDRSISFA
DLAKRPGGDTSKMNASATFASADGTYPNGTHLAEVEIDPATGIIKIVSYVIVDDFGVTLNPLMLAGQVHGGAM
QGIGQALMEQAVYSPTDGQLVTGTFMDYALPRASDGPSFVFETHNVPCTTNPMGVKGAGEAGAIGSCPAVV
NAIVEGLHREYKIDHIDMPATPERVWIAIREAQRRHNL
>SEQF6050||SEQF6050.1_00073
MTVTRAPADTLSVLDRPNSYIGKTVPRPNLDRLLQGRGQYVSDLELPRMAHVVFLRSPHAHAKIAAIDADAATR
MPGVISIVTGRELEAVITPWVGVLSHLKGLKSAPQHAIAVDRVCWQGEAVAAIVATSRAAAEDAMEHISVDYEE
LEAVTDMRAALDPAASVIHAALGDNLAFERTLDAGDVDLALSGSELVEADFVFGRHTGVTLEPRAVVADWNPA
EARLTIYQGTQAPHMVQNIAALHLGLREAQVRVVCKDVGGSFGIKVHIYADEMATYALSKLLRRPVKFVADRVE
SFSTDIHARDHRCRGRIGVRPDGTITAFEIDDLTGIGPYSMYPRTSAIEANQVVNLVGGPYATANYRARARVVFQ
NKNVMCQYRAVGHPIACSVTEGLVDLAAAKIGMDPVEIRRRNLIADDAYPCASPSGMKFERLSHHASLTKLLQ
MMDYDALRAEQAALRARNVLRGIGIASFIEVTNPSAAFYGVGGAKISSQDGVAVRLDAQGSVICQTSITEQGQ
GSESLTAQIVGSVLGVSMERVRVTLGDTDNTPYGGGTWASRGAGIGGEAALQAAKALRENILNVAAAILQSTPA
ELDIVDNGIVNAADGAPRIELHELARIVYFRPDTLPPGIQPELMATRHFVPRQYPFAFTNGVQASWLEVDTETG

FVQLLKHWVVEDCGTIINPQLVDEQIRGGVVQGLGAALFEKCIYDERGQLTNANMADYLVPMSGEMPDIEIGH
VVSPTQESELGAKGAGEAGTAGAAAAVANAVNDALRPFGATITEIPLTPQVILTALGRI
>SEQF6050||SEQF6050.1_00168
MKASNSYIGRPMERVEDLRLLRGRGTYVADVNRPNQLYAVILRSSTAHGLIKSIDSSAALALPGVHRVLTGKDLG
DNVPRIPLRLQPLPQLETFHQPVIADGKVRYVGEPVAVVIADSAAMAEDALEHIILDIEPLPAVANREHAEAKRS
VLFEDHGSNVAITWKAFRGDADDAFKNADYIRRETFKVQRHAAMFMEPRGFVAEWDAVAGKLTVWGAAKT
AWHNRRTLAAQLDLPIEAVDLIEVDVGGGFGSRGEFYPEDYLIPAAARIAGRPVKWTEDRREHLMSANHARD
MECDVEFATTRDGRFIGLRGQIWSDIGAYVRTNGSVGPRNIAQYMCGPYCMEHVDLKSSMLTTNKTPCGTYR
GPGRYETDFVRERMIDLAAQDLKIDRIELRRRNLVADAQMPYPLPSITPYDSSTELDSGDYHTVFDRCLKEFDWE
EKKKLSGKLIDGYYHGIAIGSFIEGGAAGPKEEVRLVLETDGALSVYMGSSSVGQGLETIMAQIAADAMEMPYH
KITILHGSTAYVKDGYGAYHSRSTVMGGSAILLGAEKLKELIRKTAAERLGCKPEEVIVDSERASFDGNQLSFADLS
EAPLEVEAEFFNKKYTWAYGTQAAHVAVDPGTGHVKVIEVMSVEDVGRMINPLTLHGQAIGSMVQGLGGAFL
EHLVYDDEGQLLTGSFADYLLPTASDFPKLDSVTLELRPCPNNPLGAKGAGEGGLIPVGGLMANAVADALSHLN
VQPMQLPLSPPRIWQLVEEAEASHAKT
>SEQF6050||SEQF6050.1_06889
MGVEGIGARVVRKEDRRFITGQGRYVDDVKMVGMSYAHFIRSPHAHAKVKGIDSAEAMKMPGVIAVLTGQQ
IVDDKVGNLICGWAITSKDGSPMKMGAWPAMAPETVRFVGQAVAVVIAETKNQARDAAEAVVVDYEELPAA
ADIKAAIKPGAPQLHPEAPGNIVYDWHLGDEAAVKDAFSKAANVVTLDLTNNRLVPNAMEPRAAVAHYDQAE
EHYTLYTTSQNPHVARLVLSAFYNIAPEHKLRVIAPDVGGGFGSKIYIYPEEMVALWASKKVGRPVKWTGDRSEA
FLTDAHGRDHVSKAEMAFDKDNKILGLRVKTHANFGAYMSLFSSAVPTYLYATLLSGQYVIPAIYAEVIGVYTNTT
PVDAYRGAGRPEASYLIERMMETAARQLKVDPTELRKKNFITQFPYQTPVIMAYDIGDFHASIDAAMKAIDYAG
FPARKAKAKADGKLRGIGISCYIEACGIAPSKAVGSLGAGVGLWESAEIRVNPVGTIEVLTGSHSHGQGHETTFC
QIIAERLGVPISQVSIVHGDTDKVQFGMGTYGSRSLAVGGTAIVKAMEKVEAKAKKIAAHALEASESDIVIENGE
FKVTGTDKSIALPMVALAAYTAHNLPDGMEPGLKETAFYDPTNFTFPAGAYICELEVDPGTGKTSFVNFVAADD
FGRLINPMIVEGQVHGGLAQGIGQALLEGAIYDDTGQLVTASFMDYTMPRADDLPSFQLSHTTTLCPGNPLGV
KGCGEAGAIGASAAVINAITDAISNNKLEMPATPDRVWHAIHG
>SEQF6051||SEQF6051.1_00861
MQDHTPPASLENAIALQKHGVGQPVRRKEDDTLVRGKGKYTDDFSLPGQATCWMVRSSHAHGLIKGIDTAAA
KAMPGVLGVWTGADLAAAGYNPFTCGLPLKNRDGSPLKQTNRPALVTDKVRFVGDPVAFVVAETAAQARDA
AEAVEVDIEPLPAVTDAAEAAKPGAPQLYDDIPDNVALDYHYGDTAKIEAAFAGAAHVTKLDIVNTRVAVVSME
PRVALAHYDKKTERFTLQVPTQGVSGNKAILARLLNVPADKVRILTGNVGGSFGMKNLNYPEYTCIAHAARELG
RPVKWLDERSTSFLSDSQGRAQLIHAELALDADGKFLAVRLSGYGNLGAYITGVAPGPLSLNTGKNLASVYRTPL
LGVDIKTVLTNTTLMGAYRGAGRPEANYYMERLIDAAADEMGINRFTLRKRNFIKPSQLPFPAASGVTYDSGDF
AGVFQKALEISDYDNFAKRKKESKKSGKLRGIAVGSYLEVTAPPSGELGKISFEPDGSVKLTTGTLDYGQGHATPF
AQVLSDQLGVPFEKITLEQGDSDLVRFGNGTGGSRSITATGQAIVEASALVVEKGKKAAAHILEASEADIEFGQG
RFTIAGTDRSIGIMELAERMRAGKMPEGTPETLDVDHATKETASTFPNGCHVAEVEIDPDTGVTRIVRYSAVND
FGVVVNPMIVAGQLHGGVAQGIGQALMEEISYDGSGQPITGSFMDYALPRAGDVPSMLVGDHPSPAKSNPL
GTKGCGEAGCAGSLVCIVNAVVDALSEYGIKHINMPLTPERVWRAIQDAKAKAA
>SEQF6051||SEQF6051.1_06340
MNILPGNMRFGAGQPVKRLEDQRLVTGKGHFIDDKPQDGALWLHVLRSPHAHANIKSIDAKAALEMPGVKA
VYTGADLVKDDIGTLPTLAIFKRPDGSPMTVPPRRLLAHEVVRYAGEGVAAVVATSRVLAQTAAEAIEIDYEVLPS
VVDPVEAIKPGAPAVWPEAPDNIVAAMSYGDAAKVEEAFANAAHKVSLDLVSQRLVPSAMEPRSTIAEIEKKTG
RLILHVQSQTPGSTRDLLAESILKRPKDSVRVLVGDIGGGFGQKTSLYPEDGIVAYAATKLNEKIRWRGDRTDEFV
GGTHGRDLTSTGEFALDAKGRVLAYRVRSIGGTGAYSSGTANIIPLVLGPFVQTGVYDLPLVHFEVKSVMTHTAPV

GAYRGAGRPEAVFIVERLFDAAARQIGMDPRTIRKVNYIKPAQLPYTNAVGQVYDSGAFAHMLERASDLADW
NGFAARKKAAKKKGLLYGRGLTSYIEWTGGRAHTEKVSLHATAEGRVILWSGTMAMGQGLATTYTQMVADTL
GIAMDKIDVVQGDTDLATGFGSVGSRSLFVGGTAVAVSTNDMINKARDKASNLLEASVEDIEYRDGFLTVVGTD
RRISLFEIAAKESGAKLSVESEGNVDGPSWPNGTHICEVEIDPETGVTRVVRYTTVDDVGVAVNPMLVAGQVH
GGVVQGIGQALYEGVAYSEEGQLLTASYQDYCIPRADDIPPITVTLDGSAPCKTNPLGAKGCGESGAIGGPPCITN
GVMDALSEVGITQLNTPLTPAKIWQAIQDAKVGAA
>SEQF6051||SEQF6051.1_01462
MAAPIKFGVGQSVRRKEDDALIRGRGRYTDDVAPSPALHALMLRSPHAHATYTIDAGKARGMPGVALILTAAD
VAELGGLPCLFNLETDPFTAPPYPILARDEVRHVGDAVAFVVADTVDHARDAIEAIDVKWTPLPAAVGLVNAVK
KDAPQVWPDKPGNVLFDVSIGDKKAAEDAFAKAHAVAEITIVNPRVITNFMETRAAVAEYDAKKDHLTLTIGSQ
GSHRLREILCDMILKMPKENMRVICPDVGGGFGTKLFPYREYALIAVAARKLKKSIKWTAERSDHFMGDAQGR
DNLTTAKMALAEDGKFLGMDVDLMGDMGAYLSTFAPYIPHGGAGMLPGLYDIQAFHCRVRTVFTNTVPVDA
YRGAGRPEAAYVIERLVDAAARKLGMTPDAIRRKNFIPPKSLPYTTATGKVYDSGDFVAHMKRAMEIANWKEF
PKRAKAARKDGLVRGIGMASYVEVCGTMGEETANVALDANGDISVLIGTQSSGQGHQTAYAQIVAEQFGVPPE
RVHVLQGDTDKIATGLGTGGSASIPSGGVSVQRATHDLGNKLKELAAQALEAGAGDLEIADGRIRIAGTDRSISF
ADLAKRPGGDTSKMNASATFASADGTYPNGTHLAEVEIDPATGIIKIVSYVIVDDFGVTLNPLMLAGQVHGGA
MQGIGQALMEQAVYSPTDGQLVTGTFMDYALPRASDGPSFVFETHNVPCTTNPMGVKGAGEAGAIGSCPAV
VNAIVEGLHREYKIDHIDMPATPERVWIAIREAQRRHNL
>SEQF6051||SEQF6051.1_07263
MTVTRARTDTLSVLDRPNSYIGKTVPRPNLDRLLQGRGQYVSDLELPRMAHVVFLRSPHAHAKIVAIDADAAR
RMPGVISIVTGRELEAVITPWVGVLSHLKGLKSAPQHAIAVDRVCWQGEAVAAIVATSRAAAEDAMEHISVDYE
ELEPVTDMRAALDPAAPVIHAALGDNLAFERMLDAGDVDHALSGSELVEADFVFGRHTGVTLEPRAVVADWY
AAEARLTIYQGTQAPHMVQNIAALHLGLREAQVRVVCKDVGGSFGIKVHIYADEMATYALSKLLRRPVKFVADR
VESFNTDIHARDHRCRGRIGVRPDGTITAFEIDDLTGIGPYSMYPRTSAIEANQVVNLVGGPYVTANYRARARVV
FQNKNMMCQYRAVGHPIACSVTEGLVDLAAAKIGMDPVEIRRRNLIADDAYPCASPSGMKFEQLSHHASLTKL
LQMMDYDALRAEQAALRARNVHRGIGIASVIEVTNPSAAFYGVGGAKISSQDGVAVRLDAQGSVICQTSITEQ
GQGSESLTAQIVGSVLGVSMERVRVILGDTDNTPYGGGTWASRGAGIGGEAALQAAKALRENILNVAAAILQST
PAELDIVDNGIVNTADGAPRIELHELARIVYFRPDTLPPGIQPELMATRHFVPRQYPFAFTNGVQASWLEVDTET
GFVKLLKHWVVEDCGTIINPQLVDEQIRGGVVQGLGAALFEKCIYDERGQLTNANMADYLVPMSGEMPDIEV
GHVVSPTQESELGAKGAGEAGTAGAAAAVANAVNDALRPFDATITEIPLTPQVILTALRRI
>SEQF6051||SEQF6051.1_05978
MGVEGIGARVVRKEDRRFITGQGRYVDDVKMVGMSYAHFIRSPHAHARVKGIDSAEAMKMPGVIAVLTGQQ
IVDDKVGNLICGWAITSKDGSPMKMGAWPAMAPETVRFVGQAVAVVIAETKNQARDAAEAVVVDYEELPAA
ADIKAAIKPGAPQLHPEALGNIVYDWHLGDEAAVKDAFSKAANVVTLDLTNNRLVPNAMEPRAAVAHYDQAE
EHYTLYTTSQNPHVARLVLSAFYNIAPEHKLRVIAPDVGGGFGSKIYIYPEEMVALWASKKVGRPVKWTGDRSEA
FLTDAHGRDHVSKAEMAFDKDNKILGLRVKTHANFGAYMSLFSSAVPTYLYATLLSGQYVIPAIYAEVIGVYTNTT
PVDAYRGAGRPEASYLIERMMETAARQLKVDPTELRKKNFITQFPYQTPVIMAYDIGDFHASIDAAMKAIDYAG
FPARKAKAKADGKLRGIGISCYIEACGIAPSKAVGSLGAGVGLWESAEIRVNPVGTIEVLTGSHSHGQGHETTFC
QIIAERLGVPISQVSIVHGDTDKVQFGMGTYGSRSLAVGGTAIVKAMEKVEAKAKKIAAHALEASESDIVIENGE
FKVTGTDKSIALPMVALAAYTAHNLPDGMEPGLKETAFYDPTNFTFPAGAYICELEVDPGTGKTSFVNFVAADD
FGRLINPMIVEGQVHGGLAQGIGQALLEGAIYDDTGQLVTASFMDYTMPRADDLPSFQLSHTTTLCPGNPLGV
KGCGEAGAIGASAAVINAITDAIGNNKLEMPATPDRVWHAIHG
>SEQF6051||SEQF6051.1_07983
MSSDWRVSPSDVVRPRNVGAPIKRTEDPRLLTGNGEYTADRKPDRLLHLAFLRSGQPHARIVRIDAADARMAP

GVVAVFAAEDIADDFKPVVPFSRMPNYYATPILPLASGKVRYVGEAVVAVIATSRYLAEDALDLIDVDYEPLGALA
CAELALADNAPLLHEAAGTNVLIAREFRKGDVTADLAAATVRVGGRFEMTRKAPLAMEPRSYVAEYDKGRDAI
TLYTSSNIPGIVRDAVSESLGVPGHRMRVVAPDVGGSFGSKGSLYPEELLICIAARKLRRSVKWTADRLEDVSSSS
QAFAEIVDAEMGFDANGIATSLQADVIGDVGAYSIYPWTCGLEPVQVASFLPGPYKIGSYRGAVRGVATCKPPT
GPYRGVGRPISTFVAERLMDLGAAALGIDPLEIRRRNLVRAEEFPYRIASGIIWDKAGFIECLDAAAEAAGYDRLR
KEQAEARTQGRLFGIGIASYAELTGIGSRIAVAPGMPINTGSETAKITIDSTGAITAAFGVASHGQGLETTLAQIVA
DDLGARFEDVRVIQGDSDEVPMSTGTYASRSAVLGGGAAKHASKILREKIKRVASHLLEANANDIEVTEGQAVV
LGTDRAVSFKQIAKAVYSDMKTLPVEAREELSATYTYDPVNGTTAAATHIAAVEIDPATCFVRILKFVVAEDCGRII
NPMIVDGQVHGGVAQGIGAALFEELIYDEDGQLLTASLVDYMIPSAPEVPVMDVVHVESESAVAGGFRGMGE
GGTIGAPAAIANAIADALSPLDIGVSILPMTPERIFRLMELAKIKRKS
>SEQF6052||SEQF6052.1_06958
MAAPIKFGVGQSVRRKEDDALIRGRGRYTDDVAPSPALHALMLRSPHAHATYTIDAGKARGMPGVALILTAAD
VADLGGLPCLFNLETDPFTAPPYPILAKDEVRHVGDAVAFVVADTVDHARDAIEAIDVKWTPLPAAVGLVNAVK
KDAPQVWPDKPGNVLFDVSIGDKKAAEDAFAKAHAVAEITIVNPRVITNFMETRAAVAEYDAKKDHLTLTIGSQ
GSHRLREILCDMILKMPKENMRVICPDVGGGFGTKLFPYREYALISVAARKLKKSIKWTAERSDHFMGDAQGR
DNLTTAKMALAEDGKFLGMDVDLMGDMGAYLSTFAPYIPHGGAGMLPGLYDIQAFHCRVRTVFTNTVPVDA
YRGAGRPEAAYVIERLVDAAARKLGTTPDAIRRKNFIPPKSLPYTTATGKVYDSGDFVAHMKRAMEIANWKEFP
KRAKAARKDGLVRGIGMASYVEVCGTMGEETANVALDPNGDISVLIGTQSSGQGHQTAYAQIVAEQFGVPPER
VHVLQGDTDKIATGLGTGGSASIPSGGVSVQRATHDLGNKLKELAAQALEAGAGDLEIADGRIRIAGTDRSISFA
DLAKRPGGDTSKMNASATFASADGTYPNGTHLAEVEIDPATGIIKIVSYVIVDDFGVTLNPLMLAGQVHGGAM
QGIGQALMEQAVYSPADGQLVTGTFMDYALPRASDGPSFVFETHNVPCTTNPMGVKGAGEAGAIGSCPAVV
NAIVEGLHREYKIDHIDMPATPERIWIAIREAQRRHNL
>SEQF6052||SEQF6052.1_04383
MQDHTPPASLENAIALQKYGVGQPVRRKEDDTLVRGKGKYTDDFSLPGQAYCWMVRSSHAHGLIKGIDTAAA
KAMPGVLGVWTGADLAAAGYNPFTCGLPLKNRDGSPLKQTNRPALVTDKVRFVGDPVAFVVAETAAQARDA
AEAVEVDIEPLPAVTDAAEAAKPGAPQLYDDIPDNVALDYHYGDTAKIEAAFAGAAHVTKLDIVNTRVAVVSME
PRVALAHYDKKTERFTLQVPTQGVSGNKAILARLLNVPADKVRILTGNVGGSFGMKNLNYPEYTCIAHAARELG
RPVKWLDERSTSFLSDSQGRAQLIHAELALDADGKFLAVRLSGYGNLGAYITGVAPGPLSLNTGKNLASVYRTPL
LGVDIKTVLTNTTLMGAYRGAGRPEANYYMERLIDAAADEMGINRFTLRKRNFIKPSQLPFPAASGVTYDSGDF
AAVFQKALEISDYDNFAKRKKESKKSGKLRGIAVGSYLEVTAPPSGELGKISFEPDGSVKLTTGTLDYGQGHATPFA
QVLSDQLGVPFEKITLEQGDSDLVRFGNGTGGSRSITATGQAIVEASALVVEKGKKAAAHMLEASEADIEFGAGR
FTIAGTDRSIGIMELAERMRAGKMPEGTPETLDVDHATKETASTFPNGCHVAEVEIDPDTGVTRIVRYSAVNDF
GVVVNPMIVAGQLHGGVAQGIGQALMEEVSYDASGQPITGSFMDYALPRAGDVPSMLVGDHPSPAKSNPLG
TKGCGEAGCAGSLVCIVNAVVDALSDYGIKHINMPLTPERVWRAIQDAKAKAA
>SEQF6052||SEQF6052.1_05846
MNILPGNMRFGAGQPVKRLEDQRLVTGKGHFIDDKPQDGALWLHVLRSPHAHANIKSIDAKTALEMPGVKAV
YTGADLVKDDIGTLPTLAIFKRPDGSPMTVPPRRLLAHEVVRYAGEGVAAVVATSRVLAQTAAEAIEIDYEVLPSV
VDPVEAIKPGAPAVWPEAPDNIVAAMSYGDAAKVEEAFANAAHKVSLDLVSQRLVPSAMEPRSTIAEIEKKTGR
LILHVQSQTPGSTRDLLAESILKRPKDSVRVLVGDIGGGFGQKTSLYPEDGIVAYAATKLNEKIRWRGDRTDEFVG
GTHGRDLTSTGEFALDAKGRVLAYRVRSIGGTGAYSSGTANIIPLVLGPFVQTGVYDLPLVHFEVKSVMTHTAPVG
AYRGAGRPEAVFIVERLFDAAARQIGMDPRTIRKVNYIKPAQLPYTNAVGQVYDSGAFAHMLERASDLADWN
GFAARKKAAKKKGLLYGRGLTSYIEWTGGRAHTEKVSLHATAEGRVILWSGTMAMGQGLATTYTQMVADTLGI
SMDKIDVVQGDTDLATGFGSVGSRSLFVGGTAVAVSTNDMINKARDKASNLLEASVEDIEYRDGFLTVVGTDR
RISLFEIAAKENGAKLSVESEGNVDGPSWPNGTHICEVEIDPETGVTRVVRYTTVDDVGVAVNPMLVTGQVHG

GVVQGIGQALYEGVAYSEEGQLLTASYQDYCIPRADDIPPITVTLDGSAPCKTNPLGAKGCGESGAIGGPPCITNG
VMDALSEVGITQLNTPLTPAKIWQAIRDAKVGAA
>SEQF6052||SEQF6052.1_05001
MTVTRAPADTLSVLDRPNSYIGKTVPRPNLDRLLQGRGQYVSDLELPRMAHVVFLRSPHAHAKIAAIDADAATR
MPGVISIVTGRELEAVITPWVGVLSHLKGLKSAPQHAIAVDRVCWQGEAVAAIVATSRAAAEDAMEHISVDYEE
LEAVTDMRAALDPAASVIHAALGDNLAFERTLDAGDVDLALSGSELVEADFVFGRHTGVTLEPRAVVADWNPA
EARLTIYQGTQAPHMVQNIAALHLGLREAQVRVVCKDVGGSFGIKVHIYADEMATYALSKLLRRPVKFVADRVE
SFNTDIHARDHRCRGRIGVRPDGTITAFEIDDLTGIGPYSMYPRTSAIEANQVVNLVGGPYATANYRARARVVFQ
NKNVMCQYRAVGHPIACSVTEGLVDLAAAKIGMDPVEIRRRNLIADDAYPCASPSGMKFERLSHHASLTKLLQ
MMDYDALRAEQAALRARNVHRGIGIASFIEVTNPSAAFYGVGGAKISSQDGVAVRLDAQGSVICQTSITEQGQ
GSESLTAQIVGSVLGVSMERVRVTLGDTDNTPYGGGTWASRGAGIGGEAALQAAKALRENILNVAAAILQSTPA
ELDIVDNGVVNAADGAPRIDLNELARIVYFRPDTLPPGIQPELMATRHFVPRQYPFAFTNGVQASWLEVDTET
GFVQLLKHWVVEDCGTIINPQLVDEQIRGGVVQGLGAALFEKCIYDERGQLTNANMADYLVPMSGEMPDIEIG
HVVSPTQESELGAKGAGEAGTAGAAAAVANAVNDALRPFGATITEIPLTPQVILTALGRI
>SEQF6052||SEQF6052.1_01218
MNNETRPHSSDVLRPRNVGAAIKRTEDPRLLTGRGEYSADRKPDRLLHVAFLRSGQPHALITRIDTVAAQEAPG
VIAVLTAEGIAGDFKPVIPFSRMPNYYATPIVPLAIGKVRYVGEAVAAVIATSRYLAEDALELIEVDYEPLGAIARAEL
AVVDDAVLLHEEAGTNVLISREFKKGDVVADLKAAAVRVSGRFEMTRKAPLAMEPRSYTAEYDKRRDAVTLYTS
SNIPGIVRDAICESLDLPGHRLRVVAPDVGGSFGSKGSLYPEELLICIAARKLGRSLKWTADRLEEVSSSSQAFAEIV
DADMGFDANGIATSLQADVIGDVGAYSIYPWTCGLEPVQVVSFLPGPYKIASYRGSVRGVATCKPPTGPYRGVG
RPISTFVAERLMDLGAKALGLDPLEIRRRNLIRAEEFPYRIASGIIWDKTGFVECLEAAAEVVGYEQLRAQQADAR
KHGRLFGIGIASYAELTGIGSRIAVAPGMPINTGSETAKITIDSTGAITAAFGVASHGQGLETTLAQIVADDLGARF
EDVRVIQGDSDEVPMSTGTYASRSAVLGGGAAKQASRIVQHKIKRVASHLLEANQDDIEVLDGKAVVIGTDRAV
TFKQVAKAVYSDMKTLPVEAREELTATYTYDPINGTTAAATHIAVVEVDPATCFVKILKYVVAEDCGRIINPMIVV
GQVHGGVAQGIGAALFEELVYDDDGQLLSASLVDYVIPSAPEVPAMDIVHIESESAVAGGFRGMGEGGTIGAP
AAIANAIADALSPLGIDVSILPMTPERIFKLMEQTRLNARGKADE
>SEQF6052||SEQF6052.1_03485
MGVEGIGARVVRKEDRRFITGQGRYVDDVKMVGMSYAHFIRSPHAHAKVKGIDSAEAMKMPGVIAVLTGQQ
IVDDKVGNLICGWAITSKDGSPMKMGAWPAMAPETVRFVGQAVAVVIAETKNQARDAAEAVVVDYEELPAA
ADIKAAIKPGAPQLHPEAPGNIVYDWHLGDEAAVKDAFSKAANVVTLDLTNNRLVPNAMEPRAAVAHYDQAE
EHYTLYTTSQNPHVARLVLSAFYNIAPEHKLRVIAPDVGGGFGSKIYIYPEEMVALWASKKVGRPVKWTGDRSEA
FLTDAHGRDHVSKAEMAFDKDNKILGLRVKTHANFGAYMSLFSSAVPTYLYATLLSGQYVIPAIYAEVIGVYTNTT
PVDAYRGAGRPEASYLIERMMETAARQLKVDPTELRKKNFITQFPYQTPVIMAYDIGDFHASIDAAMKAIDYAG
FPARKAKAKADGKLRGIGISCYIEACGIAPSKAVGSLGAGVGLWESAEIRVNPVGTIEVLTGSHSHGQGHETTFC
QIIAERLGVPISQVSIVHGDTDKVQFGMGTYGSRSLAVGGTAIVKAMEKVEAKAKKIAAHALEASESDIVIENGE
FKVTGTDKSIALPMVALAAYTAHNLPDGMEPGLKETAFYDPTNFTFPAGAYICELEVDPGTGKTSFVNFVAADD
FGRLINPMIVEGQVHGGLAQGIGQALLEGAIYDDTGQLVTASFMDYTMPRADDLPSFQLSHTTTLCPGNPLGV
KGCGEAGAIGASAAVINAITDAISNNKLEMPATPDRVWHAIHG
>SEQF6317||SEQF6317.1_00380
MTAEVTDRPATTEIGRDRRRKEDQRLITGRTRWTDNIVLPGMLHMAMVRSPFAHARIMSVDTSEAASATNVV
AVYSGADLDESVGVLINAWPVSPDQVAPVHPPMPADRVTFAGEIVALVVARSAAEARDAAELIDVDYEELPAAL
DLKEAAEDTVLAHPDLGTNKSALWTFDSAEAGTGGDVEKAIATARQDGIVIEREYRQQRLIPAFMEPRSVVVDP
TGEQLTMWTATQIPHIVRFALAATTGVPESKIRVIAPDVGGGFGGKLQVTPEEWLAWWVARQLGRPVKYTETR
SESLMAAHHGRDQWQRLTLAADKQGKVTGLKVDLLADLGSYVALIGGGVPVLGAFMFNAIYKFPAYHFSMQS

VLTNKAWTDAYRGAGRPEATFGIERIMDELAHELGEDPLVVRERNWITHEEFPFTSVAGLEYDSGNYEAATDLA
KQMFGYDELREEQRRRRESKDPVQLGIGISTFTEMCGLAPSRVLGSLDYGAGGWEHASVRMLATGKVEVVVG
VSPHGQGHETAFSQIVADRLGVPFEDVEILHGDTQISHKGLDTYGSRSLVVGGEAVVRAADKVIDKARRYAAHV
LEANPDDLEFTAGRFGVAGTDQSVGITDIAAAAFASHDYPDDIEPGIDAEATFDPVNFNYPHGTHLSAIEIDTET
GEVRLRKYTCVDDVGQVINPLIVEGQIHGGLVQGISQALWEEAVYDDSGTLVSGSFVDYLVPTAADTISFDTATT
TTAATGNTLGTKGVGEAGTIASTPCIVNGVVDALRIFGVDDVQMPCTPERVWQAIRSGSGGEDTTGAAAPHF
DEATGDQGQTDRMAGADGPTGPAMSTEGEVR
>SEQF6318||SEQF6318.1_03141
MTSRAMSRLEDANLVTGKGSFLDDLDPIPGTLVAAIVRSPNAHAKITSVDLQRARNHPGVAAVIGPEEVLAALK
PFPLSLRAPMDYYPTGTDKVRYVGEPVAVVVATDRYTAEDAGDLVDISYELEPVVVDAMKALTPDSPLLHERSKT
NVASDRTFNFGEVDEAFADAAHVVEGEYYFPRYSSTPMECYNVVANWVDGPQHPEVTVWSNFHGPFSMVP
VLCGAMGLTTSQLRLHVPADNGGSFGIKAGIYPYIALMALASRHAGRPVRWSEDRSEHLLASSAGSDRHMKVR
TAIDAAGIIQAIDIDFVENVGAYLRPPEPATLYRCFGNMTGAYGIEHVRLRSRAVVTNKCPTGLNRGFGGQQLYFA
LERIMDKAAKAVGLDRLEFRKRNLIGAESFPYATPTGGIYDSGNYQKVVEHLEDNSDYQALLARQAEARARGEL
VGTGVALIVEPSGTNIGYVGLATPAENRPPGRGKSGSTEHVHLSVDPQGIVGITLGTVPQGQGHATVARQVVAR
ELGLPEEQVVPTVVMDTATTAWTITSGSYSSRFSPLLTSALVDGAREIGERIKRAAALMLDTTADLLELHQGTVRV
MGDPEKSVSFKHAAGLIHWDPGRLPGGEEATLEVNVAFTPPQAQAPSRTDQVNSSLCYGFVADLVSVRIDPET
LEITIDDVVSVHDAGTILNEQLFTGQVHGAMVQALGGAMYEEMSYSDSGQPVSTFMDYLVPTSAESMFPFRM
DHIETPSPLTRLGAKGAGEGASMSFPVALANAVEDAVSEHGVEINSLPVHPSVLHDLLYPND
>SEQF6318||SEQF6318.1_01900
MTAEVTDRPATTEIGRDRRRKEDQRLITGRTRWTDNIVLPGMLHMAMVRSPFAHARIASVDTSEAASATNVVA
VVYSGADLDESAGVLINAWPVSPDQVAPVHPPMPADRVTFAGEIVALVVARSAAEARDAAELIDVDYEELPAALD
LKEAAEDTVLAHPDLGTNKSALWTFDSAEAGTGGDVEEAIATARQDGIVIEREYRQQRLIPAFMEPRSVVVDPT
GEQLTMWTATQIPHIVRFALAATTGVPESKIRVIAPDVGGGFGGKLQVTPEEWLAWWVARQLGRPVKYTETRS
ESLMAAHHGRDQWQRLTLAADKQGKVTGLKVDLLADLGSYVALIGGGVPVLGAFMFNAIYKFPAYHFSMQSV
LTNKAWTDAYRGAGRPEATFGIERIMDDLAHELGEDPLVVRERNWITHEEFPFTSVAGLEYDSGNYEAATDLAK
QMFGYDELREEQRRRRESKDPVQLGIGISTFTEMCGLAPSRVLGSLDYGAGGWEHASVRMLATGKVEVVVGV
SPHGQGHETAFSQIVADRLGVPFEDVEILHGDTQISHKGLDTYGSRSLVVGGEAVVRAADKVIDKARRYAAHVL
EANPDDLEFTAGRFGVAGTDQSVGITDIAAAAFASHDYPDDIEPGIDAEATFDPVNFNYPHGTHLSAIEIDTETG
EVRLRKYTCVDDVGQVINPLIVEGQIHGGLVQGISQALWEEAVYDDSGTLVSGSFVDYLVPTAADTISFDTATTST
AATGNTLGTKGVGEAGTIASTPCIVNGVVDALRIFGVDDVQMPCTPERVWQAIRSGSGGEDTTGAAAPHFDE
ATGDQGQTDRMAGADGPTGPAMSTEGEVR
>SEQF7913||SEQF7913.1_02479
MDATDKTPIRPVTQPLTEMPASLVDHVSGSRLEDPPLVTGQGRFLDDLDPLPGTLVAAIVRSPHAHAKIRKVDLD
RARAHPGVAAVIGPEEVLETLKPFPLSLRTNMPYYPTGTDRVRYVGEPVAVVVATDRYTAEDAAELVRVDYEVLP
AVVDTKKAEESGSVTLHEGADSNVASDRSFSFGAVDDAFARAEHVVSAEYRFPRYSSTPMECYSVVAEWRDES
TGPQITAYANFHGPFSIVPILAGALGITTAQLRLIVPADNGGSFGIKSGMYPYIALMALASRHAGRPVRWTEDRIE
HLLSSSAGSDRIMRFQAAVSEDGSVGALDVDFQENVGAYLRPPEPATLYRCFGNITGAYRIDAVRLRSRAVVTNK
MPTGLNRGFGGQQLYFGLERLMDKVASTVGKDPLEVRRINLVPTSAFPYATPTGGIYDSGDYEKAFVMLTESAQ
YEALRAEQRAARDRGEIMGIGVAAIVEPSATNIGYIALATPQDERVAGRDKSGSTEHVRVSVDPQGVVSVLLGT
VPQGQGHATVARQIVADQLGLPLEQVVPKVDMDTATTPWTITTGSYSSRFSPLLTSALVEATDRIAATIKVAASV
LLAADPAELELAEGRVRVAGDPERSVLFRHAAGLIHWDPGSLPDAFEAQLNAEAAFTPPESTAASRTDQINSSLC
YGFVVELAVVRIDPETFEIAIEKVVSVHDPGTVLNPVLLEGQIHGAMAHGLGGAMYEEMFYSESGQPTSTFMD
YLVPTSAEATFPLVTEHLETPSPLSRLGAKGAGEGSSMSFPVLVANAVADALAPHGVDITSLPLHGNVIHGLLSSQ

N
>SEQF7923||SEQF7923.1_00425
MTTEHTGAEVGAGSRQGVGARVPRKEDARHLHGKGNFVADMAMPGLCEVAFLRSPLAHARITDVRVPESVR
DKVVLRSMMGDARDIAADSTLPTYQPSVQPPLASGKVRFVGEPVAMTFAPTRAEAEDHAELVEVDYDDLPVY
ADVAGAQQATSDLVHEHWRDNVFVTLNADRDFDEHAARAEVVVRRKIDLARQCMVPMEGKAVLAYWDHQ
ADQLVVISATQVPHMIRSVLAQCLDLEQGRVRVVSPDVGGAFGYKCVLQQEELCVAWLAKTYKRPFRFIEDRRE
HLTAGANSREHHYEMTAYADKRGKLLALDARITIDGGAYSVWPFTIGLEPDQAVGNLPGPYGFRGYRCETRCVA
TNKPGFVPYRGRTGVCFAIELTMDAVAREVGREPWEVRLENLVQPEQMPYVNVTNKHFDSGDYPASLRKALE
MIDIDGVRTRQARGEADGRRIGVGVATYTEQAAHGTSVFAAWGTPVIPGFDQATVRVTPDGGLEVRVGVHSH
GQGMETTFAQIAHEILGIDVARIKVLHGDTGQTPFSTGTYASRSLVMSGGAVSQACKRLLPRMRHIAAHMLGV
ADDAVTLQDGIYRAGEKSVATGDVADAWYLRPQLLPPDVDPAGLEVNVGYKPKVDTGCFTYASHAAVVAVDP
DTGAVEILDYVVVEDCGTMINPMVVEGQTIGGIAQGIGTAFYEETPYDANGQPLASTLADYMLPGPTEVPNM
RLHHFETPSPHTEFGAKGMGEGGAIAPPAVLFNAVNDALRGLGAAELSRTPLTPIRVLQAIAQGAGEAA
>SEQF7923||SEQF7923.1_03491
MTDATSPGLGRSAPRIEDDALLRGQARFLDDIEVEGVLHACFVRSPHAHARLVSIDLSAARAVPGVAAVYGARD
LFGQLTSWRMPLGFPLAALPDDTTPFVLAEREVAFVGEAIAVVVADSRHIAEDAAARVAIEYEVLGAVVDCRDA
LRPDAPLVRGELASNILQQYTLAYGDCETAFAQAHRVLEDDFWVHRGCAHPMEGRGVLARMDRATDTLTVWS
STQMAHELHYTLALMLGQPEDRLRVVTPDVGGGFGAKFMIYPEEMAIPAAARKLGRPVKWVEDRREHFTTSI
QERDQYWKVAMAIDDQGHVLGIRGNFVHDNGAYTPQGTNVPYNAASSMTGPYVVPAFSLDVSVAYTNKVPV
ATVRGAGYPQAAFVMERMMDRVAAELGIDPGECRRRNLIGPAKIPYTKPLKSRAGMPLTIDSGDFPALQACAL
QASDYDGFAVRRDAALARGRWRGIAVANSVEPTGRGPFEVARVRVQPSGQVSIYTGALAMGQGIKTTLAQIC
AGHLGVPVAAVEVQAGDTAYVGYGMGGFASRQAIMAGSAVDQAAAQVRRQALETAAAVLKAEAETLELADG
EVRAPDGQSVSLARLAMLRKGVPGYALVSPGDPGLDATAYFHCDAQTYAGASHVCEVEVDPATGAIEIVRYVAA
QDSGRIINPQLAEGQVHGGVVHGIGNALFEWMGYDAAGQPLSTTFAEYLLPTAPEVPPIEVVFQPSPTPLNPL
GVKGVGECATIPVAVAVVGAVEHAVAHCGVRVTEFPLTPVRLLELLTQAEARAAAPTDSGEFVDGVH
>SEQF7924||SEQF7924.1_02786
MTTEHTGAEVGAGSRQGVGARVPRKEDARHLHGKGNFVADMAMPGLCEVAFLRSPLAHARITDVRVPESVR
DKVVLRSMMGDARDIAADSTLPTYQPSVQPPLASGKVRFVGEPVAMTFAPTRAEAEDHAELVEVDYDDLPVY
ADVAGAQQATSDLVHEHWRDNVFVTLNADRDFDEHAARAEVVVRRKIDLARQCMVPMEGKAVLAYWDHQ
ADQLVVISATQVPHMIRSVLAQCLDLEQGRVRVVSPDVGGAFGYKCVLQQEELCVAWLAKTYKRPFRFIEDRRE
HLTAGANSREHHYEMTAYADKRGKLLALDARITIDGGAYSVWPFTIGLEPGQAVGNLPGPYGFRGYRCETRCVA
TNKPGFVPYRGRTGVCFAIELTMDAVAREVGREPWEVRLENLVQPEQMPYVNVTNKHFDSGDYPASLRKALE
MIDIDGVRTRQARGEADGRRIGVGVATYTEQAAHGTSVFAAWGTPVIPGFDQATVRVTPDGGLEVRVGVHSH
GQGMETTFAQIAHEILGIDVARIKVLHGDTGQTPFSTGTYASRSLVMSGGAVSQACKRLLPRMRHIAAHMLGV
ADDAVTLQDGIYRAGEKSVATGDVADAWYLRPQLLPPDVDPAGLEVNVGYKPKVDTGCFTYASHAAVVAVDP
DTGAVEILDYVVVEDCGTMINPMVVEGQTIGGIAQGIGTAFYEETPYDANGQPLASTLADYMLPGPTEVPNM
RLHHFETPSPHTEFGAKGMGEGGAIAPPAVLFNAVNDALRGLGAAELSRTPLTPIRVLQAIAQGAGEAA
>SEQF7924||SEQF7924.1_03274
MTDATSPGLGRSAPRIEDDALLRGQARFLDDIEVEGVLHACFVRSPHAHARLVSIDLSAARAVPGVAAVYGARD
LFGQLTSWRMPLGFPLAALPDDTTPFVLAEREVAFVGEAIAVVVADSRHIAEDAAARVAIEYEVLGAVVDCRDA
LRPDAPLVRGELASNILQQYTLAYGDCETAFAQAHRVLEDDFWVHRGCAHPMEGRGVLARMDRATDTLTVWS
STQMAHELHYTLALMLGQPEDRLRVVTPDVGGGFGAKFMIYPEEMAIPAAARKLGRPVKWVEDRREHFTTSI
QERDQYWKVAMAIDDQGHVLGIRGNFVHDNGAYTPQGTNVPYNAASSMTGPYVVPAFSLDVSVAYTNKVPV
ATVRGAGYPQAAFVMERMMDRVAAELGIDPGECRRRNLIGPAKIPYTKPLKSRAGMPLTIDSGDFPALQACAL

QASDYDGFAVRRDAALARGRWRGIAVANSVEPTGRGPFEVARVRVQPSGQVSIYTGALAMGQGIKTTLAQIC
AGHLGVPVAAVEVQAGDTAYVGYGMGGFASRQAIMAGSAVDQAAAQVRRQALETAAAVLKAEAETLELADG
EVRAPDGQSVSLARLAMLRKGVPGYALVSPGDPGLDATAYFHCDAQTYAGASHVCEVEVDPATGAIEIVRYVAA
QDSGRIINPQLAEGQVHGGVVHGIGNALFEWMGYDAAGQPLSTTFAEYLLPTAPEVPPIEVVFQPSPTPLNPL
GVKGVGECATIPVAVAVVGAVEHAVAHCGVRVTEFPLTPVRLLELLTQAEARAAAPTDSGEFVDGVH
>SEQF7925||SEQF7925.1_03664
MTTEHTGAEVGAGSRQGVGARVPRKEDARHLHGKGNFVADMAMPGLCEVAFLRSPLAHARITDVRVPESVR
DKVVLRSMMGDARDIAADSTLPTYQPSVQPPLASGKVRFVGEPVAMTFAPTRAEAEDHAELVEVDYDDLPVY
ADVAGAQQATSDLVHEHWRDNVFVTLNADRDFDEHAARAEVVVRRKIDLARQCMVPMEGKAVLAYWDHQ
ADQLVVISATQVPHMIRSVLAQCLDLEQGRVRVVSPDVGGAFGYKCVLQQEELCVAWLAKTYKRPFRFIEDRRE
HLTAGANSREHHYEMTAYADKRGKLLALDARITIDGGAYSVWPFTIGLEPGQAVGNLPGPYGFRGYRCETRCVA
TNKPGFVPYRGRTGVCFAIELTMDAVAREVGREPWEVRLENLVQPEQMPYVNVTNKHFDSGDYPASLRKALE
MIDIDGVRTRQARGEADGRRIGVGVATYTEQAAHGTSVFAAWGTPVIPGFDQATVRVTPDGGLEVRVGVHSH
GQGMETTFAQIAHEILGIDVARIKVLHGDTGQTPFSTGTYASRSLVMSGGAVSQACKRLLPRMRHIAAHMLGV
ADDAVTLQDGIYRAGEKSVATGDVADAWYLRPQLLPPDVDPAGLEVNVGYKPKVDTGCFTYASHAAVVAVDP
DTGAVEILDYVVVEDCGTMINPMVVEGQTIGGIAQGIGTAFYEETPYDANGQPLASTLADYMLPGPTEVPNM
RLHHFETPSPHTEFGAKGMGEGGAIAPPAVLFNAVNDALRGLGAAELSRTPLTPIRVLQAIAQGAGEAA
>SEQF7925||SEQF7925.1_03177
MTDATSPGLGRSAPRIEDDALLRGQARFLDDIEVEGVLHACFVRSPHAHARLVSIDLSAARAVPGVAAVYGARD
LFGQLTSWRMPLGFPLAALPDDTTPFVLAEREVAFVGEAIAVVVADSRHIAEDAAARVAIEYEVLGAVVDCRDA
LRPDAPLVRGELASNILQQYTLAYGDCETAFAQAHRVLEDDFWVHRGCAHPMEGRGVLARMDRATDTLTVWS
STQMAHELHYTLALMLGQPEDRLRVVTPDVGGGFGAKFMIYPEEMAIPAAARKLGRPVKWVEDRREHFTTSI
QERDQYWKVAMAIDDQGHVLGIRGNFVHDNGAYTPQGTNVPYNAASSMTGPYVVPAFSLDVSVAYTNKVPV
ATVRGAGYPQAAFVMERMMDRVAAELGIDPGECRRRNLIGPAKIPYTKPLKSRAGMPLTIDSGDFPALQACAL
QASDYDGFAVRRDAALARGRWRGIAVANSVEPTGRGPFEVARVRVQPSGQVSIYTGALAMGQGIKTTLAQIC
AGHLGVPVAAVEVQAGDTAYVGYGMGGFASRQAIMAGSAVDQAAAQVRRQALETAAAVLKAEAETLELADG
EVRAPDGQSVSLARLAMLRKGVPGYALVSPGDPGLDATAYFHCDAQTYAGASHVCEVEVDPATGAIEIVRYVAA
QDSGRIINPQLAEGQVHGGVVHGIGNALFEWMGYDAAGQPLSTTFAEYLLPTAPEVPPIEVVFQPSPTPLNPL
GVKGVGECATIPVAVAVVGAVEHAVAHCGVRVTEFPLTPVRLLELLTQAEARAAAPTDSGEFVDGVH
>SEQF7926||SEQF7926.1_03564
MTTEHTGAEVGAGSRQGVGARVPRKEDARHLHGKGNFVADMAMPGLCEVAFLRSPLAHARITDVRVPESVR
DKVVLRSMMGDARDIAADSTLPTYQPSVQPPLASGKVRFVGEPVAMTFAPTRAEAEDHAELVEVDYDDLPVY
ADVAGAQQATSDLVHEHWRDNVFVTLNADRDFDEHAARAEVVVRRKIDLARQCMVPMEGKAVLAYWDHQ
ADQLVVISATQVPHMIRSVLAQCLDLEQGRVRVVSPDVGGAFGYKCVLQQEELCVAWLAKTYKRPFRFIEDRRE
HLTAGANSREHHYEMTAYADKRGKLLALDARITIDGGAYSVWPFTIGLEPDQAVGNLPGPYGFRGYRCETRCVA
TNKPGFVPYRGRTGVCFAIELTMDAVAREVGREPWEVRLENLVQPEQMPYVNVTNKHFDSGDYPASLRKALE
MIDIDGVRTRQARGEADGRRIGVGVATYTEQAAHGTSVFAAWGTPVIPGFDQATVRVTPDGGLEVRVGVHSH
GQGMETTFAQIAHEILGIDVARIKVLHGDTGQTPFSTGTYASRSLVMSGGAVSQACKRLLPRMRHIAAHMLGV
ADDAVTLQDGIYRAGEKSVATGDVADAWYLRPQLLPPDVDPAGLEVNVGYKPKVDTGCFTYASHAAVVAVDP
DTGAVEILDYVVVEDCGTMINPMVVEGQTIGGIAQGIGTAFYEETPYDANGQPLASTLADYMLPGPTEVPNM
RLHHFETPSPHTEFGAKGMGEGGAIAPPAVLFNAVNDALRGLGAAELSRTPLTPIRVLQAIAQGAGEAA
>SEQF7926||SEQF7926.1_03468
MTDATSPGLGRSAPRIEDDALLRGQARFLDDIEVEGVLHACFVRSPHAHARLVSIDLSAARAVPGVAAVYGARD
LFGQLTSWRMPLGFPLAALPDDTTPFVLAEREVAFVGEAIAVVVADSRHIAEDAAARVAIEYEVLGAVVDCRDA

LRPDAPLVRGELASNILQQYTLAYGDCETAFAQAHRVLEDDFWVHRGCAHPMEGRGVLARMDRATDTLTVWS
STQMAHELHYTLALMLGQPEDRLRVVTPDVGGGFGAKFMIYPEEMAIPAAARKLGRPVKWVEDRREHFTTSI
QERDQYWKVAMAIDDQGHVLGIRGNFVHDNGAYTPQGTNVPYNAASSMTGPYVVPAFSLDVSVAYTNKVPV
ATVRGAGYPQAAFVMERMMDRVAAELGIDPGECRRRNLIGPAKIPYTKPLKSRAGMPLTIDSGDFPALQACAL
QASDYDGFAVRRDAALARGRWRGIAVANSVEPTGRGPFEVARVRVQPSGQVSIYTGALAMGQGIKTTLAQIC
AGHLGVPVAAVEVQAGDTAYVGYGMGGFASRQAIMAGSAVDQAAAQVRRQALETAAAVLKAEAETLELADG
EVRAPDGQSVSLARLAMLRKGVPGYALVSPGDPGLDATAYFHCDAQTYAGASHVCEVEVDPATGAIEIVRYVAA
QDSGRIINPQLAEGQVHGGVVHGIGNALFEWMGYDAAGQPLSTTFAEYLLPTAPEVPPIEVVFQPSPTPLNPL
GVKGVGECATIPVAVAVVGAVEHAVAHCGVRVTEFPLTPVRLLELLTQAEARAAAPTDSGEFVDGVH
>SEQF7927||SEQF7927.1_03554
MTTEHTGAEVGAGSRQGVGARVPRKEDARHLHGKGNFVADMAMPGLCEVAFLRSPLAHARITDVRVPESVR
DKVVLRSMMGDARDIAADSTLPTYQPSVQPPLASGKVRFVGEPVAMTFAPTRAEAEDHAELVEVDYDDLPVY
ADVAGAQQATSDLVHEHWRDNVFVTLNADRDFDEHAARAEVVVRRKIDLARQCMVPMEGKAVLAYWDHQ
ADQLVVISATQVPHMIRSVLAQCLDLEQGRVRVVSPDVGGAFGYKCVLQQEELCVAWLAKTYKRPFRFIEDRRE
HLTAGANSREHHYEMTAYADKRGKLLALDARITIDGGAYSVWPFTIGLEPDQAVGNLPGPYGFRGYRCETRCVA
TNKPGFVPYRGRTGVCFAIELTMDAVAREVGREPWEVRLENLVQPEQMPYVNVTNKHFDSGDYPASLRKALE
MIDIDGVRTRQARGEADGRRIGVGVATYTEQAAHGTSVFAAWGTPVIPGFDQATVRVTPDGGLEVRVGVHSH
GQGMETTFAQIAHEILGIDVARIKVLHGDTGQTPFSTGTYASRSLVMSGGAVSQACKRLLPRMRHIAAHMLGV
ADDAVTLQDGIYRAGEKSVATGDVADAWYLRPQLLPPDVDPAGLEVNVGYKPKVDTGCFTYASHAAVVAVDP
DTGAVEILDYVVVEDCGTMINPMVVEGQTIGGIAQGIGTAFYEETPYDANGQPLASTLADYMLPGPTEVPNM
RLHHFETPSPHTEFGAKGMGEGGAIAPPAVLFNAVNDALRGLGAAELSRTPLTPIRVLQAIAQGAGEAA
>SEQF7927||SEQF7927.1_03459
MTDATSPGLGRSAPRIEDDALLRGQARFLDDIEVEGVLHACFVRSPHAHARLVSIDLSAARAVPGVAAVYGARD
LFGQLTSWRMPLGFPLAALPDDTTPFVLAEREVAFVGEAIAVVVADSRHIAEDAAARVAIEYEVLGAVVDCRDA
LRPDAPLVRGELASNILQQYTLAYGDCETAFAQAHRVLEDDFWVHRGCAHPMEGRGVLARMDRATDTLTVWS
STQMAHELHYTLALMLGQPEDRLRVVTPDVGGGFGAKFMIYPEEMAIPAAARKLGRPVKWVEDRREHFTTSI
QERDQYWKVAMAIDDQGHVLGIRGNFVHDNGAYTPQGTNVPYNAASSMTGPYVVPAFSLDVSVAYTNKVPV
ATVRGAGYPQAAFVMERMMDRVAAELGIDPGECRRRNLIGPAKIPYTKPLKSRAGMPLTIDSGDFPALQACAL
QASDYDGFAVRRDAALARGRWRGIAVANSVEPTGRGPFEVARVRVQPSGQVSIYTGALAMGQGIKTTLAQIC
AGHLGVPVAAVEVQAGDTAYVGYGMGGFASRQAIMAGSAVDQAAAQVRRQALETAAAVLKAEAETLELADG
EVRAPDGQSVSLARLAMLRKGVPGYALVSPGDPGLDATAYFHCDAQTYAGASHVCEVEVDPATGAIEIVRYVAA
QDSGRIINPQLAEGQVHGGVVHGIGNALFEWMGYDAAGQPLSTTFAEYLLPTAPEVPPIEVVFQPSPTPLNPL
GVKGVGECATIPVAVAVVGAVEHAVAHCGVRVTEFPLTPVRLLELLTQAEARAAAPTDSGEFVDGVH
>SEQF7928||SEQF7928.1_00656
MTDATSPGLGRSAPRIEDDALLRGQARFLDDIEVEGVLHACFVRSPHAHARLVSIDLSAARAVPGVAAVYGARD
LFGQLTSWRMPLGFPLAALPDDTTPFVLAEREVAFVGEAIAVVVADSRHIAEDAAARVAIEYEVLGAVVDCRDA
LRPDAPLVRGELASNILQQYTLAYGDCETAFAQAHRVLEDDFWVHRGCAHPMEGRGVLARMDRATDTLTVWS
STQMAHELHYTLALMLGQPEDRLRVVTPDVGGGFGAKFMIYPEEMAIPAAARKLGRPVKWVEDRREHFTTSI
QERDQYWKVAMAIDDQGHVLGIRGNFVHDNGAYTPQGTNVPYNAASSMTGPYVVPAFSLDVSVAYTNKVPV
ATVRGAGYPQAAFVMERMMDRVAAELGIDPGECRRRNLIGPAKIPYTKPLKSRAGMPLTIDSGDFPALQACAL
QASDYDGFAVRRDAALARGRWRGIAVANSVEPTGRGPFEVARVRVQPSGQVSIYTGALAMGQGIKTTLAQIC
AGHLGVPVAAVEVQAGDTAYVGYGMGGFASRQAIMAGSAVDQAAAQVRRQALETAAAVLKAEAETLELADG
EVRAPDGQSVSLARLAMLRKGVPGYALVSPGDPGLDATAYFHCDAQTYAGASHVCEVEVDPATGAIEIVRYVAA
QDSGRIINPQLAEGQVHGGVVHGIGNALFEWMGYDAAGQPLSTTFAEYLLPTAPEVPPIEVVFQPSPTPLNPL

GVKGVGECATIPVAVAVVGAVEHAVAHCGVRVTEFPLTPVRLLELLTQAEARAAAPTDSGEFVDGVH
>SEQF7928||SEQF7928.1_00559
MTTEHTGAEVGAGSRQGVGARVPRKEDARHLHGKGNFVADMAMPGLCEVAFLRSPLAHARITDVRVPESVR
DKVVLRSMMGDARDIAADSTLPTYQPSVQPPLASGKVRFVGEPVAMTFAPTRAEAEDHAELVEVDYDDLPVY
ADVAGAQQATSDLVHEHWRDNVFVTLNADRDFDEHAARAEVVVRRKIDLARQCMVPMEGKAVLAYWDHQ
ADQLVVISATQVPHMIRSVLAQCLDLEQGRVRVVSPDVGGAFGYKCVLQQEELCVAWLAKTYKRPFRFIEDRRE
HLTAGANSREHHYEMTAYADKRGKLLALDARITIDGGAYSVWPFTIGLEPDQAVGNLPGPYGFRGYRCETRCVA
TNKPGFVPYRGRTGVCFAIELTMDAVAREVGREPWEVRLENLVQPEQMPYVNVTNKHFDSGDYPASLRKALE
MIDIDGVRTRQARGEADGRRIGVGVATYTEQAAHGTSVFAAWGTPVIPGFDQATVRVTPDGGLEVRVGVHSH
GQGMETTFAQIAHEILGIDVARIKVLHGDTGQTPFSTGTYASRSLVMSGGAVSQACKRLLPRMRHIAAHMLGV
ADDAVTLQDGIYRAGEKSVATGDVADAWYLRPQLLPPDVDPAGLEVNVGYKPKVDTGCFTYASHAAVVAVDP
DTGAVEILDYVVVEDCGTMINPMVVEGQTIGGIAQGIGTAFYEETPYDANGQPLASTLADYMLPGPTEVPNM
RLHHFETPSPHTEFGAKGMGEGGAIAPPAVLFNAVNDALRGLGAAELSRTPLTPIRVLQAIAQGAGEAA
>SEQF7929||SEQF7929.1_00503
MTDATSPGLGRSAPRIEDDALLRGQARFLDDIEVEGVLHACFVRSPHAHARLVSIDLSAARAVPGVAAVYGARD
LFGQLTSWRMPLGFPLAALPDDTTPFVLAEREVAFVGEAIAVVVADSRHIAEDAAARVAIEYEVLGAVVDCRDA
LRPDAPLVRGELASNILQQYTLAYGDCETAFAQAHRVLEDDFWVHRGCAHPMEGRGVLARMDRATDTLTVWS
STQMAHELHYTLALMLGQPEDRLRVVTPDVGGGFGAKFMIYPEEMAIPAAARKLGRPVKWVEDRREHFTTSI
QERDQYWKVAMAIDDQGHVLGIRGNFVHDNGAYTPQGTNVPYNAASSMTGPYVVPAFSLDVSVAYTNKVPV
ATVRGAGYPQAAFVMERMMDRVAAELGIDPGECRRRNLIGPAKIPYTKPLKSRAGMPLTIDSGDFPALQACAL
QASDYDGFAVRRDAALARGRWRGIAVANSVEPTGRGPFEVARVRVQPSGQVSIYTGALAMGQGIKTTLAQIC
AGHLGVPVAAVEVQAGDTAYVGYGMGGFASRQAIMAGSAVDQAAAQVRRQALETAAAVLKAEAETLELADG
EVRAPDGQSVSLARLAMLRKGVPGYALVSPGDPGLDATAYFHCDAQTYAGASHVCEVEVDPATGAIEIVRYVAA
QDSGRIINPQLAEGQVHGGVVHGIGNALFEWMGYDAAGQPLSTTFAEYLLPTAPEVPPIEVVFQPSPTPLNPL
GVKGVGECATIPVAVAVVGAVEHAVAHCGVRVTEFPLTPVRLLELLTQAEARAAAPTDSGEFVDGVH
>SEQF7929||SEQF7929.1_00407
MTTEHTGAEVGAGSRQGVGARVPRKEDARHLHGKGNFVADMAMPGLCEVAFLRSPLAHARITDVRVPESVR
DKVVLRSMMGDARDIAADSTLPTYQPSVQPPLASGKVRFVGEPVAMTFAPTRAEAEDHAELVEVDYDDLPVY
ADVAGAQQATSDLVHEHWRDNVFVTLNADRDFDEHAARAEVVVRRKIDLARQCMVPMEGKAVLAYWDHQ
ADQLVVISATQVPHMIRSVLAQCLDLEQGRVRVVSPDVGGAFGYKCVLQQEELCVAWLAKTYKRPFRFIEDRRE
HLTAGANSREHHYEMTAYADKRGKLLALDARITIDGGAYSVWPFTIGLEPDQAVGNLPGPYGFRGYRCETRCVA
TNKPGFVPYRGRTGVCFAIELTMDAVAREVGREPWEVRLENLVQPEQMPYVNVTNKHFDSGDYPASLRKALE
MIDIDGVRTRQARGEADGRRIGVGVATYTEQAAHGTSVFAAWGTPVIPGFDQATVRVTPDGGLEVRVGVHSH
GQGMETTFAQIAHEILGIDVARIKVLHGDTGQTPFSTGTYASRSLVMSGGAVSQACKRLLPRMRHIAAHMLGV
ADDAVTLQDGIYRAGEKSVATGDVADAWYLRPQLLPPDVDPAGLEVNVGYKPKVDTGCFTYASHAAVVAVDP
DTGAVEILDYVVVEDCGTMINPMVVEGQTIGGIAQGIGTAFYEETPYDANGQPLASTLADYMLPGPTEVPNM
RLHHFETPSPHTEFGAKGMGEGGAIAPPAVLFNAVNDALRGLGAAELSRTPLTPIRVLQAIAQGAGEAA
>SEQF7930||SEQF7930.1_03559
MTTEHTGAEVGAGSRQGVGARVPRKEDARHLHGKGNFVADMAMPGLCEVAFLRSPLAHARITDVRVPESVR
DKVVLRSMMGDARDIAADSTLPTYQPSVQPPLASGKVRFVGEPVAMTFAPTRAEAEDHAELVEVDYDDLPVY
ADVAGAQQATSDLVHEHWRDNVFVTLNADRDFDEHAARAEVVVRRKIDLARQCMVPMEGKAVLAYWDHQ
ADQLVVISATQVPHMIRSVLAQCLDLEQGRVRVVSPDVGGAFGYKCVLQQEELCVAWLAKTYKRPFRFIEDRRE
HLTAGANSREHHYEMTAYADKRGKLLALDARITIDGGAYSVWPFTIGLEPDQAVGNLPGPYGFRGYRCETRCVA
TNKPGFVPYRGRTGVCFAIELTMDAVAREVGREPWEVRLENLVQPEQMPYVNVTNKHFDSGDYPASLRKALE

MIDIDGVRTRQARGEADGRRIGVGVATYTEQAAHGTSVFAAWGTPVIPGFDQATVRVTPDGGLEVRVGVHSH
GQGMETTFAQIAHEILGIDVARIKVLHGDTGQTPFSTGTYASRSLVMSGGAVSQACKRLLPRMRHIAAHMLGV
ADDAVTLQDGIYRAGEKSVATGDVADAWYLRPQLLPPDVDPAGLEVNVGYKPKVDTGCFTYASHAAVVAVDP
DTGAVEILDYVVVEDCGTMINPMVVEGQTIGGIAQGIGTAFYEETPYDANGQPLASTLADYMLPGPTEVPNM
RLHHFETPSPHTEFGAKGMGEGGAIAPPAVLFNAVNDALRGLGAAELSRTPLTPIRVLQAIAQGAGEAA
>SEQF7930||SEQF7930.1_00480
MTDATSPGLGRSAPRIEDDALLRGQARFLDDIEVEGVLHACFVRSPHAHARLVSIDLSAARAVPGVAAVYGARD
LFGQLTSWRMPLGFPLAALPDDTTPFVLAEREVAFVGEAIAVVVADSRHIAEDAAARVAIEYEVLGAVVDCRDA
LRPDAPLVRGELASNILQQYTLAYGDCETAFAQAHRVLEDDFWVHRGCAHPMEGRGVLARMDRATDTLTVWS
STQMAHELHYTLALMLGQPEDRLRVVTPDVGGGFGAKFMIYPEEMAIPAAARKLGRPVKWVEDRREHFTTSI
QERDQYWKVAMAIDDQGHVLGIRGNFVHDNGAYTPQGTNVPYNAASSMTGPYVVPAFSLDVSVAYTNKVPV
ATVRGAGYPQAAFVMERMMDRVAAELGIDPGECRRRNLIGPAKIPYTKPLKSRAGMPLTIDSGDFPALQACAL
QASDYDGFAVRRDAALARGRWRGIAVANSVEPTGRGPFEVARVRVQPSGQVSIYTGALAMGQGIKTTLAQIC
AGHLGVPVAAVEVQAGDTAYVGYGMGGFASRQAIMAGSAVDQAAAQVRRQALETAAAVLKAEAETLELADG
EVRAPDGQSVSLARLAMLRKGVPGYALVSPGDPGLDATAYFHCDAQTYAGASHVCEVEVDPATGAIEIVRYVAA
QDSGRIINPQLAEGQVHGGVVHGIGNALFEWMGYDAAGQPLSTTFAEYLLPTAPEVPPIEVVFQPSPTPLNPL
GVKGVGECATIPVAVAVVGAVEHAVAHCGVRVTEFPLTPVRLLELLTQAEARAAAPTDSGEFVDGVH
>SEQF7931||SEQF7931.1_03557
MTTEHTGAEVGAGSRQGVGARVPRKEDARHLHGKGNFVADMAMPGLCEVAFLRSPLAHARITDVRVPESVR
DKVVLRSMMGDARDIAADSTLPTYQPSVQPPLASGKVRFVGEPVAMTFAPTRAEAEDHAELVEVDYDDLPVY
ADVAGAQQATSDLVHEHWRDNVFVTLNADRDFDEHAARAEVVVRRKIDLARQCMVPMEGKAVLAYWDHQ
ADQLVVISATQVPHMIRSVLAQCLDLEQGRVRVVSPDVGGAFGYKCVLQQEELCVAWLAKTYKRPFRFIEDRRE
HLTAGANSREHHYEMTAYADKRGKLLALDARITIDGGAYSVWPFTIGLEPDQAVGNLPGPYGFRGYRCETRCVA
TNKPGFVPYRGRTGVCFAIELTMDAVAREVGREPWEVRLENLVQPEQMPYVNVTNKHFDSGDYPASLRKALE
MIDIDGVRTRQARGEADGRRIGVGVATYTEQAAHGTSVFAAWGTPVIPGFDQATVRVTPDGGLEVRVGVHSH
GQGMETTFAQIAHEILGIDVARIKVLHGDTGQTPFSTGTYASRSLVMSGGAVSQACKRLLPRMRHIAAHMLGV
ADDAVTLQDGIYRAGEKSVATGDVADAWYLRPQLLPPDVDPAGLEVNVGYKPKVDTGCFTYASHAAVVAVDP
DTGAVEILDYVVVEDCGTMINPMVVEGQTIGGIAQGIGTAFYEETPYDANGQPLASTLADYMLPGPTEVPNM
RLHHFETPSPHTEFGAKGMGEGGAIAPPAVLFNAVNDALRGLGAAELSRTPLTPIRVLQAIAQGAGEAA
>SEQF7931||SEQF7931.1_03461
MTDATSPGLGRSAPRIEDDALLRGQARFLDDIEVEGVLHACFVRSPHAHARLVSIDLSAARAVPGVAAVYGARD
LFGQLTSWRMPLGFPLAALPDDTTPFVLAEREVAFVGEAIAVVVADSRHIAEDAAARVAIEYEVLGAVVDCRDA
LRPDAPLVRGELASNILQQYTLAYGDCETAFAQAHRVLEDDFWVHRGCAHPMEGRGVLARMDRATDTLTVWS
STQMAHELHYTLALMLGQPEDRLRVVTPDVGGGFGAKFMIYPEEMAIPAAARKLGRPVKWVEDRREHFTTSI
QERDQYWKVAMAIDDQGHVLGIRGNFVHDNGAYTPQGTNVPYNAASSMTGPYVVPAFSLDVSVAYTNKVPV
ATVRGAGYPQAAFVMERMMDRVAAELGIDPGECRRRNLIGPAKIPYTKPLKSRAGMPLTIDSGDFPALQACAL
QASDYDGFAVRRDAALARGRWRGIAVANSVEPTGRGPFEVARVRVQPSGQVSIYTGALAMGQGIKTTLAQIC
AGHLGVPVAAVEVQAGDTAYVGYGMGGFASRQAIMAGSAVDQAAAQVRRQALETAAAVLKAEAETLELADG
EVRAPDGQSVSLARLAMLRKGVPGYALVSPGDPGLDATAYFHCDAQTYAGASHVCEVEVDPATGAIEIVRYVAA
QDSGRIINPQLAEGQVHGGVVHGIGNALFEWMGYDAAGQPLSTTFAEYLLPTAPEVPPIEVVFQPSPTPLNPL
GVKGVGECATIPVAVAVVGAVEHAVAHCGVRVTEFPLTPVRLLELLTQAEARAAAPTDSGEFVDGVH
>SEQF7932||SEQF7932.1_03463
MTDATSPGLGRSAPRIEDDALLRGQARFLDDIEVEGVLHACFVRSPHAHARLVSIDLSAARAVPGVAAVYGARD
LFGQLTSWRMPLGFPLAALPDDTTPFVLAEREVAFVGEAIAVVVADSRHIAEDAAARVAIEYEVLGAVVDCRDA

LRPDAPLVRGELASNILQQYTLAYGDCETAFAQAHRVLEDDFWVHRGCAHPMEGRGVLARMDRATDTLTVWS
STQMAHELHYTLALMLGQPEDRLRVVTPDVGGGFGAKFMIYPEEMAIPAAARKLGRPVKWVEDRREHFTTSI
QERDQYWKVAMAIDDQGHVLGIRGNFVHDNGAYTPQGTNVPYNAASSMTGPYVVPAFSLDVSVAYTNKVPV
ATVRGAGYPQAAFVMERMMDRVAAELGIDPGECRRRNLIGPAKIPYTKPLKSRAGMPLTIDSGDFPALQACAL
QASDYDGFAVRRDAALARGRWRGIAVANSVEPTGRGPFEVARVRVQPSGQVSIYTGALAMGQGIKTTLAQIC
AGHLGVPVAAVEVQAGDTAYVGYGMGGFASRQAIMAGSAVDQAAAQVRRQALETAAAVLKAEAETLELADG
EVRAPDGQSVSLARLAMLRKGVPGYALVSPGDPGLDATAYFHCDAQTYAGASHVCEVEVDPATGAIEIVRYVAA
QDSGRIINPQLAEGQVHGGVVHGIGNALFEWMGYDAAGQPLSTTFAEYLLPTAPEVPPIEVVFQPSPTPLNPL
GVKGVGECATIPVAVAVVGAVEHAVAHCGVRVTEFPLTPVRLLELLTQAEARAAAPTDSGEFVDGVH
>SEQF7932||SEQF7932.1_03558
MTTEHTGAEVGAGSRQGVGARVPRKEDARHLHGKGNFVADMAMPGLCEVAFLRSPLAHARITDVRVPESVR
DKVVLRSMMGDARDIAADSTLPTYQPSVQPPLASGKVRFVGEPVAMTFAPTRAEAEDHAELVEVDYDDLPVY
ADVAGAQQATSDLVHEHWRDNVFVTLNADRDFDEHAARAEVVVRRKIDLARQCMVPMEGKAVLAYWDHQ
ADQLVVISATQVPHMIRSVLAQCLDLEQGRVRVVSPDVGGAFGYKCVLQQEELCVAWLAKTYKRPFRFIEDRRE
HLTAGANSREHHYEMTAYADKRGKLLALDARITIDGGAYSVWPFTIGLEPDQAVGNLPGPYGFRGYRCETRCVA
TNKPGFVPYRGRTGVCFAIELTMDAVAREVGREPWEVRLENLVQPEQMPYVNVTNKHFDSGDYPASLRKALE
MIDIDGVRTRQARGEADGRRIGVGVATYTEQAAHGTSVFAAWGTPVIPGFDQATVRVTPDGGLEVRVGVHSH
GQGMETTFAQIAHEILGIDVARIKVLHGDTGQTPFSTGTYASRSLVMSGGAVSQACKRLLPRMRHIAAHMLGV
ADDAVTLQDGIYRAGEKSVATGDVADAWYLRPQLLPPDVDPAGLEVNVGYKPKVDTGCFTYASHAAVVAVDP
DTGAVEILDYVVVEDCGTMINPMVVEGQTIGGIAQGIGTAFYEETPYDANGQPLASTLADYMLPGPTEVPNM
RLHHFETPSPHTEFGAKGMGEGGAIAPPAVLFNAVNDALRGLGAAELSRTPLTPIRVLQAIAQGAGEAA
>SEQF7933||SEQF7933.1_00481
MTDATSPGLGRSAPRIEDDALLRGQARFLDDIEVEGVLHACFVRSPHAHARLVSIDLSAARAVPGVAAVYGARD
LFGQLTSWRMPLGFPLAALPDDTTPFVLAEREVAFVGEAIAVVVADSRHIAEDAAARVAIEYEVLGAVVDCRDA
LRPDAPLVRGELASNILQQYTLAYGDCETAFAQAHRVLEDDFWVHRGCAHPMEGRGVLARMDRATDTLTVWS
STQMAHELHYTLALMLGQPEDRLRVVTPDVGGGFGAKFMIYPEEMAIPAAARKLGRPVKWVEDRREHFTTSI
QERDQYWKVAMAIDDQGHVLGIRGNFVHDNGAYTPQGTNVPYNAASSMTGPYVVPAFSLDVSVAYTNKVPV
ATVRGAGYPQAAFVMERMMDRVAAELGIDPGECRRRNLIGPAKIPYTKPLKSRAGMPLTIDSGDFPALQACAL
QASDYDGFAVRRDAALARGRWRGIAVANSVEPTGRGPFEVARVRVQPSGQVSIYTGALAMGQGIKTTLAQIC
AGHLGVPVAAVEVQAGDTAYVGYGMGGFASRQAIMAGSAVDQAAAQVRRQALETAAAVLKAEAETLELADG
EVRAPDGQSVSLARLAMLRKGVPGYALVSPGDPGLDATAYFHCDAQTYAGASHVCEVEVDPATGAIEIVRYVAA
QDSGRIINPQLAEGQVHGGVVHGIGNALFEWMGYDAAGQPLSTTFAEYLLPTAPEVPPIEVVFQPSPTPLNPL
GVKGVGECATIPVAVAVVGAVEHAVAHCGVRVTEFPLTPVRLLELLTQAEARAAAPTDSGEFVDGVH
>SEQF7933||SEQF7933.1_03561
MTTEHTGAEVGAGSRQGVGARVPRKEDARHLHGKGNFVADMAMPGLCEVAFLRSPLAHARITDVRVPESVR
DKVVLRSMMGDARDIAADSTLPTYQPSVQPPLASGKVRFVGEPVAMTFAPTRAEAEDHAELVEVDYDDLPVY
ADVAGAQQATSDLVHEHWRDNVFVTLNADRDFDEHAARAEVVVRRKIDLARQCMVPMEGKAVLAYWDHQ
ADQLVVISATQVPHMIRSVLAQCLDLEQGRVRVVSPDVGGAFGYKCVLQQEELCVAWLAKTYKRPFRFIEDRRE
HLTAGANSREHHYEMTAYADKRGKLLALDARITIDGGAYSVWPFTIGLEPDQAVGNLPGPYGFRGYRCETRCVA
TNKPGFVPYRGRTGVCFAIELTMDAVAREVGREPWEVRLENLVQPEQMPYVNVTNKHFDSGDYPASLRKALE
MIDIDGVRTRQARGEADGRRIGVGVATYTEQAAHGTSVFAAWGTPVIPGFDQATVRVTPDGGLEVRVGVHSH
GQGMETTFAQIAHEILGIDVARIKVLHGDTGQTPFSTGTYASRSLVMSGGAVSQACKRLLPRMRHIAAHMLGV
ADDAVTLQDGIYRAGEKSVATGDVADAWYLRPQLLPPDVDPAGLEVNVGYKPKVDTGCFTYASHAAVVAVDP
DTGAVEILDYVVVEDCGTMINPMVVEGQTIGGIAQGIGTAFYEETPYDANGQPLASTLADYMLPGPTEVPNM

RLHHFETPSPHTEFGAKGMGEGGAIAPPAVLFNAVNDALRGLGAAELSRTPLTPIRVLQAIAQGAGEAA
>SEQF7934||SEQF7934.1_03564
MTTEHTGAEVGAGSRQGVGARVPRKEDARHLHGKGNFVADMAMPGLCEVAFLRSPLAHARITDVRVPESVR
DKVVLRSMMGDARDIAADSTLPTYQPSVQPPLASGKVRFVGEPVAMTFAPTRAEAEDHAELVEVDYDDLPVY
ADVAGAQQATSDLVHEHWRDNVFVTLNADRDFDEHAARAEVVVRRKIDLARQCMVPMEGKAVLAYWDHQ
ADQLVVISATQVPHMIRSVLAQCLDLEQGRVRVVSPDVGGAFGYKCVLQQEELCVAWLAKTYKRPFRFIEDRRE
HLTAGANSREHHYEMTAYADKRGKLLALDARITIDGGAYSVWPFTIGLEPDQAVGNLPGPYGFRGYRCETRCVA
TNKPGFVPYRGRTGVCFAIELTMDAVAREVGREPWEVRLENLVQPEQMPYVNVTNKHFDSGDYPASLRKALE
MIDIDGVRTRQARGEADGRRIGVGVATYTEQAAHGTSVFAAWGTPVIPGFDQATVRVTPDGGLEVRVGVHSH
GQGMETTFAQIAHEILGIDVARIKVLHGDTGQTPFSTGTYASRSLVMSGGAVSQACKRLLPRMRHIAAHMLGV
ADDAVTLQDGIYRAGEKSVATGDVADAWYLRPQLLPPDVDPAGLEVNVGYKPKVDTGCFTYASHAAVVAVDP
DTGAVEILDYVVVEDCGTMINPMVVEGQTIGGIAQGIGTAFYEETPYDANGQPLASTLADYMLPGPTEVPNM
RLHHFETPSPHTEFGAKGMGEGGAIAPPAVLFNAVNDALRGLGAAELSRTPLTPIRVLQAIAQGAGEAA
>SEQF7934||SEQF7934.1_00480
MTDATSPGLGRSAPRIEDDALLRGQARFLDDIEVEGVLHACFVRSPHAHARLVSIDLSAARAVPGVAAVYGARD
LFGQLTSWRMPLGFPLAALPDDTTPFVLAEREVAFVGEAIAVVVADSRHIAEDAAARVAIEYEVLGAVVDCRDA
LRPDAPLVRGELASNILQQYTLAYGDCETAFAQAHRVLEDDFWVHRGCAHPMEGRGVLARMDRATDTLTVWS
STQMAHELHYTLALMLGQPEDRLRVVTPDVGGGFGAKFMIYPEEMAIPAAARKLGRPVKWVEDRREHFTTSI
QERDQYWKVAMAIDDQGHVLGIRGNFVHDNGAYTPQGTNVPYNAASSMTGPYVVPAFSLDVSVAYTNKVPV
ATVRGAGYPQAAFVMERMMDRVAAELGIDPGECRRRNLIGPAKIPYTKPLKSRAGMPLTIDSGDFPALQACAL
QASDYDGFAVRRDAALARGRWRGIAVANSVEPTGRGPFEVARVRVQPSGQVSIYTGALAMGQGIKTTLAQIC
AGHLGVPVAAVEVQAGDTAYVGYGMGGFASRQAIMAGSAVDQAAAQVRRQALETAAAVLKAEAETLELADG
EVRAPDGQSVSLARLAMLRKGVPGYALVSPGDPGLDATAYFHCDAQTYAGASHVCEVEVDPATGAIEIVRYVAA
QDSGRIINPQLAEGQVHGGVVHGIGNALFEWMGYDAAGQPLSTTFAEYLLPTAPEVPPIEVVFQPSPTPLNPL
GVKGVGECATIPVAVAVVGAVEHAVAHCGVRVTEFPLTPVRLLELLTQAEARAAAPTDSGEFVDGVH
>SEQF7935||SEQF7935.1_03464
MTDATSPGLGRSAPRIEDDALLRGQARFLDDIEVEGVLHACFVRSPHAHARLVSIDLSAARAVPGVAAVYGARD
LFGQLTSWRMPLGFPLAALPDDTTPFVLAEREVAFVGEAIAVVVADSRHIAEDAAARVAIEYEVLGAVVDCRDA
LRPDAPLVRGELASNILQQYTLAYGDCETAFAQAHRVLEDDFWVHRGCAHPMEGRGVLARMDRATDTLTVWS
STQMAHELHYTLALMLGQPEDRLRVVTPDVGGGFGAKFMIYPEEMAIPAAARKLGRPVKWVEDRREHFTTSI
QERDQYWKVAMAIDDQGHVLGIRGNFVHDNGAYTPQGTNVPYNAASSMTGPYVVPAFSLDVSVAYTNKVPV
ATVRGAGYPQAAFVMERMMDRVAAELGIDPGECRRRNLIGPAKIPYTKPLKSRAGMPLTIDSGDFPALQACAL
QASDYDGFAVRRDAALARGRWRGIAVANSVEPTGRGPFEVARVRVQPSGQVSIYTGALAMGQGIKTTLAQIC
AGHLGVPVAAVEVQAGDTAYVGYGMGGFASRQAIMAGSAVDQAAAQVRRQALETAAAVLKAEAETLELADG
EVRAPDGQSVSLARLAMLRKGVPGYALVSPGDPGLDATAYFHCDAQTYAGASHVCEVEVDPATGAIEIVRYVAA
QDSGRIINPQLAEGQVHGGVVHGIGNALFEWMGYDAAGQPLSTTFAEYLLPTAPEVPPIEVVFQPSPTPLNPL
GVKGVGECATIPVAVAVVGAVEHAVAHCGVRVTEFPLTPVRLLELLTQAEARAAAPTDSGEFVDGVH
>SEQF7935||SEQF7935.1_03560
MTTEHTGAEVGAGSRQGVGARVPRKEDARHLHGKGNFVADMAMPGLCEVAFLRSPLAHARITDVRVPESVR
DKVVLRSMMGDARDIAADSTLPTYQPSVQPPLASGKVRFVGEPVAMTFAPTRAEAEDHAELVEVDYDDLPVY
ADVAGAQQATSDLVHEHWRDNVFVTLNADRDFDEHAARAEVVVRRKIDLARQCMVPMEGKAVLAYWDHQ
ADQLVVISATQVPHMIRSVLAQCLDLEQGRVRVVSPDVGGAFGYKCVLQQEELCVAWLAKTYKRPFRFIEDRRE
HLTAGANSREHHYEMTAYADKRGKLLALDARITIDGGAYSVWPFTIGLEPDQAVGNLPGPYGFRGYRCETRCVA
TNKPGFVPYRGRTGVCFAIELTMDAVAREVGREPWEVRLENLVQPEQMPYVNVTNKHFDSGDYPASLRKALE

MIDIDGVRTRQARGEADGRRIGVGVATYTEQAAHGTSVFAAWGTPVIPGFDQATVRVTPDGGLEVRVGVHSH
GQGMETTFAQIAHEILGIDVARIKVLHGDTGQTPFSTGTYASRSLVMSGGAVSQACKRLLPRMRHIAAHMLGV
ADDAVTLQDGIYRAGEKSVATGDVADAWYLRPQLLPPDVDPAGLEVNVGYKPKVDTGCFTYASHAAVVAVDP
DTGAVEILDYVVVEDCGTMINPMVVEGQTIGGIAQGIGTAFYEETPYDANGQPLASTLADYMLPGPTEVPNM
RLHHFETPSPHTEFGAKGMGEGGAIAPPAVLFNAVNDALRGLGAAELSRTPLTPIRVLQAIAQGAGEAA
>SEQF7936||SEQF7936.1_00479
MTDATSPGLGRSAPRIEDDALLRGQARFLDDIEVEGVLHACFVRSPHAHARLVSIDLSAARAVPGVAAVYGARD
LFGQLTSWRMPLGFPLAALPDDTTPFVLAEREVAFVGEAIAVVVADSRHIAEDAAARVAIEYEVLGAVVDCRDA
LRPDAPLVRGELASNILQQYTLAYGDCETAFAQAHRVLEDDFWVHRGCAHPMEGRGVLARMDRATDTLTVWS
STQMAHELHYTLALMLGQPEDRLRVVTPDVGGGFGAKFMIYPEEMAIPAAARKLGRPVKWVEDRREHFTTSI
QERDQYWKVAMAIDDQGHVLGIRGNFVHDNGAYTPQGTNVPYNAASSMTGPYVVPAFSLDVSVAYTNKVPV
ATVRGAGYPQAAFVMERMMDRVAAELGIDPGECRRRNLIGPAKIPYTKPLKSRAGMPLTIDSGDFPALQACAL
QASDYDGFAVRRDAALARGRWRGIAVANSVEPTGRGPFEVARVRVQPSGQVSIYTGALAMGQGIKTTLAQIC
AGHLGVPVAAVEVQAGDTAYVGYGMGGFASRQAIMAGSAVDQAAAQVRRQALETAAAVLKAEAETLELADG
EVRAPDGQSVSLARLAMLRKGVPGYALVSPGDPGLDATAYFHCDAQTYAGASHVCEVEVDPATGAIEIVRYVAA
QDSGRIINPQLAEGQVHGGVVHGIGNALFEWMGYDAAGQPLSTTFAEYLLPTAPEVPPIEVVFQPSPTPLNPL
GVKGVGECATIPVAVAVVGAVEHAVAHCGVRVTEFPLTPVRLLELLTQAEARAAAPTDSGEFVDGVH
>SEQF7936||SEQF7936.1_03558
MTTEHTGAEVGAGSRQGVGARVPRKEDARHLHGKGNFVADMAMPGLCEVAFLRSPLAHARITDVRVPESVR
DKVVLRSMMGDARDIAADSTLPTYQPSVQPPLASGKVRFVGEPVAMTFAPTRAEAEDHAELVEVDYDDLPVY
ADVAGAQQATSDLVHEHWRDNVFVTLNADRDFDEHAARAEVVVRRKIDLARQCMVPMEGKAVLAYWDHQ
ADQLVVISATQVPHMIRSVLAQCLDLEQGRVRVVSPDVGGAFGYKCVLQQEELCVAWLAKTYKRPFRFIEDRRE
HLTAGANSREHHYEMTAYADKRGKLLALDARITIDGGAYSVWPFTIGLEPDQAVGNLPGPYGFRGYRCETRCVA
TNKPGFVPYRGRTGVCFAIELTMDAVAREVGREPWEVRLENLVQPEQMPYVNVTNKHFDSGDYPASLRKALE
MIDIDGVRTRQARGEADGRRIGVGVATYTEQAAHGTSVFAAWGTPVIPGFDQATVRVTPDGGLEVRVGVHSH
GQGMETTFAQIAHEILGIDVARIKVLHGDTGQTPFSTGTYASRSLVMSGGAVSQACKRLLPRMRHIAAHMLGV
ADDAVTLQDGIYRAGEKSVATGDVADAWYLRPQLLPPDVDPAGLEVNVGYKPKVDTGCFTYASHAAVVAVDP
DTGAVEILDYVVVEDCGTMINPMVVEGQTIGGIAQGIGTAFYEETPYDANGQPLASTLADYMLPGPTEVPNM
RLHHFETPSPHTEFGAKGMGEGGAIAPPAVLFNAVNDALRGLGAAELSRTPLTPIRVLQAIAQGAGEAA
>SEQF7937||SEQF7937.1_03472
MTDATSPGLGRSAPRIEDDALLRGQARFLDDIEVEGVLHACFVRSPHAHARLVSIDLSAARAVPGVAAVYGARD
LFGQLTSWRMPLGFPLAALPDDTTPFVLAEREVAFVGEAIAVVVADSRHIAEDAAARVAIEYEVLGAVVDCRDA
LRPDAPLVRGELASNILQQYTLAYGDCETAFAQAHRVLEDDFWVHRGCAHPMEGRGVLARMDRATDTLTVWS
STQMAHELHYTLALMLGQPEDRLRVVTPDVGGGFGAKFMIYPEEMAIPAAARKLGRPVKWVEDRREHFTTSI
QERDQYWKVAMAIDDQGHVLGIRGNFVHDNGAYTPQGTNVPYNAASSMTGPYVVPAFSLDVSVAYTNKVPV
ATVRGAGYPQAAFVMERMMDRVAAELGIDPGECRRRNLIGPAKIPYTKPLKSRAGMPLTIDSGDFPALQACAL
QASDYDGFAVRRDAALARGRWRGIAVANSVEPTGRGPFEVARVRVQPSGQVSIYTGALAMGQGIKTTLAQIC
AGHLGVPVAAVEVQAGDTAYVGYGMGGFASRQAIMAGSAVDQAAAQVRRQALETAAAVLKAEAETLELADG
EVRAPDGQSVSLARLAMLRKGVPGYALVSPGDPGLDATAYFHCDAQTYAGASHVCEVEVDPATGAIEIVRYVAA
QDSGRIINPQLAEGQVHGGVVHGIGNALFEWMGYDAAGQPLSTTFAEYLLPTAPEVPPIEVVFQPSPTPLNPL
GVKGVGECATIPVAVAVVGAVEHAVAHCGVRVTEFPLTPVRLLELLTQAEARAAAPTDSGEFVDGVH
>SEQF7937||SEQF7937.1_03568
MTTEHTGAEVGAGSRQGVGARVPRKEDARHLHGKGNFVADMAMPGLCEVAFLRSPLAHARITDVRVPESVR
DKVVLRSMMGDARDIAADSTLPTYQPSVQPPLASGKVRFVGEPVAMTFAPTRAEAEDHAELVEVDYDDLPVY

ADVAGAQQATSDLVHEHWRDNVFVTLNADRDFDEHAARAEVVVRRKIDLARQCMVPMEGKAVLAYWDHQ
ADQLVVISATQVPHMIRSVLAQCLDLEQGRVRVVSPDVGGAFGYKCVLQQEELCVAWLAKTYKRPFRFIEDRRE
HLTAGANSREHHYEMTAYADKRGKLLALDARITIDGGAYSVWPFTIGLEPDQAVGNLPGPYGFRGYRCETRCVA
TNKPGFVPYRGRTGVCFAIELTMDAVAREVGREPWEVRLENLVQPEQMPYVNVTNKHFDSGDYPASLRKALE
MIDIDGVRTRQARGEADGRRIGVGVATYTEQAAHGTSVFAAWGTPVIPGFDQATVRVTPDGGLEVRVGVHSH
GQGMETTFAQIAHEILGIDVARIKVLHGDTGQTPFSTGTYASRSLVMSGGAVSQACKRLLPRMRHIAAHMLGV
ADDAVTLQDGIYRAGEKSVATGDVADAWYLRPQLLPPDVDPAGLEVNVGYKPKVDTGCFTYASHAAVVAVDP
DTGAVEILDYVVVEDCGTMINPMVVEGQTIGGIAQGIGTAFYEETPYDANGQPLASTLADYMLPGPTEVPNM
RLHHFETPSPHTEFGAKGMGEGGAIAPPAVLFNAVNDALRGLGAAELSRTPLTPIRVLQAIAQGAGEAA
>SEQF7938||SEQF7938.1_00480
MTDATSPGLGRSAPRIEDDALLRGQARFLDDIEVEGVLHACFVRSPHAHARLVSIDLSAARAVPGVAAVYGARD
LFGQLTSWRMPLGFPLAALPDDTTPFVLAEREVAFVGEAIAVVVADSRHIAEDAAARVAIEYEVLGAVVDCRDA
LRPDAPLVRGELASNILQQYTLAYGDCETAFAQAHRVLEDDFWVHRGCAHPMEGRGVLARMDRATDTLTVWS
STQMAHELHYTLALMLGQPEDRLRVVTPDVGGGFGAKFMIYPEEMAIPAAARKLGRPVKWVEDRREHFTTSI
QERDQYWKVAMAIDDQGHVLGIRGNFVHDNGAYTPQGTNVPYNAASSMTGPYVVPAFSLDVSVAYTNKVPV
ATVRGAGYPQAAFVMERMMDRVAAELGIDPGECRRRNLIGPAKIPYTKPLKSRAGMPLTIDSGDFPALQACAL
QASDYDGFAVRRDAALARGRWRGIAVANSVEPTGRGPFEVARVRVQPSGQVSIYTGALAMGQGIKTTLAQIC
AGHLGVPVAAVEVQAGDTAYVGYGMGGFASRQAIMAGSAVDQAAAQVRRQALETAAAVLKAEAETLELADG
EVRAPDGQSVSLARLAMLRKGVPGYALVSPGDPGLDATAYFHCDAQTYAGASHVCEVEVDPATGAIEIVRYVAA
QDSGRIINPQLAEGQVHGGVVHGIGNALFEWMGYDAAGQPLSTTFAEYLLPTAPEVPPIEVVFQPSPTPLNPL
GVKGVGECATIPVAVAVVGAVEHAVAHCGVRVTEFPLTPVRLLELLTQAEARAAAPTDSGEFVDGVH
>SEQF7938||SEQF7938.1_03560
MTTEHTGAEVGAGSRQGVGARVPRKEDARHLHGKGNFVADMAMPGLCEVAFLRSPLAHARITDVRVPESVR
DKVVLRSMMGDARDIAADSTLPTYQPSVQPPLASGKVRFVGEPVAMTFAPTRAEAEDHAELVEVDYDDLPVY
ADVAGAQQATSDLVHEHWRDNVFVTLNADRDFDEHAARAEVVVRRKIDLARQCMVPMEGKAVLAYWDHQ
ADQLVVISATQVPHMIRSVLAQCLDLEQGRVRVVSPDVGGAFGYKCVLQQEELCVAWLAKTYKRPFRFIEDRRE
HLTAGANSREHHYEMTAYADKRGKLLALDARITIDGGAYSVWPFTIGLEPDQAVGNLPGPYGFRGYRCETRCVA
TNKPGFVPYRGRTGVCFAIELTMDAVAREVGREPWEVRLENLVQPEQMPYVNVTNKHFDSGDYPASLRKALE
MIDIDGVRTRQARGEADGRRIGVGVATYTEQAAHGTSVFAAWGTPVIPGFDQATVRVTPDGGLEVRVGVHSH
GQGMETTFAQIAHEILGIDVARIKVLHGDTGQTPFSTGTYASRSLVMSGGAVSQACKRLLPRMRHIAAHMLGV
ADDAVTLQDGIYRAGEKSVATGDVADAWYLRPQLLPPDVDPAGLEVNVGYKPKVDTGCFTYASHAAVVAVDP
DTGAVEILDYVVVEDCGTMINPMVVEGQTIGGIAQGIGTAFYEETPYDANGQPLASTLADYMLPGPTEVPNM
RLHHFETPSPHTEFGAKGMGEGGAIAPPAVLFNAVNDALRGLGAAELSRTPLTPIRVLQAIAQGAGEAA
>SEQF7939||SEQF7939.1_00483
MTDATSPGLGRSAPRIEDDALLRGQARFLDDIEVEGVLHACFVRSPHAHARLVSIDLSAARAVPGVAAVYGARD
LFGQLTSWRMPLGFPLAALPDDTTPFVLAEREVAFVGEAIAVVVADSRHIAEDAAARVAIEYEVLGAVVDCRDA
LRPDAPLVRGELASNILQQYTLAYGDCETAFAQAHRVLEDDFWVHRGCAHPMEGRGVLARMDRATDTLTVWS
STQMAHELHYTLALMLGQPEDRLRVVTPDVGGGFGAKFMIYPEEMAIPAAARKLGRPVKWVEDRREHFTTSI
QERDQYWKVAMAIDDQGHVLGIRGNFVHDNGAYTPQGTNVPYNAASSMTGPYVVPAFSLDVSVAYTNKVPV
ATVRGAGYPQAAFVMERMMDRVAAELGIDPGECRRRNLIGPAKIPYTKPLKSRAGMPLTIDSGDFPALQACAL
QASDYDGFAVRRDAALARGRWRGIAVANSVEPTGRGPFEVARVRVQPSGQVSIYTGALAMGQGIKTTLAQIC
AGHLGVPVAAVEVQAGDTAYVGYGMGGFASRQAIMAGSAVDQAAAQVRRQALETAAAVLKAEAETLELADG
EVRAPDGQSVSLARLAMLRKGVPGYALVSPGDPGLDATAYFHCDAQTYAGASHVCEVEVDPATGAIEIVRYVAA
QDSGRIINPQLAEGQVHGGVVHGIGNALFEWMGYDAAGQPLSTTFAEYLLPTAPEVPPIEVVFQPSPTPLNPL

GVKGVGECATIPVAVAVVGAVEHAVAHCGVRVTEFPLTPVRLLELLTQAEARAAAPTDSGEFVDGVH
>SEQF7939||SEQF7939.1_03547
MTTEHTGAEVGAGSRQGVGARVPRKEDARHLHGKGNFVADMAMPGLCEVAFLRSPLAHARITDVRVPESVR
DKVVLRSMMGDARDIAADSTLPTYQPSVQPPLASGKVRFVGEPVAMTFAPTRAEAEDHAELVEVDYDDLPVY
ADVAGAQQATSDLVHEHWRDNVFVTLNADRDFDEHAARAEVVVRRKIDLARQCMVPMEGKAVLAYWDHQ
ADQLVVISATQVPHMIRSVLAQCLDLEQGRVRVVSPDVGGAFGYKCVLQQEELCVAWLAKTYKRPFRFIEDRRE
HLTAGANSREHHYEMTAYADKRGKLLALDARITIDGGAYSVWPFTIGLEPDQAVGNLPGPYGFRGYRCETRCVA
TNKPGFVPYRGRTGVCFAIELTMDAVAREVGREPWEVRLENLVQPEQMPYVNVTNKHFDSGDYPASLRKALE
MIDIDGVRTRQARGEADGRRIGVGVATYTEQAAHGTSVFAAWGTPVIPGFDQATVRVTPDGGLEVRVGVHSH
GQGMETTFAQIAHEILGIDVARIKVLHGDTGQTPFSTGTYASRSLVMSGGAVSQACKRLLPRMRHIAAHMLGV
ADDAVTLQDGIYRAGEKSVATGDVADAWYLRPQLLPPDVDPAGLEVNVGYKPKVDTGCFTYASHAAVVAVDP
DTGAVEILDYVVVEDCGTMINPMVVEGQTIGGIAQGIGTAFYEETPYDANGQPLASTLADYMLPGPTEVPNM
RLHHFETPSPHTEFGAKGMGEGGAIAPPAVLFNAVNDALRGLGAAELSRTPLTPIRVLQAIAQGAGEAA
>SEQF7940||SEQF7940.1_00503
MTDATSPGLGRSAPRIEDDALLRGQARFLDDIEVEGVLHACFVRSPHAHARLVSIDLSAARAVPGVAAVYGARD
LFGQLTSWRMPLGFPLAALPDDTTPFVLAEREVAFVGEAIAVVVADSRHIAEDAAARVAIEYEVLGAVVDCRDA
LRPDAPLVRGELASNILQQYTLAYGDCETAFAQAHRVLEDDFWVHRGCAHPMEGRGVLARMDRATDTLTVWS
STQMAHELHYTLALMLGQPEDRLRVVTPDVGGGFGAKFMIYPEEMAIPAAARKLGRPVKWVEDRREHFTTSI
QERDQYWKVAMAIDDQGHVLGIRGNFVHDNGAYTPQGTNVPYNAASSMTGPYVVPAFSLDVSVAYTNKVPV
ATVRGAGYPQAAFVMERMMDRVAAELGIDPGECRRRNLIGPAKIPYTKPLKSRAGMPLTIDSGDFPALQACAL
QASDYDGFAVRRDAALARGRWRGIAVANSVEPTGRGPFEVARVRVQPSGQVSIYTGALAMGQGIKTTLAQIC
AGHLGVPVAAVEVQAGDTAYVGYGMGGFASRQAIMAGSAVDQAAAQVRRQALETAAAVLKAEAETLELADG
EVRAPDGQSVSLARLAMLRKGVPGYALVSPGDPGLDATAYFHCDAQTYAGASHVCEVEVDPATGAIEIVRYVAA
QDSGRIINPQLAEGQVHGGVVHGIGNALFEWMGYDAAGQPLSTTFAEYLLPTAPEVPPIEVVFQPSPTPLNPL
GVKGVGECATIPVAVAVVGAVEHAVAHCGVRVTEFPLTPVRLLELLTQAEARAAAPTDSGEFVDGVH
>SEQF7940||SEQF7940.1_00407
MTTEHTGAEVGAGSRQGVGARVPRKEDARHLHGKGNFVADMAMPGLCEVAFLRSPLAHARITDVRVPESVR
DKVVLRSMMGDARDIAADSTLPTYQPSVQPPLASGKVRFVGEPVAMTFAPTRAEAEDHAELVEVDYDDLPVY
ADVAGAQQATSDLVHEHWRDNVFVTLNADRDFDEHAARAEVVVRRKIDLARQCMVPMEGKAVLAYWDHQ
ADQLVVISATQVPHMIRSVLAQCLDLEQGRVRVVSPDVGGAFGYKCVLQQEELCVAWLAKTYKRPFRFIEDRRE
HLTAGANSREHHYEMTAYADKRGKLLALDARITIDGGAYSVWPFTIGLEPDQAVGNLPGPYGFRGYRCETRCVA
TNKPGFVPYRGRTGVCFAIELTMDAVAREVGREPWEVRLENLVQPEQMPYVNVTNKHFDSGDYPASLRKALE
MIDIDGVRTRQARGEADGRRIGVGVATYTEQAAHGTSVFAAWGTPVIPGFDQATVRVTPDGGLEVRVGVHSH
GQGMETTFAQIAHEILGIDVARIKVLHGDTGQTPFSTGTYASRSLVMSGGAVSQACKRLLPRMRHIAAHMLGV
ADDAVTLQDGIYRAGEKSVATGDVADAWYLRPQLLPPDVDPAGLEVNVGYKPKVDTGCFTYASHAAVVAVDP
DTGAVEILDYVVVEDCGTMINPMVVEGQTIGGIAQGIGTAFYEETPYDANGQPLASTLADYMLPGPTEVPNM
RLHHFETPSPHTEFGAKGMGEGGAIAPPAVLFNAVNDALRGLGAAELSRTPLTPIRVLQAIAQGAGEAA
>SEQF7941||SEQF7941.1_00480
MTDATSPGLGRSAPRIEDDALLRGQARFLDDIEVEGVLHACFVRSPHAHARLVSIDLSAARAVPGVAAVYGARD
LFGQLTSWRMPLGFPLAALPDDTTPFVLAEREVAFVGEAIAVVVADSRHIAEDAAARVAIEYEVLGAVVDCRDA
LRPDAPLVRGELASNILQQYTLAYGDCETAFAQAHRVLEDDFWVHRGCAHPMEGRGVLARMDRATDTLTVWS
STQMAHELHYTLALMLGQPEDRLRVVTPDVGGGFGAKFMIYPEEMAIPAAARKLGRPVKWVEDRREHFTTSI
QERDQYWKVAMAIDDQGHVLGIRGNFVHDNGAYTPQGTNVPYNAASSMTGPYVVPAFSLDVSVAYTNKVPV
ATVRGAGYPQAAFVMERMMDRVAAELGIDPGECRRRNLIGPAKIPYTKPLKSRAGMPLTIDSGDFPALQACAL

QASDYDGFAVRRDAALARGRWRGIAVANSVEPTGRGPFEVARVRVQPSGQVSIYTGALAMGQGIKTTLAQIC
AGHLGVPVAAVEVQAGDTAYVGYGMGGFASRQAIMAGSAVDQAAAQVRRQALETAAAVLKAEAETLELADG
EVRAPDGQSVSLARLAMLRKGVPGYALVSPGDPGLDATAYFHCDAQTYAGASHVCEVEVDPATGAIEIVRYVAA
QDSGRIINPQLAEGQVHGGVVHGIGNALFEWMGYDAAGQPLSTTFAEYLLPTAPEVPPIEVVFQPSPTPLNPL
GVKGVGECATIPVAVAVVGAVEHAVAHCGVRVTEFPLTPVRLLELLTQAEARAAAPTDSGEFVDGVH
>SEQF7941||SEQF7941.1_03560
MTTEHTGAEVGAGSRQGVGARVPRKEDARHLHGKGNFVADMAMPGLCEVAFLRSPLAHARITDVRVPESVR
DKVVLRSMMGDARDIAADSTLPTYQPSVQPPLASGKVRFVGEPVAMTFAPTRAEAEDHAELVEVDYDDLPVY
ADVAGAQQATSDLVHEHWRDNVFVTLNADRDFDEHAARAEVVVRRKIDLARQCMVPMEGKAVLAYWDHQ
ADQLVVISATQVPHMIRSVLAQCLDLEQGRVRVVSPDVGGAFGYKCVLQQEELCVAWLAKTYKRPFRFIEDRRE
HLTAGANSREHHYEMTAYADKRGKLLALDARITIDGGAYSVWPFTIGLEPDQAVGNLPGPYGFRGYRCETRCVA
TNKPGFVPYRGRTGVCFAIELTMDAVAREVGREPWEVRLENLVQPEQMPYVNVTNKHFDSGDYPASLRKALE
MIDIDGVRTRQARGEADGRRIGVGVATYTEQAAHGTSVFAAWGTPVIPGFDQATVRVTPDGGLEVRVGVHSH
GQGMETTFAQIAHEILGIDVARIKVLHGDTGQTPFSTGTYASRSLVMSGGAVSQACKRLLPRMRHIAAHMLGV
ADDAVTLQDGIYRAGEKSVATGDVADAWYLRPQLLPPDVDPAGLEVNVGYKPKVDTGCFTYASHAAVVAVDP
DTGAVEILDYVVVEDCGTMINPMVVEGQTIGGIAQGIGTAFYEETPYDANGQPLASTLADYMLPGPTEVPNM
RLHHFETPSPHTEFGAKGMGEGGAIAPPAVLFNAVNDALRGLGAAELSRTPLTPIRVLQAIAQGAGEAA
>SEQF7942||SEQF7942.1_02523
MTDATSPGLGRSAPRIEDDALLRGQARFLDDIEVEGVLHACFVRSPHAHARLVSIDLSAARAVPGVAAVYGARD
LFGQLTSWRMPLGFPLAALPDDTTPFVLAEREVAFVGEAIAVVVADSRHIAEDAAARVAIEYEVLGAVVDCRDA
LRPDAPLVRGELASNILQQYTLAYGDCETAFAQAHRVLEDDFWVHRGCAHPMEGRGVLARMDRATDTLTVWS
STQMAHELHYTLALMLGQPEDRLRVVTPDVGGGFGAKFMIYPEEMAIPAAARKLGRPVKWVEDRREHFTTSI
QERDQYWKVAMAIDDQGHVLGIRGNFVHDNGAYTPQGTNVPYNAASSMTGPYVVPAFSLDVSVAYTNKVPV
ATVRGAGYPQAAFVMERMMDRVAAELGIDPGECRRRNLIGPAKIPYTKPLKSRAGMPLTIDSGDFPALQACAL
QASDYDGFAVRRDAALARGRWRGIAVANSVEPTGRGPFEVARVRVQPSGQVSIYTGALAMGQGIKTTLAQIC
AGHLGVPVAAVEVQAGDTAYVGYGMGGFASRQAIMAGSAVDQAAAQVRRQALETAAAVLKAEAETLELADG
EVRAPDGQSVSLARLAMLRKGVPGYALVSPGDPGLDATAYFHCDAQTYAGASHVCEVEVDPATGAIEIVRYVAA
QDSGRIINPQLAEGQVHGGVVHGIGNALFEWMGYDAAGQPLSTTFAEYLLPTAPEVPPIEVVFQPSPTPLNPL
GVKGVGECATIPVAVAVVGAVEHAVAHCGVRVTEFPLTPVRLLELLTQAEARAAAPTDSGEFVDGVH
>SEQF7942||SEQF7942.1_03430
MTTEHTGAEVGAGSRQGVGARVPRKEDARHLHGKGNFVADMAMPGLCEVAFLRSPLAHARITDVRVPESVR
DKVVLRSMMGDARDIAADSTLPTYQPSVQPPLASGKVRFVGEPVAMTFAPTRAEAEDHAELVEVDYDDLPVY
ADVAGAQQATSDLVHEHWRDNVFVTLNADRDFDEHAARAEVVVRRKIDLARQCMVPMEGKAVLAYWDHQ
ADQLVVISATQVPHMIRSVLAQCLDLEQGRVRVVSPDVGGAFGYKCVLQQEELCVAWLAKTYKRPFRFIEDRRE
HLTAGANSREHHYEMTAYADKRGKLLALDARITIDGGAYSVWPFTIGLEPDQAVGNLPGPYGFRGYRCETRCVA
TNKPGFVPYRGRTGVCFAIELTMDAVAREVGREPWEVRLENLVQPEQMPYVNVTNKHFDSGDYPASLRKALE
MIDIDGVRTRQARGEADGRRIGVGVATYTEQAAHGTSVFAAWGTPVIPGFDQATVRVTPDGGLEVRVGVHSH
GQGMETTFAQIAHEILGIDVARIKVLHGDTGQTPFSTGTYASRSLVMSGGAVSQACKRLLPRMRHIAAHMLGV
ADDAVTLQDGIYRAGEKSVATGDVADAWYLRPQLLPPDVDPAGLEVNVGYKPKVDTGCFTYASHAAVVAVDP
DTGAVEILDYVVVEDCGTMINPMVVEGQTIGGIAQGIGTAFYEETPYDANGQPLASTLADYMLPGPTEVPNM
RLHHFETPSPHTEFGAKGMGEGGAIAPPAVLFNAVNDALRGLGAAELSRTPLTPIRVLQAIAQGAGEAA
>SEQF7943||SEQF7943.1_03476
MTDATSPGLGRSAPRIEDDALLRGQARFLDDIEVEGVLHACFVRSPHAHARLVSIDLSAARAVPGVAAVYGARD
LFGQLTSWRMPLGFPLAALPDDTTPFVLAEREVAFVGEAIAVVVADSRHIAEDAAARVAIEYEVLGAVVDCRDA

LRPDAPLVRGELASNILQQYTLAYGDCETAFAQAHRVLEDDFWVHRGCAHPMEGRGVLARMDRATDTLTVWS
STQMAHELHYTLALMLGQPEDRLRVVTPDVGGGFGAKFMIYPEEMAIPAAARKLGRPVKWVEDRREHFTTSI
QERDQYWKVAMAIDDQGHVLGIRGNFVHDNGAYTPQGTNVPYNAASSMTGPYVVPAFSLDVSVAYTNKVPV
ATVRGAGYPQAAFVMERMMDRVAAELGIDPGECRRRNLIGPAKIPYTKPLKSRAGMPLTIDSGDFPALQACAL
QASDYDGFAVRRDAALARGRWRGIAVANSVEPTGRGPFEVARVRVQPSGQVSIYTGALAMGQGIKTTLAQIC
AGHLGVPVAAVEVQAGDTAYVGYGMGGFASRQAIMAGSAVDQAAAQVRRQALETAAAVLKAEAETLELADG
EVRAPDGQSVSLARLAMLRKGVPGYALVSPGDPGLDATAYFHCDAQTYAGASHVCEVEVDPATGAIEIVRYVAA
QDSGRIINPQLAEGQVHGGVVHGIGNALFEWMGYDAAGQPLSTTFAEYLLPTAPEVPPIEVVFQPSPTPLNPL
GVKGVGECATIPVAVAVVGAVEHAVAHCGVRVTEFPLTPVRLLELLTQAEARAAAPTDSGEFVDGVH
>SEQF7943||SEQF7943.1_03572
MTTEHTGAEVGAGSRQGVGARVPRKEDARHLHGKGNFVADMAMPGLCEVAFLRSPLAHARITDVRVPESVR
DKVVLRSMMGDARDIAADSTLPTYQPSVQPPLASGKVRFVGEPVAMTFAPTRAEAEDHAELVEVDYDDLPVY
ADVAGAQQATSDLVHEHWRDNVFVTLNADRDFDEHAARAEVVVRRKIDLARQCMVPMEGKAVLAYWDHQ
ADQLVVISATQVPHMIRSVLAQCLDLEQGRVRVVSPDVGGAFGYKCVLQQEELCVAWLAKTYKRPFRFIEDRRE
HLTAGANSREHHYEMTAYADKRGKLLALDARITIDGGAYSVWPFTIGLEPDQAVGNLPGPYGFRGYRCETRCVA
TNKPGFVPYRGRTGVCFAIELTMDAVAREVGREPWEVRLENLVQPEQMPYVNVTNKHFDSGDYPASLRKALE
MIDIDGVRTRQARGEADGRRIGVGVATYTEQAAHGTSVFAAWGTPVIPGFDQATVRVTPDGGLEVRVGVHSH
GQGMETTFAQIAHEILGIDVARIKVLHGDTGQTPFSTGTYASRSLVMSGGAVSQACKRLLPRMRHIAAHMLGV
ADDAVTLQDGIYRAGEKSVATGDVADAWYLRPQLLPPDVDPAGLEVNVGYKPKVDTGCFTYASHAAVVAVDP
DTGAVEILDYVVVEDCGTMINPMVVEGQTIGGIAQGIGTAFYEETPYDANGQPLASTLADYMLPGPTEVPNM
RLHHFETPSPHTEFGAKGMGEGGAIAPPAVLFNAVNDALRGLGAAELSRTPLTPIRVLQAIAQGAGEAA
>SEQF7944||SEQF7944.1_03462
MTDATSPGLGRSAPRIEDDALLRGQARFLDDIEVEGVLHACFVRSPHAHARLVSIDLSAARAVPGVAAVYGARD
LFGQLTSWRMPLGFPLAALPDDTTPFVLAEREVAFVGEAIAVVVADSRHIAEDAAARVAIEYEVLGAVVDCRDA
LRPDAPLVRGELASNILQQYTLAYGDCETAFAQAHRVLEDDFWVHRGCAHPMEGRGVLARMDRATDTLTVWS
STQMAHELHYTLALMLGQPEDRLRVVTPDVGGGFGAKFMIYPEEMAIPAAARKLGRPVKWVEDRREHFTTSI
QERDQYWKVAMAIDDQGHVLGIRGNFVHDNGAYTPQGTNVPYNAASSMTGPYVVPAFSLDVSVAYTNKVPV
ATVRGAGYPQAAFVMERMMDRVAAELGIDPGECRRRNLIGPAKIPYTKPLKSRAGMPLTIDSGDFPALQACAL
QASDYDGFAVRRDAALARGRWRGIAVANSVEPTGRGPFEVARVRVQPSGQVSIYTGALAMGQGIKTTLAQIC
AGHLGVPVAAVEVQAGDTAYVGYGMGGFASRQAIMAGSAVDQAAAQVRRQALETAAAVLKAEAETLELADG
EVRAPDGQSVSLARLAMLRKGVPGYALVSPGDPGLDATAYFHCDAQTYAGASHVCEVEVDPATGAIEIVRYVAA
QDSGRIINPQLAEGQVHGGVVHGIGNALFEWMGYDAAGQPLSTTFAEYLLPTAPEVPPIEVVFQPSPTPLNPL
GVKGVGECATIPVAVAVVGAVEHAVAHCGVRVTEFPLTPVRLLELLTQAEARAAAPTDSGEFVDGVH
>SEQF7944||SEQF7944.1_03558
MTTEHTGAEVGAGSRQGVGARVPRKEDARHLHGKGNFVADMAMPGLCEVAFLRSPLAHARITDVRVPESVR
DKVVLRSMMGDARDIAADSTLPTYQPSVQPPLASGKVRFVGEPVAMTFAPTRAEAEDHAELVEVDYDDLPVY
ADVAGAQQATSDLVHEHWRDNVFVTLNADRDFDEHAARAEVVVRRKIDLARQCMVPMEGKAVLAYWDHQ
ADQLVVISATQVPHMIRSVLAQCLDLEQGRVRVVSPDVGGAFGYKCVLQQEELCVAWLAKTYKRPFRFIEDRRE
HLTAGANSREHHYEMTAYADKRGKLLALDARITIDGGAYSVWPFTIGLEPDQAVGNLPGPYGFRGYRCETRCVA
TNKPGFVPYRGRTGVCFAIELTMDAVAREVGREPWEVRLENLVQPEQMPYVNVTNKHFDSGDYPASLRKALE
MIDIDGVRTRQARGEADGRRIGVGVATYTEQAAHGTSVFAAWGTPVIPGFDQATVRVTPDGGLEVRVGVHSH
GQGMETTFAQIAHEILGIDVARIKVLHGDTGQTPFSTGTYASRSLVMSGGAVSQACKRLLPRMRHIAAHMLGV
ADDAVTLQDGIYRAGEKSVATGDVADAWYLRPQLLPPDVDPAGLEVNVGYKPKVDTGCFTYASHAAVVAVDP
DTGAVEILDYVVVEDCGTMINPMVVEGQTIGGIAQGIGTAFYEETPYDANGQPLASTLADYMLPGPTEVPNM

RLHHFETPSPHTEFGAKGMGEGGAIAPPAVLFNAVNDALRGLGAAELSRTPLTPIRVLQAIAQGAGEAA
>SEQF7945||SEQF7945.1_03464
MTDATSPGLGRSAPRIEDDALLRGQARFLDDIEVEGVLHACFVRSPHAHARLVSIDLSAARAVPGVAAVYGARD
LFGQLTSWRMPLGFPLAALPDDTTPFVLAEREVAFVGEAIAVVVADSRHIAEDAAARVAIEYEVLGAVVDCRDA
LRPDAPLVRGELASNILQQYTLAYGDCETAFAQAHRVLEDDFWVHRGCAHPMEGRGVLARMDRATDTLTVWS
STQMAHELHYTLALMLGQPEDRLRVVTPDVGGGFGAKFMIYPEEMAIPAAARKLGRPVKWVEDRREHFTTSI
QERDQYWKVAMAIDDQGHVLGIRGNFVHDNGAYTPQGTNVPYNAASSMTGPYVVPAFSLDVSVAYTNKVPV
ATVRGAGYPQAAFVMERMMDRVAAELGIDPGECRRRNLIGPAKIPYTKPLKSRAGMPLTIDSGDFPALQACAL
QASDYDGFAVRRDAALARGRWRGIAVANSVEPTGRGPFEVARVRVQPSGQVSIYTGALAMGQGIKTTLAQIC
AGHLGVPVAAVEVQAGDTAYVGYGMGGFASRQAIMAGSAVDQAAAQVRRQALETAAAVLKAEAETLELADG
EVRAPDGQSVSLARLAMLRKGVPGYALVSPGDPGLDATAYFHCDAQTYAGASHVCEVEVDPATGAIEIVRYVAA
QDSGRIINPQLAEGQVHGGVVHGIGNALFEWMGYDAAGQPLSTTFAEYLLPTAPEVPPIEVVFQPSPTPLNPL
GVKGVGECATIPVAVAVVGAVEHAVAHCGVRVTEFPLTPVRLLELLTQAEARAAAPTDSGEFVDGVH
>SEQF7945||SEQF7945.1_03560
MTTEHTGAEVGAGSRQGVGARVPRKEDARHLHGKGNFVADMAMPGLCEVAFLRSPLAHARITDVRVPESVR
DKVVLRSMMGDARDIAADSTLPTYQPSVQPPLASGKVRFVGEPVAMTFAPTRAEAEDHAELVEVDYDDLPVY
ADVAGAQQATSDLVHEHWRDNVFVTLNADRDFDEHAARAEVVVRRKIDLARQCMVPMEGKAVLAYWDHQ
ADQLVVISATQVPHMIRSVLAQCLDLEQGRVRVVSPDVGGAFGYKCVLQQEELCVAWLAKTYKRPFRFIEDRRE
HLTAGANSREHHYEMTAYADKRGKLLALDARITIDGGAYSVWPFTIGLEPDQAVGNLPGPYGFRGYRCETRCVA
TNKPGFVPYRGRTGVCFAIELTMDAVAREVGREPWEVRLENLVQPEQMPYVNVTNKHFDSGDYPASLRKALE
MIDIDGVRTRQARGEADGRRIGVGVATYTEQAAHGTSVFAAWGTPVIPGFDQATVRVTPDGGLEVRVGVHSH
GQGMETTFAQIAHEILGIDVARIKVLHGDTGQTPFSTGTYASRSLVMSGGAVSQACKRLLPRMRHIAAHMLGV
ADDAVTLQDGIYRAGEKSVATGDVADAWYLRPQLLPPDVDPAGLEVNVGYKPKVDTGCFTYASHAAVVAVDP
DTGAVEILDYVVVEDCGTMINPMVVEGQTIGGIAQGIGTAFYEETPYDANGQPLASTLADYMLPGPTEVPNM
RLHHFETPSPHTEFGAKGMGEGGAIAPPAVLFNAVNDALRGLGAAELSRTPLTPIRVLQAIAQGAGEAA
>SEQF7946||SEQF7946.1_03461
MTDATSPGLGRSAPRIEDDALLRGQARFLDDIEVEGVLHACFVRSPHAHARLVSIDLSAARAVPGVAAVYGARD
LFGQLTSWRMPLGFPLAALPDDTTPFVLAEREVAFVGEAIAVVVADSRHIAEDAAARVAIEYEVLGAVVDCRDA
LRPDAPLVRGELASNILQQYTLAYGDCETAFAQAHRVLEDDFWVHRGCAHPMEGRGVLARMDRATDTLTVWS
STQMAHELHYTLALMLGQPEDRLRVVTPDVGGGFGAKFMIYPEEMAIPAAARKLGRPVKWVEDRREHFTTSI
QERDQYWKVAMAIDDQGHVLGIRGNFVHDNGAYTPQGTNVPYNAASSMTGPYVVPAFSLDVSVAYTNKVPV
ATVRGAGYPQAAFVMERMMDRVAAELGIDPGECRRRNLIGPAKIPYTKPLKSRAGMPLTIDSGDFPALQACAL
QASDYDGFAVRRDAALARGRWRGIAVANSVEPTGRGPFEVARVRVQPSGQVSIYTGALAMGQGIKTTLAQIC
AGHLGVPVAAVEVQAGDTAYVGYGMGGFASRQAIMAGSAVDQAAAQVRRQALETAAAVLKAEAETLELADG
EVRAPDGQSVSLARLAMLRKGVPGYALVSPGDPGLDATAYFHCDAQTYAGASHVCEVEVDPATGAIEIVRYVAA
QDSGRIINPQLAEGQVHGGVVHGIGNALFEWMGYDAAGQPLSTTFAEYLLPTAPEVPPIEVVFQPSPTPLNPL
GVKGVGECATIPVAVAVVGAVEHAVAHCGVRVTEFPLTPVRLLELLTQAEARAAAPTDSGEFVDGVH
>SEQF7946||SEQF7946.1_03557
MTTEHTGAEVGAGSRQGVGARVPRKEDARHLHGKGNFVADMAMPGLCEVAFLRSPLAHARITDVRVPESVR
DKVVLRSMMGDARDIAADSTLPTYQPSVQPPLASGKVRFVGEPVAMTFAPTRAEAEDHAELVEVDYDDLPVY
ADVAGAQQATSDLVHEHWRDNVFVTLNADRDFDEHAARAEVVVRRKIDLARQCMVPMEGKAVLAYWDHQ
ADQLVVISATQVPHMIRSVLAQCLDLEQGRVRVVSPDVGGAFGYKCVLQQEELCVAWLAKTYKRPFRFIEDRRE
HLTAGANSREHHYEMTAYADKRGKLLALDARITIDGGAYSVWPFTIGLEPDQAVGNLPGPYGFRGYRCETRCVA
TNKPGFVPYRGRTGVCFAIELTMDAVAREVGREPWEVRLENLVQPEQMPYVNVTNKHFDSGDYPASLRKALE

MIDIDGVRTRQARGEADGRRIGVGVATYTEQAAHGTSVFAAWGTPVIPGFDQATVRVTPDGGLEVRVGVHSH
GQGMETTFAQIAHEILGIDVARIKVLHGDTGQTPFSTGTYASRSLVMSGGAVSQACKRLLPRMRHIAAHMLGV
ADDAVTLQDGIYRAGEKSVATGDVADAWYLRPQLLPPDVDPAGLEVNVGYKPKVDTGCFTYASHAAVVAVDP
DTGAVEILDYVVVEDCGTMINPMVVEGQTIGGIAQGIGTAFYEETPYDANGQPLASTLADYMLPGPTEVPNM
RLHHFETPSPHTEFGAKGMGEGGAIAPPAVLFNAVNDALRGLGAAELSRTPLTPIRVLQAIAQGAGEAA
>SEQF7947||SEQF7947.1_03913
MTTEHTGAEVGAGSRQGVGARVPRKEDARHLHGKGNFVADMAMPGLCEVAFLRSPLAHARITDVRVPESVR
DKVVLRSMMGDARDIAADSTLPTYQPSVQPPLASGKVRFVGEPVAMTFAPTRAEAEDHAELVEVDYDDLPVY
ADVAGAQQATSDLVHEHWRDNVFVTLNADRDFDEHAARAEVVVRRKIDLARQCMVPMEGKAVLAYWDHQ
ADQLVVISATQVPHMIRSVLAQCLDLEQGRVRVVSPDVGGAFGYKCVLQQEELCVAWLAKTYKRPFRFIEDRRE
HLTAGANSREHHYEMTAYADKRGKLLALDARITIDGGAYSVWPFTIGLEPDQAVGNLPGPYGFRGYRCETRCVA
TNKPGFVPYRGRTGVCFAIELTMDAVAREVGREPWEVRLENLVQPEQMPYVNVTNKHFDSGDYPASLRKALE
MIDIDGVRTRQARGEADGRRIGVGVATYTEQAAHGTSVFAAWGTPVIPGFDQATVRVTPDGGLEVRVGVHSH
GQGMETTFAQIAHEILGIDVARIKVLHGDTGQTPFSTGTYASRSLVMSGGAVSQACKRLLPRMRHIAAHMLGV
ADDAVTLQDGIYRAGEKSVATGDVADAWYLRPQLLPPDVDPAGLEVNVGYKPKVDTGCFTYASHAAVVAVDP
DTGAVEILDYVVVEDCGTMINPMVVEGQTIGGIAQGIGTAFYEETPYDANGQPLASTLADYMLPGPTEVPNM
RLHHFETPSPHTEFGAKGMGEGGAIAPPAVLFNAVNDALRGLGAAELSRTPLTPIRVLQAIAQGAGEAA
>SEQF7947||SEQF7947.1_03818
MTDATSPGLGRSAPRIEDDALLRGQARFLDDIEVEGVLHACFVRSPHAHARLVSIDLSAARAVPGVAAVYGARD
LFGQLTSWRMPLGFPLAALPDDTTPFVLAEREVAFVGEAIAVVVADSRHIAEDAAARVAIEYEVLGAVVDCRDA
LRPDAPLVRGELASNILQQYTLAYGDCETAFAQAHRVLEDDFWVHRGCAHPMEGRGVLARMDRATDTLTVWS
STQMAHELHYTLALMLGQPEDRLRVVTPDVGGGFGAKFMIYPEEMAIPAAARKLGRPVKWVEDRREHFTTSI
QERDQYWKVAMAIDDQGHVLGIRGNFVHDNGAYTPQGTNVPYNAASSMTGPYVVPAFSLDVSVAYTNKVPV
ATVRGAGYPQAAFVMERMMDRVAAELGIDPGECRRRNLIGPAKIPYTKPLKSRAGMPLTIDSGDFPALQACAL
QASDYDGFAVRRDAALARGRWRGIAVANSVEPTGRGPFEVARVRVQPSGQVSIYTGALAMGQGIKTTLAQIC
AGHLGVPVAAVEVQAGDTAYVGYGMGGFASRQAIMAGSAVDQAAAQVRRQALETAAAVLKAEAETLELADG
EVRAPDGQSVSLARLAMLRKGVPGYALVSPGDPGLDATAYFHCDAQTYAGASHVCEVEVDPATGAIEIVRYVAA
QDSGRIINPQLAEGQVHGGVVHGIGNALFEWMGYDAAGQPLSTTFAEYLLPTAPEVPPIEVVFQPSPTPLNPL
GVKGVGECATIPVAVAVVGAVEHAVAHCGVRVTEFPLTPVRLLELLTQAEARAAAPTDSGEFVDGVH
>SEQF7948||SEQF7948.1_03577
MTTEHTGAEVGAGSRQGVGARVPRKEDARHLHGKGNFVADMAMPGLCEVAFLRSPLAHARITDVRVPESVR
DKVVLRSMMGDARDIAADSTLPTYQPSVQPPLASGKVRFVGEPVAMTFAPTRAEAEDHAELVEVDYDDLPVY
ADVAGAQQATSDLVHEHWRDNVFVTLNADRDFDEHAARAEVVVRRKIDLARQCMVPMEGKAVLAYWDHQ
ADQLVVISATQVPHMIRSVLAQCLDLEQGRVRVVSPDVGGAFGYKCVLQQEELCVAWLAKTYKRPFRFIEDRRE
HLTAGANSREHHYEMTAYADKRGKLLALDARITIDGGAYSVWPFTIGLEPDQAVGNLPGPYGFRGYRCETRCVA
TNKPGFVPYRGRTGVCFAIELTMDAVAREVGREPWEVRLENLVQPEQMPYVNVTNKHFDSGDYPASLRKALE
MIDIDGVRTRQARGEADGRRIGVGVATYTEQAAHGTSVFAAWGTPVIPGFDQATVRVTPDGGLEVRVGVHSH
GQGMETTFAQIAHEILGIDVARIKVLHGDTGQTPFSTGTYASRSLVMSGGAVSQACKRLLPRMRHIAAHMLGV
ADDAVTLQDGIYRAGEKSVATGDVADAWYLRPQLLPPDVDPAGLEVNVGYKPKVDTGCFTYASHAAVVAVDP
DTGAVEILDYVVVEDCGTMINPMVVEGQTIGGIAQGIGTAFYEETPYDANGQPLASTLADYMLPGPTEVPNM
RLHHFETPSPHTEFGAKGMGEGGAIAPPAVLFNAVNDALRGLGAAELSRTPLTPIRVLQAIAQGAGEAA
>SEQF7948||SEQF7948.1_03481
MTDATSPGLGRSAPRIEDDALLRGQARFLDDIEVEGVLHACFVRSPHAHARLVSIDLSAARAVPGVAAVYGARD
LFGQLTSWRMPLGFPLAALPDDTTPFVLAEREVAFVGEAIAVVVADSRHIAEDAAARVAIEYEVLGAVVDCRDA

LRPDAPLVRGELASNILQQYTLAYGDCETAFAQAHRVLEDDFWVHRGCAHPMEGRGVLARMDRATDTLTVWS
STQMAHELHYTLALMLGQPEDRLRVVTPDVGGGFGAKFMIYPEEMAIPAAARKLGRPVKWVEDRREHFTTSI
QERDQYWKVAMAIDDQGHVLGIRGNFVHDNGAYTPQGTNVPYNAASSMTGPYVVPAFSLDVSVAYTNKVPV
ATVRGAGYPQAAFVMERMMDRVAAELGIDPGECRRRNLIGPAKIPYTKPLKSRAGMPLTIDSGDFPALQACAL
QASDYDGFAVRRDAALARGRWRGIAVANSVEPTGRGPFEVARVRVQPSGQVSIYTGALAMGQGIKTTLAQIC
AGHLGVPVAAVEVQAGDTAYVGYGMGGFASRQAIMAGSAVDQAAAQVRRQALETAAAVLKAEAETLELADG
EVRAPDGQSVSLARLAMLRKGVPGYALVSPGDPGLDATAYFHCDAQTYAGASHVCEVEVDPATGAIEIVRYVAA
QDSGRIINPQLAEGQVHGGVVHGIGNALFEWMGYDAAGQPLSTTFAEYLLPTAPEVPPIEVVFQPSPTPLNPL
GVKGVGECATIPVAVAVVGAVEHAVAHCGVRVTEFPLTPVRLLELLTQAEARAAAPTDSGEFVDGVH
>SEQF7949||SEQF7949.1_00313
MTTEHTGAEVGAGSRQGVGARVPRKEDARHLHGKGNFVADMAMPGLCEVAFLRSPLAHARITDVRVPESVR
DKVVLRSMMGDARDIAADSTLPTYQPSVQPPLASGKVRFVGEPVAMTFAPTRAEAEDHAELVEVDYDDLPVY
ADVAGAQQATSDLVHEHWRDNVFVTLNADRDFDEHAARAEVVVRRKIDLARQCMVPMEGKAVLAYWDHQ
ADQLVVISATQVPHMIRSVLAQCLDLEQGRVRVVSPDVGGAFGYKCVLQQEELCVAWLAKTYKRPFRFIEDRRE
HLTAGANSREHHYEMTAYADKRGKLLALDARITIDGGAYSVWPFTIGLEPDQAVGNLPGPYGFRGYRCETRCVA
TNKPGFVPYRGRTGVCFAIELTMDAVAREVGREPWEVRLENLVQPEQMPYVNVTNKHFDSGDYPASLRKALE
MIDIDGVRTRQARGEADGRRIGVGVATYTEQAAHGTSVFAAWGTPVIPGFDQATVRVTPDGGLEVRVGVHSH
GQGMETTFAQIAHEILGIDVARIKVLHGDTGQTPFSTGTYASRSLVMSGGAVSQACKRLLPRMRHIAAHMLGV
ADDAVTLQDGIYRAGEKSVATGDVADAWYLRPQLLPPDVDPAGLEVNVGYKPKVDTGCFTYASHAAVVAVDP
DTGAVEILDYVVVEDCGTMINPMVVEGQTIGGIAQGIGTAFYEETPYDANGQPLASTLADYMLPGPTEVPNM
RLHHFETPSPHTEFGAKGMGEGGAIAPPAVLFNAVNDALRGLGAAELSRTPLTPIRVLQAIAQGAGEAA
>SEQF7949||SEQF7949.1_03390
MTDATSPGLGRSAPRIEDDALLRGQARFLDDIEVEGVLHACFVRSPHAHARLVSIDLSAARAVPGVAAVYGARD
LFGQLTSWRMPLGFPLAALPDDTTPFVLAEREVAFVGEAIAVVVADSRHIAEDAAARVAIEYEVLGAVVDCRDA
LRPDAPLVRGELASNILQQYTLAYGDCETAFAQAHRVLEDDFWVHRGCAHPMEGRGVLARMDRATDTLTVWS
STQMAHELHYTLALMLGQPEDRLRVVTPDVGGGFGAKFMIYPEEMAIPAAARKLGRPVKWVEDRREHFTTSI
QERDQYWKVAMAIDDQGHVLGIRGNFVHDNGAYTPQGTNVPYNAASSMTGPYVVPAFSLDVSVAYTNKVPV
ATVRGAGYPQAAFVMERMMDRVAAELGIDPGECRRRNLIGPAKIPYTKPLKSRAGMPLTIDSGDFPALQACAL
QASDYDGFAVRRDAALARGRWRGIAVANSVEPTGRGPFEVARVRVQPSGQVSIYTGALAMGQGIKTTLAQIC
AGHLGVPVAAVEVQAGDTAYVGYGMGGFASRQAIMAGSAVDQAAAQVRRQALETAAAVLKAEAETLELADG
EVRAPDGQSVSLARLAMLRKGVPGYALVSPGDPGLDATAYFHCDAQTYAGASHVCEVEVDPATGAIEIVRYVAA
QDSGRIINPQLAEGQVHGGVVHGIGNALFEWMGYDAAGQPLSTTFAEYLLPTAPEVPPIEVVFQPSPTPLNPL
GVKGVGECATIPVAVAVVGAVEHAVAHCGVRVTEFPLTPVRLLELLTQAEARAAAPTDSGEFVDGVH
>SEQF7950||SEQF7950.1_03561
MTTEHTGAEVGAGSRQGVGARVPRKEDARHLHGKGNFVADMAMPGLCEVAFLRSPLAHARITDVRVPESVR
DKVVLRSMMGDARDIAADSTLPTYQPSVQPPLASGKVRFVGEPVAMTFAPTRAEAEDHAELVEVDYDDLPVY
ADVAGAQQATSDLVHEHWRDNVFVTLNADRDFDEHAARAEVVVRRKIDLARQCMVPMEGKAVLAYWDHQ
ADQLVVISATQVPHMIRSVLAQCLDLEQGRVRVVSPDVGGAFGYKCVLQQEELCVAWLAKTYKRPFRFIEDRRE
HLTAGANSREHHYEMTAYADKRGKLLALDARITIDGGAYSVWPFTIGLEPDQAVGNLPGPYGFRGYRCETRCVA
TNKPGFVPYRGRTGVCFAIELTMDAVAREVGREPWEVRLENLVQPEQMPYVNVTNKHFDSGDYPASLRKALE
MIDIDGVRTRQARGEADGRRIGVGVATYTEQAAHGTSVFAAWGTPVIPGFDQATVRVTPDGGLEVRVGVHSH
GQGMETTFAQIAHEILGIDVARIKVLHGDTGQTPFSTGTYASRSLVMSGGAVSQACKRLLPRMRHIAAHMLGV
ADDAVTLQDGIYRAGEKSVATGDVADAWYLRPQLLPPDVDPAGLEVNVGYKPKVDTGCFTYASHAAVVAVDP
DTGAVEILDYVVVEDCGTMINPMVVEGQTIGGIAQGIGTAFYEETPYDANGQPLASTLADYMLPGPTEVPNM

RLHHFETPSPHTEFGAKGMGEGGAIAPPAVLFNAVNDALRGLGAAELSRTPLTPIRVLQAIAQGAGEAA
>SEQF7950||SEQF7950.1_03465
MTDATSPGLGRSAPRIEDDALLRGQARFLDDIEVEGVLHACFVRSPHAHARLVSIDLSAARAVPGVAAVYGARD
LFGQLTSWRMPLGFPLAALPDDTTPFVLAEREVAFVGEAIAVVVADSRHIAEDAAARVAIEYEVLGAVVDCRDA
LRPDAPLVRGELASNILQQYTLAYGDCETAFAQAHRVLEDDFWVHRGCAHPMEGRGVLARMDRATDTLTVWS
STQMAHELHYTLALMLGQPEDRLRVVTPDVGGGFGAKFMIYPEEMAIPAAARKLGRPVKWVEDRREHFTTSI
QERDQYWKVAMAIDDQGHVLGIRGNFVHDNGAYTPQGTNVPYNAASSMTGPYVVPAFSLDVSVAYTNKVPV
ATVRGAGYPQAAFVMERMMDRVAAELGIDPGECRRRNLIGPAKIPYTKPLKSRAGMPLTIDSGDFPALQACAL
QASDYDGFAVRRDAALARGRWRGIAVANSVEPTGRGPFEVARVRVQPSGQVSIYTGALAMGQGIKTTLAQIC
AGHLGVPVAAVEVQAGDTAYVGYGMGGFASRQAIMAGSAVDQAAAQVRRQALETAAAVLKAEAETLELADG
EVRAPDGQSVSLARLAMLRKGVPGYALVSPGDPGLDATAYFHCDAQTYAGASHVCEVEVDPATGAIEIVRYVAA
QDSGRIINPQLAEGQVHGGVVHGIGNALFEWMGYDAAGQPLSTTFAEYLLPTAPEVPPIEVVFQPSPTPLNPL
GVKGVGECATIPVAVAVVGAVEHAVAHCGVRVTEFPLTPVRLLELLTQAEARAAAPTDSGEFVDGVH
>SEQF7951||SEQF7951.1_03485
MTDATSPGLGRSAPRIEDDALLRGQARFLDDIEVEGVLHACFVRSPHAHARLVSIDLSAARAVPGVAAVYGARD
LFGQLTSWRMPLGFPLAALPDDTTPFVLAEREVAFVGEAIAVVVADSRHIAEDAAARVAIEYEVLGAVVDCRDA
LRPDAPLVRGELASNILQQYTLAYGDCETAFAQAHRVLEDDFWVHRGCAHPMEGRGVLARMDRATDTLTVWS
STQMAHELHYTLALMLGQPEDRLRVVTPDVGGGFGAKFMIYPEEMAIPAAARKLGRPVKWVEDRREHFTTSI
QERDQYWKVAMAIDDQGHVLGIRGNFVHDNGAYTPQGTNVPYNAASSMTGPYVVPAFSLDVSVAYTNKVPV
ATVRGAGYPQAAFVMERMMDRVAAELGIDPGECRRRNLIGPAKIPYTKPLKSRAGMPLTIDSGDFPALQACAL
QASDYDGFAVRRDAALARGRWRGIAVANSVEPTGRGPFEVARVRVQPSGQVSIYTGALAMGQGIKTTLAQIC
AGHLGVPVAAVEVQAGDTAYVGYGMGGFASRQAIMAGSAVDQAAAQVRRQALETAAAVLKAEAETLELADG
EVRAPDGQSVSLARLAMLRKGVPGYALVSPGDPGLDATAYFHCDAQTYAGASHVCEVEVDPATGAIEIVRYVAA
QDSGRIINPQLAEGQVHGGVVHGIGNALFEWMGYDAAGQPLSTTFAEYLLPTAPEVPPIEVVFQPSPTPLNPL
GVKGVGECATIPVAVAVVGAVEHAVAHCGVRVTEFPLTPVRLLELLTQAEARAAAPTDSGEFVDGVH
>SEQF7951||SEQF7951.1_03581
MTTEHTGAEVGAGSRQGVGARVPRKEDARHLHGKGNFVADMAMPGLCEVAFLRSPLAHARITDVRVPESVR
DKVVLRSMMGDARDIAADSTLPTYQPSVQPPLASGKVRFVGEPVAMTFAPTRAEAEDHAELVEVDYDDLPVY
ADVAGAQQATSDLVHEHWRDNVFVTLNADRDFDEHAARAEVVVRRKIDLARQCMVPMEGKAVLAYWDHQ
ADQLVVISATQVPHMIRSVLAQCLDLEQGRVRVVSPDVGGAFGYKCVLQQEELCVAWLAKTYKRPFRFIEDRRE
HLTAGANSREHHYEMTAYADKRGKLLALDARITIDGGAYSVWPFTIGLEPDQAVGNLPGPYGFRGYRCETRCVA
TNKPGFVPYRGRTGVCFAIELTMDAVAREVGREPWEVRLENLVQPEQMPYVNVTNKHFDSGDYPASLRKALE
MIDIDGVRTRQARGEADGRRIGVGVATYTEQAAHGTSVFAAWGTPVIPGFDQATVRVTPDGGLEVRVGVHSH
GQGMETTFAQIAHEILGIDVARIKVLHGDTGQTPFSTGTYASRSLVMSGGAVSQACKRLLPRMRHIAAHMLGV
ADDAVTLQDGIYRAGEKSVATGDVADAWYLRPQLLPPDVDPAGLEVNVGYKPKVDTGCFTYASHAAVVAVDP
DTGAVEILDYVVVEDCGTMINPMVVEGQTIGGIAQGIGTAFYEETPYDANGQPLASTLADYMLPGPTEVPNM
RLHHFETPSPHTEFGAKGMGEGGAIAPPAVLFNAVNDALRGLGAAELSRTPLTPIRVLQAIAQGAGEAA
>SEQF7952||SEQF7952.1_03463
MTDATSPGLGRSAPRIEDDALLRGQARFLDDIEVEGVLHACFVRSPHAHARLVSIDLSAARAVPGVAAVYGARD
LFGQLTSWRMPLGFPLAALPDDTTPFVLAEREVAFVGEAIAVVVADSRHIAEDAAARVAIEYEVLGAVVDCRDA
LRPDAPLVRGELASNILQQYTLAYGDCETAFAQAHRVLEDDFWVHRGCAHPMEGRGVLARMDRATDTLTVWS
STQMAHELHYTLALMLGQPEDRLRVVTPDVGGGFGAKFMIYPEEMAIPAAARKLGRPVKWVEDRREHFTTSI
QERDQYWKVAMAIDDQGHVLGIRGNFVHDNGAYTPQGTNVPYNAASSMTGPYVVPAFSLDVSVAYTNKVPV
ATVRGAGYPQAAFVMERMMDRVAAELGIDPGECRRRNLIGPAKIPYTKPLKSRAGMPLTIDSGDFPALQACAL

QASDYDGFAVRRDAALARGRWRGIAVANSVEPTGRGPFEVARVRVQPSGQVSIYTGALAMGQGIKTTLAQIC
AGHLGVPVAAVEVQAGDTAYVGYGMGGFASRQAIMAGSAVDQAAAQVRRQALETAAAVLKAEAETLELADG
EVRAPDGQSVSLARLAMLRKGVPGYALVSPGDPGLDATAYFHCDAQTYAGASHVCEVEVDPATGAIEIVRYVAA
QDSGRIINPQLAEGQVHGGVVHGIGNALFEWMGYDAAGQPLSTTFAEYLLPTAPEVPPIEVVFQPSPTPLNPL
GVKGVGECATIPVAVAVVGAVEHAVAHCGVRVTEFPLTPVRLLELLTQAEARAAAPTDSGEFVDGVH
>SEQF7952||SEQF7952.1_03559
MTTEHTGAEVGAGSRQGVGARVPRKEDARHLHGKGNFVADMAMPGLCEVAFLRSPLAHARITDVRVPESVR
DKVVLRSMMGDARDIAADSTLPTYQPSVQPPLASGKVRFVGEPVAMTFAPTRAEAEDHAELVEVDYDDLPVY
ADVAGAQQATSDLVHEHWRDNVFVTLNADRDFDEHAARAEVVVRRKIDLARQCMVPMEGKAVLAYWDHQ
ADQLVVISATQVPHMIRSVLAQCLDLEQGRVRVVSPDVGGAFGYKCVLQQEELCVAWLAKTYKRPFRFIEDRRE
HLTAGANSREHHYEMTAYADKRGKLLALDARITIDGGAYSVWPFTIGLEPDQAVGNLPGPYGFRGYRCETRCVA
TNKPGFVPYRGRTGVCFAIELTMDAVAREVGREPWEVRLENLVQPEQMPYVNVTNKHFDSGDYPASLRKALE
MIDIDGVRTRQARGEADGRRIGVGVATYTEQAAHGTSVFAAWGTPVIPGFDQATVRVTPDGGLEVRVGVHSH
GQGMETTFAQIAHEILGIDVARIKVLHGDTGQTPFSTGTYASRSLVMSGGAVSQACKRLLPRMRHIAAHMLGV
ADDAVTLQDGIYRAGEKSVATGDVADAWYLRPQLLPPDVDPAGLEVNVGYKPKVDTGCFTYASHAAVVAVDP
DTGAVEILDYVVVEDCGTMINPMVVEGQTIGGIAQGIGTAFYEETPYDANGQPLASTLADYMLPGPTEVPNM
RLHHFETPSPHTEFGAKGMGEGGAIAPPAVLFNAVNDALRGLGAAELSRTPLTPIRVLQAIAQGAGEAA
>SEQF7953||SEQF7953.1_03483
MTDATSPGLGRSAPRIEDDALLRGQARFLDDIEVEGVLHACFVRSPHAHARLVSIDLSAARAVPGVAAVYGARD
LFGQLTSWRMPLGFPLAALPDDTTPFVLAEREVAFVGEAIAVVVADSRHIAEDAAARVAIEYEVLGAVVDCRDA
LRPDAPLVRGELASNILQQYTLAYGDCETAFAQAHRVLEDDFWVHRGCAHPMEGRGVLARMDRATDTLTVWS
STQMAHELHYTLALMLGQPEDRLRVVTPDVGGGFGAKFMIYPEEMAIPAAARKLGRPVKWVEDRREHFTTSI
QERDQYWKVAMAIDDQGHVLGIRGNFVHDNGAYTPQGTNVPYNAASSMTGPYVVPAFSLDVSVAYTNKVPV
ATVRGAGYPQAAFVMERMMDRVAAELGIDPGECRRRNLIGPAKIPYTKPLKSRAGMPLTIDSGDFPALQACAL
QASDYDGFAVRRDAALARGRWRGIAVANSVEPTGRGPFEVARVRVQPSGQVSIYTGALAMGQGIKTTLAQIC
AGHLGVPVAAVEVQAGDTAYVGYGMGGFASRQAIMAGSAVDQAAAQVRRQALETAAAVLKAEAETLELADG
EVRAPDGQSVSLARLAMLRKGVPGYALVSPGDPGLDATAYFHCDAQTYAGASHVCEVEVDPATGAIEIVRYVAA
QDSGRIINPQLAEGQVHGGVVHGIGNALFEWMGYDAAGQPLSTTFAEYLLPTAPEVPPIEVVFQPSPTPLNPL
GVKGVGECATIPVAVAVVGAVEHAVAHCGVRVTEFPLTPVRLLELLTQAEARAAAPTDSGEFVDGVH
>SEQF7953||SEQF7953.1_03579
MTTEHTGAEVGAGSRQGVGARVPRKEDARHLHGKGNFVADMAMPGLCEVAFLRSPLAHARITDVRVPESVR
DKVVLRSMMGDARDIAADSTLPTYQPSVQPPLASGKVRFVGEPVAMTFAPTRAEAEDHAELVEVDYDDLPVY
ADVAGAQQATSDLVHEHWRDNVFVTLNADRDFDEHAARAEVVVRRKIDLARQCMVPMEGKAVLAYWDHQ
ADQLVVISATQVPHMIRSVLAQCLDLEQGRVRVVSPDVGGAFGYKCVLQQEELCVAWLAKTYKRPFRFIEDRRE
HLTAGANSREHHYEMTAYADKRGKLLALDARITIDGGAYSVWPFTIGLEPDQAVGNLPGPYGFRGYRCETRCVA
TNKPGFVPYRGRTGVCFAIELTMDAVAREVGREPWEVRLENLVQPEQMPYVNVTNKHFDSGDYPASLRKALE
MIDIDGVRTRQARGEADGRRIGVGVATYTEQAAHGTSVFAAWGTPVIPGFDQATVRVTPDGGLEVRVGVHSH
GQGMETTFAQIAHEILGIDVARIKVLHGDTGQTPFSTGTYASRSLVMSGGAVSQACKRLLPRMRHIAAHMLGV
ADDAVTLQDGIYRAGEKSVATGDVADAWYLRPQLLPPDVDPAGLEVNVGYKPKVDTGCFTYASHAAVVAVDP
DTGAVEILDYVVVEDCGTMINPMVVEGQTIGGIAQGIGTAFYEETPYDANGQPLASTLADYMLPGPTEVPNM
RLHHFETPSPHTEFGAKGMGEGGAIAPPAVLFNAVNDALRGLGAAELSRTPLTPIRVLQAIAQGAGEAA
>SEQF7954||SEQF7954.1_03465
MTDATSPGLGRSAPRIEDDALLRGQARFLDDIEVEGVLHACFVRSPHAHARLVSIDLSAARAVPGVAAVYGARD
LFGQLTSWRMPLGFPLAALPDDTTPFVLAEREVAFVGEAIAVVVADSRHIAEDAAARVAIEYEVLGAVVDCRDA

LRPDAPLVRGELASNILQQYTLAYGDCETAFAQAHRVLEDDFWVHRGCAHPMEGRGVLARMDRATDTLTVWS
STQMAHELHYTLALMLGQPEDRLRVVTPDVGGGFGAKFMIYPEEMAIPAAARKLGRPVKWVEDRREHFTTSI
QERDQYWKVAMAIDDQGHVLGIRGNFVHDNGAYTPQGTNVPYNAASSMTGPYVVPAFSLDVSVAYTNKVPV
ATVRGAGYPQAAFVMERMMDRVAAELGIDPGECRRRNLIGPAKIPYTKPLKSRAGMPLTIDSGDFPALQACAL
QASDYDGFAVRRDAALARGRWRGIAVANSVEPTGRGPFEVARVRVQPSGQVSIYTGALAMGQGIKTTLAQIC
AGHLGVPVAAVEVQAGDTAYVGYGMGGFASRQAIMAGSAVDQAAAQVRRQALETAAAVLKAEAETLELADG
EVRAPDGQSVSLARLAMLRKGVPGYALVSPGDPGLDATAYFHCDAQTYAGASHVCEVEVDPATGAIEIVRYVAA
QDSGRIINPQLAEGQVHGGVVHGIGNALFEWMGYDAAGQPLSTTFAEYLLPTAPEVPPIEVVFQPSPTPLNPL
GVKGVGECATIPVAVAVVGAVEHAVAHCGVRVTEFPLTPVRLLELLTQAEARAAAPTDSGEFVDGVH
>SEQF7954||SEQF7954.1_03561
MTTEHTGAEVGAGSRQGVGARVPRKEDARHLHGKGNFVADMAMPGLCEVAFLRSPLAHARITDVRVPESVR
DKVVLRSMMGDARDIAADSTLPTYQPSVQPPLASGKVRFVGEPVAMTFAPTRAEAEDHAELVEVDYDDLPVY
ADVAGAQQATSDLVHEHWRDNVFVTLNADRDFDEHAARAEVVVRRKIDLARQCMVPMEGKAVLAYWDHQ
ADQLVVISATQVPHMIRSVLAQCLDLEQGRVRVVSPDVGGAFGYKCVLQQEELCVAWLAKTYKRPFRFIEDRRE
HLTAGANSREHHYEMTAYADKRGKLLALDARITIDGGAYSVWPFTIGLEPDQAVGNLPGPYGFRGYRCETRCVA
TNKPGFVPYRGRTGVCFAIELTMDAVAREVGREPWEVRLENLVQPEQMPYVNVTNKHFDSGDYPASLRKALE
MIDIDGVRTRQARGEADGRRIGVGVATYTEQAAHGTSVFAAWGTPVIPGFDQATVRVTPDGGLEVRVGVHSH
GQGMETTFAQIAHEILGIDVARIKVLHGDTGQTPFSTGTYASRSLVMSGGAVSQACKRLLPRMRHIAAHMLGV
ADDAVTLQDGIYRAGEKSVATGDVADAWYLRPQLLPPDVDPAGLEVNVGYKPKVDTGCFTYASHAAVVAVDP
DTGAVEILDYVVVEDCGTMINPMVVEGQTIGGIAQGIGTAFYEETPYDANGQPLASTLADYMLPGPTEVPNM
RLHHFETPSPHTEFGAKGMGEGGAIAPPAVLFNAVNDALRGLGAAELSRTPLTPIRVLQAIAQGAGEAA
>SEQF7955||SEQF7955.1_00717
MTDATSPGLGRSAPRIEDDALLRGQARFLDDIEVEGVLHACFVRSPHAHARLVSIDLSAARAVPGVAAVYGARD
LFGQLTSWRMPLGFPLAALPDDTTPFVLAEREVAFVGEAIAVVVADSRHIAEDAAARVAIEYEVLGAVVDCRDA
LRPDAPLVRGELASNILQQYTLAYGDCETAFAQAHRVLEDDFWVHRGCAHPMEGRGVLARMDRATDTLTVWS
STQMAHELHYTLALMLGQPEDRLRVVTPDVGGGFGAKFMIYPEEMAIPAAARKLGRPVKWVEDRREHFTTSI
QERDQYWKVAMAIDDQGHVLGIRGNFVHDNGAYTPQGTNVPYNAASSMTGPYVVPAFSLDVSVAYTNKVPV
ATVRGAGYPQAAFVMERMMDRVAAELGIDPGECRRRNLIGPAKIPYTKPLKSRAGMPLTIDSGDFPALQACAL
QASDYDGFAVRRDAALARGRWRGIAVANSVEPTGRGPFEVARVRVQPSGQVSIYTGALAMGQGIKTTLAQIC
AGHLGVPVAAVEVQAGDTAYVGYGMGGFASRQAIMAGSAVDQAAAQVRRQALETAAAVLKAEAETLELADG
EVRAPDGQSVSLARLAMLRKGVPGYALVSPGDPGLDATAYFHCDAQTYAGASHVCEVEVDPATGAIEIVRYVAA
QDSGRIINPQLAEGQVHGGVVHGIGNALFEWMGYDAAGQPLSTTFAEYLLPTAPEVPPIEVVFQPSPTPLNPL
GVKGVGECATIPVAVAVVGAVEHAVAHCGVRVTEFPLTPVRLLELLTQAEARAAAPTDSGEFVDGVH
>SEQF7955||SEQF7955.1_00621
MTTEHTGAEVGAGSRQGVGARVPRKEDARHLHGKGNFVADMAMPGLCEVAFLRSPLAHARITDVRVPESVR
DKVVLRSMMGDARDIAADSTLPTYQPSVQPPLASGKVRFVGEPVAMTFAPTRAEAEDHAELVEVDYDDLPVY
ADVAGAQQATSDLVHEHWRDNVFVTLNADRDFDEHAARAEVVVRRKIDLARQCMVPMEGKAVLAYWDHQ
ADQLVVISATQVPHMIRSVLAQCLDLEQGRVRVVSPDVGGAFGYKCVLQQEELCVAWLAKTYKRPFRFIEDRRE
HLTAGANSREHHYEMTAYADKRGKLLALDARITIDGGAYSVWPFTIGLEPDQAVGNLPGPYGFRGYRCETRCVA
TNKPGFVPYRGRTGVCFAIELTMDAVAREVGREPWEVRLENLVQPEQMPYVNVTNKHFDSGDYPASLRKALE
MIDIDGVRTRQARGEADGRRIGVGVATYTEQAAHGTSVFAAWGTPVIPGFDQATVRVTPDGGLEVRVGVHSH
GQGMETTFAQIAHEILGIDVARIKVLHGDTGQTPFSTGTYASRSLVMSGGAVSQACKRLLPRMRHIAAHMLGV
ADDAVTLQDGIYRAGEKSVATGDVADAWYLRPQLLPPDVDPAGLEVNVGYKPKVDTGCFTYASHAAVVAVDP
DTGAVEILDYVVVEDCGTMINPMVVEGQTIGGIAQGIGTAFYEETPYDANGQPLASTLADYMLPGPTEVPNM

RLHHFETPSPHTEFGAKGMGEGGAIAPPAVLFNAVNDALRGLGAAELSRTPLTPIRVLQAIAQGAGEAA
>SEQF7956||SEQF7956.1_03557
MTTEHTGAEVGAGSRQGVGARVPRKEDARHLHGKGNFVADMAMPGLCEVAFLRSPLAHARITDVRVPESVR
DKVVLRSMMGDARDIAADSTLPTYQPSVQPPLASGKVRFVGEPVAMTFAPTRAEAEDHAELVEVDYDDLPVY
ADVAGAQQATSDLVHEHWRDNVFVTLNADRDFDEHAARAEVVVRRKIDLARQCMVPMEGKAVLAYWDHQ
ADQLVVISATQVPHMIRSVLAQCLDLEQGRVRVVSPDVGGAFGYKCVLQQEELCVAWLAKTYKRPFRFIEDRRE
HLTAGANSREHHYEMTAYADKRGKLLALDARITIDGGAYSVWPFTIGLEPDQAVGNLPGPYGFRGYRCETRCVA
TNKPGFVPYRGRTGVCFAIELTMDAVAREVGREPWEVRLENLVQPEQMPYVNVTNKHFDSGDYPASLRKALE
MIDIDGVRTRQARGEADGRRIGVGVATYTEQAAHGTSVFAAWGTPVIPGFDQATVRVTPDGGLEVRVGVHSH
GQGMETTFAQIAHEILGIDVARIKVLHGDTGQTPFSTGTYASRSLVMSGGAVSQACKRLLPRMRHIAAHMLGV
ADDAVTLQDGIYRAGEKSVATGDVADAWYLRPQLLPPDVDPAGLEVNVGYKPKVDTGCFTYASHAAVVAVDP
DTGAVEILDYVVVEDCGTMINPMVVEGQTIGGIAQGIGTAFYEETPYDANGQPLASTLADYMLPGPTEVPNM
RLHHFETPSPHTEFGAKGMGEGGAIAPPAVLFNAVNDALRGLGAAELSRTPLTPIRVLQAIAQGAGEAA
>SEQF7956||SEQF7956.1_03461
MTDATSPGLGRSAPRIEDDALLRGQARFLDDIEVEGVLHACFVRSPHAHARLVSIDLSAARAVPGVAAVYGARD
LFGQLTSWRMPLGFPLAALPDDTTPFVLAEREVAFVGEAIAVVVADSRHIAEDAAARVAIEYEVLGAVVDCRDA
LRPDAPLVRGELASNILQQYTLAYGDCETAFAQAHRVLEDDFWVHRGCAHPMEGRGVLARMDRATDTLTVWS
STQMAHELHYTLALMLGQPEDRLRVVTPDVGGGFGAKFMIYPEEMAIPAAARKLGRPVKWVEDRREHFTTSI
QERDQYWKVAMAIDDQGHVLGIRGNFVHDNGAYTPQGTNVPYNAASSMTGPYVVPAFSLDVSVAYTNKVPV
ATVRGAGYPQAAFVMERMMDRVAAELGIDPGECRRRNLIGPAKIPYTKPLKSRAGMPLTIDSGDFPALQACAL
QASDYDGFAVRRDAALARGRWRGIAVANSVEPTGRGPFEVARVRVQPSGQVSIYTGALAMGQGIKTTLAQIC
AGHLGVPVAAVEVQAGDTAYVGYGMGGFASRQAIMAGSAVDQAAAQVRRQALETAAAVLKAEAETLELADG
EVRAPDGQSVSLARLAMLRKGVPGYALVSPGDPGLDATAYFHCDAQTYAGASHVCEVEVDPATGAIEIVRYVAA
QDSGRIINPQLAEGQVHGGVVHGIGNALFEWMGYDAAGQPLSTTFAEYLLPTAPEVPPIEVVFQPSPTPLNPL
GVKGVGECATIPVAVAVVGAVEHAVAHCGVRVTEFPLTPVRLLELLTQAEARAAAPTDSGEFVDGVH
>SEQF7957||SEQF7957.1_00481
MTDATSPGLGRSAPRIEDDALLRGQARFLDDIEVEGVLHACFVRSPHAHARLVSIDLSAARAVPGVAAVYGARD
LFGQLTSWRMPLGFPLAALPDDTTPFVLAEREVAFVGEAIAVVVADSRHIAEDAAARVAIEYEVLGAVVDCRDA
LRPDAPLVRGELASNILQQYTLAYGDCETAFAQAHRVLEDDFWVHRGCAHPMEGRGVLARMDRATDTLTVWS
STQMAHELHYTLALMLGQPEDRLRVVTPDVGGGFGAKFMIYPEEMAIPAAARKLGRPVKWVEDRREHFTTSI
QERDQYWKVAMAIDDQGHVLGIRGNFVHDNGAYTPQGTNVPYNAASSMTGPYVVPAFSLDVSVAYTNKVPV
ATVRGAGYPQAAFVMERMMDRVAAELGIDPGECRRRNLIGPAKIPYTKPLKSRAGMPLTIDSGDFPALQACAL
QASDYDGFAVRRDAALARGRWRGIAVANSVEPTGRGPFEVARVRVQPSGQVSIYTGALAMGQGIKTTLAQIC
AGHLGVPVAAVEVQAGDTAYVGYGMGGFASRQAIMAGSAVDQAAAQVRRQALETAAAVLKAEAETLELADG
EVRAPDGQSVSLARLAMLRKGVPGYALVSPGDPGLDATAYFHCDAQTYAGASHVCEVEVDPATGAIEIVRYVAA
QDSGRIINPQLAEGQVHGGVVHGIGNALFEWMGYDAAGQPLSTTFAEYLLPTAPEVPPIEVVFQPSPTPLNPL
GVKGVGECATIPVAVAVVGAVEHAVAHCGVRVTEFPLTPVRLLELLTQAEARAAAPTDSGEFVDGVH
>SEQF7957||SEQF7957.1_03565
MTTEHTGAEVGAGSRQGVGARVPRKEDARHLHGKGNFVADMAMPGLCEVAFLRSPLAHARITDVRVPESVR
DKVVLRSMMGDARDIAADSTLPTYQPSVQPPLASGKVRFVGEPVAMTFAPTRAEAEDHAELVEVDYDDLPVY
ADVAGAQQATSDLVHEHWRDNVFVTLNADRDFDEHAARAEVVVRRKIDLARQCMVPMEGKAVLAYWDHQ
ADQLVVISATQVPHMIRSVLAQCLDLEQGRVRVVSPDVGGAFGYKCVLQQEELCVAWLAKTYKRPFRFIEDRRE
HLTAGANSREHHYEMTAYADKRGKLLALDARITIDGGAYSVWPFTIGLEPDQAVGNLPGPYGFRGYRCETRCVA
TNKPGFVPYRGRTGVCFAIELTMDAVAREVGREPWEVRLENLVQPEQMPYVNVTNKHFDSGDYPASLRKALE

MIDIDGVRTRQARGEADGRRIGVGVATYTEQAAHGTSVFAAWGTPVIPGFDQATVRVTPDGGLEVRVGVHSH
GQGMETTFAQIAHEILGIDVARIKVLHGDTGQTPFSTGTYASRSLVMSGGAVSQACKRLLPRMRHIAAHMLGV
ADDAVTLQDGIYRAGEKSVATGDVADAWYLRPQLLPPDVDPAGLEVNVGYKPKVDTGCFTYASHAAVVAVDP
DTGAVEILDYVVVEDCGTMINPMVVEGQTIGGIAQGIGTAFYEETPYDANGQPLASTLADYMLPGPTEVPNM
RLHHFETPSPHTEFGAKGMGEGGAIAPPAVLFNAVNDALRGLGAAELSRTPLTPIRVLQAIAQGAGEAA
>SEQF7958||SEQF7958.1_00409
MTTEHTGAEVGAGSRQGVGARVPRKEDARHLHGKGNFVADMAMPGLCEVAFLRSPLAHARITDVRVPESVR
DKVVLRSMMGDARDIAADSTLPTYQPSVQPPLASGKVRFVGEPVAMTFAPTRAEAEDHAELVEVDYDDLPVY
ADVAGAQQATSDLVHEHWRDNVFVTLNADRDFDEHAARAEVVVRRKIDLARQCMVPMEGKAVLAYWDHQ
ADQLVVISATQVPHMIRSVLAQCLDLEQGRVRVVSPDVGGAFGYKCVLQQEELCVAWLAKTYKRPFRFIEDRRE
HLTAGANSREHHYEMTAYADKRGKLLALDARITIDGGAYSVWPFTIGLEPDQAVGNLPGPYGFRGYRCETRCVA
TNKPGFVPYRGRTGVCFAIELTMDAVAREVGREPWEVRLENLVQPEQMPYVNVTNKHFDSGDYPASLRKALE
MIDIDGVRTRQARGEADGRRIGVGVATYTEQAAHGTSVFAAWGTPVIPGFDQATVRVTPDGGLEVRVGVHSH
GQGMETTFAQIAHEILGIDVARIKVLHGDTGQTPFSTGTYASRSLVMSGGAVSQACKRLLPRMRHIAAHMLGV
ADDAVTLQDGIYRAGEKSVATGDVADAWYLRPQLLPPDVDPAGLEVNVGYKPKVDTGCFTYASHAAVVAVDP
DTGAVEILDYVVVEDCGTMINPMVVEGQTIGGIAQGIGTAFYEETPYDANGQPLASTLADYMLPGPTEVPNM
RLHHFETPSPHTEFGAKGMGEGGAIAPPAVLFNAVNDALRGLGAAELSRTPLTPIRVLQAIAQGAGEAA
>SEQF7958||SEQF7958.1_00505
MTDATSPGLGRSAPRIEDDALLRGQARFLDDIEVEGVLHACFVRSPHAHARLVSIDLSAARAVPGVAAVYGARD
LFGQLTSWRMPLGFPLAALPDDTTPFVLAEREVAFVGEAIAVVVADSRHIAEDAAARVAIEYEVLGAVVDCRDA
LRPDAPLVRGELASNILQQYTLAYGDCETAFAQAHRVLEDDFWVHRGCAHPMEGRGVLARMDRATDTLTVWS
STQMAHELHYTLALMLGQPEDRLRVVTPDVGGGFGAKFMIYPEEMAIPAAARKLGRPVKWVEDRREHFTTSI
QERDQYWKVAMAIDDQGHVLGIRGNFVHDNGAYTPQGTNVPYNAASSMTGPYVVPAFSLDVSVAYTNKVPV
ATVRGAGYPQAAFVMERMMDRVAAELGIDPGECRRRNLIGPAKIPYTKPLKSRAGMPLTIDSGDFPALQACAL
QASDYDGFAVRRDAALARGRWRGIAVANSVEPTGRGPFEVARVRVQPSGQVSIYTGALAMGQGIKTTLAQIC
AGHLGVPVAAVEVQAGDTAYVGYGMGGFASRQAIMAGSAVDQAAAQVRRQALETAAAVLKAEAETLELADG
EVRAPDGQSVSLARLAMLRKGVPGYALVSPGDPGLDATAYFHCDAQTYAGASHVCEVEVDPATGAIEIVRYVAA
QDSGRIINPQLAEGQVHGGVVHGIGNALFEWMGYDAAGQPLSTTFAEYLLPTAPEVPPIEVVFQPSPTPLNPL
GVKGVGECATIPVAVAVVGAVEHAVAHCGVRVTEFPLTPVRLLELLTQAEARAAAPTDSGEFVDGVH
>SEQF7959||SEQF7959.1_00480
MTDATSPGLGRSAPRIEDDALLRGQARFLDDIEVEGVLHACFVRSPHAHARLVSIDLSAARAVPGVAAVYGARD
LFGQLTSWRMPLGFPLAALPDDTTPFVLAEREVAFVGEAIAVVVADSRHIAEDAAARVAIEYEVLGAVVDCRDA
LRPDAPLVRGELASNILQQYTLAYGDCETAFAQAHRVLEDDFWVHRGCAHPMEGRGVLARMDRATDTLTVWS
STQMAHELHYTLALMLGQPEDRLRVVTPDVGGGFGAKFMIYPEEMAIPAAARKLGRPVKWVEDRREHFTTSI
QERDQYWKVAMAIDDQGHVLGIRGNFVHDNGAYTPQGTNVPYNAASSMTGPYVVPAFSLDVSVAYTNKVPV
ATVRGAGYPQAAFVMERMMDRVAAELGIDPGECRRRNLIGPAKIPYTKPLKSRAGMPLTIDSGDFPALQACAL
QASDYDGFAVRRDAALARGRWRGIAVANSVEPTGRGPFEVARVRVQPSGQVSIYTGALAMGQGIKTTLAQIC
AGHLGVPVAAVEVQAGDTAYVGYGMGGFASRQAIMAGSAVDQAAAQVRRQALETAAAVLKAEAETLELADG
EVRAPDGQSVSLARLAMLRKGVPGYALVSPGDPGLDATAYFHCDAQTYAGASHVCEVEVDPATGAIEIVRYVAA
QDSGRIINPQLAEGQVHGGVVHGIGNALFEWMGYDAAGQPLSTTFAEYLLPTAPEVPPIEVVFQPSPTPLNPL
GVKGVGECATIPVAVAVVGAVEHAVAHCGVRVTEFPLTPVRLLELLTQAEARAAAPTDSGEFVDGVH
>SEQF7959||SEQF7959.1_03562
MTTEHTGAEVGAGSRQGVGARVPRKEDARHLHGKGNFVADMAMPGLCEVAFLRSPLAHARITDVRVPESVR
DKVVLRSMMGDARDIAADSTLPTYQPSVQPPLASGKVRFVGEPVAMTFAPTRAEAEDHAELVEVDYDDLPVY

ADVAGAQQATSDLVHEHWRDNVFVTLNADRDFDEHAARAEVVVRRKIDLARQCMVPMEGKAVLAYWDHQ
ADQLVVISATQVPHMIRSVLAQCLDLEQGRVRVVSPDVGGAFGYKCVLQQEELCVAWLAKTYKRPFRFIEDRRE
HLTAGANSREHHYEMTAYADKRGKLLALDARITIDGGAYSVWPFTIGLEPDQAVGNLPGPYGFRGYRCETRCVA
TNKPGFVPYRGRTGVCFAIELTMDAVAREVGREPWEVRLENLVQPEQMPYVNVTNKHFDSGDYPASLRKALE
MIDIDGVRTRQARGEADGRRIGVGVATYTEQAAHGTSVFAAWGTPVIPGFDQATVRVTPDGGLEVRVGVHSH
GQGMETTFAQIAHEILGIDVARIKVLHGDTGQTPFSTGTYASRSLVMSGGAVSQACKRLLPRMRHIAAHMLGV
ADDAVTLQDGIYRAGEKSVATGDVADAWYLRPQLLPPDVDPAGLEVNVGYKPKVDTGCFTYASHAAVVAVDP
DTGAVEILDYVVVEDCGTMINPMVVEGQTIGGIAQGIGTAFYEETPYDANGQPLASTLADYMLPGPTEVPNM
RLHHFETPSPHTEFGAKGMGEGGAIAPPAVLFNAVNDALRGLGAAELSRTPLTPIRVLQAIAQGAGEAA
>SEQF7960||SEQF7960.1_03560
MTTEHTGAEVGAGSRQGVGARVPRKEDARHLHGKGNFVADMAMPGLCEVAFLRSPLAHARITDVRVPESVR
DKVVLRSMMGDARDIAADSTLPTYQPSVQPPLASGKVRFVGEPVAMTFAPTRAEAEDHAELVEVDYDDLPVY
ADVAGAQQATSDLVHEHWRDNVFVTLNADRDFDEHAARAEVVVRRKIDLARQCMVPMEGKAVLAYWDHQ
ADQLVVISATQVPHMIRSVLAQCLDLEQGRVRVVSPDVGGAFGYKCVLQQEELCVAWLAKTYKRPFRFIEDRRE
HLTAGANSREHHYEMTAYADKRGKLLALDARITIDGGAYSVWPFTIGLEPDQAVGNLPGPYGFRGYRCETRCVA
TNKPGFVPYRGRTGVCFAIELTMDAVAREVGREPWEVRLENLVQPEQMPYVNVTNKHFDSGDYPASLRKALE
MIDIDGVRTRQARGEADGRRIGVGVATYTEQAAHGTSVFAAWGTPVIPGFDQATVRVTPDGGLEVRVGVHSH
GQGMETTFAQIAHEILGIDVARIKVLHGDTGQTPFSTGTYASRSLVMSGGAVSQACKRLLPRMRHIAAHMLGV
ADDAVTLQDGIYRAGEKSVATGDVADAWYLRPQLLPPDVDPAGLEVNVGYKPKVDTGCFTYASHAAVVAVDP
DTGAVEILDYVVVEDCGTMINPMVVEGQTIGGIAQGIGTAFYEETPYDANGQPLASTLADYMLPGPTEVPNM
RLHHFETPSPHTEFGAKGMGEGGAIAPPAVLFNAVNDALRGLGAAELSRTPLTPIRVLQAIAQGAGEAA
>SEQF7960||SEQF7960.1_00480
MTDATSPGLGRSAPRIEDDALLRGQARFLDDIEVEGVLHACFVRSPHAHARLVSIDLSAARAVPGVAAVYGARD
LFGQLTSWRMPLGFPLAALPDDTTPFVLAEREVAFVGEAIAVVVADSRHIAEDAAARVAIEYEVLGAVVDCRDA
LRPDAPLVRGELASNILQQYTLAYGDCETAFAQAHRVLEDDFWVHRGCAHPMEGRGVLARMDRATDTLTVWS
STQMAHELHYTLALMLGQPEDRLRVVTPDVGGGFGAKFMIYPEEMAIPAAARKLGRPVKWVEDRREHFTTSI
QERDQYWKVAMAIDDQGHVLGIRGNFVHDNGAYTPQGTNVPYNAASSMTGPYVVPAFSLDVSVAYTNKVPV
ATVRGAGYPQAAFVMERMMDRVAAELGIDPGECRRRNLIGPAKIPYTKPLKSRAGMPLTIDSGDFPALQACAL
QASDYDGFAVRRDAALARGRWRGIAVANSVEPTGRGPFEVARVRVQPSGQVSIYTGALAMGQGIKTTLAQIC
AGHLGVPVAAVEVQAGDTAYVGYGMGGFASRQAIMAGSAVDQAAAQVRRQALETAAAVLKAEAETLELADG
EVRAPDGQSVSLARLAMLRKGVPGYALVSPGDPGLDATAYFHCDAQTYAGASHVCEVEVDPATGAIEIVRYVAA
QDSGRIINPQLAEGQVHGGVVHGIGNALFEWMGYDAAGQPLSTTFAEYLLPTAPEVPPIEVVFQPSPTPLNPL
GVKGVGECATIPVAVAVVGAVEHAVAHCGVRVTEFPLTPVRLLELLTQAEARAAAPTDSGEFVDGVH
>SEQF7961||SEQF7961.1_03601
MTTEHTGAEVGAGSRQGVGARVPRKEDARHLHGKGNFVADMAMPGLCEVAFLRSPLAHARITDVRVPESVR
DKVVLRSMMGDARDIAADSTLPTYQPSVQPPLASGKVRFVGEPVAMTFAPTRAEAEDHAELVEVDYDDLPVY
ADVAGAQQATSDLVHEHWRDNVFVTLNADRDFDEHAARAEVVVRRKIDLARQCMVPMEGKAVLAYWDHQ
ADQLVVISATQVPHMIRSVLAQCLDLEQGRVRVVSPDVGGAFGYKCVLQQEELCVAWLAKTYKRPFRFIEDRRE
HLTAGANSREHHYEMTAYADKRGKLLALDARITIDGGAYSVWPFTIGLEPDQAVGNLPGPYGFRGYRCETRCVA
TNKPGFVPYRGRTGVCFAIELTMDAVAREVGREPWEVRLENLVQPEQMPYVNVTNKHFDSGDYPASLRKALE
MIDIDGVRTRQARGEADGRRIGVGVATYTEQAAHGTSVFAAWGTPVIPGFDQATVRVTPDGGLEVRVGVHSH
GQGMETTFAQIAHEILGIDVARIKVLHGDTGQTPFSTGTYASRSLVMSGGAVSQACKRLLPRMRHIAAHMLGV
ADDAVTLQDGIYRAGEKSVATGDVADAWYLRPQLLPPDVDPAGLEVNVGYKPKVDTGCFTYASHAAVVAVDP
DTGAVEILDYVVVEDCGTMINPMVVEGQTIGGIAQGIGTAFYEETPYDANGQPLASTLADYMLPGPTEVPNM

RLHHFETPSPHTEFGAKGMGEGGAIAPPAVLFNAVNDALRGLGAAELSRTPLTPIRVLQAIAQGAGEAA
>SEQF7961||SEQF7961.1_03506
MTDATSPGLGRSAPRIEDDALLRGQARFLDDIEVEGVLHACFVRSPHAHARLVSIDLSAARAVPGVAAVYGARD
LFGQLTSWRMPLGFPLAALPDDTTPFVLAEREVAFVGEAIAVVVADSRHIAEDAAARVAIEYEVLGAVVDCRDA
LRPDAPLVRGELASNILQQYTLAYGDCETAFAQAHRVLEDDFWVHRGCAHPMEGRGVLARMDRATDTLTVWS
STQMAHELHYTLALMLGQPEDRLRVVTPDVGGGFGAKFMIYPEEMAIPAAARKLGRPVKWVEDRREHFTTSI
QERDQYWKVAMAIDDQGHVLGIRGNFVHDNGAYTPQGTNVPYNAASSMTGPYVVPAFSLDVSVAYTNKVPV
ATVRGAGYPQAAFVMERMMDRVAAELGIDPGECRRRNLIGPAKIPYTKPLKSRAGMPLTIDSGDFPALQACAL
QASDYDGFAVRRDAALARGRWRGIAVANSVEPTGRGPFEVARVRVQPSGQVSIYTGALAMGQGIKTTLAQIC
AGHLGVPVAAVEVQAGDTAYVGYGMGGFASRQAIMAGSAVDQAAAQVRRQALETAAAVLKAEAETLELADG
EVRAPDGQSVSLARLAMLRKGVPGYALVSPGDPGLDATAYFHCDAQTYAGASHVCEVEVDPATGAIEIVRYVAA
QDSGRIINPQLAEGQVHGGVVHGIGNALFEWMGYDAAGQPLSTTFAEYLLPTAPEVPPIEVVFQPSPTPLNPL
GVKGVGECATIPVAVAVVGAVEHAVAHCGVRVTEFPLTPVRLLELLTQAEARAAAPTDSGEFVDGVH
>SEQF7962||SEQF7962.1_03574
MTTEHTGAEVGAGSRQGVGARVPRKEDARHLHGKGNFVADMAMPGLCEVAFLRSPLAHARITDVRVPESVR
DKVVLRSMMGDARDIAADSTLPTYQPSVQPPLASGKVRFVGEPVAMTFAPTRAEAEDHAELVEVDYDDLPVY
ADVAGAQQATSDLVHEHWRDNVFVTLNADRDFDEHAARAEVVVRRKIDLARQCMVPMEGKAVLAYWDHQ
ADQLVVISATQVPHMIRSVLAQCLDLEQGRVRVVSPDVGGAFGYKCVLQQEELCVAWLAKTYKRPFRFIEDRRE
HLTAGANSREHHYEMTAYADKRGKLLALDARITIDGGAYSVWPFTIGLEPDQAVGNLPGPYGFRGYRCETRCVA
TNKPGFVPYRGRTGVCFAIELTMDAVAREVGREPWEVRLENLVQPEQMPYVNVTNKHFDSGDYPASLRKALE
MIDIDGVRTRQARGEADGRRIGVGVATYTEQAAHGTSVFAAWGTPVIPGFDQATVRVTPDGGLEVRVGVHSH
GQGMETTFAQIAHEILGIDVARIKVLHGDTGQTPFSTGTYASRSLVMSGGAVSQACKRLLPRMRHIAAHMLGV
ADDAVTLQDGIYRAGEKSVATGDVADAWYLRPQLLPPDVDPAGLEVNVGYKPKVDTGCFTYASHAAVVAVDP
DTGAVEILDYVVVEDCGTMINPMVVEGQTIGGIAQGIGTAFYEETPYDANGQPLASTLADYMLPGPTEVPNM
RLHHFETPSPHTEFGAKGMGEGGAIAPPAVLFNAVNDALRGLGAAELSRTPLTPIRVLQAIAQGAGEAA
>SEQF7962||SEQF7962.1_03478
MTDATSPGLGRSAPRIEDDALLRGQARFLDDIEVEGVLHACFVRSPHAHARLVSIDLSAARAVPGVAAVYGARD
LFGQLTSWRMPLGFPLAALPDDTTPFVLAEREVAFVGEAIAVVVADSRHIAEDAAARVAIEYEVLGAVVDCRDA
LRPDAPLVRGELASNILQQYTLAYGDCETAFAQAHRVLEDDFWVHRGCAHPMEGRGVLARMDRATDTLTVWS
STQMAHELHYTLALMLGQPEDRLRVVTPDVGGGFGAKFMIYPEEMAIPAAARKLGRPVKWVEDRREHFTTSI
QERDQYWKVAMAIDDQGHVLGIRGNFVHDNGAYTPQGTNVPYNAASSMTGPYVVPAFSLDVSVAYTNKVPV
ATVRGAGYPQAAFVMERMMDRVAAELGIDPGECRRRNLIGPAKIPYTKPLKSRAGMPLTIDSGDFPALQACAL
QASDYDGFAVRRDAALARGRWRGIAVANSVEPTGRGPFEVARVRVQPSGQVSIYTGALAMGQGIKTTLAQIC
AGHLGVPVAAVEVQAGDTAYVGYGMGGFASRQAIMAGSAVDQAAAQVRRQALETAAAVLKAEAETLELADG
EVRAPDGQSVSLARLAMLRKGVPGYALVSPGDPGLDATAYFHCDAQTYAGASHVCEVEVDPATGAIEIVRYVAA
QDSGRIINPQLAEGQVHGGVVHGIGNALFEWMGYDAAGQPLSTTFAEYLLPTAPEVPPIEVVFQPSPTPLNPL
GVKGVGECATIPVAVAVVGAVEHAVAHCGVRVTEFPLTPVRLLELLTQAEARAAAPTDSGEFVDGVH
>SEQF7963||SEQF7963.1_00408
MTTEHTGAEVGAGSRQGVGARVPRKEDARHLHGKGNFVADMAMPGLCEVAFLRSPLAHARITDVRVPESVR
DKVVLRSMMGDARDIAADSTLPTYQPSVQPPLASGKVRFVGEPVAMTFAPTRAEAEDHAELVEVDYDDLPVY
ADVAGAQQATSDLVHEHWRDNVFVTLNADRDFDEHAARAEVVVRRKIDLARQCMVPMEGKAVLAYWDHQ
ADQLVVISATQVPHMIRSVLAQCLDLEQGRVRVVSPDVGGAFGYKCVLQQEELCVAWLAKTYKRPFRFIEDRRE
HLTAGANSREHHYEMTAYADKRGKLLALDARITIDGGAYSVWPFTIGLEPDQAVGNLPGPYGFRGYRCETRCVA
TNKPGFVPYRGRTGVCFAIELTMDAVAREVGREPWEVRLENLVQPEQMPYVNVTNKHFDSGDYPASLRKALE

MIDIDGVRTRQARGEADGRRIGVGVATYTEQAAHGTSVFAAWGTPVIPGFDQATVRVTPDGGLEVRVGVHSH
GQGMETTFAQIAHEILGIDVARIKVLHGDTGQTPFSTGTYASRSLVMSGGAVSQACKRLLPRMRHIAAHMLGV
ADDAVTLQDGIYRAGEKSVATGDVADAWYLRPQLLPPDVDPAGLEVNVGYKPKVDTGCFTYASHAAVVAVDP
DTGAVEILDYVVVEDCGTMINPMVVEGQTIGGIAQGIGTAFYEETPYDANGQPLASTLADYMLPGPTEVPNM
RLHHFETPSPHTEFGAKGMGEGGAIAPPAVLFNAVNDALRGLGAAELSRTPLTPIRVLQAIAQGAGEAA
>SEQF7963||SEQF7963.1_00504
MTDATSPGLGRSAPRIEDDALLRGQARFLDDIEVEGVLHACFVRSPHAHARLVSIDLSAARAVPGVAAVYGARD
LFGQLTSWRMPLGFPLAALPDDTTPFVLAEREVAFVGEAIAVVVADSRHIAEDAAARVAIEYEVLGAVVDCRDA
LRPDAPLVRGELASNILQQYTLAYGDCETAFAQAHRVLEDDFWVHRGCAHPMEGRGVLARMDRATDTLTVWS
STQMAHELHYTLALMLGQPEDRLRVVTPDVGGGFGAKFMIYPEEMAIPAAARKLGRPVKWVEDRREHFTTSI
QERDQYWKVAMAIDDQGHVLGIRGNFVHDNGAYTPQGTNVPYNAASSMTGPYVVPAFSLDVSVAYTNKVPV
ATVRGAGYPQAAFVMERMMDRVAAELGIDPGECRRRNLIGPAKIPYTKPLKSRAGMPLTIDSGDFPALQACAL
QASDYDGFAVRRDAALARGRWRGIAVANSVEPTGRGPFEVARVRVQPSGQVSIYTGALAMGQGIKTTLAQIC
AGHLGVPVAAVEVQAGDTAYVGYGMGGFASRQAIMAGSAVDQAAAQVRRQALETAAAVLKAEAETLELADG
EVRAPDGQSVSLARLAMLRKGVPGYALVSPGDPGLDATAYFHCDAQTYAGASHVCEVEVDPATGAIEIVRYVAA
QDSGRIINPQLAEGQVHGGVVHGIGNALFEWMGYDAAGQPLSTTFAEYLLPTAPEVPPIEVVFQPSPTPLNPL
GVKGVGECATIPVAVAVVGAVEHAVAHCGVRVTEFPLTPVRLLELLTQAEARAAAPTDSGEFVDGVH
>SEQF7964||SEQF7964.3_03553
MTTEHTGAEVGAGSRQGVGARVPRKEDARHLHGKGNFVADMAMPGLCEVAFLRSPLAHARITDVRVPESVR
DKVVLRSMMGDARDIAADSTLPTYQPSVQPPLASGKVRFVGEPVAMTFAPTRAEAEDHAELVEVDYDDLPVY
ADVAGAQQATSDLVHEHWRDNVFVTLNADRDFDEHAARAEVVVRRKIDLARQCMVPMEGKAVLAYWDHQ
ADQLVVISATQVPHMIRSVLAQCLDLEQGRVRVVSPDVGGAFGYKCVLQQEELCVAWLAKTYKRPFRFIEDRRE
HLTAGANSREHHYEMTAYADKRGKLLALDARITIDGGAYSVWPFTIGLEPDQAVGNLPGPYGFRGYRCETRCVA
TNKPGFVPYRGRTGVCFAIELTMDAVAREVGREPWEVRLENLVQPEQMPYVNVTNKHFDSGDYPASLRKALE
MIDIDGVRTRQARGEADGRRIGVGVATYTEQAAHGTSVFAAWGTPVIPGFDQATVRVTPDGGLEVRVGVHSH
GQGMETTFAQIAHEILGIDVARIKVLHGDTGQTPFSTGTYASRSLVMSGGAVSQACKRLLPRMRHIAAHMLGV
ADDAVTLQDGIYRAGEKSVATGDVADAWYLRPQLLPPDVDPAGLEVNVGYKPKVDTGCFTYASHAAVVAVDP
DTGAVEILDYVVVEDCGTMINPMVVEGQTIGGIAQGIGTAFYEETPYDANGQPLASTLADYMLPGPTEVPNM
RLHHFETPSPHTEFGAKGMGEGGAIAPPAVLFNAVNDALRGLGAAELSRTPLTPIRVLQAIAQGAGEAA
>SEQF7964||SEQF7964.3_00480
MTDATSPGLGRSAPRIEDDALLRGQARFLDDIEVEGVLHACFVRSPHAHARLVSIDLSAARAVPGVAAVYGARD
LFGQLTSWRMPLGFPLAALPDDTTPFVLAEREVAFVGEAIAVVVADSRHIAEDAAARVAIEYEVLGAVVDCRDA
LRPDAPLVRGELASNILQQYTLAYGDCETAFAQAHRVLEDDFWVHRGCAHPMEGRGVLARMDRATDTLTVWS
STQMAHELHYTLALMLGQPEDRLRVVTPDVGGGFGAKFMIYPEEMAIPAAARKLGRPVKWVEDRREHFTTSI
QERDQYWKVAMAIDDQGHVLGIRGNFVHDNGAYTPQGTNVPYNAASSMTGPYVVPAFSLDVSVAYTNKVPV
ATVRGAGYPQAAFVMERMMDRVAAELGIDPGECRRRNLIGPAKIPYTKPLKSRAGMPLTIDSGDFPALQACAL
QASDYDGFAVRRDAALARGRWRGIAVANSVEPTGRGPFEVARVRVQPSGQVSIYTGALAMGQGIKTTLAQIC
AGHLGVPVAAVEVQAGDTAYVGYGMGGFASRQAIMAGSAVDQAAAQVRRQALETAAAVLKAEAETLELADG
EVRAPDGQSVSLARLAMLRKGVPGYALVSPGDPGLDATAYFHCDAQTYAGASHVCEVEVDPATGAIEIVRYVAA
QDSGRIINPQLAEGQVHGGVVHGIGNALFEWMGYDAAGQPLSTTFAEYLLPTAPEVPPIEVVFQPSPTPLNPL
GVKGVGECATIPVAVAVVGAVEHAVAHCGVRVTEFPLTPVRLLELLTQAEARAAAPTDSGEFVDGVH
>SEQF7965||SEQF7965.1_03469
MTDATSPGLGRSAPRIEDDALLRGQARFLDDIEVEGVLHACFVRSPHAHARLVSIDLSAARAVPGVAAVYGARD
LFGQLTSWRMPLGFPLAALPDDTTPFVLAEREVAFVGEAIAVVVADSRHIAEDAAARVAIEYEVLGAVVDCRDA

LRPDAPLVRGELASNILQQYTLAYGDCETAFAQAHRVLEDDFWVHRGCAHPMEGRGVLARMDRATDTLTVWS
STQMAHELHYTLALMLGQPEDRLRVVTPDVGGGFGAKFMIYPEEMAIPAAARKLGRPVKWVEDRREHFTTSI
QERDQYWKVAMAIDDQGHVLGIRGNFVHDNGAYTPQGTNVPYNAASSMTGPYVVPAFSLDVSVAYTNKVPV
ATVRGAGYPQAAFVMERMMDRVAAELGIDPGECRRRNLIGPAKIPYTKPLKSRAGMPLTIDSGDFPALQACAL
QASDYDGFAVRRDAALARGRWRGIAVANSVEPTGRGPFEVARVRVQPSGQVSIYTGALAMGQGIKTTLAQIC
AGHLGVPVAAVEVQAGDTAYVGYGMGGFASRQAIMAGSAVDQAAAQVRRQALETAAAVLKAEAETLELADG
EVRAPDGQSVSLARLAMLRKGVPGYALVSPGDPGLDATAYFHCDAQTYAGASHVCEVEVDPATGAIEIVRYVAA
QDSGRIINPQLAEGQVHGGVVHGIGNALFEWMGYDAAGQPLSTTFAEYLLPTAPEVPPIEVVFQPSPTPLNPL
GVKGVGECATIPVAVAVVGAVEHAVAHCGVRVTEFPLTPVRLLELLTQAEARAAAPTDSGEFVDGVH
>SEQF7965||SEQF7965.1_03565
MTTEHTGAEVGAGSRQGVGARVPRKEDARHLHGKGNFVADMAMPGLCEVAFLRSPLAHARITDVRVPESVR
DKVVLRSMMGDARDIAADSTLPTYQPSVQPPLASGKVRFVGEPVAMTFAPTRAEAEDHAELVEVDYDDLPVY
ADVAGAQQATSDLVHEHWRDNVFVTLNADRDFDEHAARAEVVVRRKIDLARQCMVPMEGKAVLAYWDHQ
ADQLVVISATQVPHMIRSVLAQCLDLEQGRVRVVSPDVGGAFGYKCVLQQEELCVAWLAKTYKRPFRFIEDRRE
HLTAGANSREHHYEMTAYADKRGKLLALDARITIDGGAYSVWPFTIGLEPDQAVGNLPGPYGFRGYRCETRCVA
TNKPGFVPYRGRTGVCFAIELTMDAVAREVGREPWEVRLENLVQPEQMPYVNVTNKHFDSGDYPASLRKALE
MIDIDGVRTRQARGEADGRRIGVGVATYTEQAAHGTSVFAAWGTPVIPGFDQATVRVTPDGGLEVRVGVHSH
GQGMETTFAQIAHEILGIDVARIKVLHGDTGQTPFSTGTYASRSLVMSGGAVSQACKRLLPRMRHIAAHMLGV
ADDAVTLQDGIYRAGEKSVATGDVADAWYLRPQLLPPDVDPAGLEVNVGYKPKVDTGCFTYASHAAVVAVDP
DTGAVEILDYVVVEDCGTMINPMVVEGQTIGGIAQGIGTAFYEETPYDANGQPLASTLADYMLPGPTEVPNM
RLHHFETPSPHTEFGAKGMGEGGAIAPPAVLFNAVNDALRGLGAAELSRTPLTPIRVLQAIAQGAGEAA
>SEQF7966||SEQF7966.1_03469
MTDATSPGLGRSAPRIEDDALLRGQARFLDDIEVEGVLHACFVRSPHAHARLVSIDLSAARAVPGVAAVYGARD
LFGQLTSWRMPLGFPLAALPDDTTPFVLAEREVAFVGEAIAVVVADSRHIAEDAAARVAIEYEVLGAVVDCRDA
LRPDAPLVRGELASNILQQYTLAYGDCETAFAQAHRVLEDDFWVHRGCAHPMEGRGVLARMDRATDTLTVWS
STQMAHELHYTLALMLGQPEDRLRVVTPDVGGGFGAKFMIYPEEMAIPAAARKLGRPVKWVEDRREHFTTSI
QERDQYWKVAMAIDDQGHVLGIRGNFVHDNGAYTPQGTNVPYNAASSMTGPYVVPAFSLDVSVAYTNKVPV
ATVRGAGYPQAAFVMERMMDRVAAELGIDPGECRRRNLIGPAKIPYTKPLKSRAGMPLTIDSGDFPALQACAL
QASDYDGFAVRRDAALARGRWRGIAVANSVEPTGRGPFEVARVRVQPSGQVSIYTGALAMGQGIKTTLAQIC
AGHLGVPVAAVEVQAGDTAYVGYGMGGFASRQAIMAGSAVDQAAAQVRRQALETAAAVLKAEAETLELADG
EVRAPDGQSVSLARLAMLRKGVPGYALVSPGDPGLDATAYFHCDAQTYAGASHVCEVEVDPATGAIEIVRYVAA
QDSGRIINPQLAEGQVHGGVVHGIGNALFEWMGYDAAGQPLSTTFAEYLLPTAPEVPPIEVVFQPSPTPLNPL
GVKGVGECATIPVAVAVVGAVEHAVAHCGVRVTEFPLTPVRLLELLTQAEARAAAPTDSGEFVDGVH
>SEQF7966||SEQF7966.1_03565
MTTEHTGAEVGAGSRQGVGARVPRKEDARHLHGKGNFVADMAMPGLCEVAFLRSPLAHARITDVRVPESVR
DKVVLRSMMGDARDIAADSTLPTYQPSVQPPLASGKVRFVGEPVAMTFAPTRAEAEDHAELVEVDYDDLPVY
ADVAGAQQATSDLVHEHWRDNVFVTLNADRDFDEHAARAEVVVRRKIDLARQCMVPMEGKAVLAYWDHQ
ADQLVVISATQVPHMIRSVLAQCLDLEQGRVRVVSPDVGGAFGYKCVLQQEELCVAWLAKTYKRPFRFIEDRRE
HLTAGANSREHHYEMTAYADKRGKLLALDARITIDGGAYSVWPFTIGLEPDQAVGNLPGPYGFRGYRCETRCVA
TNKPGFVPYRGRTGVCFAIELTMDAVAREVGREPWEVRLENLVQPEQMPYVNVTNKHFDSGDYPASLRKALE
MIDIDGVRTRQARGEADGRRIGVGVATYTEQAAHGTSVFAAWGTPVIPGFDQATVRVTPDGGLEVRVGVHSH
GQGMETTFAQIAHEILGIDVARIKVLHGDTGQTPFSTGTYASRSLVMSGGAVSQACKRLLPRMRHIAAHMLGV
ADDAVTLQDGIYRAGEKSVATGDVADAWYLRPQLLPPDVDPAGLEVNVGYKPKVDTGCFTYASHAAVVAVDP
DTGAVEILDYVVVEDCGTMINPMVVEGQTIGGIAQGIGTAFYEETPYDANGQPLASTLADYMLPGPTEVPNM

RLHHFETPSPHTEFGAKGMGEGGAIAPPAVLFNAVNDALRGLGAAELSRTPLTPIRVLQAIAQGAGEAA
>SEQF7967||SEQF7967.1_03585
MTTEHTGAEVGAGSRQGVGARVPRKEDARHLHGKGNFVADMAMPGLCEVAFLRSPLAHARITDVRVPESVR
DKVVLRSMMGDARDIAADSTLPTYQPSVQPPLASGKVRFVGEPVAMTFAPTRAEAEDHAELVEVDYDDLPVY
ADVAGAQQATSDLVHEHWRDNVFVTLNADRDFDEHAARAEVVVRRKIDLARQCMVPMEGKAVLAYWDHQ
ADQLVVISATQVPHMIRSVLAQCLDLEQGRVRVVSPDVGGAFGYKCVLQQEELCVAWLAKTYKRPFRFIEDRRE
HLTAGANSREHHYEMTAYADKRGKLLALDARITIDGGAYSVWPFTIGLEPDQAVGNLPGPYGFRGYRCETRCVA
TNKPGFVPYRGRTGVCFAIELTMDAVAREVGREPWEVRLENLVQPEQMPYVNVTNKHFDSGDYPASLRKALE
MIDIDGVRTRQARGEADGRRIGVGVATYTEQAAHGTSVFAAWGTPVIPGFDQATVRVTPDGGLEVRVGVHSH
GQGMETTFAQIAHEILGIDVARIKVLHGDTGQTPFSTGTYASRSLVMSGGAVSQACKRLLPRMRHIAAHMLGV
ADDAVTLQDGIYRAGEKSVATGDVADAWYLRPQLLPPDVDPAGLEVNVGYKPKVDTGCFTYASHAAVVAVDP
DTGAVEILDYVVVEDCGTMINPMVVEGQTIGGIAQGIGTAFYEETPYDANGQPLASTLADYMLPGPTEVPNM
RLHHFETPSPHTEFGAKGMGEGGAIAPPAVLFNAVNDALRGLGAAELSRTPLTPIRVLQAIAQGAGEAA
>SEQF7967||SEQF7967.1_03487
MTDATSPGLGRSAPRIEDDALLRGQARFLDDIEVEGVLHACFVRSPHAHARLVSIDLSAARAVPGVAAVYGARD
LFGQLTSWRMPLGFPLAALPDDTTPFVLAEREVAFVGEAIAVVVADSRHIAEDAAARVAIEYEVLGAVVDCRDA
LRPDAPLVRGELASNILQQYTLAYGDCETAFAQAHRVLEDDFWVHRGCAHPMEGRGVLARMDRATDTLTVWS
STQMAHELHYTLALMLGQPEDRLRVVTPDVGGGFGAKFMIYPEEMAIPAAARKLGRPVKWVEDRREHFTTSI
QERDQYWKVAMAIDDQGHVLGIRGNFVHDNGAYTPQGTNVPYNAASSMTGPYVVPAFSLDVSVAYTNKVPV
ATVRGAGYPQAAFVMERMMDRVAAELGIDPGECRRRNLIGPAKIPYTKPLKSRAGMPLTIDSGDFPALQACAL
QASDYDGFAVRRDAALARGRWRGIAVANSVEPTGRGPFEVARVRVQPSGQVSIYTGALAMGQGIKTTLAQIC
AGHLGVPVAAVEVQAGDTAYVGYGMGGFASRQAIMAGSAVDQAAAQVRRQALETAAAVLKAEAETLELADG
EVRAPDGQSVSLARLAMLRKGVPGYALVSPGDPGLDATAYFHCDAQTYAGASHVCEVEVDPATGAIEIVRYVAA
QDSGRIINPQLAEGQVHGGVVHGIGNALFEWMGYDAAGQPLSTTFAEYLLPTAPEVPPIEVVFQPSPTPLNPL
GVKGVGECATIPVAVAVVGAVEHAVAHCGVRVTEFPLTPVRLLELLTQAEARAAAPTDSGEFVDGVH
>SEQF7968||SEQF7968.1_03568
MTTEHTGAEVGAGSRQGVGARVPRKEDARHLHGKGNFVADMAMPGLCEVAFLRSPLAHARITDVRVPESVR
DKVVLRSMMGDARDIAADSTLPTYQPSVQPPLASGKVRFVGEPVAMTFAPTRAEAEDHAELVEVDYDDLPVY
ADVAGAQQATSDLVHEHWRDNVFVTLNADRDFDEHAARAEVVVRRKIDLARQCMVPMEGKAVLAYWDHQ
ADQLVVISATQVPHMIRSVLAQCLDLEQGRVRVVSPDVGGAFGYKCVLQQEELCVAWLAKTYKRPFRFIEDRRE
HLTAGANSREHHYEMTAYADKRGKLLALDARITIDGGAYSVWPFTIGLEPDQAVGNLPGPYGFRGYRCETRCVA
TNKPGFVPYRGRTGVCFAIELTMDAVAREVGREPWEVRLENLVQPEQMPYVNVTNKHFDSGDYPASLRKALE
MIDIDGVRTRQARGEADGRRIGVGVATYTEQAAHGTSVFAAWGTPVIPGFDQATVRVTPDGGLEVRVGVHSH
GQGMETTFAQIAHEILGIDVARIKVLHGDTGQTPFSTGTYASRSLVMSGGAVSQACKRLLPRMRHIAAHMLGV
ADDAVTLQDGIYRAGEKSVATGDVADAWYLRPQLLPPDVDPAGLEVNVGYKPKVDTGCFTYASHAAVVAVDP
DTGAVEILDYVVVEDCGTMINPMVVEGQTIGGIAQGIGTAFYEETPYDANGQPLASTLADYMLPGPTEVPNM
RLHHFETPSPHTEFGAKGMGEGGAIAPPAVLFNAVNDALRGLGAAELSRTPLTPIRVLQAIAQGAGEAA
>SEQF7968||SEQF7968.1_00480
MTDATSPGLGRSAPRIEDDALLRGQARFLDDIEVEGVLHACFVRSPHAHARLVSIDLSAARAVPGVAAVYGARD
LFGQLTSWRMPLGFPLAALPDDTTPFVLAEREVAFVGEAIAVVVADSRHIAEDAAARVAIEYEVLGAVVDCRDA
LRPDAPLVRGELASNILQQYTLAYGDCETAFAQAHRVLEDDFWVHRGCAHPMEGRGVLARMDRATDTLTVWS
STQMAHELHYTLALMLGQPEDRLRVVTPDVGGGFGAKFMIYPEEMAIPAAARKLGRPVKWVEDRREHFTTSI
QERDQYWKVAMAIDDQGHVLGIRGNFVHDNGAYTPQGTNVPYNAASSMTGPYVVPAFSLDVSVAYTNKVPV
ATVRGAGYPQAAFVMERMMDRVAAELGIDPGECRRRNLIGPAKIPYTKPLKSRAGMPLTIDSGDFPALQACAL

QASDYDGFAVRRDAALARGRWRGIAVANSVEPTGRGPFEVARVRVQPSGQVSIYTGALAMGQGIKTTLAQIC
AGHLGVPVAAVEVQAGDTAYVGYGMGGFASRQAIMAGSAVDQAAAQVRRQALETAAAVLKAEAETLELADG
EVRAPDGQSVSLARLAMLRKGVPGYALVSPGDPGLDATAYFHCDAQTYAGASHVCEVEVDPATGAIEIVRYVAA
QDSGRIINPQLAEGQVHGGVVHGIGNALFEWMGYDAAGQPLSTTFAEYLLPTAPEVPPIEVVFQPSPTPLNPL
GVKGVGECATIPVAVAVVGAVEHAVAHCGVRVTEFPLTPVRLLELLTQAEARAAAPTDSGEFVDGVH
>SEQF7969||SEQF7969.1_03470
MTDATSPGLGRSAPRIEDDALLRGQARFLDDIEVEGVLHACFVRSPHAHARLVSIDLSAARAVPGVAAVYGARD
LFGQLTSWRMPLGFPLAALPDDTTPFVLAEREVAFVGEAIAVVVADSRHIAEDAAARVAIEYEVLGAVVDCRDA
LRPDAPLVRGELASNILQQYTLAYGDCETAFAQAHRVLEDDFWVHRGCAHPMEGRGVLARMDRATDTLTVWS
STQMAHELHYTLALMLGQPEDRLRVVTPDVGGGFGAKFMIYPEEMAIPAAARKLGRPVKWVEDRREHFTTSI
QERDQYWKVAMAIDDQGHVLGIRGNFVHDNGAYTPQGTNVPYNAASSMTGPYVVPAFSLDVSVAYTNKVPV
ATVRGAGYPQAAFVMERMMDRVAAELGIDPGECRRRNLIGPAKIPYTKPLKSRAGMPLTIDSGDFPALQACAL
QASDYDGFAVRRDAALARGRWRGIAVANSVEPTGRGPFEVARVRVQPSGQVSIYTGALAMGQGIKTTLAQIC
AGHLGVPVAAVEVQAGDTAYVGYGMGGFASRQAIMAGSAVDQAAAQVRRQALETAAAVLKAEAETLELADG
EVRAPDGQSVSLARLAMLRKGVPGYALVSPGDPGLDATAYFHCDAQTYAGASHVCEVEVDPATGAIEIVRYVAA
QDSGRIINPQLAEGQVHGGVVHGIGNALFEWMGYDAAGQPLSTTFAEYLLPTAPEVPPIEVVFQPSPTPLNPL
GVKGVGECATIPVAVAVVGAVEHAVAHCGVRVTEFPLTPVRLLELLTQAEARAAAPTDSGEFVDGVH
>SEQF7969||SEQF7969.1_03566
MTTEHTGAEVGAGSRQGVGARVPRKEDARHLHGKGNFVADMAMPGLCEVAFLRSPLAHARITDVRVPESVR
DKVVLRSMMGDARDIAADSTLPTYQPSVQPPLASGKVRFVGEPVAMTFAPTRAEAEDHAELVEVDYDDLPVY
ADVAGAQQATSDLVHEHWRDNVFVTLNADRDFDEHAARAEVVVRRKIDLARQCMVPMEGKAVLAYWDHQ
ADQLVVISATQVPHMIRSVLAQCLDLEQGRVRVVSPDVGGAFGYKCVLQQEELCVAWLAKTYKRPFRFIEDRRE
HLTAGANSREHHYEMTAYADKRGKLLALDARITIDGGAYSVWPFTIGLEPDQAVGNLPGPYGFRGYRCETRCVA
TNKPGFVPYRGRTGVCFAIELTMDAVAREVGREPWEVRLENLVQPEQMPYVNVTNKHFDSGDYPASLRKALE
MIDIDGVRTRQARGEADGRRIGVGVATYTEQAAHGTSVFAAWGTPVIPGFDQATVRVTPDGGLEVRVGVHSH
GQGMETTFAQIAHEILGIDVARIKVLHGDTGQTPFSTGTYASRSLVMSGGAVSQACKRLLPRMRHIAAHMLGV
ADDAVTLQDGIYRAGEKSVATGDVADAWYLRPQLLPPDVDPAGLEVNVGYKPKVDTGCFTYASHAAVVAVDP
DTGAVEILDYVVVEDCGTMINPMVVEGQTIGGIAQGIGTAFYEETPYDANGQPLASTLADYMLPGPTEVPNM
RLHHFETPSPHTEFGAKGMGEGGAIAPPAVLFNAVNDALRGLGAAELSRTPLTPIRVLQAIAQGAGEAA
>SEQF7970||SEQF7970.1_03563
MTTEHTGAEVGAGSRQGVGARVPRKEDARHLHGKGNFVADMAMPGLCEVAFLRSPLAHARITDVRVPESVR
DKVVLRSMMGDARDIAADSTLPTYQPSVQPPLASGKVRFVGEPVAMTFAPTRAEAEDHAELVEVDYDDLPVY
ADVAGAQQATSDLVHEHWRDNVFVTLNADRDFDEHAARAEVVVRRKIDLARQCMVPMEGKAVLAYWDHQ
ADQLVVISATQVPHMIRSVLAQCLDLEQGRVRVVSPDVGGAFGYKCVLQQEELCVAWLAKTYKRPFRFIEDRRE
HLTAGANSREHHYEMTAYADKRGKLLALDARITIDGGAYSVWPFTIGLEPDQAVGNLPGPYGFRGYRCETRCVA
TNKPGFVPYRGRTGVCFAIELTMDAVAREVGREPWEVRLENLVQPEQMPYVNVTNKHFDSGDYPASLRKALE
MIDIDGVRTRQARGEADGRRIGVGVATYTEQAAHGTSVFAAWGTPVIPGFDQATVRVTPDGGLEVRVGVHSH
GQGMETTFAQIAHEILGIDVARIKVLHGDTGQTPFSTGTYASRSLVMSGGAVSQACKRLLPRMRHIAAHMLGV
ADDAVTLQDGIYRAGEKSVATGDVADAWYLRPQLLPPDVDPAGLEVNVGYKPKVDTGCFTYASHAAVVAVDP
DTGAVEILDYVVVEDCGTMINPMVVEGQTIGGIAQGIGTAFYEETPYDANGQPLASTLADYMLPGPTEVPNM
RLHHFETPSPHTEFGAKGMGEGGAIAPPAVLFNAVNDALRGLGAAELSRTPLTPIRVLQAIAQGAGEAA
>SEQF7970||SEQF7970.1_03467
MTDATSPGLGRSAPRIEDDALLRGQARFLDDIEVEGVLHACFVRSPHAHARLVSIDLSAARAVPGVAAVYGARD
LFGQLTSWRMPLGFPLAALPDDTTPFVLAEREVAFVGEAIAVVVADSRHIAEDAAARVAIEYEVLGAVVDCRDA

LRPDAPLVRGELASNILQQYTLAYGDCETAFAQAHRVLEDDFWVHRGCAHPMEGRGVLARMDRATDTLTVWS
STQMAHELHYTLALMLGQPEDRLRVVTPDVGGGFGAKFMIYPEEMAIPAAARKLGRPVKWVEDRREHFTTSI
QERDQYWKVAMAIDDQGHVLGIRGNFVHDNGAYTPQGTNVPYNAASSMTGPYVVPAFSLDVSVAYTNKVPV
ATVRGAGYPQAAFVMERMMDRVAAELGIDPGECRRRNLIGPAKIPYTKPLKSRAGMPLTIDSGDFPALQACAL
QASDYDGFAVRRDAALARGRWRGIAVANSVEPTGRGPFEVARVRVQPSGQVSIYTGALAMGQGIKTTLAQIC
AGHLGVPVAAVEVQAGDTAYVGYGMGGFASRQAIMAGSAVDQAAAQVRRQALETAAAVLKAEAETLELADG
EVRAPDGQSVSLARLAMLRKGVPGYALVSPGDPGLDATAYFHCDAQTYAGASHVCEVEVDPATGAIEIVRYVAA
QDSGRIINPQLAEGQVHGGVVHGIGNALFEWMGYDAAGQPLSTTFAEYLLPTAPEVPPIEVVFQPSPTPLNPL
GVKGVGECATIPVAVAVVGAVEHAVAHCGVRVTEFPLTPVRLLELLTQAEARAAAPTDSGEFVDGVH
>SEQF7971||SEQF7971.1_03473
MTDATSPGLGRSAPRIEDDALLRGQARFLDDIEVEGVLHACFVRSPHAHARLVSIDLSAARAVPGVAAVYGARD
LFGQLTSWRMPLGFPLAALPDDTTPFVLAEREVAFVGEAIAVVVADSRHIAEDAAARVAIEYEVLGAVVDCRDA
LRPDAPLVRGELASNILQQYTLAYGDCETAFAQAHRVLEDDFWVHRGCAHPMEGRGVLARMDRATDTLTVWS
STQMAHELHYTLALMLGQPEDRLRVVTPDVGGGFGAKFMIYPEEMAIPAAARKLGRPVKWVEDRREHFTTSI
QERDQYWKVAMAIDDQGHVLGIRGNFVHDNGAYTPQGTNVPYNAASSMTGPYVVPAFSLDVSVAYTNKVPV
ATVRGAGYPQAAFVMERMMDRVAAELGIDPGECRRRNLIGPAKIPYTKPLKSRAGMPLTIDSGDFPALQACAL
QASDYDGFAVRRDAALARGRWRGIAVANSVEPTGRGPFEVARVRVQPSGQVSIYTGALAMGQGIKTTLAQIC
AGHLGVPVAAVEVQAGDTAYVGYGMGGFASRQAIMAGSAVDQAAAQVRRQALETAAAVLKAEAETLELADG
EVRAPDGQSVSLARLAMLRKGVPGYALVSPGDPGLDATAYFHCDAQTYAGASHVCEVEVDPATGAIEIVRYVAA
QDSGRIINPQLAEGQVHGGVVHGIGNALFEWMGYDAAGQPLSTTFAEYLLPTAPEVPPIEVVFQPSPTPLNPL
GVKGVGECATIPVAVAVVGAVEHAVAHCGVRVTEFPLTPVRLLELLTQAEARAAAPTDSGEFVDGVH
>SEQF7971||SEQF7971.1_03568
MTTEHTGAEVGAGSRQGVGARVPRKEDARHLHGKGNFVADMAMPGLCEVAFLRSPLAHARITDVRVPESVR
DKVVLRSMMGDARDIAADSTLPTYQPSVQPPLASGKVRFVGEPVAMTFAPTRAEAEDHAELVEVDYDDLPVY
ADVAGAQQATSDLVHEHWRDNVFVTLNADRDFDEHAARAEVVVRRKIDLARQCMVPMEGKAVLAYWDHQ
ADQLVVISATQVPHMIRSVLAQCLDLEQGRVRVVSPDVGGAFGYKCVLQQEELCVAWLAKTYKRPFRFIEDRRE
HLTAGANSREHHYEMTAYADKRGKLLALDARITIDGGAYSVWPFTIGLEPDQAVGNLPGPYGFRGYRCETRCVA
TNKPGFVPYRGRTGVCFAIELTMDAVAREVGREPWEVRLENLVQPEQMPYVNVTNKHFDSGDYPASLRKALE
MIDIDGVRTRQARGEADGRRIGVGVATYTEQAAHGTSVFAAWGTPVIPGFDQATVRVTPDGGLEVRVGVHSH
GQGMETTFAQIAHEILGIDVARIKVLHGDTGQTPFSTGTYASRSLVMSGGAVSQACKRLLPRMRHIAAHMLGV
ADDAVTLQDGIYRAGEKSVATGDVADAWYLRPQLLPPDVDPAGLEVNVGYKPKVDTGCFTYASHAAVVAVDP
DTGAVEILDYVVVEDCGTMINPMVVEGQTIGGIAQGIGTAFYEETPYDANGQPLASTLADYMLPGPTEVPNM
RLHHFETPSPHTEFGAKGMGEGGAIAPPAVLFNAVNDALRGLGAAELSRTPLTPIRVLQAIAQGAGEAA
>SEQF7972||SEQF7972.1_03556
MTTEHTGAEVGAGSRQGVGARVPRKEDARHLHGKGNFVADMAMPGLCEVAFLRSPLAHARITDVRVPESVR
DKVVLRSMMGDARDIAADSTLPTYQPSVQPPLASGKVRFVGEPVAMTFAPTRAEAEDHAELVEVDYDDLPVY
ADVAGAQQATSDLVHEHWRDNVFVTLNADRDFDEHAARAEVVVRRKIDLARQCMVPMEGKAVLAYWDHQ
ADQLVVISATQVPHMIRSVLAQCLDLEQGRVRVVSPDVGGAFGYKCVLQQEELCVAWLAKTYKRPFRFIEDRRE
HLTAGANSREHHYEMTAYADKRGKLLALDARITIDGGAYSVWPFTIGLEPDQAVGNLPGPYGFRGYRCETRCVA
TNKPGFVPYRGRTGVCFAIELTMDAVAREVGREPWEVRLENLVQPEQMPYVNVTNKHFDSGDYPASLRKALE
MIDIDGVRTRQARGEADGRRIGVGVATYTEQAAHGTSVFAAWGTPVIPGFDQATVRVTPDGGLEVRVGVHSH
GQGMETTFAQIAHEILGIDVARIKVLHGDTGQTPFSTGTYASRSLVMSGGAVSQACKRLLPRMRHIAAHMLGV
ADDAVTLQDGIYRAGEKSVATGDVADAWYLRPQLLPPDVDPAGLEVNVGYKPKVDTGCFTYASHAAVVAVDP
DTGAVEILDYVVVEDCGTMINPMVVEGQTIGGIAQGIGTAFYEETPYDANGQPLASTLADYMLPGPTEVPNM

RLHHFETPSPHTEFGAKGMGEGGAIAPPAVLFNAVNDALRGLGAAELSRTPLTPIRVLQAIAQGAGEAA
>SEQF7972||SEQF7972.1_00482
MTDATSPGLGRSAPRIEDDALLRGQARFLDDIEVEGVLHACFVRSPHAHARLVSIDLSAARAVPGVAAVYGARD
LFGQLTSWRMPLGFPLAALPDDTTPFVLAEREVAFVGEAIAVVVADSRHIAEDAAARVAIEYEVLGAVVDCRDA
LRPDAPLVRGELASNILQQYTLAYGDCETAFAQAHRVLEDDFWVHRGCAHPMEGRGVLARMDRATDTLTVWS
STQMAHELHYTLALMLGQPEDRLRVVTPDVGGGFGAKFMIYPEEMAIPAAARKLGRPVKWVEDRREHFTTSI
QERDQYWKVAMAIDDQGHVLGIRGNFVHDNGAYTPQGTNVPYNAASSMTGPYVVPAFSLDVSVAYTNKVPV
ATVRGAGYPQAAFVMERMMDRVAAELGIDPGECRRRNLIGPAKIPYTKPLKSRAGMPLTIDSGDFPALQACAL
QASDYDGFAVRRDAALARGRWRGIAVANSVEPTGRGPFEVARVRVQPSGQVSIYTGALAMGQGIKTTLAQIC
AGHLGVPVAAVEVQAGDTAYVGYGMGGFASRQAIMAGSAVDQAAAQVRRQALETAAAVLKAEAETLELADG
EVRAPDGQSVSLARLAMLRKGVPGYALVSPGDPGLDATAYFHCDAQTYAGASHVCEVEVDPATGAIEIVRYVAA
QDSGRIINPQLAEGQVHGGVVHGIGNALFEWMGYDAAGQPLSTTFAEYLLPTAPEVPPIEVVFQPSPTPLNPL
GVKGVGECATIPVAVAVVGAVEHAVAHCGVRVTEFPLTPVRLLELLTQAEARAAAPTDSGEFVDGVH
>SEQF8251||SEQF8251.1_02329
MNTYQPTAKNGVGQIGRSQSRVEDAALLRGLGRYADDVAVPPGTLHAAIVRSPHAHARITSVDVDAALAMKG
VHGVLTGEDIKRWANPFPVGVRAPMEHWCLAVDKARYVGEPVCVVIAEDRYLAEDALDAVRVEYEPLPPIIDP
EAAAADGAPVLHEAVGSNVVNERHFHYGEPEQAFETAPHRVTIKVHYPRNSCTPIECYVVLGQYLPATGTYDVL
SNFQGPYALHSVMARALNVPGNRLRLRTPPDSGGSFGIKQGVFPYVVLMGLAARKVGAPVKWVEDRLEHLQ
ASSSATNRVCEIQAAVESDGRVTALHYDQIDDCGGYLRAPEPATFYRMHGNLSGAYAIRNLSVRNRVVLTNKVP
SGLNRGFGGGQVYFALERLMHEVATQLGLDPLQVIRRNLVPAGVFPYRAAAGALLDSGDYPATVDLAVREGGL
DELLQRREQARAEGRLYGIGYTAVVEPSISNMGYITTAMTPEERRKSGPKNGAVSTATVSVDPLGGVTVHISSTP
QGQGHQTVVAQIISQVLGVALDSINVNVELDTGKDAWSIASGNYSSRFAGAVAGSVYKAAVRIRERMANIAAS
MWDVPVDQVRFANGKVFVEDGPNQAFHRIAGSTHWSPGLLPESEEGGLRETAFWTPPQLTAPDDNDCVNSS
LCYGFIFDYCGLEVDRVTGEVHIDRYVTCHDAGRILNPMLVDGQIRGGFTQALGVALMEEFAYGEDGSFLSGTFA
DYLVPTAPEAVEPLILHMETPSPFTPLGAKGVGEGNNMSTPVCIANAMADALGRSDIKLPLTPSRVRTMIGIDEP
PPPEGVTLEAPRVAGGSALQAQDSVEIPAPPQAVFDALLDPETLKAIIPGCHALELESENHYRADVTVGVGMIRA
RFAARVGLTDLEPPHSLRLSGSGNSPMGSATGSAKVTFVELENGHTRLDYAYDAAVSGKVAAVGGRMLQSASK
VIIGQIFTRLALRLTGKPVETSLWQRLRALLGLGGAK
>SEQF8259||SEQF8259.1_02703
MNMYQPTATAGVGHIGRSQARVEDTALLRGLGRYADDVATPPGTLHAAIVRSPHAHARIVSVDAEAALAMKG
VHGVLTGDDVKRWANPFPVGVRAPMEHWCLAVDKARYVGEPVCVVIADDRYLAEDALDAVRVEYEPLTPIID
PEAAAEDNAPVLHEAVGSNVVNERCFRYGDPEQAFETAHRQVSIKVHYPRNSCTPIECYVVLGQYLPATGTYDV
LSNFQGPYALHSVMARALNVPGNRLRLRTPPDSGGSFGIKQGVFPYVVLMGLAARKVGAPVKWVEDRLEHLQ
ASSSATNRVCEISAAVQSDGRVVALHYDQIDDCGGYLRAPEPATFYRMHGNLSGAYAIRNLSVRNRVVLTNKVP
SGLNRGFGGGQVYFALERLMHEVAVQLGLDPLQVIRRNLVPAGVFPYRAAAGALLDSGDYPATVDLAVREGGL
DELLRRREQARAEGRLYGVGYTAVVEPSISNMGYITTAMTPDERRKSGPKNGAVSTATVSIDPLGGVTVHVSSTP
QGQGHQTVVAQIVADVLGVALQSINVNVELDTGKDAWSIASGNYSSRFAGAVAGAVYNAAVKIRERMATIAAS
MWNVPADQVRFASGKVFVEDGPNQPFHRIAGSTHWSPGLLPESEGGGLRETAFWTPPQLTAPDDNDCINSSL
CYGFIFDYCALEIDKVTGEVRIDRYVTCHDAGRILNPMLVDGQIRGGFTQALGVALMEEFAYGEDGSFLSGTFAD
YLVPTAPEAVEPLILHMETPSPFTPLGAKGVGEGNNMSTPVCIANAMADALGRSDIKLPLTPSRVRTMIGIDEPP
PPEGVAPETPRVAGGSALQAQDSVEIPAPPQAVYDALLDPETLKAIIPGCHALELESENHYRADVTVGVGMIRA
RFAARVGLTDLEPPHSLRLSGSGNSPMGSATGSAKVTFVELENGHTRLDYAYDAAVSGKVAAVGGRMLQSASK
VIIGQIFARLALRLTGKPVETSLWQRLRALLGGGQ
>SEQF8259||SEQF8259.1_02059

MTSTPDRSSRKGIGASLTRKEDDRFLRGQGNYVGNIRLPGMRDVAFVRSPVAHGRITEIEKPAGQEGDIYTADD
LVGVKPILAASGLPGFKKSVQPVLASEKVRHVGEMIAMCIADTRAEAEDLAGETFVEFDELPAVVDMLAGRDP
DAALLHEHWGDNVFLETHIDADLEDIKARAAIHVHRRLRTARQSMAPMEGRGVVAYWDKRLAQLVVYTSAQ
MPHINRTGLAECLGLDEGSVRVISPDVGGGFGYKGILLPEEVCVAWLAMHLGHPVRWIEDRREQLVANTNCRE
HHYDIHGYADRDGRLLAVDCEAHVDSGAYSAYPFSACLEAAQVASILPGPYKMERYRCRTWSVATNKPPILPYR
GVARTGVCYAIETIMDAVAVEAGLEPYEVRLRNLVQPQEMPYDNITNKHFDSGDYPEAVRRAIRAIDLPAVRQR
QRQGEADGRLIGFGLAVFCEQSAHGTSVYHGWGIPMVPGREPAGLRLTPDGILEVRAGVHSHGQSMETTLAQ
IANEVLGIDPQRVRVVLGDTGATPYSTGTWGSRSIVMAGSAVGQGCKELRDRLLHVGAWLLNEPLDTVRWED
GGVVGSSGRFELRELTHIWYLQPQRLPVDVHVGGLEVHTTYQAQRDSGAFSYACHAVVVAVDPELGKTEILDYA
IVEDGGVLINPMVVDGQVYGGAAQGIGTALYEEMAYSEEGQPLASTLADYILPGATEVPSIRIEHMESPAPYTEF
GQKGIGESGAIGSAAALASAVNDALRPLGAEVTRMPLSPRAVLEALAQRGTVTAATARFEEALV
>SEQF8604||SEQF8604.1_03541
MGASDFSKLPYIGEALKRKEDYRFLTGAGQYTDDVVLAAQCHAVFVRSPYAHAKINSINVDAAKAAPGVLGVFT
GADVAADNINGLPCGWLITSTNGEPMKEPPHPILAQGKVRYVGDHVAMVVAHTQQQARDAAELVEVDYDVL
PAVVNVADAASGAVAGAVVHDIAQDNHCFKWAIGDKGGVDAAFANAAHVTKLDLVNNRLIPNAMEPRAAIG
SYNRASDEYTLYVSNQNPHVERLLMTAFVMGLPEHKVRVIAPDVGGGFGSKIYLYAEDVCLTWASKKLNRNIK
WVADRSEAFLSDAHGRDHVSHAEMAMDKDGKFLALRVHTHANLGAYLSTFASAVPTILYATLLAGQYSTPQVY
VEVDSWFTNTAPVDAYRGAGRPEATYLLERLVTRCAWEMGLSQDEIRRRNFIQTFPYQTPVALQYDTGDFHAC
MDGANKLADVAGFEQRKAASAAKGLLRGIGYSSYIEACGIAPSNIAGALGARAGLFECGEVRVHPTGSVTVFTG
SHSHGQGHETTFAQVVAARLGIPVENVDIVHGDTGRVPFGMGTYGSRSISVGGAAIMKALDKIEAKAKKIAAH
LMEASDADIDFANGEFTVRGTDKKIPFGQVALTAYVPHNYPLDKLEPGLNETAFYDPTNFTFPAGTYICEVEIDPQ
TGVTRVDKFTAVDDFGTIINPMIVEGQVHGGLVQGIGQALLENCVYDRETGQLLTGSFMDYAMPRADDFPEFK
LGHVCTPCTHNPLGTKGCGEAGAIGSPPAVINAVLDALRPLGVKDFDMPASPHRVWEAIQSAKA
>SEQF8605||SEQF8605.1_00364
MGASDFSKLPYIGEALKRKEDYRFLTGAGQYTDDVVLAAQCHAVFVRSPHAHAKINSINIDAAKAAPGVLGVFT
GADVAADNINGLPCGWLITSTNGEPMKEPPHPILAQGKVRYVGDHVAMVVAHTQQQARDAAELVEVDYDVL
PAVVNVADAASGAVAGAVVHDIAQDNHCFKWAIGDKGGVDAAFANAAHVTKLDLVNNRLIPNAMEPRAAIG
SYNRASDEYTLYVSNQNPHVERLLMTAFVMGLPEHKVRVIAPDVGGGFGSKIYLYAEDVCLTWASKKLNRNIK
WVADRSEAFLSDAHGRDHVSHAEMAMDKDGKFLALRVHTHANLGAYLSTFASAVPTILYATLLAGQYSTPQVY
VEVDSWFTNTAPVDAYRGAGRPEATYLLERLVTRCAWEMGLSQDEIRRRNFIQTFPYQTPVALQYDTGDFHAC
MDGANKLADVAAFEQRKAASAAKGLLRGIGYSSYIEACGIAPSNIAGALGARAGLFECGEVRVHPTGSVTVFTG
SHSHGQGHETTFAQVVAARLGIPVENVDIVHGDTGRVPFGMGTYGSRSISVGGAAIMKALDKIEAKAKKIAAH
LMEASDADIDFANGEFTVRGTDKKIPFGQVALTAYVPHNYPLDKLEPGLNETAFYDPTNFTFPAGTYICEVEIDPQ
TGVTRVDKFTAVDDFGTIINPMIVEGQVHGGLVQGIGQALLENCVYDRETGQLLTGSFMDYAMPRADDFPEFK
LGHVCTPCTHNPLGTKGCGEAGAIGSPPAVINAVLDALRPLGVKDFDMPASPHRVWEAIQQAKA
>SEQF8608||SEQF8608.1_03944
MGASDFSKLPHIGEAVLRREDDRFLTGAGQYTDDITLGAQAYAVFVRSPHAHARLRSVNVDAAKAAPGVVEVL
TGADVAAAGINGLPCGWLITSTNGEPMKEPPHPILALDTVRYVGDQVAMVVAETLQQARDAAELVEVDYAVLP
AVVNVADAAGGKKPGAVVHDIAPDNRCYQWAIGDKAAVDAVFAGAAHVTRLDLVNNRLIPNAMEPRVAIGS
YNRAMDEYTLYVANQNPHVERLLMTAFVMGLPESKVRVIAPDVGGGFGSKIFLYAEDVCLTWAAKKLNRNIKW
TADRSESFLTDAHGRDHVSHAEMAMDADGKFLAMRVHTDANLGAYLSTFASAVPTILYATLLAGQYTTPQIHIE
VDAWFTNTSPVDAYRGAGRPEATYLLERLVSRCAWEMNLSQAEIRRRNFITSFPYQTPVALQYDIGDYQACMD
QAEQLADVAGFAARRAASEARGLKRGLGYSSYIEACGLAPSNIAGALGARAGLFECGEVRVHPTGSVTVFTGSH
SHGQGHETTFAQVVAARLGIPVEHVEIVHGDTGRVPFGMGTYGSRSISVGGAAIMKALDKIEAKAKKIAAHLM

EASDADIDFAGGEFTVRGTDRKLPFAQIALTAYVPHNYPLDKLEPGLDETAFYDPTNFTFPAGTYICEVEVDPATG
VVRVDRFSAVDDFGTIINPMIVEGQVHGGLAQGIGQALLENCVYDRDSGQLLTGSFMDYAMPRADDLPDFKL
GTVCTPCTHNPLGTKGCGEAGAIGSPPAVINAVLDALHSLGVKDLDMPASPHRVWEAIDAARP
>SEQF8609||SEQF8609.1_04113
MGASDFSKLPHIGEAVLRREDDRFLTGAGQYTDDITMGAQAYAVFVRSPHAHARLRSVNIDAAKAAVGVIGVLT
GADVAAAGINGLPCGWLITSTNGEPMKEPPHPILALDTVRYVGDQVAMVVAETLQQARDAAELVEVDYEVLP
AVVNVADAAGGKKPGAVVHDIAPDNRCYQWAIGDKAAVDAVFAGAAHVTRLDLVNNRLIPNAMEPRVAIGS
YNRAMDEYTLYVANQNPHVERLLMTAFVMGLPESKVRVIAPDVGGGFGSKIFLYAEDVCLTWAAKKLNRNIKW
TADRSESFLTDAHGRDHVSHAEMAMDADGKFLAMRVHTDANLGAYLSTFASAVPTILYATLLAGQYTTPQIHIE
VDAWFTNTSPVDAYRGAGRPEATYLLERLVSRCAWEMNLSQAEIRRRNFITSFPYQTPVALQYDIGDYKACMD
QAEQLADVAGFAARRAASEAKGLKRGLGYSSYIEACGLAPSNIAGALGARAGLFECGEVRVHPTGSVTVFTGSH
SHGQGHETTFAQVVAARLGIPVEHVEIVHGDTGRVPFGMGTYGSRSISVGGAAIMKALDKIEAKAKKIAAHLM
EASDADIDFAGGEFTVRGTDRKLPFAQIALTAYVPHNYPLDKLEPGLDETAFYDPTNFTFPAGTYICEVEVDPATG
VVRVDRFSAVDDFGTIINPMIVEGQVHGGLAQGIGQALLENCVYDRDSGQLLTGSFMDYAMPRADDLPDFKL
GTVCTPCTHNPLGTKGCGEAGAIGSPPAVINAVLDALHSLGVKDLDMPASPHRVWEAIDAARP
>SEQF8610||SEQF8610.1_04116
MGASDFSKLPHIGEAVLRREDDRFLTGAGQYTDDITLGAQAYAVFVRSPHAHARLRSVNVDAAKAAPGVVEVL
TGADVAAAGINGLPCGWLITSTNGEPMKEPPHPILALDTVRYVGDQVAMVVAETLQQARDAAELVEVDYAVLP
AVVNVADAAGGKKPGAVVHDIAPDNRCYQWAIGDKAAVDAVFAGAAHVTRLDLVNNRLIPNAMEPRVAIGS
YNRAMDEYTLYVANQNPHVERLLMTAFVMGLPESKVRVIAPDVGGGFGSKIFLYAEDVCLTWAAKKLNRNIKW
TADRSESFLTDAHGRDHVSHAEMAMDADGKFLAMRVHTDANLGAYLSTFASAVPTILYATLLAGQYTTPQIHIE
VDAWFTNTSPVDAYRGAGRPEATYLLERLVSRCAWEMNLSQAEIRRRNFITSFPYQTPVALQYDIGDYQACMD
QAEQLADVAGFAARRAASEARGLKRGLGYSSYIEACGLAPSNIAGALGARAGLFECGEVRVHPTGSVTVFTGSH
SHGQGHETTFAQVVAARLGIAVEHVEIVHGDTGRVPFGMGTYGSRSISVGGAAIMKALDKIEAKAKKIAAHLM
EASDADIDFAGGEFTVRGTDRKLPFAQIALTAYVPHNYPLDKLEPGLDETAFYDPTNFTFPAGTYICEVEVDPATG
VVRVDRFSAVDDFGTIINPMIVEGQVHGGLAQGIGQALLENCVYDRDSGQLLTGSFMDYAMPRADDLPDFKL
GTVCTPCTHNPLGTKGCGEAGAIGSPPAVINAVLDALHSLGVKDLDMPASPHRVWEAIDAARP
>SEQF8611||SEQF8611.1_02607
MGASDFSKLPHIGEPLLRREDERFLTGAGQYTDDITLGAQTYAVFVRSPHAHARLRSVNIDAAKAAEGVIGVLTG
ADVAAAGINGLPCGWLITSTNGEPMKEPPHPILALDTVRYVGDQVAMVVAETLQQARDAAELVEVDYEVLPA
VVNVADAAGGKKPGAVVHDIAPDNHCYKWAIGDKAAVDAVFAGAAHVTRLDLVNNRLIPNAMEPRVAIGSY
NRAMDEYTLYVANQNPHVERLLMTAFVMGLPESKVRVIAPDVGGGFGSKIFLYAEDVCLTWAAKKLNRNIKWT
ADRSESFLTDAHGRDHVSHAEMAMDADGKFLAMRVHTDANLGAYLSTFASAVPTILYATLLAGQYTTPQIHIEV
DAWFTNTSPVDAYRGAGRPEATYLLERLVSRCAWEMNLSQAEIRRRNFITSFPYQTPVALQYDIGDYKACMDQ
AEQLADVAGFAARRAASEAKGLRRGLGYSSYIEACGLAPSNIAGALGARAGLFECGEVRVHPTGSVTVFTGSHS
HGQGHETTFAQVVAARLGIPVEHVEIVHGDTGRVPFGMGTYGSRSISVGGAAIMKALDKIEAKAKKIAAHLME
ASDADIDFAGGEFTVRGTDRKLPFAQIALTAYVPHNYPLDKLEPGLDETAFYDPTNFTFPAGTYICEVEVDLATGV
VRVDRFSAVDDFGTIINPMIVEGQVHGGLAQGIGQALLENCVYDRDSGQLLTGSFMDYAMPRADDLPDFKLG
TVCTPCTHNPLGTKGCGEAGAIGSPPAVINAVLDALHSLGVKDLDMPASPHRVWEAIDAARP
>SEQF8612||SEQF8612.1_02256
MGASDFSKLPHIGEPLLRREDERFLTGAGQYTDDITLGAQTYAVFVRSPHAHARLRSVNIDAAKVAEGVIGVLTG
ADVAAAGINGLPCGWLITSTNGEPMKEPPHPILALDTVRYVGDQVAMVVAETLQQARDAAELVEVDYEVLPA
VVNVADAAGGKKSGAVVHDIAPDNHCYKWAIGDKAAVDAVFAGAAHVTRLDLINNRLIPNAMEPRVAIGSYN
RAMDEYTLYVANQNPHVERLLMTAFVMGLPESKVRVIAPDVGGGFGSKIFLYAEDVCLTWAAKKLNRNIKWTA

DRSESFLTDAHGRDHVSHAEMAMDANGKFLAMRVHTDANLGAYLSTFASAVPTILYATLLAGQYTTPQIHIEVD
AWFTNTSPVDAYRGAGRPEATYLLERLVSRCAWEMNLSQAEIRRRNFITSFPYQTPVALQYDIGDYQACMDQA
EQLADVAGFAARRAASEAIGLKRGLGYSSYIEACGLAPSNIAGALGARAGLFECGEVRVHPTGSVTVFTGSHSHG
QGHETTFAQVVAARLGIPVEHVEIVHGDTGRVPFGMGTYGSRSISVGGAAIMKALDKIEAKAKKIAAHLMEAS
DADIDFAGGEFTVRGTDRKLPFAQIALTAYVPHNYPLDKLEPGLDETAFYDPTNFTFPAGTYICEVEVDPATGVVR
VDRFSAVDDFGTIINPMIVEGQVHGGLAQGIGQALLENCVYDRDSGQLLTGSFMDYAMPRADDLPDFKLGTV
CTPCTHNPLGTKGCGEAGAIGSPPAVINAVLDALHSLGVKDLDMPASPHRVWEAIDAARP
>SEQF8613||SEQF8613.1_01479
MGASDFSKLPHIGEPLLRREDERFLTGAGQYTDDITLGAQTYAVFVRSPHAHARLRSVNIDAAKAAEGVIGVLTG
ADVAAAGINGLPCGWLITSTNGEPMKEPPHPILALDTVRYVGDQVAMVVAETLQQARDAAELVEVDYEVLPA
VVNVADAAGGKKPGAVVHDIAPDNHCYKWAIGDKAAVDAVFAGAAHVTRLDLVNNRLIPNAMEPRVAIGSY
NRAMDEYTLYVANQNPHVERLLMTAFVMGLPESKVRVIAPDVGGGFGSKIFLYAEDVCLTWAAKKLNRNIKWT
ADRSESFLTDAHGRDHVSHAEMAMDADGKFLAMRVHTDANLGAYLSTFASAVPTILYATLLAGQYTTPQIHIEV
DAWFTNTSPVDAYRGAGRPEATYLLERLVSRCAWEMNLSQAEIRRRNFITSFPYQTPVALQYDIGDYKACMDQ
AEQLADVAGFAARRAASEAKGLRRGLGYSSYIEACGLAPSNIAGALGARAGLFECGEVRVHPTGSVTVFTGSHS
HGQGHETTFAQVVAARLGIPVEHVEIVHGDTGRVPFGMGTYGSRSISVGGAAIMKALDKIEAKAKKIAAHLME
ASDADIDFAGGEFTVRGTDRKLPFAQIALTAYVPHNYPLDKLEPGLDETAFYDPTNFTFPAGTYICEVEVDLATGV
VRVDRFSAVDDFGTIINPMIVEGQVHGGLAQGIGQALLENCVYDRDSGQLLTGSFMDYAMPRADDLPDFKLG
TVCTPCTHNPLGTKGCGEAGAIGSPPAVINAVLDALHSLGVKDLDMPASPHRVWEAIDAARP
>SEQF8614||SEQF8614.1_04922
MNARMHPELREDLGTQGNEYGSPSARAFGKSMPRKEDERLLRGDGKFVDDVQYAHQLEMAVLRCPFPHARI
RSVDIEAALALPGVRHILTAQSVRDLSDPLTVLRPVPNAPSLPYYALAQDVALYEGQPIVSVVATSRAVAEDALELV
DVDYEPLPHVSDALASLAEGAPVLHPGSMKSNLMARQTDAAGDATARMQEAVHVIEDTMRVNRVTPLPMET
RGIVVSWRPGARELLVNLSTQTPHLVRKQLAESLRLSESCIQVIARDVGGGFGQKLGAFPEDVLACLHAMALRR
PVKWIEDRMEHFRASTHGRESVHRFRLAADKEGNFIGIINDYVTDIGGWNSPFGSAQLSSVVFTGPYKVPDAS
VTREVVLTNKTPVGAYRGYGQPEVNFALEVLVDRMARKMGRDPFDLRLQNLLQPQDLPWTTPSGAVYDSGD
YPRSLRMAAEAVSYESHRTAPRVPRADGRIVGIGFSSFVERTGYASARFLAKRGSQFGAHESVTLRANRSGGVD
VYTGVSSIGQSGETAIAQLCSEALGIAYEHICVHSGDTATSPLNTGAFASRTLIAVAGAVREAAKAFQAKVLRLAA
WTLETSPDELCVDGEVVHFKAGSTQSLPLSQVFTRAIVGQGIPADESPGLEVTSHYEPPDAAFAFGAAAAVVAV
DPITGEFQIERFLIVHDCGVVVNPKVVDGQVRGALVQGLGAALGEELRYDEETGQLVSGSMLDYFVPIAADVPP
IDMLHTEVPSPVTPYGVRGVGEVGTIPPGAAIVNAICDALHDQGVEISSLPITPEGVWRAMERAKAQQSGVDA
>SEQF8614||SEQF8614.1_00184
MGASDFSKLPHIGEAVLRREDDRFLTGAGQYTDDITLGAQAYAVFVRSPHAHARLRSVNVDAAKAAPGVVEVL
TGADVAAAGINGLPCGWLITSTNGEPMKEPPHPILALDTVRYVGDQVAMVVAETLQQARDAAELVEVDYAVLP
AVVNVADAAGGKKPGAVVHDIAPDNRCYQWAIGDKAAVDAVFAGAAHVTRLDLVNNRLIPNAMEPRVAIGS
YNRAMDEYTLYVANQNPHVERLLMTAFVMGLPESKVRVIAPDVGGGFGSKIFLYAEDVCLTWAAKKLNRNIKW
TADRSESFLTDAHGRDHVSHAEMAMDADGKFLAMRVHTDANLGAYLSTFASAVPTILYATLLAGQYTTPQIHIE
VDAWFTNTSPVDAYRGAGRPEATYLLERLVSRCAWEMNLSQAEIRRRNFITSFPYQTPVALQYDIGDYQACMD
QAEQLADVAGFAARRAASEARGLKRGLGYSSYIEACGLAPSNIAGALGARAGLFECGEVRVHPTGSVTVFTGSH
SHGQGHETTFAQVVAARLGIPVEHVEIVHGDTGRVPFGMGTYGSRSISVGGAAIMKALDKIEAKAKKIAAHLM
EASDADIDFAGGEFTVRGTDRKLPFAQIALTAYVPHNYPLDKLEPGLDETAFYDPTNFTFPAGTYICEVEVDPATG
VVRVDRFSAVDDFGTIINPMIVEGQVHGGLAQGIGQALLENCVYDRDSGQLLTGSFMDYAMPRADDLPDFKL
GTVCTPCTHNPLGTKGCGEAGAIGSPPAVINAVLDALHSLGVKDLDMPASPHRVWEAIDAARP
>SEQF8615||SEQF8615.1_03632

MGASDFSKLPHIGEPLLRREDERFLTGAGQYTDDITLGAQTYAVFVRSPHAHARLRSVNIDAAKAAEGVIGVLTG
ADVAAAGINGLPCGWLITSTNGEPMKEPPHPILALDTVRYVGDQVAMVVAETLQQARDAAELVEVDYEVLPA
VVNVADAAGGKKSGAVVHDIAPDNHCYKWAIGDKAAVDAVFAGAAHVTRLDLVNNRLIPNAMEPRVAIGSY
NRAMDEYTLYVANQNPHVERLLMTAFVMGLPESKVRVIAPDVGGGFGSKIFLYAEDVCLTWAAKKLNRNIKWT
ADRSESFLTDAHGRDHVSHAEMAMDANGKFLAMRVHTDANLGAYLSTFASAVPTILYATLLAGQYTTPQIHIEV
DAWFTNTSPVDAYRGAGRPEATYLLERLVSRCAWEMNLSQAEIRRRNFITSFPYQTPVALQYDIGDYQACMDQ
AEQLADVAGFAARRAASEARGLKRGLGYSSYIEACGLAPSNIAGALGARAGLFECGEVRVHPTGSVTVFTGSHS
HGQGHETTFAQVVAARLGIPVEHVEIVHGDTGRVPFGMGTYGSRSISVGGAAIMKALDKIEAKAKKIAAHLME
ASDADIDFAGGEFTVRGTDRKLPFAQIALTAYVPHNYPLDKLEPGLDETAFYDPTNFTFPAGTYICEVEVDPATGV
VRVDRFSAVDDFGTIINPMIVEGQVHGGLAQGIGQALLENCVYDRDSGQLLTGSFMDYAMPRADDLPDFKLG
TVCTPCTHNPLGTKGCGEAGAIGSPPAVINAVLDALHSLGVKDLDMPASPHRVWEAIDAARP
>SEQF8616||SEQF8616.1_04941
MGASDFSKLPHIGEPLLRREDERFLTGAGQYTDDITLGAQTYAVFVRSPHAHARLRSVNIDAAKAAEGVIGVLTG
ADVAAAGINGLPCGWLITSTNGEPMKEPPHPILALDTVRYVGDQVAMVVAETLQQARDAAELVEVDYEVLPA
VVNVADAAGGKKSGAVVHDIAPDNHCYKWAIGDKAAVDAVFAGAAHVTRLDLVNNRLIPNAMEPRVAIGSY
NRAMDEYTLYVANQNPHVERLLMTAFVMGLPESKVRVIAPDVGGGFGSKIFLYAEDVCLTWAAKKLNRNIKWT
ADRSESFLTDAHGRDHVSHAEMAMDANGKFLAMRVHTDANLGAYLSTFASAVPTILYATLLAGQYTTPQIHIEV
DAWFTNTSPVDAYRGAGRPEATYLLERLVSRCAWEMNLSQAEIRRRNFITSFPYQTPVALQYDIGDYQACMDQ
AEQLADVAGFAARRAASEARGLKRGLGYSSYIEACGLAPSNIAGALGARAGLFECGEVRVHPTGSVTVFTGSHS
HGQGHETTFAQVVAARLGIPVEHVEIVHGDTGRVPFGMGTYGSRSISVGGAAIMKALDKIEAKAKKIAAHLME
ASDADIDFAGGEFTVRGTDRKLPFAQIALTAYVPHNYPLDKLEPGLDETAFYDPTNFTFPAGTYICEVEVDPATGV
VRVDRFSAVDDFGTIINPMIVEGQVHGGLAQGIGQALLENCVYDRDSGQLLTGSFMDYAMPRADDLPDFKLG
TVCTPCTHNPLGTKGCGEAGAIGSPPAVINAVLDALHSLGVKDLDMPASPHRVWEAIDAARP
>SEQF8617||SEQF8617.1_03830
MGASDFSKLPHIGEAVLRREDDRFLTGAGQYTDDITLGAQAYAIFVRSPHAHARLRSVNVDAAKAAPGVVGVLT
GADVAAAGINGLPCGWLITSTNGEPMKEPPHPILALDTVRYVGDQVAMVVAETLQQARDAAELVEVDYEVLP
AVVNVADAAGGKKPGAVVHEIAPDNRCYQWAIGDKAAVDAVFAGAAHVTRLDLVNNRLIPNAMEPRVAIGSY
NRAMDEYTLYVANQNPHVERLLMTAFVMGLPESKVRVIAPDVGGGFGSKIFLYAEDVCLTWAAKKLNRNIKWT
ADRSESFLTDAHGRDHVSHAEMAMDADGKFLAMRVHTDANLGAYLSTFASAVPTILYATLLAGQYTTPQIHIEV
DAWFTNTSPVDAYRGAGRPEATYLLERLVSRCAWEMNLSQAEIRRRNFITSFPYQTPVALQYDTGDYKACMDQ
AEQLADVAGFAARRAVSEAKGLKRGLGYSSYIEACGLAPSNIAGALGARAGLFECGEVRVHPTGSVTVFTGSHSH
GQGHETTFAQVVAARLGIPVEHVEIVHGDTGRVPFGMGTYGSRSISVGGAAIMKALDKIEAKAKKIAAHLMEA
SDADIDFAGGEFTVRGTDRKLPFAQIALTAYVPHNYPLDKLEPGLDETAFYDPTNFTFPAGTYICEVEVDPATGVV
RVDRFSAVDDFGTIINPMIVEGQVHGGLAQGIGQALLENCVYDRDSGQLLTGSFMDYAMPRADDLPDFKLGT
VCTPCTHNPLGTKGCGEAGAIGSPPAVINAVLDALHSLGVKDLDMPASPHRVWEAIDAARP
>SEQF8618||SEQF8618.2_03966
MNARMHPELREDLGTQGNEYGSPSARAFGKSMPRKEDERLLRGDGKFVDDVQYAHQLEMAVLRCPFPHARI
RSVDIEAALALPGVRHILTAQSVRDLSDPLTVLRPVPNAPSLPYYALAQDVALYEGQPIVSVVATSRAVAEDALELV
DVDYEPLPHVSDALASLAEGAPVLHPGSMKSNLMARQTDAAGDATARMQEAVHVIEDTMRVNRVTPLPMET
RGIVVSWRPGARELLVNLSTQTPHLVRKQLAESLRLSESCIQVIARDVGGGFGQKLGAFPEDVLACLHAMALRR
PVKWIEDRMEHFRASTHGRESVHRFRLAADKEGNFIGIINDYVTDIGGWNSPFGSAQLSSVVFTGPYKVPDAS
VTREVVLTNKTPVGAYRGYGQPEVNFALEVLVDRMARKMGRDPFDLRLQNLLQPQDLPWTTPSGAVYDSGD
YPRSLRMAAEAVSYESHRTAPRVPRADGRIVGIGFSSFVERTGYASARFLAKRGSQFGAHESVTLRANRSGGVD
VYTGVSSIGQSGETAIAQLCSEALGIAYEHICVHSGDTATSPLNTGAFASRTLIAVAGAVREAAKAFQAKVLRLAA

WTLETSPDELCVDGEVVHFKAGSTQSLPLSQVFTRAIVGQGIPADESPGLEVTSHYEPPDAAFAFGAAAAVVAV
DPITGEFQIERFLIVHDCGVVVNPKVVDGQVRGALVQGLGAALGEELRYDEETGQLVSGSMLDYFVPIAADVPP
IDMLHTEVPSPVTPYGVRGVGEVGTIPPGAAIVNAICDALHDQGVEISSLPITPEGVWRAMERAKAQQSGVDA
>SEQF8618||SEQF8618.2_01044
MGASDFSKLPHIGEAVLRREDDRFLTGAGQYTDDITLGAQAYAVFVRSPHAHARLRSVNVDAAKAAPGVVEVL
TGADVAAAGINGLPCGWLITSTNGEPMKEPPHPILALDTVRYVGDQVAMVVAETLQQARDAAELVEVDYAVLP
AVVNVADAAGGKKPGAVVHDIAPDNRCYQWAIGDKAAVDAVFAGAAHVTRLDLVNNRLIPNAMEPRVAIGS
YNRAMDEYTLYVANQNPHVERLLMTAFVMGLPESKVRVIAPDVGGGFGSKIFLYAEDVCLTWAAKKLNRNIKW
TADRSESFLTDAHGRDHVSHAEMAMDADGKFLAMRVHTDANLGAYLSTFASAVPTILYATLLAGQYTTPQIHIE
VDAWFTNTSPVDAYRGAGRPEATYLLERLVSRCAWEMNLSQAEIRRRNFITSFPYQTPVALQYDIGDYQACMD
QAEQLADVAGFAARRAASEARGLKRGLGYSSYIEACGLAPSNIAGALGARAGLFECGEVRVHPTGSVTVFTGSH
SHGQGHETTFAQVVAARLGIPVEHVEIVHGDTGRVPFGMGTYGSRSISVGGAAIMKALDKIEAKAKKIAAHLM
EASDADIDFAGGEFTVRGTDRKLPFAQIALTAYVPHNYPLDKLEPGLDETAFYDPTNFTFPAGTYICEVEVDPATG
VVRVDRFSAVDDFGTIINPMIVEGQVHGGLAQGIGQALLENCVYDRDSGQLLTGSFMDYAMPRADDLPDFKL
GTVCTPCTHNPLGTKGCGEAGAIGSPPAVINAVLDALHSLGVKDLDMPASPHRVWEAIDAARP
>SEQF8619||SEQF8619.1_02151
MGASDFSKLPHIGEAVLRREDDRFLTGAGQYTDDITLGAQAYAVFVRSPHAHARLRSVNVDAAKAAPGVVGVL
TGADVAAAGINGLPCGWLITSTNGEPMKEPPHPILALDTVRYVGDQVAMVVAETLQQARDAAELVEVDYAVLP
AVVNVADAAGGKKPGAVVHDIAPDNRCYQWAIGDKAAVDAVFAGAAHVTRLDLVNNRLIPNAMEPRVAIGS
YNRAMDEYTLYVANQNPHVERLLMTAFVMGLPESKVRVIAPDVGGGFGSKIFLYAEDVCLTWAAKKLNRNIKW
TADRSESFLTDAHGRDHVSHAEMAMDADGKFLAMRVHTDANLGAYLSTFASAVPTILYATLLAGQYTTPQIHIE
VDAWFTNTSPVDAYRGAGRPEATYLLERLVSRCAWEMNLSQAEIRRRNFITSFPYQTPVALQYDIGDYQACMD
QAEQLADVAGFATRRAASEARGFKRGLGYSSYIEACGLAPSNIAGALGARAGLFECGEVRVHPTGSVTVFTGSH
SHGQGHETTFAQVVAARLGIPVEHVEIVHGDTGRVPFGMGTYGSRSISVGGAAIMKALDKIEAKAKKIAAHLM
EVSDADIDFAGGEFTVRGTDRKLPFAQIALTAYVPHNYPLDKLEPGLDETAFYDPTNFTFPAGTYICEVEVDPATG
VVRVDRFSAVDDFGTIINPMIVEGQVHGGLAQGIGQALLENCVYDRDSGQLLTGSFMDYAMPRADDLPDFKL
GTVCTPCTHNPLGTKGCGEAGAIGSPPAVINAVLDALHSLGVKDLDMPASPHRVWEAIDAARP
>SEQF8620||SEQF8620.1_01184
MGASDFSKLPHIGEAVLRREDDRFLTGAGQYTDDITLGAQAYAIFVRSPHAHARLRSVNVDAAKAAPGVVGVLT
GADVAAAGINGLPCGWLITSTNGEPMKEPPHPILALDTVRYVGDQVAMVVAETLQQARDAAELVEVDYEVLP
AVVNVADAAGGKKPGAVVHEIAPDNRCYQWAIGDKAAVDAVFAGAAHVTRLDLVNNRLIPNAMEPRVAIGSY
NRAMDEYTLYVANQNPHVERLLMTAFVMGLPESKVRVIAPDVGGGFGSKIFLYAEDVCLTWAAKKLNRNIKWT
ADRSESFLTDAHGRDHVSHAEMAMDADGKFLAMRVHTDANLGAYLSTFASAVPTILYATLLAGQYTTPQIHIEV
DAWFTNTSPVDAYRGAGRPEATYLLERLVSRCAWEMNLSQAEIRRRNFITSFPYQTPVALQYDTGDYKACMDQ
AEQLADVAGFAARRAVSEAKGLKRGLGYSSYIEACGLAPSNIAGALGARAGLFECGEVRVHPTGSVTVFTGSHSH
GQGHETTFAQVVAARLGIPVEHVEIVHGDTGRVPFGMGTYGSRSISVGGAAIMKALDKIEAKAKKIAAHLMEA
SDADIDFAGGEFTVRGTDRKLPFAQIALTAYVPHNYPLDKLEPGLDETAFYDPTNFTFPAGTYICEVEVDPATGVV
RVDRFSAVDDFGTIINPMIVEGQVHGGLAQGIGQALLENCVYDRDSGQLLTGSFMDYAMPRADDLPDFKLGT
VCTPCTHNPLGTKGCGEAGAIGSPPAVINAVLDALHSLGVKDLDMPASPHRVWEAIDAARP
>SEQF8621||SEQF8621.1_04082
MGASDFSKLPHIGEPLLRREDERFLTGAGQYTDDITLGAQTYAVFVRSPHAHARLRSVNIDAAKAAEGVIGVLTG
ADVAAAGINGLPCGWLITSTNGEPMKEPPHPILALDTVRYVGDQVAMVVAETLQQARDAAELVEVDYEVLPA
VVNVADAAGGKKPGAVVHDIAPDNHCYKWAIGDKAAVDAVFAGAAHVTRLDLVNNRLIPNAMEPRVAIGSY
NRAMDEYTLYVANQNPHVERLLMTAFVMGLPESKVRVIAPDVGGGFGSKIFLYAEDVCLTWAAKKLNRNIKWT

ADRSESFLTDAHGRDHVSHAEMAMDADGKFLAMRVHTDANLGAYLSTFASAVPTILYATLLAGQYTTPQIHIEV
DAWFTNTSPVDAYRGAGRPEATYLLERLVSRCAWEMNLSQAEIRRRNFITSFPYQTPVALQYDIGDYKACMDQ
AEQLADVAGFAARRAASEAKGLRRGLGYSSYIEACGLAPSNIAGALGARAGLFECGEVRVHPTGSVTVFTGSHS
HGQGHETTFAQVVAARLGIPVEHVEIVHGDTGRVPFGMGTYGSRSISVGGAAIMKALDKIEAKAKKIAAHLME
ASDADIDFAGGEFTVRGTDRKLPFAQIALTAYVPHNYPLDKLEPGLDETAFYDPTNFTFPAGTYICEVEVDLATGV
VRVDRFSAVDDFGTIINPMIVEGQVHGGLAQGIGQALLENCVYDRDSGQLLTGSFMDYAMPRADDLPDFKLG
TVCTPCTHNPLGTKGCGEAGAIGSPPAVINAVLDALHSLGVKDLDMPASPHRVWEAIDAARP
>SEQF8622||SEQF8622.1_03827
MGASDFSKLPHIGEAVLRREDDRFLTGSGQYTDDITLGAQAYAVFVRSPHAHARLRSVNVDAAKAAPGVVGVL
TGADVAAAGINGLPCGWLITSTNGEPMKEPPHPILALDTVRYVGDQVAMVVAETLQQARDAAELVEVDYAVLP
AVVNVADAAGGKKPGAVVHDIAPDNRCYQWAIGDKAAVDAVFAGAAHVTRLDLVNNRLIPNAMEPRVAIGS
YNRAMDEYTLYVANQNPHVERLLMTAFVMGLPESKVRVIAPDVGGGFGSKIFLYAEDVCLTWAAKKLNRNIKW
TADRSESFLTDAHGRDHVSHAEMAMDADGKFLAMRVHTDANLGAYLSTFASAVPTILYATLLAGQYTTPQIHIE
VDAWFTNTSPVDAYRGAGRPEATYLLERLVSRCAWEMNLSQAEIRRRNFITSFPYQTPVALQYDTGDYKACMD
QAEQLADVAGFATRRAASEARGLKRGLGYSSYIEACGLAPSNIAGALGARAGLFECGEVRVHPTGSVTVFTGSHS
HGQGHETTFAQVVAARLGIPVEHVEIVHGDTGRVPFGMGTYGSRSISVGGAAIMKALDKIEAKAKKIAAHLME
ASDADIDFAGGEFTVRGTDRKLPFAQIALTAYVPHNYPLDKLEPGLDETAFYDPSNFTFPAGTYICEVEVDPATGV
VRVDRFSAVDDFGTIINPMIVEGQVHGGLAQGIGQALLENCVYDRDSGQLLTGSFMDYAMPRADDLPDFKLG
TVCTPCTHNPLGTKGCGEAGAIGSPPAVINAVLDALHSLGVKDLDMPASPHRVWEAIDAARP
>SEQF8622||SEQF8622.1_05388
MAKSDVAELKPRLMGVSISRKEDDAFLTGRAQYLADIRLPGMLHAAILRSPHAHAKILSIDTSAARAFPGVHGA
WTGDDIIKLCTGIPGSQQINGFVTTVQPLLAHQIVRYAGEALVIVVAQNRRTAEDALELIDVEYEELPVIANIDAA
MAEGAPLANEGVPGNLVFRHSHSSDDLQNVFAQAAVTVEGEFHNNRVSASPMEPRGYIARHEWTTGQMTL
WSATQMPALVRTMAAILLNFPEQNLEVITPHVGGGFGQKAHLHPEELLVCVLTKELGAPVAWFEDRQENLLSA
THAKHQVNQMALACDANGKFLGIRARGLTDGGAYNSMPWTQLVEAHVGLRVLTGVYKVPRMQDEAIAVTT
NKCSIGAYRGVGFQAPHTAREMLIEKAARKLGISPFEIRRRNVILDQDFPYTTPSGLTIREGTFLKSIDELERMVDY
PRFLERQKEARKQGRYLGLGLSVFNEISGIGTRALSYLNTPTTTHDTATVRMDATGKVTVTTGLVAAGQGHWTT
LAQIAADAFGVSTEDVVVCAGSTNHASSSGTWASRGAVFAAGTIGRAADIVRQRLLQLAAHLLEASVEDLVMD
KGMIHVAGVPAKGMPLAQVAGAVYFAEATHPPGFEPSLEATAAFDPADVVLANGAHAAIVEVDIETGIVKVERV
FAIEDCGQMINPMIVEGQIRGGVGQAIGSVLLEELVHDSNGQLLTTTFMDYLLPTSVDVPDIEIHHLCTPSALVP
GGVKGMGESSMISAPAALACAVNDALAPLGVEFQRFPISPERVLSTLAIKRASGSEHAAA
>SEQF8717||SEQF8717.1_01981
MTTPAAHDDRIGNGGNNGGNNGSKTSGIGARLRRKEDDRFLRGRGDYVANLRMVGMRDVAFVRSPLAHAR
IRGIEKPEGLADAVFTLADLDGVRPIVADSGLAGFKSSAQPVLADAKVRQVGEMIAMCVAPTRAQAEDIAAQV
FVDFDELPAVVDMLAAREPGAPLVHEHWGDNVFLSTAVGTDRDNEAALAAVRASAPIHVVRTLRTARQSMAP
MEGRGVVAWWDRRLAQLVVVTAAQMPHINRTGLAGCLGLDEGQVRVISPDVGGGFGYKGILLPEEVCVAWL
AMRLGRPVRWIEDRREQLTANANCREHAYRIEAWADRDGRLLAVDCEATVDSGCYSSYPFSACLEAAQVGSILP
GPYMMERYRCRTWSVATNKPPILPYRGVARTGVCFAIESVMDAIAVEAGLEPHQVRLRNLVPPERMPYDNITG
KHFDSGDYPECVRRAVAAIDLPGLRARQQRGEPDGRRIGVGFAVFCEQGAHGTSVYHGWGIPMVPGREPAVV
RLTPDGVLEIRAGVHSHGQGMETTLAQIAHEVLGVDTGQVRVLLGDTAITPYSTGTWGSRSIVMAGGAVGQA
CKQLRSRLLRIGAHLLGMPEPAASWQDGAVVAGGSRATLAELAHVWYRQPQKLPADVDPAGLEVMTTYQATR
DTGTFSYACHAVALAVDTELGKIELLDYAIVEDGGVLINPMVVDGQVYGGTAQGIGTALYEEMRYSEDGQPLAS
TLADYLLPGATEVPAIRIDHMETPAPYTEFGQKGIGESGAIGSPAVIVNAVNDALRPLGAELRELPASPRAILAALA
NARAGDAGHSGHARPMPSSPAEVVA

>SEQF8717||SEQF8717.1_02412
MNAPETPRLVGASVKRKEDYRYLTGIGQYTDDIVQPQQSYGYFVRSPYAHARIRSIDTREAMASPGVIGVFTGD
DMAADKVGGLPCGWLIHSIDGSPMKEPPHPVLAQGKVRHVGDQVALVVAETLQQARDAAEKIEVDYEELPAV
VRTADATSATTLVHDDVPANTCYVWGHGDRAATDAAFAKAAHVTTLEIVNNRLVPNAIEPRAVNASYNRQDD
SYTLYVASQNPHVERLLMGAFVLGLPESRLRVIAPDVGGGFGSKIFLYPEDVALTWASKKVKRPIKWTAERSESFL
TDAHGRDHVTRAELALDADGKFLAMRVHTTANMGAYLSTFASSVPTILYATLLAGQYATPAIYAEVTAVFTNTAP
VDAYRGAGRPEATYVVERLVETAARELGIDPAELRRRNFIRSFPYATPVGLTYDTGDYEPCLQRAQELADVAGFPA
RRDEAQRRGKLRGLGYSCYIEACGLAPSNIAGALGARAGLFEVGEIRVHPTGTVTVFTGSHSHGQGHETTFAQV
VADRLGIPLDAVEVVHGDTGRVPFGMGTYGSRSLAVGGSAIVKALDKIEAKARQIAAHLLEASADDIEFSNGVFR
VAGTDRSKTFGEVALSAYVPHNYPLDRLEPGLNENAFYDPTNFTYPSGAYVCEVEVDPDTGETRVVKFTAVDDF
GNIINPMIVEGQVHGGIGQGLGQAMLEQCVYDGDSGQLLTGSYMDYAMPRAADLPDFTVETAQGTPCTHNP
LGVKGCGEAGAIGSPPAFINALIDALSPLGVSDLQMPATPHRVWQAIHAARQGDGALGASPAAGQTADQTAD
QTATAARTI
>SEQF8718||SEQF8718.1_04905
MNAPETPRLVGASVKRKEDYRYLTGIGQYTDDIVQPQQSYGYFVRSPYAHARIRSIDTREAMASPGVIGVFTGE
DMAADKVGGLPCGWLIHSIDGSPMKEPPHPVLAQGKVRHVGDQVALVVAETLQQARDAAEKIEVDYEELPAV
VRTADATSAATLVHDDVPANTCYVWGHGDRAATDAAFAKAAHVTTLEIVNNRLVPNAIEPRAVNASYNRQDD
SYTLYVASQNPHVERLLMGAFVLGLPESRLRVIAPDVGGGFGSKIFLYPEDVALTWASKKVKRPIKWTAERSESFL
TDAHGRDHVTRAELALGADGKFLAMRVHTTANMGAYLSTFASSVPTILYATLLAGQYATPAIYAEVTAVFTNTAP
VDAYRGAGRPEATYVVERLVETAARELGIDPAELRRRNFIRSFPYATPVGLTYDTGDYEPCLRRAQELADVAGFPA
RRDEAQRRGKLRGLGYSCYIEACGLAPSNIAGALGARAGLFEVGEIRVHPTGTVTVFTGSHSHGQGHETTFAQV
VADRLGIPLDAVEVVHGDTGRVPFGMGTYGSRSLAVGGSAIVKALDKIEAKARQIAAHLLEASADDIEFSNGVFR
VAGTDRSKTFGEVALSAYVPHNYPLERLEPGLNENAFYDPTNFTYPSGAYVCEVEVDPDTGETQVVKFTAVDDF
GNIINPMIVEGQVHGGIGQGLGQAMLEQCVYDGDSGQLLTGSYMDYAMPRAADLPDFTVETAQGTPCTHNP
LGVKGCGEAGAIGSPPAFINALIDALSPLGVRDLQMPATPHRVWQAIHAARQGDGALGASPAAGQTADQTATA
ARTI
>SEQF8718||SEQF8718.1_01237
MTTPAAHDDRIGNGGNNGGNNGSKTSGIGARLRRKEDDRFLRGRGDYVANLRMVGMRDVAFVRSPLAHAR
IRGIEKPEGLADAVFTLADLDGVRPIVADSGLAGFKSSAQPVLADAKVRQVGEMIAMCVAPTRAQAEDIAAQV
FVDFDELPAVVDMLAAREPGAPLVHEHWGDNVFLSTAVGTDRDNEAALAAVRASAPIHVVRTLRTARQSMAP
MEGRGVVAWWDRRLAQLVVVTAAQMPHINRTGLAGCLGLDEGQVRVISPDVGGGFGYKGILLPEEVCVAWL
AMRLGRPVRWIEDRREQLTANANCREHVYRIEAWADRDGRLLAVDCEATVDSGCYSSYPFSACLEAAQVGSIL
PGPYMMERYRCRTWSVATNKPPILPYRGVARTGVCFAIESVMDAIAVEAGLEPHQVRLRNLVPPERMPYDNIT
GKHFDSGDYPECVRRAVAAIDLPGLRARQQRGEPDGRRIGVGFAVFCEQGAHGTSVYHGWGIPMVPGREPA
VVRLTPDGVLEIRAGVHSHGQGMETTLAQIAHEVLGVDTGQVRVLLGDTAITPYSTGTWGSRSIVMAGGAVG
QACKQLRSRLLRIGAHLLGMPEPAASWQDGAVVAGGSRATLAELAHVWYRQPQKLPADVDPAGLEVMTTYQ
AARDTGTFSYACHAVALAVDTELGKVELLDYAIVEDGGVLINPMVVDGQVYGGTAQGIGTALYEEMRYSEDGQ
PLASTLADYLLPGATEVPAIRIDHMETPAPYTEFGQKGIGESGAIGSPAAIVNAINDALRPLGAELRELPASPRAILA
ALANARAGDAGHSGHARPMPSSPAEVVA
>SEQF8719||SEQF8719.1_04744
MNAPETQRLVGASVKRKEDYRYLTGIGQYTDDIVQPQQSYGYFVRSPYAHARIRSIDTREAMASPGVIGVFTGE
DMAADKVGGLPCGWLIHSIDGSPMKEPPHPVLAQGKVRHVGDQVALVVAETLQQARDAAEKIEVDYEELPAV
VRTADATSATALVHDDVPANTCYVWGHGDRAATDAAFAKAAHVTTLEIVNNRLVPNAIEPRAVNASYNRMDD
SYTLYVASQNPHVERLLMGAFVLGLPESRLRVIAPDVGGGFGSKIFLYPEDVALTWASKKVKRPIKWTAERSESFL

TDAHGRDHVTRAELALDAGGKFLAMRVHTTANMGAYLSTFASSVPTILYATLLAGQYATPAIYAEVTAVFTNTAP
VDAYRGAGRPEATYVVERLVETAARELGIDPAELRRRNFIRSFPYATPVGLTYDTGDYEPCLQRAQELADVAGFPA
RRDEAQRRGKLRGLGYSCYIEACGLAPSNIAGALGARAGLFEVGEIRVHPTGTVTVFTGSHSHGQGHETTFAQV
VADRLGIPLDAVEVVHGDTGRVPFGMGTYGSRSLAVGGSAIVKALDKIETKARQIAAHLLEASADDIEFSNGVFR
VAGTDRSKTFGEVALSAYVPHNYPLDRLEPGLNENAFYDPTNFTYPSGAYVCEVEVDPDTGETQVVKFTAVDDF
GNIINPMIVEGQVHGGIGQGLGQAMLEQCVYDGDSGQLLTGSYMDYAMPRAADLPDFTVETAQGTPCTHNP
LGVKGCGEAGAIGSPPAFINALIDALSPLGVRDLQMPATPHRVWQAIHAARQGGGTQGASPAAGQAANQTTG
QTATAARTI
>SEQF8720||SEQF8720.1_03314
MNAPETPRLVGASVKRKEDYRYLTGIGQYTDDIVQPQQSYGYFVRSPYAHARIRSIDTREAMASPGVIGVFTGD
DMAADKVGGLPCGWLIHSIDGSPMKEPPHPVLAQGKVRHVGDQVALVVAETLQQARDAAEKIEVDYEELPAV
VRTADATSATTLVHDDVPANTCYVWGHGDRAATDAAFEKAAHVTTLEIVNNRLVPNAIEPRAVNASYNRQDDS
YTLYVASQNPHVERLLMGAFVLGLPESRLRVIAPDVGGGFGSKIFLYPEDVALTWASKKVKRPIKWTAERSESFLT
DAHGRDHVTRAELALDADGKFLAMRVHTTANMGAYLSTFASSVPTILYATLLAGQYATPAIYAEVTAVFTNTAPV
DAYRGAGRPEATYVVERLVETAARELGIDPAELRRRNFIRSFPYATPVGLTYDTGDYEPCLQRAQELADVAGFPAR
RDEAQRRGKLRGLGYSCYIEACGLAPSNIAGALGARAGLFEVGEIRVHPTGTVTVFTGSHSHGQGHETTFAQVV
ADRLGIPLDAVEVVHGDTGRVPFGMGTYGSRSLAVGGSAIVKALDKIEAKARQIAAHLLEASADDIEFSNGVFRV
AGTDRSKTFGEVALSAYVPHNYPLDRLEPGLNENAFYDPTNFTYPSGAYVCEVEVDPDTGETQVVKFTAVDDFG
NIINPMIVEGQVHGGIGQGLGQAMLEQCVYDGDSGQLLTGSYMDYAMPRAADLPDFTVETAQGTPCTHNPL
GVKGCGEAGAIGSPPAFINALIDALSPLGVRDIQMPATPHRVWQAIHAARQGDGAPGASPAATQTANQTATAA
RTI
>SEQF8720||SEQF8720.1_04525
MTTPWANKDSGEDSGKDSGIGARLRRKEDDRFLRGRGDYVANLRMVGMRDVAFVRSPLAHARIRGIEKPDG
LADAVFTLADLDGVRPIVANSGLAGFKSSAQPVLADGKVRQVGEMIAMCVAPTRAQAEDIAAQVFVDFDELPA
VVDMLAARAPGAPLVHDHWGDNVFLETAVGFDADNEAALAAIRASAPIHVARTLRTARQSMAPMEGRGVVA
WWDRRLAQLVVVTAAQMPHINRTGLAGCLGLDEGQVRVISPDVGGGFGYKGILLPEEVCVAWLAMRLGRPV
RWIEDRREQLTANANCREHAYRIEAWADRDGRLLAVDCEATVDSGCYSSYPFSACLEAAQVGSILPGPYMMER
YRCRTWSVATNKPPILPYRGVARTGVCFAIESVMDAIAVEAGLEPHQVRLRNLVPPERMPFDNITGKHFDSGDY
PECVRRAVAAIDLPGLRARQQRGEPDGRRIGVGFAVFCEQGAHGTSVYHGWGIPMVPGREPAVVRLTPDGVL
EVRAGVHSHGQSMETTLAQIAHEVLGVDTDQVRVLLGDTAITPYSTGTWGSRSIVMAGGAVGQACKQLRSRL
LRIGAHLLGMPEPAVRWQDGAVVGDGGRVTLAELARVWYLQPQKLPADVDPAGLEVMTTYQATRDTGTFSY
ACHAVAVAVDTELGKVELLDYAIVEDGGVLINPMVVDGQVYGGAAQGIGTALYEEMRYSEDGQPLASTLADYLL
PGATEVPAIRIDHMETPAPYTEFGQKGIGESGAIGSPAAIVNAINDALRPLGAELRELPASPRAILAALETARAAPA
SQSSAMPSSAEEVAA
>SEQF8721||SEQF8721.1_02776
MNAPETPRLVGASVKRKEDYRYLTGIGQYTDDIVQPQQSYGYFVRSPYAHARIRSIDTREAMASPGVIGVFTGD
DMAADKVGGLPCGWLIHSIDGSPMKEPPHPVLAQGKVRHVGDQVALVVAETLQQARDAAEKIEVDYEELPAV
VRTADATSATTLVHDDVPANTCYVWGHGDRAATDAAFEKAAHVTTLEIVNNRLVPNAIEPRAVNASYNRQDDS
YTLYVASQNPHVERLLMGAFVLGLPESRLRVIAPDVGGGFGSKIFLYPEDVALTWAAKKVKRPIKWTAERSESFLT
DAHGRDHVTRAELALDADGKFLAMRVHTTANMGAYLSTFASSVPTILYATLLAGQYATPAIYAEVTAVFTNTAPV
DAYRGAGRPEATYVVERLVETAARELGIDPAELRRRNFIRSFPYATPVGLTYDTGDYEPCLQRAQELADVAGFPAR
RDEAQRRGKLRGLGYSCYIEACGLAPSNIAGALGARAGLFEVGEIRVHPTGTVTVFTGSHSHGQGHETTFAQVV
ADRLGIPLDAVEVVHGDTGRVPFGMGTYGSRSLAVGGSAIVKALDKIEAKAKQIAAHLLEASADDIEFGNGVFR
VAGTDRSKTFGEVALSAYVPHNYPLDRLEPGLNENAFYDPTNFTYPSGAYVCEVEVDPDTGETQVVKFTAVDDF

GNIINPMIVEGQVHGGIGQGLGQAMLEQCVYDGDSGQLLTGSYMDYAMPRAADLPDFTVETAQGTPCTHNP
LGVKGCGEAGAIGSPPAFINALIDALSPLGVRDLQMPATPHRVWQAIHAAGQNAGTQAGTQPTQARTI
>SEQF8721||SEQF8721.1_01165
MTTPWSSEENGKDSGKGRDKDSGIGARLRRKEDDRFLRGRGDYVANLRMVGMRDVAFVRSPLAHGRIRGIE
KPDGLADAVFTLADLDGVRPIVANSGLAGFKPSAQPVLADGKVRQVGEMIAMCVAPTRAQAEDIAAQVFVDF
DELPAVVDMLAAREPGAPLVHDHWGDNVFLETAVGFDADNEAALAAIRASAPIHVARTLRTARQSMAPMEG
RGVVAWWDRRLAQLVVVTAAQMPHINRTGLAGCLGLDEGQVRVISPDVGGGFGYKGILLPEEVCVAWLAMR
LGRPVRWIEDRREQLTANANCREHAYRIDAWADRDGRLLAVDCEATVDSGCYSSYPFSACLEAAQVGSILPGPY
MMARYRCRTWSVATNKPPILPYRGVARTGVCFAIESVMDAIAVQAGLEPYEVRLRNLVPPERMPFDNITGKHF
DSGDYPECVRRAVAAIDLPGLRARQQRGEPDGRRIGVGLAVFCEQGAHGTSVYHGWGIPMVPGREPAVVRLT
PDGVLEVRAGVHSHGQSMETTLAQIAHEVLGVDTDQVRVLLGDTAITPYSTGTWGSRSIVMAGGAVGQACK
QLRSRLLRIGAHLLGMPEPAVRWQDGAVVADGGGRVTLAELARVWYLQPQKLPADADPAGLEVMTTYQATR
DTGTFSYACHAVAVAVDTELGKVELLDYAIVEDGGVLINPMVVDGQVYGGAAQGIGTALYEEMRYSDDGQPLA
STLADYLLPGATEVPAIRIDHMETPAPYTEFGQKGIGESGAIGSPAAIVNAINDALRPLGAELRELPASPRAILAALE
TARAAQAGQSSPMPSSAEEVAA
>SEQF8722||SEQF8722.1_00122
MNAPETPRLVGASVKRKEDYRYLTGIGQYTDDIVQPQQSYGYFVRSPYAHARIRSIDTREAMASPGVIGVFTGE
DMAADKVGGLPCGWLIHSIDGSPMKEPPHPVLAQGKVRHVGDQVALVVAETLQQARDAAEKIEVDYEELPAV
VRTADAASATTLVHDDVPANTCYVWGHGDRAATDAAFAKAAHVTTLEIVNNRLVPNAIEPRAVNASYNRQDD
SYTLYVASQNPHVERLLMGAFVLGLPESRLRVIAPDVGGGFGSKIFLYPEDVALTWASKKVKRPIKWTAERSESFL
TDAHGRDHVTRAELALDGDGKFLAMRVHTTANMGAYLSTFASSVPTILYATLLAGQYATPAIYAEVTAVFTNTAP
VDAYRGAGRPEATYVVERLVETAARELGIDPAELRRRNFIRSFPYATPVGLTYDTGDYEPCLQRAQELADVAGFPA
RRDEAQRRGKLRGLGYSCYIEACGLAPSNIAGALGARAGLFEVGEIRVHPTGTVTVFTGSHSHGQGHETTFAQV
VADRLGIPLDAVEVVHGDTGRVPFGMGTYGSRSLAVGGSAIVKALDKIEAKARQIAAHLLEASADDIEFSNGVFR
VAGTDRSKTFGEVALSAYVPHNYPLERLEPGLNENAFYDPTNFTYPSGAYVCEVEVDPDTGETQVVKFTAVDDF
GNIINPMIVEGQVHGGIGQGLGQAMLEQCVYDGDSGQLLTGSYMDYAMPRAADLPDFTVETAQGTPCTHNP
LGVKGCGEAGAIGSPPAFINALIDALSPLGVRDLQMPATPHRVWQAIHAAGQAAGNGATPARTI
>SEQF8723||SEQF8723.1_04939
MNAPETPRLVGASVKRKEDYRYLTGIGQYTDDIVQPQQSYGYFVRSPYAHARIRSIDTREAMASPGVIGVFTGD
DMAADKVGGLPCGWLIHSIDGSPMKEPPHPVLAQGKVRHVGDQVALVVAETLQQARDAAEKIEVDYEELPAV
VRTADATSATTLVHDDVPANTCYVWGHGDRAATDAAFEKAAHVTTLEIVNNRLVPNAIEPRAVNASYNRQDDS
YTLYVASQNPHVERLLMGAFVLGLPESRLRVIAPDVGGGFGSKIFLYPEDVALTWASKKVKRPIKWTAERSESFLT
DAHGRDHVTRAELALDADGKFLAMRVHTTANMGAYLSTFASSVPTILYATLLAGQYATPAIYAEVTAVFTNTAPV
DAYRGAGRPEATYVVERLVETAARELGIDPAELRRRNFIRSFPYATPVGLTYDTGDYEPCLQRAQELADVAGFPAR
RDEAQRRGKLRGLGYSCYIEACGLAPSNIAGALGARAGLFEVGEIRVHPTGTVTVFTGSHSHGQGHETTFAQVV
ADRLGIPLDAVEVVHGDTGRVPFGMGTYGSRSLAVGGSAIVKALDKIEAKARQIAAHLLEASADDIEFSNGVFRV
AGTDRSKTFGEVALSAYVPHNYPLDRLEPGLNENAFYDPTNFTYPSGAYVCEVEVDPDTGETQVVKFTAVDDFG
NIINPMIVEGQVHGGIGQGLGQAMLEQCVYDGDSGQLLTGSYMDYAMPRAADLPDFTVETAQGTPCTHNPL
GVKGCGEAGAIGSPPAFINALIDALSPLGVRDLQMPATPHRVWQAIHAARQAGGTQTATQATAARTI
>SEQF8723||SEQF8723.1_04751
MTTPWANKDSGKDSGKGRDNCRDKDSGIGARLRRKEDDRFLRGRGDYVANLRMVGMRDVAFVRSPLAHAR
IRGIEKPDGLADAVFTLADLDGVRPIVANSGLAGFKSSAQPVLADGKVRQVGEMIAMCVAPTRAQAEDIAAQV
FVDFDELPAVVDMLAARAPGAPLVHDHWGDNVFLETAVGFDADNEAALAAIRASAPIHVARTLRTARQSMAP
MEGRGVVAWWDRRLAQLVVVTAAQMPHINRTGLAGCLGLDEGQVRVISPDVGGGFGYKGILLPEEVCVAWL

AMRLGRPVRWIEDRREQLTANANCREHAYRIEAWADRDGRLLAVDCEATVDSGCYSSYPFSACLEAAQVGSILP
GPYMMERYRCRTWSVATNKPPILPYRGVARTGVCFAIESVMDAIAVEAGLEPHQVRLRNLVPPERMPFDNITG
KHFDSGDYPECVRRAVAAIDLPGLRARQQRGEPDGRRIGVGLAVFCEQGAHGTSVYHGWGIPMVPGREPAVV
RLTPDGVLEVRAGVHSHGQSMETTLAQIAHEVLGVDTDQVRVLLGDTAITPYSTGTWGSRSIVMAGGAVGQA
CKQLRSRLLRIGAHLLGMPEPAVRWQDGAVVGDGGRVTLAELARVWYLQPQKLPADVDPAGLEVMTTYQAT
RDTGTFSYACHAVAVAVDTELGKVELLDYAIVEDGGVLINPMVVDGQVYGGAAQGIGTALYEEMRYSEDGQPL
ASTLADYLLPGATEVPAIRIDHMETPAPYTEFGQKGIGESGAIGSPAAIVNAINDALRPLGAELRELPASPRAILAAL
ETARAAPASQSSAMPSSAEEVTA
>SEQF8723||SEQF8723.1_04932
MNAPETPRLVGASVKRKEDYRYLTGIGQYTDDIVQPQQSYGYFVRSPYAHARIRSIDTREAMASPGVIGVFTGD
DMAADKVGGLPCGWLIHSIDGSPMKEPPHPVLAQGKVRHVGDQVALVVAETLQQARDAAEKIEVDYEELPAV
VRTADATSATTLVHDDVPANTCYVWGHGDRAATDAAFEKAAHVTTLEIVNNRLVPNAIEPRAVNASYNRQDDS
YTLYVASQNPHVERLLMGAFVLGLPESRLRVIAPDVGGGFGSKIFLYPEDVALTWASKKVKRPIKWTAERSESFLT
DAHGRDHVTRAELALDADGKFLAMRVHTTANMGAYLSTFASSVPTILYATLLAGQYATPAIYAEVTAVFTNTAPV
DAYRGAGRPEATYVVERLVETAARELGIDPAELRRRNFIRSFPYATPVGLTYDTGDYEPCLQRAQELADVAGFPAR
RDEAQRRGKLRGLGYSCYIEACGLAPSNIAGALGARAGLFEVGEIRVHPTGTVTVFTGSHSHGQGHETTFAQVV
ADRLGIPLDAVEVVHGDTGRVPFGMGTYGSRSLAVGGSAIVKALDKIEAKARQIAAHLLEASADDIEFSNGVFRV
AGTDRSKTFGEVALSAYVPHNYPLDRLEPGLNENAFYDPTNFTYPSGAYVCEVEVDPDTGETQVVKFTAVDDFG
NIINPMIVEGQVHGGIGQGLGQAMLEQCVYDGDSGQLLTGSYMDYAMPRAADLPDFTVETAQGTPCTHNPL
GVKGCGEAGAIGSPPAFINALIDALSPLGVRDLQMPATPHRVWQAIHAARQAGGTQTATQATAARTI
>SEQF8724||SEQF8724.1_01121
MNAPETPRLVGASVKRKEDYRYLTGIGQYTDDIVQPQQSYGYFVRSPYAHARIRSIDTREAMASPGVIGVFTGD
DMAADKVGGLPCGWLIHSIDGSPMKEPPHPVLAQGKVRHVGDQVALVVAETLQQARDAAEKIEVDYEELPAV
VRTADATSAATLVHDDVPANTCYVWGHGDRAATDAAFAKAAHVTTLEIVNNRLVPNAIEPRAVNASYNRQDD
SYTLYVASQNPHVERLLMGAFVLGLPESRLRVIAPDVGGGFGSKIFLYPEDVALTWASKKVKRPIKWTAERSESFL
TDAHGRDHVTRAELALDADGKFLAMRVHTTANMGAYLSTFASSVPTILYATLLAGQYATPAIYAEVTAVFTNTAP
VDAYRGAGRPEATYVVERLVETAARELGIDPAELRRRNFIRSFPYATPVGLTYDTGDYEPCLQRAQELADVAGFPA
RRDEAQRRGKLRGLGYSCYIEACGLAPSNIAGALGARAGLFEVGEIRVHPTGTVTVFTGSHSHGQGHETTFAQV
VADRLGIPLDAVEVVHGDTGRVPFGMGTYGSRSLAVGGSAIVKALDKIEAKARQIAAHLLEASADDIEFSNGVFR
VAGTDRSKTFGEVALSAYVPHNYPLDRLEPGLNENAFYDPTNFTYPSGAYVCEVEVDPDTGETRVVKFTAVDDF
GNIINPMIVEGQVHGGIGQGLGQAMLEQCVYDGDSGQLLTGSYMDYAMPRAADLPDFTVETAQGTPCTHNP
LGVKGCGEAGAIGSPPAFINALIDALSPLGVSDLQMPATPHRVWQAIHAARQGDGALGASPAAGQTADQTATA
ARTI
>SEQF8724||SEQF8724.1_02238
MTTPAAHDDRIGNGGNNGGNNGSKTSGIGARLRRKEDDRFLRGRGDYVANLRMVGMRDVAFVRSPLAHAR
IRGIEKPEGLADAVFTLADLDGVRPIVADSGLAGFKSSAQPVLADAKVRQVGEMIAMCVAPTRAQAEDIAAQV
FVDFDELPAVVDMLAAREPGAPLVHEHWGDNVFLSTAVGTDRDNEAALAAVRASAPIHVVRTLRTARQSMAP
MEGRGVVAWWDRRLAQLVVVTAAQMPHINRTGLAGCLGLDEGQVRVISPDVGGGFGYKGILLPEEVCVAWL
AMRLGRPVRWIEDRREQLTANANCREHVYRIEAWADRDGRLLAVDCEATVDSGCYSSYPFSACLEAAQVGSIL
PGPYMMERYRCRTWSVATNKPPILPYRGVARTGVCFAIESVMDAIAVEAGLEPHQVRLRNLVPPERMPYDNIT
GKHFDSGDYPECVRRAVAAIDLPGLRARQQRGEPDGRRIGVGFAVFCEQGAHGTSVYHGWGIPMVPGREPA
VVRLTPDGVLEIRAGVHSHGQGMETTLAQIAHEVLGVDTGQVRVLLGDTAITPYSTGTWGSRSIVMAGGAVG
QACKQLRSRLLRIGAHLLGMPEPAASWQDGAVVAGGSRATLAELAHVWYRQPQKLPADVDPAGLEVMTTYQ
ATRDTGTFSYACHAVALAVDTELGKVELLDYAIVEDGGVLINPMVVDGQVYGGTAQGIGTALYEEMRYSEDGQP

LASTLADYLLPGATEVPAIRIDHMETPAPYTEFGQKGIGESGAIGSPAAIVNAINDALRPLGAELRELPASPRAILAA
LANARAGDAGHSGHARPMPSSPAEVVA
>SEQF8725||SEQF8725.1_02146
MTTPAAHDDRIGNGGNNGGNNGSKTSGIGARLRRKEDDRFLRGRGDYVANLRMVGMRDVAFVRSPLAHAR
IRGIEKPEGLADAVFTLADLDGVRPIVADSGLAGFKSSAQPVLADAKVRQVGEMIAMCVAPTRAQAEDIAAQV
FVDFDELPAVVDMLAAREPGAPLVHEHWGDNVFLSTAVGTDRDNEAALAAVRASAPIHVVRTLRTARQSMAP
MEGRGVVAWWDRRLAQLVVVTAAQMPHINRTGLAGCLGLDEGQVRVISPDVGGGFGYKGILLPEEVCVAWL
AMRLGRPVRWIEDRREQLTANANCREHVYRIEAWADRDGRLLAVDCEATVDSGCYSSYPFSACLEAAQVGSIL
PGPYMMERYRCRTWSVATNKPPILPYRGVARTGVCFAIESVMDAIAVEAGLEPHQVRLRNLVPPERMPYDNIT
GKHFDSGDYPECVRRAVAAIDLPGLRARQQRGEPDGRRIGVGFAVFCEQGAHGTSVYHGWGIPMVPGREPA
VVRLTPDGVLEIRAGVHSHGQGMETTLAQIAHEVLGVDTGQVRVLLGDTAITPYSTGTWGSRSIVMAGGAVG
QACKQLRSRLLRIGAHLLGMPEPAASWQDGAVVAGGSRATLAELAHVWYRQPQKLPADVDPAGLEVMTTYQ
ATRDTGTFSYACHAVALAVDTELGKVELLDYAIVEDGGVLINPMVVDGQVYGGTAQGIGTALYEEMRYSEDGQP
LASTLADYLLPGATEVPAIRIDHMETPAPYTEFGQKGIGESGAIGSPAAIVNAINDALRPLGAELRELPASPRAILAA
LANARAGDAGHSGHARPMPSSPAEVVA
>SEQF8725||SEQF8725.1_04807
MNAPETPRLVGASVKRKEDYRYLTGIGQYTDDIVQPQQSYGYFVRSPYAHARIRSIDTREAMASPGVIGVFTGD
DMAADKVGGLPCGWLIHSIDGSPMKEPPHPVLAQGKVRHVGDQVALVVAETLQQARDAAEKIEVDYEELPAV
VRTADATSAATLVHDDVPANTCYVWGHGDRAATDAAFAKAAHVTTLEIVNNRLVPNAIEPRAVNASYNRQDD
SYTLYVASQNPHVERLLMGAFVLGLPESRLRVIAPDVGGGFGSKIFLYPEDVALTWASKKVKRPIKWTAERSESFL
TDAHGRDHVTRAELALDADGKFLAMRVHTTANMGAYLSTFASSVPTILYATLLAGQYATPAIYAEVTAVFTNTAP
VDAYRGAGRPEATYVVERLVETAARELGIDPAELRRRNFIRSFPYATPVGLTYDTGDYEPCLQRAQELADVAGFPA
RRDEAQRRGKLRGLGYSCYIEACGLAPSNIAGALGARAGLFEVGEIRVHPTGTVTVFTGSHSHGQGHETTFAQV
VADRLGIPLDAVEVVHGDTGRVPFGMGTYGSRSLAVGGSAIVKALDKIEAKARQIAAHLLEASADDIEFSNGVFR
VAGTDRSKTFGEVALSAYVPHNYPLDRLEPGLNENAFYDPTNFTYPSGAYVCEVEVDPDTGETRVVKFTAVDDF
GNIINPMIVEGQVHGGIGQGLGQAMLEQCVYDGDSGQLLTGSYMDYAMPRAADLPDFTVETAQGTPCTHNP
LGVKGCGEAGAIGSPPAFINALIDALSPLGVSDLQMPATPHRVWQAIHAARQGDGALGASPAAGQTADQTATA
ARTI
>SEQF8732||SEQF8732.1_05343
MSKFGIGQNVKRVEDVRFITGRGRYVDDIHLSRECIGHVVLSPMAHAKILSIDVSEALKVKGVIAVLTGQDVEAD
GLGPMIPNFMPQDFGLSKEGYRTQRPILVADRVRYVGDRVAFVVAETLEAAQEAAEVMEIEYEPLAAVPNKMS
SLETGAPAVWDACQDNVCFTIEMGNEAKTEAVFNQAVHRVAVSLKNNRVAANPLEPRAAIGCYDASDDSYTLY
TTSQSPHAVREQLAENVFHISQGRLRVISPDVGGGFGVKSDSYPEDALVLWASKRCGRPVRWLLTRSDMLVGD
FHARDEVVEGELALDGNGKILGMRAHSVHWLGAYVAPTGAIAPWFTIRYSPGVYDIQNLWITAKGAFSNTAPT
HVYRGPGRAEGNFLIERLMDEAAAVIGIRQDEIRRINAISSDAMPYVTPTGSTYDSGEFLTLMARCQKLADWTG
YEERRAASQKRGMLRGRSVIFYIEHAGIFNDRMELRFDPSGSVTIVSGLHSHGQGHATTFAQMVSEWLGVPFD
SIRFLQGDTDKVAFGRGTYSARSSLLASVALKSASGKIVEKARVRAAEFLEVEPEDVDFEDGVFKTRGSNKSLLISD
VAKSLFQPVHLDEKHGLGLEAIGVASSDIPNYPNGCHICEVDIDPETGRILIDRYSVVDDVGHAINPMICEGQIVG
GIAQGLGQAMLEEIVYDEEGQILTGSFMDYAMPRASDMSHIMAELAEFPCKSNPLGVKGVGESGTIGAPPAIA
NAVIDALRGVGVTNIDMPFTPHKVWHAIRAAKAS
>SEQF8742||SEQF8742.1_05619
MSGHDHFVELRPKLVGKRVKRTEDPRFLAGVGQYVDDMNPSGTLHVALRRSDQPHARIIGIDIGEAFSVPGVV
AIYDASDLESDIKPAIPTSRMPGYYATPIWPLARGKVRYVGEPVIAVVAESRYAAEDALEHITIEYEALPFAIRQVDA
VKDDAPLLHEEAATNTIIRREFKRGDVDAAFEGAAVIVKGRFQMTRKTAVAMENRSYLAEWGERKQSLTLYTSS

NIPGVIRDVLAGCLDLPGSRLRVVAPDVGGSFGGKGSLYGEEILVCALSRKLKRPVKFISDRLEDLSATSQAFDELIE
AELAVTEDGMLIGLRADVIGDVGAYSIYPWTAALETVQVVSFLPGPYRMEHYRGRIRGVLTPKPPTGPYRGVGR
PSSTFAMERLIEMAARKLGMDPVEFRRRNLVNAEEFPYRTASGIIWDKSAFQECLQGACEHVDYPNLVRERDE
ARKAGRWVGIGLASYAELTGIGSRISVAPGMPINTGTETSKIEIDATGSITAAFGVSSHGQGLETTLAQVIVDELGC
KLEDIDVKHGDSSLVPMSSGTYASRSAVLGGGAATLAARAVKAKVLRAAAYLMEQATEDLEIHDGIVSSRNSNLT
MTLKEVASAVYTQMGRIPRDQREDLSASETYDPYLGTACSSTHLAMVEVDTETYGVKILRYVVAEDCGRIINPMI
VDGQVQGAVAQGIGAALLEEIVHDDQGQAVTASLADYLVPVATSVPNIGIVHIEADLPNTIGGFRGMGEGGTI
GAPAAIANAVSDALAHLGVTVETLPVTPERIFQMIREKRQTSKEATDAP
>SEQF8754||SEQF8754.1_00902
MYADVKKNTHSTQGWVGRSLERHEDAALLMGQGRYVDDFPIAPNTLHAAVVRASHAHALITEIDTTRALAVP
GVRHVITGEQIRAISDPFLTVLKQPIDQWALAVERVRYVGEAVAVVLADDRYIAEDGAALVDILYEPLPVVVDPVA
AVRSDAPLLHPAAGSNQLSVREFVYGEPDAAFARAERTISVDIEYPRSSYTPMECFAVVAEYRPGDKSFDVLANF
QGPFSAHPVMAKALRVPGSRLRLRIPPDSGGSFGIKLSVFPYIVLMGLAARISGRPVKWIEDRLEHLQAANSGP
NRITRLDAAVEPDGRVTALRFDNLEDYGAYLRAPMPGPLYRMHGATTGAYDIRHLAMVNRVVVTNKMPAALV
RGFGGPQLYLALERLMQRIAVELGLDPLEVIRRNLISADAFPYRTPAGALYDSGDYQKAIEIATGDGRLTDLLRRR
DEARAAGKLYGIGYAAVVEPGMSNMGYLSTILPEKIRQQQGPKNGAVSMVRVNVDAVGGVSMTADVTVQG
QGHATALSQIVADQLGLHPRDISVNLEIDTQKDAWSIAAGSYSCRFSPGTAVAAHLAGKQVRDKVARIAAGLFET
TEDDVGFGGGRVFSIAQPAKSMPFGRAAGTAHWSPVLVPDTDQPGISETGIWAPQELAPPTGGDEINTSLTYG
FVFDMCGIEIDPLTMQVKVDRYVSMHDAGKLINPLLAEGQIRGAFVQGLATALYEEIVYDDKTGALQTGTFAEYL
VPTACEVPQIEIIHMETPSPFTPLGAKGLAEGNCMSTPVCIANAVADALSLRDVRLPITPNRLNAVLATNEGPAPT
LTSNVVDAAAMEQEFAEATIAGSGTLTVATEAGHIWDTLLDPQTLKNIIPGCHTVTALRPDHFQAEVSLGVGPVR
GRFIADVYLSDLVPQRQAKLSGELRGPLGKAEGKGRIILSLTPDGTRIDYAYGVDVGGKVASVGGRMIRRAADMI
IGQFFERLVALSGDGDATPTKKTRSGLMAWLVGLFGGQK
>SEQF8755||SEQF8755.1_02439
MYADVKKNTHSTQGWVGRSLERHEDAALLMGQGRYVDDFPIAPNTLHAAVVRASHAHALITAIDTTRALAVP
GVRHVITGEQIRAISDPFLTVLKQPIDQWALAVERVRYVGEAVAVVLADDRYIAEDGAALVDILYEPLPVVVDPVA
AVRSDAPLLHPAAGSNQLSVREFVYGEPDAAFARAERTISVDIEYPRSSYTPMECFAVVAEYRPGDKSFDVLANF
QGPFSAHPVMAKALRVPGSRLRLRIPPDSGGSFGIKLSVFPYIVLMGLAARISGRPVKWIEDRLEHLQAANSGP
NRITRLDAAVEPDGRVTALRFDNLEDYGAYLRAPMPGPLYRMHGATTGAYDIRHLAMVNRVVVTNKMPAALV
RGFGGPQLYLALERLMQRIAVELGLDPLEVIRRNLISVDAFPYRTPAGALYDSGDYQKAIEIATGDGRLTDLLRRRD
EARAAGKLYGIGYAAVVEPGMSNMGYLSTILPEEIRQQQGPKNGAVSMVRVNVDAVGGVSMTADVTVQGQ
GHATALSQIVADQLGLHPRDISVNLEIDTQKDAWSIAAGSYSCRFSPGTAVAAHLAGKQVRDKVARIAAGLFKTT
EDDVGFGGGRVFSIAQPAKSMPFGRAAGTAHWSPVLVPDTDQPGISETGIWAPQELAPPTGGDEINTSLTYGF
VFDMCGIEIDPLTMQVKVDRYVSMHDAGKLINPLLAEGQIRGAFVQGLATALYEEIVYDDKTGALQTGTFAEYLV
PTACEVPQIEIIHMETPSPFTPLGAKGLAEGNCMSTPVCIANAVADALSLRDVRLPITPNRLNAVLATNEGPAPTL
TSNVVDAAAMEKEFAEATIAGSGTLTVATEAGNIWDTLLNPQTLKNIIPGCHTVTALRPDHFQAEVSLGVGPVR
GRFIADVYLSDLVPQRQAKLSGELRGPLGKAEGKGRIILSLTPDGTRIDYAYGVDVGGKVASVGGRMIRRAADMI
IGQFFERLVALSGDGDATPTKKTRSGLMAWLVGLFGGQK
>SEQF8758||SEQF8758.1_02534
MYADVKKNTHSTQGWVGRSLERHEDAALLMGQGRYVDDFPIAPNTLHAAVVRASHAHALITAIDTTRALAVP
GVRHVITGEQIRAISDPFLTVLKQPIDQWALAVERVRYVGEAVAVVLADDRYIAEDGAALVDILYEPLPVVVDPVA
AVRSDAPLLHPAAGSNQLSVREFVYGEPDAAFARAERTISVDIEYPRSSYTPMECFAVVAEYRPGDKSFDVLANF
QGPFSAHPVMAKALRVPGSRLRLRIPPDSGGSFGIKLSVFPYIVLMGLAARISGRPVKWIEDRLEHLQAANSGP
NRITRLDAAVEPDGRVTALRFDNLEDYGAYLRAPMPGPLYRMHGATTGAYDIRHLAMVNRVVVTNKMPAALV

RGFGGPQLYLALERLMQRIAVELGLDPLEVIRRNLISVDAFPYRTPAGALYDSGDYQKAIEIATGDGRLTDLLRRRD
EARAAGKLYGIGYAAVVEPGMSNMGYLSTILPEEIRQQQGPKNGAVSMVRVNVDAVGGVSMTADVTVQGQ
GHATALSQIVADQLGLHPRDISVNLEIDTQKDAWSIAAGSYSCRFSPGTAVAAHLAGKQVRDKVARIAAGLFKTT
EDDVGFGGGRVFSIAQPAKSMPFGRAAGTAHWSPVLVPDTDQPGISETGIWAPQELAPPTGGDEINTSLTYGF
VFDMCGIEIDPLTMQVKVDRYVSMHDAGKLINPLLAEGQIRGAFVQGLATALYEEIVYDDKTGALQTGTFAEYLV
PTACEVPQIEIIHMETPSPFTPLGAKGLAEGNCMSTPVCIANAVADALSLRDVRLPITPNRLNAVLATNEGPAPTL
TSNVVDAAAMEKEFAEATIAGSGTLTVATEAGNIWDTLLNPQTLKNIIPGCHTVTALRPDHFQAEVSLGVGPVR
GRFIADVYLSDLVPQRQAKLSGELRGPLGKAEGKGRIILSLTPDGTRIDYAYGVDVGGKVASVGGRMIRRAADMI
IGQFFERLVALSGDGDATPTKKTRSGLMAWLVGLFGGQK
>SEQF8760||SEQF8760.1_00983
MYADVKKNTHSTQGWVGRSLERHEDAALLMGQGRYVDDFPIAPNTLHAAVVRASHAHALITEIDTTRALAVP
GVRHVITGEQIRAISDPFLTVLKQPIDQWALAVERVRYVGEAVAVVLADDRYIAEDGAALVDILYEPLPVVVDPVA
AVRSDAPLLHPAAGSNQLSVREFVYGEPDAAFARAERTISVDIEYPRSSYTPMECFAVVAEYRPGDKSFDVLANF
QGPFSAHPVMAKALRVPGSRLRLRIPPDSGGSFGIKLSVFPYIVLMGLAARISGRPVKWIEDRLEHLQAANSGP
NRITRLDAAVEPDGRVTALRFDNLEDYGAYLRAPMPGPLYRMHGATTGAYDIRHLAMVNRVVVTNKMPAALV
RGFGGPQLYLALERLMQRIAVELGLDPLEVIRRNLISADAFPYRTPAGALYDSGDYQKAIEIATGDGRLTDLLRRR
DEARAAGKLYGIGYAAVVEPGMSNMGYLSTILPEKIRQQQGPKNGAVSMVRVNVDAVGGVSMTADVTVQG
QGHATALSQIVADQLGLHPRDISVNLEIDTQKDAWSIAAGSYSCRFSPGTAVAAHLAGKQVRDKVARIAAGLFET
TEDDVGFGGGRVFSIAQPAKSMPFGRAAGTAHWSPVLVPDTDQPGISETGIWAPQELAPPTGGDEINTSLTYG
FVFDMCGIEIDPLTMQVKVDRYVSMHDAGKLINPLLAEGQIRGAFVQGLATALYEEIVYDDKTGALQTGTFAEYL
VPTACEVPQIEIIHMETPSPFTPLGAKGLAEGNCMSTPVCIANAVADALSLRDVRLPITPNRLNAVLATNEGPAPT
LTSNVVDAAAMEQEFAEATIAGSGTLTVATEAGHIWDTLLDPQTLKNIIPGCHTVTALRPDHFQAEVSLGVGPVR
GRFIADVYLSDLVPQRQAKLSGELRGPLGKAEGKGRIILSLTPDGTRIDYAYGVDVGGKVASVGGRMIRRAADMI
IGQFFERLVALSGDGDATPTKKTRSGLMAWLVGLFGGQK
>SEQF8762||SEQF8762.1_00893
MYADVKKNTHSTQGWVGRSLERHEDAALLMGQGRYVDDFPIAPNTLHAAVVRASHAHALITEIDTTRALAVP
GVRHVITGEQIRAISDPFLTVLKQPIDQWALAVERVRYVGEAVAVVLADDRYIAEDGAALVDILYEPLPVVVDPVA
AVRSDAPLLHPAAGSNQLSVREFVYGEPDAAFARAERTISVDIEYPRSSYTPMECFAVVAEYRPGDKSFDVLANF
QGPFSAHPVMAKALRVPGSRLRLRIPPDSGGSFGIKLSVFPYIVLMGLAARISGRPVKWIEDRLEHLQAANSGP
NRITRLDAAVEPDGRVTALRFDNLEDYGAYLRAPMPGPLYRMHGATTGAYDIRHLAMVNRVVVTNKMPAALV
RGFGGPQLYLALERLMQRIAVELGLDPLEVIRRNLISADAFPYRTPAGALYDSGDYQKAIEIATGDGRLTDLLRRR
DEARAAGKLYGIGYAAVVEPGMSNMGYLSTILPEKIRQQQGPKNGAVSMVRVNVDAVGGVSMTADVTVQG
QGHATALSQIVADQLGLHPRDISVNLEIDTQKDAWSIAAGSYSCRFSPGTAVAAHLAGKQVRDKVARIAAGLFET
TEDDVGFGGGRVFSIAQPAKSMPFGRAAGTAHWSPVLVPDTDQPGISETGIWAPQELAPPTGGDEINTSLTYG
FVFDMCGIEIDPLTMQVKVDRYVSMHDAGKLINPLLAEGQIRGAFVQGLATALYEEIVYDDKTGALQTGTFAEYL
VPTACEVPQIEIIHMETPSPFTPLGAKGLAEGNCMSTPVCIANAVADALSLRDVRLPITPNRLNAVLATNEGPAPT
LTSNVVDAAAMEQEFAEATIAGSGTLTVATEAGHIWDTLLDPQTLKNIIPGCHTVTALRPDHFQAEVSLGVGPVR
GRFIADVYLSDLVPQRQAKLSGELRGPLGKAEGKGRIILSLTPDGTRIDYAYGVDVGGKVASVGGRMIRRAADMI
IGQFFERLVALSGDGDATPTKKTRSGLMAWLVGLFGGQK
>SEQF8765||SEQF8765.1_01828
MYADVKKNTHSTQGWVGRSLERHEDAALLMGQGRYVDDFPIAPNTLHAAVVRASHAHALITEIDTTRALAVP
GVRHVITGEQIRAISDPFLTVLKQPIDQWALAVERVRYVGEAVAVVLADDRYIAEDGAALVDILYEPLPVVVDPVA
AVRSDAPLLHPAAGSNQLSVREFVYGEPDAAFARAERTISVDIEYPRSSYTPMECFAVVAEYRPGDKSFDVLANF
QGPFSAHPVMAKALRVPGSRLRLRIPPDSGGSFGIKLSVFPYIVLMGLAARISGRPVKWIEDRLEHLQAANSGP

NRITRLDAAVEPDGRVTALRFDNLEDYGAYLRAPMPGPLYRMHGATTGAYDIRHLAMVNRVVVTNKMPAALV
RGFGGPQLYLALERLMQRIAVELGLDPLEVIRRNLISADAFPYRTPAGALYDSGDYQKAIEIATGDGRLTDLLRRR
DEARAAGKLYGIGYAAVVEPGMSNMGYLSTILPEKIRQQQGPKNGAVSMVRVNVDAVGGVSMTADVTVQG
QGHATALSQIVADQLGLHPRDISVNLEIDTQKDAWSIAAGSYSCRFSPGTAVAAHLAGKQVRDKVARIAAGLFET
TEDDVGFGGGRVFSIAQPAKSMPFGRAAGTAHWSPVLVPDTDQPGISETGIWAPQELAPPTGGDEINTSLTYG
FVFDMCGIEIDPLTMQVKVDRYVSMHDAGKLINPLLAEGQIRGAFVQGLATALYEEIVYDDKTGALQTGTFAEYL
VPTACEVPQIEIIHMETPSPFTPLGAKGLAEGNCMSTPVCIANAVADALSLRDVRLPITPNRLNAVLATNEGPAPT
LTSNVVDAAAMEQEFAEATIAGSGTLTVATEAGHIWDTLLDPQTLKNIIPGCHTVTALRPDHFQAEVSLGVGPVR
GRFIADVYLSDLVPQRQAKLSGELRGPLGKAEGKGRIILSLTPDGTRIDYAYGVDVGGKVASVGGRMIRRAADMI
IGQFFERLVALSGDGDATPTKKTRSGLMAWLVGLFGGQK
>SEQF8768||SEQF8768.1_00538
MSGHDHFVELRPKLVGKRVKRTEDPRFLAGVGQYVDDMNPSGTLHVALRRSDQPHARIIGIDIGEAFSVPGVV
AIYDASDLESDIKPAIPTSRMPGYYATPIWPLARGKVRYVGEPVIAVVAESRYAAEDALEHITIEYEALPFAIRQVDA
VKDDAPLLHEEAATNTIIRREFKRGDVDAAFEGAAVIVKGRFQMTRKTAVAMENRSYLAEWKERKQSLTLYTSS
NIPGVIRDVLAGCLDLPGSRLRVVAPDVGGSFGGKGSLYGEEILVCALSRKLKRPVKFISDRLEDLSATSQAFDELIE
AELAVTEDGMLIGLRADVIGDVGAYSIYPWTAALETVQVVSFLPGPYRMEHYRGRIRGVLTPKPPTGPYRGVGR
PSSTFAMERLIEMAARKLGMDPVEFRRRNLVNAEEFPYRTASGIIWDKSAFQECLQGACEHVDYPNLVRERDE
ARKAGRWVGIGLASYAELTGIGSRISVAPGMPINTGTETSKIEIDATGSITAAFGVSSHGQGLETTLAQVIVDELGC
KLEDIDVKHGDSSLVPMSSGTYASRSAVLGGGAATLAARAVKAKVLRAAAYLMEQATEDLEIHDGIVSSRNSNLT
MTLKEVASAVYTQMGRIPRDQREDLSASETYDPYLGTACSSTHLAMVEVDTETYGVKILRYVVAEDCGRIINPMI
VDGQVQGAVAQGIGAALLEEIVHDDQGQAVTASLADYLVPVATSVPNIGIVHIEADLPNTIGGFRGMGEGGTI
GAPAAIANAVSDALAHLGVTVETLPVTPERIFQMIREKRQTSKEASDAP
>SEQF8769||SEQF8769.1_01067
MYADVKKNTHSTQGWVGRSLERHEDAALLMGQGRYVDDFPIAPNTLHAAVVRASHAHALITEIDTTRALAVP
GVRHVITGEQIRAISDPFLTVLKQPIDQWALAVERVRYVGEAVAVVLADDRYIAEDGAALVDILYEPLPVVVDPVA
AVRSDAPLLHPAAGSNQLSVREFVYGEPDAAFARAERTISVDIEYPRSSYTPMECFAVVAEYRPGDKSFDVLANF
QGPFSAHPVMAKALRVPGSRLRLRIPPDSGGSFGIKLSVFPYIVLMGLAARISGRPVKWIEDRLEHLQAANSGP
NRITRLDAAVEPDGRVTALRFDNLEDYGAYLRAPMPGPLYRMHGATTGAYDIRHLAMVNRVVVTNKMPAALV
RGFGGPQLYLALERLMQRIAVELGLDPLEVIRRNLISADAFPYRTPAGALYDSGDYQKAIEIATGDGRLTDLLRRR
DEARAAGKLYGIGYAAVVEPGMSNMGYLSTILPEKIRQQQGPKNGAVSMVRVNVDAVGGVSMTADVTVQG
QGHATALSQIVADQLGLHPRDISVNLEIDTQKDAWSIAAGSYSCRFSPGTAVAAHLAGKQVRDKVARIAAGLFET
TEDDVGFGGGRVFSIAQPAKSMPFGRAAGTAHWSPVLVPDTDQPGISETGIWAPQELAPPTGGDEINTSLTYG
FVFDMCGIEIDPLTMQVKVDRYVSMHDAGKLINPLLAEGQIRGAFVQGLATALYEEIVYDDKTGALQTGTFAEYL
VPTACEVPQIEIIHMETPSPFTPLGAKGLAEGNCMSTPVCIANAVADALSLRDVRLPITPNRLNAVLATNEGPAPT
LTSNVVDAAAMEQEFAEATIAGSGTLTVATEAGHIWDTLLDPQTLKNIIPGCHTVTALRPDHFQAEVSLGVGPVR
GRFIADVYLSDLVPQRQAKLSGELRGPLGKAEGKGRIILSLTPDGTRIDYAYGVDVGGKVASVGGRMIRRAADMI
IGQFFERLVALSGDGDATPTKKTRSGLMAWLVGLFGGQK
>SEQF8797||SEQF8797.1_04206
MLHTTSLVGDAGTLVGQSVARVEDATLLTGNGRYLDDLPQRKDTRHIAFLRADQAHAKIISIDTKDARALPGVV
AVLTGDDVAALTRSMTVGVKADVEAWPMARDRVRYVGEPVAMVVAIDRYVAEDAVDLIRVEYETMPAVVDP
MEALAPGAEVLHPSLGGNLISERSFRYGDPDVAFAEAAHRVSISVTYPRNSCTPMECYGVVAEYLPSEDIFDITA
NFQGPFSIHTVIARSLGVPGNRLRLRTPPDSGGSFGVKQGVFPYVILAGVAAKVAGYPVKWIEDRLEHLSASVSA
TNRVTTLSAAVDGTGRIQALEWDQVEDCGAHLRAPEPATLYRMHGNMTGAYAIRNVSIRNRVVLTNKTPTGLN
RGFGGPQVYFALERLVHKIALELGIDPLELIRRNLVPTGDFPYRTATGALLDSGDYSKALYTAAHAGKLDKLLALRD

AARAEGRLYGVGYCVAVEPSVSNMGYITTVLTPDQRAKAGPKNGAQAVGTITVDPMGTVTVKTASVPQGQG
HRTVLAQVVGDILGLPMGDIRAIVDVDTSRDPWSIAAGNYSSRFAAAVAGAVSLAAQQLRGKLAKMAAQQLG
CGPEAITFAGGKVFDRENPAAAIPFGRLAGTSHWAPGTLPEGIGQAISETVFWSPPELTAPNEKDEVNSSLCHGF
IFDFCGVEIDRLTGQVRIDRYVSMHDCGRILHPGMVDGQIRGAFLQAVGAALYEEFVYAEDGSFESGTLADYLIP
TVMETPDIQILHFETPSPFTPLGAKGVAEGNCMSTPVCIANAVADALSCENITLPITPSKVLAMIGGEERPPSKPI
AKPVKAKAKPGERALTGEGEADVSSPREAVWHMLLEAETLAAIIPGAHGVQKLSPDHFTATVTLGVGPVKDKYE
ADVRLSDIDHLNSVTLTGAVTGRLGSGGGTGVIRLSDLPGGGTRLHYSYVATVGGKVASVGGRLLDGAARVIIG
QFFQSLARHAGGGGTGRPSFLKRIFLLFRGRR
>SEQF8798||SEQF8798.1_04232
MLHTTSLVGDAGTLVGQSVARVEDATLLTGNGRYLDDLPQRKDTRHIAFLRADQAHAKIISIDTKDARALPGVV
AVLTGDDVAALTRSMTVGVKADVEAWPMARDRVRYVGEPVAMVVAIDRYVAEDAVDLIRVEYETMPAVVDP
MEALAPGAEVLHPSLGGNLISERSFRYGDPDVAFAEAAHRVSISVTYPRNSCTPMECYGVVAEYLPSEDIFDITA
NFQGPFSIHTVIARSLGVPGNRLRLRTPPDSGGSFGVKQGVFPYVILAGVAAKVAGYPVKWIEDRLEHLSASVSA
TNRVTTLSAAVDGTGRIQALEWDQVEDCGAHLRAPEPATLYRMHGNMTGAYAIRNVSIRNRVVLTNKTPTGLN
RGFGGPQVYFALERLVHKIALELGIDPLELIRRNLVPTGDFPYRTATGALMDSGDYSKALYTAAHAGKLDKLLALR
DAARAEGRLYGVGYCVAVEPSVSNMGYITTVLTPDQRAKAGPKNGAQAVGTITVDPMGTVTVKTASVPQGQG
HRTVLAQVVGDILGLPMGDIRAIVDVDTSRDPWSIAAGNYSSRFAAAVAGAVSLAAQQLRGKLAKMAAQQLG
CGPEAITFAGGKVFDRENPAAAIPFGRLAGTSHWAPGTLPEGIGQAISETVFWSPPELTAPNEKDEVNSSLCHGF
IFDFCGVEIDRLTGQVRIDRYVSMHDCGRILHPGMVDGQIRGAFLQAVGAALYEEFVYAEDGSFESGTLADYLIP
TVMETPDIQILHFETPSPFTPLGAKGVAEGNCMSTPVCIANAVADALSCENITLPITPSKVLAMIGGEERPPSKPI
AKPVKAKAKPGERALTGEGEADVSSPREAVWHMLLEAETLAAIIPGAHGVQKLSPDHFTATVTLGVGPVKDKYE
ADVRLSDIDHLNSVTLTGAVTGRLGSGGGTGVIRLSDLPGGGTRLHYSYVATVGGKVASVGGRLLDGAARVIIG
QFFQSLARHAGGGGTGRPSFLKRIFLLFRGRR
>SEQF8800||SEQF8800.1_03772
MLHTTSLVGDAGTLVGQSVARVEDATLLTGNGRYLDDLPQRKDTRHIAFLRADQAHAKIISIDTKDARALPGVV
AVLTGDDVAALTRSMTVGVKADVEAWPMARDRVRYVGEPVAMVVAIDRYVAEDAVDLIRVEYETMPAVVDP
MEALAPGAEVLHPSLGGNLISERSFRYGDPDVAFAEAAHRVSISVTYPRNSCTPMECYGVVAEYLPSEDIFDITA
NFQGPFSIHTVIARSLGVPGNRLRLRTPPDSGGSFGVKQGVFPYVILAGVAAKVAGYPVKWIEDRLEHLSASVSA
TNRVTTLSAAVDGTGRIQALEWDQVEDCGAHLRAPEPATLYRMHGNMTGAYAIRNVSIRNRVVLTNKTPTGLN
RGFGGPQVYFALERLVHKIALELGIDPLELIRRNLVPTGDFPYRTATGALLDSGDYSKALYTAAHAGKLDKLLALRD
AARAEGRLYGVGYCVAVEPSVSNMGYITTVLTPDQRAKAGPKNGAQAVGTITVDPMGTVTVKTASVPQGQG
HRTVLAQVVGDILGLPMGDIRAIVDVDTSRDPWSIAAGNYSSRFAAAVAGAVSLAAQQLRGKLAKMAAQQLG
CGPEAITFAGGKVFDRENPAAAIPFGRLAGTSHWAPGTLPEGIGQAISETVFWSPPELTAPNEKDEVNSSLCHGF
IFDFCGVEIDRLTGQVRIDRYVSMHDCGRILHPGMVDGQIRGAFLQAVGAALYEEFVYAEDGSFESGTLADYLIP
TVMETPDIQILHFETPSPFTPLGAKGVAEGNCMSTPVCIANAVADALSCENITLPITPSKVLAMIGGEERPPSKPI
AKPVKAKAKPGERALTGEGEADVSSPREAVWHMLLEAETLAAIIPGAHGVQKLSPDHFTATVTLGVGPVKDKYE
ADVRLSDIDHLNSVTLTGAVTGRLGSGGGTGVIRLSDLPGGGTRLHYSYVATVGGKVASVGGRLLDGAARVIIG
QFFQSLARHAGGGGTGRPSFLKRIFLLFRGRR
>SEQF8801||SEQF8801.1_01039
MLHTTSLVGDAGTLVGQSVARVEDATLLTGNGRYLDDLPQRKDTRHIAFLRADQAHAKIISIDTKDARALPGVV
AVLTGDDVAALTRSMTVGVKADVEAWPMARDRVRYVGEPVAMVVAIDRYVAEDAVDLIRVEYETMPAVVDP
MEALAPGAEVLHPSLGGNLISERSFRYGDPDVAFAEAAHRVSISVTYPRNSCTPMECYGVVAEYLPSEDIFDITA
NFQGPFSIHTVIARSLGVPGNRLRLRTPPDSGGSFGVKQGVFPYVILAGVAAKVAGYPVKWIEDRLEHLSASVSA
TNRVTTLSAAVDGTGRIQALEWDQVEDCGAHLRAPEPATLYRMHGNMTGAYAIRNVSIRNRVVLTNKTPTGLN

RGFGGPQVYFALERLVHKIALELGIDPLELIRRNLVPTGDFPYRTATGALLDSGDYSKALYTAAHAGKLDKLLALRD
AARAEGRLYGVGYCVAVEPSVSNMGYITTVLTPDQRAKAGPKNGAQAVGTITVDPMGTVTVKTASVPQGQG
HRTVLAQVVGDILGLPMGDIRAIVDVDTSRDPWSIAAGNYSSRFAAAVAGAVSLAAQQLRGKLAEMAAQQLG
CGPEAITFAGGKVFDRENPAAAIPFGRLAGTSHWAPGTLPEGIGQAISQTVFWSPPELTAPNEKDEVNSSLCHG
FIFDFCGVEIDRLTGQVRIDRYVSMHDCGRILHPGMVDGQIRGAFLQAVGAALYEEFVYAEDGSFESGTLADYLI
PTVMETPDIQILHFETPSPFTPLGAKGVAEGNCMSTPVCIANAVADALSCENITLPITPSKVLAMIGGEERPPSKP
IAKPVKAKAKPGERALTGEGEADVSSPREAVWHMLLEAETLAAIIPGAHGVQKLSPDHFTATVTLGVGPVKDKY
EADVRLSDIDHLNSVTLTGAVTGRLGSGGGTGVIRLSDLPGGGTRLHYSYVATVGGKVASVGGRLLDGAARVIIS
QFFQSLARHAGGGGTVRPSFLKRIFLLFRGRR
>SEQF8803||SEQF8803.1_04094
MLHTTSLVGDAGTLVGQSVARVEDATLLTGNGRYLDDLPQRKDTRHIAFLRADQAHAKIISIDTKDARALPGVV
AVLTGDDVAALTRSMTVGVKADVEAWPMARDRVRYVGEPVAMVVAIDRYVAEDAVDLIRVEYETMPAVVDP
MEALAPGAEVLHPSLGGNLISERSFRYGDPDVAFAEAAHRVSISVTYPRNSCTPMECYGVVAEYLPSEDIFDITA
NFQGPFSIHTVIARSLGVPGNRLRLRTPPDSGGSFGVKQGVFPYVILAGVAAKVAGYPVKWIEDRLEHLSASVSA
TNRVTTLSAAVDGTGRIQALEWDQVEDCGAHLRAPEPATLYRMHGNMTGAYAIRNVSIRNRVVLTNKTPTGLN
RGFGGPQVYFALERLVHKIALELGIDPLELIRRNLVPTGDFPYRTATGALLDSGDYSKALYTAAHAGKLDKLLALRD
AARAEGRLYGVGYCVAVEPSVSNMGYITTVLTPDQRAKAGPKNGAQAVGTITVDPMGTVTVKTASVPQGQG
HRTVLAQVVGDILGLPMGDIRAIVDVDTSRDPWSIAAGNYSSRFAAAVAGAVSLAAQQLRGKLAEMAAQQLG
CGPEAITFAGGKVFDRENPAAAIPFGRLAGTSHWAPGTLPEGIGQAISQTVFWSPPELTAPNEKDEVNSSLCHG
FIFDFCGVEIDRLTGQVRIDRYVSMHDCGRILHPGMVDGQIRGAFLQAVGAALYEEFVYAEDGSFESGTLADYLI
PTVMETPDIQILHFETPSPFTPLGAKGVAEGNCMSTPVCIANAVADALSCENITLPITPSKVLAMIGGEERPPSKP
IAKPVKAKAKPGERALTGEGEADVSSPREAVWHMLLEAETLAAIIPGAHGVQKLSPDHFTATVTLGVGPVKDKY
EADVRLSDIDHLNSVTLTGAVTGRLGSGGGTGVIRLSDLPGGGTRLHYSYVATVGGKVASVGGRLLDGAARVIIS
QFFQSLARHAGGGGTVRPSFLKRIFLLFRGRR
>SEQF8854||SEQF8854.1_02599
MAKFGLSQSLRRVEDPRLLKGEGRYTDDLAVAGTATGYLLRSPHAHATILSIDTADARAMPGVLAIYTAEDLAAD
GIGPLPHAGSLKNRDGSRSANAPRFLLASGKVRHVGDPVAFVVAESLDAAKDAAEAILVDYDPLPACTDLAAAN
EPGQPQVFDDVPENRVFDWEIGDKAKTDALFDSAAHVTRLTVVNNRIVVASMEGRAALAEYDAATERFTLQA
TSQGAWHLKDMLAEHVFKLPKEKFRVVTHDVGGGFGMKLFCYVEYGLACYAARKLGRPVKWTAERSEAFLSD
THGRDNITLGELALDKDGRFLALRTRNLANMGAYLSQFAGFIPTAAGTKVLASVYGFQAIHAHVIGALTHTTPVD
AYRGAGRPESNYLVERLIDAAARELGIDRAELRRRNMVPPSAMPHTTPVGQKYDSGDFAQVLDEALRRADWA
GFPARQAEARARGKRRGIGLAYYLEATGGDPSERAEVRFTEDGHVEVLVGTQSTGQGHETAYTMIIAERLGVPV
DRIRVRQGDSDEIPTGGGTGGARSLYSEGTALLATASTVLERGRQAAGEALEASPADIEFTTTGALDGGGRFSIAG
TDRGIGILELAARQRAAAARGEQATLLDAAEVAEVPFGTFPNGCHIAEVEVDPETGLIGIARYLVVDDVGHAINP
LIVRGQVHGGVAQGVGQALHEHTVYDPESGQLLSASFMDYALPRAEDLPAIEVDLVEIPCETNPLGVKGAGEA
GAVGSPPAVMNALVDALAPDGVTHLDMPATPERVWRALGQALSRAA
>SEQF8854||SEQF8854.1_02800
MPDTNMPDPSRMRFAVGQPVTRREDPMLLRGEGHYTDDLQAEGQAHAWMVRSPYAHGVINGIDTAEAKS
MPGVLAVYTGADLAEYGPIRCAMPLKNRDGSPLASPVRPALATDRVRYVGDPVAIVIAETPAEARDAAEAVML
DIDPLDAVTEASAAAAPGAPQLYDDVPGNVVLDWHSGDAEKVAAAFATAAHRVKLPLRNNRIVVAAMEPRAA
IAEYDAESGRYTLRACSQGVFGLRRQIAEDILRVPVEKVHVLTTQVGGSFGMKGTVYPEYPGMFHAARLLGRPV
KWTDERSGSFVSDQHGRDHETVAELALDAEGRFLAVRLTSFANMGAYLTTVGPLMGTMGFVKNVQSNYATPL
IEVDTKSVFTNTSPVGAYRGAGRPEGNYYMERLVEAAAAQIGMDPLELRRRNHIRPEQMPYDAASGSRYDGG
EFTALMEEALRQADWDGFAARREESASRGLLRGRGLGNYLECTAPAGKEHGGVRFEEDGGVTIITGTLDYGQG

HWTPFAQLLGSRLGVPFERIRLIQGDSDLLKHGGGTGGSRSMMSSGTAIIEASEKVIEKGREAASHLLEAAVADIE
FDPENGRFAIAGTDRGIGILELAARMRAAPELPEDLRSLDVTHVTEDKPMAYPNGCHVAEVEVDPETGHVHLV
RYLAVSDFGVEVNPLMVQGQVHGGVAQGIGQALMERVSYDEGGQILSASFMDYALPRAEDLPPILTASRPVPA
KTNPVGAKGCGEAGCAGSLPAVMNALADALRAAGAEPVDMPATPEKVWMALQRASRG
>SEQF8855||SEQF8855.1_00350
MAKFGLSQSLRRVEDPRLLKGEGRYTDDLAVAGTATGYLLRSPHAHATILSIDTADARAMPGVLAIYTAEELAAD
GIGPLPHAGSLKNRDGSRSANAPRFLLASGKVRHVGDPVAFVVAESLDAAKDAAEAILVDYDPLPACTDLATAN
EPGQPQVFDDVPENRVFDWEIGDKAKTDALFDSAAHVTRLTVVNNRIVVASMEGRAALAEYDAATERFTLQA
TSQGAWHLKDMLAEHVFKLPKEKFRVVTHDVGGGFGMKLFCYVEYGLACYAARKLGRPVKWTAERSEAFLSD
THGRDNITLGELALDKDGRFLALRTRNLANMGAYLSQFAGFIPTAAGTKVLASVYGFQAIHAHVIGALTHTTPVD
AYRGAGRPESNYLVERLIDAAARELGIDRAELRRRNMVPPSAMPHTTPVGQKYDSGDFAQVLDEALRRADWA
GFPARQAEARARGKRRGIGLAYYLEATGGDPSERAEVRFTEDGHVEVLVGTQSTGQGHETAYTMIIAERLGVPV
DRIRVRQGDSDEIPTGGGTGGARSLYSEGTALLATASTVLERGRQAAGEALEASPADIEFTTTGALDGGGRFSIAG
TDRGIGILELAARQRAAAARGEQATLLDAAEVAEVPFGTFPNGCHIAEVEVDPETGLIGIARYLVVDDVGHAINP
LIVRGQVHGGVAQGVGQALHEHTVYDPESGQLLSASFMDYALPRAEDLPAIEVDLVEIPCETNPLGVKGAGEA
GAVGSPPAVMNALVDALAPDGVTHLDMPATPERVWRALGQALSRAA
>SEQF8855||SEQF8855.1_00554
MPDTNMPDPSRMRFAVGQPVTRREDPMLLRGEGHYTDDLQAEGQAHAWMVRSPYAHGVINGIDTAEAKS
MPGVLAVYTGADLAEYGPIRCAMPLKNRDGSPLASPVRPALATDRVRYVGDPVAIVIAETPAEARDAAEAVML
DIDPLDAVTEASAAAAPGAPQLYDDVPGNVVLDWHSGDAEKVATAFATAAHRVKLPLRNNRIVVAAMEPRAAI
AEYDAESGRYTLRACSQGVFGLRRQIAEDILRVPVEKVHVLTTQVGGSFGMKGTVYPEYPGMFHAARLLGRPV
KWTDERSGSFVSDQHGRDHETVAELALDAEGRFLAVRLTSFANMGAYLTTVGPLMGTMGFVKNVQSNYATPL
IEVDTKSVFTNTSPVGAYRGAGRPEGNYYMERLVEAAAAQIGMDPLELRRRNHIRPEQMPYDAASGSRYDGG
EFTALMEEALRQADWDGFAARREESASRGLLRGRGLGNYLECTAPAGKEHGGVRFEEDGGVTIITGTLDYGQG
HWTPFAQLLGSRLGVPFERIRLIQGDSDLLKHGGGTGGSRSMMSSGTAIIEASEKVIEKGREAASHLLEAAVADIE
FDPENGRFAIAGTDRGIGILELAARMRAAPELPEDLRSLDVTHVTEDKPMAYPNGCHVAEVEVDPETGHVHLV
RYLAVSDFGVEVNPLMVQGQVHGGVAQGIGQALMERVSYDEGGQILSASFMDYALPRAEDLPPILTASRPVPA
KTNPVGAKGCGEAGCAGSLPAVMNALADALRAAGAEPVDMPATPEKVWMALQRTSRG
>SEQF8856||SEQF8856.1_02864
MPDTNMPDPSRMRFAVGQPVTRREDPMLLRGEGHYTDDLQAEGQAHAWMVRSPYAHGVINGIDTAEAKS
MPGVLAVYTGADLAEYGPIRCAMPLKNRDGSPLASPVRPALATDRVRYVGDPVAIVIAETPAEARDAAEAVML
DIDPLDAVTEASAAAAPGAPQLYDDVPGNVVLDWHSGDAEKVAAAFATAAHRVKLPLRNNRIVVAAMEPRAA
IAEYDAESGRYTLRACSQGVFGLRRQIAEDILRVPVEKVHVLTTQVGGSFGMKGTVYPEYPGMFHAARLLGRPV
KWTDERSGSFVSDQHGRDHETVAELALDAEGRFLAVRLTSFANMGAYLTTVGPLMGTMGFVKNVQSNYATPL
IEVDTKSVFTNTSPVGAYRGAGRPEGNYYMERLVEAAAAQIGMDPLELRRRNHIRPEQMPYDAASGSRYDGG
EFTALMEEALRQADWDGFAARREESASRGLLRGRGLGNYLECTAPAGKEHGGVRFEEDGGVTIITGTLDYGQG
HWTPFAQLLGSRLGVPFERIRLIQGDSDLLKHGGGTGGSRSMMSSGTAIIEASEKVIEKGREAASHLLEAAVADIE
FDPENGRFAIAGTDRGIGILELAARMRAAPELPEDLSSLDVTHVTEDKPMAYPNGCHVAEVEVDPETGHVHLV
RYLAVSDFGVEVNPLMVQGQVHGGVAQGIGQALMERVSYDEGGQILSASFMDYALPRAEDLPPILTASRPVPA
KTNPVGAKGCGEAGCAGSLPAVMNALADALRAAGAEPVDMPATPEKVWMALQRASRG
>SEQF8856||SEQF8856.1_04088
MAKFGLSQSLRRVEDPRLLKGEGRYTDDLAVAGTATGYLLRSPHAHATILSIDTADARAMPGVLAIYTAEDLAAD
GIGPLPHAGSLKNRDGSRSANAPRFLLASGKVRHVGDPVAFVVAESLDAAKDAAEAILVDYDPLPACTDLAAAN
EPGQPQVFDDVPENRVFDWEIGDKAKTDALFDSAAHVTRLTVVNNRIVVASMEGRAALAEYDAATERFTLQA

TSQGAWHLKDMLAEHVFKLPKEKFRVVTHDVGGGFGMKLFCYVEYGLACYAARKLGRPVKWTAERSEAFLSD
THGRDNITLGELALDKDGRFLALRTRNLANMGAYLSQFAGFIPTAAGTKVLASVYGFQAIHAHVIGALTHTTPVD
AYRGAGRPESNYLVERLIDAAARELGIDRAELRRRNMVPPSAMPHTTPVGQKYDSGDFAQVLDEALRRADWA
GFPARQAEARARGKRRGIGLAYYLEATGGDPSERAEVRFTEDGHVEVLVGTQSTGQGHETAYTMIIAERLGVPV
DRIRVRQGDSDEIPTGGGTGGARSLYSEGTALLATASTVLERGRQAAGEALEASPADIEFTTTGALDGGGRFSIAG
TDRGIGILELAARQRAAAARGEQATLLDAAEVAEVPFGTFPNGCHIAEVEVDPETGLIGIARYLVVDDVGHAINP
LIVRGQVHGGVAQGVGQALHEHTVYDPESGQLLSASFMDYALPRAEDLPAIEVDLVEIPCETNPLGVKGAGEA
GAVGSPPAVMNALVDALAPDGVTHLDMPATPERVWRALGQALSRAA
>SEQF8857||SEQF8857.1_04683
MAKFGLSQSLRRVEDPRLLKGEGRYTDDLAVAGTATGYLLRSPHAHATILSIDTADARAMPGVLAIYTAEDLAAD
GIGPLPHAGSLKNRDGSRSANAPRFLLASGKVRHVGDPVAFVVAESLDAAKDAAEAILVDYDPLPACTDLAAAN
EPGQPQVFDDVPENRVFDWEIGDKAKTDALFDSAAHVTRLTVVNNRIVVASMEGRAALAEYDAATERFTLQA
TSQGAWHLKDMLAEHVFKLPKEKFRVVTHDVGGGFGMKLFCYVEYGLACYAARKLGRPVKWTAERSEAFLSD
THGRDNITLGELALDKDGRFLALRTRNLANMGAYLSQFAGFIPTAAGTKVLASVYGFQAIHAHVIGALTHTTPVD
AYRGAGRPESNYLVERLIDAAARELGIDRAELRRRNMVPPSAMPHTTPVGQKYDSGDFAQVLDEALRRADWA
GFPARQAEARARGKRRGIGLAYYLEATGGDPSERAEVRFTEDGHVEVLVGTQSTGQGHETAYTMIIAERLGVPV
DRIRVRQGDSDEIPTGGGTGGARSLYSEGTALLATASTVLERGRQAAGEALEASPADIEFTTTGALDGGGRFSIAG
TDRGIGILELAARQRAAAARGEQATLLDAAEVAEVPFGTFPNGCHIAEVEVDPETGLIGIARYLVVDDVGHAINP
LIVRGQVHGGVAQGVGQALHEHTVYDPESGQLLSASFMDYALPRAEDLPAIEVDLVEIPCETNPLGVKGAGEA
GAVGSPPAVMNALVDALAPDGVTHLDMPATPERVWRALGQALSRAA
>SEQF8857||SEQF8857.1_03218
MPDTNMPDPSRMRFAVGQPVTRREDPMLLRGEGHYTDDLQAEGQAHAWMVRSPYAHGVINGIDTAEAKS
MPGVLAVYTGADLAEYGPIRCAMPLKNRDGSPLASPVRPALATDRVRYVGDPVAIVIAETPAEARDAAEAVML
DIDPLDAVTEASAAAAPGAPQLYDDVPGNVVLDWHSGDAEKVAAAFATAAHRVKLPLRNNRIVVAAMEPRAA
IAEYDAESGRYTLRACSQGVFGLRRQIAEDILRVPVEKVHVLTTQVGGSFGMKGTVYPEYPGMFHAARLLGRPV
KWTDERSGSFVSDQHGRDHETVAELALDAEGRFLAVRLTSFANMGAYLTTVGPLMGTMGFVKNVQSNYATPL
IEVDTKSVFTNTSPVGAYRGAGRPEGNYYMERLVEAAAAQIGMDPLELRRRNHIRPEQMPYDAASGSRYDGG
EFTALMEEALRQADWDGFAARREESASRGLLRGRGLGNYLECTAPAGKEHGGVRFEEDGGVTIITGTLDYGQG
HWTPFAQLLGSRLGVPFERIRLIQGDSDLLKHGGGTGGSRSMMSSGTAIIEASEKVIEKGREAASHLLEAAVADIE
FDPENGRFAIAGTDRGIGILELAARMRAAPELPEDLRSLDVTHVTEDKPMAYPNGCHVAEVEVDPETGHVHLV
RYLAVSDFGVEVNPLMVQGQVHGGVAQGIGQALMERVSYDEGGQILSASFMDYALPRAEDLPPILTASRPVPA
KTNPVGAKGCGEAGCAGSLPAVMNALADALRAAGAEPVDMPATPEKVWMALQRASRG
>SEQF8858||SEQF8858.1_04416
MAKFGLSQSLRRVEDPRLLKGEGRYTDDLAVAGTATGYLLRSPHAHATILSIDTADARAMPGVLAIYTAEDLAAD
GIGPLPHAGSLKNRDGSRSANAPRFLLASGKVRHVGDPVAFVVAESLDAAKDAAEAILVDYDPLPACTDLAAAN
EPGQPQVFDDVPENRVFDWEIGDKAKTDALFDSAAHVTRLTVVNNRIVVASMEGRAALAEYDAATERFTLQA
TSQGAWHLKDMLAEHVFKLPKEKFRVVTHDVGGGFGMKLFCYVEYGLACYAARKLGRPVKWTAERSEAFLSD
THGRDNITLGELALDKDGRFLALRTRNLANMGAYLSQFAGFIPTAAGTKVLASVYGFQAIHAHVIGALTHTTPVD
AYRGAGRPESNYLVERLIDAAARELGIDRAELRRRNMVPPSAMPHTTPVGQKYDSGDFAQVLDEALRRADWA
GFPARQAEARARGKRRGIGLAYYLEATGGDPSERAEVRFTEDGHVEVLVGTQSTGQGHETAYTMIIAERLGVPV
DRIRVRQGDSDEIPTGGGTGGARSLYSEGTALLATASTVLERGRQAAGEALEASPADIEFTTTGALDGGGRFSIAG
TDRGIGILELAARQRAAAARGEQATLLDAAEVAEVPFGTFPNGCHIAEVEVDPETGLIGIARYLVVDDVGHAINP
LIVRGQVHGGVAQGVGQALHEHTVYDPESGQLLSASFMDYALPRAEDLPAIEVDLVEIPCETNPLGVKGAGEA
GAVGSPPAVMNALVDALAPDGVTHLDMPATPERVWRALGQALSRAA

>SEQF8858||SEQF8858.1_04616
MPDTNMPDPSRMRFAVGQPVTRREDPMLLRGEGHYTDDLQAEGQAHAWMVRSPYAHGVINGIDTAEAKS
MPGVLAVYTGADLAEYGPIRCAMPLKNRDGSPLASPVRPALATDRVRYVGDPVAIVIAETPAEARDAAEAVML
DIDPLDAVTEASAAAAPGAPQLYDDVPGNVVLDWHSGDAEKVAAAFATAAHRVKLPLRNNRIVVAAMEPRAA
IAEYDAESGRYTLRACSQGVFGLRRQIAEDILRVPVEKVHVLTTQVGGSFGMKGTVYPEYPGMFHAARLLGRPV
KWTDERSGSFVSDQHGRDHETVAELALDAEGRFLAVRLTSFANMGAYLTTVGPLMGTMGFVKNVQSNYATPL
IEVDTKSVFTNTSPVGAYRGAGRPEGNYYMERLVEAAAAQIGMDPLELRRRNHIRPEQMPYDAASGSRYDGG
EFTALMEEALRQADWDGFAARREESASRGLLRGRGLGNYLECTAPAGKEHGGVRFEEDGGVTIITGTLDYGQG
HWTPFAQLLGSRLGVPFERIRLIQGDSDLLKHGGGTGGSRSMMSSGTAIIEASEKVIEKGREAASHLLEAAVADIE
FDPENGRFAIAGTDRGIGILELAARMRAAPELPEDLRSLDVTHVTEDKPMAYPNGCHVAEVEVDPETGHVHLV
RYLAVSDFGVEVNPLMVQGQVHGGVAQGIGQALMERVSYDEGGQILSASFMDYALPRAEDLPPILTASRPVPA
KTNPVGAKGCGEAGCAGSLPAVMNALADALRAAGAEPVDMPATPEKVWMALQRASRG
>SEQF8859||SEQF8859.1_00731
MAKFGLSQSLRRVEDPRLLKGEGRYTDDLAVAGTATGYLLRSPHAHATILSIDTADARAMPGVLAIYTAEDLAAD
GIGPLPHAGSLKNRDGSRSANAPRFLLASGKVRHVGDPVAFVVAESLDAAKDAAEAILVDYDPLPACTDLAAAN
EPGQPQVFDDVPENRVFDWEIGDKAKTDALFDSAAHVTRLTVVNNRIVVASMEGRAALAEYDAATERFTLQA
TSQGAWHLKDMLAEHVFKLPKEKFRVVTHDVGGGFGMKLFCYVEYGLACYAARKLGRPVKWTAERSEAFLSD
THGRDNITLGELALDKDGRFLALRTRNLANMGAYLSQFAGFIPTAAGTKVLASVYGFQAIHAHVIGALTHTTPVD
AYRGAGRPESNYLVERLIDAAARELGIDRAELRRRNMVPPSAMPHTTPVGQKYDSGDFAQVLDEALRRADWA
GFPARQAEARARGKRRGIGLAYYLEATGGDPSERAEVRFTEDGHVEVLVGTQSTGQGHETAYTMIIAERLGVPV
DRIRVRQGDSDEIPTGGGTGGARSLYSEGTALLATASTVLERGRQAAGEALEASPADIEFTTTGALDGGGRFSIAG
TDRGIGILELAARQRAAAARGEQATLLDAAEVAEVPFGTFPNGCHIAEVEVDPETGLIGIARYLVVDDVGHAINP
LIVRGQVHGGVAQGVGQALHEHTVYDPESGQLLSASFMDYALPRAEDLPAIEVDLVEIPCETNPLGVKGAGEA
GAVGSPPAVMNALVDALAPDGVTHLDMPATPERVWRALGQALSRAA
>SEQF8859||SEQF8859.1_00931
MPDTNMPDPSRMRFAVGQPVTRREDPMLLRGEGHYTDDLQAEGQAHAWMVRSPYAHGVINGIDTAEAKS
MPGVLAVYTGADLAEYGPIRCAMPLKNRDGSPLASPVRPALATDRVRYVGDPVAIVIAETPAEARDAAEAVML
DIDPLDAVTEASAAAAPGAPQLYDDVPGNVVLDWHSGDAEKVAAAFATAAHRVKLPLRNNRIVVAAMEPRAA
IAEYDAESGRYTLRACSQGVFGLRRQIAEDILRVPVEKVHVLTTQVGGSFGMKGTVYPEYPGMFHAARLLGRPV
KWTDERSGSFVSDQHGRDHETVAELALDAEGRFLAVRLTSFANMGAYLTTVGPLMGTMGFVKNVQSNYATPL
IEVDTKSVFTNTSPVGAYRGAGRPEGNYYMERLVEAAAAQIGMDPLELRRRNHIRPEQMPYDAASGSRYDGG
EFTALMEEALRQADWDGFAARREESASRGLLRGRGLGNYLECTAPAGKEHGGVRFEEDGGVTIITGTLDYGQG
HWTPFAQLLGSRLGVPFERIRLIQGDSDLLKHGGGTGGSRSMMSSGTAIIEASEKVIEKGREAASHLLEAAVADIE
FDPENGRFAIAGTDRGIGILELAARMRAAPELPEDLRSLDVTHVTEDKPMAYPNGCHVAEVEVDPETGHVHLV
RYLAVSDFGVEVNPLMVQGQVHGGVAQGIGQALMERVSYDEGGQILSASFMDYALPRAEDLPPILTASRPVPA
KTNPVGAKGCGEAGCAGSLPAVMNALADALRAAGAEPVDMPATPEKVWMALQRASRG
>SEQF9511||SEQF9511.1_03347
MPDTTMPDPTRMRFAVGQPVTRREDPMLLRGEGRYTDDLRAEGQVHAWMVRSPYAHGVINGIDTAEAKS
MPGVLAIYTGADLAEYGPIKCAMPLKNRDGSPLSSPVRPALAMDRVRYVGDPVAIVIAETALQARDAAEAVML
DIDPLDAVTEASAAAAAPGAPQLYDGVPNNIVLDWHSGDAEKVAAAFAGAAHRVRLSLRNNRIVVAAMEPRS
AIAEYDPATGRYTLRACSQGVFGLRRQIAEDILKVPVEKVHVLTTQVGGSFGMKGTVYPEYPGMLHGAKLLGRP
VKWTDERSGSFVSDQHGRDHETVAELALDAEGHFLAVRLTSFANMGAYLTTVGPLMGTMGFVKNVQSNYAT
PLIEVDTKSVFTNTSPVGAYRGAGRPEGNYYMERLVEAAAAQIGIDPLELRRRNHIQPGQMPYSTAAGTQYDG
GEFTALMEEALRKADWDGFAARREDSAARGMLRGRGLGNYLECTAPAGKEHGGVRFEEDGGVTIITGTLDYG

QGHWTPFAQLLGSRLGIPFDRIRLIQGDSDLLKHGGGTGGSRSMMSSGTAIIEASEKVIEKGREAASHLLEAAVA
DIEFDPENGRFAIAGTDRGIGILELAARMRSAPELPEDLRSLDVTHVTEDKPMAYPNGCHVAEVEVDPETGHVR
LVRYLAVSDFGVEVNPMMVQGQVHGGVAQGIGQALMERVSYDEGGQILSASFMDYALPRAEDLPPILTASRP
VPARTNPVGAKGCGEAGCAGSLPAVMNALADALRSAGAGPVDMPATPEKVWMALQRASRG
>SEQF9512||SEQF9512.1_01557
MPDTTMPDPTRMRFAVGQPVTRREDPMLLRGEGRYTDDLQAEGQAYAWMVRSPYAHGVINGIDTAEAKSM
PGVLAIYTGADLAEYGPIKCAMPLKNRDGSPLSSPVRPALAMDRVRYVGDPVAIVIAETPAEARDAAEAVMLDI
DPLDAVTEASAAAAPGAPQLYDDVPGNVVLDWHSGDAEKVAAAFAGAAHRVKLSLRNNRVVVAAMEPRAAI
AEYDAESGRYTLRACSQGVFGLRRQIAEDILKVPVEKVHVLTTQVGGSFGMKGTVYPEYPGMFHAARLLGRPV
KWTDERSGSFVSDQHGRDHETVAELALDAEGRFLAVRLTSFANMGAYLTTVGPLMGTMGFVKNIQSNYATPLI
EVDTKSVFTNTSPVGAYRGAGRPEGNYYMERLVEAAAAQIGMDPLELRRRNHIQPGQMPYSTAAGTQYDGG
EFTALMEEALRRADWDGFAARREESASRGLLRGRGLGNYLECTAPAGKEHGGVRFEEDGGVTIITGTLDYGQG
HWTPFAQLLGSRLGVPFDRIRLIQGDSDLLKHGGGTGGSRSMMSSGTAIIEASEKVIEKGREAASHLLEAAVADI
EFDPENGRFAIAGTDRGIGILEVAARMRTAPELPEELRSLDVTHVTEDKPMAYPNGCHVAEVEVDPETGHVHLV
RYLAVSDFGVEVNPMMVQGQVHGGVAQGIGQALMERVSYDEGGQILSASFMDYALPRAEDLPPILTASRPVP
AKTNPVGAKGCGEAGCAGSLPAVMNALADALRAAGAGPVDMPATPEKVWMALQRASRG
>SEQF9512||SEQF9512.1_01356
MAKFGLSQSLRRVEDPRLLKGEGRYTDDLAVAGTATGYLLRSPHAHATILSIDTADARAMPGVLAIYTAEDLAAD
GLGPLPHAGSLKNRDGTRSANAPRFLLASGKVRHVGDPVAFVVAESLGAAKDAAEAILVDYDPLPACTDLATAN
DPGQPLVFDEVPENRVFDWAIGDKAKTDALFGAAAHVTRLTVVNNRVVVASMEGRAALAEYDPATERFTLQA
TSQGAWHLKDMLAEHVFKLPKEKFRVVTHDVGGGFGMKLFCYVEYGLACYAARRLGRPVKWTAERSEAFVSD
THGRDNITLGELALDRDGKFLALRTRNLANMGAYLSQFAGFIPTAAGTKVLASVYGFQAIYANVIGALTHTTPVD
AYRGAGRPESNYLVERLIDAAARELGIDRAELRRRNMVPPAAMPHTTPVGQKYDSGDFARVLDEALERAGWA
GFPARQAEARARGKRRGIGLAYYLEATGGDPSERAEVRFTEDGHVEVLVGTQSTGQGHETAYTMIIAERLGVPV
DRIRVRQGDSDEIPTGGGTGGARSLYSEGTALLATASTVLERGRQAAGEALEAAPADIEFTTTGALDGGGRFAIA
GTDRGIGILELAARQRAAVAKGESATLLDAAEVAEVPFGTFPNGCHIAEVEVDPETGLIEIARYLVVDDVGHAINP
LIVRGQVHGGVAQGVGQALHERTVYDPESGQLLSASFMDYALPRAEDLPAIEVDLVEIPCETNPLGVKGAGEAG
AVGSPPAVMNALVDALAADGVTHLDMPATPERVWQALGQSMARAA
>SEQF9513||SEQF9513.1_01107
MPDTTMPDPTRMRFAVGQPVTRREDPMLLRGEGRYTDDLQAEGQAYAWMVRSPYAHGVINGIDTAEARSM
PGVLAIYTGADLAEYGPIKCAMPLKNRDGSPLSSPVRPALAMDRVRYVGDPVAIVIAGTPAEARDAAEAVMLDI
DPLDAVTEASAAAAPGAPQLYDGVPGNVVLDWHSGDAEKVAAAFAGAAHRVKLSLRNNRIVVAAMEPRAAI
AEYDAESGRYTLRACSQGVFGLRRQIAEDILKVPVEKVHVLTTQVGGSFGMKGTVYPEYPGMFHAARLLGRPV
KWTDERSGSFVSDQHGRDHETVAELALDAEGRFLAVRLTSFANMGAYLTTVGPLMGTMGFVKNVQSNYATPL
IEVDTKSVFTNTSPVGAYRGAGRPEGNYYMERLVEAAAAQIGMDPLELRRRNHIQPGQMPYSTAAGTQYDGG
EFTALMEEALRQADWDGFAARREDSASRGLLRGRGLGNYLECTAPAGKEHGGVRFDEDGGVTIITGTLDYGQG
HWTPFAQLLGSRLGVPFDRIRLIQGDSDLLKHGGGTGGSRSMMSSGTAIIEASEKVIEKGREAASHLLEAAVADI
EFDPGNGRFAIAGTDRGIGILELAARMRTAPELPEELRSLDVTHVTEDKPMAYPNGCHVAEVEVDPETGHVHLV
RYLAVSDFGVEVNPLMVQGQVHGGVAQGIGQALMERVSYDEGGQILSASFMDYALPRAEDLPPILTASRPVPA
KTNPVGAKGCGEAGCAGSLPAVMNALADALRSAGAEPVDMPATPEKVWMALQRASRG
>SEQF9513||SEQF9513.1_00905
MAKFGLSQSLRRVEDPRLLKGEGRYTDDLAVAGTVTGYLLRSPHAHATILSIDTAEARAMPGVLAIYTAEDLAAD
GLGPLPHAGSLKNRDGSRSANAPRFLLASGKVRHVGDPVAFVVAESLDAAKDAAEAILVDYDPLPACTDLATAN
DPGQPLVFDEVPENRVFDWAIGDKAKTDALFDAAAHVTRLTVVNNRVVVASMEGRAALAEYDAATERFTLQA

TSQGAWHLKDMLAEHVFKLPKDRFRVVTHDVGGGFGMKLFCYVEYGLACYAARRLGRPVKWTAERSEAFVS
DTHGRDNITLGELALDRDGKFLALRTRNLANMGAYLSQFAGFIPTAAGTKVLASVYGFQAIYANVIGVLTHTTPV
DAYRGAGRPESNYLVERLIDAAARELGIDRAELRRRNMVPPSAMPHTTPVGQKYDSGDFARVLDEALERAGW
AGFPARQAEARARGKRRGIGLAYYLEATGGDPSERAEVRFTEDGPNGNSMVEVLVGTQSTGQGHETAYTMIIA
ERLGVSVDRIRVRQGDSDEIPTGGGTGGARSLYSEGTALLATASTVLERGRQAASEALEAAPADIEFTTTGALDG
GGRFTIAGTDRGIGILELAARQRAAVAKGESATLLDAAEVAEVPFGTFPNGCHVAEVEVDPETGLIEIARYLVVDD
VGHAINPLIVRGQVHGGVAQGVGQALHERTVYDPESGQLLSASFMDYALPRAEDLPPIEVDLVEIPCETNPLGV
KGAGEAGAVGSPPAVMNALVDALAADGVTHLDMPATPERVWQALGQSMARAA
>SEQF9514||SEQF9514.1_02148
MPDTTMPDPTRMRFAVGQPVTRREDPMLLRGEGRYTDDLQAEGQAYAWMVRSPYAHGVINGIDTAEARSM
PGVLAIYTGADLAEYGPIKCAMPLKNRDGSPLSSPVRPALAMDRVRYVGDPVAIVIAGTPAEARDAAEAVMLDI
DPLDAVTEASAAAAPGAPQLYDGVPGNVVLDWHSGDAEKVAAAFAGAAHRVKLSLRNNRIVVAAMEPRAAI
AEYDAESGRYTLRACSQGVFGLRRQIAEDILKVPVEKVHVLTTQVGGSFGMKGTVYPEYPGMFHAARLLGRPV
KWTDERSGSFVSDQHGRDHETVAELALDAEGRFLAVRLTSFANMGAYLTTVGPLMGTMGFVKNVQSNYATPL
IEVDTKSVFTNTSPVGAYRGAGRPEGNYYMERLVEAAAAQIGMDPLELRRRNHIQPGQMPYSTAAGTQYDGG
EFTALMEEALRQADWDGFAARREDSASRGLLRGRGLGNYLECTAPAGKEHGGVRFDEDGGVTIITGTLDYGQG
HWTPFAQLLGSRLGVPFDRIRLIQGDSDLLKHGGGTGGSRSMMSSGTAIIEASEKVIEKGREAASHLLEAAVADI
EFDPGNGRFAIAGTDRGIGILELAARMRTAPELPEELRSLDVTHVTEDKPMAYPNGCHVAEVEVDPETGHVHLV
RYLAVSDFGVEVNPLMVQGQVHGGVAQGIGQALMERVSYDEGGQILSASFMDYALPRAEDLPPILTASRPVPA
KTNPVGAKGCGEAGCAGSLPAVMNALADALRSAGAEPVDMPATPEKVWMALQRASRG
>SEQF9514||SEQF9514.1_02352
MAKFGLSQSLRRVEDPRLLKGEGRYTDDLAVAGTVTGYLLRSPHAHATILSIDTAEARAMPGVLAIYTAEDLAAD
GLGPLPHAGSLKNRDGSRSANAPRFLLASGKVRHVGDPVAFVVAESLDAAKDAAEAILVDYDPLPACTDLATAN
DPGQPLVFDEVPENRVFDWAIGDKAKTDALFDAAAHVTRLTVVNNRVVVASMEGRAALAEYDAATERFTLQA
TSQGAWHLKDMLAEHVFKLPKDRFRVVTHDVGGGFGMKLFCYVEYGLACYAARRLGRPVKWTAERSEAFVS
DTHGRDNITLGELALDRDGKFLALRTRNLANMGAYLSQFAGFIPTAAGTKVLASVYGFQAIYANVIGVLTHTTPV
DAYRGAGRPESNYLVERLIDAAARELGIDRAELRRRNMVPPSAMPHTTPVGQKYDSGDFARVLDEALERAGW
AGFPARQAEARARGKRRGIGLAYYLEATGGDPSERAEVRFTEDGPNGNSMVEVLVGTQSTGQGHETAYTMIIA
ERLGVSVDRIRVRQGDSDEIPTGGGTGGARSLYSEGTALLATASTVLERGRQAASEALEAAPADIEFTTTGALDG
GGRFTIAGTDRGIGILELAARQRAAVAKGESATLLDAAEVAEVPFGTFPNGCHVAEVEVDPETGLIEIARYLVVDD
VGHAINPLIVRGQVHGGVAQGVGQALHERTVYDPESGQLLSASFMDYALPRAEDLPPIEVDLVEIPCETNPLGV
KGAGEAGAVGSPPAVMNALVDALAADGVTHLDMPATPERVWQALGQSMARAA
>SEQF9734||SEQF9734.1_01392
MTGTVDTRYAGARVPRVEDTRLLTGRGTYVDDIQRPGMLHACFVRSPFARATVNGIDTAAALALPGVQAVFTA
ADINPDVREAWHAVAGKDIADTPRPPLADGEVKFVGDPVALVVAASRRLAEDAADLVDVDYTPLPALADFRKAI
GAEVVVHQAYPDNVAGGMGGMPPDEDVFANAAHVVEERIYQQMYVPVPMETRGIVVEWTAGTSELTVWG
SSQTPHELRAFAARLLGIPAQGVRVIVRDTGGGFGQKVVPMREDMCIMLAARKVPGPLKWIEDRRENLMAA
GQSRHVDGTVRMAFDADGRILAADIDFIQDIGAYPTPYPVLTTAAIGMFFPGPYRVPKASFNYKTVFSNTPGLH
AYRGPWQYETLTREMLLDIAARKIGMDPVELRRINILRGDEMPFFNPNGMPYDNCAPADTFEQAVKILDHEGF
RKAQEQALAHGRYIGLGFSAYIEPTGAATGHLATEGATIRMESTGKINVYVNGGSAGNSIETTVVQLTADALGAN
IDDVSTIQGDTAVTPYGAGTQGSRSGPMTAGAVHEAGSILRGQIVAIAAHILGVDADDIALGDSTATVRSDPDRS
VSFADIAYRSYYEPAQLGGVPPTLEATARFSSQAMIHWANATHVCTCEVDIVTGQVSLTRYIVSEDVGPMINPNV
VEGQVAGGTVQGIGGALLEHLAYDDAGNPVASTFVDYLLPTATEIPPIEFGHVEIPGPGVGGYKGAGEGGAIGS
PPAVINAINDALAPLGVTVTSMPATPAAIVDLIEAAAQRKDR

>SEQF9737||SEQF9737.1_04584
MTGTVDTRYAGARVPRVEDTRLLTGRGTYVDDIQRPGMLHACFVRSPFARATVNGIDTAAALALPGVQAVFTA
ADINPDVREAWHAVAGKDIADTPRPPLADGEVKFVGDPVALVVAASRRLAEDAADLVDVDYTPLPALADFRKAI
GAEVVVHQAYPDNVAGGMGGMPPDEEVFANAAHVVEERIYQQMYVPVPMETRGIVVEWTAGTSELTVWG
SSQTPHELRAFAARLLGIPAQGVRVIVRDTGGGFGQKVVPMREDMCIMLAARKVPGPLKWIEDRRENLMSAG
QSRHVDGTVRMAFDADGRILAADIDFIQDIGAYPTPYPVLTTAAIGMFFPGPYRVPKASFNYKTVFSNTPGLHA
YRGPWQYETLTREMLLDIAARKIGMDPVELRRINILRGDEMPFFNPNGMPYDNCAPADTFEQAVKILDHEGFR
KAQEQALAHGRYIGLGFSAYIEPTGAATGHLATEGATIRMESTGKINVYVNGGSAGNSIETTVVQLTADALGANI
DDVSTIQGDTAVTPYGAGTQGSRSGPMTAGAVHEAGSILRGQIVAIAAHILGVDADDIALGDSTATVRSDPGRS
VSFADIAYRSYYEPAQLGGVPPTLEATARFNSQAMIHWANATHVCTCEVDIVTGQVSLTRYIVSEDVGPMINPN
VVEGQVAGGTVQGIGGALLEHLAYDEAGNPVASTFVDYLLPTATEIPPIEFGHVEIPGPGVGGYKGAGEGGAIGS
PPAVINAINDALAPLGVTVTSMPATPAAIVDLIEAAAQRKDR
>SEQF9739||SEQF9739.1_00691
MTGTVDTRYAGARVPRVEDTRLLTGRGTYVDDIQRPGMLHACFVRSPFARATVNGIDTAAALALPGVQAVFTA
ADINPDVREAWHAVAGKDIADTPRPPLADGEVKFVGDPVALVVAASRRLAEDAADLVDVDYTPLPALADFRKAI
GAEVVVHQAYPDNVAGGMGGMPPDEEVFANAAHVVEERIYQQMYVPVPMETRGIVVEWTAGTSELTVWG
SSQTPHELRAFAARLLGIPAQGVRVIVRDTGGGFGQKVVPMREDMCIMLAARKVPGPLKWIEDRRENLMSAG
QSRHVDGTVRMAFDADGRILAADIDFIQDIGAYPTPYPVLTTAAIGMFFPGPYRVPKASFNYKTVFSNTPGLHA
YRGPWQYETLTREMLLDIAARKIGMDPVELRRINILRGDEMPFFNPNGMPYDNCAPADTFEQAVKILDHEGFR
KAQEQALAHGRYIGLGFSAYIEPTGAATGHLATEGATIRMESTGKINVYVNGGSAGNSIETTVVQLTADALGANI
DDVSTIQGDTAVTPYGAGTQGSRSGPMTAGAVHEAGSILRGQIVAIAAHILGVDADDIALGDSTATVRSDPDRS
VSFADIAYRSYYEPAQLGGVPPTLEATARFNSQAMIHWANATHVCTCEVDIVTGQVSLTRYIVSEDVGPMINPN
VVEGQVAGGTVQGIGGALLEHLAYDDAGNPVASTFVDYLLPTATEIPPIEFGHVEIPGPGVGGYKGAGEGGAIG
SPPAVINAINDALAPLGVTVTSMPATPAAIVDLIEAAAQRKDR
>SEQF9740||SEQF9740.1_03063
MTGTVDTRYAGARVPRVEDTRLLTGRGTYVDDIQRPGMLHACFVRSPFARATVNGIDTAAALALPGVQAVFTA
ADINPDVREAWHAVAGKDIADTPRPPLADGEVKFVGDPVALVVAASRRLAEDAADLVDVDYTPLPALADFRKAI
GAEVVVHQAYPDNVAGGMGGMPPDEDVFANAAHVVKERIYQQMYVPVPMETRGIVVEWTAGTSELTVWG
SSQTPHELRAFAARLLGIPAQGVRVIVRDTGGGFGQKVVPLREDMCIMLAARKVPGPLKWIEDRRENLMSAG
QSRHVDGTVRMAFDADGRILAADIDFIQDIGAYPTPYPVLTTAAIGMFFPGPYRVPKASFNYKTVFSNTPGLHA
YRGPWQYETLTREMLLDIAARKIGMDPVELRRINILRGDEMPFFNPNGMPYDNCAPADTFEQAVKILDHEGFR
KAQEQALAHGRYIGLGFSAYIEPTGAATGHLATEGATIRMESTGKINVYVNGGSAGNSIETTVVQLTADALGANI
DDVSTIQGDTAVTPYGAGTQGSRSGPMTAGAVHEAGSILRGQIVAIAAHILGVDADDIALGDSTATVRSDPDRS
VSFADIAYRSYYEPAQLGGVPPTLEATARFNSQAMIHWANATHVCTCEVDIVTGQVSLTRYIVSEDVGPMINPN
VVEGQVAGGTVQGIGGALLEHLAYDDAGNPVASTFVDYLLPTATEIPPIEFGHVEIPGPGVGGYKGAGEGGAIG
SPPAVINAINDALAPLGVTVTSMPATPAAIVDLIEAAAQRKDR
>SEQF9741||SEQF9741.1_03853
MTGTVDTRYAGARVPRVEDTRLLTGRGTYVDDIQRPGMLHACFVRSPFARATVNGIDTAAALALPGVQAVFTA
ADINPDVREAWHAVAGKDIADTPRPPLADGEVKFVGDPVALVVAASRRLAEDAADLVDVDYTPLPALADFRKAI
GAEVVVHQAYPDNVAGGMGGMPPDEDVFANAAHVVKERIYQQMYVPVPMETRGIVVEWTAGTSELTVWG
SSQTPHELRAFAARLLGIPAQGVRVIVRDTGGGFGQKVVPLREDMCIMLAARKVPGPLKWIEDRRENLMSAG
QSRHVDGTVRMAFDADGRILAADIDFIQDIGAYPTPYPVLTTAAIGMFFPGPYRVPKASFNYKTVFSNTPGLHA
YRGPWQYETLTREMLLDIAARKIGMDPVELRRINILRGDEMPFFNPNGMPYDNCAPADTFEQAVKILDHEGFR
KAQEQALAHGRYIGLGFSAYIEPTGAATGHLATEGATIRMESTGKINVYVNGGSAGNSIETTVVQLTADALGANI

DDVSTIQGDTAVTPYGAGTQGSRSGPMTAGAVHEAGSILRGQIVAIAAHILGVDADDIALGDSTATVRSDPDRS
VSFADIAYRSYYEPAQLGGVPPTLEATARFNSQAMIHWANATHVCTCEVDIVTGQVSLTRYIVSEDVGPMINPN
VVEGQVAGGTVQGIGGALLEHLAYDDAGNPVASTFVDYLLPTATEIPPIEFGHVEIPGPGVGGYKGAGEGGAIG
SPPAVINAINDALAPLGVTVTSMPATPAAIVDLIEAAAQRKDR
>SEQF9742||SEQF9742.1_00397
MTGTVDTRYAGARVPRVEDTRLLTGRGTYVDDIQRPGMLHACFVRSPFARATVNGIDTAAALALPGVQAVFTA
ADINPDVREAWHAVAGKDIADTPRPPLADGEVKFVGDPVALVVAASRRLAEDAADLVDVDYTPLPALADFRKAI
GAEVVVHQAYPDNVAGGMGGMPPDEEVFANAAHVVEERIYQQMYVPVPMETRGIVVEWTAGTSELTVWG
SSQTPHELRAFAARLLGIPAQGVRVIVRDTGGGFGQKVVPMREDMCIMLAARKVPGPLKWIEDRRENLMSAG
QSRHVDGTVRMAFDADGRILAADIDFIQDIGAYPTPYPVLTTAAIGMFFPGPYRVPKASFNYKTVFSNTPGLHA
YRGPWQYETLTREMLLDIAARKIGMDPVELRRINILRGDEMPFFNPNGMPYDNCAPADTFEQAVKILDHEGFR
KAQEQALAHGRYIGLGFSAYIEPTGAATGHLATEGATIRMESTGKINVYVNGGSAGNSIETTVVQLTADALGANI
DDVSTIQGDTAVTPYGAGTQGSRSGPMTAGAVHEAGSILRGQIVAIAAHILGVDADDIALGDSTATVRSDPGRS
VSFADIAYRSYYEPAQLGGVPPTLEATARFNSQAMIHWANATHVCTCEVDIVTGQVSLTRYIVSEDVGPMINPN
VVEGQVAGGTVQGIGGALLEHLAYDEAGNPVASTFVDYLLPTATEIPPIEFGHVEIPGPGVGGYKGAGEGGAIGS
PPAVINAINDALAPLGVTVTSMPATPAAIVDLIEAAAQRKDR
>SEQF9743||SEQF9743.1_00413
MTGTVDTRYAGARVPRVEDTRLLTGRGTYVDDIQRPGMLHACFVRSPFARATVNGIDTAAALALPGVQAVFTA
ADINPDVREAWHAVAGKDIADTPRPPLADGEVKFVGDPVALVVAASRRLAEDAADLVDVDYTPLPALADFRKAI
GAEVVVHQAYPDNVAGGMGGMPPDEDVFANAAHVVKERIYQQMYVPVPMETRGIVVEWTAGTSELTVWG
SSQTPHELRAFAARLLGIPAQGVRVIVRDTGGGFGQKVVPLREDMCIMLAARKVPGPLKWIEDRRENLMSAG
QSRHVDGTVRMAFDADGRILAADIDFIQDIGAYPTPYPVLTTAAIGMFFPGPYRVPKASFNYKTVFSNTPGLHA
YRGPWQYETLTREMLLDIAARKIGMDPVELRRINILRGDEMPFFNPNGMPYDNCAPADTFEQAVKILDHEGFR
KAQEQALAHGRYIGLGFSAYIEPTGAATGHLATEGATIRMESTGKINVYVNGGSAGNSIETTVVQLTADALGANI
DDVSTIQGDTAVTPYGAGTQGSRSGPMTAGAVHEAGSILRGQIVAIAAHILGVDADDIALGDSTATVRSDPDRS
VSFADIAYRSYYEPAQLGGVPPTLEATARFNSQAMIHWANATHVCTCEVDIVTGQVSLTRYIVSEDVGPMINPN
VVEGQVAGGTVQGIGGALLEHLAYDDAGNPVASTFVDYLLPTATEIPPIEFGHVEIPGPGVGGYKGAGEGGAIG
SPPAVINAINDALAPLGVTVTSMPATPAAIVDLIEAAAQRKDR
>SEQF9896||SEQF9896.1_02386
MNLYQPDPVTQHSGHIGARQTRVEDAALLRGLGCYADDAATPPGTLHAAIVRSPHPHARITAVDFSKALLMKG
VHGVLIGEDIKRWALPFPVGVRQPMEHWCIAVDKVRYVGEPVAVVIAESRYLAEDALEGVRVDYEPLPPIIDPE
AATTEQAPVLHEAVGSNVVNERHFRYGEPEQAFAQAPHRVALKVSFPRSSCTPIECYVVLAQYERATGIYDVLA
NFQGPYALHTVMARALNVPSNRLRLRTPKDSGGSFGIKQGVFPYVVMMGLAARKVGAPVKWVEDRLEHLQG
ASSATNRVTEIEAAVQADGRITALRYDQIDDCGAYLRAPEPATFYRMHGNLTGAYAIRNLLVRNRVVLTNKTPTGL
NRGFGGPQVYFALERLLQHIAVQLDLDPLEVIRRNLVPADAFPYRAAAGALLDSGNYQAGIDLAVKDGGLDELL
RRRDQARGEGRLYGIGYAAVIEPSISNMGYITTAMMPEERRKAGPKNGAVSTATINVGPLGDVSVHVSSTPQG
QGHQTAVAQIVADVLGVALESIVVNVELDTQKDAWSIASGNYSSRFAGAVAGVVYQAALKVRDRLAAIAAAQL
QAKPEDIRFGGGKIFVANGGPSAAFHRIAGATHWTPGLLPQGEPGGLRETAFWSPPQLMAPDDDDLINSSLCY
GFVFDICGLEIDRVTGAIHLDRYVTCHDAGRLLNPALVDGQIRGGFTQGLGAALMEEFAYGEDGSFLSGTFADYL
VPTAPEVLEPVILHLETPSPFTPLGAKGVGEGNNMSTPVCIANAVADALGRADIRLPLTPSKVRSMIGIDEPPRPA
GMQQDDAFAAEPAGGSALRANDAVVIPAPPQQVFDTLLDPATLAAIIPGCHALELQGENRYRADVTVGVGMI
RARFEAKVALSELDPPHSLRLSGSGTSSMGSAEGHARVRFVALENGHTRLEYQYQVAVSGKVAAVGSRMMQG
ASKVIIGQIFTRLSQRVSGQAITAGWWSRLRALFAGLLGKGGAQ
>SEQF9897||SEQF9897.1_02605

MNLYQPDPVTQHSGHIGARQTRVEDAALLRGLGCYADDAATPPGTLHAAIVRSPHPHARITAVDFSKALLMKG
VHGVLIGEDIKRWAQPFPVGVRQPMEHWCIAVDKVRYVGEPVAVVIAESRYLAEDALEGVRVDYEPLPPIIDPE
AATTEQAPVLHEAVGSNVVNERHFRYGEPEQAFAQAPHRVSLKVSFPRSSCTPIECYVVLAQYERATGIYDVLAN
FQGPYALHTVMARALNVPSNRLRLRTPKDSGGSFGIKQGVFPYVVMMGLAARKVGAPVKWVEDRLEHLQGA
SSATNRVTEIEAAVQADGRITALRYDQIDDCGAYLRAPEPATFYRMHGNLTGAYAIRNLLVRNRVVLTNKTPTGL
NRGFGGPQVYFALERLLQHIAVQLDLDPLEVIRRNLVPADAFPYRAAAGALLDSGNYQAGIDLAVKDGGLDELL
RRRDQARGEGRLYGIGYAAVIEPSISNMGYITTAMMPEERRKAGPKNGAVSTATINVGPLGDVSVHVSSTPQG
QGHQTAVAQIVADVLGVALESIVVNVELDTQKDAWSIASGNYSSRFAGAVAGVVYQAALKVRDRLAAIAAAQL
QAKPEDIRFGGGKIFVANGGPSAAFHRIAGATHWTPGLLPQGEPGGLRETAFWSPPQLMAPDDDDLINSSLCY
GFVFDICGLEIDRVTGAIHLDRYVTCHDAGRLLNPALVDGQIRGGFTQGLGAALMEEFAYGEDGSFLSGTFADYL
VPTAPEVLEPVILHLETPSPFTPLGAKGVGEGNNMSTPVCIANAVADALGRADIRLPLTPSKVRSMIGIDEPPRPA
GMQQDDAFAAEPAGGSALRANDAVVIPAPPQQVFDTLLDPATLAAIIPGCHALELQGENRYRADVTVGVGMI
RARFEAKVALSELDPPHSLRLSGSGTSSMGSAEGHARVRFVALENGHTRLEYQYQVAVSGKVAAVGSRMMQG
ASKVIIGQIFTRLSQRVSGQAIAAGWWPRLRALLASLLGKGGAQ
>SEQF9899||SEQF9899.1_02037
MNLYQPDPVTQHSGHIGARQTRVEDAALLRGLGCYADDAATPPGTLHAAIVRSPHPHARITAVDFSKALLMKG
VHGVLIGEDIKRWALPFPVGVRQPMEHWCIAVDKVRYVGEPVAVVIAESRYLAEDALEGVRVDYEPLPPIIDPE
AATTEQAPVLHEAVGSNVVNERHFRYGEPEQAFAQAPHRVSLKVSFPRSSCTPIECYVVLAQYERATGIYDVLAN
FQGPYALHTVMARALNVPSNRLRLRTPKNSGGSFGIKQGVFPYVVMMGLAARKVGAPVKWVEDRLEHLQGA
SSATNRVTEIEAAVQADGRITALRYDQIDDCGAYLRAPEPATFYRMHGNLTGAYAIRNLLVRNRVVLTNKTPTGL
NRGFGGPQVYFALERLLQHIAVQLDLDPLEVIRRNLVPADAFPYRAAAGALLDSGNYQAGIDLAVKDGGLDELL
RRRDQARGEGRLYGIGYAAVIEPSISNMGYITTAMMPEERRKAGPKNGAVSTATINVGPLGDVSVHVSSTPQG
QGHQTAVAQIVADVLGVALESIVVNVELDTQKDAWSIASGNYSSRFAGAVAGVVYQAALKVRDRLAVIAAAQL
QAKPEDIRFGGGKIFVANGGPSAAFHRIAGATHWTPGLLPQGEPGGLRETAFWSPPQLMAPDDGDLINSSLCY
GFVFDICGLEIDRVTGAIHIDRYVTCHDAGRLLNPALVDGQIRGGFTQGLGAALMEEFAYGEDGSFLSGTFADYL
VPTAPEVLEPVILHLETPSPFTPLGAKGVGEGNNMSTPVCIANAVADALGRADIRLPLTPSKVRSMIGIDEPPRPA
GMQQDDAFAAESAGGSALRANDAVVIPAPPQQVFDTLLDPATLAAIIPGCHALELQGENRYRADVTVGVGMI
RARFEAKVALSELDPPHSLRLSGSGTSSMGSAEGHARVRFVALENGHTRLEYQYQVAVSGKVAAVGSRMMQG
ASKVIIGQIFTRLSQRVSGQAIAAGWWPRLRALLARLLGKGGAQ
>SEQF9900||SEQF9900.1_02559
MNLYQPDPVTQHSGHIGARQTRVEDAALLRGLGCYADDAATPPGTLHAAIVRSPHPHARITAVDFSKALLMKG
VHGVLIGEDVKRWALPFPVGVRQPMEHWCIAVDKVRYVGEPVAVVIAESRYLAEDALECVRVDYEPLPPIIDPE
AATTEQAPVLHEAVGSNVVNERHFRYGEPEQAFAQAPHRVALKVSFPRSSCTPIECYVVLAQYERATGIYDVLA
NFQGPYALHTVMARALNVPSNRLRLRTPKDSGGSFGIKQGVFPYVVMMGLAARKVGAPVKWVEDRLEHLQG
ASSATNRVTEIEAAVQADGRITALRYDQIDDCGAYLRAPEPATFYRMHGNLTGAYAIRNLLVRNRVVLTNKTPTGL
NRGFGGPQVYFALERLLQHIAVQLDLDPLEVIRRNLVPADAFPYRAAAGALLDSGNYQAGIDLAVKDGGLDELL
RRRDQARGEGRLYGIGYAAVIEPSISNMGYITTAMTPEERRKAGPKNGAVSTATINVGPLGDVSVHVSSTPQGQ
GHQTAVAQIVADVLGVALESIVVNVELDTQKDAWSIASGNYSSRFAGAVAGVVYQAALKVRDRLAAIAAAQLQ
AKPEDIRFGGGKIFVANGGPSAAFHRIAGATHWTPGLLPQGEPGGLRETAFWSPPQLMAPDDGDLINSSLCYG
FVFDICGLEIDRVTGAIHLDRYVTCHDAGHLLNPALVDGQIRGGFTQGLGAALMEEFAYGEDGSFLSGTFADYLV
PTAPEVLEPVILHLETPSPFTPLGAKGVGEGNNMSTPVCIANAVADALGRADIRLPLTPSKVRSMIGIDEPPRPAG
MQQDDAFAAEPAGGSALRANDAVVIPAPPQQVFDTLLDPATLAAIIPGCHALELQGENRYRADVTVGVGMIR
ARFEAKVALSELDPPHSLRLSGSGTSSMGSAEGHARVRFVALENGHTRLEYQYQVAVSGKVAAVGSRMMQGA
SKVIIGQIFTRLSQRVSGQAIAAGWWSRLRALFAGLFGKGGAQ

>SEQF9901||SEQF9901.1_03881
MNLYQPDPVTQHSGHIGARQTRVEDAALLRGLGCYADDAATPPGTLHAAIVRSPHPHARITAVDFSKALLMKG
VHGVLIGEDIKRWAQPFPVGVRQPMEHWCIAVDKVRYVGEPVAVVIAESRYLAEDALEGVRVEYEPLPPIIDPE
AATTEQAPVLHEAVGSNVVNERHFRYGEPEQAFAQAPHRVSLKVSFPRSSCTPIECYVVLAQYERATGIYDVLAN
FQGPYALHTVMARALNVPSNRLRLRTPKDSGGSFGIKQGVFPYVVMMGLAARKVGAPVKWVEDRLEHLQGA
SSATNRVTEIEAAVQADGRITALRYDQIDDCGAYLRAPEPATFYRMHGNLTGAYAIRNLLVRNRVVLTNKTPTGL
NRGFGGPQVYFALERLLQHIAVQLDLDPLEVIRRNLVPADAFPYRAAAGALLDSGNYQAGIDLAVKDGGLDELL
RRRDQARGEGRLYGIGYAAVIEPSISNMGYITTAMTPEERRKAGPKNGAVSTATINVGPLGDVSVHVSSTPQGQ
GHQTAVAQIVADVLGVALESIVVNVELDTQKDAWSIASGNYSSRFAGAVAGVVYQAALKVRDRLAAIAAAQLQ
AKPEDIRFGGGKIFVANGGPSAAFHRIAGATHWTPGLLPQGEPGGLRETAFWSPPQLMAPDDDDLINSSLCYG
FVFDICGLEIDRVTGAIHLDRYVTCHDAGRLLNPALVDGQIRGGFTQGLGAALMEEFAYGEDGSFLSGTFADYLV
PTAPEVLEPVILHLETPSPFTPLGAKGVGEGNNMSTPVCIANAVADALGRADIRLPLTPSKVRSMIGIDEPPRPAG
MLQDDAFAAESAGGSALRANDAVVIPAPPQQVFDTLLDPATLAAIIPGCHALELQGENRYRADVTVGVGMIRA
RFEAKVALSELDPPHSLRLSGSGTSSMGSAEGHARVRFVALENGHTRLEYQYQVAVSGKVAAVGSRMMQGAS
KVIIGQIFTRLSQRVSGQAIAAGWWPRLRALLASLLGKGGAQ
>SEQF9902||SEQF9902.1_04261
MNLYQPDPVTQHSGHIGARQTRVEDAALLRGLGCYADDAATPPGTLHAAIVRSPHPHARITAVDFSKALLMKG
VHGVLIGEDVKRWALPFPVGVRQPMEHWCIAVDKVRYVGEPVAVVIAESRYLAEDALECVRVDYEPLPPIIDPE
AATTEQAPVLHEAVGSNVVNERHFRYGEPEQAFAQAPHRVALKVSFPRSSCTPIECYVVLAQYERATGIYDVLA
NFQGPYALHTVMARALNVPSNRLRLRTPKDSGGSFGIKQGVFPYVVMMGLAARKVGAPVKWVEDRLEHLQG
ASSATNRVTEIEAAVQADGRITALRYDQIDDCGAYLRAPEPATFYRMHGNLTGAYAIRNLLVRNRVVLTNKTPTGL
NRGFGGPQVYFALERLLQHIAVQLDLDPLEVIRRNLVPADAFPYRAAAGALLDSGNYQAGIDLAVKDGGLDELL
RRRDQARGEGRLYGIGYAAVIEPSISNMGYITTAMTPEERRKAGPKNGAVSTATINVGPLGDVSVHVSSTPQGQ
GHQTAVAQIVADVLGVALESIVVNVELDTQKDAWSIASGNYSSRFAGAVAGVVYQAALKVRDRLAAIAAAQLQ
AKPEDIRFGGGKIFVANGGPSAAFHRIAGATHWTPGLLPQGEPGGLRETAFWSPPQLMAPDDGDLINSSLCYG
FVFDICGLEIDRVTGAIHLDRYVTCHDAGHLLNPALVDGQIRGGFTQGLGAALMEEFAYGEDGSFLSGTFADYLV
PTAPEVLEPVILHLETPSPFTPLGAKGVGEGNNMSTPVCIANAVADALGRADIRLPLTPSKVRSMIGIDEPPRPAG
MQQDDAFAAEPAGGSALRANDAVVIPAPPQQVFDTLLDPATLAAIIPGCHALELQGENRYRADVTVGVGMIR
ARFEAKVALSELDPPHSLRLSGSGTSSMGSAEGHARVRFVALENGHTRLEYQYQVAVSGKVAAVGSRMMQGA
SKVIIGQIFTRLSQRVSGQAIAAGWWSRLRALFAGLFGKGGAQ
>SEQF9905||SEQF9905.1_04342
MNLYQPDPVTQHSGHIGARQTRVEDAALLRGLGCYADDAATPPGTLHAAIVRSPHPHARITAVDFSKALLMKG
VHGVLIGEDIKRWAQPFPVGVRQPMEHWCIAVDKVRYVGEPVAVVIAESRYLAEDALEGVRVEYEPLPPIIDPE
AATTEQAPVLHEAVGSNVVNERHFRYGEPEQAFAQAPHRVSLKVSFPRSSCTPIECYVVLAQYERATGIYDVLAN
FQGPYALHTVMARALNVPSNRLRLRTPKDSGGSFGIKQGVFPYVVMMGLAARKVGAPVKWVEDRLEHLQGA
SSATNRVTEIEAAVQADGRITALRYDQIDDCGAYLRAPEPATFYRMHGNLTGAYAIRNLLVRNRVVLTNKTPTGL
NRGFGGPQVYFALERLLQHIAVQLDLDPLEVIRRNLVPADAFPYRAAAGALLDSGNYQAGIDLAVKDGGLDELL
RRRDQARGEGRLYGIGYAAVIEPSISNMGYITTAMTPEERRKAGPKNGAVSTATINVGPLGDVSVHVSSTPQGQ
GHQTAVAQIVADVLGVALESIVVNVELDTQKDAWSIASGNYSSRFAGAVAGVVYQAALKVRDRLAAIAAAQLQ
AKPEDIRFGGGKIFVANGGPSAAFHRIAGATHWTPGLLPQGEPGGLRETAFWSPPQLMAPDDDDLINSSLCYG
FVFDICGLEIDRVTGAIHLDRYVTCHDAGRLLNPALVDGQIRGGFTQGLGAALMEEFAYGEDGSFLSGTFADYLV
PTAPEVLEPVILHLETPSPFTPLGAKGVGEGNNMSTPVCIANAVADALGRADIRLPLTPSKVRSMIGIDEPPRPAG
MLQDDAFAAESAGGSALRANDAVVIPAPPQQVFDTLLDPATLAAIIPGCHALELQGENRYRADVTVGVGMIRA
RFEAKVALSELDPPHSLRLSGSGTSSMGSAEGHARVRFVALENGHTRLEYQYQVAVSGKVAAVGSRMMQGAS

KVIIGQIFTRLSQRVSGQAIAAGWWPRLRALLASLLGKGGAQ
>SEQF9907||SEQF9907.1_02498
MNLYQPDPVTQHSGHIGARQTRVEDAALLRGLGCYADDAATPPGTLHAAIVRSPHPHARITAVDFSKALLMKG
VHGVLIGEDIKRWAQPFPVGVRQPMEHWCIAVDKVRYVGEPVAVVIAESRYLAEDALEGVRVEYEPLPPIIDPE
AATTEQAPVLHEAVGSNVVNERHFRYGEPEQAFAQAPHRVSLKVSFPRSSCTPIECYVVLAQYERATGIYDVLAN
FQGPYALHTVMARALNVPSNRLRLRTPKDSGGSFGIKQGVFPYVVMMGLAARKVGAPVKWVEDRLEHLQGA
SSATNRVTEIEAAVQADGRITALRYDQIDDCGAYLRAPEPATFYRMHGNLTGAYAIRNLLVRNRVVLTNKTPTGL
NRGFGGPQVYFALERLLQHIAVQLDLDPLEVIRRNLVPADAFPYRAAAGALLDSGNYQAGIDLAVKDGGLDELL
RRRDQARGEGRLYGIGYAAVIEPSISNMGYITTAMTPEERRKAGPKNGAVSTATINVGPLGDVSVHVSSTPQGQ
GHQTAVAQIVADVLGVALESIVVNVELDTQKDAWSIASGNYSSRFAGAVAGVVYQAALKVRDRLAAIAAAQLQ
AKPEDIRFGGGKIFVANGGPSAAFHRIAGATHWTPGLLPQGEPGGLRETAFWSPPQLMAPDDDDLINSSLCYG
FVFDICGLEIDRVTGAIHLDRYVTCHDAGRLLNPALVDGQIRGGFTQGLGAALMEEFAYGEDGSFLSGTFADYLV
PTAPEVLEPVILHLETPSPFTPLGAKGVGEGNNMSTPVCIANAVADALGRADIRLPLTPSKVRSMIGIDEPPRPAG
MLQDDAFAAESAGGSALRANDAVVIPAPPQQVFDTLLDPATLAAIIPGCHALELQGENRYRADVTVGVGMIRA
RFEAKVALSELDPPHSLRLSGSGTSSMGSAEGHARVRFVALENGHTRLEYQYQVAVSGKVAAVGSRMMQGAS
KVIIGQIFTRLSQRVSGQAIAAGWWPRLRALLASLLGKGGAQ
>SEQF9908||SEQF9908.1_01948
MNLYQPDPVTQHSGHIGARQTRVEDAALLRGLGCYADDAATPPGTLHAAIVRSPHPHARITAVDFSKALLMKG
VHGVLIGEDIKRWAQPFPVGVRQPMEHWCIAVDKVRYVGEPVAVVIAESRYLAEDALEGVRVEYEPLPPIIDPE
AATTEQAPVLHEAVGSNVVNERHFRYGEPEQAFAQAPHRVSLKVSFPRSSCTPIECYVVLAQYERATGIYDVLAN
FQGPYALHTVMARALNVPSNRLRLRTPKDSGGSFGIKQGVFPYVVMMGLAARKVGAPVKWVEDRLEHLQGA
SSATNRVTEIEAAVQADGRITALRYDQIDDCGAYLRAPEPATFYRMHGNLTGAYAIRNLLVRNRVVLTNKTPTGL
NRGFGGPQVYFALERLLQHIAVQLDLDPLEVIRRNLVPADAFPYRAAAGALLDSGNYQAGIDLAVKDGGLDELL
RRRDQARGEGRLYGIGYAAVIEPSISNMGYITTAMTPEERRKAGPKNGAVSTATINVGPLGDVSVHVSSTPQGQ
GHQTAVAQIVADVLGVALESIVVNVELDTQKDAWSIASGNYSSRFAGAVAGVVYQAALKVRDRLAAIAAAQLQ
AKPEDIRFGGGKIFVANGGPSAAFHRIAGATHWTPGLLPQGEPGGLRETAFWSPPQLMAPDDDDLINSSLCYG
FVFDICGLEIDRVTGAIHLDRYVTCHDAGRLLNPALVDGQIRGGFTQGLGAALMEEFAYGEDGSFLSGTFADYLV
PTAPEVLEPVILHLETPSPFTPLGAKGVGEGNNMSTPVCIANAVADALGRADIRLPLTPSKVRSMIGIDEPPRPAG
MLQDDAFAAESAGGSALRANDAVVIPAPPQQVFDTLLDPATLAAIIPGCHALELQGENRYRADVTVGVGMIRA
RFEAKVALSELDPPHSLRLSGSGTSSMGSAEGHARVRFVALENGHTRLEYQYQVAVSGKVAAVGSRMMQGAS
KVIIGQIFTRLSQRVSGQAIAAGWWPRLRALLASLLGKGGAQ
>SEQF9909||SEQF9909.1_00052
MNLYQPDPVTQHSGHIGARQTRVEDAALLRGLGCYADDAATPPGTLHAAIVRSPHPHARITAVDFSKALLMKG
VHGVLIGEDIKRWAQPFPVGVRQPMEHWCIAVDKVRYVGEPVAVVIAESRYLAEDALEGVRVEYEPLPPIIDPE
AATTEQAPVLHEAVGSNVVNERHFRYGEPEQAFAQAPHRVSLKVSFPRSSCTPIECYVVLAQYERATGIYDVLAN
FQGPYALHTVMARALNVPSNRLRLRTPKDSGGSFGIKQGVFPYVVMMGLAARKVGAPVKWVEDRLEHLQGA
SSATNRVTEIEAAVQADGRITALRYDQIDDCGAYLRAPEPATFYRMHGNLTGAYAIRNLLVRNRVVLTNKTPTGL
NRGFGGPQVYFALERLLQHIAVQLDLDPLEVIRRNLVPADAFPYRAAAGALLDSGNYQAGIDLAVKDGGLDELL
RRRDQARGEGRLYGIGYAAVIEPSISNMGYITTAMTPEERRKVGPKNGAVSTATINVGPLGDVSVHVSSTPQGQ
GHQTAVAQIVADVLGVALESIVVNVELDTQKDAWSIASGNYSSRFAGAVAGVVYQAALKVRDRLAAIAAAQLQ
AKPEDIRFGGGKIFVANGGPSAAFHRIAGATHWTPGLLPQGEPGGLRETAFWSPPQLMAPDDDDLINSSLCYG
FVFDICGLEIDRVTGAIHLDRYVTCHDAGRLLNPALVDGQIRGGFTQGLGAALMEEFAYGEDGSFLSGTFADYLV
PTAPEVLEPVILHLETPSPFTPLGAKGVGEGNNMSTPVCIANAVADALGRADIRLPLTPSKVRSMIGIDEPPRPAG
MQQDDAFAAEPAGGSALRANDAVVIPAPPQQVFDTLLDPATLAAIIPGCHALELQGENRYRADVTVGVGMIR

ARFEAKVALSELDPPHSLRLSGSGTSSMGSAEGHARVRFVALENGHTRLEYQYQVAVSGKVAAVGSRMMQGA
SKVIIGQIFTRLSQRVSGQAITAGWWSRLRALFAGLFGKGGAQ
>HRGM_Genome_0179||HRGM_Genome_0179_CDS_02860
MMTTTQQQQERKFKYIGKKNKPVEDYRFVQGLGTYVADIKIDGMHHIALIPSPYAHAKIISIDTSDALKLPGVIA
AVTGKELAQHVKPLKQYLDIPGVHWYPLAVDYAKYAGEWVVAIVAESRYIAEDAAELVKIKYEPLPFVTDPEEAM
KEDAPAVHPNHPKNVMWQRKFVWGEVERDFAEADDKIEFRCRWNRNSTVPLETFGVVTKWDEGRQILDVW
ASIQMPQYAEQIAEALQIPLNNVRVHYDVDVGGSYGVKRGIKHTVLVAYISRQYRVPTKLIEDRLENMRGSDMH
GPDRIFDVQVAFDNKGVIKSLKIRTIDDEGAYPGRSPLQMGKPIGAIVGAYKIKSVEYEGVAVTTNKTGQVAVRG
FGQSPTNYAIEMAVNKVANKLNLSPIEIRQRNFIQHHEFPYKIPSGTTYDSGNYPVVLEKAMKLANYEQLLKWQ
TAERKKGRIIGIGVGTCMEPGGGNALFEPLLNPKNDKTTFPEGCQVKVDQTGKVTANIAFSSSGQGHQTLVSTIL
AEEFDIVRDEIRVVYSDSLSALPSQSPVASRMAIVLGGATSGAAKKIKQKMMKIASHNLEEPLENLYWEGSTIKTT
TDEQKKLTWNEIVRIAHITYHKMPEDMEPGLQAQFVLEVPTGGTLPTPDGKVQMYPCYSFSAHIPVIEIDRDTG
KISFLNYYIADDCGTVINPDIVKGMVVGGVAHGIGAALYEQFSYDSNGQMISQTFMDYLLPSTMEVPHIDIVKH
CTPSPLTSMGQKGVGEGGYMSAPAAIMNAVNDALSPFNVEMTSVPVTPMDVLKKINQVEGDF
>HRGM_Genome_0200||HRGM_Genome_0200_CDS_03026
MNAPVVDAGTGIGASVRRKEDYRFTTGSGKYTDDVQMVGQTYACFLRSPHAHAAIRSIDKEQALQAPGVVAV
LTGEDLAADKVGGLPCGWLITDVNGQPMKEPPHPALAQGKVRYVGDPVAVVIADSYLQARDAAELIDVDYEVL
PAVVDGRAALQPGAPALHELAPDNKCYTWSIGDKAAVDEAFRRAHHVTKLDIHNNRLVPNAIEPRAALGHYNR
ADDSYTLYVANQNPHVERLLLTAFVLGLPEHKVRVIAPDVGGGFGSKIFLYGEEVVVTWASKKVNRPVKWTADR
SEAFLADAHGRDHATQAELALDQDGKFLAMRVHTVANLGAYLSTFAPSIPTILYATLLAGQYTTPAIYCEVDGVFT
NTTPVDAYRGAGRPEAAYVVERLVSAAAREMNIDQADIRRRNFITEFPYQTPVALQYDTGDFHACLDGAMELA
DVAGFPARRAEAERRGKLRGLGYSTYIEACGLAPSNIAGALGARAGLYEVGEIRVHPTGSVTVFTGSHSHGQGH
ETTFAQIVATRLGVPFEQVDIVHGDTGRVPFGMGTYGSRSLAVGGSAIMKALDKIEAKGKKIAAHLLEAADSDIE
FKDGQFTVAGTDRSVPFAQVAFSAYVPHNYPLDKLEPGLNETAFYDPTNFTYPGGAHICEVEVDPETGAVQIVG
MTAVDDFGKIVNPMIVEGQVHGGLAQGIGQALVENCVYDPESGQLLSGSYMDYAMPRAGDLPSFKVGNRVT
PCTHNPLGAKGCGEAGAIGSPPAVINAVIDALAPLGVTDIDMPATPERVWRAIQSARPAAQQAA
>HRGM_Genome_0335||HRGM_Genome_0335_CDS_00503
MSGRPGGTGRLVGTSVRRQEDPRLLTGRGRFTDDVHLPGMLYAHFVRSTVASGSVVSLDLSAVREVPGVVAAF
DAADLGLGDITARLDRPAGEFVPTAMPVLARDRVRFAGEPLAVVVARDPYAAEDGTEAAKVVHDTRPAVTRDT
QALAPGAPLVHDAAAANTLLDVTMFATEGIDSVFDRAHCVVDVETRTGRQNALPLETRGAVAAWDDRDRQL
VLHTSTQVPHQVRTVAARCLGLDERAVRVLVPDMGGGFGLKCVVGREEIAVAAAALRLGRPVKWTEDRKEALT
ASFLAREQHYRVRAAFDAEGRMLGLDADIVCDMGAYSCYPFTAGIEPLMAAAEMPGVYRLPAYRARARAVTT
NKAPSAPYRGVSRPQFVLVVERLFERAARELGLDPVEIRRRNLITEFPYTGVNNITYDPGSYRESLDLCERTLREEG
WYAARDTAAAEGRHLGIGYACFSERTGYGSAAFAQRKMEVVPGFDLAEVRMDTSGALTVTSGTMSHGQSHET
TMAQIAADELGLPLEKVRLHQGDTERVAYGWGTFASRSIAVGGSAVRRAAAELGRKLRHLAAARWGVPPDEV
VLGPGAVRRADGTAELSHTELARIAYLATHLLPEGMEPGLTATASFDGVGDGTFSNATHGVVVELHEGTGQVEIL
RYVCVEDCGVAVNPQVVEGQCRGGIAQGIAGALFEEITYDAQGEPSATGFMDYKVPTALEIPDVTVRHLETPCA
FTETGAKGAGEGGTIGAPAAVLNAVNDALRPTGVELNDIPVTPQTIAHALEQHS
>HRGM_Genome_0376||HRGM_Genome_0376_CDS_02768
MASMIGMRVKRKEDPKLITGNGNFTDDIKLPGMLYAAFLRSTHAHARIKRIDVSQAVELPGVVAVYTGEDLTGK
IKSVPTSWYVPGCNLKAKDRSPLAVDKVLYVGEGVAMVVAEDRYTAYDALNAIEVEYEELKAVTGQEAALKDGA
PLVHEDVKNNLAFLWKAGDVPDEAFTNAEVVVRERYYEQRVVPNPMETRAAVAQYNSGSGDMTVWCTSQN
PHIHRMVYAEVLGIPESKLRIIAPDVGGGFGAKIGVYADEAVVAYAARHLKRPVKWMEDRKEHFMATNHARDE
VIEVELAGKRDGTMTALRVRNTANMGAYLSTMGAGVPTICFGLMVTGAYAIPQAAVEVYGVYTNTTPTDAYRG

AGKPESAYQIERAVDAFAREIGMDPVEIRRKNFVPKEKFPYDTAMAVTYDSGDYMLTLDKALEIADYEELRREQE
ALRKQGRYLGIGLSTYVELCGLGPSKVAGAIGLQFGQWENATVRVHPSGKVTVLTGASPHGQGEDTTFAQVVA
DKFGVPLEDIEVLHGDTQMIPMGWGTYGSRTTPVGGAAVAIAAERVTEKAKKIAAHELEVSTEDLEFSDGIFQV
KGVPGHQRTFQEIARSANMAWNLPEGMEPALEAQSFFDPSNFVYPFGAHICVVEVDSNTGQIELKRYIAVDDV
GRVINPMIAEGQVHGGLAQGIGQALWEGAVYEENGQLISGTFMDYTMPKADFFPVLETAFTETPSPVNPLGA
KGVGETGATASPPAVVNAVLDALRPFGITHLDMPLTPEKVWRAMQKGRKEA
>HRGM_Genome_0384||HRGM_Genome_0384_CDS_04736
MTSFQPATESQTGHIGARQTRIEDAALLRGLGCYADDAAIPPGTLHAAIIRSPHAHARITSVDFSAALLMKGVHG
VLVGEDVKRWALPFPVGVRQPMEHWCVAVDKVRYVGEPVAVVIAESRYLAEDAIEGVRVEYEPLPPIIDPELAT
ADQAPILHEAVGSNVVNERHFRYGEPEQAFEQAPHKVSLKVKFPRSSCTPIECYVVLAQYERATGIYDVLANFQ
GPYALHTVMARALNVPGNRLRLRTPKDSGGSFGIKQGVFPYVVMMGLASRKVGAPVKWVEDRLEHLQGASS
ATNRVTEIEAAVEADGRITALRYDQIEDCGAYLRAPEPATFYRMHGNLTGAYAIRNVQVRNRVVLTNKTPSGLNR
GFGGPQVYFALERLLQHIAVQLKLDPLDVIRRNLVPTDAFPYQAAAGALLDSGNYQAGIALAAADGGLDDLLRR
RNQARAEGRIYGIGYAAVIEPSISNMGYITTAMTPEERRKAGPKNGAVATATINVGPLGDVSVHVSSTPQGQGH
QTTVAQVVAEVLGVALESIVVNVELDTQKDAWSIASGNYSSRFAGAVAGAVYKAALKIRDRLAAIAAEQLQASP
EDIRFAGGKIFVVNGGAVAPFHRIAGATHWSPGLLPGGESGGLRETAFWSPPQLVAPDDQDQVNSSLCYGFIF
DICGLEIDRMTGEIHIDRYVTCHDAGRLLNPALVDGQIRGGFTQGLGAALMEEFAYGEDGSFLSGTFADYLVPTA
PEVIEPVILHMDTPSPFTPLGAKGVGEGNNMSTPVCIANAVADALGRSDIRLPLTPSKVRTLIGIDEPPRPAGME
ADDNLDAAAGGPALRANDSVVIPASPQQVFDTLLDPQTLAAIIPGCHDLVLDGENRYRADVTVGVGMIRARFE
AKVALSDLDPPHSLRLSGSGSSSMGSAQGQAQVRFVELENGHTRLEYQYQVAVSGKVAAVGGRMLQGASKVII
GQIFTRLSQRVSGQAISTGWWARLRASLGALFGKGGAQ
>HRGM_Genome_0422||HRGM_Genome_0422_CDS_02639
MTAVEDRAQAPAAGPAKEVGQARRRKEDARLITGRTTWTDNLVLPGMLHLAIVRSPVAHATITGVDVEAARSA
PGVVAVLTGRDLADEQGSIPCAWPVTPDMVNPGHPSVAVDEVNHVGEAVAVIVARSKAAAQDAVELVDVDY
DQLPVVLDMEQALGEAPQLCHDHLDSNESFRWVFDAGEAGTGEDTDRAFADAEVVVSRRFVQQRLIPAFME
PRATVVQPQGDNYTMWSSTQVPHILRIMLAMVTGVPEHKLRVIAPDVGGGFGGKLQVTPEEVITLLVAKRLGK
PIKWAETRSESLMSAHHGRDQIQYVDIAADRDGTVRGLKVHILSDMGAYLRLVSPGVPILGAFMYPGIYKFPAY
RFDCQGVFTNKTPTDAYRGAGRPEATFAVERIMDELAVELGMDPLELRRKNWIRAEEFPFTTVAGLEYDSGDY
DEATRQALELLGYDDLRAEQRRRRESGDPVQLGIGFSTFTEMCGLAPSRVLGSLDYGAGGWEHASIRMLPTGK
VEVVTGSTPHGQGHETAWSQLVADELGVPFEDVEVLHGDTAISPRGLDTYGSRSLVVGGAAVVRASQKVVAKA
RAVAAHLLEAAEEDLQFEGGTFSVRGTPGTGKTIQEIALATFAAHDFPEGIEPSIDADATFDPVNFSFPHGTHICA
AEVDTETGMVRIRKYACVDDVGTIVNPLIVEGQVHGGLAQGIAQALYEEAVYDADGNLTTGTFVDYLVPSAADL
PHFDTGNTVHVAPGNPIGAKGVGEAGCIASTPAVVNAALDAVRHLGVSDIRMPLTPERVWRALQSGGDGGD
RATAGTNAYGGAQTETTAGVSTPESSLGGDR
>HRGM_Genome_0429||HRGM_Genome_0429_CDS_02131
MAQVVGRPLKRKEDPKLITGHGQFTDDIKLPGLLFAAFKRSQYAHAKIKSIDVSKAMQFPGVVAVLTGKDVQDK
IKPIRSIWLVPGCDLIPKDRMPLAVDKVNYAGEAVALVVADSRYTARDALELIDVVYEELPAVVQQEAAISESAPVI
YHDVPNNIAFTWKAGDVPEEIFANAEVVVKQRLVEQRVIANPMETRGAVAQYNPGSGDLTIWCTTQNPHIHR
MTYADALGIPETKLRIIAPDMGGGFGAKIAVYTEEAVVGLAAMVLKQPIKWVEDRQEHFLATSHARDQVITTEL
AGTKDGKILAVRAKNIANLGAYLSTMGAGVPTIDFGLMITGAYNIPHASAETIGVYTNTTPTDAYRGAGKPEASF
QIERIINIFADKIGMDPVEVRKKNLVASVPYTNAMGCNYDSGDYLGTFAKALEAIDYAGLRKEQEKARKKGRLLGI
GISTYVELCGLGPSKVAGAIGFGGGLWENSTIRVHPSGKVTVFTGASPHGQGVGTTFAQVVAEKFGIPVEDIELV
SGDTLMISMGWGTYGSRTTPVGGGAVANAAERIIEKAKIIAAHELAVTQDELQFDNGVFSVKGQPEKQRTFQEI
AWSANNAWNLPDGVEPALESQSFFDPPDFVYPFGAHICVVEVDANTGLIELKRYLCIDDVGRIINPMIATGQVH

GGLAQGIGQALWEQTVYSEKGQLLSGTFMDYTMPKASFFPRFESSFVESPSPVNPLGAKGVGETGTTAAPSAIV
NAVIDALAPYGVKDLAMPLTPEKVWKAIHKRGDA
>HRGM_Genome_1533||HRGM_Genome_1533_CDS_00177
MNAPAEPNNHLIGASVKRKEDFRFLTGAGQYTDDVVQAHQSYAVFLRSPYAHARIKHINTDAARNHPGVLAVL
TGDDLAADKVNGLPCGWLIHSIDGTPMKEPPHPVLAQGKVRHVGDQVALVVAESVKIAKDAVEMIDVEYDEL
PAVVDTATADTAGTAVHDDVPNNTCYTWGHGDKAATDAAFARAAHVTRLDIVNNRLIPNAIEPRAVNASYSRQ
DDSYTLYVANQNPHVERLLMSAFVLGLTEAKVRVIAPDVGGGFGSKIFLYPEDVALTWASKKVGRPIKWTAERSE
SFLTDAHGRDHVTHAELALDAQGNFLAMRVHTTANMGAYLSTFASSVPTILYATLLAGQYKTPAIYAEVKAVFTN
TAPVDAYRGAGRPEATYVVERLVETAAHELQIDPAELRRRNFIRTFPYATPVGLTYDTGDYEPCLDRAIELADVKGF
AARRDASRAKGRLRGMGYSCYIEACGLAPSNIAGALGARAGLFEAGEIRVHPTGSVTVFTGSHSHGQGHETTFA
QVVADRLGVPIDNIEIVHGDTGRIPFGMGTYGSRSIAVGGSAIMKALDKIEAKAKKIAAHLLEASAEDIEFKDGVF
RVAGTDRTKTFGEVALTAYVPHNYPLDKLEPGLDENAFYDPTNFTYPAGAYICEVEVDPDTGEVHIDRFVAVDDF
GNIINPMIVEGQVHGGLGQGIGQALLEACVYDENGQLLTGSYMDYAMPRANDLPSFTVETAKGTPCTHNPLG
VKGCGEAGAIGSPPALINAIVDALAPLGVKDIQMPATPHRVWQTIQAAKA
>HRGM_Genome_1533||HRGM_Genome_1533_CDS_03341
MKRFDAGETTGTAQETQQTTGQPYVGRPMQRVEDAAILTGRGRYADDLGVKPGTLHAAILRSPHAHAELGLID
FAAALKAPGVRAVLTGADLPAWSKPFVVGVKAPMEQWALAMDRVRYVGEPVAVVVAESRALAEDALDLVRV
DYRVLPPVVSIEAAIADAAPQLHSGLGSNVASDRHFRYGDPEAAFATAPHRVSLTVHYPRNTCTPIECGVVIAEHL
PGNEGYQVTSNFMGPFSLHAVMAMALQVSANHLRHIAPRDSGGSFGVKQAVFPYVVLMCLASRKAGAPVK
WVEDRLEHLSAATSATARLSTIEAAVEADGRIVALDYDQLEDCGGYLRAPEPATFYRMHGVLTGAYAIPNLRVRN
RVVLTNKTPTGLVRGFGGPQVYFALERLVQRISIELNLDPLDVYRRNFVPSNAFPYRAAAGALLDSGNYQLAMS
RALETGAYDELKRRRDIARAEGRLYGIGFAAIVEPSVSNMGYITTATPAEARRKAGPKNGAIASATVSVDLLGGVV
VTIASTPAGQGHMTVCAQVVADVLGIDPAEVIVNVEFDTHKDAWSVAAGNYSSRFAGAVAGTVHLAATRVRD
KLARIVASQLDCDPAELIFAEGRITRRDAPETAVVFARAASNAPHWSPQLLPAGEEPGLRETVFWSPPNLDAPDE
QDRINTSACYGFAFDLCGLEIDRATGRVRIDRYVTAHDAGKLLNPALADGQIRGAFAQGLGAALMEEFRYGPDG
SFQSGTLADYLLPTTCEVPDPMIVHLETPSPFTPLGAKGLGEGNNMSTPPCIANAVADALGVRDIRLPLTPAKVM
AMIGLEDPPPSRLELAETATAAATGGKERSKGAKALSARGTVDLDAPPEAVFAVLLDPQALAQVVPGCHVLEPIG
DNRYRADVTVGVGMIKARYEAEIALSDLEPPHRLRLSGAGLSSLGSARGSGMVELAPHEGGTRLTYDYEAEVSG
KVAAVGGRMLEGAAKVVLRQLFESLGRQAGGKPVRKPGWIARLLALFGARR
>HRGM_Genome_1582||HRGM_Genome_1582_CDS_02214
MNMHQSTVEAATGHIGRRQARVEDAALLRGLGRYADDVATPPGTLHAAIVRSPHAHARVISVDASAALTMPG
VHGVLTGEDAKRWANPFPVGVRAPMEHWCLAVDKVRYVGEPVCVVIADDRYLAEDALDAVKVEYEPLPAIVD
PEAATAEDAPVLHEAVGSNVVNERHFRYGEPEEAFAKAARRVAIKVHYPRNSCTPIECYVVLGQYQPATGTYEVL
SNFQGPYALHSVMARALNVPGNRLRLRTPPDSGGSFGIKQGVFPYIVLMGLAARKVGAPVKWVEDRLEHLQA
SSSATNRVCEIEAAVEADGRVLALKYDQIDDCGGYLRAPEPATFYRMHGNLTGAYAIRNLQVRNRVVLTNKVPS
GLNRGFGGGQVYFALERLMHQVAVELGLDPLEVIRRNLVPAGAFPYRAAAGALLDSGDYPAAIELAVRDGGLD
ELLRRREQARAEGRIYGIGYTAAVEPSISNMGYITTAMTPEERRKAGPKNGAVSTATVSVGPLGDVSVHVSSTPQ
GQGHQTVVAQVVAEVLGVALDGISVNVELDTGKDAWSIASGNYSSRFAGAVAGSVYNAAMRIRERMAGIAA
AMWQVPAEDVRFAGGKVFVEGGPSQPFHRIAGATHWSPGLLPEGELGGLRETAFWSPPQLTAPSDEDCINSSL
CYGFIFDICAVEIDRVTGEVHIDRYVTCHDAGRILNPMLVDGQIRGGFTQALGVALMEEFAYANDGSFLSGTFAD
YLVPTAPEAVEPVILHMETPSPFTPLGAKGVGEGNNMSTPVCIANAVADALGRSDIRLPLTPSRVRTMIGIDEPP
APEGMVLDAPRVAGGSSLQAEDSVEIQASPQQVYDALLDPETLKAIIPGCHALELESENHYRADVTVGVGMIR
ARFAARVGLTDLDPPHSLKLSGSGNSPMGSATGSARVRFVELENGHTRLEYVYDAAVSGKVAAVGGRMLQSAS
KVIIGQIFTRLAQRLTGAPVRSGLWQRLLGLFGKGGAQ

>HRGM_Genome_1715||HRGM_Genome_1715_CDS_03218
MGESKVDGGYAHIGRPLVRVEDARHLAGGGAFVADIQAPGCLEVAFCRSLVAHGRLKGVTLPPELPAGTFWTA
ADIAGLALPIYCALLRPEFNGAPYPLLADGKVRFAGEPVVLALGATRAEAEERAEQVQLDIEPLAAVVDAWDEVE
HPGVPLHDHLDSNLVMRIGRALGDAQAIIDDPAARGLRQVTRRLSMGRVLASPLECRGCLAYPDPAGGVVVH
VSSQRPHLIRTFLAEQIPGLTEADIRVVVPDVGGGFGSKSNLYPEEVLVTALAWKLRRPVRWIEDRYEHFVASNH
SRQHEQRITAYFDDTGRIHALDALVVVDAGAYSAKTSTGAIEANMATNVMLGPYDIRNYRFEAVSIHTNKSPLGP
YRGVGRPGGCFAMERIIEEVARELGADPLDVRRANLISPAQMPYVSATGLAYDSGNYREVVDAAGRFVAENWS
AARQADDGGRVGVGYAMLVEQAAHGAEEWRRRGSPLIYGHESARATLNMDGTLVIEVGTLSHGQGHFTSLA
QIAAEITTLPVSAIRIRQGDTSAAPYGLGSVASRSIVMAGGAVAMACRDIVGKARAIHAVLHETDDAQIEVRDG
ACVSPQGTTSFGDLARVAYVGVSKLPRSISPGFSFQADYRPSVETGTFSYTLHAARVSVDPDTGFVRILDYLVVED
CGTVVNPLILDGQIRGGVAQGIGQALYEEMIYDSNGQPQTVTFGDYAVPSAVEVPPISILHFSTPSPHSEFGMKG
MGEGGAVAPPAAIANAVRDALADLGVDVCRTPITPDYLLRQLHEAGRGGTR
>HRGM_Genome_1715||HRGM_Genome_1715_CDS_01962
MTDPGKFRYIGKHRRAVEHRRFVTGQGRYAADIQLPDVLHVAIVASPHAHARIRSIDASGALAMPGVHAVLTG
EELCAHTDAMLPGVDAPQVKRFPLAHGVARYAGEWVAAVVADTRALAEDAAEAVIVEYEPIDHVVDPLAAME
PGAPQVHPLHGSNVIFRRVFNWGEVDEHFAQAPHKLSYRVHWARSATVPIETFAVSAWWNDATGILDVWASI
QMPKYPDLLARALRLPGNAVRVHYDVDVGGSYGVKRGIKHSVLVGYLARKLGRPVRFIEDRLENMRGGDMQ
GPDRVFDVTLAFDGDGIVKSMRMRAVDDIGAYSGRAPLQLGKPVGAIVGPYRIASVEYDAVSVMTNKTPQEA
VRGFGQSPTNYAIETGMDKVARFLGLDRVELRRRNLIGKDEFPYLIPSGTTYDSGDYATVLGKALDAASLEQIQR
QRDELRKRGLLAGIGVSTCLEPSGGNSAFEPLFNPKNTTTTWMDSCLVRIDLSGSVTALMGTSTSGQAHETLVS
TVVGEILQREPDGIRVLHADSLNALPSNSPVGSRMAIMLGGAAAGAAKKLRAKLLRIAAHNLGLPEEQLSYDGG
DVVAKSDSQRRMSWDNLVEIAHRMFHKLPEGTEPGLQEKFVWEVPTGGKMPTEDGRVQMYPCHSFESHVV
LVSIDPDTGKATFHRYVCGHDCGVMISPDVVHGMTYGGIAHGLGAAMMEKFVFSEEGQLLAGTFMDYLLPSA
LEVPAVTIVDHCTPSPLTEFGQKGSGEAGYLGSPAAIASAINDALAPVGASIDALPMTPQAIWNALRRASGGQR
VSPAHSEIPEMEKA
>HRGM_Genome_1715||HRGM_Genome_1715_CDS_01933
MHAERQLTAIPGMGASPGRIEDETLLRGKACFLDDVPVPDALHACFVRSPHAHARLRGIDAGQARALPGVVAV
YTRQDLFGHITSWRMPLGFPLAALPEDTTPFALAGDEVAFVGEAYAVVVARTRHEAEDAAAAVQAEFEVLPAV
ADCRQAVRGDAPRVRTELASNVLQDYTLAYGDCDAAFARADIVLDDEFQVHRGCGHPMEGRGVVAALDPAT
GMLTVWSSTQMAHELHYTIAHMLDQPEDQLRVITPDVGGGFGAKFMIYPEEIVVPAVARALGRPVKWVEDR
RENFLTTIQERDQFWRISVAADRDGRMLGVRGGFVHDHGAYTPQGTNVPYNAASSMTGPYVVPAFSLHVQV
AYTNKVPVATIRGAGYPQAAFVMERMMDRLASAAGIDRLECRRRNLIPPEKIPYTKPLKSRAGVPLVIDSGDFPA
LQATAARAIDYEGFARRQEESARAGLLRGIAIANSVKPTGRGPYESARVRVHPSGRIVVHTGALAMGQGIKTTLA
QLCAAHLGVEPQAVEVSAGDTGLSGYGMGGFASRQAMMAGSAVSEAAAKVRDKALATAAAVLGVSADELM
LSQGEVHTPDFARRVPLARLAMLLKGVPGYALPVPGDPGLDATVHFHCDAQAYAGSSHACEVEVDPLTGAIHV
RRYVAVQDSGNLINPQIAHGQVHGGVVHGIGNALFEWMGYDDNAQPLTTTFAEYLLPTAPEVPPIEVIFQPSP
TRLNPLGVKGIGECATLAVAAAVIGAVEHALDGHGVRITEFPLTPVRLAELLDPPARDTTAPSANP
>HRGM_Genome_1715||HRGM_Genome_1715_CDS_04969
MDSRLVGQPRPRAEDQRLVRGQGRYTDDVNLPGQAYGAFVRSTHAHAIIRGIDIEAAAQAPGVIAVLRGQDYL
DDGLRGMDHIPNPADAIAFKERAFQHSLTGSIFNQRHLPLVVDRVRHVGEPVVLVVARSMREARDACEMVQI
DYEPLPAVVDAVQAMEDGAPQLWDGAPRNLCFQARVGDEAAVREAFARAHCVVRREFRNSRVVNCQMEPR
SALGSFDASSGEYLLISGSQGVTRQQMCIADALGISPAKLRVVSPDVGGGFGPRSCVNPDQLAVLWAARRVGQ
PVKWTSDRNEAFLSDYQGRDQVIRAAIAFDAQGRILGIDNEIIGNIGAHTVSYVPLANGSRIMTTVYHVPAATAL
LSAVLTNTPPTGPYRGAGRPEATHVMERMLDIAADEMGMDRIELRRRNLIQPAQMPYRSPMGLTYDSGSFAA

NMERALVVSQWSEAEQRKRDARARGKLLGTGLANYVESPVGAPRERIEITIGTDGSVDFVVGTQSTGQGHETS
FVQVLADELGVPFEAVTMRTGDSRFVKVGGGSHSDRSMRLVGHLLVQAGERLRSTALSAAASMWGLQVQDL
CYEDGRVRDQAGQHVAGLGEIAAHIGREGMPGNPEERRFFAEAEFNGRIAAHPTGTAVCELEIDPETGDVEIVR
YTSIDDVGRPINPLIVEGQVHGGLAQGIGQAFFEDYHVDPESGQIFSGSYMDYGVARAGAIPRRFDVELTEDRT
HGNALGVKGGGESGITPATAAIFNALADALRGITREELPMPATPAAIWSAIQRAQGVQP
>HRGM_Genome_1715||HRGM_Genome_1715_CDS_03750
MSDASVLATADAPFVGRRQERVEDAALLSGRGAYGDDLPVKPGTLHAAILRSPHAHARVLGMDTGAAEAHPG
VRAVLTGADVKRWSQPFVVGVKSPMEHWSLAQDRVRYVGEPVAVVVAEDRYVAEDALELIRVDYEVLPVAVEI
EAAIAGDAPVLHPAVGSNIVSDRSFSYGEPDAAFASAEHRVQLTVHYPRNSCTPIEGAVVIAEHLAGDDGYEVSS
NFMGPYSLHSVMALALKIPGTRLRHKLPRDSGGSFGVKQSVFPYVVLMCLASRKAGAPVKWVEDRLEHLVAAT
SATSRLCTIEAAVDGEGRISALSYDQFDDVGGYLRAPEPATFYRMHGCLTGAYAIPNLKVRNRVVLTNRTPAGLVR
GFGGPQVYFALERLVQRIAQQLGIDPLTVYRRNFVPPEAFPYRAAAGALLDSGNYPLALEMAERQGGLEELLRR
RDVARAEGRLYGIGYAAIVEPSISNMGYISTVMTAEQRAKAGPKNGAIASATVGIDPLGGVTVVVASSPAGQGH
MTVCAQVVADVFGLLPQQVAVNVDVDTQKDAWSVAAGNYSSRFAGAVAGTVHLAASRLRDKVARIVAHQLR
CDPADVVFAGGKVGLPDAPGKMLPFARAASTAHWAPGQLPEGESPSLRVTEFWTPPELTAPDEQDRVNTSAA
YGFAFDICAVEIDRDTGRVRIDRYVTTHDAGKLLNPALADGQIRGAFAQGLGAALMEEFRYAGDGSFQSGTFAD
YLVPTTCEVPDPVIVHLETPSPFTPLGAKGLGEGNNMSTPVCVANAVADALAPIRGACDVRLPLTPLKIMELIGFD
DPPPSAGAQAASAPPASAAAPGGGSGKALSATGSVQIAAAPEVVFNVLLDPQALAKVVPGCHSLEAVGENRYR
ADVTVGVGMIKARYAAHIELSELDPPHSLRLAGSGSSSLGAARGSGKVRLEPSEGGTRLVYDYSAEVSGKVAAV
GSRMLEGAAKIVLAQLFEQLGRQASGQPTPAAPRRWWRKLLALFGWKGAA
>HRGM_Genome_1928||HRGM_Genome_1928_CDS_00398
MTTIESRPPSPEDLADNAQQPCGHGRMMRKEDPRFIRGRGTYVDDVALPGMLHLAILRSPYAHARIVRIDVTA
AQAHPKVKAVVTGADLAAKGLAWMPTLANDVQAVLATDKTRFQGQEVAFVVAEDRYSARDACELVDVDYEP
RDPVVDARTALDPSAPVIRTDLEGKSDNHIFDWETGDAAATEAVFAKADVVVQQEIVYPRVHPAPMETCGAVA
DLDPVTGKLTLWTTSQAPHAHRTLYALVAGLPEHKIRVISPDIGGGFGNKVPIYPGYVCAIVASLLLDKPVKWME
DRSENLTSTGFARDYIMVGEIAANRDGKILAIRSNVLADHGAFNAQAAPAKYPAGFFGVFTGSYDIEAAYCHMT
AVYTNKAPGGVAYACSFRITEAVYFVERLVDCLAFELKMDPAELRLRNLLRPNQFPYQSKTGWVYDSGDYETTM
RKAMNMIGYEALRAEQKQRRARGELMGIGMSFFTEAVGAGPRKDMDILGLGMADGCELRVHPTGKAVLRLS
VQTQGQGHETTFAQIVAEELGIAPDDIEVVHGDTDQTPFGLGTYGSRSTPVSGGAAALVARKVRDKAKIIASGM
LEVSVADLQWEKGKFHVKGDPSAAVTIADIAMRAHGAGDLPEGIEGGLDAEVCYNPSNLTYPYGAYFCVVDIDP
GTAVVKVRRFLAVDDCGTRINPMIIEGQVHGGIVDGIGMALMEMIAFDEDGNCLGGSLMDYLIPTALEVPHLE
TGHTVTPSPHHPIGAKGIGESATVGSPPAVVNAVVDALAPFGVRHADMPLTPSRVWEAMQGRATPPI
>HRGM_Genome_1933||HRGM_Genome_1933_CDS_04532
MNKEFRADELALQRFGIGQAVPRSEDPRLIRGGGCYSDDVSLPSQVHGVFVRSPHAHGIIQGLDASAAREMPG
VLAVITGEDLESAGYKDLPCAMALKGRDGKSLRKPAHPSLPRDRVRYLGEPVALVIAETALEARDAAEAIVLDIEPL
PAATTPAEALAADAPSIHDDVPGNLALDYHYGDARRVEEAFARAAHVTRLQLSNNRVVVSPLEPRSALAEYDAG
EERWTLHVGSQGVFGLRNTLATEILGVEPEKLRVLTRNVGGSFGMKASPFPEYVALLHAARALARPVKWTDQR
SESFLSDHQGRDAESTAELALDAEGRFLAVRISGLANMGAYLTQVSPLFSTINIAKNVVSVYRTPEVEVSVRCMFT
NTTPIGAYRGAGRPEGNYYMERLIDSAAAEMGLDRLELRRRNHIQPQEMPYAAASGMTYDSGEFTSLLDRAVE
AADWSGFEERRRESQERGKLRGIGVGQYLEVTAPPMKEMGGIRFEPDGTVTIITGTLDYGQGHATPFAQVLHS
KLGIPFERIRLLQGDSDELIAGGGTGGSKSIMASGTAILEASDKVIEKGRQIAASILEASSADIEFSRGWFTIAGTDR
AVHIMDVAQRLQAGLVLPDDVPHTLDVQHVHENSPSAFPNGCHIAEVEVDAETGTVEVVRYSMVNDFGTLIN
PMLVEGQLHGGVVQALGQALLERTVYDADGQLLTGSYMDYALPRADDAPAFAFESRPVPATTNPLGVKGCGE
AGCAGGLPAIMNAVVDALRPLGVRHVDMPATPQRVWEAIRAVS

>HRGM_Genome_1933||HRGM_Genome_1933_CDS_07554
MTEAGIGAPVRRKEDHRFITGQGRYVDDLNRSGQAYAYFVRSPHAHARIAGIDTSAARTMPGVLGIFTGDDLA
ADKVGGLICGWMIHSKDGSPMKAGPHPALAQGKVRYVGDHVAVVVAETLAQARDAAEAIVVDYDPLPAVVD
PATAQKAEAQVHEAAPDNTVFRWHLGDKSAVDQAFEQAKHVTKLDLVNNRLVPNAIEPRAAIGEYDAGTDSY
TLYTTSQNPHVARLVLSAFIGIAPEHKLRVVAPDVGGGFGSKIFIYAEETVCVWAAKKVKRPVKWTADRTEAFLS
DAHGRDHVTHAELALDESGKCIGLRVSTIANLGAYLSTFSSSVPTYLYAPLLSGQYDIPAIYCEVDAVYTNTAPVDA
YRGAGRPEATFVLERIMEKAARELQMDPAKFRKRNYITRFPHQTPVIMAYDIGDYQASLNKALEIADYKNLGKR
KRESARNGKLRGIGFSSYIEACGIAPSQAVGALGAGVGLWESAEVRVNPTGSVEVLTGSHSHGQGHETTFAQLV
SDRLGISIDQVTIVHGDTDKVQFGMGTYGSRSGAVGMSAIAKAIDKVIAKGKKVAAYVLEAGEGDIEFRDGRFS
VAGTDRSLAFGEVALQAYIAHKFAGQDLEPGLKEGAFYDPTNFTFPAGVHICEVEVDPETGVSTIDRWVAVDDF
GVVINPMIVEGQVHGGIAQGIGQALLEGAVYDGSGQLVTASYMDYCMPRAVDLPSFEVGMTVTPCPSNPLGI
KGCGEAGAIAAPPAVINAITDALGHEDLQMPATPQNVWRAAQKATLPMAAE
>HRGM_Genome_1933||HRGM_Genome_1933_CDS_05685
MLMWGPYVRELKIGRSIARSEDFRLVRGQGRFTDDLAFSGEAFLYVLRSPHAAARIRSIDVEAARKTPGVLAVLT
GEDCIRDRLGVIPSRVQRHLPDGSPHVAPPYRALAVNAVHHVGDAVAAVVATSLPAAKDAAECIVVDYEPLPAV
TRTRQASKPGTPPVWDEAPNNICFVYELGDRAGVDAAFSRAAHVARLEFDISRVSANPLEARNAIGLYDSGSGR
YTLFAGLQSPHSLRTDIARILGVAEADLRVVAYDCGGAFGMKEGVYPEMILVLWAARKVGRPVRWQSDRSEAF
LADHQARDNASTVELALDAGGRFLALRIRTVANLGAYLNSYGTHSPTNNLGGLAGTYTTPFIHASVTGIFSNTNP
TSPYRGAGRPEASYAIERVIDVAAVQIGIDRIELRRRNMIPPSAMPFRTGLIYTYDSGAFEENMDRALLLSDWAN
FETRRLEASKRGLLRGIGLASVIEIAGGPVTQPAEEYAEIRFDPGGGVTLFTGTHAQGQGHETTFRQIACDLLGVA
EESVRVTFGDTDQVPHGRGTVGSRSTSVVAASLHKAAEKIVARGKTIAAHMLEVSSDDVEFQDGRFVVSGTDR
SVSLIDVARAAFSPERLPKGIEAGFAESATVVPSAPTFPNGCHVAEVEIDPETGRTRLVGYWVIDDVGRVVNPALV
KGQMHGGIAQGAGQALCEEIVHDADTGQPLTASFLDYYMPRCDDLPSLVVEANEVLATTNPFGIKGAGEAGTV
GSLPAVMNAVNDALAGFGIYDFDPPASPFRVWQAMRSAREKDVAGRTGSSAVNARAIWDDDQR
>HRGM_Genome_1933||HRGM_Genome_1933_CDS_01620
MATAKFGVGQPMRRVEDRRLLTGGGKYTDDYTPEGCLRAIVVRSPHAHARFTITDAATARAMPDVRLILTADE
VSHLGPVPCLAPVPNADRTQSHLAHIPVLARGAVKHVGDAVAFVVADTLEQARDAAEALEIDYEPLPAVSDSRS
ALEAGAPLVWSEAPGNVAFDTALGDEAAVDAAFAAADRVVSLTIENNRVVSNFMETRGVVAEYDTRNESFTLT
LSSQGVHSLRDTLAKEILKIDSDKVRVVTGDVGGGFGTKTFMYREYPLAAEAARRLGRAVKWVADRSDHFTGD
SQGRDNVSTGSVALDAAGRFLAMRFDIIGNLGAYLSQFGPFIPYLGATMMTGVYRTPLIHVRVRGVYTNTVPV
DAYRGAGRPEAAYLLERLVDHAARETGIAPEEIRRRNFVRPDEMPYTTPVGERTYDTGDFNAHMTRALEQAD
WAGFEDRARASRQAGRLRGIGLATYIECTAWGEGEDVKVQLEQDGTITVYSGTQSNGQGHATAYAQFVSQHL
DLPLEQIRVMQGDTVQVATGNGTGGSRSIPVGGISVSVASRDLAAKLKDLASEHLEAGVSDLEIGEGAVRVAGT
DRRITFAELAQLPRATDERRTGSGDFTPPDATYPNGTHIAEVEVDPETGTTRIARYTIVDDFGMTVNPLLLEGQV
HGGITQGVGQALLERTVYDAEGQLLTASFMDYCVPRAEDVPLFHFETLNVPSTTNPLGIKGAGEAGSIGSCPAV
ANAVVDALHRASGIRHIDMPATPARVFAALRSARAQMPVA
>HRGM_Genome_1937||HRGM_Genome_1937_CDS_02977
MAINQTELRPMGKSIHRKEDPRFIRGQGKYLDDIVLPNMLYMSIVRSPYAHAKIKSINTEAAKQVPGVKLIITGED
LAKMNLAWMPTMNGDVQMVLATNKVLFQYQEVVAVVAETRGAAVDAAQLVEVEYEALPVIVDPFRALQPD
APVLRDDREKKSNHIWHWEAGDQESTEEIFKNAPVVVSQDVRFQRVHPSPLEPCGCIADYKKGTGRLTWYVTS
QAPHAHRTILAMISGLPEHKIQVISPDVGGGFGNKVPVYPGYVCALVASIQLGIPVKWVETRTENIASTGFARDY
HMTSEIAADENGKVLALRVKTIADHGAFDAAADPTKFPAGLFSIVTGSYDFKQAFVEVDGVYTNKAPGGVAYR
CSFRVTEAAYLIERTMDILARKLGMDPAELRLLNFIRKDQFPYQSPTGWTYDSGDYEKTFRLALEKIGYHELRKEQ
AEKRARGELMGIGISTFTEIVGAGPSHSFDIMGIKMFDSAEIRVHPTGKVIARLGVRHQGQGHETTFAQIIGEEL

GLTADDVLIEEGDTDTAPYGLGTYASRSTPTAGGAAALCARRIRDKARKIAAHLLEVGDEDISWNGESFEVKGTP
SRKVTMGEVALAAYTNVPEGMEPGLEATYYYDPPNLTFPHGAYIAVVDIDKGTGVVKVRRFLAVDDCGNVINP
MIVEGQIHGGLTEGFAIAFMQDIPFDEDGNCLAPNWMDYLVPTSLDTPNWETDRTITPSPHHPIGAKGVGESP
NVGSPAAFVNAVVDALEPFGVEHIDMPIFPWKVWEILRQNGVAE
>HRGM_Genome_1937||HRGM_Genome_1937_CDS_01640
MAGLVGARIKRKEDPRLITGNGNFTDDIKLPGMLHAAFLRSPYAHARIKRIDVSEALKLPGVKAVFTGADIAKKM
KAVPAMWFVTGADLKPKPRSPLAVDKVLYAGEAVAVAVAEDRYTARDALDLIVVEYEELPAVVGQEAAMQEGA
PLVHEDVPNNLALVWKAGEIPDEVFEQAEVVVRQRLYNQRVIPNPMETRAAVAQYNPGSGEMTIWCTTQNP
HIHRMVYSEVLGIPESKLRIIAPDVGGGFGAKIATYTEEAVVGCVARELKQPVKWAEERSEHFKATTHARDEVIEI
ELAGTRDGIMTALRVKNTANLGAYLSTFGAGCPTIDFGLMVTGAYTIPRAAVVTYGVYTNTTPTDAYRGAGKPE
ATFQIERAVDLFAREIGMDPVEVRRKNMVQKEAFPFSNPMGCVYDSGDYHLTLDKALEMIDYQALRQEQEELR
KQGRYIGIGVSTYVELCGFGPSKVAGAIGFQGGVWENSTVRVHPSGKVNVYTGTSPHGQGTETAFSQVVAEKL
GIPMEDVEIIHGDTQSISMGWGTYGSRTTAVGGAAVAIATDRVIEKAKKIAAHELEVAEEDLEFSEGIFQVAGVP
GQARTFQEMAKSANFAWNLPEGMEPSLEAQSFFDPSNFVYPFGAHICVVEVDGSTGQVELKRYVAVDDVGR
VINPMLAEGQVHGGVAQGVGQALWEGAVYGENGQLLSGTFMDYTMPKARFFPTIETAFTETPSPVNPLGAK
GIGETGTTAAPPAVANAVMDALRPFGIKDLDMPLTPEKVWKAMRNGGSGE
>HRGM_Genome_1937||HRGM_Genome_1937_CDS_01535
MTIGTSVKRVEDPRLVRGKGRYVGDIQLPGQCEAVIVRSQHAHANLRAIRTQQAANRPDVLLVLTVNDLPDDL
PPIPMRLSPEGSLELALQYPLAKDKVRYVGEPVALIVAKNRYAAEDAAELVEIDYEPLAPVASVDQALSADAGLLH
PAVGSNDLFVIHSQKGSVEEKLKESPHVLQEELYVQRHTGVPLETRGLLAVYDPNRTSGKLTVYGTTKVVHFNRQ
ILARLLKQDESQIRCVETDVGGGFGPRGEFYPEDFLIPYAAMQIGRPVRWIEDRLEHLVATNHSRQQKHRVTVG
FDEEGRILALRDEIFVDTGAYIRTHGVTVPALTQAMLPGPYDIEALELTTHVVATNKTPTGTYRGPGRFEGTFVRE
RVMDMIAKTLGRDPLKVREINVIRPEQMPYSNGISALGQEVELDSGDYPRILAHVRRVMNWDGFAARKAEAA
ASGRLLGMGLAMFVEKSGLGPWEWAEVELLENGEACCKTGLADVGQGVKTMLAQVCSDRLGLPYTKIRVVH
GDTAAVPKGNGSFATRGTVMGGTAAWQAADALRSKMLQTAAGLLQIETAQLGLLGESVVMQATGQTLLSFAQ
LLEECRRQGIRLREEYTFSAAHMTYPYGVHAAEVEVDQDTGRIKINRYYLAYDVGRAINPLLVKGQLVGGMAQG
LGGALFEKLYYDETGQLLSGTFMDYLLPSSMEVPDVEVEILEWAPSLLNPLGVKGAGEGGTVAVAPAVANAVCH
ALQDYGLAITSLPIRPELIRQAIRQGKNRPKGAEVLS
>HRGM_Genome_1937||HRGM_Genome_1937_CDS_01534
MKWFGKPIKRKIDPRLLRGGGRYIADITVQGTLSAAFLRSPHAHARIADISLEQARGLPGVVAVYGPDDAIDFPF
LPVLFPHANLTPVTQRPLNRTVHHVGEPVAMVVAETRYIAEDALDLIDVRYEPLPAVAQLQDAIGADAPLAHEH
LDSNLAARFTQSIGDAKAAMQAADVVVTQQFQIGRVSCLPIETRGLLAVWREEGTNPALEVYAATQNQHEMR
SILARLLDLPERQVRVIAPDVGGAFGAKAPFYVEDFLVCWAARRLGAPVRWVEDRMEHMMSCIHEREQIHEA
SLGVSADGKILAVTDAMLANTGAYVPWGIIVPLMTSTLIPGPYKVPNYFCDVQVLYTNTVPLAPFRGAGRPQAA
LILNRLLDQAAQKLNLDPIEIRHRNLIQADEFPYHTGLLSRDGSPQVYDSGDYARLLDEAVKRSRYGDWRQKQEE
CRRLGRHIGIGTAVAIENTGFGSFEGATVRVETTGEVTVLTGAASQGQGHETTLAQIAAEVLDVPLPQVTVCQG
DTSLISYGTGTFASRIATITGTAIYKAAQAVKEKALAIAACRLQAEVEMLELSQGYIRLKNDPDCCISLAEAAREAKG
LYPGTTYSLPVSPGLEVTEYFAPPAAAVTSMADIAVVEVDPASCEIKILQYTSVHDSGRLINPLVVNGQIHGGIVN
GVGTALYEEVVYDRQGQLLTSTLMDYLVPTSCESPELTVAHIETPSPLNPLGIKGTGESGTIPVPAAIQSAVEDALR
PWKIPIGRIPVKPSYISQLLGSRSAAAAVDRGGAE
>HRGM_Genome_4330||HRGM_Genome_4330_CDS_01933
MTVTGAPTQTLSVLDRPNSYIGKTVPRPNLDRLLQGRGQYVSDLELPRMAHVVFLRSPHAHARIVAIDADAAR
RMSGVISIVTGPELEAVVTPWVGVLSHLKGLKSAPQHAIAVDRVCWQGEAVAAIVATSRAAAEDAMEHVAVD
YEELEAVTDMRTALDPATPVIHASLGDNLAFERTFNAGDVDHALAESEVVEADFIFGRHTGVTLEPRAVVADW

NAAEARLTIYQGTQAPHMVQNIAALHLGLSEAQVRVVCKDVGGSFGIKVHIYADEMATYALSRLLRRPVKFVAD
RIESFNTDIHARDHQCRGRIGVRPDGNITAFEIDDLTGIGPYSMYPRTSAIEANQVVNLVGGPYVTPNYRARARV
VFQNKNVMCQYRAVGHPIACSVTEGLIDLAAARIGMDPVEIRRRNLIPDDAYPCASPSGMKFEQLSHHASLAK
LLQMMDYDALRAEQAALRTRNVHRGIGVATFIEVTNPSAAFYGVGGAKISSQDGVAVRLDAQGSVICQTSITE
QGQGSESLTAQIVGSVLGVSMDRVRVILGDTDNTPYGGGTWASRGAGIGGEAALQAAKALRQNILDVAAVILQ
STPAALDISDNRIVNADDGAPRIELSELARTVYFRPDTLPQGIQPELMATRHFVPRQYPFAFTNGVQASWLEVD
TETGFVTLLKHWVVEDCGTIVNPQLVDEQIRGGVVQGFGAALFEKCIYDERGQLTNASMADYLVPMSGEMPD
IEVGHVVSPTQESELGAKGAGEAGTAGAAAAVANAVNDALRPFGATITEIPLTPQVILTALGRI
>HRGM_Genome_4330||HRGM_Genome_4330_CDS_02140
MNILPGNMRFGAGQPVKRLEDQRLVTGKGHFIDDKPQDGTLWLHVLRSPHAHANIKSIDVKAALEMPGVKA
VYTGADLVKDDIGTLPTLAIFKRPDGSPMTVPPRRLLAHEVVRYAGEGVAAVVATSRTLAQTAAEAIEIDYEVLPS
VVDPVEAVKPGAPVVWPEAPDNIVAAMSYGDAAKVEAAFASAAHKVSLDLISQRLVPSAMEPRSTIAEIEKKTG
RLILHVQSQTPGSTRDLLAESILKRPKDSVRVLVGDIGGGFGQKPSLYPEDGIVAYAATKLNETIRWRGDRTDEFV
GGTHGRDLTSTGEFALDAKGRVLAYRVRSIGGTGAYSSGTANIIPLVLGPFVQTGVYDLPLVHFEVKSVMTHTAPV
GAYRGAGRPEAVFIVERLFDAAARQIGMDPRTIRKVNYIKPAQLPYTNAVGQVYDSGAFAHMLERASDLADW
NGFAARKKAAKKKGLLYGRGLTSYIEWTGGRAPTENVSLHATAEGRIVLHSGTMAMGQGLATTYTQMISDALGI
GMDKIDVIQGDTDLATGFGSVGSRSLFVGGTAVAVSANDMINKARDKASHLLEASVGDIEYRDGFLTVVGTDR
RISLFEIARKESGAKLSVESEGNVDGPSWPNGTHICEVEIDPETGVTRVVRYTTVDDVGVAVNPMLVTGQVHG
GVVQGIGQALYEGVAYSEEGQLLTASYQDYCIPRASDVPPMSVTLDPSAPCKTNPLGAKGCGESGAIGGPPCITN
GVMDALSEVGITQLNTPLTPVKIWQAIRDAKVGAA
>HRGM_Genome_4330||HRGM_Genome_4330_CDS_03322
MAAPIKFGVGQSVRRKEDDALIRGKGRYTDDVAPSPALHALMLRSPHAHATYTIDAGKARGMPGVALILTAAD
VAELGGLPCLFNLETDPFIAPPYPILAKDEVRHVGDAVAFVVADTVDHARDAIEAIDVKWTPLPAVAGLVNAVKK
GAPQVWPDKPGNVLFDVSIGDKGAAEAAFAKAHAVAEITIVNPRVITNFMETRAAVAEYDAKKDHLTLTIGSQ
GSHRLREILCDMILKMPKENMRVICPDVGGGFGTKLFPYREYALISVAARKLKKSVKWTAERSDHFMGDAQGR
DNLTTAKMALAEDGKFLGMDVDLMGDMGAYLSTFAPYIPHGGAGMLPGLYDIQAFHCRVRTVFTNTVPVDA
YRGAGRPEAAYVIERLVDAAARKLGMTPDAIRRKNFISPKSLPYTTATGKVYDSGDFVAHMKRAMEIANWKEFP
KRAKAAKKDGLVRGIGMASYVEVCGTMGEETANVALDPNGDISILIGTQSSGQGHQTAYAQIVAEQFGVPPER
VHVLQGDTDKIATGLGTGGSASIPSGGVSVQRATHELGNKLKELAAQALEAGAGDLEIADGRIRIAGTDRSVSFA
DLAKRPGGDTSKMNASATFASADGTYPNGTHLAEVEIDPSTGIIKIVSYVIVDDFGVTLNPLMLAGQVHGGAM
QGIGQALMEQAVYSPTDGQLVTGTFMDYAMPRAADGPSFVFETHNVPCTTNPLGVKGAGEAGAIGSCPAVV
NAIVEGLHREFGIDHIDMPATPERVWIAIREAQRRHTL
>HRGM_Genome_4330||HRGM_Genome_4330_CDS_05536
MQDHTPPASLDNAIALQKYGVGQPVRRKEDDTLVRGKGRYTDDFSLPGQAICWMVRSSHAHGLIKGIDTEAA
KAMPGVLGVWTGADLAAAGYKPFTCGLPLKNRDGSPLLQTNRPALATDKVRFVGDPVAFVVAETAAQARDAA
EAVEVDIEPLPAVTDAAEASKPGAPQLYDNIPNNVALDYHYGDTAKIEAAFAAAAHVTKLDIVNTRVAVVSMEP
RVALAHYDKKTERFTLQVPTQGVSGNKAIMARLLNVPADKVRILTGNVGGSFGMKNLSYPEYTCIAHAARELGR
PVKWLDERSTSFLSDSQGRAQLIHAELALDAEGKFLAVRLSGYGNLGAYITGVAPGPLSLNTGKNLASVYRTPLL
GVDIKTVLTNTTLMGAYRGAGRPEANYYMERLIDAAADEMGISRVTLRKRNFIKPSQLPFPAASGVTYDSGDFA
GVFQKALEISDYENFAKRKKESRKNGKLRGIAVGSYLEVTAPPSGELGKITFEPDGSVKLTTGTLDYGQGHATPFA
QVLSDQLGVPFENITLEQGDSDLVRFGNGTGGSRSITATGQAIVEASALVVEKGKKAAAHMLEASEADIEFGQG
RFTIAGTDRSIGIMELAERMRAGKMPEGAPDTLDVDHATKETASTFPNGCHVAEVEIDPDTGVTRIVRYSAVND
FGVVVNPMIVAGQLHGGVAQGIGQALMEEVSYDASGQPITGSFMDYALPRAGDVPPMEVGDHPSPAKSNPL
GTKGCGEAGCAGSLVCVVNAVVDALSEYGIKHINMPLTPEKVWRAIQDAKANAA

>HRGM_Genome_4331||HRGM_Genome_4331_CDS_01528
MAAPIKFGVGQSVLRKEDDALIRGKGRYTDDLAPSAALHALVLRSPHAHATFTIDVAAARLLPGVALILTAEDTAE
LGGLPCLFNLETDPFTAPPYPILAKEEVRHVGDAIAFVVADSLDHARDAIEAIKVEWTTLPAVTGVVNAIKPDAP
QVWAEHKGNVLFDVSIGDKAAVDAAFAKAHAVAEIRIVNPRVVASFMETRAAVCEYDTKRDHLTLTAGSQGSH
RLRDILCQNVLKMPTEKMRVICPDVGGGFGTKLFPYREYALLAVAARRLRKSVRWAADRSEHFMGDAQGRDN
VTIARMALAEDGKFLAMDVDLMGDMGAYLSTFAPYIPHGGAGMLPGLYDIRAFHCRVRTIFTHTVPVDAYRG
AGRPEAAYVIERLVDACARKLDMTVDAVRRKNFIPPRALPYKTATGKVYDSGDFSAHLKRAMEIAEWKEFPKRA
KAAKKHGLIRGIGLASYVEVCGTMGEETANVRLDPNGDVTILIGTQSSGQGHQTAYAQIVAEQFGLPPERIHIHQ
GDTEEIATGLGTGGSASIPSGGVSVQRATHELGDKLKQIAAEALEAGLGDLEIADGVIRVAGTDRAISFADLAKRA
GADPAKLNGSATFASADGTYPNGTHVAEVEIDPATGIIRIVNYVIVDDFGVTLNPLLLAGQVHGGAVQGIGQAL
MEQVVYSNSDAQLVTGSFMDYALPRAADAPSFTFETHNVPCKTNPMGVKGAGEAGAIGSCPAVVNAIVEGL
WREYKIDHIDMPATPERVWIAIREQHRRHSL
>HRGM_Genome_4331||HRGM_Genome_4331_CDS_02204
MTAPLTPLDRPNSYIGRSVPRPNAKRLLAGRGRYVTDIRLPRMLHAAFLRSPHAHARIISIDADAARALAGVHLIA
TGADLARICTPWTGTLDHFKGMTSEPQLPLPLDRVVWAGQAVVAVVADSRALAEDALELISVDYEELPAVVDL
DEARQPGTPRANPASQSNICFRTQLDSGGVDEVFVAAAHVIEQQFSFGRHTPVTLEPRAIVADYEPDSGMLTV
HHATQTPYQFQDLYSRHYNIPEARVRVIAPDIGGSFGMKLHVYHEDMAVVGLSMMLGRPVKYVADRIESFVSD
IHARDHRVQARMAIDADGKILAMDVHDATAIGAFSTYPRTSVVEGNQVIRLIGAPYGFTSYRAALEVVFQNKV
QTSQYRAVGHPIACAVTERMVDIAAGRAGLDPLAMRDRNVIADDAYPLVSPTGYKFEALSHQACLRRLREIMD
YDALRGEQAELRRRGVHRGIGIATFVEITNPSPAFYGVGGARISSQDGATISLTPTGEVRCAISVTEQGQGTEAIIG
QIVADQLGLAQEHVKVITGDTEVTPHGGATWACRGAGIGGETALQAARRLKANILAIAALVLQEQAAALDIVDG
QVVSAASRQPRLDVAEIARIAYFRSDTLPPGTQAQLTVSHHFAPQGYPFAFTNGIQGCSLEVDVDTGFVKLLKHY
VVEDCGRIINPMLVDEQLRGGVVQGLGAALFEECRYGETGQLLNGSLADYLVPMAMEMPDIVIDHVETPTADT
ILGAKGCGEAGTAAASACVLNAVNDALTPFGASINTIPITPARILKALNRY
>HRGM_Genome_4331||HRGM_Genome_4331_CDS_06248
MGMEGIGASVVRKEDKRFITGKGRYVDDIKLLGMTYAHFIRSPHAHAKVKSIDSSAAEAMPGVVAVLTGRQIV
DDKVGNLICGWAVTSKDGSPMRMGAWPAMAPETVRFVGQAVAVVIAESKNLARDAAEAVVVEYEELPAVAD
MPSALKPGAPQLHPEAPGNVVYDWHIGDEAAVNAAFSKAANVVSLELTNNRLAPNAMEPRAAIADYDAAEE
HFTLYTTSQNPHVARLVLSAFYNIAPEHKLRVIAPDVGGGFGSKIFIYPEEMVALWASKKVGRPVKWTGDRTEAF
LTDAHGRDHISKAEMAFDADNKILGLRVKTHANFGAYMSLFSSSVPTYLYATLLSGQYNIPAIYAEVMGVYTNTT
PVDAYRGAGRPEASYLLERLVETAARQLKVDPAELRRKNFITQFPHQTPVIMAYDTGDFNASLDAAMKAIDYAG
FPARKAAAKAQGKLRGIGLSCYIEACGIAPSKAVGSLGAGVGLWESAEIRVNPVGTIEVLTGSHSHGQGHETTFC
QLIAERLGVPISQVSIVHGDTDKVQFGMGTYGSRSAAVGLTAILKAMEKVEAKAKKIAAHALEASEGDIIIENGEF
KVTGTDKSIALPMVALAAYTAHNLPDGMEPGLKESAFYDPTNFTFPAGTYICELEVDQATGKTSFVNFVAADDF
GRLINPMIVEGQVHGGLVQGIGQALLEHAVYDSNGQPVTASFMDYAMPRADDVPSFKLSHTTTLCPGNPLGV
KGCGEAGAIGASAAVINAITDAIGNNKLEMPATPDRVWHAIHGNA
>HRGM_Genome_4331||HRGM_Genome_4331_CDS_03679
MNDMTPTREQREAALEGMGCRRKRVEDIRFTQGKGNYVDDVKLPGMLHGDFVRSPHAHARVKAIHTEEALK
VPGVLAVITAETLKTVNLAWMPTLAGDVQMVLADGKVLFQNQEVAFVVATDRYAADDGVSKVVVEYEPLPVL
VDPFKAMDPDAPVLREDLAGKTVGAHGPRKHHNHIFEWSVGDKELTDAAFKKAEVTIKEMISYHRTHPSPLET
CQCVCAFDKIKGELTIWGTFQAPHVIRTVVSLIAKIPEQKIHVIAPDIGGGFGNKVGAYPGYICAAVASIVTGKPVK
WVEDRIENLTATAFARDYHMTTEIAATKDGKVTGLRVHVLADHGGFDACADPSKWPAGFFNIVTGSYDFPVAH
LSVDGVYTNKAPGGVAYRCSFRVTEAAYCIERAMDILAQKLGMDPAELRLKNFVKPEQFPYHSALGWEYDSGD
YHTAMRKMMEAVDYAGLRKEQAEKRAAFKRGETREIMGIGVSFFTEIVGAGPSKNCDILGIAMFDSCEIRLHPT

GAGIARMGTKSQGQGHETTWAQIIASEIGIPADNIMVEEGNTDTAPYGLGTYGSRSTPVAGAAIAMAARKIKA
KAQMIAAYKLEVHEDDLEWDIDGFRVKGLPEKVMSMKDICWAAYNSVPPGMEPGLEAVSYYDPPNMTYPFG
AYLCVMDIDVDTGVYKVRRFYALDDCGTRINPMIIEGQIHGGLTEAFAIAMGQEIRYDSEGNVVTGSFMDFFM
PTAVETPHWETDFTVTPSPHHPIGAKGVGESPNVGGVPAFSNAVNDAFAFLGATHIQMPHDYWRNWQAAK
NLGVVA
>HRGM_Genome_4331||HRGM_Genome_4331_CDS_02045
MQDHTSPASLENAIALQKFGVGQPVRRKEDDTLVRGHGKYTDDFNLPGQAYAWIVRSSHAHGIIKGIDTTAAK
GMPGVLGVWTGADLAAAAYGPFTCGLPLKSRDGSPLLQTNRPALATDKVRFVGDPIAFVVAETAAQARDAAE
AVGLDIEPLPAVTNAEEATRPGAPQLYDHIPNNVALDYHYGDTAKIEEAFAKAAHVTRLDIVNTRVAVVSMEPRV
ALAAFDGKTERFTLQVPTQGVSGNKATLAKILNVAPDKVRILTTNVGGSFGMKNVSYPEYVCILHAARELGRPV
KWRDERTTAFLSDSQGRDQIIHGELALDADGKFLAVRLSGYGNLGAYIMGVAPLPLSLNTGKNLASVYKTPLLGV
DIKTVLTNVTLMGAYRGAGRPEANYFMERLIDTAAREMGIDRLTIRKRNFIKPSQMPFPAASGVTYDSGDFAAV
FEKALAVSDYAGFAKRKRESRKNGKLRGIAVGSYLEVTAPPSGELGKITFDADGGVTLTTGTLDYGQGHATPFAQ
VLSAQLGVPFEKIRLEQSDSDLVRFGNGTGGSRSITATGQAIVESSQLVIAKGKQAAAHLLEASEADIEFAGGQFTI
AGTDRSIDIMELARRMREGKMPEGVPASLDVDHNSTATESTFPNGCHVCEVEIEPDTGVAQIVAYTAINDFGTVI
NPMIVAGQLHGGVAQGIGQAMMEHLRYDESGQPITGSLMDYALPRASDVPMMTVDNHPVPAKSNPLGTKG
CGEAGCAGSMASVINAVIDALSDYGVKHLDMPLTTEKIWRAIQDGKAKSAA
>HRGM_Genome_4331||HRGM_Genome_4331_CDS_05793
MGQVIGIGAAPKRKEDQRFLTGRGNYVSDIKRPGMTAGVFIRSPHAHAVLRGIDKSAALAAPGVVAVLTGEDV
ATDGLGSLPCGWGISDAKGVPMKEPPFPMLAQGKVRFVGDMVAFVIAETLEQAKSAAELVNVNYEVLPSVVG
VLDAVRPDAPQLFDDVPNNVCCDWELGDKAAVEAAFRKAAHVAKLGLVNNRLIGNPMEPRAAIAEYDPGKG
HHTLWTTSQFPHVVRFLMSALVLKLPEHKVRVVAPDVGGGFGVKQFHYGEEAVVTWAAKRVLRPVKWVADR
SEGYLSDRHGRDHVTEAELALDENGKFLALRVKTLANMGGYLSTFGPNIPTNLYAPLLGGVYTTPAIYCNVKVVF
TNTVPVDAYRGAGRPEATFVVERIVDVAAAEMGIDRVELRRRNMIPKEAYPYQTPVLVQYDSGDPMGCLDGAL
VAADVKNFGVRKAESARNGKFRGLGFSTYVEACGLAPSRFAGRLGARGGLYESATVRVHPAGQVTVMIGTHN
HGQGHETTFAQIVCEKLGVPFENVDIVFGDTDRVQFGMGTYGSRSLVVGGAALSKAADKVIVKGKKIAAHLLEA
SEQDIHFEAGKFSVAGTDRVKTIEEIAGAAYVPHNYPLEILEPGLEEQAYYDPVNFTYPGGCHIAEVEIDPETGIVT
LVNYTAMDDVGTVINPMIVEGQLHGGIVQGVGQALYENAVYDESSGQLVSGSLMDYCVPRADHLPMMKVAT
HSTLCTHTPMGVKGCGEVGTIGSPAAVINAVVDALAHLGVTHVDMPATPNRIWRLLQNASLPVAAE
>HRGM_Genome_4331||HRGM_Genome_4331_CDS_05163
MNILPNNMRFGAGQPVKRLEDQRLLTGKGQFIDDKPDDGALWLYVLRSPHAHAKILSIDTAAAREIAGVQAIYT
GADLVADDVGTIPTLAIFKRPDGKPMTVPPRRLLAHEVVRYTGEAVAAVVATSRVIAQEAAEAIVVDYEVLPAVT
DPVQALEPGAPVVWAEAPDNIVAAMSYGDAAKVEEAFASAPHKVSLDIVSQRLVPSAMEPRSTIAEIEKKTGRL
LLYVQSQTPASTRDVLAEAVLKRPKESVRVLVGDIGGGFGQKTNLYPEDGIVAYAATKLAKKIRWRAERTDEFVG
GTHGRDLTSTAEFALDAKGKVLAYRVRSIGATGAYSSGGAANIIPLVLGPFVQTGVYDLPLVHFEVKTVMTHTAPVG
AYRGAGRPEAVFIVERLFDTAARQIGIDPRTIRKANYIKPAQLPYTNAVGQVYDSGAFAHMLERASKLADWDGF
NARKREAKKRGMLYGRGLTSYIEWTGGRAHTEKVSLHATSEGRVILHSGTMAMGQGLQTTYTQMISDTLGIPL
DKIDVVQGDTDLATGFGSVGSRSLFVGGTAVAVSSNDLMTKAREKASNLLETAVEDIEYRDGFLTVVGTDRRISL
FEIAQKEQGAKLSVDSEGEVDGPSWPNGTHICEVEIDPETGISRVVRYTTVDDVGVAVNPMLVAGQIHGGVAQ
GIGQALYENVAYDADGQLLTATYQDYCIPRADDVPPIVVTLDDSAPCKTNPLGSKGCGESGAIGGPPCVTNGVM
DALFELGITNLQTPLTPEKVWAAIRDAKAKKAAA
>HRGM_Genome_4332||HRGM_Genome_4332_CDS_00327
MVEQGVGARLLRKEDDRLMRGRGEFVGDIRLPGMRDVAFVRSPLAHARIKGIRIPPEYRDSVFIAADLEGVKPI
RAVSGLPGFKVSEQPILAFEKVRQVGELIAMCVADTRAEAEDIAAAVEVDFEELPAVHDMLLAKRPDSALLHEH

WGDNIFLETFVDVNMEAAYDAPIKITREISTARQSMAPMEGRGTVAMWHKRMDQLVLYTGNQQPHIVRNGL
SECLGLEQIQVRIVSPDVGGGFGYKGIVLTEDVCLGWLAKRCGYPVRWIEDRREHLTAGANCREHHYNITVYAD
RDGKLRGVECEATVDSGAYSSYPFSACLEAAQVASILPGPYDFPSYRCRTWSVATNKCPILPYRGVARTGVCFAIE
LMMDAIARETGLEPYEVRLKNLVQPEQMPFDNITKKHFDSGDYPESLRQALAKIDLPALRERQKKPEADGRLIG
VGLSVYCEQGAHGTSVYAGWGIPMVPGHEQATARMTPDGGLELRVGVHSHGQSMETTLPQVAHEILGIDTA
KIKLVHGDTEYTPYSTGSWGSRCAVMAGGAVATASRELGKLVLGIGAHLLQTDISNVKLRDGVVVGPSGSVTLQ
EVAYTWYRRPQDLPPSVDPRGLEVTIGYKPVRDSGTFSYASHIAVVAVDPEMGDIELLDYVIVEDGGKLINPMV
VDGQIYGGLAQGIGTALYEEMNFDTSGQPLASTFADYLLPGPTEVPEPKLGHMETLAPYTEFGVKGLGEGGAIA
PPGAIGNAVNDALKSLGAEIRHSPITPRRVLEAIEEAKSTARAKAGEGVLA
>HRGM_Genome_4332||HRGM_Genome_4332_CDS_00379
MHNVGDDLATRPKMVGARVRRTEDPRLLTGNGNYVDDRQSAGMLHVAFRRSDHSHARIVSINCEAARRSPG
VIGIFTGADLEGRIKPLLATSRMANYHATPLVALAQGKVRYVGEPVVAVVAESRYLAEDAIELIEIDFEALPVVIDPE
AAVQPDAPLLHKEIGTNVLLSREFKRGDVDAEMENAVVRVSGRFRMRRKTPLAMENRSYVAEYSAGQDALTLY
SATQIPGIIRDALSEALDMPGNRLRVIAPDVGGGFGGKASLYPEELFVTFAARHLGRAVKWTSDRLEDLASTSQG
FDEIIDAELGFDKAGLAVALRADVIGDVGAYSIYPWTAVLEPVQVVSFLPGPYKIQHYRGRVRGVATSKPPMGPY
RGVGRPASTFVTERLMDMAARKFNVDPKLLRLKNMIQPDEFPHKIGSGIVWDQCSFTECLEAACERIGYATLRE
RQVKARAEGRWFGIGMACYAELTGIGSRISVAPGMPINTGTETAKILIDSTGAVTVAVGTAAHGQGHETTFAQV
VAEHLGARLQDIRVIHGDSAAVPNATGSYASRSAVLAGGAATLAAQDVKEKVLRAASHLLEASVADLTIEDGRIA
VAGTDRAMTFRELARAVYSEMGRIPSDQREELSSTKTYDPVFGTAATAAHVAVLEIDPETYQVKLERFLVAEDCG
KLINPMIVDGQVHGGVAQGIGAALYEEVIYNDEGQNVTASLVDYVVPSASEVPSIDVVHIEAASKTTLGGFRGM
GEGGTIGAPAAVANAISDALASLNIDVFELPVTPERLFRLIENAKAHS
>HRGM_Genome_4332||HRGM_Genome_4332_CDS_02293
MDFQGVGASLLRKEDDRFLRGRGQYVGDLKLPGLKDVAFVRSPLAHAKMRGVRIPPEFQDRVFTASDLTDVKP
IVAASGLPGFKYSEQPLLAHEKVRQVGELVAMCIGNTRAEAEDIAAAVELDLDELPVVSEMLTARRPGAPLVHD
HWGDNVFLETYIDVNMEAAYDAPIKVTREIRTARQCMAPLEGRATVAYWDKRLDQLVLHTGTQQPHIIRTGISE
CLGLDQSKVRVISPDVGGGFGYKALMPPEDVCLAWLAMHCGHPVRWIEDRREHLTASANCREHHYIITAYADR
DGTLRGIECEATVDSGAYSSYPFSACLEAAQIASILPGPYDFPSYRCRTWSVATNKCPILPYRGVARTGVCYAMEL
MVDAVAREAGLDPLEVRLKNLVKPEQMPFDNITKKHFDSGDYPEALKRAMARIDLDAIRKQQENTHRLIGIGLS
IYCEQGAHGTSVYSGWGIPMIPGHEQATARVTPDGGLELRIGVHSHGQSMETTLPQIAHEILGIETAKIKLVHGD
TEYTPYSTGTWGSRSAVMAGGAVSRASQEIAGLIKGIGAHLLQTDVANVRLEDGCVIGPSGSVTVKEVAHTWY
RRPQDLPASVDPRGLETTIGYKPVRDSGTFSYAAHIAVVAVDPEMGDIEILDYVIVEDGGKLINPMVVDGQIYGG
LAQGIGTALYEEMPFDPSAQPLASTFADYLLPGPTEVPAPQVEHMETLSPYTEFGVKGLGEGGAIAPPAAIGNAV
NDALKSLGVELLCCPMTPRRVLEAIHRAKGNA
>HRGM_Genome_4332||HRGM_Genome_4332_CDS_02623
MTQPAAAEVELNRPWVGRSIQRVEDFALLMGRGRFIDDLGTRPGTLHAAILRSPHAHADIVAIDTSAALKSRGV
AAVLVGEDVKKLTSSLVVGVKAPVECWPIAVGRVRYVGEPVAVVVASDRYLAEDAIDLVEVQYSVRPAVIDPLVA
VRADAPVLHDGFSGNVASDRRFRYGDPEKAFAEAAHRISIDIKYPRNSCTPIETYGLIADYDPAENAFDVLANFQ
GPFSIHAVISRALKVPGNRLRLRTPPDSGGSFGVKQGIFPYIVLIAAASRVVGRPVKWIEDRLEHLTASVSATNRAT
TLAAAVTAEGKILALDWDQVEDCGAHLRAPEPATLYRMHGNLTGAYDIRHVAVRNRVVVTNKMPTGLNRGFG
GPQVYFALERLLHRIAIELGLDPLEVIRLNLVPADAFPYRTATGALLDSGNYQEALIRGARDGQLAELKARRDQAR
AEGRLYGIGYTAVVEPSVSNMGYITTVLTAEERRKAGPKNGAQATATIGLDPVGGVTVHVASVPQGQGHRTVLS
QVVADVFGLLPQDIRVNTEIDTAKDAWSIASGNYASRFAPAVAGTAKLAAQRLAAKLARIAASQLNVDASDIVFA
GGSVGSKHNPENKISFSRLAALSHWSPGSLPDDVGDTIRETVFWTPPELTPPDDHDLINSSLCHGFIFDFCGVEI
DRTTLQTRIDRYVTMHDCGTILHPGMVDGQIRGGFVQALGAALYEEYAYGPDGSYLTGTLADYLLPTTTEVPEP

VILHMETPSPFTPLGSKGVGEGNCMSTPVCIANAVADALDVKDLTLPLVPARLAEIVRGAEPSAPAGRAVSGPKA
GGNDRRLRGEGNASVNVPPERVWDMLLDPETLRAVVPGCQSVEKVSDTHFRADVTLGIGPVTGRYRANVML
SDLDPPHAVTLSGSAEGVLGFGGGEGRITLTPDGNGGTTMHYVYHAAIGGKVASIGGRLLDGATKVIIGQFFSA
LARQAEGGGSGRDGLSLVVRLKRLIGIGS
>HRGM_Genome_4332||HRGM_Genome_4332_CDS_02229
MSVEGIGARVTRKEDKRFITGKGKYTDDVRLHGMTYASFVRSPHAHAKIKSINVDAAKAMPGVVDVLTGQQLV
DDKIGNLICGWMIHSKDGSPMKMGAWPAMAPETVRFVGNAVAVVIAETRNQARDAAEAVEVTYQELPAVA
DIRSAIAPGAPQLHPEAPGNVIYDWSIGDEAATGEAFKKAANVVAMDITNNRLVPNAMEPRAAVAEYDSAEEH
FTLYTTSQNPHVARLVLSAFYNVAAENKLRVIAPDVGGGFGSKIFIYPEEMVALWASKRTGRPVKWTSDRTEAFL
TDAHGRDHITKAEMAFDKDNKIIGLRVKTHANLGAYMSLFSSSVPTYLYATLLSGQYNIPNIYAEVISVYTNTTPV
DAYRGAGRPEASFVMERMMETAARQLKVDPAELRRKNFITSFPHQTPVIMAYDAGDFNASLDAALKAIDYAGF
PARKAKAKAEGKLRGIGFSCYIEACGIAPSKAVGSLGAGVGLWESCEVRVNPVGTIEILTGSHSHGQGHETTFAQ
VVADRLGIPISQVSILHGDTDKVQFGMGTYGSRSGAVGMSAIVKAMEKVEAKAKKIVAHQLEASENDIVIENGE
FKVTGTDKAIALPMVALAAYTAHNLPDGMEPGLKETAFYDPTNFTFPAGAYVCEVDVDPGTGKTDIVNFVAAD
DFGRLINPMIVEGQVHGGLAQGIGQAMLEGAVYDKSGQLVTASFMDYAMPRADDLPSFKVSHTMTPCPSNP
LGIKGCGEAGAIGSTPAVINAITDAIGNNKLEMPASPDRVWHAIHQQQAAE
>HRGM_Genome_4332||HRGM_Genome_4332_CDS_00007
MLDHPSRVSSDNDIALQKFGVGQPVRRKEDDTLVRGKGTYTDDISLPGQAYAWIVRSTHAHGIIKGIDVEAARA
MPGVLGVWTGKDLAAAGYNPFTVGLPMKNRDGSPLLQTNRLPLMTDKVRFVGDPVAFVVAETLAQARDAG
EAVVVDIEPLPSVTSAEDAAKPGAPLLYDHIPSNVVLDYHAGDDAALDAAFARAAHVTRLDIVNTRLAVVAMEP
RAALASYDKKSQRFTIEIPTQGVAGNRTSLAKTLNVPNDKVHLRTHHVGGSFGMKNANYPEYICILHAARELGR
AVKWTDERSTSFLSDSHGRAQDVHCELALAADGAFLGVRVRGFGNLGAYITGVAPLPLSLNIAKNINSVYRMPL
IGVDIKCVLTNTTLMGAYRGAGRPEANYFMERLIDRAADEMGIDRLAMRKRNFIKPAQIPYAAANGLTYDSGDF
PGVFSKAVEQADVAGFAKRKKESRKRGKLRGIAVGSYLEITAPPSGELGKIVFEADGTVRLITGTLDYGQGHATPF
AQVLAAQLGVPFDAIKLTQGDSDIVHTGNGTGGSRSITASGAAIVASSRLVIEKGKQAAAYVLEASEGDMEFANG
RFTIAGTDRGIGIMELAAKLRNSKMPEGVPSSLDVDHTVQGVPSTFPNGCHVAEVEVDPDTGNVQIVRYSGVN
DFGTVVNPMIVAGQIHGGVAQGIGQALMECVTYDDNGQPVTGSFMDYALPRAGDIPSMTLGSHPSPATSNP
LGTKGCGEAGCAGSLATIVNAVLDALSDEGVTSIDMPLTSEKVWRAIQDGRKAKSA
>HRGM_Genome_4332||HRGM_Genome_4332_CDS_00881
MNDSSMKQRGLSVLDRPNSYIGKTVPRPNLGRLMQGRGQYVSDLTLPRMAHVVFLRSPYAHARILKIDATEAK
AMPGVIAVVTGAELAKVISPWVGVLSHLKGLKSAPQHAIAVDRACWQGEAVAAIVATSRALAEDAAELVSVDY
EELVPVTDMRTALDPATPVIHPNLGDNLAFERNLDAGAVDQAFRESDEVVEAEFVFGRHTGVTLEPRAVLADW
NSGDERLTVYLGTQAPHMVQNIAAKHLGLDEAQVRVVCKDVGGSFGIKVHIYADEMATLALSKLLRRPIKFVAD
RVESFNTDIHARDHVCKARIGVSKNGTINAFEIDDVTGIGPYSMYPRTSAIEANQVVNLVGGPYTTPNYRARTR
VVFQNKNVTCQYRGVGHPIACSITEGLVDLAAQRIGMDPFEIRRRNLIRDDAYPCGSPSGLKFEALSHHASLDKL
YAMMNYGALRAEQAALRKQGIYRGIGIASFIEVTNPSAAFYGVGGARISSQDGVAVRLDATGRVICQTSITEQG
QGSESLTAQIVGSVLGVSMERVRVILGDTDNTPYGGGTWASRGAGIGGEAALQAAKVLRNNILDVAAAILQAK
PADLDIVDNAVVDVHGGQQRIELHELARIVYFRPDTLPPGFQPELMATRHFVPREYPFAFTNGIQASWLEVDTR
TGFIKLLRHWVVEDCGTIINPQLVDEQIRGGVVQGLGAALYEHCIYDERGQLTNANMADYLVPMSGEMPDIDV
RHIVSPTLDGELGAKGAGEAGTAAAAAVVANAVNDALSPFNVTVTEIPLTPRVILTALGRI
>HRGM_Genome_4332||HRGM_Genome_4332_CDS_00390
MTEAAAIRYMGQPLRRREDFKFITGKGRYTDDMKSPGMLHMAILRSPHAHAVIKHVDLSTAQSAPGVHLVLS
GADLVGKMGPIEPNWVIPGTKVPDRPVVAVDRVRFVGECVAVVVAETQALAHDAVGLIEVDYETLPAVIDEEA
AIREGAPQLHDNVPKNITTLYKIGGGDYKKAASEADHVIKLRVINNRLIPTCMETRSILAEPNVDGTLTVNIQSQV

PHMHRRWIADTVRIPEHQLRIVAPDIGGGFGAKMHLYPEELLCPYLARQLGVPVKWWESRSESHQSTNHGRA
HTETIEVAFRNDGKILGLRVETLGNVGAYLSNMASGGPTVNTVNFGTGTYKIDNYEAFSRVVVTNTVPVDAYRG
YGRPEGGYIAERAIDAVARHLKLDQVEVRNRNFIQRADFPHRPYNGPAVIYDSGNYQGLLTKALEVFKYDERIAE
RDQLRSQGRYRGIGVAAYTHMCGMAPSRRLSLMGFNRGGWESARVSIDSSGRATIFSGSMSQGHGHNTSLA
QIAADVLQIPIESIDIVQGDTRQVQAGHGTFNSRSMAVGGSSVHVTSQRIVAKARKIAASMLEVDEKDVSYRAG
EFSVPGTDIAPLSFGKIARMAYVGHKLPDGMEPGLDETTFYDPAGMGSPSGIHMAYIEVDPETGMVDILDYVA
VDDVGTIINPLLAAGQIHGSVVQGIAQALYEEVSYDPDTGQLMTGSLLDYAVPRAEHVPNIRSSFQETPSPTNPI
GVKGVGESGSIAAPPCMVHAVLDALSPFEILHLDMPMTPPRIWSAVQQARAGVTQ
>HRGM_Genome_4332||HRGM_Genome_4332_CDS_03971
MAPVKFGVGQSVLRKEDDALIRGKGRYTDDYTPAAAMHALVLRSPHAHAKFKIDVSRARGLPGVGAILTADDI
QDLGSLPCLFNLPDEPFTGPPYPILARDVVRHVGDAVAFVVADTVEQARDAIEAIEVEWTALPAAIGLVNAVKKD
APQVWPDHAGNILFDTSIGDKKAAEAAFAKAHAVAEVTIVNPRIVTNYMETRAVVCEYDAKRDHLTLTIGSQGS
HRLRDILCQNVLNIPVEKMRVICPDVGGGFGTKLFPYREYALAAVAARKLKKTVKWTAERADHFVGDAQGRDN
VTTARMALAEDGKFLGMDVDLMGDMGAYLSTFGPYIPHGGAGMLPGLYDLQAFHCRVRTVFTNTVPVDAYR
GAGRPEAAYVVERLVDAAARKLGMTPDAIRRKNFVSPRAMPYTTATGKIYDSGDFAAHMKRAMDVAEWKEF
PRRAKAAKKQGLVRGIGMGTYVEVCGTMGEETAQVRLDPDGDVTILIGTQSSGQGHQTAYAQLVAEQFGIAPE
RVHIHQGDTDEIPTGLGTGGSSSIPSGGVSVERATRKLGENLKEIAADALETGAGDLEFRDGAIRIVGTDRTISFA
DLAKRADPSRLNASATFSSADGTYPNGTHIAEVEIDPATGVIHVVNYVIVDDFGVTLNPLLLAGQGHGGTMQGI
GQALMEAAVYDAADGQLVTGTFMDYALPRAADGPSIKFETHNVPCKTNPLGVKGAGEAGAIGSCPAVVNAIID
GLYREYGIDHIDMPATAERVWMAIAKSRREHRL
>HRGM_Genome_4332||HRGM_Genome_4332_CDS_03434
MNILPSNLRFGAGQSVKRLEDQRLLTGQGHFIDDKAKDGALWLYVLRSPHAHARITAIDTSTALAMPGVAAVYT
GADLVADDIGTIPTLPIFKRPDGSPMAAPPRRLLAHDVVRFAGEPVAAVLAPSRADAQTAAEAIVVDYEILPAVV
SPADALAPGAPVVWPDAPDNIAAAMSYGDAAATEAAFAKAKHVVSLDITSQRLVPSALEPRSTMAEVDKKTGR
LTLHVQSQTPTATRDILSDVVLKRPKDSVHVMVGDIGGGFGHKVNLYPEDGIVAYAATKLKRTVRWRGDRIDDFI
GGSHGRDLTSTGEFALDEKGRVLAFRVRSLGGTGAYLTGAGVIIPLVLGPFVATGVYDLPLVHFDIKAVLTHTAPTG
PYRGAGRPEAVFIVERLMDAAARQIGMDPRQIRKVNYIKPSQLPYKNAVGEVYDSGAFAHMMERASKLADW
DGFSARKKAAKKKGLLYGRGLTSYIEWTGGRVHTEKVTLSATAEGRIVLQSGTQAMGQGLQTSYSQMVAGALGI
PLDRIDVIQGDTDKATGGGSVGSRSLFVGGTATVVSAGDLIQKAREKASHVLEASVGDIEYGDGVLTVVGTDRR
VSLFDLAKDEKDSRLSVESKGEVDGPTWPNGTHICEVEIDPETGVTRVVRYTTVDDVGVAINPMLVTGQVHGG
VAQGIGQALYEGVVYDSSGQLLTASYQDYCIPRASDMPHIDVTLDDSAPCKTNPLGAKGCGESGAIGGPPCIVN
GVMDALSDLGITSLQTPLTPVKIWNAIQQAKATKAA
>HRGM_Genome_4807||HRGM_Genome_4807_CDS_00279
MGASDFSKLPHIGESLKRKEDYRFLTGAGQYTDDVVLAAQSHAVFVRSPHAHAKINSINIDAAKAAPGVLGVFT
GADVAADNINGLPCGWLITSTNGEPMKEPPHPILAQGKVRYVGDHVAMVVALTQQQARDAAELVEVDYDVL
PAVVNVADAASGAVAGAALHDIAPDNHCFKWAIGDKGAVDAAFANAAHVTKLDLINNRLIPNAMEPRAAIGS
YNRASDEYTLYVSNQNPHVERLLMTAFVMGLPEHKVRVIAPDVGGGFGSKIYLYAEDVCLTWAAKKLNRNIKW
VADRSEAFLSDAHGRDHVSHAEMAMDKDGKFLAMRVHTHANLGAYLSTFASAVPTILYATLLAGQYTTPQVYV
EVDSWFTNTAPVDAYRGAGRPEATYLLERLVTRCGWEMGLGQDEIRKRNFITSWPYQTPVALQYDVGDYHAC
MTQAQQLADVAGFAARKAASEAKGLKRGIGYSSYIEACGIAPSNIAGALGARAGLFECGEVRVHPTGSVTVFTG
SHSHGQGHETTFAQVVAARLGIPVENVDIVHGDTGRVPFGMGTYGSRSISVGGAAIMKALDKIEAKAKKIAAH
LMEASDADIDFSGGEFTVRGTDKKIPFGQVALTAYVPHNYPLDKLEPGLNETAFYDPTNFTFPAGTYICEVEIDPQ
TGVTRVDKFTAVDDFGTIINPMIVEGQVHGGLVQGIGQALLENCVYDRETGQLLTGSFMDYAMPRADDFPEFK
LGHVCTPCTHNPLGTKGCGEAGAIGSPPAVINAVLDALRPLGVKDFDMPASPHRVWEAIQSAKA

>HRGM_Genome_4808||HRGM_Genome_4808_CDS_00010
MHSEPSLAGAARQAWVGQRLERLEDDALLNGRGAYADDLGTRPGTLHAAVLRSPHPHARLLAVHTQAALALP
GVRAVLTGADVQAWAQPFVVGVKQPMQHWALAVDRVRHVGEPVAVVVAEDRYLAEDALDLLRVDYEPLPV
VSDIEHALREDACPLHDGVGSNVVSDRAFRYGDPDSAFSQPGVRTVRTTVHYPRNSCSPMECAVVIAEHLPGD
EGYDVTSNFMGPFSLHAVMAMALKVPGNKLRHRVPRDSGGSFGVKQAVFPYVVLMCLASRKAGAPVKWVE
DRLEHLSAATSATARLTTIEAAVTPEGRVLALRYDQVDEVGAYLRAPEPATFYRMHGALTGAYAIDHLQVRNRVV
VCNKTPTGLVRGFGGPQVFYALERLMDRIAVELAIDPVPLRLRNYVPAQAFPYTAAAGAVLDSGDYVRLTEMA
MAQADALQLPERQRAARAAGKLYGIGVAAIVEPSVSNMGYISTVLTAEQRAKAGPKNGAIASATVAIDLLGGVN
VTIASAPAGQGHMTVCAQVVADALGLHPSAVVVNVEFDTAKDAWSVAAGNYSSRFAGAVAGTVHLAAQRLR
DKLARIAAAQWGCDSADIGFEGGQIFNRRQPAQSQPFTRLAASPHWAPALLPEGETPGLRETAFWTPETLKAP
DSADRVNTSASYGFVFDVCGLEIDPATGAVRVDRYVTAHDAGRLLNPALADGQIRGAFAQGLGAALLEEFRYSP
DGSFQSGTLADYLMPTTCETPDPVIVHLETPSPFTPLGAKGLGEGNNMSTPVCIANAFADALRPVRDVADVRLP
LTPDRVLAHLQTEDPPARHPVAKAPAPAALRDGLSLAAQGSVDIAAPPARVFEVLLDPVALARVIPGCHALQSD
GPNRYRADVTVGVGLIKARYEARITLSDIDAPRSLRLAGVGSSSLGTGAGDGSVRLEETAGGTRLHYDYSAQVGG
KVAMVGSRMLESAARLIVAQLFESLGRQASGGVAARASWWQRLLQRLGVRS
>HRGM_Genome_4808||HRGM_Genome_4808_CDS_02054
MGASDFSKLPYIGEALKRKEDYRFLTGAGQYTDDVVLAAQCHAVFVRSPYAHAKINSINVDAAKAAPGVLGVFT
GADVAADNINGLPCGWLITSTNGEPMKEPPHPILAQGKVRYVGDHVAMVVAHTQQQARDAAELVEVDYEVL
PAVVNVADAASGAVAGAVVHDIAQDNHCFKWAIGDKGGVDAAFANAAHVTKLDLVNNRLIPNAMEPRAAIG
SYNRASDEYTLYVSNQNPHVERLLMTAFVMGLPEHKVRVIAPDVGGGFGSKIYLYAEDVCLTWASKKLNRNIK
WVADRSEAFLSDAHGRDHVSHAEMAMDKDGKFLALRVHTHANLGAYLSTFASAVPTILYATLLAGQYSTPQVY
VEVDSWFTNTAPVDAYRGAGRPEATYLLERLVTRCAWEMGLSQDEIRRRNFIQTFPYQTPVALQYDTGDFNAC
MDGANKLADVAGFEQRKAASAAKGLLRGIGYSSYIEACGIAPSNIAGALGARAGLFECGEVRVHPTGSVTVFTG
SHSHGQGHETTFAQVVAARLGIPVENVDIVHGDTGRVPFGMGTYGSRSISVGGAAIMKALDKIEAKAKKIAAH
LMEASDADIDFANGEFTVRGTDKKIPFGQVALTAYVPHNYPLDKLEPGLNETAFYDPTNFTFPAGTYICEVEIDPQ
TGVTRVDKFTAVDDFGTIINPMIVEGQVHGGLVQGIGQALLENCVYDRETGQLLTGSFMDYAMPRADDFPEFK
LGHVCTPCTHNPLGTKGCGEAGAIGSPPAVINAVLDALRPLGVKDFDMPASPHRVWEAIQSAKA
>HRGM_Genome_5019||HRGM_Genome_5019_CDS_00529
MGVEGIGSRVLRKEDKRFLTGKGRYTDDMVVPGMKHAVFVRSPHAHAKITGISTGAALKMPGVIGVLTGKELK
ADGIGNLICGWMIQSKDGTPMKMGAWSPLATDTVRYVGDAVAIVVADTKGEARDAAEAVEIAYEELPVVVSA
VEALKPGAPQVHPEAPGNLIFDWDIGDEKATADAFARAAHVTTLDIDNNRLVPNAMEPRAALGQYDAAEDHY
TCWTTSQNPHVARLVMSAFYNVAPENKLRVIAPDVGGGFGSKIFIYPEEIVCLWASKKTGVPVKWVADRTESFL
CDAHGRDHATHAQIAFDKDHKILGLKVETIANLGAYMSLFSSSVPTYLYATLLSGQYDIPAIHANVKTVYTNTAPV
DAYRGAGRPEASYVLERIMETAARELKISPAELRRRNFIRAFPHQTPVIMAYDAGDFDATLDASMKAADYAGFE
KRRTEAAGRGKLRGIGMSCYIEACGIAPSAAVGALGAGVGLWESAEVRVNAVGTIEILTGSHSHGQGHETTFAQ
LAAERFGVPLDSISIVHGDTDKVQMGMGTYGSRSGAVGMSAVVKAMDKVEAKAKKIAAHLLEADEADIEIKD
GNVRVAGTDKSLPWFQVALASYTAHNLPSGMEPGLKETAFYDPSNFTFPAGCYICEVEIDPETGTTKIVQFVAA
DDFGKIINPMIVEGQVHGGLAQGIGQALLEGTHYDESGQLLTASYMDYTMPRAQDLPSFNIMTTETPCPSNPL
GIKGCGEAGAIGSPPAVMNAITDAIRNNSLSMPATPQKVWAAIQAAK
>HRGM_Genome_5143||HRGM_Genome_5143_CDS_03893
MTKYVGQSVKRKEDYRLVTGTGQFVGDIRMANMVEAVFVRSTHAHATIKGIDISEAQALEGVHAILTGMDIDG
IIKPLVQFESHCALPPNLEEAINPTIHYCYEDVLAKNKVMYVGQPLAVVVAENRYLAEDAADLIKVEYETLPVVVD
PFKSLEKGAPLIQEHLQNNVQSDFHVTTGNAVQSLKDADHILKARIQTPRVSSNPLETRGVVSIYENRTDQLHVY
SSTQLPFEIRTYIARLLDLVEQQIRVTAPDVGGGFGPKGGVHPEEIIIPYLSKLLKRPVKWIEDRLEHLTSARHSRDQ

THDVEVAYNLDGTILGIKDHFIIDSGAVNYFGLTCAYNSAYHLRGAYKIPNYDVTCQIVLTNKTPNVPFRGAGRPE
VVFVMDRIIDMVARRLKKDPVEVMRKNMIQAEDMPYDQGILVKDGAKLIYDSGDYPAALDQGLKMVDYEGF
REQQKELKKQGKLVGVGISTYVEGTGAGPFESAYVTIDGSGQVVAYLGASPQGQGHETVFSQICADELMLSPNE
ITIRVGDTTNLAFGAGTYASRSAVNAGSAIQVASAKLREKVLAVAAAMLEVETADLQMEKGKVFGKTEPEKCVT
YKEISEAARPGNRCKVPVGMEPGLQVTHYFVPPTVTFSSSVHIAMVEVDKETGFVELKGYSVVHDAGKVINPMI
VDGQVQGGIAQGIGAALYEEVVYDEKGQLLTGSYMDYLLPTSMEIPSVEKSHQEFLSTRNPLGIKGVGEGGAISP
PAAIANAVVDALDPLEITINQLPISPSKLRNWIKEAETKKYKIEIS
>CABIYS02||gene_3900|GeneMark.hmm|792_aa|+|387531|389909
MTKYVGQSVKRKEDYRLVTGTGQFVGDIRMANMVEAVFVRSTHAHATIKGIDISEAQALEGVHAILTGMDIDG
IIKPLVQFESHCALPPNLEEAINPTIHYCYEDVLAKNKVMYVGQPLAVVVAENRYLAEDAADLIKVEYETLPVVVD
PFKSLEKGAPLIQEHLQNNVQSDFHVTTGNAVQSLKDADHILKARIQTPRVSSNPLETRGVVSIYENRTDQLHVY
SSTQLPFEIRTYIARLLDLVEQQIRVTAPDVGGGFGPKGGVHPEEIIIPYLSKLLKRPVKWIEDRLEHLTSARHSRDQ
THDVEVAYNLDGTILGIKDHFIIDSGAVNYFGLTCAYNSAYHLRGAYKIPNYDVTCQIVLTNKTPNVPFRGAGRPE
VVFVMDRIIDMVARRLKKDPVEVMRKNMIQAEDMPYDQGILVKDGAKLIYDSGDYPAALDQGLKMVDYEGF
REQQKELKKQGKLVGVGISTYVEGTGAGPFESAYVTIDGSGQVVAYLGASPQGQGHETVFSQICADELMLSPNE
ITIRVGDTTNLAFGAGTYASRSAVNAGSAIQVASAKLREKVLAVAAAMLEVETADLQMEKGKVFGKTEPEKCVT
YKEISEAARPGNRCKVPVGMEPGLQVTHYFVPPTVTFSSSVHIAMVEVDKETGFVELKGYSVVHDAGKVINPMI
VDGQVQGGIAQGIGAALYEEVVYDEKGQLLTGSYMDYLLPTSMEIPSVEKSHQEFLSTRNPLGIKGVGEGGAISP
PAAIANAVVDALDPLEITINQLPISPSKLRNWIKEAETKKYKIEIS
>CABKLU02||gene_279|GeneMark.hmm|795_aa|-|28827|31214
MGASDFSKLPHIGESLKRKEDYRFLTGAGQYTDDVVLAAQSHAVFVRSPHAHAKINSINIDAAKAAPGVLGVFT
GADVAADNINGLPCGWLITSTNGEPMKEPPHPILAQGKVRYVGDHVAMVVALTQQQARDAAELVEVDYDVL
PAVVNVADAASGAVAGAALHDIAPDNHCFKWAIGDKGAVDAAFANAAHVTKLDLINNRLIPNAMEPRAAIGS
YNRASDEYTLYVSNQNPHVERLLMTAFVMGLPEHKVRVIAPDVGGGFGSKIYLYAEDVCLTWAAKKLNRNIKW
VADRSEAFLSDAHGRDHVSHAEMAMDKDGKFLAMRVHTHANLGAYLSTFASAVPTILYATLLAGQYTTPQVYV
EVDSWFTNTAPVDAYRGAGRPEATYLLERLVTRCGWEMGLGQDEIRKRNFITSWPYQTPVALQYDVGDYHAC
MTQAQQLADVAGFAARKAASEAKGLKRGIGYSSYIEACGIAPSNIAGALGARAGLFECGEVRVHPTGSVTVFTG
SHSHGQGHETTFAQVVAARLGIPVENVDIVHGDTGRVPFGMGTYGSRSISVGGAAIMKALDKIEAKAKKIAAH
LMEASDADIDFSGGEFTVRGTDKKIPFGQVALTAYVPHNYPLDKLEPGLNETAFYDPTNFTFPAGTYICEVEIDPQ
TGVTRVDKFTAVDDFGTIINPMIVEGQVHGGLVQGIGQALLENCVYDRETGQLLTGSFMDYAMPRADDFPEFK
LGHVCTPCTHNPLGTKGCGEAGAIGSPPAVINAVLDALRPLGVKDFDMPASPHRVWEAIQSAKA
>CABKLW02||gene_2129|GeneMark.hmm|795_aa|-|2269|4656
MGASDFSKLPYIGEALKRKEDYRFLTGAGQYTDDVVLAAQCHAVFVRSPYAHAKINSINVDAAKAAPGVLGVFT
GADVAADNINGLPCGWLITSTNGEPMKEPPHPILAQGKVRYVGDHVAMVVAHTQQQARDAAELVEVDYEVL
PAVVNVADAASGAVAGAVVHDIAQDNHCFKWAIGDKGGVDAAFANAAHVTKLDLVNNRLIPNAMEPRAAIG
SYNRASDEYTLYVSNQNPHVERLLMTAFVMGLPEHKVRVIAPDVGGGFGSKIYLYAEDVCLTWASKKLNRNIK
WVADRSEAFLSDAHGRDHVSHAEMAMDKDGKFLALRVHTHANLGAYLSTFASAVPTILYATLLAGQYSTPQVY
VEVDSWFTNTAPVDAYRGAGRPEATYLLERLVTRCAWEMGLSQDEIRRRNFIQTFPYQTPVALQYDTGDFNAC
MDGANKLADVAGFEQRKAASAAKGLLRGIGYSSYIEACGIAPSNIAGALGARAGLFECGEVRVHPTGSVTVFTG
SHSHGQGHETTFAQVVAARLGIPVENVDIVHGDTGRVPFGMGTYGSRSISVGGAAIMKALDKIEAKAKKIAAH
LMEASDADIDFANGEFTVRGTDKKIPFGQVALTAYVPHNYPLDKLEPGLNETAFYDPTNFTFPAGTYICEVEIDPQ
TGVTRVDKFTAVDDFGTIINPMIVEGQVHGGLVQGIGQALLENCVYDRETGQLLTGSFMDYAMPRADDFPEFK
LGHVCTPCTHNPLGTKGCGEAGAIGSPPAVINAVLDALRPLGVKDFDMPASPHRVWEAIQSAKA
>CABKLW02||gene_10|GeneMark.hmm|999_aa|-|8563|11562

LHSEPSLAGAARQAWVGQRLERLEDDALLNGRGAYADDLGTRPGTLHAAVLRSPHPHARLLAVHTQAALALP
GVRAVLTGADVQAWAQPFVVGVKQPMQHWALAVDRVRHVGEPVAVVVAEDRYLAEDALDLLRVDYEPLPV
VSDIEHALREDACPLHDGVGSNVVSDRAFRYGDPDSAFSQPGVRTVRTTVHYPRNSCSPMECAVVIAEHLPGD
EGYDVTSNFMGPFSLHAVMAMALKVPGNKLRHRVPRDSGGSFGVKQAVFPYVVLMCLASRKAGAPVKWVE
DRLEHLSAATSATARLTTIEAAVTPEGRVLALRYDQVDEVGAYLRAPEPATFYRMHGALTGAYAIDHLQVRNRVV
VCNKTPTGLVRGFGGPQVFYALERLMDRIAVELAIDPVPLRLRNYVPAQAFPYTAAAGAVLDSGDYVRLTEMA
MAQADALQLPERQRAARAAGKLYGIGVAAIVEPSVSNMGYISTVLTAEQRAKAGPKNGAIASATVAIDLLGGVN
VTIASAPAGQGHMTVCAQVVADALGLHPSAVVVNVEFDTAKDAWSVAAGNYSSRFAGAVAGTVHLAAQRLR
DKLARIAAAQWGCDSADIGFEGGQIFNRRQPAQSQPFTRLAASPHWAPALLPEGETPGLRETAFWTPETLKAP
DSADRVNTSASYGFVFDVCGLEIDPATGAVRVDRYVTAHDAGRLLNPALADGQIRGAFAQGLGAALLEEFRYSP
DGSFQSGTLADYLMPTTCETPDPVIVHLETPSPFTPLGAKGLGEGNNMSTPVCIANAFADALRPVRDVADVRLP
LTPDRVLAHLQTEDPPARHPVAKAPAPAALRDGLSLAAQGSVDIAAPPARVFEVLLDPVALARVIPGCHALQSD
GPNRYRADVTVGVGLIKARYEARITLSDIDAPRSLRLAGVGSSSLGTGAGDGSVRLEETAGGTRLHYDYSAQVGG
KVAMVGSRMLESAARLIVAQLFESLGRQASGGVAARASWWQRLLQRLGVRS
>CABKQE02||gene_3329|GeneMark.hmm|1011_aa|+|226789|229824
MKRFDAGETTGTAQETQQTTGQPYVGRPMQRVEDAAILTGRGRYADDLGVKPGTLHAAILRSPHAHAELGLID
FAAALKAPGVRAVLTGADLPAWSKPFVVGVKAPMEQWALAMDRVRYVGEPVAVVVAESRALAEDALDLVRV
DYRVLPPVVSIEAAIADAAPQLHSGLGSNVASDRHFRYGDPEAAFATAPHRVSLTVHYPRNTCTPIECGVVIAEHL
PGNEGYQVTSNFMGPFSLHAVMAMALQVSANHLRHIAPRDSGGSFGVKQAVFPYVVLMCLASRKAGAPVK
WVEDRLEHLSAATSATARLSTIEAAVEADGRIVALDYDQLEDCGGYLRAPEPATFYRMHGVLTGAYAIPNLRVRN
RVVLTNKTPTGLVRGFGGPQVYFALERLVQRISIELNLDPLDVYRRNFVPSNAFPYRAAAGALLDSGNYQLAMS
RALETGAYDELKRRRDIARAEGRLYGIGFAAIVEPSVSNMGYITTATPAEARRKAGPKNGAIASATVSVDLLGGVV
VTIASTPAGQGHMTVCAQVVADVLGIDPAEVIVNVEFDTHKDAWSVAAGNYSSRFAGAVAGTVHLAATRVRD
KLARIVASQLDCDPAELIFAEGRITRRDAPETAVVFARAASNAPHWSPQLLPAGEEPGLRETVFWSPPNLDAPDE
QDRINTSACYGFAFDLCGLEIDRATGRVRIDRYVTAHDAGKLLNPALADGQIRGAFAQGLGAALMEEFRYGPDG
SFQSGTLADYLLPTTCEVPDPMIVHLETPSPFTPLGAKGLGEGNNMSTPPCIANAVADALGVRDIRLPLTPAKVM
AMIGLEDPPPSRLELAETATAAATGGKERSKGAKALSARGTVDLDAPPEAVFAVLLDPQALAQVVPGCHVLEPIG
DNRYRADVTVGVGMIKARYEAEIALSDLEPPHRLRLSGAGLSSLGSARGSGMVELAPHEGGTRLTYDYEAEVSG
KVAAVGGRMLEGAAKVVLRQLFESLGRQAGGKPVRKPGWIARLLALFGARR
>CABKQE02||gene_179|GeneMark.hmm|792_aa|+|185762|188140
MNAPAEPNNHLIGASVKRKEDFRFLTGAGQYTDDVVQAHQSYAVFLRSPYAHARIKHINTDAARNHPGVLAVL
TGDDLAADKVNGLPCGWLIHSIDGTPMKEPPHPVLAQGKVRHVGDQVALVVAESVKIAKDAVEMIDVEYDEL
PAVVDTATADTAGTAVHDDVPNNTCYTWGHGDKAATDAAFARAAHVTRLDIVNNRLIPNAIEPRAVNASYSRQ
DDSYTLYVANQNPHVERLLMSAFVLGLTEAKVRVIAPDVGGGFGSKIFLYPEDVALTWASKKVGRPIKWTAERSE
SFLTDAHGRDHVTHAELALDAQGNFLAMRVHTTANMGAYLSTFASSVPTILYATLLAGQYKTPAIYAEVKAVFTN
TAPVDAYRGAGRPEATYVVERLVETAAHELQIDPAELRRRNFIRTFPYATPVGLTYDTGDYEPCLDRAIELADVKGF
AARRDASRAKGRLRGMGYSCYIEACGLAPSNIAGALGARAGLFEAGEIRVHPTGSVTVFTGSHSHGQGHETTFA
QVVADRLGVPIDNIEIVHGDTGRIPFGMGTYGSRSIAVGGSAIMKALDKIEAKAKKIAAHLLEASAEDIEFKDGVF
RVAGTDRTKTFGEVALTAYVPHNYPLDKLEPGLDENAFYDPTNFTYPAGAYICEVEVDPDTGEVHIDRFVAVDDF
GNIINPMIVEGQVHGGLGQGIGQALLEACVYDENGQLLTGSYMDYAMPRANDLPSFTVETAKGTPCTHNPLG
VKGCGEAGAIGSPPALINAIVDALAPLGVKDIQMPATPHRVWQTIQAAKA
>CABKRK02||gene_2835|GeneMark.hmm|804_aa|-|247019|249433
MMTTTQQQQERKFKYIGKKNKPVEDYRFVQGLGTYVADIKIDGMHHIALIPSPYAHAKIISIDTSDALKLPGVIA
AVTGKELAQHVKPLKQYLDIPGVHWYPLAVDYAKYAGEWVVAIVAESRYIAEDAAELVKIKYEPLPFVTDPEEAM

KEDAPAVHPNHPKNVMWQRKFVWGEVERDFAEADDKIEFRCRWNRNSTVPLETFGVVTKWDEGRQILDVW
ASIQMPQYAEQIAEALQIPLNNVRVHYDVDVGGSYGVKRGIKHTVLVAYISRQYRVPTKLIEDRLENMRGSDMH
GPDRIFDVQVAFDNKGVIKSLKIRTIDDEGAYPGRSPLQMGKPIGAIVGAYKIKSVEYEGVAVTTNKTGQVAVRG
FGQSPTNYAIEMAVNKVANKLNLSPIEIRQRNFIQHHEFPYKIPSGTTYDSGNYPVVLEKAMKLANYEQLLKWQ
TAERKKGRIIGIGVGTCMEPGGGNALFEPLLNPKNDKTTFPEGCQVKVDQTGKVTANIAFSSSGQGHQTLVSTIL
AEEFDIVRDEIRVVYSDSLSALPSQSPVASRMAIVLGGATSGAAKKIKQKMMKIASHNLEEPLENLYWEGSTIKTT
TDEQKKLTWNEIVRIAHITYHKMPEDMEPGLQAQFVLEVPTGGTLPTPDGKVQMYPCYSFSAHIPVIEIDRDTG
KISFLNYYIADDCGTVINPDIVKGMVVGGVAHGIGAALYEQFSYDSNGQMISQTFMDYLLPSTMEVPHIDIVKH
CTPSPLTSMGQKGVGEGGYMSAPAAIMNAVNDALSPFNVEMTSVPVTPMDVLKKINQVEGDF
>CABKRR02||gene_3030|GeneMark.hmm|799_aa|+|39296|41695
MNAPVVDAGTGIGASVRRKEDYRFTTGSGKYTDDVQMVGQTYACFLRSPHAHAAIRSIDKEQALQAPGVVAV
LTGEDLAADKVGGLPCGWLITDVNGQPMKEPPHPALAQGKVRYVGDPVAVVIADSYLQARDAAELIDVDYEVL
PAVVDGRAALQPGAPALHELAPDNKCYTWSIGDKAAVDEAFRRAHHVTKLDIHNNRLVPNAIEPRAALGHYNR
ADDSYTLYVANQNPHVERLLLTAFVLGLPEHKVRVIAPDVGGGFGSKIFLYGEEVVVTWASKKVNRPVKWTADR
SEAFLADAHGRDHATQAELALDQDGKFLAMRVHTVANLGAYLSTFAPSIPTILYATLLAGQYTTPAIYCEVDGVFT
NTTPVDAYRGAGRPEAAYVVERLVSAAAREMNIDQADIRRRNFITEFPYQTPVALQYDTGDFHACLDGAMELA
DVAGFPARRAEAERRGKLRGLGYSTYIEACGLAPSNIAGALGARAGLYEVGEIRVHPTGSVTVFTGSHSHGQGH
ETTFAQIVATRLGVPFEQVDIVHGDTGRVPFGMGTYGSRSLAVGGSAIMKALDKIEAKGKKIAAHLLEAADSDIE
FKDGQFTVAGTDRSVPFAQVAFSAYVPHNYPLDKLEPGLNETAFYDPTNFTYPGGAHICEVEVDPETGAVQIVG
MTAVDDFGKIVNPMIVEGQVHGGLAQGIGQALVENCVYDPESGQLLSGSYMDYAMPRAGDLPSFKVGNRVT
PCTHNPLGAKGCGEAGAIGSPPAVINAVIDALAPLGVTDIDMPATPERVWRAIQSARPAAQQAA
>CABKSN02||gene_490|GeneMark.hmm|775_aa|+|757069|759396
VGTSVRRQEDPRLLTGRGRFTDDVHLPGMLYAHFVRSTVASGSVVSLDLSAVREVPGVVAAFDAADLGLGDITA
RLDRPAGEFVPTAMPVLARDRVRFAGEPLAVVVARDPYAAEDGTEAAKVVHDTRPAVTRDTQALAPGAPLVH
DAAAANTLLDVTMFATEGIDSVFDRAHCVVDVETRTGRQNALPLETRGAVAAWDDRDRQLVLHTSTQVPHQ
VRTVAARCLGLDERAVRVLVPDMGGGFGLKCVVGREEIAVAAAALRLGRPVKWTEDRKEALTASFLAREQHYR
VRAAFDAEGRMLGLDADIVCDMGAYSCYPFTAGIEPLMAAAEMPGVYRLPAYRARARAVTTNKAPSAPYRGV
SRPQFVLVVERLFERAARELGLDPVEIRRRNLITEFPYTGVNNITYDPGSYRESLDLCERTLREEGWYAARDTAAA
EGRHLGIGYACFSERTGYGSAAFAQRKMEVVPGFDLAEVRMDTSGALTVTSGTMSHGQSHETTMAQIAADEL
GLPLEKVRLHQGDTERVAYGWGTFASRSIAVGGSAVRRAAAELGRKLRHLAAARWGVPPDEVVLGPGAVRRA
DGTAELSHTELARIAYLATHLLPEGMEPGLTATASFDGVGDGTFSNATHGVVVELHEGTGQVEILRYVCVEDCGV
AVNPQVVEGQCRGGIAQGIAGALFEEITYDAQGEPSATGFMDYKVPTALEIPDVTVRHLETPCAFTETGAKGAG
EGGTIGAPAAVLNAVNDALRPTGVELNDIPVTPQTIAHALEQHS
>CABKST02||gene_2852|GeneMark.hmm|782_aa|+|1210|3558
MASMIGMRVKRKEDPKLITGNGNFTDDIKLPGMLYAAFLRSTHAHARIKRIDVSQAVELPGVVAVYTGEDLTGK
IKSVPTSWYVPGCNLKAKDRSPLAVDKVLYVGEGVAMVVAEDRYTAYDALNAIEVEYEELKAVTGQEAALKDGA
PLVHEDVKNNLAFLWKAGDVPDEAFTNAEVVVRERYYEQRVVPNPMETRAAVAQYNSGSGDMTVWCTSQN
PHIHRMVYAEVLGIPESKLRIIAPDVGGGFGAKIGVYADEAVVAYAARHLKRPVKWMEDRKEHFMATNHARDE
VIEVELAGKRDGTMTALRVRNTANMGAYLSTMGAGVPTICFGLMVTGAYAIPQAAVEVYGVYTNTTPTDAYRG
AGKPESAYQIERAVDAFAREIGMDPVEIRRKNFVPKEKFPYDTAMAVTYDSGDYMLTLDKALEIADYEELRREQE
ALRKQGRYLGIGLSTYVELCGLGPSKVAGAIGLQFGQWENATVRVHPSGKVTVLTGASPHGQGEDTTFAQVVA
DKFGVPLEDIEVLHGDTQMIPMGWGTYGSRTTPVGGAAVAIAAERVTEKAKKIAAHELEVSTEDLEFSDGIFQV
KGVPGHQRTFQEIARSANMAWNLPEGMEPALEAQSFFDPSNFVYPFGAHICVVEVDSNTGQIELKRYIAVDDV
GRVINPMIAEGQVHGGLAQGIGQALWEGAVYEENGQLISGTFMDYTMPKADFFPVLETAFTETPSPVNPLGA

KGVGETGATASPPAVVNAVLDALRPFGITHLDMPLTPEKVWRAMQKGRKEA
>CABKSW02||gene_4523|GeneMark.hmm|998_aa|-|29421|32417
MTSFQPATESQTGHIGARQTRIEDAALLRGLGCYADDAAIPPGTLHAAIIRSPHAHARITSVDFSAALLMKGVHG
VLVGEDVKRWALPFPVGVRQPMEHWCVAVDKVRYVGEPVAVVIAESRYLAEDAIEGVRVEYEPLPPIIDPELAT
ADQAPILHEAVGSNVVNERHFRYGEPEQAFEQAPHKVSLKVKFPRSSCTPIECYVVLAQYERATGIYDVLANFQ
GPYALHTVMARALNVPGNRLRLRTPKDSGGSFGIKQGVFPYVVMMGLASRKVGAPVKWVEDRLEHLQGASS
ATNRVTEIEAAVEADGRITALRYDQIEDCGAYLRAPEPATFYRMHGNLTGAYAIRNVQVRNRVVLTNKTPSGLNR
GFGGPQVYFALERLLQHIAVQLKLDPLDVIRRNLVPTDAFPYQAAAGALLDSGNYQAGIALAAADGGLDDLLRR
RNQARAEGRIYGIGYAAVIEPSISNMGYITTAMTPEERRKAGPKNGAVATATINVGPLGDVSVHVSSTPQGQGH
QTTVAQVVAEVLGVALESIVVNVELDTQKDAWSIASGNYSSRFAGAVAGAVYKAALKIRDRLAAIAAEQLQASP
EDIRFAGGKIFVVNGGAVAPFHRIAGATHWSPGLLPGGESGGLRETAFWSPPQLVAPDDQDQVNSSLCYGFIF
DICGLEIDRMTGEIHIDRYVTCHDAGRLLNPALVDGQIRGGFTQGLGAALMEEFAYGEDGSFLSGTFADYLVPTA
PEVIEPVILHMDTPSPFTPLGAKGVGEGNNMSTPVCIANAVADALGRSDIRLPLTPSKVRTLIGIDEPPRPAGME
ADDNLDAAAGGPALRANDSVVIPASPQQVFDTLLDPQTLAAIIPGCHDLVLDGENRYRADVTVGVGMIRARFE
AKVALSDLDPPHSLRLSGSGSSSMGSAQGQAQVRFVELENGHTRLEYQYQVAVSGKVAAVGGRMLQGASKVII
GQIFTRLSQRVSGQAISTGWWARLRASLGALFGKGGAQ
>CABKTE02||gene_2608|GeneMark.hmm|833_aa|-|931412|933913
MTAVEDRAQAPAAGPAKEVGQARRRKEDARLITGRTTWTDNLVLPGMLHLAIVRSPVAHATITGVDVEAARSA
PGVVAVLTGRDLADEQGSIPCAWPVTPDMVNPGHPSVAVDEVNHVGEAVAVIVARSKAAAQDAVELVDVDY
DQLPVVLDMEQALGEAPQLCHDHLDSNESFRWVFDAGEAGTGEDTDRAFADAEVVVSRRFVQQRLIPAFME
PRATVVQPQGDNYTMWSSTQVPHILRIMLAMVTGVPEHKLRVIAPDVGGGFGGKLQVTPEEVITLLVAKRLGK
PIKWAETRSESLMSAHHGRDQIQYVDIAADRDGTVRGLKVHILSDMGAYLRLVSPGVPILGAFMYPGIYKFPAY
RFDCQGVFTNKTPTDAYRGAGRPEATFAVERIMDELAVELGMDPLELRRKNWIRAEEFPFTTVAGLEYDSGDY
DEATRQALELLGYDDLRAEQRRRRESGDPVQLGIGFSTFTEMCGLAPSRVLGSLDYGAGGWEHASIRMLPTGK
VEVVTGSTPHGQGHETAWSQLVADELGVPFEDVEVLHGDTAISPRGLDTYGSRSLVVGGAAVVRASQKVVAKA
RAVAAHLLEAAEEDLQFEGGTFSVRGTPGTGKTIQEIALATFAAHDFPEGIEPSIDADATFDPVNFSFPHGTHICA
AEVDTETGMVRIRKYACVDDVGTIVNPLIVEGQVHGGLAQGIAQALYEEAVYDADGNLTTGTFVDYLVPSAADL
PHFDTGNTVHVAPGNPIGAKGVGEAGCIASTPAVVNAALDAVRHLGVSDIRMPLTPERVWRALQSGGDGGD
RATAGTNAYGGAQTETTAGVSTPESSLGGDR
>CABKTQ02||gene_2086|GeneMark.hmm|779_aa|+|357658|359997
MAQVVGRPLKRKEDPKLITGHGQFTDDIKLPGLLFAAFKRSQYAHAKIKSIDVSKAMQFPGVVAVLTGKDVQDK
IKPIRSIWLVPGCDLIPKDRMPLAVDKVNYAGEAVALVVADSRYTARDALELIDVVYEELPAVVQQEAAISESAPVI
YHDVPNNIAFTWKAGDVPEEIFANAEVVVKQRLVEQRVIANPMETRGAVAQYNPGSGDLTIWCTTQNPHIHR
MTYADALGIPETKLRIIAPDMGGGFGAKIAVYTEEAVVGLAAMVLKQPIKWVEDRQEHFLATSHARDQVITTEL
AGTKDGKILAVRAKNIANLGAYLSTMGAGVPTIDFGLMITGAYNIPHASAETIGVYTNTTPTDAYRGAGKPEASF
QIERIINIFADKIGMDPVEVRKKNLVASVPYTNAMGCNYDSGDYLGTFAKALEAIDYAGLRKEQEKARKKGRLLGI
GISTYVELCGLGPSKVAGAIGFGGGLWENSTIRVHPSGKVTVFTGASPHGQGVGTTFAQVVAEKFGIPVEDIELV
SGDTLMISMGWGTYGSRTTPVGGGAVANAAERIIEKAKIIAAHELAVTQDELQFDNGVFSVKGQPEKQRTFQEI
AWSANNAWNLPDGVEPALESQSFFDPPDFVYPFGAHICVVEVDANTGLIELKRYLCIDDVGRIINPMIATGQVH
GGLAQGIGQALWEQTVYSEKGQLLSGTFMDYTMPKASFFPRFESSFVESPSPVNPLGAKGVGETGTTAAPSAIV
NAVIDALAPYGVKDLAMPLTPEKVWKAIHKRGDA
>CABMGL01||gene_1661|GeneMark.hmm|782_aa|-|71652|74000
MAGLVGARIKRKEDPRLITGNGNFTDDIKLPGMLHAAFLRSPYAHARIKRIDVSEALKLPGVKAVFTGADIAKKM
KAVPAMWFVTGADLKPKPRSPLAVDKVLYAGEAVAVAVAEDRYTARDALDLIVVEYEELPAVVGQEAAMQEGA

PLVHEDVPNNLALVWKAGEIPDEVFEQAEVVVRQRLYNQRVIPNPMETRAAVAQYNPGSGEMTIWCTTQNP
HIHRMVYSEVLGIPESKLRIIAPDVGGGFGAKIATYTEEAVVGCVARELKQPVKWAEERSEHFKATTHARDEVIEI
ELAGTRDGIMTALRVKNTANLGAYLSTFGAGCPTIDFGLMVTGAYTIPRAAVVTYGVYTNTTPTDAYRGAGKPE
ATFQIERAVDLFAREIGMDPVEVRRKNMVQKEAFPFSNPMGCVYDSGDYHLTLDKALEMIDYQALRQEQEELR
KQGRYIGIGVSTYVELCGFGPSKVAGAIGFQGGVWENSTVRVHPSGKVNVYTGTSPHGQGTETAFSQVVAEKL
GIPMEDVEIIHGDTQSISMGWGTYGSRTTAVGGAAVAIATDRVIEKAKKIAAHELEVAEEDLEFSEGIFQVAGVP
GQARTFQEMAKSANFAWNLPEGMEPSLEAQSFFDPSNFVYPFGAHICVVEVDGSTGQVELKRYVAVDDVGR
VINPMLAEGQVHGGVAQGVGQALWEGAVYGENGQLLSGTFMDYTMPKARFFPTIETAFTETPSPVNPLGAK
GIGETGTTAAPPAVANAVMDALRPFGIKDLDMPLTPEKVWKAMRNGGSGE
>CABMGL01||gene_1556|GeneMark.hmm|774_aa|-|12879|15203
MTIGTSVKRVEDPRLVRGKGRYVGDIQLPGQCEAVIVRSQHAHANLRAIRTQQAANRPDVLLVLTVNDLPDDL
PPIPMRLSPEGSLELALQYPLAKDKVRYVGEPVALIVAKNRYAAEDAAELVEIDYEPLAPVASVDQALSADAGLLH
PAVGSNDLFVIHSQKGSVEEKLKESPHVLQEELYVQRHTGVPLETRGLLAVYDPNRTSGKLTVYGTTKVVHFNRQ
ILARLLKQDESQIRCVETDVGGGFGPRGEFYPEDFLIPYAAMQIGRPVRWIEDRLEHLVATNHSRQQKHRVTVG
FDEEGRILALRDEIFVDTGAYIRTHGVTVPALTQAMLPGPYDIEALELTTHVVATNKTPTGTYRGPGRFEGTFVRE
RVMDMIAKTLGRDPLKVREINVIRPEQMPYSNGISALGQEVELDSGDYPRILAHVRRVMNWDGFAARKAEAA
ASGRLLGMGLAMFVEKSGLGPWEWAEVELLENGEACCKTGLADVGQGVKTMLAQVCSDRLGLPYTKIRVVH
GDTAAVPKGNGSFATRGTVMGGTAAWQAADALRSKMLQTAAGLLQIETAQLGLLGESVVMQATGQTLLSFAQ
LLEECRRQGIRLREEYTFSAAHMTYPYGVHAAEVEVDQDTGRIKINRYYLAYDVGRAINPLLVKGQLVGGMAQG
LGGALFEKLYYDETGQLLSGTFMDYLLPSSMEVPDVEVEILEWAPSLLNPLGVKGAGEGGTVAVAPAVANAVCH
ALQDYGLAITSLPIRPELIRQAIRQGKNRPKGAEVLS
>CABMGL01||gene_3010|GeneMark.hmm|745_aa|+|179395|181632
MLYMSIVRSPYAHAKIKSINTEAAKQVPGVKLIITGEDLAKMNLAWMPTMNGDVQMVLATNKVLFQYQEVVA
VVAETRGAAVDAAQLVEVEYEALPVIVDPFRALQPDAPVLRDDREKKSNHIWHWEAGDQESTEEIFKNAPVVV
SQDVRFQRVHPSPLEPCGCIADYKKGTGRLTWYVTSQAPHAHRTILAMISGLPEHKIQVISPDVGGGFGNKVPV
YPGYVCALVASIQLGIPVKWVETRTENIASTGFARDYHMTSEIAADENGKVLALRVKTIADHGAFDAAADPTKFP
AGLFSIVTGSYDFKQAFVEVDGVYTNKAPGGVAYRCSFRVTEAAYLIERTMDILARKLGMDPAELRLLNFIRKDQ
FPYQSPTGWTYDSGDYEKTFRLALEKIGYHELRKEQAEKRARGELMGIGISTFTEIVGAGPSHSFDIMGIKMFDS
AEIRVHPTGKVIARLGVRHQGQGHETTFAQIIGEELGLTADDVLIEEGDTDTAPYGLGTYASRSTPTAGGAAALCA
RRIRDKARKIAAHLLEVGDEDISWNGESFEVKGTPSRKVTMGEVALAAYTNVPEGMEPGLEATYYYDPPNLTFP
HGAYIAVVDIDKGTGVVKVRRFLAVDDCGNVINPMIVEGQIHGGLTEGFAIAFMQDIPFDEDGNCLAPNWMD
YLVPTSLDTPNWETDRTITPSPHHPIGAKGVGESPNVGSPAAFVNAVVDALEPFGVEHIDMPIFPWKVWEILRQ
NGVAE
>CABMGL01||gene_1555|GeneMark.hmm|782_aa|-|10534|12882
MKWFGKPIKRKIDPRLLRGGGRYIADITVQGTLSAAFLRSPHAHARIADISLEQARGLPGVVAVYGPDDAIDFPF
LPVLFPHANLTPVTQRPLNRTVHHVGEPVAMVVAETRYIAEDALDLIDVRYEPLPAVAQLQDAIGADAPLAHEH
LDSNLAARFTQSIGDAKAAMQAADVVVTQQFQIGRVSCLPIETRGLLAVWREEGTNPALEVYAATQNQHEMR
SILARLLDLPERQVRVIAPDVGGAFGAKAPFYVEDFLVCWAARRLGAPVRWVEDRMEHMMSCIHEREQIHEA
SLGVSADGKILAVTDAMLANTGAYVPWGIIVPLMTSTLIPGPYKVPNYFCDVQVLYTNTVPLAPFRGAGRPQAA
LILNRLLDQAAQKLNLDPIEIRHRNLIQADEFPYHTGLLSRDGSPQVYDSGDYARLLDEAVKRSRYGDWRQKQEE
CRRLGRHIGIGTAVAIENTGFGSFEGATVRVETTGEVTVLTGAASQGQGHETTLAQIAAEVLDVPLPQVTVCQG
DTSLISYGTGTFASRIATITGTAIYKAAQAVKEKALAIAACRLQAEVEMLELSQGYIRLKNDPDCCISLAEAAREAKG
LYPGTTYSLPVSPGLEVTEYFAPPAAAVTSMADIAVVEVDPASCEIKILQYTSVHDSGRLINPLVVNGQIHGGIVN
GVGTALYEEVVYDRQGQLLTSTLMDYLVPTSCESPELTVAHIETPSPLNPLGIKGTGESGTIPVPAAIQSAVEDALR

PWKIPIGRIPVKPSYISQLLGSRSAAAAVDRGGAE
>CABMGP01||gene_7211|GeneMark.hmm|787_aa|+|156133|158496
MTEAGIGAPVRRKEDHRFITGQGRYVDDLNRSGQAYAYFVRSPHAHARIAGIDTSAARTMPGVLGIFTGDDLA
ADKVGGLICGWMIHSKDGSPMKAGPHPALAQGKVRYVGDHVAVVVAETLAQARDAAEAIVVDYDPLPAVVD
PATAQKAEAQVHEAAPDNTVFRWHLGDKSAVDQAFEQAKHVTKLDLVNNRLVPNAIEPRAAIGEYDAGTDSY
TLYTTSQNPHVARLVLSAFIGIAPEHKLRVVAPDVGGGFGSKIFIYAEETVCVWAAKKVKRPVKWTADRTEAFLS
DAHGRDHVTHAELALDESGKCIGLRVSTIANLGAYLSTFSSSVPTYLYAPLLSGQYDIPAIYCEVDAVYTNTAPVDA
YRGAGRPEATFVLERIMEKAARELQMDPAKFRKRNYITRFPHQTPVIMAYDIGDYQASLNKALEIADYKNLGKR
KRESARNGKLRGIGFSSYIEACGIAPSQAVGALGAGVGLWESAEVRVNPTGSVEVLTGSHSHGQGHETTFAQLV
SDRLGISIDQVTIVHGDTDKVQFGMGTYGSRSGAVGMSAIAKAIDKVIAKGKKVAAYVLEAGEGDIEFRDGRFS
VAGTDRSLAFGEVALQAYIAHKFAGQDLEPGLKEGAFYDPTNFTFPAGVHICEVEVDPETGVSTIDRWVAVDDF
GVVINPMIVEGQVHGGIAQGIGQALLEGAVYDGSGQLVTASYMDYCMPRAVDLPSFEVGMTVTPCPSNPLGI
KGCGEAGAIAAPPAVINAITDALGHEDLQMPATPQNVWRAAQKATLPMAAE
>CABMGP01||gene_1546|GeneMark.hmm|773_aa|+|553827|556148
VATAKFGVGQPMRRVEDRRLLTGGGKYTDDYTPEGCLRAIVVRSPHAHARFTITDAATARAMPDVRLILTADEV
SHLGPVPCLAPVPNADRTQSHLAHIPVLARGAVKHVGDAVAFVVADTLEQARDAAEALEIDYEPLPAVSDSRSA
LEAGAPLVWSEAPGNVAFDTALGDEAAVDAAFAAADRVVSLTIENNRVVSNFMETRGVVAEYDTRNESFTLTLS
SQGVHSLRDTLAKEILKIDSDKVRVVTGDVGGGFGTKTFMYREYPLAAEAARRLGRAVKWVADRSDHFTGDSQ
GRDNVSTGSVALDAAGRFLAMRFDIIGNLGAYLSQFGPFIPYLGATMMTGVYRTPLIHVRVRGVYTNTVPVDAY
RGAGRPEAAYLLERLVDHAARETGIAPEEIRRRNFVRPDEMPYTTPVGERTYDTGDFNAHMTRALEQADWAG
FEDRARASRQAGRLRGIGLATYIECTAWGEGEDVKVQLEQDGTITVYSGTQSNGQGHATAYAQFVSQHLDLPLE
QIRVMQGDTVQVATGNGTGGSRSIPVGGISVSVASRDLAAKLKDLASEHLEAGVSDLEIGEGAVRVAGTDRRIT
FAELAQLPRATDERRTGSGDFTPPDATYPNGTHIAEVEVDPETGTTRIARYTIVDDFGMTVNPLLLEGQVHGGIT
QGVGQALLERTVYDAEGQLLTASFMDYCVPRAEDVPLFHFETLNVPSTTNPLGIKGAGEAGSIGSCPAVANAVV
DALHRASGIRHIDMPATPARVFAALRSARAQMPVA
>CABMGP01||gene_4322|GeneMark.hmm|784_aa|+|223748|226102
MNKEFRADELALQRFGIGQAVPRSEDPRLIRGGGCYSDDVSLPSQVHGVFVRSPHAHGIIQGLDASAAREMPG
VLAVITGEDLESAGYKDLPCAMALKGRDGKSLRKPAHPSLPRDRVRYLGEPVALVIAETALEARDAAEAIVLDIEPL
PAATTPAEALAADAPSIHDDVPGNLALDYHYGDARRVEEAFARAAHVTRLQLSNNRVVVSPLEPRSALAEYDAG
EERWTLHVGSQGVFGLRNTLATEILGVEPEKLRVLTRNVGGSFGMKASPFPEYVALLHAARALARPVKWTDQR
SESFLSDHQGRDAESTAELALDAEGRFLAVRISGLANMGAYLTQVSPLFSTINIAKNVVSVYRTPEVEVSVRCMFT
NTTPIGAYRGAGRPEGNYYMERLIDSAAAEMGLDRLELRRRNHIQPQEMPYAAASGMTYDSGEFTSLLDRAVE
AADWSGFEERRRESQERGKLRGIGVGQYLEVTAPPMKEMGGIRFEPDGTVTIITGTLDYGQGHATPFAQVLHS
KLGIPFERIRLLQGDSDELIAGGGTGGSKSIMASGTAILEASDKVIEKGRQIAASILEASSADIEFSRGWFTIAGTDR
AVHIMDVAQRLQAGLVLPDDVPHTLDVQHVHENSPSAFPNGCHIAEVEVDAETGTVEVVRYSMVNDFGTLIN
PMLVEGQLHGGVVQALGQALLERTVYDADGQLLTGSYMDYALPRADDAPAFAFESRPVPATTNPLGVKGCGE
AGCAGGLPAIMNAVVDALRPLGVRHVDMPATPQRVWEAIRAVS
>CABMGP01||gene_5421|GeneMark.hmm|807_aa|-|172054|174477
MLMWGPYVRELKIGRSIARSEDFRLVRGQGRFTDDLAFSGEAFLYVLRSPHAAARIRSIDVEAARKTPGVLAVLT
GEDCIRDRLGVIPSRVQRHLPDGSPHVAPPYRALAVNAVHHVGDAVAAVVATSLPAAKDAAECIVVDYEPLPAV
TRTRQASKPGTPPVWDEAPNNICFVYELGDRAGVDAAFSRAAHVARLEFDISRVSANPLEARNAIGLYDSGSGR
YTLFAGLQSPHSLRTDIARILGVAEADLRVVAYDCGGAFGMKEGVYPEMILVLWAARKVGRPVRWQSDRSEAF
LADHQARDNASTVELALDAGGRFLALRIRTVANLGAYLNSYGTHSPTNNLGGLAGTYTTPFIHASVTGIFSNTNP
TSPYRGAGRPEASYAIERVIDVAAVQIGIDRIELRRRNMIPPSAMPFRTGLIYTYDSGAFEENMDRALLLSDWAN

FETRRLEASKRGLLRGIGLASVIEIAGGPVTQPAEEYAEIRFDPGGGVTLFTGTHAQGQGHETTFRQIACDLLGVA
EESVRVTFGDTDQVPHGRGTVGSRSTSVVAASLHKAAEKIVARGKTIAAHMLEVSSDDVEFQDGRFVVSGTDR
SVSLIDVARAAFSPERLPKGIEAGFAESATVVPSAPTFPNGCHVAEVEIDPETGRTRLVGYWVIDDVGRVVNPALV
KGQMHGGIAQGAGQALCEEIVHDADTGQPLTASFLDYYMPRCDDLPSLVVEANEVLATTNPFGIKGAGEAGTV
GSLPAVMNAVNDALAGFGIYDFDPPASPFRVWQAMRSAREKDVAGRTGSSAVNARAIWDDDQR
>CABMIL01||gene_389|GeneMark.hmm|773_aa|-|451149|453470
MMRKEDPRFIRGRGTYVDDVALPGMLHLAILRSPYAHARIVRIDVTAAQAHPKVKAVVTGADLAAKGLAWMP
TLANDVQAVLATDKTRFQGQEVAFVVAEDRYSARDACELVDVDYEPRDPVVDARTALDPSAPVIRTDLEGKSD
NHIFDWETGDAAATEAVFAKADVVVQQEIVYPRVHPAPMETCGAVADLDPVTGKLTLWTTSQAPHAHRTLYAL
VAGLPEHKIRVISPDIGGGFGNKVPIYPGYVCAIVASLLLDKPVKWMEDRSENLTSTGFARDYIMVGEIAANRDG
KILAIRSNVLADHGAFNAQAAPAKYPAGFFGVFTGSYDIEAAYCHMTAVYTNKAPGGVAYACSFRITEAVYFVER
LVDCLAFELKMDPAELRLRNLLRPNQFPYQSKTGWVYDSGDYETTMRKAMNMIGYEALRAEQKQRRARGEL
MGIGMSFFTEAVGAGPRKDMDILGLGMADGCELRVHPTGKAVLRLSVQTQGQGHETTFAQIVAEELGIAPDD
IEVVHGDTDQTPFGLGTYGSRSTPVSGGAAALVARKVRDKAKIIASGMLEVSVADLQWEKGKFHVKGDPSAAV
TIADIAMRAHGAGDLPEGIEGGLDAEVCYNPSNLTYPYGAYFCVVDIDPGTAVVKVRRFLAVDDCGTRINPMIIE
GQVHGGIVDGIGMALMEMIAFDEDGNCLGGSLMDYLIPTALEVPHLETGHTVTPSPHHPIGAKGIGESATVGS
PPAVVNAVVDALAPFGVRHADMPLTPSRVWEAMQGRATPPI
>CABMKW01||gene_5003|GeneMark.hmm|786_aa|+|111142|113502
MDSRLVGQPRPRAEDQRLVRGQGRYTDDVNLPGQAYGAFVRSTHAHAIIRGIDIEAAAQAPGVIAVLRGQDYL
DDGLRGMDHIPNPADAIAFKERAFQHSLTGSIFNQRHLPLVVDRVRHVGEPVVLVVARSMREARDACEMVQI
DYEPLPAVVDAVQAMEDGAPQLWDGAPRNLCFQARVGDEAAVREAFARAHCVVRREFRNSRVVNCQMEPR
SALGSFDASSGEYLLISGSQGVTRQQMCIADALGISPAKLRVVSPDVGGGFGPRSCVNPDQLAVLWAARRVGQ
PVKWTSDRNEAFLSDYQGRDQVIRAAIAFDAQGRILGIDNEIIGNIGAHTVSYVPLANGSRIMTTVYHVPAATAL
LSAVLTNTPPTGPYRGAGRPEATHVMERMLDIAADEMGMDRIELRRRNLIQPAQMPYRSPMGLTYDSGSFAA
NMERALVVSQWSEAEQRKRDARARGKLLGTGLANYVESPVGAPRERIEITIGTDGSVDFVVGTQSTGQGHETS
FVQVLADELGVPFEAVTMRTGDSRFVKVGGGSHSDRSMRLVGHLLVQAGERLRSTALSAAASMWGLQVQDL
CYEDGRVRDQAGQHVAGLGEIAAHIGREGMPGNPEERRFFAEAEFNGRIAAHPTGTAVCELEIDPETGDVEIVR
YTSIDDVGRPINPLIVEGQVHGGLAQGIGQAFFEDYHVDPESGQIFSGSYMDYGVARAGAIPRRFDVELTEDRT
HGNALGVKGGGESGITPATAAIFNALADALRGITREELPMPATPAAIWSAIQRAQGVQP
>CABMKW01||gene_3250|GeneMark.hmm|790_aa|+|299399|301771
MGESKVDGGYAHIGRPLVRVEDARHLAGGGAFVADIQAPGCLEVAFCRSLVAHGRLKGVTLPPELPAGTFWTA
ADIAGLALPIYCALLRPEFNGAPYPLLADGKVRFAGEPVVLALGATRAEAEERAEQVQLDIEPLAAVVDAWDEVE
HPGVPLHDHLDSNLVMRIGRALGDAQAIIDDPAARGLRQVTRRLSMGRVLASPLECRGCLAYPDPAGGVVVH
VSSQRPHLIRTFLAEQIPGLTEADIRVVVPDVGGGFGSKSNLYPEEVLVTALAWKLRRPVRWIEDRYEHFVASNH
SRQHEQRITAYFDDTGRIHALDALVVVDAGAYSAKTSTGAIEANMATNVMLGPYDIRNYRFEAVSIHTNKSPLGP
YRGVGRPGGCFAMERIIEEVARELGADPLDVRRANLISPAQMPYVSATGLAYDSGNYREVVDAAGRFVAENWS
AARQADDGGRVGVGYAMLVEQAAHGAEEWRRRGSPLIYGHESARATLNMDGTLVIEVGTLSHGQGHFTSLA
QIAAEITTLPVSAIRIRQGDTSAAPYGLGSVASRSIVMAGGAVAMACRDIVGKARAIHAVLHETDDAQIEVRDG
ACVSPQGTTSFGDLARVAYVGVSKLPRSISPGFSFQADYRPSVETGTFSYTLHAARVSVDPDTGFVRILDYLVVED
CGTVVNPLILDGQIRGGVAQGIGQALYEEMIYDSNGQPQTVTFGDYAVPSAVEVPPISILHFSTPSPHSEFGMKG
MGEGGAVAPPAAIANAVRDALADLGVDVCRTPITPDYLLRQLHEAGRGGTR
>CABMKW01||gene_1964|GeneMark.hmm|791_aa|+|95231|97606
MHAERQLTAIPGMGASPGRIEDETLLRGKACFLDDVPVPDALHACFVRSPHAHARLRGIDAGQARALPGVVAV
YTRQDLFGHITSWRMPLGFPLAALPEDTTPFALAGDEVAFVGEAYAVVVARTRHEAEDAAAAVQAEFEVLPAV

ADCRQAVRGDAPRVRTELASNVLQDYTLAYGDCDAAFARADIVLDDEFQVHRGCGHPMEGRGVVAALDPAT
GMLTVWSSTQMAHELHYTIAHMLDQPEDQLRVITPDVGGGFGAKFMIYPEEIVVPAVARALGRPVKWVEDR
RENFLTTIQERDQFWRISVAADRDGRMLGVRGGFVHDHGAYTPQGTNVPYNAASSMTGPYVVPAFSLHVQV
AYTNKVPVATIRGAGYPQAAFVMERMMDRLASAAGIDRLECRRRNLIPPEKIPYTKPLKSRAGVPLVIDSGDFPA
LQATAARAIDYEGFARRQEESARAGLLRGIAIANSVKPTGRGPYESARVRVHPSGRIVVHTGALAMGQGIKTTLA
QLCAAHLGVEPQAVEVSAGDTGLSGYGMGGFASRQAMMAGSAVSEAAAKVRDKALATAAAVLGVSADELM
LSQGEVHTPDFARRVPLARLAMLLKGVPGYALPVPGDPGLDATVHFHCDAQAYAGSSHACEVEVDPLTGAIHV
RRYVAVQDSGNLINPQIAHGQVHGGVVHGIGNALFEWMGYDDNAQPLTTTFAEYLLPTAPEVPPIEVIFQPSP
TRLNPLGVKGIGECATLAVAAAVIGAVEHALDGHGVRITEFPLTPVRLAELLDPPARDTTAPSANP
>CABMKW01||gene_3789|GeneMark.hmm|1007_aa|+|26388|29411
MSDASVLATADAPFVGRRQERVEDAALLSGRGAYGDDLPVKPGTLHAAILRSPHAHARVLGMDTGAAEAHPG
VRAVLTGADVKRWSQPFVVGVKSPMEHWSLAQDRVRYVGEPVAVVVAEDRYVAEDALELIRVDYEVLPVAVEI
EAAIAGDAPVLHPAVGSNIVSDRSFSYGEPDAAFASAEHRVQLTVHYPRNSCTPIEGAVVIAEHLAGDDGYEVSS
NFMGPYSLHSVMALALKIPGTRLRHKLPRDSGGSFGVKQSVFPYVVLMCLASRKAGAPVKWVEDRLEHLVAAT
SATSRLCTIEAAVDGEGRISALSYDQFDDVGGYLRAPEPATFYRMHGCLTGAYAIPNLKVRNRVVLTNRTPAGLVR
GFGGPQVYFALERLVQRIAQQLGIDPLTVYRRNFVPPEAFPYRAAAGALLDSGNYPLALEMAERQGGLEELLRR
RDVARAEGRLYGIGYAAIVEPSISNMGYISTVMTAEQRAKAGPKNGAIASATVGIDPLGGVTVVVASSPAGQGH
MTVCAQVVADVFGLLPQQVAVNVDVDTQKDAWSVAAGNYSSRFAGAVAGTVHLAASRLRDKVARIVAHQLR
CDPADVVFAGGKVGLPDAPGKMLPFARAASTAHWAPGQLPEGESPSLRVTEFWTPPELTAPDEQDRVNTSAA
YGFAFDICAVEIDRDTGRVRIDRYVTTHDAGKLLNPALADGQIRGAFAQGLGAALMEEFRYAGDGSFQSGTFAD
YLVPTTCEVPDPVIVHLETPSPFTPLGAKGLGEGNNMSTPVCVANAVADALAPIRGACDVRLPLTPLKIMELIGFD
DPPPSAGAQAASAPPASAAAPGGGSGKALSATGSVQIAAAPEVVFNVLLDPQALAKVVPGCHSLEAVGENRYR
ADVTVGVGMIKARYAAHIELSELDPPHSLRLAGSGSSSLGAARGSGKVRLEPSEGGTRLVYDYSAEVSGKVAAV
GSRMLEGAAKIVLAQLFEQLGRQASGQPTPAAPRRWWRKLLALFGWKGAA
>CABMKW01||gene_1993|GeneMark.hmm|813_aa|-|119958|122399
MTDPGKFRYIGKHRRAVEHRRFVTGQGRYAADIQLPDVLHVAIVASPHAHARIRSIDASGALAMPGVHAVLTG
EELCAHTDAMLPGVDAPQVKRFPLAHGVARYAGEWVAAVVADTRALAEDAAEAVIVEYEPIDHVVDPLAAME
PGAPQVHPLHGSNVIFRRVFNWGEVDEHFAQAPHKLSYRVHWARSATVPIETFAVSAWWNDATGILDVWASI
QMPKYPDLLARALRLPGNAVRVHYDVDVGGSYGVKRGIKHSVLVGYLARKLGRPVRFIEDRLENMRGGDMQ
GPDRVFDVTLAFDGDGIVKSMRMRAVDDIGAYSGRAPLQLGKPVGAIVGPYRIASVEYDAVSVMTNKTPQEA
VRGFGQSPTNYAIETGMDKVARFLGLDRVELRRRNLIGKDEFPYLIPSGTTYDSGDYATVLGKALDAASLEQIQR
QRDELRKRGLLAGIGVSTCLEPSGGNSAFEPLFNPKNTTTTWMDSCLVRIDLSGSVTALMGTSTSGQAHETLVS
TVVGEILQREPDGIRVLHADSLNALPSNSPVGSRMAIMLGGAAAGAAKKLRAKLLRIAAHNLGLPEEQLSYDGG
DVVAKSDSQRRMSWDNLVEIAHRMFHKLPEGTEPGLQEKFVWEVPTGGKMPTEDGRVQMYPCHSFESHVV
LVSIDPDTGKATFHRYVCGHDCGVMISPDVVHGMTYGGIAHGLGAAMMEKFVFSEEGQLLAGTFMDYLLPSA
LEVPAVTIVDHCTPSPLTEFGQKGSGEAGYLGSPAAIASAINDALAPVGASIDALPMTPQAIWNALRRASGGQR
VSPAHSEIPEMEKA
>CABSQF01||gene_523|GeneMark.hmm|781_aa|-|41401|43746
MGVEGIGSRVLRKEDKRFLTGKGRYTDDMVVPGMKHAVFVRSPHAHAKITGISTGAALKMPGVIGVLTGKELK
ADGIGNLICGWMIQSKDGTPMKMGAWSPLATDTVRYVGDAVAIVVADTKGEARDAAEAVEIAYEELPVVVSA
VEALKPGAPQVHPEAPGNLIFDWDIGDEKATADAFARAAHVTTLDIDNNRLVPNAMEPRAALGQYDAAEDHY
TCWTTSQNPHVARLVMSAFYNVAPENKLRVIAPDVGGGFGSKIFIYPEEIVCLWASKKTGVPVKWVADRTESFL
CDAHGRDHATHAQIAFDKDHKILGLKVETIANLGAYMSLFSSSVPTYLYATLLSGQYDIPAIHANVKTVYTNTAPV
DAYRGAGRPEASYVLERIMETAARELKISPAELRRRNFIRAFPHQTPVIMAYDAGDFDATLDASMKAADYAGFE

KRRTEAAGRGKLRGIGMSCYIEACGIAPSAAVGALGAGVGLWESAEVRVNAVGTIEILTGSHSHGQGHETTFAQ
LAAERFGVPLDSISIVHGDTDKVQMGMGTYGSRSGAVGMSAVVKAMDKVEAKAKKIAAHLLEADEADIEIKD
GNVRVAGTDKSLPWFQVALASYTAHNLPSGMEPGLKETAFYDPSNFTFPAGCYICEVEIDPETGTTKIVQFVAA
DDFGKIINPMIVEGQVHGGLAQGIGQALLEGTHYDESGQLLTASYMDYTMPRAQDLPSFNIMTTETPCPSNPL
GIKGCGEAGAIGSPPAVMNAITDAIRNNSLSMPATPQKVWAAIQAAK
>CABTAY01||gene_2074|GeneMark.hmm|791_aa|-|36647|39022
MQDHTSPASLENAIALQKFGVGQPVRRKEDDTLVRGHGKYTDDFNLPGQAYAWIVRSSHAHGIIKGIDTTAAK
GMPGVLGVWTGADLAAAAYGPFTCGLPLKSRDGSPLLQTNRPALATDKVRFVGDPIAFVVAETAAQARDAAE
AVGLDIEPLPAVTNAEEATRPGAPQLYDHIPNNVALDYHYGDTAKIEEAFAKAAHVTRLDIVNTRVAVVSMEPRV
ALAAFDGKTERFTLQVPTQGVSGNKATLAKILNVAPDKVRILTTNVGGSFGMKNVSYPEYVCILHAARELGRPV
KWRDERTTAFLSDSQGRDQIIHGELALDADGKFLAVRLSGYGNLGAYIMGVAPLPLSLNTGKNLASVYKTPLLGV
DIKTVLTNVTLMGAYRGAGRPEANYFMERLIDTAAREMGIDRLTIRKRNFIKPSQMPFPAASGVTYDSGDFAAV
FEKALAVSDYAGFAKRKRESRKNGKLRGIAVGSYLEVTAPPSGELGKITFDADGGVTLTTGTLDYGQGHATPFAQ
VLSAQLGVPFEKIRLEQSDSDLVRFGNGTGGSRSITATGQAIVESSQLVIAKGKQAAAHLLEASEADIEFAGGQFTI
AGTDRSIDIMELARRMREGKMPEGVPASLDVDHNSTATESTFPNGCHVCEVEIEPDTGVAQIVAYTAINDFGTVI
NPMIVAGQLHGGVAQGIGQAMMEHLRYDESGQPITGSLMDYALPRASDVPMMTVDNHPVPAKSNPLGTKG
CGEAGCAGSMASVINAVIDALSDYGVKHLDMPLTTEKIWRAIQDGKAKSAA
>CABTAY01||gene_1553|GeneMark.hmm|769_aa|+|245989|248298
MAAPIKFGVGQSVLRKEDDALIRGKGRYTDDLAPSAALHALVLRSPHAHATFTIDVAAARLLPGVALILTAEDTAE
LGGLPCLFNLETDPFTAPPYPILAKEEVRHVGDAIAFVVADSLDHARDAIEAIKVEWTTLPAVTGVVNAIKPDAP
QVWAEHKGNVLFDVSIGDKAAVDAAFAKAHAVAEIRIVNPRVVASFMETRAAVCEYDTKRDHLTLTAGSQGSH
RLRDILCQNVLKMPTEKMRVICPDVGGGFGTKLFPYREYALLAVAARRLRKSVRWAADRSEHFMGDAQGRDN
VTIARMALAEDGKFLAMDVDLMGDMGAYLSTFAPYIPHGGAGMLPGLYDIRAFHCRVRTIFTHTVPVDAYRG
AGRPEAAYVIERLVDACARKLDMTVDAVRRKNFIPPRALPYKTATGKVYDSGDFSAHLKRAMEIAEWKEFPKRA
KAAKKHGLIRGIGLASYVEVCGTMGEETANVRLDPNGDVTILIGTQSSGQGHQTAYAQIVAEQFGLPPERIHIHQ
GDTEEIATGLGTGGSASIPSGGVSVQRATHELGDKLKQIAAEALEAGLGDLEIADGVIRVAGTDRAISFADLAKRA
GADPAKLNGSATFASADGTYPNGTHVAEVEIDPATGIIRIVNYVIVDDFGVTLNPLLLAGQVHGGAVQGIGQAL
MEQVVYSNSDAQLVTGSFMDYALPRAADAPSFTFETHNVPCKTNPMGVKGAGEAGAIGSCPAVVNAIVEGL
WREYKIDHIDMPATPERVWIAIREQHRRHSL
>CABTAY01||gene_5891|GeneMark.hmm|795_aa|+|15898|18285
MGQVIGIGAAPKRKEDQRFLTGRGNYVSDIKRPGMTAGVFIRSPHAHAVLRGIDKSAALAAPGVVAVLTGEDV
ATDGLGSLPCGWGISDAKGVPMKEPPFPMLAQGKVRFVGDMVAFVIAETLEQAKSAAELVNVNYEVLPSVVG
VLDAVRPDAPQLFDDVPNNVCCDWELGDKAAVEAAFRKAAHVAKLGLVNNRLIGNPMEPRAAIAEYDPGKG
HHTLWTTSQFPHVVRFLMSALVLKLPEHKVRVVAPDVGGGFGVKQFHYGEEAVVTWAAKRVLRPVKWVADR
SEGYLSDRHGRDHVTEAELALDENGKFLALRVKTLANMGGYLSTFGPNIPTNLYAPLLGGVYTTPAIYCNVKVVF
TNTVPVDAYRGAGRPEATFVVERIVDVAAAEMGIDRVELRRRNMIPKEAYPYQTPVLVQYDSGDPMGCLDGAL
VAADVKNFGVRKAESARNGKFRGLGFSTYVEACGLAPSRFAGRLGARGGLYESATVRVHPAGQVTVMIGTHN
HGQGHETTFAQIVCEKLGVPFENVDIVFGDTDRVQFGMGTYGSRSLVVGGAALSKAADKVIVKGKKIAAHLLEA
SEQDIHFEAGKFSVAGTDRVKTIEEIAGAAYVPHNYPLEILEPGLEEQAYYDPVNFTYPGGCHIAEVEIDPETGIVT
LVNYTAMDDVGTVINPMIVEGQLHGGIVQGVGQALYENAVYDESSGQLVSGSLMDYCVPRADHLPMMKVAT
HSTLCTHTPMGVKGCGEVGTIGSPAAVINAVVDALAHLGVTHVDMPATPNRIWRLLQNASLPVAAE
>CABTAY01||gene_3736|GeneMark.hmm|806_aa|-|55417|57837
MNDMTPTREQREAALEGMGCRRKRVEDIRFTQGKGNYVDDVKLPGMLHGDFVRSPHAHARVKAIHTEEALK
VPGVLAVITAETLKTVNLAWMPTLAGDVQMVLADGKVLFQNQEVAFVVATDRYAADDGVSKVVVEYEPLPVL

VDPFKAMDPDAPVLREDLAGKTVGAHGPRKHHNHIFEWSVGDKELTDAAFKKAEVTIKEMISYHRTHPSPLET
CQCVCAFDKIKGELTIWGTFQAPHVIRTVVSLIAKIPEQKIHVIAPDIGGGFGNKVGAYPGYICAAVASIVTGKPVK
WVEDRIENLTATAFARDYHMTTEIAATKDGKVTGLRVHVLADHGGFDACADPSKWPAGFFNIVTGSYDFPVAH
LSVDGVYTNKAPGGVAYRCSFRVTEAAYCIERAMDILAQKLGMDPAELRLKNFVKPEQFPYHSALGWEYDSGD
YHTAMRKMMEAVDYAGLRKEQAEKRAAFKRGETREIMGIGVSFFTEIVGAGPSKNCDILGIAMFDSCEIRLHPT
GAGIARMGTKSQGQGHETTWAQIIASEIGIPADNIMVEEGNTDTAPYGLGTYGSRSTPVAGAAIAMAARKIKA
KAQMIAAYKLEVHEDDLEWDIDGFRVKGLPEKVMSMKDICWAAYNSVPPGMEPGLEAVSYYDPPNMTYPFG
AYLCVMDIDVDTGVYKVRRFYALDDCGTRINPMIIEGQIHGGLTEAFAIAMGQEIRYDSEGNVVTGSFMDFFM
PTAVETPHWETDFTVTPSPHHPIGAKGVGESPNVGGVPAFSNAVNDAFAFLGATHIQMPHDYWRNWQAAK
NLGVVA
>CABTAY01||gene_5248|GeneMark.hmm|777_aa|-|79604|81937
MNILPNNMRFGAGQPVKRLEDQRLLTGKGQFIDDKPDDGALWLYVLRSPHAHAKILSIDTAAAREIAGVQAIYT
GADLVADDVGTIPTLAIFKRPDGKPMTVPPRRLLAHEVVRYTGEAVAAVVATSRVIAQEAAEAIVVDYEVLPAVT
DPVQALEPGAPVVWAEAPDNIVAAMSYGDAAKVEEAFASAPHKVSLDIVSQRLVPSAMEPRSTIAEIEKKTGRL
LLYVQSQTPASTRDVLAEAVLKRPKESVRVLVGDIGGGFGQKTNLYPEDGIVAYAATKLAKKIRWRAERTDEFVG
GTHGRDLTSTAEFALDAKGKVLAYRVRSIGATGAYSSGAANIIPLVLGPFVQTGVYDLPLVHFEVKTVMTHTAPVG
AYRGAGRPEAVFIVERLFDTAARQIGIDPRTIRKANYIKPAQLPYTNAVGQVYDSGAFAHMLERASKLADWDGF
NARKREAKKRGMLYGRGLTSYIEWTGGRAHTEKVSLHATSEGRVILHSGTMAMGQGLQTTYTQMISDTLGIPL
DKIDVVQGDTDLATGFGSVGSRSLFVGGTAVAVSSNDLMTKAREKASNLLETAVEDIEYRDGFLTVVGTDRRISL
FEIAQKEQGAKLSVDSEGEVDGPSWPNGTHICEVEIDPETGISRVVRYTTVDDVGVAVNPMLVAGQIHGGVAQ
GIGQALYENVAYDADGQLLTATYQDYCIPRADDVPPIVVTLDDSAPCKTNPLGSKGCGESGAIGGPPCVTNGVM
DALFELGITNLQTPLTPEKVWAAIRDAKAKKAAA
>CABTAY01||gene_2240|GeneMark.hmm|791_aa|+|216618|218993
MTAPLTPLDRPNSYIGRSVPRPNAKRLLAGRGRYVTDIRLPRMLHAAFLRSPHAHARIISIDADAARALAGVHLIA
TGADLARICTPWTGTLDHFKGMTSEPQLPLPLDRVVWAGQAVVAVVADSRALAEDALELISVDYEELPAVVDL
DEARQPGTPRANPASQSNICFRTQLDSGGVDEVFVAAAHVIEQQFSFGRHTPVTLEPRAIVADYEPDSGMLTV
HHATQTPYQFQDLYSRHYNIPEARVRVIAPDIGGSFGMKLHVYHEDMAVVGLSMMLGRPVKYVADRIESFVSD
IHARDHRVQARMAIDADGKILAMDVHDATAIGAFSTYPRTSVVEGNQVIRLIGAPYGFTSYRAALEVVFQNKV
QTSQYRAVGHPIACAVTERMVDIAAGRAGLDPLAMRDRNVIADDAYPLVSPTGYKFEALSHQACLRRLREIMD
YDALRGEQAELRRRGVHRGIGIATFVEITNPSPAFYGVGGARISSQDGATISLTPTGEVRCAISVTEQGQGTEAIIG
QIVADQLGLAQEHVKVITGDTEVTPHGGATWACRGAGIGGETALQAARRLKANILAIAALVLQEQAAALDIVDG
QVVSAASRQPRLDVAEIARIAYFRSDTLPPGTQAQLTVSHHFAPQGYPFAFTNGIQGCSLEVDVDTGFVKLLKHY
VVEDCGRIINPMLVDEQLRGGVVQGLGAALFEECRYGETGQLLNGSLADYLVPMAMEMPDIVIDHVETPTADT
ILGAKGCGEAGTAAASACVLNAVNDALTPFGASINTIPITPARILKALNRY
>CABTAY01||gene_6355|GeneMark.hmm|779_aa|+|9703|12042
MEGIGASVVRKEDKRFITGKGRYVDDIKLLGMTYAHFIRSPHAHAKVKSIDSSAAEAMPGVVAVLTGRQIVDDK
VGNLICGWAVTSKDGSPMRMGAWPAMAPETVRFVGQAVAVVIAESKNLARDAAEAVVVEYEELPAVADMPS
ALKPGAPQLHPEAPGNVVYDWHIGDEAAVNAAFSKAANVVSLELTNNRLAPNAMEPRAAIADYDAAEEHFTL
YTTSQNPHVARLVLSAFYNIAPEHKLRVIAPDVGGGFGSKIFIYPEEMVALWASKKVGRPVKWTGDRTEAFLTD
AHGRDHISKAEMAFDADNKILGLRVKTHANFGAYMSLFSSSVPTYLYATLLSGQYNIPAIYAEVMGVYTNTTPVD
AYRGAGRPEASYLLERLVETAARQLKVDPAELRRKNFITQFPHQTPVIMAYDTGDFNASLDAAMKAIDYAGFPA
RKAAAKAQGKLRGIGLSCYIEACGIAPSKAVGSLGAGVGLWESAEIRVNPVGTIEVLTGSHSHGQGHETTFCQLI
AERLGVPISQVSIVHGDTDKVQFGMGTYGSRSAAVGLTAILKAMEKVEAKAKKIAAHALEASEGDIIIENGEFKVT
GTDKSIALPMVALAAYTAHNLPDGMEPGLKESAFYDPTNFTFPAGTYICELEVDQATGKTSFVNFVAADDFGRLI

NPMIVEGQVHGGLVQGIGQALLEHAVYDSNGQPVTASFMDYAMPRADDVPSFKLSHTTTLCPGNPLGVKGC
GEAGAIGASAAVINAITDAIGNNKLEMPATPDRVWHAIHGNA
>CABTKA01||gene_2199|GeneMark.hmm|991_aa|+|521726|524701
MNMHQSTVEAATGHIGRRQARVEDAALLRGLGRYADDVATPPGTLHAAIVRSPHAHARVISVDASAALTMPG
VHGVLTGEDAKRWANPFPVGVRAPMEHWCLAVDKVRYVGEPVCVVIADDRYLAEDALDAVKVEYEPLPAIVD
PEAATAEDAPVLHEAVGSNVVNERHFRYGEPEEAFAKAARRVAIKVHYPRNSCTPIECYVVLGQYQPATGTYEVL
SNFQGPYALHSVMARALNVPGNRLRLRTPPDSGGSFGIKQGVFPYIVLMGLAARKVGAPVKWVEDRLEHLQA
SSSATNRVCEIEAAVEADGRVLALKYDQIDDCGGYLRAPEPATFYRMHGNLTGAYAIRNLQVRNRVVLTNKVPS
GLNRGFGGGQVYFALERLMHQVAVELGLDPLEVIRRNLVPAGAFPYRAAAGALLDSGDYPAAIELAVRDGGLD
ELLRRREQARAEGRIYGIGYTAAVEPSISNMGYITTAMTPEERRKAGPKNGAVSTATVSVGPLGDVSVHVSSTPQ
GQGHQTVVAQVVAEVLGVALDGISVNVELDTGKDAWSIASGNYSSRFAGAVAGSVYNAAMRIRERMAGIAA
AMWQVPAEDVRFAGGKVFVEGGPSQPFHRIAGATHWSPGLLPEGELGGLRETAFWSPPQLTAPSDEDCINSSL
CYGFIFDICAVEIDRVTGEVHIDRYVTCHDAGRILNPMLVDGQIRGGFTQALGVALMEEFAYANDGSFLSGTFAD
YLVPTAPEAVEPVILHMETPSPFTPLGAKGVGEGNNMSTPVCIANAVADALGRSDIRLPLTPSRVRTMIGIDEPP
APEGMVLDAPRVAGGSSLQAEDSVEIQASPQQVYDALLDPETLKAIIPGCHALELESENHYRADVTVGVGMIR
ARFAARVGLTDLDPPHSLKLSGSGNSPMGSATGSARVRFVELENGHTRLEYVYDAAVSGKVAAVGGRMLQSAS
KVIIGQIFTRLAQRLTGAPVRSGLWQRLLGLFGKGGAQ
>CABTWY01||gene_3444|GeneMark.hmm|769_aa|-|4303|6612
MAAPIKFGVGQSVRRKEDDALIRGKGRYTDDVAPSPALHALMLRSPHAHATYTIDAGKARGMPGVALILTAAD
VAELGGLPCLFNLETDPFIAPPYPILAKDEVRHVGDAVAFVVADTVDHARDAIEAIDVKWTPLPAVAGLVNAVKK
GAPQVWPDKPGNVLFDVSIGDKGAAEAAFAKAHAVAEITIVNPRVITNFMETRAAVAEYDAKKDHLTLTIGSQ
GSHRLREILCDMILKMPKENMRVICPDVGGGFGTKLFPYREYALISVAARKLKKSVKWTAERSDHFMGDAQGR
DNLTTAKMALAEDGKFLGMDVDLMGDMGAYLSTFAPYIPHGGAGMLPGLYDIQAFHCRVRTVFTNTVPVDA
YRGAGRPEAAYVIERLVDAAARKLGMTPDAIRRKNFISPKSLPYTTATGKVYDSGDFVAHMKRAMEIANWKEFP
KRAKAAKKDGLVRGIGMASYVEVCGTMGEETANVALDPNGDISILIGTQSSGQGHQTAYAQIVAEQFGVPPER
VHVLQGDTDKIATGLGTGGSASIPSGGVSVQRATHELGNKLKELAAQALEAGAGDLEIADGRIRIAGTDRSVSFA
DLAKRPGGDTSKMNASATFASADGTYPNGTHLAEVEIDPSTGIIKIVSYVIVDDFGVTLNPLMLAGQVHGGAM
QGIGQALMEQAVYSPTDGQLVTGTFMDYAMPRAADGPSFVFETHNVPCTTNPLGVKGAGEAGAIGSCPAVV
NAIVEGLHREFGIDHIDMPATPERVWIAIREAQRRHTL
>CABTWY01||gene_2008|GeneMark.hmm|796_aa|+|20807|23197
MTVTGAPTQTLSVLDRPNSYIGKTVPRPNLDRLLQGRGQYVSDLELPRMAHVVFLRSPHAHARIVAIDADAAR
RMSGVISIVTGPELEAVVTPWVGVLSHLKGLKSAPQHAIAVDRVCWQGEAVAAIVATSRAAAEDAMEHVAVD
YEELEAVTDMRTALDPATPVIHASLGDNLAFERTFNAGDVDHALAESEVVEADFIFGRHTGVTLEPRAVVADW
NAAEARLTIYQGTQAPHMVQNIAALHLGLSEAQVRVVCKDVGGSFGIKVHIYADEMATYALSRLLRRPVKFVAD
RIESFNTDIHARDHQCRGRIGVRPDGNITAFEIDDLTGIGPYSMYPRTSAIEANQVVNLVGGPYVTPNYRARARV
VFQNKNVMCQYRAVGHPIACSVTEGLIDLAAARIGMDPVEIRRRNLIPDDAYPCASPSGMKFEQLSHHASLAK
LLQMMDYDALRAEQAALRTRNVHRGIGVATFIEVTNPSAAFYGVGGAKISSQDGVAVRLDAQGSVICQTSITE
QGQGSESLTAQIVGSVLGVSMDRVRVILGDTDNTPYGGGTWASRGAGIGGEAALQAAKALRQNILDVAAVILQ
STPAALDISDNRIVNADDGAPRIELSELARTVYFRPDTLPQGIQPELMATRHFVPRQYPFAFTNGVQASWLEVD
TETGFVTLLKHWVVEDCGTIVNPQLVDEQIRGGVVQGFGAALFEKCIYDERGQLTNASMADYLVPMSGEMPD
IEVGHVVSPTQESELGAKGAGEAGTAGAAAAVANAVNDALRPFGATITEIPLTPQVILTALGRI
>CABTWY01||gene_2224|GeneMark.hmm|795_aa|+|23871|26258
LSKASNPLKPAEISELKDPAMNILPGNMRFGAGQPVKRLEDQRLVTGKGHFIDDKPQDGTLWLHVLRSPHAHA
NIKSIDVKAALEMPGVKAVYTGADLVKDDIGTLPTLAIFKRPDGSPMTVPPRRLLAHEVVRYAGEGVAAVVATSR

TLAQTAAEAIEIDYEVLPSVVDPVEAVKPGAPVVWPEAPDNIVAAMSYGDAAKVEAAFASAAHKVSLDLISQRL
VPSAMEPRSTIAEIEKKTGRLILHVQSQTPGSTRDLLAESILKRPKDSVRVLVGDIGGGFGQKPSLYPEDGIVAYAA
TKLNETIRWRGDRTDEFVGGTHGRDLTSTGEFALDAKGRVLAYRVRSIGGTGAYSSGTANIIPLVLGPFVQTGVY
DLPLVHFEVKSVMTHTAPVGAYRGAGRPEAVFIVERLFDAAARQIGMDPRTIRKVNYIKPAQLPYTNAVGQVYD
SGAFAHMLERASDLADWNGFAARKKAAKKKGLLYGRGLTSYIEWTGGRAPTENVSLHATAEGRIVLHSGTMA
MGQGLATTYTQMISDALGIGMDKIDVIQGDTDLATGFGSVGSRSLFVGGTAVAVSANDMINKARDKASHLLEA
SVGDIEYRDGFLTVVGTDRRISLFEIARKESGAKLSVESEGNVDGPSWPNGTHICEVEIDPETGVTRVVRYTTVD
DVGVAVNPMLVTGQVHGGVVQGIGQALYEGVAYSEEGQLLTASYQDYCIPRASDVPPMSVTLDPSAPCKTNPL
GAKGCGESGAIGGPPCITNGVMDALSEVGITQLNTPLTPVKIWQAIRDAKVGAA
>CABTWY01||gene_5736|GeneMark.hmm|790_aa|+|5262|7634
MQDHTPPASLDNAIALQKYGVGQPVRRKEDDTLVRGKGRYTDDFSLPGQAICWMVRSSHAHGLIKGIDTEAA
KAMPGVLGVWTGADLAAAGYKPFTCGLPLKNRDGSPLLQTNRPALATDKVRFVGDPVAFVVAETAAQARDAA
EAVEVDIEPLPAVTDAAEASKPGAPQLYDNIPNNVALDYHYGDTAKIEAAFAAAAHVTKLDIVNTRVAVVSMEP
RVALAHYDKKTERFTLQVPTQGVSGNKAIMARLLNVPADKVRILTGNVGGSFGMKNLSYPEYTCIAHAARELGR
PVKWLDERSTSFLSDSQGRAQLIHAELALDAEGKFLAVRLSGYGNLGAYITGVAPGPLSLNTGKNLASVYRTPLL
GVDIKTVLTNTTLMGAYRGAGRPEANYYMERLIDAAADEMGISRVTLRKRNFIKPSQLPFPAASGVTYDSGDFA
GVFQKALEISDYENFAKRKKESRKNGKLRGIAVGSYLEVTAPPSGELGKITFEPDGSVKLTTGTLDYGQGHATPFA
QVLSDQLGVPFENITLEQGDSDLVRFGNGTGGSRSITATGQAIVEASALVVEKGKKAAAHMLEASEADIEFGQG
RFTIAGTDRSIGIMELAERMRAGKMPEGAPDTLDVDHATKETASTFPNGCHVAEVEIDPDTGVTRIVRYSAVND
FGVVVNPMIVAGQLHGGVAQGIGQALMEEVSYDASGQPITGSFMDYALPRAGDVPPMEVGDHPSPAKSNPL
GTKGCGEAGCAGSLVCVVNAVVDALSEYGIKHINMPLTPEKVWRAIQDAKANAA
>CABTXE01||gene_4041|GeneMark.hmm|766_aa|+|3365|5665
MAPVKFGVGQSVLRKEDDALIRGKGRYTDDYTPAAAMHALVLRSPHAHAKFKIDVSRARGLPGVGAILTADDI
QDLGSLPCLFNLPDEPFTGPPYPILARDVVRHVGDAVAFVVADTVEQARDAIEAIEVEWTALPAAIGLVNAVKKD
APQVWPDHAGNILFDTSIGDKKAAEAAFAKAHAVAEVTIVNPRIVTNYMETRAVVCEYDAKRDHLTLTIGSQGS
HRLRDILCQNVLNIPVEKMRVICPDVGGGFGTKLFPYREYALAAVAARKLKKTVKWTAERADHFVGDAQGRDN
VTTARMALAEDGKFLGMDVDLMGDMGAYLSTFGPYIPHGGAGMLPGLYDLQAFHCRVRTVFTNTVPVDAYR
GAGRPEAAYVVERLVDAAARKLGMTPDAIRRKNFVSPRAMPYTTATGKIYDSGDFAAHMKRAMDVAEWKEF
PRRAKAAKKQGLVRGIGMGTYVEVCGTMGEETAQVRLDPDGDVTILIGTQSSGQGHQTAYAQLVAEQFGIAPE
RVHIHQGDTDEIPTGLGTGGSSSIPSGGVSVERATRKLGENLKEIAADALETGAGDLEFRDGAIRIVGTDRTISFA
DLAKRADPSRLNASATFSSADGTYPNGTHIAEVEIDPATGVIHVVNYVIVDDFGVTLNPLLLAGQGHGGTMQGI
GQALMEAAVYDAADGQLVTGTFMDYALPRAADGPSIKFETHNVPCKTNPLGVKGAGEAGAIGSCPAVVNAIID
GLYREYGIDHIDMPATAERVWMAIAKSRREHRL
>CABTXE01||gene_903|GeneMark.hmm|797_aa|-|28917|31310
MNDSSMKQRGLSVLDRPNSYIGKTVPRPNLGRLMQGRGQYVSDLTLPRMAHVVFLRSPYAHARILKIDATEAK
AMPGVIAVVTGAELAKVISPWVGVLSHLKGLKSAPQHAIAVDRACWQGEAVAAIVATSRALAEDAAELVSVDY
EELVPVTDMRTALDPATPVIHPNLGDNLAFERNLDAGAVDQAFRESDEVVEAEFVFGRHTGVTLEPRAVLADW
NSGDERLTVYLGTQAPHMVQNIAAKHLGLDEAQVRVVCKDVGGSFGIKVHIYADEMATLALSKLLRRPIKFVAD
RVESFNTDIHARDHVCKARIGVSKNGTINAFEIDDVTGIGPYSMYPRTSAIEANQVVNLVGGPYTTPNYRARTR
VVFQNKNVTCQYRGVGHPIACSITEGLVDLAAQRIGMDPFEIRRRNLIRDDAYPCGSPSGLKFEALSHHASLDKL
YAMMNYGALRAEQAALRKQGIYRGIGIASFIEVTNPSAAFYGVGGARISSQDGVAVRLDATGRVICQTSITEQG
QGSESLTAQIVGSVLGVSMERVRVILGDTDNTPYGGGTWASRGAGIGGEAALQAAKVLRNNILDVAAAILQAK
PADLDIVDNAVVDVHGGQQRIELHELARIVYFRPDTLPPGFQPELMATRHFVPREYPFAFTNGIQASWLEVDTR
TGFIKLLRHWVVEDCGTIINPQLVDEQIRGGVVQGLGAALYEHCIYDERGQLTNANMADYLVPMSGEMPDIDV

RHIVSPTLDGELGAKGAGEAGTAAAAAVVANAVNDALSPFNVTVTEIPLTPRVILTALGRI
>CABTXE01||gene_8|GeneMark.hmm|798_aa|+|7590|9986
VIFSGFFMLDHPSRVSSDNDIALQKFGVGQPVRRKEDDTLVRGKGTYTDDISLPGQAYAWIVRSTHAHGIIKGID
VEAARAMPGVLGVWTGKDLAAAGYNPFTVGLPMKNRDGSPLLQTNRLPLMTDKVRFVGDPVAFVVAETLA
QARDAGEAVVVDIEPLPSVTSAEDAAKPGAPLLYDHIPSNVVLDYHAGDDAALDAAFARAAHVTRLDIVNTRLA
VVAMEPRAALASYDKKSQRFTIEIPTQGVAGNRTSLAKTLNVPNDKVHLRTHHVGGSFGMKNANYPEYICILH
AARELGRAVKWTDERSTSFLSDSHGRAQDVHCELALAADGAFLGVRVRGFGNLGAYITGVAPLPLSLNIAKNIN
SVYRMPLIGVDIKCVLTNTTLMGAYRGAGRPEANYFMERLIDRAADEMGIDRLAMRKRNFIKPAQIPYAAANG
LTYDSGDFPGVFSKAVEQADVAGFAKRKKESRKRGKLRGIAVGSYLEITAPPSGELGKIVFEADGTVRLITGTLDYG
QGHATPFAQVLAAQLGVPFDAIKLTQGDSDIVHTGNGTGGSRSITASGAAIVASSRLVIEKGKQAAAYVLEASEG
DMEFANGRFTIAGTDRGIGIMELAAKLRNSKMPEGVPSSLDVDHTVQGVPSTFPNGCHVAEVEVDPDTGNV
QIVRYSGVNDFGTVVNPMIVAGQIHGGVAQGIGQALMECVTYDDNGQPVTGSFMDYALPRAGDIPSMTLGS
HPSPATSNPLGTKGCGEAGCAGSLATIVNAVLDALSDEGVTSIDMPLTSEKVWRAIQDGRKAKSA
>CABTXE01||gene_324|GeneMark.hmm|798_aa|+|7668|10064
VVFRLPSKEIGMVEQGVGARLLRKEDDRLMRGRGEFVGDIRLPGMRDVAFVRSPLAHARIKGIRIPPEYRDSVFI
AADLEGVKPIRAVSGLPGFKVSEQPILAFEKVRQVGELIAMCVADTRAEAEDIAAAVEVDFEELPAVHDMLLAKR
PDSALLHEHWGDNIFLETFVDVNMEAAYDAPIKITREISTARQSMAPMEGRGTVAMWHKRMDQLVLYTGNQ
QPHIVRNGLSECLGLEQIQVRIVSPDVGGGFGYKGIVLTEDVCLGWLAKRCGYPVRWIEDRREHLTAGANCREH
HYNITVYADRDGKLRGVECEATVDSGAYSSYPFSACLEAAQVASILPGPYDFPSYRCRTWSVATNKCPILPYRGVA
RTGVCFAIELMMDAIARETGLEPYEVRLKNLVQPEQMPFDNITKKHFDSGDYPESLRQALAKIDLPALRERQKKP
EADGRLIGVGLSVYCEQGAHGTSVYAGWGIPMVPGHEQATARMTPDGGLELRVGVHSHGQSMETTLPQVA
HEILGIDTAKIKLVHGDTEYTPYSTGSWGSRCAVMAGGAVATASRELGKLVLGIGAHLLQTDISNVKLRDGVVVG
PSGSVTLQEVAYTWYRRPQDLPPSVDPRGLEVTIGYKPVRDSGTFSYASHIAVVAVDPEMGDIELLDYVIVEDGG
KLINPMVVDGQIYGGLAQGIGTALYEEMNFDTSGQPLASTFADYLLPGPTEVPEPKLGHMETLAPYTEFGVKGL
GEGGAIAPPGAIGNAVNDALKSLGAEIRHSPITPRRVLEAIEEAKSTARAKAGEGVLA
>CABTXE01||gene_376|GeneMark.hmm|779_aa|+|59936|62275
MVGARVRRTEDPRLLTGNGNYVDDRQSAGMLHVAFRRSDHSHARIVSINCEAARRSPGVIGIFTGADLEGRIK
PLLATSRMANYHATPLVALAQGKVRYVGEPVVAVVAESRYLAEDAIELIEIDFEALPVVIDPEAAVQPDAPLLHKEI
GTNVLLSREFKRGDVDAEMENAVVRVSGRFRMRRKTPLAMENRSYVAEYSAGQDALTLYSATQIPGIIRDALSE
ALDMPGNRLRVIAPDVGGGFGGKASLYPEELFVTFAARHLGRAVKWTSDRLEDLASTSQGFDEIIDAELGFDKA
GLAVALRADVIGDVGAYSIYPWTAVLEPVQVVSFLPGPYKIQHYRGRVRGVATSKPPMGPYRGVGRPASTFVTE
RLMDMAARKFNVDPKLLRLKNMIQPDEFPHKIGSGIVWDQCSFTECLEAACERIGYATLRERQVKARAEGRWF
GIGMACYAELTGIGSRISVAPGMPINTGTETAKILIDSTGAVTVAVGTAAHGQGHETTFAQVVAEHLGARLQDIR
VIHGDSAAVPNATGSYASRSAVLAGGAATLAAQDVKEKVLRAASHLLEASVADLTIEDGRIAVAGTDRAMTFREL
ARAVYSEMGRIPSDQREELSSTKTYDPVFGTAATAAHVAVLEIDPETYQVKLERFLVAEDCGKLINPMIVDGQVH
GGVAQGIGAALYEEVIYNDEGQNVTASLVDYVVPSASEVPSIDVVHIEAASKTTLGGFRGMGEGGTIGAPAAVA
NAISDALASLNIDVFELPVTPERLFRLIENAKAHS
>CABTXE01||gene_2661|GeneMark.hmm|996_aa|-|10180|13170
MTQPAAAEVELNRPWVGRSIQRVEDFALLMGRGRFIDDLGTRPGTLHAAILRSPHAHADIVAIDTSAALKSRGV
AAVLVGEDVKKLTSSLVVGVKAPVECWPIAVGRVRYVGEPVAVVVASDRYLAEDAIDLVEVQYSVRPAVIDPLVA
VRADAPVLHDGFSGNVASDRRFRYGDPEKAFAEAAHRISIDIKYPRNSCTPIETYGLIADYDPAENAFDVLANFQ
GPFSIHAVISRALKVPGNRLRLRTPPDSGGSFGVKQGIFPYIVLIAAASRVVGRPVKWIEDRLEHLTASVSATNRAT
TLAAAVTAEGKILALDWDQVEDCGAHLRAPEPATLYRMHGNLTGAYDIRHVAVRNRVVVTNKMPTGLNRGFG
GPQVYFALERLLHRIAIELGLDPLEVIRLNLVPADAFPYRTATGALLDSGNYQEALIRGARDGQLAELKARRDQAR

AEGRLYGIGYTAVVEPSVSNMGYITTVLTAEERRKAGPKNGAQATATIGLDPVGGVTVHVASVPQGQGHRTVLS
QVVADVFGLLPQDIRVNTEIDTAKDAWSIASGNYASRFAPAVAGTAKLAAQRLAAKLARIAASQLNVDASDIVFA
GGSVGSKHNPENKISFSRLAALSHWSPGSLPDDVGDTIRETVFWTPPELTPPDDHDLINSSLCHGFIFDFCGVEI
DRTTLQTRIDRYVTMHDCGTILHPGMVDGQIRGGFVQALGAALYEEYAYGPDGSYLTGTLADYLLPTTTEVPEP
VILHMETPSPFTPLGSKGVGEGNCMSTPVCIANAVADALDVKDLTLPLVPARLAEIVRGAEPSAPAGRAVSGPKA
GGNDRRLRGEGNASVNVPPERVWDMLLDPETLRAVVPGCQSVEKVSDTHFRADVTLGIGPVTGRYRANVML
SDLDPPHAVTLSGSAEGVLGFGGGEGRITLTPDGNGGTTMHYVYHAAIGGKVASIGGRLLDGATKVIIGQFFSA
LARQAEGGGSGRDGLSLVVRLKRLIGIGS
>CABTXE01||gene_2252|GeneMark.hmm|804_aa|-|41345|43759
MCNCPRCSRDQGSPYGKVGIMSVEGIGARVTRKEDKRFITGKGKYTDDVRLHGMTYASFVRSPHAHAKIKSIN
VDAAKAMPGVVDVLTGQQLVDDKIGNLICGWMIHSKDGSPMKMGAWPAMAPETVRFVGNAVAVVIAETR
NQARDAAEAVEVTYQELPAVADIRSAIAPGAPQLHPEAPGNVIYDWSIGDEAATGEAFKKAANVVAMDITNNR
LVPNAMEPRAAVAEYDSAEEHFTLYTTSQNPHVARLVLSAFYNVAAENKLRVIAPDVGGGFGSKIFIYPEEMVAL
WASKRTGRPVKWTSDRTEAFLTDAHGRDHITKAEMAFDKDNKIIGLRVKTHANLGAYMSLFSSSVPTYLYATLLS
GQYNIPNIYAEVISVYTNTTPVDAYRGAGRPEASFVMERMMETAARQLKVDPAELRRKNFITSFPHQTPVIMAY
DAGDFNASLDAALKAIDYAGFPARKAKAKAEGKLRGIGFSCYIEACGIAPSKAVGSLGAGVGLWESCEVRVNPV
GTIEILTGSHSHGQGHETTFAQVVADRLGIPISQVSILHGDTDKVQFGMGTYGSRSGAVGMSAIVKAMEKVEAK
AKKIVAHQLEASENDIVIENGEFKVTGTDKAIALPMVALAAYTAHNLPDGMEPGLKETAFYDPTNFTFPAGAYVC
EVDVDPGTGKTDIVNFVAADDFGRLINPMIVEGQVHGGLAQGIGQAMLEGAVYDKSGQLVTASFMDYAMPR
ADDLPSFKVSHTMTPCPSNPLGIKGCGEAGAIGSTPAVINAITDAIGNNKLEMPASPDRVWHAIHQQQAAE
>CABTXE01||gene_3483|GeneMark.hmm|776_aa|-|12714|15044
MNILPSNLRFGAGQSVKRLEDQRLLTGQGHFIDDKAKDGALWLYVLRSPHAHARITAIDTSTALAMPGVAAVYT
GADLVADDIGTIPTLPIFKRPDGSPMAAPPRRLLAHDVVRFAGEPVAAVLAPSRADAQTAAEAIVVDYEILPAVV
SPADALAPGAPVVWPDAPDNIAAAMSYGDAAATEAAFAKAKHVVSLDITSQRLVPSALEPRSTMAEVDKKTGR
LTLHVQSQTPTATRDILSDVVLKRPKDSVHVMVGDIGGGFGHKVNLYPEDGIVAYAATKLKRTVRWRGDRIDDFI
GGSHGRDLTSTGEFALDEKGRVLAFRVRSLGGTGAYLTGAGVIIPLVLGPFVATGVYDLPLVHFDIKAVLTHTAPTG
PYRGAGRPEAVFIVERLMDAAARQIGMDPRQIRKVNYIKPSQLPYKNAVGEVYDSGAFAHMMERASKLADW
DGFSARKKAAKKKGLLYGRGLTSYIEWTGGRVHTEKVTLSATAEGRIVLQSGTQAMGQGLQTSYSQMVAGALGI
PLDRIDVIQGDTDKATGGGSVGSRSLFVGGTATVVSAGDLIQKAREKASHVLEASVGDIEYGDGVLTVVGTDRR
VSLFDLAKDEKDSRLSVESKGEVDGPTWPNGTHICEVEIDPETGVTRVVRYTTVDDVGVAINPMLVTGQVHGG
VAQGIGQALYEGVVYDSSGQLLTASYQDYCIPRASDMPHIDVTLDDSAPCKTNPLGAKGCGESGAIGGPPCIVN
GVMDALSDLGITSLQTPLTPVKIWNAIQQAKATKAA
>CABTXE01||gene_387|GeneMark.hmm|781_aa|-|73461|75806
MGQPLRRREDFKFITGKGRYTDDMKSPGMLHMAILRSPHAHAVIKHVDLSTAQSAPGVHLVLSGADLVGKMG
PIEPNWVIPGTKVPDRPVVAVDRVRFVGECVAVVVAETQALAHDAVGLIEVDYETLPAVIDEEAAIREGAPQLH
DNVPKNITTLYKIGGGDYKKAASEADHVIKLRVINNRLIPTCMETRSILAEPNVDGTLTVNIQSQVPHMHRRWIA
DTVRIPEHQLRIVAPDIGGGFGAKMHLYPEELLCPYLARQLGVPVKWWESRSESHQSTNHGRAHTETIEVAFRN
DGKILGLRVETLGNVGAYLSNMASGGPTVNTVNFGTGTYKIDNYEAFSRVVVTNTVPVDAYRGYGRPEGGYIAE
RAIDAVARHLKLDQVEVRNRNFIQRADFPHRPYNGPAVIYDSGNYQGLLTKALEVFKYDERIAERDQLRSQGRY
RGIGVAAYTHMCGMAPSRRLSLMGFNRGGWESARVSIDSSGRATIFSGSMSQGHGHNTSLAQIAADVLQIPIE
SIDIVQGDTRQVQAGHGTFNSRSMAVGGSSVHVTSQRIVAKARKIAASMLEVDEKDVSYRAGEFSVPGTDIAP
LSFGKIARMAYVGHKLPDGMEPGLDETTFYDPAGMGSPSGIHMAYIEVDPETGMVDILDYVAVDDVGTIINPLL
AAGQIHGSVVQGIAQALYEEVSYDPDTGQLMTGSLLDYAVPRAEHVPNIRSSFQETPSPTNPIGVKGVGESGSIA
APPCMVHAVLDALSPFEILHLDMPMTPPRIWSAVQQARAGVTQ

>CABTXE01||gene_2315|GeneMark.hmm|775_aa|-|22589|24916
MMDFQGVGASLLRKEDDRFLRGRGQYVGDLKLPGLKDVAFVRSPLAHAKMRGVRIPPEFQDRVFTASDLTDV
KPIVAASGLPGFKYSEQPLLAHEKVRQVGELVAMCIGNTRAEAEDIAAAVELDLDELPVVSEMLTARRPGAPLVH
DHWGDNVFLETYIDVNMEAAYDAPIKVTREIRTARQCMAPLEGRATVAYWDKRLDQLVLHTGTQQPHIIRTGI
SECLGLDQSKVRVISPDVGGGFGYKALMPPEDVCLAWLAMHCGHPVRWIEDRREHLTASANCREHHYIITAYA
DRDGTLRGIECEATVDSGAYSSYPFSACLEAAQIASILPGPYDFPSYRCRTWSVATNKCPILPYRGVARTGVCYAM
ELMVDAVAREAGLDPLEVRLKNLVKPEQMPFDNITKKHFDSGDYPEALKRAMARIDLDAIRKQQENTHRLIGIG
LSIYCEQGAHGTSVYSGWGIPMIPGHEQATARVTPDGGLELRIGVHSHGQSMETTLPQIAHEILGIETAKIKLVH
GDTEYTPYSTGTWGSRSAVMAGGAVSRASQEIAGLIKGIGAHLLQTDVANVRLEDGCVIGPSGSVTVKEVAHT
WYRRPQDLPASVDPRGLETTIGYKPVRDSGTFSYAAHIAVVAVDPEMGDIEILDYVIVEDGGKLINPMVVDGQI
YGGLAQGIGTALYEEMPFDPSAQPLASTFADYLLPGPTEVPAPQVEHMETLSPYTEFGVKGLGEGGAIAPPAAIG
NAVNDALKSLGVELLCCPMTPRRVLEAIHRAKGNA
>ACNO01||EEN84262.1
MSTPVKPAKRNGAKHPASDHSTADHSVADDGKFFGKPIPREEDTRLLSGQGRYLDDLGHNALIAAFVRSPHAH
ARIIDIDIDQALEVPGVHAIYTYEDLLADTPEMAENLPLLIPHPGIIAPRNGYPLAKDEVKHVGEAIAMVVADNRY
IAEDACAKIDVTYEALKPVVGINVARTADNAVHADVPDNVAAHLQHGFGDLDAELAAAPHRLTLDLEIERSASM
PMEGKGVYARWDGDENTLTFWTSTQTSTSARAAIAARLGMALNKVHCIAPDVGGGFGVKIVHPWPEEVMIT
WAARRLGQAGISCEVKWVEDRREHFISSAHERGQIQKVDIGFDDDGRLLAFDFTFWHDNGAYLPYGIIVMINT
ATQVLGPYKPKSFRVNAYSLYTNTVIVTPYRGAGRPQAVFAMERSMDAIAKYLKKDKIAVREANFIRPEDMPYD
FGLMFQDGRPLIYDTGDYQAGIDKLKKLIDWDGFPEYQRKTRAEGRSVGIGIGAYVEGTGPGPYEGAHVVVETS
GRVKAATGLTTQGQGHQTAFAQIVADDLGVKVSDVEIVTGDTRRFGYAVGTFASRGAVMSGSAFHVAAQMVA
EKAKKIASGILDLPETELELREGHVCKIGTEAGVEGTSVPLSVVAVLSNPLRYAFDRESKLATGFAKTDTDMSKPPI
PEGEQPGLEATGYYSPPSSTFASGVHAAIVETDPVTAEITVRRYVVIHDCGNVINPRIVEGQVMGAVAQGIGGAL
YERIVYDEHGQMLNASYMDFLMPFVTEMPDSLEMDHTVTPSGLNPLGMKGAGEAGVIPTSAVIAAAIEDAEG
ISITSMPISPSELFELRLAHAGSSSEENA
>ACUF01P||EFP64797.1
MNAPAEPNNHLIGASVKRKEDFRFLTGAGQYTDDVVQAHQSYAVFLRSPYAHARIKHINTDAARNHPGVLAVL
TGDDLAADKVNGLPCGWLIHSIDGTPMKEPPHPVLAQGKVRHVGDQVALVVAESVKIAKDAVEMIDVEYDEL
PAVVDTATADTAGTAVHDDVPNNTCYTWGHGDKAATDAAFARAAHVTRLDIVNNRLIPNAIEPRAVNASYSRQ
DDSYTLYVANQNPHVERLLMSAFVLGLTEAKVRVIAPDVGGGFGSKIFLYPEDVALTWASKKVGRPIKWTAERSE
SFLTDAHGRDHVTHAELALDAQGNFLAMRVHTTANMGAYLSTFASSVPTILYATLLAGQYKTPAIYAEVKAVFTN
TAPVDAYRGAGRPEATYVVERLVETAAHELQIDPAELRRRNFIRTFPYATPVGLTYDTGDYEPCLDRAIELADVKGF
AARRDASRAKGRLRGMGYSCYIEACGLAPSNIAGALGARAGLFEAGEIRVHPTGSVTVFTGSHSHGQGHETTFA
QVVADRLGVPIDNIEIVHGDTGRIPFGMGTYGSRSIAVGGSAIMKALDKIEAKAKKIAAHLLEASAEDIEFKDGVF
RVAGTDRTKTFGEVALTAYVPHNYPLDKLEPGLDENAFYDPTNFTYPAGAYICEVEVDPDTGEVHIDRFVAVDDF
GNIINPMIVEGQVHGGLGQGIGQALLEACVYDENGQLLTGSYMDYAMPRANDLPSFTVETAKGTPCTHNPLG
VKGCGEAGAIGSPPALINAIVDALAPLGVKDIQMPATPHRVWQTIQAAKA
>ACUF01P||EFP64456.1
MKRFDAGETTGTAQETQQTTGQPYVGRPMQRVEDAAILTGRGRYADDLGVKPGTLHAAILRSPHAHAELGLID
FAAALKAPGVRAVLTGADLPAWSKPFVVGVKAPMEQWALAMDRVRYVGEPVAVVVAESRALAEDALDLVRV
DYRVLPPVVSIEAAIADAAPQLHSGLGSNVASDRHFRYGDPEAAFATAPHRVSLTVHYPRNTCTPIECGVVIAEHL
PGNEGYQVTSNFMGPFSLHAVMAMALQVSANHLRHIAPRDSGGSFGVKQAVFPYVVLMCLASRKAGAPVK
WVEDRLEHLSAATSATARLSTIEAAVEADGRIVALDYDQLEDCGGYLRAPEPATFYRMHGVLTGAYAIPNLRVRN
RVVLTNKTPTGLVRGFGGPQVYFALERLVQRISIELNLDPLDVYRRNFVPSNAFPYRAAAGALLDSGNYQLAMS

RALETGAYDELKRRRDIARAEGRLYGIGFAAIVEPSVSNMGYITTATPAEARRKAGPKNGAIASATVSVDLLGGVV
VTIASTPAGQGHMTVCAQVVADVLGIDPAEVIVNVEFDTHKDAWSVAAGNYSSRFAGAVAGTVHLAATRVRD
KLARIVASQLDCDPAELIFAEGRITRRDAPETAVVFARAASNAPHWSPQLLPAGEEPGLRETVFWSPPNLDAPDE
QDRINTSACYGFAFDLCGLEIDRATGRVRIDRYVTAHDAGKLLNPALADGQIRGAFAQGLGAALMEEFRYGPDG
SFQSGTLADYLLPTTCEVPDPMIVHLETPSPFTPLGAKGLGEGNNMSTPPCIANAVADALGVRDIRLPLTPAKVM
AMIGLEDPPPSRLELAETATAAATGGKERSKGAKALSARGTVDLDAPPEAVFAVLLDPQALAQVVPGCHVLEPIG
DNRYRADVTVGVGMIKARYEAEIALSDLEPPHRLRLSGAGLSSLGSARGSGMVELAPHEGGTRLTYDYEAEVSG
KVAAVGGRMLEGAAKVVLRQLFESLGRQAGGKPVRKPGWIARLLALFGARR
>ADMS01P||EFF75750.1
MGARVPRKEDARHLHGKGNFVADMAMPGLSEVAFLRSPLAHARITAVRVADQIAGNVFLRQAMPDARDIVA
DSTLPTYQASAQPPLASGKVRFVGEPVAMVFAPTRAEAEDYAELIEVDYDDLPVYADVDSAQAAQGDLLHEQ
WRDNVFVTLNADKQFDEHAAQAEVVVRRKIDLARQCMVPMEGKAVLAYWDHQADQLVVVSATQVPHMIR
SVLAQCLDLEQGRVRVVSPDVGGAFGYKCVLQQEELCVAWLAKTFKRPFRFIEDRREHLTAGANSREHHYEMV
AYADRRGRLLALDAKITIDGGAYSVWPFTIGLEPGQAIGNLPGPYAFKGYRCVTRAVATNKPGFVPYRGVARTGV
CFAMELTMDAIAREVGREPWEVRLENLVQGEQMPFVNVTGKHLDSGDYPASLQQAMDMIDVAAVRERQRQ
GEADGRLIGVGLATYTEQAAHGTSVFAAWGTPVIPGFDQATARVTPDGGLELRVGVHSHGQGMETTFAQIAN
EILGVDVGSIKLLHGDTGQTPFSTGTYASRSLVMSGGAVSQACKRLLPRLTHIGAHLLQADPASVSWNQDRLEA
GGKSVSVKDVADAWYLRPQLLPPDVDPAGLEVTVGYKPKVDTGCFTYATHAAVVAVDPGTGGVEILDYVVVED
CGTMINPMVVEGQTIGGVAQGIGTAFYEETPYDENGQPLASTLADYMLPGATEVPNMRLHHFETPSPHTEFG
AKGMGEGGAIAPPAVLFNAVNDALRPLGAAELLRTPLSPTRVLAAIAQGVAAQAKQTVQPATAQVMA
>ADNV01||EFG76654.1
MTHDVQGAPAVPVTPAPRYAGTRVQRVEDGRLLTGRGSFVDDISRPGMLHACFVRSPFARARINGIDASAALE
LPGVRAVFTAADLNPDVHEAWHAVAGKDVPDTPRPPLAEGEAKFVGDPVALIVAESRYVAEDALELVDVDYEPL
PAVTDFTRAQSSDVLVHDAYPDNVAGGMGGAPPDEELFADAPCVAKEHIYQQIYAPVPIETRGLVVEWTAATG
ELTMWASTQTPHELRAFCARLLGIAAQRVRVIMRDTGGGFGQKVVPMREDMCIMLAARKVPAALKWIEDRR
ENLMSAGQARHVDGTGRMAFDGEGNILAADIDFVQDIGAYPTPYPVLTTAAIGMFFPGPYRVPKASFNYKTVF
SNTSGLAAYRGPWQYETLAREVLLDIAARKMDIDPVELRRRNLLRRDEMPYVNPNGMPYDHVAPIDTFEQAV
KILDHEGFRKEQAEALAQGRYLGLGFSAYIEPTGAATGHLATEGATIRMEPTGKINVYVNGGSSGNSIETTVVQLT
ADALGADIDDVATIQGDTAITPYGAGTQGSRSAPMTAGAVNEAGAILREKIVALAAHHLKVAESEVDLAFSVASV
RGDPSKSVSFGELAYVAHYSPQQLPPGLSANLEATARFNSTNPIHWSNATHACTCEVDVATGKVTLLRYIVSEDV
GPMINPSVVEGQIAGGTVQGIGGALMEDMVYDDDGNPLATTFVDYLLPTATEVPPIEFGHVEIPGPGPGGYKG
AGEGGAIGSTPAVINAINDALAPLGATVTRLPASPASIAALLEEGKR
>ADNV01||EFG76645.1
MTTIESRPPSPEDTADNDQKPCGHGRMLRKEDPRFIRGRGTYVDDVALPGMLHLAILRSPYAHARIASIDTTAA
LAHPKVKAVVTGADLAEKGLAWMPTLSNDVQAVLATDKVRFQGQEVAFVVAEDRYSARDALELIDVDYDPLD
PVVDVRRALDPSAPVIRTDLENKTDNHIFDWETGDAAATEAVFAKADVVIKQEMVYPRVHPAPMETCGAVAD
LDPVTGKLTLWTTSQAPHAHRTLYALVAGLPEHKIRVISPDIGGGFGNKVPIYPGYVCAIVGSLLLGKPVKWMED
RSENLTSTSFARDYIMVGEIAATKDGKILALRSNVLADHGAFNGQAAPTKYPAGFYGVFTGSYDLEAAYCHMTAV
YTNKAPGGVAYACSFRITEAVYFVERLVDCLAFELKMDPAEVRLRNLLKPNQFPYTTKTGWTYDSGDYETTMRK
AMDMLGYDALRAEQKERRARGELMGIGMSFFTEAVGAGPRKDMDILGLGMADGCELRVHPTGKAVVRLSV
QTQGQGHETTFAQIVAEELGIPPDDIDVVHGDTDQTPFGLGTYGSRSTPVSGAAAALVARKVRDKAKIIASGML
EVSVADLEWEKGSFHVKGDPSASVTIADIAMRAHGAGDLPEGIEGGLDAEVCYNPSNLTYPYGAYFCVVDVDP
GTAVVKVRRFLAVDDCGTRINPMIIEGQVHGGIVDGIGMALMEMIAFDDDGNCLGGSLMDYLIPTAVEVPHLE
TGHTVTPSPHHPIGAKGIGESATVGSPPAVVNAVVDALAPFGVRHADMPLTPSRVWEAMQGRARPPI

>ADNW02||EGD22908.1
MTTLDHPQTDPDRPVNDGKPCGHGRMLRKEDPRFIRGQGRYVDDVQLPGMLHLAILRSPVAHARVVRIDTT
AAQAHPKVAAVVTGVDLAEKGLAWMPTLSNDVQAVLATDKVRFQGQEVAFVVAEDRYSARDALELIDVEYEIL
DPVIDARRALAADAPVIRTDLDGKTDNHCFDWETGDSAATAAVFDRADVVVRQEMVYPRVHPAPMETCGAV
ADYDAVDGKLTLWSTSQAPHAHRTLYALVAGLPEHKIRVVSPDIGGGFGNKVPIYPGYVCAIVGSLLTGKPVKW
MEDRSENLTSTGFARDYIMVGEIAATREGKILAIRTDVLADHGAFNGTAAPVKYPAGFFGVFTGSYDLEAAYCH
MTAVYTNKAPGGVAYACSFRITEAVYLVERLVDCLAFELDLDPAELRLRNLLRPEQFPYTSKTGWKYDSGDYATT
MRKAMDMIGYDDLRREQAERRRRGELMGIGMSFFTEAVGAGPRKDMDILGLGMADGCELTVHPTGKAVVR
LSVQTQGQGHETTFAQIVAEELGIPPEDIDVVHGDTDNTPFGLGTYGSRSTPVSGAAAALVARKVRDKARIIASG
MLEVSVADLEWEKGNFHVKGDPSAAVTIQDIAMRSYGAGDLPDGIEGGLEAQICYNPENLTYPYGAYFCVVDV
DPGTGQVSVRRFLAVDDCGTRINPMIIEGQVHGGITDGIGMALMEIVAFDEDGNCLGGSLMDYLIPTAREVPH
LETGYTVTPSPHHPIGAKGIGESATVGSPPAVVNAVVDALKPFGVRHADMPLTPSRVWEAMQGRATPPI
>ADNW02||EGD23478.1
MSVTKPQPAERPATDAASDVASEPDRYFGKPIPRSEDARLLSGNGRYLDDLGHNALIAAFVRSPHAHARITGIDT
DAASELAGVHAIYTYEDLEADSAGMAENLPLLIPHPAITAPRNGYPLAKDEVNHVGEAIAMVVADNRYIAEDAC
ALIEVGYEMLPPVVGIDVARRAENAVHADVPDNVAAHLQHGFGDLDAELASAPHRLTLDLEIERSASMPMEGK
GVYARWDDDEQALTFWTSTQTSTSARAAIAARLNMAHNKVHCIAPDVGGGFGVKIVHPWPEEVMVTWAAR
QLGRAGISSEVKWVEDRREHFVSSAHERGQLQQVTVGFDDEGRLLAFDFTFWHDNGAYLPYGIIVMLNTSTQ
VLGPYKPRSFRVDAYSLYTNTVLVTPYRGAGRPQAVFAMERSMDAIAQYLGKDVLAVREANLIRPEEMPYDFGL
MFQDGRPLIYDTGDYQAGIDKLKELIDWDGFPEYKRRAEAQGRTVGIGVGAYVEGTGPGPYEGAHVLVETSGK
VKAATGLTTQGQGHQTSFAQIVADDLGVPVSDVEIVTGDTRRFGYAVGTFASRGAVMSGSAFHVAAQMVAEK
ARKIAGEHLHLDPGDLELRGGHVCRKGTEPGAAGTSIPLGVVAVLSNPLRYAFDESSRLATGFASTETDMTVPPV
REGEQPGLEATGYYSPPTSTFASGVHAAIVETDPVTAEIHVRKYAVVHDCGNVINPRIVEGQVQGAVAQGIGGA
LYERIVYDEHGQMLNASYMDFLMPFVTEMPDSLDMDHTVTPSGLNPLGMKGAGEAGVIPTSAVIAAAIEDAE
GIPIRSMPISPSELFELRLTHAASQSEENE
>ADVL01||EFH09652.1
MAKFGLSQAVRRIEDPRLLKGHGRYTDDISAAGQLHGYVLRSPHAHARILSIDTAEAKAIPGVHAILTGQDWLAE
GLGQIPCAIPLKNRDGSPRAETPRYGLAVEKVRHVGDPVAFIVADSLAIARDAAEAVLVDYDILPACTDLATATDP
GQPQLWDSAPNNLCFDWETGDKAATEALFAKAAHVTKLEVVNNRIVVASMEGRAALAEYDAGSEKFTLHAGT
QGSWLVKDLLAKVVFNLPPEKFRVVTPDVGGGFGMKLYLYAEYALCCWAARKLGRPVKWAAERMEAFQSDT
QGRANLTLGEIALDAEGKFLALRTRNIADMGGYLSTFAPFIPTGAGTKVLASVYGFQAIYANVLGVLTNTVPVDA
YRGAGRPESNYLVERLIDAAAHELGIDRIELRRRNMVPPSAMPHRTPVGQNYDSGDFATVLDAALAKADWAG
FEARRAESEKRGRKRGIGMAYYLEATGGDASERAEMRFAQDGMVEVLVGTQSTGQGHETAYAMITSHQLGIPL
EKIRVVQGDSDEIPTGGGTGGARSLYSEGTALLATAATVIEKGKQAAGEALEAAPADIEFTGGRFAIAGTDRGIGIL
ELAMAQRERAARGETATLLDAAEIAAIPAHTFPNGCHIAELEVDPDTGHITASRYIVVDDVGHALNPLIVRGQVH
GGVAQGYGQAVMERTAYDPETGQLISASLNDYALPRASDLPDIEVELMEIPCETNPLGVKGAGEAGAVGSPPAV
MNALVDALRGSGIHHLDMPATPEVVWRALQEAKRAA
>ADVL01||EFH10400.1
MTPNITPWEAKSMNVTLPHEGIGASVRRKEDLRFLSGRGQYTDDMNRPGQLHAWILRSPHAHARIDGIDTAA
AAAMPGVAAVYTARDIEAAGLGGIPCGWQVTGKGGKVMAEPMHPVLASGKVRHVGDPVAVVIAESKAQAR
DAAEAIEVSYTTLPALSTMAAALAPGAAALHDDAADNLCFDWEIGDAAATDAAFARAHKVVRFETTNNRLVPN
AMEPRAALGDYDPNSGEHTLITTSQNPHVIRLLMGAFVLKVPEHKLRVVAPDVGGGFGSKIYHYAEEAIVTWA
AAQLKRPVKWTAERTESFLSDAHGRDHVTTAELALDQDGIFLGLRVSTLANMGAYLSTFAPAVPTYLSATLLAGV
YRTPAIHGQVKAVFTSTVPVDAYRGAGRPETTYLLERLVDVAAKEMGMDRLEIRRRNFIRPDQFPYQTPVALQY

DSGDYEATLKGAMEASDYAGFEARRAEAKARGKLRGIGVSTYLEACGIAPSKLVGSLGARAGLYEVANIRVHPTG
SISVFTGTHSHGQGHETTFAQLVSEQLGVPLSQVEIVHGDTSKIPFGMGTYGSRSLAVGGAAMVKAMDKIIAKG
RKIAAHLLEASVEDIEFDRGNFRVAGTDRAKTLTDISMAAYVPHNYPIDEIEPGLEETAFYDPKNFTYPGGCHICE
VEIDPDTGEVAVVNFTACDDVGRVINPMIVEGQIQGGLAQGIGQALIEHTAYDADGQLLTASFNDYAMPRAAD
LPGFAVSTATTLCTHNPLGVKGCGEVGAIGSPPAVINAVVDALRDHGVTHVEMPATPLKIWSIIHGKGAAHAQP
QAAE
>ADVL01||EFH10474.1
MADSLPFDPSRLKFGIGQPVPRNEDPILLQGQGRYTDDLQLPGQLWCAIVRSPYAHAVLKGVDTSAALDVPGV
VAVYTGQDLQAAGYGHLRCTIPLQGLVNIERPALAVDRLRFVGDPFACVIAETREAAKDGAEAVYAEVETLPAVT
EASAAAAPDAPQLYDHIPGNQVLDFHYGDAEKVRDAFASAAHVARLDLRNNRVVVCAMEPRSAIAEYAPDEG
RYTLHLGCQGVFGLRNQIANDLLKVPVEKLRILTGHVGGSFGMKASAYPEYVCLLHAAKQLGRPVKWTDERSGS
FLSDMHGRDHEVTAELALDAEGRFLAVRLTSLANMGGYLATVGPLMGTMNFVRNIQSNYATPLIEVSTRCLVT
NTTPISAYRGAGRPEGNYFMERLIETAAPAMGLDPLELRRRNFIRPAQMPYAAASGSRYDSGDFEGLLARAMR
EADWDGFAARKAASAAKGLLRGRGVGNFLECTAPPAKEMGGLRFEEDGGVTIITGTLDYGQGHWTPFAQVLH
QTLGVPFDRIRLVQGDSDQLIAGGGTGGSKSLMASGAAIIEASAKVVEKARLAAAHLLEAAEEDLDFEPHAEGG
ARFVIAGTDRAIGLLELAARLRQATGLPEGVPDSLDVQHVFQEAPMAYPNGCHIVELEVDPETGTVRFDRYVTV
NDFGVIVNPLLVEGQAHGGIVQGIGQALMERTAYSEDGQLLSGSYSDYALPRASDVPSFHFASHATPAQTNPLG
AKGCGEAGCAGSLPAVMNALVDALSGRGVTHIDMPATPEVIWRALNQAA
>AGER01||EPD69083.1
MTTTVPADGHRTAFVDNDQKPCGHGRMLRKEDPRFVRGRGRYVDDLQLPGMLHLAILRSPLAHARLVSVDTS
LAEAHPKVRLVVTGAMLAEKGLAWMPTLSGDVQAVLATDKVRFQGQEVAFVVAEDRYAARDALELIDVEYEPL
DPVIDVRTALAPDAPVIRDDLEGKQDNHCFDWETGDAAETDAVFARADVVVKEDIVYPRVHPAPMETCGAVA
DYDAVDGKLTLWSTTQAPHAHRTLYAIVAGLPEHKIRVVSPDIGGGFGNKVPIYPGYVCAIVGSLLTGKPVKWM
EDRSENLITTGFARDYIMRGEIAATREGKILAVRTHVLADHGAFNGTAAPVKYPAGFFGVFTGSYDIEAAYCKMT
AAYTNKAPGGVAYACSFRITEAVYLIERIVDCLAYELGMDPVELRMQNLIRPEQFPYRTKTGWVYDSGNYAPTM
ELAAQLAGYAELRAEQAEKRARGELMGIGVSFFTEAVGAGPRKDMDILGLGMADGCELRVHPTGKAVVRLSV
QSQGQGHETTFAQIVAEELGIPPQDIEVVHGDTDQTPFGLGTYGSRSTPVSGAAAALVARKVRDKARLIASSML
EVSVADLEWEKGSFQVAGDPKASVTIQDIAMRAHGAGDLPDGVEGGLEAQICYNPENLTYPHGAYICVVDIDP
GTAKVTVRRFVAVDDCGTRINPMIIEGQVHGGLTDGVGMALMEMIAFDEDGNCLSGSLMDYLIPTSLEVPDW
ETGFTVTPSPHHPIGAKGVGESATVGSPPAIVNAVVDALKPFGVRHADMPLTPSRVWEAMQGRPTPPI
>AGER01||EPD68165.1
MTDQATEREVGRARPRKEDARLITGQTNWTDNISVNGLLHMAILRSPMAHARITRVDVSPALERPGVIAAFSG
ADLAEGLGSLPCAWPVTEDIVLPDHPPIAVDEVRHAGDPVALVVARDRYAAADALEAIEVDYEPLPPVLDLEAAL
AQDSPLVHSDKGTNRCYVWPLKTGEAFDSVRERAEVTLTRRYHHQRLIPNAMEPRAVVVTPLAASGEYTLYSST
QVPHIVRVMMAVVTGIPEHKIRVVAPDVGGGFGSKLQVYGEEALALAVARRIGRPVKWTESRSEGYLATHHGR
GMIQDIEIAANRDGKLLGLKVDLLVDMGAYLMLVTPGIPILGAFMYPAIYKMDSYDFTCTGVFTTRTPTDAYRG
AGRPEATYAIERIMDDLAVELGLDPVELRRRNWIGHEEFPYTSIAGLTYDSGNYEAATDKALSLFDYDKLRAEQT
DRNRRGDSVRLGIGVSTYTEMCGLAPSRVLRDLRYAAGGWEAASIRMLPTGKVEVVTGTSPHGQGHVTCWS
QIAADVLGVPFEDVEVVHGDTKASPQGMDTYGSRSLVVGGTAVHSAAEKVVAKARKVAAHLLEASEQDLDFT
DGVFSVKGSPDARKTIQEVAFETFTSHDLPDGFEPTINAEHLIDPDNFSYPHGTHLCAVEVDTETGQTHIRSYVC
VDDVGKVINPMIVEGQVHGGLAQGIAQALYEEAVYDDEGNLVSGTMTDYLVPSAADLPEFTTERTETPATSNPL
GVKGVGEAGTIASTPAVVNAIVDALRPLGVQDIRMPCTPERVWRAVREAQA
>AGEZ01||EHM01367.1
MPDSLPPDSSRLKFGIGQPVPRNEDPILLQGKGRYTDDLQLPGQLWCVMVRSPYAHGTLNGIDISAAKEVPGV

VGVYTGADLAEYGTLHCMMPAPGLTDIQRPVLAMDKVRFVGDPIAFVVAETKEAAKDGAEAVFADIDPLPAVT
EASAAAAPDAPLLYDDIPGNQVQDFHFGDSAKVAEAFASAAHVTRLDIRNNRIVVCAMEPRSAIGEYDPENGR
YTLHLGSQGVFGMRAAIAKELLKVPVEQLRILTGHVGGSFGMKAGAYPEYLCLLHAAKVLGRPVKWTDERTGSF
LSDYHGRDHEVKAELALDAQGKFLAVRLTAFANIGAYLSPVGPLMGTSGFMRNVQSNYTTPLIEIATRSILTNTPP
VTAYRGAGRPEGNYFMERLIEAAALEMGLDAVELRRRNHIKADQFPYDAASGSKYDGGEFTALMEKALNAAD
WQGFEARKAESKARGKLRGRGIGNFLECTAPPAKEMGGLRFNEDGTVTIITGTLDYGQGHWTPFAQVLHQTL
GIPFDKIKLLQGDSDKLIAGGGTGGSKSLMASGAAIVEASAKVVDKARIAAAHMLEAAEADIEFDPHAEGGGRF
SIAGTDRGIGIMQLAAQIRQASDLPPEVPDTLDVDHIFDQAPMAFPNGCHVVELEVDPETGVVQFDRYVTVN
DFGTIVNPMLVQGQAHGGIVQGIGQALMERTNYSEDGQFLSGSYSDYAVPRAVDVPSFEFISHGVPAKTNPLG
VKGCGEAGCAGSLPAVMNALVDALSEFGVKHIDMPATPEVVWRAIQQHRQG
>AGEZ01||EHL97359.1
MAKFGLSQPLRRIEDPRLLKGSGRYTDDISVPRQLHGYLLRSPHAHARIVAIDTSAALALPGVHAVLTGQDWLD
DGLGEIPCAIPLKNRDGSPRAETPRYGLAVGTVRHVGDPVAFIVADTVQAARDAAEAVMVDYDVLPSATDLATA
SESGQPQIWEGAPNNICFDWEAGDKAATEAAFAQAAHVTKLTVVNNRIVVNSMEGRAALAEYDAAQQKFTLY
AGTQGSWLVKDLLAKSVFHLPPEKFRVVTPDVGGGFGMKLYLYGEYALCCWAARRLGRPVKWTSERMEAFQS
DTQGRANLTTGELALDKDGKFLALRTLNLADMGGYLSTFAPFIPCGAGTKVLASVYGFRAIYANVLGILTNTVPV
DAYRGAGRPESNYLVERLIDAAARELGIDRIELRRRNMVPPSAMPHRTPVGQNYDSGDFAQVLDKALEKSGWA
GFAARKAASAAKGRKRGIGMAYYLEATGGDPSERAEVRFAADGMVDVLVGTQSTGQGHETAYAMITSHQLGI
PMEKIRILQGDSDEIPVGGGTGGARSLYSEGTALLATAATVIEKGKQAAGEALEASPSDIEFSSGLFTIAGTDRGIGI
LELAAAQRARAAKGEAATQLDAAEVAAVPHGTYPNGCHIAEVEVDPETGHTDILRYIVVDDVGHALNPLIVRG
QVHGGVAQGIGQAVMERTAYDPETGQLLSASLNDYALPRAADLPDIEVELVEIPCETNPLGVKGAGEAGAVGSP
PAVINALVDALADEGVTHLDMPATPEVVWRALHAAA
>AGWX01||EKS36714.1
MNDSSMKQRGLSVLDRPNSYIGKTVPRPNLGRLMQGRGQYVSDLTLPRMAHVVFLRSPYAHARILKIDATEAK
AMPGVIAVVTGVELAKVISPWVGVLSHLKGLKSAPQHAIAVDRACWQGEAVAAIIATSRALAEDAAELVSVDYE
ELVPVTDMRTALDPATPVIHPDLGDNLAFERNLDAGAVDQAFRESDEVVEAEFVFGRHTGVTLEPRAVLADWN
SGDERLTVYLGTQAPHMVQNIAAKHLGLDEPQVRVVCKDVGGSFGIKVHIYADEMATLALSKLLRRPIKFVADR
VESFNTDIHARDHVCKARIGVSKNGTINAFEIDDVTGIGPYSMYPRTSAIEANQVVNLVGGPYTTQNYRARTRV
VFQNKNVTCQYRGVGHPIACSITEGLVDLAAQRIGMDPFEIRRRNLIRDDAYPCGSPSGLKFEALSHHASLDKLY
AMMNYGALRAEQAALRKQGIYRGIGIASFIEVTNPSAAFYGVGGARISSQDGVAVRLDATGRVICQTSITEQGQ
GSESLTAQIVGSVLGVSMERVRVILGDTDNTPYGGGTWASRGAGIGGEAALQAAKVLRNNILDVAAAILQAKPA
DLDIVDNAVVDVHGGQQRIELHELARIVYFRPDTLPPGFQPELMATRHFVPREYPFFAFTNGIQASWLEVDTRTG
FIKLLKHWVVEDCGTIINPQLVDEQIRGGVVQGLGAALYEHCIYDERGQLTNANMADYLVPMSGEMPDIDVGH
IVSPTLDGELGAKGAGEAGTAAAAAVVANAVNDALSPFNVTVTEIPLTPRVILTALGRI
>AGWX01||EKS34453.1
MSVEGIGARVTRKEDKRFITGKGKYTDDVRLHGMTYASFVRSPHAHAKIKSINVDAAKAMPGVVDVLTGQQLV
DDKIGNLICGWMIHSKDGSPMKMGAWPAMAPETVRFVGNAVAVVIAETRNQARDAAEAVEVTYQELPAVA
DIRSAIAPGAPQLHPEAPGNVIYDWSIGDEAATGEAFKKAANVVAMDITNNRLVPNAMEPRAAVAEYDSAEEH
FTLYTTSQNPHVARLVLSAFYNVAAENKLRVIAPDVGGGFGSKIFIYPEEMVALWASKRTGRPVKWTSDRTEAFL
TDAHGRDHITRAEMAFDKDNKIIGLRVKTHANLGAYMSLFSSSVPTYLYATLLSGQYNIPNIYAEVVSVYTNTTPV
DAYRGAGRPEASFVMERMMETAARQLKVDPAELRRKNFITSFPHQTPVIMAYDAGDFNASLDAALKAIDYAGF
PARKAKAKAEGKLRGIGFSCYIEACGIAPSKAVGSLGAGVGLWESCEVRVNPVGTIEILTGSHSHGQGHETTFAQ
VVADRLGIPIGQVSIVHGDTDKVQFGMGTYGSRSGAVGMSAIVKAMEKVEAKAKKIVAHQLEASENDIVIENG
EFKVTGTDKAIALPMVALAAYTAHNLPDGMEPGLKETAFYDPTNFTFPAGAYVCEVDVDPGTGKTDIVNFVAA

DDFGRLINPMIVEGQVHGGLAQGIGQAMLEGAVYDKSGQLVTASFMDYAMPRADDLPSFKVSHTMTPCPSN
PLGIKGCGEAGAIGSTPAVINAITDAIGNNKLEMPASPDRVWHAIHQQQAAE
>AGWX01||EKS41128.1
MTQPAAAEVELNRPWVGRSIQRVEDFALLMGRGRFIDDLGTRPGTLHAAILRSPHAHADIVAIDTSAALKSRGV
AAVLVGEDVKKLTSSLVVGVKAPVECWPIAVGRVRYVGEPVAVVVASDRYLAEDAIDLVEVQYSVRPAVIDPLVA
VRADAPVLHDGFSGNVASDRRFRYGDPEKAFAEAAHRISIDIKYPRNSCTPIETYGLIADYDPAENAFDVLANFQ
GPFSIHAVISRALKVPGNRLRLRTPPDSGGSFGVKQGIFPYIVLIAAASRVVGRPVKWIEDRLEHLTASVSATNRAT
TLAAAVTAEGKILALDWDQVEDCGAHLRAPEPATLYRMHGNLTGAYDIRHVAVRNRVVVTNKMPTGLNRGFG
GPQVYFALERLLHRIAIELGLDPLEVIRLNLVPADAFPYRTATGALLDSGNYQEALIRGARDGQLAELKARRDQAR
AEGRLYGIGYTAVVEPSVSNMGYITTVLTAEERRKAGPKNGAQATATIGLDPVGGVTVHVASVPQGQGHRTVLS
QVVADVFGLLPQDIRVNTEIDTAKDAWSIASGNYASRFAPAVAGTAKLAAQRLAAKLARIAASQLNVDASDIVFA
GGSVGSKHNPENKISFSRLAALSHWSPGSLPDDVGDTIRETVFWTPPELTPPDDHDLINSSLCHGFIFDFCGVEI
DRTTLQTRIDRYVTMHDCGTILHPGMVDGQIRGGFVQALGAALYEEYAYGPDGSYLTGTLADYLLPTTTEVPEP
VILHMETPSPFTPLGSKGVGEGNCMSTPVCIANAVADALDVKDLTLPLVPARLAEIVRGAEPSAPAGRAVSGPKA
GGNDRRLRGEGNASVNVPPERVWDMLLDPETLRAVVPGCQSVEKVSDTHFRADVTLGIGPVTGRYRANVML
SDLDPPHAVTLSGSAEGVLGFGGGEGRITLTPDGNGGTTMHYVYHAAIGGKVASIGGRLLDGATKVIIGQFFSA
LARQAEGGGSGRGGLSLVVRLKRLIGIGS
>AGWX01||EKS41669.1
MVEQGVGARLLRKEDDRLMRGRGEFVGDIRLPGMRDVAFVRSPLAHARIKGIRIPPEYRDTVFIAADLEGVKPI
RAVSGLPGFKVSEQPILAFEKVRQVGELIAMCVADTRAEAEDIAAAVEVDFEELPAVHDMLLAKRPDSALLHEH
WGDNIFLETFVDVNMEAAYDAPIKITREISTARQSMAPMEGRGTVAMWHKRMDQLVLYTGNQQPHIVRNGL
SECLGLEQIQVRIVSPDVGGGFGYKGIVLTEDVCLGWLAKRCGYPVRWIEDRREHLTAGANCREHHYNITVYAD
RDGKLRGVECEATVDSGAYSSYPFSACLEAAQVASILPGPYDFPSYRCRTWSVATNKCPILPYRGVARTGVCFAIE
LMMDAIARETGLEPYEVRLKNLVQPEQMPFDNITKKHFDSGDYPESLRQALAKIDLPALRERQKKPEADGRLIG
VGLSVYCEQGAHGTSVYAGWGIPMVPGHEQATARMTPDGGLELRVGVHSHGQSMETTLPQVAHEILGIDTA
KIKLVHGDTEYTPYSTGSWGSRCAVMAGGAVATASRELGKLVLGIGAHLLQTDISNVKLSDGVVVGPSGSVTLQ
EVAYTWYRRPQDLPPSVDPRGLEVTIGYKPVRDSGTFSYASHIAVVAVDPEMGDIELLDYVIVEDGGKLINPMV
VDGQIYGGLAQGIGTALYEEMNFDTSGQPLASTFADYLLPGPTEVPEPKLGHMETLAPYTEFGVKGLGEGGAIA
PPGAIGNAVNDALKSLGAEIRHSPITPRRVLEAIEEAKSTARAKAGEGVLA
>AGWX01||EKS39526.1
MLDHPSRVSSDNDIALQKFGVGQPVRRKEDDTLVRGKGTYTDDISLPGQAYAWIVRSTHAHGIIKGIDVEAARA
MPGVLGVWTGKDLAAAGYNPFTVGLPMKNRDGSPLLQTNRLPLMTDKVRFVGDPVAFVVAETLAQARDAG
EAVVVDIEPLPSVTSAEDAAKPGAPLLYDHIPSNVVLDYHAGDDAALDAAFARAAHVTRLDIVNTRLAVVAMEP
RAALASYDKKSQRFTIEIPTQGVAGNRTSLAKTLNVPNDKVHLRTHHVGGSFGMKNANYPEYICILHAARELGR
AVKWTDERSTSFLSDSHGRAQDVHCELALAADGAFLGVRVRGFGNLGAYITGVAPLPLSLNIAKNINSVYRMPL
IGVDIKCVLTNTTLMGAYRGAGRPEANYFMERLIDRAAEEMGIDRLAMRKRNFIKPAQIPYAAANGLTYDSGDF
PGVFSKAVEQADVAGFAKRRKESRKRGKLRGIAVGSYLEITAPPSAELGKIVFEADGTVRLITGTLDYGQGHATPF
AQVLAAQLGVPFDAIKLTQGDSDIVHTGNGTGGSRSITASGAAIVASSRLVIEKGKRAAAHVLEASEGDMEFAN
GRFTIAGTDRGIGIMELAAKLRNSKMPEGVPSSLDVDHTVQGVPSTFPNGCHVAEVEVDPDTGNVQIVRYSGV
NDFGTVVNPMIVAGQIHGGVAQGIGQALMECVTYDDNGQPVTGSFMDYALPRAGDIPSMTLGSHPSPATSN
PLGTKGCGEAGCAGSLATIVNAVLDALSDEGVTSIDMPLTSEKVWRAIQDGRKAKKSA
>AGWX01||EKS41732.1
MTEAAAIRYMGQPLRRREDFKFITGKGRYTDDMKSPGMLHMAILRSPHAHAVIKHVDLSTAQSAPGVHLVLS
GADLVGKMGSIEPNWVIPGTKVPDRPVVAVDRVRFVGECVAVVVAETQALAHDAVGLIEVDYETLPAVIDEEA

AIREGAPQLHDNVPKNITTLYKIGGGDYKKAASEADHVIKLRVINNRLIPTCMETRSILAEPNVDGTLTVNIQSQV
PHMHRRWIADTVRIPEHQLRIVAPDIGGGFGAKMHLYPEELLCPYLARQLGVPVKWWESRSESHQSTNHGRA
HTETIEVAFRNDGKILGLRVETLGNVGAYLSNMASGGPTVNTVNFGTGTYKIDNYEAFSRVVVTNTVPVDAYRG
YGRPEGGYIAERAIDAVARHLKLDQVEVRKRNFIQRADFPHRPYNGPAVIYDSGNYQGLLAKALEVFKYDERIAE
RDQLRSQGRYRGIGVAAYTHMCGMAPSRRLSLMGFNRGGWESARVSIDSSGRATIFSGSMSQGHGHNTSLA
QIAADVLQIPIESIDIVQGDTRQVQAGHGTFNSRSMAVGGSSVHVTSQRIVAKARKIAASMLEVDEKDVSYRAG
EFSVPGTDIAPLSFGKIARMAYVGHKLPDGMEPGLDETTFYDPAGMGSPSGIHMAYIEVDPETGMVDILDYVA
VDDVGTIINPLLAAGQIHGGVVQGIAQALYEEVSYDPDTGQLMTGSLLDYAVPRAEHVPNIRSSFQETPSPTNPI
GVKGVGESGSIAAPPCMVHAVLDALSPFEILHLDMPMTPPRIWSAVQQARAGVTQ
>AGWX01||EKS41594.1
MDFQGVGASLLRKEDDRFLRGRGQYVGDLKLPGLKDVAFVRSPLAHAKIRGVRIPPEFQDRVFTASDLTDVKPI
VAASGLPGFKHSEQPLLAHEKVRQVGELVAMCIGGTRAEAEDIAGAVELDLDELPVVSEMLTARRPGAPLVHD
HWGDNVFLETYIDVNMEAAYDAPIKVTREIRTARQCMAPLEGRATVAYWDKRLDQLVLYTGTQQPHIIRTGISE
CLGLDQSKVRVISPDVGGGFGYKALMPPEDVCLAWLAMHCGHPVRWIEDRREHLTASANCREHHYVITAYAD
RDGTLRGIECEATVDSGAYSSYPFSACLEAAQIASILPGPYDFPSYRCRTWSVATNKCPILPYRGVARTGVCYAME
LMVDAVAREAGLDPLEVRLKNLVKPEQMPFDNITKKHFDSGDYPEALKRAMARIDLDAIRKQQENTHRLIGIGL
SIYCEQGAHGTSVYSGWGIPMIPGHEQATARVTPDGGLELRIGVHSHGQSMETTLPQIAHEILGIETPKIKLVHG
DTEYTPYSTGTWGSRSAVMAGGAVSRASQEIAGLIKGIGAHLLQTDVANVRLEDGCVIGPSGSVTVKEVAHTW
YRRPQDLPASVDPRGLETTIGYKPVRDSGTFSYAAHIAVVAVDPEMGDIEILDYVIVEDGGKLINPMVVDGQIYG
GLAQGIGTALYEEMPFDPSAQPLASTFADYLLPGPTEVPAPQVEHMETLSPYTEFGVKGLGEGGAIAPPAAIGNA
VNDALKSLGVELLCCPMTPRRVLEAIHRAKGTRSVRCT
>AGWX01||EKS36363.1
MAPVKFGVGQSVLRKEDDALIRGKGRYTDDYTPAAAMHALVLRSPHAHAKFKIDVSRARGLPGVGAILTADDI
QELGSLPCLFNLPDEPFTGPPYPILARDVVRHVGDAIAFVVADTVEQARDAIEAIEVEWTALPAAIGLVNAVKKD
APQVWPDHAGNILFDTSIGDKKAAEAAFAKAHAVAEVTIVNPRIVTNYMETRAVVCEYDAKRDHLTLTIGSQGS
HRLRDILCQNVLNIPVEKMRVICPDVGGGFGTKLFPYREYALAAVAARKLKKTVKWTAERADHFVGDAQGRDN
VTTARMALAEDGKFIGMDVDLMGDMGAYLSTFGPYIPHGGAGMLPGLYDLQAFHCRVRTVFTNTVPVDAYR
GAGRPEAAYVVERLVDAAARKLGMTPDAIRRKNFISPRAMPYTTATGKIYDSGDFAAHMKRAMDIAEWKEFP
RRAKAAKKQGLVRGIGMGTYVEVCGTMGEETAQVRLDPDGDVTILIGTQSSGQGHQTAYAQLVAEQFGIAPER
VHIHQGDTDEIPTGLGTGGSSSIPSGGVSVERATRKLGENLKEIAADALETGVGDLEFRDGAIRIVGTDRTISFADL
AKRADPSKLNASATFSSADGTYPNGTHIAEVEIDPATGVIHVVNYVIVDDFGVTLNPLLLAGQVHGGTMQGIG
QALMEAAVSGAADGQLVTGTFMDYALPRAADGPSIKFETHNVPCKTNPLGVKGAGEAGAIGSCPAVVNAIIDG
LYREYGIDHIDMPATAERVWMAIAKSRREHRL
>AGWX01||EKS41721.1
MHNVGDDLATRPKMVGARVRRTEDPRLLTGNGNYVDDRQSAGMLHVAFRRSDHSHARIVSINCEAARRSPG
VIGIFTGADLEGRIKPLIATSRMANYHATPLVALAQGKVRYVGEPVVAVVAESRYLAEDAIELIEIDFEALPVVIDPE
AAVQPDSPLLHKEIGTNVLLSREFKRGDVDAEMENAVVRVSGRFRMRRKTPLAMENRSYVAEYSAGQDALTLY
SATQIPGIIRDALSEALDMPGNRLRVIAPDVGGGFGGKASLYPEELFVTFAARHLGRAVKWTSDRLEDLASTSQG
FDEIIDAELGFDKAGFAVALRADVIGDVGAYSIYPWTAVLEPVQVVSFLPGPYKIQHYRGRVRGVATSKPPMGPY
RGVGRPASTFVTERLMDMAARKFNVDPKLLRLKNMIQPDEFPHKIGSGIVWDQCSFTECLEAACERIGYATLRE
RQAQARAEGRWFGIGMACYAELTGIGSRISVAPGMPINTGTETAKILIDSTGAVTVAVGTAAHGQGHETTFAQV
VAEHLGARLQDIRVIHGDSAAVPNATGSYASRSAVLAGGAATLAAQDVKEKVLRAASHLLEASVADLTIEDGRIA
VAGTDRAMTFRELARAVYSEMGRIPSDQREELSSTKTYDPVFGTAATAAHVAVLEIDPETYQVKLERFLVAEDCG
KLINPMIVDGQVHGGVAQGIGAALYEEVIYNDEGQNVTASLVDYVVPSASEVPSIDVVHIEAASKTTLGGFRGM

GEGGTIGAPAAVANAISDALASLNIDVFELPVTPERLFRLIENAKAHS
>AGWX01||EKS34429.1
MNILPSNLRFGAGQSVKRLEDQRLLTGQGHFIDDKAQDGALWLYVLRSPHAHARITAIDTSAALAMPGVAAVY
TGADLVADDIGTIPTLPIFKRPDGSPMAAPPRRLLAHDVVRFAGEPVAAVLAPSRADAQTAAEAIVVDYEILPAV
VSPADALAPGAPVVWPDAPDNIAAAMSYGDAAATEAAFAKAKHVVSLDITSQRLVPSALEPRSTMAEVDKKT
GRLTLHVQSQTPTATRDILSDVVLKRPKDSVHVMVGDIGGGFGHKVNLYPEDGIVAYAATKLKRTVRWRGDRID
DFIGGSHGRDLTSTGEFALDEKGRVLAFRVRSLGGTGAYLTGAGVIIPLVLGPFVATGVYDLPLVHFDIKAVLTHTA
PTGPYRGAGRPEAVFIVERLMDAAARQIGMDPRQIRKVNYIKPSQLPYKNAVGEVYDSGAFAHMMERASKLA
DWDGFNARKKAAKKKGLLYGRGLTSYIEWTGGRVHTEKVTLSATAEGRIVLQSGTQAMGQGLQTSYSQMVAG
ALGIPLDRIDVIQGDTDKATGGGSVGSRSLFVGGTATVVSAGDLIQKAREKASHVLEASVGDIEYGDGVLTVVGT
DRRVSLFDLAKDEKDSRLSVESKGEVDGPTWPNGTHICEVEIDPETGVTRVVRYTTVDDVGVAINPMLVTGQV
HGGVAQGIGQALYEGVVYDSSGQLLTASYQDYCIPRASDMPHIDVTLDDSAPCKTNPLGAKGCGESGAIGGPP
CIVNGVMDALSDLGITSLQTPLTPVKIWNAIQQAKATKAA
>AGWY01||EKS33756.1
MSAEGIGARVTRKEDKRFITGKGKYTDDVRLFGMTYASFVRSPHAHAKIKSIKVDAAKAMPGVVDVLTGQQIV
DDKVGNLICGWMIHSKDGSPMKMGAWPAMAPEVVRFVGNAVAVVIAETRNQARDAAEAVEVVYEELPAAA
DIRAAIAAGAPQLHPEAPGNVIYDWSIGDEAATNDAFKKAANVVAMDITNNRLVPNAMEPRSAVAEYDSAED
HFTLYTTSQNPHVARLVLSAFYNIAPENKLRVIAPDVGGGFGSKIFIYPEEMVALWASKRTGRPVKWTADRTEAF
LTDAHGRDHLTKAEMAFDKDNKIIGLRVKTHANLGAYMSLFSSSVPTYLYATLLSGQYNIPNIYAEVISVYTNTTPV
DAYRGAGRPEASFVMERLMETAARQLKVDPAELRRKNFITSFPHQTPVIMAYDAGDFNASLDAALKAIDYAGFP
ARKEKAKKEGKLRGIGFSCYIEACGIAPSKAVGSLGAGVGLWESCEVRVNPVGTIEILTGSHSHGQGHETTFAQV
VADRLGIPIGQVSIVHGDTDKVQFGMGTYGSRSGAVGMSAIVKAMEKVEAKAKKIVAHQLEASENDIVIEEGQ
FKVAGTDKAIALPMVALAAYTAHNLPDGMEPGLKETAFYDPTNFTFPAGAYVCEVDVDPGTGKTDIINFVAADD
FGRLINPMIVEGQVHGGIAQGVGQALLEGAVYDKSGQLVTASFMDYAMPRADDLPSFKISHTMTPCPSNPLGI
KGCGEAGAIGSTPAVINAITDAIGTNNLEMPASPSRVWHAIHQQQAAE
>AGWY01||EKS35603.1
MAPVKFGVGQSVLRKEDDALIRGKGRYTDDYTPAAALHALVLRSPHAHAKFKLDATKARALPGVAAILTPDDVK
DLGGLPCLFNLPDEPFTGPPYPILARDVVRHVGDAVAFVVAETVEQARDALEAIEVEWTPLPAVVGLVNAVKKG
APQVWPDHTGNVLFDTSIGDKKATEAAFAKAHAVAEISIVNPRIVTNYMETRAVVCEYDAKRDHLTLTIGSQGS
HRLRDILCQNVLNIPVEKMRVICPDVGGGFGTKLFPYREYALAAVAARKLKKTVKWTAERADHFVGDAQGRDN
VTTARMALAEDGKFLGMDVDLMGDMGAYLSTFGPYIPHGGAGMLPGLYDLQAFHCRVRTVFTNTVPVDAYR
GAGRPEAAYVVERLVDAAARKLGMAPDAIRRKNFIQPKAMPYKTATGKIYDSGDFAAHMKRAMDVAEWKEF
PRRAKAAKKQGLVRGIGLGTYVEVCGTMGEETAQVRLDSDGDITILIGTQSSGQGHQTAYAQIVAEQFGIAPER
VHIHQGDTDEIATGLGTGGSSSIPSGGVSVERATRTLGTNLKDIAADVLETGAGDLEFSDGAIRIVGTDRTVSFAD
LAKRAGADPSKLTASSTFSSADGTYPNGTHIAEVEIDPSTGVIHVVNYVIVDDFGVTLNPLLLAGQVHGGTMQGI
GQALMEAAVYDATDGQLVTGTFMDYALPRAADGPSIKFETHNVPCKTNPLGVKGAGEAGAIGSCPAVVNAIID
GLYRDYGIGHIDMPATAERVWMAIENSRRVHRM
>AGWY01||EKS31922.1
MKKPETPSAELRPKLVGQRIKRTEDPRLLTGAGRYVDDMTPPGTLHVALRRSDQPHARIAGIDVGEAFSIPGVV
AIFDATDIEGDLKPAIPTSKMKNYYATPIWPLARGKVRYVGEPVVAVLAESRYAAEDALEHISIKYEPLPFAIRQVD
AVADDAPLLHDEAGTNVIISREFARGDVDKAITDAPVTVKGVFRMTRKTAVSMENRSYLAEWDRRRLALTLHSS
TNIPGIIRDVLAGCLDLPGNRMRVVAPDVGGSFGGKGSLYHEEMLVCVLARKLGRPVKYVADRLEDLSATSQAF
DELMEAELALDHDGKLIGLRADVIGDIGAYSIYPWTGALEPVQVVSFLPGPYRLEHYRGRIRGVLTPKPPTGPYR
GVGRPSSTFAMERLIEMAARKIGMDPVEIRRRNLVTAEEFPYRTGSGIIWDKSAFQECLQGAVDLADYPALVRE

RDKARQEGRWVGIGLSSYAELTGIGSRISVAPGMPINTGTETSSIRIDATGAITAAFGISSHGQGLETTLAQVIADE
MGCKMHDIEVQHGDSSLVPMSSGTFASRSAVIAGGAATMSARVVKAKVLKAAAYLMEVPVDDLEASDGIVRA
RSSNASMTFKEVAHAVYSQMGRIPRELREDLVASETYDPYLGTACSSTHLVMVEIDPQTFGVTIKRYVVAEDCG
RIINPMIVDGQVHGAVAQGIGAALLEELVYDEKGQFLTASLADYLVPVATNVPDIGIVHIEAELPNNIGGFRGMG
EGGTIGAPAAIANAVSDALSPLGIEIDTLPATPERIFRLVEAARSGGGVV
>AGWY01||EKS42388.1
MLDHPSGLSAENDIALQKFGVGQPVRRKEDDTLVRGKGTYTDDINLPGQTYAWIVRSTHAHGRIKSIDTEAAKA
MPGVLGVWTGKDLAGYTPFSVGLPLKNRDGSPLLQTNRLPLMTDKVRFVGDPVAFVVAETLAQARDAGEAVV
VDIEPLPSVTSAEDAAAPGAPLLYDHIPSNVVLDYHSGDDAALDAAFASAAHVTRLGIVNTRLAVVAMEPRAGV
ASYDKKTQRFTIEVPTQGVSGNRLSLAKTLNVPPDKVHLRTRNVGGSFGMKNTNYPEYICILHAARELGRAVKW
TDERSTSFLSDSHGRAQDVHCELALTADGTFLGVRVRGFGNLGAYITGVAPLPLSLNIAKNINSVYRMPLIGVDIK
CVLTNTTLMGAYRGAGRPEANYFMERLIDRAADEMGLDRLALRKRNFIKPSQIPYAAANGLTYDSGDFPGVFA
QAVEQADLAGFAKRKKESRKRGKLRGIAVGSYLEITAPPSAELGKIVFEADGSVRLITGTLDYGQGHATPFAQVLV
AQLGVPFDAVKLTQGDSDIVHTGNGTGGSRSITASGAAIVEASKLVIEKGRQAAAHLLEASESDIEFADGQFTIAG
TDRSIGIMDLAAKLRDTKMPDGVPASLDVDHTVQGVPSTFPNGCHVAEVEIDPETGVVQIVRYNGVNDFGTV
VNPMIVAGQIHGGVAQGIGQALMECVTYDDNGQPVTGSFMDYALPRAGDIPSMTLSNHPSPATSNPLGTKG
CGEAGCAGSLATIVNAVLDALADEGVTSIDMPLTSEKVWHAIQDGRKAKAA
>AGWY01||EKS33737.1
MNILPANLRFGAGQSVKRLEDQRLLTGQGHFVDDKPQDGGLWLYVLRSPHAHARIKAIDTGAALAMEGVAAV
YTGADLVADNIGTIPTLPIFKRPDGSSMQAPPRRLLAHEIVRFAGEPVAAVLAASRADAQTAAEAIAIDYEILPAVV
APADALAPGAPVVWPDAPDNIAASMSYGDATATEAAFAKAKHVVSLDLTSQRLVPSALEPRSTMAEIDKKTGRL
TLHVQSQTPTATRDILADVVLKRPKDSVHVLVGDIGGGFGHKVNLYPEDGIVAYAAQKLKRTVRWRGDRLDDFV
GGHHGRDLTSTGEFALDEKGRVLAFRVRSLGGVGAYLTGAGVIIPLVLGPFVATGVYDLPLIHFDIKAVLTHTAPTG
PYRGAGRPEAVFIVERLMDAAARQIGMDPRQIRKVNYIKPSQLPYKNAVGETYDSGAFAHMLERASKLADWD
GFNARKKAAKKKGLLYGRGLTSYIEWTGGRVHTEKVTLHATAEGRVILQSGTQAMGQGLQTSYSQMVAGALGI
PLDRIDVIQGDTDKATGGGSVGSRSLFVGGTAAVVSAGDLIQKAREKASHVLEASVGDIEYGDGMLTVVGTDRR
VSLFDLAKDEEDARLTVNSQGDVDGPTWPNGTHICEVEIDPETGITRVVRYTTVDDVGIAINPMLVTGQVHGG
VAQGIGQALYEAVVYDPSGQLLTASYQDYCIPRASDMPHIDVTLDDSAPCKTNPLGAKGCGESGAIGGPPCVVN
GVMDALSEFGITSLQTPLTPVKIWDAIQQAKATKAA
>AGWZ01||EKS29350.1
MSGFPNNYVGRSILRVEDERLLQGKGCFVDDMHLPDCLYAVIVRSQIAHGYLRHIDSSAALALDGVVSVITANDI
PKPIGVIPIRIAPIEGGDTFRQPVIAHDKVRYVGEPVAVVLASTQAIAEDAAELVFVEIEEIEPVPSVEVAGSDQSA
RLFEVANTNTAAKYSASRGDTDAAFGSADCVLRETFAIQRHTAMPMEMRGLLAKWDVLTDHLTVWGAAKVP
FFNRNVLANLLGLPKTGIDLIELDVGGGFGVRGEFYPEDFLIPFAARLVGRPVKWQEDRREHMMATNHSRETYC
DVEIACRKNGEILGIRGSVTADIGAYMGTTGGILASRTAQFLAGPYRVRNVSFEVSAVVTNKTPAGSYRGPGRFE
STFFRERLFDMVAKRLNIDPLAFRQTNLIRSDELPFDIGQLVPYEGPASYDHGDYLKLLNDCAQAIGWKDKDAIR
GKENDGRYHGLGISCFVDSSGAGPKENARIRIEPNGNVTVFIGSCALGQGIETALAQICADELRIPMERIKVLHGS
TTLLDEGFGSFHSRSIIMGGNAIADAAGHLIAKIKSIAAEAWSVAPDHVAYEDGFIRDGDRMLSLAEIVRLAGNS
VESEGTFGTKNKPFGYGTHAAHVAVDIATGHVEVLDYVAMEDVGRMVNPMIVHGQKIGAIVQGLGGVFLEQL
VYDDRAQLMTGSLADYLMPTATDFRHVRAFALDLTRTTRNPLGFKGAGEDAIAPVAGVIGNAIADALRDFDVE
PKDLPITPPKLWALINSRGDESKTRSASL
>AGWZ01||EKS30140.1
MTGNSTAVADVNVGQRPVARWTGQSLPRDEDRQLLTGRGCYIDDLGVSTRTAEGAILRSPHGHARLLGIDTSK
ALALPGVHAVVTAKELQRFGSPLVAGLKIPVECWPLAVDKVRYYGEPVAMVVADTRYIAEDALDLIEVNYEPLPA

VIDPQEALAADAPVLHEKLGSNLANERSYVYGDPDDAFAKAAHRVGIDITYPRNACTPIETYGVIADYEPGTDSY
DVIANFQGPFSLHAVMARALNVPGNRLRLRTPGDSGGSFGVKQAVFPYVVLTAIAARVAGRPVKWIEDRLEHL
MASAVATNRETTLTAAVAQDGRILALDWDQLEDTGAHLRAPEPATLYRMHGNMTGAYAIEHLRIRNRIVLTNK
MPTGLNRGFGGPQVYFALERLVQKIALALKLDPLDVLRRNLIPAKAFPYRTASGALYDSGDYPTAVATAIDEGGLA
ELKARQETARREGRLYGIGYAAVVEPSVSNMGYITAVLTPEQRAKAGPKNGAQATSTIGIDAVGSVAVHVASTPQ
GQGHRTVLSQVVADEFGLKPSDIRVITELDTARDSWSIASGNYSSRFAAAVAGATQIAATRLRGKLAAVAARLLD
ADLDAIVFENGRIHAGDPAKSLPFARVASSSHWAPAELPQGIDQTIRETVFWSPPQLTAPTEDDRVNSSLCHGFI
FDICAVEIDRATCEVKIDRYVTMHDCGRILHPGMVAGQVTGSFAHALGAALYEESSYAPDGGLSSGTLADYLIPT
MMEVPELRILHHESPSPFTPLGAKGVGEGNCMSTPVCIANAIADALGVEDITLPVTPAKLAPHVHGAEPEPKNP
PVVQKASAAKAGERALTGSGKAVVAASRQQIWNMLLDPATLMSVIPGAHNVEKVGESEFHADVTLGIGPVKG
RYKAKIKLSDMNEPESVTLSGGTEGALGSGRGTGFITLTEADGKTTVAYTYEAAVGGKVASIGGRLLDGAAKVVI
GQFFEALANKASGKRGSPLMSAIRRLLALMGVGR
>AGYX01||EPD37115.1
MGASDFSKLPHIGEPVKRKEDYRFLTGAGQYTDDIALAAQAHAVFVRSPHAHARVRSVSTDAAKAAPGVIGVLT
GADVAADKINGLPCGWLITSTNGEPMKEPPHPILALDTVRYVGDQVAMVVAETLEQARDAAELVEVDYDPLPA
VVLVADAAAGSVPGAVVHDIAPDNHCYKWAIGDKAAVDAVFAGAAHIAQLDLVNNRLVPNAMEPRAAIGSYS
RANDEYTLYVANQNPHVERLLMTAFVMGLPEHKVRVIAPDVGGGFGSKIFLYAEDVCLTWGARKLNRSIKWTA
DRSESFLTDAHGRDHVSHAEMAMDASGKFLAMRVHTDANLGAYLSTFASAVPTILYATLLAGQYATPQVYVEV
DAWFTHTAPVDAYRGAGRPEATYLLERLVSRCAWQLNLSQAEIRRRNFVTSFPYQTPVALQYDVGDYGACMDK
AEALADVAGFAARRADSEARGLRRGLGYSSYIEACGLAPSNIAGALGARAGLFECGEVRVHPTGSVTVFTGSHS
HGQGHETTFAQVVAARLGIPVENVDIVHGDTGRVPFGMGTYGSRSISVGGAAIMKALDKIEAKAKKIAAHLME
ASDADIDFAGGEFTVRGTDKKIPFAQIALTAYVPHNYPLDKLEPGLNETAFYDPTNFTFPAGTYICEVEVDPATGV
VRVDRFSAVDDFGTIINPMIVEGQVHGGVAQGIGQALLENCVYDRETGQLLTGSFMDYAMPRADDFPEFKLG
TVCTPCTHNPLGTKGCGEAGAIGSPPAVINAVLDALHPLGVRDLDMPASPHRVWSAIDAAATS
>AGYY01||EPD42064.1
MGASDFSKLPHIGEPVKRKEDYRFLTGAGQYTDDIALAAQAHAVFVRSPHAHARVRSVSTDAAKAAPGVIGVLT
GADVAADKINGLPCGWLITSTNGEPMKEPPHPILALDTVRYVGDQVAMVVAETLEQARDAAELVEVDYDPLPA
VVLVADAAAGSVPGAVVHDIAPDNHCYKWAIGDKAAVDAVFAGAAHIAQLDLVNNRLVPNAMEPRAAIGSYS
RANDEYTLYVANQNPHVERLLMTAFVMGLPEHKVRVIAPDVGGGFGSKIFLYAEDVCLTWGARKLNRSIKWTA
DRSESFLTDAHGRDHVSHAEMAMDASGKFLAMRVHTDANLGAYLSTFASAVPTILYATLLAGQYATPQVYVEV
DAWFTHTAPVDAYRGAGRPEATYLLERLVSRCAWQLNLSQAEIRRRNFVTSFPYQTPVALQYDVGDYGACMDK
AEALADVAGFAARRADSEARGLRRGLGYSSYIEACGLAPSNIAGALGARAGLFECGEVRVHPTGSVTVFTGSHS
HGQGHETTFAQVVAARLGIPVENVDIVHGDTGRVPFGMGTYGSRSISVGGAAIMKALDKIEAKAKKIAAHLME
ASDADIDFAGGEFTVRGTDKKIPFAQIALTAYVPHNYPLDKLEPGLNETAFYDPTNFTFPAGTYICEVEVDPATGV
VRVDRFSAVDDFGTIINPMIVEGQVHGGVAQGIGQALLENCVYDRETGQLLTGSFMDYAMPRADDFPEFKLG
TVCTPCTHNPLGTKGCGEAGAIGSPPAVINAVLDALHPLGVRDLDMPASPHRVWSAIDAAATS
>AGZI01||EKU82455.1
MHTTSPRSLERQAPAPFVGRSLQRIEDVSLLTGRGQFGDDVGVKPGTLHAAIVRAPHAHALINGIDSTRAEQAP
GVRAVLTGDDLADWSKPFVVGVKASMEMWALARERVRYAGEPVAVVVADTRYLAEDAADLLVVDYTRLDPV
TSIDAAIAPDAPVLHDGVGSNVASDRSFRYGDPEAAFARAAHTVELSVRYPRNSCTPIECAVVVADYLSPEEGYD
VASNFMGPFSLHTVMALALRVPGHKLRHRTFRDSGGSFGVKHAVFQYAVLMCLASRKAGAPVKWVEDRLEHL
GGLTSATSRQSTIRAAVEADGRITALDYDQFDDVGGYLRAPEPATFYRMHGCLTGAYAIPNLAVRNRVVLTNKTP
AGLVRGFGGPQVYFALERLMQRISVELGIDPVDLYRRNFIPADAFPYQAAAGALIDSGNYQRALELVAGEGGLA
ALYAKRERARAEGRLYGIGFAAIIEPSISNMGYITTVLPKETRAKVGPKNGAIASATVAIDPLGGVNVTIASAPAGQ

GHRTVCAQVVADVLGVDPDQVAVNVEFDTQKDAWSVAAGNYSSRFAGAVAGTVHLAAMRIRDKLAAIAAPQ
LGADAADIVFAGGAVHVKDDPARALPFPRMAASPHWAPALLPEGVGLGLRETAFWSAPQLGAPDDDDRVNT
SAAYGFAFDVCGVEIDRHTGRVTIDKYVTTHDAGKILNPALADGQIRGAFAQGLGAALMEEFRYGEDGSFQSGT
FADYLVPTTCEVPEPHIIHLETPSPFTPLGAKGLGEGNNMSTPPCIGNAVADALGVADIVLPLTPSKVMALIGADE
PPPSAAVRQAMPAPAPAAGGKGGKALSARGEVVLAAPPEAVFRVLLDPVALAKVIPGCNALEPAGEHRYRADV
SVGVGMIKARYAAEVALSELDPPRSLRLAGSGLSAVGSASGSGLVTLEPADGGTLLRYDYSAEVSGKVAAVGSR
MLEGAAKIVLGQLFEQLGREAGGQGALPQASADQGRGLWRRLLALFGVTR
>AGZU01||EKU74355.1
MNAPPISAQRFIGQRVPRKEDGRLLTGRGSFVDDIILPGMLHAAFVRSPIARGTIRSIDTDVARAQPGVHAVLTQ
ADLAPFGVTMLSFFLGPVEVAMTPLADGRVAYVGHPVALVIADDRYLAEDAASLVVVDYAEEAAVVTLDDARL
GPRVHPDTDDNVAALMGEEEADAALEALLAGAPHLVSQSIRHQRIAQSPMETRGVVASLQGESELLVHITCAG
PQLVARWLTLALDRPGLSVRVIAKDVGGSFGLKNHPWMEEVSAILAAMLLRRPVKWIEDRIENLTAANQAREQ
EMTLRAAFDTDGRLVASHADYALNNGAYPMGADANIAVHMFLWAAYNMPAYSFVSRGWYSNTPGLAAYRG
PWAIETLARETLLDRAARQIGIDPVEIRRRNLCTAADQPSVTPLGIPREDITPAQCLEKLLEVVDVPAFRAEQAAA
RAQGRYLGLGLAAYIEPTGAAGSIAVMTGELAQLRIEPTGRVVAVMSVHSQGHGTQTTMAQCIADQLGVPIED
VTIFDGDSSRGGFSPGAGGSRQGVIGGGAAIRAGRLLADKVKVVAAHLLNASPQAISLADGMVHVAGAPEMR
RTLREIAEIAYGEPGRLPPGMETGLEAQYRYDPPPMTFTSAAHACIVEVDADTGFVTIQRWVSSEDCGVMINPA
VVEGQIAGGLAQAIGSVLLEDAARDAQGNPTAATFKDYALPTIFDVPDFEYVHADTPSQAEGGFRGVGEGGCII
GPPTLVNAIADALAPFGEVAVDLPLTPDKLMTVIEGQPWPERPVSRFHPDHRAPEVNTPAPMAPPPPPITPAAP
VGIDGAWKLVLATPMGPQPMVAHFQVMGDRVTGRLEADQGSQEFAGTIAGNQVAWEMKVTKPMAITLKY
ALLFDGDSVSGKCKMGLFGTAKVRGERA
>AQFP01||ENA27827.1
MTSFQPATESQTGHIGARQTRIEDAALLRGLGCYADDAAIPPGTLHAAIIRSPHAHARITSVDFSAALLMKGVHG
VLVGEDVKRWALPFPVGVRQPMEHWCVAVDKVRYVGEPVAVVIAESRYLAEDAIEGVRVEYEPLPPIIDPELAT
ADQAPILHEAVGSNVVNERHFRYGEPEQAFEQAPHKVSLKVKFPRSSCTPIECYVVLAQYERATGIYDVLANFQ
GPYALHTVMARALNVPGNRLRLRTPKDSGGSFGIKQGVFPYVVMMGLASRKVGAPVKWVEDRLEHLQGASS
ATNRVTEIEAAVEADGRITALRYDQIEDCGAYLRAPEPATFYRMHGNLTGAYAIRNVQVRNRVVLTNKTPSGLNR
GFGGPQVYFALERLLQHIAVQLKLDPLDVIRRNLVPTDAFPYQAAAGALLDSGNYQAGIALAAADGGLDDLLRR
RNQARAEGRIYGIGYAAVIEPSISNMGYITTAMTPEERRKAGPKNGAVATATINVGPLGDVSVHVSSTPQGQGH
QTTVAQVVAEVLGVALESIVVNVELDTQKDAWSIASGNYSSRFAGAVAGAVYKAALKIRDRLAAIAAEQLQASP
EDIRFAGGKIFVVNGGAVAPFHRIAGATHWSPGLLPGGESGGLRETAFWSPPQLVAPDDQDQVNSSLCYGFIF
DICGLEIDRMTGEIHIDRYVTCHDAGRLLNPALVDGQIRGGFTQGLGAALMEEFAYGEDGSFLSGTFADYLVPTA
PEVIEPVILHMDTPSPFTPLGAKGVGEGNNMSTPVCIANAVADALGRSDIRLPLTPSKVRTLIGIDEPPRPAGME
ADDNLDAAAGGPALRANDSVVIPASPQQVFDTLLDPQTLAAIIPGCHDLVLDGENRYRADVTVGVGMIRARFE
AKVALSDLDPPHSLRLSGSGSSSMGSAQGQAQVRFVELENGHTRLEYQYQVAVSGKVAAVGGRMLQGASKVII
GQIFTRLSQRVSGQAISTGWWARLRASLGALFGKGGAQ
>ATCE01||EPD96890.1
MSGRPGGTGRLVGTSVRRQEDPRLLTGRGRFTDDVHLPGMLYAHFVRSTVASGSVVSLDLSAVREVPGVVAAF
DAADLGLGDITARLDRPAGEFVPTAMPVLARDRVRFAGEPLAVVVARDPYAAEDGTEAAKVVHDTRPAVTRDT
QALAPGAPLVHDAAAANTLLDVTMFATEGIDSVFDRAHCVVDVETRTGRQNALPLETRGAVAAWDDRDRQL
VLHTSTQVPHQVRTVAARCLGLDERAVRVLVPDMGGGFGLKCVVGREEIAVAAAALRLGRPVKWTEDRKEALT
ASFLAREQHYRVRAAFDAEGRMLGLDADIVCDMGAYSCYPFTAGIEPLMAAAEMPGVYRLPAYRARARAVTT
NKAPSAPYRGVSRPQFVLVVERLFERAARELGLDPVEIRRRNLITEFPYTGVNNITYDPGSYRESLDLCERTLREEG
WYAARDTAAAEGRHLGIGYACFSERTGYGSAAFAQRKMEVVPGFDLAEVRMDTSGALTVTSGTMSHGQSHET

TMAQIAADELGLPLEKVRLHQGDTERVAYGWGTFASRSIAVGGSAVRRAAAELGRKLRHLAAARWGVPPDEV
VLGPGAVRRADGTAELSHTELARIAYLATHLLPEGMEPGLTATASFDGVGDGTFSNATHGVVVELHEGTGQVEIL
RYVCVEDCGVAVNPQVVEGQCRGGIAQGIAGALFEEITYDAQGEPSATGFMDYKVPTALEIPDVTVRHLETPCA
FTETGAKGAGEGGTIGAPAAVLNAVNDALRPTGVELNDIPVTPQTIAHALEQHS
>AWSJ01||ERI11424.1
MASMIGMRVKRKEDPKLITGNGNFTDDIKLPGMLYAAFLRSTHAHARIKRIDVSQAVELPGVVAVYTGEDLTGK
IKSVPTSWYVPGCNLKAKDRSPLAVDKVLYVGEGVAMVVAEDRYTAYDALNAIEVEYEELKAVTGQEAALKDGA
PLVHEDVKNNLAFLWKAGDVPDEAFTNAEVVVRERYYEQRVVPNPMETRAAVAQYNSGSGDMTVWCTSQN
PHIHRMVYAEVLGIPESKLRIIAPDVGGGFGAKIGVYADEAVVAYAARHLKRPVKWMEDRKEHFMATNHARDE
VIEVELAGKRDGTMTALRVRNTANMGAYLSTMGAGVPTICFGLMVTGAYAIPQAAVEVYGVYTNTTPTDAYRG
AGKPESAYQIERAVDAFAREIGMDPVEIRRKNFVPKEKFPYDTAMAVTYDSGDYMLTLDKALEIADYEELRREQE
ALRKQGRYLGIGLSTYVELCGLGPSKVAGAIGLQFGQWENATVRVHPSGKVTVLTGASPHGQGEDTTFAQVVA
DKFGVPLEDIEVLHGDTQMIPMGWGTYGSRTTPVGGAAVAIAAERVTEKAKKIAAHELEVSTEDLEFSDGIFQV
KGVPGHQRTFQEIARSANMAWNLPEGMEPALEAQSFFDPSNFVYPFGAHICVVEVDSNTGQIELKRYIAVDDV
GRVINPMIAEGQVHGGLAQGIGQALWEGAVYEENGQLISGTFMDYTMPKADFFPVLETAFTETPSPVNPLGA
KGVGETGATASPPAVVNAVLDALRPFGITHLDMPLTPEKVWRAMQKGRKEA
>N221356||gene_270930|GeneMark.hmm|791_aa|+|12589|14964
MGANEFAKLPHIGESLKRKEDERFLTGVGQYTDDINQANQKYAAFVRSPHAHAAIKSVDTSAADKMPGVVRVF
TGKDTEGKMGGLPCGWLISNPDGSPMKEPPHPILAHAKVRYVGDHVAMVVADTPQQAKDAAEAVVVDYEP
LGAVIDMRTAKTAPALHDEAPDNHCYKWTLGDKAAVDEAFAKAAHVTQLDITNNRLIPNAMEPRAANASYNR
ATDEYVLYVANQNPHVERLLMTAFVLQLPEHKVRVIAPDVGGGFGSKIFLYAEDVALTWGAKQLNCAIKWTAER
SESFLSDAHGRDHISHAEMAMDKDGKFLAMRVHTDANLGAYLSTFASSVPTILYGTLLAGQYATPQIYVEVDG
WFTSTAPVDAYRGAGRPEATYLVERLVTRCAWDMGLPQDEIRRRNFITSFPYQTPVALQYDIGDYKACMDQAQ
ELADVKGYEARKAASEAKGLKRGIGYSSYIEACGLAPSNIAGALGARAGLFECGEVRVHPTGSVTVFTGSHSHGQ
GHETTFAQLVAARLGLDPNQVDVVHGDTGRVPFGMGTYGSRSLSVGGTAIMKALDKIEAKAKKIAAHLMEAS
DADIDFANGEFSVKGTDKKIPFAQVALTAYVPHNYPLDKLEPGLNETAFYDPTNFTYPAGTYICEVEVDPDTGVVR
VDRFSAVDDFGVIINPMIVEGQVHGGLVQGMGQALMEHGIYDTETGQLMTGSFMDYTMPRAADFPEFKLG
HVCTPCTHNPIGSKGCGEAGAIGSPPAVINAVLDALKPLGVTELDMPATPSRVWTAIQEARAA
>N221356||gene_213823|GeneMark.hmm|777_aa|-|24934|27267
MNTVAPAVARFGSGQAVKRMEDDALLQGQGRYTNDLAQAGDGHLVFVRSPYPHATIGTIDSAAAEAMPGVR
AVITGAQLAAQGVAPMPTGAPFKRADGSDCASPPRRVLAHERARFVGEAVALVVADTLQQARDAAEQVMVD
YEELPPVPTLAAALRADVSLTPAAPDNISCEARHGDAAACDAAFASAAHVVKLDITHQRLAALTLEPRAVRAWD
EAGRMQVHISSQMPTGVRAAVAAGLQVRPDDVRVRVTDVGGGFGMKTGAYPEDVAVALAARATGRPVTWT
GDRSEEFLSTTHGRDVQAHAELALAADGKILGLRVHSHANVGAYATGTGVAIQLLIGPWVQTSVYHVPVIDFHF
RAMLTNQAPTGAYRGAGRPEAIFNIERLMDEAARQTGIDRVTLRRRNFIDPAAMPYKNPMGQTYDVGRFEHV
MDQALPLADWDGFAARAAESKARGLWRGLGIATFLEWTGGNVFEERVTVDVKGDGFIEVYSAVNQMGQGIA
TSLAQLVVDAFGVPIEKVRVVLGDTDRGNGFGSAGSRSLFTGGSAMRSGADATLIEAQQLAAKSLEASADDIRY
EAGRFTVVGTDHGIGLFELAAKQEGGHIFVDHTHTVSGPTWPNGCHISEVEVDPATGAVAIVAYASVNDVGRV
VNPMIVRGQLDGGAVQGIGQALTEQIVYDGDTGQLMTGSLMDYAAPRAQDLQTMFKTEMDESTPCKNNPL
GVKGVGELGTIGATPSIVNAVADALARAGRADVAPHLQMPLSPGRLWGLLNGESIKI
>N22886||gene_378674|GeneMark.hmm|830_aa|+|89735|92227
MTATTEKPTHEIGKARKRKEDRRLITGRTRWTDNIQLPGMLHLAMVRSPFAHAKITKVDTAGAKAAPGVVDVI
TGEDVKDIQGGLPCAWPITPDQKAPVHPAIAVDRVAFSGEVVAVIVARSAVAARDAAELVDVDYEQLPAILDLRE
AADDTVLAHPDLGTNKSAFWQFDSANAGTGGDVEEAIAKARTDGIVIERDYRQQRLIPAFMEPRSTVVDPTGE

QFVMWSATQVPHILRLMLALTLGVDENKVRVIAPDVGGGFGGKLQVTPEEVITFLAARRTGKPCKYTETRSESL
LAAHHGRDQWQHLTLAADKDGKVTALKVDLLADMGAYLGLVTPGVPILGAFMFNAIYKFPAYQFNCTNLFTN
KTWTDAYRGAGRPEATFAIERLMDELAVEVGVDPMEIREKNWITHEEFPYTSVCGLEYDSGNYELATARAKELF
DYDGLRAEQKQRRESGDRVQLGLGISTFTEMCGLAPSRVLGSLDYGAGGWEHAQVRMLPTGKVEVVTGSSAH
GQGHETAWSQLVADKLGVAFEDVEVLHGDTQISPKGLDTYGSRSLVVGGSAVVMAADKVIEKAKPIAAHMLEA
SADDVEFEAGSFGVRGAGEGAKTLTIGEVALATFAAHDLPDGVEPTLDSDAVFDPENFSFPHGTHLCAVEVDTE
TGQVTMRKYVCVDDVGTIVNPLIVEGQIHGGLVQGIAQALWEEAVYDDQGTLVSGSFVDYTLPTTADTIDFITD
NTVTPSTTNAIGAKGVGEAGCIASTPAVVNAVVDAVRHFGIDDIQMPCAPMRVWKAIQGAGGAHATPAQEQ
PHFAEGAPNQDPPGDGADNEKGAGQ
