# Supplementary material for: Genomic and Metagenomic Insights into the Distribution of Nicotine-degrading Enzymes in Human Microbiota
Source: Curr Genomics. 2024 Mar 20;25(3):226–35. doi: 10.2174/0113892029302230240319042208 (PMC11288164; doi:10.2174/0113892029302230240319042208)
Supplement: Supplementary file 1 [file CG-25-226_SD1.zip › CG-25-226_SD1/Xie MS Suppl file 3.pdf]

>GCA_001404355||gene_3630|GeneMark.hmm|414_aa|+|1490|2734
VETKSYKISKHIVWEAYKKVKANKGAAGVDNINIEKFEENIKDNLYKLWNRLSSGSYFPPPVRAVEIPKKNGGTRL
LGVPTVEDRIAQMVVRMYFEPSVDKVFYKDSYGYRPNKNAIEALGVIRERCWKYDWVLEFDIKGLFDNIDHKLL
MKAVKKHTEEKWVILYIERWLKVPFKMSDGRIVERNTGTPQGGVISPVLANLFLHYTFDKWMELHFPQCPWA
RYADDAVAHCKSKAQAQLLLMKLGKRFQECGLELHPDKTKIIYCKDDFRKQDEEITSFDFLGYTFRPRRAKSKKG
KFFINFSPAVSNKATKSMRQVIRNWRIQLKPDKSIIDISNMFNPVIRGWINYYGNFYKSELYKVLRHMNKALVQ
WARRKYKKLARGRKKAERWLGKLAKNMPKLFAHWQIGILPTTG
>GCA_001404455||gene_1021|GeneMark.hmm|418_aa|-|78934|80190
MSEAKQFDISKKAVIAAFQAVKENAGSYGADEQTIKEFEEHLNNNLYKLWNRMASGSYFPKPVRAVAIPKKNG
GIRILGIPTVEDRIAQMVAKMYFEPLVEPMFYNDSYGYRPNKSAIQAVGQARERCFKRDWVLELDIKGLFDNIK
HGYLMYMVEKHTQIKWLILYIKRWLTVPFIMSDGSVAERRSGTPQGGVISPVLANLFLHYVFDDFMTKAYPNI
WWERYADDGVLHCQSYKQAAFIKQKLEERFQQFGLELNKEKTRIVYCKDNRRPQNYSCTQFTFLGYTFRPRLN
KNKEGKFFVGFTPAVSEKAKTAMKQKIREWKIQLKADLSLKDIGNMINKVVQGWINYYTHYYKSEFYEVLRYIN
QCLIKWVRRSYKKKNTRSRAEHWLGAVARRDRNLFAHWKFGILPSVGEGAV
>GCA_001404755||gene_1375|GeneMark.hmm|418_aa|+|560|1816
MSEAKQFDISKKAVIAAFQAVKENAGSYGADEQTIKEFEEHLNNNLYKLWNRMASGSYFPKPVRAVAIPKKNG
GIRILGIPTVEDRIAQMVAKMYFEPLVEPMFYNDSYGYRPNKSAIQAVGQARERCFKRDWVLELDIKGLFDNIK
HGYLMYMVEKHTQIKWLILYIKRWLTVPFIMSDGSVAERRSGTPQGGVISPVLANLFLHYVFDDFMTKAYPNI
WWERYADDGVLHCQSYKQAAFIKQKLEERFQQFGLELNKEKTRIVYCKDNRRPQNYSCTQFTFLGYTFRPRLN
KNKEGKFFVGFTPAVSEKAKTAMKQKIREWKIQLKADLSLKDIGNMINKVVQGWINYYTHYYKSEFYEVLRYIN
QCLIKWVRRSYKKKNTRSRAEHWLGAVARRDRNLFAHWKFGILPSVGEGAV
>GCA_001404775||gene_2437|GeneMark.hmm|418_aa|+|20942|22198
MSEAKQFDISKKAVIAAFQAVKENAGSYGADEQTIKEFEEHLNNNLYKLWNRMASGSYFPKPVRAVAIPKKNG
GIRILGIPTVEDRIAQMVAKMYFEPLVEPMFYNDSYGYRPNKSAIQAVGQARERCFKRDWVLELDIKGLFDNIK
HGYLMYMVEKHTQIKWLILYIKRWLTVPFIMSDGSVAERRSGTPQGGVISPVLANLFLHYVFDDFMTKAYPNI
WWERYADDGVLHCQSYKQAAFIKQKLEERFQQFGLELNKEKTRIVYCKDNRRPQNYSCTQFTFLGYTFRPRLN
KNKEGKFFVGFTPAVSEKAKTAMKQKIREWKIQLKADLSLKDIGNMINKVVQGWINYYTHYYKSEFYEVLRYIN
QCLIKWVRRSYKKKNTRSRAEHWLGAVARRDRNLFAHWKFGILPSVGEGAV
>GCA_001405055||gene_1552|GeneMark.hmm|430_aa|-|49693|50985
MQNDNAKPISISKQLVYDAFLRVKANRGSAGIDKVTLEDYEKNLRGNLYKLWNRMSSGSYFPPSVKLVEIPKSTG
GKRPLGIPTVSDRVAQMAVVMLITPSIEPCFHEDSYAYRPHRSAHDAVGKARERCWKYAWVLDMDISKFFDTI
DHELLLKALKRHTQEKWVLMYIERWLKVPYEKSDGSQVDRALGVPQGSVIGPVLANLFLHYTFDKWMEKNFP
RVPFERYADDTICHCHSLKQAEYMQAMIQQRFECCRLRLNEEKTKIVYCKSSRQKECYPNVTFDFLGFTFQPRES
VDKYGNRFTGFLPAISRKSMKRINETMRSWHLNRHSNLTLEHLASDINPIVRGWMTYYGKFYPTRLKWFMQTL
NGRLARWVMCKFERYRHRFYPAQEWLARIAEKEGLIFYHWKCGALPRFTNKEKVSSQLIMVK
>AF15-25_scaffold||gene_3154|GeneMark.hmm|420_aa|-|2490|3752
MNEAKPFVIDKRLVWEAYHKVKENKGSAGIDKVDQKTFDKEMSKNLYKIWNRMSSGCYFPKAVKLVEIPKSNG
GTRPLGIPTIEDRIAQQVVVSVLTPILEPIFKEDSYGYRPGKGAHQAIAKAKERCYVNPWVLDMDISKFFDTINHE
LLMKAVRKHTEEKWVLLYIERWLKVPYQTSKGEVIERTMGVPQGSVIGPVLANLFLHYVFDEWMSRNYPTIPFE
RYADDTICHCVSDKQAQFLKAVLMKRFEECGLKLNEEKTKIVYCKDSNRRGDSEHTSFDFLGFTFRPRGARNRKT
GQNFTAFLPAISKKSMKRIKEAVRAWKLNRKTFACLLDISNEVDTQISGWMNYYMKFGRSEFRKVLNYINERLT
RWVMRKYKRFSKGRKFDRAYDWLVEYAAHNRNEFSHWVKGFVPYPRLG
>AF17-5AC_scaffold||gene_2863|GeneMark.hmm|414_aa|-|37719|38963
MIETKPYEISKWAVYIAYERVKANKGSYGVDEQSIEDFEKNLKNNLYKIWNRMSSGSYFPQPVKAVSVPKKNGGI

RVLGIPTVEDRIAQMTAKLYFEPCVEPLFLEDSYGYRPGKSAIQALSVTRKRCWHRDWVLEYDIKGLFDNIRHDY
LLEMVRRHTPHKWILLYVERWLTTPFQLEDGTLQSRTSGTPQGGVISLVLANLFLHYAFDSFMAKEYPKAWWE
RYADDGVLHCKSSQQAMYMKSVLRERFRLFGLELNEEKTRIVYCKDADRTEDYSEISLDSLGYTFRPRLARNKHG
NIFLNFLPAMSAKAIKAMKEEVRRWKLQLKVSKSLTDLANILNSQIQGWISYYGHFYKSELIYLLRYINQCLIKWV
RRKYKKFNHRRRAEYWLGRIARRDNNLFAHWRYGVLPTAG
>AF19-29_scaffold||gene_3897|GeneMark.hmm|418_aa|-|1110|2366
MSEAKQFDISKKAVIAAFQAVKENAGSYGADEQTIKEFEEHLNNNLYKLWNRMASGSYFPKPVRAVAIPKKNG
GIRILGIPTVEDRIAQMVAKMYFEPLVEPMFYNDSYGYRPNKSAIQAVGQARERCFKRDWVLELDIKGLFDNIK
HGYLMYMVEKHTQIKWLILYIKRWLTVPFIMSDGSVAERRSGTPQGGVISPVLANLFLHYVFDDFMTKAYPNI
WWERYADDGVLHCQSYKQAAFIKQKLEERFQQFGLELNKEKTRIVYCKDNRRPQNYSCTQFTFLGYTFRPRLN
KNKEGKFFVGFTPAVSEKAKTAMKQKIREWKIQLKADLSLKDIGNMINKVVQGWINYYTHYYKSEFYEVLRYIN
QCLIKWVRRSYKKKNTRSRAEHWLGAVARRDRNLFAHWKFGILPSVGEGAV
>AF19-9LB_scaffold||gene_1524|GeneMark.hmm|413_aa|-|303792|305033
MSESKQYEIPKKVVIEAYKRVKANKGSAGIDGIDFERFEKKLNNNLYKIWNRMSSGSYFPSPVLSVEIPKKAGGTR
RLGIPTITDRIAQMVARMYVEPVVEPMFCDDSYGYRPNKSAIDAIATARKRCWRYDYVIELDVKGLFDNINHELL
MRVVLKHVKEEWICLYIKRWLETPFITREGQVIERLSGTPQGGVISPVLANMYLHYVFDMWMKRNFPQAPFE
RYADDGVIHCRTKEEAFVIKKKLAARFAECKLELHPVKTRVVYCKDKDRTRNEELTEFDFLGYTFKAVYIMCKDGK
VRYNFIASVSKTSSKSFRDKIKAMEVHKRTGCKIDIIAEILNPLIRGWMNYFGKFNPSAMKGTLQCIDRRLVKWA
MCKYKNFRGKRGRAEKWLCTVRQREPKLFAHWSNLYSYC
>AF21-3_scaffold||gene_4010|GeneMark.hmm|393_aa|-|1110|2291
MSGSDPNVVIKEFEEHLNNNLYKLWNRMASGSYFPKPVRAVAIPKKNGGTRILGIPTVEDRIAQMVAKMYFEP
LVEPMFYKDSYGYRPNKSAIQAVGQARERCFKRDWVLELDIKGLFDNIKHGYLMYMVEKHTQIKWLILYIKRWL
TVPFIMSDGSVAERRSGTPQGGVISPVLANLFLHYVFDDFMTKAYPNIWWERYADDGVLHCQSYKQAVFIKQK
LEERFQQFGLELNKEKTRIVYCKDNRRSQNYSCTQFTFLGYTFRPRLNKNKEGKFFVGFTPAVSEKAKTAMKQKI
REWKIQLKADLSLKDIGNMINKVVQGWINYYTHYYKSEFYEVLRYINQCLIKWVRRSYKKKNTRSRAEHWLGAV
ARRDRNLFAHWKFGILPSVGEGAV
>AF22-1_scaffold||gene_3281|GeneMark.hmm|420_aa|+|12359|13621
MNAANPFVIDKRLVWEAYHKVKENKGSAGIDKVDQKTFDKEMSKNLYKIWNRMSSGCYFPKAVKLVEMPKS
NGGTRPLGIPAIEDRIAQQVVVSVLTPILEPIFKEDSYGYRPGKGAHQAIAKAKERCYVTPWVLDMDISKFFDTIN
HELLMKAIRKHTEEKWVLLYIERWLKVPYQTSKGEVIERTMGVPQGSVIGPVLANLFLHYVFDEWMSRNYPTIP
FERYADDTICHCVSEKQAQFLKAVLMKRFEECGLKLNEEKTKIVYCKDSNRRGDSEHTSFDFLGFTFRPRGARNR
KTGQNFTAFLPAISKKSMKRIKEAVRAWKLNRKTFACLLDISNEVDTQISGWMNYYMKFGRSEFRKVLNYINERL
TRWVMRKYKRFSKGRKFDRAYDWLVEYATHNRNEFSHWVKGFVPYPRLD
>AF31-11BH_scaffold||gene_4713|GeneMark.hmm|410_aa|+|4214|5446
MQRKSFEIPKALVWASYLDVRRNKGAPGCDGQTLKMFDQQRDGNLYKIWNRLCSGTWFPPPVLEKRIPKPN
GKERILGIPTVSDRIAQGAIKLFMEEKLDPIFHADSYGYRPGKSAHDALKQCAIRCWRYSWILEVDISAFFDHVRH
DLVLKALEHHGMPKWVILYCRRWMEAPMQSCENGELITRTRGTPQGGVISPLLANLFLHYAFDLWMEREYRG
VPFERYADDIVVHCSRMSDATRLKNRLSERFSEVGLVLNAGKTNIAYIDTFKRRNVATSFTFLGYDFKVRTLKNFK
GELYRKCMPGASNAAMRKITETIKKWRIHRSTAESLLDFARRYNAIVRGWIEYYGKFWSRNFNYRLWSAMQSR
LLKWMQSKYRLSNRRAQRKLTLVRKEYPKLFVHWYLLRASNE
>AF33-11BH_scaffold||gene_3120|GeneMark.hmm|418_aa|-|57504|58760
MSEAKQFDISKKAVIAAFQAVKENAGSYGADEQTIKEFEEHLNNNLYKLWNRMASGSYFPKPVRAVAIPKKNG
GIRILGIPTVEDRIAQMVAKMYFEPLVEPMFYNDSYGYRPNKSAIQAVGQARERCFKRDWVLELDIKGLFDNIK
HGYLMYMVEKHTQIKWLILYIKRWLTVPFIMSDGSVAERRSGTPQGGVISPVLANLFLHYVFDDFMTKAYPNI

WWERYADDGVLHCQSYKQAAFIKQKLEERFQQFGLELNKEKTRIVYCKDNRRPQNYSCTQFTFLGYTFRPRLN
KNKEGKFFVGFTPAVSEKAKTAMKQKIREWKIQLKADLSLKDIGNMINKVVQGWINYYTHYYKSEFYEVLRYIN
QCLIKWVRRSYKKKNTRSRAEHWLGAVARRDRNLFAHWKFGILPSVGEGAV
>AF36-16BH_scaffold||gene_2113|GeneMark.hmm|422_aa|-|146575|147843
MMQHQIAKPFTIDKHVIMAAWKRVRENKGSAGIDNVSISDYETNLGTHLYKLWNRMSSGSYFPNAVKLVEIPK
SSGGTRPLGIPTVGDRIAQMAVVLLIEARLEEIFHPNSYGYRPNRSAHDAIGQARERCWRYNWVLDMDISKFFD
TIDHDLLMKAVERHVQERWILLYIRRWLKVPYATITGECIERTMGVPQGSVIGPILANLYLHYTFDKWMSIYHPN
VPFERYADDTICHCSSLEEAQRLKASIVERFAACKLKLNEEKTRIVYCKDGKRRGKYPEITFDFLGYTFQPRGQRNR
NGQVFNGYAPAISKKSKKRITEKMRGWHLSRRVQIKLSDIASEINAEVRGWINYYGKFYGSLLKAFLQSINLKLAR
WAERKYKRFRRKPNDAYKWLVKVASKSPNLFYHWQYGVKPNRLKSFG
>AF38-4_scaffold||gene_3421|GeneMark.hmm|420_aa|+|19646|20908
MNEAKPFVIDKRLVWEAYHKVKENKGSAGIDKVDQKTFDKEMSKNLYKIWNRMSSGCYFPKAVKLVEIPKSNG
GTRPLGIPTIEDRIAQQVVVSVLTPILEPIFKEDSYGYRPGKGAHQAIAKAKERCYVNPWVLDMDISKFFDTINHD
LLMKAVRKHTEEKWVLLYIERWLKVPYQTSKGEVIERTMGVPQGSVIGPVLANLFLHYVFDEWMSRNYPTIPFE
RYADDTICHCVSEKQAQFLKAVLMKRFEECGLKLNEEKTKIVYCKDSNRRGDSEHTSFDFLGFTFRPRSARNRKT
GQNFTAFLPAISKKSLKRIKEAVRAWKLNRKTFACLLDISNEVDTQISGWMNYYMKFGRSEFRKVLNYINERLTR
WVMRKYKRFSKGKKFSRAYEWLVEYAVHNRNEFSHWAKGFVPYPRLG
>AM16-13_scaffold||gene_4602|GeneMark.hmm|422_aa|-|90|1358
MKDAKSFEISKQLVMEAYKRVKANRGTSGIDDVTIADFESDLKGNLYKIWNRMCSGSYLPPAVKLVEIPKSNGGK
RPLGIPTVGDRVAQMVVVMTIEPEIEPHFHEDSYAYRPKKSALDAVEKAKDRCYTFHWVLDLDIKGFFDNIDHE
LLIRALERHVKCKWAMMYIKRWLSVPYQLKDGTQVERTKGVPQGSVVGPILANLFLHYTFDEWMRRNHSNIS
FERYADDTICHCVSQKQAEFIHRAIKKRFAECKLELNEEKTKIVYCKRNHRNIEYECIQFDFLGYTFRPRRSIDTHGE
VFLNFSPAVSKKARTKIWETIRDWNQKYWVQMKLEDIAKQINPIIQGWINYYGKFNPGVLKEVLKRINLKLSRW
IRDKFKGFRKFKTQAIHRLGDIALKNPDLFAHWSWGVKPTASPRNRARV
>AM21-17_scaffold||gene_4155|GeneMark.hmm|414_aa|-|78|1322
MQEAKPYSISKKAVIAAYQRVKANKGTYGVDEQSIEDFERKLNNNLYKIWNRMSSGTYFPKPVKAVAIPKKNGG
TRILGVPTVEDRIAQMVAKLYFEPCVEPIFYEDSYGYRPNKSAIQALEATRTRCWRKDWVLEFDIRGLFDNIRHDY
LMEMVKKHTKEKWIILYIQRWLTAPFQMEDGTIVERKSGTPQGGVISPVLANLFLHYVFDDFMVKEFPTIPWA
RYADDGIAHCVSQKQAKYLRRRLGQRFQSYGLELNQEKTRIVYCKDDDRRGNHENTSFDFLGYTFRPRHAKNR
YGKFFTNFLPAISEKAKKAIRKEVRGWKLQLKSDKDLYDIANMFNRQIQGWINYYTHFYKSEIYDVLRYINGCLVK
WVRRKYKKRKARRKAEHWLGEIAKRDRNLFAHWKFGILPAAG
>AM23-12_scaffold||gene_1164|GeneMark.hmm|414_aa|-|148562|149806
MIETKPYEISKWAVYIAYERVKANKGSYGVDEQSIEDFEKNLKNNLYKIWNRMSSGSYFPQPVKAVSVPKKNGGI
RVLGIPTVEDRIAQMTAKLYFEPCVEPLFLEDSYGYRPGKSAIQALSVTRKRCWHRDWVLEYDIKGLFDNIRHDY
LLEMVRRHTPHKWILLYVERWLTTPFQLEDGTLQSRTSGTPQGGVISLVLANLFLHYAFDSFMAKEYPKAWWE
RYADDGVLHCKSSQQAMYMKSVLRERFRLFGLELNEEKTRIVYCKDADRTEDYSEISLDSLGYTFRPRLARNKHG
NIFLNFLPAMSAKAIKAMKEEVRRWKLQLKVSKSLTDLANILNSQIQGWISYYGHFYKSELIYLLRYINQCLIKWV
RRKYKKFNHRRRAEYWLGRIARRDNNLFAHWRYGVLPTAG
>AM23-12_scaffold||gene_567|GeneMark.hmm|430_aa|-|140654|141946
MQNDNAKPISISKQLVYDAFLRVKANRGSAGIDKVTLEDYEKNLRGNLYKLWNRMSSGSYFPPSVKLVEIPKSTG
GKRPLGIPTVSDRVAQMAVVMLITPSIEPCFHEDSYAYRPHRSAHDAVGKARERCWKYAWVLDMDISKFFDTI
DHELLLKALKRHTQEKWVLMYIERWLKVPYEKSDGSQVDRALGVPQGSVIGPVLANLFLHYTFDKWMEKNFP
RVPFERYADDTICHCHSLKQAEYMQAMIQQRFECCRLRLNEEKTKIVYCKSSRQKECYPNVTFDFLGFTFQPRES
VDKYGNRFTGFLPAISRKSMKRINETMRSWHLNRHSNLTLEHLASDINPIVRGWMTYYGKFYPTRLKWFMQTL

NGRLARWVMCKFERYRHRFYPAQEWLARIAEKEGLIFYHWKCGALPRFTNKEKVSSQLIMVK
>AM29-12LB_scaffold||gene_3815|GeneMark.hmm|418_aa|-|1348|2604
MSEAKQFDISKKAVIAAFQAVKENAGSYGADEQTIKEFEEHLNNNLYKLWNRMASGSYFPKPVRAVAIPKKNG
GIRILGIPTVEDRIAQMVAKMYFEPLVEPMFYNDSYGYRPNKSAIQAVGQARERCFKRDWVLELDIKGLFDNIK
HGYLMYMVEKHTQIKWLILYIKRWLTVPFIMSDGSVAERRSGTPQGGVISPVLANLFLHYVFDDFMTKAYPNI
WWERYADDGVLHCQSYKQAAFIKQKLEERFQQFGLELNKEKTRIVYCKDNRRPQNYSCTQFTFLGYTFRPRLN
KNKEGKFFVGFTPAVSEKAKTAMKQKIREWKIQLKADLSLKDIGNMINKVVQGWINYYTHYYKSEFYEVLRYIN
QCLIKWVRRSYKKKNTRSRAEHWLGAVARRDRNLFAHWKFGILPSVGEGAV
>AM29-12LB_scaffold||gene_1189|GeneMark.hmm|418_aa|+|554|1810
MSEAKQFDISKKAVIAAFQAVKENAGSYGADEQTIKEFEEHLNNNLYKLWNRMASGSYFPKPVRAVAIPKKNG
GIRILGIPTVEDRIAQMVAKMYFEPLVEPMFYNDSYGYRPNKSAIQAVGQARERCFKRDWVLELDIKGLFDNIK
HGYLMYMVEKHTQIKWLILYIKRWLTVPFIMSDGSVAERRSGTPQGGVISPVLANLFLHYVFDDFMTKAYPNI
WWERYADDGVLHCQSYKQAAFIKQKLEERFQQFGLELNKEKTRIVYCKDNRRPQNYSCTQFTFLGYTFRPRLN
KNKEGKFFVGFTPAVSEKAKTAMKQKIREWKIQLKADLSLKDIGNMINKVVQGWINYYTHYYKSEFYEVLRYIN
QCLIKWVRRSYKKKNTRSRAEHWLGAVARRDRNLFAHWKFGILPSVGEGAV
>AM30-15AC_scaffold||gene_3665|GeneMark.hmm|418_aa|-|2914|4170
MSEAKQFDISKKAVIAAFQAVKENAGSYGVDEQTIKEFEEHLNNNLYKLWNRMASGSYFPKPVRAVAIPKKNG
GTRILGIPTVEDRIAQMVAKMYFEPLVEPMFYKDSYGYRPNKSAIQAVGQARERCFKRDWVLELDIKGLFDNIK
HGYLMYMVEKHTQIKWLILYIKRWLTVPFIMSDGSVAERRSGTPQGGVISPVLANLFLHYVFDDFMTKAYPNI
WWERYADDGVLHCQSYKQAVFIKQKLEERFQQFGLELNKEKTRIVYCKDNRRSQNYSCTQFTFLGYTFRPRLNK
NKEGKFFVGFTPAVSEKAKTAMKQKIREWKIQLKADLSLKDIGNMINKVVQGWINYYTHYYKSEFYEVLRYINQ
CLIKWVRRSYKKKNTRSRAEHWLGAVARRDRNLFAHWKFGILPSVGEGAV
>AM32-17LB_scaffold||gene_2312|GeneMark.hmm|418_aa|+|606|1862
MSEAKQFDISKKAVIAAFQAVKENAGSYGVDEQTIKEFEEHLNNNLYKLWNRMASGSYFPKPVRAVEIPKKNG
GTRILGIPTVEDRIAQMVAKMYFEPLVEPMFYNDSYGYRPNKSAIQAVGQAREKCFKRDWVLELDIKGLFDNIK
HGYLMYMVEKHTQIKWLILYIKRWLTVPFIMSDGSVAERRSGTPQGGVISPVLANLFLHYVFDDFMTKAYPNI
WWERYADDGVLHCQSYKQAVFIKQKLEERFQQFGLELNKEKTRIVYCKDDRRSRNYSCTQFTFLGYTFRPRLNK
NKEGKFFVGFTPAVSEKAKTAMKQKIRGWKIQLKADLSLKDIGNMINKVVQGWINYYTHYYKSEFYEVLRYINQ
CLIKWVRRSYKKKNTRSRAEHWLGAVARRDRNLFAHWKFGILPSVGEGAV
>AM34-19LB_scaffold||gene_545|GeneMark.hmm|420_aa|-|136546|137808
MNEAKPFVIDKRLVWEAYHKVKENKGSAGIDKVDQKTFDKEMSKNLYKIWNRMSSGCYFPKAVKLVEIPKSNG
GTRPLGIPTIEDRIAQQVVVSVLTPILEPIFKEDSYGYRPGKGAHQAVAKAKERCYVNPWVLDMDISKFFDTINHE
LLMKAVRKHTGEKWVLLYIERWLKVPYQTLKGEVIERTMGVPQGSVIGPVLANLFLHYVFDEWMSRNYPTIPFE
RYADDTICHCVSEKQAQFLKAVLMKRFEECGLKLNEEKTKIVYCKDSNRRGDSEHTSFDFLGFTFRPRGARNRKT
GQNFTAFLPAISRKSMKRIKEAVRAWKLNRKTFACLLDISNEVDTQISGWMNYYMKFGRSEFRKVLNYINERLT
RWVMRKYKRFSKGKKLGRAYEWLVEYAAHNRNEFSHWVKGFVPYPRLG
>AM34-19LB_scaffold||gene_426|GeneMark.hmm|420_aa|-|114|1376
MNEAKPFVIDKRLVWEAYHKVKENKGSAGIDKVDQKTFDKEMSKNLYKIWNRMSSGCYFPKAVKLVEIPKSNG
GTRPLGIPTIEDRIAQQVVVSVLTPILEPIFKEDSYGYRPGKGAHQAVAKAKERCYVNPWVLDMDISKFFDTINHE
LLMKAVRKHTGEKWVLLYIERWLKVPYQTLKGEVIERTMGVPQGSVIGPVLANLFLHYVFDEWMSRNYPTIPFE
RYADDTICHCVSEKQAQFLKAVLMKRFEECGLKLNEEKTKIVYCKDSNRRGDSEHTSFDFLGFTFRPRGARNRKT
GQNFTAFLPAISRKSMKRIKEAVRAWKLNRKTFACLLDISNEVDTQISGWMNYYMKFGRSEFRKVLNYINERLT
RWVMRKYKRFSKGKKLGRAYEWLVEYAAHNRNEFSHWVKGFVPYPRLG
>AM34-9LB_scaffold||gene_3076|GeneMark.hmm|418_aa|+|20711|21967

MSEAKQFDISKKAVIAAFQAVKENAGSYGADEQTIKEFEEHLNNNLYKLWNRMASGSYFPKPVRAVAIPKKNG
GIRILGIPTVEDRIAQMVAKMYFEPLVEPMFYNDSYGYRPNKSAIQAVGQARERCFKRDWVLELDIKGLFDNIK
HGYLMYMVEKHTQIKWLILYIKRWLTVPFIMSDGSVAERRSGTPQGGVISPVLANLFLHYVFDDFMTKAYPNI
WWERYADDGVLHCQSYKQAAFIKQKLEERFQQFGLELNKEKTRIVYCKDNRRPQNYSCTQFTFLGYTFRPRLN
KNKEGKFFVGFTPAVSEKAKTAMKQKIREWKIQLKADLSLKDIGNMINKVVQGWINYYTHYYKSEFYEVLRYIN
QCLIKWVRRSYKKKNTRSRAEHWLGAVARRDRNLFAHWKFGILPSVGEGAV
>AM36-17_scaffold||gene_3311|GeneMark.hmm|418_aa|-|59228|60484
MSEAKQFDISKKAVIAAFQAVKENAGSYGADEQTIKEFEEHLNNNLYKLWNRMASGSYFPKPVRAVAIPKKNG
GIRILGIPTVEDRIAQMVAKMYFEPLVEPMFYNDSYGYRPNKSAIQAVGQARERCFKRDWVLELDIKGLFDNIK
HGYLMYMVEKHTQIKWLILYIKRWLTVPFIMSDGSVAERRSGTPQGGVISPVLANLFLHYVFDDFMTKAYPNI
WWERYADDGVLHCQSYKQAAFIKQKLEERFQQFGLELNKEKTRIVYCKDNRRPQNYSCTQFTFLGYTFRPRLN
KNKEGKFFVGFTPAVSEKAKTAMKQKIREWKIQLKADLSLKDIGNMINKVVQGWINYYTHYYKSEFYEVLRYIN
QCLIKWVRRSYKKKNTRSRAEHWLGAVARRDRNLFAHWKFGILPSVGEGAV
>AM42-23AC_scaffold||gene_3449|GeneMark.hmm|420_aa|+|2771|4033
MNEAKPFVIDKRLVWEAYHKVKENKGSAGIDKVDQKTFDKEMSKNLYKIWNRMSSGCYFPKAVKLVEIPKSNG
GTRPLGIPTIEDRIAQQVAVSVLTPILEPIFKEDSYGYRPGKGAHQAVAKAKERCYVNPWVLDMDISKFFDTINHE
LLMKAVRKHTEEKWVLLYIERWLKVPYQTLKGEVIERTMGVPQGSVIGPVLANLFLHYVFDEWMSRNYPTIPFE
RYADDTICHCVSEKQAQFLKAVLMKRFEECGLKLNEEKTKIVYCKDSNRRGDSEHTSFDFLGFTFRPRGARNRKT
GQNFTAFLPAISKKSMKRIKEAVRAWKLNRKTFACLLDISNEVDTQISGWMNYYMKFGRSEFRKVLNYINERLT
RWVMRKYKRFSKGKKFSKAYDWLVEYAAHNRNEFSHWVKGFVPYPRLG
>AM42-24_scaffold||gene_2541|GeneMark.hmm|420_aa|-|9513|10775
MNEAKPFVIDKRLVWEAYHKVKENKGSAGIDKVDQKTFDKEMSKNLYKIWNRMSSGCYFPKAVKLVEIPKSNG
GTRPLGIPTIEDRIAQQVVVSVLTPILEPIFKEDSYGYRPGKGAHQAIAKAKERCYVTPWVLDMDISKFFDTINHEL
LMKAIRKHTEEKWVLLYIERWLKVPYQTSKGEVIERTMGVPQGSVIGPVLANLFLHYVFDEWMSRNYPTIPFER
YADDTICHCVSEKQAQFLKAVLMKRFEECGLKLNEEKTKIVYCKDSNRRGDSEHISFDFLGFTFRPRGARNRKTG
QNFTAFLPAISKKSMKRIKEAARAWKLNRKTFACLLDISNEVDTQISGWMNYYMKFGRSEFRKVLNYINERLTR
WVMRKYKRFSKGKKFSKAYDWLVEYAAHNRNEFSHWVKGFVPYPRLG
>AM44-16_scaffold||gene_2009|GeneMark.hmm|421_aa|-|240834|242099
MQEAKPFQIDKRIIFEAFKKVKSNGGSPGIDGIEMSAYEQNLGSNFYRLWNRMSSGSYMPKAVKLVEILKSNGG
KRPLGIPSVEDRIAQMAVVNVIEPLVEPYFHKDSFGYRPHRSAHDAIAKAERRCWKYAWVLDIDISKFFDTIDHG
LLMKAVEKHIKTKWILLYIKRWLTVPYQGNDGAIVKRHMGVPQGSVIGPILANQFLHYTFDKWMSYKYPHVPF
ERYADDCVCHCGTLAQAEYIKDRLGERFAECKLTFNEEKTKIVFCKTSNRSSEHYHCTSFDYLGFTFRPRAAKDKR
KNVLFTSYLPAISNKSESRIHETIKSWNLKRLHNRSLRFVAAYINDVVRGWISYYGKFGKTEFWKVMCHLNRSIAY
WAKTKYKRLRRRGVISAHYWLAYIAQKEPNLFYHWQVGYIPYARQKK
>AM50-15BH_scaffold||gene_3272|GeneMark.hmm|418_aa|-|1427|2683
MSEAKQFDISKKAVIAAFQAVKENAGSYGADEQTIKEFEEHLNNNLYKLWNRMASGSYFPKPVRAVAIPKKNG
GIRILGIPTVEDRIAQMVAKMYFEPLVEPMFYNDSYGYRPNKSAIQAVGQARERCFKRDWVLELDIKGLFDNIK
HGYLMYMVEKHTQIKWLILYIKRWLTVPFIMSDGSVAERRSGTPQGGVISPVLANLFLHYVFDDFMTKAYPNI
WWERYADDGVLHCQSYKQAAFIKQKLEERFQQFGLELNKEKTRIVYCKDNRRPQNYSCTQFTFLGYTFRPRLN
KNKEGKFFVGFTPAVSEKAKTAMKQKIREWKIQLKADLSLKDIGNMINKVVQGWINYYTHYYKSEFYEVLRYIN
QCLIKWVRRSYKKKNTRSRAEHWLGAVARRDRNLFAHWKFGILPSVGEGAV
>AM54-14NS_scaffold||gene_4145|GeneMark.hmm|418_aa|-|172|1428
MSEAKQFDISKKAVIAAFQAVKENAGSYGADEQTIKEFEEHLNNNLYKLWNRMASGSYFPKPVRAVAIPKKNG
GIRILGIPTVEDRIAQMVAKMYFEPLVEPMFYNDSYGYRPNKSAIQAVGQARERCFKRDWVLELDIKGLFDNIK

HGYLMYMVEKHTQIKWLILYIKRWLTVPFIMSDGSVAERRSGTPQGGVISPVLANLFLHYVFDDFMTKAYPNI
WWERYADDGVLHCQSYKQAAFIKQKLEERFQQFGLELNKEKTRIVYCKDNRRPQNYSCTQFTFLGYTFRPRLN
KNKEGKFFVGFTPAVSEKAKTAMKQKIREWKIQLKADLSLKDIGNMINKVVQGWINYYTHYYKSEFYEVLRYIN
QCLIKWVRRSYKKKNTRSRAEHWLGAVARRDRNLFAHWKFGILPSVGEGAV
>OF01-4LB_scaffold||gene_1790|GeneMark.hmm|414_aa|-|354409|355653
MQEAKPYSISKKAVIAAYQRVKANKGTYGVDEQSIEDFERKLNNNLYKIWNRMSSGTYFPKPVKAVAIPKKNGG
TRILGVPTVEDRIAQMVAKLYFEPCVEPIFYEDSYGYRPNKSAIQALEATRTRCWRKDWVLEFDIRGLFDNIRHDY
LMEMVKKHTKEKWIILYIQRWLTAPFQMEDGTIVERKSGTPQGGVISPVLANLFLHYVFDDFMVKEFPTIPWA
RYADDGIAHCVSQKQAKYLRRRLEQRFQSYGLELNQEKTRIVYCKDDDRRGNHENTSFDFLGYTFRPRDAKNRY
GKFFTNFLPAISEKAKKAIRKEVRGWKLQLKSDKDLYDIANMFNRQIQGWINYYTHFYKSEIYDVLRYINGCLVK
WVRRKYKKRKARRKAEHWLGEIAKRDRNLFAHWKFGILPAAG
>OF02-3AC_scaffold||gene_4435|GeneMark.hmm|414_aa|-|78|1322
MQETKSYNISKQAVYQAFLKVKANKGTFGVDEESIEAYEYKLKDNLYKLWNRLSSGSYFPKPVKAVSIPKKSGGLR
VLGIPTVEDRIAQMVAKMYFEPVVERLFYEDSYGYRPNKSAIQAIEKTRVRCWKRDWVLEFDIKGLFDNIRHDY
LIEMVKRYTQEKWIILYVERWLKAPFQREDGSTVSRKAGTPQGGVISPVLANLFLHYTFDDFMEKEFPNIQWAR
YADDGITHCVSLKQAKYLKKRLEERFRIFGLELHPDKTKIVYCRDSDRMGNYPITTFDFLGFTFRPRGAKNKYGKC
FTNFLPAVSDKAKKAIRKEVRNWRLQLKADKKLEDLANMFNSKIQGWMNYYMKFYKSEMYSLLRYINQCLVK
WVRRKYKKRQARRKAEHWLGEIAKRERNLFAHWKIGILPSAG
>OM02-11AC_scaffold||gene_4747|GeneMark.hmm|410_aa|+|513|1745
MQRKSFEIPKALVWASYLDVRRNKGAPGCDGQTLKMFDQQRDGNLYKIWNRLCSGTWFPPPVLEKRIPKPN
GKERILGIPTVSDRIAQGAIKLFMEEKLDPIFHADSYGYRPGKSAHDALKQCAIRCWRYSWILEVDISAFFDHVRH
DLVLKALEHHGMPKWVILYCRRWMEAPMQSCENGELITRTRGTPQGGVISPLLANLFLHYAFDLWMEREYRG
VPFERYADDIVVHCSRMSDATRLKNRLSERFSEVGLVLNAGKTNIAYIDTFKRRNVATSFTFLGYDFKVRTLKNFK
GELYRKCMPGASNAAMRKITETIKKWRIHRSTAESLLDFARRYNAIVRGWIEYYGKFWSRNFNYRLWSAMQSR
LLKWMQSKYRLSNRRAQRKLTLVRKEYPKLFVHWYLLRASNE
>OM02-12AC_scaffold||gene_4685|GeneMark.hmm|410_aa|+|513|1745
MQRKSFEIPKALVWASYLDVRRNKGAPGCDGQTLKMFDQQRDGNLYKIWNRLCSGTWFPPPVLEKRIPKPN
GKERILGIPTVSDRIAQGAIKLFMEEKLDPIFHADSYGYRPGKSAHDALKQCAIRCWRYSWILEVDISAFFDHVRH
DLVLKALEHHGMPKWVILYCRRWMEAPMQSCENGELITRTRGTPQGGVISPLLANLFLHYAFDLWMEREYRG
VPFERYADDIVVHCSRMSDATRLKNRLSERFSEVGLVLNAGKTNIAYIDTFKRRNVATSFTFLGYDFKVRTLKNFK
GELYRKCMPGASNAAMRKITETIKKWRIHRSTAESLLDFARRYNAIVRGWIEYYGKFWSRNFNYRLWSAMQSR
LLKWMQSKYRLSNRRAQRKLTLVRKEYPKLFVHWYLLRASNE
>OM02-28_scaffold||gene_4644|GeneMark.hmm|410_aa|+|513|1745
MQRKSFEIPKALVWASYLDVRRNKGAPGCDGQTLKMFDQQRDGNLYKIWNRLCSGTWFPPPVLEKRIPKPN
GKERILGIPTVSDRIAQGAIKLFMEEKLDPIFHADSYGYRPGKSAHDALKQCAIRCWRYSWILEVDISAFFDHVRH
DLVLKALEHHGMPKWVILYCRRWMEAPMQSCENGELITRTRGTPQGGVISPLLANLFLHYAFDLWMEREYRG
VPFERYADDIVVHCSRMSDATRLKNRLSERFSEVGLVLNAGKTNIAYIDTFKRRNVATSFTFLGYDFKVRTLKNFK
GELYRKCMPGASNAAMRKITETIKKWRIHRSTAESLLDFARRYNAIVRGWIEYYGKFWSRNFNYRLWSAMQSR
LLKWMQSKYRLSNRRAQRKLTLVRKEYPKLFVHWYLLRASNE
>TF09-4AC_scaffold||gene_4650|GeneMark.hmm|413_aa|+|557|1798
MSESKQYEIPKRVVVEAYKRVKANKGSVGIDGIDFDIFEKKLNNNLYKIWNRMSSGSYFPSPVLAVEIPKKAGGT
RRLGIPTIADRIAQMIARMYIEPAVEPMFCEDSYGYRPNKSAIEAIAVTRKRCWRYDYVIELDVKGLFDNINHELL
MRVVEKHVKESWICLYVKRWMETPFVTKERAAIERKSGTPQGGVISPVLANMFLHYVFDMWMKRKFPQAPF
ERYADDGIVHCRTKEEAICIRQSLAKRFEECKLELHPTKTRIVYCKDEDRRKEEELTEFDFLGYTFKARYIKCRDGKL

RYNFIASVSKVSAKAFRTKVKEMELHRRTGCKIDILAEMLNPMVRGWMNYFGKYNPSAMKDTLLCIERRLVKW
AMCKYKKFRGRRRKAEEWLCTLRKREPKLFAHWSMIYSYC
>TF11-2AC_scaffold||gene_3405|GeneMark.hmm|418_aa|+|3498|4754
MSEAKQFDISKKAVIAAFQAVKENAGSYGADEQTIKEFEEHLNNNLYKLWNRMASGSYFPKPVRAVAIPKKNG
GIRILGIPTVEDRIAQMVAKMYFEPLVEPMFYNDSYGYRPNKSAIQAVGQARERCFKRDWVLELDIKGLFDNIK
HGYLMYMVEKHTQIKWLILYIKRWLTVPFIMSDGSVAERRSGTPQGGVISPVLANLFLHYVFDDFMTKAYPNI
WWERYADDGVLHCQSYKQAAFIKQKLEERFQQFGLELNKEKTRIVYCKDNRRPQNYSCTQFTFLGYTFRPRLN
KNKEGKFFVGFTPAVSEKAKTAMKQKIREWKIQLKADLSLKDIGNMINKVVQGWINYYTHYYKSEFYEVLRYIN
QCLIKWVRRSYKKKNTRSRAEHWLGAVARRDRNLFAHWKFGILPSVGEGAV
>TM09-4_scaffold||gene_367|GeneMark.hmm|418_aa|-|68186|69442
MSEAKQFDISKKAVIAAFQAVKENAGSYGADEQTIKEFEEHLNNNLYKLWNRMASGSYFPKPVRAVAIPKKNG
GIRILGIPTVEDRIAQMVAKMYFEPLVEPMFYNDSYGYRPNKSAIQAVGQARERCFKRDWVLELDIKGLFDNIK
HGYLMYMVEKHTQIKWLILYIKRWLTVPFIMSDGSVAERRSGTPQGGVISPVLANLFLHYVFDDFMTKAYPNI
WWERYADDGVLHCQSYKQAAFIKQKLEERFQQFGLELNKEKTRIVYCKDNRRPQNYSCTQFTFLGYTFRPRLN
KNKEGKFFVGFTPAVSEKAKTAMKQKIREWKIQLKADLSLKDIGNMINKVVQGWINYYTHYYKSEFYEVLRYIN
QCLIKWVRRSYKKKNTRSRAEHWLGAVARRDRNLFAHWKFGILPSVGEGAV
>scaffold_AF03-15||gene_2331|GeneMark.hmm|414_aa|+|20924|22168
MQETKSYNISKQAVYQAFLKVKANKGTFGVDEESIEAYEYKLKDNLYKLWNRLSSGSYFPKPVKAVSIPKKSGGLR
VLGIPTVEDRIAQMVAKMYFEPVVERLFYEDSYGYRPNKSAIQAIEKTRVRCWKRDWVLEFDIKGLFDNIRHDY
LIEMVKRYTQEKWIILYVERWLKAPFQREDGSTVSRKAGTPQGGVISPVLANLFLHYTFDDFMEKEFPNIQWAR
YADDGITHCVSLKQAKYLKKRLEERFRIFGLELHPDKTKIVYCRDSDRMGNYPITTFDFLGFTFRPRGAKNKYGKC
FTNFLPAVSDKAKKAIRKEVRNWRLQLKADKKLEDLANMFNSKIQGWMNYYMKFYKSEMYSLLRYINQCLVK
WVRRKYKKRQARRKAEHWLGEIAKRERNLFAHWKIGILPSAG
>scaffold_AF05-12B||gene_4840|GeneMark.hmm|410_aa|+|702|1934
MQRKSFEIPKALVWASYLDVRRNKGAPGCDGQTLKMFDQQRDGNLYKIWNRLCSGTWFPPPVLEKRIPKPN
GKERILGIPTVSDRIAQGAIKLFMEEKLDPIFHADSYGYRPGKSAHDALKQCAIRCWRYSWILEVDISAFFDHVRH
DLVLKALEHHGMPKWVILYCRRWMEAPMQSCENGELITRTRGTPQGGVISPLLANLFLHYAFDLWMEREYRG
VPFERYADDIVVHCSRMSDATRLKNRLSERFSEVGLVLNAGKTNIAYIDTFKRRNVATSFTFLGYDFKVRTLKNFK
GELYRKCMPGASNAAMRKITETIKKWRIHRSTAESLLDFARRYNAIVRGWIEYYGKFWSRNFNYRLWSAMQSR
LLKWMQSKYRLSNRRAQRKLTLVRKEYPKLFVHWYLLRASNE
>scaffold_AF102-62||gene_2389|GeneMark.hmm|420_aa|+|37362|38624
MNEAKPFVIDKRLVWEAYHKVKENKGSAGIDKVDQKTFDKEMSKNLYKIWNRMSSGCYFPKAVKLVEIPKSNG
GTRPLGIPTIEDRIAQQVVVSVLTPILEPIFKEDSYGYRPGKGAHQAIAKAKERCYVNPWVLDMDISKFFDTINHD
LLMKAVRKHTEEKWVLLYIERWLKVPYQTSKGEVIERTMGVPQGSVIGPVLANLFLHYVFDEWMSRNYPTIPFE
RYADDTICHCVSEKQAQFLKAVLMKRFEECGLKLNEEKTKIVYCKDSNRRGDSEHTSFDFLGFTFRPRSARNRKT
GQNFTAFLPAISKKSLKRIKEAVRAWKLNRKTFACLLDISNEVDTQISGWMNYYMKFGRSEFRKVLNYINERLTR
WVMRKYKRFSKGKKFSRAYEWLVEYAVHNRNEFSHWAKGFVPYPRLG
>scaffold_AF17-4AC||gene_2658|GeneMark.hmm|414_aa|-|37694|38938
MIETKPYEISKWAVYIAYERVKANKGSYGVDEQSIEDFEKNLKNNLYKIWNRMSSGSYFPQPVKAVSVPKKNGGI
RVLGIPTVEDRIAQMTAKLYFEPCVEPLFLEDSYGYRPGKSAIQALSVTRKRCWHRDWVLEYDIKGLFDNIRHDY
LLEMVRRHTPHKWILLYVERWLTTPFQLEDGTLQSRTSGTPQGGVISLVLANLFLHYAFDSFMAKEYPKAWWE
RYADDGVLHCKSSQQAMYMKSVLRERFRLFGLELNEEKTRIVYCKDADRTEDYSEISLDSLGYTFRPRLARNKHG
NIFLNFLPAMSAKAIKAMKEEVRRWKLQLKVSKSLTDLANILNSQIQGWISYYGHFYKSELIYLLRYINQCLIKWV
RRKYKKFNHRRRAEYWLGRIARRDNNLFAHWRYGVLPTAG

>scaffold_AF42-20||gene_1518|GeneMark.hmm|400_aa|-|12966|14168
MLTPKGAYGIDEQTIEMFEENYKNNMYKLWNRMSSGTYFPKPVKAVDIPKKNGGTRTLGIPTVEDRIAQMVAK
IYFEPNVESIFYEDSYGYRPNKSAIQAIEITRQRCWKRDWVLEFDIKGLFDNIRHDYLMEMVKRHTKEEWIILYIQ
RWLTTPFQLSDGKLVERNSGTPQGGVISPVLANLFLHYTFDDFMTKEFPNVPWARYADDGIAHCVSLKQAKYL
QRRLIERFKAFGLELNLEKTRIVYCKDDERRGDYTHTSFDFLGYTFRPRRSKNRYGRYFINFSPAISNKAKKAIRKN
VKSWKIQLKVDKNINDLSNMFNKRIQGWINYYGHFYKSEMYGVLRYVNQKLKYWVRRKYKKLNSSRRAERWL
GQIALREPNLFAHWKMGILPAVSNGSRMS
>scaffold_AF45-12||gene_3450|GeneMark.hmm|424_aa|+|120|1394
MMKEAKPFVIDKTLIYKAYLKVKENKGSAGVDSVSIEDYEKDLGNCLYKLWNRMSSGSYMPNAVRLVEIPKPGG
GKRPLGIPTVEDRIAQQAAVLFIEPSIDPCFDQDSYGYRPNRSASDAIAKARERCFKFGWVLDMDISKFFDTIDH
DLLMKAVECHVREKWVLLYIRRWLKVPYRTSKGETIERTMGVPQGSVIGPVLANLFLHYTFDKWMRIHYPNIPF
ERYADDTICHCVSKAQAEYLKEVLTCRFEQCRLKLNAEKTKIVQCPTSTRKKVEGYEASFDFLGYTFQCRKSWNR
KQCQCFTSFLPAISKKSVKKLHEKMKEWKLHSHLDWKLQQVGIEIESQVRGWYNYYNKFGKTEFVKVMNHLN
MVLAYWIRRKYKRFHRKPIVKALIWLQEIASKDRSLFYHWQRGQTPRLCLYTKS
>scaffold_AF54-44A||gene_4558|GeneMark.hmm|414_aa|+|1772|3016
MQETKSYNISKQAVYQAFLKVKANKGTFGVDEESIEAYEYKLKDNLYKLWNRLSSGSYFPKPVKAVSIPKKSGGLR
VLGIPTVEDRIAQMVAKMYFEPVVERLFYEDSYGYRPNKSAIQAIEKTRVRCWKRDWVLEFDIKGLFDNIRHDY
LIEMVKRYTQEKWIILYVERWLKAPFQREDGSTVSRKAGTPQGGVISPVLANLFLHYTFDDFMEKEFPNIQWAR
YADDGITHCVSLKQAKYLKKRLEERFRIFGLELHPDKTKIVYCRDSDRMGNYPITTFDFLGFTFRPRGAKNKYGKC
FTNFLPAVSDKAKKAIRKEVRNWRLQLKADKKLEDLANMFNSKIQGWMNYYMKFYKSEMYSLLRYINQCLVK
WVRRKYKKRQARRKAEHWLGEIAKRERNLFAHWKIGILPSAG
>scaffold_AF73-11pH9TA||gene_2303|GeneMark.hmm|414_aa|+|217721|218965
VKEGKSFQITQNEVLSAYKAVKANKGAGGVDRVDIEMFEKNWKNRLYTLWNRMSSGTYFPKPVRGVEIPKKN
GKVRLLGIPTIEDRVAQMVLRNRLEPHIEPIFYEDSYGYRPNKSALDAVGIARERCYRMKWVIEFDIVGLFDNINH
EYLMKFVNYHSKEKWVNLYIERCLKAPIVMPDGAVEEREKGTPQGGVISPLLSGLYMHYAFDRWITREFPMCK
WERYADDGIIHCVSRKQAEYVLDMLKKRMQMCGLEIHPEKSKIVYCQRNNEKIDGEITSFTFLGYSFRPRLTKNR
NGQYFMGFTPAVSAESATAFREEIRVEIQNSNTTDIVAVSKRLNPIIRGWYNYFGRYCPSEAFRKGINYVNLKLVR
WLEGTRKSVRRSLTKAQHLLHRIAMSNPEMFYHWKVGYMPVK
>scaffold_AF98-24||gene_2235|GeneMark.hmm|418_aa|-|56390|57646
MSEAKQFDISKKAVIAAFQAVKENAGSYGADEQTIKEFEEHLNNNLYKLWNRMASGSYFPKPVRAVAIPKKNG
GIRILGIPTVEDRIAQMVAKMYFEPLVEPMFYNDSYGYRPNKSAIQAVGQARERCFKRDWVLELDIKGLFDNIK
HGYLMYMVEKHTQIKWLILYIKRWLTVPFIMSDGSVAERRSGTPQGGVISPVLANLFLHYVFDDFMTKAYPNI
WWERYADDGVLHCQSYKQAAFIKQKLEERFQQFGLELNKEKTRIVYCKDNRRPQNYSCTQFTFLGYTFRPRLN
KNKEGKFFVGFTPAVSEKAKTAMKQKIREWKIQLKADLSLKDIGNMINKVVQGWINYYTHYYKSEFYEVLRYIN
QCLIKWVRRSYKKKNTRSRAEHWLGAVARRDRNLFAHWKFGILPSVGEGAV
>scaffold_AF98-51||gene_2837|GeneMark.hmm|418_aa|+|2454|3710
MSEAKQFDISKKAVIAAFQAVKENAGSYGVDEQTIKEFEEHLNNNLYKLWNRMASGSYFPKPVRAVAIPKKNG
GTRILGIPTVEDRIAQMVAKMYFEPLVEPMFYKDSYGYRPNKSAIQAVGQARERCFKRDWVLELDIKGLFDNIK
HGYLMYMVEKHTQIKWLILYIKRWLTVPFIMSDGSVAERRSGTPQGGVISPVLANLFLHYVFDDFMTKAYPNI
WWERYADDGVLHCQSYKQAVFIKQKLEERFQQFGLELNKEKTRIVYCKDNRRSQNYSCTQFTFLGYTFRPRLNK
NKEGKFFVGFTPAVSEKAKTAMKQKIREWKIQLKADLSLKDIGNMINKVVQGWINYYTHYYKSEFYEVLRYINQ
CLIKWVRRSYKKKNTRSRAEHWLGAVARRDRNLFAHWKFGILPSVGEGAV
>scaffold_AM110-110||gene_4192|GeneMark.hmm|440_aa|-|78|1400
MNVERRGSVGQSASQPNCKQEEAAGEQTKPFQVSKLHVVEAYRRVKANAGAAGVDNQTLKDFERDLKGNLY

KIWNRLSSGSWMPPPVRAVEIPKKDGSKRLLGIPTVSDRIAQMTVLVTFEPLVERYFLNDSYGYRHGKSALDAIA
VTRKRCWQYDWYLEFDIKGLFDNIPHDLLLRAVDKHCADKWVRLSIRRWLTAPVQMPDGTLKERNKGTPQGG
VISPVLANLFLHYVFDKWLSLLYPEIPWCRYADDGLIHCGSKQQAEELLNKLAKPFQECGLELHPEKTKIVYCKDS
ERQANHETVQFNFLGYTFRARRARNQRRGNLFTSFSLAVSNSAQKDMIGKLRKLRLRRRVEMSLEDIAKRLNP
MISGWLNYYAKYYKSAMKKVCRYINLTLIAWARKKYKTLRYKKTKACQLMERLSKEKLELFAHWKAGPGSAFA
>scaffold_AM110-183||gene_1945|GeneMark.hmm|423_aa|+|1303|2574
MTQKQGAKPFDIDRWKLYYAYQRVNQNRGGSGVDNVTLEKYNSNLKRNLYKLWNRMSSGSYVPKPVRLVQIP
KPAGGTRPLGIPTVEDRIAQMLVVEMIEPEIEKIFHEDSYGYRPNRSAHDALGRARERCWKYAWVLDMDISKFF
DTIDHQLLMKAVRLHVKERWIILYIERWLKVPYQNADKSLIERTCGVPQGSVIGPILANLFLHYCFDRWMQIHH
PEIPFERYADDTVCHCRSQREAESLYEELIIRFKSCKLSLNEEKTKIVYCKSSRRKENHSNVTFDFLGHTFRPCKTM
HKSSREAFTGFQPRISMKATTKIRATMRSWNLKSKSHTPLDCIAHMVNPILRGWVNYYGKYGGKSFQKLLGYF
DLLLARWAKAKYKTFRRKPMYVILKWLGNVADRDAVFYHWQIGLKPAKGTIKL
>scaffold_AM113-178||gene_3249|GeneMark.hmm|440_aa|-|39343|40665
MNVERRGSVGQSASQPNCKQEEAAGEQTKPFQVSKLHVVEAYRRVKANAGAAGVDNQTLKDFERDLKGNLY
KIWNRLSSGSWMPPPVRAVEIPKKDGSKRLLGIPTVSDRIAQMTVLVTFEPLVERYFLNDSYGYRHGKSALDAIA
VTRKRCWQYDWYLEFDIKGLFDNIPHDLLLRAVDKHCADKWVRLSIRRWLTAPVQMPDGTLKERNKGTPQGG
VISPVLANLFLHYVFDKWLSLLYPEIPWCRYADDGLIHCGSKQQAEELLNKLAKPFQECGLELHPEKTKIVYCKDS
ERQANHETVQFNFLGYTFRARRARNQRRGNLFTSFSLAVSNSAQKDMIGKLRKLRLRRRVEMSLEDIAKRLNP
MISGWLNYYAKYYKSAMKKVCRYINLTLIAWARKKYKTLRYKKTKACQLMERLSKEKLELFAHWKAGPGSAFA
>scaffold_AM63-24T6NA||gene_4551|GeneMark.hmm|413_aa|+|1564|2805
MERGNPRADVKGVFQxxxxIKANKGTFGVDEESIETYECKLKDNLYKLWNRLSSGSYFPKPVKAVSIPKKNGGLR
VLGIPTVEDRIAQMVAKMYFEPAVERLFYEDSYGYRPNKSAIQAIEKTRVRCWKRDWVLEFDIKGLFDNIRHDYL
IEMVKRHTQEKWIILYVERWLKTPFQMEDGSTVSREAGTPQGGVISPVLANLFLHYTFDDFMEKEFPNIQWAR
YADDGITHCVSLKQAKYLKKRLEERFKIFGLELHPDKTKIVYCKDSDRTGNYPITTFDFLGFTFRPRGAKNKYGKHF
TNFLPAVSDKAKKAIRKEVRNWRLQLKADKKLEDLANMFNSKIQGWMNYYMKFYKSEMYSILRYINQCLVKW
VRRKYKKRQARRKAEHWLGEIAKRERSLFAHWKIGILPSAG
>scaffold_AM63-24T6NA||gene_4563|GeneMark.hmm|414_aa|+|569|1813
MQETKSYNISKQAVYQAFLKVKANKGTFGVDEESIEAYEYKLKDNLYKLWNRLSSGSYFPKPVKAVSIPKKSGGLR
VLGIPTVEDRIAQMVAKMYFEPVVERLFYEDSYGYRPNKSAIQAIEKTRVRCWKRDWVLEFDIKGLFDNIRHDY
LIEMVKRYTQEKWIILYVERWLKAPFQREDGSTVSRKAGTPQGGVISPVLANLFLHYTFDDFMEKEFPNIQWAR
YADDGITHCVSLKQAKYLKKRLEERFRIFGLELHPDKTKIVYCRDSDRMGNYPITTFDFLGFTFRPRGAKNKYGKC
FTNFLPAVSDKAKKAIRKEVRNWRLQLKADKKLEDLANMFNSKIQGWMNYYMKFYKSEMYSLLRYINQCLVK
WVRRKYKKRQARRKAEHWLGEIAKRERNLFAHWKIGILPSAG
>scaffold_AM64-12MHA||gene_4542|GeneMark.hmm|414_aa|-|1781|3025
MQETKSYNISKQAVYQAFLKVKANKGTFGVDEESIEAYEYKLKDNLYKLWNRLSSGSYFPKPVKAVSIPKKSGGLR
VLGIPTVEDRIAQMVAKMYFEPVVERLFYEDSYGYRPNKSAIQAIEKTRVRCWKRDWVLEFDIKGLFDNIRHDY
LIEMVKRYTQEKWIILYVERWLKAPFQREDGSTVSRKAGTPQGGVISPVLANLFLHYTFDDFMEKEFPNIQWAR
YADDGITHCVSLKQAKYLKKRLEERFRIFGLELHPDKTKIVYCRDSDRMGNYPITTFDFLGFTFRPRGAKNKYGKC
FTNFLPAVSDKAKKAIRKEVRNWRLQLKADKKLEDLANMFNSKIQGWMNYYMKFYKSEMYSLLRYINQCLVK
WVRRKYKKRQARRKAEHWLGEIAKRERNLFAHWKIGILPSAG
>scaffold_OF20-1A||gene_3277|GeneMark.hmm|414_aa|+|1772|3016
MQETKSYNISKQAVYQAFLKVKANKGTFGVDEESIEAYEYKLKDNLYKLWNRLSSGSYFPKPVKAVSIPKKSGGLR
VLGIPTVEDRIAQMVAKMYFEPVVERLFYEDSYGYRPNKSAIQAIEKTRVRCWKRDWVLEFDIKGLFDNIRHDY
LIEMVKRYTQEKWIILYVERWLKAPFQREDGSTVSRKAGTPQGGVISPVLANLFLHYTFDDFMEKEFPNIQWAR

YADDGITHCVSLKQAKYLKKRLEERFRIFGLELHPDKTKIVYCRDSDRMGNYPITTFDFLGFTFRPRGAKNKYGKC
FTNFLPAVSDKAKKAIRKEVRNWRLQLKADKKLEDLANMFNSKIQGWMNYYMKFYKSEMYSLLRYINQCLVK
WVRRKYKKRQARRKAEHWLGEIAKRERNLFAHWKIGILPSAG
>scaffold_OF23-5pH10A||gene_4294|GeneMark.hmm|414_aa|-|678|1922
MQETKSYNISKQAVYQAFLKVKANKGTFGVDEESIEAYEYKLKDNLYKLWNRLSSGSYFPKPVKAVSIPKKSGGLR
VLGIPTVEDRIAQMVAKMYFEPVVERLFYEDSYGYRPNKSAIQAIEKTRVRCWKRDWVLEFDIKGLFDNIRHDY
LIEMVKRYTQEKWIILYVERWLKAPFQREDGSTVSRKAGTPQGGVISPVLANLFLHYTFDDFMEKEFPNIQWAR
YADDGITHCVSLKQAKYLKKRLEERFRIFGLELHPDKTKIVYCRDSDRMGNYPITTFDFLGFTFRPRGAKNKYGKC
FTNFLPAVSDKAKKAIRKEVRNWRLQLKADKKLEDLANMFNSKIQGWMNYYMKFYKSEMYSLLRYINQCLVK
WVRRKYKKRQARRKAEHWLGEIAKRERNLFAHWKIGILPSAG
>scaffold_OF25-24MHA||gene_4035|GeneMark.hmm|420_aa|-|14577|15839
MNEAKPFVIDKRLVWEAYHKVKENKGSAGIDKVDQKTFDKEMSKNLYKIWNRMSSGCYFPKAVKLVEIPKSNG
GTRPLGIPTIEDRIAQQVVVSVLTPILEPIFKEDSYGYRPGKGAHQAIAKAKERCYVNPWVLDMDISKFFDTINHD
LLMKAVRKHTEEKWVLLYIERWLKVPYQTSKGEVIERTMGVPQGSVIGPVLANLFLHYVFDEWMSRNYPTIPFE
RYADDTICHCVSEKQAQFLKAVLMKRFEECGLKLNEEKTKIVYCKDSNRRGDSEHTSFDFLGFTFRPRSARNRKT
GQNFTAFLPAISKKSLKRIKEAVRAWKLNRKTFACLLDISNEVDTQISGWMNYYMKFGRSEFRKVLNYINERLTR
WVMRKYKRFSKGKKFSRAYEWLVEYAVHNRNEFSHWAKGFVPYPRLG
>scaffold_OM18-28A||gene_3990|GeneMark.hmm|414_aa|+|1756|3000
MQETKSYNISKQAVYQAFLKVKANKGTFGVDEESIEAYEYKLKDNLYKLWNRLSSGSYFPKPVKAVSIPKKSGGLR
VLGIPTVEDRIAQMVAKMYFEPVVERLFYEDSYGYRPNKSAIQAIEKTRVRCWKRDWVLEFDIKGLFDNIRHDY
LIEMVKRYTQEKWIILYVERWLKAPFQREDGSTVSRKAGTPQGGVISPVLANLFLHYTFDDFMEKEFPNIQWAR
YADDGITHCVSLKQAKYLKKRLEERFRIFGLELHPDKTKIVYCRDSDRMGNYPITTFDFLGFTFRPRGAKNKYGKC
FTNFLPAVSDKAKKAIRKEVRNWRLQLKADKKLEDLANMFNSKIQGWMNYYMKFYKSEMYSLLRYINQCLVK
WVRRKYKKRQARRKAEHWLGEIAKRERNLFAHWKIGILPSAG
>scaffold_UN03-31||gene_3781|GeneMark.hmm|413_aa|-|558|1799
MTKPFNIPKALIWEAFKKVKENGGAPGVDHESIEQFEKHLKNNLYKLWNRLCSGSYFPPPVKAVPIPKKSGGVRI
LGIPTVADRVAQTAVKLLLEPKIDPLFHPNSYGYRPGRSAHDAIAIVRRRSWDYDWVVEFDIKGLFDNIDHDLLM
RALKKHCEIPWILLYVQRWLKAPMQHINGHLLERNRGTPQGGVVSPLLANLFMHYAFDMWITKHLQSVRFCR
YADDGVIHCRSLSQAKLVLQKIDARFRECGLELHPDKTKIVYCQDINRRKAYPDVQFTFLGYTFRPRKAVDKYKRV
YVNFSPAVSRDALKAMRQTIRKWHLHLMCNRELSDLSAIFNPILQGWQQYYGRFHGSAMSAIWQHMNAYLI
RWMRRKYKNLARHKRRARYALGRLARDFPNAFVHWKMGCLPSVG
>SRS011084||gene_23176|GeneMark.hmm|414_aa|+|13616|14860
MIETKPYEISKWAVYIAYERVKANKGSYGVDEQSIEDFEKNLKNNLYKIWNRMSSGSYFPQPVKAVSVPKKNGGI
RVLGIPTVEDRIAQMTAKLYFEPCVEPLFLEDSYGYRPGKSAIQALSVTRKRCWHRDWVLEYDIKGLFDNIRHDY
LLEMVRRHTPHKWILLYVERWLTTPFQLEDGTLQSRTSGTPQGGVISLVLANLFLHYAFDSFMAKEYPKAWWE
RYADDGVLHCKSSQQAMYMKSVLRERFRLFGLELNEEKTRIVYCKDADRTEDYSEISLDSLGYTFRPRLARNKHG
NIFLNFLPAMSAKAIKAMKEEVRRWKLQLKVSKSLTDLANILNSQIQGWISYYGHFYKSELIYLLRYINQCLIKWV
RRKYKKFNHRRRAEYWLGRIARRDNNLFAHWRYGVLPTAG
>SRS011084||gene_137644|GeneMark.hmm|430_aa|-|170|1462
MQNDNAKPISISKQLVYDAFLRVKANRGSAGIDKVTLEDYEKNLRGNLYKLWNRMSSGSYFPPSVKLVEIPKSTG
GKRPLGIPTVSDRVAQMAVVMLITPSIEPCFHEDSYAYRPHRSAHDAVGKARERCWKYAWVLDMDISKFFDTI
DHELLLKALKRHTQEKWVLMYIERWLKVPYEKSDGSQVDRALGVPQGSVIGPVLANLFLHYTFDKWMEKNFP
RVPFERYADDTICHCHSLKQAEYMQAMIQQRFECCRLRLNEEKTKIVYCKSSRQKECYPNVTFDFLGFTFQPRES
VDKYGNRFTGFLPAISRKSMKRINETMRSWHLNRHSNLTLEHLASDINPIVRGWMTYYGKFYPTRLKWFMQTL

NGRLARWVMCKFERYRHRFYPAQEWLARIAEKEGLIFYHWKCGALPRFTNKEKVSSQLIMVK
>SRS011134||gene_370816|GeneMark.hmm|420_aa|+|1688|2950
MNEAKPFVIDKRLVWEAYHKVKENKGSAGIDKVDQKTFDKEMSKNLYKIWNRMSSGCYFPKAVKLVEIPKSNG
GTRPLGIPSIEDRIAQQVVVSVLTPILEPIFKEDSYGYRPGKGAHQAIAKAKERCYVNPWVLDMDISKFFDTINHE
LLMKAVRKHTEEKWILLYIERWLKVPYQTSKGEVIERTMGVPQGSVIGPVLANLFLHYVFDEWMSRNYPTIPFE
RYADDTICHCVSEKQAQFLKAVLMKRFEECGLKLNEEKTKIVYCKDSNRRGDSEHTSFDFLGFTFRPRGARNRKT
GQNFTAFLPAISKKSMKRIKEAVRAWKLNRKTFACLLDISNEVDTQISGWMNYYMKFGRSEFRKVLNYINERLT
RWVMRKYKRFSKGRKFNRAYDWLVEYAAHNRNEFSHWVKGFVPYPRLG
>SRS011239||gene_220565|GeneMark.hmm|422_aa|+|191|1459
MKDAKSFEISRHLVMEAYKRVKANKGAAGVDDVSIADFESNLKSNLYKIWNRMSSGSYFPPAVKLVEIPKSNGG
KRPLGIPTIGDRVAQMVVVMTIEPGIEPYFHEDSYAYRPNRSALDAVRKAKERSYTFHWVLDLDIKGFFDNIDHG
LLIKALERHVKCEWAMLYIKRWLSVPYQLKDGTQIERTKGVPQGSVVGPILANLFLHYVFDEWMRRNHSNISFE
RYADDTICHCVSLKQAEFILRAIRKRFAECKLELNEDKTKIVYCKKNHRDIPYECIQFDFLGYTFRPRRSIDANGEVF
LNFSPAISKKARTKIWEAIQNWNSNHWVPMELEDIAKEINPVIQGWINYYGQHNPRILKEVLQHVNDRLVRW
GRRKFKGLRKRKTATVHRLGDIALQKPNLFAHWAWGVKPTASEKNRKRK
>SRS011271||gene_288530|GeneMark.hmm|422_aa|+|688|1956
MKDAKSFEISRHLVMEAYKRVKANKGAAGVDDVSIADFESNLKSNLYKIWNRMSSGSYFPPAVKLVEIPKSNGG
KRPLGIPTIGDRVAQMVVVMTIEPGIEPYFHEDSYAYRPNRSALDAVRKAKERSYTFHWVLDLDIKGFFDNIDHG
LLIKALERHVKCEWAMLYIKRWLSVPYQLKDGTQIERTKGVPQGSVVGPILANLFLHYVFDEWMRRNHSNISFE
RYADDTICHCVSLKQAEFILRAIRKRFAECKLELNEDKTKIVYCKKNHRDIPYECIQFDFLGYTFRPRRSIDANGEVF
LNFSPAISKKARTKIWEAIQNWNSNHWVPMELEDIAKEINPVIQGWINYYGQHNPRILKEVLQHVNDRLVRW
GRRKFKGLRKRKTATVHRLGDIALQKPNLFAHWAWGVKPTASEKNRKRK
>SRS011405||gene_145846|GeneMark.hmm|421_aa|-|88|1353
MQEAKPFQIDKRIIFESFKKVKFNRGSSGIDGIEMTTYEQNLGSNLYRLWNRMSSGSYMPKAVKLVEIPKSNGG
KRPLGIPTIEDRIAQMAVVNVIEPLIEPCFHEDSFGYRPHRSAHDAIAKAERRCWKYAWVLDIDISKFFDTIDHGL
LMKAVEKHINIKWILLYIKRWLTVPYQRSDGEIVKRDMGVPQGSVIGPILANLFLHYTFDKWMSYKYPHIPFERY
ADDCVCHCSTLAQAEYIKERLGERFTECKLKFNEEKTKIVFCKMSSRSSKHYHCTSFDYLGFTFRSRAAKDKRNN
VLFTSYLPAISKKSVSRIHETIKSWNLKRLHNRSLRFVAAYINDVVRGWINYYEKFGKTEFWKVMCHLNRSIAYW
AKTKYKRLRRRGVISAHYWLAYIAQKEPNLFYHWQVGYVPYARQKK
>SRS011529||gene_80475|GeneMark.hmm|421_aa|-|368|1633
MQEAKPFQIDKRIIFEAFKKVKSNGGSPGIDGIEMSAYEQNLGSNFYRLWNRMSSGSYMPKAVKLVEILKSNGG
KRPLGIPSVEDRIAQMAVVNVIEPLVEPYFHKDSFGYRPHRSAHDAIAKAERRCWKYAWVLDIDISKFFDTIDHG
LLMKAVEKHIKTKWILLYIKRWLTVPYQGNDGAIVKRHMGVPQGSVIGPILANQFLHYTFDKWMSYKYPHVPF
ERYADDCVCHCGTLAQAEYIKDRLGERFAECKLTFNEEKTKIVFCKTSNRSSEHYHCTSFDYLGFTFRPRAAKDKR
KNVLFTSYLPAISNKSESRIHETIKSWNLKRLHNRSLRFVAAYINDVVRGWISYYGKFGKTEFWKVMCHLNRSIAY
WAKTKYKRLRRRGVISAHYWLAYIAQKEPNLFYHWQVGYIPYARQKK
>SRS012273||gene_378785|GeneMark.hmm|414_aa|-|1264|2508
MDETKPFKISKQIVKIAFDRVKENKGTYGIDEQSIADFEENLKDNLYKIWNRMSSGTYFPKAVKAVAIPKKNGGT
RILGIPTVEDRVAQMVAKIYFEPNVEKIFYEDSYGYRPNKSAIQAVGVLRERCWRKDWVVDFDITGLFDNIRHDY
LIEMVKKHTDEQWIILYIERWLKTPFKMQDGTIVERTAGTPQGGVISPVLANLFLHYVFDDFMSKEFPNIPWVR
YADDGALNCVSIKQAKYIIKVLDKRFKAFGLELNLSKTRIVYCKDDDRNGNYENTSFDFLGYTFKPRSAKNKHGK
MFRSFLPAMSDKAQKAIRKEIKSWKLQLKVDKTINDITEIYNSKIQGWINYYAHYYKSEIYGVLKYINRCLIKWVR
RKYKKKNTRKRAIDLLMKIARRDNCLFAHWKFGILPTAG
>SRS012969||gene_62711|GeneMark.hmm|422_aa|-|566|1834

MKDAKSFEISRHLVMEAYKRVKANKGAAGVDDVSIADFESNLKSNLYKIWNRMSSGSYFPPAVKLVEIPKSNGG
KRPLGIPTIGDRVAQMVVVMTIEPGIEPYFHEDSYAYRPNRSALDAVRKAKERSYTFHWVLDLDIKGFFDNIDHG
LLIKALERHVKCEWAMLYIKRWLSVPYQLKDGTQIERTKGVPQGSVVGPILANLFLHYVFDEWMRRNHSNISFE
RYADDTICHCVSLKQAEFILRAIRKRFAECKLELNEDKTKIVYCKKNHRDIPYECIQFDFLGYTFRPRRSIDANGEVF
LNFSPAISKKARTKIWEAIQNWNSNHWVPMELEDIAKEINPVIQGWINYYGQHNPRILKEVLQHVNDRLVRW
GRRKFKGLRKRKTATVHRLGDIALQKPNLFAHWAWGVKPTASEKNRKRK
>SRS013098||gene_353728|GeneMark.hmm|430_aa|+|685|1977
MQNGNAKPISISKQLVYDAFLRVKANRGSAGIDKVTLEDYEKNLRGNLYKLWNRMSSGSYFPPSVKLVEIPKSTG
GKRPLGIPTVSDRVAQMAIVMLITPSIEPCFHEDSYAYRPHRSAHDAVGKARERCWKYAWVLDMDISKFFDTID
HELLLKALKRHTQEKWVLMYIERWLKVPYEKADGSQVDRALGVPQGSVIGPVLANLFLHYTFDKWMEKSFPR
VPFERYADDTICHCHSLKQAEYMQAMIQQRFECCRLRLNEEKTKIVYCKSSRQKGRYPNVTFDFLGFTFQPRES
VDKYGSRFTGFLPAISRKSMKRINETIRSWHLNRHSNLTLEHLASDINPIVRGWMTYYGKFYPTRLKWFMQTLN
GRLARWIMCKFERYRHRFYPAQEWLARIAEKEGLIFYHWKCGVLPRFTNKEKVSSQLIMVK
>SRS013800||gene_74409|GeneMark.hmm|422_aa|-|370|1638
MKDAKSFEISRHLVMEAYKRVKANKGAAGVDEVSIADFENNLKSNLYKIWNRMSSGSYLPPAVKLVEIPKSNGG
KRPLGIPTVGDRVAQMVVVMTIEPGIEPYFHEDSYAYRPNRSALDAVRKAKERSYTFHWVLDLDIKGFFDNIDH
ELLIKALERHVKCKWAILYIKRWLSVPYQLKDGTQKERTKGVPQGSVVGPILANLFLHYVFDEWMRRNHSNISFE
RYADDTICHCVSLKQAEFILRAIRKRFAECKLELNEDKTKIVYCKKNHRDIPYECIQFDFLGYTFRPRRSIDANGEVF
LNFSPAISKKARTKIWEAIQNWNSNHWVPMELEDIAKEINPVIQGWINYYGQHNPRILKEVLQHVNDRLVRW
GRRKFKGLRKRKTATVHRLGDIALQKPNLFAHWAWGVKPTASERNRKRK
>SRS013951||gene_12116|GeneMark.hmm|430_aa|+|302|1594
MQNDNAKPISISKQLVYDAFLRVKANRGSAGIDKVTLEDYEKNLRGNLYKLWNRMSSGSYFPPSVKLVEIPKSTG
GKRPLGIPTVSDRVAQMAIVMLITPSIEPCFHEDSYAYRPHRSAHDAVGKARERCWKYAWVLDMDISKFFDTID
HELLLKALKRHTQEKWVLMYIERWLKVPYEKADGSQVDRALGVPQGSVIGPVLANLFLHYTFDKWMEKSFPR
VPFERYADDTICHCHSLKQAEYMQAMIQQRFECCRLRLNEEKTKIVYCKSSRQKEFYPNVTFDFLGFTFQPRESV
DKYGNRFTGFLPAISRKSMKRINETIRSWHLNRHSNLTLEHLASDINPIVRGWMTYYGKFYPTRLKWFMQTLN
GRLARWVMCKFERYRHRFYPAQEWLARIAEKEGLIFYHWKCGVLPRFTNKEKVSSQLIMVK
>SRS014313||gene_40141|GeneMark.hmm|414_aa|+|13941|15185
MQTTKPYNISKKAVVMAYRRVKANKGTYGIDEQSIEDFEKNLQDNLYKLWNRMSSGTYFPKPVKAVAIPKKNG
GKRILGIPTVEDRIAQMVAKLYFEPNVERIFYEDSYGYRPNKSAIQALEVTRKRCWRKDWVLEFDIKGLFDNINH
DILLKMVEKHTKEKWVLLYIRRWLITPFQMNDGDIVERPSGTPQGGVISPVLANLFLHHVFDDFMSKEFPSIPW
ARYADDGIAHCVSLKQAKYLLKRLQGRFKQFGLELNLDKTRIVYCKDEDRKGDYENTSFDFLGYTFRPRRAKKKY
GKYFTSFLPAMSNKAKKAIRKEVSGWKLQLKSDKSINDLAHMFNSKIQGWINYYTHFYKSEIYDVLRYINKCLIK
WVRRKFKKRKSNRRAERWLGDIARRDNKLFAHWKFGILPSVG
>SRS014459||gene_109454|GeneMark.hmm|423_aa|-|5665|6936
MTQKQGAKPFDIDRWKLYYAYQRVNQNRGGSGVDNVTLEKYNSNLKRNLYKLWNRMSSGSYVPKPVRLVQIP
KPAGGTRPLGIPTVEDRIAQMLVVEMIEPEIEKIFHEDSYGYRPNRSAHDALGRARERCWKYAWVLDMDISKFF
DTIDHQLLMKAVRLHVKERWIILYIERWLKVPYQNADKSLIERTCGVPQGSVIGPILANLFLHYCFDRWMQIHYP
EIPFERYADDTVCHCRSQREAESLYEELIIRFKSCKLSLNEEKTKIVYCKSSRRKENHSNVTFDFLGHTFRPCKTMH
KSSREAFTGFQPRISMKATTKIRATMRSWNLKSKSHTPLDCIAHMVNPILRGWVNYYGKYGGKSFQKLLGYFDL
LLARWAKAKYKTFRRKPMYVILKWLGNVADRDAVFYHWQIGLKPAKGTIKL
>SRS014613||gene_48346|GeneMark.hmm|430_aa|+|22443|23735
MQNDNAKPISISKQLVYDAFLRVKANRGSAGIDKVTLEDYEKNLRGNLYKLWNRMSSGSYFPPSVKLVEIPKSTG
GKRPLGIPTVSDRVAQMAVVMLITPSIEPCFHEDSYAYRPHRSAHDAVGKARERCWKYAWVLDMDISKFFDTI

DHELLLKALKRHTQEKWVLMYIERWLKVPYEKSDGSQVDRALGVPQGSVIGPVLANLFLHYTFDKWMEKNFP
RVPFERYADDTICHCHSLKQAEYMQAMIQQRFECCRLRLNEEKTKIVYCKSSRQKECYPNVTFDFLGFTFQPRES
VDKYGNRFTGFLPAISRKSMKRINETMRSWHLNRHSNLTLEHLASDINPIVRGWMTYYGKFYPTRLKWFMQTL
NGRLARWVMCKFERYRHRFYPAQEWLARIAEKEGLIFYHWKCGALPRFTNKEKVSSQLIMVK
>SRS014923||gene_68216|GeneMark.hmm|430_aa|-|775|2067
MQNDNAKPISISKQLVYDAFLRVKANRGSAGIDKVTLEDYEKNLRGNLYKLWNRMSSGSYFPPSVKLVEIPKSTG
GKRPLGIPTVSDRVAQMAVVMLITPSIEPCFHEDSYAYRPHRSAHDAVGKARERCWKYAWVLDMDISKFFDTI
DHELLLKALKRHTQEKWVLMYIERWLKVPYEKSDGSQVDRALGVPQGSVIGPVLANLFLHYTFDKWMEKNFP
RVPFERYADDTICHCHSLKQAEYMQAMIQQRFECCRLRLNEEKTKIVYCKSSRQKECYPNVTFDFLGFTFQPRES
VDKYGNRFTGFLPAISRKSMKRINETMRSWHLNRHSNLTLEHLASDINPIVRGWMTYYGKFYPTRLKWFMQTL
NGRLARWVMCKFERYRHRFYPAQEWLARIAEKEGLIFYHWKCGALPRFTNKEKVSSQLIMVK
>SRS015065||gene_99768|GeneMark.hmm|423_aa|-|5626|6897
MTQKQGAKPFDIDRWKLYYAYQRVNQNRGGSGVDNVTLEKYNSNLKRNLYKLWNRMSSGSYVPKPVRLVQIP
KPAGGTRPLGIPTVEDRIAQMLVVEMIEPEIEKIFHEDSYGYRPNRSAHDALGRARERCWKYAWVLDMDISKFF
DTIDHQLLMKAVRLHVKERWIILYIERWLKVPYQNADKSLIERTCGVPQGSVIGPILANLFLHYCFDRWMQIHYP
EIPFERYADDTVCHCRSQREAESLYEELIIRFKSCKLSLNEEKTKIVYCKSSRRKENHSNVTFDFLGHTFRPCKTMH
KSSREAFTGFQPRISMKATTKIRATMRSWNLKSKSHTPLDCIAHMVNPILRGWVNYYGKYGGKSFQKLLGYFDL
LLARWAKAKYKTFRRKPMYVILKWLGNVADRDAVFYHWQIGLKPAKGTIKL
>SRS015095||gene_110120|GeneMark.hmm|423_aa|-|17932|19203
MTQKQGAKPFDIDRWKLYYAYQRVNQNRGGSGVDNVTLEKYNSNLKRNLYKLWNRMSSGSYVPKPVRLVQIP
KPAGGTRPLGIPTVEDRIAQMLVVEMIEPEIEKIFHEDSYGYRPNRSAHDALGRARERCWKYAWVLDMDISKFF
DTIDHQLLMKAVRLHVKERWIILYIERWLKVPYQNADKSLIERTCGVPQGSVIGPILANLFLHYCFDRWMQIHYP
EIPFERYADDTVCHCRSQREAESLYEELIIRFKSCKLSLNEEKTKIVYCKSSRRKENHSNVTFDFLGHTFRPCKTMH
KSSREAFTGFQPRISMKATTKIRATMRSWNLKSKSHTPLDCIAHMVNPILRGWVNYYGKYGGKSFQKLLGYFDL
LLARWAKAKYKTFRRKPMYVILKWLGNVADRDAVFYHWQIGLKPAKGTIKL
>SRS015264||gene_92828|GeneMark.hmm|430_aa|-|383|1675
MQNDNAKPISISKQLVYDAFLRVKANRGSAGIDKVTLEDYEKNLRGNLYKLWNRMSSGSYFPPSVKLVEIPKSTG
GKRPLGIPTVSDRVAQMAVVMLITPSIEPCFHEDSYAYRPHRSAHDAVGKARERCWKYAWVLDMDISKFFDTI
DHELLLKALKRHTQEKWVLMYIERWLKVPYEKSDGSQVDRALGVPQGSVIGPVLANLFLHYTFDKWMEKNFP
RVPFERYADDTICHCHSLKQAEYMQAMIQQRFECCRLRLNEEKTKIVYCKSSRQKECYPNVTFDFLGFTFQPRES
VDKYGNRFTGFLPAISRKSMKRINETMRSWHLNRHSNLTLEHLASDINPIVRGWMTYYGKFYPTRLKWFMQTL
NGRLARWVMCKFERYRHRFYPAQEWLARIAEKEGLIFYHWKCGALPRFTNKEKVSSQLIMVK
>SRS015280||gene_79234|GeneMark.hmm|430_aa|-|147|1439
MQNDNAKPISISKQLVYDAFLRVKANRGSAGIDKVTLEDYEKNLRGNLYKLWNRMSSGSYFPPSVKLVEIPKSTG
GKRPLGIPTVSDRVAQMAVVMLITPSIEPCFHEDSYAYRPHRSAHDAVGKARERCWKYAWVLDMDISKFFDTI
DHELLLKALKRHTQEKWVLMYIERWLKVPYEKSDGSQVDRALGVPQGSVIGPVLANLFLHYTFDKWMEKNFP
RVPFERYADDTICHCHSLKQAEYMQAMIQQRFECCRLRLNEEKTKIVYCKSSRQKECYPNVTFDFLGFTFQPRES
VDKYGNRFTGFLPAISRKSMKRINETMRSWHLNRHSNLTLEHLASDINPIVRGWMTYYGKFYPTRLKWFMQTL
NGRLARWVMCKFERYRHRFYPAQEWLARIAEKEGLIFYHWKCGALPRFTNKEKVSSQLIMVK
>SRS015431||gene_222796|GeneMark.hmm|418_aa|+|2215|3471
MSEAKQFDISKKAVIAAFQAVKENAGSYGADEQTIKEFEEHLNNNLYKLWNRMASGSYFPKPVRAVAIPKKNG
GIRILGIPTVEDRIAQMVAKMYFEPLVEPMFYNDSYGYRPNKSAIQAVGQARERCFKRDWVLELDIKGLFDNIK
HGYLMYMVEKHTQIKWLILYIKRWLTVPFIMSDGSVAERRSGTPQGGVISPVLANLFLHYVFDDFMTKAYPNI
WWERYADDGVLHCQSYKQAAFIKQKLEERFQQFGLELNKEKTRIVYCKDNRRPQNYSCTQFTFLGYTFRPRLN

KNKEGKFFVGFTPAVSEKAKTAMKQKIREWKIQLKADLSLKDIGNMINKVVQGWINYYTHYYKSEFYEVLRYIN
QCLIKWVRRSYKKKNTRSRAEHWLGAVARRDRNLFAHWKFGILPSVGEGAV
>SRS015578||gene_88282|GeneMark.hmm|421_aa|+|3040|4305
MQEAKPFQIDKRIIFEAFKKVKFNRGSSGIDGIEMTTYEQNLGSNLYRLWNRMSSGSYMPKAVKLVEIPKSNGG
KRPLGIPTIEDRIAQMAVVNVIEPLIEPCFHEDSFGYRPHRSAHDAIAKAERRCWKYAWVLDIDISKFFDTIDHGL
LMKAVEKHINIKWILLYIKRWLTVPYQRSDGEIVKRDMGVPQGSVIGPILANLFLHYTFDKWMSYKYPHIPFERY
ADDCVCHCSTLAQAEYIKERLGERFTECKLKFNEEKTKIVFCKMSSRSSKHYHCTSFDYLGFTFRSRAAKDKRNN
VLFTSYLPAISKKSVSRIHETIKSWNLKRLHNRSLRFVAAYINDVVRGWINYYEKFGKTEFRKVMCHLNRSIAYWA
KTKYKRLRRRGVISAHYWLAYIAQKEPNLFYHWQVGYVPYARQKK
>SRS015579||gene_20342|GeneMark.hmm|430_aa|+|1545|2837
MQNDNAKPISISKQLVYDAFLRVKANRGSAGIDKVTLEDYEKNLRGNLYKLWNRMSSGSYFPPSVKLVEIPKSTG
GKRPLGIPTVSDRVAQMAVVMLITPSIEPCFHEDSYAYRPHRSAHDAVGKARERCWKYAWVLDMDISKFFDTI
DHELLLKALKRHTQEKWVLMYIERWLKVPYEKSDGSQVDRALGVPQGSVIGPVLANLFLHYTFDKWMEKNFP
RVPFERYADDTICHCHSLKQAEYMQAMIQQRFECCRLRLNEEKTKIVYCKSSRQKECYPNVTFDFLGFTFQPRES
VDKYGNRFTGFLPAISRKSMKRINETMRSWHLNRHSNLTLEHLASDINPIVRGWMTYYGKFYPTRLKWFMQTL
NGRLARWVMCKFERYRHRFYPAQEWLARIAEKEGLIFYHWKCGALPRFTNKEKVSSQLIMVK
>SRS015663||gene_19109|GeneMark.hmm|421_aa|+|1334|2599
MQEAKPFQIDKRIIFESFKKVKFNRGSSGIDGIEMTTYEQNLGSNLYRLWNRMSSGSYMPKAVKLVEIPKSNGG
KRPLGIPTIEDRIAQMAVVNVIEPLIEPCFHEDSFGYRPHRSAHDAIAKAERRCWKYAWVLDIDISKFFDTIDHGL
LMKAVEKHINIKWILLYIKRWLTVPYQRSDGEIVKRDMGVPQGSVIGPILANLFLHYTFDKWMSYKYPHIPFERY
ADDCVCHCSTLAQAEYIKERLGERFTECKLKFNEEKTKIVFCKMSSRSSKHYHCTSFDYLGFTFRSRAAKDKRNN
VLFTSYLPAISKKSVSRIHETIKSWNLKRLHNRSLRFVAAYINDVVRGWINYYEKFGKTEFWKVMCHLNRSIAYW
AKTKYKRLRRRGVISAHYWLAYIAQKEPNLFYHWQVGYVPYARQKK
>SRS015794||gene_9671|GeneMark.hmm|422_aa|-|2863|4131
MKDAKSFEISRHLVMEAYKRVKANKGAAGVDDVSIADFESNLKSNLYKIWNRMSSGSYFPPAVKLVEIPKSNGG
KRPLGIPTIGDRVAQMVVVMTIEPGIEPYFHEDSYAYRPNRSALDAVRKAKERSYTFHWVLDLDIKGFFDNIDHG
LLIKALERHVKCEWAMLYIKRWLSVPYQLKDGTQIERTKGVPQGSVVGPILANLFLHYVFDEWMRRNHSNISFE
RYADDTICHCVSLKQAEFILRAIRKRFAECKLELNEDKTKIVYCKKNHRDIPYECIQFDFLGYTFRPRRSIDANGEVF
LNFSPAISKKARTKIWEAIQNWNSNHWVPMELEDIAKEINPVIQGWINYYGQHNPRILKEVLQHVNDRLVRW
GRRKFKGLRKRKTATVHRLGDIALQKPNLFAHWAWGVKPTASEKNRKRK
>SRS015816||gene_30|GeneMark.hmm|422_aa|-|5272|6540
MKDAKSFEISRHLVMEAYKRVKANKGAAGVDDVSIADFESNLKSNLYKIWNRMSSGSYFPPAVKLVEIPKSNGG
KRPLGIPTIGDRVAQMVVVMTIEPGIEPYFHEDSYAYRPNRSALDAVRKAKERSYTFHWVLDLDIKGFFDNIDHG
LLIKALERHVKCEWAMLYIKRWLSVPYQLKDGTQIERTKGVPQGSVVGPILANLFLHYVFDEWMRRNHSNISFE
RYADDTICHCVSLKQAEFILRAIRKRFAECKLELNEDKTKIVYCKKNHRDIPYECIQFDFLGYTFRPRRSIDANGEVF
LNFSPAISKKARTKIWEAIQNWNSNHWVPMELEDIAKEINPVIQGWINYYGQHNPRILKEVLQHVNDRLVRW
GRRKFKGLRKRKTATVHRLGDIALQKPNLFAHWAWGVKPTASEKNRKRK
>SRS015960||gene_71268|GeneMark.hmm|430_aa|+|19685|20977
MQNDNAKPISISKQLVYDAFLRVKANRGSAGIDKVTLEDYEKNLRGNLYKLWNRMSSGSYFPPSVKLVEIPKTTG
GKRPLGIPTVSDRVAQMAVVMLITPSIEPCFHEDSYAYRPHRSAHDAVGKARERCWKYAWVLDMDISKFFDTI
DHELLLKALKRHTQEKWVLMYIERWLKVPYEKSDGSQVDRALGVPQGSVIGPVLANLFLHYTFDKWMEKNFP
RVPFERYADDTICHCHSLKQAEYMQAMIQQRFECCRLRLNEEKTKIVYCKSSRQKECYPNVTFDFLGFTFQPRES
VDKYGNRFTGFLPAISRKSMKRINETMRSWHLNRHSNLTLEHLASDINPIVRGWMTYYGKFYPTRLKWFMQTL
NGRLARWVMCKFERYRHRFYPAQEWLARIAEKEGLIFYHWKCGVLPRFTNKEKVSSQLIMVK

>SRS015960||gene_179060|GeneMark.hmm|421_aa|+|557|1822
MQEAKPFQIDKRIIFESFKKVKFNRGSSGIDGIEMTTYEQNLGSNLYRLWNRMSSGSYMPKAVKLVEIPKSNGG
KRPLGIPTIEDRIAQMAVVNVIEPLIEPCFHEDSFGYRPHRSAHDAIAKAERRCWKYAWVLDIDISKFFDTIDHGL
LMKAVEKHINIKWILLYIKRWLTVPYQRSDGEIVKRDMGVPQGSVIGPILANLFLHYTFDKWMSYKYPHIPFERY
ADDCVCHCSTLAQAEYIKERLGERFTECKLKFNEEKTKIVFCKMSSRSSKHYHCTSFDYLGFTFRSRAAKDKRNN
VLFTSYLPAISKKSVSRIHETIKSWNLKRLHNRSLRFVAAYINDVVRGWINYYEKFGKTEFWKVMCHLNRSIAYW
AKTKYKRLRRRGVISAHYWLAYIAQKEPNLFYHWQVGYVPYARQKK
>SRS016335||gene_169679|GeneMark.hmm|430_aa|+|533|1825
MQNDNAKPISISKQLVYDAFLRVKANRGSAGIDKVTLEDYEKNLRGNLYKLWNRMSSGSYFPPSVKLVEIPKSTG
GKRPLGIPTVSDRVAQMTVVMLITPSIEPCFHEDSYAYRPHRSAHDAVGKARERCWKYAWVLDMDISKFFDTID
HELLLKALKRHTQEKWVLMYIERWLKVPYEKSDGSQVDRALGVPQGSVIGPVLANLFLHYTFDKWMEKNFPR
VPFERYADDTICHCHSLKQAEYMQAMIQQRFECCRLRLNEEKTKIVYCKSSRQKECYPNVTFDFLGFTFQPRESV
DKYGNRFTGFLPAISRKSMKRINETMRSWHLNRHSNLTLEHLASDINPIVRGWMTYYGKFYPTRLKWFMQTLN
GRLASWVMCKFERYRHRFYPAQEWLARIAEKEGLIFYHWKCGVLPRFTNKEKVSSQLIMVK
>SRS016495||gene_21089|GeneMark.hmm|414_aa|+|28007|29251
VKEGKSFQITQNEVLSAYKAVKANKGAGGVDRVDFEMFEKNWKNRLYILWNRMASGTYFPKPVRGVEIPKRN
GKVRLLGIPTIEDRVAQMVLRNRLEPHIEPISYEDSYGYRPNKSALDAVGMARERCYRMKWVIEFDIVGLFDNIN
HEYLMKFVKHHSKEKWVNLYIERCLKAPIVMPDGTVKEREKGTPQGGVISPLLSGLYMHYAFDRWITREFPMC
KWERYSDDGGIHCVSKKQAEYVLDMLKKRMRMCGLEIHPEKSKIVYCQRNNEKIDGEITSFTFLGYCFRPRLTKS
RNGQYFMGFTPAVSAGSATVFREKIRREIQNSNTTDIVALSKRLNPIIRGWYNYFGKYCPSEAFRKGINYVNLKLV
RWLEGTRKSVRRSLTKAQHLLHRIAMSTPELFYHWKVGYMPVK
>SRS016495||gene_75066|GeneMark.hmm|413_aa|+|1053|2294
MNKSKQYEIPKRTVIEAYKRVKANKGSAGIDGMDFEKFEERLNNNLYKIWNRMSSGSYFPSPVMAVEIPKKSEG
TRRLGIPTIADRIAQMVARTYVERAVEPMFCEDSYGYRPHKSALDAVEKTRKRCWKYDYVIELDVKGLFDNIDHE
LLMRVVRRHVKEPWICLYIERWLKSPFVLPDGSRIERESGTPQGGVISPVLANMFLRYVFDMWMKRNFPQAP
FERYADDGVVHCRTKEEAFYIKEKLAKRFEECKLELHPVKTRIVYCKDKDRTKEEELTEFDFLGYTFKAVYIKCKDG
VMRNNFIASVSKTAAKDFRDKIKALEIHKRTGCKIGMIAELLNPMIRGWMNYFGKFNPSAMKSTLQCIERRLIK
WAMCKYKSFRGRRQRAEKWLSSIRKREPKLFAHWSRMYSYC
>SRS016517||gene_75741|GeneMark.hmm|413_aa|+|817|2058
MNKSKQYEIPKRTVIEAYKRVKANKGSAGIDGMDFEKFEERLNNNLYKIWNRMSSGSYFPSPVMAVEIPKKSEG
TRRLGIPTIADRIAQMVARTYVERAVEPMFCEDSYGYRPHKSALDAVEKTRKRCWKYDYVIELDVKGLFDNIDHE
LLMRVVRRHVKEPWICLYIERWLKSPFVLPDGSRIERESGTPQGGVISPVLANMFLRYVFDMWMKRNFPQAP
FERYADDGVVHCRTKEEAFYIKEKLAKRFEECKLELHPVKTRIVYCKDKDRTKEEELTEFDFLGYTFKAVYIKCKDG
VMRNNFIASVSKTAAKDFRDKIKALEIHKRTGCKIGMIAELLNPMIRGWMNYFGKFNPSAMKSTLQCIERRLIK
WAMCKYKSFRGRRQRAEKWLSSIRKREPKLFAHWSRMYSYC
>SRS016629||gene_28659|GeneMark.hmm|421_aa|-|603|1868
MQEAKPFQIDKRIIFEAFKKVKSNGGSPGIDGIEMSAYEQNLGSNFYRLWNRMSSGSYMPKAVKLVEILKSNGG
KRPLGIPSVEDRIAQMAVVNVIEPLVEPYFHKDSFGYRPHRSAHDAIAKAERRCWKYAWVLDIDISKFFDTIDHG
LLMKAVEKHIKTKWILLYIKRWLTVPYQGNDGAIVKRHMGVPQGSVIGPILANQFLHYTFDKWMSYKYPHVPF
ERYADDCVCHCGTLAQAEYIKDRLGERFAECKLTFNEEKTKIVFCKTSNRSSEHYHCTSFDYLGFTFRPRAAKDKR
KNVLFTSYLPAISNKSESRIHETIKSWNLKRLHNRSLRFVAAYINDVVRGWISYYGKFGKTEFWKVMCHLNRSIAY
WAKTKYKRLRRRGVISAHYWLAYIAQKEPNLFYHWQVGYIPYARQKK
>SRS016989||gene_29156|GeneMark.hmm|430_aa|+|10346|11638
MQNDNAKPISISKQLVYDAFLRVKANRGSAGIDKVTLEDYEKNLRGNLYKLWNRMSSGSYFPPSVKLVEIPKSTG

GKRPLGIPTVSDRVAQMAIVMLITPSIEPCFHEDSYAYRPHRSAHDAVGKARERCWKYAWVLDMDISKFFDTID
HELLLKALKRHTQEKWVLMYIERWLKVPYEKADGSQVDRALGVPQGSVIGPVLANLFLHYTFDKWMEKSFPR
VPFERYADDTICHCHSLKQAEYMQAMIQQRFECCRLRLNEEKTKIVYCKSSRQKEFYPNVTFDFLGFTFQPRESV
DKYGNRFTGFLPAISRKSMKRINETIRSWHLNRHSNLTLEHLASDINPIVRGWMTYYGKFYPTRLKWFMQTLN
GRLARWVMCKFERYRHRFYPAQEWLARIAEKEGLIFYHWKCGVLPRFTNKEKVSSQLIMVK
>SRS017103||gene_267912|GeneMark.hmm|420_aa|-|324|1586
MNEAKPFVIDKRLVWEAYHKVKENKGSAGIDKVDQKTFDKEMSKNLYKIWNRMSSGCYFPKAVKLVEIPKSNG
GTRPLGIPTIEDRIAQQVVVSVLTPILEPIFKEDSYGYRPGKGAHQAIAKAKERCYVNPWVLDMDISKFFDTINHD
LLMKAVRKHTEEKWVLLYIERWLKVPYQTSKGEVIERTMGVPQGSVIGPVLANLFLHYVFDEWMSRNYPTIPFE
RYADDTICHCVSEKQAQFLKAVLMKRFEECGLKLNEEKTKIVYCKDSNRRGDSEHTSFDFLGFTFRPRSARNRKT
GQNFTAFLPAISKKSLKRIKEAVRAWKLNRKTFACLLDISNEVDTQISGWMNYYMKFGRSEFRKVLNYINERLTR
WVMRKYKRFSKGKKFSRAYEWLVEYAVHNRNEFSHWAKGFVPYPRLG
>SRS017191||gene_180269|GeneMark.hmm|418_aa|+|855|2111
MSEAKQFDISKKAVIAAFQAVKENAGSYGADEQTIKEFEEHLNNNLYKLWNRMASGSYFPKPVRAVAIPKKNG
GIRILGIPTVEDRIAQMVAKMYFEPLVEPMFYNDSYGYRPNKSAIQAVGQARERCFKRDWVLELDIKGLFDNIK
HGYLMYMVEKHTQIKWLILYIKRWLTVPFIMSDGSVAERRSGTPQGGVISPVLANLFLHYVFDDFMTKAYPNI
WWERYADDGVLHCQSYKQAAFIKQKLEERFQQFGLELNKEKTRIVYCKDNRRPQNYSCTQFTFLGYTFRPRLN
KNKEGKFFVGFTPAVSEKAKTAMKQKIREWKIQLKADLSLKDIGNMINKVVQGWINYYTHYYKSEFYEVLRYIN
QCLIKWVRRSYKKKNTRSRAEHWLGAVARRDRNLFAHWKFGILPSVGEGAV
>SRS017307||gene_80536|GeneMark.hmm|420_aa|-|487|1749
MNEAKPFVIDKRLVWEAYHKVKENKGSAGIDKVDQKTFDKEMSKNLYKIWNRMSSGCYFPKAVKLVEIPKSNG
GTRPLGIPTIEDRIAQQVVVSVLTPILEPIFKEDSYGYRPGKGAHQAIAKAKERCYVNPWVLDMDISKFFDTINHD
LLMKAVRKHTEEKWVLLYIERWLKVPYQTSKGEVIERTMGVPQGSVIGPVLANLFLHYVFDEWMSRNYPTIPFE
RYADDTICHCVSEKQAQFLKAVLIKRFEECGLKLNEEKTKIVYCKDSNRRGDSEHTSFDFLGFTFRPRSARNRKTG
QNFTAFLPAISKKSLKRIKEAVRAWKLNRKTFACLLDISNEVDTQISGWMNYYMKFGRSEFRKVLNYINERLTRW
VMRKYKRFSKGKKFSRAYEWLVEYAVHNRNEFSHWAKGFVPYPRLG
>SRS017521||gene_135177|GeneMark.hmm|418_aa|-|10445|11701
MSEAKQFDISKKAVIAAFQAVKENAGSYGADEQTIKEFEEHLNNNLYKLWNRMASGSYFPKPVRAVAIPKKNG
GIRILGIPTVEDRIAQMVAKMYFEPLVEPMFYNDSYGYRPNKSAIQAVGQARERCFKRDWVLELDIKGLFDNIK
HGYLMYMVEKHTQIKWLILYIKRWLTVPFIMSDGSVAERRSGTPQGGVISPVLANLFLHYVFDDFMTKAYPNI
WWERYADDGVLHCQSYKQAAFIKQKLEERFQQFGLELNKEKTRIVYCKDNRRPQNYSCTQFTFLGYTFRPRLN
KNKEGKFFVGFTPAVSEKAKTAMKQKIREWKIQLKADLSLKDIGNMINKVVQGWINYYTHYYKSEFYEVLRYIN
QCLIKWVRRSYKKKNTRSRAEHWLGAVARRDRNLFAHWKFGILPSVGEGAV
>SRS017521||gene_76669|GeneMark.hmm|430_aa|+|30719|32011
MQNDNAKPISISKQLVYDAFLRVKANRGSAGIDKVTLEDYEKNLRGNLYKLWNRMSSGSYFPPSVKLVEIPKSTG
GKRPLGIPTVSDRVAQMAIVMLITPSIEPCFHEDSYAYRPHRSAHDAVGKARERCWKYAWVLDMDISKFFDTID
HELLLKALKRHTQEKWVLMYIERWLKVPYEKADGSQVDRALGVPQGSVIGPVLANLFLHYTFDKWMEKSFPR
VPFERYADDTICHCHSLKQAEYMQAMIQQRFECCRLRLNEEKTKIVYCKSSRQKEFYPNVTFDFLGFTFQPRESV
DKYGNRFTGFLPAISRKSMKRINETIRSWHLNRHSNLTLEHLASDINPIVRGWMTYYGKFYPTRLKWFMQTLN
GRLARWVMCKFERYRHRFYPAQEWLARIAEKEGLIFYHWKCGVLPRFTNKEKVSSQLIMVK
>SRS018541||gene_23703|GeneMark.hmm|430_aa|-|4258|5550
MQNDNAKPISISKQLVYDAFLRVKANRGSAGIDKVTLEDYEKNLRGNLYKLWNRMSSGSYFPPSVKLVEIPKSTG
GKRPLGIPTVSDRVAQMTVVMLITPSIEPCFHEDSYAYRPHRSAHDAVGKARERCWKYAWVLDMDISKFFDTID
HELLLKALKRHTQEKWVLMYIERWLKVPYEKSDGSQVDRALGVPQGSVIGPVLANLFLHYTFDKWMEKNFPR

VPFERYADDTICHCHSLKQAEYMQAMIQQRFECCRLRLNEEKTKIVYCKSSRQKECYPNVTFDFLGFTFQPRESV
DKYGNRFTGFLPAISRKSMKRINETMRSWHLNRHSNLTLEHLASDINPIVRGWMTYYGKFYPTRLKWFMQTLN
GRLASWVMCKFERYRHRFYPAQEWLARIAEKEGLIFYHWKCGVLPRFTNKEKVSSQLIMVK
>SRS018623||gene_32615|GeneMark.hmm|418_aa|-|162|1418
MSEAKQFDISKKAVIAAFQAVKENAGSYGADEQTIKEFEEHLNNNLYKLWNRMASGSYFPKPVRAVAIPKKNG
GIRILGIPTVEDRIAQMVAKMYFEPLVEPMFYNDSYGYRPNKSAIQAVGQARERCFKRDWVLELDIKGLFDNIK
HGYLMYMVEKHTQIKWLILYIKRWLTVPFIMSDGSVAERRSGTPQGGVISPVLANLFLHYVFDDFMTKAYPNI
WWERYADDGVLHCQSYKQAAFIKQKLEERFQQFGLELNKEKTRIVYCKDNRRPQNYSCTQFTFLGYTFRPRLN
KNKEGKFFVGFTPAVSEKAKTAMKQKIREWKIQLKADLSFKDIGNMINKVVQGWINYYTHYYKSEFYEVLRYIN
QCLIKWVRRSYKKKNTRSRAEHWLGAVARRDRNLFAHWKFGILPSVGEGAV
>SRS018817||gene_49699|GeneMark.hmm|430_aa|+|2876|4168
MQNDNAKPISISKQLVYDAFLRVKANRGSAGIDKVTLEDYEKNLRGNLYKLWNRMSSGSYFPPSVKLVEIPKSTG
GKRPLGIPTVSDRVAQMAVVMLITPSIEPCFHEDSYAYRPHRSAHDAVGKARERCWKYAWVLDMDISKFFDTI
DHELLLKALKRHTQEKWVLMYIERWLKVPYEKSDGSQVDRALGVPQGSVIGPVLANLFLHYTFDKWMEKNFP
RVPFERYADDTICHCHSLKQAEYMQAMIQQRFECCRLRLNEEKTKIVYCKSSRQKECYPNVTFDFLGFTFQPRES
VDKYGNRFTGFLPAISRKSMKRINETMRSWHLNRHSNLTLEHLASDINPIVRGWMTYYGKFYPTRLKWFMQTL
NGRLARWVMCKFERYRHRFYPAQEWLARIAEKEGLIFYHWKCGVLPRFTNKEKVSSQLIMVK
>SRS019068||gene_284738|GeneMark.hmm|418_aa|+|1356|2612
MSEAKQFDISKKAVIAAFQAVKENAGSYGADEQTIKEFEEHLNNNLYKLWNRMASGSYFPKPVRAVAIPKKNG
GIRILGIPTVEDRIAQMVAKMYFEPLVEPMFYNDSYGYRPNKSAIQAVGQARERCFKRDWVLELDIKGLFDNIK
HGYLMYMVEKHTQIKWLILYIKRWLTVPFIMSDGSVAERRSGTPQGGVISPVLANLFLHYVFDDFMTKAYPNI
WWERYADDGVLHCQSYKQAAFIKQKLEERFQQFGLELNKEKTRIVYCKDNRRPQNYSCTQFTFLGYTFRPRLN
KNKEGKFFVGFTPAVSEKAKTAMKQKIREWKIQLKADLSLKDIGNMINKVVQGWINYYTHYYKSEFYEVLRYIN
QCLIKWVRRSYKKKNTRSRAEHWLGAVARRDRNLFAHWKFGILPSVGEGAV
>SRS019161||gene_99911|GeneMark.hmm|430_aa|-|319|1611
MQNDNAKPISISKQLVYDAFLRVKANRGSAGIDKVTLEDYEKNLRGNLYKLWNRMSSGSYFPPSVKLVEIPKSTG
GKRPLGIPTVSDRVAQMAIVMLITPSIEPCFHEDSYAYRPHRSAHDAVGKARERCWKYAWVLDMDISKFFDTID
HELLLKALKRHTQEKWVLMYIERWLKVPYEKADGSQVDRALGVPQGSVIGPVLANLFLHYTFDKWMEKSFPR
VPFERYADDTICHCHSLKQAEYMQAMIQQRFECCRLRLNEEKTKIVYCKSSRQKEFYPNVTFDFLGFTFQPRESV
DKYGNRFTGFLPAISRKSMKRINETIRSWHLNRHSNLTLEHLASDINPIVRGWMTYYGKFYPTRLKWFMQTLN
GRLARWVMCKFERYRHRFYPAQEWLARIAEKEGLIFYHWKCGVLPRFTNKEKVSSQLIMVK
>SRS019286||gene_33505|GeneMark.hmm|430_aa|+|23470|24762
MQNDNAKPISISKQLVYDAFLRVKANRGSAGIDKVTLEDYEKNLRGNLYKLWNRMSSGSYFPPSVKLVEIPKSTG
GKRPLGIPTVSDRVAQMAVVMLITPSIEPCFHEDSYAYRPHRSAHDAVGKARERCWKYAWVLDMDISKFFDTI
DHELLLKALKRHTQEKWVLMYIERWLKVPYEKSDGSQVDRALGVPQGSVIGPVLANLFLHYTFDKWMEKNFP
RVPFERYADDTICHCHSLKQAEYMQAMIQQRFECCRLRLNEEKTKIVYCKSSRQKECYPNVTFDFLGFTFQPRES
VDKYGNRFTGFLPAISRKSMKRINETMRSWHLNRHSNLTLEHLASDINPIVRGWMTYYGKFYPTRLKWFMQTL
NGRLARWVMCKFERYRHRFYPAQEWLARIAEKEGLIFYHWKCGALPRFTNKEKVSSQLIMVK
>SRS019496||gene_185389|GeneMark.hmm|423_aa|+|6590|7861
MQEAKPKPFQIDKRIIFESFKKVKFNRGSSGIDGIEMTTYEQNLGSNLYRLWNRMSSGSYMPKAVKLVEIPKSN
GGKRPLGIPTIEDRIAQMAVVNVIEPLIEPCFHEDSFGYRPHRSAHDAIAKAERRCWKYAWVLDIDISKFFDTIDH
GLLMKAVEKHINIKWILLYIKRWLTVPYQRSDGEIVKRDMGVPQGSVIGPILANLFLHYTFDKWMSYKYPHIPFE
RYADDCVCHCSTLAQAEYIKERLGERFTECKLKFNEEKTKIVFCKMSSRSSKHYHCTSFDYLGFTFRSRAAKDKRN
NVLFTSYLPAISKKSVSRIHETIKSWNLKRLHNRSLRFVAAYINDVVRGWINYYEKFGKTEFWKVMCHLNRSIAY

WAKTKYKRLRRRGVISAHYWLAYIAQKEPNLFYHWQVGYVPYARQKK
>SRS019601||gene_69072|GeneMark.hmm|430_aa|-|213|1505
MQNDNAKPISISKQLVYDAFLRVKANRGSAGIDKVTLEDYEKNLRGNLYKLWNRMSSGSYFPPSVKLVEIPKSTG
GKRPLGIPTVSDRVAQMAVVMLITPSIEPCFHEDSYAYRPHRSAHDAVGKARERCWKYAWVLDMDISKFFDTI
DHELLLKALKRHTQEKWVLMYIERWLKVPYEKSDGSQVDRALGVPQGSVIGPVLANLFLHYTFDKWMEKNFP
RVPFERYADDTICHCHSLKQTEYMQAMIQQRFECCRLRLNEEKTKIVYCKSSRQKECYPNVTFDFLGFTFQPRES
VDKYGNRFTGFLPAISRKSMKRINETMRSWHLNRHSNLTLEHLASDINPIVRGWMTYYGKFYPTRLKWFMQTL
NGRLARWVMCKFERYRHRFYPAQEWLARIAEKEGLIFYHWKCGALPRFTNKEKVSSQLIMVK
>SRS019638||gene_81493|GeneMark.hmm|430_aa|-|2|1294
MQNDNAKPISISKQLVYDAFLRVKANRGSAGIDKVTLEDYEKNLRGNLYKLWNRMSSGSYFPPSVKLVEIPKSTG
GKRPLGIPTVSDRVAQMAVVMLITPSIEPCFHEDSYAYRPHRSAHDAVGKARERCWKYAWVLDMDISKFFDTI
DHELLLKALKRHTQEKWVLMYIERWLKVPYEKSDGSQVDRALGVPQGSVIGPVLANLFLHYTFDKWMEKNFP
RVPFERYADDTICHCHSLKQTEYMQAMIQQRFECCRLRLNEEKTKIVYCKSSRQKECYPNVTFDFLGFTFQPRES
VDKYGNRFTGFLPAISRKSMKRINETMRSWHLNRHSNLTLEHLASDINPIVRGWMTYYGKFYPTRLKWFMQTL
NGRLARWVMCKFERYRHRFYPAQEWLARIAEKEGLIFYHWKCGALPRFTNKEKVSSQLIMVK
>SRS020508||gene_16249|GeneMark.hmm|422_aa|-|3069|4337
MMQHQVTKPFTIDKHLIMNAWKRVKENKGSVGIDNVSTDDYESNLGKNLYKLWNRMSSGSYFPEAVKLVDIP
KSSGGTRPLGIPTVGDRIAQMSVVLLIEDRLEAIFHADSYGYRPNRSAHDAIGKARERCWHYNWVLDMDISKFF
DTINHDLLMKAVERHVQEKWILLYIRRWLEVPYATLTGERIERRMGVPQGSVIGPVLANLYLHYTFDKWMSLYH
PTIPFERYADDTICHCNSLEEAQMLKASIVERFAACKLKLNEEKTRIVYCKDGKRRREYKDITFDFLGYTFQPRGQR
NKQGQVFNGYAPAISRKSKKRIAETMRGWHLNRRVQLKLSDIAVEINAEVRGWMNYYGKFYGSQLKAFLQCIN
LKLARWAERKYKRFRRKPNDAYKWLVRVASKNPALFYHWQHGVKPNRLKPFG
>SRS020622||gene_8844|GeneMark.hmm|431_aa|+|515|1810
MQNGNAKPISISKQLVYDAFLRVKANRGSAGIDKVTLEDYEKNLRGNLYKLWNRMSSGSYFPPSVKLVEIPKSTG
GKRPLGIPTVSDRVAQMAIVMLITPSIEPCFHEDSYAYRPHRSAHDAVGKARERCWKYAWVLDMDISKFFDTID
HELLLKALKRHTQEKWVLMYIERWLKVPYEKADGSQVDRALGVPQGSVIGPVLANLFLHYTFDKWMEKSFPR
VPFERYADDTICHCHSLKQAEYMQAMIQQRFECCRLRLNEEKTKIVYCKSSRQKGRYPNVTFDFLGFTFQPRES
VDKYGSRFTGFLPAISRKSMKRINETIRSWHLNRHSNLTLEHLASDINPIVRGWMTYYGKFYPTRLKWWFMQT
LNGRLARWIMCKFERYRHRFYPAQEWLARIAEKEGLIFYHWKCGVLPRFTNKEKVSSQLIMVK
>SRS020869||gene_53740|GeneMark.hmm|421_aa|+|641|1906
MQEAKPFQIDKRIIFESFKKVKFNRGSSGIDGIEMTTYEQNLGSNLYRLWNRMSSGSYMPKAVKLVEIPKSNGG
KRPLGIPTIEDRIAQMAVVNVIEPLIEPCFHEDSFGYRPHRSAHDAIAKAERRCWKYAWVLDIDISKFFDTIDHGL
LMKAVEKHINIKWILLYIKRWLTVPYQRSDGEIVKRDMGVPQGSVIGPILANLFLHYTFDKWMSYKYPHIPFERY
ADDCVCHCSTLAQAEYIKERLGERFTECKLKFNEEKTKIVFCKMSSRSSKHYHCTSFDYLGFTFRSRAAKDKRNN
VLFTSYLPAISKKSVSRIHETIKSWNLKRLHNRSLRFVAAYINDVVRGWINYYEKFGKTEFWKVMCHLNRSIAYW
AKTKYKRLRRRGVISAHYWLAYIAQKEPNLFYHWQVGYVPYARQKK
>SRS021153||gene_29082|GeneMark.hmm|422_aa|-|502|1770
MMQHQVTKPFTIDKYLIMNAWKRVKENKGSAGIDNVSTEDYESNLGKNLYKLWNRMSSGSYFPEAVKLVDIP
KPSGGTRPLGIPTVGDRIAQMSVVLLIEERLEAIFHADSYGYRPNRSAHDAIEKARERCWHYNWVLDMDISKFF
DTIDHDLLMKAVERHVQEKWILLYIRRWLKVPYATLTGERIERKMGVPQGSVIGPVLANLYLHYTFDKWMSLYH
PTIPFERYADDTICHCNSLKEAQMLKASIVERFAACKLRLNEEKTRIVYCKDGKRRGEYKEITFDFLGYTFQPRGQ
RNKQGQVFNGYAPAISRKSKKRITEKMRGWHLNRRVQLKLSDIAVEINAEVRGWMNYYGKFYGSQLKAxLQCI
NLKLARWAERKYKRFRRKPNDAYKWLVRVASKNPALFYHWQHGVKPNRLKPFG
>SRS021219||gene_17753|GeneMark.hmm|420_aa|-|1126|2388

MNEAKPFVIDKRLVWEAYHKVKENKGSAGIDKVDQKTFDKEMSKNLYKIWNRMSSGCYFPKAAKLVEIPKSNG
GTRPLGIPTIEDRIAQQVVVSVLTPILEPIFKEDxYGYRPGKGAHQAIAKAKERCYVNPWVLDMDISKFFDTINHE
LLMKAVRKHTEEKWVLLYIERWLKVPYQTSKGEVIERTMGVPQGSVIGPVLANLFLHYVFDEWMSRNYPTIPFE
RYADDTICHCVSEKQAQFLKAVLMKRFEECGLKLNEEKTKIVYCKDSNRRGDSEHTSFDFLGFTFRPRSARNRKT
GQNFTAFLPAISKKSMKRIKEAVRAWKLNHKTFACLLDISNEVDTQISGWMNYYMKFGRSEFRKVLNYINERLT
RWVMRKYKRFSKGRKFGRAYDWLVEYAAYNRNEFSHWVKGYVPYPRLG
>SRS021219||gene_30322|GeneMark.hmm|422_aa|+|699|1967
MMQHQVTKPFTIDKYLIMNAWKRVKENKGSAGIDNVSTEDYESNLGKNLYKLWNRMSSGSYFPEAVKLVDIP
KPSGGTRPLGIPTVGDRIAQMSVVLLIEERLEAIFHADSYGYRPNRSAHDAIEKARERCWHYNWVLDMDISKFF
DTIDHDLLMKAVERHVQEKWILLYIRRWLKVPYATLTGERIERKMGVPQGSVIGPVLANLYLHYTFDKWMSLYH
PTIPFERYADDTICHCNSLKEAQMLKASIVERFAACKLRLNEEKTRIVYCKDGKRRGEYKEITFDFLGYTFQPRGQ
RNKQGQVFNGYAPAISRKSKKRITEKMRGWHLNRRVQLKLSDIAVEINAEVRGWMNYYGKFYGSQLKAFLQCI
NLKLARWAERKYKRFRRKPNDAYKWLVRVASKNPALFYHWQHGVKPNRLKPFG
>SRS021484||gene_131020|GeneMark.hmm|430_aa|-|1145|2437
MQNDNAKPISISKQLVYDAFLRVKANRGSAGIDKVTLEDYEKNLRGNLYKLWNRMSSGSYFPPSVKLVEIPKSTG
GKRPLGIPTVSDRVAQMAVVMLITPSIEPCFHEDSYAYRPHRSAHDAVGKARERCWKYAWVLDMDISKFFDTI
DHELLLKALKRHTQEKWVLMYIERWLKVPYEKSDGSQVDRALGVPQGSVIGPVLANLFLHYTFDKWMEKNFP
RVPFERYADDTICHCHSLKQAEYMQAMIQQRFECCRLRLNEEKTKIVYCKSSRQKECYPNVTFDFLGFTFQPRES
VDKYGNRFTGFLPAISRKSMKRINETMRSWHLNRHSNLTLEHLASDINPIVRGWMTYYGKFYPTRLKWFMQTL
NGRLARWVMCKFERYRHRFYPAQEWLARIAEKEGLIFYHWKCGALPRFTNKEKVSSQLIMVK
>SRS021484||gene_101472|GeneMark.hmm|414_aa|+|1834|3078
MIETKPYEISKWAVYIAYERVKANKGSYGVDEQSIEDFEKNLKNNLYKIWNRMSSGSYFPQPVKAVSVPKKNGGI
RVLGIPTVEDRIAQMTAKLYFEPCVEPLFLEDSYGYRPGKSAIQALSVTRKRCWHRDWVLEYDIKGLFDNIRHDY
LLEMVRRHTPHKWILLYVERWLTTPFQLEDGTLQSRTSGTPQGGVISLVLANLFLHYAFDSFMAKEYPKAWWE
RYADDGVLHCKSSQQAMYMKSVLRERFRLFGLELNEEKTRIVYCKDADRTEDYSEISLDSLGYTFRPRLARNKHG
NIFLNFLPAMSAKAIKAMKEEVRRWKLQLKVSKSLTDLANILNSQIQGWISYYGHFYKSELIYLLRYINQCLIKWV
RRKYKKFNHRRRAEYWLGRIARRDNNLFAHWRYGVLPTAG
>SRS021948||gene_257442|GeneMark.hmm|430_aa|-|159|1451
MQNDNAKPISISKQLVYDAFLRVKANRGSAGIDKVTLEDYEKNLRGNLYKLWNRMSSGSYFPPSVKLVEIPKSTG
GKRPLGIPTVSDRVAQMAVVMLITPSIEPCFHEDSYAYRPHRSAHDAVGKARERCWKYAWVLDMDISKFFDTI
DHELLLKALKRHTQEKWVLMYIERWLKVPYEKSDGSQVDRALGVPQGSVIGPVLANLFLHYTFDKWMEKNFP
RVPFERYADDTICHCHSLKQTEYMQAMIQQRFECCRLRLNEEKTKIVYCKSSRQKECYPNVTFDFLGFTFQPRES
VDKYGNRFTGFLPAISRKSMKRINETMRSWHLNRHSNLTLEHLASDINPIVRGWMTYYGKFYPTRLKWFMQTL
NGRLARWVMCKFERYRHRFYPAQEWLARIAEKEGLIFYHWKCGALPRFTNKEKVSSQLIMVK
>SRS022137||gene_109883|GeneMark.hmm|418_aa|-|2022|3278
MSEAKQFDISKKAVIAAFQAVKENAGSYGADEQTIKEFEEHLNNNLYKLWNRMASGSYFPKPVRAVAIPKKNG
GIRILGIPTVEDRIAQMVAKMYFEPLVEPMFYNDSYGYRPNKSAIQAVGQARERCFKRDWVLELDIKGLFDNIK
HGYLMYMVEKHTQIKWLILYIKRWLTVPFIMSDGSVAERRSGTPQGGVISPVLANLFLHYVFDDFMTKAYPNI
WWERYADDGVLHCQSYKQAAFIKQKLEERFQQFGLELNKEKTRIVYCKDNRRPQNYSCTQFTFLGYTFRPRLN
KNKEGKFFVGFTPAVSEKAKTAMKQRIREWKIQLKADLSLKDIGNMINKVVQGWINYYTHYYKSEFYEVLRYIN
QCLIKWVRRSYKKKNTRSRAEHWLGAVARRDRNLFAHWKFGILPSVGEGAV
>SRS022609||gene_190274|GeneMark.hmm|420_aa|+|1058|2320
MNEAKPFVIDKRLVWEAYHKVKENKGSAGIDKVDQKTFDKEMSKNLYKIWNRMSSGCYFPKAVKLVEIPKSNG
GTRPLGIPSIEDRIAQQVVVSVLTPILEPIFKEDSYGYRPGKGAHQAIAKAKERCYVNPWVLDMDISKFFDTINHE

LLMKAVRKHTEEKWILLYIERWLKVPYQTSKGEVIERTMGVPQGSVIGPVLANLFLHYVFDEWMSRNYPTIPFE
RYADDTICHCVSEKQAQFLKAVLMKRFEECGLKLNEEKTKIVYCKDSNRRGDSEHTSFDFLGFTFRPRGARNRKT
GQNFTAFLPAISKKSMKRIKEAVRAWKLNRKTFACLLDISNEVDTQISGWMNYYMKFGRSEFRKVLNYINERLT
RWVMRKYKRFSKGRKFNRAYDWLVEYAAHNRNEFSHWVKGFVPYPRLG
>SRS022713||gene_48129|GeneMark.hmm|421_aa|-|5266|6531
MQEAKPFQIDKRIIFEAFKKVKSNGGSPGIDGIEMSAYEQNLGSNFYRLWNRMSSGSYMPKAVKLVEILKSNGG
KRPLGIPSVEDRIAQMAVVNVIEPLVEPYFHKDSFGYRPHRSAHDAIAKAERRCWKYAWVLDIDISKFFDTIDHG
LLMKAVEKHIKTKWILLYIKRWLTVPYQGNDGAIVKRHMGVPQGSVIGPILANQFLHYTFDKWMSYKYPHVPF
ERYADDCVCHCGTLAQAEYIKDRLGERFAECKLTFNEEKTKIVFCKTSNRSSEHYHCTSFDYLGFTFRPRAAKDKR
KNVLFTSYLPAISNKSESRIHETIKSWNLKRLHNRSLRFVAAYINDVVRGWISYYGKFGKTEFWKVMCHLNRSIAY
WAKTKYKRLRRRGVISAHYWLAYIAQKEPNLFYHWQVGYIPYARQKK
>SRS022713||gene_75571|GeneMark.hmm|422_aa|+|880|2148
MKDAKSFEISRHLVMEAYKRVKANKGAAGVDDVSIADFESNLKSNLYKIWNRMSSGSYFPPAVKLVEIPKSNGG
KRPLGIPTIGDRVAQMVVVMTIEPGIEPYFHEDSYAYRPNRSALDAVRKAKERSYTFHWVLDLDIKGFFDNIDHG
LLIKALERHVKCEWAMLYIKRWLSVPYQLKDGTQIERTKGVPQGSVVGPILANLFLHYVFDEWMRRNHSNISFE
RYADDTICHCVSLKQAEFILRAIRKRFAECKLELNEDKTKIVYCKKNHRDIPYECIQFDFLGYTFRPRRSIDANGEVF
LNFSPAISKKARTKIWEAIQNWNSNHWVPMELEDIAKEINPVIQGWINYYGQHNPRILKEVLQHVNDRLVRW
GRRKFKGLRKRKTATVHRLGDIALQKPNLFAHWAWGVKPTASEKNRKRK
>SRS023526||gene_100417|GeneMark.hmm|418_aa|-|237|1493
MSEAKQFDISKKAVIAAFQAVKENAGSYGVDEQTIKEFEEHLNNNLYKLWNRMASGSYFPKPVRAVAIPKKNG
GIRILGIPTVEDRIAQMVAKMYFEPLVEPMFYNDSYGYRPNKSAIQAVGQARERCFKRDWVLELDIKGLFDNIK
HGYLMYMVEKHTQIKWLILYIKRWLTVPFIMSDGSVAERRSGTPQGGVISPVLANLFLHYVFDDFMTKAYPNI
WWERYADDGVLHCQSYKQAVFIKQKLEERFQQFGLELNKEKTRIVYCKDNRRSQNYSCTQFTFLGYTFRPRLNK
NKEGKFFVGFTPAVSEKAKTAMKQKIREWKIQLKADLSLKDIGNMINKVVQGWINYYTHYYKSEFYEVLRYINQ
CLIKWVRRSYKKKNTRSRAEHWLGAVARRDRNLFAHWKFGILPSVGEGAV
>SRS023526||gene_30216|GeneMark.hmm|422_aa|+|1931|3199
MKDAKSFEISRHLVMEAYKRVKANKGAAGVDDVSIADFESNLKSNLYKIWNRMSSGSYFPPAVKLVEIPKSNGG
KRPLGIPTIGDRVAQMVVVMTIEPGIEPYFHEDSYAYRPNRSALDAVRKAKERSYTFHWVLDLDIKGFFDNIDHG
LLIKALERHVKCEWAMLYIKRWLSVPYQLKDGTQIERTKGVPQGSVVGPILANLFLHYVFDEWMRRNHSNISFE
RYADDTICHCVSLKQAEFILRAIRKRFAECKLELNEDKTKIVYCKKNHRDIPYECIQFDFLGYTFRPRRSIDANGEVF
LNFSPAISKKARTKIWEAIQNWNSNHWVPMELEDIAKEINPVIQGWINYYGQHNPRILKEVLQHVNDRLVRW
GRRKFKGLRKRKTATVHRLGDIALQKPNLFAHWAWGVKPTASEKNRKRK
>SRS024009||gene_37018|GeneMark.hmm|421_aa|-|2125|3390
MQEAKPFQIDKRIIFESFKKVKFNRGSSGIDGIEMTTYEQNLGSNLYRLWNRMSSGSYMPKAVKLVEIPKSNGG
KRPLGIPTIEDRIAQMAVVNVIEPLIEPCFHEDSFGYRPHRSAHDAIAKAERRCWKYAWVLDIDISKFFDTIDHGL
LMKAVEKHINIKWILLYIKRWLTVPYQRSDGEIVKRDMGVPQGSVIGPILANLFLHYTFDKWMSYKYPHIPFERY
ADDCVCHCSTLAQAEYIKERLGERFTECKLKFNEEKTKIVFCKMSSRSSKHYHCTSFDYLGFTFRSRAAKDKRNN
VLFTSYLPAISKKSVSRIHETIKSWNLKRLHNRSLRFVAAYINDVVRGWINYYEKFGKTEFWKVMCHLNRSIAYW
AKTKYKRLRRRGVISAHYWLAYIAQKEPNLFYHWQVGYVPYARQKK
>SRS024331||gene_242407|GeneMark.hmm|420_aa|-|196|1458
MNEAKPFVIDKRLVWEAYHKVKENKGSAGIDKVDQKTFDKEMSKNLYKIWNRMSSGCYFPKAVKLVEIPKSNG
GTRPLGIPTIEDRIAQQVVVSVLTPILEPIFKEDSYGYRPGKGAHQAIAKAKERCYVNPWVLDMDISKFFDTINHD
LLMKAVRKHTEEKWVLLYIERWLKVPYQTSKGEVIERTMGVPQGSVIGPVLANLFLHYVFDEWMSRNYPTIPFE
RYADDTICHCVSEKQAQFLKAVLMKRFEECGLKLNEEKTKIVYCKDSNRRGDSEHTSFDFLGFTFRPRSARNRKT

GQNFTAFLPAISKKSLKRIKEAVRAWKLNRKTFACLLDISNEVDTQISGWMNYYMKFGRSEFRKVLNYINERLTR
WVMRKYKRFSKGKKFSRAYEWLVEYAVHNRNEFSHWAKGFVPYPRLG
>SRS024388||gene_446|GeneMark.hmm|430_aa|-|14762|16054
MQNGNAKPISISKQLVYDAFLRVKANRGSAGIDKVTLEDYEKNLRGNLYKLWNRMSSGSYFPPSVKLVEIPKSTG
GKRPLGIPTVSDRVAQMAIVMLITPSIEPCFHEDSYAYRPHRSAHDAVGKARERCWKYAWVLDMDISKFFDTID
HELLLKALKRHTQEKWVLMYIERWLKVPYEKADGSQVDRALGVPQGSVIGPVLANLFLHYTFDKWMEKSFPR
VPFERYADDTICHCHSLKQAEYMQAMIQQRFECCRLRLNEEKTKIVYCKSSRQKGRYPNVTFDFLGFTFQPRES
VDKYGSRFTGFLPAISRKSMKRINETIRSWHLNRHSNLTLEHLASDINPIVRGWMTYYGKFYPTRLKWFMQTLN
GRLARWIMCKFERYRHRFYPAQEWLARIAEKEGLIFYHWKCGVLPRFTNKEKVSSQLIMVK
>SRS024435||gene_69676|GeneMark.hmm|422_aa|+|544|1812
MKDAKSFEISRHLVMEAYKRVKANKGAAGVDEVSIADFENNLKSNLYKIWNRMSSGSYLPPAVKLVEIPKSNGG
KRPLGIPTVGDRVAQMVVVMTIEPGIEPYFHEDSYAYRPNRSALDAVRKAKERSYTFHWVLDLDIKGFFDNIDH
ELLIKALERHVKCKWAILYIKRWLSVPYQLKDGTQKERTKGVPQGSVVGPILANLFLHYVFDEWMRRNHSNISFE
RYADDTICHCVSLKQAEFILRAIRKRFAECKLELNEDKTKIVYCKKNHRDIPYECIQFDFLGYTFRPRRSIDANGEVF
LNFSPAISKKARTKIWEAIQNWNSNHWVPMELEDIAKEINPVIQGWINYYGQHNPRILKEVLQHVNDRLVRW
GRRKFKGLRKRKTATVHRLGDIALQKPNLFAHWAWGVKPTASERNRKRK
>SRS042231||gene_107567|GeneMark.hmm|430_aa|+|4232|5524
MQNDNAKPISISKQLVYDAFLRVKANRGSAGIDKVTLEDYEKNLRGNLYKLWNRMSSGSYFPPSVKLVEIPKSTG
GKRPLGIPTVSDRVAQMAVVMLITPSIEPCFHEDSYAYRPHRSAHDAVGKARERCWKYAWVLDMDISKFFDTI
DHELLLKALKRHTQEKWVLMYIERWLKVPYEKSDGSQVDRALGVPQGSVIGPVLANLFLHYTFDKWMEKNFP
RVPFERYADDTICHCHSLKQAEYMQAMIQQRFECCRLRLNEEKTKIVYCKSSRQKECYPNVTFDFLGFTFQPRES
VDKYGNRFTGFLPAISRKSMKRINETMRSWHLNRHSNLTLEHLASDINPIVRGWMTYYGKFYPTRLKWFMQTL
NGRLARWVMCKFERYRHRFYPAQEWLARIAEKEGLIFYHWKCGALPRFTNKEKVSSQLIMVK
>SRS042690||gene_59474|GeneMark.hmm|430_aa|-|777|2069
MQNDNAKPISISKQLVYDAFLRVKANRGSAGIDKVTLEDYEKNLRGNLYKLWNRMSSGSYFPPSVKLVEIPKSTG
GKRPLGIPTVSDRVAQMAVVMLITPSIEPCFHEDSYAYRPHRSAHDAVGKARERCWKYAWVLDMDISKFFDTI
DHELLLKALKRHTQEKWVLMYIERWLKVPYEKSDGSQVDRALGVPQGSVIGPVLANLFLHYTFDKWMEKNFP
RVPFERYADDTICHCHSLKQAEYMQAMIQQRFECCRLRLNEEKTKIVYCKSSRQKECYPNVTFDFLGFTFQPRES
VDKYGNRFTGFLPAISRKSMKRINETMRSWHLNRHSNLTLEHLASDINPIVRGWMTYYGKFYPTRLKWFMQTL
NGRLARWVMCKFERYRHRFYPAQEWLARIAEKEGLIFYHWKCGALPRFTNKEKVSSQLIMVK
>SRS043701||gene_19991|GeneMark.hmm|418_aa|-|165|1421
MSEAKQFDISKKAVIAAFQAVKENAGSYGADEQTIKEFEEHLNNNLYKLWNRMASGSYFPKPVRAVAIPKKNG
GIRILGIPTVEDRIAQMVAKMYFEPLVEPMFYNDSYGYRPNKSAIQAVGQARERCFKRDWVLELDIKGLFDNIK
HGYLMYMVEKHTQIKWLILYIKRWLTVPFIMSDGSVAERRSGTPQGGVISPVLANLFLHYVFDDFMTKAYPNI
WWERYADDGVLHCQSYKQAAFIKQKLEERFQQFGLELNKEKTRIVYCKDNRRPQNYSCTQFTFLGYTFRPRLN
KNKEGKFFVGFTPAVSEKAKTAMKQKIREWKIQLKADLSLKDIGNMINKVVQGWINYYTHYYKSEFYEVLRYIN
QCLIKWVRRSYKKKNTRSRAEHWLGAVARRDRNLFAHWKFGILPSVGEGAV
>SRS044535||gene_116224|GeneMark.hmm|430_aa|-|317|1609
MQNDNAKPISISKQLVYDAFLRVKANRGSAGIDKVTLEDYEKNLRGNLYKLWNRMSSGSYFPPSVKLVEIPKSTG
GKRPLGIPTVSDRVAQMAIVMLITPSIEPCFHEDSYAYRPHRSAHDAVGKARERCWKYAWVLDMDISKFFDTID
HELLLKALKRHTQEKWVLMYIERWLKVPYEKADGSQVDRALGVPQGSVIGPVLANLFLHYTFDKWMEKSFPR
VPFERYADDTICHCHSLKQAEYMQAMIQQRFECCRLRLNEEKTKIVYCKSSRQKEFYPNVTFDFLGFTFQPRESV
DKYGNRFTGFLPAISRKSMKRINETIRSWHLNRHSNLTLEHLASDINPIVRGWMTYYGKFYPTRLKWFMQTLN
GRLARWVMCKFERYRHRFYPAQEWLARIAEKEGLIFYHWKCGVLPRFTNKEKVSSQLIMVK

>SRS045004||gene_106215|GeneMark.hmm|421_aa|+|2443|3708
MQEAKPFQIDKRIIFEAFKKVKSNGGSPGIDGIEMSAYEQNLGSNFYRLWNRMSSGSYMPKAVKLVEILKSNGG
KRPLGIPSVEDRIAQMAVVNVIEPLVEPYFHKDSFGYRPHRSAHDAIAKAERRCWKYAWVLDIDISKFFDTIDHG
LLMKAVEKHIKTKWILLYIKRWLTVPYQGNDGAIVKRHMGVPQGSVIGPILANQFLHYTFDKWMSYKYPHVPF
ERYADDCVCHCGTLAQAEYIKDRLGERFAECKLTFNEEKTKIVFCKTSNRSSEHYHCTSFDYLGFTFRPRAAKDKR
KNVLFTSYLPAISNKSESRIHETIKSWNLKRLHNRSLRFVAAYINDVVRGWISYYGKFGKTEFWKVMCHLNRSIAY
WAKTKYKRLRRRGVISAHYWLAYIAQKEPNLFYHWQVGYIPYARQKK
>SRS045645||gene_80254|GeneMark.hmm|421_aa|+|25697|26962
MQEAKPFQIDKRIIFEAFKKVKSNGGSPGIDGIEMSAYEQNLGSNFYRLWNRMSSGSYMPKAVKLVEILKSNGG
KRPLGIPSVEDRIAQMAVVNVIEPLVEPYFHKDSFGYRPHRSAHDAIAKAERRCWKYAWVLDIDISKFFDTIDHG
LLMKAVEKHIKTKWILLYIKRWLTVPYQGNDGAIVKRHMGVPQGSVIGPILANQFLHYTFDKWMSYKYPHVPF
ERYADDCVCHCGTLAQAEYIKDRLGERFAECKLTFNEEKTKIVFCKTSNRSSEHYHCTSFDYLGFTFRPRAAKDKR
KNVLFTSYLPAISNKSESRIHETIKSWNLKRLHNRSLRFVAAYINDVVRGWISYYGKFGKTEFWKVMCHLNRSIAY
WAKTKYKRLRRRGVISAHYWLAYIAQKEPNLFYHWQVGYIPYARQKK
>SRS046502||gene_58174|GeneMark.hmm|420_aa|+|1664|2926
MNEAKPFVIDKRLVWEAYHKVKENKGSAGIDKVDQKTFDKEMSKNLYKIWNRMSSGCYFSKAVKLVEIPKSNG
GTRPLGIPTIEYRIAQQVVVSVLTPILEPIFKEDSYGYRPGKGAHQAIAKAKERCYVTPWVLDMDISKFFDTINHEL
LMKAIRKHTEEKWVLLYIERWLKVPNQTSKGEVIERTMGVPQGSVIGPVLANLFLHYVFDEWMSRNYPTIPFER
YADATICHCVSEKQARFLKAVLMKRFEEYGLKLNEEKTKIVYCKDSNRRGDSEHTSFNFLGFTFRPRGARNRKTG
QNFTAFLPAISNKSMKRIKEAIRAWKLNRKTFACLLDISTEVDTQISGWMNYYMKFGRSEFRKVLNYINERLTRW
VMRKYKRFSKGKKFSKAYEWLVEYAAHNRNEFSHWVKGFVPYPRLD
>SRS046712||gene_39263|GeneMark.hmm|418_aa|-|151|1407
MSEAKQFDISKKAVIAAFQAVKENAGSYGADEQTIKEFEEHLNNNLYKLWNRMASGSYFPKPVRAVAIPKKNG
GIRILGIPTVEDRIAQMVAKMYFEPLVEPMFYNDSYGYRPNKSAIQAVGQARERCFKRDWVLELDIKGLFDNIK
HGYLMYMVEKHTQIKWLILYIKRWLTVPFIMSDGSVAERRSGTPQGGVISPVLANLFLHYVFDDFMTKAYPNI
WWERYADDGVLHCQSYKQAAFIKQKLEERFQQFGLELNKEKTRIVYCKDNRRPQNYSCTQFTFLGYTFRPRLN
KNKEGKFFVGFTPAVSEKAKTAMKQKIREWKIQLKADLSLKDIGNMINKVVQGWINYYTHYYKSEFYEVLRYIN
QCLIKWVRRSYKKKNTRSRAEHWLGAVARRDRNLFAHWKFGILPSVGEGAV
>SRS047014||gene_275543|GeneMark.hmm|430_aa|-|398|1690
MQNDNAKPISISKQLVYDAFLRVKANRGSAGIDKVTLEDYEKNLRGNLYKLWNRMSSGSYFPPSVKLVEIPKSTG
GKRPLGIPTVSDRVAQMTVVMLITPSIEPCFHEDSYAYRPHRSAHDAVGKARERCWKYAWVLDMDISKFFDTID
HELLLKALKRHTQEKWVLMYIERWLKVPYEKSDGSQVDRALGVPQGSVIGPVLANLFLHYTFDKWMEKNFPR
VPFERYADDTICHCHSLKQAEYMQAMIQQRFECCRLRLNEEKTKIVYCKSSRQKECYPNVTFDFLGFTFQPRESV
DKYGNRFTGFLPAISRKSMKRINETMRSWHLNRHSNLTLEHLASDINPIVRGWMTYYGKFYPTRLKWFMQTLN
GRLASWVMCKFERYRHRFYPAQEWLARIAEKEGLIFYHWKCGVLPRFTNKEKVSSQLIMVK
>SRS047433||gene_108935|GeneMark.hmm|420_aa|-|13453|14715
MNEAKPFVIDKRLVWEAYHKVKENKGSAGIDKVDQKTFDKEMSKNLYKIWNRMSSGCYFSKAVKLVEIPKSNG
GTRPLGIPTIEYRIAQQVVVSVLTPILEPIFKEDSYGYRPGKGAHQAIAKAKERCYVTPWVLDMDISKFFDTINHEL
LMKAIRKHTEEKWVLLYIERWLKVPNQTSKGEVIERTMGVPQGSVIGPVLANLFLHYVFDEWMSRNYPTIPFER
YADATICHCVSEKQARFLKAVLMKRFEEYGLKLNEEKTKIVYCKDSNRRGDSEHTSFNFLGFTFRPRGARNRKTG
QNFTAFLPAISNKSMKRIKEAIRAWKLNRKTFACLLDISTEVDTQISGWMNYYMKFGRSEFRKVLNYINERLTRW
VMRKYKRFSKGKKFSKAYEWLVEYAAHNRNEFSHWVKGFVPYPRLD
>SRS047741||gene_98448|GeneMark.hmm|376_aa|-|103|1233
YKIWNRMSSGSYLPPAVKLVEIPKSNGGKRPLGIPTVGDRVAQMVVVMTIEPGIEPYFHEDSYAYRPNRSALDA

VRKAKERSYTFHWVLDLDIKGFFDNIDHELLIKALERHVKCKWAILYIKRWLSVPYQLKDGTQKERTKGVPQGSV
VGPILANLFLHYVFDEWMRRNHSNISFERYADDTICHCVSLKQAEFILRAIRKRFAECKLELNEDKTKIVYCKKNH
RDIPYECIQFDFLGYTFRPRRSIDANGEVFLNFSPAISEKARTKIWEAIQNWNSNHWVPMELEDIAKEINPVIQG
WINYYGQHNPRILKEVLQHVNDRLVRWGRRKFKGLRKRKTATVHRLGDIALQKPNLFAHWAWGVKPTASERN
RKRK
>SRS048060||gene_40803|GeneMark.hmm|368_aa|+|2837|3943
MSSGSYVPKPVRLVQIPKPAGGTRPLGIPTVEDRIAQMLVVEMIEPEIEKIFHEDSYGYRSNRSAHDALGRARER
CWKYAWVLDMDISKFFDSIDHQLLMKAVRLHVKERWIILYIERWLKVPYQNADKSLIERTCGVPQGSVIGPILA
NLFLHYCFDRWMQIHHPEIPFERYADDTVCHCRSQREAESLYEELIIRFKSCKLSLNEEKTKIVYCKSSRRKENHSN
VTFDFLGHTFRPCKTMHKSSREAFTGFQPRISMKATTKIKATMRSWNLKSKSHTPLDCIAQMVNPILRGWVNY
YGKYGGKSFQKLLGYFDLLLARWAKAKYKTFRRKPMYVILKWLGNIADRNAIFYHWQIGLKPAKGTIKL
>SRS048164||gene_102606|GeneMark.hmm|422_aa|-|19240|20508
MNEAKPFVIDKRLVWEAYHKVKENKGSAGIDKVDQKTFDKEMSKNLYKIWNRMSSGCYFPKAVKLVEIPKSNG
GTRPLGIPTIEDRIAQQVAVSVLTPILEPIFKEDSYGYRPGKGAHQAIAKAKERCYVNPWVLDMDISKFFDTINHE
LLMKAVRKHTEEKWVLLYIERWLKVPYQTSKGEVIERTMGVPQGSVIGPVLANLFLHYVFDEWMSRNYPTIPFE
RYADDTICHCVSEKQAQFLKAVLMKRFEECGLKLNEEKTKIVYCKDSNRRGDSEHISFDFLGFTFRPRGARNRKT
GQNFTAFLPAISKKSMNRIKEAVRAWKQNHKTFACLLDISNEVDTQISGWMNYYMKFGxSEFRKVLNYINERLT
RWVMRKYKRFSKGRKLGRAYDWLVEYAAHNRNESTLKSKACLKCLYFTKI
>SRS048164||gene_241968|GeneMark.hmm|421_aa|-|131|1396
MQEAKPFQIDKRIIFESFKKVKFNRGSSGIDGIEMTTYEQNLGSNLYRLWNRMSSGSYMPKAVKLVEIPKSNGG
KRPLGIPTIEDRIAQMAVVNVIEPLIEPCFHEDSFGYRPHRSAHDAIAKAERRCWKYAWVLDIDISKFFDTIDHGL
LMKAVEKHINIKWILLYIKRWLTVPYQRSDGEIVKRDMGVPQGSVIGPILANLFLHYTFDKWMSYKYPHIPFERY
ADDCVCHCSTLAQAEYIKERLGERFTECKLKFNEEKTKIVFCKMSSRSSKHYHCTSFDYLGFTFRSRAAKDKRNN
VLFTSYLPAISKKSVSRIHETIKSWNLKRLHNRSLRFVAAYINDVVRGWINYYEKFGKTEFWKVMCHLNRSIAYW
AKTKYKRLRRRGVISAHYWLAYIAQKEPNLFYHWQVGYVPYARQKK
>SRS048262||gene_180820|GeneMark.hmm|421_aa|-|241|1506
MQEAKPFQIDKRIIFESFKKVKFNRGSSGIDGIEMTTYEQNLGSNLYRLWNRMSSGSYMPKAVKLVEIPKSNGG
KRPLGIPTIEDRIAQMAVVNVIEPLIEPCFHEDSFGYRPHRSAHDAIAKAERRCWKYAWVLDIDISKFFDTIDHGL
LMKAVEKHINIKWILLYIKRWLTVPYQRSDGEIVKRDMGVPQGSVIGPILANLFLHYTFDKWMSYKYPHIPFERY
ADDCVCHCSTLAQAEYIKERLGERFTECKLKFNEEKTKIVFCKMSSRSSKHYHCTSFDYLGFTFRSRAAKDKRNN
VLFTSYLPAISKKSVSRIHETIKSWNLKRLHNRSLRFVAAYINDVVRGWINYYEKFGKTEFWKVMCHLNRSIAYW
AKTKYKRLRRRGVISAHYWLAYIAQKEPNLFYHWQVGYVPYARQKK
>SRS048262||gene_75345|GeneMark.hmm|422_aa|-|19084|20352
MNEAKPFVIDKRLVWEAYHKVKENKGSAGIDKVDQKTFDKEMSKNLYKIWNRMSSGCYFPKAVKLVEIPKSNG
GTRPLGIPTIEDRIAQQVAVSVLTPILEPIFKEDSYGYRPGKGAHQAIAKAKERCYVNPWVLDMDISKFFDTINHE
LLMKAVRKHTEEKWVLLYIERWLKVPYQTSKGEVIERTMGVPQGSVIGPVLANLFLHYVFDEWMSRNYPTIPFE
RYADDTICHCVSEKQAQFLKAVLMKRFEECGLKLNEEKTKIVYCKDSNRRGDSEHISFDFLGFTFRPRGARNRKT
GQNFTAFLPAISKKSMNRIKEAVRAWKQNHKTFACLLDISNEVDTQISGWMNYYMKFGRSEFRKVLNYINERLT
RWVMRKYKRFSKGRKLGRAYDWLVEYAAHNRNESTLKSKACLKCLYFTKI
>SRS049164||gene_62054|GeneMark.hmm|430_aa|-|553|1845
MQNDNAKPISISKQLVYDAFLRVKANRGSAGIDKVTLEDYEKNLRGNLYKLWNRMSSGSYFPPSVKLVEIPKSTG
GKRPLGIPTVSDRVAQMTVVMLITPSIEPCFHEDSYAYRPHRSAHDAVGKARERCWKYAWVLDMDISKFFDTID
HELLLKALKRHTQEKWVLMYIERWLKVPYEKSDGSQVDRALGVPQGSVIGPVLANLFLHYTFDKWMEKNFPR
VPFERYADDTICHCHSLKQAEYMQAMIQQRFECCRLRLNEEKTKIVYCKSSRQKECYPNVTFDFLGFTFQPRESV

DKYGNRFTGFLPAISRKSMKRINETMRSWHLNRHSNLTLEHLASDINPIVRGWMTYYGKFYPTRLKWFMQTLN
GRLASWVMCKFERYRHRFYPAQEWLARIAEKEGLIFYHWKCGVLPRFTNKEKVSSQLIMVK
>SRS049402||gene_176569|GeneMark.hmm|430_aa|+|583|1875
MQNDNAKPISISKQLVYDAFLRVKANRGSAGIDKVTLEDYEKNLRGNLYKLWNRMSSGSYFPPSVKLVEIPKSTG
GKRPLGIPTVSDRVAQMAIVMLITPSIEPCFHEDSYAYRPHRSAHDAVGKARERCWKYAWVLDMDISKFFDTID
HELLLKALKRHTQEKWVLMYIERWLKVPYEKADGSQVDRALGVPQGSVIGPVLANLFLHYTFDKWMEKSFPR
VPFERYADDTICHCHSLKQAEYMQAMIQQRFECCRLRLNEEKTKIVYCKSSRQKEFYPNVTFDFLGFTFQPRESV
DKYGNRFTGFLPAISRKSMKRINETIRSWHLNRHSNLTLEHLASDINPIVRGWMTYYGKFYPTRLKWFMQTLN
GRLARWVMCKFERYRHRFYPAQEWLARIAEKEGLIFYHWKCGVLPRFTNKEKVSSQLIMVK
>SRS049900||gene_57328|GeneMark.hmm|418_aa|-|2654|3910
MSEAKQFDISKKAVIAAFQAVKENAGSYGADEQTIKEFEEHLNNNLYKLWNRMASGSYFPKPVRAVAIPKKNG
GIRILGIPTVEDRIAQMVAKMYFEPLVEPMFYNDSYGYRPNKSAIQAVGQARERCFKRDWVLELDIKGLFDNIK
HGYLMYMVEKHTQIKWLILYIKRWLTVPFIMSDGSVAERRSGTPQGGVISPVLANLFLHYVFDDFMTKAYPNI
WWERYADDGVLHCQSYKQAVFIKQKLEERFQQFGLELNKEKTRIVYCKDDRRSRNYSCTQFTFLGYTFRPRLNK
NKEGKFFVGFTPAVSEKAKTAMKQKIRGWKIQLKADLSLKDIGNMINKVVQGWINYYTHYYKSEFYEVLRYINQ
CLIKWVRRSYKKKNTRSRAEHWLGAVARRDRNLFAHWKFGILPSVGEGAV
>SRS050299||gene_121944|GeneMark.hmm|430_aa|+|3105|4397
MQNDNAKPISISKQLVYDAFLRVKANRGSAGIDKVTLEDYEKNLRGNLYKLWNRMSSGSYFPPSVKLVEIPKSTG
GKRPLGIPTVSDRVAQMAVVMLITPSIEPCFHEDSYAYRPHRSAHDAVGKARERCWKYAWVLDMDISKFFDTI
DHELLLKALKRHTQEKWVLMYIERWLKVPYEKSDGSQVDRALGVPQGSVIGPVLANLFLHYTFDKWMEKNFP
RVPFERYADDTICHCHSLKQAEYMQAMIQQRFECCRLRLNEEKTKIVYCKSSRQKECYPNVTFDFLGFTFQPRES
VDKYGNRFTGFLPAISRKSMKRINETMRSWHLNRHSNLTLEHLASDINPIVRGWMTYYGKFYPTRLKWFMQTL
NGRLARWVMCKFERYRHRFYPAQEWLARIAEKEGLIFYHWKCGALPRFTNKEKVSSQLIMVK
>SRS050422||gene_271909|GeneMark.hmm|418_aa|+|1329|2585
MSEAKQFDISKKAVIAAFQAVKENAGSYGADEQTIKEFEEHLNNNLYKLWNRMASGSYFPKPVRAVAIPKKNG
GIRILGIPTVEDRIAQMVAKMYFEPLVEPMFYNDSYGYRPNKSAIQAVGQARERCFKRDWVLELDIKGLFDNIK
HGYLMYMVEKHTQIKWLILYIKRWLTVPFIMSDGSVAERRSGTPQGGVISPVLANLFLHYVFDDFMTKAYPNI
WWERYADDGVLHCQSYKQAAFIKQKLEERFQQFGLELNKEKTRIVYCKDNRRPQNYSCTQFTFLGYTFRPRLN
KNKEGKFFVGFTPAVSEKAKTAMKQKIREWKIQLKADLSLKDIGNMINKVVQGWINYYTHYYKSEFYEVLRYIN
QCLIKWVRRSYKKKNTRSRAEHWLGAVARRDRNLFAHWKFGILPSVGEGAV
>SRS050925||gene_263577|GeneMark.hmm|430_aa|-|88|1380
MQNDNAKPISISKQLVYDAFLRVKANRGSAGIDKVTLEDYEKNLRGNLYKLWNRMSSGSYFPPSVKLVEIPKSTG
GKRPLGIPTVSDRVAQMAVVMLITPSIEPCFHEDSYAYRPHRSAHDAVGKARERCWKYAWVLDMDISKFFDTI
DHELLLKALKRHTQEKWVLMYIERWLKVPYEKSDGSQVDRALGVPQGSVIGPVLANLFLHYTFDKWMEKNFP
RVPFERYADDTICHCHSLKQAEYMQAMIQQRFECCRLRLNEEKTKIVYCKSSRQKECYPNVTFDFLGFTFQPRES
VDKYGNRFTGFLPAISRKSMKRINETMRSWHLNRHSNLTLEHLASDINPIVRGWMTYYGKFYPTRLKWFMQTL
NGRLARWVMCKFERYRHRFYPAQEWLARIAEKEGLIFYHWKCGALPRFTNKEKVSSQLIMVK
>SRS051031||gene_83632|GeneMark.hmm|369_aa|+|39|1148
MSSGSYMPKAVKLVEIPKSNGGKRPLGIPTIEDRIAQMAVVNVIEPLIEPCFHEDSFGYRPHRSAHDAIAKAERR
CWKYAWVLDIDISKFFDTIDHGLLMKAVEKHINIKWILLYIKRWLTVPYQRSDGEIVKRDMGVPQGSVIGPILAN
LFLHYTFDKWMSYKYPHIPFERYADDCVCHCSTLAQAEYIKERLGERFTECKLKFNEEKTKIVFCKMSSRSSKHYH
CTSFDYLGFTFRSRAAKDKRNNVLFTSYLPAISKKSVSRIxETIKSWNLKRLHNRSLRFVAAYINDVVRGWINYYEK
FGKTEFWKVMCHLNRSIAYWAKTKYKRLRRRGVISAHYWLAYIAQKEPNLFYHWQVGYVPYARQKK
>SRS052027||gene_69717|GeneMark.hmm|430_aa|-|508|1800

MQNDNAKPISISKQLVYDAFLRVKANRGSAGIDKVTLEDYEKNLRGNLYKLWNRMSSGSYFPPSVKLVEIPKSTG
GKRPLGIPTVSDRVAQMAIVMLITPSIEPCFHEDSYAYRPHRSAHDAVGKARERCWKYAWVLDMDISKFFDTID
HELLLKALKRHTQEKWVLMYIERWLKVPYEKADGSQVDRALGVPQGSVIGPVLANLFLHYTFDKWMEKSFPR
VPFERYADDTICHCHSLKQAEYMQAMIQQRFECCRLRLNEEKTKIVYCKSSRQKEFYPNVTFDFLGFTFQPRESV
DKYGNRFTGFLPAISRKSMKRINETIRSWHLNRHSNLTLEHLASDINPIVRGWMTYYGKFYPTRLKWFMQTLN
GRLARWVMCKFERYRHRFYPAQEWLARIAEKEGLIFYHWKCGVLPRFTNKEKVSSQLIMVK
>SRS052576||gene_5298|GeneMark.hmm|422_aa|-|2666|3934
MMQHQVTKPFTIDKHLIMNAWKRVKENKGSVGIDNVSTDDYESNLGKNLYKLWNRMSSGSYFPEAVKLVDIP
KSSGGTRPLGIPTVGDRIAQMSVVLLIEDRLEAIFHADSYGYRPNRSAHDAIGKARERCWHYNWVLDMDISKFF
DTINHDLLMKAVERHVQEKWILLYIRRWLEVPYATLTGERIERRMGVPQGSVIGPVLANLYLHYTFDKWMSLYH
PTIPFERYADDTICHCNSLEEAQMLKASIVERFAACKLKLNEEKTRIVYCKDGKRRREYKDITFDFLGYTFQPRGQR
NKQGQVFNGYAPAISRKSKKRIAETMRGWHLNRRVQLKLSDIAVEINAEVRGWMNYYGKFYGSQLKAFLQCIN
LKLARWAERKYKRFRRKPNDAYKWLVRVASKNPALFYHWQHGVKPNRLKPFG
>SRS054753||gene_23094|GeneMark.hmm|430_aa|-|60932|62224
MQNDNAKPISISKQLVYDAFLRVKANRGSAGIDKVTLEDYEKNLRGNLYKLWNRMSSGSYFPPSVKLVEIPKSTG
GKRPLGIPTVSDRVAQMAIVMLITPSIEPCFHEDSYAYRPHRSAHDAVGKARERCWKYAWVLDMDISKFFDTID
HELLLKALKRHTQEKWVLMYIERWLKVPYEKADGSQVDRALGVPQGSVIGPVLANLFLHYTFDKWMEKSFPR
VPFERYADDTICHCHSLKQAEYMQAMIQQRFECCRLRLNEEKTKIVYCKSSRQKEFYPNVTFDFLGFTFQPRESV
DKYGNRFTGFLPAISRKSMKRINETIRSWHLNRHSNLTLEHLASDINPIVRGWMTYYGKFYPTRLKWFMQTLN
GRLARWVMCKFERYRHRFYPAQEWLARIAEKEGLIFYHWKCGVLPRFTNKEKVSSQLIMVK
>SRS055966||gene_15707|GeneMark.hmm|422_aa|+|1140|2408
MMQHQVTKPFTIDKHLIMNAWKRVKENKGSVGIDNVSTDDYESNLGKNLYKLWNRMSSGSYFPEAVKLVDIP
KSSGGTRPLGIPTVGDRIAQMSVVLLIEDRLEAIFHADSYGYRPNRSAHDAIGKARERCWHYNWVLDMDISKFF
DTINHDLLMKAVERHVQEKWILLYIRRWLEVPYATLTGERIERRMGVPQGSVIGPVLANLYLHYTFDKWMSLYH
PTIPFERYADDTICHCNSLEEAQMLKASIVERFAACKLKLNEEKTRIVYCKDGKRRREYKDITFDFLGYTFQPRGQR
NKQGQVFNGYAPAISRKSKKRIAETMRGWHLNRRVQLKLSDIAVEINAEVRGWVNYYGKFYGSQLKAFLQCIN
LKLARWAERKYKRFRRKPNDAYKWLVRVASKNPALFYHWQHGVKPNRLKPFG
>SRS055982||gene_164849|GeneMark.hmm|418_aa|+|1071|2327
MSEAKQFDISKKAVIAAFQAVKENAGSYGADEQTIKEFEEHLNNNLYKLWNRMASGSYFPKPVRAVAIPKKNG
GIRILGIPTVEDRIAQMVAKMYFEPLVEPMFYNDSYGYRPNKSAIQAVGQARERCFKRDWVLELDIKGLFDNIK
HGYLMYMVEKHTQIKWLILYIKRWLTVPFIMSDGSVAERRSGTPQGGVISPVLANLFLHYVFDDFMTKAYPNI
WWERYADDGVLHCQSYKQAAFIKQKLEERFQQFGLELNKEKTRIVYCKDNRRPQNYSCTQFTFLGYTFRPRLN
KNKEGKFFVGFTPAVSEKAKTAMKQRIREWKIQLKADLSLKDIGNMINKVVQGWINYYTHYYKSEFYEVLRYIN
QCLIKWVRRSYKKKNTRSRAEHWLGAVARRDRNLFAHWKFGILPSVGEGAV
>SRS055982||gene_23866|GeneMark.hmm|422_aa|+|4697|5965
MMQHQVTKPFTIDKHVIMNAWKRVKENKGSAGIDNISTEDYESNLGKNLYKLWNRMSSGSYFPKAVKLVDIP
KPSGGTRPLGIPTVGDRIAQMSVVLLIESRLEAIFHMDSYGYRPNRSAHDAIGKARERCWRYNWVLDMDISKFF
DTIDHALLMKAVERHVQERWILLYIRRWLKVPYATVTGECIERSMGIPQGSVIGPVLANLYLHYTFDKWMSLYH
PTIPFERYADDTICHCNSLEEAQRLKTSIVERFAACKLRLNEEKTRIVYCKDGKRRGEYKEITFDFLGYTFQPRGQR
NKQGQVFNGYAPAISKKSKKRITEKIRGWHLNSRVQLKLSDIAMEINAEVRGWMNYYGKFYGSQLKAFLQCVN
LKLARWAERKYKRFRRKPNDAYKWLVWIASKNPTLFYHWQHGVKPNRLKPFG
>SRS056259||gene_79138|GeneMark.hmm|430_aa|-|410|1702
MQNDNAKPISISKQLVYDAFLRVKANRGSAGIDKVTLEDYEKNLRGNLYKLWNRMSSGSYFPPSVKLVEIPKTTG
GKRPLGIPTVSDRVAQMAVVMLITPSIEPCFHEDSYAYRPHRSAHDAVGKARERCWKYAWVLDMDISKFFDTI

DHELLLKALKRHTQEKWVLMYIERWLKVPYEKSDGSQVDRALGVPQGSVIGPVLANLFLHYTFDKWMEKNFP
RVPFERYADDTICHCHSLKQAEYMQAMIQQRFECCRLRLNEEKTKIVYCKSSRQKECYPNVTFDFLGFTFQPRES
VDKYGNRFTGFLPAISRKSMKRINETMRSWHLNRHSNLTLEHLASDINPIVRGWMTYYGKFYPTRLKWFMQTL
NGRLARWVMCKFERYRHRFYPAQEWLARIAEKEGLIFYHWKCGVLPRFTNKEKVSSQLIMVK
>SRS056259||gene_98217|GeneMark.hmm|421_aa|+|2598|3863
MQEAKPFQIDKRIIFESFKKVKFNRGSSGIDGIEMTTYEQNLGSNLYRLWNRMSSGSYMPKAVKLVEIPKSNGG
KRPLGIPTIEDRIAQMAVVNVIEPLIEPCFHEDSFGYRPHRSAHDAIAKAERRCWKYAWVLDIDISKFFDTIDHGL
LMKAVEKHINIKWILLYIKRWLTVPYQRSDGEIVKRDMGVPQGxVIGPILANLFLHYTFDKWMSYKYPHIPFERY
ADDCVCHCSTLAQAEYIKERLGERFTECKLKFNEEKTKIVFCKMSSRSSKHYHCTSFDYLGFTFRSRAAKDKRNN
VLFTSYLPAISKKSVSRIHETIKSWNLKRLHNRSLRFVAAYINDVVRGWINYYEKFGKTEFWKVMCHLNRSIAYW
AKTKYKRLRRRGVISAHYWLAYIAQKEPNLFYHWQVGYVPYARQKK
>SRS057478||gene_40330|GeneMark.hmm|422_aa|+|833|2101
MKDAKSFEISRHLVMEAYKRVKANKGAAGVDDVSIADFESNLKSNLYKIWNRMSSGSYFPPAVKLVEIPKSNGG
KRPLGIPTIGDRVAQMVVVMTIEPGIEPYFHEDSYAYRPNRSALDAVRKAKERSYTFHWVLDLDIKGFFDNIDHG
LLIKALERHVKCEWAMLYIKRWLSVPYQLKDGTQIERTKGVPQGSVVGPILANLFLHYVFDEWMRRNHSNISFE
RYADDTICHCVSLKQAEFILRAIRKRFAECKLELNEDKTKIVYCKKNHRDIPYECIQFDFLGYTFRPRRSIDANGEVF
LNFSPAISKKARTKIWEAIQNWNSNHWVPMELEDIAKEINPVIQGWINYYGQHNPRILKEVLQHVNDRLVRW
GRRKFKGLRKRKTATVHRLGDIALQKPNLFAHWAWGVKPTASEKNRKRK
>SRS057478||gene_8991|GeneMark.hmm|421_aa|-|18872|20137
MQEAKPFQIDKRIIFEAFKKVKSNGGSPGIDGIEMSAYEQNLGSNFYRLWNRMSSGSYMPKAVKLVEILKSNGG
KRPLGIPSVEDRIAQMAVVNVIEPLVEPYFHKDSFGYRPHRSAHDAIAKAERRCWKYAWVLDIDISKFFDTIDHG
LLMKAVEKHIKTKWILLYIKRWLTVPYQGNDGAIVKRHMGVPQGSVIGPILANQFLHYTFDKWMSYKYPHVPF
ERYADDCVCHCGTLAQAEYIKDRLGERFAECKLTFNEEKTKIVFCKTSNRSSEHYHCTSFDYLGFTFRPRAAKDKR
KNVLFTSYLPAISNKSESRIHETIKSWNLKRLHNRSLRFVAAYINDVVRGWISYYGKFGKTEFWKVMCHLNRSIAY
WAKTKYKRLRRRGVISAHYWLAYIAQKEPNLFYHWQVGYIPYARQKK
>SRS063040||gene_318939|GeneMark.hmm|418_aa|+|101|1357
MSEAKQFDISKKAVIAAFQAVKENAGSYGADEQTIKEFEEHLNNNLYKLWNRMASGSYFPKPVRAVAIPKKNG
GIRILGIPTVEDRIAQMVAKMYFEPLVEPMFYNDSYGYRPNKSAIQAVGQARERCFKRDWVLELDIKGLFDNIK
HGYLMYMVEKHTQIKWLILYIKRWLTVPFIMSDGSVAERRSGTPQGGVISPVLANLFLHYVFDDFMTKAYPNI
WWERYADDGVLHCQSYKQAAFIKQKLEERFQQFGLELNKEKTRIVYCKDNRRPQNYSCTQFTFLGYTFRPRLN
KNKEGKFFVGFTPAVSEKAKTAMKQKIREWKIQLKADLSLKDIGNMINKVVQGWINYYTHYYKSEFYEVLRYIN
QCLIKWVRRSYKKKNTRSRAEHWLGAVARRDRNLFAHWKFGILPSVGEGAV
>SRS063127||gene_26050|GeneMark.hmm|422_aa|-|32193|33461
MMQHQVTKPFTIDKHLIMNAWKRVKENKGSVGIDNVSTDDYESNLGKNLYKLWNRMSSGSYFPEAVKLVDIP
KSSGGTRPLGIPTVGDRIAQMSVVLLIEDRLEAIFHADSYGYRPNRSAHDAIGKARERCWHYNWVLDMDISKFF
DTINHDLLMKAVERHVQEKWILLYIRRWLEVPYATLTGERIERRMGVPQGSVIGPVLANLYLHYTFDKWMSLYH
PTIPFERYADDTICHCNSLEEAQMLKASIVERFAACKLKLNEEKTRIVYCKDGKRRREYKDITFDFLGYTFQPRGQR
NKQGQVFNGYAPAISRKSKKRIAETMRGWHLNRRVQLKLSDIAVEINAEVRGWVNYYGKFYGSQLKAFLQCIN
LKLARWAERKYKRFRRKPNDAYKWLVRVASKNPALFYHWQHGVKPNRLKPFG
>SRS064276||gene_73543|GeneMark.hmm|430_aa|+|22282|23574
MQNDNAKPISISKQLVYDAFLRVKANRGSAGIDKVTLEDYEKNLRGNLYKLWNRMSSGSYFPPSVKLVEIPKSTG
GKRPLGIPTVSDRVAQMAVVMLITPSIEPCFHEDSYAYRPHRSAHDAVGKARERCWKYAWVLDMDISKFFDTI
DHELLLKALKRHTQEKWVLMYIERWLKVPYEKSDGSQVDRALGVPQGSVIGPVLANLFLHYTFDKWMEKNFP
RVPFERYADDTICHCHSLKQAEYMQAMIQQRFECCRLRLNEEKTKIVYCKSSRQKECYPNVTFDFLGFTFQPRES

VDKYGNRFTGFLPAISRKSMKRINETMRSWHLNRHSNLTLEHLASDINPIVRGWMTYYGKFYPTRLKWFMQTL
NGRLARWVMCKFERYRHRFYPAQEWLARIAEKEGLIFYHWKCGALPRFTNKEKVSSQLIMVK
>SRS065397||gene_14118|GeneMark.hmm|430_aa|+|2364|3656
MQNDNAKPISISKQLVYDAFLRVKANRGSAGIDKVTLEDYEKNLRGNLYKLWNRMSSGSYFPPSVKLVEIPKSTG
GKRPLGIPTVSDRVAQMAVVMLITPSIEPCFHEDSYAYRPHRSAHDAVGKARERCWKYAWVLDMDISKFFDTI
DHELLLKALKRHTQEKWVLMYIERWLKVPYEKSDGSQVDRALGVPQGSVIGPVLANLFLHYTFDKWMEKNFP
RVPFERYADDTICHCHGLKQAEYMQAMIQQRFECCRLRLNEEKTKIVYCKSSRQKECYPNVTFDFLGFTFQPRE
SVDKYGNRFTGFLPAISRKSMKRINETMRSWHLNRHSNLTLEHLASDINPIVRGWMTYYGKFYPTRLKWFMQT
LNGRLARWVMCKFERYRHRFYPAQEWLARIAEKEGLIFYHWKCGALPRFTNKEKVSSQLIMVK
>SRS065504||gene_133230|GeneMark.hmm|430_aa|-|2200|3492
MQNDNAKPISISKQLVYDAFLRVKANRGSAGIDKVTLEDYEKNLRGNLYKLWNRMSSGSYFPPSVKLVEIPKSTG
GKRPLGIPTVSDRVAQMAVVMLITPSIEPCFHEDSYAYRPHRSAHDAVGKARERCWKYAWVLDMDISKFFDTI
DHELLLKALKRHTQEKWVLMYIERWLKVPYEKSDGSQVDRALGVPQGSVIGPVLANLFLHYTFDKWMEKNFP
RVPFERYADDTICHCHSLKQTEYMQAMIQQRFECCRLRLNEEKTKIVYCKSSRQKECYPNVTFDFLGFTFQPRES
VDKYGNRFTGFLPAISRKSMKRINETMRSWHLNRHSNLTLEHLASDINPIVRGWMTYYGKFYPTRLKWFMQTL
NGRLARWVMCKFERYRHRFYPAQEWLARIAEKEGLIFYHWKCGALPRFTNKEKVSSQLIMVK
>SRS075821||gene_102681|GeneMark.hmm|422_aa|+|4111|5379
MKDAKSFEISRHLVMEAYKRVKANKGAAGVDEVSIADFENNLKSNLYKIWNRMSSGSYLPPAVKLVEIPKSNGG
KRPLGIPTVGDRVAQMVVVMTIEPGIEPYFHEDSYAYRPNRSALDAVRKAKERSYTFHWVLDLDIKGFFDNIDH
ELLIKALERHVKCKWAILYIKRWLSVPYQLKDGTQKERTKGVPQGSVVGPILANLFLHYVFDEWMRRNHSNISFE
RYADDTICHCVSLKQAEFILRAIRKRFAECKLELNEDKTKIVYCKKNHRDIPYECIQFDFLGYTFRPRRSIDANGEVF
LNFSPAISKKARTKIWEAIQNWNSNHWVPMELEDIAKEINPVIQGWINYYGQHNPRILKEVLQHVNDRLVRW
GRRKFKGLRKRKTATVHRLGDIALQKPNLFAHWAWGVKPTASERNRKRK
>SRS075821||gene_86377|GeneMark.hmm|420_aa|+|8704|9966
MNEAKPFVIDKRLVWEAYHKVKENKGSAGIDKVDQKTFDKEMSKNLYKIWNRMSSGCYFPKAVKLVEIPKSNG
GTRPLGIPTIEDRIAQQVVVSVLTPILEPIFKEDSYGYRPGKGAHQAIAKAKERCYVNPWVLDMDISKFFDTINHD
LLMKAVRKHTEEKWVLLYIERWLKVPYQTSKGEVIERTMGVPQGSVIGPVLANLFLHYVFDEWMSRNYPTIPFE
RYADDTICHCVSEKQAQFLKAVLMKRFEECGLKLNEEKTKIVYCKDSNRRGDSEHTSFDFLGFTFRPRSARNRKT
GQNFTAFLPAISKKSLKRIKEAVRAWKLNRKTFACLLDISNEVDTQISGWMNYYMKFGRSEFRKVLNYINERLTR
WVMRKYKRFSKGKKFSRAYEWLVEYAVHNRNEFSHWAKGFVPYPRLG
>SRS076804||gene_55651|GeneMark.hmm|430_aa|+|7516|8808
MQNDNAKPISISKQLVYDAFLRVKANRGSAGIDKVTLEDYEKNLRGNLYKLWNRMSSGSYFPPSVKLVEIPKTTG
GKRPLGIPTVSDRVAQMAVVMLITPSIEPCFHEDSYAYRPHRSAHDAVGKARERCWKYAWVLDMDISKFFDTI
DHELLLKALKRHTQEKWVLMYIERWLKVPYEKSDGSQVDRALGVPQGSVIGPVLANLFLHYTFDKWMEKNFP
RVPFERYADDTICHCHSLKQAEYMQAMIQQRFECCRLRLNEEKTKIVYCKSSRQKECYPNVTFDFLGFTFQPRES
VDKYGNRFTGFLPAISRKSMKRINETMRSWHLNRHSNLTLEHLASDINPIVRGWMTYYGKFYPTRLKWFMQTL
NGRLARWVMCKFERYRHRFYPAQEWLARIAEKEGLIFYHWKCGVLPRFTNKEKVSSQLIMVK
>SRS077086||gene_132987|GeneMark.hmm|430_aa|+|4232|5524
MQNDNAKPISISKQLVYDAFLRVKANRGSAGIDKVTLEDYEKNLRGNLYKLWNRMSSGSYFPPSVKLVEIPKSTG
GKRPLGIPTVSDRVAQMAVVMLITPSIEPCFHEDSYAYRPHRSAHDAVGKARERCWKYAWVLDMDISKFFDTI
DHELLLKALKRHTQEKWVLMYIERWLKVPYEKSDGSQVDRALGVPQGSVIGPVLANLFLHYTFDKWMEKNFP
RVPFERYADDTICHCHSLKQAEYMQAMIQQRFECCRLRLNEEKTKIVYCKSSRQKECYPNVTFDFLGFTFQPRES
VDKYGNRFTGFLPAISRKSMKRINETMRSWHLNRHSNLTLEHLASDINPIVRGWMTYYGKFYPTRLKWFMQTL
NGRLARWVMCKFERYRHRFYPAQEWLARIAEKEGLIFYHWKCGALPRFTNKEKVSSQLIMVK

>SRS077117||gene_133730|GeneMark.hmm|430_aa|-|297|1589
MQNDNAKPISISKQLVYDAFLRVKANRGSAGIDKVTLEDYEKNLRGNLYKLWNRMSSGSYFPPSVKLVEIPKSTG
GKRPLGIPTVSDRVAQMAVVMLITPSIEPCFHEDSYAYRPHRSAHDAVGKARERCWKYAWVLDMDISKFFDTI
DHELLLKALKRHTQEKWVLMYIERWLKVPYEKSDGSQVDRALGVPQGSVIGPVLANLFLHYTFDKWMEKNFP
RVPFERYADDTICHCHSLKQAEYMQAMIQQRFECCRLRLNEEKTKIVYCKSSRQKECYPNVTFDFLGFTFQPRES
VDKYGNRFTGFLPAISRKSMKRINETMRSWHLNRHSNLTLEHLASDINPIVRGWMTYYGKFYPTRLKWFMQTL
NGRLARWVMCKFERYRHRFYPAQEWLARIAEKEGLIFYHWKCGALPRFTNKEKVSSQLIMVK
>SRS077127||gene_10448|GeneMark.hmm|430_aa|+|6592|7884
MQNDNAKPISISKQLVYDAFLRVKANRGSAGIDKVTLEDYEKNLRGNLYKLWNRMSSGSYFPPSVKLVEIPKSTG
GKRPLGIPTVSDRVAQMAVVMLITPSIEPCFHEDSYAYRPHRSAHDAVGKARERCWKYAWVLDMDISKFFDTI
DHELLLKALKRHTQEKWVLMYIERWLKVPYEKSDGSQVDRALGVPQGSVIGPVLANLFLHYTFDKWMEKNFP
RVPFERYADDTICHCHSLKQAEYMQAMIQQRFECCRLRLNEEKTKIVYCKSSRQKECYPNVTFDFLGFTFQPRES
VDKYGNRFTGFLPAISRKSMKRINETMRSWHLNRHSNLTLEHLASDINPIVRGWMTYYGKFYPTRLKWFMQTL
NGRLARWVMCKFERYRHRFYPAQEWLARIAEKEGLIFYHWKCGALPRFTNKEKVSSQLIMVK
>SRS077194||gene_237424|GeneMark.hmm|430_aa|+|795|2087
MQNDNAKPISISKQLVYDAFLRVKANRGSAGIDKVTLEDYEKNLRGNLYKLWNRMSSGSYFPPSVKLVEIPKSTG
GKRPLGIPTVSDRVAQMAIVMLITPSIEPCFHEDSYAYRPHRSAHDAVGKARERCWKYAWVLDMDISKFFDTID
HELLLKALKRHTQEKWVLMYIERWLKVPYEKADGSQVDRALGVPQGSVIGPVLANLFLHYTFDKWMEKSFPR
VPFERYADDTICHCHSLKQAEYMQAMIQQRFECCRLRLNEEKTKIVYCKSSRQKEFYPNVTFDFLGFTFQPRESV
DKYGNRFTGFLPAISRKSMKRINETIRSWHLNRHSNLTLEHLASDINPIVRGWMTYYGKFYPTRLKWFMQTLN
GRLARWVMCKFERYRHRFYPAQEWLARIAEKEGLIFYHWKCGVLPRFTNKEKVSSQLIMVK
>SRS077194||gene_129539|GeneMark.hmm|422_aa|+|1837|3105
MKDAKSFEISRHLVMEAYKRVRANKGAAGVDDVSIADFESNLKSNLYKIWNRMSSGSYFPPAVKLVEIPKSNGG
KRPLGIPTIGDRVAQMVVVMTIESGIEPYFHEDSYAYRPNRSALDAVRKAKERSYTFHWVLDLDIKGFFDNIDHG
LLIKALERHVKCEWAMLYIKRWLSVPYQLKDGTQIERTKGVPQGSVVGPILANLFLHYVFDEWMRRNHSNISFE
RYADDTICHCVSLKQAEFILRAIRKRFAECKLELNEDKTKIVYCKKNHRDTPYECIQFDFLGYTFRPRRSIDANGEVF
LNFSPAISKKARTKIWEAIQNWNSNHWVPMELEDIAKEINPVIQGWINYYGQHNPRILKEVLQHVNDRLVRW
GRRKFKGLRKRKTATVHRLGDIALQKPNLFAHWAWGVKPTASERNRKRK
>SRS077194||gene_62655|GeneMark.hmm|420_aa|+|1664|2926
MNEAKPFVIDKRLVWEAYHKVKENKGSAGIDKVDQKTFDKEMSKNLYKIWNRMSSGCYFSKAVKLVEIPKSNG
GTRPLGIPTIEYRIAQQVVVSVLTPILEPIFKEDSYGYRPGKGAHQAIAKAKERCYVTPWVLDMDISKFFDTINHEL
LMKAIRKHTEEKWVLLYIERWLKVPNQTSKGEVIERTMGVPQGSVIGPVLANLFLHYVFDEWMSRNYPTIPFER
YADATICHCVSEKQARFLKAVLMKRFEEYGLKLNEEKTKIVYCKDSNRRGDSEHTSFNFLGFTFRPRGARNRKTG
QNFTAFLPAISNKSMKRIKEAIRAWKLNRKTFACLLDISTEVDTQISGWMNYYMKFGRSEFRKVLNYINERLTRW
VMRKYKRFSKGKKFSKAYEWLVEYAAHNRNEFSHWVKGFVPYPRLD
>SRS077454||gene_71039|GeneMark.hmm|430_aa|+|22528|23820
MQNDNAKPISISKQLVYDAFLRVKANRGSAGIDKVTLEDYEKNLRGNLYKLWNRMSSGSYFPPSVKLVEIPKSTG
GKRPLGIPTVSDRVAQMAVVMLITPSIEPCFHEDSYAYRPHRSAHDAVGKARERCWKYAWVLDMDISKFFDTI
DHELLLKALKRHTQEKWVLMYIERWLKVPYEKSDGSQVDRALGVPQGSVIGPVLANLFLHYTFDKWMEKNFP
RVPFERYADDTICHCHSLKQAEYMQAMIQQRFECCRLRLNEEKTKIVYCKSSRQKECYPNVTFDFLGFTFQPRES
VDKYGNRFTGFLPAISRKSMKRINETMRSWHLNRHSNLTLEHLASDINPIVRGWMTYYGKFYPTRLKWFMQTL
NGRLARWVMCKFERYRHRFYPAQEWLARIAEKEGLIFYHWKCGALPRFTNKEKVSSQLIMVK
>SRS077849||gene_57461|GeneMark.hmm|422_aa|-|838|2106
MKDAKSFEISRHLVMEAYKRVKANKGAAGVDDVSIADFESNLKSNLYKIWNRMSSGSYFPPAVKLVEIPKSNGG

KRPLGIPTIGDRVAQMVVVMTIEPGIEPYFHEDSYAYRPNRSALDAVRKAKERSYTFHWVLDLDIKGFFDNIDHG
LLIKALERHVKCEWAMLYIKRWLSVPYQLKDGTQIERTKGVPQGSVVGPILANLFLHYVFDEWMRRNHSNISFE
RYADDTICHCVSLKQAEFILRAIRKRFAECKLELNEDKTKIVYCKKNHRDIPYECIQFDFLGYTFRPRRSIDANGEVF
LNFSPAISKKARTKIWEAIQNWNSNHWVPMELEDIAKEINPVIQGWINYYGQHNPRILKEVLQHVNDRLVRW
GRRKFKGLRKRKTATVHRLGDIALQKPNLFAHWAWGVKPTASEKNRKRK
>SRS097889||gene_80683|GeneMark.hmm|418_aa|-|317|1573
MSEAKQFDISKKAVIAAFQAVKENAGSYGADEQTIKEFEEHLNNNLYKLWNRMASGSYFPKPVRAVAIPKKNG
GIRILGIPTVEDRIAQMVAKMYFEPLVEPMFYNDSYGYRPNKSAIQAVGQARERCFKRDWALELDIKGLFDNIK
HGYLMYMVEKHTQIKWLILYIKRWLTVPFIMSDGSVAERRSGTPQGGVISPVLANLFLHYVFDDFMTKAYPNI
WWERYADDGVLHCQSYKQAAFIKQKLEERFQQFGLELNKEKTRIVYCKDNRRPQNYSCTQFTFLGYTFRPxLNK
xxEGKFFVGFTPAVSEKAKTAMKQKIREWKIQLKADLSLKDIGNMINKVVQGWINYYTHYYKSEFYEVLRYINQC
LIKWVRRSYKKKNTRSRAEHWLGAVARRDRNLFAHWKFGILPSVGEGAV
>SRS097889||gene_260270|GeneMark.hmm|431_aa|+|3|1298
PELFSFENKSTLKRGGTYAETKPYSISKRAVIAAYERVKVNKGTYGVDEQSIEDFERKLNNNLYKIWNRMSSGSYF
PKPVKAVATPQKNGRTRILGIPTVEDRIAQMVVKLYLEPSVEPIFYDDSYGYRPNKSAIQAIEVTRTRCWRKDWV
LEFDIKGLFDNIRHDYLMDMVKRHTKEEWILLYIQRWLIAPFQMEDGTIVPRTSGTPQGGVISPVLANLFLHYVF
DDFMVKEFPSIPWARYADDGIAHCVSLKQAKYLQRRLQERFVRFGLELNMEKTRIVYCKDDNRKGKHEHTSFA
FLGYTFRLGQAKNKNGKFFTNFLPAMSEKTKKAIRKVVRGWKLQFKPDKDLWDTANIINKQIQRWINYYTHFYK
SEIYEVLRHINRRLVYWVRRKYKNRNTRKRAEYWLGEIAKRDKNLFAHWKFGILPLAG
>SRS098514||gene_28121|GeneMark.hmm|418_aa|+|1331|2587
MSEAKQFDISKKAVIAAFQAVKENAGSYGADEQTIKEFEEHLNNNLYKLWNRMASGSYFPKPVRAVAIPKKNG
GIRILGIPTVEDRIAQMVAKMYFEPLVEPMFYNDSYGYRPNKSAIQAVGQARERCFKRDWVLELDIKGLFDNIK
HGYLMYMVEKHTQIKWLILYIKRWLTVPFIMSDGSVAERRSGTPQGGVISPVLANLFLHYVFDDFMTKAYPNI
WWERYADDGVLHCQSYKQAAFIKQKLEERFQQFGLELNKEKTRIVYCKDNRRPQNYSCTQFTFLGYTFRPRLN
KNKEGKFFVGFTPAVSEKAKTAMKQKIREWKIQLKADLSLKDIGNMINKVVQGWINYYTHYYKSEFYEVLRYIN
QCLIKWVRRSYKKKNTRSRAEHWLGAVARRDRNLFAHWKFGILPSVGEGAV
>SRS098571||gene_49373|GeneMark.hmm|420_aa|-|493|1755
MNEAKPFVIDKRLVWEAYHKVKENKGSAGIDKVDQKTFDKEMSKNLYKIWNRMSSGCYFPKAVKLVEIPKSNG
GTRPLGIPTIEDRIAQQVVVSVLTPILEPIFKEDSYGYRPGKGAHQAIAKAKERCYVNPWVLDMDISKFFDTINHD
LLMKAVRKHTEEKWVLLYIERWLKVPYQTSKGEVIERTMGVPQGSVIGPVLANLFLHYVFDEWMSRNYPTIPFE
RYADDTICHCVSEKQAQFLKAVLMKRFEECGLKLNEEKTKIVYCKDSNRRGDSEHTSFDFLGFTFRPRSARNRKT
GQNFTAFLPAISKKSLKRIKEAVRAWKLNRKTFACLLDISNEVDTQISGWMNYYMKFGRSEFRKVLNYINERLTR
WVMRKYKRFSKGKKFSRAYEWLVEYAVHNRNEFSHWAKGFVPYPRLG
>SRS098571||gene_100344|GeneMark.hmm|422_aa|+|3692|4960
MKDAKSFEISRHLVMEAYKRVKANKGAAGVDEVSIADFENNLKSNLYKIWNRMSSGSYLPPAVKLVEIPKSNGG
KRPLGIPTVGDRVAQMVVVMTIEPGIEPYFHEDSYAYRPNRSALDAVRKAKERSYTFHWVLDLDIKGFFDNIDH
ELLIKALERHVKCKWAILYIKRWLSVPYQLKDGTQKERTKGVPQGSVVGPILANLFLHYVFDEWMRRNHSNISFE
RYADDTICHCVSLKQAEFILRAIRKRFAECKLELNEDKTKIVYCKKNHRDIPYECIQFDFLGYTFRPRRSIDANGEVF
LNFSPAISKKARTKIWEAIQNWNSNHWVPMELEDIAKEINPVIQGWINYYGQHNPRILKEVLQHVNDRLVRW
GRRKFKGLRKRKTATVHRLGDIALQKPNLFAHWAWGVKPTASERNRKRK
>SRS098881||gene_68741|GeneMark.hmm|430_aa|-|898|2190
MQNDNAKPISISKQLVYDAFLRVKANRGSAGIDKVTLEDYEKNLRGNLYKLWNRMSSGSYFPPSVKLVEIPKSTG
GKRPLGIPTVSDRVAQMAVVMLITPSIEPCFHEDSYAYRPHRSAHDAVGKARERCWKYAWVLDMDISKFFDTI
DHELLLKALKRHTQEKWVLMYIERWLKVPYEKSDGSQVDRALGVPQGSVIGPVLANLFLHYTFDKWMEKNFP

RVPFERYADDTICHCHSLKQAEYMQAMIQQRFECCRLRLNEEKTKIVYCKSSRQKECYPNVTFDFLGFTFQPRES
VDKYGNRFTGFLPAISRKSMKRINETMRSWHLNRHSNLTLEHLASDINPIVRGWMTYYGKFYPTRLKWFMQTL
NGRLARWVMCKFERYRHRFYPAQEWLARIAEKEGLIFYHWKCGVLPRFTNKEKVSSQLIMVK
>SRS100021||gene_118747|GeneMark.hmm|418_aa|+|2387|3643
MSEAKQFDISKKAVIAAFQAVKENAGSYGADEQTIKEFEEHLNNNLYKLWNRMASGSYFPKPVRAVAIPKKNG
GIRILGIPTVEDRIAQMVAKMYFEPLVEPMFYNDSYGYRPNKSAIQAVGQARERCFKRDWVLELDIKGLFDNIK
HGYLMYMVEKHTQIKWLILYIKRWLTVPFIMSDGSVAERRSGTPQGGVISPVLANLFLHYVFDDFMTKAYPNI
WWERYADDGVLHCQSYKQAVFIKQKLEERFQQFGLELNKEKTRIVYCKDDRRSRNYSCTQFTFLGYTFRPRLNK
NKEGKFFVGFTPAVSEKAKTAMKQKIREWKIQLKADLSLKDIGNMINKVVQGWINYYTHYYKSEFYEVLRYINQ
CLIKWVRRSYKKKNTRSRAEHWLGAVARRDRNLFAHWKFGILPSVGEGAV
>SRS101433||gene_72615|GeneMark.hmm|422_aa|+|4704|5972
MMQHQVTKPFTIDKHVIMNAWKRVKENKGSAGIDNISTEDYESNLGKNLYKLWNRMSSGSYFPKAVKLVDIP
KPSGGTRPLGIPTVGDRIAQMSVVLLIESRLEAIFHMDSYGYRPNRSAHDAIGKARERCWRYNWVLDMDISKFF
DTIDHALLMKAVERHVQERWILLYIRRWLKVPYATVTGECIERSMGIPQGSVIGPVLANLYLHYTFDKWMSLYH
PTIPFERYADDTICHCNSLEEAQRLKTSIVERFAACKLRLNEEKTRIVYCKDGKRRGEYKEITFDFLGYTFQPRGQR
NKQGQVFNGYAPAISKKSKKRITEKIRGWHLNSRVQLKLSDIAMEINAEVRGWMNYYGKFYGSQLKAFLQCVN
LKLARWAERKYKRFRRKPNDAYKWLVWIASKNPTLFYHWQHGVKPNRLKPFG
>SRS103987||gene_21909|GeneMark.hmm|430_aa|+|1450|2742
MQNDNAKPISISKQLVYDAFLRVKANRGSAGIDKVTLEDYEKNLRGNLYKLWNRMSSGSYFPPSVKLVEIPKSTG
GKRPLGIPTVSDRVAQMAIVMLITPSIEPCFHEDSYAYRPHRSAHDAVGKARERCWKYAWVLDMDISKFFDTID
HELLLKALKRHTQEKWVLMYIERWLKVPYEKADGSQVDRALGVPQGSVIGPVLANLFLHYTFDKWMEKSFPR
VPFERYADDTICHCHSLKQAEYMQAMIQQRFECCRLRLNEEKTKIVYCKSSRQKEFYPNVTFDFLGFTFQPRESV
DKYGNRFTGFLPAISRKSMKRINETIRSWHLNRHSNLTLEHLASDINPIVRGWMTYYGKFYPTRLKWFMQTLN
GRLARWVMCKFERYRHRFYPAQEWLARIAEKEGLIFYHWKCGVLPRFTNKEKVSSQLIMVK
>SRS104036||gene_33295|GeneMark.hmm|420_aa|+|2675|3937
MNEAKPFVIDKRLVWEAYHKVKENKGSAGIDKVDQKTFDKEMSKNLYKIWNRMSSGCYFPKAVKLVEIPKFNG
GTRPLGIPTIEYRIAQQVVVSVLTPILEPIFKEDSYGYRPGKGVHQAIAKAKERCYVTPWVLDMDISKFFDTINHEL
LMKAIRKHTEEKWVLLYIERWLKVPNQTSKGEVIERTMGVPQGSVIGPVLANLFLHYVFDEWMSRNYPTIPFER
YADATICHCVSEKQARFLKAILMKRFEEYGLKLNEEKTKIVYCKDSNRRGDSEHTSFNFLGFTFRPRGARNRKTG
QNFTAFLPAISNKSMKRIKEAIRAWKQNRKTFACLLDISTEVDTQISGWMNYYMKFGRSEFRKVLNYINERLTR
WVMRKYKRFSKGKKFSKAYEWLVEYAAHNRNEFSHWVKGFVPYPRLD
>SRS1041037||gene_17972|GeneMark.hmm|399_aa|-|304|1503
KENKGSAGIDKVDQKTFDKEMSKNLYKIWNRMSSGCYFPKAVKLVEIPKSNGGTRPLGIPTIEDRIAQQVVVSVL
TPILEPIFKEDSYGYRPGKGAHQAIAKAKERCYVNPWVLDMDISKFFDTINHDLLMKAVRKHTEEKWVLLYIER
WLKVPYQTSKGEVIERTMGVPQGSVIGPVLANLFLHYVFDEWMSRNYPTIPFERYADDTICHCVSEKQAQFLKA
VLMKRFEECGLKLNEEKTKIVYCKDSNRRGDSEHTSFDFLGFTFRPRSARNRKTGQNFTAFLPAISKKSLKRIKEAV
RAWKLNRKTFACLLDISNEVDTQISGWMNYYMKFGRSEFRKVLNYINERLTRWVMRKYKRFSKGKKFSRAYE
WLVEYAVHNRNEFSHWAKGFVPYPRLG
>SRS1041130||gene_84605|GeneMark.hmm|422_aa|-|5474|6742
MKDAKSFEISRHLVMEAYKRVRANKGAAGVDDVSIADFESNLKSNLYKIWNRMSSGSYFPPAVKLVEIPKSNGG
KRPLGIPTIGDRVAQMVVVMTIEPGIEPYFHEDSYAYRPNRSALDAVRKAKERSYTFHWVLDLDIKGFFDNIDHG
LLIKALERHVKCEWAMLYIKRWLSVPYQLKDGTQIERTKGVPQGSVVGPILANLFLHYVFDEWMRRNHSNISFE
RYADDTICHCVSLKQAEFILRAIRKRFAECKLELNEDKTKIVYCKKNHRDIPYECIQFDFLGYTFRPRRSVDANGEV
FLNFSPAISKKARTKIWEAIQNWNSNHWVPMELEDIAKEINPVIQGWINYYGQHNPRILKEVLQHVNDRLVR

WGRRKFKGLRKRKTATVHRLGDIALQKPNLFAHWAWGVKPTASERNRKRK
>SRS1041132||gene_46150|GeneMark.hmm|430_aa|-|9187|10479
MQNGNAKPISISKQLVYDAFLRVKANRGSAGIDKVTLEDYEKNLRGNLYKLWNRMSSGSYFPPSVKLVEIPKSTG
GKRPLGIPTVSDRVAQMAIVMLITPSIEPCFHEDSYAYRPHRSAHDAVGKARERCWKYAWVLDMDISKFFDTID
HELLLKALKRHTQEKWVLMYIERWLKVPYEKADGSQVDRALGVPQGSVIGPVLANLFLHYTFDKWMEKSFPR
VPFERYADDTICHCHSLKQAEYMQAMIQQRFECCRLRLNEEKTKIVYCKSSRQKGRYPNVTFDFLGFTFQPRES
VDKYGSRFTGFLPAISRKSMKRINETIRSWHLNRHSNLTLEHLASDINPIVRGWMTYYGKFYPTRLKWFMQTLN
GRLARWIMCKFERYRHRFYPAQEWLARIAEKEGLIFYHWKCGVLPRFTNKEKVSSQLIMVK
>SRS1041136||gene_186252|GeneMark.hmm|422_aa|+|997|2265
MMQHQVTKPFTIDKYLIMNAWKRVKENKGSAGIDNVSTEDYESNLGKNLYKLWNRMSSGSYFPEAVKLVDIP
KPSGGTRPLGIPTVGDRIAQMSVVLLIEERLEAIFHADSYGYRPNRSAHDAIEKARERCWHYNWVLDMDISKFF
DTIDHDLLMKAVERHVQEKWILLYIRRWLKVPYATLTGERIERKMGVPQGSVIGPVLANLYLHYTFDKWMSLYH
PTIPFERYADDTICHCNSLKEAQMLKASIVERFAACKLRLNEEKTRIVYCKDGKRRGEYKEITFDFLGYTFQPRGQ
RNKQGQVFNGYAPAISRKSKKRITEKMRGWHLNRRVQLKLSDIAVEINAEVRGWMNYYGKFYGSQLKAFLQCI
NLKLARWAERKYKRFRRKPNDAYKWLVRVASKNPALFYHWQHGVKPNRLKPFG
>SRS1041137||gene_134854|GeneMark.hmm|421_aa|+|1108|2373
MQEAKPFQIDKRIIFESFKKVKFNRGSSGIDGIEMTTYEQNLGSNLYRLWNRMSSGSYMPKAVKLVEIPKSNGG
KRPLGIPTIEDRIAQMAVVNVIEPLIEPCFHEDSFGYRPHRSAHDAIAKAERRCWKYAWVLDIDISKFFDTIDHGL
LMKAVEKHINIKWILLYIKRWLTVPYQRSDGEIVKRDMGVPQGSVIGPILANLFLHYTFDKWMSYKYPHIPFERY
ADDCVCHCSTLAQAEYIKERLGERFTECKLKFNEEKTKIVFCKMSSRSSKHYHCTSFDYLGFTFRSRAAKDKRNN
VLFTSYLPAISKKSVSRIHETIKSWNLKRLHNRSLRFVAAYINDVVRGWINYYEKFGKTEFWKVMCHLNRSIAYW
AKTKYKRLRRRGVISAHYWLAYIAQKEPNLFYHWQVGYVPYARQKK
>SRS104311||gene_130324|GeneMark.hmm|418_aa|-|25225|26481
MSEAKQFDISKKAVIAAFQAVKENAGSYGADEQTIKEFEEHLNNNLYKLWNRMASGSYFPKPVRAVAIPKKNG
GIRILGIPTVEDRIAQMVAKMYFEPLVEPMFYNDSYGYRPNKSAIQAVGQARERCFKRDWVLELDIKGLFDNIK
HGYLMYMVEKHTQIKWLILYIKRWLTVPFIMSDGSVAERRSGTPQGGVISPVLANLFLHYVFDDFMTKAYPNI
WWERYADDGVLHCQSYKQAAFIKQKLEERFQQFGLELNKEKTRIVYCKDNRRPQNYSCTQFTFLGYTFRPRLN
KNKEGKFFVGFTPAVSEKAKTAMKQKIREWKIQLKADLSLKDIGNMINKVVQGWINYYTHYYKSEFYEVLRYIN
QCLIKWVRRSYKKKNTRSRAEHWLGAVARRDRNLFAHWKFGILPSVGEGAV
>SRS104311||gene_60407|GeneMark.hmm|430_aa|-|6598|7890
MQNDNAKPISISKQLVYDAFLRVKANRGSAGIDKVTLEDYEKNLRGNLYKLWNRMSSGSYFPPSVKLVEIPKTTG
GKRPLGIPTVSDRVAQMAVVMLITPSIEPCFHEDSYAYRPHRSAHDAVGKARERCWKYAWVLDMDISKFFDTI
DHELLLKALKRHTQEKWVLMYIERWLKVPYEKSDGSQVDRALGVPQGSVIGPVLANLFLHYTFDKWMEKNFP
RVPFERYADDTICHCHSLKQAEYMQAMIQQRFECCRLRLNEEKTKIVYCKSSRQKECYPNVTFDFLGFTFQPRES
VDKYGNRFTGFLPAISRKSMKRINETMRSWHLNRHSNLTLEHLASDINPIVRGWMTYYGKFYPTRLKWFMQTL
NGRLARWVMCKFERYRHRFYPAQEWLARIAEKEGLIFYHWKCGVLPRFTNKEKVSSQLIMVK
>SRS104485||gene_23761|GeneMark.hmm|383_aa|-|1|1149
MSEAKQFDISKKAVIAAFQAVKENAGSYGADEQTIKEFEEHLNNNLYKLWNRMASGSYFPKPVRAVAIPKKNG
GIRILGIPTVEDRIAQMVAKMYFEPLVEPMFYNDSYGYRPNKSAIQAVGQARERCFKRDWVLELDIKGLFDNIK
HGYLMYMVEKHTQIKWLILYIKRWLTVPFIMSDGSVAERRSGTPQGGVISPVLANLFLHYVFDDFMTKAYPNI
WWERYADDGVLHCQSYKQAVFIKQKLEERFQQFGLELNKEKTRIVYCKDDRRSRNYSCTQFTFLGYTFRPRLNK
NKEGKFFVGFTPAVSEKAKTAMKQKIRGWKIQLKADLSLKDIGNMINKVVQGWINYYTHYYKSEFYEVLRYINQ
CLIKWVRRSYKKKNT
>SRS104636||gene_129573|GeneMark.hmm|430_aa|+|23477|24769

MQNDNAKPISISKQLVYDAFLRVKANRGSAGIDKVTLEDYEKNLRGNLYKLWNRMSSGSYFPPSVKLVEIPKSTG
GKRPLGIPTVSDRVAQMAVVMLITPSIEPCFHEDSYAYRPHRSAHDAVGKARERCWKYAWVLDMDISKFFDTI
DHELLLKALKRHTQEKWVLMYIERWLKVPYEKSDGSQVDRALGVPQGSVIGPVLANLFLHYTFDKWMEKNFP
RVPFERYADDTICHCHSLKQAEYMQAMIQQRFECCRLRLNEEKTKIVYCKSSRQKECYPNVTFDFLGFTFQPRES
VDKYGNRFTGFLPAISRKSMKRINETMRSWHLNRHSNLTLEHLASDINPIVRGWMTYYGKFYPTRLKWFMQTL
NGRLARWVMCKFERYRHRFYPAQEWLARIAEKEGLIFYHWKCGALPRFTNKEKVSSQLIMVK
>SRS104693||gene_74250|GeneMark.hmm|423_aa|-|88|1359
MQEAKPKPFQIDKRIIFESFKKVKFNRGSSGIDGIEMTTYEQNLGSNLYRLWNRMSSGSYMPKAVKLVEIPKSN
GGKRPLGIPTIEDRIAQMAVVNVIEPLIEPCFHEDSFGYRPHRSAHDAIAKAERRCWKYAWVLDIDISKFFDTIDH
GLLMKAVEKHINIKWILLYIKRWLTVPYQRSDGEIVKRDMGVPQGSVIGPILANLFLHYTFDKWMSYKYPHIPFE
RYADDCVCHCSTLAQAEYIKERLGERFTECKLKFNEEKTKIVFCKMSSRSSKHYHCTSFDYLGFTFRSRAAKDKRN
NVLFTSYLPAISKKSVSRIHETIKSWNLKRLHNRSLRFVAAYINDVVRGWINYYEKFGKTEFWKVMCHLNRSIAY
WAKTKYKRLRRRGVISAHYWLAYIAQKEPNLFYHWQVGYVPYARQKK
>SRS1054929||gene_28956|GeneMark.hmm|421_aa|+|1595|2860
MQEAKPFQIDKRIIFEAFKKVKSNGGSPGIDGIEMSAYEQNLGSNFYRLWNRMSSGSYMPKAVKLVEILKSNGG
KRPLGIPSVEDRIAQMAVVNVIEPLVEPYFHKDSFGYRPHRSAHDAIAKAERRCWKYAWVLDIDISKFFDTIDHG
LLMKAVEKHIKTKWILLYIKRWLTVPYQGNDGAIVKRHMGVPQGSVIGPILANQFLHYTFDKWMSYKYPHVPF
ERYADDCVCHCGTLAQAEYIKDRLGERFAECKLTFNEEKTKIVFCKTSNRSSEHYHCTSFDYLGFTFRPRAAKDKR
KNVLFTSYLPAISNKSESRIHETIKSWNLKRLHNRSLRFVAAYINDVVRGWISYYGKFGKTEFWKVMCHLNRSIAY
WAKTKYKRLRRRGVISAHYWLAYIAQKEPNLFYHWQVGYIPYARQKK
>SRS1055043||gene_115261|GeneMark.hmm|430_aa|-|24278|25570
MQNDNAKPISISKQLVYDAFLRVKANRGSAGIDKVTLEDYEKNLRGNLYKLWNRMSSGSYFPPSVKLVEIPKSTG
GKRPLGIPTVSDRVAQMAVVMLITPSIEPCFHEDSYAYRPHRSAHDAVGKARERCWKYAWVLDMDISKFFDTI
DHELLLKALKRHTQEKWVLMYIERWLKVPYEKSDGSQVDRALGVPQGSVIGPVLANLFLHYTFDKWMEKNFP
RVPFERYADDTICHCHSLKQAEYMQAMIQQRFECCRLRLNEEKTKIVYCKSSRQKECYPNVTFDFLGFTFQPRES
VDKYGNRFTGFLPAISRKSMKRINETMRSWHLNRHSNLTLEHLASDINPIVRGWMTYYGKFYPTRLKWFMQTL
NGRLARWVMCKFERYRHRFYPAQEWLARIAEKEGLIFYHWKCGALPRFTNKEKVSSQLIMVK
>SRS1055067||gene_20053|GeneMark.hmm|430_aa|+|547|1839
MQNDNAKPISISKQLVYDAFLRVKANRGSAGIDKVTLEDYEKNLRGNLYKLWNRMSSGSYFPPSVKLVEIPKSTG
GKRPLGIPTVSDRVAQMAVVMLITPSIEPCFHEDSYAYRPHRSAHDAVGKARERCWKYAWVLDMDISKFFDTI
DHELLLKALKRHTQEKWVLMYIERWLKVPYEKSDGSQVDRALGVPQGSVIGPVLANLFLHYTFDKWMEKNFP
RVPFERYADDTICHCHSLKQAEYMQAMIQQRFECCRLRLNEEKTKIVYCKSSRQKECYPNVTFDFLGFTFQPRES
VDKYGNRFTGFLPAISRKSMKRINETMRSWHLNRHSNLTLEHLASDINPIVRGWMTYYGKFYPTRLKWFMQTL
NGRLARWVMCKFERYRHRFYPAQEWLARIAEKEGLIFYHWKCGALPRFTNKEKVSSQLIMVK
>SRS1055099||gene_136920|GeneMark.hmm|430_aa|-|81|1373
MQNDNAKPISISKQLVYDAFLRVKANRGSAGIDKVTLEDYEKNLRGNLYKLWNRMSSGSYFPPSVKLVEIPKSTG
GKRPLGIPTVSDRVAQMAVVMLITPSIEPCFHEDSYAYRPHRSAHDAVGKARERCWKYAWVLDMDISKFFDTI
DHELLLKALKRHTQEKWVLMYIERWLKVPYEKSDGSQVDRALGVPQGSVIGPVLANLFLHYTFDKWMEKNFP
RVPFERYADDTICHCHSLKQAEYMQAMIQQRFECCRLRLNEEKTKIVYCKSSRQKECYPNVTFDFLGFTFQPRES
VDKYGNRFTGFLPAISRKSMKRINETMRSWHLNRHSNLTLEHLASDINPIVRGWMTYYGKFYPTRLKWFMQTL
NGRLARWVMCKFERYRHRFYPAQEWLARIAEKEGLIFYHWKCGALPRFTNKEKVSSQLIMVK
>SRS140492||gene_92345|GeneMark.hmm|416_aa|-|1560|2810
MTKTKAFNIDKSLVVSAYRRVKTSAGAAGIDKQSLADFDKRLVDNLYKIWNRLSSGSYFPPAVKAVAIPKKLGGER
ILGIPTVSDRIAQTVVKLAFEPQVEPHFLADSYGYRPNKSALDAIGVTRKRCWYYDWVLEFDIKGLFDNIPHELIM

KAVDKHNPARWVKLYIQRWLTAPMVMSDGEVRARTMGTPQGGVISPLLANLFMHYVFDKWLAKYYPKVPW
YRYADDGILHCHSEAEATEMREVLRKRFSECGLEMHPEKTRVIYCKDGSRKGDYEHTMFDFLGYTFRRRVVKNV
KRNSLFVSFTPAASKSALKAMRREIKATGIRKRVDLSIEQIAKWINPKLNGWINYYGRYTCSELYSVFRYINKALVR
WGRKKYKMLSRYKTRASKFLEEMAKRSPQLFAHWRLKMRGGLV
>SRS140492||gene_77776|GeneMark.hmm|430_aa|-|24315|25607
MQNDNAKPISISKQLVYDAFLRVKANRGSAGIDKVTLEDYEKNLRGNLYKLWNRMSSGSYFPPSVKLVEIPKSTG
GKRPLGIPTVSDRVAQMAIVMLITPSIEPCFHEDSYAYRPHRSAHDAVGKARERCWKYAWVLDMDISKFFDTID
HELLLKALKRHTQEKWVLMYIERWLKVPYEKADGSQVDRALGVPQGSVIGPVLANLFLHYTFDKWMEKSFPR
VPFERYADDTICHCHSLKQAEYMQAMIQQRFECCRLRLNEEKTKIVYCKSSRQKEFYPNVTFDFLGFTFQPRESV
DKYGNRFTGFLPAISRKSMKRINETIRSWHLNRHSNLTLEHLASDINPIVRGWMTYYGKFYPTRLKWFMQTLN
GRLARWVMCKFERYRHRFYPAQEWLARIAEKEGLIFYHWKCGVLPRFTNKEKVSSQLIMVK
>SRS142503||gene_210001|GeneMark.hmm|418_aa|+|1331|2587
MSEAKQFDISKKAVIAAFQAVKENAGSYGADEQTIKEFEEHLNNNLYKLWNRMASGSYFPKPVRAVAIPKKNG
GIRILGIPTVEDRIAQMVAKMYFEPLVEPMFYNDSYGYRPNKSAIQAVGQARERCFKRDWVLELDIKGLFDNIK
HGYLMYMVEKHTQIKWLILYIKRWLTVPFIMSDGSVAERRSGTPQGGVISPVLANLFLHYVFDDFMTKAYPNI
WWERYADDGVLHCQSYKQAAFIKQKLEERFQQFGLELNKEKTRIVYCKDNRRPQNYSCTQFTFLGYTFRPRLN
KNKEGKFFVGFTPAVSEKAKTAMKQKIREWKIQLKADLSLKDIGNMINKVVQGWINYYTHYYKSEFYEVLRYIN
QCLIKWVRRSYKKKNTRSRAEHWLGAVARRDRNLFAHWKFGILPSVGEGAV
>SRS142599||gene_119398|GeneMark.hmm|430_aa|-|84|1376
MQNDNAKPISISKQLVYDAFLRVKANRGSAGIDKVTLEDYEKNLRGNLYKLWNRMSSGSYFPPSVKLVEIPKSTG
GKRPLGIPTVSDRVAQMAVVMLITPSIEPCFHEDSYAYRPHRSAHDAVGKARERCWKYAWVLDMDISKFFDTI
DHELLLKALKRHTQEKWVLMYIERWLKVPYEKSDGSQVDRALGVPQGSVIGPVLANLFLHYTFDKWMEKNFP
RVPFERYADDTICHCHSLKQAEYMQAMIQQRFECCRLRLNEEKTKIVYCKSSRQKECYPNVTFDFLGFTFQPRES
VDKYGNRFTGFLPAISRKSMKRINETMRSWHLNRHSNLTLEHLASDINPIVRGWMTYYGKFYPTRLKWFMQTL
NGRLARWVMCKFERYRHRFYPAQEWLARIAEKEGLIFYHWKCGALPRFTNKEKVSSQLIMVK
>SRS142618||gene_206839|GeneMark.hmm|430_aa|+|877|2169
MQNDNAKPISISKQLVYDAFLRVKANRGSAGIDKVTLEDYEKNLRGNLYKLWNRMSSGSYFPPSVKLVEIPKSTG
GKRPLGIPTVSDRVAQMAVVMLITPSIEPCFHEDSYAYRPHRSAHDAVGKARERCWKYAWVLDMDISKFFDTI
DHELLLKALKRHTQEKWVLMYIERWLKVPYEKSDGSQVDRALGVPQGSVIGPVLANLFLHYTFDKWMEKNFP
RVPFERYADDTICHCHSLKQAEYMQAMIQQRFECCRLRLNEEKTKIVYCKSSRQKECYPNVTFDFLGFTFQPRES
VDKYGNRFTGFLPAISRKSMKRINETMRSWHLNRHSNLTLEHLASDINPIVRGWMTYYGKFYPTRLKWFMQTL
NGRLARWVMCKFERYRHRFYPAQEWLARIAEKEGLIFYHWKCGALPRFTNKEKVSSQLIMVK
>SRS142712||gene_21105|GeneMark.hmm|418_aa|+|1994|3250
MSEAKQFDISKKAVIAAFQAVKENAGSYGADEQTIKEFEEHLNNNLYKLWNRMASGSYFPKPVRAVAIPKKNG
GIRILGIPTVEDRIAQMVAKMYFEPLVEPMFYNDSYGYRPNKSAIQAVGQARERCFKRDWVLELDIKGLFDNIK
HGYLMYMVEKHTQIKWLILYIKRWLTVPFIMSDGSVAERRSGTPQGGVISPVLANLFLHYVFDDFMTKAYPNI
WWERYADDGVLHCQSYKQAVFIKQKLEERFQQFGLELNKEKTRIVYCKDDRRSRNYSCTQFTFLGYTFRPRLNK
NKEGKFFVGFTPAVSEKAKTAMKQKIRGWKIQLKADLSLKDIGNMINKVVQGWINYYTHYYKSEFYEVLRYINQ
CLIKWVRRSYKKKNTRSRAEHWLGAVARRDRNLFAHWKFGILPSVGEGAV
>SRS142890||gene_69162|GeneMark.hmm|430_aa|+|21779|23071
MQNDNAKPISISKQLVYDAFLRVKANRGSAGIDKVTLEDYEKNLRGNLYKLWNRMSSGSYFPPSVKLVEIPKSTG
GKRPLGIPTVSDRVAQMTVVMLITPSIEPCFHEDSYAYRPHRSAHDAVGKARERCWKYAWVLDMDISKFFDTID
HELLLKALKRHTQEKWVLMYIERWLKVPYEKSDGSQVDRALGVPQGSVIGPVLANLFLHYTFDKWMEKNFPR
VPFERYADDTICHCHSLKQAEYMQAMIQQRFECCRLRLNEEKTKIVYCKSSRQKECYPNVTFDFLGFTFQPRESV

DKYGNRFTGFLPAISRKSMKRINETMRSWHLNRHSNLTLEHLASDINPIVRGWMTYYGKFYPTRLKWFMQTLN
GRLASWVMCKFERYRHRFYPAQEWLARIAEKEGLIFYHWKCGVLPRFTNKEKVSSQLIMVK
>SRS142921||gene_178781|GeneMark.hmm|430_aa|+|23220|24512
MQNDNAKPISISKQLVYDAFLRVKANRGSAGIDKVTLEDYEKNLRGNLYKLWNRMSSGSYFPPSVKLVEIPKSTG
GKRPLGIPTVSDRVAQMTVVMLITPSIEPCFHEDSYAYRPHRSAHDAVGKARERCWKYAWVLDMDISKFFDTID
HELLLKALKRHTQEKWVLMYIERWLKVPYEKSDGSQVDRALGVPQGSVIGPVLANLFLHYTFDKWMEKNFPR
VPFERYADDTICHCHSLKQAEYMQAMIQQRFECCRLRLNEEKTKIVYCKSSRQKECYPNVTFDFLGFTFQPRESV
DKYGNRFTGFLPAISRKSMKRINETMRSWHLNRHSNLTLEHLASDINPIVRGWMTYYGKFYPTRLKWFMQTLN
GRLASWVMCKFERYRHRFYPAQEWLARIAEKEGLIFYHWKCGVLPRFTNKEKVSSQLIMVK
>SRS143417||gene_128438|GeneMark.hmm|430_aa|+|22293|23585
MQNDNAKPISISKQLVYDAFLRVKANRGSAGIDKVTLEDYEKNLRGNLYKLWNRMSSGSYFPPSVKLVEIPKSTG
GKRPLGIPTVSDRVAQMAVVMLITPSIEPCFHEDSYAYRPHRSAHDAVGKARERCWKYAWVLDMDISKFFDTI
DHELLLKALKRHTQEKWVLMYIERWLKVPYEKSDGSQVDRALGVPQGSVIGPVLANLFLHYTFDKWMEKNFP
RVPFERYADDTICHCHSLKQAEYMQAMIQQRFECCRLRLNEEKTKIVYCKSSRQKECYPNVTFDFLGFTFQPRES
VDKYGNRFTGFLPAISRKSMKRINETMRSWHLNRHSNLTLEHLASDINPIVRGWMTYYGKFYPTRLKWFMQTL
NGRLARWVMCKFERYRHRFYPAQEWLARIAEKEGLIFYHWKCGVLPRFTNKEKVSSQLIMVK
>SRS143598||gene_53183|GeneMark.hmm|423_aa|-|125|1396
MQEAKPKPFQIDKRIIFESFKKVKFNRGSSGIDGIEMTTYEQNLGSNLYRLWNRMSSGSYMPKAVKLVEIPKSN
GGKRPLGIPTIEDRIAQMAVVNVIEPLIEPCFHEDSFGYRPHRSAHDAIAKAERRCWKYAWVLDIDISKFFDTIDH
GLLMKAVEKHINIKWILLYIKRWLTVPYQRSDGEIVKRDMGVPQGSVIGPILANLFLHYTFDKWMSYKYPHIPFE
RYADDCVCHCSTLAQAEYIKERLGERFTECKLKFNEEKTKIVFCKMSSRSSKHYHCTSFDYLGFTFRSRAAKDKRN
NVLFTSYLPAISKKSVSRIHETIKSWNLKRLHNRSLRFVAAYINDVVRGWINYYEKFGKTEFWKVMCHLNRSIAY
WAKTKYKRLRRRGVISAHYWLAYIAQKEPNLFYHWQVGYVPYARQKK
>SRS143722||gene_69873|GeneMark.hmm|430_aa|-|473|1765
MQNDNAKPISISKQLVYDAFLRVKANRGSAGIDKVTLEDYEKNLRGNLYKLWNRMSSGSYFPPSVKLVEIPKSTG
GKRPLGIPTVSDRVAQMAVVMLITPSIEPCFHEDSYAYRPHRSAHDAVGKARERCWKYAWVLDMDISKFFDTI
DHELLLKALKRHTQEKWVLMYIERWLKVPYEKSDGSQVDRALGVPQGSVIGPVLANLFLHYTFDKWMEKNFP
RVPFERYADDTICHCHSLKQAEYMQAMIQQRFECCRLRLNEEKTKIVYCKSSRQKECYPNVTFDFLGFTFQPRES
VDKYGNRFTGFLPAISRKSMKRINETMRSWHLNRHSNLTLEHLASDINPIVRGWMTYYGKFYPTRLKWFMQTL
NGRLARWVMCKFERYRHRFYPAQEWLARIAEKEGLIFYHWKCGALPRFTNKEKVSSQLIMVK
>SRS143780||gene_226287|GeneMark.hmm|414_aa|-|1072|2316
MQETKPYSISKRAVIAAYEKVKANKGTYGVDEQSIEDFERKLNNNLYKIWNRMSSGSYFPKPVKAVAIPKKNGG
TRILGIPTVEDRIAQMVVKLYLEPSVEPIFYDDSYGYRPNKSAIQAIEVTRTRCWRKDWVLEFDIKGLFDNIRHDY
LMDMVKRHTKEEWILLYIQRWLIAPFQMEDGTIVPRTSGTPQGGVISPVLANLFLHYVFDDFMVKEFPSIPWA
RYADDGIAHCVSLKQAKYLQRRLQERFVGFGLELNMEKTRIVYCKDDDRKGKHEHTSFDFLGYTFRPRHAKNKY
GKFFTNFLPAMSEKAKKAIRKVVRGWKLQFKPDKDLWDIANMFNKQIQGWINYYTHFYKSEIYEVLRYINGRLV
YWVRRKYKNRNSRKRAEYWLGEIAKRDRNLFAHCKFGILPSAG
>SRS143876||gene_18664|GeneMark.hmm|430_aa|-|105630|106922
MQNDNAKPISISKQLVYDAFLRVKANRGSAGIDKVTLEDYEKNLRGNLYKLWNRMSSGSYFPPSVKLVEIPKSTG
GKRPLGIPTVSDRVAQMAIVMLITPSIEPCFHEDSYAYRPHRSAHDAVGKARERCWKYAWVLDMDISKFFDTID
HELLLKALKRHTQEKWVLMYIERWLKVPYEKADGSQVDRALGVPQGSVIGPVLANLFLHYTFDKWMEKSFPR
VPFERYADDTICHCHSLKQAEYMQAMIQQRFECCRLRLNEEKTKIVYCKSSRQKEFYPNVTFDFLGFTFQPRESV
DKYGNRFTGFLPAISRKSMKRINETIRSWHLNRHSNLTLEHLASDINPIVRGWMTYYGKFYPTRLKWFMQTLN
GRLARWVMCKFERYRHRFYPAQEWLARIAEKEGLIFYHWKCGVLPRFTNKEKVSSQLIMVK

>SRS144007||gene_103285|GeneMark.hmm|418_aa|-|162|1418
MSEAKQFDISKKAVIAAFQAVKENAGSYGADEQTIKEFEEHLNNNLYKLWNRMASGSYFPKPVRAVAIPKKNG
GIRILGIPTVEDRIAQMVAKMYFEPLVEPMFYNDSYGYRPNKSAIQAVGQARERCFKRDWVLELDIKGLFDNIK
HGYLMYMVEKHTQIKWLILYIKRWLTVPFIMSDGSVAERRSGTPQGGVISPVLANLFLHYVFDDFMTKAYPNI
WWERYADDGVLHCQSYKQAAFIKQKLEERFQQFGLELNKEKTRIVYCKDNRRPQNYSCTQFTFLGYTFRPRLN
KNKEGKFFVGFTPAVSEKAKTAMKQKIREWKIQLKADLSLKDIGNMINKVVQGWINYYTHYYKSEFYEVLRYIN
QCLIKWVRRSYKKKNTRSRAEHWLGAVARRDRNLFAHWKFGILPSVGEGAV
>SRS144135||gene_159116|GeneMark.hmm|430_aa|-|219|1511
MQNDNAKPISISKQLVYDAFLRVKANRGSAGIDKVTLEDYEKNLRGNLYKLWNRMSSGSYFPPSVKLVEIPKSTG
GKRPLGIPTVSDRVAQMAVVMLITPSIEPCFHEDSYAYRPHRSAHDAVGKARERCWKYAWVLDMDISKFFDTI
DHELLLKALKRHTQEKWVLMYIERWLKVPYEKSDGSQVDRALGVPQGSVIGPVLANLFLHYTFDKWMEKNFP
RVPFERYADDTICHCHSLKQAEYMQAMIQQRFECCRLRLNEEKTKIVYCKSSRQKECYPNVTFDFLGFTFQPRES
VDKYGNRFTGFLPAISRKSMKRINETMRSWHLNRHSNLTLEHLASDINPIVRGWMTYYGKFYPTRLKWFMQTL
NGRLARWVMCKFERYRHRFYPAQEWLARIAEKEGLIFYHWKCGVLPRFTNKEKVSSQLIMVK
>SRS144362||gene_142422|GeneMark.hmm|418_aa|-|1029|2285
MSEAKQFDISKKAVIAAFQAVKENAGSYGADEQTIKEFEEHLNNNLYKLWNRMASGSYFPKPVRAVAIPKKNG
GIRILGIPTVEDRIAQMVAKMYFEPLVEPMFYNDSYGYRPNKSAIQAVGQARERCFKRDWALELDIKGLFDNIK
HGYLMYMVEKHTQIKWLILYIKRWLTVPFIMSDGSVAERRSGTPQGGVISPVLANLFLHYVFDDFMTKAYPNI
WWERYADDGVLHCQSYKQAAFIKQKLEERFQQFGLELNKEKTRIVYCKDNRRPQNYSCTQFTFLGYTFRPRLN
KNKEGKFFVGFTPAVSEKAKTAMKQKIREWKIQLKADLSLKDIGNMINKVVQGWINYYTHYYKSEFYEVLRYIN
QCLIKWVRRSYKKKNTRSRAEHWLGAVARRDRNLFAHWKFGILPSVGEGAV
>SRS144506||gene_197038|GeneMark.hmm|420_aa|+|1557|2819
MNEAKPFVIDKRLVWEAYHKVKENKGSAGINKVDQKTFDKEMSKNLYKIWNRMSSGCYFSKAVKLVEIPKSNG
GTRPLGIPTIEYRIAQQVVVSVLTPILEPIFKEDSYGYRPGKGVHQAIAKAKERCYVTPWVLDMDISKFFDTINHEL
LMKAIRKHTEEKWVLLYIERWLKVPNQTSKGEVIERTMGVPQGSVIGPVLANLFLHYVFDEWMSRNYPTIPFER
YADATICHCVSEKQARFLKAVLMKRFEEYGLKLNEEKTKIVYCKDSNRRGDSEHTSFNFLGFTFRPRGARNRKTG
QNFTAFLPAISNKSMKRIKEAIRAWKQNRKTFACLLDISTEVDTQISGWMNYYMKFGRSEFRKVLNYINERLTR
WVMRKYKRFSKGKKFSKAYEWLVEYAAHNRNEFSHWVKGFVPYPRLD
>SRS144537||gene_185215|GeneMark.hmm|420_aa|+|2473|3735
MNEAKPFVIDKRLVWEAYHKVKENKGSAGIDKVDQKTFDKEMSKNLYKIWNRMSSGCYFPKAVKLVEIPKSNG
GTRPLGIPTIEDRIAQQVVVSVLTPILEPIFKEDSYGYRPGKGAHQAIAKAKERCYVNPWVLDMDISKFFDTINHE
LLMKAVRKHTGEKWVLLYIERWLKVPYQTSKGEVIERTMGVPQGSVIGPVLANLFLHYVFDEWMSRNYPTIPF
ERYADDTICHCVSEKQAQFLKAVLIKRFEECGLKLNEEKTKIVYCKDSNRRGDSEHTSFDFLGFTFRPRGARNRKT
GQNFTAFLPAISKKSMKRIKESVRAWKLNRKTFACLLDISNEVDTQISGWMNYYMKFGRSEFRKVLNYINERLTR
WVMRKYKRFSKGKKLGKAYDWLVEYAAHNRNEFSHWVKGFVPYPRLG
>SRS145308||gene_34924|GeneMark.hmm|422_aa|+|756|2024
MMQHQVTKPFTIDKHLIMNAWKRVKENKGSVGIDNVSTDDYESNLGKNLYKLWNRMSSGSYFPEAVKLVDIP
KSSGGTRPLGIPTVGDRIAQMSVVLLIEDRLEAIFHADSYGYRPNRSAHDAIGKARERCWHYNWVLDMDISKFF
DTINHDLLMKAVERHVQEKWILLYIRRWLEVPYATLTGERIERRMGVPQGSVIGPVLANLYLHYTFDKWMSLYH
PTIPFERYADDTICHCNSLEEAQMLKASIVERFAACKLKLNEEKTRIVYCKDGKRRREYKDITFDFLGYTFQPRGQR
NKQGQVFNGYAPAISRKSKKRIAETMRGWHLNRRVQLKLSDIAVEINAEVRGWMNYYGKFYGSQLKAFLQCIN
LKLARWAERKYKRFRRKPNDAYKWLVRVASKNPALFYHWQHGVKPNRLKPFG
>SRS146764||gene_250883|GeneMark.hmm|430_aa|+|327|1619
MQNDNAKPISISKQLVYDAFLRVKANRGSAGIDKVTLEDYEKNLRGNLYKLWNRMSSGSYFPPSVKLVEIPKSTG

GKRPLGIPTVSDRVAQMAIVMLITPSIEPCFHEDSYAYRPHRSAHDAVGKARERCWKYAWVLDMDISKFFDTID
HELLLKALKRHTQEKWVLMYIERWLKVPYEKADGSQVDRALGVPQGSVIGPVLANLFLHYTFDKWMEKSFPR
VPFERYADDTICHCHSLKQAEYMQAMIQQRFECCRLRLNEEKTKIVYCKSSRQKEFYPNVTFDFLGFTFQPRESV
DKYGNRFTGFLPAISRKSMKRINETIRSWHLNRHSNLTLEHLASDINPIVRGWMTYYGKFYPTRLKWFMQTLN
GRLARWVMCKFERYRHRFYPAQEWLARIAEKEGLIFYHWKCGVLPRFTNKEKVSSQLIMVK
>SRS146812||gene_410105|GeneMark.hmm|430_aa|-|808|2100
MQNDNAKPISISKQLVYDAFLRVKANRGSAGIDKVTLEDYEKNLRGNLYKLWNRMSSGSYFPPSVKLVEIPKSTG
GKRPLGIPTVSDRVAQMAVVMLITPSIEPCFHEDSYAYRPHRSAHDAVGKARERCWKYAWVLDMDISKFFDTI
DHELLLKALKRHTQEKWVLMYIERWLKVPYEKSDGSQVDRALGVPQGSVIGPVLANLFLHYTFDKWMEKNFP
RVPFERYADDTICHCHSLKQAEYMQAMIQQRFECCRLRLNEEKTKIVYCKSSRQKECYPNVTFDFLGFTFQPRES
VDKYGNRFTGFLPAISRKSMKRINETMRSWHLNRHSNLTLEHLASDINPIVRGWMTYYGKFYPTRLKWFMQTL
NGRLARWVMCKFERYRHRFYPAQEWLARIAEKEGLIFYHWKCGVLPRFTNKEKVSSQLIMVK
>SRS146813||gene_100906|GeneMark.hmm|430_aa|-|378|1670
MQNDNAKPISISKQLVYDAFLRVKANRGSAGIDKVTLEDYEKNLRGNLYKLWNRMSSGSYFPPSVKLVEIPKSTG
GKRPLGIPTVSDRVAQMAVVMLITPSIEPCFHEDSYAYRPHRSAHDAVGKARERCWKYAWVLDMDISKFFDTI
DHELLLKALKRHTQEKWVLMYIERWLKVPYEKSDGSQVDRALGVPQGSVIGPVLANLFLHYTFDKWMEKNFP
RVPFERYADDTICHCHGLKQAEYMQAMIQQRFECCRLRLNEEKTKIVYCKSSRQKECYPNVTFDFLGFTFQPRE
SVDKYGNRFTGFLPAISRKSMKRINETMRSWHLNRHSNLTLEHLASDINPIVRGWMTYYGKFYPTRLKWFMQT
LNGRLARWVMCKFERYRHRFYPAQEWLARIAEKEGLIFYHWKCGALPRFTNKEKVSSQLIMVK
>SRS146832||gene_95320|GeneMark.hmm|430_aa|-|213|1505
MQNDNAKPISISKQLVYDAFLRVKANRGSAGIDKVTLEDYEKNLRGNLYKLWNRMSSGSYFPPSVKLVEIPKSTG
GKRPLGIPTVSDRVAQMAVVMLITPSIEPCFHEDSYAYRPHRSAHDAVGKARERCWKYAWVLDMDISKFFDTI
DHELLLKALKRHTQEKWVLMYIERWLKVPYEKSDGSQVDRALGVPQGSVIGPVLANLFLHYTFDKWMEKNFP
RVPFERYADDTICHCHGLKQAEYMQAMIQQRFECCRLRLNEEKTKIVYCKSSRQKECYPNVTFDFLGFTFQPRE
SVDKYGNRFTGFLPAISRKSMKRINETMRSWHLNRHSNLTLEHLASDINPIVRGWMTYYGKFYPTRLKWFMQT
LNGRLARWVMCKFERYRHRFYPAQEWLARIAEKEGLIFYHWKCGALPRFTNKEKVSSQLIMVK
>SRS147022||gene_17471|GeneMark.hmm|421_aa|+|3556|4821
MQEAKPFQIDKRIIFESFKKVKFNRGSSGIDGIEMTTYEQNLGSNLYRLWNRMSSGSYMPKAVKLVEIPKSNGG
KRPLGIPTIEDRIAQMAVVNVIEPLIEPCFHEDSFGYRPHRSAHDAIAKAERRCWKYAWVLDIDISKFFDTIDHGL
LMKAVEKHINIKWILLYIKRWLTVPYQRSDGEIVKRDMGVPQGSVIGPILANLFLHYTFDKWMSYKYPHIPFERY
ADDCVCHCSTLAQAEYIKERLGERFTECKLKFNEEKTKIVFCKMSSRSSKHYHCTSFDYLGFTFRSRAAKDKRNN
VLFTSYLPAIGKKSVSRIHETIKSWNLKRLHNRSLRFVAAYINDVVRGWINYYEKFGKTEFWKVMCHLNRSIAYW
AKTKYKRLRRRGVISAHYWLAYIAQKEPNLFYHWQVGYVPYARQKK
>SRS147139||gene_153607|GeneMark.hmm|430_aa|-|1685|2977
MQNDNAKPISISKQLVYDAFLRVKANRGSAGIDKVTLEDYEKNLRGNLYKLWNRMSSGSYFPPSVKLVEIPKSTG
GKRPLGIPTVSDRVAQMAVVMLITPSIEPCFHEDSYAYRPHRSAHDAVGKARERCWKYAWVLDMDISKFFDTI
DHELLLKALKRHTQEKWVLMYIERWLKVPYEKSDGSQVDRALGVPQGSVIGPVLANLFLHYTFDKWMEKNFP
RVPFERYADDTICHCHSLKQAEYMQAMIQQRFECCRLRLNEEKTKIVYCKSSRQKECYPNVTFDFLGFTFQPRES
VDKYGNRFTGFLPAISRKSMKRINETMRSWHLNRHSNLTLEHLASDINPIVRGWMTYYGKFYPTRLKWFMQTL
NGRLARWVMCKFERYRHRFYPAQEWLARIAEKEGLIFYHWKCGALPRFTNKEKVSSQLIMVK
>SRS147346||gene_366985|GeneMark.hmm|418_aa|+|623|1879
MSEAKQFDISKKAVIAAFQAVKENAGSYGADEQTIKEFEEHLNNNLYKLWNRMASGSYFPKPVRAVAIPKKNG
GIRILGIPTVEDRIAQMVAKMYFEPLVEPMFYNDSYGYRPNKSAIQAVGQARERCFKRDWVLELDIKGLFDNIK
HGYLMYMVEKHTQIKWLILYIKRWLTVPFIMSDGSVAERRSGTPQGGVISPVLANLFLHYVFDDFMTKAYPNI

WWERYADDGVLHCQSYKQAAFIKQKLEERFQQFGLELNKEKTRIVYCKDNRRPQNYSCTQFTFLGYTFRPRLN
KNKEGKFFVGFTPAVSEKAKTAMKQKIREWKIQLKADLSLKDIGNMINKVVQGWINYYTHYYKSEFYEVLRYIN
QCLIKWVRRSYKKKNTRSRAEHWLGAVARRDRNLFAHWKFGILPSVGEGAV
>SRS147652||gene_7978|GeneMark.hmm|423_aa|+|15300|16571
MTQKQGAKPFDIDRWKLYYAYKRVNQNRGGSGVDNVTLEKYNSNLKRNLYKLWNRMSSGSYVPKPVRLVQM
PKPAGGTRPLGIPTVEDRIAQMLVVEMIEPEIEKIFHEDSYGYRPDRSAHDALGRARERCWKYAWVLDMDISKF
FDTIDHQLLMKAVKLHVKERWIILYIERWLKVPYQNADKSLIERTCGVPQGSVIGPILANLFLHYSFDRWMQIHH
PEIPFERYADDTVCHCHSKQEAESLYEELIIRFKSCKLSLNEEKTKIVYCKSSRRNENHSNVTFDFLGHTFRPCKTIH
KSSRKAFTGFQPRISMKATTKIRATMRSWNLKSKSHTPLDCIAQMVNPILRGWANYYGKYGGKSFQKLLRYFDL
LLAKWAKAKYKTFRRKPMYVILKWLGNIAERDAIFYHWQIGLKPAKGTIKL
>SRS148319||gene_56653|GeneMark.hmm|422_aa|-|78|1346
MMQHQVTKPFTIDKYLIMNAWKRVKENKGSAGIDNVSTEDYESNLGKNLYKLWNRMSSGSYFPEAVKLVDIP
KPSGGTRPLGIPTVGDRIAQMSVVLLIEERLEAIFHADSYGYRPNRSAHDAIEKARERCWHYNWVLDMDISKFF
DTIDHDLLMKAVERHVQEKWILLYIRRWLKVPYATLTGERTERKMGVPQGSVIGPVLANLYLHYTFDKWMSLYH
PTIPFERYADDTICHCNSLKEAQMLKASIVERFAACKLRLNEEKTRVVYCKDGKRRGEYKEITFDFLGYTFQPRGQ
RNKQGQVFNGYAPAISRKSKKRITEKMRGWHLNRRVQLKLSDIAVEINAEVRGWMNYYGKFYGSQLKAFLQCI
NLKLARWAERKYKRFRRKPNDAYKWLVRVASKNPALFYHWQHGVKPNRLKPFG
>SRS148721||gene_201492|GeneMark.hmm|418_aa|+|13486|14742
MSEAKQFDISKKAVIAAFQAVKENAGSYGADEQTIKEFEEHLNNNLYKLWNRMASGSYFPKPVRAVAIPKKNG
GIRILGIPTVEDRIAQMVAKMYFEPLVEPMFYNDSYGYRPNKSAIQAVGQARERCFKRDWVLELDIKGLFDNIK
HGYLMYMVEKHTQIKWLILYIKRWLTVPFIMSDGSVAERRSGTPQGGVISPVLANLFLHYVFDDFMTKAYPNI
WWERYADDGVLHCQSYKQAAFIKQKLEERFQQFGLELNKEKTRIVYCKDNRRPQNYSCTQFTFLGYTFRPRLN
KNKEGKFFVGFTPAVSEKAKTAMKQKIREWKIQLKADLSLKDIGNMINKVVQGWINYYTHYYKSEFYEVLRYIN
QCLIKWVRRSYKKKNTRSRAEHWLGAVARRDRNLFAHWKFGILPSVGEGAV
>SRS150029||gene_79026|GeneMark.hmm|420_aa|-|13509|14771
MNEAKPFVIDKRLVWEAYHKVKENKGSAGIDKVDQKTFDKEMSKNLYKIWNRMSSGCYFSKAVKLVEIPKSNG
GTRPLGIPTIEYRIAQQVVVSVLTPILEPIFKEDSYGYRPGKGAHQAIAKAKERCYVTPWVLDMDISKFFDTINHEL
LMKAIRKHTEEKWVLLYIERWLKVPNQTSKGEVIERTMGVPQGSVIGPVLANLFLHYVFDEWMSRNYPTIPFER
YADATICHCVSEKQARFLKAVLMKRFEEYGLKLNEEKTKIVYCKDSNRRGDSEHTSFNFLGFTFRPRGARNRKTG
QNFTAFLPAISNKSMKRIKEAIRAWKLNRKTFACLLDISTEVDTQISGWMNYYMKFGRSEFRKVLNYINERLTRW
VMRKYKRFSKGKKFSKAYEWLVEYAAHNRNEFSHWVKGFVPYPRLD
>SRS886701||gene_85133|GeneMark.hmm|418_aa|+|3002|4258
MSEAKQFDISKKAVIAAFQAVKENAGSYGADEQTIKEFEEHLNNNLYKLWNRMASGSYFPKPVRAVAIPKKNG
GIRILGIPTVEDRIAQMVAKMYFEPLVEPMFYNDSYGYRPNKSAIQAVGQARERCFKRDWVLELDIKGLFDNIK
HGYLMYMVEKHTQIKWLILYIKRWLTVPFIMSDGSVAERRSGTPQGGVISPVLANLFLHYVFDDFMTKAYPNI
WWERYADDGVLHCQSYKQAAFIKQKLEERFQQFGLELNKEKTRIVYCKDNRRPQNYSCTQFTFLGYTFRPRLN
KNKEGKFFVGFTPAVSEKAKTAMKQRIREWKIQLKADLSLKDIGNMINKVVQGWINYYTHYYKSEFYEVLRYIN
QCLIKWVRRSYKKKNTRSRAEHWLGAVARRDRNLFAHWKFGILPSVGEGAV
>SRS893230||gene_56205|GeneMark.hmm|422_aa|+|946|2214
MKDAKSFEISRHLVMEAYKRVRANKGAAGVDDVSIADFESNLKSNLYKIWNRMSSGSYFPPAVKLVEIPKSNGG
KRPLGIPTIGDRVAQMVVVMTIEPGIEPYFHEDSYAYRPNRSALDAVRKAKERSYTFHWVLDLDIKGFFDNIDHG
LLIKALERHVKCEWAMLYIKRWLSVPYQLKDGTQIERTKGVPQGSVVGPILANLFLHYVFDEWMRRNHSNISFE
RYADDTICHCVSLKQAELILRAIRKRFAECKLELNEDKTKIVYCKKNHRDIPYECIQFDFLGYTFRPRRSVDANGEV
FLNFSPAISKKARTKIWEAIQNWNSNHWVPMELEDIAKEINPVIQGWINYYGQHNPRILKEVLQHVNDRLVR

WGRRKFKGLRKRKTATVHRLGDIALQKPNLFAHWAWGVKPTASERNRKRK
>SRS893231||gene_86853|GeneMark.hmm|428_aa|+|608|1894
MQNDNAKPISISKQLVYDAFLRVKANRGSAGIDKVTLEDYEKNLRGNLYKLWNRMSSGSYFPPSVKLVEIPKSTG
GKRPLGIPTVSDRVAQMAVVMLITPSIEPCFHEDSYAYRPHRSAHDAVGKARERCWKYAWVLDISKFFDTIDHE
LLLKALKRHTQEKWVLMYIERWLKVPYEKSDGSQVDRALGVPQGSVIGPVLANLFLHYTFDKWMEKNFPRVP
FERYADDTICHCHSLKQAEYMQAMIQQRFECCRLRLNEEKTKIVYCKSSRQKECYPNVTFDFLGFTFQPRESVDK
YGNRFTGFLPAISRKSMKRINETMRSWHLNRHSNLTLEHLASDINPIVRGWMTYYGKFYPTRLKWFMQTLNG
RLARWVMCKFERYRHRFYPAQEWLARIAEKEGLIFYHWKCGALPRFTNKEKVSSQLIMVK
>SRS893327||gene_51830|GeneMark.hmm|418_aa|-|692|1948
MSEAKQFDISKKAVIAAFQAVKENAGSYGADEQTIKEFEEHLNNNLYKLWNRMASGSYFPKPVRAVAIPKKNG
GIRILGIPTVEDRIAQMVAKMYFEPLVEPMFYNDSYGYRPNKSAIQAVGQARERCFKRDWVLELDIKGLFDNIK
HGYLMYMVEKHTQIKWLILYIKRWLTVPFIMSDGSVAERRSGTPQGGVISPVLANLFLHYVFDDFMTKAYPNI
WWERYADDGVLHCQSYKQAVFIKQKLEERFQQFGLELNKEKTRIVYCKDNRRPQNYSCTQFTFLGYTFRPRLNK
NKEGKFFVGFTPAVSEKAKTAMKQKIRGWKIQLKADLSLKDIGNMINKVVQGWINYYTHYYKSEFYEVLRYINQ
CLIKWVRRSYKKKNTRSRAEHWLGAVARRDRNLFAHWKFGILPSVGEGAV
>SRS893366||gene_26345|GeneMark.hmm|430_aa|-|42|1334
MQNDNAKPISISKQLVYDAFLRVKANRGSAGIDKVTLEDYEKNLRGNLYKLWNRMSSGSYFPPSVKLVEIPKSTG
GKRPLGIPTVSDRVAQMAIVMLITPSIEPCFHEDSYAYRPHRSAHDAVGKARERCWKYAWVLDMDISKFFDTID
HELLLKALKRHTQEKWVLMYIERWLKVPYEKADGSQVDRALGVPQGSVIGPVLANLFLHYTFDKWMEKSFPR
VPFERYADDTICHCHSLKQAEYMQAMIQQRFECCRLRLNEEKTKIVYCKSSRQKEFYPNVTFDFLGFTFQPRESV
DKYGNRFTGFLPAISRKSMKRINETIRSWHLNRHSNLTLEHLASDINPIVRGWMTYYGKFYPTRLKWFMQTLN
GRLARWVMCKFERYRHRFYPAQEWLARIAEKEGLIFYHWKCGVLPRFTNKEKVSSQLIMVK
>SRS893380||gene_21109|GeneMark.hmm|418_aa|-|479|1735
MSEAKQFDISKKAVIAAFQAVKENAGSYGADEQTIKEFEEHLNNNLYKLWNRMASGSYFPKPVRAVAIPKKNG
GIRILGIPTVEDRIAQMVAKMYFEPLVEPMFYNDSYGYRPNKSAIQAVGQARERCFKRDWALELDIKGLFDNIK
HGYLMYMVEKHTQIKWLILYIKRWLTVPFIMSDGSVAERRSGTPQGGVISPVLANLFLHYVFDDFMTKAYPNI
WWERYADDGVLHCQSYKQAAFIKQKLEERFQQFGLELNKEKTRIVYCKDNRRPQNYSCTQFTFLGYTFRPRLN
KNKEGKFFVGFTPAVSEKAKTAMKQKIREWKIQLKADLSLKDIGNMINKVVQGWINYYTHYYKSEFYEVLRYIN
QCLIKWVRRSYKKKNTRSRAEHWLGAVARRDRNLFAHWKFGILPSVGEGAV
>SRS971275||gene_99712|GeneMark.hmm|430_aa|-|1403|2695
MQNDNAKPISISKQLVYDAFLRVKANRGSAGIDKVTLEDYEKNLRGNLYKLWNRMSSGSYFPPSVKLVEIPKSTG
GKRPLGIPTVSDRVAQMAIVMLITPSIEPCFHEDSYAYRPHRSAHDAVGKARERCWKYAWVLDMDISKFFDTID
HELLLKALKRHTQEKWVLMYIERWLKVPYEKADGSQVDRALGVPQGSVIGPVLANLFLHYTFDKWMEKSFPR
VPFERYADDTICHCHSLKQAEYMQAMIQQRFECCRLRLNEEKTKIVYCKSSRQKEFYPNVTFDFLGFTFQPRESV
DKYGNRFTGFLPAISRKSMKRINETIRSWHLNRHSNLTLEHLASDINPIVRGWMTYYGKFYPTRLKWFMQTLN
GRLARWVMCKFERYRHRFYPAQEWLARIAEKEGLIFYHWKCGVLPRFTNKEKVSSQLIMVK
>SRS971275||gene_330923|GeneMark.hmm|422_aa|-|279|1547
MKDAKSFEISRHLVMEAYKRVKANKGAAGVDDVSIADFESNLKSNLYKIWNRMSSGSYFPPAVKLVEIPKSNGG
KRPLGIPTIGDRVAQMVVVMTIEPGIEPYFHEDSYAYRPNRSALDAVRKAKERSYTFHWVLDLDIKGFFDNIDHG
LLIKALERHVKCEWAMLYIKRWLSVPYQLKDGTQIERTKGVPQGSVVGPILANLFLHYVFDEWMRRNHSNISFE
RYADDTICHCVSLKQAEFILRAIRKRFAECKLELNEDKTKIVYCKKNHRDIPYECIQFDFLGYTFRPRRSIDANGEVF
LNFSPAISKKARTKIWEAIQNWNSNHWVPMELEDIAKEINPVIQGWINYYGQHNPRILKEVLQHVNDRLVRW
GRRKFKGLRKRKTATVHRLGDIALQKPNLFAHWAWGVKPTASEKNRKRK
>SEQF1087||SEQF1087.1_05559

MDRAKPYTIPKREVWEAYKRVRANQGAAGIDGQTIADFEADLRNNLYKLWNRLASGSYFPPPVRRVDIPKSDG
KTRPLGIPTVADRVAQMVVKRHLEPVVEPEFHPDSYGYRPGKSALDAISVARQRCWRYNWVLDLDIKAFFDSIE
PDLLMRAVRKHTDCPWVLLYIERWLKAPVQMPDGNLVARERGTPQGGVISPLLASLFLHYAFDMWMCRNFP
DIPFERYADDAICHCRSEDQAMALQNALDARFTDCGLTLHPDKTKIVYCRDESRRGTHPVYKFDFLGYTFRPRLV
SKKAGGMGVSFGPAASPTALKAIRGTIRSWSLHLRSDKALDDLARMFNSYIRGWINYYGRFCPSALQPTLWSVE
RYLARWASGKYKSLRRHKRRSRHWLLRIAQRQPRLFAHWPLLHGYGRTMGAG
>SEQF1188||SEQF1188.2_00017
MCVATREPPGCDGQTLKMFDQQRDGNLYKIWNRLCSGTWFPPPVLEKRIPKSNGKERILGIPTVSDRIAQGAIK
LFMEEKLDPIFHADSYGYRPGKSAHDALKQCAIRCWRYSWILEVDISAFFDHVKHDLVLKALEHHGMPKWVILY
CRRWMEAPMQSCENGELITRTRGTPQGGVISPLLANLFLHYAFDLWMEREYRGVPFERYADDIVVHCSRMSD
ATRLKNRLSERFSEVGLVLNAGKTNIAYIDTFKRRNVATSFTFLGYDFKVRTLKNFKGERYRKCMPGASNAAMRK
ITETIKKWRIHRSTAESLLDFARRYNAIVRGWIEYYGKFWSRNFNYRLWSAMQSRLLKWMQSKYRLSNRKAQR
KLTLVRKEYPKLFVHWYLLRASNE
>SEQF1199||SEQF1199.1_00258
MTKTKAFNIDKSLVVSAYRRVKTSAGAAGIDKQSLADFDKRLVDNLYKIWNRLSSGSYFPPAVKAVAIPKKLGGER
ILGIPTVSDRIAQTVVKLAFEPQVEPHFLADSYGYRPNKSALDAIGVTRKRCWYYDWVLEFDIKGLFDNIPHELIM
KAVDKHNPARWVKLYIQRWLTAPMVMSDGEVRARTMGTPQGGVISPLLANLFMHYVFDKWLAKYYPKVPW
YRYADDGILHCHSEAEATEMREVLRKRFSECGLEMHPEKTRVIYCKDGSRKGDYEHTMFDFLGYTFRRRVVKNV
KRNSLFVSFTPAASKSALKAMRREIKATGIRKRVDLSIEQIAKWINPKLNGWINYYGRYTCSELYSVFRYINKALVR
WGRKKYKMLSRYKTRASKFLEEMAKRSPQLFAHWRLKMRGGLV
>SEQF1200||SEQF1200.1_04999
MQRKSFEIPKALVWASYLDVRRNKGAPGCDGQTLKMFDQQRDGNLYKIWNRLCSGTWFPPPVLEKRIPKPN
GKERILGIPTVSDRIAQGAIKLFMEEKLDPIFHADSYGYRPGKSAHDALKQCAIRCWRYSWILEVDISAFFDHVRH
DLVLKALEHHGMPKWVILYCRRWMEAPMQSCENGELITRTRGTPQGGVISPLLANLFLHYAFDLWMEREYRG
VPFERYADDIVVHCSRMSDATRLKNRLSERFSEVGLVLNAGKTNIAYIDTFKRRNVATSFTFLGYDFKVRTLKNFK
GELYRKCMPGASNAAMRKITETIKKWRIHRSTAESLLDFARRYNAIVRGWIEYYGKFWSRNFNYRLWSAMQPR
LLKWMQSKYRLSNRRAQRKLTLVRKEYPKLFVHWYLLRASNE
>SEQF1200||SEQF1200.1_00335
MQRKSFEIPKALVWASYLDVRRNKGAPGCDGQTLKMFDQQRDGNLYKIWNRLCSGTWFPPPVLEKRIPKPN
GKERILGIPTVSDRIAQGAIKLFMEEKLDPIFHADSYGYRPGKSAHDALKQCAIRCWRYSWILEVDISAFFDHVRH
DLVLKALEHHGMPKWVILYCRRWMEAPMQSCENGELITRTRGTPQGGVISPLLANLFLHYAFDLWMEREYRG
VPFERYADDIVVHCSRMSDATRLKNRLSERFSEVGLVLNAGKTNIAYIDTFKRRNVATSFTFLGYDFKVRTLKNFK
GELYRKCMPGASNAAMRKITETIKKWRIHRSTAESLLDFARRYNAIVRGWIEYYGKFWSRNFNYRLWSAMQSR
LLKWMQSKYRLSNRRAQRKLTLVRKEYPKLFVHWYLLRASNE
>SEQF1218||SEQF1218.2_00252
MTKTKAFNIDKSLVVSAYRRVKTSAGAAGIDKQSLADFDKRLVDNLYKIWNRLSSGSYFPPAVKAVAIPKKLGGER
ILGIPTVSDRIAQTVVKLAFEPQVEPHFLADSYGYRPNKSALDAIGVTRKRCWYYDWVLEFDIKGLFDNIPHELIM
KAVDKHNPARWVKLYIQRWLTAPMVMSDGEVRARTMGTPQGGVISPLLANLFMHYVFDKWLAKYYPKVPW
YRYADDGILHCHSEAEATEMREVLRKRFSECGLEMHPEKTRVIYCKDGSRKGDYEHTMFDFLGYTFRRRVVKNV
KRNSLFVSFTPAASKSALKAMRREIKATGIRKRVDLSIEQIAKWINPKLNGWINYYGRYTCSELYSVFRYINKALVR
WGRKKYKMLSRYKTRASKFLEEMAKRSPQLFAHWRLKMRGGLV
>SEQF1429||SEQF1429.1_02408
MNVERRGSVGQSASQPNCKQEEAAGEQTKPFQVSKLHVVEAYRRVKANAGAAGVDNQTLKDFERDLKGNLY
KIWNRLSSGSWMPPPVRAVEIPKKDGSKRLLGIPTVSDRIAQMTVLVTFEPLVERYFLNDSYGYRHGKSALDAIA

VTRKRCWQYDWYLEFDIKGLFDNIPHDLLLRAVDKHCADKWVRLSIRRWLTAPVQMPDGTLKERNKGTPQGG
VISPVLANLFLHYVFDKWLSLLYPEIPWCRYADDGLIHCGSKQQAEELLNKLAKPFQECGLELHPEKTKIVYCKDS
ERQANHETVQFNFLGYTFRARRARNQRRGNLFTSFSLAVSNSAQKDMIGKLRKLRLRRRVEMSLEDIAKRLNP
MISGWLNYYAKYYKSAMKKVCRYINLTLIAWARKKYKTLRYKKTKACQLMERLSKEKLELFAHWKAGPGSAFA
>SEQF1433||SEQF1433.1_03914
MQRKSFEIPKALVWASYLDVRRNKGAPGCDGQTLKMFDQQRDGNLYKIWNRLCSGTWFPPPVLEKRIPKSNG
KERILGIPTVSDRIAQGAIKLFMEEKLDPIFHADSYGYRPGKSAHDALKQCAIRCWRYSWILEVDISAFFDHVRHD
LVLKALEHHGMPKWAILYCRRWMEAPMQSCENGELITRTRGTPQGGVISPLLANLFLHYAFDLWMEREYRGV
PFERYADDIVVHCSRMSDATRLKNRLSERFSEVGLVLNAGKTNIAYIDTFKRRNVATSFTFLGYDFKVRTLKNFKG
ELYRKCMPGASNAAMRKITETIKKWRIHRSTAESLLDFARRYNAIVRGWIGYYAKFWSRNFNYRLWSAMQSRL
LKWMQSKYRLSNRKAQRKLTLVRKEYPKLFVHWYLLRASNE
>SEQF2325||SEQF2325.2_00336
MGQAKPYDIPKRWVWEAYKRVKANRGAAGVDEQSIEVFEADLQSNLYKLWNRMSSGSYFPPPVKRVQIDKR
DGGKRPLGIPTVSDRVAQAVVKGYLEPDLEKHFHPDSFGYRPGKSALDAVGVARQRCWRHPFVLDLDIRAYFD
SISHELLLKAIRKHTDCAWVLLYIERWLKAPVQLEDGTLEPREKGTPQGSVVSPLMANLFLHYTFDMWMRRNH
PSIPFERYADDILCHCDSERQAQQLKEALAKRFAECGLELHPDKTKIVYCKDDDRRGDYPEQKFDFLGYTFRARRS
KNRWGKHFVNFSPGVSNAATKAIRQEIRAWQLRCRVDKRIDDLARMFNPIIRGWMNYYGRYYKSALYPTLRHL
DRCLARWAMSKYKRLRRHRRRAEHWVRDIACRTPTLLAHWPMLHKTAAGR
>SEQF2382||SEQF2382.1_01654
MSGVSSAKPYDIAKRTVWDAYQQVRANRGAAGIDDETIADFERDLSKNLYKLWNRMSSGSYFPPPVKQVEIPK
ASGGTRKLGVPTVGDRVAQTVVKLLIEPELDSIFHSDSYGYRPGRSAKQAVAITRERCWRYDWVVEFDIKAAFD
QINHGLLMKAVRLHIKEDWILLYIERWLVAPFETDHGMRVRRERGTPQGGVISPLLMNLFMHYAFDTWMQRT
SPNCPFARYADDAVVHCRSRKQAEYVMRSIASRLAACGLTMHPEKSKVVYCKDSNRRAGYPHVSFTFLGFTFRP
RKALSKQDQLFTSFLPGASADALKRMRQAVRRWRLNRQTHVTLVDVARLYNPVIQGWSQYYGSFYRTAMLGIF
QHIDRALERWARRKYKALHRRKRRISQWLDKMRTVVPRLFHHWRVTGQQGWITGAV
>SEQF2423||SEQF2423.2_02894
MSTAKSYSISKLTVWEAYQRVKANRGAAGIDEQSIAQFEQKLQRNLYKLWNRMSSGSYFPPPVRQVEIPKQSGG
KRKLGIPTVADRVAQTAVKLLIEPRLDCLFHSDSYGYRPGKSAKQAVEITRKRCWNMNWVVEFDIKGAFDHIDH
ELLLKAVRHHVKDDWILLYIERWLKAPFETADGVQVPRESGTPQGGVVSPLLMNLFMHYAFDTWMQRTFPGC
PFARYADDAVVHCRIEKHACEVMAAIKARLEACLLTMHPEKSKIVYCKDSNRKAPYPTTQFTFLGFTFRPQEAW
GNHGHRFTSFLPAASNDALKRMRQQTRGWNIHRQTPASLFELSKQYNATLRGWWNYYGTFYRTAMRKVFNH
FDLKLQRWARQKYKPLAGHKRRSADWLNRMKKACPSLFEHWNVFGNADRLGNGSRMS
>SEQF2423||SEQF2423.2_01450
MSTAKSYSISKLTVWEAYQRVKANRGAAGIDEQSIAQFEQKLQRNLYKLWNRMSSGSYFPPPVRQVEIPKQSGG
KRKLGIPTVADRVAQTAVKLLIEPRLDCLFHSDSYGYRPGKSAKQAVEITRKRCWNMNWVVEFDIKGAFDHIDH
ELLLKAVRHHVKDDWILLYIERWLKAPFETADGVQVPRESGTPQGGVVSPLLMNLFMHYAFDTWMQRTFPGC
PFARYADDAVVHCRSEKQACEVMAAIKARLEACLLTMHPEKSKIVYCKDSNRKAAYPTTQFTFLGFTFRPREAW
GNHGRRFTSFLPAASNDALKRMRQQTRGWNIQRQTPASLFELSKQYNATLRGWWNYYGTFYRTAMRKVFNH
FDLKLQRWARQKYKPLAGHKRRSADWLNRMKKACPSLFVHWNVFGNADRLGNGSRMS
>SEQF2423||SEQF2423.2_01089
MSTAKSYSISKLTVWEAYQRVKANRGAAGIDEQSIAQFEQKLQRNLYKLWNRMSSGSYFPPPVRQVEIPKQSGG
KRKLGIPTVADRVAQTAVKLLIEPRLDCLFHSDSYGYRPGKSAKQAVEITRKRCWNMNWVVEFDIKGAFDHIDH
ELLLKAVRHHVKDDWILLYIERWLKAPFETADGVQVPRESGTPQGGVVSPLLMNLFMHYAFDTWMQRTFPGC
PFARYADDAVVHCRSEKQACEVMAAIKARLEACLLTMHPEKSKIVYCKDSNRKAAYPTTQFTFLGFTFRPREAW

GNHGRRFTSFLPAASNDALKRMRQQTRGWNIQRQTPASLFELSKQYNATLRGWWNYYGTFYRTAMRKVFNH
FDLKLQRWARQKYKPLAGHKRRSADWLNRMKKACPSLFVHWNVFGNADRLGNGSRMS
>SEQF2423||SEQF2423.2_03353
MSTAKSYSISKLTVWEAYQRVKANRGAAGIDEQSIAQFEQKLQRNLYKLWNRMSSGSYFPPPVRQVEIPKQSGG
KRKLGIPTVADRVAQTAVKLLIEPRLDCLFHSDSYGYRPGKSAKQAVEITRKRCWNMNWVVEFDIKGAFDHIDH
ELLLKAVRHHVKDDWILLYIERWLKAPFETADGVQVPRESGTPQGGVVSPLLMNLFMHYAFDTWMQRTFPGC
PFARYADDAVVHCRSEKQACEVMAAIKARLEACLLTMHPEKSKIVYCKDSNRKAAYPTTQFTFLGFTFRPREAW
GNHGRRFTSFLPAASNDALKRMRQQTRGWNIQRQTPASLFELSKQYNATLRGWWNYYGTFYRTAMRKVFNH
FDLKLQRWARQKYKPLAGHKRRSADWLNRMKKACPSLFVHWNVFGNADRLGNGSRMS
>SEQF2513||SEQF2513.1_04084
MSDVSSAKPYDIAKRTVWDAYQQVRANRGAAGIDDETIADFERDLSKNLYKLWNRMSSGSYFPPPVKQVEIPK
ASGGTRKLGVPTVGDRVAQTVVKLLIEPELDSIFHSDSYGYRPGRSAKQAVAITRERCWRYDWVVEFDIKAAFD
QINHGLLMKAVRLHIKEDWILLYIERWLVAPFETDDGMRVPRERGTPQGGVISPLLMNLFMHYAFDTWMQRT
SPNCPFARYADDAVVHCRSRKQAEYVMRSIASRLAACGLTMHPEKSKVVYCKDSNRRAGYPHVSFTFLGFTFRP
RKALSKQDQLFTSFLPGASADALKRMRQAVRRWRLNRQTHVTLVDVARLYNPVIQGWWQYYGSFYRTAMLGI
FQHIDRALERWARRKYKALHRRKRRISQWLDKMRTVVPRLFHHWRVTGQQGWITGAV
>SEQF2697||SEQF2697.1_03300
MSGVSSAKPYDIAKRTVWDAYQQVRANRGAAGIDDETIADFERDLSKNLYKLWNRMSSGSYFPPPVKQVEIPK
ASGGTRKLGVPTVGDRVAQTVVKLLIEPELDSIFHSDSYGYRPGRSAKQAVAITRERCWRYDWVVEFDIKAAFD
QINHGLLMKAVRLHIKEDWILLYIERWLVAPFETDDGMRVPRERGTPQGGVLSPLLMNLFMHYAFDTWMQR
TSPNCPFARYADDAVVHCRSRKQAEYVMRSIASRLAACGLTMHPEKSKVVYCKDSNRRAGYPHVSFTFLGFTFR
PRKALSKQDQLFTSFLPGASADALKRMRQAVRRWRLNRQTHVTLVDVARLYNPVIQGWWQYYGSFYRTAML
GIFQHIDRALERWARRKYKALHRRKRRISQWLDKMRTVVPRLFHHWRVTGQQGWITGAV
>SEQF2884||SEQF2884.1_03305
MTKPFNIPKALIWEAFKKVKENGGAAGIDHESIEQFEHHLKGNLYKLWNRLCSGSYFPPPVKGVPIPKKSGGVR
MLGIPTVADRVAQTAVKLILEPQIDPLFHPNSYGYRPGRSAHDAIAVVRRRSWEYDWVVEFDIKGLFDNIDHNL
LMRALKKHCEIPWILLYVERWLKAPMQNVDGQVLERNHGTPQGGVISPLLANLFMHYAFDMWITKNLASVR
FCRYADDGVIHCRSLSQAKLVLQKIGARFRECGLELHPDKTKIVYCQDVNRRQAYPDVQFTFLGYTFRPRKAVDK
YKRVYVNFSPAVSRDALKTMRQTIRKWHLHLMCNRELSDLSAIFNPILQGWQQYYGRFHGSAMSTIWQHMN
AYLIRWMRRKYKNLARHKRRARYALGRLARDFPNAFVHWKMGCLPSVG
>SEQF2884||SEQF2884.1_03182
MTKPFNIPKALIWEAFKKVKENGGAAGIDHESIEQFEHHLKGNLYKLWNRLCSGSYFPPPVKGVPIPKKSGGVR
MLGIPTVADRVAQTAVKLILEPQIDPLFHPNSYGYRPGRSAHDAIAVVRRRSWEYDWVVEFDIKGLFDNIDHNL
LMRALKKHCEIPWILLYVERWLKAPMQNVDGQVLERNHGTPQGGVISPLLANLFMHYAFDMWITKNLASVR
FCRYADDGVIHCRSLSQAKLVLQKIGARFRECGLELHPDKTKIVYCQDVNRRQAYPDVQFTFLGYTFRPRKAVDK
YKRVYVNFSPAVSRDALKTMRQTIRKWHLHLMCNRELSDLSAIFNPILQGWQQYYGRFHGSAMSTIWQHMN
AYLIRWMRRKYKNLARHKRRARYALGRLARDFPNAFVHWKMGCLPSVG
>SEQF2956||SEQF2956.1_01668
MSAAKSYSISKLTVWEAFQRVKANRGAAGIDEQSIAQFEQKLQRNLYKVWNRMSSGSYFPPPVRQVEIPKQSG
CKRKLGIPTVADRVAQTAIKLLIEPSLDCLFHPDSYGYRPGKSAKQAVEITRRRCWNINWVVEFDIKGAFDHIDHE
LLLKAVKHHIKDEWILLYIERWLKAPFETADGVQVPRESGTPQGGVISPLLMNLFMHYAFDAWMQRTFPGCPF
ARYADDAVVHCRSEKQACEVMAAIKARLEVCLLTMHPEKSKIVYCKDSNRKAAYPTTQFTFLGFTFRPREAWGN
NGRRFTSFLPGASNEALKRMRQRTRSWNIQRQTPASLLELSKQYNAILRGWWNYYGAFYKTVMRKVFNHFDL
KLQRWARQKYKPLAGHKRRSVDWLNRMKKACPSLFVHWHVYGNEFRPGNGSRMS

>SEQF3749||SEQF3749.1_00630
MSGVTSAKPYGIAKRTVWKAYQQVKANRGAAGIDDETIADFERDLSKNLYKLWNRMSSGSYFPPPVKQVEIPK
ASGGTRKLGVPTVSDRVAQTVVKLLIEPELDPIFHPDSYGYRPGRSAKQAVAVTRDRCWRYDWVVEFDIKAAFD
QIDHGRLMKAVRTHIKEDWILLYIERWLVAPFATEDGARVPRERGTPQGGVVSPILMNLFMHYAFDTWMQRT
SPNCPFARYADDAVVHCRSRKQAEYVMRSIASRLADCGLTMHPEKSKVVYCKDSNRTEQHSNVSFTFLGFTFRP
RKAHSKRDQLFTSFLPGASDDALRRMRQAVRRWRLNRQTHVTLADVARLYNPVIQGWWQYYGAFYRTAML
GIFQHIDRALERWARRKYKALHRRKAASVGWLDKMQGAAPLLFHHWRVAGAQVG
>SEQF3775||SEQF3775.1_05734
MSGVSSAKPYDIAKRTVWDAYQQVRANRGAAGIDDETIADFERDLSKNLYKLWNRMSSGSYFPPPVKQVEIPK
ASGGTRKLGVPTVGDRVAQTVVKLLIEPELDSIFHSDSYGYRPGRSAKQAVAITRERCWRYDWVVEFDIKAAFD
QINHGLLMKAVRLHIKEDWILLYIERWLVAPFETDHGMRVRRERGTPQGGVISPLLMNLFMHYAFDTWMQRT
SPNCPFARYADDAVVHCRSRKQAEYVMRSIASRLAACGLTMHPEKSKVVYCKDSNRRAGYPHVSFTFLGFTFRP
RKALSKQDQLFTSFLPGASADALKRMRQAVRRWRLNRQTHVTLVDVARLYNPVIQGWSQYYGSFYRTAMLGIF
QHIDRALERWARRKYKALHRRKRRISQWLDKMRTVVPRLFHHWRVTGQQGWITGAV
>SEQF3777||SEQF3777.1_02653
MSGVSSAKPYDIAKRTVWDAYQQVRANRGAAGIDDETIADFERDLSKNLYKLWNRMSSGSYFPPPVKQVEIPK
ASGGTRKLGVPTVGDRVAQTVVKLLIEPELDSIFHSDSYGYRPGRSAKQAVAITRERCWRYDWVVEFDIKAAFD
QINHGLLMKAVRLHIKEDWILLYIERWLVAPFETDDGMRVPRERGTPQGGVLSPLLMNLFMHYAFDTWMQR
TSPNCPFARYADDAVVHCRSRKQAEYVMRSIASRLAACGLTMHPEKSKVVYCKDSNRRAGYPHVSFTFLGFTFR
PRKALSKQDQLFTSFLPGASADALKRMRQAVRRWRLNRQTHVTLVDVARLYNPVIQGWWQYYGSFYRTAML
GIFQHIDRALERWARRKYKALHRRKRRISQWLDKMRTVVPRLFHHWRVTGQQGWITGAV
>SEQF3781||SEQF3781.3_00175
MSGVSSAKPYDIAKRTVWDAYQQVRANRGAAGIDDETIADFERDLSKNLYKLWNRMSSGSYFPPPVKQVEIPK
ASGGTRKLGVPTVGDRVAQTVVKLLIEPELDSIFHSDSYGYRPGRSAKQAVAITRERCWRYDWVVEFDIKAAFD
QINHGLLMKAVRLHIKEDWILLYIERWLVAPFETDDGMRVPRERGTPQGGVLSPLLMNLFMHYAFDTWMQR
TSPNCPFARYADDAVVHCRSRKQAEYVMRSIASRLAACGLTMHPEKSKVVYCKDSNRRAGYPHVSFTFLGFTFR
PRKALSKQDQLFTSFLPGASADALKRMRQAVRRWRLNRQTHVTLVDVARLYNPVIQGWWQYYGSFYRTAML
GIFQHIDRALERWARRKYKALHRRKRRISQWLDKMRTVVPRLFHHWRVTGQQGWITGAV
>SEQF4085||SEQF4085.1_02455
MDKAKPFCISKREVWEAYQQVKANRGAAGVDGQSMADFEADLKNNLHRIWNRMSSGSYMPPPVLRVDIPK
AGGAGTRPLGIPTVSDRIAETVVKRYLEPLVEPVFHRDSYGYRPGRSAHQALDATRQRCWEYEWVLDLDIKNFF
GSIEWDLMMRAVRRHTDCAWALLYIERWLKAAVQMPDGSVVHPDRGTPQGGVVSPLLANLFLHYAFDRW
MQKHNPGVPFERYADDVICHCKSEAQARELRQGLETRLTECKPDLHPEKTKIVYCKRANRPASYPLCQFDFLGYT
FRPRSARNRMGKLTVGFLPAVSNKAAKAMRQQLRRRSALHRRDLSLSELADRTRPTLLGWIQYYGRFYRSALG
QVLLAVDAALVRWAQRKYKHLRGRKVRAAAWLRDIKSRQPSLFAHWAVETTVGR
>SEQF4087||SEQF4087.1_06761
MDKAKPFCISKREVWEAYQQVKANRGAAGVDGQSMADFEADLKNNLHRIWNRMSSGSYMPPPVLRVDIPK
AGGAGTRPLGIPTVSDRIAETVVKRYLEPLVEPVFHRDSYGYRPGRSAHQALDATRQRCWEYEWVLDLDIKNFF
GSIEWDLMMRAVRRHTDCAWALLYIERWLKAAVQMPDGSVVHPDRGTPQGGVVSPLLANLFLHYAFDRW
MQKHNPGVPFERYADDVICHCKSEAQARELRQGLETRLTECKPDLHPEKTKIVYCKRANRPASYPLCQFDFLGYT
FRPRSARNRMGKLTVGFLPAVSNKAAKAMRQQLRRRSALHRRDLSLSELADRTRPTLLGWIQYYGRFYRSALG
QVLLAVDAALVRWAQRKYKHLRGRKVRAAAWLRDIKSRQPSLFAHWAVETTVGR
>SEQF4115||SEQF4115.1_06802
MDKAKPFCISKREVWEAYQQVKANRGAAGVDGQSMADFEADLKNNLHRIWNRMSSGSYMPPPVLRVDIPK

AGGAGTRPLGIPTVSDRIAETVVKRYLEPLVEPVFHRDSYGYRPGRSAHQALDATRQRCWEYEWVLDLDIKNFF
GSIEWDLMMRAVRRHTDCAWALLYIERWLKAAVQMPDGSVVHPDRGTPQGGVVSPLLANLFLHYAFDRW
MQKHNPGVPFERYADDVICHCKSEAQARELRQGLETRLTECKPDLHPEKTKIVYCKRANRPASYPLCQFDFLGYT
FRPRSARNRMGKLTVGFLPAVSNKAAKAMRQQLRRRSALHRRDLSLSELADRTRPTLLGWIQYYGRFYRSALG
QVLLAVDAALVRWAQRKYKHLRGRKVRAAAWLRDIKSRQPSLFAHWAVETTVGR
>SEQF5829||SEQF5829.1_03727
MTKTKAFNIDKSLVVSAYRRVKTSAGAAGIDKQSLADFDKRLVDNLYKIWNRLSSGSYFPPAVKAVAIPKKLGGER
ILGIPTVSDRIAQTVVKLAFEPQVEPHFLADSYGYRPNKSALDAIGVTRKRCWYYDWVLEFDIKGLFDNIPHELIM
KAVDKHNPARWVKLYIQRWLTAPMVMSDGEVRARTMGTPQGGVISPLLANLFMHYVFDKWLAKYYPKVPW
YRYADDGILHCHSEAEATEMREVLRKRFSECGLEMHPEKTRVIYCKDGSRKGDYEHTMFDFLGYTFRRRVVKNV
KRNSLFVSFTPAASKSALKAMRREIKATGIRKRVDLSIEQIAKWINPKLNGWINYYGRYTCSELYSVFRYINKALVR
WGRKKYKMLSRYKTRASKFLEEMAKRSPQLFAHWRLKMRGGLV
>SEQF5876||SEQF5876.1_03911
MTKPFNIPKALIWEAFKKVKENGGAPGVDHESIEQFEKHLKNNLYKLWNRLCSGSYFPPPVKAVPIPKKSGGVRI
LGIPTVADRVAQTAVKLLLEPKIDPLFHPNSYGYRPGRSAHDAIAIVRRRSWDYDWVVEFDIKGLFDNIDHDLLM
RALKKHCEIPWILLYVQRWLKAPMQHINGHLLERNRGTPQGGVVSPLLANLFMHYAFDMWITKHLQSVRFCR
YADDGVIHCRSLSQAKLVLQKIDARFRECGLELHPDKTKIVYCQDINRRKAYPDVQFTFLGYTFRPRKAVDKYKRV
YVNFSPAVSRDALKAMRQTIRKWHLHLMCNRELSDLSAIFNPILQGWQQYYGRFHGSAMSAIWQHMNAYLI
RWMRRKYKNLARHKRRARYALGRLARDFPNAFVHWKMGCLPSVG
>SEQF5876||SEQF5876.1_03965
MTKPFNIPKALIWEAFKKVKENGGAPGVDHESIEQFEKHLKNNLYKLWNRLCSGSYFPPPVKAVPIPKKSGGVRI
LGIPTVADRVAQTAVKLLLEPKIDPLFHPNSYGYRPGRSAHDAIAIVRRRSWDYDWVVEFDIKGLFDNIDHDLLM
RALKKHCEIPWILLYVQRWLKAPMQHINGHLLERNRGTPQGGVVSPLLANLFMHYAFDMWITKHLQSVRFCR
YADDGVIHCRSLSQAKLVLQKIDARFRECGLELHPDKTKIVYCQDINRRKAYPDVQFTFLGYTFRPRKAVDKYKRV
YVNFSPAVSRDALKAMRQTIRKWHLHLMCNRELSDLSAIFNPILQGWQQYYGRFHGSAMSAIWQHMNAYLI
RWMRRKYKNLARHKRRARYALGRLARDFPNAFVHWKMGCLPSVG
>SEQF5876||SEQF5876.1_04089
MTKPFNIPKALIWEAFKKVKENGGAAGIDHESIEQFEHHLKGNLYKLWNRLCSGSYFPPPVKGVPIPKKSGGVR
MLGIPTVADRVAQTAVKLILEPQIDPLFHPNSYGYRPGRSAHDAIAVVRRRSWEYDWVVEFDIKGLFDNIDHNL
LMRALKKHCEIPWILLYVERWLKAPMQNVDGQVLERNHGTPQGGVISPLLANLFMHYAFDMWITKNLASVR
FCRYADDGVIHCRSLSQAKLVLQKIGARFRECGLELHPDKTKIVYCQDVNRRQAYPDVQFTFLGYTFRPRKAVDK
YKRVYVNFSPAVSRDALKAMRQTIRKWHLHLMCNRELSDLSAIFNPILQGWQQYYGRFHGSAMSAIWQHMN
AYLIRWMRRKYKNLARHKRRARYALGRLARDFPNAFVHWKMGCLPSVG
>SEQF5876||SEQF5876.1_03812
MTKPFNIPKALIWEAFKKVKENGGAPGVDHESIEQFEKHLKNNLYKLWNRLCSGSYFPPPVKAVPIPKKSGGVRI
LGIPTVADRVAQTAVKLLLEPKIDPLFHPNSYGYRPGRSAHDAIAIVRRRSWDYDWVVEFDIKGLFDNIDHDLLM
RALKKHCEIPWILLYVQRWLKAPMQHINGHLLERNRGTPQGGVVSPLLANLFMHYAFDMWITKHLQSVRFCR
YADDGVIHCRSLSQAKLVLQKIDARFRECGLELHPDKTKIVYCQDINRRKAYPDVQFTFLGYTFRPRKAVDKYKRV
YVNFSPAVSRDALKAMRQTIRKWHLHLMCNRELSDLSAIFNPILQGWQQYYGRFHGSAMSAIWQHMNAYLI
RWMRRKYKNLARHKRRARYALGRLARDFPNAFVHWKMGCLPSVG
>SEQF5876||SEQF5876.1_00776
MTKPFNIPKALIWEAFKKVKENGGAAGIDHESIEQFEHHLKGNLYKLWNRLCSGSYFPPPVKGVPIPKKSGGVR
MLGIPTVADRVAQTAVKLILEPQIDPLFHPNSYGYRPGRSAHDAIAVVRRRSWEYDWVVEFDIKGLFDNIDHNL
LMRALKKHCEIPWILLYVERWLKAPMQNVDGQVLERNHGTPQGGVISPLLANLFMHYAFDMWITKNLASVR

FCRYADDGVIHCRSLSQAKLVLQKIGARFRECGLELHPDKTKIVYCQDVNRRQAYPDVQFTFLGYTFRPRKAVDK
YKRVYVNFSPAVSRDALKAMRQTIRKWHLHLMCNRELSDLSAIFNPILQGWQQYYGRFHGSAMSAIWQHMN
AYLIRWMRRKYKNLARHKRRARYALGRLARDFPNAFVHWKMGCLPSVG
>SEQF5876||SEQF5876.1_02914
MTKPFNIPKALIWEAFKKVKENGGAPGVDHESIEQFEKHLKNNLYKLWNRLCSGSYFPPPVKAVPIPKKSGGVRI
LGIPTVADRVAQTAVKLLLEPKIDPLFHPNSYGYRPGRSAHDAIAIVRRRSWDYDWVVEFDIKGLFDNIDHDLLM
RALKKHCEIPWILLYVQRWLKAPMQHINGHLLERNRGTPQGGVVSPLLANLFMHYAFDMWITKHLQSVRFCR
YADDGVIHCRSLSQAKLVLQKIDARFRECGLELHPDKTKIVYCQDINRRKAYPDVQFTFLGYTFRPRKAVDKYKRV
YVNFSPAVSRDALKAMRQTIRKWHLHLMCNRELSDLSAIFNPILQGWQQYYGRFHGSAMSAIWQHMNAYLI
RWMRRKYKNLARHKRRARYALGRLARDFPNAFVHWKMGCLPSVG
>SEQF5876||SEQF5876.1_02034
MTKPFNIPKALIWEAFKKVKENGGAPGVDHESIEQFEKHLKNNLYKLWNRLCSGSYFPPPVKAVPIPKKSGGVRI
LGIPTVADRVAQTAVKLLLEPKIDPLFHPNSYGYRPGRSAHDAIAIVRRRSWDYDWVVEFDIKGLFDNIDHDLLM
RALKKHCEIPWILLYVQRWLKAPMQHINGHLLERNRGTPQGGVVSPLLANLFMHYAFDMWITKHLQSVRFCR
YADDGVIHCRSLSQAKLVLQKIDARFRECGLELHPDKTKIVYCQDINRRKAYPDVQFTFLGYTFRPRKAVDKYKRV
YVNFSPAVSRDALKAMRQTIRKWHLHLMCNRELSDLSAIFNPILQGWQQYYGRFHGSAMSAIWQHMNAYLI
RWMRRKYKNLARHKRRARYALGRLARDFPNAFVHWKMGCLPSVG
>SEQF5876||SEQF5876.1_03931
MTKPFNIPKALIWEAFKKVKENGGAPGVDHESIEQFEKHLKNNLYKLWNRLCSGSYFPPPVKAVPIPKKSGGVRI
LGIPTVADRVAQTAVKLLLEPKIDPLFHPNSYGYRPGRSAHDAIAIVRRRSWDYDWVVEFDIKGLFDNIDHDLLM
RALKKHCEIPWILLYVQRWLKAPMQHINGHLLERNRGTPQGGVVSPLLANLFMHYAFDMWITKHLQSVRFCR
YADDGVIHCRSLSQAKLVLQKIDARFRECGLELHPDKTKIVYCQDINRRKAYPDVQFTFLGYTFRPRKAVDKYKRV
YVNFSPAVSRDALKAMRQTIRKWHLHLMCNRELSDLSAIFNPILQGWQQYYGRFHGSAMSAIWQHMNAYLI
RWMRRKYKNLARHKRRARYALGRLARDFPNAFVHWKMGCLPSVG
>SEQF5879||SEQF5879.1_02652
MTKPFNIPKALIWEAFKKVKENGGAPGVDHESIEQFEKHLKNNLYKLWNRLCSGSYFPPPVKAVPIPKKSGGVRI
LGIPTVADRVAQTAVKLLLEPKIDPLFHPNSYGYRPGRSAHDAIAIVRRRSWDYDWVVEFDIKGLFDNIDHDLLM
RALKKHCEIPWILLYVQRWLKAPMQHINGHLLERNRGTPQGGVVSPLLANLFMHYAFDMWITKHLQSVRFCR
YADDGVIHCRSLSQAKLVLQKIDARFRKCGLELHPDKTKIVYCQDINRRKAYPDVQFTFLGYTFRPRKAVDKYKRV
YVNFSPAVSRDALKAMRQTIRKWHLHLMCNRELSDLSAIFNPILQGWQQYYGRFHGSAMSAIWQHMNAYLI
RWMRRKYKNLARHKRRARYALGRLARDFPNAFVHWKMGCLPSVG
>SEQF5879||SEQF5879.1_02675
MTKPFNIPKALIWEAFKKVKENGGAPGVDHESIEQFEKHLKNNLYKLWNRLCSGSYFPPPVKAVPIPKKSGGVRI
LGIPTVADRVAQTAVKLLLEPKIDPLFHPNSYGYRPGRSAHDAIAIVRRRSWDYDWVVEFDIKGLFDNIDHDLLM
RALKKHCEIPWILLYVQRWLKAPMQHINGHLLERNRGTPQGGVVSPLLANLFMHYAFDMWITKHLQSVRFCR
YADDGVIHCRSLSQAKLVLQKIDARFRKCGLELHPDKTKIVYCQDINRRKAYPDVQFTFLGYTFRPRKAVDKYKRV
YVNFSPAVSRDALKAMRQTIRKWHLHLMCNRELSDLSAIFNPILQGWQQYYGRFHGSAMSAIWQHMNAYLI
RWMRRKYKNLARHKRRARYALGRLARDFPNAFVHWKMGCLPSVG
>SEQF5882||SEQF5882.1_01301
MTKPFNIPKALIWEAFKKVKENGGAAGIDHESIEQFEHHLKGNLYKLWNRLCSGSYFPPPVKGVPIPKKSGGVR
MLGIPTVADRVAQTAVKLILEPQIDPLFHPNSYGYRPGRSAHDAIAVVRRRSWEYDWVVEFDIKGLFDNIDHNL
LMRALKKHCEIPWILLYVERWLKAPMQNVDGQVLERNHGTPQGGVISPLLANLFMHYAFDMWITKNFASVR
FCRYADDGVIHCRSLSQAKLVLQKIGARFRECGLELHPDKTKIVYCQDVNRRQAYPDVQFTFLGYTFRPRKAVDK
YKRVYVNFSPAVSRDALKTMRQTIRKWHLHLMCNRELSDLSAIFNPILQGWQQYYGRFHGSAMSAIWQHMN

AYLIRWMRRKYKNLARHKRRARYALGRLARDFPNAFVH
>SEQF5894||SEQF5894.1_03350
MTKPFNIPKALIWEAFKKVKENGGAAGIDHESIEQFEHHLKGNLYKLWNRLCSGSYFPPPVKGVPIPKKSGGVR
MLGIPTVADRVAQTAVKLILEPQIDPLFHPNSYGYRPGRSAHDAIAVVRRRSWEYDWVVEFDIKGLFDNIDHNL
LMRALKKHCEIPWILLYVERWLKAPMQNVDGQVLERNHGTPQGGVISPLLANLFMHYAFDMWITKNFASVR
FCRYADDGVIHCRSLSQAKLVLQKIGARFRECGLELHPDKTKIVYCQDVNRRQAYPDVQFTFLGYTFRPRKAVDK
YKRVYYVNFSPAVSRDALKTMRQTIRKWHLHLMCNRELSDLSAIFNPILQGWQQYYGRFHGSAMSAIWQHMN
AYLIRWMRRKYKNLARHKRRARYALGRLARDFPNAFVH
>SEQF5903||SEQF5903.1_00708
MTKPFNIPKALIWEAFKKVKENGGAPGVDHESIEQFEKHLKNNLYKLWNRLCSGSYFPPPVKAVPIPKKSGGVRI
LGIPTVADRVAQTAVKLLLEPKIDPLFHPNSYGYRPGRSAHDAIAIVRRRSWDYDWVVEFDIKGLFDNIDHDLLM
RALKKHCEIPWILLYVQRWLKAPMQHINGHLLERNRGTPQGGVVSPLLANLFMHYAFDMWITKHLQSVRFCR
YADDGVIHCRSLSQAKLVLQKIDARFRKCGLELHPDKTKIVYCQDINRRKAYPDVQFTFLGYTFRPRKAVDKYKRV
YVNFSPAVSRDALKAMRQTIRKWHLHLMCNRELSDLSAIFNPILQGWQQYYGRFHGSAMSAIWQHMNAYLI
RWMRRKYKNLARHKRRARYALGRLARDFPNAFVHWKMGCLPSVG
>SEQF5909||SEQF5909.1_00673
MTKPFNIPKALIWEAFKKVKENGGAAGIDHESIEQFEHHLKGNLYKLWNRLCSGSYFPSPVKGVPIPKKSGGVR
MLGIPTVADRVAQTAVKLILEPRIDPLFHPNSYGYRPGRSAHDAIAMVRRRSWEYDWVVEFDIKGLFDNIDHNL
LMRALKKHCEIPWILLYVERWLKAPMQNVDGQVLERNRGTPQGGVISPLLANLFMHYAFDMWITKNLASVRF
CRYADDGVIHCRSLSQAKLVLQKIGARFRECGLELHPDKTKIVYCQDVNRRQAYPDVQFTFLGYTFRPRKAVDKY
KRVYVNFSPAVSRDALKAMRQTIRKWHLHLMCNRELSDLSAIFNPILQGWQQYYGRFHGSAMSAIWQHMNA
YLIRWMRRKYKNLARHKRRARYALGRLARDFPNAFVHWKMGCLPSVG
>SEQF6037||SEQF6037.1_05235
MNSGKPLPITKRMVWEAYKLVKKKGKAAGVDGQSLEDFAGDLENHLYRLWNRLASGSYFPPPVRRVEIPKTDG
GVRPLGIPTVADRIAQMVVKRCLEPEVDGEFDPDSYGYRPGKSAHQAIEQARKRCWQHDWVVDLDIKSFFDTI
DHELLMRAVYRHTKADWIRLYIERWLKAPVEMPDGSVQARTTGTPQGGVVSPILANLFLHYVFDVWMKRSYP
HIPFERYADDVICHCRTRQEAEELKSALERRFADCHLLLHPEKTKVVYCADSNRRRSYPHTHFDFLGFSFRPRMA
KNRWGRIFTCFLPGVSPNSLKEMRARIRGWRLPQHSPLPLEDIARSLNPVLRGWDQYYGRFYPTELRKLYEYFD
ERLGAWLRCKYKQLKSHRGRSLRKLNEMAKQNPKLFVHWQKLGRATVG
>SEQF6037||SEQF6037.1_00701
MNQPGKPFNIDKREVYEAYLQVRSNGGAAGVDGVTIEQFESDLKSNLYKIWNRMSSGAYFPPPVRAVSIPKKS
GGQRILGVPTVADRVAQTVVKQLIEPALDAIFLADSYGYRPGKSALDAVGVTRQRCWKYDWVLEFDIKGLFDNI
DHELLLRAVRKHVTCAWALLYIERWLTAPMVQEDGTVIERSRGTPQGGVVSPILANLFMHYAFDLWMARMFP
DLRWCRYADDGLVHCRNEMEAQSVREALQARLAKCRLELHPTKTRIVYCKDDRRRGKSETVMFDFLGYCFRPR
SVLGPHSQKMFCGFTPAVSKPALNAMRATVRGLKLRRRTEVTLDDIARELNPMVRGWIAYYGQYTRSALYPLAR
YINQTLAIWLKRKYKRFHHRLGRARLFLEKIAREKRRLFVHWQLGDGGKLA
>SEQF6039||SEQF6039.1_02631
MGQAKPYDIPKRWVWEAYKRVKANRGAAGVDEQSIEVFEADLQSNLYKLWNRMSSGSYFPPPVKRVQIDKR
DGGKRPLGIPTVSDRVAQAVVKGYLEPDLEKHFHPDSFGYRPGKSALDAVGVARQRCWRHPFVLDLDIRAYFD
SISHELLLKAIRKHTDCAWVLLYIERWLKAPVQLEDGTLEPREKGTPQGSVVSPLMANLFLHYTFDMWMRRNH
PSIPFERYADDILCHCDSERQAQQLKEALAKRFAECGLELHPDKTKIVYCKDDDRRGDYPEQKFDFLGYTFRARRS
KNRWGKHFVNFSPGVSNAATKAIRQEIRAWQLRCRVDKRIDDLARMFNPIIRGWMNYYGRYYKSALYPTLRHL
DRCLARWAMSKYKRLRRHRRRAEHWVRDIACRTPTLLAHWPMLHKTAAGR
>SEQF6042||SEQF6042.1_03173

MGQAKPYDIPKRWVWEAYKRVKANRGAAGVDEQSIEVFEADLQSNLYKLWNRMSSGSYFPPPVKRVQIDKR
DGGKRPLGIPTVSDRVAQAVVKGYLEPDLEKHFHPDSFGYRPGKSALDAVGVARQRCWRHPFVLDLDIRAYFD
SISHELLLKAIRKHTDCAWVLLYIERWLKAPVQLEDGTLEPREKGTPQGSVVSPLMANLFLHYTFDMWMRRNH
PSIPFERYADDILCHCDSERQAQQLKEALAKRFAECGLELHPDKTKIVYCKDDDRRGDYPEQKFDFLGYTFRARRS
KNRWGKHFVNFSPGVSNAATKAIRQEIRAWQLRCRVDKRIDDLARMFNPIIRGWMNYYGRYYKSALYPTLRHL
DRCLARWAMSKYKRLRRHRRRAEHWVRDIACRTPTLLAHWPMLHKTAAGR
>SEQF6045||SEQF6045.1_08878
MGQAKPYDIPKRWVWEAYKRVKANRGAAGVDEQSIEVFEADLQSNLYKLWNRMSSGSYFPPPVKRVQIDKR
DGGKRPLGIPTVSDRVAQAVVKGYLEPDLEKHFHPDSFGYRPGKSALDAVGVARQRCWRHPFVLDLDIRAYFD
SISHELLLKAIRKHTDCAWVLLYIERWLKAPVQLEDGTLEPREKGTPQGSVVSPLMANLFLHYTFDMWMRRNH
PSIPFERYADDILCHCDSERQAQQLKEALAKRFAECGLELHPDKTKIVYCKDDDRRGDYPEQKFDFLGYTFRARRS
KNRWGKHFVNFSPGVSNAATKAIRQEIRAWQLRCRVDKRIDDLARMFNPIIRGWMNYYGRYYKSALYPTLRHL
DRCLARWAMSKYKRLRRHRRRAEHWVRDIACRTPTLLAHWPMLHKTAAGR
>SEQF6045||SEQF6045.1_09009
MDEQSIEVFEADLQSNLYKLWNRMSSGSYFPPPVKRVQIDKRDGGKRPLGIPTVSDRVAQAVVKGYLEPDLEK
HFHPDSFGYRPGKSALDAVGVARQRCWRHPFVLDLDIRAYFDSISHELLLKAIRKHTDCAWVLLYIERWLKAPV
QLEDGTLEPREKGTPQGSVVSPLMANLFLHYTFDMWMRRNHPSIPFERYADDILCHCDSERQAQQLKEALAK
RFAECGLELHPDKTKIVYCKDDDRRGDYPEQKFDFLGYTFRARRSKNRWGKHFVNFSPGVSNAATKAIRQEIRA
WQLRCRVDKRIDDLARMFNPIIRGWMNYYGRYYKSALYPTLRHLDRCLARWAMSKYKRLRRHRRRAEHWVR
DIACRTPTLLAHWPMLHKTAAGR
>SEQF6045||SEQF6045.1_08446
MGQAKPYDIPKRWVWEAYKRVKANRGAAGVDEQSIEVFEADLQSNLYKLWNRMSSGSYFPPPVKRVQIDKR
DGGKRPLGIPTVSDRVAQAVVKGYLEPDLEKHFHPDSFGYRPGKSALDAVGVARQRCWRHPFVLDLDIRAYFD
SISHELLLKAIRKHTDCAWVLLYIERWLKAPVQLEDGTLEPREKGTPQGSVVSPLMANLFLHYTFDMWMRRNH
PSIPFERYADDILCHCDSERQAQQLKEALAKRFAECGLELHPDKTKIVYCKDDDRRGDYPEQKFDFLGYTFRARRS
KNRWGKHFVNFSPGVSNAATKAIRQEIRAWQLRCRVDKRIDDLARMFNPIIRGWMNYYGRYYKSALYPTLRHL
DRCLARWAMSKYKRLRRHRRRAEHWVRDIACRTPTLLAHWPMLHKTAAGR
>SEQF6047||SEQF6047.1_09305
MGQAKPYDIPKRWVWEAYKRVKANRGAAGVDEQSIEVFEADLQSNLYKLWNRMSSGSYFPPPVKRVQIDKR
DGGKRPLGIPTVSDRVAQAVVKGYLEPDLEKHFHPDSFGYRPGKSALDAVGVARQRCWRHPFVLDLDIRAYFD
SISHELLLKAIRKHTDCAWVLLYIERWLKAPVQLEDGTPEPREKGTPQGSVVSPLMANLFLHYAFDMWMRRNH
PSIPFERYADDILCHCDSERQAQELKEALAKRFAECGLELHPDKTKIVYCKDDDRRGDYPEQKFDFLGYTFRARRS
KNRWGKHFVNFSPGVSNAATKAIRQEIRAWQLRCRVDKRIDDLARMFNPIIRGWMNYYGRYYKSALYPTLRHL
DRCLARWAMSKYKRLRRHRRRAEHWVREIACRTPTLLAHWPMLHKTAAGR
>SEQF6047||SEQF6047.1_05395
MNQPGKPFNIDKREVYEAYLQVRSNGGAAGVDGVTIEQFESDLKSNLYKIWNRMSSGAYFPPSVRAVSIPKKS
GGQRILGVPTVADRVAQTVVKQLIEPALDAIFLADSYGYRPGKSALDAVGVTRQRCWKYDWVLEFDIKALFDNI
DHELLLRAVRKHVTCAWALLYIERWLTAPMVQEDGTVIERSRGTPQGGVVSPILANLFMHYAFDLWMARMFP
DLRWCRYADDGLVHCRNEMEAQSIREALQARLAKCRLELHPTKTRIVYCKDDRRRGKSETVMFDFLGYCFRPRS
VLGPHSQKMFCGFTPAVSKPALNAMRATVRGLKLRRRTEVTLDDIARELNPMVRGWIAYYGQYTRSALYPLARY
INQTLAIWLKRKYKRFHHRLGRARLFLEKIAREKRRLFVHWQLGDGGKLA
>SEQF6047||SEQF6047.1_09423
MGQAKPYDIPKRWVWEAYKRVKANRGAAGVDEQSIEVFEADLQSNLYKLWNRMSSGSYFPPPVKRVQIDKR
DGGKRPLGIPTVSDRVAQAVVKGYLEPDLEKHFHPDSFGYRPGKSALDAVGVARQRCWRHPFVLDLDIRAYFD

SISHELLLKAIRKHTDCAWVLLYIERWLKAPVQLEDGTPEPREKGTPQGSVVSPLMANLFLHYAFDMWMRRNH
PSIPFERYADDILCHCDSERQAQELKEALAKRFAECGLELHPDKTKIVYCKDDDRRGDYPEQKFDFLGYTFRARRS
KNRWGKHFVNFSPGVSNAATKAIRQEIRAWQLRCRVDKRIDDLARMFNPIIRGWMNYYGRYYKSALYPTLRHL
DRCLARWAMSKYKRLRRHRRRAEHWVREIACRTPTLLAHWPMLHKTAAGR
>SEQF6048||SEQF6048.1_09477
MNSGKPLPITKRMVWEAYKLVKKKGKAAGVDGQSLEDFAGDLENHLYRLWNRLASGSYFPPPVRRVEIPKAG
GGVRPLGIPTVADRIAQMVVKRCLEPELDGEFDPDSYGYRPGKSAHQAIEQARKRCWQHDWVVDLDIKSFFD
TIDHELLMRAVYRHTKADWIRLYIERWLKAPVEMPDGSVQARTTGTPQGGVVSPILANLFLHYVFDVWMKGS
YPHIPFERYADDVICHCRTRQEAEELKSALERRFADCHLLLHPAKTKVVYCADSNRRRSYPHIHFDFLGFSFRPRM
AKNRWGRIFTCFLPGVSPNSLKEMRARIRGWRLPQHSPLPLEDIARSLNPVLRGWDQYYGRFYPTELRRLYEYF
DERLGAWLRCKYKQLKGHRGRSLRKLNEMAKQNPKLFVHWQKLGRATVG
>SEQF6048||SEQF6048.1_09472
MNQPGKPFNIDKREVYEAYLQVRSNGGAAGVDGVTIEQFESDLKSNLYKIWNRMSSGAYFPPPVRAVSIPKKS
GGQRILGVPTVADRVAQTVVKQLIEPALDAIFLADSYGYRPGKSALDAVGVTRQRCWKYDWVLEFDIKGLFDNI
DHELLLRAVRKHVTCAWALLYIERWLTAPMVQEDGTVIERSRGTPQGGVVSPILANLFMHYAFDLWMARMFP
DLRWCRYADDGLVHCRNEMEAQSVREALQARLAKCRLELHPTKTRIVYCKDDRRRGKSETVMFDFLGYCFRPR
SVLGPHSQKMFCGFTPAVSKPALNAMRATVRGLKLRRRTEVTLDDIARELNPMVRGWIAYYGQYTRSALYPLAR
YINQTLAIWLKRKYKRFHHRLGRARLFLEKIAREKRRLFVHWQLGDGGKLA
>SEQF6050||SEQF6050.1_06214
MGQAKPYDIPKRWVWEAYKRVKANRGAAGVDEQSIEVFEADLQSNLYKLWNRMSSGSYFPPPVKRVQIDKR
DGGKRPLGIPTVSDRVAQAVVKGYLEPDLEKHFHPDSFGYRPGKSALDAVGVARQRCWRHPFVLDLDIRAYFD
SISHELLLKAIRKHTDCAWVLLYIERWLKAPVQLEDGTLEPREKGTPQGSVVSPLMANLFLHYTFDMWMRRNH
PSIPFERYADDILCHCDSERQAQQLKEALAKRFAECGLELHPDKTKIVYCKDDDRRGDYPEQKFDFLGYTFRARRS
KNRWGKHFVNFSPGVSNAATKAIRQEIRAWQLRCRVDKRIDDLARMFNPIIRGWMNYYGRYYKSALYPTLRHL
DRCLARWAMSKYKRLRRHRRRAEHWVRDIACRTPTLLAHWPMLHKTAAGR
>SEQF7745||SEQF7745.1_06031
MSSGSYFPPPVKAVEIPKASGGIRRLGVPTVSDRIAQTVVKLLIEPKLDALFHPDSYGYRPGRSAKQAIAITRERC
WRYDWVVEFDIKAAFDHIDHELLMKAVRTHIKEDWILLYIERWLVAPFEAADGVRIQRERGTPQGGVISPMLM
NLFMHYAFDAWMQRNSPNCPFARYADDAVVHCRSQRQAEHVMRSIASRLAVCGLTMHPEKSKIVYCKDSNR
RAGYPHVSFTFLGFTFRPRKAIGQQNKLFTSFLPGVSAQALKRMRRAVREWRVSRQTHVTLAAVARLYNPVIQ
GWWQYYGAFYRTAMLGIFRHIDSALKRWAGRKYKILHGRKRRISEWLDTVHKAAPRLFYHWQVTEQQVG
>SEQF7752||SEQF7752.1_01163
MSGVSSAKPYDIAKRTVWDAYQQVRANRGAAGIDDETIADFERDLSKNLYKLWNRMSSGSYFPPPVKQVEIPK
ASGGTRKLGVPTVGDRVAQTVVKLLIEPELDSIFHSDSYGYRPGRSAKQAVAITRERCWRYDWVVEFDIKAAFD
QINHGLLMKAVRLHIKEDWILLYIERWLVAPFETDDGMRVPRERGTPQGGVLSPLLMNLFMHYAFDTWMQR
TSPNCPFARYADDAVVHCRSRKQAEYVMRSIASRLAACGLTMHPEKSKVVYCKDSNRRAGYPHVSFTFLGFTFR
PRKALSKQDQLFTSFLPGASADALKRMRQAVRRWRLNRQTHVTLVDVARLYNPVIQGWWQYYGSFYRTAML
GIFQHIDRALERWARRKYKALHRRKRRISQWLDKMRTVVPRLFHHWRVTGQQGWITGAV
>SEQF7755||SEQF7755.1_01051
MSGVSSAKPYDIAKRTVWDAYQQVRANRGAAGIDDETIADFERDLSKNLYKLWNRMSSGSYFPPPVKQVEIPK
ASGGTRKLGVPTVGDRVAQTVVKLLIEPELDSIFHSDSYGYRPGRSAKQAVAITRERCWRYDWVVEFDIKAAFD
QINHGLLMKAVRLHIKEDWILLYIERWLVAPFETDDGMRVPRERGTPQGGVLSPLLMNLFMHYAFDTWMQR
TSPNCPFARYADDAVVHCRSRKQAEYVMRSIASRLAACGLTMHPEKSKVVYCKDSNRRAGYPHVSFTFLGFTFR
PRKALSKQDQLFTSFLPGASADALKRMRQAVRRWRLNRQTHVTLVDVARLYNPVIQGWWQYYGSFYRTAML

GIFQHIDRALERWARRKYKALHRRKRRISQWLDKMRTVVPRLFHHWRVTGQQGWITGAV
>SEQF7758||SEQF7758.1_06355
MSGVSSAKPYDIAKRTVWDAYQQVRANRGAAGIDDETIADFERDLSKNLYKLWNRMSSGSYFPPPVKQVEIPK
ASGGTRKLGVPTVGDRVAQTVVKLLIEPELDSIFHSDSYGYRPGRSAKQAVAITRERCWRYDWVVEFDIKAAFD
QINHGLLMKAVRLHIKEDWILLYIERWLVAPFETDDGMRVPRERGTPQGGVISPLLMNLFMHYAFDTWMQRT
SPNCPFARYADDAVVHCRSRKQAEYVMRSIASRLAACGLTMHPEKSKVVYCKDSNRRAGYPHVSFTFLGFTFRP
RKALSKQDQLFTSFLPGASADALKRMRQAVRRWRLNRQTHVTLVDVARLYNPVIQGWWQYYGSFYRTAMLGI
FQHIDRALERWARRKYKALHRRKRRISQWLDKMRTVVPRLFHHWRVTGQQGWITGAV
>SEQF7766||SEQF7766.2_01057
MSGVSSAKPYDIAKRTVWDAYQQVRANRGAAGIDDETIADFERDLSKNLYKLWNRMSSGSYFPPPVKQVEIPK
ASGGTRKLGVPTVGDRVAQTVVKLLIEPELDSIFHSDSYGYRPGRSAKQAVAITRERCWRYDWVVEFDIKAAFD
QINHGLLMKAVRLHIKEDWILLYIERWLVAPFETDDGMRVPRERGTPQGGVLSPLLMNLFMHYAFDTWMQR
TSPNCPFARYADDAVVHCRSRKQAEYVMRSIASRLAACGLTMHPEKSKVVYCKDSNRRAGYPHVSFTFLGFTFR
PRKALSKQDQLFTSFLPGASADALKRMRQAVRRWRLNRQTHVTLVDVARLYNPVIQGWWQYYGSFYRTAML
GIFQHIDRALERWARRKYKALHRRKRRISQWLDKMRTVVPRLFHHWRVTGQQGWITGAV
>SEQF7776||SEQF7776.2_03491
MSGVSSAKPYDIAKRTVWDAYQQVRANRGAAGIDDETIADFERDLSKNLYKLWNRMSSGSYFPPPVKQVEIPK
ASGGTRKLGVPTVGDRVAQTVVKLLIEPELDSIFHSDSYGYRPGRSAKQAVAITRERCWRYDWVVEFDIKAAFD
QINHGLLMKAVRLHIKEDWILLYIERWLVAPFETDDGMRVPRERGTPQGGVLSPLLMNLFMHYAFDTWMQR
TSPNCPFARYADDAVVHCRSRKQAEYVMRSIASRLAACGLTMHPEKSKVVYCKDSNRRAGYPHVSFTFLGFTFR
PRKALSKQDQLFTSFLPGASADALKRMRQAVRRWRLNRQTHVTLVDVARLYNPVIQGWWQYYGSFYRTAML
GIFQHIDRALERWARRKYKALHRRKRRISQWLDKMRTVVPRLFHHWRVTGQQGWITGAV
>SEQF7776||SEQF7776.2_05387
MSGVSSAKPYDIAKRTVWDAYQQVRANRGAAGIDDETIADFERDLSKNLYKLWNRMSSGSYFPPPVKQVEIPK
ASGGTRKLGVPTVGDRVAQTVVKLLIEPELDSIFHSDSYGYRPGRSAKQAVAITRERCWRYDWVVEFDIKAAFD
QINHGLLMKAVRLHIKEDWILLYIERWLVAPFETDDGMRVPRERGTPQGGVLSPLLMNLFMHYAFDTWMQR
TSPNCPFARYADDAVVHCRSRKQAEYVMRSIASRLAACGLTMHPEKSKVVYCKDSNRRAGYPHVSFTFLGFTFR
PRKALSKQDQLFTSFLPGASADALKRMRQAVRRWRLNRQTHVTLVDVARLYNPVIQGWWQYYGSFYRTAML
GIFQHIDRALERWARRKYKALHRRKRRISQWLDKMRTVVPRLFHHWRVTGQQGWITGAV
>SEQF8267||SEQF8267.1_01554
MSGVSSAKPYDIAKRTVWDAYQQVRANRGAAGIDDETIADFERDLSKNLYKLWNRMSSGSYFPPPVKQVEIPK
ASGGTRKLGVPTVGDRVAQTVVKLLIEPELDSIFHSDSYGYRPGRSAKQAVAITRERCWRYDWVVEFDIKAAFD
QINHGLLMKAVRLHIKEDWILLYIERWLVAPFETDDGMRVPRERGTPQGGVLSPLLMNLFMHYAFDTWMQR
TSPNCPFARYADDAVVHCRSRKQAEYVMRSIASRLAACGLTMHPEKSKVVYCKDSNRRAGYPHVSFTFLGFTFR
PRKALSKQDQLFTSFLPGASADALKRMRQAVRRWRLNRQTHVTLVDVARLYNPVIQGWWQYYGSFYRTAML
GIFQHIDRALERWARRKYKALHRRKRRISQWLDKMRTVVPRLFHHWRVTGQQGWITGAV
>SEQF8908||SEQF8908.1_00060
MTKPFNIPKALIWEAFKKVKENGGAPGVDHESIEQFEKHLKNNLYKLWNRLCSGSYFPPPVKAVPIPKKSGGVRI
LGIPTVADRVAQTAVKLLLEPKIDPLFHPNSYGYRPGRSAHDAIAIVRRRSWDYDWVVEFDIKGLFDNIDHDLLM
RALKKHCEIPWILLYVQRWLKAPMQHINGHLLERNRGTPQGGVVSPLLANLFMHYAFDMWITKHLQSVRFCR
YADDGVIHCRSLSQAKLVLQKIDARFRECGLELHPDKTKIVYCQDINRRKAYPDVQFTFLGYTFRPRKAVDKYKRV
YVNFSPAVSRDALKAMRQTIRKWHLHLMCNRELSDLSAIFNPILQGWQQYYGRFHGSAMSAIWQHMNAYLI
RWMRRKYKNLARHKRRARYALGRLARDFPNAFVHWKMGCLPSVG
>SEQF9059||SEQF9059.1_01542

MTKPFNIPKALIWEAFKKVKENGGAAGIDHESIEQFEHHLKGNLYKLWNRLCSGSYFPPPVKGVPIPKKSGGVR
MLGIPTVADRVAQTAVKLILEPQIDPLFHPNSYGYRPGRSAHDAIAVVRRRSWEYDWVVEFDIKGLFDNIDHNL
LMRALKKHCEIPWILLYVERWLKAPMQNVDGQVLERNHGTPQGGVISPLLANLFMHYAFDMWITKNLASVR
FCRYADDGVIHCRSLSQAKLVLQKIGARFRECGLELHPDKTKIVYCQDVNRRQAYPDVQFTFLGYTFRPRKAVDK
YKRVYVNFSPAVSRDALKTMRQTIRKWHLHLMCNRELSDLSAIFNPILQGWQQYYGRFHGSAMSTIWQHMN
AYLIRWMRRKYKNLARHKRRARYALGRLARDFPNAFVHWKMGCLPSVG
>SEQF9059||SEQF9059.1_03252
MTKPFNIPKALIWEAFKKVKENGGAAGIDHESIEQFEHHLKGNLYKLWNRLCSGSYFPPPVKGVPIPKKSGGVR
MLGIPTVADRVAQTAVKLILEPQIDPLFHPNSYGYRPGRSAHDAIAVVRRRSWEYDWVVEFDIKGLFDNIDHNL
LMRALKKHCEIPWILLYVERWLKAPMQNVDGQVLERNHGTPQGGVISPLLANLFMHYAFDMWITKNLASVR
FCRYADDGVIHCRSLSQAKLVLQKIGARFRECGLELHPDKTKIVYCQDVNRRQAYPDVQFTFLGYTFRPRKAVDK
YKRVYVNFSPAVSRDALKTMRQTIRKWHLHLMCNRELSDLSAIFNPILQGWQQYYGRFHGSAMSTIWQHMN
AYLIRWMRRKYKNLARHKRRARYALGRLARDFPNAFVHWKMGCLPSVG
>SEQF9059||SEQF9059.1_03362
MTKPFNIPKALIWEAFKKVKENGGAAGIDHESIEQFEHHLKGNLYKLWNRLCSGSYFPPPVKGVPIPKKSGGVR
MLGIPTVADRVAQTAVKLILEPQIDPLFHPNSYGYRPGRSAHDAIAVVRRRSWEYDWVVEFDIKGLFDNIDHNL
LMRALKKHCEIPWILLYVERWLKAPMQNVDGQVLERNHGTPQGGVISPLLANLFMHYAFDMWITKNLASVR
FCRYADDGVIHCRSLSQAKLVLQKIGARFRECGLELHPDKTKIVYCQDVNRRQAYPDVQFTFLGYTFRPRKAVDK
YKRVYVNFSPAVSRDALKTMRQTIRKWHLHLMCNRELSDLSAIFNPILQGWQQYYGRFHGSAMSTIWQHMN
AYLIRWMRRKYKNLARHKRRARYALGRLARDFPNAFVHWKMGCLPSVG
>SEQF9059||SEQF9059.1_03123
MTKPFNIPKALIWEAFKKVKENGGAAGIDHESIEQFEHHLKGNLYKLWNRLCSGSYFPPPVKGVPIPKKSGGVR
MLGIPTVADRVAQTAVKLILEPQIDPLFHPNSYGYRPGRSAHDAIAVVRRRSWEYDWVVEFDIKGLFDNIDHNL
LMRALKKHCEIPWILLYVERWLKAPMQNVDGQVLERNHGTPQGGVISPLLANLFMHYAFDMWITKNLASVR
FCRYADDGVIHCRSLSQAKLVLQKIGARFRECGLELHPDKTKIVYCQDVNRRQAYPDVQFTFLGYTFRPRKAVDK
YKRVYVNFSPAVSRDALKTMRQTIRKWHLHLMCNRELSDLSAIFNPILQGWQQYYGRFHGSAMSTIWQHMN
AYLIRWMRRKYKNLARHKRRARYALGRLARDFPNAFVHWKMGCLPSVG
>SEQF9063||SEQF9063.1_03231
MTKPFNIPKALIWEAFKKVKENGGAAGIDHESIEQFEHHLKGNLYKLWNRLCSGSYFPPPVKGVPIPKKSGGVR
MLGIPTVADRVAQTAVKLILEPQIDPLFHPNSYGYRPGRSAHDAIAVVRRRSWEYDWVVEFDIKGLFDNIDHNL
LMRALKKHCEIPWILLYVERWLKAPMQNVDGQVLERNHGTPQGGVISPLLANLFMHYAFDMWITKNLASVR
FCRYADDGVIHCRSLSQAKLVLQKIGARFRECGLELHPDKTKIVYCQDVNRRQAYPDVQFTFLGYTFRPRKAVDK
YKRVYVNFSPAVSRDALKAMRQTIRKWHLHLMCNRELSDLSAIFNPILQGWQQYYGRFHGSAMSAIWQHMN
AYLIRWMRRKYKNLARHKRRARYALGRLARDFPNAFVHWKMGCLPSVG
>SEQF9066||SEQF9066.1_02910
MTKPFNIPKALIWEAFKKVKENGGAAGIDHESIEQFEHHLKGNLYKLWNRLCSGSYFPPPVKGVPIPKKSGGVR
MLGIPTVADRVAQTAVKLILEPQIDPLFHPNSYGYRPGRSAHDAIAVVRRRSWEYDWVVEFDIKGLFDNIDHNL
LMRALKKHCEIPWILLYVERWLKAPMQNVDGQVLERNHGTPQGGVISPLLANLFMHYAFDMWITKNLASVR
FCRYADDGVIHCRSLSQAKLVLQKIGVRFRECGLELHPDKTKIVYCQDVNRRQAYPDVQFTFLGYTFRPRKAVDK
YKRVYVNFSPAVSRDALKTMRQTIRKWHLHLMCNRELSDLSAIFNPILQGWQQYYGRFHGSAMSAIWQHMN
AYLIRWMRRKYKNLARHKRRARYALGRLARDFPNAFVHWKMGCLPSVG
>SEQF9066||SEQF9066.1_01990
MTKPFNIPKALIWEAFKKVKENGGAAGIDHESIEQFEHHLKGNLYKLWNRLCSGSYFPPPVKGVPIPKKSGGVR
MLGIPTVADRVAQTAVKLILEPQIDPLFHPNSYGYRPGRSAHDAIAVVRRRSWEYDWVVEFDIKGLFDNIDHNL

LMRALKKHCEIPWILLYVERWLKAPMQNVDGQVLERNHGTPQGGVISPLLANLFMHYAFDMWITKNLASVR
FCRYADDGVIHCRSLSQAKLVLQKIGVRFRECGLELHPDKTKIVYCQDVNRRQAYPDVQFTFLGYTFRPRKAVDK
YKRVYVNFSPAVSRDALKTMRQTIRKWHLHLMCNRELSDLSAIFNPILQGWQQYYGRFHGSAMSAIWQHMN
AYLIRWMRRKYKNLARHKRRARYALGRLARDFPNAFVHWKMGCLPSVG
>SEQF9072||SEQF9072.1_01702
MTKPFNIPKALIWEAFKKVKENGGAAGIDHESIEQFEHHLKGNLYKLWNRLCSGSYFPPPVKGVPIPKKSGGVR
MLGIPTVADRVAQTAVKLILEPQIDPLFHPNSYGYRPGRSAHDAIAVVRRRSWEYDWVVEFDIKGLFDNIDHNL
LMRALKKHCEIPWILLYVERWLKAPMQNVDGQVLERNHGTPQGGVISPLLANLFMHYAFDMWITKNLASVR
FCRYADDGVIHCRSLSQAKLVLQKIGARFRECGLELHPDKTKIVYCQDVNRRQAYPDVQFTFLGYTFRPRKAVDK
YKRVYVNFSPAVSRDALKAMRQTIRKWHLHLMCNRELSDLSAIFNPILQGWQQYYGRFHGSAMSAIWQHMN
AYLIRWMRRKYKNLARHKRRARYALGRLARDFPNAFVHWKMGCLPSVG
>SEQF9076||SEQF9076.1_00055
MTKPFNIPKALIWEAFKKVKENGGAAGIDHESIEQFEHHLKGNLYKLWNRLCSGSYFPPPVKGVPIPKKSGGVR
MLGIPTVADRVAQTAVKLILEPQIDPLFHPNSYGYRPGRSAHDAIAVVRRRSWEYDWVVEFDIKGLFDNIDHNL
LMRALKKHCEIPWILLYVERWLKAPMQNVDGQVLERNHGTPQGGVISPLLANLFMHYAFDMWITKNLASVR
FCRYADDGVIHCRSLSQAKLVLQKIGARFRECGLELHPDKTKIVYCQDVNRRQAYPDVQFTFLGYTFRPRKAVDK
YKRVYVNFSPAVSRDALKTMRQTIRKWHLHLMCNRELSDLSAIFNPILQGWQQYYGRFHGSAMSTIWQHMN
AYLIRWMRRKYKNLARHKRRARYALGRLARDFPNAFVHWKMGCLPSVG
>SEQF9076||SEQF9076.1_00268
MTKPFNIPKALIWEAFKKVKENGGAAGIDHESIEQFEHHLKGNLYKLWNRLCSGSYFPPPVKGVPIPKKSGGVR
MLGIPTVADRVAQTAVKLILEPQIDPLFHPNSYGYRPGRSAHDAIAVVRRRSWEYDWVVEFDIKGLFDNIDHNL
LMRALKKHCEIPWILLYVERWLKAPMQNVDGQVLERNHGTPQGGVISPLLANLFMHYAFDMWITKNLASVR
FCRYADDGVIHCRSLSQAKLVLQKIGARFRECGLELHPDKTKIVYCQDVNRRQAYPDVQFTFLGYTFRPRKAVDK
YKRVYVNFSPAVSRDALKTMRQTIRKWHLHLMCNRELSDLSAIFNPILQGWQQYYGRFHGSAMSTIWQHMN
AYLIRWMRRKYKNLARHKRRARYALGRLARDFPNAFVHWKMGCLPSVG
>SEQF9076||SEQF9076.1_00180
MTKPFNIPKALIWEAFKKVKENGGAAGIDHESIEQFEHHLKGNLYKLWNRLCSGSYFPPPVKGVPIPKKSGGVR
MLGIPTVADRVAQTAVKLILEPQIDPLFHPNSYGYRPGRSAHDAIAVVRRRSWEYDWVVEFDIKGLFDNIDHNL
LMRALKKHCEIPWILLYVERWLKAPMQNVDGQVLERNHGTPQGGVISPLLANLFMHYAFDMWITKNLASVR
FCRYADDGVIHCRSLSQAKLVLQKIGARFRECGLELHPDKTKIVYCQDVNRRQAYPDVQFTFLGYTFRPRKAVDK
YKRVYVNFSPAVSRDALKTMRQTIRKWHLHLMCNRELSDLSAIFNPILQGWQQYYGRFHGSAMSTIWQHMN
AYLIRWMRRKYKNLARHKRRARYALGRLARDFPNAFVHWKMGCLPSVG
>SEQF9077||SEQF9077.1_00051
MTKPFNIPKALIWEAFKKVKENGGAAGIDHESIEQFEHHLKGNLYKLWNRLCSGSYFPPPVKGVPIPKKSGGVR
MLGIPTVADRVAQTAVKLILEPQIDPLFHPNSYGYRPGRSAHDAIAVVRRRSWEYDWVVEFDIKGLFDNIDHNL
LMRALKKHCEIPWILLYVERWLKAPMQNVDGQVLERNHGTPQGGVISPLLANLFMHYAFDMWITKNLASVR
FCRYADDGVIHCRSLSQAKLVLQKIGARFRECGLELHPDKTKIVYCQDVNRRQAYPDVQFTFLGYTFRPRKAVDK
YKRVYVNFSPAVSRDALKTMRQTIRKWHLHLMCNRELSDLSAIFNPILQGWQQYYGRFHGSAMSTIWQHMN
AYLIRWMRRKYKNLARHKRRARYALGRLARDFPNAFVHWKMGCLPSVG
>SEQF9078||SEQF9078.1_00032
MTKPFNIPKALIWEAFKKVKENGGAAGIDHESIEQFEHHLKGNLYKLWNRLCSGSYFPPPVKGVPIPKKSGGVR
MLGIPTVADRVAQTAVKLILEPQIDPLFHPNSYGYRPGRSAHDAIAVVRRRSWEYDWVVEFDIKGLFDNIDHNL
LMRALKKHCEIPWILLYVERWLKAPMQNVDGQVLERNHGTPQGGVISPLLANLFMHYAFDMWITKNLASVR
FCRYADDGVIHCRSLSQAKLVLQKIGARFRECGLELHPDKTKIVYCQDVNRRQAYPDVQFTFLGYTFRPRKAVDK

YKRVYVNFSPAVSRDALKTMRQTIRKWHLHLMCNRELSDLSAIFNPILQGWQQYYGRFHGSAMSTIWQHMN
AYLIRWMRRKYKNLARHKRRARYALGRLARDFPNAFVHWKMGCLPSVG
>SEQF9078||SEQF9078.1_03312
MTKPFNIPKALIWEAFKKVKENGGAAGIDHESIEQFEHHLKGNLYKLWNRLCSGSYFPPPVKGVPIPKKSGGVR
MLGIPTVADRVAQTAVKLILEPQIDPLFHPNSYGYRPGRSAHDAIAVVRRRSWEYDWVVEFDIKGLFDNIDHNL
LMRALKKHCEIPWILLYVERWLKAPMQNVDGQVLERNHGTPQGGVISPLLANLFMHYAFDMWITKNLASVR
FCRYADDGVIHCRSLSQAKLVLQKIGARFRECGLELHPDKTKIVYCQDVNRRQAYPDVQFTFLGYTFRPRKAVDK
YKRVYVNFSPAVSRDALKTMRQTIRKWHLHLMCNRELSDLSAIFNPILQGWQQYYGRFHGSAMSTIWQHMN
AYLIRWMRRKYKNLARHKRRARYALGRLARDFPNAFVHWKMGCLPSVG
>SEQF9080||SEQF9080.1_03217
MTKPFNIPKALIWEAFKKVKENGGAAGIDHESIEQFEHHLKGNLYKLWNRLCSGSYFPPPVKGVPIPKKSGGVR
MLGIPTVADRVAQTAVKLILEPQIDPLFHPNSYGYRPGRSAHDAIAVVRRRSWEYDWVVEFDIKGLFDNIDHNL
LMRALKKHCEIPWILLYVERWLKAPMQNVDGQVLERNHGTPQGGVISPLLANLFMHYAFDMWITKNLASVR
FCRYADDGVIHCRSLSQAKLVLQKIGARFRECGLELHPDKTKIVYCQDVNRRQAYPDVQFTFLGYTFRPRKAVDK
YKRVYVNFSPAVSRDALKTMRQTIRKWHLHLMCNRELSDLSAIFNPILQGWQQYYGRFHGSAMSTIWQHMN
AYLIRWMRRKYKNLARHKRRARYALGRLARDFPNAFVHWKMGCLPSVG
>SEQF9084||SEQF9084.1_00535
MTKPFNIPKALIWEAFKKVKENGGAAGIDHESIEQFEHHLKGNLYKLWNRLCSGSYFPPPVKGVPIPKKSGGVR
MLGIPTVADRVAQTAVKLILEPQIDPLFHPNSYGYRPGRSAHDAIAVVRRRSWEYDWVVEFDIKGLFDNIDHNL
LMRALKKHCEIPWILLYVERWLKAPMQNVDGQVLERNHGTPQGGVISPLLANLFMHYAFDMWITKNLASVR
FCRYADDGVIHCRSLSQAKLVLQKIGARFRECGLELHPDKTKIVYCQDVNRRQAYPDVQFTFLGYTFRPRKAVDK
YKRVYVNFSPAVSRDALKTMRQTIRKWHLHLMCNRELSDLSAIFNPILQGWQQYYGRFHGSAMSAIWQHMN
AYLIRWMRRKYKNLARHKRRARYALGRLARDFPNAFVHWKMGCLPSVG
>SEQF9896||SEQF9896.1_00010
MSTAKSYSISKLTVWEAYQRVKANRGAAGIDEQSIAQFEQKLQRNLYKLWNRMSSGSYFPPPVRQVEIPKQSGG
KRKLGIPTVADRVAQTAVKLLIEPRLDCLFHSDSYGYRPGKSAKQAVEITRKRCWNMNWVVEFDIKGAFDHIDH
ELLLKAVRHHVKDDWILLYIERWLKAPFETADGVQVPRESGTPQGGVVSPLLMNLFMHYAFDTWMQRTFPGC
PFARYADDAVVHCRSEKQACEVMAAIKARLEACLLTMHPEKSKIVYCKDSNRKAAYPTTQFTFLGFTFRPREAW
GNHGRRFTSFLPAASNDALKRMRQQTRGWNIQRQTPASLFELSKQYNATLRGWWNYYGTFYRTAMRKVFNH
FDLKLQRWARQKYKPLAGHKRRSADWLNRMKKACPSLFVHWNVFGNADRLGNGSRMS
>SEQF9896||SEQF9896.1_03361
MSTAKSYSISKLTVWEAYQRVKANRGAAGIDEQSIAQFEQKLQRNLYKLWNRMSSGSYFPPPVRQVEIPKQSGG
KRKLGIPTVADRVAQTAVKLLIEPRLDCLFHSDSYGYRPGKSAKQAVEITRKRCWNMNWVVEFDIKGAFDHIDH
ELLLKAVRHHVKDDWILLYIERWLKAPFETADGVQVPRESGTPQGGVVSPLLMNLFMHYAFDTWMQRTFPGC
PFARYADDAVVHCRSEKQACEVMAAIKARLEACLLTMHPEKSKIVYCKDSNRKAAYPTTQFTFLGFTFRPREAW
GNHGRRFTSFLPAASNDALKRMRQQTRGWNIQRQTPASLFELSKQYNATLRGWWNYYGTFYRTAMRKVFNH
FDLKLQRWARQKYKPLAGHKRRSADWLNRMKKACPSLFVHWNVFGNADRLGNGSRMS
>SEQF9896||SEQF9896.1_01729
MSTAKSYSISKLTVWEAYQRVKANRGAAGIDEQSIAQFEQKLQRNLYKLWNRMSSGSYFPPPVRQVEIPKQSGG
KRKLGIPTVADRVAQTAVKLLIEPRLDCLFHSDSYGYRPGKSAKQAVEITRKRCWNMNWVVEFDIKGAFDHIDH
ELLLKAVRHHVKDDWILLYIERWLKAPFETADGVQVPRESGTPQGGVVSPLLMNLFMHYAFDTWMQRTFPGC
PFARYADDAVVHCRSEKQACEVMAAIKARLEACLLTMHPEKSKIVYCKDSNRKAAYPTTQFTFLGFTFRPREAW
GNHGRRFTSFLPAASNDALKRMRQQTRGWNIQRQTPASLFELSKQYNATLRGWWNYYGTFYRTAMRKVFNH
FDLKLQRWARQKYKPLAGHKRRSADWLNRMKKACPSLFVHWNVFGNADRLGNGSRMS

>SEQF9896||SEQF9896.1_01457
MSTAKSYSISKLTVWEAYQRVKANRGAAGIDEQSIAQFEQKLQRNLYKLWNRMSSGSYFPPPVRQVEIPKQSGG
KRKLGIPTVADRVAQTAVKLLIEPRLDCLFHSDSYGYRPGKSAKQAVEITRKRCWNMNWVVEFDIKGAFDHIDH
ELLLKAVRHHVKDDWILLYIERWLKAPFETADGVQVPRESGTPQGGVVSPLLMNLFMHYAFDTWMQRTFPGC
PFARYADDAVVHCRSEKQACEVMAAIKARLEACLLTMHPEKSKIVYCKDSNRKAAYPTTQFTFLGFTFRPREAW
GNHGRRFTSFLPAASNDALKRMRQQTRGWNIQRQTPASLFELSKQYNATLRGWWNYYGTFYRTAMRKVFNH
FDLKLQRWARQKYKPLAGHKRRSADWLNRMKKACPSLFVHWNVFGNADRLGNGSRMS
>SEQF9896||SEQF9896.1_01096
MSTAKSYSISKLTVWEAYQRVKANRGAAGIDEQSIAQFEQKLQRNLYKLWNRMSSGSYFPPPVRQVEIPKQSGG
KRKLGIPTVADRVAQTAVKLLIEPRLDCLFHSDSYGYRPGKSAKQAVEITRKRCWNMNWVVEFDIKGAFDHIDH
ELLLKAVRHHVKDDWILLYIERWLKAPFETADGVQVPRESGTPQGGVVSPLLMNLFMHYAFDTWMQRTFPGC
PFARYADDAVVHCRSEKQACEVMAAIKARLEACLLTMHPEKSKIVYCKDSNRKAAYPTTQFTFLGFTFRPREAW
GNHGRRFTSFLPAASNDALKRMRQQTRGWNIQRQTPASLFELSKQYNATLRGWWNYYGTFYRTAMRKVFNH
FDLKLQRWARQKYKPLAGHKRRSADWLNRMKKACPSLFVHWNVFGNADRLGNGSRMS
>SEQF9896||SEQF9896.1_02905
MSTAKSYSISKLTVWEAYQRVKANRGAAGIDEQSIAQFEQKLQRNLYKLWNRMSSGSYFPPPVRQVEIPKQSGG
KRKLGIPTVADRVAQTAVKLLIEPRLDCLFHSDSYGYRPGKSAKQAVEITRKRCWNMNWVVEFDIKGAFDHIDH
ELLLKAVRHHVKDDWILLYIERWLKAPFETADGVQVPRESGTPQGGVVSPLLMNLFMHYAFDTWMQRTFPGC
PFARYADDAVVHCRSEKQACEVMAAIKARLEACLLTMHPEKSKIVYCKDSNRKAAYPTTQFTFLGFTFRPREAW
GNHGRRFTSFLPAASNDALKRMRQQTRGWNIQRQTPASLFELSKQYNATLRGWWNYYGTFYRTAMRKVFNH
FDLKLQRWARQKYKPLAGHKRRSADWLNRMKKACPSLFVHWNVFGNADRLGNGSRMS
>SEQF9897||SEQF9897.1_02453
MSTAKSYSISKLTVWEAYQRVKANRGAAGIDEQSIAQFEQKLQRNLYKLWNRMSSGSYFPPPVRQVEIPKQSGG
KRKLGIPTVADRVAQTAVKLLIEPRLDCLFHSDSYGYRPGKSAKQAVEITRKRCWNMNWVVEFDIKGAFDHIDH
ELLLKAVRHHVKDDWILLYIERWLKAPFETADGVQVPRESGTPQGGVVSPLLMNLFMHYAFDTWMQRTFPGC
PFARYADDAVVHCRSEKQACEVMAAIKARLEACLLTMHPEKSKIVYCKDSNRKAAYPTTQFTFLGFTFRPREAW
GNHGRRFTSFLPAASNDALKRMRQQTRGWNIQRQTPASLFELSKQYNATLRGWWNYYGTFYRTAMRKVFNH
FDLKLQRWARQKYKPLAGHKRRSADWLNRMKKACPSLFVHWNVFGNADRLGNGSRMS
>SEQF9899||SEQF9899.1_00532
MISAKPYGLAKRTVWEAYQQVKANRGAAGVDDETIADFEQGLSKNLYKLWNRMSSGSYFPPPVKQVEIPKAS
GGMRKLGVPTVSDRVAQTVVKLLIEPELDSIFHPDSYGYRPGRSAKQAVAITRERCWRYDWVVEFDIKAAFDQI
DHGLLMKAVRAHIKEDWILLYIERWLVAPFETKDGVCVPRDRGTPQGGVVSPILMNLFMHYAFDMWMQRTS
ANCPFARYADDAVVHCRSRKQAEYMMRTIASRLADCGLTMHPEKSKIVYCKDSNRTEQHLHVSFTFLGFMFRP
RKALSKEGRLFTSFLPGASEGALKRMRQTVRRWRLNSQTHVTLADVARLYNPVIQGWWHYYGSFYRTSMLGIF
QHIDRALERWARRKYKALHRRKRRGSRWVEKMRMAAPRLFHHWRVAGQQGWITGAV
>HRGM_Genome_0046||HRGM_Genome_0046_CDS_02603
MSVERRGCVKQPEPASQLSQCGRRKACGQAKPFAISKWEVQAAFDKVKANQGGAGIDGVTLEVFERNLKNNL
YKIWNRLSSGTYFPPSVKAVNIPKKTGGVRTLGIPTVGDRVAQMVIKERLEAIIEPCFLSDSYGYRPAKSAIQAIGV
TRKRCWEYNWVLEFDIKGLFDNIRHDLLLKAVEKHVANGSQIQGQDLSWLTFYIKRWLVSPLQQDDGNIILRDR
GTPQGGVVSPLLANLFLHYVFDKWMQREFPENPWCRYADDGLVHAITKGKAESLYERIKRRLEECGLELHPEKT
KIIYCKDDKRKGTYLHTSFDFLGYTFRQRRCRKCSDNTFFNSFIPAVSMMAMKAMRRRIRELKIRQKSYYSLEELS
RWLNPIVQGWISYYGQYCRSALDPVFRHINKTLVRWARRKFKTLKRHKSRTIGLFDRLSVKCPKLFAHWRFGSA
RTFA
>HRGM_Genome_0081||HRGM_Genome_0081_CDS_03915

MDKAKPFSISKAIVWKAYQRFKANGGAAGVDRQSIEEFEKDLTGNLYKLWNRMASGSYFPPPVRRVVIPKSGG
DTRPLGIPTVTDRIAQMVAKLVLEPGVEPCFHPDSYGYRPERSAIDVVGVARQRCWKYDWVVDMDIRAFFDS
MDHDLLMRAVRKHTSCRWVLLYIERWLKAPVQLQDGTVQPRDRGTPQGGVISPLLANLFLHYAFDLWMKRT
HPDKPFERYADDVIVHCRSLDASRSLMREIDQRLSECGLTLHPGETKVIYCKDRSRKADYPVISFDFLGYRFQPHC
AQRRDGSLFLNFLLPAVSPKAARTMRGSIRSWKIHRWTQLTIKELANSFNPVLRGWINYYGKFYKSKLVPILDQL
NYSLVRWARRKYKRLGSASQAATWLKRVVAQLPRLFPHWTITHTGMAGR
>HRGM_Genome_1111||HRGM_Genome_1111_CDS_01511
MSESKQYEIPKKVVIEAYKRVKANKGSAGIDGIDFERFEKKLNNNLYKIWNRMSSGSYFPSPVLSVEIPKKAGGTR
RLGIPTITDRIAQMVARMYVEPVVEPMFCDDSYGYRPNKSAIDAIATARKRCWRYDYVIELDVKGLFDNINHELL
MRVVLKHVKEEWICLYIKRWLETPFITREGQVIERLSGTPQGGVISPVLANMYLHYVFDMWMKRNFPQAPFE
RYADDGVIHCRTKEEAFVIKKKLAARFAECKLELHPVKTRVVYCKDKDRTRNEELTEFDFLGYTFKAVYIMCKDGK
VRYNFIASVSKTSSKSFRDKIKAMEVHKRTGCKIDIIAEILNPLIRGWMNYFGKFNPSAMKGTLQCIDRRLVKWA
MCKYKNFRGKRGRAEKWLCTVRQREPKLFAHWSNLYSYC
>HRGM_Genome_1238||HRGM_Genome_1238_CDS_02644
MQETKPYSISKRAVIAAYERVKANKGTYGVDDQSIQDFERKLNNNLYKIWNRMSSGSYFPKPVKAVAIPKKNGG
TRLLGIPTVEDRIAQMVVKLYFEPNVESIFYEDSYGYRPNKSAIQALDVTRTRCWRKDWVLEFDIKGLFDHIRHD
YLMEMVKRHTKEEWIPLYIERWLVAPFQMEDGTLVPRTSGTPQGGVISPVLANLFLHYVFDDFMAKEFPSIPW
ARYADDGIAHCASLKQAKYLQRRLEERFMRFGLELNLDKTRIVYCKDDDRKGNHEYTSFDFLGYTFRPRHAMNK
YGKFFTNFLPAMSEKAKKSIRKTVRKWKLQHKPDKELRDLANMFNSQIQGWINYYTHFYKSEIYDVLRYINQRL
VYWVRRKYKKRNARRRAEYWLGEIAKRDRTLFAHWKFGILPSVG
>HRGM_Genome_1338||HRGM_Genome_1338_CDS_01676
MSEAKQFDISKKAVIAAFQAVKENAGSYGADEQTIKEFEEHLNNNLYKLWNRMASGSYFPKPVRAVAIPKKNG
GIRILGIPTVEDRIAQMVAKMYFEPLVEPMFYNDSYGYRPNKSAIQAVGQARERCFKRDWALELDIKGLFDNIK
HGYLMYMVEKHTQIKWLILYIKRWLTVPFIMSDGSVAERRSGTPQGGVISPVLANLFLHYVFDDFMTKAYPNI
WWERYADDGVLHCQSYKQAAFIKQKLEERFQQFGLELNKEKTRIVYCKDNRRPQNYSCTQFTFLGYTFRPRLN
KNKEGKFFVGFTPAVSEKAKTAMKQKIREWKIQLKADLSLKDIGNMINKVVQGWINYYTHYYKSEFYEVLRYIN
QCLIKWVRRSYKKKNTRSRAEHWLGAVARRDRNLFAHWKFGILPSVGEGAV
>HRGM_Genome_1350||HRGM_Genome_1350_CDS_00200
MSEAKQFDISKKAVIAAFQAVKENAGSYGADEQTIKEFEEHLNNNLYKLWNRMASGSYFPKPVRAVAIPKKNG
GIRILGIPTVEDRIAQMVAKMYFEPLVEPMFYNDSYGYRPNKSAIQAVGQARERCFKRDWVLELDIKGLFDNIK
HGYLMYMVEKHTQIKWLILYIKRWLTVPFIMSDGSVAERRSGTPQGGVISPVLANLFLHYVFDDFMTKAYPNI
WWERYADDGVLHCQSYKQAAFIKQKLEERFQQFGLELNKEKTRIVYCKDNRRPQNYSCTQFTFLGYTFRPRLN
KNKEGKFFVGFTPAVSEKAKTAMKQKIREWKIQLKADLSLKDIGNMINKVVQGWINYYTHYYKSEFYEVLRYIN
QCLIKWVRRSYKKKNTRSRAEHWLGAVARRDRNLFAHWKFGILPSVGEGAV
>HRGM_Genome_1401||HRGM_Genome_1401_CDS_04193
MQEAKPYSISKKAVIAAYQRVKANKGTYGVDEQSIEDFERKLNNNLYKIWNRMSSGTYFPKPVKAVAIPKKNGG
TRILGVPTVEDRIAQMVAKLYFEPCVEPIFYEDSYGYRPNKSAIQALEATRTRCWRKDWVLEFDIRGLFDNIRHDY
LMEMVKKHTKEKWIILYIQRWLTAPFQMEDGTIVERKSGTPQGGVISPVLANLFLHYVFDDFMVKEFPTIPWA
RYADDGIAHCVSQKQAKYLRRRLGQRFQSYGLELNQEKTRIVYCKDDDRRGNHENTSFDFLGYTFRPRHAKNR
YGKFFTNFLPAISEKAKKAIRKEVRGWKLQLKSDKDLYDIANMFNRQIQGWINYYTHFYKSEIYDVLRYINGCLVK
WVRRKYKKRKARRKAEHWLGEIAKRDRNLFAHWKFGILPAAG
>HRGM_Genome_1421||HRGM_Genome_1421_CDS_03545
MQHQVTKPFTIDKYLIMNAWKRVKENKGSAGIDNVSTEDYESNLGKNLYKLWNRMSSGSYFPEAVKLVDIPKP
SGGTRPLGIPTVGDRIAQMSVVLLIEERLEAIFHADSYGYRPNRSAHDAIEKARERCWHYNWVLDMDISKFFDT

IDHDLLMKAVERHVQEKWILLYIRRWLKVPYATLTGERIERKMGVPQGSVIGPVLANLYLHYTFDKWMSLYHPTI
PFERYADDTICHCNSLKEAQMLKASIVERFAACKLRLNEEKTRIVYCKDGKRRGEYKEITFDFLGYTFQPRGQRNK
QGQVFNGYAPAISRKSKKRITEKMRGWHLNRRVQLKLSDIAVEINAEVRGWMNYYGKFYGSQLKAFLQCINLK
LARWAERKYKRFRRKPNDAYKWLVRVASKNPALFYHWQHGVKPNRLKPFG
>HRGM_Genome_1421||HRGM_Genome_1421_CDS_04332
MQNDNAKPISISKQLVYDAFLRVKANRGSAGIDKVTLEDYEKNLRGNLYKLWNRMSSGSYFPPSVKLVEIPKSTG
GKRPLGIPTVSDRVAQMAVVMLITPSIEPCFHEDSYAYRPHRSAHDAVGKARERCWKYAWVLDMDISKFFDTI
DHELLLKALKRHTQEKWVLMYIERWLKVPYEKSDGSQVDRALGVPQGSVIGPVLANLFLHYTFDKWMEKNFP
RVPFERYADDTICHCHSLKQAEYMQAMIQQRFECCRLRLNEEKTKIVYCKSSRQKECYPNVTFDFLGFTFQPRES
VDKYGNRFTGFLPAISRKSMKRINETMRSWHLNRHSNLTLEHLASDINPIVRGWMTYYGKFYPTRLKWFMQTL
NGRLARWVMCKFERYRHRFYPAQEWLARIAEKEGLIFYHWKCGALPRFTNKEKVSSQLIMVK
>HRGM_Genome_1423||HRGM_Genome_1423_CDS_01737
MQHQVTKPFTTDKHLIMNAWKRVKENKGSGGIDNVSIEDYESNLGKHLYKLWNRMSSGSYFPEAVKLVDIPKS
SGGTRPLGIPTVGDRIAQMSVVLLIEDRLESIFHADSYGYRPNRSAHDAVGKARERCWHHNWVLDMDISKFFD
TIDHDLLMKAVERHVQEKWILLYIRRWLKVPYATLTGERIERKMGVPQGSVIGPVLANLYLHYTFDKWMSLYHP
TIPFERYADDTICHCNSLEEAHMLKVSIVERFAACKLRLNEEKTRIVYCKDGKRRGEYKEITFDFLGYTFQPRGQR
NKQGQVFNGYAPAISRKSKKRITEKMRGWHLNRRVQLKLSDIAVEINAEVRGWMNYYGKFYGSQLKAFLQCIN
LKLARWAERKYKRFRRKPNDAYKWLVRVASRNPALFYHWQHGVKPNRLKPFG
>HRGM_Genome_1580||HRGM_Genome_1580_CDS_05618
MSGVSSAKPYDIAKRTVWDAYQQVRANRGAAGIDDETIADFERDLSKNLYKLWNRMSSGSYFPPPVKQVEIPK
ASGGTRKLGVPTVGDRVAQTVVKLLIEPELDSIFHSDSYGYRPGRSAKQAVAITRERCWRYDWVVEFDIKAAFD
QINHGLLMKAVRLHIKEDWILLYIERWLVAPFETDDGMRVPRERGTPQGGVLSPLLMNLFMHYAFDTWMQR
TSPNCPFARYADDAVVHCRSRKQAEYVMRSIASRLAACGLTMHPEKSKVVYCKDSNRRAGYPHVSFTFLGFTFR
PRKALSKQDQLFTSFLPGASADALKRMRQAVRRWRLNRQTHVTLVDVARLYNPVIQGWWQYYGSFYRTAML
GIFQHIDRALERWARRKYKALHRRKRRISQWLDKMRTVVPRLFHHWRVTGQQGWITGAV
>HRGM_Genome_1718||HRGM_Genome_1718_CDS_04631
MQEAKPYSISKKAVIAAYQRVKANKGTYGVDEQSIEDFERKLNNNLYKIWNRMSSGTYFPKPVKAVAIPKKNGG
TRILGVPTVEDRIAQMVAKLYFEPCVEPIFYEDSYGYRPNKSAIQALEATRTRCWRKDWVLEFDIRGLFDNIRHDY
LMEMVKKHTKEKWIILYIQRWLTAPFQMEDGTIVERKSGTPQGGVISPVLANLFLHYVFDDFMVKEFPTIPWA
RYADDGIAHCVSQKQAKYLRRRLEQRFQSYGLELNQEKTRIVYCKDDDRRGNHENTSFDFLGYTFRPRHAKNRY
GKFFTNFLPAISEKAKKAIRKEVRGWKLQLKSDKDLYDIANMFNRQIQGWINYYTHFYKSEIYDVLRYINGCLVK
WVRRKYKKRKARRKAEHWLGEIAKRDRNLFAHWKFGILPAAG
>HRGM_Genome_1718||HRGM_Genome_1718_CDS_00950
MQEAKPYSISKKAVIAAYQRVKANKGTYGVDEQSIEDFERKLNNNLYKIWNRMSSGTYFPKPVKAVAIPKKNGG
TRILGVPTVEDRIAQMVAKLYFEPCVEPIFYEDSYGYRPNKSAIQALEATRTRCWRKDWVLEFDIRGLFDNIRHDY
LMEMVKKHTKEKWIILYIQRWLTAPFQMEDGTIVERKSGTPQGGVISPVLANLFLHYVFDDFMVKEFPTIPWA
RYADDGIAHCVSQKQAKYLRRRLEQRFQSYGLELNQEKTRIVYCKDDDRRGNHENTSFDFLGYTFRPRHAKNRY
GKFFTNFLPAISEKAKKAIRKEVRGWKLQLKSDKDLYDIANMFNRQIQGWINYYTHFYKSEIYDVLRYINGCLVK
WVRRKYKKRKARRKAEHWLGEIAKRDRNLFAHWKFGILPAAG
>HRGM_Genome_1718||HRGM_Genome_1718_CDS_05488
MQEAKPYSISKKAVIAAYQRVKANKGTYGVDEQSIEDFERKLNNNLYKIWNRMSSGTYFPKPVKAVAIPKKNGG
TRILGVPTVEDRIAQMVAKLYFEPCVEPIFYEDSYGYRPNKSAIQALEATRTRCWRKDWVLEFDIRGLFDNIRHDY
LMEMVKKHTKEKWIILYIQRWLTAPFQMEDGTIVERKSGTPQGGVISPVLANLFLHYVFDDFMVKEFPTIPWA
RYADDGIAHCVSQKQAKYLRRRLEQRFQSYGLELNQEKTRIVYCKDDDRRGNHENTSFDFLGYTFRPRHAKNRY

GKFFTNFLPAISEKAKKAIRKEVRGWKLQLKSDKDLYDIANMFNRQIQGWINYYTHFYKSEIYDVLRYINGCLVK
WVRRKYKKRKARRKAEHWLGEIAKRDRNLFAHWKFGILPAAG
>HRGM_Genome_1718||HRGM_Genome_1718_CDS_05188
MQEAKPYSISKKAVIAAYQRVKANKGTYGVDEQSIEDFERKLNNNLYKIWNRMSSGTYFPKPVKAVAIPKKNGG
TRILGVPTVEDRIAQMVAKLYFEPCVEPIFYEDSYGYRPNKSAIQALEATRTRCWRKDWVLEFDIRGLFDNIRHDY
LMEMVKKHTKEKWIILYIQRWLTAPFQMEDGTIVERKSGTPQGGVISPVLANLFLHYVFDDFMVKEFPTIPWA
RYADDGIAHCVSQKQAKYLRRRLEQRFQSYGLELNQEKTRIVYCKDDDRRGNHENTSFDFLGYTFRPRHAKNRY
GKFFTNFLPAISEKAKKAIRKEVRGWKLQLKSDKDLYDIANMFNRQIQGWINYYTHFYKSEIYDVLRYINGCLVK
WVRRKYKKRKARRKAEHWLGEIAKRDRNLFAHWKFGILPAAG
>HRGM_Genome_1841||HRGM_Genome_1841_CDS_02451
METRHGTKYRQLHIEDYLREIPAEQGKVTGVYAHEWITGNPDTNTDFWTDNLLDTILRSDNLNAAYKRVKANK
GSAGIDGMDFEKFEKRLNNNLYKIWNRMSSGSYFPSPVMAVEIPKKSGGTRRLGIPTIADRIAQMVARAYVERA
VEPMFCEDSYGYRPHKSALDAVEKTRKRCWKYDYVIELDVKGLFDNIDHELLMRVVRRHVKEPWICLYIERWLK
SPFVLPDGSRIERESGTPQGGVISPVLANMFLHYVFDMWMKRNFPQAPFERYADDGVVHCRTKEEALYIKKKL
VKRFEECKLELHPVKTRIVYCKDKDRTKEEELAEFDFLGYTFKAVYIKCKDGVMRNNFIASVSKTAAKGFRDKIKAL
EIHKRTGCKIDMIAELLNPMIRGWMNYFGKFNPSAMKNTLQCIECRLIKWAMCKYKSFRGRRQRAEKWLSSIR
KREPKLFAHWSRMYSYC
>HRGM_Genome_1843||HRGM_Genome_1843_CDS_00633
METRHGTKYRQLHIEDYLREIPAEQGKVTGVYAHEWITGNPDTNTDFWTDNLLDTILRSDNLNAAYKRVKANK
GSAGIDGMDFDKFEKRLNNNLYKIWNRMSSGSYFPSPVMAVEIPKKSGGTRRLGIPTIADRIAQMVARAYVER
AVEPMFCEDSYGYRPHKSALDAVEKTRKRCWKYDYVIELDVKGLFDNIDHELLMRVVRRHVKEPWICLYIERWL
KSPFVLPDGSRIERESGTPQGGVISPVLANMFLHYVFDMWMKRNFPQAPFERYADDGVVHCRTKEEALYIKKK
LVKRFEECKLELHPVKTRIVYCKDKDRTKEEELAEFDFLGYTFKAVYIKCKDGVMRNNFIASVSKTAAKGFRDKIKA
LEIHKRTGCKIDMIAELLNPMIRGWMNYFGKFNPSAMKNTLQCIECRLIKWAMCKYKSFRGRRQRAEKWLSSI
RKREPKLFAHWSRMYSYC
>HRGM_Genome_2053||HRGM_Genome_2053_CDS_04411
MQETKSYNISKQAVYQAFLKVKANKGTFGVDEESIEAYEYKLKDNLYKLWNRLSSGSYFPKPVKAVSIPKKSGGLR
VLGIPTVEDRIAQMVAKMYFEPVVERLFYEDSYGYRPNKSAIQAIEKTRVRCWKRDWVLEFDIKGLFDNIRHDY
LIEMVKRYTQEKWIILYVERWLKAPFQREDGSTVSRKAGTPQGGVISPVLANLFLHYTFDDFMEKEFPNIQWAR
YADDGITHCVSLKQAKYLKKRLEERFRIFGLELHPDKTKIVYCRDSDRMGNYPITTFDFLGFTFRPRGAKNKYGKC
FTNFLPAVSDKAKKAIRKEVRNWRLQLKADKKLEDLANMFNSKIQGWMNYYMKFYKSEMYSLLRYINQCLVK
WVRRKYKKRQARRKAEHWLGEIAKRERNLFAHWKIGILPSAG
>HRGM_Genome_2542||HRGM_Genome_2542_CDS_01778
MNEAKPFVIDKRLVWEAYHKVKENKGSAGIDKVDQKTFDKEMSKNLYKIWNRMSSGCYFPKAVKLVEIPKSNG
GTRPLGIPTIEDRIAQQVVVSVLTPILEPIFKEDSYGYRPGKGAHQAIAKAKERCYVNPWVLDMDISKFFDTINHE
LLMKAIRKHAEEKWVLLYIERWLKVPYQTSKGEVIERTMGVPQGSVIGPVLANLFLHYVFDEWMSRNYPTIPFE
RYADDTICHCVSEKQAQFLKAVLMKRFEECGLKLNEEKTKIVYCKDSNRRGDSEHTSFDFLGFTFRPRGARNRKT
GQNFTAFLPAISKKSMKRIKEAVRAWNLNRKTFVCLLDISNEVDTQISGWMNYYMKFGRSEFRKVLNYINERLT
RWVMRKYKRFSKGKKFSRAYEWLVEHAVHNRNEFSHWAKGFVPYPRLG
>HRGM_Genome_2547||HRGM_Genome_2547_CDS_02143
MNEAKPFVIDKRLVWEAYHKVKENKGSAGIDKVDQKTFDKEMSKNLYKIWNRMSSGCYFPKAVKLVEIPKSNG
GTRPLGIPTIEDRIAQQVVVSVLTPILEPIFKEDSYGYRPGKGAHQAIAKAKERCYVNPWVLDMDISKFFDTINHE
LLMKAIRKHTEEKWVLLYIERWLKVPYQTSKGEVIERTMGVPQGSVIGPVLANLFLHYVFDEWMSRNYPTIPFE
RYADDTICHCVSEKQAQFLKAVLMKRFEECGLKLNEEKTKIVYCKDSNRRGDSEHTSFDFLGFTFRPRGARNRKT

GQNFTAFLPAISKKSMKRIKEAVRAWKLNRKTFACLLDISNEVDTQISGWMNYYMKFGRSEFRKVLNYINERLT
RWVMRKYKRFSKGKKFSKAYDWLVEYAAHNRNEFSHWVKGFVPYPRLG
>HRGM_Genome_2566||HRGM_Genome_2566_CDS_02350
MNEAKPFVIDKRLVWEAYHKVKENKGSAGIDKVDQKTFDKEMSKNLYKIWNRMSSGCYFPKAVKLVEIPKSNG
GTRPLGIPTIEDRIAQQVVVSVLTPILEPIFKEDSYGYRPGKGAHQAIAKAKERCYVNPWVLDMDISKFFDTINHD
LLMKAVRKHTEEKWVLLYIERWLKVPYQTSKGEVIERTMGVPQGSVIGPVLANLFLHYVFDEWMSRNYPTIPFE
RYADDTICHCVSEKQAQFLKAVLMKRFEECGLKLNEEKTKIVYCKDSNRRGDSEHTSFDFLGFTFRPRSARNRKT
GQNFTAFLPAISKKSLKRIKEAVRAWKLNRKTFACLLDISNEVDTQISGWMNYYMKFGRSEFRKVLNYINERLTR
WVMRKYKRFSKGKKFSRAYEWLVEYAVHNRNEFSHWAKGFVPYPRLG
>HRGM_Genome_2588||HRGM_Genome_2588_CDS_02566
MNEAKPFVIDKRLVWEAYHKVKENKGSAGIDKVDQKTFDKEMSKNLYKIWNRMSSGCYFPKAVKLVEIPKSNG
GTRPLGIPTIEDRIAQQVVVSVLTPILEPIFKEDSYGYRPGKGAHQAIAKAKERCYVTPWVLDMDISKFFDTINHEL
LMKAIRKHTEEKWVLLYIERWLKVPYQTSKGEVIERTMGVPQGSVIGPVLANLFLHYVFDEWMSRNYPTIPFER
YADDTICHCVSEKQAQFLKAVLMKRFEECGLKLNEEKTKIVYCKDSNRRGDSEHISFDFLGFTFRPRGARNRKTG
QNFTAFLPAISKKSMKRIKEAARAWKLNRKTFACLLDISNEVDTQISGWMNYYMKFGRSEFRKVLNYINERLTR
WVMRKYKRFSKGKKFSKAYDWLVEYAAHNRNEFSHWVKGFVPYPRLG
>HRGM_Genome_2589||HRGM_Genome_2589_CDS_02361
MNEAKPFVIDKRLVWEAYHKVKENKGSAGIDKVDQKTFDKEMSKNLYKIWNRMSSGCYFPKAVKLLEIPKSNG
GTRPLGIPTIEDRIAQQVVVSVLIPILEPIFKEDSFGYRPDKGAHQAIAKAKERCYVNPWVLDMDISKFFDTINHD
LLMKAVRKHTEEKWVLLYIERWLKVPYQTSKGEVIERTMGVPQGSVIGPVLANLFLHYVFDEWMSRNYPTIPFE
RYADDTICHCVSEIQAQFLKAVLMKRFEECGLKLNEEKTKIVYCKDSNRRGNSEHTSFDFLGFTFRPRGARNRKT
GQNFTAFLPAISKKSMKRIKEAIRAWKLNRKTFACLLDISNEVDTQISGWMNYYMKFGMSEFRKVLNYINERLT
RWVMCKYKRFSKGKKLGTAYDWLVEYAAHNRNEFSHWVKGFVPYPRLG
>HRGM_Genome_3353||HRGM_Genome_3353_CDS_02743
MQEAKPYNISKKAVFAAYEKVKANKGTYGVDGKSIEMFERNLKNNLYKIWNRMSSGTYFPKPVKAVAIPKKNG
GTRTLGIPTVEDRIAQMVVKLYFEPGVEPIFYEDSYGYRPNKSAIQALEVTRVRCWRRDWVLEFDIRGLFDNIRH
EDLMKMVKKHTQEKWIHLYIQRWMLAPFQMEDGTLVERTAGTPQGGVISPVLANLCLHYVFDDYMEREFKTI
PWARYADDGIAHCVSRKQAKYLQRRLQERFQKFGLELNLEKTRIVYCKDDDRKGNHEHTSFDFLGYTFRPRHAK
NKYGKYFTNFLPAISEKAKKSIRKKVRGWKLQLKSDKDLYDIANMFNGQIQGWINYYTHFYKTEIYEVLQYINGCL
VKWVRRKYKKRKARRKAEHWLGQIAKRDKRLFAHWKLGILPSAG
>HRGM_Genome_4864||HRGM_Genome_4864_CDS_01618
MSESKQYEIPKRAVIEAYKKVKANKGSAGIDGIDFEAFEKKLNNNLYKIWNRMSSGSYFPSPVLAVEIPKKTGGTR
TLGIPTIADRIAQMVARMYVEPVVEPMFCEDSYGYRPNKSAIDAVGTARKRCWRYDYVIELDVKGLFDNIDHEL
LMRVVERHVKETWVCMYIKRWLEAPFVTREGHKIERKSGTPQGGVISPVLANMFLHYVFDMWMKRNFPQA
PFERYADDGVIHCRTKEEAISIKEKVAVRFEECRLELHPIKTRIVYCKDKDRTRKEELTKFDFLGYTFKAVYIMCKDG
KVRHNFIASVSNASAKNFRNKVKGMGIHKKTGCKIDIIAEILNPLIRGWMNYFGKFNPSAMKGTLQCIERRIVK
WAMCKYKNFRGRRRRAEKWLCTVRQREPKLFAHWSNLYSYC
>HRGM_Genome_5105||HRGM_Genome_5105_CDS_01672
MNKSKQYEIPKRAVINAYKRVKANKGSAGIDGMDFEKFEEKLNNNLYKIWNRMSSGSYFPSPVMAVEIPKKSG
GTRRLGIPTITDRIAQMVARMYVEPAVEPMFCEDSYGYRPNKSAIDAIETTRKRCWKYDYAIELDVKGLFDNIDH
ELLMKVVHRHVKEPWICMYIERWLKTPFVLQDGQVIERSAGTPQGGVISPVLANMFLHYVFDMWMKRNFP
QAPFERYADDGVVHCSTKEEALYIKERLAKRFEECKLELHPIKTRIVYCKDKDRTRNEELTEFDFLGYTFKAVYIKCK
DGVMRNNFIASVSKTAAKGFRDKIKALEIHKKTGCKIDIIAEMLNPMIRGWMNYFGKFNPSAMKYTLQCIERRL
VKWAMCKYKSFRGRRQRAEKWLSSIRKREPKLFAHWGRMYSYC

>HRGM_Genome_5202||HRGM_Genome_5202_CDS_03579
MSESKQYEIPKKVVIEAYKRVKANKGSAGIDGIDFERFEKKLNNNLYKIWNRMSSGSYFPSPVLSVEISKKAGGTR
RLGIPTITDRIAQMVARMYVEPVVEPMFCNDSYGYRPNKSAIDAIATARKRCWRYDYVIELDVKGLFDNINHELL
MRVVLKHVKEEWICLYIKRWLETPFITREGQVIERLSGTPQGGVISPVLANMYLHYVFDMWMKRNFPQAPFE
RYADDGVIHCRTKEEAFVIKKKLAARFAECKLELHPVKTRIVYCKDKDRTRNEELTEFDFLGYTFKAVYIMCKDGK
VRYNFIASVSKTSSKSFRDKIKAMEVHKRTGCKIDIIAEILNPLIRGWMNYFGKFNPSAMKGTLQCIDRRLVKWA
MCKYKNFRGKRGRAEKWLCTVRQREPKLFAHWSNLYSYC
>HRGM_Genome_5373||HRGM_Genome_5373_CDS_00364
MNEAKPFKVEKRLVYEAYKRVKSNKGSAGIDGVEMEKFEENLSDNLYKLWNRMSSGTYFPKAVKLVEIPKSNGG
KRPLGIPTIEDRIAQMAAVMEMMPELDKIFHEDSYGYRPGKSAHDAVAKADERCWSYNWVLDMDISKFFDTI
DHELLMKAVHRHVTEKWILLYIERWLKVPYKTKEGAVIERTMGVPQGSVIGPVLANLFLHYTFDKWMQINFPTI
PFERYADDSVCHCRTKKQAEYLKERLKARFAECKLKLNEEKTKIVYCKDSNRHDKGDNESFDYLGFTFRPRSARN
KQTFQVFTVFKPAISRKSGMKIRETIRGWKFMHNYHIELADIAKAINPVVRGWVNYYSKFGKTEFRRVMDYLN
QRLVLWAAKKYKRFKRLRKRAGNWLAMVATYNKTLFSHWAEGYIPYQISR
>CABIVX02||gene_3879|GeneMark.hmm|416_aa|-|59508|60758
LDKAKPFSISKAIVWKAYQRFKANGGAAGVDRQSIEEFEKDLTGNLYKLWNRMASGSYFPPPVRRVVIPKSGGD
TRPLGIPTVTDRIAQMVAKLVLEPGVEPCFHPDSYGYRPERSAIDVVGVARQRCWKYDWVVDMDIRAFFDSM
DHDLLMRAVRKHTSCRWVLLYIERWLKAPVQLQDGTVQPRDRGTPQGGVISPLLANLFLHYAFDLWMKRTHP
DKPFERYADDVIVHCRSLDASRSLMREIDQRLSECGLTLHPGETKVIYCKDRSRKADYPVISFDFLGYRFQPHCAQ
RRDGSLFLNFLLPAVSPKAARTMRGSIRSWKIHRWTQLTIKELANSFNPVLRGWINYYGKFYKSKLVPILDQLNYS
LVRWARRKYKRLGSASQAATWLKRVVAQLPRLFPHWTITHTGMAGR
>CABIXU02||gene_4266|GeneMark.hmm|430_aa|+|542|1834
MQNDNAKPISISKQLVYDAFLRVKANRGSAGIDKVTLEDYEKNLRGNLYKLWNRMSSGSYFPPSVKLVEIPKSTG
GKRPLGIPTVSDRVAQMAVVMLITPSIEPCFHEDSYAYRPHRSAHDAVGKARERCWKYAWVLDMDISKFFDTI
DHELLLKALKRHTQEKWVLMYIERWLKVPYEKSDGSQVDRALGVPQGSVIGPVLANLFLHYTFDKWMEKNFP
RVPFERYADDTICHCHSLKQAEYMQAMIQQRFECCRLRLNEEKTKIVYCKSSRQKECYPNVTFDFLGFTFQPRES
VDKYGNRFTGFLPAISRKSMKRINETMRSWHLNRHSNLTLEHLASDINPIVRGWMTYYGKFYPTRLKWFMQTL
NGRLARWVMCKFERYRHRFYPAQEWLARIAEKEGLIFYHWKCGALPRFTNKEKVSSQLIMVK
>CABIXU02||gene_3470|GeneMark.hmm|422_aa|+|26020|27288
MMQHQVTKPFTIDKYLIMNAWKRVKENKGSAGIDNVSTEDYESNLGKNLYKLWNRMSSGSYFPEAVKLVDIP
KPSGGTRPLGIPTVGDRIAQMSVVLLIEERLEAIFHADSYGYRPNRSAHDAIEKARERCWHYNWVLDMDISKFF
DTIDHDLLMKAVERHVQEKWILLYIRRWLKVPYATLTGERIERKMGVPQGSVIGPVLANLYLHYTFDKWMSLYH
PTIPFERYADDTICHCNSLKEAQMLKASIVERFAACKLRLNEEKTRIVYCKDGKRRGEYKEITFDFLGYTFQPRGQ
RNKQGQVFNGYAPAISRKSKKRITEKMRGWHLNRRVQLKLSDIAVEINAEVRGWMNYYGKFYGSQLKAFLQCI
NLKLARWAERKYKRFRRKPNDAYKWLVRVASKNPALFYHWQHGVKPNRLKPFG
>CABJAP02||gene_2821|GeneMark.hmm|414_aa|-|5206|6450
MQEAKPYNISKKAVFAAYEKVKANKGTYGVDGKSIEMFERNLKNNLYKIWNRMSSGTYFPKPVKAVAIPKKNG
GTRTLGIPTVEDRIAQMVVKLYFEPGVEPIFYEDSYGYRPNKSAIQALEVTRVRCWRRDWVLEFDIRGLFDNIRH
EDLMKMVKKHTQEKWIHLYIQRWMLAPFQMEDGTLVERTAGTPQGGVISPVLANLCLHYVFDDYMEREFKTI
PWARYADDGIAHCVSRKQAKYLQRRLQERFQKFGLELNLEKTRIVYCKDDDRKGNHEHTSFDFLGYTFRPRHAK
NKYGKYFTNFLPAISEKAKKSIRKKVRGWKLQLKSDKDLYDIANMFNGQIQGWINYYTHFYKTEIYEVLQYINGCL
VKWVRRKYKKRKARRKAEHWLGQIAKRDKRLFAHWKLGILPSAG
>CABJCM02||gene_1524|GeneMark.hmm|413_aa|-|303792|305033
MSESKQYEIPKKVVIEAYKRVKANKGSAGIDGIDFERFEKKLNNNLYKIWNRMSSGSYFPSPVLSVEIPKKAGGTR

RLGIPTITDRIAQMVARMYVEPVVEPMFCDDSYGYRPNKSAIDAIATARKRCWRYDYVIELDVKGLFDNINHELL
MRVVLKHVKEEWICLYIKRWLETPFITREGQVIERLSGTPQGGVISPVLANMYLHYVFDMWMKRNFPQAPFE
RYADDGVIHCRTKEEAFVIKKKLAARFAECKLELHPVKTRVVYCKDKDRTRNEELTEFDFLGYTFKAVYIMCKDGK
VRYNFIASVSKTSSKSFRDKIKAMEVHKRTGCKIDIIAEILNPLIRGWMNYFGKFNPSAMKGTLQCIDRRLVKWA
MCKYKNFRGKRGRAEKWLCTVRQREPKLFAHWSNLYSYC
>CABJFU02||gene_2541|GeneMark.hmm|420_aa|-|9513|10775
MNEAKPFVIDKRLVWEAYHKVKENKGSAGIDKVDQKTFDKEMSKNLYKIWNRMSSGCYFPKAVKLVEIPKSNG
GTRPLGIPTIEDRIAQQVVVSVLTPILEPIFKEDSYGYRPGKGAHQAIAKAKERCYVTPWVLDMDISKFFDTINHEL
LMKAIRKHTEEKWVLLYIERWLKVPYQTSKGEVIERTMGVPQGSVIGPVLANLFLHYVFDEWMSRNYPTIPFER
YADDTICHCVSEKQAQFLKAVLMKRFEECGLKLNEEKTKIVYCKDSNRRGDSEHISFDFLGFTFRPRGARNRKTG
QNFTAFLPAISKKSMKRIKEAARAWKLNRKTFACLLDISNEVDTQISGWMNYYMKFGRSEFRKVLNYINERLTR
WVMRKYKRFSKGKKFSKAYDWLVEYAAHNRNEFSHWVKGFVPYPRLG
>CABKOW02||gene_1708|GeneMark.hmm|418_aa|+|1839690|1840946
MSEAKQFDISKKAVIAAFQAVKENAGSYGADEQTIKEFEEHLNNNLYKLWNRMASGSYFPKPVRAVAIPKKNG
GIRILGIPTVEDRIAQMVAKMYFEPLVEPMFYNDSYGYRPNKSAIQAVGQARERCFKRDWALELDIKGLFDNIK
HGYLMYMVEKHTQIKWLILYIKRWLTVPFIMSDGSVAERRSGTPQGGVISPVLANLFLHYVFDDFMTKAYPNI
WWERYADDGVLHCQSYKQAAFIKQKLEERFQQFGLELNKEKTRIVYCKDNRRPQNYSCTQFTFLGYTFRPRLN
KNKEGKFFVGFTPAVSEKAKTAMKQKIREWKIQLKADLSLKDIGNMINKVVQGWINYYTHYYKSEFYEVLRYIN
QCLIKWVRRSYKKKNTRSRAEHWLGAVARRDRNLFAHWKFGILPSVGEGAV
>CABKWG02||gene_2591|GeneMark.hmm|414_aa|+|1165011|1166255
MQETKPYSISKRAVIAAYERVKANKGTYGVDDQSIQDFERKLNNNLYKIWNRMSSGSYFPKPVKAVAIPKKNGG
TRLLGIPTVEDRIAQMVVKLYFEPNVESIFYEDSYGYRPNKSAIQALDVTRTRCWRKDWVLEFDIKGLFDHIRHD
YLMEMVKRHTKEEWIPLYIERWLVAPFQMEDGTLVPRTSGTPQGGVISPVLANLFLHYVFDDFMAKEFPSIPW
ARYADDGIAHCASLKQAKYLQRRLEERFMRFGLELNLDKTRIVYCKDDDRKGNHEYTSFDFLGYTFRPRHAMNK
YGKFFTNFLPAMSEKAKKSIRKTVRKWKLQHKPDKELRDLANMFNSQIQGWINYYTHFYKSEIYDVLRYINQRL
VYWVRRKYKKRNARRRAEYWLGEIAKRDRTLFAHWKFGILPSVG
>CABLBP02||gene_4744|GeneMark.hmm|414_aa|+|547|1791
MQEAKPYSISKKAVIAAYQRVKANKGTYGVDEQSIEDFERKLNNNLYKIWNRMSSGTYFPKPVKAVAIPKKNGG
TRILGVPTVEDRIAQMVAKLYFEPCVEPIFYEDSYGYRPNKSAIQALEATRTRCWRKDWVLEFDIRGLFDNIRHDY
LMEMVKKHTKEKWIILYIQRWLTAPFQMEDGTIVERKSGTPQGGVISPVLANLFLHYVFDDFMVKEFPTIPWA
RYADDGIAHCVSQKQAKYLRRRLEQRFQSYGLELNQEKTRIVYCKDDDRRGNHENTSFDFLGYTFRPRHAKNRY
GKFFTNFLPAISEKAKKAIRKEVRGWKLQLKSDKDLYDIANMFNRQIQGWINYYTHFYKSEIYDVLRYINGCLVK
WVRRKYKKRKARRKAEHWLGEIAKRDRNLFAHWKFGILPAAG
>CABLBP02||gene_5303|GeneMark.hmm|414_aa|-|80|1324
MQEAKPYSISKKAVIAAYQRVKANKGTYGVDEQSIEDFERKLNNNLYKIWNRMSSGTYFPKPVKAVAIPKKNGG
TRILGVPTVEDRIAQMVAKLYFEPCVEPIFYEDSYGYRPNKSAIQALEATRTRCWRKDWVLEFDIRGLFDNIRHDY
LMEMVKKHTKEKWIILYIQRWLTAPFQMEDGTIVERKSGTPQGGVISPVLANLFLHYVFDDFMVKEFPTIPWA
RYADDGIAHCVSQKQAKYLRRRLEQRFQSYGLELNQEKTRIVYCKDDDRRGNHENTSFDFLGYTFRPRHAKNRY
GKFFTNFLPAISEKAKKAIRKEVRGWKLQLKSDKDLYDIANMFNRQIQGWINYYTHFYKSEIYDVLRYINGCLVK
WVRRKYKKRKARRKAEHWLGEIAKRDRNLFAHWKFGILPAAG
>CABLBP02||gene_5622|GeneMark.hmm|414_aa|-|80|1324
MQEAKPYSISKKAVIAAYQRVKANKGTYGVDEQSIEDFERKLNNNLYKIWNRMSSGTYFPKPVKAVAIPKKNGG
TRILGVPTVEDRIAQMVAKLYFEPCVEPIFYEDSYGYRPNKSAIQALEATRTRCWRKDWVLEFDIRGLFDNIRHDY
LMEMVKKHTKEKWIILYIQRWLTAPFQMEDGTIVERKSGTPQGGVISPVLANLFLHYVFDDFMVKEFPTIPWA

RYADDGIAHCVSQKQAKYLRRRLEQRFQSYGLELNQEKTRIVYCKDDDRRGNHENTSFDFLGYTFRPRHAKNRY
GKFFTNFLPAISEKAKKAIRKEVRGWKLQLKSDKDLYDIANMFNRQIQGWINYYTHFYKSEIYDVLRYINGCLVK
WVRRKYKKRKARRKAEHWLGEIAKRDRNLFAHWKFGILPAAG
>CABLBP02||gene_957|GeneMark.hmm|414_aa|-|408773|410017
MQEAKPYSISKKAVIAAYQRVKANKGTYGVDEQSIEDFERKLNNNLYKIWNRMSSGTYFPKPVKAVAIPKKNGG
TRILGVPTVEDRIAQMVAKLYFEPCVEPIFYEDSYGYRPNKSAIQALEATRTRCWRKDWVLEFDIRGLFDNIRHDY
LMEMVKKHTKEKWIILYIQRWLTAPFQMEDGTIVERKSGTPQGGVISPVLANLFLHYVFDDFMVKEFPTIPWA
RYADDGIAHCVSQKQAKYLRRRLEQRFQSYGLELNQEKTRIVYCKDDDRRGNHENTSFDFLGYTFRPRHAKNRY
GKFFTNFLPAISEKAKKAIRKEVRGWKLQLKSDKDLYDIANMFNRQIQGWINYYTHFYKSEIYDVLRYINGCLVK
WVRRKYKKRKARRKAEHWLGEIAKRDRNLFAHWKFGILPAAG
>CABMET01||gene_4435|GeneMark.hmm|414_aa|-|78|1322
MQETKSYNISKQAVYQAFLKVKANKGTFGVDEESIEAYEYKLKDNLYKLWNRLSSGSYFPKPVKAVSIPKKSGGLR
VLGIPTVEDRIAQMVAKMYFEPVVERLFYEDSYGYRPNKSAIQAIEKTRVRCWKRDWVLEFDIKGLFDNIRHDY
LIEMVKRYTQEKWIILYVERWLKAPFQREDGSTVSRKAGTPQGGVISPVLANLFLHYTFDDFMEKEFPNIQWAR
YADDGITHCVSLKQAKYLKKRLEERFRIFGLELHPDKTKIVYCRDSDRMGNYPITTFDFLGFTFRPRGAKNKYGKC
FTNFLPAVSDKAKKAIRKEVRNWRLQLKADKKLEDLANMFNSKIQGWMNYYMKFYKSEMYSLLRYINQCLVK
WVRRKYKKRQARRKAEHWLGEIAKRERNLFAHWKIGILPSAG
>CABMLY01||gene_5706|GeneMark.hmm|422_aa|+|528|1796
MSGVSSAKPYDIAKRTVWDAYQQVRANRGAAGIDDETIADFERDLSKNLYKLWNRMSSGSYFPPPVKQVEIPK
ASGGTRKLGVPTVGDRVAQTVVKLLIEPELDSIFHSDSYGYRPGRSAKQAVAITRERCWRYDWVVEFDIKAAFD
QINHGLLMKAVRLHIKEDWILLYIERWLVAPFETDDGMRVPRERGTPQGGVLSPLLMNLFMHYAFDTWMQR
TSPNCPFARYADDAVVHCRSRKQAEYVMRSIASRLAACGLTMHPEKSKVVYCKDSNRRAGYPHVSFTFLGFTFR
PRKALSKQDQLFTSFLPGASADALKRMRQAVRRWRLNRQTHVTLVDVARLYNPVIQGWWQYYGSFYRTAML
GIFQHIDRALERWARRKYKALHRRKRRISQWLDKMRTVVPRLFHHWRVTGQQGWITGAV
>CABMMR01||gene_2562|GeneMark.hmm|461_aa|-|65651|67036
METRHGTKYRQLHIEDYLREIPAEQGKVTGVYAHEWITGNPDTNTDFWTDNLLDTILRSDNLNAAYKRVKANK
GSAGIDGMDFEKFEKRLNNNLYKIWNRMSSGSYFPSPVMAVEIPKKSGGTRRLGIPTIADRIAQMVARAYVERA
VEPMFCEDSYGYRPHKSALDAVEKTRKRCWKYDYVIELDVKGLFDNIDHELLMRVVRRHVKEPWICLYIERWLK
SPFVLPDGSRIERESGTPQGGVISPVLANMFLHYVFDMWMKRNFPQAPFERYADDGVVHCRTKEEALYIKKKL
VKRFEECKLELHPVKTRIVYCKDKDRTKEEELAEFDFLGYTFKAVYIKCKDGVMRNNFIASVSKTAAKGFRDKIKAL
EIHKRTGCKIDMIAELLNPMIRGWMNYFGKFNPSAMKNTLQCIECRLIKWAMCKYKSFRGRRQRAEKWLSSIR
KREPKLFAHWSRMYSYC
>CABMNM01||gene_2586|GeneMark.hmm|451_aa|+|100907|102262
MSVERRGCVKQPEPASQLSQCGRRKACGQAKPFAISKWEVQAAFDKVKANQGGAGIDGVTLEVFERNLKNNL
YKIWNRLSSGTYFPPSVKAVNIPKKTGGVRTLGIPTVGDRVAQMVIKERLEAIIEPCFLSDSYGYRPAKSAIQAIGV
TRKRCWEYNWVLEFDIKGLFDNIRHDLLLKAVEKHVANGSQIQGQDLSWLTFYIKRWLVSPLQQDDGNIILRDR
GTPQGGVVSPLLANLFLHYVFDKWMQREFPENPWCRYADDGLVHAITKGKAESLYERIKRRLEECGLELHPEKT
KIIYCKDDKRKGTYLHTSFDFLGYTFRQRRCRKCSDNTFFNSFIPAVSMMAMKAMRRRIRELKIRQKSYYSLEELS
RWLNPIVQGWISYYGQYCRSALDPVFRHINKTLVRWARRKFKTLKRHKSRTIGLFDRLSVKCPKLFAHWRFGSA
RTFA
>CABRZV01||gene_2344|GeneMark.hmm|420_aa|+|5368|6630
MNEAKPFVIDKRLVWEAYHKVKENKGSAGIDKVDQKTFDKEMSKNLYKIWNRMSSGCYFPKAVKLLEIPKSNG
GTRPLGIPTIEDRIAQQVVVSVLIPILEPIFKEDSFGYRPDKGAHQAIAKAKERCYVNPWVLDMDISKFFDTINHD
LLMKAVRKHTEEKWVLLYIERWLKVPYQTSKGEVIERTMGVPQGSVIGPVLANLFLHYVFDEWMSRNYPTIPFE

RYADDTICHCVSEIQAQFLKAVLMKRFEECGLKLNEEKTKIVYCKDSNRRGNSEHTSFDFLGFTFRPRGARNRKT
GQNFTAFLPAISKKSMKRIKEAIRAWKLNRKTFACLLDISNEVDTQISGWMNYYMKFGMSEFRKVLNYINERLT
RWVMCKYKRFSKGKKLGTAYDWLVEYAAHNRNEFSHWVKGFVPYPRLG
>CABSHI01||gene_2153|GeneMark.hmm|420_aa|-|16939|18201
MNEAKPFVIDKRLVWEAYHKVKENKGSAGIDKVDQKTFDKEMSKNLYKIWNRMSSGCYFPKAVKLVEIPKSNG
GTRPLGIPTIEDRIAQQVVVSVLTPILEPIFKEDSYGYRPGKGAHQAIAKAKERCYVNPWVLDMDISKFFDTINHE
LLMKAIRKHTEEKWVLLYIERWLKVPYQTSKGEVIERTMGVPQGSVIGPVLANLFLHYVFDEWMSRNYPTIPFE
RYADDTICHCVSEKQAQFLKAVLMKRFEECGLKLNEEKTKIVYCKDSNRRGDSEHTSFDFLGFTFRPRGARNRKT
GQNFTAFLPAISKKSMKRIKEAVRAWKLNRKTFACLLDISNEVDTQISGWMNYYMKFGRSEFRKVLNYINERLT
RWVMRKYKRFSKGKKFSKAYDWLVEYAAHNRNEFSHWVKGFVPYPRLG
>CABSUY01||gene_1643|GeneMark.hmm|413_aa|+|24557|25798
MSESKQYEIPKRAVIEAYKKVKANKGSAGIDGIDFEAFEKKLNNNLYKIWNRMSSGSYFPSPVLAVEIPKKTGGTR
TLGIPTIADRIAQMVARMYVEPVVEPMFCEDSYGYRPNKSAIDAVGTARKRCWRYDYVIELDVKGLFDNIDHEL
LMRVVERHVKETWVCMYIKRWLEAPFVTREGHKIERKSGTPQGGVISPVLANMFLHYVFDMWMKRNFPQA
PFERYADDGVIHCRTKEEAISIKEKVAVRFEECRLELHPIKTRIVYCKDKDRTRKEELTKFDFLGYTFKAVYIMCKDG
KVRHNFIASVSNASAKNFRNKVKGMGIHKKTGCKIDIIAEILNPLIRGWMNYFGKFNPSAMKGTLQCIERRIVK
WAMCKYKNFRGRRRRAEKWLCTVRQREPKLFAHWSNLYSYC
>CABTDH01||gene_1703|GeneMark.hmm|413_aa|+|4463|5704
MNKSKQYEIPKRAVINAYKRVKANKGSAGIDGMDFEKFEEKLNNNLYKIWNRMSSGSYFPSPVMAVEIPKKSG
GTRRLGIPTITDRIAQMVARMYVEPAVEPMFCEDSYGYRPNKSAIDAIETTRKRCWKYDYAIELDVKGLFDNIDH
ELLMKVVHRHVKEPWICMYIERWLKTPFVLQDGQVIERSAGTPQGGVISPVLANMFLHYVFDMWMKRNFP
QAPFERYADDGVVHCSTKEEALYIKERLAKRFEECKLELHPIKTRIVYCKDKDRTRNEELTEFDFLGYTFKAVYIKCK
DGVMRNNFIASVSKTAAKGFRDKIKALEIHKKTGCKIDIIAEMLNPMIRGWMNYFGKFNPSAMKYTLQCIERRL
VKWAMCKYKSFRGRRQRAEKWLSSIRKREPKLFAHWGRMYSYC
>CABTHO01||gene_1703|GeneMark.hmm|422_aa|-|40518|41786
MMQHQVTKPFTTDKHLIMNAWKRVKENKGSGGIDNVSIEDYESNLGKHLYKLWNRMSSGSYFPEAVKLVDIP
KSSGGTRPLGIPTVGDRIAQMSVVLLIEDRLESIFHADSYGYRPNRSAHDAVGKARERCWHHNWVLDMDISKF
FDTIDHDLLMKAVERHVQEKWILLYIRRWLKVPYATLTGERIERKMGVPQGSVIGPVLANLYLHYTFDKWMSLY
HPTIPFERYADDTICHCNSLEEAHMLKVSIVERFAACKLRLNEEKTRIVYCKDGKRRGEYKEITFDFLGYTFQPRGQ
RNKQGQVFNGYAPAISRKSKKRITEKMRGWHLNRRVQLKLSDIAVEINAEVRGWMNYYGKFYGSQLKAFLQCI
NLKLARWAERKYKRFRRKPNDAYKWLVRVASRNPALFYHWQHGVKPNRLKPFG
>CABTSZ01||gene_3620|GeneMark.hmm|413_aa|+|2296|3537
MSESKQYEIPKKVVIEAYKRVKANKGSAGIDGIDFERFEKKLNNNLYKIWNRMSSGSYFPSPVLSVEISKKAGGTR
RLGIPTITDRIAQMVARMYVEPVVEPMFCNDSYGYRPNKSAIDAIATARKRCWRYDYVIELDVKGLFDNINHELL
MRVVLKHVKEEWICLYIKRWLETPFITREGQVIERLSGTPQGGVISPVLANMYLHYVFDMWMKRNFPQAPFE
RYADDGVIHCRTKEEAFVIKKKLAARFAECKLELHPVKTRIVYCKDKDRTRNEELTEFDFLGYTFKAVYIMCKDGK
VRYNFIASVSKTSSKSFRDKIKAMEVHKRTGCKIDIIAEILNPLIRGWMNYFGKFNPSAMKGTLQCIDRRLVKWA
MCKYKNFRGKRGRAEKWLCTVRQREPKLFAHWSNLYSYC
>CABTTT01||gene_375|GeneMark.hmm|433_aa|-|966|2267
LAEFKVQLLNREEETMNEAKPFKVEKRLVYEAYKRVKSNKGSAGIDGVEMEKFEENLSDNLYKLWNRMSSGTYF
PKAVKLVEIPKSNGGKRPLGIPTIEDRIAQMAAVMEMMPELDKIFHEDSYGYRPGKSAHDAVAKADERCWSYN
WVLDMDISKFFDTIDHELLMKAVHRHVTEKWILLYIERWLKVPYKTKEGAVIERTMGVPQGSVIGPVLANLFLH
YTFDKWMQINFPTIPFERYADDSVCHCRTKKQAEYLKERLKARFAECKLKLNEEKTKIVYCKDSNRHDKGDNESF
DYLGFTFRPRSARNKQTFQVFTVFKPAISRKSGMKIRETIRGWKFMHNYHIELADIAKAINPVVRGWVNYYSKF

GKTEFRRVMDYLNQRLVLWAAKKYKRFKRLRKRAGNWLAMVATYNKTLFSHWAEGYIPYQISR
>JACOOU01||MBC5671097.1
MQEAKPYSISKKAVIAAYQRVKANKGTYGVDEQSIEDFERKLNNNLYKIWNRMSSGTYFPKPVKAVAIPKKNGG
TRILGVPTVEDRIAQMVAKLYFEPCVEPIFYEDSYGYRPNKSAIQALEATRTRCWRKDWVLEFDIRGLFDNIRHDY
LMEMVKKHTKEKWIILYIQRWLTAPFQMEDGTIVERKSGTPQGGVISPVLANLFLHYVFDDFMVKEFPTIPWA
RYADDGIAHCVSQKQAKYLRRRLEQRFQSYGLELNQEKTRIVYCKDDDRRGNHENTSFDFLGYTFRPRDAKNRY
GKFFTNFLPAISEKAKKAIRKEVRGWKLQLKSDKDLYDIANMFNRQIQGWINYYTHFYKSEIYDVLRYINGCLVK
WVRRKYKKRKARRKAEHWLGEIAKRDRNLFAHWKFGILPAAG
>JAHZDV01||gene_1780|GeneMark.hmm|420_aa|-|3636|4898
MNEAKPFVIDKRLVWEAYHKVKENKGSAGIDKVDQKTFDKEMSKNLYKIWNRMSSGCYFPKAVKLVEIPKSNG
GTRPLGIPTIEDRIAQQVVVSVLTPILEPIFKEDSYGYRPGKGAHQAIAKAKERCYVNPWVLDMDISKFFDTINHE
LLMKAIRKHAEEKWVLLYIERWLKVPYQTSKGEVIERTMGVPQGSVIGPVLANLFLHYVFDEWMSRNYPTIPFE
RYADDTICHCVSEKQAQFLKAVLMKRFEECGLKLNEEKTKIVYCKDSNRRGDSEHTSFDFLGFTFRPRGARNRKT
GQNFTAFLPAISKKSMKRIKEAVRAWNLNRKTFVCLLDISNEVDTQISGWMNYYMKFGRSEFRKVLNYINERLT
RWVMRKYKRFSKGKKFSRAYEWLVEHAVHNRNEFSHWAKGFVPYPRLG
>DXOD01||gene_205|GeneMark.hmm|418_aa|+|4471|5727
MSEAKQFDISKKAVIAAFQAVKENAGSYGADEQTIKEFEEHLNNNLYKLWNRMASGSYFPKPVRAVAIPKKNG
GIRILGIPTVEDRIAQMVAKMYFEPLVEPMFYNDSYGYRPNKSAIQAVGQARERCFKRDWVLELDIKGLFDNIK
HGYLMYMVEKHTQIKWLILYIKRWLTVPFIMSDGSVAERRSGTPQGGVISPVLANLFLHYVFDDFMTKAYPNI
WWERYADDGVLHCQSYKQAAFIKQKLEERFQQFGLELNKEKTRIVYCKDNRRPQNYSCTQFTFLGYTFRPRLN
KNKEGKFFVGFTPAVSEKAKTAMKQKIREWKIQLKADLSLKDIGNMINKVVQGWINYYTHYYKSEFYEVLRYIN
QCLIKWVRRSYKKKNTRSRAEHWLGAVARRDRNLFAHWKFGILPSVGEGAV
>DXOP01||gene_4264|GeneMark.hmm|414_aa|+|600|1844
MQEAKPYSISKKAVIAAYQRVKANKGTYGVDEQSIEDFERKLNNNLYKIWNRMSSGTYFPKPVKAVAIPKKNGG
TRILGVPTVEDRIAQMVAKLYFEPCVEPIFYEDSYGYRPNKSAIQALEATRTRCWRKDWVLEFDIRGLFDNIRHDY
LMEMVKKHTKEKWIILYIQRWLTAPFQMEDGTIVERKSGTPQGGVISPVLANLFLHYVFDDFMVKEFPTIPWA
RYADDGIAHCVSQKQAKYLRRRLGQRFQSYGLELNQEKTRIVYCKDDDRRGNHENTSFDFLGYTFRPRHAKNR
YGKFFTNFLPAISEKAKKAIRKEVRGWKLQLKSDKDLYDIANMFNRQIQGWINYYTHFYKSEIYDVLRYINGCLVK
WVRRKYKKRKARRKAEHWLGEIAKRDRNLFAHWKFGILPAAG
>DXSC01||gene_659|GeneMark.hmm|461_aa|+|44063|45448
METRHGTKYRQLHIEDYLREIPAEQGKVTGVYAHEWITGNPDTNTDFWTDNLLDTILRSDNLNAAYKRVKANK
GSAGIDGMDFDKFEKRLNNNLYKIWNRMSSGSYFPSPVMAVEIPKKSGGTRRLGIPTIADRIAQMVARAYVER
AVEPMFCEDSYGYRPHKSALDAVEKTRKRCWKYDYVIELDVKGLFDNIDHELLMRVVRRHVKEPWICLYIERWL
KSPFVLPDGSRIERESGTPQGGVISPVLANMFLHYVFDMWMKRNFPQAPFERYADDGVVHCRTKEEALYIKKK
LVKRFEECKLELHPVKTRIVYCKDKDRTKEEELAEFDFLGYTFKAVYIKCKDGVMRNNFIASVSKTAAKGFRDKIKA
LEIHKRTGCKIDMIAELLNPMIRGWMNYFGKFNPSAMKNTLQCIECRLIKWAMCKYKSFRGRRQRAEKWLSSI
RKREPKLFAHWSRMYSYC
>DXTV01||gene_2340|GeneMark.hmm|420_aa|-|2528|3790
MNEAKPFVIDKRLVWEAYHKVKENKGSAGIDKVDQKTFDKEMSKNLYKIWNRMSSGCYFPKAVKLVEIPKSNG
GTRPLGIPTIEDRIAQQVVVSVLTPILEPIFKEDSYGYRPGKGAHQAIAKAKERCYVNPWVLDMDISKFFDTINHD
LLMKAVRKHTEEKWVLLYIERWLKVPYQTSKGEVIERTMGVPQGSVIGPVLANLFLHYVFDEWMSRNYPTIPFE
RYADDTICHCVSEKQAQFLKAVLMKRFEECGLKLNEEKTKIVYCKDSNRRGDSEHTSFDFLGFTFRPRSARNRKT
GQNFTAFLPAISKKSLKRIKEAVRAWKLNRKTFACLLDISNEVDTQISGWMNYYMKFGRSEFRKVLNYINERLTR
WVMRKYKRFSKGKKFSRAYEWLVEYAVHNRNEFSHWAKGFVPYPRLG

>ACFX02||EFE14684.1
METRHGTKYRQLHIEDYLREIPAEQGKVTGVYAHEWITGNPDTNTDFWTDNLLDTILRSDNLNAAYKRVKANK
GSAGIDGMDFEKFEKRLNNNLYKIWNRMSSGSYFPSPVMAVEIPKKSGGTRRLGIPTIADRIAQMVARAYVERA
VEPMFCEDSYGYRPHKSALDAVEKTRKRCWKYDYVIELDVKGLFDNIDHELLMRVVRRHVKEPWICLYIERWLK
SPFVLPDGSRIERESGTPQGGVISPVLANMFLHYVFDMWMKRNFPQAPFERYADDGVVHCRTKEEALYIKKKL
VKRFEECKLELHPVKTRIVYCKDKDRTKEEELAEFDFLGYTFKAVYIKCKDGVMRNNFIASVSKTAAKGFRDKIKAL
EIHKRTGCKIDMIAELLNPMIRGWMNYFGKFNPSAMKNTLQCIECRLIKWAMCKYKSFRGRRQRAEKWLSSIR
KREPKLFAHWSRMYSYC
>ACII02||EES77665.1
MSEAKQFDISKKAVIAAFQAVKENAGSYGADEQTIKEFEEHLNNNLYKLWNRMASGSYFPKPVRAVAIPKKNG
GIRILGIPTVEDRIAQMVAKMYFEPLVEPMFYNDSYGYRPNKSAIQAVGQARERCFKRDWALELDIKGLFDNIK
HGYLMYMVEKHTQIKWLILYIKRWLTVPFIMSDGSVAERRSGTPQGGVISPVLANLFLHYVFDDFMTKAYPNI
WWERYADDGVLHCQSYKQAAFIKQKLEERFQQFGLELNKEKTRIVYCKDNRRPQNYSCTQFTFLGYTFRPRLN
KNKEGKFFVGFTPAVSEKAKTAMKQKIREWKIQLKADLSLKDIGNMINKVVQGWINYYTHYYKSEFYEVLRYIN
QCLIKWVRRSYKKKNTRSRAEHWLGAVARRDRNLFAHWKFGILPSVGEGAV
>ACTQ01P||EHP62963.1
MTKTKAFNIDKSLVVSAYRRVKTSAGAAGIDKQSLADFDKRLVDNLYKIWNRLSSGSYFPPAVKAVAIPKKLGGER
ILGIPTVSDRIAQTVVKLAFEPQVEPHFLADSYGYRPNKSALDAIGVTRKRCWYYDWVLEFDIKGLFDNIPHELIM
KAVDKHNPARWVKLYIQRWLTAPMVMSDGEVRARTMGTPQGGVISPLLANLFMHYVFDKWLAKYYPKVPW
YRYADDGILHCHSEAEATEMREVLRKRFSECGLEMHPEKTRVIYCKDGSRKGDYEHTMFDFLGYTFRRRVVKNV
KRNSLFVSFTPAASKSALKAMRREIKATGIRKRVDLSIEQIAKWINPKLNGWINYYGRYTCSELYSVFRYINKALVR
WGRKKYKMLSRYKTRASKFLEEMAKRSPQLFAHWRLKMRGGLV
>ACUD01||KEJ86832.1
MQNDNAKPISISKQLVYDAFLRVKANRGSAGIDKVTLEDYEKNLRGNLYKLWNRMSSGSYFPPSVKLVEIPKSTG
GKRPLGIPTVSDRVAQMAVVMLITPSIEPCFHEDSYAYRPHRSAHDAVGKARERCWKYAWVLDMDISKFFDTI
DHELLLKALKRHTQEKWVLMYIERWLKVPYEKSDGSQVDRALGVPQGSVIGPVLANLFLHYTFDKWMEKNFP
RVPFERYADDTICHCHSLKQTEYMQAMIQQRFECCRLRLNEEKTKIVYCKSSRQKECYPNVTFDFLGFTFQPRES
VDKYGNRFTGFLPAISRKSMKRINETMRSWHLNRHSNLTLEHLASDINPIVRGWMTYYGKFYPTRLKWFMQTL
NGRLARWVMCKFERYRHRFYPAQEWLARIAEKEGLIFYHWKCGALPRFTNKEKVSSQLIMVK
>ACWK01||EHF03938.1
MSESKQYEIPKKVVIEAYKRVKANKGSAGIDGIDFERFEKKLNNNLYKIWNRMSSGSYFPSPVLSVEISKKAGGTR
RLGIPTITDRIAQMVARMYVEPVVEPMFCNDSYGYRPNKSAIDAIATARKRCWRYDYVIELDVKGLFDNINHELL
MRVVLKHVKEEWICLYIKRWLETPFITREGQVIERLSGTPQGGVISPVLANMYLHYVFDMWMKRNFPQAPFE
RYADDGVIHCRTKEEAFVIKKKLAARFAECKLELHPVKTRIVYCKDKDRTRNEELTEFDFLGYTFKAVYIMCKDGK
VRYNFIASVSKTSSKSFRDKIKAMEVHKRTGCKIDIIAEILNPLIRGWMNYFGKFNPSAMKGTLQCIDRRLVKWA
MCKYKNFRGKRGRAEKWLCTVRQREPKLFAHWSNLYSYC
>ADBY01||EFE93402.1
MIILNKVKPYLIDKTIIWRAWLAVKANKGSAGVDGMTIEAFEHNLARNLYKIWNRLSSGCYMPPPVKRVEIPKS
DGKTRPLGIPTVSDRVAQMAVKMILEPQWDPLFSDSSFGYRPGKSAHDAVAQAKANCWKYEWVIDLDIRGFF
DNLDHALLLKAVDHLHPAPWVRLCIVRWLKAEIIFPDGHRHSPEKGTPQGGVISPLLANLFLHYTQDKWLEKHY
PNNSWERYADDSIIHCRSRREAGLLLSQLRERMKACGLELHPEKTRIVNCHPLTRRKNDGHYSFDFLGFTFRRRA
ARKIAGGLFTGFLPAISNKAQKAIVRTFRAWNIQRLTSLSAEEIADRINPQLRGWINYYGKFYPSEMNRLWRILD
WRLVKWVRCRYKQYRWHQSRASEVLERIRQRNKSLFAHWKFMIRKG
>ADLJ01||EHE95238.1

MKEGKTFHISQNEVLNAYKAVKANKGAGGVDGIELEEFDKNWKNRLYVLWNRMSSGCYFPKPVRGVEIPKKN
GKVRLLGIPTIEDRVAQMVLRNHIEPFVEPVFHEDSYGYRPGKSALDAVETARKRCFQMRWVIEFDIVGLFNNIE
HDKLMRLVENHCKEKWVSLYVKRCLKAPVQMPDGTVCEKNSGTPQGGVISPVLANLFMHYGFDNWMNRKF
PNCPWERYADDGLIHCVSRKQAEFVLEMLKEQMQRVGLTIHPEKSKIVFCQRNNEEVPEDVETSFVFLGYCFRP
RLVKSGEGKYFMGFTPAVSSDAGKVFREKIKEGIEQQNSTDIVALSERLNPIIRGWMNYFTKFTPSEAFRQGINY
VNLTLVRWLRKTRRKARRSYQKAQRLLHQIAMSNIEMFYHWKVGYIPVK
>ADTJ01P||EFJ73270.1
MTKTKAFNIDKSLVVSAYRRVKTSAGAAGIDKQSLADFDKRLVDNLYKIWNRLSSGSYFPPAVKAVAIPKKLGGER
ILGIPTVSDRIAQTVVKLAFEPQVEPHFLADSYGYRPNKSALDAIGVTRKRCWYYDWVLEFDIKGLFDNIPHELIM
KAVDKHNPARWVKLYIQRWLTAPMVMSDGEVRARTMGTPQGGVISPLLANLFMHYVFDKWLAKYYPKVPW
YRYADDGILHCHSEAEATEMREVLRKRFSECGLEMHPEKTRVIYCKDGSRKGDYEHTMFDFLGYTFRRRVVKNV
KRNSLFVSFTPAASKSALKAMRREIKATGIRKRVDLSIEQIAKWINPKLNGWINYYGRYTCSELYSVFRYINKALVR
WGRKKYKMLSRYKTRASKFLEEMAKRSPQLFAHWRLKMRGGLV
>AXTI01||ESD28825.1
MTKTKAFNIDKSLVVSAYRRVKTSAGAAGIDKQSLADFDKRLVDNLYKIWNRLSSGSYFPPAVKAVAIPKKLGGER
ILGIPTVSDRIAQTVVKLAFEPQVEPHFLADSYGYRPNKSALDAIGVTRKRCWYYDWVLEFDIKGLFDNIPHELIM
KAVDKHNPARWVKLYIQRWLTAPMVMSDGEVRARTMGTPQGGVISPLLANLFMHYVFDKWLAKYYPKVPW
YRYADDGILHCHSEAEATEMREVLRKRFSECGLEMHPEKTRVIYCKDGSRKGDYEHTMFDFLGYTFRRRVVKNV
KRNSLFVSFTPAASKSALKAMRREIKATGIRKRVDLSIEQIAKWINPKLNGWINYYGRYTCSELYSVFRYINKALVR
WGRKKYKMLSRYKTRASKFLEEMAKRSPQLFAHWRLKMRGGLV
>AXTM01||ESD54286.1
MTKTKAFNIDKSLVVSAYRRVKTSAGAAGIDKQSLADFDKRLVDNLYKIWNRLSSGSYFPPAVKAVAIPKKLGGER
ILGIPTVSDRIAQTVVKLAFEPQVEPHFLADSYGYRPNKSALDAIGVTRKRCWYYDWVLEFDIKGLFDNIPHELIM
KAVDKHNPARWVKLYIQRWLTAPMVMSDGEVRARTMGTPQGGVISPLLANLFMHYVFDKWLAKYYPKVPW
YRYADDGILHCHSEAEATEMREVLRKRFSECGLEMHPEKTRVIYCKDGSRKGDYEHTMFDFLGYTFRRRVVKNV
KRNSLFVSFTPAASKSALKAMRREIKATGIRKRVDLSIEQIAKWINPKLNGWINYYGRYTCSELYSVFRYINKALVR
WGRKKYKMLSRYKTRASKFLEEMAKRSPQLFAHWRLKMRGGLV
>AXTN01||ESD59453.1
MTKTKAFNIDKSLVVSAYRRVKTSAGAAGIDKQSLADFDKRLVDNLYKIWNRLSSGSYFPPAVKAVAIPKKLGGER
ILGIPTVSDRIAQTVVKLAFEPQVEPHFLADSYGYRPNKSALDAIGVTRKRCWYYDWVLEFDIKGLFDNIPHELIM
KAVDKHNPARWVKLYIQRWLTAPMVMSDGEVRARTMGTPQGGVISPLLANLFMHYVFDKWLAKYYPKVPW
YRYADDGILHCHSEAEATEMREVLRKRFSECGLEMHPEKTRVIYCKDGSRKGDYEHTMFDFLGYTFRRRVVKNV
KRNSLFVSFTPAASKSALKAMRREIKATGIRKRVDLSIEQIAKWINPKLNGWINYYGRYTCSELYSVFRYINKALVR
WGRKKYKMLSRYKTRASKFLEEMAKRSPQLFAHWRLKMRGGLV
>AXTT01||ESD90243.1
MTKTKAFNIDKSLVVSAYRRVKTSAGAAGIDKQSLADFDKRLVDNLYKIWNRLSSGSYFPPAVKAVAIPKKLGGER
ILGIPTVSDRIAQTVVKLAFEPQVEPHFLADSYGYRPNKSALDAIGVTRKRCWYYDWVLEFDIKGLFDNIPHELIM
KAVDKHNPARWVKLYIQRWLTAPMVMSDGEVRARTMGTPQGGVISPLLANLFMHYVFDKWLAKYYPKVPW
YRYADDGILHCHSEAEATEMREVLRKRFSECGLEMHPEKTRVIYCKDGSRKGDYEHTMFDFLGYTFRRRVVKNV
KRNSLFVSFTPAASKSALKAMRREIKATGIRKRVDLSIEQIAKWINPKLNGWINYYGRYTCSELYSVFRYINKALVR
WGRKKYKMLSRYKTRASKFLEEMAKRSPQLFAHWRLKMRGGLV
>AXTU01||ESD93268.1
MTKTKAFNIDKSLVVSAYRRVKTSAGAAGIDKQSLADFDKRLVDNLYKIWNRLSSGSYFPPAVKAVAIPKKLGGER
ILGIPTVSDRIAQTVVKLAFEPQVEPHFLADSYGYRPNKSALDAIGVTRKRCWYYDWVLEFDIKGLFDNIPHELIM

KAVDKHNPARWVKLYIQRWLTAPMVMSDGEVRARTMGTPQGGVISPLLANLFMHYVFDKWLAKYYPKVPW
YRYADDGILHCHSEAEATEMREVLRKRFSECGLEMHPEKTRVIYCKDGSRKGDYEHTMFDFLGYTFRRRVVKNV
KRNSLFVSFTPAASKSALKAMRREIKATGIRKRVDLSIEQIAKWINPKLNGWINYYGRYTCSELYSVFRYINKALVR
WGRKKYKMLSRYKTRASKFLEEMAKRSPQLFAHWRLKMRGGLV
>AXUI01||ESA89085.1
MTKTKAFNIDKSLVVSAYRRVKTSAGAAGIDKQSLADFDKRLVDNLYKIWNRLSSGSYFPPAVKAVAIPKKLGGER
ILGIPTVSDRIAQTVVKLAFEPQVEPHFLADSYGYRPNKSALDAIGVTRKRCWYYDWVLEFDIKGLFDNIPHELIM
KAVDKHNPARWVKLYIQRWLTAPMVMSDGEVRARTMGTPQGGVISPLLANLFMHYVFDKWLAKYYPKVPW
YRYADDGILHCHSEAEATEMREVLRKRFSECGLEMHPEKTRVIYCKDGSRKGDYEHTMFDFLGYTFRRRVVKNV
KRNSLFVSFTPAASKSALKAMRREIKATGIRKRVDLSIEQIAKWINPKLNGWINYYGRYTCSELYSVFRYINKALVR
WGRKKYKMLSRYKTRASKFLEEMAKRSPQLFAHWRLKMRGGLV
>LSNZ01||KXG99286.1
MTKTKAFNIDKSLVVSAYRRVKTSAGAAGIDKQSLADFDKRLVDNLYKIWNRLSSGSYFPPAVKAVAIPKKLGGER
ILGIPTVSDRIAQTVVKLAFEPQVEPHFLADSYGYRPNKSALDAIGVTRKRCWYYDWVLEFDIKGLFDNIPHELIM
KAVDKHNPARWVKLYIQRWLTAPMVMSDGEVRARTMGTPQGGVISPLLANLFMHYVFDKWLAKYYPKVPW
YRYADDGILHCHSEAEATEMREVLRKRFSECGLEMHPEKTRVIYCKDGSRKGDYEHTMFDFLGYTFRRRVVKNV
KRNSLFVSFTPAASKSALKAMRREIKATGIRKRVDLSIEQIAKWINPKLNGWINYYGRYTCSELYSVFRYINKALVR
WGRKKYKMLSRYKTRASKFLEEMAKRSPQLFAHWRLKMRGGLV
>LTGI01||OFL07873.1
MQHQVTKPFTIDKHVIMNAWKRVKENKGSAGIDNISTEDYESNLGKNLYKLWNRMSSGSYFPKAVKLVDIPKP
SGGTRPLGIPTVGDRIAQMSVVLLIESRLEAIFHMDSYGYRPNRSAHDAIGKARERCWRYNWVLDMDISKFFD
TIDHALLMKAVERHVQERWILLYIRRWLKVPYATVTGECIERSMGIPQGSVIGPVLANLYLHYTFDKWMSLYHPT
IPFERYADDTICHCNSLEEAQRLKTSIVERFAACKLRLNEEKTRIVYCKDGKRRGEYKEITFDFLGYTFQPRGQRNK
QGQVFNGYAPAISKKSKKRITEKIRGWHLNSRVQLKLSDIAMEINAEVRGWMNYYGKFYGSQLKAFLQCVNLK
LARWAERKYKRFRRKPNDAYKWLVWIASKNPTLFYHWQHGVKPNRLKPFG
>LTHV01||OFL89816.1
MQETKSYNISKQAVYQAFLKVKANKGTFGVDEESIEAYEYKLKDNLYKLWNRLSSGSYFPKPVKAVSIPKKSGGLR
VLGIPTVEDRIAQMVAKMYFEPVVERLFYEDSYGYRPNKSAIQAIEKTRVRCWKRDWVLEFDIKGLFDNIRHDY
LIEMVKRYTQEKWIILYVERWLKAPFQREDGSTVSRKAGTPQGGVISPVLANLFLHYTFDDFMEKEFPNIQWAR
YADDGITHCVSLKQAKYLKKRLEERFRIFGLELHPDKTKIVYCRDSDRMGNYPITTFDFLGFTFRPRGAKNKYGKC
FTNFLPAVSDKAKKAIRKEVRNWRLQLKADKKLEDLANMFNSKIQGWMNYYMKFYKSEMYSLLRYINQCLVK
WVRRKYKKRQARRKAEHWLGEIAKRERNLFAHWKIGILPSAG
>LTKO01||OFN62243.1
MQETKSYNISKQAVYQAFLKVKANKGTFGVDEESIEAYEYKLKDNLYKLWNRLSSGSYFPKPVKAVSIPKKSGGLR
VLGIPTVEDRIAQMVAKMYFEPVVERLFYEDSYGYRPNKSAIQAIEKTRVRCWKRDWVLEFDIKGLFDNIRHDY
LIEMVKRYTQEKWIILYVERWLKAPFQREDGSTVSRKAGTPQGGVISPVLANLFLHYTFDDFMEKEFPNIQWAR
YADDGITHCVSLKQAKYLKKRLEERFRIFGLELHPDKTKIVYCRDSDRMGNYPITTFDFLGFTFRPRGAKNKYGKC
FTNFLPAVSDKAKKAIRKEVRNWRLQLKADKKLEDLANMFNSKIQGWMNYYMKFYKSEMYSLLRYINQCLVK
WVRRKYKKRQARRKAEHWLGEIAKRERNLFAHWKIGILPSAG
>LTQO01||OHP40731.1
MSGVSSAKPYDIAKRTVWDAYQQVRANRGAAGIDDETIADFERDLSKNLYKLWNRMSSGSYFPPPVKQVEIPK
ASGGTRKLGVPTVGDRVAQTVVKLLIEPELDSIFHSDSYGYRPGRSAKQAVAITRERCWRYDWVVEFDIKAAFD
QINHGLLMKAVRLHIKEDWILLYIERWLVAPFETDDGMRVPRERGTPQGGVLSPLLMNLFMHYAFDTWMQR
TSPNCPFARYADDAVVHCRSRKQAEYVMRSIASRLAACGLTMHPEKSKVVYCKDSNRRAGYPHVSFTFLGFTFR

PRKALSKQDQLFTSFLPGASADALKRMRQAVRRWRLNRQTHVTLVDVARLYNPVIQGWWQYYGSFYRTAML
GIFQHIDRALERWARRKYKALHRRKRRISQWLDKMRTVVPRLFHHWRVTGQQGWITGAV
>LTQP01||OHP35192.1
MSGVSSAKPYDIAKRTVWDAYQQVRANRGAAGIDDETIADFERDLSKNLYKLWNRMSSGSYFPPPVKQVEIPK
ASGGTRKLGVPTVGDRVAQTVVKLLIEPELDSIFHSDSYGYRPGRSAKQAVAITRERCWRYDWVVEFDIKAAFD
QINHGLLMKAVRLHIKEDWILLYIERWLVAPFETDDGMRVPRERGTPQGGVLSPLLMNLFMHYAFDTWMQR
TSPNCPFARYADDAVVHCRSRKQAEYVMRSIASRLAACGLTMHPEKSKVVYCKDSNRRAGYPHVSFTFLGFTFR
PRKALSKQDQLFTSFLPGASADALKRMRQAVRRWRLNRQTHVTLVDVARLYNPVIQGWWQYYGSFYRTAML
GIFQHIDRALERWARRKYKALHRRKRRISQWLDKMRTVVPRLFHHWRVTGQQGWITGAV
>LTWH01||OFQ82631.1
MSGVSSAKPYDIAKRTVWDAYQQVRANRGAAGIDDETIADFERDLSKNLYKLWNRMSSGSYFPPPVKQVEIPK
ASGGTRKLGVPTVGDRVAQTVVKLLIEPELDSIFHSDSYGYRPGRSAKQAVAITRERCWRYDWVVEFDIKAAFD
QINHGLLMKAVRLHIKEDWILLYIERWLVAPFETDDGMRVPRERGTPQGGVLSPLLMNLFMHYAFDTWMQR
TSPNCPFARYADDAVVHCRSRKQAEYVMRSIASRLAACGLTMHPEKSKVVYCKDSNRRAGYPHVSFTFLGFTFR
PRKALSKQDQLFTSFLPGASADALKRMRQAVRRWRLNRQTHVTLVDVARLYNPVIQGWWQYYGSFYRTAML
GIFQHIDRALERWARRKYKALHRRKRRISQWLDKMRTVVPRLFHHWRVTGQQGWITGAV
>LTZR01||OFU65971.1
MSAAKSYSISKLTVWEAFQRVKANRGAAGIDEQSIAQFEQKLQRNLYKVWNRMSSGSYFPPPVRQVEIPKQSG
CKRKLGIPTVADRVAQTAIKLLIEPSLDCLFHPDSYGYRPGKSAKQAVEITRRRCWNINWVVEFDIKGAFDHIDHE
LLLKAVKHHIKDEWILLYIERWLKAPFETADGVQVPRESGTPQGGVISPLLMNLFMHYAFDAWMQRTFPGCPF
ARYADDAVVHCRSEKQACEVMAAIKARLEVCLLTMHPEKSKIVYCKDSNRKAAYPTTQFTFLGFTFRPREAWGN
NGRRFTSFLPGASNEALKRMRQRTRSWNIQRQTPASLLELSKQYNAILRGWWNYYGAFYKTVMRKVFNHFDL
KLQRWARQKYKPLAGHKRRSVDWLNRMKKACPSLFVHWHVYGNEFRPGNGSRMS
>LUAL01||OFV23721.1
MSAAKSYSISKLTVWEAFQRVKANRGAAGIDEQSIAQFEQKLQRNLYKVWNRMSSGSYFPPPVRQVEIPKQSG
CKRKLGIPTVADRVAQTAIKLLIEPSLDCLFHPDSYGYRPGKSAKQAVEITRRRCWNINWVVEFDIKGAFDHIDHE
LLLKAVKHHIKDEWILLYIERWLKAPFETADGVQVPRESGTPQGGVISPLLMNLFMHYAFDAWMQRTFPGCPF
ARYADDAVVHCRSEKQACEVMAAIKARLEVCLLTMHPEKSKIVYCKDSNRKAAYPTTQFTFLGFTFRPREAWGN
NGRRFTSFLPGASNEALKRMRQRTRSWNIQRQTPASLLELSKQYNAILRGWWNYYGAFYKTVMRKVFNHFDL
KLQRWARQKYKPLAGHKRRSVDWLNRMKKACPSLFVHWHVYGNEFRPGNGSRMS
>LWOL01||OFT75245.1
MQETKSYNISKQAVYQAFLKVKANKGTFGVDEESIEAYEYKLKDNLYKLWNRLSSGSYFPKPVKAVSIPKKSGGLR
VLGIPTVEDRIAQMVAKMYFEPVVERLFYEDSYGYRPNKSAIQAIEKTRVRCWKRDWVLEFDIKGLFDNIRHDY
LIEMVKRYTQEKWIILYVERWLKAPFQREDGSTVSRKAGTPQGGVISPVLANLFLHYTFDDFMEKEFPNIQWAR
YADDGITHCVSLKQAKYLKKRLEERFRIFGLELHPDKTKIVYCRDSDRMGNYPITTFDFLGFTFRPRGAKNKYGKC
FTNFLPAVSDKAKKAIRKEVRNWRLQLKADKKLEDLANMFNSKIQGWMNYYMKFYKSEMYSLLRYINQCLVK
WVRRKYKKRQARRKAEHWLGEIAKRERNLFAHWKIGILPSAG
>LWON01||OFT69792.1
MSAAKSYSISKLTVWEAFQRVKANRGAAGIDEQSIAQFEQKLQRNLYKVWNRMSSGSYFPPPVRQVEIPKQSG
CKRKLGIPTVADRVAQTAIKLLIEPSLDCLFHPDSYGYRPGKSAKQAVEITRRRCWNINWVVEFDIKGAFDHIDHE
LLLKAVKHHIKDEWILLYIERWLKAPFETADGVQVPRESGTPQGGVISPLLMNLFMHYAFDAWMQRTFPGCPF
ARYADDAVVHCRSEKQACEVMAAIKARLEVCLLTMHPEKSKIVYCKDSNRKAAYPTTQFTFLGFTFRPREAWGN
NGRRFTSFLPGASNEALKRMRQRTRSWNIQRQTPASLLELSKQYNAILRGWWNYYGAFYKTVMRKVFNHFDL
KLQRWARQKYKPLAGHKRRSVDWLNRMKKACPSLFVHWHVYGNEFRPGNGSRMS

>N22001||gene_312462|GeneMark.hmm|418_aa|+|1825|3081
MSEAKQFDISKKAVIAAFQAVKENAGSYGADEQIIKEFEEHLNNNLYKLWNRMASGSYFPKPVRAVAIPKKNGG
IRILGIPTVEDRIAQMVAKMYFEPLVEPMFYNDSYGYRPNKSAIQAVGQARERCFKRDWVLELDIKGLFDNIKH
GYLMYMVEKHTQIKWLILYIKRWLTVPFIMSDGSVAERRSGTPQGGVISPVLANLFLHYVFDDFMTKAYPNIW
WERYADDGVLHCQSYKQAAFIKQKLEERFQQFGLELNKEKTRIVYCKDNRRPQNYSCTQFTFLGYTFRPRLNKN
KEGKFFVGFTPAVSEKAKTAMKQKIREWKIQLKADLSLKDIGNMINKVVQGWINYYTHYYKSEFYEVLRYINQCL
IKWVRRSYKKKNTRSRAEHWLGAVARRDRNLFAHWKFGILPSVGEGAV
>N22001||gene_72541|GeneMark.hmm|420_aa|+|817|2079
MNAANPFVIDKRLVWEAYHKVKENKGSAGIDKVDQKTFDKEMSKNLYKIWNRMSSGCYFPKAVKLVEIPKSN
GGTRPLGIPAIEDRIAQQVVVSVLTPILEPIFKEDSYGYRPGKGAHQAIAKAKERCYVTPWVLDMDISKFFDTINH
ELLMKAIRKHTEEKWVLLYIERWLKVPYQTSKGEVIERTMGVPQCSVIGPVLANLFLHYVFDEWMSRNYPTIPF
ERYADDTICHCVSEKQARFLKAVLMKRFEECGLKLNEEKTKIVYCKDSNRRGDSEHTSFDFLGFTFRPRGARNRK
TGQNFTAFLPAISKKSMKRIKEAVRAWKLNRKTFACLLDISNEVDTQISGWMNYYMKFGRSEFRKVLNYINERLT
RWVMRKYKRFSKGRKFDRAYDWLVEYATHNRNEFSHWVKGFVPYPRLD
>N22010||gene_11673|GeneMark.hmm|410_aa|+|453|1685
MQRKSFEIPKALVWASYLDVRRNKGAPGCDGQTLKMFDQQRDGNLYKIWNRLCSGTWFPPPVLEKRIPKPN
GKERILGIPTVSDRIAQGAIKLFMEEKLDPIFHADSYGYRPGKSAHDALKQCAIRCWRYSWILEVDISAFFDHVRH
DLVLKALEHHGMPKWVILYCRRWMEAPMQSCENGELITRTRGTPQGGVISPLLANLFLHYAFDLWMEREYRG
VPFERYADDIVVHCSRMSDATRLKNRLSERFSEVGLVLNAGKTNIAYIDTFKRRNVATSFTFLGYDFKVRTLKNFK
GELYRKCMPGASNAAMRKITETIKKWRIHRSTAESLLDFARRYNAIVRGWIEYYGKFWSRNFNYRLWSAMQSR
LLKWMQSKYRLSNRRAQRKLTLVRKEYPKLFVHWYLLRASNE
>N22012||gene_81998|GeneMark.hmm|412_aa|-|2|1237
MQEAKPFQIDKRIIFESFKKVKFNRGSSGIDGIEMTTYEQNLGSNLYRLWNRMSSGSYMPKAVKLVEIPKSNGG
KRPLGIPTIEDRIAQMAVVNVIEPLIEPCFHEDSFGYRPHRSAHDAIAKAERRCWKYAWVLDIDISKFFDTIDHGL
LMKAVEKHINIKWILLYIKRWLTVPYQRSDGEIVKRDMGVPQGSVIGPILANLFLHYTFDKWMSYKYPHIPFERY
ADDCVCHCSTLAQAEYIKERLGERFTECKLKFNEEKTKIVFCKMSSRSSKHYHCTSFDYLGFTFRSRAAKDKRNN
VLFTSYLPAISKKSVSRIHETIKSWNLKRLHNRSLRFVAAYINDVVRGWINYYEKFGKTEFWKVMCHLNRSIAYW
AKTKYKRLRRRGVISAHYWLAYIAQKEPNLFYHWQVG
>N221202||gene_107046|GeneMark.hmm|413_aa|-|1371|2612
MSESKQYEIPKKVVIEAYKRVKANKGSAGIDGIDFERFEKKLNNNLYKIWNRMSSGSYFPSPVLSVEISKKAGGTR
RLGIPTITDRIAQMVARMYVEPVVEPMFCNDSYGYRPNKSAIDAIATARKRCWRYDYVIELDVKGLFDNINHELL
MRVVLKHVKEEWICLYIKRWLETPFITREGQVIERLSGTPQGGVISPVLANMYLHYVFDMWMKRNFPQAPFE
RYADDGVIHCRTKEEAFVIKKKLAARFAECKLELHPVKTRIVYCKDKDRTRNEELTEFDFLGYTFKAVYIMCKDGK
VRYNFIASVSKTSSKSFRDKIKAMEVHKRTGCKIDIIAEILNPLIRGWMNYFGKFNPSAMKGTLQCIDRRLVKWA
MCKYKNFRGKRGRAEKWLCTVRQREPKLFAHWSNLYSYC
>N221207||gene_26911|GeneMark.hmm|416_aa|+|609|1859
MTKTKAFNIDKSLVVSAYRRVKTSAGAAGIDKQSLADFDKRLVDNLYKIWNRLSSGSYFPPAVKAVAIPKKLGGER
ILGIPTVSDRIAQTVVKLAFEPQVEPHFLADSYGYRPNKSALDAIGVTRKRCWYYDWVLEFDIKGLFDNIPHELIM
KAVDKHNPARWVKLYIQRWLTAPMVMSDGEVRARTMGTPQGGVISPLLANLFMHYVFDKWLAKYYPKVPW
YRYADDGILHCHSEAEATEMREVLRKRFSECGLEMHPEKTRVIYCKDGSRKGDYEHTMFDFLGYTFRRRVVKNV
KRNSLFVSFTPAASKSALKAMRREIKATGIRKRVDLSIEQIAKWINPKLNGWINYYGRYTCSELYSVFRYINKALVR
WGRKKYKMLSRYKTRASKFLEEMAKRSPQLFAHWRLKMRGGLV
>N221223||gene_67653|GeneMark.hmm|436_aa|+|24226|25536
MQNDNAKPISISKQLVYDAFLRVKANRGSAGIDKVTLEDYEKNLRGNLYKLWNRMSSGSYFPPSVKLVEIPKSTG

GKRPLGIPTVSDRVAQMAVVMLITPSIEPCFHEDSYAYRPHRSAHDAVGKARERCWKYAWVLDMDISKFFDTI
DHELLLKALKRHTQEKWVLMYIERWLKVPYEKSDGSQVDRALGVPQGSVIGPVLANLFLHYTFDKWMEKNFP
RVPFERYADDTICHCHSLKQAEYMQAMIQQRFECCRLRLNEEKTKIVYCKSSRQKECYPNVTFDFLGFTFQPRES
VDKYGNRFTGFLPAISRKSMKRINETMRSWHLNRHSNLTLEHLASDINPIVRGWMTYYGKFYPTRLKWFMQTL
NGRLARWVMCKFERYRHRFYPAQEWLARIAEKEGLIFYHWKCGALPRFINWELTFSLLVMWRSAKIYQ
>N221228||gene_63446|GeneMark.hmm|430_aa|-|57696|58988
MQNDNAKPISISKQLVYDAFLRVKANRGSAGIDKVTLEDYEKNLRGNLYKLWNRMSSGSYFPPSVKLVEIPKTTG
GKRPLGIPTVSDRVAQMAVVMLITPSIEPCFHEDSYAYRPHRSAHDAVGKARERCWKYAWVLDMDISKFFDTI
DHELLLKALKRHTQEKWVLMYIERWLKVPYEKSDGSQVDRALGVPQGSVIGPVLANLFLHYTFDKWMEKNFP
RVPFERYADDTICHCHSLKQAEYMQAMIQQRFECCRLRLNEEKTKIVYCKSSRQKECYPNVTFDFLGFTFQPRES
VDKYGNRFTGFLPAISRKSMKRINETMRSWHLNRHSNLTLEHLASDINPIVRGWMTYYGKFYPTRLKWFMQTL
NGRLARWVMCKFERYRHRFYPAQEWLARIAEKEGLIFYHWKCGVLPRFTNKEKVSSQLIMVK
>N221229||gene_243973|GeneMark.hmm|430_aa|+|2083|3375
MQNDNAKPISISKQLVYDAFLRVKANRGSAGIDKVTLEDYEKNLRGNLYKLWNRMSSGSYFPPSVKLVEIPKSTG
GKRPLGIPTVSDRVAQMAVVMLITPSIEPCFHEDSYAYRPHRSAHDAVGKARERCWKYAWVLDMDISKFFDTI
DHELLLKALKRHTQEKWVLMYIERWLKVPYEKSDGSQVDRALGVPQGSVIGPVLANLFLHYTFDKWMEKNFP
RVPFERYADDTICHCHSLKQAEYMQAMIQQRFECCRLRLNEEKTKIVYCKSSRQKECYPNVTFDFLGFTFQPRES
VDKYGNRFTGFLPAISRKSMKRINETMRSWHLNRHSNLTLEHLASDINPIVRGWMTYYGKFYPTRLKWFMQTL
NGRLARWVMCKFERYRHRFYPAQEWLARIAEKEGLIFYHWKCGVLPRFTNKEKVSSQLIMVK
>N221229||gene_172302|GeneMark.hmm|418_aa|-|204|1460
MSEAKQFDISKKAVIAAFQAVKENAGSYGADEQIIKEFEEHLNNNLYKLWNRMASGSYFPKPVRAVAIPKKNGG
IRILGIPTVEDRIAQMVAKMYFEPLVEPMFYNDSYGYRPNKSAIQAVGQARERCFKRDWVLELDIKGLFDNIKH
GYLMYMVEKHTQIKWLILYIKRWLTVPFIMSDGSVAERRSGTPQGGVISPVLANLFLHYVFDDFMTKAYPNIW
WERYADDGVLHCQSYKQAAFIKQKLEERFQQFGLELNKEKTRIVYCKDNRRPQNYSCTQFTFLGYTFRPRLNKN
KEGKFFVGFTPAVSEKAKTAMKQKIREWKIQLKADLSLKDIGNMINKVVQGWINYYTHYYKSEFYEVLRYINQCL
IKWVRRSYKKKNTRSRAEHWLGAVARRDRNLFAHWKFGILPSVGEGAV
>N221230||gene_152949|GeneMark.hmm|418_aa|-|56|1312
MSEAKQFDISKKAVIAAFQAVKENAGSYGADEQIIKEFEEHLNNNLYKLWNRMASGSYFPKPVRAVAIPKKNGG
IRILGIPTVEDRIAQMVAKMYFEPLVEPMFYNDSYGYRPNKSAIQAVGQARERCFKRDWVLELDIKGLFDNIKH
GYLMYMVEKHTQIKWLILYIKRWLTVPFIMSDGSVAERRSGTPQGGVISPVLANLFLHYVFDDFMTKAYPNIW
WERYADDGVLHCQSYKQAAFIKQKLEERFQQFGLELNKEKTRIVYCKDNRRPQNYSCTQFTFLGYTFRPRLNKN
KEGKFFVGFTPAVSEKAKTAMKQKIREWKIQLKADLSLKDIGNMINKVVQGWINYYTHYYKSEFYEVLRYINQCL
IKWVRRSYKKKNTRSRAEHWLGAVARRDRNLFAHWKFGILPSVGEGAV
>N221235||gene_266432|GeneMark.hmm|418_aa|+|1398|2654
MSEAKQFDISKKAVIAAFQAVKENAGSYGADEQTIKEFEEHLNNNLYKLWNRMASGSYFPKPVRAVAIPKKNG
GIRILGIPTVEDRIAQMVAKMYFEPLVEPMFYNDSYGYRPNKSAIQAVGQARERCFKRDWVLELDIKGLFDNIK
HGYLMYMVEKHTQIKWLILYIKRWLTVPFIMSDGSVAERRSGTPQGGVISPVLANLFLHYVFDDFMTKAYPNI
WWERYADDGVLHCQSYKQAAFIKQKLEERFQQFGLELNKEKTRIVYCKDNRRPQNYSCTQFTFLGYTFRPRLN
KNKEGKFFVGFTPAVSEKAKTAMKQKIREWKIQLKADLSLKDIGNMINKVVQGWINYYTHYYKSEFYEVLRYIN
QCLIKWVRRSYKKKNTRSRAEHWLGAVARRDRNLFAHWKFGILPSVGEGAV
>N221235||gene_349772|GeneMark.hmm|430_aa|+|17018|18310
MQNDNAKPISISKQLVYDAFLRVKANRGSAGIDKVTLEDYEKNLRGNLYKLWNRMSSGSYFPPSVKLVEIPKSTG
GKRPLGIPTVSDRVAQMAVVMLITPSIEPCFHEDSYAYRPHRSAHDAVGKARERCWKYAWVLDMDISKFFDTI
DHELLLKALKRHTQEKWVLMYIERWLKVPYEKSDGSQVDRALGVPQGSVIGPVLANLFLHYTFDKWMEKNFP

RVPFERYADDTICHCHSLKQAEYMQAMIQQRFECCRLRLNEEKTKIVYCKSSRQKECYPNVTFDFLGFTFQPRES
VDKYGNRFTGFLPAISRKSMKRINETMRSWHLNRHSNLTLEHLASDINPIVRGWMTYYGKFYPTRLKWFMQTL
NGRLARWVMCKFERYRHRFYPAQEWLARIAEKEGLIFYHWKCGALPRFTNKEKVSSQLIMVK
>N221236||gene_231001|GeneMark.hmm|368_aa|-|22970|24076
MSSGSYVPKPVRLVQIPKPAGGTRPLGIPTVEDRIAQMLVVEMIEPEIEKIFHEDSYGYRPNRSAHDALGRARER
CWKYAWVLDMDISKFFDTIDHQLLMKAVRLHVKERWIILYIERWLKVPYQNADKSLIERTCGVPQGSVIGPILA
NLFLHYCFDRWMQIHYPEIPFERYADDTVCHCRSQREAESLYEELIIRFKSCKLSLNEEKTKIVYCKSSRRKENHSN
VTFDFLGHTFRPCKTMHKSSREAFTGFQPRISMKATTKIRATMRSWNLKSKSHTPLDCIAHMVNPILRGWVNY
YGKYGGKSFQKLLGYFDLLLARWAKAKYKTFRRKPMYVILKWLGNVADRDAVFYHWQIGLKPAKGTIKL
>N221236||gene_56589|GeneMark.hmm|419_aa|-|1894|3153
MNAANPFVIDKRLVWEAYHKVKENKGSAGIDKVDQKTFDKEMSKNLYKKWNRMSSGCYFPKAVKLVEIPKSN
GGTRPLGIPAIEDRIAQQVVSVLTPILEPIFKEDSYGYRPGKGAHQAIAKAKERCYVTPWVLDMDISKFFDTINHE
LLMKAIRKHTEEKWVLLYIERWLKVPNQTSKGEVIERTMGVPQGSVIGPVLANLFLHYVFDEWMSRNYPTIPFE
RYADDTICHCVSEKQARFLKAVLMKRFEECGLKLNEEKTKIVYCKDSNRRGDSEHTSFDFLGFTFRPRGARNRKT
GQNFTAFLPAISNKSMKRIKEAVRAWKLNRKTFACLLDISTEVDMQISGWMNYYMKFGRSEFRKVLNYINERLT
RWVMRKYKRFSKGKKFSKAYEWLVEYAAHNRNEFSHWVKGFVPYPRLD
>N221238||gene_4784|GeneMark.hmm|418_aa|+|560|1816
MSEAKQFDISKKAVIAAFQAVKENAGSYGVDEQTIKEFEEHLNNNLYKLWNRMASGSYFPKPVRAVEIPKKNG
GTRILGIPTVEDRIAQMVAKMYFEPLVEPMFYNDSYGYRPNKSAIQAVGQARERCFKRDWVLELDIKGLFDNIK
HGYLMYMVEKHTQIKWLILYIKRWLTVPFIMSDGSVAERRSGTPQGGVISPVLANLFLHYVFDDFMTKAYPNI
WWERYADDGVLHCQSYKQAVFIKQKLEERFQQFGLELNKEKTRIVYCKDDRRSRNYSCTQFTLLGYTFRPRLNK
NKEGKFFVGFTPAVSEKAKTAMKQKIRGWKIQLKADLSLKDIGNMINKVVQGWINYYTHYYKSEFYEVLRYINQ
CLIKWVRRSYKKKNTRSRAEHWLGAVARRDRNLFAHWKFGILPSVGEGAV
>N221244||gene_83188|GeneMark.hmm|430_aa|-|38705|39997
MQNDNAKPISISKQLVYDAFLRVKANRGSAGIDKVTLEDYEKNLRGNLYKLWNRMSSGSYFPPSVKLVEIPKSTG
GKRPLGIPTVSDRVAQMAIVMLITPSIEPCFHEDSYAYRPHRSAHDAVGKARERCWKYAWVLDMDISKFFDTID
HELLLKALKRHTQEKWVLMYIERWLKVPYEKADGSQVDRALGVPQGSVIGPVLANLFLHYTFDKWMEKSFPR
VPFERYADDTICHCHSLKQAEYMQAMIQQRFECCRLRLNEEKTKIVYCKSSRQKEFYPNVTFDFLGFTFQPRESV
DKYGNRFTGFLPAISRKSMKRINETIRSWHLNRHSNLTLEHLASDINPIVRGWMTYYGKFYPTRLKWFMQTLN
GRLARWVMCKFERYRHRFYPAQEWLARIAEKEGLIFYHWKCGVLPRFTNKEKVSSQLIMVK
>N221244||gene_175513|GeneMark.hmm|420_aa|+|568|1830
MNEAKPFVIDKRLVWEAYHKVKENKGSAGIDKVDQKTFDKEMSKNLYKIWNRMSSGCYFPKAVKLVEIPKSNG
GTRPLGIPTIEDRIAQQVVVSVLTPILEPIFKEDSYGYRPGKGAHQAIAKAKERCYVNPWVLDMDISKFFDTINHD
LLMKAVRKHTEEKWVLLYIERWLKVPYQTSKGEVIERTMGVPQGSVIGPVLANLFLHYVFDEWMSRNYPTIPFE
RYADDTICHCVSEKQAQFLKAVLMKRFEECGLKLNEEKTKIVYCKDSNRRGDSEHTSFDFLGFTFRPRSARNRKT
GQNFTAFLPAISKKSLKRIKEAVRAWKLNRKTFACLLDISNEVDTQISGWMNYYMKFGRSEFRKVLNYINERLTR
WVMRKYKRFSKGKKFSRAYEWLVEYAVHNRNEFSHWAKGFVPYPRLG
>N221244||gene_183255|GeneMark.hmm|414_aa|+|1714|2958
MQETKSYNISKQAVYQAFLKVKANKGTFGVDEESIEAYEYKLKDNLYKLWNRLSSGSYFPKPVKAVSIPKKSGGLR
VLGIPTVEDRIAQMVAKMYFEPVVERLFYEDSYGYRPNKSAIQAIEKTRVRCWKRDWVLEFDIKGLFDNIRHDY
LIEMVKRYTQEKWIILYVERWLKAPFQREDGSTVSRKAGTPQGGVISPVLANLFLHYTFDDFMEKEFPNIQWAR
YADDGITHCVSLKQAKYLKKRLEERFRIFGLELHPDKTKIVYCRDSDRMGNYPITTFDFLGFTFRPRGAKNKYGKC
FTNFLPAVSDKAKKAIRKEVRNWRLQLKADKKLEDLANMFNSKIQGWMNYYMKFYKSEMYSLLRYINQCLVK
WVRRKYKKRQARRKAEHWLGEIAKRERNLFAHWKIGILPSAG

>N221246||gene_21066|GeneMark.hmm|430_aa|+|7628|8920
MQNDNAKPISISKQLVYDAFLRVKANRGSAGIDKVTLEDYEKNLRGNLYKLWNRMSSGSYFPPSVKLVEIPKSTG
GKRPLGIPTVSDRVAQMTVVMLITPSIEPCFHEDSYAYRPHRSAHDAVGKARERCWKYAWVLDMDISKFFDTID
HELLLKALKRHTQEKWVLMYIERWLKVPYEKSDGSQVDRALGVPQGSVIGPVLANLFLHYTFDKWMEKNFPR
VPFERYADDTICHCHSLKQAEYMQAMIQQRFECCRLRLNEEKTKIVYCKSSRQKECYPNVTFDFLGFTFQPRESV
DKYGNRFTGFLPAISRKSMKRINETMRSWHLNRHSNLTLEHLASDINPIVRGWMTYYGKFYPTRLKWFMQTLN
GRLASWVMCKFERYRHRFYPAQEWLARIAEKEGLIFYHWKCGVLPRFTNKEKVSSQLIMVK
>N221247||gene_38413|GeneMark.hmm|423_aa|-|154|1425
MTQKQGAKPFDIDRWKLYYAYQRVNQNRGGSGVDNVTLEKYNSNLKRNLYKLWNRMSSGSYVPKPVRLVQIP
KPAGGTRPLGIPTVEDRIAQMLVVEMIEPEIEKIFHEDSYGYRPNRSAHDALGRARERCWKYAWVLDMDISKFF
DTIDHQLLMKAVRLHVKERWIILYIERWLKVPYQNADKSLIERTCGVPQGSVIGPILANLFLHYCFDRWMQIHH
PEIPFERYADDTVCHCRSQREAESLYEELIIRFKSCKLSLNEEKTKIVYCKSSRKKENHSNVTFDFLGHTFRPCKTMH
KSSREAFTGFQPRISMKATTKIRATMRSWNLKSKSHTPLDCIAHMVNPILRGWVNYYGKYGGKSFQKLLGYFDL
LLARWAKAKYKTFRRKPMYVILKWLGNVADRDAVFYHWQIGLKPAKGTIKL
>N221247||gene_213731|GeneMark.hmm|418_aa|+|14119|15375
MSEAKQFDISKKAVIAAFQAVKENAGSYGADEQTIKEFEEHLNNNLYKLWNRMASGSYFPKPVRAVAIPKKNG
GIRILGIPTVEDRIAQMVAKMYFEPLVEPMFYNDSYGYRPNKSAIQAVGQARERCFKRDWVLELDIKGLFDNIK
HGYLMYMVEKHTQIKWLILYIKRWLTVPFIMSDGSVAERRSGTPQGGVISPVLANLFLHYVFDDFMTKAYPNI
WWERYADDGVLHCQSYKQAAFIKQKLEERFQQFGLELNKEKTRIVYCKDNRRPQNYSCTQFTFLGYTFRPRLN
KNKEGKFFVGFTPAVSEKAKTAMKQKIREWKIQLKADLSLKDIGNMINKVVQGWINYYTHYYKSEFYEVLRYIN
QCLIKWVRRSYKKKNTRSRAEHWLGAVARRDRNLFAHWKFGILPSVGEGAV
>N221248||gene_106096|GeneMark.hmm|418_aa|-|570|1826
MSEAKQFDISKKAVIAAFQAVKENAGSYGADEQTIKEFEEHLNNNLYKLWNRMASGSYFPKPVRAVAIPKKNG
GIRILGIPTVEDRIAQMVAKMYFEPLVEPMFYNDSYGYRPNKSAIQAVGQARERCFKRDWVLELDIKGLFDNIK
HGYLMYMVEKHTQIKWLILYIKRWLTVPFIMSDGSVAERRSGTPQGGVISPVLANLFLHYVFDDFMTKAYPNI
WWERYADDGVLHCQSYKQAAFIKQKLEERFQQFGLELNKEKTRIVYCKDNRRPQNYSCTQFTFLGYTFRPRLN
KNKEGKFFVGFTPAVSEKAKTAMKQKIREWKIQLKADLSLKDIGNMINKVVQGWINYYTHYYKSEFYEVLRYIN
QCLIKWVRRSYKKKNTRSRAEHWLGAVARRDRNLFAHWKFGILPSVGEGAV
>N221250||gene_30292|GeneMark.hmm|422_aa|-|819|2087
MMQHQVTKPFTIDKHLIMNAWKRVKENKGSVGIDNVSTDDYESNLGKNLYKLWNRMSSGSYFPEAVKLVDIP
KSSGGTRPLGIPTVSDRIAQMSVVLLIEDRLEAIFHADSYGYRPNRSAHDAIGKARERCWHYNWVLDMDISKFF
DTINHDLLMKAVERHVQEKWILLYIRRWLEVPYATLTGERIERRMGVPQGSVIGPVLANLYLHYTFDKWMSLYH
PTIPFERYADDTICHCNSLEEAQMLKASIVERFAACKLKLNEEKTRIVYCKDGKRRREYKDITFDFLGYTFQPRGQR
NKQGQVFNGYAPAISRKSKKRIAETMRGWHLNRRVQLKLSDIAVEINAEVRGWMNYYGKFYGSQLKAFLQCIN
LKLARWAERKYKRFRRKPNDAYKWLVRVASKNPALFYHWQHGVKPNRLKPFG
>N221250||gene_48490|GeneMark.hmm|418_aa|+|3002|4258
MSEAKQFDISKKAVIAAFQAVKENAGSYGADEQTIKEFEEHLNNNLYKLWNRMASGSYFPKPVRAVAIPKKNG
GIRILGIPTVEDRIAQMVAKMYFEPLVEPMFYNDSYGYRPNKSAIQAVGQARERCFKRDWVLELDIKGLFDNIK
HGYLMYMVEKHTQIKWLILYIKRWLTVPFIMSDGSVAERRSGTPQGGVISPVLANLFLHYVFDDFMTKAYPNI
WWERYADDGVLHCQSYKQAAFIKQKLEERFQQFGLELNKEKTRIVYCKDNRRPQNYSCTQFTFLGYTFRPRLN
KNKEGKFFVGFTPAVSEKAKTAMKQKIREWKIQLKADLSLKDIGNMINKVVQGWINYYTHYYKSEFYEVLRYIN
QCLIKWVRRSYKKKNTRSRAEHWLGAVARRDRNLFAHWKFGILPSVGEGAV
>N221256||gene_348806|GeneMark.hmm|418_aa|-|22483|23739
MSEAKQFDISKKAVIAAFQAVKENAGSYGADEQTIKEFEEHLNNNLYKLWNRMASGSYFPKPVRAVAIPKKNG

GIRILGIPTVEDRIAQMVAKMYFEPLVEPMFYNDSYGYRPNKSAIQAVGQARERCFKRDWVLELDIKGLFDNIK
HGYLMYMVEKHTQIKWLILYIKRWLTVPFIMSDGSVAERRSGTPQGGVISPVLANLFLHYVFDDFMTKAYPNI
WWERYADDGVLHCQSYKQAAFIKQKLEERFQQFGLELNKEKTRIVYCKDNRRPQNYSCTQFTFLGYTFRPRLN
KNKEGKFFVGFTPAVSEKAKTAMKQKIREWKIQLKADLSLKDIGNMINKVVQGWINYYTHYYKSEFYEVLRYIN
QCLIKWVRRSYKKKNTRSRAEHWLGAVARRDRNLFAHWKFGILPSVGEGAV
>N221256||gene_161062|GeneMark.hmm|420_aa|-|632|1894
MNEAKPFVIDKRLVWEAYHKVKENKGSAGIDKVDQKTFDKEMSKNLYKIWNRMSSGCYFPKAVKLVEIPKSNG
GTRPLGIPTIEDRIAQQVVVSVLTPILEPIFKEDSYGYRPGKGAHQAIAKAKERCYVNPWVLDMDISKFFDTINHE
LLMKAVRKHTEEKWILLYIERWLKVPYQTSKGEVIERTMGVPQGSVIGPVLANLFLHYVFDEWMSRNYPTIPFE
RYADDTICHCVSEKQAQFLKAVLMKRFEECGLKLNEEKTKIVYCKDSNRRGDSEHTSFDFLGFTFRPRGARNRKT
GQNFTAFLPAISKKSMKRIKEAVRAWKLNRKTFACLLDISNEVDTQISGWMNYYMKFGRSEFRKVLNYINERLT
RWVMRKYKRFSKGRKFNRAYDWLVEYAAHNRNEFSHWVKGFVPYPRLG
>N221256||gene_108168|GeneMark.hmm|416_aa|+|1285|2535
MTKTKAFNIDKSLVVSAYRRVKTSAGAAGIDKQSLADFDKRLVDNLYKIWNRLSSGSYFPPAVKAVAIPKKLGGER
ILGIPTVSDRIAQTVVKLAFEPQVEPHFLADSYGYRPNKSALDAIGVTRKRCWYYDWVLEFDIKGLFDNIPHELIM
KAVDKHNPARWVKLYIQRWLTAPMVMSDGEVRARTMGTPQGGVISPLLANLFMHYVFDKWLAKYYPKVPW
YRYADDGILHCHSEAEATEMREVLRKRFSECGLEMHPEKTRVIYCKDGSRKGDYEHTMFDFLGYTFRRRVVKNV
KRNSLFVSFTPAASKSALKAMRREIKATGIRKRVDLSIEQIAKWINPKLNGWINYYGRYTCSELYSVFRYINKALVR
WGRKKYKMLSRYKTRASKFLEEMAKRSPQLFAHWRLKMRGGLV
>N221260||gene_41818|GeneMark.hmm|418_aa|-|1712|2968
MSEAKQFDISKKAVIAAFQAVKENAGSYGADEQTIKEFEEHLNNNLYKLWNRMASGSYFPKPVRAVAIPKKNG
GIRILGIPTVEDRIAQMVAKMYFEPLVEPMFYNDSYGYRPNKSAIQAVGQARERCFKRDWVLELDIKGLFDNIK
HGYLMYMVEKHTQIKWLILYIKRWLTVPFIMSDGSVAERRSGTPQGGVISPVLANLFLHYVFDDFMTKAYPNI
WWERYADDGVLHCQSYKQAAFIKQKLEERFQQFGLELNKEKTRIVYCKDNRRPQNYSCTQFTFLGYTFRPRLN
KNKEGKFFVGFTPAVSEKAKTAMKQKIREWKIQLKADLSLKDIGNMINKVVQGWINYYTHYYKSEFYEVLRYIN
QCLIKWVRRSYKKKNTRSRAEHWLGAVARRDRNLFAHWKFGILPSVGEGAV
>N221264||gene_354672|GeneMark.hmm|418_aa|-|3079|4335
MSEAKQFDISKKAVIAAFQAVKENAGSYGADEQTIKEFEEHLNNNLYKLWNRMASGSYFPKPVRAVAIPKKNG
GIRILGIPTVEDRIAQMVAKMYFEPLVEPMFYNDSYGYRPNKSAIQAVGQARERCFKRDWVLELDIKGLFDNIK
HGYLMYMVEKHTQIKWLILYIKRWLTVPFIMSDGSVAERRSGTPQGGVISPVLANLFLHYVFDDFMTKAYPNI
WWERYADDGVLHCQSYKQAAFIKQKLEERFQQFGLELNKEKTRIVYCKDNRRPQNYSCTQFTFLGYTFRPRLN
KNKEGKFFVGFTPAVSEKAKTAMKQKIREWKIQLKADLSLKDIGNMINKVVQGWINYYTHYYKSEFYEVLRYIN
QCLIKWVRRSYKKKNTRSRAEHWLGAVARRDRNLFAHWKFGILPSVGEGAV
>N221267||gene_197170|GeneMark.hmm|418_aa|+|27523|28779
MSEAKQFDISKKAVIAAFQAVKENAGSYGVDEQTIKEFEEHLNNNLYKLWNRMASGSYFPKPVRAVEIPKKNG
GTRILGIPTVEDRIAQMVAKMYFEPLVEPMFYNDSYGYRPNKSAIQAVGQAREKCFKRDWVLELDIKGLFDNIK
HGYLMYMVEKHTQIKWLILYIKRWLTVPFIMSDGSVAERRSGTPQGGVISPVLANLFLHYVFDDFMTKAYPNI
WWERYADDGVLHCQSYKQAVFIKQKLEERFQQFGLELNKEKTRIVYCKDDRRSRNYSCTQFTFLGYTFRPRLNK
NKEGKFFVGFTPAVSEKAKTAMKQKIRGWKIQLKADLSLKDIGNMINKVVQGWINYYTHYYKSEFYEVLRYINQ
CLIKWVRRSYKKKNTRSRAEHWLGAVARRDRNLFAHWKFGILPSVGEGAV
>N221270||gene_32230|GeneMark.hmm|418_aa|+|25418|26674
MSEAKQFDISKKAVIAAFQAVKENAGSYGVDEQTIKEFEEHLNNNLYKLWNRMASGSYFPKPVRAVEIPKKNG
GTRILGIPTVEDRIAQMVAKMYFEPLVEPMFYNDSYGYRPNKSAIQAVGQAREKCFKRDWVLELDIKGLFDNIK
HGYLMYMVEKHTQIKWLILYIKRWLTVPFIMSDGSVAERRSGTPQGGVISPVLANLFLHYVFDDFMTKAYPNI

WWERYADDGVLHCQSYKQAVFIKQKLEERFQQFGLELNKEKTRIVYCKDDRRSRNYSCTQFTFLGYTFRPRLNK
NKEGKFFVGFTPAVSEKAKTAMKQKIRGWKIQLKADLSLKDIGNMINKVVQGWINYYTHYYKSEFYEVLRYINQ
CLIKWVRRSYKKKNTRSRAEHWLGAVARRDRNLFAHWKFGILPSVGEGAV
>N221276||gene_2111|GeneMark.hmm|418_aa|-|1454|2710
MSEAKQFDISKKAVIAAFQAVKENAGSYGADEQIIKEFEEHLNNNLYKLWNRMASGSYFPKPVRAVAIPKKNGG
IRILGIPTVEDRIAQMVAKMYFEPLVEPMFYNDSYGYRPNKSAIQAVGQARERCFKRDWVLELDIKGLFDNIKH
GYLMYMVEKHTQIKWLILYIKRWLTVPFIMSDGSVAERRSGTPQGGVISPVLANLFLHYVFDDFMTKAYPNIW
WERYADDGVLHCQSYKQAAFIKQKLEERFQQFGLELNKEKTRIVYCKDNRRPQNYSCTQFTFLGYTFRPRLNKN
KEGKFFVGFTPAVSEKAKTAMKQKIREWKIQLKADLSLKDIGNMINKVVQGWINYYTHYYKSEFYEVLRYINQCL
IKWVRRSYKKKNTRSRAEHWLGAVARRDRNLFAHWKFGILPSVGEGAV
>N221281||gene_41730|GeneMark.hmm|420_aa|+|4251|5513
MNEAKPFVIDKRLVWEAYHKVKENKGSAGIDKVDQKTFDKEMSKNLYKIWNRMSSGCYFPKAVKLVEIPKSNG
GTRPLGIPTIEDRIAQQVVVSVLTPILEPIFKEDSYGYRPGKGAHQAIAKAKERCYVNPWVLDMDISKFFDTINHD
LLMKAVRKHTEEKWVLLYIERWLKVPYQTSKGEVIERTMGVPQGSVIGPVLANLFLHYVFDEWMSRNYPTIPFE
RYADDTICHCVSEKQAQFLKAVLMKRFEECGLKLNEEKTKIVYCKDSNRRGDSEHTSFDFLGFTFRPRSARNRKT
GQNFTAFLPAISKKSLKRIKEAVRAWKLNRKTFACLLDISNEVDTQISGWMNYYMKFGRSEFRKVLNYINERLTR
WVMRKYKRFSKGKKFSRAYEWLVEYAVHNRNEFSHWAKGFVPYPRLG
>N221283||gene_33391|GeneMark.hmm|418_aa|-|162|1418
MSEAKQFDISKKAVIAAFQAVKENAGSYGADEQTIKEFEEHLNNNLYKLWNRMASGSYFPKPVRAVAIPKKNG
GIRILGIPTVEDRIAQMVAKMYFEPLVEPMFYNDSYGYRPNKSAIQAVGQARERCFKRDWVLELDIKGLFDNIK
HGYLMYMVEKHTQIKWLILYIKRWLTVPFIMSDGSVAERRSGTPQGGVISPVLANLFLHYVFDDFMTKAYPNI
WWERYADDGVLHCQSYKQAAFIKQKLEERFQQFGLELNKEKTRIVYCKDNRRPQNYSCTQFTFLGYTFRPRLN
KNKEGKFFVGFTPAVSEKAKTAMKQKIREWKIQLKADLSLKDIGNMINKVVQGWINYYTHYYKSEFYEVLRYIN
QCLIKWVRRSYKKKNTRSRAEHWLGAVARRDRNLFAHWKFGILPSVGEGAV
>N221283||gene_93251|GeneMark.hmm|423_aa|-|32724|33995
MTQKQGAKPFDIDRWKLYYAYQRVNQNRGGSGVDNVTLEKYNSNLKRNLYKLWNRMSSGSYVPKPVRLVQIP
KPAGGTRPLGIPTVEDRIAQMLVVEMIEPEIEKIFHEDSYGYRPNRSAHDALGRARERCWKYAWVLDMDISKFF
DTIDHQLLMKAVRLHVKERWIILYIERWLKVPYQNADKSLIERTCGVPQGSVIGPILANLFLHYCFDRWMQIHH
PEIPFERYADDTVCHCRSQREAESLYEELIIRFKSCKLSLNEEKTKIVYCKSSRKKENHSNVTFDFLGHTFRPCKTMH
KSSREAFTGFQPRISMKATTKIRATMRSWNLKSKSHTPLDCIAHMVNPILRGWVNYYGKYGGKSFQKLLGYFDL
LLARWAKAKYKTFRRKPMYVILKWLGNVADRDAVFYHWQIGLKPAKGTIKL
>N221284||gene_233359|GeneMark.hmm|430_aa|-|1031|2323
MQNDNAKPISISKQLVYDAFLRVKANRGSAGIDKVTLEDYEKNLRGNLYKLWNRMSSGSYFPPSVKLVEIPKSTG
GKRPLGIPTVSDRVAQMAVVMLITPSIEPCFHEDSYAYRPHRSAHDAVGKARERCWKYAWVLDMDISKFFDTI
DHELLLKALKRHTQEKWVLMYIERWLKVPYEKSDGSQVDRALGVPQGSVIGPVLANLFLHYTFDKWMEKNFP
RVPFERYADDTICHCHSLKQAEYMQAMIQQRFECCRLRLNEEKTKIVYCKSSRQKECYPNVTFDFLGFTFQPRES
VDKYGNRFTGFLPAISRKSMKRINETMRSWHLNRHSNLTLEHLASDINPIVRGWMTYYGKFYPTRLKWFMQTL
NGRLARWVMCKFERYRHRFYPAQEWLARIAEKEGLIFYHWKCGALPRFTNKEKVSSQLIMVK
>N221290||gene_98781|GeneMark.hmm|422_aa|+|716|1984
MMQHQVTKPFTIDKYLIMNAWKRVKENKGSAGIDNVSTEDYESNLGKNLYKLWNRMSSGSYFPEAVKLVDIP
KPSGGTRPLGIPTVGDRIAQMSVVLLIEERLEAIFHADSYGYRPNRSAHDAIEKARERCWHYNWVLDMDISKFF
DTIDHDLLMKAVERHVQEKWILLYIRRWLKVPYATLTGERIERKMGVPQGSVIGPVLANLYLHYTFDKWMSLYH
PTIPFERYADDTICHCNSLKEAQMLKASIVERFAACKLRLNEEKTRIVYCKDGKRRGEYKEITFDFLGYTFQPRGQ
RNKQGQVFNGYAPAISRKSKKRITEKMRGWHLNRRVQLKLSDIAVEINAEVRGWMNYYGKFYGSQLKAFLQCI

NLKLARWAERKYKRFRRKPNDAYKWLVRVASKNPALFYHWQHGVKPNRLKPFG
>N221290||gene_114488|GeneMark.hmm|418_aa|+|1373|2629
MSEAKQFDISKKAVIAAFQAVKENAGSYGADEQTIKEFEEHLNNNLYKLWNRMASGSYFPKPVRAVAIPKKNG
GIRILGIPTVEDRIAQMVAKMYFEPLVEPMFYNDSYGYRPNKSAIQAVGQARERCFKRDWVLELDIKGLFDNIK
HGYLMYMVEKHTQIKWLILYIKRWLTVPFIMSDGSVAERRSGTPQGGVISPVLANLFLHYVFDDFMTKAYPNI
WWERYADDGVLHCQSYKQAAFIKQKLEERFQQFGLELNKEKTRIVYCKDNRRPQNYSCTQFTFLGYTFRPRLN
KNKEGKFFVGFTPAVSEKAKTAMKQKIREWKIQLKADLSLKDIGNMINKVVQGWINYYTHYYKSEFYEVLRYIN
QCLIKWVRRSYKKKNTRSRAEHWLGAVARRDRNLFAHWKFGILPSVGEGAV
>N221293||gene_29225|GeneMark.hmm|420_aa|-|230|1492
MNEAKPFVIDKRLVWEAYHKVKENKGSAGIDKVDQKTFDKEMSKNLYKIWNRMSSGCYFPKAVKLVEIPKSNG
GTRPLGIPTIEDRIAQQVVVSVLTPILEPIFKEDSYGYRPGKGAHQAIAKAKERCYVNPWVLDMDISKFFDTINHD
LLMKAVRKHTEEKWVLLYIERWLKVPYQTSKGEVIERTMGVPQGSVIGPVLANLFLHYVFDEWMSRNYPTIPFE
RYADDTICHCVSEKQAQFLKAVLMKRFEECGLKLNEEKTKIVYCKDSNRRGDSEHTSFDFLGFTFRPRSARNRKT
GQNFTAFLPAISKKSLKRIKEAVRAWKLNRKTFACLLDISNEVDTQISGWMNYYMKFGRSEFRKVLNYINERLTR
WVMRKYKRFSKGKKFSRAYEWLVEYAVHNRNEFSHWAKGFVPYPRLG
>N221295||gene_58302|GeneMark.hmm|418_aa|+|677|1933
MSEAKQFDISKKAVIAAFQAVKENAGSYGVDEQTIKEFEEHLNNNLYKLWNRMASGSYFPKPVRAVEIPKKNG
GTRILGIPTVEDRIAQMVAKMYFEPLVEPMFYNDSYGYRPNKSAIQAVGQARERCFKRDWVLELDIKGLFDNIK
HGYLMYMVEKHTQIKWLILYIKRWLTVPFIMSDGSVAERRSGTPQGGVISPVLANLFLHYVFDDFMTKAYPNI
WWERYADDGVLHCQSYKQAVFIKQKLEERFQQFGLELNKEKTRIVYCKDDRRSRNYSCTQFTLLGYTFRPRLNK
NKEGKFFVGFTPAVSEKAKTAMKQKIRGWKIQLKADLSLKDIGNMINKVVQGWINYYTHYYKSEFYEVLRYINQ
CLIKWVRRSYKKKNTRSRAEHWLGAVARRDRNLFAHWKFGILPSVGEGAV
>N221296||gene_393313|GeneMark.hmm|418_aa|-|2905|4161
MSEAKQFDISKKAVIAAFQAVKENAGSYGADEQIIKEFEEHLNNNLYKLWNRMASGSYFPKPVRAVAIPKKNGG
IRILGIPTVEDRIAQMVAKMYFEPLVEPMFYNDSYGYRPNKSAIQAVGQARERCFKRDWVLELDIKGLFDNIKH
GYLMYMVEKHTQIKWLILYIKRWLTVPFIMSDGSVAERRSGTPQGGVISPVLANLFLHYVFDDFMTKAYPNIW
WERYADDGVLHCQSYKQAVFIKQKLEERFQQFGLELNKEKTRIVYCKDNRRPQNYSCTQFTFLGYTFRPRLNKN
KEGKFFVGFTPAVSEKAKTAMKQKIREWKIQLKADLSLKDIGNMINKVVQGWINYYTHYYKSEFYEVLRYINQCL
IKWVRRSYKKKNTRSRAEHWLGAVARRDRNLFAHWKFGILPSVGEGAV
>N221296||gene_216041|GeneMark.hmm|420_aa|+|19540|20802
MNEAKPFVIDKRLVWEAYHKVKENKGSAGIDKVDQKTFDKEMSKNLYKIWNRMSSGCYFPKAVKLVEIPKSNG
GTRLLGIPTIEDRIAQQVVVSVLTPILEPIFKEDSYGYRPGKGAHQAIAKAKERCYVNPWVLDMDISKFFDTINHE
LLMKAVRKHTEEKWILLYIERWLKVSYQTSKGEVIERTMGVPQGSVIGPVLANLFLHYVFDEWMSRNYPTIPFES
YADDTICHCVSEKQAQFLKAVLMKRFEECGLKLNEEKTKIVYCKDSNRRGDSEHTSFDFLGFTFRPRGARNRKTG
QNFTAFLPAISKKSMKRIKEAVRAWKLNRKTFACLLDISNEVDTQISGWMNYYMKFGRSEFRKVLNYINERLTR
WVMRKYKRFSKGRKFNRAYDWLVEYAAHNRNEFSHWVKGFVPYPRLG
>N221296||gene_402457|GeneMark.hmm|422_aa|+|700|1968
MKDAKSFEISRHLVMEAYKRVKANKGAAGVDEVSIADFENNLKSNLYKIWNRMSSGSYLPPAVKLVEIPKSNGG
KRPLGIPTVGDRVAQMVVVMTIEPGIEPYFHEDSYAYRPNRSALDAVRKAKERSYTFHWVLDLDIKGFFDNIDH
ELLIKALERHVKCKWAILYIKRWLSVPYQLKDGTQKERTKGVPQGSVVGPILANLFLHYVFDEWMRRNHSNISFE
RYADDTICHCVSLKQAEFILRAIRKRFAECKLELNEDKTKIVYCKKNHRDIPYECIQFDFLGYTFRPRRSIDANGEVF
LNFSPAISKKARTKIWEAIQNWNSNHWVPMELEDIAKEINPVIQGWINYYGQHNPRILKEVLQHVNDRLVRW
GRRKFKGLRKRKTATVHRLGDIALQKPNLFAHWAWGVKPTASERNRKRK
>N221300||gene_135316|GeneMark.hmm|418_aa|+|1764|3020

MSEAKQFDISKKAVIAAFQAVKENAGSYGVDEQTIKEFEEHLNNNLYKLWNRMASGSYFPKPVRAVAIPKKNG
GTRILGIPTVEDRIAQMVAKMYFEPLVEPMFYKDSYGYRPNKSAIQAVGQARERCFKRDWVLELDIKGLFDNIK
HGYLMYMVEKHTQIKWLILYIKRWLTVPFIMSDGSVAERRSGTPQGGVISPVLANLFLHYVFDDFMTKAYPNI
WWERYADDGVLHCQSYKQAVFIKQKLEERFQQFGLELNKEKTRIVYCKDNRRSQNYSCTQFTFLGYTFRPRLNK
NKEGKFFVGFTPAVSEKAKTAMKQKIREWKIQLKADLSLKDIGNMINKVVQGWINYYTHYYKSEFYEVLRYINQ
CLIKWVRRSYKKKNTRSRAEHWLGAVARRDRNLFAHWKFGILPSVGEGAV
>N221300||gene_65760|GeneMark.hmm|430_aa|-|242|1534
MQNGNAKPISISKQLVYDAFLRVKANRGSAGIDKVTLEDYEKNLRGNLYKLWNRMSSGSYFPPSVKLVEIPKSTG
GKRPLGIPTVSDRVAQMAIVMLITPSIEPCFHEDSYAYRPHRSAHDAVGKARERCWKYAWVLDMDISKFFDTID
HELLLKALKRHTQEKWVLMYIERWLKVPYEKADGSQVDRALGVPQGSVIGPVLANLFLHYTFDKWMEKSFPR
VPFERYADDTICHCHSLKQAEYMQAMIQQRFECCRLRLNEEKTKIVYCKSSRQKGRYPNVTFDFLGFTFQPRES
VDKYGSRFTGFLPAISRKSMKRINETIRSWHLNRHSNLTLEHLASDINPIVRGWMTYYGKFYPTRLKWFMQTLN
GRLARWIMCKFERYRHRFYPAQEWLARIAEKEGLIFYHWKCGVLPRFTNKEKVSSQLIMVK
>N221304||gene_136183|GeneMark.hmm|418_aa|+|17693|18949
MSEAKQFDISKKAVIAAFQAVKENAGSYGADEQTIKEFEEHLNNNLYKLWNRMASGSYFPKPVRAVAIPKKNG
GIRILGIPTVEDRIAQMVAKMYFEPLVEPMFYNDSYGYRPNKSAIQAVGQARERCFKRDWVLELDIKGLFDNIK
HGYLMYMVEKHTQIKWLILYIKRWLTVPFIMSDGSVAERRSGTPQGGVISPVLANLFLHYVFDDFMTKAYPNI
WWERYADDGVLHCQSYKQAAFIKQKLEERFQQFGLELNKEKTRIVYCKDNRRPQNYSCTQFTFLGYTFRPRLN
KNKEGKFFVGFTPAVSEKAKTAMKQKIREWKIQLKADLSLKDIGNMINKVVQGWINYYTHYYKSEFYEVLRYIN
QCLIKWVRRSYKKKNTRSRAEHWLGAVARRDRNLFAHWKFGILPSVGEGAV
>N221305||gene_102155|GeneMark.hmm|418_aa|+|1929|3185
MSEAKQFDISKKAVIAAFQAVKENAGSYGVDEQTIKEFEEHLNNNLYKLWNRMASGSYFPKPVRAVEIPKKNG
GTRILGIPTVEDRIAQMVAKMYFEPLVEPMFYNDSYGYRPNKSAIQAVGQAREKCFKRDWVLELDIKGLFDNIK
HGYLMYMVEKHTQIKWLILYIKRWLTVPFIMSDGSVAERRSGTPQGGVISPVLANLFLHYVFDDFMTKAYPNI
WWERYADDGVLHCQSYKQAVFIKQKLEERFQQFGLELNKEKTRIVYCKDDRRSRNYSCTQFTFLGYTFRPRLNK
NKEGKFFVGFTPAVSEKAKTAMKQKIRGWKIQLKADLSLKDIGNMINKVVQGWINYYTHYYKSEFYEVLRYINQ
CLIKWVRRSYKKKNTRSRAEHWLGAVARRDRNLFAHWKFGILPSVGEGAV
>N221305||gene_16170|GeneMark.hmm|413_aa|-|11482|12723
MSESKQYEIPKRVVVEAYKRVKANKGSAGIDGIDFDIFEKKLNNNLYKIWNRMSSGSYFPSPVLAVEIPKKAGGT
RRLGIPTIADRIAQMIARMYIEPAVEPMFCEDSYGYRPNKSAIEAIAVTRKRCWRYDYVIELDVKGLFDNINHELL
MRVVEKHVKESWICLYVKRWMETPFVTKERAAIERKSGTPQGGVISPVLANMFLHYVFDMWMKRKFPQAPF
ERYADDGIVHCRTKEEAICIRQSLAKRFEECKLELHPTKTRIVYCKDEDRRKEEELTEFDFLGYTFKARYIKCRDGKL
RYNFIASVSKVSAKAFRTKVKEMELHRRTGCKIDILAEMLNPMVRGWMNYFGKYNPSAMKDTLLCIERRLVKW
AMCKYKKFRGRRRKAEEWLCTLRKREPKLFAHWSMIYSYC
>N221305||gene_142615|GeneMark.hmm|416_aa|+|602|1852
MTKTKAFNIDKSLVVSAYRRVKTSAGAAGIDKQSLADFDKRLVDNLYKIWNRLSSGSYFPPAVKAVAIPKKLGGER
ILGIPTVSDRIAQTVVKLAFEPQVEPHFLADSYGYRPNKSALDAIGVTRKRCWYYDWVLEFDIKGLFDNIPHELIM
KAVDKHNPARWVKLYIQRWLTAPMVMSDGEVRARTMGTPQGGVISPLLANLFMHYVFDKWLAKYYPKVPW
YRYADDGILHCHSEAEATEMREVLRKRFSECGLEMHPEKTRVIYCKDGSRKGDYEHTMFDFLGYTFRRRVVKNV
KRNSLFVSFTPAASKSALKAMRREIKATGIRKRVDLSIEQIAKWINPKLNGWINYYGRYTCSELYSVFRYINKALVR
WGRKKYKMLSRYKTRASKFLEEMAKRSPQLFAHWRLKMRGGLV
>N221308||gene_217622|GeneMark.hmm|421_aa|+|3001|4266
MQEAKPFQIDKRIIFESFKKVKFNRGSSGIDGIEMTTYEQNLGSNLYRLWNRMSSGSYMPKAVKLVEIPKSNGG
KRPLGIPTIEDRIAQMAVVNVIEPLIEPCFHEDSFGYRPHRSAHDAIAKAERRCWKYAWVLDIDISKFFDTIDHGL

LMKAVEKHINIKWILLYIKRWLTVPYQRSDGEIVKRDMGVPQGSVIGPILANLFLHYTFDKWMSYKYPHIPFERY
ADDCVCHCSTLAQAEYIKERLGERFTECKLKFNEEKTKIVFCKMSSRSSKHYHCTSFDYLGFTFRSRAAKDKRNN
VLFTSYLPAISKKSVSRIHETIKSWNLKRLHNRSLRFVAAYINDVVRGWINYYEKFGKTEFWKVMCHLNRSIAYW
AKTKYKRLRRRGVISAHYWLAYIAQKEPNLFYHWQVGYVPYARQKK
>N221316||gene_12333|GeneMark.hmm|418_aa|-|68|1324
MSEAKQFDISKKAVIAAFQAVKENAGSYGADEQTIKEFEEHLNNNLYKLWNRMASGSYFPKPVRAVAIPKKNG
GIRILGIPTVEDRIAQMVAKMYFEPLVEPMFYNDSYGYRPNKSAIQAVGQARERCFKRDWVLELDIKGLFDNIK
HGYLMYMVEKHTQIKWLILYIKRWLTVPFIMSDGSVAERRSGTPQGGVISPVLANLFLHYVFDDFMTKAYPNI
WWERYADDGVLHCQSYKQAAFIKQKLEERFQQFGLELNKEKTRIVYCKDNRRPQNYSCTQFTFLGYTFRPRLN
KNKEGKFFVGFTPAVSEKAKTAMKQKIREWKIQLKADLSLKDIGNMINKVVQGWINYYTHYYKSEFYEVLRYIN
QCLIKWVRRSYKKKNTRSRAEHWLGAVARRDRNLFAHWKFGILPSVGEGAV
>N221319||gene_189872|GeneMark.hmm|420_aa|-|8027|9289
MNEAKPFVIDKRLVWEAYHKVKENKGSAGIDKVDQKTFDKEMSKNLYKIWNRMSSGCYFPKAVKLVEIPKSNG
GTRPLGIPTIEDRIAQQVVVSVLTPILEPIFKEDSYGYRPGKGAHQAIAKAKERCYVNPWVLDMDISKFFDTINHD
LLMKAVRKHTEEKWVLLYIERWLKVPYQTSKGEVIERTMGVPQGSVIGPVLANLFLHYVFDEWMSRNYPTIPFE
RYADDTICHCVSEKQAQFLKAVLMKRFEECGLKLNEEKTKIVYCKDSNRRGDSEHTSFDFLGFTFRPRSARNRKT
GQNFTAFLPAISKKSLKRIKEAVRAWKLNRKTFACLLDISNEVDTQISGWMNYYMKFGRSEFRKVLNYINERLTR
WVMRKYKRFSKGKKFSRAYEWLVEYAVHNRNEFSHWAKGFVPYPRLG
>N221319||gene_107074|GeneMark.hmm|419_aa|-|35664|36923
MNAANPFVIDKRLVWEAYHKVKENKGSAGIDKVDQKTFDKEMSKNLYKIWNRMSSGCYFPKAVKLVEIPKSN
GGTRPLGIPAIEDRIAQQVVSVLTPILEPIFKEDSYGYRPGKGAHQAIAKAKERCYVTPWVLDMDISKFFDTINHE
LLMKAIRKHTEEKWVLLYIERWLKVPNQTSKGEVIERTMGVPQGSVIGPVLANLFLHYVFDEWMSRNYPTIPFE
RYADDTICHCVSEKQARFLKAVLMKRFEEYGLKLNEEKTKIVYCKDSNRRGDSEHTSFNFLGFTFRPRGARNRKT
GQNFTAFLPAISNKSMKRIKEAVRAWKLNRKTFACLLDISTEVDTQISGWMNYYMKFGRSEFRKVLNYINERLTR
WVMRKYKRFSKGKKFSKAYEWLVEYAAHNRNEFSHWVKGFVPYPRLD
>N221319||gene_389357|GeneMark.hmm|414_aa|+|847|2091
MIETKPYEISKWAVYIAYERVKANKGSYGVDEQSIEDFEKNLKNNLYKIWNRMSSGSYFPQPVKAVSVPKKNGGI
RVLGIPTVEDRIAQMTAKLYFEPCVEPLFLEDSYGYRPGKSAIQALSVTRKRCWHRDWVLEYDIKGLFDNIRHDY
LLEMVRRHTPHKWILLYVERWLTTPFQLEDGTLQSRTSGTPQGGVISLVLANLFLHYAFDSFMAKEYPKAWWE
RYADDGVLHCKSSQQAMYMKSVLRERFRLFGLELNEEKTRIVYCKDADRTEDYSEISLDSLGYTFRPRLARNKHG
NIFLNFLPAMSAKAIKAMKEEVRRWKLQLKVSKSLTDLANILNSQIQGWISYYGHFYKSELIYLLRYINQCLIKWV
RRKYKKFNHRRRAEYWLGRIARRDNNLFAHWRYGVLPTAG
>N221320||gene_44076|GeneMark.hmm|418_aa|+|560|1816
MSEAKQFDISKKAVIAAFQAVKENAGSYGADEQTIKEFEEHLNNNLYKLWNRMASGSYFPKPVRAVAIPKKNG
GIRILGIPTVEDRIAQMVAKMYFEPLVEPMFYNDSYGYRPNKSAIQAVGQARERCFKRDWVLELDIKGLFDNIK
HGYLMYMVEKHTQIKWLILYIKRWLTVPFIMSDGSVAERRSGTPQGGVISPVLANLFLHYVFDDFMTKAYPNI
WWERYADDGVLHCQSYKQAAFIKQKLEERFQQFGLELNKEKTRIVYCKDNRRPQNYSCTQFTFLGYTFRPRLN
KNKEGKFFVGFTPAVSEKAKTAMKQKIREWKIQLKADLSLKDIGNMINKVVQGWINYYTHYYKSEFYEVLRYIN
QCLIKWVRRSYKKKNTRSRAEHWLGAVARRDRNLFAHWKFGILPSVGEGAV
>N221323||gene_180394|GeneMark.hmm|410_aa|+|573|1805
MQRKSFEIPKALVWASYLDVRRNKEAPGCDGQTLKMFDQQRDGNLYKIWNRLCSGTWFPPPVLEKRIPKSNG
KERILGIPTVSDRIAQGAIKLFMEEKLDPIFHVDSYGYRPDKSAHDALKQCAIRCWRYSWILEVDISAFFDHVRHD
LVLKALEHHGMPKWVILYCRRGMEAPMQSCENGELITRTRGTPQGGVISPLLANLFLHYAFDLWMEREYRGVP
FERYADDIVVHCSRMSDATRLKNRLSERFSEVGLVLNAGKTNIAYIDTFKRRNVATSFTFLGYDFKVCTLKNFKGEL

YRKCMPGTSNAAMRKITETIKKWRIHRLTAESLLDFARRYNAIVRGWIEYYGKFWSRNFNYRLWSAMQSRLLK
WMQSKYRLSNRKAQRKLTLVRKEYPKLFVHWYLLRASNE
>N221335||gene_178721|GeneMark.hmm|420_aa|-|6797|8059
MNEAKPFVIDKRLVWEAYHKVKENKGSAGIDKVDQKTFDKEMSKNLYKIWNRMSSGCYFPKAVKLVEIPKSNG
GTRPLGIPTIEDRIAQQVVVSVLTPILEPIFKEDSYGYRPGKGAHQAIAKAKERCYVNPWVLDMDISKFFDTINHD
LLMKAVRKHTEEKWVLLYIERWLKVPYQTSKGEVIERTMGVPQGSVIGPVLANLFLHYVFDEWMSRNYPTIPFE
RYADDTICHCVSEKQAQFLKAVLMKRFEECGLKLNEEKTKIVYCKDSNRRGDSEHTSFDFLGFTFRPRSARNRKT
GQNFTAFLPAISKKSLKRIKEAVRAWKLNRKTFACLLDISNEVDTQISGWMNYYMKFGRSEFRKVLNYINERLTR
WVMRKYKRFSKGKKFSRAYEWLVEYAVHNRNEFSHWAKGFVPYPRLG
>N221335||gene_159575|GeneMark.hmm|418_aa|+|409|1665
MSEAKQFDISKKAVIAAFQAVKENAGSYGVDEQTIKEFEEHLNNNLYKLWNRMASGSYFPKPVRAVEIPKKNG
GTRILGIPTVEDRIAQMVAKMYFEPLVEPMFYNDSYGYRPNKSAIQAVGQARERCFKRDWVLELDIKGLFDNIK
HGYLMYMVEKHTQIKWLILYIKRWLTVPFIMSDGSVAERRSGTPQGGVISPVLANLFLHYVFDDFMTKAYPNI
WWERYADDGVLHCQSYKQAVFIKQKLEERFQQFGLELNKEKTRIVYCKDDRRSRNYSCTQFTFLGYTFRPRLNK
NKEGKFFVGFTPAVSEKAKTAMKQKIRGWKIQLKADLSLKDIGNMINKVVQGWINYYTHYYKSEFYEVLRYINQ
CLIKWVRRSYKKKNTRSRAEHWLGAVARRDRNLFAHWKFGILPSVGEGAV
>N221339||gene_429150|GeneMark.hmm|418_aa|+|2740|3996
MSEAKQFDISKKAVIAAFQAVKENAGSYGVDEQTIKEFEEHLNNNLYKLWNRMASGSYFPKPVRAVAIPKKNG
GTRILGIPTVEDRIAQMVAKMYFEPLVEPMFYKDSYGYRPNKSAIQAVGQARERCFKRDWVLELDIKGLFDNIK
HGYLMYMVEKHTQIKWLILYIKRWLTVPFIMSDGSVAERRSGTPQGGVISPVLANLFLHYVFDDFMTKAYPNI
WWERYADDGVLHCQSYKQAVFIKQKLEERFQQFGLELNKEKTRIVYCKDNRRSQNYSCTQFTFLGYTFRPRLNK
NKEGKFFVGFTPAVSEKAKTAMKQKIREWKIQLKADLSLKDIGNMINKVVQGWINYYTHYYKSEFYEVLRYINQ
CLIKWVRRSYKKKNTRSRAEHWLGAVARRDRNLFAHWKFGILPSVGEGAV
>N221339||gene_461640|GeneMark.hmm|416_aa|-|26315|27565
MTKTKAFNIDKSLVVSAYRRVKTSAGAAGIDKQSLADFDKRLVDNLYKIWNRLSSGSYFPPAVKAVAIPKKLGGER
ILGIPTVSDRIAQTVVKLAFEPQVEPHFLADSYGYRPNKSALDAIGVTRKRCWYYDWVLEFDIKGLFDNIPHELIM
KAVDKHNPARWVKLYIQRWLTAPMVMSDGEVRARTMGTPQGGVISPLLANLFMHYVFDKWLAKYYPKVPW
YRYADDGILHCHSEAEATEMREVLRKRFSECGLEMHPEKTRVIYCKDGSRKGDYEHTMFDFLGYTFRRRVVKNV
KRNSLFVSFTPAASKSALKAMRREIKATGIRKRVDLSIEQIAKWINPKLNGWINYYGRYTCSELYSVFRYINKALVR
WGRKKYKMLSRYKTRASKFLEEMAKRSPQLFAHWRLKMRGGLV
>N221356||gene_133641|GeneMark.hmm|421_aa|+|17904|19169
MQEAKPFQIDKRIIFEAFKKVKSNGGSPGIDGIEMSAYEQNLGSNFYRLWNRMSSGSYMPKAVKLVEILKSNGG
KRPLGIPSVEDRIAQMAVVNVIEPLVEPYFHKDSFGYRPHRSAHDAIAKAERRCWKYAWVLDIDISKFFDTIDHG
LLMKAVEKHIKTKWILLYIKRWLTVPYQGNDGAIVKRHMGVPQGSVIGPILANQFLHYTFDKWMSYKYPHVPF
ERYADDCVCHCGTLAQAEYIKDRLGERFAECKLTFNEEKTKIVFCKTSNRSSEHYHCTSFDYLGFTFRPRAAKDKR
KNVLFTSYLPAISNKSESRIHETIKSWNLKRLHNRSLRFVAAYINDVVRGWISYYGKFGKTEFWKVMCHLNRSIAY
WAKTKYKRLRRRGVISAHYWLAYIAQKEPNLFYHWQVGYIPYARQKK
>N221358||gene_110241|GeneMark.hmm|430_aa|-|2196|3488
MQNDNAKPISISKQLVYDAFLRVKANRGSAGIDKVTLEDYEKNLRGNLYKLWNRMSSGSYFPPSVKLVEIPKSTG
GKRPLGIPTVSDRVAQMAVVMLITPSIEPCFHEDSYAYRPHRSAHDAVGKARERCWKYAWVLDMDISKFFDTI
DHELLLKALKRHTQEKWVLMYIERWLKVPYEKSDGSQVDRALGVPQGSVIGPVLANLFLHYTFDKWMEKNFP
RVPFERYADDTICHCHSLKQAEYMQAMIQQRFECCRLRLNEEKTKIVYCKSSRQKECYPNVTFDFLGFTFQPRES
VDKYGNRFTGFLPAISRKSMKRINETMRSWHLNRHSNLTLEHLASDINPIVRGWMTYYGKFYPTRLKWFMQTL
NGRLARWVMCKFERYRHRFYPAQEWLARIAEKEGLIFYHWKCGALPRFTNKEKVSSQLIMVK

>N221358||gene_51709|GeneMark.hmm|413_aa|+|717|1958
MSESKQYEIPKRVVVEAYKRVKANKGSAGIDGIDFDIFEKKLNNNLYKIWNRMSSGSYFPSPVLAVEIPKKAGGT
RRLGIPTIADRIAQMIARMYIEPAVEPMFCEDSYGYRPNKSAIEAIAVTRKRCWRYDYVIELDVKGLFDNINHELL
MRVVEKHVKESWICLYVKRWMETPFVTKERAAIERKSGTPQGGVISPVLANMFLHYVFDMWMKRKFPQAPF
ERYADDGIVHCRTKEEAICIRQSLAKRFEECKLELHPTKTRIVYCKDEDRRKEEELTEFDFLGYTFKARYIKCRDGKL
RYNFIASVSKVSAKAFRTKVKEMELHRRTGCKIDILAEMLNPMVRGWMNYFGKYNPSAMKDTLLCIERRLVKW
AMCKYKKFRGRRRKAEEWLCTLRKREPKLFAHWSMIYSYC
>N221360||gene_70603|GeneMark.hmm|430_aa|+|16969|18261
MQNDNAKPISISKQLVYDAFLRVKANRGSAGIDKVTLEDYEKNLRGNLYKLWNRMSSGSYFPPSVKLVEIPKSTG
GKRPLGIPTVSDRVAQMAVVMLITPSIEPCFHEDSYAYRPHRSAHDAVGKARERCWKYAWVLDMDISKFFDTI
DHELLLKALKRHTQEKWVLMYIERWLKVPYEKSDGSQVDRALGVPQGSVIGPVLANLFLHYTFDKWMEKNFP
RVPFERYADDTICHCHSLKQAEYMQAMIQQRFECCRLRLNEEKTKIVYCKSSRQKECYPNVTFDFLGFTFQPRES
VDKYGNRFTGFLPAISRKSMKRINETMRSWHLNRHSNLTLEHLASDINPIVRGWMTYYGKFYPTRLKWFMQTL
NGRLARWVMCKFERYRHRFYPAQEWLARIAEKEGLIFYHWKCGALPRFTNKEKVSSQLIMVK
>N221361||gene_20613|GeneMark.hmm|430_aa|+|16970|18262
MQNDNAKPISISKQLVYDAFLRVKANRGSAGIDKVTLEDYEKNLRGNLYKLWNRMSSGSYFPPSVKLVEIPKSTG
GKRPLGIPTVSDRVAQMAVVMLITPSIEPCFHEDSYAYRPHRSAHDAVGKARERCWKYAWVLDMDISKFFDTI
DHELLLKALKRHTQEKWVLMYIERWLKVPYEKSDGSQVDRALGVPQGSVIGPVLANLFLHYTFDKWMEKNFP
RVPFERYADDTICHCHSLKQAEYMQAMIQQRFECCRLRLNEEKTKIVYCKSSRQKECYPNVTFDFLGFTFQPRES
VDKYGNRFTGFLPAISRKSMKRINETMRSWHLNRHSNLTLEHLASDINPIVRGWMTYYGKFYPTRLKWFMQTL
NGRLARWVMCKFERYRHRFYPAQEWLARIAEKEGLIFYHWKCGALPRFTNKEKVSSQLIMVK
>N221361||gene_3117|GeneMark.hmm|418_aa|-|86|1342
MSEAKQFDISKKAVIAAFQAVKENAGSYGADEQTIKEFEEHLNNNLYKLWNRMASGSYFPKPVRAVAIPKKNG
GIRILGIPTVEDRIAQMVAKMYFEPLVEPMFYNDSYGYRPNKSAIQAVGQARERCFKRDWVLELDIKGLFDNIK
HGYLMYMVEKHTQIKWLILYIKRWLTVPFIMSDGSVAERRSGTPQGGVISPVLANLFLHYVFDDFMTKAYPNI
WWERYADDGVLHCQSYKQAAFIKQKLEERFQQFGLELNKEKTRIVYCKDNRRPQNYSCTQFTFLGYTFRPRLN
KNKEGKFFVGFTPAVSEKAKTAMKQKIREWKIQLKADLSLKDIGNMINKVVQGWINYYTHYYKSEFYEVLRYIN
QCLIKWVRRSYKKKNTRSRAEHWLGAVARRDRNLFAHWKFGILPSVGEGAV
>N221361||gene_116981|GeneMark.hmm|413_aa|+|34668|35909
MSESKQYEIPKKVVIEAYKRVKANKGSAGIDGIDFERFEKKLNNNLYKIWNRMSSGSYFPSPVLSVEISKKAGGTR
RLGIPTITDRIAQMVARMYVEPVVEPMFCNDSYGYRPNKSAIDAIATARKRCWRYDYVIELDVKGLFDNINHELL
MRVVLKHVKEEWICLYIKRWLETPFITREGQVIERLSGTPQGGVISPVLANMYLHYVFDMWMKRNFPQAPFE
RYADDGVIHCRTKEEAFVIKKKLAARFAECKLELHPVKTRIVYCKDKDRTRNEELTEFDFLGYTFKAVYIMCKDGK
VRYNFIASVSKTSSKSFRDKIKAMEVHKRTGCKIDIIAEILNPLIRGWMNYFGKFNPSAMKGTLQCIDRRLVKWA
MCKYKNFRGKRGRAEKWLCTVRQREPKLFAHWSNLYSYC
>N221363||gene_7722|GeneMark.hmm|430_aa|-|67823|69115
MQNDNAKPISISKQLVYDAFLRVKANRGSAGIDKVTLEDYEKNLRGNLYKLWNRMSSGSYFPPSVKLVEIPKSTG
GKRPLGIPTVSDRVAQMAVVMLITPSIEPCFHEDSYAYRPHRSAHDAVGKARERCWKYAWVLDMDISKFFDTI
DHELLLKALKRHTQEKWVLMYIERWLKVPYEKSDGSQVDRALGVPQGSVIGPVLANLFLHYTFDKWMEKNFP
RVPFERYADDTICHCHSLKQAEYMQAMIQQRFECCRLRLNEEKTKIVYCKSSRQKECYPNVTFDFLGFTFQPRES
VDKYGNRFTGFLPAISRKSMKRINETMRSWHLNRHSNLTLEHLASDINPIVRGWMTYYGKFYPTRLKWFMQTL
NGRLARWVMCKFERYRHRFYPAQEWLARIAEKEGLIFYHWKCGALPRFTNKEKVSSQLIMVK
>N221366||gene_270507|GeneMark.hmm|430_aa|-|119438|120730
MQNDNAKPISISKQLVYDAFLRVKANRGSAGIDKVTLEDYEKNLRGNLYKLWNRMSSGSYFPPSVKLVEIPKSTG

GKRPLGIPTVSDRVAQMAVVMLITPSIEPCFHEDSYAYRPHRSAHDAVGKARERCWKYAWVLDMDISKFFDTI
DHELLLKALKRHTQEKWVLMYIERWLKVPYEKSDGSQVDRALGVPQGSVIGPVLANLFLHYTFDKWMEKNFP
RVPFERYADDTICHCHSLKQAEYMQAMIQQRFECCRLRLNEEKTKIVYCKSSRQKECYPNVTFDFLGFTFQPRES
VDKYGNRFTGFLPAISRKSMKRINETMRSWHLNRHSNLTLEHLASDINPIVRGWMTYYGKFYPTRLKWFMQTL
NGRLARWVMCKFERYRHRFYPAQEWLARIAEKEGLIFYHWKCGALPRFTNKEKVSSQLIMVK
>N221373||gene_46398|GeneMark.hmm|421_aa|+|25738|27003
MQEAKPFQIDKRIIFEAFKKVKSNGGSPGIDGIEMSAYEQNLGSNFYRLWNRMSSGSYMPKAVKLVEILKSNGG
KRPLGIPSVEDRIAQMAVVNVIEPLVEPYFHKDSFGYRPHRSAHDAIAKAERRCWKYAWVLDIDISKFFDTIDHG
LLMKAVEKHIKTKWILLYIKRWLTVPYQGNDGAIVKRHMGVPQGSVIGPILANQFLHYTFDKWMSYKYPHVPF
ERYADDCVCHCGTLAQAEYIKDRLGERFAECKLTFNEEKTKIVFCKTSNRSSEHYHCTSFDYLGFTFRPRAAKDKR
KNVLFTSYLPAISNKSESRIHETIKSWNLKRLHNRSLRFVAAYINDVVRGWISYYGKFGKTEFWKVMCHLNRSIAY
WAKTKYKRLRRRGVISAHYWLAYIAQKEPNLFYHWQVGYIPYARQKK
>N221376||gene_41973|GeneMark.hmm|420_aa|+|1706|2968
MNEAKPFVIDKRLVWEAYHKVKENKGSAGIDKVDQKTFDKEMSKNLYKIWNRMSSGCYFSKAVKLVEIPKSNG
GTRPLGIPTIEYRIAQQVVVSVLTPILEPIFKEDSYGYRPGKGAHQAIAKAKERCYVTPWVLDMDISKFFDTINHEL
LMKAIRKHTEEKWVLLYIERWLKVPNQTSKGEVIERTMGVPQGSVIGPVLANLFLHYVFDEWMSRNYPTIPFER
YADATICHCVSEKQARFLKAVLMKRFEEYGLKLNEEKTKIVYCKDSNRRGDSEHTSFNFLGFTFRPRGARNRKTG
QNFTAFLPAISNKSMKRIKEAIRAWKLNRKTFACLLDISTEVDTQISGWMNYYMKFGRSEFRKVLNYINERLTRW
VMRKYKRFSKGKKFSKAYEWLVEYAAHNRNEFSHWVKGFVSYPRLD
>N221376||gene_244270|GeneMark.hmm|418_aa|-|764|2020
MSEAKQFDISKKAVIAAFQAVKENAGSYGADEQIIKEFEEHLNNNLYKLWNRMASGSYFPKPVRAVAIPKKNGG
IRILGIPTVEDRIAQMVAKMYFEPLVEPMFYNDSYGYRPNKSAIQAVGQARERCFKRDWVLELDIKGLFDNIKH
GYLMYMVEKHTQIKWLILYIKRWLTVPFIMSDGSVAERRSGTPQGGVISPVLANLFLHYVFDDFMTKAYPNIW
WERYADDGVLHCQSYKQAAFIKQKLEERFQQFGLELNKEKTRIVYCKDNRRPQNYSCTQFTFLGYTFRPRLNKN
KEGKFFVGFTPAVSEKAKTAMKQKIREWKIQLKADLSLKDIGNMINKVVQGWINYYTHYYKSEFYEVLRYINQCL
IKWVRRSYKKKNTRSRAEHWLGAVARRDRNLFAHWKFGILPSVGEGAV
>N221395-1||gene_100709|GeneMark.hmm|416_aa|+|953|2203
MTKTKAFNIDKSLVVSAYRRVKTSAGAAGIDKQSLADFDKRLVDNLYKIWNRLSSGSYFPPAVKAVAIPKKLGGER
ILGIPTVSDRIAQTVVKLAFEPQVEPHFLADSYGYRPNKSALDAIGVTRKRCWYYDWVLEFDIKGLFDNIPHELIM
KAVDKHNPARWVKLYIQRWLTAPMVMSDGEVRARTMGTPQGGVISPLLANLFMHYVFDKWLAKYYPKVPW
YRYADDGILHCHSEAEATEMREVLRKRFSECGLEMHPEKTRVIYCKDGSRKGDYEHTMFDFLGYTFRRRVVKNV
KRNSLFVSFTPAASKSALKAMRREIKATGIRKRVDLSIEQIAKWINPKLNGWINYYGRYTCSELYSVFRYINKALVR
WGRKKYKMLSRYKTRASKFLEEMAKRSPQLFAHWRLKMRGGLV
>N221395-2||gene_160235|GeneMark.hmm|416_aa|-|239|1489
MTKTKAFNIDKSLVVSAYRRVKTSAGAAGIDKQSLADFDKRLVDNLYKIWNRLSSGSYFPPAVKAVAIPKKLGGER
ILGIPTVSDRIAQTVVKLAFEPQVEPHFLADSYGYRPNKSALDAIGVTRKRCWYYDWVLEFDIKGLFDNIPHELIM
KAVDKHNPARWVKLYIQRWLTAPMVMSDGEVRARTMGTPQGGVISPLLANLFMHYVFDKWLAKYYPKVPW
YRYADDGILHCHSEAEATEMREVLRKRFSECGLEMHPEKTRVIYCKDGSRKGDYEHTMFDFLGYTFRRRVVKNV
KRNSLFVSFTPAASKSALKAMRREIKATGIRKRVDLSIEQIAKWINPKLNGWINYYGRYTCSELYSVFRYINKALVR
WGRKKYKMLSRYKTRASKFLEEMAKRSPQLFAHWRLKMRGGLV
>N221397||gene_125563|GeneMark.hmm|430_aa|-|132|1424
MQNDNAKPISISKQLVYDAFLRVKANRGSAGIDKVTLEDYEKNLRGNLYKLWNRMSSGSYFPPSVKLVEIPKSTG
GKRPLGIPTVSDRVAQMAVVMLITPSIEPCFHEDSYAYRPHRSAHDAVGKARERCWKYAWVLDMDISKFFDTI
DHELLLKALKRHTQEKWVLMYIERWLKVPYEKSDGSQVDRALGVPQGSVIGPVLANLFLHYTFDKWMEKNFP

RVPFERYADDTICHCHSLKQAEYMQAMIQQRFECCRLRLNEEKTKIVYCKSSRQKECYPNVTFDFLGFTFQPRES
VDKYGNRFTGFLPAISRKSMKRINETMRSWHLNRHSNLTLEHLASDINPIVRGWMTYYGKFYPTRLKWFMQTL
NGRLARWVMCKFERYRHRFYPAQEWLARIAEKEGLIFYHWKCGALPRFTNKEKVSSQLIMVK
>N221397||gene_286747|GeneMark.hmm|420_aa|-|6580|7842
MNEAKPFVIDKRLVWEAYHKVKENKGSAGIDKVDQKTFDKEMSKNLYKIWNRMSSGCYFPKAVKLVEIPKSNG
GTRPLGIPTIEDRIAQQVVVSVLTPILEPIFKEDSYGYRPGKGAHQAIAKAKERCYVNPWVLDMDISKFFDTINHD
LLMKAVRKHTEEKWVLLYIERWLKVPYQTSKGEVIERTMGVPQGSVIGPVLANLFLHYVFDEWMSRNYPTIPFE
RYADDTICHCVSEKQAQFLKAVLMKRFEECGLKLNEEKTKIVYCKDSNRRGDSEHTSFDFLGFTFRPRSARNRKT
GQNFTAFLPAISKKSLKRIKEAVRAWKLNRKTFACLLDISNEVDTQISGWMNYYMKFGRSEFRKVLNYINERLTR
WVMRKYKRFSKGKKFSRAYEWLVEYAVHNRNEFSHWAKGFVPYPRLG
>N221419||gene_48062|GeneMark.hmm|420_aa|+|50044|51306
MNEAKPFVIDKRLVWEAYHKVRENKGSAGIDKVDQKTFDKEMSKNLYKIWNRMSSGCYFPKAVKLVEIPKSNG
GTRPLGIPTIEDRIAQQVVVSVLPPILEPIFKEDSYGYRPGKGAQQAIAKAKERCYVNPWVLDMDISKFFDTINH
DLLMKAVRKHTEEKWVLLYIERWLKVPYQTSKGEVIERTMGVPQGSVIGPVLANLFLHYVFDEWMSRNYPTIP
FERYADDTICHCVSEKQAQFLKAVLMKRFEECGLKLNEEKTKIVYCKDSNRRGDSEHTSFDFLGFTFRPRSARNR
KTGQNFTAFLPAISKKSLKRIKDAVRAWKLNRKTFACLLDISNEVDTQISGWMNYYMKFGRSEFRKVLNYINERL
TRWVMRKYKRFSKGKKFSRAYEWLVEYAVHNRNEFSHWAKGFVLHPRLG
>N22886||gene_284265|GeneMark.hmm|418_aa|+|409|1665
MSEAKQFDISKKAVIAAFQAVKENAGSYGVDEQTIKEFEEHLNNNLYKLWNRMASGSYFPKPVRAVEIPKKNG
GTRILGIPTVEDRIAQMVAKMYFEPLVEPMFYNDSYGYRPNKSAIQAVGQAREKCFKRDWVLELDIKGLFDNIK
HGYLMYMVEKHTQIKWLILYIKRWLTVPFIMSDGSVAERRSGTPQGGVISPVLANLFLHYVFDDFMTKAYPNI
WWERYADDGVLHCQSYKQAVFIKQKLEERFQQFGLELNKEKTRIVYCKDDRRSRNYSCTQFTFLGYTFRPRLNK
NKEGKFFVGFTPAVSEKAKTAMKQKIRGWKIQLKADLSLKDIGNMINKVVQGWINYYTHYYKSEFYEVLRYINQ
CLIKWVRRSYKKKNTRSRAEHWLGAVARRDRNLFAHWKFGILPSVGEGAV
>N22988||gene_176581|GeneMark.hmm|416_aa|+|1160|2410
MTKTKAFNIDKSLVVSAYRRVKTSAGAAGIDKQSLADFDKRLVDNLYKIWNRLSSGSYFPPAVKAVAIPKKLGGER
ILGIPTVSDRIAQTVVKLAFEPQVEPHFLADSYGYRPNKSALDAIGVTRKRCWYYDWVLEFDIKGLFDNIPHELIM
KAVDKHNPARWVKLYIQRWLTAPMVMSDGEVRARTMGTPQGGVISPLLANLFMHYVFDKWLAKYYPKVPW
YRYADDGILHCHSEAEATEMREVLRKRFSECGLEMHPEKTRVIYCKDGSRKGDYEHTMFDFLGYTFRRRVVKNV
KRNSLFVSFTPAASKSALKAMRREIKATGIRKRVDLSIEQIAKWINPKLNGWINYYGRYTCSELYSVFRYINKALVR
WGRKKYKMLSRYKTRASKFLEEMAKRSPQLFAHWRLKMRGGLV
>N22988||gene_83449|GeneMark.hmm|393_aa|+|5075|6256
MSGSDPNVVIKEFEEHLNNNLYKLWNRMASGSYFPKPVRAVAIPKKNGGTRILGIPTVEDRIAQMVAKMYFEP
LVEPMFYKDSYGYRPNKSAIQAVGQARERCFKRDWVLELDIKGLFDNIKHGYLMYMVEKHTQIKWLILYIKRWL
TVPFIMSDGSVAERRSGTPQGGVISPVLANLFLHYVFDDFMTKAYPNIWWERYADDGVLHCQSYKQAVFIKQK
LEERFQQFGLELNKEKTRIVYCKDNRRSQNYSCTQFTFLGYTFRPRLNKNKEGKFFVGFTPAVSEKAKTAMKQKI
REWKIQLKADLSLKDIGNMINKVVQGWINYYTHYYKSEFYEVLRYINQCLIKWVRRSYKKKNTRSRAEHWLGAV
ARRDRNLFAHWKFGILPSVGEGAV
>N22990||gene_3795|GeneMark.hmm|420_aa|-|3763|5025
MNEAKPFVIDKRLVWEAYHKVKENKGSAGIDKVDQRTFDKEMSKNLYKIWNRMSSGCYFPKAVKLVEIPKSNG
GTRPLGIPTIEDRIAQQVVVSVLTPILEPIFKEDSYGYRPGKGAHQAIAKAKERCYVNPWVLDMDISKFFDTINHE
LLMKAIRKHAEEKWVLLYIERWLKVPYQTSKGEVIERTMGVPQGSVIGPVLANLFLHYVFDEWMSRNYPTIPFE
RYADDTICHCVSEKQAQFLKAVLMKRFEECGLKLNEEKTKIVYCKDSNRRGDSEHTSFDFLGFTFRPRGARNRKT
GQNFTAFLPAISKKSMKRIKEAVRAWNLNRKTFVCLLDISNEVDTQISGWMNYYMKFGRSEFRKVLNYINERLT

RWVMRKYKRFSKGKKFSRAYEWLVEHAVHNRNEFSHWAKGFVPYPRLG
>N22990||gene_57839|GeneMark.hmm|420_aa|-|3654|4916
MNEAKPFVIDKRLVWEAYHKVKENKGSAGIDKVDQKTFDKEMSKNLYKIWNRMSSGCYFPKAVKLVEIPKSNG
GTRPLGIPTIEDRIAQQVVVSVLTPILEPIFKEDSYGYRPGKGAHQAIAKAKERCYVNPWVLDMDISKFFDTINHE
LLMKAVRKHTEEKWILLYIERWLKVPYQTSKGEVIERTMGVPQGSVIGPVLANLFLHYVFDEWMSRNYPTIPFE
RYADDTICHCVSEKQAQFLKAVLMKRFEECGLKLNEEKTKIVYCKDSNRRGDSEHTSFDFLGFTFRPRGARNRKT
GQNFTAFLPAISKKSMKRIKEAVRAWKLNRKTFACLLDISNEVDTQISGWMNYYMKFGRSEFRKVLNYINERLT
RWVMRKYKRFSKGRKFNRAYDWLVEYAAHNRNEFSHWVKGFVPYPRLG
>N22R02||gene_179999|GeneMark.hmm|430_aa|-|84|1376
MQNDNAKPISISKQLVYDAFLRVKANRGSAGIDKVTLEDYEKNLRGNLYKLWNRMSSGSYFPPSVKLVEIPKSTG
GKRPLGIPTVSDRVAQMAVVMLITPSIEPCFHEDSYAYRPHRSAHDAVGKARERCWKYAWVLDMDISKFFDTI
DHELLLKALKRHTQEKWVLMYIERWLKVPYEKSDGSQVDRALGVPQGSVIGPVLANLFLHYTFDKWMEKNFP
RVPFERYADDTICHCHSLKQAEYMQAMIQQRFECCRLRLNEEKTKIVYCKSSRQKECYPNVTFDFLGFTFQPRES
VDKYGNRFTGFLPAISRKSMKRINETMRSWHLNRHSNLTLEHLASDINPIVRGWMTYYGKFYPTRLKWFMQTL
NGRLARWVMCKFERYRHRFYPAQEWLARIAEKEGLIFYHWKCGALPRFTNKEKVSSQLIMVK
>N22R03||gene_125131|GeneMark.hmm|421_aa|+|1417|2682
MQEAKPFQIDKRIIFESFKKVKFNRGSSGIDGIEMTTYEQNLGSNLYRLWNRMSSGSYMPKAVKLVEIPKSNGG
KRPLGIPTIEDRIAQMAVVNVIEPLIEPCFHEDSFGYRPHRSAHDAIAKAERRCWKYAWVLDIDISKFFDTIDHGL
LMKAVEKHINIKWILLYIKRWLTVPYQRSDGEIVKRDMGVPQGSVIGPILANLFLHYTFDKWMSYKYPHIPFERY
ADDCVCHCSTLAQAEYIKERLGERFTECKLKFNEEKTKIVFCKMSSRSSKHYHCTSFDYLGFTFRSRAAKDKRNN
VLFTSYLPAISKKSVSRIHETIKSWNLKRLHNRSLRFVAAYINDVVRGWINYYEKFGKTEFWKVMCHLNRSIAYW
AKTKYKRLRRRGVISAHYWLAYIAQKEPNLFYHWQVGYVPYARQKK
>N22R04||gene_236306|GeneMark.hmm|410_aa|-|5476|6708
MQRKSFEIPKALVWASYLDVRRNKEAPGCDGQTLKMFDQQRDGNLYKIWNRLCSGTWFPPPVLEKRIPKSNG
KERILGIPTVSDRIAQGAIKLFMEEKLDPIFHVDSYGYRPDKSAHDALKQCAIRCWRYSWILEVDISAFFDHVRHD
LVLKALEHHGMPKWVILYCRRRMEAPMQSCENGELITRTRGTPQGGVISPLLANLFLHYAFDLWMEREYRGVP
FERYADDIVVHCSRMSDATRLKNRLSERFSEVGLVLNAGKTNIAYIDTFKRRNVATSFTFLGYDFKVCTLKNFKGEL
YRKCMPGTSNAAMRKITETIKKWRIHRLTAESLLDFARRYNAIVRGWIEYYGKFWSRNLNYRLWSAMQSRLLK
WMQSKYRLSNRKAQRKLTLVRKEYPKLFVHWYLLRASNE
>N22R08||gene_156294|GeneMark.hmm|430_aa|-|119438|120730
MQNDNAKPISISKQLVYDAFLRVKANRGSAGIDKVTLEDYEKNLRGNLYKLWNRMSSGSYFPPSVKLVEIPKSTG
GKRPLGIPTVSDRVAQMAVVMLITPSIEPCFHEDSYAYRPHRSAHDAVGKARERCWKYAWVLDMDISKFFDTI
DHELLLKALKRHTQEKWVLMYIERWLKVPYEKSDGSQVDRALGVPQGSVIGPVLANLFLHYTFDKWMEKNFP
RVPFERYADDTICHCHSLKQAEYMQAMIQQRFECCRLRLNEEKTKIVYCKSSRQKECYPNVTFDFLGFTFQPRES
VDKYGNRFTGFLPAISRKSMKRINETMRSWHLNRHSNLTLEHLASDINPIVRGWMTYYGKFYPTRLKWFMQTL
NGRLARWVMCKFERYRHRFYPAQEWLARIAEKEGLIFYHWKCGALPRFTNKEKVSSQLIMVK
>N22R09||gene_127760|GeneMark.hmm|422_aa|-|4425|5693
MKDAKSFEISRHLVMEAYKRVKANKGAAGVDEVSIADFENNLKSNLYKIWNRMSSGSYLPPAVKLVEIPKSNGG
KRPLGIPTVGDRVAQMVVVMTIEPGIEPYFHEDSYAYRPNRSALDAVRKAKERSYTFHWVLDLDIKGFFDNIDH
ELLIKALERHVKCKWAILYIKRWLSVPYQLKDGTQKERTKGVPQGSVVGPILANLFLHYVFDEWMRRNHSNISFE
RYADDTICHCVSLKQAEFILRAIRKRFAECKLELNEDKTKIVYCKKNHRDIPYECIQFDFLGYTFRPRRSIDANGEVF
LNFSPAISKKARTKIWEAIQNWNSNHWVPMELEDIAKEINPVIQGWINYYGQHNPRILKEVLQHVNDRLVRW
GRRKFKGLRKRKTATVHRLGDIALQKPNLFAHWAWGVKPTASERNRKRK
>N22R10||gene_92911|GeneMark.hmm|430_aa|+|20244|21536

MQNDNAKPISISKQLVYDAFLRVKANRGSAGIDKVTLEDYEKNLRGNLYKLWNRMSSGSYFPPSVKLVEIPKSTG
GKRPLGIPTVSDRVAQMAVVMLITPSIEPCFHEDSYAYRPHRSAHDAVGKARERCWKYAWVLDMDISKFFDTI
DHELLLKALKRHTQEKWVLMYIERWLKVPYEKSDGSQVDRALGVPQGSVIGPVLANLFLHYTFDKWMEKNFP
RVPFERYADDTICHCHSLKQAEYMQAMIQQRFECCRLRLNEEKTKIVYCKSSRQKECYPNVTFDFLGFTFQPRES
VDKYGNRFTGFLPAISRKSMKRINETMRSWHLNRHSNLTLEHLASDINPIVRGWMTYYGKFYPTRLKWFMQTL
NGRLARWVMCKFERYRHRFYPAQEWLARIAEKEGLIFYHWKCGALPRFTNKEKVSSQLIMVK
>N22R13||gene_31198|GeneMark.hmm|413_aa|+|609|1850
MSESKQYEIPKRVVVEAYKRVKANKGSAGIDGIDFDIFEKKLNNNLYKIWNRMSSGSYFPSPVLAVEIPKKAGGT
RRLGIPTIADRIAQMIARMYIEPAVEPMFCEDSYGYRPNKSAIEAIAVTRKRCWRYDYVIELDVKGLFDNINHELL
MRVVEKHVKESWICLYVKRWMETPFVTKERAAIERKSGTPQGGVISPVLANMFLHYVFDMWMKRKFPQAPF
ERYADDGIVHCRTKEEAICIRQSLAKRFEECKLELHPTKTRIVYCKDEDRRKEEELTEFDFLGYTFKARYIKCRDGKL
RYNFIASVSKVSAKAFRTKVKEMELHRRTGCKIDILAEMLNPMVRGWMNYFGKYNPSAMKDTLLCIERRLVKW
AMCKYKKFRGRRRKAEEWLCTLRKREPKLFAHWSMIYSYC
>N22R13||gene_142468|GeneMark.hmm|422_aa|+|13063|14331
MMQHQVTKPFTIDKHLIMNAWKRVKENKGSVGIDNVSTDDYESNLGKNLYKLWNRMSSGSYFPEAVKLVDIP
KSSGGTRPLGIPTVGDRIAQMSVVLLIEDRLEAIFHADSYGYRPNRSAHDAIGKARERCWHYNWVLDMDISKFF
DTINHDLLMKAVERHVQEKWILLYIRRWLEVPYATLTGERIERRMGVPQGSVIGPVLANLYLHYTFDKWMSLYH
PTIPFERYADDTICHCNSLEEAQMLKASIVERFAACKLKLNEEKTRIVYCKDGKRRREYKDITFDFLGYTFQPRGQR
NKQGQVFNGYAPAISRKSKKRIAETMRGWHLNRRVQLKLSDIAVEINAEVRGWMNYYGKFYGSQLKAFLQCIN
LKLARWAERKYKRFRRKPNDAYKWLVRVASKNPALFYHWQHGVKPNRLKPFG
>N22R15||gene_366123|GeneMark.hmm|418_aa|-|5683|6939
MSEAKQFDISKKAVIAAFQAVKENAGSYGADEQTIKEFEEHLNNNLYKLWNRMASGSYFPKPVRAVAIPKKNG
GIRILGIPTVEDRIAQMVAKMYFEPLVEPMFYNDSYGYRPNKSAIQAVGQARERCFKRDWVLELDIKGLFDNIK
HGYLMYMVEKHTQIKWLILYIKRWLTVPFIMSDGSVAERRSGTPQGGVISPVLANLFLHYVFDDFMTKAYPNI
WWERYADDGVLHCQSYKQAAFIKQKLEERFQQFGLELNKEKTRIVYCKDNRRPQNYSCTQFTFLGYTFRPRLN
KNKEGKFFVGFTPAVSEKAKTAMKQKIREWKIQLKADLSLKDIGNMINKVVQGWINYYTHYYKSEFYEVLRYIN
QCLIKWVRRSYKKKNTRSRAEHWLGAVARRDRNLFAHWKFGILPSVGEGAV
>N22R15||gene_113853|GeneMark.hmm|422_aa|+|627|1895
MKDAKSFEISRHLVMEAYKRVKANKGAAGVDEVSIADFENNLKSNLYKIWNRMSSGSYLPPAVKLVEIPKSNGG
KRPLGIPTVGDRVAQMVVVMTIEPGIEPYFHEDSYAYRPNRSALDAVRKAKERSYTFHWVLDLDIKGFFDNIDH
ELLIKALERHVKCKWAILYIKRWLSVPYQLKDGTQKERTKGVPQGSVVGPILANLFLHYVFDEWMRRNHSNISFE
RYADDTICHCVSLKQAEFILRAIRKRFAECKLELNEDKTKIVYCKKNHRDIPYECIQFDFLGYTFRPRRSIDANGEVF
LNFSPAISKKARTKIWEAIQNWNSNHWVPMELEDIAKEINPVIQGWINYYGQHNPRILKEVLQHVNDRLVRW
GRRKFKGLRKRKTATVHRLGDIALQKPNLFAHWAWGVKPTASERNRKRK
>N22R16||gene_11842|GeneMark.hmm|409_aa|+|843|2072
MKESKQYNISKKVVLEAYKKVKFNRGSAGVDGVDFEKFEENLKDNLYKIWNRMSSGSYFPSPVLAVEIPKKNGG
TRTLGIPTISDRIAQMIARMYLEPKVEPIFHKDSYGYRPNKSAIDAVGKVRERCWRYDYVIEFDIKGLFDNIDHELL
MKAVELHTEEKWMKLYIRRWLTAPFVTKKGRVIERNSGTPQGGVISPVLANIFLHYAFDIWMETNYSMAPFAR
YADDAVIHCKSEKQAKEIKESLTLRMKQCKLELHPDKTRIIYCKDKDRTKDYPITQFDFLGYTYRAVYIKCRDGKLR
NNFIASASKKACKSLRNKIKDMELHKMTGSNINIIAKIINPIVRGWINYFNKYNPSAIKYTIECIQRRIVRWAMCK
YKHLRGRRQRAEKWLLEVKTREPKLFAHWSYR
>N22R19||gene_406061|GeneMark.hmm|420_aa|-|1984|3246
MNEAKPFVIDKRLVWEAYHKVKENKGSAGIDKVDQKTFDKEMSKNLYKIWNRMSSGCYFPKAVKLVEIPKSNG
GTRPLGIPTIEDRIAQQVVVSVLTPILEPIFKEDSYGYRPGKGAHQAIAKAKERCYVNPWVLDMDISKFFDTINHD

LLMKAVRKHTEEKWVLLYIERWLKVPYQTSKGEVIERTMGVPKGAVIGPVLANLFLHYVFDEWMSRNYPTIPFE
RYADDTICHCVSEKQAQFLKAVLMKRFEECGLKLNEEKTKIVYCKDSNRRGDSEHTSFDFLGFTFRPRSARNRKT
GQNFTAFLPAISKKSLKRIKEAVRAWKLNRKTFACLLDISNEVDTQISGWMNYYMKFGRSEFRKVLNYINERLTR
WVMRKYKRFSKGKKFSRAYEWLVEYAVHNRNEFSHWAKGFVPYPRLG
>N22R27||gene_94318|GeneMark.hmm|420_aa|+|804|2066
MNEAKPFVIDKRLVWEAYHKVKENKGSAGIDKVDQKTFDKEMSKNLYKIWNRMSSGCYFPKAVKLVEIPKSNG
GTRPLGIPTIEDRIAQQVVVSVLTPILEPIFKEDSYGYRPGKGAHQAIAKAKERCYVNPWVLDMDISKFFDTINHE
LLMKAIRKHTEEKWVLLYIERWLKVPYQTSKGEVIERTMGVPQGSVIGPVLANLFLHYVFDEWMSRNYPTIPFE
RYADDTICHCVSEKQAQFLKAVLMKRFEECGLKLNEEKTKIVYCKDSNRRGDSEHTSFDFLGFTFRPRGARNRKT
GQNFTAFLPAISKKSMKRIKEAVRAWKLNRKTFACLLDISNEVDTQISGWMNYYMKFGRSEFRKVLNYINERLT
RWVMRKYKRFSKGKKFSKAYDWLVEYAAHNRNEFSHWVKGFVPYPRLG
>N22R28||gene_196660|GeneMark.hmm|418_aa|+|3412|4668
MSEAKQFDISKKAVIAAFQAVKENAGSYGADEQTIKEFEEHLNNNLYKLWNRMASGSYFPKPVRAVAIPKKNG
GIRILGIPTVEDRIAQMVAKMYFEPLVEPMFYNDSYGYRPNKSAIQAVGQARERCFKRDWVLELDIKGLFDNIK
HGYLMYMVEKHTQIKWLILYIKRWLTVPFIMSDGSVAERRSGTPQGGVISPVLANLFLHYVFDDFMTKAYPNI
WWERYADDGVLHCQSYKQAAFIKQKLEERFQQFGLELNKEKTRIVYCKDNRRPQNYSCTQFTFLGYTFRPRLN
KNKEGKFFVGFTPAVSEKAKTAMKQKIREWKIQLKADLSLKDIGNMINKVVQGWINYYTHYYKSEFYEVLRYIN
QCLIKWVRRSYKKKNTRSRAEHWLGAVARRDRNLFAHWKFGILPSVGEGAV
>N22R29||gene_155834|GeneMark.hmm|410_aa|-|1062|2294
MQRKSFEIPKALVWASYLDVRRNKGAPGCDGQTLKMFDQQRDGNLYKIWNRLCSGTWFPPPVLEKRIPKPN
GKERILGIPTVSDRIAQGAIKLFMEEKLDPIFHADSYGYRPGKSAHDALKQCAIRCWRYSWILEVDISAFFDHVRH
DLVLKALEHHGMPKWVILYCRRWMEAPMQSCENGELITRTRGTPQGGVISPLLANLFLHYAFDLWMEREYRG
VPFERYADDIVVHCSRMSDATRLKNRLSERFSEVGLVLNAGKTNIAYIDTFKRRNVATSFTFLGYDFKVRTLKNFK
GELYRKCMPGASNAAMRKITETIKKWRIHRSTAESLLDFARRYNAIVRGWIEYYGKFWSRNFNYRLWSAMQSR
LLKWMQSKYRLSNRRAQRKLTLVRKEYPKLFVHWYLLRASNE
>N22R29||gene_30601|GeneMark.hmm|440_aa|+|1216|2538
MNVERRGSVGQSASQPNCKQEEAAGEQTKPFQVSKLHVVEAYRRVKANAGAAGVDNQTLKDFERDLKGNLY
KIWNRLSSGSWMPPPVRAVEIPKKDGSKRLLGIPTVSDRIAQMTVLVTFEPLVERYFLNDSYGYRHGKSALDAIA
VTRKRCWQYDWYLEFDIKGLFDNIPHDLLLRAVDKHCADKWVRLSIRRWLTAPVQMPDGTLKERNKGTPQGG
VISPVLANLFLHYVFDKWLSLLYPEIPWCRYADDGLIHCGSKQQAEELLNKLAKPFQECGLELHPEKTKIVYCKDS
ERQANHETVQFNFLGYTFRARRARNQRRGNLFTSFSLAVSNSAQKDMIGKLRKLRLRRRVEMSLEDIAKRLNP
MISGWLNYYAKYYKSAMKKVCRYINLTLIAWARKKYKTLRYKKTKACQLMERLSKEKLELFAHWKAGPGSAFA
>N22R29||gene_145941|GeneMark.hmm|418_aa|+|3147|4403
MSEAKQFDISKKAVIAAFQAVKENAGSYGVDEQTIKEFEEHLNNNLYKLWNRMASGSYFPKPVRAVEIPKKNG
DTRILGIPTVEDRIAQMVAKMYFEPLVEPMFYNDSYGYRPNKSAIQAVGQARERCFKRDWVLELDIKGLFDNIK
HGYLMYMVEKHTQIKWLILYIKRWLTVPFIMSDGSVAERRSGTPQGGVISPVLANLFLHYVFDDFMTKAYPNI
WWERYADDGVLHCQSYKQAAFIKQKLEERFQQFGLELNKEKTRIVYCKDNRRPQNYSCTQFTFLGYTFRPRLN
KNKEGKFFVGFTPAVSEKAKTAMKQKIREWKIQLKADLSLKDIGNMINKVVQGWINYYTHYYKSEFYEVLRYIN
QCLIKWVRRSYKKKNTRSRAEHWLGAVARRDRNLFAHWKFGILPSVGEGAV
>N22R32||gene_159375|GeneMark.hmm|418_aa|+|1373|2629
MSEAKQFDISKKAVIAAFQAVKENAGSYGADEQTIKEFEEHLNNNLYKLWNRMASGSYFPKPVRAVAIPKKNG
GIRILGIPTVEDRIAQMVAKMYFEPLVEPMFYNDSYGYRPNKSAIQAVGQARERCFKRDWVLELDIKGLFDNIK
HGYLMYMVEKHTQIKWLILYIKRWLTVPFIMSDGSVAERRSGTPQGGVISPVLANLFLHYVFDDFMTKAYPNI
WWERYADDGVLHCQSYKQAAFIKQKLEERFQQFGLELNKEKTRIVYCKDNRRPQNYSCTQFTFLGYTFRPRLN

KNKEGKFFVGFTPAVSEKAKTAMKQKIREWKIQLKADLSLKDIGNMINKVVQGWINYYTHYYKSEFYEVLRYIN
QCLIKWVRRSYKKKNTRSRAEHWLGAVARRDRNLFAHWKFGILPSVGEGAV
>N22R32||gene_52428|GeneMark.hmm|430_aa|+|477|1769
MQNDNAKPISISKQLVYDAFLRVKANRGSAGIDKVTLEDYEKNLRGNLYKLWNRMSSGSYFPPSVKLVEIPKSTG
GKRPLGIPTVSDRVAQMAVVMLITPSIEPCFHEDSYAYRPHRSAHDAVGKARERCWKYAWVLDMDISKFFDTI
DHELLLKALKRHTQEKWVLMYIERWLKVPYEKSDGSQVDRALGVPQGSVIGPVLANLFLHYTFDKWMEKNFP
RVPFERYADDTICHCHSLKQAEYMQAMIQQRFECCRLRLNEEKTKIVYCKSSRQKECYPNVTFDFLGFTFQPRES
VDKYGNRFTGFLPAISRKSMKRINETMRSWHLNRHSNLTLEHLASDINPIVRGWMTYYGKFYPTRLKWFMQTL
NGRLARWVMCKFERYRHRFYPAQEWLARIAEKEGLIFYHWKCGALPRFTNKEKVSSQLIMVK
>N22R33||gene_39109|GeneMark.hmm|416_aa|-|956|2206
MTKTKAFNIDKSLVVSAYRRVKTSAGAAGIDKQSLADFDKRLVDNLYKIWNRLSSGSYFPPAVKAVAIPKKLGGER
ILGIPTVSDRIAQTVVKLAFEPQVEPHFLADSYGYRPNKSALDAIGVTRKRCWYYDWVLEFDIKGLFDNIPHELIM
KAVDKHNPARWVKLYIQRWLTAPMVMSDGEVRARTMGTPQGGVISPLLANLFMHYVFDKWLAKYYPKVPW
YRYADDGILHCHSEAEATEMREVLRKRFSECGLEMHPEKTRVIYCKDGSRKGDYEHTMFDFLGYTFRRRVVKNV
KRNSLFVSFTPAASKSALKAMRREIKATGIRKRVDLSIEQIAKWINPKLNGWINYYGRYTCSELYSVFRYINKALVR
WGRKKYKMLSRYKTRASKFLEEMAKRSPQLFAHWRLKMRGGLV
>N22R34||gene_116522|GeneMark.hmm|418_aa|+|665|1921
MSEAKQFDISKKAVIAAFQAVKENAGSYGADEQTIKEFEEHLNNNLYKLWNRMASGSYFPKPVRAVAIPKKNG
GIRILGIPTVEDRIAQMVAKMYFEPLVEPMFYNDSYGYRPNKSAIQAVGQARERCFKRDWVLELDIKGLFDNIK
HGYLMYMVEKHTQIKWLILYIKRWLTVPFIMSDGSVAERRSGTPQGGVISPVLANLFLHYVFDDFMTKAYPNI
WWERYADDGVLHCQSYKQAAFIKQKLEERFQQFGLELNKEKTRIVYCKDNRRPQNYSCTQFTFLGYTFRPRLN
KNKEGKFFVGFTPAVSEKAKTAMKQKIREWKIQLKADLSLKDIGNMINKVVQGWINYYTHYYKSEFYEVLRYIN
QCLIKWVRRSYKKKNTRSRAEHWLGAVARRDRNLFAHWKFGILPSVGEGAV
>N22R34||gene_38537|GeneMark.hmm|413_aa|+|31799|33040
MSESKQYEIPKKVVIEAYKRVKANKGSAGIDGIDFERFEKKLNNNLYKIWNRMSSGSYFPSPVLSVEISKKAGGTR
RLGIPTITDRIAQMVARMYVEPVVEPMFCNDSYGYRPNKSAIDAIATARKRCWRYDYVIELDVKGLFDNINHELL
MRVVLKHVKEEWICLYIKRWLETPFITREGQVIERLSGTPQGGVISPVLANMYLHYVFDMWMKRNFPQAPFE
RYADDGVIHCRTKEEAFVIKKKLAARFAECKLELHPVKTRIVYCKDKDRTRNEELTEFDFLGYTFKAVYIMCKDGK
VRYNFIASVSKTSSKSFRDKIKAMEVHKRTGCKIDIIAEILNPLIRGWMNYFGKFNPSAMKGTLQCIDRRLVKWA
MCKYKNFRGKRGRAEKWLCTVRQREPKLFAHWSNLYSYC
>N22R37||gene_292041|GeneMark.hmm|440_aa|+|488|1810
MNVERRGSVGQSASQPNCKQEEAAGEQTKPFQVSKLHVVEAYRRVKANAGAAGVDNQTLKDFERDLKGNLY
KIWNRLSSGSWMPPPVRAVEIPKKDGSKRLLGIPTVSDRIAQMTVLVTFEPLVERYFLNDSYGYRHGKSALDAIA
VTRKRCWQYDWYLEFDIKGLFDNIPHDLLLRAVDKHCADKWVRLSIRRWLTAPVQMPDGTLKERNKGTPQGG
VISPVLANLFLHYVFDKWLSLLYPEIPWCRYADDGLIHCGSKQQAEELLNKLAKPFQECGLELHPEKTKIVYCKDS
ERQANHETVQFNFLGYTFRARRARNQRRGNLFTSFSLAVSNSAQKDMIGKLRKLRLRRRVEMSLEDIAKRLNP
MISGWLNYYAKYYKSAMKKVCRYINLTLIAWARKKYKTLRYKKTKACQLMERLSKEKPELFAHWKAGPGSAFA
>R10H001||gene_180274|GeneMark.hmm|418_aa|-|514|1770
MSEAKQFDISKKAVIAAFQAVKENAGSYGADEQTIKEFEEHLNNNLYKLWNRMASGSYFPKPVRAVAIPKKNG
GIRILGIPTVEDRIAQMVAKMYFEPLVEPMFYNDSYGYRPNKSAIQAVGQARERCFKRDWVLELDIKGLFDNIK
HGYLMYMVEKHTQIKWLILYIKRWLTVPFIMSDGSVAERRSGTPQGGVISPVLANLFLHYVFDDFMTKAYPNI
WWERYADDGVLHCQSYKQAAFIKQKLEERFQQFGLELNKEKTRIVYCKDNRRPQNYSCTQFTFLGYTFRPRLN
KNKEGKFFVGFTPAVSEKAKTAMKQKIREWKIQLKADLSLKDIGNMINKVVQGWINYYTHYYKSEFYEVLRYIN
QCLIKWVRRSYKKKNTRSRAEHWLGAVARRDRNLFAHWKFGILPSVGEGAV

>R10H006||gene_71375|GeneMark.hmm|416_aa|-|71157|72407
MTKTKAFNIDKSLVVSAYRRVKTSAGAAGIDKQSLADFDKRLVDNLYKIWNRLSSGSYFPPAVKAVAIPKKLGGER
ILGIPTVSDRIAQTVVKLAFEPQVEPHFLADSYGYRPNKSALDAIGVTRKRCWYYDWVLEFDIKGLFDNIPHELIM
KAVDKHNPARWVKLYIQRWLTAPMVMSDGEVRARTMGTPQGGVISPLLANLFMHYVFDKWLAKYYPKVPW
YRYADDGILHCHSEAEATEMREVLRKRFSECGLEMHPEKTRVIYCKDGSRKGDYEHTMFDFLGYTFRRRVVKNV
KRNSLFVSFTPAASKSALKAMRREIKATGIRKRVDLSIEQIAKWINPKLNGWINYYGRYTCSELYSVFRYINKALVR
WGRKKYKMLSRYKTRASKFLEEMAKRSPQLFAHWRLKMRGGLV
>R10H010||gene_32241|GeneMark.hmm|420_aa|+|14361|15623
MNEAKPFVIDKRLVWEAYHKVKENKGSAGIDKVDQKTFDKEMSKNLYKIWNRMSSGCYFPKAVKLVEIPKSNG
GTRPLGIPTIEDRIAQQVVVSVLTPILEPIFKEDSYGYRPGKGAHQAIAKAKERCYVNPWVLDMDISKFFDTINHD
LLMKAVRKHTEEKWVLLYIERWLKVPYQTSKGEVIERTMGVPQGSVIGPVLANLFLHYVFDEWMSRNYPTIPFE
RYADDTICHCVSEKQAQFLKAVLMKRFEECGLKLNEEKTKIVYCKDSNRRGDSEHTSFDFLGFTFRPRSARNRKT
GQNFTAFLPAISKKSLKRIKEAVRAWKLNRKTFACLLDISNEVDTQISGWMNYYMKFGRSEFRKVLNYINERLTR
WVMRKYKRFSKGKKFSRAYEWLVEYAVHNRNEFSHWAKGFVPYPRLG
>R10H010||gene_57816|GeneMark.hmm|418_aa|-|68|1324
MSEAKQFDISKKAVIAAFQAVKENAGSYGADEQTIKEFEEHLNNNLYKLWNRMASGSYFPKPVRAVAIPKKNG
GIRILGIPTVEDRIAQMVAKMYFEPLVEPMFYNDSYGYRPNKSAIQAVGQARERCFKRDWVLELDIKGLFDNIK
HGYLMYMVEKHTQIKWLILYIKRWLTVPFIMSDGSVAERRSGTPQGGVISPVLANLFLHYVFDDFMTKAYPNI
WWERYADDGVLHCQSYKQAAFIKQKLEERFQQFGLELNKEKTRIVYCKDNRRPQNYSCTQFTFLGYTFRPRLN
KNKEGKFFVGFTPAVSEKAKTAMKQKIREWKIQLKADLSLKDIGNMINKVVQGWINYYTHYYKSEFYEVLRYIN
QCLIKWVRRSYKKKNTRSRAEHWLGAVARRDRNLFAHWKFGILPSVGEGAV
>R10H016||gene_233630|GeneMark.hmm|421_aa|-|24338|25603
MQEAKPFQIDKRIIFESFKKVKFNRGSSGIDGIEMTTYEQNLGSNLYRLWNRMSSGSYMPKAVKLVEIPKSNGG
KRPLGIPTIEDRIAQMAVVNVIEPLIEPCFHEDSFGYRPHRSAHDAIAKAERRCWKYAWVLDIDISKFFDTIDHGL
LMKAVEKHINIKWILLYIKRWLTVPYQRSDGEIVKRDMGVPQGSVIGPILANLFLHYTFDKWMSYKYPHIPFERY
ADDCVCHCSTLAQAEYIKERLGERFTECKLKFNEEKTKIVFCKMSSRSSKHYHCTSFDYLGFTFRSRAAKDKRNN
VLFTSYLPAISKKSVSRIHETIKSWNLKRLHNRSLRFVAAYINDVVRGWINYYEKFGKTEFWKVMCHLNRSIAYW
AKTKYKRLRRRGVISAHYWLAYIAQKEPNLFYHWQVGYVPYARQKK
>R10H017||gene_26645|GeneMark.hmm|421_aa|+|342|1607
MQEAKPFQIDKRIIFEAFKKVKSNGGSPGIDGIEMSAYEQNLGSNFYRLWNRMSSGSYMPKAVKLVEILKSNGG
KRPLGIPSVEDRIAQMAVVNVIEPLVEPYFHKDSFGYRPHRSAHDAIAKAERRCWKYAWVLDIDISKFFDTIDHG
LLMKAVEKHIKTKWILLYIKRWLTVPYQGNDGAIVKRHMGVPQGSVIGPILANQFLHYTFDKWMSYKYPHVPF
ERYADDCVCHCGTLAQAEYIKDRLGERFAECKLTFNEEKTKIVFCKTSNRSSEHYHCTSFDYLGFTFRPRAAKDKR
KNVLFTSYLPAISNKSESRIHETIKSWNLKRLHNRSLRFVAAYINDVVRGWISYYGKFGKTEFWKVMCHLNRSIAY
WAKTKYKRLRRRGVISAHYWLAYIAQKEPNLFYHWQVGYIPYARQKK
>R10H018||gene_125558|GeneMark.hmm|410_aa|-|335|1567
MQRKSFEIPKALVWASYLDVRRNKGAPGCDGQTLKMFDQQRDGNLYKIWNRLCSGTWFPPPVLEKRIPKSNG
KERILGIPTVSDRIAQGAIKLFMEEKLDPIFHADSYGYRPGKSAHDALKQCAIRCWRYSWILEVDISAFFDHVRHD
LVLKALEHHGMPKWAILYCRRWMEAPMQSCENGELITRTRGTPQGGVISPLLANLFLHYAFDLWMEREYRGV
PFERYADDIVVHCSRMSDATRLKNRLSERFSEVGLVPNAGKTNIAYIDTFKRRNVATSFTFLGYDFKVRALKNFKG
ELYRKCMPGASNAAMRKITETIKKWRIHRSTAESLLDFARRYNAIVRGWIGYYAKFWSRNFNYRLWSAMQSRL
LKWMQSKYRLSNRKAQRKLTLVRKEYPKLFVHWYLLRASNE
>R10H022||gene_87626|GeneMark.hmm|422_aa|-|20274|21542
MKDAKSFEISRHLVMEAYKRVKANKGAAGVDDVSIADFESNLKSNLYKIWNRMSSGSYFPPAVKLVEIPKSNGG

KRPLGIPTIGDRVAQMVVVMTIEPGIEPYFHEDSYAYRPNRSALDAVRKAKERSYTFHWVLDLDIKGFFDNIDHE
LLIKALERHVKCKWAMLYIKRWLSVPYQLKDGTQIERTKGVPQGSVVGPILANLFLHYVFDEWMRRNHSNISFE
RYADDTICHCVSLKQAEFILRAIRKRFAECKLELNEDKTKIVYCKKNHRDIPYECIQFDFLGYTFRPRRSIDVNGEVF
LNFSPAISKKARTKIWEAIQNWNSNHWVPMELEDIAKEINPVIQGWINYYGQHNPRILKEVLQHVNDRLVRW
GRRKFKGLRKRKTATVHRLGDIALQKPNLFAHWAWGVKPTASERNRKRK
>R10H022||gene_94074|GeneMark.hmm|414_aa|-|266|1510
MQTTKPYNISKKAVVMAYRRVKANKGTYGIDEQSIEDFEKNLQNNLYKLWNRMSSGTYFPKPVKAVAIPKKNG
GKRILGIPTVEDRIAQMVAKLYFEPNVERIFYEDSYGYRPNKSAIQALEVTRKRCWRKDWVLEFDIKGLFDNINH
DILLKMVEKHTNEKWVLLYIRRWLITPFQMNDGDIVERPSGTPQGGVISPVLANLFLHYVFDDFMSKEFPSIPW
ARYADDGIAHCVSLKQAKYLLKRLQGRFKQFGLELNLDKTRIVYCKDEDRKGDYENTSFDFLGYTFRPRRAKNKY
GKYFTSFLPAMSNKAKKAIRKEVSGWKLQLKSDKSINDLAHMFNSKIQGWINYYTHFYKSEIYDVLRYINKCLIK
WVRRKFKKRKSNRRAERWLGDIARRDNKLFAHWKFGILPSVG
>R10H024||gene_164886|GeneMark.hmm|418_aa|-|813|2069
MSEAKQFDISKKAVIAAFQAVKENAGSYGADEQTIKEFEEHLNNNLYKLWNRMASGSYFPKPVRAVAIPKKNG
GIRILGIPTVEDRIAQMVAKMYFEPLVEPMFYNDSYGYRPNKSAIQAVGQARERCFKRDWALELDIKGLFDNIK
HGYLMYMVEKHTQIKWLILYIKRWLTVPFIMSDGSVAERRSGTPQGGVISPVLANLFLHYVFDDFMTKAYPNI
WWERYADDGVLHCQSYKQAAFIKQKLEERFQQFGLELNKEKTRIVYCKDNRRPQNYSCTQFTFLGYTFRPRLN
KNKEGKFFVGFTPAVSEKAKTAMKQKIREWKIQLKADLSLKDIGNMINKVVQGWINYYTHYYKSEFYEVLRYIN
QCLIKWVRRSYKKKNTRSRAEHWLGAVARRDRNLFAHWKFGILPSVGEGAV
>R10H025||gene_69378|GeneMark.hmm|418_aa|+|1589|2845
MSEAKQFDISKKAVIAAFQAVKENAGSYGADEQTIKEFEEHLNNNLYKLWNRMASGSYFPKPVRAVAIPKKNG
GIRILGIPTVEDRIAQMVAKMYFEPLVEPMFYNDSYGYRPNKSAIQAVGQARERCFKRDWVLELDIKGLFDNIK
HGYLMYMVEKHTQIKWLILYIKRWLTVPFIMSDGSVAERRSGTPQGGVISPVLANLFLHYVFDDFMTKAYPNI
WWERYADDGVLHCQSYKQAAFIKQKLEERFQQFGLELNKEKTRIVYCKDNRRPQNYSCTQFTFLGYTFRPRLN
KNKEGKFFVGFTPAVSEKAKTAMKQKIREWKIQLKADLSLKDIGNMINKVVQGWINYYTHYYKSEFYEVLRYIN
QCLIKWVRRSYKKKNTRSRAEHWLGAVARRDRNLFAHWKFGILPSVGEGAV
>R10H027||gene_50707|GeneMark.hmm|418_aa|-|790|2046
MSEAKQFDISKKAVIAAFQAVKENAGSYGADEQTIKEFEEHLNNNLYKLWNRMASGSYFPKPVRAVAIPKKNG
GIRILGIPTVEDRIAQMVAKMYFEPLVEPMFYNDSYGYRPNKSAIQAVGQARERCFKRDWVLELDIKGLFDNIK
HGYLMYMVEKHTQIKWLILYIKRWLTVPFIMSDGSVAERRSGTPQGGVISPVLANLFLHYVFDDFMTKAYPNI
WWERYADDGVLHCQSYKQAAFIKQKLEERFQQFGLELNKEKTRIVYCKDNRRPQNYSCTQFTFLGYTFRPRLN
KNKEGKFFVGFTPAVSEKAKTAMKQKIRGWKIQLKADLSLKDIGNMINKVVQGWINYYTHYYKSEFYEVLRYIN
QCLIKWVRRSYKKKNTRSRAEHWLGAVARRDRNLFAHWKFGILPSVGEGAV
>R10H029||gene_152499|GeneMark.hmm|410_aa|+|584|1816
MQRKSFEIPKALVWASYLDVRRNKGAPGCDGQTLKMFDQQRDGNLYKIWNRLCSGTWFPPPVLEKRIPKPN
GKERILGIPTVSDRIAQGAIKLFMEEKLDPIFHADSYGYRPGKSAHDALKQCAIRCWRYSWILEVDISAFFDHVRH
DLVLKALEHHGMPKWVILYCRRWMEAPMQSCENGELITRTRGTPQGGVISPLLANLFLHYAFDLWMEREYRG
VPFERYADDIVVHCSRMSDATRLKNRLSERFSEVGLVLNAGKTNIAYIDTFKRRNVATSFTFLGYDFKVRTLKNFK
GELYRKCMPGASNAAMRKITETIKKWRIHRSTAESLLDFARRYNAIVRGWIEYYGKFWSRNFNYRLWSAMQSR
LLKWMQSKYRLSNRRAQRKLTLVRKEYPKLFVHWYLLRASNE
>R10H030||gene_361231|GeneMark.hmm|418_aa|-|182|1438
MSEAKQFDISKKAVIAAFQAVKENAGSYGADEQTIKEFEEHLNNNLYKLWNRMASGSYFPKPVRAVAIPKKNG
GIRILGIPTVEDRIAQMVAKMYFEPLVEPMFYNDSYGYRPNKSAIQAVGQARERCFKRDWVLELDIKGLFDNIK
HGYLMYMVEKHTQIKWLILYIKRWLTVPFIMSDGSVAERRSGTPQGGVISPVLANLFLHYVFDDFMTKAYPNI

WWERYADDGVLHCQSYKQAAFIKQKLEERFQQFGLELNKEKTRIVYCKDNRRPQNYSCTQFTFLGYTFRPRLN
KNKEGKFFVGFTPAVSEKAKTAMKQKIREWKIQLKADLSLKDIGNMINKVVQGWINYYTHYYKSEFYEVLRYIN
QCLIKWVRRSYKKKNTRSRAEHWLGAVARRDRNLFAHWKFGILPSVGEGAV
>R10H033||gene_297743|GeneMark.hmm|407_aa|+|3|1226
KAVIAAFQAVKENAGSYGADEQTIKEFEEHLNNNLYKLWNRMASGSYFPKPVRAVEIPKKNGGTRILGIPTVED
RIAQMVAKMYFEPLVEPMFYNDSYGYRPNKSAIQAVGQARERCFKRDWVLELDIKGLFDNIKHGYLMYMVEK
HTQIKWLILYIKRWLTVPFIMSDGSVAERRSGTPQGGVISPVLANLFLHYVFDDFMTKAYPNIWWERYADDGVL
HCQSYKQAAFIKQKLEERFQQFGLELNKEKTRIVYCKDNRRPQNYSCTQFTFLGYTFRPRLNKNKEGKFFVGFTP
AVSEKAKTAMKQKIREWKIQLKADLSLKDIGNMINKVVQGWINYYTHYYKSEFYEVLRYINQCLIKWVRRSYKK
KNTRSRAEHWLGAVARRDRNLFAHWKFGILPSVGEGAV
>R10H034||gene_135684|GeneMark.hmm|420_aa|+|2922|4184
MNEAKPFVIDKRLVWEAYHKVKENKGSAGIDKVDQKTFDKEMSKNLYKIWNRMSSGCYFPKVVKLVEIPKSNG
GTRPLGIPTIEDRIAQQVVVSVLTPILEPIFKEDSYGYRPGKGAHQAIAKAKERCYVNPWVLDMDISKFFDTINHE
LLMKAVRKHTEEKWVLLYIERWLKVPYQTSKGEVIERTMGVPQGSVIGPVLANLFLHYVFDEWMSRNYPTIPFE
RYADDTICHCVSEKQAQFLKAVLMKRFGECGLKLNEEKTKIVYCKDSNRRGDSEHTSFDFLGFTFRPRGARNRKT
GQNFTAFLPAISKKSMKRIKESVRAWKLNRKTFACLLDISNEVDTQISGWMNYYMKFGRSEFRKVLNYINERLTR
WVMRKYKRFSKGKKLGKAYDWLVEYAAHNRNEFSHWVKGFVPYPRLG
>R10H035||gene_71651|GeneMark.hmm|418_aa|-|756|2012
MSEAKQFDISKKAVIAAFQAVKENAGSYGADEQTIKEFEEHLNNNLYKLWNRMASGSYFPKPVRAVAIPKKNG
GIRILGIPTVEDRIAQMVAKMYFEPLVEPMFYNDSYGYRPNKSAIQAVGQARERCFKRDWVLELDIKGLFDNIK
HGYLMYMVEKHTQIKWLILYIKRWLTVPFIMSDGSVAERRSGTPQGGVISPVLANLFLHYVFDDFMTKAYPNI
WWERYADDGVLHCQSYKQAVFIKQKLEERFQQFGLELNKEKTRIVYCKDNRRSQNYSCTQFTFLGYTFRPRLNK
NKEGKFFVGFTPAVSEKAKTAMKQKIRGWKIQLKADLSLKDIGNMINKVVQGWINYYTHYYKSEFYEVLRYINQ
CLIKWVRRSYKKKNTRSRAEHWLGAVARRDRNLFAHWKFGILPSVGEGAV
>R10H036||gene_87745|GeneMark.hmm|420_aa|-|641|1903
MNEAKPFVIDKRLVWEAYHKVKENKGSAGIDKVDQKTFDKEMSKNLYKIWNRMSSGCYFPKAVKLVEIPKSNG
GTRPLGIPTIEDRIAQQVVVSVLTPILEPIFKEDSYGYRPGKGAHQAVAKAKERCYVNPWVLDMDISKFFDTINHE
LLMKAVRKHTEEKWVLLYIERWLKVPYQTLKGEVIERTMGVPQGSVIGPVLANLFLHYVFDEWMSRNYPTIPFE
RYADDTICHCVSEKQAQFLKAVLMKRFEECGLKLNEEKTKIVYCKDSNRRGDSEHTSFDFLGFTFRPRGARNRKT
GQNFTAFLPAISRKSMKRIKEAVRAWKLNRKTFACLLDISNEVDTQISGWMNYYMKFGRSEFRKVLNYINERLT
RWVMRKYKRFSKGKKLGRAYEWLVEYAAHNRNEFSHWVKGFVPYPRLG
>R10H037||gene_129923|GeneMark.hmm|418_aa|-|3313|4569
MSEAKQFDISKKAVIAAFQAVKENAGSYGADEQTIKEFEEHLNNNLYKLWNRMASGSYFPKPVRAVAIPKKNG
GIRILGIPTVEDRIAQMVAKMYFEPLVEPMFYNDSYGYRPNKSAIQAVGQARERCFKRDWVLELDIKGLFDNIK
HGYLMYMVEKHTQIKWLILYIKRWLTVPFIMSDGSVAERRSGTPQGGVISPVLANLFLHYVFDDFMTKAYPNI
WWERYADDGVLHCQSYKQAAFIKQKLEERFQQFGLELNKEKTRIVYCKDNRRPQNYSCTQFTFLGYTFRPRLN
KNKEGKFFVGFTPAVSEKAKTAMKQKIREWKIQLKADLSLKDIGNMINKVVQGWINYYTHYYKSEFYEVLRYIN
QCLIKWVRRSYKKKNTRSRAEHWLGAVARRDRNLFAHWKFGILPSVGEGAV
>R10H039||gene_143960|GeneMark.hmm|422_aa|-|2627|3895
MKDAKSFEISKQLVMEAYKRVKANRGTSGIDDVTIADFESDLKGNLYKIWNRMCSGSYLPPAVKLVEIPKSNGGK
RPLGIPTVGDRVAQMVVVMTIEPEIEPHFHEDSYAYRPKKSALDAVEKAKDRCYTFHWVLDLDIKGFFDNIDHE
LLIRALERHVKCKWAMMYIKRWLSVPYQLKDGTQVERTKGVPQGSVVGPILANLFLHYTFDEWMRRNHSNIS
FERYADDTICHCVSQKQAEFIHRAIKKRFAECKLELNEEKTKIVYCKRNHRNIEYECIQFDFLGYTFRPRRSIDTHGE
VFLNFSPAVSKKARTKIWETIRDWNQKYWVQMKLEDIAKQINPIIQGWINYYGKFNPGVLKEVLKRINLKLSRW

IRDKFKGFRKFKTQAIHRLGDIALKNPDLFAHWSWGVKPTASPRNRARV
>R10H040||gene_59725|GeneMark.hmm|430_aa|-|529|1821
MQNDNAKPISISKQLVYDAFLRVKANRGSAGIDKVTLEDYEKNLRGNLYKLWNRMSSGSYFPPSVKLVEIPKSTG
GKRPLGIPTVSDRVAQMAVVMLITPSIEPCFHEDSYAYRPHRSAHDAVGKARERCWKYAWVLDMDISKFFDTI
DHELLLKALKRHTQEKWVLMYIERWLKVPYEKSDGSQVDRALGVPQGSVIGPVLANLFLHYTFDKWMEKNFP
RVPFERYADDTICHCHSLKQAEYMQAMIQQRFECCRLRLNEEKTKIVYCKSSRQKECYPNVTFDFLGFTFQPRES
VDKYGNRFTGFLPAISRKSMKRINETMRSWHLNRHSNLTLEHLASDINPIVRGWMTYYGKFYPTRLKWFMQTL
NGRLARWVMCKFERYRHRFYPAQEWLARIAEKEGLIFYHWKCGVLPRFTNKEKVSSQLIMVK
>R10H041||gene_90121|GeneMark.hmm|420_aa|-|2228|3490
MNEAKPFVIDKRLVWEAYHKVKENKGSAGIDKVDQKTFDKEMSKNLYKIWNRMSSGCYFPKAVKLVEIPKSNG
GTRPLGIPTIEDRIAQQVVVSVLTPILEPIFKEDSYGYRPGKGAHQAIAKAKERCYVNPWVLDMDISKFFDTINHE
LLMKAIRKHTEEKWVLLYIERWLKVPYQTSKGEVIERTMGVPQGSVIGPVLANLFLHYVFDEWMSRNYPTIPFE
RYADDTICHCVSEKQAQFLKAVLMKRFEECGLKLNEEKTKIVYCKDSNRRGDSEHTSFDFLGFTFRPRGARNRKT
GQNFTAFLPAISKKSMKRIKEAVRAWKLNRKTFACLLDISNEVDTQISGWMNFYMKFGRSEFRKVLNYINERLTR
WVMRKYKRFSKGKKFSKAYDWLVEYAAHNRNEFSHWVKGFVPYPRLG
>R10H041||gene_29090|GeneMark.hmm|420_aa|+|570|1832
MNEAKPFVIDKRLVWEAYHKVKENKGSAGIDKVDQKTFDKEMSKNLYKIWNRMSSGCYFPKAVKLVEIPKSNG
GTRPLGIPTIEDRIAQQVVVSVLTPILEPIFKEDSYGYRPGKGAHQAVAKAKERCYVNPWVLDMDISKFFDTINHE
LLMKAVRKHTGEKWVLLYIERWLKVPYQTLKGEVIERTMGVPQGSVIGPVLANLFLHYVFDEWMSRNYPTIPFE
RYADDTICHCVSEKQAQFLKAVLMKRFEECGLKLNEEKTKIVYCKDSNRRGDSEHTSFDFLGFTFRPRGARNRKT
GQNFTAFLPAISRKSMKRIKEAVRAWKLNRKTFACLLDISNEVDTQISGWMNYYMKFGRSEFRKVLNYINERLT
RWVMRKYKRFSKGKKLGRAYEWLVEYAAHNRNEFSHWAKGFVPYPRLG
>R10H044||gene_278960|GeneMark.hmm|418_aa|+|468|1724
MSEAKQFDISKKAVIAAFQAVKENAGSYGADEQTIKEFEEHLNNNLYKLWNRMASGSYFPKPVRAVAIPKKNG
GIRILGIPTVEDRIAQMVAKMYFEPLVEPMFYNDSYGYRPNKSAIQAVGQARERCFKRDWVLELDIKGLFDNIK
HGYLMYMVEKHTQIKWLILYIKRWLTVPFIMSDGSVAERRSGTPQGGVISPVLANLFLHYVFDDFMTKAYPNI
WWERYADDGVLHCQSYKQAAFIKQKLEERFQQFGLELNKEKTRIVYCKDNRRPQNYSCTQFTFLGYTFRPRLN
KNKEGKFFVGFTPAVSEKAKTAMKQKIREWKIQLKADLSLKDIGNMINKVVQGWINYYTHYYKSEFYEVLRYIN
QCLIKWVRRSYKKKNTRSRAEHWLGAVARRDRNLFAHWKFGILPSVGEGAV
>R10H045||gene_155286|GeneMark.hmm|410_aa|+|2|1234
LLDTILRSDNLNAAYKRVKANKGSAGIDGMDFEKFEKRLNNNLYKIWNRMSSGSYFPSPVMAVEIPKKSGGTRR
LGIPTIADRIAQMVARAYVERAVEPMFCEDSYGYRPHKSALDAVEKTRKRCWKYDYVIELDVKGLFDNIDHELL
MRVVRRHVKEPWICLYIERWPKSPFVLPDGSRIERESGTPQGGVISPVLANMFLHYVFDMWMKRNFPQAPFE
RYADDGVVHCRTKEEALYIKKKLVKRFEECKLELHPVKTRIVYCKDKDRTKEEELTEFDFLGYTFKAVYIKCKDGVM
RNNFIASVSKTAAKGFRDKIKALEIHKRTGCKIDMIAELLNPMIRGWMNYFGKFNPSAMKNTLQCIECRLIKWA
MCKYKSFRGRRQRAEKWLSSIRKREPKLFAHWSRMYSYC
>R10H046||gene_74205|GeneMark.hmm|413_aa|+|450|1691
MNKSKQYEIPKRAVIEAYKRVKANKGSAGIDGIDFEKFEERLNNNLYKIWNRMSSGSYFPSPVMAVEIPKKSGGT
RRLGIPTIADRIAQMVARAYVERAVEPMFCEDSYGYRPHKSALDAVEKTRKRCWKYDYVIELDVKGLFDNIDHEL
LMRVVRRHVKEPWICLYIERWLKSPFILVDGSRMERESGTPQGGVISPVLANMFLHYVFDMWMKRNFPQAP
FERYADDGVVHCRTKEEALYIKEKLVKRFEECKLELHPVKTRIVYCKDKDRTKEEELTEFDFLGYTFKAVYIKCKDGV
MRNNFIASVSKTAAKGFRDKIKVLEIHKRTGSKIDMIAELLNPMIRGWMNYFGKFNPSAMKNTLQCIERRLIKW
AMCKYKSFRGRRRRAEKWLSSIRKREPKLFAHWSRMYPYC
>R10H046||gene_10754|GeneMark.hmm|418_aa|+|3243|4499

MSEAKQFDISKKAVIAAFQAVKENAGSYGADEQTIKEFEEHLNNNLYKLWNRMASGSYFPKPVRAVAIPKKNG
GIRILGIPTVEDRIAQMVAKMYFEPLVEPMFYNDSYGYRPNKSAIQAVGQARERCFKRDWVLELDIKGLFDNIK
HGYLMYMVEKHTQIKWLILYIKRWLTVPFIMSDGSVAERRSGTPQGGVISPVLANLFLHYVFDDFMTKAYPNI
WWERYADDGVLHCQSYKQAAFIKQKLEERFQQFGLELNKEKTRIVYCKDNRRPQNYSCTQFTFLGYTFRPRLN
KNKEGKFFVGFTPAVSEKAKTAMKQKIREWKIQLKADLSLKDIGNMINKVVQGWINYYTHYYKSEFYEVLRYIN
QCLIKWVRRSYKKKNTRSRAEHWLGAVARRDRNLFAHWKFGILPSVGEGAV
>R10H047||gene_42252|GeneMark.hmm|418_aa|+|10175|11431
MSEAKQFDISKKAVIAAFQAVKENAGSYGADEQTIKEFEEHLNNNLYKLWNRMASGSYFPKPVRAVAIPKKNG
GIRILGIPTVEDRIAQMVAKMYFEPLVEPMFYNDSYGYRPNKSAIQAVGQARERCFKRDWVLELDIKGLFDNIK
HGYLMYMVEKHTQIKWLILYIKRWLTVPFIMSDGSVAERRSGTPQGGVISPVLANLFLHYVFDDFMTKAYPNI
WWERYADDGVLHCQSYKQAAFIKQKLEERFQQFGLELNKEKTRIVYCKDNRRPQNYSCTQFTFLGYTFRPRLN
KNKEGKFFVGFTPAVSEKAKTAMKQKIREWKIQLKADLSLKDIGNMINKVVQGWINYYTHYYKSEFYEVLRYIN
QCLIKWVRRSYKKKNTRSRAEHWLGAVARRDRNLFAHWKFGILPSVGEGAV
>R10H048||gene_256261|GeneMark.hmm|422_aa|-|492|1760
MKDAKSFEISRHLVMEAYKKVKANKGAAGVDDISIADFESNLKSNLYKIWNRMSSGSYLPPAVKLVEIPKSNGGK
RPLGIPTVGDRVAQMVVVMTIEPSIEPYFHEDSYAYRPNRSVLDAVRKAKERSYTFHWVLDLDIKGFFDNIDHEL
LIKALERHVKCKWAMLYIKRWLSVPYQLKDGTQIERTKGVPQGSVVGPILANLFLHYVFDEWMKRNHSNISFER
YADDTICHCVSLKQAEFILRAIKKRFAECKLELNEEKTKIVYCKKNHRDIPYECIQFDFLGYTFRPRRSIDENGEVFL
NFSPAISKKARTKIWETIQNWNSNHWIPMELEDIAKEINPVIQGWINYYGQHNPRILKEVLQHINDRLVRWGR
RKFKGLRKRKTATVHKLGDIALQKPNLFAHWAWGVKPTASERNRKRK
>R10H049||gene_231215|GeneMark.hmm|418_aa|-|1266|2522
MSEAKQFDISKKAVIAAFQAVKENAGSYGADEQTIKEFEEHLNNNLYKLWNRMASGSYFPKPVRAVAIPKKNG
GIRILGIPTVEDRIAQMVAKMYFEPLVEPMFYNDSYGYRPNKSAIQAVGQARERCFKRDWVLELDIKGLFDNIK
HGYLMYMVEKHTQIKWLILYIKRWLTVPFIMSDGSVAERRSGTPQGGVISPVLANLFLHYVFDDFMTKAYPNI
WWERYADDGVLHCQSYKQAAFIKQKLEERFQQFGLELNKEKTRIVYCKDNRRPQNYSCTQFTFLGYTFRPRLN
KNKEGKFFVGFTPAVSEKAKTAMKQKIREWKIQLKADLSLKDIGNMINKVVQGWINYYTHYYKSEFYEVLRYIN
QCLIKWVRRSYKKKNTRSRAEHWLGAVARRDRNLFAHWKFGILPSVGEGAV
>R10H053||gene_180493|GeneMark.hmm|421_aa|+|3210|4475
MQEAKPFQIDKRIIFEAFKKVKFNRGSSGIDGIEMTTYEQNLGSNLYRLWNRMSSGSYMPKAVKLVEIPKSNGG
KRPLGIPTIEDRIAQMAVVNVIEPLIEPCFHEDSFGYRPHRSAHDAIAKAERRCWKYAWVLDIDISKFFDTIDHGL
LMKAVEKHINIKWILLYIKRWLTVPYQRSDGEIVKRDMGVPQGSVIGPILANLFLHYTFDKWMSYKYPHIPFERY
ADDCVCHCSTLAQAEYIKERLGERFTECKLKFNEEKTKIVFCKMSSRSSKHYHCTSFDYLGFTFRSRAAKDKRNN
VLFTSYLPAISKKSVSRIHETIKSWNLKRLHNRSLRFVAAYINDVVRGWINYYEKFGKTEFWKVMCHLNRSIAYW
AKTKYKRLRRRGVISAHYWLAYIAQKEPNLFYHWQVGYVPYARQKK
>R10H057||gene_167048|GeneMark.hmm|413_aa|-|107|1348
MNESKQYDIPKKAVIEAYKRVKANKGSAGIDGINFEKFEGKLNNNLYKIWNRMSSGSYFPSPVMAVEIPKKTGG
VRRLGIPTITDRVAQMVARMYVESAVEPMFCDDSYGYRPNKSALDAIEMTRKRCWRYDYVIELDVKGLFDNID
HELLIRVVRRHVKEVWICMYIERWLKTPFVLKNGEVIERNAGTPQGGVISPVLANMFLHYVFDMWMKRNFP
QAPFERYADDGVIHCKTKEEALFIKECLVKRFAECKLELHPIKTRIVYCKDKDRTRDEDLTEFDFLGYTFKAVYIMCK
DGKRRSNFIASVSKTSAKTFRDKIKSLEIHKKTGCKINMIAEILNPLLRGWINYFGKFNPSAMKYTLQCIERRLVKW
AMCKYKNFRGRRRRAEKWLLSVRKREPKLFAHWSKMYSYC
>R10H059||gene_127463|GeneMark.hmm|414_aa|+|136|1380
MQTTKPYNISKKAVVMAYRRVKANKGTYGIDEQSIEDFEKNLQDNLYKLWNRMSSGTYFPKPVKAVAIPKKNG
GKRILGIPTVEDRIAQMVAKLYFEPNVERIFYEDSYGYRPNKSAIQALEVTRKRCWRKDWVLEFDIKGLFDNINH

DILLKMVEKHTKEKWVLLYIRRWLITPFQMNDGDIVERPSGTPQGGVISPVLANLFLHYVFDDFMSKEFPSIPW
ARYADDGIAHCVSLKQAKYLLKRLQGRFKQFGLELNLDKTRIVYCKDEDRKGDYENTSFDFLGYTFRPRGAKNKY
GKYFTSFLPAMSNKAKKAIRKEVSGWKLQLKSDKSINDLAHMFNSKIQGWINYYTHFYKSEIYDVLRYINKCLIK
WVRRKFKKRKSNRRAERWLGDIARRDNKLFAHWKFGILPSVG
>R10H061||gene_233900|GeneMark.hmm|421_aa|+|569|1834
MQEAKPFQIDKRIIFEAFKKVKFNRGSSGIDGIEMTTYEQNLGSNLYRLWNRMSSGSYMPKAVKLVEIPKSNGG
KRPLGIPTIEDRIAQMAVVNVIEPLMEPCFHEDSFGYRPHRSAHDAIAKAERRCWKYAWVLDIDISKFFDTIDHG
LLMKAVEKHINIKWILLYIKRWLTVPYQRSDGEIVKRDMGVPQGSVIGPILANLFLHYTFDKWMSYKYPHIPFER
YADDCVCHCSTLAQAEYIKERLGERFTECKLKFNEEKTKIVFCKMSSRSSKHYHCTSFDYLGFTFRSRAAKDKRNN
VLFTSYLPAISKKSVSRIHETIKSWNLKRLHNRSLRFVAAYINDVVRGWINYYEKFGKTEFWKVMCHLNRSIAYW
AKTKYKRLRRRGVISAHYWLAYIAQKEPNLFYHWQVGYVPYARQKK
>R10H062||gene_394720|GeneMark.hmm|430_aa|-|132|1424
MQNDNAKPISISKQLVYDAFLRVKANRGSAGIDKVTLEDYEKNLRGNLYKLWNRMSSGSYFPPSVKLVEIPKSTG
GKRPLGIPTVSDRVAQMAVVMLITPSIEPCFHEDSYAYRPHRSAHDAVGKARERCWKYAWVLDMDISKFFDTI
DHELLLKALKRHTQEKWVLMYIERWLKVPYEKSDGSQVDRALGVPQGSVIGPVLANLFLHYTFDKWMEKNFP
RVPFERYADDTICHCHSLKQAEYMQAMIQQRFECCRLRLNEEKTKIVYCKSSRQKECYPNVTFDFLGFTFQPRES
VDKYGNRFTGFLPAISRKSMKRINETMRSWHLNRHSNLTLEHLASDINPIVRGWMTYYGKFYPTRLKWFMQTL
NGRLARWVMCKFERYRHRFYPAQEWLARIAEKEGLIFYHWKCGALPRFTNKEKVSSQLIMVK
>R10H062||gene_283039|GeneMark.hmm|422_aa|-|1852|3120
MMQHQVTKPFTIDKYLIMNAWKRVKENKGSAGIDNVSTEDYESNLGKNLYKLWNRMSSGSYFPEAVKLVDIP
KPSGGTRPLGIPTVGDRIAQMSVVLLIEERLEAIFHADSYGYRPNRSAHDAIEKARERCWHYNWVLDMDISKFF
DTIDHDLLMKAVERHVQEKWILLYIRRWLKVPYATLTGERIERKMGVPQGSVIGPVLANLYLHYTFDKWMSLYH
PTIPFERYADDTICHCNSLKEAQMLKASIVERFAACKLRLNEEKTRIVYCKDGKRRGEYKEITFDFLGYTFQPRGQ
RNKQGQVFNGYAPAISRKSKKRITEKMRGWHLNRRVQLKLSDIAVEINAEVRGWMNYYGKFYGSQLKAFLQCI
NLKLARWAERKYKRFRRKPNDAYKWLVRVASKNPALFYHWQHGVKPNRLKPFG
>R10H063||gene_20385|GeneMark.hmm|421_aa|+|2425|3690
MQEAKPFQIDKRIIFEAFKKVKSNGGSPGIDGIEMSAYEQNLGSNFYRLWNRMSSGSYMPKAVKLVEILKSNGG
KRPLGIPSVEDRIAQMAVVNVIEPLVEPYFHKDSFGYRPHRSAHDAIAKAERRCWKYAWVLDIDISKFFDTIDHG
LLMKAVEKHIKTKWILLYIKRWLTVPYQGNDGAIVKRHMGVPQGSVIGPILANQFLHYTFDKWMSYKYPHVPF
ERYADDCVCHCGTLAQAEYIKDRLGERFAECKLTFNEEKTKIVFCKTSNRSSEHYHCTSFDYLGFTFRPRAAKDKR
KNVLFTSYLPAISNKSESRIHETIKSWNLKRLHNRSLRFVAAYINDVVRGWISYYGKFGKTEFWKVMCHLNRSIAY
WAKTKYKRLRRRGVISAHYWLAYIAQKEPNLFYHWQVGYIPYARQKK
>R10H064||gene_51678|GeneMark.hmm|418_aa|-|709|1965
MSEAKQFDISKKAVIAAFQAVKENAGSYGADEQTIKEFEEHLNNNLYKLWNRMASGSYFPKPVRAVAIPKKNG
GIRILGIPTVEDRIAQMVAKMYFEPLVEPMFYNDSYGYRPNKSAIQAVGQARERCFKRDWVLELDIKGLFDNIK
HGYLMYMVEKHTQIKWLILYIKRWLTVPFIMSDGSVAERRSGTPQGGVISPVLANLFLHYVFDDFMTKAYPNI
WWERYADDGVLHCQSYKQAAFIKQKLEERFQQFGLELNKEKTRIVYCKDNRRPQNYSCTQFTFLGYTFRPRLN
KNKEGKFFVGFTPAVSEKAKTAMKQKIREWKIQLKADLSLKDIGNMINKVVQGWINYYTHYYKSEFYEVLRYIN
QCLIKWVRRSYKKKNTRSRAEHWLGAVARRDRNLFAHWKFGILPSVGEGAV
>R10H065||gene_109682|GeneMark.hmm|413_aa|+|504|1745
MSESKQYEIPKKVVIEAYKRVKANKGSAGIDGIDFERFEKKLNNNLYKIWNRMSSGSYFPSPVLSVEISKKAGGTR
RLGIPTITDRIAQMVARMYVEPVVEPMFCNDSYGYRPNKSAIDAIATARKRCWRYDYVIELDVKGLFDNINHELL
MRVVLKHVKEEWICLYIKRWLETPFITREGQVIERLSGTPQGGVISPVLANMYLHYVFDMWMKRNFPQAPFE
RYADDGVIHCRTKEEAFVIKKKLAARFAECKLELHPVKTRIVYCKDKDRTRNEELTEFDFLGYTFKAVYIMCKDGK

VRYNFIASVSKTSSKSFRDKIKAMEVHKRTGCKIDIIAEILNPLIRGWMNYFGKFNPSAMKGTLQCIDRRLVKWA
MCKYKNFRGKRGRAEKWLCTVRQREPKLFAHWSNLYSYC
>R10H068||gene_78871|GeneMark.hmm|371_aa|+|2|1117
NNNLYKIWNRMSSGSYFPSPVMAVEIPKKSGGTRRLGIPTIADRIAQMVARAYVERAVEPMFCEDSYGYRPHK
SALDAVEKTRKRCWKYDYVIELDVKGLFDNIDHELLMRVVRRHVKEPWICLYIERWLKSPFVLPDGSRIERESGT
PQGGVISPVLANMFLHYVFDMWMKRNFPQAPFERYADDGVVHCRTKEEALYIKKKLVKRFEECKLELHPVKTR
IVYCKEKDRTKEEELAEFDFLGYTFKAVYIKCKDGVMRNNFIASVSKTAAKGFRDKIKALEIHKRTGCKIDMIAELL
NPMIRGWMNYFGKFNPSAMKNTLQCIECRLIKWAMCKYKSFRGRRQRAEKWLSSIRKREPKLFAHWSRMYS
YC
>R10H070||gene_232460|GeneMark.hmm|440_aa|+|488|1810
MNVERRGSVGQSASQPNCKQEEAAGEQTKPFQVSKLHVVEAYRRVKANAGAAGVDNQTLKDFERDLKGNLY
KIWNRLSSGSWMPPPVRAVEIPKKDGSKRLLGIPTVSDRIAQMTVLVTFEPLVERYFLNDSYGYRHGKSALDAIA
VTRKRCWQYDWYLEFDIKGLFDNIPHDLLLRAVDKHCADKWVRLSIRRWLTAPVQMPDGTLKERNKGTPQGG
VISPVLANLFLHYVFDKWLSLHYPEIPWCRYADDGLIHCGSKQQAEELLNKLAKPFQECGLELHPEKTKIVYCKDS
ERQANHETVQFNFLGYTFRARRARNQRRGNLFTSFSLAVSNSAQKDMIGKLRKLRLRRRVEMSLEDIAKRLNP
MISGWLNYYAKYYKSAMKKVCRYINLTLIAWARKKYKTLRYKKTKACQLMERLSKEKPELFAHWKAGPGSAFA
>R10H070||gene_97112|GeneMark.hmm|418_aa|+|3055|4311
MSEAKQFDISKKAVIAAFQAVKENAGSYGADEQTIKEFEEHLNNNLYKLWNRMASGSYFPKPVRAVAIPKKNG
GIRILGIPTVEDRIAQMVAKMYFEPLVEPMFYNDSYGYRPNKSAIQAVGQARERCFKRDWVLELDIKGLFDNIK
HGYLMYMVEKHTQIKWLILYIKRWLTVPFIMSDGSVAERRSGTPQGGVISPVLANLFLHYVFDDFMTKAYPNI
WWERYADDGVLHCQSYKQAAFIKQKLEERFQQFGLELNKEKTRIVYCKDNRRPQNYSCTQFTFLGYTFRPRLN
KNKEGKFFVGFTPAVSEKAKTAMKQKIREWKIQLKADLSLKDIGNMINKVVQGWINYYTHYYKSEFYEVLRYIN
QCLIKWVRRSYKKKNTRSRAEHWLGAVARRDRNLFAHWKFGILPSVGEGAV
>R10H070||gene_84745|GeneMark.hmm|414_aa|-|2535|3779
MQEAKPYSISKKAVIAAYQRVKANKGTYGVDEQSIEDFERKLNNNLYKIWNRMSSGTYFPKPVKAVAIPKKNGG
TRILGVPTVEDRIAQMVAKLYFEPCVEPIFYEDSYGYRPNKSAIQALEATRTRCWRKDWVLEFDIRGLFDNIRHDY
LMEMVKKHTKEKWIILYIQRWLTAPFQMEDGTIVERKSGTPQGGVISPVLANLFLHYVFDDFMVKEFPTIPWA
RYADDGIAHCVSQKQAKYLRRRLGQRFQSYGLELNQEKTRIVYCKDDDRRGNHENTSFDFLGYTFRPRHAKNR
YGKFFTNFLPAISEKAKKAIRKEVRGWKLQLKSDKDLYDIANMFNRQIQGWINYYTHFYKSEIYDVLRYINGCLVK
WVRRKYKKRKARRKAEHWLGEIAKRDRNLFAHWKFGILPAAG
>R10H073||gene_177937|GeneMark.hmm|418_aa|+|672|1928
MSEAKQFDISKKAVIAAFQAVKENAGSYGVDEQTIKEFEEHLNNNLYKLWNRMASGSYFPKPVRAVAIPKKNG
GIRILGIPTVEDRIAQMVAKMYFEPLVEPMFYNDSYGYRPNKSAIQAVGQARERCFKRDWVLELDIKGLFDNIK
HGYLMYMVEKHTQIKWLILYIKRWLTVPFIMSDGSVAERRSGTPQGGVISPVLANLFLHYVFDDFMTKAYPNI
WWERYADDGVLHCQSYKQAAFIKQKLEERFQQFGLELNKEKTRIVYCKDNRRPQNYSCTQFTFLGYTFRPRLN
KNKEGKFFVGFTPAVSEKAKTAMKQKIRGWKIQLKADLSLKDIGNMINKVVQGWINYYTHYYKSEFYEVLRYIN
QCLIKWVRRSYKKKNTRSRAEHWLGAVARRDRNLFAHWKFGILPSVGEGAV
>R10H074||gene_24770|GeneMark.hmm|430_aa|-|5942|7234
MQNDNAKPISISKQLVYDAFLRVKANRGSAGIDKVTLEDYEKNLRGNLYKLWNRMSSGSYFPPSVKLVEIPKSTG
GKRPLGIPTVSDRVAQMAVVMLITPSIEPCFHEDSYAYRPHRSAHDAVGKARERCWKYAWVLDMDISKFFDTI
DHELLLKALKRHTQEKWVLMYIERWLKVPYEKADGSQVDRALGVPQGSVIGPVLANLFLHYTFDKWMEKNFP
RVPFERYADDTICHCHSLKQAEYMQAMIQQRFECCRLRLNEEKTKIVYCKSSRQKECYPNVTFDFLGFTFQPRES
VDKYGNRFTGFLPAISRKSMKRINETMRSWHLNRHSNLTLEHLASDINPIVRGWMTYYGKFYPTRLKWFMQTL
NGRLARWVMCKFERYRHRFYPAQEWLARIAEKEGLIFYHWKCGALPRFTNKEKVSSQLIMVK

>R10H076||gene_38745|GeneMark.hmm|414_aa|-|2535|3779
MQTTKPYNISKKAVVMAYRRVKANKGTYGIDEQSIEDFEKNLQDNLYKLWNRMSSGTYFPKPVKAVAIPKKNG
GKRILGIPTVEDRIAQMVAKLYFEPNVERIFYEDSYGYRPNKSAIQALEVTRKRCWRKDWVLEFDIKGLFDNINH
DILLKMVEKHTKEKWVLLYIRRWLITPFQMNDGDIVERPSGTPQGGVISPVLANLFLHYVFDDFMSKEFPSIPW
ARYADDGIAHCVSLKQAKYLLKRLQGRFKQFGLELNLDKTRIVYCKDEDRKGDYENTSFDFLGYTFRPRRAKNKY
GKYFTSFLPAMSNKAKKAIRKEVSGWKLQLKSDKSINDLAHMFNSKIQGWINYYTHFYKSEIYDVLRYINKCLIK
WVRRKFKKRKSNRRAERWLGDIARRDNKLFAHWKFGILPSVG
>R10H076||gene_91198|GeneMark.hmm|418_aa|-|654|1910
MSEAKQFDISKKAVIAAFQAVKENAGSYGVDEQTIKEFEEHLNNNLYKLWNRMASGSYFPKPVRAVAIPKKNG
GIRILGIPTVEDRIAQMVAKMYFEPLVEPMFYKDSYGYRPNKSAIQAVGQARERCFKRDWVLELDIKGLFDNIKH
GYLMYMVEKHTQIKWLILYIKRWLTVPFIMSDGSVAERRSGTPQGGVISPVLANLFLHYVFDDFMTKAYPNIW
WERYADDGVLHCQSYKQAAFIKQKLEERFQQFGLELNKEKTRIVYCKDNRRSQNYSCTQFTFLGYTFRPRLNKN
KEGKFFVGFTPAVSEKDKTAMKQKIRGWKIQLKADLSLKDIGNMINKVVQGWINYYTHYYKSEFYEVLRYINQC
LIKWVRRSYKKKNTRSRAEHWLGAVARRDRNLFAHWKFGILPSVGEGAV
>R10H079||gene_27692|GeneMark.hmm|418_aa|-|20351|21607
MSEAKQFDISKKAVIAAFQAVKENAGSYGADEQTIKEFEEHLNNNLYKLWNRMASGSYFPKPVRAVAIPKKNG
GIRILGIPTVEDRIAQMVAKMYFEPLVEPMFYNDSYGYRPNKSAIQAVGQARERCFKRDWVLELDIKGLFDNIK
HGYLMYMVEKHTQIKWLILYIKRWLTVPFIMSDGSVAERRSGTPQGGVISPVLANLFLHYVFDDFMTKAYPNI
WWERYADDGVLHCQSYKQAAFIKQKLEERFQQFGLELNKEKTRIVYCKDNRRPQNYSCTQFTFLGYTFRPRLN
KNKEGKFFVGFTPAVSEKAKTAMKQKIREWKIQLKADLSLKDIGNMINKVVQGWINYYTHYYKSEFYEVLRYIN
QCLIKWVRRSYKKKNTRSRAEHWLGAVARRDRNLFAHWKFGILPSVGEGAV
>R10H082||gene_74182|GeneMark.hmm|420_aa|-|11403|12665
MNEAKPFVIDKRLVWEAYHKVRENKGSAGIDKVDQKTFDKEMSKNLYKIWNRMSSGCYFPKAVKLVEIPKSNG
GTRPLGIPTIEDRIAQQVVVSVLPPILEPIFKEDSYGYRPGKGAQQAIAKAKERCYVNPWVLDMDISKFFDTINH
DLLMKAVRKHTEEKWVLLYIERWLKVPYQTSKGEVIERTMGVPQGSVIGPVLANLFLHYVFDEWMSRNYPTIP
FERYADDTICHCVSEKQAQFLKAVLMKRFEECGLKLNEEKTKIVYCKDSNRRGDSEHTSFDFLGFTFRPRSARNR
KTGQNFTAFLPAISKKSLKRIKEAVRAWKLNRKTFACLLDISNEVDTQISGWMNYYMKFGRSEFRKVLNYINERL
TRWVMRKYKRFSKGKKFSRAYEWLVEYAVHNRNEFSHWAKGFVLHPRLG
>R10H083||gene_167509|GeneMark.hmm|418_aa|-|68|1324
MSEAKQFDISKKAVIAAFQAVKENAGSYGADEQTIKEFEEHLNNNLYKLWNRMASGSYFPKPVRAVAIPKKNG
GIRILGIPTVEDRIAQMVAKMYFEPLVEPMFYNDSYGYRPNKSAIQAVGQARERCFKRDWVLELDIKGLFDNIK
HGYLMYMVEKHTQIKWLILYIKRWLTVPFIMSDGSVAERRSGTPQGGVISPVLANLFLHYVFDDFMTKAYPNI
WWERYADDGVLHCQSYKQAAFIKQKLEERFQQFGLELNKEKTRIVYCKDNRRPQNYSCTQFTFLGYTFRPRLN
KNKEGKFFVGFTPAVSEKAKTAMKQKIREWKIQLKADLSLKDIGNMINKVVQGWINYYTHYYKSEFYEVLRYIN
QCLIKWVRRSYKKKNTRSRAEHWLGAVARRDRNLFAHWKFGILPSVGEGAV
>R10H084||gene_61865|GeneMark.hmm|430_aa|+|584|1876
MQNDNAKPISISKQLVYDAFLRVKANRGSAGIDKVTLEDYEKNLRGNLYKLWNRMSSGSYFPPSVKLVEIPKSTG
GKRPLGIPTVSDRVAQMAVVMLITPSIEPCFHEDSYAYRPHRSAHDAVGKARERCWKYAWVLDMDISKFFDTI
DHELLLKALKRHTQEKWVLMYIERWLKVPYEKSDGSQVDRALGVPQGSVIGPVLANLFLHYTFDKWMEKNFP
RVPFERYADDTICHCHSLKQAEYMQAMIQQRFECCRLRLNEEKTKIVYCKSSRQKECYPNVTFDFLGFTFQPRES
VDKYGNRFTGFLPAISRKSMKRINETMRSWHLNRHSNLTLEHLASDINPIVRGWMTYYGKFYPTRLKWFMQTL
NGRLARWVMCKFERYRHRFYPAQEWLARIAEKEGLIFYHWKCGALPRFTNKEKVSSQLIMVK
>R10H086||gene_61528|GeneMark.hmm|418_aa|+|572|1828
MSEAKQFDISKKAVIAAFQAVKENAGSYGADEQTIKEFEEHLNNNLYKLWNRMASGSYFPKPVRAVAIPKKNG

GIRILGIPTVEDRIAQMVAKMYFEPLVEPMFYNDSYGYRPNKSAIQAVGQARERCFKRDWVLELDIKGLFDNIK
HGYLMYMVEKHTQIKWLILYIKRWLTVPFIMSDGSVAERRSGTPQGGVISPVLANLFLHYVFDDFMTKAYPNI
WWERYADDGVLHCQSYKQAAFIKQKLEERFQQFGLELNKEKTRIVYCKDNRRPQNYSCTQFTFLGYTFRPRLN
KNKEGKFFVGFTPAVSEKAKTAMKQKIREWKIQLKADLSLKDIGNMINKVVQGWINYYTHYYKSEFYEVLRYIN
QCLIKWVRRSYKKKNTRSRAEHWLGAVARRDRNLFAHWKFGILPSVGEGAV
>R10H087||gene_266920|GeneMark.hmm|418_aa|-|182|1438
MSEAKQFDISKKAVIAAFQAVKENAGSYGADEQTIKEFEEHLNNNLYKLWNRMASGSYFPKPVRAVAIPKKNG
GIRILGIPTVEDRIAQMVAKMYFEPLVEPMFYNDSYGYRPNKSAIQAVGQARERCFKRDWVLELDIKGLFDNIK
HGYLMYMVEKHTQIKWLILYIKRWLTVPFIMSDGSVAERRSGTPQGGVISPVLANLFLHYVFDDFMTKAYPNI
WWERYADDGVLHCQSYKQAAFIKQKLEERFQQFGLELNKEKTRIVYCKDNRRPQNYSCTQFTFLGYTFRPRLN
KNKEGKFFVGFTPAVSEKAKTAMKQKIREWKIQLKADLSLKDIGNMINKVVQGWINYYTHYYKSEFYEVLRYIN
QCLIKWVRRSYKKKNTRSRAEHWLGAVARRDRNLFAHWKFGILPSVGEGAV
>R10H088||gene_150895|GeneMark.hmm|391_aa|-|3|1175
MQNDNAKPISISKQLVYDAFLRVKANRGSAGIDKVTLEDYEKNLRGNLYKLWNRMSSGSYFPPSVKLVEIPKSTG
GKRPLGIPTVSDRVAQMAVVMLITPSIEPCFHEDSYAYRPHRSAHDAVGKARERCWKYAWVLDMDISKFFDTI
DHELLLKALKRHTQEKWVLMYIERWLKVPYEKSDGSQVDRALGVPQGSVIGPVLANLFLHYTFDKWMEKNFP
RVPFERYADDTICHCHSLKQAEYMQAMIQQRFECCRLRLNEEKTKIVYCKSSRQKECYPNVTFDFLGFTFQPRES
VDKYGNRFTGFLPAISRKSMKRINETMRSWHLNRHSNLTLEHLASDINPIVRGWMTYYGKFYPTRLKWFMQTL
NGRLARWVMCKFERYRHRFYPAQ
>R10H091||gene_43974|GeneMark.hmm|421_aa|+|568|1833
MQEAKPFQIDKRIIFEAFKKVKFNRGSSGIDGIEMTTYEQNLGSNLYRLWNRMSSGSYMPKAVKLVEIPKSNGG
KRPLGIPTIEDRIAQMAVVNVIEPLIEPCFHEDSFGYRPHRSAHDAIAKAERRCWKYAWVLDIDISKFFDTIDHGL
LMKAVEKHINIKWILLYIKRWLTVPYQRSDGEIVKRDMGVPQGSVIGPILANLFLHYTFDKWMSYKYPHIPFERY
ADDCVCHCSTLAQAEYIKERLGERFTECKLKFNEEKTKIVFCKMSSRSSKHYHCTSFDYLGFTFRTRAAKDKRNN
VLFTSYLPAISKKSVSRIHETIKSWNLKRLHNRSLRFVAAYINDVVRGWINYYEKFGKTEFWKVMCHLNRSIAYW
AKTKYKRLRRRGVISAHYWLAYIAQKEPNLFYHWQVGYVPYARQKK
>R10H091||gene_190110|GeneMark.hmm|401_aa|-|675|1880
NLNAAYKRVKANKGSAGIDGMDFEKFEKRLNNNLYKIWNRMSSGSYFPSPVMAVEIPKKSGGTRRLGIPTIADR
IAQMVARAYVERAVEPMFCEDSYGYRPHKSALDAVEKTRKRCWKYDYVIELDVKGLFDNIDHELLMRVVRRHV
KEPWICLYIERWLKSPFVLPDGSRIERESGTPQGGVISPVLANMFLHYVFDMWMKRNFPQAPFERYADDGVV
HCRTKEEALYIKKKLVKRFEECKLELHPVKTRIVYCKDKDRTKEEELAEFDFLGYTFKAVYIKCKDGVMRNNFIASV
SKTAAKGFRDKIKALEIHKRTGCKIDMIAELLNPMIRGWMNYFGKFNPSAMKNTLQCIECRLIKWAMCKYKSFR
GRRQRAEKWLSSIRKREPKLFAHWSRMYSYC
>R10H095||gene_66886|GeneMark.hmm|420_aa|-|53|1315
MNEAKPFVIDKRLVWEAYHKVKENKGSAGIDKVDQKTFDKEMSKNLYKIWNRMSSGCYFPKAVKLVEIPKSNG
GTRPLGIPTIEDRIAQQVVVSVLTPILEPIFKEDSYGYRPGKGAHQAVAKAKERCYVNPWVLDMDISKFFDTINHE
LLMKAVRKHTGEKWVLLYIERWLKVPYQTLKGEVIERTMGVPQGSVIGPVLANLFLHYVFDEWMSRNYPTIPFE
RYADDTICHCVSEKQAQFLKAVLMKRFEECGLKLNEEKTKIVYCKDSNRRGDSEHTSFDFLGFTFRPRGARNRKT
GQNFTAFLPAISRKSMKRIKEAVRAWKLNRKTFACLLDISNEVDTQISGWMNYYMKFGRSEFRKVLNYINERLT
RWVMRKYKRFSKGKKLGRAYEWLVEYAAHNRNEFSHWVKGFVPYPRLG
>R10H096||gene_11704|GeneMark.hmm|418_aa|-|68|1324
MSEAKQFDISKKAVIAAFQAVKENAGSYGADEQTIKEFEEHLNNNLYKLWNRMASGSYFPKPVRAVAIPKKNG
GIRILGIPTVEDRIAQMVAKMYFEPLVEPMFYNDSYGYRPNKSAIQAVGQARERCFKRDWVLELDIKGLFDNIK
HGYLMYMVEKHTQIKWLILYIKRWLTVPFIMSDGSVAERRSGTPQGGVISPVLANLFLHYVFDDFMTKAYPNI

WWERYADDGVLHCQSYKQAAFIKQKLEERFQQFGLELNKEKTRIVYCKDNRRPQNYSCTQFTFLGYTFRPRLN
KNKEGKFFVGFTPAVSEKAKTAMKQKIRGWKIQLKADLSLKDIGNMINKVVQGWINYYTHYYKSEFYEVLRYIN
QCLIKWVRRSYKKKNTRSRAEHWLGAVARRDRNLFAHWKFGILPSVGEGAV
>R10H097||gene_78011|GeneMark.hmm|418_aa|+|560|1816
MSEAKQFDISKKAVIAAFQAVKENAGSYGADEQTIKEFEEHLNNNLYKLWNRMASGSYFPKPVRAVAIPKKNG
GIRILGIPTVEDRIAQMVAKMYFEPLVEPMFYNDSYGYRPNKSAIQAVGQARERCFKRDWVLELDIKGLFDNIK
HGYLMYMVEKHTQIKWLILYIKRWLTVPFIMSDGSVAERRSGTPQGGVISPVLANLFLHYVFDDFMTKAYPNI
WWERYADDGVLHCQSYKQAAFIKQKLEERFQQFGLELNKEKTRIVYCKDNRRPQNYSCTQFTFLGYTFRPRLN
KNKEGKFFVGFTPAVSEKAKTAMKQKIREWKIQLKADLSLKDIGNMINKVVQGWINYYTHYYKSEFYEVLRYIN
QCLIKWVRRSYKKKNTRSRAEHWLGAVARRDRNLFAHWKFGILPSVGEGAV
>R10H098||gene_162896|GeneMark.hmm|422_aa|-|221|1489
MKDAKSFEISRHLVMEAYKRVKANKGAAGVDDVSIADFESNLKSNLYKIWNRMSSGSYFPPAVKLVEIPKSNGG
KRPLGIPTIGDRVAQMVVVMTIEPGIEPYFHEDSYAYRPNRSALDAVRKAKERSYTFHWVLDLDIKGFFDNIDHG
LLIKALERHVKCEWAMLYIKRWLSVPYQLKDGTQIERTKGVPQGSVVGPILANLFLHYVFDEWMRRNHSNISFE
RYADDTICHCVSLKQAEFILRAIRKRFAECKLELNEDKTKIVYCKKNHRDIPYECIQFDFLGYTFRPRRSIDANGEVF
LNFSPAISKKARTKIWEAIQNWNSNHWVPMELEDIAKEINPVIQGWINYYGQHNPRILKEVLQHVNDRLVRW
GRRKFKGLRKRKTATVHRLGDIALQKPNLFAHWAWGVKPTASEKNRKRK
>R10H099||gene_330768|GeneMark.hmm|421_aa|+|568|1833
MQEAKPFQIDKRIIFESFKKVKFNRGSSGIDGIEMTTYEQNLGSNLYRLWNRMSSGSYMPKAVKLVEIPKSNGG
KRPLGIPTIEDRIAQMAVVNVIEPLIEPCFHEDSFGYRPHRSAHDAIAKAERRCWKYAWVLDIDISKFFDTIDHGL
LMKAVEKHINIKWILLYIKRWLTVPYQRSDGEIVKRDMGVPQGSVIGPILANLFLHYTFDKWMSYKYPHIPFERY
ADDCVCHCSTLAQAEYIKERLGERFTECKLKFNEEKTKIVFCKMSSRSSKHYHCTSFDYLGFTFRSRAAKDKRNN
VLFTSYLPAISKKSVSRIHETIKSWNLKRLHNRSLRFVAAYINDVVRGWINYYEKFGKTEFWKVMCHLNRSIAYW
AKTKYKRLRRRGVISAHYWLAYIAQKEPNLFYHWQVGYVPYARQKK
>R10H101||gene_176123|GeneMark.hmm|367_aa|+|857|1960
MGSGCYFPPSVKLVEIPKSTGGKRPLGIPTVSDRVAQMVIVMLITPSIESYFHEDSYAYRPHRSALDAVGKARECC
WKYAWVLDMDISKFFDTIDHELLLKALKRHTQEKWVLMYIERWLNVPYEKGDGSQVDRVLRVPQDSVIGPILA
NLFLHYIFDKWMEKNFPRVPFERYADDTICHCHSLKQAEYMQAMIQQRFESCRLRLNEEKTKIVYCKSSRQKEC
YPNVTFDFLGFTFQPRESVDKYGNRFMGFLPVISQKSMKRINETIHSWNLNRHSNLTLERLAADINPIVRGWM
TYYGKFYPTRLKWFMQTLNGRLARWVICNSSVIDIVSILLKNGLPVLQRKRDLSFTIGNVAFCQDLPIKKR
>R10H103||gene_167740|GeneMark.hmm|421_aa|-|393|1658
MQEAKPFQIDKRIIFESFKKVKFNRGSSGIDGIEMTTYEQNLGSNLYRLWNRMSSGSYMPKAVKLVEIPKSNGG
KRPLGIPTIEDRIAQMAVVNVIEPLIEPCFHEDSFGYRPHRSAHDAIAKAERRCWKYAWVLDIDISKFFDTIDHGL
LMKAVEKHINIKWILLYIKRWLTVPYQRSDGEIVKRDMGVPQGSVIGPILANLFLHYTFDKWMSYKYPHIPFERY
ADDCVCHCSTLAQAEYIKERLGERFTECKLKFNEEKTKIVFCKMSSRSSKHYHCTSFDYLGFTFRSRAAKDKRNN
VLFTSYLPAISKKSVSRIHETIKSWNLKRLHNRSLRFVAAYINDVVRGWINYYEKFGKTEFWKVMCHLNRSIAYW
AKTKYKRLRRRGVISAHYWLAYIAQKEPNLFYHWQVGYVPYARQKK
>R10H104||gene_110214|GeneMark.hmm|420_aa|-|241|1503
MNEAKPFVIDKRLVWEAYHKVKENKGSAGIDRVDQKTFDKEMSKNLYKIWNRMSSGCYFPKAVKLVEIPKSNG
GTRPLGIPTIEDRIAQQVVVSVLTPILEPIFKEDSYGYRPGKGAHQAIAKAKERCYVNPWVLDMDISKFFDTINHD
LLMKAVRKHTEEKWVLLYIERWLKVPYQTSKSEVIERTMGVPQGSVIGPVLANLFLHYVFDEWMSRNYPTIPFE
RYADDTICHCVSEKQARFLKAVLMKRFEECGLKLNEEKTKIVYCKDSNRRGDSEHTSFDFLGFTFRPRGARNRKT
GQNFTAFLPAISKKSMKRIKEAVRAWKLNRKTFACLLDISNEVDTQISGWMNYYMKFGRSEFRKVLNYINERLT
RWVMRKYKRFSKGKKFSKAYDWLVEYAAHNRNEFSHWVKGFVPYPRLG

>R10H105||gene_18404|GeneMark.hmm|420_aa|+|14223|15485
MNEAKPFVIDKRLVWEAYHKVKENKGSAGIDKVDQKTFDKEMSKNLYKIWNRMSSGCYFPKAVKLVEIPKSNG
GTRPLGIPTIEDRIAQQVVVSVLTPILEPIFKEDSYGYRPGKGAHQAIAKAKERCYVNPWVLDMDISKFFDTINHD
LLMKAVRKHTEEKWVLLYIERWLKVPYQTSKGEVIERTMGVPQGSVIGPVLANLFLHYVFDEWMSRNYPTIPFE
RYADDTICHCVSEKQAQFLKAVLMKRFEECGLKLNEEKTKIVYCKDSNRRGDSEHTSFDFLGFTFRPRSARNRKT
GQNFTAFLPAISKKSLKRIKEAVRAWKLNRKTFACLLDISNEVDTQISGWMNYYMKFGRSEFRKVLNYINERLTR
WVMRKYKRFSKGKKFSRAYEWLVEYAVHNRNEFSHWAKGFVPYPRLG
>R10H107||gene_305933|GeneMark.hmm|418_aa|+|56|1312
MSEAKQFDISKKAVIAAFQAVKENAGSYGVDEQTIKEFEEHLNNNLYKLWNRMASGSYFPKPVRAVAIPKKNG
GTRILGIPTVEDRIAQMVAKMYFEPLVEPMFYNDSYGYRPNKSAIQAVRQARERCFKRDWVLELDIKGLFDNIK
HGYLMYMVEKHTQIKWLILYIKRWLTVPFIMSDGSVAERRSGTPQGGVISPVLANLFLHYVFDDFMTKAYPNI
WWERYADDGVLHCQSYKQAVFIKQKLEERFQQFGLELNKEKTRIVYCKDNRRSQNYSCTQFTFLGYTFRPRLNK
NKEGKFFVGFTPAVSEKAKTAMKQKIREWKIQLKAELSLKDIGNMINKVVQGWINYYTHYYKSEFYKVLRYINQ
CLIKWGRRSYKKKNTRSRAEHWLGAVARRDRNLFVHWKFGILPSVGEGAV
>R10H110||gene_201574|GeneMark.hmm|418_aa|+|2014|3270
MSEAKQFDISKKAVIAAFQAVKENAGSYGADEQTIKEFEEHLNNNLYKLWNRMASGSYFPKPVRAVAIPKKNG
GIRILGIPTVEDRIAQMVAKMYFEPLVEPMFYNDSYGYRPNKSAIQAVGQARERCFKRDWVLELDIKGLFDNIK
HGYLMYMVEKHTQIKWLILYIKRWLTVPFIMSDGSVAERRSGTPQGGVISPVLANLFLHYVFDDFMTKAYPNI
WWERYADDGVLHCQSYKQAAFIKQKLEERFQQFGLELNKEKTRIVYCKDNRRPQNYSCTQFTFLGYTFRPRLN
KNKEGKFFVGFTPAVSEKAKTAMKQKIREWKIQLKADLSLKDIGNMINKVVQGWINYYTHYYKSEFYEVLRYIN
QCLIKWVRRSYKKKNTRSRAEHWLGAVARRDRNLFAHWKFGILPSVGEGAV
>R10H114||gene_980|GeneMark.hmm|414_aa|-|999|2243
MQETKPYSISKRAVIAAYEKVKANKGTYGVDEQSIEDFEKRLNNNLYKIWNRMSSGSYFPKPVKAVAIPKKNGG
TRILGIPTVEDRIAQMVVKVYFEPNVESIFYEDSYGYRPNKSAIQAIEVTRTRCWRKDWVLEFDIKGLFDNIRHDY
LMEMVKRHTKEEWILLYIQRWLVAPFQMEDGTLAPRTSGTPQGGVISPVLANLFLHYVFDDFMVKEFPSIPWA
RYADDGIAHCASMKQAKYLQRRLQERFVSFGLELNLEKTRIVYCKDDDRKGNHECTSFDFLGYTFRPRHAKNKY
GKYFTNFLPAMGEKAKKAIRKVVRGWKLQYKPDKDLWDIANMFNKQIQGWINYYTHFYKSEIYEVLRYINGRL
VYWVRRKYKNRNSRKRAEYWLGEIAKRDRNLFAHWKFGILPSAG
>R10H115||gene_192131|GeneMark.hmm|430_aa|+|3640|4932
MQNDNAKPISISKQLVYDAFLRVKANRGSAGIDKVTLEDYEKNLRGNLYKLWNRMSSGSYFPPSVKLVEIPKSTG
GKRPLGIPTVSDRVAQMAVVMLITPSIEPCFHEDSYAYRPHRSAHDAVGKARERCWKYAWVLDMDISKFFDTI
DHELLLKALKRHTQEKWVLMYIERWLKVPYEKSDGSQVDRALGVPQGSVIGPVLANLFLHYTFDKWMEKNFP
RVPFERYADDTICHCHSLKQAEYMQAMIQQRFECCRLRLNEEKTKIVYCKSSRQKECYPNVTFDFLGFTFQPRES
VDKYGNRFTGFLPAISRKSMKRINETMRSWHLNRHSNLTLEHLASDINPIVRGWMTYYGKFYPTRLKWFMQTL
NGRLARWVMCKFERYRHRFYPAQEWLARIAEKEGLIFYHWKCGALPRFTNKEKVSSQLIMVK
>R10H115||gene_16988|GeneMark.hmm|420_aa|+|4416|5678
MNEAKPFVIDKRLVWEAYHKVKENKGSAGIDKVDQQTFDKEMSKNLYKIWNRMSSGCYFPKAVKLVEIPKSNG
GTRPLGIPTIEDRIAQQVVVSVLTPILEPIFKEDSYGYRPGKGAHQAIAKAKERCYVNPWVLDMDISKFFDTINHE
LLMKAVRKHTEEKWVLLYIERWLKVPYQTSKGEVIERTMGVPQGSVIGPVLANLFLHYVFDEWMSRNYPTIPFE
RYADDTICHCVSDKQAQFLKAVLMKRFEECGLKLNEEKTKIVYCKDSNRRGDSEHTSFDFLGFTFRPRGARNRKT
GQNFTAFLPAISKKSMKRIKEAVRAWKLNRKTFACLLDISNEVDTQISGWMNYYMKFGRSEFRKVLNYINERLT
RWVMRKYKRFSKGRKFDRAYDWLVEYAAHNRNEFSHWVKGFVPYPRLG
>R10H115||gene_140243|GeneMark.hmm|421_aa|-|89|1354
MQEAKPFQIDKRIIFESFKKVKFNRGSSGIDGIEMTTYEQNLGSNLYRLWNRMSSGSYMPKAVKLVEIPKSNGG

KRPLGIPTIEDRIAQMAVVNVIEPLIEPCFHEDSFGYRPHRSAHDAIAKAERRCWKYAWVLDIDISKFFDTIDHGL
LMKAVEKHINIKWILLYIKRWLTVPYQRSDGEIVKRDMGVPQGSVIGPILANLFLHYTFDKWMSYKYPHIPFERY
ADDCVCHCSTLAQAEYIKERLGERFTECKLKFNEEKTKIVFCKMSSRSSKHYHCTSFDYLGFTFRSRAAKDKRNN
VLFTSYLPAISKKSVSRIHETIKSWNLKRLHNRSLRFVAAYINDVVRGWINYYEKFGKTEFWKVMCHLNRSIAYW
AKTKYKRLRRRGVISAHYWLAYIAQKEPNLFYHWQVGYVPYARQKK
>R10H117||gene_92385|GeneMark.hmm|418_aa|+|595|1851
MSEAKQFDISKKAVIAAFQAVKENAGSYGADEQTIKEFEEHLNNNLYKLWNRMASGSYFPKPVRAVAIPKKNG
GIRILGIPTVEDRIAQMVAKMYFEPLVEPMFYNDSYGYRPNKSAIQAVGQARERCFKRDWVLELDIKGLFDNIK
HGYLMYMVEKHTQIKWLILYIKRWLTVPFIMSDGSVAERRSGTPQGGVISPVLANLFLHYVFDDFMTKAYPNI
WWERYADDGVLHCQSYKQAAFIKQKLEERFQQFGLELNKEKTRIVYCKDNRRPQNYSCTQFTFLGYTFRPRLN
KNKEGKFFVGFTPAVSEKAKTAMKQKIREWKIQLKADLSLKDIGNMINKVVQGWINYYTHYYKSEFYEVLRYIN
QCLIKWVRRSYKKKNTRSRAEHWLGAVARRDRNLFAHWKFGILPSVGEGAV
>R10H118||gene_219695|GeneMark.hmm|420_aa|+|9665|10927
MNEAKPFVIDKRLVWEAYHKVKENKGSAGIDKVDQKTFDKEMSKNLYKIWNRMSSGCYFPKAVKLVEIPKSNG
GTRPLGIPTIEDRIAQQVVVSVLTPILEPIFKEDSYGYRPGKGAHQAIAKAKERCYVNPWVLDMDISKFFDTINHD
LLMKAVRKHTEEKWVLLYIERWLKVPYQTSKGEVIERTMGVPQGSVIGPVLANLFLHYVFDEWMSRNYPTIPFE
RYADDTICHCVSEKQAQFLKAVLMKRFEECGLKLNEEKTKIVYCKDSNRRGDSEHTSFDFLGFTFRPRSARNRKT
GQNFTAFLPAISKKSLKRIKEAVRAWKLNRKTFACLLDISNEVDTQISGWMNYYMKFGRSEFRKVLNYINERLTR
WVMRKYKRFSKGKKFSRAYEWLVEYAVHNRNEFSHWAKGFVPYPRLG
>R10H124||gene_81892|GeneMark.hmm|420_aa|-|6034|7296
MNEAKPFVIDKRLVWEAYHKVKENKGSAGIDKVDQKTFDKEMSKNLYKIWNRMSSGCYFPKAVKLVEIPKSNG
GTRPLGIPTIEDRIAQQVVVSVLTPILEPIFKEDSYGYRPGKGAHQAIAKAKERCYVNPWVLDMDISKFFDTINHD
LLMKAVRKHTEEKWVLLYIERWLKVPYQTSKGEVIERTMGVPQGSVIGPVLANLFLHYVFDEWMSRNYPTIPFE
RYADDTICHCVSEKQAQFLKAVLMKRFEECGLKLNEEKTKIVYCKDSNRRGDSEHTSFDFLGFTFRPRSARNRKT
GQNFTAFLPAISKKSLKRIKEAVRAWKLNRKTFACLLDISNEVDTQISGWMNYYMKFGRSEFRKVLNYINERLTR
WVMRKYKRFSKGKKFSRAYEWLVEYAVHNRNEFSHWAKGFVPYPRLG
>R10H128||gene_124863|GeneMark.hmm|410_aa|-|8535|9767
MQRKSFEIPKALVWASYLDVRRNKGAPGCDGQTLKMFDQQRDGNLYKIWNRLCSGTWFPPPVLEKRIPKPN
GKERILGIPTVSDRIAQGAIKLFMEEKLDPIFHADSYGYRPGKSAHDALKQCAIRCWRYSWILEVDISAFFDHVRH
DLVLKALEHHGMPKWVILYCRRWMEAPMQSCENGELITRTRGTPQGGVISPLLANLFLHYAFDLWMEREYRG
VPFERYADDIVVHCSRMSDATRLKNRLSERFSEVGLVLNAGKTNIAYIDTFKRRNVATSFTFLGYDFKVRTLKNFK
GELYRKCMPGASNAAMRKITETIKKWRIHRSTAESLLDFARRYNAIVRGWIEYYGKFWSRNFNYRLWSAMQSR
LLKWMQSKYRLSNRRAQRKLTLVRKEYPKLFVHWYLLRASNE
>R10H128||gene_189113|GeneMark.hmm|416_aa|+|1003|2253
MTKTKAFNIDKSLVVSAYRRVKTSAGAAGIDKQSLADFDKRLVDNLYKIWNRLSSGSYFPPAVKAVAIPKKLGGER
ILGIPTVSDRIAQTVVKLAFEPQVEPHFLADSYGYRPNKSALDAIGVTRKRCWYYDWVLEFDIKGLFDNIPHELIM
KAVDKHNPARWVKLYIQRWLTAPMVMSDGEVRARTMGTPQGGVISPLLANLFMHYVFDKWLAKYYPKVPW
YRYADDGILHCHSEAEATEMREVLRKRFSECGLEMHPEKTRVIYCKDGSRKGDYEHTMFDFLGYTFRRRVVKNV
KRNSLFVSFTPAASKSALKAMRREIKATGIRKRVDLSIEQIAKWINPKLNGWINYYGRYTCSELYSVFRYINKALVR
WGRKKYKMLSRYKTRASKFLEEMAKRSPQLFAHWRLKMRGGLV
>R10H128||gene_132964|GeneMark.hmm|423_aa|+|568|1839
MQEAKPKPFQIDKRIIFESFKKVKFNRGSSGIDGIEMTTYEQNLGSNLYRLWNRMSSGSYMPKAVKLVEIPKSN
GGKRPLGIPTIEDRIAQMAVVNVIEPLIEPCFHEDSFGYRPHRSAHDAIAKAERRCWKYAWVLDIDISKFFDTIDH
GLLMKAVEKHINIKWILLYIKRWLTVPYQRSDGEIVKRDMGVPQGSVIGPILANLFLHYTFDKWMSYKYPHIPFE

RYADDCVCHCSTLAQAEYIKERLGERFTECKLKFNEEKTKIVFCKMSSRSSKHYHCTSFDYLGFTFRSRAAKDKRN
NVLFTSYLPAISKKSVSRIHETIKSWNLKRLHNRSLRFVAAYINDVVRGWINYYEKFGKTEFWKVMCHLNRSIAY
WAKTKYKRLRRRGVISAHYWLAYIAQKEPNLFYHWQVGYVPYARQKK
>R2H12||gene_21514|GeneMark.hmm|420_aa|+|880|2142
MNEAKPFVIDKRLVWEAYHKVKENKGSAGIDKVDQQTFDKEMSKNLYKIWNRMSSGCYFPKAVKLVEIPKSNG
GTRPLGIPTIEDRIAQQVVVSVLTPILEPIFKEDSYGYRPGKGAHQAIAKAKERCYVNPWVLDMDISKFFDTINHE
LLMKAVRKHTEEKWVLLYIERWLKVPYQTSKGEVIERTMGVPQGSVIGPVLANLFLHYVFDEWMSRNYPTIPFE
RYADDTICHCVSDKQAQFLKAVLMKRFEECGLKLNEEKTKIVYCKDSNRRGDSEHTSFDFLGFTFRPRGARNRKT
GQNFTAFLPAISKKSMKRIKEAVRAWKLNRKTFACLLDISNEVDTQISGWMNYYMKFGRSEFRKVLNYINERLT
RWVMRKYKRFSKGRKFDRAYDWLVEYAAHNRNEFSHWVKGFVPYPRLG
>R2H13||gene_32372|GeneMark.hmm|420_aa|-|1528|2790
MNEAKPFVIDKRLVWEAYHKVKENKGSAGIDKVDQKTFDKEMSKNLYKIWNRMSSGCYFSKAVKLVEIPKSNG
GTRPLGIPTIEYRIAQQVVVSVLTPILEPIFKEDSYGYRPGKGAHQAIAKAKERCYVTPWVLDMDISKFFDTINHEL
LMKAIRKHTEEKWVLLYIERWLKVPNQTSKGEVIERTMGVPQGSVIGPVLANLFLHYVFDEWMSRNYPTIPFER
YADATICHCVSEKQARFLKAVLMKRFEEYGLKLNEEKTKIVYCKDSNRRGDSEHTSFNFLGFTFRPRGARNRKTG
QNFTAFLPAISNKSMKRIKEAIRAWKLNRKTFACLLDISTEVDTQISGWMNYYMKFGRSEFRKVLNYINERLTRW
VMRKYKRFSKGKKFSKAYEWLVEYAAHNRNEFSHWVKGFVSYPRLD
>R2H19||gene_27846|GeneMark.hmm|416_aa|+|705|1955
MTKTKAFNIDKSLVVSAYRRVKTSAGAAGIDKQSLADFDKRLVDNLYKIWNRLSSGSYFPPAVKAVAIPKKLGGER
ILGIPTVSDRIAQTVVKLAFEPQVEPHFLADSYGYRPNKSALDAIGVTRKRCWYYDWVLEFDIKGLFDNIPHELIM
KAVDKHNPARWVKLYIQRWLTAPMVMSDGEVRARTMGTPQGGVISPLLANLFMHYVFDKWLAKYYPKVPW
YRYADDGILHCHSEAEATEMREVLRKRFSECGLEMHPEKTRVIYCKDGSRKGDYEHTMFDFLGYTFRRRVVKNV
KRNSLFVSFTPAASKSALKAMRREIKATGIRKRVDLSIEQIAKWINPKLNGWINYYGRYTCSELYSVFRYINKALVR
WGRKKYKMLSRYKTRASKFLEEMAKRSPQLFAHWRLKMRGGLV
>R2H2||gene_15836|GeneMark.hmm|420_aa|-|116|1378
MNEAKPFVIDKRLVWEAYHKVKENKGSAGIDKVDQKTFDKEMSKNLYKIWNRMSSGCYFPKAVKLVEIPKSNG
GTRPLGIPTIEDRIAQQVVVSVLTPILEPIFKEDSYGYRPGKGAHQAIAKAKERCYVNPWVLDMDISKFFDTINHE
LLMKAVRKHTEEKWVLLYIERWLKVPYQTSKGEVIERTMGVPQGSVIGPVLANLFLHYVFDEWMSRNYPTIPFE
RYADDTICHCVSEKQAQFLKAVLMKRFEECGLKLNEEKTKIVYCKDSNRRGDSEHTSFDFLGFTFRPRSARNRKT
GQNFTAFLPAISKKSMKRIKEAIRAWKLNRKTFACLLDISNEVDTQISGWMNYYMKFGRSEFRKVLNYINERLTR
WVMRKYKRFSKGRKFDRAYDWLVEYAAHNRNEFSHWVKGFVPYPRLG
>R2H20||gene_57386|GeneMark.hmm|420_aa|+|4455|5717
MNEAKPFVIDKRLVWEAYHKVKENKGSAGIDKVDQQTFDKEMSKNLYKIWNRMSSGCYFPKAVKLVEIPKSNG
GTRPLGIPTIEDRIAQQVVVSVLTPILEPIFKEDSYGYRPGKGAHQAIAKAKERCYVNPWVLDMDISKFFDTINHE
LLMKAVRKHTEEKWVLLYIERWLKVPYQTSKGEVIERTMGVPQGSVIGPVLANLFLHYVFDEWMSRNYPTIPFE
RYADDTICHCVSDKQAQFLKAVLMKRFEECGLKLNEEKTKIVYCKDSNRRGDSEHTSFDFLGFTFRPRGARNRKT
GQNFTAFLPAISKKSMKRIKEAVRAWKLNRKTFACLLDISNEVDTQISGWMNYYMKFGRSEFRKVLNYINERLT
RWVMRKYKRFSKGRKFDRAYDWLVEYAAHNRNEFSHWVKGFVPYPRLG
>R2H21||gene_73468|GeneMark.hmm|420_aa|+|817|2079
MNEAKSFVIDKRLVWEAYHKVKENKGSAGIDKVDQKTFDKEMSKNLYKIWNRMSSGCYFPKAVKLVEIPKSNG
GTRPLGIPTIEDRIAQQVVVSVLIPILEPIFKEDSYGYRPGKGAHQAIAKAKERCYVNPWVLDMDISKFFDTINHEL
LMKAIRKHTEEKWVLLYIERWLKVPNQTSKGEVIERTMGVPQGSVIGPVLANLFLHYVFDEWMSRNYPTIPFER
YADDTICHCVSEKQAQFLKAVLMKRFEECGLKLNEEKTKIVYCKDSNRRGDSEHTSFDFLGFTFRPRGARNRKTG
QNFTAFLPAISKKSMKRIKEAVRAWKLNRKTFACLLDISNEVDTQISGWMNYYMKFGRSEFRKVLNYINERLTR

WVMRKYKRFSKGRKFDRAYDWLVEYATHNRNEFSHWVKGFVPYPRLD
>R2H22||gene_20006|GeneMark.hmm|420_aa|-|3623|4885
MNEAKSFVIDKRLVWEAYHKVKENKGSAGIDKVDQKTFDKEMSKNLYKIWNRMSSGCYFPKAVKLVEIPKSNG
GTRPLGIPTIEDRIAQQVVVSVLIPILEPIFKEDSYGYRPGKGAHQAIAKAKERCYVNPWVLDMDISKFFDTINHEL
LMKAIRKHTEEKWVLLYIERWLKVPNQTSKGEVIERTMGVPQGSVIGPVLANLFLHYVFDEWMSRNYPTIPFER
YADDTICHCVSEKQAQFLKAVLMKRFEECGLKLNEEKTKIVYCKDSNRRGDSEHTSFDFLGFTFRPRGARNRKTG
QNFTAFLPAISKKSMKRIKEAVRAWKLNRKTFACLLDISNEVDTQISGWMNYYMKFGRSEFRKVLNYINERLTR
WVMRKYKRFSKGRKFDRAYDWLVEYATHNRNEFSHWVKGFVPYPRLD
>R2H23||gene_148311|GeneMark.hmm|420_aa|-|16813|18075
MNEAKPFVIDKRLVWEAYHKVKENKGSAGIDKVDQKTFDKEMSKNLYKIWNRMSSGCYFPKAVKLVEIPKSNG
GTRPLGIPTIEDRIAQQVVVSVLTPILEPIFKEDSYGYRPGKGAHQAIAKAKERCYVNPWVLDMDISKFFDTINHE
LLMKAVRKHTEEKWVLLYIERWLKVPYQTSKGEVIERTMGVPQGSVIGPVLANLFLHYVFDEWMSRNYPTIPFE
RYADDTICHCVSEKQAQFLKAVLMKRFEECGLKLNEEKTKIVYCKDSNRRGDSEHTSFDFLGFTFRPRSARNRKT
GQNFTAFLPAISKKSMKRIKEAIRAWKLNRKTFACLLDISNEVDTQISGWMNYYMKFGRSEFRKVLNYINERLTR
WVMRKYKRFSKGRKFDRAYDWLVEYAAHNRNEFSHWVKGFVPYPRLG
>R2H23||gene_103739|GeneMark.hmm|423_aa|+|703|1974
MTQKQGAKPFDIDRWKLYYAYQRVNQNRGGSGVDNVTLEKYNSNLKRNLYKLWNRMSSGSYVPKPVRLVQIP
KPAGGTRPLGIPTVEDRIAQMLVVEMIEPEIEKIFHEDSYGYRPNRSAHDALGRARERCWKYAWVLDMDISKFF
DTIDHQLLMKAVRLHVKERWIILYIERWLKVPYQNADKSLIERTCGVPQGSVIGPILANLFLHYCFDRWMQIHYP
EIPFERYADDTVCHCRSQREAESLYEELIIRFKSCKLSLNEEKTKIVYCKSSRRKENHSNVTFDFLGHTFRPCKTMH
KSSREAFTGFQPRISMKATTKIRATMRSWNLKSKSHTPLDCIAHMVNPILRGWVNYYGKYGGKSFQKLLGYFDL
LLARWAKAKYKTFRRKPMYVILKWLGNVADRDAVFYHWQIGLKPAKGTIKL
>R2H24||gene_105016|GeneMark.hmm|420_aa|+|573|1835
MNEAKPFVIDKRLVWEAYHKVKENKGSAGIDKVDQQTFDKEMSKNLYKIWNRMSSGCYFPKAVKLVEIPKSNG
GTRPLGIPTIEDRIAQQVVVSVLTPILEPIFKEDSYGYRPGKGAHQAIAKAKERCYVNPWVLDMDISKFFDTINHE
LLMKAVRKHTEEKWVLLYIERWLKVPYQTSKGEVIERTMGVPQGSVIGPVLANLFLHYVFDEWMSRNYPTIPFE
RYADDTICHCVSDKQAQFLKAVLMKRFEECGLKLNEEKTKIVYCKDSNRRGDSEHTSFDFLGFTFRPRGARNRKT
GQNFTAFLPAISKKSMKRIKEAVRAWKLNRKTFACLLDISNEVDTQISGWMNYYMKFGRSEFRKVLNYINERLT
RWVMRKYKRFSKGRKFDRAYDWLVEYAAHNRNEFSHWVKGFVPYPRLG
>R2H28||gene_16069|GeneMark.hmm|421_aa|+|2310|3575
MAEAKPFVIDKWAVHNAFLKVKENKGSSGIDGISIEQYEKKLSMHLYKLWNRMSSGTYFPKPVKLVEIPKANGG
TRPLGIPTVEDRVAQMVVVMTINERLEAIFHHDSYAYRPNRSTSDAIAVARQRCMRYSWVLDMDISKFFDTIDH
ELLMKAVRKHVSEKWILLYIERWLKVPYQTKDGKTIERYKGVPQGSVVGPVLANLFLTYVFDLWMKKNYPTIPFE
RYADDTICHCWTQQQAEELKSSLIRRFEDCKLKLNEEKTKIVYCKDSNRKGNYAETSFDFLGFTFRGRAARNSKT
HQVFTAFLPAMSKKAYEKKKEEIRSWKYLRNYHLDIEDLSVEMEDKVRGWILYYTKFGKTEFYKLTNFLNQRIVC
WARRKYKRFKGKKSKAHEWLVQLSQTKQWLFYHWQVGYVPYPYINKNK
>R2H29||gene_40174|GeneMark.hmm|423_aa|+|576|1847
MTQKQGAKPFDIDRWKLYYAYQRVNQNRGGSGVDNVTLEKYNSNLKRNLYKLWNRMSSGSYVPKPVRLVQIP
KPAGGTRPLGIPTVEDRIAQMLVVEMIEPEIEKIFHEDSYGYRPNRSAHDALGRARERCWKYAWVLDMDISKFF
DTIDHQLLMKAVRLHVKERWIILYIERWLKVPYQNADKSLIERTCGVPQGSVIGPILANLFLHYCFDRWMQIHYP
EIPFERYADDTVCHCRSQREAESLYEELIIRFKSCKLSLNEEKTKIVYCKSSRRKENHSNVTFDFLGHTFRPCKTMH
KSSREAFTGFQPRISMKATTKIRATMRSWNLKSKSHTPLDCIAHMVNPILRGWVNYYGKYGGKSFQKLLGYFDL
LLARWAKAKYKTFRRKPMYVILKWLGNVADRDAVFYHWQIGLKPAKGTIKL
>R2H29||gene_35495|GeneMark.hmm|420_aa|-|12524|13786

MNEAKPFVIDKRLVWEAYHKVKENKGSAGIDKVDQQTFDKEMSKNLYKIWNRMSSGCYFPKAVKLVEIPKSNG
GTRPLGIPTIEDRIAQQVVVSVLTPILEPIFKEDSYGYRPGKGAHQAIAKAKERCYVNPWVLDMDISKFFDTINHE
LLMKAVRKHTEEKWVLLYIERWLKVPYQTSKGEVIERTMGVPQGSVIGPVLANLFLHYVFDEWMSRNYPTIPFE
RYADDTICHCVSEKQAQFLKAVLMKRFEECGLKLNEEKTKIVYCKDSNRRGDSEHTSFDFLGFTFRPRGARNRKT
GQNFTAFLPAISKKSMKRIKEAVRAWKLNRKTFACLLDISNEVDTQISGWMNYYMKFGRSEFRKVLNYINERLT
RWVMRKYKRFSKGRKFDRAYDWLVEYAAHNRNEFSHWVKGFVPYPRLG
>R2H30||gene_134946|GeneMark.hmm|430_aa|-|336|1628
MQNDNAKPISISKQLVYDAFLRVKANRGSAGIDKVTLEDYEKNLRGNLYKLWNRMSSGSYFPPSVKLVEIPKSTG
GKRPLGIPTVSDRVAQMAVVMLITPSIEPCFHEDSYAYRPHRSAHDAVGKARERCWKYAWVLDMDISKFFDTI
DHELLLKALKRHTQEKWVLMYIERWLKVPYEKSDGSQVDRALGVPQGSVIGPVLANLFLHYTFDKWMEKNFP
RVPFERYADDTICHCHSLKQAEYMQAMIQQRFECCRLRLNEEKTKIVYCKSSRQKECYPNVTFDFLGFTFQPRES
VDKYGNRFTGFLPAISRKSMKRINETMRSWHLNRHSNLTLEHLASDINPIVRGWMTYYGKFYPTRLKWFMQTL
NGRLARWVMCKFERYRHRFYPAQEWLARIAEKEGLIFYHWKCGALPRFTNKEKVSSQLIMVK
>R2H31||gene_114135|GeneMark.hmm|420_aa|+|3469|4731
MNEAKPFVIDKRLVWEAYHKVKENKGSAGIDKVDQKTFDKEMSKNLYKIWNRMSSGCYFPKAVKLVEIPKSNG
GTRPLGIPTIEDRIAQQVVVSVLTPILEPIFKEDSYGYRPGKGAHQAIAKAKERCYVNPWVLDMDISKFFDTINHE
LLMKAIRKHTEEKWVLLYIERWLKVPYQTSKGEVIERTMGVPQGSVIGPVLANLFLHYVFDEWMSRNYPTIPFE
RYADDTICHCVSEKQAQFLKAVLMKRFEECGLKLNEEKTKIVYCKDSNRRGNSEHTSFDFLGFTFRPRSARNRKT
GQNFTAFLPAISKKSMKRIKEAVRAWKLNRKTFACLLDISNEVDTQISGWMNYYMKFGRSEFRKVLNYINERLT
RWVMRKYKRFSKGKKLGRAYEWLVEYAAHNRNEFSHWVKGFVPYPRLG
>R2H32||gene_54728|GeneMark.hmm|423_aa|+|7004|8275
MTQKQGAKPFDIDRWKLYYAYQRVNQNRGGSGVDNVTLEKYNSNLKRNLYKLWNRMSSGSYVPKPVRLVQIP
KPAGGTRPLGIPTVEDRIAQMLVVEMIEPEIEKIFHEDSYGYRPNRSAHDALGRARERCWKYAWVLDMDISKFF
DTIDHQLLMKAVRLHVKERWIILYIERWLKVPYQNADKSLIERTCGVPQGSVIGPILANLFLHYCFDRWMQIHYP
EIPFERYADDTVCHCRSQREAESLYEELIIRFKSCKLSLNEEKTKIVYCKSSRRKENHSNVTFDFLGHTFRPCKTMH
KSSREAFTGFQPRISMKATTKIRATMRSWNLKSKSHTPLDCIAHMVNPILRGWVNYYGKYGGKSFQKLLGYFDL
LLARWAKAKYKTFRRKPMYVILKWLGNVADRDAVFYHWQIGLKPAKGTIKL
>R2H35||gene_16466|GeneMark.hmm|421_aa|-|16953|18218
MQEAKPFQIDKRIIFEAFKKVKSNGGSPGIDGIEMSAYEQNLGSNFYRLWNRMSSGSYMPKAVKLVEILKSNGG
KRPLGIPSVEDRIAQMAVVNVIEPLVEPYFHKDSFGYRPHRSAHDAIAKAERRCWKYAWVLDIDISKFFDTIDHG
LLMKAVEKHIKTKWILLYIKRWLTVPYQGNDGAIVKRHMGVPQGSVIGPILANQFLHYTFDKWMSYKYPHVPF
ERYADDCVCHCGTLAQAEYIKDRLGERFAECKLTFNEEKTKIVFCKTSNRSSEHYHCTSFDYLGFTFRPRAAKDKR
KNVLFTSYLPAISNKSESRIHETIKSWNLKRLHNRSLRFVAAYINDVVRGWISYYGKFGKTEFWKVMCHLNRSIAY
WAKTKYKRLRRRGVISAHYWLAYIAQKEPNLFYHWQVGYIPYARQKK
>R2H36||gene_92983|GeneMark.hmm|420_aa|-|2256|3518
MNAANPFVIDKRLVWEAYHKVKENKGSAGIDKVDQKTFDKEMSKNLYKIWNRMSSGCYFPKAVKLVEMPKS
NGGTRPLGIPAIEDRIAQQVVVSVLTPILEPIFKEDSYGYRPGKGAHQAIAKAKERCYVTPWVLDMDISKFFDTIN
HELLMKAIRKHTEEKWVLLYIERWLKVPYQTSKGEVIERTMGVPQGSVIGPVLANLFLHYVFDEWMSRNYPTIP
FERYADDTICHCVSEKQARFLKAVLMKRFEECGLKLNEEKTKIVYCKDSNRRGDSEHTSFDFLGFTFRPRGARNR
KTGQNFTAFLPAISKKSMKRIKEAVRAWKLNRKTFACLLDISNEVDTQISGWMNYYMKFGRSEFRKVLNYINERL
TRWVMRKYKRFSKGKKFSKAYEWLVEYAAHNRNEFSHWAKGFVPYPRLD
>R2H40||gene_95375|GeneMark.hmm|420_aa|-|2781|4043
MNEAKSFVIDKRLVWEAYHKVKENKGSAGIDKVDQKTFDKEMSKNLYKIWNRMSSGCYFPKAVKLVEIPKSNG
GTRPLGIPTIEDRIAQQVVVSVLIPILEPIFKEDSYGYRPGKGAHQAIAKAKERCYVNPWVLDMDISKFFDTINHEL

LMKAIRKHTEEKWVLLYIERWLKVPNQTSKGEVIERTMGVPQGSVIGPVLANLFLHYVFDEWMSRNYPTIPFER
YADDTICHCVSEKQAQFLKAVLMKRFEECGLKLNEEKTKIVYCKDSNRRGDSEHTSFDFLGFTFRPRGARNRKTG
QNFTAFLPAISKKSMKRIKEAVRAWKLNRKTFACLLDISNEVDTQISGWMNYYMKFGRSEFRKVLNYINERLTR
WVMRKYKRFSKGRKFDRAYDWLVEYATHNRNEFSHWVKGFVPYPRLD
>R2H42||gene_39495|GeneMark.hmm|420_aa|+|790|2052
MNEAKPFVIDKRLVWEAYHKVKENKGSAGIDKVDQQTFDKEMSKNLYKIWNRMSSGCYFPKAVKLVEIPKSNG
GTRPLGIPTIEDRIAQQVVVSVLTPILEPIFKEDSYGYRPGKGAHQAIAKAKERCYVNPWVLDMDISKFFDTINHE
LLMKAVRKHTEEKWVLLYIERWLKVPYQTSKGEVIERTMGVPQGSVIGPVLANLFLHYVFDEWMSRNYPTIPFE
RYADDTICHCVSDKQAQFLKAVLMKRFEECGLKLNEEKTKIVYCKDSNRRGDSEHTSFDFLGFTFRPRGARNRKT
GQNFTAFLPAISKKSMKRIKEAVRAWKLNRKTFACLLDISNEVDTQISGWMNYYMKFGRSEFRKVLNYINERLT
RWVMRKYKRFSKGRKFDRAYDWLVEYAAHNRNEFSHWVKGFVPYPRLG
>R2H44||gene_92900|GeneMark.hmm|420_aa|+|816|2078
MNEAKSFVIDKRLVWEAYHKVKENKGSAGIDKVDQKTFDKEMSKNLYKIWNRMSSGCYFPKAVKLVEIPKSNG
GTRPLGIPTIEDRIAQQVVVSVLIPILEPIFKEDSYGYRPGKGAHQAIAKAKERCYVNPWVLDMDISKFFDTINHEL
LMKAIRKHTEEKWVLLYIERWLKVPNQTSKGEVIERTMGVPQGSVIGPVLANLFLHYVFDEWMSRNYPTIPFER
YADDTICHCVSEKQAQFLKAVLMKRFEECGLKLNEEKTKIVYCKDSNRRGDSEHTSFDFLGFTFRPRGARNRKTG
QNFTAFLPAISKKSMKRIKEAVRAWKLNRKTFACLLDISNEVDTQISGWMNYYMKFGRSEFRKVLNYINERLTR
WVMRKYKRFSKGRKFDRAYDWLVEYATHNRNEFSHWVKGFVPYPRLD
>R2H54||gene_219774|GeneMark.hmm|422_aa|+|544|1812
MKDAKSFEISRHLVMEAYKRVKANKGAAGVDEVSIADFENNLKSNLYKIWNRMSSGSYLPPAVKLVEIPKSNGG
KRPLGIPTVGDRVAQMVVVMTIEPGIEPYFHEDSYAYRPNRSALDAVRKAKERSYTFHWVLDLDIKGFFDNIDH
ELLIKALERHVKCKWAILYIKRWLSVPYQLKDGTQKERTKGVPQGSVVGPILANLFLHYVFDEWMRRNHSNISFE
RYADDTICHCVSLKQAEFILRAIRKRFAECKLELNEDKTKIVYCKKNHRDIPYECIQFDFLGYTFRPRRSIDANGEVF
LNFSPAISKKARTKIWEAIQNWNSNHWVPMELEDIAKEINPVIQGWINYYGQHNPRILKEVLQHVNDRLVRW
GRRKFKGLRKRKTATVHRLGDIALQKPNLFAHWAWGVKPTASERNRKRK
>R2H55||gene_72349|GeneMark.hmm|420_aa|+|1707|2969
MNEAKPFVIDKRLVWEAYHKVKENKGSAGIDKVDQKTFDKEMSKNLYKIWNRMSSGCYFPKAVKLVEIPKSNG
GTRPLGIPTIEDRIAQQVVVSVLIPILEPIFKEDSYGYRPGKGAHQAIAKAKERCYVNPWVLDMDISKFFDTINHEL
LMKAIRKHAEEKWVLLYIERWLKVPYQTSKGEVIERTMGVPQGSVIGPVLANLFLHYVFDEWMSRNYPTIPFER
YADDTICHCVSEKQAQFLKAVLMKRFEECGLKLNEEKTKIVYCKDSNRRGDSEHTSFDFLGFTFRPRGARNRKTG
QNFTAFLPAISKKSMKRIKEAVRAWKLNRKTFACLLDISNEVDTQISGWMNYYMKFGRSEFRKVLNYINERLTR
WVMRKYKRFSKGKKFSKAYEWLVEYAAHNRNEFSHRVKGFVPYPRLD
>R2H56||gene_184395|GeneMark.hmm|420_aa|-|12153|13415
MNAANPFVIDKRLVWEAYHKVKENKGSAGIDKVDQKTFDKEMSKNLYKIWNRMSSGCYFPKAVKLVEIPKSN
GGTRPLGIPAIEDRIAQQVVVSVLTPILEPIFKEDSYGYRPGKGAHQAIAKAKERCYVTPWVLDMDISKFFDTINH
ELLMKAIRKHTEEKWVLLYIERWLKVPYQTSKGEVIERTMGVPQCSVIGPVLANLFLHYVFDEWMSRNYPTIPF
ERYADDTICHCVSEKQARFLKAVLMKRFEECGLKLNEEKTKIVYCKDSNRRGDSEHTSFDFLGFTFRPRGARNRK
TGQNFTAFLPAISKKSMKRIKEAVRAWKLNRKTFACLLDISNEVDTQISGWMNYYMKFGRSEFRKVLNYINERLT
RWVMRKYKRFSKGRKFDRAYDWLVEYATHNRNEFSHWVKGFVPYPRLD
>R2H62||gene_7823|GeneMark.hmm|430_aa|+|547|1839
MQNDNAKPISISKQLVYDAFLRVKANRGSAGIDKVTLEDYEKNLRGNLYKLWNRMSSGSYFPPSVKLVEIPKSTG
GKRPLGIPTVSDRVAQMAVVMLITPSIEPCFHEDSYAYRPHRSAHDAVGKARERCWKYAWVLDMDISKFFDTI
DHELLLKALKRHTQEKWVLMYIERWLKVPYEKSDGSQVDRALGVPQGSVIGPVLANLFLHYTFDKWMEKNFP
RVPFERYADDTICHCHSLKQAEYMQAMIQQRFECCRLRLNEEKTKIVYCKSSRQKECYPNVTFDFLGFTFQPRES

VDKYGNRFTGFLPAISRKSMKRINETMRSWHLNRHSNLTLEHLASDINPIVRGWMTYYGKFYPTRLKWFMQTL
NGRLARWVMCKFERYRHRFYPAQEWLARIAEKEGLIFYHWKCGALPRFTNKEKVSSQLIMVK
>R2H66||gene_68566|GeneMark.hmm|391_aa|-|819|1994
NKGSYGVDEQSIEDFEKNLKNNLYKIWNRMSSGSYFPQPVKAVSVPKKNGGIRVLGIPTVEDRIAQMTAKLYFE
PCVEPLFLEDSYGYRPGKSAIQALSVTRKRCWHRDWVLEYDIKGLFDNIRHDYLLEMVRRHTPHKWILLYVERW
LTTPFQLEDGTLQSRTSGTPQGGVISLVLANLFLHYAFDSFMAKEYPKAWWERYADDGVLHCKSSQQAMYMK
SVLRERFRLFGLELNEEKTRIVYCKDADRTEDYSEISLDSLGYTFRPRLARNKHGNIFLNFLPAMSAKAIKAMKEEV
RRWKLQLKVSKSLTDLANILNSQIQGWISYYGHFYKSELIYLLRYINQCLIKWVRRKYKKFNHRRRAEYWLGRIAR
RDNNLFAHWRYGVLPTAG
>R2H66||gene_83753|GeneMark.hmm|422_aa|-|4600|5868
MMQHQIAKPFTIDKHVIMAAWKRVRENKGSAGIDNVSISDYETNLGTHLYKLWNRMSSGSYFPNAVKLVEIPK
SSGGTRPLGIPTVGDRIAQMAVVLLIEARLEEIFHPNSYGYRPNRSAHDAIGQARERCWRYNWVLDMDISKFFD
TIDHDLLMKAVERHVQERWILLYIRRWLKVPYATITGECIERTMGVPQGSVIGPILANLYLHYTFDKWMSIYHPN
VPFERYADDTICHCSSLEEAQRLKASIVERFAACKLKLNEEKTRIVYCKDGKRRGKYPEITFDFLGYTFQPRGQRNR
NGQVFNGYAPAISKKSKKRITEKMRGWHLSRRVQIKLSDIASEINAEVRGWINYYGKFYGSLLKAFLQSINLKLAR
WAERKYKRFRRKPNDAYKWLVKVASKSPNLFYHWQYGVKPNRLKSFG
>R2H67||gene_45116|GeneMark.hmm|421_aa|-|261|1526
MQEAKPFQIDKRIIFEAFKKVKSNGGSPGIDGIEMSAYEQNLGSNFYRLWNRMSSGSYMPKAVKLVEILKSNGG
KRPLGIPSVEDRIAQMAVVNVIEPLVEPYFHKDSFGYRPHRSAHDAIAKAERRCWKYAWVLDIDISKFFDTIDHG
LLMKAVEKHIKTKWILLYIKRWLTVPYQGNDGAIVKRHMGVPQGSVIGPILANQFLHYTFDKWMSYKYPHVPF
ERYADDCVCHCGTLAQAEYIKDRLGERFAECKLTFNEEKTKIVFCKTSNRSSEHYHCTSFDYLGFTFRPRAAKDKR
KNVLFTSYLPAISNKSESRIHETIKSWNLKRLHNRSLRFVAAYINDVVRGWISYYGKFGKTEFWKVMCHLNRSIAY
WAKTKYKRLRRRGVISAHYWLAYIAQKEPNLFYHWQVGYIPYARQKK
>R2H68||gene_40425|GeneMark.hmm|420_aa|-|6040|7302
MNEAKPFVIDKRLVWEAYHKVKENKGSAGIDKVDQQTFDKEMSKNLYKIWNRMSSGCYFPKAVKLVEIPKSNG
GTRPLGIPTIEDRIAQQVVVSVLTPILEPIFKEDSYGYRPGKGAHQAIAKAKERCYVNPWVLDMDISKFFDTINHE
LLMKAVRKHTEEKWVLLYIERWLKVPYQTSKGEVIERTMGVPQGSVIGPVLANLFLHYVFDEWMSRNYPTIPFE
RYADDTICHCVSDKQAQFLKAVLMKRFEECGLKLNEEKTKIVYCKDSNRRGDSEHTSFDFLGFTFRPRGARNRKT
GQNFTAFLPAISKKSMKRIKEAVRAWKLNRKTFACLLDISNEVDTQISGWMNYYMKFGRSEFRKVLNYINERLT
RWVMRKYKRFSKGRKFDRAYDWLVEYAAHNRNEFSHWVKGFVPYPRLG
>R2H7||gene_36280|GeneMark.hmm|430_aa|-|1329|2621
MQNDNAKPISISKQLVYDAFLRVKANRGSAGIDKVTLEDYEKNLRGNLYKLWNRMSSGSYFPPSVKLVEIPKSTG
GKRPLGIPTVSDRVAQMAVVMLITPSIEPCFHEDSYAYRPHRSAHDAVGKARERCWKYAWVLDMDISKFFDTI
DHELLLKALKRHTQEKWVLMYIERWLKVPYEKSDGSQVDRALGVPQGSVIGPVLANLFLHYTFDKWMEKNFP
RVPFERYADDTICHCHSLKQAEYMQAMIQQRFECCRLRLNEEKTKIVYCKSSRQKECYPNVTFDFLGFTFQPRES
VDKYGNRFTGFLPAISRKSMKRINETMRSWHLNRHSNLTLEHLASDINPIVRGWMTYYGKFYPTRLKWFMQTL
NGRLARWVMCKFERYRHRFYPAQEWLARIAEKEGLIFYHWKCGALPRFTNKEKVSSQLIMVK
>R2H71||gene_30852|GeneMark.hmm|420_aa|-|9183|10445
MNEAKPFVIDKRLVWEAYHKVKENKGSAGIDKVDQQTFDKEMSKNLYKIWNRMSSGCYFPKAVKLVEIPKSNG
GTRPLGIPTIEDRIAQQVVVSVLTPILEPIFKEDSYGYRPGKGAHQAIAKAKERCYVNPWVLDMDISKFFDTINHE
LLMKAVRKHTEEKWVLLYIERWLKVPYQTSKGEVIERTMGVPQGSVIGPVLANLFLHYVFDEWMSRNYPTIPFE
RYADDTICHCVSDKQAQFLKAVLMKRFEECGLKLNEEKTKIVYCKDSNRRGDSEHTSFDFLGFTFRPRGARNRKT
GQNFTAFLPAISKKSMKRIKEAVRAWKLNRKTFACLLDISNEVDTQISGWMNYYMKFGRSEFRKVLNYINERLT
RWVMRKYKHFSKGRKFDRAYDWLVEYAAHNRNEFSHWVKGFVPYPRLG

>R2H72||gene_92591|GeneMark.hmm|420_aa|+|567|1829
MNEAKPFVIDKRLVWEAYHKVKENKGSAGIDKVDQQTFDKEMSKNLYKIWNRMSSGCYFPKAVKLVEIPKSNG
GTRPLGIPTIEDRIAQQVVVSVLTPILEPIFKEDSYGYRPGKGAHQAIAKAKERCYVNPWVLDMDISKFFDTINHE
LLMKAVRKHTEEKWVLLYIERWLKVPYQTSKGEVIERTMGVPQGSVIGPVLANLFLHYVFDEWMSRNYPTIPFE
RYADDTICHCVSDKQAQFLKAVLMKRFEECGLKLNEEKTKIVYCKDSNRRGDSEHTSFDFLGFTFRPRGARNRKT
GQNFTAFLPAISKKSMKRIKEAVRAWKLNRKTFACLLDISNEVDTQISGWMNYYMKFGRSEFRKVLNYINERLT
RWVMRKYKRFSKGRKFDRAYDWLVEYAAHNRNEFSHWVKGFVPYPRLG
>R2H73||gene_17825|GeneMark.hmm|420_aa|-|21051|22313
MNEAKPFVIDKRLVWEAYHKVKENKGSAGIDKVDQQTFDKEMSKNLYKIWNRMSSGCYFPKAVKLVEIPKSNG
GTRPLGIPTIEDRIAQQVVVSVLTPILEPIFKEDSYGYRPGKGAHQAIAKAKERCYVNPWVLDMDISKFFDTINHE
LLMKAVRKHTEEKWVLLYIERWLKVPYQTSKGEVIERTMGVPQGSVIGPVLANLFLHYVFDEWMSRNYPTIPFE
RYADDTICHCVSDKQAQFLKAVLMKRFEECGLKLNEEKTKIVYCKDSNRRGDSEHTSFDFLGFTFRPRGARNRKT
GQNFTAFLPAISKKSMKRIKEAVRAWKLNRKTFACLLDISNEVDTQISGWMNYYMKFGRSEFRKVLNYINERLT
RWVMRKYKRFSKGRKFDRAYDWLVEYAAHNRNEFSHWVKGFVPYPRLG
>R2H74||gene_230307|GeneMark.hmm|420_aa|-|442|1704
MNEAKPFVIDKRLVWEAYHKVKENKGSAGIDKVDQQTFDKEMSKNLYKIWNRMSSGCYFPKAVKLVEIPKSNG
GTRPLGIPTIEDRIAQQVVVSVLTPILEPIFKEDSYGYRPGKGAHQAIAKAKERCYVNPWVLDMDISKFFDTINHE
LLMKAVRKHTEEKWVLLYIERWLKVPYQTSKGEVIERTMGVPQGSVIGPVLANLFLHYVFDEWMSRNYPTIPFE
RYADDTICHCVSDKQAQFLKAVLMKRFEECGLKLNEEKTKIVYCKDSNRRGDSEHTSFDFLGFTFRPRGARNRKT
GQNFTAFLPAISKKSMKRIKEAVRAWKLNRKTFACLLDISNEVDTQISGWMNYYMKFGRSEFRKVLNYINERLT
RWVMRKYKRFSKGRKFDRAYDWLVEYAAHNRNEFSHWVKGFVPYPRLG
>R2H79||gene_31630|GeneMark.hmm|420_aa|-|130|1392
MNEAKPFVIDKRLVWEAYHKVKENKGSAGIDKVDQQTFDKEMSKNLYKIWNRMSSGCYFPKAVKLVEIPKSNG
GTRPLGIPTIEDRIAQQVVVSVLTPILEPIFKEDSYGYRPGKGAHQAIAKAKERCYVNPWVLDMDISKFFDTINHE
LLMKAVRKHTEEKWVLLYIERWLKVPYQTSKGEVIERTMGVPQGSVIGPVLANLFLHYVFDEWMSRNYPTIPFE
RYADDTICHCVSDKQAQFLKAVLMKRFEECGLKLNEEKTKIVYCKDSNRRGDSEHTSFDFLGFTFRPRGARNRKT
GQNFTAFLPAISKKSMKRIKEAVRAWKLNRKTFACLLDISNEVDTQISGWMNYYMKFGRSEFRKVLNYINERLT
RWVMRKYKRFSKGRKFDRAYDWLVEYAAHNRNEFSHWVKGFVPYPRLG
>R2H80||gene_6529|GeneMark.hmm|420_aa|+|1973|3235
MNAANPFVIDKRLVWEAYHKVKENKGSAGIDKVDQKTFDKEMSKNLYKIWNRMSSGCYFPKAVKLVEMPKS
NGGTRPLGIPAIEDRIAQQVVVSVLTPILEPIFKEDSYGYRPGKGAHQAIAKAKERCYVTPWVLDMDISKFFDTIN
HELLMKAIRKHTEEKWVLLYIERWLKVPYQTSKGEVIERTMGVPQGSVIGPVLANLFLHYVFDEWMSRNYPTIP
FERYADDTICHCVSEKQAQFLKAVLMKRFEECGLKLNEEKTKIVYCKDSNRRGDSEHTSFDFLGFTFRPRGARNR
KTGQNFTAFLPAISKKSMKRIKEAVRAWKLNRKTFACLLDISNEVDTQISGWMNYYMKFGRSEFRKVLNYINERL
TRWVMRKYKRFSKGRKFDRAYDWLVEYATHNRNEFSHWVKGFVPYPRLD
>R2H81||gene_148037|GeneMark.hmm|420_aa|+|3618|4880
MNEAKPFVIDKRLVWEAYHKVKENKGSAGIDKVDQKTFDKEMSKNLYKIWNRMSSGCYFPKAVKLVEIPKSNG
GTRPLGIPTIEDRIAQQVVVSVLTPILEPIFKEDSYGYRPGKGAHQAIAKAKERCYVNPWVLDMDISKFFDTINHE
LLMKAIRKHTEEKWVLLYIERWLKVPYQTSKGEVIERTMGVPQGSVIGPVLANLFLHYVFDEWMSRNYPTIPFE
RYADDTICHCVSEKQAQFLKAVLMKRFEECGLKLNEEKTKIVYCKDSNRRGNSEHTSFDFLGFTFRPRSARNRKT
GQNFTAFLPAISKKSMKRIKEAVRAWKLNRKTFACLLDISNEVDTQISGWMNYYMKFGRSEFRKVLNYINERLT
RWVMRKYKRFSKGKKLGRAYEWLVEYAAHNRNEFSHWVKGFVPYPRLG
>R2H82||gene_114528|GeneMark.hmm|420_aa|-|12321|13583
MNEAKSFVIDKRLVWEAYHKVKENKGSAGIDKVDQKTFDKEMSKNLYKIWNRMSSGCYFPKAVKLVEIPKSNG

GTRPLGIPTIEDRIAQQVVVSVLIPILEPIFKEDSYGYRPGKGAHQAIAKAKERCYVNPWVLDMDISKFFDTINHEL
LMKAIRKHTEEKWVLLYIERWLKVPNQTSKGEVIERTMGVPQGSVIGPVLANLFLHYVFDEWMSRNYPTIPFER
YADDTICHCVSEKQAQFLKAVLMKRFEECGLKLNEEKTKIVYCKDSNRRGDSEHTSFDFLGFTFRPRGARNRKTG
QNFTAFLPAISKKSMKRIKEAVRAWKLNRKTFACLLDISNEVDTQISGWMNYYMKFGRSEFRKVLNYINERLTR
WVMRKYKRFSKGRKFDRAYDWLVEYATHNRNEFSHWVKGFVPYPRLD
>R2H85||gene_42956|GeneMark.hmm|420_aa|-|13081|14343
MNAANPFVIDKRLVWEAYHKVKENKGSAGIDKVDQKTFDKEMSKNLYKIWNRMSSGCYFPKAVKLVEIPKSN
GGTRPLGIPAIEDRIAQQVVVSVLTPILEPIFKEDSYGYRPGKGAHQAIAKAKERCYVTPWVLDMDISKFFDTINH
ELLMKAIRKHTEEKWVLLYIERWLKVPYQTSKGEVIERTMGVPQCSVIGPVLANLFLHYVFDEWMSRNYPTIPF
ERYADDTICHCVSEKQARFLKAVLMKRFEECGLKLNEEKTKIVYCKDSNRRGDSEHTSFDFLGFTFRPRGARNRK
TGQNFTAFLPAISKKSMKRIKEAVRAWKLNRKTFACLLDISNEVDTQISGWMNYYMKFGRSEFRKVLNYINERLT
RWVMRKYKRFSKGRKFDRAYDWLVEYATHNRNEFSHWVKGFVPYPRLD
>R2H87||gene_49803|GeneMark.hmm|430_aa|-|18394|19686
MQNDNAKPISISKQLVYDAFLRVKANRGSAGIDKVTLEDYEKNLRGNLYKLWNRMSSGSYFPPSVKLVEIPKTTG
GKRPLGIPTVSDRVAQMAVVMLITPSIEPCFHEDSYAYRPHRSAHDAVGKARERCWKYAWVLDMDISKFFDTI
DHELLLKALKRHTQEKWVLMYIERWLKVPYEKSDGSQVDRALGVPQGSVIGPVLANLFLHYTFDKWMEKNFP
RVPFERYADDTICHCHSLKQAEYMQAMIQQRFECCRLRLNEEKTKIVYCKSSRQKECYPNVTFDFLGFTFQPRES
VDKYGNRFTGFLPAISRKSMKRINETMRSWHLNRHSNLTLEHLASDINPIVRGWMTYYGKFYPTRLKWFMQTL
NGRLARWVMCKFERYRHRFYPAQEWLARIAEKEGLIFYHWKCGVLPRFTNKEKVSSQLIMVK
>R2H88||gene_217264|GeneMark.hmm|420_aa|+|616|1878
MNEAKPFVIDKRLVWEAYHKVKENKGSAGIDKVDQKTFDKEMSKNLYKIWNRMSSGCYFPKAVKLVEIPKSNG
GTRPLGIPTIEDRIAQQVVVSVLTPILEPIFKEDSYGYRPGKGAHQAIAKAKERCYVNPWVLDMDISKFFDTINHD
LLMKAVRKHTEEKWVLLYIERWLKVPYQTSKGEVIERTMGVPQGSVIGPVLANLFLHYVFDEWMSRNYPTIPFE
RYADDTICHCVSEKQAQFLKAVLMKRFEECGLKLNEEKTKIVYCKDSNRRGDSEHTSFDFLGFTFRPRSARNRKT
GQNFTAFLPAISKKSLKRIKEAVRAWKLNRKTFACLLDISNEVDTQISGWMNYYMKFGRSEFRKVLNYINERLTR
WVMRKYKRFSKGKKFSRAYEWLVEYAVHNRNEFSHWAKGFVPYPRLG
>R2H89||gene_85753|GeneMark.hmm|430_aa|-|6876|8168
MQNDNAKPISISKQLVYDAFLRVKANRGSAGIDKVTLEDYEKNLRGNLYKLWNRMSSGSYFPPSVKLVEIPKSTG
GKRPLGIPTVSDRVAQMAVVMLITPSIEPCFHEDSYAYRPHRSAHDAVGKARERCWKYAWVLDMDISKFFDTI
DHELLLKALKRHTQEKWVLMYIERWLKVPYEKSDGSQVDRALGVPQGSVIGPVLANLFLHYTFDKWMEKNFP
RVPFERYADDTICHCHSLKQAEYMQAMIQQRFECCRLRLNEEKTKIVYCKSSRQKECYPNVTFDFLGFTFQPRES
VDKYGNRFTGFLPAISRKSMKRINETMRSWHLNRHSNLTLEHLASDINPIVRGWMTYYGKFYPTRLKWFMQTL
NGRLARWVMCKFERYRHRFYPAQEWLARIAEKEGLIFYHWKCGALPRFTNKEKVSSQLIMVK
>R2H92||gene_59721|GeneMark.hmm|417_aa|+|2562|3815
MNAANPFVIDKRLVWEAYHKVKENKGSAGIDKVDQKTFDKEMSKNLYKIWNRMSSGCYFPKAVKLVEMPKS
NGGTRPLGIPAIEDRIAQQVVVSVLTPILEPIFKEDSYGYRPGKGAHQAIAKAKERCYVTPWVLDMDISKFFDTIN
HELLMKAIRKHTEEKWVLLYIERWLKVPYQTSKGEVIERTMGGSVIGPVLANLFLHYVFDEWMSRNYPTIPFERY
ADDTICHCVSEKQARFLKAVLMKRFEECGLKLNEEKTKIVYCKDSNRRGDSEHTSFDFLGFTFRPRGARNRKTG
QNFTAFLPAISKKSMKRIKEAVRAWKLNRKTFACLLDISNEVDTQISGWMNYYMKFGRSEFRKVLNYINERLPR
WVMRKYKRFSKGKKFSKAYEWLVEYAAHNRNEFSHWVKGFVPYPRLD
>R2H99||gene_159733|GeneMark.hmm|420_aa|+|4387|5649
MNEAKPFVIDKRLVWEAYHKVKENKGSAGIDKVDQQTFDKEMSKNLYKIWNRMSSGCYFPKAVKLVEIPKSNG
GTRPLGIPTIEDRIAQQVVVSVLTPILEPIFKEDSYGYRPGKGAHQAIAKAKERCYVNPWVLDMDISKFFDTINHE
LLMKAVRKHTEEKWVLLYIERWLKVPYQTSKGEVIERTMGVPQGSVIGPVLANLFLHYVFDEWMSRNYPTIPFE

RYADDTICHCVSEKQAQFLKAVLMKRFEECGLKLNEEKTKIVYCKDSNRRGDSEHTSFDFLGFTFRPRGARNRKT
GQNFTAFLPAISKKSMKRIKEAVRAWKLNRKTFACLLDISNEVDTQISGWMNYYMKFGRSEFRKVLNYINERLT
RWVMRKYKRFSKGRKFDRAYDWLVEYAAHNRNEFSHWVKGFVPYPRLG
>R2nH100||gene_155889|GeneMark.hmm|430_aa|-|22045|23337
MQNDNAKPISISKQLVYDAFLRVKANRGSAGIDKVTLEDYEKNLRGNLYKLWNRMSSGSYFPPSVKLVEIPKSTG
GKRPLGIPTVSDRVAQMTVVMLITPSIEPCFHEDSYAYRPHRSAHDAVGKARERCWKYAWVLDMDISKFFDTID
HELLLKALKRHTQEKWVLMYIERWLKVPYEKSDGSQVDRALGVPQGSVIGPVLANLFLHYTFDKWMEKNFPR
VPFERYADDTICHCHSLKQAEYMQAMIQQRFECCRLRLNEEKTKIVYCKSSRQKECYPNVTFDFLGFTFQPRESV
DKYGNRFTGFLPAISRKSMKRINETMRSWHLNRHSNLTLEHLASDINPIVRGWMTYYGKFYPTRLKWFMQTLN
GRLASWVMCKFERYRHRFYPAQEWLARIAEKEGLIFYHWKCGVLPRFTNKEKVSSQLIMVK
>R2nH13||gene_145774|GeneMark.hmm|430_aa|+|3689|4981
MQNDNAKPISISKQLVYDAFLRVKANRGSAGIDKVTLEDYEKNLRGNLYKLWNRMSSGSYFPPSVKLVEIPKSTG
GKRPLGIPTVSDRVAQMAVVMLITPSIEPCFHEDSYAYRPHRSAHDAVGKARERCWKYAWVLDMDISKFFDTI
DHELLLKALKRHTQEKWVLMYIERWLKVPYEKSDGSQVDRALGVPQGSVIGPVLANLFLHYTFDKWMEKNFP
RVPFERYADDTICHCHSLKQAEYMQAMIQQRFECCRLRLNEEKTKIVYCKSSRQKECYPNVTFDFLGFTFQPRES
VDKYGNRFTGFLPAISRKSMKRINETMRSWHLNRHSNLTLEHLASDINPIVRGWMTYYGKFYPTRLKWFMQTL
NGRLARWVMCKFERYRHRFYPAQEWLARIAEKEGLIFYHWKCGALPRFTNKEKVSSQLIMVK
>R2nH15||gene_40442|GeneMark.hmm|423_aa|+|810|2081
MTQKQGAKPFDIDRWKLYYAYQRVNQNRGGSGVDNVTLEKYNSNLKRNLYKLWNRMSSGSYVPKPVRLVQIP
KPAGGTRPLGIPTVEDRIAQMLVVEMIEPEIEKIFHEDSYGYRPNRSAHDALGRARERCWKYAWVLDMDISKFF
DTIDHQLLMKAVRLHVKERWIILYIERWLKVPYQNADKSLIERTCGVPQGSVIGPILANLFLHYCFDRWMQIHYP
EIPFERYADDTVCHCRSQREAESLYEELIIRFKSCKLSLNEEKTKIVYCKSSRRKENHSNVTFDFLGHTFRPCKTMH
KSSREAFTGFQPRISMKATTKIRATMRSWNLKSKSHTPLDCIAHMVNPILRGWVNYYGKYGGKSFQKLLGYFDL
LLARWAKAKYKTFRRKPMYVILKWLGNVADRDAVFYHWQIGLKPAKGTIKL
>R2nH16||gene_38705|GeneMark.hmm|416_aa|+|1060|2310
MTKTKAFNIDKSLVVSAYRRVKTSAGAAGIDKQSLADFDKRLVDNLYKIWNRLSSGSYFPPAVKAVAIPKKLGGER
ILGIPTVSDRIAQTVVKLAFEPQVEPHFLADSYGYRPNKSALDAIGVTRKRCWYYDWVLEFDIKGLFDNIPHELIM
KAVDKHNPARWVKLYIQRWLTAPMVMSDGEVRARTMGTPQGGVISPLLANLFMHYVFDKWLAKYYPKVPW
YRYADDGILHCHSEAEATEMREVLRKRFSECGLEMHPEKTRVIYCKDGSRKGDYEHTMFDFLGYTFRRRVVKNV
KRNSLFVSFTPAASKSALKAMRREIKATGIRKRVDLSIEQIAKWINPKLNGWINYYGRYTCSELYSVFRYINKALVR
WGRKKYKMLSRYKTRASKFLEEMAKRSPQLFAHWRLKMRGGLV
>R2nH18||gene_33330|GeneMark.hmm|421_aa|+|568|1833
MQEAKPFQIDKRIIFESFKKVKFNRGSSGIDGIEMTTYEQNLGSNLYRLWNRMSSGSYMPKAVKLVEIPKSNGG
KRPLGIPTIEDRIAQMAVVNVIEPLIEPCFHEDSFGYRPHRSAHDAIAKAERRCWKYAWVLDIDISKFFDTIDHGL
LMKAVEKHINIKWILLYIKRWLTVPYQRSDGEIVKRDMGVPQGSVIGPILANLFLHYTFDKWMSYKYPHIPFERY
ADDCVCHCSTLAQAEYIKERLGERFTECKLKFNEEKTKIVFCKMSSRSSKHYHCTSFDYLGFTFRSRAAKDKRNN
VLFTSYLPAISKKSVSRIHETIKSWNLKRLHNRSLRFVAAYINDVVRGWINYYEKFGKTEFWKVMCHLNRSIAYW
AKTKYKRLRRRGVISAHYWLAYIAQKEPNLFYHWQVGYVPYARQKK
>R2nH22||gene_107808|GeneMark.hmm|421_aa|+|1103|2368
MQEAKPFQIDKRIIFESFKKVKFNRGSSGIDGIEMTTYEQNLGSNLYRLWNRMSSGSYMPKAVKLVEIPKSNGG
KRPLGIPTIEDRIAQMAVVNVIEPLIEPCFHEDSFGYRPHRSAHDAIAKAERRCWKYAWVLDIDISKFFDTIDHGL
LMKAVEKHINIKWILLYIKRWLTVPYQRSDGEIVKRDMGVPQGSVIGPILANLFLHYTFDKWMSYKYPHIPFERY
ADDCVCHCSTLAQAEYIKERLGERFTECKLKFNEEKTKIVFCKMSSRSSKHYHCTSFDYLGFTFRSRAAKDKRNN
VLFTSYLPAISKKSVSRIHETIKSWNLKRLHNRSLRFVAAYINDVVRGWINYYEKFGKTEFWKVMCHLNRSIAYW

AKTKYKRLRRRGVISAHYWLAYIAQKEPNLFYHWQVGYVPYARQKK
>R2nH25||gene_92105|GeneMark.hmm|417_aa|-|2699|3952
MNEAKPFVIDKRLVWEAYHKVKENKGSSGIDKVDQKTFDKEMSKNLYKIWNRMSSGCYFPKAVKLVEIPKSNG
GTRPLGIPTIEDRIAQQVVVSVLIPILEPIFKEDSYGYRPGKGAHQAIAKAKERCYVNPWVLDMDISKFFDTINHEL
LMKAIRKHAEEKWVLLYIERWLKVPYQTSKGEVIERTMGGSVIGPVLANLFLHYVFDEWMSRNYPTIPFERYAD
DTISHCVSEKQARFLKAVLMKRFEECGLKLNEEKTKIVYCKDSNRRGDSEHTSFDFLGFTFRPRGARNRKTGQNF
TAFLPAISKKSMKRIKEAVRAWKLNRKTFACLLDISNEVDTQISGWMNYYMKFGRSEFRKVLNYINERLTRWVM
RKYKRFSKGKKFSKAYEWLVEYAAHNRNEFSHWVKGFVPYPRLD
>R2nH28||gene_24615|GeneMark.hmm|421_aa|+|19781|21046
MQEAKPFQIDKRIIFESFKKVKFNRGSSGIDGIEMTTYEQNLGSNLYRLWNRMSSGSYMPKAVKLVEIPKSNGG
KRPLGIPTIEDRIAQMAVVNVIEPLIEPCFHEDSFGYRPHRSAHDAIAKAERRCWKYAWVLDIDISKFFDTIDHGL
LMKAVEKHINIKWILLYIKRWLTVPYQRSDGEIVKRDMGVPQGSVIGPILANLFLHYTFDKWMSYKYPHIPFERY
ADDCVCHCSTLAQAEYIKERLGERFTECKLKFNEEKTKIVFCKMSSRSSKHYHCTSFDYLGFTFRSRAAKDKRNN
VLFTSYLPAISKKSVNRIHETIKSWNLKRLHNRSLRFVAAYINDVVRGWINYYEKFGKTEFWKVMCHLNRSIAYW
AKTKYKRLRRRGVISAHYWLAYIAQKEPNLFYHWQVGYVPYARQKK
>R2nH32||gene_213218|GeneMark.hmm|422_aa|+|2877|4145
MMQHQIAKPFTIDKHVIMAAWKRVRENKGSAGIDNVSISDYETNLGTHLYKLWNRMSSGSYFPNAVKLVEIPK
SSGGTRPLGIPTVGDRIAQMAVVLLIEARLEEIFHPNSYGYRPNRSAHDAIGQARERCWRYNWVLDMDISKFFD
TIDHDLLMKAVERHVQERWILLYIRRWLKVPYATITGECIERTMGVPQGSVIGPILANLYLHYTFDKWMSIYHPN
VPFERYADDTICHCSSLEEAQRLKASIVERFAACKLKLNEEKTRIVYCKDGKRRGKYPEITFDFLGYTFQPRGQRNR
NGQVFNGYAPAISKKSKKRITEKMRGWHLSRRVQIKLSDIASEINAEVRGWINYYGKFYGSLLKAFLQSINLKLAR
WAERKYKRFRRKPNDAYKWLVKVASKSPNLFYHWQYGVKPNRLKSFG
>R2nH43||gene_103366|GeneMark.hmm|420_aa|-|12578|13840
MNEAKPFVIDKRLVWEAYHKVKENKGSAGIDKVDQQTFDKEMSKNLYKIWNRMSSGCYFPKAVKLVEIPKSNG
GTRPLGIPTIEDRIAQQVVVSVLTPILEPIFKEDSYGYRPGKGAHQAIAKAKERCYVNPWVLDMDISKFFDTINHE
LLMKAVRKHTEEKWVLLYIERWLKVPYQTSKGEVIERTMGVPQGSVIGPVLANLFLHYVFDEWMSRNYPTIPFE
RYADDTICHCVSDKQAQFLKAVLMKRFEECGLKLNEEKTKIVYCKDSNRRGDSEHTSFDFLGFTFRPRGARNRKT
GQNFTAFLPAISKKSMKRIKEAVRAWKLNRKTFACLLDISNEVDTQISGWMNYYMKFGRSEFRKVLNYINERLT
RWVMRKYKRFSKGRKFDRAYDWLVEYAAHNRNEFSHWVKGFVPYPLLG
>R2nH44||gene_177381|GeneMark.hmm|423_aa|+|800|2071
MTQKQGAKPFDIDRWKLYYAYQRVNQNRGGSGVDNVTLEKYNSNLKRNLYKLWNRMSSGSYVPKPVRLVQIP
KPAGGTRPLGIPTVEDRIAQMLVVEMIEPEIEKIFHEDSYGYRPNRSAHDALGRARERCWKYAWVLDMDISKFF
DTIDHQLLMKAVRLHVKERWIILYIERWLKVPYQNADKSLIERTCGVPQGSVIGPILANLFLHYCFDRWMQIHH
PEIPFERYADDTVCHCRSQREAESLYEELIIRFKSCKLSLNEEKTKIVYCKSSRRKENHSNVTFDFLGHTFRPCKTM
HKSSREAFTGFQPRISMKATTKIRATMRSWNLKSKSHTPLDCIAHMVNPILRGWVNYYGKYGGKSFQKLLGYF
DLLLARWAKAKYKTFRRKPMYVILKWLGNVADRDAVFYHWQIGLKPAKGTIKL
>R2nH45||gene_102789|GeneMark.hmm|430_aa|-|339|1631
MQNDNAKPISISKQLVYDAFLCVKANRGSAGIDKVTLEDYEKNLRGNLYKLWNRMSSGSYFPPSVKLVEIPKSTG
GKRPLGIPTVSDRVAQMAVVMLITPSIEPCFHEDSYAYRPHRSAHDAVGKARERCWKYAWVLDMDISKFFDTI
DHELLLKALKRHTQEKWVLMYIERWLKVPYEKSDGSQVDRALGVPQGSVIGPVLANLFLHYTFDKWMEKNFP
RVPFERYADDTICHCHSLKQAEYMQAMIQQRFECCRLRLNEEKTKIVYCKSSRQKECYPNVTFDFLGFTFQPRES
VDKYGNRFTGFLPAISRKSMKRINETMRSWHLNRHSNLTLEHLASDINPIVRGWMTYYGKFYPTRLKWFMQTL
NGRLARWVMCKFERYRHRFYPAQEWLARIAEKEGLIFYHWKCGVLPRFTNKEKVSSQLIMVK
>R2nH45||gene_36175|GeneMark.hmm|418_aa|+|1373|2629

MSEAKQFDISKKAVIAAFQAVKENAGSYGADEQTIKEFEEHLNNNLYKLWNRMASGSYFPKPVRAVAIPKKNG
GIRILGIPTVEDRIAQMVAKMYFEPLVEPMFYNDSYGYRPNKSAIQAVGQARERCFKRDWVLELDIKGLFDNIK
HGYLMYMVEKHTQIKWLILYIKRWLTVPFIMSDGSVAERRSGTPQGGVISPVLANLFLHYVFDDFMTKAYPNI
WWERYADDGVLHCQSYKQAAFIKQKLEERFQQFGLELNKEKTRIVYCKDNRRPQNYSCTQFTFLGYTFRPRLN
KNKEGKFFVGFTPAVSEKAKTAMKQKIREWKIQLKADLSLKDIGNMINKVVQGWINYYTHYYKSEFYEVLRYIN
QCLIKWVRRSYKKKNTRSRAEHWLGAVARRDRNLFAHWKFGILPSVGEGAV
>R2nH64||gene_18255|GeneMark.hmm|410_aa|-|407|1639
MQRKSFEIPKALVWASYLDVRRNKGAPGCDGQTLKMFDQQRDGNLYKIWNRLCSGTWFPPPVLEKRIPKPN
GKERILGIPTVSDRIAQGAIKLFMEEKLDPIFHADSYGYRPGKSAHDALKQCAIRCWRYSWILEVDISAFFDHVRH
DLVLKALEHHGMPKWVILYCRRWMEAPMQSCENGELITRTRGTPQGGVISPLLANLFLHYAFDLWMEREYRG
VPFERYADDIVVHCSRMSDATRLKNRLSERFSEVGLVLNAGKTNIAYIDTFKRRNVATSFTFLGYDFKVRTLKNFK
GELYRKCMPGASNAAMRKITETIKKWRIHRSTAESLLDFARRYNAIVRGWIEYYGKFWSRNFNYRLWSAMQSR
LLKWMQSKYRLSNRRAQRKLTLVRKEYPKLFVHWYLLRASNE
>R2nH70||gene_112012|GeneMark.hmm|420_aa|-|2303|3565
MNEAKPFVIDKRLVWEAYHKVKENKGSAGIDKVDQKTFDKEMSKNLYKIWNRMSSGCYFPKAVKLVEIPKSNG
GTRPLGIPTIEDRIAQQVVVSVLIPILEPIFKEDSYGYRPGKGAHQAIAKAKERCYVNPWVLDMDISKFFDTINHEL
LMKAIRKHAEEKWVLLYIERWLKVPYQTSKGEVIERTMGVPQGSVIGPVLANLFLHYVFDEWMSRNYPTIPFER
YADDTICHCVSEKQAQFLKAVLMKRFEECGLKLNEEKTKIVYCKDSNRRGDSEHTSFDFLGFTFRPRGARNRKTG
QNFTAFLPAISKKSMKRIKEAVRAWKLNHKTFACLLDISNEVDTQISGWMNYYMKFGRSEFRKVLNYINERLTR
WVMRKYKRFSKGRKFDRAYDWLVEYATHNRNEFSHWVKGFAPYPRLG
>R2nH78||gene_32290|GeneMark.hmm|420_aa|-|3645|4907
MNEAKPFVIDKRLVWEAYHKVKENKGSAGIDKVDQKTFDKEMSKNLYKIWNRMSSGCYFPKAVKLVEIPKSNG
GTRPLGIPTIEDRIAQQVVVSVLTPILEPIFKEDSYGYRPGKGAHQAIAKAKERCYVNPWVLDMDISKFFDTINHE
LLMKAVRKHTEEKWVLLYIERWLKVPYQTSKGEVIERTMGVPQGSVIGPVLANLFLHYVFDEWMSRNYPTIPFE
RYADDTICHCVSEKQAQFLKAVLMKRFEECGLKLNEEKTKIVYCKDSNRRGDSEHTSFDFLGFTFRPRSARNRKT
GQNFTAFLPAISKKSMKRIKEAIRAWKLNRKTFACLLDISNEVDTQISGWMNYYMKFGRSEFRKVLNYINERLTR
WVMRKYKRFSKGRKFDRAYDWLVEYAAHNRNEFSHWVKGFVPYPRLG
>R2nH87||gene_314652|GeneMark.hmm|420_aa|+|3517|4779
MNEAKPFVIDKRLVWEAYHKVKENKGSAGIDKVDQKTFDKEMSKNLYKIWNRMSSGCYFPKAVKLVEIPKSNG
GTRPLGIPTIEDRIAQQVVVSVLTPILEPIFKEDSYGYRPGKGAHQAVAKAKERCYVNPWVLDMDISKFFDTINHE
LLMKAVRKHTEEKWVLLYTERWLKVPYQTLKGEVIERMMGVPQGSVIGPVLANLFLHYVFDEWMSRNYPTIP
FERYADDTICHCVSEKQAQFLKAVLMKRFEECGLKLNEEKTKIVYCKDSNRRGDSEHASFDFLGFTFRPRGARNR
KTGQNFTAFLPAISRKSMKRIKEAVRAWKLNRKTFACLLDISNEVDTQISGWMNYYMKFGRSEFRKVLNYINERL
TRWVMRKYKRFSKGKKLGRAYEWLVEYAAHNRNEFSHWVKGFVPYPRLG
>R2nH92||gene_114961|GeneMark.hmm|420_aa|-|213|1475
MNEAKPFVIDKRLVWEAYHKVKENKGSAGIDKVDQKTFDKEMSKNLYKIWNRMSSGCYFPKAVKLVEIPKSNG
GTRPLGIPTIEDRIAQQVVVSVLTPILEPIFKEDSYGYRPGKGAHQAVAKAKERCYVNPWVLDMDISKFFDTINHE
LLMKAVRKHTEEKWVLLYTERWLKVPYQTLKGEVIERMMGVPQGSVIGPVLANLFLHYVFDEWMSRNYPTIP
FERYADDTICHCVSEKQAQFLKAVLMKRFEECGLKLNEEKTKIVYCKDSNRRGDSEHASFDFLGFTFRPRGARNR
KTGQNFTAFLPAISRKSMKRIKEAVRAWKLNRKTFACLLDISNEVDTQISGWMNYYMKFGRSEFRKVLNYINERL
TRWVMRKYKRFSKGKKLGRAYEWLVEYAAHNRNEFSHWVKGFVPYPRLG
>R3AE1236||gene_44502|GeneMark.hmm|418_aa|-|1419|2675
MSEAKQFDISKKAVIAAFQAVKENAGSYGVDEQTIKEFEEHLNNNLYKLWNRMASGSYFPKPVRAVEIPKKNG
GTRILGIPTVEDRIAQMVAKMYFEPLVEPMFYNDSYGYRPNKSAIQAVGQARERCFKRDWVLELDIKGLFDNIK

HGYLMYMVEKHTQIKWLILYIKRWLTVPFIMSDGSVAERRSGTPQGGVISPVLANLFLHYVFDDFMTKAYPNI
WWERYADDGVLHCQSYKQAVFIKQKLEERFQQFGLELNKEKTRIVYCKDDRRSRNYSCTQFTFLGYTFRPRLNK
NKEGKFFVGFTPAVSEKAKTAMKQKIRGWKIQLKADLSLKDIGNMINKVVQGWINYYTHYYKSEFYEVLRYINQ
CLIKWVRRSYKKKNTRSRAEHWLGAVARRDRNLFAHWKFGILPSVGEGAV
>R3AE1237||gene_37826|GeneMark.hmm|418_aa|-|65|1321
MSEAKQFDISKKAVIAAFQAVKENAGSYGADEQTIKEFEEHLNNNLYKLWNRMASGSYFPKPVRAVAIPKKNG
GIRILGIPTVEDRIAQMVAKMYFEPLVEPMFYNDSYGYRPNKSAIQAVGQARERCFKRDWVLELDIKGLFDNIK
HGYLMYMVEKHTQIKWLILYIKRWLTVPFIMSDGSVAERRSGTPQGGVISPVLANLFLHYVFDDFMTKAYPNI
WWERYADDGVLHCQSYKQAAFIKQKLEERFQQFGLELNKEKTRIVYCKDNRRPQNYSCTQFTFLGYTFRPRLN
KNKEGKFFVGFTPAVSEKAKTAMKQKIREWKIQLKADLSLKDIGNMINKVVQGWINYYTHYYKSEFYEVLRYIN
QCLIKWVRRSYKKKNTRSRAEHWLGAVARRDRNLFAHWKFGILPSVGEGAV
>R3AE1238||gene_36766|GeneMark.hmm|420_aa|+|566|1828
MNEAKPFVIDKRLVWEAYHKVKENKGSAGIDKVDQKTFDKEMSKNLYKIWNRMSSGCYFPKAVKLVEIPKSNG
GTRPLGIPTIEDRIAQQVVVSVLTPILEPIFKEDSYGYRPGKGAHQAIAKAKERCYVNPWVLDMDISKFFDTINHD
LLMKAVRKHTEEKWVLLYIERWLKVPYQTSKGEVIERTMGVPQGSVIGPVLANLFLHYVFDEWMSRNYPTIPFE
RYADDTICHCVSEKQAQFLKAVLMKRFEECGLKLNEEKTKIVYCKDSNRRGDSEHTSFDFLGFTFRPRSARNRKT
GQNFTAFLPAISKKSLKRIKEAVRAWKLNRKTFACLLDISNEVDTQISGWMNYYMKFGRSEFRKVLNYINERLTR
WVMRKYKRFSKGKKFSRAYEWLVEYAVHNRNEFSHWAKGFVPYPRLG
>R3AE1238||gene_492187|GeneMark.hmm|418_aa|+|2381|3637
MSEAKQFDISKKAVIAAFQAVKENAGSYGADEQTIKEFEEHLNNNLYKLWNRMASGSYFPKPVRAVAIPKKNG
GIRILGIPTVEDRIAQMVAKMYFEPLVEPMFYNDSYGYRPNKSAIQAVGQARERCFKRDWVLELDIKGLFDNIK
HGYLMYMVEKHTQIKWLILYIKRWLTVPFIMSDGSVAERRSGTPQGGVISPVLANLFLHYVFDDFMTKAYPNI
WWERYADDGVLHCQSYKQAVFIKQKLEERFQQFGLELNKEKTRIVYCKDNRRPQNYSCTQFTFLGYTFRPRLNK
NKEGKFFVGFTPAVSEKAKTVMKQKIREWKIQLKADLSLKDIGNMINKVVQGWINYYTHYYKSEFYEVLRYINQ
CLIKWVRRSYKKKNTRSRAEHWLGAVARRDRNLFAHWKFGILPSVGEGAV
>R3AE1239||gene_517223|GeneMark.hmm|418_aa|+|998|2254
MSEAKQFDISKKAVIAAFQAVKENAGSYGADEQTIKEFEEHLNNNLYKLWNRMASGSYFPKPVRAVAIPKKNG
GIRILGIPTVEDRIAQMVAKMYFEPLVEPMFYNDSYGYRPNKSAIQAVGQARERCFKRDWVLELDIKGLFDNIK
HGYLMYMVEKHTQIKWLILYIKRWLTVPFIMSDGSVAERRSGTPQGGVISPVLANLFLHYVFDDFMTKAYPNI
WWERYADDGVLHCQSYKQAVFIKQKLEERFQQFGLELNKEKTRIVYCKDNRRSQNYSCTQFTFLGYTFRPRLNK
NKEGKFFVGFTPAVSEKAKTAMKQKIRGWKIQLKADLSLKDIGNMINKVVQGWINYYTHYYKSEFYEVLRYINQ
CLIKWVRRSYKKKNTRSRAEHWLGAVARRDRNLFAHWKFGILPSVGEGAV
>R3AE1239||gene_517849|GeneMark.hmm|430_aa|-|11064|12356
MQNDNAKPISISKQLVYDAFLRVKANRGSAGIDKVTLEDYEKNLRGNLYKLWNRMSSGSYFPPSVKLVEIPKSTG
GKRPLGIPTVSDRVAQMTVVMLITPSIEPCFHEDSYAYRPHRSAHDAVGKARERCWKYAWVLDMDISKFFDTID
HELLLKALKRHTQEKWVLMYIERWLKVPYEKSDGSQVDRALGVPQGSVIGPVLANLFLHYTFDKWMEKNFPR
VPFERYADDTICHCHSLKQAEYMQAMIQQRFECCRLRLNEEKTKIVYCKSSRQKECYPNVTFDFLGFTFQPRESV
DKYGNRFTGFLPAISRKSMKRINETMRSWHLNRHSNLTLEHLASDINPIVRGWMTYYGKFYPTRLKWFMQTLN
GRLASWVMCKFERYRHRFYPAQEWLARIAEKEGLIFYHWKCGVLPRFTNKEKVSSQLIMVK
>R3AE1240||gene_275034|GeneMark.hmm|376_aa|+|2|1132
NNNLYKLWNRMASGSYFPKPVRAVAIPKKNGGIRILGIPTVEDRIAQMVAKMYFEPLVEPMFYNDSYGYRPNK
SAIQAVGQARERCFKRDWVLELDIKGLFDNIKHGYLMYMVEKHTQIKWLILYIKRWLTVPFIMSDGSVAERRSG
TPQGGVISPVLANLFLHYVFDDFMTKAYPNIWWERYADDGVLHCQSYKQAAFIKQKLEERFQQFGLELNKEKT
RIVYCKDNRRPQNYSCTQFTFLGYTFRPRLNKNKEGKFFVGFTPAVSEKAKTAMKQKIREWKIQLKADLSLKDIG

NMINKVVQGWINYYTHYYKSEFYEVLRYINQCLIKWVRRSYKKKNTRSRAEHWLGAVARRDRNLFAHWKFGIL
PSVGEGAV
>R3AE1242||gene_119606|GeneMark.hmm|418_aa|+|3715|4971
MSEAKQFDISKKAVIAAFQAVKENAGSYGADEQTIKEFEEHLNNNLYKLWNRMASGSYFPKPVRAVAIPKKNG
GIRILGIPTVEDRIAQMVAKMYFEPLVEPMFYNDSYGYRPNKSAIQAVGQARERCFKRDWVLELDIKGLFDNIK
HGYLMYMVEKHTQIKWLILYIKRWLTVPFIMSDGSVAERRSGTPQGGVISPVLANLFLHYVFDDFMTKAYPNI
WWERYADDGVLHCQSYKQAAFIKQKLEERFQQFGLELNKEKTRIVYCKDNRRPQNYSCTQFTFLGYTFRPRLN
KNKEGKFFVGFTPAVSEKAKTAMKQKIREWKIQLKADLSLKDIGNMINKVVQGWINYYTHYYKSEFYEVLRYIN
QCLIKWVRRSYKKKNTRSRAEHWLGAVARRDRNLFAHWKFGILPSVGEGAV
>R3AE1243||gene_375482|GeneMark.hmm|418_aa|+|1829|3085
MSEAKQFDISKKAVIAAFQAVKENAGSYGADEQTIKEFEEHLNNNLYKLWNRMASGSYFPKPVRAVAIPKKNG
GIRILGIPTVEDRIAQMVAKMYFEPLVEPMFYNDSYGYRPNKSAIQAVGQARERCFKRDWVLELDIKGLFDNIK
HGYLMYMVEKHTQIKWLILYIKRWLTVPFIMSDGSVAERRSGTPQGGVISPVLANLFLHYVFDDFMTKAYPNI
WWERYADDGVLHCQSYKQAAFIKQKLEERFQQFGLELNKEKTRIVYCKDNRRPQNYSCTQFTFLGYTFRPRLN
KNKEGKFFVGFTPAVSEKAKTAMKQKIREWKIQLKADLSLKDIGNMINKVVQGWINYYTHYYKSEFYEVLRYIN
QCLIKWVRRSYKKKNTRSRAEHWLGAVARRDRNLFAHWKFGILPSVGEGAV
>R5GH004||gene_228801|GeneMark.hmm|418_aa|-|65|1321
MSEAKQFDISKKAVIAAFQAVKENAGSYGVDEQTIKEFEEHLNNNLYKLWNRMASGSYFPKPVRAVEIPKKNG
GTRILGIPTVEDRIAQMVAKMYFEPLVEPMFYNDSYGYRPNKSAIQAVGQARERCFKRDWVLELDIKGLFDNIK
HGYLMYMVEKHTQIKWLILYIKRWLTVPFIMSDGSVAERRSGTPQGGVISPVLANLFLHYVFDDFMTKAYPNI
WWERYADDGVLHCQSYKQAVFIKQKLEERFQQFGLELNKEKTRIVYCKDDRRSRNYSCTQFTFLGYTFRPRLNK
NKEGKFFVGFTPAVSEKAKTAMKQKIRGWKIQLKADLSLKDIGNMINKVVQGWINYYTHYYKSEFYEVLRYINQ
CLIKWVRRSYKKKNTRSRAEHWLGAVARRDRNLFAHWKFGILPSVGEGAV
>R5GH007||gene_22617|GeneMark.hmm|420_aa|+|567|1829
MNEAKPFVIDKRLVWEAYHKVKENKGSAGIDKVDQKTFDKEMSKNLYKIWNRMSSGCYFPKAVKLVEIPKSNG
GTRPLGIPTIEDRIAQQVVVSVLTPILEPIFKEDSYGYRPGKGAHQAVAKAKERCYVNPWVLDMDISKFFDTINHE
LLMKAVRKHTEEKWVLLYIERWLKVPYQTLKGEVIERTMGVPQGSVIGPVLANLFLHYVFDEWMSCNYPTIPFE
RYADDTICHCVSEKQAQFLKAVLMKRFEECGLKLNEEKTKIVYCKDSNRRGDSEHTSFDFLGFTFRPRGARNRKT
GQNFTAFLPAISRKSMKRIKEAVRAWKLNRKTFACLLDISNEVDTQISGWMNYYMKFGRSEFRKVLNYINERLT
RWVMRKYKRFSKGKKLGRAYEWLVEYAAHNRNEFSHWVKGFVPYPRLG
>R5GH008||gene_119400|GeneMark.hmm|422_aa|-|7338|8606
MMQHQVTKPFTIDKYLIMNAWKRVKENKGSAGIDNVSTEDYESNLGKNLYKLWNRMSSGSYFPEAVKLVDIP
KPSGGTRPLGIPTVGDRIAQMSVVLLIEERLEAIFHADSYGYRPNRSAHDAIEKARERCWHYNWVLDMDISKFF
DTIDHDLLMKAVERHVQEKWILLYIRRWLKVPYATLTGERIERKMGLPQGSVIGPVLANLYLHYTFDKWMSLYH
PTIPFERYADDTICHCNSLKEAQMLKASIVERFAACKLRLNEEKTRIVYCKDGKRRGEYKEITFDFLGYTFQPRGQ
RNKQGQVFNGYAPAISRKSKKRITEKMRGWHLNRRVQLKLSDIAVEINAEVRGWMNYYGKFYGSQLKAFLQCI
NLKLARWAERKYKRFRRKPNDAYKWLVRVASKNPALFYHWQHGVKPNRLKPFG
>R5GH010||gene_4134|GeneMark.hmm|418_aa|-|705|1961
MSEAKQFDISKKAVIAAFQAVKENAGSYGADEQTIKEFEEHLNNNLYKLWNRMASGSYFPKPVRAVAIPKKNG
GIRILGIPTVEDRIAQMVAKMYFEPLVEPMFYNDSYGYRPNKSAIQAVGQARERCFKRDWVLELDIKGLFDNIK
HGYLMYMVEKHTQIKWLILYIKRWLTVPFIMSDGSVAERRSGTPQGGVISPVLANLFLHYVFDDFMTKAYPNI
WWERYADDGVLHCQSYKQAAFIKQKLEERFQQFGLELNKEKTRIVYCKDNRRPQNYSCTQFTFLGYTFRPRLN
KNKEGKFFVGFTPAVSEKAKTAMKQKIREWKIQLKADLSLKDIGNMINKVVQGWINYYTHYYKSEFYEVLRYIN
QCLIKWVRRSYKKKNTRSRAEHWLGAVARRDRNLFAHWKFGILPSVGEGAV

>R5GH014||gene_73563|GeneMark.hmm|423_aa|-|32949|34220
MNEAKPFEIDKRSVYEAYKAVRSNKGSAGIDNIGMEAYVKNMGNNLFKLWNRMSSGCYFPKAVKLVEIPKSNG
GTRPLGIPTIEDRIAQMTVVLAITPRIDPLFHEDSYGYRPSKSAHDAVAKAKERCMKYAWVLDMDISKFFDTINH
ELLMKAVRKHVDEKWILLYIERWLKVPYQTKDGTMIERIMGVPQGSVIGPLLANLFLHYVFDMWMSNNYPSIP
FERYADDCVCHCVSEKQVIHLRIALKRRFEECGLKLNEDKTKIVYCKDSNRKGNSDNTSFDYLGFTFRPRGARNS
KTGQIFTAFLPAISKKAMMRIKSEIRDWNLNRRLQIGIEQIAEDICPKVRGWLNYYSPFGRTEIGKVMKYLNQKL
SRWVMRKYKRFAKGHRRGHAFDWLVRRALYDKSLFVHWAEGYVPYPRVYKLK
>R5GH019||gene_3883|GeneMark.hmm|430_aa|+|198|1490
MQNDNAKPISISKQLVYDAFLRVKANRGSAGIDKVTLEDYEKNLRGNLYKLWNRMSSGSYFPPSVKLVEIPKSTG
GKRPLGIPTVSDRVAQMAVVMLITPSIEPCFHEDSYAYRPHRSAHDAVGKARERCWKYAWVLDMDISKFFDTI
DHELLLKALKRHTQEKWVLMYIERWLKVPYEKSDGSQVDRALGVPQGSVIGPVLANLFLHYTFDKWMEKNFP
RVPFERYADDTICHCHSLKQAEYMQAMIQQRFECCRLRLNEEKTKIVYCKSSRQKECYPNVTFDFLGFTFQPRES
VDKYGNRFTGFLPAISRKSMKRINETMRSWHLNRHSNLTLEHLASDINPIVRGWMTYYGKFYPTRLKWFMQTL
NGRLARWVMCKFERYRHRFYPAQEWLARIAEKEGLIFYHWKCGALPRFTNKEKVSSQLIMVK
>R5GH020||gene_385100|GeneMark.hmm|420_aa|+|422|1684
MNEAKPFVIDKRLVWEAYHKVKENKGSAGIDKVDQKTFDKEMSKNLYKIWNRMSSGCYFPKAVKLVEIPKSNG
GTRPLGIPTIEDRIAQQVVVSVLTPILEPIFKEDSYGYRPGKGAHQAIAKAKERCYVNPWVLDMDISKFFDTINHD
LLMKAVRKHTEEKWVLLYIERWLKVPYQTSKGEVIERTMGVPQGSVIGPVLANLFLHYVFDEWMSRNYPTIPFE
RYADDTICHCVSEKQAQFLKAVLMKRFEECGLKLNEEKTKIVYCKDSNRRGDSEHTSFDFLGFTFRPRSARNRKT
GQNFTAFLPAISKKSLKRIKEAVRAWKLNRKTFACLLDISNEVDTQISGWMNYYMKFGRSEFRKVLNYINERLTR
WVMRKYKRFSKGKKFSRAYEWLVEYAVHNRNEFSHWAKGFVPYPRLG
>R5GH022||gene_41625|GeneMark.hmm|410_aa|+|439|1671
MQRKSFEIPKALVWASYLDVRRNKGAPGCDGQTLKMFDQQRDGNLYKIWNRLCSGTWFPPPVLEKRIPKPN
GKERILGIPTVSDRIAQGAIKLFMEEKLDPIFHADSYGYRPGKSAHDALKQCAIRCWRYSWILEVDISAFFDHVRH
DLVLKALEHHGMPKWVILYCRRWMEAPMQSCENGELITRTRGTPQGGVISPLLANLFLHYAFDLWMEREYRG
VPFERYADDIVVHCSRMSDATRLKNRLSERFSEVGLVLNAGKTNIAYIDTFKRRNVATSFTFLGYDFKVRTLKNFK
GELYRKCMPGASNAAMRKITETIKKWRIHRSTAESLLDFARRYNAIVRGWIEYYGKFWSRNFNYRLWSAMQSR
LLKWMQSKYRLSNRRAQRKLTLVRKEYPKLFVHWYLLRASNE
>R5GH022||gene_93775|GeneMark.hmm|418_aa|+|409|1665
MSEAKQFDISKKAVIAAFQAVKENAGSYGVDEQTIKEFEEHLNNNLYKLWNRMASGSYFPKPVRAVEIPKKNG
GTRILGIPTVEDRIAQMVAKMYFEPLVEPMFYNDSYGYRPNKSAIQAVGQARERCFKRDWVLELDIKGLFDNIK
HGYLMYMVEKHTQIKWLILYIKRWLTVPFIMSDGSVAERRSGTPQGGVISPVLANLFLHYVFDDFMTKAYPNI
WWERYADDGVLHCQSYKQAVFIKQKLEERFQQFGLELNKEKTRIVYCKDDRRSRNYSCTQFTFLGYTFRPRLNK
NKEGKFFVGFTPAVSEKAKTAMKQKIRGWKIQLKADLSLKDIGNMINKVVQGWINYYTHYYKSEFYEVLRYINQ
CLIKWVRRSYKKKNTRSRAEHWLGAVARRDRNLFAHWKFGILPSVGEGAV
>R5GH023||gene_413955|GeneMark.hmm|418_aa|-|1221|2477
MSEAKQFDISKKAVIAAFQAVKENAGSYGADEQTIKEFEEHLNNNLYKLWNRMASGSYFPKPVRAVAIPKKNG
GIRILGIPTVEDRIAQMVAKMYFEPLVEPMFYNDSYGYRPNKSAIQAVGQARERCFKRDWVLELDIKGLFDNIK
HGYLMYMVEKHTQIKWLILYIKRWLTVPFIMSDGSVAERRSGTPQGGVISPVLANLFLHYVFDDFMTKAYPNI
WWERYADDGVLHCQSYKQAAFIKQKLEERFQQFGLELNKEKTRIVYCKDNRRPQNYSCTQFTFLGYTFRPRLN
KNKEGKFFVGFTPAVSEKAKTAMKQKIREWKIQLKADLSLKDIGNMINKVVQGWINYYTHYYKSEFYEVLRYIN
QCLIKWVRRSYKKKNTRSRAEHWLGAVARRDRNLFAHWKFGILPSVGEGAV
>R5GH023||gene_116476|GeneMark.hmm|430_aa|-|1842|3134
MQNDNAKPISISKQLVYDAFLRVKANRGSAGIDKVTLEDYEKNLRGNLYKLWNRMSSGSYFPPSVKLVEIPKSTG

GKRPLGIPTVSDRVAQMAVVMLITPSIEPCFHEDSYAYRPHRSAHDAVGKARERCWKYAWVLDMDISKFFDTI
DHELLLKALKRHTQEKWVLMYIERWLKVPYEKSDGSQVDRALGVPQGSVIGPVLANLFLHYTFDKWMEKNFP
RVPFERYADDTICHCHSLKQAEYMQAMIQQRFECCRLRLNEEKTKIVYCKSSRQKECYPNVTFDFLGFTFQPRES
VDKYGNRFTGFLPAISRKSMKRINETMRSWHLNRHSNLTLEHLASDINPIVRGWMTYYGKFYPTRLKWFMQTL
NGRLARWVMCKFERYRHRFYPAQEWLARIAEKEGLIFYHWKCGALPRFTNKEKVSSQLIMVK
>R5GH024||gene_177655|GeneMark.hmm|420_aa|-|671|1933
MNEAKPFVIDKRLVWEAYHKVKENKGSAGIDKVDQKTFDKEMSKNLYKIWNRMSSGCYFPKAVKLVEIPKSNG
GTRPLGIPTIEDRIAQQVVVSVLTPILEPIFKEDSYGYRPGKGAHQAIAKAKERCYVNPWVLDMDISKFFDTINHD
LLMKAVRKHTEEKWVLLYIERWLKVPYQTSKGEVIERTMGVPQGSVIGPVLANLFLHYVFDEWMSRNYPTIPFE
RYADDTICHCVSEKQAQFLKAVLMKRFEECGLKLNEEKTKIVYCKDSNRRGDSEHTSFDFLGFTFRPRSARNRKT
GQNFTAFLPAISKKSLKRIKEAVRAWKLNRKTFACLLDISNEVDTQISGWMNYYMKFGRSEFRKVLNYINERLTR
WVMRKYKRFSKGKKFSRAYEWLVEYAVHNRNEFSHWAKGFVPYPRLG
>R5GH024||gene_69637|GeneMark.hmm|430_aa|-|7369|8661
MQNDNAKPISISKQLVYDAFLRVKANRGSAGIDKVTLEDYEKNLRGNLYKLWNRMSSGSYFPPSVKLVEIPKSTG
GKRPLGIPTVSDRVAQMAVVMLITPSIEPCFHEDSYAYRPHRSAHDAVGKARERCWKYAWVLDMDISKFFDTI
DHELLLKALKRHTQEKWVLMYIERWLKVPYEKSDGSQVDRALGVPQGSVIGPVLANLFLHYTFDKWMEKNFP
RVPFERYADDTICHCHSLKQAEYMQAMIQQRFECCRLRLNEEKTKIVYCKSSRQKECYPNVTFDFLGFTFQPRES
VDKYGNRFTGFLPAISRKSMKRINETMRSWHLNRHSNLTLEHLASDINPIVRGWMTYYGKFYPTRLKWFMQTL
NGRLARWVMCKFERYRHRFYPAQEWLARIAEKEGLIFYHWKCGALPRFTNKEKVSSQLIMVK
>R5GH028||gene_166996|GeneMark.hmm|430_aa|+|713|2005
MQNDNAKPISISKQLVYDAFLRVKANRGSAGIDKVTLEDYEKNLRGNLYKLWNRMSSGSYFPPSVKLVEIPKSTG
GKRPLGIPTVSDRVAQMAVVMLITPSIEPCFHEDSYAYRPHRSAHDAVGKARERCWKYAWVLDMDISKFFDTI
DHELLLKALKRHTQEKWVLMYIERWLKVPYEKSDGSQVDRALGVPQGSVIGPVLANLFLHYTFDKWMEKNFP
RVPFERYADDTICHCHSLKQAEYMQAMIQQRFECCRLRLNEEKTKIVYCKSSRQKECYPNVTFDFLGFTFQPRES
VDKYGNRFTGFLPAISRKSMKRINETMRSWHLNRHSNLTLEHLASDINPIVRGWMTYYGKFYPTRLKWFMQTL
NGRLARWVMCKFERYRHRFYPAQEWLARIAEKEGLIFYHWKCGALPRFTNKEKVSSQLIMVK
>R5GH029||gene_225512|GeneMark.hmm|417_aa|+|1685|2938
MNAANPFVIDKRLVWEAYHKVKENKGSAGIDKVDQKTFDKEMSKNLYKIWNRMSSGCYFPKAVKLVEMPKS
NGGTRPLDIPAIEDRIAQQVVVSVLTPILEPIFKEDSYGYRPGKGAHQAIAKAKERCYVTPWVLDMDISKFFDTIN
HELLMKAIRKHTEEKWVLLYIERWLKVPYQTSKGEVIERTMGGSVIGPVLANLFLHYVFDEWMSRNYPTIPFERY
ADDTICHCVSEKQAQFLKAVLMKRFEECGLKLNEEKTKIVYCKDSNRRGDSEHTSFDFLGFTFRPRGARNRKTG
QNFTAFFPAISKKSMKRIKEAVRAWKLNRKTFACLLDISNEVDTQISGWMNYYMKFGRSEFRKVLNYINERLSR
WVMRKYKCFSKGKKLGRAYEWLVEHAVHNRNEFSHWVKGFVPYPRLD
>R5GH030||gene_259798|GeneMark.hmm|430_aa|-|88|1380
MQNDNAKPISISKQLVYDAFLRVKANRGSAGIDKVTLEDYEKNLRGNLYKLWNRMSSGSYFPPSVKLVEIPKSTG
GKRPLGIPTVSDRVAQMAVVMLITPSIEPCFHEDSYAYRPHRSAHDAVGKARERCWKYAWVLDMDISKFFDTI
DHELLLKALKRHTQEKWVLMYIERWLKVPYEKSDGSQVDRALGVPQGSVIGPVLANLFLHYTFDKWMEKNFP
RVPFERYADDTICHCHSLKQAEYMQAMIQQRFECCRLRLNEEKTKIVYCKSSRQKECYPNVTFDFLGFTFQPRES
VDKYGNRFTGFLPAISRKSMKRINETMRSWHLNRHSNLTLEHLASDINPIVRGWMTYYGKFYPTRLKWFMQTL
NGRLARWVMCKFERYRHRFYPAQEWLARIAEKEGLIFYHWKCGALPRFTNKEKVSSQLIMVK
>R5GH032||gene_25464|GeneMark.hmm|418_aa|+|2840|4096
MSEAKQFDISKKAVIAAFQAVKENAGSYGADEQTIKEFEEHLNNNLYKLWNRMASGSYFPKPVRAVAIPKKNG
GIRILGIPTVEDRIAQMVAKMYFEPLVEPMFYNDSYGYRPNKSAIQAVGQARERCFKRDWVLELDIKGLFDNIK
HGYLMYMVEKHTQIKWLILYIKRWLTVPFIMSDGSVAERRSGTPQGGVISPVLANLFLHYVFDDFMTKAYPNI

WWERYADDGVLHCQSYKQAAFIKQKLEERFQQFGLELNKEKTRIVYCKDNRRPQNYSCTQFTFLGYTFRPRLN
KNKEGKFFVGFTPAVSEKAKTAMKQKIREWKIQLKADLSLKDIGNMINKVVQGWINYYTHYYKSEFYEVLRYIN
QCLIKWVRRSYKKKNTRSRAEHWLGAVARRDRNLFAHWKFGILPSVGEGAV
>R5GH033||gene_145303|GeneMark.hmm|418_aa|-|182|1438
MSEAKQFDISKKAVIAAFQAVKENAGSYGVDEQTIKEFEEHLNNNLYKLWNRMASGSYFPKPVRAVEIPKKNG
GTRILGIPTVEDRIAQMVAKMYFEPLVEPMFYNDSYGYRPNKSAIQAVGQARERCFKRDWVLELDIKGLFDNIK
HGYLMYMVEKHTQIKWLILYIKRWLTVPFIMSDGSVAERRSGTPQGGVISPVLANLFLHYVFDDFMTKAYPNI
WWERYADDGVLHCQSYKQAVFIKQKLEERFQQFGLELNKEKTRIVYCKDDRRSRNYSCTQFTFLGYTFRPRLNK
NKEGKFFVGFTPAVSEKAKTAMKQKIRGWKIQLKADLSLKDIGNMINKVVQGWINYYTHYYKSEFYEVLRYINQ
CLIKWVRRSYKKKNTRSRAEHWLGAVARRDRNLFAHWKFGILPSVGEGAV
>R5GH034||gene_111036|GeneMark.hmm|423_aa|-|4277|5548
MNEAKPFEIDKRSVYEAYKAVRSNKGSAGIDNIGMEAYVKNMGNNLFKLWNRMSSGCYFPKAVKLVEIPKSNG
GTRPLGIPTIEDRIAQMTVVLAITPRIDPLFHEDSYGYRPSKSAHDAVAKAKERCMKYAWVLDMDISKFFDTINH
ELLMKAVRKHVDEKWILLYIERWLKVPYQTKDGTMIERIMGVPQGSVIGPLLANLFLHYVFDMWMSNNYPSIP
FERYADDCVCHCVSEKQVIHLRIALKRRFEECGLKLNEDKTKIVYCKDSNRKGNSDNTSFDYLGFTFRPRGARNS
KTGQIFTAFLPAISKKAMMRIKSEIRDWNLNRRLQIGIEQIAEDICPKVRGWLNYYSPFGRTEIGKVMKYLNQKL
SRWVMRKYKRFAKGHRRGHAFDWLVRRALYDKSLFVHWAEGYVPYPRVYKLK
>R5GH034||gene_77149|GeneMark.hmm|418_aa|-|709|1965
MSEAKQFDISKKAVIAAFQAVKENAGSYGADEQTIKEFEEHLNNNLYKLWNRMASGSYFPKPVRAVAIPKKNG
GIRILGIPTVEDRIAQMVAKMYFEPLVEPMFYNDSYGYRPNKSAIQAVGQARERCFKRDWVLELDIKGLFDNIK
HGYLMYMVEKHTQIKWLILYIKRWLTVPFIMSDGSVAERRSGTPQGGVISPVLANLFLHYVFDDFMTKAYPNI
WWERYADDGVLHCQSYKQAAFIKQKLEERFQQFGLELNKEKTRIVYCKDNRRPQNYSCTQFTFLGYTFRPRLN
KNKEGKFFVGFTPAVSEKAKTAMKQKIREWKIQLKADLSLKDIGNMINKVVQGWINYYTHYYKSEFYEVLRYIN
QCLIKWVRRSYKKKNTRSRAEHWLGAVARRDRNLFAHWKFGILPSVGEGAV
>R5GH035||gene_190668|GeneMark.hmm|418_aa|+|3251|4507
MSEAKQFDISKKAVIAAFQAVKENAGSYGADEQTIKEFEEHLNNNLYKLWNRMASGSYFPKPVRAVAIPKKNG
GIRILGIPTVEDRIAQMVAKMYFEPLVEPMFYNDSYGYRPNKSAIQAVGQARERCFKRDWVLELDIKGLFDNIK
HGYLMYMVEKHTQIKWLILYIKRWLTVPFIMSDGSVAERRSGTPQGGVISPVLANLFLHYVFDDFMTKAYPNI
WWERYADDGVLHCQSYKQAAFIKQKLEERFQQFGLELNKEKTRIVYCKDNRRPQNYSCTQFTFLGYTFRPRLN
KNKEGKFFVGFTPAVSEKAKTAMKQKIREWKIQLKADLSLKDIGNMINKVVQGWINYYTHYYKSEFYEVLRYIN
QCLIKWVRRSYKKKNTRSRAEHWLGAVARRDRNLFAHWKFGILPSVGEGAV
>R5GH036||gene_235752|GeneMark.hmm|422_aa|-|2362|3630
MKDAKSFEISRHLVMEAYKRVKANKGAAGVDEVSIADFENNLKSNLYKIWNRMSSGSYLPPAVKLVEIPKSNGG
KRPLGIPTVGDRVAQMVVVMTIEPGIEPYFHEDSYAYRPNRSALDAVRKAKERSYTFHWVLDLDIKGFFDNIDH
ELLIKALERHVKCKWAILYIKRWLSVPYQLKDGTQKERTKGVPQGSVVGPILANLFLHYVFDEWMRRNHSNISFE
RYADDTICHCVSLKQAEFILRAIRKRFAECKLELNEDKTKIVYCKKNHRDIPYECIQFDFLGYTFRPRRSIDANGEVF
LNFSPAISKKARTKIWEAIQNWNSNHWVPMELEDIAKEINPVIQGWINYYGQHNPRILKEVLQHVNDRLVRW
GRRKFKGLRKRKTATVHRLGDIALQKPNLFAHWAWGVKPTASERNRKRK
>R5GH037||gene_265434|GeneMark.hmm|402_aa|-|2|1207
MSEAKQFDISKKAVIAAFQAVKENAGSYGADEQTIKEFEEHLNNNLYKLWNRMASGSYFPKPVRAVAIPKKNG
GIRILGIPTVEDRIAQMVAKMYFEPLVEPMFYNDSYGYRPNKSAIQAVGQARERCFKRDWVLELDIKGLFDNIK
HGYLMYMVEKHTQIKWLILYIKRWLTVPFIMSDGSVAERRSGTPQGGVISPVLANLFLHYVFDDFMTKAYPNI
WWERYADDGVLHCQSYKQAAFIKQKLEERFQQFGLELNKEKTRIVYCKDNRRPQNYSCTQFTFLGYTFRPRLN
KNKEGKFFVGFTPAVSEKAKTAMKQKIREWKIQLKADLSLKDIGNMINKVVQGWINYYTHYYKSEFYEVLRYIN

QCLIKWVRRSYKKKNTRSRAEHWLGAVARRDRNLF
>R5GH040||gene_338753|GeneMark.hmm|414_aa|-|21971|23215
MIETKPYEISKWAVYIAYERVKANKGSYGVDEQSIEDFEKNLKNNLYKIWNRMSSGSYFPQPVKAVSVPKKNGGI
RVLGIPTVEDRIAQMTAKLYFEPCVEPLFLEDSYGYRPGKSAIQALSVTRKRCWHRDWVLEYDIKGLFDNIRHDY
LLEMVRRHTPHKWILLYVERWLTTPFQLEDGTLQSRTSGTPQGGVISLVLANLFLHYAFDSFMAKEYPKAWWE
RYADDGVLHCKSSQQAMYMKSVLRERFRLFGLELNEEKTRIVYCKDADRTEDYSEISLDSLGYTFRPRLARNKHG
NIFLNFLPAMSAKAIKAMKEEVRRWKLQLKVSKSLTDLANILNSQIQGWISYYGHFYKSELIYLLRYINQCLIKWV
RRKYKKFNHRRRAEYWLGRIARRDNNLFAHWRYGVLPTAG
>R5GH040||gene_385910|GeneMark.hmm|420_aa|+|14270|15532
MNEAKPFVIDKRLVWEAYHKVKENKGSAGIDKVDQKTFDKEMSKNLYKIWNRMSSGCYFPKAVKLVEIPKSNG
GTRPLGIPTIEDRIAQQVVVSVLTPILEPIFKEDSYGYRPGKGAHQAIAKAKERCYVNPWVLDMDISKFFDTINHD
LLMKAVRKHTEEKWVLLYIERWLKVPYQTSKGEVIERTMGVPQGSVIGPVLANLFLHYVFDEWMSRNYPTIPFE
RYADDTICHCVSEKQAQFLKAVLMKRFEECGLKLNEEKTKIVYCKDSNRRGDSEHTSFDFLGFTFRPRSARNRKT
GQNFTAFLPAISKKSLKRIKEAVRAWKLNRKTFACLLDISNEVDTQISGWMNYYMKFGRSEFRKVLNYINERLTR
WVMRKYKRFSKGKKFSRAYEWLVEYAVHNRNEFSHWAKGFVPYPRLG
>R5GH040||gene_172241|GeneMark.hmm|401_aa|-|29344|30549
VKIAFERVKANKGTYGIDEQTIADFEENLKDNLYKIWNRMSSGTYFPKPVKAVAIPKKSGGTRILGIPTVEDRVAQ
MVAKIYFEPKVEELFYEDSYGYRPNKSAIDAIGILRKRCWKKDWVVDFDIKGLFDNIRHDYLIEMVKRHTNEQWI
ILYIERWLKTPFKMQDGTIVERTAGTPQGGVISPVLANLFMHYVFDDFMSKEFPTIQWVRYADDGVLNCVSLK
QAKYIIKVLDKRFKTCGLELNLEKTKIVYCKDDNRKGKYGNTKFDFLGYTFKIRSAENNKGQIFNSFIPAMSDKAQ
KDIRKELRSYRIQRQVDKSLSDIANMFNSKIQGWINYYSHYYKTEVNKILDYFNSILVKWVMRKYKTIKSKKRAIK
WLAEIAQRDTTLFAHWKFGILPMAR
>R5GH040||gene_275357|GeneMark.hmm|430_aa|+|2077|3369
MQNDNAKPISISKQLVYDAFLRVKANRGSAGIDKVTLEDYEKNLRGNLYKLWNRMSSGSYFPPSVKLVEIPKSTG
GKRPLGIPTVSDRVAQMAVVMLITPSIEPCFHEDSYAYRPHRSAHDAVGKARERCWKYAWVLDMDISKFFDTI
DHELLLKALKRHTQEKWVLMYIERWLKVPYEKSDGSQVDRALGVPQGSVIGPVLANLFLHYTFDKWMEKNFP
RVPFERYADDTICHCHSLKQAEYMQAMIQQRFECCRLRLNEEKTKIVYCKSSRQKECYPNVTFDFLGFTFQPRES
VDKYGNRFTGFLPAISRKSMKRINETMRSWHLNRHSNLTLEHLASDINPIVRGWMTYYGKFYPTRLKWFMQTL
NGRLARWVMCKFERYRHRFYPAQEWLARIAEKEGLIFYHWKCGALPRFTNKEKVSSQLIMVK
>R5GH042||gene_114243|GeneMark.hmm|418_aa|-|23289|24545
MSEAKQFDISKKAVIAAFQAVKENAGSYGADEQTIKEFEEHLNNNLYKLWNRMASGSYFPKPVRAVAIPKKNG
GIRILGIPTVEDRIAQMVAKMYFEPLVEPMFYNDSYGYRPNKSAIQAVGQARERCFKRDWVLELDIKGLFDNIK
HGYLMYMVEKHTQIKWLILYIKRWLTVPFIMSDGSVAERRSGTPQGGVISPVLANLFLHYVFDDFMTKAYPNI
WWERYADDGVLHCQSYKQAAFIKQKLEERFQQFGLELNKEKTRIVYCKDNRRPQNYSCTQFTFLGYTFRPRLN
KNKEGKFFVGFTPAVSEKAKTAMKQKIREWKIQLKADLSLKDIGNMINKVVQGWINYYTHYYKSEFYEVLRYIN
QCLIKWVRRSYKKKNTRSRAEHWLGAVARRDRNLFAHWKFGILPSVGEGAV
>R5GH043||gene_114668|GeneMark.hmm|418_aa|-|1135|2391
MSEAKQFDISKKAVIAAFQAVKENAGSYGADEQTIKEFEEHLNNNLYKLWNRMASGSYFPKPVRAVAIPKKNG
GIRILGIPTVEDRIAQMVAKMYFEPLVEPMFYNDSYGYRPNKSAIQAVGQARERCFKRDWVLELDIKGLFDNIK
HGYLMYMVEKHTQIKWLILYIKRWLTVPFIMSDGSVAERRSGTPQGGVISPVLANLFLHYVFDDFMTKAYPNI
WWERYADDGVLHCQSYKQAAFIKQKLEERFQQFGLELNKEKTRIVYCKDNRRPQNYSCTQFTFLGYTFRPRLN
KNKEGKFFVGFTPAVSEKAKTAMKQKIREWKIQLKADLSLKDIGNMINKVVQGWINYYTHYYKSEFYEVLRYIN
QCLIKWVRRSYKKKNTRSRAEHWLGAVARRDRNLFAHWKFGILPSVGEGAV
>R5GH044||gene_113591|GeneMark.hmm|420_aa|-|6099|7361

MNEAKPFVIDKRLVWEAYHKVKENKGSAGIDKVDQKTFDKEMSKNLYKIWNRMSSGCYFPKAVKLVEIPKSNG
GTRPLGIPTIEDRIAQQVVVSVLTPILEPIFKEDSYGYRPGKGAHQAIAKAKERCYVNPWVLDMDISKFFDTINHD
LLMKAVRKHTEEKWVLLYIERWLKVPYQTSKGEVIERTMGVPQGSVIGPVLANLFLHYVFDEWMSRNYPTIPFE
RYADDTICHCVSEKQAQFLKAVLMKRFEECGLKLNEEKTKIVYCKDSNRRGDSEHTSFDFLGFTFRPRSARNRKT
GQNFTAFLPAISKKSLKRIKEAVRAWKLNRKTFACLLDISNEVDTQISGWMNYYMKFGRSEFRKVLNYINERLTR
WVMRKYKRFSKGKKFSRAYEWLVEYAVHNRNEFSHWAKGFVPYPRLG
>R5GH045||gene_3813|GeneMark.hmm|423_aa|+|530|1801
MTQKQGAKPFDIDRWKLYYAYQRVNQNRGGSGVDNVTLEKYNSNLKRNLYKLWNRMSSGSYVPKPVRLVQIP
KPAGGTRPLGIPTVEDRIAQMLVVEMIEPEIEKIFHEDSYGYRPNRSAHDALGRARERCWKYAWVLDMDISKFF
DTIDHQLLMKAVRLHVKERWIILYIERWLKVPYQNADKSLIERTCGVPQGSVIGPILANLFLHYCFDRWMQIHYP
EIPFERYADDTVCHCRSQREAESLYEELIIRFKSCKLSLNEEKTKIVYCKSSRRKENHSNVTFDFLGHTFRPCKTMH
KSSREAFTGFQPRISMKATTKIRATMRSWNLKSKSHTPLDCIAHMVNPILRGWVNYYGKYGGKSFQKLLGYFDL
LLARWAKAKYKTFRRKPMYVILKWLGNVADRDAVFYHWQIGLKPAKGTIKL
>R5GH046||gene_27497|GeneMark.hmm|418_aa|+|554|1810
MSEAKQFDISKKAVIAAFQAVKENAGSYGVDEQTIKEFEEHLNNNLYKLWNRMASGSYFPKPVRAVEIPKKNG
GTRILGIPTVEDRTAQMVAKMYFEPLVEPMFYNDSYGYRPNKSAIQAVGQARERCFKRDWVLELDIKGLFDNIK
HGYLMYMVEKHTQIKWLILYIKRWLTVPFIMSDGSVAERRSGTPQGGVISPVLANLFLHYVFDDFMTKAYPNI
WWERYADDGVLHCQSYKQAVFIKQELEERFQQFGLELNKEKTRIVYCKDNRRSQNYSCTQFTFLGYTFRPRLNK
NKEGKFFVGFTPAVSEKAKTAMKQKIRGWKIQLKADLSLKDIGNMINKVVQGWINYYTHYYKSEFYEVLRYINQ
CLIKWVRRSYKKKNTRSRAEHWLGAVARRDRNLFAHWKFGILPSVGEGAV
>R5GH048||gene_29088|GeneMark.hmm|421_aa|-|88|1353
MQEAKPFQIDKRIIFESFKKVKFNRGSSGIDGIEMTTYEQNLGSNLYRLWNRMSSGSYMPKAVKLVEIPKSNGG
KRPLGIPTIEDRIAQMAVVNVIEPLIEPCFHEDSFGYRPHRSAHDAIAKAERRCWKYAWVLDIDISKFFDTIDHGL
LMKAVEKHINIKWILLYIKRWLTVPYQRSDGEIVKRDMGVPQGSVIGPILANLFLHYTFDKWMSYKYPHIPFERY
ADDCVCHCSTLAQAEYIKERLGERFTECKLKFNEEKTKIVFCKMSSRSSKHYHCTSFDYLGFTFRSRAAKDKRNN
VLFTSYLPAISKKSVSRIHETIKSWNLKRLHNRSLRFVAAYINDVVRGWINYYEKFGKTEFWKVMCHLNRSIAYW
AKTKYKRLRRRGVISAHYWLAYIAQKEPNLFYHWQVGYVPYARQKK
>R5GH048||gene_68832|GeneMark.hmm|418_aa|-|299|1555
MSEAKQFDISKKAVIAAFQAVKENAGSYGADEQTIKEFEEHLNNNLYKLWNRMASGSYFPKPVRAVAIPKKNG
GIRILGIPTVEDRIAQMVAKMYFEPLVEPMFYNDSYGYRPNKSAIQAVGQARERCFKRDWVLELDIKGLFDNIK
HGYLMYMVEKHTQIKWLILYIKRWLTVPFIMSDGSVAERRSGTPQGGVISPVLANLFLHYVFDDFMTKAYPNI
WWERYADDGVLHCQSYKQAAFIKQKLEERFQQFGLELNKEKTRIVYCKDNRRPQNYSCTQFTFLGYTFRPRLN
KNKEGKFFVGFTPAVSEKAKTAMKQKIREWKIQLKADLSLKDIGNMINKVVQGWINYYTHYYKSEFYEVLRYIN
QCLIKWVRRSYKKKNTRSRAEHWLGAVARRDRNLFAHWKFGILPSVGEGAV
>R5GH050||gene_84865|GeneMark.hmm|418_aa|+|3178|4434
MSEAKQFDISKKAVIAAFQAVKENAGSYGVDEQTIKEFEEHLNNNLYKLWNRMASGSYFPKPVRAVEIPKKNG
GTRILGIPTVEDRTAQMVAKMYFEPLVEPMFYNDSYGYRPNKSAIQAVGQARERCFKRDWVLELDIKGLFDNIK
HGYLMYMVEKHTQIKWLILYIKRWLTVPFIMSDGSVAERRSGTPQGGVISPVLANLFLHYVFDDFMTKAYPNI
WWERYADDGVLHCQSYKQAVFIKQELEERFQQFGLELNKEKTRIVYCKDNRRSQNYSCTQFTFLGYTFRPRLNK
NKEGKFFVGFTPAVSEKAKTAMKQKIRGWKIQLKADLSLKDIGNMINKVVQGWINYYTHYYKSEFYEVLRYINQ
CLIKWVRRSYKKKNTRSRAEHWLGAVARRDRNLFAHWKFGILPSVGEGAV
>R5GH051||gene_252306|GeneMark.hmm|410_aa|-|366|1598
MQRKSFEIPKALVWASYLDVRRNKGAPGCDGQTLKMFDQQRDGNLYKIWNRLCSGTWFPPPVLEKRIPKPN
GKERILGIPTVSDRIAQGAIKLFMEEKLDPIFHADSYGYRPGKSAHDALKQCAIRCWRYSWILEVDISAFFDHVRH

DLVLKALEHHGMPKWVILYCRRWMEAPMQSCENGELITRTRGTPQGGVISPLLANLFLHYAFDLWMEREYRG
VPFERYADDIVVHCSRMSDATRLKNRLSERFSEVGLVLNAGKTNIAYIDTFKRRNVATSFTFLGYDFKVRTLKNFK
GELYRKCMPGASNAAMRKITETIKKWRIHRSTAESLLDFARRYNAIVRGWIEYYGKFWSRNFNYRLWSAMQSR
LLKWMQSKYRLSNRRAQRKLTLVRKEYPKLFVHWYLLRASNE
>R5GH052||gene_255444|GeneMark.hmm|420_aa|-|188|1450
MNEAKPFVIDKRLVWEAYHKVKENKGSAGIDKVDQKTFDKEMSKNLYKIWNRMSSGCYFSKAVKLVEIPKSNG
GTRPLGIPTIEYRIAQQVVVSVLTPILEPIFKEDSYGYRPGKGAHQAIAKAKERCYVTPWVLDMDISKFFDTINHEL
LMKAIRKHTEEKWVLLYIERWLKVPNQTSKGEVIERTMGVPQGSVIGPVLANLFLHYVFDEWMSRNYPTIPFER
YADATICHCVSEKQARFLKAVLMKRFEEYGLKLNEEKTKIVYCKDSNRRGDSEHTSFNFLGFTFRPRGARNRKTG
QNFTAFLPAISNKSMKRIKEAIRAWKLNRKTFACLLDISTEVDTQISGWMNYYMKFGRSEFRKVLNYINERLTRW
VMRKYKRFSKGKKFSKAYEWLVEYAAHNRNEFSHWVKGFVPYPRLD
>R5GH052||gene_153458|GeneMark.hmm|430_aa|+|608|1900
MQNDNAKPISISKQLVYDAFLRVKANRGSAGIDKVTLEDYEKNLRGNLYKLWNRMSSGSYFPPSVKLVEIPKSTG
GKRPLGIPTVSDRVAQMAVVMLITPSIEPCFHEDSYAYRPHRSAHDAVGKARERCWKYAWVLDMDISKFFDTI
DHELLLKALKRHTQEKWVLMYIERWLKVPYEKSDGSQVDRALGVPQGSVIGPVLANLFLHYTFDKWMEKNFP
RVPFERYADDTICHCHSLKQAEYMQAMIQQRFECCRLRLNEEKTKIVYCKSSRQKECYPNVTFDFLGFTFQPRES
VDKYGNRFTGFLPAISRKSMKRINETMRSWHLNRHSNLTLEHLASDINPIVRGWMTYYGKFYPTRLKWFMQTL
NGRLARWVMCKFERYRHRFYPAQEWLARIAEKEGLIFYHWKCGALPRFTNKEKVSSQLIMVK
>R5GH053||gene_282488|GeneMark.hmm|448_aa|+|1747|3093
VKRAIERWKERRAYSIQALRISQLEHKEELMQEAKRFNISKRTVIAAFQRVKKNAGAYGVDKQSIKDFEEHLNNN
LYKIWNRMTSGSYFPKPVRAVSIPKKSGGTRILGIPTVEDRIAQMVAKMYFEPLVEPMFYEDAYGYRSGKSAIQA
VGVTRERCFKQDWVLELDIKGLFDNIRHDYLMYMVKKHTQEKWLLLYIQRWLTVPFQMEDETVVQRESGTPQ
GGVISPILANLFLHYVFDDFMAKVYPHITWERYADDGVLHCQSYKQAIYIKSVLEERFRQFGLELNQEKTSIVYCK
DDRRKLNYECTSFTFLGYTFRARRNKSRNGAYFVGFTPAVSEKAKTAMKQVIRKWKLQLKTNMSLKDIADTINRI
VQGWINYYTHYYKTEFYEVLRYINNCLVKWVRRTYKKRNTRSRAEHWLGKIAKRDRNLFAHWKFGITPAVGEG
AV
>R5GH053||gene_130931|GeneMark.hmm|383_aa|+|1|1152
KEFEEHLNNNLYKLWNRMASGSYFPKPVRAVAIPKKNGGTRILGIPTVEDRIAQMVAKMYFEPLVEPMFYNDS
YGYRPNKSAIQAVGQARERCFKRDWVLELDIKGLFDNIKHGYLMYMVEKHTQIKWLILYIKRWLTVPFIMSDGS
VAERRSGTPQGGVISPVLANLFLHYVFDDFMTKAYPNIWWERYADDGVLHCQSYKQAVFIKQELEERFQQFGL
ELNKEKTRIVYCKDNRRSQNYSCTQFTFLGYTFRPRLNKNKEGKFFVGFTPAVSEKAKTAMKQKIREWKIQLKAD
LSLKDIGNMINKVVQGWINYYTHYYKSEFYEVLRYINQCLIKWVRRSYKKKNTRSRAEHWLGAVARRDRNLFAH
WKFGILPSVGEGAV
>R5GH055||gene_308874|GeneMark.hmm|418_aa|-|154|1410
MSEAKQFDISKKAVIAAFQAVKENAGSYGVDEQTIKEFEEHLNNNLYKLWNRMASGSYFPKPVRAVAIPKKNG
GTRILGIPTVEDRIAQMVAKMYFEPLVEPMFYNDSYGYRPNKSAIQAVGQARERCFKRDWVLELDIKGLFDNIK
HGYLMYMVEKHTQIKWLILYIKRWLTVPFIMSDGSVAERRSGTPQGGVISPVLANLFLHYVFDDFMTKAYPNI
WWERYAYDGVLRCQSYKQAVFIKQKLEERFQQFGLELNKEKTRIVYCKDDRRSRNYSCTQFTLLGYTFRPRLNK
NKEGKFFVGFTPAVSEKAKTAMKQKIRGWKIQLKADLSLKDIGNMINKVVQGWINYYTHYYKSEFYEVLRYINQ
CLIKWVRRSYKKKNTRSRAEHWLGAVARRDRNLFAHWKFGILPSVGEGAV
>R5GH056||gene_236816|GeneMark.hmm|418_aa|-|709|1965
MSEAKQFDISKKAVIAAFQAVKENAGSYGADEQTIKEFEEHLNNNLYKLWNRMASGSYFPKPVRAVAIPKKNG
GIRILGIPTVEDRIAQMVAKMYFEPLVEPMFYNDSYGYRPNKSAIQAVGQARERCFKRDWVLELDIKGLFDNIK
HGYLMYMVEKHTQIKWLILYIKRWLTVPFIMSDGSVAERRSGTPQGGVISPVLANLFLHYVFDDFMTKAYPNI

WWERYADDGVLHCQSYKQAAFIKQKLEERFQQFGLELNKEKTRIVYCKDNRRPQNYSCTQFTFLGYTFRPRLN
KNKEGKFFVGFTPAVSEKAKTAMKQKIREWKIQLKADLSLKDIGNMINKVVQGWINYYTHYYKSEFYEVLRYIN
QCLIKWVRRSYKKKNTRSRAEHWLGAVARRDRNLFAHWKFGILPSVGEGAV
>R5GH058||gene_75172|GeneMark.hmm|422_aa|-|55|1323
HVQEETMTKTKAFNIDKSLVVSAYRRVKTSAGAAGIDKQSLADFDKRLVDNLYKIWNRLSSGSYFPPAVKAVAIP
KKLGGERILGIPTVSDRIAQTVVKLAFEPQVEPHFLADSYGYRPNKSALDAIGVTRKRCWYYDWVLEFDIKGLFD
NIPHELIMKAVDKHNPARWVKLYIQRWLTAPMVMSDGEVRARTMGTPQGGVISPLLANLFMHYVFDKWLAK
YYPKVPWYRYADDGILHCHSEAEATEMREVLRKRFSECGLEMHPEKTRVIYCKDGSRKGDYEHTMFDFLGYTFR
RRVVKNVKRNSLFVSFTPAASKSALKAMRREIKATGIRKRVDLSIEQIAKWINPKLNGWINYYGRYTCSELYSVFR
YINKALVRWGRKKYKMLSRYKTRASKFLEEMAKRSPQLFAHWRLKMRGGLV
>R5GH060||gene_328810|GeneMark.hmm|378_aa|-|1|1134
MMQHQIAKPFTIDKHVIMAAWKRVRENKGSAGIDNVSISDYETNLGTHLYKLWNRMSSGSYFPNAVKLVEIPK
SSGGTRPLGIPTVGDRIAQMAVVLLIEARLEEIFHPNSYGYRPNRSAHDAIGQARERCWRYNWVLDMDISKFFD
TIDHDLLMKAVERHVQERWILLYIRRWLKVPYATITGECIERTMGVPQGSVIGPILANLYLHYTFDKWMSIYHPN
VPFERYADDTICHCSSLEEAQRLKASIVERFAACKLKLNEEKTRIVYCKDGKRRGKYPEITFDFLGYTFQPRGQRNR
NGQVFNGYAPAISKKSKKRITEKMRGWHLSRRVQIKLSDIASEINAEVRGWINYYGKFYGSLLKAFLQSINLKLAR
WAE
>R5GH063||gene_146581|GeneMark.hmm|414_aa|+|560|1804
VETKSYKISKHIVWEAYKKVKANKGAAGVDNINIEKFEENIKDNLYKLWNRLSSGSYFPPPVRAVEIPKKNGGTRL
LGVPTVEDRIAQMVVRMYFEPSVDKVFYKDSYGYRPNKNAIEALGVIRERCWKYDWVLEFDIKGLFDNIDHKLL
MKAVKKHTEEKWVILYIERWLKVPFKMSDGRIVERNTGTPQGGVISPVLANLFLHYTFDKWMELHFPQCPWA
RYADDAVAHCKSKAQAQLLLMKLGKRFQECGLELHPDKTKIIYCKDDFRKQDEEITSFDFLGYTFRPRRAKSKKG
KFFINFSPAVSNKATKSMRQVIRNWRIQLKPDKSIIDISNMFNPVIRGWINYYGNFYKSELYKVLRHMNKALVQ
WARRKYKKLARGRKKAERWLGKLAKNMPKLFAHWQIGILPTTG
>R5GH063||gene_66223|GeneMark.hmm|418_aa|-|1176|2432
MSEAKQFDISKKAVIAAFQAVKENAGSYGADEQTIKEFEEHLNNNLYKLWNRMASGSYFPKPVRAVAIPKKNG
GIRILGIPTVEDRIAQMVAKMYFEPLVEPMFYNDSYGYRPNKSAIQAVGQARERCFKRDWVLELDIKGLFDNIK
HGYLMYMVEKHTQIKWLILYIKRWLTVPFIMSDGSVAERRSGTPQGGVISPVLANLFLHYVFDDFMTKAYPNI
WWERYADDGVLHCQSYKQAAFIKQKLEERFQQFGLELNKEKTRIVYCKDNRRPQNYSCTQFTFLGYTFRPRLN
KNKEGKFFVGFTPAVSEKAKTAMKQKIREWKIQLKADLSLKDIGNMINKVVQGWINYYTHYYKSEFYEVLRYIN
QCLIKWVRRSYKKKNTRSRAEHWLGAVARRDRNLFAHWKFGILPSVGEGAV
>R5GH065||gene_36971|GeneMark.hmm|418_aa|-|614|1870
MSEAKQFDISKKAVIAAFQAVKENAGSYGADEQTIKEFEEHLNNNLYKLWNRMASGSYFPKPVRAVAIPKKNG
GIRILGIPTVEDRIAQMVAKMYFEPLVEPMFYNDSYGYRPNKSAIQAVGQARERCFKRDWVLELDIKGLFDNIK
HGYLMYMVEKHTQIKWLILYIKRWLTVPFIMSDGSVAERRSGTPQGGVISPVLANLFLHYVFDDFMTKAYPNI
WWERYADDGVLHCQSYKQAAFIKQKLEERFQQFGLELNKEKTRIVYCKDNRRPQNYSCTQFTFLGYTFRPRLN
KNKEGKFFVGFTPAVSEKAKTAMKQKIREWKIQLKADLSLKDIGNMINKVVQGWINYYTHYYKSEFYEVLRYIN
QCLIKWVRRSYKKKNTRSRAEHWLGAVARRDRNLFAHWKFGILPSVGEGAV
>R5GH067||gene_154035|GeneMark.hmm|418_aa|-|372|1628
MSEAKQFDISKKAVIAAFQAVKENAGSYGADEQTIKEFEEHLNNNLYKLWNRMASGSYFPKPVRAVAIPKKNG
GIRILGIPTVEDRIAQMVAKMYFEPLVEPMFYNDSYGYRPNKSAIQAVGQARERCFKRDWVLELDIKGLFDNIK
HGYLMYMVEKHTQIKWLILYIKRWLTVPFIMSDGSVAERRSGTPQGGVISPVLANLFLHYVFDDFMTKAYPNI
WWERYADDGVLHCQSYKQAAFIKQKLEERFQQFGLELNKEKTRIVYCKDNRRPQNYSCTQFTFLGYTFRPRLN
KNKEGKFFVGFTPAVSEKAKTAMKQKIREWKIQLKADLSLKDIGNMINKVVQGWINYYTHYYKSEFYEVLRYIN

QCLIKWVRRSYKKKNTRSRAEHWLGAVARRDRNLFAHWKFGILPSVGEGAV
>R5GH071||gene_5968|GeneMark.hmm|414_aa|+|3744|4988
VETKSYKISKHIVWEAYKKVKANKGAAGVDNINIEKFEENIKDNLYKLWNRLSSGSYFPPPVRAVEIPKKNGGTRL
LGVPTVEDRIAQMVVRMYFEPSVDKVFYKDSYGYRPNKNAIEALGVIRERCWKYDWVLEFDIKGLFDNIDHKLL
MKAVKKHTEEKWVILYIERWLKVPFKMSDGRIVERNTGTPQGGVISPVLANLFLHYTFDKWMELHFPQCPWA
RYADDAVAHCKSKAQAQLLLMKLGKRFQECGLELHPDKTKIIYCKDDFRKQDEEITSFDFLGYTFRPRRAKSKKG
KFFINFSPAVSNKATKSMRQVIRNWRIQLKPDKSIIDISNMFNPVIRGWINYYGNFYKSELYKVLRHMNKALVQ
WARRKYKKLARGRKKAERWLGKLAKNMPKLFAHWQIGILPTTG
>R5GH072||gene_302320|GeneMark.hmm|426_aa|-|2|1279
MQNDNAKPISISKQLVYDAFLRVKANRGSAGIDKVTLEDYEKNLRGNLYKLWNRMSSGSYFPPSVKLVEIPKSTG
GKRPLGIPTVSDRVAQMAVVMLITPSIEPCFHEDSYAYRPHRSAHDAVGKARERCWKYAWVLDMDISKFFDTI
DHELLLKALKRHTQEKWVLMYIERWLKVPYEKSDGSQVDRALGVPQGSVIGPVLANLFLHYTFDKWMEKNFP
RVPFERYADDTICHCHSLKQAEYMQAMIQQRFECCRLRLNEEKTKIVYCKSSRQKECYPNVTFDFLGFTFQPRES
VDKYGNRFTGFLPAISRKSMKRINETMRSWHLNRHSNLTLEHLASDINPIVRGWMTYYGKFYPTRLKWFMQTL
NGRLARWVMCKFERYRHRFYPAQEWLARIAEKEGLIFYHWKCGALPRFTNKEKVSSQL
>R5GH072||gene_490047|GeneMark.hmm|406_aa|-|2|1219
MSEAKQFDISKKAVIAAFQAVKENAGSYGVDEQTIKEFEEHLNNNLYKLWNRMASGSYFPKPVRAVEIPKKNG
GTRILGIPTVEDRIAQMVAKMYFEPLVEPMFYNDSYGYRPNKSAIQAVGQAREKCFKRDWVLELDIKGLFDNIK
HGYLMYMVEKHTQIKWLILYIKRWLTVPFIMSDGSVAERRSGTPQGGVISPVLANLFLHYVFDDFMTKAYPNI
WWERYADDGVLHCQSYKQAVFIKQKLEERFQQFGLELNKEKTRIVYCKDNRRPQNYSCTQFTFLGYTFRPRLNK
NKEGKFFVGFTPAVSEKAKTAMKQKIREWKIQLKADLSLKDIGNMINKVVQGWINYYTHYYKSEFYEVLRYINQ
CLIKWVRRSYKKKNTRSRAEHWLGAVARRDRNLFAHWK
>R5GH072||gene_127577|GeneMark.hmm|414_aa|-|2513|3757
VETKSYKISKHIVWEAYKKVKANKGAAGVDNINIEKFEENIKDNLYKLWNRLSSGSYFPPPVRAVEIPKKNGGTRL
LGVPTVEDRIAQMVVRMYFEPSVDKVFYKDSYGYRPNKNAIEALGVIRERCWKYDWVLEFDIKGLFDNIDHKLL
MKAVKKHTEEKWVILYIERWLKVPFKMSDGRIVERNTGTPQGGVISPVLANLFLHYTFDKWMELHFPQCPWA
RYADDAVAHCKSKAQAQLLLMKLGKRFQECGLELHPDKTKIIYCKDDFRKQDEEITSFDFLGYTFRPRRAKSKKG
KFFINFSPAVSNKATKSMRQVIRNWRIQLKPDKSIIDISNMFNPVIRGWINYYGNFYKSELYKVLRHMNKALVQ
WARRKYKKLARGRKKAERWLGKLAKNMPKLFAHWQIGILPTTG
>R5GH075||gene_67784|GeneMark.hmm|420_aa|+|61|1323
MNEAKPFVIDKRLVWEAYHKVKENKGSAGIDKVDQQTFDKEMSKNLYKIWNRMSSGCYFPKAVKLVEIPKSNG
GTRPLGIPTIEDRIAQQVVVSVLTPILEPIFKEDSYGYRPGKGAHQAIAKAKERCYVNPWVLDMDISKFFDTINHE
LLMKAVRKHTEEKWVLLYIERWLKVPYQTSKGEVIERTMGVPQGSVIGPVLANLFLHYVFDEWMSRNYPTIPFE
RYADDTICHCVSDKQAQFLKAVLMKRFEECGLKLNEEKTKIVYCKDSNRRGDSEHTSFDFLGFTFRPRGARNRKT
GQNFTAFLPAISKKSMKRIKEAVRAWKLNRKTFACLLDISNEVDTQISGWMNYYMKFGRSEFRKVLNYINERLT
RWVMRKYKRFSKGRKFDRAYDWLVEYATHNRNEFSHWVKGFVPYPRLD
>R5GH077||gene_5330|GeneMark.hmm|418_aa|+|2841|4097
MSEAKQFDISKKAVIAAFQAVKENAGSYGADEQTIKEFEEHLNNNLYKLWNRMASGSYFPKPVRAVAIPKKNG
GIRILGIPTVEDRIAQMVAKMYFEPLVEPMFYNDSYGYRPNKSAIQAVGQARERCFKRDWVLELDIKGLFDNIK
HGYLMYMVEKHTQIKWLILYIKRWLTVPFIMSDGSVAERRSGTPQGGVISPVLANLFLHYVFDDFMTKAYPNI
WWERYADDGVLHCQSYKQAAFIKQKLEERFQQFGLELNKEKTRIVYCKDNRRPQNYSCTQFTFLGYTFRPRLN
KNKEGKFFVGFTPAVSEKAKTAMKQKIREWKIQLKADLSLKDIGNMINKVVQGWINYYTHYYKSEFYEVLRYIN
QCLIKWVRRSYKKKNTRSRAEHWLGAVARRDRNLFAHWKFGILPSVGEGAV
>R5GH077||gene_193924|GeneMark.hmm|414_aa|+|14578|15822

MIETKPYEISKWAVYIAYERVKANKGSYGVDEQSIEDFEKNLKNNLYKIWNRMSSGSYFPQPVKAVSVPKKNGGI
RVLGIPTVEDRIAQMTAKLYFEPCVEPLFLEDSYGYRPGKSAIQALSVTRKRCWHRDWVLEYDIKGLFDNIRHDY
LLEMVRRHTPHKWILLYVERWLTTPFQLEDGTLQSRTSGTPQGGVISLVLANLFLHYAFDSFMAKEYPKAWWE
RYADDGVLHCKSSQQAMYMKSVLRERFRLFGLELNEEKTRIVYCKDADRTEDYSEISLDSLGYTFRPRLARNKHG
NIFLNFLPAMSAKAIKAMKEEVRRWKLQLKVSKSLTDLANILNSQIQGWISYYGHFYKSELIYLLRYINQCLIKWV
RRKYKKFNHRRRAEYWLGRIARRDNNLFAHWRYGVLPTAG
>R5GH077||gene_72869|GeneMark.hmm|421_aa|+|697|1962
MQEAKPFQIDKRIIFESFKKVKFNRGSSGIDGIEMTTYEQNLGSNLYRLWNRMSSGSYMPKAVKLVEIPKSNGG
KRPLGIPTIEDRIAQMAVVNVIEPLIEPCFHEDSFGYRPHRSAHDAIAKAERRCWKYAWVLDIDISKFFDTIDHGL
LMKAVEKHINIKWILLYIKRWLTVPYQRSDGEIVKRDMGVPQGSVIGPILANLFLHYTFDKWMSYKYPHIPFERY
ADDCVCHCSTLAQAEYIKERLGERFTECKLKFNEEKTKIVFCKMSSRSSKHYHCTSFDYLGFTFRSRAAKDKRNN
VLFTSYLPAISKKSVSRIHETIKSWNLKRLHNRSLRFVAAYINDVVRGWINYYEKFGKTEFWKVMCHLNRSIAYW
AKTKYKRLRRRGVISAHYWLAYIAQKEPNLFYHWQVGYVPYARQKK
>R5GH078||gene_72430|GeneMark.hmm|418_aa|+|695|1951
MSEAKQFDISKKAVIAAFQAVKENAGSYGADEQTIKEFEEHLNNNLYKLWNRMASGSYFPKPVRAVEIPKKNG
GTRILGIPTVEDRIAQMVAKMYFEPLVEPMFYNDSYGYRPNKSAIQAVGQARERCFKRDWVLELDIKGLFDNIK
HGYLMYMVEKHTQIKWLILYIKRWLTVPFIMSDGSVAERRSGTPQGGVISPVLANLFLHYVFDDFMTKAYPNI
WWERYADDGVLHCQSYKQAVFIKQKLEERFQQFGLELNKEKTRIVYCKDDRRSRNYSCTQFTFLGYTFRPRLNK
NKEGKFFVGFTPAVSEKAKTAMKQKIRGWKIQLKADLSLKDIGNMINKVVQGWINYYTHYYKSEFYEVLRYINQ
CLIKWVRRSYKKKNTRSRAEHWLGAVARRDRNLFAHWKFGILPSVGEGAV
>R5GH079||gene_25465|GeneMark.hmm|430_aa|-|131|1423
MQNDNAKPISISKQLVYDAFLRVKANRGSAGIDKVTLEDYEKNLRGNLYKLWNRMSSGSYFPPSVKLVEIPKSTG
GKRPLGIPTVSDRVAQMAVVMLITPSIEPCFHEDSYAYRPHRSAHDAVGKARERCWKYAWVLDMDISKFFDTI
DHELLLKALKRHTQEKWVLMYIERWLKVPYEKSDGSQVDRALGVPQGSVIGPVLANLFLHYTFDKWMEKNFP
RVPFERYADDTICHCHSLKQAEYMQAMIQQRFECCRLRLNEEKTKIVYCKSSRQKECYPNVTFDFLGFTFQPRES
VDKYGNRFTGFLPAISRKSMKRINETMRSWHLNRHSNLTLEHLASDINPIVRGWMTYYGKFYPTRLKWFMQTL
NGRLARWVMCKFERYRHRFYPAQEWLARIAEKEGLIFYHWKCGALPRFTNKEKVSSQLIMVK
>R5GH082||gene_52804|GeneMark.hmm|418_aa|+|409|1665
MSEAKQFDISKKAVIAAFQAVKENAGSYGVDEQTIKEFEEHLNNNLYKLWNRMASGSYFPKPVRAVEIPKKNG
GTRILGIPTVEDRIAQMVAKMYFEPLVEPMFYNDSYGYRPNKSAIQAVGQAREKCFKRDWVLELDIKGLFDNIK
HGYLMYMVEKHTQIKWLILYIKRWLTVPFIMSDGSVAERRSGTPQGGVISPVLANLFLHYVFDDFMTKAYPNI
WWERYADDGVLHCQSYKQAAFIKQKLEERFQQFGLELNKEKTRIVYCKDDRRSRNYSCTQFTFLGYTFRPRLNK
NKEGKFFVGFTPAVSEKAKTAMKQKIREWKIQLKADLSLKDIGNMINKVVQGWINYYTHYYKSEFYEVLRYINQ
CLIKWVRRSYKKKNTRSRAEHWLGAVARRDRNLFAHWKFGILPSVGEGAV
>R5GH083||gene_188189|GeneMark.hmm|420_aa|+|9745|11007
MNEAKPFVIDKRLVWEAYHKVKENKGSAGIDKVDQKTFDKEMSKNLYKIWNRMSSGCYFPKAVKLVEIPKSNG
GTRPLGIPTIEDRIAQQVVVSVLTPILEPIFKEDSYGYRPGKGAHQAIAKAKERCYVNPWVLDMDISKFFDTINHD
LLMKAVRKHTEEKWVLLYIERWLKVPYQTSKGEVIERTMGVPQGSVIGPVLANLFLHYVFDEWMSRNYPTIPFE
RYADDTICHCVSEKQAQFLKAVLMKRFEECGLKLNEEKTKIVYCKDSNRRGDSEHTSFDFLGFTFRPRSARNRKT
GQNFTAFLPAISKKSLKRIKEAVRAWKLNRKTFACLLDISNEVDTQISGWMNYYMKFGRSEFRKVLNYINERLTR
WVMRKYKRFSKGKKFSRAYEWLVEYAVHNRNEFSHWAKGFVPYPRLG
>R5GH085||gene_266407|GeneMark.hmm|420_aa|+|14264|15526
MNEAKPFVIDKRLVWEAYHKVKENKGSAGIDKVDQKTFDKEMSKNLYKIWNRMSSGCYFPKAVKLVEIPKSNG
GTRPLGIPTIEDRIAQQVVVSVLTPILEPIFKEDSYGYRPGKGAHQAIAKAKERCYVNPWVLDMDISKFFDTINHD

LLMKAVRKHTEEKWVLLYIERWLKVPYQTSKGEVIERTMGVPQGSVIGPVLANLFLHYVFDEWMSRNYPTIPFE
RYADDTICHCVSEKQAQFLKAVLMKRFEECGLKLNEEKTKIVYCKDSNRRGDSEHTSFDFLGFTFRPRSARNRKT
GQNFTAFLPAISKKSLKRIKEAVRAWKLNRKTFACLLDISNEVDTQISGWMNYYMKFGRSEFRKVLNYINERLTR
WVMRKYKRFSKGKKFSRAYEWLVEYAVHNRNEFSHWAKGFVPYPRLG
>R5GH086||gene_241508|GeneMark.hmm|421_aa|-|455|1720
MQEAKPFQIDKRIIFEAFKKVKFNRGSSGIDGIEMTTYEQNLGSNLYRLWNRMSSGSYMPKAVKLVEIPKSNGG
KRPLGIPTIEDRIAQMAVVNVIEPLIEPCFHEDSFGYRPHRSAHDAIAKAERRCWKYAWVLDIDISKFFDTIDHGL
LMKAVEKHINIKWILLYIKRWLTVPYQRSDGEIVKRDMGVPQGSVIGPILANLFLHYTFDKWMSYKYPHIPFERY
ADDCVCHCSTLAQAEYIKERLGERFTECKLKFNEEKTKIVFCKMSSRSSKHYHCTSFDYLGFTFRSRAAKDKRNN
VLFTSYLPAISKKSVSRIHETIKSWNLKRLHNRSLRFVAAYINDVVRGWINYYEKFGKTEFWKVMCHLNRSIAYW
AKTKYKRLRRRGVISAHYWLAYIAQKEPNLFYHWQVGYVPYARQKK
>R5GH086||gene_73689|GeneMark.hmm|418_aa|+|11843|13099
MSEAKQFDISKKAVIAAFQAVKENAGSYGVDEQTIKEFEEHLNNNLYKLWNRMASGSYFPKPVRAVEIPKKNG
GTRILGIPTVEDRTAQMVAKMYFEPLVEPMFYNDSYGYRPNKSAIQAVGQARERCFKRDWVLELDIKGLFDNIK
HGYLMYMVEKHTQIKWLILYIKRWLTVPFIMSDGSVAERRSGTPQGGVISPVLANLFLHYVFDDFMTKAYPNI
WWERYADDGVLHCQSYKQAVFIKQELEERFQQFGLELNKEKTRIVYCKDNRRSQNYSCTQFTFLGYTFRPRLNK
NKEGKFFVGFTPAVSEKAKTAMKQKIRGWKIQLKADLSLKDIGNMINKVVQGWINYYTHYYKSEFYEVLRYINQ
CLIKWVRRSYKKKNTRSRAEHWLGAVARRDRNLFAHWKFGILPSVGEGAV
>R5GH087||gene_426309|GeneMark.hmm|418_aa|-|1475|2731
MSEAKQFDISKKAVIAAFQAVKENAGSYGVDEQTIKEFEEHLNNNLYKLWNRMASGSYFPKPVRAVEIPKKNG
GTRILGIPTVEDRIAQMVAKMYFEPLVEPMFYNDSYGYRPNKSAIQAVGQARERCFKRDWVLELDIKGLFDNIK
HGYLMYMVEKHTQIKWLILYIKRWLTVPFIMSDGSVAERRSGTPQGGVISPVLANLFLHYVFDDFMTKAYPNI
WWERYADDGVLHCQSYKQAVFIKQKLEERFQQFGLELNKEKTRIVYCKDNRRPQNYSCTQFTFLGYTFRPRLNK
NKEGKFFVGFTPAVSEKAKTAMKQKIRGWKIQLKADLSLKDIGNMINKVVQGWINYYTHYYKSEFYEVLRYINQ
CLIKWVRRSYKKKNTRSRAEHWLGAVARRDRNLFAHWKFGILPSVGEGAV
>R5GH089||gene_306281|GeneMark.hmm|401_aa|+|2787|3992
VKIAFERVKANKGTYGIDEQTIADFEENLKDNLYKIWNRMSSGTYFPKPVKAVAIPKKSGGTRILGIPTVEDRVAQ
MVAKIYFEPKVEELFYEDSYGYRPNKSAIDAIGILRKRCWKKDWVVDFDIKGLFDNIRHDYLIEMVKRHTNEQWI
ILYIERWLKTPFKMQDGTIVERTAGTPQGGVISPVLANLFMHYVFDDFMSKEFPTIQWVRYADDGVLNCVSLK
QAKYIIKVLDKRFKTCGLELNLEKTKIVYCKDDNRKGKYGNTKFDFLGYTFKIRSAENNKGQIFNSFIPAMSDKAQ
KDIRKELRSYRIQRQVDKSLSDIANMFNSKIQGWINYYSHYYKTEVNKILDYFNSILVKWVMRKYKTIKSKKRAIK
WLAEIAQRDTTLFAHWKFGILPMAR
>R5GH089||gene_130935|GeneMark.hmm|418_aa|+|461|1717
MSEAKQFDISKKAVIAAFQAVKENAGSYGADEQTIKEFEEHLNNNLYKLWNRMASGSYFPKPVRAVEIPKKNG
GTRILGIPTVEDRIAQMVAKMYFEPLVEPMFYNDSYGYRPNKSAIQAVGQARERCFKRDWVLELDIKGLFDNIK
HGYLMYMVEKHTQIKWLILYIKRWLTVPFIMSDGSVAERRSGTPQGGVISPVLANLFLHYVFDDFMTKAYPNI
WWERYADDGVLHCQSYKQAVFIKQKLEERFQQFGLELNKEKTRIVYCKDDRRSRNYSCTQFTFLGYTFRPRLNK
NKEGKFFVGFTPAVSEKAKTAMKQKIRGWKIQLKADLSLKDIGNMINKVVQGWINYYTHYYKSEFYEVLRYINQ
CLIKWVRRSYKKKNTRSRAEHWLGAVARRDRNLFAHWKFGILPSVGEGAV
>R5GH090||gene_5032|GeneMark.hmm|422_aa|+|2759|4027
MMQHQVTKPFTIDKHLIMNAWKRVKENKGSVGIDNVSTDDYESNLGKNLYKLWNRMSSGSYFPEAVKLVDIP
KSSGGTRPLGIPTVGDRIAQMSVVLLIEDRLEAIFHADSYGYRPNRSAHDAIGKARERCWHYNWVLDMDISKFF
DTINHDLLMKAVERHVQEKWILLYIRRWLEVPYATLTGERIERRMGVPQGSVIGPVLANLYLHYTFDKWMSLYH
PTIPFERYADDTICHCNSLEEAQMLKASIVERFAACKLKLNEEKTRIVYCKDGKRRREYKDITFDFLGYTFQPRGQR

NKQGQVFNGYAPAISRKSKKRIAETMRGWHLNRRVQLKLSDIAVEINAEVRGWMNYYGKFYGSQLKAFLQCIN
LKLARWAERKYKRFRRKPNDAYKWLVRVASKNPALFYHWQHGVKPNRLKPFG
>R5GH090||gene_155395|GeneMark.hmm|420_aa|-|7512|8774
MNEAKPFVIDKRLVWEAYHKVKENKGSAGIDKVDQKTFDKEMSKNLYKIWNRMSSGCYFPKAVKLVEIPKSNG
GTRPLGIPTIEDRIAQQVVVSVLTPILEPIFKEDSYGYRPGKGAHQAIAKAKERCYVNPWVLDMDISKFFDTINHD
LLMKAVRKHTEEKWVLLYIERWLKVPYQTSKGEVIERTMGVPQGSVIGPVLANLFLHYVFDEWMSRNYPTIPFE
RYADDTICHCVSEKQAQFLKAVLMKRFEECGLKLNEEKTKIVYCKDSNRRGDSEHTSFDFLGFTFRPRSARNRKT
GQNFTAFLPAISKKSLKRIKEAVRAWKLNRKTFACLLDISNEVDTQISGWMNYYMKFGRSEFRKVLNYINERLTR
WVMRKYKRFSKGKKFSRAYEWLVEYAVHNRNEFSHWAKGFVPYPRLG
>R5GH090||gene_52614|GeneMark.hmm|421_aa|+|3250|4515
MQEAKPFQIDKRIIFEAFKKVKSNGGSPGIDGIEMSAYEQNLGSNFYRLWNRMSSGSYMPKAVKLVEILKSNGG
KRPLGIPSVEDRIAQMAVVNVIEPLVEPYFHKDSFGYRPHRSAHDAIAKAERRCWKYAWVLDIDISKFFDTIDHG
LLMKAVEKHIKTKWILLYIKRWLTVPYQGNDGAIVKRHMGVPQGSVIGPILANQFLHYTFDKWMSYKYPHVPF
ERYADDCVCHCGTLAQAEYIKDRLGERFAECKLTFNEEKTKIVFCKTSNRSSEHYHCTSFDYLGFTFRPRAAKDKR
KNVLFTSYLPAISNKSESRIHETIKSWNLKRLHNRSLRFVAAYINDVVRGWISYYGKFGKTEFWKVMCHLNRSIAY
WAKTKYKRLRRRGVISAHYWLAYIAQKEPNLFYHWQVGYIPYARQKK
>R5GH092||gene_95207|GeneMark.hmm|418_aa|+|3250|4506
MSEAKQFDISKKAVIAAFQAVKENAGSYGADEQTIKEFEEHLNNNLYKLWNRMASGSYFPKPVRAVAIPKKNG
GIRILGIPTVEDRIAQMVAKMYFEPLVEPMFYNDSYGYRPNKSAIQAVGQARERCFKRDWVLELDIKGLFDNIK
HGYLMYMVEKHTQIKWLILYIKRWLTVPFIMSDGSVAERRSGTPQGGVISPVLANLFLHYVFDDFMTKAYPNI
WWERYADDGVLHCQSYKQAAFIKQKLEERFQQFGLELNKEKTRIVYCKDNRRPQNYSCTQFTFLGYTFRPRLN
KNKEGKFFVGFTPAVSEKAKTAMKQKIREWKIQLKADLSLKDIGNMINKVVQGWINYYTHYYKSEFYEVLRYIN
QCLIKWVRRSYKKKNTRSRAEHWLGAVARRDRNLFAHWKFGILPSVGEGAV
>R5GH092||gene_218229|GeneMark.hmm|420_aa|+|795|2057
MNEAKPFVIDKRLVWEAYHKVKENKGSAGIDKVDQKTFDKEMSKNLYKIWNRMSSGCYFPKAVKLVEIPKSNG
GTRPLGIPTIEDRIAQQVVVSVLTPILEPIFKEDSYGYRPGKGAHQAIAKAKERCYVNPWVLDMDISKFFDTINHD
LLMKAVRKHTEEKWVLLYIERWLKVPYQTSKGEVIERTMGVPQGSVIGPVLANLFLHYVFDEWMSRNYPTIPFE
RYADDTICHCVSEKQAQFLKAVLMKRFEECGLKLNEEKTKIVYCKDSNRRGDSEHTSFDFLGFTFRPRSARNRKT
GQNFTAFLPAISKKSLKRIKEAVRAWKLNRKTFACLLDISNEVDTQISGWMNYYMKFGRSEFRKVLNYINERLTR
WVMRKYKRFSKGKKFSRAYEWLVEYAVHNRNEFSHWAKGFVPYPRLG
>R5GH092||gene_137969|GeneMark.hmm|422_aa|+|3891|5159
MKDAKSFEISRHLVMEAYKRVKANKGAAGVDEVSIADFENNLKSNLYKIWNRMSSGSYLPPAVKLVEIPKSNGG
KRPLGIPTVGDRVAQMVVVMTIEPGIEPYFHEDSYAYRPNRSALDAVRKAKERSYTFHWVLDLDIKGFFDNIDH
ELLIKALERHVKCKWAILYIKRWLSVPYQLKDGTQKERTKGVPQGSVVGPILANLFLHYVFDEWMRRNHSNISFE
RYADDTICHCVSLKQAEFILRAIRKRFAECKLELNEDKTKIVYCKKNHRDIPYECIQFDFLGYTFRPRRSIDANGEVF
LNFSPAISKKARTKIWEAIQNWNSNHWVPMELEDIAKEINPVIQGWINYYGQHNPRILKEVLQHVNDRLVRW
GRRKFKGLRKRKTATVHRLGDIALQKPNLFAHWAWGVKPTASERNRKRK
>R5GH093||gene_64200|GeneMark.hmm|381_aa|-|1|1143
MQRKSFEIPKALVWASYLDVRRNKGAPGCDGQTLKMFDQQRDGNLYKIWNRLCSGTWFPPPVLEKRIPKPN
GKERILGIPTVSDRIAQGAIKLFMEEKLDPIFHADSYGYRPGKSAHDALKQCAIRCWRYSWILEVDISAFFDHVRH
DLVLKALEHHGMPKWVILYCRRWMEAPMQSCENGELITRTRGTPQGGVISPLLANLFLHYAFDLWMEREYRG
VPFERYADDIVVHCSRMSDATRLKNRLSERFSEVGLVLNAGKTNIAYIDTFKRRNVATSFTFLGYDFKVRTLKNFK
GELYRKCMPGASNAAMRKITETIKKWRIHRSTAESLLDFARRYNAIVRGWIEYYGKFWSRNFNYRLWSAMQSR
LLKWMQSKYRLSN

>R5GH093||gene_5477|GeneMark.hmm|370_aa|+|3|1115
LWNRMASGSYFPKPVRAVAIPKKNGGIRILGIPTVEDRIAQMVAKMYFEPLVEPMFYNDSYGYRPNKSAIQAV
GQARERCFKRDWVLELDIKGLFDNIKHGYLMYMVEKHTQIKWLILYIKRWLTVPFIMSDGSVAERRSGTPQGG
VISPVLANLFLHYVFDDFMTKAYPNIWWERYADDGVLHCQSYKQAAFIKQKLEERFQQFGLELNKEKTRIVYCK
DNRRPQNYSCTQFTFLGYTFRPRLNKNKEGKFFVGFTPAVSEKAKTAMKQKIREWKIQLKADLSLKDIGNMINK
VVQGWINYYTHYYKSEFYEVLRYINQCLIKWVRRSYKKKNTRSRAEHWLGAVARRDRNLFAHWKFGILPSVGE
GAV
>R5GH094||gene_157809|GeneMark.hmm|430_aa|+|4915|6207
MQNDNAKPISISKQLVYDAFLRVKANRGSAGIDKVTLEDYEKNLRGNLYKLWNRMSSGSYFPPSVKLVEIPKSTG
GKRPLGIPTVSDRVAQMTVVMLITPSIEPCFHEDSYAYRPHRSAHDAVGKARERCWKYAWVLDMDISKFFDTID
HELLLKALKRHTQEKWVLMYIERWLKVPYEKSDGSQVDRALGVPQGSVIGPVLANLFLHYTFDKWMEKNFPR
VPFERYADDTICHCHSLKQAEYMQAMIQQRFECCRLRLNEEKTKIVYCKSSRQKECYPNVTFDFLGFTFQPRESV
DKYGNRFTGFLPAISRKSMKRINETMRSWHLNRHSNLTLEHLASDINPIVRGWMTYYGKFYPTRLKWFMQTLN
GRLASWVMCKFERYRHRFYPAQEWLARIAEKEGLIFYHWKCGVLPRFTNKEKVSSQLIMVK
>R5GH094||gene_62079|GeneMark.hmm|418_aa|-|65|1321
MSEAKQFDISKKAVIAAFQAVKENAGSYGVDEQTIKEFEEHLNNNLYKLWNRMASGSYFPKPVRAVEIPKKNG
GTRILGIPTVEDRIAQMVAKMYFEPLVEPMFYNDSYGYRPNKSAIQAVGQARERCFKRDWVLELDIKGLFDNIK
HGYLMYMVEKHTQIKWLILYIKRWLTVPFIMSDGSVAERRSGTPQGGVISPVLANLFLHYVFDDFMTKAYPNI
WWERYADDGVLHCQSYKQAVFIKQKLEERFQQFGLELNKEKTRIVYCKDDRRSRNYSCTQFTFLGYTFRPRLNK
NKEGKFFVGFTPAVSEKAKTAMKQKIRGWKIQLKADLSLKDIGNMINKVVQGWINYYTHYYKSEFYEVLRYINQ
CLIKWVRRSYKKKNTRSRAEHWLGAVARRDRNLFAHWKFGILPSVGEGAV
>R5GH095||gene_97639|GeneMark.hmm|420_aa|-|6360|7622
MNEAKPFVIDKRLVWEAYHKVKENKGSAGIDKVDQKTFDKEMSKNLYKIWNRMSSGCYFPKAVKLVEIPKSNG
GTRPLGIPTIEDRIAQQVVVSVLTPILEPIFKEDSYGYRPGKGAHQAIAKAKERCYVNPWVLDMDISKFFDTINHD
LLMKAVRKHTEEKWVLLYIERWLKVPYQTSKGEVIERTMGVPQGSVIGPVLANLFLHYVFDEWMSRNYPTIPFE
RYADDTICHCVSEKQAQFLKAVLMKRFEECGLKLNEEKTKIVYCKDSNRRGDSEHTSFDFLGFTFRPRSARNRKT
GQNFTAFLPAISKKSLKRIKEAVRAWKLNRKTFACLLDISNEVDTQISGWMNYYMKFGRSEFRKVLNYINERLTR
WVMRKYKRFSKGKKFSRAYEWLVEYAVHNRNEFSHWAKGFVPYPRLG
>R5GH095||gene_181429|GeneMark.hmm|421_aa|-|99|1364
MQEAKPFQIDKRIIFESFKKVKFNRGSSGIDGIEMTTYEQNLGSNLYRLWNRMSSGSYMPKAVKLVEIPKSNGG
KRPLGIPTIEDRIAQMAVVNVIEPLIEPCFHEDSFGYRPHRSAHDAIAKAERRCWKYAWVLDIDISKFFDTIDHGL
LMKAVEKHINIKWILLYIKRWLTVPYQRSDGEIVKRDMGVPQGSVIGPILANLFLHYTFDKWMSYKYPHIPFERY
ADDCVCHCSTLAQAEYIKERLGERFTECKLKFNEEKTKIVFCKMSSRSSKHYHCTSFDYLGFTFRSRAAKDKRNN
VLFTSYLPAISKKSVSRIHETIKSWNLKRLHNRSLRFVAAYINDVVRGWINYYEKFGKTEFWKVMCHLNRSIAYW
AKTKYKRLRRRGVISAHYWLAYIAQKEPNLFYHWQVGYVPYARQKK
>R5GH097||gene_376529|GeneMark.hmm|413_aa|+|7692|8933
MSESKQYEIPKKVVIEAYKRVKANKGSAGIDGIDFERFEKKLNNNLYKIWNRMSSGSYFPSPVLSVEIPKKAGGTR
RLGIPTITDRIAQMVARMYVEPVVEPMFCDDSYGYRPNKSAIDAIATARKRCWRYDYVIELDVKGLFDNINHELL
MRVVLKHVKEEWICLYIKRWLETPFITREGQVIERLSGTPQGGVISPVLANMYLHYVFDMWMKRNFPQAPFE
RYADDGVIHCRTKEEAFVVKKKLAARFAECKLELHPVKTRVVYCKDKDRTRNEELTEFDFLGYTFKAVYIMCKDG
KVRYNFIASVSKTSSKSFRDKIKAMEVHKRTGCKIDIIAEILNPLIRGWMNYFGKFNPSAMKGTLQCIDRRLVKW
AMCKYKNFRGKRGRAEKWLCTVRQREPKLFAHWSNLYSYC
>R5GH099||gene_355897|GeneMark.hmm|420_aa|-|537|1799
MNEAKPFVIDKRLVWEAYHKVKENKGSAGIDKVDQKTFDKEMSKNLYKIWNRMSSGCYFPKAVKLVEIPKSNG

GTRPLGIPTIEDRIAQQVVVSVLTPILEPIFKEDSYGYRPGKGAHQAIAKAKERCYVNPWVLDMDISKFFDTINHD
LLMKAVRKHTEEKWVLLYIERWLKVPYQTSKGEVIERTMGVPQGSVIGPVLANLFLHYVFDEWMSRNYPTIPFE
RYADDTICHCVSEKQAQFLKAVLMKRFEECGLKLNEEKTKIVYCKDSNRRGDSEHTSFDFLGFTFRPRSARNRKT
GQNFTAFLPAISKKSLKRIKEAVRAWKLNRKTFACLLDISNEVDTQISGWMNYYMKFGRSEFRKVLNYINERLTR
WVMRKYKRFSKGKKFSRAYEWLVEYAVHNRNEFSHWAKGFVPYPRLG
>R5GH100||gene_270714|GeneMark.hmm|420_aa|-|2685|3947
MNEAKPFVIDKRLVWEAYHKVKENKGSAGIDKVDQKTFDKEMSKNLYKIWNRMSSGCYFPKAVKLVEIPKSNG
GTRPLGIPTIEDRIAQQVVVSVLTPILEPIFKEDSYGYRPGKGAHQAIAKAKERCYVNPWVLDMDISKFFDTINHD
LLMKAVRKHTEEKWVLLYIERWLKVPYQTSKGEVIERTMGVPQGSVIGPVLANLFLHYVFDEWMSRNYPTIPFE
RYADDTICHCVSEKQAQFLKAVLMKRFEECGLKLNEEKTKIVYCKDSNRRGDSEHTSFDFLGFTFRPRSARNRKT
GQNFTAFLPAISKKSLKRIKEAVRAWKLNRKTFACLLDISNEVDTQISGWMNYYMKFGRSEFRKVLNYINERLTR
WVMRKYKRFSKGKKFSRAYEWLVEYAVHNRNEFSHWAKGFVPYPRLG
>R5GH103||gene_11474|GeneMark.hmm|430_aa|+|379|1671
MQNDNAKPISISKQLVYDAFLRVKANRGSAGIDKVTLEDYEKNLRGNLYKLWNRMSSGSYFPPSVKLVEIPKSTG
GKRPLGIPTVSDRVAQMAVVMLITPSIEPCFHEDSYAYRPHRSAHDAVGKARERCWKYAWVLDMDISKFFDTI
DHELLLKALKRHTQEKWVLMYIERWLKVPYEKSDGSQVDRALGVPQGSVIGPVLANLFLHYTFDKWMEKNFP
RVPFERYADDTICHCHSLKQAEYMQAMIQQRFECCRLRLNEEKTKIVYCKSSRQKECYPNVTFDFLGFTFQPRES
VDKYGNRFTGFLPAISRKSMKRINETMRSWHLNRHSNLTLEHLASDINPIVRGWMTYYGKFYPTRLKWFMQTL
NGRLARWVMCKFERYRHRFYPAQEWLARIAEKEGLIFYHWKCGALPRFTNKEKVSSQLIMVK
>R5GH104||gene_151360|GeneMark.hmm|430_aa|+|547|1839
MQNDNAKPISISKQLVYDAFLRVKANRGSAGIDKVTLEDYEKNLRGNLYKLWNRMSSGSYFPPSVKLVEIPKSTG
GKRPLGIPTVSDRVAQMAVVMLITPSIEPCFHEDSYAYRPHRSAHDAVGKARERCWKYAWVLDMDISKFFDTI
DHELLLKALKRHTQEKWVLMYIERWLKVPYEKSDGSQVDRALGVPQGSVIGPVLANLFLHYTFDKWMEKNFP
RVPFERYADDTICHCHSLKQAEYMQAMIQQRFECCRLRLNEEKTKIVYCKSSRQKECYPNVTFDFLGFTFQPRES
VDKYGNRFTGFLPAISRKSMKRINETMRSWHLNRHSNLTLEHLASDINPIVRGWMTYYGKFYPTRLKWFMQTL
NGRLARWVMCKFERYRHRFYPAQEWLARIAEKEGLIFYHWKCGALPRFTNKEKVSSQLIMVK
>R5GH106||gene_180862|GeneMark.hmm|418_aa|+|1785|3041
MSEAKQFDISKKAVIAAFQAVKENAGSYGADEQTIKEFEEHLNNNLYKLWNRMASGSYFPKPVRAVAIPKKNG
GIRILGIPTVEDRIAQMVAKMYFEPLVEPMFYNDSYGYRPNKSAIQAVGQARERCFKRDWVLELDIKGLFDNIK
HGYLMYMVEKHTQIKWLILYIKRWLTVPFIMSDGSVAERRSGTPQGGVISPVLANLFLHYVFDDFMTKAYPNI
WWERYADDGVLHCQSYKQAAFIKQKLEERFQQFGLELNKEKTRIVYCKDNRRPQNYSCTQFTFLGYTFRPRLN
KNKEGKFFVGFTPAVSEKAKTAMKQKIREWKIQLKADLSLKDIGNMINKVVQGWINYYTHYYKSEFYEVLRYIN
QCLIKWVRRSYKKKNTRSRAEHWLGAVARRDRNLFAHWKFGILPSVGEGAV
>R5GH107||gene_98352|GeneMark.hmm|418_aa|-|182|1438
MSEAKQFDISKKAVIAAFQAVKENAGSYGVDEQTIKEFEEHLNNNLYKLWNRMASGSYFPKPVRAVEIPKKNG
GTRILGIPTVEDRIAQMVAKMYFEPLVEPMFYNDSYGYRPNKSAIQAVGQAREKCFKRDWVLELDIKGLFDNIK
HGYLMYMVEKHTQIKWLILYIKRWLTVPFIMSDGSVAERRSGTPQGGVISPVLANLFLHYVFDDFMTKAYPNI
WWERYADDGVLHCQSYKQAVFIKQKLEERFQQFGLELNKEKTRIVYCKDDRRSRNYSCTQFTFLGYTFRPRLNK
NKEGKFFVGFTPAVSEKAKTAMKQKIRGWKIQLKADLSLKDIGNMINKVVQGWINYYTHYYKSEFYEVLRYINQ
CLIKWVRRSYKKKNTRSRAEHWLGAVARRDRNLFAHWKFGILPSVGEGAV
>R5GH109||gene_68624|GeneMark.hmm|418_aa|-|2665|3921
MSEAKQFDISKKAVIAAFQAVKENAGSYGVDEQTIKEFEEHLNNNLYKLWNRMASGSYFPKPVRAVEIPKKNG
GTRILGIPTVEDRTAQMVAKMYFEPLVKPMFYNDSYGYRPNKSAIQAVGQARERCFKRDWVLELDIKGLFDNIK
HGYLMYMVEKHTQIKWLILYIKRWLTVPFIMSDGSVAERRSGTPQGGVISPVLANLFLHYVFDDFMTKAYPNI

WWERYADDGVLHCQSYKQAVFIKQELEERFQQFGLELNKEKTRIVYCKDNRRSQNYSCTQFTFLGYTFRPRLNK
NKEGKFFVGFTPAVSEKAKTAMKQKIRGWKIQLKADLSLKDIGNMINKVVQGWINYYTHYYKSEFYEVLRYINQ
CLIKWVRRSYKKKNTRSRAEHWLGAVARRDRNLFAHWKFGILPSVGEGAV
>R5GH112||gene_67006|GeneMark.hmm|420_aa|-|688|1950
MNEAKPFVIDKRLVWEAYHKVKENKGSAGIDKVDQKTFDKEMSKNLYKIWNRMSSGCYFPKAVKLVEIPKSNG
GTRPLGIPTIEDRIAQQVVVSVLTPILEPIFKEDSYGYRPGKGAHQAVAKAKERCYVNPWVLDMDISKFFDTINHE
LLMKAVRKHTEEKWVLLYIERWLKVPYQTLKGEVIERTMGVPQGSVIGPVLANLFLHYVFDEWMSRNYPTIPFE
RYADDTICHCVSEKQAQFLKAVLMKRFEECGLKLNEEKTKIVYCKDSNRRGDSEHTSFDFLGFTFRPRGARNRKT
GQNFTAFLPAISRKSMKRIKEAVRAWKLNRKTFACLLDISNEVDTQISGWMNYYMKFGRSEFRKVLNYINERLT
RWVMRKYKRFSKGKKLGRAYEWLVEYAAHNRNEFSHWVKGFVPYPRLG
>R5GH114||gene_124897|GeneMark.hmm|410_aa|-|1488|2720
MQRKSFEIPKALVWASYLDVRRNQGAPGCDGQTLKMFDQQRDGNLYKIWNRLCLGTWFPPPVLEKRIPKPN
GKERILGIPTVSDRIAQGAIKLFMEEKLDPIFHADSYGFRPGKSAHDALKQCAIRCWRYSWILEVDISAFFDHVRH
DLVLKALEHHGMPKWVILYCRRWMEAPMQSCENGELITRTRGTPQGGVISPLLANLFFHYAFDLWMEREYRG
VPFERYADDIVVHCSRMSDATRLKNRLSERFSEVGLVLNAGKTNIAYIDTFKRRNVATSFTFLGYDFKVRTLKNFK
GELYRKCMPGASNAAMRKITETIKKWRIHRSIAESLLDFARRYNAIVRGWIEYYGKFWSRNFNYRLWSAMQSR
LLKWMQSKYRLSNRNAQRKLTLIRKEYPKLFVHWYLLRASNE
>R5GH117||gene_89947|GeneMark.hmm|418_aa|+|982|2238
MSEAKQFDISKKAVIAAFQAVKENAGSYGADEQTIKEFEEHLNNNLYKLWNRMASGSYFPKPVRAVAIPKKNG
GIRILGIPTVEDRIAQMVAKMYFEPLVEPMFYNDSYGYRPNKSAIQAVGQARERCFKRDWVLELDIKGLFDNIK
HGYLMYMVEKHTQIKWLILYIKRWLTVPFIMSDGSVAERRSGTPQGGVISPVLANLFLHYVFDDFMTKAYPNI
WWERYADDGVLHCQSYKQAAFIKQKLEERFQQFGLELNKEKTRIVYCKDNRRPQNYSCTQFTFLGYTFRPRLN
KNKEGKFFVGFTPAVSEKAKTAMKQKIREWKIQLKADLSLKDIGNMINKVVQGWINYYTHYYKSEFYEVLRYIN
QCLIKWVRRSYKKKNTRSRAEHWLGAVARRDRNLFAHWKFGILPSVGEGAV
>R5GH117||gene_75685|GeneMark.hmm|421_aa|+|606|1871
MQEAKPFQIDKRIIFESFKKVKFNRGSSGIDGIEMTTYEQNLGSNLYRLWNRMSSGSYMPKAVKLVEIPKSNGG
KRPLGIPTIEDRIAQMAVVNVIEPLIEPCFHEDSFGYRPHRSAHDAIAKAERRCWKYAWVLDIDISKFFDTIDHGL
LMKAVEKHINIKWILLYIKRWLTVPYQRSDGEIVKRDMGVPQGSVIGPILANLFLHYTFDKWMSYKYPHIPFERY
ADDCVCHCSTLAQAEYIKERLGERFTECKLKFNEEKTKIVFCKMSSRSSKHYHCTSFDYLGFTFRSRAAKDKRNN
VLFTSYLPAISKKSVSRIHETIKSWNLKRLHNRSLRFVAAYINDVVRGWINYYEKFGKTEFWKVMCHLNRSIAYW
AKTKYKRLRRRGVISAHYWLAYIAQKEPNLFYHWQVGYVPYARQKK
>R5GH119||gene_263588|GeneMark.hmm|420_aa|-|573|1835
MNEAKPFVIDKRLVWEAYHKVKENKGSAGIDKVDQKTFDKEMSKNLYKIWNRMSSGCYFPKAVKLVEIPKSNG
GTRPLGIPTIEDRIAQQVVVSVLTPILEPIFKEDSYGYRPGKGAHQAIAKAKERCYVNPWVLDMDISKFFDTINHD
LLMKAVRKHTEEKWVLLYIERWLKVPYQTSKGEVIERTMGVPQGSVIGPVLANLFLHYVFDEWMSRNYPTIPFE
RYADDTICHCVSEKQAQFLKAVLMKRFEECGLKLNEEKTKIVYCKDSNRRGDSEHTSFDFLGFTFRPRSARNRKT
GQNFTAFLPAISKKSLKRIKEAVRAWKLNRKTFACLLDISNEVDTQISGWMNYYMKFGRSEFRKVLNYINERLTR
WVMRKYKRFSKGKKFSRAYEWLVEYAVHNRNEFSHWAKGFVPYPRLG
>R5GH119||gene_43747|GeneMark.hmm|418_aa|-|726|1982
MSEAKQFDISKKAVIAAFQAVKENAGSYGADEQTIKEFEEHLNNNLYKLWNRMASGSYFPKPVRAVAIPKKNG
GIRILGIPTVEDRIAQMVAKMYFEPLVEPMFYNDSYGYRPNKSAIQAVGQARERCFKRDWVLELDIKGLFDNIK
HGYLMYMVEKHTQIKWLILYIKRWLTVPFIMSDGSVAERRSGTPQGGVISPVLANLFLHYVFDDFMTKAYPNI
WWERYADDGVLHCQSYKQAAFIKQKLEERFQQFGLELNKEKTRIVYCKDNRRPQNYSCTQFTFLGYTFRPRLN
KNKEGKFFVGFTPAVSEKAKTAMKQKIREWKIQLKADLSLKDIGNMINKVVQGWINYYTHYYKSEFYEVLRYIN

QCLIKWVRRSYKKKNTRSRAEHWLGAVARRDRNLFAHWKFGILPSVGEGAV
>R5GH121||gene_212867|GeneMark.hmm|420_aa|+|1685|2947
MNAANPFVIDKRLVWEAYHKVKENKGSAGIDKVDQKTFDKEMSKNLYKIWNRMSSGCYFPKAVKLVEMPKS
NGGTRPLGIPAIEDRIAQQVVVSVLTPILEPIFKEDSYGYRPGKGAHQAIAKAKERCYVTPWVLDMDISKFFDTIN
HELLMKAIRKHTEEKWVLLYIERWLKVPYQTSKGEVIERTMGVPQGSVIGPVLANLFLHYVFDEWMSRNYPTIP
FERYADDTICHCVSDKQAQFLKAVLMKRFEECGLKLNEEKTKIVYCKDSNRRGDSEHTSFDFLGFTFRPRGARNR
KTGQNFTAFLPAISKKSMKRIKEAVRAWKLNRKTFACLLDISNEVDTQISGWMNYYMKFGRSEFRKVLNYINERL
TRWVMRKYKRFSKGRKFDRAYDWLVEYATHNRNEFSHWVKGFVPYPRLD
>R5GH122||gene_190838|GeneMark.hmm|420_aa|-|12110|13372
MNEAKPFVIDKRLVWEAYHKVKENKGSAGIDKVDQKTFDKEMSKNLYKIWNRMSSGCYFPKAVKLVEIPKSNG
GTRPLGIPTIEDRIAQQVVVSVLTPILEPIFKEDSYGYRPGKGAHQAVAKAKERCYVNPWVLDMDISKFFDTINHE
LLMKAVRKHTEEKWVLLYIERWLKVPYQTLKGEVIERTMGVPQGSVIGPVLANLFLHYVFDEWMSCNYPTIPFE
RYADDTICHCVSEKQAQFLKAVLMKRFEECGLKLNEEKTKIVYCKDSNRRGDSEHTSFDFLGFTFRPRGARNRKT
GQNFTAFLPAISRKSMKRIKEAVRAWKLNRKTFACLLDISNEVDTQISGWMNYYMKFGRSEFRKVLNYINERLT
RWVMRKYKRFSKGKKLGRAYEWLVEYAAHNRNEFSHWVKGFVPYPRLG
>R5GH123||gene_192313|GeneMark.hmm|418_aa|+|672|1928
MSEAKQFDISKKAVIAAFQAVKENAGSYGADEQTIKEFEEHLNNNLYKLWNRMASGSYFPKPVRAVAIPKKNG
GIRILGIPTVEDRIAQMVAKMYFEPLVEPMFYNDSYGYRPNKSAIQAVGQARERCFKRDWVLELDIKGLFDNIK
HGYLMYMVEKHTQIKWLILYIKRWLTVPFIMSDGSVAERRSGTPQGGVISPVLANLFLHYVFDDFMTKAYPNI
WWERYADDGVLHCQSYKQAAFIKQKLEERFQQFGLELNKEKTRIVYCKDNRRPQNYSCTQFTFLGYTFRPRLN
KNKEGKFFVGFTPAVSEKAKTAMKQKIREWKIQLKADLSLKDIGNMINKVVQGWINYYTHYYKSEFYEVLRYIN
QCLIKWVRRSYKKKNTRSRAEHWLGAVARRDRNLFAHWKFGILPSVGEGAV
>R5GH124||gene_75194|GeneMark.hmm|418_aa|+|3961|5217
MSEAKQFDISKKAVIAAFQAVKENAGSYGADEQTIKEFEEHLNNNLYKLWNRMASGSYFPKPVRAVAIPKKNG
GIRILGIPTVEDRIAQMVAKMYFEPLVEPMFYNDSYGYRPNKSAIQAVGQARERCFKRDWVLELDIKGLFDNIK
HGYLMYMVEKHTQIKWLILYIKRWLTVPFIMSDGSVAERRSGTPQGGVISPVLANLFLHYVFDDFMTKAYPNI
WWERYADDGVLHCQSYKQAAFIKQKLEERFQQFGLELNKEKTRIVYCKDNRRPQNYSCTQFTFLGYTFRPRLN
KNKEGKFFVGFTPAVSEKAKTAMKQKIREWKIQLKADLSLKDIGNMINKVVQGWINYYTHYYKSEFYEVLRYIN
QCLIKWVRRSYKKKNTRSRAEHWLGAVARRDRNLFAHWKFGILPSVGEGAV
>R5GH126||gene_150222|GeneMark.hmm|420_aa|+|397|1659
MNEAKPFVIDKRLVWEAYHKVKENKGSAGIDKVDQKTFDKEMSKNLYKIWNRMSSGCYFPKAVKLVEIPKSNG
GTRPLGIPTIEDRIAQQVVVSVLTPILEPIFKEDSYGYRPGKGAHQAIAKAKERCYVNPWVLDMDISKFFDTINHD
LLMKAVRKHTEEKWVLLYIERWLKVPYQTSKGEVIERTMGVPQGSVIGPVLANLFLHYVFDEWMSRNYPTIPFE
RYADDTICHCVSEKQAQFLKAVLMKRFEECGLKLNEEKTKIVYCKDSNRRGDSEHTSFDFLGFTFRPRSARNRKT
GQNFTAFLPAISKKSLKRIKEAVRAWKLNRKTFACLLDISNEVDTQISGWMNYYMKFGRSEFRKVLNYINERLTR
WVMRKYKRFSKGKKFSRAYEWLVEYAVHNRNEFSHWAKGFVPYPRLG
>R5GH127||gene_64464|GeneMark.hmm|420_aa|-|195|1457
MNEAKPFVIDKRLVWEAYHKVKENKGSAGIDKVDQKTFDKEMSKNLYKIWNRMSSGCYFPKAVKLVEIPKSNG
GTRPLGIPTIEDRIAQQVVVSVLTPILEPIFKEDSYGYRPGKGAHQAIAKAKERCYVNPWVLDMDISKFFDTINHE
LLMKAVRKHTEEKWVLLYIERWLKVPYQTSKGEVIERTMGVPQGSVIGPVLANLFLHYVFDEWMSRNYPTIPFE
RYADDTICHCVSEKQAQFLKAVLMKRFGECGLKLNEEKTKIVYCKDSNRRGDSEHTSFDFLGFTFRPRGARNRKT
GQNFTAFLPAISKKSMKRIKESVRAWKLNRKTFACLLDISNEVDTQISGWMNYYMKFGRSEFRKVLNYINERLTR
WVMRKYKRFSKGKKLGKAYDWLVEYAAHNRNEFSHWVKGFVPYPRLG
>R5GH127||gene_170686|GeneMark.hmm|430_aa|+|584|1876

MQNDNAKPISISKQLVYDAFLRVKANRGSAGIDKVTLEDYEKNLRGNLYKLWNRMSSGSYFPPSVKLVEIPKSTG
GKRPLGIPTVSDRVAQMAVVMLITPSIEPCFHEDSYAYRPHRSAHDAVGKARERCWKYAWVLDMDISKFFDTI
DHELLLKALKRHTQEKWVLMYIERWLKVPYEKSDGSQVDRALGVPQGSVIGPVLANLFLHYTFDKWMEKNFP
RVPFERYADDTICHCHSLKQAEYMQAMIQQRFECCRLRLNEEKTKIVYCKSSRQKECYPNVTFDFLGFTFQPRES
VDKYGNRFTGFLPAISRKSMKRINETMRSWHLNRHSNLTLEHLASDINPIVRGWMTYYGKFYPTRLKWFMQTL
NGRLARWVMCKFERYRHRFYPAQEWLARIAEKEGLIFYHWKCGALPRFTNKEKVSSQLIMVK
>R5GH128||gene_382521|GeneMark.hmm|430_aa|+|2341|3633
MQNDNAKPISISKQLVYDAFLRVKANRGSAGIDKVTLEDYEKNLRGNLYKLWNRMSSGSYFPPSVKLVEIPKSTG
GKRPLGIPTVSDRVAQMAVVMLITPSIEPCFHEDSYAYRPHRSAHDAVGKARERCWKYAWVLDMDISKFFDTI
DHELLLKALKRHTQEKWVLMYIERWLKVPYEKSDGSQVDRALGVPQGSVIGPVLANLFLHYTFDKWMEKNFP
RVPFERYADDTICHCHSLKQAEYMQAMIQQRFECCRLRLNEEKTKIVYCKSSRQKECYPNVTFDFLGFTFQPRES
VDKYGNRFTGFLPAISRKSMKRINETMRSWHLNRHSNLTLEHLASDINPIVRGWMTYYGKFYPTRLKWFMQTL
NGRLARWVMCKFERYRHRFYPAQEWLARIAEKEGLIFYHWKCGALPRFTNKEKVSSQLIMVK
>R5GH129||gene_18189|GeneMark.hmm|422_aa|-|83|1351
MMQHQVTKPFTIDKYLIMNAWKRVKENKGSAGIDNVSTEDYESNLGKNLYKLWNRMSSGSYFPEAVKLVDIP
KPSGGTRPLGIPTVGDRIAQMSVVLLIEERLEAIFHADSYGYRPNRSAHDAIEKARERCWHYNWVLDMDISKFF
DTIDHDLLMKAVERHVQEKWILLYIRRWLKVPYATLTGERIERKMGLPQGSVIGPVLANLYLHYTFDKWMSLYH
PTIPFERYADDTICHCNSLKEAQMLKASIVERFAACKLRLNEEKTRIVYCKDGKRRGEYKEITFDFLGYTFQPRGQ
RNKQGQVFNGYAPAISRKSKKRITEKMRGWHLNRRVQLKLSDIAVEINAEVRGWMNYYGKFYGSQLKAFLQCI
NLKLARWAERKYKRFRRKPNDAYKWLVRVASKNPALFYHWQHGVKPNRLKPFG
>R5GH130||gene_234700|GeneMark.hmm|430_aa|-|120|1412
MQNDNAKPISISKQLVYDAFLRVKANRGSAGIDKVTLEDYEKNLRGNLYKLWNRMSSGSYFPPSVKLVEIPKSTG
GKRPLGIPTVSDRVAQMAVVMLITPSIEPCFHEDSYAYRPHRSAHDAVGKARERCWKYAWVLDMDISKFFDTI
DHELLLKALKRHTQEKWVLMYIERWLKVPYEKSDGSQVDRALGVPQGSVIGPVLANLFLHYTFDKWMEKNFP
RVPFERYADDTICHCHSLKQAEYMQAMIQQRFECCRLRLNEEKTKIVYCKSSRQKECYPNVTFDFLGFTFQPRES
VDKYGNRFTGFLPAISRKSMKRINETMRSWHLNRHSNLTLEHLASDINPIVRGWMTYYGKFYPTRLKWFMQTL
NGRLARWVMCKFERYRHRFYPAQEWLARIAEKEGLIFYHWKCGALPRFTNKEKVSSQLIMVK
>R5GH130||gene_316685|GeneMark.hmm|418_aa|+|743|1999
MSEAKQFDISKKAVIAAFQAVKENAGSYGADEQTIKEFEEHLNNNLYKLWNRMASGSYFPKPVRAVAIPKKNG
GIRILGIPTVEDRIAQMVAKMYFEPLVEPMFYNDSYGYRPNKSAIQAVGQARERCFKRDWVLELDIKGLFDNIK
HGYLMYMVEKHTQIKWLILYIKRWLTVPFIMSDGSVAERRSGTPQGGVISPVLANLFLHYVFDDFMTKAYPNI
WWERYADDGVLHCQSYKQAAFIKQKLEERFQQFGLELNKEKTRIVYCKDNRRPQNYSCTQFTFLGYTFRPRLN
KNKEGKFFVGFTPAVSEKAKTAMKQKIREWKIQLKADLSLKDIGNMINKVVQGWINYYTHYYKSEFYEVLRYIN
QCLIKWVRRSYKKKNTRSRAEHWLGAVARRDRNLFAHWKFGILPSVGEGAV
>R5GH133||gene_305606|GeneMark.hmm|422_aa|+|623|1891
MMQHQVTKPFTIDKHLIMNAWKRVKENKGSVGIDNVSTDDYESNLGKNLYKLWNRMSSGSYFPEAVKLVDIP
KSSGGTRPLGIPTVGDRIAQMSVVLLIEDRLEAIFHADSYGYRPNRSAHDAIGKARERCWHYNWVLDMDISKFF
DTINHDLLMKAVERHVQEKWILLYIRRWLEVPYATLTGERIERRMGVPQGSVIGPVLANLYLHYTFDKWMSLYH
PTIPFERYADDTICHCNSLEEAQMLKASIVERFAACKLKLNEEKTRIVYCKDGKRRREYKDITFDFLGYTFQPRGQR
NKQGQVFNGYAPAISRKSKKRIAETMRGWHLNRRVQLKLSDIAVEINAEVRGWMNYYGKFYGSQLKAFLQCIN
LKLARWAERKYKRFRRKPNDAYKWLVRVASKNPALFYHWQHGVKPNRLKPFG
>R5GH134||gene_54206|GeneMark.hmm|420_aa|+|2200|3462
MNEAKPFVIDKRLVWEAYHKVKENKGSAGIDKVDQQTFDKEMSKNLYKIWNRMSSGCYFPKAVKLVEIPKSNG
GTRPLGIPTIEDRIAQQVVVSVLTPILEPIFKEDSYGYRPGKGAHQAIAKAKERCYVNPWVLDMDISKFFDTINHE

LLMKAVRKHTEEKWVLLYIERWLKVPYQTSKGEVIERTMGVPQGSVIGPVLANLFLHYVFDEWMSRNYPTIPFE
RYADDTICHCVSDKQAQFLKAVLMKRFEECGLKLNEEKTKIVYCKDSNRRGDSEHTSFDFLGFTFRPRGARNRKT
GQNFTAFLPAISKKSMKRIKEAVRAWKLNRKTFACLLDISNEVDTQISGWMNYYMKFGRSEFRKVLNYINERLT
RWVMRKYKRFSKGRKFDRAYDWLVEYAAHNRNEFSHWVKGFVPYPRLG
>R5GH136||gene_261002|GeneMark.hmm|420_aa|-|265|1527
MNEEKSFVIDKRLVWEAYHKVKENKGSAGIDKVDQKTFDKEMSKNLYKIWNRMSSGCYFSKAVKLVEIPKSNG
GTRPLGIPTIEYRIVQQVVVSVLTPILEPIFKEDSYGYRPGKGAHQAIAKAKERCYVTPWVLDMDISKFFDTINHEL
LMKAIRKHTEEKWVLLYIERWLKVPYQTSKGEVIERTMGVPQGSVIGPVLANLFLHYVFDEWMSRNYPTIPFER
YADDTICHCVSEKQARFLKAVLMKRFEECGLKLNEEKTKIVYCKDSNRRGDSEHTSFDFLGFTFRPRGARNRKTG
QNFTAFLPAISNKSMKRIKEAVRAWKLNRKTFACLLDISNEVDTQISGWMNYYMKFGRSEFRKVLNYINERLTR
WVMRKYKRFSKGKKFSKAYEWLVEYAAHNRNEFSHWVKGFVPYPRLD
>R5GH137||gene_294163|GeneMark.hmm|421_aa|-|784|2049
MQEAKPFQIDKRIIFEAFKKVKSNGGSPGIDGIEMSAYEQNLGSNFYRLWNRMSSGSYMPKAVKLVEILKSNGG
KRPLGIPSVEDRIAQMAVVNVIEPLVEPYFHKDSFGYRPHRSAHDAIAKAERRCWKYAWVLDIDISKFFDTIDHG
LLMKAVEKHIKTKWILLYIKRWLTVPYQGNDGAIVKRHMGVPQGSVIGPILANQFLHYTFDKWMSYKYPHVPF
ERYADDCVCHCGTLAQAEYIKDRLGERFAECKLTFNEEKTKIVFCKTSNRSSEHYHCTSFDYLGFTFRPRAAKDKR
KNVLFTSYLPAISNKSESRIHETIKSWNLKRLHNRSLRFVAAYINDVVRGWISYYGKFGKTEFWKVMCHLNRSIAY
WAKTKYKRLRRRGVISAHYWLAYIAQKEPNLFYHWQVGYIPYARQKK
>R5GH137||gene_241876|GeneMark.hmm|388_aa|-|3|1166
MSEAKQFDISKKAVIAAFQAVKENAGSYGADEQTIKEFEEHLNNNLYKLWNRMASGSYFPKPVRAVAIPKKNG
GIRILGIPTVEDRIAQMVAKMYFEPLVEPMFYNDSYGYRPNKSAIQAVGQARERCFKRDWVLELDIKGLFDNIK
HGYLMYMVEKHTQIKWLILYIKRWLTVPFIMSDGSVAERRSGTPQGGVISPVLANLFLHYVFDDFMTKAYPNI
WWERYADDGVLHCQSYKQAAFIKQKLEERFQQFGLELNKEKTRIVYCKDNRRPQNYSCTQFTFLGYTFRPRLN
KNKEGKFFVGFTPAVSEKAKTAMKQKIREWKIQLKADLSLKDIGNMINKVVQGWINYYTHYYKSEFYEVLRYIN
QCLIKWVRRSYKKKNTRSRAE
>R5GH138||gene_80400|GeneMark.hmm|418_aa|+|1351|2607
MSEAKQFDISKKAVIAAFQAVKENAGSYGADEQTIKEFEEHLNNNLYKLWNRMASGSYFPKPVRAVAIPKKNG
GIRILGIPTVEDRIAQMVAKMYFEPLVEPMFYNDSYGYRPNKSAIQAVGQARERCFKRDWVLELDIKGLFDNIK
HGYLMYMVEKHTQIKWLILYIKRWLTVPFIMSDGSVAERRSGTPQGGVISPVLANLFLHYVFDDFMTKAYPNI
WWERYADDGVLHCQSYKQAAFIKQKLEERFQQFGLELNKEKTRIVYCKDNRRPQNYSCTQFTFLGYTFRPRLN
KNKEGKFFVGFTPAVSEKAKTAMKQKIREWKIQLKADLSLKDIGNMINKVVQGWINYYTHYYKSEFYEVLRYIN
QCLIKWVRRSYKKKNTRSRAEHWLGAVARRDRNLFAHWKFGILPSVGEGAV
>R5GH139||gene_26562|GeneMark.hmm|418_aa|+|447|1703
MSEAKQFDISKKAVIAAFQAVKENAGSYGADEQTIKEFEEHLNNNLYKLWNRMASGSYFPKPVRAVAIPKKNG
GIRILGIPTVEDRIAQMVAKMYFEPLVEPMFYNDSYGYRPNKSAIQAVGQARERCFKRDWVLELDIKGLFDNIK
HGYLMYMVEKHTQIKWLILYIKRWLTVPFIMSDGSVAERRSGTPQGGVISPVLANLFLHYVFDDFMTKAYPNI
WWERYADDGVLHCQSYKQAAFIKQKLEERFQQFGLELNKEKTRIVYCKDNRRPQNYSCTQFTFLGYTFRPRLN
KNKEGKFFVGFTPAVSEKAKTAMKQKIREWKIQLKADLSLKDIGNMINKVVQGWINYYTHYYKSEFYEVLRYIN
QCLIKWVRRSYKKKNTRSRAEHWLGAVARRDRNLFAHWKFGILPSVGEGAV
>R5GH140||gene_124070|GeneMark.hmm|418_aa|-|182|1438
MSEAKQFDISKKAVIAAFQAVKENAGSYGADEQTIKEFEEHLNNNLYKLWNRMASGSYFPKPVRAVAIPKKNG
GIRILGIPTVEDRIAQMVAKMYFEPLVEPMFYNDSYGYRPNKSAIQAVGQARERCFKRDWVLELDIKGLFDNIK
HGYLMYMVEKHTQIKWLILYIKRWLTVPFIMSDGSVAERRSGTPQGGVISPVLANLFLHYVFDDFMTKAYPNI
WWERYADDGVLHCQSYKQAVFIKQKLEERFQQFGLELNKEKTRIVYCKDNRRPQNYSCTQFTFLGYTFRPRLNK

NKEGKFFVGFTPAVSEKAKTAMKQKIREWKIQLKADLSLKDIGNMINKVVQGWINYYTHYYKSEFYEVLRYINQ
CLIKWVRRSYKKKNTRSRAEHWLGAVARRDRNLFAHWKFGILPSVGEGAV
>R5GH141||gene_248227|GeneMark.hmm|420_aa|+|1737|2999
MNEAKPFVIDKRLVWEAYHKVKENKGSVGIDKVDQKTFDKEMSKNLYKIWNRMSSGCYFPKAVKLVEIPKSNG
GTRPLGIPTIEDRIAQQVVVSVLIPILEPIFKEDSYGYRPGKGAHQAIAKAKERCYVNPWVLDMDISKFFDTINHEL
LMKAIRKHAEEKWVLLYIERWLKVPYQTSKGEVIERTMGVPQGSVIGPVLANLFLHYVFDEWMSRNYPTIPFER
YADDTICHCVSEKQAQFLKAVLMKRFEECGLKLNEEKTKIVYCKDSNRRGDSEHTSFDFLGFTFRPRGARNRKTG
QNFTAFLPAISKKSMKRIKEAVRAWKLNRKTFACLLDISNEVDTQISGWMNYYMKFGRSEFRKVLNYINERLTR
WVMRKYKRFSKGKKFSKAYEWLVEYAAHNRNEFSHWVKGFVPYPRLD
>R5GH142||gene_231467|GeneMark.hmm|418_aa|+|1878|3134
MSEAKQFDISKKAVIAAFQAVKENAGSYGADEQTIKEFEEHLNNNLYKLWNRMASGSYFPKPVRAVAIPKKNG
GIRILGIPTVEDRIAQMVAKMYFEPLVEPMFYNDSYGYRPNKSAIQAVGQARERCFKRDWVLELDIKGLFDNIK
HGYLMYMVEKHTQIKWLILYIKRWLTVPFIMSDGSVAERRSGTPQGGVISPVLANLFLHYVFDDFMTKAYPNI
WWERYADDGVLHCQSYKQAAFIKQKLEERFQQFGLELNKEKTRIVYCKDNRRPQNYSCTQFTFLGYTFRPRLN
KNKEGKFFVGFTPAVSEKAKTAMKQKIREWKIQLKADLSLKDIGNMINKVVQGWINYYTHYYKSEFYEVLRYIN
QCLIKWVRRSYKKKNTRSRAEHWLGAVARRDRNLFAHWKFGILPSVGEGAV
>R5GH143||gene_18126|GeneMark.hmm|421_aa|-|6841|8106
MQEAKPFQIDKRIIFEAFKKVKSNGGSPGIDGIEMSAYEQNLGSNFYRLWNRMSSGSYMPKAVKLVEILKSNGG
KRPLGIPSVEDRIAQMAVVNVIEPLVEPYFHKDSFGYRPHRSAHDAIAKAERRCWKYAWVLDIDISKFFDTIDHG
LLMKAVEKHIKTKWILLYIKRWLTVPYQGNDGAIVKRHMGVPQGSVIGPILANQFLHYTFDKWMSYKYPHVPF
ERYADDCVCHCGTLAQAEYIKDRLGERFAECKLTFNEEKTKIVFCKTSNRSSEHYHCTSFDYLGFTFRPRAAKDKR
KNVLFTSYLPAISNKSESRIHETIKSWNLKRLHNRSLRFVAAYINDVVRGWISYYGKFGKTEFWKVMCHLNRSIAY
WAKTKYKRLRRRGVISAHYWLAYIAQKEPNLFYHWQVGYIPYARQKK
>R5GH143||gene_297232|GeneMark.hmm|430_aa|+|2563|3855
MQNDNAKPISISKQLVYDAFLRVKANRGSAGIDKVTLEDYEKNLRGNLYKLWNRMSSGSYFPPSVKLVEIPKSTG
GKRPLGIPTVSDRVAQMAVVMLITPSIEPCFHEDSYAYRPHRSAHDAVGKARERCWKYAWVLDMDISKFFDTI
DHELLLKALKRHTQEKWVLMYIERWLKVPYEKSDGSQVDRALGVPQGSVIGPVLANLFLHYTFDKWMEKNFP
RVPFERYADDTICHCHSLKQAEYMQAMIQQRFECCRLRLNEEKTKIVYCKSSRQKECYPNVTFDFLGFTFQPRES
VDKYGNRFTGFLPAISRKSMKRINETMRSWHLNRHSNLTLEHLASDINPIVRGWMTYYGKFYPTRLKWFMQTL
NGRLARWVMCKFERYRHRFYPAQEWLARIAEKEGLIFYHWKCGALPRFTNKEKVSSQLIMVK
>R5GH144||gene_114753|GeneMark.hmm|418_aa|-|1103|2359
MSEAKQFDISKKAVIAAFQAVKENAGSYGADEQTIKEFEEHLNNNLYKLWNRMASGSYFPKPVRAVAIPKKNG
GIRILGIPTVEDRIAQMVAKMYFEPLVEPMFYNDSYGYRPNKSAIQAVGQARERCFKRDWVLELDIKGLFDNIK
HGYLMYMVEKHTQIKWLILYIKRWLTVPFIMSDGSVAERRSGTPQGGVISPVLANLFLHYVFDDFMTKAYPNI
WWERYADDGVLHCQSYKQAAFIKQKLEERFQQFGLELNKEKTRIVYCKDNRRPQNYSCTQFTFLGYTFRPRLN
KNKEGKFFVGFTPAVSEKAKTAMKQKIREWKIQLKADLSLKDIGNMINKVVQGWINYYTHYYKSEFYEVLRYIN
QCLIKWVRRSYKKKNTRSRAEHWLGAVARRDRNLFAHWKFGILPSVGEGAV
>R5GH148||gene_82451|GeneMark.hmm|420_aa|+|79774|81036
MNEAKPFVIDKRLVWEAYHKVKENKGSAGIDKVDQKTFDKEMSKNLYKIWNRMSSGCYFPKAVKLVEIPKSNG
GTRPLGIPTIEDRIAQQVVVSVLTPILEPIFKEDSYGYRPGKGAHQAIAKAKERCYVNPWVLDMDISKFFDTINHE
LLMKAVRKHTEEKWVLLYIERWLKVPYQTLKGEVIERTMGVPQGSVIGPVLANLFLHYVFDEWMSRNYPTIPFE
RYADDTICHCVSEKQAQFLKAVLMKRFEECGLKLNEEKTKIVYCKDSNRRGDSEHTSFDFLGFTFRPRGARNRKT
GQNFTAFLPAISKKSMKRIKEAVRAWKLNRKTFACLLDISNEVDTQISGWMNYYMKFGRSEFRKVLNYINERLT
RWVMRKYKRFSKGKKFSKAYEWLVEYAAHNRNEFSHWVKGFVPYPRLG

>R5GH151||gene_299276|GeneMark.hmm|421_aa|-|382|1647
MQEAKPFQIDKRIIFEAFKKVKFNRGSSGIDGIEMTTYEQNLGSNLYRLWNRMSSGSYMPKAVKLVEIPKSNGG
KRPLGIPTIEDRIAQMAVVNVIEPLIEPCFHEDSFGYRPHRSAHDAIAKAERRCWKYAWVLDIDISKFFDTIDHGL
LMKAVEKHINIKWILLYIKRWLTVPYQRSDGEIVKRDMGVPQGSVIGPILANLFLHYTFDKWMSYKYPHIPFERY
ADDCVCHCSTLAQAEYIKERLGERFTECKLKFNEEKTKIVFCKMSSRSSKHYHCTSFDYLGFTFRSRAAKDKRNN
VLFTSYLPAISKKSVSRIHETIKSWNLKRLHNRSLRFVAAYINDVVRGWINYYEKFGKTEFWKVMCHLNRSIAYW
AKTKYKRLRRRGVISAHYWLAYIAQKEPNLFYHWQVGYVPYARQKK
>R5GH153||gene_7740|GeneMark.hmm|418_aa|-|709|1965
MSEAKQFDISKKAVIAAFQAVKENAGSYGADEQTIKEFEEHLNNNLYKLWNRMASGSYFPKPVRAVAIPKKNG
GIRILGIPTVEDRIAQMVAKMYFEPLVEPMFYNDSYGYRPNKSAIQAVGQARERCFKRDWVLELDIKGLFDNIK
HGYLMYMVEKHTQIKWLILYIKRWLTVPFIMSDGSVAERRSGTPQGGVISPVLANLFLHYVFDDFMTKAYPNI
WWERYADDGVLHCQSYKQAAFIKQKLEERFQQFGLELNKEKTRIVYCKDNRRPQNYSCTQFTFLGYTFRPRLN
KNKEGKFFVGFTPAVSEKAKTAMKQKIREWKIQLKADLSLKDIGNMINKVVQGWINYYTHYYKSEFYEVLRYIN
QCLIKWVRRSYKKKNTRSRAEHWLGAVARRDRNLFAHWKFGILPSVGEGAV
>R5GH154||gene_139630|GeneMark.hmm|418_aa|+|3788|5044
MSEAKQFDISKKAVIAAFQAVKENAGSYGADEQTIKEFEEHLNNNLYKLWNRMASGSYFPKPVRAVAIPKKNG
GIRILGIPTVEDRIAQMVAKMYFEPLVEPMFYNDSYGYRPNKSAIQAVGQARERCFKRDWVLELDIKGLFDNIK
HGYLMYMVEKHTQIKWLILYIKRWLTVPFIMSDGSVAERRSGTPQGGVISPVLANLFLHYVFDDFMTKAYPNI
WWERYADDGVLHCQSYKQAAFIKQKLEERFQQFGLELNKEKTRIVYCKDNRRPQNYSCTQFTFLGYTFRPRLN
KNKEGKFFVGFTPAVSEKAKTAMKQKIREWKIQLKADLSLKDIGNMINKVVQGWINYYTHYYKSEFYEVLRYIN
QCLIKWVRRSYKKKNTRSRAEHWLGAVARRDRNLFAHWKFGILPSVGEGAV
>R5GH155||gene_191957|GeneMark.hmm|422_aa|-|9370|10638
MKDAKSFEISRHLVMEAYKKVKANKGAAGVDDISIADFESNLKSNLYKIWNRMSSGSYLPPAVKLVEIPKSNGGK
RPLGIPTVGDRVAQMVVVMTIEPSIEPYFHEDSYAYRPNRSALDAVRKAKERSYTFHWVLDLDIKGFFDNIDHEL
LIKALERHVKCKWAMLYIKRWLSVPYQLKDGTQIERTKGVPQGSVVGPILANLFLHYVFDEWMKRNHSNISFER
YADDTICHCVSLKQAEFILRAIKKRFAECKLELNEEKTKIVYCKKNHRDIPYECIQFDFLGYTFRPRRSIDENGEVFL
NFSPAISKKARTKIWETIQNWNSNHWIPMELEDIAKEINPVIQGWINYYGQHNPRILKEVLQHINDRLVRWGR
RKFKGLRKRKTATVHKLGDIALQKPNLFAHWAWGVKPTASERNRKRK
>R5GH157||gene_235811|GeneMark.hmm|423_aa|+|2575|3846
MTQKQGAKPFDIDRWKLYYAYQRVNQNRGGSGVDNVTLEKYNSNLKRNLYKLWNRMSSGSYVPKPVRLVQIP
KPAGGTRPLGIPTVEDRIAQMLVVEMIEPEIEKIFHEDSYGYRPNRSAHDALGRARERCWKYAWVLDMDISKFF
DTIDHQLLMKAVRLHVKERWIILYIERWLKVPYQNADKSLIERTCGVPQGSVIGPILANLFLHYCFDRWMQIHYP
EIPFERYADDTVCHCRSQREAESLYEELIIRFKSCKLSLNEEKTKIVYCKSSRRKENHSNVTFDFLGHTFRPCKTMH
KSSREAFTGFQPRISMKATTKIRATMRSWNLKSKSHTPLDCIAHMVNPILRGWVNYYGKYGGKSFQKLLGYFDL
LLARWAKAKYKTFRRKPMYVILKWLGNVADRDAVFYHWQIGLKPAKGTIKL
>R5GH158||gene_47411|GeneMark.hmm|419_aa|+|523|1782
MNAAKPFVIDKRLVWEAYHKVKENKGSAGIDKVDQKTFDKEMSKNLYKIWNRMSSGCYFPKAVKLVEIPKSNG
GTRPLGIPAIEDRIAQQVVSVLTPILEPIFKEDSYGYRPGKGAHQAIAKAKERCYVTPWVLGMDISKFFDTINHELL
MKAIRKHTEEKWVLLYIERWLKVPNQTSKGEVIERTMGVPQGSVIGPVLANLFLHYVFDEWMSRNYPTIPFERY
ADDTICHCVSEKQARFLKAVLMKRFEECGLKLNEEKTKIVYCKDSNRRGDSEHTSFDFLGFTFRPRGARNRKTG
QNFTAFLPAISNKSMKRIKEAVRAWKLNRKTFACLLYISTEVDTQISGWMNYYMKFGRSEFRKVLNYINERLTR
WVMRKYKRFSKGKKFSKAYEWLVEYAAHNRNEFSHWVKGFVPYPRLD
>R5GH161||gene_180944|GeneMark.hmm|371_aa|+|2|1117
KLWNRMASGSYFPKPVRAVAIPKKNGGIRILGIPTVEDRIAQMVAKMYFEPLVEPMFYNDSYGYRPNKSAIQAV

GQARERCFKRDWVLELDIKGLFDNIKHGYLMYMVEKHTQIKWLILYIKRWLTVPFIMSDGSVAERRSGTPQGG
VISPVLANLFLHYVFDDFMTKAYPNIWWERYADDGVLHCQSYKQAAFIKQKLEERFQQFGLELNKEKTRIVYCK
DNRRPQNYSCTQFTFLGYTFRPRLNKNKEGKFFVGFTPAVSEKAKTAMKQKIREWKIQLKADLSLKDIGNMINK
VVQGWINYYTHYYKSEFYEVLRYINQCLIKWVRRSYKKKNTRSRAEHWLGAVARRDRNLFAHWKFGILPSVGE
GAV
>R5GH163||gene_174513|GeneMark.hmm|391_aa|-|202|1377
MNEAKPFVIDKRLVWEAYHKVKENKGSAGIDKVDQKTFDKEMSKNLYKIWNRMSSGCYFPKAVKLVEIPKSNG
GTRPLGIPTIEDRIAQQVVVSVLTPILEPIFKEDSYGYRPGKGAHQAIAKAKERCYVNPWVLDMDISKFFDTINHD
LLMKAVRKHTEEKWVLLYIERWLKVPYQTSKSEVIERTMGVPQGSVIGPVLANLFLHYVFDEWMSRNYPTIPFE
RYADDTICHCVSEKQARFLKAVLMKRFEECGLKLNEEKTKIVYCKDSNRRGDSEHTSFDFLGFTFRPRGARNRKT
GQNFTAFLPAISKKSMKRIKEAVRAWKLNRKTFACLLDISNEVDTQISGWMNYYMKFGRSEFRKVLNYINERLT
RWVMRKYKRFSKGKKFSKA
>R5GH163||gene_86866|GeneMark.hmm|420_aa|-|399|1661
MNEAKPFVIDKRLVWEAYHKVKENKGSAGIDKVDQQTFDKEMSKNLYKIWNRMSSGCYFPKAVKLVEIPKSNG
GTRPLGIPTIEDRIAQQVVVSVLTPILEPIFKEDSYGYRPGKGAHQAIAKAKERCYVNPWVLDMDISKFFDTINHE
LLMKAVRKHTEEKWVLLYIERWLKVPYQTSKGEVIERTMGVPQGSVIGPVLANLFLHYVFDEWMSRNYPTIPFE
RYADDTICHCVSDKQAQFLKAVLMKRFEECGLKLNEEKTKIVYCKDSNRRGDSEHTSFDFLGFTFRPRGARNRKT
GQNFTAFLPAISKKSMKRIKEAVRAWKLNRKTFACLLDISNEVDTQISGWMNYYMKFGRSEFRKVLNYINERLT
RWVMRKYKRFSKGRKFDRAYDWLVEYAAHNRNEFSHWVKGFVPYPRLG
>R5GH165||gene_23947|GeneMark.hmm|420_aa|-|12914|14176
MNEAKPFVIDKRLVWEAYHKVKENKGSAGIDKVDQKTFDKEMSKNLYKIWNRMSSGCYFPKAVKLVEIPKSNG
GTRPLGIPTIEDRIAQQVVVSVLTPILEPIFKEDSYGYRPGKGAHQAIAKAKERCYVNPWVLDMDISKFFDTINHD
LLMKAVRKHTEEKWVLLYIERWLKVPYQTSKGEVIERTMGVPQGSVIGPVLANLFLHYVFDEWMSRNYPTIPFE
RYADDTICHCVSEKQAQFLKAVLMKRFEECGLKLNEEKTKIVYCKDSNRRGDSEHTSFDFLGFTFRPRSARNRKT
GQNFTAFLPAISKKSLKRIKEAVRAWKLNRKTFACLLDISNEVDTQISGWMNYYMKFGRSEFRKVLNYINERLTR
WVMRKYKRFSKGKKFSRAYEWLVEYAVHNRNEFSHWAKGFVPYPRLG
>R5GH165||gene_22576|GeneMark.hmm|415_aa|+|669|1916
MKEGKTFHISQNEVLNAYKAVKANKGAGGVDGIELEEFDKNWKNRLYVLWNRMSSGCYFPKPVRGVEIPKKN
GKVRLLGIPTIEDRVAQMVLRNHIEPFVEPVFHEDSYGYRPGKSALDAVETARKRCFQMRWVIEFDIVGLFDNIE
HDKLMRLVENHCKEKWVSLYVKRCLKAPVQMPDGTVCEKNSGTPQGGVISPVLANLFMHYGFDNWMNRKF
PNCPWERYADDGLIHCVSRKQAEFVLEMLKEQMQRVGLTIHPEKSKIVFCQRNNEEVPEDVETSFVFLGYCFRP
RLVKSGEGKYFMGFTPAVSSDAGKVFREKIKEGIEQQNSTDIVALSERLNPIIRGWINYFTKFTPSEAFRQGINYV
NLTLVRWLRKTRRKARRSYQKAQRLLHQIAMSNIEMFYHWKVGYIPVK
>R5GH169||gene_304682|GeneMark.hmm|410_aa|-|214|1446
MQRKSFEIPKALVWASYLDVRRNKGAPGCDGQTLKMFDQQRDGNLYKIWNRLCSGTWFPPPVLEKRIPKPN
GKERILGIPTVSDRIAQGAIKLFMEEKLDPIFHADSYGYRPGKSAHDALKQCAIRCWRYSWILEVDISAFFDHVRH
DLVLKALEHHGMPKWVILYCRRWMEAPMQSCENGELITRTRGTPQGGVISPLLANLFLHYAFDLWMEREYRG
VPFERYADDIVVHCSRMSDATRLKNRLSERFSEVGLVLNAGKTNIAYIDTFKRRNVATSFTFLGYDFKVRTLKNFK
GELYRKCMPGASNAAMRKITETIKKWRIHRSTAESLLDFARRYNAIVRGWIEYYGKFWSRNFNYRLWSAMQSR
LLKWMQSKYRLSNRRAQRKLTLVRKEYPKLFVHWYLLRASNE
>R5GH169||gene_167358|GeneMark.hmm|420_aa|+|1089|2351
MNEAKPFVIDKRLVWEAYHKVKENKGSAGIDKVDQKTFDKEMSKNLYKIWNRMSSGCYFPKAVKLVEIPKSNG
GTRPLGIPTIEDRIAQQVVVSVLTPILEPIFKEDSYGYRPGKGAHQAIAKAKERCYVNPWVLDMDISKFFDTINHD
LLMKAVRKHTEEKWVLLYIERWLKVPYQTSKGEVIERTMGVPQGSVIGPVLANLFLHYVFDEWMSRNYPTIPFE

RYADDTICHCVSEKQAQFLKAVLMKRFEECGLKLNEEKTKIVYCKDSNRRGDSEHTSFDFLGFTFRPRSARNRKT
GQNFTAFLPAISKKSLKRIKEAVRAWKLNRKTFACLLDISNEVDTQISGWMNYYMKFGRSEFRKVLNYINERLTR
WVMRKYKRFSKGKKFSRAYEWLVEYAVHNRNEFSHWAKGFVPYPRLG
>R5GH171||gene_69637|GeneMark.hmm|418_aa|+|962|2218
MSEAKQFDISKKAVIAAFQAVKENAGSYGADEQTIKEFEEHLNNNLYKLWNRMASGSYFPKPVRAVAIPKKNG
GIRILGIPTVEDRIAQMVAKMYFEPLVEPMFYNDSYGYRPNKSAIQAVGQARERCFKRDWVLELDIKGLFDNIK
HGYLMYMVEKHTQIKWLILYIKRWLTVPFIMSDGSVAERRSGTPQGGVISPVLANLFLHYVFDDFMTKAYPNI
WWERYADDGVLHCQSYKQAAFIKQKLEERFQQFGLELNKEKTRIVYCKDNRRPQNYSCTQFTFLGYTFRPRLN
KNKEGKFFVGFTPAVSEKAKTAMKQKIREWKIQLKADLSLKDIGNMINKVVQGWINYYTHYYKSEFYEVLRYIN
QCLIKWVRRSYKKKNTRSRAEHWLGAVARRDRNLFAHWKFGILPSVGEGAV
>R5GH171||gene_16362|GeneMark.hmm|410_aa|+|136|1368
MQRKSFEIPKALVWASYLDVRRNKGAPGCDGQTLKMFDQQRDGNLYKIWNRLCSGTWFPPPVLEKRIPKPN
GKERILGIPTESDRIAQGAIKLFMEEKLDPIFHADSYGYRPGKSAHDALKQCAIRCWRYSWILEVDISAFFDHVRH
DLVLKALEHHGMPKWVILYCRRWMEAPMQSCENGELITRTRGTPQGGVISPLLANLFLHYAFDLWMEREYRG
VPFERYADDIVVHCSRMSDATRLKNRLSERFSEVGLVLNAGKTNIAYIDTFKRRNVATSFTFLGYDFKVRTLKNFK
GELYRKCMPGASNAAMRKITETIKKWRIHRSTAESLLDFARRYNAIVRGWIEYYGKFWSRNFNYRLWSAMQSR
LLKWMQSKYRLSNRRAQRKLTLVRKEYPKLFVHWYLLRASNE
>R5GH174||gene_273458|GeneMark.hmm|430_aa|+|547|1839
MQNDNAKPISISKQLVYDAFLRVKANRGSAGIDKVTLEDYEKNLRGNLYKLWNRMSSGSYFPPSVKLVEIPKSTG
GKRPLGIPTVSDRVAQMAVVMLITPSIEPCFHEDSYAYRPHRSAHDAVGKARERCWKYAWVLDMDISKFFDTI
DHELLLKALKRHTQEKWVLMYIERWLKVPYEKSDGSQVDRALGVPQGSVIGPVLANLFLHYTFDKWMEKNFP
RVPFERYADDTICHCHSLKQAEYMQAMIQQRFECCRLRLNEEKTKIVYCKSSRQKECYPNVTFDFLGFTFQPRES
VDKYGNRFTGFLPAISRKSMKRINETMRSWHLNRHSNLTLEHLASDINPIVRGWMTYYGKFYPTRLKWFMQTL
NGRLARWVMCKFERYRHRFYPAQEWLARIAEKEGLIFYHWKCGALPRFTNKEKVSSQLIMVK
>R5GH176||gene_161881|GeneMark.hmm|421_aa|-|12336|13601
MQEAKPFQIDKRIIFESFKKVKFNRGSSGIDGIEMTTYEQNLGSNLYRLWNRMSSGSYMPKAVKLVEIPKSNGG
KRPLGIPTIEDRIAQMAVVNVIEPLIEPCFHEDSFGYRPHRSAHDAIAKAERRCWKYAWVLDIDISKFFDTIDHGL
LMKAVEKHINIKWILLYIKRWLTVPYQRSDGEIVKRDMGVPQGSVIGPILANLFLHYTFDKWMSYKYPHIPFERY
ADDCVCHCSTLAQAEYIKERLGERFTECKLKFNEEKTKIVFCKMSSRSSKHYHCTSFDYLGFTFRSRAAKDKRNN
VLFTSYLPAISKKSVNRIHETIKSWNLKRLHNRSLRFVAAYINDVVRGWINYYEKFGKTEFWKVMCHLNRSIAYW
AKTKYKRLRRRGVISAHYWLAYIAQKEPNLFYHWQVGYVPYARQKK
>R5GH177||gene_69451|GeneMark.hmm|418_aa|-|2567|3823
MSEAKQFDISKKAVIAAFQAVKENAGSYGADEQTIKEFEEHLNNNLYKLWNRMASGSYFPKPVRAVAIPKKNG
GIRILGIPTVEDRIAQMVAKMYFEPLVEPMFYNDSYGYRPNKSAIQAVGQARERCFKRDWVLELDIKGLFDNIK
HGYLMYMVEKHTQIKWLILYIKRWLTVPFIMSDGSVAERRSGTPQGGVISPVLANLFLHYVFDDFMTKAYPNI
WWERYADDGVLHCQSYKQAAFIKQKLEERFQQFGLELNKEKTRIVYCKDNRRPQNYSCTQFTFLGYTFRPRLN
KNKEGKFFVGFTPAVSEKAKTAMKQKIREWKIQLKADLSLKDIGNMINKVVQGWINYYTHYYKSEFYEVLRYIN
QCLIKWVRRSYKKKNTRSRAEHWLGAVARRDRNLFAHWKFGILPSVGEGAV
>R5GH178||gene_99587|GeneMark.hmm|423_aa|+|386|1657
MTQKQGAKPFDIDRWKLYYAYQRVNQNRGGSGVDNVTLEKYNSNLKRNLYKLWNRMSSGSYVPKPVRLVQIP
KPAGGTRPLGIPTVEDRIAQMLVVEMIEPEIEKIFHEDSYGYRPNRSAHDALGRARERCWKYAWVLDMDISKFF
DTIDHQLLMKAVRLHVKERWIILYIERWLKVPYQNADKSLIERTCGVPQGSVIGPILANLFLHYCFDRWMQIHYP
EIPFERYADDTVCHCRSQREAESLYEELIIRFKSCKLSLNEEKTKIVYCKSSRRKENHSNVTFDFLGHTFRPCKTMH
KSSREAFTGFQPRISMKATTKIRATMRSWNLKSKSHTPLDCIAHMVNPILRGWVNYYGKYGGKSFQKLLGYFDL

LLARWAKAKYKTFRRKPMYVILKWLGNVADRDAVFYHWQIGLKPAKGTIKL
>R5GH179||gene_73838|GeneMark.hmm|416_aa|+|683|1933
MTKTKAFNIDKSLVVSAYRRVKTSAGAAGIDKQSLADFDKRLVDNLYKIWNRLSSGSYFPPAVKAVAIPKKLGGER
ILGIPTVSDRIAQTVVKLAFEPQVEPHFLADSYGYRPNKSALDAIGVTRKRCWYYDWVLEFDIKGLFDNIPHELIM
KAVDKHNPARWVKLYIQRWLTAPMVMSDGEVRARTMGTPQGGVISPLLANLFMHYVFDKWLAKYYPKVPW
YRYADDGILHCHSEAEATEMREVLRKRFSECGLEMHPEKTRVIYCKDGSRKGDYEHTMFDFLGYTFRRRVVKNV
KRNSLFVSFTPAASKSALKAMRREIKATGIRKRVDLSIEQIAKWINPKLNGWINYYGRYTCSELYSVFRYINKALVR
WGRKKYKMLSRYKTRASKFLEEMAKRSPQLFAHWRLKMRGGLV
>R5GH180||gene_203395|GeneMark.hmm|413_aa|+|629|1870
MSESKQYEIPKRVVVEAYKRVKANKGSAGIDGIDFDIFEKKLNNNLYKIWNRMSSGSYFPSPVLAVEIPKKAGGT
RRLGIPTIADRIAQMIARMYIEPAVEPMFCEDSYGYRPNKSAIEAIAVTRKRCWRYDYVIELDVKGLFDNINHELL
MRVVEKHVKESWICLYVKRWMETPFVTKERAAIERKSETPQGGVISPVLANMFLHYVFDMWMKRKFPQAPF
ERYADDGIVHCRTKEEAICIRQSLAKRFEECKLELHPTKTRIVYCKDEDRRKEEELTEFDFLGYTFKARYIKCRDGKL
RYNFIASVSKVSAKAFRTKVKEMELHRRTGCKIDILAEMLNPMVRGWMNYFGKYNPSAMKDTLLCIERRLVKW
AMCKYKKFRGRRRKAEEWLCTLRKREPKLFAHWSMIYSYC
>R5GH181||gene_189868|GeneMark.hmm|418_aa|-|141|1397
MSEAKQFDISKKAVIAAFQAVKENAGSYGADEQTIKEFEEHLNNNLYKLWNRMASGSYFPKPVRAVAIPKKNG
GIRILGIPTVEDRIAQMVAKMYFEPLVEPMFYNDSYGYRPNKSAIQAVGQARERCFKRDWVLELDIKGLFDNIK
HGYLMYMVEKHTQIKWLILYIKRWLTVPFIMSDGSVAERRSGTPQGGVISPVLANLFLHYVFDDFMTKAYPNI
WWERYADDGVLHCQSYKQAAFIKQKLEERFQQFGLELNKEKTRIVYCKDNRRPQNYSCTQFTFLGYTFRPRLN
KNKEGKFFVGFTPAVSEKAKTAMKQKIREWKIQLKADLSLKDIGNMINKVVQGWINYYTHYYKSEFYEVLRYIN
QCLIKWVRRSYKKKNTRSRAEHWLGAVARRDRNLFAHWKFGILPSVGEGAV
>R5GH182||gene_193320|GeneMark.hmm|418_aa|-|146|1402
MSEAKQFDISKKAVIAAFQAVKENAGSYGEDEQTIKEFEEHLNNNLYKLWNRMASGSYFPKPVRAVAIPKKNG
GIRILGIPTVEDRIAQMVAKMYFEPLVEPMFYNDSYGYRPNKSAIQAVGQARERCFKRDWVLELDIKGLFDNIK
HGYLMYMVEKHTQIKWLILYIKRWLTVPFIMSDGSVAERRSGTPQGGVISPVLANLFLHYVFDDFMTKAYPNI
WWERYADDGVLHCQSYKQAAFIKQKLEERFQQFGLELNKEKTRIVYCKDNRRPQNYSCTQFTFLGYTFRPRLN
KNKEGKFFVGFTPAVSEKAKTAMKQKIREWKIQLKADLSLKDIGNMINKVVQGWINYYTHYYKSEFYEVLRYIN
QCLIKWVRRSYKKKNTRSRAEHWLGAVARRDRNLFAHWKFGILPSVGEGAV
>R5GH184||gene_202337|GeneMark.hmm|418_aa|+|1802|3058
MSEAKQFDISKKAVIAAFQAVKENAGSYGVDEQTIKEFEEHLNNNLYKLWNRMASGSYFPKPVRAVEIPKKNG
GTRILGIPTVEDRIAQMVAKMYFEPLVEPMFYNDSYGYRPNKSAIQAVGQARERCFKRDWVLELDIKGLFDNIK
HGYLMYMVEKHTQIKWLILYIKRWLTVPFIMSDGSVAERRSGTPQGGVISPVLANLFLHYVFDDFMTKAYPNI
WWERYADDGVLHCQSYKQAVFIKQKLEERFQQFGLELNKEKTRIVYCKDNRRSQNYSCTQFTFLGYTFRPRLNK
NKEGKFFVGFTPAVSEKAKTAMKQKIRGWKIQLKADLSLKDIGNMINKVVQGWINYYTHYYKSEFYEVLRYINQ
CLIKWVRRSYKKKNTRSRAEHWLGAVARRDRNLFAHWKFGILPSVGEGAV
>R5GH185||gene_333802|GeneMark.hmm|418_aa|+|1351|2607
MSEAKQFDISKKAVIAAFQAVKENAGSYGADEQTIKEFEEHLNNNLYKLWNRMASGSYFPKPVRAVAIPKKNG
GIRILGIPTVEDRIAQMVAKMYFEPLVEPMFYNDSYGYRPNKSAIQAVGQARERCFKRDWVLELDIKGLFDNIK
HGYLMYMVEKHTQIKWLILYIKRWLTVPFIMSDGSVAERRSGTPQGGVISPVLANLFLHYVFDDFMTKAYPNI
WWERYADDGVLHCQSYKQAAFIKQKLEERFQQFGLELNKEKTRIVYCKDNRRPQNYSCTQFTFLGYTFRPRLN
KNKEGKFFVGFTPAVSEKAKTAMKQKIREWKIQLKADLSLKDIGNMINKVVQGWINYYTHYYKSEFYEVLRYIN
QCLIKWVRRSYKKKNTRSRAEHWLGAVARRDRNLFAHWKFGILPSVGEGAV
>R5GH186||gene_224103|GeneMark.hmm|410_aa|+|287|1519

MQRKSFEIPKALVWASYLDVRRNKGAPGCDGQTLKMFDQQRDGNLYKIWNRLCSGTWFPPPVLEKRIPKPN
GKERILGIPTVSDRIAQGAIKLFMEEKLDPIFHADSYGYRPGKSAHDALKQCAIRCWRYSWILEVDISAFFDHVRH
DLVLKALEHHGMPKWVILYCRRWMEAPMQSCENGELITRTRGTPQGGVISPLLANLFLHYAFDLWMEREYRG
VPFERYADDIVVHCSRMSDATRLKNRLSERFSEVGLVLNAGKTNIAYIDTFKRRNVATSFTFLGYDFKVRTLKNFK
GELYRKCMPGASNAAMRKITETIKKWRIHRSTAESLLDFARRYNAIVRGWIEYYGKFWSRNFNYRLWSAMQSR
LLKWMQSKYRLSNRRAQRKLTLVRKEYPKLFVHWYLLRASNE
>R5GH188||gene_48541|GeneMark.hmm|420_aa|-|6863|8125
MNEAKPFVIDKRLVWEAYHKVKENKGSAGIDKVDQKTFDKEMSKNLYKIWNRMSSGCYFPKAVKLVEIPKSNG
GTRPLGIPTIEDRIAQQVVVSVLTPILEPIFKEDSYGYRPGKGAHQAIAKAKERCYVNPWVLDMDISKFFDTINHD
LLMKAVRKHTEEKWVLLYIERWLKVPYQTSKGEVIERTMGVPQGSVIGPVLANLFLHYVFDEWMSRNYPTIPFE
RYADDTICHCVSEKQAQFLKAVLMKRFEECGLKLNEEKTKIVYCKDSNRRGDSEHTSFDFLGFTFRPRSARNRKT
GQNFTAFLPAISKKSLKRIKEAVRAWKLNRKTFACLLDISNEVDTQISGWMNYYMKFGRSEFRKVLNYINERLTR
WVMRKYKRFSKGKKFSRAYEWLVEYAVHNRNEFSHWAKGFVPYPRLG
>R5GH189||gene_43313|GeneMark.hmm|418_aa|+|15413|16669
MSEAKQFDISKKAVIAAFQAVKENAGSYGADEQTIKEFEEHLNNNLYKLWNRMASGSYFPKPVRAVAIPKKNG
GIRILGIPTVEDRIAQMVAKMYFEPLVEPMFYNDSYGYRPNKSAIQAVGQARERCFKRDWVLELDIKGLFDNIK
HGYLMYMVEKHTQIKWLILYIKRWLTVPFIMSDGSVAERRSGTPQGGVISPVLANLFLHYVFDDFMTKAYPNI
WWERYADDGVLHCQSYKQAAFIKQKLEERFQQFGLELNKEKTRIVYCKDNRRPQNYSCTQFTFLGYTFRPRLN
KNKEGKFFVGFTPAVSEKAKTAMKQKIREWKIQLKADLSLKDIGNMINKVVQGWINYYTHYYKSEFYEVLRYIN
QCLIKWVRRSYKKKNTRSRAEHWLGAVARRDRNLFAHWKFGILPSVGEGAV
>R5GH193||gene_339391|GeneMark.hmm|417_aa|-|1471|2724
MNEAKPFVIDKRLVWEAYHKVKENKGSSGIDKVDQKTFDKEMSKNLYKIWNRMSSGCYFPKAVKLVEIPKSNG
GTRPLGIPTIEDRIAQQVVVSVLIPILEPIFKEDSYGYRPGKGAHQAIAKAKERCYVNPWVLDMDISKFFDTINHEL
LMKAIRKHAEEKWVLLYIERWLKVPYQTSKGEVIERTMGGSVIGPVLANLFLHYVFDEWMSRNYPTIPFERYAD
DTISHCVSEKQARFLKAVLMKRFEECGLKLNEEKTKIVYCKDSNRRGDSEHTSFDFLGFTFRPRGARNRKTGQNF
TAFLPAISKKSMKRIKEAVRAWKLNRKTFACLLDISNEVDTQISGWMNYYMKFGRSEFRKVLNYINERLTRWVM
RKYKRFSKGKKFSKAYEWLVEYAAHNRNEFSHWVKGFVPYPRLD
>R5GH194||gene_176389|GeneMark.hmm|423_aa|+|573|1844
MTQKQGAKPFDIDRWKLYYAYQRVNQNRGGSGVDNVTLEKYNSNLKRNLYKLWNRMSSGSYVPKPVRLVQIP
KPAGGTRPLGIPTVEDRIAQMLVVEMIEPEIEKIFHEDSYGYRPNRSAHDALGRARERCWKYAWVLDMDISKFF
DTIDHQLLMKAVRLHVKERWIILYIERWLKVPYQNADKSLIERTCGVPQGSVIGPILANLFLHYCFDRWMQIHH
PEIPFERYADDTVCHCRSQREAESLYEELIIRFKSCKLSLNEEKTKIVYCKSSRRKENHSNVTFDFLGHTFRPCKTM
HKSSREAFTGFQPRISMKATTKIRATMRSWNLKSKSHTPLDCIAHMVNPILRGWVNYYGKYGGKSFQKLLGYF
DLLLARWAKAKYKTFRRKPMYVILKWLGNVADRDAVFYHWQIGLKPAKGTIKL
>R5GH194||gene_154440|GeneMark.hmm|420_aa|-|7648|8910
MNEAKPFVIDKRLVWEAYHKVKENKGSAGIDKVDQKTFDKEMSKNLYKIWNRMSSGCYFPKAVKLVEIPKSNG
GTRPLGIPTIEDRIAQQVVVSVLTPILEPIFKEDSYGYRPGKGAHQAIAKAKERCYVNPWVLDMDISKFFDTINHD
LLMKAVRKHTEEKWVLLYIERWLKVPYQTSKGEVIERTMGVPQGSVIGPVLANLFLHYVFDEWMSRNYPTIPFE
RYADDTICHCVSEKQAQFLKAVLMKRFEECGLKLNEEKTKIVYCKDSNRRGDSEHTSFDFLGFTFRPRSARNRKT
GQNFTAFLPAISKKSLKRIKEAVRAWKLNRKTFACLLDISNEVDTQISGWMNYYMKFGRSEFRKVLNYINERLTR
WVMRKYKRFSKGKKFSRAYEWLVEYAVHNRNEFSHWAKGFVPYPRLG
>R5GH195||gene_250287|GeneMark.hmm|418_aa|-|369|1625
MSEAKQFDISKKAVIAAFQAVKENAGSYGADEQTIKEFEEHLNNNLYKLWNRMASGSYFPKPVRAVAIPKKNG
GIRILGIPTVEDRIAQMVAKMYFEPLVEPMFYNDSYGYRPNKSAIQAVGQARERCFKRDWVLELDIKGLFDNIK

HGYLMYMVEKHTQIKWLILYIKRWLTVPFIMSDGSVAERRSGTPQGGVISPVLANLFLHYVFDDFMTKAYPNI
WWERYADDGVLHCQSYKQAAFIKQKLEERFQQFGLELNKEKTRIVYCKDNRRPQNYSCTQFTFLGYTFRPRLN
KNKEGKFFVGFTPAVSEKAKTAMKQKIREWKIQLKADLSLKDIGNMINKVVQGWINYYTHYYKSEFYEVLRYIN
QCLIKWVRRSYKKKNTRSRAEHWLGAVARRDRNLFAHWKFGILPSVGEGAV
>R5GH196||gene_349403|GeneMark.hmm|420_aa|-|115|1377
MNEAKPFVIDKRLVWEAYHKVKENKGSAGIDKVDQKTFDKEMSKNLYKIWNRMSSGCYFPKAVKLVEIPKSNG
GTRPLGIPTIEDRIAQQVVVSVLTPILEPIFKEDSYGYRPGKGAHQAIAKAKERCYVNPWVLDMDISKFFDTINHE
LLMKAVRKHTEEKWVLLYIERWLKVPYQTSKGEVIERTMGVPQGSVIGPVLANLFLHYVFDEWMSRNYPTIPFE
RYADDTICHCVSEKQAQFLKTVLMKRFGECGLKLNEEKTKIVYCKDSNRRGDSEHTSFDFLGFTFRPRGARNRKT
GQNFTAFLPAISKKSMKRIKESVRAWKLNRKTFACLLDISNEVDTQISGWMNYYMKFGRSEFRKVLNYINERLTR
WVMRKYKRFSKGKKLGKAYDWLVEYAAHNRNEFSHWVKGFVPYPRLG
>R5GH197||gene_2045|GeneMark.hmm|407_aa|-|1|1221
MQRKSFEIPKALVWASYLDVRRNKGAPGCDGQTLKMFDQQRDGNLYKIWNRLCSGTWFPPPVLEKRIPKPN
GKERILGIPTVSDRIAQGAIKLFMEEKLDPIFHADSYGYRPGKSAHDALKQCAIRCWRYSWILEVDISAFFDHVRH
DLVLKALEHHGMPKWVILYCRRGMEAPMQSCENGELITRTRGTPQGGVISPLLANLFLHYAFDLWMEREYRG
VPFERYADDIVVHCSRMSDATRLKNRLSERFSEVGLVLNAGKTNIAYIDTFKRRNVATSFTFLGYDFKVRTLKNFK
GELYRKCMPGTSNAAMRKITETIKKWRIHRSTAESLLDFARRYNAIVRGWIEYYGKFWSRNFNYRLWSAMQSR
LLKWMQSKYRLSNRRAQRKLTLVRKEYPKLFVHWYLLRA
>R5GH198||gene_276509|GeneMark.hmm|423_aa|+|41427|42698
MTQKQGAKPFDIDRWKLYYAYQRVNQNRGGSGVDNVTLEKYNSNLKRNLYKLWNRMSSGSYVPKPVRLVQIP
KPAGGTRPLGIPTVEDRIAQMLVVEMIEPEIEKIFHEDSYGYRPNRSAHDALGRARERCWKYAWVLDMDISKFF
DTIDHQLLMKAVRLHVKERWIILYIERWLKVPYQNADKSLIERTCGVPQGSVIGPILANLFLHYCFDRWMQIHYP
EIPFERYADDTVCHCRSQREAESLYEELIIRFKSCKLSLNEEKTKIVYCKSSRRKENHSNVTFDFLGHTFRPCKTMH
KSSREAFTGFQPRISMKATTKIRATMRSWNLKSKSHTPLDCIAHMVNPILRGWVNYYGKYGGKSFQKLLGYFDL
LLARWAKAKYKTFRRKPMYVILKWLGNVADRDAVFYHWQIGLKPAKGTIKL
>R5GH201||gene_333028|GeneMark.hmm|420_aa|-|5503|6765
MNEAKPFVIDKRLVWEAYHKVKENKGSAGIDKVDQKTFDKEMSKNLYKIWNRMSSGCYFPKAVKLVEIPKSNG
GTRPLGIPTIEDRIAQQVVVSVLTPILEPIFKEDSYGYRPGKGAHQAIAKAKERCYVNPWVLDMDISKFFDTINHE
LLMKAVRKHTEEKWVLLYIERWLKVPYQTSKGEVIERTMGVPQGSVIGPVLANLFLHYVFDEWMSRNYPTIPFE
RYADDTICHCVSEKQAQFLKAVLMKRFGECGSKLNEEKTKIVYCKDSNRRGDSEHTSFDFLGFTFRPRGARNRKT
GQNFTAFLPAISKKSMKRIKESVRAWKLNRKTFACLLDISNEVDTQISGWMNYYMKFGRSEFRKVLNYINERLTR
WVMRKYKRFSKGKKLGKAYDWLVEYAAHNRNEFSHWVKGFVPYPRLG
>R5GH202||gene_4385|GeneMark.hmm|414_aa|+|7444|8688
MQEAKPYSISKKAVIAAYQRVKANKGTYGVDEQSIEDFERKLNNNLYKIWNRMSSGTYFPKPVKAVAIPKKNGG
TRILGVPTVEDRIAQMVAKLYFEPCVEPIFYEDSYGYRPNKSAIQALEATRTRCWRKDWVLEFDIRGLFDNIRHDY
LMEMVKKHTKEKWIILYIQRWLTAPFQMEDGTIVERKSGTPQGGVISPVLANLFLHYVFDDFMVKEFPTIPWA
RYADDGIAHCVSQKQAKYLRRRLEQRFQSYGLELNQEKTRIVYCKDDDRRGNHENTSFDFLGYTFRPRHAKNRY
GKFFTNFLPAISEKAKKAIRKEVRGWKLQLKSDKDLYDIANMFNRQIQGWINYYTHFYKSEIYDVLRYINGCLVK
WVRRKYKKRKARRKAEHWLGEIAKRDRNLFAHWKFGILPAAG
>R5GH203||gene_206570|GeneMark.hmm|410_aa|-|84|1316
MQRKSFEIPKALVWASYLDVRRNKGAPGCDGQTLKMFDQQRDGNLYKIWNRLCSGTWFPPPVLEKRIPKPN
GKERILGIPTVSDRIAQGAIKLFMEEKLDPIFHADSYGYRPGKSAHDALKQCAIRCWRYSWILEVDISAFFDHVRH
DLVLKALEHHGMPKWVILYCRRWMEAPMQSCENGELITRTRGTPQGGVISPLLANLFLHYAFDLWMEREYRG
VPFERYADDIVVHCSRMSDATRLKNRLSERFSEVGLVLNAGKTNIAYIDTFKRRNVATSFTFLGYDFKVRTLKNFK

GELYRKCMPGASNAAMRKITETIKKWRIHRSTAESLLDFARRYNAIVRGWIEYYGKFWSRNFNYRLWSAMQSR
LLKWMQSKYRLSNRRAQRKLTLVRKEYPKLFVHWYLLRASNE
>R5GH204||gene_17690|GeneMark.hmm|420_aa|-|17639|18901
MNEAKPFVIDKRLVWEAYHKVKENKGSAGIDKVDQKTFDKEMSKNLYKIWNRMSSGCYFPKAVKLVEIPKSNG
GTRPLGIPTIEDRIAQQVVVSVLTPILEPIFKEDSYGYRPGKGAHQAIAKAKERCYVNPWVLDMDISKFFDTINHE
LLMKAIRKHTEEKWVLLYLERWLKVPYQTSKGEVIERTMGVPQGSVIGPVLANLFLHYVFDEWMSRNYPTIPFE
RYADDTICHCVSEKQAQFLKAALMKRFEECGLKLNEEKTKIVYCKDSNRRGDSEHTSFDFLGFTFRPRGARNRKT
GQNFTAFLPAISKKSMKRIKEAVRAWKLNHKTFACLLDISNEVDTQISGWMNYYMKFGRSEFRKVLKYINERLTR
WVMRKYKRFSKGRKFDRAYDWLVEYAAHNRNEFSHWVKGFVPYPRLG
>R5GH205||gene_19487|GeneMark.hmm|420_aa|+|485|1747
MNEAKPFVIDKRLVWEAYHKVKENKGSAGIDKVDQKTFDKEMSKNLYKIWNRMSSGCYFPKAVKLVEIPKSNG
GTRPLGIPTIEDRIAQQVVVSVLTPILEPIFKEDSYGYRPGKGAHQAIAKAKERCYVNPWVLDMDISKFFDTINHD
LLMKAVRKHTEEKWVLLYIERWLKVPYQTSKGEVIERTMGVPQGSVIGPVLANLFLHYVFDEWMSRNYPTIPFE
RYADDTICHCVSEKQAQFLKAVLMKRFEECGLKLNEEKTKIVYCKDSNRRGDSEHTSFDFLGFTFRPRSARNRKT
GQNFTAFLPAISKKSLKRIKEAVRAWKLNRKTFACLLDISNEVDTQISGWMNYYMKFGRSEFRKVLNYINERLTR
WVMRKYKRFSKGKKFSRAYEWLVEYAVHNRNEFSHWAKGFVPYPRLG
>R5GH209||gene_251735|GeneMark.hmm|418_aa|+|4451|5707
MSEAKQFDISKKAVIAAFQAVKENAGSYGADEQTIKEFEEHLNNNLYKLWNRMASGSYFPKPVRAVAIPKKNG
GIRILGIPTVEDRIAQMVAKMYFEPLVEPMFYNDSYGYRPNKSAIQAVGQARERCFKRDWVLELDIKGLFDNIK
HGYLMYMVEKHTQIKWLILYIKRWLTVPFIMSDGSVAERRSGTPQGGVISPVLANLFLHYVFDDFMTKAYPNI
WWERYADDGVLHCQSYKQAAFIKQKLEERFQQFGLELNKEKTRIVYCKDNRRPQNYSCTQFTFLGYTFRPRLN
KNKEGKFFVGFTPAVSEKAKTAMKQKIREWKIQLKADLSLKDIGNMINKVVQGWINYYTHYYKSEFYEVLRYIN
QCLIKWVRRSYKKKNTRSRAEHWLGAVARRDRNLFAHWKFGILPSVGEGAV
>R5GH213||gene_4022|GeneMark.hmm|418_aa|-|481|1737
MSEAKQFDISKKAVIAAFQAVKENAGSYGADEQTIKEFEEHLNNNLYKLWNRMASGSYFPKPVRAVEIPKKNG
GTRILGIPTVEDRIAQMVAKMYFEPLVEPMFYNDSYGYRPNKSAIQAVGQARERCFKRDWVLELDIKGLFDNIK
HGYLMYMVEKHTQIKWLILYIKRWLTVPFIMSDGSVAERRSGTPQGGVISPVLANLFLHYVFDDFMTKAYPNI
WWERYADDGVLHCQSYKQAAFIKQKLEERFQQFGLELNKEKTRIVYCKDNRRPQNYSCTQFTFLGYTFRPRLN
KNKEGKFFVGFTPAVSEKAKTAMKQKIRGWKIQLKADLSLKDIGNMINKVVQGWINYYTHYYKSEFYEVLRYIN
QCLIKWVRRSYKKKNTRSRAEHWLGAVARRDRNLFAHWKFGILPSVGEGAV
>R5GH213||gene_262213|GeneMark.hmm|420_aa|+|1684|2946
MNEAKSFVIDKRLVWEAYHKVKENKGSAGIDKVDQKTFDKEMSKNLYKIWNRMSSGCYFPKAVKLVEIPKSNG
GTRPLGIPTIEYRIAQQVVVSVLTPILEPIFKEDSYGYRPGKGAHQAIAKAKERCYVTPWVLDMDISKFFDTINHEL
LMKAIRKHTEEKWVLLYIERWLKVPNQTSKGEVIERTMGVPQGSVIGPVLANLFLHYVFDEWMSRNYPTIPFER
YADDTISHCVSEKQAQFLKAVLMKRFEECGLKLNEEKTKIVYCKDSNRRGDSEHTSFDFLGFTFRPRGARNRKTG
QNFTAFLPAISKKSMKRIKEAVRAWKLNRKTFACLLDISNEVDTQISGWMNYYMKFGRSEFRKVLNYINERLTR
WVMRKYKRFSKGKKFSKAYEWLVEYAAHNRNEFSHWVKGFVPYPRLD
>R5GH214||gene_138993|GeneMark.hmm|418_aa|+|2498|3754
MSEAKQFDISKKAVIAAFQAVKENAGSYGADEQTIKEFEEHLNNNLYKLWNRMASGSYFPKPVRAVAIPKKNG
GIRILGIPTVEDRIAQMVAKMYFEPLVEPMFYNDSYGYRPNKSAIQAVGQARERCFKRDWVLELDIKGLFDNIK
HGYLMYMVEKHTQIKWLILYIKRWLTVPFIMSDGSVAERRSGTPQGGVISPVLANLFLHYVFDDFMTKAYPNI
WWERYADDGVLHCQSYKQAAFIKQKLEERFQQFGLELNKEKTRIVYCKDNRRPQNYSCTQFTFLGYTFRPRLN
KNKEGKFFVGFTPAVSEKAKTAMKQKIREWKIQLKADLSLKDIGNMINKVVQGWINYYTHYYKSEFYEVLRYIN
QCLIKWVRRSYKKKNTRSRAEHWLGAVARRDRNLFAHWKFGILPSVGEGAV

>R5GH215||gene_196340|GeneMark.hmm|418_aa|+|569|1825
MSEAKQFDISKKAVIAAFQAVKENAGSYGVDEQTIKEFEEHLNNNLYKLWNRMASGSYFPKPVRAVEIPKKNG
GTRILGIPTVEDRIAQMVAKMYFEPLVEPMFYNDSYGYRPNKSAIQAVGQARERCFKRDWVLELDIKGLFDNIK
HGYLMYMVEKHTQIKWLILYIKRWLTVPFIMSDGSVAERRSGTPQGGVISPVLANLFLHYVFDDFMTKAYPNI
WWERYADDGVLHCQSYKQAAFIKQKLEERFQQFGLELNKEKTRIVYCKDDRRSRNYSCTQFTFLGYTFRPRLNK
NKEGKFFVGFTPAVSEKAKTAMKQKIRGWKIQLKADLSLKDIGNMINKVVQGWINYYTHYYKSEFYEVLRYINQ
CLIKWVRRSYKKKNTRSRAEHWLGAVARRDRNLFAHWKFGILPSVGEGAV
>R5GH217||gene_11837|GeneMark.hmm|420_aa|+|4033|5295
MNEAKPFVIDKRLVWEAYHKVKENKGSAGIDKVDQKTFDKEMSKNLYKIWNRMSSGCYFPKAVKLVEIPKSNG
GTRPLGIPTIEDRIAQQVVVSVLTPILEPIFKEDSYGYRPGKGAHQAVAKAKERCYVNPWVLDMDISKFFDTINHE
LLMKAVRKHTEEKWVLLYIERWLKVPYQTLKGEVIERTMGVPQGSVIGPVLANLFLHYVFDEWMSRNYPTIPFE
RYADDTICHCVSEKQAQFLKAVLMKRFEECELKLNEEKTKIVYCKDSNRSGDSEHTSFDFLGFTFRPRGARNRKT
GQNFTAFLPAISRKSMKRIKEAVRAWKLNRKTFACLLDISNEVDTQISGWMNYYMKFGRSEFRKVLNYINERLP
RWVMRKYKRFSKGKKLGRAYEWLVEYAAHNRNEFSHWVKGFVPYPRLG
>R5GH220||gene_38347|GeneMark.hmm|415_aa|+|637|1884
MKEGKTFHISQNEVLNAYKAVKANKGAGGVDGIELEEFDKNWKNRLYVLWNRMSSGCYFPKPVRGVEIPKKN
GKVRLLGIPTIEDRVAQMVLRNHIEPFVEPVFHEDSYGYRPGKSALDAVERARKRCFQMRWVIEFDIVGLFDNIE
HDKLMRLVENHCKEKWVSLYVKRCLKAPVQMLDGTVCEKNSGTPQGGVISPVLANLFMHYGFDNWMNRKF
PNCPWERYADDGLIHCVSRKQAEFVLEMLKEQMQRVGLTIHPEKSKIVFCQRNNEEVPEDVETSFVFLGYCFRP
RLVKSGQGKYFMGFTPAVSSDAGKVFREKIKEGIEQQNSTDIVALSERLNPIIRGWINYFTKFTPSEAFRQGINYV
NLTLVRWLRKTRRKARRSYQKAQRLLHQIAMSNIEMFYHWKVGYIPVK
>R5GH220||gene_177130|GeneMark.hmm|413_aa|+|607|1848
MSESKQYEIPKKVVIEAYKRVKANKGSAGIDGIDFERFEKKLNNNLYKIWNRMSSGSYFPSPVLSVEIPKKAGGTR
RLGIPTITDRIAQMVARMYVEPVVEPMFCDDSYGYRPNKSAIDAIATARKRCWRYDYVIELDVKGLFDNINHELL
MRVVLKHVKEEWICLYIKRWLETPFITREGQVIERLSGTPQGGVISPVLANMYLHYVFDMWMKRNFPQAPFE
RYADDGVIHCRTKEEAFVIKKKLAARFAECKLELHPVKTRVVYCKDKDRTRNEELTEFDFLGYTFKAVYIMCKDGK
VRYNFIASVSKTSSKSFRDKIKAMEVHKRTGCKIDIIAEILNPLIRGWMNYFGKFNPSAMKGTLQCIDRRLVKWA
MCKYKNFRGKRGRAEKWLCTVRQREPKLFAHWSNLYSYC
>R5GH220||gene_166572|GeneMark.hmm|418_aa|+|3219|4475
MSEAKQFDISKKAVIAAFQAVKENAGSYGADEQTIKEFEEHLNNNLYKLWNRMASGSYFPKPVRAVAIPKKNG
GIRILGIPTVEDRIAQMVAKMYFEPLVEPMFYNDSYGYRPNKSAIQAVGQARERCFKRDWVLELDIKGLFDNIK
HGYLMYMVEKHTQIKWLILYIKRWLTVPFIMSDGSVAERRSGTPQGGVISPVLANLFLHYVFDDFMTKAYPNI
WWERYADDGVLHCQSYKQAAFIKQKLEERFQQFGLELNKEKTRIVYCKDNRRPQNYSCTQFTFLGYTFRPRLN
KNKEGKFFVGFTPAVSEKAKTAMKQKIREWKIQLKADLSLKDIGNMINKVVQGWINYYTHYYKSEFYEVLRYIN
QCLIKWVRRSYKKKNTRSRAEHWLGAVARRDRNLFAHWKFGILPSVGEGAV
>R5GH221||gene_211076|GeneMark.hmm|420_aa|+|1684|2946
MNEAKSFVIDKRLVWEAYHKVKENKGSAGIDKVDQKTFDKEMSKNLYKIWNRMSSGCYFPKAVKLVEIPKSNG
GTRPLGIPTIEDRIAQQVVVSVLIPILEPIFKEDSYGYRPGKGAHQAIAKAKERCYVNPWVLDMDISKFFDTINHEL
LMKAIRKHTEEKWVLLYIERWLKVPNQTSKGEVIERTMGVPQGSVIGPVLANLFLHYVFDEWMSRNYPTIPFER
YADDTICHCVSEKQAQFLKAVLMKRFEECGLKLNEEKTKIVYCKDSNRRGDSEHTSFDFLGFTFRPRGARNRKTG
QNFTAFLPAISKKSMKRIKEAVRAWKLNRKTFACLLDISNEVDTQISGWMNYYMKFGRSEFRKVLNYINERLTR
WVMRKYKRFSKGRKFDRAYDWLVEYATHNRNEFSHWVKGFVPYPRLD
>R5GH223||gene_72175|GeneMark.hmm|418_aa|-|182|1438
MSEAKQFDISKKAVIAAFQAVKENAGSYGADEQTIKEFEEHLNNNLYKLWNRMASGSYFPKPVRAVAIPKKNG

GIRILGIPTVEDRIAQMVAKMYFEPLVEPMFYNDSYGYRPNKSAIQAVGQARERCFKRDWVLELDIKGLFDNIK
HGYLMYMVEKHTQIKWLILYIKRWLTVPFIMSDGSVAERRSGTPQGGVISPVLANLFLHYVFDDFMTKAYPNI
WWERYADDGVLHCQSYKQAAFIKQKLEERFQQFGLELNKEKTRIVYCKDNRRPQNYSCTQFTFLGYTFRPRLN
KNKEGKFFVGFTPAVSEKAKTAMKQKIREWKIQLKADLSLKDIGNMINKVVQGWINYYTHYYKSEFYEVLRYIN
QCLIKWVRRSYKKKNTRSRAEHWLGAVARRDRNLFAHWKFGILPSVGEGAV
>R5GH224||gene_191324|GeneMark.hmm|420_aa|-|1074|2336
MNEAKPFVIDKRLVWEAYHKVKENKGSAGIDKVDQKTFDKEMSKNLYKIWNRMSSGCYFPKAVKLVEIPKSNG
GTRPLGIPTIEDRIAQQVVVSVLTPILEPIFKEDSYGYRPGKGAHQAIAKAKERCYVNPWVLDMDISKFFDTINHD
LLMKAVRKHTEEKWVLLYIERWLKVPYQTSKGEVIERTMGVPQGSVIGPVLANLFLHYVFDEWMSRNYPTIPFE
RYADDTICHCVSEKQAQFLKAVLMKRFEECGLKLNEEKTKIVYCKDSNRRGDSEHTSFDFLGFTFRPRSARNRKT
GQNFTAFLPAISKKSLKRIKEAVRAWKLNRKTFACLLDISNEVDTQISGWMNYYMKFGRSEFRKVLNYINERLTR
WVMRKYKRFSKGKKFSRAYEWLVEYAVHNRNEFSHWAKGFVPYPRLG
>R5GH228||gene_276197|GeneMark.hmm|420_aa|+|1684|2946
MNEAKPFVIDKRLVWEAYHKVKENKGSAGIDKVDQKTFDKEMSKNLYKIWNRMSSGCYFPKAVKLVEMPKSN
GGTRPLGIPAIEDRIAQQVVVSVLTPILEPIFKEDSYGYRPGKGAHQAIAKAKERCYVTPWVLDMDISKFFDTINH
ELLMKAIRKHTEEKWVLLYIERWLKVPYQTSKGEVIERTMGVPQGSVIGPVLANLFLHYVFDEWMSRNYPTIPF
ERYADDTICHCVSEKQAQFLKAVLMKRFEECGLKLNEEKTKIVYCKDSNRRGDSEHTSFDFLGFTFRPRGARNRK
TGQNFTAFLPAISKKSMKRIKEAVRAWKLNRKTFACLLDISNEVDTQISGWMNYYMKFGRSEFRKVLNYINERLT
RWVMRKYKRFSKGRKFDRAYDWLVEYATHNRNEFSHWVKGFVPYPRLD
>R5GH229||gene_32096|GeneMark.hmm|420_aa|+|3316|4578
MNEAKPFVIDKRLVWEAYHKVKENKGSAGIDKVDQKTFDKEMSKNLYKIWNRMSSGCYFPKAVKLVEIPKSNG
GTRPLGIPTIEDRIAQQVVVSVLTPILEPIFKEDSYGYRPGKGAHQAIAKAKERCYVNPWVLDMDISKFFDTINHE
LLMKAVRKHTEEKWVLLYIERWLKVPYQTSKGEVIERTMGVPQGSVIGPVLANLFLHYVFDEWMSRNYPTIPFE
RYADDTICHCVSEKQAQFLKAVLMKRFGECGLKLNEEKTKIVYCKDSNRRGDSEHTSFDFLGFTFRPRGARNRKT
GQNFTAFLPAISKKSMKRIKESVRAWKLNRKTFACLLDISNEVDTQISGWMNYYMKFGRSEFRKVLNYINERLTR
WVMRKYKRFSKGKKLGKAYDWLVEYAAHNRNEFSHWVKGFVPYPRLG
>R5GH229||gene_52721|GeneMark.hmm|410_aa|+|515|1747
MQRKSFEIPKALVWASYLDVRRNKGAPGCDGQTLKMFDQQRDGNLYKIWNRLCSGTWFPPPVLEKRIPKPN
GKERILGIPTVSDRIAQGAIKLFMEEKLDPIFHADSYGYRPGKSAHDALKQCAIRCWRYSWILEVDISAFFDHVRH
DLVLKALEHHGMPKWVILYCRRWMEAPMQSCENGELITRTRGTPQGGVISPLLANLFLHYAFDLWMEREYRG
VPFERYADDIVVHCSRMSDATRLKNRLSERFSEVGLVLNAGKTNIAYIDTFKRRNVATSFTFLGYDFKVRTLKNFK
GELYRKCMPGASNAAMRKITETIKKWRIHRSTAESLLDFARRYNAIVRGWIEYYGKFWSRNFNYRLWSAMQSR
LLKWMQSKYRLSNRRAQRKLTLVRKEYPKLFVHWYLLRASNE
>R5GH232||gene_289023|GeneMark.hmm|418_aa|-|514|1770
MSEAKQFDISKKAVIAAFQAVKENAGSYGVDEQTIKEFEEHLNNNLYKLWNRMASGSYFPKPVRAVEIPKKNG
GTRILGIPTVEDRIAQMVAKMYFEPLVEPMFYNDSYGYRPNKSAIQAVGQARERCFKRDWVLELDIKGLFDNIK
HGYLMYMVEKHTQIKWLILYIKRWLTVPFIMSDGSVAERRSGTPQGGVISPVLANLFLHYVFDDFMTKAYPNI
WWERYADDGVLHCQSYKQAVFIKQKLEERFQQFGLELNKEKTRIVYCKDDRRSRNYSCTQFTFLGYTFRPRLNK
NKEGKFFVGFTPAVSEKAKTAMKQKIRGWKIQLKADLSLKDIGNMINKVVQGWINYYTHYYKSEFYEVLRYINQ
CLIKWVRRSYKKKNTRSRAEHWLGAVARRDRNLFAHWKFGILPSVGEGAV
>R5GH233||gene_193417|GeneMark.hmm|418_aa|+|745|2001
MSEAKQFDISKKAVIAAFQAVKENAGSYGADEQTIKEFEEHLNNNLYKLWNRMASGSYFPKPVRAVAIPKKNG
GIRILGIPTVEDRIAQMVAKMYFEPLVEPMFYNDSYGYRPNKSAIQAVGQARERCFKRDWVLELDIKGLFDNIK
HGYLMYMVEKHTQIKWLILYIKRWLTVPFIMSDGSVAERRSGTPQGGVISPVLANLFLHYVFDDFMTKAYPNI

WWERYADDGVLHCQSYKQAAFIKQKLEERFQQFGLELNKEKTRIVYCKDNRRPQNYSCTQFTFLGYTFRPRLN
KNKEGKFFVGFTPAVSEKAKTAMKQKIREWKIQLKADLSLKDIGNMINKVVQGWINYYTHYYKSEFYEVLRYIN
QCLIKWVRRSYKKKNTRSRAEHWLGAVARRDRNLFAHWKFGILPSVGEGAV
>R5GH234||gene_19744|GeneMark.hmm|418_aa|+|1376|2632
MSEAKQFDISKKAVIAAFQAVKENAGSYGADEQTIKEFEEHLNNNLYKLWNRMASGSYFPKPVRAVAIPKKNG
GIRILGIPTVEDRIAQMVAKMYFEPLVEPMFYNDSYGYRPNKSAIQAVGQARERCFKRDWVLELDIKGLFDNIK
HGYLMYMVEKHTQIKWLILYIKRWLTVPFIMSDGSVAERRSGTPQGGVISPVLANLFLHYVFDDFMTKAYPNI
WWERYADDGVLHCQSYKQAAFIKQKLEERFQQFGLELNKEKTRIVYCKDNRRPQNYSCTQFTFLGYTFRPRLN
KNKEGKFFVGFTPAVSEKAKTAMKQKIREWKIQLKADLSLKDIGNMINKVVQGWINYYTHYYKSEFYEVLRYIN
QCLIKWVRRSYKKKNTRSRAEHWLGAVARRDRNLFAHWKFGILPSVGEGAV
>R5OH003||gene_90128|GeneMark.hmm|382_aa|-|1428|2576
MTAPRGAFEKDLKNNLYKIWNRMSSGSYFPPPVRAVEMPKPSGGVRVLGVPTVADRVAQTVVAEKLEKRVEPI
FHPDSYGYRPGRSALDAVGQCRRRCWNRAWVVDLDIARFFDEVDHQLLLKAVAGHAPEPWVLLYISRWLKAP
IQHGDGTIAQRSRGTPQGSAVSPVLANLFLHYAFDMWMARRFPTVQFERYVDDVVVHCVTERQAREVREAV
EGRLARVGLRMHPDKTRIVYCRTQKRKGDHPEVSFDFLGYTFRPRAARDGKGGIFTSFLPAISKSALKRLSARVRS
WRLHLRTGSTLTGLARTINPIVRGWMQYYGRFYRTALYPLLKTHQRLLGALAAEEVQTAADLQEGQSGLEEGDP
SVPLLLSHWAWVQSFW
>R5OH003||gene_39089|GeneMark.hmm|430_aa|+|534|1826
MSGSVKLAGSSVRSSGSAVKPFDVPKWLVMEAWEKVRSDKGAPGVDGAAVEDFEKDLRASLYKIWNRMSSG
SYFPSPVREVRIPKPDGGIRVLGVPTVAGRLAQTVVAMVLEHRAERVFHPGSYGYRPGRGAIDAVRACRRRCW
ENDWVIDLDIQAFFDTVPWDLVCRAVGAVCDLPWVMLYVRRWLKAPLQHSDGTLTERERGTPQGSAVSPVL
ANLFMHYAFDTWMARSYPGIVFERYADDVVIHCKSLNQARVVLTAVEERMGQVGLGLHPRKTRIVYCKDANR
PGSWEHTGFTFLGYEFRERTVKGRHGLFRSFSPAVSRTALKRMSTQVRSWRLHRWVTATAGDLAAQINPVLRG
WMSYYGVFHPSALYPLLKRVNSYLIRWLRGKYRKLRVSWPKTMRKWYTGVKKAPNYFVHWAWVTEPGPVW
>R5OH004||gene_7757|GeneMark.hmm|421_aa|+|2497|3762
VTPRPSKTAGKAFDIPKALVVEAWERVRSNKGAPGVDGEAIEDFEKDLKNNLYKIWNRMSSGSYFPPPVRQVRI
PKPDGGIRVLGVPTVADRLAQTVVAQVLERRAEPVFHRDSYGYRPGRGAIDAVAACRRRCWENNWVIDMDI
QAFFDTVPWDLVCRAVETVCDLPWVMLYVRRWLKAPAQQGDGTLTERRRGTPQGSAVSPVLANLFMHYALD
AWLTRNFPGVVFERYADDVVIHCKSLEQARTVLAAVTERMRRVGLRLHPGKTRIVYCKDANRTGSWEHTEFTF
LGYEFRERTVKGRHGLFRSFSPAVSRAALKRMSAAVRSWRLHRWVTATAGDLAARVNPVVRGWMCYYGAFH
PSALYPLLRRINSYMVRWLRGKYRRLRASWARTMRKWYAGVKKAPGYFVHWAWVTEPGPVW
>R5OH004||gene_94629|GeneMark.hmm|416_aa|+|618|1868
MSGPQLSGKPFDISKQEVWRAYQKVKANKGAPGVDDCSVEAFEKDLKNNLYKIWNRMSSGSYFPPPVRAVE
MPKPSGGVRVLGVPTVADRVAQTVVAEKLEKRVEPIFHPDSYGYRPGRSALDAVGQCRRRCWNRAWVVDLDI
ARFFDEVDHQLLLKAVAGHAPEPWVLLYISRWLKAPIQHGDGTIAQRSRGTPQGSAVSPVLANLFLHYAFDM
WMARRFPTVQFERYVDDVVVHCVTERQAREVREAVEGRLARVGLRMHPDKTRIVYCRTQKRKGDHPEVSFD
FLGYTFRPRAARDGKGGIFTSFLPAISKSALKRLSARVRSWRLHLRTGSTLTGLARTINPIVRGWMQYYGRFYRTA
LYPLLKRINAYLVRWLRKKYKRLRTFKKAKAAWRRVTRQCPLLLSHWAWVQSFW
>R5OH006||gene_134534|GeneMark.hmm|430_aa|-|1061|2353
MSGSVKLAGSSVRSSGSAVKPFDVPKWLVMEAWEKVRSNKGAPGVDGAAVEDFEKDLRANLYKIWNRISSGS
YFPSPVREVRIPKPDGGIRVLGVPTVADRLAQTVVAMVLEHRAERVFHPGSYGYRPGRGAIDAVRACRRRCWE
NDWVIDLDIQAFFDTVPWDLVCRAVGAVCDLPWVMLYVRRWLKAPLQHSDGTLTERERGTPQGSAVSPVLA
NLFMHYAFDTWMARSYPGIVFERYADDVVIHCKSLNQARVVLTAVEERMGQVGLGLHPRKTRIVYCKDANRP
GSWEHTGFTFLGYEFRERTVKGRHGLFRSFSPAVSRTALKRMSTQVRSWRLHRWVTATAGDLAAQINPVLRG

WMSYYGVFQPSALYPLLKRVNSYLIRWLRGKYRKLRVSWSKTMRKWYTGVKKAPNYFVHWAWVTEPGPVW
>R5OH010||gene_265742|GeneMark.hmm|416_aa|+|1883|3133
MSGPQLSGKPFDISKQEVRRAYQKVKANKGAPGVDDCSVEAFEKDLKNNLYKIWNRMSSGSYFPPPVRAVEM
PKPSGGVRVLGVPTVADRVAQTVVAEKLEKRVEPIFHPDSYGYRPGRSALDAVGQCRRRCWNRAWVVDLDIA
RFFDEVDHQLLLKAVAGHAPEPWVLLYISRWLKAPIQHGDGTITQRSRGTPQGSAVSPVLANLFLHYAFDMW
MARRFPTVQFERYVDDVVVHCVTERQAREVREAVEGRLARVGLRMHPDKTRIVYCRTQKRRGDHPEVSFDFL
GYTFRPRAARDGKGGIFTSFLPAISKSALKRLSARVRSWRLHLRTGSTLTGLARTINPIVRGWMQYYGRFYRTALY
PLLKRINAYLVRWLRKKYKRLRTFKKAKAAWRRVTRQCPLLLSHWAWVQSFW
>R5OH012||gene_169793|GeneMark.hmm|382_aa|+|888|2036
MTAPRGAFEKDLKNNLYKIWNRMSSGSYFPPPVRAVEMPKPSGGVRVLGVPTVADRVAQTVVAEKLEKRVEPI
FHPDSYGYRPGRSALDAVGQCRRRCWNRARVVDLDIARFFDEVDHQLLLKAVAGHAPEPWVLLYISRWLKAPI
QHGDGTIAQRSRGTPQGSAVSPVLANLFLHYAFDMWMARRFPTVQFERYVDDVVVHCVTERQAREVREAVE
GRLARVGLRMHPDKTRIVYCRTQKRKGDHPEVSFDFLGYTFRPRAARDGKGGIFTSFLPAISKSALKRLSARVRS
WRLHLRTGSTLTGLARTINPIVRGWMQYYGRFYRTALYPLLKTHQRLLGALAAEEVQTAADLQEGQSGLEEGDP
SVPLLLSHWAWVQSFW
>R5OH016||gene_312236|GeneMark.hmm|416_aa|+|1297|2547
MSGPQLSGKPFDISKQEVWRAYQKVKANKGAPGVDDCSVEAFEKDLKNNLYKIWNRMSSGSYFPPPVRAVE
MPKPSGGVRVLGVPTVADRVAQTVVAEKLEKRVEPIFHPDSYGYRPGRSALDAVGQCRRRCWNRAWVVDLDI
ARFFDEVDHQLLLKAVAGHAPEPWVLLYISRWLKAPIQHGDGTIAQRSRGTPQGSAVSPVLANLFLHYAFDM
WMARRFPTVQFERYVDDVVVHCVTERQAREVREAVEGRLARVGLRMHPDKTRIVYCRTQKRKGDHPEVSFD
FLGYTFRPRAARDGKGGIFTSFLPAISKSALKRLSARVRSWRLHLRTGSTLTGLARTINPIVRGWMQYYGRFYRTA
LYPLLKRINAYLVRWLRKKYKRLRTFKKAKAAWRRVTRQCPLLLSHWAWVQSFW
>R5OH018||gene_96268|GeneMark.hmm|416_aa|+|1301|2551
MSGPQLSGKPFDISKQEVWRAYQKVKANKGAPGVDDCSVEAFEKDLKNNLYKIWNRMSSGSYFPPPVRAVE
MPKPSGGVRVLGVPTVADRVAQTVVAEKLEKRVEPIFHPDSYGYRPGRSALDAVGQCRRRCWNRAWVVDLDI
ARFFDEVDHQLLLKAVAGHAPEPWVLLYISRWLKAPIQHGDGTIAQRSRGTPQGSAVSPVLANLFLHYAFDM
WMARRFPTVQFERYVDDVVVHCVTERQAREVREAVEGRLARVGLRMHPDKTRIVYCRTQKRKGDHPEVSFD
FLGYTFRPRAARDGKGGIFTSFLPAISKSALKRLSARVRSWRLHLRTGSTFTGLARTINPIVRGWMQYYGQFYRTA
LYPLLKRINAYLVRWLRKKYKRLRTFKKAKAAWRRVTRQCPLLLSHWAWVQSFW
>R5OH022||gene_71035|GeneMark.hmm|388_aa|+|4099|5265
MSGSVKLAGSSVRSSGSAVKPFDVPKWLVMAAWEKVRSNKGAPGVDGAAVEDFEKDLRANLYKIWNRMSS
GSYFPSPVREVRIPKPDGGIRVLGVPTVAGRLAQTVVAMVLEHRAERVFHPGSYGYRPGRGAIDAVRACRRRC
WENDWVIDLDIQAFFDTVPWDLVCRAVGAVCDLPWVMLYVRRWLKAPLQHSDGTLTERERGTPQGSAVSPV
LANLFMHYAFDTWMARSYPGIVFERYSDDVVIHCKSLNQARVVLTAVEERMGQVGLGLHPRKTRIVYCKDANR
PGSWEHTGFTFLGYEFRERTVKGRHGLFRSFSPAVSRTTLKRMSTQVRSWRLHRWVTATAGDLAAQINPVLRG
WMSYYGVFQPSALYPGGRQSGWVRAAES
>R5OH024||gene_118244|GeneMark.hmm|422_aa|+|1698|2966
MSRSLSEVPGGKSFDISKQLVMDAWQRVKSDRGALGVDECSVEEFEEDLAGNLYKIWNRMFSGSCFPPAVRM
VEIPRPQAGTRVLGVPTVADRVAQTAAMVLEKAAEPVFHPDSYGYRPGRGAIDAVAACRRRCWSRSWVIDL
DIEAFFDSVPWDLVCKAVGAVGAPGWVMLYVKRWLAAPLQHPDGAVVERVRGTPQGSAVSPVLANLFMHY
AFDAWLARAFPGVVFERYADDAVIHCRSLTQARGVLAALEARMKQVGLRLHPRRTRIVYCKDANRPGCYEHTG
FTFLGYEFTERTVKGRHGLFRSFSPAASKTALKTMSARLRSWRLHRWVRATARDLAEHINPIMRGWMSYYGAF
HPSALYPLLKRVSSYLIRWLRGKYRRLRRSWRATMRKWYGGIKAAPGYFVHWAWITGPGPLW
>R5OH024||gene_40840|GeneMark.hmm|421_aa|+|709|1974

VAPRPSKTAGKAFDIPKALVVEAWERVRSNKGAPGADGAAIEDFERDLQANLYKIWNRMSSGSYFPPPVRQVR
IPKPDGGIRVLGVPTVADRLAQTVVALVLERRAEPVFHQGSYGYRPGRGAIDAVAACRRRCWESSWVIDMDIQ
AFFDTVPWDLVCRAVATVCDLPWVMLYVRRWLKAPAQQGDGTLTERWRGTPQGSAVSPVLANLFMHYALD
AWLARNFPGVVFERYADDVVIHCKSLEQARAVLAAVAERMRQVGLRLHPRKTRIVYCKDANRTGSWEHTEFTF
LGYEFRERTVKGRHGLFRSFSPAVSKAALKRMSATVRSWRLHRWVTATVSDLAAHVNPVVRGWMRYYGAFHP
SALYPLLRRINSYLVRWLRGKYRRLRASWARTMRKWYTGVKKAPSYFAHWAWVTEPGPVW
>R5OH029||gene_630|GeneMark.hmm|416_aa|+|995|2245
MSGPQLSGKPFDISKQEVWRAYQKVKANKGAPGVDDCSVEAFEKDLKNNLYKIWNRMSSGSYFPPPVRAVE
MPKPSGGVRVLGVPTVADRVAQTVVAEKLEKRVEPIFHPDSYGYRPGRSALDAVGQCRRRCWNRAWVVDLDI
ARFFDEVDHQLLLKAVAGHAPEPWVLLYISRWLKAPIQHGDGTIAQRSRGTPQGSAVSPVLANLFLHYAFDM
WMARRFPTVQFERYVDDVVVHCVTERQAREVREAVEGRLARVGLRMHPDKTRIVYCRTQKRKGDHPEVSFD
FLGYTFRPRAARDGKGGIFTSFLPAISKSALKRLSARVRSWRLHLRTGSTLTGLARTINPIVRGWMQYYGRFYRTA
LYPLLKTHQRLLGALAAEEVQTAADLQEGQSGLEEGDPSVPLLLSHWAWVQSFW
>R5OH031||gene_458068|GeneMark.hmm|430_aa|-|973|2265
MSGSVKLAGSSVRSSGSAVKPFDVPKWLVMAAWEKVRSNKGAPGVDGAAVEDFEKDLRANLYKIWNRMSS
GSYFPSPVREVRIPKPDGGIRVLGVPTVADRLAQTVVAMVLEHRAERVFHPGSYGYRPGRGAIDAVRSCRRRC
WENDWVIDLDIQAFFDTVPWDLVCRAVGAVCDLPWVMLYVRRWLKAPLQHSDGTLTERERGTPQGSAVSPV
LANLFMHYAFDTWMARSYPGIVFERYADDVVIHCKSLNQARVVLTAVEERMGQVGLGLHPRKTRIVYCKDAN
RPGSWEHIGFTFLGYEFRERTVKGRHGLFRSFSPAVSRTALKRMSTQVRSWRLHRWVTAMAGDLAAQINPVLR
GWMSYYGVFHPSALYPLLKRVNSYLIRWLRGKYRKLRVSWSKTMRKWYTGVKKAPNYFVHWAWVTEPGPV
W
>R5OH031||gene_119851|GeneMark.hmm|421_aa|+|1400|2665
VAPRPSKTAGKAFDIPKALVVEAWERVRNNKGAPGVDGAAIEDFERDLQANLYKIWNRMSSGSYFPPPVRQV
RIPKPDGGIRVLGVPTVADRLAQTVVALVLERRAEPVFHQGSYGYRPGRGAIDAVAACRRRCWESNWVIDMDI
QAFFDTVPWDLVCRAVATVCDLPWVMLYVRRWLKAPAQQGDGTLTERWRGTPQGSAVSPVVANLFMHYAL
DAWLARNFPGVVFERYADDVVIHCKSLEQARAVLAAVAERMRQVGLRLHPRKTRIVYCKDANRTGSWEHTEF
TFLGYEFRERTVKGRHGLFRSFSPAVSRAALKRMSATVRSWRLHRWVTATVRDLAAHVNPVVRGWMRYYGAF
HPSALYPLLRRINSYLVRWLRGKYRRLRASWARTMRKWYTGVKKAPSYFAHWAWVTEPGPVW
>R5OH031||gene_271515|GeneMark.hmm|363_aa|-|2|1090
MTAPRGAFEKDLKNNLYKIWNRMSSGSYFPPPVRAVEMPKPSGGVRVLGVPTVADRVAQTVVAEKLEKRVEPI
FHPDSYGYRPGRSALDAVGQCRRRCWNRAWVVDLDIARFFDEVDHQLLLKAVAGHAPEPWVLLYISRWLKAP
IQHGDGTIAQRSRGTPQGSAVSPVLANLFLHYAFDMWMARRFPTVQFERYVDDVVVHCVTERQAREVREAV
EGRLARVGLRMHPDKTRIVYCRTQKRKGDHPEVSFDFLGYTFRPRAARDGKGGIFTSFLPAISKSALKRLSARVRS
WRLHLRTGSTLTGLARTINPIVRGWMQYYGRFYRTALYPLLKRINAYLVRWLRKKYKRLRTFKKAKAAWRR
>R5OH034||gene_153869|GeneMark.hmm|416_aa|+|587|1837
MSGPQLSGKPFDISKQEVWRAYQKVKANKGAPGVDDCSVEAFEKDLKNNLYKIWNRMSSGSYFPPPVRAVE
MPKPSGGVRVLGVPTVADRVAQTVVAEKLEKRVEPIFHPDSYGYRPGRSALDAVGQCRRRCWNRAWVVDLDI
ARFFDEVDHQLLLKAVAGHAPEPWVLLYISRWLKAPIQHGDGTIAQRSRGTPQGSAVSPVLANLFLHYAFDM
WMARRFPTVQFERYVDDVVVHCVTERQAREVREAVEGRLARVGLRMHPDKTRIVYCRTQKRKGDHPEVSFD
FLGYTFRPRAARDGKGGIFTSFLPAISKSALKRLSARVRSWRLHLRTGSTLTGLARTINPIVRGWMQYYGRFYRTA
LYPLLKRINAYLVRWLRKKYKRLRTFKKAKAAWRRVTRQCPLLLSHWAWVQSFW
>R5OH036||gene_118280|GeneMark.hmm|420_aa|-|64|1326
VQATAKPLPISKRQVWEAYRQVKANGGAAGIDGQTVEAFDEDMANNLYKLWNRLASGSYMPPAVKRVDIPK
AGGGTRPLGVPTVADRIAQTVIRQMLEPIVEPLFHEDSYGYRPGKSAHQALAQTRRRCWRYAWVVEIDIKGFF

DNIDHALLLKAVRHHTRERWVVMYIERWLRAPVQMPDGTIQQREKGTPQGGVISPLLANLFLHYAFDMWM
QRHHGDVPFERYADDAVCHCHSQVRAQSLIDHLRERFAQCGLELHPQKTRVVFCKDSNRRGDYPDTSFDFLGF
TFRPRLSRGRDGRLFVGFNPAVSAKAAKSIRQEVRSWRLQLRSDKALDDLARMFNAKIRGWVNYYGAFYKSAL
YSTLRKIDFKLVLWATRKFKRLRGRRRRARHWLARIARRNPQLFAHWPLLWGQASMGRAG
>R5OH036||gene_126182|GeneMark.hmm|416_aa|-|1043|2293
MSGPQLSGKPFDISKQEVWRAYQKVKANKGAPGVDDCSVEAFEKDLKNNLYKIWNRMSSGSYFPPPVRAVE
MPKPSGGVRVLGVPTVADRVAQTVVAEKLEKRVEPIFHPDSYGYRPGRSALDAVGQCRRRCWNRAWVVDLDI
ARFFDEVDHQLLLKAVAGHAPEPWVLLYISRWLKAPIQHGDGTIAQRSRGTPQGSAVSPVLANLFLHYAFDM
WMARRFPTVQFERYVDDVVVHCVTERQAREVREAVEGRLARVGLRMHPDKTRIVYCRTQKRRGDHPEVSFD
FLGYTFRPRAARDGKGGIFTSFLPAISKSALKRLSARVRSWRLHLRTGSTLAGLARTINPIVRGWMQYYGRFYRTA
LYPLLKRINAYLVRWLRKKYKRLRTFKKAKAAWRRVTRQCPLLLSHWAWVQSFW
>R5OH038||gene_47404|GeneMark.hmm|421_aa|+|2574|3839
VAPRPSKTAGKAFDIPKALVVEAWERVRNNKGAPGVDGAAIEDFERDLQANLYKIWNRMSSGSYFPPPVRQV
RIPKPDGGIRVLGVPTVADRLAQTVVALVLERRAEPVFHQGSYGYRPGRGAIDAVAACRRRCWESSWVIDMDI
QAFFDTVPWDLVCRAVAMVCDLPWVMLYVRRWLKAPAQQGDGTLTERWRGTPQGSAVSPVLANLFMHYA
LDAWLARNFPGVVFERYADDVVIHCKSLEQARAVLAAVTERMRQVGLRLHPRKTRIVYCKDANRTGSWEHTEF
TFLGYEFRERTVKGRHGLFRSFSPAVSRAALKRMSATVRSWRLHRWVTATVSDLAAHVNPVVRGWMRYYGAF
HPSALYPLLRRINSYLVRWLRGKYRRLRASWARTMRKWYTGVKKAPSYFAHWAWVTEPGPVW
>R5OH040||gene_106439|GeneMark.hmm|430_aa|-|8946|10238
MSGSVKLAGSSVRSSGSAVKPFDVPKWLVMAAWEKVRSNKGAPGVDGAAVEDFEKDLRANLYKIWNRMSS
GSYFPSPVREVRIPKPDGGIRVLGVPTVADRLAQTVVAMVLEHRAERVFHPGSYGYRPGRGAIDAVRSCRRRC
WENDWVIDLDIQAFFDTVPWDLVCRAVGAVCDLPWVMLYVRRWLKAPLQHSDGTLTERERGTPQGSAVSPV
LANLFMHYAFDTWMARSYPGIVFERYADDVVIHCESLNQARVVLTAVEERMGQVGLGLHPRKTRIVYCKDANR
PGSWEHIGFTFLGYEFRERTVKGRHGLFRSFSPAVSRTALKRMSTQVRSWRLHRWVTAMAGDLAAQINPVLRG
WMSYYGVFHPSALYPLLKRVNSYLIRWLRGKYRKLRVSWSKTMRKWYTGVKKAPNYFVHWAWVTEPGPVW
>R5OH042||gene_86823|GeneMark.hmm|430_aa|-|778|2070
MSGSVKLAGSSVRSSGSAVKPFDVPKWLVMEAWEKVRSNKGAPGVDGAAVEDFEKDLRANLYKIWNRISSGS
YFPSPVREVRIPKPDGGIRVLGVPTVADRLAQTVVAMVLEHRAERVFHPGSYGYRPGRGAIDAVRACRRRCWE
NDWVIDLDIQAFFDTVPWDLVCRAVGAVCDLPWVMLYVRRWLKAPLQHSDGTLTERERGTPQGSAVSPVLA
NLFMHYAFDTWMARSYPGIVFERYADDVVIHCKSLNQARVVLTAVEERMGQVGLGLHPRKTRIVYCKDANRP
GSWEHTGFTFLGYEFRKRTVKGRHGLFRSFSPAVSRTALKRMSTQVRSWRLHRWVTATAGDLAAQINPVLRG
WMSYYGVFHPSALYPLLKRVNSYLIRWLRGKYRKLRVSWSKTMRKWYTGVKKAPNYFVHWAWVTEPGPVW
>R5OH044||gene_4914|GeneMark.hmm|416_aa|-|211|1461
MSGPQLSGKPFDISKQEVWRAYQKVKANKGAPGVDDCSVEAFEKDLKNNLYKIWNRMSSGSYFPPPVRAVE
MPKPSGGVRVLGVPTVADRVAQTVVAEKLEKRVEPIFHPDSYGYRPGRSALDAVGQCRRRCWNRAWVVDLDI
ARFFDEVDHQLLLKAVAGHAPEPWVLLYISRWLKAPIQHGDGTIAQRSRGTPQGSAVSPVLANLFLHYAFDM
WMARRFPTVQFERYVDDVVVHCVTERQAREVREAVEGRLARVGLRMHPDKTRIVYCRTQKRKGDHPEVSFD
FLGYTFRPRAARDGKGGIFTSFLPAISKSALKRLSARVRSWRLHLRTGSTLTGLARTINPIVRGWMQYYGRFYRTA
LYPLLKRINAYLVRWLRKKYKRLRTFKKAKAAWRRVTRQCPLLLSHWAWVQSFW
>R5OH044||gene_70094|GeneMark.hmm|421_aa|-|736|2001
VTPRPSKTAGKAFDIPKALVVEAWERVRSNKGAPGVDGEAIEDFEKDLKNNLYKIWNRMSSGSYFPPPVRQVRI
PKPDGGIRVLGVPTVADRLAQTVVAQVLERRAEPVFHRDSYGYRPGRGAIDAVAACRRRCWENNWVIDMDI
QAFFDTVPWDLVCRAVETVCDLPWVMLYVRRWLKAPAQQGDGTLTERRRGTPQGSAVSPVLANLFMHYALD
AWLTRNFPGVVFERYADDVVIHCKSLEQARTVLAAVTERMRRVGLRLHPGKTRIVYCKDANRTGSWEHTEFTF

LGYEFRERTVKGRHGLFRSFSPAVSRAALKRMSAAVRSWRLHRWVTATAGDLAARVNPVVRGWMCYYGAFH
PSALYPLLRRINSYMVRWLRGKYRRLRASWARTMRKWYAGVKKAPGYFVHWAWVTEPGPVW
>R5OH046||gene_107295|GeneMark.hmm|416_aa|+|1017|2267
MSGPQLSGKPFDISKQEVWRAYQKVKANKGAPGVDDCSVEAFEKDLKNNLYKIWNRMSSGSYFPPPVRAVEI
PKPSGGVRVLGVPTVADRVAQTVVAEKLEKRVEPIFHPDSYGYRPGRSALDAVGQCRRRCWNRAWVVDLDIA
RFFDEVDHQLLLKAVAGHAPEPWVLLYISRWLKAPIQHGDGTIAQRSRGTPQGSAVSPVLANLFLHYAFDMW
MARRFPTVQFERYVDDVVVHCVTERQAREVREAVEGRLARVGLRMHPDKTQIVYCRTQKRRGDHPEVSFDFL
GYTFRPRAARDGKGGIFTSFLPAISKSALKRLSARVRSWRLHLRTGSTFTGLARTINPIVRGWMQYYGRFYRTALY
PLLKRINAYLVRWLRKKYKRLRTFKKAKAAWRRVTRQCPLLLSHWAWVQSFW
>R5OH047||gene_225114|GeneMark.hmm|430_aa|+|2498|3790
MSGSVKLAGSSVRSSGSAVKPFDVPKWLVMAAWEKVRSNKGAPGVDGAAVEDFEKDLRANLYKIWNRMSS
GSYFPSPVREVRIPKPDGGIRVLGVPTVAGRLAQTVVAMVLEHRAERVFHPGSYGYRPGRGAIDAVRSCRRRC
WENDWVIDLDIQAFFDTVPWDLVCRVVGAVCDLPWVMLYVRRWLKAPLQHSDGTLTERERGTPQGSAVSPV
LANLFMHYAFDTWMARSYPGIVFERYADDVVIHCESLNQARVVLTAVEERMGQVGLGLHPRKTRIVYCKDANR
PGSWEHTGFTFLGYEFRERTVKGRHGLFRSFSPAVSRTALKRMSTQVRSWRLHRWVTATAGDLAAQINPVLRG
WMSYYGVFHPSALYPLLKRVNSYLIRWLRGKYRKLRVSWSKTMRKWYTGVKKAPNYFVHWAWVTEPGPVW
>R5OH048||gene_236773|GeneMark.hmm|430_aa|+|888|2180
MSGSVKLAGSSVRSSGSAVKPFDVPKWLVMAAWEKVRSNKGAPGVDGAAVEDFEKDLRANLYKIWNRMSS
GSYFPSPVREVRIPKPDGGIRVLGVPTVADRLAQTVVAMVLEHRAERVFHPGSYGYRPGRGAIDAVRSCRRRC
WENDWVIDLDIQAFFDTVPWDLVCRAVGAVCDLPWVMLYVRRWLKAPLQHSDGTLTERERGTPQGSAVSPV
LANLFMHYAFDTWMARSYPGIVFERYADDVVIHCESLNQARVVLTAVEERMGQVGLGLHPRKTRIVYCKDANR
PGSWEHTGFTFLGYEFRERTVKGRHGLFRSFSPAVSRTALKRMSTQVRSWRLHRWVTATAGDLAAQINPVLRG
WMSYYGVFHPSALYPLLKRVNSYLIRWLRGKYRKLRVSWSKTMRKWYTGVKKAPNYFVHWAWVTEPGPVW
>R5OH048||gene_325690|GeneMark.hmm|421_aa|+|610|1875
VAPRPSKTAGKAFDIPKALVVEAWERVRSNKGAPGADGAAIEDFERDLQANLYKIWNRMSSGSYFPPPVRQVR
IPKPDGGIRVLGVPTVADRLAQTVVALVLERRAEPVFHQGSYGYRPGRGAIDAVAACRRRCWESSWVIDMDIQ
AFFDTVPWDLVCRAVETVCDLPWVMLYVRRWLMAPAQQGDGTLTERWRGTPQGSAVSPVLANLFMHYALD
AWLARNFAGVVFERYADDVVIHCKSLEQARAVLAAVAERMRQVGLRLHPRKTRIVYCKDANRTGSWEHTEFTF
LGYEFRERTVKGRHGLFRSFSPAVSKAALKRMSATVRSWRLHRWVTATVSDLAAHVNPVVRGWMRYYGAFHP
SALYPLLRRINSYLVRWLRGKYRRLRASWARTMRKWYTGVKKAPNYFAHWAWVTEPGPVW
>R5OH048||gene_105895|GeneMark.hmm|416_aa|-|147|1397
MSGPQLSGKPFDISKQEVWRAYQKVKANKGAPGVDDCSVEAFEKDLKNNLYKIWNRMSSGSYFPPPVRAVE
MPKPSGGVRVLGVPTVADRVAQTVVAEKLEKRVEPIFHPDSYGYRPGRSALDAVGQCRRRCWNRAWVVDLDI
ARFFDEVDHQLLLKAVAGHAPEPWVLLYISRWLKAPIQHGDGTIAQRSRGTPQGSAVSPVLANLFLHYAFDM
WMARRFPTVQFERYVDDVVVHCVTERQAREVREAVEGRLARVGLRMHPDKTRIVYCRTQKRKGDHPEVSFD
FLGYTFRPRAARDGKGGIFTSFLPAISKSALKRLSARVRSWRLHLRTGSTLTGLARTINPIVRGWMQYYGRFYRTA
LYPLLKRINAYLVRWLRKKYKRLRTFKKAKAAWRRATRQCPLLLSHWAWVQSFW
>R5OH050||gene_28647|GeneMark.hmm|430_aa|+|575|1867
MSGSVKLAGSSVRSSGSAVKPFDVPKWLVMEAWEKVRSDKGAPGVDGAAVEDFEKDLRASLYKIWNRMSSG
SYFPSPVREVRIPKPDGGIRVLGVPTVAGRLAQTVVAMVLEHRAERVFHPGSYGYRPGRGAIDAVRACRRRCW
ENDWVIDLDIQAFFDTVPWDLVCRAVGAVCDLPWVMLYVRRWLKAPLQHSDGTLTERERGTPQGSAVSPVL
ANLFMHYAFDTWMARSYPGIVFERYADDVVIHCKSLNQARVVLTAVEERMGQVGLGLHPRKTRIVYCKDANR
PGSWEHTGFTFLGYEFRERTVKGRHGLFRSFSPAVSRMALKRMSTQVRSWRLHRWVTATAGDLAAQINPVLR
GWMSYYGVFHPSALYPLLKRVNSYLIRWLRGKYRKLRVSWSKTMRKWYTGVKKAPNYFVHWAWVTEPGPV

W
>R5OH050||gene_23285|GeneMark.hmm|382_aa|+|966|2114
MTAPRGAFEKDLKNNLYKIWNRMSSGSYFPPPVRAVEMPKPSGGVRVLGVPTVADRVAQTVVAEKLEKRVEPI
FHPDSYGYRPGRSALDAVGQCRRRCWNRAWVVDLDIARFFDEVDHQLLLKAVAGHAPEPWVLLYISRWLKAP
IQHGDGTIAQRSRGTPQGSAVSPVLANLFLHYAFDMWMARRFPTVQFERYVDDVVVHCVTERQAREVREAV
EGRLARVGLRMHPDKTRIVYCRTQKRKGDHPEVSFDFLGYTFRPRAARDGKGGIFTSFLPAISKSALKRLSARVRS
WRLHLRTGSTLTGLARTINPIVRGWMQYYGRFYRTALYPLLKRINAYLVRWLRKKYKRLRTFKKAKAAWRRVTR
QCPLLLSHWAWVQSFW
>R5OH052||gene_113837|GeneMark.hmm|416_aa|-|429|1679
MSGPQLSGKPFDISKQEVWRAYQKVKANKGAPGVDDCSVEAFEKDLKNNLYKIWNRMSSGSYFPPPVRAVE
MPKPSGGVRVLGVPTVADRVAQTVVAEKLEKRVEPIFHPDSYGYRPGRSALDAVGQCRRRCWNRAWVVDLDI
ARFFDEVDHQLLLKAVAGHAPEPWVLLYISRWLKAPIQHGDGTIAQRSRGTPQGSAVSPVLANLFLHYAFDM
WMARRFPTVQFERYVDDVVVHCVTERQAREVREAVEGRLARVGLRMHPDKTRIVYCRTQKRKGDHPEVSFD
FLGYTFRPRAARDGKGGIFTSFLPAISKSALKRLSARVRSWRLHLRTGSTFTGLARTINPIVRGWMQYYGRFYRTA
LYPLLKRINAYLVRWLRKKYKRLRTFKKAKAAWRRVTRQCPLLLSHWAWVQSFW
>R5OH053||gene_155433|GeneMark.hmm|430_aa|+|4849|6141
MSGSVSLVGVAPRPSKTAGKAFDIPKALVVEAWERVRSNKGAPGADGAAIEDFERDLQANLYKIWNRMSSGSY
FPPPVRQVRIPKPDGGIRVLGVPTVADRLAQTVVALVLERRAEPVFHQGSYGYRPGRGAIDAVAACRRRCWESS
WVIDMDIQAFFDTVPWDLVCRAVETVCDLPWVMLYVRRWLMAPAQQGDGTLTERWRGTPQGSAVSPVLA
NLFMHYALDAWLARNFAGVVFERYADDVVIHCKSLEQARAVLAAVAERMRQVGLRLHPRKTRIVYCKDANRT
GSWEHTEFTFLGYEFRERTVKGRHGLFRSFSPAVSKAALKRMSATVRSWRLHRWVTATVSDLAAHVNPVVRG
WMRYYGAFHPSALYPLLRRINSYLVRWLRGKYRRLRASWARTMRKWYTGVKKAPNYFAHWAWVTEPGPVW
>R5OH055||gene_102246|GeneMark.hmm|430_aa|+|814|2106
MSGSVKLAGSSVRSSGSAVKPFDVPKWLVMEAWEKVRSNKGAPGVDGAAVEDFEKDLRANLYKIWNRISSGS
YFPSPVREVRIPKPDGGIRVLGVPTVADRLAQTVVAMVLEHRAERVFHPGSYGYRPGRGAIDAVRACRRRCWE
NDWVIDLDIQAFFDTVPWDLVCRAVGAVCDLPWVMLYVRRWLKAPLQHSDGTLTERERGTPQGSAVSPVLA
NLFMHYAFDTWMARSYPGIVFERYADDVVIHCKSLNQARVVLTAVEERMGQVGLGLHPRKTRIVYCKDANRP
GSWEHTGFTFLGYEFRERTVKGRHGLFRSFSPAVSRTALKRMSTQVRSWRLHRWVTATAGDLAAQINPVLRG
WMSYYGVFQPSALYPLLKRVNSYLIRWLRGKYRKLRVSWSKTMRKWYTGVKKAPNYFVHWAWVTEPGPVW
>R5OH055||gene_118691|GeneMark.hmm|416_aa|-|782|2032
MSGPQLSGKPFDISKQEVWRAYQKVKANKGAPGVDDCSVEAFEKDLKNNLYKIWNRMSSGSYFPPPVRAVE
MPKPSGGVRVLGVPTVADRVAQTVVAEKLEKRVEPIFHPDSYGYRPGRSALDAVGQCRRRCWNRAWVVDLDI
ARFFDEVDHQLLLKAVAGHAPEPWVLLYISRWLKAPIQHGDGTIAQRSRGTPQGSAVSPVLANLFLHYAFDM
WMARRFPTVQFERYVDDVVVHCVTERQAREVREAVEGRLARVGLRMHPDKTRIVYCRTQKRKGDHPEVSFD
FLGYTFRPRAARDGKGGIFTSFLPAISKSALKRLSARVRSWRLHLRTGSTLTGLARTINPIVRGWMQYYGRFYRTA
LYPLLKRINAYLVRWLRKKYKRLRTFKKAKAAWRRVTRQCPLLLSHWAWVQSFW
>R5OH057||gene_210919|GeneMark.hmm|416_aa|-|359|1609
MSGPQLSGKPFDISKQEVWRAYQKVKANKGAPGVDDCSVEAFEKDLKNNLYKIWNRMSSGSYFPPPVRAVE
MPKPSGGVRVLGVPTVADRVAQTVVAEKLEKRVEPIFHPDSYGYRPGRSALDAVGQCRRRCWNRAWVVDLDI
ARFFDEVDHQLLLKAVAGHAPEPWVLLYISRWLKAPIQHGDGTIAQRSRGTPQGSAVSPVLANLFLHYAFDM
WMARRFPTVQFERYVDDVVVHCVTERQAREVREAVEGRLARVGLRMHPDKTRIVYCRTQKRKGDHPEVSFD
FLGYTFRPRAARDGKGGIFTSFLPAISKSALKRLSARVRSWRLHLRTGSTLTGLARTINPIVRRWMQYYGRFYRTA
LYPLLKRINAYLVRWLRKKYKRLRTFKKAKAAWRRVTRQCPLLLSHWAWVQSFW
>R5OH057||gene_4894|GeneMark.hmm|430_aa|+|534|1826

MSGSVKLAGSSVRSSGSAVKPFDVPKRLVMEAWEKVRSNKGAPGVDGAAVEDFEKDLRANLYKIWNWMSSG
SYFPSPVREVRIPKPDGGIRVLGVPTVAGRLAQTVVAMVLEHRAERVFHPGSYGYRPGRGAIDAVRACRRRCW
ENDWVIDLDIQAFFDTVPWDLVCRAVGAVCDLPWVMLYVRRWLKAPLQHSDGTLTERERGTPQGSAVSPVL
ANLFMHYAFDTWMARSYPGIVFERYADDVVIHCKSLNQARVVLTAVEERMGQVGLGLHPRKTRIVYCKDANR
PGSWEHTGFTFLGYEFRERTVKGRHGLFRSFSPAVSRTALKRMSTQVRSWRLHRWVTATAGDLAAQINPVLRG
WMSYYGVFQPSALYPLLKRVNSYLIRWLRGKYRKLRVSWSKTMRKWYTGVKKAPNYFVHWAWVTEPGPVW
>R5OH057||gene_103646|GeneMark.hmm|421_aa|-|354|1619
VTPRPSKTAGKAFDIPKALVVEAWERVRSNKGAPGVDGEAIEDFEKDLKNNLYKIWNRMSSGSYFPPPVRQVRI
PKPDGGIRVLGVPTVADRLAQTVVAQVLERRAEPVFHRDSYGYRPGRGAIDAVAACRRRCWENNWVIDMDI
QAFFDTVPWDLVCRAVETVCDLPWVMLYVRRWLKAPAQQGDGTLTERRRGTPQGSAVSPVLANLFMHYALD
AWLTRNFPGVVFERYADDVVIHCKSLEQARTVLAAVTERMRRVGLRLHPGKTRIVYCKDANRTGSWEHTEFTF
LGYEFRERTVKGRHGLFRSFSPAVSRAALKRMSAAVRSWRLHRWVTATAGDLAARVNPVVRGWMCYYGAFH
PSALYPLLRRINSYMVRWLRGKYRRLRASWARTMRKWYAGVKKAPGYFVHWAWVTEPGPVW
>R5OH058||gene_188211|GeneMark.hmm|363_aa|+|19485|20576
MSSGSYFPPPVRQVRIPKPDGGIRVLGVPTVADRLAQTVVALVLERRAEPVFHQGSYGYRPGRGAIDAVAACRR
RCWESSWVIDMDIQAFFDTVPWDLVCRAVETVCDLPWVMLYVRRWLMAPAQQGDGTLTERWRGTPQGSA
VSPVLANLFMHYALDAWLARNFAGVVFERYADDVVIHCKSLEQARAVLAAVAERMRQVGLRLHPRKTRIVYCK
DANRTGSWEHTEFTFLGYEFRERTVKGRHGLFRSFSPAVSKAALKRMSATVRSWRLHRWVTATVSDLAAHVNP
VVRGWMRYYGAFHPSALYPLLRRINSYLVRWLRGKYRRLRASWARTMRKWYTGVKKAPNYFAHWAWVTEP
GPVW
>R5OH059||gene_209151|GeneMark.hmm|422_aa|+|1283|2551
MSRSLSEVPGGKSFDISKQLVMDAWQRVKSDRGALGVDECSVEEFEEDLAGNLYKIWNRMFSGSCFPPAVRM
VEIPRPQAGTRVLGVPTVADRVAQTAAAMVLEKAAEPVFHPDSYGYRPGRGAIDAVAACRRRCWSRSWVIDL
DIEAFFDSVPWDLVCKAVGAVGAPGWVMLYVKRWLAAPLQHPDGAVVERVRGTPQGSAVSPVLANLFMHY
AFDAWLARAFPGVVFERYADDAVIHCRSLTQARGVLAALEARMKQVGLRLHPRRTRIVYCKDANRPGCYEHTG
FTFLGYEFTERTVKGRHGLFRSFSPAASKTALKTMSARLRSWRLHRWVRATARDLAEHINPIMRGWMSYYGAF
HPSALYPLLKRVSSYLIRWLRGKYRRLRRSWRATMRKWYGGIKAAPGYFVHWAWITGPGPLW
>R5OH060||gene_19613|GeneMark.hmm|416_aa|-|3060|4310
MSGPQLSGKPFDISKQEVWRAYQKVKANKGAPGVDDCSVEAFEKDLKNNLYKIWNRMSSGSYFPPPVRAVE
MPKPSGGVRVLGVPTVADRVAQTVVAEKLEKRVEPIFHPDSYGYRPGRSALDAVGQCRRRCWNRAWVVDLDI
ARFFDEVDHQLLLKAVAGHAPEPWVLLYISRWLKAPIQHGDGTIAQRSRGTPQGSAVSPVLANLFLHYAFDM
WMARRFPTVQFERYVDDVVVHCVTERQAREVREAVEGRLARVGLRMHPDKTRIVYCRTQKRKGDHPEVSFD
FLGYTFRPRAARDGKGGIFTSFLPAISKSALKRLSARVRSWRLHLRTGSTLTGLARTINPIVRGWMQYYGRFYRTA
LYPLLKRINAYLVRWLRKKYKRLRTFKKAKAAWRRVTRQCPLLLSHWAWVQSFW
>R5OH061||gene_117207|GeneMark.hmm|416_aa|+|1511|2761
MSGPQLSGKPFDISKQEVWRAYQKVKANKGAPGVDDCSVEAFEKDLKNNLYKIWNRMSSGSYFPPPVRAVE
MPKPSGGVRVLGVPTVADRVAQTVVAEKLEKRVEPIFHPDSYGYRPGRSALDAVGQCRRRCWNRAWVVDLDI
ARFFDEVDHQLLLKAVAGHAPEPWVLLYISRWLKAPIQHGDGTIAQRSRGTPQGSAVSPVLANLFLHYAFDM
WMARRFPTVQFERYVDDVVVHCVTERQAREVREAVEGRLARVGLRMHPDKTRIVYCRTQKRRGDHPEVSFD
FLGYTFRPRAARDGKGGIFTSFLPAISKSALKRLSARVRSWRLHLRTGSTLAGLARTINPIVRGWMQYYGRFYRTA
LYPLLKRINAYLVRWLRKKYKRLRTFKKAKAAWRRVTRQCPLLLSHWAWVQSFW
>R5OH061||gene_88543|GeneMark.hmm|430_aa|-|1056|2348
MSGSVKLAGSSVRSSGSAVKPFDVPKWLVMAAWEKVRSNKGAPGVDGAAVEDFEKDLRANLYKIWNRMSS
GSYFPSPVREVRIPKPDGGIRVLGVPTVADRLAQTVVAMVLEHRAERVFHPGSYGYRPGRGAIDAVRSCRRRC

WENDWVIDLDIQAFFDTVPWDLVCRAVGAVCDLPWVMLYVRRWLKAPLQHSDGTLTERERGTPQGSAVSPV
LANLFMHYAFDTWMARSYPGIVFERYADDVVIHCESLNQARVVLTAVEERMGQVGLGLHPRKTRIVYCKDANR
PGSWEHIGFTFLGYEFRERTVKGRHGLFRSFSPAVSRTALKRMSTQVRSWRLHRWVTAMAGDLAAQINPVLRG
WMSYYGVFHPSALYPLLKRVNSYLIRWLRGKYRKLRVSWSKTMRKWYTGVKKAPNYFVHWAWVTEPGPVW
>R5OH064||gene_50021|GeneMark.hmm|382_aa|+|1121|2269
MTAPRGAFEKDLKNNLYKIWNRMSSGSCFPPPVRAVEMPKPSGGVRVLGVPTVADRVAQTVVAEKLEKRVEPI
FHPDSYGYRPGRSALDAVGQCRRRCWNRAWVVDLDIARFFDEVDHQLLLKAVAGHAPEPWVLLYISRWLKAP
IQHGDGTIAQRSRGTPQGSAVSPVLANLFLHYAFDMWMARRFPTVQFERYVDDVVVHCVTERQAREVREAV
EGRLARVGLRMHPDKTRIVYCRTQKRKGDHPEVSFDFLGYTFRPRAARDGKGGIFTSFLPAISKSALKRLSARVRS
WRLHLRTGSTFTGLARTINPIVRGWMQYYGRFYRTALYPLLKRINAYLVRWLRKKYKRLRTFKKAKAAWRRVTR
QCPLLLSHWAWVQSFW
>R5OH070||gene_152487|GeneMark.hmm|360_aa|-|303|1385
MSSGSYFPPPVRAVEIPKPSGGVRVLGVPTVADRVAQTVVAEKLEKRVEPIFHPDSYGYRPGRSALDAVGQCRR
RCWNRAWVVDLDIARFFDEVDHQLLLKAVAGHAPEPWVLLYISRWLKAPIQHGDGTIAQRSRGTPQGSAVSP
VLANLFLHYAFDMWMARRFPTVQFERYVDDVVVHCVTERQAREVREAVEGRLARVGLRMHPDKTRIVYCRT
QKRRGDHPEVSFDFLGYTFRPRAARDGKGGIFTSFLPAISKSALKRLSARVRSWRLHLRTGSTFTGLARTINPIVR
GWMQYYGRFYRTALYPLLKRINAYLVRWLRKKYKRLRTFKKAKAAWRRVTRQCPLLLSHWAWVQSFW
>R5OH070||gene_331273|GeneMark.hmm|421_aa|-|1173|2438
VAPRPSKTAGKAFDIPKALVVEAWERVRSNKGAPGADGAAIEDFERDLQANLYKIWNRMSSGSYFPPPVRQVR
IPKPDGGIRVLGVPTVADRLAQTVVALVLERRAEPVFHQGSYGYRPGRGAIDAVAACRRRCWESSWVIDMDIQ
AFFDTVPWDLVCRAVETVCDLPWVMLYVRRWLMAPAQQGDGTLTERWRGTPQGSAVSPVVANLFMHYALD
AWLARNFAGVVFERYADDVVIHCKSLEQARAVLAAVAERMRQVGLRLHPRKTRIVYCKDANRTGSWEHTEFTF
LGYEFRERTVKGRHGLFRSFSPAVSKAALKRMSATVRSWRLHRWVTATVSDLAAHVNPVVRGWMRYYGAFHP
SALYPLLRRINSYLVRWLRGKYRRLRASWARTMRKWYAGVKKAPGYFVHWAWVTEPGPVW
>R5OH073||gene_68209|GeneMark.hmm|421_aa|+|625|1890
VTPRPSKTAGKAFDIPKALVVEAWERVRSNKGAPGVDGEAIEDFEKDLKNNLYKIWNRMSSGSYFPPPVRQVRI
PKPDGGIRVLGVPTVADRLAQTVVAQVLERRAEPVFHRDSYGYRPGRGAIDAVAACRRRCWENNWVIDMDI
QAFFDTVPWDLVCRAVETVCDLPWVMLYVRRWLKAPAQQGDGTLTERRRGTPQGSAVSPVLANLFMHYALD
AWLTRNFPGVVFERYADDVVIHCKSLEQARTVLAAVTERMRRVGLRLHPGKTRIVYCKDANRTGSWEHTEFTF
LGYEFRERTVKGRHGLFRSFSPAVSRAALKRMSAAVRSWRLHRWVTATAGDLAARVNPVVRGWMCYYGAFH
PSALYPLLRRINSYMVRWLRGKYRRLRASWARTMRKWYAGVKKAPGYFVHWAWVTEPGPVW
>R5OH073||gene_190485|GeneMark.hmm|416_aa|-|1415|2665
MSGPQLSGKPFDISKQEVWRAYQKVKANKGAPGVDDCSVEAFEKDLKNNLYKIWNRMSSGSYFPPPVRAVE
MPKPSGGVRVLGVPTVADRVAQTVVAEKLEKRVEPIFHPDSYGYRPGRSALDAVGQCRRRCWNRAWVVDLDI
ARFFDEVDHQLLLKAVAGHAPEPWVLLYISRWLKAPIQHGDGTIAQRSRGTPQGSAVSPVLANLFLHYAFDM
WMARRFPTVQFERYVDDVVVHCVTERQAREVREAVEGRLARVGLRMHPDKTRIVYCRTQKRRGDHPEVSFD
FLGYTFRPRAARDGKGGIFTSFLPAISKSALKRLSARVRSWRLHLRTGSTLAGLARTINPIVRGWMQYYGRFYRTA
LYPLLKRINAYLVRWLRKKYKRLRTFKKAKAAWRRVTRQCPLLLSHWAWVQSFW
>R5OH079||gene_139489|GeneMark.hmm|430_aa|+|9277|10569
MSGSVKLAGSSVRSSGSAVKPFDVPKWLVMAAWEKVRSNKGAPGVDGAAVEDFEKDLRANLYKIWNRMSS
GSYFPSPVREVRIPKPDGGIRVLGVPTVADRLAQTVVAMVLEHRAERVFHPGSYGYRPGRGAIDAVRSCRRRC
WENDWVIDLDIQAFFDTVPWDLVCRAVGAVCDLPWVMLYVRRWLKAPLQHSDGTLTERERGTPQGSAVSPV
LANLFMHYAFDTWMARSYPGIVFERYADDVVIHCESLNQARVVLTAVEERMGQVGLGLHPRKTRIVYCKDANR
PGSWEHIGFTFLGYEFRERTVKGRHGLFRSFSPAVSRTALKRMSTQVRSWRLHRWVTAMAGDLAAQINPVLRG

WMSYYGVFHPSALYPLLKRVNSYLIRWLRGKYRKLRVSWSKTMRKWYTGVKKAPNYFVHWAWVTEPGPVW
>R5OH085||gene_211189|GeneMark.hmm|366_aa|-|2219|3319
WNRMSSGSYFPSPVREVRIPKPDGGIRVLGVPTVADRLAQTVVAMVLEHRAERVFHPGSYGYRPGRGAIDAV
RSCRRRCWENDWVIDLDIQAFFDTVPWDLVCRAVGAVCDLPWVMLYVRRWLKAPLQHSDGTLTERERGTPQ
GSAVSPVLANLFMHYAFDTWMARSYPGIVFERYADDVVIHCESLNQARVVLTAVEERMGQVGLGLHPRKTRIV
YCKDANRPGSWEHTGFTFLGYEFRERTVKGRHGLFRSFSPAVSRTALKRMSTQVRSWRLHRWVTATAGDLAAQ
INPVLRGWMSYYGVFHPSALYPLLKRVNSYLIRWLRGKYRKLRVSWSKTMRKWYTGVKKAPNYFVHWAWVT
EPGPVW
>R5OH085||gene_21744|GeneMark.hmm|416_aa|+|978|2228
MSGPQLSGKPFDISKQEVWRAYQKVKANKGAPGVDDCSVEAFEKDLKNNLYKIWNRMSSGSYFPPPVRAVE
MPKPSGGVRVLGVPTVADRVAQTVVAEKLEKRVEPIFHPDSYGYRPGRSALDAVGQCRRRCWNRAWVVDLDI
ARFFDEVDHQLLLKAVAGHAPEPWVLLYISRWLKAPIQHDDGTIAQRSRGTPQGSAVSPVLANLFLHYAFDM
WMARRFPTVQFERYVDDVVVHCVTERQAREVREAVEGRLARVGLRMHPDKTRIVYCRTQKRKGDHPEVSFD
FLGYTFRPRAARDGKGGIFTSFLPAISKSALKRLSARVRSWRLHLRTGSTLAGLARTINPIVRGWMQYYGRFYRTA
LYPLLKRINAYLVRWLRKKYKRLRTFKKAKAAWRRVTRQCPLLLSHWAWVQSFW
>R5OH088||gene_164822|GeneMark.hmm|420_aa|+|757|2019
VRATAKPLPISKRQVWEAYRQVKANGGAAGIDGQTVEAFDEDMANNLYKLWNRLASGSYMPPAVKRVDIPKA
GGGMRPLGVPTVADRIAQTVIRQMLEPIVEPLFHEDSYGYRPGRSAHQALAQTRRRCWRYAWVVEIDIKGFFD
NIDHALLLKAVRHHTRERWVVMYIERWLRAPVQMPDGTIQQREKGTPQGGVISPLLANLFLHYAFDMWMQ
RHHGDVPFERYADDAVCHCHSQVRAQSLIDQLHERFAQCGLELHPQKTRVVYCKDEDRRGNYSDTSFDFLGFT
FRPRLSKNRYGKIFVNFSPAISVKAAKSIRQEVRSWRLQLRSDKALDDLARMFNAKIRGWVNYYGAFYKSALYPT
LRQIDRKLVLWATRKFKRLRGHRRRASHWLARIARRHTRLFAHWPLLWGQVSMGRAG
>R5OH088||gene_433751|GeneMark.hmm|430_aa|-|612|1904
MSGSVKLAGSSVRSSGSAVKPFDVPKWLVMEAWEKVRSDKGAPGVDGAAVEDFEKDLRANLYKIWNRMSSG
SYFPSPVREVRIPKPDGGIRVLGVPTVADRLAQTVVAMVLEHRAERVFHPGSYGYRPGRGAIDAVRSCRRRCW
ENDWVIDLDIQAFFDTVPWDLVCRVVGAVCDLPWVMLYVRRWLKAPLQHSDGTLTERERGTPQGSAVSPVL
ANLFMHYAFDTWMARSYPGIVFERYADDVVIHCKSLNQARVVLTAVEERMGQIGLGLHPRKTRIVYCKDANRP
GSWEHTGFTFLGYEFRERTVKGRHGLFRSFSPAVSRTALKRMSTQVRSWRLHRWVTATAGDLAAQINPVLRG
WMSYYGVFQPSALYPLLKRVNSYLIRWLRGKYRKLRVSWSKTMRKWYTGVKKAPNYFVHWAWVTEPGPVW
>R5OH097||gene_273241|GeneMark.hmm|416_aa|-|359|1609
MSGPQLSGKPFDISKQEVWRAYQKVKANKGAPGVDDCSVEAFEKDLKNNLYKIWNRMSSGSYFPPPVRAVE
MPKPSGGVRVLGVPTVADRVAQTVVAEKLEKRVEPIFHPDSYGYRPGRSALDAVGQCRRRCWNRAWVVDLDI
ARFFDEVDHQLLLKAVAGHAPEPWVLLYISRWLKAPIQHGDGTIAQRSRGTPQGSAVSPVLANLFLHYAFDM
WMARRFPTVQFERYVDDVVVHCVTERQAREVREAVEGRLARVGLRMHPDKTRIVYCRTQKRKGDHPEVSFD
FLGYTFRPRAARDGKGGIFTSFLPAISKSALKRLSARVRSWRLHLRTGSTLTGLARTINPIVRGWMQYYGRFYRTA
LYPLLKRINAYLVRWLRKKYKRLRTFKKAKAAWRRVTRQCPLLLSHWAWVQSFW
>R5OH100||gene_91789|GeneMark.hmm|382_aa|+|190|1338
MTAPRGAFEKDLKNNLYKIWNRMSSGSYFPPPVRAVEMPKPSGGVRVLGVPTVADRVAQTVVAEKLEKRVEPI
FHPDSYGYRPGRSALDAVGQCRRRCWNRAWVVDLDIARFFDEVDHQLLLKAVAGHAPEPWVLLYISRWLKAP
IQHGDGTIAQRSRGTPQGSAVSPVLANLFLHYAFDMWMARRFPTVQFERYVDDVVVHCVTERQAREVREAV
EGRLARVGLRMHPDKTRIVYCRTQKRKGDHPEVSFDFLGYTFRPRAARDGKGGIFTSFLPAISKSALKRLSARVRS
WRLHLRTGSTFTGLARTINPIVRGWMQYYGRFYRTALYPLLKRINAYLVRWLRKKYKRLRTFKKAKAAWRRVTR
QCPLLLSHWAWVQSFW
>R5OH106||gene_334804|GeneMark.hmm|430_aa|-|356|1648

MSGSVKLAGSSVRSSGSAVKPFDVPKWLVMAAWEKVRSNKGAPGVDGAAVEDFEKDLRANLYKIWNRMSS
GSYFPSPVREVRIPKPDGGIRVLGVPTVADRLAQTVVAMVLEHRAERVFHPGSYGYRPGRGAIDAVRACRRRC
WENDWVIDLDIQAFFDTVPWDLVCRVVGAVCDLPWVMLYVRRWLKAPLQHSDGTLTERERGTPQGSAVSPV
LANLFMHYAFDTWMARSYPGIVFERYADDVVIHCKSLNQARVVLTAVEERMGQVGLGLHPRKTRIVYCKDAN
RPGSWEHTGFTFLGYEFRERTVKGRHGLFRSFSPAVSRTALKRMSTQVRSWRLHRWVTATAGDLAAQINPVLR
GWMSYYGVFQPSALYPLLKRVNSYLIRWLRGKYRKLRVSWSKTMRKWYTGVKKAPNYFVHWAWVTEPGPV
W
>R5OH106||gene_200064|GeneMark.hmm|416_aa|-|603|1853
MSGPQLSGKPFDISKQEVWRAYQKVKANKGAPGVDDCSVEAFEKDLKNNLYKIWNRMSSGSYFPPPVRAVE
MPKPSGGVRVLGVPTVADRVAQTVVAEKLEKRVEPIFHPDSYGYRPGRSALDAVGQCRRRCWNRAWVVDLDI
ARFFDEVDHQLLLKAVAGHAPEPWVLLYISRWLKAPIQHGDGTIAQRSRGTPQGSAVSPVLANLFLHYAFDM
WMARRFPTVQFERYVDDVVVHCVTERQAREVREAVEGRLARVGLRMHPDKTRIVYCRTQKRRGDHPEVSFD
FLGYTFRPRAARDGKGGIFTSFLPAISKSALKRLSARVRSWRLHLRTGSTLAGLARTINPIVRGWMQYYGRFYRTA
LYPLLKRINAYLVRWLRKKYKRLRTFKKAKAAWRRVTRQCPLLLSHWAWVQSFW
>R5OH109||gene_26930|GeneMark.hmm|416_aa|-|854|2104
MSGPQLSGKPFDISKQEVWRAYQKVKANKGAPGVDDCSVEAFEKDLKNNLYKIWNRMSSGSYFPPPVRAVE
MPKPSGGVRVLGVPTVADRVAQTVVAEKLEKRVEPIFHPDSYGYRPGRSALDAVGQCRRRCWNRAWVVDLDI
ARFFDEVDHQLLLKAVAGHAPEPWVLLYISRWLKAPIQHGDGTIAQRSRGTPQGSAVSPVLANLFLHYAFDM
WMARRFPTVQFERYVDDVVVHCVTERQAREVREAVEGRLARVGLRMHPDKTRIVYCRTQKRKGDHPEVSFD
FLGYTFRPRAARDGKGGIFTSFLPAISKSALKRLSARVRSWRLHLRTGSTFTGLARTINPIVRGWMQYYGRFYRTA
LYPLLKRINAYLVRWLRKKYKRLRTFKKAKAAWRRVTRQCPLLLSHWAWVQSFW
>R5OH109||gene_42818|GeneMark.hmm|430_aa|+|713|2005
MSGSVKLAGSSVGSSGSAVKPFDVPKRLVMEAWEKVRSNKGAPGVDGAAVEDFEKDLRANLYKIWNRMSSG
SYFPSPVREVRIPKPDGGIRVLGVPMVADRLAQTVVAMVLEHRAERVFHPGSYGYRPGRGAIDAVRACRRRC
WENDWVIDLDIQAFFDTVPWDLVCRAVGAVCDLPWVMLYVRRWLKAPLQHSDGTLTERERGTPQGSAVSPV
LANLFMHYAFDTWMARSYPGIVFERYADDVVIHCKSLNQARVVLTAVEERMGQVGLGLHPRKTRIVYCKDAN
RPGSWEHTGFTFLGYEFRERTVKGRHGLFRSFSPAVSRTALKRMSTQVRSWRLHRWVTATAGDLAAQINPVLR
GWMSYYGVFQPSALYPLLKRVNSYLIRWLRGKYRKLRVSWSKTMRKWYTGVKKAPNYFVHWAWVTEPGPV
W
>R5OH112||gene_35244|GeneMark.hmm|416_aa|+|587|1837
MSGPQLSGKPFDISKQEVWRAYQKVKANKGAPGVDDCSVEAFEKDLKNNLYKIWNRMSSGSYFPPPVRAVEI
PKPSGGVRVLGVPTVADRVAQTVVAEKLEKRVEPIFHPDSYGYRPGRSALDAVGQCRRRCWNRAWVVDLDIA
RFFDEVDHQLLLKAVAGHAPEPWVLLYISRWLKAPIQHGDGTIAQRSRGTPQGSAVSPILANLFLHHALDMW
MARRFPTVQFERYVDDVVVHCVTERQAREVREAVEGRLARVGLRMHPDKTRIVYCRTQKRRGDHPEVSFDFL
GYTFRPRAARDGKGGIFTSFLPAISKSALKRLSARVRSWRLHLRTGSTLTGLARTINPIVRGWMQYYGRFYRTALY
PLLKRINAYLVRWLRKKYKRLRTFKKAKAAWRRVTRQCPLLLSHWAWVQSFW
>R5OH115||gene_326322|GeneMark.hmm|430_aa|+|986|2278
MSGSVKLAGSSVRSSGSAVKPFDVPKWLVMEAWEKVRSNKGAPGVDGAAVEDFEKDLRANLYKIWNRMSSG
SYFPSPVREVRIPKPDGGIRVLGVPTVAGRLAQTVVAMVLEHRAERVFHPGSYGYRPGRGAIDAVRACRRRCW
ENDWVIDLDIQAFFDTVPWDLVCRAVGAVCDLPWVMLYVRRWLKAPLQHSDGTLTERERGTPQGSAVSPVL
ANLFMHYAFDTWMARSYPGIVFERYADDVVIHCKSLNQARVVLTAVEERMGQVGLGLHPRKTRIVYCKDANR
PGSWEHTGFTFLGYEFRERTVKGRHGLFRSFSPAVSRTALKRMSTQVRSWRLHRWVTATAGDLAAQINPVLRG
WMSYYGVFQPSALYPLLKRVNSYLIRWLRGKYRKLRVSWSKTMRKWYTGVKKAPNYFVHWAWVTEPGPVW
>R5OH118||gene_80003|GeneMark.hmm|430_aa|+|9288|10580

MSGSVKLAGSSVRSSGSAVKPFDVPKWLVMEAWEKVRSDKGAPGVDGAAVEDFEKDLRASLYKIWNRMSSG
SYFPSPVREVRIPKPDGGIRVLGVPTVAGRLAQTVVAMVLEHRAERVFHPGSYGYRPGRGAIDAVRACRRRCW
ENDWVIDLDIQAFFDTVPWDLVCRVVGAVCDLPWVMLYVRRWLKAPLQHSDGTLTERERGTPQGSAVSPVL
ANLFMHYAFDTWMARSYPGIVFERYADDVVIHCKSLNQARVVLTAVEERMGQIGLGLHPRKTRIVYCKDANRP
GLWEHTGFTFLGYEFRERTVKGRHGLFRSFSPAVSRTALKRMSTQVRSWRLHRWVTATAGDLAAQINPVLRGW
MSYYGVFQPSALYPLLKRVNSYLIRWLRGKYRKLRVSWSKTMRKWYTGVKKAPNYFVHWAWVTEPGPVW
>R5OH121||gene_37630|GeneMark.hmm|421_aa|-|331|1596
VTPRPSKTAGKAFDIPKALVVEAWERVRSNKGAPGVDGEAIEDFEKDLKNNLYKIWNRMSSGSYFPPPVRQVRI
PKPDGGIRVLGVPTVADRLAQTVVAQVLERRAEPVFHRDSYGYRPGRGAIDAVAACRRRCWENNWVIDMDI
QAFFDTVPWDLVCRAVETVCDLPWVMLYVRRWLKAPAQQGDGTLTERRRGTPQGSAVSPVLANLFMHYALD
AWLTRNFPGVVFERYADDVVIHCKSLEQARTVLAAVTERMRRVGLRLHPGKTRIVYCKDANRTGSWEHTEFTF
LGYEFRERTVKGRHGLFRSFSPAVSRAALKRMSAAVRSWRLHRWVTATAGDLAARVNPVVRGWMCYYGAFH
PSALYPLLRRINSYMVRWLRGKYRRLRASWARTMRKWYAGVKKAPGYFVHWAWVTEPGPVW
>R5OH124||gene_142934|GeneMark.hmm|382_aa|-|6658|7806
MTAPRGAFEKDLKNNLYKIWNRMSSGSYFPPPVRAVEMPKPSGGVRVLGVPTVADRVAQTVVAEKLEKRVEPI
FHPDSYGYRPGRSALDAVGQCRRRCWNRAWVVDLDIARFFDEVDHQLLLKAVAGHAPEPWVLLYISRWLKAP
IQHGDGTIAQRSRGTPQGSAVSPVLANLFLHYAFDMWMARRFPTVQFERYVDDVVVHCVTERQAREVREAV
EGRLARVGLRMHPDKTRIVYCRTQKRKGDHPEVSFDFLGYTFRPRAARDGKGGIFTSFLPAISKSALKRLSARVRS
WRLHLRTGSTLTGLARTINPIVRGWMQYYGRFYRTALYPLLKRINAYLVRWLRKKYKRLRTFKKAKAAWRRVTR
QCPLLLSHWAWVQSFW
>R5OH124||gene_161372|GeneMark.hmm|430_aa|-|510|1802
MSGSVKLAGSSVRSSGSAVKPFDVPKWLVMAAWEKVRSNKGAPGVDGAAVEDFEKDLRANLYKIWNRMSS
GSYFPSPVREVRIPKPDGGIRVLGVPTVADRLAQTVVAMVLEHRAERVFHPGSYGYRPGRGAIDAVRSCRRRC
WENDWVIDLDIQAFFDTVPWDLVCRAVGAVCDLPWVMLYVRRWLKAPLQHSDGTLTERERGTPQGSAVSPV
LANLFMHYAFDTWMARSYPGIVFERYADDVVIHCESLNQARVVLTAVEERMGQVGLGLHPRKTRIVYCKDANR
PGSWEHTGFTFLGYEFRERTVKGRHGLFRSFSPAVSRTALKRMSTQVRSWRLHRWVTATAGDLAAQINPVLRG
WMSYYGVVHPSALYPLLKRVNSYLIRWLRGKYRKLRVSWSKTMRKWYTGVKKAPNYFVHWAWVTEPGPVW
>R5OH127||gene_12410|GeneMark.hmm|430_aa|-|99|1391
MSGSVKLAGSSVRSSGSAVKPFDVPKWLVMEAWEKVRSNKGAPGVDGAAVEDFEKDLRANLYKIWNRISSGS
YFPSPVREVRIPKPDGGIRVLGVPTVADRLAQTVVAMVLEHRAERVFHPGSYGYRPGRGAIDAVRACRRRCWE
NDWVIDLDIQAFFDTVPWDLVCRAVGAVCDLPWVMLYVRRWLKAPLQHSDGTLTERERGTPQGSAVSPVLA
NLFMHYAFDTWMARSYPGIVFERYADDVVIHCKSLNQARVVLTAVEERMGQVGLGLHPRKTRIVYCKDANRP
GSWEHTGFTFLGYEFRERTVKGRHGLFRSFSPAVSRTALKRMSTQVRSWRLHRWVTATAGDLAAQINPVLRG
WMSYYGVFQPSALYPLLKRVNSYLIRWLRGKYRKLRVSWSKTMRKWYTGVKKAPNYFVHWAWVTEPGPVW
>R5OH130||gene_82939|GeneMark.hmm|405_aa|-|1015|2232
MSGPQLSGKPFDISKQEVWRAYQKVKANKGAPGVDDCSVEAFEKDLKNNLYKIWNRMSSGSYFPPPVRAVE
MPKPSGGVRVLGVPTVADRVAQTVVAEKLEKRVEPIFHPDSYGYRPGRSALDAVGQCRRRCWNRAWVVDLDI
ARFFDEVDHQLLLKAVAGHAPEPWVLLYISRWLKAPIQHGDGTIAQRSRGTPQGSAVSPVLANLFLHYAFDM
WMARRFPTVQFERYVDDVVVHCVTERQAREVREAVEGRLARVGLRMHPDKTRIVYCRTQKRKGDHPEVSFD
FLGYTFRPRAARDGKGGIFTSFLPAISKSALKRLSARVRSWRLHLRTGSTLTGLARTINPIVRGWMQYYGRFYRTA
LYPLLKRINAYLVRWLRKKYKRLRTFKKAKAAWRRVTRQCPSS
>R5OH130||gene_463247|GeneMark.hmm|420_aa|+|758|2020
VRATAKPLPISKRQVWEAYRQVKANGGAAGIDGQTVEAFDEDMANNLYKLWNRLASGSYMPPAVKRVDIPKV
GGGMRPLGVPTVADRIAQTVIRQMLEPIVEPLFHEDSYGYRPGKSAHQALAQTRRRCWRYAWVVEIDIKGFFD

NIDHALLLKAVRHHTRERWVVMYIERWLRAPVQMPDGTIQQREKGTPQGGVISPLLANLFLHYAFDMWMR
RHHGDVPFERYADDAVCHCHSQARARSLIDQLRERFAQCGLELHPQKTRVVYCKDENRRGNYPDTSFDFLGFT
FRPRLSKNRYGKIFVNFSPAVSVKAAKSIRQEVRSWRLQLRSDKALDDLARMFNAKIWGWVNYYGAFYKSALYS
TLRKIDFKLVLWATRKFKRLRGRRRRARHWLARIARRNPQLFAHWPLLWGQASMGRAG
>R5OH130||gene_97696|GeneMark.hmm|430_aa|-|461|1753
MSGSVKLAGSSVRSSGSAVKPFDVPKWLVMEAWEKVRSDKGAPGVDGAAVEDFEKDLRASLYKIWNRMSSG
SYFPSPVREVRIPKPDGGIRVLGVPTVAGRLAQTVVAMVLEHRAERVFHPGSYGYRPGRGAIDAVRACRRRCW
ENDWVIDLDIQAFFDTVPWDLVCRVVGAVCDLPWVMLYVRRWLKAPLQHSDGTLTERERGTPQGSAVSPVL
ANLFMHYAFDTWMARSYPGIVFERYADDVVIHCKSLNQARVVLTAVEERMGQIGLGLHPRKTRIVYCKDANRP
GSWEHTGFTFLGYEFRERTVKGRHGLFRSFSPAVSRTALKRMSTQVRSWRLHRWVTATAGDLAAQINPVLRG
WMSYYGVFQPSALYPLLKRVNSYLIRWLRGKYRKLRVSWSKTMRKWYTGVKKAPNYFVHWAWVTEPGPVW
>R5OH136||gene_164516|GeneMark.hmm|416_aa|+|587|1837
MSGPQLSGKPFDISKQEVWRAYQKVKANKGAPGVDDCSVEAFEKDLKNNLYKIWNRMSSGSYFPPPVRAVE
MPKPSGGVRVLGVPTVADRVAQTVVAEKLEKRVEPIFHPDSYGYRPGRSALDAVGQCRRRCWNRAWVVDLDI
ARFFDEVDHQLLLKAVAGHAPEPWVLLYISRWLKAPIQHGDGTIAQRSRGTPQGSAVSPVLANLFLHYAFDM
WMARRFPTVQFERYVDDVVVHCVTERQAREVREAVEGRLARVGLRMHPDKTRIVYCRTQKRKGDHPEVSFD
FLGYTFRPRAARDGKGGIFTSFLPAISKSALKRLSARVRSWRLHLRTGSTLTGLARTINPIVRGWMQYYGRFYRTA
LYPLLKRINAYLVRWLRKKYKRLRTFKKAKAAWRRVTRQCPLLLSHWAWVQSFW
>R5OH139||gene_66966|GeneMark.hmm|430_aa|-|763|2055
MSGSVKLAGSSVRSSGSAVKPFDVPKWLVMEAWEKVRSDKGAPGVDGAAVEDFEKDLRASLYKIWNRMSSG
SYFPSPVREVRIPKPDGGIRVLGVPTVADRLAQTVVAMVLEHRAERVFHPGSYGYRPGRGAIDAVRACRRRCW
ENDWVIDLDIQAFFDTVPWDLVCRVVGAVCDLPWVMLYVRRWLKAPLQHSDGTLTERERGTPQGSAVSPVL
ANLFMHYAFDTWMARSYPGIVFERYADDVVIHCKSLNQARVVLTAVEERMGQIGLGLHPRKTRIVYCKDANRP
GSWEHTGFTFLGYEFRERTVKGRHGLFRSFSPAVSRTALKRMSTQVRSWRLHRWVTATAGDLAAQINPVLQG
WMSYYGVFQPSALYPLLKRVNSYLIRWLRGKYRKLRVSWPKTMRKWYTGVKKAPNYFVHWAWVTEPGPVW
>R5OH139||gene_189837|GeneMark.hmm|421_aa|+|1531|2796
VTPRPSKTAGKAFDIPKALVVEAWERVRSNKGAPGVDGEAIEDFEKDLKNNLYKIWNRMSSGSYFPPPVRQVRI
PKPDGGIRVLGVPTVADRLAQTVVAQVLERRAEPVFHRDSYGYRPGRGAIDAVAACRRRCWENNWVIDMDI
QAFFDTVPWDLVCRAVETVCDLPWVMLYVRRWLKAPAQQGDGTLTERRRGTPQGSAVSPVLANLFMHYALD
AWLTRNFPGVVFERYADDVVIHCKSLEQARTVLAAVTERMRRVGLRLHPGKTRIVYCKDANRTGSWEHTEFTF
LGYEFRERTVKGRHGLFRSFSPAVSRAALKRMSAAVRSWRLHRWVTATAGDLAARVNPVVRGWMCYYGAFH
PSALYPLLRRINSYMVRWLRGKYRRLRASWARTMRKWYAGVKKAPGYFVHWAWVTEPGPVW
>R5OH142||gene_52244|GeneMark.hmm|382_aa|-|26|1174
MTAPRGAFEKDLKNNLYKIWNRMSSGSYFPPPVRAVEMPKPSGGVRVLGVPTVADRVAQTVVAEKLEKRVEPI
FHPDSYGYRPGRSALDAVGQCRRRCWNRAWVVDLDIARFFDEVDHQLLLKAVAGHAPEPWVLLYISRWLKAP
IQHGDGTIAQRSRGTPQGSAVSPVLANLFLHYAFDMWMARRFPTVQFERYVDDVVVHCVTERQAREVREAV
EGRLARVGLRMHPDKTRIVYCRTQKRKGDHPEVSFDFLGYTFRPREARDGKGGIFTSFLPAISKSALKRLSARVRS
WRLHLRTGSTLTGLARTINPIVRGWMQYYGRFYRTALYPLLKRINAYLVRWLRKKYKRLRTFKKAKAAWRRVTR
QCPLLLSHWAWVQSFW
>R5OH145||gene_56483|GeneMark.hmm|416_aa|+|3266|4516
MSGPQLSGKPFDISKQEVWRAYQKVKANKGAPGVDDCSVEAFEKDLKNNLYKIWNRMSSGSYFPPPVRAVE
MPKPSGGVRVLGVPTVADRVAQTVVAEKLEKRVEPIFHPDSYGYRPGRSALDAVGQCRRRCWNRAWVVDLDI
ARFFDEVDHQLLLKAVAGHAPEPWVLLYISRWLKAPIQHGDGTIAQRSRGTPQGSAVSPVLANLFLHYAFDM
WMARRFPTVQFERYVDDVVVHCVTERQAREVREAVEGRLARVGLRMHPDKTRIVYCRTQKRRGDHPEVSFD

FLGYTFRPRAARDGKGGIFTSFLPAISKSALKRLSARVRSWRLHLRTGSTLAGLARTINPIVRGWMQYYGRFYRTA
LYPLLKRINAYLVRWLRKKYKRLRTFKKAKAAWRRVTRQCPLLLSHWAWVQSFW
>R5OH148||gene_282308|GeneMark.hmm|430_aa|+|7650|8942
MSGSVKLAGSSVRSSGSAVKPFDVPKWLVMAAWEKVRSNKGAPGVDGAAVEDFEKDLRANLYKIWNRMSS
GSYFPSPVREVRIPKPDGGIRVLGVPTVADRLAQTVVAMVLEHRAERVFHPGSYGYRPGRGAIDAVRSCRRRC
WENDWVIDLDIQAFFDTVPWDLVCRAVGAVCDLPWVMLYVRRWLKAPLQHSDGTLTERERGTPQGSAVSPV
LANLFMHYAFDTWMARSYPGIVFERYADDVVIHCESLNQARVVLTAVEERMGQVGLGLHPRKTRIVYCKDANR
PGSWEHIGFTFLGYEFRERTVKGRHGLFRSFSPAVSRTALKRMSTQVRSWRLHRWVTAMAGDLAAQINPVLRG
WMSYYGVFHPSALYPLLKRVNSYLIRWLRGKYRKLRVSWSKTMRKWYTGVKKAPNYFVHWAWVTEPGPVW
>R5OH149||gene_408269|GeneMark.hmm|363_aa|-|66|1157
MSSGSYFPPLVRQVRIPKPDGGIRVLGVPTVADRLAQTVVALVLERRAEPVFHQGSYGYRPGRGAIDAVAACRR
RCWESSWVIDMDIQAFFDTVPWDLVCRAVATVCDLPWVMLYVRRWLKAPAQQGDGTLTERWRGTPQGSA
VSPVLANLFMHYALDAWLARNFAGVVFERYADDVVIHCKSLEQARAVLAAVAERMRQVGLRLHPRKTRIVYCK
DANRTGSWEHTEFTFLGYEFGERTVKGRHGLFRSFSPAVSRAALKRMSATVRSWRLHRWVTATVSDLAAHVN
PVVRGWMRYYGAFHPSALYPLLRRINSYLVRWLRGKYRRLRASWARTMRKWYTGVKKAPGYFAHWAWVTEP
GPVW
>R5OH151||gene_227219|GeneMark.hmm|363_aa|-|932|2023
MSSGSYFPPLVRQVRIPKPDGGIRVLGVPTVADRLAQTVVALVLERRAEPVFHQGSYGYRPGRGAIDAVAACRR
RCWESSWVIDMDIQAFFDTVPWDLVCRAVATVCDLPWVMLYVRRWLKAPAQQGDGTLTERWRGTPQGSA
VSPVLANLFMHYALDAWLARNFAGVVFERYADDVVIHCKSLEQARAVLAAVAERMRQVGLRLHPRKTRIVYCK
DANRTGSWEHTEFTFLGYEFGERTVKGRHGLFRSFSPAVSRAALKRMSATVRSWRLHRWVTATVSDLAAHVN
PVVRGWMRYYGAFHPSALYPLLRRINSYLVRWLRGKYRRLRASWARTMRKWYTGVKKAPGYFAHWAWVTEP
GPVW
>R5OH153||gene_114515|GeneMark.hmm|420_aa|-|197|1459
VQATAKPLPISKRQVWEAYRQVRANGGAAGIDGQTVEAFDEDMANNLYRLWNRLASGSYMPPAVKRVDIPK
AGGGTRPLGVPTVADRIAQTVIRQMLEPIVEPLFHEDSYGYRPGKSAHQALAQTRRRCWRYAWVVEIDIKGFF
DNIDHALLLKAVRHHTRERWVVMYIERWLRAPVQMPDGTIQQREKGTPQGGVISPLLANLFLHYAFDMWM
RRHHGDVPFERYADDAVCHCHSQARARSLIDQLRERFAQCGLELHPQKTRVVYCKDEDRRGNYPDTSFDFLGF
TFRPRLSKNRYGKIFVNFSPAVSVKAAKSIRQEVRSWRLQLRSDKALDDLARMFNAKIWGWVNYYGAFYKSALY
PTLRQIDRKLVLWATRKFKRLRGHRRRARHWLARIACRTPRLFAHWSLLWGQASMGRAG
>R5OH154||gene_10249|GeneMark.hmm|421_aa|+|316|1581
VAPRPSKTAGKAFDIPKALVVEAWERVGSNKGAPGVDGAAIEDFERDLQANLYKIWNRMSSGSYFPPPVRQV
WIPKPDGGIRVLGVPTVADRLAQTVVALVLERRAEPVFHQGSYGYRPGRGAIDAVAACRRRCWESSWVIDMDI
QAFFDTVPWDLVCRAVETVCDLPWVMLYVRRWLMAPAQQGDGTLTERWRGTPQGSAVSPVLANLFMHYAL
DAWLARNFAGVVFERYADDVVIHCKSLEQARAVLAAVAERMRQVGLRLHPRKTRIVYCKDANRTGSWEHTEF
TFLGYEFRERTVKGRHGLFRSFSPAVSKAALKRMSATVCSWRLHRWVTATVSDLAAHVNPVVRGWMRYYGAF
HPSALYPLLRRINSYLVRWLRGKYRRLRASWARTMRKWYTGVKKAPNYFAHWAWVTEPGPVW
>R5OH154||gene_236740|GeneMark.hmm|420_aa|-|2330|3592
VQATAKPLPISKRQVWEAYRQVRANGGAAGIDGQTVEAFDEDMANNLYRLWNRLASGSYMPPAVKRVDIPK
AGGGMRPLGVPTVSDRIAQTVIRQMLEPIVEPLFHEDSYGYRLGKSAHQALAQTRRRCWRYAWVVEIDIKGFF
DNIDHALLLKAVRHHTRERWVVMYIERWLRAPVQMPDGTIQQREKGTPQGGVISPLLANLFLHYAFDMWM
RRHHGEVPFERYADDVVCHCHSQARAQSLIDQLRERFAQCGLELHPQKTRVVYCKDSNRRGDYPDTSFDFLGY
TFRPRLSRGRDGRLFVGFNPAVSARAAKSIRQEVRSWRLQLRSDKALDDLARMFNAKIRGWVNYYGAFYKSAL
YSTLRKIDFKLVLWATRKFKRLRGRRRRARHWLARIARRNPQLFAHWPLLWGQASMGRAG

>R5OH157||gene_242402|GeneMark.hmm|430_aa|+|533|1825
MSGSVKLAGSSVRSSGSAVKPFDVPKWLVMEAWEKVRSNKGAPGVDGAAVEDFEKDLRANLYKIWNRMSSG
SYFPSPVREVRIPKPDGGIRVLGVPTVADRLAQTVVAMVLEHRAERVFHPGSYGYRPGRGAIDAVRACRRRCW
ENDWVIDLDIQAFFDTVPWDLVCRAVGAVCDLPWVMLYVRRWLKAPLQHSDGTLTERERGTPQGSAVSPVL
ANLFMHYAFDTWMARSYPGIVFERYADDVVIHCKSLNQARVVLTAVEERMGQVGLGLHPRKTRIVYCKDANR
PGSWAHTGFTFLGYEFRERTVKGRHGLFRSFSPAVSRTALKRMSTQVRSWRLHRWVTATAGDLAAQINPVLRG
WMSYYGVFHPSALYPLLKRVNSYLIRWLRGKYRKLRVSWSKTMRKWYTGVKKAPNYFVHWAWVTEPGPVW
>R5OH157||gene_97117|GeneMark.hmm|416_aa|+|705|1955
MSGPQLSGKPFDISKQEVWRAYQKVKANKGAPGVDDCSVEAFEKDLKNNLYKIWNRMSSGSYFPPPVRAVE
MPKPSGGVRVLGVPTVADRVAQTVVAEKLEKRVEPIFHPDSYGYRPGRSALDAVGQCRRRCWNRAWVVDLDI
ARFFDEVDHQLLLKAVAGHAPEPWVLLYISRWLKAPIQHGDGTIAQRSRGTPQGSAVSPVLANLFLHYAFDM
WMARRFPTVQFERYVDDVVVHCVTERQAREVREAVEGRLARVGLRMHPDKTRIVYCRTQKRKGDHPEVSFD
FLGYTFRPRAARDGKGGIFTSFLPAISKSALKRLSARVRSWRLHLRTGSTLTGLARTINPIVRGWMQYYGRFYRTA
LYPLLKRINAYLVRWLRKKYKRLRTFKKAKAAWRRVTRQCPLLLSHWAWVQSFW
>R5OH160||gene_110845|GeneMark.hmm|416_aa|+|1024|2274
MSGPQLSGKPFDISKQEVWRAYQKVKANKGAPGVDDCSVEAFEKDLKNNLYKIWNRMSSGSYFPPPVRAVE
MPKPSGGVRVLGVPTVADRVAQTVVAEKLEKRVEPIFHPDSYGYRPGRSALDAVGQCRRRCWNRAWVVDLDI
ARFFDEVDHQLLLKAVAGHAPEPWVLLYISRWLKAPIQHGDGTIAQRSRGTPQGSAVSPVLANLFLHYAFDM
WMARRFPTVQFERYVDDVVVHCVTERQAREVREAVEGRLARVGLRMHPDKTRIVYCRTQKRKGDHPEVSFD
FLGYTFRPRAARDGKGGIFTSFLPAISKSALKRLSARVRSWRLHLRTGSTLTGLARTINPIVRGWMQYYGRFYRTA
LYPLLKRINAYLVRWLRKKYKRLRTFKKAKAAWRRVTRQCPLLLSHWAWVQSFW
>R5OH161||gene_84349|GeneMark.hmm|430_aa|-|127|1419
MSGSVKLAGSSVRSSGSAVKPFDVPKRLVMEAWEKVRSNKGAPGVDGAAVEDFEKDLRANLYKIWNWMSSG
SYFPSPVREVRIPKPDGGIRVLGVPTVAGRLAQTVVAMVLEHRAERVFHPGSYGYRPGRGAIDAVRACRRRCW
ENDWVIDLDIQAFFDTVPWDLVCRAVGAVCDLPWVMLYVRRWLKAPLQHSDGTLTERERGTPQGSAVSPVL
ANLFMHYAFDTWMARSYPGIVFERYSDDVVIHCKSLNQARVVLTAVEERMGQVGLGLHPRKTRIVYCKDANR
PGSWEHTGFTFLGYEFRERTVKGRHGLFRSFSPAVSRTTLKRMSTQVRSWRLHRWVTATAGDLAAQINPVLRG
WMSYYGVFQPSALYPLLKRVNSYLIRWLRGKYRKLRVSWSKTMRKWYTGVKKAPNYFVHWAWVTEPGPVW
>R5OH161||gene_313316|GeneMark.hmm|416_aa|+|595|1845
MSGPQLSGKPFDISKQEVWRAYQKVKANKGAPGVDDCSVEAFEKDLKNNLYKIWNRMSSGSYFPPPVRAVE
MPKPSGGVRVLGVPTVADRVAQTVVAEKLEKRVEPIFHPDSYGYRPGRSALDAVGQCRRRCWNRAWVVDLDI
ARFFDEVDHQLLLKAVAGHAPEPWVLLYISRWLKAPIQHGDGTIAQRSRGTPQGSAVSPVLANLFLHYAFDM
WMARRFPTVQFERYVDDVVVHCVTERQAREVREAVEGRLARVGLRMHPDKTRIVYCRTQKRRGDHPEVSFD
FLGYTFRPRAARDGKGGIFTSFLPAISKSALKRLSARVRSWRLHLRTGSTLAGLARTINPIVRGWMQYYGRFYRTA
LYPLLKRINAYLVRWLRKKYKRLRTFKKAKAAWRRVTRQCPLLLSHWAWVQSFW
>R5OH166||gene_85399|GeneMark.hmm|421_aa|-|83|1348
VAPRPSRTAGKAFDIPKALVVEAWERVRSNKGAPGVDGVAIEDFERDLQANLYKIWNRMSSGSYFPPPVRQVR
IPKPDGGIRVLGVLTVADRLAQTVVALVLERRAEPVFHQGSYGYRPGRGAIDAVAACRRRCWESSWVIDMDIQ
AFFDTVPWDLVCRAVETVCDLPWVMLYVRRWLMAPAQQGDGTLTERWRGTPQGSAVSPVLANLFMHYALD
AWLARNFAGVVFERYADDVVIHCKSLEQARAVLAAVAERMRQVGLRLHPSKTRIVYCKDANRTGSWEHTEFTF
LGYEFRERTVKGRHGLFRSFSPAVSRAALKRMSATVRSWRLHRWVTATVSDLAAHVNPVVRGWMRYYGAFHP
SALYPLLRRINSYLVRWLRGKYRRLRASWARTMRKWYTGVKKAPSYFAHWAWVTEPGPVW
>R5OH167||gene_151549|GeneMark.hmm|430_aa|+|1018|2310
MSGSVKLAGSSVRSSGSAVKPFDVPKWLVMAAWEKVRSNKGAPGVDGAAVEDFEKDLRANLYKIWNRMSS

GSYFPSPVREVRIPKPDGGIRVLGVPTVADRLAQTVVAMVLEHRAERVFHPGSYGYRPGRGAIDAVRSCRRRC
WENDWVIDLDIQAFFDTVPWDLVCRAVGAVCDLPWVMLYVRRWLKAPLQHSDGTLTERERGTPQGSAVSPV
LANLFMHYAFDTWMARSYPGIVFERYADDVVIHCESLNQARVVLTAVEERMGQVGLGLHPRKTRIVYCKDANR
PGSWEHTGFTFLGYEFRERTVKGRHGLFRSFSPAVSRTALKRMSTQVRSWRLHRWVTATAGDLAAQINPVLRG
WMSYYGVFHPSALYPLLKRVNSYLIRWLRGKYRKLRVSWSKTMRKWYTGVKKAPNYFVHWAWVTEPGPVW
>R5OH167||gene_241820|GeneMark.hmm|421_aa|-|112|1377
VAPRPSRTAGKAFDIPKALVVEAWERVRSNKGAPGVDGVAIEDFERDLQANLYKIWNRMSSGSYFPPPVRQVR
IPKPDGGIRVLGVLTVADRLAQTVVALVLERRAEPVFHQGSYGYRPGRGAIDAVAACRRRCWESSWVIDMDIQ
AFFDTVPWDLVCRAVETVCDLPWVMLYVRRWLMAPAQQGDGTLTERWRGTPQGSAVSPVLANLFMHYALD
AWLARNFAGVVFERYADDVVIHCKSLEQARAVLAAVAERMRQVGLRLHPSKTRIVYCKDANRTGSWEHTEFTF
LGYEFRERTVKGRHGLFRSFSPAVSRAALKRMSATVRSWRLHRWVTATVSDLAAHVNPVVRGWMRYYGAFHP
SALYPLLRRINSYLVRWLRGKYRRLRASWARTMRKWYTGVKKAPSYFAHWAWVTEPGPVW
>R5OH169||gene_221748|GeneMark.hmm|447_aa|+|3|1346
VRDVFVLVNQEDWGGIGMSGSVKLAGSSVRSSGSAVKPFDVPKWLVMAAWEKVRSNKGAPGVDGAAVEDF
EKDLRANLYKIWNRMSSGSYFPSPVREVRIPKPDGGIRVLGVPTVADRLAQTVVAMVLEHRAERVFHPGSYGY
RPGCGAIDAVRSCRRRCWENDWVIDVDIQAFFDTVPWDLVCRAVGAVCDLPWVMLYVRRWLKAPLQHSDG
TLTERERGTPQGSAVSPVLANLFMHYAFDTWMARSYPGIVFERYADDVVIHCESLNQARVVLTAVEERMGQV
GLGLHPRKTRIVYCKDANRPGSWEHTGFTFLGYEFRERTVKGRHGLFRSFSPAVLRTALKRMSTQVRSWRLHR
WVTATAGDLAAQINPVLRGWMSYYGVFHPSALYPLLKRVNSYLIRWLRGKYRKLRVSWSKTMRKWYTGVKKA
PNYFVHWAWVTEPGPVW
>R5OH169||gene_336543|GeneMark.hmm|420_aa|-|764|2026
VRATAKPLPISKRQVWEAYRQVKANGGAAGIDGQTVEAFDEDMANNLYKLWNRLASGSYMPPAVKRVDIPKV
GGGMRPLGVPTVADRIAQTVIRQMLEPIVEPLFHEDSYGYRPGKSAHQALAQTRRRCWRYAWVVEIDIKGFFD
NIDHALLLKAVRHHTRERWVVMYIERWLRAPVQMPDGTIQQREKGTPQGGVISPLLANLFLHYAFDMWMR
RHHGDVPFERYADDAVCHCHSQARARSLIDQLRERFAQCGLELHPQKTRVVYCKDENRRGNYPDTSFDFLGFT
FRPRLSKNRYGKIFVNFSPAVSVKAAKSIRQEVRSWRLQLRSDKALDDLARMFNAKIRGWVNYYGAFYKSALYS
TLRKIDFKLVLWATRKFKRLRGRRRRARHWLARIARRNPQLFAHWPLLWGQASMGRAG
>R5OH172||gene_167580|GeneMark.hmm|430_aa|+|671|1963
MSGSVKLAGSSVRSSGSAVKPFDVPKWLVMAAWEKVRSNKGAPGVDGAAVEDFEKDLRANLYKIWNRMSS
GSYFPSPVREVRIPKPDGGIRVLGVPTVADRLAQTVVAMVLEHRAERVFHPGSYGYRPGRGAIDAVRSCRRRC
WENDWVIDLDIQAFFDTVPWDLVCRAVGAVCDLPWVMLYVRRWLKAPLQHSDGTLTERERGTPQGSAVSPV
LANLFMHYAFDTWMARSYPGIVFERYADDVVIHCESLNQARVVLTAVEERMGQVGLGLHPRKTRIVYCKDANR
PGSWEHTGFTFLGYEFRKRTVKGRHGLFRSFSPAVSRTALKRMSTQVRSWRLHRWVTATAGDLAAQINPVLRG
WMSYYGVFHPSALYPLLKRVNSYLIRWLRGKYRKLRVSWSKTMRKWYTGVKKAPNYFVHWAWVTEPGPVW
>R5OH172||gene_11093|GeneMark.hmm|360_aa|-|1516|2598
MSSGSYFPPPVRAVEMPKPSGGVRVLGVPTVADRVAQTVVAEKLEKRVEPIFHPDSYGYRPGRSALDAVGQCR
RRCWNRAWVVDLDIARFFDEVDHQLLLKAVAGHAPEPWVLLYISRWLKAPIQHGDGTIAQRSRGTPQGSAVS
PVLANLFLHYAFDMWMARRFPTVQFERYVDDVVVHCVTERQAREVREAVEGRLARVGLRMHPDKTRIVYCR
TQKRKGDHPEVSFDFLGYTFRPRAARDGKGGIFTSFPPAISKSALKRLSARVRSWRLHLRTGSTFTGLARTINPIV
RGWMQYYGRFYRTALYPLLKRINAYLVRWLRKKYKRLRTFKKAKAAWRRVTRQCPLLLSHWAWVQSFW
>R5OH173||gene_108868|GeneMark.hmm|416_aa|+|1506|2756
MSGPQLSGKPFDISKQEVWRAYQKVKANKGAPGVDDCSVEAFEKDLKNNLYKIWNRMSSGSYFPPPVRAVE
MPKPSGGVRVLGVPTVADRVAQTVVAEKLEKRVEPIFHPDSYGYRPGRSALDAVGQCRRRCWNRAWVVDLDI
ARFFDEVDHQLLLKAVAGHAPEPWVLLYISRWLKAPIQHGDGTIAQRSRGTPQGSAVSPVLANLFLHYAFDM

WMARRFPTVQFERYVDDVVVHCVTERQAREVREAVEGRLARVGLRMHPDKTRIVYCRTQKRKGDHPEVSFD
FLGYTFRPRAARDGKGGIFTSFLPAISKSALKRLSARVRSWRLHLRTGSTLTGLARTINPIVRGWMQYYGRFYRTA
LYPLLKRINAYLVRWLRKKYKRLRTFKKAKAAWRRVTRQCPLLLSHWAWVQSFW
>R5OH174||gene_176884|GeneMark.hmm|416_aa|-|719|1969
MSGPQLSGKPFDISKQEVWRAYQKVKANKGAPGVDDCSVEAFEKDLKNNLYKIWNRMSSGSYFPPPVRAVE
MPKPSGGVRVLGVPTVADRVAQTVVAEKLEKRVEPIFHPDSYGYRPGRSALDAVGQCRRRCWNRAWVVDLDI
ARFFDEVDHQLLLKAVAGHAPEPWVLLYISRWLKAPIQHGDGTIAQRSRGTPQGSAVSPVLANLFLHYAFDM
WMARRFPTVQFERYVDDVVVHCVTERQAREVREAVEGRLARVGLRMHPDKTRIVYCRTQKRKGDHPEVSFD
FLGYTFRPRAARDGKGGIFTSFLPAISKSALKRLSARVRSWRLHLRTGSTFTGLARTINPIVRGWMQYYGQFYRTA
LYPLLKRINAYLVRWLRKKYKRLRTFKKAKAAWRRVTRQCPLLLSHWAWVQSFW
>R5OH175||gene_159846|GeneMark.hmm|421_aa|-|290|1555
VAPRPSKTAGKAFDIPKALVVEAWERVRNNKGAPGVDGAAIEDFERDLQANLYKIWNRMSSGSYFPPPVRQV
RIPKPDGGIRVLGVPTVADRLAQTVVALVLERRAEPVFHQGSYGYRPGRGAIDAVAACRRRCWESNWVIDMDI
QAFFDTVPWDLVCRAVATVCDLPWVMLYVRRWLKAPAQQGDGTLTERWRGTPQGSAVSPVLANLFMHYAL
DAWLARNFPGVVFERYADDVVIHCKSLEQARAVLAAVAERMRQVGLRLHPRKTRIVYCKDANRTGSWEHTEF
TFLGYEFRERTVKGRHGLFRSFSPAVSRAALKRMSATVRSWRLHRWVTATVSDLAAHVNPVVRGWMRYYGAF
HPSALYPLLRRINSYLVRWLRGKYRRLRASWATTMRKWYTGVKKAPGYFAHWAWVTEPGPVW
>R5OH176||gene_100451|GeneMark.hmm|421_aa|+|712|1977
VTPRPSKTAGKAFDIPKALVVEAWERVRSNKGAPGVDGEAIEDFEKDLKNNLYKIWNRMSSGSYFPPPVRQVRI
PKPDGGIRVLGVPTVADRLAQTVVAQVLERRAEPVFHRDSYGYRPGRGAIDAVAACRRRCWENNWVIDMDI
QAFFDTVPWDLVCRAVETVCDLPWVMLYVRRWLKAPAQQGDGTLTERRRGTPQGSAVSPVLANLFMHYALD
AWLTRNFPGVVFERYADDVVIHCKSLEQARTVLAAVTERMRRVGLRLHPGKTRIVYCKDANRTGSWEHTEFTF
LGYEFRERTVKGRHGLFRSFSPAVSRAALKRMSAAVRSWRLHRWVTATAGDLAARVNPVVRGWMCYYGAFH
PSALYPLLRRINSYMVRWLRGKYRRLRASWARTMRKWYAGVKKAPGYFVHWAWVTEPGPVW
>R5OH176||gene_22936|GeneMark.hmm|416_aa|-|50|1300
MSGPQLSGKPFDISKQEVWRAYQKVKANKGAPGVDDCSVEAFEKDLKNNLYKIWNRMSSGSYFPPPVRAVE
MPKPSGGVRVLGVPTVADRVAQTVVAEKLEKRVEPIFHPDSYGYRPGRSALDAVGQCRRRCWNRAWVVDLDI
ARFFDEVDHQLLLKAVAGHAPEPWVLLYISRWLKAPIQHGDGTIAQRSRGTPQGSAVSPVLANLFLHYAFDM
WMARRFPTVQFERYVDDVVVHCVTERQAREVREAVEGRLARVGLRMHPDKTRIVYCRTQKRKGDHPEVSFD
FLGYTFRPRAARDGKGGIFTSFLPAISKSALKRLSARVRSWRLHLRTGSTLTGLARTINPIVRGWMQYYGRFYRTA
LYPLLKRINAYLVRWLRKKYKRLRTFKKAKAAWRRVTRQCPLLLSHWAWVQSFW
>R5OH180||gene_274533|GeneMark.hmm|416_aa|+|936|2186
MSGPQLSGKPFDISKQEVWRAYQKVKANKGAPGVDDCSVEAFEKDLKNNLYKIWNRMSSGSYFPPPVRAVE
MPKPSGGVRVLGVPTVADRVAQTVVAEKLEKRVEPIFHPDSYGYRPGRSALDAVGQCRRRCWNRAWVVDLDI
ARFFDEVDHQLLLKAVAGHAPEPWVLLYISRWLKAPIQHGDGTIAQRSRGTPQGSAVSPVLANLFLHYAFDM
WMARRFPTVQFERYVDDVVVHCVTERQAREVREAVEGRLARVGLRMHPDKTRIVYCRTQKRRGDHPEVSFD
FLGYTFRPRAARDGKGGIFTSFLPAISKSALKRLSARVRSWRLHLRTGSTLTGLARTINPIVRGWMQYYGRFYRTA
LYPLLKRINAYLVRWLRKKYKRLRTFKKAKAAWRRVTRQCPLLLSHWAWVQSFW
>R5OH180||gene_176518|GeneMark.hmm|430_aa|-|94|1386
MSGSVKLAGSSVRSSGSAVKPFDVPKWLVMAAWEKVRSNKGAPGVDGAAVEDFEKDLRANLYKIWNRMSS
GSYFPSPVREVRIPKPDGGIRVLGVPTVADRLAQTVVAMVLEHRAERVFHPGSYGYRPGRGAIDAVRSCRRRC
WENDWVIDLDIQAFFDTVPWDLVCRAVGAVCDLPWVMLYVRRWLKAPLQHSDGTLTERERGTPQGSAVSPV
LANLFMHYAFDTWMARSYPGIVFERYADDVVIHCESLNQARVVLTAVEERMGQVGLGLHPRKTRIVYCKDANR
PGSWEHTGFTFLGYEFRERTVKGRHGLFRSFSPAVSRTALKRMSTQVRSWRLHRWVTATAGDLAAQINPVLRG

WMSYYGVVHPSALYPLLKRVNSYLIRWLRGKYRKLRVSWSKTMRKWYTGVKKAPNYFVHWAWVTEPGPVW
>R5OH186||gene_536|GeneMark.hmm|382_aa|-|592|1740
MTAPRGAFEKDLKNNLYKIWNRMSSGSYFPPPVRAVEMPKPSGGVRVLGVPTVADRVAQTVVAEKLEKRVEPI
FHPDSYGYRPGRSALDAVGQCRRRCWNRAWVVDLDIARFFDEVDHQLLLKAVAGHAPEPWVLLYISRWLKAP
IQHGDGTIAQRSRGTPQGSAVSPVLANLFLHYAFDMWMARRFPTVQFERYVDDVVVHCVTERQAREVREAV
EGRLARVGLRMHPDKTRIVYCRTQKRKGDHPEVSFDFLGYTFRPRAARDGKGGIFTSFLPAISKSALKRLSARVRS
WRLHLRTGSTLTGLARTINPIVRGWMQYYGRFYRTALYPLLKRINAYLVRWLRKKYKRLRTFKKAKAAWRRVTR
QCPLLLSHWAWVQSFW
>R5OH189||gene_55474|GeneMark.hmm|421_aa|+|1328|2593
VAPRPSKTAGKAFDIPKALVVEAWERVRSNKGAPGADGAAIEDFERDLQANLYKIWNRMSSGSYFPPPVRQVR
IPKPDGGIRVLGVPTVADRLAQTVVALVLERRAEPVFHQGSYGYRPGRGAIDAVAACRRRCWESSWVIDMDIQ
AFFDTVPWDLVCRAVETVCDLPWVMLYVRRWLMAPAQQGDGTLTERWRGTPQGSAVSPVLANLFMHYALD
AWLARNFAGVVFERYADDVVIHCKSLEQARAVLAAVAERMRQVGLRLHPRKTRIVYCKDANRTGSWEHTEFTF
LGYEFRERTVKGRHGLFRSFSPAVSKAALKRMSATVRSWRLHRWVTATVSDLAAHVNPVVRGWMRYYGAFHP
SALYPLLRRINSYLVRWLRGKYRRLRASWARTMRKWYTGVKKAPNYFAHWAWVTEPGPVW
>R5OH190||gene_72503|GeneMark.hmm|416_aa|+|3421|4671
MSGPQLSGKPFDISKQEVWRAYQKVKANKGAPGVDDCSVEAFEKDLKNNLYKIWNRMSSGSYFPPPVRAVE
MPKPSGGVRVLGVPTVADRVAQTVVAEKLEKRVEPIFHPDSYGYRPGRSALDAVGQCRRRCWNRAWVVDLDI
ARFFDEVDHQLLLKAVAGHAPEPWVLLYISRWLKAPIQHGDGTIAQRSRGTPQGSAVSPVLANLFLHYAFDM
WMARRFPTVQFERYVDDVVVHCVTERQAREVREAVEGRLARVGLRMHPDKTRIVYCRTQKRKGDHPEVSFD
FLGYTFRPRAARDGKGGIFTSFLPAISKSALKRLSARVRSWRLHLRTGSTFTGLARTINPIVRGWMQYYGRFYRTA
LYPLLKRINAYLVRWLRKKYKRLRTFKKAKAAWRRVTRQCPLLLSHWAWVQSFW
>R5OH191||gene_144931|GeneMark.hmm|416_aa|+|618|1868
MSGPQLSGKPFDISKQEVWRAYQKVKANKGAPGVDDCSVEAFEKDLKNNLYKIWNRMSSGSYFPPPVRAVE
MPKPSGGVRVLGVPTVADRVAQTVVAEKLEKRVEPIFHPDSYGYRPGRSALDAVGQCRRRCWNRAWVVDLDI
ARFFDEVDHQLLLKAVAGHAPEPWVLLYISRWLKAPIQHGDGTIAQRSRGTPQGSAVSPVLANLFLHYAFDM
WMARRFPTVQFERYVDDVVVHCVTERQAREVREAVEGRLARVGLRMHPDKTRIVYCRTQKRKGDHPEVSFD
FLGYTFRPRAARDGKGGIFTSFLPAISKSALKRLSARVRSWRLHLRTGSTLTGLARTINPIVRGWMQYYGRFYRTA
LYPLLKRINAYLVRWLRKKYKRLRTFKKAKAAWRRVTRQCPLLLSHWAWVQSFW
>R5OH192||gene_74247|GeneMark.hmm|430_aa|+|60|1352
MSGSVKLAGSSVRSSGSAVKPFDVPKWLVMAAWEKVRSNKGAPGADGAAVEDFEKDLRASLYKIWNRMSSG
SYFPSPVREVRIPKPDGGIRVLGVPTVAGRLAQTVVAMVLEHRAERVFHPGSYGYRPGRGAIDAVRACRRRCW
ENDWVIDLDIQAFFDTVPWDLVCRVVGAVCDLPWVMLYVRRWLKAPLQHSDGTLTERERGTPQGSAVSPVL
ANLFMHYAFDTWMARSYPGIVFERYADDVVIHCKSLNQARVVLTAVEERMGQVGLGLHPRKTRIVYCKDANR
PGSWEHTGFTFLGYEFRERTVKGRHGLFRSFSPAVSRTALKRMSTQVRSWRLHRWVTATAGDLAAQINPVLRG
WMSYYGVFHPSALYPLLKRVNSYLIRWLRGKYRKLRVSWSKTMRKWYTGVKKAPNYFVHWAWVTEPGPVW
>R5OH192||gene_64410|GeneMark.hmm|382_aa|+|1009|2157
MTAPRGAFEKDLKNNLYKIWNRMSSGSYFPPPVRAVEMPKPSGGVRVLGVPTVADRVAQTVVAEKLEKRVEPI
FHPDSYGYRPGRSALDAVGQCRRRCWNRAWVVDLDIARFFDEVDHQLLLKAVAGHAPEPWVLLYISRWLKAP
IQHGDGTIAQRSRGTPQGSAVSPVLANLFLHYAFDMWMARRFPTVQFERYVDDVVVHCVTERQAREVREAV
EGRLARVGLRMHPDKTRIVYCRTQKRKGDHPEVSFDFLGYTFRPRAARDGKGGIFTSFLPAISKSALKRLSARVRS
WRLHLRTGSTLTGLARTINPIVRGWMQYYGRFYRTALYPLLKRINAYLVRWLRKKYKRLRTFKKAKAAWRRVTR
QCPLLLSHWAWVQSFW
>R5OH194||gene_76530|GeneMark.hmm|430_aa|+|985|2277

MSGSVKLAGSSVRSSGSAVKPFDVPKWLVMAAWEKVRSNKGAPGVDGAAVEDFEKDLRANLYKIWNRMSS
GSYFPSPVREVRIPKPDGGIRVLGVPTVADRLAQTVVAMVLEHRAERVFHPGSYGYRPGRGAIDAVRSCRRRC
WENDWVIDLDIQAFFDTVPWDLVCRAVGAVCDLPWVMLYVRRWLKAPLQHSDGTLTERERGTPQGSAVSPV
LANLFMHYAFDTWMARSYPGIVFERYADDVVIHCESLNQARVVLTAVEERMGQVGLGLHPRKTRIVYCKDANR
PGSWEHTGFTFLGYEFRERTVKGRHGLFRSFSPAVSRTALKRMSTQVRSWRLHRWVTATAGDLAAQINPVLRG
WMSYYGVVHPSALYPLLKRVNSYLIRWLRGKYRKLRVSWSKTMRKWYTGVKKAPNYFVHWAWVTEPGPVW
>R5OH198||gene_249640|GeneMark.hmm|416_aa|+|2424|3674
MSGPQLSGKPFDISKQEVWRAYQKVKANKGAPGVDDCSVEAFEKDLKNNLYKIWNRMSSGSYFPPPVRAVEI
PKPSGGVRVLGVPTVADRVAQTVVAEKLEKRVEPIFHPDSYGYRPGRSALDAVGQCRRRCWNRAWVVDLDIA
RFFDEVDHQLLLKAVAGHAPEPWVLLYISRWLKAPIQHGDGTIAQRSRGTPQGSAVSPVLANLFLHYAFDMW
MARRFPTVQFERYVDDVVVHCVTERQAREVREAVEGRLARVGLRMHPDKTRIVYCRTQKRRGDHPEVSFDFL
GYTFRPRAARDGKGGIFTSFLPAISKSALKRLSARVRSWRLHLRTGSTFTGLARTINPIVRGWMQYYGRFYRTALY
PLLKRINAYLVRWLRKKYKRLRTFKKAKAAWRRVTRQCPLLLSHWAWVQSFW
>R5OH202||gene_156648|GeneMark.hmm|430_aa|+|840|2132
MSGSVKLAGSSVRSSGSAVKPFDVPKWLVMAAWEKVRSNKGAPGVDGAAVEDFEKDLRANLYKIWNRMSS
GSYFPSPVREVRIPKPDGGIRVLGVPTVAGRLAQTVVAMVLEHRAERVFHPGSYGYRPGCGAIDAVRSCRRRC
WENDWVIDVDIQAFFDTVPWDLVCRAVGAVCDLPWVMLYVRRWLKAPLQHSDGTLTERERGTPQGSAVSP
VLANLFMHYAFDTWMARSYPGIVFERYADDVVIHCESLNQARVVLTAVEERMGQVGLGLHPRKTRIVYCKDA
NRPGSWEHTGFTFLGYEFRERTVKGRHGLFRSFSPAVSRTALKRMSTQVRSWRLHRWVTATAGDLAAQINPVL
RGWMSYYGVFHPSALYPLLKRVNSYLIRWLRGKYRKLRVSWSKTMRKWYTGVKKAPNYFVHWAWVTEPGPV
W
>R5OH205||gene_162149|GeneMark.hmm|416_aa|-|3088|4338
MSGPQLSGKPFDISKQEVWRAYQKVKANKGAPGVDDCSVEAFEKDLKNNLYKIWNRMSSGSYFPPPVRAVE
MPKPSGGVRVLGVPTVADRVAQTVVAEKLEKRVEPIFHPDSYGYRPGRSALDAVGQCRRRCWNRAWVVDLDI
ARFFDEVDHQLLLKAVAGHAPEPWVLLYISRWLKAPIQHGDGTIAQRSRGTPQGSAVSPVLANLFLHYAFDM
WMARRFPTVQFERYVDDVVVHCVTERQAREVREAVEGRLARVGLRMHPDKTRIVYCRTQKRKGDHPEVSFD
FLGYTFRPRAARDGKGGIFTSFLPAISKSALKRLSARVRSWRLHLRTGSTLTGLARTINPIVRGWMQYYGRFYRTA
LYPLLKRINAYLVRWLRKKYKRLRTFKKAKAAWRRVTRQCPLLLSHWAWVQSFW
>R5OH206||gene_65978|GeneMark.hmm|430_aa|-|658|1950
MSGSVKLAGSSVRSSGSAVKPFDVPKWLVMEAWEKVRSDKGAPGVDGAAVEDFEKDLRASLYKIWNRMSSG
SYFPSPVREVRIPKPDGGIRVLGVPTVAGRLAQTVVAMVLEHRAERVFHPGSYGYRPGRGAIDAVRACRRRCW
ENDWVIDLDIQAFFDTVPWDLVCRVVGAVCDLPWVMLYVRRWLKAPLQHSDGTLTERERGTPQGSAVSPVL
ANLFMHYAFDTWMARSYPGIVFERYADDVVIHCKSLNQARVVLTAVEERMGQVGLGLHPRKTRIVYCKDANR
PGSWEHTGFTFLGYEFRERTVKGRHGLFRSFSPAVSRMALKRMSTQVRSWRLHRWVTATAGDLAAQINPVLR
GWMSYYGVFHPSALYPLLKRVNSYLIRWLRGKYRKLRVSWSKTMRKWYTGVKKAPNYFVHWAWVTEPGPV
W
>R5OH206||gene_81016|GeneMark.hmm|416_aa|+|1667|2917
MSGPQLSGKPFDISKQEVWRAYQKVKANKGAPGVDDCSVEAFEKDLKNNLYKIWNRMSSGSYFPPPVRAVE
MPKPSGGVRVLGVPTVADRVAQTVVAEKLEKRVEPIFHPDSYGYRPGRSALDAVGQCRRRCWNRAWVVDLDI
ARFFDEVDHQLLLKAVAGHAPEPWVLLYISRWLKAPIQHGDGTIAQRSRGTPQGSAVSPVLANLFLHYAFDM
WMARRFPTVQFERYVDDVVVHCVTERQAREVREAVEGRLARVGLRMHPDKTRIVYCRTQKRKGDHPEVSFD
FLGYTFRPRAARDGKGGIFTSFLPAISKSALKRLSARVRSWRLHLRTGSTLTGLARTINPIVRGWMQYYGRFYRTA
LYPLLKRINAYLVRWLRKKYKRLRTFKKAKAAWRRVTRQCPLLLSHWAWVQSFW
>R5OH208||gene_94156|GeneMark.hmm|416_aa|+|1330|2580

MSGPQLSGKPFDISKQEVWRAYQKVKANKGAPGVDDCSVEAFEKDLKNNLYKIWNRMSSGSYFPPPVRAVE
MPKPSGGVRVLGVPTVADRVAQTVVAEKLEKRVEPIFHPDSYGYRPGRSALDAVGQCRRRCWNRAWVVDLDI
ARFFDEVDHQLLLKAVAGHAPEPWVLLYISRWLKAPIQHDDGTIAQRSRGTPQGSAVSPVLANLFLHYAFDM
WMARRFPTVQFERYVDDVVVHCVTERQAREVREAVEGRLARVGLRMHPDKTRIVYCRTQKRRGDHPEVSFD
FLGYTFRPRAARDGKGGIFTSFLPAISKSALKRLSARVRSWRLHLRTGSTLAGLARTINPIVRGWMQYYGRFYRTA
LYPLLKRINAYLVRWLRKKYKRLRTFKKAKAAWRRVTRQCPLLLSHWAWVQSFW
>R5OH210||gene_160768|GeneMark.hmm|416_aa|-|188|1438
MSGPQLSGKPFDISKQEVWRAYQKVKANKGAPGVDDCSVEAFEKDLKNNLYKIWNRMSSGSYFPPPVRAVE
MPKPSGGVRVLGVPTVADRVAQTVVAEKLEKRVEPIFHPDSYGYRPGRSALDAVGQCRRRCWNRAWVVDLDI
ARFFDEVDHQLLLKAVAGHAPEPWVLLYISRWLKAPIQHGDGTIAQRSRGTPQGSAVSPVLANLFLHYAFDM
WMARRFPTVQFERYVDDVVVHCVTERQAREVREAVEGRLARVGLRMHPDKTRIVYCRTQKRKGDHPEVSFD
FLGYTFRPREARDGKGGIFTSFLPAISKSALKRLSARVRSWRLHLRTGSTLTGLARTINPIVRGWMQYYGRFYRTAL
YPLLKRINAYLVRWLRKKYKRLRTFKKAKAAWRRVTRQCPLLLSHWAWVQSFW
>R5OH213||gene_15324|GeneMark.hmm|430_aa|-|389|1681
MSGSVKLAGSSVRSSGSAVKPFDVPKWLVMAAWEKVRSNKGAPGVDGAAVEDFEKDLRANLYKIWNRMSS
GSYFPSPVREVRIPKPDGGIRVLGVPTVADRLAQTVVAMVLEHRAERVFHPGSYGYRPGRGAIDAVRSCRRRC
WENDWVIDLDIQAFFDTVPWDLVCRAVGAVCDLPWVMLYVRRWLKAPLQHSDGTLTERERGTPQGSAVSPV
LANLFMHYAFDTWMARSYPGIVFERYADDVVIHCESLNQARVVLTAVEERMGQVGLGLHPRKTRIVYCKDANR
PGSWEHTGFTFLGYEFRERTVKGRHGLFRSFSPAVSRTALKRMSTQVRSWRLHRWVTATAGDLAAQINPVLRG
WMSYYGVVHPSALYPLLKRVNSYLIRWLRGKYRKLRVSWSKTMRKWYTGVKKAPNYFVHWAWVTEPGPVW
>R5OH213||gene_158429|GeneMark.hmm|416_aa|+|3476|4726
MSGPQLSGKPFDISKQEVWRAYQKVKANKGAPGVDDCSVEAFEKDLKNNLYKIWNRMSSGSYFPPPVRAVE
MPKPSGGVRVLGVPTVADRVAQTVVAEKLEKRVEPIFHPDSYGYRPGRSALDAVGQCRRRCWNRAWVVDLDI
ARFFDEVDHQLLLKAVAGHAPEPWVLLYISRWLKAPIQHGDGTIAQRSRGTPQGSAVSPVLANLFLHYAFDM
WMARRFPTVQFERYVDDVVVHCVTERQAREVREAVEGRLARVGLRMHPDKTRIVYCRTQKRRGDHPEVSFD
FLGYTFRPRAARDGKGGIFTSFLPAISKSALKRLSARVRSWRLHLRTGSTLAGLARTINPIVRGWMQYYGRFYRTA
LYPLLKRINAYLVRWLRKKYKRLRTFKKAKAAWRRVTRQCPLLLSHWAWVQSFW
>R5OH217||gene_41571|GeneMark.hmm|416_aa|+|587|1837
MSGPQLSGKPFDISKQEVWRAYQKVKANKGAPGVDDCSVEAFEKDLKNNLYKIWNRMSSGSYFPPPVRAVE
MPKPSGGVRVLGVPTVADRVAQTVVAEKLEKRVEPIFHPDSYGYRPGRSALDAVGQCRRRCWNRAWVVDLDI
ARFFDEVDHQLLLKAVAGHAPEPWVLLYISRWLKAPIQHGDGTIAQRSRGTPQGSAVSPVLANLFLHYAFDM
WMARRFPTVQFERYVDDVVVHCVTERQAREVREAVEGRLARVGLRMHPDKTRIVYCRTQKRRGDHPEVSFD
FLGYTFRPRAARDGKGGIFTSFLPAISKSALKRLSARVRSWRLHLRTGSTLAGLARTINPIVRGWMQYYGRFYRTA
LYPLLKRINAYLVRWLRKKYKRLRTFKKAKAAWRRVTRQCPLLLSHWAWVQSFW
>R5OH219||gene_40818|GeneMark.hmm|421_aa|+|565|1830
VTPRPSKTAGKAFDIPKALVVEAWERVRSNKGAPGVDGEAIEDFEKDLKNNLYKIWNRMSSGSYFPPPVRQVRI
PKPDGGIRVLGVPTVADRLAQTVVAQVLERRAEPVFHRDSYGYRPGRGAIDAVAACRRRCWENNWVIDMDI
QAFFDTVPWDLVCRAVETVCDLPWVMLYVRRWLKAPAQQGDGTLTERRRGTPQGSAVSPVLANLFMHYALD
AWLTRNFPGVVFERYADDVVIHCKSLEQARTVLAAVTERMRRVGLRLHPGKTRIVYCKDANRTGSWEHTEFTF
LGYEFRERTVKGRHGLFRSFSPAVSRAALKRMSAAVRSWRLHRWVTATAGDLAARVNPVVRGWMCYYGAFH
PSALYPLLRRINSYMVRWLRGKYRRLRASWARTMRKWYAGVKKAPGYFVHWAWVTEPGPVW
>R5OH220||gene_199880|GeneMark.hmm|421_aa|+|1571|2836
VAPRPSKTAGKAFDIPKALVVEAWERVRNNKGAPGVDGAAIEDFERDLQANLYKIWNRMSSGSYFPPPVRQV
RIPKPDGGIRVLGVPTVADRLAQTVVALVLERRAEPVFHQGSYGYRPGRGAIDAVAACRRRCWESSWVIDMDI

QAFFDTVPWDLVCRAVAMVCDLPWVMLYVRRWLKAPAQQGDGTLTERWRGTPQGSAVSPVLANLFMHYA
LDAWLARNFPGVVFERYADDVVIHCKSLEQARAVLAAVTERMRQVGLRLHPRKTRIVYCKDANRTGSWEHTEF
TFLGYEFRERTVKGRHGLFRSFSPAVSRAALKRMSATVRSWRLHRWVTATVSDLAAHVNPVVRGWMRYYGAF
HPSALYPLLRRINSYLVRWLRGKYRRLRASWARTMRKWYTGVKKAPSYFAHWAWVTEPGPVW
>R5OH221||gene_99957|GeneMark.hmm|421_aa|+|688|1953
VAPRPSKTAGKAFDIPKALVVEAWERVRNNKGAPGVDGAAIEDFERDLQANLYKIWNRMSSGSYFPPPVRQV
RIPKPDGGIRVLGVPTVADRLAQTVVALVLERRAEPVFHQGSYGYRPGRGAIDAVAACRRRCWESSWVIDMDI
QAFFDTVPWDLVCRAVETVCDLPWVMLYVRRWLKAPAQQGDGTLTERWRGTPQGSAVSPVLANLFMHYAL
DAWLARNFAGVVFERYADDVVIHCKSLEQARAVLAAVTERMRQVGLRLHPRKTRIVYCKDANRTGSWEHTEF
TFLGYEFRERTVKGRHGLFRSFSPAVSRAALKRMSATVRSWRLHRWVTATVSDLAAHVNPVVRGWMRYYGAF
HPSALYPLLRRINSYLVRWLRGKYRRLRASWARTMRKWYTGVKKAPSYFAHWAWVTEPGPVW
>R5OH225||gene_42764|GeneMark.hmm|363_aa|+|984|2075
MSSGSYFPPLVRQVRIPKPDGGIRVLGVPTVADRLAQTVVALVLERRAEPVFHQGSYGYRPGRGAIDAVAACRR
RCWESSWVIDMDIQAFFDTVPWDLVCRAVETVCDLPWVMLYVRRWLKAPAQQGDGTLTERWRGTPQGSA
VSPVVANLFMHYALDAWLARNFAGVVFERYADDVVIHCKSLEQARAVLAAVAERIRQVGLRLHPRKTRIVYCKD
ANRTGSWEHTEFTFLGYEFRERTVKGRHGLFRSFSPAVSRAALKRVSATVRSWRLHRWVTATVSDLAAHVNPV
VRGWMRYYGAFHPSALYPLLRRINSYLVRWLRGKYRRLRASWARTMRKWYTGVKKAPNYFAHWAWVTEPG
PVW
>R5OH225||gene_26046|GeneMark.hmm|416_aa|-|3082|4332
MSGPQLSGKPFDISKQEVWRAYQKVKANKGAPGVDDCSVEAFEKDLKNNLYKIWNRMSSGSYFPPPVRAVE
MPKPSGGVRVLGVPTVADRVAQTVVAEKLEKRVEPIFHPDSYGYRPGRSALDAVGQCRRRCWNRAWVVDLDI
ARFFDEVDHQLLLKAVAGHAPEPWVLLYISRWLKAPIQHGDGTIAQRSRGTPQGSAVSPVLANLFLHYAFDM
WMARRFPTVQFERYVDDVVVHCVTERQAREVREAVEGRLARVGLRMHPDKTRIVYCRTQKRKGDHPEVSFD
FLGYTFRPRAARDGKGGIFTSFLPAISKSALKRLSARVRSWRLHLRTGSTLTGLARTINPIVRGWMQYYGRFYRTA
LYPLLKRINAYLVRWLRKKYKRLRTFKKAKAAWRRVTRQCPLLLSHWAWVQSFW
>R5OH235||gene_169575|GeneMark.hmm|382_aa|+|720|1868
MTAPRGAFEKDLKNNLYKIWNRMSSGSYFPPPVRAVEMPKPSGGVRVLGVPTVADRVAQTVVAEKLEKRVEPI
FHPDSYGYRPGRSALDAVGQCRRRCWNRAWVVDLDIARFFDEVDHQLLLKAVAGHAPEPWVLLYISRWLKAP
IQHGDGTIAQRSRGTPQGSAVSPVLANLFLHYAFDMWMARRFPTVQFERYVDDVVVHCVTERQAREVREAV
EGRLARVGLRMHPDKTRIVYCRTQKRKGDHPEVSFDFLGYTFRPRAARDGKGGIFTSFLPAISKSALKRLSARVRS
WRLHLRTGSTLTGLARTINPIVRGWMQYYGRFYRTALYPLLKRINAYLVRWLRKKYKRLRTFKKAKAAWRRVTR
QCPLLLSHWAWVQSFW
>R5OH235||gene_239615|GeneMark.hmm|430_aa|-|2320|3612
MSGSVKLAGSSVRSSGSAVKPFDVPKWLVMEAWEKVRSDKGAPGVDGAAVEDFEKDLRANLYKIWNRMSSG
SYFPSPVREVRIPKPDGGIRVLGVPTVAGRLAQTVVAMVLEHRAERVFHPGSYGYRPGRGAIDAVRACRRRCW
ENDWVIDLDIQAFFDTVPWDLVCRAVGAVCDLPWVMLYVRRWLKAPLQHSDGTLTERERGTPQGSAVSPVL
ANLFMHYAFDTWMARSYPGIVFERYSDDVVIHCKSLNQARVVLTAVEERMGQVGLGLHPRKTRIVYCKDANR
PGSWEHTGFTFLGYEFRERTVKGRHGLFRSFSPAVSRTALKRMSTQVRSWRLHRWVTATAGDLAAQINPVLRG
WMSYYGVFQPSALYPLLKRVNSYLIRWLRGKYRKLRVSWSKTMRKWYTGVKKAPNYFVHWAWVTEPGPVW
>R5OH238||gene_67384|GeneMark.hmm|416_aa|+|807|2057
MSGPQLSGKPFDISKQEVWRAYQKVKANKGAPGVDDCSVEAFEKDLKNNLYKIWNRMSSGSYFPPPVRAVE
MPKPSGGVRVLGVPTVADRVAQTVVAEKLEKRVEPIFHPDSYGYRPGRSALDAVGQCRRRCWNRAWVVDLDI
ARFFDEVDHQLLLKAVAGHAPEPWVLLYISRWLKAPIQHGDGTIAQRSRGTPQGSAVSPVLANLFLHYAFDM
WMARRFPTVQFERYVDDVVVHCVTERQAREVREAVEGRLARVGLRMHPDKTRIVYCRTQKRKGDHPEVSFD

FLGYTFRPRAARDGKGGIFTSFLPAISKSALKRLSARVRSWRLHLRTGSTLTGLARTINPIVRGWMQYYGRFYRTA
LYPLLKRINAYLVRWLRKKYKRLRTFKKAKAAWRRVTRQCPLLLSHWAWVQSFW
>R5OH241||gene_196332|GeneMark.hmm|430_aa|-|12469|13761
MSGSVKLAGSSVRSSGSAVKPFDVPKWLVMAAWEKVRSNKGAPGVDGAAVEDFEKDLRANLYKIWNRMSS
GSYFPSPVREVRIPKPDGGIRVLGVPTVADRLAQTVVAMVLEHRAERVFHPGSYGYRPGRGAIDAVRSCRRRC
WENDWVIDLDIQAFFDTVPWDLVCRAVGAVCDLPWVMLYVRRWLKAPLQHSDGTLTERERGTPQGSAVSPV
LANLFMHYAFDTWMARSYPGIVFERYADDVVIHCESLNQARVVLTAVEERMGQVGLGLHPRKTRIVYCKDANR
PGSWEHTGFTFLGYEFRERTVKGRHGLFRSFSPAVSRTALKRMSTQVRSWRLHRWVTAMAGDLAAQINPVLR
GWMSYYGVFHPSALYPLLKRVNSYLIRWLRGKYRKLRVSWSKTMRKWYTGVKKAPNYFVHWAWVTEPGPV
W
>R5OH243||gene_222133|GeneMark.hmm|360_aa|+|1202|2284
MSSGSYFPPPVRAVEMPKPSGGVRVLGVPTVADRVAQTVVAEKLEKRVEPIFHPDSYGYRPGRSALDAVGQCR
RRCWNRAWVVDLDIARFFDEVDHQLLLKAVAGHAPEPWVLLYISRWLKAPIQHGDGTIAQRSRGTPQGSAVS
PVLANLFLHYAFDMWMARRFPTVQFERYVDDVVVHCVTERQAREVREAVEGRLARVGLRMHPDKTRIVYCR
TQKRKGDHPEVSFDFLGYTFRPRAARDGKGGIFTSFLPAISKSALKRLSARVRSWRLHLRTGSTFTGLARTINPIVR
GWMQYYGRFYRTALYPLLKRINAYLVRWLRKKYKRLRTFKKAKAAWRRVTRQCPLLLSHWAWVQSFW
>R5OH245||gene_144391|GeneMark.hmm|430_aa|-|91|1383
MSGSVKLAGSSVRSSGSAVKPFDVPKWLVMAAWEKVRSNKGAPGVDGAAVEDFEKDLRANLYKIWNRMSS
GSYFPSPVREVRIPKPDGGIRVLGVPTVADRLAQTVVAMVLEHRAERVFHPGSYGYRPGRGAIDAVRSCRRRC
WENDWVIDLDIQAFFDTVPWDLVCRAVGAVCDLPWVMLYVRRWLKAPLRHSDGTLTERERGTPQGSAVSPV
LANLFMHYAFDTWMARSYPGIVFERYADDVVIHCESLNQARVVLTAVEERMGQVGLGLHPRKTRIVYCKDANR
PGSWEHTGFTFLGYEFRERTVKGRHGLFRSFSPAVSRTALKRMSTQVRSWRLHRWVTATAGDLAAQINPVLRG
WMSYYGVFHPSALYPLLKRVNSYLIRWLRGKYRKLRVSWSKTMRKWYTGVKKAPNYFVHWAWVTEPGPVW
>R5OH245||gene_91089|GeneMark.hmm|420_aa|-|194|1456
VRATAKPLPISKRQVWEAYRQVKANGGAAGIDGQTVEAFDEDMANNLYKLWNRLASGSYMPPAVKRVDIPKA
GGGMRPLGVPTVADRIAQTVIRQMLEPIVEPLFHEDSYGYRPGRSAHQALAQTRRRCWRYAWVVEIDIKGFFD
NIDHALLLKAVRHHTRERWVVMYIERWLRAPVQMPDGTIQQREKGTPQGGVISPLLANLFLHYAFDMWMQ
RHHGDVPFERYADDAVCHCHSQVRAQSLIDQLHERFAQCGLELHPQKTRVVYCKDEDRRGNYSDTSFDFLGFT
FRPRLSKNRYGKIFVNFSPAISVKAAKSIRQEVRSWRLQLRSDKALDDLARMFNAKIRGWVNYYGAFYKSALYPT
LRQIDRKLVLWATRKFKRLRGHRRRASHWLARIARRHTRLFAHWPLLWGQASMGRAG
>R5OH249||gene_148204|GeneMark.hmm|421_aa|+|2057|3322
VAPRPSKTAGKAFDIPKALVVEAWERVRSNKGAPGADGAAIEDFERDLQANLYKIWNRMSSGSYFPPPVRQVR
IPKPDGGIRVLGVPTVADRLAQTVVALVLERRAEPVFHQGSYGYRPGRGAIDAVAACRRRCWESSWVIDMDIQ
AFFDTVPWDLVCRAVETVCDLPWVMLYVRRWLMAPAQQGDGTLTERWRGTPQGSAVSPVLANLFMHYALD
AWLARNFAGVVFERYADDVVIHCKSLEQARAVLAAVAERMRQVGLRLHPRKTRIVYCKDANRTGSWEHTEFTF
LGYEFRERTVKGRHGLFRSFSPAVSKAALKRMSATVRSWRLHRWVTATVSDLAAHVNPVVRGWMRYYGAFHP
SALYPLLRRINSYLVRWLRGKYRRLRASWARTMRKWYTGVKKAPNYFAHWAWVTEPGPVW
>R5OH249||gene_46592|GeneMark.hmm|416_aa|+|1069|2319
MSGPQLSGKPFDISKQEVWRAYQKVKANKGAPGVDDCSVEAFEKDLKNNLYKIWNRMSSGSYFPPPVRAVE
MPKPSGGVRVLGVPTVADRVAQTVVAEKLEKRVEPIFHPDSYGYRPGRSALDAVGQCRRRCWNRAWVVDLDI
ARFFDEVDHQLLLKAVAGHAPEPWVLLYISRWLKAPIQHGDGTIAQRSRGTPQGSAVSPVLANLFLHYAFDM
WMARRFPTVQFERYVDDVVVHCVTERQAREVREAVEGRLARVGLRMHPDKTRIVYCRTQKRRGDHPEVSFD
FLGYTFRPRAARDGKGGIFTSFLPAISKSALKRLSARVRSWRLHLRTGSTLAGLARTINPIVRGWMQYYGRFYRTA
LYPLLKRINAYLVRWLRKKYKRLRTFKKAKAAWRRVTRQCPLLLSHWAWVQSFW

>R5OH251||gene_184841|GeneMark.hmm|403_aa|-|1|1209
MSGPQLSGKPFDISKQEVRRAYQKVKANKGAPGVDDCSVEAFEKDLKNNLYKIWNRMSSGSYFPPPVRAVEIP
KPSGGVRVLGVPTVADRVAQTVVAEKLEKRVEPIFHPDSYGYRPGRSALDAVGQCRRRCWNRAWVVDLDIAR
FFDEVDHQLLLKAVAGHAPEPWVLLYISRWLKAPIQHGDGTITQRSRGTPQGSAVSPVLANLFLHYAFDMWM
ARRFPTVQFERYVDDVVVHCVTERQAREVREAVEGRLARVGLRMHPDKTRIVYCRTQKRRGDHPEVSFDFLGY
TFRPRAARDGKGGIFTSFLPAISKSALKRLSARVRSWRLHLRTGSTLTGLARTINPIVRGWMQYYGRFYRTALYPLL
KRINAYLVRWLRKKYKRLRTFKKAKAAWRRVTRQCP
>R5OH257||gene_78927|GeneMark.hmm|388_aa|-|1764|2930
GQTVEAFDEDMANNLYKLWNRLASGSYMPPAVKRVDIPKAGGGMRPLGVPTVADRIAQTVIRQMLEPIVEPL
FHEDSYGYRPGKSAHQALAQTRRRCWRYAWVVEIDIKGFFDNIDHALLLKAVRHHTRERWVVMYIERWLRAP
VQMPDGTIQQREKGTPQGGVISPLLANLFLHYAFDMWMRRHHGDVPFERYADDAVCHCHSQARARSLIDQL
RERFAQCGLELHPQKTRVVYCKDEDRRGNYPDTSFDFLGFTFRPRLSKNRYGKIFVNFSPAVSVKAAKSIRQEVR
SWRLQLRSDKALDDLARMFNAKIWGWVNYYGAFYKSALYPTLRQIDRKLVLWATRKFKRLRGHRRRSRHWLA
RIACRTPRLFAHWSLLWGQASMGRAG
>R5OH259||gene_83323|GeneMark.hmm|422_aa|+|1246|2514
MSRSLSEVPGGKSFDISKQLVMDAWQRVKSDRGALGVDECSVEEFEEDLAGNLYKIWNRMFSGSCFPPAVRM
VEIPRPQAGTRVLGVPTVADRVAQTAAAMVLEKAAEPVFHPDSYGYRPGRGAIDAVAACRRRCWSRSWVIDL
DIEAFFDSVPWDLVCKAVGAVGAPGWVMLYVKRWLAAPLQHPDGAVVERVRGTPQGSAVSPVLANLFMHY
AFDAWLARAFPGVVFERYADDAVIHCRSLTQARGVLAALEARMKQVGLRLHPRRTRIVYCKDANRPGCYEHTG
FTFLGYEFTERTVKGRHGLFRSFSPAASKTALKTMSARLRSWRLHRWVRATARDLAEHINPIMRGWMSYYGAF
HPSALYPLLKRVSSYLIRWLRGKYRRLRRSWRATMRKWYGGIKAAPGYFVHWAWITGPGPLW
>R5OH261||gene_268653|GeneMark.hmm|349_aa|-|1280|2329
MSSGSYFPPPVRAVEMPKPSGGVRVLGVPTVADRVAQTVVAEKLEKRVEPIFHPDSYGYRPGRSALDAVGQCR
RRCWNRAWVVDLDIARFFDEVDHQLLLKAVAGHAPEPWVLLYISRWLKAPIQHGDGTIAQRSRGTPQGSAVS
PVLANLFLHYAFDMWMARRFPTVQFERYVDDVVVHCVTERQAREVREAVEGRLARVGLRMHPDKTRIVYCR
TQKRKGDHPEVSFDFLGYTFRPRAARDGKGGIFTSFLPAISKSALKRLSARVRSWRLHLRTGSTLTGLARTINPIVR
GWMQYYGRFYRTALYPLLKRINAYLVRWLRKKYKRLRTFKKAKAAWRRVTRQCPSS
>R5OH261||gene_243879|GeneMark.hmm|430_aa|+|301|1593
MSGSVKLAGSSVRSSGSAVKPFDVPKWLVMAAWEKVRSNKGAPGVDGAAVEDFEKDLRANLYKIWNRMSS
GSYFPSPVREVRIPKPDGGIRVLGVPTVADRLAQTVVAMVLEHRAERVFHPGSYGYRPGRGAIDAVRSCRRRC
WENDWVIDLDIQAFFDTVPWDLVCRAVGAVCDLPWVMLYVRRWLKAPLQHSDGTLTERERGTPQGSAVSPV
LANLFMHYAFDTWMARSYPGIVFERYADDVVIHCESLNQARVVLTAVEERMGQVGLGLHPRKTRIVYCKDANR
PGSWEHTGFTFLGYEFRERTVQGRHGLFRSFSPAVSRTALKRMSTQVRSWRLHRWVTATAGDLAAQINPVLRG
WMSYYGVFHPSALYPLLKRVNSYLIRWLRGKYRKLRVSWSKTMRKWYTGVKKAPNYFVHWAWVTEPGPVW
>R5OH265||gene_248869|GeneMark.hmm|421_aa|-|1466|2731
VAPRPSKTAGKAFDIPKALVVEAWERVRSNKGAPGADGAAIEDFERDLQANLYKIWNRMSSGSYFPPPVRQVR
IPKPDGGIRVLGVPTVADRLAQTVVALVLERRAEPVFHQGSYGYRPGRGAIDAVAACRRRCWESSWVIDMDIQ
AFFDTVPWDLVCRAVETVCDLPWVMLYVRRWLMAPAQQGDGTLTERWRGTPQGSAVSPVLANLFMHYALD
AWLARNFAGVVFERYADDVVIHCKSLEQARAVLAAVAERMRQVGLRLHPRKTRIVYCKDANRTGSWEHTEFTF
LGYEFRERTVKGRHGLFRSFSPAVSKAALKRMSATVRSWRLHRWVTATVSDLAAHVNPVVRGWMRYYGAFHP
SALYPLLRRINSYLVRWLRGKYRRLRASWARTMRKWYTGVKKAPNYFAHWAWVTEPGPVW
>R5OH267||gene_158960|GeneMark.hmm|416_aa|+|7253|8503
MSGPQLSGKPFDISKQEVWRAYQKVKANKGAPGVDDCSVEAFEKDLKNNLYKIWNRMSSGSYFPPPVRAVE
MPKPSGGVRVLGVPTVADRVAQTVVAEKLEKRVEPIFHPDSYGYRPGRSALDAVGQCRRRCWNRAWVVDLDI

ARFFDEVDHQLLLKAVAGHAPEPWVLLYISRWLKAPIQHGDGTIAQRSRGTPQGSAVSPVLANLFLHYAFDM
WMARRFPTVQFERYVDDVVVHCVTERQAREVREAVEGRLARVGLRMHPDKTRIVYCRTQKRKGDHPEVSFD
FLGYTFRPRAARDGKGGIFTSFLPAISKSALKRLSARVRSWRLHLRTGSTLTGLARTINPIVRGWMQYYGRFYRTA
LYPLLKRINAYLVRWLRKKYKRLRTFKKAKAAWRRVTRQCPLLLSHWAWVQSFW
>R5OH268||gene_124641|GeneMark.hmm|430_aa|-|1569|2861
MSGSVSLVGVAPRPSKTAGKAFDIPKALVVEAWERVRSNKGAPGADGAAIEDFERDLQANLYKIWNRMSSGSY
FPPPVRQVRIPKPDGGIRVLGVPTVADRLAQTVVALVLERRAEPVFHQGSYGYRPGRGAIDAVAACRRRCWESS
WVIDMDIQAFFDTVPWDLVCRAVETVCDLPWVMLYVRRWLMVPAQQGDGTLTERWRGTPQGSAVSPVLA
NLFMHYALDAWLARNFAGVVFERYADDVVIHCKSLEQARAVLAAVAERMRQVGLRLHPRKTRIVYCKDANRT
GSWEHTEFTFLGYEFRERTVKGRHGLFRSFSPAVSKAALKRMSATVRSWRLHRWVTATVSDLAAHVNPVVRG
WMRYYGAFHPSALYPLLRRINSYLVRWLRGKYRRLRASWARTMRKWYTGVKKAPNYFAHWAWVTEPGPVW
>R5OH272||gene_414562|GeneMark.hmm|421_aa|+|143|1408
VAPRPSKTAGKAFDVPKWLVMEAWEKVRSNKGAPGVDGAAVEDFEKDLRANLYKIWNRMSSGSYFPSPVRE
VRIPKPDGGIRVLGVPTVADRLAQTVVAMVLEHRAERVFHPGSYGYRPGRGAIDAVRACRRRCWENDWVIDL
DIQAFFDTVPWDLVCRAVGAVCDLPWVMLYVRRWLKAPLQHSDGTLTERERGTPQGSAVSPVLANLFMHYA
FDTWMARSYPGIVFERYADDVVIHCKSLNQARVVLTAVEERMGQVGLGLHPRKTRIVYCKDANRPGSWEHTG
FTFLGYEFRERTVKGRHGLFRSFSPAVSRTALKRMSTQVRSWRLHRWVTATAGDLAAQINPVLRGWMSYYGVF
QPSALYPLLKRVNSYLIRWLRGKYRKLRVSWSKTMRKWYTGVKKAPNYFVHWAWVTEPGPVW
>R5OH273||gene_95017|GeneMark.hmm|430_aa|+|833|2125
MSGSVKLAGSSVRSSGSAVKPFDVPKWLVMEAWEKVRSDKGAPGVDGAAVEDFEKDLRASLYKIWNRMSSG
SYFPSPVREVRIPKPDGGIRVLGVPTVAGRLAQTVVAMVLEHRAERVFHPGSYGYRPGRGAIDAVRACRRRCW
ENDWVIDLDIQAFFDTVPWDLVCRAVGAVCDLPWVMLYVRRWLKAPLQHSDGTLTERERGTPQGSAVSPVL
ANLFMHYAFDTWMARSYPGIVFERYADDVVIHCKSLDQARVVLTAVEERMGQVGLGLHPRKTRIVYCKDANR
PGSWEHTGFTFLGYEFRERTVKGRHGLFRSFSPAVSRTALKRMSTQVRSWRLHRWVTATAGDLAAQINPVLQG
WMSYYGVFQPSALYPLLKRVNSYLIRWLRGKYRKLRVSWPKTMRKWYTGVKKAPNYFVHWAWVTEPGPVW
>R5OH273||gene_164961|GeneMark.hmm|353_aa|+|2|1063
IWNRMSSGSYFPPPVRAVEMPKPSGGVRVLGVPTVADRVAQTVVAEKLEKRVEPIFHPDSYGYRPGRSALDAV
GQCRRRCWNRAWVVDLDIARFFDEVDHQLLLKAVAGHAPEPWVLLYISRWLKAPIQHGDGTIAQRSRGTPQ
GSAVSPVLANLFLHYAFDMWMARRFPTVQFERYVDDVVVHCVTERQAREVREAVEGRLARVGLRMHPDKTR
IVYCRTQKRKGDHPEVSFDFLGYTFRPRAARDGKGGIFTSFLPAISKSALKRLSARVRSWRLHLRTGSTFTGLARTI
NPIVRGWMQYYGQFYRTALYPLLKRINAYLVRWLRKKYKRLRTFKKAKAAWRRVTRQCPSS
>R5OH276||gene_80986|GeneMark.hmm|430_aa|+|3157|4449
MSGSVKLAGSSVRSSGSAVKPFDVPKWLVMAAWEKVRSNKGAPGVDGAAVEDFEKDLRANLYKIWNRMSS
GSYFPSPVREVRIPKPDGGIRVLGVPTVADRLAQTVVAMVLEHRAERVFHPGSYGYRPGRGAIDAVRSCRRRC
WENDWVIDLDIQAFFDTVPWDLVCRAVGAVCDLPWVMLYVRRWLKAPLQHSDGTLTERERGTPQGSAVSPV
LANLFMHYAFDTWMARSYPGIVFERYADDVVIHCESLNQARVVLTAVEERMGQVGLGLHPRKTRIVYCKDANR
PGSWEHTGFTFLGYEFRERTVKGRHGLFRSFSPAVSRTALKRMSTQVRSWRLHRWVTATAGDLAAQINPVLRG
WMSYYGVFHPSALYPLLKRVNSYLIRWLRGKYRKLRVSWSKTMRKWYTGVKKAPNYFVHWAWVTEPGPVW
>R5OH277||gene_123663|GeneMark.hmm|430_aa|+|564|1856
MSGSVKLAGSSVRSSGSAVKPFDVPKWLVMAAWEKVRSNKGAPGVDGAAVEDFEKDLRANLYKIWNRMSS
GSYFPSPVREVRIPKPDGGIRVLGVPTVADRLAQTVVAMVLEHRAERVFHPGSYGYRPGRGAIDAVRSCRRRC
WENDWVIDLDIQAFFDTVPWDLVCRAVGAVCDLPWVMLYVRRWLKAPLQHSDGTLTERERGTPQGSAVSPV
LANLFMHYAFDTWMARSYPGIVFERYADDVVIHCESLNQARVVLTAVEERMGQVGLGLHPRKTRIVYCKDANR
PGSWEHTGFTFLGYEFRERTVKGRHGLFRSFSPAVSRTALKRMSTQVRSWRLHRWVTATAGDLAAQINPVLRG

WMSYYGVFHPSALYPLLKRVNSYLIRWLRGKYRKLRVSWSKTMRKWYTGVKKAPNYFVHWAWVTEPGPVW
>R5OH280||gene_248008|GeneMark.hmm|410_aa|+|2|1234
PFDVPKWLVMEAWEKVRSDKGAPGVDGAAVEDFEKDLRASLYKIWNRMSSGSYFPSPVREVRIPKPDGGIRVL
GVPTVAGRLAQTVVAMVLEHRAERVFHPGSYGYRPGRGAIDAVRACRRRCWENDWVIDLDIQAFFDTVPWD
LVCRVVGAVCDLPWVMLYVRRWLKAPLQHSDGTLTERERGTPQGSAVSPVLANLFMHYAFDTWMARSYPGI
VFERYADDVVIHCKSLNQARVVLTAVEERMGQVGLGLHPRKTRIVYCKDANRPGSWEHTGFTFLGYEFRERTVK
GRHGLFRSFSPAVSRMALKRMSTQVRSWRLHRWVTATAGDLAAQINPVLRGWMSYYGVFHPSALYPLLKRVN
SYLIRWLRGKYRKLRVSWSKTMRKWYTGVKKAPNYFVHWAWVTEPGPVW
>R5OH281||gene_313886|GeneMark.hmm|430_aa|-|426|1718
MSGSVKLAGSSVRSSGSAVKPFDVPKWLVMEAWEKVRSDKGAPGVDGAAVEDFEKDLRASLYKIWNRMSSG
SYFPSPVREVRIPKPDGGIRVLGVPTVAGRLAQTVVAMVLEHRAERVFHPGSYGYRPGRGAIDAVRACRRRCW
ENDWVIDLDIQAFFDTVPWDLVCRVVGAVCDLPWVMLYVRRWLKAPLQHSDGTLTERERGTPQGSAVSPVL
ANLFMHYAFDTWMARSYPGIVFERYADDVVIHCKSLNQARVVLTAVEERMGQVGLGLHPRKTRIVYCKDANR
PGSWEHTGFTFLGYEFRERTVKGRHGLFRSFSPAVSRMALKRMSTQVRSWRLHRWVTATAGDLAAQINPVLR
GWMSYYGVFHPSALYPLLKRVNSYLIRWLRGKYRKLRVSWSKTMRKWYTGVKKAPNYFVHWAWVTEPGPV
W
>R5OH283||gene_136771|GeneMark.hmm|382_aa|+|1072|2220
MTAPRGAFEKDLKNNLYKIWNRMSSGSYFPPPVRAVEMPKPSGGVRVLGVPTVADRVAQTVVAEKLEKRVEPI
FHPDSYGYRPGRSALDAVGQCRRRCWNRAWVVDLDIARFFDEVDHQLLLKAVAGHAPEPWVLLYISRWLKAP
IQHGDGTIAQRSRGTPQGSAVSPVLANLFLHYAFDMWMARRFPTVQFERYVDDVVVHCVTERQAREVREAV
EGRLARVGLRMHPDKTRIVYCRTQKRRGDHPEVSFDFLGYTFRPRAARDGKGGIFTSFLPAISKSALKRLSARVRS
WRLHLRTGSTLTGLARTINPIVRGWMQYYGRFYRTALYPLLKRINAYLVRWLRKKYKRLRTFKKAKAAWRRVTR
QCPLLLSHWAWVQSFW
>R5OH285||gene_103848|GeneMark.hmm|433_aa|-|3|1301
MSGPQLSGKPFDISKQEVWRAYQKVKANKGAPGVDDCSVEAFEKDLKNNLYKIWNRMSSGSYFPPPVRAVE
MPKPSGGVRVLGVPTVADRVAQTVVAEKLEKRVEPIFHPDSYGYRPGRSALDAVGQCRRRCWNRAWVVDLDI
ARFFDEVDHQLLLKAVAGHAPEPWVLLYISRWLKAPIQHGDGTIAQRSRGTPQGSAVSPVLANLFLHYAFDM
WMARRFPTVQFERYVDDVVVHCVTERQAREVREAVEGRLARVGLRMHPDKTRIVYCRTQKRKGDHPEVSFD
FLGYTFRPRAARDGKGGIFTSFLPAISKSALKRLSARVRSWRLHLRTGSTLTGLARTINPIVRGWMQYYGRFYRTA
LYPLLKTHQRLLGALAAEEVQTAADLQEGQSGLEEGDPSVPPPPEPLGLGPIILVTKDDKSRVTGDCYARF
>R5OH289||gene_796|GeneMark.hmm|360_aa|+|722|1804
MSSGSYFPPPVRAVEMPKPSGGVRVLGVPTVADRVAQTVVAEKLEKRVEPIFHPDSYGYRPGRSALDAVGQCR
RRCWNRAWVVDLDIARFFDEVDHQLLLKAVAGHAPEPWVLLYISRWLKAPIQHGDGTIAQRSRGTPQGSAVS
PVLANLFLHYAFDMWMARRFPTVQFERYVDDVVVHCVTERQAREVREAVEGRLARVGLRMHPDKTRIVYCR
TQKRRGDHPEVSFDFLGYTFRPRAARDGKGGIFTSFLPAISKSALKRLSARVRSWRLHLRTGSTFTGLARTINPIVR
GWMQYYGRFYRTALYPLLKRINAYLVRWLRKKYKRLRTFKKAKAAWRRVTRQCPLLLSHWAWVQSFW
>R5OH295||gene_237754|GeneMark.hmm|416_aa|+|1000|2250
MSGPQLSGKPFDISKQEVWRAYQKVKANKGAPGVDDCSVEAFEKDLKNNLYKIWNRMSSGSYFPPPVRAVE
MPKPSGGVRVLGVPTVADRVAQTVVAEKLEKRVEPIFHPDSYGYRPGRSALDAVGQCRRRCWNRAWVVDLDI
ARFFDEVDHQLLLKAVAGHAPEPWVLLYISRWLKAPIQHGDGTIAQRSRGTPQGSAVSPVLANLFLHYAFDM
WMARRFPTVQFERYVDDVVVHCVTERQAREVREAVEGRLARVGLRMHPDKTRIVYCRTQKRKGDHPEVSFD
FLGYTFRPRAARDGKGGIFTSFLPAISKSALKRLSARVRSWRLHLRTGSTFTGLARTINPIVRGWMQYYGRFYRTA
LYPLLKRINAYLVRWLRKKYKRLRTFKKAKAAWRRVTRQCPLLLSHWAWVQSFW
>R5OH295||gene_319532|GeneMark.hmm|430_aa|-|1626|2918

MSGSVKLAGSSVRSSGSAVKPFDVPKWLVMAAWEKVRSNKGAPGVDGAAVEDFEKDLRANLYKIWNRMSS
GSYFPSPVREVRIPKPDGGIRVLGVPTVADRLAQTVVAMVLEHRAERVFHPGSYGYRPGRGAIDAVRSCRRRC
WENDWVIDLDIQAFFDTVPWDLVCRAVGAVCDLPWVMLYVRRWLKAPLQHSDGTLTERERGTPQGSAVSPV
LANLFMHYAFDTWMARSYPGIVFERYADDVVIHCESLNQARVVLTAVEERMGQVGLGLHPRKTRIVYCKDANR
PGSWEHTGFTFLGYEFRERTVKGRHGLFRSFSPAVSRTALKRMSTQVRSWRLHRWVTATAGDLAAQINPVLRG
WMSYYGVVHPSALYPLLKRVNSYLIRWLRGKYRKLRVSWSKTMRKWYTGVKKAPNYFVHWAWVTEPGPVW
