# Supplementary material for: Genomic and Metagenomic Insights into the Distribution of Nicotine-degrading Enzymes in Human Microbiota
Source: Curr Genomics. 2024 Mar 20;25(3):226–35. doi: 10.2174/0113892029302230240319042208 (PMC11288164; doi:10.2174/0113892029302230240319042208)
Supplement: Supplementary file 1 [file CG-25-226_SD1.zip › CG-25-226_SD1/Xie MS Suppl file 4.pdf]

>SEQF8252||SEQF8252.1_03737
MQNKSFTRIEAIRKANADPTYVNDRIYRLMFKEDLYIAAYEKIKSKPGNMTAGADGTTLDEFSIRTIKNIIEKMKS
ESFSFRGARKVLIPKANGKTRALSVAPPTDKVVQEVMRMILEAIFEPTFSSNSHGFRSGRSCHTALKQIRKNWSG
VTWIIEGDIKGCFDNINHEKLIRELAKRITDERFINLVRKALNAGYFEDGAFFSGDLGTPQGSIISPILANIFLHQLD
RKAEKIIEENEIGEEDKKALNPEYRKVVKRRAYLQKTLERKEGTEREAAIAEIRALNNQSLTMSPNLITSNGFIRVK
YVRYADDWVIGVNGPRKLAETIRADIGDTLSTMGLELSMEKTHIRHAKTESATFLGTTFRVGSATPKIMKVSRG
GRIFKKRVAGWTPLMYAPIGEIIKRLSAKGFCDPKGNPTAIKKWIYLDDTQIVEQVGAVWRGICNYYSFVDGFAK
LSRIQFILQHAVAKTLAAKHRSSRSKVFSKHGANLRFRVRNEAGEVVKTVSFPLVKSWKSSPDRFKTNEVDINFLE
RNLRLRTRSKLGSLCVICGSGDRVAMHHVKHIRKLGKEVKGFNRVMAILNRKQIPVCHECHHKIHSGKYDGISL
NQFALPHVAAA
>HRGM_Genome_1933||HRGM_Genome_1933_CDS_08276
MAEFDLRRLESIARLNANPTWVNRDLYRLLFKEDLYLAAYERIKSKPGNMTKGVDGTTLDGMNMPHVEAVIEA
MRSESFTFAPARRTYIPKKNGKLRPLGIANPREKLVQEAIRMILEAIYDSPFGPTFSDKSYGFRQKRGTHNALREIR
TRWTGVRWIIEGDIMSFFDNIEHDRLIELLRKRIKDERFLNLIRKALNAGVLDQGDFTATTSGTPQGSVVSPILAN
VYLHELDIKVTEIVARETKGKGAKPNPAYRSLVNKLYAGRKKGTITAEEVKAIQKAMRELSPYAQDDPDFIRVHYV
RYADDWVIGIIGSRELAERTRSEIAEWLKRELRLELNIGKTHIRHAATEEAFFLGTRLSCAGRRNDSLVKHAPKPGY
KAVKCRLPAGTVHLKAPINDIVAKLHQNGFCTKEGHPLSKRAWAVLDDDQIISRFNAVLDGILNYYSFTDNFAR
MRRVQYILQFSAAKTLSHRHRMKSIRRAFAKYGYNLTTRLRNKDGEERVVSMRLRKSFEGTPFNFKGLSNPGAG
TEIRALKFGIRTKSSLLKDCAICGATDGVEMHHVRHIRKMGQEVKGFTRIMAAINRKQIPTCKKCHTDIHAGRYD
GISLTEVALRMA
>CABMGP01||gene_7897|GeneMark.hmm|614_aa|-|24870|26714
MAEFDLRRLESIARLNANPTWVNRDLYRLLFKEDLYLAAYERIKSKPGNMTKGVDGTTLDGMNMPHVEAVIEA
MRSESFTFAPARRTYIPKKNGKLRPLGIANPREKLVQEAIRMILEAIYDSPFGPTFSDKSYGFRQKRGTHNALREIR
TRWTGVRWIIEGDIMSFFDNIEHDRLIELLRKRIKDERFLNLIRKALNAGVLDQGDFTATTSGTPQGSVVSPILAN
VYLHELDIKVTEIVARETKGKGAKPNPAYRSLVNKLYAGRKKGTITAEEVKAIQKAMRELSPYAQDDPDFIRVHYV
RYADDWVIGIIGSRELAERTRSEIAEWLKRELRLELNIGKTHIRHAATEEAFFLGTRLSCAGRRNDSLVKHAPKPGY
KAVKCRLPAGTVHLKAPINDIVAKLHQNGFCTKEGHPLSKRAWAVLDDDQIISRFNAVLDGILNYYSFTDNFAR
MRRVQYILQFSAAKTLSHRHRMKSIRRAFAKYGYNLTTRLRNKDGEERVVSMRLRKSFEGTPFNFKGLSNPGAG
TEIRALKFGIRTKSSLLKDCAICGATDGVEMHHVRHIRKMGQEVKGFTRIMAAINRKQIPTCKKCHTDIHAGRYD
GISLTEVALRMA
