# Supplementary material for: Genomic and Metagenomic Insights into the Distribution of Nicotine-degrading Enzymes in Human Microbiota
Source: Curr Genomics. 2024 Mar 20;25(3):226–35. doi: 10.2174/0113892029302230240319042208 (PMC11288164; doi:10.2174/0113892029302230240319042208)
Supplement: Supplementary file 1 [file CG-25-226_SD1.zip › CG-25-226_SD1/Xie MS Suppl file 5.pdf]

>SEQF8741||SEQF8741.1_00296
MTEKIYDAIVVGAGFSGLVAARELSAQGRSVLIIEARHRLGGRTHVVNFLGRPVEIGGAGVHWCQPHVFAEMQ
RYGFGFKEAPLADLDKAYMVFADGQKIDVPPSTFDEEYTTAFEKFCSRSRELFPRPYSPLDNHEVSNLDGVSARD
HLESLGLNELQLASMNAELTLYGGAPTTELSYPSFVKFHALASWDTITFTDSEKRYHVQGGTNALCQAIFDDCRA
DSEFGVPVEAVAQTDNGVTVTLADKRVFRALTCVLTLPTKVYADVRFEPPLPPEKRAFIEHAEMADGAELYVHV
RQNLGNTFTFCDDPNPFNAVQTYAYDDELGTILKITIGRQSLINLENFDAIAAEIRKIHGDVEVLEALPYNWAMD
EYARTSYPAMRKGWFSRYKDMAKPENRLFFAGSATADGWHEYIDGAIESGIRVGREIRHFMKATA
>SEQF8748||SEQF8748.1_03514
MTEKIYDAIVVGAGFSGLVAARELSAQGRSVLIIEARHRLGGRTHVVNFLGRPVEIGGAGVHWCQPHVFAEMQ
RYGFGFKEAPLADLDKAYMVFADGQKIDVPPSTFDEEYTTAFEKFCSRSRELFPRPYSPLDNHEVSNLDGVSARD
HLESLGLNELQLASMNAELTLYGGAPTTELSYPSFVKFHALASWDTITFTDSEKRYHVQGGTNALCQAIFDDCRA
DSEFGVPVEAVAQTDNGVTVTLADKRVFRALTCVLTLPTKVYADVRFEPPLPPEKRAFIEHAEMADGAELYVHV
RQNLGNTFTFCDDPNPFNAVQTYAYDDELGTILKITIGRQSLINLENFDAIAAEIRKIHGDVEVLEALPYNWAMD
EYARTSYPAMRKGWFSRYKDMAKPENRLFFAGSATADGWHEYIDGAIESGIRVGREIRHFMKATA
>SEQF8770||SEQF8770.1_03353
MTEKIYDAIVVGAGFSGLVAARELSAQGRSVLIIEARHRLGGRTHVVNFLGRPVEIGGAGVHWCQPHVFAEMQ
RYGFGFKEAPLADLDKAYMVFADGQKIDVPPSTFDEEYTTAFEKFCSRSRELFPRPYSPLDNHEVSNLDGVSARD
HLESLGLNELQLASMNAELTLYGGAPTTELSYPSFVKFHALASWDTITFTDSEKRYHVQGGTNALCQAIFDDCRA
DSEFGVPVEAVAQTDNGVTVTLADKRVFRALTCVLTLPTKVYADVRFEPPLPPEKRAFIEHAEMADGAELYVHV
RQNLGNTFTFCDDPNPFNAVQTYAYDDELGTILKITIGRQSLINLENFDAIAAEIRKIHGDVEVLEALPYNWAMD
EYARTSYPAMRKGWFSRYKDMAKPENRLFFAGSATADGWHEYIDGAIESGIRVGREIRHFMKATA
